# De novo assembly and transcriptome analysis of *Plasmodium gallinaceum* identifies the Rh5 interacting protein (*ripr*), and reveals a lack of EBL and RH gene family diversification

**DOI:** 10.1186/s12936-015-0814-0

**Published:** 2015-08-05

**Authors:** Elvin J Lauron, Han Xian Aw Yeang, Samantha M Taffner, Ravinder N M Sehgal

**Affiliations:** Department of Molecular Microbiology, Washington University School of Medicine, St. Louis, MO USA; Rheumatology Division, Washington University School of Medicine, St. Louis, MO USA; Department of Biology, San Francisco State University, San Francisco, CA USA

**Keywords:** *Plasmodium*, Transcriptome, Avian malaria, Invasion, Erythrocytes, *ripr*

## Abstract

**Background:**

Malaria parasites that infect birds can have narrow or broad host-tropisms. These differences in host specificity make avian malaria a useful model for studying the evolution and transmission of parasite assemblages across geographic ranges. The molecular mechanisms involved in host-specificity and the biology of avian malaria parasites in general are important aspects of malaria pathogenesis that warrant further examination. Here, the transcriptome of the malaria parasite *Plasmodium gallinaceum* was characterized to investigate the biology and the conservation of genes across various malaria parasite species.

**Methods:**

The *P. gallinaceum* transcriptome was annotated and KEGG pathway mapping was performed. The *ripr* gene and orthologous genes that play critical roles in the purine salvage pathway were identified and characterized using bioinformatics and phylogenetic methods.

**Results:**

Analysis of the transcriptome sequence database identified essential genes of the purine salvage pathway in *P. gallinaceum* that shared high sequence similarity to *Plasmodium falciparum* when compared to other mammalian *Plasmodium* spp. However, based on the current sequence data, there was a lack of orthologous genes that belonged to the erythrocyte-binding-like (EBL) and reticulocyte-binding-like homologue (RH) family in *P. gallinaceum*. In addition, an orthologue of the Rh5 interacting protein (*ripr*) was identified.

**Conclusions:**

These findings suggest that the pathways involved in parasite red blood cell invasion are significantly different in avian *Plasmodium* parasites, but critical metabolic pathways are conserved throughout divergent *Plasmodium* taxa.

**Electronic supplementary material:**

The online version of this article (doi:10.1186/s12936-015-0814-0) contains supplementary material, which is available to authorized users.

## Background

A key determinant of host species susceptibility and host-specificity in malaria infections is the successful interaction between host erythrocyte receptors and parasite invasion ligands [1–4]. For mammalian malaria species, the invasion ligands that are implicated in host-specificity are relatively well characterized and can be grouped into two gene families, the erythrocyte-binding-like (EBL) and reticulocyte-binding-like homologue (RH) genes [5]. EBL and RH genes have been difficult to identify in avian malaria species since the host erythrocytes are nucleated, which in turn leads to methodological challenges for sequencing genetic material from these parasites [6, 7]. Therefore, the invasion pathways and ligands involved in avian malaria parasite host-specificity remain poorly understood. However, insight into the invasion process of avian malaria parasites was recently gained from the sequenced transcriptomes of *Plasmodium relictum*, a host generalist avian malaria parasite [7], and *Plasmodium gallinaceum*, a parasite of domestic chickens (*Gallus gallus*) [6].

Sequencing of the *P. gallinaceum* transcriptome has led to the identification of several orthologous genes of mammalian *Plasmodium* spp., two of which are critical for these parasites to invade host erythrocytes, the apical membrane antigen-1 (*ama*-*1*) and the rhoptry neck protein 2 (*RON2*) [6]. Additionally, an orthologue of the merozoite apical erythrocyte binding-ligand (*maebl*) gene, a member of the EBL family, was also identified and is currently the only known EBL gene expressed in avian *Plasmodium* [8]. These invasion genes are fairly conserved and show greater sequence similarity to *Plasmodium falciparum* genes, in comparison to other mammalian *Plasmodium* spp. The genotypic and biological similarities between *P. falciparum* and *P. gallinaceum* [9–13] suggest that knowledge gained from *P. gallinaceum* may be applicable to *P. falciparum* or vice versa, and thus highlights the importance of *P. gallinaceum* as a *Plasmodium* model. Furthermore, orthologous genes of EBL and RH receptors are encoded in the *Gallus gallus* genome [14–16]. Among these host receptors are glycophorin C, complement component receptor 1 and basigin, which bind to the EBL/RH proteins EBA-140, Rh4 and Rh5, respectively [3, 14, 15]. It is plausible that avian *Plasmodium* also utilize these diverse invasion pathways. However, mammalian *Plasmodium* spp. are generally host-specific as opposed to avian *Plasmodium* spp.

The host range of avian malaria parasites can include multiple avian host species or can be restricted to a single avian host species [17]. Investigating the genetic determinants of host-specificity in avian malaria parasites may lead to a better understanding of the molecular mechanisms that augment host switching or zoonotic malaria. However, little is known regarding the molecular mechanisms of avian malaria pathogenesis. The aim of this study was to elucidate key features of avian malaria parasite biology by characterizing the *P. gallinaceum* transcriptome and by identifying orthologous genes that may contribute to the host-specificity of these *Plasmodium* parasites.

## Methods

### Sequencing and assembly of the *Plasmodium gallinaceum* transcriptome

RNA from *P. gallinaceum*-infected chick blood was obtained for transcriptome sequencing and analysis as previously described [6]. In brief, White Leghorn chickens were infected with erythrocytes containing *P. gallinaceum* and infections were verified by PCR amplification of the *P. gallinaceum cytochrome b* gene and microscopy. Total RNA was extracted from infected chick blood, and cDNA libraries were prepared for sequencing on the HiSeq2000 platform. Raw reads were deposited in the NCBI sequence read archive (accession No. SRR1611148). To remove chicken sequences, the total reads were mapped to the *Gallus gallus* (chicken) genome using Bowtie [18]. The unmapped reads were collected for de novo assembly with or without quality trimming using Trimmomatic/Trinity [19] (Additional files 1, 2, 3, 4); paired end Trimmomatic parameters used were: *LEADING*:5 *TRAILING*:5 *SLIDINGWINDOW*:4:15 *MINLEN*:36. Lowly supported Trinity transcripts (FPKM <1) were removed using the Trinity RSEM utility and the remaining transcripts were clustered with CD-HIT-EST [20] using a sequence similarity threshold of 97%. Subsequently, a BLASTx query with the assembled *P. gallinaceum* transcriptomes was performed against the non-redundant protein database to remove any remaining chicken sequences. Queries with hits matching *Gallus gallus* sequences were removed using a custom BLAST parser script. The de novo transcriptome assemblies (before and after filtering) were evaluated using RSEM-EVAL [21].

### Transcriptome characterization and analyses

Transcripts from the filtered assemblies were annotated using Blast2Go [22]. The Kyoto Encyclopedia of Genes and Genomes (KEGG) pathways were inferred and analysed through the KEGG database [23]. A database of *P. falciparum* EBL and RH gene sequences (Additional file 5) was generated and a tBLASTx query with the assembled *P. gallinaceum* transcripts was performed. A minimum query coverage of 60% and an E value cut-off of 1 × 10^−10^ were chosen for identifying putative orthologues. tBLASTx query of the EBL and RH gene sequences were also performed against the *P. gallinaceum* genome.

### Phylogenetic analyses and characterization of *Plasmodium gallinaceum* orthologues

Sequences were aligned using MUSCLE in SEAVIEW [24]. For the maximum likelihood (ML) and Bayesian inference analysis, the Modeltest Version 3.7 [25] was used to determine the most appropriate nucleotide and amino acid substitution model based on the Akaike Information Criterion of the orthologous genes. The GTR + I + G model was selected for both the *ripr* and adenosine deaminase (*ada*) alignments. The GTR + G model was selected for both the hypoxanthine–guanine phosphoribosyl transferase (*hgprt*) and purine nucleoside phosphorylase (*pnp*) alignments. The GTR + I model was selected for the nucleoside transporter 1 (*nt1*) alignment. The WAG + I + G, LG + G, LG + G, WAG + G and CPREV + G amino acid substitution models were selected for the amino acid sequence alignments of *ripr*, *ada*, *hgprt*, *pnp*, and *nt1*, respectively. ML methods were implemented in RAxML [26] using 1,000 rapid bootstrap inferences. A Bayesian approach, as implemented in Beast v1.8.0 [27], was used to estimate posterior probabilities of the tree branches. The non-synonymous substitutions per non-synonymous sites divided by the synonymous substitutions per synonymous sites (d_N_/d_S_) ratio was analysed in an alignment of multiple *Plasmodium* spp. *ripr* gene sequences (see Additional file 5 for the gene IDs and accession numbers) using a sliding window method, as applied in DNAsp [28]. The variance of d_N_ and d_S_ (d_N_–d_S_) and standard errors were calculated by the Nei and Gojobori method with the Jukes and Cantor correction, and the significance was assessed using a two-tailed Z test with 1,000 bootstrap pseudosamples in MEGA v5.2.2 [29]. Genetic distances were calculated in MEGA v5.2.2.

## Results

### Characterization of the *Plasmodium gallinaceum* transcriptome

Without quality trimming prior to de novo assembly, the de novo transcriptome assembly resulted in 44,256 contigs (including alternatively spliced isoforms) with N50 of 1125 nt and a mean sequence length of 772 nt. After filtering out contigs that were not well supported based on the abundance estimations, transcripts that are redundant or highly similar, and transcripts with BLAST hits matching chicken sequences, the resulting assembly had 17,832 transcripts, with N50 of 1348 nt and a mean sequence length of 893 nt. Filtered assemblies resulted in better RSEM-EVAL scores (Additional file 6) and were used for functional annotation.

**Fig. 1:**
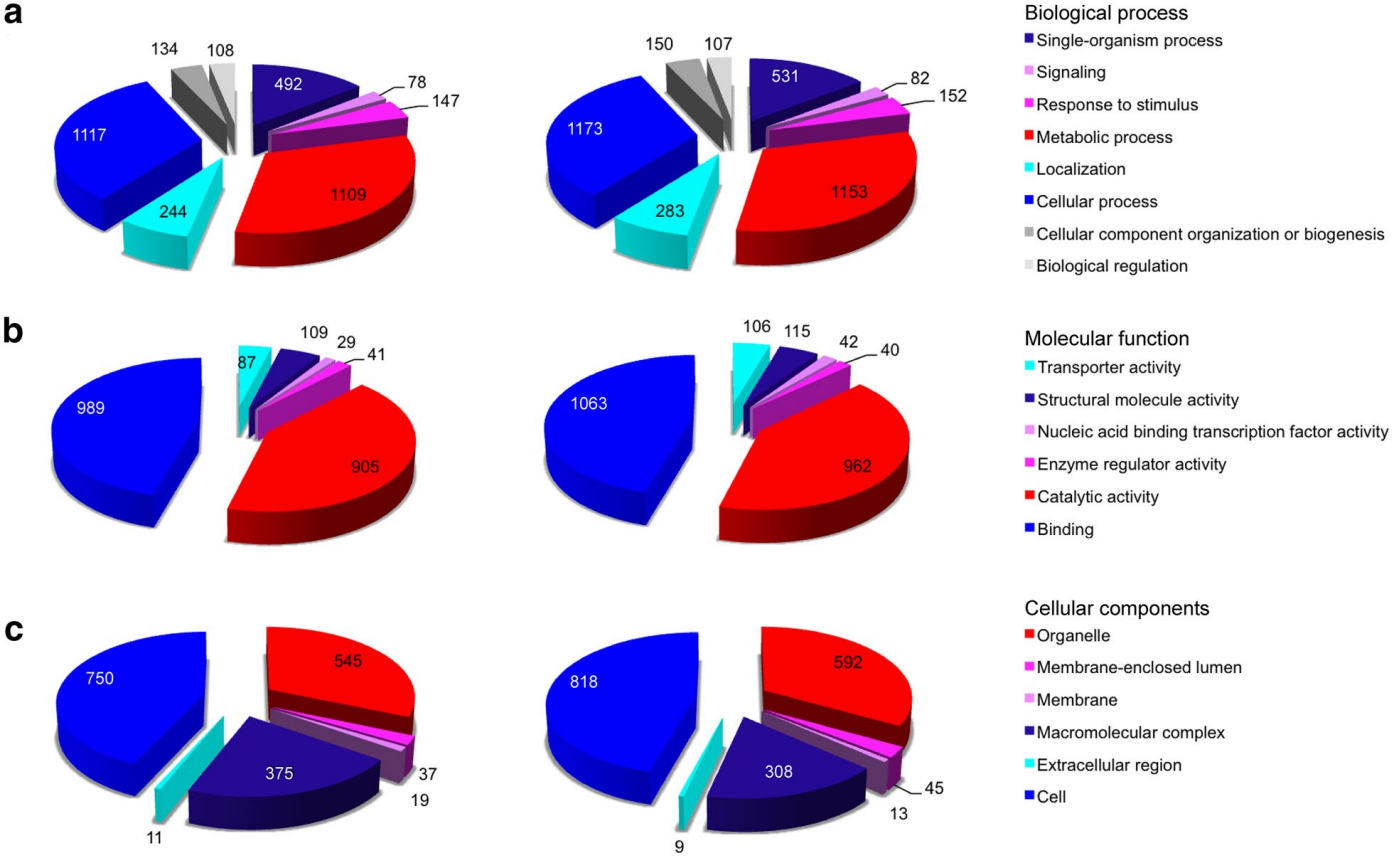
Functional annotations of the assembled sequences without (*left*) and with (*right*) quality trimming from the blood stages of *Plasmodium gallinaceum.* The *pie charts* show the number of sequences that are grouped into three general categories: biological process (**a**), molecular function (**b**) and cellular components (**c**).

A total of 8,550 sequences were annotated with Blast2go (Additional files 7, 8, 9). GO terms were assigned to contigs and the sequences were grouped based on biological processes, molecular functions, and cellular components (Fig. 1). Analysis of all the sequences identified GO annotations for 3,429 sequences in biological processes, 2,160 sequences in molecular functions and 1,737 sequences in cellular components. The sequences associated with cellular components were represented by several sub-categories: cell (43%), organelle (31%) and macromolecular complex (21%). Similarly, the sequences associated with biological processes were distributed primarily across several sub-categories: cellular processes (33%), metabolic processes (32%), single-organism processes (14%), and localization (7%). In contrast, the majority of sequences for molecular components were mainly associated with two sub-categories, catalytic activity (42%) and binding (46%) components.

**Fig. 2:**
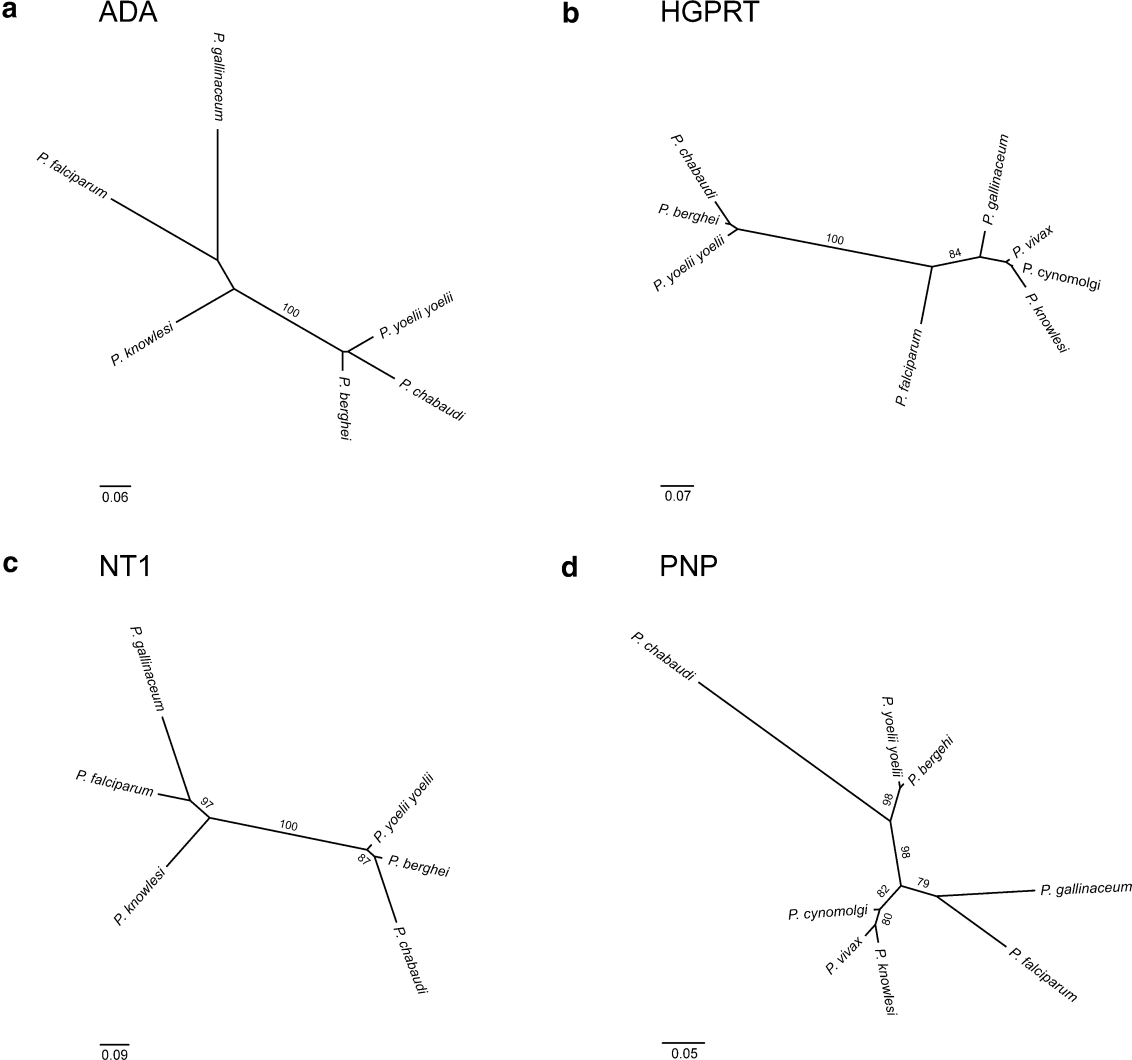
Phylogeny of *Plasmodium* spp. based on the amino acid sequences of **a** adenosine deaminase (ADA), **b** hypoxanthine–guanine phosphoribosyl transferase (HGPRT), **c** nucleoside transporter 1 (NT1), and **d** purine nucleoside phosphorylase (PNP). See Additional file 5 for accession numbers.

In addition, the *P. gallinaceum* transcriptome was further characterized by KEGG pathway analysis, which resulted in a total 909 sequences mapping to 71 KEGG pathways (Additional file 10). Thiamine, pyrimidine and purine metabolism comprised the greatest amount of mapped sequences amongst the assigned KEGG pathways. These findings and the fact that *Plasmodium* parasites are purine auxotrophs prompted a search in the *P. gallinaceum* transcriptome assembly for genes that play critical roles in purine salvage. A partial cDNA sequence encoding a *P. gallinaceum* version of the purine salvage enzyme PNP and full-length cDNA sequences encoding the enzymes ADA, HGPRT and NT1 were identified. Phylogenetic analyses consistently showed, with strong posterior probabilities and bootstrap support, that the identified orthologous *P. gallinaceum* genes are most similar to the *P. falciparum* genes in these analyses (Fig. 2; Additional file 11).

Quality trimming of the raw sequence reads prior to de novo assembly generated a higher number of contigs, 233,882 in total, with N50 of 532 nt and a mean sequence length of 474 nt. The resulting filtered transcriptome had 32,549 contigs with N50 of 950 nt and a mean sequence length of 678 nt. Quality trimming and filtering of the transcriptome assembly did not significantly change the annotation/mapping and KEGG pathway result (Fig. 1, Additional files 12, 13, 14, 15).

### Identification and characterization of *ripr* in *Plasmodium gallinaceum*

To identify orthologous EBL and RH genes, BLAST searches were performed against *P. falciparum* EBL and RH gene sequences using the *P. gallinaceum* transcriptome sequences as queries. There were no significant BLAST hits that met the selected criteria. However, a partial cDNA sequence, with a length of 3,006 base pairs (bp), encoding an orthologue of *ripr* (sequence comp28469_c1_seq2, Additional file 1) was identified in *P. gallinaceum*. To confirm that the *ripr* sequence is present in the *P. gallinaceum* genome, a BLAST search against the *P. gallinaceum* genome was performed using the identified partial cDNA sequence of *ripr* as a query. The search resulted with a significant hit comprising 100% query coverage and identity to nucleotide (nt) positions 1101-1495 of the contig Pg_2265551.c000013273. The identified genomic sequence was then used to annotate the full-length *ripr* gene of *P. gallinaceum*. Furthermore, 6,207 *P. gallinaceum* RNA-seq reads mapped to the full-length *P. gallinaceum ripr* sequence with coverage across the entire gene.

**Fig. 3:**
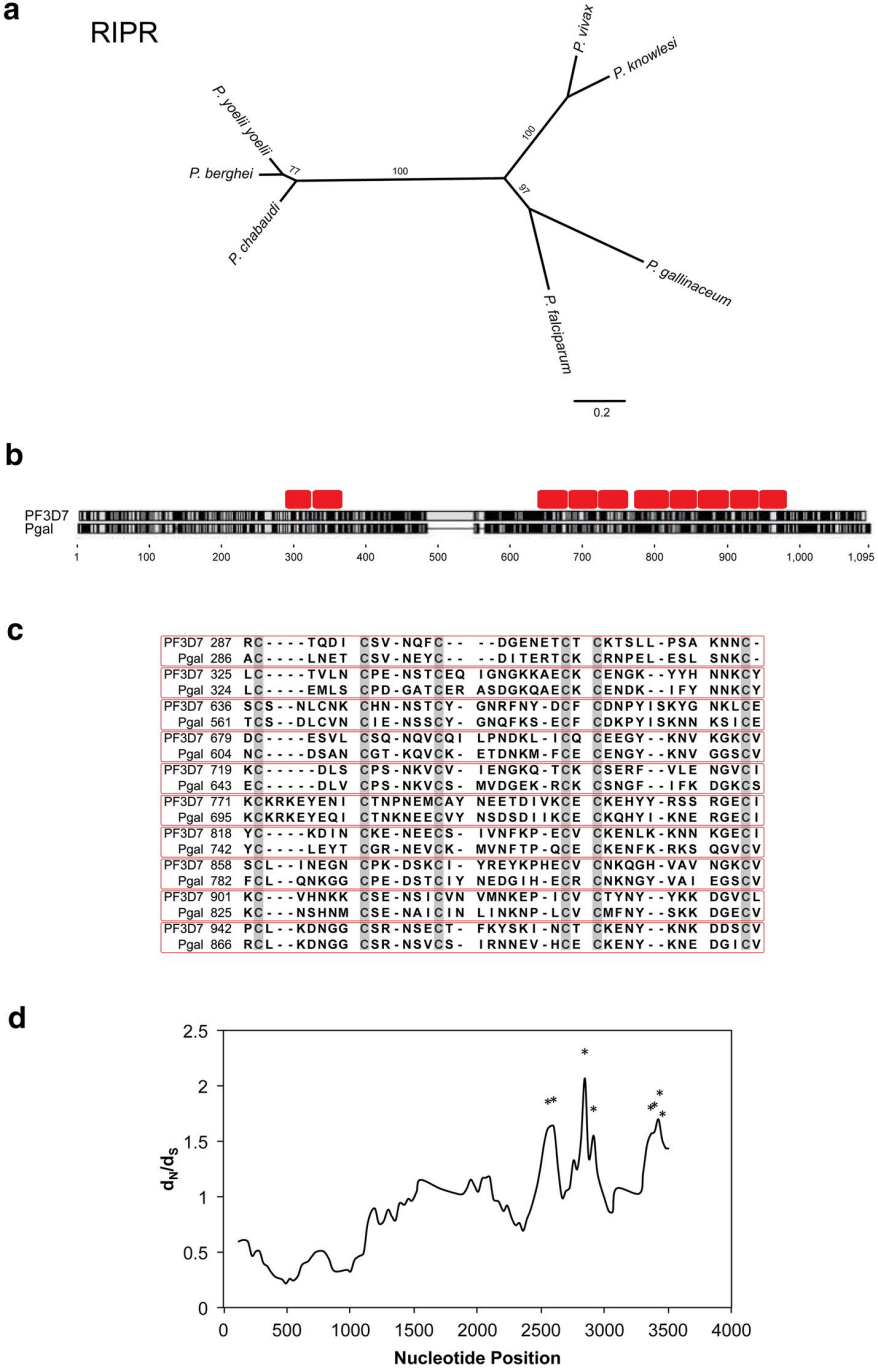
Comparison of *Plasmodium* *rip.* **a** Phylogeny of the *Plasmodium* based on amino acid sequences of RIPR. **b** Schematic of *P. falciparum* and *P. gallinaceum* RIPR amino acid alignment. Conserved regions are represented by *black blocks*. Variable regions are represented by *grey blocks*. Gaps in the alignment are represented by *horizontal black lines*. Epidermal growth factor-like domains are represented by *red blocks*. **c** Alignment of *P. falciparum* and *P. gallinaceum* epidermal growth factor-like domains. Individual epidermal growth factor-like domains are outlined in *red* and are ordered according to location, with the domain located closest to the N-terminus positioned at the *top* and the domain located closest to the C-terminus positioned at the *bottom* of the alignment. **d** Sliding window plot of d_N_/d_S_ of *ripr*. The window length is 180 bp with a step size of 90 bp. *Asterisks* indicate regions with a significant excess of non-synonymous substitutions (P ≤ 0.05).

The full-length gene and the translated amino acid sequence of *P. gallinaceum ripr* are 3,051 bp and 1,016 amino acids in length, respectively, and share 69% nucleotide and 54% amino acid sequence identity in a pair-wise comparison to *P. falciparum*. Phylogenetic analysis of *P. gallinaceum* and mammalian *Plasmodium* spp*. ripr* shows that *P. gallinaceum ripr* is most similar to that of *P. falciparum* (Fig. 3a). Sixty-six amino acids at positions 482–547 and eight amino acids at positions 554–561 (relative to *P. falciparum* RIPR) were absent from *P. gallinaceum* RIPR (Fig. 3b). Ninety cysteine residues were present in *P. gallinaceum* RIPR, 87 of which are conserved between *P. falciparum and P. gallinaceum*. The ten EGF-like domains of RIPR are also present and highly conserved in *P. gallinaceum* RIPR (Fig. 3c); as observed with *P. falciparum* RIPR, two EGF-like domains were clustered at the N-terminus, whereas the remaining eight EGF-like domains were clustered around the C-terminus in *P. gallinaceum* RIPR. d_N_/d_S_ methods were applied to orthologous *Plasmodiumripr* sequences to assess diversifying selection on the *ripr* gene. There were no significant differences between d_N_ and d_S_ when analysing the entire coding region of *ripr* (d_N_ = 0.50 ± 0.01, d_S_ = 0.67 ± 0.03, P > 0.05). However, sliding window analysis of d_N_/d_S_ revealed significant signatures of diversifying selection throughout the region encoding the C-terminus of RIPR (Fig. 3d; Additional file 16). In contrast to the C-terminus, the d_N_/d_S_ ratios were below 1.0 throughout the region encoding the N-terminus, suggesting that the N-terminus of RIPR is under strong purifying selection.

## Discussion

Malaria parasites pose a significant threat to avian wildlife populations, and the emergence of avian malaria in naïve populations can have major ecological consequences [30]. The emergence of avian malaria is attributed to malaria parasites’ diverse host-specificity and geographic dispersal ranges that are facilitated by its avian hosts [31–33]. Large geographic dispersal ranges increase the probability for parasites to encounter new potential hosts, therefore increasing the chances for host-switching events [17, 33–36]. Indeed, host switching is a common phenomena that appears to be a distinct feature of avian malaria parasites [34, 37–40], i.e., mammalian malaria parasites are generally host-specific, whereas avian malaria parasites have a broad host-specificity. One aim of this study was to identify orthologous genes that may play a role in host-specificity, and to subsequently assess the evolution of these genes between mammalian and avian *Plasmodium*.

Based on the currently available sequence data, avian *Plasmodium* appear to lack the majority of EBL and RH genes that have been implicated in *Plasmodium* host-specificity, including *eba*-*175*, *eba*-*181*, *eba*-*140*, *Rh1*, *Rh2a*, *Rh2b*, *Rh4,Rh5* [2–5, 41–45]. The only identified gene that exhibits clear orthology was *maebl* [8]. It is important to note that this finding may be attributed to several possibilities. For instance, the technical challenges and limitations for obtaining sequence data from avian *Plasmodium* parasites may have resulted in insufficient sequence coverage or sequencing that is not deep enough to detect these genes. An anticipated high quality draft genome, currently being produced as part of the Wellcome Trust Sanger Institute parasite genomics project, may help to resolve these uncertainties. Therefore, these findings do not rule out the presence of EBL and RH orthologues in avian *Plasmodium*. However, the number of invasion-associated genes also differ when comparing the genomes of *Plasmodium reichenowi* and *P. falciparum* [5]; only three of six Rh gene orthologues (*Rh2b*, *Rh4* and *Rh5*) were found in *P. reichenowi* [5], and differences in the presence or absence of orthologous RBL and EBL genes were also observed between *P. reichenowi*, *Plasmodium knowlesi* and *Plasmodium cynomolgi*, *Plasmodium vivax*, and *P. falciparum* [46–51]. Moreover, the *var* gene family, which encode immunovariant adhesion proteins, seems to be restricted to primate malaria species [5].

Considering these findings, significant differences between EBL and RBL genes of avian and mammalian *Plasmodium* species are unlikely haphazard. Rather, it is speculated that avian *Plasmodium* spp. likely lack members of the EBL and RH family, and that the evolutionary shift of *Plasmodium* into mammals and the differences in host cell biology (nucleated erythrocytes in birds *versus* anucleated erythrocytes in mammals) led to the gain and diversification of the genes that modulate red blood cell invasion. In spite of this possibility, *ripr* is conserved and expressed in *P. gallinaceum*, suggesting that this gene is of ancient origin. Surprisingly, an orthologue of the RIPR binding partner RH5 was not identified, possibly due to low levels of *rh5* transcripts in the sequenced blood stages of *P. gallinaceum*. However, an orthologue of *rh5* appears to be absent from the *P. gallinaceum* genome as well. In the case of the latter, the putative avian *Plasmodium* RIPR may not be a functional counterpart of the mammalian *Plasmodium* spp. RIPR, despite being relatively conserved at the amino acid sequence level. The region encoding the N-terminus of RIPR lacks sequence diversity and appears to have evolved under purifying selection. Conversely, comparisons of the region encoding the RIPR C-terminus revealed evidence for diversifying selection. In this comparison, the observed nucleotide substitutions at the C-terminus may be indicative of interspecies sequence or structural specificity for the respective RH5 binding partners since this region directly interacts with RH5 [52]. Sequencing *ripr* from other avian *Plasmodium* spp. will thus be important to further assess the diversity of *ripr* and the selective pressures acting on *ripr* in avian malaria parasites. Taken together, these findings suggest that avian *Plasmodium* may express a novel set of invasion ligands or highly divergent EBL/RH genes that are currently undetectable.

Contrary to the results with EBL/RH genes, BLAST searches for essential genes involved in the purine salvage pathway detected highly conserved orthologues of ADA, HGPRT, NT1, and PNP in *P. gallinaceum*, highlighting the importance of the *Plasmodium* purine salvage pathway throughout the intra-erythrocytic growth of *Plasmodium*. In the intra-erythrocytic stages, *Plasmodium* parasites grow rapidly and require the salvage of host cell purines to synthesize nucleic acids [53, 54]. To carry out the purine salvage pathway, purine nucleosides must be transported into the parasites. This process is mediated by NT1, an essential purine transporter [55]. Transport of purine nucleosides results in adenosine being converted to hypoxanthine by ADA and PNP [56]. HGPRT can then convert hypoxanthine to inosine monophosphate (IMP), which is used as the precursor for the synthesis of purine nucleotides during nucleic acid synthesis [57]. These critical enzymes are highly expressed and abundant in *Plasmodium* [56–58]. Inhibiting these enzymes is lethal to *Plasmodium* [56, 59, 60], which therefore provides promising targets for anti-malarial drugs. Interestingly, *Plasmodium* purine salvage pathways were observed in initial investigations of the avian malaria parasite *Plasmodium lophurae* [61]. Thus, the conservation and expression of these enzymes that are essential to this pathway was expected, and affirmed that the de novo assembly generated *P. gallinaceum* transcripts of good quality.

## Conclusions

Phylogenetic analysis of the purine salvage enzymes position *P. gallinaceum* closest to *P. falciparum*, and are consistent with several studies based on an assortment of genes [6, 13, 62]. The results here provide sequence data from an additional five nuclear genes to support this hypothesis. However, the theory that human *P. falciparum* originated from a lateral transfer of an avian parasite, as originally proposed [9], is contentious [63, 64], and additional sequencing from other avian *Plasmodium* species will be necessary to firmly resolve these relationships. Nevertheless, the genetic and biochemical similarities between *P. falciparum* and *P. gallinaceum* are evident, as demonstrated by genomic, biochemical and proteomic approaches [6, 8, 12, 13, 65, 66]. It is clear that improved methods for obtaining pure genetic material of avian malaria will be important for future genomic studies and would advance the field rapidly. As demonstrated previously [6] and reported here, establishing the *P. gallinaceum* transcriptome database will serve as a valuable fundamental resource for understanding avian malaria parasite biology.

## Additional files

Additional file 1:
*Plasmodium gallinaceum* de novo transcriptome assembly without quality trimming and no subsequent filtering of transcripts. Additional file 2:
*Plasmodium gallinaceum* de novo transcriptome assembly without quality trimming and with subsequent filtering of transcripts. Additional file 3:
*Plasmodium gallinaceum* de novo transcriptome assembly with quality trimming and no subsequent filtering of transcripts. Additional file 4:
*Plasmodium gallinaceum* de novo transcriptome assembly with quality trimming and with subsequent filtering of transcripts. Additional file 5: Genes and the corresponding GenBank Accession numbers and Gene IDs for the parasite taxa used in the study. Additional file 6: RSEM-eval score of various assemblies. Additional file 7: Annotation file for the assembly without quality trimming. Additional file 8: Mapping file for the assembly without quality trimming. Additional file 9: Sequence table file for the assembly without quality trimming. Additional file 10: KEGG pathway file for the assembly without quality trimming. Additional file 11: Phylogeny of *Plasmodium* genes. Additional file 12: Annotation file for the assembly with quality trimming. Additional file 13: Mapping file for the assembly with quality trimming. Additional file 14: Sequence table file for the assembly with quality trimming. Additional file 15: KEGG pathway file for the assembly with quality trimming. Additional file 16: Pairwise comparisons of genetic distances, and the proportion of nonsynonymous differences per nonsynonymous site and synonymous differences per synonymous site between *Plasmodium* *species*.
