# Supplementary material for: De novo assembly and transcriptome analysis of Plasmodium gallinaceum identifies the Rh5 interacting protein (ripr), and reveals a lack of EBL and RH gene family diversification
Source: Malar J. 2015 Aug 5;14:296. doi: 10.1186/s12936-015-0814-0 (PMC4524024; doi:10.1186/s12936-015-0814-0)
Supplement: Additional file 5: — Genes and the corresponding GenBank Accession numbers and Gene IDs for the parasite taxa used in the study. [file 12936_2015_814_MOESM5_ESM.docx]

| ***Plasmodium spp.*** | **Gene** | **Gene ID** | **Accession No.** |
| --- | --- | --- | --- |
| *P. falciparum* | EBA175 | PF3D7_0731500 |  |
| *P. falciparum* | EBA181 | PF3D7_0102500 |  |
| *P. falciparum* | EBA140 | PF3D7_1301600 |  |
| *P. falciparum* | RH1 | PF3D7_0402300 |  |
| *P. falciparum* | RH2a | PF3D7_1335400 |  |
| *P. falciparum* | RH2b | PF3D7_1335300 |  |
| *P. falciparum* | RH4 | PF3D7_0424200 |  |
| *P. falciparum* | RH5 | PF3D7_0424100 |  |
| *P. falciparum* | RIPR | PF3D7_0323400 |  |
| *P. berghei* | RIPR | PBANKA_12150 |  |
| *P. chabaudi* | RIPR | PCHAS_121580 |  |
| *P. knowlesi* | RIPR | PKH_081690 |  |
| *P. vivax* | RIPR | PVX_095055 |  |
| *P. yoelii yoelii* | RIPR | PY17X_1218300 |  |
| *P. gallinaceum* | RIPR |  | KP164517 |
| *P. gallinaceum* | ADA |  | KP164513 |
| *P. gallinaceum* | HGPRT |  | KP164514 |
| *P. gallinaceum* | NT1 |  | KP164515 |
| *P. gallinaceum* | PNP |  | KP164516 |
| *P. gallinaceum* | ADA |  | KP164513 |

**Additional File 5**
