# Supplementary material for: De novo assembly and transcriptome analysis of Plasmodium gallinaceum identifies the Rh5 interacting protein (ripr), and reveals a lack of EBL and RH gene family diversification
Source: Malar J. 2015 Aug 5;14:296. doi: 10.1186/s12936-015-0814-0 (PMC4524024; doi:10.1186/s12936-015-0814-0)
Supplement: Additional file 6: — RSEM-eval score of various assemblies. [file 12936_2015_814_MOESM6_ESM.docx]

|  | **Non-filtered Score** | **Filtered Score** |
| --- | --- | --- |
| **Non-trimmed** | -15457083083.96 | -16451193243.99 |
| **Quality trimmed** | -15078436705.85 | -16246584779.19 |
| **Additional File 6** |  |  |
