# Supplementary material for: De novo assembly and transcriptome analysis of Plasmodium gallinaceum identifies the Rh5 interacting protein (ripr), and reveals a lack of EBL and RH gene family diversification
Source: Malar J. 2015 Aug 5;14:296. doi: 10.1186/s12936-015-0814-0 (PMC4524024; doi:10.1186/s12936-015-0814-0)
Supplement: Additional file 16: — Pairwise comparisons of genetic distances, and the proportion of nonsynonymous differences per nonsynonymous site and synonymous differences per synonymous site between Plasmodium species. [file 12936_2015_814_MOESM16_ESM.pdf]

Table 1

	P. falciparum	*P. gallinaceum*	*P. berghei*	*P. chabaudi*	*P. knowlesi*	*P. vivax*	*P. yoelii yoelii*
P. falciparum							
P. gallinaceum	0.31						
P. berghei	0.38	0.39					
P. chabaudi	0.39	0.39	0.13				
P. knowlesi	0.41	0.44	0.47	0.47			
P. vivax	0.44	0.46	0.49	0.49	0.21		
P. yoelii yoelii	0.37	0.39	0.09	0.12	0.47	0.49	

Uncorrected ("p") distance matrix.

Table 2

(a)

2467-2667 nt, P = 0.01	*P. falciparum*	*P. gallinaceum*	*P. berghei*	*P. chabaudi*	*P. knowlesi*	*P. vivax*	*P. yoelii yoelii*
P. falciparum		0.40 (0.17)	0.30 (0.13)	0.23 (0.10)	0.26 (0.10)	0.31 (0.10)	0.20 (0.10)
P. gallinaceum	0.21 (0.05)		0.39 (0.14)	0.40 (0.18)	0.29 (0.12)	0.26 (0.10)	0.41 (0.16)
P. berghei	0.35 (0.07)	0.44 (0.09)		0.14 (0.08)	0.34 (0.12)	0.34 (0.11)	0.06 (0.05)
P. chabaudi	0.39 (0.07)	0.34 (0.07)	0.15 (0.04)		0.28 (0.10)	0.26 (0.10)	0.11 (0.06)
P. knowlesi	0.54 (0.09)	0.62 (0.11)	0.67 (0.11)	0.69 (0.13)		0.04 (0.03)	0.29 (0.11)
P. vivax	0.62 (0.10)	0.68 (0.11)	0.80 (0.12)	0.80 (0.14)	0.19 (0.05)		0.30 (0.11)
P. yoelii yoelii	0.37 (0.07)	0.36 (0.07)	0.07 (0.03)	0.15 (0.04)	0.71 (0.12)	0.83 (0.12)	

(b)

2497-2697 nt, P < 0.01	*P. falciparum*	*P. gallinaceum*	*P. berghei*	*P. chabaudi*	*P. knowlesi*	*P. vivax*	*P. yoelii yoelii*
P. falciparum		0.36 (0.17)	0.36 (0.15)	0.27 (0.12)	0.30 (0.11)	0.35 (0.12)	0.24 (0.11)
P. gallinaceum	0.27 (0.06)		0.39 (0.14)	0.40 (0.18)	0.28 (0.11)	0.25 (0.10)	0.41 (0.16)
P. berghei	0.41 (0.08)	0.56 (0.12)		0.15 (0.08)	0.39 (0.14)	0.38 (0.13)	0.07 (0.05)
P. chabaudi	0.45 (0.08)	0.47 (0.09)	0.15 (0.04)		0.31 (0.11)	0.29 (0.11)	0.12 (0.07)
P. knowlesi	0.60 (0.11)	0.71 (0.14)	0.80 (0.14)	0.86 (0.16)		0.05 (0.04)	0.32 (0.12)
P. vivax	0.67 (0.12)	0.74 (0.13)	0.84 (0.15)	0.89 (0.17)	0.20 (0.05)		0.34 (0.12)
P. yoelii yoelii	0.42 (0.08)	0.47 (0.09)	0.09 (0.04)	0.16 (0.05)	0.86 (0.14)	0.89 (0.15)	

(c)

2728-2916 nt, P < 0.01	*P. falciparum*	*P. gallinaceum*	*P. berghei*	*P. chabaudi*	*P. knowlesi*	*P. vivax*	*P. yoelii yoelii*
P. falciparum		0.17 (0.10)	0.27 (0.15)	0.19 (0.11)	0.20 (0.09)	0.28 (0.12)	0.27 (0.15)
P. gallinaceum	0.38 (0.08)		0.29 (0.14)	0.21 (0.11)	0.15 (0.08)	0.24 (0.10)	0.29 (0.14)
P. berghei	0.39 (0.08)	0.51 (0.10)		0.04 (0.05)	0.24 (0.11)	0.31 (0.14)	0.00 (0.00)
P. chabaudi	0.42 (0.08)	0.50 (0.09)	0.07 (0.03)		0.17 (0.09)	0.24 (0.11)	0.04 (0.05)
P. knowlesi	0.48 (0.10)	0.60 (0.12)	0.75 (0.15)	0.80 (0.15)		0.07 (0.06)	0.24 (0.11)
P. vivax	0.66 (0.11)	0.69 (0.12)	0.71 (0.12)	0.75 (0.13)	0.18 (0.04)		0.31 (0.14)
P. yoelii yoelii	0.39 (0.08)	0.46 (0.09)	0.03 (0.02)	0.06 (0.03)	0.75 (0.14)	0.71 (0.11)	

(d)

2758-2946 nt, P < 0.01	*P. falciparum*	*P. gallinaceum*	*P. berghei*	*P. chabaudi*	*P. knowlesi*	*P. vivax*	*P. yoelii yoelii*
P. falciparum		0.05 (0.05)	0.14 (0.09)	0.09 (0.06)	0.18 (0.09)	0.25 (0.12)	0.14 (0.09)
P. gallinaceum	0.33 (0.07)		0.19 (0.10)	0.13 (0.08)	0.11 (0.07)	0.18 (0.09)	0.19 (0.10)
P. berghei	0.33 (0.07)	0.48 (0.09)		0.04 (0.04)	0.18 (0.09)	0.23 (0.11)	0.00 (0.00)
P. chabaudi	0.32 (0.06)	0.46 (0.08)	0.06 (0.02)		0.13 (0.07)	0.18 (0.09)	0.04 (0.04)
P. knowlesi	0.41 (0.08)	0.50 (0.10)	0.74 (0.16)	0.69 (0.14)		0.07 (0.06)	0.18 (0.09)
P. vivax	0.59 (0.11)	0.68 (0.10)	0.72 (0.12)	0.72 (0.12)	0.19 (0.05)		0.23 (0.11)
P. yoelii yoelii	0.32 (0.07)	0.46 (0.08)	0.02 (0.02)	0.06 (0.03)	0.71 (0.15)	0.69 (0.11)	

(e)

2818-3036 nt, P < 0.01	*P. falciparum*	*P. gallinaceum*	*P. berghei*	*P. chabaudi*	*P. knowlesi*	*P. vivax*	*P. yoelii yoelii*
P. falciparum		0.19 (0.11)	0.17 (0.10)	0.12 (0.08)	0.36 (0.15)	0.45 (0.19)	0.17 (0.09)
P. gallinaceum	0.31 (0.06)		0.29 (0.14)	0.21 (0.11)	0.14 (0.08)	0.30 (0.12)	0.29 (0.13)
P. berghei	0.38 (0.06)	0.50 (0.08)		0.09 (0.07)	0.42 (0.19)	0.41 (0.17)	0.00 (0.00)
P. chabaudi	0.34 (0.06)	0.47 (0.07)	0.04 (0.02)		0.36 (0.17)	0.36 (0.16)	0.09 (0.07)
P. knowlesi	0.54 (0.10)	0.53 (0.10)	0.73 (0.15)	0.70 (0.14)		0.12 (0.06)	0.41 (0.18)
P. vivax	0.69 (0.13)	0.69 (0.11)	0.74 (0.14)	0.71 (0.13)	0.30 (0.06)		0.40 (0.17)
P. yoelii yoelii	0.38 (0.07)	0.48 (0.08)	0.05 (0.02)	0.07 (0.03)	0.71 (0.15)	0.72 (0.14)	

(f)

3277-3456 nt, P = 0.03	*P. falciparum*	*P. gallinaceum*	*P. berghei*	*P. chabaudi*	*P. knowlesi*	*P. vivax*	*P. yoelii yoelii*
P. falciparum		0.12 (0.08)	0.44 (0.21)	0.46 (0.22)	0.14 (0.08)	0.15 (0.07)	0.43 (0.20)
P. gallinaceum	0.28 (0.07)		0.27 (0.13)	0.27 (0.14)	0.22 (0.11)	0.18 (0.09)	0.27 (0.13)
P. berghei	0.39 (0.08)	0.49 (0.10)		0.05 (0.05)	0.33 (0.14)	0.30 (0.14)	0.00 (0.00)
P. chabaudi	0.43 (0.09)	0.54 (0.10)	0.09 (0.03)		0.46 (0.20)	0.49 (0.21)	0.11 (0.05)
P. knowlesi	0.63 (0.10)	0.59 (0.11)	0.68 (0.14)	0.68 (0.14)		0.11 (0.07)	0.33 (0.14)
P. vivax	0.57 (0.09)	0.57 (0.11)	0.76 (0.16)	0.71 (0.15)	0.27 (0.07)		0.33 (0.14)
P. yoelii yoelii	0.41 (0.09)	0.56 (0.11)	0.04 (0.02)	0.07 (0.03)	0.71 (0.15)	0.81 (0.17)	

(g)

3307-3486 nt, P < 0.01	*P. falciparum*	*P. gallinaceum*	*P. berghei*	*P. chabaudi*	*P. knowlesi*	*P. vivax*	*P. yoelii yoelii*
P. falciparum		0.12 (0.07)	0.31 (0.14)	0.40 (0.18)	0.20 (0.10)	0.20 (0.09)	0.31 (0.14)
P. gallinaceum	0.30 (0.08)		0.29 (0.14)	0.23 (0.12)	0.18 (0.09)	0.19 (0.09)	0.29 (0.14)
P. berghei	0.42 (0.08)	0.52 (0.11)		0.09 (0.07)	0.30 (0.12)	0.31 (0.12)	0.00 (0.00)
P. chabaudi	0.41 (0.07)	0.53 (0.10)	0.10 (0.03)		0.35 (0.14)	0.40 (0.16)	0.09 (0.07)
P. knowlesi	0.65 (0.11)	0.65 (0.13)	0.69 (0.14)	0.69 (0.13)		0.13 (0.07)	0.29 (0.12)
P. vivax	0.66 (0.12)	0.64 (0.12)	0.91 (0.21)	0.86 (0.19)	0.30 (0.07)		0.33 (0.13)
P. yoelii yoelii	0.44 (0.08)	0.59 (0.12)	0.04 (0.02)	0.07 (0.03)	0.72 (0.15)	0.97 (0.22)	

(h)

3337-3522 nt, P < 0.01	*P. falciparum*	*P. gallinaceum*	*P. berghei*	*P. chabaudi*	*P. knowlesi*	*P. vivax*	*P. yoelii yoelii*
P. falciparum		0.10 (0.06)	0.18 (0.09)	0.22 (0.11)	0.21 (0.10)	0.31 (0.12)	0.17 (0.09)
P. gallinaceum	0.37 (0.08)		0.26 (0.14)	0.18 (0.11)	0.22 (0.10)	0.27 (0.11)	0.26 (0.14)
P. berghei	0.45 (0.08)	0.51 (0.10)		0.08 (0.06)	0.24 (0.11)	0.30 (0.12)	0.26 (0.00)
P. chabaudi	0.42 (0.08)	0.49 (0.09)	0.09 (0.03)		0.26 (0.12)	0.36 (0.13)	0.08 (0.06)
P. knowlesi	0.65 (0.10)	0.57 (0.11)	0.62 (0.10)	0.64 (0.10)		0.13 (0.07)	0.24 (0.11)
P. vivax	0.69 (0.12)	0.58 (0.10)	0.81 (0.16)	0.81 (0.16)	0.26 (0.06)		0.32 (0.13)
P. yoelii yoelii	0.43 (0.09)	0.55 (0.11)	0.04 (0.02)	0.09 (0.03)	0.69 (0.12)	0.82 (0.16)	

(i)

3367-3555 nt, P < 0.01	*P. falciparum*	*P. gallinaceum*	*P. berghei*	*P. chabaudi*	*P. knowlesi*	*P. vivax*	*P. yoelii yoelii*
P. falciparum		0.11 (0.07)	0.20 (0.10)	0.29 (0.14)	0.31 (0.13)	0.44 (0.18)	0.14 (0.08)
P. gallinaceum	0.40 (0.09)		0.27 (0.15)	0.24 (0.12)	0.23 (0.11)	0.30 (0.13)	0.22 (0.12)
P. berghei	0.50 (0.10)	0.51 (0.11)		0.09 (0.07)	0.27 (0.13)	0.42 (0.17)	0.04 (0.05)
P. chabaudi	0.39 (0.07)	0.44 (0.08)	0.10 (0.04)		0.33 (0.14)	0.43 (0.17)	0.13 (0.09)
P. knowlesi	0.76 (0.14)	0.73 (0.14)	0.67 (0.12)	0.65 (0.11)		0.14 (0.08)	0.20 (0.10)
P. vivax	0.70 (0.13)	0.67 (0.12)	0.83 (0.18)	0.80 (0.17)	0.22 (0.06)		0.33 (0.14)
P. yoelii yoelii	0.48 (0.10)	0.51 (0.11)	0.03 (0.02)	0.12 (0.04)	0.68 (0.12)	0.78 (0.16)	

dN and dS values in regions of *ripr* with significant variance in dN-dS (a-i) between pairs of *Plasmodium* species. Nucleotide positions and

p-values are indicated at the top left corner of the tables. Values beneath and above the diagonal are pairwise dN and dS values, respectively. Values in brackets are standard errors.
