# Supplementary material for: De novo assembly and transcriptome analysis of Plasmodium gallinaceum identifies the Rh5 interacting protein (ripr), and reveals a lack of EBL and RH gene family diversification
Source: Malar J. 2015 Aug 5;14:296. doi: 10.1186/s12936-015-0814-0 (PMC4524024; doi:10.1186/s12936-015-0814-0)
Supplement: Additional file 2: — Plasmodium gallinaceum de novo transcriptome assembly without quality trimming and with subsequent filtering of transcripts. [file 12936_2015_814_MOESM2_ESM.doc]

>c9_g1_i1 len=219 path=[489:0-218]

GGAAACATTAACAAGAAATCACACTGGTGAGGTTTGCAGAAAGCAGAGAAGGAGCAAAGT

CAAAATCAGCTGAGCAGCTGCTGAGATTTGGCTTGCTCCAACAGCAGGTGTTGGGCTTAC

AGCAGGCAGAGGGGCTTTGGGCACAGAGGGAATTTGCTGTGGGGCTCTTCCCAAACTGGA

GCTGAGCTGCTCCCACAGCTTTGGCTCCCATTGCCCGGC

>c24_g1_i1 len=205 path=[1:0-204]

AGATGGAAAATATCCCCTTTCAGCATCAGAGGCTACAACATACTTATCTATAACCCCATT

ATCATGTACCACTTCTGCTTCCCTGCCACTGCCCTCCCATAGGTCACATGCTTGTAGCTT

GTAGGCAACAGACCCTTTGACAGGGATCCCAAACTGCTTGCTTCATGCTTTATTCTGGGA

GACAGTGGAGGTGGAGCTGAGCAGT

>c27_g1_i1 len=203 path=[155:0-202]

AGCATGGGGAGAGAAAATGACATTCAATATTTTCTACCTCATAGGACTACTACCACTCTC

TGGAATGTCATCTGTCATGCTCGAACTATACCCCACTGCATCCACTTATTAGTGAGCTTA

ACTTTTCGAACATCCATGCAAACCCAAAAAACATTGTGAACCAATAGGTTTTAAGAACCA

TAACCTGGAGTATAATTGCGTGT

>c35_g1_i1 len=205 path=[155:0-204]

GGATTGTTCAGTCTGGAGGAGGCTCAGGGGAGACCTGATCGCTCTCTACAGCTGCCCACA

AGGAGGCTGTGGTGAGATGGGGTGTCGTAGGGTTGTCTGCTTGCGCTGTGAGGATCTGTG

CTTGAAGAGCGTTGTTAATTTTAAAGCAGTACTGTGGTTTTGTTTGTCTTATCCTTTTTT

TAAGGGAAGTGCTCAAAATAGCAGA

>c43_g1_i1 len=293 path=[1:0-146 148:147-292]

TTTTTTTTTTTTATTTAAAAATAATTGTTGATTATGTTTCATTAATATATATTCATTACA

TTCATATTGTAAAATTTATTTACCACATCATAACGACATTATAACTTTATCAAAATAAAA

TATTCATTAATTCAGTACTATAATTATTTTTAAAATGGGAATAAAAAAAATGTTTTGTAG

ATATGATAATGATACTTAAACTTTTTCAAAAATAATAACATTAAATAATTATGAAATTTA

TGAATTTAATAATAGAACCAGAAAAATAATTGTGATAAATTTTCTTTTTTATA

>c63_g1_i1 len=245 path=[229:0-244]

TGTTTTCTTAAAAAAAAAAAAAATACGAGAAAGAGATATAATCCAAACAAACACGTCATC

ACAGCAATCAGCAGAGGAAGGCAATTACAATGTTAAAAGCCTTCGTGCCCGCTGACCCAT

CCTAGAACCGCGCCGGCGGCTCCGTGCATCCATGGCAGAGCTGAAAAGTTCCCTACAAAA

GTTGGTAAGGCAGACGAAGGCCGAGCAGAAACAAGAGCGTGGCGGCTGAGAAATGATCAT

CCCCT

>c68_g1_i1 len=384 path=[155:0-383]

ATTAACTTTATTCAAGGAATTATAAAAGACAATGAAAAGAAAATTAATAAAACAAATGCA

TAAATTTCATTAAAAAAAAAAAAATTAAAGTATCTCTAAAAAAATAACATAAAATAAGAA

TATCACAATATTAAAAATATCTTTAAAAAACTGATAAATTTTTTCTTTTTTCATACTGTA

CAATTTAGAATATTCACTTTCACAAGTATTTTCTGTTTACAACTAAAATTTTGAGTCTCT

TCTATTTTATACTATAGAAAATTTCAAAAGATAAATATTCTACTTTATTAATAGATACAA

ATTATAACCAGTAAACCATTATTCATATAGATATAAAAATTCAACGTGATATTCCTATTT

TTTTTAGATTGTATTATTAGACTA

>c71_g1_i1 len=288 path=[420:0-287]

ATAAACAAAGCTTAATTCACATTACATAGAACTCTAAATATACTTAAAGTTTTTAGAACA

ACTATTCCCAAACCCACAGAGAAAGACTCACCAATAACAGTACAGAGTTAAATCGATTAA

TAGAATATTGGAATGTATCACAGAAGTTTTCCAAGAGACTGTTTCCCATCAAAAATACTG

TTTGAAAAATGTATTTATTCTTACTACATTTTTGTAAGTACAATTTAGAAAAAGATGAGT

TGTGGTGGGTCAGGATGGGACTGGGAGGACCCTAAGGAGCCACCAGCA

>c74_g1_i1 len=237 path=[535:0-236]

GCTTGTGTCTGCTTTTTCTTCATAGTGCATAATTGAAATCCTCATGGGAGATTTTGTGCT

GGTACCTCAGAATGTGGTTTTGAAGTCTGCTGGCATAGCTAATGGCTGTTAGCTTGTGCT

TTCAGATGCTGAATTTCCTAACTTTAAGTAATAACTCGAGCCATCTACCACCTCTACCCT

AGCAGAATTATTAAAAACAGATGTTGCAGGTTTGCACCGGTATTATGCCGTTCCTGT

>c74_g2_i1 len=276 path=[15:0-275]

CACAAGCAAACAGATACAATCTCCAACACCACAACCCCAGCACATGCTCAGGAAGTACAG

AGAATACTAATGAAGAATCACCATCAGCACCAATCAAAGCAACAGCGCTCTTTCTTCTGA

GCGTGAAGATTTTAAGAGTTCGGTTCTGCAGAGCTCATCTAAAAATGGCTTTGTTTGGTT

CTTCCTTTGTGGTCTCACCTGAAATCGTTCAGCTCTACCAGTAAACAGATGGCTTTTTAT

AATTGCCACTGCTGTACATTTTGTCACGGGTCGTTT

>c78_g1_i1 len=241 path=[219:0-240]

AGGGGTCACACATCAACAAAACTGCCCTACAGTAAGCACTACACAAAAAACCAATCAAAC

AAACAGCAGAAGGAGTTGCAAACCTGAAGATCTTGCAGTCCCAGTACTATAGAAGGAACT

GCAGGTGGATGTAATGAGACATAAAAGCCCAAGGTGAAAGCAAAGCTACTGGCCAAGAGG

GTAAGGAACGATACCAGGCACCAGCTGCTCAGATATTGCCTGCTAAAAACTGACAATATC

T

>c83_g1_i1 len=213 path=[191:0-34 226:35-212]

ATAAACAAAGGGTGCTGAAAGATGCGACTGTGAACAATGTGCTTGTGTGTGATCATCCTG

TTCTGTCCTTTGTGGAACTTTCCCAGTTGTATTCCACTGGTTTTGGAAAGTTGCATTTGT

GCTGTGGATGAGAACACATCTCCCAGGGCACTTCTGGCTTAGTTAAATCCATGTCTTCCC

TCTCGTTCAATAGCTTTCAAAAATTCCTCCTCT

>c83_g1_i2 len=274 path=[381:0-95 226:96-273]

GAACTACAGACTAATCAAGGGTGGGATTTAAAATACACGATGTTTTTTTCTGTGACTTGA

TGGATTAAGCATTGTGCAATTATAGAAGAACAAAAAAATGTGCTTGTGTGTGATCATCCT

GTTCTGTCCTTTGTGGAACTTTCCCAGTTGTATTCCACTGGTTTTGGAAAGTTGCATTTG

TGCTGTGGATGAGAACACATCTCCCAGGGCACTTCTGGCTTAGTTAAATCCATGTCTTCC

CTCTCGTTCAATAGCTTTCAAAAATTCCTCCTCT

>c86_g1_i1 len=269 path=[1:0-109 111:110-268]

AATCCTGCGCATTACATCATAGCTGCCTGTGCTGCATTTCCAATTCAGTGTGTGCTAGAA

ACATTTGGAAAGCATCCTAAGGAGGTATGTACTGCCTTATGTGAGTTGAAGTGTGAATGT

GGAACTTTGGATTTGAGTGTTGCTTTCTGTGTATAAGATGCATCAAATGAACATTCACTA

TCACATATCAAAAGCACCTTTTTTTTCTCGAACAGTTAAAGTGCATAAGGGTTGTTTTTT

TTTTTAAACATAGAGTTATTTGCTTTCAG

>c105_g1_i1 len=227 path=[1:0-50 52:51-226]

AGCTGCTCAGGGACCAGATGGGCACCGGGCAGCGGGCGGTGAGACACCGCACTGTGCACC

ACTTGTTGTATACATTTACATATATAGATAGACAGGTTTGGATTTGCGCCCACTTTTGTA

CACAGATCTGTGCCTCATGTTCGATGTCTTTCAAAGAGATGGTGGTGAGGTGCTTCCCGG

AGTGGTTGCCCTGTCCCTGGAGATGTTCAAGGCCAGCTTGGATGGGG

>c106_g1_i1 len=233 path=[199:0-232]

TTTTTATTTTCCTTTTTTTTTAATATGGATGAATTAAAAGAAATTATATATGAATTGAAA

AAACTAAAAAAAAATTTAAGAAATAAAGTTGTAAATATAAATTACTATGAATTTATAAAT

AATAATTATTATATTGAAAAAAAAATTATTTATAATTTATATCAAATAATAAAGAGCTAT

TATTATTTACTAACTTTTTTTAGTAAAACAAAAAAGAAAAAGAAAAAAGAAAA

>c125_g1_i1 len=246 path=[242:0-223 841:224-245]

ACTCAAAGCCTTGACTGCAGGCACGTTTGGGTTGGAATCCAAACAGCAGAGGCTTCCATT

GCAGCACCCACCAACTGGGCTCCGAGCGGTGCTCCTGTACAGACCACCTCCTTTCCATGG

GACCTGTCCTGCATGCCTCTGCTGGCCAGCACAACCCTTCCCTGGGCAGGTTCTTATCTC

CTACTGATGGAGAAACAGAACAGCCTTTCAGAAACGTCCAGCACTTGGAAGGCATGTCTC

CCAAGT

>c128_g1_i1 len=310 path=[1:0-309]

GGTCACAAATAAAATGATGAGCAGCATCACAATCTTCCCTTCTGAGCCAACAGTTCCAAG

GCCCAGTACAGACACAGCAGGAAAGGAAAGGCATATTACCACCAGCAACATCAGGGTTGC

TCCAAATGAACCCTCCCCGATAACGAACAGCCTCCCCCAGCCTTTTGAGGTCTCAGTTAA

TAGAAGCGCCCTGAAATTATCTGAGACCGACAGAAGTGGAGATGCGGTGCTGAGAAGTAG

AACAGAAACGGTGGTGTCCAGGAGCAGCATTGTGATAAAGACTTCTGAGCCCCCCGAGAG

GAGCGGTGAG

>c136_g1_i1 len=230 path=[208:0-109 318:110-229]

CTATTCCCCCTTGTCCTGTCACTGTCTACCTCTGTAAAAACCTCAGTCTCACTCCTGCTT

ATAAGCTTCCTTTAAGTATTGAAAGGCTACAATGAAGTGTCCCTGGAACCTTCTCTCCTC

AAACCTAAATAAGCCCAGCTCCCTAAGCCTATCTCCACAGGTGAGATGCCATCCTCTGGA

TCTACTCTAACAGCTTCACATTTTTGCTGTGCTGTGGGCCCCAGGCCTGG

>c139_g1_i1 len=231 path=[1:0-230]

AGGCCGCTGGGGGAGGTGGTGGAGAGTGGAGAGGGTAGAAAGGAAACAACAGTGGAACAA

GAGAAAGAGAACTTCAGGGGAACAGCCTCTTCCCTTCTACAGCTCTTGCGAAAGCTCAAT

TGCTTGGAAACCTGTGGGCACTGACTCAGCAGGAGAATCACCAGAATAGGTAGGAGCTGG

AGATGAATAGGCCAAGCTGGGGGGCAGCCTTCCAGCAGGATGGGTGGCTCT

>c142_g1_i1 len=310 path=[506:0-232 139:233-309]

TAGTGGTTTCTGTGGTATACAGAAGTGAAAACAAAGAGAAAGCAAAGTCATTCAAATTGA

ATTTAACAATTTGTTTTTGATAACGTCTTTAAGGTTTTACTGCTTAAGCTTACCTATTTT

CTTTATAATATTAATATATGCAAAATAGCAGACAAAACCCATATAGTAACTACCAGATTT

AACTTAGAATATAAAAAATAAATCATTAGAAATTCATTTGGAAAATATTTTTATGCTGCT

TTGTGCAGACACCAAGGTGAAAAGCTCCTCTCCAATATGAGCATTGCTTTTAGCACTTGT

CATCCAAGCT

>c142_g1_i2 len=310 path=[506:0-232 738:233-309]

TAGTGGTTTCTGTGGTATACAGAAGTGAAAACAAAGAGAAAGCAAAGTCATTCAAATTGA

ATTTAACAATTTGTTTTTGATAACGTCTTTAAGGTTTTACTGCTTAAGCTTACCTATTTT

CTTTATAATATTAATATATGCAAAATAGCAGACAAAACCCATATAGTAACTACCAGATTT

AACTTAGAATATAAAAAATAAATCATTAGAAATTCATTTGGAAAATATTTTTAGCTGCTT

AGATTTGTGTTTGAATTTGCTAATTCTCCACATTTCAGCCTTGAAAGCTTGGATGACAAG

TGCTAAAAGC

>c151_g1_i1 len=772 path=[1:0-118 120:119-771]

TTACACAATGTTATGTTAGTAATATTATTTTAAGTTTCAAAAGATTTGTCATCTTGAAAA

ATATTTTATATTTTCTATTTATGTTATTTAATTAACGTAGTTGATAAAAACAAAAAAAGA

AATAATTTCACGGTTTAAATTATATTATTCTAAATGAAAAGTATAAATAATAAAAAATTT

GTTAAGAGTTTTTTATAATTTTTTTTTAATTAAAAAGAAAATTGTATTTTTTTATTAGAG

TTTTAGAAAGGGAATAATACTATTTAATTATACTCTAAAAAGAAATCCCAAAATAAAAAT

ACGGAGTTTTTTCCATTGTATCTTTTTCTTTTTTTTTCATTTTATTTTATGTATTTAATT

ATAATTTAATATTCCAAAAATATATTACTATTTTACATCTTTTACAAATAAAAAAAATAA

TGAAAAAAAATGTTATTAGGCAATTATTAATATCCACTTAAGAAAGTTAATATATTACTA

TAAAATAAATTATTAATGAAAAAAATAAAAAGGCTTACAAATCATTTTCTTATACTTCAC

ACAAAAGTTTTCCGTAAAATAATTGAATGGTATCATTTCATATTTATGAAATAGAAATTT

ACTTGTATTAATTTATTAATTATTATAGTGAAAGTAAAATTTTTTAATTAAAAATTACAA

ATAAGAATAATACATATCGTTGAAGTTATTCATAATGCATAAATCTTCCTAAAATATGAT

AAAAAAATACTAAAGCTTAAACGTTTTTTAATTACTTTAGACTAATATTAAT

>c181_g1_i1 len=307 path=[486:0-178 178:179-306]

CGTCGCACTGGCAGGGGGAAGGGAAGGGAAGGCGGGGGGTTACGCACCAGGGCAGAGCCG

GGATCCCTTCTCCACCCCGCGAGCCCGCGGGGAGGGGAGAGAGAAGGGAGGAGGGGGGGG

AGCGTTCTCCTCCTCCTTCCTCCTCCCCCTTTTTCACCCTTCCTCCTCCTCCTTCTTTCC

CCAAAGTTAAACTTCCTGCTCCCGGCCCGGCTGGGAAGGGGACGCGCAGCCCGGCCGGAC

TCAGCCCTCGACACACACAACAGCTGGAGCTGTCAAAACTTCCTCCGCTCGGCCTCGGCC

CCCGCCG

>c195_g1_i1 len=227 path=[16:0-226]

AATAAAAATGTTTGTACATGTATATATTTTTTTTGTCACTTTGCTAAAGAAACAAAGTAG

TGGTTGTAATGTATCCCTAACTTAGTTTGGAGAACAAACTTAATGAATGCTGTGTTTGAG

GTATCTAAATGTGTGCAGGTTATTTTGAAGGACAGAGTCCTCCTCATGCTGTACTACAGC

ATGGAATATGTTGAATAAGTTAGTAAGTTCATAGAACCCCTTTGAGT

>c211_g1_i1 len=295 path=[285:0-294]

CTGAGGTGTAAGCAGCATAATCACTCGCACAGAGACGCCCTCAGCTAGAGGTAGAACGAG

AATACAGCTCACCCATCAAGAGGCCCAACCCTCTGCTTTGGGCACGAGGCTTAGAAATAA

CATGGTGCTTTGCAACCTGGAAGTTATTTTCAGCAAGGGTGATACCCTTAGTTAAAGTGC

CTGGTTTTCCATCCCTTTTCTGGCTTCTCAAACTGTCCTGTCTCCATCTCTACGCAACCT

ATGGGAGGAACATGAACATGGCAACAGTTGCAGGGTTTGTTTGAAGGTGGGCAAA

>c217_g1_i1 len=304 path=[5:0-103 318:104-303]

TTTAAAAAATAAAAAAAAAATAAAAAATTAAATTATTTTATAAAAACTTTTTGATTTTTA

TATGGAAGCATATTGTAATTTAAGCAATTAAGACTCTTAAAAAACTTACTAAAAAATTAA

GTAAATATATGTATTTCACAAAACTAAAAAATTAAAAGAACAAAATACATATAATTTAAA

AAAAAAATTAAGTATGAAATATTATAAAACATAATATAAATACTTTAAATAAAAGATATA

AAAAAAATATATAGTACAACATATATATTAACATTTTATAATCTTTTCAAAAAAAAAAAA

AAAA

>c219_g1_i1 len=367 path=[19:0-247 644:248-366]

CTTTGTGTTTGAGGGGAAGTGTGCGGAGGCGCCCGCGGGCGCGTCCAGCCCCAGCCCCGA

AGTGTCCTGCCTGCCTCCTCCCGCCGCGTCGCTGCTCGGGAAGCTGGCACCGTCCTGCCG

CCTCTTCAGCTGCTTAAGGCCATCGTGTCAATTGCCTGCTCCAGCTTACTTCTCAGCCGC

TGCCTCCTGGCCTGCTCGTCCTTCTCTAACGCGGCTAAAATCTCATCCTTGTACTTGGTG

ATGTAGGAGTAGATCTCGTGCAGCGCACTCATGCTGTTGAACTGGCTGAGGTGTAACCGT

GACTGCTCTGCCAGGTAGGCGCTCATGTCCTGGTCACTAATTGCTGGCATTTTAGCAATA

TCTGCAT

>c219_g1_i2 len=315 path=[19:0-247 267:248-314]

CTTTGTGTTTGAGGGGAAGTGTGCGGAGGCGCCCGCGGGCGCGTCCAGCCCCAGCCCCGA

AGTGTCCTGCCTGCCTCCTCCCGCCGCGTCGCTGCTCGGGAAGCTGGCACCGTCCTGCCG

CCTCTTCAGCTGCTTAAGGCCATCGTGTCAATTGCCTGCTCCAGCTTACTTCTCAGCCGC

TGCCTCCTGGCCTGCTCGTCCTTCTCTAACGCGGCTAAAATCTCATCCTTGTACTTGGTG

ATGTAGGAAAAGACTCTCCCTCCAACAAACTACTGTACGCCAAGGACATCCCCAACTACA

AGAGCTGGGTAGAAA

>c226_g1_i1 len=262 path=[420:0-261]

GAAGGACATTTGCAAAACATTCCTGGAAATAAACATCAGCAGTTTAATCGTAAAGAAAAA

AACAAGCAGAAAGCACAAGGTACGCAACGCCACAAATCATTGGGGCAGGGAAAATAAAAA

AGGAAAAGAAAACAAATTAACCATTCAATCCCTCCCCGTACCCTTTCCCCCCAAAAACAT

GAACACTTTACCTCAGGAATCACATTGCAAATATGACCTGCAGAAAGAACGTAGCCCCCA

TCACCATCACCACCTCAAACAC

>c227_g1_i1 len=217 path=[195:0-216]

AAAGGAGCCAGGCCATGAATTCAGGTTGCTGTTAAGGAAGCTGGAGTTGTTTTTAACTTG

ATGTTCCTGGTTGTTGCACCTGACTTACTCTGTGTGGGAAGAAGCCTCTTTGCAGATAAA

TGTCTTCTACTGCACTTGAGTCGGTTGTCTGAAACACCGACTCTGCTCTGCTGCTGCAGG

AAAGTGGTTTTTTCCCCCTCTGTCTTGGGCTTGTTGA

>c229_g1_i1 len=352 path=[512:0-192 24:193-351]

TTTCCAGATAGAATATTTCCTTGAGTGAAAATAATTTTTTTTAAAATTGACACATTAATT

TCCTGAAGAAAACATTAAGCAAAAATCCTTTTCACTTCTAATACTGCTCAACAGTAATGA

ACATTCACATTATTCAGGGCAAGAGTACAGATTGTACTTATTTTAATTTGAAACTACTAT

GTAAGTAACACTAAATTTTTGTATACTTTACAGTAATGTTATATACAGTTGTGTTTCTCC

TCGGACATCTGCTTAAATTATACATTGATTTAATAATGTGCTCTTAATAAAATATTCCTG

TCTTATATAGCTTATGTAGCAAAGGCAGTACTTGCCCTTCAGCAAGGTTATT

>c243_g1_i1 len=366 path=[215:0-365]

GGTTCTGGAAAAGTAATGAGAGAAGGAGGTCTGAGACACAGGCTACAAAGGGGAATGAAA

ATCATGCAGAGATCAAGGGGCAAAAAGCTGTCCGCTGTCATATCAGTGTAATCCAGAATT

TGGGGAGGAAAGTATTTATATCCAATTACTTCTTTCTCTTTGAAGACTTCTTCTAGGATG

ATGTAGATTAATTTAAGATAGATTCTATAAGCTACTATTCAGTCAAACAAAGGGAAATCT

GAAAAACCATCAAAACATGTTATGTTATATCATGCATGGGAAGACAGGTTGTCAAAACTT

CTTTTAAGTAACAGCTGAAATTGTACAATGAATGCTGTAGGGGCTCTCTGTGTCTGCAGC

TCAGTG

>c247_g1_i1 len=222 path=[1:0-221]

GAATGCTCTTTGCTAGACGGTATGAGGTCTATGGTGCCAGCCAAATAAATCATAGCAGCA

CAGAGAAGGGGTCCAAACCTGACCAGAAACAAGCAATTCCGCTCCACCAGAACTGAGGAG

CTCCTTTGCATCACTTGAAAAGAAAGAGGGGAGAAAAGGAACTGGGGAAAAAACATGAGC

TCTTCAAGCACGGAGTCCCTTGAAGCTGACACCACAACCTTT

>c270_g1_i1 len=212 path=[155:0-181 337:182-211]

ATCAGACCTTCCCTTTGTGTTTCCCTGCGCACGTTAACAAATAGAAACGACTAATAATAT

TATAAACAAATATCAGACCCTCGCAGCCTCTCGGGTTTCGGCAGGGATTCGTGAGAGAGA

GTAGCTGGGGCTTCACAATGGTGAAGGACTGCGGTAATGCGCCGGATCACACTGACTGTT

GTTATTTTTATAGCTGTTCCCGCACAGGCTAC

>c270_g1_i2 len=213 path=[155:0-181 397:182-212]

ATCAGACCTTCCCTTTGTGTTTCCCTGCGCACGTTAACAAATAGAAACGACTAATAATAT

TATAAACAAATATCAGACCCTCGCAGCCTCTCGGGTTTCGGCAGGGATTCGTGAGAGAGA

GTAGCTGGGGCTTCACAATGGTGAAGGACTGCGGTAATGCGCCGGATCACACTGACTGTT

GTGTCACTGTAGCCTGTGCGGGAACAGCTATAA

>c279_g1_i1 len=252 path=[230:0-79 310:80-251]

AACCTGTTCCAGTGCGTCACCACACTCTGGGTGAAAAACTTCCTGATATCTAGTCTAAAT

CTGCCCTCTTCAAGTTTAAAGCCATTTCCTCTCATCCTGTTGCTATTACATGGGGCAAGA

GGCTGACCCCCTCCTTACCACAACCACCTTTCAGGGAGTTGTAGGGAGCGATAAGGTATC

CCCTGAGACTCCTGCAGACTGCACAATTCCAGCTCTACCCATATGGCTTATGCTCCAGAC

CCCCCTCAAACA

>c279_g1_i2 len=206 path=[459:0-33 310:34-205]

TATCTATCCCGAACCTCTCCTGGTGCAACTTATGGCCATTTCCTCTCATCCTGTTGCTAT

TACATGGGGCAAGAGGCTGACCCCCTCCTTACCACAACCACCTTTCAGGGAGTTGTAGGG

AGCGATAAGGTATCCCCTGAGACTCCTGCAGACTGCACAATTCCAGCTCTACCCATATGG

CTTATGCTCCAGACCCCCCTCAAACA

>c290_g1_i1 len=206 path=[1:0-114 282:115-205]

TGTGCCATCTGAGGAACTTCAGGAAATTTGCAGTCTTGGTGCTATTGCAGTCTTTACAGG

TTAAGTTGAATAGGAGGGAGCTTAATTATTCTTGGGAGCTGTGGGGAACTGTGTTTGAAT

GAGGTATGCGTGCTTAAACAGAAGTCTCGGAGTATATGTAGTGGTAATGTGTTGACTGGC

TGTTGTGTTTTGAGTTTGGGGCTGCA

>c298_g1_i1 len=235 path=[200:0-191 560:192-234]

ATATATATTTTTATATAAAAATGTGTGCCTTATGAATTAACAGAATTTATAATATTTTTT

TTTATTATTTCTTTTATAACATGTTTCTTCCATATGAATAACCTTCAAAAAAAATAAAGT

TAGGCATAAATGATATCCATTAAAAAATAAAAGTTCAAAGAATATTAAGCAAAACTGAAA

TAAGATTAAACTTTACAACTTAAAATATATATAATATTTAAATGGTCATACAATA

>c302_g1_i1 len=222 path=[1:0-181 183:182-221]

AGGAGGGACACAGGTAGAAATCTTAAAACTGCAGCCAGCTAAAAGGCTCCGTGGTCTCCA

TCACAGGGCACTCTAATTCCCACCTTCCCAAAATTTGCACACGTTATCTAAACGCACTCA

ATTATCTCAGTGCAAGTTGGGGATATGGATTCCAGCAGGGTATGAGAGGTGGTCATAGCT

CTCTGTTCATTTCCAGATGTTCCTGCAAAGCCTCTCGTGCTC

>c302_g1_i2 len=223 path=[1:0-181 263:182-196 278:197-222]

AGGAGGGACACAGGTAGAAATCTTAAAACTGCAGCCAGCTAAAAGGCTCCGTGGTCTCCA

TCACAGGGCACTCTAATTCCCACCTTCCCAAAATTTGCACACGTTATCTAAACGCACTCA

ATTATCTCAGTGCAAGTTGGGGATATGGATTCCAGCAGGGTATGAGAGGTGGTCATAGCT

CTTTGTTCAGAGAGGGCAGAGCACGAGAGGCTTTGCAGGAACA

>c322_g1_i1 len=220 path=[349:0-219]

CATGATGGTGTTTATCCGGTGAGCTATTGTTAACACTGTGCTCTCTTTGAATTGAGTTCT

CAGGGCAGACTGAATCTGAAGGTCAGTTTCTATATCTATTGCTGCTGTGGCCTCATCTAG

AATGAGGACTTTGGCCTTCTGAAGAAGTGCTCTGGCCAGGCAAACCAGCTGCTTCTGACC

AGTGCTGGGGAATGACAGTTATCTTCATTCTAAGGTCGTG

>c326_g1_i1 len=211 path=[60:0-59 258:60-210]

GGACAGCCTGGTCTGGTGGCTGGTGATCCTGGCACAGTGGTATTGAAATTTGATGATATT

TGAGGTCCTTTTCAACCCTGGCCATTCCATGATACAATTACTAGCACAATGTCAGCACAG

CCAGACTGTCAAGTATGAACATGTCAGCAGCAGTCTCAAACATTAGGATGGCCAAACTCT

GACAGCAAATGCTTGTCCAGTCAAACACAAG

>c333_g1_i1 len=233 path=[1:0-190 480:191-232]

GCTTAATGGTGGTCTGAGTGTTCTATTGAAAAGAAAATACAAGATTCTGAAATATTATTA

TTACAAACAGCTTTATAAACTCATTGAAAATAAAATGGGTAGAATGCCCACTAAACATCT

TTTAAATAAACAATTCCAAAGTCAATTGAATTTCCAGTTATTTTAAGTACTTTCTTACTT

TTGAGATCTTGCAATAATTTTAATTTTAGGAATGTATACTTTAAAGCTCTATA

>c338_g1_i1 len=221 path=[167:0-220]

GCATGTCAATCACAGAACAATTGGAAATGTGGCTCAGACGAAAATGAGACACAATAGCTG

AACAACAAAGAAAACTAGTCAGAAGCACGTAAATGAAATTTACCAGTACTGCTGCTTTAG

AGATATAAAATGGATTATCACTGAAATGATGTAATATGCAGGTAACAAATGCAACATTTG

TTTTTTATCTTATTAATCTTATATTTTATCTGATACCATAA

>c345_g1_i1 len=217 path=[565:0-19 567:20-216]

GGAGTACAGCACAGTCACTACTCTGGTGCTGCTAAAGCACTGTAGAAGGGTTGTTGCAAG

GAGTGTACATGTACTGTGGAAGATGAAGGTAAGTTTGTGAAGTCTAAAAGATGTATGTGA

ATGTCTGCAGCTAAAACTTTAATATATGCTAATGTAAAGCATTCAAATGTACTGATGAGT

CAATAAAGAGAGATGGACTTAAAAAAAAAAAAAAAAA

>c345_g1_i2 len=275 path=[1:0-77 567:78-274]

TGTGCTGTACTCCAGCCACATAGCTGACTGTACTTCAGTAGTCCTAAATCTGGATAAGTG

TTGTGCTTTCGCTGGTGGCTCTGGTGCTGCTAAAGCACTGTAGAAGGGTTGTTGCAAGGA

GTGTACATGTACTGTGGAAGATGAAGGTAAGTTTGTGAAGTCTAAAAGATGTATGTGAAT

GTCTGCAGCTAAAACTTTAATATATGCTAATGTAAAGCATTCAAATGTACTGATGAGTCA

ATAAAGAGAGATGGACTTAAAAAAAAAAAAAAAAA

>c346_g1_i1 len=241 path=[219:0-240]

AAGCGATTCGTTACGCATTCCAATGGTGAAACATCTAACATCTTAGAGTGTGTAAAAACG

TGTGCATGAGAAGGATTCAAATGTCTGGAAGTCTGAAACAGCAAAAGACTAAGTCTAGAA

AGAAGAACGAGGGCACAGCAAATCTTGCATGGAGCGCTCTCCTGGAAAGCAGACAGAAAA

GGAGCTGGAGACTGTAATGGTCCACAACAGCTACTTCTCTGTCCTGGGAACAGACTGCCA

G

>c346_g2_i1 len=297 path=[437:0-296]

GCTTGACAAGGACGAACGTCACTGCACCCCCTGGCCATGCTGCTGTTTCTCTATGTACCT

GTCCAGCTTTGAATGGAGAGAAGCTTTTCCTTTACAACACCTCATGAGGAAGCAGTCCAT

CTTGTCTGGGGAGCAATTAGTTGCTGTCCTTTGGGCTCAGGATCTTTCATTCCTTATGCC

TGATGGCAGAGATGGAACAAGATAATCCTGTTGTTTCAGCCCAAGTGTGTCTTTTTTATA

AGCCTGCAGAACAAAAAAAAAACCCATGGAATTGAAGTTAAGATGGAATAATGGATG

>c348_g1_i1 len=225 path=[1:0-224]

GGTGCTGGTGCCACTGAGGAGCGAATACTCAAGAACTGTTCCTAAACAACTCCATGTCCT

CACACAGCTGGCAGCAAGCACACTTGGGGCTTGCTTAGAGCCACTGCAGCAGATTGAGAT

CTGTGGGTCTACCCTGAGACAGCTGCCTGCAGGATCCAGCAGATGTGAGGTTGGCAACGG

TGGCATACAGGGAAGCCAGCCCCCAAGGAGAGACAGTGCAAGGAA

>c354_g1_i1 len=361 path=[339:0-360]

GCAAAACTGAAAGCTTGCTTTTACATTAAACGTATTTTCATATACTCATTTTCAATGGAA

CCGAACATCTCAGCTGCGACTCCTCCACACTTCTCTAGTTCGATGTTAATGGAACTCACG

ATGTCTCAAGATATCACATACGATCAACCATATGTGGTAGATCACCCATTAATTCACTCG

AATGTTAGGCTAATGTAGAAATGCTTATGCTAAGTTCATTAACTTTGGTACAGCATTCTT

TTGTGCTATCTAATCTGATTCAAAATAAAAGCTATTATTGTAATGTTCTCTTCTGCTGTC

ATCTGGAACAGTCTTTGGGTATGTCAGATAACAACTAGAAGCCACTTCTGTCTTGTTAGT

T

>c356_g1_i1 len=240 path=[372:0-239]

GTGAGCAAGCAGCGATGTTTATTCCAGCTGGGTTAACTGATTGCAGACTTCTATGTTGTG

ATGGTAAGAAGAAAAGGGTAAGAAGAGGGTAAGAGAACAGTGCTGAGATGCAGACCTGAC

AAAGTGAAAATGGAAATGGTGGTAAGAAACAAGCCTCTGGTTGTGTTAGTGGATGGGCTA

TCTGGCTACAGGTGCCGCTTTCAGGAAGATCAACTTAGACTTTGCAACTGATAAGACTGA

>c359_g1_i1 len=275 path=[81:0-274]

AGGGGGACCTTGGCTGAAAGAAACCTTATGACAGACTTGGAAAGTGTTTTTCTCCAGTAG

ACTAAAATAATGAGGGAAAGAAAAACCACTCGGAAAGGAGAGGGAGGTCATATATTCCAT

TGTTCACTTCACCTTGTTTTTTGCCAAGTAGTAGTTCCCCCTCCCAGATTTCCACTCCGA

ATGCCTCTCCAAGCTGTGGTGGTGGGTCGCTCCTTTGGTATTTTCACCACCATGATAAAG

TTTACCCTCATTTTTCTGAGGCAAGTTTTCGGGCT

>c360_g1_i1 len=201 path=[1:0-200]

AATATTTCATAAAAGTCTTTAAATGAATAGTTAATAAATGATGATTAAAGTAATTCTTAC

AAAAAATAAAATCAATAGATACATTTATGTATACTCTCATTAATTTGTCTATTAAATACT

CTGCAAATTCCTTTAAACACAAAGCTAATAATTTCAATGGTAAAGATGTTACTTATTCAC

ATAGATTAACAAAATAATTTG

>c363_g1_i1 len=201 path=[349:0-24 22:25-200]

CTGCAGAGCTGACCTGTGCTTCAAGACTGAAGTCCTGCCTGGCCACAGCCTGCCCATGCT

GCTGATTTAGCTGCCTGTGATGTGCAAACTGGAAAATAATACGAACAATGAATAACTGTA

TGTACCAACCTCACCCAAAAGTACTTGAGATAGGACTGTGACAAGCCCTGCTTGTGATCT

CACCCCCACAGACCATCTGTA

>c369_g1_i1 len=221 path=[260:0-177 438:178-220]

TGATGCATATCAAGAAATGTTTCATAAAGACAGAATAGCAGAACTGAAAAAATATCTGTC

ATCAACAATTTAAAAAAAAAGTTGAAAACTCGAACGTATTCTATTGTAAAATCTAGTTGT

GTGGTAGCAAATCTTACTGCGAAAAGATAAAAACCACTTACTAGTGATGAGTTTATTACT

CAGTGTATGGAGAGCATGGAAGATGTAACGTGTCCTCATTA

>c371_g1_i1 len=232 path=[210:0-231]

GTTGTCCTGGGCGCAGCAGCTGCCAAGCAGGTTAAATGAGGAATGTACAAAGAGAGCAGT

GGGAGCACGGGGCCGGACAGACAGACACGGCCACACGCTCCTCGCCCACCATCGGACCCC

ACAGCACTGTCTCCCCCCCCGAGGAGCGCACCGTCAAAGACACAACGCCCAGCTCCCTGG

ATGCAGACCCCCTGATGGCCATGCTGCTGAAGCTCCAGGAAGCCGCCACCTA

>c381_g1_i1 len=295 path=[297:0-106 404:107-294]

TATACATTTAGGAAAACTTAAAATTAAATACAATAATTAATACGTTGTAATATATAATTA

TATATTTTTATAAGACTGTTTTAAGTTTAAATACTACACAGGAAAGGTGCATAATTAAAA

AAAATTCTATTATTTTATAAAAGTTTATATGTTGATATACTCATTAATACAATTATTCTC

ATTAATAATTTTTATCTTTTTCTATATATTACGAAAAATAATAAAATTAAAACATGATGA

GAATTAAAAAATTAATATTAATTAAAAATATGTTAATAAAATTATTTATAATTAT

>c381_g1_i2 len=206 path=[593:0-17 404:18-205]

AAAAATCAAACACAAAGTTGCATAATTAAAAAAAATTCTATTATTTTATAAAAGTTTATA

TGTTGATATACTCATTAATACAATTATTCTCATTAATAATTTTTATCTTTTTCTATATAT

TACGAAAAATAATAAAATTAAAACATGATGAGAATTAAAAAATTAATATTAATTAAAAAT

ATGTTAATAAAATTATTTATAATTAT

>c384_g1_i1 len=321 path=[1:0-320]

ATTTTTTTTCACTTATTACAAATAACATAATTAAATTTATTTATATAATAAAATTACTAA

TAATGTAAAATAAAGTGATATGAAATTTTTATTAACAAATAATGAAAAATTAGAATTTTT

TCTTCTAAGAAGAAAGCTTACATTAGTATAATAAAATTAAAGTAAATTTTATATAAAATT

TTTTTCTCTTTGTTCATTTTTATAGTAATTAGAATTATTAAAGTACTCACTTTACTAATT

CATTCTTATGTGATTTATTAATTAGTTGCTTGGTTACTTAAATCATATTTTAAAAAATTA

AATTAATAAAGAGCATGTAGA

>c387_g1_i1 len=319 path=[12:0-271 285:272-318]

TTTTTTTTTTTTTTATAAAAATATTCTTTAAAGCATATATTGCTTTTTATTTAGAAAAAG

AAAAAGTTCCTTTTTGTTATTATGTAACTTTTAAATGATTCCTAGCGCAAAACCAATGAA

TAATGTTAAAATTTTTTTTTGTAACAAAAATAAATACAAAAGAAAAAAATATATGAAAAG

AAATTTAATACATAATGGAATATGAAGGTGAAATTTTTCATTTAACTGAATACCAAATAA

AAAAAAGCATTAAAGCTTTCAATTACTTAGATAAAAAAAAAAAGGGCTACTAAAATTTGA

GTATATATAAAAAAAAAAA

>c391_g1_i1 len=213 path=[1:0-40 42:41-212]

TAGGGCTCAGCTCTACATGCAAGTAAGCCTTGCTAATGCCCGTGCATGGCAGGCCACGCC

AGCCTCCCCACGCCATCCCTGAGGTCACCTCCTGCCATTCTGCTCATGCAAACTCCAGCA

TCCATGCAGTGCTGGCACATCATGCTCTGGGGGTCCCGCTCGTCCATCCCAGGCTTTGCT

AAATCCATCCATGAGCCATTCCAGCCCTATAAA

>c392_g1_i1 len=277 path=[255:0-141 397:142-276]

GGTAACTTTGTGTTTGAGCCAGGGTTAATGAAGTGTAATGGAGATATAGTATGAGCATTG

CAGTTTCCTTATTAACAGATTAAAAAGGTGTTTAATTACAGTGGGAATATTGCAGCTAGA

AGTTTATACATTTCACACCTGTTTGAAAGATTTTTTTTTTAAACTAGCCTATTAAATTAA

TATAGAAGTTAAATATAAAAGTTACCTTTTCGTTGTTGTTCGTACTTTTTGTTTCTCCAT

TGCTTTGCACTTTAGGTTCTACATGGCAACAAGAAAC

>c399_g1_i1 len=279 path=[1:0-208 210:209-278]

CACAGAACCTGGGCACACTCTGCTCCCCACGCCGTGCAGTGCTCACGGTGAGAATTGCAA

CTGGGTTTGCACTGACTTGCAGAACACGAAGCATTACAAAGCCCTGGTAGGCGTTAAAGT

TGAAATAATTAATATTTTTAATGAAAGGAACAGTTCATACCAGACCTACAAGGAAGCCAA

ACATATCGCTGTTGTGAATATATCACAGAACAAAAGAGAAAAATAAAATGGACAGATACA

ATCAAATTCTGTTTGCTGTCAGAGCAGGTTGTTCAGGTT

>c400_g1_i1 len=350 path=[332:0-221 554:222-349]

TAACTTTGTGTTTGACGGGTTTGTGGTTTCTTGTTTGTTTTTAAATTTCACACGCTTGAT

CCATTTACTTTTGCATCAAAGACCTACACACGGTTTTTCTAAACCAGGCATTACCTGCTG

CTCTCACTGTTAATTAAAGTACAGATTATAATATCATTCAATCCACTGTACTGATATGCC

AACTCAAAAGCTTCTTAAAACCTGATTCCACAGGGAGAAAAATGTTTTTAAAAAAATACC

TGCTCTGTGTAAGCACTGCTGTACAACAACTGAAACATCAACCTGCTATCACCACTGCTT

TCCTCAAAAATCCAGACCATGGCATCCTATAAGCCCCTATGAAGAAAACT

>c402_g1_i1 len=465 path=[443:0-464]

AGGAACCAATGAGAAACATGAGCCTCTTATGCAATTGAAACCTCTCTAAGCAGATAATAC

GAAGCAAACAGCTTTTACACATAAAATAATTTTATGGTGAAAAAGATCCCTTTGCATTGT

CTACTGCTGTGTTATCTCTATAGCCTCATACAATCTGGATTCATTTTTAGGCACCATCAA

AGCTTTTCATTATGCGGATTCAAACATCCAATTACACTCGTGTTCTCCTGAACATTTATG

TTGTCTCAGTGTTTGCTTTTTTATGCTTATGGCATCCATGTCTGTATTGGAAGCTGTGAA

ACCAAAGTCTTAGCATGGACAACGTTCTGTGCGTGGCTTCCTGCATTTTAGACCGCGTTT

ACAGGAAGAAGGTTCTTTGGAAGCATTTCTGGTGTTGAACAAAAAACCAGAAACTGAATA

TCAGGTTTGCTGTGGAACTGAGATGAAACTAGCCTTTATGCAAGT

>c417_g1_i1 len=249 path=[501:0-20 296:21-248]

TCATATTTCTCATATATATCAGAAAATTTTTTATTGATATAATCTACAATATAAGAATGA

TAAAAATAAAGTAAAGAATACAAGATTTTTAAATTTTTTAAAAGAACTAAGAATTTTAGC

AATGAACTATTTTTTAATTTATCGATTTTTGAAAAAAACAAATTTTTATCTGTTAAAATT

AAATAATTTTTTAGTGTTTTAAGAAAAAAGAAATTTTTTTTAAGTTTATTTTATTTTTTT

TTTTATTGT

>c417_g1_i2 len=273 path=[251:0-44 296:45-272]

AATAAAAATATCAAATATGTTATTTATACACATTTTTTTAAAGTTGAAAATTTTTTATTG

ATATAATCTACAATATAAGAATGATAAAAATAAAGTAAAGAATACAAGATTTTTAAATTT

TTTAAAAGAACTAAGAATTTTAGCAATGAACTATTTTTTAATTTATCGATTTTTGAAAAA

AACAAATTTTTATCTGTTAAAATTAAATAATTTTTTAGTGTTTTAAGAAAAAAGAAATTT

TTTTTAAGTTTATTTTATTTTTTTTTTTATTGT

>c420_g1_i1 len=313 path=[295:0-145 4:146-149 447:150-312]

ACTTTGTGTTTGATTGTGGAATATAAAATAAGTAAAATAATTGTCCAAATGGTATTTTAG

GATAACAAAAGAAAATGCCTTATAGAAATAATTAAAAACATAATGGAGATAAAAAGATTA

TTTAATAATCTTTGAATAGAAAAAAGAATAAAAAAAAAAAACAAAATAAATTACATACAA

TAAAGAAAAAGGAAAGAATTATTAAAATTGTAATATTTAGACCTTTTAGGAACTATAAAT

TTAATAATTATTATCCTTATAATTATATGATATAAAAAGAAAGTGTATGCATTTTAATTA

AAAATTATTATGT

>c421_g1_i1 len=223 path=[4:0-222]

ATGAACTTTTTTTTTTTTTTTACTTATTATTTTCATTTCTCGTTTTTTATTAATTTACTA

TCTGAAAATATTTATAAAAATTCATATCTCTTTTTATAATGGTAATAAAATTTTATTCAT

AATGAAGTATTTTGTTTTTATCTTAATATATGAGAACATAATAACTATGATATAGAACAA

TAAAATATGCTCAAACAATTACATTTAAAAAAATATCTTTAAA

>c421_g2_i1 len=330 path=[407:0-329]

AAAAAAAAAAAAAAAGCATTCTTTAATTAATATGAAAATATTTATATTTTTTGCATTCAT

AAATTCATTTTACTATTTAGCATCCAAAAAATGGGAAATTGTATGAAAATAGTTATATTT

CATTTAATCACCTTATATTAAGAAACATTTTTAGATACTTAATTTTTTGGATAAAAAAAA

AAAAAGTATTATAGAAATGTAAATAAAAGTACTAACCTTCAAAATTAATACTAATATTTT

ATAATTAAAAAATTCTAAAGTAAAAAAGAAAATAATGATATTAAAATATGAAGGTAAAAA

AACTTCCATATAAGGAAATTTTTTTATTTA

>c424_g1_i1 len=322 path=[1:0-321]

GGGATGTTAGAACCTGCAATAAACTATTTGGATCATTCACGTATCTGAGTCAGATGCCAC

TTAAAGTTAAGATAATTAAGCAGGCGTAATGTATAAAGCAACTTTTCTGGTAGGAATTTG

CTTTGTAGGCTGTGAAGGATGGAAAAGTAATGTACATTTACAAATAGTATGGAAACTAGC

TAATGTAACTAATTCCTCTGATTTCTGGGTATACATCCAATTATCGAGGAGCATGCAAAC

AGGGTATCCATTAGCAGGAGTATTGTTGAACCTGACAGAAATAGGTAGGGTTGGCAACCC

CAACAGTCCAGTGTGCTTTGAT

>c433_g1_i1 len=249 path=[1:0-248]

AAAAAAAAATATATATATATATACGTGTAACATTTTAAATGAATATATATTTTCTTATAT

TAACTTTAGGTAATCACATCTGGATTTAAAGTAATAAATTGAAAAAGAAAATATACATTC

ATATTTCTAAATAGAAAATTAAAATAAACATTGTAAATTAATGACGTAACAATATATATG

AAAGAAAATAAAAATTTTATCATAAAAATTTGTTATTTTATTTTAAAGTAATAAAGAACA

TATATTTAT

>c437_g1_i1 len=258 path=[1:0-162 164:163-257]

AGAGTAAACTGCTATTGCAACTGAAAGCGTTTGATGGCTGTCTGGTTTGTTACTCACAAG

GAACTCACAAGTCTTCATTCACCTGTTTGCTTTTGGTTTTGTCCTTTTTCTTTTTGTCAG

GGCCACTTGTTCTTTTGCAAAGCCTGATCAGACAGTAGGGATTGTATGTGAAGAAGTGTG

TTTTATACGCTGGGCAACGTTTTGCCCTCTGTTTTATAGGCCTGGCTTTGCACAGTACTG

AACTTCTTAAAGATGTGG

>c455_g1_i1 len=203 path=[411:0-25 203:26-202]

GTCTTCTAATTATTTTTTCTTCTATGAATATTTCTTTTACATCTCATAGTAATATAACAT

ATTTTTCATATTTTAAAATAAAAATTCTTTAATAAAAAGATAAATAAAAAGATTACTTAA

GTATTTCATATATTTTGACATAATTATGATAGATATAAAAGTAAGAAAAAGTAAAAAGTA

AATTGGGAAATGTAGATTTTTTT

>c464_g1_i1 len=217 path=[415:0-216]

TTTTTTATTTTATTTTATTTTTTCAACAAATAAAAATAAATTTTGTGTATTTTTCAAAAA

AAAAATGTTCTATATTATTATTCTGTTTTATTTTTTTCTACTAATAATTAATAAATTTTT

TTTATAATCTTGTCTTATTAATAAGATATACTCTTGTATAAAAAACGAAAAAATAAACTT

TAAAAATATATGTCCATAATAAAAAAAAAAAAAAAAA

>c486_g1_i1 len=278 path=[256:0-175 432:176-277]

GTTACGTATGTTGAAACCAAACTAGAAGCAACCTATGAGTTTAATTCTATTACCTATCAG

GCCATTAAGTAAAGTTTATATGCATATGATTATATGTCTTTGTCTGTAGTATGCATATGT

AATTACATAATATACAAATACTTTATGTTCATTCTTACTCTCCTTTATTAATGCTGTTTT

CTTTTATGTTTAAATCATGTTTATGTCCTATGATATGTCGTCTGTTGCTGCATCAGAGTT

AGAAGGTTTACAGTGCCAGAGAAGTCTAGGTTACAATG

>c504_g1_i1 len=415 path=[485:0-170 22:171-414]

TTTTTTTGTTTGTGCTTACATTTTCTATATTTCTTTATAGCATTTATATGTAATTTAATG

AAAAGTTCGCAATATTTATTATTGTTTGTGTTTTCTATGATTATTATTGCAGCTTATTAA

AATAAATTATATATCGACAATGTTTTATAAATTATCTTAAAAATTATAAGCTTTTTTTTT

TTTATTGTCTTACAAAAAAATTGAAATATTTGTTTTTTATTTTTCATACATTGTGATACT

TGTGAAAAAATATTTAATAAATCATAGCCTTATAAGAATTTATATTTAAGTGTTTATGTA

ATTGAATGAAAAATTTAAAAAAAGAAATTTTTAATAATTTATGTGTGTGATTAAGATATA

TATATATATGCAAATAATTTTTTTTTTTTCGTTTGTATGTAATTTTTTATACAAA

>c509_g1_i1 len=230 path=[208:0-2 211:3-229]

GAAATTAGATTTTTTAATACATGGTAAGATTTATTAAAATAAAAAAAATACATATATATT

TTTATGAGTAAAAAATATATTTTAATATTATTGTATAACTTTTGATAAGTTTTATATAAT

ATGTTAATATTATTAAATTGTCTAATAATTAAACATATATTTTTATAATTAAAGTATATA

TACCATATAATTTTCATTTTAATAACATAAAAAAAAAAATAAATAAATAA

>c514_g1_i1 len=226 path=[12:0-225]

AAAGAAAAAAAAATATATATTTATATACTACATAAAAATGCATCTATACAATTTTTCTTA

TATTGTAGATGGATCAACAAATATGAATCATAATTGATACATTTAATGTTGCAATTTTTT

TTATAGTTTTATTTAAATAATTAATTTTTTTTCTCTTTGTTTAAATATAAAGATCGATTA

AAAAATTATAAATATAACTAGAAAAAACAAAAAAAAAGAAAAAAGA

>c524_g1_i1 len=207 path=[185:0-206]

AGCACAAAGAATTATTTTTAAAGGTTTATATTTATACAAAAAATAAATACAAAATTTTAT

ATTTTGATTACATATAAATACAATTTTTTGGTAGATTTTAATAGATGTTTTAAGATTAGT

TATGCATACATAATTTTTAGTATATATTTTTAAGATCTTTATTTTATTTATTTTATGAAT

TAATAGGCAAAATATTAAAAGACAAAG

>c529_g1_i1 len=316 path=[374:0-273 831:274-276 25:277-315]

ACTGAAAGGACAGAAGGCAATGGGCACAAATTGAAATACAAGAAATTCCATTTAAGTATA

AGAAAATGCTTTAAACTGTGAAGGTGGTTGAACAGTGGCACAGGGTGCCCAGAAAGATTG

TAGAGTCTCCATTCCAGGAGGTACTGAAAACCTGACTTAGCTCTTAGTAACCTGCTCTGA

GTAGGTAGACTGGACTAGACAATATCCACAAGTCCCTTTCAACTCCAGTGATATATTTAT

TAACCATTGCAAAGTAAGTTTTACTCCATCTGTTGCTTTTCATACAAACACTTAGTACAG

ACTAACGTATGTCATC

>c529_g1_i2 len=358 path=[374:0-273 831:274-276 650:277-357]

ACTGAAAGGACAGAAGGCAATGGGCACAAATTGAAATACAAGAAATTCCATTTAAGTATA

AGAAAATGCTTTAAACTGTGAAGGTGGTTGAACAGTGGCACAGGGTGCCCAGAAAGATTG

TAGAGTCTCCATTCCAGGAGGTACTGAAAACCTGACTTAGCTCTTAGTAACCTGCTCTGA

GTAGGTAGACTGGACTAGACAATATCCACAAGTCCCTTTCAACTCCAGTGATATATTTAT

TAACCATTGCAAAGTAAGTTTTACTCCATCTGTTGCTGTCATGTATTGCAAGTCATGTAA

GACTTAGCCCTGTACACTTTCCCAAGACTGTAAGATGACATACGTTAGTCTGTACTAA

>c533_g1_i1 len=280 path=[1:0-279]

GAAGTGATCAATAAATAAGAAATCACATTTTGTCTCAAACAAATAACTTTGAACATCTTG

AAAACTTCTCAGACTTCTGCCACAGGGAGCTTTATAATGCACATCCAGTGATTTTGAAAG

GCAGTCTGCTTTCGCATGGCGTCTTTGAAAGTGAAACAGTATTGGTATCTTAAGAGGATT

TTCACCCTTGTAGGAGCTTACTGCTCTGTTGGAGAGACATGCACTGGAGCACTTGTGGAT

CCGATAGCGGGGAAGTATTTAAATATACCCGCGTCAAACA

>c537_g1_i1 len=300 path=[278:0-117 587:118-299]

AAAGCTGAGTGCCGGTCAACAATAGGAGGAGGAGCAGAGGCTGGAGCCTGCTCTGTGGCT

GCAGGCTCTCTCGGTTCCCAGGCGTGCCCACAGGCTGAGCGTAGCCAGAGGACACTTGGA

GCAAACGCTGGTGCTCGCACGGCCGCAGGAGCGCTGGGGACAGACGGAGACGGGGCTGGC

AGTGGGGCTGGAGGCTGCCGCTGTTCCTGGTGCGTCAGACCTCGCCAGTTTCAGCACTTT

GCGAATGTGTCAAACTTTTTATTCTGCTCTTTTGAGTCTATTGCAAAAAAAAAAAAAAAA

>c539_g1_i1 len=319 path=[1:0-141 466:142-318]

GGAAATGGCCGGGAGCCGCGGGCTGAGCCCCAAAGCCGCTCCGCTCGGCCGGCCCCGAGC

CCCAGCGCCTGTTCCCCGCCCGTTTGCTTCCTCTCCCCCCCCTTCCTTCTCCCTCTCTTT

CTCCCCCCTCTCTGCTTCCCCCTTTTTTTTTCCCCTCCTTCTTCCCCCTCTCTCGCTCGC

TTTCCCTCTCGCTCTCTCTCTCTGCCCGCCATCTTTTGAAAGGGAAAAAAAAAAATTCGG

AGCGAGGGAAAAGAAGGGCTGTAGGCGCTCTTAGAAAATAAGAAAGGAGGAGAAAAAAAA

AGAAAGAGGAAAGAAAGAA

>c541_g1_i1 len=235 path=[315:0-206 657:207-211 663:212-234]

ACTGGATGCAAAATAGAAAAAACAGAGGGTCAAGTCATACCTCTTGTATTTTATCACCAA

TTGTAAGAATCAGGGAAATTCAGGCTATCAGGAATTATGAACATCCTACAGGTGGCATAC

CAGTTTTAAATCTTAAAAAATTACTACTAAGGACAGACATAAATGAGTAAACTTCGACCT

ACTGTCAGTACTGAGTCAGAAGACTCATTTGATTAGAAAGCCAAAGTTGTAATCA

>c542_g1_i1 len=227 path=[1:0-179 181:180-226]

CTCTCTGCACAGTGGACATTCTGTCTACAAAAGGAAAGAGATATTCTAAATTGCTGTATT

TAAAAGGTTAAAGAAAAACATCCAACTATGTTAAACAACAACTAGAAAATGAGCTTCTGT

AACTACACGTTTTTTCAGTCCAGTCTGCATTGCATGCTTAACCAGGTAAAGCTCTGAAAG

GTAACCCCACTCAAACACAAAGTTAATTTGTACTACTGGATATCTGA

>c544_g1_i1 len=231 path=[150:0-115 426:116-121 433:122-230]

GCAAGGACTCCTGAGCAAACATAACTATCCCGTTAAAGCAAGAGCTCCAAAGGAGTACAA

TCATGAGGGAGATGGGAAGCTCACCTTGTGGGATGCCTACTGATCCATTCAAGAATCATA

TTGACTTTTCTGGAAAGATTGATTGGTATTCTTGGAAACTTGAACGTTTTCTTTCACTTG

TATGATTTTGAACGTTAACCTTTCATTTCAGCTTGCTTGTAAGCAAATACA

>c545_g1_i1 len=256 path=[239:0-154 394:155-172 528:173-214 453:215-255]

TATGAGCATATGAGCATGTAGCTGCTCATAACTGATTTCAAAGTCATCAGCTTCTTCTCA

AACATCAAGACCTATTTCCTTTATTTTCTTACTCACTTTTTAGCAAGTAATTGCTTATAT

ACGTGCTTAGTGCTTAAGTTATTAGTTATACTGCACAAATTATTAAGTTAAATTGGGCTT

AATTAAAAGTCAAATTTAAAACTTGTTGAGGAACTCAAATTATATCATGGCATTTTTCTT

TTAATTAAGCCCAATT

>c546_g1_i1 len=208 path=[1:0-207]

GGACATCTTGACAAAATCTCTCTCTTTTTATCAGAAATGATACTCGTTCAGGTAATACAT

TCATAAAACCATGGAGAACGAGAAACCTTTGCCATGCTTGAAAAGAAGCGCACAGTAACT

GTACTTACAGTGCTATGTGTGCCTGTAGCTCCGACAGGAGCCCCTCCAAATGAGATGCTG

TAACATCAGGCTTAACCCCACATCAACT

>c547_g1_i1 len=209 path=[325:0-82 222:83-208]

ATTGTTTTTTCCCCACACTGGTTCAACTCCCTGAACCAGCTCTCCAGGTGGCAGAGTGCC

AACGCAGAGAGACTGGGAATGTATGGCACTGAGCTGTGGGAATTTTCTTGCCCTTAATGT

GACAAATGTTAACCAGGTCACTTTGTGTTACCAATGATTATTAAAACTATGCTTAATGAT

GTATAAATTGTTAAGTTTTAGGAACATCA

>c555_g1_i1 len=231 path=[1:0-230]

TGTTAACACTGGGAAACAAGTAAGCAAACCTCTACCACAGACAGAGGCATCTGCAGGACA

CAGTTGTTTAGAACTTAACAAATGCACAAATATAAACACTTACATACAACCACAATTCTG

AATCTCTTTTTATTCAGCATGCATAATAAAAGCAATCACTTATTATGATGGAAGAGAAGT

TCAAGCCTCCTGTATACAAAAAAGATTTACCTACAGCCAGTCTTATTAATG

>c561_g1_i1 len=225 path=[475:0-224]

GGAAGATGCAGTAACATTTTCAACAAAAACTTTAGTCGTTAAAAGATCACAACGATTAAT

TAACTCTTTACAAACATTATCAATATACACATTGGGAATATTGTTATTTGGTATAATATT

TGGTTGTCTCATTAAAACACAATAATATTCTTCAGTTATAGCAGTATGTACTCCTAAGGT

AAGTACAGTTTCAGGATAAGGATTTAAATTTTTTTAAACGTCATA

>c565_g1_i1 len=228 path=[206:0-227]

GGAGTTCCACACTTAACTCATACAGATCACACAGCATCTGCAGAACTGCCCTGCTAGGGA

ACATAAACATAATATACGCAGAGTAACAGAGGAGATATTAAGAGGAATTACAGCAAGATA

AATACTTATGAAAGTTATTTCACGCAGCTACTGCTGCACATCCAGAAGAAAGGAGTTTCT

TTGGATGAGAATTGAGAGCTTGCTGTGCTAGAGCAAGTGTAGCTTTGG

>c575_g1_i1 len=284 path=[1:0-197 199:198-283]

TTACTTTCCCCCTTCTATTTTGTTAAACAGATCCTTGAAGAACATTAAACATTTAGAAAT

CAAAATCATATCAATCACAAACATTGCATATGACTAAAATTGCATAATGTACACATATTA

GAGTGATAGGCTCATTTCAAACATCTAGCTAGAACAGAAGTTACTTTTTAAGCAGATGAG

TGAGATTTTAATATTAAAAGTGGAGTATTTGACTGGTTTCTCAAATCTCAATAGAGATTA

AAGTAATGATATCTCATGGTGAGATATATGCTTTGCATATAAAG

>c577_g1_i1 len=253 path=[231:0-252]

CTGCCTGTTGTAAGGCAAATCAGGTAAAACACACAGAACAAGGTGCTCGAAATTGCTGAG

TGCAAAAACGCGATGAAATATGCATGTGCTGGTTAACTTTGCTGTTGTCATCAGCAAGAA

CAAAAGTAATTATTTCTGTTTGCACGGCTAATCAAAGCCTCTTGTTTAACAGATGGAAAA

CAAAACTGATTACATCCTGTGTGAGTGCAGGTTGTAAGACAAGCGTACTTAGCAGGGATT

CAAACTCTTAAAG

>c579_g1_i1 len=242 path=[1:0-150 816:151-155 320:156-241]

GATATGCACATGCTGACAGGCACTGTCACACATCAAGAAGCAGTTCAGTTTATGCCAGCT

AGAGAAAGCAGTATCCTAACTCCCTGCAAGAAGGGAAAATAGAAGGCATGGTAGATGGAA

AACCTAAAAAGAATTAACGCCCTATGTTTTTTACCATTCTTTTAACCATTCTGCAGCTTT

CTTTTTGGTCTTTTCAAGACTTGTGTATGTAGTATTTCTACTTGTCTGGAATGATGTTAT

GT

>c579_g1_i2 len=405 path=[1:0-150 152:151-318 320:319-404]

GATATGCACATGCTGACAGGCACTGTCACACATCAAGAAGCAGTTCAGTTTATGCCAGCT

AGAGAAAGCAGTATCCTAACTCCCTGCAAGAAGGGAAAATAGAAGGCATGGTAGATGGAA

AACCTAAAAAGAATTAACGCCCTATGTTTTTAGTAATTAGAACACCTTATAGCCTCAAAG

TGGCTCCCCTGTTTCACTTCTAGAGAATGATGCATTGTGGAAGAAGATGACTAGACTTAA

TGAGTTACCTTAGGTAGCTCAGGAACAGGCACTACCCCTGTATACCTCCACAGGTTACAT

CAGCAGGGGTTTCTCCATGTTCTTTTAACCATTCTGCAGCTTTCTTTTTGGTCTTTTCAA

GACTTGTGTATGTAGTATTTCTACTTGTCTGGAATGATGTTATGT

>c583_g1_i1 len=201 path=[256:0-132 559:133-139 396:140-200]

GTGGCAACATAAGGCTACAACACTGTTTCTATCACAAGACAAGCTTGAGTCAGCGACTGC

CTTTGAAACATCTGCGGTGTTGGCTAAAATAGACCAGATTTGATGTACATTGAAGGAGTT

GTGAAAATAGCCCAGAATAGGAAGAGGGAACAGGGAATATCAGAGAAGAGGAGAAGAAAA

AAAAAACATTGGAGTAAACCA

>c594_g1_i1 len=365 path=[637:0-364]

ATTTTTTTTTATAGTCTCTCTGCAAGGGAGTAAGTGCCAAATTAATGAATGGTAATGAAA

TGAGAGAAACTCAGAGCTCATGGATGGATCTGACAGGTGGAATTTGTGATTGAAGTGTCT

GTTCCCAGTCACTCTTAGTGTTGTCTAGCACAACCTGTGTTGTTTCATGAGTTGTTGGAG

CAACTGATTTTTGCATATGGGTGAAGCCACATCATTTTGTAAGTGACAATTTGCCAAATT

ATACACTCTTCAGTTAAGCAGGGCTGGGTAGATTTTGCTTGCAGGCTTCCAGCAGTGTTG

CTTCCTTTATCTAGCCAATACTCCATTGTAAGAATCGGTGGTATTAGAATCACAGAATCA

TAGAA

>c596_g1_i1 len=284 path=[43:0-283]

ATGCTTGTGTGTGCAGGCACATCTGTGTATTTTCTTCTTGCTCTGATTCTTGCCTTGTTT

CACAGTTTGTTTCACAGCAGTTTTTGAAAAGCACCTTTCAAGAGATGTGCTGACTTCTAA

AAAGATGACCTTTGTATCTTTGGCCTCACACTGTACATTCAAACCACCAGACCTTTTCAA

TGGTTGGAGTCCTTTCACTTAGTTGCACTCTCCAGGAGATGTGAATAATCTAGCAGGATA

AAACTGTATTTTTTTAAACACTTTAAACTGGTGCACAGATGATA

>c603_g1_i1 len=207 path=[220:0-148 18:149-206]

CAACATCTCTGCTACTGAGAAGGAAAATAAGGTCTCCATCATATTTTGTATCTAATTGTT

GCATATCAATATTTGTTTTTACAATAATGGGTGAAACATAAGCATTTAAGAAACTGTGAC

ATACTGAAACTAGGAAAGCTGCAGAAGGATTGTCTACTGAAATGGATGATTTTTTCAACA

GCTAAAATAAGCTTGGCTTCCAAAATG

>c604_g1_i1 len=220 path=[161:0-219]

ACTTGCTTAGCCTCTGAGCATAGCATTCATGCTAGCAATAAGGACTCTTCTGCAAATACT

CAGCCCGTGTAGTACAGATCGTCCCTCACTAGGTAACGTTCTGGCAGCATTAATAGAGGA

ATTTGGTTTTAAATGTTTCATGTTCTGGAAGTATGGTGCCTATAGACATTTTGGATCAGG

GCAAGCTCTTGTCTTGGCATTACGAAACACCACTCTAACA

>c611_g1_i1 len=270 path=[472:0-17 474:18-269]

CATCACTCTCATCACAGATATCATTCCTCCCTCCACCCCCAGACTTACTTTGCAGGTTCT

AAACGTTGAAGTCAGCAGCTCTGAACATGAAACGCATTTCATATTTGCAGTTGAAAAATG

ATCTTGCATTGTGCTTCTGGGGTGAAATGTAACCTCAGTCCTCTCTCTTTGACAGTAGAC

TCAATAGACTTTCTTCATTTCTTTTTGTTGCTCTTTCCCCCGGAAATGTGAATTTAAGGG

AGTTCTTACTTTAGATTTGATTTTTTGATA

>c611_g1_i2 len=381 path=[39:0-128 474:129-380]

GTTGAATTTGGAGTATTGTTTTGTTTAGAAGTGGGAGTGCATCCATTCCTTTTTGACGTG

CATAGAGCTTTAGAAAATGTCACTCCTCTGAGTTTTTGCAAATGATTTCAAGTGGAAGTG

TGCTGCTGATATCATTCCTCCCTCCACCCCCAGACTTACTTTGCAGGTTCTAAACGTTGA

AGTCAGCAGCTCTGAACATGAAACGCATTTCATATTTGCAGTTGAAAAATGATCTTGCAT

TGTGCTTCTGGGGTGAAATGTAACCTCAGTCCTCTCTCTTTGACAGTAGACTCAATAGAC

TTTCTTCATTTCTTTTTGTTGCTCTTTCCCCCGGAAATGTGAATTTAAGGGAGTTCTTAC

TTTAGATTTGATTTTTTGATA

>c619_g1_i1 len=209 path=[1:0-208]

GTATTCTATCTCCTTCAAACTTTTTCTCCTGGACCTGAAAAGCTACAACATATTTATTGC

TACAGACACCAAAAAGTAAAATTTAAAAAAGTATAATGCACATCAACTTCTTAAGAGAAA

TTAAATGAAGACATTCCACGATATTTCTGAAACAGATGCTGTCTGGTTTCTGGAAATAGA

TTTCAACCTACCTGTGACATTACTAGCAT

>c630_g1_i1 len=300 path=[39:0-45 335:46-299]

GAAAATTTGCCCATATATCTCATTATGGTTAAAAATTAAAATAATAAGTATTTTCTGTAT

ATATTTTTTAATATTTAGGTTTTGTAATTTTTAAAAACATATAACATACATAAAATTTAA

TATGATAATATTTAAAAAGGAAAAATTAACGCTTTTTATAAGCATAGTAAAAATGAAAAA

AAATTTGCCAAATTAATGACATAAAAAAAAAAAAAATACTGAGAATAAAAGAGTTTATCA

TTAATATATTTGAATAAATTATATTACTGTAAAGATATTTCAAAATAAATATATATAATT

>c638_g1_i1 len=203 path=[129:0-82 117:83-95 321:96-202]

GATCGTCAAAGCAACAAAATTAATAAGCAATATGCCATCTCTGTTACCTTCATTAACTAG

CTGAATTGAATACTGGCTCATAGCAGAACAAAAACAAAATGCTCAGAGTAATCAGACAGC

CCAGCTCTCTGAGTGCTTTAAACAGTAGGCTATTTTTAATCATCTGTCTCCTAATTGAAA

TGCTGAAGGGAGAGGACTGACAG

>c645_g1_i1 len=240 path=[41:0-60 278:61-239]

TTGAATTTATTCACCATACACTCCTACAGCGAATTGGAGAAAAAAGTTATGCATCTAACA

TATCTATGTTCCAGTAGGAACTTACTGTTGAAATATTACTCAGAATAATATTAAATTATA

TATAATAACAGTAACAATAAATTAAGCATATTTTGAAGACTAAATAATGCATTCTAACTA

TACTCTAATGTAGAAGTATTTTGCAAGTAAAAACAAAAATCTGTTGTCTTACATGAAGAA

>c649_g1_i1 len=229 path=[240:0-121 34:122-228]

GACGAAACACTATCAGTATGGCAATTCTAATACCACCTTGAAAGTCACCAAATTACCATA

CTGATAGTGTTTCGTCTGAGCAATATTTCTGATGGCTTTGGAAGCATGCTGATGTTTAAT

TAAATTTGAAAGCTGTCTTTCCTGTATATGGCCTACCTGTTTTCTACACAGGTACCTCTT

GACAGTGAATAGGGTCAGATATGCTTACACAGATGGAAAAGAAATATAT

>c652_g1_i1 len=279 path=[358:0-174 549:175-175 550:176-278]

AAAAAAAATAAATAAAACAGAAAATACAAAAAATACATTTCATTAAAAAAATAAAAACAA

GTATTAAAACAAAATTACATAAATGTATATAAAACATAAAATTACATAAATTCGAAAATT

CTGTTAGATGAATATACTACGTAAATACTCGTAAACATTATGTTAAATGTTCTGCAAATA

TTTAGTAAAATATTTTTTACTAAAAGAAATACATATAAATTATATATATGAAATTATTTG

TAACATTATGAATTTTATATATATAATTTTATAAAATTT

>c657_g1_i1 len=368 path=[454:0-66 520:67-73 33:74-367]

ATGTATATAAAAGTAATTTAGATTATTATAATAAAAAAAATAAAATTCACATATTTTTGG

ATATAATAGATTTATTTTTTAATATTTATCATAATTTGCTTTTTATTAAAATGTGTTTCA

AAAAAAAAAGAATTGTTTTTATAAACAAAAAAAAGCATTATAAAAAATACCTATTAATTT

TAATTTTTGTACTATTTTTTAGTGTAACGAATAAAAAAGGAAATAAGAAAAGTATGCTAA

TATTTTATAAAAATAATATACTTAATAATAATAATTTTATAATAAAAAGGAAAATTGATG

AAATAATTTATAGTATAAATATGAAAAATTATCAAAAGTTTTTAAACAATGTTTCTTGCA

TAAAAATT

>c659_g1_i1 len=260 path=[58:0-55 114:56-259]

GTCTTATTGGAACACTCAAGCATTAGAGGTTTCAGCTGTTCACTAGAGAGAGTTAAGAGA

GTGGGACATCAAAGACAACACTGGTACACCAAAGACAACATTACAGAGTAATATTCAACT

CACAGAACTTAGACTAGTGAAATTAAACAAGGAGCATAGTGGAAATCTCTTATACTGCAA

CAAAAGTCAAGAAAGAACATTTAATCCTGTTCTTAAGTCTCTGGAAGAGAAGTTGTTAAT

CAAAGAGGAGGGTGAAAATG

>c659_g1_i2 len=218 path=[309:0-13 114:14-217]

GTAACTTTGTGTTTGAGAGTGGGACATCAAAGACAACACTGGTACACCAAAGACAACATT

ACAGAGTAATATTCAACTCACAGAACTTAGACTAGTGAAATTAAACAAGGAGCATAGTGG

AAATCTCTTATACTGCAACAAAAGTCAAGAAAGAACATTTAATCCTGTTCTTAAGTCTCT

GGAAGAGAAGTTGTTAATCAAAGAGGAGGGTGAAAATG

>c669_g1_i1 len=237 path=[264:0-169 25:170-236]

CAAGTCTACGTACAGCTGGTATCGCAACAGTGAGCCTTTATCACCGGACACAAAATCAAA

TGCCAAATTCCAGAATTCATCCTACAGCTTGAATCCCACCACAGGCACTCTGGTTTTTCA

TGCTGTGCACAAAGGTGACACGGGCCGTTACTCCTGCATTGCAACAAACGATGCTGGCTT

TGCCAAGTGTGAGGAGCAGGAGATGGAAGTCTATGACCTCAACATTGGTGGGATAAT

>c675_g1_i1 len=216 path=[8:0-86 95:87-215]

TTTTTTCTTTTTCATCTTCTTCTTTTCCTTCCTCTTTTACTATTCTTCTTCCACTTCTTC

TTCCCTATTTTCATTTTCCATTTTCTTCCCCAATTTCTTTTCCATTTTTCCTTAGCCTTC

TTTTCTTTTTTCCTGTATTTGCTTTTTTTCCTATTTTTCCCATTTTTTCCTTTTCTTTTT

CTCTCCCCCCTTTTCCTATTTCTATAGTTTAGGTAA

>c677_g1_i1 len=218 path=[500:0-217]

AAAAAAAAAATATCTTAAAAATGAAAAAATATTTTCATTATAGATATATTTAAAAATGGG

AAAGAACTATTACCAAAAAAAAGAAAAATAATATTATATAATGAAGGTTTTTTTTTTTCA

TATATAACAATGCAATAATAAAATATTTAACTAAAAATATAACTAAAAATATTTAAATAT

ATAACCCTGTGAAGTATTAGAAAAAAAAAAAAAAAAAA

>c681_g1_i1 len=204 path=[213:0-203]

AACTTTGTGTTTGAATTGTTAAAAAAAAAAAAATTTAAAAAGAATAAAAAAAAGTTATGT

TCTTATATGAAACATAAAATATGCAGTGTATTTCATATATGATGTAATAGTATATTTTTT

TTAATAATAAAAGTTAAAACAAATCATAAGCAAAATAAGAGAATACTTAATAAAAGATGA

AATATTTTTATTAATTAAAAAACA

>c690_g1_i1 len=259 path=[1:0-258]

GATTATGACATTCCTTTTGTTATTCAACATGTAATTAAAAATTATTTTAATGTGTTATAT

ATATATTTTTAGTTACCATTTAACTCTTGCATGTCCAAATACATACTAATGAAAATAGAA

ATACATTATTAAAACAATGCTTCTCAGTAGAGCATGTATTAGAAAAAAAAAAAGAGAAAT

GGACAAATAAAATTAAAGCAAAGAATTAAATGTAAATTAAAATATTAAATTGATATTAGT

ATTTTGTCTTTTTTCATTA

>c693_g1_i1 len=212 path=[217:0-211]

ATTAATTTTATTTAATATTTTTAAAGAAAAATCTATGCTTTCAATGTTTTTATTTTTGTG

TTTAAAATAATTATATAAATTTAAATATGTTTTTTAATACTTACTTATTTTTATTTATTT

AAAAGCATTAATTTTGAATTTCTAAACTTTATTCGTAACATGGTAATTAAAATATATTTA

TTTAAAAATCTATGATTAATGGGGATCTGTGT

>c694_g1_i1 len=366 path=[394:0-365]

TTTATCTGCCTCCCAGCGGAAGATTAATAACAGCAAAATCCATGACAAGTGCAAAGATAA

AGGTGATCATTATCGTTATTATTGGTATTATGTTGTTTTCTCCGTGTGCTGTTAACAGAG

AAATTGTGCTTAATGGAAGAAGGCAGATAAAGGCTGTTTATTCCTATTTAGGGAGGTTAA

GGGTGAATATGTAGACATGTATCTACCTTGAGTAATCTGGGTGTGGGTTTTAGAATGGGG

AACCCCAAGCCTTGAAACCCAATCCAAGCCTCTTCACAAGACAGACATTGCTTCTCAATA

TGCTACTAGACGTTAATTAACGTCTTTTGTTTGCTGATCATTCGCTCTCAATGAGAAGCC

AGTAGA

>c695_g1_i1 len=202 path=[1:0-201]

AGATAAAATTTAAAATATTGAGGTTTGGAAAATGTGCATGCAGAAAACTTCACAGAAGGC

AGTAACCATGGATAAAACTAAACTCTTTTCTCATCGTCTGACAATTAAATAGCTGGCTTG

GAGTTTTAAAGAGTTTTCCAGAATAGTTCCTTTCTCTGTTTGCAAACTGCTCTGTAATTG

TACTAAATCAGCTCTGTGGTTA

>c703_g1_i1 len=206 path=[155:0-205]

CGTTTCTCCTTTGGGAGTGTAAATCCTGCAAGGAAATCTGTGTGTGGCTTCAGCCAGTTG

CAGCGGCCTATTGCTCAAGGCTGTTCAGAGTTGTGTGACACAGCGGGTGTCACCATGCTT

GTTCTTGTACTTGTTCGAGCATTGGTTGCCGTTGCTCTTTTGTGAGAGGAGGAAACGGCT

TGCAACGCAGCGGGTGGAAGCCATGG

>c707_g1_i1 len=258 path=[171:0-257]

AACTCTATCAAATTGGATGCCTTTTCCTGAAAACCCATCTGCTCCACATGAACTCACTCA

GACATATTTACATTCTTTGAAACTGAAGATTTCCAAGTCAACCTCGGTCACCTGCAGGAT

CTTTGAAATGCCTCCTCTTCTGCTCCTGCCAAAGAGCGCCTTCCTGCATTTCCCATTCCC

GGCTCCGCTGGCCGTGCAGTCATCAGTAAGTTCTGGCCAAGTTATCCTATGTAACTTTTA

AGATACTTATGTCTCAAT

>c710_g1_i1 len=242 path=[1:0-183 185:184-241]

CCAACAGCTGAAGAGATTTGTTGCTTTTTGGACATTAGTCGTCGTAAGGATTGTTGCTGG

GCTTGCCGGAGAAGGAAGATCTGTGTGGAAAAAGGACCCCAACTCCAGTCCTTGACTCAT

GCTAGATGCACGTATTTCTATTAGAGGTCTTCTCCTGTAGAATGGCACCTGTGGCTGCTT

TCACACCTACCTGACATCAGGCCTTTTTCTTTTTTTTCTTCTTCCAAGCTGTGCAGTTCT

AG

>c711_g1_i1 len=201 path=[333:0-200]

GTAGAAGGGAAATGCCTGCCTCATGGAACCGCGGATTCAGCTGTCAAAAAATGTCGTCTC

TGCTAGACTTTTGAGGGAGGCAGACCAGGTTGTGCATTTACCAGACATTTCATTATTGCC

CTCAGTTGAAACAGTAGTTGATGTGAAGTGCTGTGTTAAACACCATTATCATGTTGTTGA

TATTCTGTAGTCTTTGAATTT

>c719_g1_i1 len=220 path=[1:0-154 156:155-219]

TTCTTTTTTTTTTTTTTTATTATTTATGTGTTAATTTTAAAAAAAAAGCTTGTTTCATAA

TTAAACACAGAAATACAATAAAAAATGAAAATAAAAATATTACATAAAAAAAATATTTTT

TTGCATAAAAATAAATTTGTTATAAATCTATTAAATTTTAATCAATGGATTGTTTTCATA

TTTTTGTTGTTATTTGTTTATTTTTTTAATAAAATATTAT

>c723_g1_i1 len=310 path=[288:0-309]

CTCAGCGCTCCAGAAACCAACCTGCAGCCAAATGCGAGAAGTCAAAAGTCAGACACAAAT

CAGCAAATAGCTCCCCTGCTTCGTGTAATTGAATTGCTTGTTAGTAGGTAAAGCAGTGCT

TACCATATGCTTCTGTAATGTGTAGATGTGCTGCTGCTCTGGAAGCTTGACTCCAAATTG

CAGTGTTTAACCACAGCACACAGTTACCATGGAACGGTGCGTGCAGGTCTGATCTCTGCA

GCCCATTTCCAGCCAGAGACCCTCCTGGTGTCTCTGCTTCTGCTCCTCGTCCTCAAACAC

AAAGTTTAGG

>c735_g1_i1 len=262 path=[220:0-148 127:149-261]

TATATATATACATTTATATATATATACAGTTATATATATTTTTTTTTCGTATATATTGCT

TTATTAAATATGAATGCACATGCTATTTGAGTTATAACATCTATATTATTTTATTATAAG

TATTGAGAAAATTTATTATAATATAATGTGAAAAAGAAAATAAAGCAAAAAAAATAGTTT

ATAAATATTTATAAAAATGCAAAAGGTGTTTTAAAAGACTAAAAAATTATTTTATTAAAT

TTTAGCTATTTAATATTATATA

>c747_g1_i1 len=422 path=[447:0-421]

GCAAAGTTTTTTAAATATATAAAAAAATCAAGTAACTAAAAAGTTCATTATTTTTTAAAT

TTTATATTTAATAAATTAACATTTTTTATGCAAGAAAAAAAAGCATATTAAAAATTAATT

TCTAATAAATAAAAAGAAAAAAGGTGTTAAACAATTTAAAATGTATTTGATTAGGTTTTA

TTACATTTTTCTAGTTTAATAAATACAACTAGCTTTAATAAATATATGAACAGACATGTT

TTTATTAAACGAAATATAATTTTTTCCTTTTATTATATAACTATATTAAATTTTAAAAAA

AATATCATTTTTTTTTATTAAATAAAATATATGAAATATAGGAAATATATGAAATATAGC

TTTTAATAACTATGAATTCTATATTTAATTTATTTTCTATAAAAATATAAATAAAAGAAA

AA

>c751_g1_i1 len=267 path=[313:0-266]

AGGTGGCAATTCTCCCCACCTATTTATATAGGGCTGTCTGAAGACAGGGTGGGTACTAAA

GCACTTGTTAATCTCTCTTCATGTATGAGAAGCAGAACTTACCATACTGTTAGGCAGAGC

ATAGAAAACATGAGGCAAAATGTTTATTTTCCAAGAGCAATTAGGTGCTATTGAGGTTCT

TCCTGGTTTTGGGCCCTGCTTCTAAAGGAACGGGCTAGTTTGGCAAGTGCTGGCCTATCC

CGTGCATCATGATATTTGTAACACTAT

>c763_g1_i1 len=237 path=[343:0-236]

AGGTACTGCGTAACTCACGGTACTGTAGAGCACGTTCTAGCACAGAGGGGTACCAACATA

CACATACCTACTGCAGAGTATTTGTTCAGATAAATAGTCTTTGAAATGTAGTATCACAGT

AATTCAGCTAATCGGGGCTCCAGTGGTAACAAGCTTATAGTATGCTCCTTCCATTGCCAT

TAGTTCATCATGAGTGCCCCTTTCAATGATGATTCCTTGTGACATCACAGCAATGAT

>c767_g1_i1 len=220 path=[198:0-219]

GCCGCGGGTGGGATTCGGTCGCTGTGCAGCTTAACGTGTGCTGAGACAGCTGGGAGTCTG

ACCTGAGAAGCGATGGAGAAAATGAATAGAGCTTGAGAGCGTTACGGTTCACGGGAGGAC

AGAGAACATTGCGGTGTGCATCTAAAAGTGGGGATGGGAGAGGAACAGGCAAGGCGAGGC

TCTGTGGGGACATTGCCTCTTTGAGGAGTTCTGCAGCCTA

>c772_g1_i1 len=341 path=[666:0-18 162:19-340]

TAGGTAACATTGTGTTTGAGTGGGTGGGTAATAGAGCAAGGATCATCGTGGTGGGGAGAT

GCAAAGCATATAAAAACACAAAGCTATAGATAAAGTTTCCCCTGTAAAATCAGGTTCTAT

AACCTATCAAGAAAAAGTACTATCTACAGTAAATGTCATGATCAGGCTATGACATCTGCT

TAGAGATGTGTCAGCTTAGAGAGAAAAGAAAGGTGTCTTTTTTCCTCTCTCAGAACGAAC

AGAAGCCTATGCAATGAGAAATAACACCAGAGAGGATTTAACATCATACACAGCTTTCTC

TACAAACTTATGAACATTCCTAGTAGAAAACCCAACTCACA

>c772_g1_i2 len=483 path=[1:0-160 162:161-482]

GTAACTTTGTGTTTGACCGTGGAAGATATGAAGCAACTGGTTTATAAACCAGTTGAGGGC

AGCTAAGTTGGTCAGTCAATATTAACATCCCAGTATTTATCCACACTCATAAGGATCAAC

AAATAATACTTCTTATCTCTGGGGATATCGCATTAGAAATGGTGGGTGGGTAATAGAGCA

AGGATCATCGTGGTGGGGAGATGCAAAGCATATAAAAACACAAAGCTATAGATAAAGTTT

CCCCTGTAAAATCAGGTTCTATAACCTATCAAGAAAAAGTACTATCTACAGTAAATGTCA

TGATCAGGCTATGACATCTGCTTAGAGATGTGTCAGCTTAGAGAGAAAAGAAAGGTGTCT

TTTTTCCTCTCTCAGAACGAACAGAAGCCTATGCAATGAGAAATAACACCAGAGAGGATT

TAACATCATACACAGCTTTCTCTACAAACTTATGAACATTCCTAGTAGAAAACCCAACTC

ACA

>c799_g1_i1 len=203 path=[314:0-131 691:132-132 447:133-202]

AAGCTACAAGAAACAGTTTAAAGTGGAGCATAAAGCCTAGAAAAGTGTTCTACATCTATG

TCTTCCCTACAGAAGTCATTTCAGAGCACACAGAGTCTACCTGGGTCATGAGCTACTGGT

TTTCAGCCAGTGAGAATATTGTTATGTTCCATGAACGACATCTGCAAACAGTAATGAAGA

GGAGCAGACCTCTCGCCAGTAGG

>c803_g1_i1 len=244 path=[418:0-243]

AAATGTTTATATTCTTAAATTTCTGTTGTAGTTCTTGATCTTTCTTGTTTTGCTGCTGTC

TTAATTCATGGAAGTCCTGTTGAAGTGATGCCAACAAAAGGTTCTGCTTTTAAACAGAAG

CATTATGAAGTAATGTAGTTGCACTGATAATTTTTTTTTAGGCTTCCACACTACCATCCT

TATTAATGTTACCAAAATAGTTCTTTTTCCTTTCATCTTTTTCCTTGCCTCAAACACAAA

GTTA

>c815_g1_i1 len=207 path=[1:0-206]

AAAAAAAAAAAAAAATATAATTATCGTATTATATATATTAAAAACTAATTTTAGAATTAA

TACAAAAAAAAGAAAACAAAATATATAGATTAATTTTACGTAAATTTTCATTCTATTTTA

AATTAATATTATTAATTTAAATATTTAATTTGCAAAAATAAACAGTTGTGAGAAATTTTT

ATTGTTTATTTAAAATTAAAAATGTAG

>c820_g1_i1 len=281 path=[343:0-280]

TTAAAATTTTTTTTTTTTTAAACTATATGAGAAAATTCTTCAAAATAAATAATAATTTTA

ATTTCTTATTATCATAAATGAGAAAATGAATTGTATTTTGGCAGAATTTACAAGATGTAA

TTTAAAAAAAATTTATAAGAAATGTATTGACACTTTAATTGTTATTTTATTTAAAAATAA

CAATTTTAATAGAAGATATGTATTTTTAAAAATAATATTGTGATAAATCAGAAGTTTTAT

TTTTTTTGTTTTTTCGTTTTTATTTTTTTACACGAATATTA

>c824_g1_i1 len=207 path=[343:0-206]

TAAATTTAATTTGTATGAATCTTTGATATAAAATAAATAAAAATCTATATGCATAAATAC

TATCTTCTTAATATTTTAACGCTCATTTTACTTAGAACAATAGTCATATTTTTAATAAAA

GTGAGTTTTTATTATTTATATTTTTTTAGTATAATACATAAATGCAGTTTTTCCAACTAA

CAATATGTGAAATATAATATTATCTTG

>c825_g1_i1 len=234 path=[366:0-233]

GATCGTACAAGCACTCAGTGTCAATAAACACCACCCATGCAGAGCAGATCATTAGCACAT

GAACAAGTGCACAGCATGTGTTGCCAACTGTACAGTAGTCCATAATATCCATTATGGATG

ACAATGAAATCAAAATGGTCCAATTATTTATGTCAGATTCATTTAATTCTCACTTCTTGA

CTAAATACTGTTCTCAGCAAATTTATCTGTGACCCACTCTTATTTTGTGCTACA

>c827_g1_i1 len=246 path=[1:0-22 24:23-245]

TAGAATGATACTTAATAGAAGAATTTTCCTAAGATTACTTTTTGTTGTATGTGTAGTGTA

TTACATTTATTATTAAAAAAAATAAGAGGTAGATTTAAGCAATTATAGAATTTTAAAAAC

TTATATTAGTATTTATTAAGTTTATAGAAATCTTTTACTTGCATTATATATAATTAATAT

GAAAATATACATTGTGTTTTTATTTTATTAAAATCATATATTAAATTTTTTTGAAACCAA

ATTTGT

>c827_g1_i2 len=287 path=[511:0-63 24:64-286]

TATTATATGCTTTTAAAAAAAAAAAAATGTTCCATATTATTTCCTTTTCTTATTGCATTT

ATAGTTTTCCTAAGATTACTTTTTGTTGTATGTGTAGTGTATTACATTTATTATTAAAAA

AAATAAGAGGTAGATTTAAGCAATTATAGAATTTTAAAAACTTATATTAGTATTTATTAA

GTTTATAGAAATCTTTTACTTGCATTATATATAATTAATATGAAAATATACATTGTGTTT

TTATTTTATTAAAATCATATATTAAATTTTTTTGAAACCAAATTTGT

>c840_g1_i1 len=236 path=[207:0-235]

GGTAAAAATTCTATAAGGATGACCTGCACAAAAAGAAAAAAAAACAGGTATTAAATGACA

GTAGCAACCCTATTACTTATCACAATGACAACTCACTTTTTTTGTTTCCTCTTGGTTCTT

TACTAGTTACGATTTTCTTCCTCCAAAACGTTCTATTTAACTTGATTTTTGTACCTGGTG

GAAGTATTATTACCTGATGGACTTCAGAATAAAACCAAACCAAAAAAAGTGAGTTG

>c845_g1_i1 len=465 path=[687:0-464]

AATTTATATTTTTTCTTTTTTTTTAGCATACTTATTTCAATTTACGTATTATATTTGTAT

ACTTTAAATGCATAGGAAAATTTAGTCTATTTTACATCCATTCTTATTGTAATAATAAAG

TCACTAATACTGTTTATTAAGCAGTAAGTTAAAAAAAAATACTTTTTTGTAAGAAATAAT

AAGTAAAATAAAAACAATTATCATCTACATTCTAAAAATATATTAAGAGGTAAATATATT

TTTAATTCTCTCATTTAAAATGAGTAGGACTCTTTATAGTACTTGTTTTAGTATAATAAT

TTTGCTTACATGTCTTATATACACAGGTTAAACATTATATACTTTATATTATTTGTTCCC

TTTGAAATTAAAAATAAATCTATAATATCATTTATCTACTAAATATGTATATTAATTTAG

CAGAATTAAGAACTTATAAAAAAAATATTTCATTTTAATATTAAT

>c853_g1_i1 len=291 path=[568:0-184 150:185-290]

TGGCCTCGAACGCCTCCAGGGATGAGGTCATCTTAACCTTGGTACTGATGAATAAAGGCA

CAGTTCAAATTCTTTTCTTCATCAGCATAAGCCAAAATCAACTTCATCAATGCAAATACC

AGATCAAAATCTGACACAAGCGTCTAATCTTCGCAACAGAAAAACACCTAGTTTTGAAGA

TACCGAAGCATTCTTCTCAGCAACTTCTCTTCCACACAATGACACCACAAGACTGGGAAG

ATGAAGCACACTCAGGTGCCAGTGGGCACGTCTCTACAACTCTGGAGCATG

>c853_g1_i2 len=255 path=[1:0-148 150:149-254]

TAAGAAGCTTTGAAACTTTACTGTCAGGACTTTGCAGCCCTTGAGATTGTAGAACAACAT

GGCTCTTTCTTTTAGTTTGAAATACTATAGTTAGTAACACACACACAAATGGTGCTTCAA

CCATTATTCACTTCTGCTGTCTCTTCCGAAAGCATTCTTCTCAGCAACTTCTCTTCCACA

CAATGACACCACAAGACTGGGAAGATGAAGCACACTCAGGTGCCAGTGGGCACGTCTCTA

CAACTCTGGAGCATG

>c862_g1_i1 len=239 path=[125:0-199 62:200-238]

TTGAGCAACCTGGTCTAGAGGGATATGTACCTGCCTATAGTAGAGGGGTTGTGTCGACAG

TTGATGACTGACTTTCATCTGACATAAGAAGAGGTCATGCTTAGTATAGGTATCTCAGAG

AATTAAAACCACACTCGCTGAACAGCCTGAAAGGCCACAGAAGTTTCTTTAGACCAGTGA

TTGATCTGCAAGGACCGCAGGACATCAGTGTTTTCTGCCATCTTAGCAGCCTTCTGGTA

>c865_g1_i1 len=231 path=[53:0-230]

GATAGTCTTATTACAATAAGTTCTTGTTGCCCAGGAACTACAACCAATTAATACAGCTCA

TTTTGTTTTGTTTTGATTTTACATTGTTGTTATTATTTAATACGTTCTACATATTCAAAA

TGTATAGAGGAGATATAATCCTCTGAATTTCTGATTTGCATCACAAGAGCTCTGAAATCT

GTTCTCAGAGACCTCCTTCAGGACATAAGACTGGTAACTTGCTGCTCCATG

>c881_g1_i1 len=210 path=[460:0-209]

GAGAAAGCGCAGTACCTCACACAGAGCACTGCTCTTGGTTATTTCAGACTTGACACAAAT

CCATTCTGTTGCTCTTGCACTGGTTCTTAATTTGTCAGACTTTTCTCTCCCAATGCAATG

TATTGAGAAGGTGTCAAGTATTATTGATTCCATTGATTCTATGATAAATAACATTGTAGA

AGGAAATAATAGCAAATTATTGGTACTAAT

>c899_g1_i1 len=249 path=[1:0-248]

TGCCATCTGCCTAGCCTGGCACAGGATTTGCCTTCTTTCTGAGAGCCATTTTTTGAGGCG

CATATTTAAGAAGTCATTGAGAAGACATATTTTAAAGTTGAAGGAAGAGAAACAACTCAT

CCTCAACTCAATAGTGGAAGATTGTCATTTTGTTACAAGTGCCGTTAATGCATTTTGATT

TTCTCCCTTTTTGATAAGAATGAAGTCTTTTTAATCTGAAGGGAGAGGTAAAAAGCAGGA

ATTTCATTG

>c912_g1_i1 len=220 path=[101:0-62 163:63-219]

GTGAGCACAGACTCTTTCCAATAGAGCTGTTAAATTCTAAGTTGCATTAAGATAATCTTA

CTCAAACCAGCACCAGAGGTTTCAACACCTCACTCCTACAGCTCTGACAAATGCAGTGAC

AGCTCATGCCTTCCAGGTAAATCTGGTAAATGAGATCAATTTCTTTTTTTAGCACCAGGG

CACATCACAACTCACCACAAAGGAATTCAGAGGTGTGAAG

>c913_g1_i1 len=311 path=[315:0-20 336:21-310]

AAAAAAATTATTACATCTCTTAATTCTAAAACCAAGTGTTCAACATAAACAAAGCATTTC

ATTGCATTCAAATAGAAATTTAAAATAAAAAAGTTATTTTTTATTGTTACAAAGCTTAAA

AAAAAATGTTTCAACTCCTGTAAGAAGTAAGAGATGTAATAATTTTTTTATCTTTCTTTT

ATATATAATGTGTATTTCACTTAAAATTTATTTAAATTAATCTCAATTTTTTAATTCTTT

TTCATTTACTAGATTTTTACTATTTTTCCTCTATTTTTTCATAATGTGAAACCTAAAGAT

TTCCTTATTTA

>c929_g1_i1 len=317 path=[31:0-56 30:57-316]

AAGAATTTGTATTATATTATTGATAAATTTTTTAAATTGATTGCATAATTCATTTATAAA

GAATACATATACGTATATTAAATATAAAGTTACAAACTGGCAATTTATTTGTATAATTTA

CTTATAATTATCCCAATTTTTTTAATTCTTATGTAGAAAAATTAAGAAACATTTTTTTTT

ATATACCATTTTGTTATTGTTGTATACATTTTATTTATTAAACAATAAGTAAAAAACGAA

ATTTAATATAGAAAAACACTAGAATATGTTTCAAATTGATAACTAAAATTTTTTAATGTT

TCTAAAAAAAAAAAAAA

>c938_g1_i1 len=288 path=[422:0-287]

AAAAAAAAAAAAAGTGAAATAGTAAAAAAATGGGATAGAAATTAATAAAAAAGAAAAAGT

TTGAATAAAAAAATTATTGCTTTGTATTAAAATTTTTTATATTTTTTTTTTTATATGTTA

TATATATATAGTATTAATTAAAATAAAAGAAAATTTACATAAATATAGTGTTTCATAGAA

ATAAGTGTTTAATGAACAATTGTAAAAATTTATTATACATAAAAAAGCTTAAAGTTTAAT

TTATATAGTTTTAAAAATTATATGCTTTTATTTTTTCTTTTAATTTTA

>c978_g1_i1 len=212 path=[390:0-211]

TGCTGTCCTCTACCATTTCCATTCGTCACAAAACATGACAAAGGTTTAGTGGCACTTTGT

CTCCATGGTCATTTCCAGCTTCTTCAGAGTTGCATCTCTTGTCCCGAAATGCACCAGACA

ATAAAGGTGGATTTTTTGATGGGAAGACACAGTTCCCGTTAGGTGCAAAGCCAGCGGTGT

CTGGCTCAGCTTTAAGCCATGGGAGCGAGCAG

>c985_g1_i1 len=246 path=[410:0-245]

ACGAGGTATTAATGAGTTGCAGTTAGATTCACCTTTATTCAGTTACTTCATTCCTTACGC

TGGCTGCCAAAAGAGTTCCTAGAAGACCTTTACAAAGGTCTACTTGCAGTCTTTATTTAA

TGATTTCAGAACAATTCTCAAACTTTAAACAATCTCCTGCCTGTAGGAGGATTGATTGTA

TTATTTGCTATTTAAAAAATGCTTACTCAAAAGCATAGGTTAAGAGACTATTCCATGTTA

ATAGAA

>c986_g1_i1 len=260 path=[238:0-259]

AAAAAAAATAAGAAAATATTTTAAAATAAAATATATATAGTTTTTTGTTATGTTTTTTTT

TTTTAATAATGCTTAATTTTAACTTGTTAAATTAATATGTAATAGTTTTAGAATCAAACT

AATCTTTTACATTTTTATATATTTATTTTTCTTTTTTTGTTTATTTAATTTATTCTACTT

CCACATATATCATATTAATTTACTTATTAAATAATTATCCATTTTTTTTCTTTAATAGAA

TTTTAATAAAAGTATTTTTG

>c995_g1_i1 len=241 path=[155:0-240]

AAGCTCAGGTTCTTTTTTTACTGCTCAGAAATAGATGTGTTCCTGCTGCTGCTTGTGCTC

TTTATTGAAGTTTTAAATGACAGTGTACCCCTTGCACTTCTAGTTCTTCCGTAATGTTTC

ATTTGAACTAGAAGCATACTTCCTAGTTTGAGCTTATCTTTGATGAAGTGAGCTTTATCC

CTTGTTCTTTGTTAACCCTTTTCAGTTATTCTTTTTTGTGGGAGAGTATAAGGATAAATA

T

>c998_g1_i1 len=220 path=[198:0-219]

CAGGAAGTCAACTTTAAAGCAGAGAACAAAAAAGCAGCATCAGAAGAAATAAAACAGCCT

CACTATCTAGCTGGCTAAGTTAATTGTCTCTGCATCCTCCCTGATAACATTTCTCAGCAT

TTTTGATTAGAGAAGTTGAATTTTTTTCCTAAGAGAATAAAGATCATCTTGCTCAACATT

TTCACAATTACTTTAATAGGATCAGTGTCTTTCACATTAG

>c1008_g1_i1 len=226 path=[231:0-117 448:118-225]

GAGTGAAGATTCTTAAATTGGCAGTTATGAAGTATGCGATGAAATAATTTTGATGCCTTT

CTGAGTGAAGGTTTCTGTGAAATTCAAAGTACTTTTTCTTTAATGGAAATGGTTTATCTG

AGGAGGGTTTCTGTTCTGGCAGTTCCTCTAGAAAAGAAATGATATAAACTGCAGTTTTAG

TACTCTGACACTTTAATGGTCAGGATTTCCTGATGTCATGCTGTAA

>c1013_g1_i1 len=217 path=[195:0-216]

AAAAAAAAAAAAAAATAAAAGAAACATATAATGGTAAACACGAATTATATTTTATAAATT

ACAAAGATATAAAGGAAGAAAAACATAAGCAACTAAATTTCATATATCACGATAATAAAA

TAAAAAAAAGTGACAGTAAAATTATATGTAAAAAAAGTTTTTTAAAAACATTAAAATTAA

TGGATAGAATATATAATAAAAATAATGAAGAAGAAAT

>c1017_g1_i1 len=379 path=[579:0-308 25:309-378]

TATATATATATTTATATATAGAAAGATATACAAATATCTTTCTTTATTTTTAAATATATT

TAATTACCTTTAGAATGAATATATTTGAAAAACAAAATATAATAAAATAAATAATAGATT

ATTAAAACTGTATATTATTATATTTTTATTTGTAACATTATTCTCATATAATGTCTCTAT

TATACGTTTCTTTCTTTACACATAGATTTTTATTTTTTTTTTCTGTATTTTTTCTATATC

TTTTATAAATATTGCATAAATATTACAAGCATATATGCACATGTATATGCCTATAAATTT

AACTCTTTTTTTTTTTAAGTTATATTACTATGATAGGAATTAAACATTCTTTTCTTTTTT

AATTATAGATGTTAATGTA

>c1017_g1_i2 len=373 path=[579:0-308 887:309-372]

TATATATATATTTATATATAGAAAGATATACAAATATCTTTCTTTATTTTTAAATATATT

TAATTACCTTTAGAATGAATATATTTGAAAAACAAAATATAATAAAATAAATAATAGATT

ATTAAAACTGTATATTATTATATTTTTATTTGTAACATTATTCTCATATAATGTCTCTAT

TATACGTTTCTTTCTTTACACATAGATTTTTATTTTTTTTTTCTGTATTTTTTCTATATC

TTTTATAAATATTGCATAAATATTACAAGCATATATGCACATGTATATGCCTATAAATTT

AACTCTTTTAATTTTTATCTTGTTATTCTGTTGATTTTATAAGTGCATTATAATGATGCA

TCTTTTTATTCTC

>c1020_g1_i1 len=356 path=[384:0-355]

ATGGGTAACGCATTTTTTTTTTTTAAATTTAGAATTTACAAAATATTATCTAATTTTTAC

ACCTTTCTTAAGGCTTACCTAGATTCATTATTAATAATAGATAGGTGTAAATTATATTTA

TTTTAAAATTTTCTCAATATTTTTTCATTTTTCTCAGTTTTTCTAATCATCCATTTAAAA

ATAATAAATAAGAAATATTTATATCAATAAATTTTTTTATTTTTTCTATTTCTTTCATAT

TATAGGTAATTATATATGTTTCATTTTGATATGTATGATTATAAGTATTTTTTTAATTAT

AACAACACATGTTATTTTTTCAAGTGTTATATAAAAATCTTTTGAGAAAAAAAAAA

>c1028_g1_i1 len=329 path=[87:0-224 86:225-328]

TTTTTTTTTTTTTTAATATTCAAATAAAACTATATATTTTTGAATAATTTCTTTTAAAAT

AATTTGATATTCATTTGAATTTTTTTAATTTCATGATGATTAAATGCAAATAAAGATTCT

ATAATATTTAAAAAAAATTGTAAAATTAAAATTTTTTTTTGTCGCCTTTTTTATATTTTA

ATGAACAATTGAATCATCTAACATATAAACAAGAAAAAATATAAATATATTTTCCATTCT

CAATAAAAAAAAAAATGATAATCTAAAAGTGTTAAAAGAATTATTTTATAATGTTAAAAA

TATTTCTGTAATATTTAAAATTAAAAAAA

>c1030_g1_i1 len=276 path=[1:0-119 381:120-275]

TGTTCCTTTTCAGTTATGGCTGCCAGAAAGCCTGCACAAATGCGTCACTGATAAAATACC

AGGTGAAATAAAACCAATACAGTTTGATGTGGTATTATCTGATTAAAAGCAACTCACTCA

CAGCAATCTTGAAATAGTTGCTGCCGCCAAGTTTTGCATCCCCAGGCAAAGGCTGAACCC

CCTCCTTTCCCATTTAGGATGCATGCAGAACTGTGTCTCAGTAGAATTCACTAGTGTATA

TTTAAAATTTCTAGATCCTGTGTAGGGTTTTGATGG

>c1031_g1_i1 len=236 path=[1:0-125 127:126-235]

CGTGGGCTGCGACTGATAGGATCATTTAGGAGGAAGGCACGTTTTGCTAGAGCAGCCCTG

AACACCTCAGTCATCACTCTGAAAAATCTCACATTTGAGGATGTGTCCTGTTACAGATGC

ATCTTCAGTGTGTACCCTCATGGCTCTTTCAGCAGCAAAGCTATGTGCCTCAACATCCAA

AAAAGTGGAAACACGAACAAATCAGAAGTCAAAACGTTGGATATGGGTTCCCCCAG

>c1034_g1_i1 len=247 path=[259:0-164 500:165-246]

ATCATTTTGTAATTTTCCACTTCTGTGTGTTTACGTCTGCAGCTTGATTATAGTCAAATG

TAGCATATTCATAGACATTTAAGGCAACAGATGTTTTAATAAGAAAAAGAATAGTCAGTT

GTAAATGTTCCAAAATGATGCTTTAAAAGCACAAAAAATTCTGTCACTGGACACGCTTTC

CAGTGTCTGCCATGGCACCGTTCCTAAAAGATGGTTAGGAAAATGAGAGGCACATTCAGA

GCCTGCA

>c1035_g1_i1 len=234 path=[53:0-233]

TCCTCCACGAAACAAAGCAGTGAGAGGCTAAAATACTCAGAATACCTTTTTGTCCAATCT

CTCCCGCAACACATATTTTGTTTTTCTTTTCAAATCCAATTAAAGAAAATGGAAAACTCA

TTATAACACATTTGCCAGTAAGGCAGCATTAGAAATCCCACCGTCACAAGCTAAAAAGCA

GCGCTGTAATGATGAGACCACAAATTCTACGAACAAGGAGCATCTCCTTCCAAG

>c1042_g1_i1 len=243 path=[300:0-175 80:176-242]

GAGAGCAAGCAGAAACAATAGCTTTCCTAAATTAGCAGAAGCATGTCTGTAATTTTAAAT

TTTGAAAATGCAGATGGCTACACATGAAAAGCAAACTTCCAGAAATTAAGCTTACCATAT

GGTCAGAAAAGACAGGAGATCACCTAGTAAATTTCTGGTATATCACTGGGAAAAAACATT

TCCACATTAGTTACCTTTTTGCAGGGCACACGTTACTTGGTTCCAAGTTATGCCCACAAC

ATC

>c1045_g1_i1 len=356 path=[1:0-131 450:132-165 241:166-355]

TGACCGTGGTGGAAATAGCCGTTTGCGATTCTAGTTTGTGAGTGTGAGAGCAGCTAGGTT

TCTTTCTGCTTTCCCACAGGTTATGTGTAAAAAGACTTAGATACAGTGCAGCAAAGCCCA

TTTCCCTGTTGTAAGGGACTTGATGAGGAGCAGCCCTGGGGATGCCCTTCTGCCTCCACC

TTGAATCTCATACAACTCCAGGCCTGCTCCTCATCAAGTCCCTTACAACAGGGAAATGGG

CTTTGCTGCACTGTATCTAAGTCTTTTTACACATAACCTGTGGGAAAGCAGAAAGAAACC

TAGCTGCTCTCACACTCACAAACTAGAATCGCAAACGGCTATTTCCACCACGGTCA

>c1046_g1_i1 len=273 path=[491:0-272]

GAAAATTAATATATAATTAATTAAAATAACACTTTTTTAAATACACATTTACATATTATT

TCTGTAATGTAATTCTTTATATTTTTATCTTTTTTTTAATATATGTAATCATTTTGAAAA

ATATATGGTGAAAAATATGGGGAATTCAAAGATTTTTAACTTTTCATAGTTCATTTTTAA

ATTAAATAGCTTATTTTTTTAAAACTTCAAAATTCTTAATTTTTTTTGCAAATAGAGTAA

CTTTTATATGTGATAAATTAAAAATTAAATAAT

>c1074_g1_i1 len=303 path=[1:0-276 688:277-289 688:290-302]

GAAAAACAGGGCAGGCACTTTGAAGTTGGCTACTCCGTCAGCAGCTCGTGGTTCACGTTT

GCATGTGTTGTCACAATCGAGAGGCTACAGATTAACATGTTCCTTTGATGCACACAAATA

ACTGCTCCAAATATTGCAGTTTGTTTTGCAAAACATGCATGAAGACCCCACACTTTTTAA

ATCTTCTGTAGGAGAGAAGGAACACAAAAGCAGCCAGAAATGGAAGCATTCACAAACATC

AGTATAAGGCCCTTTAACATTTATTCAAAGTAACGTGTTAAACCCCTTCATTAAACCCCT

TCA

>c1083_g1_i1 len=438 path=[654:0-437]

ACAGAGGTGTTGAAAAATATAATTGCAATGTCCTCTGATACTGCCTTATACTGCAAGCCA

CTATTTCCCTCAAAAAGCAATGACAGCTGATGGCGATACCTAGCTCATCCCATACAGAAG

CTATTTTCCAAGCCCATCTTCTTGTCCAGATACAATTTTTCTTTAAATAATTATTTTATT

TGAACAGTCTTTTGACAAGCGTTGACAACCACAAAGCATAATAGTAAACACTTCAACCTA

CCTTCCTCTTCCTCTTTTCCTATGCTCAGGCTGTTTTCAAGGCATAAGTTACTTATAACC

TTCAACTATTTATTAAATAATACAGTAAATAATACAATACAATAATACAATAATACATTA

AAACATGTCATCTTTTACTTGATCCTTATTGCCAGAAAAAAAAAAACAAGATTTGTTTAG

AAATAATGAAAACTAACA

>c1097_g1_i1 len=205 path=[515:0-204]

AAAAAAAGAAAAGAAAAAATAGTATTCTTTAATATTCTATTTAAACAAATAAACTCATAA

AATTATATATAAATATATATAACTTAAAAAATTATATATTTACTACAAAAGCCTAAATAC

ATATACAAATGTTGTTAAAACATAAAGAATAAAAACAATATATTTTTTTCATTGATTTCA

TGTTAAATCTATAGAAAAAAAAAAA

>c1110_g1_i1 len=232 path=[210:0-231]

CAGCGCTGGAGTTATTTCACTGCCTGCAGTACAGGGGCTGGTAAAACTGCCATTAGGATA

CAGAGCAACTAAAGGTCTGATAACTGCTGTCCTGGGCAGCCTGGATCTCATTTCCCCATT

GCAGCCTCACTTTGTGCCTGTCCTCCTCGCCCATAGGAGGCACGGAGTTGTCACAGCACT

CAGCACATGAGCTGCAGGCAGCGTTGTCCATCACACTGAAGGTAGAAGCAAG

>c1122_g1_i1 len=232 path=[364:0-231]

GACTTTGGTGAGCCAGGAGGGCTCTGACTTGACATGGTTTTGCTCAGGTTTGAGATGGGG

AGTCCTGGAGGTCTACAAGCACTGGCCCCTCCATGCTGCAGCTGCACGGCTGTGCCTGAG

GTAGCTTTGCCAGCCAGAATGTCTGTGTCTTCAAAGAATTCAAAACTGCTGAGATCACCC

CATGATGAAGGATCCTGAAACAACGTGGGAGGCACGGAACCATAAGACAGTA

>c1123_g1_i1 len=217 path=[1:0-170 172:171-216]

GTTTAAAAGAATTAGCAGATGAGGTATTTCTTTTTTTTAAGTATTACATTGCTTCTCTTC

ATACATATATATTAATTTTTTTTTTAGATAATAATAGTTTATATATAAAGTTTTTTTATT

GTTATGATTATTTTTTTTGTTTTATTTCATTTTATTTTTGATATATATTTTTTTTATGTA

TATATTTAAAATGTAGGAGTATTCTTATAAAGATTTG

>c1127_g1_i1 len=347 path=[1:0-346]

AAAAAAAAAAAAAAAGTATTAAAAAGAAAAAAGAAATATTAAATTTTTGCTAATAAGAAA

AAGAAATTCTATTAGATTACATTGTAACTTATATAAATTTAGAATACTTATTATTGTTTT

TCATATATTAGACTTTAGACTAAAGAAAAAAATATATAAATAACAAAATTAGAAAAATTT

TAGAAGTTCTTTTAAACATAAAAATATAAATATATGTGCATATGTTAGATTTGCTATTTT

ACCATTATTTTACATTTTAACTTATATTTTAAACGAAGTTTATATATTTACTTTTGTATT

TTTTTTTTTTTTTTCTGAAAATTCAGGTATAAGTGTAAGTGTTAGAG

>c1140_g1_i1 len=469 path=[370:0-367 738:368-468]

CTTTGTGTTTGAGGTGTCACTGTGACATGAATGGAATTAAGACTTAATCACTCTGAAGCT

TAACACAGGCTTAAGGTGCTTCAGAATTAGGATATAGAAGATGCAGCCAATGTTACTGAC

AGCACCACAACTCTGTTCAGCAGCATCCCTCAAATTTGGTGTCTCAGCTTATGCTTTACT

AAGAACCTGAAAGGAGCAACACATTTTAAATAATTCTGACCAAAGTTTTTTACATAGGTG

TTCAAAATTCATTAACAAACTTCGTGAAAAATCAACCATAAACCAGCATTGATTTGCTTT

TTCCCCAGCTTCTCAACTTAAGGCATAACTTATTCTTGACATCAGCTCCTGCAATTATTC

AACGTGATCAATTAGGCCAGTCTTATGTTCATGTTTGCCATTTAAAGTGATTTGAAAGTG

GCATAATAGGTCTATCACTGGTAATACTGAAAGACCAAGGTATCCTTTT

>c1140_g1_i2 len=298 path=[739:0-196 738:197-297]

AGAACAACTCAAGCTGGAATCAGTGCTTCTCTCGAACCTATGGATGAGCTGCATAAGGCT

TCCTCTGTACAAATTTTTGCATCTGAACCTTATTTTGGTGCTTTTCTTCCCTTGAAAGCA

CCTGACGTACTAGGTCTTGTTTTATTCAGGGGCTATGAAGAAGCATAGGATATCTAATGC

CATTGTTGCTCATAAAACAATTAGGCCAGTCTTATGTTCATGTTTGCCATTTAAAGTGAT

TTGAAAGTGGCATAATAGGTCTATCACTGGTAATACTGAAAGACCAAGGTATCCTTTT

>c1142_g1_i1 len=215 path=[1:0-214]

GGCAAATCTTAGTGGGAGATGCTGGAGGTTTAGTGCTTCAGAAATTCAGTTTCACACACA

TGGAAACAACATTCCTTACAACCTGACAACTTTAAGTGACTTATCTGGCCTTCATCGCAC

TACTTAGAGCTCTGCAGTGTTTGATAGAAGTGCAATCTGAAGCTACTGAAATTAAGAAAT

ACAAAATCCAGTAAGAAATTTTTCCTTTGTTTCCT

>c1144_g1_i1 len=211 path=[402:0-126 603:127-135 165:136-210]

ACTTCACTACAGTAACCTGAAAGAACAGCAGCCAAAGTTGTGGGTTCTTCCAAAAGGATC

CAATCTCACCTCCATAGTTCTTTATGTTGCTTTCTAGATTGTCTGGAGCTTTTTTTAGCT

ATGGTTATAAATGAGAGAATCTTGGACACTACTAGTGATGTTTGGACTCGCTCAGACTCC

TGCTTCATTTGCTTTTAAATGTGTGGATTTT

>c1144_g1_i2 len=211 path=[402:0-126 603:127-135 537:136-210]

ACTTCACTACAGTAACCTGAAAGAACAGCAGCCAAAGTTGTGGGTTCTTCCAAAAGGATC

CAATCTCACCTCCATAGTTCTTTATGTTGCTTTCTAGATTGTCTGGAGCTTTTTTTAGCT

ATGGTTATAAATGAGAAGAATGTAGGAGAAGTATAGAAGTGTTCACAATCTTCAGATGTA

ACTTTAAAAATCCACACATTTAAAAGCAAAT

>c1144_g1_i3 len=208 path=[33:0-123 603:124-132 165:133-207]

TTCCAGGGCTGCTTCGGAGAGTGCCAGAAAGCCTTATGAGTTTCTCTGAAGGTGGTACAA

GAAAAAGGCATTTTAACTTTCTGATGTTGCCAGCCAACCTCACACAGCCAGGTCATCTTG

CCAGTAAATGAGAGAATCTTGGACACTACTAGTGATGTTTGGACTCGCTCAGACTCCTGC

TTCATTTGCTTTTAAATGTGTGGATTTT

>c1156_g1_i1 len=539 path=[517:0-415 933:416-538]

AAGAAATCTGAGATATCTTCCTTGCAGAGGAAGCATGTGCTACTAGTTCCAATGTTTTTA

CTTAACAAAAAGCAAATCAACCACAACTATGCTATTTTCCCTTTTGCTACCTTCCTTTGA

CTATTCTCATCTTTTTACTCCTTTTCTGTTCCAAAAAAACAGATGTGTACTGAAGAAGCA

GATGTAGAACAGGAAGAGGAGGTAGAATAAAAGCACAGAAAAGCAAGGAAAAAGCCTGTT

GCTTTTTTATCCCCCACTTCCAAGAAGTGCTGAGTGCCTATCAGGACACACAGGAATCGT

AGGTCTGAAGGAAGAACCATGAGGGTAATCTTCTACAGGACTCTAAGCCCTGGAATTTTC

AGAAACAACACAGCTCAACAGTAGAAGACTAAGTACAATCAACTAAAATCCATCTAAGTT

CCTCATTATGCTCTTCAAAATGCAGGTCAATGCTTTCAAAATCGCTCTTAGCTGCTGAGG

GGACAGGTGACTGGCTGCATCTGTAGCCTCCACATGCAGTAACTATATCAATCCATGGG

>c1164_g1_i1 len=359 path=[42:0-39 82:40-80 253:81-178 82:179-219 253:220-317 82:318-358]

GGATCCTATAAGGGGCTATAGGGATAGTATGGGGTCCTATAGGGGAATAAGGACGTTATG

GGTACTCTATGGGGTGATAGGAAGCCTTTGAGTCCTACCGGGGTAGGGGGACCCTATAAG

GGCATATAGGGGCGTTACGGGACCCTATAAGGGGTTATAGGGATAGTATGGGGTCCTACA

GGGGAATAAGGACGTTATGGGTACTCTATGGGGTGATAGGAAGCCTTTGAGTCCTACCGG

GGTAGGGGGACCCTATAAGGGCATATAGGGGCGTTACGGGACCCTATAAGGGGTTATAGG

GATAGTATGGGGTCCTACAGGGGAATAAGGACGTTATGGGTACTCTATGGGGTGATAGG

>c1164_g1_i2 len=293 path=[251:0-14 253:15-112 82:113-153 253:154-251 82:252-292]

CCGAGTCCTCCCGGGAAGCCTTTGAGTCCTACCGGGGTAGGGGGACCCTATAAGGGCATA

TAGGGGCGTTACGGGACCCTATAAGGGGTTATAGGGATAGTATGGGGTCCTACAGGGGAA

TAAGGACGTTATGGGTACTCTATGGGGTGATAGGAAGCCTTTGAGTCCTACCGGGGTAGG

GGGACCCTATAAGGGCATATAGGGGCGTTACGGGACCCTATAAGGGGTTATAGGGATAGT

ATGGGGTCCTACAGGGGAATAAGGACGTTATGGGTACTCTATGGGGTGATAGG

>c1168_g1_i1 len=430 path=[408:0-429]

GATTTTGCAGAGCTCCATTTCTAACATTCATGGGACACTGGGCAACCTGGCCTTGTACCA

GATCCCAAATTCCTGATCCCCCAAATTACCAGAACCAGACCTGACCACAGTGGTACACTC

CAGTAATACTGCTGCTTGTGTTGTGGCCATGGAATCATAGCAAGCTTTATTCACTGCAGA

GTGAGAAACAATTTATCCTTGGGCTGTGCTCACTGTTGATTCAGAATAAGCAGAATTAGC

TGAAGGTGGAGGAAGGAGGAGCAGGCAGGGTGCTGGCGTTAAGGCTTATCCCATGCTGGG

TTCTGCAGAACAGCAGTTTCCAGGAAATTCCTCTGTGAAGGCCAGGCCCGAGTCATTGCC

TGTGGAAGTCAGAGAGGTGTTCACCACTGTTAATTGTCGGGTGTCTTCTGATGGTTCTGC

ATGTCAGGAT

>c1174_g1_i1 len=260 path=[238:0-259]

TATATAGGAGAAAATAACTTATTTGAAGATAATGTTACTATAATAAATAATACAAATAAA

AATATGTACATTGGTAATTATAATATTTTTAGGTCAGGAACATGTATAACAGATTCTCTT

AAAATAGGAAATAAAAACTATTTTGATTATAAATGTAAAGTGTACATATAATATATATAC

AAGTATTCTTTTTTCTTTATTATTCTTTATTTTTTTCCTTTTGGAAATATAATAAGAAAT

AATTTAAATAAAAAAAAATA

>c1177_g1_i1 len=206 path=[1:0-205]

AGGTTTGAAGGATTACCTGCTTTGATCACATGGGCTCACATGGAGGCTTGTCTCCAGCCT

CCTGTCTGCTGACAGGCATCTCATTTGGAGGCAGCTGCTCACATCCTGCTCGTGGCCTTT

GCAGTGATGTTAGCAGAAGGATCTCCCAAGATTTACCTCATACTAAACCTAAACCTAGTC

ACAGTGTGTTACGACGTAAATCTTGG

>c1184_g1_i1 len=288 path=[266:0-287]

ACTATATCCCACAGGACAGCCGGTGTGCATTCCCAGGCAATCCTAACAAAGGCCATGAGC

TGGGTTATGAAGAAATGTGGTTGGGCAGCTTTCTGCACCAGAACACAGCTACCCTGAGAA

GCACAGCATTGCATGTCACAGAACCGTTAATAAAGTTTATTTTAGAAACTGTGGACGTGA

GCAAATGCAGAAGATTTGCTGGAAAGATATGAGATTTTTAATGCTACTTCTGCACTCCAT

GCTCAGCAAAGGGGTAATAACTTCAATTGCCACTGAACCATGAGGACA

>c1185_g1_i1 len=212 path=[388:0-211]

AAGAGTGGTGAAATCCTTGCTATTAAGCACTAATAACCGCCTCGTGTAAAGTACCTGTGA

AAAATGTCTGCATAACTGACTGCTTTAATGATACACACCAGGCACAGACTTCACTGACAT

ATGGTTGCACGTTCTACACGTTGCAGATTTAGGATTTCAATCTGCATGTAGGTACAGCGG

CCACGTTAACTCACTGAACCATCCAGGCAAAG

>c1190_g1_i1 len=223 path=[1:0-222]

CATCACGGCCACAGCCTCTCATTTTGTTTGTGTACCACTGGAAAAGAGCTTTGAGTGTTC

ATTCACCAGCCTTCCTTGCTGAAGAACTTGCAATGATATCTACCTGTATTTCAGCTTCTG

TGTCAATGGTGATACACTTAGCACTGGAATGGCAGGAGTTTTCCATTTGTTGCTGAGCAG

AAACCTGTGCACATCTTGTGTGCAGTTCTGGTGTAAGTTAGAG

>c1190_g2_i1 len=346 path=[724:0-345]

AATGAGAGGCTGTGGCCGTGATGCAGAACAGTCTGAGCGTGGAGGATGGACAAACAGGCC

ATGGCTGTGTGATTTGATACCGCAGAGCTGATCCCCTTCTCCACAAGAAATTGCAGTCTT

ATTTTCCATTGTAATATTACAGATTCTGAGACAGTTGGTACAGATGCAGTGAGACTGTTC

CTCGCCTTCTCAGGAAGCCCTGCAGGAAAGCTTGGACAAATAGCAGCAGTTCAGACTGAT

CTCCTTCCAATCCCACGTGCACTATCAAAAATGCACCTTTAGTAAAACAGTGCAGATCTG

AGGCTCCTGACCTAGGTGAGGATTCTGCAAGACACTCAAACACAAA

>c1191_g1_i1 len=358 path=[336:0-256 709:257-257 710:258-357]

CTGGACTGCTGTTTAAGCTTCCTCAGGAGTGCTGTAACTGAACAGAGGTTAAAGATAAGG

TAAAACTATGCTACTTGGCTAACTGCAGCTTAGTGCTGCCATGCAACAGATTTTAAAAAA

TAAAAAATAATTGACGTTAATAAGGCATTTATTCAGACTTCGGAGTAATGCACTTCAAGT

TACAGTAATTATAAATAACTCAATATGATAGAAACTAGTAGCTTTTGTTCCTGTAGTAAG

GATATTTAGCAAACTGCAAAAAGAAAAAAAGCTTTTTGTCCATTTCAAGGGGTCGCAGAA

TGATGTGCATTCCTAAGATGCTCCCTCCTCAAATTCTAGAAATTCAGTCAGATAACGG

>c1192_g1_i1 len=217 path=[195:0-216]

TGATGCGACAGCAGTGCATGATCCCCTGGAACATGGCTTGCCTTTCACATTTCTGTTCCC

ACTGCTGAAACACACCGCCCACTACCTCACTATGCTCACATCCATTGTGTGGTCTCCATC

AGTGTTTAGCAAGTGTTGATGAATGCCAGTGGGTGCCATTTTCTCTCCGTGGAGGAATTC

ACTTCCACGCTTTTACTTCACGTGCACTTCCATGTCA

>c1200_g1_i1 len=228 path=[206:0-77 284:78-103 310:104-227]

ACTTTGTGTTTGAGTGGGAAGGAGTGGCAGGCTAGAACTGCTTTGAGGCATACTTTCTCA

CTTGATATTTGATTTGAATGGGTGCCTAGAATTTGGGTTTTGAAACACAATAACACTCTG

CGGGGCTTCCTTGGAAACTGTAGCAGTACTTTAGAAAATGATTTTGCCTTCTCTAGATAC

AAAGGAAGACTTGCTGCCTAGGTGATTCAGCCGCAAATAGCTGTAGTA

>c1215_g1_i1 len=300 path=[429:0-248 76:249-299]

AGTTTACAGAGCTGGAGTGACATACTCCGATCTGTAGCGGTGATGGTGACATCCTGCTGC

TAAAAGTAGAAACCTGGCCATGACTGTCTTCCCTGGCCATGCCTGTCCTCTGGAATTATC

AGTGAAGCTGTTTAATGAGTTCCAGGCAGCTATACCTGTGCAAACTGAGCCAAGGCCATG

GCTGTACACAAACACTAACCAGCCCTAACTGGAAAACAAGGAAGCCTTTTGCAGTGGAGC

CAAGAGGAGTTTGACCGACAGGGTGATAATGTTTTGGCAGGATGGAAGGAGAGAAACCCA

>c1218_g1_i1 len=389 path=[1403:0-388]

TATAAATTAATATATATATATATTTTTATATTATTTTTTCTAATTAAAAATGTTATGTCT

TATACCTTATCGAAACCATAAAATATAATTTACACTCTAACATTTAAATTTTCTCTAATC

CATTATTCAGGTAGCAGTAAATTAGATTAAGCCATTTTTTCATGATCATTTCTGTACCTC

ATATAGAGTAACAAAAAATGTCTTTAATATTTTCTATTTTAGTCTTCTGGATCTTAAAAT

TTTTATAAAGTTTTAAAAATTTGAATTTAAAATAATAGTATAAAATGGAAAATACATTTA

TTTTAATTTGTATTAATTTCATTTTCTTAACATTCTATAATTGTGGTTCTTTTATTTTAT

CCAAAAATAAAAAATTATATTAATATATA

>c1218_g2_i1 len=513 path=[520:0-482 1002:483-512]

ATAAAAAATTATATTAATATATATAAAAAAAAAAAAGCAGCTCAAATTTTAAAACCACAT

AGAACATCGGAGATTATAAAAAAAACATAAAAATAATGAGGGATCACAAACTTAAAGGTT

CTTGGTAACTTCGTGTTCATTATAAAGCTAATTTACATTTTTAAAATTCATTTAAACTTA

AAAAAAGTATTTAGTAAGAAATTATATTAAAATTATATACTTCAAAATTTTACTTTAATA

TCTTAAAATTCTTATAAATACATTTTAAGATACTAGACATACTTTTTATTAAAAAAAAAT

AAATATATTTTTGTAATTATAAATTTTAAATAACATTTACAAATCTTCAAATTCAGTATT

TCTTAACTAAACATTTTAATTTACATATTTTTGTTTGTAAAAGTTATTTAATTTTTTCAA

ATTTTTATTTTAAATACATTGTCTTTTGAAGAAAATTTGATAAAAAATTTATACTGGTTT

TAAAGTTTTAATATTTATTTATATAAGTAATAA

>c1219_g1_i1 len=227 path=[399:0-226]

CTTCTTATCTCTTCCCTCTCCCTCTCTTGGCTGCTGCAGCAGGCAGAGCCCGCCTGTATT

CGGATAAGTTCTGTTGTTCCCCATTGCAGAGGAGCTCCCTGCAGTGGGTGAGGGCTGGCT

GCGGTTAGCCGGGCAGCAGCTCCCATGCAGTCACACACCTCTGACCTGGTCGGTTTCCTC

TCACCTTTTGGCCTTCATTTACAAACATGCAAGGGTAGAAAGACTGG

>c1224_g1_i1 len=217 path=[365:0-29 201:30-216]

ACGGCTCAGCTGAGCCTTTCTGAAAACTGTCCCCAATTTAACTTCATAGCGAATCAGTCT

GGCCTCGAGCACTCCAAGGATCCCAACTACATGTTCAGGGTAAGCATGCATGCACATCTG

CTGAGTGTTAGCAGTTTGTCCTTGAGGAATTCTCCCAAGCAGCCCAGCCACAGGCCACCT

CATACTGACGTCTCAAACAAGAAGAATCACATGCAGA

>c1224_g1_i2 len=205 path=[183:0-17 201:18-204]

CAGACCAAGACCTACTTCCCCCAATTTAACTTCATAGCGAATCAGTCTGGCCTCGAGCAC

TCCAAGGATCCCAACTACATGTTCAGGGTAAGCATGCATGCACATCTGCTGAGTGTTAGC

AGTTTGTCCTTGAGGAATTCTCCCAAGCAGCCCAGCCACAGGCCACCTCATACTGACGTC

TCAAACAAGAAGAATCACATGCAGA

>c1226_g1_i1 len=406 path=[498:0-309 17:310-333 831:334-350 58:351-405]

GTTTGATTTTTTTTTTTTTAGATATAAATGCAAACAGCAAAACTTGGTTTATTGCACACA

GCTCTGAAACTTCAACAGTACAAAGGAACACCGTTCTAAACAAGTTTGTACACGGAAAGG

TGAAAAATGCTCACATCTAAAATCCACCCTTTTGTTTTTTTAATCCATGATGATTATTTT

TGCTCGAGCAATGCAGTATTCAGCGCGTCCGTACAACTGCTTCTAATACAGATGCACAAA

ATTGAGCGCACAGTAAAAATCCTTGGGACGTGGCTGGGTGCACTGCTGCCATGGCCTTGG

GTTTGTATGCTTTGATAAAAGGAAGAAATTGAACATCTGAGAGACACAACCAGTTTTGTA

ACCATACTCCTCCTGATACCAGAATCCCCTACCACAGTCTCCACAG

>c1232_g1_i1 len=349 path=[1:0-348]

TCCAGTTGTACAACACCGCACCGTGAATTGTGAAGACATCTTTTCAGTGGTCTGTGGGGA

CAGGGCAAGGGGAAACGGCCATAAACAGGAGCATAGAATGTTCTGCACCAGTACGTGAAG

GAACTTCTTCACAGTATGTGACAGAGCTCTGGAACGGGCTGCCCAGGGGAGGACGGGGCG

GGCGCCGTGCCTGAGGACCACGTATGGTTGGGCACCGTGGTCAGGTTGGATGGAGCCGAT

GTGGGGGGGGTGCTGGTGGGGGCCGTGGCCATGCTGCTGGTGCGGCTGCGCTGCTGCGGG

AAGCCCCGCTTCCAACGGCTGGACGAGGTGCCCATGGTGCGTGGGGGGG

>c1235_g1_i1 len=267 path=[1:0-202 811:203-207 816:208-266]

TGTGAGCTGTGTGGCTGTGACGTGTGCTGGCACTTGGTATTTTTAGAGACACTTCTTTTT

TTTTAGCAGAGTAAATTTAAAATTAAACAGCATCCTTGTATGGCTGCATCCTCCCATCTC

CGGGAGGTACCTTCTGGAGCTGATGTAGTTCAGCAGCGATGAAACAGATGCCTTAAAGAG

TAGAAATGACTTAGGAAAGGCCAGCGTTGGATTTGGCTCTCGTTTAAGTGTTGCACGAGT

TACGACACCCCGCCGTTCCAACGCTGG

>c1236_g1_i1 len=298 path=[405:0-234 648:235-245 659:246-297]

CTCTCCGGCCGTGGCCTGATTCTCTTATTTCTGGAGTGGAATGTGAGTAAGTTACTCTCG

AAAAAAGGAATCCTCAAGAGTGAGCTACATGGCCTCAACGAGCACGACGCAGATGCTCTG

CAGAGCTCACTAGTCCTCCTGTGTGTCCATGCAGTGGTTTCTTTCCCCACTAAGCAACTC

ACGGTTGCTTTGTACAGCCTCCAGGAAAGTGGTCCAATATAAATTCTCCATATATTAGGT

AGGTTCAATAATTGAGCAATAATTCATGATATTTCTGGATCATTGAACCTACCTAATA

>c1238_g1_i1 len=370 path=[348:0-369]

CAGCATCAGCACTCGAAGTTCTCCCAGCTGAGTGAGCTGCAGGGGCCACGGCTGGGCACA

GCTGACACAGCCTGGCCAGGCAGAAGTACAGCAGGAGTAAAACCTCACGCAGTGTCCTTG

CTCCACCTCACAACCATTTGCACCAGCGAGTTGTCAAAGCTGGATTTTCTGATGCCAACA

ATAAACTGTTTTCTCCATCTGCAAAACTCTGCTTACAGAAGTTTTTAATTTCATAAGTTT

TTCTTCTGTTTACTTGCTGAGTTCTGTGGGCTGCACAGATCCTTCAGTATTAGAAAGTCT

CATTCCCTTCCCTCCCATTTCCAGATGGTTTGTGTGCAAAGCTGCAGATTCACCTCTCTG

CCAGGCTGAG

>c1239_g1_i1 len=393 path=[299:0-274 620:275-392]

TAGACGGAGGGTCAAAATCAACAGAAATAACTGCGGAGTACTGAAAGCTAGGATTTTCAA

TCCATAGCTATTCCTTAAAATGAAAGAGAAAAACAAACCAACCAACCCTACGGAGTGTAG

TTGGAAAAACAACCATGTGACCAGCGTAGATGAAATGAGTCACAGGCACATCAAACACTG

AAAGAATTTCCTGTCTGCAGACTGCAGGCTTCTCTGCAAAGCAAATGAGTAAGACAGCAC

CAATTTCTGCACAGACCAAAACTAAACTTACGTCAGTTAACTTCTCCATGCAGAGTACAG

AACATAAATCTGTGTGGCCAAACATTTCCTTGTGTGCGCAGAGGAGGTGATACTGAGCCA

GGACATCTTCTCATGCAGTTCATTGTTGCTGTA

>c1239_g1_i2 len=321 path=[299:0-274 574:275-320]

TAGACGGAGGGTCAAAATCAACAGAAATAACTGCGGAGTACTGAAAGCTAGGATTTTCAA

TCCATAGCTATTCCTTAAAATGAAAGAGAAAAACAAACCAACCAACCCTACGGAGTGTAG

TTGGAAAAACAACCATGTGACCAGCGTAGATGAAATGAGTCACAGGCACATCAAACACTG

AAAGAATTTCCTGTCTGCAGACTGCAGGCTTCTCTGCAAAGCAAATGAGTAAGACAGCAC

CAATTTCTGCACAGACCAAAACTAAACTTACGTCAAAAAACTAGATCTTTATTTTTCAGC

TACGAAATCTCATACTCTGTA

>c1243_g1_i1 len=300 path=[486:0-299]

GAGGCTGGTCACAAGTGGTGTCCCCGTGGGGTCTATCTTGGGACCAGTGCTCCTCAACAT

CTTTATCAATGACAAAGGTGATGGGATTGAGTGCGCTCTCAGTAAATTTGCAGATGACAC

CAAGCTGAATCGTGCAACTGACAAAACAGAAGGAAGGGTTGCCATCCACAGGGACCTGAA

CAAGCCCTAAAGGTGGGCCCATAGGAATGTAATGAGGTTCAACAAAGTCAAGAGCACAGC

CTTGTACTTGGGTTGGGGAAATCCCAGATACGTTTACATTCTGGGAAAAGAACTCACTGA

>c1247_g1_i1 len=273 path=[1:0-272]

GGTAAACATGACGGGTTGGTCCTACAGTGTTAGAACAGTAAATACTAATTAAACAAGTGG

TTCCTAAACTGGGGTTTACAGACTATGATTAAGCTGTGGAGAGAGTCCAGGAAGCATACT

TAAATAATAACGGTGCATAAACTCAACAGTCAAGAGCGTTTGATTAAGGTGAGCTGTGGC

TCATTAGAAACTTTAAGAGTAGATGGTTGTCCCTCAATGCAAAAAGATTGGAATGATACT

GAAGCAGACAACATACAAGACAAAGACACGCCG

>c1253_g1_i1 len=211 path=[4:0-148 153:149-210]

TGTGTTTGATTGGTCATTTTTATTCCATTTTTTCATTTTTGTTTCATTTCACGTATTTCG

TTTTTTAATATTTATTTCATTGTTATTTCTTCCCTTTGTTTTTAACCGTATTCATTTTTC

ATATTACTCATTTCTTACCTTATGTTTTATTCCCTACACTTTCATTTTCTTCTTCCATCC

TTCCCTTCTCCCACCCCACTCAAACACAAAG

>c1266_g1_i1 len=208 path=[1:0-207]

TATATATATATATATATATATGAGAAAATAAAGTGAAATTATCTTAATTTTTGAGAAATA

ACAACGAATCATACATTTTCTATATATGAAATATTAATAGAAAACATAATAATTAATATA

AAAAAAAATAAAGTTATAGTTTTTGTAAAATATATAACATAAATTGATGGAATTATTTAA

AAGAGAAAGAAATAAAAAAAAAAAAAAA

>c1266_g2_i1 len=269 path=[371:0-268]

AAAAAAAAAATAAAATAAAAAAATGAAAGATAATTATATATTTTAAGACTTCCAATAAAA

ATTTTACAATTATTCTTTTATAAAATAATAATAGAAAATAATATAATTTATATATGAATA

TTAATAAGTATAGCATAATAATTAATATATATATAAAGTGGAATTAATTCTATTTTGAAG

AAGTATTATCAAAAAGTGATAAAGTTAATGTATATGAAATTTAACAAGAATTACAATAAA

GTTACATAAACATATATATATATATATAT

>c1269_g1_i1 len=211 path=[1:0-210]

AGTTTTCGGGTGATGAAGGAGCCGCGGGCTGTTGGTGGAGAGAAGAGAGTCATAAGAATG

TAGCTGGGATTGCCCAAGGGGCAGTGCTCAGCGCCCGATGTTGTGCTGCACAGTTGGAGC

GGCAGCCAAGGTTCAGATCGGAGGTCCTTGGGGTTGTATTTTAGGCCCTTTCTAAACCCG

GTGTTTGACGGGGTGGTGTAGCTGCTGGGAG

>c1269_g2_i1 len=230 path=[377:0-229]

GGGGTGGTGTAGCTGCTGGGAGAGGCAGGTGCACAGAGGAGCCTGCCATTTCACGGCTCT

GTGCTGCTGTGTTTGCTGTGGGTGGGGTGGCCTCTGGGGGCGGAAACTTCCAATGCCTCA

GGTTGTGCTGCAGGCTGTGCTTTCCTCTTGAGTGTGCCAACATTCTTTTCCCATTCTTTG

AGTGTTTTGCTGTTTGCAGTGCAAACTTCAGTGGCGATCAACAAGCATAA

>c1273_g1_i1 len=241 path=[1:0-240]

GGTGTCCATAGGATTGATGTGCAGAATACTGGCTGCTTGCTCACTATTACTTGAACTCGT

TTGAAGTCATTTTATTCTTTTGAACTTAGAAGAGTAGCCTTGACTTTTCCAGTATCAGGA

TGTTATCTGTCATCATCAGTATCTACTTCTGCAAGAGACAGGGATTACTTGTGAGAATGT

GAGCTGTTTGTGGGTGCCTTCAGGTATGTGGGGGAAGCGTAGGGAAGAGATGGAGCCTGA

C

>c1275_g1_i1 len=244 path=[398:0-243]

AGTGGTATTCCACCTTCTCTTATCACAGTACAAATTAAAGACTTCCTTAACGGGCCGGCT

GACGTCAATGTCGATGTATCGAAACCTTTGAAAGCAAATCTGAGCTTTGTCAAACTTGAT

CAACTAAAAGAGTTTTTGAAGAAAATTATGACCACAAATGACAACAAGCCCGGGAAGGAG

ACTGCTCCATCAACTGGTACTCATCCAGATGAGGCTGTCGCTTCAGGAGCCAAACACTCC

AGTA

>c1286_g1_i1 len=270 path=[671:0-12 437:13-269]

ACATCAGGACCCAGGTCGCTCCTAGGAATTAACAACAACAAAGCTCATTTGTGGGAACAG

CATTACAAAAGCATGGCAATTCCAAATCCTAATGACTGTTGTGTAAGGAGGAGTCAAGAC

AGAGCATCCGTTTCAGAAGAAACCAGCTTAACAAACAGAACCTCAACACATCAGATTTCT

CCCTTGAAGGACGCCAGGCTGCTTCCATGAGCTGTGTGTACCTTGACCCTTTAGGAAGTT

ACACGCCTGTATGGGAACTGTTGAATCACA

>c1286_g1_i2 len=358 path=[336:0-100 437:101-357]

AAAATCTGCAACAGAGCAGTCAGGTAGGTTCACGGCCTTCCCTGGGAGCTGTCTGCCTGC

ATGGAGGCTTTTGTGGCTCTGTGTGGCCGCAGAGAGGATCTGGTCGCTCCTAGGAATTAA

CAACAACAAAGCTCATTTGTGGGAACAGCATTACAAAAGCATGGCAATTCCAAATCCTAA

TGACTGTTGTGTAAGGAGGAGTCAAGACAGAGCATCCGTTTCAGAAGAAACCAGCTTAAC

AAACAGAACCTCAACACATCAGATTTCTCCCTTGAAGGACGCCAGGCTGCTTCCATGAGC

TGTGTGTACCTTGACCCTTTAGGAAGTTACACGCCTGTATGGGAACTGTTGAATCACA

>c1289_g1_i1 len=272 path=[289:0-230 519:231-271]

GAAGGAGTGGAGAAGGTGCTCAAAGCACTGAAGCAGAGGTGCCCCCTGCAGCTAATGGAA

GATAGCATGGTAAAGCAGTATTTCTCTGCAATCCTACAATTGTTAAAACATTATTTTAAT

ATCAGAGACAAGGGGCATATATTTATACAAGTTTACTTATATACTCATACCTTTTAAAAA

AAACCAATTTTCAATTTATTTTCAATTGCTTTGTTTCTTCTATCTTTATATGTCAAATAA

AATACTATTTTAAATGGCAGCACTTAAATTTG

>c1292_g1_i1 len=453 path=[1:0-363 1278:364-369 371:370-452]

CCGCGCGGTTGGCCCCCGGCCACGCGCTATTCCTGGAGCCGCTTCACGCACTTTCCCTGC

CCCCCCGTTCCCAAACGTCGCTCTTATTTTGAGCGCTGCGCGCTATTTTCAGAGTCGCTT

CACGCACTTTCTCCCCTCCCCAACCCCGTTCCCAAGCGTCGCACCGCGCTTTATTCACAG

CCGCCCCGGTTCCCCGTCGAAGCCAACGCGGTTCCCCCGCCCCGAGGCCGGCGGGAGGGA

GAGGACGTGCGGCGGTCGGGCCGGGCCTCCCCGCGGGCTCCCCCCTTCCTCGGGCCGGGA

AATGCCGCAACTCTCCTCCATCAGCGCCCGCTCCTCGCCCGGGGCTTGACAGATCGCGGC

GCGGCCCCGCCACAGGTTGCAAAAAACGCAGCCGCATCGGGGACGGAATAACAACAACCG

GAACGCGGGGAGAACGCAATCATCATAGTAATA

>c1292_g1_i2 len=488 path=[1:0-363 1278:364-369 704:370-487]

CCGCGCGGTTGGCCCCCGGCCACGCGCTATTCCTGGAGCCGCTTCACGCACTTTCCCTGC

CCCCCCGTTCCCAAACGTCGCTCTTATTTTGAGCGCTGCGCGCTATTTTCAGAGTCGCTT

CACGCACTTTCTCCCCTCCCCAACCCCGTTCCCAAGCGTCGCACCGCGCTTTATTCACAG

CCGCCCCGGTTCCCCGTCGAAGCCAACGCGGTTCCCCCGCCCCGAGGCCGGCGGGAGGGA

GAGGACGTGCGGCGGTCGGGCCGGGCCTCCCCGCGGGCTCCCCCCTTCCTCGGGCCGGGA

AATGCCGCAACTCTCCTCCATCAGCGCCCGCTCCTCGCCCGGGGCTTGACAGATCGCGGC

GCGGCCCCGCGCTGTCTTTCTTTTCCACCGCACGAAGCCGCCCCGCAACCTCCTCTCGGG

GCCTTTTCCGCATGGTTTCCATTCCCTTTTGCTTTTGTATTTTTATTACTATGATGATTG

CGTTCTCC

>c1292_g1_i3 len=208 path=[614:0-83 1278:84-89 704:90-207]

TTTATTGAATCTTCTTTTATATTTTTATTCCCCTCGTTTTTATTTCCCTTTCTCCTCTCT

TTCTCTCTTCTACCCCCCCTCCCTCCCCGCGCTGTCTTTCTTTTCCACCGCACGAAGCCG

CCCCGCAACCTCCTCTCGGGGCCTTTTCCGCATGGTTTCCATTCCCTTTTGCTTTTGTAT

TTTTATTACTATGATGATTGCGTTCTCC

>c1296_g1_i1 len=299 path=[277:0-298]

GGAGAGGTGCGGGCGGCGGGCCCCGTTAACAAAGGCCCCGGGCAGGCGCCGCTCTCGGGG

AAGGGCATTCCCCGCGCCCGGCCCGCATCCATCCCCCCGCCCGTGGCCTGCGGGGACCCG

CGCCCAGCGCAGGGGCCGGGGCCGAGGAGGCACTGCGAGGAGCTGCGGCCCAATCCTCTT

CCGGTAGGAAATCATCTGACACCCGCGGAGCCGGGCTCAAAGTCGGGGATGCGCGCCTGG

TATTCCGCGCCGACCCTCATGCCTACATCTGCAACAGCAAAGGAAAAACATGGATACAA

>c1297_g1_i1 len=220 path=[31:0-166 30:167-219]

TTAGATTTTTTGGTTTTTTTTCCATTTCAAAAATATGACTTAAGCAATCGAGAACCACAT

ATATGAATAAGTACTCTTAACACTTGTACATTTGTATTGCACTTGTAGAATGTCTTAATC

TGAAATATTCAGTGGCAAAGGTATTTTATTATTGACTAGGAAAAAAGCTGAAAGATAATT

TCTGCTTTGCTTGTTTACAGCACCTCCGTTTACAACATGG

>c1301_g1_i1 len=238 path=[33:0-40 16:41-237]

CTAAACTCCTGCGGCAGGACAAAAAGACTGTTCACACCTACTTGGAAAAGTTTCTGACAG

AACAGATGATGGAGAGAAGAGAAGACGTGTGGCTCCCACTGATTTCCTATAGGAACTCAC

TGGTTACAGGCGGCGAAGATGACAGAATGTCTGTTAACAGTGGGAGTAGCAGCAGCAAAA

CCTCTTCAGTGAGAAATAAGAAAGGACGGCCACCGCTACACAAAAAGAGAGTAGAAGA

>c1306_g1_i1 len=390 path=[363:0-272 88:273-389]

TGGGAGGAAGTATTTATATCCTGATACTGACTGCAGTAAAATGGCGAATAAACTAAACTT

GCAGAACTGTTTGTGCACAGAGAGAGAGGCTGTATTTTCATTACAGAAACCTGTTGATGC

AGATCAAAAGAAGCTTCACATGGCAGTCTCATTGGGCACTAATCTGGAAGATAGCGTTTG

AAGCAAGAAGGCATCCCAATCACCTTTCTGGTCTAATAACTTATTTTCTTTATAATTGCC

GTGTTTTGATTAATAGCTTCTCTTTGCACACCCCCACACCCACTGCTGCATCACGTACTC

CTCTGTGTTTCTTGGAAGTACCATTTTGCTATCTGATACTGATGAATTTGAAGTAGAGAA

CATGCTGGAATTGGGGGAGTTTGCAATTGG

>c1306_g1_i2 len=204 path=[1:0-86 88:87-203]

TGTGTCCTCAGCTAACTCCACTAAACTGTGATTTCACAGAGTTCAGGCAACTGCTTGTTT

AAAAACCTTGACCAGATTCCACACATACCACACCCACTGCTGCATCACGTACTCCTCTGT

GTTTCTTGGAAGTACCATTTTGCTATCTGATACTGATGAATTTGAAGTAGAGAACATGCT

GGAATTGGGGGAGTTTGCAATTGG

>c1307_g1_i1 len=209 path=[1:0-208]

AAATCCCCTCCCTGCCCCCCACTCCCCTAAGGAAAAGCTCTGGGACTCTGAATCATTTTA

AGGAAGTTTGCCTGCTTTCAACTGAAAAAGAAGCGAGTTTATTGTACATATTTAATGGTA

GTCAGATGCTTAAAGACTTGGTCATTAATCTGGAGGCAATTGGAAGAAAAAAGAAACAAA

GACACCCCCCACCCCCATCAAACACAAAG

>c1314_g1_i1 len=292 path=[357:0-127 38:128-291]

CTGCACTTATATATCTAAGCTTTCAAAGTTTTCTAAAGTTTTTTAACACTGAAATCAGAC

TAATTCAGGTTATAAGGAACCCCTGAAATTACGTGGTACAACTCCAGCTCAAAGCAGGGC

CAACTCCTAAGTATTTTTCTCTACCAGATCCACATAAATACATATCATATACTTGTCACA

AAAATGGTGTTCCATGTACTGATAAGGTCTTTGAGGATTTTTCTGTGCTGTGTTGTGCAT

TTTTCTGTGCCTTCTTTTATACAAGTGTTGGCATTAAACTTGTGCCATGATG

>c1314_g1_i2 len=201 path=[1:0-36 38:37-200]

AGTTTTGCTATTATCTTTACCATTGGCAGTATCTCCCAAGTATTTTTCTCTACCAGATCC

ACATAAATACATATCATATACTTGTCACAAAAATGGTGTTCCATGTACTGATAAGGTCTT

TGAGGATTTTTCTGTGCTGTGTTGTGCATTTTTCTGTGCCTTCTTTTATACAAGTGTTGG

CATTAAACTTGTGCCATGATG

>c1319_g1_i1 len=224 path=[353:0-25 2:26-223]

GAAGACCCCGAGGAGCAAGGAAGCGCGCCAGAAGAGAAGGCAGAACCCAAGGGGTAAGTG

TGGGGGAAACCATAAAATGAACTCTAGGCTCTTTAGCTGCCGGCGCACGCAGTCAGTGAG

AAGACTGTACAAGTGCTCTGACTGTACAGAAAGCTTCAAATGGAAATCCAGTCTCACCTA

CGATGAGCACATCCATACGAGGGAGAGGTCCTTTAAGTTCTGAG

>c1321_g1_i1 len=204 path=[9:0-21 31:22-203]

TTTATAATTTTTTTTCTTTTTATTCGCAAAGTTATTATACACATTTCTGTATATTATAAC

TAATCGTTTGTATAATATTATATAAATTTCTTTTAGGTTTTTACCTTACTGTCTTTATAT

TATTAATTTGATTTAGTTCAATTTTTTTTAGTATTTTTACTTGTTTTAGTTCAATTTTTT

ATTTTGGTATGTTCAAACACAAAG

>c1324_g1_i1 len=275 path=[253:0-274]

ATTAGAGGAGCTGGTTATATTCAGGAAGTGCTTAAATTCCCAGCCATATCTTGCTTAGCA

AGAACTTTGTGATAACAGCGCTGTACTGCTCTCAGCTAGTGTGCCTGTAAAGAGCATGAT

TGCTTACAGAGCTGTACTGTTGTAGATGCAATTACATCAGCATAAAATATATCAAACCGG

TATAATTATTTTATTGGACTAGATTGTTCTTTTATGATCACCTGTATCTGTTACAACTGC

ATCCATCCTAAGGAACATTTGCTCTCTGTCAAAAT

>c1325_g1_i1 len=204 path=[182:0-203]

TCTGCCAGCTGCCAAATGGAACTGCCTTTCTAATCCTCTTCCTACCCTGCCAGGTTAATC

TGGGATTTGCCTGCCTATTCTGTGAGGAATTAAAGAAACTCCCTAATCAGGAGGACCTCC

TTCCTTACGCAGCCTGAGAGTGTGGGGGGTCCCCTGTGCACACAATCTCCCTTTGACCCA

GCTGTGCATGGAGCATTCTGGTGG

>c1327_g1_i1 len=587 path=[901:0-355 1257:356-586]

TTTTTTTTTAAGGAAAAAAATTTTTTTTTCAAAAGGATAATAATTACATAAAATTTTTTA

CTTTTTATAGATTGAAAGATATAATTATAAAGCGTTATAAGAATAAAAAAATAAATTATA

CTAAAATTTTTCAGTACTTAAAAAAAAAAAAAGAAGGAAAATAATATATTTAGAAAAAAA

AGATAACAAATAAATATTTTAGAAAATATGAAAATAACAATGTTTAAACAATTCTTGGTG

TTAAGTAAAACGCATATTTCATTTTTAAAAAATAAACGATAATTTTATCTTATACAATAT

TATATAGAATATTTAAAAATTTAATAAATTACATATTATACTTTTCTTTTTTTTTCAAAT

AATTATTCTATTGATCTAACATTTTTCTATATCCATTTAACTCAAATACTTTAATCAAGT

CTTTCATTAGATTATTATTTAATCCTTTTATACCATATTTTGAAAAAACTAGTGATAAAT

CATTTGATAAATTCTCTGCTCCACTTTCAACATCAGTTAAATCGTAGTTAGGGTTAAGAT

TAGGATTATTTTTAAGTAAATTTCTTAATAATGCTTCATCTTCTTTT

>c1327_g1_i2 len=358 path=[1618:0-126 1257:127-357]

AGTAATTGGAAGTATCATTCTTTAAGTTATATTTTAATATCTTATAAAAATAAAAAAGGA

AAAAAGAGTTCTATTTTTTTAATTCAAATATAAAGTTATCTATAATTTTTATTTTTATAT

AATGTTTAAATAATTATTCTATTGATCTAACATTTTTCTATATCCATTTAACTCAAATAC

TTTAATCAAGTCTTTCATTAGATTATTATTTAATCCTTTTATACCATATTTTGAAAAAAC

TAGTGATAAATCATTTGATAAATTCTCTGCTCCACTTTCAACATCAGTTAAATCGTAGTT

AGGGTTAAGATTAGGATTATTTTTAAGTAAATTTCTTAATAATGCTTCATCTTCTTTT

>c1328_g1_i1 len=304 path=[282:0-110 393:111-113 667:114-303]

AAAAAAATAAAAAAAAAAATTAAATGGAGAAAATTTTATATTATATTTAAATATGTGCTA

AAATAATAAAAATAAAATCATGAAAACTATAAAGTATTTAACTTATATCTATAAAAAAAA

AGAAAAAACATATATTATAAATATTTTACTGATATTTGTATAATAACTATTTTTAATAGC

ATGTAATACTTTTTCATATATTTCATTAATTAACTCTTTATCTAAGTCTTCATATAAAAT

ATATAAATAATTTAAACATAACAAAAAATTATAGCTATTAGAATATAAAAAATAATTATA

TTTT

>c1328_g1_i2 len=293 path=[563:0-99 393:100-102 667:103-292]

TAAAATAATATTTTATAATAACTAAATCTTTACAATCTAAAAAATTTGGAAAAAATTCTT

TTATATCCTTTTCACATAAATTTACAATATATTTTTTGTTTAAAAAAAAAGAAAAAACAT

ATATTATAAATATTTTACTGATATTTGTATAATAACTATTTTTAATAGCATGTAATACTT

TTTCATATATTTCATTAATTAACTCTTTATCTAAGTCTTCATATAAAATATATAAATAAT

TTAAACATAACAAAAAATTATAGCTATTAGAATATAAAAAATAATTATATTTT

>c1332_g1_i1 len=282 path=[617:0-281]

TTAATTATTAAAAAAAAAAAATAAGATAAATTTATTTTTAATGAATTAACATAAATACTA

TTCAAATTTATACAAATTCCATTTAAAATGGTTATTATTATATATTGTTCATTTCAAAAT

ATCGTCAAGTGAGGATATATTAAATATTAATTTAACATATTATAATGTTATATCAATATC

AGAAATTCTCATATTTATATAAATTTTTTATTGTATTTTACTCAAAGAAGATATTTGCTT

TCTTTAATTTTATTAAATCTAATATACAAATTTAAAATGTGA

>c1335_g1_i1 len=260 path=[954:0-92 287:93-259]

AACTTTGTGTTTGAATGGGGGAGATCTGGGAAAATGGGATATAGAAGATACTTAGGGACA

CGGGGGATATAGAGGACACCTGGGGATATACGGGCCTCTGGGGAACCTCTGCCAGTGTCT

GACCAACATCATGGGAGCCTTTTGGTTTGTTCTTTTTTTTTTTTCTTTCCCTCAAGATCT

AACCAGAATTTCCCATGCTGCAACTTCTATATTGTTTCTCACCCGTTAAGAGCCTGGCTC

CTGACTGGCAAATTGTCTTT

>c1335_g1_i2 len=453 path=[1:0-285 287:286-452]

AGGTTCAACCTCTACTGCCATACTAGCACCAAGGCAGCAACGCAGCTGGTGACTGAGAAG

TATTCTCTGATAGATATGAGCTAAATAGCTCTTTTGATACTCAGGGCACTGCTGTCACTT

TATAAATTTAAGTAATTTGTGCCAGAAATAGCCTCTGGAGATCACCTCAGGAGATCTGGG

TCCATCATCTGATCGAAGCAACGCCAGCTTAGATCACGTTCACGTGATCTACGCTCAGTG

CCTTGTCCAGACAAGTTCAGAATGCTTCAAGAGTGGAAATTACACAGCCTCTGGGGAACC

TCTGCCAGTGTCTGACCAACATCATGGGAGCCTTTTGGTTTGTTCTTTTTTTTTTTTCTT

TCCCTCAAGATCTAACCAGAATTTCCCATGCTGCAACTTCTATATTGTTTCTCACCCGTT

AAGAGCCTGGCTCCTGACTGGCAAATTGTCTTT

>c1336_g1_i1 len=366 path=[1:0-251 253:252-281 283:282-365]

GAAGTCAGTCATTTTGGCTTTCTGTTTGAAAAGAATCAATCATGAGGCTGTGCTACATAT

CCATGCAAGTTATAAAACTCAGCACTTCAGAATGTTCCATAATTAACTCTGACCACCTCC

AAACTGGGTCTGGCTACTCATCTGGAATTAATTCACTACAGAGGACCTCAATGGGATGTA

GTGATTCAAGAAAAAAAGGAAATTACAAGAATGACAAAATCAACCTTTACAGTACAAAGA

CAAGTCAAAGCTAATAGACAGCAATTTTTGATCTATACTTCAACCACTTTTAATCCTAGC

AAGAGTAAATACATAATTTATTATACTAAACCTAATTTTCACGAGTAGAAGCAGAATGAA

GTGCAT

>c1337_g1_i1 len=210 path=[217:0-209]

TGCTGTGTTTCCCCACTGCATGTGCATATTCATTACCCAGTTTCTCATCAGGTTCAGAAT

ATTCTCTGAGTACAACTAGTACTGACAAATTAACCAGAATTCATCTTAATGGAGTTAATT

GGTTGTAATCAAATCCTAAGCATATTTGCAATGATAAAGTGAAGATGGAATAGTGAAGGG

TAACAGAAGTGTTTCCATGCAGAAGTGAAG

>c1338_g1_i1 len=249 path=[1:0-91 93:92-248]

ATATTCCTTTTTTTTTTTACGTCTTTTTATTTTTCTTTTTATATAATTCATATTTATATT

ATTTTTTGTTAAAATTTTGGAGTTTTCATATAATATTATAATGTATATTTCTCTTTTTTT

TCCACTACATATGTTTGTATATTAATTTTTTTTATAACTTTATTTAAAGAAACCAAAATT

TCAAGAAAATTAATGATAGTTGCTTATTATTAAAATTTAGGACAATTCTAATGAATTATT

GATATTTTG

>c1346_g1_i1 len=201 path=[193:0-200]

GTGCTGGTCTTGGTGCGTGTTCTTTTCCGCTCGGGACCTTCAGTGGCTTTGGCTTCGTGG

ACTTCTTGAGTTGGAGGTTGGGGTCTTCTGCTTTGCATTCGCGGTGTCCTTTCGGGTGGT

GTTCACTGCCCGCTCTTGTGGTCTGGTCTCATGGGCGCGCGCGTCCGCTGTCTCCTGGAG

TCGGCCCTTCTTTGGGGCTTG

>c1351_g1_i1 len=209 path=[159:0-208]

ATAGAACAGGTAACAGGTCAAAAATTCACTCCTAGAAGTAACTTAATGAGAATCCTTTTC

AATTGGTTCTTTGCTCCATAAAGAATATCTCCATATTGAAGAAGATAAGCGTGGATATGC

AATATCATACCTTACTATACTCACCCATATTAATTGAACATTTCCTGTACAAGTCTATAC

TAAGTTTCTCCCAGTCAAACACAAAGTTA

>c1353_g1_i1 len=279 path=[293:0-122 427:123-278]

AGATACATGGAAATTAGCAAACAGAAAAGTAAGCACTCCTTACTCCTCTAAAAGGGTACT

GATCACCCCAGCAGTTGCTCTAAAACAATGCTTGTAGGCTCTTTTTCTTGGACTTATTTG

CTGCATTGATAAAAGTAGGAGGTCTGTCTTTTGATTTTTATTTTTAATATTCTTTTTGTT

TAATGCTAGGTTTTTAGTGTCTGTATTCAGATTCTTTGTGATTTAATATAAACAATACTT

TGTTACAATCTAGAGTCTTCTGGATTGTAACAAAGTATT

>c1355_g1_i1 len=284 path=[686:0-283]

GGCCCGGCCGGGGGACGGGAGGCACGGGGACGAGGATACAGGGACACGGGTTGGCCTCTG

GGGCTCTTCCTATGGCCTTGCCGTCCTGGGAGCTGCTGGCTCTGGGGCTGGGATGTGCTG

CAGCACAGACAGTGGGTGCTGCGCAGTGGGGGCGTGGCATGGGGATGGTGTGACCCACAT

GCAGCCCCAGGAGCTCCTGAAATGTGCCAACCTCAGCAAACAGTGCTGTTTACACCACAT

TTGTAAGTATGTTCCTTTCTTGTTTTTGTTGTTTTTTTTTCAAT

>c1355_g2_i1 len=235 path=[213:0-234]

GGGGTCCCTCCCGGCTGCAGCGTGTGGCCTCTATGGCCTTGTGCCGTGTGGTGTCCCCAT

GCCGTGCCCCAGCTGTGACCAACAGCAACCAGCAGGGCAGGCGAAGGCGCTGCGACGCTC

CGTGCCTCTCCAGTTTTCACCTTTCTGCCCTAAATTCCTGGGTTTTGCAGCTTTTTGGAG

CTGGAGCTCGGCTGCATCTGAGGGATGTGTACCTGGGCAGGAGTCAAAGACAGCC

>c1356_g1_i1 len=343 path=[321:0-342]

ACCTTTCAGTTAAGGAGAAATTCAGACTGACCCAGAACATTCCTCGCTAATACCAGATGC

CACAGATTCTGGGACAGAACTGAGAGAATTTATTACATTCCAGAGCAAAACAGTACAGCT

GCCTATTCTAAGCAGTCACCCACAGCTCATTCCTGATGGGAAGAGAACTGGATGTTTGTA

TATGTTAGCTCAGGGAAGAGTCAGCACAGGAAGGGGAAGAACTTCTGAACACAGCATTCT

GGGTGGCCCCACCTTCTCAGGGAGATTATTTATGAACTGTGATGCTTGTTCGTGGCTGAA

TCCTGATACATTAAATCTTCTGGTGTAATAGGGCAAGAAAACA

>c1365_g1_i1 len=398 path=[899:0-26 72:27-50 96:51-397]

CCTATCTTACCAGAGAAAAATGTCCCTTGCTTCACAATCATGCCTTATTCATGGCATAGT

TTTTTTGCAGTATATACGTTATTGAATAACTATTGTCAAGGATGAAATACAGAAAGAGTA

AAATTTTATCAATAATCTCTGACAAACGCCTTGAGAACACCCTAAGAACCACAACCATTT

CCATCAGATCAGACTGATGCAAAAGGCTTTGATTTTACATTTTCTTTAATGCTATCCTTG

CAATTTTTCTTCCAGGGCATGTGACATCCCCTGTATAGCTAAGCAAATGTTATTGACCAT

AGGTTTACTTTTTTAGTTATCTTCCAAAGTCTTCTTGGAAATAGTTCAAAAGGCACTCTG

AAGTGCAGAAAGTAGAATTTGAAAAGGAAATATTTGTT

>c1368_g1_i1 len=336 path=[314:0-335]

GGACTCAGAGCTCCTGGAACAGCTTTCTCCTTTTGAGTATCTGAATAAAAACAGCCATAA

ACTGCATAAGCACCCATTCAGGCTTTCAAGTTTTTTCTGTTTTCTCTTTTCATATTGATG

TCATGTATTTCGTGGTTTTGTTTGTTTGTTTTGCATAGACTGAGAGCCTTGTACTTTCAT

GCTCAAGTATAATTCCCATTATGACAACTATTTGATTGGCTAAGGCACCAATAATACTAA

CAGAACCCTTTTGTGAAGATTTTCAGCAGTAGAGCTTGAAGTCTGTAATAATATAATAAG

GCAAGCAGGAAACAATAAAAATGAAAAGAGAAAACA

>c1374_g1_i1 len=344 path=[1:0-343]

GTTAGGTGTGAGTGCCGGCAATGGTGGCAGTAGAGCTGCCGGATTAGCTCATGTCTTAAT

TGTTCAGGAGAGAGAAATAGAGGGCACCCAGATTACCAGCCTAAGTGCAAATTGGTGTTT

CCGCTGTTGGAAACATCTGTTGTAAATTAACTCCGTGACACCAGTAAGAAAACCCTTCAT

TTGTATCTGTTTTTCTTTCCCTTCCATTCTTATTTTTAAAAGGAAATGTTAGCCAATCCT

GTAAACACCACCTGCAAGAACCAGCAGAGGAGTTCAAGGTTCTCAGAACCTAGGCTGTGG

GCATGGATGATTAGATTTTCTAAGAGAAGAAATTTGCTTCGCAG

>c1378_g1_i1 len=276 path=[476:0-275]

TTCAATATTTCCCTTCCCTGAAATTATTTTTCTTTCTTTAAGACAGAATGGGTAACCAGG

TAAATGCTGTTTAATATTATAAAAATAGCATGCAGTTGTTTTATTTGCAAAACAAACTAC

TATCAATCTGGAGTTCTATATGTTCGTATCTTCACTTGGTAATTTAAAACCTACTTCAAA

TAAAAATACTAAAACTCAGTGAGTCTTTTAAGCTGTGAATGTTACTTAATGAGACACATT

AAACAACTGATATTAATATGAAAAGAAACAAACAGA

>c1379_g1_i1 len=243 path=[467:0-242]

AGCAGAGTTTCGATTACCCAGAACAGAACCAGATGCCTTTTAATATGGGGAAAAAAAAAC

CCACATCCTGGAGCTGATTTGCCGACAGCCTTTTCCTGTTCCAGGGATATCAATGGCCAG

GATGCAGAGACTTGTCTTCAGTTTGTTTAAATGCGTTACTTGCTACTCAAGTCCTTCAAA

AAATATTTAACCTGAATGTGAATAATGCTGAAGCTTAACAGAAAGTACACACAGCCTCAA

ACA

>c1380_g1_i1 len=214 path=[155:0-213]

ATGGGGTGGGGGAGTGGGGAGGGATAGCATTTGGAGATATACCTAATGTTCAATGAGGAG

TTACTAGGTGCAGCACACCAACATGGCACATGTATACATATGTCACAAACCTGCACGTTG

TGCACATGTACCCTAGAACTTAAAGTATAATAATAATAAATAAACCATAAAAAATAAAAA

ATAATCCCACTTCAACTAAAATTTAAAAATAAGG

>c1386_g1_i1 len=245 path=[223:0-244]

ATCTCTGTTTGATTTTACTGGGCTAGGTATTTCACAAGTAGTCTGCGTGGCTTGATCCTG

TGTTTTATGTCCTGCAGGTCTTGTTGCTTGCTGCAGTGCACTACTTGAATTCTTCATCTT

TGACCCATCGTTTACTCTTTGATTGTCTCTAATGAAGAAAGAGCCGTCATCATTTCTTTC

TATCCAGAGTATGTCAATATGTAGTTTTATGGGTACTAGCTCACATGCCTTGAGATTTTC

ACGTG

>c1386_g2_i1 len=219 path=[641:0-218]

AAAGCGGCTCTTGCTGAAGTCCAGATGGAAAAAGATGCCCTGCTTCATCGTATAAAGAAG

ATGAATGTTGATTATCAGTAGGACAGTGTTCTGTTGGTACGAGGCATTGCCAAGCACTTC

AGGTGACTGCAACATCTGAAGAACTGTTTGCGAATTGCTGGAAGACATGCTTATTTGTTT

TTGGTCACAAACAAAAGTTTTCTCATCACAAACAAAATG

>c1387_g1_i1 len=229 path=[479:0-228]

TGAGTTGTTATTGGAAGGGGTGAGGCAATTTTACTTCTTTTTTTTTTATCTGCTATTACT

CTGTAGGGTTTCTATCTTATTAGAAGGCACCCTCGCTACCTTCTTCTGCCTTACAACATT

ATGTCTGCAGGCACCAAAAGCATTATTACTTCAACTCTTTATTCCCTATTTAATATATTA

ACTACTCTTTTGCTAATAAATGATACTTCATTTACTTTCCTATCTGGAG

>c1390_g1_i1 len=565 path=[1514:0-505 3061:506-564]

AATAAATAAAAATATAATAAAATTTTAATTTTGAAAAGATAATGCATAACTTTTGTTTTT

ATGCATATTTCATAAGACAAATGAAATGCCATATTTTTAGGAAGAATCCTCATAAGTATA

CTTGTATATTGATTCATTTTTATACTTTTTCCTATTAATATAAAAATTTACTAGTATATT

TCTATATTTTTTTTTTAAGTTATATTATAATGTAATAGAGCAATGAAATATCTTGTACAA

TACAGAAAAATCCTTATTTTTATTTGCTGTTCATCCTTTCTTTGAACTTGTAATTTTGAA

TTATACTTTTCAATAACATAAATTTATTTTTCATTATTTTAATGTTTTAAATTTATTTTT

TTTCATTTTCCCATTGTTCCACTGATATGTTAACAAATTCATCCCATTCTTTCATTATTT

TAATCTTTTTCTTTTCAAATGATTTTTCTTTTTTCATAAATGATTGAACAATTACTTCTA

AAGAATATTCTCTTTTTATCTTTTTCTTGAAACTCTTCGTATCTTTCATATCTTCTTTCC

ACATTTTTTTAGAGAACATTTCAAA

>c1390_g2_i1 len=1031 path=[1:0-971 3061:972-1030]

TGGAAAATAATTTTGTTAATTTTATACTTAATTTTAATTTTATTAAAGCATAATTTTTTT

TTTTAAACATTATATGAAAGAAATTAGAATTATGATTTTTTAAAATAATTTTTTACCTGA

AAAATTTCTACAATTCTACATTTCTACATTTTATATATTATTTTATTCGTTATATATCTT

ATTATGATTTTTTTAAAGTATTAATTATTAATTTTATAAAAAAACAACCATTATATAATG

AATTACTCAAAAAATAAATTAAAATAAAATTAAAGTATTTTTATATTACTATATGTTAAT

TTGTATTTTAAAAATACATTAATATGACTTTTTATAATGACAAGGGGAGAAAATTGCCAA

GAACATTATCTTTTAGTGATATGTATACATTAGTGTTCTTTATTAACTATCTTAGAAATA

AAAATGAAGCATTTAAAAGAATAAATCTAGTAAATTCAAAGTTTTATAGATTATATTCAA

TTTTATTTATTATTTCCTTATATTTTTTGCTACAACGTATTTCTAAACGAAAGGATAAAA

CATGTTTGGGATTATATTTCAATAATTTAAATTTAAGAGAATTATCTGAACAGCTCAGCA

AAGAGTATAGAGAATCTGTAGAAGATTTTCCATGTTTGAGATTATATTTCAATAAATTAA

ATTTAAGAGAATCAACTGAACATCTAAGTAAAGAATGTAGAAAGTATTACTTAGAGAGTA

GTGAAAGTATGTATGCAAAAAGGAAAATCTATGAGTTAGATCTCTTTCCAGATATTGAAA

CAGACGAAGAAAAATCCAAGAGACTAAAGGAAATTTGGTATAAAAATTGCCTTAAAGAAA

GAGCAAGTTATTGGCAAAAAAGGATGAAATACTGGAATGAAATAGAAGAAAACATTTATA

TTGATTGGTATTTTGGACCTAAAACTAAAAAAATGGATAAAGAGTGGAAAGATAAGAAGT

GGAATGAATGGTTTGAAACTCTTCGTATCTTTCATATCTTCTTTCCACATTTTTTTAGAG

AACATTTCAAA

>c1398_g1_i1 len=226 path=[295:0-225]

ATGCTAAGCAGATCAACAACCTAGGAAAAGTAATCCCTTCCTTCCTTATAAACTTGATAC

CTCCTCAAAATAAAAAGTTTGCCCAGAGTTTTCAGAGCTGTCAAGCACATTCCACAGCTA

TGGCTGTCTGTAATGCAGAAGGTTGTGGAGATCTGCACAACCAATACAATGAATAACAAA

TTGGGTCAAAGTAGATCCGTGTTCCTGAGAAATAGCAGTTCCTACC

>c1404_g1_i1 len=223 path=[393:0-96 25:97-222]

GTTGTTTGTGCGGTAGTAATGTGCTTGTGGTGCTGTGGCTCTGTGATGGGGCAAACCTTT

TGTGGAGATTGATTGTGGGACTTGGGGCCGGGCCTGGATGGCTTCTCCTGTGTCTATGTA

TGTGTGTTTTGTGGTGCACATTTCAAGATGTGCAGGACCTGGTGATCGGCAAGGCTGCTG

TGTTTGGCTTGAGGGTAGGCTTCAGTTGTGTCCTTGGTTGGTT

>c1405_g1_i1 len=212 path=[187:0-189 422:190-211]

GATTAACTGAGTTTGGGATTAACTGGAAACTTCCTCTGCATAGCAACCACAGCTACAACA

CAGTGACATATAACACAGGGTGCACGAGCCTTGAGGCCAAGTCCCACCTGGGAGTCCAAC

AGGCAAAAGCTGTCCTTGCTTTGCACAGAAACCAACAGGTAAAGGACCTGAATATGCTCT

TACCCCTATTAAGCTCCTGGCTTGGAGCTTAA

>c1407_g1_i1 len=442 path=[47:0-344 23:345-441]

GTGAAAACAGATTTCTGAACTACTGGCTCCACAAGATAAAGGCAGAGCCACTAAACACGC

AGCAAACAGCAATGCCTGATTCTCCTTCAGAAACACCTGACATAAGCATATCTCCAAAGA

GAGCAATGAAGTTTTTACCCCAGCACCTGTGGTCTGAAATGCATGTTTCCTGACTCCTAG

TCCTGCTGCATGCTCAGACAGGCTGAGTTCCATGCCTTAGGCCTTTCTTACGTTGTTCCA

AAACGGAGTTGGAGGTGAAATAGTTCCATGCTCTCACCGTGCACAACAGGCTTATCTGCT

GCTTAAAGGAATTCAAGACAACATCCAGCATATAATGGAATGGGATGTCTAACCCTTCAT

CTTGAAGCTTGGCTCATGTGCAAAAAGGAAGGTGGACATTGTGGTTCTAGAGGGAGCCTA

TAATAGAATCATAGAATGGCCT

>c1416_g1_i1 len=401 path=[461:0-400]

GCTGGCTCTCTCCTCTGCTATTTATTATTGAAGTTCTCTGCAAACTTTGTTCCAAATATC

TGATACTGCTGTGATTTAGAAAGAGAAGGAAATACAACCAAACGAATTGATGAATTGGAT

CAAGTAGCAAAGGGAGAAGTGGAAGGCAAAAGTCACTTCTATATGACCTTATAGAAATGT

GCACAAATTTTACTTGAATTCAGGGAGTTCTATGGATGCAAATGGCAATACTTATATAAG

GTAAATGCAAAGTTTATAATCCAGACTTCCATTAGGAGTTGACTTGACAAATGTGGCTTG

CATTTCTTAAAGACTAACTCTTCATGTTTTAGTGACCGATAAAAAATTAATATGAGTCCT

TCAGTTTTGTTTTTAACCTCACAGTATATGGGCACCAGTCC

>c1416_g2_i1 len=475 path=[869:0-474]

GGTCCCTTGCAACATAGGTGCAAAGTTGCAGTTTTTGCTTTAGACACAGCTGACATCATC

TCTGCATTGTTATGCCTCCTGTATGTGCACAGCACGATCTAGAAAGTGAGTTATAATGGA

GACAATCCCACTGCAAGGCTGATAGAAAAGTAAAAGCTGTGCACAAAAGAACAGTATACA

CTACATACATTAGAGATGCATTGCATTTCTGTTTGTGTTCACATGATTTCCAGGTGATGA

GTTTTATTAGTACAGACTTTTGTTTAAAAATTCTTTAAGATAGCTCAGAAGCAAAAATAC

TCCCAATTTAGAGCCAGGCTGGAAAAAAAGAGATCACTGTGTATGTGGACAGCCAGCACT

ACTTGGTTCTGCCCTGTACTGTAATGCTGAGAACAACCAGAATGGGATAGTTAGCTAGAG

GGCAAATGGAGAGATACAAAATGTGCTGAGTACACACACACACACACACGAGGAG

>c1418_g1_i1 len=223 path=[1509:0-222]

TTGTACCATTAAGCGTTATACACTTCACTTTAAAATCAATGCCGATCGTGGCCAGGTAGG

AAGAGGAGGAGAAGGCGGCAGCCGCGTTGGGCTCCGTGTACCTCCGCACGATGGAGGTTT

TCCCCACGCAGGACTCCCCGGCCATCACGACTTTCATCAGCAGCGCCGGCCTCCCCTCGG

CGCCCCGTCCCGGCACCGCCATGGCGGGCGGGAGGCACGGAGG

>c1418_g2_i1 len=677 path=[655:0-676]

ACGCTTAATGGTACAAGAGTGAAACTTCAAATATGGGATACTGCTGGCCAGGAACGATTC

CATACACTCAGTACAAGCTATTTTCGTGGAGCACAAGGCTTTGTTCTTGTATATGACATC

ACAAATCTGGACAGTTTTCAAGGTGTAACAAGCTGGATGAAAGACATACATGAGAAAGCA

GGTGATGAAGTGGACATAGTACTGCTGGGCAACAAGTGTGATAAGGAATCAGAACGTGTA

GTTCCAAAACATAAAGGAGAAAAGCTTGCTTGGGAGCATGGGATACCCTTTTTCGAAACC

AGTGCCAAAGATAACGTGAACATTGAGGATGCATTTTCAGTTTTGACTAAGGAAATACTG

GAAAAGAAGTCCTGGGTATTATATGACTTAGATGTTGTGGATCTAAATGAAAGTAAGAAG

AGAACCTGCTGTGCATTCTGAAATATCATTTATTATTTCATTTCCTCTTCAGAGAATATT

AACTATTACAAGGGAACATAACAATTTTCTTAAAGGGAGTCTGTAATCCCAAAGCACAGA

ATTTAGGCATCCTTTTTATTACAGAAACCTTGCTGCCAAATATTTTGTCGTTTATATTTT

TTTATTTGTAATGTGAAGTTAGAGCAGTTATCTGACTTTTTGCATACAAATGGTTGCACA

GGAGAAATCTAAATGTA

>c1421_g1_i1 len=575 path=[563:0-574]

AAAAAAAAGAAAAAAATAATATTTTTAATTTGTTAAATTATTTGAATGAAAAAAAAAATC

AGAAAATTTTGGAAAAAATAATATTTAAAAGTTTAATAATAAAAATAAAAATTCCAAGTA

TAACCATATTTATAGAAAAGAAAATTGTAATTGAAGATAAATTCATCATTTTAGCATATC

CTTTAAATGCTATTCATAAGAAAAAAATAAAAAAAGATTTAAAAAATAATACTTCTATGA

ATAACAATGAGGATATATTGGAAACATATGATTCTTTTTTTAAATTTTTATCAAAAAATA

ATGTTTCTTCTTTTAATAACGAGAACAACTATTTACAATTCATATTTTATTTTAGTGGAT

TAGGTTTTTGCTTTAAATCGTATAAAATAGAAGATTTATTAAAAAAGCACGAAATCCTAT

TTTTAAAAATTTATAACAAATTTGTTTCATATATTTCATATATATGTATTCAATATAAAG

AATATTTTATGAATTCCTATTTATTTTTTATATTTTTTCAAATTTATAATTTAAAAATTC

ATATAAATCTTTTAAAAAAGAAAAGAGAAATAAAA

>c1422_g1_i1 len=641 path=[803:0-640]

GAGCTCCAAGGCCCCCAAGATGGCGATGCTGGCCGAGCCCCGTAGAAGACAGAAGTGGTC

CGTGGATCCACGAAACAGTGCCTGGAGTAAAGATGAATCTAAATTTGGACAGAGGATGCT

GGAAAAGATGGGCTGGTCCAAAGGAAAGGGTCTTGGAGCTCAGGAGCAAGGAAATACAGA

GCATATCAAGGTTCAAGTGAAAAACAACATGCTGGGGTTAGGAGCATCCATCAAGTACGA

GGACAGCTGGATTGCTCATCAGGATGACTTCAACCAGCTCCTAGCTGAACTGAATGATTG

CCATGGACAGGGTGAAACAGAATCCTCAGTGACTAATCAGAAGAAGACATTTAATCTTGA

GGAAAAATCAAAATCCTCTAAGAAGCGTGTCCATTATATGAAGTTTGCAAAAGGTAAGGA

TCTCTCGTTGCGCAGTGAGGATGACCTGTCCTGCATATTTGGGAAGCGACAGAAGAGTAT

GAAGACACAGGACGAGGCTACCGATGCTGAGTCTCAGGAAGAGAAGCATGGAGGCTGCTG

GCTCGCTGAGTCTACAGACAACATCAATACTGTGAAGAGTGTTCTCACTGTCCAGGAATA

TTTTGCCAAGCGCATGGCAAAACTGAAAGGATCCCAGAATG

>c1423_g1_i1 len=246 path=[378:0-245]

GAACTCTTCTCTATGTAGGCAGAGAGAAGATGCTATAAGTAAATTTTTGATGGAAGAATT

GAATTACAGGTTTGTTACATATGTCATTAAACTAATCTAATACCAAAACTTTGCCTGCAG

GTCCCACAGCTTCCTGGCTTTTCCTGGCTAAAGCCCTGTCTTTCAGCATCTGATATTGTG

TACATTGGGTTGAGGGATGTGGATCCTGCTGAATACTATATTTTGAAAAACTATGACATC

CAGTAT

>c1425_g1_i1 len=242 path=[56:0-53 110:54-241]

CACCATCTGCCATGATACATCTGATTTTTGAGAAAAGGAAAGCCAGGCAGCAGACACAAC

ACAGTGGCAAACAAGTTGCTGTTATCTGTAAGGCAGTACACTGGCTGTGTAATGTGACGC

TGACATGAAAGTCACAAAGCACTGAAAGAGAACTTTTTCTCTGGGCTGAACTTTTTGGCT

GGTTCAGCATATTGTGCTAATGAGAAGGAAAGCTGGTGCTTTGTGTTTGAGGTGCAGGAT

AA

>c1425_g1_i2 len=267 path=[111:0-78 110:79-266]

AAGCAAAATATAGTAATACAGCAAATTATTTGGCTAATTTTTCTTTTGTTAGGCTCCCAT

ATTTGGTCCCGTTGCTTCCCACAACACAGTGGCAAACAAGTTGCTGTTATCTGTAAGGCA

GTACACTGGCTGTGTAATGTGACGCTGACATGAAAGTCACAAAGCACTGAAAGAGAACTT

TTTCTCTGGGCTGAACTTTTTGGCTGGTTCAGCATATTGTGCTAATGAGAAGGAAAGCTG

GTGCTTTGTGTTTGAGGTGCAGGATAA

>c1434_g1_i1 len=297 path=[298:0-271 25:272-296]

AGGAGGACTTCAGTAAATTTATTTTAGATCTGAGTGTAGCCCCATTCAGAACATATGAGC

TCCAGTCAGTGACGCTGCATGGAAAGCTCAGCTCTTGTTGTTGGCTCCTGTCATAGAGGT

TTTCTGTTGGGAAGATCCTGCACAATTTAAAATTATTTAAATATACATATTTTTAGGAGA

CTTGTGTATGCCTTGCTTGTTGGGATAGAACAGTGGGTTCAATTATGGAGGAGTATCTTA

AATCATCCATAATATTCTTATAAGAACAAACAAAAAATAATCCACGGCTTTTCTAAT

>c1435_g1_i1 len=266 path=[244:0-265]

CAGGAAATTAAGTAAGGGCAAGAGCCAGAATAAAAAAGATTTCTGTAGTGTTAGTTCTCA

GAACTCAGCTTGAGTCACACGGAAGGCTCTGATTTTGAGTGTTATGTACATTCTCCATGA

AAAATCAGGCTCTGTGAAGGTGTCTCTAAGCAAGTTCACATGCTGCTTTCCAAACTGTTC

AGTAGGGATCCTGAAGTAACAAGAAGGATGAAGTAACAGTGACACTGTTTCTTTAAATAT

ATGTAGGATTTCAGAACTTCTAGGAG

>c1440_g1_i1 len=254 path=[47:0-188 485:189-199 496:200-253]

CCTGTGTTTCAGCCTGACCCAGAACAGAGAAGTCATGCCCAGTTAATAGACGATTATTTG

AAACTGCTTCAGACTGCATTTTTCAGGAAGCAGCAGCATTGTAGTTCAAGATAAAGTAGG

ACTTCCCTCTTCTCAGACAAGCAAGACAAGATTTAGTAAAGACAACAGCATACAATGTGC

CAGTTTACTTACAAGTAAGTCACTCTAGCAAGCAGTACTCCTCCTGCCTACTGGAGTATG

ACTTACTTGTAAGT

>c1449_g1_i1 len=205 path=[363:0-17 399:18-204]

TTCAGACAGAAACAAATGCTGGCTAGTGGAGGAGAAAGCCAGGAGTTCTGCTGTCTTCCC

AGACCTCTGAAGAGGGGTTGATAATGCAGTCAGAGCACAAATAAGTCTGCTGCATTATGG

ATGATTTCACAACTCTGCTGTTTCTTCTGCAAAGGCTGTTACAGTGGGCAATTTCATCAA

ATCATAGAACCACAGAATGGCCTGG

>c1449_g1_i2 len=350 path=[183:0-147 384:148-162 399:163-349]

CCAGGCCATTCTGTGGTTCTATGATTTGATGAAATTGCCCACTGTAACAGCCTTTGCAGA

AGAAACAGCAGAGTTGTGAAATCATCCATAATGCAGCAGACTTATTTGTGCTCTGACTGC

ATTATCAACCCCTCTTCAGAGGTCTGGGAAGACAGCAGAACTCCTGGCTAGTGGAGGAGA

AAGCCAGGAGTTCTGCTGTCTTCCCAGACCTCTGAAGAGGGGTTGATAATGCAGTCAGAG

CACAAATAAGTCTGCTGCATTATGGATGATTTCACAACTCTGCTGTTTCTTCTGCAAAGG

CTGTTACAGTGGGCAATTTCATCAAATCATAGAACCACAGAATGGCCTGG

>c1451_g1_i1 len=201 path=[179:0-200]

AGCTCTATGAAGGGACTGCAACTGCCATTGAAATTCCTACGTCTTGCTCGTGGTTGGCTG

AAACCAGACAGCGAGTTCCAGAGGTACCAAAGGGAGGAACAGAGAGGCAAACAACACAGT

CACACAGGCTGGGCTCCCCTCAGAACCTGGTGATAAACGCTGAAAGGAAGATCCTTATCA

GCTTCCCCTGCTCACAGCAAT

>c1454_g1_i1 len=554 path=[5:0-553]

GTTATGTCATCTAAGTCTGGCACTTCAATTTTTTTTTTTTTAATTTATATTTTAAATAAA

AAATATCAATTTTTATGTATGTTCATAAAAACACATTTCTTAACTTTTCATTTAAGATAA

AAAAAGTTATACATATATATATGTATTTATATTTATTTATTTGGAAAATAATTTATAATG

ATAATAATTGAATGTAACAAAATTGTTAAAGGTCACATGGTTATATTTTTTTAATAAAAA

ACCAAATATTTTACATTTCTAAAATGCATTTAAAATCATTATATATATAAAAAAGGAATC

ATTTATATATGTTAAAATTCTAAAACATTGAAGGGAAATGTATATTTTCTAATTAATATT

CATACTTTAAAATTACAGAAAAATGAAATATTCTTTATATTTTATATATGATAAGAAATT

TTAATAAAGGAATCACATATTAAATTTAATATATATTTTTTATCAAAGTAATTTAGAGAT

TGTTATAAAATGGATGATATTCAAATACTTTTTCTTTTTCACATGTAAAATAAATAAATT

TTTAAAATATCTAA

>c1455_g1_i1 len=342 path=[155:0-341]

AGCAGTAGGGAACTTGACAGCTAGCTCTTAGTTAGAAGGGGTGAACTGGGCAAACTGTGT

TTGAGAGGATAGATGTGGAGCACCAGCAGAGGACAGCGAGCGCCTGCAGAGGACGGGAGC

ATCTGCAGAGAACAGGGAGCGCCTGCGGCGGACGGGAGCGCCATCGGAGTATAGATGCAG

CGCGCCGGCGGAGATTAGAGGAAGCACACCTGCGGAGGTTACTAGCGCGCTGTCAGAGGA

TAGAAGTGGAGTGCCATTGGAGGACAGCGAGCACCTGCGGAGGACAGGAGCGCCAGCAGA

AGATAGAGGAAGTGCCACATGCGCTTTCCCTCATCCTAATTT

>c1457_g1_i1 len=291 path=[2:0-214 217:215-290]

ATATATATATATATATATTGTAACCAAAATATGTAAATAAATAAATATATAAAAACATAA

AAATAATATCATAATAAACATAAATGAATATATATCTATTATATTCCATGATATTTGTTT

TTAATCACCTATTTTATAATTTATTTACTTATAGTTACATGTGTATAATTTTTTTATATG

AATGTGTAAATTTCATAGAGATATATTTTATTTTTATATTTGCATTACTAAATATATAAT

GTATTTACAAAAGTCTAAATTAAATATACATAGAAATTAAAAAAAAAAAAA

>c1460_g1_i1 len=382 path=[360:0-77 778:78-381]

GCTTGTTATATAGCTATTTTTCCAAGGCTTTAAACTAGCTATATAACAAGCTACAATTTA

CCAAACTATGACAACATATCATTTGAATGAATGTTTGTGTTGATGATTGATTCTACTAAA

TGACAGCTCAGAAAACCTTACTGTTTCAGTGAGTTGATGGATAAGTTCAGTGTCTTTGAA

ATAACTGTTTACAACATGAATTTACTGAAAAGTAGTTCATGATCAACTAACTAGTAGGAG

AAGCTAATGGTCTGAGATAGGTAATTCCCATCTCTCACGTGATGAAGGTTTTGTTTCTTG

TTTTTCAACAAGGATGATTGAAATACAGACTAGGAGAACAAAGAATCAGTTCATTTGTAG

ACTGCTACTAACACCATATAAT

>c1460_g1_i2 len=323 path=[776:0-18 778:19-322]

CTGGAATTATATGGTGTTATCATTTGAATGAATGTTTGTGTTGATGATTGATTCTACTAA

ATGACAGCTCAGAAAACCTTACTGTTTCAGTGAGTTGATGGATAAGTTCAGTGTCTTTGA

AATAACTGTTTACAACATGAATTTACTGAAAAGTAGTTCATGATCAACTAACTAGTAGGA

GAAGCTAATGGTCTGAGATAGGTAATTCCCATCTCTCACGTGATGAAGGTTTTGTTTCTT

GTTTTTCAACAAGGATGATTGAAATACAGACTAGGAGAACAAAGAATCAGTTCATTTGTA

GACTGCTACTAACACCATATAAT

>c1461_g1_i1 len=208 path=[1:0-168 435:169-207]

GGGGAGGTGCAGATGCCACCCACGGGCCCAACTAACACCCTAACCCCAAATGATCTCTTG

GATCTTACACATCCACAGCTCCTCCCCACATCTCTTTTTCAGTCCAGAAGAATTAACGCG

AAGCCGCAGTATTACTGATTCATATGGAGATAGAAGCAGAGATTTTTTGCATAAACATCA

GCAGAAAGCACAGGTCTCGAAGCAGACA

>c1464_g1_i1 len=219 path=[155:0-218]

AATACCAGGGACACTGTAATAGAATCACAAGATCACTGCACTAGAAATATATTGCAGTTG

TGCGCGCATCACGTTAAGCACCTCAAAGCACTGGGATAGTACAGAAATTATGCCACAACC

ACGTGTAATTTTGATCTTCCTACTGAAATACATCACTAAGATACAGAATAATTAACTGCT

CACTGAGAAAAATCAAAGAAATGACCTTGAGAAACAAAA

>c1465_g1_i1 len=306 path=[1:0-305]

AAGGGATGCTCTTTGAAATTAGGAGCCAGGGCTTTGATTAGTGCAAGTAATGATAATAGC

AGTTGTATACGAGGAACAGCAGAAGGGCATGTGAGCTACTCCAGGGCAAGACAGATTTCT

GAAATCCTGCCTTTGCTGAAGCCCCTGTAACATCTGCAGTTTTATTTTCTTTCTCTCACT

GCTGAGAAAATGAGAAATCCTCTGCCTGTTTTCTTTTCTTTCTCACTAATAGTTTAACAG

TGGTAGAAAATAGTGATAGGAAAGTGCAACTTGCTTTCACATCCTCTGCTGTTTTAAAGG

ATTCTG

>c1466_g1_i1 len=675 path=[53:0-674]

TTTTTTTTTTTTTTTACTATGAACATTTTATAAAGATAAAATGAATAGATTTATTCTGCA

TATTATTTTTTATTTTATAATATTAATCTTATTTACATACTTGTGTATGATAAAAATATT

ATTTTATATTAAGATTTTTTTTAATATTATTAAATAACTATTCTTAAAAAATGTGACTTT

TATTTATATAAAATGAAACTATGAATTTATGCAAATATTAGAATATTCGTAAGCATATTT

CATGAAAAGTATGGTAGTTAATATATTAACATTTTCTTCAAATTAAAGATAGTAGAATAT

ATCAAAAATATTGTTAAAAGAAGGAAAAAAATGTTTCTTGATATAATACCACACTCATCA

CTATGTAAAAAACAAATTATAAAGTAATGCTTTATTATAAAAATAAAACATATATTTTAT

ATTTAGCATATTTTCATTTATATATGAAAACATAATTAAAATCATGAAGAACTAAAATAT

ACTCATGTATATAAAATAAAGAAGTAAATATTTTTTTAAAAAACTTTTTGTAAAATTGTG

AATATATTTCTAAAAGAAAAATTCTCATTTAATGTATATAGTTGTTAAACCTATTTATTT

TTTTTATTTATTCTTCTATTTTTTCTTTTTAGCATACTTAATAAAAAAATTGTCTTGAAA

AAAAAAAAAAAAAAA

>c1467_g1_i1 len=469 path=[1:0-390 392:391-468]

ACAAAGGTGTGACTGACTTTATACAACAGCTGCATGCTGCCATGCCCTGTTGCATTGATG

TTCAGTGGCTGGAGATGTTTGATAGCAGCAGTGCTGAAAACTTCCATTTCCCTTATCTAC

AAGGGACTGTTGTGGCAAGGAAGACAGCCTGATTTGCACAGTGGCTGCACAAGCTCTAGC

CATGCAGCAGCGTGTGAGAGCTCCTCAGGGCACCTTAAATGAGAATGCTGTGGCTCCCTG

GGCAGGAACTGGGCTTCCTAAATACTGCTCATTCCTTGCACTGTTCTCTATCCTTTATTC

TGTTCAGTCCATAGTGCTGTCCAGAGAACTTAAAAATAAATAGTGTAAAAGCACACCACT

TGCTTTCCCTACAGTAGGGGGTATCAAGCAGCTCTAGGAATGGTGTATCTGGAGTTGGGG

AGGCATTTGTTTTTGAACAAAGCCAAATCTAGAGTTTCTATAAACCTAT

>c1475_g1_i1 len=417 path=[395:0-416]

GATTAGATTAAGCAGCCCTCATGGCAGTTGAGAACGGTGGGTTTAAACTTCCAGTGGCAA

AGGAAGAATTAAACACTGGGAACTAAGTGTGGATGATAAAAGATGCCACATTTATAAATG

TAAAGAAAGAGCAGGATGGAGGTGAGGGAAGCTTCAGACTCTTCCTAGTACCCCTGGTTA

AGAGACTAAAGTACCTGAAAGATGTCCAACCTGCGCAGTGAATTCTATCGCCACTATTAC

CATTGGAAACTGTAAGAATGAATGGGACCTGGCTACAGGTCTGATGTCAGCACTGCAGAG

GTGCTTCTAAGATTCCTCAGGTGGCAAAGAGCGTTGGCTACAACATCAGGAGTTGCTTAG

GATCAATTAGCAGCACTAGTGCTGAAATGAAAGAATACTGCCTCCTCAAACACAAAG

>c1476_g1_i1 len=234 path=[223:0-233]

TGTTTGAGCGTGGTTGGTTTGTTCTTAACTGCCATCGAGAAGACTGAGGTCAATCTTGAA

ATGCTGATGAATGAGATTTTTCCTTACGCTTTCAGTAATTTTTAAGTGACTTCAAACATC

TTCAGTTCATAGCGTGATAAAAAGATATTTCAGAGGCAAGTGGTACTATGGTAGTATTTA

TTATTTTTTGGTAAGTTACAAAGATGGAATCTCCATTGGATTTAAATTAAACAA

>c1480_g1_i1 len=233 path=[1:0-232]

ACAGCTATGTGCACACAAGCAAGGCACAGTTAAGTTAGCGTGGGGATTTTGAGCAAAGCA

GTTACTCTAATATTAAACTGCTAACACATACCTTTTAGCCTATGGTAAGTGCATGCAAAA

TAGCAGTTAGCACGTGGTAATTACTGCTTAGAAATCCACATTTATCCTTAAGTAACTTCT

TTTTCTGTGGCCATCCTGTGGGTATCTTTTCTCTGTTATCTATTTCCCAATTG

>c1484_g1_i1 len=206 path=[338:0-205]

AATATATATTAAAAGAGGTACTTCCAAGTGCTTTTCCACTTTATTTCTGCTTCAAGGTGG

AGAAAGAACTTGGAAAGACCTCCCAGCATTACACACATATTGTGTATATATTTATGTCAT

TACATAGATTGTCCATATCCGGTCTGATTGCTTTCAAATTTGGATCCATTTTTCCCCCAT

TTACTACCCAAATCAAACACAAAGTT

>c1486_g1_i1 len=306 path=[284:0-305]

AAAAAGAAAACAAAAAAAAAAAAATGAAATAAATACAGTAAAATCAATTTAAATAAATAA

ATATATATATGTTAAAACTAAAACAAGAACAACAAAAAAAAATTTATTAAGAATGAAACA

AAGAAAAAAGTATATATTCTTGTACATCTCAAATATAAATTTAAGAAAAAGTATTGTTTT

CGCATACACACAGTTATTTTATATTAATAAAAATTTTTTGTTTTTTTAATAAGTTCATTA

CTTCAAAAATATTTATTTTTTATATGCTAAAAAAAATTTCAATAAATTTTCTTAAAAAAA

AAAAAA

>c1489_g1_i1 len=210 path=[255:0-209]

CAGTTGTTTTAATCCCGATGCCAAGCCTTTTGGTTAGGAAGAATTTTTTTGACTGAGTTG

AAAGTCTATCACAAGTACACAGAAAGACCTAGGGCGAGAACAAAGGTGCAGGGCAGAAGC

TAGTTTATTCAGGCTTGTCAAGTTAAATCTTGTAAAGTATGTACACATTTAATTGTTTTC

ATCACTTATATATGTTCTAAGTGTGGAGGG

>c1495_g1_i1 len=230 path=[1:0-229]

ACAGCCACACAGGTTTCCTGCAGCACCTATTCCATGGCTCCAGCTGCTGGCAACCTGTGT

GGCTGTGTGCCCATGCTGTCTCTGGCTCTTGGCTGTATATTGATACATCTTCAAGTTAAT

CCAATTGTTTTTTTTTAAAAGGAAAGCTGTATTTCCGAAAACGAATAGAAAGACCTGAGT

GGCAATTGTCATCTCAGTTCCTTTCATCTCTCTTGTCCCCGGGGCTTAAA

>c1502_g1_i1 len=277 path=[1:0-129 131:130-276]

AGGACAGCCATCATGATAAATTATGATGGCTGTCCTTCAGCACCGTATTAGGTACAATAT

TAAGAAGTGGCTTGTCAGCATCAGACAGCTTTTGTAGCCTGTTAAGAACTGAGCTCCAAA

ATCCATTGAGGTAGCTAAGACAAGTACAAAATGTGAACTAAGGACCTCTGAATTTTCTTT

CACCTTTGTGCTTTTGTTCTTTCCTCTTTCTTTTTTTTTTTGCCTTTTTTTTGTCCAATA

AGAATTTTTCAGTGAGTCATGACGTATCCTGTTTATG

>c1506_g1_i1 len=398 path=[1:0-304 306:305-397]

GCCCGGGACAGCTCCTCCTGGGGGCCTGAATGGATCACCACCAGCACCAGCACAGCTGGC

ATGCATGTGAACAGGTTCCTTATGTTAAAGAGGGTCTCATCCACGTAATCCCACTGCATG

AGCTGCTTCAAAGTGGTGGCTTGCCAAACCATAAAAACCAGATGCGGTTGAACCTCTTTC

TCTTCCTCTTTTTCCTCTACCTGCTGTAGTTCAGTCCTCCCATCACCTGCACTGAACAGG

TCTTGAGCAAAATCCAGCAGCACTGAGGGGGTTCTCACATGGACATCCTTCATCTCCATC

ACCAGCAGCTCGCCGTTCTGCATCCCCAGCATGTTCAGTAGGTTATGGAACTCCCAGCTG

GTTTGGTCTGTAAGGGAGGAGCAGGCGGTGGGCTCCTC

>c1506_g1_i2 len=399 path=[1:0-304 495:305-331 525:332-398]

GCCCGGGACAGCTCCTCCTGGGGGCCTGAATGGATCACCACCAGCACCAGCACAGCTGGC

ATGCATGTGAACAGGTTCCTTATGTTAAAGAGGGTCTCATCCACGTAATCCCACTGCATG

AGCTGCTTCAAAGTGGTGGCTTGCCAAACCATAAAAACCAGATGCGGTTGAACCTCTTTC

TCTTCCTCTTTTTCCTCTACCTGCTGTAGTTCAGTCCTCCCATCACCTGCACTGAACAGG

TCTTGAGCAAAATCCAGCAGCACTGAGGGGGTTCTCACATGGACATCCTTCATCTCCATC

ACCAGTCATTGCCTCCAGCATAGAATCTGAATGTGCAGCAAAAAATAATTGCACAGGAAG

AAGCAATGGCATCAGAGGAGCCCACCGCCTGCTCCTCCC

>c1514_g1_i1 len=321 path=[372:0-258 25:259-280 47:281-320]

AAAAATTTGATGTCGCCATTTTGCTTCTCATGAATTTTCAGGACACGATGGACTATAGGA

ATTTCCCTGCCTTCTATCCTAAAGACCACAATTTCTCCCACTCTGATTGGATCTTCAATT

CGGTTTGTTAGGAACAGGAGATCTCCTCTGTGAAACGCAGGCTCCATGCTTCCACTCAGA

ACCACCACGATGGGGCTCTCGCTGCCCGTCACCACCATCAGCCCCTTCCAGATCATGAGG

GCGGACGAGACGATCATGCCGAAGTTCAGCACCTGGTAGTAGAGCTGCCGCTTGTTCATG

CGCCGCACATCGTCCAGAAAG

>c1514_g1_i2 len=333 path=[372:0-258 630:259-292 664:293-332]

AAAAATTTGATGTCGCCATTTTGCTTCTCATGAATTTTCAGGACACGATGGACTATAGGA

ATTTCCCTGCCTTCTATCCTAAAGACCACAATTTCTCCCACTCTGATTGGATCTTCAATT

CGGTTTGTTAGGAACAGGAGATCTCCTCTGTGAAACGCAGGCTCCATGCTTCCACTCAGA

ACCACCACGATGGGGCTCTCGCTGCCCGTCACCACCATCAGCCCCTTCCAGATCATGAGG

GCGGACGAGACGATCATGCGGAAGTGGCGCGGGGTCGCCGCCGTGGTGCCCGCGATGCTG

TCGCTGGACTTTCTGGACGATGTGCGGCGCATG

>c1516_g1_i1 len=219 path=[1:0-218]

AGGAGTTTCTTTTTCCTCACTGGGAAGTGTGTCAAAGTCAGGAGGGTGAACGCATTGGAT

GTGTTCAGGGCCACAGGAGCAAGGTGTGTGAGGGGAGCAATGGCTGTGTTTCATCAGCAG

AGGTAGCAGCGTGGTGTTCAGGCATTTCTCCAAGGACCTGGGGAGAGTTGTGGCTTTCCC

CCTTCATTCAAAACCCCCTCAAACACCTTGCTCCTGTGG

>c1521_g1_i1 len=250 path=[412:0-12 414:13-249]

ATCTTTATTATAAAAAATGAAAGCTGACAACTTGGAAAGCAGCTTGGCAAAGATGGTCAA

CAGGCTCCAGGGCTGCATTTTGCAGAACAATGCCAGTACGCTGAGGGAGGTGAGCCTTAC

CCCCTTCTCCTTGGCACTTCATTCTGATAAGAAATACATAGAGTGCAGGGTTCAGTTCTG

TGCTACCCAATACAGGACGGCACGAAAATACTGGATCAAGTCCAGTGAAAGGCAACAAAG

ATGATTAAGG

>c1521_g1_i2 len=302 path=[107:0-64 414:65-301]

GAAAAATTGGATGCACCTGGGGACCTGGGCAGGAATAGCCCTAGGAACCGGTACACACTG

GGATAAAAATGAAAGCTGACAACTTGGAAAGCAGCTTGGCAAAGATGGTCAACAGGCTCC

AGGGCTGCATTTTGCAGAACAATGCCAGTACGCTGAGGGAGGTGAGCCTTACCCCCTTCT

CCTTGGCACTTCATTCTGATAAGAAATACATAGAGTGCAGGGTTCAGTTCTGTGCTACCC

AATACAGGACGGCACGAAAATACTGGATCAAGTCCAGTGAAAGGCAACAAAGATGATTAA

GG

>c1526_g1_i1 len=259 path=[237:0-258]

GCAATGGAAGATAGGTTGGTATTCCCTTCTTTCACCCCATCTTTTTAATTCCCTGGCAAC

ACTTCCTTCGCAACAATGGTTCTCAAAATTAGACCATGGAAAATGTATTAAAAATAGATA

CAAAGAATAATCAGTACCTCAAACAAAGCCAAGTTTTAATGAGCTGAAGATGGAAAAATA

CTATTCCACTGAAACTCGAAGGAAAAACTCATACGGTCATATAGGCTGTATGACAAGAGC

AAAGTAACTCGTGCTTTTA

>c1527_g1_i1 len=206 path=[19:0-154 174:155-205]

TTCCCCCTTCAGCAGAGATGAACTCCGATCCGGTTGAGCAGTGACAGTTGAGTAAGTGGA

ATCATTTTATGGAAGACATTTGAGCAGTATACCTGATGTAGTTGTCAGGTAGGAGCTGTG

TGGTCACATTGCCCATTTTGCATCAGTAGGCTGCTAAATACATCTGGAAATTTCTTAGGA

GTCAAATGCATCACGGAAAGGGGGGG

>c1545_g1_i1 len=227 path=[53:0-226]

ATAAAACGGAGCACTCTCTCATAACCTGTGGAACCGTTCTTGTGGAAGCTGGCGGTACCA

TCACGCCCCCGCGCGGTTGTTTCTCTCCGCCCCTCGCGCCTCCCCCTGCTTCCGGCCGCC

ACATCTCAGCAGCGCTCGAGCGCCCCCGTGCGGCCGGTTTGCGCATGCTCACTGTGTGCA

GCGCTGGGGTCGCATTTCCGCTTCCGGGCGGCGTGGGGAGGAGAGCG

>c1547_g1_i1 len=395 path=[790:0-178 113:179-394]

CATAAGTTTCTTTAATAATTATTAATCTTGAAATATTTTAAACAGTTTTTGAAATTCAAA

AAATATGAAAACAAAGACAAATGTTACTTCAAAAAATTTTATGAAAAATACTTTGTGAGA

AAATTTTCATTATAATTAACAATAAATTTCAAGTCTGTATAAATTTCTTTTAATACCATT

AAAATAAAAAAAAAAATTCTAAATTTCTATATGAAACCCTCTTCTCATTTATGAAATTTT

CTTTAGCATTTAGTATCACTTTTTTAATTTAAAAAAAATGGACGTGCTACTATATTTTTA

ATAAAAAATATTGAAATTTCTAATATTAAGAAAAAAGGAAAGAGTTTTTATATATATATA

TATTTAGCATATTTCATATTATAAATTCATTTAAA

>c1547_g1_i2 len=328 path=[1:0-111 113:112-327]

TATTATTTTAAGTTTAGATATATTGAATAAAAGAAAACAAAACAAATATTTAGTGAGGTT

GATTTATGAAATAAAAAAACATAAAAATATTTCATATAAATAAACAAAAGTCTAAAATAA

AAAAAAAAATTCTAAATTTCTATATGAAACCCTCTTCTCATTTATGAAATTTTCTTTAGC

ATTTAGTATCACTTTTTTAATTTAAAAAAAATGGACGTGCTACTATATTTTTAATAAAAA

ATATTGAAATTTCTAATATTAAGAAAAAAGGAAAGAGTTTTTATATATATATATATTTAG

CATATTTCATATTATAAATTCATTTAAA

>c1552_g1_i1 len=239 path=[265:0-211 477:212-238]

ATAAGGCCACATTCTGTAACCTGCCTGCTTTCTAGAGGAATGAAGGGTTCCTCTTCCACC

TCATCCCTTCCCAGCTATACTGCCGTATCAGCATCAACTTGAAGCCTCCACATGGGAAAA

GCCATCTCTCCTGAGGATTCTCAAGAAGAGCCGAAAGCACCGCCAGCTGAGGCAACCAAC

AGAGCACAACCTACACCAAGTTACAACACTTCCTGTAAGGTCAGCCACTGCTCTGCAAA

>c1552_g1_i2 len=240 path=[265:0-211 531:212-239]

ATAAGGCCACATTCTGTAACCTGCCTGCTTTCTAGAGGAATGAAGGGTTCCTCTTCCACC

TCATCCCTTCCCAGCTATACTGCCGTATCAGCATCAACTTGAAGCCTCCACATGGGAAAA

GCCATCTCTCCTGAGGATTCTCAAGAAGAGCCGAAAGCACCGCCAGCTGAGGCAACCAAC

AGAGCACAACCTACACCAAGTTACAACACTTCGTATTTGCAGAGCAGTGGCTGACCTTAC

>c1560_g1_i1 len=223 path=[235:0-222]

GGTACATCTTGGTAAATAGTTTGTGGTGTTCTGCTTTCTATATATTCTTTATATTTTCTC

TCTTCATGATTATATGATCTCAGATAGCTTCTTCCCCCCTTTTTTTTTCCCCAAGCAGTA

TTTACTCTGCAACTTCCACATCTTTATATCCTAGTTTCTTCTGTTACCCTCATTGAACTT

CATCCAGAATAATGCCTGTGTTGTTCGATGGGGTTGGATCTCT

>c1570_g1_i1 len=239 path=[276:0-178 12:179-238]

GTGTTAATAGACAAAGACAGTGCGCTGTAGGATATTATTACTGTTGTTAAGAGAATATAA

AAGACACATGTCCTTTTGTAGGAGCAGTGAGCCTTTATCCTGCAGGAGTAGCTGAAGTGG

TATGAAACATTAGTAGAAACTGCATGTAAGCAACGTAATTTTTGCTGATATTAACTTATC

TGAAACAGTTATGGGCAGCATGCAGGTCTCTGTAGAGCATTTAGGTAACTTTGTGTTTG

>c1573_g1_i1 len=218 path=[196:0-217]

GAGCCATCTCTGCAGAGCTGCTTGAAAGAGAGCTGAGATGGCTCTTTTGGGACCAGCTGG

GTTCCAAGAGCAAACGACCACAATAGCAGCTTTTGTACCAAATGTAATGCCAAGAAGACC

CAGGAGATCCATGCAAGGCCTCTCCTCCGCATTTGGGCAGATCCCAAGGCCAGCTCAGAC

AGTCCCAGATACTTATTGCTTTCCCTCCCGTTCAGAAA

>c1574_g1_i1 len=208 path=[1:0-207]

ATGAATGCAATCCTATAATGAACCATTTCACGTTGATTGGAAATGATTAGCCTTTTACTC

AGGCTATTCAGAATCAGGTACTGAATAGCTGTTAAATGTATTTCACGTGGAAGTGTTTTA

CAAATGGATTCTGGAAATACACAGTTTATGCCCCTAATTTGTGCTACTAACTAAAAGATA

AATAATTTGTTAGATTTAAATCAAATAT

>c1578_g1_i1 len=313 path=[291:0-277 569:278-312]

CAATTTTGTTATACTCTGAATATAAAGTAGATGAGCTCTATTGATGAGCTCTATTGATGA

GCTCTCATAAACTCATATGAATTATAAATCTATTAATATATCTATGTCATAAACTGTTCA

GGTTGAGCTGATATTTCCATTACAAAAACACAGCTATTTTCCTGTAGCTCTTATGGTTTT

GCCTGTTGGGATACACTTAGGGCTGAAATTCAACATATGCATCTTAGTCTGAATACTAAG

TCATTTCAAAAAGAAGCTAAGATTTTTTACAGTTACTTCAAAAATAACATGTTTAGGCAA

ATAAAATTTCTGA

>c1582_g1_i1 len=244 path=[280:0-126 522:127-243]

ACTAGATGATCTTAAATGTCCCTTCCAACCAAAACCATTCTATCATTCTATAACACTGAT

CTGGGTGGAGAGAAGGATCAGCACAATAGCAGTTGTGTAAACACTACACCAGTCACTCAG

TGTGTCAGAGCTTATTTGGTTCTTTCTATTTAAAAAGTAAAATACCTTATTTATTCCTTA

CTGTTATCTTACTAATTATTTATAATACTTCAGATATTAAAATGACTCTCTGTAATGCCC

GCTA

>c1590_g1_i1 len=352 path=[330:0-351]

AAAAAAACAAAAAAAAAAAAATGTAACCTTCATCACATCACATTGATAAAACAACGTGCA

CACAAAGTGCTTTACAGAACTTGTGCAGAATACATTCCATTTAGAAGCACCAATGCATGT

CAATGGTGACTAATGTTACCTCATGGACTTGTGCACCGACTCATTTATCATCAGCCACTT

TTATATTAAGAGATGATTACAACTTATTTTTCCTTTATTCTCTTTATCCAAATGAATCAA

ACCATCCGTGCTAACAGCTACACATCTCTACTGCAGGATGAGCGTCTCCATAAAGGAAGC

AGATAGTACAGTAAATATGGCATTTTAAAAGCAGGATTCGCAGAAAATCTGA

>c1602_g1_i1 len=382 path=[522:0-333 1078:334-381]

AATTAATTTTTATTTAAAAAAAATTAAAATTTTATTAAAATGAAATTAACCAATTTAAAA

AAATACATCTAAAATAATTTGAAGTTGAAAAAAATCAAGTATATACTTATATATACACCT

ATTTAAGCACATATTAACAGAAAGTTAAATTATAACATTTAACATTTTTTAAAATATAAA

TTAAAAAATTAATTATTAATTAAAGTGTAAGAGTGTATAAATATTTATATAGATATGAAC

TAAAAAAAAAAAAAAGAATAAGATAGTATGAATAGAATTAAAAAATTACATGTATTTTTA

ATAAAAAAAACACTTTTTTAATTTTACATAATTTCAATTACTTTCCGTAAATAATGCTTA

TATTGAAATTATGTAAAATTAA

>c1602_g2_i1 len=221 path=[3:0-172 1078:173-220]

AAAGAAAAAAAAAAAAATTATTATTAAATTTCTATTCATATTAAATTATTAAATATTGTT

AGGATTTTATAATTTCTATTATTAATATTATATAGTTATTATTAAAAATATATATTCTTT

TAAATATGATTAGTTTTACATATAGAAAATGTTTTTCTTGATATTTTTGATAACAATTAC

TTTCCGTAAATAATGCTTATATTGAAATTATGTAAAATTAA

>c1609_g1_i1 len=365 path=[1:0-41 43:42-364]

AGGGATACAGTATTTTGTGCAGAGGCGCCCATCATTGAAGACGCTGATAGTTGACAGTTA

CAGTGAAGTTCTTTTATGATCCTAAGAGACTTATGTCGCTTCATGTTTGTCTATCTAGTA

TTCCTCTTGGGATTTTCCACAGCTGTGGTGACTTTAATTGAAGATGACAATGAGGGGCAG

GACACAAATAGCTCTGAATATGCCCGATGCAGCCATACGAAACGAGGCCGCACATCCTAT

AACAGTCTGTATTATACCTGCTTGGAACTTTTCAAGTTCACTATTGGGATGGGAGACCTG

GAGTTTACAGAGAACTACAGGTTCAAGTCTGTGTTTGTCATCCTTTTGGTTCTCTATGTC

AACCT

>c1609_g1_i2 len=336 path=[399:0-12 43:13-335]

CTGTGGTGAGGATGCTGATAGTTGACAGTTACAGTGAAGTTCTTTTATGATCCTAAGAGA

CTTATGTCGCTTCATGTTTGTCTATCTAGTATTCCTCTTGGGATTTTCCACAGCTGTGGT

GACTTTAATTGAAGATGACAATGAGGGGCAGGACACAAATAGCTCTGAATATGCCCGATG

CAGCCATACGAAACGAGGCCGCACATCCTATAACAGTCTGTATTATACCTGCTTGGAACT

TTTCAAGTTCACTATTGGGATGGGAGACCTGGAGTTTACAGAGAACTACAGGTTCAAGTC

TGTGTTTGTCATCCTTTTGGTTCTCTATGTCAACCT

>c1610_g1_i1 len=241 path=[219:0-240]

AACAAACTCAAAGCATGCTTTATGATCATTTATCCAGTATAAAAGATTATTTACTAGAAC

AGGGTCATCACAAAAGACTGCTGTAAAAACAGTTCAAATTACCTAAAAACTTAGATTCCA

CAGTAAACAATACTCTAGCAAAAAACCTACTGTACTGTTTTTATAAGCAAAAGGAAAAAG

GCCAATCTGACAGCTGCTGTTTATAAAACAGCTGGAATGCTTTCAAGTAAAAACTCCCAA

T

>c1616_g1_i1 len=239 path=[1:0-238]

AGGGGGATGGGAAGAACTCCGGTTCAATGTAGTTAAATATGTGGTGTGCTAAGTATGAAT

AAATTGTGACTATTTTAAACAAGTTCATATATTCTGTGGCTGGCTCAGGAGAGTATCAGG

AATGGTATCACAACACTGAGCATACAGGTAAATGACTTTGTGCGATTGTTCTGCCATTGT

TCTACCTTAACAGTAAGATGAACATAAAGGCGTTTGCGTGGTCCTTTTGTTTGTTGATG

>c1621_g1_i1 len=290 path=[233:0-289]

ACGTTGGGTCTTCCGAGTTGGGTGCAGATGGAAGAGGGAAGTCGAGGCTGCCTTGATCTT

CCCCTGTAGATGGGAGTTAAACGTATGGACCATTAAGCGTTGATTAACGACGAGTAGTTG

ATAAACGTGCTTTAAGCAGTGGCATCAGACACTACGTTCCTTAGAATTAGAGATCTGATT

TAACGAGACAAACTGTGACATGCACAAAAAGAAGACGTTTGTTTTTATTTTTCACTGCCA

GCTTAAATTTTTCTACAATTCGCATGTTTGAGGGTTTTTTAATTGTATGT

>c1626_g1_i1 len=252 path=[384:0-251]

TTATATTAGGAATTTAACAGTATTCAAAACTTGTTTTAGTGGGTTTTTAATTTTTTAATT

TTTATTGTATTTAATTTTCAATATTCTACCCCCAGAATCTATGATTTGTGGCATTTTTGG

CTATTTGTAAGATGATCTTTCTGTGACTGAAATCTCTAAACATTCCTTCCAGAGGTAAAA

CCTTTTATTCTGACAACTAAAAAGAACCCAAGGCTTCTTAAGGAGGAAAAAAACATCTGC

CAATCCCCATGA

>c1628_g1_i1 len=222 path=[200:0-86 287:87-221]

ACAATCTACCCTCTCTTCCTGATGTGAGTATACTATCCATACCAATCTGTTACAGATTCT

ATTTTGAAGCATGCTTTAATGGTTTTGAAAAGTAAAAATCCTTTTTGTTTTGAAATTCTT

CTGCTGGTTTATCAAAATGAGAAAATTCCAGAAAGTGATGCAGGATTGCCTACATATTTT

TGGCAGTATACAGGACTGTTCTTGGAAACAGCCATTAAGAGG

>c1629_g1_i1 len=284 path=[31:0-144 15:145-283]

GGAATCAAAGTTAATCTGGAAAGAACCTAGCAAGTCCTTCCAACAGATCTTTGCTAGGTC

TTCTCCATAATATTCCTTCATTATAGGCATAGATCGGTGTTTACACCGTACGTGTTTAGT

CAAGTTTCCTGAAGCATTTGAGTATGACAATAGAAGAATGCTTCCATCCATGTAACAGTG

AATCTTAGAAGCTGTATTTCCATAAAACAAGATAATTTTTGGAAATGAGTAATTACTAGA

GAGTCTGAATGACTGAATGACAGAAAGTCAAATCATTCTTTCCT

>c1631_g1_i1 len=206 path=[398:0-26 215:27-205]

AGAATAAACACTAAATAACTATACTCGTAATGTTCATGCCAATGTAAAATGTTCCATTTA

AGCTGCAATTCTTATTATTGAAATAATAATAACAATATATTGAAAAAATAATAACAATAA

TAATCTAGCTTCACCAGAAACACCATAAACATAATCTTCATATCTTTAAGCTGGATATTA

GGAGGAATTTTTTCACCCAGAGGGTG

>c1631_g1_i2 len=208 path=[186:0-28 215:29-207]

TTTAAATTTCCATTACGAGTATAGTTACTTAATGTTCATGCCAATGTAAAATGTTCCATT

TAAGCTGCAATTCTTATTATTGAAATAATAATAACAATATATTGAAAAAATAATAACAAT

AATAATCTAGCTTCACCAGAAACACCATAAACATAATCTTCATATCTTTAAGCTGGATAT

TAGGAGGAATTTTTTCACCCAGAGGGTG

>c1632_g1_i1 len=283 path=[1:0-189 191:190-282]

CTTTTTTGCACTGGAAAGGAAAATGAAGAATTTTCTTTTCCTTTGCCACAAGAATGGTCT

TACAGAATTCCAACCATTAGATCCAGAAGACTAAATATAGAAAAAGCAGAAGAGTTGTGG

GTAGGAAAGGAACCAATTCAATAGCTTGAAAATTGAGAGTTGGAGATGGCAGGGAGAAGA

ATCAAGTTAGAGTTATTTGCGAATGTGTGGGGAAAATATGATAATAGAGAGCCAGATATT

TCTGCACCAAGGGCAAGCATTTGTGGAGTGCATGGTGAGACAG

>c1642_g1_i1 len=208 path=[186:0-207]

CACCCTCTTGCTCTCTGTGATCAGCATACTGCGATTTCAAACCACATGAGCAGGAAAGCC

TGCCTGCAGGCACTGTTCCAGGCCGGGATCGGGATATACAAAACTTGGAAGTGGCTCTGT

AGTGGTTACTCAGCACCGGCACAGGCACTTCTGCCTGCAACCAGCAGGCTGCAGCAGATC

CACATGTAGATCCTCCCGTTTAATACGG

>c1643_g1_i1 len=266 path=[161:0-265]

AATGTAACATTTTGCTGAAGAGAATTTTACAAAAATATGCCATGACATTTCCAGATGCTA

ACATTTACAATTCAGAATCACTTATTTTCCAGAAAAATAAGTATATGTTAAAATAACAGT

TTATTCACATATGAAGTCAATTTCCACATTGCTTTAGAGGTAGAACAGATAACACTGTCT

TTATTTTGCCTTATTCTATAAGTAGGAACGCTGTAACCTCTGTAACCTGGCCTACTTTTA

GTATTTCTATTTCATGCATTTTGGAG

>c1646_g1_i1 len=328 path=[33:0-146 379:147-327]

ATTATTCATATACATTACAACATAATGTATTCACAAATAAATATATATACATATATTTTT

TTTTGTATGTTCATAAAATGACCAAAATACAATAAAAATTTAAATCCAAAATTTACACTT

TTTATAAATTTCTTATAAGTTTTTAACAATATATCATTAAATTTTGTGGTTTGCTATTTT

TTTAATATATACATATATATATATCTGTTAAGTAAGCTTGAATAAACAATAAAGTTTATA

TCCATTGGGCTTGCAACTTAACTGAATGTTAGACTGTTCATTTCAAAATAGCCTTATGTT

TTGACACCTGTTGTCTAAGACTACTGCT

>c1656_g1_i1 len=241 path=[373:0-240]

AGAGAGAAAAACACGTATGTGTTATAGCACAAGTGAAATTTTGTTTCCCATTGCATTTTT

CCCCTCCTACAACGAGGGTCAGAAGAAGGGTTTCAGTGTCAGAAGGAGGTTTCTAATGTC

TGTGTTTAAAGAAAAACACCGAAGAGCAATAAGCCATGTTAGCTGAAACAATCCATGTTA

CATAAAGGGGCAGAGCCTCCGGGGGATCAGCGCGTGGCATTAGCAGTGCCAGTAAGCCTT

T

>c1662_g1_i1 len=206 path=[184:0-205]

ATAATATCCAGCTCACACACATTTCCAAAGTATCGATCCAGAAGTTCTACATATCGATGA

ACGACCTCTAGCGTCAGGAGCTCGTTATCCTGGTCTTCTATTGCACAACAGAAATACAAA

CTAGCATATCTAGGAAAATAAAGACAAAAACATGAGTGTGAGCAGCTCAGTGCTCTGCTC

ATAGAAGTTACGCAAGCGATAGTTAA

>c1664_g1_i1 len=234 path=[500:0-233]

GCTGTCCCTTCATGTGAAACGTTTAGTGGAAATTGCTCTGGAGGAGAAAACGGGAGAAGT

CCGGAGCAGCATCTGGAAGTCTGACACTGAAGTTTCTTCAAAGGAGCTATTGCAGAGAGA

TCAGTAATTGTCAGATGGTTCTGGAGATAAAGCCTCTATTTGATCTTAATGATGGAATTA

ATCTAAAAAGAAAGATATGATCTTACATGAAAAGTTGGACACGCGACCTATGAG

>c1672_g1_i1 len=220 path=[198:0-219]

AGTTAAAACGAATCCAGCATCTTTTCTAAATTTTAAAGAAAAATAGGAGTGAGCAAAAAC

GCCTTAGAAATTTAACTTTTATTGTTGAGGACTAATTGCACATTAGCAAAGCAAGAAAAT

ATTAAGCTATCAGATAAGGAAAAAGTAGTCTGCAGAATACTTGGTCCATGTAGCTCTTTT

GAAACCCTCATTTGACCAAAAACTTGCCAAGAAAAAAATG

>c1677_g1_i1 len=201 path=[333:0-200]

ATATCTGATGTTTCTAGAACTGTTAAATGCTCTGCATTTCTACATTGAAGACTTTGTAAG

AAGATACACAAAGACCTGAGGTATTGCTGTAAATGTCACAAAATACATCCAGATTTATTT

CAGCATGGAAAAGGTGATGGTCACTGTCTGCACCGTAACTCTGTGTGTGCTGAGCTTCTG

AAGCCTAAATGTGACTGTCAG

>c1683_g1_i1 len=238 path=[1:0-237]

GTAAAAGGTAGATGTTAGCTGAAGTGAGGTGGATACAGAGAATGATAGTACAGTCATCAT

TACTTACATTTGCAGTATGATGAACCAGTATGTCTCAAAGCAATTGATATTATATAGCAT

TTATATGAGCTTATTAAATAGCTGTTGCACTTAATTGGAAATCTATGAGTTTTTTTTGTG

GTGACTGTTGATGCGCATCTTTAATTTACAGCTTCATAATGCTTCAGTACAAACTGGA

>c1689_g1_i1 len=252 path=[639:0-13 641:14-251]

AAATTATCAAATAATTAGGAAGCAATTACAAAAAAAAAATCCAGAAAATCAGACATACAT

GGCAGATCTGTTTTGTTCTTTTAAATCTAGTAGATTAGGTGTACTAGATCTGCTTCACAT

TGAGACCGTTCTGAAAATGGAAACTTAAAACTTGCAAATGACACTCCTCCTTATTTAACA

ATCTACAAATGTATTTGGTTTTCTATACTACAGAAAAGAGCAGCTGTATGACAAAATACT

AGGATGAAATGA

>c1689_g1_i2 len=316 path=[13:0-77 641:78-315]

AAGCAGAGTGCCTAGAAAAGAAATGCAAATGAATTACTAAGCAAGATTAGGTAGTGACAG

ACTCTACTCATAGACAAATTAGGAAGCAATTACAAAAAAAAAATCCAGAAAATCAGACAT

ACATGGCAGATCTGTTTTGTTCTTTTAAATCTAGTAGATTAGGTGTACTAGATCTGCTTC

ACATTGAGACCGTTCTGAAAATGGAAACTTAAAACTTGCAAATGACACTCCTCCTTATTT

AACAATCTACAAATGTATTTGGTTTTCTATACTACAGAAAAGAGCAGCTGTATGACAAAA

TACTAGGATGAAATGA

>c1707_g1_i1 len=249 path=[227:0-248]

CATTGCTCTCCCTCTGCCTGCGCCCTTTGTGCTTTAGGGAGGGGCAGAATCTCCCAGAGG

AGTCACTGGCAGCGCACGCAGGGAATGGATGATGCTTTGGTTATGGTGCAGTTCTGCTCG

TTGCAGTGCTCCCCTGCTGAACGCAGCAGAGGCGGCTGTCGGGTTTCACAGTTCTGGAGG

TCTGCCCAGTAAGCTGACCAGGGAGAATGCACTCATTTACTTATGTTAACTCTCAGAAGC

CCTCACCAG

>c1712_g1_i1 len=203 path=[181:0-202]

GGAACGTGTGCTGACTCAGCAGTGCAATGACTGCACTGAGATACCCAATGGCCTGTGATG

GTACGACACTGGCATCATTCATTCAGTGCAGTGTTCTCCTGGACTTGGTGAGGGACACAC

TGGTCTCACATGGCAGTAACATCTGAAGGACCAGTTATTACAAACAGTGTTTTGAACCAG

AAGATGATGAGCACAGCCTCCTG

>c1720_g1_i1 len=534 path=[512:0-361 874:362-533]

AACATCTCTTAAAAATATAATAAAAAAATTAATAAAAATAATTTTAAAGAAAAAATAGTA

AAACTATTTTTTTATATTTTTGGAGTTAAATTTTTTAATTTATTTTAAATAATAATCTAC

TTAATTTTAAATTTTTTTGTAAAATTTATAATTCCTTTTTTTTTCTTTATTAGATTTACA

AATAAACAATAAAAAAATCCTACATTAAATATTTCATTATATGCGCAGAAATTTTTTTTT

CATTTTTCTCAGTTTATTTTTCTTTTAGCCTTAAACTGTGAAAATGTAAATTTTGTAAAT

CATACAACTTAAATATGTAAAAACATATTTTTATCAATTTGTAAGACCATATATTACTTT

TTTTAATTATTAATTAAAATTATAAAAGTTCACATAATTTTAAGACAGAAAAAACGCCAA

TGTAATTAAATTATTATTATGTATACTTCTGATTTTTGTTTATTAGGTAATTTTTATGTC

TTACTATTCATTAAAAACAAATTATATATAACTTAAAAATACTTTAAAAAAAAT

>c1720_g1_i2 len=474 path=[512:0-361 1046:362-473]

AACATCTCTTAAAAATATAATAAAAAAATTAATAAAAATAATTTTAAAGAAAAAATAGTA

AAACTATTTTTTTATATTTTTGGAGTTAAATTTTTTAATTTATTTTAAATAATAATCTAC

TTAATTTTAAATTTTTTTGTAAAATTTATAATTCCTTTTTTTTTCTTTATTAGATTTACA

AATAAACAATAAAAAAATCCTACATTAAATATTTCATTATATGCGCAGAAATTTTTTTTT

CATTTTTCTCAGTTTATTTTTCTTTTAGCCTTAAACTGTGAAAATGTAAATTTTGTAAAT

CATACAACTTAAATATGTAAAAACATATTTTTATCAATTTGTAAGACCATATATTACTTT

TTATACTTTAATGTAATAATTTAGTATGACAACTCTTAAAATTTTAAAATTAATAAACAT

GTTCTTTTAATTAAAATATAGCTTAAAATTTAAATAACTTTACTAGTTACTTAT

>c1727_g1_i1 len=251 path=[521:0-250]

GCGGCTTGCTTTTCACTGCTGGTTTCCTGGAAGATAAGCTAGCTGCCCTTAGAGCTCCTT

TTCTTTGCAGTAAAGGAAGAATATCAGGACAGCACATCCCTGCAGAAGAGCTGGCTTGTG

TGCACCCATCTGACAGCAGTGCACGTGGTCTTGTTAGTGTTTAGAGTCCTTCACCTTTTC

CTGTTGTCTTTCTAAAATAGTTTTATGATCTCGGTGCCTCATACTCTTTGGAGCTGTATT

AGCCTCATACT

>c1729_g1_i1 len=316 path=[331:0-199 19:200-315]

AAAAAAAACTATAATTTAAAAAAAAATATTATTGTCTATATGCTAATCACTATTTTTCTG

TCTTTTTATAAAATACGTAAATTTTTTCTAAAGTAGAAATAATAAATTATTTAATATTCT

TAAAAAGAAACTATAAAAAACTCTTTGTGTAATTATATGATGGTGTATATACAGAATGGA

TATATAAATAAAGTAAATAAAAATATTCCAATTTATGCACTGATAATATTTTTCCAGTAA

AATTCTCAGTTGATTTAACTTAATGTTATTTATATATTTTTTTCATTATATTTTCCTCAG

TTTAAATATAATATAA

>c1732_g1_i1 len=264 path=[5:0-263]

CCTTCAGATAAACAGAACTGATATTTAATGATCTAAAAAGAATTTCACACTGATTGAGAA

ATGACCACTACATCTTCTTAAAGTTTTAGTCTTTTACTAACCTCAGGTCTGCATGAAGCA

CTAACACTGGTTCTGGGATTAAGGTAACTAATGGTAGAGACAGTTGTTGAATGAGTAACA

CTTTATTGTATTGACCTTCGTTTCTCAGCATGAACAAGTAGTACTGAATGGCAGTGGTGT

GTTTTGTACCCTGTACTGTCTTTC

>c1735_g1_i1 len=229 path=[207:0-154 362:155-228]

TCATCATCTTCCTGCATTTCTTTGGTATAGCCACTGGCAGCAATCTCTATGTCAAGAGAA

CTCTCTCTTCTGATGTCTTTGAATTCTGGGAAGAGCTCACTCTCATAGTTGAAGCGTTTC

TTAAAGAACTCCTTAATACAGTTAACATCTCTGTCAAAACTTGCCAATCACGGTTTGATT

CATGGGGATTTCAATGAATTCAATCTCATATTGGATAACGATGACCATG

>c1741_g1_i1 len=314 path=[306:0-279 664:280-281 632:282-313]

TTTTTTGCAGTTCCAGTGGCTTGGGGTTTCTCCCCAGAGCTAGAGATTACATTACAAATT

TTCTCATATCTCTCAGCAAGAGACATCAATAGGCTGCTATATTTTGTAGCACAACAGCTC

CATCTTTCAAAGAACTTTAAATATTATTCCAGTGGGGCACAGTCACTACAGGAAAAGGTA

GGATACTGTACAGAGGTATTGTCACCTAAGTTCACAGTTTTGCAACCAAGTGTTTACAGA

AACTCATTATTCATCAATGCTCTAGGTAAATTCTTGAGGCTGGGCTGAATGAAAATTGTA

ATGCTTTTTAGTAG

>c1741_g1_i2 len=304 path=[306:0-279 664:280-281 665:282-303]

TTTTTTGCAGTTCCAGTGGCTTGGGGTTTCTCCCCAGAGCTAGAGATTACATTACAAATT

TTCTCATATCTCTCAGCAAGAGACATCAATAGGCTGCTATATTTTGTAGCACAACAGCTC

CATCTTTCAAAGAACTTTAAATATTATTCCAGTGGGGCACAGTCACTACAGGAAAAGGTA

GGATACTGTACAGAGGTATTGTCACCTAAGTTCACAGTTTTGCAACCAAGTGTTTACAGA

AACTCATTATTCATCAATGCTCTAGGTAAATTCTTGAGGCTGTTTTGCTTCCCCCTCCTC

CACC

>c1753_g1_i1 len=203 path=[478:0-20 70:21-202]

GCTGCCCGAAGTGCCCCGCGGAGCAGCAGTCCTCCGTGCTCACCGAAGGGAAGCTCCGAC

AGAAAGCTTACTGTTTGCATCGGTCTGAATGCAGTCATCGTGCTTCTGAATAAAGTTACT

TTTTGAGATGGAAGTTTTGAGAGAATCCGAAATAGGAACATCGCCGCCAAAATGGTGCTG

GCCTCTCATTGATCGTTCTCCGG

>c1753_g1_i2 len=251 path=[1:0-68 70:69-250]

CCCTTGGCCCCGCCCTCGCCCGCCGCGTGCAAGCCGTGCGGGTGCGCTCCCGTGGCCGTG

CGGGTGCGCAGCAGCAGTCCTCCGTGCTCACCGAAGGGAAGCTCCGACAGAAAGCTTACT

GTTTGCATCGGTCTGAATGCAGTCATCGTGCTTCTGAATAAAGTTACTTTTTGAGATGGA

AGTTTTGAGAGAATCCGAAATAGGAACATCGCCGCCAAAATGGTGCTGGCCTCTCATTGA

TCGTTCTCCGG

>c1755_g1_i1 len=214 path=[433:0-69 22:70-93 46:94-213]

TGGCCTGGCACATCAGAACCACAAGAATATAAGGCACTGGTGGACACCGGTGTGCAGTGC

ACTCTGATGTCCTTGAGTCATGAAGGGACAGAATTGATTAATATTTCTGGAGTGACTGGG

GGTTTCCAAGAATTGACAGTGCTGAAGGCTGAAATAAGCCTCAGTGGTAAAGACTGGCAA

AAGAACCCTGTTGTGACTGGCTCAGGGGCTCCCT

>c1756_g1_i1 len=206 path=[26:0-173 326:174-205]

AAAATGGGTGAAATTTGGGGAAAAACAGGAGTTATGAACCGAAACTTGGGTAAAATGGGT

GGATACGGCCTGAAATTTGGGGGAAATGGGTGGATTCGGCCCGAAATTTGGGGAAAATGG

GTAAATGTGAGCCGAAATTTGGGGAAAATGGGTGGATATGGCCCGAAATTTGGGGAAAAT

GGATGGATATGGCCCAAAATTTGGGG

>c1756_g1_i2 len=349 path=[26:0-173 25:174-174 26:175-348]

AAAATGGGTGAAATTTGGGGAAAAACAGGAGTTATGAACCGAAACTTGGGTAAAATGGGT

GGATACGGCCTGAAATTTGGGGGAAATGGGTGGATTCGGCCCGAAATTTGGGGAAAATGG

GTAAATGTGAGCCGAAATTTGGGGAAAATGGGTGGATATGGCCCGAAATTTGGGTAAAAT

GGGTGAAATTTGGGGAAAAACAGGAGTTATGAACCGAAACTTGGGTAAAATGGGTGGATA

CGGCCTGAAATTTGGGGGAAATGGGTGGATTCGGCCCGAAATTTGGGGAAAATGGGTAAA

TGTGAGCCGAAATTTGGGGAAAATGGGTGGATATGGCCCGAAATTTGGG

>c1760_g1_i1 len=226 path=[2:0-138 141:139-225]

TTTTTGTTTGTTTGTTTTTAATGAATACGGATTCCTTGTATGATCTTTTAAAGCAAATGA

CTCTTCTTCTCCTCTTGGCTTATACAAAATTATCTGCACAACCTCATCCTTAATACTCAT

AGCCACCCATGCTCATGTCTTTTCCTTTTCTTCTCTGCCACCTTTATCTGCCATATCTCC

CTAGTTCTCTTCCCTCTCAAACACAAAGAGAAACAAAAAGAAAACA

>c1767_g1_i1 len=297 path=[174:0-173 456:174-296]

ATCACTGACTACTTAAAATTACAGCACATTTTTGCTACTTGGGCTAAAGATACCATCACT

GGGTATCTACCTGGGCAAATGGTAGAGAAAATTCCTAACTAAATTGTAAAATCACTTTCA

AAGCAGAGGAAAAAATAAAGCATTTTCCAAGCTTTTCTCTGAAGTGCTTTGGATTGGGGG

AGCGTTATCTGTCTAAGTACATGCATCTGCCTTTCTTCTACCTATTTTCTTCCCCCCAAA

TCCATCCGAGTTGTAAGAATTTAAAACATCACATGCACAGAAAATAGAAGTAAAAAG

>c1779_g1_i1 len=205 path=[1:0-204]

AGGGGTGAGGGGCTCCATCACCACAGGGCGCAATAATGAAGTCTGTAGAGTGTCTTAAAG

GTTTCTGAAGATGACAGTGGGCTCACAGAAAGTGAATCCAGAAAGTAGTGATTCTGTGTT

AGCTTCTACCAGCTGCTTCTATCTATGCCAGAAGAAAGATTTTTCAATTAACCTAGCCAT

CCTGCTTTTGCGTCTCAGGTGCAGT

>c1787_g1_i1 len=209 path=[419:0-208]

ATTTATTTAATACAAATAAATTAAATATGCACTTTAAAAAAAAAATCAATATCTATTAAA

GGTATAATATTCTCATAATATAAAAAATTACATATCCAGTATATCTGATATAGAATCATT

TAAATGATGCATTATTTAATCTAAGTAAATATAAATGTAATAAATACTTTAAGGGAGAAT

ATGATTATATAATTCTTGTCTATTAGACA

>c1787_g2_i1 len=206 path=[210:0-205]

AAAGAAAAAAAAGAAAACGAAACAGTTTATTTTTTAAACATATAGAAAAATTTTATTAGT

ATAACAAGAAATATGAATCTTATAAAAATATTTATAATAAAAATCATAAAACAAATATTT

GTAAAAACATTATATTCTAATTTATCAACTAATAAATTAAATAAATAAAAGAGTTTATAT

TTTTAAAATATTTACGTATAATCTAA

>c1790_g1_i1 len=290 path=[268:0-124 576:125-289]

AAATAATCACCTTCAAGAAGACACAGCACTGAGATAAACATATTATCTCCATGGTTCCGT

TCCCATCCTGATTTTTTTAGAGTATTTTCAGAAGGAGCCAAGACAGCACAGTGCAGGGGG

AGGGAGATGCCAGGCGATGTAACTTCTCAGTTTGATCCTGATATTACAAGCTGGATGCAA

ACCTTCCCAGAAATTTGAGCAAGCAGTTCTCAGCAGCGTTGAAGTACTGGCACTGAAGGT

GGCTGCAAACACAGCATTTCATGCTTTCATGGAATGGCTTGGGTTGGAAG

>c1793_g1_i1 len=300 path=[278:0-45 324:46-299]

ACTACAGAACCCTTCCTGTTCTTTTCACTTTGCGCTATGATGAACTACAATGAGAACACA

CGCACACATGCAAACACATAGTTACAACACCATAGTAGAAGGGAGAGAACTTACCATAAA

CCAGGTTTTCACCTTATTATATCCTGAGACACACGGTCGGTCTTAATTACCATTTCTAAC

CTTCAAACACGATACTCACACACAAAGCCAAGTTTATAGTACAAACAGCGTGTGTATATA

TAGAAGTACATGCGCACCTGTCCTCCTTAGAACTTTGAAAGCATAAGCCAATTTTAACCA

>c1793_g1_i2 len=275 path=[576:0-20 324:21-274]

ATTAGACATCAGAGAATCCACACAATGAGAACACACGCACACATGCAAACACATAGTTAC

AACACCATAGTAGAAGGGAGAGAACTTACCATAAACCAGGTTTTCACCTTATTATATCCT

GAGACACACGGTCGGTCTTAATTACCATTTCTAACCTTCAAACACGATACTCACACACAA

AGCCAAGTTTATAGTACAAACAGCGTGTGTATATATAGAAGTACATGCGCACCTGTCCTC

CTTAGAACTTTGAAAGCATAAGCCAATTTTAACCA

>c1796_g1_i1 len=270 path=[350:0-166 103:167-269]

CACAAAATGTTTAACAATGTAAGTGTTAATGTCCATAGGAAGCTCATATGTGCCAGAAAT

ACAGGGCACTGTTGAACAGAAGTACTCAGACACAGCCCTCAGAGCTGGAGAAGACGAGGG

AAACGGCTCTGCCAAAAACACACTGAGTGGAAAGAAAGTAAAAAGAAAAAATAGAGCCTG

AGTTATACTTTCTCCACAGTCCACTGGGATATTAGGTGCAGGAAGTGATTGCTGTAGACG

ATGGGAGGCACAGTAACTCCTGAGTTTGTA

>c1801_g1_i1 len=397 path=[73:0-249 72:250-396]

GTAACTTGTGTTTGAGGGGAAGAAGGTGTGGAGGGAGGAAAAGAAGAAAGGAAAAAAATA

AAATGCAAAGACATACCTGATCTTCGAACTAATCTGAAGGAAAATATGCGTTTGTTTTAG

CTCAGCATTTGTAAATTGAAGTTATTACAATTCAGGTGAAGAATTGATGTACTTCCATAT

TCTATGTCAAAAAATAAAAGAAGTGTAATCTTACTCTGCAGGCTAGCTTACCAAGTCAGG

AATTTGTGGCTGATGTTCTGAAGAACAAACTTATACTTCTGTGAAACATGGAGTCTTTAA

AAAAAATGATATGTAAATATTAAGCAAGTTGTTGAATTCTTTTTTGAGACTCCTAAGACA

AGACCATACCTGCTCATACTGTCAGATGGGAGAGAAA

>c1805_g1_i1 len=205 path=[1:0-204]

GGCATTTCACTGAAGGCTGCAATTTCCAGCTCCCCTCAGTGCTGTGCATTACTCATTATC

CCTGACCTTGAAACGGGCAGTGCTCCCTCACTGGCATGCAGCTTGGCCTGGTTGCAAGCT

ACTTCTCCTCCAAGCTGGGGCAGCACAGCTCATAGGGGCTGACACGTGGCCAGGCAGGAG

CAGCAGGGGAACGTGTGGTACCCAG

>c1809_g1_i1 len=201 path=[1:0-200]

AGTAGATAAAGTGACAGAAAGGGTAAGTCCTTAATGTGTCTACTGTTACCGGTCTTTCAA

ACATACCTTAACATTTTCATTACAAACAAAACCTTGTAAACTATCAGAATGTCCTTTTGC

AGAGGCATACGCAAGTTACATGGTTATAAACTGAATGGTGGGGTAGATTGCACCTTGAGT

AAATTCACAATGACACAGAAG

>c1809_g2_i1 len=350 path=[684:0-349]

GAAAGACAAGAATTCTAGGAAAGCTCTCTGTGTTAATGAATTCCACTATTAGCACCAACA

TCTACTAACAGGTAAAACATGAGTAACTTACTATTTCTGAATTATGTATCAAATAACTAC

GCTGAATAAAAAAATAACATTGACACCGAAGTGCTGGGGATGGCTAATTCACCAGAGGGA

CCTCAGTAGGTTGGAGACATGGGCTGACCTGCATCTCATGAAGTTCAGCAAGGGCAGTAC

GAAGTCCTGTACTAGGGGAGGAGCAGCCCTGAGCACCAGTACGTGGTGGGGGCCACCCAG

CCAGAAAGCAGCTTTGCAGGAAGGGGCCTGGGGTCCTGGTGGGCACCAAG

>c1811_g1_i1 len=365 path=[155:0-364]

GGCAGATGAGAGGGATCATTAAAGGCAACAAACGTGTTGTGAGAGGAATGTTTGAAGACA

CTGTCAGCAGGATTAGCCTGGTGATGCCAGCTGACCCCAGAATTAACATTACACAGGGAA

CTCCTTGGCTCTTGGAGGAGTTCCTCAGAAGTGGTGTACCAGGCCACACCATGGTGCATT

GCCTCCATCTAGCACTGTCTTCTCAAGGCCATGTGTTTAACCTGGTGAGACAGAGGGGTA

TGACTAGACCCATCTCAGGCTGATATGGCACGCTGACCAGGATCATCTGAGCTCATCAGT

AAGCAGCGATCTGAGCAATCAGTAAGCAGGCCAGCAGGCTTGACTTAGCATTGGCCATTC

TTATC

>c1819_g1_i1 len=231 path=[363:0-230]

AACTGGCTGCCCTCGTACTCTGCAGCTGGGATGGAACAACCACCTGCAACAGTTAAGGCA

GGGGACAGATCAGCAAGGCATCTTGGTGAATTTTATGCCTAAACATAAGATCACACACTT

TGGATTTATGCCTAGGCATAAGTCAGCAGCGTGCCTTGGCAGCGAGAAAGCACTACACAC

AAAGATGAAGATGACAGTTCAAAGTATATTGATGAATATACGTGATGATGA

>c1825_g1_i1 len=307 path=[1:0-306]

TGTTGTTTAGCTGCAGATGTGTCCAAGCAGAGTTCAGATGGATAGACAGAATGTTTTTAT

GTCCAATAGAACGTGCTATTCATACACCTCTTCCCTAAACCAGGATAACCATTTTACATC

CTATCACCATCCACACATGGGCATGCTCATGCTGAGCTCAGCAAAGCTGTACGGCTGAAG

CCTACAGAATACAGCCATCTGCTCCAGCTCACAAGTAAGGCACAGAGCATATAGCATCTT

AGTCATATTTCTCTCCTGGAAATGAGCTCTTAAAAACCACCACATTCTGTGCGCAGTTTG

GAAGAAA

>c1835_g1_i1 len=265 path=[217:0-154 22:155-264]

AAAAATACTTATGTAATTTTTATCTATAAAAATTTTTATTTTTTCTATGGCCAATAATAT

TAATTTTCTTTATATATGAAATATCATATATTCTAATTGAATTTTTTTTTTTTCTTTTCA

ATTTGAATATTCTATTAAGAATAATTATATAAAACTATGTTTTAACTTTACTTCTATTAT

TATATGTATTCATTTTTATGAATAAATTTAAAATAGTATAAATATTGTATATTTTATAAT

CAAAATTTTTTGTTTTTTTTTTTTT

>c1838_g1_i1 len=229 path=[1:0-228]

GAAGAAACAGTAGCGGGCAGGCACCAGCAATGACTGCTTCCAAGTTGAAAGTGGCATTGT

TAAGTGTGCCACTGTTACACATAGTCATGAAGTAATTTCCACTTTGTTTCTTCTTTTCTC

TTTTTGTTTCTTCTGTTTGTCTTGCAATGTCTGTACGTGTTACATTACTGTTATGTTACT

GGAAGAGAGTTAAGACATGACAATTTGCTGATGCAGGTATTTTATCTCT

>c1840_g1_i1 len=216 path=[1:0-215]

TATGCACACTGAAACTGATCTTTACCTTCTATGAACTACTGCATCCTTGAAACAGTATTA

ATTAGTACCAACCAGTCACTGAGGTACAAATTAACAGCCTGTCTTTTCAGCCTCTGCAGT

AACAAGACCAGCAGAGATTGTTTTTGCATAAATGTATGAGGGAAAGTATAAAATGGGTCA

TGTACTATATGTTTTCAGAGAATTATACTACTCCTG

>c1841_g1_i1 len=203 path=[1:0-202]

GAAAAAAAAAACAGGGAATAAGCACATGCCTTTACGTACCTTATCTTAGTCATCTCACTT

GTGCAGCAGGCAGGTCAGTGCATGAGGTGAAAAAGTTTGGTTACGTTTGCCTGAGAGTTT

ACAGATAATCGTTTAAAAAATACACAAACAACAAAAGCACAACAGGATGAACTAACAGAA

TATGAGTATATCATTTCCTATTT

>c1841_g2_i1 len=213 path=[551:0-212]

TACTTTTCTCCAAATAGTCTTCAAAATACACCAGACTGGTCCAAAAAAATACATATATTT

CTGTTGACTTTTAAGAAAGTTTTTCTCATATATTTAAAATACTGGAACAACAAAAACACA

GGTCTCTGGAAAACAGTGTTCTGAGAAGCAATCTTTGTAATTTAATGGTATACTCAAATT

CTGGATGAAAGAATCCCAGTCTACTATGAGGTA

>c1854_g1_i1 len=220 path=[221:0-195 25:196-219]

AAAGGAATAAATCTCCCACAAGTAAAGGCAAGAGGAGATATGGTTTCTAAATGAATGATT

CATTGTTGGAGGGCGCCGCTGTGATTTGCTGGAGTTGCTATGATACCTCAGCAGCAATGC

CAACTTGTAATAGTCCTCAACGCCTTCTGGAAGCTCAACAAGCCGAAAAGGCACTGCTGA

GGGCAATTATTTATTTTTAAACAGGACAAGTCTATTTACA

>c1854_g1_i2 len=220 path=[221:0-195 416:196-219]

AAAGGAATAAATCTCCCACAAGTAAAGGCAAGAGGAGATATGGTTTCTAAATGAATGATT

CATTGTTGGAGGGCGCCGCTGTGATTTGCTGGAGTTGCTATGATACCTCAGCAGCAATGC

CAACTTGTAATAGTCCTCAACGCCTTCTGGAAGCTCAACAAGCCGAAAAGGCACTGCTGA

GGGCAATTATTTATTTGTAAATAGACTTGTCCTGTTTAAA

>c1854_g2_i1 len=233 path=[439:0-232]

TGTGGGAGATTTATTCCTTTCAGGAGGAAAATTTATATCCACGAGGCAGACATCTGATAC

ACAATACCTAATAGAGTGTGTACAATGGCTTTGGAGGGATTGCTCGGGCATTGCCTTTAA

CCAAGACAGTAGTTTTAAAGAAATCTCAAAGCACTGGTAGCCACTTGGATGGCTGCCACT

TCTCAAAGCTGCTTCCCTTCAACCTCTGTATTTTGCCTGTTTCACTGGAAGGA

>c1857_g1_i1 len=239 path=[217:0-238]

TAAGGCTATTGATTACTCACAAAAAAACCACACAGGCTTATTCCAGTGTGAGATGTATTA

GTGTTTCCCAAAGACAAGACTGCATTTGGCAAAAACCATGTCTTGAAAATACAAAGGCAG

AAGAAACTGATTTTAAGCTATGGCTCTGTTTCTTCCCCAAATAATGTTCCTCAGAGTGTG

CCTCTGAAGCAGCCAGAGAGGTTAGGTTAGTTCTGACTTCTCTATCTTAACAAATACTG

>c1858_g1_i1 len=340 path=[318:0-153 472:154-339]

GTGCGGATTCTGATAACCACAGGTACACTAATTGCTGCTACTTGATGAGGAGGTAACACA

TACAAGATCTCCCTTCTGATTCAAGTAGCACAGGTTTATAATTACTTTTTTCTGGATACA

AAGTGTAATAGATGGCTGAGATATAGTGATATTCTACTGTCCCGTGTCGGAATGCTTACT

AGTATGTATTTTACACCATTCCCCCAACCTCAGCCCTCAAACAATGAGGGGTGAGGTACC

AGAATTGTACCAGAAGCAGCAGGCTTACCGTGCTGCATGCCCTGCTCTTGGGACATCCTG

GAGGAAGCAGAGTCACTGGGCTGCTGGCCGTCAGTCAAAC

>c1858_g1_i2 len=203 path=[652:0-16 472:17-202]

ACACTGAAGTTTATTTGTACTGTCCCGTGTCGGAATGCTTACTAGTATGTATTTTACACC

ATTCCCCCAACCTCAGCCCTCAAACAATGAGGGGTGAGGTACCAGAATTGTACCAGAAGC

AGCAGGCTTACCGTGCTGCATGCCCTGCTCTTGGGACATCCTGGAGGAAGCAGAGTCACT

GGGCTGCTGGCCGTCAGTCAAAC

>c1859_g1_i1 len=964 path=[1633:0-963]

ATATATTAAGTTGATAACCTTACTTAATAACTATGAAAATATTACTGAATCTAATAATAT

TTTAAATGCTCCCTATTTTAAAAATCCTAATTTAGTTTTATATAAATATTTTCAATTTTT

TAATGAGAAAAATGCAAAAAAGAAGCTATTAAATTTGTTAATGTTGCTGTATTCAAGAAA

TATAAGAGAGTACCAAGACCAAAAAAACTTTTTTTACAACTCATCTATATATGTTCATAA

TTTTGTTTTCAGAAAGTTTTTTTATTTTCACTCAAGTTTTTATTTTAAAATAAAAGAAAA

TAAAGAAACAGTAAATGTGATAAATAAATATAACTTATATACTGATTTCTTTTTGTATTC

ATTGGATTTTTATTTATTTCATTTATCTAAAAATTGTAACATTCTAAGCATTGAAAATTT

AAATATCCTATCAAATATATATTCAAATATAAATATAACTAGGGGATATGAATATCATTT

ACATTTAGATGATAATATAAATGGAAAATTTTATAAATACGATAAAGTAGATAAAGTTAC

TACCTATAAGGATAGTAACAATAATAAATACGAAACGTTTGAAGATAATACTGAAAAAAA

TATTGATACAAATAACCAGATTTCAACTGCGTTCGATAATAATTTAAGTAATAAAATATG

CTTATTATATATATATATTTCTAAGAGCATAATTTATCAAACCCATACATACATATTGAA

TAAAAGGAAAGTGTTTCAAAAAGAAATTTCCATTAAAGAAATTATGTTAAATAATGTTAA

TGCTTGTAAGAACATTTTAAATAAAATTTTAAATAAAAGCATAAAAGAGCAGTATATTAA

ATATAAAATTTTAAAAAAAAAACATAAAAAACTTTTTAATTAGTAAGTATATATAAAATC

TTCACATAAAAAAAATTTTACAAGTCTTAATAAATTATACAAATATATTATTAAAAAAAA

AGAA

>c1862_g1_i1 len=254 path=[232:0-253]

TGTGGTTTAGTTACAAATCCTCAATTTACCGAAGGCTTTCTAGGAATGTTATTTAGGACA

AGTGGCTGTTTTTCATCTGTAGCTCTTCTTTTGGAAATTGCAAGCATATAGGTGCTGTTC

AAGCATTTCTTTAGTTGGTGCCATGGAAAACACCATTGTTCCAAACAATTATTTCTTCAT

TTTTTCCTTTGCTTTTAGTGGGTTTGCTTCCAGTAACCTCAAATTCAGTGCAGAAGAAAA

GGAAGCATAAAGCA

>c1863_g1_i1 len=259 path=[272:0-258]

AAAAAAAAAAAAAAAAATACTTATGCTTTACAACAAAAATTTTCAATTATTCACTTTTAA

AGAATGAAAGCTTTTATTAAAAGTTCTTATAAAGATATCCTGTATTGTGAAATATTTTTT

ATAACTTTTCAAATTATTTTTTTTTCTACAATACTGTTAAAAGAAATTCATTTTTATTTA

AACATTTAAAAATTATTTTTAAATTATTGTTAATAATATAGTATTTAAGCATGATAATAT

ACATAAACATTAATATTTA

>c1864_g1_i1 len=241 path=[227:0-240]

CTGCCACCCTTCCTTGCTCTCAGCTAATTGCTTCTGTAGCCGCAGCGCTCTGTCCCGTTG

TGCTTCTGCCTCGGCCAGCTGCTTGCTCAGGCTGCACACCCGCACCTGCAGCTCCTCCTT

ATCCCGCTGGGCATCCTGCAGCAGCTGCCCAGCCTGGCGCACGTCGCCAGCTGTCTGCTC

GAGGATGGCGTCCACACTGGCCAGGCTGCGCACCCGCGCCTTGATCTCCTCGGCCAGGCG

G

>c1875_g1_i1 len=218 path=[195:0-217]

AGTTTGGTTTGTTTATTAACTTGTACCTGTCTACTCATTGGGCTCCAGGTCAGAGAATAA

GAGTTAAGCCAGTCCGACACTCATTCACAGCTAATTGCTCTTACTGTGGAGCACCACCAC

CTTGTCCAGACTGTAGTTCTCCCTTTTGGAAGACCTCAGCTACATCAGGATCCTTCTCTT

CCTGCCCAGTGGTGACACTTTCCTCTGCTCTGGCATGG

>c1879_g1_i1 len=261 path=[155:0-260]

AGGCAGAAATCAAACAAGATTCAACTGAAGCAATAGAAACCACAATGATTTACCCCAAGT

AATACTCTGGTCCAGAACCTCAACTTGCATGTATTAAACCTGAAAGTTAGGCTGTATTAC

ATACCACACTTCCATGTAATCAATAGCAAAAGGAGTGCATAACTTCCACAGAAGCAACTT

GCCACTCCAATACAAATGCCATGCCAACCACATGGTACACTGAAGACTGTCTGAAAATGT

GCCAACACCACTATTAAGAGT

>c1885_g1_i1 len=221 path=[262:0-134 397:135-178 440:179-220]

ATTTTTTTTTTTTTCTATTTTTGACATTTTAATAAACTTTGATTTGAAAACGGAGGCTCG

TGAGCAAACACTGATATAAAATGTATTTTTAGGACACAAACTGAAAGCGTTTGGAGTTTT

CCAACCACAGGTGCTACAGAGCTCTAACAGCAAGGGGCAACAGCCAAAAATACATCTTAG

GAGCTGAAGGTAGGAAGGCTTTTCACCTAGAAGCACAAAGG

>c1886_g1_i1 len=583 path=[892:0-232 3:233-582]

AATAATTACATTTCTTTAATTATCTTTATTATATTCACATTTCATTTCCCTTATTTTTAA

AAATAAAATAAGTAATTTCATTATCGAATAATAAAATATTAAAAAAATAGAAAATATATA

TTTTAAAGAATTTGTTTATGTTTTGGTTTATTTAGTATTTGCTTTCAATTTTGAAAACAA

AAGTATAATTATTTTTTCTTTCAAATTTGTACTATAAATAATCTATATTTTATTAAAAAT

TTTAAAATTTCATTTGGCTTTATGATATATAATTGTAATTTTTTTACAATATGTTTTCAT

TTGGCATTATTAATATTTGGTTACTATTTAAATATATAGTTTTATGTGTAATTATTTGTA

TTATATATCAATATTCCTTTTATTGCATTTATTAGAAATATATATTCCTTTTATATTGAA

ATGTATATAACTTGCTATAAAATCTATTGTCAAATATGAGATATAGTATATAAATTTCTA

ATTATAAATAACTAAATTATAATATTCTTCAACATTATATTATAAAATGACCTAAATATA

TACAATTATTTCTTATATATATATTATATAAATTATACTTTTA

>c1889_g1_i1 len=333 path=[535:0-332]

CGCAACCCGAAGCTGCACTCCGCCATGTTCATCTTCATCGCCAACCTCGCCTTCTCCGAC

CTGCTGGCCGGCCTGGCCTTCATGGCCAACGTCCTCCTCTCGGGCTCCGCCACCTTCAAC

CTCACCCCGGTGCAGTGGTTCGTCCGCGAGGGGACGGCCTTCGCCACGTTGGCCGCTTCG

GTCTTCAGCCTGTTGGCCATCGCCATCGAGCGCCACGTGGCCATCACCCGCGTGAAGGTG

TACGGCGGCGACAAAGGCTGCCGGATGGTGCTGCTGATCGGCGCCTGCTGGTTGGTGGCG

GCGGCCGTGGCCAGCCTGCCCATCATGGGCTGG

>c1894_g1_i1 len=340 path=[582:0-339]

ATAACATGAGTCCATGTAGTTCAGTGATGCTTCTCCAATAGGAGCCAGACATCCGAGAAA

CAGGTTGACAGATGTAGATGGCATTTTACAAAGCACTTTGAAGGAACTCAAAGGGGAAAC

AACTGCATTGTTAGCTGCAGCACGCAAACTCTTGTTCTAAATTGCCTCTGTGTTCAAGGC

TAAGAGAGCAGAGAGTGTAGGAGCACATTTTAAACATATTTTTTTAGGTACTGAGGAATT

ACAGATTTATAATCATGATGTCTGTTCCAGGACAATTACTAAATCAGAAGGTATAGTTCA

GACCGAAAAAATCAGCAACAGGCAAAATCAACTCATTAAG

>c1898_g1_i1 len=284 path=[13:0-159 173:160-283]

TTACAAGAGGAAATCAAACACAAAAGCCCTGTTCATCCTGAAACCATGGGATTTTGTAAC

CCCCTAGATGGCAATATGAAGGAATCTTCCAGCATTGCTTCTTTGCTGTGTGAAGAGGGA

GAAGAGGCACTATAGACTGAAACATGAGGGGCTTCAGCTGGCTGATCTGTGGGTGTTTGT

GGTACTCTGAAGTAAGAGCTGGGGTGCTGCAAAGTCTCTTCTGTCCTATGAATATGTAGT

AGGTGCTTGGCTGTTGATGGCTCCCCAGCTCAGCATGTTGGCTG

>c1900_g1_i1 len=211 path=[300:0-144 112:145-210]

TCAGAATTCCAGGATTACCTTCAAATCCTGACTCGTACGTTAACAGATGTTCTTTCTCTA

TACCTTCTGGTTTCCAGACCTCCAACACACTCAAGTTCCAAAATGGCAAAATAATAAAAA

AAAACTCTATAATAACAGCATGAACACTTTTAAATTTCTCTCAAAAAAGAATAAAGGAAA

AAAAATGAAACTTCAGTACTGGAAGACAATA

>c1901_g1_i1 len=553 path=[531:0-552]

AAAATTAAAAAAAAAAAAAAGGTATTTTAAGAAAAGTTCTGATAAAATAATTCAAAATAA

CAAAAATGAAAAGAATTTTTCTCATAATAAAAAAGAATATCATACTATTAAAAATTCTCT

TTTACAAAGGAGCATAAATAAGGATGTTGATAAAAAATTTAAACTAAATAATTTAAAAAA

ACATACATTTTTAATATTTAATAAAAATTATGCAAAAAAGAATAATGCAAACATAAATGG

AAAAAAAAATATTGTTATTAATGAAAATATAATTTTTGATAAAAAAGAAAATGTTATAGA

AAAATATAATTTTAATATAAAAAATAATAATGAAATAGAATATATTTTAAGTTATATGTG

TAATAATACTAATTCTCAATTAAATTACAATACAAAGAGTTTAATTAATTTTATTAAATT

TGAAAATGTAAAAACCAACTTAATAACTATGAAATTTTTTAATATACTTATGAGAAAACA

TTTGTATGGTTATATAAATAATGAAAGTAATAATTATCAGAATAATAACAATAATAATAA

TATTGATAGTAGT

>c1903_g1_i1 len=340 path=[308:0-49 358:50-277 742:278-289 754:290-339]

TGTCCAGAGAGGACAAATCCCAACTTCTCATGTTAGAATCTGCTTCAGGCCAAGAACATT

GTTTGCGACTCACGTGGTTGCTTATGCTGTTGTGCAAACAGCATGTAAATGAATCGAAAT

GTCTTATCCATAATGAAAATATTTCATGCTTTTTTAATCTATTAGATATGGAAATATTTT

TTGAAACTGAAAATGCAGTCAGTAGAATTTAAATTGAAACGTAATACATGTAAAATATAT

TTTAGTTGATAATTTTGTAAAATGCACTTTTTTTGTGTCTCTTCCCTGGTTTCCATCTGT

AAACACTGAAATACAGATCTGGGAAACCAGGGAAGAGACA

>c1903_g1_i2 len=341 path=[407:0-50 358:51-278 742:279-290 754:291-340]

AGTTGGGATTTGTCCTCTCTGGACACAGCAGAGCCCATTCTATGCCCAGGGCAAGAACAT

TGTTTGCGACTCACGTGGTTGCTTATGCTGTTGTGCAAACAGCATGTAAATGAATCGAAA

TGTCTTATCCATAATGAAAATATTTCATGCTTTTTTAATCTATTAGATATGGAAATATTT

TTTGAAACTGAAAATGCAGTCAGTAGAATTTAAATTGAAACGTAATACATGTAAAATATA

TTTTAGTTGATAATTTTGTAAAATGCACTTTTTTTGTGTCTCTTCCCTGGTTTCCATCTG

TAAACACTGAAATACAGATCTGGGAAACCAGGGAAGAGACA

>c1904_g1_i1 len=262 path=[240:0-261]

GCCTAGGCCTGAAGTCTAACTGCCAGGATAACCATGGCTAAAGATAATTCTGTGCTCACC

GCGAGCTACCAAAGCCAGGACACAGCAGCACCAGCAGTAGGAGACTCCCGCAGAGATGGC

GTCCGAGCACGAGCCCGCACTGCAGGTAGGAGCCCTGCACTGACTGCTTTCCTGCAGTTA

CTCATTTCCCTTACAGTACCAGGATTTCCAAAACAAGACGTGCACAAAGAACAGCTTCTC

AGAAATCAAACTTTCTAGTAGG

>c1907_g1_i1 len=276 path=[37:0-192 18:193-275]

CTAGACAAATAATATAATTTAAATATTTTATATGGAATTTAATTATCATAATTTCTTGTG

ACTAAAAAATATATAAGTCATGAAGTGAAGTTAAATTCTATAAAAATCTAAGAACATTTT

AAAGAACATAATATATGTCATCACGTTATAATTTATTATGTAAGTTTTATATTTGATAAA

ATAATTTTATTTCTTTGAGTGTTGAATAAAATTGATGTTAATAAAACTACATATTTTAGC

AAGTCTTTAATTTACCTTAAAATAATTCATCTAAAT

>c1910_g1_i1 len=262 path=[251:0-239 554:240-261]

TTTTGTTTGTTTGTTTGTTTTTAAAGTCCATGCAATCTGCCTCAAAACCTAGGCTGTCCT

AAAGCAGATGTCCTAAGATGTCCTGAAGCAATTACCACAACACAGTTAGCACATAAGAAA

TGTTAGTCCCACAGTTCAAAGGCCTGCCAGAATCTTCCCTTACTGCTTTTCCATCTTTGA

TTAATCAAATCACTTCTCTAGGAAATTCTCCCTCTCTGACTATGCCTGTTTCTTCATTTA

TTTGAGTTTGCAGAAGTTTATA

>c1910_g1_i2 len=263 path=[251:0-239 556:240-262]

TTTTGTTTGTTTGTTTGTTTTTAAAGTCCATGCAATCTGCCTCAAAACCTAGGCTGTCCT

AAAGCAGATGTCCTAAGATGTCCTGAAGCAATTACCACAACACAGTTAGCACATAAGAAA

TGTTAGTCCCACAGTTCAAAGGCCTGCCAGAATCTTCCCTTACTGCTTTTCCATCTTTGA

TTAATCAAATCACTTCTCTAGGAAATTCTCCCTCTCTGACTATGCCTGTTTCTTCATTTA

TAAACTTCTGCAAACTCAAATAA

>c1914_g1_i1 len=288 path=[266:0-287]

GATTCCCAGAACAGAAATAAATCCCAGGCTTGATAAAAAGACCAAGAAGAGTATAAATTC

TACCCACTGATATTAAGGAAAGATGAGGAGGTTAGGTCCTGCAGCTCACTGGATCTGCAG

TCAAGAGATGAAATCCCACAGTTAAAATTTGTTGGAGTTCAAGACATCATCAGGCACTGC

CAATTTACTACAGAGTTCTGTTCTTAATTTATTTAATAAAAACAACCACACCCTTTTTCC

ATTTGTGCATTAGTTAAGTAAAATCACTACCAAGAGGTTTGTGATCCT

>c1917_g1_i1 len=284 path=[420:0-283]

TGAATCTGTTCAGTACTCAAAACTTCTCCTGTAAACCCTCCTTCCCCCCCACTCCATCTC

AACGCCTGCTTCCTTCTCGATACTGTAAGCATAGAAGGTGAATAAAGATGGTCTCAAATC

GCTTTACCAAGTGGGAAGAGCTGCAAGATAACAGCTGTCTGCTGAGTCAAAATATATTGG

TGGAGAAGAGTTAAATCGGAGCTGGTAGCACAAATACCCCATATCGTTCATACCTGGGTC

TGCTACCTCCTCTCTCAGTGTAATCTTTAAGGTTGGTGGATAAG

>c1919_g1_i1 len=365 path=[1:0-47 49:48-364]

ATCAGCAAGTTTTTCCCAAGGAACTTCCACATGGCAGTCCAGACAGCAGGGCAGATCCTG

GAGGTTTGAAACCACATTCATGTGGTGATAATTTTTCTGCATCTTGGCTTATGTGGGTGG

GTGAGTGAGAGAGTGAGTGAGTGAGAAGTCGAATAGAGTTGGTACAGTGCTTGGTGTGAA

GGACGTGCTGCACCGCCTCCTGGCATCCACATCACAGTTGGCAGACCCACTGCATTCAGT

TCTGCTGGATTTCCAGTCAGCCAAGGCCTTCAAGTCGTGCCTAGAAAGGATTTGCATGCA

AAAAATGACCTGTGCAGGAGAAGTCACGCATTCAGCTGCCCAGTCTTCCGAGCACTCATC

CGTAG

>c1919_g1_i2 len=441 path=[685:0-123 49:124-440]

ATCCTACAGCTTTTTATGTCACTGCTGCTCAGTGAATGGTGCCACTGACCCTTGCAGACA

TGGTGAGCCCTCTGTACGGCGGAGCAGAAGTGACATAAAAAGCTGTAGGATTGGCTATAG

AGTCGGGCAGATCCTGGAGGTTTGAAACCACATTCATGTGGTGATAATTTTTCTGCATCT

TGGCTTATGTGGGTGGGTGAGTGAGAGAGTGAGTGAGTGAGAAGTCGAATAGAGTTGGTA

CAGTGCTTGGTGTGAAGGACGTGCTGCACCGCCTCCTGGCATCCACATCACAGTTGGCAG

ACCCACTGCATTCAGTTCTGCTGGATTTCCAGTCAGCCAAGGCCTTCAAGTCGTGCCTAG

AAAGGATTTGCATGCAAAAAATGACCTGTGCAGGAGAAGTCACGCATTCAGCTGCCCAGT

CTTCCGAGCACTCATCCGTAG

>c1921_g1_i1 len=289 path=[354:0-198 11:199-288]

AGTTTCATAGTTGCAGGTTCTTTTTGCAACACTGAAGAATCTTTGCCACAAGACTCAAGG

TTCTCATCTGGAAGTTTTAACTCCTGTGCTTCTTTCCTTTTACCTCTTCTTGGTCTTTGC

ATTTCCAAAGTTGAAACAGTTTCATCTTTTTTCTCATCATTATGTTTTGAAACGTTTTCT

TCTACCGCGCAAGCATTTGTTCTCCTTCCTCTTCCTAGATTTTTAGCTACTGAAGTTGAA

GTTCTGACCTCTCCCATCTCTTCTTGGAAGCTCTTTTTCTCCAGACCTT

>c1923_g1_i1 len=293 path=[41:0-152 331:153-292]

ATCAGACGTTGTTGGAGACTACAGACAGCACAGATCAGTGGTAGGAATTATATCTCAGCC

TGTAACTTCTGGGAGTCAGCTATATATTGCCCTTGGCAACACTAAGCTTCTGCTAATAGA

TGAGAAGAGACTCATGAGAGCGGAGGGTACTTAAAAATTAGAATTAATCATTAAAGTGTT

TTGGCTAAAGGCTGCATGCCTGAGTTTGTGCAGTAGATTGGTGCAGCCGGAGCACTGGTT

AACCAGAGCAGCCTTTCAGGCTTTATGTTTCATTCTCCAAATTGACCATCTCG

>c1928_g1_i1 len=254 path=[232:0-253]

GACAGTTTGCTGGGCGTCAGCATCCTGGGGGAGGGAAGATGAGCAGCAATTTTTCTGAAG

TCTGCAGCAGAAACAAACTGTACGTTCTGCTCCTCTCTGCTCTGCGCAGCCCTCTGTGGG

GCAGCCAGCTCTCTGCTGTTTACAAGCCCATCTGCTTCTGTTGCTTTTCTTCACCTGAAG

TTACAGCACTCCGTTCAGGATATTTCTCGCAGTTGTGGTGGGAACTGAAAAAGCAGGTTC

TTGGAGGAGCTTTG

>c1934_g1_i1 len=231 path=[78:0-75 154:76-230]

AGAAGCAGTCACCGTTGGTCTCCGGTAACAATGCTGCCTGCTGGCAGACTGATAGCTCTG

CCTGAGAGCTCAGCACGAACTCCATCTTTTTTTAGTTCACTCTTTTTGCTTGTTTCTGTA

GTCAGCTGCCTGCAGTAAGTGAAGATGCATCTATTTCATTGCATTCCTGTTCTTTTGGCA

ATTTTCCCCGGATTTTCATGTCTGTTCTATGAGATAGGATAGGTAATCTAG

>c1937_g1_i1 len=291 path=[1:0-290]

ACATCCATGTCTTGGATGTTAAAGGCATTTTGAAGACTTTTTAAAGTCTTCTGTTAAAGG

GATTTTGAAGACTTTTTCCCCTAATAAGCGCAGTGTTCTTGTATGTGAAAATTATAGTTA

GACCAGTTGCTATGGAGTTGTTTAGATTAAGTAAAGCAGCATTTATTAATAAAATGTGTA

TTCAACTAAAGTGGGAGGTATTTAAGATTGCCTGGATTGAGGATTAGCCTCATCTGTGAG

GGTGGAGGAGGAAGTTCTAAGTGTGCACAGCTGTGCAGGCACGCACACACA

>c1939_g1_i1 len=233 path=[211:0-145 357:146-232]

ACCAGCCAAGAGTTTTGGGAGGAGAGAAAAGCATAGCAGAATCTAGATTTGAATGGAGTG

ACATGCAGGTAGGCTACATGGTGTGCTATTGATAACTGCTTCAGGAGTCTGTGTTCTGTG

CTTATTTACTGGAGAGAGATAACCATTCTTATTTTACTTACTAGCAATTCCTCCCCGTGT

GTTTTGAGTCATTCATAGCTAGATTTAATGTCTCAGTGCTAACATATTCCACT

>c1941_g1_i1 len=234 path=[490:0-187 259:188-233]

ATTTTCCAGACAGAGATAATGATTTAAACTTTGAAGAAAAACCAGAAAAACTCTACAGGA

AAATTAACACTAAAAGGTACTGAAATACTCATTGCATAATTGCAGCTGCCAGGGGAATAA

TATGTTATTTTTTCCTTAAAATTGAAAGAATCAATACTAGAAAGCATTGTATTTCAAATT

ATCCCAATGGTACTAATGAGAAGTTTAACAGTGACGTAACATATATACATATTG

>c1949_g1_i1 len=208 path=[355:0-21 192:22-207]

TGCCCCAAGCCTGTAGCACTGCCTTGGGGCAGTGGTTGGAATACTGTGTAGAGGAAATGG

ACCTGGAGTCAACACCCAGCTGAACGTGAGCCAGCAGCATGCACAGGTGGCAAAGGCAGC

CAATGGCATCAGAAATAGTGTTGTCAGCAGGAACAGGGAAGCGATCACCCCTCTGTACTC

AGACTGGTGAGGCTGCATCTCCAGTACT

>c1959_g1_i1 len=344 path=[389:0-274 689:275-279 68:280-343]

CTGTGCTAGGCACTCGTTAACTGCCATTATCCTTAAGGGGGGAGGCAAGTACTTAGAATG

ACATAACACAACCAAAGCCTATTTTTTTTTTTTTACCCACTTAATTATTTCCTTCAGTCT

CCCTTTCTCACAACAAGAATACCTTTCTTTCTTTGTTGTTTTTTTCCTTTTTTGTTGTTT

TTTGTTTGTTTGTTTCCCCCCAAGCATTCCACTATTTTTATTACAAAGACTTCAAGAACC

AGGCTACTACATCACACTGCTTAGCGAAATGCTACTTTTTTTGAGAATTACTTTAATGAA

AAATAAAGGAACACTGCAATATATTGTTCTGAGTAAGGTTCTCT

>c1959_g1_i2 len=306 path=[389:0-274 689:275-279 668:280-305]

CTGTGCTAGGCACTCGTTAACTGCCATTATCCTTAAGGGGGGAGGCAAGTACTTAGAATG

ACATAACACAACCAAAGCCTATTTTTTTTTTTTTACCCACTTAATTATTTCCTTCAGTCT

CCCTTTCTCACAACAAGAATACCTTTCTTTCTTTGTTGTTTTTTTCCTTTTTTGTTGTTT

TTTGTTTGTTTGTTTCCCCCCAAGCATTCCACTATTTTTATTACAAAGACTTCAAGAACC

AGGCTACTACATCACACTGCTTAGCGAAATGCTACTTTTTAAGAGAACCTTACTCAGAAC

AATATA

>c1960_g1_i1 len=227 path=[161:0-158 320:159-226]

ATGCCTCTCTTTGTTTTTAATTCTTAACCCTGCAGTTTCACAGTAGGGACATCATCAGCT

TAATTTAATGATGTATTGGAGTCAACAAGTATGGTTATTGGGTTGGTTTGTCGTTTGCTT

TTTAAAACATTTATTACTGCGATCGTATTAAAACTAATGAGTTACATTTCCAGGGGTACA

GACACTGAAGTGAAATATCCCCTCACGAGCACACTAACTCATTAGTT

>c1963_g1_i1 len=232 path=[1:0-129 482:130-231]

ACATAAACCCATACCCTGTAGGCCAAGGACCATGTCCTCTGGTGTGTTTGTTCAGTGGTT

AGGTGTCTGCGCTACTACAGGACAAGCTGTATTGGAATACAGACTCTATAGCAACATTTA

CCCCATTCTTAAGACCACATTTTAGTATTAGGTTACAATTTAATTCCTATGGTTAAGCAT

GCATGAAAGGAAATTGCTTGTAAAGTTACAAACCTTTTCGTGCAGCGTTACA

>c1966_g1_i1 len=204 path=[35:0-110 34:111-203]

GAACTCTCAAGCGTAGGATGTGTGCTACAGTAACATGGCAGGACTTACTGACAGCCTGAG

GGCCTTTGTGGCCCAATGAAAGGAGTTTGGTAGTCAATCTTGCCACAGTTCATGAACATG

AAAGAAAAGTAATGTGACTTTGTGAAGAATCATAGAATGGTCTGGGTTCAAACTCGACCA

GTGGTCCTTTTCAACCCAGACCAT

>c1967_g1_i1 len=222 path=[29:0-120 28:121-221]

ATGGCAACTACTGAGAATAATCAAGTAATTAAAGAATCTTTAAAAAATATATTTTGTGAT

TTTATTCAAGATACATATGATTCTTAATACATTATTATACTTTTAAAATAAATGTGTAAT

TAATTTTTAATTTTGTACCTTTTATGTACCAAATTTTGATTTTATTCTTCATTTTTTATT

TTGGGAATTATTATATTTCCAATACCAGCTGAAGAGGAAAAA

>c1968_g1_i1 len=214 path=[35:0-213]

AAAAAAAAAAAAAAAAACTATTTTCGATTATGATAATTCATATATAAATATATATAATTT

TAAAAAGAAAAGAATAAAGAGTTAGTTTGCTATATATTATTAAAAACAAATATATATTTA

TATGAGTAAAATAGAGACAAATATTTATAATTTGAGATGAGAGATGATAATTGTTTATGA

ATATGAATAAAAGTTAATAAAATAATAAAAATTT

>c1969_g1_i1 len=238 path=[216:0-237]

AAATTTTATTTAATTATTTTAAAAGAGAAAAAATTAATTTAAAATGTAAGTTAAAAATAT

TAGAAAACATTTATTTTTCTTCAAAATTACATTTCATATTACCTCATGCAAATAATGATC

TGAATTATTCAAAGTTTTATTAAAATAATATCTACATAAAATAATAGTTGTAAATTAAAA

ATGGTCATTTAATTTTAAAAATTTAACATTTTTACATTACAAAATCTCCAAATTGATA

>c1971_g1_i1 len=221 path=[49:0-38 32:39-220]

TTTTTTTTTTTTTATTAAATTTTTATCTTCCTATTAAGTTAAATTTTTTTTGTTAACATG

AACGTATATTTCTTTTTAATATACATTAATTATTACTATTCTATTTATTTTTTTTTATAT

GATAAGATTTATTTTGAGATATTTTGACATAGGAACTTTTTTAAATAAAAAAATAATAAC

AAAATAAAATGTGTTACATATATATAAAAAAAAAAAAAAAA

>c1973_g1_i1 len=252 path=[267:0-251]

GATATAATTTGACTAATCTATGGAAAAGACGTTAAGAGAAGCCAGTTCACAGCTGTTGCT

TTAGATGAGTTGATTTCCACAGCCAAGCAATAAACAGGCCAGCACTGCTTGTGGAAAGCA

TTTAGCATAATGAAAAGCATCAGCATTAAGGGGTACCTCTCAGCTCCTCAAGGCTAAGTT

TTGAAATGTATACCTGCTGCTTTTTGATAGGGCGAAATGAAACATAGTATGGAAAAATAC

ACACCTGAAAGT

>c1977_g1_i1 len=280 path=[258:0-279]

ATGAAAGAACAATTTGATAATCAATTGATAATAATTAATACCTGCATCCCCCTTGACTGA

CCTAAAAGCATCATGAAGCAGAAGTGAAATCCAAGGACTTCCAGACCTAAACACCTTGAC

ATGCCACAGCACACACAGCAGCAAGCTTGTGGAGCTAGGCAGCAGGGAGAACCTATCCAG

AAGGACCCTCCACTACAGGGAGATGATGAGGAGATGCACTGGTTGCTGGTGTCAATGCAT

GAACAGAAGTAGTCCTGAGACTGCAACGCTGAGCTCAAGT

>c1979_g1_i1 len=452 path=[1:0-451]

GGACAGTTGTTGGAACCTCTAATGAATAGGTGCACAGAAAAGGTTTGATGTCTGTTTTAA

CAGGTGACTCCCTGGCAGCGTGTGTCCTCCATGCAGATGGCCCATTCCTTAGCTTGTCCC

TGGCAACTGGAAAGACAATATGGATGTAGATTGGACAGCCAGACTGTGAGTGTTCCTTGC

AGATAGCCAGCTGCCAAGTCTCAAAAAATTAAAGAAAACCCAGGCTGGAGTTGGAACAGC

AAACACTGACTTGGACAAGTCAGAGTTTCCACTCACAGTTCTTTAATCAGGCTCAGCATA

CAGGTGGATTGCAAGGTAAGGAATAACACATCATCAGCATGAGGTCTGGGTAATCCAGGA

CACAGTGACCATGATGTCCTGGTCAGTCCATTATCTCAGGCTTCTCTTGTTCAGTTCCTG

GCATGCTAGATCTGGACAACCACTTTTTCTTT

>c1981_g1_i1 len=233 path=[1:0-99 101:100-232]

AGTCTCTCAAAATTGGGCACTTAATACATTTTCTAATTTTGCTCTTCTGTGTTTGTTGTT

GGTTTTTCTTCCCTGCATTAACTCTTCTTCCTTTGGTCATCATGGTGATTTACTTTCAGA

ATACAGTGCGAGAAATAAGGTCAAATCCTTCTCTCAGCTCAGTTTGGACTTTTGAGAGCT

TAAGAGGCCCGGAGTGCCAACTGTGACTGCTGGGAGATTCAAGGTCACATCCT

>c1982_g1_i1 len=210 path=[1:0-50 52:51-209]

GTCACCAGGCAGAAGCTCCATTCTCTGGATACCATACTGTCCTCGAATGATCTTGTTCCA

GATGAGGACAGGGGTTGGGATGCCAATGACTTCACAGCTCAGGTAGATCTGTGCTCCTGT

CACATTCCAGATGTCCTTAGGAGGGGTCACGATGGAGGGTCCTTGCTCGCAGGCTCCCTT

GCTGCGCCGCCGGGCTGGAATGCGTGAAGA

>c1991_g1_i1 len=201 path=[155:0-200]

ATTGCACACATCAGAGATGCTGACAATGAAATAGATATTTTTTTAAGACTCTATAAGGAT

TTTGACAAATTCAGATTACTGGAAAGCAAAGGCTTTGGATGCTGGTTCCTCTAGAATTTA

AGCCCCAAAATCTCTATGGTCAAATCTCTTCACGGGTGCGTTTTCCTTACCGAAGTCCAG

GAGTTACTTGCAGGCAGGATA

>c1995_g1_i1 len=242 path=[379:0-49 21:50-241]

GAGGGATAACACTAACACTGGTCTAGAAGAACCCAGGAGATGATGTGATGTGGATTAGTG

ATCGATTGAAGATTTTCAGGTGAAAATGAGTCATTCGAGCTGTTCTCATGGCAAAACCAA

AATCACCTCTTGCTGCAAGCTGAATTTCTCATCTTGGAATCTTGGATCTTGGAAGAAAGG

GCTTATCCAGCAGCCCCCGAGCAAGCAGTGAACTTGACAACTGGAAGTAGAAAAGGGGCA

CA

>c1995_g1_i2 len=212 path=[1:0-19 21:20-211]

TTAATAAAAAAGAGAGCATCTGGATTAGTGATCGATTGAAGATTTTCAGGTGAAAATGAG

TCATTCGAGCTGTTCTCATGGCAAAACCAAAATCACCTCTTGCTGCAAGCTGAATTTCTC

ATCTTGGAATCTTGGATCTTGGAAGAAAGGGCTTATCCAGCAGCCCCCGAGCAAGCAGTG

AACTTGACAACTGGAAGTAGAAAAGGGGCACA

>c1999_g1_i1 len=225 path=[42:0-138 34:139-224]

TATAAATATATATATATGTGTTATACATAAAATTATAAATAAAAAAAGACCTTGAAAAAT

TAAAAATAAGATAAAATAAGAAATTGAAAAAGAAATTATACAATATTTATATTTACAAAT

TCATAAAAATTACAAATATATTCAAATAAGAAAAAATGATACATTGAAGAAAAAAAATGA

TTAAAATAAGTTGGATTAGTTATTCAGAATAATAATAATAATAAT

>c2001_g1_i1 len=244 path=[649:0-57 153:58-243]

AGCTAAAGCCTTGAATGTAAATACAAGGACAATAAAAGTACGTATATGGTGTAGATAAAA

TGGAGTATTTGATTTTTGCCCTTTATAAAATGGGAGTTCTTGTGTTTCAAGTCAGTTTCA

CTGTTGAGAATGGCGGACATTTTCTGTGCTCATTCCGTTCTGGCCAGCTGATTTCTGCTT

TGGTTTTGTCACCAAATAGCATTGTTTCCACGCCTTCTGAATCCCTTCCCTTAACTTCCA

GCTT

>c2001_g1_i2 len=302 path=[96:0-56 153:57-242 19:243-301]

TGTATTTACATTCAAGGCTTTAGCTTAAATATAGAAAGCTTTTGTTGCAGGCTTAACAAT

GGAGTATTTGATTTTTGCCCTTTATAAAATGGGAGTTCTTGTGTTTCAAGTCAGTTTCAC

TGTTGAGAATGGCGGACATTTTCTGTGCTCATTCCGTTCTGGCCAGCTGATTTCTGCTTT

GGTTTTGTCACCAAATAGCATTGTTTCCACGCCTTCTGAATCCCTTCCCTTAACTTCCAG

CTTTGTCTAATTATCTTTTTCTTCCATACGTCTTCCAGGTGTGTGTTGGGAAACACTACC

AT

>c2003_g1_i1 len=260 path=[159:0-158 531:159-259]

AGAGAACAGTGATCTGGAGAGTATGGCATGGTGCTGTTATTAAGGAGAGGAGGATGTCGG

TTTAGCATATATGTAACTGTATAGTTGGAAGTAGAGAGTATTATGCAGAACAAATCAAGT

TTAAGTAAACAACTCAATATAGAGAGCAAGTCAAAAGAGTTCTGTAGAAGATTGTCTTTG

ATATGGAATCATTGCCTCTGAGCACAACGTGCACTATGGCTGAAGTATGTCTGAGTGTCA

TAGAATCATAGAATCACCAA

>c2007_g1_i1 len=202 path=[1:0-201]

TTTTCAGCATACCTCAAGAAGAGAACCACTGAACTGACATAACTTAAGCAACACCAGGGC

ACAGGGATACCCACAGCACAATGCAGCACACTCACTGACTTATCATAAGCGAATGACATG

AGAATTGAGATAAAAATGTTACTTATTAGGTACCATACAGATACCTGCTAACACTGCAGG

ATTAACATTGGAGAGCAGATGG

>c2009_g1_i1 len=436 path=[414:0-246 889:247-435]

AATTGGCTGTACTCTCCCACAGAATGAACTGCTTTATGATATTTGACAAGGGGCGAGTAT

ATTCAAATGCTTCTCCTTCTGATTCCTGTAAGTTGAATTTTGGGCCAAAGTATTTTTGTG

TTGAATATTAAGGGAATCCATCATACCTTTGGAAGGAATGTATGAAAGTTATGTATGCAG

ATAAAGGATCCCACAAAACAAAATTTTAAAACCACATTAATTTGATACAATTTAAGCAGA

TGCTAAGAGGAATATTTCATTTAAAGATGGACTGTAGCCAGGAAGCATGTAGTCAAATGG

AGAGACTTAACAACGGATGTCCACTGCTAGAAGTGTCCTAGTTTTCTCATGAATCTGTTG

TTTTGCCTTTGCATTATTACAGCATTATGAGGTAAAAAGGGCCTGAGCCCTTATTGTGGT

CTCTGTGGGTCTTGTG

>c2012_g1_i1 len=344 path=[1:0-98 100:99-343]

ATAAACACACACAACTGCTCATGAAAAAGAAAGAAAAAAAGGAAATGAATTCAGATAATG

TTTTCCACACAACGAAGAGACTATTTGGAAAGATTGCCTTTACTTGACGGGAAAGCACTA

GCCTTTGCAACCAGAAGAACTTGTTACACAGGCTTTAAACACAGTAGGTGGTAATAGGGA

ACTTCCCTTTAAAATGAGCTGTGGCACACATTGAACATTTTAAACACAAATAAAGAAGAT

ACATATTTGGTAATAAAAAACAAACGTTTTAAATGCTGCCTTATTTAAGGTTCCGAATCG

AAAGAAAGTATTCCTTGATGCATTGTCATGTGATCACTAAAATA

>c2012_g1_i2 len=266 path=[643:0-20 100:21-265]

CCTAAACTTTGTGTTTGAGCCTTACTTGACGGGAAAGCACTAGCCTTTGCAACCAGAAGA

ACTTGTTACACAGGCTTTAAACACAGTAGGTGGTAATAGGGAACTTCCCTTTAAAATGAG

CTGTGGCACACATTGAACATTTTAAACACAAATAAAGAAGATACATATTTGGTAATAAAA

AACAAACGTTTTAAATGCTGCCTTATTTAAGGTTCCGAATCGAAAGAAAGTATTCCTTGA

TGCATTGTCATGTGATCACTAAAATA

>c2014_g1_i1 len=245 path=[502:0-54 57:55-244]

GTAGAGCCATCACTCGTAGTGTTACAACTTCAATAAGCTGATGTGCTTCTGAAACAAACA

AATTAATTCTTAGAGACTGTATAATTTTTGCAGAAATACAATATGTAATAAAGTTACGCT

TCTTCAAAGAGGTAGTTCTGGAATATTACAAATATTAAAAAGTGCTGCAACTTGATTGCC

CTACCTGAAAATCAGTGAAAACTATATAGGAATATATAATAAGGAGAAAATCCAGGGAAA

GTTAT

>c2014_g1_i2 len=246 path=[1:0-55 57:56-245]

AGTAGATGTAGTTTGAAAGCTTGCAACTTCTAGTTTATTAGATCTATATCTTTATAAAAC

AAATTAATTCTTAGAGACTGTATAATTTTTGCAGAAATACAATATGTAATAAAGTTACGC

TTCTTCAAAGAGGTAGTTCTGGAATATTACAAATATTAAAAAGTGCTGCAACTTGATTGC

CCTACCTGAAAATCAGTGAAAACTATATAGGAATATATAATAAGGAGAAAATCCAGGGAA

AGTTAT

>c2017_g1_i1 len=299 path=[641:0-45 140:46-105 48:106-298]

GGAGAGTTATACTGTATTAAGGAAATGAAGGTTTTTGCTTGTTTGATTTTATTTATTTTT

AACATTTTGGCAAAGTAGGATGCTTGAAGTAGTTCCAAAAGACAAAGAAACTGTGCTCTG

GTATTCTTGTATTTAATTGCAATAGAAACAGCGAGGCACCAGGGAGCTTTGATCAGAGGA

GTGTGGGGAGAGGGAAGGGTCACCTTGCAAGGTTTAAGTTGGACTTCTAGAAAGTTAAGT

GATGTATCCAAATGCAGGGAATATGTCATGTCTGTCCATGTCCTGTGACCGAGTTAGAT

>c2017_g1_i2 len=216 path=[25:0-22 48:23-215]

GGCAAAGTATCCAAGAAAAAAAGGAAACTGTGCTCTGGTATTCTTGTATTTAATTGCAAT

AGAAACAGCGAGGCACCAGGGAGCTTTGATCAGAGGAGTGTGGGGAGAGGGAAGGGTCAC

CTTGCAAGGTTTAAGTTGGACTTCTAGAAAGTTAAGTGATGTATCCAAATGCAGGGAATA

TGTCATGTCTGTCCATGTCCTGTGACCGAGTTAGAT

>c2017_g1_i3 len=299 path=[94:0-45 140:46-105 48:106-298]

TTTCCTTAATACAGTATAACTCTCCTCTGTCAGCAAGTGCTGTTTTTTTTATTTATTTTT

AACATTTTGGCAAAGTAGGATGCTTGAAGTAGTTCCAAAAGACAAAGAAACTGTGCTCTG

GTATTCTTGTATTTAATTGCAATAGAAACAGCGAGGCACCAGGGAGCTTTGATCAGAGGA

GTGTGGGGAGAGGGAAGGGTCACCTTGCAAGGTTTAAGTTGGACTTCTAGAAAGTTAAGT

GATGTATCCAAATGCAGGGAATATGTCATGTCTGTCCATGTCCTGTGACCGAGTTAGAT

>c2022_g1_i1 len=267 path=[155:0-266]

AGCATAAAGAGTTCGAATGGGTTGTTTCCATTTCTCCTCTTTATTAATTCAGCTGCTTCT

GTTCAGTCACTGCAAAGAGCAGATCACAGCTTAGGCATTATTTCAAAGGGTATCCCTTTT

GTAAATGATCAGATCTGTGTAAATGCATGCAATGATATTGCAAATTGTTGCTACGTTTTT

AAAACCAATAAGTTCCAATTATGTTTTCTTCATTATCATCTTTATAGGTTAGCTAATCAG

CACTTAATTATTATTGAAAATGCTGTG

>c2026_g1_i1 len=270 path=[389:0-109 3:110-269]

ATTGTCAGTCAGAACAGATAAGATAAAATATACCCGCTCAGTGGTATATTTTGCTTTGGT

TTATTCTGGTCAGAAGGACCTTCTCAACTCAGAATGGGAGGATTACTCATTTCTGACTGA

CAATGAGGAAGACTTCCTCTTTTCTCTTTTGGTGCCAAGATCCCTCACCTCTGTTTCTGT

GTGTGTGGAGGGAAGGGAATCCATTCCAATAGGTGTTTCTACACATCTGCTTTCATGTGC

AGTAATGTTCTGACCTCTTTTAAAAACAAG

>c2027_g1_i1 len=640 path=[37:0-579 672:580-639]

GCTGGGGTGGGTGAGCACTGTGATCCCAGTGCGCCAAGAGCAGTGCGTGGGGTTGGGCTG

CAAACACATCGCCCTGGGTGTGCTGGCTGTGATGGGGAAGGGATGTTCCATGCGCACGGA

GCCCTCCCCAGAGCCCCAAAGCTCTGACGTGGGTCAGCCCATGGACTCTGTCACCAACTT

GGAACTATGCTGAGCCCCAAAGCCAGCAGTTCTATGGCCAAAGCTGCAGCTGTGGGAGCT

GCAGAGGAGCAGTGCCGGTGCAGCAGCCCACAGCACAGCTGTGTGCCGTCACACATTGCG

TCCGCCAGGCCGTCATCGAGCAGAGAGCGAAACCAAAGGGCTGGGAAGGGATGTGTGCAG

CGAGGAGCTGTGCTGGAGGGCTGTGCTTTGGGATGTGCCATTGCTCCAGAAGGGTTTTGT

TGCTGTCCCGGAGCTGGAATGCTCTCCTTGCTCCGGCAGGCAGTGTGGGCTCAGGGGATG

AATTGCTCCTCCAAGGGCTGCAGTTCTCTCCTATGAGCACAGAGCCTGCGTGGCATTGCC

GTGGCATCCATAGGACGGCGCCGTGAGGGCTTTGGGGAGCAGCTGTTGGCTCCTTCCGAC

CTTAATGGAGAGGTGGAAAGGGTTTTGTTTTGTTTTGTTT

>c2030_g1_i1 len=209 path=[1:0-55 57:56-208]

AGCTGTGGCTCAAGAACTGCTCGAATTAGAGATGATATCATCAAGTTTATTGCCCATATC

AGGATGTGGCCAGCTGAGCATTTTTTCATATGGTTAACCTCTCAGAACCAACAAAAACTT

CTTCCCTATATTCATATACCCCTCTTGGTAGGCTACGTGCTTTGGTCAACAACTCCTGAC

TACTCTGAAAGCTATTCTTCAGATTTCTA

>c2031_g1_i1 len=215 path=[1:0-48 50:49-214]

GCTCTGAGGGTCAGACCAACTGTCTATGGTCATGGGATGGAGTGTGATGGTTCTCCCCTG

CCTCTCAGCCCGGCCCTCCTCCCATACCCCTCTTGGGTGATAGCCATCAGGATGGATGTG

TCTGGTTGTGACAGCAGAACCAGGCTAGGGTGGATGGAGGGGTGATGGTCAGCAACGCCA

ACCCGACACGGCTGGGATGCAGAGATGAGCAGTAG

>c2032_g1_i1 len=279 path=[1:0-278]

GAAGTAATTCACGTTGGATACAACTTCCTTTTTAACTACAAATCACTCAGTCACACAAAG

CAAAGCATCCTCAGGGATGGGGTGTTCTATATTTTAGGGTGGTTAATTTGGAAAGACAGG

TCTTTGGTATCCAGGCAGTATTTTCTCAACAGCACATCTCAAAGCGGTTTCCGAAGCTTG

GTAAGAACAAGGATATGTGGGGTCAGATGAACCCTGGAGTGAGTGCCTGGTTTTCAGTCA

CGCATTCCAGATCTTAGCTCAAATATTTTTGTGTTGGGT

>c2038_g1_i1 len=222 path=[109:0-108 365:109-221]

AATTTGGAAAAAAATAAAATTATATACAAAAGGTATTGAAAATTATTATATTTATTTTCT

TGAAATTTTTAGAAGTTGACTAAATTTATAAGTGCATCGAAGTGCATAAAAAATAAAAAA

TATATATAGTAGAAATAAATTACACAAAAATATAAAAAAAAAAGTTAATAGAAATTAGAA

CATACAGATATAAATAAATTAATATATAAGAGACAAAAGCAC

>c2039_g1_i1 len=218 path=[196:0-217]

AGAAACAAGGTCAGTCTTATGTACACCAAAACAGAGGTAGAGGTGGGGGAAACTGGGAAA

ATATGTTCAGTGCCTTCCGTTGACCTTTCACTATCTCATGCTCTTGAGTAATGACTCCCC

TGCCTGTTGCAGGTTTCAATTTCAACCTCTCGCTGATTTCATTTTTTTTTAAGAAAATAG

TAACTCATCCAGAATTTGGTCCTAATGTTACTAGGTTT

>c2040_g1_i1 len=1422 path=[1612:0-770 2383:771-856 2469:857-1421]

TTTTATTTTATTTTTTTTTTTTGTTGTTTATTTTATATAAAAGTATATTATATGTTTGAT

TTTTTTTTTTTGTAGTTCATAAAGAAAAAAAAAAAAAATTCATTTAATATGAGTTTAAAT

TTCAGCATAGCTAATGTAGTATACGTAAAGAATCTCTCAACAGATGTATCAGAAAAAGAT

ATAAAAGAAAAATTTGAATCTTGTGATGAAATAATTAATATTACTTTCAAAAACTTTCCA

GGTAAAAATCAGAAATATTGTCAAATAGAATTTAAAAGTTCTGAAGGAATAACAAAAGCT

TCAAGATTAAATGGAGAACTTTTATTAAATGTTCCAATGGTTGTAACAGTTATTGAACCA

GTCTCACATAATTTCAATGAAATATCTAATAATGAAAATGAAAAAAATTTAAATTCAGAT

TTGAGGAATATTCATATTAATAATAATATACAAAATAATATAACAAACCAGAATGTACAA

AATATTTTATTACAAAAACAACTAATTTCAGAACAAAAAAAAGGATTAGTTGATTTTCAA

AATTCATTAAATGCAAAAAATAATAAATTTGATGTTTTTTCAAAAATAGTTTATATGGAA

AATATTCCTGAAAATTTTGATGAAGAGGATATAAAGAAATTTTTTAAAAATGTAGGAAAA

ACAACAAACTATAAAATTCAATATAATGAACATAAAAAATTATATACTGCTTTTGTTGAA

TTTAAAAATGAAGAACATGCAAAAGCTGCTTTAAATTTGAATGGAAACAAAGTAGGAAAA

AATGAGATATGTATAAGAGATGCATATAGCTTAATAAATGAAAAAGATATTTTAAAAAAT

AATTTTTCACTTTGTAGTAATAGTAAAGATGATATAAATGTGTCAAATAAACAAACAGTT

AATGAAAAAGTAGAAAAAGTTTTAGCATTAAAAGAAAAGCTTACATTGAAATTATGTGCA

ATGTATAAACCAAATTTGCTTTTAGTAAATAACTTAGTAAAAGCTAACCCTTTTTTACTT

AATACAAATATAGAAAATGAGGAAAATAAAAATATAGAAAAATGCGTAGATGAAACAAAA

AAAGAAATAATAAACAATTCTAATGATATTAAATTAGTTAAAAGTGATATTCCAACAGAT

AAAAGTTGTGAAGAAGAAAAGAAAATGAAAAAAAGAAAAAATTCTAAAAGTAAATATAGC

AATTCTATTTTAAGTGGAAAAAGCTATTCAGATAATAGTAGCAGATCAAGTAGTTACGAT

GATAGTTATAGAAGGAGTAGTAAAACCAGCTCAAAGAAAAAAAAAATTTTAAAAAAACAT

AAGAGTAGTAAAAGGAGGAAAAAATATAAAAGTCGCTCAAAAAGTTCATATTATTATAGC

AGAAGTAATTCAAGAAGCTCATATGAATCAGAAAAATTGAGG

>c2043_g1_i1 len=242 path=[192:0-17 400:18-74 210:75-241]

AATTTCTACTAAAATTTATTGTTTTAGCTTTTAAAAATATTTTATATGAATTCATAATTT

AATACAATAATTTATTTGTTTGCTAAATGTCCTTCAAAGAGTGGATACAATATTTTAACA

GTATTTCAATTTATGTAACTTAAGAATTTTAAGGAATAAATTGTTACGAAAATAATATTA

GCAAAAACATGCTAATTAACACAATTTAAAAAATCTTCTTTTAATTTTAAACTACAAATA

AT

>c2046_g1_i1 len=364 path=[53:0-363]

TAACTTTGTGTTTGAGCAGGGCATGAAATGGTCTGGAAAAAAGAAATAGGTCATAAAAGG

AACAGCGTTCATCTTATTTTTGTATCACAAAGAATTTCAAGGCAATAGACTTCACTACAA

TCCGTAACAAAAACAGTAGAGTGGCTTTATCATCACACCCCAGATGTTCCACTGTGCACA

GCATGATTCCTCCCACTCCCAGCAAAGATGCATGCACCAACTGCATTCAGTTCCTTCAGC

CTCTCTCGTGATAAAGACTGTAACCCTCAAAGTTTTAAGGTATGAGGGCTTTGTCACTGC

TGTTTTGTGGGTTTTCTGCTGTTTGTTTTGTTGGGGGTTTTTTGTTTGTTTTTAATCTTT

AGTG

>c2047_g1_i1 len=214 path=[35:0-77 463:78-85 343:86-213]

TTTTTTTTTTTTTTTTCAATACTTAATTTAATCTTTATTAACGTTGTGCAAAATATAAGC

AAGTACATGTAACATCATATTTTTTACTATAATTAATATGGATGGTGTATATGAGTTAAT

TGTAGCATTTAACTATTAAAAAAATATAATTATGATAAAAAGAGATAATTTTACGCATTT

GTTTATATTAAAAAATTATTCGAATTTTGTTATG

>c2049_g1_i1 len=240 path=[218:0-173 392:174-239]

GCTAGATCAGCAGCTATGAGTACAAGAACACACAACAACAAAGCACAGACCTTATCTGTT

ATCAGAGACAAAAGCCATGACATGTCAAACACAGTCAAGGTGAAAAACATTTTTTTATTT

TCAGCTCCTGACCTCAGTTTTACTATTTCTAAGTTGGGTAACTGTTTACATATATCACCT

TTAGAACTTTAGAGAAACATCAATTTGTTCTTTTAGCATACTTAATTAAAACTAGTCTGT

>c2050_g1_i1 len=210 path=[155:0-209]

AAAGGTGTAATTTTGAAAATGCTGACTGTCATGCTCAGATGTCATTCATACTGCACAGGG

GATTCCCAAAGAATGATGTGGGGGGAAAAAAAGACAGTAGGAAGTCCATATGGAGAAGTT

GTTCTTAAGAATTTGCAGAATCATAGTAATTTTGTGCCCTCTTTCCTTCCGTGGTGCCTA

ACTATCGCTTAGGAAGCTACTTAAGGAAAA

>c2054_g1_i1 len=501 path=[2971:0-452 3424:453-500]

ATTAATGAAAGAAAAAGAGAAAAAAAAATGAAAGAAATATATGAAAGAAGGCAAAAAAAG

CTAAAAAGGGAAGAAGAAAAAAGAAATAAAATATTGTTAAAAGAACAAAAAAAAAAGTTA

AGAGAAGAAAAAAAAAAAATGAAGGAAGAAGAAAGGTTAAGAAAAATAGAAGAAAAAATA

AAATTAAAGAAATTAAAACAGTTGAAACCAATTATAGAAAAAATAAATAAATATCGAAGT

TCATTAAAAAGTAAATTTAATAAAGAAGCACTCCATTTCATAAAAAATAAAGAAATATTC

AAAAAGGATAATAAAGATTATCTTGATTACTTAAATTCTTTTGATAAACCATCAGAAAAT

ATCTTTAGTCTTATCAAAGCATCTATAGAAGAAAAGCCATTAACTAAGAGAGAAAGAGTT

ATAGAATTGATAAAAAAAAACAAAAATGTACATATTAATACCAACTTATACCTTGATATA

AATTCTGAAGATGATGTAGAA

>c2054_g2_i1 len=2566 path=[25:0-30 25:31-61 55:62-2501 5945:2502-2517 3424:2518-2565]

TATCTCTTTTCTCTATCTTTCTATCTCTTTTTATCTCTTTTCTCTATCTTTCTATCTCTT

TTATCTCTTTCTCTCTATCTTTATATCTCTTTTATCTCTTGCTCTCTATCTTTATATCTC

CTTTTCTATCTCTCTTTCTATATTTTTATATCTCCAATTATATGTCTTTATATATCTCTC

TCTATCTCTATCTTTATATCTCCTATTCTATATCTTTATATATCTCTCTCTCTATCTCTT

TCTCATTCTCTATCTATCGATTTATCTATCTATCTATCTATCCATTAGTATATATAAATA

TATCCTTTTCAATTTTACTTTTCGAAAAATATTATATATATAAAATATATTTGAAATTTT

TATATTTTTATCATTTTATAAACCTTTTTTTGATTTTATTGGCTTTTCTTGAAAAATTTG

AATAATAATAATCTTCCAAAATCTTAATTAAAAAATGTTCATCATCTATATCTATATTTA

TATTTTCTTTTAAAAAAAGTTCTTTATCTTCTTCATTAGACAAAAAATTTATTAAATCAT

TATCATTACATGAATACATTTGTTTTAATTGAAAATATTTCTCATTATATACATCTTCAT

ATAATTTTTTAAAGTTTATATATGAATTATGCATATAAGAATTTTTGAATTCATTTGGAG

AATTGTTATCTTTATTAATTGCATTGTCATTCAATGTATTTTCATTATATGTGTATAAAC

TTTTATCTTTATGATGTTTTTCTGCATTATGTTCACTGTTATTCTTTTTAATGTTTTTTT

TTTGGAATTCCTTAATTTTTAAATTAATAAATGACTCACAAGAATCATCTACTACTTTAG

AACAATGTTTTTTTTCTTCTTTTTTAATTTTATCATTTTCATCATTTCTCAGTTCACAAT

AAAAATCATTTAATGTATCATCACATTTTTCTTGGTTTGTATTATTATTGATATAAGTTA

CTTCATCATAATAATTATCTATGTTGTTATGATTATTATCTTTATTAATTATATTTGTAT

CTTTAATAATTAGATTTTTGTATCGGTCTACTATATTATTAACTGTATTATTTGACATAC

TAAGAGTGCTATTATTGCATTTACAATTATCGTTATTACAGGAAATCTTATCATTATTCT

TCATACATTTGTTATCACAATGATTATATCCATCCATTTTTATATAATTTTTATTTTTAT

TAATTTGCTTCCCATTATTTTGACTGTCATCACTGTATTTATAGTCGTTCTGAATTATTC

TTTTACCTTTTTCTTCGTCTATTTTTTCATAAATATTTTTCTTAAAGATATTAATATTAT

CGTGAATGTTGTTTTTATTATCATCGTTTTTATTACTATTTATACTACCGTTTTTGCAGA

TATATAACTTATTTTCAAAAGCGCTATCTTTTTTTTGTTCTTTATTATAATCTTTAGTAT

TATTATCATAACTTATATCTAAACAGTTATTACTATTATTTGAAGTGCTATAAGACTTAA

TACCAAAAGAACTCATACTACATTTATTTTTATTATGAAGTTTGTTATTTATATTATTTT

CTACGTTTAAATATTCATTAGAATTATTTTTATTATAATTCTTATTTAAAAAGATATTTT

GATATATTAAGTTTTTGTGTTCTGATGAACTCCTATTATAAAAATCTTCATACATAAATG

TCTTATTTTCATTTAAATCTATAGCCAATAGTAGTGCTAGTTTTCTTGAATTAAGCCATC

CATAAATGCTGATATTGAAATAATAATTTTTTTTATTTAATTGATGAGGAACATATCTTG

ATATTTTCCATGCATTTTTTTTATTATCATATTGTAAATTCGGTACTTTTGTATCAAAAT

AATTATTTAAAATATTGTTTTCTGCAATTAAATAATCACAAATAGCTATATATTTAGCGA

TAATATATCCATATTTTTGTTCTGAATAATATTTTTTTTCTCTTATGTACCTATTATTTT

GTTTTTTCATAATTTCTATTTTCCAACAGCTCAATTTATTTACCCACATAATTTTGTTTT

TGAAATATTGATCAAATAATGGATTTTTTAATATATGATTATAAAAATTAATCATATTAA

TAGAATTATAGATTTTCTGATGATCTTTTGATTCACTACTTAATTTTGAATATGGAAAAG

TACAGCGTATATTCCTTTTTTCATCATTTTCTTTTTGAATATTAGATTCATATTTGACGG

AAAATTCATTATTTTTTTTTTGTTCGATATTTCTCATTAATTCTTGTGAATCAGCATTAT

CCTTTTCATTATTTATTTGATTATTTTTAATTTGAATTATTTCTTTAAAATGATTATATT

TAGAAAAATTATTTAGTATACAGTTTATTCTTTCATTTTTATAATTTTCTGCTAATTCTT

TTGCCTTTTTGAAACCATATTCATTAATGGAAAATTTCTTTCTATAAGTTCTTCCATAGA

ATACCCAAGAAACTATCCAATTTCCTTTATTATTAGAATTTTCTACATCATCTTCAGAAT

TAATACCAACTTATACCTTGATATAAATTCTGAAGATGATGTAGAA

>c2055_g1_i1 len=518 path=[1109:0-324 220:325-331 1446:332-517]

TGAAAAAGCCACAGTGAAAACTGGTTGTGTTGTGTTGGAATAATTTCTGTAAATTTCTAT

ACTGAGATAGCAGATCACACTAATTTTGATGAAATTGAGGTGTCAGAACAGATTATTCCT

TTATTTTTAGCTTTGTAATGGAATCCAGAGGTGGCTAAATATTCATAAACACAGGTATAG

GTTGGTAGATACTCCATTTGGAGTGTGAACAGACTTTAATACTTATGATCTTATGATTAG

CATATTCTTGATCCTAAAGTTGAATTCACAGGAGGAAGTTTGTCAAAACCATACAATCGC

TTCAGGGTGAAATGCAAATTAAGGATCAACAAGGAAGAGTGCTTTGGATATAGGCAAATA

ATCCTTGGGAAGGTGGTAGGGAGAGTCCTTCTGGAAGACATTTCCAAACACATGAAGGAC

AAGCAGGGGATCAGGAGTTGTCAGCATGGATTTACAAGGAGGAAATGATACTTGACCAAC

CTCATTACCCTCTGCAATGAGGTGACTAACTTGGTGGA

>c2055_g1_i2 len=412 path=[1:0-218 220:219-225 1446:226-411]

GGAAAGAATCTGGATTTAAAAATGAACTTCATTATGATACTGGTTTATTTGTATGTGCTT

ATGAAAGTTTGGATCTCGGAGCAGTCTATTTTCAAACAGGAGGTCTGACACATATCAGGG

CTTCACACCTACTAGAAAGACAGCACAGCACAGCTCAAGCAATCTGGGATATTTCTTGAG

TGCACTGATGATGACTTCCTGGCATGGGTAGTTAAGGAGTCAACAAGGAAGAGTGCTTTG

GATATAGGCAAATAATCCTTGGGAAGGTGGTAGGGAGAGTCCTTCTGGAAGACATTTCCA

AACACATGAAGGACAAGCAGGGGATCAGGAGTTGTCAGCATGGATTTACAAGGAGGAAAT

GATACTTGACCAACCTCATTACCCTCTGCAATGAGGTGACTAACTTGGTGGA

>c2061_g1_i1 len=201 path=[431:0-200]

GGCACTTCTGTCCCTGTGTCAAAGGAGGAATCTAACCATGAAGAAGGGCTGAAATAACAG

AACAAAACGCAGGTTAGTTCCATTACCTCAGAGAGAGGGGAGCCTGGGTTAGTGGCAGAT

TGCACGCAATCTACAGCCTCAGTCTGACAGGATTGGGGGTCTGCTAATTCAGAAAAGGGA

ATACTGGCAAATACCGCAGAA

>c2064_g1_i1 len=647 path=[1107:0-149 5:150-646]

ATCTGAGCTCTTGTCTTGGCAGATGCTCTGCGTAATTCCTGCTAGAGCTCAGGGGGAGTG

GGGTGCCTGCAGCACCCATGGGGCACACCTGGGGTAGATGTGCTGTAAGCCTGCCTCACA

GCCACGCAAGCAGGACACAGATCTGTGCACATCCTGCAGGAAAGACCAGAAGCCAGCATC

GAGGTTGTGGCCGTTTCCTCCAGTGAAGAAGCAGATGGCTCCAGGAATTGGGTGTCCTGC

TTTTAGTAACATGTTTGTCTTTAAACCAAAAGCCTCTGTGCTTTCTTCAAAAAATAATAA

TAATCCCCTCAAAACTCTTCAAAACACTCAGCACGTTGGGCACAGCTCCAGATTTCCTGC

TGCAGCAAAGGCTCCTTGTCCTCTGCGGGCTGACCCTGCAGCTGCAGGCTGATGCTGCTC

CCAAATCTGGGGCTGGGAAGACTCCACATAGCCAGCATGTCTGGCTCCCCCTGGATCTGC

TGTCACCAGGCTACCCCTCGGGTCTGGGTTAAGTCCTTCTTGTTTAGGTTGAGGTTCTCT

TCCATGGCAGCCTGTTGGTCACTGTGAGGTCTGTCCTGTATTGTGTCTTGCACAGGTCAG

AGCCCTGCTGGGGTGGTGCTCGCGTGCTATGTCTTCTGTTGCTTCCT

>c2065_g1_i1 len=4014 path=[1:0-4013]

AAAAATAAAATAATAATAAATATATTATAAAAAAAAAGTATACTTCGATATAAAAACTTA

ATAATTTTATATAAATATGTCATAATATAATATATAATGGTAATTTATGTATTAATCAAA

AATTAGTAATACTGCTTAAAAGTTTCCTCTACAAATTTTTTTAGATCAAACCCTTTATCT

TTTCCTGTTGACCCAAAAGCCTTTTTTTTATTTCCTCCTGAATGCCCCCCTACTAATTTC

ATTATTTCATTCATAAATATATCTGCTTGTATATTTTTATTTTTTAAAGAATCTCTTATT

TCAAAAACACAATATGTATTGTTTTCATCACAAATAATAAAAAAATAACTTAAATCTTTA

TTGTTTTTAGTGTACGATTGAACAATCTTCTCCAAAACTTTTTGATTTCCCTTTATATTA

TTAAAAAAATTAACATCTAACAAATTGTTATGTTTGTTTTCTAATGAATATAATTTCCCA

ATACTCATAGCTTTGTTGTATAATTCTTTTTGCATATTTTTTGCTTTTTCAATAATACTT

TTTTCTATTTTTTCAAGATCTTGTAATATGTAATTTTTTTTAATATATGGCAAAAATTTA

TCTTCTTTTAAAATTCTTTTATAATTTTGTAAATCTGTTAATTTATCATTTGGTTCCTCA

AAAATATTTTTATATTTAATATATAAATTATTAAATTTATTTTCCACTTCTTCAGCTTTT

TTATTAGTTACTGCTGTAATCCTATATATACCTTTTCCTATGCTTTCTTCTGATGTAATA

ATAAATTTTTTTATATACTTAGTATTTGCAATATGCGTTCCTCCACATAATTCTATAGAA

CATAAATAGGTATAATTTACATCTAAATTATTTAAAATTTTATTTACATCTTTATCTATA

AAAACAACATTTACTTTATCTGCATAATCTTCTTCAAATATAGCTCTAATACCTTTTATT

TTTCTGCTTTCAGTTAAATCCATATTTTTAACAGAAACATTTAATTCTTCTTTTATTAAA

TTATTTATTTCTTCTTCTATTTTACTTATTAATTCAATATTTATATTTCCAAAAAAGGAA

AAATCAAATCTTAGTTTTTCTTCATCTACTAAAGAGCCCTTCTGTTCACAACTAAAAATG

GAACAAGAGTCATCGTCTTTACTGATACTACATCCTTCATCGTTAGTAATTCCATTTTTT

TGATTACTGCATTTTATATTTTCTTCTTTTGTAAAGATGTTTTCTTCTTTATCTTTAAAA

TTTTCATTTTGCATATTTTCACTGTTTTTTATAGTTATGTGATCATTTAAAACTTTTCTT

ATAGTAAAATTTAGTAAGTGAGTAGCTGTGTGATTACAAGCTATAAGTTTTCTTCTTTGA

AAATCTACTACTGTTTCCACTTCATCCTTTGCATGAATGCTTCCTTCTAGTAATATACCA

ATATGTAGTATATAGTCATTAATTTTTTGAACATTTAAAACCTGGAACTTCATCTTACAA

TTTTGAATAAATCCAATATCGTATATTTGTCCTCCATTTTCGTAGTAGAAATTTGTTTCT

TTTAAAATTAAAGCATATTTTTTATCATTACTTGTAAAAGATATATTATCTAAAAAATTT

GCTCCATCATATATAATTTGAACGGAAGATTTTAATTTAAAATCATTTTCTATTTCATTA

TTATTCCATTTATATTTATGATCGTCAATGGTAGCAGATATGTTATATTTTTTTTTCAAT

TCATGTGATTTTTCCACAGGTATATCAAAAAATTTAGTTATTTTAAAATTATTAGTATCT

GAAACTAACTGATGCTTTTTAAATAAATTATTAAATTCTTCTATATTTAGTTTAAAATTC

TTTTCTTCGCACATTATTTCTATTAAATCTACTGGAAACCCGTAAGATGTATACAAATCA

AAAGCATCTTTTCCACTAAATGAGTTGTTACTACTTTTTTTAATAATCTTATGGAATTGA

TCTACTCCTTTTTCTAAAGTTTTATTGAATAGTATTTCTTCCTGTTTGATAGCATTTTTT

ATATAATTTACTTTTTTTTCATTTTTCAAGTCCTTAAAAGAGTTCCCTAATGTTTGACAA

ACAGAGTCTACTAATTTATAAAACCATAAAACATTACTTTTAATATTAAAAACTTGCTTT

CCAAATCTTATAGCTCTTCTTATGATCCTTCGGATAACATAGTTTCTTCCTTCGTTACTT

GGAAGACATCCATCACATATGGCAATAGTGACACATCTAATATGATCACTTATAACTCTA

TAAGCAGTATCAATTTTATCAACATCTTCTTCATTTATTTTTCCTTCATAATCTGGAAGA

TAATTAAATATTTCTTTTATTTGTTTAAAAATTGGTTGAAATAGATCCGTATCATAATTA

CTATTAACATTTTGTAAAATAGAGGTGATTCTTTCTAATCCCATTCCTGTATCAATACAA

GGGAAAGGTAATTTATTCATATTTTTATTTTCATCTTTATTATATTGCATAAAAACTATA

TTCCATATTTCTAAAACACTTGGGTCATCTTTATTAACTAAATTTGATGCATCTCTATTC

CCAATCCTATCATAATGAATTTCAGAACATGGACCACATGGTCCCGTTTCTGCCATTTCC

CAAAAATTTTCTTTAATTCCAAATGGTAAAATACGACTTTCATCTAAATATTTTGTCCAT

ATTTTTTTTGTTTCTTTATCTTCTGGACATGCAGGTAAATTTGGATCTCCTCCATAATAT

GTTACATATAATCTATCTGGATTTATTTTATAAATATTAGTTAATAAATCCCAAGCATAT

TCAATACTTTCTTCTTTAAAATAATCCCCAAAACTCCAGTTACCTAACATTTCAAAAAAT

GTATGATGATATACATCTTTACCTACATCATCTAAATCATTATGTTTTCCACCTGCTCTT

ATGCATTTCTGTGTATCAACTGCTCGTTTAAGTTTTCCTAAATCACTATTTTTATCTACA

TTCCCTAAAAATATCTTTTTAAATTGATTCATGCCTGCATTAGTGAACAATAAAGTGTTG

TCATTGTAAGGCACTACTGAAGAACTTTCTACTATAGTATGATTTTTTTCATTAAAATAT

TTTATAAAATTATTTCTAACTTCTTCTGAAGACATATATCTAGGATTCCCATTTTTCGTA

TTTTCTTCGTTACATCTTCTATTCATATTATTCAAAGAATAATTGTTTCCTTCATTTTCT

TCATCATACCTTTTATCAAAGTTATTCATGCTACAAATATTTTTCATCATATATGATTTT

GCTCCCAACTTACTTTCTATTAATCTATATGTTTTAAAACTGTTTTTAACATATAGAGAA

TTTATGTAATTCAAATTTTTTATATATAAATGTTTTTTAATCTTTGTGAAATCAGTAATT

CGTACTTTTTTGTTAATACCTTTAAAAATCATAAAGAATTTTTTTTCTTTTTTATCTAAA

GGAAACAGGTTATTTACATTCAATAAGACATTTATATCAAATAAATTATTTTTTTTTGAT

ATAAAAGGAAAACTATATGTCTTTTCTACTTTTTGTTTTATTAAATTTTTTTTTTTTCTA

CTTTTTAAAAAAAATATTCTATTTTTATATTTTTCTGATAAATTGTTTACTTCATTAAGA

GTTTTTATATTTCTGCAAATTCCTAAGATGAGTTTTTCTTTATTAGATACAAATAAGTAT

AATAAATAAAAAAATAAAAAAGTCATATATTTATCTAACAAAAATTATATATAAGATAAA

TCATTGTATATAATTATATATATGAAAATCACTGCCAATTCAAGTAATATAAAAAAATAT

ATCTATAAGAAATTGCAATTAATGAAAAAATATATTTCTATAATAAACAAAAAGTAAAAA

GTTTGCCAATATATTCAAAGTAATTTAATATTGGTGGGTCATTATATTCTTAAAAATAAT

ACTTATAAATTTATAATATTCCCTTAATTACAAAAAAAAAAAAAAAAAACGCGA

>c2070_g1_i1 len=296 path=[173:0-295]

ATAAATTTTTTTTTTTTTTTTTTCATATTCTACATATAAACGAATACAAATATGATACTA

TTTTATAAATATATAGGATATTGAAAAATGTAAACAAATGTTAATCATTACAATGATTTA

TTATCATGACCTTTAGATTGTCATCAAATTATAATTTTTATAAATACAGATTTTAAATGG

TTTTTCACAAATATACATAAAAAAATGACTAACTTTACGTTTTTTTTAATAAATTATACA

TTTAAATAATAAATATCAGAGAAAATGTTATATTTGCATTTACATATTTAAAATAA

>c2072_g1_i1 len=201 path=[179:0-200]

TGTTCAACAATTTTGTTGATCTAGCTTTATGAAGCTCAGTATTTAGACTGTATCTTAGGA

GCAGAAACTATCAGCACTTCCACAGTGACATCTGTAGGACACGACATCAAGAGCATCTGA

AAGATAAAGGCATTCTGAAGCTTGGAAAGAGAACATAAGTCTATCATAAGTATTTCCTGT

ATTTAGGGAGCTCTCTTCTGG

>c2073_g1_i1 len=352 path=[700:0-138 16:139-199 77:200-351]

AAAGTTTTTTTTCTTTATTTTTTATCATCATTATAAAAACATATTTAAAGAAATATAAAA

AAATTCAAATACAAAAAAAATATAATTAGGAAAAAAAAAATTAAAAAATCAATAGGTGTT

TAAGCGATATACTTATTTAGAAGGGTGCAATAATATTAATTCATAATAGAAATTTATTTC

ATTTGTTAAAATGAGTATGCATATATGTAAATATTTCTTTCACTTATTTAATTCCATATT

AGCGGTTGTTTTTAATTATCTAAACTTAAATCTTAAATATTTCTTATTTTATCCTATTTT

TTTCCATAATTTATCTTGTTATGCACTAGATATATATATAAATATATATATA

>c2073_g1_i2 len=229 path=[548:0-76 77:77-228]

ATAAAAAATAAAGAAAAAAAACTTTTTATTTTTGCCTTTTTTGTGATATTATTTATTCTC

ATTTTTGTTATACTTTAATATATGTAAATATTTCTTTCACTTATTTAATTCCATATTAGC

GGTTGTTTTTAATTATCTAAACTTAAATCTTAAATATTTCTTATTTTATCCTATTTTTTT

CCATAATTTATCTTGTTATGCACTAGATATATATATAAATATATATATA

>c2074_g1_i1 len=246 path=[207:0-245]

GAGTGTGACCTGGATTTTAGTTGTGATTGGTGATGTAGGCTGTGTGGCTGGGGATGCATT

GGTTTATTGTCTTTGGGAGCTTGTACAGACCCTACCCAGGTGTGTGGCAGTGTGCCCAAA

GCCCTGCAGCTTGAGGCTGGGCTTGGAGCGGTAGTGCTTCTGTGCCGAGTTCTGCTGCTG

CACACCTCTGTCCTCCATCTTGTGTCTGCACAGGCTTCTCCCTAACACATCTCACTTCAT

TTCTTC

>c2076_g1_i1 len=220 path=[198:0-219]

ACTCAAGCTTCCTAAAGCATTTCAGTCACATGCTTGCATTACCCTGCAATTTCAAGCGCT

GCTAGCATGTCTTCCGTAATAATCCGAGCACAGGATATCACTCAGCAGTTACAAAAAGGA

ACCTGTGGTCAGGTCAAGGGTTCAACACACGGTTCTGTGCTGCCTGAGTGAGTGGGGGAG

TTGATAACAGTACTAGACTGTCAGTGCTGTTTGTATTCAG

>c2077_g1_i1 len=288 path=[376:0-164 81:165-174 28:175-287]

ATATAGTCTGGCAGTATCACAGAGATAAGCTAATTGTGTTATTTCCTGTCTGTAGTAAGC

CACAGGAGATCTGTGATCAAAAAGTAGTGTATTGGTAATCTGACAGCTTTAAGTGAAGGA

ATTCACATCCTCGTGCTTTAGAGGAAGATTTTATTGGTATTTGTGTTTGAGTCCCTGTTA

AAAGTAAGGTGTGAAAATCAGTAAAGTCTGCTATTATGGAATAATACATAAAATATCAAG

GAAAAAAAAAAAACAACAGAATTTAACAATATGCAAAAACTTATCAAA

>c2077_g1_i2 len=221 path=[376:0-164 540:165-220]

ATATAGTCTGGCAGTATCACAGAGATAAGCTAATTGTGTTATTTCCTGTCTGTAGTAAGC

CACAGGAGATCTGTGATCAAAAAGTAGTGTATTGGTAATCTGACAGCTTTAAGTGAAGGA

ATTCACATCCTCGTGCTTTAGAGGAAGATTTTATTGGTATTTGTGGTGTCCTTTTTAGAG

GAAATGGTGATAGACTTTTGATAAGTTTTTGCATATTGTTA

>c2078_g1_i1 len=355 path=[374:0-304 727:305-354]

GAAGTCAATACAGGAAATTAACCCAGAAAACGCTGCTCAGAGACAGTGGGCTGGAACCAT

CTCAGTGCCACGGCCACAAGGCAGAAGTGGGGGCCATGCTGACAACAGGAGCAGTGCTGA

GTGGTGATGAGCAGCGTTAAGCACATCCTCAGCACAGAAATGGGAACACAGCACTACAGC

TGGATGGCAGGCTTGGCACAGAAATTCAGCCACCAGGAATTCATGTAAAGGCACACGAGG

GATGGGGAGGTGATCTGAGAGTCAGCGTGGCTTCACCAAGGGCAGGTCATGCCCAATCCA

TCTGGTGGCCTTCTGTGATGCAGCGATGGCACATAGTTCTCTGAGGAGGACACAT

>c2079_g1_i1 len=760 path=[674:0-364 22:365-518 1190:519-629 180:630-741 1327:742-759]

ACTTTTATAAGATTAAGAAATTATTACTAATGATGGATACTGGAATCAATCTAAGGCATT

CTTTTGTGATCCTGGTGAATGGAGCTGGTATCTCTATTTGCAGATGTAATACAGAAAGTA

TTGTCAATATAAAGAAGGATATCACTACTCAGGGATCGCGTGATTTATAGTATAAGAATA

GTGGAAGAGGTAAAACACGGTTATTAACATGTAAAAAACATGTACGTGAACATGTACAGT

ATACTGCTTTGAGTTCTAGGCAGTCATAGTGAGACAGTGTTAAGTTTCAATAGATGAATG

CTACCTAGAGGATAGTTGGCCGAGTTTCCAAAGGTGCACTAACAATGGCAGTGTTGTTTC

TGAGGCAAAAAGCTATTCACCACTAATTGAGCTCCATTTGTAAAATTGAGCTAGCAGAAG

TGTTTGTTTGGTATGAATTTAGATTCTTGTTGATTTGGATGAGATTTGTCCCACTGCTAG

ATAGCTGCTTTGTTGAACAATTGCACTTGGACAATTTAACAGATAACTGAAAACGTGCTC

CTAGTAGAGTAATGGTAGAGTTGTTCTGCACTTGAACCAGTTTTGCAACTCTGAGGCTTT

AAAGGGCTGTAGAGGTCAAGCATTTATATATTAAATTGTCCAAGTGCAATTGTTCAACAA

AGCAGCTATCTAGCAGTGGGACAAATCTCATCCAAATCAACAAGAATCTAAATTCATACC

AAACAAACACTTCTGCTAGCTCAATTTTACAAATGGAGCT

>c2080_g1_i1 len=379 path=[497:0-10 @69@!:11-355 514:356-378]

ATAGTAAAATTATAGTAAAATTAACAAAAAAGATAAAATTAAAAAAAGATGCAAACAATT

ACTATATAAATAATTTTTAAAACAGAAAAAAATAAAAATTTTGACAAAATATAATTTTTT

TTTTTTCTACACATCAACTAATTTCAATATATTTTAATACATAAATTTTACAGTGTATTT

TGTTCTTCTTTAAGTTCTATTTTTATAACTTAAAAAATTGGTTACCACTTTTTTATTAAT

TTTCATAATTCAAATTAAATATTAAGAAAAAATTTTGAATGCTTCTTATTTTTTTTAAAT

TATAAATCAATATCTTTCTCTACATTTTTTAAAAGATTCCATTTCTTAATCCTTTTTTTC

CATTTCCAATTAGCTTTAT

>c2087_g1_i1 len=275 path=[284:0-274]

TACCATTTCCTAAATACAAAATATAGCTTTCCAAAAAAATGAAACACACATTAGATATAA

ACCATCCAAAACTTGAACGATTGAAAACTGTATTCAAAATTAAATGACCCAGAGCAGGTC

TCTTTCTGAAAAGTTCCCTTCATATCGGCAAGAAGCCTTACTACTGAAAACGTAATTCAA

TGCCTCTCAATGAGAGAGAGAAAGAGAGAGACAGAAAGAGAAAATGAACGTTATGACAGA

CCGACAAACAGGAAAAATCACTCAAACACAAAGTT

>c2088_g1_i1 len=243 path=[17:0-14 32:15-242]

GTGTGATCAGCACTGTCTGGGCTGGAGGCAGGGCTCAGGGGACAGTGCAGTGCTAGCTCA

AGGCATGGCAGATAAGCTTAAGGGTTCTGAGACATTGGCTAAACACAGTTTTGTGAGCAA

AGAAAACTACGCAGCCATGCTTTCCATGGCGATAAAGGAATTGGAGAATGGGTTTCAAGA

TTGCCAAAAAAATCACCCATTTTCTGGTTTATTTGAGGCCCAATTTGCAGTCAACATAAA

TAC

>c2088_g1_i2 len=323 path=[33:0-94 32:95-322]

GGCCCCCTTGTTCTTGTTTTAGTGCCAACAATTAAAAGAGTTTCAAATATAAAACTTTCA

GCAGCGTGCTATTAAGCAGGCATGTATTGACCCCATCTGGGCTGGAGGCAGGGCTCAGGG

GACAGTGCAGTGCTAGCTCAAGGCATGGCAGATAAGCTTAAGGGTTCTGAGACATTGGCT

AAACACAGTTTTGTGAGCAAAGAAAACTACGCAGCCATGCTTTCCATGGCGATAAAGGAA

TTGGAGAATGGGTTTCAAGATTGCCAAAAAAATCACCCATTTTCTGGTTTATTTGAGGCC

CAATTTGCAGTCAACATAAATAC

>c2090_g1_i1 len=328 path=[337:0-147 658:148-327]

GTCAGTGCTGTCAGACAGACATGTCTCTGCACAGACTCTGAACACTTATGTGCTGAGCAG

GACTCCATGGAAACACCAGATCCAGACAGGGGACTTCTCTGTGCAGGATGAGACTGAGGA

GCGTGCAAGGCAGGAGGAGCATTTTGTCGTCTCCATCCTCTATCACAAATAGCCTGTGAA

GACTTGAGGATGCTAAAATAACACAACAATAATTCCATTCAAAGTTCAGCATTCAACTTG

AAGATTAAGCTTCAGAATTACAAAGTTGCATATAAATCCATTCCACATTTACAATTCTGT

CTTACTGTGTTTGAGAGAGGGTGGAAGA

>c2092_g1_i1 len=397 path=[470:0-351 42:352-396]

GCAATAGGGGGCCTGGGCTGCCCGGGGGGGGCAATGGAGGTGGAGGGGCGAAGGGTGCGG

CCAATGGGGGGTCCAGTTCCATGGACAGCCCCCCCGGCAGCAGCCCCCCTCCTTGCTCCA

TGAGGCCGGGCCCGATGCCCACGGGGATGTCGAGGGGCTGCACCCCGTAAGGGGGGGCGA

AGGCGGCCTCGGGGGGGGCCCCGGGGGGGTCCCCGAGTTCGTCGAAGTCGGCGACAGCCA

AAGAGGGGTCGGAATCCAAAGAGATGGGGGGGATCTCAAACTCCTCGTCCCCCAGGCTGG

GGGTGTGGAAGGTCTCAGCGCCCGTCAGGAAGTGGTGAGCCGCCCCCGTGATGGCCGCGG

CCTGGCCGGGCGGCGAAACCATGGAGTTTCCCGGCGG

>c2092_g1_i2 len=425 path=[470:0-351 888:352-367 836:368-379 42:380-424]

GCAATAGGGGGCCTGGGCTGCCCGGGGGGGGCAATGGAGGTGGAGGGGCGAAGGGTGCGG

CCAATGGGGGGTCCAGTTCCATGGACAGCCCCCCCGGCAGCAGCCCCCCTCCTTGCTCCA

TGAGGCCGGGCCCGATGCCCACGGGGATGTCGAGGGGCTGCACCCCGTAAGGGGGGGCGA

AGGCGGCCTCGGGGGGGGCCCCGGGGGGGTCCCCGAGTTCGTCGAAGTCGGCGACAGCCA

AAGAGGGGTCGGAATCCAAAGAGATGGGGGGGATCTCAAACTCCTCGTCCCCCAGGCTGG

GGGTGTGGAAGGTCTCAGCGCCCGTCAGGAAGTGGTGAGCCGCCCCCGTGATGGCCAAAT

AGTTGTCACTTCCGGTCCCCGGCCGCGGCCTGGCCGGGCGGCGAAACCATGGAGTTTCCC

GGCGG

>c2092_g1_i3 len=396 path=[470:0-351 888:352-367 67:368-395]

GCAATAGGGGGCCTGGGCTGCCCGGGGGGGGCAATGGAGGTGGAGGGGCGAAGGGTGCGG

CCAATGGGGGGTCCAGTTCCATGGACAGCCCCCCCGGCAGCAGCCCCCCTCCTTGCTCCA

TGAGGCCGGGCCCGATGCCCACGGGGATGTCGAGGGGCTGCACCCCGTAAGGGGGGGCGA

AGGCGGCCTCGGGGGGGGCCCCGGGGGGGTCCCCGAGTTCGTCGAAGTCGGCGACAGCCA

AAGAGGGGTCGGAATCCAAAGAGATGGGGGGGATCTCAAACTCCTCGTCCCCCAGGCTGG

GGGTGTGGAAGGTCTCAGCGCCCGTCAGGAAGTGGTGAGCCGCCCCCGTGATGGCCAAAT

AGTTGTCATTGCCGCCGGGAAACTCCATGGTTTCGC

>c2093_g1_i1 len=564 path=[569:0-177 14:178-563]

ATTTAATGAATTGAATATTTTTCCACTGACATTATAAATACATTTATTAATTATATTCTA

GATTATTAATTAATGGTTTATAATTTATTTTTTTATAATTAAAAATGGAATTATAAAATG

TGGGAACATTTTGGCAATTTAAGTGGTACCACTCATCCTTACATTTTTTTACATATTTCC

TAAACCAGCCTTCTATTACCTTACAAACCTTAGACAACTCTTTTTTTTTTTTCTTGAAAA

AAGTAGAGCACTCTTTATCATTAGGACGGCTTTCCCATAATAATGTATACTCTGCAAGAA

CTTTATCATTGAAATCTTTTATATCATCCGATATTTTATTATACCAGTGATTGAAATTTT

CTTGTTCCCACTCTCTTCCAAATAAAGAATATCTTACTCCTAAATCAGAACATACATTTT

CCCAATCAAATAACATCCTTTTTATGAGTAAATCTTTTATATTATTAAAATGTTTATTAT

ATTTTCCATATGACTTATCCGTGTTTAAAATTTTCTTTTTAATTTCTGCTTCTTCCTCTG

TTAGAATTCTTAAATATTCTTTAT

>c2093_g1_i2 len=399 path=[1:0-12 14:13-398]

AAACACAAGGTTACCTAAACCAGCCTTCTATTACCTTACAAACCTTAGACAACTCTTTTT

TTTTTTTCTTGAAAAAAGTAGAGCACTCTTTATCATTAGGACGGCTTTCCCATAATAATG

TATACTCTGCAAGAACTTTATCATTGAAATCTTTTATATCATCCGATATTTTATTATACC

AGTGATTGAAATTTTCTTGTTCCCACTCTCTTCCAAATAAAGAATATCTTACTCCTAAAT

CAGAACATACATTTTCCCAATCAAATAACATCCTTTTTATGAGTAAATCTTTTATATTAT

TAAAATGTTTATTATATTTTCCATATGACTTATCCGTGTTTAAAATTTTCTTTTTAATTT

CTGCTTCTTCCTCTGTTAGAATTCTTAAATATTCTTTAT

>c2096_g1_i1 len=563 path=[53:0-562]

TGACACCTTGGTGGGTGGCAATGTAGGTAGATACCAAGGCTCTCCTTGCAGGACATTTTT

ACTGCTTCTTCCTGCATTGCCACCCACCAAGGTGTCAGTGTCATCCCTGCTCACTGCCAC

CTCAAGGACCACTGCCACTCACTGTCTTAAAAGTAGAGTCAAACAGGGTTAAGTCTTAGC

CTTCATAGAATTTAAAGGCTTGTCACTCTTATGGCTATTTGGAATGAAGAGTATAATTTT

CTGTTGATGACTGATGTAGCATTAGTGATGAACATGGCAAACAATAAGGCATTTGCACTG

CTGCAAAACTGCATTTTGTACTGGACTCTAAGAGAGACTATGGAGTGGCATCTGTGAGAT

GACACCAGAAATAAAATGGAATTTCTCTATCCCCAAACACTAAGCCTCTCCTTTTTCCAA

GAACCAAGCCAACACTGGCAAAGTGTAACAGGGCATCACACTGAGCAAAATTCACTAAAA

ATACAGCTAACAGAAAACTGACTTTAGTGACACAAAGGCTTTAACTGACCAGGAAAAACT

ACTGATCGCTTTTACTTCATTAA

>c2098_g1_i1 len=490 path=[1:0-363 365:364-384 365:385-405 365:406-426 365:427-447 365:448-468 365:469-489]

TATTTTTGGACATCTGTTTTGTCCCTACTTTCATTGTTGCTAAATACCTGCAGTATCCAT

ACCTTATCTATACCAGCCGTCCTACTGCTGTTGCTGGTAGAGACAAGCAACAGTGGGAAA

GCAAAAGACAAATTTACTATAAAAATGAACCTTGGCACACCCTTCCCACTCTGATCCCTT

TCCTGATCTGAGACAGATGACTCCTGTGGCTCTGCCCCACCGTAGACTTGCTGCAATCAC

TGCACAAATGTCCCATAGCACACTGCTGTCAGCACTGACTTGAAGGAAGAAACAAACTAC

GTTCCTAAAAGCATAATGATCCCAAGACCACTGCTCACAAACACACACATTTTTATTGAA

ATTTGAAAAGAAAAGAGAGAAAAAGGAAAAGAAAAGAGAGAAAAAGGAAAAGAAAAGAGA

GAAAAAGGAAAAGAAAAGAGAGAAAAAGGAAAAGAAAAGAGAGAAAAAGGAAAAGAAAAG

AGAGAAAAAG

>c2103_g1_i1 len=219 path=[399:0-218]

CATAGATGAGCACTGACCCCCTTTCCATCAGCTCCTGCATCACTGGATGGAGAGCCATCC

ACTAGAGAGCAGTGTCAGGCAGTCCACAGTGCCTACAAGCCACACGTCACATTTACAGCT

GGCTCTAAATGCACCTAAGCATCCTAACAGCCAAAATAACATGCGTGTGGAGGAAAGAAC

CTGATATTTGGAGTGCAGTGTCTCTTTAGGGAGGAACAG

>c2106_g1_i1 len=302 path=[1:0-272 751:273-275 277:276-301]

TGAGTACTGCGATCCCAGTTAAAATGCAGGGAACTGAGATAAAAGACATACTGCAGAGGC

ATGCCAGTTCACTCAATCAGCATCGCTTGCGTGAAGGCTCTGATTTATAACAGTAGCACT

ATGGAGGATTGATACTCAGCAAATGTGCTTTTCTTTCTGGAAGAGCATTAAAGAGTTCAA

CAGGAGCACTGCTTCCAGGACAACGTACAGCTGCACAGAGCTCCAGTGCAGCCTGCAGCT

TCCCAGCTGATACTCTCAGCAGGTTTTCAGCGCCAAACTGGAAGTGTCTGAACAACCTAC

AG

>c2110_g1_i1 len=254 path=[357:0-253]

TTATTACATTTTCTTCCCATTTGAGGGGAAGAGTGAGAAAGTGGTGAGATGGAGTTCAGT

TAGCCATCAGTGTGAAACCACCACACCACGTCTACAGCTGTCCTTGACTTTTCACATAGA

AGAAATCTGGAATACTGTTCACCAATTCCTCAAAAATGTCTGCGTGATGAAACAGCCACC

ACAGTGGCATTGGTGTATCTTGAATATTCATAGTGCCTGTAATCCACGTGTTGCATTTGG

GGAGACAGTAACAG

>c2110_g2_i1 len=201 path=[179:0-200]

GGGAGACAGTAACAGGGTTATTAGGACCTTCATGATAAAGATAGGAATCAGGGGATTTTG

TGTGTGCAGAGACCAGACAAAAAAGCTCAGCATAATAAATAAAAAACCTCTATAAAGAGC

TGAAATTCCAGAACAGAAACTATCTTAATTTCTGATAAAAGTAAGGGAGTAATGATGAAG

AGATTAACTACAGTGTCTAAG

>c2112_g1_i1 len=209 path=[187:0-208]

AGATGCTCTTCCAGGCAAGGACCAGATCACCGCGGGTTCCCTTCAACCGCTCCCTGTCTG

AACCTGTGCCGCACTCTGCAAACAGGAATGCCAAGCCCTTTCTGCAGGCGGTGCGCTCTG

TGGATTGTGAGCAGCACAGCTATTTTGTCCGTGGCATCTTCTCAACTCTCTCAAGTGGCT

TTTCAAAGATAATAAATCGAAGGGGTGAG

>c2120_g1_i1 len=235 path=[213:0-234]

GTGGTGTTACAGTACAACTACATATTGTTTCTTATTAAGGCTGATACATAAAACTACCTA

GAACCCTTCCAAAATTTCTCATGTATGTATGTATTTGTAAAAATTAAGACTTAATTTGGA

AAAGAAACTATTTTCCTGGGTATACAATACACAACTTTTTCCAGGAACCCTAGGTAAGGC

ACAACTGTAGAAGTACTGTAACACCACTACAGAGTAACCAAATAACGTTCACATT

>c2124_g1_i1 len=232 path=[1:0-231]

GCAGAACGGCCAGACTTAACAGAAATCCCTAATGCAGAATTAGGAGGAGCAATTTAATCC

AGTTGCAGTATGTTTTTCTGGGTATGTAAGTGAGGTTCCACACAGACACGTGCTTTGCAG

TGGTGCTTCTATGAGCTGCTAAAGGGAACTGGCTTGGCAGAAAGCATACTCATGTTTTTT

AATCTTTCTGCAGTTAGCCCTCTGTCAGCCTTGTTCCCTGAATCAGTTTGTT

>c2132_g1_i1 len=300 path=[181:0-299]

ACATTTAAGAAATTTAATAAAGTCCCTCAGAATGTAGACTGTACAAAGTGGGAAAACAAA

AGGTTTTGGATGAGATGTATGTTTAATAAGCTGTGCATTAATAAATTGAAGGATTTCTTA

TTTCTGTTATGCATACTATATTATCACAACCTTGCGCACGCATAGTGGTTCCCGAGGTAC

CACTTTCAAACTGTGTTGATTTTACACTGTTAAAATCACTGTTTTTCACTTTTAAAATTT

GCAAGGTCGTGCCTTAAACACGTTAAACACGCACAGAAATACCTTCTTAAAATAACAGGT

>c2134_g1_i1 len=205 path=[383:0-204]

ATGGCCCTGAACTCCAAGGCTGAGGCTATTTGCAAGCACAGGGAGCCTTGCTCAAGGTCA

TGTAGTGCCTGAATGGCAGAGCCAAGAACAGTCCAGGATTCCCAGCTCTCCACCCTGCAG

CACCTTCAATTGCTGGATCGTGCTTCCTTTGAGAAATATCAGGGCTTTAACATTCTCTCC

ATTAAAATACTCCAGGGCAAGTCAA

>c2147_g1_i1 len=264 path=[407:0-121 24:122-263]

GTGTGCTGTTCTGTGTCATCAGAAACAGCTCTTGGCATTGAAATATCAGAGTTTGGAAGG

GTTAATAGCTTTCCACTCCCTTTCCTTTAGATATGCCATATGCAATATACTAGGGAGGGA

TACTACACTGTTCCATACAGTAGCCAGCATCAGAAGACAATGACTGACACAAACTCACTG

TGGGAAAACATTGCTTAAAATGATAGAAGTGTTTGCTGACATCCTTGGAGAAGTGTGAGG

TAACAATAAAACTACACAAATTAG

>c2152_g1_i1 len=334 path=[1:0-255 257:256-333]

GGAGACTGTCAAAAGTGTTGAGATCCCGCCCTCTTCTCCCACGTACATCTGCTCTGGGCT

AAAATACAGCCCAAACCATTGGGTAGAATGAGACAGGGATGTCCTTCCTGCTCCTTCAGG

CAGAGAAACCCTCACCGTCTTTCCTTGGCCACGGATGGCATGCAGCAGCCACCAGAGCAG

GGGGCACTGCTGGTGGCACCTGTATGGGCTCAGCACGATGCACCCTGACCCTGGGGATGC

CTCTCAGGCAGCGTTGATCCAGTTCACTTGGACATGGCTGTAGGAAGAGAGGAAAAAATG

GCATTTCAGACAAGTTAATGGGCTCCCATTTTGA

>c2153_g1_i1 len=221 path=[63:0-194 281:195-220]

GTGTTTGATGTGGGATTTCTTCTGGAGATTTGACTTAATATTTCCCCTTACATGTTGAAA

ATGTTGGTGCTGGAAAAGGGTTGGCTCTTGTTCTATAGTCTTGCCACGGGTTGACTGAAT

TACCAGAATTTACGAAGATCTCAGTTGCTACAGCTGGAAACCAAGCAGTTGAAAACTTTT

CTTTTTCCCCTATAGATTTTCAACTGAATTCTCACCAGATT

>c2158_g1_i1 len=226 path=[1:0-225]

ATATATTGAAAGAAATATACCACTATATGATGATATATTAAATAAGAATAGTGAGTCATT

ATTTAAAAAATTAGATATTTTCGATGAAGATGAAAAAGTAGATAACTGTGAACAAGGATA

TGATATAGTAGATAGTATATGTGAGTATTTCACTTATTTAATGTCTTTAACATTAATCAA

ATGATAAATGTTTCCATAAATAAAATATGTTATTAAAAAAAATAAA

>c2159_g1_i1 len=275 path=[545:0-274]

GCGGACCACCTCGCGGTCGTAGTTGAGCTCCTTGGCGATCTCGGTGATCTCCTGGCCCGT

GGGCAGAGCGTTCTTCTCGAAGTAGGCGTTGAGCGCCTCGATGGCCTGCTGGGTGAAGGA

CGTGCGGCGCTTGCGCTTCTTGGAGGGCTCCCCGCCCACGAACTCCATCAGGTTCTGCTG

ACCCTCCTGGTTGCGCAGCTCCGCCTCGCTCAGCCACTTCTCCAGCACCGGCAAAAAGAG

CGGCCCCCCCGAGACCCCCACCAAGAGTGAGGTGC

>c2162_g1_i1 len=320 path=[1:0-319]

AAGATGTGTGTTAATTCCACACTACCCACTGGTCATCACATAGAGCGAGCAGTACCAGAG

AGAAGACTGGACTATCTGAACCAATTCAGTTTGGCCTGTTTTTTTTTCCCCCAACCATTT

TCTGACTTAATGGCCTGAGAGCACCTCAGTGACTCCACTGATTAGCTGGAGCAACCCTGT

GGCAAATGCAAATCCAGTCAGAACAGCTAGCTTTTACAAAAGTTAGCTTCTAAGTGAAGT

TTCAATTCTATTTAATTAAAGACATACCCTATATTCATGGGTGGTAATAGCAATACCATA

ATTTCCACTCAATTTTCAGA

>c2163_g1_i1 len=231 path=[155:0-230]

AACGGATGCCACAGATGTTCAGAACAAGAATGGAAGGCAATATTGGCCCCAGACGTTCGA

GCTGAGCACAGTCCAAAAAGGTGCAGCTACCCGGGGCTCACTCTGCAAGGCAAGCATCTC

AAAGATGCTATCTCAGATTAAATGCTGTCTTTATCCAAGGGCAGCATCGCTCCTACTTGG

TGTCCATTTCCTGACAACATGAAAGGCTTTTCAAATTAACATGATGAACGA

>c2165_g1_i1 len=617 path=[1:0-300 302:301-616]

CTAATGCAGAAACAACATTTGATTCCCACAGGCTCTAAGCACCCATCAGCAATGAAAATA

AGAAATCAGCAGTTTCACTACCTTTTAGTAACTTCAGTTCATAGTTTTGGCTTCTTCTCT

CCTTTGGGGAAAGAAGATTTTCTGGCTTATGAGGGAAATTCATCAGCTGTTTTTCATGCA

CTTTCATAGCCTGGAGCTTGAAAACATAAGGAAGAAGACCGTAACACGAAGAAAGTGTAG

AATATCGCCTTTGTGGCTTTCAGCAGTAACCATAACATCTCTGTCTCTCACGATTTTGTT

TAGCAAAGATGAAAACTGCAAAAGTCAGCGCGTTTCCCCCACAGCTGCACTCCCCACCAT

GCACAACTGCTTTGTTTCCATCTCCAAGCCTGGAAGAGACAGTGATGCTGAGCACCAAAG

CTCCTTTTCTTAGAACTTGCTGGGACATTGTGCTTCTTGGAGGAGACTGTGTAACACCAA

GGGAAACAGTGAAAGGACACTGTCTTTATCCAATTCCCCCTGCAGTGATTAAAACGTAAT

ATTTTGATAAAATATTTTGATAGATATTTTAATAAATGACCAGATTTGAAAAACAACCCA

TTCCTGGTTGTCACTGA

>c2165_g1_i2 len=337 path=[1210:0-20 302:21-336]

ACAGTGACACTGACGGACTTCAGCAAAGATGAAAACTGCAAAAGTCAGCGCGTTTCCCCC

ACAGCTGCACTCCCCACCATGCACAACTGCTTTGTTTCCATCTCCAAGCCTGGAAGAGAC

AGTGATGCTGAGCACCAAAGCTCCTTTTCTTAGAACTTGCTGGGACATTGTGCTTCTTGG

AGGAGACTGTGTAACACCAAGGGAAACAGTGAAAGGACACTGTCTTTATCCAATTCCCCC

TGCAGTGATTAAAACGTAATATTTTGATAAAATATTTTGATAGATATTTTAATAAATGAC

CAGATTTGAAAAACAACCCATTCCTGGTTGTCACTGA

>c2167_g1_i1 len=244 path=[222:0-243]

AAAATAAGAAAAAAGGAATAAAAGCTGAGGTAGAGAAGAGGTATAGGGGGAGCATTCCTT

ATGGGAGATGTAAGGTTTGGATAGAGAGGAAAGTACAGGGATTAAATGATAAAGAACTGC

TGTGAAAAGTGGTGAAGGTTAACGTACATAATGTTAAACAAGTTACAACATCAGGATCAA

TATCCTTCTAACTCTGTATATATATTTCTATATCCAAAGACTAGATAAATCTAGAACACA

CTAA

>c2176_g1_i1 len=275 path=[499:0-274]

ATTGTCTTTTTCAGTAACAAGAGACAGTAAAATAAAACTTGGTGAAAACTGAAAAGCTGC

ATGCAGTTCAGAACACTTAACCAGAAATTGTTACTGTTATTAGTTAAAAAAGCAGAAGGA

ATTTATTTTAAAGCAGTATGACTTAAATTGCAGCTGAGTCACTAATTCTATAATGTTTCC

TGTTTGGAGATGGCAAAATTGGCAGGCAGCAGCTAAAGAAGAAATCACTGCTTCCATTTT

ATAGCCAACACACAAGACCACCTCTGATTTTTATG

>c2178_g1_i1 len=520 path=[645:0-179 328:180-519]

GGGGTTGGAGCTTCATGATCCTTGTGGTCCTTTCCAACCTGGGCCCTTCTGTGTGATTCT

GTGCTCAGAGTCCTGAAAGAACCTCTGGTCTGCTCAGCTCTGCCTGCAGTTCCACCCTGT

CTTCTGCACTGAAAGAGCTGAAGCAACTGTTTCAGCTAAAATTAAAATAAAGGAAGCTTA

TCAGATCTGTTGAACAGTAATATTCTACTAACAGAAGATCAGGATTACTCCAATAAAAAG

AAAAGCATGGGCAATCATATGCCAGTGAACACTGATAAATGAGAATACTAAAAGCAATCG

TATCAACCAGACGTATCCAGAAAGGAAGAATCTCATCAGATCATTTTAACCTGTGACCTC

AGATGTTTGAGCAGCATTTAAGATGAAAGCATGCACAGAAGTTGCATAATGCAGGGTAGC

AAACATAAGACAGAATATGGGAATACTGAAGTCCAGTTTTTTTTCCGGTCTATTTGTTAA

AGCTGTTTAATGCTCTTAACAGTCCCTAGAAGCTAAACCT

>c2182_g1_i1 len=271 path=[177:0-270]

TGAGTAGTGCATAATTCATTTATTTAATGCTAATAGTCCCACATATAAAAAATATTTATG

TAAAAAGTATATAAAAAAAATTATTAATATTTTTTTTAGTATAATAATTATTAAATAAAG

GAAATTTACAAAAAAAAGCATATGAATATATAATTACAATAAATAACTTGAGAGAAGATT

TTTAATTGTAATTCTCATTTCCTATTTATAAAATGATGACTTTACTGAATTTAATATATA

CAAAGACATCATACATAATAGAAAATAAAAA

>c2190_g1_i1 len=207 path=[185:0-206]

ACAGTCTGACGAATGGCCATCAACAATCCAGTTTTTCCCCCTTTAAAAATATCTAATTCT

TCTTTTGAAAACTCTGACGCATAAAATCTACTATTTGCTGTATTTGTTTTCAGACTCCTC

ATACTGTACTGCTTCGGTATCTCAGTCTTTAAAGTGAAAACATGATTTTTTCCTTCCTTT

CAAGCGAAAGAAGTGGAAGTTTGGCAG

>c2193_g1_i1 len=248 path=[412:0-215 179:216-247]

CTACCACTCCCCTGCAACAGCAAAGGTACTTCACTTTGGGTCACTCTGCTCCTTCCAAAT

GGGAGGAGCCTCAAGGGCATCCAGGCAGGTTTCCTCCCAAAGAGCACATGTGACAACCAT

AAAACAATTAGATGGGCAGTGTGCAACCAAGGGTCAGGAATACCCCTCCCATAAAACCAC

TGGATAATATAGACTGGGCCAAGCTATGGCTATGACTCCCTTGGGTCTCCACAGTGTCTC

CCCATTGC

>c2193_g1_i2 len=249 path=[412:0-215 627:216-248]

CTACCACTCCCCTGCAACAGCAAAGGTACTTCACTTTGGGTCACTCTGCTCCTTCCAAAT

GGGAGGAGCCTCAAGGGCATCCAGGCAGGTTTCCTCCCAAAGAGCACATGTGACAACCAT

AAAACAATTAGATGGGCAGTGTGCAACCAAGGGTCAGGAATACCCCTCCCATAAAACCAC

TGGATAATATAGACTGGGCCAAGCTATGGCTATGACAGAGCATAGCAATGGGGAGACACT

GTGGAGACC

>c2196_g1_i1 len=295 path=[1:0-294]

GCTTATAAATGATTGTTCAGGACTTTACTGTTGTAGGAAACAAATACACAAACAGCCCTG

AGGAAGTTCCTGTCCTAGGTGTGTTGTGGACTGGGGCATAAATAGTAAGCAGGAACAAAA

TCAGGCAGAGTCTGAGTGAGATACTAATACACAGAAACTGTTGAGTTGTTCACATGACAA

AAAATGTTATAATCTTGAACATTTAAAAAACAAACCACTAGACATATCATTAGCACAGAC

ACCATCTCAAAGCAGTCTCTCAAACAATACAAAGTCAGATCTCATCAAGGACCCA

>c2198_g1_i1 len=1469 path=[1494:0-1468]

AAAAAAAAGAAAAAAACAGAATAACATATAATTACCTCTATGAATAAAATATAAGAAAAA

AAGCATATTATATATATGTGTAAATAATTTATACAATATAGTAAATTTTTTTTTCTATTT

TTTATATTTTTAAAAATGTTATATTATATTAAAAAATTTAACACAGTTTTTTTATTTTAT

GTTTTAATAATAATTAAAACAATTAAAGGAGATGAAGAATATATTTCTCCTAATGATTTA

GGTAATAAAATATCTGGATTTTTAGGATATAAATGTAATTTTTCAACAAAAGGTGTTCAT

AATTTAGAACCAGATATACTTGAATATAGATCTGTTTTTTGTAGTGTAAATTCATATTTC

ATATATGACAAGATAAGGTTAATGATTCCTAAAAATGATAAAACATCAAAGTTTAAAATT

TTGCCTGGGAGTTGTTTTCAAAAGGTGTATGTTGATTTTGATAAAAGAGTTGAAACAGAT

ATTTCTGATCTGGGTTTAGTAGAATATGAAGTTAATGAAGATGATTCTTCTCCTGATTAT

ACAGAAAAAACTATAACAATATCACCATTTAATAATACAGATGTAGAATTTTTTTGTTTT

TGTGACAATACTGGTAAAGTTGTATCAGATGTTGAAGGAAGGAGTGCTTTAGTATATGTC

CACGTATTAAAATATCCACATGATATAATATCTATTAACTTAACAAATGATTCTTTTCCT

TATCTAAAAAATCCCAAAAGTAAAGAAGATTTTGAACATAATAGTTTAGAAGTTGAATTA

AAATCAGGTGAACTTTTAGTATTAGCTTGTGAATTAATAGATAAAGAATGCTTTCAAAAA

CCTAAATATAATGACTTATATAAAAGTAATAAAATTATTTATCACAAAAAATTTGCAATA

TTCAAAGCTCCTTCATACACTAAAGCTGGAGACATGGATGCTGCATGCACATGTAAGGCA

GATTCTATAGTTTACAGTATAATAGCAAAACCAAAAGAAGAAAAAAAAATGATACATGGA

TGTGATTTTTCTACTGATGATGCTAGTGAGCAATTTTCAAATAGTGTTAATTTAAAAAAA

GCTGAGGACAATACACCAGTAAATTGTGAAATTGATTTAAAAGAAAAAACTTATAATCAT

TTAATAGGTTTAAAATGTCCAGGTGAAATTATACCAGATTGTTTTTTTCAAGTATATCAA

CAAGAAAAAGAACAATTAGAACCATCAAAAATAGTTTATTTAGATGCACACTTAAATGTT

GGACATATTGAATATTTTGAAGATAGTAAAGTAGAAGATAAAGTAAAAATTTTTGGTATA

GTTGGAAGTGTACCAAAAGACACAACTTTCGTCTGTTTGTGTAAGAATGGTAAAAAAACT

GGATATATGACAGTTAAAATAGCATCAGGTTATTATGGATTTTTGGCTAAAATTTTCATA

TTTTTAATTGTTATAATATTATTATACGT

>c2199_g1_i1 len=220 path=[197:0-219]

TTTTTTTTATATTATTATAAATATTATCAACTTTTCAAATGATATTTTTTTTATGTAATA

AATATTTAATGACATTGTATTTTTAATAAAAAATTATTAATGAAAAAGTGTCATAAATAC

GTGTAACAAGAAGATTTTTATATTATTAGTAAAATTTGTAAATGATCCTTATTAACTATA

CATAAAGACAATTGTTAAAATATGTTGAAAAAAAAAAAAA

>c2208_g1_i1 len=279 path=[388:0-252 25:253-278]

TATATATATATATATATATTTAATATATGTATAATTATATCATATGTGTATTTATAATTT

ATTATAAATTACATTATTTTTTGAGAAGAAAGTTATCAACTGTTTGATTTCATAGGTTTA

AAAGTTTAAAACTTGCTAATACATAAATTATAACCATAAAAAAAAAATATATATGTGTTG

ATATTTTTTCATTATTTCATTTCATGAATCTTTACATTTAAAGATAATATCTATGTGAGC

AAAAAAAAAAAAATACTATATATACATATATATATATAT

>c2208_g1_i2 len=316 path=[388:0-252 640:253-315]

TATATATATATATATATATTTAATATATGTATAATTATATCATATGTGTATTTATAATTT

ATTATAAATTACATTATTTTTTGAGAAGAAAGTTATCAACTGTTTGATTTCATAGGTTTA

AAAGTTTAAAACTTGCTAATACATAAATTATAACCATAAAAAAAAAATATATATGTGTTG

ATATTTTTTCATTATTTCATTTCATGAATCTTTACATTTAAAGATAATATCTATGTGAGC

AAAAAAAAAAAAAATACTATATATGTGAAATAAAAAATATTTTGAAATTTTATTTTTATA

CATAAAAAAATATATG

>c2210_g1_i1 len=228 path=[337:0-102 235:103-227]

TGAATTCTGCTTCCTTTGGATCTGTTTGCAAATTAAGCAGTTTCGTACATGCAATGAAGA

TGCATATATAACAAGTAGCATGTGTAACTGTATTTGGTTTCTCCAGGAATCAAATTGTTC

ATCAGTTTCTTCCTTGGAAGGGGAGGAAAGAGGATCAGTTTCAGGGACTTTGATAATGAC

ATGACTGTCAAGTAACAGACTTCAGTAGGGCTGGAGTAAAAACAGCTA

>c2219_g1_i1 len=236 path=[249:0-235]

TAAAAAAAAAAAAAAAGACAGCAATTACTTTTATCAGCCAGTAATCTAAAAGTTCGGTCT

ATATCAAAAAGGAAATAATTTCTCCTTTTCCTTTTCAGCTACAGTTAGGTGTTTAGTGAT

GGATTTCTGCTTTCACGTTTCTATTTTTATGCCTCAGCTGGCCTAATTATTACCAGTGTC

CTCTGGAGCCCTTAGTAATAATTCTTATTGGCAGCAGTAATACTCTCTGCTGAAGA

>c2238_g1_i1 len=214 path=[241:0-79 24:80-213]

CTTTGTGTTTGAGTGGTTTCAATCTAAAAGAAGGGATATTTATATTAGATTCTATAAAGT

GATGAGGCACTGTCTCAAGAGCAGCTGTGGGTGCCCCATCCCTGAAGGTACTCCAGGCCA

GGTTGGATGGAGCCCTGGACAACCTGGTCTACTCCCAGTTCTGGAGGTTGGTGGCCCTGC

ATGTGACAGAGGGTTGGAGCTTCGTGATCCTTGG

>c2244_g1_i1 len=786 path=[43:0-612 42:613-785]

GACATTATTTCTATAAATAAACATGACAGACATTAAGGGTGAGGAATGTTATCAGGGTTC

TGTTTCATCTTGCCTCCTAGTAAGTAAGTAAACTGTCTTCCCAGAAGAGTAATGAAACTC

AGATCAGTGTTCTCTGATCACCTTTCCTGAATCACTTGATCATTCTCTTGGATGGAAAGG

AAAGTTTGGAATCATAACCTGCTCTACTATTAGAGCCTGATTGAAGTTCCTGTCCTTTAT

CGTTGTTACATGTGTCTCAGCCTTCAGACCAACTCCATTCTCTGCAAAAAGCTTTGTTGA

TTGTTTTGAAGAGAACACTGACACCTAAATCCCACCTTCAAAGTGTAAAAACCAAAATAT

GCTCCAAGGACTAGAACTTGAACAGACTCTTTTTCCATGTCACTGTTTGCTTATTTTTCT

TCAGTGTTTTCTTATTTTTCTGAAGTTTTCTGTGAACATTTTTTTCAATCTCACTTCACA

ATCATGAAAAAAGCTTCAATCTGAAATATTCTCTTGTTTTTTTAATAGCATGTTAGTAAT

TGTATATGGTTTACAGTAGAACAGTAGTAGTAAGTATTTGAAGGACTTTGACACAAATTA

ATTAGTAATTGATGATGCAGCCCTACTTTCCTATATGTTGTCATCTTCTTAATGCAAATA

CTACTTTTAAGGTCCTGTATAGCTACTAGTGAAGCAAAATGTGTATTTTGGATTGTATTG

AATTTTGGAGCTTAATTGAGGAGGCAATCTTTGGAGTATTTACAGCAAACTAAGAAAAAA

AATATG

>c2254_g1_i1 len=505 path=[337:0-504]

GCTGTTGCATGAGCTTAGAACATTTTTTACAGATCTTGATTTTCCCCTAAACTAAAGAGT

TGTATCTGTTTCTACATTACACCTGCAGAGGTGCAAAGCAACTTAAGAGTTGAGGGCAGA

AATACCTGCAGCTCCTACACACCAGGGAGACCCAAAACCAACCGCCTCACTGTCTGCACA

GAACAGTTCTTTCAGGAAAGACCAAATAGTAGCAGTCCTCATGGAGTTGAGCATTACAGC

ACTGACACCAGAGAGGAGTTTTCATCATAACCATCTCGTATTTCCCGATTACTTATAGAA

AAGAGGTATTGCCTAGAAGTAACCAGGAGCCATTCAGGTCTGTATCACTCTGATCAGACA

AGGATAATCTGAAATACCAGCTAAGCAGTAGTAGGTTCTTTCACTTCCATTAAATGGAGT

TGGACTCAATGATCCCTGTGGGTCCCTTCCAAAACAGGGTATTCTATGATATTATGCAAA

GCCACTACAAAATTTAGAAAGGTAT

>c2264_g1_i1 len=227 path=[359:0-226]

AGGACACTGAATTTCAACAGTGTTTTTGGGCACTGTGAAACAAAACACTTCTATATAGAA

GATGTAGGTGGGATGTGCTCTGAGGGCAGAAGTGCCTTTTACTCTAAAGAAACTTGTGAT

GTTGCCAAGACTTTGGATGAGAAACTTTAAACTGGATCCTTTTTCCACAAGGTTGTGTTG

TGCTTATACTGCCCTGCTCAAGACACCCCATGAAGGGCACTGTGGAA

>c2267_g1_i1 len=451 path=[41:0-184 20:185-450]

TTGTGTTTGATTTTGCAGAAATGAATAGGTTTAAGCTGAGACCTGAAACTCAGATGGCTA

GCATGGTTCTTACTGTTATGGTGAGATATATTGTCATGAAACTTACTGGTGATTATGATG

AGAATCAAGGGCCAGGATTTGAGATGGACCTTGTCAAACTCCGGTCTTGACAGCTATCAG

GTAGTTTGAAATTAGTTCAAGTCCTTTGAGTGCAAGGAAAGCTAGGATTGAGCTTAAGTT

TGGGCATTGAATCGTGTCTATTTTTGCTAGATTTTAGTCCAGTGTATGCTGGAGGAACTG

ATACTTAAATATATGCTTTTGTTAGTTTTGTCACACTTGGATCCCTTATGTTAGTTATAG

CTCATAGGTGTGGAAGAGCTTAAGGTTCACTTCTGAATCCATGGGCATGCTCACTTCTAC

CACTGGCCTTGAAAAGATGTCATCTTATTAG

>c2270_g1_i1 len=208 path=[155:0-207]

GAAAGTACCTGGTAATAAAATTTTAATACACGTATTATAAATTCTATTACCAGGTACTTT

CTTGCTAAGACATGTCTGTGCAAAGCAGTGGAGTACAGGAGAGTTTAGCTGATGCAATTT

ACACACAAAAATGAAGCCTTTTGTATTGTTGCCCAGATGCAAATTGGAAATACTGTGATG

GCTGTTGTGGATAATAACATAAGGGCTT

>c2279_g1_i1 len=213 path=[310:0-212]

ACCAGCAGCTGGCCGACTTCAAGGAAAAAAAAAGGAAAAGAGAAGACCCGGCGGAGCTCC

TCTGTGCGGCAGCGGGATGGGGCACGGCGGGGCGAAGGGAGCAACAATGCCATCATGTTT

TTGAGACAGGAAGCCTCCGAGTTTGCCCTGAATGAAGAACTTCCTGTGTTTAAACTTGCA

CGAATATCCGCTGACAAGCTCGCTCAAACACAA

>c2288_g1_i1 len=291 path=[185:0-245 113:246-290]

AATATGAGCACTAAAATACATTCTTCCAAAATACTTCAGTTTTGCTACATGTATTGAGAG

GACACTGCAAAGTCTCCAAACACACAATTTTCATCAGAGGATTCTTCAAACAGTCAACCA

CATTTACAGGTACATATTTTTGGTCAATTAGCTCTTGAAAACTCTCAAAACACCCATATG

GAGCAGAACAGCATTGTCTGTGGGTAACATTCCAACAGATTTAATATTTTAAATTATCCA

ATGAGAAAAATTGAGTTTTCTGCACCAGTTTTCATTTCCAAGATCTCTCTA

>c2291_g1_i1 len=300 path=[278:0-299]

GTCGTGCAAAGCCCGTGCCCCTCCGTGTGGGCACTGCAGCTCAGATGTGAGACCCACGGC

TTTGGTGAGGGTCAGGAGAGGCACAGCACTGCCGCCAGCACAGCCCCGTTCTGACATCAA

CTCAGTGGCCTCAGCAGTGAGCTCTCAAATGCTGGATGCTGCTCTTGCCTGGTGCTTCCT

CTGGATGCGGTGTGACATCGGAGCGCGTCCCCTTTGTGCCTGCAGCTCAGGGCTCTGCTT

TGGAGCACGCGAGGCAGCTCCGGGCAGGCATGCAGCACTCTGGCACTGGCAGCCACATAG

>c2293_g1_i1 len=261 path=[203:0-260]

GTGGGAGTGTAGCAAGAGATGTTTTGTAATCATAAAAGCAACCAACAGCATTGTAAAGGC

TGCAAAGCAGAGACTGGTGAGAATGTGTAGCTCCAACATTCAGCATGAATTGGCAAAGCA

GGCATGAACGTAAACACTGTTTCTGAAAATCTTGTACTGGAAAGAAGGCAAAATCTGTGC

TACAGACAAGAACCACAGCATGAAGTGAAGTTAAAATGGGGAGTTAAGAGTACCAGCATC

AGACGTGCTACGAGACAGCTT

>c2296_g1_i1 len=284 path=[225:0-35 112:36-283]

GGGGGTTGCTGCGGCCGCCGCAGGCCCGGCACCGCGAGCTGAACGTTGAGCCGCCCGTGC

GGTGTTGCTGACATGTAGGAGCGCTGCTGAATCCCCGCCGCTTTCCTGTGGGTCAGCTCT

TTGTTTCTGTTTTGCTTTGGTTGGTGGGTTGGTTGTTTTTACCTATTCGTACAGTTTCCC

TGTTCGGTTCGCTCCTGAATTTTGCTTTTCGGAAGGTTGGGCTTGTGAAGGCCTTAGTGT

TAACAGCTGCAGTTAGCTCGCTCGTGCTGACCGTTCAGGCTGCT

>c2296_g1_i2 len=359 path=[1:0-110 112:111-358]

GCAGCCCACGGGCCGTACGGAGCTCTCACACAGCTTTATTTTACATCTGGGGTAATTAAA

GGCCAGCAAACTGCACCAAGCTGCCCTCACTCCATCTCTTCCCACAGAAGCAGCTGAACG

TTGAGCCGCCCGTGCGGTGTTGCTGACATGTAGGAGCGCTGCTGAATCCCCGCCGCTTTC

CTGTGGGTCAGCTCTTTGTTTCTGTTTTGCTTTGGTTGGTGGGTTGGTTGTTTTTACCTA

TTCGTACAGTTTCCCTGTTCGGTTCGCTCCTGAATTTTGCTTTTCGGAAGGTTGGGCTTG

TGAAGGCCTTAGTGTTAACAGCTGCAGTTAGCTCGCTCGTGCTGACCGTTCAGGCTGCT

>c2297_g1_i1 len=252 path=[1:0-217 219:218-251]

GCTGGACTTCCAGTAACTGTTAGATGACAGTAAGTGTTCTTCAAAGTCTATTTGTTGTCC

TCTGAAAGGAAGCTCTTCTTTCTGTCAGCAGAAAGTTGTTCTATTTGATCCATGTCGTTC

TTTAACACTTTTTAGAGGAGATGAGACAGATACTCAGACATTCCCTTCGCAGAATGCATT

GGTAATCCTTTTGCTCACAGATGCATCTGGAGCATTTCCAGTGGTTCTGCACAGCTTTGG

CAGTGATTGCAG

>c2297_g1_i2 len=252 path=[1:0-217 286:218-251]

GCTGGACTTCCAGTAACTGTTAGATGACAGTAAGTGTTCTTCAAAGTCTATTTGTTGTCC

TCTGAAAGGAAGCTCTTCTTTCTGTCAGCAGAAAGTTGTTCTATTTGATCCATGTCGTTC

TTTAACACTTTTTAGAGGAGATGAGACAGATACTCAGACATTCCCTTCGCAGAATGCATT

GGTAATCCTTTTGCTCACAGATGCATCTGGAGCATTTCTGAGGAGTCCTGCAATCACTGC

CAAAGCTGTGCA

>c2302_g1_i1 len=206 path=[338:0-205]

GTAACTTTGTGTTTGAAATCCGAATTGATATCTCTCTCCAGGTAAGTGTTCTGTACATGC

CCTGGAGGACAAACTGCTACTTGTGTGCATGCTGCTTACAGTATGCAATGCACTATTATT

CTATTACTAGACTTTAGTACATTTCATCTGGGCTTCAATTTATTCTTGCATCATTTTTGT

TTCACTAGTAAGAAGAGTACAGGTAT

>c2304_g1_i1 len=289 path=[267:0-288]

AAGATCTATATGAAAATGGAGTACCAGACAGCAAAATCCATCAAAGACTTTATTTAAGTA

GGTGTATAGTATTAAGATCTTACAAATCCCATGATGTAGATCAGAAGAAAGTATTTACGT

AGACATTATTTTAGTGGCAGTACATCAGGACAGGAAGAAAGGAAACAGAGGAATAATTAC

AGAAGTAGGAAAAGTAGTTTTAGCACCAAGTCTTGTCACTTTGCCTGGAGTTGGTTTTTC

TTTTAATTGCATAATATTTAGCAGCTTTTAGAGAGAATAGCTTGCATAT

>c2315_g1_i1 len=279 path=[297:0-278]

GGGTGGGTCTGAGGCTCCTCTCAGTGAATAGATTTTGCTCTTGAGCTCTCAGCAGCTTAG

GTCTCTCCAGGGACCAAATGTCTGCCTTCAAGAGAAATGGCCACACTCTGTCCCACTGAC

TGCAGACCCACCCCTAAAAGACTGGAATAATGGTTCTTATGACTAATATAAAACTATGAG

ATACGGAGAAAATAAGGTTATTGCCAGGGATGCAAAGGGAAGAGTCAGCTCATGAAAAGA

GCTCAACTGGTTGGACTGACACAGCAAAGTTGTTCCATT

>c2327_g1_i1 len=460 path=[657:0-198 46:199-350 442:351-459]

ACCTTATTTTCCCCATCACCCAATGTGCTCTGCTGAACTAAGATGTTTTTCCTTACAGCA

GCAGTACTCAAAAGCACCCTTGGTCAATTTTGTAATTCTCTTGTAATACCAAGAGAGTAT

AACCAGCATTTCCTCTGTGCCCATACATCCACTGTTAGCAGTGGAACGGGGGGAGAAGCA

GCTATGGGCCAACACCACCAAGCAGTCCCAGAATGAAATACCCAAATCCCCCTCTTTAAA

TAATGCAGGGAGATCCATTACACGATTAGCTCCCCTTTTAAACAAGCGCCAGCTTCATTT

TTTCTTTTCTTATTAATATTTTCCTGGCTCTTACACAGTTATTTAAGCTTATTCCTTGTT

TGTTTCATATCAGGGTGATTCTGCTCAACAGATTATTGATATCACGTCCCTTGTTAATTT

TGATTACTTTTAATAGGCTAATGTAAAGCTATTAGAAGTA

>c2333_g1_i1 len=214 path=[192:0-213]

AGACAGAAGGAAGCAGACATGTGAGTATTACACTGTCTCTATGCTCACTGACGTAATTTG

AACTCAGAAGAGTTCTTCTTATTCCCTGGTTTGTTCCTGAGATGTGAATAAATGTACTAA

GGAAAAGAAAACAAACTTCTAAGCAAGCGGTCAGCATGCAGCTTGCATCTCAGTCTTAAA

CTTCTTTGATTTCCAGATCAGTTTCCTAAGTAGT

>c2334_g1_i1 len=252 path=[230:0-251]

AGTGATATTTATGGGCCTTTTGGAGACCTTAAAACTATAAAATCACGCAGTCGCTGAGGT

TGGAAAAGACCGCTACGATCATCTAGTCCAACTGCCAACTCATCAGACATGCAGTCAGGA

GAGGGATCGACCCTTACTGACTACAGCACCTCTTAAATATCTCCTGAGTTTGGGTATTAA

TGGGAATTGAAACCATTTGATAGTTTTCTTACCTTTCCCCCCCTCCTGTGTGCTGTTGAG

AATCGCCCTACG

>c2335_g1_i1 len=204 path=[1:0-203]

GAACGCGCAGAAGTTGTCAGACTTGATTTTTTTTAATGTAAATGCTAGCAGCAAGTAATT

TGACGATTCCAGTTTTCCAATCCGATGGTTAAGAAAAGTCATTTGGATACTGCATCCAAC

TCAAGGAGGCAAATAAGCTACACATGGAAGAATACCTGAGAGAAGCATTGTTTGCTTTGT

TCTTGACATTCTTTCTAGCCATCT

>c2335_g2_i1 len=222 path=[363:0-221]

TTTAGATTAGACCAAGAAGCTAATCTTTCCAGGATAAGCCATGGAAGTGTGCATGTTCTG

CCAAGTTAAGTCTGAAAAGCAAATGATCTATTGTAGGTGGGAGTCTTCTCTTGGCTCTAT

GTTCACACCCATCCTTATCCTGGAAAGATCAGCTTCTTGGTCTAATCTAAATCGTTCATT

TTACAAGGCTTGCGGATAAGAATATGAAAGATTTAGATTAGA

>c2337_g1_i1 len=214 path=[346:0-213]

AACTGAGATCAAGCTCAAGAGACGGTTGGGGCTGACCAAAGGAAGGGGCGTCTGCATGGC

AACAGTAAGTCTCCTTTGGCTTCAACTACCCCTGTAGCACTGTGCTGCACAGACACAAGA

AGTAGCGTTTATGAAGGACTTAAATTATCTCTCCTCCTATTGAATTAGGAGGTCTAAAGG

TGAACCTCGCTCTGGCATTTTAGCTTACAGTTTG

>c2343_g1_i1 len=245 path=[155:0-244]

AATTACAATAAAATGAACATTTGCAAATAATAGTAGATTTATAGTAATTTTTTAAAAAAA

GTGTAAAGGTAATCAATAAAAGAAATGAATATATAAATGATTTAAATTGAATTTACTTTT

TTTCTTCTCATTTAAATTCATGGAATATTCAAACTGTTGTTTAATTAATCAAATAAGTTT

CAAATAAATTAATATATTATGCATTGATTCATTAAATTTTTATATATTCTATTTATTTAT

CTTAT

>c2347_g1_i1 len=278 path=[256:0-277]

GATTTTCCTAGAGAACCCAAGCAGATCACATGCTGTCTGTGATTACTGCTCTACGTGTAG

CACAGTACATCAAAGGAGTATGCAATATCCATACAGAACTCCTGAAGCTCATCCTGTGCT

TAAGTGTGAAGTTCAAACACACTTAAGCACAGGATGCAGAGGTGCAGACTCCCAAAAAGT

TTTTCTCCTAAAAGGTACAAATACCACATTTGGCCATCTGAGAAAAAAATGCATCCAAAT

ATATTTGAAAGCAAGAAAGCCATTAAAAAAAAAAATAA

>c2348_g1_i1 len=272 path=[1:0-247 249:248-271]

ATAAAATAAAATAAAAAAAAAATATTATATATACTTTTAATTCAAAGGTAAGCCAAGTTT

CATGTAATTTTCTTCTATATATATTTAAATATAAAAATATTGATAGAAAATCATATGAAG

GAAATTTTTTTAAATTATATAGAAATGAATTTTAAAGAATTATTTATTTTTCATCTTTTT

CTTTAATGATTGCAACCATATAAATTCTTTTTGTATGAAGATTAATATACTTCATTTAAC

AAAAATTAATTAGATATAATTTAAAATATTAA

>c2348_g1_i2 len=339 path=[1:0-247 613:248-338]

ATAAAATAAAATAAAAAAAAAATATTATATATACTTTTAATTCAAAGGTAAGCCAAGTTT

CATGTAATTTTCTTCTATATATATTTAAATATAAAAATATTGATAGAAAATCATATGAAG

GAAATTTTTTTAAATTATATAGAAATGAATTTTAAAGAATTATTTATTTTTCATCTTTTT

CTTTAATGATTGCAACCATATAAATTCTTTTTGTATGAAGATTAATATACTTCATTTAAC

AAAAATTAGTATTAGAAAAAGAAAATAAAATTAAATTATAATTTAAAGATAAAAAGATAT

ATAAATAAAAATAATATATATTTACATTATATATATATA

>c2353_g1_i1 len=261 path=[559:0-260]

AGCATGGTGCTTGCAGTTTTAAATATTGTGGATATTAGTCATGCATCAGAAAACGATCTT

TGGTTTTTACTGATTCAACTGTGTTGAAATCAAAACATTGCAGCAGCGGAAAAAAAAAAT

AATTGTTTTTATTTCATGTAAAGTTCTGAAGGGATCAATTTCAAATCCTGCTTATGATAT

GAAAAATATTAAAAAAAAGAAATAACGAAAATTATAAAATAAAATATATACTTATATATA

TAAAAAATAAAAAACTTATAT

>c2356_g1_i1 len=389 path=[155:0-388]

GAGCTCCCCATCTTCGTGGGGAGAGCTGCCAGCAGCCAGATGAAGTCCAAAAGCTCCATT

AAGGCCACGGGCTGAAAGGTCTGACAACCAGGAGGAGGACTTGAGCTGATCTTGATCAAA

TCTGTGCAAGGATGGTTGCTTTCCTCTGTCCCCAGGTGCCGGTGGCTTATGGCTGAGGCT

CTGCCTCATACTGCTTCTCTGTGGCCGTGTAAAAGTCCTCAAGGACGGACTGAAGGTACT

CAAAGGTCGGGCGCTCCTCGGGTTTGTTTCGCCAGCATCTCAGGATGATGCTGTAGAGCT

CAGCTGGACACATGTCCGGGCAGGGCATGCGGTAACCCTGCTCCAGGTTGCGAATCACCT

CCGGGTTGGTCATCCCTGGATAAGGGATC

>c2366_g1_i1 len=268 path=[404:0-267]

TTCTAAAAGCGTTTACTGCTTTTACTGCTGAAATTTTTCTGGTTGACTGATAAACTGTTG

CTTTCAAAGAAATGTCTCTTGCACCAGCACATCTCATTCCTGCATGGCAGTAGTAAGCTT

CAAAGTCCACTACTTCATGTGTGCTTGCTTTTACCTACCTCTTTCTTGCATGTATCACTA

ATGTTCCTTGGCAACTTTTGATGGTCAGACTTCATCTTTCCTATCCAGAACATACAGAAC

TACAGAACAGCACAGTCCCCAGCACAGA

>c2367_g1_i1 len=228 path=[1:0-227]

TGCTTTTCTAAAATATATTATTTTGCCCCACTCAGGACCCTGTGTTTCTATTTTCTGATT

GGGTGAGGGGTCAGGATATGCTATTTTAGAAAAGTAATGTTGTTTTTTTGCAGCAGCAGA

CTGACACCATTCTGCAGTTTATACAGTTCCACAACTCACTAGGGTGTGGTGTGCCACATT

TCTACTTTATATAAAGAGATACAAGCAATATGCGTGGAACTAACATAG

>c2378_g1_i1 len=256 path=[1:0-255]

GGCCTGGTGCAGGCCATCTCCTGCCACACATCAGAGTGCAGCAACAGCAAAGGGTTTCCT

CACTTGGGCGGTGCAAACTCTAGGAGACTGTCACAACTGCTTCAGAAACGCATGTGTAAA

AGTAGGCCAGGCATAATCCGCGACACAAGGAAGTGTTAGAACTCCACAAGCAGAGAGACA

AGGGGGAGGGAGACTTCTCCAGGGAAGATTTCCTTTCCTCCCTTTCCTGAGGGGCGATGG

TTGTGTCTGGACACTG

>c2382_g1_i1 len=487 path=[465:0-332 798:333-486]

AATAGGAAGTAATAGAAAAAGTTAGAGACAATACCAGACCAGCAAAACATATGCAGAAGG

GGAGAGAGGAAGGGAGAATGGGGACAGGAATGACAGCACAAAAAACAGGAAATGCATCTG

TATAAAACCAAGGTCTGGTTTTACACAAGTTTCTGTTTTGTTTTTTAAACACAAAAGTTA

AGTTTTTCAAAGAATTCCAGTTATCTACAAGCATACACAGTTCAGCTACACAGTAACACA

GCAATTAATTGGCACAAGAATACATCCACCCTCAGCTTAGATAACCAGATTTTCTGCACT

CACCACAGGCCCATCTGAGCACACAAAAGGAATGTGTAAATCCTTGGGCTTTCCCCCTCA

GAACATGATGGGGACGGCTCACACAGCCCTCTTTCACCTTGTCAGCCAGTCAGACCATAC

AGATGGCTCTTACACATTTTGACTCGTAAGTTCTGGAGACACGAGCTTCCCTTAGCAAGG

GAAGTTG

>c2383_g1_i1 len=269 path=[247:0-268]

GGAGCTCTCTTTGCTTTTGGACCAAAACCCCCAAAATCAGCAGCAGCAGCAAAGCTGTTG

ATACACACTGATCACAGAAACAAAACCATTCCCCTTAGAGGATGCTAGTATGCTGTTGAG

CAACAAAGGCACTGCATATAGCTGATCTCGAAAATAAATTCCAGCTAAATTCATTAAAAC

CTTGAAGGAGGGGGAGAGTGGAGGTGAAACAAGTCAGCCAGCCCAACTCCAGGTGTCATT

TATCATTCTCCCCCCTTCAAACACAAAGT

>c2385_g1_i1 len=405 path=[13:0-23 583:24-404]

TATTCTGTGATTCTGTGATTCTGTGAAAGGACACCAGGGGAAGTTCTAAAAATGCTTCCA

TTTCATCAAATACTGGTGATTTCTTGAAATCTAGAGAAGTATTTAAATTTGGGAATAAAT

TTGGAACCCCACCAGCCACAGGTTCTCTCAAGTGACATTTTTGCAGGCAGACAAATAAAA

AAGATGCAGAGTAAAACGAAATTTCCCTTCCTTTTATAAGAAAAAAGCCCTATCAATTAC

ACTGGCCGTCTCACATACAGTCCTGGAGGAGCAGGATGCTTTCAAACTGCCAACCCTTTC

CAGATTTAGGAATCAGATGTCATCTTATGAGGATGTGAGCCAAGAGCAGGATTTACCACC

CAGCACCTCCTGCAGGAGGCCTGTACCCAGAGCCGGGGTCCTGAG

>c2388_g1_i1 len=226 path=[1:0-15 17:16-225]

GTCTCGCTGAGATGAGCTTCCCCATCCAGACCACCTCAATTGGGTTTCCAGGCCGTGGCA

CTGCCATCTCTCTGAGTACCTCTCCCTACCCGCCTCTCTGCCACCAGATTTGTCATCCTG

GGTGCACTGACCCCTCTGCAGGGACCCCCGCTGTCTGCCGCCCTCCTCGAGCCAAGCATT

TCACAAAGTCCTCTTTCTTTTTCAGGTCAACGCACAATGAGATGGA

>c2388_g1_i2 len=430 path=[441:0-219 17:220-429]

GTGCTCCGCTCGGTTTGGGGTTGGCGTTAGGATGAGGAGAGCTTCCCCTTCCTGCATGGT

GCTGCTGAGCGGTGGTTGGGCCCCTTTGCTTCCTCCTCTCCCTTACAACCACTCATCCAT

GGAGTACATGATGTGTTGGGTGCTGCTGCCATCCCCGGCACTGCATTGTGTGCTCTCGTG

CTGCTGCTCCCCCTAACCACAGCCCTTCCAGCAGCGAGCCCTTCCCCATCCAGACCACCT

CAATTGGGTTTCCAGGCCGTGGCACTGCCATCTCTCTGAGTACCTCTCCCTACCCGCCTC

TCTGCCACCAGATTTGTCATCCTGGGTGCACTGACCCCTCTGCAGGGACCCCCGCTGTCT

GCCGCCCTCCTCGAGCCAAGCATTTCACAAAGTCCTCTTTCTTTTTCAGGTCAACGCACA

ATGAGATGGA

>c2397_g1_i1 len=604 path=[1405:0-18 299:19-35 316:36-603]

ATGAAGAAGAGTTAAAAGATGCAAAAACTAGGATTATTGCATCTTTTAACTCTTCTTCAT

TTATCATTCTATGTAATTCTTCTCTAGCATCATCAATTCTTTCTCTATCATTACTATCAA

CAACAAAAATTAAACCGTCTGTATTTGAATAATAATGCCTCCATAATGGTCTTATCTTAT

CTTGTCCTCCAACATCCCATACAGTAAAGGAAATATTGCGAAATTCAACAGTTTCAACAT

TGAAACCTATTGTAGGAATTGTTGTAACAACTTCCCCTAATTTTACTTTGTACAAAATTG

TTGTTTTTCCTGCAGCATCTAACCCCACCATTAAAATACGAACATCTTTTTTTTGAAATA

AACGATTAAATAACCTACTTACATATAATCCCATCTTAACTTTGTATGATAATTATCTCA

AAGAAATAACTTTATAAAAAAAATAATAAATTAGCAAAAAAACTATTCAAAAAAATAAAT

TAGTCATATAAATAAATTTAAAAGATATATATATATATAAATATTAAATATTAAAAAAAT

TTATTTATTTATTTTATTTATATATTTTTTTTTTTTAATTAGGAAAAGTAATAAAAGCAG

GTGA

>c2397_g1_i2 len=883 path=[1:0-297 299:298-314 316:315-882]

AAAAAAAAAAAAAATAAAAGGAAACTTTATTTCAGTAATCGTAAGAAAATATAATTAGTA

GTTCAATATATTCCTTGATAACTTTTTCCTTTTTCATATTTTATTAATAATAATTAATGA

CAATTTTTTACTTAGCATTATTTAAATGTGTAGTAAGCCAGTCAAAACCTTCGTATAACC

CATCTCCTCTTGTAGCACAAGTTGATTGAATGAACCAATTTCTCTCTCTAATAGTGTTTA

AATGTAATTTTTCAGTAACTTCAGCTGCTGACATAGCATTTGGTAAATCTTGTTTATTTG

CAAAAACTAGGATTATTGCATCTTTTAACTCTTCTTCATTTATCATTCTATGTAATTCTT

CTCTAGCATCATCAATTCTTTCTCTATCATTACTATCAACAACAAAAATTAAACCGTCTG

TATTTGAATAATAATGCCTCCATAATGGTCTTATCTTATCTTGTCCTCCAACATCCCATA

CAGTAAAGGAAATATTGCGAAATTCAACAGTTTCAACATTGAAACCTATTGTAGGAATTG

TTGTAACAACTTCCCCTAATTTTACTTTGTACAAAATTGTTGTTTTTCCTGCAGCATCTA

ACCCCACCATTAAAATACGAACATCTTTTTTTTGAAATAAACGATTAAATAACCTACTTA

CATATAATCCCATCTTAACTTTGTATGATAATTATCTCAAAGAAATAACTTTATAAAAAA

AATAATAAATTAGCAAAAAAACTATTCAAAAAAATAAATTAGTCATATAAATAAATTTAA

AAGATATATATATATATAAATATTAAATATTAAAAAAATTTATTTATTTATTTTATTTAT

ATATTTTTTTTTTTTAATTAGGAAAAGTAATAAAAGCAGGTGA

>c2398_g1_i1 len=1306 path=[1344:0-910 15:911-1305]

TATATATTTATATATAATTTTTTTATCTTTTTTTTTCTAAGAATAAAACACTTTTTGTCT

TGATATTTATTATTTCTTTTTATTACATAATTCCAAATCTAATTGCTCTAGCTTTTAACT

TTTCACTTTCTTTCATTAAGCCAAATCTTTCTGCTCTTTTCTTTTTTGTTTCTACATCAT

TCAGTTTATTCATATTCAATCCAAATCGTTCTGCTCTTTTTCTTTTATTTTCTTCTTCCA

TTTTATGAGTAATAATACTAAATCTTTTAGCTCTAGACTCAAGAACTTCATCTGTAGAAA

CAAAACCAAATCTTTTTTTTCTTAATTCTCTTTTTTTTTCTTCTGATATGTAAGTATCTT

TATCATCTTTTGAAGTAATATTTTTTTCTAAAGTATTTTTATTGGATAAAGATGATTCAG

AAAATGTTATTTGTGGAATAACAGTTTTACTTTTTTTATCATTATCATTTAAAAAATTAT

TCTTTTCTTTTTGAGTATCTTCTATATTTTTAGATACACAAGTTTTTTCATTATTTTTTA

TTTTATCATTTTCGTAATATTTGGAATTCTCTCCATTATTAGTAGTTAATATATTTTTTA

TATAATCATTCATTTTTTTTAAATTTCCTTTATTTAATTCTTCTTTATTATTTATTGATA

TATATTTTTTTTTATTTTCCTTATTTTCTTTTACTCCTATATTGGTTTCCATATTATTTT

TATCTTCATCAAATTTTTCTTTTGTTGGAGATTTAACATTTATATCATTGTTATTTTTTA

TGCCTCCTTTTTCTTCTACAAATAATTCAACATTCTCTCTATTGATACTATTATCATTTT

TATCTATTATATATGATGATGATTTTACATTATCTTTTTGTTCATATTTTAATAATCTTT

CTAATAATTCATTTTTTTTTCCATGAGATGGCAATCCTCTTTTGGCTAATAAATCTTTTA

ATTCTGAGCACTTTAAATTTTGATAATTCATTTTTTTTTCTTTCTTTTAGTTTACAAAGT

TAATAAAATTTTCTTTTAATTCCTTTTCAGTTTAATATTAGCTTCATTAAAGCATTTTAT

TTCCTTATTTTTTATATGCATATGAACACTTTTTTCTTAAAAATATTCAATTACAAAAAA

TAAAAAAAATATAGATTATACATATATAATATAAATGAAGCTGAACTTTTGATTAATTTT

TGTATTTTCACTTAATGCATATAAGATTAATTTTCTTTTCAAATGTATTTTGTTAATTTT

CAATTGTATTTTGTTAAAACTTAAAAAAATAAAACTTCATTTTCAT

>c2402_g1_i1 len=263 path=[1:0-262]

GCATACCCTGAACCTAAATCCTTGTCTACTAATTTTGGTCCATGTCCAGCTGTTACTGAT

TCACTACTTGGCTGAATGGAAGTAGTCAAAGAAAGATTATCACCAGATCCAAATGAATTC

TCTTCAACTGTAGAAAAATTAAGTATTGAATCATACTTGTCCTCTTCAGCTTTCATGTTA

TCATCACTGACTTGTGTTGACCGTATGACAAGATTTGGCATTGTCTGCACACTTATTATA

TCTTCAGGCATTGACTTGGAGTG

>c2406_g1_i1 len=404 path=[428:0-351 1049:352-356 784:357-403]

GCCTGGCCTTGAAATGAATAGCAGCTTCTACTAAAATGATGAATTACATGCTCTTCTAGG

TAAATTACATGAAATTATGGTGAACCCCTCTTTCTCAACCATAATTCAGTACACATGAAT

GTGTTCGACTACTGCTGTTATGAGGAACGTTGTCTTGTTACTAATCTGCAGAATGTCAAT

TTGCTGCAAGAGGATAAAATACATTTATCGTTTCTAGTTTCACACAAGAGTGAATTATTT

CAATTTCATTCATTTTCACTCAAAAGAAATAATACAGATTTCCCATATTGAAACAATCTG

TTTCCCTTTCATTTACACACTTATCTACTACGGCAAGATAAAATATGACCTAAGAAGACA

TTATCACTGATTATTCCAGTTTTTATTAAATTATAAAGCATATG

>c2415_g1_i1 len=1882 path=[15:0-1077 1093:1078-1881]

TTACATTTTTTATATTAAATTTATTATCCTATGGAAAATTAAGTAATATTATTTTTAAAA

AATTTTTTAATATTTAAGGTTTTATGATTCTTCATCATTCAGAATTTCTTCTTTCATAAC

TTCTTTGAGTTGAATATAAATTTCGTAATAATCATCTCCTTTTAGCATATCTTTAACGAA

GTAACAATTAACTTTAATTCTAATTTGTTCTAATGCTCCTTTTATAACACCACATAATAA

ACTACAATAATTTAGAGAAGATAATGATTTTGGTAATTCAACAAAATCACTTAATGGATT

ACTGTCAAAAATTATAGAAAAAATATTTGATTCTCTATTTATACAATTAACAGTTCCACT

TATTCCTAGAAACATTTTAAATGCTACTTTAGCTATAACTTCTACAGTTTCTTCAAAATT

TTCGCAAAATGAAATATCAGATTTAGCTAGAAATTCTTCAATTAACCTTATACCAATATT

ATATCCCATTTTTTCTAATTGTTCATTAACTTCATCTACTAATTCTAAATCTTTTAACAA

TTGGCTTACTAAAGCCCCATATGTTAATGATAACAATTCCGAATTCACTTTTTCTAATTT

TGAAAAAACGGCTTCTCCATGTTTTTGATATTTATCTTTTGACATTTTTTTTTTTTAAAC

TATACAATGTTGTTTTTTTGCATAATTTTGTGTATAAAGTAATACACAATTTTCCTTTTT

TTGAAAAATTATTATTTTGTAAATTTTAAAAAAATAAAAACAGATGAGTGAAAAAAATTA

TTCTTTATATATTTGTGTTAAAAAAAAATTATCACATATAATAAACACAAAAGAATATTT

AAATCATACAAAAATCATCTAGTATTTGTTTTTTTTCATAAAATAAATTATGATTCTTCA

TCTGTTATTATATTTTTATTTCATGTATATTCTGAAATTATAATTATTTTTTTACATGGG

TATGCTTCTAATATAAAAAAATTATAAACTTTTAAAACTTACATTTTTTTTTAACAAATA

ATGAATAAGTTTTATTCTTGTAATTATATGTAATCATTGAAGTTTATTATTTAGTTTAAG

TAAAAATTTTTATTTTTTCTTATCCTAATGAATATGAATATTTAGGTTTAGGTATATGTA

AACATATAAAATTTAGTGGGTTAAATAAAGAAGGTAAAATAAAAGGGAAATGGAATAATA

ATGATGATAAAGAAAAAGATTTGAAGCAAATATTTAGAAGTTATACACCTATATATATAG

ATAAAAAAAATAAGCATGTTCATTTTGTTATTCGAATATATTATCCAGATGATAATTATG

TAGATGGTGGAAAAATGAGTATGCAATTAAACAATTTAAAATATAATGACAAATTAGAAA

TAACAGGTCCTTTTGGTTTATTAGAATATAAAGGAAATAATGAATTTGTACATTTATCAA

AAACTATAAAAAATAAAAAACATATAATTATGATTGCTGGTGGTACCGGTATGACACCTT

TTTTTCGATTAATTAATCATCTACTTTTAACAGAAAATAAAGAACATAGCAATATAATTT

ATATGACATTTATTTATGCAAATAGAAATGAAGAAGAAATTTTACTAAAGTCAATCTTTG

ATGAATATGAAAGAAAATATGAAAACATTAAAATAGTTTATAGTATAGACAAATGCTTAG

ATGAAACAAAAAAAAATACATTTGAACACATTGGATTTATAAATGAAGAATTATTAAAAA

AATATGTTTTAAAGTATCAGAAGTTAGATATAAAAATTGAAAATGAAGACACACTAATTC

TATTATGTGGTCCTCCACCTATGACTACTTTTCTTCAAAAAATGCTAAAAGATCTAAATC

AATTAGAAAATATTATAACTCT

>c2415_g1_i2 len=1072 path=[4087:0-267 1093:268-1071]

AAAAAAAAATATATATATATATAGAATTTGTATTAATGGGAAACATTAATTTATTAATTG

CAAATTTTATTAGTCTGATAATTATATCTATGTCGATAGCATTTTTAAGTTTTTTTAGAA

AGAAGGATTGTGCAAAAAACTTATTTCACATATATTCAGAAAAACATGCTTATGAAGAAT

TAGATGATAAAAATGAAAAAACAAATGTGTTTTTAAATAAAAAAAAACAAATTTTAAAAT

TACATAAGATTATAAAATTAACAAAAACAGTAAAAATTTTTATTTTTTCTTATCCTAATG

AATATGAATATTTAGGTTTAGGTATATGTAAACATATAAAATTTAGTGGGTTAAATAAAG

AAGGTAAAATAAAAGGGAAATGGAATAATAATGATGATAAAGAAAAAGATTTGAAGCAAA

TATTTAGAAGTTATACACCTATATATATAGATAAAAAAAATAAGCATGTTCATTTTGTTA

TTCGAATATATTATCCAGATGATAATTATGTAGATGGTGGAAAAATGAGTATGCAATTAA

ACAATTTAAAATATAATGACAAATTAGAAATAACAGGTCCTTTTGGTTTATTAGAATATA

AAGGAAATAATGAATTTGTACATTTATCAAAAACTATAAAAAATAAAAAACATATAATTA

TGATTGCTGGTGGTACCGGTATGACACCTTTTTTTCGATTAATTAATCATCTACTTTTAA

CAGAAAATAAAGAACATAGCAATATAATTTATATGACATTTATTTATGCAAATAGAAATG

AAGAAGAAATTTTACTAAAGTCAATCTTTGATGAATATGAAAGAAAATATGAAAACATTA

AAATAGTTTATAGTATAGACAAATGCTTAGATGAAACAAAAAAAAATACATTTGAACACA

TTGGATTTATAAATGAAGAATTATTAAAAAAATATGTTTTAAAGTATCAGAAGTTAGATA

TAAAAATTGAAAATGAAGACACACTAATTCTATTATGTGGTCCTCCACCTATGACTACTT

TTCTTCAAAAAATGCTAAAAGATCTAAATCAATTAGAAAATATTATAACTCT

>c2417_g1_i1 len=348 path=[1:0-347]

GGGACAAATAGGAAAAATGAACAGATGAGGACAAGGGGAAGTCATCTGCTCTCCCCACTG

CCAGAGCAGACCTGGTGGAGCTGTCCAGGGGAGGAGGAGAACCCAGAGCATGGTGAGAAA

TCAGTGGCAGCAGGTTCTGCTCTCTCAGGGCAGATGAAGGAAGGGAGCAGCCAGCTTGAC

ATAGGTGTTAGAAAGGTGCACAAGCCTCCTGCCTGCACTTGGTTGCAGGTCTCTTCCCTT

GTCTCCTCGCTGGCTTAGAAATGCACTGCCCTGCTTCTCAAGGGACTCATTCAGATCCCT

GGTGGTTGGACTTTGTGATCTTAGAGATCTTTTCCAACCTTAATGATT

>c2421_g1_i1 len=318 path=[296:0-62 359:63-317]

AGTGAGAACAAATGTTCCAATTGCTGGGTTAATTTGACCTTACTAGAGGGGAAAAGAAAC

TGAAACCACGTGTTCCAAGTATCCTCTCAGGATTAGGGATCAGAGAAAAACAAATCAAAA

TTATCTGCAGAAAAGATGGAGAGTGATACTGTCCGTTACTCCATAAAAGTGGGGTATTTG

TGGGCTGAATGAGTGTGGAGTCGCTGTGTTTTGTACGAATCTACTTGCAAAAGGCATGAG

ATAGCAGTTTGTCTGCCACTTGCTCTGCTTCAGGCTCCTGATTCTCCATCAGGCTACAAT

GGTGCGCATTCATTTGCT

>c2421_g1_i2 len=336 path=[672:0-80 359:81-335]

AAAAAAAAAAAAAAACAAAACACATGGGAATCATTGCTAAAAAGAACAAAGAGTTTTCTG

TTGTGTCAATTGAAAAACTTTAACCACGTGTTCCAAGTATCCTCTCAGGATTAGGGATCA

GAGAAAAACAAATCAAAATTATCTGCAGAAAAGATGGAGAGTGATACTGTCCGTTACTCC

ATAAAAGTGGGGTATTTGTGGGCTGAATGAGTGTGGAGTCGCTGTGTTTTGTACGAATCT

ACTTGCAAAAGGCATGAGATAGCAGTTTGTCTGCCACTTGCTCTGCTTCAGGCTCCTGAT

TCTCCATCAGGCTACAATGGTGCGCATTCATTTGCT

>c2422_g1_i1 len=210 path=[223:0-209]

GATAAACAATCATTTTCAGGTCACCTGATGTAGCCCGTGTAAGACAGCAGGTATGCCTGT

AATGACTCCTCCTTGCCAGAAAATGCCTGGCGTGGTGAGAGCTTACTCCCAGCTTTCCAA

ATCAGGCAAAGAAAACACAGGGAATGCTGCAACCGTATCCACTACAACTACTCTATGCCT

GTAAACAATCAATAATACAGTCTATCAGAA

>c2423_g1_i1 len=210 path=[155:0-209]

AAAAAAAGCCTAATTATGTTTATGATATTGGAAAAAATAAGAGAAATGAAACTTTAAAAA

AATATAAGGACTCAATTTTTAAGGATCTACAAAAAGAAATCAAGAATAATTCAAATAAAA

ATAATAAAAAGAAAGTTGTACTTAAAATGTTACACAATAAATTAAATAATTTTGAATTAG

ATAAATTCAATGATTATAAATATATTAATT

>c2424_g1_i1 len=210 path=[188:0-209]

ACCATTGCACAGCATATATGCAGCAAGGCACAGACCTCCCATTCCCCCAGCTTTCCTACA

ACTACAGTCTCTCTGGGACACGCAAACACAGTGCGAAGACTCGGCCTAACCCTGCACAAA

AACAGAACAAAATGGAGACCCAGGAGCAGTGGAGAGGAGGAAGACAAGGAAGGAAGGCCA

GGGAGGGCAAAACCCTGTTGCAGCCCATGG

>c2432_g1_i1 len=230 path=[351:0-229]

ATTGACTGCGGGTCTCTTGTAAGAATATCTAAATGTAGGCTGTTGAATAATCCCTTTGGG

GGCCCTGTAACTATTTTTGTGCCTGGATATGCGTCATCAGTGGGTATTTTCTCTGGTGAC

TAGCTTACATCAGTAGGTCTCCTACCATACACTCTATCAGCAAGTGACTTATGATTGATG

GGAGTCACGGCCGCGCTTGGGCATTTATCTGTGACCTCAAATAATGGACA

>c2435_g1_i1 len=253 path=[231:0-119 351:120-252]

TGGCAAAGTGATGTCAGAAGGACAGTGCACTGGCAATGAGTCAGCGTTCTACTGCTAAGC

GCAGCATCACGTGCTTGCCCACACGTCTGAAAATAAAACTCTTCCAAAAGAACTCACTTT

GCCAATGCCTCCCCGGGGAGAGAAGAAAGCTGTTTTATTTTTGGGCTGTAGTTGTACGTG

AACCGAGGTGGAATCAATGTCCCGGAGTAACAAAGTTAAGCTTGAGCTGTGAGCCTCCAA

CCTGCGTTCCGTA

>c2435_g1_i2 len=215 path=[231:0-119 484:120-214]

TGGCAAAGTGATGTCAGAAGGACAGTGCACTGGCAATGAGTCAGCGTTCTACTGCTAAGC

GCAGCATCACGTGCTTGCCCACACGTCTGAAAATAAAACTCTTCCAAAAGAACTCACTTT

TCAGTTACTGCCATTTTATTAGGAGGAAAATTACGCTGAATACAAAATACTTTCTCACAA

AGAGAGAAAGGTAAAAAAAAATCTCTCTGAAATTA

>c2438_g1_i1 len=1205 path=[1:0-934 3167:935-937 939:938-1204]

TTGTGTTTGAATCGTTTAAAGCTCTTTTATTTTTAGAGGGAGTATTATTATTAGTAAGAA

GCAATTGAATTTCATTTAATAAATAGGAAATATCGGAATGTCTTAAAGGAAATATTCTCA

TAGCGGTAAAATTAACTCTTTCTATTCCATTATAAACTTTCTGATTAACACGAACAACAA

CTTTATATTCTTTATATAAAAATTCATAATTAAATAAATAATCTAAAGTATCATCATCAA

GATTTTTTAATTCTTCTGCTTTTTTTCCTAATAAATTATATCCTTCATCAGAAAAAGCTC

TTAAATTAATTGACCCAGAAAAATCCATAAAAAGAAAATTAAAAGTATAATTATAAAAAG

GTTTGACATTATTTTCATCACAATTCATACATGAATAAACAACATTATCTTCTTCATTAG

TATTATTATAATTACTATCTAAATTATTATCTTGTCCACTTGATAACAATTTTTTCTTAC

ATTTCGTGCAGGCAGGATAATAAAATCTGTGTTCATTTTCTTTACTTTTCCAATAGATTC

TTTTAATTCTTCCTACTAAGGTATAAGTACCTGATATTTTAGATAATACATCGTTAACAT

CACTTAATGCATAATATTTATCTGCATTTTGAGATACATCATTAGATAAAATATTTCTCA

TATTTGTAAATTGTTCATATTTACCAGTAGTATTATACCACTGTAAAATTTTTGCACATT

TTCCTTTTAATTCTTCATTTAAAGTTTCCATTTTTTCAATAGAACTTGTAACACCAAATG

TACCATTTTTCATATCATTCCAATTTCTTATACTAATATTAGTAAAAATGTATATTTCAT

TTTCTTGAATTTCATCCTTAATTAAATTTACATTGGTATCCCAAAAGGAAATTTCAAATG

AATGTTTCGTATCATCAACAATAATAATATTTTGCTTTGTTATATCATTTCCTTGTTTTG

TTTTTAAGTTACTTATTGCACCAATATGTTTTACTATACCTATTAAATCAGCAGTAAATG

GAGTTTCTTTTGTTGCAATTTTTATATCTCTTAAATTAACTAGTGATATTTTTTTTTGAA

TTTTTATTTCTCCATCATCTTTTACTTCATGAATTTCACTATCTTCATTAAAAGTTAATT

CATATTTATGTTTTACTGTATTATATTTTGGATTAGCTATTGATACTCTTCCTTTCGAAA

AAATA

>c2438_g1_i2 len=1247 path=[1:0-934 3167:935-937 3170:938-1246]

TTGTGTTTGAATCGTTTAAAGCTCTTTTATTTTTAGAGGGAGTATTATTATTAGTAAGAA

GCAATTGAATTTCATTTAATAAATAGGAAATATCGGAATGTCTTAAAGGAAATATTCTCA

TAGCGGTAAAATTAACTCTTTCTATTCCATTATAAACTTTCTGATTAACACGAACAACAA

CTTTATATTCTTTATATAAAAATTCATAATTAAATAAATAATCTAAAGTATCATCATCAA

GATTTTTTAATTCTTCTGCTTTTTTTCCTAATAAATTATATCCTTCATCAGAAAAAGCTC

TTAAATTAATTGACCCAGAAAAATCCATAAAAAGAAAATTAAAAGTATAATTATAAAAAG

GTTTGACATTATTTTCATCACAATTCATACATGAATAAACAACATTATCTTCTTCATTAG

TATTATTATAATTACTATCTAAATTATTATCTTGTCCACTTGATAACAATTTTTTCTTAC

ATTTCGTGCAGGCAGGATAATAAAATCTGTGTTCATTTTCTTTACTTTTCCAATAGATTC

TTTTAATTCTTCCTACTAAGGTATAAGTACCTGATATTTTAGATAATACATCGTTAACAT

CACTTAATGCATAATATTTATCTGCATTTTGAGATACATCATTAGATAAAATATTTCTCA

TATTTGTAAATTGTTCATATTTACCAGTAGTATTATACCACTGTAAAATTTTTGCACATT

TTCCTTTTAATTCTTCATTTAAAGTTTCCATTTTTTCAATAGAACTTGTAACACCAAATG

TACCATTTTTCATATCATTCCAATTTCTTATACTAATATTAGTAAAAATGTATATTTCAT

TTTCTTGAATTTCATCCTTAATTAAATTTACATTGGTATCCCAAAAGGAAATTTCAAATG

AATGTTTCGTATCATCAACAATAATAATATTTTGCTTTTTTAAATAATTTCCTTATTTTA

AAGAAATATATCATTTTATTAATGAAATATTCAAAATGAGTGAAAACGACTTGCTCTTTC

GCATTAGCCAAATAACAACATATGTGAGTAAATGGATAATAAAAGCCAAAGTTGTAAATA

AAACAAAATTATCAACTTTTAAAAATAATAATAGTTTTTTTAGTATAGATATAACAGATG

TACACGGAGATTCTATTTCTTGTAAATTTTGGGGTAGTTCAGCAGATAAATGGTTTAATA

ATATAGAATTAAAGAAAGTATATATTTTTTCGAAAGGAAGAGTATCA

>c2440_g1_i1 len=1291 path=[1424:0-1208 2633:1209-1290]

AATGAATATTGAAAATATATTTCTCCATTTTAAACAACTTTTCATCATTTTTTTTTTCAA

ATTTATAATTAAAAATGTAAATAATTCAATGAAAAAATTATTGAATACAATTAAAAATGA

ATATTGAAAATAAAATGATGAAAAGTTGTTTTAAATGGAGAAATATATTTTCTTTTCTTA

AAGAGAAAGAAAAAGAAAAAGTTTTATTACATAATTGCATTCAGACGTTGTTATCTGATC

CTATAGTTGTTTTATATACAGACGATGATTATATATGTACAGGATCTTTAACGGGAAAAG

TATGCTTATACAAAATAACAAAGAAAGAAAATAAGGAAGTGAATACAGAGATTTCCTTAA

TTGATAATAAAAAGAAGATAGAATCAGATGAGCAAATTTTAAATGATATGAACAAAAATG

AATTTAAAGAAATAAATAACAATTCAAATAATGAAGATGTATTGAGAAATAAGAAAGAAA

TAAATAATAATGAAATAATTGAAAAATACCATATAATATGTATGTTTAGGAATAATAGTA

TTAAGTCTATATATATAGATAAACAATATGTTTACATTTATTATGAAAACTGTATTGATG

TTTTTTCTATAAAAACATATCAATTTATAAAAAAGGTAAACTTAGAAGTTTCTAATAATA

AACAAGTAATATACTATGAGAACCTTATTTTATTTAATAATTCAAAAAATTTATTTATCT

ACGATATTACAAATAATTTTATGAGTTATTTTTACAAAAACTTTTTAAAAAATATAGTTA

TATTTGATTTCAATATAAATTATTTATTATGTTACAAAAACAATTTTGGGAAATGTCACA

TTCGTGTTTTACAAATTTCTACAAATTCACAAAAGTGTGATCATGAATTAATTTTTTGCG

TAGATTTTTCTTCATCTAAATTTATAAGTCATGGGAAATTTTTAGATGATGAAAAAATAA

TTGTAGCTAAAAATGGAAACAGACTAATAATATTTGATTTTAAAAAGGCAATAGATATGT

ATAGAATAAAAAAATTAAAGAAAAAAATTTTAGACATTTATAAATCCGTTGAGAATATGC

TATTTATATTAGTAGAGAATCAAATACTAATTTTTAATTTATCCACTTTTTTATTTTTTA

AATGTGTCCAATTACCAGTTGGTCTATTTTATTTTAAATGGTCATATTTTATTAGATATA

AAAATAATAAAATAATTTTTTCATGTGATAAAGGAATTTATTTTATTGATTATCAATTTG

AAGAAAATGATTAAATTCAAAATTACTTGTT

>c2440_g1_i2 len=216 path=[11:0-133 2633:134-215]

TAAAAAAAAAAAAATAAAATAATAGTATATTTAATAACATTAGATATATATGAGAAGGTT

TGATAAATTGTTTTGGAATTAATATAATTATATATATTACCAGTATTAAAAACAGAGCAA

CATATATATTACATAAATAATTTTTTCATGTGATAAAGGAATTTATTTTATTGATTATCA

ATTTGAAGAAAATGATTAAATTCAAAATTACTTGTT

>c2445_g1_i1 len=1068 path=[1:0-1067]

AAAAAAAAAAGTAAAAAAAAAAAATATCCACATAATAAATCATTTAAAAATAATAATATA

GAAAATGCGAATGCTTATATGTTAAAAAATGAAAACTATGAAGAAAATACAAATTGTGAT

ATTTACGAAAATCAAATAATACAACAAAATTCTTGCCATGAGCATAATAGCGAAGAAATC

AATAACATTAGTTTAAAAGCAGCATATATTCATGCCATTAGTGATTTAATTCAAAATATA

GGTGTTATGATTGCATCTGTAATTATTTGGTACAATCCCAAATATTCTATTACAGACCCT

ATTTGCTCTATTATTTTTTGTTTTATTGTTTTTTCAACTACCATATCGGTAATCAAAGAA

GTTTTAAATGTGCTAATGGAAGGAACTCCTGTTAGCATTAATTTAATTGATTTAAAAAAT

GATCTATTAAAAATACCAGGTGTTATAGATGTTCATGATTTACATGTTTGGTCATTATCT

ATAGGAAAACCAGCTTTAGCTTGTCACATAGTTGCACACAAAAATAATTCACACAATGTT

TTGAACGATGCAACTTTATTGTGTCAGTATAAATACAAAATTTTGCACACTACTGTTCAA

ACTGATTATCCATCAAATGTTTCCAATTGTGAGACTTATGCACATCTTAAATGTTCAAAT

TTAAAAACAAATACGTAATAATAATAGCTGAATATATATGCATATGTGTATCATTTTGAA

AACTTATTTGTTTAATTTAATTTGTGAATATCATGACTATTTTTATGGTTTATATGAGTA

GAATTTATCTATAATACAATCATTTGTACCATAATTTAATTTGCACACTTATATTTACAT

TTTAATTAAAGATATTTATAAATTATCATAAAAAATATTTTGTGATGTTTAATTATATAC

ATATGTAACCATAAAAAAGTGACATAATTAAACGCATAGAAAAATGAACTTATGAAAAAA

TAAAATTTTGATGTAATATTTTTTAACTTTTAGTTTGTCAATATTTACTAACAAAATAAG

AAGAAAATGATATATGCATTATATTTTATTTATTTTATTTTATTTTTT

>c2445_g2_i1 len=1318 path=[3386:0-1317]

ATTTTTATTTTTTTTTTTTTTATTTTACTTTTTTTTTTTTTTATTAGATCATCGTTATTA

TTTACATGATCATTTATAACTACTTGAAAATTATCATTATTTTCATTACTGTTTTCAAAA

TTGCCTTTCTTAATATCATTTTTTGATTCTTCTAATGAGTTTCCTATTGATTTTTCTTTA

TGAGAATTTTCATTATCATTTTTATTCAAGTTTTCATAATAATCAAAAGTAACATAATTA

CATGAAATTCCATTAATTTTATTGTTTAATACATTTTTATTCATTGTAGTATCATTATTT

TCTAAAGCAATATCACTATTTATATTTTTGTATCTTTTATTTTTTGATTGAATTTTTGAA

TTACTTACACAAGTTTTATTATCCTTATGATTATGTGTCCCATCATTATGACTACATTTT

CTATTGTCAAAAAATCCAAATCCATGTGTATGAACTTTTAAAATATATGCGATGAATATA

TTTGCTAGAGTACTTACAAATGATGTAACAAACATTATATATCCATTAACAACTTTAACA

TTAAATAATCTGAATATGGCACTATATAAAATATAAGCAGATAATGCCCATATAAAAAAA

ATAGAAAATAAAGCTCCTATAATTTCGGCTCTATGATATCCAAATGACATATCAACATTT

CCTTCAAAATTTGAGACATAAATAGAAAAAAGATTTAACCCAAAAGAAAGTAAATCACAA

AATAGGTGAGAAGCATCTGTCATTAAAGATAAAGAATTAGATACAATTGCAGCAATAATT

TCAATTATCATAAATATGACGCATATTATACTTGCAATAATTAATTTTTTAGTAGCTCTC

TTTTGAGATTTATCATATAAACGAGACATTTTCTCTGCATCATTTACATTCATATCTTGT

TCAAGTAATGATTTATTCATTTTAATTTTACTTTATTATTTTATATTTCTATTTTATGAA

AATACATTTAGCTAAATGTGAGAAATTTTTATTTCCTCATAAAATGTGTACCTTCATATA

GAGATTTATTCTGACCATTTATATATATTTTACATTTAATTATTTTTTATTCTATCAATT

CTTCAAACTATAAACAATAGAATATTATTAATACACCCTTATAATTTTAATAGGTCATTT

ATTTTGGTACTATTATATGAAAGAAAAATATATAATTACCAAAAAAAATATATTTAAAAA

AATTATGCTTTAAACATATTCTTAAAAATAAAAAAAAGTTGCTTATGTTCTAACTAAAAA

AATTATATAAAAAATAAAAATGAGATAAATAAAAGACAAGAAAAAAAAAAAAAAAAAA

>c2446_g1_i1 len=227 path=[1:0-226]

GAGTGTGGTGCTCCCAGCTCTGGGTTTGCTGCCTTCATCCCACGCTGCATGCAAGCCATC

GTGCTGTTCCTGCTGGGAACCTGCCTGGCTTTGAAGTGGTATTGCTTGCAGGTGGTGGTG

GGACCGTGGGGGGAAAGGGGAACAGAGGCACGCAGGGGGCAGCCCCTCAGCTCTGCAAGC

AGAGCGTGCTTGTGCTGTGCTGCTGATGGAAGGAGCCAACCTTCCAA

>c2458_g1_i1 len=244 path=[1:0-101 370:102-243]

GACTCTGACAGAATTTTGTCTCTGAACAGACTGGCAGCCATTCTACATCTGCATTTATCA

GGAGGTGTATGACGACAGCCTAGGATGGAAAGTCTCTCACTCCTCTTTGACAGTAATTTA

CAGATAGCAATTTTTCCTAGGTCACGTGATTATTTCTCTAAGACTCTGTCCTCCAGACTG

TCACTTTTGTTTAAGAGGAGGAAGACGTAGACCTTGGAAAACAATCTGATCTCAACATTT

TTGC

>c2459_g1_i1 len=318 path=[47:0-252 487:253-269 504:270-317]

CAAGAATGCTGAGGAGTATTCTCCAGGGGGATTTTAAACTACAGTCCCCTTGCTGGTGGT

TTCCTACCTGCATCAGAGGCGGTGGCTCTGAGCTCATCCCAGGCACGAGCCCTCCCCAAG

GGCCACCAGCTATATTCTCAAAGAATGGATGTATGGGTGAACCATGGATGTAGTACACTG

TCATCCACATTGTCAGCTGGACAACTGCTTCCTTCAGAACATCTCTTCCTGGTTTTGATG

GCTGACAAACCATTTTAAAACCCAGATAACTTGTTAATGACACAGTACACATTAAAAAGT

TATCTGGGTTTTAAAATG

>c2461_g1_i1 len=209 path=[155:0-208]

ACGTGGTGGCCTGTGCAGCTTTGCAGAGAAGGAGGGGACAAACCTTTTGACATCAGCATC

TTCTCAGTTTGCATTTGGCCGCAAGTGATGCGGGTTTTGCAGCAAACCGAGGAGAAGACC

TGAATTATTCTTGTGGTGCAGCACTGCCCCATTGCATGTCACTGCTGTCCTAATGTTGCT

GAGACATTGTGGGGTGGAGATGAGGCCCA

>c2463_g1_i1 len=320 path=[339:0-211 21:212-319]

TGCTATTATGGAAGAAATGAGATGTAAAAATAAGAAAAAATATAGACTAAATAAAAAATT

TAACTTTTAATAACATATATTTTTTTTTTGTATAATGAGCTTATTAAAGATATGTTTTAT

ATGATGAATTTCTTTATAAACATACATTTTTTATAATTCCAATTAAATATATTCATGATA

TTATGAAAATTGTAGTATTCATAAAGTTAAATATTATCCATTTATGAATTTTTAGAAATT

ATTTTATATGATTATATATAATTAAACACATAATTTTTTTCTATTTTTTTTTTTAGCATT

GTTAGATTCCCTTATGGGGA

>c2469_g1_i1 len=289 path=[215:0-288]

AGAGAGGAGCCAGGGAGCGCAGTGCCTGTGCCACAGCCATCTGCAGGACTCTGGGGTACA

GACACCCACTGGGACCCAGAGGTGCTGCATCCTCACTGCAGTTTGTTCTGCACATTGATG

TGCATTGCAGCAACCCTCATAGCTCTTCTGGTGCTGGTGGTTAAAGTAGGAATGACTCAT

GTGACCCACATTGTGTTTGGATACTGCAAGTATGCAGACAGAGCCCTGTGGCAGCCCATG

GTGGGACAGCACCCACAGCCGGGGGGGAAATCAGGAGAGTCAGAGATAA

>c2474_g1_i1 len=238 path=[1:0-205 207:206-237]

CCAAATAGCTCTTCCAGAACAGCATGACCACAATACCAAAGCTTTAACAGGCGATACACT

AGGAAGTCCAGAAGAATAACAGCTTGGTTTCTTGATGATCTGTTTTCCTACTTCCTGCCA

GCACAGAGATAAAAGTACTTAAACCAGAAGGAATGGATCAAAGCTACATCTGTTAGGAGA

TGGCTGTACTTTAGCAGCTGGTGAAGGATCTTAAACTAGGCCCTGAATACATGTGCAG

>c2474_g1_i2 len=237 path=[1:0-205 269:206-236]

CCAAATAGCTCTTCCAGAACAGCATGACCACAATACCAAAGCTTTAACAGGCGATACACT

AGGAAGTCCAGAAGAATAACAGCTTGGTTTCTTGATGATCTGTTTTCCTACTTCCTGCCA

GCACAGAGATAAAAGTACTTAAACCAGAAGGAATGGATCAAAGCTACATCTGTTAGGAGA

TGGCTGTACTTTAGCAGCTGGTGAAGTGATACCTGCACATGTATTCAGGGCCTAGTT

>c2476_g1_i1 len=231 path=[267:0-171 438:172-230]

TCTTAAACAAGCCTAGAGAAGCAGATTATCAGTTTTGTATACATTACTAACATCACTACA

ATTTCAGAGAGAGCTGCACACGGAAGGAAAATAGCAGCTCATGGCTAATTGCTGAGATTA

ATATGCACTTATCAGTATATTAAGAATCTGGGTTTGCACTACAATTCTCTTTGTCATTAT

TGCTCTCAAAAACCAGAACGGAAATGTATCGTTTTATCTTACTTTCCTAGA

>c2476_g1_i2 len=231 path=[267:0-171 25:172-230]

TCTTAAACAAGCCTAGAGAAGCAGATTATCAGTTTTGTATACATTACTAACATCACTACA

ATTTCAGAGAGAGCTGCACACGGAAGGAAAATAGCAGCTCATGGCTAATTGCTGAGATTA

ATATGCACTTATCAGTATATTAAGAATCTGGGTTTGCACTACAATTCTCTTTTTCATTAA

TTGCTACACTTGTCCTGTTTTGTTTCTCTAGGAAAGTAAGATAAAACGATA

>c2476_g2_i1 len=241 path=[749:0-240]

GGGAATCCACTGCTGTCACACATAAGCTACCAGGCCTGGCAATAAATAAAGCAGGTAAAG

TGCAATGGTTTTAATAGGAAAGCATTCAAACTATCTGATCAAAGGGTGCCTCCTACTCTT

TCGTTCTGTGACTCTGTTGGCTGAATGCAGGCTGTATTATAGAAGATGTTCATGTCAGTT

CTGTTAGCTTGTATGTCAACAAAAATTGGTACATTAAGACATGGGGTTTGGTGATTATGT

C

>c2478_g1_i1 len=204 path=[1:0-203]

AGATGAATATGAAAATGAAGAAAGCCTGTGCTTGAAATTGATGCTAAATATAAAACTCTT

ACATCAGGTGATGATTGTGGTTTAGTCAGTTGTGTATGAAGTAAGAAGCATTAACAGTTT

TCCTGTAGTGAAATATCATCCAGATTCCTGATGTTCTTAAGTGTCTAGAATAAGAGTGGC

AATTTTGTTTTTAAGACCCTCCTG

>c2481_g1_i1 len=1009 path=[44:0-353 34:354-1008]

AAAAAAAAAAAAAATTAATAAAAGTAAAAAATTTTTATTTATTTAATTGATTTTTCAAAA

AGAAAAAAGGAAAAGTAATAATAATAATTTAAATAGCATAAGTTATTTAGAAATTGAATA

TATATATAAACATATATGAAGAAAATTATTATTGACAGTAGATATTATTATAAAAAATAA

AAAAAAAAAATTTTAATTTTAAAATAAATTAGTTTTTTTCATAATTCTCATAAATTCCTC

TTCATTGATTTCTCCATCACCATCTCTATCAGCTTCATCTATCATTTCTTGAATTTCTTC

ATCAGTTATATTTTCTCCAAGTTCTTTTGCAACCCGCTTCAGATTTTTGAAAGATATTTT

TCCTGTTTCATCATCATCAAACAAACGAAATGCTTTTAATATTTCTTCTTTTGGATCCCT

TTCACTCATTTTTATAGTCATAATGTCTAAAAAGTCATTAAAATCAATAGTGCCTGATCC

ATCTTTATCAACATCAGATATTATTTTACGTATATCTTCTTTTTTAGGTTCAAATCCTAA

AGCTCTCATTGCCACTTTTAATTCTTTTGCATCTATTCTCCCTGTTCCATTAGTGTCAAA

TAAATCAAAAGCTTCTTTAATTTCTAATTTTTGTTCCTCATTTAATTCGTTTCTTTTATT

ACGATTATTAGCGTTTCTTACCATGTTTTGATTTTTTCTATTCATTTTAATTATCGTTTA

TATATTTATAAATATATATACATATGAGTATATATATTATTTTCCTCGTTTTTTCTAATA

TTTTATGTGTATTTTAACAAATTTTTGATTATATGTATTTTTTACAAAATTTAAGATTTG

TATTATTTTTAATCTAATATCTTCTATTTTATTTAATCTTATATTTTTTATTTTATTATA

TCGTAATATTTTATATTCAATGTAATTTTAAGTAAATATTTGTTTTCAAAGAACAAAATG

TAATTATTATTATTGTATTATTCTGTTAATATATATATATATATATTTA

>c2483_g1_i1 len=393 path=[471:0-392]

GGGCGGCTCAGGCCGTGCCCGGGCTCGTGGCGCCACCTGCCGGCAGCCGTGCCGGGACCG

TCGGTTTCCCGCAGCCCTTCGTGTGGTGACGCACCCGCGCTCCTTAACGGCCTCCGCTAA

AAGGTGGTCCCCAGCAAACGGTCGCTCCAGCACAGAAAATGCTACGGGAAAGAGCCCCGA

CCAATATGATTAGCATCTGTTAATTCTGTTATAAATTTTTTTCAAGGTCAAGTAAAATAG

CACTGAAATTAGAAGTGATACAAATGACTGAGTAGATTCAATTTAAACAATTCATTTGCA

GGGGATGAAATGCTGAAGAGAGGTTTGTTGACTTCGAAGGTGGTGTGTTTGCACACGACA

CAGCACGGGATGGACGAAGCGATGGGAAAGCAA

>c2483_g2_i1 len=233 path=[237:0-205 442:206-232]

GACGAAGCGATGGGAAAGCAAGCCCGGACCTGAGGCTGCGACAGCAAATCACACCTCCGT

GGAAAACGCTGTTCTGTCCCTTCGGGGCATCTGAGCCTCCTCCAGGGAGCTGGAAGCGCC

CTCTGCACCGAGCCCGGAACTGCACAGGCCCTACAAACCCAACCGCTCGGGAATGGCTCT

ACGTACCAGTGTGCCGAGATTACCACGCTTCTGATGCCAGCTAAAGGCATGCA

>c2485_g1_i1 len=209 path=[155:0-182 338:183-208]

AACCCAAGCCATTCTGTGATTCTCTGTAGTAATTTAGAGCTCAAATATTTTAGTGCTGGA

GAGTGGAACATTACTCAGAGTAACTTGCTTATATGACCAATTTCACATTGTGTGTACTTT

TGCATGTCATTTGTCTCTTTATGCTATTAATGAGGCCACTTCAGTTCTCTTCATTCTTTA

CAGTAATAGTCTTTCAAAGTCTGGAAGAT

>c2488_g1_i1 len=203 path=[281:0-60 504:61-174 454:175-202]

ATACCCTTAAAGAAAAGCACTGCACATTTCAACAACATTACCACTTAAATAGCTTCACAT

TAGCCTAATTGTGGTACAGTGGTTACCTAGTCTTACAAACATATTGCTGCTATAACAAGA

AATACTTGGGGTCATCTGGAAATGAATTACACATATCAAAACAACAAGAAAATGACCCAG

TATTCTTATCTCCTGCACTTCCT

>c2490_g1_i1 len=349 path=[327:0-5 333:6-348]

TTATTTTCCATTTTTAACCTACTGGCGACAGGTAACATTTGGCCATTTTGTTTAATTTAC

TATTTCCTTCTTTTCCCTCCTCCCTTCTGTCCTCATCATGTAGGTAGAGCTGTGGGCTTT

GTTTTTTTCCAAGTACCTAAAAGGATATAGTGACAGGTAAAGAGAGAAGCTCAGATATTA

GCCTCACTCTGACTTCAAATAGTTAATTTTTTAATTCTTGCTTTTTCTTCCAGTTGCCAT

TCGACAACCCATATCACAGTCAAAGCCCTGTAATATTCACAAATAAATGATATTTTACAA

ATGCCTATTCCAACAGTCAATGTATTTTGAACCTGAGAGAAACCAACAT

>c2491_g1_i1 len=246 path=[378:0-245]

AACTTCATCATAGCCCTGGGATTTCCTCTGATGTCATGCTTACAAGACCTCTGGAATTCA

GATGCTAAATGTTGTGCTAAAGCTTCTGTGAAGTACACTCCGCCAATGCCATCATCTGTG

TCTGTAGCAAGCACACGATATATTCCACTGTTTACTTCTATAACTGTGATAGACAGCGAT

GTTCCACCAAGCTTATAAACCAACACGTTGCTTTTCCCAGTGGGTGAATCTTGGCCAATT

CCGTAA

>c2493_g1_i1 len=246 path=[1:0-245]

CGGCAGGGAAACAAAACAGACGGGGCCTGTGAGGAACACAGCACAGAGGTCAATTTGTCA

GAGCCTGACCCCCCTATTGCTGCAACATCCTCATGTTTTCCCTCCTGTGATGACTTCCAA

ACGTTTTTCTGTCTTGCACACAGTGCTGGAGCTGCCCTGGGCTGTGTAGGATCAGGAAGA

TTGGGCTCCTCTGTCCCCACAGAGTAAAATTGAAATCATAACCCTACAGGATTAGGTTAT

CTGCAG

>c2494_g1_i1 len=295 path=[1:0-57 59:58-72 74:73-294]

GACACAACAACATTTCAAAGCCTTTTATCTTGCATTCTGGTTAAAAATCCAGACCACGCC

TGCCCCCACGGTAGCAGGGTAGAAAGGAAGCTTACGATGAACAAGCGCAGGCTGAGGGAT

CTCTTTTGTATTCTCCCGCCACCCGCCGCGTCACACCTGAGGCGCGGGCAGGTGGACCCG

GCCGAGTCCCGCCCCACATCAGCAGGGCCGGCGGGACCGCCAGGATGGATGTTCCTATCT

CACTGTTTTTGACCGAACAGAGCCGACTTTAATGCAGGTCACGTTAAACTAGGAT

>c2495_g1_i1 len=207 path=[1:0-206]

ATTTACCTGTGGGAAAGCAGAAGTCAGCTTGGGGAGGATATGTAGGTGAGGCCCTCTGTA

TCTCTGCAGCGCAGGGAGATGTGGTGGCAATGAGATTAGCCAAAACAGGGTTTGAGAAAA

ACGAGCATCTCTCCAGGTTAGCCCAGAAGGATGGCAAGTGAAGAGTCCTTCCTTTGTTGC

AGACTCAGGTCACTTCTATGCTGCAGT

>c2512_g1_i1 len=950 path=[1:0-896 1003:897-924 1031:925-949]

GCTTCATGGTGATGGTGCCGAAGATCTCCTCGCCGGTCTTCACGGTCAGGTAGTCCTCCA

TGTAGAACACCGTCTGCTTCCAGTGCGTGTACGGAGACTCCGGGCTGGTGGAGAAGCCGG

TGCGCTTATGGCAGCGCGTGAACTCGATGTTGAAGTAGGCGACGAGCGCGTGGACGTAAT

CATTGCGCTTCACCTGCAGGCAGAACGGCGCCGTGAACGTCAGCTCCTCCACCTTCACCG

TGTAGATGTCCACCTCCTTAATGAGGCAGGCGTTGGTAACGAGCTGCTTGGGGTCCACCA

CGTCCACCAGGGGCTCCTTGATGGCCACGTCCTTAATGCACGACATGTCGAAGCCGTACA

CGTTCTCCCACCAGTGGATTTTGTAGTCCTTATACTGCCGGTCCTCAATGGCCGTCACAT

ACAGCGTGGCGCGGTCGGGGAAGATGAGGCCGTCGGGGGTCAGCCACTTGTCGCGCGCGT

AGATGACGGTGTTGAGCATGGACTCGTAGAACAGGCAGTAGCCCATCCACTCACTGATGA

TGATGTCCACTTTGTCCACGGGCAGCTCCACCTCCTCCACCTTCCCCTTAATGATGGACA

CCACATGGTCCAATTTGTTGGCTTTGACGATCTTGACGGCGTAATCGGAGATGCTGGAGC

ACTCGATCCCGATGACCCTCCGGGCCCCAGCCTTGGCAGCAAACATGCATAGGATGCCGG

TGCCGCTGCCCACGTCCAGCACCACTTTGTCCTTGAACAGATGGCGGTTGTGGAACATGG

AGTTGCGGTAGGTGAGCGTGCGCACCTCATCCTTCAGCATCTCCTCATGGATGCCGAAGT

GCGCGTAGGAGTCGAAGTAGTAATCCTTGGAGGTCATCTCCTCTGCCGACGGCTTGGAGC

TGCTCTCCGCGGGGGGGCACGAAACCTCCATGATGCAGTTCGCCGCCTCC

>c2512_g1_i2 len=947 path=[1:0-896 1003:897-924 2046:925-946]

GCTTCATGGTGATGGTGCCGAAGATCTCCTCGCCGGTCTTCACGGTCAGGTAGTCCTCCA

TGTAGAACACCGTCTGCTTCCAGTGCGTGTACGGAGACTCCGGGCTGGTGGAGAAGCCGG

TGCGCTTATGGCAGCGCGTGAACTCGATGTTGAAGTAGGCGACGAGCGCGTGGACGTAAT

CATTGCGCTTCACCTGCAGGCAGAACGGCGCCGTGAACGTCAGCTCCTCCACCTTCACCG

TGTAGATGTCCACCTCCTTAATGAGGCAGGCGTTGGTAACGAGCTGCTTGGGGTCCACCA

CGTCCACCAGGGGCTCCTTGATGGCCACGTCCTTAATGCACGACATGTCGAAGCCGTACA

CGTTCTCCCACCAGTGGATTTTGTAGTCCTTATACTGCCGGTCCTCAATGGCCGTCACAT

ACAGCGTGGCGCGGTCGGGGAAGATGAGGCCGTCGGGGGTCAGCCACTTGTCGCGCGCGT

AGATGACGGTGTTGAGCATGGACTCGTAGAACAGGCAGTAGCCCATCCACTCACTGATGA

TGATGTCCACTTTGTCCACGGGCAGCTCCACCTCCTCCACCTTCCCCTTAATGATGGACA

CCACATGGTCCAATTTGTTGGCTTTGACGATCTTGACGGCGTAATCGGAGATGCTGGAGC

ACTCGATCCCGATGACCCTCCGGGCCCCAGCCTTGGCAGCAAACATGCATAGGATGCCGG

TGCCGCTGCCCACGTCCAGCACCACTTTGTCCTTGAACAGATGGCGGTTGTGGAACATGG

AGTTGCGGTAGGTGAGCGTGCGCACCTCATCCTTCAGCATCTCCTCATGGATGCCGAAGT

GCGCGTAGGAGTCGAAGTAGTAATCCTTGGAGGTCATCTCCTCTGCCGACGGCTTGGAGC

TGCTCTCCGCGGGGGGGCACGAAACTTCATCCCGCGCCGTCTGCAGG

>c2516_g1_i1 len=220 path=[198:0-63 262:64-84 283:85-219]

AATATGAAATAATTTTATTTTATTTTTTACATGCATCTATTAACAATTATTCTAAAAAGG

TGTATAAATCCTTTATTAATCAATGTTAAAAAAGTAATTTATTATTTCTCTGTTTAATTA

ATTTTGTAGTATATTGATTTATTAATATGATTTGGAAAGACTTGATTAGAATATTAAACA

GGAAAATTATAAAGAGTTTATACAATTTCTTATACCAATT

>c2518_g1_i1 len=259 path=[155:0-258]

GTTTCCAATTTTAGCAGAGCATTATTCTGTCTGAGCTGTGAGGAGGTAGGTAGCATGCAG

TCTAAAAGAATGAGGAAAAAAAACCAACCAGGGTATTTATCAGGACAGTTGCTAAGTTTG

TCTTAGTGGAAAAAGGAAGCATCCTCAACTTAGGCTCTGATATATCGAAAATGCCATGCT

TCCTTCCTGTGTGTGGTAATACGTAATTTCCATTAGAAACGTGGTCACCTCTATAGTGGT

CTTTAATACTTCCACAACA

>c2528_g1_i1 len=203 path=[1:0-69 71:70-202]

AACCCCTCAACAAGTGCTGATTAGTAATTCTTGTAAGTGAAGAAAAGTGCAGTGTAAAAG

GACTGTGTCTGGACAGAGAACTGAACAGGTGATGGCAGGGGGTTTCTCTTCCTGCCCTCA

TCTCAGGGGAGCCATGAGGCTGGCTGGATGCACAGGGCAATGTGTGCTGTGTGGATGAAA

GTGCTGTGCGTTGGGCTGCCGTG

>c2529_g1_i1 len=246 path=[1:0-135 137:136-245]

GGAACTGTATAGTAGCAACAGTAAGTGAAAGGTGAGCTGCATCTTCTGTGTGCTCCTGTC

ATTGTTGTGGCACTTCTTCAGAGTACTTATTCTCAACTTGAAATCAGTTGGTGTACCTCC

CAAAAGGTTTCTGCCTTGCATGTTCCTTAAAGTCCTTGGTCAAATTAGGCTTGTGACCTC

CAGATCAAAGATACAGTTATGGCTTTAATAGTCTCCTTCAGTCATTTCCAGAAAGAGACA

AAAATT

>c2538_g1_i1 len=554 path=[1:0-249 251:250-553]

GGGCAGGAGGATTCAATGCTTTTGTGAGTATCCTGGGCCTTTCTGTTTGTTGCTTTGCAT

GCTGTGAGTAGTCTTGTCCCTAGCCTAACCTCCTCCCTTTTCCCCAGCAAAATCAACACA

GATTGAGAGTTAACCGATGCCAGGGACTCTTCAGTCTTGATACATCTCGTGTCTTTTCCT

GTCCTGATCATCTGACCAGATTGTACAGTAACGGGCCTTAGTGGAGGCCCTTTCTTTCCC

CAATGGCAGGAAGGGCTGAGCTCAGTGAATTTGCAGTCCAGTTTGCTCTGCTATTTTCAA

AGCTCGTTAATGAGTTGCAGCTGCAGATGGTGTTTTTCACTGGGCCCTGCCAATATGCCT

CCCATACAGCCAGGAGTAGGAATTGCTGCTTTTTGCAAAATGAATTCTCAATTTATAAAG

TATGTTCAGATTCTGGATTTCATTATGTGTTGGCCTGTTCCCCTCACGCCACATGGCTCA

CTTGCAACATCAGTTAAATCTCCTCCTACCCTCCTCCCTTGCCTGGATCAGCTGATGAAA

CTGTGTAGAAATCA

>c2538_g1_i2 len=321 path=[1080:0-16 251:17-320]

CTGCCATTGGGGAAAGAAAGGGCTGAGCTCAGTGAATTTGCAGTCCAGTTTGCTCTGCTA

TTTTCAAAGCTCGTTAATGAGTTGCAGCTGCAGATGGTGTTTTTCACTGGGCCCTGCCAA

TATGCCTCCCATACAGCCAGGAGTAGGAATTGCTGCTTTTTGCAAAATGAATTCTCAATT

TATAAAGTATGTTCAGATTCTGGATTTCATTATGTGTTGGCCTGTTCCCCTCACGCCACA

TGGCTCACTTGCAACATCAGTTAAATCTCCTCCTACCCTCCTCCCTTGCCTGGATCAGCT

GATGAAACTGTGTAGAAATCA

>c2540_g1_i1 len=208 path=[454:0-207]

GTGGCCGGACACAGGAGAGAGCAAGAGCAGGTTGGGTTGCTCTTTCCAGAAGAACAAGTC

TGGAAGAACAAAGCTGGAAGGCGTGGGGTGGGGAGAAACAGTGGCATCTGGAAGACCACG

GGGTGCTATCTGCAGCAAAGCATCGTACGTGCATCAGCAGTAAGCCCGGTGTGCAGGGGA

GGAGAGCCAGGTGGCTGCAGCCAGGCTG

>c2541_g1_i1 len=234 path=[212:0-192 405:193-233]

AGAAAGGAAGGCCTATGAGGTGTGCCTGAGGCAAGTGGGGTCTCATTGCTCCCTACACCT

ATGGGAGGCTGCAGTGAGGAGGTGTTGGTCCTTTTCTCAGGTAACATGTGGTAAGGATGT

GAGGCAACATCTTCAAGTGACATGAGGGGAGGTTTAGATTGAATATCAAGAGGGATTTCT

TCCCATATAAGATCGCCAAGCATTGGAACTGGCTGCCCAGGGAGGAGGTGGAGT

>c2541_g1_i2 len=312 path=[212:0-192 446:193-311]

AGAAAGGAAGGCCTATGAGGTGTGCCTGAGGCAAGTGGGGTCTCATTGCTCCCTACACCT

ATGGGAGGCTGCAGTGAGGAGGTGTTGGTCCTTTTCTCAGGTAACATGTGGTAAGGATGT

GAGGCAACATCTTCAAGTGACATGAGGGGAGGTTTAGATTGAATATCAAGAGGGATTTCT

TCCCATATAAGATTACCAAGCCCAACCACCAACCTGACTCACCAAATCCCATCGCTAAAC

CACATCCCTTAGTGCTACATCCACATTACTCAAATACCTGCAGGGATGGTGACTCCACCT

CCTCCCTGGGCA

>c2549_g1_i1 len=319 path=[1131:0-42 878:43-318]

CCCACCACTTATCACGTTTGCAGAGAATGTATCCCGCTCCCACCTTCTGGTTTGTGACAG

CTTCTTTTCCAAACTCTGACAGTCCTCTTCAAGCTTTTGCTTTTCCTTATTCAGAGACTC

AATATTATTTTGCAGAGTGCGCATTTGAGAAACCAGATGTTCATTTTCCTGACGGTCCTT

TTGAAGTAGTTGTTCCTGCCAGTCTACTTCCCCTTGCTGACAATGAAATAATCTCTGAAT

ATTCTCCAATTTTAACTTCTCTTCTAGAACTGAAACATGTTCTGGAAATGTTGGAGATAG

AAAATAATGAACTTCAGGG

>c2549_g1_i2 len=588 path=[566:0-311 878:312-587]

CAAACACAGAAAAGTTTCTATCTTGACACATTAAATATTTTCTCTGCTGGTAGATATTTG

AGCATGCGGGTAACATCATATAATTGTTACATAAACTACATCTTTCAACGTAATTTCTTT

TAAACTAGCTTAAAAACTGCACTTTCATCTTTACCTTGAAGATCTTTTTGTACAACAGTG

ATGTCTCCATGCAGTGACTTTTTTTGTTTATTCAGTACATCTATCTCCTGCTGCAGGTTT

TTAATATCCAATTCCCTTTTTTCTACATTGGCCAACGCAGTTTTGATGCTGTCTTCAGTA

GCAGTCAAGTCTCTTCTGGTTTGTGACAGCTTCTTTTCCAAACTCTGACAGTCCTCTTCA

AGCTTTTGCTTTTCCTTATTCAGAGACTCAATATTATTTTGCAGAGTGCGCATTTGAGAA

ACCAGATGTTCATTTTCCTGACGGTCCTTTTGAAGTAGTTGTTCCTGCCAGTCTACTTCC

CCTTGCTGACAATGAAATAATCTCTGAATATTCTCCAATTTTAACTTCTCTTCTAGAACT

GAAACATGTTCTGGAAATGTTGGAGATAGAAAATAATGAACTTCAGGG

>c2550_g1_i1 len=272 path=[1:0-271]

TTTCAATTAATGCTAATTCTTCTTTTCCTGTTAAAAAATGCAATCAGACTTGAGTTGGAG

CTCAGGACCTCATCAATGAGGTGCAGCCTGTGAAGAAAATGAATGCAAGCACAGACTTTC

TTTGTAAAGAATCATCAAATCCCCAAGGTTTTGAACCCCCAGTGACAAAATCTGGTGGAC

TTCAAAGGGTTTTGATGGGCTTACAAGGGCTTTGTTTGACCTCTGGAGAGACAGTGAGAT

TCCAGCCAGGTCTGGAGTTGATTTAAGCTGAA

>c2554_g1_i1 len=246 path=[219:0-168 56:169-245]

TTTTAAGTGATTTTAAGTAACTCTCATATGCATCCCAAAATGGATCCAAGTGCAAGAGGA

CAACAGCACACATGGTTTTCTCCAGAGAAGGAGGGTTTGGAATTATAATTCTCCAAGCCT

TATGCTCTTCTTTCTCATCATGACAGGCTTGGAAATGAAGTATTGCCTTGCTGCAAGATT

TGGTAGTATTTAGCAGCAGAGCCGAAGTAGGGTACATGCTGAAATAGTGTTTTGTTCTAG

GATATA

>c2555_g1_i1 len=255 path=[747:0-254]

GCCCAGCCCTGCACCCATCAGACGCTGTCCTCTTTGCTGCCCTAACAAGCATTTGGGCTT

CAGGTGCCCAGGGGGGAGCAGAACTGCAAGCTCTGAGATTCAAGGGGATTGAAAAGAGGG

AAAAAAGGGCAACTGTTTGTCACAGCCCCCAGGAACAGGAATCGGTCTGTGTGCCAGGAG

GCTGGAGTCTTGTCATTTGAATTTCAAAGGCTCTCAGGAAAGGCTACCTCAAGACACTTT

CCCAAGCTGCTCTGA

>c2555_g2_i1 len=396 path=[374:0-395]

TTATGTGAGGGTGTTGGGATGTGGGGGGGCTGCTCCTCACTGTTCGTTAAAGAGGAGAAA

CCCAACCGCTGCGGCCCGTGCCGGCAGAGTGCCACCAGGACAGCAGCTGGCGGTGAGGGC

AAGCTTCTGCAGGTGCAGGGAAACACAAGTCCCCCATCTGTTCCTCCCGCTTCCTGCCCC

TGCTGCTGGCAGCGTGGCTGTTTGTCCTGCTGCAGCTTTCTGTGGCTGTGGCTGAGCGTC

CTCAAGCAGGCTGAGAGGCTGGGCTGCCACCCAGTCACTGTGTGTTACTGGAGGGAATAG

TCTCGACAGAGATGGGTTGCGCAGGGTCATCCTATTGTACTTGTGATGATGGCAATTCAT

AGTTGAAGCACCTGATTTGTTAACCTTATAAAAAGG

>c2571_g1_i1 len=227 path=[1:0-226]

ATTACTCAATGAAAAACATTACTGAAGACTTTTCCATTCTCAGTGCAGCAGCTGGAGCAA

AACAGGACAGAGGCCTATTGCCAGCCAACAACTCGTGAGATAGCTGAAACCTTCATTTCT

TCTATGAAAGACGCAATACTACCATTGCAGTATTTTCAGATGTTCACATAGAAATGTGAT

ATCTGATATTAATCTATTTTGTTACTTATTTCAAAATATCCCTTATA

>c2572_g1_i1 len=201 path=[1:0-60 62:61-200]

CCCCCATTTAACTGAAAATCAAGCGTTTTCTCTTCGCTAATACCCCATTTAACTAAAAAA

CACGTGTTTTCTCTTCGAGAATCCCCCATGTAAGCGAAAAGCACACGTTTTCTCTTTGGA

AATCCTTGTTTTAAGCAAAAATCACGCATTTTCTCTTCGAGCATTCCCCATTTAACCCAA

AAATCACGTTTTTTCTCGTCG

>c2574_g1_i1 len=415 path=[393:0-359 753:360-363 757:364-414]

TGGCTGCTTTGCAAAGGGGGAAAGCAGAGCACAGCTTCTCTGGGACAGAAGGGTCCTTTG

GGTTAAATTCAGGCTGCAGCAGCCAGGAGGGAGATGGGGTTCAAAGGAGCAACTGGATAC

TTCCCGTGTGGTCTGCAGCTGCCTGCTCTCTGGGCAAGGCAGAGGGGATGTTGCATGGGC

AGCTGCAATATGTGGCCAGAGTGCACGGCGTGGACAGACACCCAGCACTGCAGGAGCTGG

GAGCACGCGGGGACCCATCGCTGCTCCATCTGCACCAAGGAGTGGTGCCCAGAGGGGATG

AATCAGCAGCACAGTGGGGTGCAGCACAGGGGTCCCCATCCCAGACTGTCCCTCTGCATG

GGGACACTGCGCTCACCTTGTGCCCTCCCCCAGGGACTGGAGAGTGCAGGAACAG

>c2582_g1_i1 len=243 path=[3:0-167 171:168-192 171:193-217 171:218-242]

GTAACTTTGTGTTTGATAGGGTCTGTAGGTGCTGTGTTATTGTATGAACCCTGGCACTGT

GTTCTTGTAGGGTCTCCCAATGCTGTGTTATTGTAGGGTCTCCCAGGGATGTGTTATTGT

AGGGTCTCATGGCACAGTGTTCCTGTAGGATCTCCTGGCTCTTGTCATAGGGTCTATTAT

AGGGTCTTGTTACAGGGTCTATTATAGGGTCTTGTTACAGGGTCTATTATAGGGTCTTGT

TAC

>c2586_g1_i1 len=375 path=[507:0-374]

ATTTGCCTGTTCAAAATTCTCAGCATAGTGACCATAAAGCTCCAATTTCTGACAGAAATT

CTCCAGTCCTAGTCCTAAATTGCCCCTCTCCAGGTGAAGCGACAGAACTTGGCTGGCAGA

ATATATGGATTCCAGGGGTCCAAATATGGTTTCCATTACGGCAGGTTTCAGTGTCCCTTT

GGCCTTTAAAATTGTCACAAAGAACGTCACCACCAAATCCAGCTGCTCGAGGTATCTGTG

CTCCGTTTCCAGCAGCTCCTTGGCTGTGCGGCTGCGCTTTCTCTCCCAGCGAGCCCTCTG

CTCTTCCACTGTGTTTGCTCCAGCCATGGGTGCAGGGGGCGGAGCTCGGTTTGAACGCGG

AGCGGCGGCTGCGGG

>c2589_g1_i1 len=303 path=[494:0-102 214:103-302]

TCCCAACTCCCTTTGTATTTACTGATGCATAATTGAGAACTGCCTCTGTGATGATACGTT

GGGAATAAATTGTGAAAAACACGTAATTAGGAAGAGGAAGTATACAAATGTGAAATGGGT

TAGGGGCAACATGCAGAGGTAAGACAGAAAAGAATAAAAGGAATTTCCTTGATTTAAGTA

TTAGTGTTTCTATACCTTGCTTGACATTCCTTATTTCTACCTAAAGTTCATACATTGCAG

TGCAGGTGTATTCTTTATCAATATAAGTATAAATTCAGACCTAATCTTGCATTTTCTTTT

CAG

>c2589_g1_i2 len=218 path=[196:0-17 214:18-217]

TGTGAGGAAGAGAATTAGACAAATGTGAAATGGGTTAGGGGCAACATGCAGAGGTAAGAC

AGAAAAGAATAAAAGGAATTTCCTTGATTTAAGTATTAGTGTTTCTATACCTTGCTTGAC

ATTCCTTATTTCTACCTAAAGTTCATACATTGCAGTGCAGGTGTATTCTTTATCAATATA

AGTATAAATTCAGACCTAATCTTGCATTTTCTTTTCAG

>c2591_g1_i1 len=296 path=[1:0-56 58:57-295]

AGCAAAGGAACAAGCTACTGAGAGAGACTCTGAAACATCATTCTCTTTCAAGTCCTACCT

GGTTAAAGCCCTCAGCAATCTGGCGTGCAAGCAAGGGTGAGCAAGAGGTTGATTTAGATG

ACTGCCCAAGGTTCTTTCCAACTTGAATTTATTTTGTAATCTTTGAATCATCCAGGGCAG

TGAGCAAACCACTCTTCTAGGCTAAGAGCCTCCAATGAGTTACTCAGAATAAAAATCAGT

TCCTGAAGACTGCTGTGCTACTTCTGCTGCCATAAGGGGAGACAGGGACATTGCCA

>c2591_g1_i2 len=259 path=[567:0-19 58:20-258]

AATAAGATATTCTGGGTATGCCTGGTTAAAGCCCTCAGCAATCTGGCGTGCAAGCAAGGG

TGAGCAAGAGGTTGATTTAGATGACTGCCCAAGGTTCTTTCCAACTTGAATTTATTTTGT

AATCTTTGAATCATCCAGGGCAGTGAGCAAACCACTCTTCTAGGCTAAGAGCCTCCAATG

AGTTACTCAGAATAAAAATCAGTTCCTGAAGACTGCTGTGCTACTTCTGCTGCCATAAGG

GGAGACAGGGACATTGCCA

>c2597_g1_i1 len=209 path=[265:0-51 317:52-176 28:177-208]

GCCCAGACCTCCTCCGTACCGACGCTCAGCACTGCCCCACATCTTCCAACCCCACAGCCA

GCCCTTCTATGCTCCAAAGTATCACTTCTAGCCCACGGTCCCTAGCACTGCCTCCTACAG

CCTTCCACTCTACAGCTACCCTTCTAATGCCTCCAAAGTACTGCTCCTACTTTCAATACA

CAAAAACTCTGCCTTACAGTCTTCCTCCT

>c2598_g1_i1 len=211 path=[189:0-210]

AACACTTCTGCTGTGCCATTTCATTTCAGATGGACAGAAGCTATGCTCGTTATTCTCAGC

TTTACTTACATGTTAACCCTAGATTTAAGCACCGCCTGGGAGGAGCTGAACATTTCTTTA

AAAAGCTGCCAGCACATTTCTATAGGTCCTGGTTTTGGAAGAGAATGGTTGTACGTACTG

ATGTTTAGGTAATGGCTGCCAGAACAGATGG

>c2599_g1_i1 len=220 path=[352:0-219]

AAGCACCTGCACAGTGAAATCTCAAGGGTGCTGGTGCATAAAATAAATGGTTAATAATGA

GCAGGACAAAAATGATGTCCCTCCAACTAGGGCATGTTCGGCACCCACTAAGAGACAGTC

ACATTGCTTGAATTTGGGCTAGAAATGTTTTGTTAGTTCACATTTCTTGCTTGTTTGCTG

CTGCCAGTGCCTCCATCTGCTGAAAACCAAGACTAATGTG

>c2600_g1_i1 len=212 path=[335:0-211]

GCCACTAAGTGAAACCAGTAGAAGACAGTTTAAAAAACAGAATTAAGAAAGCCTATAAGT

GGCAGAATTCTCTGCCAAAGGACAGTGTAAACTAAAAAAAAAAAGTTTCAGGGGGATACC

AGAGAGAAAAACAACAACCATCACAACCACAGAAGAGATCAGGCCCAAGTTAATCAACAG

AGAAGTCAAGCAGGAATTCTCTGCAACAGGAA

>c2604_g1_i1 len=225 path=[334:0-93 25:94-224]

AATTCGAAGTATGGGAATGTATTTTAATATCATGGAACAAACATGTATCACAGTGAATCC

ACACTGCTGCTCATTCTGTAGGCACACTGTGGCTCCCTACAGGTCAGTTTGTTATTCTTG

AGCTGAAAAAGTGCAACATGTAAAGGGGAATAATGAAATTCATTTCAGGGGATTTTTTTT

AAAGCAAAATGTTTTATCTCTTGTTAGGAAATCCTCACTTCCCAA

>c2604_g1_i2 len=226 path=[334:0-93 427:94-225]

AATTCGAAGTATGGGAATGTATTTTAATATCATGGAACAAACATGTATCACAGTGAATCC

ACACTGCTGCTCATTCTGTAGGCACACTGTGGCTACCTACAGGTCAGCATTGTCCAAAAT

AAATTTTGCACAGATTCTTTTTTTTAAGATCCATCACCATTAGTTTCAGGATGGAAATTA

TGAAAAATTATGTCTCTTCCTTTGGGAAGTGAGGATTTCCTAACAA

>c2612_g1_i1 len=246 path=[256:0-180 568:181-187 575:188-245]

TTTTTTTTTTTTTTTTACAATAATTTCTGTATAACTCTTTTTCATATATTTAAATAATAC

AAAACATTCTTAATAACTTTTATATTAGTATATTTTTTTATATTCGTATAATTTTTTTTT

TCAAAAAAAACATATATATTTATACACACAAAAATATACACACATATATTATGCACATAT

ATATACACAATTTTAAATATGTATAATATTATGCATATGTATATATAATTAAATTTGTGT

ATATAT

>c2616_g1_i1 len=241 path=[219:0-240]

GAGTGGTCTCTCATTGAGATGTAGCCTTGAGTTGTTGTTCCACAGTGACATGGAAGTAGC

CGAAAGCTGGGTGAAATGGTGATGCGACAAACCATGCTAACAGGCTGTGCTCTGTGTTAG

GGAGCGGTGGCTGCTTAACAACAGCGACTGAGGAATCTAGGTTCTATTTTAAAACACCCT

GAGGAAGCAAGGGAGCAATGTTCCTTTTCCTGAGTATGCTTAGGTTACCACAATACCAAT

C

>c2616_g2_i1 len=620 path=[437:0-619]

GATTTTTTTTTTTTTTGTTGTTAAGATTTATTACCTAGGTCTTACAGCAGTAACGCTATT

GAAGCAGTTAAGACACAAAAGTAGTTTCTGAAAAGTACTTCACAGGCGTTAAGTTGATTC

CTTCTCTTTGCATGCTGTATCTATAGCTACAGAAGCCGCCTGGTGTACATCAGTGTTAGC

TGGGTTGGTATCATCTAAGTGCTCTGACTTGTTGCCTTCTACTGTGACTTGAGCAGCATC

AGGCTTTGCTTCAGTAGAGCTTCCATTTGTATATTCAACCCTCTCAAAGAGTATCAGTAA

CTCACAGTGCTTGGTCTGAGGGAAGAGATCCACAGCCATTGCTTTAACGGGACGGAAGGC

GGCTCCTTTGACTCGGTTGGAAGGGGCCCGGCAAAGGTCCACAAAGTTATTCATTGCAGC

TCTAGGATTGCAGGACACATAGATAAGCTTCTTCAGCTGTTCAGCTCTTCTAATTGCAAG

AATTACCCTGGAATCTAGACAAAGAAAAAACTGGGTGTGAGTGTCAACAGACCAGAAAAA

TTCCAGCATATACTCCATCAAGAACAAAACCGGTGTGGTATTCCACCATTTCAAGTTACA

GTTACAGCCTCCCACTTTAT

>c2619_g1_i1 len=240 path=[1:0-239]

TATTGAAGTTTCTTTGTTTGAAGTGCTCTCTTAGTCAAACCTGTGACTAGTGCAGCCCAG

CCAGGACCCCCACAGGCTAGATATAAAACTGATAGCTTGTGTCCCAGGATGCCGTCAGTT

TGCTTTGTACTGCACACACATTGGTAATTTTTACTCCCAGTCTGAGAATTCAGCTCATCT

CTTGAGAACCACACAAAACAAAAACCCAAAGCCCTTACCTCCAGCAAAAGTCAGATTAGG

>c2623_g1_i1 len=208 path=[11:0-207]

CTTTCTTTTTTTTTCTTCTTTATTTTCTTAAATTGCAATAATAAACTTTTTCCTTTTAAA

ACTTAATATTTCTTGTATTTTTAATTTAATGATTTTTTAAGAATATATTTTACTTATAAA

AATAGATCATTAAAATACAATTTATATATATTTGTGAATACATTTATAATATATAAATTT

ATTTTATATACATATGTATATTTAAAAA

>c2625_g1_i1 len=264 path=[1:0-263]

AATGCTGTGAATTTTGCTGAGCAGCCTTTCAAGGTCAGGGAGTTTCTTCAGATGCTCAGT

AATTTCAGTCAGTTTAGCTGGCACCGCCAGAAGGTCCTCAACAGCATCTAAACGGTCATT

AATGGATGTAGGATTACAAATACAGCAAAAAAAAAAATTGCCGACTTTTTGTCTGCGCTG

GAGGGGTTTAAAGTAATGAATGAAATTGTCGATGCCATGGAGGAAGTTGCCAGCGACTTC

AAATCTCAAGTCCTTAAGCAGTTA

>c2628_g1_i1 len=409 path=[391:0-408]

ATATATATATATATATATATTACTATTATTGTTTACCAAATTACACATATATTTTTTTAC

TTTAAATATTTTATTATTATTTTAATTTATGTGTAGTAATTTATTTACGCATCAGTCATA

AAAGTTTTTTTTTTATTTTATATTAATGTTCATATAGAGTTGATACTTCTTATCTTTTTT

TAAGTTTTTACTTCAATTTTTTTAAATATTACTTTATTATTAACAATTTATATAAATTTA

TTTTATGTTTGTTTAATAACTTTATATTTTCTAATAATTACATTTTTAAATAAAATAAAT

AAAAACTCATTAACTTTTATTTTTTTTTTAATTATGTAAAACTTATTTTCCATAATAAAT

GATTCTGTTTTTTTATTTTTATTATCTTTTTATTTTTATATTTAATATT

>c2631_g1_i1 len=230 path=[208:0-44 477:45-229]

GTCCAGGCTCCAATTTTGGCTCCTAGCAAAAAATCATCAGCTTTTCTTTGGACAGGAAGC

ATTGTTTTATCAATGAGTCTCCACATAAGTACCAGTGTCAGAGCAACTCACAGCAGGCCC

TGTGGGAAGCTCAAGGAAGTCAAGAGCTGAGCATTCAGAACGGCTGACCTGGTGCTGTAC

CTCAGCAGAGCAGAGGATGAATGAAGACCGAACGCCTCTGAGAGGCAGGA

>c2632_g1_i1 len=239 path=[1:0-238]

AAAAAAAAAAAAAAAAGAATATTAGTAAATAAGCTTCAATAAAAATTTATTAATATAAAA

TGAATGAAGAAAAATTTTTTTATATTAATATAGAATTTTATAATGATATATTTATCTAAT

TTACTATATTTTTTTTTTTTTTAAAGAGAATATAAAATGCAATTATATTAGTATCATATT

TTATTTATTTTTATGATAATGAATTAATAAAATTACCAGTGAATTGTTTGTTGAAAGAA

>c2637_g1_i1 len=306 path=[1:0-305]

ATTATTTTTTAAAAGGTGTAAAATAAATATAAAGTAAATTTTTTTTTATATTTTTAATAG

GATGATTAAAATTTAACTTTTTTAATTAGTGTTGAAAAATGGGCATCTATTAAAATTTTT

TTTTTATTTGGGGAAAAATATATATAAATATAAATACATGTATGAAATTAGCTTTAGTAA

AAGAATAATTTATTATATCTCTTTTTTTTTTTTTTAAATATATTCATATAATAAAAATAT

ATATTAAGCTAAAATTTAAAATTATTAAAGGCTTCAAATAATAAAATAATATATAAGAAA

AATATA

>c2644_g1_i1 len=308 path=[636:0-16 42:17-307]

AACTTTGTGTTTGAGTGGGTGTAGAACGTAGACCCTCCAAATGCAGCATACAGGAATAAA

AGGGAACTCTCTTTTTTTCCATACTGTATATAGAACCAGGAACAGCTGACAAACAGTGCT

GCTGAAGAAGGGGTTATTTGTAGATGGGGTTTTTAATGCCTGACATTTCACAGCTATCTG

ACCATTTTGAAAGTTATCTAGTTTCAGAGAATGTTGTACAACAAGAATTGTAGGCATTTG

TAAAAGCACTTAGGTGTTTACAGAGCTAGTGAGAGTTTATTAAATGTGCTGTAGAGATAC

ATCAGGTT

>c2644_g1_i2 len=332 path=[1:0-40 42:41-331]

GTAACTTTGTGTTTGAGTGGTTAACTTTGCTATCTGGAGAAGGTGTAGAACGTAGACCCT

CCAAATGCAGCATACAGGAATAAAAGGGAACTCTCTTTTTTTCCATACTGTATATAGAAC

CAGGAACAGCTGACAAACAGTGCTGCTGAAGAAGGGGTTATTTGTAGATGGGGTTTTTAA

TGCCTGACATTTCACAGCTATCTGACCATTTTGAAAGTTATCTAGTTTCAGAGAATGTTG

TACAACAAGAATTGTAGGCATTTGTAAAAGCACTTAGGTGTTTACAGAGCTAGTGAGAGT

TTATTAAATGTGCTGTAGAGATACATCAGGTT

>c2645_g1_i1 len=363 path=[1:0-190 192:191-362]

TCGTTTGGGCGCTGTGTGTTTTATGGACGCGTTCGCCTGCTTGTTTGGCGAGATTGGAGG

CAGAAGCGGGGCAGAGCCCCGTGTTCTCTGCCTGCGCCTTCGAGCAGAGGTGTAGCCTCG

GAAATACGCCCGGCTGCGTGGAACCGGGACTGGCTTCCTGCTGGGGCCGTCACACCGCCC

GACCCCGGGTTGGGCTCCTCGGGGACTTGAAAGCCTCCCCGTTGTGTTTCTGTCTGTCAA

TTAGGTAGGCAAAGGGCCGCTTTAAAAGCGCTCACGTTGCTTTGAAGGCGCGATTTTAAT

TTAATCTGGAGCGAAACAACCCGGAGCTGTTGGGGCAGAGTCAGGGCTGTGGTGCTGGGA

TGG

>c2646_g1_i1 len=216 path=[1:0-148 425:149-215]

ACTACAGTGGACCACAGACAAAAGTAAGTTTTATTCACATCCTATTCCTTGCAAAACAGA

GCAATTTCCTCAATCAAGCTCTATTCCTAATCCTATAGGAATAGTCTCCTATTCCTTGAT

TTTGCCCATATCCGTCTGGATTTACTTTTACTATTGATGCGTGTGGACTCTTTTGTATGG

GTGAGGCTAAAGATTTAAAGGTTGGTCAAGAAATTG

>c2648_g1_i1 len=294 path=[1:0-133 135:134-293]

AACAACTAACAAAGCCATCAAACAAGACAAAAGATGAAACAAGACAATTAATTCCCAGTC

TCTAATAGAGAAATCTAAAAACTGTTCTTATGCAACTACAGTATCCATAGCATCTGTAAA

ATAACATTTTTAAAAAAAATCTAGAAATAGTTTTATGTAGCAAGTGGGAAAAAAAAGCCT

TCACAATATCCTTGCTGATATCAGCAAACCTATTAGTAATGCAATCAATGAACAGAAAAC

TCACGACACCTGGCCAAACCGAAGGCCACTACATCCCATCTCTAACAGCAATAA

>c2650_g1_i1 len=208 path=[1:0-32 34:33-207]

GCAGTGTTTGCAAAGGATGTACCACTTCTCCTCTTGGAAGCACTTGAAAGGACCTGAACT

CCATGGGGCTGGGCCACGTGGGGAGCACTGCTGGAGCTGAGCGGTGATGGGGCCAGGCAG

CTCGCATTTGTTATGGTCTTGGACAAAGTTTTGACTGTTCTTGGAGGTAATTTTATAGGG

GACTGGGATTCTGAAGACCACATACAGT

>c2650_g1_i2 len=207 path=[403:0-31 34:32-206]

CCATTTCTCCTCTAGGGAAGTGTGGAGAGGGGTTGGAAGCACTTGAAAGGACCTGAACTC

CATGGGGCTGGGCCACGTGGGGAGCACTGCTGGAGCTGAGCGGTGATGGGGCCAGGCAGC

TCGCATTTGTTATGGTCTTGGACAAAGTTTTGACTGTTCTTGGAGGTAATTTTATAGGGG

ACTGGGATTCTGAAGACCACATACAGT

>c2651_g1_i1 len=1681 path=[1:0-1680]

AAAAAAAAATAATAATAAGATAAACAATGAAGTTATGAAGTTAATAAATAAAGAAGTGAT

AATTGATTATTGTACCAACCAAAAATCAAATGCTAAATTAATTTTAAATAGATATTTAAA

TATAATTAAATATTTGATGAAAGAAAATTTAAATTTAAAGAAAATAATAGAAAGGAACAA

ACTAGAAGATGCTCAAAAGGATGATATTATCATTAAACATGAAAATGAATTAAAAAAAAA

AAATGATATTATCAATGATTTAATAAATAAATTAGAAGGAGACATATTAAAAGAAAATAT

AAAAATATGTGATAATTCAGAACCACATGAAAGTGAAATGTTAATTCAAGAAAATGATAA

TTCTACAAAAGTAAAATCAAAAAAATTAATAGATCTAATTAAAAAAAATAACTCTATTAA

TAGTGAAGTTAAGCAAAACATATATCTTACAAATTGCCATCTAAATGATAATATAAAAGA

AAATGATAATTATATAGGAAATTCATCACTTAATTATGAAAATGAAAATTCTAAATTTAC

ACAATATAATGAGAAAAATTATATAAAGAAATACTTAAAAGATGAAAACAATAATTCCAT

ATTAAAAAAAAAAAATTATTCATCTGAATATACACTCATTAACATTAACAAGCCTATTGA

AAAAAAACATTATGAAAGTGAACTTCATCAGATATATACATATAATAGTTTCAATAAAGA

AAAAAATTTAGGAAAAAAAAAATCATCAATATTTATTAGGGAAGAAAGAAATGAATTATT

AAAAGATGAGAAATTAAACCAGAAAGGAAATAACAACTTGAAAGAAAACAGTTTAAAATT

TTTTGTAAATAACAACAATAAATTGCATGATGATGATGAATGTATTGATGAATTGAATTA

TCTTATAAATAATAAATCTAGTATTGAAAAATTTGAAAATTATTCTAAAAAATATGAAAT

GGAGTATAAGAACATCAATGCTCAAAATGATTTTATTAAGGAGGAATTGTATTATTCAAA

CCTATTTCAAAATGAAAAAAAATTATCTAAAACTCCTATGGAGAACAAAGAATATTATAT

GGAAAACTCAAAAATGAGTGATACTTATGTAAAAAAAAAAAGTATAAATATTACGATGGA

TAACTTTGATGAAAAAAAAGAAAATATTGACAAAAATCCCAAAGAATTACCAAATAAACA

AATTCATGATAATTCTTATTATGCAAAAGTTAAAGATGAGAACAATATATATATAAACAG

TGGAGATGAAAGTAATACAAATATAGAAAATAGAACTAATAAAAATCTAGTAAACAAAAA

CATGATATACGTAAATAATGATATAAGAATAAATAATAAATTAAATGAGAACGATTATGG

TGACATTTACAATAAAAAAAATTCTAATTTGTCGGAAAAAGAAAATAAAAGTGATTTAGA

AGATGCTAATTACGAAGATTTAGAAAATATTATGTCTACTATATTGAAACTTCGAAAGAA

AAGCTAAATGAAATAAATATACATACGTTTATGGGTATGTATAACAATATTTTCTGTTTG

CTTATTTTTACATCTCTTTTTTTATTCTTTTAAATACTTAAACCATTTAAAAAAATATCA

TATTATTTCTTCTTTCGAAAAGCATTTATTACATTATGTTTTTCTTTAAACTTTTGTTTT

T

>c2655_g1_i1 len=229 path=[211:0-228]

AACTTTGTGTTTGAGGGGTGAGATATTAAAGGTAACTTTTAAAGTGTGTTACACTTCCAG

GTTATTCACTTAAGTTCATTTTGATGCCTGAGTGCTGTGATTACTGGTTGGGGTGAGTGC

TAGGATGCTAGGCACTAAGTTGCTTTACCTCATCTTTAATTCTCTTTCTGTACCTAAGCT

TTCTAAATGTGATTCCACAATTTTCAGCAGCCCGAGGGAACTGGCATGA

>c2656_g1_i1 len=247 path=[1:0-160 505:161-246]

AAAAAAATAAAATAAAATAAGATAAGATAAAATATATATTATTTAGGAAAATTAAAAAAG

AAAATAAAATTTTTACATATAAAAAAAAAAATGAAATGAAATGCGAACAATATAAGTTTT

CTTGATAAATTACACATGAACTAAATAAAAAAACATTTATTTTTTCCTTTTAATCACAAA

TAATATTTAAGTAAAATATTAATATATATTTTAAACTATCAAATATTAAAGCAATTTTTA

AAGAAAA

>c2662_g1_i1 len=202 path=[180:0-201]

GACCTAAACTAACTAAATCTGTGTATTCTGCCTTTTTGGGTAAAGAGGGCTGGGAGAAGG

GCTCATTGTTAGATTTCGACAGGCCCACATGGCAGGCCCGGGCTCCTGCTGTTGGCTTTG

TGGCAAAGTGCTCTGTTGTATCACACTGAGCCTTGATTCCTGGCACAAGAGGGGTGTTGA

GGAGAACGTCTCGCTGAGTTAG

>c2664_g1_i1 len=202 path=[180:0-201]

GGTCTGCAGTATTGCTGGCAGACAGGAGGAAATGGACATTCCACCTAGTAGCAGAGAGCA

GTTTAGTGAGGATCCTTCCTTCACATCATCCATCGCAGACAGGCACTGAGGTGAGAAAGA

GAATAACAAATTATAGAGTCGTGCAACGCACCAAGACCTGTAGAAGAGAAGATTTGCTAA

ACTGGAACAGCTTAACTAAGTG

>c2665_g1_i1 len=307 path=[285:0-221 507:222-306]

GGCCATGAAACTGGACTCCTGACGTAAGTTAAATACACTTCACGTGTTTTATCAACATCC

AGTTGTTTATATTCTCTAAGCTTTTTGAGGTAGAGTTTGATCTTATTTCTTACTTCTCGA

GATAGCGTCAAGCAGAAGCTATTGTGAGCTAGTGGAGATAGCTGAACATAGTGTAAAAGC

TAAGCAAAGAATTTGCCTTTTAATATTATTTGCTCTGTTTCTGTCTTTCCCTTCCCATTC

TTCTTCAAATCACTAATTTAACAGTACAGAATTACAGAATTATTGTGGTTGGAAAAGGCT

TCTAAGA

>c2667_g1_i1 len=236 path=[214:0-235]

GCCCCCCCGGCACCCCCATCATGCCCAGCCCCGGTGATTCCACCAACTCCAGTGAGAACA

TGTACACCATGATGAACCCCATCGGGCCGGCGGGGACCCGGCCAAATTTCCCAATGGGGC

CGGGGCCGGAGGGGCCCATGAGCGGGATGAGCGCAATGGAGCCCCATCACATGAACGGAT

CCTTAGGCTCTGGGGACATGGATGGGCTGCCCAAGAGCTCTCCCAGTAACATGGCG

>c2669_g1_i1 len=239 path=[1:0-89 358:90-238]

ATATCAATATCATCTTTTGCACTGACGTTATTCAGTCAGCGCTCCATATGGTTCGCGATA

ACCGCCTTTACAAGAGCCCCTCAGGGCCGGGGCCCGCGGCCTCGCTGGGCCCGGGCGGCG

CAGGCAGGGCGGGCCGCGCTGAGGAGCGGTGGCCGCTTCGCTTGCCGTCTCTCTCGGCCC

GTTCCCGGCCCCGCGTGGCGGCGGGTCGGCTCTCGGTCGCCGGCTAAAAATAGCGCCTG

>c2670_g1_i1 len=236 path=[1:0-26 28:27-235]

AAAAAAGTAAAAAAGGAAAGTCAATTAAGAAAGAAATCTGGTTGGTCTGATTTTTAATCA

GCTTCATCAAAAAGATGCTTCAGTTGAAAAATGCCTTTGGACTTTTTCAAGCTCTTTTTC

ATGCAGATAAAAATCAAGAAAATTTCAAAACTAATTATGAACCTCCCGCTCCCCCCCAAA

AAAACCAATGACCAAAAAACCACTAAATCATGGAATCATAGAATCACTAAGGTTGG

>c2670_g1_i2 len=228 path=[446:0-18 28:19-227]

GTGAAGAATGTGAAAATTTAGAAAGAAATCTGGTTGGTCTGATTTTTAATCAGCTTCATC

AAAAAGATGCTTCAGTTGAAAAATGCCTTTGGACTTTTTCAAGCTCTTTTTCATGCAGAT

AAAAATCAAGAAAATTTCAAAACTAATTATGAACCTCCCGCTCCCCCCCAAAAAAACCAA

TGACCAAAAAACCACTAAATCATGGAATCATAGAATCACTAAGGTTGG

>c2671_g1_i1 len=210 path=[260:0-166 524:167-170 25:171-183 38:184-209]

TGCTCCATCTGCAGCAATGTTATCTGTTTTTAAAAAAATAATAAAGACTCAGTAAAAGAA

GTACCTAGGTATCAACATCAAGTGCACAAATCTACAGTGTAGCTTTCTGAATACAGCAAT

ACATAGTTGTTCACACAATGTTAGTTGAAAAAACATCAAATAGGCTACAAAGAAAACACA

CCCCCAAACTGAAGTTAAGCCTTAGTAATT

>c2673_g1_i1 len=275 path=[253:0-192 446:193-274]

TTGAGCAACCACAGTGCAAACAGTTACAAGAAATGTAATTAAGAACACAAGAACTCAGGC

TAGCTAGCCTCACAGCCTGGGAAAAGCTGTCACAAACCAGGAGGCCAACTACAGCAGGGA

AGGTTTGGACCACAGCTTTTGATATGCACTTACTGTCTTCCAAATTTATGTGCAGCCTCA

GACAATTGTAGACTGCTGCTGAAACTGAACACCATCCATTTCGCCTCAAGTAACCAAAAG

GACCACAGGCATACTGCACACCAATATGTCATAAA

>c2674_g1_i1 len=260 path=[1:0-212 214:213-259]

GCATCCTTCTTCACTTTCTTTTTTATTCCTTCCTTCTTTTAACCTGGTAGCTTTTTATCC

TGGTATGATTAAAACAGGTAACCAAATGTAATGCAAATGCAGCAGTTTAGAGATGCAGTT

TCTGCTTTTTATCAGCTGTCACCAATCTCCAATTCGCATACGTTGAAAGGATGGTTTTAT

TTCTTGAAAAATGTTATAGTATAACAGCAGTTTGAGTCTTTTAACATTAATATTTCCAGA

GAAGGAAGGTCCGGAGAAAA

>c2675_g1_i1 len=224 path=[202:0-150 353:151-223]

TGCTACTGAGCATAGAAAGGTACTCTCTCTTGCTCCTAGGCTGATCTCTCTATTTTTACA

GTTGCCAGATTGGAAAGCTAGAGATTCAGGATGGCGATTGAGCCTGGGTCCAGTTTGTTT

CTGGATCTAGCTAACTGTTGTTTTGTGTTTTCTTCCGTTTTAGAGTTTTCTCTGTCTAAT

TCACTTTTGTTAATTTCTTTAAGTTATGGAGCAGCACCTGTGAA

>c2676_g1_i1 len=254 path=[232:0-103 336:104-253]

ATATATATATATATCTATATGTATTTTGTCAGAAATTTTTTATATAAACAAGTGAATATT

AAGAAATAATACATTTATATATAATACAGAATGTCGAATTAAATTTTAATTTTTAAACAA

AGAAAATTTCTTAGTTCTTGTTTATTGTTGCTAAGTTTATTTGTTTTTGTACTTTAGATC

TTTTAAATTTATATTTTTTTATCTTTTTTTCTTTTCATTTTTTATATTTCATTTTCTATA

AATATATATATATA

>c2677_g1_i1 len=260 path=[238:0-221 460:222-259]

TTGTAGGCATACTTAAAAATATCTTTATATAACAAAAATATAAAAGAAAAAAGAAAAAAT

GTGACAATTTAAAAAGAAATTAAATTGAATATTGATAGAATAAAATATAAAAAGATACAT

GAAGTATTATTATTTTGAAAGCAAGTAATATAACATTTTGAGGAAAAAAGATAAAATAAA

GAAAAATTAAATTTGAAATAGCCTTTATAATATATATTTTACTTTTTTTGTTTTACCATC

TTCATTTTTACTCATTAAAT

>c2683_g1_i1 len=287 path=[356:0-227 641:228-235 650:236-286]

TCCTTCCCACGCAGTTTAAGGCCTGGAGTAAGACAAAGGACACAGCCATCAGCTGTTGTG

CACTCGATCATTCACAGCTTTAAAATCTTTGCTATAAAAGAGAGAGCGTCCAGAAAAGAA

GACACGGAAAACAGATTCAGGAAGAATCATACTTCAGTTCATAGGAGCATGAGAAGGTAC

TAGAAAGGCTCCAATTTGCCCATGCATTCACTGCGTTTCTAAAAAATGGTATCTAAAGAG

GAACAGGAGAGCCACATCAACACTAGTGGATGCAAGATACTTTCCTG

>c2683_g1_i2 len=287 path=[356:0-227 59:228-286]

TCCTTCCCACGCAGTTTAAGGCCTGGAGTAAGACAAAGGACACAGCCATCAGCTGTTGTG

CACTCGATCATTCACAGCTTTAAAATCTTTGCTATAAAAGAGAGAGCGTCCAGAAAAGAA

GACACGGAAAACAGATTCAGGAAGAATCATACTTCAGTTCATAGGAGCATGAGAAGGTAC

TAGAAAGGCTCCAATTTGCCCATGCATTCACTGCGTTTCTAAAAAATGATATGCTGTGGT

GCTTGAAGGTCTGATGTTAACTCAGGAAAGTATCTTGCATCCACTAG

>c2684_g1_i1 len=237 path=[215:0-236]

TGGCAGTTACTCTCAGAAAAGTAAGCATCGTTGCTCTGTGCCATTTAAAGGTTGCCCACG

TCGTAGAAGCTTGCTAGGCCTTGTAAGGTACATAATTCATCAAATACCCTCCAATTTCCT

ATTTTTGTGTTTCTCTTTCAATTCAATGCATCTCTGTAACTCTAAACTGTAGACCATATG

GGTATTTGTTATTCATAAGGCCATAATGCAAATCTATGGAATACTTCCATCTGTTGG

>c2685_g1_i1 len=235 path=[1204:0-9 2388:10-19 1223:20-234]

TTTTTTTTTTTTTTTTTTGAAACATGAATGAATTAGAAAATATAACATATATTAAAAATA

TTCAAGAGAGATTGAAAGATAAGGAAGATAAATTAGCAGATGCAAGTAGTGAGAATATAG

ATTCTATTCTTATAAAAGAAATTAATGAAGATTTAAATTTATTGAAAAACGTAAAGATAA

ATAAAAATATTTTAAAATCAACTAAAATTGGAATAACTGTTAATAAATTTACAAA

>c2685_g1_i2 len=969 path=[2354:0-20 71:21-770 2388:771-780 830:781-968]

AAAATGCAACAACAGATGGAAATTTTAAACATGTTACAAAAGTAGTCATTGGTTCATCGC

TACTTCTAGTTTGTAGTTGATGGTATATAGTATCATAACCTTTGCATTTATAACATTGAA

ATTCTCCTTTTCTAGATTTTTTTAGAAGAATATTTTTAACATCCCAATCTGATTGGCAAG

CTATTAAACTTTCTTGTAAACATTTTTCTCTTTCATTTTTCTTCTCATCACTTGCCATAT

CTTGAGAATTCATTGTTACTAATTCTTTTGGAGAAATAATTTCTTTATATATTTTTTCGT

TAAAATTAGGGTTTTTTTTATCACTTAAATTAAATTTAATTGATTTCAATTGCATATTAT

ATTCTCTTTGTGAGTTCCTTTTCTGAATGTAATATTTTTGTAACTCATTTTCTATATCAT

ATATAATTTCATTTAATTTTTTTCTATCAATAATATATAGCAGATTATCACTTGATCCTA

TTAGAAATGCTTTGAATAAAAATTGTTTTGCTTTATCTCTGTGAACATCATTGTGTAGTT

TTCCTTCATAATTCCATTCATTAAGTGTTTTTAAATCAGTAATTTTATGATCTTTAATAT

TTTTATCATTATTAAAATTAGAGTTATTATTTTTCCCATCAGTATTTATGCCATTAGTAT

CATCATTTTGTTTTTCTACATTATTTTCATAGCAATTATTTTGTTCTTCTTTGTCATTAT

TCTCTGAGTTATCTAATGAATAATTTTTATTAGAATCATTTTGAGCTTTTATTTTTTTTG

ATTCATATTCATCCGACCGAGTATCACCACTTTCAACAATTTCAGATTTTCTTTTTTTAA

TATTCTCTATATTTTTAATATTAGAAATTTTTTCATTTAAAGCTATATTTTTCCATTTTT

CTACAAGTTCTTTTGCTAAATTTTGTATTTCTTCATTATTTATTTTTGTAAATTTATTAA

CAGTTATTC

>c2685_g1_i3 len=996 path=[2354:0-20 71:21-770 2388:771-780 1223:781-995]

AAAATGCAACAACAGATGGAAATTTTAAACATGTTACAAAAGTAGTCATTGGTTCATCGC

TACTTCTAGTTTGTAGTTGATGGTATATAGTATCATAACCTTTGCATTTATAACATTGAA

ATTCTCCTTTTCTAGATTTTTTTAGAAGAATATTTTTAACATCCCAATCTGATTGGCAAG

CTATTAAACTTTCTTGTAAACATTTTTCTCTTTCATTTTTCTTCTCATCACTTGCCATAT

CTTGAGAATTCATTGTTACTAATTCTTTTGGAGAAATAATTTCTTTATATATTTTTTCGT

TAAAATTAGGGTTTTTTTTATCACTTAAATTAAATTTAATTGATTTCAATTGCATATTAT

ATTCTCTTTGTGAGTTCCTTTTCTGAATGTAATATTTTTGTAACTCATTTTCTATATCAT

ATATAATTTCATTTAATTTTTTTCTATCAATAATATATAGCAGATTATCACTTGATCCTA

TTAGAAATGCTTTGAATAAAAATTGTTTTGCTTTATCTCTGTGAACATCATTGTGTAGTT

TTCCTTCATAATTCCATTCATTAAGTGTTTTTAAATCAGTAATTTTATGATCTTTAATAT

TTTTATCATTATTAAAATTAGAGTTATTATTTTTCCCATCAGTATTTATGCCATTAGTAT

CATCATTTTGTTTTTCTACATTATTTTCATAGCAATTATTTTGTTCTTCTTTGTCATTAT

TCTCTGAGTTATCTAATGAATAATTTTTATTAGAATCATTTTGAGCTTTTATTTTTTTTG

AAACATGAATGAATTAGAAAATATAACATATATTAAAAATATTCAAGAGAGATTGAAAGA

TAAGGAAGATAAATTAGCAGATGCAAGTAGTGAGAATATAGATTCTATTCTTATAAAAGA

AATTAATGAAGATTTAAATTTATTGAAAAACGTAAAGATAAATAAAAATATTTTAAAATC

AACTAAAATTGGAATAACTGTTAATAAATTTACAAA

>c2686_g1_i1 len=208 path=[98:0-95 194:96-207]

GTTGGTTCCAGCAGAAGAAATCCTTAGGAGAAGGAAAGGGATGAGTTTTGTGGAGCAGCA

ATGACAGGATGAGTAACAAGTGTTTATTTAGACAGCTTTTTTTAGCATTCATTAAATCTC

AAGTGGATAGCTGTGGGCTTTTAGAAATGTCTTTACTATTACTGTATGGAAAAATATCCT

TGCTCTAATAGACAACAGGCGGTATGCC

>c2687_g1_i1 len=275 path=[286:0-274]

CTAAGCAAATCCTAGATACATTGAGGGAATCTGGTCAAATCTAGCACAGTGAGAAAGAAC

AGGAAGGAGCATTCTTCTAGCTGCGCTTATCAGTCTTGAAGTTTATTACCATCCTGACAA

ACAAGGATAATTTCATAACAGCTGAAAGAAATTGAACAGTCTGAAACTTGATACCATTCC

CTCTGCAGAGCATTAGAAAACCTTAAATATTGAAATTTGAACTACATGTTGTAAAAGAAA

TGTGCAGGAATCAGCTTCTCTGCGTTGCTGAGACA

>c2688_g1_i1 len=271 path=[1:0-270]

ATAATAATAATAATAATTTTAATCTTAAGAGTGAAACAGAAAATGGGATTGTTAAAAGTT

GGGTTACTGATAAAGATAAACAAAATAGTTTCATTAATAGAAGAAGAAAAAAAGAAAGAA

ATAGGAATTATGAATTAAAAAATAATACTATAGAAAATGAAAATTATTCAAAAAATTTAA

AATTATTTTGTAAATTAAAAAAAAAATATTTGCATATTAAAAAAAAGAATAAAATCTTAT

TTGAAACTAAAAAAGAAAAAAAGAAAAAAAA

>c2688_g2_i1 len=243 path=[497:0-242]

ATATGTATTTGTTTTGTGGTATTACGTAATAATATAAAAATAAATATAGAAATATAGAAA

AAATAAATAATGCTATATTAATTAATATATAAATTATTAAGAAATATAAGAGGCGCTTAA

TTTTTAAATTGGGTATAGAATTTATACAAAAATTTTTTATTTGAATATATTGTCTGTTCA

TACAATTAATTATATTTATTATAGATTCTGATATTAAATCAAAAAAAGTTTTATGAGAAG

GGT

>c2689_g1_i1 len=232 path=[125:0-66 34:67-124 249:125-231]

CGAGTTTTTTAAACACATGAGCTTAATCAACTGGAGCTTAATCAACTGGCTGTTCCACGG

GGCTGCCAAAAGCACCTGCGAAATCCTTGGGCGCTGCTGAAGTCCCTGTGTGCTGATTAG

GCTCTTGTGTTTAAAAAACTCGTGGTGAATACGCTTCAATTCTGAATTGGAGAGATCTTC

CCCAATTATTTTCTCCTAACGCGCTGATCGTAAACTGTTTGCTCTTACTCTG

>c2692_g1_i1 len=219 path=[407:0-111 25:112-158 72:159-218]

AAAAGGAGCCGTTTAGCCACCGCAGACCTGAGCTGGCCTCGTGCAATCCAGAAGCAGCTC

GCAGCAGTGGCCCGGCGCTAGAAGAACATCCAAGTGCTGCACCTCCCTCACTCTCGGTGT

TTCTGAGTGAGAACTGCCAGGTGCGGCCTGCGCCCCGCAGACGGGTTCCTCCTTCCTGCT

GGAGGAGATGCTCCCGGTTGCAGGTTGCCCGGTGAGAGG

>c2692_g1_i2 len=220 path=[407:0-111 518:112-219]

AAAAGGAGCCGTTTAGCCACCGCAGACCTGAGCTGGCCTCGTGCAATCCAGAAGCAGCTC

GCAGCAGTGGCCCGGCGCTAGAAGAACATCCAAGTGCTGCACCTCCCTCACTTCGCTCAA

GAAGTCGTCCTCTTTTTCCCCTGCGTATATTAAAGTCACCCCTCGGCCACACGTGAGGCT

CTTTGCTGTAAGCGACCTCTCACCGGGCAACCTGCAACCG

>c2699_g1_i1 len=214 path=[378:0-213]

ATAAGACTTAAAAACATGTTCGTTGGGAACATGCAGCTCAAAAATGGTCAAGATTATTTG

AAAGCATCTCCTCAGACTTCTGTAAACAAAAATGACAGCGATAAGTTTCTTGCCAGCCCC

ACTCACTTATCTGTGGTGATTTATAAGCTGTCCTAAATACTCTCCTTTTCTACAAAGCTA

TCCTTTTTCTTGTTCACATGAACTGTTTATATAG

>c2703_g1_i1 len=314 path=[849:0-288 1137:289-313]

TCAACCCAGGCCATTCTATGATGATTCTATGATAATAATGTATTATAAGAAGACATGAGG

AGTAACCCAGAGAAAATAGACAGCTATTTGTTCATATAGTCATAATGCCCTCCACACTGG

TTTGGAAAGACAAATCTATCAGCAGTGGAGTAAAAGAAATGTTTTGTGAGTTGACAACAG

ACATCAAATCAGATCCCTCACTATCCATCATTAGTAATTTAAAATTATTGGCTAATTTAA

GCATACATAGCAGACCTAGATCCTTAGTCTTCAGGAAAGGATCTGATATCGAATGATAAA

TGGATCATCATAGC

>c2703_g1_i2 len=391 path=[849:0-288 25:289-303 40:304-390]

TCAACCCAGGCCATTCTATGATGATTCTATGATAATAATGTATTATAAGAAGACATGAGG

AGTAACCCAGAGAAAATAGACAGCTATTTGTTCATATAGTCATAATGCCCTCCACACTGG

TTTGGAAAGACAAATCTATCAGCAGTGGAGTAAAAGAAATGTTTTGTGAGTTGACAACAG

ACATCAAATCAGATCCCTCACTATCCATCATTAGTAATTTAAAATTATTGGCTAATTTAA

GCATACATAGCAGACCTAGATCCTTAGTCTTCAGGAAAGGATCTGATATTGAATATTATG

TTGACATCTCTGCTGTGGCACTGAGGATAAATGTTGGCAGCTTGGCAACACCGTAAGGCA

GATAATGCTATGATGATCCATTTATCATTCG

>c2703_g2_i1 len=480 path=[105:0-392 40:393-479]

TGGTGATTCTATGATTCTATGATTAGTCTCTTTTATTTATTCCATTTACACTATGTAATA

GGTGGTACCCACTGAGATGTTTCAGCTTCTCATCAGAAAGTCATGGTTACCATTCATGAG

CAGTATTCCTGTGACTGCACAACAGAAAGAGTTAGGTAGGCGATGCTGAAAAGAAGTCTG

ACTAATTTCACAACAGCGCTAAAGCTTTGTCACCAAGTGATATTATTGGGTAGTGAAAGT

GTCGTCAGATAACAGGTTTGGTATTGCTTCTTAAGAATCAGAACCATTTTGTTGGGGTTG

CCAGTGCTGATAGCTCTTTGTTCTGCTGTTTTGGTTTCAAACACATGCGTCACTGTGCTA

AGAATCCGGAGTGGGTATTGAATATTATGTTGGCATCTCTGCTGTGGCACTGAGGATAAA

TGTTGGCAGCTTGGCAACACCGTAAGGCAGATAATGCTATGATGATCCATTTATCATTCG

>c2712_g1_i1 len=229 path=[429:0-228]

TCTCTGAAGAGACAGAACGATAGTGTCTTAAGGGTTCTGAAAGTAGCTGTGGTAGCAAAC

GTGCTTCGGAGAAAAGCACGAGCCCTTGGTATTTCTCCTGTGTACCTTCAGAGTCAGGAC

AACCAAGTCAGACCAAGTGAAGCCAATCAGGCTTGGTTTTGTTCGACACTCAGATGAGCA

GTCTTCAATGAAAGCCACAAAATGTTCAATGAAGCCCTCAAACACAAAG

>c2713_g1_i1 len=1678 path=[61:0-23 168:24-41 185:42-1521 1664:1522-1560 1664:1561-1599 1664:1600-1638 1664:1639-1677]

TCTTCATATTTCTTTTCATTTTCAACATTTTTATTTTTTAGTTCTTCATATTTCTTTTCA

TTCTCATCATTTCTATTTTTTAATTTTTCATTTTCATGTTTTAATTCTATTAATTCCTTT

TCTTTTCCTTTATCTCCATTATTTCCAGAGTTATATATAAAAGATGATTCTATGTTTACA

TTGTTATTTATTTCTTGTCTTAATTGATCAATTTCTTCAATAGCTTTGTTTTTTTCTTGA

ATAATTTTATTTATATACGTTTCTTTTTCATTTAATGATTTTTCTATTTCCTTAATTATA

TTATCTTTTTGATTAATTAAATATTGCATTTTATTTATTTTTTTGTTATATTCTATACAG

TAATTACTTTCATCATTTTTATTTTCATCCATCGTAAGTTCCTCTTTATTTTTAATTAAT

GAATTTAAACTTAATTTATCTTGATTTATATCTTCATAATTATTTATTGTGGGTTTTTCC

AGTTCTTTCATTATATTATTTTCTTCCATCAAATTATTTACTTTATTTTCATGACTTATA

AAACTACACTTGCACTTCTTTGAATTATTTTGTAAAATATTTAAGTCTTCTGTAATTTTT

TTAACTGTTTCTTCTTTTATTTTAATTTCGCTCTGCAAATTTTCAATAATATTAATATAA

TAATTTTTATGTTCGCTTGATAATATTCCTTTATCTTCATTTTCTTTATTTAATTCATCT

ACATTGATTCTCGTATTTACTTCTTTCATATTTTCAATAGATTTATCACATATATATTTT

TTAGTAATATCTTCTATATACTTTATTAAAGTTTCATCATCATTGATGTTTAATATATTT

TTTATACGTAACCACTTTTTCACACAATTATTATATTCATTTTTTACATTAGTTACTTCA

CTATTTAAAATATCTACTTTTTTATTATAAAAATGTATTATTTCTTCATTACATCTTAAC

TTAGATATATTGTTTAAATCTTCTCTTAAATTATTAATAAGAGTATCTTTTTCCATTATT

TTACTTAGAAGGATTTTATTTTTACATATTAAATCATTATTATTTTCATAATCATTATTA

TTTTCATAAACATTATTATTTTCTTCCGTATATTTTTTCTGTATTGATAGATTTTCATTA

CAATTGAAATTTATATTATTTAGACCTTTTTTTTTTTCTTTTATGTTTAAAAAAAAAGAG

GAATATTTATCAAACTTTTTTTTAACATTTGCAAAAATTTCATTTTTTTCATTATTTGAA

AAATTTTCATTATTTTTTTTTAATTTATCAAGAAATTTAACATTATCATTTAGGATACTT

TCTTCTATAACTTCATGAGTGTTTTTATTAATTAAATTATCTGTTGTAGTTTCATTCATT

AAAGTACTAGATATATTTACATTGCTTAAAGAATTAAATGAATCTTCATTAATCGAATTA

CTTGTTTTATCTAGATCTATTAAACCACTTTCTATTTTATCATTATTTAAATTATTATTT

ACATTTTCATTATTTAAAATAATATTTATATTTTCATTATTTAAATCATTATTTAAAGTA

TTATTTATATTTTCATTATTTAAATCATTATTTAAAGTATTATTTATATTTTCATTATTT

AAATCATTATTTAAAGTATTATTTATATTTTCATTATTTAAATCATTATTTAAAGTAT

>c2728_g1_i1 len=327 path=[305:0-326]

AGGGCTCTTACTGGCTATAAAACCACTTACCTGCCTAACACCACAAAAGGTGGCTTTAAA

ACTCTGCAGAGATTAAACAGGGCAGACACTGCTTCAAGCCAGGAGAACAATGGGACCGGT

CTGCTGCCTGTTCCCTTAATGGAGACAGGAGGCAGCAGAGCCAGCACAATTGAAAACCAC

CACATCAATGTGTATTTTCAGCTCTCACACTATGGCAATGTAACAGAGAGTAGTAGCTCT

CCAGTAGACAGACAACTTCCATAAGAACATACAACTTTTTTTTTTAAGATGGCAGACAAA

ACACATCGATTTCTTACCTATTCTTGT

>c2729_g1_i1 len=263 path=[247:0-262]

GCTGGGGTCCCCTCCTGTGGGCATGGGGGAAATCAGAGGTTCCCCTTTCTTTGCAGTTGG

TTAGAAATGTATGCGTTGGAAAATGGAGAAGGACTTAAATATGTTGTCTTGGTGATGGAT

CTGAATGTTTGCTGCTCAGTTTGTGGTGAAAGGAGATACGTTTACCATCTGCTGAGGGTG

ACTGATGGCTCCCGGGGATGCGCTGCGCTTCCTGAATGCTGAACAGCAACGTCTGAGCTC

TGCTCATGGCCATGGGAAGGAGC

>c2732_g1_i1 len=266 path=[53:0-265]

AGCTCAGAATTCACAGATCTGCAGCAGAATGGAAATTTTTTCTTCTTTGTGAAAGGGACT

TTCCAACCAATGAAGCTGGTCTTAGTGGTGTGGTCATCTCATGGGTTCTTAAGTGCATGT

TGAAACCCAGCATGTTTCCAGTATGTTCCATGTCAATGCTTTTCCAGCTGTCTCTATTTT

CTCTTCTGTTTCTCCTCCTTTCCCCATTCCTCTTTCCCCAGCACGTGTGCAATGCATGTT

GCCCCCATCAGAGGGGCTTCTCTGAG

>c2735_g1_i1 len=250 path=[1:0-249]

GAGTTGCTAATTGAGCAGATGTCGGTCACAGAGCGTATTGCTCCCTGCTTGGAGTGCCTA

GAAAGGTCACAGGCCAAGGATTCAGTCTGACTCCCCTTGGGGTAACCAGATTTTTCTCCT

GGGTTTGGACAGATTATCAGCAAGCACGCATTGGCACCCTGACTCATTCCAGAGTGAAAA

AGCAGATGGGCAGCTGCCTAGCGGCCTGTACTGAACTTGTAAATATTTAAGACAAATTTA

GAGTTACCAG

>c2744_g1_i1 len=349 path=[327:0-348]

GGAATTCCTGCTAAGAATTCATTAGTTCTGTCCAGTAAGACTACCCATGTAATTTCCGCA

AGGACTCTTCGGTCTAAACTTAAGTTCTAACATCTACTAAGCCTGTACAAACTTGTTTCA

GATTCAAAGCCACATCTGTCTCTCCTTCAGAGTGTCAATCAGCTCCTCCCCAGTCATACC

ATGATACCTTGCACAGATTTGCCTTATTATGCAAGTAAGATACCAACTTGCTACAATTTG

GGGGAGAGGCATTTACAAAACACAGGCATACACCCCAGCATACTAACTAGTAAGAAGAAC

AGAGGGAATAAGAAAGCAAGAATTACCTTCTCTTCTGAAGTAAACACTA

>c2749_g1_i1 len=212 path=[331:0-69 25:70-211]

CTCTCCGATGAAGAAATTCAGGCAACGTAAAGCCTGAACCAATGTTTCTTCAGCCTTTTA

CATTTCTGAAAGCAGAGGCTCCACACAGCACCTCAACAGCTGCCTTACGGCTGAAGCATA

TCCAGACATTGTGTAGAGATGCACTCTAACTTAGCAGTCTCAAAATAACCCTATCCTGAT

GCAGGTTCAGCTGAGAGGTAAAGCAGTGGACA

>c2749_g1_i2 len=212 path=[331:0-69 400:70-211]

CTCTCCGATGAAGAAATTCAGGCAACGTAAAGCCTGAACCAATGTTTCTTCAGCCTTTTA

CATTTCTGAAGTTATGATTTCCTAGCTTTTTGCAGCTTAAGGACAACTGTAGTGGAGTGG

GTGCTTTGTGTACTTCTTACTGTGCTTTAGGAAAGCAAGCAGTAATGGTACCTTCTCTAT

TCGACATTGTCCACTGCTTTACCTCTCAGCTG

>c2753_g1_i1 len=705 path=[47:0-531 578:532-532 30:533-704]

TAAAAAAAAAAAAACATATGAAAATAAAAATAAATATATATGATTTAAAAAAAAAATATT

AATATATTGAAATCTTTCAATGTTTATAAAAACTTATTATCCTTATTAATAGAAATCATT

ATTGCTACATTTTATTTATTTCTATCTTCTTATTTTTTTTTTTACTCTTTTAGTAAAATA

TAATTTTTTCTTTTCTGATGACCGGTTTTAGAAAAAATAACTTTTTTAGACAGTGTTGGA

AATATATAGGCTAATCTTTTTTTTGAAAGACCAATAACATATGACATTGTATCATTCTTA

TTTAAAGAAAAAGGTGGAGATTTATAATCTCTAACATATTTGAAAAGTTTTACATTTTCA

TCAAGTACATTTTTATTAATTGAATTATCCATATCAGTTAAACTTACACTTTTTTCAATT

TTTATTTCATTTTTTTCATTCCCTTCATCTAAATTTTTTTCTGTTTGTTGATATTTAATT

TTTTCATTAAGTGATGAGCCTTCATTAATTTGTAATGATTGTCTCATAAAAATTTTTTTT

TTTGTACTAGAAAATTCTTCTTTGTTATCTTTTGTAAGTGATTTCTTTACTTCTTTTGGA

ATATTATTTTTTAATTTTTCTAATTTTTTTAATTTTAATATATGCTTTTTTATTAAGAAT

TTACTTAAACTAATTTTTTTTTTTGTATTATTATTATTATTATTA

>c2754_g1_i1 len=356 path=[463:0-302 19:303-355]

GCGGCGGGCAGCAGATCCTGCACGTCATCCACACCATCCCTTCCGTCAACCTGCCCAGCA

AGATGGGCAACCTGCAGACCATCCCCGTGGTCGTGCAGTCGCTGCCCGTCGTCTACACCA

CCATGCCGACCGATGGCGTCACCGCCATCACCGTCCCGCTCATCGGGGGTGACAGCAAGA

ACACTGGCTCCGTGAAAATGGACCCGGGCGCCATGTACCCCATGGACATGCCGAGTGACA

GTGAGGACAGCGCCTCGGAGAGCGGCCCTGCGCCCTTCCAGGACCTGCAGCGAGAGTCCG

CCACGCCGCGCCTGCCCCGCTCCGACTCCCCCGACCTGAAGAAGCGGCGGATCCAC

>c2758_g1_i1 len=294 path=[109:0-293]

GATGTCTTCAGCTGTTGTTCAAATATATGCAGCAGATCGCAATGCCATGTGGTCAAAGAA

ATGCTGTGGTGTAGCTTGCCTTGTAAAGGACAATCCGCAGAGGTCATATTTTATCAGAAT

ATATGACATTAAGGATGGGAAATTATTGTGGGAGCAGGAGCTGTACAATAACTTTGTATA

TAATAGTCCTAGAGGATATTTTCATACCTTCGCTGGAGATACATGTCAAGTTGGTCTTAA

CTTTGCTAATGAAGAAGAGGCTAAACTATTCCGCAAAACAGTAACAGATTTACT

>c2764_g1_i1 len=247 path=[53:0-74 127:75-246]

GAATGTCCTGTAGAATATGCAGGTTTTGGTAGACAAGGTTTTCTTTACTTATTTTAGACT

CTTCATAGTCTGATTACCAAATGCTGGAGAGTTCACTCTGTGCTAGGTTGTTGTGATAAC

CAAGCAATGAAACATATCACACCAATTTCATGCCAGCTGCAAAATAACACTATATTATTG

GAATGAAGTAGTTTGTGTGTGTGCTGATATATATATCTTGCTAAGACAGAAGGATACTTG

ACAGTTA

>c2767_g1_i1 len=234 path=[212:0-233]

AGTGACAGATTTGCTCCAAATTAGCTGTTATGTGGACCAAAATGTCTGTTACCAACACAA

CTTGTAGGGCTGCATATGTTGAAAGCAACACACAGTAACACACAAGCTTTGGCCTTGAAG

ATTTCAAGGATTTCAAAGAGGTGAATTGAAAGGCTTTGAGAGCTTATCAGTGAGATAGCT

AAGAAAAGATCAAAAGAACAGAACAATTTAAGTTCCTGACTTGGTGAGAGAGAT

>c2772_g1_i1 len=278 path=[319:0-194 903:195-201 520:202-277]

AATGTTGATGTCTGGAATCAGGAGGGGTTTAAAATACCAGAGCAGCAAATCCCCCAGCAC

AGGACTTACTTCATCCACGGGAAGAAATACACTGGGCTTTCAAGAGTTTAATTGTTTAGT

TTAGCCAAAACACTGCAAGGTGTTCAACATAAAATGGTTTCTGAATGTGATCTTAACTAA

ACAAAGCAACTTTTCTTAAAGGAGTTAAGTATAAAGATGATATCTGTTACCAAGCAAATC

TTTGCCTGCAACTTGGCCAGTCATCCAAATATTAGACA

>c2772_g1_i2 len=244 path=[319:0-194 903:195-201 25:202-243]

AATGTTGATGTCTGGAATCAGGAGGGGTTTAAAATACCAGAGCAGCAAATCCCCCAGCAC

AGGACTTACTTCATCCACGGGAAGAAATACACTGGGCTTTCAAGAGTTTAATTGTTTAGT

TTAGCCAAAACACTGCAAGGTGTTCAACATAAAATGGTTTCTGAATGTGATCTTAACTAA

ACAAAGCAACTTTTCTTAAAGGGAGATGTTAGCAACATCTGTCTAATATTTGGATGACTG

GCCA

>c2772_g1_i3 len=220 path=[750:0-136 903:137-143 520:144-219]

AGAAAACCTGCTGCCTGCGGCTCGAGGCGGTGCTCGGCGGAGGGAGCGCAGCCGGGAGGC

ACGGGGAGACCGAGCTGAGAAACCAGATCAGAGTAAGAATTACTAATTCCTTATTTTTCG

TGATGAAGTTCCTCCTTTTAAAGGAGTTAAGTATAAAGATGATATCTGTTACCAAGCAAA

TCTTTGCCTGCAACTTGGCCAGTCATCCAAATATTAGACA

>c2774_g1_i1 len=260 path=[155:0-259]

AGAATTGTAATAAAAATATTGAAAATAATTCAACCACCGGGCATAAGTATATTGGAAAAC

AAGCTTCAGTCATCTTGCTGGTAAGAATGATAAATTTTAGCTGGAACTGTAGTATATACT

GAGCAGAGCTCTGCAAGTACACACAAGAAGGTGCTATCCTTTCTGAAAACTGTATGCTAA

TATAACGTGAGTATTCCTGCAGTTTTGTTCATAGATGATGGATCAACGTAGATTTATGTC

TCTTTCCAAAAAATGAAGGT

>c2781_g1_i1 len=252 path=[625:0-251]

AAAAGGTACACATATTTTTAATTTATGAGAAGATTTCATGGTTGTATATTTTATTATTAT

AACAAAAATTACAATATTTACTATTTAAAAATGAAAATAATATTTACTTAAGAGATTTGT

GAAGTTGAAAAATTAAAAAAATAGTAAAACTTTTTTATTTTATAAAATACAAACATCACA

ATTTATTATTTTTTCCTCTTTAAAGTATATGACAGTCAAAAATATTTATATACATATATT

GCTCATTTATTA

>c2792_g1_i1 len=316 path=[189:0-315]

GGGGGTTGAGGTATTTCTCCACAGAGAGCCAAAATGGCGGCGGCGGCTTCGCGGCCCACT

TCCGCCTCCCGCCAAACCCCTTTTCTTTCCACGGCGCATGCGCGGAGCCCGCACTGCGGG

CACCGCGCAGGCGCGCTCTCCGCTCCGCCCGTACTTCCGCCAGCTCTCCGGCGTCACTTC

CGGTCAGCTGGCGGCACCGCGCATGCGCGGCGCCCCCACTTCCGGCGCCTCGCAGCGGCT

CTCCCTGAGGCGATTAAAACGGGTTTTATTTCTCATCCTCCGCTCCGCGGCTCCGCGACG

GCGGCACTTTTGCGTC

>c2793_g1_i1 len=333 path=[380:0-203 36:204-332]

TATTTTTTTAATTTGTTTTTGTCACTTTAATTCAAAAAAAGAAATATTATAGCAATTACC

TTTTTTAGATTCATAAAAAAAGAAATAATTCCTTTATAATTACTTTAAATAGTATTATAT

CCTATTTAGACCTACTTTTAAAAATATTTCTTATATACAAATTTGAAATAATATTCATAA

GATATGCATACTAAAAATGGATAAGTTAGCCTACATATAAATATGAAGTTTGACACATTT

TTAAAAAAAATTTAAGATTCTTATTTATTTTCTTATTTTGTTTAATGTTTTAATTCGGAA

TAATTTTATTTTTGTATTTTTCTTCCAAATTAA

>c2798_g1_i1 len=269 path=[247:0-268]

AGGCAGGAGAGTACTTCTGCAAGGCTGTCAGTGATGGAGGTTCAGCAAAGTCCCAGCCTG

CCACGCTTTCTGTAATAGGTAAGAGAAAACGTGTGTGATGTGCTCCAAGCAGCAGGGGTA

CAGAAAGTGCTCAGAAATTACAACACATGCATGAAAGGAAAGGTGCAGGAAAAATATTTG

GGTGTTATTTTATGCACCCATTAGCAGATAAGCCTGAGTGTGCTGGTTATCAGCCAGATA

CTCACTACAAACCGGAGCTCTGGGTTTCA

>c2805_g1_i1 len=511 path=[556:0-432 44:433-510]

AAAGCTCTTGAAAACCTGAAGGTTTGGTGAAAGTGTACTGAAAGAATTTGAACGGGTCCC

CAGCCATCTGACAGAGGAGCTGCATCTCTTCTCACTGGATGATCTGGTGAAGATCAAACG

AGGGCAGTTACTGCCCCTTCTTAAAGAAATTCTGAAGAGCTCCACCTCTCATGTTGATGG

CTGTGAGCTCTGTCAAGCAAAGGGATTTATCTGTGAATTCTGTCAGAGTGCAGATCTGCT

CTTCGCATTTCAGATTTCCAAATGCAAAAGGTGCACAGAGTGCAAAGCATGCTTTCACAA

AGCCTGTTTCAAGGCTGGAGGCTGCCCCAAGTGTCTGCGGATCGCAGCTCGGAGGACGCT

TTCAGAAACTCCATTGCAGGTGCCTCTGTGAAGCTGGATTCCTCTGTTGGCTGCTCAGAG

CGAAGTCTTCAGGTGCATGACTAAGAATTAAATAATGTTGTTTTAGACATTACTATTTTT

AATGCATGTTTCCTTTAGGGGAAAAAAAAGA

>c2810_g1_i1 len=205 path=[155:0-204]

AAGGGTCCTTGTCTTATGAAAGGTTCTTGTCTTAAGAAATATAGTGAAGACCCCAGTCAT

AATCATGCACACTGTCACCTTATGTCCTCTGGGACAAGCACACTGCTGCCCCGAATATAG

TTCAACATCTGCTTCGTAGGCTCCCCATGCAGCCCACTGTCATTGACCGTGGCCCGCAGT

TGTGACAACAACTTCCACTCAAGAG

>c2814_g1_i1 len=224 path=[291:0-223]

GGTGTTGTCATTGCAGAAAAGTTAAGGCATGATCAAGTGCCAAGAGGCTACAAGTCCTAT

TTCACCTTCTTCACAGATTTTTATTAATATTTCCAAAAGTCACAGCCACATTTTGAACAA

ATTTTACATCTCACTGAATTAGAACAACAGCTTCTATTATGAGCTAAATCTCTCCCAGGC

AAATCCCTGCCCCCAGGCAAACAGCCTGGTTATTTTATTTCTAG

>c2816_g1_i1 len=255 path=[1:0-96 506:97-254]

TGCTAAAAATATATTAGAAATTTTTTCAATGATTACAAAAGAAATAAAAACATTTATTAT

AATAACAAAAATAAGAAGTTTAATTAATTTTGAACTATTAATATTAAAATGAAAATATAA

TAAATAAATAAATACATGTTAATTTATGTATTATAATTTTTATGTATGATTATTTCTTGA

TATTCAATAATAAAATATTAGGAAAAAGAATAATTAAATTTGTCTTATTTGTACTAAAAA

AAAAAAAATAAAATA

>c2821_g1_i1 len=355 path=[812:0-354]

CGCTCACGGGCCCCGCCGAGGACGCGCCGCCCCGCCCGGCCGAGCCCCCCCCGGCCGCTC

CTCTCCTGGCGCAGGAGGACAGCCAGCAGTACTTCCCTTCCGAGGACGATGTGCTGGAGA

GCGAGCTCTTCATGGAGAGGGACTTCCTCGAGTTCTGCAGCCGGATCGCCTGAGCCCGGC

GGGGCCGCGGGCGAGCGCTTTATTTATTCCTCGGTCGCCTCTCTCCGCGTTTTTGGCAGG

AGGCGGCGGCCGCTTTTAGTACCGCGCGTGTGGACGAACAGCGGGAGCTCCCGCGGCTCT

CTTGTAAATAATAAAGAAAAAACTTTTCTGCAGGCCAAAAAAAAAAAAAAAAAAA

>c2821_g2_i1 len=239 path=[241:0-238]

AGCGCCCCGCGGGCGTTCTCCAGCACCCCCAGGTACGAGTCCATGTCCAGCGGGTCCATC

TCGCAGTCCGACCCGCCGCTCTCGGTGCCCACCGAGTCCGAGCGCAGGTGCATCAGCTGG

TACTTCTCCCGCATCAGGAACTGGTTGGTGTTGCGGGGCGCCCGCATGCCCGGCGCCCGC

CGGCCCAGCAAGTTGACGGGCCGCAGGGAGAGCAGCACCCGCGGCCGGTACGGACGGTG

>c2822_g1_i1 len=336 path=[343:0-47 15:48-335]

CGATTGCGTTGCTGCTTCCGAGCGTCCTCCCGGCGCTGCCCTCTTATCGCGGGCTGCGAT

CGCCCGTGTGCTTAGCTTTCCTCCTGATTCCTTTATAAAGCGCTGCTCTCGGACCTGTAG

GTTGCTTGTGGATGAGCCATCTTAACGAAGGAGAGGGGATCTCTCTCCCCGTGTAACCTC

TCTGAGCGTTTCACAGGTAGCAGACGTTCAGGCTTTCTCATTTCGATGCGCATCTCAGCT

CACAGAACTTGGCTGTACGGCACGCGCTGCCTAAAATCATCACCGGGGCTGCGTGTCAGG

AGCTGTAATCTGTGTGAACCTCAGCTCCTAATTAAT

>c2822_g1_i2 len=302 path=[1:0-13 15:14-301]

GCGAGTGGGCCATGGCGGGCTGCGATCGCCCGTGTGCTTAGCTTTCCTCCTGATTCCTTT

ATAAAGCGCTGCTCTCGGACCTGTAGGTTGCTTGTGGATGAGCCATCTTAACGAAGGAGA

GGGGATCTCTCTCCCCGTGTAACCTCTCTGAGCGTTTCACAGGTAGCAGACGTTCAGGCT

TTCTCATTTCGATGCGCATCTCAGCTCACAGAACTTGGCTGTACGGCACGCGCTGCCTAA

AATCATCACCGGGGCTGCGTGTCAGGAGCTGTAATCTGTGTGAACCTCAGCTCCTAATTA

AT

>c2835_g1_i1 len=248 path=[155:0-247]

GTGGAAAGGGGTTGGCATCAAATAATATATAATTAGACTGAAACAATTGATATGATGTAA

CTGGAGCTGAATCATTCAAGTTGTATCCCATAATTTTAGTAAAGTTGTTATCAAAAATCA

TAACCAAGAAAACTTTCCAAACCAAATAAGGGTTTAGAGAGACAAAATCAGTTTATTGCA

GCACTAGGTCTGTGGAGGATTCCTCCTAGCATGCAGGCCAAGAGATGAAAGCACACTCAT

TATATACA

>c2849_g1_i1 len=483 path=[813:0-482]

TTATGTTGTCTGTTTTAAAGAGATGAACCACCACACACTGAGTTGTCCTCAGGCCAAAAC

ACAGGAACAACTCTCCCGAGGACATAATAAAGGCTCTGTGTGTCCTCACCTATCACTGTC

AAGCAATCTGCCCATGCTGAAGGTGTTCTTTCCAACTTCAGAAGACAGCATTAATTAAAT

GTTGGTAAGCAGGATCTACAGTAAGCAGCACCCTTCTGGTTGCTGCTCTTCTTACACCGT

AAGCGCCTGCATGACAAGACAGTACCATCAGTGAACACTACAAGCTTTAGTTGCTGAAGT

GGCAAGAGATCCCAGCACAGAGAACTTGTGTTGCTTTAAAACAAATAAGCACTTTAAACA

TCATAGCTCCTAAATCCTGAAGATTGTCATTTGCAGTGATATGCTTTTCTAGTACATGTT

AGTCAGAAGGATCTTTCCCAATATGCTGCCTCTGATTTATTTACTTTCTCGTGGTAGGAG

GAG

>c2851_g1_i1 len=511 path=[2:0-510]

AAGAAAAAGAAAAAGAAAAAGAACACATAGATGATGAGGAAGAGAAAGAAGAAAATTTTA

CTTTAGATGAATATGTGCATTATGACTACTCTAAATATCAAGAAAATTTTCTAAAGTTTT

ATAGTAATAATAAGGATAATGATGGAAATGATCTGTATTTTGATGACAATAATAATATAT

ATTTAATGAATATTACAAGAAATATTAATAAAAATTGTAATAAATTAATACAGTTACAAA

ATAAAATATTATTAAATGTAAATAAATTACAGAATTATGACAAAAATAGATTAATGTTAT

TTTATAAATTTAACGAGCTAATAAGTACATATATTGAACCTCATAATGTTGTTGTTAATT

TAAATAATTCAAAAGAAGTTTATAAATTAGATAAATTATATGATAAGGGTAGAAAAATTA

ATTTATCTAACAATATTTTATTTTCTGTTACTACTGATAATGAAAAAGAAATGAAAAATA

TAGAAAATAAAAAATTGCATAATATTATAAA

>c2854_g1_i1 len=724 path=[1:0-281 283:282-723]

CAATTGCAAACAAGGTTTATGGTAAACCTTTTGAAGAGGCACTGAGATGAAGAAGAAATG

TATTTCCTGCCTCTCAAAACATCCTAAAAAGTGATGGATTAGAAACCTTAGTGCCTACAG

ATCTAGTGCCTTCCTGGTTCAACATGAGTCTAATGCTGTATTAGAATGACTACTGTCAGT

GTATCTCCAGGGTCTGCAAAGGATGCGACTCAGACTTGAGCTCTTGGCCTGGTGGTGCTT

GCAGAGGAGATCTGGCAGAGCCCTGGCTCTCCTCCATACACACTGACCCCTGGCTTCATT

GCTTGTCCCCTCTGATCATCTCCCCTTGGGAGAGGTGAGGGGATAGCAAACATGGATTGC

CTGTGTTGTAGCTGGTAGTATGTCAGCCTGCTCAAACACATGGGTCCAGACTCATGCCCA

AGTTAGCTTATCAACAAAGCTTATGAGCTCGAACCAATCCCTTCATTAATATACATGTAC

AGTTTCCAAAATGGAGCTAGTCCACATCTGCCTTAGAGTACGGATAATGGAGGCTGGATT

TGTATGATGTAATTGGATACAACATTGATAGAACCAGTAGCCCTCAGTCATCGGGGTACA

TCAGTAAGTCAAAGAAAGGAAACCGCTCACTTGGCTTAAGACTCGGCACATACTCACTCT

GCACCAGAGCTGGCAAAAAGTAAAGACTGAAAGATCCCTCGAGAGTGTATTCATTGACCT

CCCA

>c2858_g1_i1 len=214 path=[576:0-213]

GTTGCAAGAAAAGTGCTTGGTTATAAAGTTTTATGTGTGCTTATCTCCATTTCTCAGCCT

CGTTGTAGCTCTGTCTGGTGAATTTGACAGGAACAGGCTGAACTGAAGGTATACAGAAGT

ACAAGGACGAGGGCACGTGTGACTGTAAAAACCCTAAGGAACAAGAGGAGAATTACTGTG

AATAAGAAGTCTTGTGAATCTCAAGAGTGTTAAG

>c2858_g2_i1 len=215 path=[1:0-214]

ATAAATGGCTTAGATTTCCCTAGGATACGGGGCCCCACCAGCACAGGGCAGTCATTTGCC

ACCTGATCCATAAGTTGCCCATCATTCATGCCATACATATCACTGATCTCTGCCTCCAAA

GTTCTTCCTCATCTACACTTGCTGTAAATACAAAAATACCAAAAGTGCATCTCCTGATGA

AAAGCACTGCCCAGTTACTTCCAGTACACCTCACA

>c2860_g1_i1 len=312 path=[1:0-230 232:231-311]

CAGCTCCATTATGATTCAGCCCAAAGTTGACAATATATTGAACTCATTCCCTTCATATTT

TTTTGATCCATTAGTATCCCCAAATACTCCACTTAATACTGTACATACCACTCAGTACTG

CACATACCACTCAGTTTTCAAAAAGGAATACTACAGCATTTCAAAAATCTTAGTTTTATA

GAATCCATGCAACATGTTATTCTGTTTAGTGTTGTATGAGTATTAACTACGCACATTCAT

AGAGGACTTCCAGATTTTCCATTTAACTTTTGAGAGTAAAGATCACAACAGGGTCTGCGT

GCTTTACAAATA

>c2861_g1_i1 len=313 path=[261:0-312]

GTTCTAGCTTTTTATAAATACGATGGACAAGACTTGACTACATAATTAACCATCTAGTAG

TTTGCATTGGAAGGGACTTTCAAGATCATCAGTTCAGTTGTTTGCCTGATCTAGAAATAA

ATTAATTTTGGTAAAAGATTATGCCTACTCTTGAATACTGCGATAAGTATATTTTAAAAA

GTACACTCTTTACGTACTGAAACGTCTTTTTTTCCTGAAGGTTACCAAGGGACTTAGAGA

GACCTGACTAGAGAAGTGTTGCAGCAGTCGACTGTATAGTTATGCCCATAGCTCCCTGAC

AGTTGCTTCTCAT

>c2863_g1_i1 len=218 path=[114:0-111 226:112-127 331:128-217]

GCGGACGGCCTGGCTCCCGACGTCCCGCTGCCTGCTGAAGGCTCTGAGCACATGGAGCTT

TCCCCCGAGGTCAAGCCCATCCACCTGTCTCCGTCCAAAATGCTAAAACCCATCCCAACA

GCAGCAGAAGAGGAGTCGTTGGAAGAGAGGGTGAAATCGGAAGTACAAATGGAGGAAAGC

CAAGAGAGCATCTGCGAGCAGGACATGGGGGCTCTGAA

>c2864_g1_i1 len=317 path=[449:0-316]

AAGGAGAGGAAGTTTTCAATTCTTTCTACATTTGGGAAGAAATCTGAAAGAAACAGTGTA

ATTCTTCAATCTAGCTTTTGATCTTTATAATCATATAGTTTACTATTTTTAATTCACATT

CAGGTAACTTGGATTCCATTTTGAGGATGGAAAACAGAATACATAGTGTGGTTTTAAAAA

TGTCTTCAAAGTGAAGTTTGACTCATGAAACTACTGATTCTTGTTTTATTGCTCAAAATG

TCTGAAGTTGCTAACTAGATTCACCGAAAACAATTCCTTAAAACCCAATCCCTACAGCAG

CTATAAAAAACAAACCA

>c2869_g1_i1 len=1369 path=[1274:0-1248 2523:1249-1272 2523:1273-1296 2523:1297-1320 2523:1321-1344 2523:1345-1368]

TTTTTTTTTTTTTTTTTAATTTTTCTTTCCTCATATTTTCATTATCAATTTCTTCCCATT

GCTCTTCATCATTTAAGTCAACGTATATTATATCAGCTTCATCATTATTATTTTTCGTAG

AATTTTTATGTTCATTTTTTTTTTCATTTTTAAATGAATTTAAATAGTTTTTTGTGTTAT

TTTTTAAAAAGTTATCTTTATTTTCAGGGAAAAAATTCTTATTTTTTTTCAAAAATAAAG

AATTTTTAATTGTTTTATTACTAGTAGTATTTTTATTTTTGTTAATTTTTATTAGACTTT

CGTTACTATTAAAAATTTTATCATTTTCACTACGAAAATCTGTCTTTAAATGATTCTTTT

TCGTTTTAAAATTTATGTTTGTTTGTACAAGAGATATACTATCTTCGTTTAATTTTTTAT

CATTCTCATTAGTTGTATCATTGACATCTTTCCTGTTATCTTCACAACTATTTGCTTTAT

CATTGCTATTATTTAAATCTTTTTCATACAGACTGAGATTATCATCATTACTGCTATGAA

AATTATCATAATCATCTTTGTTAGTATTACAATCATAACTAACTTTATCGTCATAATAGT

TACTATTTATAATTTTTACACTGTTACTATCATAATCGTTATTCTTTTTACTACTATTGC

TATTATCAACCGTACTATCATTATTACTTAAACTTATATCATTATTTTTAATAGGATTAC

TATTATCATTTAAACTACTGTAACTATTATGAATAATATAATCATCATTATTTATACTAT

TATTAACAGATATACCCTCTTTAAATATATCATTAGGAACATTAATAAAAGAACAATCCT

TGTTATTCGAGCTATTATCACAATTTTTAGCATTATTGTTATAATCTGAATATTTATCAT

TATTTTCATTATTAAATATGCCTTCATCTTTTTGTAAAAAATTTCTTTTATCTTTTAAAT

TCATTTTATTACTAAGTAAATTATTAGCACCTTTCATACTATCTCTTAAGAATGTTAATA

AATTTCCATCGTATGTATTCACTTTTTTCTTTTCAGAGTTATTCCCTTTAAAATTATCAA

TATCATTATAGTCTTTTTCATATTCATTTGTATCTAAATGATTATATATATAATTTGAAA

AAGAGTTTTTTAATTTTAAGTCACACTCTTTTTCTTTATTATTATTATTAATACTAATAT

TAATAATATCAGTAATATTATTACTATTTTTAGTAATATCATTATTATAATTATTTTCAC

TAATATTGTTATTATTATTTTCACTAATATTGTTATTATTATTTTCACTAATATTGTTAT

TATTATTTTCACTAATATTGTTATTATTATTTTCACTAATATTGTTATT

>c2869_g2_i1 len=330 path=[2860:0-329]

AAAATTAAAAAAAAAAAAAATAATGACAGTTTTAAAAGAAAATGTTGAAACATCAAAGGC

AAATAATGAAAAAGATAGCATTGATAAAATGTCATTAAGAAAAAAAGATAATATGCATAA

TAAAGATAAAAAAAAGACTAAAAAAACGAATGAATACAATAAACATGACGAATTAAAAGA

ATTAGGTGAAGAATTGAATAAGCAAATAAATAAGTTAGAAAAAGAACAAAATAAAGTAAA

AAAATTAGAATATGAATTAATAGCAAAAAGCGCTGAAATAGAATTAGAAAGAGAAGAAAT

GAAAAACAAAATGGAAGAAGAAAAAAAAAA

>c2871_g1_i1 len=209 path=[187:0-161 349:162-208]

ACTTCTCCTTTCAAAATAAACTAACACAGTGACTGCTTGAAATTCAGAAAATACTTCTGT

ATTCTGACTTCCTTACTTATCTTCCCCTTCATCAATCTTCTCTTGCTTGTGTCAAATAAA

CACCGTTTTGTTTTTAAAAATAATAAGCGTACCTTACAAATCTGAATTTTTCTGCTACTC

AGTGATTGACTTGTCCTTCAAGCTCAGCA

>c2872_g1_i1 len=450 path=[43:0-360 491:361-449]

AAAAAAAAAAAAAAAAAAACGTTAAAGTAATGGAAATTATACATATAATTCCATAAGTAT

ATTATTGTCAAACGTAATTATATTTTAAATTTTCTACATGATTAAAATTTGTAGCTTTAA

ATGAAAATTTACATTAAAATATTATATCATTAAAATTTTTATAATAGCTACAATAATGTT

TCATTTAAAATGTAGATTTCTTTAAAATGTTATAAAAGATACTCAAACTTTATATATATG

CGTTTTATTGAATGTTTAAAGAAATAAATTTCACAACTAATAAATTTTTTGAAAAAATAA

TATTTTATAATTTATTTGATTTTTATAAACTGTAGTACAATCCTGATTATTACATAAAAC

TAATTTTTTATATAAATAATAAATTCATTTAAATAAATTTAAAATCTAACTTATAAAAAA

AAAATTTTTTCTTCAAAATATATATATATA

>c2876_g1_i1 len=256 path=[71:0-130 201:131-231 46:232-255]

TCTATGGTTCTACAGCAGGGATGGGTTGGTCTTGATGATCGTGGAGAACTGCTTCACCCC

ACTGATTCCATGGGTGGGGACTGGTGGGGTTGGGTGACCTTGGAGGTCTCCTCAACCTCA

ACTCTATGGTTCTACGGTGGCAATGGGTTGGGGGTGGGCTCGGTGACCTCAGAAGCCTCC

TCCAACCCACTCACCCCAATGGACGGAGGGGGGTCTCCACGCCGGGACTCACCTCGTTGC

GCGAGAGGTCGAGGAT

>c2880_g1_i1 len=204 path=[182:0-203]

CATAACTCCAAGCTCTTGCACACTGCATCACTCAGCATGAGTTTGGCAGCACACCTTCTG

TACACACTGGATACAGTAATCATCTCTACATCAATGACTACTTGTTATTGAATAGACATC

CTTTTTGAAGAAGCTCATGAACTACTACTTGTATACATTTTGAATCGGGGAACTTCTGCA

CAATGTTATCATTTCAAATGTGAG

>c2887_g1_i1 len=237 path=[123:0-179 122:180-236]

CAACCTTGGTGATTCTATGATTCCATGATTCTATGACAAGTGATTTAACTGGGCTACGCC

TATTTATTTAGTGAATATAAATTTATCAATCAATAATTCATTATACCTAAGTTGGTGCTT

TTTCACCATGGTCCTCTACTAGTGAATGCAGCAGTATCTAACCCTTGATCCTTCAATTCT

TCCTTTTCTTGATCCCTTAACCAAGGGCAGTAGCAACAGAGGGAAGAATTGAAGGAT

>c2893_g1_i1 len=356 path=[325:0-82 325:83-165 25:166-219 79:220-287 79:288-355]

CTGCAGCAAGGCATGATGGGAGCTGTAGTCCTTCCATAAGGCATCACAGAGGCATGCTGG

GAGTTGTAGTCCAGCACTGGGCCCTGCAGCAAGGCATGATGGGAGCTGTAGTCCTTCCAT

AAGGCATCACAGAGGCATGCTGGGAGTTGTAGTCCAGCACTGGGCCGTGCAGCAAGGCAT

GATGGGAGTTGTAGTCCAGCATTGAACCTTACCACAAAGGATGCTGGGAGTTGTAGTCCT

TCAATAGGGCAGCACTGAGGCATGATGGGAGCCGTAGTCCAACAAGGCATGCTGGGAGTT

GTAGTCCTTCAATAGGGCAGCACTGAGGCATGATGGGAGCCGTAGTCCAACAAGGC

>c2897_g1_i1 len=315 path=[159:0-314]

AAAGCAAACAATTTCGCTACGAGATTCCCCCTTTGAACCCAAAAATCATGCATTTCCTCT

ACAACAATCTCCCATGTAAATGAAAATCATGCATTTTCTCTTCAAAAACCCCCATTTAAC

TCGAAAAAACGCAATTTCGCTTCGGAAATCCCCCATGTCACCCAAAAATGATGCATTTTC

TCTTCAAGAATCTCTGACCTATCCCAAAAGTCACGCATTTTCTTTTCAAAAATCCCCCAT

TTAATCCAAAAACCATGCATTTTCTCTTGGAAAAATACACAATCAAAAATCACCACTTTT

CTCTAAACGAAAACA

>c2900_g1_i1 len=225 path=[41:0-72 20:73-224]

GGTTTTTTCTCTAGGTATTTTGATGTAATTTTCCAACTACAAGGATAAAGCTCTTCCCAT

TTTATAGTGTTCTGGGGAGAAATTGTGAGGAGTATGCACAGAACTTGAAAAAAAAATGCT

TGAAAAATTTGTTTAGGGATAAGGAGGTAGCACTTGGTTGAAATAAATTGCCATTAATGC

TCAAATGTATACTTAACAAAACTTTGCTAGAATACTGACTGGACA

>c2901_g1_i1 len=401 path=[123:0-400]

TACGCCGGGATCTTCCACTTCCAGGTGTGCTCCTTCACTCCCCAAAGCCCCCAGAATGGG

ATTTTTGGGGGGCTCATTCAGGTGTTTTCCACCCCAAAATGGGGTTTATCTCTCTGTGGG

GCGACGCCCGGTGCCGCGGGCGCCTTGAAGCGATGAGTCACCCTCTGGGGTTGGGCACTG

CAGGGTGGGGGCCCAGAAATGGGTGTTTCCCCCTCAAAATAACCCCACATTTATCCTGGG

GACAGGGATCCGCGGCTGCTTAATGAGGTTCTGGCTGTTAATTATTGCTGGAGTGGAAAA

ATAGGGCGGGTTTTAGTTAATTACTGCGCTTACGGCAGCAAAGAGGGCTCAGCTCCCCTT

GTAGGGGAAAAGGGGAGAACACAGTGTGCCCCACAGCTTGG

>c2902_g1_i1 len=242 path=[1:0-241]

AGCTGACTCCCAGCTATAGCTACATTATTATTATCTTTTAACAAAGAAACATCTTATTGC

ATACTAGATTCGTATTTTACTACCAGAATTGTTCTATTTTTTTTTTTAACATATTTCTTT

TTTAAAACATTCATATCTTTGTCACATGAAAAACACACATCTACACTTGTCATTACTCAA

TATCCATTTAGAGTATGCTGGGAAAAAAAAAAAACGAACAATTCTGGTAGTGAAATACGA

AT

>c2905_g1_i1 len=247 path=[1:0-246]

TGCGTGACGGGGAGAAGCGCTGCCACAGCCGGCACGCCAGCGCCGGCACCGGGATGCACC

GCGGTGCGAGCACCGGGGCCATGAACAACATCCGCAGCACTCTGCTCAACTCCACGTCCG

ACTCCGACCTGGTGCGGTACCGCGCCATCAGCCGCATCCCGCAGATCACCCTCAACTTTG

TTGACTTCAAGGCGGACGCCTTCCTGGGGGCGCCGTCCGGAGAGAAGGAGATCATCGACC

CCACCAA

>c2908_g1_i1 len=243 path=[409:0-242]

TTAAAGCGCTGGTTGTGTTTCCAAGTTGTTCAGTGAGCAAAGAGCTATTAGATCCACGTG

TTTAACTAATACAAAACTAAACAGAAAAGCAGTTTTGCTTGATGTACCCCACCCTGTGAA

TTATGCTGGCTTATGGAGAAAAATACTTTTACTTCCTTGATAGGTGGGTGGAGTTAGTCG

AGTGAGTGGAAAGGAATTTAGGATGTGTCTAAGCAGACCAGAATGAGGAACTCAAACACA

AAG

>c2909_g1_i1 len=222 path=[41:0-196 294:197-198 296:199-221]

GTTGCAAGCGATCTCCCAATGTCCACACAGTGACTAATTCAAATCCCAGCTTTCTCCCAG

AGCGTGTGTTCTGTCTGTGCCCACCAGACAGCACACCATGCTGAATGTGTTACTACACAC

ATATGCTGTTAGGTAAAGAATTTCCTTTTTCTGTTGTCAGAGGATGATGTGTTTTCCGTG

CTGTATTGTATCTGTATATGAGGCTGCAAGTGAAGCCTCATA

>c2913_g1_i1 len=330 path=[308:0-329]

GGGTTTCCATGGGGCTCAGCTCGCACCCCAGCCCCTCATTTCTCCCTCCCCACAGCAATG

CTTGCTGCCTGCCTTCCCCCCCGGCCTCCCTCTGGGTCCTTGTGACAGGCTTAGGGATCC

TGGGACCCTGATGCTGGAATGGCATTTTGGCCTAGCTGGAATTGTTTATAAGCACAGAGC

TTTGATGCTCTGTGAGCAAGGCCTCTGGGGAGCTGAGAAGGGCTATTTTTCATGTGATGC

TTTTGGAGTATGCTATGGGAAAAGGTCAAGGGACGCAGTGGGAATGAGTGGGTCACATAC

TCAATGGTTTAGGGCACGACGACAGCATTG

>c2914_g1_i1 len=218 path=[1:0-217]

AGGAGGTCTGCAAGAACAGAGCAGCACAGAGCCATGCTCCTGCCAAGGAAAGCTTCACAA

AATTCAAGTTCAACAGCAACGTTAGCCCCTGTTAATTAACTTAAGAACAACCCTCTTAGG

AAGGCAAAGCCTGCGCATCACCTGCCAAGCACCTCGTGCTGCCAAACCCTCAGCCCGTTG

CCCTGCTGAGAGGGGGGGGAGGGAGGCTGAGCTTGCTA

>c2915_g1_i1 len=349 path=[197:0-287 98:288-348]

ACTTTAAGCACATTATGATCTTCAGAGATAATCAACCAGTAATTTCTTTCTAAATTTCCT

AATTTTGAATTCTGATTTTTGGATTTTAAATATTATCACATTCATAGGTAAATAAAATAC

TTCAGAAAGACCAATTACTACTTCATGGTGCATCAGACTATTTAGACAATTCGTGACCTT

TTAACACGGTGAGCAGTTGCATCATTATTATGTGAAATTATCCTGTAAAATACTTGTCAT

ACACCTCATGCATACATTTATGAGCAGAGAAAACGTGAGAGGAATTCCTGAAAACTGTCC

AGTTTTTAAGAATTCCTTGCAATGAGTAATACGTTGTAGTAGGACTGAA

>c2919_g1_i1 len=213 path=[379:0-212]

GGGTGGCTTCAGGGCGTCCACCGCTTCGTGGCGAGGGACAAAGTGGTGACAGCGCTGTCC

CAGCGCGGGCTGTACCGGGGCACCCAGGACCACGCCATGACGCTGCCTATATGCAGCCGC

TCGGGGGACGTCATCGAGTACCTCCTCAAGGACCAATGGTTCCTGCGGTGTCAGGAGATG

GCGCAGCGCGCCCGTGAGGCTGTGGTTTCGGGG

>c2920_g1_i1 len=408 path=[155:0-379 535:380-407]

AGGGAGTGGGGGAAGCAGTTGGCAGTTGAAATCTAGCAATAAATAAATAAATACAGAAGA

AGAGATGATCCGTATCAGAGCACAACCTACCATCAGTACTGCAGCCTCAGGTGTACAGAA

TTACTGATGCTGAAAACGAACTCCAATAAAGGCAGGTGTCTGCAAAAATAGCCAGTACTC

TTGCTCAAGCCCTTATCAGACATGACCAACCTCGTTAGGCAGTTATACAGGTATCCACTC

ATTTGGTTGCAGATGAAAAGCAAGGCATATAGCTTTGTCTCCCCTTCAGAATCTCAGGGA

GCTTAGAAAAGAAACAACACAATAAGAGAGCAATGAAAGCTATAAGATGTAAGGCTGTGA

TAAGTCAAAGCCTTCTTCTGATGTCCTCTCCTCAGAGGCTGGACTACT

>c2922_g1_i1 len=233 path=[511:0-232]

GACAATTTGAAAGACAGAAATTGTACTGACCATTGCTACTCTGATTCTTTTATACTATTT

TCTAGAATTCTTTGAACTTTCTGTGAAATAATTGGTTCTTCTAATGAAACTTTTTCCTGT

AACCAGGGTTTACTTAACAGCTTAATGTCTTAAAGATAAGTAAGAGGATCTGAAACATTG

CAATTGAAGGGCCATTTACAGTGAGGGCATATCTGATGAATGAAGAATTATTT

>c2923_g1_i1 len=262 path=[67:0-66 44:67-261]

CAATGCACAAAAAGATGCTTCAGGTAAGCCTGGGCCTTATGCTGCCTCACAGCAGTCCTC

TGGGCCAGGACTGGAGTTGTGGGTTTCCTTGCAGAGCTCTGGGTGTTCCCCATTTCTGCA

GCTGGTGAGGATGAGGGGGCCCGCATGGTGGTTGGAGGACGAAACTCAGTGAACCCAGAG

TGTCTACACCCAGTTCTCAGCACAGGAGAGAAGGAATAAAGTTCCTGCTGCCTTTCAGGG

GATGCATTTAGCTTGTTAGCTT

>c2924_g1_i1 len=362 path=[340:0-361]

GCACTTGTACAATACACCACCCTGTGCTTTGGTCACCTCTGTTTAGGTCATGAAGTCCAA

TAACAGTAACCATCCACAAGGCCATTTTGTTCAATTCTTCTGAGATAATCCTCTCCTATA

ATTTTCTAGGGGAACTTTCTTACAGAAAAAATGTCATTCTTACAGACTGTCAGGACAGAG

CTGTATAGCAGCTTACACAAAATCAAAACGTTTAAAAATGTTCTCCTGCTAGAAAGCCAA

AGAATGTCTTAAAGTTACTTTAAGATATTTTCACCGTGCTTCCACTAATACAGCATCATT

AGTAAAACCTAACTGAAATTAATATTAGCTCTTATGGTGAACAACCAAAAAATTGTTCTT

TA

>c2928_g1_i1 len=435 path=[64:0-434]

TATTTTCTTTTTTTAATATATTTTACAACAAATTTCATATGAAAATAAAAATTAATAAAA

TTTTAGATACATTGTTTTTTGTCATTTTATGAATTCATAATACTCTGTAAATGTCTGTTA

CAGTTTATTCTATATGTATAAAATATAAAAAAAAGTAAATGTTATAAAGTATATGTCACT

TCTTTTTATCATTATAACTTATGATATTTGAAAAAGTTCCTAAAAGAATGTTGTGTCTTG

AAATATATAAAAATAATATGTAATGATTATTTCATTTTAATAAGTCATATTTAATATTTT

AAAAAAAAGAATATTCCATAAGATGTAAACTAATTGTAAAATAATGTTTTATGTTGCAAA

ATATTATGAGAATTAATATTTCTAAGAAAAATAAAAATGGTTAAATTATTAGTTTGAATA

ATGATATAATAATAT

>c2930_g1_i1 len=272 path=[635:0-234 375:235-271]

CTGACAACTGGCACAGCTAAATTCAGGACAAGCAAAGACTAAACCCCCATTGTGATCTTT

TGCCCTGTGACTAAAGCAGTGCTATCTTACAAACACAGTGAACATGTGCAAACAGTGACA

ATGTCAGCGTGGGGATCAGCCAAGGAGACGCTTTGTAAATCAGCATTTGCTCTGCCTTCA

TGGGAACCCCACTCAACATCCTTACAAGTTGGATGCTGCAGACCTTGCAATTTCAGCTAT

TAAAACAGATAGTGGCAGTTCGGGGGAAACTG

>c2930_g1_i2 len=271 path=[635:0-234 869:235-270]

CTGACAACTGGCACAGCTAAATTCAGGACAAGCAAAGACTAAACCCCCATTGTGATCTTT

TGCCCTGTGACTAAAGCAGTGCTATCTTACAAACACAGTGAACATGTGCAAACAGTGACA

ATGTCAGCGTGGGGATCAGCCAAGGAGACGCTTTGTAAATCAGCATTTGCTCTGCCTTCA

TGGGAACCCCACTCAACATCCTTACAAGTTGGATGCTGCAGACCTTGCAATTTCATCTCT

TACCACCAGTTTCCCCCGAACTGCCACTATC

>c2939_g1_i1 len=207 path=[185:0-206]

TTTTTCCCATAGTTTATAATTTAATTATTAGATCCAGAAGTCATAATAAGCCAATTGATC

ATATGTTCTCTACACATACATAAACAAAATATATAAGAAAATACAATCATATATAATCTT

ACTTTGTATGATAGATACATACATATATATAACTTTATAGTAGAGGACTATCCTTTGGTT

TTTTTTACATAGCTTAGCTTATTGAGA

>c2941_g1_i1 len=222 path=[444:0-221]

AGAAGAAGTGCTGGAAGGAGCCCTAGCTATGCTGATCGACCATCCTTGCCTGACAGGCGC

TGCAGTTTCACATTCCCAAGTGCTCTGTGCAATCAGATGGATGGTTCCGGATGGAAACCA

AACTCAGCCCTTTGCTGCTGCCAGACCACTTCCTTCAGCATGTTCTTTCATGCATCAAAA

ACTCCAGCAAAGCCCTGGCAATTGCTTTGCAGGACAGAACAT

>c2943_g1_i1 len=236 path=[155:0-235]

AGTTGGCAGGGTTGATGCCAACAATTAGACTGAAGATGTAAAACAGTGACAGGAGAAGAA

AAGCACCTTGCTAAGAGATAAAGTTCTTCAGTCAACTAAAGTGAAATATCAGTTTAATTG

GATACCAGGGGTTTTCCAAGCTTTACTCATACCTTTCTCAGACCTCTTTCTCACCATAGG

AGACCAGAGAGGAAAAACAGTTTTGAGCTTGAGGAAACAGCTGTTCTCATAAGTGT

>c2944_g1_i1 len=240 path=[218:0-239]

TGCTTTTAAAAGTCCGCGGTTCTTGAAAACATGTGTCCTTTTGAAACTTACAGGTATGTC

TATGTCCCACTGGAAGTTTTAAAAGGAAACCTAATCTGCTATAAACTTGTGCAAAAAATA

TCTAGATAGCCATATATTAAGCATTTGATGCTATATTCCATGCTATTATGGGACCATACA

TTTGTTAAGCAGGTAACTACGTATTAGCAAGTTCCTGTACCTTGACATACAGACCTAAAA

>c2945_g1_i1 len=253 path=[81:0-191 80:192-252]

CTTCTTGCTTTTTTCAGGGTCAGAATCTCAGTGGTTCAAGCCAAGCTCCATTTGCGTGAA

TAGGATGCAGGATTTGCATGAAATATAACCATAGAATCACAGAATACCCCAAGTCAAATG

GAACCCATAAGGATCATTATGTCCAACCCCTGGGTCCAAGCGGGACCACCCAAAGTTCAA

AGCATGTTTCAGAAAAGTATGTGGTGCTTTCCTTGTGTGATCTTTCTTTGTTAATCAGAG

GAAAATTAAAAAG

>c2949_g1_i1 len=234 path=[155:0-233]

AAAAGGAAAAAAAAGAAAAAAAAAGAGCAGAATAACGAAACACTGGGATCAAAGTCAATG

GGAGCAGGCGCTGCCGGGGGCTGCGCGGAGGGGCGGAGGGAAGCTGAGGGCTTCTCATGC

TTTGTGGGAACGGTTCTTGGGGAAGCCGACAACGACACGATAACCCGGAATCTGTATTTA

CACACAAAGATTCTTATTGCACTTGTATTTTTTATATTAAAGTTTGCATGATTT

>c2951_g1_i1 len=244 path=[1:0-243]

AAAGAAAAGGAAAAGAAAAGGAAAGGTGCAAAGATAACCCTCTTTCCTATGTTGCCTCTA

ATTCCTATTAAAGACATTTTGTATTGTTGAACTTTACTGGGACAACATATGTGTTTCGAA

GCACTAAAACACAGTGCCTGAGTACGAATACTGCATAGAATCTGAGTGTTTGAGTGCTTT

TTAGAGATCACTCAGTCAGTGGAATGAGTATCTGAATTTATTCTGTAAGTATAAATGTCG

TGGT

>c2951_g2_i1 len=305 path=[443:0-304]

GTCACAGGAGGCAGTGACTGCACCTGGGGGACAGCAATGGCTCTGCAGCGAAGGGGAACA

GGAGCTTGATTTGTCTGTCCTGTCCTTTGGGCAGATCTGCTATAGTTATACTTTCACTGG

CACCACTGAGAAGAGTTTGGCCATATCTCCCCTCATCCCTCTGCCTCTATTTGTGCACAC

TGATAAAATTTCCCTGGAGTTTTCTTTCCTGAAAGCTAAATAGTCCTAACTCTTGCAGCC

TTTCCTCACCTGCTGGGTGCTTCAAACCCTTAATCGTCTTTGTGTCTGTTTAAAAAAAAA

AAAAA

>c2952_g1_i1 len=1814 path=[1855:0-1154 16:1155-1813]

GTAACTTTGTGTTTGAGGGTCATCATATGCTTTTATATTCATAACGTTACCTTTAGAATC

TTCACATTCTATTTCTTGATCTGGTTTAAAAATATTTAGTTGTGTTTGTTTTTTTAATTT

TTCATATAAATTTAAAGCATCTTCAATTTTTGTAATTTCTTTAAAATGTAGTGTATTTGG

TATCTTTAAGCATTTCATTCCAAATGAATGTCTCCATTCATAAAAATGTTTTTCAAACAT

AGCTCTACCAAAATAAGAATAATTTCCACAAATTTCACATTTATATTCTTTTGATAATCC

ATGTAATTTATATAACCAATATGGTATTGGTTTATTATCATAACCTAGTGGTAAATTTAG

AGGATTATATATTGGTTTGTCTTCTTCATTTTCTTCTTCATCATTACTACCTATTTCTGA

GAATTCAGAAGTATCATCATTTGAAGAATACATAGAAGAAGATAAATTATGTGTAAGATC

AATATTATTATCATTGTTGTTAATATCATCATTTATACTTAATATATATTCATTTTTTTT

TTCTGTTTTTTTTCTTTTTTGCAATTCATCTACAGTATATGCTTGTTTTCTTTGTATATT

TTGTATTGTTTTTTGAAAAAGTTTATTTAGTAAATTCTTATATAATTCAATTAAATATTC

GCATTTTGCAATTTCTCTATCTTCTTTTTCTATTTTTTTTTTAATTTCTTCTTCCTGTTC

CAATGTATACTCATTCTTTAAATATTTTTTTAAATCATCATCATATTTTTTACTTTTTAA

ATAACTTTTATATGTTCCTTCCGATGCATACAACTTATTGTTTATATTACAATAAAAATC

TAAATCATAGGTGTATTTTTCCCAACCTTTCACTTCTTTATTTTCGAATTTTTCATTGAA

ATTTTTTTCATATTGTACATAAGTCTTTTTACAATCTACTAGTATATTTATTTTTGCAAA

ATAATTCATTAAATAATCTAACAAACTTTCTAAATAACTTTTATATTCTTCATTTCTATA

TTTACAATATCTAGGTATATAATAAAATCTTGTAAAATTGTGTAAGTATGTTACTATATC

CATTTCTTTAAATTCACTTGTTTTATTAAAAGCACTTTTTTCATTTAAATTTTTTTTTTT

TAATCTAGCCATTTCTTTTTTTTTGAACATACTTAATCTAAATTCTTTAATTCTTTTTAT

ATTTATAAAATCATTATAAAATTTGTTCATATCAACATATTTCCCTTTTTTTTCTAATGG

AGAAAACATTTCTTTTAAATTGTTATTTTTTAAAGCTTCATTTTTATATGATTTATAACT

TTTCACTTCTATTTTTTTAATATTAGTTTTTTTATGATAATCTCTTATATATTTAATTCT

TTCATAATAATTCTTCCATACATCATTTTTTTCATCATTAGTCTTCCCTGCAATGAATTG

CATTTCTTCCTTTTTTAAATTATCTTCATCATTATAAAATTGTAAAAGTGTTTTTGATTT

TTTCTGAATCTTTTCTACTAAATAATTTATAGAATAATCATATAAAATATTTTTCTTACT

ATTTTTTACTTTTTCTTCAATTAATTCTGCTAAGGCTTTTTCTATTAATTCAATTTCTTC

ATGTAAGTATCTAATTTGCTCAATTAAAAATTGTGACATTTCCTCAAAAAAAAAAAATTA

AATGATTCGTATGTGTATTTTAATAAAAAATATTATTTAATTCATAATTTATTTAAATTT

TTACATAATACAATGATTAAAAATTTATATATTTTTTAAATCATTAAAATAAATACAATT

ATACAATTATACAA

>c2958_g1_i1 len=214 path=[314:0-213]

TTGTAATATTGATAGTAAATCCTATCTTTTTTTTAATACATCTAAATCATTTATGAATTG

AAACATTTTATACAGAGTTTAATGAATTACAAAATTATAAAAAATGAAAAAAAATGACTA

TACTTCTCTAAAAACAATATGAATAAAATTTTAATGAGTTTTCTATCTTCTATACAATGT

CTTATAGCAATAAACTACAATTAAAATCATTTAA

>c2962_g1_i1 len=300 path=[645:0-91 257:92-111 184:112-140 25:141-299]

CCATATGGGTCCTCTGGGTTCCCTTTTGGGGTCCTATGGCTCCCACTGTCCCCAGTGACC

CCCTAGCAGCGACATGAGCTCACTTTTGGGGTCCTGTGTGTCCCCCTATGGTTCCCCTGG

GCTCCCTTTTGGGGTCCTATGTCTCCCAATGACCCCACAGCAGCACAGTGGGCTCCATTT

TGGGGTCCTGTGGGTCCCCATATGGGTCTCCCGGGCTCCCTTTTGGGGCCCTATGTGTGC

CGCTGTCCCCCTAGCAGCACCATTAGCTCACTTTAGGGGCCCTATGTGTCCCCCTATGGG

>c2974_g1_i1 len=418 path=[1:0-417]

ATGGTGTTATGATAATTCTTTAATGCATTTATTTTCACAGACTTCTATTTCGTGTAATTG

CATATATTTTAGCCATCTTTCAGAAATTTTGTAGTTAAACTTTCCTTGGAAAGGTCAACC

CTCAGAAGACATAACCTTGTTTTTTTTTCCTTGTGCAGATGTCTAAGGATTGGGGCATAA

ATAAATCAGTGCAAGCTTTTGATGTTTTGAGCAGTGGTGGTGAATGAGGGGAGGTGTTGT

TCTGAGAGCTGCTGTGGTGGTGACAGTGCAATGCAAAGGCTATGCAGAAAAATGCAGCAA

GTAACTTAGAGCAAACTTTCATTATCTTTCCTTGCTTAAAAATGCTGTTTGCTTTCATAA

CTGTAACTTGTAAATGCAGTGCTTTGGAGCTCAACTCAAAATGGAAGTGCATTAAATA

>c2975_g1_i1 len=639 path=[155:0-638]

TTCATAGTTATATTATTTGAATTTTGTTTATAATATAATATCTCATTTATGTTTTTTGTT

ATTTTCATACCATTATTATTTACTTTATTTGTGACTTTAAACTTCTCATTAAAACCATTT

TTCTTTTCAGTGTTTGGTATATAATTTATCTCCTCATTAAAAGAACAAGGAGATGAATTA

TTTTTTTTTAAGATTTTATCTTTTTTTTTAGATAATCCATTTTTTTCGCTATGTATTATA

TTATTATTACCTGAACTATATATATCTTTTTCATTGAACTTTAATATGTCTACAGATTCT

TCACTATAAGTTGATAAATTATTCTTTTCACTATTATTACTTTTTTCGGTATATAATATA

TTTTTTTCACTAGTTTTGATAATATTTTTGCAATTATTTATATATTTTGTAGGTTGATCA

CAAAGTTCATATGGATTTTCATTTATTATCTGTGAATTAGACTTGTTAATTTCGTTACTA

AACACATTATTTTTAACTTCATTAACTTCTTTATTATGACTTTCTTTTTTATATTTTTCC

TGCATCTTTACAATTATTTGTTTTATTCCTTTTCTCAAAGAAAGTTGTTTTTATATATAT

AACATAATATGTTATTATAATGAATTATTTATTATATAG

>c2977_g1_i1 len=269 path=[247:0-268]

GTGGGGGCATTTACAGGAACCTGGAAGCGTTTCTATAAGCATTCCTATCAAGAAAGGAGC

TTCATAAGTCAGAATTTGGGGTTTTTTTTCAGATAAAGTAGTTTCATGGATTGTCTTCAG

TGGTTTCCCTTTGTATAAAAAAAGAAACTAAAAAATATTCATTATTGTTTCATGATGGCA

TGATCTGAACAGTTTCAGACCAATCTTTAATTAGCTCAACTCTTTTTGTATCTGATAGTG

ACATCTTTTGTTTATGTTGAAGTAGTGCA

>c3006_g1_i1 len=350 path=[155:0-349]

AGAAACCTCTTAAATAAAACACTGAGAAAATTTTTATTCCAGAATCACTTTGCAATGTAA

CCAGAATGGTACTTCTCCTTTGCTGCAACATTATTTTGTGGTACTAACTCAAAAGTTCAA

ACCATTTTCATTTCTTTATTACCAGTAGCAATCTAGGAAATGCTGCCATCAATAGTAAAT

CTACATGAAGTCACACTCCTCCAGTGCATTTTGTGCATAGCAATCAGTTCATAACGCAAT

CCTGCGTTGTTTAAAATTCAGTTTATTCTTCAATTACTCTGCTAGCTCAACTCTAATTTA

TTATATTTTCTGGTACATTTGATTTTGGATATTATGACATCCTGGCTCAT

>c3016_g1_i1 len=514 path=[492:0-513]

TGCTGCTGGCGCTGGGGCTCGCCGCCGCCGCCAAGTCCCCGTACCAGCAGGTCCTGCAGC

ACAGCCGCCTCCGCGGCCGGCAGCACGGCCCCAACGTCTGTGCTGTGCAGAAGCTGATTG

GCACCAACAGGAAATACTTCACCAACTGCAAGCAGTGGTACCAGAGGAAGATCTGCGGGA

AGGCAACAGTGATCAGCTACGAGTGCTGTCCTGGATATGAAAAAGTTCCTGGAGAAAAAG

GCTGCCCAGCTGCACTGCCACTCGCAAACATCTACGAGACCCTGGGAGTGGTGGGCTCTG

CCACCACACAGCTCTACTCCGACCGCTCCAACCTGAGGCCAGAGATCGAAGGACCTGGAA

CTTTTACTATTTTTGCCCCAAGTAACGAGGCCTGGGCTTCCTTGTCTGCTGAAACACTGG

ATTCCTTGGTCAGCAACGTCAACATTGAGCTGCTCAATGCCCTCCGCTACCACATGGTGA

ACAAACGGGTCCTCACGGATGACCTGAAGCATGG

>c3020_g1_i1 len=224 path=[1:0-223]

AAAGACAGTTTAAATCTTTCTTAACTACAGTGTAATATAGAAAAATTTAAACTGTCTTTT

CTTGTTACATGCTTCTTTCCGATTGTCCTTAAAACAGCATCAGTTACCAAAAAGTACAAA

CTAATTGTCTAGCTGTGTTCCATGTTTATTTACAGCCTCTTGGCCCCACACAGCTAAATC

ATTTGAATTGTGCAGTTTATTAGCAACAGATTACGGGTGCTTTT

>c3021_g1_i1 len=252 path=[1:0-67 69:68-141 143:142-159 69:160-233 143:234-251]

ATTGGGTCTCCCGTTGGGTCCCCCATTGGGCCCCCCGTTGGGTCCCCATTGGGTCTCCAT

CAGGTCCCCCATTGGGTCTCCCATTGGGTCCCCATTGGGTCCCCTATTGAGTCCCCATCA

GGTCCCCCATTGGGTCCACATTGGGTCCCCATTGGGTCATCCATTGGGTCTCCCATTGGG

TCCCCATTGGGTCCCCTATTGAGTCCCCATCAGGTCCCCCATTGGGTCCACATTGGGTCC

CCATTGGGTCAT

>c3022_g1_i1 len=254 path=[21:0-189 211:190-253]

TTTTTTTTATTTTATTTTTTTTATCTATGTAAATATGTTACAAGTAAAAATATTTAGATT

TTATCACATTTCGTAAAAATATATCTTCACAAAAATTTATCAAAAAAAAAAAATTTCAAA

CTAAAAGCACATTTTTTTTAAAAAAACATTTAAAATTAAAATATATATTAACGGTTTATT

TAATTTATTCAAAGAAAATTTTGAGAAAGATTTTTTATATAAAATAGTATACAATTACAA

AAAAAAAAAAAAAA

>c3023_g1_i1 len=322 path=[53:0-321]

AAGTTTTTTATTATTAATTTTTAATTCATAAAAATAATTTTTTTAATTAATAAATATTTT

ATTATGATATATAAAATTATTCACTAAAAAATATAGATATAAAATAAAAAGGAATATAAA

CTTATTTTTTCCTGTATTTTTAATCAGAATCATAATATGATTTCATAAAAAATTAAGAGT

ATGTGAAATGACTTTTAAAATATTATGTATTTATATATGTATCAACAATAAAAAAGAGGA

AAATTTTAAATAATATTTTGAAAAATTAAACAAAAAAAAGAAGAAAAAAATGATAACAAA

ATACAAAGACAAACTAATTATA

>c3024_g1_i1 len=746 path=[1:0-745]

AAAAAAAAAAAAAAAAAAAGAATTTATGAGTATTGCATATATTTCTTTTTTTTGTAAAGT

TAATAGATCTAGATAAAACAACTTAATCTTTTATAGAATTATTTAATTTATATAATTTTT

AGTAAGATGAAAAATGATAAAATAAAGTTAGTAAGTTTTGAAGGGGATGAATTTATTGTA

GATAAATATACTGCTTCGATGTCCACTGTAATAATGAATATATTAGAAGTTATGACTGAA

GAAGAAGATACAATTCCTCTTCCTAATATAAAAACTCAGATTTTAAAAAAAATTATTGAA

TACATGGAATATCATATACAAAATCCAGCAGACGAAATACCAAAGCCATTAAGTACATCA

AACTTACAAGATGTTGTATCTAGTTGGGATTACGATTTTGTTAATACTGACAAGGAAACT

TTATACGAACTTATTGAAGCATCTAATTATTTAGATATAAAGCCACTTTTAGATTTAACT

TGTGGTAAAATTGCTTCTATGATGAAAGATAAAACAACTGAAGAGATCAGAGCAGAATTT

GATATAGTTAATGATTTTACAAGAGAAGAAGAAAAACAAATAAGGGAAGAAAACAGATGG

TGTGGTGATATATAATTTGCAAATTTTTTTTTTTTATCATATATAAACTAGAATAATAAT

ATAAATTTAGAGAATGGTTTTTTGCTTATGTTTCGCTTATTTTTTCCGCAAGTAAACAAG

GGCATTTTTTTTTTTTTTTTTTTTTT

>c3026_g1_i1 len=221 path=[218:0-151 519:152-197 521:198-220]

GAGGCGGTGGGTGGTGCGTTTCAGCAGTGCTGACAGTGACAGCAGCCACCTCTGCTGGTG

CAGCTTTGTAGGAGCGTGGCATAGAGCTCTGGGTCATCGCTGGCAAAAGCACACAGCCAA

TGGTGGTAGCTGTGCCGAAAACTGGTGTTTTGTGGCTGAGAGTTTGCACTAACAAATGGT

GTCATTGGGCTTTTTGTGCCTGTGGTGGTTTCCACGGGCAC

>c3036_g1_i1 len=201 path=[219:0-200]

CCGAATAGTAGGGCTTGCACTGTGCTTCAGCCACTACCTGGATTTGTTCATCACGGGCGT

CATCGGCCTCAACGTGATCACCATGGCTATGGAGCACTACCAGCAGCCGAAGGTTCTTGA

CGAAGCCCTGAAGATTTGCAATTACATCTTCACTGTTATCTTTGTCCTGGAGTCTGTTTC

CAAGCTCATTGCCTTTGGGTT

>c3037_g1_i1 len=211 path=[156:0-39 25:40-170 156:171-210]

TGATGTGGGATGATAACATTACCTGAAGATGAAGTCAAGGAAAGCATTAGAGATGGCACT

GGAGAAGCTACAGACAAGAATAGTTGAGATTTCATCCTATTTAAAGTCTTGTGGGAATCT

GCATTGTCAGTGTACTCATACATGAACCAGGAAAAAGTATGAGATGTGGGATGATGTGGG

ATGATAACATTACCTGAAGATGAAGTCAAGG

>c3038_g1_i1 len=257 path=[347:0-256]

ACGCTGTCACCCGAGGGATGCCACCCCACTCCTGCAAGCATCGCACCCCAAACGCCAGCG

AGGTTCCCCCTTTGTGCGCCAAGAAAGGCATCCCCGGGGGGTCACCTGTGTACTGTGAGT

TGAGTTCCTCCCAGGCAATGCAGAGAGATCTCTCATTGAGCCTGCTGTCTTTGTCATGTA

TTTATTTTAAAGTTTGCCCCTTTGATGTCTGACTTTGCGGCTTGTTTTATGCCAGAAGGC

TCGGAGCAGCCTGTGTA

>c3040_g1_i1 len=283 path=[261:0-174 436:175-282]

TGTCCAGGCTAGGGAGCGTGTTAAATATACCTACTGAATTCAGCTTTAGTTCTGTTTTAA

AGAAAATTAATAAATAAATCATTTGTGCTGTTTTTATTCCATGGCTGTTTTGGAGTAGGG

GGTGAGAGGGAAAGGAGGAGCGAGAGAGCACCTGCAGGTGCAACAACAGGTAAGAAGAAG

ACAGCAAGCATGTTCTAGTGTGGGGTATAAAGACAGCGGGGATATATTCATAGCATATGT

ATGTGTGCTGCAGCGGGGAGGTAAGTTGGGCTGAATTTGTTGA

>c3046_g1_i1 len=242 path=[270:0-241]

AAAAAAAAAAAAAAGTTAAAAATTCACAAATAATTTGAGATCTTTTAAAATAGAAAATTA

TTTTAACTTAGTTGTGAAGCACATATATGTAAAATATATTATTTTTCTAAATATCTTTTT

TAACTTAATTATTTTTTAATAAAAATTTATATTTTTTCGTTATTTCCTTTTTTTCTTTTT

TCAAAATTGTTTTTTCCTATCATCTTTTTTTTTTACATTATTATACACACTTTCAGAAGT

AA

>c3047_g1_i1 len=241 path=[1:0-143 145:144-240]

GACAAAGATGGAATGAGCTTGGGGTTAAGACTGAAGAGATATTTTTTCCCCTGAATAACA

AATACCTATTTACAAATAGTTCTAACCAGTCATGAAAATCATGATCTCAATGAGGTCACT

GAGGAAAGTGTTAGGGGACTAACCTTTCTGTTTCTTAAAATTAGTAAGAAAACTTGTTGA

CTAATTTCAGAGGCAGCCAGTTATAGCGTGTACCATATACCATATGTAGTGTACATAGTA

T

>c3048_g1_i1 len=685 path=[907:0-684]

AATAAAAAAAATTTAATAAATAATAAAATGAAAAAAAAAAAATAAAGAGAATAAGAAAAG

CATAAGTGAAAGATATTTATTTTTAAAAGCATAATTTTTTTTTTCTTTTAATCATTAGTT

GGTTAAAAAGCCAATTCATTGAAGAATCTATTCCGTATCCTGTTTTAGATGAACATTCCG

CTAAATGGCAATTTTTATCAACAATTTGATCTATTTTCAGTTCTTTTCTAATATGTTCTA

ATAGCATGCAATTTTTTTTATCTTGTTTACTTGCTAAAATTAAAAAATCATCTATATGAC

TAAAATCTTGTAAAATTTTATAAAATCCCTTTTTAGCTATTTTTAGTCTTCCCCTGTCAG

ATACATCAATGAAATAAATAACAGCATCAATGTCTATCCTATTAATAGACACGTCATCTT

CTATATTATATCTGGAATCCCATATGATTACTGAAAATTCTTCAAAATCAATTTTTTCAA

CAAAAAGACCTTCTGTTGGTTTTACTTTTAAAAATTTATTATATAATAAATATTTTACGA

AAGTTGTTTTTCCACTTTGAGCTGGACCACAGATTCTTATTTGAAAAATAATTTTTCTGT

TAAATAATTTATTACAACAATCTCTAAAAAAAGTTATTATTTTATTACCCATTATAATTA

AACATATATATATATATATATATGT

>c3051_g1_i1 len=287 path=[499:0-286]

ATTATTAAAAATTAATATGTATGCTATATAATGTTTTGAATACCTTAAATTGTATATTAT

CATGTTCATTATTAACATTATAATATAAATAAAAATTCATTTACACGAAATTAATTAAGG

AGAACCAGTAAAAATACAAATTAATTTACGTTTCCCTTTTTATAAGAATATTTTACTTCC

TTTTTGTTAATGAAGAAATATTACAACATATATTAGCAATTGTATTTTATATTTGGTATT

GCAGTAATTTATTATATGTTCAAATACGTTTAACGTATATGGAACAG

>c3058_g1_i1 len=405 path=[383:0-404]

AACAGAACTCAAAAGGCAACTTACCATGTAGTCATGTAGTTTGCTGGTGGAAACACAACA

GAAACTAGGAGTTAAGGTTACTTCTACAGTAACTTTATATCAGTAACTTTAACTTGTAAT

TCCATGTATTTTGTTCTGGAGTAAATTGCCACAGCAAGGAATTTAATAGCTGCAAGCCAT

CCACCTTCTTCAGAAGTCAAGGAATGTTGTCAAGAGGGATAACAACAATTATTTCACCAT

GGCAATTTCAAAAAAAGAGAGGAAAAAAAAAAGCATGAATTAAACTCATCTGTCCACTGC

TTCCTTCTGATTCTGAAAGAGGCAAGGACCTGTGTTGTGATCCTCTTGATAGGAGAACAC

TTCCTCTAAGGGCCCCAAGTTTTCCTCAAGCTCATGCTTTCAGAG

>c3072_g1_i1 len=291 path=[1:0-290]

CTCCACTTGTACAGGAGTGAATTGGCAGATGTCAGTGAGCTGTGATACAGGCAAAAGCTG

AACCTAGGTGAGAAGAGTTGAGAGTAGGAAGAAAAGCAGATATGAAGCAGAAAGGTTGGT

TGGTTGTTTCCCGTTCATTTAAACTTTCCAGGTAAACATGACAAGTTTCACTTGAATGTG

TAATATGTAATATGTGACATGGATACCTTCTGCTCAGGGGAGAAGGCTCTGGGGCTCCAG

GGAGACCTTACAGCAGCCTTCCACTGTGTAAAAGGGGCTTAGAAAGGGGGG

>c3073_g1_i1 len=265 path=[429:0-264]

GCAGCAAACTGGGCAGAAGCTGTTTTCCCCGGGAAAGCACCTTGCAGCAGGCAAGCTGTC

AAATGCAGCCAGAGGGACTGCTGCAGGTGTGCCCACGTGCATCCAGGTGCAGCACAGAGA

CACTCACCGGGTGGCAGCACCCGCACTTGTTGTGTGCCCACGTGTGGGTCTGCAGCTGTC

ACCTGTGCATTTCCTACACATATCTGCAGAGCTGCATGAGGCCAGGCGGAGGTCCCACAG

CACCTGAAGCCAGCTTCTTTCAGAA

>c3074_g1_i1 len=1303 path=[8:0-1302]

TAATTTATCAAATAGTACTATAAATAAGATATACAATCTATATTTTAAAAAAGAAAAAAA

CACAAGTGTTTTGAAAAGAAATATAGAAGAAAATATAAATGATAAAAGGGATAATTCAGA

AGAAAATAAAATGAATGAAAATAAAGAAAATAGTCATGGAACTATAGAAAAAAAAAAATC

ATTAGATCAATTAAAATTTGAAAAAATATTTTTTGAAAAGCATAATATTTACGTAATGAG

AGCAGAATTACATAAGGATACGGAGTATCGGTTAAACAAAAATAATATAGATAAAAATTT

TAAGTCATTAAAAAATATATGTGATAATTATTATTCTGGTTTTAACAAATTATATAATCA

TTACTCAAGCATACGTGAAGAATTTATATTAACATATTTAGGAAAAGAAAATTACAAATT

GTATAGAAAAATTGTATCTGATTATTTCTCTTCTAATAAGCATTCATATAAAGAATTTCT

ACAAGTACCTTGTGAAGAATTAGCATCAACAATTTTTGATTTCATTTTTGAAAATTCAAA

TGAATTAACTGTAGAAATGGTAAAAAATTATAATGAAGAATTATTGGGAAAAGTAAAACA

ACTTAATATTTTTGAATTAAAATATTTATTAAATGATCTTATTAAGTTGAAAAATAAATT

ATATATATATCCTTATACTTATAAAACAAAAACAGGAAGAATGAATTTACTTTTAAATAA

TCAGAAACCAATAAAAAGAGAACACATAATAAATTTAAATATAGATAATAAAATAAAAGA

GGAATTATTAAAAAAATATATGAATATTATTTATCCTATTGATTATATAAGGAAATTGAT

TTTAATAAAAAAAAATTCTAAAAATAAAGAAAATGAAAAAATGCCAATTATTTATATATT

AGCAGAAGAAACTATAAGAATTTACGAAAATTCTGCAAAAGATGTAGAACCTTTTAGTGA

TGAACAAATAAAAAATTTGAAGTCATATGTTTTGAAGAAAAAAAAAAATATCATTTTAGA

TATCCATAATAATGAAGAGGAAAATTTAAATAGCGCTTTATTTGATTCAGAAAATAGTCA

AATTAGAAAAATTCTTTATATGATAAAGGAAGACTTAGATAAACAGAAAAAGAAAAATAA

GATAGATGAAGTTGATAAAGAATACGAAGAAGAACAAAAAAACTATATAGATGCAACTGA

AATTCATAAAAATATACCTGAATTAGAAGAAATGAATAAATTAACAGAAGATATGTTTTA

TATGAAGATCCCTTTTGTTGAATAAAATGTCTTTTTTTAATTT

>c3074_g2_i1 len=1688 path=[4310:0-1687]

AAAAAAAAAAAAAAAAAAATAGCTTTTCATTAAAATGTTAAATTTTTCTGTATTTTACTT

TTTTATGGTCTTATGTATTTTAAAATATAATTATGCTTTTAAGAATAAGAAAAACATATT

TTTATTTTTGAATAAATCTATTAAAAATAAGCCTATAATTAGAAAATATAAGTGTAGGAG

ATATTTAAATAAAACGAATGATATTAAAAACATCAATATAGGAGAATTAAAAATAGGAAA

AATATATGATATTATAAGTAATGATATATATATTCAGTTAAAAGATAGTAATCATCATGT

TATTATAAAAAGAGAAAATAATTATTTATTGGAAAACGAACTTTTATATGAATATTTGTT

TAATTCTAATTTAATTGATAATATTTTATATAATAAATTGATGAAAAAAATTACAAAAAA

AAATGAGGAGAAAAATGAAATAAATGAATGTGTAGTTTTAATTAAAGAAATAAATGAAAA

AAATGAAATAATTGGTGAGCTATATACTAATGAAATAAGTAAAAGAAAAGAGAAAATTTA

TGATAAATTGAATGAGATAAAAAATTTAGAAAAAAAAATTTTCATAAAAATTCTAAAAAA

TGTAAGAAATAAATATTTTACAGTTCTTATACATGATTGTATAAAAGGTTTTTTGTTATA

TGATGAAGAAGAAGATATGTTTGATTCGATATTTTCTCAGAAGTTAAGTGCATACATAAT

TGATGTAAATAAAAAGCTTGAATATGTTTTTTTATCAATGAAAAAATTATCTCAAAACAC

TTTAAATGACAAGTTATTGAATTTCCAACATACTATTATATTAGAAAACCTATTTGAGAA

TAATTATTTTAAAGCAGAAATTTCTGATTTTTGCAATGATGGGAATAATGTAATAGTAAA

AATATTTGATAATAAAATAAATTCGATAAAAGTAAAGATAAAATCACATAATATAATCAA

CACAAGAAATATTTTGAGGAATTTTGATTATATGAAAAATAAAGTTATAGATAATCAATT

TAATAAATATAATGAAAATACAGAGGATTCATATGATGACGATAAGATTAACATGAAAAA

GGAAGATTTCGATGAAAAAAATATTAATAAAAGAAATAAAGTTGTTTCAAAAAAAGATTA

TAATGAAAAACTTAAAAAATTTAAAGAAGATTTCTATGGTTGTAAAAATCCAGATGCATT

AAAATATATAGATATGGATAATTATATTTATAAAAAAGAAAAATTACTTTATGTTAGAAT

TATAAATAAAACATCAGATAAAAACATGTATGAAGGAAGTATGGTAAATTCAGATATCAT

AAATCAACACATATATGATATTATAAAAAATATAGCTAACGATGAAAATAAAGTTTCTTA

TTATGATGAAAAATTAAGATATCCATCTAAAGTTCTAGGATTTTTTAATAAGTTTGTTAT

ACTATCTACAAGAATATTGAACACTGATAGCAATAAAGAAGGAAATAATAATAGTAATAT

CGATGAAAATACTAAAATTTATAGTAATAGTAACAATAATAAACATATCAAAGATGTAAT

CACACTGATAGAGAAGCGTTATATAGATTATGAGAATTTAAAGGAAGGAGATATTTTTTT

TTGTAGATTTGAAAGCGTAAAAAGACAAAGTATTCGTAATGTACTTAATTTATCAAATAG

TACTATAA

>c3082_g1_i1 len=257 path=[235:0-256]

CAATGGAGATTCCAATAGAAACAGATATTTAAGAAGGCAATTTAGAGTAATGCAAAGGAA

AGAATCCTACATGTCATCAGTATTTTTACTGATAACCAGGGATTACCAGAATGCTGTAAA

AGAAATTGAGATATGAGCTTACCTTCATCTTAGTACAAATATAATTATTTCTAAGCCCAT

TGTTCAATGTAAGGTTGAAAAGAAAGCACACAGATTCCACCCTCCACGCCTTCCCCAGCC

TGACCTCAAACACAAAG

>c3083_g1_i1 len=239 path=[1:0-238]

AGTCCTATGTCTTGGGATGAAGAACTGAGAACTTCAGGAGTTCATTTGAACTCAATAATG

AGCAGCAGCTTCTCTCCTGCAGATAATTTGATATAGTTCTTAATGGAAATGCTGATAAAT

AACAGCTCCTTGCTTTTTTTCAATGTAAATAGCCAGATTTTTGAACTAGTGTATGAGAGC

AAAGAAAAACTGGCTGTTATCCTGTGAATGCTAGGAGGATTTCAGAGGGAAAAAGGAGG

>c3083_g2_i1 len=291 path=[433:0-267 769:268-290]

CCCTCTGGTCTTTTCACAGGACAGATCTGACTTCAATTACAAAGTGGCAGCAGTCCAAGC

AAGTGCGCTCTGCCCAATGCAGGCACAGCCTCTAAAAACAAGCCTCAGGAATACGCTCTG

TGACCTTTGAAATGTGGGGGTAACTCAACCCCCCCCACGTGAGAAAGACAGAGCCATGTT

AAAAAAAGTAAAGCTTTATGATGAGCATTTGATTACTTCTAATATTGAATAACCTCTACT

ACTAACTTCAGAGCATTCACTAAATATTTTTTTGAAGAGTGCACAAAGAAT

>c3085_g1_i1 len=291 path=[1:0-249 580:250-290]

AAAGCCACAGATATCACACTTGATCATTAATTCTAGCTGATAAAGATCTTATCAAAGAGT

TTATGAAATGCCTTACAAAAGGATACAAATATACTTATCTAGCCATTCCTTGGAATGTTA

GCTTTAGACATTGTCATTTTAGGTAATTATCACTTTTCTAGTATCTACCAAACCACACTG

CAAAGCTGAAATGATAAAAGACTTTTATAAAACATTTATTAAGATAACCAAACCCTCCAG

TTCATTTTTTGTTCTTAGGTTTTGCTCTGTTAAAAAGACCTAAATACGAAG

>c3085_g1_i2 len=301 path=[1:0-249 251:250-300]

AAAGCCACAGATATCACACTTGATCATTAATTCTAGCTGATAAAGATCTTATCAAAGAGT

TTATGAAATGCCTTACAAAAGGATACAAATATACTTATCTAGCCATTCCTTGGAATGTTA

GCTTTAGACATTGTCATTTTAGGTAATTATCACTTTTCTAGTATCTACCAAACCACACTG

CAAAGCTGAAATGATAAAAGACTTTTATAAAACATTTATTAAGATAACCAAACCCTCCAG

TTCATTTTTTATAGACTACTTGGAAAGCAGGAAACAAGAAATTGTGTTACAGTGACTTTT

A

>c3085_g1_i3 len=290 path=[1:0-249 659:250-289]

AAAGCCACAGATATCACACTTGATCATTAATTCTAGCTGATAAAGATCTTATCAAAGAGT

TTATGAAATGCCTTACAAAAGGATACAAATATACTTATCTAGCCATTCCTTGGAATGTTA

GCTTTAGACATTGTCATTTTAGGTAATTATCACTTTTCTAGTATCTACCAAACCACACTG

CAAAGCTGAAATGATAAAAGACTTTTATAAAACATTTATTAAGATAACCAAACCCTCCAG

TTCATTTTTTTTCTACTCAGGAAATCTTCGTATTTAGGTCTTTTTAACAG

>c3086_g1_i1 len=271 path=[1:0-270]

ATTCAGGTAAAAAAAAAGTTTGAAGAAAAGAAGAATAGGAGCGTATTGTAATGTCTGGAC

AGGTTGTAACCTTTAAAGAACCATTATACACATAGAACAGCATACAGTTCTCAGGTTTCA

CTTCTTACCTTACTTGCTTTACTTCCTTACGTATGGATTTGGATAGGGTAAATGCCTTCT

TCATTATTTTTTTTATGGCCACTGTTCTACCATCACTGAAACAGAATTAAATGCTGTTCA

GAGAATATTAGGCCAAATACATAATTTCATA

>c3086_g2_i1 len=266 path=[740:0-265]

GAGCAGAACATCATGAAGGCTGCCTTTGGGGCAGTACTCTGTCACTATGGCAACATTTGG

TATTTCTATACAACCACCAATGAATTTGCAGAGATTAGGATGGTCAAGTTCCCTGTAAAA

ATGAGCAGAAATCACAGCACACAAATAATTTTCATGGATCTCCTTCCTACTTTCTGTACA

GTAACTGGAAAACTAGAAGCCTTGTCTCAAAAAGTAAAGAGAAAAGTAAAACAGCTGTAA

AAAGATTTCAATCTAGTGTCAAAGAA

>c3092_g1_i1 len=371 path=[51:0-49 101:50-55 107:56-370]

CAGTAGAGCACCAGTCTCAAAGAGATCTGTTAAAAAAAAAAAAAGTAATTTTTCATAGGA

TTTTTCTAGTTTGTAATGTTTTGAGGGTTTTTGCTTTCAGGAACATATTACTTCTTAGGT

GTTTATGACATTTGACATTAGTGTGTTTTGGTTTGGTCCAGAAGTTTACCAGCACCTACC

TGTCTGTCCTTGGTAGTGACTGTGTTTGTAAATTCGTTGATACTCGTATATTTTGCTAAT

GTCATTTTTATGTTGGTTTTGTGTTTTTATTATTGAAAATAACCTATGGATTTTCACAGT

CATAGCAGAACTATTGCCGTAGCAATTTCAGCAGTTGTCAATCCTCTCTCTGATGGATCT

CTGTCTGATGT

>c3092_g1_i2 len=372 path=[744:0-50 101:51-56 107:57-371]

TCTCTTTGAGACTGGTGCTCTACTGGCATAAGGAGGTTGACTCTAAAAATGTTTCATAGG

ATTTTTCTAGTTTGTAATGTTTTGAGGGTTTTTGCTTTCAGGAACATATTACTTCTTAGG

TGTTTATGACATTTGACATTAGTGTGTTTTGGTTTGGTCCAGAAGTTTACCAGCACCTAC

CTGTCTGTCCTTGGTAGTGACTGTGTTTGTAAATTCGTTGATACTCGTATATTTTGCTAA

TGTCATTTTTATGTTGGTTTTGTGTTTTTATTATTGAAAATAACCTATGGATTTTCACAG

TCATAGCAGAACTATTGCCGTAGCAATTTCAGCAGTTGTCAATCCTCTCTCTGATGGATC

TCTGTCTGATGT

>c3093_g1_i1 len=255 path=[337:0-173 207:174-254]

TTTAAAATTGTAATCTTATCTGTGTCAGAGCAAGACTTTACTATCCCAGGTACATTTCTT

TGCCAAAGAGGGCAGTGCAGGAAAGGACAGAAAGGTGGAGGTGTGAATTACCCATAATCA

AAAGAGGCATTGTATACCCACTAAGCATTCAAGACAAACGTTCAATGAAAAATTGTTTTC

ATATTTTACTAGATTTCAGTTGGCTGTCTGCTGCATTTTTAGCACCCAGCATTAGAAAGT

TGCCCCTTAGAGGAG

>c3096_g1_i1 len=229 path=[31:0-60 30:61-228]

AAGTGCATGAGGTGCTCTCACCAATCTTATTCTGCTCCCAGTGAGACCTCTGAGGTCCTG

CTGTGGCTGCTGCCACCTTCCCCATGTGAGAGGAACCCAGAGAGAAGCATTCTCTTCTGT

GTCTGCTTGCACTGTGGGATTATCAGGAGTCAGAAATAATAGGAATGCACTGCATGGCTC

TAAATTAATAGATCTGACTTTGAATACACTTGGGAGCAGACACAGAAGA

>c3099_g1_i1 len=244 path=[169:0-243]

CTCCAGATGCTCCGTCAGGTCACAGCCGAATGCGCTCGTGGCTCCCTTCCTCTTGAGTTT

CTGCTTGGCTCCCTTGTTCTTCATGAGGTTAGTCCCCACTGCCACCATTGCCTGCCCACA

TCTCGCCAGCTCGGATGCGGGCACCCATCCACCAGCCGCTTCCCCTCGATGCGAAACCTT

CCGGGAGTGAGCTATGATCGCTGCTGGGCAGCCTCCTTGCACCCTGACCTTCCCCTGCCA

CCGG

>c3101_g1_i1 len=226 path=[204:0-225]

ACCTCGTGCTCTCTTAACAGAGTTTTGAAGGCACCATTGTATGGGAGAGCCAGGATCTTC

AGGGCCTTGTTTCAAGAAACCTTCACAAAGTGATGGTGAATGACGGAGGGGGTGTTTTCA

GAGTCATTACAGCAGGGGAAGGGCTGTTGCCACATGAACTCACAGAAGGTGTGGAGGGGA

TAGCAGGTGGTTTCATCTACACTATTCAGGGTAAGTCTGCAGACAG

>c3107_g1_i1 len=380 path=[122:0-119 242:120-379]

CATACTACCCTAACTCTAACAACGCTCTCTGCTCAGCCATTGACATTACATGTAAACTTT

TAGATGGCAGGAGCTTGTTAGCAACCTTTTCCCATGGACTGACTTGGAAAAAATCCTAGC

AAAAAGTCCTGGCTTTGACCATTAAAACAAGATCAATACAGACAGATTTAATAGATTGGT

TGTGATTTTAAGGACTCACATTAAAGAAAAGGGAGCTGAGAAAAGCATACTAAGAGCTAT

ATTGACAAATAAGTTATTTATTTGAAGTTGTGAATTAGAAAAGAAAAGTTTAGTAAATGA

AAACATTGCAATATGTTAAAACCAAGAAAGGACATACAAAAGCAGAGAAAGAACAACACA

ACTGCCCTTAAATGGCCCAG

>c3107_g1_i2 len=293 path=[512:0-32 242:33-292]

GTAGACAGAAAATAGAGAAGAAAAAAGAAAAAAAAAAAGTCCTGGCTTTGACCATTAAAA

CAAGATCAATACAGACAGATTTAATAGATTGGTTGTGATTTTAAGGACTCACATTAAAGA

AAAGGGAGCTGAGAAAAGCATACTAAGAGCTATATTGACAAATAAGTTATTTATTTGAAG

TTGTGAATTAGAAAAGAAAAGTTTAGTAAATGAAAACATTGCAATATGTTAAAACCAAGA

AAGGACATACAAAAGCAGAGAAAGAACAACACAACTGCCCTTAAATGGCCCAG

>c3114_g1_i1 len=2403 path=[35:0-2402]

AAAAAAAAAAAAAAAAAATACTAATGTATCCTATTAGATAATTCCATTTTTAATAAATCT

AATCTCTTTCGAACTTTTCTAGTTCTTGATAAAAAAAATTTTATTTTATATATATATACT

CTCCTTTTCCAATAATTTATCATGATTTCATCTTGGTTGTAACAACCTCTCTGAATAACT

TTAGATGTATTTGAACAGTTGGGGCAAAACCAAAAGTTTTTTAATTTGGTCTCTTTGAAA

ATAGAATTAGGTGAAAAACAATAAGAATGCCAACCATTCGTACAACAAGCATAATCACAC

ATAATTAATTCTCCTCCATTCATACATTTATAACATAAAACATCATGTTTATAAAAAGTT

AAGTAGTAAATGTAGTCACATAAAATCTGAATAATTAAGTTTTTATCAATCGTACTATGA

TGTAAATCATAATTTATTTGTTTCATTATGTACTGCAAAACATAACTAAGAAAAAAAAGA

AAACTTGCGGTACTTAATTTTATTTTATTATAATTATTTATATTTATAGTTTTTTCTTCT

AATATTTTTTTAGTTATAGATATATGATTCAGTACATATTTTAAATAATTTTTTAAATAA

ATTATTCTTCTACTCTGAATATATAAATATTTTTTTTTATGGTACATAATTATTTCACTA

TTTGTATTTTCTTTTCCATTTTCTATTGAATCTCTCATATTTGTTTTTCCATTAAGTAGC

TTATATTCTTCATCATTAAAATTCTCACTGTTGTTTTTACATATATTGTTTATATCATTA

TTATTTTCTTTAATATCATTGCTTTTTATATAGTTATTATCTATATCGTTACTTTTAATA

TTACAAATTGCATCTTTATTAAAGGTAAGGTTCAATTCTAAGTTTATATTATTATCTATT

TCATTAATTGCCTTATTTATGGAATTAACAAAGATGTATTTATTAAATATATCAGTATCT

TCATTATTTGCTACTGTTCCAGACAGTAAATTTTTTTTATCTTCATTAATTAATATGTCA

TTCTTATTTTCATTTAAAAAAGAGTGAAAATCTATATTTGTAGAATTAGTTAAATCATTC

AATAGAAAAATTTTAGATTTTTCAGTATTAATAAATAATTCTCTCAGCATAAAAAACAAA

AAGCGATAATACTTACTTATGATCGCTGATAAATAATTACCGAATAAAAATTTGGGTTTT

ACTAAATTAAATTTTTTTTTTATATTTACTAAATTTAATTCTATTGTATTATAATTATAA

AAAATAGCATCGTTAATTTTTATTATATATTTGTCAATATACAAATCTAAATTACTATTA

AATTTTTTAAAAAATATATTCTTCAAATTTTTCTTATTTTCATTTAAATCATCATTGTAT

GTGTCTAAGATGCAATTTCTATCATTATGAATATTATCGGCAGCTTTATAATTTTTTTTG

ATATGTTCATCGAAATATAAATCATTATTTATATGCTTTAAATTTATGCCAGCATATGTG

TTTTCGAGAGTATATAAAGGTTTGCAACTTTCATTATTTGTAATATGTTCACTACCTAAA

GAATATTCTTCTGATTGAATTAAATCGCTTTTGCAATTATTATTTCTATTATTAGGGGAT

TGATGTTCATTAGTATGAATGATTATATCTACATTTTTATTATATTCAGACTTGTTTATG

TAATTATTATTGCAGTCATCATTAGCACTATTTACTGTTTTTTCCTTATAATTATTTAAA

GGCTCATTAGAAAAATTATCATTTATATAACATATTTTATTATTTTTTTTTTTTTTCTCA

TTTTCATTTTTTTCTTGAGAATTTCTGCAAGAATTGTCTCTTTCTAAATAATCAAAAAAA

TTTTGAATTTTATTTAAAGAATGAATTAAAAATTTATATTCTTTTTTTATCACTGAAATA

GGAATGTTAGATAAGCTTTCATCATCTGCAGAACTTTCATAATCTGTTATTGCTTCATCA

CTACCATCAGTTAAGTAAAAACCAAGTAAAGAATTCTGCATTTTTGGTAAAGGTATATTA

GTAAATGGGCATGTATTATTAGGAAAAAAAAATACATCCGTATTATAATATTTTTCATCA

AAACTTTTTAATTTTAATCCAATATGTGCTATATCTTCAGCTGCTCGTATACCTGTATCA

TAAGCGCCATCTACACATTGAAAATAACTTTTTGATATATATTCTCCACAGAAAACTACT

TTTGGTTTATTAATAGGATGAGGAGATCTTAAATAGATTAAATCATTATCGTTACAATTA

ACATATGGATATGCATAAGAACCAAAAGAAAAATTATCATCTTGCCATTTTGTTACAATA

TAGTCAACTAATATAGGCAATCTTTTGCCACTTTTTTCAAACATTTTGTTTAATGTATAT

AAA

>c3114_g2_i1 len=771 path=[3159:0-770]

AATAATAATAATAATAATTTTATTAATACTGATCACAAAGAAGATGATTTAGATAACACT

AATTATTATAAAAAAAACCTTTTTAATAAAGATATTCAAAAACATAATGAATACGATAAA

AATGTTCAAAGAAAAAATATTTTTGATTCAAATATTCAAAATGAAATAAATATTGATGAA

AGTATTCAAAAAGAAAATGATTCTTATAAAAGTTGTAAAATTAACGAAAATACAAAAAAA

ATTCAAAATTATAAAAGTTGTTATAAAGCGTGTGACAATAAGGGTAATTATAATGTTTTT

GTTAGATGTAAGAGCTATGATAATGCATCAAAAAGCAGAAATAAAAGTTTTCATGGTATA

TATGATTTAAAAAATATCGATTATTTAAATATTAATATATACGCAAAATATGTTATAGTA

GCCTTACCATTAGGTTCATTAATAGATAATGATAAAAATAGCAGTAGGAAAACATGCTTA

AAATTTGATCCAGAGCTACACCCGCTAAAAATAAAAGCTCTCAATCATTATAAAATGGGT

AATCATAATAAAATTATATTGAGATTTTATCCATATAGTTTTACATGGCCATTTGATAGT

TTACAGTTAAATTGTTTAGATCAAAAATTTCAATTTCTTAATTTGCATGCATATGGAAAA

GTTGGTTGTGTTTTAGTTCATTGTTTTCCTCCATGGTCATCAACACATGGTTATATAAAA

AAGGAATATTTTATTGTAAATGAATGTTTATATACATTAAACAAAATGTTT

>c3118_g1_i1 len=272 path=[51:0-235 372:236-242 350:243-271]

AAAAAAATGCCCATACCTCTATTATGTTAGCACAGACAATAAGTTAAGTTTTCAATTATG

TCAGCTTTTGTCTTTTGCCAGTCCAGTTACGCGCTTTAGAAACATACAGTTCCAGTAATT

ATGAAATGCTGTGAAAAACAGCTTGATACTGATGTATTTACTCATTAAAATTGTGTGTAT

GTAACAATAGAGTGTTGAATGGAAGTTTGAAATTTCAAACATATGTCATTATTGGGTTAA

CAGGGAGAGCCTTTGAACATCAAAAGCAGTGT

>c3118_g1_i2 len=272 path=[51:0-235 372:236-242 293:243-271]

AAAAAAATGCCCATACCTCTATTATGTTAGCACAGACAATAAGTTAAGTTTTCAATTATG

TCAGCTTTTGTCTTTTGCCAGTCCAGTTACGCGCTTTAGAAACATACAGTTCCAGTAATT

ATGAAATGCTGTGAAAAACAGCTTGATACTGATGTATTTACTCATTAAAATTGTGTGTAT

GTAACAATAGAGTGTTGAATGGAAGTTTGAAATTTCAAACATATGTCATTATTGGGTTAA

CAGAACAACACTGCTTTTGATGTTCAAAGGCT

>c3119_g1_i1 len=206 path=[392:0-180 209:181-205]

TAAGAATTCTGCAGGAGTTACCTGTCAAAGAATTTGGTCCTGTATTAGCTAAGGATATTA

AAGGGGGGCTCATGACTTGGGCAAGTAGAATTGACCCAACATTTCCTCAGTACTAAACAT

TTAAATGTTAAAAAGAAGATGAATATCAACAGTTAATTCTCTAGAGTTTAATTTTTTTGT

GCAACGTCTATATCATGCGAATCAAA

>c3120_g1_i1 len=201 path=[1:0-16 18:17-200]

AAAAAAAAAAAGATACTCATATATATATTCATTAAAACACTTTTGAATACCCTTTTTAAA

TTACTACATGTAATACAAAAATAATTTATATGTTTAAATATGTTGATGTTTATAATTATG

TATATTTATATATTTTTAATAAGAATTAAATAGTTTCCTTTTCCACATTTGTTTTACATT

ATTCTTTTTTTTTTTTTTTTT

>c3121_g1_i1 len=315 path=[1549:0-16 1548:17-98 1647:99-170 1647:171-242 1647:243-314]

TAGGTAACTTTGTGTTTGAGGTGGAGGATATGGAGGTAGTTTTGGTAGAGGTGGATACAG

ATCTGGTGGTGGTGGAATGGGAGGTGGAAGAGGTGGATACAGAGGTGGATATAGAGGAGG

AAGAGAAGGTGGCTACAGAGGGGGAAACAGAGGAGGAGGATACAGAAGCGGCAGAGGTGG

ATATAGAGGAGGAAGAGAAGGTGGCTACAGAGGGGGAAACAGAGGAGGAGGATACAGAAG

CGGCAGAGGTGGATATAGAGGAGGAAGAGAAGGTGGCTACAGAGGGGGAAACAGAGGAGG

AGGATACAGAAGCGG

>c3121_g1_i2 len=1071 path=[775:0-772 1548:773-854 1647:855-926 1647:927-998 1647:999-1070]

AAAAAAAAAAAAAAGGAATTAAAACTCAAAAATAAATATACCTTTAGTTGTTTTAATTAA

AAAAGATATATATAAATATATATATTTATTCTGTATATTGCAATTAAAGTAAAAAATATA

ATTGTTTGACAATAACTGAGGGAAACATTTTTGTTACCGCAAAAATTAAAAAAATTTAAA

AAAAGAATAACTAATTTTTATTTTTAAATAATACTTAATAAAAATGAAGAAAGATAGAGA

ACCAATAGATGAAGATGAAATGAGAATAACATCAACTGGGAGAATGACCAACTATGTTAA

TTATGCTGCTAAACTTCTTGGGGAAGAAGAAAAAAAAAGTATAAAAATAAAAGCCACTGG

AAATGCAATTGCAAAAGCAGTAACTTTAACAGAAATCGTTAAAAGAAGATTTAAAGGATT

ACATCAAATTACAAAATGTGGAAGCACTATTATAACAGATCAGTATGTTAGTGGACAAGA

TAATAACGAACATGTTGTTCAAGAAAAGACTGTTTCTTTTATTGAAATTTTATTATCAAG

AGATCAATTAGATACTAAAGATGCAGGTTATCAACCACCCTTAGACGAGAAATATGTTAA

AGAAATGACACCTGAAGAAATTGTTAATTCTAGAGGATTTAGAGGTAGAGGATTTAGACC

AAGATATTACAGAGGCTTTAGAGGAGGAAGAGGTAGTGGATTTTTAAGAAGAGGTGGATA

TAGAGGATTTTCAGAAAGAATGTATGAAGGAAGAAATAGTTTTAGAGGTGGTAGAGGTGG

AGGATATGGAGGTAGTTTTGGTAGAGGTGGATACAGATCTGGTGGTGGTGGAATGGGAGG

TGGAAGAGGTGGATACAGAGGTGGATATAGAGGAGGAAGAGAAGGTGGCTACAGAGGGGG

AAACAGAGGAGGAGGATACAGAAGCGGCAGAGGTGGATATAGAGGAGGAAGAGAAGGTGG

CTACAGAGGGGGAAACAGAGGAGGAGGATACAGAAGCGGCAGAGGTGGATATAGAGGAGG

AAGAGAAGGTGGCTACAGAGGGGGAAACAGAGGAGGAGGATACAGAAGCGG

>c3128_g1_i1 len=212 path=[190:0-211]

GCCCCTTCTGTTATTTTGCCTTAACTTGCTGTCTGAAGACAGGAAGCTGTTTGGGCCAGA

ATTTTCTGCCTTTCTCTGTAAATCTCTCTTCTGAAAGGCTTGTAGGCTTGGGAGTAGGAG

TGCCTGCATGCTGAGGAGTTTTGAAAACCTCATCTGTGTTAGTGCTGGGTATAAGCTCAC

CAGATTAACCTCCTGGGAATAATTTTTAAGTT

>c3129_g1_i1 len=233 path=[365:0-232]

GTAGTCACAAAAGAACAGGCACAGTTTAAGTACAGGCATTATTAGAAAACGCCAAAAAGA

AATCAAACCCAACAAGACAAAATTCTGGTGCTAGCTATGCACTTATCTTTTATTATTGAT

ACTGTATGAAGAGATTCCATCTAAAGTAAAATACAATAATAGAAGACAGCTGTACATGTA

ATAGAAAAACCACAAACGTTTTGTAATTCGCATGACAAGGGAGCTGGAACTAG

>c3131_g1_i1 len=27117 path=[27127:0-21082 34:21083-27116]

TTTTTTTTTTTTTTTTTTTTTTATGTTATTAATTCTTTTATTTTAAATATTTTAAGGATT

AAAGTATAAATATATTTTAATGGAAAAACAAAAATTAGTATAGTAGAATAATTATTATTA

AATATAAATATTATTCTATAATTTATTGTTAATTTAATATATTATTAAAATAACTTTTAA

TGATGTAAACTTTATATGTTTAATTTTATAAATCTTATTAATTACCATTTTTTATACTCT

ACATATTTTTTTTTCTACTCCGTAATTTGCCAATCTATGTTTAACTACATATTAAATTTT

CTGTCTCACATAATAATAAATAAAAATGAATAATTTATATTCTCTCTATGAAAATATAAG

TGAAAATGATAAAAATTTAATTTCAGAAAAATTAAAAAATATACTAAACGATTTTCAAGA

TAAAAATAAAAATTATGTTTCTGAATTTGTTGTTGTCAGTAGAACAAGATTTTTTGAATC

TATTGACAGCGATGGAGAATTTTTACTGTTGCAATCTGCTTCTCGTATAATAAGCAGTTA

TGAAAATATTATACGTTTATTACATAAAGCAAAAGTATACTTGGAATTTGTTAAATGTTT

AAAATTGAGTAATTATGATTTAAATAATAACAAAAATTGTGAAGAAAATATTTTAAAATT

TATTAATTATTTAGAGTCAAAAAAAACGAAGTATTATTATCATATATGTGGAATGCTAAT

ACAGCTTATTATAACTTCCCTCAAAAAAAAAGATAACTCTACTGATAATAAATCAAACGA

AAATGAATATGAAAAACATAATATATTTGATAGTTACGAAAAAAATAATACTAAAAACTA

TGAAAAAATTCATGATTCGACTTTGAATGAGGCACATAAAATAAAGGAAAATATTGATAC

ATGGGAAACTAATCATAATACTGAAAAAAGTATTATTGAACCTTTTGATGAATTAGAAGA

ATATGGTGATTATCTATATGATGTTTTAAATCAAATGGACAATAAATCATGGAAAGATAA

AAAATATACAGAATATTTAAAAATGATTAGTTTAAACGATGCAATCATAAAAGATGAAAA

TAGTAAAACACAAATCTCTGAAAGTAGTAGTTTCACCAATATTTCAACCTTAACAAATAA

TATGATTTTACTACCAAAATACTTAGAAAATGTAAGAAATACCATATATAATCATCAAAA

TAAATCGATAATAGAAATGAAATCGATGATTCAACATGCAATAAATGATAATATTAAAAT

TCCATTATTTAAAACTGGTAAAATTATTAGTGAAATGCTTCCATCTCCAAATTATGAAAA

ATATTGGTCCATAACACAATATGATATTTCAAATAAGCTAGACACTTCAATGATACAAGA

TATGGTGTTCAAATTTTTTAATATAAATAAAAGTAAGACGTATGGTAGTGTCACAAATAG

TTATCATATTAATTATTATGATATTTTTAAAATTCTTTTAAAAGGAGTAAATTCAATAAA

AAATGAAAAAGAAGAAATACATATATTTCTTAAGCATTTATTGAATAAAACAAAAAAAAA

ATATTCATATACCTGCATTAAAGAATCAAGAGATATGCAATTAAGCACTGAAGGTAAATT

TGATAATTGTCTATTACCATTACATATATATAGTAATTCTCATGAAATATGTAAAGAAGT

ATTAAAATATGCTATTTGTAACAATGATTTAGTATTAACATTAAAAATAGTCGATACCTT

ATTAACAATAACAGTATATGAAAACTCTTTAAAAAATTTGATACATTTCTTAAATACAGT

AGCAGAATACATAATTATACCTTATTTAAAATACGAAATGAAAACATACCATAAAAGTGT

TAATGAAAGACCCACTTGTAATAAAATTGATACAAAAACTTATGAAACAAAATTAAAAGT

TGAAAATAATAACATTAATAACGAATATTGTAAAAACTATACTAATTTAAAGAAATTAAT

TTGTAAAATTCCATTGCATGAATATGAATCACTAAAACTTTTAGAGTTACTTTCTTTACA

ATACCCAATGTTTTTGAAAATTCATGATAAACCTCAAAGATGTTTAAAAGGAATTCCTAA

GCCATGTGAATATTATAGTAATATACTTCCTACTTATGGATCTTATATATTTCAAATTGT

TGTTTCTTTTGAAATAGATAATTCTATAAAAGTATTAGATGAACTAGGATCCAAATGCAA

AATATTGTTTCAATTAACTGAAGATGATAATAATGAAGATCAATTAAAAAATAATTCAAG

TGATAACATAAATAAATATAAGGAATATAACTTTTTAGAAATTGATTTTCAGCAAAACTA

TATGTATACTAAATCTACTAAATCAAGGAGCGCAATAAATTATATGAGAGAAAAGCAATT

TGAAAAAATAAACTATGATAAATCAAATCAAATGAAAAACATTAAGAATAATGATGAAAA

AAAATACAAAAATAATATTTTAAACAATGATTTTATAATAAATGAGAAACATAAAATAAA

AAGTAGTGAAAGGAATAAACATATAAATGTATATGATGTGACAAATATGAACACAATTGT

GTTTGATATATTGATTGATCAAGGATGCTTTATTGATGAAGGAAATATATATGACTTTCA

AAACTATCATGGAAAAATAGTAATTTATTCAAACGGAAAACATTTATTATCAACTAATAT

AAAAAAAATTGGTTCATTTGAAAACCATGTTGTATCCAATAGTAATATAAAACTAAAAGT

ATTTACTTCTCGCAGTTGCAGCTTTGAGTTTCCAAAAGAGTGCATTTGGTCTTCCGCATT

TGTTGATGATTTAGTAAATAGAAAATTAATCCCAGAAATAAACGAAAATTTTCTTCTTAA

AAATCCTATTAGTATAATAGAACTTTACAATAAGGTATCTACTTCATTAAATCGTTATAT

ATATCGCTTTTGTGATTGGAAAGATAACGATTATAAAAAAATAATTGAAAGAGAGACATC

ATATACATTGTATAATAACATAGATATTTATACATTTGAAGAACTAGTTAAATATATAGA

AAACAATAATAAAATTCTTAAAGCATTATTTAAATTAACAAAAAATTGCCTAATAGAAAA

AGATAATAATAATTTCAACAAAGATTCAAATGAATTTTATGCAGATCAATTTGATTATTA

TTTTAGAAAATACAGAGAAAATTTATATGAATGGAATCTAATATCTGATACTCAAAATTG

TGCATTAAGCGTTTTAAAATTATATTTAAAGGACAATTTAAACACTGAGAACAGAATAGA

AAATAATGAAAACTTTTATTCTTTGTCAGAAAGATTAATGAAATCCTTTTTAAAAATAAT

TAAACTAAAAAGTCCTTTCATAAATAGGGATGATTTTTATATAATTAAAAATTATGAAAT

AGAAGAATTGGATCTAAACGGATGCTTATGGTTATTAAGGAGAAATGTGTTTCAAATACT

TCTGCAAGCAGATAAAATAATTATAAGTAACATATTTATGAGAATAAGTATTGAAACCAT

TTTAAATCTACTTAAATTTTTTTCTGATAACAATAGCATGGATTCACAAATGATACTGAA

ATTATTGAAATCTCTAGAAAATAAAGGAATTTTAGAAATAATGATGAAGCGAATGCTAAG

TGCTCCATCTAAAAATTATTCGGGTATCTATAAATATGTAATCATGTATCCAAATGATAG

TTACTTAAAACCTCAAAACATGAATTCAAGTGTATTTTTTTCTTCTTCTTCTAATTCCAA

TATTTATAATACTAGTTTTGATAATTTAAACGAAGTTAATTTAAGCAGTAACAAAGATGA

AAAAAGAAAGAAAAGAAAATATTATGAAGAAAACAAACATGAAATAGAAAGATATGAAAA

AAGAAAAAAAAATATTAAATCTAGAATTATTGATGCCACCCATTTTGGTCTTTTTGTAGA

AGAGTTAATTAGTCTAATAGAGCTTCAAACATATAATGCCATTAATATACCAGTTGTACA

GAATAATGAAGAAAACGATTTTTTAATGGAAAATAAAACTTGTGATGTTATTCATAAATT

GTGTACATTTTATGAGGATGAATCAAAAAATGAAATTTATCTATATTATAATTATTTTGA

TATATATATTAAGATAATAAGTGATATGAAACAAAAAAATAATAATGTAAGTAATGCAAG

TGAGGTAGCATCTATTTTTTTAAGAGAAATTGTTTTAATGCTTACCACATATATACTAAC

TATATCAAGTGAATATGAATCTGATAAAGCTTTTTTATATCAACCTGTTATTCCGTTTTA

TAACTTGCATAATGAAATGTCAAATATAAAAATTGAGAATAAAGAAAAAAATAACTTACA

AGAAGGACATTTAAAATGTGATGAAAAACAGATGTTTGTAAAAGATTGCTTTAATAAATC

ATATAATTCAATGAATCAACTTGATAATACTTTGAAATGTGATTTTGATGATATAAATAG

TTGTAATAATGGGTATAGTAGTTATGAAAATCAAGATGATGCTCTGGAAAATGGGAATGG

ATATTCATTAAAATTTAATAATTATGATAAAAACGGAAAATCATGGATTCCGTTAATTTC

TAACCAAATACAAAGTTCAGTAGATGATTTATCATTTCACTCTGATGATTCATGCTCACA

TTTGTATAAGAATAAAAATAAATTATCATCTTGCATTCAAAATAGCTTAGAAAGCCTAAA

TCACATAGGAGAAAATACAAATAAAAAAATGAAAAATGAGTCGCATATTAGCAAAACTAG

TTCTACAGTTTTATCTGAATATAACTTTAGCTCTGATGAAACTGAAGAATTTTATTTTAG

AAAAAATTTATTAGATTTTAACGAAACAAATAGAGATTCATACAACTTTAAAAGTCAATT

AAATGAAATTATATATAGAATTTCAAAAAAGAAAAAATTTGATAAAGATAGAAAGAAACT

TAAATATCTTAAAAAAAGAATGAAAAAGTATAAAAACGATTCAAAAAAATTAAACTTTAT

TTTTTCTCTTTTAGAAGAAATATTAAAAATTATGCTAGGAGTTGGTTTACGTGTTTTGTC

TAGTATTTCATATCATTATTTATATGAAAAAAACGGTATGCAAAAATTTATATATATATT

TGGATCAAAAATAATAAGAAATGACATAAATGAAAAAAATTCATTTCTTCAATTATGTGA

AAATATGATAAAACCTAAAAGTTTAACATTATTACAAGCATGCAATTTCATCATAAGTGG

AATAATTCCCTGCTTAGGTTATGTTACAACCCTATTTGTTGATAAGAAAAGCAAAAAATA

TTCTTATATAAAGGAATTAAACAACTATTTATCTTTAAAAATGGCTTTAAATATTTGGCC

AATGTTTTTGAAAATAACTTTGCAAGGTCTGTTAACTTCAAGACATTTATCCTCTGAAAT

GTCTGTTCACAAAGATAATTTGCATCCAATAGCAGAATGCTGCATAAGTATTACTAGTTT

AGCTACGCATTTTTTAACATTCTTTACAATCATGACTGATAATTTAAGCAAATATGAAAT

ATATATTAACATGAGAAAAAATATGAAAATGTCCACTTATCATCCAATACCCATGATTTT

AATTAATTCAGCAAAATATATTCGCATTTCATCAAAAAAAAATAAATATTATGATTTTGA

AAAGGCCATAAGAACTATATTGTTACAACTTACTGATTGTTCTGTATGTGATTATGAAAA

TTATGTTTTTAATCACTACATGATTCCTCAAAGCATTTCAAATTTTTTATCAGCAACAAA

GGATACAAATGAGATAACTTTAATTAATGATCCACTATCCGATAAATTAACAACTAAATC

AACCTACGATGATAATATTATAAATAAAAATAATTCATTGGAAAATGAATTTATTAAAAC

ATATCAATCAACATATGAAGAAGAAACTACTAAAAGTACTTATAAAAAAGGAAATTTGAA

ACAAAAAATAGAAAAAAAGAATGCAACAAATGAAAAATTTAATATGTGTAATAAAAAAAG

CATTGAATTTTTAAAGGCTTTAATGAACAACGATAAAGATGCCATAAAATATTTAGGATC

ATTTTATCGTGTATTTCACAAAAATTTTGAAAGAGCTCAAGTGGCGTTTGTGGCTGTGTT

TTTGTATCTTATAGGATATAATCATAACCCAGTATGTGATATTGATGACAATTCTAAAAA

TGCTGATAACATAGAAAAAAGCGAAAATATATCAGTCAACGAAACACTGGAACATAAGAA

AATAAACTTAGCTTGCAATTTAGCTAGACAGGGAGTTGTAGATCTTATATCTAAAAGTCA

GACACAATTTCAGAAAAAAAACCTCGAATATAAGTTATCCTCTAAATCAAAAATCGATGA

GAAAAAACAAAATGAGAAAAGTTTAGAATATCTCGAATCAGATAATAAAACATTAGATGA

AATGGAATTCAAAACACATTCAAATAATGATTTGAAATATTCATTAAATACAAGGAACAA

TTGCACAAATTCTGGAAATAGTAATAATCAAATAGAATTTCTCTTAGATAAAAAAAATAA

GCAGAATAATTTTTCTTTTGAACATAAAAATGATGATTACACTTCAAGTAATTTAAGTAA

GGAGGACATAAATTATTTAAACGATGCATTACCTAATACGGAAAAGTGTAGTGATAAAAG

TAATGAATTTATAACAATGGATTTAATAAAAAAAAAAAGAGAAAAACAAATAGATAAAAT

ATTAAAAAAATGTGAATGGATAATAAATACATACCCTTTTGGTTTATGCTCTATAAATGT

AAGAACAAATTTTTCTCATGAATGGGATGAGCATTTTGTATTTATGCGTAGAAATTCCTA

TGACATACCTATTACTGTTAGAAAAATCTATTCAGATAGAAGCAATTACCATATGATTCA

AGAAGCTGCCACCATGCCAACATTCACTACATGTTTGAAAGCAAATGTTTCTTCTTTGAG

TTATTTGGATCAATTATTATGTCATCAAAATAAAATTGATGAAAAATTGGAATATATGCA

ATTTAGTACAGAAAATTTTTTTGGTAAAGCAACAACGAAAAACCTATACCTTCTAAAAAG

ATTATTAAAAACATACAAAAGAAATGTAAATATAAAAGAACCTAACAATGCAGATATTAA

ATATGATAAAAAAATTCTTGAAAATCATAATCAATTAAATGCCTATTTGTGTAATAACAA

ACATACAAACTTTTCAAATAGTTGTCCTAGTTGTGACAGCGGAAATTATTATAATGATGA

ATCAAATTATTATATAAATTATAATTTTTCTGAGAAGAACAATGGTAATTCTAATAATTC

ATTAGTGTTAAAGTGCAATAGTAGTAAAAATAACATTAAAAATAATGGAGAATTATGGTT

AAATTCACGAATGACTAAAAATTATATTTTTCAGCAAGATAATGATGCACAAATAGAAGA

ACTCAATTCAAACATTTATTATAAATCTGATTGTTTTCTTCTTCCACTTAAATCGCTTGA

TGAAAATGATTATATAATTAGGAATATGCATAATACAAAATGGGATAATAGTACAAGCCA

TATTACAAAATATACACATTCAGTATTTGATTATGATGAAAATATATCTGATCTTAATTT

ATCCGAGATTAATAACAAAGGCAATGAAATTAATAAATGTCAAAAAGAATCATCTGTGAA

TAGACAAGAATTTTTACATGAATATGAAGAAGAAATGAAAAATCTACAACCTTCAAACAA

AAAAAAAAGTTTTGCATCATTTGATTATTTCAAAAATAAATATCATCATATAAATTCAAA

GGATATAATTATAAATAATATGTTCGAACAAAGTATTGATCCTAAAGATTTATTACAATT

AAAGGAGATATTAAATTTTTTAATTAATGGACCCGATCTTGAAACAGTACTAAATATTAT

AAGAGTTGAACAGTTAACATCTTTATGTAGATATTTAATAAGTAGAAGTTTAAAATCAAT

GTTGTGTGCAGGAATTTTTGGAGGAATTAAAAATCCAATAATAAATATAAGCAATAAAAA

CTGTTTTTCCAACAAGAGGTCACAAAAAGGATATAATTCTAATTCAGATGATATCAAAAA

GAAAAAAAAAAGTGATTATAACGAAAAAAATACAAACATAAATGATCAACATAAGTTAAA

TGAAAAAGATCAAGATGAATCACATATATCAAATAATACTAAAAAAGAAAAATATTCTTC

ATTTAGGAAAAATAAGTTCCTTATTTTGTGTGAAAATGATAAAACTAATTTACTTAGAGA

TCAAAGAAAATATAAAAACCTTGATTTAAATCAAAAATATAATAAAAATGATAAAAGAAA

AAAAAGTATTTTTGAGCATTGGAGACAAAAATCTACAAAAAGTATGCAATGGCCAGTTAT

ATTAGATTTACTCGTTCCATCTTCAAGTAGGGGAACATATAATGAATGCAACAGGATAAT

TTCTAATGTCGAACCATCTTTATTTTCTGTGCAAAATTATGGATGGATGCTCTTAAAATT

ATTGCATATTATAATTATAAAATTCCGAAATATAACACCTTTGAAAAATATGATTAATGA

AATGAACAATAATAATAAAATTAGTAATTTTAACAAAGAAAATTTAAAAGATGAATTTTC

ATATTGTTACTGGTTCACTAGGCCTATGGAATATGCTCTTTCTTTTACATTAACTGATAT

TACTAAAAGTATTTGTATTTGGTATTTGAGATACAACCGCAAAATGATAGTGAATTCTTC

TAATTTTTCTCAAATTATAAAAAATTCGATGAATACACTATGGATTGAAAGTGTCCTAAG

GTTAATAGCTTTGCAGATATGTTTAAGAGTAAAACCTTTAAACGACAGCAGATATGTAGT

ACCATTCTTATATCATATGCTTACAATCTGCCAACCCTTGTTAAGAGAAGTGCATATAAG

TAGTGAATATCTGAAACGTCATTTATTCGGAGGAAATAAAAAGTTATTTTTTAATGATCA

GAATAATTTCGTTTTCAAAGTAAAATGTTCTTATTGTAACAAATCTATATCCAGACAAGC

TAAAAATAAAACACCAAAAATTTCTCATATCAGCAAAAATTTTAAACATATAAGAAAAGG

AAATTCTGTTGAAATGTTATCTACTAAGAGAACTTGTAAAAATGGAGTAGAAAATAATAT

ACTTGATGTAAAGGGGTATATACATTCAGAACACATACAGAAAAAACAAAAAAAAATAAA

GCAAAAAATTGTTAAAATGAAAAATTATAACTCATCAGAAGAACATGAAAATGAAGTTAT

ACTTGACAATGTAATAACAGGAATTGTTATATACCATGCAATTATAGAAGAAAATGCTCC

TGTAAATAAATTGAAGATTGTACAGAATCTAAAAGGTTTTCAAATAAAAAAAGAATATTT

GTATAAAAACAAATATTGGGCTCTTTGCACTCAGCAATATAAAAATTCGTCCTTATACAC

TTTACCTTTAAGAAATCTTAACAGGTCTATAAAACATACGAATAAAAATAGTTTAGTGAA

TATCAATAAATTTGATTCTTGTTACTGGTTTATCAGTGGAGATCCAGTTTATAATATTTA

TGGTAATGCTAAAACTATATCAGATGCGCAATTAGAAATATGTATTGATAATAGTTACGA

TATGAATGTAAGATCTTTTCTTAAAAAAAGCAATTATGAATTAGGGTTTTCAATTAATAC

AGAGTCAAATGACACAATGAATATTTGGATAAAAAGAAAATCAACTATTATAAATAAAGA

GTTAATAGAATATAGTGACCCTAATGCAATTAGTTCTGTAGTCAATTTACAACAAAAGGA

AAAAATAACTGAAAATGAAGAAACGCCATTAAAAAGTAAATTTAATATTTGTAGCAAAAA

AAAGGATACGTTAAATAATAATAAGAATAATAAATTCCTAAATTATGAAAATGAAGATCA

AGAATATTTATCTGAAAATTTTTGCGAATGTAAAAAACTTTCAACAGATCAAGATTTTTC

TAATGATAATAATGATAATTCTTATAAAAACACGCAATCTGAAAGTTGCAGAAATGATGC

TAGTATAAATTATGATCAGAATGATAACTTGAATTATAAAAAGCTAAAATTAATAAGTTG

GAATTTGATTAAAACAATTCTTTGGGGAATATTGGTTTCTGGAGAAAATATGGAAATTGA

TAATACAGATCAAGTAGTAACGGAAAGTACTACTAAATATATTAAAAAGAAAATATGTAA

CATTCATGATGAAATGGATGACAATTATAATTATAAGAATTATAAATCTCAATTCCATTC

GCTTCAACGAAAAAGCCTCATAGAATCCGTATTAAAAATGATTATGTCAGTTTTAATTAA

ATTATGCAATATTGTAAAAATATATAATGATTTAACAAAAAATCAAAAAGGTTCATTTTA

TATTACTATTAATAACACAAAAATAAACAAACAACAAATTCAAGATTGTGAGAAAATGAT

TACTGATTTTATGTATTCATTAATTAATTGTGTTCGAAACTCACCAACTGCTGCAGTTAC

ACTTCTTAAATGTTTCCTCTTTGGTCAAGACAATCTTACTTATCGTTTTAAAAAATTGAA

CAAACTAAACATAAATTGTGATTTATATAAGCTAATTACTGAATGCATAAAAAAGGCTCC

CAATGACTCTGATGAAATAACTGTTTTTTTTAATAGATATTTTGATAAAAATATATTAAA

TAATTATAATATTGATCAAAAATATTACACTCTAAACCAAGAAGAAAATATGAAGTGTAC

CATAGATAGTCCCTTAAAGAACAGTGTTTATAATATGAATAGCAACAATGTTAATATATC

TTCAAAAGAAAATATTAAATATAATAGTTCTATAAAAAATAAAGAAAAAGATACAAAAAT

ATCAAATTTTTTTCTTAAATATTGTGACTCTAATATAAGAAAAAATTATAACCAAAGTAA

AATATATGGCTTTTTTTTATTAAGATGCTTACCTCCTATTAAAATGTTTCCAAAACAATT

AATTGTTCAAGAAACAAAAAAATTATTTTATAGTGAAATGAACTTTATGAATGAAAACAA

TGAATCTAAGATTCATGAAAAAGGTGATAAAAAAATGTTAAACATCATTGAAATGCCTCA

AAATAATGACTTACAAATAAATTCTAGTTCCAAAGAAAATATTTCTCCGAATGATTATCA

TATGGATGGAAGTATAACTAATAATGAAGGAAATTCTCAGTGCAATAGTACAAATAATAT

ATTGGGATTAATACCAAGAATATCTTATAGAAATCATCCTTTTGATGAATTACCTCAATC

TGAATTTTTTATGTCTAGTAGACCTTCTGATGATTTCGAAGTTCATATGGAAGGAAGAGT

TTTGAGAGTTCTACGTGGTTTAAATTTAGGAGGAGTCGTTTTATGCTTAGCACCTCTGAC

ATTTCCAGCTTTAAATATTGGACCAAATGAAGTAAATGACATAATTAATTTTCGTATCCA

AACAAGAGGACGTTTTGGTGTAACTGTTGCACCAGCTGAATGTGTTTTATCAAATGTTTC

TGAAGTATTCGACAGAAATGATGTTGTCGGATTCCGTACTAATATGTGTCCTCCATTTAG

ACATGATGTATTTGCAGCAAATGTACAATATGAACTAGGAGACTTATTAAGTGTAAAATT

TAATGTAGAAGATCAACAAAATCAAAAATGCTTGAGAACAGAACTTATGGTTTCAGGCGT

TAGTATTGGATCAGTATTAGAAGTTCTTTTTGAAGCAGACTCTCAAATTGAATTTGAAAG

AAGAATGAATTTGATATTTATTTTTCAAGATCCATTGACTATTGTATATGAAGGGCCAAC

ATTTACAATGGAGTATGTAGACCCAAGCAAAAGTAATTCAAATAATTCAAGAAATGAAGA

AAAAAATGTAGATGAGGAATATTTAAATGAATTTTATCCATTTTCTGGATATGATAAAAA

AAGTATAAGGAAACAATTAAAAAAAATGAAGTTAAAAAAAAAAGAATTCTATGAATCAGT

AAAACTCTATCAATCAATTCTTGAATCAGATAATGAAATATTAAAAGATCTGAAAAGTGA

AGTTTTAATAAGGATGAACTCCACATTAACTAGCATAGCTCCATATTTAAGATATAGTTC

ATCACACTATATATTTTCCGAAAATGAGTATCCATGTATTATTGATTTTATACAATCTAA

AACGAAGGAATTTGCACCTCTAAATAATATTTCACTTGAAGAAAAAAGGAAAAGTTTTGT

AAATATGTACTTCAAGAAGATCCTATCTACAAAGGACAAAAATGAATTCATAAAAGAAAA

AATAGATAATTCATCTCTTAATGATAAAAATAAAAATAATAAAACTGATGATCCCATAAA

AAAAAAATATAATGAAAATCAGGATTTATCTTATGGAGAACAGCAAAATTATAATATCAA

TTATTTTAAAAATATGAATAGCTTAGATAAAAACAATAATTATGATAATTCAAATGAATC

TCATGAATATATTATAGAAAAAAATAAAATAACTCAAGATAAAAGTGATCATATTTCATA

TGTACATCCACACTCTTCGTTAAATATGAATATATACAACAAATACAAAGATAATATAAA

TATAAATAAATCAATAAGAGAGGTTTTATCTATAATATGCATTTTGGGGTTAAACATAAA

TGGAAAACCTAAAGAAAAAAATAGAGACCATCGTTTTCATGGTAAAAAATCTTTTCTTGA

TGAACTTCCTTGGTTATCAAGCTTATCAGATCTGAAAAATTCAGTAACATATAGTTTAGA

TAGAAAATGCATTGAACTATTCTTTTGCAATTTGGTTGAAATTATATGTAGTAAGGTAGT

TTTAAGTGCAATCTTAAATTTTATTACTCACAAGATGAATAAAAACGATTGTGATGATGA

TAATAGCAAAGATAAAAATAGCAGTAATAGTTTAAAAGCTAATAGAAAAGATAAAAAAAA

ATTCAAAAATTTAAATACATTAGAATCAAAAGATATTTCAGATACAGAAAACAATATTTA

TAATCAAAATAATATATATAAGCAAAATATTAGTAATAGCACAAATACCTTAAAAACAAA

TAGCGGAAACGATAATGGATGCCAGAATAATAGTGCTACATCAGCTAATGCAAACAGTAA

TAACTGTAATTCATTAATGAATTCTATCTTTATAGAAGAAAATGGGTCATTAGAAGAAGA

TGAAACAAATGAAGCTTATAAAAATAATATGGAAAATTCATACAAAGAAAAGTCAATAGA

GAGAAATTTAAAAGATAAAGGTTTAAATTTAAAAAATAGTTCATTTGGAGATGGGATGTA

TAAATATTCCTTTTTTGAATATAGTTTGTGTCTAATAGGAACAAAAGCAATTATGTGTTG

CTGTAATTTATTGAGAACACTACTAGAAATGAATTGTGATGGATCATACGAACCAAATGA

AACAGTTTATAGATCATTTAAAAAACTGCTAAATTTTATTAAAATTTTCCTTTTTGTTGA

TATAGGAATTAGTGATGTTTTAATTAGATACTTTGCTGAAAAAAATTTAATAGACGTTGA

TTTTTTATTGAGTAAGCACAAAGGGAAAAATTTTCAAAATTATAATTCATCTTCATTTGT

AAATAGTTTTGGGAATAAAACTGCACAAAATACAAAACTTTTTTCTAATAATTCGAAGGA

TGATTCTATTTATAGCAAAGATACTAACTTTTCACCTTATTATTCACCAAAATGTAATAA

CTTTACTAAGGAAAATACAAAAAATGAATACATAAACATAGTAGAAATAGCAAATATTTG

TGGTATTCCATCTGATTTTCTACAATATTTTAATATATCTATTCCAAAAATTAAAAATCA

ACAGGTAATAACACAAAATGATACTTTTGACTTATGGATTTATATCATGAATCTTTTAAT

TTGCTTCAATAGCCAGCACAGATATTCATTATGCTTCCCTTACACTTCCAATAATTTTAA

TCCAAACTCCATTAAAAATACACTATTCGATTTTGAATATACTAATTCATACTTTTCCAA

TAGGATTGTACGTAAAACAGTTAAATTTTATGCATCTTGGTTGTTAAAAAAGTATTTAAT

AGATATAAGTTGTGTTATGGCATTGTTATTACCAAAACTTTTCATTGATGAATTAAAAAC

AAGTTTAACAAAAATAAATGAAAAAAAATCAAAAGAAGAAATAAATAAAATATATATCGA

TAGTAATGATAATAAAAATGATAATTTTAATCCAAGTAATACTATGAAGGCAAACATAAA

TAACATGGAGAAAAATGAATGTTTTTCAAAACAAGCAAATAAAAATTTTGAAACACTAAA

TAATACAAATGATAGAATATATAAATCCATTAATGAAAAAAATGATAATGAATACGAATT

TGTTAATTGCAATTATCGTGATATTAAAGAAAACAAATCATTGAAAGAAGAAAATAGAGA

AGAAAATAAGCATAAAGTTTTATGGAGCAATGATGACTGCACACAAAATAAAGGAGAAAG

TTTAAATATTAAAATTATACATTGCAAAAAAATTAAAAGAAATAAAAAATTACACAACCT

AGGTCTATTATTTAATTCTGAATTAAGGAATAACGAAGAAATGTTAAATATATTTAGCAT

AATGTATCTGTTTCGCGTTTGTATTTATTATCTTAGTAGAAGCAAAGATTTCAGAAAAGA

AGATTATTGGCAAGGATTTCAAGGACAATTAAGAAATGCAAAAATTAAAGAAAATGTTTA

TAAATTACTTAATGAAAAAAGTTCCACTTCTTCTTTGTTGTACGGATGGCAAAGAATATT

TTATCAATGCGTAGTTACATGGGAAGATAAAAACTTAGTAGAACAATTGTTACTAATAAT

TCAGTCTGAATTATTATATCATTTAATGGGATCAATAGCTCAAACTATGTCACCGAAAAA

AGTTAAAGTTGAACAATATTATGCTATTTCACCTTCTGTTCATGTTGCTCATTGGATGCT

AGATACTTTTGTTAGACACCCTTTGTGTCCATCCAAAATTAGATCATGTTGTATTACACC

AATAACAATTACCTCTTTATTTGGATTAATTATGAATGGAAATAGACTACCAATATTTTT

AGGTATAGACGTATGTAGATTAGTTTACTGGTCCTCTATAGTTTCTATATGGCAAAATAG

CAAGATTCATAGAAAATATAGGAGATTAAGCAACATAGATATGTTAACTCAATTTAATGT

TTGCACCGAATTTAACACTGATAACGATGATATTCAAGATACATCAAATAGAAATTATAT

TAAAAAGAAGAAAGAAAAAATTAAATATACAGAAGAAAAACTTAAAAGAATAAAATGCAA

TTATTACTCTTTTTTAAGTTACATATTTCAGTGTGTTAATACTTGTGGGTCATCAATTTT

TAACCAAATTATGAATATAAATAATGCAAATAATTATAAAAATAATACTAGCAAAAATAA

AGTCAATGACAATAATAATAACAATATTAGAAATAATAATGATCATGTTAATAACATAAT

AGATAATTTAAACAATAATATTAATGATATCCTTAACTGTATACAGAATTCAACTAATAA

TAGGGTGTTAAACAATAATCTTAATGTAGTTAGTAGTATAAGTAATGAAACCATTTATAA

TTTAAATATGGCAAATAGAAGCAGCCCTAATATTTCTTCAAACAATATTAACAATACTTT

ACAAGAAAATGTAGAAGCAGTTAATACATGTAATGGCAATAATTCAACTACTTCTAATAG

TTTATACATTAATCCAACTTGTGTAAATGGAAAAAATGAAAAGAAAACTTCATTTAACTA

TTCTAATTTTGATATATCTGAAAAAAAAGAACAAGGAAAATATAACTCTATAAAAATTAA

CGCGGCTTTATTAATTCATTATTTTGCGTGTGCCGCATATTATATTAAAAAAGTTGATAT

ATATAATGAGATGCATTACTATAACGAAAATATGCATATGGATTTTAAACAGTATTGCTT

ATACTTGTATGTTGTTGATCATATATCAAAGATATATTCTAAATATACAAAAAATAGTAA

TATTAAAAGTTGTGTAAAAGATGATCGAACAAACAAAGAATGCAGTTTCTCTAACGTAAT

TAAAGAGGACCAAAATTGCAAGTCATCATCTATTTATGATTATGTGAATAAGGAAAAAAC

AAAAAGTTATTCATTAAACAATGAAAATTTTAGAAGTGATTCTTCTAATATATGTAATAA

AAATGATTTTCTTTTAAATTATGAAAATATAGAAAAAAAAAGTAGCAAAAATATTTTAGA

TGATGATTACGAAGGGCATTACCCGGAAATATTTGTTGATTATGTACTTCAATTACCAAA

AAAAGTTTCTGCAAATGTTATAATTGATATTAATGAAAAAATACATTACATATCTTCTTC

TAATTCAGACATCATTAAATTAAAAATAAAAGATATATTTCCTTTACCAAAAAAAAAAGA

GGAATCTTTTATTGAAAAATCAAATTATATGATAGACAGTCATAATGAAGGCGATAATAG

AGATGTAATAGAATATAATAATGTTTCTCTTAATGATTATAACAGCATTGAAAAAGCAAA

AAAATTAAATAAAACTGAAAAGGAGATAATTAAAAAGATATGCAGAAAAAAATTTAGACA

TCCAAATATAATTTTTAGTGCCGACATCGATTTCAATTGTATTTTAGATGATGTTAAACT

TAATAGTCTTCAAGAATTTCTATACCTTGAAGTACAATCTTTAGATACGGATTTAGATTT

TGACAAGACAGGAAAAATTAATTTAGGTTCTTACATTGAATTAACTGATATATATAATTT

ATCATATCCATTTTTTTCATTGGGTAGTTTGCCATTATATTCAGGATCTTACACTTCTTC

ATTTACTATATGTCTAGCATTAACTGATTATAACATAGATAAATTACAAGGAAATTTGAA

TATTATTCCTGATATAGAAATTGGAGTATTGAATGTAAAAAGGCATTTTGAAGTAAATGA

TGATATATTATTAAATGACTATGATAAAAATGACTATAATGTAGTATGTGGGTATAAGAA

GGAAAATATACCAGTAAATAAACAACATTACGAAAGGAATGTTTCTTATAAAGATAACCA

TAGAGAAAATGTTAATATAATTTACAACAATAAAACTTATTCAAAAGTTGAGAGAACAAT

GGATAAAACATTAAAATATTCTTCTTGTTTATCAAAAAATACGGAAAAAAAAGATTTTAT

CAATAGAACAAAAAGAAACGAATATAATTATGACTATTCTTTGAAGAAGCGAGCTTACAA

TCTTCGTAATATAAATAAATATAATAAGAAGTCTATAGAAAATTGTTTAAGTAATAAAAA

AATTGATAAGAACAAAAAATCCATAAAAAATTATGAGACGGAAGATGATAATAAAAATAA

AAGTATTAAAAATGGTTTATATAATAATATATATTCTGAAACATTATCTGATATTGCCCC

TATTAATGGAAATAATTATTTTGCTGAAAGCAACAAAGTAAGTAAAAAAATAAACAATAT

GATCACTCTAAATGTTCTAAATGGTACAGTTAACACAACGGGATTATTTTCTGATAATTC

TATTGAGAAAGTAAATCCAATAATTTTGAATAAAATATCAGATTACATAAATATTACAGT

GAAGATAATTGGTAAACAAGTAATATGGTTTGCAAATAATATATTAATTTTTCGTAAAGA

ATTTGGTGCAGAAATGAGCGAATTTGTGCCTTTAATAAAGATCTTTCCTAAAAAATCGCC

ATCTATAGATAAATTTAACAATTTTAACAACGTATCTTTGCACATTAAGTTTAATGAAAT

GACACAAAAAACTTTCATTACTCCTTTATCAAAATTTTACTTTAAATTTGAAAATATTCA

TAAAATGAAATATAAAAATGATTGTACAAATGTACATGAATATTGGAAAAAAAACTTATG

GAAACCTAAATGTTTTATTTTACCTGATAATTCAACAACAATAACAACAATAAGGAAATT

AATGCCTTTAATAATACCACAATCATCTAATAAGATATTAATAAAATATAATAACCATAA

GCAAGAACCAGATGATTGTACGAATAATTTTTCTAACACTATGTTTTTTACACCCAATGT

TCAAATTAAAAGTGCACCAATAAATTTTACATATTATATATATTGCAAGCATGTACAAGG

AGAAAAATGGAAATTTTTATCATTAGAAAGTATTAATAATATAAAAAATATATCAATTAA

AAGTTATTATGGTTATATAAATTTATATAAACAATCGAACATAAATAAGATAGATTTAAA

TGGACTAAACAACTGCGAAAAGAAATTTGCAAAAACGAATTTGAACATTCATAATCTATT

AGATACACAAAAGCCTTATACTAAAATTTTAGAAACAAATTCAAAGAAAGATTTACTAAA

AGAAAGTTCAAATAAAATAATTAAGGGTGATTTAAATTTTCAGATTCATTTAAATAAAAT

AAATAAAAATCAAGACGTGGACATAAACACTAAAAGTAATTTAGACATTGCAAATAATTT

ATTTTTAAAAAATGAAAACAAATGTAATGAAAAAAATAAGAAAAATAGAACTAGTAATAT

AAGCTCAAAAGTAAATTATTATGAATTAGATAATAAAAATAGAACACATTATACCTCTTA

TTATCAAGAATTTATTCCAGAAGGCAATACAGAAAATTATATTCATAGTAACTATATTAA

TGATTACATTTCGACAAATAATGAAAATAATTTGCATGTAAAATATAAATTTAGTAATTT

TCCTAAAATTAAAAAGGATCTAGTTCACAACATAAAATATAGCTCAATTCAAGAAATTAA

TGATTCCTCACTTTACAATGTAGAAGCTATATATGATATGAAAAAACAAAAGCAAATTTT

TATTGATTCAAATAATAAAAAGAAATATATATACAATAAAGATAATCCTAAAAAAAGTCA

AATAAATGATAATAATTGTGAATTTATAAGTAAAATTTATAATACACATTATAAGAATGA

TAGGTGCATGAACAGAGATTTGTCAAAAAAAGACGAATATAATGAAGTTGATAAAATTAC

AGATAATTATAATTTTAACGAATTAAATGACTATTCCAGTAATTGTTGTATGTATTATAT

AGGTTGCAAAGAATTTAAGTTGAGTATAGATTCATATGATTCTATCTTTCCTGCTGAACC

TTTTATTTTTAATTGCACTGTTAATTTTAATGTATATGATAGTCATAAAAAGGGAAATAT

ATATGAAAGTTCTATTGGTATTATATGGGGTATTTATCGTTGGCTATGGACAAGTAATGG

AAATTTTATTTGTCCCTTGAAACTTCCATTATGTACATCAGAACTTCCATCAGATAAAAT

ACAAGAATGCGAAATAAATGTTGTTGGATTTAAATCTAATGATAATTTATGCATAGAATT

TCAACCTTTATATCAAAGTTTATTTTTTTATAAGAATGGAAATTATGAATTTGGATTTAA

ATTTGATACTTTTTATAATTATAGAAATAAGGAAAATATATCAATGAATGAAAAAAGTAA

AGATATAAAAATATCAGAAGTAAGGAATGTATCAAAAATATCATGTTCTAGTGGCATGAG

CAATATAAATTTCAATGATTTGAGTATTAAGACAAATATAGATAATAGATATAGAAGTAC

TATAAATGATAAGAATGTTATACAGAAGGATGTATTAAAAATTCACACTGAAACTGATAA

AAATGATAATAATAATAATATCTATAATTCACATATGGATTTAAAAAGCTCTTTCAATTC

ATTATATAGTTCCAAAGTAAATAATTCAAATACATGTTCTATTTCTAATGAACAAAAATG

TAATAAGCGAATAAATGAAAAAAATAATGTAAAAACAAAAGAAAAAAAATTAAATATGTA

TTTAAGTAATTCTGATAATATAATGGAGGAAAACCAGAATGAAACTGTAATAAAAATTAA

AAATCATGTAAATATGATTAAATTATATAACTCCTTATTAATAAATGATTGCACAAGTGT

TTCTTCTATATTAACCAATAATCCAAATTTATTGTTAAAATCAAGTGAAGGTCAACCTGA

AATATCATTTGAACATTATGAAATAAACAAGGAAGAATTTCCGTATGAACTTATTACATC

CATGAAAAATGTTAAATATGATAAACATTTAACACCCTTAATGCTAGCATGTAAAATAGG

GTGTGAAGAGTGTGTACAAAATATTATAGAGATAGGTAAAGTAAACCCTAATAAATATTG

CTATAAAATGGAAAAAGAAACTCCTCTAATGGTTGCAGCACAATACGGATATAGTAGAAT

TATATGCATGCTAGTTTGTATGTATGGTGTACAAATAAATAAAAAAGATTCAAAAGGAAA

TACTGCTTTGCATAAAGTTTTATTGAATTCTTGCAATCATGGGAAAAAGAAAAATTCTAC

TTTTAAAATTGTTCAACTACTGTTAAAATTAGGTGCTGATATAACTATTAAAAACAAAAA

AGGGATTTCAGTGGAAGATTTATCGCAAACAAATCTTATAAAAGATAAAAAAGTTGAAAA

TCTTCTAAAAATGTGGACGAGATTATCCTTAAAAAGTAAAAAAGAAGATAAAGCATGCTA

TATCAATCGGGAAATTATACCTGGCCTACTATCTAATTGTTCTATAGTTGTGGGATTTCC

AGTATATTCAACTAATAATATAAATAGTGTTTCATCGGTTAAAATTAACTCATTTGTTGT

AGGAGGTGAGGAATTAGTTAATATAAAGGAATGTAAACAAATAAAAAGTTATAATAATTG

GGATATAAGAAAAAAAGAAGTAGAGTATTATAGTAGTGATATTTCGTCCAGAAGCTCTTC

AATCTCAAGGTTCTACAACTTAGAAAAAAAAATAAAGCAAGACAATGAAAAATATTATGC

TTCAAAATTTAATAAATTTCAAGGGGAGTTTTCTTATTTACAAGAAGAAAATGAAAAAAG

TAAAAATTGTATTCAAAAAAAAAACTCCTTACAGTTAAATACATTTAATGAAGATAAAAA

AAAATATGAAATAAACAATGAAGACAATACAGAATTTATAGGCAAGATAAATCAAGAAAA

TTATGTTAAAAAAAGGAATAATACGTTGTATGATTCAAAAGATTATATGAATTCATTATT

TGAAAAAAATAATAATTCTTTATATTTAAGGAAACATAGCAAAACAGAAGATGGTAATAA

TATAGACAAATCAAAATTTTCAAATAACCTTTTATTTATGCAAAAAGAATACTCCAGTGA

CACAAATATTTATATGAATGATAATTGTGTATATAAATGGAAGAATAAATATAAAATGAA

TCATAATTTACATAGTGATGAAGGAAATAAGTATCATAATAATTTGGATATAGAAAAAAA

CGAGGATACAGATAATTTAAAAAACAGAATAAATTATGATAGTATTAAATGTAATTCATC

AAAGCAGAAAAAAAAAAAATTTACAAATGACATATTAAATTATATAGTAGTTCATAAATA

TTGTAATAAAAGGTGTGTATACTCTCATAAAAATTTTATAGATGAAAAAAATTACAAAAA

TATTTTTTTAGAAAAGGAAATTAAGAATAAAGAAAATAACAAAAATAAAAGTTATAATTA

TATGAAAAAAGAAATTAAAAAAAGCTTTATATATTCTAAATTAAAAAACAATAGATTCAA

TTACCAAGATTTAAAATTCTCTCTCATAGAATTTATTAATAAATCAGGAATATTTTCTGA

TGTTTTAAAAAATGCCAATTATTTAGAATCAAAAGATGTTCCTAAATTTGATGAACTAAA

AAATACACTACTAAAAGTTAATAATGAATTAGAAATAATTAAAGGATTCAAAGTATTTTT

GGAATTATTTAATTTTATATATATGGATGAAAGATCAAAATATTACTTAAATGTTGACTT

AAAAAAAAATTTAGAAAATTCTTACAGTATAGTTGATCTAGGTTATATCCTTTTAAAAAT

AGATAGTTACTGCAATATTTTTAACAATTTTAAATTAAAATGGAAAGATAATGCACATGA

AGAATGGAAAAACAAACTTTTAAAAGGAATTGGAATATATAACGAGTTTTATAATGTTAA

ATGGATAACAGATTCTTATACTTTTTTTAAGTATGAAGAAGAAATCTTAGAATCTGCTTC

ATTTATAGACAAAATAATACAAAATGGTAACTATATGATATTTTATAATAGCATAATGAA

TATTATAGATGAAAAAAACGAATTCTTAAAAAACGGCTTTTATGATCCACAAATATTTAA

TGATGTACTTAAAAATATAGACTTTGAAAATTTAAATAGAGAAACTATTTTATATGATTT

TTATTCTAATAAAAACATTATAAAAAATATTTTTCCAGAAAATAATAAACAGGAATTTAA

TGATAACGATACAAATGAAAATAATGAGAAAAATAAAAATTACCTAAATGAGAGTTTTAT

AAATAAAAAAGTAAAAGAAAATAAAGAGTTATATCTCCCACATCATGTTGATGAAACAAA

TATTAGCATAGAAAAAAAACCTAGAAAAGACAATTCATTTGATAGATCCATGTATAAGAA

TGATTCACAGCAATATATTCCATTGTTTAAAAATTATGCTGAATTTTATAGTGATATAAA

TGATGATAATAATATAAATAATACTAATGATATTATTGAAGGTAAAGAAAGTAAATATAA

TAGTATGAATAATACTAATAAAAATATGCATAAAACACCCAATAATAATAAAAAAAGAAA

TGAAAATGATGATACATTATACAATAAAATATCAAATTGTTATGCAAGTAATAATAAAAA

AGAATTCCTAAATAATAATGAAAATGATGACATAGAAAATAAAAAAATAAAAGCAAGCTC

TACACTATCTATAGAGTTAGATGGTAACATATATAACTCGAGAGTTAATGAAGAGAAAAA

TCAAATTTATAATTGTAAAAAATTAAGGAATATTCATAGAATGTTCCCGACAAGGTATGA

GCAAGATGAAGTTACAAATAATGATTCTTCATACGAATTTTCGAGTATTTTATTTAGTAA

CAAGAACGAAAAAAATATAAATAAGTTTACAAGAGAAGAAAATAATAATTTAATTAATTA

TAATAATAAAAATTATGATATTAATACTTATAAAAATGAAAATATCTATTGCAATAACAA

AAATAGGTATATAAATGGTAAAGAACAAAATAACTTAAATAATGTTCCTAATAAATTAAA

TTTATATCCTTTTAATTACTCCAATAATCTTACTTCAAAAAATTCCTTAGATGAAAGCTT

CTATACTGAAAGCTCATACGATGAATATGAAACTGAAGAAAGTGATTTTGATTTAATGTG

TGAAATGGATTTTATATCAAAACCATGTGAAAACGAGATAATAAGAGAACAATGTAATAA

AATATGGCCACATACGATAGATACATATATACTTCATATATATGATTTAATAAGTAGTTA

TATGGATTCTCCTGAAAAAATAAGTTTATTTATTTATCATGCATTTGATATAGAAAAAAT

AAAAAAAAAGTTACCTAGTAAAACATCACAATTATGGAAAAGAATAAATATGTCTTTAGA

TGAATTTATTGAAGAAATAAAAAAAGTTCATAATTTTACAATGAGGATTATATATCAAAG

TAATTATAAGAGATATCGTATGTTACCACCAAATTTTAAAAAATATTTTGTTTTTTCTGA

TGAATGCTTAAAGAATATTACTAAAAATGAGGTAGTTATGACCATAGAATATTATAAAAA

TCATATACATGAACAAAATAAAAATTTGTTTTTAAGAATTATACAAGATCGTATAATATT

ATTGAATAAATTTAATTTTTACTTTGAAAGTTGTCTACCTATAACAGGAATATTATTCAA

TAAACCTCTAATTGAATTGGAAGAGCAAGGATTAAGGACTCTAGAATGTCCTCTTTGCTT

TTTTATGAGTAATGATAAATACTTTCCATGGTATATAAACAAAAATAAAAATGATTTCCA

TTATTGTAATAGCAATTTAATTCAAGAAAAAGACGAATTAGATAGTAATACCACTAAAAA

TACTTCTATATTAAGTATGAACAATAGTTATGTGAACGTATATATAAATGAAGGTTATAA

TTATTCTACAGAATTTAATAATTTAAAAGAAAATAAGAATAATATAACCTCAGAAAGTTC

TCAAAATCAAAAAAATAAATGCGATAATTTAGAAGACATAGAGTATGAAAAAAATAAAAA

CGGTAATTTTTCAAAAAAAAAAAGCAGAAATAATCTTCTTATAGATAAAGCAGAATCATT

ATCTAAAAACAATATACCATATAAAAATGATTGCAGTAGCTATATTAATTTTTCGAATAA

AAACAATTCAAATTCTAATAATATAAAGAAAGAATTGTTTTTCGATAATTCAGAAAATTT

ACGGAAATTAAATGAAATAATTCATTTATATAGCGATGAAAGTAAAAAAGAAAACTTAAA

TGAGATATCAACTGGTTGTGTTAAATATTGTAAAAATATATATTCTTCAAATAAAATGGT

TTTAGCAATGGGCTCATCTGATTTACCTATACAATTATCCTCATACTTATTTATTAAAAA

GCTACTTAAGCATGATACTGTACTAAAATTATGGAAAGAATGTATGAAATATATTACTAT

ATCAAGTTCAGAAAAACCAAATTTAACATTAAGAATAGATAGAGGTATGGCTGCCACAGC

AAAAAATATTTCTCACACTATGTGGTATCAAAGTACATTTTCATTATTAAATGTAGATAC

TTATAAACTTAGAGGAAAACCGAGAGATAGACCATTTATGGTTGTTTTCATAGGAGAAGG

AGCAACTGATTTTGGTGGTCCGTTTCATGAATATCTTTCATCAATTTCAAGAGAAGTTAT

GGGAAAGTTATCGGATTCACCTGATAAGAGTTTGCCAGTTTTTCCTTTGTGTATACCTTC

TCCAAATTACACACAGGCTATAGGAGTGCATCAAGACACCGTAATTATAAACCCCAAAAG

TACTCCATACATAATAAAAGAATCAGATTATGTTGGTGAAATAGATCTCATTGATAATTT

GGAGAATTACAGATGGCAAATATTACACATATATAATTCTATTAAAGATATAAAAAAAAA

ATTACAGAGATCTCATAAAAACTTATATTCAATGTATAAAAATAGTCAAGAAAGTAACTT

TTCTGAGGAAGATATAAAAAAAAACAGGGATGAAATTTATGGACAAAATTGTGTAGATAA

AATTCAAACAGAAAACTTCTCTAATTACAATAAAAGTACAAATATAAATGAAGGTGATAT

TGAAAATAATAATTTACACAATGATGAAGCCAGTAATAACAAACATATGTGCGATAACAA

TTTAAGAAAATATTATAATAATAGTAATATAAATAATGATAACAATGATGTATTATTAAA

TGAAAAATTTAATTCAAAAAAAGATAATAAAAATATAATTAGCAATGCTATGGATAAGGA

AAAGACATATCTTTTTGGTAGTAAAAAGTCAGTTACCTTTGAAAAGCTAGACGAAAACAA

TAAAATACCTTTCCACATATATAACAAATATAATTGCTTCTATGATAATTCTATATTCGA

TGATGATCTACTTGAAATTATAAATAATTCTTCCTTAAAAGCTAGAAAGGCTTTTCTAGA

TAAAATATTGAAAAAAAAAAAAAATGAAGATAATGATTCATTGCAAAAAGAAAATGAAAG

GAAAAATAAAAATATGGGAGATAATTTAATGGGTAATAATAAAAAATCGAAAGATTCAAA

TAAAAATAGCTTACATAATGTAGAATATGATGAAAAAACAATTAAAGCAATGGAATTAGC

TATGTATGAATCCTTAGGTAGAGTAATGGGAATGTGTGTTTGTATAGCATCAACATTAAA

TATATGCTTTAACCCTATAATTTGGAAGAAAATTTGTGGAATGCCTTTAGAATTACAAGA

TTTATGTGATTATGATTTTGTTGCTGTAGAAATGCTGAAAACTTTAAAAATGCTAAACTC

TGAAAAGACAAATGGGTGGAACATAGAATTAAAACAATCTTTAGGAGATATGACTTTTTT

AACAGAAGATTCTGGGGGTAATTCTATTGAGCTTATAAGAAATGGAGTTAATATACCAAT

TAATTTTGATAATCTAGAACTTTTTATTAGATTAATGACAAGATGCAAAATGAATGAATC

ATCTAAAGGAATAAAACATTTACTAAAAGGTTTTAGTTCTGTTATACCATTAGGTAGAAT

AAGATTATTATATGAGTTCAAGGAAGTTGAACATATGGTTTGTGGTGAAAGAGAAATTAA

CTTAGAAGTATTAAAGGCACACACATGGTCAAATGATTTAAGAATAAAAGATAAACTTTT

TGCAGTACTTGAAGAGTTTACCAATGAACAATTACAATCATTTCTTAGATTTGTTTCCGG

TCGCTCAAGATTACCTACAACAAAAAACGATTGGTATATGATTATTGACGTGGAAAATGT

TAATCAAAATATTAATCAAGTAGATCAAAGATTACCTACAGCTGTTACATGCGGTTTTCG

TTTATTATTACCTCAATATTCTTCTTTAGGAATTTTAAAAGAAAGATTATTGTATGCAAT

TAAAAATTGTACTGCTATTGATTTAGATGCATATGTAGTCCATGATCAAATGCAATTAAT

GTATGGAGAATAAAATTATATTGATATCTGTAACATTTTAAGAAAAAATAAAATTAAAAA

AAGTAGAGGTTTTAAATATAGATTTTTTCTTCAATTATTAAGGAATATGTATTTCTATAT

ATATTGATAAATAAAAATAATTTCTTGTTTACTGCAATAACGTATTCAAACACAAAG

>c3132_g1_i1 len=355 path=[31:0-284 454:285-354]

GCACGCCGCCACGGTTTGAATGCCATTAATGAGACTTTCCCAGCTTTCTCTTCAACAAAG

GTTTATTCCCATCTGTGAGCGGCCGTTGCGAGGTCCTGCCCGCCCCTCAGGTGCCTGTGG

GGAGAAGCGGCCGTTCGAGCGGTGAGGTGAGCGGCGCCCCCTCAGAGCACCGCGGCGGCC

TCACCTCCTCCTCCCGGCCCGGGCCCGGCGTCCGCCTTGTCGCAGGAATGGGCTCGCTGC

CTCCGCTGGGGCTTCGGGGGGGGCAGCTGGCGGCTCCTGTCCGAGGTCCCTGCGAGAGAG

AGATCCGGCATCCCACAGTGCCCCGGGAACATTGCCTGAACCCCTCCGGCATGCC

>c3132_g1_i2 len=355 path=[31:0-284 316:285-315 540:316-354]

GCACGCCGCCACGGTTTGAATGCCATTAATGAGACTTTCCCAGCTTTCTCTTCAACAAAG

GTTTATTCCCATCTGTGAGCGGCCGTTGCGAGGTCCTGCCCGCCCCTCAGGTGCCTGTGG

GGAGAAGCGGCCGTTCGAGCGGTGAGGTGAGCGGCGCCCCCTCAGAGCACCGCGGCGGCC

TCACCTCCTCCTCCCGGCCCGGGCCCGGCGTCCGCCTTGTCGCAGGAATGGGCTCGCTGC

CTCCGCTGGGGCTTCGGGGGGGGCAGCTGGCGGCTCCTGTCCGAGACGCCGCGATAGCGG

GAGGGGGCGGGGGGGCTCCAGAGGTGCGCTGGCATGCCGGAGGGGTTCAGGCAAT

>c3140_g1_i1 len=1386 path=[53:0-1385]

TTGGGCGCCATGCCTGGGTACAGCCAGCACCGCACCGCACCGCCCCGCCGGAGCTGCCCG

GCCCCACACGCGGGTCCCGTACTCACTTGCAGCCGGGCCCGGGGGCCTGAGCAGCGCCGA

CCTCCGCCGCATCCCGCGGGGGCGGCTCCAGGCCCGGCTGCGGGCTCCGTGGCGGCGCTC

GGCGGCGGCTGAGGGACGAGCGGACATCATCACCTCAGCGGGAACGGCGGGAACGGCCGC

CGCCCCCCACTACCGGAAGTGGCGCCGCTCTCGCCGGAAGTAGCGTCCAGCGCACCGGAA

GTGGCTCCCCGTCCTCGGCAGCGCTGACTGTACCCAGGCATGGCGCCCAAAGGTGGTCCC

GGCGGGCGGCAGCAGTCGGAGGAGGATCTGCTGCTGCAGGACTTCAGCCGCAACCTCTCG

GCCAAGTCCTCCGCGCTCTTCTTCGGCAACGCCTTCATCGTCTCTGCCATCCCCATCTGG

CTCTACTGGAGGATATGGCACATGGATCTGGTTCAGTCTGCCGTGCTGTACAGCGTGATG

ACCCTCATCAGTACCTACCTCGTGGCGTTTGCGTATAAGAACGTCAAGTTCGTCCTCAAG

CACAAGGTAGCACAGAAGAGAGAAGATGCCGTTTCCAAAGAAGTTACTCGCAAGCTTTCT

GAGGCAGACAACAGGAAGATGTCTCGGAAGGAGAAGGATGAAAGGATTTTATGGAAGAAA

AACGAAGTTGCAGATTATGAAGCAACAACCTTTTCCATCTTCTACAACAATACTCTCTTT

CTGGTCCTGGTCATCATTGCTTCATTTTTTGTGCTGAAGAACTTCAACCCCACTGTAAAC

TATATCCTTTCCATAAGTGCTTCGTCTGGACTGATTGCCCTGCTGTCTACAGGATCCAAG

TAGACCAAAGAAAAAGCAGTTTGTTTTTGTGGGAGGGTTCAACTTCTGCTTGAGGGTGGA

ATAAGAATAGACTTTTTTAAATAATGGGACTGCCTGAGGGTTTGGGGGGAGGTTTTCAAT

TGAAAGTTCATTTACCTTTGGTATCTTTATATGGTATTGCTCAATAACCGAAGCTAATAC

TTACTTGGTATTGAAAGTAAATGGCACGTGAGCCATTATGAAACAGCTTTTCTGAAGTAC

TTCACAGGTGTGATGGTACTGCAGACCATTAGACAGTACGAGTCAACCACAAAGGACTGC

TCCACGGCAGCACAGAACAGCTATACTCAAACCCCATAGTGGAATTCCCCTGTAAACGTA

GTGGCTATTAGAAGAGCTGCAGTCTGTGATCATAAAAATTGACAATCTATCCTTTTTCAT

TGTAACTTGCAAAGTTTTAATAAAAAGGAGACTTTTTGTCATTAAAAAAAAAAAAAAAAA

AATTAA

>c3141_g1_i1 len=221 path=[472:0-100 179:101-220]

GTATTTCCCGTTCATCTCCCTCATGGCCCTGACGTAGTCGTTGGGGTCCTTGAAGGACAC

GAAGCCGTAACCTTTGGTCTTCCCGCTGCGTTTGTCGCGGAGCCTATAGGGGCAGAAGAG

AGGAAGAAGGGGCGGCCGGAGAGGCTGAAGCGCTGCATCCGTACAGCAGCCGGGAGCAGC

TGGGAGGACCCCAGTCTGCTGGAGTGGGATGCCGACGACTT

>c3141_g1_i2 len=221 path=[472:0-100 572:101-220]

GTATTTCCCGTTCATCTCCCTCATGGCCCTGACGTAGTCGTTGGGGTCCTTGAAGGACAC

GAAGCCGTAACCTTTGGTCTTCCCGCTGCGTTTGTCGCGGATGACTTTGGCCTTCAGGAA

GGACGGGTAGCGGCCGAAGGCGCGCGCCAGGATGTCGTCGTTCACCTCATTGCCCAAATC

CCCGCAGAAGATCCGGAAGTCGTCGGCATCCCACTCCAGCA

>c3148_g1_i1 len=254 path=[386:0-253]

GGCCACATGGCGAAGAGCTCCGCTCCTCAGCCACCTTGAGGGCCCCCTGCTCAACTCACT

GCAGTTCAGCATTACCTTCCTTGTATGGAGACGAGCACAGCGCCCCTCCAGATGGGATCT

CCCCACACCCAAGTAGAGGGGACAGATCACTTTCCCAGATCTGCTGGATATATTCATGCT

CTGACAGCCCAGGATGCAGTTGGCCTTCCTTACTGCTTCACGTTCAACTTCCATGCTGAG

CATCATGAGGCTGC

>c3149_g1_i1 len=224 path=[1:0-223]

TTAAGACTCTTGCGCCCATCCTCTCCCTTCTCCTTCCCTGGACCAGCATCTCCCAGCCCA

AGCAGGACTGGAAGAAGAAGTGGAAAAATGTAGGGTCACTGCTTCAGAGGGTTTGCAGGT

GGGATACAGCAGAAGCAGGGGGGGCTGCTTGAGTGCATCCCATTTAGAAACACAACCAGG

GACAAGGATTGGGCAAGATGAGGTGCTGGGGCACAGAGGGGTGG

>c3158_g1_i1 len=284 path=[1:0-283]

GCACACTGTAAAAAGTTGTCCCTCTTTCTTAAGGAAAAAAAAACAAACCTTCACATATGA

TTTGGGAAGCTTTATATTTCTGGTAATAACCTGTAGCCACATACAGATTTGATTTCAGTA

AGCAAATTAGTGTGAAGTAAGATTGTTTTTGTACAACGTATACAGTTAAACTCATATTAA

CATATGAGTTCCTATGGGTTATAATACAGAAGATATAAGAGTATATCACTGGTGCTTACA

GCTGAAGTTTGCTAATTTTCTTACTGTTAACAAGGCTTAGGGAA

>c3161_g1_i1 len=233 path=[1:0-232]

AGCCGTACAGAGTACCGGAGCAGTACACACTGGGTGGTCATTTGCAGCCTAAGACAAAGG

AGAATTCTTCTGCATCACAGGCTGGAAAAGATGTTTCAGAAGATCATTTTGATATCTGGG

GCACCTGACATTCAACGCTGAAAAGTGGCTGCAAGGACACAGGCATTGCAGGTTCCTACG

GAAACATTTACGAAAGCAGCTTCCTGCGGCCTGGACAATGCACTCAAACACAA

>c3173_g1_i1 len=2192 path=[2170:0-2191]

TTTTTTTTTTTTTTTTTTGTGTTAACTTTTGGAATATTTTAAAAATCAAATTGAAATAAT

ATATTTAAAGAAAAAAATAAAAGAAGTAATATCAAAAAAACATTACAAAAAAAAAGAAAA

AATAATCTTTTAGGATTCATTCATAAAAAGTCAATTGAAAAAAAAGTAAACTGTTAAATT

AATTATTTACTACATATTAATTTTATTAAAATAAAAAAATTTATTTTCGTTATTTTTTAT

AAATTTCTTATCTCTTTTTAATAAAAAAATTTTATCTAGAAATTTTGTTATATAAAAATT

TATAAATTTAATAATTTTAAATTTTCCCAAAAATGGTGAATAAAAAAGTTTTGAAAGATA

ATTTATTTAACATAATAAAATACAATGTTCACAATAACTCTGCTTTTAATATATGTGGAA

AGATAAAAGTTGCAAATCCTATAGTAGAATTGGATGGAGATGAAATGACTAGAATAATAT

GGAAGGATATAAAAGATAAACTAATATTACCTTATTTAGATTTAAAAATAAAATATTTTG

ATTTGTCAATTCAAAGTAGAGATAAAACAAATGATGAAGTAACTTTGGAAGCAGCAGAGG

AAATTAAAAAACATTCTGTTGGTATAAAATGTGCAACTATTACTCCTGATGCAGCAAGAG

TAAAAGAATTTAATTTAAAGCAAATGTGGAAAAGTCCAAATGGTACCATAAGAAATATAT

TAGATGGTACTGTTTTTCGAGCACCAATACTAATTAAAAATATACCAAGACTTATACCCA

ATTGGAAGAAACCTATAATAATAGGAAGACATGCATATGCTGATCAATATAAACAGAAAT

CACTAAAAATTGATAAAACAGGAAAATTTGAAATTGTTTTTACTCCAGACGATAATTCTG

GAGTTATTAGAGAAACTATATTTAATTTTAAATCACCTGGTGTGTGCTTAGGTATGTATA

ATACAGAAGAATCTATAAGGAACTTTGCCTTATCATGCTTTCAATATGCATTAGACCTTA

AAATGCCTGTTTATATGAGCACAAAAAGTACTATACTAAAAATTTATGATGGATTATTTA

AAGATATATTTAGTGAAATTTATGAAGAGAAATTTAGAAAGTCATTTGAACAACATAATT

TATGGTATGAACATAAATTAATTGATGATATGGTTGCACAGGTTTTAAAATCGGAAGGAG

GATTTGTATGGGCATGTAAAAATTATGATGGTGATATACAATCAGATGCAGTTGCACAAG

GATATGGAAGTCTAGGATTAATGAGTTCAATCTTAATGTGTCCCGATGGAGTTACATGTG

TTGCAGAAGCTGCACATGGTACTGTCACAAAGCATTATAGATGCTACCAAAAAGGACAAA

AAACTTCAACAAATCCAATCGCCTCTATTTTTGCGTGGACTAGAGGACTTCAACAAAGAG

CCAAATTAGATAATAACCAAAAGCTACAACAATTTTGTTATGCCCTTGAAAAAGCATGCA

TTGAAACTGTTGAAGATGGGTTAATGTCTAAAGATTTAGCAGCTTGTATTAAAGGAATAA

AAAATGTTACAGAAAAGGATTATTTGTATACCGATGATTTTATAGATGCAATTAATGAAA

AACTCAAATTAAAATTACTAGTTTACCAATCAAAAAATGATTCACAAGCTACTACCACAA

AATTAAATAATGATAATTGGAATCATTATGCTCCACAGGAACATTCCTCGTAAAAAATAA

GGAATACCCTCAAGAAATAGGGGCTTTATTTTCATTTTCTTCTTATTATATATTTAAAAA

TTTATGTATGCATATATTAAACTTATTTTATTTTTATGCAATATTTATATATATACAACA

ATTATTGTTTTATATTTATGATATTCATATTGTTTTTAAATATTACTTTTATCATTTACT

AATTTTTAATTTAGAATCATGCTAATTTCTATCTTTTTATAATGTACAATTTAAAGGTTT

TATTTTATTATTAATTTAAATTTTTTTTTTAAGAAAACTAAATAACTTAAGTTTATTATA

AATATTATAGTTAAAGAGATATGTTAAAATAGCAGAAAGTCAATATGTTAATTTACTTAA

AAAAAAAATGATTATGACACTTGGATTAATTATTATCCTTAAATTTTTTAAAATAATTTC

AAAAGTAAAATTGAAAAAAAAAAAAAAAAAAA

>c3174_g1_i1 len=321 path=[3587:0-8 3329:9-239 3560:240-266 3560:267-293 3560:294-320]

ATTTATATCATTTATATCATTTAAATTTCCTAAATTATTCAAATTGTTTAAACTGTGAAC

TCTTTTTATATGATTCATATTATTTGTGCTATTTATATCGTTTAATTCTTTTAAATTATT

CATATCATTTAAATTATTCATATCATTTAAGTTATTCACATTACTTAAACTATTTACGTT

ATTTACATTATTCAAATTATTCACATTACTTAAGCTATTTACGTTATTTAGATTATTTAC

ATTATTTAAATTATTTACATTATTTATATTATTTAAATTATTTACATTATTTATATTATT

TAAATTATTTACATTATTTAT

>c3174_g2_i1 len=1491 path=[1183:0-1164 2348:1165-1179 2348:1180-1194 2348:1195-1209 3006:1210-1490]

TAACTTTGTGTTTGAGCTCGTTAAAATTATTAGAATGAGGATTGTTGTTAATACTTATAG

AATTTAAAAGATTACTTGATAAATAATCAGTATTAATTGTGCTTGAATTAGATGTAGATC

TTAATTTATCTATCATTTTTTTATTTGAATAATAATCATAATTATTTAAATCTTTTGTGT

TAGTAGTAGACGTAGATATCGAAACCATTGCTGGATTGTTTTTTTTTAAATAGTAATCAT

ATAAAATATCTTCATATTTATTAAAATTTTTATTTACCTTTTTTATATTATTTTTTTCTA

ATATATTTTCATTTAATGGATTATTCTCATTATTTAATAATTTTAAATTATAATAAGAAT

CTTTAAATTGCAATTGTGGATGTCTATTTACAATATTATCACACGAATTCTTTTCTCTTT

CAATATTATCATAATTTTCTATTTTATTTATCATGTTTTTAAACGGAAAATTGTGCTTAT

TAAAAATTTCATATTCATTGTTATTGTTATTATAATAAAGGTATTCCTTATTTATATTAT

TTATTGTACCTTTTTTCTGATACTTTTTTAAATCATAATTTAATTCATAATTTAGAGATT

CATTTGAATTTAAGAAATTTCCTTGTTCATTATTACGGTAATATTTTAATTTGGAATTAT

TATTTTCCATATATCCTTTATTTGCAATGTTTGAAAATGATTTGTTTTTATCTATTAATA

GTTTTTTATCTGATATATTTAATTTGTCATTAGTTAAACATTTTTTACTATTTTCATCAT

TTCCTATCATTAGTAAATCTTTGTGTTCACTGTTAATTATTTCACTTCCTTTTTCCAAAC

TATCAGATTTTAAATTTAATTCTTTTTTTTCTTCTATCATATTGTGAATATTTTTATAAT

ATTCTTCCTTTTTAACATCATTAATATCATTAGTATGATCTTTGTTATTAAGAGAACTAT

AATTATTTATTATAGTAAGCATAGTTTTTGTATTTAATTCACCTTCATTTTCTTCATTTG

AATTTTTTACTAAATTAAAATTTTCATTTGTTACATTTTTTATATAATTATTATATAAAT

TCACATTAGAATTATTATAACACAATGTATTTTCTCCATGATTTGCTGTCTGATAAATAT

TCATATTTTTATATTCAATATTTCCATTTATATCATTCATATTTATATCATTCATATTTA

TATCATTCATATTTATATCATTCATATTTATATTATTCATATCATTCATTATTTTTTTCT

TATTTTCTTTCATATAATTAATGTTCCCATTGATATCATCAAAATTATTTATATCTTCAT

ATACTAAATTAAAATTATTATTTGTCATTGGGTTTATATTATTTTCATCATTACTTACTT

TAATATGATTCAATTCATTACCTAAATTTATATTAGTGAAGTTCATACTTTCATTACTTA

AAATATTTATATTATTTAATTTATTTGTATTACTTGCATTTTTTACTTCAT

>c3174_g2_i2 len=1476 path=[1183:0-1164 2348:1165-1179 2348:1180-1194 3006:1195-1475]

TAACTTTGTGTTTGAGCTCGTTAAAATTATTAGAATGAGGATTGTTGTTAATACTTATAG

AATTTAAAAGATTACTTGATAAATAATCAGTATTAATTGTGCTTGAATTAGATGTAGATC

TTAATTTATCTATCATTTTTTTATTTGAATAATAATCATAATTATTTAAATCTTTTGTGT

TAGTAGTAGACGTAGATATCGAAACCATTGCTGGATTGTTTTTTTTTAAATAGTAATCAT

ATAAAATATCTTCATATTTATTAAAATTTTTATTTACCTTTTTTATATTATTTTTTTCTA

ATATATTTTCATTTAATGGATTATTCTCATTATTTAATAATTTTAAATTATAATAAGAAT

CTTTAAATTGCAATTGTGGATGTCTATTTACAATATTATCACACGAATTCTTTTCTCTTT

CAATATTATCATAATTTTCTATTTTATTTATCATGTTTTTAAACGGAAAATTGTGCTTAT

TAAAAATTTCATATTCATTGTTATTGTTATTATAATAAAGGTATTCCTTATTTATATTAT

TTATTGTACCTTTTTTCTGATACTTTTTTAAATCATAATTTAATTCATAATTTAGAGATT

CATTTGAATTTAAGAAATTTCCTTGTTCATTATTACGGTAATATTTTAATTTGGAATTAT

TATTTTCCATATATCCTTTATTTGCAATGTTTGAAAATGATTTGTTTTTATCTATTAATA

GTTTTTTATCTGATATATTTAATTTGTCATTAGTTAAACATTTTTTACTATTTTCATCAT

TTCCTATCATTAGTAAATCTTTGTGTTCACTGTTAATTATTTCACTTCCTTTTTCCAAAC

TATCAGATTTTAAATTTAATTCTTTTTTTTCTTCTATCATATTGTGAATATTTTTATAAT

ATTCTTCCTTTTTAACATCATTAATATCATTAGTATGATCTTTGTTATTAAGAGAACTAT

AATTATTTATTATAGTAAGCATAGTTTTTGTATTTAATTCACCTTCATTTTCTTCATTTG

AATTTTTTACTAAATTAAAATTTTCATTTGTTACATTTTTTATATAATTATTATATAAAT

TCACATTAGAATTATTATAACACAATGTATTTTCTCCATGATTTGCTGTCTGATAAATAT

TCATATTTTTATATTCAATATTTCCATTTATATCATTCATATTTATATCATTCATATTTA

TATCATTCATATTTATATTATTCATATCATTCATTATTTTTTTCTTATTTTCTTTCATAT

AATTAATGTTCCCATTGATATCATCAAAATTATTTATATCTTCATATACTAAATTAAAAT

TATTATTTGTCATTGGGTTTATATTATTTTCATCATTACTTACTTTAATATGATTCAATT

CATTACCTAAATTTATATTAGTGAAGTTCATACTTTCATTACTTAAAATATTTATATTAT

TTAATTTATTTGTATTACTTGCATTTTTTACTTCAT

>c3175_g1_i1 len=248 path=[226:0-247]

GGTGAGTGGAGCTGAAACCTGGCACTTAGTTCTGTTACTGCTGTCAGCAGAGTCCCCAGC

TGCAGTGCACTGATGAGAGTCGTGACAGTCTGCTTTTGCAGACATGAAAGTTGTGAGGAG

CCTGTCAGCTAGCAGAAAGCCTGTCTTGGGAAGTACTAGCCCTAGAATCTGGAGGGTAAG

ACCAGGAAACAGCACTATGCTTGAGACAGCTGTTGAGCAAGGAACGTGAGTGACACAGCT

CTGCTCCT

>c3176_g1_i1 len=4868 path=[16410:0-41 4833:42-65 4857:66-4867]

TATATATATATATATATATATATGAGTTTAAAATAATACAATAAAAAAAAAAAAAAAAAG

GAATTAAAAAAAAATATGCATATATAAATTCATTATAATAATGGAAAAATTAGACAGTAA

CGAAACAATGGTTATTACCTTAAAAAACATAAACAAAAATAAGTGGAGTTCATTATCAAA

AAAGAAAATAGATGATAAATCATGCTATTCTCCTAGAACTGAAAAATTAATTGAAATAAT

TTCACCAATTTTAGATTATTATGGATATAACAAAGAAGATGTATATAATTTTATTAGTTT

ATACAAGTTTGATATAGAGAAAATTCAATTAGAATTATGTAATATAATGGAATCTAAGGA

AGGAAAAGAAGAAGGATTATGGAAATTGGTGAAAAGCAAAAAAAGTAAGAAACCTAGTGA

AAAAAATAATAAAAAAAGTAACATAGGAAGAAAAAATCATAAAAAAATAGAGAAGTTATT

TTCAAAAGGATTTAACTCTAAAGAAAAAGATCAAAAAAAAATTAAAAGTACATCAAATGT

TGATACAGTAGGTCAAAAAAAGACTTTTTCTAGTAATAGCAAAACTAATTATTTCCCTAA

TCTAAATGATAAAAATGAAAATGTTTCAATGATACATGATGTTAATAAAAATATACAAGA

GGATAAAGATATAAAAGAAAATAAAAGGATAGAAGAAACTTTAAGCAAGTATCATAATGT

AGAAAATAAAAAATTAAGAATAGGAAATGAGGACATAAAAAATAAAAAAAGTAAAAAAAA

GAAAGGAATAAAATCAGAAGAAAAAAAAAATAGTATAAATGAAAGTTGTGATGATGATTT

TTTGAATTATTCAATGGAGGAAAAAGGAGAAAATAAAAAAAACCATAGTTTGTTTAAAGA

ATCAGATAATATAAACAATGAAAATTTTTTTAAGAATAAAGATGAATTATTAAACAAAAA

GGGTAACAAAAATAATTATTTTAATTATTTTGATAGAAAAAATCATAAAATGGAAGAAAA

TAAAAAGGGATACAAGTCAAATGAAGAACATTTCATTAAAAAAGATTTTAAAAATAAAAA

TTCTCCTAATGTGAATTTAGCAAATGGTCACAATCATAATTTTTCTAGATATAATAATTT

TAATCATCACGAGAAATATTTTAAAAATTATAATAGATATAATAAACCATTAAATGATAA

TTTGATTTCAAGGAATATAAAGCAGAGATATTCTATTCTGAATAATAAAATAAATTTGTA

CAATTCTGGTAATAATTACTTCAATAATTATACTAAAGGAAATCAAAAGAAGGGATACTT

AAGCAAGAAAAATTTTAGAAAAAAGAGAACATTTAGCAAAAATAGCATAGATCTTGAATA

TAATCCATATACAGGGCAAAAATTAAATTATGATGTAACTAACACTGAACAAAATGATAA

TAATAAAAAAGATATGAATATGAAAGCATTAAACGAAAAAACAAATTATAATGCATCTTA

TGCATCTATAGTAAATAACACTGTTAAGCAACCTCCAGTAGAAGAAAATGATAATGAAAA

CAAAAAAAATTTATCAAATGTATTGAATAATATAAATGAAGATAAAAATGAAATAAAAGA

AAAAAAAAAATCAAAATGTCAGTTAATACCTATTAAAAATACTAAACCAGAAAAATTATG

GGTATCTATAATAACAAAAGGTAGTAGCAATACATGTAATGAAATTGAGAATACGAATGA

CTCAATTATAACAAAAAAAGATGCAAATGAAAAAGAATTAAAAAAAGATCAAAATATCAA

AGAAAATAATAGTGTGAATAAAAAAGAATTATTTAACAATAATAAAACAATGAAACCAAA

GAAAAAAGATATTTCAGGAAATTTGAATATATCCAGTGAAAATATTAGGAATAGTAAATG

TAATGAAAAGTTACACAAAAATGATTTATCGAAAGAAGAAAATTTAACTTCAGATTTCAA

AGGAAATGAAAATGAAGATATTAATTTGTTGAGCTATAGTAAGATGGTTAAAAAAGGAAA

TGAATCTATTAATAAAAAACATAAAATTACAAATGATGATTCTTTCAATCCATTACATTC

TAATGAAGAAAATAAAAGTAACCTAGAGACAATAGAAAATAGAGAAAATATAGTAATGAA

AAAAGGAAATGAAAATAAAACTAAAATGGAAAAAGAAAAAAATATTGGTAACGAAAATGA

TTCAGAGAAGAAAGAAATAAATTCTAATAAAAAAATTATGGAAGAGAAAAAAGAAAAAAA

AAATGAAACAAAAATAGAGTTGGAAGAAAAAAAAAAAAAGAAGAAAAAGAAAAATGATCA

AGAGAAAAATATTTTAAATAATAATATATCTATAAAAACAAAAAGTCTCTTACCATGTGA

TTCTAAAAAAGGTAAAAATAAAATGAATAAAATATCAGAACATAATGAAAAAACGTTACC

AGAGAAGATTATATCAATTGATAAAGATGGAAAAGATAAAACTAAAATACCAATTTATAT

GCCTAATATATCCTTAATGGCAAATATAAATAAAAAGATATTTTTTGGTAATATAAATAT

AAGTGGAGATCAAATAGAAAACATTGACATTGTATCAGAAAATAAGATAGATGAAATAAA

AAATAACAGAAATAATAATACAGGTGATAATAATGATAGCAGCAGCAAAATTATGACATC

AGAAAGCAAAAAAAAACATAGTTTAAAAAAAAATTATAGTGTAGAATCATCATTAACAAT

AAATAATGACAATCATCCAACATTACATAAAGATGGGAATAGAAATTATGAAAATAGCGA

TATGAGAGAGCAAAAAGAAAAGGAAGTAAAACAGTTAGATTTAAAAAATAATAAAAACCA

AAAAAGGGAAGATAACAATAACAATGATATCTTAAATGATAACAAACAAAAATCAATTTT

TTCATCATCTAACAAAAATTCTCATATAAATTCCAAATCACAAAATATATCTCTCTCAAA

AAATGAAACATCTTCATCTTCTACTCATCAACAAATGCAAAATTCTATTAGTGCTAATCA

AAATACAAAACAATTATTAAATAACTCTTCTGTTGTATTTATTAACCAATCACCTCCTCC

ACCTCCAGATGCTTCATCATCTGTTTTAATTAATGGGAAAAATAATAAATATGTTCAAAG

TACATTGAGAAATAAAAATTATGAACAAGAGGTAGAAGATTCTAGTTCTATACAACTTAA

TCAAATTTCGCAAAAAATTTTACAGAGTCAACAAAGTTCCATAAATAATAATTTGTCATC

AAATAATAATAATAATACAGTTGCAAATATGAGTAATAAGAATACAAATACAGATGTTAA

TAATATGAATTTAATAAATATGAATATGAATAATCTTAATAATAATAACATGTACAATTC

ATATAATTCACCTCCAGGTTTAGCTAATCAATTTAATTATTCATTTACTAATTTATCTTA

TCCATACAATAATTTGCATTATAATGGATATACAACACAAGCATTGGTTCCTCAATATGG

ATTAATAAATAATCATTATAATAAAAGTAATAGTATTCATAACAATAATTTTAATTATAA

CATGAATTATGATAATTTTGATGAGAATAATTTATATGCACATAATAATAATATACATAT

GAACAGCTATGTAAATTTTATGAATGTTAATGTAAAAAAAAATCAGTTAAACACAGATGA

TGATAATTTAAGTAATTCAGGAGAAGTTTCAATTTCTGGATCTCTTCAACCTTTAAATTT

ATCACACGATAATCCAACAAATTCAAATCAAAACTCAACAAATATTAACTCGTGTGGAGA

AAATACAAATAATGGATTAAATAATTTATCTTTAAATAATCTGAACAATGTTAACCCGAA

TTTAAATATATCATGTGCAGAATTAAAAAATAAGCAACAGGTAAATAAAAATATAAATGA

ATTACAATTGCAACCACAAATTCAAACAATGAAGCAAACACATGTTCAAAATAAAAATAA

TTTAAATAATCAACATTCCAATCAACATAATCAAAATAAACATTCAGGACAGCATCAACA

AAATTTAATGAATGCTTCTATTAATCAATATTCACAAAGTCAAATACATCCAAATAATTC

TCAAAATACTGCATCATCTACCTCTTCTTCGTTAACAGGGAAAGTAGAAACTAATAATTT

AAATAATTCAACTCAAAATAATCAAAATAGTTTTATAAATAAGCAGTTGTATCATAATAG

TAATTATTATAATATAAACTCTAACAATTTTAACATACCTCCAGGATTTGCCATATCTCA

AGAAAAAGAACAAAACAATAATTATTTGAATAACAATGTAACAACAAATAATGCAAATAC

AACAGTAACTGCTTATAGAAATAATTGGAATAATATTAATGAGAAAAGTTTAAATCATTA

TAACATAAATTATAATTATAATAGAAACAATAAAAATAATTTCAATTACAGTAATAATTT

GAATTACAGTAATAGTAATGGATACAATGGAACAAGAGATAATACCAATACCTTTTTTAA

TTATAATAATTTTAGTTATGCTTTACAAACACCACCAGGTTTACAGAATTATTATCAAAG

TACACAATATCAACAACAAAGTAATTATAACAATAGAAATTACAATTATTCAGGATATAA

TAACAATTTATCAATGTGGAATAGAGAAGAATAGTTAAAAAATAACATTTTTATTTTTTC

TAATTTTTTATAATTTATTATCATTTATTTTATTATAATTTATTTTATTTTATTACACCT

TATTATAATTTATTCTATTTTCTTTTAAATTTTTTTTTTTTTTTTTAAAAAAAGAATAAA

GTTCTCAG

>c3176_g2_i1 len=6671 path=[4832:0-0 4833:1-24 16475:25-6670]

AAAAAAAAAAAAAAAAAAGGAATTATTATATAAAACATATAAGTAAAATAAATCATGTGG

TAGAAAATGAATAACAACTTTAACATAAATGTTCAGGTGGATGAAGCAGCTCTTAACAAA

CATGAAGTTGAAGTGAATGGTTATTTTGCAAAATTATATACTGGGGAAATTACAGTGAAT

ACAATGCTGGATATTATGAAAAATTTATCCTACTCCCCTAAAGGATCAAAAAATAATGAA

GTATATAAATCAATGTTGCTTATTCTATTTAATGAGTGTAGATTTTTTCCAAAATACCCT

GTTGAAGAATTAGATGTGACAGCTCAATTGTTTGGAAAATTAATTAAACATAATTTATTA

ATATCGTATGGTAATACATTAGCTGTAGCATTAAAATGTATATTAGAAGCATTAAAAAAA

GGAAAAGATTCTAAAATGTTTAATTTTGGAATAACTGCATTAGAACAATTTGAAGATTCA

CTAATATGTTATCCTAGTTTTTTATCATCCTTAATATCTATACCTAATTTAAGGCAATAT

AATGCCCAATATATAATTCACTGCAGAAATTTACTAAACTCGCTTCCTGAACACATGAGA

AACTTACCATATATAGATTCGTCTACCATATTAAAAATCAAACATTTTTCTGAAATGAGT

ACAAATAACTCTTCAAATATTAATATATCACAATTGTTAAACAGTGATTTAAACAACAAT

TTAAAAAATGTGAACAATTTAATGCTAAATTTTAACAAAAATAGAAATAATAATACTAGT

AGCATTGGTAATGTTAATAATAATGATTTTAGCAGGGGTAGTAGTTGTAGCAGTAATAAC

AATAATAACAATAACAATTTATCTAATGCTTTAGGTAATTTACTAAATAACGGTTGTAAT

AACAATAATATAAATAATGATAATTTATTATTATATATCAATGATATTTTACCAAATTGC

ACAAATAAAGAGCAATCTATCCATAATGTTAATGATATTTCATCCATAAATAGAAATGAT

AACATCTTTAAAAATAACAATGTGAATGACAACTTAAATAATAATTTAAATTTAAACAAC

TTTTCATCAATAAATAATTTAAATAATCAATTGAATATAAACATTTTAAATAACGATAAG

TCTAATAATACTAGTAGTACAAATCAGTTATATTTAAATAGGTCCTTTTTAACCTTAAAT

GAGAGTAATCAACTTAATAGAAATAGAAACATAAATATAGTAGGAAACAATTCGAATGTT

AGTAGAGAGATAAATTCAAGAAATAGTTCTTGTTTAAATGAAAATATAGTAGATACAAAT

ATAACAAATTTTATAGATAATCCAGTTAAAGTAAACATTAATGAAAATATCAAATGTGAA

GATAATGAAGACAATAATAATTCCTTTAAGGAAAAAGAACAGTTATATGGTATAATTGGA

AGCTTCTATGATCAACCTCAAAAAAGCTTACCATCTCATTTAAATTTAAGCAATATAAGT

GGGTTTGGTTTAGGACAAATTGAATGCTTAATGGATGGAACAGATTTTACAAAAAATATA

ATTGTGCCTTCTTCCTTTATAGTAGGGGAAGTATATAGTATTTTTAACACTTTATGTTTA

TTTAATATTGATGAAAAGGTGAAAATTTTAAAAAATGTAATGCAGCCAGAATATTATAGT

TGGCTTGCATTTTACATAGTAAAGTCTAGAGCATCAAAAGAAGTAAACCTACATAACTTA

TTCTTAGAGTTTATTGATAAATTAAGCTACCCATTATTTATAGATACTATAATAAATATG

ACATATGATTGTATACTTATATTATTTAAGTATATAAACGAATTAAAAGAAGTTAGTGCT

TTTAGAACTGTTTTAAAAAATTTGGGTTCTTGGTTGGGATATATAACATTGGGAAGAAAT

AGACCTCTAAAATCAAAAATATTAGATTTAAAATTAGTATTATTTGAAGCATATGAGAAA

GGTTGTTTAGTTTGTATTTTGCCAATGGTATGCAAAATCTTAGAGTCAGTAAAACTTTCA

AAAAACTTTAAACCACCTAATCCATGGACAACAACTGTCTTATGTTTATTAACTGAAATA

CATGAATTACCAAATGTTAAAACATACATAATATTTGAAGTAGAAGTATTATTTAAAAAT

TTATCAATTAATATACAAGATTATCAAAATAAAACAGCATTACTAAGTAAAAGAATACCA

TACAATAAAAAAAATGATTTTGTAATATCAAGAAATAATAACATTTCATCAATAAATACT

GAAATAAAAAATATCTCTTTAAAAGATAATATAACAAATATTAATAGCTTACATAGAGAA

GAAAATTTGAATGAAAATTTTAAATTAGAGGAATTAGAAAACGCAGGAAAAAATTTATTA

ACAAATAATAGAATGAATACAGATAAAATTCAACAAATGAATCAAAACAATCAGCAAAAT

ATAAATTTAAAAAATATAGCATCTCAATGGAATCAAAATAATGCTAATAGAATTGATCCT

CTTATGAATGATACAACATTAGTTTCATCTTTTTCACCTCCAGATTTAAATTTAATTAAT

TCTCATTTTACAAAAAAATCAGTCAACAATTTAAATAAAAATCCAAATATAATGAATGAT

TTAAATACTAATAATTTGAAGGTGTCTGATTTTTCTAATGATAATATTTCTAATAATAAA

ATAAACAATAGTAAGTTTCTGCAAAGTTTAAATAATGCAGTAATAATATCTCCTTCAATA

GCCTTATTTCAAATTCAGCCAAGTTTAAAGAAAGTAGTTCCTATAGCGGTTGATAGAGCA

ATTAGGGAAATAATTTCTGCTATACTTGAGAGATCAGTAGCAATATCATGTGTTACCACT

AGAGAAATTATATTCAAGGATTTTTGCTTAGAAAAAGACGAAAATTTAATTCGTAAAGCA

TCTCATATTATGATTGCTTCTTTAGCAGGTTCATTAGCCTTAGCTACCTGCAAAGAACCT

TTGCGAATATCTCTAACTCAACATTTAAGGCAACTGTTACAACCAACTAGTACAAAAGAT

TGCAATGACCAGGTTTTAATTGAGCAAGTAGTTCAAATTTTGAGTGCAGATAATTTAGAA

TTAGGTTGTAATTTAATCGAACAGGCAGTTATTGAAAAAGCTATAAAAGATATAAATGAA

GCGTTAGCACCTTCATTTTTAGCTAGACAAGTAGCAAGTGAAAATAGCATAACATTTAAT

GATTCTTCTTATGCTATTAACACAAAAAAGATACAAATGGAATTTGCTGAAGTTTTAAAG

TTAGATTCTCCAATAACAAATCATCAATTGCAGATATATAAAGATTTTTTAAATATAGCT

CCATTAAAAAAGTTGCATGCTGCTGCTAAAGCTTTATCGCTTATTTATAAAAATGTTAAT

GAAAATAATACAGAGAATCAACATCAAAATTTTCAAGGAAATGTTTCATTTTCAAAAGAA

AATTTGCAAAATAATCAAGAGAAAATCGCATATTCGAAAAATATGTCTCAAGCACATTTA

AATGAACAGCAAAATAATATTTCAAAAAGCGATATAAAAATAAACAAGGTGAACAATGTA

GATTATAATATGAATAATAATATAAAAAATCAACAAAGTTTTGCTTCAAAAGCAAATATA

AATGAAAATAATACACAATCAAATATACCCCTTTCAATTCCATCAGCATCTAATGTTCAG

TTTCATAAAGTTCTGAAAAAATTAGAACTTGCAACAGGTCAATTAAAAGAAGCTATTAAA

GATATTATTATGTTACCTCCAATGTTATTTAATTTAAATAAAGAAAAAAGTTATTGCAAT

ATTAGTAAAATGAGTCTTTATATTTTATATAGTTTATCAGTGGATGGAAATATATTTAAT

TTAATTAAATCTATACCAGAAATAGCTGCTTTAACACATCATAAAAATGAAACTATTATA

TCTTATTCAAACAAAATATATAAATTTCTTTTTGAAATTACTCCATACAACAGTAAGACA

CAATTAAACTATAGATTAAATGATCCTGCAGGTATTTATTTGGAGGTATTTTTATGTATA

CTAGAAAAATTAAAAAAAAAATGTCCATTATTAAAAAAACACATATCTAATTTTATAATG

CCTAATAATATTTATTCATCAAATAAGAATAATACAGAAAATAGAAATACATCATTTTTA

AATATGAATGAATCTCCTAAAGAAAACGAAATAAATAACGAATCGAAAGATGAAATAAAT

AACGTAGTAAAAAATACTTGCTTGAATAATAATTCGGATACAAATAATGAAAGCAAAGAT

GAAATGAATAAAAAAAATAATGAAGACTTATATAAATACAATGTAAATGTTATATCTGGT

CTAATTAGGTATAACCTAGTTGATATAGAAAAATACAAACAATATCTCGAAAGAGAACTC

ATTGAAGAAAAATGTTTTAATTGTACAGAGTTCGTTGTTTTATTATTAAAAAATATTTTA

TTAGATTTTCAAATTTTTAGATATAATGATTTTGAAAACATTTTTTCAATTTTAAATACA

TTTAAAGAACAGAATATCGTTCCAAATAAAATTATTTATACTAATAATTGGACACCAATG

ACTATCAATTCAGCGATTAAAAAATTAACAGAAGAATCAAAAAAAATACAAGAAAAAGAG

GATAGTATATTTTTCTCTGATTTATTAGATTTTTGTGAAGAATATATGAATAGTGATGAT

GAATATGATGAAGAAAATGAAGAAGAGAAAAAAAAAGAAAAATTGAAAAAAGGCCAAGAG

AATGAAGGTGAAAATGAAGAAGATATAAAAAAAAAGGAAGAAATAAAAGAGGAAAAAAAG

GAAAAGGAAATAAAAACACATGATAATAAGAAAAATAAAATTGAACAATATATACAGTCA

GACAATATTATAGAAAATGTAAATGAAGAAAAATTAGATAAAGTAGATTTGAATGAAAAA

GAAAAAGAAAATTTTAGTAAAATAGAAAATGATTTAATAAATAATAAAAATAAGCATGAA

ATAGAAAAAAGTAAATCCTCAAATGAAATATCTTTAAATTTAAAAAGAGAACATAATTTA

AATATTGATAATTTAATAGGTGTAAATGAAAAAAATATTGGAGAAAATAATAATAGTGAT

AATTTAGGATTGTTAAATGAAAAAAGAACAAAGGAAAATAATATAAGTGAAGATATGCAA

ATAAATAATGAAAATGAACAAAGTGAAAATTTAAGTGGAAATATTTGCATAAATCGTTTA

AATAACGATATAAATAATGCAAAGAAAAAGGAAATAAAATATAACAAGAATAATAAAAAA

AAAGATTTTAAAAGATATTTAAAAATTTTAATGGATCCTAAAAATTTAACTACATTAACA

CAAATAATTGATTTTTGTTTAAATACAGATTGGAATATATTACAAAAAAAAAAGAATAAA

AAATATTTTAAAGATAAAGGAGTAGGAGGAGTAAGAATTGTACCTAAACCACAAAAAGTT

TCAAAAGAGTATGAAAAAATTATTAGTATAGTGTTTTTTCAATGGATAAAATTAAGTAAT

TTGAGAAATACAGATAATTCAAATAAAAATTACGTTCAATTTTTTCAAAAAATATCGAAT

CAAGGTTTGTTAAGAATAGATAATAACACTGATAAATTTTTTACTGTATGTATTAATAAG

GCAGTTGAAAGAGCATGCAATATTTTAAATCCACATTCACATGAAAATGAAAAAGAGAAT

ATCACAAATACTTTAGATGAAGAATCCTTATCAAACAAAACTAATTCAAATATAACAATT

CAAGTTAATGAAAATGACATTAAAGATAATACATCATATCATAAAATAAATGCAAAAAGT

CATACTAATGATAATTATAATAAAAATAATGTAGAAACGGATGAAACAAGTAAACATATA

ATAAACGGAATAAATAAAGAATATGTAAATTGCAGTGAAATTAAAAATGAGAAATCACAT

AAAAAAGAAGAAAAAGATGTTATATTAGAATGTGTGAAAGATTCTAATGAAAATCTAAAT

GACAAAAAAAAGGAAAATGAAGAAATAATTGAAAAAAATGAAAATAATAATAATTTAATG

AAAGGAGGTAAAGAAAAACCATCCTTGCATCTAGAAGCACATAATAATGATCATATAGAT

AAAATAAATGAAAATTTGTTAGATAATGAAAGAACTTTTACAAATAATGAGTATTCAGGA

GATTCTGATGATAATTTTTCTGATATATCATCAACAAACTTAACTTTTTATTCAAATATA

TCTAACTGTGATAATTCATTTTCAGAAGATGAAAGATGTCACAGCTTTTCTATTAAAAAT

GATAGTAATGCAAAACAAATGAAAGAAAGAAAAAATGAAATTTTAAAAAAATTGTCATCA

GATATAAATGAATCTATAGGAATCGATATATCATCTGAATCAGATAAAAATATTAATAAA

AAGGAATCAGAATTAGAATATAAAAAATATACAAAGAAAGATGAAGAAAAAACTAAAAGT

CATCTTAATAAGAGCAGTACATTTTTAGAATATGATGAAAATGATAAAAAGAATAATGAA

GAGAAACAGAGTGATGATGACAAAGAAGAAGAAGTAGAAGAGGAAGAAGAAGAGGAAGAA

AATGAAGAAGG

>c3189_g1_i1 len=525 path=[15:0-524]

TTTTTTTTTATATATTTTTATTTTATTTGAATATTTGATTTAATATATAAAACGCAAATT

TGAAAAAAAATAAATAATACGTGAACTATACAAGCTAAAATATTAACACTTTGTCTAGTA

ATATGAAACAATAAAAAAAAAAAATTAGAAAAAATTTGCATTTGTCTATTTGTAAAAAAG

TCATATACAAATACACTATATAAAGAAATTAATCCACCAGAAATAAAAAAGATACTATTG

TCTATATAAAATATTTTTCTTTTTTTAAAGAAATAATCATTAGAGTCTAATAGTTGTTTT

TGTAAAAATATATTTTTTTTTTTTCCTTCCATTTTCGAAGATAAAAATTCATTGTTTTTT

TCTCCATAATGATCACTAAAACTTTTTTCATTTCTTTTTTTACACTCTAATTTATTAATA

TTTTTATATGGTAAAATCCCCTTAATCTTTAATATCATATTTTTTAAAATTTTATTACTA

TATATGTTCATTGTTGTTTTATATTATTTTCAGTTTCCATTTTTT

>c3191_g1_i1 len=421 path=[399:0-109 509:110-420]

GTACAAGAAAAACAATGCATCAACTTCACAGATAGCCTCAAAAGTGATGTTATTATTTAT

GTGGGACATGCTCTGAAATCCATCTCCCAAACCCAGAAATAGTTCTCACCTGGATACAGA

TTTTGTTACGATCGTCATATACATATATATAAAAGTGGGGAAAAAACCAACCCCCACCCC

CAACAAAAACCTATGAAATAAATGAAGAACAACATGGAAGTTGGACTGGTCATGCAAAAG

GTTTGTACAGAGGATTTCTGCTGTCAAATAATTGATAAAGCACATTCAATATTCATCCAT

CAAAATGGACCGTCTCCCCAAAGCCCGACTTCCCTTCCTGGCTTCCTGACGCTGCAGTTG

CTCTGAAGATGTCACTAAACAAACACACACCTCAAACACAAACCCAAACCCTACAACTAT

G

>c3195_g1_i1 len=228 path=[250:0-160 25:161-194 562:195-227]

ATTGCAAAGAGTTTATTTTGCAGAACTGGCAGGCTCGTATCAGTGCTCGCTTAAGGAAAA

AAACACACATTGCTGTTTTGCAATTGCTTGCGTTTGTTGACAGCCTGCGCTGAGCAGGCA

GAATTGGGATGTTTGTATAATGTAAACCTATAGCTTGTCTGATGAAGTGTAGTGTACTCT

TAGGTAGTGCATAGATTGCTTGATAAAAGGGATCAAACACAAAGTTTA

>c3195_g1_i2 len=217 path=[250:0-160 410:161-216]

ATTGCAAAGAGTTTATTTTGCAGAACTGGCAGGCTCGTATCAGTGCTCGCTTAAGGAAAA

AAACACACATTGCTGTTTTGCAATTGCTTGCGTTTGTTGACAGCCTGCGCTGAGCAGGCA

GAATTGGGATGTTTGTATAATGTAAACCTATAGCTTGTCTGTTGTTCCAGTTTGTCATAA

ATGCAGTCACCTTGTAACCCCTTTTATCAAGCAATCT

>c3197_g1_i1 len=489 path=[467:0-488]

ATATTAATATAAAAACTATTTTTTTTGATAAATGGAGTTATTTATTTCCTGAAATTTCAC

TATATCATTTTTATATATATCAGCTGCTACTTGATTAAAAGGGTCATCTGTTGAAGGTTC

ATTTAATAAAAGTAATAAACCCAATATTAACATTTGTAAATTAATAATAGGATTCCAATC

TAGTTTTAAAACATTTAAACATACATTTCCTTTTTCATCAATATTTGGATGGAATATTTT

GTTTAGACATATAATTTTTGGTGGATTAATAGGATAGCTATCCTTAAATTTTACCAGAAA

TTTAAAATTTTTGTTTTTAAAATAACCTTCTTTAGGTGTAATTGAAATACCAACTTCTCT

TAAATTATTCTCATCAATTAAAAATAATTCTACACCTTCCATTTCATCTAAATCCATTAT

TTCTTTTTGAAGTATTAATTCAAATGGTTTTAATTTTTTTATACTTTCATTATTCATTTC

GGAATTAGA

>c3214_g1_i1 len=809 path=[814:0-808]

TTTAAAATTAAAAAAAAAAAAAATGACTTAAATTTTTTATAATAAAAATAGTGTGATTCA

TACTTAATTTATTTTTTTTATGTATATGATTATTTTTTTTTAAATGATTCCTACACCAGT

TTCAAAAAAAATAAGAAGTAGATTGAATTTATCAATTCAAAAAAAACCGCATTTTTTATT

TCGACAAAATGTATTACTTGATAAACGTGAAAAGTGGGAACCAAAACTGAGAAAAGGTCA

TCCGCAATTTATAAAACAATTTGATATATTAAATAGACAAGTTACAGGTTTTCGTAAATA

TAAACAAGTTACTATTAAGAGAGAAGAAATAAAAAAAAATTATGATATTACAAATTTTCA

TGAAGTAAAATCAAAATTTAGATTTGAAATCAATGGATATTTTGGAGATAACGGGAATAT

ATTTTGTGAAGAGTTGTATAGAACTGCCAAACGAATGTTTATAGTAGGATGGATAAAATG

CAGAAAACGATTTGCCATGGGACACTTTCAAGGAGATTCTTATGCTATTTCTTATTTAAG

GCATTGGTTTGATATGTATTCATCAGACACAAATAGAATTGATAAATTAAAAATTTTTGA

TGAGAATCATGGGATACCAAATTTAGATTACTACAACATTACAATAGTAAAAGATTATAG

AAGTCCAGCAAAGAAAAAAATGCATATGATTCAGAGTCAAGATTTTTTAAAAACAAAGAA

ATTATTTAATATAAAATAAAAAAATTTATCTAACTTTTAATATGTGGTAAATAGTTATAG

TACTACTAAATAAAAAAAAAAAAAAAAAA

>c3215_g1_i1 len=237 path=[229:0-236]

CAGCTTCTTGCACTGAGGGGTCCCAGGCCCAGACACAGTACCCCAGATGGGCCTTACAAG

GGCAGAGCAGAGAGACAATCACCTCCCTTGCCCTGCTGGCAACTCCTGGAAGCCTCCCAG

ACCATCCTCCTAATTACAGCAAAGTTTAGTGGCAGGCTGCTTGCTTCCACTGACTGACGT

GACTTGCAGCTCCTTGGGGACTGAACACAAGGAGCCCTGGCAGAGTGCTGCCCAGAG

>c3217_g1_i1 len=219 path=[1:0-191 659:192-194 196:195-218]

CACGTTACCAGGAGCTGGCAGTAGAAGAAGGAAGGGGCAGAAAAGGAGGAGGGGAGGAAA

AAAGCATTAATTAAAAAGGACAGAAAAACAAATCATCATCACAGTTTGATAAAATCAGAT

TCCCCAAATCAATCTACAGATCCGACAAGAGTAGCTGGGACATTAACTTACATTAACTTA

CTTGTGCTTTCTGCTAAGGCATTTTTTGCCATAAAAGTG

>c3227_g1_i1 len=248 path=[1:0-247]

GTGTTTCTCCATCCTTTAAAATAGCAGGAATGTTAATTAGCATCTTATCTAAGTAACAGA

TATGAAAACAGTCAAATATGCTGTTAGGTACTTTAGCTGTTAAGCTCACATACAGACATC

ACACAATTGAAAAAAGAAAAGGCAGATCAAGCTTAAGCAACCACATATGTTAGCACTGCT

TTTGCTGTCACACAGAAATTGCCAATAAAAAAAAGGCTTACTTACCCTTCCTGTGAAATG

AATTTCTT

>c3232_g1_i1 len=237 path=[215:0-176 461:177-236]

GAATGGTGGGAGCCCAGGGTGACAGCTCTGGTTTCATGCAGCAGGAGTCAAACACAGTTC

TCATTCATCAGTGCATACGGAATTCATGCAGACCCAAGATACATAATCCCACAAGGCTTT

TCCAGGTACACTGCCTCAACCAAGACCATTCAAATCCAAATTTCTTGGCAAGACAAACTT

TGCTTCACACTCCTAACACTCAGCTATTAAGGGAAGCTTTGAAGAACTGTAATCAAG

>c3236_g1_i1 len=220 path=[214:0-196 521:197-219]

CCCTATTGATTTTTCTAGACTTATAGCACATGCTTGGGACAACAGGATTTTCTCCTGCCC

TGTTTTTGTGCCAACACTGCATGTGGAAAGTTCATAGTTTGCTTTTAATTTTTTTTTTTC

TGTAGTTTAAGCCTTGTCCAAATCAACCTTTTTATACATAACCTCTGGTTTTCAGATGTT

GCAGCTCCAGGTAACAATTCCCAGAGCCATCAGGGAATTG

>c3237_g1_i1 len=219 path=[179:0-218]

ACCAACCTCCCACTTTCAACAGAGAAACCCTTACCCAATACATACACAAATAATACAGAG

TATCTGTACGATTCCTAAGCAATCAGTGAGTCTGTTACAATGTTCACACAATGGTAAAAC

ACAATGCCTGATTTCTTGTTAATGAATCGACTGTCAGCATTTTAAATGCTACCAAAGGAC

TACTTCTGCAAATCAAACTTCAGTTCTTGATTTAATAAA

>c3248_g1_i1 len=512 path=[493:0-291 1008:292-305 802:306-511]

AGCAAACTATGCACACAGAATGCTGCTTCTGCAAAACTGCCATCAGGGCGCCCTGCATCC

AGGGCCCGGCCGAGCAGGGACCCCACTGCTGCCTCCCTGACACTAGCAATGCCATTGTAA

ACATGAAAGAAAGCCATTCAGATCCCCCAGTGAATAGAACAAACACACAAAGAAACTATT

TGAGATTTCTTGCTTTTAAAAGCCTGTATCAAAACTAAGAACTAAAAACGTACGGCCGGG

ATTGGAAGCCATCTAATCCAGCACTGTTATCACAGCCAGCCCCCAGGCAAGGTGGTTTTT

CCACTGGTTTCTAAGTAGCTTGTACTCATCATGGACATAGCCCATTCCCACACCAAGGGA

ATCTTTTGTTTCTTGCTGCTTTTCTGGTTATTATTAATGGCCTCTGTTGCTAGTAGTGCC

TAAATATTGCTGGAAAAGGGCGGGTGCTGGATGCTGGCCATAGTTCTCTGAACCAGAGAT

ACAGCCTAAATAGTAAAGAGAAACTACTGTTG

>c3253_g1_i1 len=323 path=[351:0-322]

ATCCTTGAGGTCCCTTCCAACCCGAGCCATTCTGTGATTCTATGATCTAATCCAGGACTT

CAACATTTTGGAATACGGTGTCTAGCAATTACTACCAAAGGTAACTGTATACTAAATGAA

AAGCTTCTATCTATCACAAATGCTAACTGCCCTCTGTGAGCAGAGCATTAGTAGGTTAGA

TTTCCAAATTCAAGACATCTCAAGTTGGTGAGCACTATCCTGAAGCACTACTGGAGTCCT

ACAAAGCCTCACTGAGGCATCCCATCCAACATGCTCTACTCACCGACGCACTGCTAAGCC

TCCCTGAGGTACAAGCTTCTGGG

>c3261_g1_i1 len=365 path=[343:0-74 418:75-364]

GGAAGGGCTATTGGACAAATCCATGCGACTACCCTTCATAAAGGAGTTTACTATGCGTTA

TAGCAACACAATCCTGTTTACTGTTTGGTGGCTGGCTGTATGTGTTACTTTTGGAAATCG

TTTTTATTCTAGAAGATTGAAATATGCCAAGAAAATGAGGCTGACTGTTTTGCAGTGAGC

TATTAACGTAAATGTAGTGCATGCGTACAATGAATGCTAGTCAAGTGATACATTTGTTGT

CTCGTGTGTGTAATTGCCTTCGCAGGCTAAAGCTAAAGAATACTGAATAGATTGCAGTTT

TTTGTGCTCCTAAGCAGCTTTTAGCACAGACGATGTTGGTATTGGTTAAAGGAGCAGATA

TACGA

>c3271_g1_i1 len=271 path=[383:0-270]

AGAGGCGATGTTTGGGGAGCCCGAATTTGTCGGCGCAGGCGGTCAGGAAGGTGCGGATGT

TCCTCAGGCACAGGAACTGTGACATTTGGGGACGGGGGCAGATGTCCCGGGGGGGCACAG

CGTTGGGCAGGAGGGCGTTGAGGAGGTGACAGAGGAGGACCCCATCCCTCAGCGCCTGCG

CCAGGTCCCACACCTGTCCCGTGGGGCTGCTGGCACGGTGACCCGCCGGGAGCACTCGTG

CTGCCACCAACCAATGGGCGCACTGCCTCCA

>c3274_g1_i1 len=249 path=[1:0-156 500:157-248]

AATAAAATAAAAAAATAAAATAAACAAATATATATAAAGTGAATAATTAATTAAGATTAA

AAAAGGAAAAAAATAGTTAAATAAATAACAATTTATGTTACGTGTATAAATATATATATA

GACATATACTATATATTTTAATTAGTAGAATATTTCAAAAAAAATATTTTCCTTAAAATC

TAAATAATAACATGTAAAAATTTATAAAATTTAAACTTATATATATACCTTGCAAAAAAA

AAAAAAAAA

>c3278_g1_i1 len=327 path=[1:0-206 1031:207-207 590:208-326]

CTTACCCATGTGGATGACAACATCAGAGTGAATCTCAGAGCTCTGACTAAGGCTGCTTTG

GAGGACTCCACTGGAGAAGAATGGATATAGATTCTTGCAGTCTTTTCTTGTACCTCAGCC

ACAAACGATTATTCCTTTGTAGTTTTCCAGCATCTAGTATTTCTATTTCTCAAATACTCC

CTCTCTGCAGCATTTCATTCTAATCCTTGTTTTAAGGAATGTTTTCAATTTGCTAAATAA

ATTATAAAAGGAAGGTTGCAAACGGCTGATTTCATTTGCAGTGACACAAATCCCAAGCAG

ATTTTTCAGTAGTGTTACAAACTATAT

>c3278_g1_i2 len=326 path=[1:0-206 1031:207-207 209:208-325]

CTTACCCATGTGGATGACAACATCAGAGTGAATCTCAGAGCTCTGACTAAGGCTGCTTTG

GAGGACTCCACTGGAGAAGAATGGATATAGATTCTTGCAGTCTTTTCTTGTACCTCAGCC

ACAAACGATTATTCCTTTGTAGTTTTCCAGCATCTAGTATTTCTATTTCTCAAATACTCC

CTCTCTGCAGCATTTCATTCTAATCCTTTTTTAATGTTGCCCTACTTTTCTGTCTTTTAG

CTTGGCTAATTTCAGGGAGAAAGACAGACAACTGATGCCAAGGAAGCCTGCAAATCACTT

TATATAGTTTGTAACACTACTGAAAA

>c3278_g1_i3 len=215 path=[494:0-94 1031:95-95 590:96-214]

CCTATCCTCACAAGACAATGCCTTCAGTAGTTTAGTTTATGATCTTTCCTGGGATTTCTC

TGTGGCAGTGGCATGGCTCTGATTGTCTCTGTGGGTGTTTTAAGGAATGTTTTCAATTTG

CTAAATAAATTATAAAAGGAAGGTTGCAAACGGCTGATTTCATTTGCAGTGACACAAATC

CCAAGCAGATTTTTCAGTAGTGTTACAAACTATAT

>c3282_g1_i1 len=213 path=[191:0-58 250:59-212]

AAGTAATACTGATACTTGTATTGATACAAGTAATTCATCAGCTTTATGAGAAATTTTAGT

ATGTTTTTTGCATAGTGTGTTCCTGTGCCTGGAATAGAGAGCAGTCATCTCACTATCATT

GCAGTCCAGAACCAATACAATGGGAGAGAGGAGAGTGGAGATTGAAGTGCTCTTGTATTT

CAGGTTTCCCTTTGATTTCCTTAAGTTTTGCAA

>c3287_g1_i1 len=242 path=[374:0-241]

TCTGTCTGAAAGACAAAAAAAATTGATGAGTCTCAGGAATTAAGGTGGAAACAGAGCCTC

ACAGAATCGTTCTCCCCTAAACTTAACCACTGATGTTTTTTTTCAATGTATCATCACTCT

CCTTTTAAAGGCTTTGCCACTTTTCTTGCTGCATCGCATGTTAAATATTTTCCCTTTGTC

CTAAGTTCTTGTGTGGCCACTTCTGGAATACTGTGTTCAGTTCTGGATTCTTCAGGAAAG

GG

>c3293_g1_i1 len=210 path=[188:0-209]

ATTATTGTGTGTGTTTTTAGCCAACCTTGCATCTAAGTCTTTTTAGCTTTGTAATCAGTG

CTTTGACTGAAAACTCTTTTGGAGTTTTGAATGTTAGGAGAACTTTTTTTTTTCCTTCAG

CCACTCTGTAAAGCTTTTTTTTCCAAAGAGTTCCAGCTTTTTCATAGGATTTGAACTACC

TCACCAGTTCATTGTATTTATTTCTTCATG

>c3320_g1_i1 len=394 path=[51:0-188 304:189-228 280:229-374 627:375-393]

GAATAGGACCAGCTAGTCTTAGTCAAGCAAAGTGCACGTTTTCAATAAAGCAGCGCCGTT

CTTCCAGTGCCTATAGCTCAAACCTCAAAAAGAAATTGATGAAATAAATACTTGAAACTG

TTTCTAACTTTCATAATGCTATCTGGGGAGTTGGTATTTTCCAAGCTATATAAGTTGGAA

AAGAACAAGCTTGCATTCCTATTTCCTAGCTTTTGTCATATGTACAATACTTGTTCTTTT

CCAACTTATATAGCTTGGAAAATACCAACTCCCCAGATAGCATTATGAAAGTTAGAAACA

GTTTCAAGTATTTATTTCATCAATTTCTTTTTGAGGTTTGAGCTATAGGCACTGGAAGAA

CGGCGCTGCTTTATTGAAAACGTGCACTTTGCTT

>c3336_g1_i1 len=219 path=[1:0-218]

AATTCTCCCCATGGGAAATAAGAAATATTATAGTGGTTGACAGAGAAGGAAGGGGAAGAA

GACACTTTCCTTGAATATTAACTTTTAACCTTAAAATGCATCTGTCTTTGGCCTCCAGAT

TGAATGAGGGATGCAAAGCCCTGCACCTTGGCCTGCGGACATAGCAAAGCACAGGCAAGG

CTGCTGCAGCCTTTGGGGAGCAGGCAGAAATGAAAGCCA

>c3342_g1_i1 len=367 path=[237:0-366]

TCTGATGAACCAAATAGTGATAACTTTAGAATTTACTGAAATAAAAGCAAGAAAAACAAT

ATCAGATTATCATTAGATCATAATAAATGTAAATGTGAGAAAACTTAATTATTTTTACAA

ATATAGTTTATTTGTATCAGTACCATGAATTTTGAATCAAAAATAATAGATTTATTATCA

AGAAAGAATATATGAGTTTCCTAAACCAATGAAATGTCTTTAGAAGATTATTTTAATTGC

TTTATAAATTTTGATAATAAAAATAAAGCTATTAAAATTACTGGGAAAAATGGCATATAC

TTGAGCTATATTATATTTTCTAATATAATGAATTAAGTAAAATTTTTAATTATTGATAAA

ATAACCA

>c3353_g1_i1 len=285 path=[1:0-259 309:260-284]

CACCTCACAAACTAACCTCCCGTAACAGGTTCCTAGTGTTAGTACAGACTTTCAAAGTAC

TTTCACATCTGTCAAAGTAATGAATTTAGCTGGGTCTTTAGATGTTTTTCATTGTTAATT

CTTAAAACCTGTAGCTTATTAGAAGAAGAGATGCTTCAATGTCTATTATTGAAGCTTCTG

CAACTTGCATTTTGTTGTGCTGCAAAAGTGGAGTGATACTCTTAATAAAATAGACAAACA

GCTCTCGATGCAAATAGACATTGAAATAGTGTTACTACTGTAAGT

>c3365_g1_i1 len=255 path=[5:0-254]

AATCTACATCATAAAAGAATTAGATAAAATTAATGAATTTTATTTATTATTAAGAAAATA

TATTACCATAATAATAATAACTTCTAACAAAAATATATCGTAAAATATTATTAAAACTGA

AAGTAAAAGAGTAATAAAAAAAAATAGATATAGTAATAGAAATAAAGTAAATATTTATTT

GTAACTAATGTAAAGAAAAGTTCACATTTATAATAATACTAAAAAGGATAAATTATTTCA

ATTTTTTAATAATTA

>c3367_g1_i1 len=317 path=[1:0-100 102:101-316]

AGTAGAATCTCTTGTAAAATGAATACTTTACCTAGAGATGTACAGTGATACTACTAGATG

TGAACAATCATAAGAAAGTAAGAACAGGTATATTTGAAACTGTGTAGAATTCCGATGCCT

CCAGTAGTGGCTTGAAACTCTGTTCCTGCATTTTTCAACTGTGAGAGAGGTGGAGGACTT

GCAAAATAAGGTTGCTAAGGATTAAAATGGAAACAAAACCATTAGATTTTGTTTGCTGTT

AACACTTTGTTGCATTTTTGGTAGTAAGCATTTGATTGAGTAAATCCTGTATTACAAAGT

TCAGATGATGAAGGTAT

>c3368_g1_i1 len=237 path=[215:0-236]

TTTTTTTTTTGTATGTGTATTCTCATATAAAAAGACAAACTATTATTTTTTTAAAAGTTT

ATGTGTATTAATCTTGTTAATATTATGTTGTTTATATTTATTTAAAATATTTATAAACAT

AAATATTCTTCTAACAGTTATTCATTGTACTATTTTTATATCTATATATGCTCATAACTA

AATACATCTCATTAAATAACATATAATTCCAATGAAAATAAATAATAAAAAAAAAAA

>c3378_g1_i1 len=272 path=[1:0-271]

ATCAAAAGGATCTGGGATAGCAGAGATGCAGGGTCTGTCTGCTGAATGTTTCTCTTCTGT

ATGCTTTCAAATCATTGGTGACGTGTGGACGACCTGAACAGTGATCCTAGATGACACAGT

GAATCTCAGTCCATGCTTTGTGCCCACTGGATGCACTGGAAGATGTCTGTGCTCATGAGG

ATGGTTGGCAACCAACAGCACCAGACTGCAGCAGATTGTCTGCACTTCAGAAGTGTCCTC

CTCGCTGAGCAGCTTGTTTTAAAAGATGTTTG

>c3382_g1_i1 len=302 path=[379:0-251 100:252-301]

TTTTTTTTTTCTTTTAATTTATATTACTTTCTTCAATGAATATGCTTTTTAATATAACAG

TACTATAAGTTTGTGTTTTTTTTTTTTAAAAAACATGTGATTAAAATAATAAATTGAAAG

AAAAATATTTGAAAAAAATTATCATTAAGAAAAACCATTTTTAATAATATTCCTAATATT

TAATTAAACAACTAAAAAAAAAAAGACAATAATATTGAATGCTATAATTTAATTAAATTA

ATTTGTTCATTTTAGGTTATTCAAAATTATATAAATTGTCTCAATAATGGTTATTCAAAA

TT

>c3385_g1_i1 len=218 path=[255:0-157 25:158-217]

TGCTTTTTATAATTTAGGAAACCTACTCACAAAGCATTTACCTTCATTTACAGGTAACAG

CTGCTGCTTGAAAATCAAACTGCTAGAGCATGTTGAAACAGGAAGTTCAAGAATATTTAT

TGTAATGAAGATGGCAATATTCAAGAAACCACTTTCATGTTTAAGTCTCCCCTCGTCAAA

TTTGTAGGCAATCCAGTAATATTTCTTCCAAGTAAAAC

>c3385_g1_i2 len=218 path=[255:0-157 412:158-217]

TGCTTTTTATAATTTAGGAAACCTACTCACAAAGCATTTACCTTCATTTACAGGTAACAG

CTGCTGCTTGAAAATCAAACTGCTAGAGCATGTTGAAACAGGAAGTTCAAGAATATTTAT

TGTAATGAAGATGGCAATATTCAAGAAACCACTTTCATATTTTTACACTCAATGTGCAGT

ACAAACTGATGGTGTTTTACTTGGAAGAAATATTACTG

>c3386_g1_i1 len=228 path=[285:0-227]

GAGTTCCACTCACCTGGAATGTTTCCACTCCGAAGCATTGCAAAGCACCGTGGCTTTGTT

GTGTAACACCTTTGGTCTCAAACACCACCTTTAACAACAAGCTCCAAGAGGAAGTATGGG

ACACGATAAGAGGCGTGCTGAGCCCGAGGGCTGCCCAGCACCGCTGGGGTTGTGCTGCTG

GCTGTGGCTGCAGCCTCTGCCCTCTCCATTCCCCGCTCCAACCCGAAG

>c3389_g1_i1 len=202 path=[1:0-139 141:140-201]

GTCTGACTCCAATGCCCTCAACACCCACAAACACACAACAAGCTAAGGAGATTAGAGAGC

ACGCCATGGCTGCGTTGCAAAATCAACCTTGGAAAGCAAGAGCTGCACTTTAGGTATAAG

AGTGCAGAATGGATGGTGAGGAAGGACTGAGAGCACAGGAACCCAAAGAAAACCTCCAGA

GCACACCTTGGGCACAGCTTGG

>c3389_g1_i2 len=224 path=[1:0-139 382:140-223]

GTCTGACTCCAATGCCCTCAACACCCACAAACACACAACAAGCTAAGGAGATTAGAGAGC

ACGCCATGGCTGCGTTGCAAAATCAACCTTGGAAAGCAAGAGCTGCACTTTAGGTATAAG

AGTGCAGAATGGATGGTGAGTCTGCTCGTGAGTCGAACAGGAAACAATAAAACAGCCTCC

AAAAATAGCTCTGGTTTCCCGTCCTCTGCGCCGTGGCATACGGG

>c3397_g1_i1 len=261 path=[239:0-260]

GGTAATTTAACCACTTAACCTCCTGCAGTATCACCAGTACAGCCCATTCCTTCATAGTGA

AGAGTGTTTAGCCTGGAATTCTTTGCTTGATGGAATTTGTCCAAACAAATGAAGTTCTTG

GTGAGGTTGGTGGTGGTGAGGACAAAGGTCTTTCACTCCAAAAACGATTTCTGCATCTTT

GTGAAGTAGCAAAGGGCTGACAGCTGTGCACTTTGGGTTGTGTTTTGCCCTTTGTATGGG

CAGAAGAGGCACTGTTATAAG

>c3398_g1_i1 len=239 path=[1:0-238]

AGGAAAAGTAAAACATCACTGAGTTCTTTACAAAGATGGAATAAAATTAATGGCTGTTTC

TGAAAGGTTGATACTGCAATGAGAGTGCAGATGTAGATATCATCAGGCGCTCTGAGCAAC

ATGGACATCAGAGCTCTGTCACTCTGTCCACTGAAAGCAGATTAAAATTTGAGATCCTGA

AGTATTGATCCATGTTGTTTCTTTTGTAGATTCTTTCTCTTTTCTTGATTAATTTTACG

>c3409_g1_i1 len=286 path=[264:0-138 583:139-139 584:140-285]

AAAAATCAAACACAAAAAAAAAAATCAAACACATCCAGAGCTGAGAGGGGTCACTGGCAC

AGCTGGTGTAGGGAAGGCATTTGGGGCTACAGCATGTTCCAACATCCCAGTGAGTGCCAG

AGATGCAGGAACAGTGCATGGGCTGCAGTATATCAGCTCCTTGCGTTGTGATGGATCTCC

ATCACTGCCCTGGGATGGCTGCATGGTATGAAGGAGGTGGGGACAGCATCGGCTGATGGA

TTCTCTTCTTCCTCCTTCACCAGCTCCTGGGGGACATCCTGATCAT

>c3410_g1_i1 len=213 path=[191:0-27 219:28-212]

ACAACTGGATGAGCAAACAAGAAGTGAGTGCGTGTATTTGAACTCTCCAGTTTGATTTTC

ATTAATGCCTGGCCTTCTGTAGTCACAAACCATTTTTAATTACTAGTGGCAGTGACTGAC

TACCACTGGACATGCACAAGGAGTAAGGTGCTGTGCAGTTGGTTACATGGCTGCCAAAGA

TGCTACGATGACAGTCAAACCAAAACCACAAAA

>c3415_g1_i1 len=342 path=[37:0-106 374:107-341]

GTACACACAACATCAGGCTGCTGCTCACAAGCATTAAATGCACTGAGGATGCAACAGAGA

GGAGGCCTGGCAGTGATGTGCAGGCATGACATAAGCTTGGAGTTAAACAGCAGCACAAGT

AGACTTTTCTGTAGCCTGTTAACAACACTAATCTTCTGTCTCAGACAGCAAATGGACAAA

ATAAGGGTATTCAGTTCCTGTTCTAAGATTCATCATGTTTTGCCTGTTAAAGCAGGCGCT

GCCAGCAGATCCAATGCCCAGTTCAGTTCCTAAACTCAGAACTTGTTTGAGAGCCTCTCT

ATTGCATCCACACATTCTGTAGATCCCTAATGAAGCAGAATT

>c3415_g1_i2 len=252 path=[372:0-16 374:17-251]

CAGACTGCCTCTGCAGGCAGCAGCACAAGTAGACTTTTCTGTAGCCTGTTAACAACACTA

ATCTTCTGTCTCAGACAGCAAATGGACAAAATAAGGGTATTCAGTTCCTGTTCTAAGATT

CATCATGTTTTGCCTGTTAAAGCAGGCGCTGCCAGCAGATCCAATGCCCAGTTCAGTTCC

TAAACTCAGAACTTGTTTGAGAGCCTCTCTATTGCATCCACACATTCTGTAGATCCCTAA

TGAAGCAGAATT

>c3422_g1_i1 len=420 path=[1:0-156 1016:157-187 261:188-419]

GGTTTAACTGTTGACAGTGGAAGGAGAAAGACATCTTCAGTCTCCTTACAGCAATGCCAA

AGAGTCTGTTCCTGATGCTGTGTAGAAAAGATTTCTTTTTAGGGAACAGTAACGTTGGTA

GGGCTTAGTCAATAGCTCATTTAAGGAAAATATATGTTGGTACTTCAGCTGACAAGAAGG

AGTGAGCAAGAGATAGTGATTGATCTACATGGATATTTCCTTTCCTTTTGAAAAGAGAAG

TGTTTCCATCAGATCACTTCTGGAGTGAAGTCTAAAACTAAGAAATCTCAGACTTGAATT

GTGGTGCTGCACAAAGCTGAAGACTGTAAGCCCTGCCACTGGTTTTCCTAGCAGTAGAAA

GTTGCAGTGAGTTCTTCTGGAGTGTTTGCCTTTTCCAGAATCATAGAATCACAGAACGGT

>c3422_g1_i2 len=492 path=[1:0-156 158:157-259 261:260-491]

GGTTTAACTGTTGACAGTGGAAGGAGAAAGACATCTTCAGTCTCCTTACAGCAATGCCAA

AGAGTCTGTTCCTGATGCTGTGTAGAAAAGATTTCTTTTTAGGGAACAGTAACGTTGGTA

GGGCTTAGTCAATAGCTCATTTAAGGAAAATATATGTGCATGCAGCTTGTTCTTTTTCCC

TGCCGGATACACCAGTATGTGGCAGTGGATGTGCAGACAGCTGCATGAAGGGACAGATGA

ACTTTGTCTGAAAGTGCTTCAGAGATAGTGATTGATCTACATGGATATTTCCTTTCCTTT

TGAAAAGAGAAGTGTTTCCATCAGATCACTTCTGGAGTGAAGTCTAAAACTAAGAAATCT

CAGACTTGAATTGTGGTGCTGCACAAAGCTGAAGACTGTAAGCCCTGCCACTGGTTTTCC

TAGCAGTAGAAAGTTGCAGTGAGTTCTTCTGGAGTGTTTGCCTTTTCCAGAATCATAGAA

TCACAGAACGGT

>c3429_g1_i1 len=210 path=[1:0-71 73:72-86 88:87-209]

GAGTCTTTCATTTTGATTTAAGAATAACCAACATTGGTTTTACTAGGTAGCAGTCACAGA

AGACTGCAGAAGGCAGGACAGTCCTGAAACTGAGGCTTTTAGTCATGAAGAATTCTCCAG

AATAACACACCTGGTAGCTTTTTTTAATGTGGTTTTGGTTTGGGATTTTGTTATAGCTTT

TAGGATTTGTTTTTTCATGTTGTTTTTGAA

>c3431_g1_i1 len=280 path=[277:0-240 576:241-279]

TAAAAAAACAAACACTTTATAACATCTGACCATGCATTTAAGTGTGGCAAGTTGGACTCC

ATTATGCAATATTCTTTGACAGCCTCTTTATGACAGGAGTCCTGTGCTTCCTAATATTTA

AACATCCAAATGTCTGCTTTGATTTTCTTTTATTAGTGCAAATCAAGGAAAAATATTAAC

CCCTGCATAGTTAAAACTCTGTATGATCATCTGCCTCTGGCTACGTTGCTATTTTTTTTA

AGACTGGTTTGCTGCTTTGTTCCTGTTTGTTAGGTCTCAA

>c3431_g1_i2 len=299 path=[277:0-240 518:241-298]

TAAAAAAACAAACACTTTATAACATCTGACCATGCATTTAAGTGTGGCAAGTTGGACTCC

ATTATGCAATATTCTTTGACAGCCTCTTTATGACAGGAGTCCTGTGCTTCCTAATATTTA

AACATCCAAATGTCTGCTTTGATTTTCTTTTATTAGTGCAAATCAAGGAAAAATATTAAC

CCCTGCATAGTTAAAACTCTGTATGATCATCTGCCTCTGGCTACGTTGCTATTTTTTTTA

ACATAGCATTTTCTTCTGCTGTGTGAATAAAATTCATCACAACTGTAGTTACATGTTTA

>c3439_g1_i1 len=308 path=[1:0-307]

TTACCACAGAACTTGGGAGACCGTTTTGTTTCTTCTGCTGCTGCCTGATGCAAAACAAGT

GGAGGAACAGAAGCCATTTGCTGCAGTGACTTCTGTTTGTTTCTTTACTGCAGTGGAGGT

TTGCGTGTAGGAAAGGAGGTATCCCAGGTGAAGACAAGAATTTAGACTGGCTTTATTACC

TTGCCTATTAGCTGAGAGATGACTTGGGAAATCTGCAGTGTCCCCAGGGAAGCAGTCCAC

CTAGGTTACTTTGTGATGCAACTGTCCTCTCTCAAAGAGATGCGTTTAGGATAGGTGTCC

TTTTGGAA

>c3458_g1_i1 len=303 path=[33:0-215 16:216-302]

TCATGCTTTGTACGTAACCAGAGGTCTGTCGTGGTTGCACTGATGTGTTTTGTTCCAAGC

ATAACACACCCAAGGAGATGGACAGAGGTGATGTGAGGAAGCTGTGGGTGCCGGTGTACA

GGAGCTGTCTGATTTAGCTTGGGGCTATACAGCACAATTCCTTAGTTATACAGGATGAAC

TCTTTTGGGAAAGAAGAAGGGTAGAAGGCTTGGGAGTGATCCGAGGAAAAGAAATGCAGC

AGAAGTCGGTTCCCTGATCGAGAATTGACAGTATATATTTGTGAGAAAGCGTTGTTGAAT

TTG

>c3459_g1_i1 len=664 path=[58:0-394 38:395-663]

AAGAGAAAAAAAAAAATTATATAAAATTAATGTTTATGATGAAAAATCAGTTAAAACATA

TTTAATAAAAAATAATATAAGTTTTAAGGAAACCGTTAATCAAATTTATGCATTTCATAA

AAATAAATATCAATTAATATTTCATGTTATATTTAGTAGATTTAAAAATATATTTTTTCT

TATTTTTCTAATTTTGTGCCTACTGCAGCAAATAAGAATTATTAGAACAAGTAATTATGT

ATATTTTGTTATTAGTTTTTACTTTTTATTTATCTTCTGCATATTAAAAGACATTCATCT

AATTATACAAAAAATAAAAAATGAAAACAACATAAATAATAAAAATTATAAAAGGGTAAC

TCATGTTGGACTAGTAGAAGTCAAATATTCCAATATAAAAGCTGTGATAATGAAAAAAAT

ATTTTTATTTCCTCAAAAAATTTTGATGGAAATACAGATTTAAAATTAAAAAAATGTTTA

AAAATAACAAGAAATTTACCAAATATATATGATATTTTTAGATTAAAAATAAAAATAATA

ATTGAAAAGCCACATATATACTCTAATAAAATGAATGGATTATTTATATTACTAAACTAC

TGTGAATTTGTTCAAAATATTTATAATGATTTAATATTATTACAAAATATTTTTTATAAA

AATG

>c3459_g1_i2 len=728 path=[58:0-394 452:395-458 38:459-727]

AAGAGAAAAAAAAAAATTATATAAAATTAATGTTTATGATGAAAAATCAGTTAAAACATA

TTTAATAAAAAATAATATAAGTTTTAAGGAAACCGTTAATCAAATTTATGCATTTCATAA

AAATAAATATCAATTAATATTTCATGTTATATTTAGTAGATTTAAAAATATATTTTTTCT

TATTTTTCTAATTTTGTGCCTACTGCAGCAAATAAGAATTATTAGAACAAGTAATTATGT

ATATTTTGTTATTAGTTTTTACTTTTTATTTATCTTCTGCATATTAAAAGACATTCATCT

AATTATACAAAAAATAAAAAATGAAAACAACATAAATAATAAAAATTATAAAAGGGTAAC

TCATGTTGGACTAGTAGAAGTCAAATATTCCAATATAAAAGTAGGAGATATCATATATCT

AGAAGAAAATGAAGAAAGTCCTGTTGATCTAATTTTACTTAAAAGCTGTGATAATGAAAA

AAATATTTTTATTTCCTCAAAAAATTTTGATGGAAATACAGATTTAAAATTAAAAAAATG

TTTAAAAATAACAAGAAATTTACCAAATATATATGATATTTTTAGATTAAAAATAAAAAT

AATAATTGAAAAGCCACATATATACTCTAATAAAATGAATGGATTATTTATATTACTAAA

CTACTGTGAATTTGTTCAAAATATTTATAATGATTTAATATTATTACAAAATATTTTTTA

TAAAAATG

>c3473_g1_i1 len=252 path=[372:0-251]

TGGTGCCTGGACCAGCTGGAGACCATCCAGACGTACCGCTCCGTCAGCGAGATGGCCTCC

AACAAGTTCAAGAGGATGCTGAACCGCGAGCTGACGCACCTCTCCGAGATGAGCCGCTCC

GGCAACCAGGTGTCCGAGTACATCTCCAACACCTTCCTGGACAAGCAGAATGATGTGGAG

ATTCCTTCTCCCACACAGAAAGACAGAGAGAAGAAGAAAAAGCAGCAGCTCATGACCCAG

ATCAGCGGAGTG

>c3474_g1_i1 len=244 path=[155:0-243]

GAAGATTTTCTAAGGAGGCAGGAAAAATGAAAGGTAAACCAAACAGTTTAATTTAACAAG

CTTAGCTAGCAGCAGTACGTCTCTGAATAAGAATTAATTGCTGGGTACGCATCAGAAAAA

CAAGTTATTTTAAAATTAGATTTTGGCCAATTAGGCTCTTAAAAATTATCTTTCTAACTC

ATATGCTAAAAATGCAGGTCACTTTATATTTTGAAGGCACAACATCTCAAGGACGTGCAG

GTTG

>c3479_g1_i1 len=917 path=[895:0-916]

TGATTGGGTCCTAACAGCTTTTAGCTGTCTTATTGGAGATAAATATCACAAGGCTTAAGC

CCATACACTATTCAGATAATTAAATCAATTTCCAAATATTCAAGAGACAAATTAAGTGGT

AAAATTGATTAAAAAAAAACAACCAAAAAATAAATACCTTCTTAAATAAAAAGAGGCATT

ATGTTGGATCACACAATGTGACTCACCACATGACCATGGGCACAGCATCCAACTTCCCTG

TGCATTCAGTAATACATCTGCAGTAGTGTAACAGCGTTACCTCAGTGCTGAGCTACATCT

CATTTCAGTCACGAATGAATAATCGACGTGTGCCTGTGCTTCAGGAACAGTCATATCCAT

GTAGAAATTACTATACCACACCAACTCTACTCATGCTGATCAGACACGTGAAGAGGGAAT

TAAGTACAAACAAGCAACCACAAATTTAAATTCTAACTCTTCACAGTAACTGAGTGCCAC

TGTATCAGTCACTCCAGTCTAGTTCAGAGATAGACTTATGATAAAGCAACTGCAGAGGCT

AATATTTACATTAGATCCACATTTCTAATATCTTTCAACTTTTCTTTTTGCTCAGAATTG

CCCATTTACAGAAGCTACATTCTAGATCTCACTTTGCAGAGTCAAACACAGCTTTCTTTG

TTGCAGCAGATTCCAGTGTCACAGCAGCTCAGCTGTGTTAAAGGCACAAAAGCATTGGTT

ACCTGTCGAGCTTCAATTTGTACACTGGAAAACAGACAGATACCGTTTCCTTACCATTCT

ACACACAAAAAGTAAGACAAGTAAATAGCACAACAGCATATCACACTTCCTTGTTCCAAA

GTTCTTTCCCAACACTAAGCCTATCCCTCTTTCCCACAGCTGTTTCATTTCTGTATCTCC

ATTGCTTCCCAAGTATG

>c3486_g1_i1 len=287 path=[1:0-286]

ATTTCTGAGCCTCAGCCTTAAAGCTCTGTACTTTCAACATGCATAGTAAGTTCAAGTCAG

TCAGAATTCAGTATTGTAATCTTTGATTCCCTTCTAACAGTACTAAGTTGTAGGAATGGG

AAGGTTTGATTATTTAATTAGATATTATTTCTTATCAAAGTTGCTAGGGGACCTATGTAA

AGGCAAAACTTAATCTATGAGGGATTTTTCTAAAAGAATTTAAATTATTTTTAACTGAAC

TATTAGTCATGAAAAAAACCCCTTCTGCAAGTTACTCTCATGATTAG

>c3491_g1_i1 len=251 path=[1:0-250]

AGTTGAATCTTTGTTTGCAACTGCGTATTTTGGTGAAATGCATTTCTGGCAAAGTAAGAG

GATTCAGTGGGTTTTAAATAGAGATGTTGCATTGGTTGGTGGTGGTTGTTGTTTTTGCTT

TTCTTTAAAGATTCTGTCTCACAAAAGTGTGCACTTTGTCAAAGAGTGAAGTGATTTGAG

GGATGATAACTTCAAAGGGACTACTTTGGATCACTGGTATACACAGCTCTGATACATCAG

AAATACTCATT

>c3503_g1_i1 len=203 path=[270:0-73 50:74-91 491:92-202]

ATGGCACCCACTGACATTCACTGAAGTTTTCTGCGCATTTATGGAAACCAAACAGTGGAC

ATGAGCACACTGATGCGGTGGATGGTGCATTTCAGCAATGCTAGATCACTTCCATTGGTG

CAGATCTTTATGAGCATGCCTTGCAGGCTCTTCTTCATCACTGCTGAAGATGTATAGCCA

CTGGTGGTGACTATGTTGAAATA

>c3509_g1_i1 len=205 path=[241:0-76 25:77-81 30:82-204]

TTGTCAAATGGTGCTTAAAAAAAAAATAAGAATTTGCTTAATATTGAGTGTTAAAAATAA

ATTAAAACAATTGGGAGGATTCATATTGTAAGAATAACTTTATATTTTAACATAATAAAA

TCAAAAATTATATATACAATTTTTTTATTTAATAATTTTAATAAAAAGTAATATAAAACT

AAATGATACATCATATTTTGTTTTG

>c3514_g1_i1 len=218 path=[155:0-217]

TACATGGAGGCGAGGTGGTTATGGCCCATTTTTGCTAACACCTTTATTTATCTAAGTGTT

ACAGTAAGCAACCTTTTTAATAAGACAACTTTTACTTACAAGCTATTTTATCTTAAATAA

GTACACTTGTATTATGCTGACACAACTACAACGTTATCTTGCATTGCTTTTCCAGCAAGT

ACAGAAAAATTATTTCTCACTGAAGTAGCTGTTTTTAA

>c3518_g1_i1 len=209 path=[1:0-96 98:97-208]

CTGTAAGCAGCTCCCTTAGTTGCACTAGGACTTTGGCATGGGAGCAAGTTGAAAGAGACT

TGTGTAAGTTGCTCTTATGAACATTTAGAGAGTATAAAAGATAATAAACTTGCAGCTAGA

TTCACCTTTGTAGTCCAGCCCTATGGACATCAGGCATTTTTTACTGGGGTCATAATTTCT

TGCAAGGGATTGTACCAGTGGGTGTACAG

>c3522_g1_i1 len=246 path=[259:0-86 18:87-245]

GGGCAGCATGATTTTGTGTTTGATGAATATGAAAAGTTCCCCGATCATATTGTAAATGTG

GAAACATGCTTTTCCTCAGTGGGGGACGAAAGGGTGTTAAGTTCTGCAAAATCCATGGTC

CTACTGAAGTCCTAGCAGCGCGGAGCAGTTGAGGTGAGAATTTGCGTGCTGATCCACTGT

GCACTTGTTGGCTCACTTCAGATGTCTGGTGCTCTTTGTTAGGATGTTGTCTTAGAGCTG

GAGTTT

>c3523_g1_i1 len=203 path=[226:0-73 23:74-202]

AAAAGAAGTCAAAGTTATGCAGTGTAAGGCTGAAAGTGCTCCAGATGTCATTGGGCCATC

GTATAGCTGTCTTTTTACACTGAAGGAGGGCAGGATGTGGGGCGGACAGATGTCAGGATC

CAAATAGTTTGTCATCACGTTGATATAAGACAATCAACATCTTGAAGGCTGTAATAGCCT

TCTATTTTATGGAAGCTATATTT

>c3525_g1_i1 len=301 path=[279:0-300]

ATTGGTACTGTGTGTCTGGCCACCTGCATCCTTAGAGACAGCTGCTCACAAGTCTGCATT

GTCCTTTGCTTTCAGGAGATTGAAAACGGTCCACAAGAGACAAATAGCACTATAACGTAT

GGATGACTTGAAGACCTAGAGTGACACAGTCAGGAAGAGGAAGGGGAGGAGAAGTTGCTC

AGATTGTGGAGTTCAGGGGTGCTGCAGCATAGAAAGGGTGGAGTACAGGCATTTGCCTGC

AACAACTACTGTGCAAGGACTGATCACTACTGGGTGAGACCCCAAGTTGGGAATAAGCAA

G

>c3528_g1_i1 len=207 path=[185:0-206]

GCCTTTATTTATCCAAATAAAAGTAAACTAGAAGAGCTAAGCATGGGAATTGCCTGTAAT

TGCCTGCTGAGCTACTAGAAATAGCTGCTCTTGGGAAACAGGAGAAGCCTTCACCATGTT

TAGGAAATACAGTATCAGCTATCTCCTGTTAGCAAAGTTTGCCATCCTGCTTGATCCACA

AATGACTTTATTTCTGAAAAGAGAGGG

>c3540_g1_i1 len=232 path=[492:0-101 593:102-231]

TCACCTGGATTCTTCCTACTTAAATACTGATTTTTTATTTCACTCATTATCAGACATTTT

CCCATATCCTTCTTCATTAATAACGACTAACAGGAAAGGGCTTATTTCTAAGAATGTGAT

GTCTCTTTCCCTTTTGATATCCCTGTAGGATAGTGGTAGCAATGGCCGTTATGCAGCAGC

ACTTTTGTGGGTTGTGCTTTTGACTTTGATGAGCCTAATTTTATGCACAAAC

>c3540_g1_i2 len=231 path=[492:0-101 179:102-230]

TCACCTGGATTCTTCCTACTTAAATACTGATTTTTTATTTCACTCATTATCAGACATTTT

CCCATATCCTTCTTCATTAATAACGACTAACAGGAAAGGGCTCATCACTGCTTGGAAATG

GAAATCACTTGGAAATCACTGACTTGGAAAATGAAGCACTGTTTTGCACAGCACTTCATC

ACCAGGCATTTTTGTAGGAGCCTTGTGTTTGTGCATAAAATTAGGCTCATC

>c3542_g1_i1 len=211 path=[1:0-210]

GCTACGCAGCCGTGAAAAGACAAACTGAACAGTAAACATGATTAGAAAAAGAGAAACGCA

GCCAAAGTGCAAACCCACCGTTACCTCCAGAACAGCACCATGCACTTACAGTCCAACCAT

TTCTAAAGAAAGGATATTTGAGAACAGGGGGCACATGCAAGTCAAAGCACGGAAAACACT

GTACACAGCAAGGACAGTTTATTCTCTTGCA

>c3543_g1_i1 len=262 path=[155:0-261]

TAAGAATACAAGCTGTGTAATTAGGGAAATACCATTTAATATATTCATATTATGTTATAT

TCCTTGACATTTAAAACCCCTTAAAATTTAATTATTATAAATGAGTTAAAAATACTAAAT

GTAATGTGTTACTCTATCTTTATATTTTTTTTTATAGTAATGAATTAAAATTTTTACTTT

ATGTGAATCATATACAAAGAAGAAATAATAATACATAATTAAATTGAATAAAAAAATATT

TTTGCAAAGATGACATTTCCTG

>c3547_g1_i1 len=314 path=[231:0-313]

GTTCTTAAATCTGGTTTCTCTTTTGATACTCTGAAGTCAGGTTATTTGGCACGTGTAACT

GCTGTGGATAAGAGGTGTTTATTTAAAAAAAAATAAATTGTATATGTGGCAGCAGCAGGG

GAGATGTCTGTTCTGAAAACATCTGAGGTGCTGGGAAGCGTCTCCAGTGGGCAAGAGAAA

TTGCATAGCTAGCACTGAGGCTGACTCCTTGTGCATGTGTGGCCATGTTTAGAAAAGCAT

CAACAGTAGGAGCTGAATTGCTAGGGAAGTGAGTGGCTAAAGGATTGGAAGTAAAGGGAA

TCAAACACAAAGTT

>c3550_g1_i1 len=219 path=[197:0-218]

GTTCTCACCAAACACCACTCTCAAGGGTGGGATTCCTCTCACCTACTTTAAATTCTTCGC

TGTTACAGCGTTTAAGAACAAATTTCTCCATTTGACAGTTGTCACCTCAGAGTTGTTTAT

TAAACCACTACTCTCCAAGACTGTACTAGCAGCAAAGCTGCCAAAGAGCAACATTTCTTT

CCTCCAACACGTACGCTGAGCAATCCAGACTGCTCCAGA

>c3551_g1_i1 len=213 path=[229:0-212]

TGCATATTTCTGTCACTGCTTGCTGTGCTTCCATTTCAGCTTTAACAAAATTCATGCGAG

CCAAACCAAAACACTGCTTCAGGAGCAGGAAGACACATTGAGATATGTGCACAAGACTTC

CGCAGCATCGCTTCTAAATGAAGTTTGCTGCTAGTCCATGACATAAAAATCTACTGCTAG

ATGCTACAGAGGAGGCTGTTCACGGATGCCCAG

>c3552_g1_i1 len=245 path=[1:0-244]

GAGAGGGATCCCAGGCAGTGCCCACAGCTCTCCCCTCCTCCCAGAATTAGGCCAAAAACC

GGCCAGAGCGCTAAAAGGGGAAGGATGGAGCTTTCAGCTCGGGATATACGTAATCACCGC

ATGGAGAGAGAACCGGCGTTCCCTTAACAGCAGATCGAAGTTACACTGTAACCTGCTTAT

CTGTACTCAGTCACTTAGGAAGGGGCAGCTGAACAGCGTGGATGGCACGTTGCAGCCTTG

CAGGC

>c3554_g1_i1 len=231 path=[1:0-104 106:105-230]

TGGCAGTCCTTTTAAACTGATATTCAGGAAGTAATGATTTGCAGTGAGTAATCAGTATGT

GTTTACAACAGTATGCATGTTGGAGCTAAAACTGTTAATGGAAATAAAAAGCCACTTGTG

AAAGCGATTCTCCTACTTTCTCACAATTACTCTGCTTTAGCTATGGCTTTAATACAATGA

CTAACAGTAAATGAAATTTTCAGTCTATTACTAGGTAGCAAGTGCAGATCA

>c3555_g1_i1 len=252 path=[1:0-251]

ATTGCATCTAACTATCCTACAGGAACACATCCATACTGTGAAATGGGCTTAAGCCATACA

TATAATAGTTATTTTATAATGCCTTTGTAATTTTTAAGATCTTACAAATACTACATATAG

TACAACACTTCCTTTCTTAAGAAGAAAAGGAAACTAATTGATACCATTTGTTTGGCAACA

ACAAGTGCACTAAAAAGTTACACCACCATCGTGGGGGTGGGGATTCCCCAGTTCTAAGTA

TTAACATTGAAA

>c3558_g1_i1 len=313 path=[43:0-159 42:160-312]

TCGGTAAGTGACGGCTGCGGGGGTGACCTCGGTGACAAAGGGGACCGTTGGTGGTGCTCT

GCAGCTGGCCAACCCCCCGGCTCTGTGCCCTTGCAGAAGCAACAGCAGAACTGCTATCTC

TCTGGGCACACTGCTGGGATGGGGTGGTGCTGAGGCCAGCGGCTAGGCCCTGTGCTTCAC

ATGGAGGTGCCCGTAAGGCCTGGTGGCTCTGGGAGCGAGGAAACACCTTCCTGGGTGTTT

TCCTCCCTTTGTGCCTCTAAAACCTGGTGGAGCACCTGGTGACTGCGATAGGTTTCCATG

TAAAAGAATTGGG

>c3562_g1_i1 len=207 path=[1:0-206]

ACAATTCATCAAAAAAGAGCCAATCCTTCTGCTAGCGCAGGTGACTTCCTTAACTACAAG

TCCGAAATGCAGATCCACTTAAGATACCCAGTAGGTCCATCTCAGAAGTGATTGCAACAG

ACATCTTCACAGAAAAACAACAATCATCTAAATTACATTCTCTTCCTCAATGGTGAGAAC

CACTCTGCACTACTTCTAATAGATATG

>c3575_g1_i1 len=277 path=[1:0-276]

ATGCCTCGCTTCCTCTACCCAGCTCTTCTGCAATTCTTGTGCTGAACTGGAGCATGAACC

TCTGACAAATGGGACAGTGTCACCCTTCATCTGCAAATGAACAACTGTATTTGTTCTTCT

TAGGCAAATGATGCAATACCTGAGGCCGTTGGCACACTGAACTGATCTCACAGTTGACTA

ATGAGGAGCCAGAGAAGCCAAAGGTGAACATGAACCATATAATGGGCTTTGATGCCTTTG

TGTGACTTCTTATGATCAAGTAGTATTACATCAGCAG

>c3576_g1_i1 len=228 path=[265:0-156 30:157-227]

CAGCCATAAAGAGAGATGGAGTAAGTGCAGTGTTGCATCTGTACCAGAGTCTAATTGAAA

TGTGCAGCTTTCAGAGGTCCCAGGAAGGGAGGGATTACAGCCCTTTTCTTGCCATTTACA

GCACAGCTATTGATAATATTGAATCCTAACTTAAGCTTTCTTTCTCCACCTTGGGAGCTA

CGTGCCCTTCTCAGAGTGCCTTTGTGGTGCATGAGATGGTGATCCTTT

>c3608_g1_i1 len=226 path=[319:0-225]

TAAAACAAGCAGAAGAAAATAGAACCCTCTGGGATTTTAGAAGGTAGAGTATCCACCTGT

ATACACGCTGGCATCACACAGATGCTTTTCACCACATGGCAGATAATGCATTAGGGAGAA

TAATTAACTTAACTTGACCTTAACTATCTCCATCACCATTCCCTGTATGTTCCTTGTATG

TTTTTAATTTGTATTGCATGTAAAGCTGTTTATGTTTGGTCACAAG

>c3609_g1_i1 len=241 path=[563:0-240]

ATTAATTGAAACACATATTACTGAAGCTGTAACTTCCTTCCTTTGCACTGATGCAAATTC

ATAACGCTTTACCTAAACAGTGGGCAGAATCGGACTGTAAACAGCTACAGAATGTGGCAG

TAAGCAGCATTTCTGGCTGAGAGGATGGGAGAGACCTTCAGTAAAATGGGTCAATGACCT

ACCTTGGTTTGTTGGTGGAAGTTGAGGTACTTCATGTTCTTCTGGGTTGTGACAAGTATG

A

>c3610_g1_i1 len=230 path=[208:0-229]

GCCTCCCCCAAAAAACAGGTCACAGAAATATTTCACTTTTTTTCTTTACATTCAGAAATG

TCTCATTATTTCCTGGAGCCTCAGGAGACAAATGCTGGCAACACAGCAACACAAAGGCTT

ACAACAAGTTCAAAGCACATTTCCCCTTCCTGAGTCAACAACAATGGAAATACATGCTGT

TTGAAAAAAGATGCAGAAATACGTACATATTTGGCTTATACTTGTTAAAG

>c3631_g1_i1 len=228 path=[380:0-227]

AGACCTAAGTGAAGATTTTTCAGTGCACGCTACATCAGACCAAGCACCACCTGACAGTCA

CCTCTTGCAAAAGGACAATACTTGTTGGGAATGGGCAACCAATTTGGCACGTTGTCTTAC

TCTTACCTACTTAAGGAAGGCCAGAAAGCACCCTCTGACAGCCCATTCAAATTCCGAGGG

TGAGTTCTCATCTCCCTTCCTTTATGACACATCACAGACTAAGAAGAG

>c3633_g1_i1 len=228 path=[206:0-227]

GGAAGTTATTGATGGATCTAATCCTGTAATTAAGTATGATTTAATTGTACAGTTTGTACA

CGATCATCTGAATTACGCAAGCGTTTACAAACCTCAGTCGGTCTGCCTTAGAATCAAATT

TATTTTGGAAAATGTATAATTTATTGATATGTTAGGGGTTAGTAGTACTTCAGAGTACCA

TAGCATCAATGCAAGGATAACATAGCTATTATTTTTTCACATAGCTAT

>c3634_g1_i1 len=256 path=[234:0-255]

ATCTTAGTCTGTGTTTTTTTTTCCTTCGTGCTTCTGAATATTTGCTCAGACTTCTCACAC

ATTCTGCAGATAGTGGAAACAAACCCTATTAAATTGACCTTGAGACTTCAGTGGTTTTGA

ATGCTGCTCACCCACCTGTGAGAAATAAAAGCACGTTCTTTCCTCAGCTATGTCAGCAAT

GAAGAATTAAATTAGAAATGTGCTCTTAGCTCTGCAGTATGGAGTTTGCTTTTCTTAAAC

AGGGAGGCTCAGTAGG

>c3646_g1_i1 len=207 path=[650:0-18 652:19-206]

AAAGCAACCAGTAAAAGGAAGGTTTATTTTGGCACCAGTAGTAGACAGCAGTAGTTTCTG

TCCTTGGGAGCTGTGACAATAACTCCTGCTATTGAGAATTCCTTGTGCGAGCACACAGTT

GTAAAAGTCCAGAAGAATTTGTCTGAACAGTGCTTTGAGGAAGGTGTTATACTAACGATG

TAGAGAACTTGGAACAGCTCCTTATAA

>c3646_g1_i2 len=319 path=[297:0-130 652:131-318]

TGTGGTTGTGTTGTATAAGACTGACTGATGAAGTCTGAGAGAAATATTTTTGCCTGTGAA

TAGCTTACTTGAAAGGAGCCATGTGTGGCATACAGAGAGGAAGATATCGATGAGTGCTCT

TTGGCCAACCCAGGTTTATTTTGGCACCAGTAGTAGACAGCAGTAGTTTCTGTCCTTGGG

AGCTGTGACAATAACTCCTGCTATTGAGAATTCCTTGTGCGAGCACACAGTTGTAAAAGT

CCAGAAGAATTTGTCTGAACAGTGCTTTGAGGAAGGTGTTATACTAACGATGTAGAGAAC

TTGGAACAGCTCCTTATAA

>c3650_g1_i1 len=499 path=[477:0-318 796:319-498]

AAAAAGGAAACTGTTATTTTCCCCATTTCAGCATAGATTAATCCTATGCATACAAAATAG

TCATCAGTAAGGAAAGCTGAGATTCAACTGACAGCACCTGGGCAGAAAGAAGAAGTGCTG

ACTGTGCACACTAAGTGGCCAAAGCTGCCCATTGGTGGTTTTAGCAGTAGCTGGGCTATG

GGATAGATGAACCCACCACAAGTTGTTGTTTCATGATTGCTACGTAGTTGTTTGATACCA

CTCTCGCTTCAATTTGCATCAGCGGGCAAGAAATGGGAAAACAGTTTGCATATGAACATT

ACAACTGCTGAGCAGCATCACACAAATCTTTCTGTTTCACTTAATGGGAAATGACAGCAA

AACAAACAACATCCTTGATTTTCCACTAGGAAACTTTCAACATAGAGTGGGGACATAGTA

GCATTTTTGACTTATTTTTTTGAGTTTCATTAATGATTTTTATTTAATGAAAAGGGCAAT

GATGAAAAAGTTGTCTCAT

>c3656_g1_i1 len=286 path=[414:0-135 549:136-181 594:182-285]

AATTTAGAGATTTGGATTTAGTACTTCAAGAGTTAATGTAAAACAGAGGATAATAAGGGA

GGTGGAATAGCCTGCTCATTTTACTTTCATGAAAAATGCTCACATTCTATATAGAGCACA

AACTATAGACTAGCATAATTTTGTACAAAAAACATTTATAAGATCAAATTCCTCGAATCT

GAGTACAGCTGAAGTAGGCAGATTGTCTTTTTGGTTTGAAGCAAATCTGTTAGTTCTGGG

CTTACTAGGTGAAAAGCAGTGCTGCAATCTTAAGTTCTTTGCCAGC

>c3656_g1_i2 len=286 path=[414:0-135 549:136-181 73:182-285]

AATTTAGAGATTTGGATTTAGTACTTCAAGAGTTAATGTAAAACAGAGGATAATAAGGGA

GGTGGAATAGCCTGCTCATTTTACTTTCATGAAAAATGCTCACATTCTATATAGAGCACA

AACTATAGACTAGCATAATTTTGTACAAAAAACATTTATAAGATCAAATTCCTCGAATCT

GAATACTGCTCAGAGGCAAAAAGCACATGCATAGAAACCACCCCCAAAATACATTTCAAC

ACAATCAGGCTGCCTTGTAAAGCTGGCAAAGAACTTAAGATTGCAG

>c3661_g1_i1 len=212 path=[518:0-211]

AGTCCCCCCAGAAGTCACTGGAAAAAAAAAGAAAAAATCAGGAAAGCCACCAAACTCCAG

CTTTGATCCTTGTCCAGGAAAGCACAGTAGCGTTTTACTCCTGTTTTCACAGCGGCTGCA

AGGGGATTTCACACCGAAAGAGAAATGCTTGTGTTCTAACCAGTCTGTCAACAGTATCTT

TCCAGCAATCTTAGAGTGGGATTAACTTCAGA

>c3662_g1_i1 len=350 path=[328:0-349]

GAGCACCTCCTTTGCCCTCTGTGCCGTACACCCTCGGGCTCCACAGGTCTTGGCCATCAG

AGCACCCTGCTGGCTCATGGTCAACTGTTGGTCAACTGCTGGTCTACTGCTGGTCAACAA

GATTCTTCTCTGCAGAGCTCCTTCCCAGCAGCTCAAACCCTAATCTGTCCTGATGCATGC

AGTGGAGGAAAAATTCTCAAAGCAGGGACAGCTCTCCTTGTCTGGTAACAAACATTGCAG

ATATGTGGAGTTCTTTAAAAGGACTATTAAATGAAAAGTAATGTAGGAAGTCATGGAATA

TAATTAGAGTAAATTTTCTGGGTCAAGAAAAAATCACTTTTATCAATTTG

>c3663_g1_i1 len=222 path=[200:0-221]

GCTCTGAGGGTAAAACAAATCATTTTCTCTCCATTCCCCTGGGATTGTCAATAAAAAGAA

TTCATCTAAATAGTTTACATGCATTTTGAAATGTTGCATCTACTAGACTTGGTTTTGTTG

TTAGTGGAAAATTGCCAGTGTGAGTGGAATATCAGCTTGAACTTGATTATAACAGTGCTA

CAGAATGTAATGCCATGCAGTGCCTGGAAAGACTACAGCCAT

>c3667_g1_i1 len=349 path=[233:0-216 455:217-348]

ATCACGGGTGTAATGCAACCTCACAGTATTAGGAGACAACAGTTCCTTCACTTTCCAGGG

AGATGAGACCCAGCATTTTCTGAGCCCCCCTAAGCAAAGTAGGAATTAATTTTGCCTTTT

CCAATGAGGCAGCAATCACAGGAGGTGGCTCACATGGCATAGCCCTTAAGGCAGGGGAAT

AACTGCTCTGATCTTACCAGGAGAAATCAGTGTTACAGAGCACGGGAGCTGAGGGGCACA

GCATCCAGCTGCTGCCATAGGAGCTCTGCTGCCAGGCTTTCTAGATAGGAAAGCTGGAAT

GTGAGCATAGAATCTGCCTTCCACAGAGCTCTGCAGTGACTGACAGATG

>c3678_g1_i1 len=226 path=[254:0-141 395:142-161 26:162-225]

AATGGTTTGGGTTGGAAGGGACCACAAAGATAATCTAGTTCCAGCCCCCTTGCCATGGGC

AAGGCTGCCAACTGCTTGATCGGGTTGCCCAGGGTCCTATCCATGGCCGTGGGCACCTCC

AGGAACAGGGCATCCCAGCTCCGGGCAGCAGTGCCAGCTCCGGGCAGCAGTGCCCTCTTA

GTGAAAAACTTCTCCCTGATATCTAATCTGAATCTTCCCAGTTTAA

>c3683_g1_i1 len=522 path=[986:0-521]

ACACATAATGTCAAAACCTCAAACACAAGGTAAGGTAAATTAGGTTCCTCCGTTCCATAA

AACAATTCTGCTCTGATAGAAACCCACATTTAAATGTAAGAAATGAGACCTTACAATTAT

TATTACTGCAACAAAAAACAAAGCATTTGTCAGTCCACTACAATTAATCACGTCAGGTGA

GCACCTTACCTGCCAGACTTTTCATAGCAGTTGTGTATCATGAGGTGTGAAAGGTAGTTT

AAAGCAGTTATTGAAATGCATACAAATGAACCCTTTCAAAATTCTACCCTAGACCAAGAA

ATTCAATCCAAAGAATATTTAATACTTTAAATAATCCAAAAGAATGCTCATAGTTTGTAC

ATAAAGAATCGGACCAACTAATAAAAAAACCAAGCACTCAAACAAAAACCTTTGGTCAAG

ATTTCTTGCTCTGTTATCACTACTGCATTCTAAACATCTTTAGAGATCACTGAAGTCAAT

AAGATAGCCAAGAAGGCCTCTAAGAATTTATGCTGTGAAAGT

>c3683_g2_i1 len=266 path=[244:0-265]

GCTGAGCAGAGAAGAAAGTGCTCTAAATTGTACCAAATCTTGACGTTCTGCTGTTCTCAC

AAGAGGGATCTAAACTTGGTCTAATTTACCTTACCTTGTTTTGAATGTGTAGTAATTACA

TCTGTTGAATTTGATAATGGATGATTTCCAGGACAGTTTTCAACAAGTAGGAATTTCAGT

TTTTGAGCACATATATGGTGTAATTACCTCTTCAAACAATGAAATAAACACCTCACCTGT

CAGTTGCCTGCATTCTCGGTTGTCTT

>c3686_g1_i1 len=223 path=[181:0-222]

TAAAGAAGTGTGAACAGCTGAGACAATAAATTGAAAAGTAACAAATGATAAAAGTGACCG

ATTCAAGTATATGGCCCATAAAGCTCCTAAGCAATGGGAAGAAATTGTATTCATAAGAGA

ATGCGATAAATACATTTCATTTGGGTCTGTTTTATCACTTCAGAAAGAAAAAAACTTTGT

GCTTCCCAACAAAAGACAAAATTATTAAGCAAGAACTCACATA

>c3688_g1_i1 len=306 path=[454:0-283 752:284-305]

AAAAAGATTGCTAGCAGTATTATGACAGAAACATAAAGAGGATTTTCACATACAAACTGT

GTAATCACCTTCAAATGCTAAGCAAAATACTAAAAAAAAACCCTTGCAAAACAAACAAAC

AAAATACACTACAAAAGAGAGACAAGATAAAGCACTTAGAGGTGTCAGTAGTGTGGCTGC

ATGAAAGCACCCCAGGGATAAAGGGATCAACCCTGCTCCTGCTGTCACATGAAAACTGCA

TGCAAATAAGTAATTCCCTGACACAGGGCTTAGATATCCAGAACACCATATCTACATCTA

TGGTGT

>c3692_g1_i1 len=202 path=[1:0-201]

GCCCAAGTGATGTCTTCAAAGTCATGCTCACTCTGAAATCACGTGGTCTCACAGCTGATG

TCTGTGTACTGGATTGTCTTCAGCAATTTGCAAACATAGAACTAAAGATAAGATATTTAA

GCAACTTTTAAACCTTGTCCTTCACTGTATGATTTTATGCTGCAGTGCTGGTATTAGGAA

TTGCTTTAGTTATACCTCAGTA

>c3698_g1_i1 len=225 path=[242:0-191 464:192-224]

AATGGTGTGGACTGAAATGGAAAATCCTTTCCATTTTGCAGGGTGAGAGAACACTCTTAG

CACCTGAGAAATGATGCCTCCCTATTCCTTTCTGAGTGCACACAATGATCTCAGTCCTCC

TGGTACTATTTTAGGTAAAGGTATCTTCTCACATCCTGTGAGGATGTAACGTGATATCAG

CAGGTTTGAAGTGATGATGATGATGATTATTATTATTACTATTAT

>c3704_g1_i1 len=244 path=[222:0-243]

TCGCCCTAATAACTAGCTATTTCATAATTAACTCTTATCCCTACTGAGATCCGTCAAGGC

TGCTCACTGATTCCCCAGGACATTTCTGCCTTTGATGTGGAAGAATGGATATTTTTCTAG

TCTTGATCAATCTGTCAGACAATGCCAAAACAGAGTACAGAGACTTATGCACTCATTTCT

ATGTGCGAGCATATTTACCTTGAGGCTTCTGCTTTGGACTGACTGATTCGCTGTCACTGC

TGGA

>c3724_g1_i1 len=234 path=[212:0-233]

TAACTTTGTGCCTGGTGTTTCTGTGGGGTTTGGGAATGGTAAATAACTATTAGGACTCAG

ATAAACTTGATGAAATACAGTTCTAGACATCTTTCAGCAACTGATTTAAGATCGTATGCA

ACTTGGCTAATTAGAAAGATGGCAATACAAACTATACCCTTCTATTTTTTACTTAAATTT

TTATACTCTCCTCTAAGTGTTAGCGTGTAAGCATGCAACACAAATAGCCTCTAG

>c3740_g1_i1 len=510 path=[559:0-467 1183:468-468 1028:469-509]

AGAGAAGCTCCACAAAGCAGAGATTGTCCATGGAGATCTGAGGCCAGAAATATTCTTTCT

GGGAGACAGGATCTGTGATCCATTTGCTAATAATGGGATGACAAGAGCTCTGAAGATAGT

GGATTTCTCTCACAGTCTGGATTTAAGGTTGCAGTCTCGAGTAAGCTTGCCCAACAGCTT

TCCAATATCTCAGACTCCTCATGGACAGCAGCTTCTGGCAAAAAGTTGCCTTCCTTATCA

GGCAGACTTGGTTGGTATAGCAGATATAGCCCATTTGATGCTGTTTGGTGAGCATGTCCA

GGTCTATCAGGAGAACTCCGTTTGGAAAATCAGCCAAAATGTATCCAAGACTGTTGGCTC

TGATTTCTGGGGTAAATTTTTCCGGCAAATTCTGAATGCAGATGGTAAGTCCACAGTGCT

TCTGCTGAGGGAGTTGAGAGAGGAAATGAGTGACATGTTTGACAGCTGTTTCCAAGAACG

TCTTTGTGAATCCTTAGTAGCGCTGGGTGA

>c3744_g1_i1 len=202 path=[295:0-201]

GCTTCACAGAAATGCAAAAATGATCCAGTTGCAGGCAAAGAAAATCTATCACGAACATAG

GCAAGCAAACAGATTGTACAGAAATTGTCTTTGGACACCAGATTACCAAGAATCTTCACT

GTTCTATTCAAAAGACAATAATTACACAGTATATATTACCTCTAAATATATGATTACATA

GCAATAATAAGCACTTGCTTTG

>c3750_g1_i1 len=223 path=[168:0-165 334:166-222]

CTTGGGGAGGGTAGAGATCTGTACAACAGAGGAACGAAAGATGCTGAAAGCAAAGCACAG

AACATTAAAAAACCCACAAGATCTATATTACTTAAGTATTGACCGTTCCATGCCCCCAAC

ACCAACACTTTTTCTCAACCACCTGCATATCATCAGCATCTGCTCGGTACGTCCATGCTG

AGAATTTACCCCTCACTAATCAAGGTAGAATGAAACAAAACCA

>c3757_g1_i1 len=282 path=[1:0-281]

TGGAGATAGAGGAAATTAGTGTAAAGTTGCAAGAGAAGTATTTGGGTCCTTTTAGACTAG

AATCCTACAGTAACGAGGTTGTGTTTCACAGCACAGAGCCGTGCTAAAATGAATAGGCAC

AGAACAAAAGAATCAGACCCAGAACTGAATATTTTATGCCCTAGCATTAACAGGCATATG

GGACACGGGGCATATCCCATTACAAAGGCTCTGACCGCTCGCCTTCCCCAGAGTGCTGAT

TGCTGTGCATAAATGAAGAGTGCATTTCGAGGTACTGTCTGG

>c3757_g2_i1 len=206 path=[723:0-183 928:184-205]

CTGGAAGCTTCCAAAGAGGGGATGTGGTGTTGCACTCCCACAGCAAGTTGCATTAGGGCT

CCATTGGCTCACTTCAGAGCAAGCCAGTACCCTTCTTTGCGTAGAACAGCCTTCTTTTTC

AACTACAACAGTAAGTCTAAACCTAACCAACTGAATTACTTAGGACCTTAACTCAGCAAA

GCATGCCTGTGTAAGCTCACTTAATG

>c3765_g1_i1 len=343 path=[563:0-139 383:140-342]

CCAAAGACCCGAAATGGAAGTGTTGACAAAATGCTCTACAGTCCCACCCGAGTCCGCTCT

AGAACAATTTTGCCAGGGGCAAAAATGAGGATCAATCTGTGCCTGATGTTGCCCGAAGAC

CCACTGCAATCATGAATGTCTTGGAAAACTTCCTCTTATTTAAAATTCTGGTTATCTGTT

AACAACTTAAAACATACTGTGAGGCAGTACAAAAAGCAAGCAGTAGACAAACTAGTTATT

TCTCCTTCCTTTTGAAAATGAAAACTTCAATCTGCTTTTTGTTTTTTGCACTCTTCCTAA

CCATTCTTTCCATCACAATAGATCCCCCTGGTCCTATTGATAA

>c3765_g1_i2 len=304 path=[282:0-100 383:101-303]

AGTCTTACCTGAGTTTGTTTTAGAACAATTTTGCTAGGGGTAAAAATGAGGATTAATTTG

TGTTTGATGTTGCTTGAAGACTTACTGTAATCATGAATGTTTTGGAAAACTTCCTCTTAT

TTAAAATTCTGGTTATCTGTTAACAACTTAAAACATACTGTGAGGCAGTACAAAAAGCAA

GCAGTAGACAAACTAGTTATTTCTCCTTCCTTTTGAAAATGAAAACTTCAATCTGCTTTT

TGTTTTTTGCACTCTTCCTAACCATTCTTTCCATCACAATAGATCCCCCTGGTCCTATTG

ATAA

>c3766_g1_i1 len=224 path=[202:0-223]

AGACGCTCGGCAGCAGCCCGCGGCGCGGCCCCGCTCGGCTCGGATCCGCTCGGACCATGT

CGCCATGTCGGAAGTGAGGAAGTTCACGAAGCGGCTCAGCAAGCCGGGCACGGCCGCCGA

GCTTCGGCAGAGCGTCTCGGAGGCGGTGCGAAGCTCCTTCGCGCTGGAAAAAACCAAAGT

GGTTGAACCCTTGGACTATGAGAACGTGACCCTCTCAAACACAA

>c3774_g1_i1 len=246 path=[224:0-245]

AGCTCTGCACGCTCTGAGGCAGCGCCCTGTCAGGCAGGCCACCTCTCGAGTCCATCAGGA

CTGTGCCACCTCCATACCACTGTATAGAACAGGCTGCTCCCTCAGACATCCCTGCTGATC

ACATCCCCAGCCACAGCTGAACAACCTGCAGTAGAGCAACAGGCTTTCCCCCCCACGTGA

GCAGCAGCTGCTAGGCATGCTGAAGAAAACAGCCAACCGCCGATGCAGAATGCCACCCAA

TTAGAT

>c3775_g1_i1 len=307 path=[285:0-306]

GTGAAGAAAAACTCCTGAGAAAGTTGTACCACCTACAGTTTGACCCACAGCTTGCTGAAA

TCAAGGGGACTTTTACTGTGATCTTCATCTTTAGAAAATAGTCTTGCTAAAAGCTAAGTG

ATATGAAGCCCTGCAAACTCATGGCCAATTGGCTAAATTTAGGGTCGAGAACGACAGTGT

AATTAAAATTCTGCTAACGCTTTTACTTCTGACATTAAGGCCATTCTCAGATTGAATTTT

AACTTCCAATTGAGCATATGTGAAAGGACATTCCAGAGGAAGTATAAAAGGAGAACGCGA

ATCTTAG

>c3780_g1_i1 len=207 path=[185:0-206]

GAAATGAATGAGCAAACACTTAAACCACTGACTTATACGTAAGAAGCAGTGCCAGTGTGA

TCAACTTCAAAAAGCAGAGCACCAAAGATGCACAGTGTCAATTAAAAACAGAAAAGCATT

TGTGAGAGTCACTGTTAGAAGGAGGTTCTGAACAGACATCGTTTTATAATGCTGAATCCA

AAGTCATTTCTGCCATCTTACTGATTA

>c3785_g1_i1 len=417 path=[549:0-416]

AATGAAAACAAATAAAATCAGTTGTTCTAATACCATAACTCGAGTGGCAAGTGTAATCTT

CTCCTTTTATCTGGTTCACGAGTTGAAAAAAAAATACAGGGTCACGTGCACTGAGTAGAG

CACAGGAATAAAGTAACTGTACTTCAGGGCTGCAGTACTATATTTTTAAGTCCAGAGTTG

ATGCAGACATAGGTAATGTTTGATTGAAGTTCCTGGGGTCATCTGTGGGTGCCTTGCGTG

AAAAAATACTGGAAGGGTTCTTCTAACGGAAGAAAGAAAAAGATGCCAGCTACATACATC

TCATCCCAGATATGTGCGAGTTGTCGTGCTTATCATTACCATTGTTCATTTGTTGCAAAG

CAGGTGTTAGCTGAGAAAAAAACATAAAAGCTGCATTTTGTATTGTTTGTTCCTCGT

>c3788_g1_i1 len=281 path=[1:0-253 255:254-280]

AGGTTGGAAAAGACCAAGATCATCTAGTCCAACTGTCAAACCTGGAGCAGGAGAAAATAA

AAGCTGTTCTATAAGAGATGAGAAAGAACACTGAAATTACAAAGAGAAGAATGCTACAAA

AACTTTTCTTCTTCCACCCTTCTCTTCATTCCTGAAAGACAGAAAATGCACTAGTATTGC

CAAAGCCATGTTGAGGCTCAAGGTATGTTACCAAACCAGCCTGTAATTACTTAGATGACC

AGATAGTCCCCATTCTCATCTCTTGCACAAATACAAGAAAT

>c3798_g1_i1 len=242 path=[43:0-39 42:40-241]

CTTTGTGTTTGAGTGTCTGCATGGCCGTTTGTCCGCCTCCGCGCCGCCCGTCTGTCTGTC

CGTCCCTCCGTCCGGCTGTCCGTCCGTCCTTTCGCCTCCGGTTCTGCCCTCCTGTCCGTC

TGTCCGCCCTTCTGTCCGTATGTCCGCCCTTCTGTCCGCGTTTCTGCCCTTCTGCCCATC

CATCCCTCCGTCCTTCTGTCCGTGTGTCCGTCCCTCCCTCCGCTCCCAGCTCAAACACAA

AG

>c3811_g1_i1 len=333 path=[311:0-332]

GTCCTTTTCAACCCAGGCCATTCGAAGATTCTATCAACTACAAGACTTTAGTTTCAGTTC

TACTTGCACATCACATGCATGTGCGCTAAGAACCAACCATCCCTTTGCTTCACAAACCCT

GGGGCAGTCTGCCCAGCAGGTCAATGGTGGAAGCTATGCCAGTGACTCAAGCAGTGCTGC

AAGATGCTCCTGGGCTTCTGAGTACAACCTGCTATGATTTAGCTCAGTGGTTGCTAGCTC

AGCACTGCTGTGCTCTGCAGGTGAGGAAACTGAGCACCAATACAGCCATGCCAGCAGCCC

TCGGGTAAGAACAGCCTATGGTGCACTTAATTT

>c3817_g1_i1 len=290 path=[609:0-19 81:20-219 281:220-233 281:234-247 281:248-261 281:262-275 281:276-289]

GATCGGGAAATACATTGCGGTACCATCTCCATTTGATTTGGGCAAGTTACTTAACCCAGA

TTTTTCACAAGCATCAATCGTTCGGATGGTTGGACTAGATGATCTTGAAGGTCCTTTCCA

ACTTGTGATTCTATGATTCTACATCTAAGCATATATAACAAAATCTGTTTCTTAACAATT

GGCCTGTTTTGAAAAATTCTGCTTTATTTGGAACTAAGCCTATCATGTCAGTGTTATCAT

GTCAGTGTTATCATGTCAGTGTTATCATGTCAGTGTTATCATGTCAGTGT

>c3817_g1_i2 len=350 path=[1:0-79 81:80-279 281:280-293 281:294-307 281:308-321 281:322-335 281:336-349]

AGAAAGAAAAATGTAAGTTCAATGACAAGAGCAGTTGAGTTTAGCAGCTCTGAGCAACAA

TCCCAACCTTGACTTCATCTTACCATCTCCATTTGATTTGGGCAAGTTACTTAACCCAGA

TTTTTCACAAGCATCAATCGTTCGGATGGTTGGACTAGATGATCTTGAAGGTCCTTTCCA

ACTTGTGATTCTATGATTCTACATCTAAGCATATATAACAAAATCTGTTTCTTAACAATT

GGCCTGTTTTGAAAAATTCTGCTTTATTTGGAACTAAGCCTATCATGTCAGTGTTATCAT

GTCAGTGTTATCATGTCAGTGTTATCATGTCAGTGTTATCATGTCAGTGT

>c3818_g1_i1 len=349 path=[327:0-348]

TTCAGTTCATAGCACAACTTCCAGCAGTGACTCGCACGACAGTGAAGAGAATTATGTATC

CATGAATCCAAATCAGTCCACTGAAGACCCAAATTTGTTCGGCAGCAACAGTCTTGATGG

AGGAAGCAGCCCCATGGTAAAACCTAAAGGAGATAAGCAAGTAGAATATTTAGATCTGGA

CCTAGATTCAGGAAAATCTACCCCACCTCGTAAGAAAAAGAGCAGCGGTTCAGGCAGCAG

TGTGGCAGATGAAAGAGTTGATTATGTTGTAGTTGACCAGCAGAAAACACTTGCACTGAA

GAGCACACGAGAGGCATGGACTGATGGGCGACAGTCTACAGAATCAGAA

>c3819_g1_i1 len=472 path=[450:0-471]

GGACCAAATCACAAATGCTCCAGTAAATCACAGTTTGGAGGTGACTTCCCTCTGAAGCTA

ACACTGAGAGGGAAGAGCCAGTGAGTTGCCTTCCTCACAATGTCCAGGAGGCACGGCCTG

TCTCTGTTTCCTTGAACAGCATCTCAAGTACTGCAGTTGGGTGGTTGGAGCCAATGAGGG

AGGGCTGCCTTCCTTCCCTACAGAAGATAGAACGACTGTGTCTTTCCTGTGCCAAGGGAA

ATGTGACAAGGATGATTTGTTGAAAGAATTGTTGATCTGCAGTTTCTCAGCCACCTTAGC

TGAACATTTGCTTGACAACATATGTGACCACTTGGGTATGTAATTAGATGCTTTTGTGAT

CTACCTACAGTTTAAATTATATTGTTCAATTTTCATTTGTATGTAAGACAGTGCTGAGGC

TGGTTGTTCAGTGGTTCCCCAAGATAGTGATGTTTCTTCTCTGAATAAGCCT

>c3825_g1_i1 len=248 path=[226:0-247]

ATCTCTAGCATGCAAACATTGAGATATTTCAATATTTTTATTTAAAATAATCCAAAGGTA

ATTGAAATACCATTAACTGTGCACTTGCATGTCTTTTACTGACAAACATTTATGGAGACA

CTTTTTGTCTCAATAAACTAGATAAACGCTTAAAATACGAAATGATTTTAAAGTTAGGAA

GTTCCAATATTAGAAACATCTGTGCATTGTGCTACTGTACAGACATCAGGCAGTTTTTAT

TTACAATA

>c3846_g1_i1 len=209 path=[341:0-208]

TAATTGCATAGAGAGAGCTGTGTATAATCCATTAAAATCATCTTATTTTTCTATGGTCTG

ATAGGAGGCACACATCTTGTTAGATGCAGATTGTGAACTTGATAATATTCTCCTTAGTTT

GTGCTATAAGACATGGAAGAAATGCTTCAGGTATCCTTACCTGCAATCTCAGTCTATTTT

AAAATGTGAATTAGGCATCTAAATTCATA

>c3852_g1_i1 len=294 path=[29:0-113 28:114-293]

GGTGGGGCACAAACACGCCCACAGGTACTGATAAGGCAACATTGGCATCTGGAGAGGTGC

TTGTGATAAGAACACCTGCAATACAGGGCTATGTGTGGGCAGTGTATTTCAAAGGAGTGT

GGTTCCTGGTAAATAATGTTTCTTTTCGTTGCCAGAAATAGTAATGTAACCGTAGGTATA

TAGTAAGAGTTACCTTGTTATCTAGGCTTTGTGCTGGGAGAAATGAACTTTCTGTGTCTT

TTGTCATTTGAGGGATTGAATAGATGTGTGTAGGCTGCCTGATAGAAGATTTTT

>c3853_g1_i1 len=211 path=[343:0-210]

CACCTAACTGATGAGATCTTCCATACCCCAGTGGGTATCTCTGGTATCGTGGGACACCAA

ATGGAGGCAGGAGATGAAATCTTTTCTCCAGCAACGGTTCAAGTCTGCAGGCAGATGGGT

CAGCGGAATGAAAGAAACTGTACACTTGGCTGCAGGCTGGGCGAACCTGACCAACATCCA

GGCCCGGCAGAACAGTACTCCTCATGGCCAG

>c3854_g1_i1 len=262 path=[540:0-261]

CTCAAACCGCTGCTCTTCGAAATTCCAGACTTCCTGACAGAGGAGGAGTGCAAACTCATC

ATCCATCTGGCACAGCTGAAGGGGCTGCAGAAGAGCCAGATCCTACCGACGGATGACTAC

GAGGAAGCCATGGAAATGATAGAAATCAGCCAGATGGACTTCTTCAATCTGCTGGACCAC

AATCAGGATGGGCAGCTGCAGCTCAAAGAGCTGACAGGGCTTCAGGATCACCCACGGTAA

CAAACCTGAGGGGGAGATAAGG

>c3859_g1_i1 len=201 path=[472:0-173 668:174-176 647:177-200]

ATTCCGTCTCCTGAAAGCTCTTTATGACAACAGACTGATACAAGAGATCAGGAAACGAGT

TCTTGAGGATGGGATTCCTTACATGGGATCCAGTGCAGGAACTAACGTTGCTACTGTCAG

CATCAACACTACCAATGACATGCCAATTGTTTATCCACCTTCCCTGCAAGCCCTAGGGTT

AATGTTAAAAGGAACTAAACC

>c3860_g1_i1 len=428 path=[406:0-145 552:146-427]

CTTTGTGTTTGAGGGGAGACGTGGGCATACTGGGCAGTACTTCTGCTGCCTGCTGGTGTC

TGTGAAATCAACACACGATCTGTTGTTGTAGGGACAGAGCCTACGCACCCATGGAATGGT

GCTGGGCATGGGGGGAAATGGAAAGCCAGGTAAGAAGCTCTTGTGAGCTGTGCTGACTGC

AGAAGCGGCATCATGTTGTAGCAGCAGAAAGGCAAGCTTAAGAAACTGCTCTTGAAGACC

AGAACTTTGTGGTTGGCTGTGCTCAAATCTTCCGCCACAGAGATGCTCTTCATGTCTTTC

TTGTCTCCTTTCTGCCAGAAGCTGATGCGAGCGGCCTTCCAGCAAAGCGCAGCCTTGCAG

AAAGGCAAGGCAGAAGCGCCCAGCGTGGCACAGCACCCAAGAGAGGTACAGACACACAGC

AGGCACCT

>c3864_g1_i1 len=270 path=[564:0-269]

TAACTTTGTGTTTGAGGATTAGATTAATGGGGTCTTTAGCCACCCACCCACGTGTGCTTT

GCTTCTTTGGCCCCAGTTTTGCAGCTGACCAGGGGGCTTCTTTGATCCATGTATCTGATG

CAGTCACACAGTCTGGTCTGGAACTAAATATATACTACATCCAGTGAGCAAGCTATTATC

AATCTATTCTGAGCAAATCTGAGTCTGCTTGTTAGCCAGAAAAAATTACCTTTTCATTCA

TTTTGATGGCTGAACAGCCACTTGCTAAAA

>c3880_g1_i1 len=231 path=[1:0-230]

GGAATTCATTCTTTTTAATACTTACATCCTGACAAATCTGAGTGGCAGTCATGGGACTAA

GAAAAGGACTCTTCTGTACTTCAGCTGCTTCTGTCAAATATCAAAAGATCTGCAGCAAAA

ACACATAGTCAAGGCTTTCTCAAAGGAAAGGTCATAACTATCCTTTCCAAGGAAAGATTA

GGTCATATCTGTGCAGTTTGGGTTTGGCTTTTTGTTTTTTGTTTTTTTTTT

>c3883_g1_i1 len=690 path=[822:0-689]

GCTTAGTACAACAAAAACTGGAGACTGAAGTCTTCTTTCATGAATCATCTCACACAACCC

GTCCCTTTTATTACTTATTAAAATCTTTTTTTTTTTTTAAGATTTTTATTGTCATATATT

TCAAAATATTTACTTGAAAAAAATAGTTCACACCTAGCTGTGAATTTATAATTTATAACA

CATTTCATTATTTCTAAAATTTAATTCTTCCAATAATATATTGTTATGTAACAAATTTGA

TCATAATTAAAATAAATTCAAATTTATGTAAATTACATTTTTATTATTGTTTTCATTAAA

ACTACCATTATACTCATAAATGTAAATAAGATTATAATACCTTGTTTTTATTTTTTTCAA

TATTTATTGCTATATAACATAAATTTTTTATCAAATATATTTTAGTGGTTGTACGTTAAA

ATTGTTTATGTTAATAACATTTTATCACTATAACGTATATCCATTATCTCAATTACATAT

TATTTATAAACATCCTGCATTAATATTAAATCAATTTATGTATCTTATGATGTAAATAGT

CATTATGCCACAATCACTTTTCGAATTGATACAAATTAAATTACATAATTTATTAATATA

AAAAACATTAAACAATTGTAATATTCGATAATCATGATATTCATCAATATAATTTGTTTA

TCATTACACTTATTTTTACCTTTATTATCA

>c3890_g1_i1 len=242 path=[584:0-18 161:19-178 611:179-191 624:192-241]

TAAAATTAAAATTATATTCTTTTATTTTTTTTTTCTCCTTAGGATTATATGGAATTTTTT

TTTTATTATATTTTCACTCTTGCATAAAAAAGAAAAATATGTTTTTCAAAATTACGAAAA

ATAGTATGGATTTTTTTTTAACACTTAGAAAAATTCTTTTCTTTTTCTTATTTAATAAAG

TATGATTATTAAAATATAAAACATCTTTGAATAAATGTTTTTAATTTTAATAATCATACT

TT

>c3898_g1_i1 len=255 path=[21:0-254]

AAATAAAGTAAAATAAATAAAGTAAAATAAAATAAGCTAAATAAAATGTAACGAGACAAA

AAATTCATGTAATTTGTATCTTTTTATTATTTTTCTATAATTTAATAAAAACAATAAATT

AAAATTATATAAAAATAATATAAAATAAAAAATATATGTTGTAGAATTTTGTTAATAATT

AATGTTACTTATATAATTTAAGTTTTTTGTAACATACACATATGGTTAATTATAATTAAT

TTTTTTTATTAGTAT

>c3898_g2_i1 len=394 path=[914:0-393]

AAGAAAGAAATATTTAGAGATATTAAACAGAAAGTTATTTTTTGAAATGATTAGAAAGAT

GAATTATTGAGAAATCAGCATACTGTTGTTTGTATATGACAGAAATGTTAATCGAAATAA

ATTCCTAAATAAAAACTATTAAATATAAAAAACATAATTTTACAACTGCATTTTTCTTCT

AATTTTAATTTCTTTATAATAATTACATTATCAAAAATAATAATACATTATGCCTATGTA

TATAAATTTCCATAGAGTTGAAACATATATAATCCTTTTTCATTTATTTGTGTAATTCAT

ATTACATTCTTCTCATGTTTAAATGTTAAATATAATTTTTTTCTTTCAAAAATATCATAA

AAAAAAAAAAATGTATCACATTAAAATTACTTTA

>c3904_g1_i1 len=235 path=[155:0-234]

GAAAAAAAGCTGGCAATAGCATCTTTTGTTTCTCCAACAAGTACAAAATCAGAATTTCTA

ATTTTCTCAAAGACTTCTTGTTGGCCAAATACAAGTCTTCAAGATGATTCTGCAGACTTT

TCATGGAAGTAAGGATGATGGGAAGCTGCCTTCAATACAGACAGACTGCTGGAGTTTTTC

TTTCCCTTCCCTCTTTCTTAGCTGGCGTATCAAGAAGGAAGGTGTGCTGGTTCCA

>c3905_g1_i1 len=257 path=[123:0-14 61:15-256]

GGTAACTTTGTGTTTGAGCCCTTACCCTGAGACATCACATGTCTTCCCAGATGCACTGCT

AGCTAAAGAGCCATCCCACAGCAGTATGACCACAGGGGCACGCAGTGTATCGAGGAAAGC

ACAGCTGTGTCTTGAAATCCTTGCCTTTAAACATAGCATAAAAGACCAAAATTGAAGGTT

ATTTAATTAGCAAAATTATGCCTGCACTATAGAAATACATTTATTTCCCTTCTACACACC

AACCCTTAGCACGCAAG

>c3905_g1_i2 len=302 path=[1:0-59 61:60-301]

TTAAATAACCTTCAATTTTGTGTTTGATGCATTAATGTGTGGTTAATGCCATTTCATTCA

GAGCCCTTACCCTGAGACATCACATGTCTTCCCAGATGCACTGCTAGCTAAAGAGCCATC

CCACAGCAGTATGACCACAGGGGCACGCAGTGTATCGAGGAAAGCACAGCTGTGTCTTGA

AATCCTTGCCTTTAAACATAGCATAAAAGACCAAAATTGAAGGTTATTTAATTAGCAAAA

TTATGCCTGCACTATAGAAATACATTTATTTCCCTTCTACACACCAACCCTTAGCACGCA

AG

>c3906_g1_i1 len=269 path=[1:0-268]

ACTAAAACCACCGACAGCTGGGACAGCAGCAGTGGTGATGGCAGCCCAGTGGAAGGCAGC

AGCGTTGCAATGGGGGGCAAAGCAACACAGAAGAAATGCGAAGTGATGGTGATGAACCTG

GAGCTAAACTACTGCTTTTTCTCCTCCTCCCCCTCCTTCCTCTCTCTGACCACGCTGCCA

CACAACCTTTAAAAAGCCATGACTAAGAGAAATGAAAAGCACTGAGAGCTACTGCACAGT

ACTAAAGCCATCCCAGATCAAACACAAAG

>c3911_g1_i1 len=228 path=[155:0-182 338:183-227]

TTTCAAGGCAGGGCTGGGGAGCGCATCCACTCAAGCATTTAATACAGGAAGCTAGAATGT

GGATAATTGTTTTCCTTTGCACCTAGGAACTGAGCATTGTCTGAAGAAGTTCCCCTGTAT

GATTTACAGTCTTACCAGATTGCTGAATTATGCCTTGTGAATTTACTGCCTAGATTACAT

TTACTGTTGAATTTTACTGTTGAATGTTCCTGGTTTTAACTAGTCTCG

>c3911_g1_i2 len=229 path=[155:0-182 428:183-228]

TTTCAAGGCAGGGCTGGGGAGCGCATCCACTCAAGCATTTAATACAGGAAGCTAGAATGT

GGATAATTGTTTTCCTTTGCACCTAGGAACTGAGCATTGTCTGAAGAAGTTCCCCTGTAT

GATTTACAGTCTTACCAGATTGCTGAATTATGCCTTGTGAATTTACTGCCTAGATTACAT

TTATTTTAGAATTATACAGAAGAACGAGACTAGTTAAAACCAGGAACAT

>c3914_g1_i1 len=214 path=[155:0-213]

CTGAATTAATCCTTTTTTGCCCTCCTCCCAAAATAAACCGTATTGGGAATAGAAGTGCAT

TCTGTGCCGATTATAACTTCTGCTTAATATGTATGCGTATACATTCACTAATCCCAGGCA

TTGCCTAATAGCTATTAAAAATACCCAGAAAATGACCTTTTTCCTATTCTTCGGTTTGGT

CCTCTTGGCAATTGCTACTTCTTGCTGTGAAGGG

>c3918_g1_i1 len=309 path=[287:0-308]

AAAATGTACTATAAGATATTCCTGGACTCCTTTTCAATTTTAACTATGTCGAGAATGATA

TTAACGAAGTCATTTTTCCAACACAAGCATGGTTCAGTCACATTCTCAGAAGTTTTATGA

CACTGGGCTTCAGTCAGTCCTCAGCATACTTTGAACAAGTCCCTGCGCTGCCACAGAAGG

GAACCATATAAGGTGCAACTGTAGTGAAACTAGGTTACCAAACAAGTTAAAATGTATTGT

GATAAAAAGCAATTTTACCTCTCAGTTCAGTTTAGGTTTCGTAGGTGGTTCTTTTCCTAC

CCTAAAGTG

>c3919_g1_i1 len=284 path=[1:0-283]

TGTGAGAAGCTTGTCTGTGACCAAGCAAGGTTATATTCCCTCAAGATCAAATTATCTCTG

GTTGGTGTTCTCACCAAGTCCAGGATTCACAGAGGATACCAGAAATGTTTCTCCTAGTTC

ATGAAGTGTCAAGGATTTGGCTATCTTTCATATGCACCATCTTGCTGAAGCAAAGCAGAA

TGGGACCATTCTTGGCTGTTTCCTTTTCTTGATGTCTCGGTCAAAAACAAAGATTTAGGG

CTTGAAATTAATTGTAAAAATGTGAAAAACTCATATAAGTAACA

>c3922_g1_i1 len=384 path=[387:0-131 193:132-383]

AAATTCTATCACAGCATTATTTGAATGCACTCTGTTCTAGAAAAGCTTAATTCCTGCAAA

TCACAGCAGTCAAGCATAGTTCATTTGACCCTTTTGGGCAGGTTTTTATAGAATAGTGGC

AGTATATATTCAAGTGTACCATACACAGCATATTTTCAAAATGTCTTCATCTTTAGAGTT

CATGTTCTGTGTAATTTTGGCTGTGCTTTCATAAGGTATTATTCTCTTGATTTAATTCTC

AGAACAATACCATTTGCAGAAGTGTAATTTTACTCTTGATTACGTTTGTGGTTTGTCTTC

CCCATGATGGTAGCTGTGTGTATTTGCATCAGTTTCTCATGAGATTGTTTTAATGGTTGC

AAGTCATACATAACTGTTCAGATC

>c3922_g1_i2 len=444 path=[1:0-191 193:192-443]

GAGAAGCAGAGCCAGCACTAATGCTGTGATAACTTTTTCAAAGCAGATTTTTAGAGAAAC

ACACAAAAGTATCTGCAGAATTTTAACTTAATCAGCCTGATGACAGAAAAGCCTCTCAGC

ATTTCTAACCCAGTCAGTACAAGAGACTACTGCACAGGCTGACTACAGAAAACTACTGTC

CAGAAAGTGAATAGTGTACCATACACAGCATATTTTCAAAATGTCTTCATCTTTAGAGTT

CATGTTCTGTGTAATTTTGGCTGTGCTTTCATAAGGTATTATTCTCTTGATTTAATTCTC

AGAACAATACCATTTGCAGAAGTGTAATTTTACTCTTGATTACGTTTGTGGTTTGTCTTC

CCCATGATGGTAGCTGTGTGTATTTGCATCAGTTTCTCATGAGATTGTTTTAATGGTTGC

AAGTCATACATAACTGTTCAGATC

>c3926_g1_i1 len=237 path=[328:0-110 438:111-213 585:214-236]

GGGAAGAATGTGTGAAAATGGGAAAAGTGTCTTCACATCAATCATGGGAAAGGTTGTTTC

TCTACAGATTTCTATCTGTGTTGATCAGTGTAAAGAAAGAATAGTCCGTATGCTGGTAGC

ACTGAGGGATCTGCATAACATTCTTCTCAACTTTGCAGTTATTCTTTCTTCATAGCTAAG

AATCACTTGCTCACCACACACCGAGGAATAATTTAGAGCAATTTCCCTTGCAATAAA

>c3928_g1_i1 len=203 path=[181:0-202]

AATGAACTGGGTTCATCATCACACTTCACATAGACTGGTAATGCTGCTGGTTATTTTAAA

TTGGCTTTAACCAAGAACTCTGAGATTTGATTGCACTGATTTGAAAATTGCTTTATTTCA

ATCAGTGGACAAGCTTCTTGTTTTTCACTAGCTCCATCTAGTGCAACACAGGGTACACTT

GTAATGTCAGGTATCTTCAGCAT

>c3929_g1_i1 len=215 path=[289:0-125 24:126-214]

GTGCTGTGAAGTAGTTTGGGATGTTCAGATTTGTTGCATTCTTACTAATGCTATTTACAC

AATTTTCCCTTTTGACTGCGTAGCCCAGGAAGGTGAAGGTCAGAGGAGTGATTACCATCT

GCAGCCCCCTCATTTCCCATTTATATGACAAAACAGCTTCTGGGTTTCAGAAGCTGACTC

CAAACCACTTCCAACCATATCTAATGAATTTCTTT

>c3937_g1_i1 len=264 path=[155:0-263]

TTTATTTGTGAACTCTTCACCTGGTTGTGCCCTGATCCCAGCAGGCTCTGTGCCTCAGCT

CTGGGCTGAGCTCCAGGGCTGCATTCACACAGCTGGGTAACGAAGTTATCATGACCAATG

TGGTATTTCAGTATTGGGAAATTGGGACCACTTTACTGCCTGTGATTAACTGATTCCATC

TCCTCTTACAGCACAAAGAGGGATTTGCAATGGTGAGTGCAGAGAAAAAATCACAACCAG

TCCTGAAACATCTTCCTTTTATTG

>c3952_g1_i1 len=239 path=[1:0-176 605:177-183 185:184-238]

CTGGGATGGGCCAGGATCACCCCTGCTGGGAGGGAGGCTTTCTCCCCCTCTGAGGCTGCT

GCAAGGACAAGTAACAAGAATATTCCTCCAGGCCCTCTCCCCACACGTGCTCCTTTCTCA

TCACACTGCTCCTACCACCCACACCGAAACCCATCAGACTGCAGTGCAGAACTGCCTCCC

TTCCTGTTAACACTCTTATCAGAGCCCACAGCTGCACTCCCCTGCTGTTGGCACAGGGA

>c3954_g1_i1 len=237 path=[153:0-236]

GGCCACGTACAAAAACATAAATGATCCAAATCCACGCTCTTCTCCCTGACGTGACTGAAC

ACTACTGTACTTTTCAGCAAAATGCCACAACAAGGGCTAATGGCAAGAGGATTTACTGGA

GGGCATGAGTCAAAATAGACCTTTCAGCTAGAGATACATAATGCAGAAATGGAAATACTT

AAGGTGATGTGGCCAAAGCATAAGGAACTGAGCAGACCATGCATCACAGCCAAGCTA

>c3957_g1_i1 len=253 path=[231:0-252]

AAGATTTAGAAGATGCTGTCCATACAACACCAGGTATTCTTTCAGGCTCCATTAACAGCA

TCACACAAAATGCCCTTCTACAGTCATACATAGCCAGAAATAAACACAAGTACGGATGGG

TTGTTAAAGAGGAGCCCACAGCACCAGCCACGTTTCTAAGTGCTCACAACCTGCACACAT

CTCCTTTTTTATTCATTTTTAAAAGCCTTTTCAACCAATTGTTCCTCCTCATCTGTTACC

ACAGCTGGGGCAT

>c3962_g1_i1 len=251 path=[383:0-250]

CTGCTCAAGGCACCCACACTGGCAGAGATGGAGGAGATGAGCATCTCCGAGGAGGAGGAC

TCACCGAGCACAGAGTCTGCCTCGGGGCTGCCCATGAAGCGGGACCGCTCCTTCTCAGAG

CAGGACCTAGCCCAGTTCCAGAGTGAGCTTGCTGGTGGCCATGCAGCACCCAAGGTGCTG

GAGTCACTGGATCCTGAGCCCCGGCCCCGATCAGGCTCTGTCCCCGCCAGCTACCACCCA

GTGCCATATGG

>c3969_g1_i1 len=374 path=[410:0-373]

TTTTATTTTTTTTTTATATTTCTCTTTTTTTCACAATTACATTTAATTTTTAACTCGTCT

AAAAATATAAATTTGTTTTAAAAAATAAAAAATAGTTACACTAATAGCTATATAATAAGC

AATGTTTGAATTATCTCATATTTTTGTTTATATTTTTTAGTTAATCTATTTTGAAAAAGA

TTATTTGTTTATGTTCAGTTATTATTCTTAAATATAATTGTACAAATAATATCTCTTACA

ATTTTAAAATGTTCTTGATCATATTTTTTTTTAATATAATCTAACTACTATTGAAGAAAT

AAATAAAAATTAATATCCTTTTTAGATTTATATTTTAAGGAAATTCTTTATTATATTCTC

TTTTCATTTCACAT

>c3974_g1_i1 len=256 path=[153:0-255]

GGAAAGTACAGAGCAAATTGAGTGTGCACCGGAAAAAGAAGATGACATGCAGTGACGTGG

CAGTCTGTCTGTCACAAGGCTAGAAAAACCGGTGACTCTGAATTCCTTGTGGGATTTGGG

GGCACTTGGTGCTGGGAAGCCACCTTTATTCTTGCATGAGGGCATCATGGCAGAAGACAA

GAGCTACTATGGTCTCTCTGAAGGCTTGATCTAGGATTTAGCTCTCTTCCTGATTCTTCT

CCTTTCTCCAGTCCCT

>c3975_g1_i1 len=526 path=[1:0-525]

AGGAAGATGCACGGTGATAGGAAGGTATGACAGAATGAAAATTCCCGATAGAAAGATACA

AGGAACAGGATGGGGATTTACCTTGGCTGACATCCTGATAATGCCCTTTCTGTGTGCGGA

GTCTTGTGTTTTTCCAACTGTAGGAAGCTGACTGTATATCTGATGTCATAGAGGACCATG

TTTTTAATGGATAAAAGTAATACATAAGTGCTACTAGGGAACAAACTTTCCATGAAGAAA

TAGTCTAGAGACTGTATATTAGCAGAAAATTAAGTTAATCATGTCACTGCTGCTGCTACT

AGTCTTAAAGCCCTTTCATCTCTTAGGATTTGCAGGTTGCAGTACATTGCCACTGATACT

CAGGATATACAGCTTGCTGCCTTAGTATTCCAATTTTGTAAATGGGGCCAGAGGAAGTTA

CATTTGTAATGCTTCCTTTCCTTTCAAAGTTATCGAGGATCTATAAACAGAGGTCATCTG

GATGGAAGCGATTACTGCATGGTAGTGTTTCATCCCGGACAAAAGG

>c3981_g1_i1 len=231 path=[238:0-156 394:157-230]

ATCTTGATGCTTTAAAACATCATCATCTTGACGGAGATACAGGTGGGGAACCAAACATGG

GCATGAGAAAATATCAGGAAAACAACTCCAGCACGATGACAACTGGTAGCACCAGAATTA

TCGTGTAAAAACTGGTGTCAGCCTATTAAAGCTTCTGTGGGCTTGATGGCATGAGGTGCT

TTGGGGTGTTTGGAAGAGGTTGAAGCCATGTCTTTTGCCGCTCTGTTTTTC

>c3981_g1_i2 len=557 path=[496:0-482 394:483-556]

ATAATCTAATCACATTAATCACAGAAATGCCAAAGCGCCATCCAAGTTCTGAAACCTCAG

CGTATGCTTTGACATTTCTGGAAAAACAATTTTTATTCAATGTCACCTACCATTTTCCTT

AACATTATTAAAGAAATGAAGATGTAAGAATACTCTGTTGGAGAAGCCAGGCCTCTAGCT

TCTCTGGTGGGTGCCAGCAAGAAAACCTACCTGGTGATTCCTACAAGTCTTTCCCCTGCC

ATATTTCATTAGGGGAATACCAGTGGAAATGCACTGCTTTGCTTTGCTCTCTTTCATGGG

CATGCAGCCCATATGGGGCTGCGGTTCTTGTGAAGCTTTGCAGTTTCTCCCTCCTTGTCC

AAGGTGCTCATAGTTTCTTATCTCTTGGCCTGTTGGGGCTATGCATGTACAGCAGTGGAT

ATCTGTGGTAATTCAGACTCATCTAAAGCTGGCTGAGATCCATAGGATCACAGGAAAACA

CCCTGGGCTTGATGGCATGAGGTGCTTTGGGGTGTTTGGAAGAGGTTGAAGCCATGTCTT

TTGCCGCTCTGTTTTTC

>c3990_g1_i1 len=259 path=[684:0-38 126:39-258]

ATTTCAGCCCATTTGCTTTTAGATAAGGGAACACACATAAGGAAAACATATTGATGTTTT

TACGTAGCTGTGCAATAAAATGTTTAGGTTAAAAATAAATCTGACTTAATAGCACCCACG

AACATAAAAAATAATGTCTAAAACCAGTGTATTATTTTTCCCCCATGATTTATGAGCATT

GACTTTTTTTATTGTGATGTGTTCAGATGGTTTAGCTTCTTTGAAGTCGCTGCTTGGGAA

TAAATTATCAAAGTGTAAA

>c3990_g1_i2 len=345 path=[1:0-124 126:125-344]

GTCAAATGTCTGGACAATACCTAATGCTGTGTCAATAACATTTACAGCAGAAAATATCCC

AAATAATACAGAAATAGAGTGCAGGAACTATTTCCACTTGGAACAAACGGAAAGCTAACA

ACATTAGGAAAACATATTGATGTTTTTACGTAGCTGTGCAATAAAATGTTTAGGTTAAAA

ATAAATCTGACTTAATAGCACCCACGAACATAAAAAATAATGTCTAAAACCAGTGTATTA

TTTTTCCCCCATGATTTATGAGCATTGACTTTTTTTATTGTGATGTGTTCAGATGGTTTA

GCTTCTTTGAAGTCGCTGCTTGGGAATAAATTATCAAAGTGTAAA

>c3993_g1_i1 len=230 path=[1:0-229]

GCACTTGGCGACATTTGCATTTGAAAGCCCCCAGTGAGATGCAGAGGGCCGCTGCATGCC

TCAGCTCTTGCTCACGTCTCCCTCCCCGTCTCGACTGCAGACTCCCCGGCCCTCAGAAGG

AGGGTTTGGCGGAGACAATGGCGAGAAATGGCCGGCTGTAAGGCCAGGTATTAGCGGCCA

TCGCCACCACGTCGGAAAGGGCTTATGAAAGAAAGCCGGGAATGGCAAAG

>c3997_g1_i1 len=226 path=[358:0-225]

AACACCAACATTTGTCCCCACATACAGCGGCCATGAGCACGCACGCCCCATGTTAGTTGG

GTTGGCTCCCACTTGGGAGCTCTTCTCTTTTGCTTTGATTCTCCCTCTTTGTTACCTTCT

GGGGTGTTTGAGCAGGTCAAGGACCGCCTGCTCTGTTGCCTGCTGGCTGCGGGGCACGAG

AACCGATAGCTCTTCTGAGCTGAGACCAGAAGCGTCCCTACATATG

>c3998_g1_i1 len=564 path=[420:0-293 1001:294-415 714:416-563]

TAATGATAAAATAGTCATAATTTCGTAATAAAATATAATAAAAAAATTTAATAAAAATAT

AATTTTTTCTACAATGAAATAAAAATATTGCAATGAAAAGTACAATAAGAATCATTCAGA

TAAGAAAATTAATGTTGTAATAATTAAAATCATAAAAAGTCGTTGAAAAAAAAATGCGGA

TACTATATACTTTTAAATATATTAGTATTTATGCTTATTATAATAAAAAAAATACATTTT

AGATTAAAATAATGAAGAAAATGAAATTTAAAATTTAAAACTTAAACATGTGATGGTAAT

ATCAAAAAATTTCTAATTTTTATAATGATTTTATTTTCATGATATAGTTGAGTAATTTAT

TTTAACGATAATAATATTTTGCAAATGATTTATTAATATTATGTTATTATTTCTTAGGTT

TAATTAAAGAAAAAATAATTAAAATTGAATAAATTTAATATTTCGATTTACATTGTTAAT

AAAGATTATTTTTAAAATTTTTGAACTTTTAAAAGAATTAAATAGTCTACAAATAAAAGT

TTAGTTGTTTATAGAAGGTATTTG

>c3998_g1_i2 len=442 path=[420:0-293 714:294-441]

TAATGATAAAATAGTCATAATTTCGTAATAAAATATAATAAAAAAATTTAATAAAAATAT

AATTTTTTCTACAATGAAATAAAAATATTGCAATGAAAAGTACAATAAGAATCATTCAGA

TAAGAAAATTAATGTTGTAATAATTAAAATCATAAAAAGTCGTTGAAAAAAAAATGCGGA

TACTATATACTTTTAAATATATTAGTATTTATGCTTATTATAATAAAAAAAATACATTTT

AGATTAAAATAATGAAGAAAATGAAATTTAAAATTTAAAACTTAAACATGTGATGGTTTA

ATTAAAGAAAAAATAATTAAAATTGAATAAATTTAATATTTCGATTTACATTGTTAATAA

AGATTATTTTTAAAATTTTTGAACTTTTAAAAGAATTAAATAGTCTACAAATAAAAGTTT

AGTTGTTTATAGAAGGTATTTG

>c3999_g1_i1 len=266 path=[33:0-140 173:141-202 338:203-218 354:219-265]

TTTAGAGCACGGGCTTCAGGATGTATTTTGTATTTTAAAACGTTACAGTCCAAGCACTTC

ATTAGTCTCATTTGATAGAAGGAACTGATCAGTTATGCATTTAATTTTCATCAGAGTGCC

TATCATATGCACTGTGTCTTCTTACTGCTTCTACCCATTTAGGTTACAACAGTTGCCCCC

CAGAAATAATTACTATGCAGTAGTAGACAAGAAATGACATTACTGCTTTTACTACTACCT

GCATTAATGTCATTTCTTGTCTACTA

>c4009_g1_i1 len=254 path=[1:0-173 493:174-253]

GGGGTAGAACTGGGTGATCTTTAAGGTCTCTCCCAACCAAAACCATTCTGTGAATAGGTA

ACCACAGCAAAGGCTTGAATTTATTGTGAAGGTTCGGTCCTGCAGGCACCTCAGAAATGC

CTGTGTGATTTGGGTTGGAAGAACTGGATGGAGACTTTTCCAAACTGCTGCTAAAGTGTT

TGACCTTCTTCACTGAGTTTAAGCAATACTTTTCCCGACCAGCTTCTGCCTTTTGTTCGC

TCTCTCATACAGAG

>c4014_g1_i1 len=288 path=[225:0-287]

GGATCTTTGGAGATCATCTAATCTCTTTTCATTTCCTTATGAAGTGTGGTAGCAAAAACA

CCAAACTTTGGCAAAGCTTTTGGCCCCCATCACAAGAAAAACTGCAACTTCGCATTTTAG

TTGCAACTAAACAGCTGTGTCAGGGTTGAAAGAACATTTAGATGTTGCAAAACTAAGCAC

TTTTTCCTCTACGCTTTGACAGCTGCCTTCGTGTTCTCAAGTCAGTGTTGAAAGGCCTTC

CTTTTTCCCCCTCTCTGTATGTTGTATTTTCTTACTATTTTCCTGAGA

>c4032_g1_i1 len=201 path=[273:0-117 18:118-200]

ATAAGCCCTTCTTCTTTTTTTTCTTTCCTTAAATTTACATGCTTATTAAAGTGATATGAT

CGAAGTATTCAATGATTTGATACACGTATCCATTATTAAAGGAGCGTGTCCCTAGGAATT

CCTGTGTGGGGCTTCAAGTACAGGGAGTACTGAGGCACATTGGTGTGATCGTATCGTTCC

GTGCGTTGGAAATCCTTTACT

>c4035_g1_i1 len=209 path=[141:0-208]

ATTTATAATTTTTTTTTATATTTAAATTACATATGAATTCGAAATAATTACATATGCATA

AACACATATATAATATATAGTTTTGAAGTAAACGGACAAATATAAAATACAAATTTATAT

ATAAGGTCATTATTAATAGTTTATATAAAATGTTTCTTTGCATTTATCAAAGTTGGTGAA

AATATTTTTTTTTTTTTTTTCTACAATAA

>c4038_g1_i1 len=585 path=[653:0-584]

TTTTTTTTTTTTTTTTTCTATGTAGCAACATGAAGAATAAGTTTGTCCTATTTATAAATA

ACTTTTAAATAATTGGTTTTTTTATTTTAGTAATTTTTTTTTATGAAAAATAATTATTGA

TTAATATACATACATACATACCTTTAGAGTGTGTTTTATGCTTAATGTGTGCCTATTTTA

ATGGAAAATAATGATATTATATAAAAAAATAAACAATGAAATTATATTAACGTATTTAAT

TTTTTGTTAATTGTATTCATAATTTATTTTCAATGGCGTATTCTAATATTATATTGTTCT

TATAAAAATATATTTTATAAACAAAAAATTATTGAAAATTTTTTATTAATAATATATACA

TCATTATGATTTTCATAATTCATTTTTTTTTTATAATTACATTTATTATTTTATTTAAAA

GTTAAAAATATTATTAAATTTAATAAATATCTACTATTTTTTTTTTAATATATAATGAGT

ACACAAAAATAAAATTAAATTAGTACATAAAACAAGATTGATATAAATATATTTTTAACA

TAATATTTTAAGAAATAAAAGAGTAATACAAAAAAAAAAAAAAAA

>c4038_g2_i1 len=243 path=[1435:0-242]

AAAAAAAAAAAAGAAGAAGAAATGCAACAATCAAAAATTACATTTATCTAAGAATTTGTC

AATTTGTGTATTTTTATATCTTAATTTCTTTTTCTAAACAATTTATCATTTAAGTAGTAT

ACATAATTATTTTTAGGTGTATGAAACAATGAAAATAATATTATTCCTACTTTTTGTTTA

TAAAATATAGTTTTTTTGTATGTGTGGATTTTTTTTTTTTTTACTGTTGAAAAAAAATAT

AGA

>c4050_g1_i1 len=210 path=[522:0-209]

AGAGAGGCCCAACATCACAGCCGTAACCACCAATGCCCAGAAGAGTTCTGGACCCTACTG

TGCAGGGAGCACTAGTTCCCAGTAGATTGGCGCACTCCCTGTTGTGCAGATGCTGGACAT

CTCTGCTAGGATCTATGTGCTGGCTTACAGTCCATCCCGTGGGAGCCACCGGCCTTTGAG

ATGACATTTAGACTCTATTGAAGTAAATGG

>c4054_g1_i1 len=214 path=[1:0-213]

TTATTAGCTTGGTTTGGAGTATAAAATTGTGATGCACCAAAAATGCTTCGAGTGGCATAT

CAGCTGCCTGGTCCAGCTTTGGACTTCCATGTGTGGAGCAGTCCAGCTGATCTTGAAACT

TCTCCAACATAAATGAGGTTCTGAAGCGTGCTGTGCGTCTGCCCCGCTCTGTCTCCTCTG

CGAGCCCTTCGATTTTATACATACCAAACACGTG

>c4061_g1_i1 len=218 path=[243:0-170 25:171-217]

CCTTTTCCAGATTTACTTTTGTTCTCTTCCCTGTTGAGTGAGCATTGCCAGAAGGAACTA

TGAAGTACCGGGCCTTCTTGAGAAGATGGATGACAAGCACTTGAAGAGAGAACAGGACTG

CTTTGTTACACCTTTGTTTTGGAAAGTTTTGGGGGCCACTCAACTCCATTTTTCCTCAGA

AATCTCAAACTCTTCATTTTTCTCACAGTGCAAAGAGC

>c4061_g1_i2 len=219 path=[243:0-170 413:171-218]

CCTTTTCCAGATTTACTTTTGTTCTCTTCCCTGTTGAGTGAGCATTGCCAGAAGGAACTA

TGAAGTACCGGGCCTTCTTGAGAAGATGGATGACAAGCACTTGAAGAGAGAACAGGACTG

CTTTGTTACACCTTTGTTTTGGAAAGTTTTGGGGGCCACTCAACTCCATTTGTCTTTTCC

CACTTTCCTCCACTGCTCTTTGCACTGTGAGAAAAATGA

>c4074_g1_i1 len=351 path=[517:0-323 840:324-350]

TCAAAAAAAAATCAAGCTCTCAAACTACTTAACGTTTCTACTCTTTCATGAATATATCTC

AAAACCTGTAGGAATAGAACAAAAAACCCTGCATATCCCATGGATTTTCACCAAATTAAA

CTCAGGTCTTAAGATTTATGCATACAAGTTGCACATTTCAGTATTGAGTCACCTGTCCTA

AGCTAACTTTTGTAGTGAAATTGTATCGGCTTCATTTTCATACACCCCTCTTCGTCATAC

ATGTCACTGATCAAACACTGTCAGTTTTATTCCTTCACAACAAAATCACCTTTCTGTCTT

GCTTCAGACAACTACCTGCATCTCTTGCTTGTATGTGCTGCATCATAAAAC

>c4076_g1_i1 len=422 path=[554:0-421]

TTTTTGTCTGCTGATAATGCTGCATAGCCAATCCAGCACCCACAGGTCTCTTGTAAGCAG

CTGGCATGGCCATCTGCCAAGGCCGTGCCCACGTTCTGTGGCAGCGAGGCCTGGAGATGC

TGTGCCCACCCCTCACCACTGCCCAAAGGTCTGCATGGTTACGCGAGGCTGTGCAGCAGC

CACTCCCCATCCCCTGCCTGGAGAGTCTGTTGCAGAGCAGCTATTTCACTGCTGATCTCC

AGTGCGCCTTCCGTGATATATGCATCTTATATTGCGGGGCATTCCTTTCAGCCTTATCAG

CATGATGGATAGTTTAAGCCATCTTGAGCTGCACTGAGGCTCCTGAAGGAGGTTGGTGCT

TCTGAATGCATCACAGGTTCAGTGGAAGCCGCACAGAGAAGCGCACAATCATTGCTTTCC

TG

>c4079_g1_i1 len=222 path=[200:0-221]

TCCCTACACCCATCATCCCACCCCACACCTATGGGCTCTCAGCTGCTTCCTCCCACCGTC

AGTTTAACTCCCAGGAGCTAACGAGCTGGAGCTGATGAGGTTTTAATGAGGAGCGAGGAG

GGGTCTCAGTTCTGTCCCTCACAGTGATGGGATGGGAGCTTTACATTGCTGCAGGGCGGG

TGGCACCGTGCATGCAGCGCTAGAGCTCTCTCCAAAGGCAAG

>c4095_g1_i1 len=2531 path=[2828:0-660 3489:661-2530]

AAAAAAAAAAATATATATATATGTGTATATATAAAATATATATAAGATAAATATGTTAAA

TTAAGAATATAAGTCATCATCTACATTATAATCTTGAGGTTCTTCATTATTTTCTTCATC

TGGCCAATCAATGATAAAGTCTTCATTTGCTCCACCAGTTTTTGTTTTATATAAGGGATC

AAATTTTAAGCGGAAATTATCATATTTAATTAAATCAGCTTGTGAAACACTTCTTCTTGC

CCCAGCTAACCCTTCTTTAAAATGATGTCTAGTAATTTCATATTTTACCGCATTTTCTTC

ATTATCTTGATTTTGGTTTTCATTATTTTTATTTTCATTGTTTTGATTTTCATTTGGATT

TAATTGTAATTTTGACTTTTTATTCATTTCTTCTGCATCTATAGCATCTCTAATGGCAGC

TCTTGCTGCTCTTTGGCATAATTCTGCTAAATCAGCACCACTAAATCCTGCAGTTTTTTG

AGCTAAAAAATCAATAGGAACATTATCTGCAACAGGGCATTTTCTTAATATAGCATTTAA

TATAGAAATTCTCGCAGCTAAGTCTGGTAATGGAATATAAATTAATTGGTCTAATCTTCC

TGGTCTTAGTAAAGCTTCATCTAATAATTCAGGTCTGTTAGTTGCACCGATAAAGAAAAG

ATTTTTTTTTGGACCTACGCCATCTATTTCTGTTAACAATTGATTCATAACACGATCTCC

TGCACCGCTACCATCTCCTAAAGTTGTTCCTCTTTGTGTTCCTATAGAATCTAATTCATC

AAAAAATAGAACACAGGGTGCAGCTGCCCTTGCTTTATCAAAAACTTCTCTAACGTTAGC

TTCAGATTCACCAAACCACATTGTTAATAATTCAGGACCTTTAATTGAAACAAAATTAGC

TGAACATTCAGAAGCTACTGCTTTAGCTAGTAATGTTTTTCCACATCCTGGAGGCCCATA

AAACAAAACACCCCTAGATGGAGACATTCCAAATTTTTCAAATTTATCAGGATGATCAAT

TGGATACAATATCATTTCTCTTAAGGTATTTTTCACTTCATCTAAACCTCCAATATCATC

CCATTTTACATTGGGAACTTCTACAACAGTTTCCCTTAGAGAAGAGGGGTTACATGTTCC

TAATGCCATGTTAAAATGATCTTGTGTTACACACATACTTTCTAAAACTTCTTTATCAAT

AATTTCATCTTCTAAATCAATTACATCCATTTTTTCACGTATACATGTTAATGCAGCTTC

TGTACATAATTGTGCTAAGTCAGCTCCAACAAAACCATGTGTATTGCTAGCTAATTCTTC

TAATTTCACGTCAGGTGATAATTTCATATTTTTTGTGTGTATTCTTAATATTTCAAATCT

TCCATTATCATCTGGTACTCCAATATCAATTTCTCTATCAAATCTTCCGAATCTTCTTAA

TGCAGGATCAATGGAATTTTGTCTATTTGTTGCTGCAATAACAACAACTTGCCCTCTACT

TTTTATACCATCCATTAATGTTAGTAATTGAGAAACCACTCTTCTTTCTACTTCTCCATT

TGTTTTTTCTCTTTTTGGTGCAATTGAATCAATTTCATCAATAAATATAATAGCAGGAGA

ATTTTTTTCTGCTTCTTCAAAAGCTCTTCTTAAATTAGCTTCAGCTTCTCCAGCCATTTT

ACTCATAACTTCTGGACCGTTTATTAAAAAAAAAAAGGCTCCCGTTTCATTTGCTACTGC

TCTTGCTATGCAAGTTTTTCCACTACCTGGGGGACCATATAATAATACACCCCTAGGTGG

TTTAACCCCCAAAGTTTTAAATAAACCTGGATGTCTTAATGGCAATTCAATCATTTCTCT

AATTTGAGCTAACTGCTTTTTACAACCTCCTATATCATCATAACCGATTTCATCTAATTT

TTCTTCATCATCTCTTTTTATAGGATCTCCTTCATAATATATTACTGTATCAGGTGAAAC

AATACAATAATCATCAGGATCCACTTCAACAACTTTAAATTCGACAGACATAAATCCTCC

TCTCACTAAAAATAAATCCCCTTTTTTAACAGGTCTATATGATTCATTAAAATAAGGTTT

TAAAAATATTTCAAATAAAGTATCTTTTGCCAATCCTTCTATTGTATCATCTATAGGTAA

CACTTGAATCTTCTTTCCATATGGAATTTCAGGACAAGGTTTCACATATACAACATCTCC

TAAGCATACTCTCAAGTTTTTTCTTGCAACTTTATTGATTCTTATTTTTCCTTCATCTAA

ATCATTATCATTTAAAATAATACAAATAGTTGAATGCCTTTTTTTTCCTTTTATTAAAAT

TGTATCCCCTCTAAAAAATTTTAATTCTTCCATTCTTTTTGTGTTTAAAGCAACCACTGA

GTTATCATCATTTGTTGCCTCTTCTACTATAAGCCTACTTAAGTTTTTTTTTTTTACTAC

TTTATTTTCACCATTATTTTCATCAGGTAAAGCTTTAAAATCAGAACTATTTTCCATTAT

GACTCATCAGA

>c4098_g1_i1 len=223 path=[1:0-222]

TAATTATTAGGACTTCTTTTCTTTAAATCTTTCAGTCATAGTAGCTGCTTGCTGAACTTC

TGAACGTGGAACATTATGGTTGTTTACTATAAGTTTTTCAGAAATGGCTTCCAAGTGACT

CCTTCTCCCCCAGCCTCATTACAATAAATAGCCTGAGGCAGCTTCTTATAGAAGAATTCA

GATTAAAGTTTAAGTCAGCCAGCCATTTCTGAAAAACTTATAG

>c4102_g1_i1 len=201 path=[1:0-200]

TCTATGCCTTTATTTAATGCTTGAGACATCCCGCCAACAAAACCTCGCTAATTCACACAT

GGGATGGAGCTGAACCTGTCCCGGTAGGTTCTGCAGGCCTGTCCCGTTCCTAGCACAGAT

CCCCATGCTCATTGCTACCTGCCAGCTGGTGATGCTGCAGGACACAACAGGGCATTGGGC

TGCAAGCTCTGCAATTACACG

>c4103_g1_i1 len=204 path=[182:0-203]

GAGGCAATCCCCGCTGTTCCCGCAGCGATGCAGGGGAGGAGGAAACCACAGGGGATGGCA

AAAAATAAAGCGAGGAAACCTGCTTGACCCAGCGAGGAACTGGCAGCGACTCACCTCGCC

GGGGAGGAAAGATGCCATTTTGTCACCTCTCCAGCGACAGGTTTGAACCCAACTCTGGGA

AGGTTCCCCAAGGAACGGGGAGAT

>c4104_g1_i1 len=225 path=[357:0-224]

GTGAAGGACTGCTCGTGGTGCAGGTGGGCGCTGGGAGACCGCGCCGCTTTCCTCCACAGC

TCCGCCCGGCTGTGAAAGGGGGGAAGGAGGAGCGCATCCCCCACCCGAGGGCTGCCGTGG

GATTCTGCGGTCTCCTGGGGCTGCTGCGGGGCACAGCTCCGGCCGGGCAGCATAGCAGTG

CATGGAGCCGTGCAGTGTGTGGGCGGTGTGCCGGGCTGCTGCAGC

>c4105_g1_i1 len=201 path=[1:0-200]

GCACTGAGGGGGCTCCCCTCCTTCTCCTCAGTGCTGCATACCGCATCCATGTCCCATGGG

ACAGCTGCAGGACTGCAGGCAGGCAGCAGTTGCAGGCTGGGGGGCAGGTGTGGGGCTGCA

GCCCCCCGCTGCACTCAGGGGCTCATTGTTGGGGCGCACTGAGGGGGCTCCCTTCTTTCT

CTTCAGTGTTCTGTACCTCAT

>c4108_g1_i1 len=205 path=[1:0-146 400:147-204]

GGACTTCTTTCTTCTTCATTAGAGAAAACACACCTGTGATCTAAAATAACTTGTAGAAGA

TGACAAATTCAAAACGAATGAAACCTAGCATTTTATCTTCTCCATCTTTCACATGCTTAA

GCTACTTGATCTCCTTAAGATCCGTTTAAACTAATATACAAATAAAGAAACACATTTCTG

GAGATTATTTATTTAATTCTAAGAC

>c4109_g1_i1 len=229 path=[81:0-170 255:171-228]

GCCAACTTCAGAAAATGAGTCAAGAAGCAAATAGAATTGTGGGTGTGAACTATTGAAAGG

ACAGGTGGCTTTTCTGTTTGTTATTTTTACAAGCAGATTGATAATGGAAGGGTTGTGGGA

GTCTGAATGCGCTGTTAGTAAGAATGAGAGCGGAGGGGATGATGGGCAGTACCAGGAGGT

TGAAACTCCCTAGGAAGGGTGAAGATCATAGAATCATAGAATAGCTTGG

>c4121_g1_i1 len=248 path=[380:0-247]

GGGGTGGGTTGTATTTTTTTTCTTCCCTGAAACTGATATTGTTGTGTTTGTTGTCAAAAT

ACTTTGTATTTTCTCTCCTGTTGTTACTTAGTCTGGCTGTGGCACCAACTTGCTTATTTG

TTACTGCACCTTCTTTTCTTTGCCACCTCCAGCTGGGAAGAAGAGGAAAGATAGTGACTT

AAGTGTTCTCCAAGGAGTGTGGGTTATAGTCTGTAGTATGAACAGGTCTTCAGCTCTGAC

ACTGTTAG

>c4129_g1_i1 len=223 path=[78:0-77 412:78-222]

ACGTATGGAGTCTTTGGCAGAGGATTTGGGGCGCTTCGCTTCTGACGGCACTTGTGTAGA

GCAAGGAGTTTGTAAAGAAGCCCTGGGCAGGAGAGGCACCTTCTCAGAAGCAGAGGAGGC

ACCAGGTGCTCCCACAGAACCAGAGGCTGATGGGAGTGTTTCATTCTGATCACTGCTTGC

ACTGTTAGCAACAGAGAGAGCTTCACACCAAAGCTTCTTCCCA

>c4131_g1_i1 len=207 path=[23:0-20 44:21-206]

ATTTAGAAATTTGAATACTGTTCACTCCGCATGAATTTTTCAGTCTAAGTTTACCTTTTT

TGATCCTGAGTGTTCAAGATGGAATGGCAAATTTCAGATAAGGTCTATGATTGCTCTCTG

TGCCCTTGCTAATTGTCCTTCATCCCACTGGTGATATGCTGGCTTTGGATTTGTATTCCA

GAGTTTGCCTTGTATTTAAGCACAGTG

>c4131_g1_i2 len=224 path=[45:0-37 44:38-223]

CAATATATGCTTATTTTCATTTCTCTTATCACACTGTGTCACTCCGCATGAATTTTTCAG

TCTAAGTTTACCTTTTTTGATCCTGAGTGTTCAAGATGGAATGGCAAATTTCAGATAAGG

TCTATGATTGCTCTCTGTGCCCTTGCTAATTGTCCTTCATCCCACTGGTGATATGCTGGC

TTTGGATTTGTATTCCAGAGTTTGCCTTGTATTTAAGCACAGTG

>c4132_g1_i1 len=245 path=[377:0-244]

GTTAAGCACTGTGCAGAGTCTGTAGCTATTAGAACACTGCATTTGCTAACTTCGACTGAT

GATCTGAGTGGAGAAATACAAAGGATGAAGATGCACCAGCTCAACCTAAGGGCACAAGAG

ACTTGGCTGACGAGCTTGGTTTCTTGCCCAGCATCAGGCTAGAAACCTCATACGCACTGC

ACTGACATTTAGTTGAGAGCTATATCAAATCAGTCTAAGGAGCAAAAGTCATGCAAGTGC

ATGCA

>c4137_g1_i1 len=263 path=[53:0-262]

ATGTTGTATTTTAGAAAGTACTTAAAGCTATTGTGGTTAGAAACCCATCTATGAGAGTCC

CAGTGTCCTATAACCTAAAGTACTTGACCGTGTTGCTGATGTGTTGTATAAGAGGTTCTG

TCTGAAATTCCATCTGTAAATTCCAGTGTTTTCCACAGCCAACCTGGTAAGAAGTAGAGC

TAAAACCAGAGCACAATAGGGTGAACGAGAGGGGTAGATGAACAGCAGTACAAAGCCAAT

GATTATATCTCTAATACATAAGG

>c4149_g1_i1 len=268 path=[183:0-230 69:231-267]

AAAAAGAAAGCAGCAACACAAAAACATGACACTGGCATCACGGGAGTCCACCATGAGGCT

ATAAAACCTATTTCCATCCAGCTTGAAAAAACAGACATGCTATAAAAATAGCATTTTTGA

GTTATGTCCAAATTTACATATGCATGACTGACACATGTTCATAAGGGTATTATTTAACAC

TTCAGAATATTACGGAGCATCACATTCTAAGATAATGATTCATGGTCAGATACAGTTGTG

TCACAAATCCAGCTACAATCTGCAAGTA

>c4153_g1_i1 len=268 path=[155:0-267]

ATTTTTAATTAAAAAAACCAACAAACAAACAAAAACATGCATCAGCTGTGACATACTTAT

TCCCAGCATTTAAGAGCACGAAAAAGTGACTCCTCCATATTGGGCAATTTACCTGTACTG

AAAAACTCAAACTTAATAATGATTCAATCTCCTAACTAAAACTACAGCACTGTTTTTTAG

GCCCTAGACAGTAACCAGTCTTTAGCCTTGCATTTTTCCCCATGCTACAAATATTACCCA

GTAGAGTAAATCAAAATAATAAGACAGA

>c4154_g1_i1 len=223 path=[1:0-184 260:185-222]

ACACCAACAGTCTGAATAATTTTATAGGAGACACCCTCCAGCAGCAGAGTGAGCGTGCAT

GCTTTATTGAGAAGTGTTCTGTGTGACACAGTTAAGGAGCTCCTTGTACTAGTGCTTTTT

GTGAGATGGATGTCATTGTGACAGGAAGGAGTGATTTTGTGTGTTGAGAACCACTGTGGT

ATGCTGCCCTCTGCTGGTGATCAGAGTGTATGTACAACTAAAC

>c4154_g1_i2 len=222 path=[1:0-184 186:185-221]

ACACCAACAGTCTGAATAATTTTATAGGAGACACCCTCCAGCAGCAGAGTGAGCGTGCAT

GCTTTATTGAGAAGTGTTCTGTGTGACACAGTTAAGGAGCTCCTTGTACTAGTGCTTTTT

GTGAGATGGATGTCATTGTGACAGGAAGGAGTGATTTTGTGTGTTGAGAACCACTGTGGT

ATGCTCCCCAGAAGCCAGTTTAGTTGTACATACACTCTGATC

>c4166_g1_i1 len=208 path=[348:0-207]

AATTATTCTACGAAATTGTCAGAATTATGCGTAACAAACTCCATTCTAACAACTTTGAAA

GCAGCATGAAAGGAAAATTAGACCAAAAAATATGAAATCTGTCTCCCAAAGACTTTCACT

GCTGTTCTACTGGTAAATATAACTTACGCTTCAAGACAAAACATGAAACTACACTCCCCA

TACTGTTACTTCAGCAAATGTGGCAATG

>c4171_g1_i1 len=282 path=[470:0-281]

ACCAACCTGGGGATGTTGTAGGGAGGTCTTGTATGGTCCAGGCGGTCCTCTTGTTGCTCC

AGAGAGTCCTTGGATGAATCCAGAAGGGTCTGGAGATGTGCCAGAAGGATCCTGTAGGGT

GCAGGGCATCTCCAGATGGGTCCCTGGTATCCTTGGATTCATTCAGAAGGGTCTGGGGAT

GTCACAGAGCGGACTTATAGGATCCAGGGGGTCCTCAGCTGGGTTCAAAGGGTCCTGTTG

TCTTCTGCAAGGGTCTGGAGATGAGCCAGAGGGGTTAAGGTA

>c4172_g1_i1 len=205 path=[131:0-117 249:118-130 400:131-204]

CACAGGGACCACTTGGTGCACTGGGAAGCTCCAGAGCAGGCCGGGGGGCGCCGTATCAGA

AGATTCCAGGTCTGCGGGGGAAGGCTTCACCAAGGAGGGATTTCCAACCACAAATCCCTC

CCCAGCGGCAGTCAGCCCTTCAGTGAGGTCTGAGAGAGGCGCACCCAGGGCCGGTACCGG

TGCGGGGAACGCAGGCGGTGCTGGG

>c4176_g1_i1 len=345 path=[545:0-73 25:74-344]

ACACTATTAGCCCGATTGACAGTGCTCATTAACCTAACCTGCGCAATCAGTCAACGAGCT

TTTAGCCCTGCTGGGATGAGCAGTTCAGAAAATTGTAGGGGAGTTAATTAAAGGGGGCTT

CTGTGAGGATCTAGAGCAAGACTTGAGCTCCTGCTCTGAACCTTCACTGCTGGTCTTTGA

CAGCTGTGCTCTCTGTGTCACAGAAGGACTGCAGTACGTATGCTGGTCTGTGCCAATGTG

TATTTATATCTGAATGTAATTCAGACCATTAGCTGCATTGTTCAGTATCTCCTTTTCATG

CAATGCAAAGTTTTTTATGGTGATGGTGGTGAGGCACTGGAACAG

>c4182_g1_i1 len=443 path=[933:0-321 1371:322-324 421:325-442]

GTTGAACTTGCATGTTTTGTTTTTTCCTATGCAGAGTTCCAATCCACAGACTGGGGCAAA

CCAGAAAAACACAGCACGAGGGCACTGCTTCTGGTTAACACAAGCATCATCTGGCTGTTG

CTATCCACAAGCCCTAAAGACACCCTAGTGTGACCCAGCACCAGGAGCAACGTGTTTCTT

ATTTGTGTTTTCATCTTGACACCTCTGCCAGGTCAAAATTCCCTGCAAACTGCAGCCTCA

GCTTTCCCTTTTGAGCAGGATCTGTTAGGGGGATCCCCAGGGTAGCACCCCAGGGCATAC

CTGACCCCTTGTGCTGTTCACGTTCACAAGCTCTTGGAGGACACAGACATATTTGAGACA

CTGTGATTACAACAGTAATTTTAGAGCATTACCGGAAAACTGAGACCCATCTCAGCTCAG

TTCTGCTCTGTACAGCTCCTGCA

>c4182_g1_i2 len=318 path=[1:0-196 1371:197-199 1256:200-317]

TTTCCTAGAATTATCACTGTTCAGAGATGCTACTTGTCACTGCTGCAAAATTTTTTGAGA

CTCTGGCAAAGTCAAGACAGGCACAAATGATTTCCATAGTGAGTACAAATGGGATTCTTC

AGTAGAAGAGCTATTTTATTTCTTTAATCTTATACTCTAGAACACATTTGCATTGGCAGA

ATTCCTAGGAAGTTACTTTCCCAAAGCAGGGGACAGATAGAGTATGGCTGTTGATGCCAG

TATGAACCCTGTAGGTCCAGGAATGAATGGTTTTGGATGGACTGCTGCCTGACTGCAGGA

GCTGTACAGAGCAGAACT

>c4182_g1_i3 len=443 path=[933:0-321 1371:322-324 1256:325-442]

GTTGAACTTGCATGTTTTGTTTTTTCCTATGCAGAGTTCCAATCCACAGACTGGGGCAAA

CCAGAAAAACACAGCACGAGGGCACTGCTTCTGGTTAACACAAGCATCATCTGGCTGTTG

CTATCCACAAGCCCTAAAGACACCCTAGTGTGACCCAGCACCAGGAGCAACGTGTTTCTT

ATTTGTGTTTTCATCTTGACACCTCTGCCAGGTCAAAATTCCCTGCAAACTGCAGCCTCA

GCTTTCCCTTTTGAGCAGGATCTGTTAGGGGGATCCCCAGGGTAGCACCCCAGGGCATAC

CTGACCCCTTGTGCTGTTCACGTTCCCAAAGCAGGGGACAGATAGAGTATGGCTGTTGAT

GCCAGTATGAACCCTGTAGGTCCAGGAATGAATGGTTTTGGATGGACTGCTGCCTGACTG

CAGGAGCTGTACAGAGCAGAACT

>c4188_g1_i1 len=423 path=[870:0-53 25:54-422]

AAAATATGTGCTAAAAACTTGGGACAACCACAGGCAGTGGAGGTCATGGTTTTTAGCACA

TATTTTGAAATCTAATTCCCTTAGTGATTCTCGCAAGCAAAACCAGCCAGATGTCTAAAC

TGTAATTTTTCAGATACTATTCTTTACTCCCTCAAATAATTACGATGTATAAGAATGTCC

TTTTATCAGAGTACTCTCACTGTTCTTGTCAAGACCTGCATGGCTCTTACTTTTGACTAG

TAGTTTTTAGTAATATAACCATAACAATTCCAGAATGAAAGATGCCCACCGTCCAGACAT

TTCACATTCAATTTACCTTGTCTTGACAAGAACAGCCCTCGCTAAGGTATCTATTATTTT

ATACAATATAAACCACAGCAGTTTTAATGCAGTAGCAGCATTGCTACAGTCACAGTAGTA

AAG

>c4194_g1_i1 len=269 path=[401:0-268]

ACAGATCCTTACTGTTTCTAGTTCTTGTGTGGTTTAGCTAGTTAGGTAGCTCTGTCTTAA

ATTGTAGAAATTCAAACAATCAGACTTGATTGATCTGTATGAGTAAGCAATAATCAGCAC

GTTCTTTTCAGGACATACGTTGTTGGTTAAGTTGACTGAAATTTGAATAATAGTGTGTCT

TAAGTCCACTGTATTTATTCCAGATACCTCTCACCATCAGTTGCAGTAGATTTTTTGTGT

AATAGAAGATTCAATAAACATACCACGTG

>c4198_g1_i1 len=228 path=[247:0-166 417:167-181 432:182-227]

TAACTTCCTTTTCTTAGTGGATTTTCTTAGCAGATTTTTGACTGTATTTTAACAGTAAAT

TGTATACGGTAAGCAGTTTTCTTAAAACCACGCTAAATAAAGTATAGCACATGCTATATA

TATTGGCAGTATTTAACAAATGAAAACTATCTCCATGTAAATCAAATACTCAATTTTCAG

AACCTGTTTTTCCTCTATTGTTAAATCAGGTTCTGAAAATTGAGTATT

>c4207_g1_i1 len=246 path=[1:0-245]

GGATTTCTGAGGCTAAAGGATGCGTGGCTTTGTGTCACATCAACTGTTCTGGTTTGGAAG

CGGTGCACCCTCTCTGCAGGTAGAGACCATGCACACCACACACACACGTTCTGATTTGCA

GGAGCAAGCAGTGCTGGTATGGAGCCCCTTTTGCTGACAATCTCACCTTCTTTAGGGCTT

TCATTGATTTAACTGTTTCAGTCGTACCTTTAATTGGAACAGGAATTCTAGGCACAAAGC

AGGCAC

>c4208_g1_i1 len=392 path=[1:0-172 550:173-391]

AAGAAGCACCTCCTTCGTTGAAGCTGAGCATACAGACAAGTGGAATGTCAAAGCTCTAAT

CTAACGTCTCCATCTCTCACTCTTTTCACCATGGGAGTGCAGCTCCAGTAACCTGGTTAC

CCTTCTGACAGTGTGCAAACCTGTTGCTTATGCATCAGCTCTACTTTCCCCAAACAGGGA

AAAAAAAAAAAACAAAGGAGCATTGCCCAGAATACTTCTTTTAGTGAGTAATCATCTCTC

AGAGCTAGCTGTGTTTTATTTTCTCCTTCTTCCTCTTCCTTCCTATATCACAGTGAGATT

TCCAGCACTCAGCATTTCTCTGCAGCTGTGATGTATCTAACACCCAGCTCTGTTCCTGGA

ATTACTCTGCTAATACCAGAGAGCTTGTTTAT

>c4216_g1_i1 len=250 path=[391:0-194 38:195-249]

TGACGGGCAGCTCAGTGATTTCAATGGTCGTAGAATCAAGGATAGACACTTCACCACTTA

TCACATACTGGTTAGGTCCAAGCTCATCTATTGTGCCTTTGAAGTTCTTGTAGCTGGGAA

GCATTGGCAGTGGTTCTTTTCCATCCAACAGACAACGGATGTTATTCACAGTCTCCCTGA

TATCAAAGTTGGGGATCTTTGAACTCAGGCTCCGATACTATGGTTTGAGGAAAGAATGGC

TTATTGGGAT

>c4218_g1_i1 len=235 path=[1:0-234]

AGAGGAGGTCAAACAGAGGGCAATGCCTGGAGGCACTGAGTTTCAGGAATGATCAGTAAT

GGCTTTGGGGGGTGAAGGGTAAGGCCAGAGGAGTTGCACTCATTTTATGGGTATAAAGCT

CTCAGAGCCTCCCAGCCCTTTCCCCATCTCTGGGCTTTGCTGCTGGTGCTTGCTCAATGT

GTTACAACACGCTGCTGCTCTGGGTCAGGGTGGTTTTTCTCCCAGTGCGTATCTG

>c4222_g1_i1 len=330 path=[1:0-329]

AGATAGCTGTTTTTTATAAAGAAACATTGTCTTTCTAAAGAAGCCTTCATTTACATACAT

AGAGAATATGCAACATAAACACAAGGGTAAGAGCTTTTAAGAAGTATTTTTATATGTATA

TCTTATTTGGAAGCAAATTTCCCCACACATCTGGCCAAATACATTTATTCTTTCTCTAAG

CTCAAACACACCAGCAGAAACACACCCTTGTCTCCTGGAAGTTCATCTGGAGCAAAACGT

TTTGGTGCACATACTTAAGGAAGAATTTATTTTTATGTAATGAACTGTTTCGGTGTGATT

TGAGTGAGGTATACATTACAAGGACATATA

>c4226_g1_i1 len=368 path=[1:0-367]

GCACCCTCAGCAGTTTGGTGCTGGCACTGAGCTGAGCAGTGCAATTGGCACAAAGGAAGG

AAGAGATCCATCCCAGACAGAAGCAGAAAGTGGACACATGAGAACCTAATGAGGTTCAAC

AAAGCTTTGTGATCTTGAAGTACCCTTCCAACCCTACAGCGCTGTGATTCCCTGGATCTG

TGACCTTCAAGACTCCTCCAAACCCAAACCCAACCCAACTATTCTCTGTTTCTGTGATTT

TCAAGGTCCCTCCCAACTCAGCCCATTCCATGGTTCTATGATCTTTCAGGACCTTTCCAG

CCCAACTCACTCCATGGTTCTGTGATCTTTCAGGACCCTTCCTACCCAACACATTCCATG

TTACTGCG

>c4226_g2_i1 len=226 path=[691:0-225]

TGTGTCATGGATAATGGTGTTGAAATCCCAGATGGACCCGAGGGGACACCACCTCTGTCC

CACTTCCACCGGGACACGGAGCCATTGACCCCATTGGCTGTATTATGGGCACACCATGAC

TTCACCCCATAGCGCTGCCACAAAGCTTTCTGCACAGTTTTCACAACTGATGGGTCTGCT

TGGAAGCCCTAAAGCAGCACAGATGTCTCCTGTTGATATCCACCCG

>c4229_g1_i1 len=326 path=[331:0-234 28:235-325]

AAAATAAAATATAAAAAAATAAAAAGTAAAATAAAATGAATTAAATAATATTAAAATAAA

AAGTAAAAATACTAAAAAATATAATGTGTGTTCTCCTTAAAAAAAACTTCTTTAGTAATC

TTAACTCCTTCAAAAAAACGTAAACAAATGTTTATATTTACATATATATGTAGTATATAA

TATTCATACCCCTTTTACTTTTTTAAAACAAATTCTACATAAAAATATTAAAAATAAAAA

ATAAAACAAATTGTTTTTACAGAATACTTACATACTATAAATTTTTATATTACATTTAAA

AAAAAAATAACAAAGTAAAATATAAT

>c4234_g1_i1 len=288 path=[266:0-287]

GAGGGAGCATAGCAAACAGCACAAAGCTTCACATCTGTGCCCAGTGCAGGTGTGTTACAG

GGTGTATTTGGGATGATGGCACCAGTCATGGATGTGAGCAGGAAAAGGGCCTTTCTGTAT

CCCCCACACATCCAGAAAGGGACAGATTGCTGCTGAATTTCAGAGTTATCCCTGCTGCTG

TTGTTAAAAGTTCAAGCTCTTTCTGCTGCAGCCTTTTGCAGCCTTCTATTCGTAGTGCTA

AACTACTTCGTTTCCTTTTTCTTTCAGTTCTTCAGAAGACTGGGTCTG

>c4235_g1_i1 len=286 path=[264:0-285]

TAAAAGAGTTAGTTCATTAAGTGAATATATATTTAAATTTAAAAAATTAAAAACAGAAAA

TAATACTTTTGAAAGTAATATATTTAGAAAATTTAATACTAATAAAAAAAATTTAGAGTT

ATCAGATTTAGACGCAGATCCTAATCTAAGTTCAGAATACGATTCTACATTTAGTGAAGC

GAGTGTTATTAAAGAAATAAAAAATGATTACAGTAACAATGAAAATAATTCAAAACATAC

TGATACATTTGGCGATGATATATATAAACATAATTCAAAAAATAAG

>c4241_g1_i1 len=259 path=[237:0-258]

TGTTTGATAGTTACAGCATAGGAGATTAAAAAACGTATGAGTTACAGGAAAACAATACTT

AAAGCTTCTCAGCTGCTTCTTACCTTTTGTTCAGTTTATTCCACTTCTGTGCCTCGACAT

TTGATTATTAAACCTTCATTTTGTAAATAATAAAAATAATACAGAAACAAAGATCTGGAA

TCTGACTAAAGGCAGATTCCTCCTGACTCCTTGAAAGCAAATGATGACAGGCTAAGAATT

ATTGTCTTGACTTCCAGAA

>c4242_g1_i1 len=210 path=[37:0-156 36:157-209]

TATAAGAGCATTAACCTTTTATTTCAGCTAAGCTGAAAGTATCTACGATGTCTGATATTG

TAAGAAAAAACTGCTTAGGAACTATACTAAGGTCAACCGTAACAACTGTTTGGTATTATG

ATCTTTTCAAGTCATTTAACACAACTGTTATCAAAACATTTCTAAACCTTCTTTATTATA

AAATGAATTGATAACAGTTGTGTTAAATGA

>c4243_g1_i1 len=317 path=[1:0-96 98:97-316]

GTAGCACTCTCCTCTCCTGCCTCGTGCCCTCATTTGTTTCCCCTCATCCCATAATCCTGT

TCTCTTGTGCTGATCTCCTGCTAAGTACGGAAATGTTCTGGAAAAGGTACCTCTGGTGAG

AATATTATTTGATAAGTGCTTTATGTTTTGTGGTGTGTTCCTGTAGAAAGTAAGTTGACC

AATTCTACTAGAATCAGTGTTTCTCTCTGCATTTTCTCCCTCCATCTGGCTGTTCCTCTC

TGTAACTGGAGCTCCTTTACCTTTCTGGTATGAGCAGCCTCTTCTCTGCAGGCTATTGTG

CTTAAGCTCAACCCTCT

>c4243_g1_i2 len=282 path=[651:0-61 98:62-281]

GAGTGCTACAGGACAGTAGAAGCCAGGGCAGGACGAGGTGGCCATTCTGTCCAGGAAAAG

CACTGGAAAAGGTACCTCTGGTGAGAATATTATTTGATAAGTGCTTTATGTTTTGTGGTG

TGTTCCTGTAGAAAGTAAGTTGACCAATTCTACTAGAATCAGTGTTTCTCTCTGCATTTT

CTCCCTCCATCTGGCTGTTCCTCTCTGTAACTGGAGCTCCTTTACCTTTCTGGTATGAGC

AGCCTCTTCTCTGCAGGCTATTGTGCTTAAGCTCAACCCTCT

>c4245_g1_i1 len=221 path=[525:0-220]

GGTCAGGAATGTAATGCAGAGCTGACAAGAACTACAATGCACTGCAGAGGCATTGAAACA

TGCTCAGGTTGTCCAGAAAGGTTTCAAAGATTTCCCCTGAGAAGGCAACAGCCCAGAAGA

CAATTGCATGTGAGCTATAGTGAGCACAACTGTGACTGCAGTGCAACTTCCCCAACGCCT

CCATCCTGCTCTGTGATGGGAATCTGATTTGACCCTTTAAT

>c4254_g1_i1 len=271 path=[187:0-270]

TGGCTTGGCTTGGCTTGCGTGATGCATCCAATACAGAGACCTTGATCTTCAAACCTGCAG

TTAATCCGTGCTCAAAATGAGCTTTTTTCCCCCAATATGGTGCTATCTCCAGACCTTCAG

ACTGAGCATGAAGAAAAAAGGCAATGAGAAACCCTCAGCTTTCAGCAGGGCACTTGTAGA

GTCATTAAGATTGGAAAAGACCGCTAAGATCATCATGTCCAACCATCGACCCATCCCCAC

GAGGCCGTGTCCCGTGTCCCATCCATCCAAA

>c4256_g1_i1 len=296 path=[1:0-295]

GGATGCCATGCAGTTACCCCGGCTGTGACAGCCCAGTGATACGCTGATTCAAATCAGCAG

GAATACACATCGCTGTATTTGAGCACCCTCAGAAACGGACGTTTTGGAAAATGCCAGATG

AAATTCAGTTATGTTTGCAAATTGTATTGCATCCCTGCCTGCACCTCTGCCTATCACTGC

ACAGAGCTGGCTGCCACACATCTGGCTTTGCATTTGTTCTTACCTTTTTGCTTTCTCCCT

GAAAGATGTCGGTGCTGTTACTGACGCCTGGCAGCCAGTGGGAATGGCTTGTGGGT

>c4256_g2_i1 len=201 path=[725:0-200]

GTAGAAACAAACATCAAAATACAGATAGCAGCTGAAACGAAAATACTCTGTGCCACTCAA

CCTCGCTGTCATCTCCCTCTAACAAAAGATGGGTTTCTTTTCTCCTGTTGTTGTGACTCC

AGAGCAGAGGAGTTGCAATGCACTGCAAACTCATACCTCGAATTAGTGCAGTTAGTATTT

TTAGTGTTACTAACATGGTGA

>c4260_g1_i1 len=249 path=[234:0-208 490:209-248]

ATTGATGCCATGGTAGAGGAGAATCGCGGTGCTCCATGTTACACGGCAAAAATGCTGGAG

GAAGACAAGCGGAGATTCCGTGAAAAATTTTGTACCATCCTGTAGGCAGCAAGGGGTCAG

TCCCAGAGCAGACATCTCCTCTGTTGTGCTGCTCCTGCTGTCTCTGTGAGGTGATGTGGG

GCACGGTGTGTGGTGATGGGACTGAAGGAACTGTCAATAAACACTGAGCCTTGCTGTCTG

AAAAAAAAA

>c4261_g1_i1 len=202 path=[169:0-201]

AAGGAGGGAGCAGAGAGGCAGCACTGAGCTCTACTCTCTGTGACAGCGACAAGAGAATGG

CATGGAGCTGTCCCAGGGGAGGATCAGGTTGGAGTTTAGGGAAGGTTCTTCACCAGGGGC

TGGTCAGGCCCTGGAACAGGTTGCCCAGGGCAGTGGGCATGGCCCCAAGCTGGCAAAGTT

CAAGAGGTGTTTGACGATGAGA

>c4265_g1_i1 len=218 path=[1:0-217]

GTAGATTTTAAGATTGGACAGATAGTTGGGAGTCACAACCTACAGAGCACGTGAGCTTTT

TTTGAAACTGAAGAATGTGGCAATTTAACTGGTAAATAAAATTTTCAGTAGGTTGAATGA

AATGTTCTTTTCTTGTTGCATCTGCCTTTTGTCTCATTAGGTACCTCTTCTGGACAGATT

GGGGTCACGTTGCTAAAATTGAACGGGCAAACTTGGAT

>c4265_g2_i1 len=276 path=[391:0-275]

GGACTGTCTCTTTGTTCCGCCCAGAGTATTTGTCCACTCTCTGTATAGATTTTGTTTGCC

AGTCAGTCCAATATATCCACCTATCCTGCTGAGTCAGAGCAAAGGGATGTGACACCTGGC

TGACCAGCACTTGTCGTAGCTTCCCATTGAGATCACAACTCTCTATGCGATCAAGATGCG

CGTCCACCCAGTATATCCTTCTGGTGTCATAATCCAAGGTCAAGCCATTTGGCCATCCTA

GGTCAGTGTTGATCAGGATTTTACGTTCAGAGCCAT

>c4273_g1_i1 len=331 path=[1:0-330]

GACAACGCTGAGATTCAACTAAATAAAGCAATGGCACAAGCTCTGATTTTTGCCCTTCTC

AGAACAGTTTGAAGTGAAGCTGAGTGACAGTCCAACTGCAAAACATGCGTTGAAATAGGA

AAGGTTTAGCTTTAAATGTAGACCGCTGCAGCAGTTATTTAACTTAAATAATTCCGGTTA

TCTCAGCGTTGATCGGCCTTTTGTGATCAAAAAGGAGCTTCCCAAGTCACTTCAAGTTTA

AAAGGCAAAGCATTCCTGTCCTGATTCCACAAGCAGAACTTCAGTGCTACTGAGCAGAAG

GCTGTGCGTATTGCTTACTGGAAAATTAGAC

>c4276_g1_i1 len=252 path=[25:0-125 25:126-251]

ACGTATTTTACCACTATTTACAAAGAAACACACTCAATCTCAGAATTCCAACTACAAAGC

CTCTTCAGCTCTCCACTGGTCTTGGTTCTTTAGCTCAGGATTGTAATTTTAGATATACAT

TGTAATACGTATTTTACCACTATTTACAAAGAAACACACTCAATCTCAGAATTCCAACTA

CAAAGCCTCTTCAGCTCTCCACTGGTCTTGGTTCTTTAGCTCAGGATTGTAATTTTAGAT

ATACATTGTAAT

>c4277_g1_i1 len=251 path=[229:0-250]

AAGGACTGTAGTTGGTAGAAAGACAAATAGTTGGTGGAGGAAGTAAAGAATCCCAGCTAC

CGTAGAAAAATACACAGTATTGTGTTCCCAGTCATGGGAGTCAGTTATGAAAATTAATAT

ATCTCTTCTCCAGACTTAGGATTTACTGACCATAACTTATTGGATATTCGTATTCCAAAA

GCCACTTTTCTTCGCTGTATTATTATATTTCATTTTCAACATGATTTCATACATGTTTAT

ATATTGCACTT

>c4285_g1_i1 len=223 path=[1:0-222]

TGCTTAATGAACCTGAATGTAGAATCCTTTGATCAAGTACTTCTTATACTAACACAGAAT

GTCTTAGCCATTGATGTAATCTGATTAATCGAGGACAATTGCTCTCCTTGGAAAACTGGC

AAGAGAAAAACTGTTCAGCTTGTTCTTAGGTGACCACATAAGGATAAAAAGAGTAAGAGG

AACATTCATGAAATGACCACTGAGAGACTCGTAAGACACTCAA

>c4299_g1_i1 len=303 path=[281:0-302]

TGCTTTATATTCACAGACACTGGCTTGGGTAACACCCACTTTCAAGATTTGGGGCAATGG

GAAAATTGCTTTGTTTTTGTCACAGCGTTGCATCAACATACGATGCTGTTGAGCAGTGTC

TGTACATACCACAGCAGTACTGCATCACACTGGCACGGTGCTACACCTGTATGAGCAGCC

ATGGTCGCTGCAGTCATTTACAATTAGTGGCAATGCTCTACGAGGTCTATGAGTGTACAC

AGCTGTGCATACTAATTAGCTGGAAGTAGAAATTCTATCCATCATAAAATGAAATGATTG

CAA

>c4301_g1_i1 len=225 path=[455:0-224]

AGGCCCAGAGCTGAGACTGACTGGCCTATAGTTCTCTGGATCTCAGGGGTCTTTTTCTTT

ACTGAAAATGGGAGTTATGCTTCCCTTTTTCCAGTTTCTGGGAACGTTACCAGACTGGCA

TGACCCTCATGATGGATAGCAGCTTGGCGGCTTCGTCATCCAATTCTCTTAGAACCTGTG

GATGGATCTTGTCAGGTCCCATGGACTTGTGCATCATTGGGTTTT

>c4307_g1_i1 len=254 path=[466:0-253]

GGAATGTCTGTATGGGGGGGACTAACAGTATGCAAGCATTTCTGATGACATTTAGTGTAA

TGCTTAATTATGTTGTCACAATTTAGGAAGACTGGGCAGCTGTAGGACTAACATAGAACC

CTTGATGGAGTGGCTTTGCACTCTTTTTTTTTAGGGGAGGGAGGGGGAAGATTCTGGCTT

AAATGGTCTTTGTAACTCAAGAAAGTAGTTGAAGCATGTTGTGCAGTCTTGCATTGGGAT

CATGTTGAGCATTT

>c4308_g1_i1 len=264 path=[1:0-263]

ATGCTAGACATTTCAGTCATTTTACTAGATGGAAAATCTGACTTTTAAAATACAAAAATA

TGTATATACTGAAAGCTAGTCAGAAATTTAGATGCAAGGTAAAAAGTACAGAAAACACAA

AATTTTACTTCCTTGATAGGATCAAGGCCAGTCAAGGCCTAACCAGCAGATCTGTAGTCT

TCCTAACCTTATGCTTATGGAGAGTGGAAGACTTCATTCAGTAACCTGATTGAAATCAGC

CAGCTCGTAAAACATGTGCTAGGT

>c4308_g2_i1 len=212 path=[483:0-211]

ACCTTGTAAATTGATCAGCAGGGCAAAAAAATGCTGGTTACCCAAAGTTATACCCTTTGT

TTGCTCAGACCTACTCCTCCTTCTTTAACTTGCTCTTATTTCCTTTCCCCTCTCATGATT

AGAAAAGATTATCTCTTACTTCTCATGCCCTTAAATGACCTCCATCCAATTCTTCATGTC

ATTCAAAATTTTCTCTCACTTGAAGCCATTAA

>c4317_g1_i1 len=228 path=[206:0-227]

GTAACTTTGTGTTTGACTCCCTGAGAGATCTAGAAACAATAAAGAAAAAAGAAAAACCCT

TATCAAGATGTATCTGTGAAGAGTAGACTACCTAAGTTTAGGTGTTCCACTCAAAAGATA

ACATGAACAAACAACAGAAGTATCAAAATGAATAAAATAAACTTCTATTCTGTTATTAAC

CCCTATAACTGGAATGAAAGTTACCACATCTGTGTCAGGGCCATGAAG

>c4318_g1_i1 len=249 path=[1:0-248]

GCCAGTTTGTGAAGTAGTATAGCTTCGTGAATATGAGTTCTCGTGGTTTTCAACTTCTGG

TGTGTTTTCAAGGTTATATCACAAAGACAAAAAAAATCAGGAGACGTTTTAAGCTTTTCC

TGTTAATTTTTTATCTGTGGTGTGAAGGGCAAACTGTTCTTTGGGCTTGTTGGAGGATAA

CAGCCCCTTTCCTTATGCAGAGAGCTTTGTAATCATGATCTTTTAACTGTGAGGTATTCC

ACTGCTGAT

>c4322_g1_i1 len=203 path=[181:0-202]

GAGAGAACAATGAAGAAACTGTTCATCATGAACAGAAAAATTGCCTTTCTGCATTTCTTG

GCCCAATTACTCACTTAACTGCAGAAGTAACTAGTGAACTGCTTATTAATTGCTTATGTT

CCATATACGCATCAGGGACCTCTATTGAGCATGAAGGTTCAACAACCAAAAAGTTTTACT

CAAGTTCTGCACACCATCCTATT

>c4333_g1_i1 len=213 path=[1:0-212]

TTCATCCCAGCCCTTGTGCTCGGGGTGACTCAGCTGTGCTCCTCCTGCAGGTAGCGGTTC

CGACACGCGGTAGTTCCCATGAACACAGAAGAAAACGCTGATTGAGAATGTGACGTGTTG

GCTCTGGGTGTGTGTGCACAGGACGGGGACGGCCTGGCCCTGATGTCACCACCTGGACTT

GTAACTTTGGATCTGTGTGTTGGCACTGCACAG

>c4334_g1_i1 len=234 path=[258:0-186 25:187-203 42:204-233]

GCTACGTAACAACAACAGCAAAAGAATGTCAGCGCAAATCCGCCGTGTGCCACAGCATTC

AACACCAGAATTGCTGCAAAGCCCCTGAGCCAGCCATGCACTTGAGTATTTGGGTAGCAT

CAATTAAAACCCCCTAACCCTCCTCCAGCAGCCCTAACAAATGTGCCCAGGGCTGCCTGG

ATGCTCCCGAAAAATACTTCTTTATCACTCATTTGCTATTGTTATGGATGGTCT

>c4334_g1_i2 len=234 path=[258:0-186 444:187-233]

GCTACGTAACAACAACAGCAAAAGAATGTCAGCGCAAATCCGCCGTGTGCCACAGCATTC

AACACCAGAATTGCTGCAAAGCCCCTGAGCCAGCCATGCACTTGAGTATTTGGGTAGCAT

CAATTAAAACCCCCTAACCCTCCTCCAGCAGCCCTAACAAATGTGCCCAGGGCTGCCTGG

ATGCTCCTGGAATTCACCACTTCTCACCCAGACCATCCATAACAATAGCAAATG

>c4337_g1_i1 len=374 path=[1:0-373]

ACAGCCTCCTTGGGCAACCTGTTTAATACTGATCAGTATCTTCTCCAAAAAGCTGTTTTC

CTTCTCTACATTCTAGTTCCTCTGCCTGCAACAGAGAGAGATGACGTCACCTTCTGAAGT

GTGCCTTTAAACAAGGATGCTTCAAACAAACACGTTTTAAGTGTAACTATTGATGTATTG

GCCGGTGCTGTGTGATGATGAAATGGGAATGTTTTCATATCAAAGAGAATTCCTAACAGA

TAACCTGAGATGAGCGGTGTTTGCAACGTAGGCAGCAGCATTTCTGGGAGATGGCACGTT

CTGGTGCTGGCAGCCCTCAGAGCAGCGCTGCTCCCAAGTGCCCGCTCACATTCGGGGCAG

GGTAGTGACAGCAG

>c4338_g1_i1 len=285 path=[1:0-284]

AAATAATAATTAATGCAAAACAAGTATAGAAAAAATATTGGGTAGGCAGGTGTGGTAGTT

CTAGTAGCTACTATTCCATGAGATCTCTTCAAAGCCAGTTCTACTCTTGGAAATATGTTT

TTATAATTTGATACCTTTAAACTTTGCAGTCCAAAGAGCCCATATATTTGAAGGAGTAAC

TGTATTACATATCTATATTGAATATATTCTTTAAAAATTCTTCTGAATTCAGACCTTGTT

TAAGCAGAGAAGGAGATTTGTCCTTAAAAAAGGTCTGAATTCAGA

>c4341_g1_i1 len=221 path=[35:0-220]

AAGCAATTATAAAAACAATATTAAAAAATAAACATACACGAAATTACTATATCTGTGAAA

AAATAAAAAAGAAAATATAAGATATTATTTCTATTATAAAATAATTATAAAAATTGCTAT

AAAAGGCATATTAAAATTTTTTTGTATCATTTTTAAATGCAATCACACTTTCATTAGTAC

TACAAGATAATAACAAAAAAGATAAATCTTATAATGAACAG

>c4345_g1_i1 len=214 path=[514:0-213]

GATGTGTTCCCACCCCGCTGCTCAGACAGATCATCTGAGAAGTATTAATCTGGGCCCTGT

CATCTCATCAAATAATGAACTTTACCCTTTGGCTGAGAGATGAAAAAAGAAGGCAGGGGG

AAGGGTGGGGAAGGTAGTGCTGTACACGCTTATCAGTGCCCAGTTGCAGCACAGCATGCC

CCTACAAGAGCTATAAATAAGTTCTTCACTTCTA

>c4353_g1_i1 len=1178 path=[959:0-958 2142:959-1177]

TGGTTGTCCCTGCTGTGCCAGTTCAAACCATGTGAAACGCACACCAGCAGCAGCTGCTGC

CTGCTGAGAGCAGTGTAAGGCTTGTGAGGTATCTGGTGGGTGTCTGAAGTAGTTCCTTCA

CCCCTAGCATCCTTGGTCTAGTGGTCACTAACTCTCCCCAGGGTGCTCAAACAATGGTGA

CCACTCAACAGAAAGCTTTGTCCTCTGTGGTATTAGCACCTGCTGAGACCAATGTGGCTA

CCCAAACACAACTTTCAAGGGAACATAGCCACCTGGGTCTCCGGTGCAGGATGTGCCAGA

GGTTTCTACTGGTGTCTGAAGGCAGTAGCAAGAATACCTGTAGGAGATGTGCCCAGGCAG

AACTGCTGAGCTTGGTGGCTGGCCTTTGGGAGGAAGTGGACAGGTTGAGGAACATTTGTG

AGCCTGTGGAAACTGGTAGAACTATACTCTGCCCTCAGTGGAACAAAAATGTTAATTAGG

CATAACAGATGACAAGGGGGATTCTGTACATGCTCTTCACCAGGCAGAAGGCATTGACTT

GAGGGATAGAGGTGAGTGGAAATAAGTCCCTGTTCAGGTCAGTGGGAAAAGCTCTTCCTT

GTCTACCTTCCCTTGCCCTTACACAACAGATATAAGGCTCTGGAGTTGAAAGGGCAGGGA

CATGATGAGGTGAATGATGGTACATCTATACAAGAGGAGTCAACAAGGTCAGGTCAGCTT

ATCCCTTTGCATCATGACAATCTCCATTTAAAAAAAAAAAAACCATGGGTTATTGCTGTA

GGGGATCAGAGCCTGATCTTGCCAACCAGACCCACCTTTTTTAGGTAAGCTTGCTGACCC

ACTGGGTTTTGGGTTAAGGATATATTCAGGAAACTTTATCCTGGTACAACCCTCAGATTA

TCATCTGCTCTTGCTTTTTTCCTGTGGGTAGTGACAGAGTCATGAGAAGTCAAAGGTAAT

CAAAAAAAACTTAAGGTTTTGGGAGATTGGAGAAGTATTCAGGAGCATGGGTAGTATTCT

TTGTCCTTTGAGTTGTAGGGAATGATGTTGTGGGGCATCATGAGGAGGACCACAAAAATA

TGATCAAAGGTAAGGAATGCCTCCCTACAAGGACAGGCTTGAAAGCATTGGGGCTGTGAA

GCTTGGAGAAGAGGAGGCTCTGGAGAGACCTGAGAGCA

>c4353_g1_i2 len=232 path=[2140:0-12 2142:13-231]

CTTAAGGTTTTGGTCAAAAAAAACTTAAGGTTTTGGGAGATTGGAGAAGTATTCAGGAGC

ATGGGTAGTATTCTTTGTCCTTTGAGTTGTAGGGAATGATGTTGTGGGGCATCATGAGGA

GGACCACAAAAATATGATCAAAGGTAAGGAATGCCTCCCTACAAGGACAGGCTTGAAAGC

ATTGGGGCTGTGAAGCTTGGAGAAGAGGAGGCTCTGGAGAGACCTGAGAGCA

>c4355_g1_i1 len=221 path=[199:0-220]

ACGAGCAAACAATCGACTGAAAAGTAATAAAGGACAAAAAAGATAAATACAAAGGAGAAG

AGAAATAAACATAATCTTTCTTATTATCATTTCATCCGAACTGCATCTACAGCAGAAACT

TTAGTCCAAGTAATGGCAGTATGCATTACTTCTAAATTTGTCATTTTGTGTACAGCATTT

GCGTACTGGCAAAAGGCGTAGTGTTAAAGGCATTTCTGTCA

>c4365_g1_i1 len=219 path=[152:0-149 302:150-218]

GCTTTTGTGGATGTGGAATCACTATTTTTTCATTGATAAAAGAAGAGGAGAGAAGTAATG

GTTTGGTAATGTTTCTTTGCATTAAGAACTGAAAGAGAGGGTAAGAGGAGAAAAGAGAAG

GAAAAGATGTCTTAATCTATTGCTTTAACAAGGGTGAAATGTAGCTAAAATGTTCCAAAC

TATGATAAATGTTGAGGAAACTAACTTGTGTTTCAGGAG

>c4369_g1_i1 len=306 path=[284:0-201 609:202-305]

GAATGAGACCAAGCGTAATGTGAAATCGCAACCCTAACAGCCAAGCTATTTCTACTCAAT

GCTATAAAATAAGACTACATAAGTGCTGTGTATTTTTAAATGCAACTACCTACAAAAACA

GAAGGAAAATTCTATGCAAAGTGCATACTTCTCACAATAGGAAATGTTTAGAAAAGATTT

GAGAAGCTGGTCTTCTATTGTTTCCCCACATTTTCATACCACGTATGTAGTGTGGAGAGT

TAACACAAAACAAAGATCAAACAACTGTCCCTCAGAGACAAGTGAGTTACCAGTATGATG

ATGAAG

>c4370_g1_i1 len=221 path=[353:0-220]

GCTGCAAGAGGCACAAGGCCAGGCCTCAGGCTTTCCACCCCACCAGCCAAAACATCTGCC

AGTACCTCATCCTCAGAGGAGGGAAACAGGCCGCTCCCTGGTGAGCACACTGCTGGGAGA

GATGTACCGACTCCCTGTGGGAGTGGGGAATGGAGCCATGTGAAATCCCCCATGCTGCCC

TGTTCTGTGTACACCACACTCCTTGCTCACGAAGCGAGGGC

>c4372_g1_i1 len=405 path=[149:0-404]

GCAATAAAAGCACAGACATTCTGAATTTCAGTACTTCCAGCTGAGAAACTCATTTGCTCT

GTGACTAAGAATGAATTACATCTCCTGTGGCTACACACCTGCCTTCTGGTGCACAGTAAT

TCTCAGCTTCCAAAACAAAGGAGAGGAGGAGCAAACAGTTAAGGAGATGCTTATACAGTT

TTGGCCATCTAAATTTCCACACAAGTATGCTCCATGTGCGAGGCCTTAAGAGAAATGGGA

GTGCACTATGACGGGAGAACAGTGATGACCAGGAAAGGGATTATTTTAAAAGACAGATGA

GGCAATTTATGAGGACTGCTCCAAAAGTAATGGCTCTTATTTTATTACGTTAGTCAGAGG

TGGATGTCAGTGGTACGGCAATAGAGGTTGAACCTTCCCATCAGT

>c4375_g1_i1 len=225 path=[203:0-224]

AATGATGTGTAAAAATCAAGTGTATTCTTCAAACACCTGACACTATTTAAGAAAGCACCC

AGTTTACTTTTGCAGAAATACTAACATAATGATAGGTTTAAAAGAGCAGACTGGTTTACC

TATCCCAGTATACACCCTGCAATACACTCAGAGAACATAAGATTGAGGCTCATCTCATGC

TATGTTACATGCACTGCATTCCAGCCTCCAGCAATTTGCCATTTG

>c4376_g1_i1 len=216 path=[1:0-215]

AGATAGCACTTGCTGTCACATTACAAGGCTGAACGCTGAGGTGATGCTACAGAAGTAGTA

GAAACATTCCAAGTCTTTGGATGCTTCTTACTGAAGGGCTGTGTGGCAGAAGAATGTGAG

GTTTTAATACAGCTGTGTTCTGAGTGCTGTGTGAGCAAAGTGATGGTGGACTTGCATTGC

TGCGATTAATATAACTTCTGTTCCTAAATAGAAGGG

>c4378_g1_i1 len=206 path=[213:0-92 411:93-205]

GGTTTAGTACGAATGTTCCTTGTTGCAGCTCCTTTCCACTAACTCTTGTCCTTTTTCAGT

ACACCTGTAAGAATGTGGCTGTATCTTCTCTACAGATAGCAGAAGACAAACATTGACTAA

CTGGAAAGATAATGGTACAGAGCATATGGTAAATGTTCAGGGAACGTGGAAGGGATTTCA

GCCAAGAAAAAGACTGAAAAAACTGC

>c4384_g1_i1 len=226 path=[204:0-225]

GACCCAAGAGCTGGTTTGTGAGGACAGCTTCTGCCAGAACCACGCTCTCGGAGCAGAGTG

GCTTTCAGCTTGCTGGCGGCTGCAGCCTGCATCTGCTTTGCAGAGGAATCAGGATAAAAC

ATCAGCAAAACCACAACCCCAAGTTTTCAGGAACAAAGTGGAAGGTCAAGACCACATTCT

CTAACTCCACGCTGTCTGCCAGCACAGCCTCCCAAGTCAGAGCACA

>c4388_g1_i1 len=228 path=[440:0-227]

CGAGCCTGGGAACCGGCTCCCTCTGTCCCTGCACAAACCGAGCTCTATTTTCTAAGACTT

TAAATGGGAGGAGCTACTGCTACAGGATGGGAAGCCTCCATGACTCGCTTAACAGCCCCA

ACGGCAGCTGTAAATAAATAAAACGGCTAACTTTGTACACCCCAGTAATTCAATTAAACC

TTGGTGTGACCACACATTAGCACAGATAAACAGAAGCAACCACAGAGG

>c4396_g1_i1 len=214 path=[192:0-213]

AGTACTGAAATCTTTTTTCCTCATTCTCAATAATAGCAGTGCAACCCTTTGAAAGGCGTT

AACAGTGGTACTGATGTACTCCTTTGGAATCTATTTTTTAGCAGAAAAAGATTTCAGTAC

TTAAAGATGTTATGTTAGTATGAGGCAGTATAGATGAAAAGTCTCTACCTAAAACAGGTG

TACAGAAAAATGGATGCTTGCCTTCTGAAACCTT

>c4412_g1_i1 len=502 path=[480:0-501]

TATGCCTTGCTGTCTCAAGCCTTTCTCTTGCTGACCTTCAGCGCATGACACACCCAGAGA

GGTCTCCCCCAAGTGGGCAAGAGGCTGCAGATTGTCTTGGTTAATTTCGTTCTGCAGGAA

GCATAAGAAGAATCATGCAAAGCGGTGGGTGTGGCCAGGAGTAGGGCAGAGGTGGAGCAA

AGGGGCCAGGCTGCTGGCTACAGCAGTTGCAAGGGTGAGCAGGGAATGCTAGGGAAATGG

GGGAACCTGCAGCAGTGAAGCTGCTCCTTCTCCCCACTCTAGCACTGTGGGATGGGAGCC

TCATCTTCTTCTGATTTCTGAGCCCTCCTACGTGCTATCTGGAGAATTTGTGTAGATAGG

TCATGCTCTCTGAAGTGATTGGCTCCTGTGCAGGCATGTTCTGCAAAATGGTGGGTGTTC

ATGCTGTGAGAGGTGGTCCTGAAATCCAGGAGATCCTGGAGTGAGGCAGGAGACATCAGA

AGGCGCCAGGAGCTCTCAGTAA

>c4413_g1_i1 len=280 path=[416:0-279]

ACCAAACACTCTAAGGGCACCACAACACACACAAAAGGAACTGCCAAGAAGAATTTAAAC

CAGCACTGCTAAAATGCTGAAGCAGAAACCTGCAGCAATGCTCCGAACTGCTAACATTTG

CCATTATAATTCTACATGTTAGAAGACAGACAGACAGGTATCTCAATATACATGTCATAT

TGTACATACAAAGGAAGATGGTTGAAAAATAAAGTAGTCTGAGTTATCTGTGCACTACAG

CAATTGTTAGTTTAGTTGATACTGAAAGGATCAATATGGA

>c4415_g1_i1 len=241 path=[219:0-240]

AAGGTTATTTGCTTTATTCAAAACCACTTTTTTCTCATTAGCTGGCAGAGACAGGGAAGG

GAGTAGAAGGAAACTTGAGTTTTGATGTGCTCCCGTCTGGGATCCTTAAAGCTGTCTTTG

GTTTTGGAATGACTGGAATCTGCCCTGGGCTCTGCAGGAGCGTAGCACAGCACGCAGAGA

GCAGCGCTTTTGTGCGAGCGCTGAAGACAAAGTGCATGTCCAGTTCCATGCAAACAGCCT

T

>c4415_g2_i1 len=228 path=[437:0-227]

TCAAGGGCACCGGAGAAGCAGCTCTGTACCACCATGCAAACAGCCTTATCTTTAGTTCAT

CGCTGCAACAGGACATATTTCCATCAGTGCTGTGCCATTCAAGATAAAAAAGGTAGTATT

CCCTTTGATATCTCAGCCGCCTTCGTGGTCACGCTCTCGAGAGCGAGGCCATTGTGTAGC

TACCTGCTGCAGCCTGCGTGTCAGGCACAGGCAGACGTGGCTGCAGTG

>c4424_g1_i1 len=357 path=[335:0-356]

GGCCGGTGGCACTGGTGGCTTTGTGCTCCCAGAGACCCCACCCAGTGATGCGCAGTCCTG

CTGCAGGCTCAGGGACAGGGCTCGCTGTGCATTCAGGGTGCGCCGTGCCTTTCCATGGGC

CATAAAGCCAATACTGCGTCGTCTGCAAGAGCTGCGTGTTGCAGCAAATCCTCCTGACCC

TGCCACCACGGCACTGCGCCGTGACCACAGAGGTGTAGCTATTTTTCCTGCTCTTTGGCT

TCATCCATTTGTCATATTAATAACATGACTCATAGCATTAGCTGCTAAACAAGACTGAAA

CCCTCTCTGCACCATCCTCTGTAGTGCACATATCCCTTCTGGTCCCAGCCCTCATCA

>c4435_g1_i1 len=201 path=[333:0-200]

AGGACAGCAATGAAAAGCTGAATGCGTGTGCACTGAAATGCATGTTGTTGTGCTGTGTGC

TGAAAACAAGGAGCTGTGTTCTTTTCAAGCAGGAATGTAGATGTCTTCTGTACACCAGGA

CAAAATTTAAAGCCCACCAGCATGAGAAAGTTTGCTATCTAAACTGAAAGAGCTATTTAT

GATTCTGGATTAATCTTTTCT

>c4440_g1_i1 len=391 path=[369:0-390]

GGTGCCTGTTTGCTCAGTAGCATGTCTAAATATTGATTTAGGAGCACTGCATCAAGCAGC

AAGTTAAATTCAGTCATTTCTACAATTTACTTTTTGACTTACCAAACCTAATCAATATCT

TTGTCTAATAGGACTGATTTGAAGCCACCACCCAGCTGACCTCAGCTGCCCGTTGGACAG

CACTATGGCACTGCCATTTTTGTCACTGCTGACTGGATTAGGCCCACGTTCCTAAAGGTC

TGTTCTGCACAACTCCCGAGTAGCAAAACATTGAGGTACCCATTCTTCATTTTGTAAAAT

TTGAGCTTCAAGTGTACTAGGAAGAAAGCAGGTTAGCTTTAAGGATTGCACTGAATTGAC

AATACATACCAAAGGTGCGTCTTGCCAGTGT

>c4445_g1_i1 len=280 path=[522:0-279]

AAAAAAAAAAAAAAGTAAAATTATTTATAGACACATAATTTTCAATTTTTGTATCATAAA

TTATTTTTTTTGTTGAAATTGTTTCGTATTCTAACACAATATATAAAAAATTAAATTAAG

TTCAAAAAGGTAATATTAAATTTACGAAATTATGAATAAATATTATGAAAAAATAAGAAG

CATGAAATGAAAGTGAATCATACAAACTATAATCATATCAATAAAATGTATAAGAAATGA

ATTATCAAAATATTAAAACAAAATAAATAAATAAATAAAT

>c4447_g1_i1 len=214 path=[284:0-30 315:31-36 94:37-213]

TTCATTTTATGTTCTGGAGGAGGGTGTTAGCTTGGTATCTCTAAAGAAAGCATTGGAATC

CAGGATGAAGAGGTGTCCTGGGTCACACACACATGGTGAATGAACTTCTTTTACATAGAA

AGCAGTGCATTTTCAAAGGCAGATATTCACGTATTCTCAGTGATGCCATGAAAATAAGGT

GACTGTTTTGGAAATGAAGGGACTGCTTGTTACA

>c4447_g1_i2 len=223 path=[48:0-45 94:46-222]

AAACTGCACAAATACAAGGTAAACTAATGGAAACAAAGAAACTTGTTCTCTAAAGAAAGC

ATTGGAATCCAGGATGAAGAGGTGTCCTGGGTCACACACACATGGTGAATGAACTTCTTT

TACATAGAAAGCAGTGCATTTTCAAAGGCAGATATTCACGTATTCTCAGTGATGCCATGA

AAATAAGGTGACTGTTTTGGAAATGAAGGGACTGCTTGTTACA

>c4447_g1_i3 len=215 path=[345:0-31 315:32-37 94:38-214]

ACCCTCCTCCAGAACATAAAATGAATGATAGATTGGTATCTCTAAAGAAAGCATTGGAAT

CCAGGATGAAGAGGTGTCCTGGGTCACACACACATGGTGAATGAACTTCTTTTACATAGA

AAGCAGTGCATTTTCAAAGGCAGATATTCACGTATTCTCAGTGATGCCATGAAAATAAGG

TGACTGTTTTGGAAATGAAGGGACTGCTTGTTACA

>c4453_g1_i1 len=216 path=[194:0-215]

TGGTCCTGGATGGTGTTCTACTATATAGAACACCATCCAGGACCAGTCGGGCTGAAATAC

AACACAAACAAGGTGCGTGCATCTCACACAAATGCACTTGGAAGGCTCAAATTAACACTG

TTTAACACTGAATTTTGAGGGCTCCCTGGAGATGCGATTATCTGAAAAGGCACCTTTTTG

CCATGGAGGCTTACAGCAAGTTGGGCACATATATGA

>c4456_g1_i1 len=292 path=[512:0-291]

GCTCAGTTATTGGAAGTGATTCAAAATGTGAAAAATAAGGTTATCATCAAGAATATGGGT

TGGAATCTCATCTCTCCTCTTGTTAGATGTATTTTAGCATATAAGCAGGAAGATGAGAAG

CGAGAACTCTGCTTGAAGATACTAGATCAGTTGGTGCAGCTGTGCAATCCAAAGGAACTT

TTTTTGGGTTTACTTGAGCAAATTGAACAGACATCAGGAGAGCAGGTGTGCCAGACTATC

ATGTTACTGCTTCAACCTTTGCAGACAGTGCTTTTGAAACTTCAGAACAAGA

>c4459_g1_i1 len=264 path=[49:0-93 24:94-263]

GAGAGCTGTGGTTGGACCCTGAGCTCAGGACCTGCTCCGTAAGGCTGAGGCCAACTGACA

GCCAGGGAGGGCCCTGCTGCAACCCTACCCCCCTCCTGGAACCCCACCATTCGCCCTGCC

CTTCTCTCACACCAGCAGAAAGAAAGCCTTTTACCTTCTCCAGGGCTTATTGATGGTTTT

GTGTCTTAGCTCTTCCACGAGCAAGAAGAGTCTGCAAATGGAAATAAAAAGCGTCTGACA

GAAACAGTAAGTAATTCCTAATGA

>c4460_g1_i1 len=247 path=[1:0-246]

ACAAAGACACAAAAAATCCCCAAGGTGTTATGAATGACTGTATTCAAAAAGAAAAAGAAA

GTTTAGACTAATTCAGCTCCCTAAAGCAAAGAAACAAATGAATACCATGAATGAGCTCAA

AGCCGTGCTTGAATGTTCTCCTGTTATATACTGCCATTAGATGATTCATGTTCTGCAGGT

TAAATTGAAGGGACTTGTGTGTTTCTTCCATGGCATTTTGTCTATGCTCTAAGACCAAAG

ACAGGTA

>c4473_g1_i1 len=332 path=[347:0-293 25:294-331]

ATTTCACAATTTGTACATCATGACACATCCCCCACACACCAGGAAATCAGCTCCACAGAC

TCCTTTAGGGCCTTGAAAGGATCCTTGCGCTTCAGTGTTAATTTTTAAATGACCATAAGC

ACTCGACCGAGCTGTAAGTAATTAAAACAAGACACAACTTTGGCAACAAACAAAATATTT

TCCCCCAAATTTGCTTAGTTGTCCCATTCATGGACCTGTGCCTTTGGAATGGGGAATGCT

TTGGGATTCCAAAGAACACAAATAACAGCAACATCAACACATTCCATTTTACTGAACGAA

ACAGTGATATACCAAAATGCTCATGATACATT

>c4474_g1_i1 len=252 path=[1:0-124 506:125-251]

AAGAAGTACAAAATACGTTACAAACACAGGAAGCTTTTGTCCCAGCTGTGCTATTAGCTT

CAAGAAACGGGAGAAAATAGTCCTCATCTCGTCTCCTGTGAAGGCGATACCACTCTGCTC

TTAACTAATGAAACTGTTTTTTTTTTTCCTAAAGATGAAAATGTTGTGAAAAAGCGCATC

TGATCAAGGTGTCCATCAATCTTCTCTGATACTCTGGAATATAAATAATGTGACCTTTCC

ATGTGTAGCCCA

>c4475_g1_i1 len=214 path=[15:0-213]

GTGGGGTTGAGCTGATGATTGGACTGGATGATCTTGGAGACCTTTTCCAACCTCAGTGAT

TCTGTAAAAGACTTCTATGAAAACGTGCAGGCTGCTTTCAGCAGAATGACAGAAGGCAAC

AAAGCCCTGCCTAACAGAGCCTGAAGGCCACTGCAGCCGTTATTTCAGCTGGAGAAAATC

CGCCCGCTCGGAGCGGATTTTCTCCAGCTGAAAT

>c4480_g1_i1 len=258 path=[1:0-257]

TACAGTCACCTATTTAAGCCACGCCCCCTTTAAGCCACGCCCCCTTTAAAGACACCCATT

AACCCATGCCCCCTTAAGCCATGCCCCCTTTAAGCCACATCCCCTTTAAAGTCACCCCCT

TAAACCACGCCCTCCATAAGCCGCACCCCTTTAATCCACGCCCCCTTTAAAGCCACCACT

TAAGCCACGCCCCAAAACCACGCCCCTTTAAAGCCACGCCCCCTTTTAAACCCACCCCAG

GCCTCCGCCCCCCTTAAA

>c4487_g1_i1 len=391 path=[345:0-328 773:329-339 784:340-390]

CTCTTTCTTTCTTCCAGCTTACAATCCTACAAATTAGAGGATTACAAAGCAAGGCTGTTC

CCAAACTTTCAATGATATCAGCTCACGACACCTTCTTTGCCAACCCCCAGTCATGGGGAT

GCTTCGAGAAGCATTGCGCTGCCACATCTCCCTGCACACAGGACCCCTCTGCCACACAGA

ACATGACCTCAGCAGGAAAGCAGCCTGGTTATTGTGAGGTTTAAAGCTTGTGCTACATGA

TACCGTCCCTCAGCATTCCCATTCAGCTGGGTTGGCTTTAAAGACTGGGGAGGGCAGGTC

GGCAAGCTTGAATTAATGGCACTAGATGGGAGAAAAACAGTTTTAGTGTGTCTTGTCAGC

CTGAAGACAAATGAAAACTGTTTTTCTCCCA

>c4487_g2_i1 len=222 path=[1:0-170 784:171-221]

TAATGGTAGATGCCATCCAAAACATGTCTTCGTGTCCCGGCTGATCCCAAACTGTTTTAT

TTTGAGGGATGTTCCTTTTTTAGGGGCAGAGAGAGGTATCACCTGGCTGATGCCTTCAGC

CAAAGCTTCTTTGTTAGTGAAGGACTTCAAAGCAGGTGGAAGGTGATGCCCTTTTAGTGT

GTCTTGTCAGCCTGAAGACAAATGAAAACTGTTTTTCTCCCA

>c4492_g1_i1 len=230 path=[189:0-229]

ATCATTACTGGTGATGAGATTTGGTGTCACCACTACTGCCAGAGTCAAAGGGCCACGGAG

CACTGACATGTGAATTCCCCACCAAAGAGAAAGCCCATGACAATAACCCTCAGCAAGACG

AGTGATGTGCACCATCTTCTGGGATGGAAAGGGGTGGGCAAACCCTCAGCTCTGACCACT

GCATTGTGATGTCGACTGAGCTGAAGGCTCAAACTTCTAAAGTCACATCA

>c4496_g1_i1 len=333 path=[493:0-332]

AAAAGTGTTAATATTCTTTGACTCAAATTATATGAAATCCAGTTTCTAGTGGAAAACAAA

CAAAAGATGGCCAACCAGACCTATTGCATATCTCAAAACGCATTGCCACCTGTACAGCTT

CTGGGGGAGATCTGTATGTTAGCAACTGCTTATATAAAGGTAATTCTGTGATGGAAGGTT

TCCTAAATCAGGTTTCTCTATAGCTTTGTTTTCCAAAATACTGTGTAACATGGAAAGATG

GCATCAGTTTCTTCTCAGACATGCAGGTACTCCACTGCACGTAGACAAAATAAAGGGGCT

TATCACAAAACCATGCAATCAAACACAAAGTTA

>c4505_g1_i1 len=259 path=[1:0-258]

TATGCGCCACTCAGAGCAGATAGCAAATGATATGGTGTAGGAAGGTAGGTGCACGTAGAA

AATCTACTTGGCGTATTGGTATGTTGGCATTTTGAATGGAGGCTTTAAAATCTGAGAACT

TTTAAGAAGTGTTTTAGAGATAGAAAAGTGTTTCAGATGGTTGGAAAAGTGACTCTCTTA

TCAGGAAAATTGAATTCAGTTTTTCTTTTTCTCAAGTAAACCAGAATGTAAATGTAAAGG

GTAAAAAAAAAAAAAAAAA

>c4511_g1_i1 len=233 path=[123:0-122 441:123-232]

GCCATAGGAAGAAGTTGTGAGCTTCTAGCCAGGAGTGGACATGCTGTTTCTGGCCGTGAC

CTCACCTTGTGTCATGAAACCCCAGGTAGAAGCTTTGTGATAAGAACAACTGCAATCTTT

TTCAGAATATAGCATACTGATTTCTAAAGAATGGGGATCCTAACAGAACTCCTGAGTTAG

TTCCTATGCCTCACCCCTCGAGTCTATTTCTGTGTTAAACTCCTAAGCAGAAG

>c4520_g1_i1 len=211 path=[371:0-210]

ACTTTATCAAATACGGCTTTGCCAACTCTAGAGGTTTTCTCCTGTGCCCTGTGGTGTGAT

TATTTCTGTAAAGCGTCACCTGCTGAAGAGAGATGCTCACGTGACAATCTCAGTTTTCAT

CTATTAGTTTACTTTTGCTTATAGACTAAAAGGAAAAGGTGCAAGATGTGACTTTTTTCC

TCGTGACAGGCTCAGAAGAGGCTGAGAAAAA

>c4524_g1_i1 len=206 path=[171:0-205]

AATATATGTATATATTTTTTTTTTCATGAGGGAATCTTAGGAGGATTTATGTTAGGAATC

CACATGTAGTTATAAATGCTGTGCAAAAGGAATAACATTATGAGGCTGTATTGAGTTATT

TGGGATAGTGATAATGAAGGGCTAGACTTAAAGAATTGCAAAATTACACTGCCATACTGA

TTTTTAGATGGCAGACTGCAATTTCA

>c4525_g1_i1 len=291 path=[1:0-290]

ACTGCCAATGAACTTTGATTAAAACCAAGCAGAAACTTTGATGTGCCTATACATTTATGG

AAAAAAAATTAACAGAGCCACATATACTGGGGATGTGTGCAACTTCAGTTCTTCAGTAAT

ATCATAATAAATCTGAGTTGGAATTTCACTAGCTTTTGGAAATCTGCTTTCAAGACAGCA

TTTCTTTAGTACAAGAAAACCCCACACGTTATTTTAAGAATAAGTTTATAAAAAACTAGA

GCAAAATCTTTTGGATGACTGCTGCTTGAGAGCCTACTTGTTATCAAGATG

>c4531_g1_i1 len=221 path=[252:0-54 306:55-220]

AATACAGAAGGAAACCAGTGGTTTGACTTTCTTAAAAATAGTACTCATCTTAAAGAAAGT

CCACTTTTGTTTCCCTACAATCCTGAGAAATCACTGCATTTTGTCAAAAGGCAAATGGAA

GAGGTCATTGATCAGTGTTTACAAAAGCCAGCTGATGTGATTGGAAAGTCAGTGCATCAA

GCATTCTGTGTGTCTCTCTACAAAACCCCTCAAACACAAAG

>c4551_g1_i1 len=220 path=[1:0-45 47:46-219]

CAGGTAATTAAAATAGATATTTCAGTGCATATTTGGTATTTCCATGGGGCTTTATGATCA

ATAAAAAGTAATTACGCCGAAAGAGAAAGTTCATTCTAAAGATATTTTACACCGAGGTAG

TTCATCCACATGAATTTTTGTATTTGTCCATTAACACCAAAATCAGATACTCAGATTTTA

CTCATGAGACTGCAAGACTCAAACTATTATTTATTTTTAA

>c4561_g1_i1 len=260 path=[385:0-259]

GAAAAGACAGCAACTGACTACTACTAGGACATCTGTAAAACACATGTGACATCTGCAGGA

AGGGAAAAGATTTAGGAAGCAATCTGAAGTGATAACTGGTCTTCATTAAGGCTATCTGCT

TTATTAAGATTTTCTGATGTGGACACCTTCTGAAACAAATATGTACTCATTAACATCATC

CTATCAGATGTTTACATGATGCTGTGTTTCTTCTTAAGAAGTATTATAGCTTAACTTGCA

CTTTCATACTGGTGTAGCTA

>c4563_g1_i1 len=264 path=[396:0-263]

TTCATACAGAAAAATAATTAATTTTGAATATTTTATAGCTTTACTATTTTTAAAAATAAT

ATTTATCAAATCCTTTTCATTTTTTGAATACTAAAGAATAAAAAAAAAGAGATAAGAGAA

AAAAAAACATGAAAATGGGTGTATTGTAATTTCATATTAACAAAAGAATTCGTTATTAAA

ATAAAGTTTTAATAAGTATTCTTTAAATATTGAAAAGATATGTTTTTCGTTTTCGTTTGT

CCCAAATTAAATTTGAGTGAGTGA

>c4568_g1_i1 len=255 path=[233:0-254]

TGGAGTGAGGGGCTGCAGGTACACCGTCAAATTAGGAGAAAATACCCTCTCCCAGCCAGG

GTGCTATCAAATATGTAAGAAGGAAATTGAGGAGAAAAAGTGTTGCCCAGGATTTTGGGG

TACAGAGTGTTATGAATGTCCAGGAGGCTCTGAAAATCCCTGCAGTAGTCATGGAACGTG

TTTGGATGGTATACAGCGAAATGGAACCTGCATCTGCAAGAATCACAAAAAGGCTTGAAG

AAAGACGAGAGGAGA

>c4570_g1_i1 len=253 path=[701:0-55 149:56-252]

GTCTGCAGAGAACAGTAATTATTTTTATAATTTTAATGTAAAAGAGATTATTTATTTTTA

TGCTGTAGCTACTTTAGCTTAGTAACTCAATCACACTACTTGAAGCAGCTCATGTGTTTT

GGATGGTATTTGAAAATAAAATGTGGTAAGAAGGGGGAAATGATCTCATAGGTCTGCTCT

AGTGATGGTGTAAAATTAAGGAAATCATGGATACCTTTCTGATATTTCTGTTCCAACCCC

CTACCACAGGCAG

>c4570_g1_i2 len=345 path=[1:0-147 149:148-344]

AAGAGATTATTTATTTTTAATGCTAAGCTTGCATAAAACTGTCAAAGAAAATTATTACAC

ATTGATTTCTGCTAGAGAATAAATACATAAAAAAATTACTGTTCTCTGCAGACTTGCAAT

ATCATTTTGTCTTGTCACAGATCTCAAATTTATGCTGTAGCTACTTTAGCTTAGTAACTC

AATCACACTACTTGAAGCAGCTCATGTGTTTTGGATGGTATTTGAAAATAAAATGTGGTA

AGAAGGGGGAAATGATCTCATAGGTCTGCTCTAGTGATGGTGTAAAATTAAGGAAATCAT

GGATACCTTTCTGATATTTCTGTTCCAACCCCCTACCACAGGCAG

>c4574_g1_i1 len=223 path=[201:0-222]

AAAGACTTCTGGAAACGTCCACATTTCTATGTCAGTAGAGAAGCTACATCAACGCAATCA

CTGACTGAGCAATACAAGTGACAACCATACAGCTGTCAAGCAATCTCAGGTTTTAACTCA

AGTAAACAGAACCTAAACCCCGTTTTACTTCTTCAAGTGATTGAACAACACAGGACAAGT

ACTTAGCTGAGAGATGCAAGGAGCTGAAATTCTACACTGCTTA

>c4576_g1_i1 len=264 path=[41:0-23 20:24-35 40:36-263]

TTCCAAATGGGGACCTGACAGAGGTATAAACTTTGCTTATAATAAACTACTTAGTAGTTG

CAGGAAAAAAAAAATAGAATTTTAGTTTGCTGAACTGGAGTGAGGGGGAACATCATAATA

ATGGTATGCATATAAGCCAAGCTGCTGAATAAACCTTGAGCACCAAAGCTGTTGAGAGTA

GTGCCATGTTTTTGGCTGAGAAGTTACCATTATGGGAGTTTTTCATTGTGTTGTAAATTC

TGCTGCCTAAACAGGACCTATTTG

>c4577_g1_i1 len=492 path=[155:0-491]

ACAGAAATCTTGCAGTGAGATACACAGTTTCTAAGTAACCTCAAATTTGCACATGTTAGA

TAGCGTACCAATTTTCAAACCAGCCTCTACTGCTACGTTGCAGTAGACTAATTTAATTTT

GCAATCCTCAGTTCAAGTAATTTACAAATAGTCCTTAAAAAGAACAAATAAAAAGATCAC

AACTTCTGGTGAATGGAAGCACACATTCTTGCTGGTAATTAGAAGGTCTGGAACTGGAAC

TTATGCAGATACTGGAAAGCAGCAGCAGCAATTAAGCCACTTGCCACAATTTCTTGAGGT

TTGCAACAGCAGTTACAAATGCCTCCGGGATTTCTGAAGTGCAGGCAGCAAGCTCGTTAT

CTCCCTATCTTCTATTTTCTCATCTTGTAGTGGGAAGAAGTGCCCTGAGGTCACTCGCTG

CTTACTTAGCCTAAACAAGGAAGTGGGCTCCGGGATTTCTTAACCTAATCCACACAGTTA

AGAGCTACAAAG

>c4581_g1_i1 len=369 path=[235:0-368]

GACATCTTTTTGGAGTCATGCATAGCCTTTGGGAACCTGTTGGTAGGGACTGGCATCCAG

CAGGCCTGCAGTGAGCAGGCATCATGGCGTATGCATAGAGAGCAAAGCGATGCAGTTATG

TCTATTTTTATACATATACCTTCCTTTTGAGTCGTGGATTTCAAAACCACAAATGGAGAT

GTGCATTTCCTAAAGAGGGAAAGACAGAAAACTCCTCAAATGCCATCCAAACGAGTGCAG

TGTACTGAGAAAATACCTAGGGGCCTTGCCAGTGGGGTGATGGCAGGAGGTCTGCTTGAG

TGCTGCTTGCCATTCTTGCTGGAGAAGTGCAGAAGTGCTCCCAGGTCCCGAAGGTTTTTG

CCTGGGAGG

>c4584_g1_i1 len=274 path=[588:0-273]

TTACTTTAAAACAACATATATATTAACATTACAATATAGCATTTTGTAATAAACCAATAC

AGTATAGCTCAAGTACCATTCCTCTCCACCACCACTAATTCTTTCTCATGGAGATAACCA

AAATAATTATGGGTAAATAAGAAGCAGTTTTAAAAAGTATGCACTGTAATAGCAGACATG

CAAGTGACTGGAAGTATGTTTCTGTTCATAAAGAACATCAAATGTTATTTCTCCAGTGCC

AACTTATACAGGTACAAGGACCACGTCACACTTG

>c4590_g1_i1 len=223 path=[201:0-222]

TGATGTTCTTGCTGTGTTTAATTGAATGTAATCTTAGTTCAGATTGCCAGCATTTACAGC

ATGTGAAGTGACCAAGTCATCAGAAAAGCATTTGTTATTTTTTTTTTTTAAACCTGGCAC

ATAAAGAAATAAATTACTGACAGCACAGAGGTCAAACTGAGAAATTGTAGTAACAGGCTC

TTTAGACTTCAGCTGGGAATTCAGAGCTTCATTCAGTTTCTTA

>c4593_g1_i1 len=235 path=[1:0-234]

TACAAGGATACGCTCTACTTCTTTAAAACAAATTTACCGATTGTGGTGTTAGAATACATT

TGCTCTGTATTTCATCTTGCAGTCATAAATCCCTCTGAAGTATCAGTAAGATCAATGGGA

CTGTTGACTGTGTAAAGATCGCAAATTTAAGCCCTCAAAGTCTGCTGCAGTACATAACAT

CTCCAGGGAGCCGCTCTATTTATGCTAGCTACACAGACATCTGTAGAAGGCATAG

>c4599_g1_i1 len=213 path=[155:0-212]

CATTCTGCACATGGGAAAGTTAGCACTGCTGAACTTGGCAACACAGAAGCACTTGAGAAA

GCTCAAGTGATGGGAATAATGTGTACCAGATGGACACTCAGCAGCAAAATGGAAACCTAC

TGTTGGATGGCTAAGGGGAGATTTTGCTGTCATACTGGAAAAGAAGCGGTGCTGGCTTGC

ATACCACATGCTGAAGTCATTGGTATATTTTGG

>c4601_g1_i1 len=214 path=[155:0-213]

GTACAAAGATGAGCAGATTTTAAATGCTTTTCCCTGGTTTCGCAAACACTGCGGTGCATC

CCAAAAATGCTGCGCACAGCCTGAGTTAAACACAAGCTGAAACCAATAAAAATCTGTAGG

ATAGCACCTCATCTAATGTGCTTTTTAAAAGTTGGATGAACTTCTTATTTGACTTAAAGG

AATGTAATCTTTTCCAGATACAACCTGGGCTTTT

>c4609_g1_i1 len=235 path=[215:0-142 214:143-234]

GAAGCCTATCCGTTAGCTGAGGCTGTGGGACCTCGGGGACTCGCAACGCTGAGAAGCCGG

GCGTCCCTTTAAAGAACCGCGTTGTGGTAACTCATCCTGAGTCACAGCCGCTTATGTAAA

CGGTTGCCATGGGAGCAGCGCATCTGAGCCGGCTGCGCCCTCCTGGGGAGGCGTACAGCC

ACCCCTTCCGCCTCCCCGCCCGGCCCCACCGCCATCCCACCGCTCAAACACAAAG

>c4616_g1_i1 len=232 path=[185:0-140 25:141-231]

AGAAGGAAGGAACTGAAATTTATTTAGGGACTGTTTCTTTAACATCTGGCAACAAACAGC

CAAAGCAACTCTGTTACTGTTTTAGGCAAGACTGTACTTTCCACAATCTGTACAAGCCCA

AGTTATAGTGTTCAGCTATTTATAATGACAGTTTTTAATATAAACTGTGCAACTGAGCAC

GTAGGTCTCTCTGACCTCATTAAAAATACTGCAGATCTTTTTAATAAAAGGA

>c4620_g1_i1 len=302 path=[578:0-34 255:35-58 279:59-301]

TGGAAGGCTGCTCTAAGGTCTCCCCACAGCCCTCTCTTCTCCAGGCTGAACAACCCCAAT

TTTCACAGCCTGTCTTCACAGCAGAGGTACCCCTGCCCTCTGATTTTCCTTGTAGTCCTC

TCTGGACCCACTCCAACAGCTTCACATCATTCTTGTGCAGGGGACCCCAGAGCTGAACAT

AGCACTGTAGGCTGGGGTCACATGAGAAACTCAGAAAAACCCTATTTTACTACCTCTCTA

TTTTACTCAGAAAACCTAAGATGCAAATACCTCACTGCATAATCTGGATTTGATCTTTTT

TT

>c4648_g1_i1 len=289 path=[1:0-288]

GGTAACTTTGTGTTTGACAGGATTGCATTGCAAATGGCTCCCTCCTCGCTTGCATTAGCC

CTAGGCACAGCCCCCATAGTGTTTGAAAGACATTTTGTGTCTCCACAAGCTTCGTGTGTT

TGTTTCATCACAGCACTGTTGCCCTCTTCTTCATTACAGCCTTCAGACTGATTCCTGTAC

AGACAGTTGTCCAGCCGCAATCTTGCCTTCCACTTTTCATGTAGTGTTGACTGTTCTGGT

CACAGTTACCCCAGCCAAGATACTTTACTGTGCTCTTTAATCCCTCTTT

>c4648_g2_i1 len=253 path=[763:0-252]

AATCCCTCGCACTGCATCTCAGCACTCTACTGCTGTTGGCTCATGTCCTCAACCTCAACA

CCTGTGACTCGTGTTGCCCTGTAGCTAGATTCCCTTAGCTCTTTCCTTCTGCAGGCTGAG

AAGACTGAGGACACTTCCTGGCTGAGGCAGAGAGAGGAAGTTTAGGCAGTGAACTCATTT

TTTTCTGCCTTCATGGGAAGCTGGGCAGCAACAGAGAGGTTTCCAAATGTTAGCAAGTCA

TAAAGAGGGATTA

>c4650_g1_i1 len=207 path=[185:0-206]

GTGCAACAGTCTGAAGTATGTTCATAGGCACTGTGCAAGGATCTTAGAGGTTTAGTGTTT

GTAAAGCCTTTGGTGATGCTTCTCCTTGCAGACCTGTTTCCCTGTGGTGGTTATAGCAAT

GGGGCACAGAAAAAACGGAAGCCATGAATATAACCCAGTGGCATATTTTTGTGTGCTTTG

ACGGTGTTATTTTATTCATGAACTGCA

>c4652_g1_i1 len=201 path=[258:0-61 538:62-75 56:76-200]

AACAAAAAAAAAAAAAAATTAATATGGTTATAGTAACCTAAATATTTTAAAATATGTGCT

CCTCAATTAATAATAATAATTATTCTTCCCATGTTATATTTTTTTTTGAATTTATATAAA

CTAAGATTTTTGTTTTATGTTCAATAGCTCTACATAAATTATGCTTACTTTTTTTAGTAA

TAAGGTTAAATTTAAAAAATT

>c4662_g1_i1 len=208 path=[186:0-207]

ATTTAAAAAAAAAAAAATTTTGTTCAACAGGCCAAAATAATATACAAGTATCGCTTTACT

TTTTTGTTCCTATGCACATGTATTTATTTCTATTTTATTTTTTTTTAATTTTTGCTTGCA

CAAATAAACAGCATAAACGCGTTTCAAATATAATAAATAATTAGATTGAATATTAAAATT

AATAAAACAATATTTATTAATAACTATA

>c4662_g2_i1 len=285 path=[371:0-284]

TATTTATTTTTTTTTTTTTTTACAAAATGTTAATAAGTTTTAAAAGATATATAATAAAAG

CACAAAAAATATGTTTATGTACTTATATTTTTTTAAACATATGTAAAATACGTATTCGTT

AATCTGTTTGTGTACTTTAATCTAGTTAGTTTCTTATTTAAACATTAGCATGATAAATGT

ATTAAAAAGAAATAATAATAAAATTATAGTACTAGGAAATATATAAAATAGCAAAATTCA

ATTTAAATTTATTAAAATTATAGTATAAGTATAAAACAAATAATT

>c4668_g1_i1 len=392 path=[53:0-391]

GCAAGTGGGTTGTCGTCTGCTCGAACCTCGGTTTTTTGTGTCCTACTGCCAACAGAGCAA

ACTTCTGCACTGCTCGGACATTTTCCATCATTTGGAGCGGGTTGCACAGGAATGCTTCAG

AGATGGCTGCTCAGGTGGCTGTCCTCTGCTAGCTTGGGATGCTGCTGAGTTCCAGGAGAT

GCTTCCGTGGCAGTCTGTCTTTTACAGTGCGAGTTACGAATGAAATTTAGTAACTAACAC

CCCGCTCCTCACCTGACCTCCAATAGACTTCCTGTAACACTGAGGAGAAGGGGTCAGAGG

AGAAGGGCCCAGGGTCACCATGAAGCTGAAGCAGCGAGTTGTGCTGTTGGCCATCCTCCT

TGTCATCTTCATTTTCACAAAAGTTTTCCTCA

>c4671_g1_i1 len=337 path=[469:0-336]

GTCGCTTCCCAGCACCATCTCTGCAGCCTTTGGGGTCCCTATTTGAGAACTGGGGGGGGG

CAGGATGGATATGGGGCTTTTCGCACCCCTACATCCACCCTAAGGTACCCCATACCTTGG

GGGTGCACTGAGACCCCAGACCCAAACCTTGGGGATGCACTTGGACTCCAAACCTATACC

CTGGGGGTGCACTTAGACCCCAAACCTAAACACTTGGATGTACTTCAGACCCCAGACCCA

AAACCCTGGGGTGCACTTGGAACCCCAACCCAAACCTTGGGGGCGCGCTCAGACCCCAAG

CCTACACCCTGGGGTGCACTTGGACCCCAAACCCAAA

>c4675_g1_i1 len=358 path=[1:0-357]

GCCACTAAAAGTGAGAAAATATCACTGACCTCCTGCAGTATTGATCAAACAGCTGAGACG

CTCAAGGCCAAAATCACTCTCTGCAGAAACAGCTGATGGAACTGTTTGATCAGATTTCTG

TGGGGTCTGCATCTACTGAAGATGATATCACAGATATGGTGTCAGTAATTCACACTGTGC

TGGAGATCTGCTCTGTCATTTCCAATATGGACCACGCTCTTCATGCCAATACATGGAAGT

TCATAATCAAGCAAAGCCTGAAGCACCATGCTCTGCTGCAGTGCCATCTGAAACACAGTG

ACATCCTCAGTGGTCTGTGCAAAGATACTTTGCTTTCTTTTCACTCCAGTTTACAACT

>c4677_g1_i1 len=337 path=[419:0-336]

AGGTAGAATGGAAGCTCTTCAGGTGAGCTTTTGCACTTCTCTAAAAACTACTATTGTTAA

GAGAATCGTTTAGTTCTCTTCATATTCTTACAACTACAGCCCATAGTAGCACAAAAGTGT

AAATTTGCAGCTAGCAGAAATACCTAAAACCTGTTTTATAGTTTCTGTTGGCTAAGAGTC

TACACATGCTGTATTATGCTTTAAAGTTCAAGCATGTCACTTTTATTTGGCAAAAGTAGA

AGTCAGCACATAAAAAAAAACTTGTTTGGAAGTAAGCTTCGTTAATTGTTGCAGAAACAT

AGTAAAGTCAAAATAATTACCCACTCAAACACAAAGT

>c4677_g2_i1 len=232 path=[210:0-231]

CAAGTAGATTTTAGGTTTTGAAAAGTTATGGGTATGTTTATCACCTAATATTTGCATGTG

CAAGACCTCAGGCATTTCTACCAGAAGTGGTTGAGATGTAATTGTGCTCAACAGTGGCAA

AAATTGTGAATATAAGGGTGAAAGTCATTTCTAGACTTGAGAATTTTTTAATAGATTTTT

CTTGAAATAATTTTTCCTCTGACTTAAATAGGCTTACGGAGTGTAGTTGAAG

>c4684_g1_i1 len=232 path=[502:0-231]

AGCAGCTGCCTGCTTGCACTGAGCACCCCTCACCCCCTGGCCCCGCTGTCCCCAGCAGGC

TCTGAGCAGGTCGCACACTGGTCCATCTCATCGTGTTGGATCCAAGGGAAGATTTTTAAG

ACAAGACCGACATCATCAGGAAACTGAGAAGCAGAGCAGGCAGGAGGTGGAGCATCGTGG

CCACGGTACCAAGAGGGGCTGAGGATGGTGGCTGAAACTTCGGCGTGACGAT

>c4690_g1_i1 len=319 path=[297:0-318]

CTGGTGGACAGTGTATTTATGTTGTAATTCTCTGAAAATGTTGCCTGATCTTTCTGGGGA

AAGAGGAGATAGGAGTTCAGTGGTGACCTTAATTCTCCTCTCTGTCCGTAGTTCAAAGGA

TTGGAATAAAACCTGTGGGACAAAGTTCAAGCCAAGCTGTTGTTGACATTGGCCTGTATG

TTTCTTGGGCATGGGCTGAGACATGGCACCGGCACTGAGCTGGGTTTCATGTTTCTGGTG

CCCACTTAGGGAGCCCTGGCTTAGCAGGATACCACTGCTGCTCTTACCCATGTGGTCCTG

ACTGACCCCAGGTCTGGAG

>c4698_g1_i1 len=278 path=[1:0-92 94:93-277]

AGCACTTAACCTCGTTCCCTAAATGTCAGGGTGCAGCAGGGGGCTGCTGCTCTCAGGCTG

TCCCGGTGACCCCTAACAAAGACATCAGTGCCATCTGACTCTTGGGTCAAAGTCAAAACC

TGACATAATCATGGCATGCAGCAAGTAGAAAAGCAGAAAAATCCCCACATAAATCAAACC

TAAACAAGCCCTCATGTCTTACTCACCAGCTGCCAAGTGAACCGGCCCTACTCTCAGACA

CCACTGGATAAAGTGACAGAGAATCCCCAGTGGAGCAT

>c4698_g1_i2 len=346 path=[256:0-117 604:118-160 94:161-345]

ATGCTCCACTGGGGATTCTCTGTCACTTTATCCAGTGGTGTCTGAGAGTAGGGCCGGTTC

ACTTGGCAGCTGGTGAGTAAGACATGAGGGCTTGTTTAGGTTTGATTTATGTGGGGATTT

TTCTGCTTTTCTACTTGCTACATGCCATGATTATGTCAGGTTCTGACTCTTGGGTCAAAG

TCAAAACCTGACATAATCATGGCATGCAGCAAGTAGAAAAGCAGAAAAATCCCCACATAA

ATCAAACCTAAACAAGCCCTCATGTCTTACTCACCAGCTGCCAAGTGAACCGGCCCTACT

CTCAGACACCACTGGATAAAGTGACAGAGAATCCCCAGTGGAGCAT

>c4699_g1_i1 len=272 path=[429:0-271]

GTTTCTACTGCTGTAGTGGAACCTTACAATTCAATTTTAACTACACATACAACACTGGAG

CATTCAGACTGCGCATTCATGGTAGATAATGAAGCCATTTATGATAGAGTGGAAAAAAGC

AGGAGAGAAGAAAACATACGTTTATTATTACCCTTTCCAAAATCATGAAATATTAAAATA

AGCATTCTAAAGCAATTATTTCACTTCACTTTGATTCATGTCATGCTATGAGAGTTTCTT

ACATTACATCAAAAAATCTAGCTAAAAAGCTA

>c4703_g1_i1 len=242 path=[220:0-101 322:102-241]

AGGGCTGTGTGTCTCGTACATATGTCTGACTCCACAGTATGGAGCTGACATACTGACTTG

AGGAGAAAAAAAAGGAAGCAGTTCTGTTTTTCCCCTTCAATCAAAAGCAGCGTAACTGTG

AACTCAATCTCTTCCTAGGAAAAAAAAACATCTATTACATTTGCACTGAGATATAAATTT

AATAAGAGGTCTAACTGTAAATTCTAGAATAATGACATAAAATTGAAGTGTTTAAAGCAT

TA

>c4716_g1_i1 len=252 path=[384:0-251]

ATTTTCACATGCCTCCCTGCAGGTAGGAGTTTAGACATGCAATTCAAGAAGTGATTGCTT

TCAGTTCAAAGACAGGCCATTTACTGCCAAGACGACCTTCCAGAATTGTGCTGAATTCAA

TTATACTTCCTTGCCTCAAGTTCAACAACACTTTCTTAAACTCATTGCTTGCCTTCAGCA

CAATATCTTTGGTTATGTCATCTACCATTATTCCAACACATCACATCAATGCTCTCATCT

TCTTCTTCATCA

>c4720_g1_i1 len=217 path=[1:0-194 571:195-216]

AAGCAGCTGGCTGAATTTGCTAGTCTGATGAGAGTCTGCCTTAGAGTACTCTGGGGTCCT

CAGCACAAGAAGGACATGGATCCGTATGAACAAGTCCATAGGAGGCCATGAGGATAATCA

GACTGCTGAAGCGCCTCTTCTATGAAGACAAAGAGAGGCATTCATTCTGGAGAAGAGACC

TTAGTCCGCCTTCTGAATGAAGCCAAGAAAAATTAAG

>c4720_g1_i2 len=275 path=[1:0-194 196:195-274]

AAGCAGCTGGCTGAATTTGCTAGTCTGATGAGAGTCTGCCTTAGAGTACTCTGGGGTCCT

CAGCACAAGAAGGACATGGATCCGTATGAACAAGTCCATAGGAGGCCATGAGGATAATCA

GACTGCTGAAGCGCCTCTTCTATGAAGACAAAGAGAGGCATTCATTCTGGAGAAGAGACC

TTAGTCCGCCTTCTGGTACATAAAGGGGGTCTAAAAGAATGCTGGAGAGGGACTTTCTAC

AAGGACATGTAGGGATAGAACAAGGGGTAATGGTG

>c4722_g1_i1 len=264 path=[1:0-263]

AGAAGAGCTGTCAATCATCTTTCCTAACAGGAAGCTTCAGACATTTCTTCACCTAACACA

AATATTTAAACTTATATTAAGAAAGTGAGCACGCAGAAAGTCCATTCTGCTATTCGTCAC

TTCTTGCAGGAGCCCTACATGGGCGCGCTGTGTACGCACGCTTTCACTTCTCTCCCAACC

CACATCTACTTGTATGAGCAGATGGTTACAGCTTTAAGCACACACGTTATTGAGGACTAT

GCTGCGTGCTCACTTTCTTAATAT

>c4725_g1_i1 len=349 path=[503:0-210 713:211-238 42:239-348]

AACTTTGTGTTTGAGGGGGTGGTCTCATTTTGGGGCCTTTTTGCCCTTCTTTTTGGGGGG

GGGCGGAGGGGCGGCCCCCACCATCGTCAGCTTTTTGGGGAGGGCAGCGGAGGCCTTCAG

GGCGACCTCCCGTTCAATGCGGAGCCGAATGCCGACCTCCAACGTCTTCTTCAGCTTCTG

CTGTTGGATCACACGCAGCTTCTTGGGGGCGATGACCCGGCCTCCTTTCCTCGGCCCCTG

CGCCCCCCCACTTCCGCCTCGCTGCATCCCGTCGTGCCTTACGCTTCCTGTTCAAATGTC

CCTGCTTCCGCTTCCTCTTCGCCAAACCGGAAGTGCTGTCATGGCGCAA

>c4725_g1_i2 len=327 path=[503:0-210 109:211-216 42:217-326]

AACTTTGTGTTTGAGGGGGTGGTCTCATTTTGGGGCCTTTTTGCCCTTCTTTTTGGGGGG

GGGCGGAGGGGCGGCCCCCACCATCGTCAGCTTTTTGGGGAGGGCAGCGGAGGCCTTCAG

GGCGACCTCCCGTTCAATGCGGAGCCGAATGCCGACCTCCAACGTCTTCTTCAGCTTCTG

CTGTTGGATCACACGCAGCTTCTTGGGGGCGTACCCCGCGCCCCCCCACTTCCGCCTCGC

TGCATCCCGTCGTGCCTTACGCTTCCTGTTCAAATGTCCCTGCTTCCGCTTCCTCTTCGC

CAAACCGGAAGTGCTGTCATGGCGCAA

>c4725_g1_i3 len=328 path=[503:0-210 713:211-238 740:239-327]

AACTTTGTGTTTGAGGGGGTGGTCTCATTTTGGGGCCTTTTTGCCCTTCTTTTTGGGGGG

GGGCGGAGGGGCGGCCCCCACCATCGTCAGCTTTTTGGGGAGGGCAGCGGAGGCCTTCAG

GGCGACCTCCCGTTCAATGCGGAGCCGAATGCCGACCTCCAACGTCTTCTTCAGCTTCTG

CTGTTGGATCACACGCAGCTTCTTGGGGGCGATGACCCGGCCTCCTTTCCTCGGCCCCTT

CGCCCCCCCCCTTTCCGCCGCCGCCGCTCTCCGCGGTTTCTTCTCCGCCGCTTTCGGTCT

CCCTTGCGCCATGACAGCACTTCCGGTT

>c4726_g1_i1 len=228 path=[440:0-227]

AAAGAAGATAGAAAATGAAAGGATACATGGAGCAAACATGACCTTAACACCAGGACTGTT

ATAATGGATGACAACTGGGAGGGGGAAAAAAATAATAGTAATAATGCTATTTCCTAAAAA

ACAAACAAACAAAAGAAAACTCAGAACTGTCTTGGAAACATTTAATTACAAGACACCATC

AAGGGAATGAAGAACAGTTCCATAGGGAAAGTAAATCAGTATTTGGTT

>c4728_g1_i1 len=297 path=[601:0-296]

GTTCTAGAACTTTGGCTTCAAAGTCAATAGCAATGGTTGAGCTAGAGCAATTCTACTGGA

AAATGTGAAGATTTTGTGAAGATTGTGAAGCCTTTGGTGATGGAAAAATCTTTACTGACC

TTGCAGTTGTACGTGTTGTACAACTGTTCCATGGTTAGGCTTATCCTTCCTGTAAAGTAA

AATGGCTACCAAAAAATCACAGAAACAAGGAATGTTGGCAGACACATGAGAAAATGGAGA

CTCTGACCTGAAACCTAAAGAGGTCTTGAGCATCATCTCTCCAAGAGATTCTGTGAT

>c4730_g1_i1 len=351 path=[1:0-350]

GTTGAAGGTGTGGTTGAGGCCGAGGAAAGCGGCCTTGCAGGGCAGCAGGGCCGTGCGAGC

CCGGTTGGCCGCGTCCACCTGCAGCACACAGAAAACCTCCCGCGTTTCGGCGCGCGGCGG

CTTCAGGTCCCGCACGCCGTAGAGGTGCACGCTGACCAACCCCGAGATGTGCTGCCCGCC

CCGCGGCAGCTCCCCGCAGCTGTAGGGCCGGAAAGCGGCCACATCCAAGCCGCTCTCCCC

GTCCTCCGGTTCGGCGAGCGCCCCGGTTTTAGCCGGCAGCTCCGGCGAGTCCCCGTCGCT

CAGGTAGCCTCCTTTGCTGGCTGCTTTGGAGCGAGGCAGAGAGGCCACGCT

>c4737_g1_i1 len=202 path=[260:0-144 418:145-147 421:148-201]

TTCCATCCTGCCACTCAATGTCCAAAATTCTGAACGTGAGGTAGACGTCGACCAAGCATC

AGCAGCGACTCAACTGCGTAACGGCGATCAAACACATCTATATCTGCCATACTTCAGTTT

AAACGACCGAAGTTAGGAGAACTTCCAAGAGCGAAGCGGGCTCTCGGGGAGGAGGGCGGG

AGGCCTCTGCTCGCTCTTGGAA

>c4738_g1_i1 len=268 path=[1:0-267]

AAAAAAACACAGAATAGTGAGAATGCCTTTTGGGGGAACCTAAAAGGAAAGAGGAGACAC

GTGACTGGAATTAGTGGATGCAGATTTAAAGTAAATCATCCCAGCTCCTGGCTGGGCTGC

TGCCTGCTGTTGCTGGGGATCCCCACGCCTCCCAGCTGCTAGTTTCCTCCCTACGCATTG

GGCTCCTCCAACCCCCAGGGCCCTGCTGTACCTCTCCTGCACCAGACCAGGCCCCAGCTC

CCTGGCATGGAGGAAGCAGCGCTCCTCG

>c4738_g2_i1 len=286 path=[754:0-285]

GAGGAAGCAGCGCTCCTCGGTACTGCAATCATCATCTCCTTGCAGTCTGCTGGGACGTCA

GGGAGCATTCACAGAACCTCACCTGAGGACATGGGCAAGCTCCTGCTGCCTTCACTAGCT

CACCAAAACCAGCCTCCACTTCCTGCAAACTGCATTCCAAGCACAGCAGCTTTAGCTGAG

CCCAGCAAATGTGAAGGCCCTGCTGCAGGAGGTGTCAGACACAATGTCCCAAGGTCAAAG

CAAAAACCTTTAAGATCAAAAGGGAGCTCTCATGTGGACTAACAAG

>c4751_g1_i1 len=209 path=[1:0-208]

AATATTAGTATATAATTTTCCAAAGAATACATTAAGGAATTTTTAAACAATTAAGAACGA

ATGTCCGCTGTTTTAATAAAATTATTAATCCAACATATGATTTATGAAATAAAGATTTCA

CATGTTATAGACTCTAAACATACTTTAAAATAATTTTATTTAAAGTATCGGCAAATGTTT

ATATCCGTACTAAATAGAAAAATAATGAA

>c4756_g1_i1 len=358 path=[379:0-215 22:216-357]

AGAAAAAAAAATGCAATAGCGTTTTTTTTTTTTTTTTGTGGATTTAAAAAAGAATTAATC

TATTCTTTAAAATTATTTCATCACTTTTTGTCATTTTTACATCATAAAACAGTTTTTATA

CAACCGATTAAATGTGTGTAACATATTTTTGGAAACGTTACATATATATTAAATTGCATA

TGAAAATTAAAATATATATAGTATATTAAATTAGCCGCAAATTAAATTATATGTATATTA

TTATTACACTCTATTTTTTATTAGCACCTTTCAGCAAAATGAAAAAAAGAAAAAAACTAT

TACTTACATATGAAATTGTATGAACACTTCTAAATGAATTTTTTTTTTTAACATAATT

>c4760_g1_i1 len=229 path=[264:0-55 16:56-202 465:203-228]

TGGTGGCAGTGCCCTGGTTGTCCCCAGCCTGCCTTGAGCGGCTGTCACTCTGCCCCCAGC

TCCCTTTCAGAGCCATATGTTGAGTTTCTTGCTCCTTTCTACAAGGCCTCTTAATGATGC

CGCTGAACAGATGTTTGAGCTGAGCCATGCCCTCCAGAGACTATTTTTGTTTAGTTTTTT

TTTCCCCAAAGCTTCTTGCAGCATGCCCAGGATCTCCTAGCAGTCCTTC

>c4761_g1_i1 len=204 path=[336:0-203]

GGTTAAGTACAGGATGCACTTTATGATACAGATGAAATCATGGATTGATTTCATATTAGA

AATGCATACGCTATCCAAAAGTTATAAAAGTGACACATAACACAACAGTGCTGTTCTCAG

CACTAAGAGATCTATAAATCTGTAGAACCACAGAAGCTTAAGGGCTACCAGAGGACATCC

AGTCCAACACTCTCAAACACAAAG

>c4763_g1_i1 len=310 path=[288:0-25 314:26-118 407:119-309]

AAAAAAAAAAATATATGTACATATATGTATGTGTTTTATAAAATATATATATATATAAAA

TATGAAAAATAAATATAAATAAAAAATTATAATAATATTTTTTCTTGTTTCAATGTCAGG

TGAGTCAGCAACTCATAAAAAAAAAAATGCTTAACTCGTTTTATCATTGATAATTATTTT

TTTTTTAGCCTCAAATAAAATATTTTTCATAACAACATAATAAATCATGAACATGTTATA

TTTAAGGCACACAAAAATGTTATAGAAAAATAAAGGAAAAAAAAAAAAAATCAAATGATC

AAACACAAAG

>c4763_g1_i2 len=217 path=[288:0-25 407:26-216]

AAAAAAAAAAATATATGTACATATATGTGAGTCAGCAACTCATAAAAAAAAAAATGCTTA

ACTCGTTTTATCATTGATAATTATTTTTTTTTTAGCCTCAAATAAAATATTTTTCATAAC

AACATAATAAATCATGAACATGTTATATTTAAGGCACACAAAAATGTTATAGAAAAATAA

AGGAAAAAAAAAAAAAATCAAATGATCAAACACAAAG

>c4773_g1_i1 len=243 path=[248:0-83 14:84-242]

ATGGCGTCACTCCAAGTAGGCGTCAAAGACGTCCAGCTCGCTGTCGGTCTCGTACAGCGC

GGAGCACGAGGGTTGGTTTGATCAAACGTTTCCTTACCGCTGTAGCTGTCGGTGACGCTA

CCACGTTGGAAATGTAAGGATTCAGCGTTTTGTAACGCTGAACTGCAGTTGGGGAGAGAA

GCACTATTTCCAGCGAGGACGTTCTGTGCCAAATGTATGTTAACAGAAACGTTCACAGTT

TAA

>c4787_g1_i1 len=355 path=[183:0-354]

TTCTCCAGGAAATGTCTTCTCTACACTTAGAGGAACTGTAGCAGTTACTGAGTGATAATG

AGGTTATTTTTGTAAGCTGGAGTTTTCACAGCTGTCTTCCCAAAGACTGAACAGTTTTGC

TATTACTTTAAAGAACAACTTAAAAAGACAGCAAAACACTTTCTTCTTCTTGCAACGTCA

GCTAGGGAATGAATGTAGAGAATATATAGTGTAGTGCACATAGGAGTAAGATGATTGTAT

GTCCTCATTAGTATATCAGATTTATATGTTATAAGTAACCAGTCAGTTGGTTGCCTCCTG

CCATGAAGGACTGCATACTTTAGCAATTTACCATGCATATGCCCAGGAGGGAAAG

>c4791_g1_i1 len=285 path=[261:0-153 153:154-284]

TTTATTACAGGATAGCAGCTCTTCTCTACTGAAATAGCCCTCACATTGTGCTATCTCCAC

ATCCATACATCCTGTGCTTCACACAGCACCTGAAGCTTTGTTTCTGACCAGTAGAACTAT

TAATTCATGTAAGTAAGCTGAAACATTAAGGTCTAGCTTTTCAGACAGGTAGTACACTGA

ACACAACTATTTTAATGTGCTTTTTACAAGCTCTGTTGCAAAAAAAAATTACCCAGAGAA

ACCCAGTTTGTCTTAGTATCAAGGGCATGCTCATACTGAACAGTA

>c4802_g1_i1 len=230 path=[1:0-229]

GGGTCTTATGGGGGATTTATGGGGACTTATAGGGGTTTATAGGGTCTTATGGGGCCTTTA

TGGGGGGTTATGGGGTCCTATGAGGTCTTAACGGGTCCTATTGGGGCTTATGGGGTTCTA

TGCGGGATTTATGGGGTCTTTATGGGGTCTTATGGGGGATTTATAGGGACTTATAGGGGT

TTATAGGACACCATAAGCCCCCATAAAGACCCCATAAGACCCTATAAACC

>c4804_g1_i1 len=237 path=[714:0-16 716:17-236]

CACACAGGCCCACGGTGGCACCAACACAGGCCCACGATGCGGAGGAACATGGGGACACCC

CGTGGGTATGGGATGTGGTGGGGCACGGAGCCGTGGGCGGCTGTGGGTGTTCCCAGGGGT

GGATATGGGGCGGTCACAGGAGGTCCTACATGGACACGGGGCGGTCACGGACGGTCCCAG

CTGCTCGTGGGTTCCAGGTGGTCACAGCACACGGGGGGGGCCGCAGGAGGACACGGG

>c4804_g1_i2 len=354 path=[134:0-133 716:134-353]

TTTTTGAGGGGTTCCCAGGGCTTTATTGGGAGGCAGCAGCAGCAACCCCGGCAGCCGTGA

CACCACAGAACCCAATGGGACCACCGGACACCCCCTAATATGCTGGCACCCATTGAAGCC

AAAACACCCCCTCAGCACCAACACAGGCCCACGATGCGGAGGAACATGGGGACACCCCGT

GGGTATGGGATGTGGTGGGGCACGGAGCCGTGGGCGGCTGTGGGTGTTCCCAGGGGTGGA

TATGGGGCGGTCACAGGAGGTCCTACATGGACACGGGGCGGTCACGGACGGTCCCAGCTG

CTCGTGGGTTCCAGGTGGTCACAGCACACGGGGGGGGCCGCAGGAGGACACGGG

>c4805_g1_i1 len=207 path=[1:0-206]

TCCACTTGACTGCAGTACCCCTCTCATTTCTACTTAGCTAGTTGTTAAGAAATGTCAGGG

TAGGCCTTTTTGTCCCTTACATCTGAAGAGGAGCATGGCTGAGTTCACCTACTTCTTCCC

TCCTAGACCTTGACCTCACAAAGCATCAATGTCCAAGTCAGCAAAGAGCACCATATAGCC

TGTCACTGGAAATTGGCTGCCATGAGG

>c4810_g1_i1 len=243 path=[375:0-242]

GAGTTGTCGATAAGACTACAAGCCAAGTTAGATGCTCAGAAATGTTGGGATTGCTGCGGA

ATAGCTGTTTGGGTGGAACTACCTGGATTCAAATGACTTTGCTGTTGCAAAGAGCGGAGA

ACACAAGTCTCACGTTGAATAACGTGGGGGTTTCCTCGGTATCACCTGGTGACGCAAACA

GCGGTGGTTGGCCAAAACATTTCAATTGCAGACTTCTAACGCTTGGGAGGGAGAGTGCAG

AGC

>c4815_g1_i1 len=243 path=[1:0-242]

CTCCAGCCTCTTCTTCCCAGTGCTGCAGTTGGAGAGCGATTCGCTCCGGCTCCCCATGGC

ATCGTCCTCTGTTGGGGAGCAGGTCAGGAGGTCGGAGTCCGATTTGGACAAGCTGCGGCT

GAGCTTGAGCTCGGCTTTGGGTTTGCTCCCCGGGTGGGCTCTGCCCGCAGCACCACCTCT

GTCCCCCTCGTCCTCGGCACCGCCGGGGTGCTGGAGCACGTGGGGCGTGCCTACAGTTTC

AGG

>c4816_g1_i1 len=208 path=[496:0-207]

CTTCTCCTCAACAGAGTGAACGCAGTCAGAACAATAAGCCCAGGCAGGGAAGTCTGGCTC

AGGATGTGCACGTGATCGGGCAAGGATGCATCATCAACATAACCCCCCTTTATATAAGTC

TAAGAACATTTACCACAGAAAAGCATTTCCTGTAAAACCTGGAGCTATTTGAAAGCCAAC

TGGTTTTCATGTTGAAAACAAAACACAA

>c4822_g1_i1 len=469 path=[1:0-468]

GGGGGGGGGGGGCACAGCCACGGCACTGACGTAAATATCCCGGTGGTTCTCATCGCTCAT

CATCATCATGTTGACCAGCAGGCCGTGCGCCGAGCGGGTGTTGAAGCCGAAGACGATGAG

CTTGGAGGAGTCGCTGGGCCATTCGCACACCGTCAGGTAGAGGTCGCTGTAGATCTGCTC

GTCCTGGAAGAGCCGCACCTGGCGCACCAGCCGCAGCTTGCCGTGCAGCCGGAACTCCCA

CCAGTACAGGTGGTAGATGTAGAAGGAGAACTCATCGTCCCCACTGCTGCTGGTGTAGGA

CAGCACGAAGCGCCCGCACTTGGAGAAGCCCAGGAAGATGTGCCCCGCACACAGGAACTC

CTCGTCCACAATGCTCTTCAATGAGATGCACGCCCGGGGGGGCAGCTTGCGGAAGAGGCG

GGGGGAGAGCTGCCCGCTGATCCGGACTCGCTCCAGCTGCCTCACGATG

>c4824_g1_i1 len=226 path=[1:0-225]

GCCAACAAGCTCTTGATTTCTTTATTCATCTCGTTTAGCAGCTGTTCCAGGGACTCAGGT

TTGTACAGGGACTGCAGGCCATCTGAAAGCCCTGGGTCTGCTGTGTCGGTGGAGGACAGC

TGAAGGCCTTCTGTATCATCAGCCTCCAGCTCTTTCCTGCGTTTACTTCTGACTTCCTCA

GGTTTTTGTGTGCTGCTCGTCATCGTTACCCGCAGAGATGGGCGCT

>c4835_g1_i1 len=235 path=[189:0-186 376:187-234]

ACAAGTTATTATTTGGAGGTGTAGGACATAGATTTGATATTGGAAAATGATAAAACAAGT

CACTTCATTCTTGTTACCTCCTCACATATAATAACTTGTGAATTGTAAAATTATGTACAT

TTTTGTAGGCTAAGTTGAAATGAAATTACTGAAAACATGATGAAGAATTGTGTTTTTAAT

GTGTTTGATGTGTGTTCTCTTTTACTTGAAGTGTAGTATGTGGTGTGTACTAGTT

>c4841_g1_i1 len=278 path=[345:0-56 45:57-277]

TTATCTTTTTCACTAATAGTTTTCCTGTTTGCTTTTTTCTTTTTTTTTCGTTATTTTTTT

GTTAGAGAGCAACTCAGATTTGATGCTTTGAGGATGTTGGGAGAGAGGGTGGAGGGCGAG

TCAAACTTCTGGTTGGAATAACCTCCAGAGTTTGGTTCAGAGAAGGATGAGCCTTTGTTT

GCTGAGAGCTCCAGGACAGGCTTCTTTGGGTGAGATTCTGAGTTTTAAGAAAGCCAGAAA

AATGTAGCTCACTTTAAAAAGAAAAAAGAAAAAAAAAA

>c4844_g1_i1 len=366 path=[344:0-365]

GTCTCCCTTAATAACACTGTGTGGAAGACAAGTCTCTCTGCACCAAAATTACTGCCCACT

TTAAAAAGGACATACAGACACTTTCTGAACTCAGATTAACAGCTGTGCTTCTGGCAGTCC

TCTCACTATCGTTTCATGAGTGCATCTTCCCAATAACATACAACTTCCAAATCTCACATA

TGCTGTATACTGGGACCACTGTCACCAGACAGCCCCAAACCCATAACTGTACATGTTTTT

CTTCTGCCAAATTATCAGATTCCATAGCTGTTAGTCAGACATGCCACCTCCCACTTCATC

AGACCAACAGTCAATCACCCTCTTCCTTATTGTTCGATCAACGTTTCTGTCCAACAGGAG

CTGAGA

>c4853_g1_i1 len=232 path=[1:0-231]

AAAGTAGTATAAGAACTTTTGAATGAAAAAACGTAATATGTCAGTTTTCAAAAGAATACT

TTCAGCCATCATCAGAATAGATTTGAGTTATGTTTAGAGTTGTGGATATTCTATCCATTT

TAATGCATCAGTATGACAGTGAGATCCTTCAGCTTTTAAAATATTGTATGATCCATGGTA

AGAAACAGAGGCAAGGTAGTTTGACTTCTTAAAGATAAATCTCATCATGAGT

>c4859_g1_i1 len=224 path=[300:0-146 23:147-223]

GAGGCTGCTGTTCTCTGTGCTCTGAAAATAAGATTCACACAAGCTGTTAAATTTCACTCT

AGAATAACACACTGGGAAAAAAACACAAAGACGGGAGACCACAAGGCATCTGTCATGGTA

CATCGTGCCAAGACACACAAACACTGGGTCAAAGGCGAGGATAGACTTAAGGCTTTAGCC

GTAATTTGGGACATGTGACTACTGCTTCCTGCTTTGTTGCATTC

>c4862_g1_i1 len=368 path=[1:0-87 89:88-367]

TGCGGCACGCCAAGTGGTTCCAGGTTTGCATTTTGTTCTTCTCTTTGTCTTTAAGTAGCA

AACGTGTAAACCTTAAACCTTAGCGGGGCTCTCGGTTCGCTGTAACGCCGGTAGCCGTGC

CGGACTTGGCGCCTGTCGGCGGGACAGCCGCTAACGAGGCACTCGTTAACGAGCCCCAGG

TCGCAAAGGCAGCCCCGCAGCCATTTAAAGGCGCAGCGGCCGCGCTGCGCTGAGGGCTCC

GCGCTGCGCTGCGTCGCATTGCCGCGTGGAATTCTGTGTCCCCTTTGCCGAAGGAGCACC

GAGCGCCGCGCGGCGTTCATTGAGTGCTGTCTGCTTCTGCTTCCCGTTCCAGGCCAGGGC

GAACGGCC

>c4862_g1_i2 len=299 path=[710:0-18 89:19-298]

GGAACCCGGGTGCACAGATCTCTCGGTTCGCTGTAACGCCGGTAGCCGTGCCGGACTTGG

CGCCTGTCGGCGGGACAGCCGCTAACGAGGCACTCGTTAACGAGCCCCAGGTCGCAAAGG

CAGCCCCGCAGCCATTTAAAGGCGCAGCGGCCGCGCTGCGCTGAGGGCTCCGCGCTGCGC

TGCGTCGCATTGCCGCGTGGAATTCTGTGTCCCCTTTGCCGAAGGAGCACCGAGCGCCGC

GCGGCGTTCATTGAGTGCTGTCTGCTTCTGCTTCCCGTTCCAGGCCAGGGCGAACGGCC

>c4871_g1_i1 len=202 path=[1:0-77 382:78-201]

GCCTTTCAGCTTTCTATGCTAGAGATATTAATCTCTTATCTGTGCATGTAAATACAAGTT

TGTAGATTGTAATAGAAGTGACAAAAGTTATTGTGTTCTTGTGTTAGGAAACAGAAATGT

TTTCAGTAAGGAGATCTGTAAAGAGTCACTGCAGAGGAAAAACTGGTCAGTAGTGAAATT

AGTTAATTAGATGTGGAAAAAA

>c4874_g1_i1 len=235 path=[297:0-234]

AGGCAGGGTGCTCCTCCTGTCAGGACCAGCTGACTGCATTTGGTTTCCTGCTGCTACCAA

TGCTATCATGTCGGCTTTGAAGACAACGGAGTGAGGTTTGCTTAGAATTTTGGGGATGTG

AGGTTTTCTCATCGCCGTTTGTCATCACAGCATCTGTGTGCATCAGAGCTTCTGCTGCAG

GAACAAGTGGAGCAGGATCTGTGCATCCCTGCCTGCTTTAGGTTCTCCTGCTTCA

>c4887_g1_i1 len=316 path=[319:0-278 598:279-315]

TAAAAATAAGTTTAACGTGTATATATGACAAAGAAACACTGAATACGAAGTTCAAGAGTT

TAAATAACAAACTACAAAACAGTTTATATAAATTATAAAGAAGATTATACATGAAAATAA

ATATATATACAATTGTAGCTAACAAAACAATAATAGTTATACTAAAAAAATAATAAATAA

TATTAAAAATTTTCCATCATTAATGAACATTTATTTAAATAAATATATATAAATATATAC

ATGTGCTTAATAGCAAGAGGCTATATTTTTTTAAAATTTTATTTTTGTTTTTTACATATT

TTATTTATTTATAATG

>c4887_g1_i2 len=303 path=[319:0-278 660:279-302]

TAAAAATAAGTTTAACGTGTATATATGACAAAGAAACACTGAATACGAAGTTCAAGAGTT

TAAATAACAAACTACAAAACAGTTTATATAAATTATAAAGAAGATTATACATGAAAATAA

ATATATATACAATTGTAGCTAACAAAACAATAATAGTTATACTAAAAAAATAATAAATAA

TATTAAAAATTTTCCATCATTAATGAACATTTATTTAAATAAATATATATAAATATATAC

ATGTGCTTAATAGCAAGAGGCTATATTTTTTTAAAATTTGAGTCCTGGCCCTAACCTTCA

GTG

>c4892_g1_i1 len=266 path=[244:0-223 468:224-232 477:233-265]

AGATGTGAATCAAGTGTGAAGTAGCTCGAAGGTCCCAGTCCAGGATCAGGCATTTTGAAA

ATACAGGATGGGAGACGGGTAAATTGCTGATGTGCTGTGCGATACAGGTCTTTTAGAAGG

GTTTCTTTCAGATTTACTGAGCTGCTCACCAGATGAAACACATTCTACTCTTGAACCTGC

AGTAATCTCAGTGGTTGTTTTGTGTATTTTTGCTCGATTCTGTAGATTCCAGTCTCAAAC

ACAAAGTTTAAGCATTAAGTTGATGT

>c4898_g1_i1 len=210 path=[388:0-209]

ACTTTGTGTTTGATTGGGTTTTTTGGTTTTTTTTGTGGTGAGGTGAAGATGCTACAAAAC

ATTTCCTTCAGTAGGCTGTGTAAAATACTCCTCAGGGCTGTGATTCTGATCATTAATTTG

GACATGTTATTGCAAGTATGCCAGTAGTTAAAAAGCTGAGTTTCTTGTTTGAGAGTTGTG

ACTAAATATGAAGTTGTTTAATCAGTTGTA

>c4905_g1_i1 len=276 path=[431:0-189 700:190-195 626:196-275]

GAAAATAAAATATTTTTTTAAAAAGACATTTTTTATATATAAAAATAATTTATTTATAAT

ATCAAAAAATGTTTTATGTAATATATCCTTACTAGTTTTCAAACATTTAAACACCTCTTC

TTTCATTATCTGTGTATTTGAATCATACAAAGCATCTTGTATATACTGATAGCACTTATT

ATTTTTCCCATTTTTTGTAACATACGAAAAAAAATAATTGTTTAATTTATTTCCTTTTCT

GTATAGTAAAGAGAATTTATTTTTAAATACCTTATT

>c4905_g1_i2 len=276 path=[431:0-189 700:190-195 124:196-275]

GAAAATAAAATATTTTTTTAAAAAGACATTTTTTATATATAAAAATAATTTATTTATAAT

ATCAAAAAATGTTTTATGTAATATATCCTTACTAGTTTTCAAACATTTAAACACCTCTTC

TTTCATTATCTGTGTATTTGAATCATACAAAGCATCTTGTATATACTGATAGCACTTATT

ATTTTTCCCATTTTTTTAAAGAAAATTTAGGTATTAAAATTTATACAAATGATTCAGCAA

TATTTGATATAAATAAGGTATTTAAAAATAAATTCT

>c4937_g1_i1 len=259 path=[264:0-0 14:1-258]

AATAATACAAAATATGATACCTATGATACCTAGCGTAATTTGCATAGAGGAGGATGATAT

TTGGAAATATTTAACACAAGTTTGATAAAAGGTTACTTCAGTCTGCCTTTCAGGTTGAGA

ATACTTGTGCTGCTGTTCTCCTAAAAACTCGGATTATACGTTTCTGTAGCACATCTGAGT

TCATACCATTACTGTGGTTACATACGTAGTCAAACACAGCACTGGTAGTGACTCCAACTG

TGCCACTAAAAAACCAAGA

>c4938_g1_i1 len=436 path=[1:0-139 141:140-435]

TCAACTTATACTTTTTAAAAAAAATAAAAATATATTTATTCTTGTTTGAACATTTTTATA

AGCTATTGATAACAATATATAGAATAAGAGACTGAAAATATAATTTTATTGTATACAATT

TTATTTGAATACTTGAATAATTTTTCAATACATTTTCCTACAATTTTTAATATCTTTTTA

TTTTTTTTTAAACATTAAAAGATATAAATTATTTCTTTTAAAATTTTTCAATATTAGAAA

GAATAATTTTTACACACAAAAAACTAAAAAAATTGAGCACCTAGTTTTTTATATTTAATG

TATTTTTGCTATTTGCATAATGCACAAAGCATATGCTTATAATAAAATTAATAAAATTAT

AATCAACTAGGAAAAAATAAAAATAATTATTCTTTATAAGAATTTAAAGAAGTTATATAT

TTTTGTTAAAAAAAAG

>c4945_g1_i1 len=411 path=[6:0-187 194:188-410]

GTAAAAAATAAATATGTTACCCCATCAAACACAAAGAATTGTTGATTGAGGCTTCTTCTA

TCTTCAAAGTCCTCCCTTACGCGGTTTGTGGAACGTGTAGGCCTCAGTAAGAAAGCAGTC

CTTTGTCCAGAATTCTTAGGCTGGGGTGAAGGGAGCATTTTTTTGCAAGGAAAATGCCTT

TATTCTAACCTGCAAACCTATCAGTTTTCAGGCTTTATCTCATACAGAAACACTTCAGTC

TCTCAACTCTTCTACTTAACCTACCAGTTGTAGGATGAGGTAAAATTAAACTGAAAGCAC

TGGCTCATTTCCATGTATGTTAGTGAAAAGTTGGTATGGCGTTATGGAGTAACATAAAGA

ATTGTTGATTGGGGGTTAGCATTACAGGTGGAGGTATCAGGAGGAAAGACG

>c4945_g1_i2 len=241 path=[644:0-17 194:18-240]

TTTCTGTGTGAGATAAAGCCTGCAAACCTATCAGTTTTCAGGCTTTATCTCATACAGAAA

CACTTCAGTCTCTCAACTCTTCTACTTAACCTACCAGTTGTAGGATGAGGTAAAATTAAA

CTGAAAGCACTGGCTCATTTCCATGTATGTTAGTGAAAAGTTGGTATGGCGTTATGGAGT

AACATAAAGAATTGTTGATTGGGGGTTAGCATTACAGGTGGAGGTATCAGGAGGAAAGAC

G

>c4945_g2_i1 len=202 path=[427:0-198 234:199-201]

CGTCTTTCCTCCTGATACCTCCACCTGTAATGCTAACCCCCAATCAACAATTCTTTATGT

TACTCCATAACGCCATACCAACTTTTCACTAACATACATGGAAATGAGCCAGTGCTTTCA

GTTTAATTTTACCTCATCCTACAACTGGTAGGTTAAGTAGAAGAGTTGAGAGACTGAAGT

GTTTCTGTATGAGATAAAGCCT

>c4948_g1_i1 len=241 path=[381:0-240]

GTGAAATGGCTTGTAATCCCACTGCAGCTCTGCCCTTAGTGACCCCTGCCTACCTGCCTG

ACAACACAGATTTAAACAGCAGCTCTCAGGGCAGCGGCCGGCACGCTGTCCCCATCAGAT

AGAATATCTCAGCACTAAAAGGAGATATGTTTCTGAGTCCAAGAGGAGTGTGTGCTGGGT

AAAAGAGAGATGTGTACAGCAAGCAAAAGCATGCATGAGCTGGGTTTCTAGAAAAAAAAA

A

>c4951_g1_i1 len=245 path=[1:0-244]

TACATACATACTTTCAAGAACGATGTCTGATGGCTGTTCTTGATGCCGTGGCTGTGAGTA

ACTACTTGTTACAGAGCTGTAAGTTAAGGTTAAGCAGACCTCTGGAGATTTAGTCCAAGG

AGAGTCCCTTAATCAAGGATTAAGGACACTGGGCTTCCAAGGAAAGGCTGAGAGAGCTGG

GACGCACTGCTCCGGGTGCAGCCTCAACAGTACTGGGGGGGAGGACCACCTCCCTCCACC

TGCTG

>c4961_g1_i1 len=222 path=[511:0-221]

AATCTCTTCTCTGTCAACCTGTAACTATATTTTAATGAAATCAGAGTTCTATAGGTAGGA

AATATTACTGAATGTTCACTGTTTAACCTAGTTTGGAGATTCTTTGGAATTGAAACTCTT

TACCTCTTAGATTATATTATTGTTAATTAGACAAACAGCTGTTGGTGGCCACCTCGTTTT

TCTAATTAACAATAATATAACCAGGGGCCAATCCATTAAAGT

>c4961_g2_i1 len=253 path=[281:0-252]

ATAAAATGCAATTAAACATATATTTGTTTGCAAAGTTGTGTTCTGGATTTTCTGATGATT

CTGAGTGTTGGGTACTTCTCTTACATGAAATGCCTGTTGCAAATGGCACCATGTTAATCA

AATAATTAGTAATGAATATTGCATATACCTTGGATTTGCGTGCATCTCATGTCTTTTAGA

CTCCATAGGTTGAGTAACAATGATGTTGAGTAACTGACTACACCTCAGGTTCTCCTGTAA

CCATATTTTAATG

>c4974_g1_i1 len=234 path=[239:0-233]

AAAAAAAAATAAAATAAAATAAATCTCACATCGTTGTTTGAATTGAGATATTAAAATCTA

AAAAATAAAATAAATTGTGTAATAATTTTATATTCATAATAAAATTAAATAATAGAAAAG

TGTAATAGACAAAAAAGAAAGTATATATATATTTTTAAATAATTTTTAAATAAATAAAAC

ATTGGTAGAGTTTAAATAAAATAGAAAATAAATAATCTAGAAAAAAAAAAAAAA

>c4979_g1_i1 len=365 path=[343:0-364]

AAAGAGGCACTTTTTCTCTCCAGAGGATTTACAACTGGCAGAAAAGCAACCTTGAAGTTG

TCAGCAGTAAGGTCTGAAAGTGCCACTGCTAGTCCTGCCAGAGCTCCTCAGAACACTGAG

ATATCCTTTCATTACAGAGGAAGGGAAATAGTTATGCCACCATATGTCCCTATCAGCCAC

TACAATTACACTGGTGAATTACTTAAACTTGTGTGAACTACAGTGCTAAAGCTGAGATGC

TCACTCAGCTCCATTATTCTGAGTGTCTGGCCTGTGCTTAAGAGCTTTTCTTATGTAACT

CATTTCAGAAAATGAGACATCTTCAAAATGTCACAGTAGTTACACAAGTTTAAGTAATTC

TCCAG

>c4983_g1_i1 len=216 path=[19:0-16 36:17-215]

AATGGAAGAGAAAATAGAAAAGTTGGTGTTGAAAATGGATCATTTTTAATTGGGTGAATG

TAGTACTTCCGTTTTTCATTTTAGTGGAATGACTTAAAGCCTACTAGCTTGTTTCTCTTT

GTTGCTGTCTTAAAATTGAATGATGAGAGAAGATGAATGATTAGACTGTAGTCTTACATT

ACTGTTACTGTTTAAGAGTGTAAGACTACAGTCTAA

>c4983_g1_i2 len=300 path=[313:0-100 36:101-299]

TAGCATTTTTCACACTTGTTTATTAATACCATTCAAAAAAAGAAATCACCTAGGTACTGT

GCAAGACATTCTTGCTAAAGCATTCCTGTCACAGACAAAAAAAAAGTTGGTGTTGAAAAT

GGATCATTTTTAATTGGGTGAATGTAGTACTTCCGTTTTTCATTTTAGTGGAATGACTTA

AAGCCTACTAGCTTGTTTCTCTTTGTTGCTGTCTTAAAATTGAATGATGAGAGAAGATGA

ATGATTAGACTGTAGTCTTACATTACTGTTACTGTTTAAGAGTGTAAGACTACAGTCTAA

>c4986_g1_i1 len=288 path=[1:0-287]

CAGTTGTTATAAAGCAAGACAACAGAGCTTGGTATGGAATAGGTTTTAACTTGAGTTAAT

GAAGCAGTGTTTCTTCAGTAAAAAACACCTTGCGAAACTTGAAACCAATTAGAAATGTGG

GTCAGGTGTTTGTTTTAGAACACTGTTTTTATATATAGCCTGGGATACAATTTTTTGGAG

GTTTAGGAAGCTATTATTTTCATTTGAAAAACTGTCCAAAGCTGATTATAGATTTCCCCA

TTACACTAGTAATTGTTTTTGTGTGTTTTCTGGTTCTCTTGTGATGTG

>c4986_g2_i1 len=350 path=[858:0-349]

AAGTTCACAAAAGAGCAACTTTCCAGCTGGCTCTTCAGATAGGTTTACTTGGTTCTCCAG

GATAAATCAGACCTATGTTTTTATCTCATATAGAAAAAACAAATACAAAAAAATAAGGCA

CCCAAGTGCCTAAGTTTCAGCCTTGCACAGGGGAATATTCTTTAGGGAATACACTTTGTA

CAGAATCTCTACATCTGTTGGATACTCTACCAAAATGATAACGTACAAAAATCAGTGTTT

TGCCAATATGGCAAATTGTTCCTCTTGGTGTGAGATCTAATTTTGCTTCACGGGCAATTA

ACAAAAAGCTCAGAAAAACAGTAGCAGCCATTTGACAGAATGAAAAAGCA

>c4987_g1_i1 len=209 path=[187:0-208]

AAGAAAAGGAGCTCACAGCCAACCATGAAATCCAGGCTGGGACGTGGGCCAGCACACAGC

TCTCACCCTACTCCGTGCCCAAGAGAGGGGGCTGTCACGGGCAGGGTGCTAGGCAGCCAC

GTTTGGGGCTGGTAGCACATACCCCATGGTGTATTAACACACGTACACACAGAGCAGTCC

CCCTGGCTGAGCATGGAGCCATCAGACTG

>c4988_g1_i1 len=247 path=[35:0-246]

TTATTTATTTTTTTTTTTTTTCCTGAAAATATTACATATAATATTTTTATAGGATGATTC

ATCATATTTTTTTTTTATTATCATTTTCAATTTATTTTTTTTTTTCTTGAATCCATTATG

ATGATAATTGTTCAATAAAATGTAACTATTTAAAAATTTTATTTTTAACCTTACAGGATA

AAAAAAATATTATATTCATATAAATATATCATAAAAATATATATATATATATAAATGATT

TTCAAAT

>c5009_g1_i1 len=297 path=[281:0-237 592:238-247 602:248-296]

TGTTTCGTAAGTGCAAAGCGATGCTACTGTGCAGTGATAAAAAAGCACATCCTGTCAAGG

CTTAAACCTTAAAAACATGGAATGCAGAATGTTCCATCAATCAATACAAGAATTAATTCA

TGTTATGTCAACAAGGACCCCTGAAAAATCCTGCTGTAGGCTTTGTTTTGTGTGTACATA

GATCTGTGTATAGGTTGATTCTAAAAACTGCTCCCACACAACTTAAGTCTTTCTTTCTTT

TTCCCCTTCCTTCTGGATCCCTGCAGTGCACAATGGGAGGAAGGAAGGGGAAAAAGA

>c5012_g1_i1 len=206 path=[1:0-26 28:27-205]

ACTTGAATTTTACCAGTGTTTGATAGGAGTTTTCCTACCACAGCGCAACTCTGTGCAACA

CTTCATCATGAATGTGTAGCCTGAGAAACACTGGTAAAATTCAAGTAGTCTGGGATGATG

GCTCAGCCCAATATGGCATTTTAATTGTGCCAAATGAAATACACGAATCAGAACAGCCAA

GCAATCAATACTTCATTAATTATTAA

>c5012_g1_i2 len=216 path=[273:0-36 28:37-215]

TTTTTTTTTTTTTTAAATAATATGCGTGTTCTCTAGAAGTTTTCCTACCACAGCGCAACT

CTGTGCAACACTTCATCATGAATGTGTAGCCTGAGAAACACTGGTAAAATTCAAGTAGTC

TGGGATGATGGCTCAGCCCAATATGGCATTTTAATTGTGCCAAATGAAATACACGAATCA

GAACAGCCAAGCAATCAATACTTCATTAATTATTAA

>c5014_g1_i1 len=367 path=[15:0-12 28:13-366]

TGCTCGAGCTGCGCTCCTGGCTCACGTTGGTACGTGACAGCATGGCAGCGGTGTCCCCTG

AGCCCTCCCACACCACGGCCAGCCCTGAGGTGGCCCTGCAGCCCCCGTGCCTTCCTGTCC

CGTTTGTCACCTCCATCCTTGCTGCTGTGCTGGGGCTCTGCCACCCATCGGCAGTGCCGG

GTGCCCTGCAATGTGCCCTCTGCTTTCATCACTTCCTGCCTCCCAAATGCCTTTGTCTTC

ATCTTTGGCATGAGATGATGCCCTTTCCAGGCTGCTCCTTCCAACACCAGAAATCCCAGC

TGCCTTTGTTAGGGCCGGGGCCACGCTTGTGCAATACCCCGTCTGCTCGCGGGGCCATAT

CAAAGAA

>c5014_g1_i2 len=416 path=[29:0-61 28:62-415]

GTCACGTACCAACGTGAGCCAGCAGCACGGAGCGCAGCTCGAGCACTGCGACTGCTCGCA

GCCTCCTGGCTCACGTTGGTACGTGACAGCATGGCAGCGGTGTCCCCTGAGCCCTCCCAC

ACCACGGCCAGCCCTGAGGTGGCCCTGCAGCCCCCGTGCCTTCCTGTCCCGTTTGTCACC

TCCATCCTTGCTGCTGTGCTGGGGCTCTGCCACCCATCGGCAGTGCCGGGTGCCCTGCAA

TGTGCCCTCTGCTTTCATCACTTCCTGCCTCCCAAATGCCTTTGTCTTCATCTTTGGCAT

GAGATGATGCCCTTTCCAGGCTGCTCCTTCCAACACCAGAAATCCCAGCTGCCTTTGTTA

GGGCCGGGGCCACGCTTGTGCAATACCCCGTCTGCTCGCGGGGCCATATCAAAGAA

>c5016_g1_i1 len=297 path=[43:0-88 42:89-296]

TAGCCACGTGGAGACAGACAGTGTTCAGGTCTGTGGGTTTTGTACTGCACCACGTGGCTG

ACTGCTGGCTTCTGCTGGGCAGTCTCTGTGCTGGTTACGTGGGCTTTTGATTGCAGTCAT

TAAAGCAGTAGGTGTTACATGAAACAAAAATGGTCTGACTTGGTAAAACTGGGTAAGTTG

TGCTTGTGTTTGGCCGTTAATGCCTACTTGCCCTTCTTCCTCTTTCAGTGGCGAACAAAC

TCCCTTGTTACCAAGCTTTATTGTGTGTAATTGTGTGGGTAAGCGTTGCGTGGTAAT

>c5016_g1_i2 len=228 path=[22:0-19 42:20-227]

AGTGTATGAGCTATTCATGAGCTGGTTACGTGGGCTTTTGATTGCAGTCATTAAAGCAGT

AGGTGTTACATGAAACAAAAATGGTCTGACTTGGTAAAACTGGGTAAGTTGTGCTTGTGT

TTGGCCGTTAATGCCTACTTGCCCTTCTTCCTCTTTCAGTGGCGAACAAACTCCCTTGTT

ACCAAGCTTTATTGTGTGTAATTGTGTGGGTAAGCGTTGCGTGGTAAT

>c5020_g1_i1 len=273 path=[1:0-272]

GCATCGTTCTTCAAATGCATACACAAACAATTTTGCAAAGTCTGTGGTGAACCTTGTAGA

TGCTATATACAAGGAACAGCTGAACACCAGGGTGGTCCTTGTTGCGGTGGAAACTTGGAC

AGACAGAGATCGCATTAATATTCACCCTGACCCACTGCAGATGCTTCATGATTTCTCCAA

GTATCGGCAGCACTACATCAAACAGCACGCTGATGCTGTGCACCTGCTCTCGAATGTGAC

ATTTCATTACAAAAGGAGCAGCTTAAGTTATTT

>c5023_g1_i1 len=206 path=[184:0-205]

AACTTTGTGTTTGAGGGTTTTGTTGTGGATGTTTTTAGGCTATTTTGCATTAAGCCACAG

TGGATCTGTGCCAGATTTTGCAATATCTTTGCATGCGTAAAGTAACTCCTATTAGACTGA

GCACTGATGAACAAGTTCTCAACTGCTGTGTATAAATTTGCCACCAAACACTGAGCAGTG

ACCTGCAGAATGTAGGAGATGCTTCA

>c5023_g2_i1 len=232 path=[611:0-195 391:196-231]

ATGGCAAAGTTATGGGTGGAGAAGAGCAAGCCTAATACTGGCCAGCCCAGACCTGTTCTG

TGGCTCTGCAAATCCCCTGGAAACTTCAGCAAGTCAGAGGTGAAAGCCCTGACTTTGTGC

TCTGAGTTGTAGCCTACCAGTGCTGTAAGTGACTTGAGCAAATTCGCTGGTCTGGTTTCA

CTGTAGTCATTAGGTTCTCTTGTAAGCATTTTCCAGAGTCCATCTAATCAAT

>c5023_g2_i2 len=232 path=[611:0-195 806:196-231]

ATGGCAAAGTTATGGGTGGAGAAGAGCAAGCCTAATACTGGCCAGCCCAGACCTGTTCTG

TGGCTCTGCAAATCCCCTGGAAACTTCAGCAAGTCAGAGGTGAAAGCCCTGACTTTGTGC

TCTGAGTTGTAGCCTACCAGTGCTGTAAGTGACTTGAGCAAATTCGCTGGTCTGGTTTCA

CTGTAGTCATTAGGTTATCAGAGAAGTATTGATTAGATGGACTCTGGAAAAT

>c5024_g1_i1 len=485 path=[50:0-139 189:140-140 32:141-484]

AAAAAAAATAATAAAATAAAAATAATTTAGAAAATCTTTTTATATAATAAAAATGTCAAC

TATTATAAACAAAATTAGTAATCTTGAAGTACCAGAGAATGGAAACAGTTCAGTTAATAT

AATTTTGGGTGTATTAAATATTTTTTTTTTCGGTATGGGTATGATAATATTAGGAATAAT

AAATAAAGATATAGATGATTTAATAATAGGTATATTACAATTTATTACACCAATAGTTGG

ATGGATATGGGCTATTATTTGGGGTGTTTTAATTATAATTAGAAATGCTAAGTAAATTGA

TATCTTAAAATATTATAAAAAAAATGTAAAAAATAAAAAAAAATGATTCTTTTTTAAATT

ATTATTCACATTCTTACCATATATATTTTAATTCTTTCATATTTTATTCTATCTTTTAAA

TTATTTTGTTAGTTCTTGATTTTTTGTAAATTATAAAATATTAAAAAATATATATTTTTA

CTTTA

>c5026_g1_i1 len=260 path=[392:0-259]

ACTACCAGATCATAAAATGAAAATAAGAAGAAGGTGTTTGCAGATATTTTAAATACATTG

CTGAAAACAAAGAAAAAGCAGATGGAGCTATGTAGGTCTCAGCTCTTTTTAAAAGCAAGA

CCTCCATAGTCTTAAATTAAATGCAAACTATGTATGCATTTGTGAAGGGTAAGGAACAGG

TCTCAGCACCGCACTGAATAAATGAAAAAAACATATGAAAAATCTAGTTACTCCAGTTTG

GGAAACTGCACTGGAAAAGA

>c5027_g1_i1 len=365 path=[343:0-364]

GAGCAGCTTCACCAGCCATGCGGTATTTAACCTCACTGAGCTGCTCTTGCACTTTCTTGA

TTCTCCGGATTTCTGGGGTATCAATTGTGGAAGCATATGGCTTTCCTCTTTCCTTATTAA

ATTTTTCTTTGTACTTGTTCTCACTGAACAGGTCCTTCATTTTATTACTGTGCTGCAGGT

AAGGTGTCAAGAACTTGGAAGTGTCCACTTTTGTCCGTATCGTCTTCTTCCTGACTGGAG

TTGCTGCACCCTCACCAGAAGTTTTTTCATACTCAATCGTACGGGTGATGGTTGTAGGAC

CTGTGAATTCTGTGCTAGTGCTGTCAGTGATCTCAGTGACCACCTTCCGAGCCTGTTCCT

CAGCT

>c5029_g1_i1 len=236 path=[285:0-163 448:164-235]

AAATGGCAACAGTTCCCTCAAAGTAACTGGCCATGAACTATATGCAGGTTGAGATTACCT

CTCTCACAGTTTTACAGATGTAACTAGGTGTACCTGGAATTTTTTTTTTTCCTTTTAAAC

CAGGAAGCATTAACTCTTTGGATTTCCTAGGATACAACCAACTTCACCAAAGTTAGTAGA

GAGTAGAACACCAAAGCAGGAATTTAAATTTGTTGTTGAGGTGTGCTTGTTCAAAG

>c5029_g1_i2 len=236 path=[285:0-163 25:164-235]

AAATGGCAACAGTTCCCTCAAAGTAACTGGCCATGAACTATATGCAGGTTGAGATTACCT

CTCTCACAGTTTTACAGATGTAACTAGGTGTACCTGGAATTTTTTTTTTTCCTTTTAAAC

CAGGAAGCATTAACTCTTTGGATTTCCTAGGATACAACCAACTTTACCAAAGTTAGTAGA

AAGGCTAGAACTTCAAATTAAGCTTTTGGGACTTTGAACAAGCACACCTCAACAAC

>c5036_g1_i1 len=212 path=[155:0-211]

TGCTGGCAAATGGAATGTTTAATTAATTAAGTCTGACCTCCTTCATTTGAGTACATATCT

GGATAAAATGATTGGCTCTACATTTGTTGGAACAACTTCTAAAAGTGCAGTTTCCAAATG

AATTAAATGCACAAAGTCAAGTATATAGTTGATGAGTTCCATTCTCAGTGTTTTCTATCT

CCAGAGATTCTTTCTGCTCTAATGAAAGCAGA

>c5038_g1_i1 len=435 path=[413:0-42 456:43-434]

GGGGAGCGGCGCTGCTCTGTGTGACTCCCTGACATCAGGACGTGTTGGTGCCCAGTGCCG

TGAAGGTGACAGACTCCCAGGCAGTTCCTGTGTTCAGGTCAATCATCTGCCTCTCCCGGT

TGCGCTCGATGCGAATCCACTTCTTCTGATACCTGGAACAAGAGGACAAGGCATCTCCAC

ACTCCATCAACCAACCTGTGTGGGCAGAGAAGAGAGTGGGGAAGGTCTGAATGATGGAGC

AACGTACTACAGCAGCAGAGGACAAGATGGGTAGCTCGCAGATGCCTGGCCTTTACCATG

GCCTCTTCCCAAAGCATCCATTGCACCTTCTGCCACAAGAAGCCTCACGCTTTACACCAT

TTTGTTATTTAAATCATTGTAAATCCCCATGCACTGCAAGCATCTAGGATTTACTGCTTG

GAAAAGCTCAGGTGT

>c5038_g1_i2 len=470 path=[825:0-77 456:78-469]

CAGGAAGGGACCGTTCCCAGTAGGGCCCTACGCAGCTGCCTACAGACCTTCCTGGAGGAT

GTTGAAGAAGATCTCCCGGTTGGTGCCCAGTGCCGTGAAGGTGACAGACTCCCAGGCAGT

TCCTGTGTTCAGGTCAATCATCTGCCTCTCCCGGTTGCGCTCGATGCGAATCCACTTCTT

CTGATACCTGGAACAAGAGGACAAGGCATCTCCACACTCCATCAACCAACCTGTGTGGGC

AGAGAAGAGAGTGGGGAAGGTCTGAATGATGGAGCAACGTACTACAGCAGCAGAGGACAA

GATGGGTAGCTCGCAGATGCCTGGCCTTTACCATGGCCTCTTCCCAAAGCATCCATTGCA

CCTTCTGCCACAAGAAGCCTCACGCTTTACACCATTTTGTTATTTAAATCATTGTAAATC

CCCATGCACTGCAAGCATCTAGGATTTACTGCTTGGAAAAGCTCAGGTGT

>c5039_g1_i1 len=291 path=[269:0-290]

AGATCTGCGGACAGCACTTCACAGCATCTCACAGGGCCAGCCAACCAGTAGCTCACCAAC

AAGACAACCCATATTAAGGCAATGTGCATAAAATAAAGAGCAGAGAGACCCAGTGTCCTC

CCCAGTCTTGCACAAAGTGGCTCAGCTCTGGGTGCCAGGTAGCCCTGTCGCATCCAACTG

CAGCTCTCAGGGTCTGGCCAGCAACTGAGTGCATTCCATGGAGACAACCAGCTTACACTG

GGCATCCTTGATAGCACAGCTCCCACTGCTCCTCTCCTCGTTTGGCACAGG

>c5040_g1_i1 len=217 path=[603:0-216]

GAAATTTCTCAGTTGGCAAGAGCAGATGAATTCAGTGCTGATCGAAACCATGAAACGTTG

TGTGTGTGTGGAGGGCACTGAACTGGCTTTACTTGCTCATAACTTCTGGTTCTGGTCTGT

CCTGTGCAGCGTTGCGTAGCTGGATATTAGGCAGGACCTGTTCTTCATCAGTGATGACTT

CCTGCAGCATTTTTTGGAGCTGTTTATTCCTGGAAAT

>c5040_g2_i1 len=298 path=[302:0-297]

GATAAGCTTTGAGTGGCAATAGGATATTTTGCTATTTTTGTAGTTACTATGAGTAACAAG

TGTAAACAGCTGTGCGGTTTCAGAGAGCAGATACTGAAGAACCCAAAATCTGCATGCTAC

AGTTGAAGCTTTCTGCAGTGATTGCCCGTGATACGTGGCCAGATGGAAAATCACAGTAAG

ATTGGACGGGTCCCCTGGGTCCGTGTGGCTCAAGCAGAGACCTGGAGTGGGTTGGCCAGG

ACCACATGGCGGTGGTTGTGGACCGCCATGATTTGCATTCGTATTCATTTTTCCGGTG

>c5042_g1_i1 len=205 path=[1:0-39 41:40-204]

GCTGGCTGGAGCAGTACCTGCCAGCAGTACCTGTGCCACAAGGGGCACGTGCACAAACCC

TTGGCTGCAAAAGAGGAGAGCCAGTGCCTGCTGCACGAGCCACGTGCTCCGTGAAGAAGA

GGAGTGAGACAGCAGCCCAGGAAGGCGGGCGGAGAAGACAGCAGAGAAGGAGCAGAGCTA

ATGAGCAGGGACCGCCTGTCCCTGG

>c5053_g1_i1 len=354 path=[332:0-110 726:111-117 734:118-353]

ATTTTATGAAGCTCTCCAATTTTCCCTTGCCATGTTCTAGGTGGAAGTTAAATGCTATCT

GAGTAAAGCAGGAGCAGACTTCTGCAAAATAGAAGAACAGATGCAATTGTGGGAATATAA

AACAAAACCTCTGAATTGTCAGAAACTAAGGAAACCTTTTAAAATTAAAAGAACACAATA

AGAACTAAATCATAATGTGAAACTGTTGTCTTCCAAGTAAATAACTTGTTGAAAAGGAAG

TTGTTCAGACTGTAATAAGTCAAGATTTTCTGCATCCTTATGATTAAAAATTATTTATAT

AAAACTGTTTTGCTGCAATTCTATTACTGTTCTTTATATTTTTTCCATCTATTG

>c5055_g1_i1 len=302 path=[305:0-134 597:135-301]

TTTTTTGTTTTTTGTTTTTTTTTGCAGTAAAATTTGCTGGGCATTGTAGACAGTTTTGTA

ATTTAGTTTTGTTTCGAAGTCACAAACTGTACTATATAATATTGCAGTTTTACTGTAAGT

GTCAAAAAAGAAAGTACTTTTGGCTCATACATGGTTTCCAGAATCGCAGCAGGAGCTGCA

GTTAGACGTGAAGATACAGTTTGTGTCTGATGTTGGTTAATTTTCAAAGATCCAGTGTTT

TTGTTCTGAGATCCTCTCTATTCTGAAAAATAGAGTAATTGTCACTCACTTTTGAAGTAA

AG

>c5057_g1_i1 len=286 path=[264:0-285]

GGCCTCATGCTGTAGGTTTGGAGGCATTTGAAAATGATTAAAGCATCCATCAGCCTTCCA

GAGCTTAACAACCGTCATTTTGAAGGGATTCAGGGCAACGTTCCACCAGATACAGTAGAA

ATAACCTCATAGGAGTTTTCTGGCTTGCAAAAGCTAAATGACAAGACAAAGTCAGTGAGG

ACCACAGACAAAGCAAGGATTTCTGGGCCACTCTTAAGGGACATATGGATGTGACAAGAA

AAAAAGCAAAGCTGATATCGAAGGGGCCTGGAGGGCCAGAGGAAAG

>c5064_g1_i1 len=217 path=[1:0-216]

GAAGTAGCAGCCTTGGAAAGGCATGTTTAAAAGCAGGAAGATTTACTCCAGTAAAAACAA

ACTGAATTGGAAGGTAAAGAAAGTATGCCGTGTGATCGTTCAGATATGTTAGGAAGACCC

TCCCTGGTATTCAGAGGTGAAAACATTACTGTCCTGCTACATGTGGGAGTTTGCATGAAA

CACTTCATATGGCATTGATGCAGTTTTAAAAGTGGGG

>c5065_g1_i1 len=304 path=[334:0-229 563:230-231 27:232-303]

TGATAGCCTGCAGCAGCCTGTATGCAAAGCTCAATGGTAGCAGTTATCCTAATCACCTAA

TTCCAGCTGATTTAGCAAATCAGTTTCTCCTTACCTTGGTGTTTAATGATTGCCAGCCCC

AGCTCACACTCCACTGCACTAACTGCACCAGCAGAAGTCATTGCAGCTCACGGAGCTTTT

TTAGAAGTAGGAAGCATAAAAAAGTGAAAGACTTTTGTTCCTTCTCTGGTGCAAAGCAAC

AAATTGCCATTAAGAAATTCTCCAGAGCTTCATTTTCTGCTCAGGGCGTAACTGTGCTTT

CTTA

>c5072_g1_i1 len=460 path=[1:0-459]

AGTCAATTTATAGAATTTCATTTCATTTTTTAAATTATCTAATTTATTTTTATTTTTTGG

TGTTAAGTTTGGGGTTGTTGTAGCACTTAAAAGAAGATATTTATCTATATTTTTTATCTT

ACTTACTTGATATTTATTTTCGTTATCTTTATAAAAATTTAATTTTTTAGAACTATTTTT

TTCCTTAAGTTTAGACATTGAAATATATTTACTTTTCCTTTTTACATGTGAAAAATTCTT

TTCTTTATAGGAACATGAATTATCAATTTTTTCTGTATGTTGATCTTCAAATAATGCACC

ATTTTTAACAAATTCAAAAATTCTTGTATATTCATTTAAATATGTTAATTTTTCCATTTT

TCATATGACAAATTTATAAAAGTAAAATATATTTTAAGTAATAAAAGTAATAAAAGAAAA

AACGAAACAAAAATATTAATAAGAAAAATTTAAGTTTTAT

>c5078_g1_i1 len=219 path=[55:0-69 54:70-218]

GAACTAAACTTATTTATTAACCTATTTTGGTTGCACGTTACAGAAAGGAAGCTATTGTGC

TGTTGGTTTGCGGGATGCTAATGGGCACTCTGGGTATAATCACTCTGTGTTGGTGTGTGA

ATAAACGCCACTGATATATGAGTTGTCTGCCCGATAGTAACTTTTTCACTTTCCTTGAAA

GAGAAGATAGAGAGAGAAAAAAAAAAAAGAAAGAAGTCT

>c5079_g1_i1 len=298 path=[1:0-297]

AATTTATGTATCTAATGTGTATTTTATATTCTACATTTATATATCTAAATTTATTTGAAA

AGTTCAACAGTAATTTTTATTTATTCATAATAGAAATCTAATGATATATAATCAAAGGAT

AATAAATCAAAAATTATGTTGATGCAAAATGGAAATAAATCTTGAGCATAGGTTTCCTCT

TTTCTATGACACATTTAACTATCAACACTTATTTTTACAGAAAATTCTACTTTTTTCGCA

TATTTAACAGTTTTGAAAAAAAAATAAAAAAATTATGATTCAAGTTTTTCCAAATAGT

>c5082_g1_i1 len=400 path=[155:0-399]

ACTGTAAACTGTTGGGCAGTTTTACTAATTAAAAACAACGCAGAGTGAACTAACTGTGGG

TCTGGATCTCCAGCTATCCCGTGGTGCCATTCTAGGCCTGACGAAGCTGTGTTTGTGCAG

CTTCAATGAGGAGTGTTGCCTTTACATTTGGCTTGGTTTCATCCCTACCTCTGAAGTATG

GGATGAAGCCTATCGTGAGGACTCACAGGTGAACATAGGGTTGACGTGAAGCATGAAATA

AAGCATTACTTGATAGACAAAGCGTTTTAAATGCACAGAAGATATGTGGCTGGCTGGGAG

GAGCTGCTTGCTGGTGCTGGAAAGCTGTTGGCAAACAGCTGCTCTTGCTCTTTCCTTTTC

CTAGTGTTTTTTGTTTTGTTCGTTTTTAATACAAGAGTAA

>c5091_g1_i1 len=203 path=[287:0-21 143:22-202]

ACCCGGTCCTTTCCCCAGGTGCTCCATCATGTTGATCTTTCTGATTGTTTCATTTGCTTG

CTGCATGTCTCTGATTTGGAGGGAGGTTGTCTTGCAGTGAGCATTTACAACAGTGTGGAC

AGGATGTTGTTATTCCTAGACCGCCTGCAATCTACCTTTCATGGTCTCCTAATTCCTTGG

CTGGCGCTTGTAGGTAGATTGCA

>c5091_g1_i2 len=323 path=[1:0-141 143:142-322]

TCTGCCTGGAGATCCCTTTTTGATGGAGCTTTCCCCTGTGAGCCTCGATCGTTGCAGACA

GGTGAACAGTCCTTTTTTCTTTCCCTTATAACACCTTTCTATTCTGTTGGCCCCTTTGTT

ATTGCACTTCTTGTAGCACCCTTCCATCATGTTGATCTTTCTGATTGTTTCATTTGCTTG

CTGCATGTCTCTGATTTGGAGGGAGGTTGTCTTGCAGTGAGCATTTACAACAGTGTGGAC

AGGATGTTGTTATTCCTAGACCGCCTGCAATCTACCTTTCATGGTCTCCTAATTCCTTGG

CTGGCGCTTGTAGGTAGATTGCA

>c5094_g1_i1 len=285 path=[263:0-197 461:198-284]

TTTTTTTTTTTTTTTTTTTGGTAGCACACACACAGAATGATTATGCGTAAGTACCAAACC

TTCTGTCTCAGCACTGCTTTAACATGGTCTATCGTTAATCCAGGTAGTCATGGGCCACGT

TTTATGTAATCATCCACACTTTCACACCACAGACACAAAGCCAACTTCTTACAAATTAAT

AAATCTTTCCTTTAATTCAGTCTGCCAAGAATGAAGATCGAATGCTGACTACTGCAGGAG

ACACAGTAATGATGCAAACCAAAGGACTAGGGATCAAACACAAAG

>c5096_g1_i1 len=227 path=[53:0-226]

TTTGGCCCTGGAGGACCCCCCTCACGCCGCCGTCCCCCCCCCACACGTGGCACCCACAGA

CAGGGGGACCCACAGCTTCGCCACGGCTGCGGTGTCACCTGCACAGAGCGCCCCGCGCCC

CGAGGACCCCCCCAATGGGACCTTGCAGCCCTCGGCTGTCAGCTGAACCCACAGCCCCCC

CCCGGGAGCTGATTGGCGGGCAGCACAGCCCCCTCTCAAACACAAAG

>c5107_g1_i1 len=247 path=[225:0-246]

TGAAATTCCTTTTAATTCTCAATGGGATGATGAAATACGAGAAGCAGGAGCTTCTTACAC

CCTACAAATCTTAGTAAGACCATATACCAATAAGTCATCTGTATGCAGACATGTGGATAC

GGATCCTTACTTAATAAATCAAATTCTATTAAAACAAGGATCGCTTGAGAGCATTTTTCT

TCTACCCCAAGGGGAACTGGATTTCACCACTGAGTCAAAACTTGGCACTGGATGTGTCAA

AAGAGTG

>c5109_g1_i1 len=616 path=[1:0-615]

GGATGAGCTTTATATAAAGAATTTTTCTTCTTGTTATGCTTAGTTAAACGAGATTTATAG

TAATCACTTTTATATATATGAAAAAGCATAGAATTATATCAAATGATCTGATAACTGATA

ATTTTCCTTCTATTTTATTTTTACGTACATAAGATTCATATAAGAAAATTCGTTTATATT

CAGCATCCATATTTGTCTTATGAGAATTAGTTAATTAAAAATTTTCATCTTTTTTTGTTA

GTAAACCTTTAGAGTCAACACAATTTATAATGTAACAAAATTGATATAATATTAACAATA

TAATTACAGTGGAACGAAAATTTTTATTGTCTCCATTTAAATTATATAAAAAATCATCAT

CTGTTTTTTTAAAATCTTTAAATATTGATAATTTAATATCATTATATATATTCTTTGATA

AAATTATATCTCCACATAAATAAAAAACTTATTTTTGAAAATTTTTTACTATCTTTTTTT

TAATATATTTAACACATTCTAAACCATTATATCTATCATAAATATTAATAAAAGAATAAG

TTAATTTATCATTTTAATTTATTCTTACATTTTCAAGTATAATAAACTTATCATCTAAAT

TCTTTATTATTCCTCT

>c5110_g1_i1 len=232 path=[364:0-231]

GCAAGAGCCAGACACCTCCTCTTCCAAACAAAGTTTCCGCCTGTCCAAGCAACGGGTTGT

CTGGGATCATGTACAGGGCCTAGGACTAAAGGCACTAAAAATGTTTGCTCTCTGCAAATA

CAGCATAATTCTTTTGACCACAAAATCAATTGAACTGTAGCCTTCCATTAATTTGAATTT

CCCATTGTGTGCTTCATGGGGATATGTCATACTTGCACACAGGACTCAACCT

>c5112_g1_i1 len=236 path=[214:0-235]

TGTATTTCACTGTAAATAAGTAGTTACAGAATCCCTCTTGACACTTTTGATTCTTGAGAA

AAGCAGTCACTGAAAACACAGAAGACACGGCTCACTGCATGAATGCGAATTTTTCAGTGT

CGATTTCTATCTCACATATTGGAAGACAGAAAAGCCATAGGGGGAAAAAAAAACACGACA

CAACAAGAATTCATAATACTCTGTACTGAGGCCACCAAGAAAGTATCCTACAGTGA

>c5121_g1_i1 len=228 path=[1:0-227]

CAAATGAAGCTTCCTGCAAATGAAGGGATCTGTTATTCCATTCATCCTTTTTCTCAGCCC

TGCACAATTTTTCATTAGCCGGTGATGCAACAGTTTGCAAACATGCTTTCCAGCTTGGTG

TAGCTACTGAAAAGCCAGTGACCTTCTGCATCCAGCTCGGGAGCAGTGTGTGCAGAGCTG

CTGCGGAGCTGAGGATGAACTTCCCATGGTGATGAAGAGGCACGAAGG

>c5121_g2_i1 len=228 path=[616:0-227]

TCTGCAGGAAGGCATGCAGTGCCGCTGGAAAGCACACACCTCCTGTCCAAACACCACGAT

GCCTGCAGGTTGCCATAGCAATGAAGAGAGCTGAGGCCATTTGGGGCTGTAATTCTGCAA

TGCACGCAGCAGTGAGGTGCCACATCCCCGTGCTCAGCACTGCTCAGACTCCAGCAGCCA

CATCCAGCCTGGGAAGGGTGTTTCACAGACACTGAGCCTGTTGTGCAG

>c5136_g1_i1 len=221 path=[225:0-220]

TCAGCTGAACCAGTGGCTCCTTGCAAAGTGGGGGCAGTTTGGGAAGGAGGGCAGAGGGGT

TTTGTTCCCTCCAGGACCTGCACAAGTGGGTCTGTCTGCAGCCAGGTGGGACCAGAGCCG

CTGGTTCAGCTGAGCGTAGAGTAAAACCTTAACGTCTGCATGGGCAGATCTTCAATCATT

GGGTACCCAGAGTTGTTTTCACGTCGTGCTGCAGAGGCATG

>c5155_g1_i1 len=298 path=[461:0-291 770:292-297]

GGGCAGGTTTTGTTCTAAAGCCACTGTTATTTTCTTAGTATCATCAGTTACATTTACTTT

ACTGCTATATAAAGTTAAGCACTTAATACTGCATGGCTCACCTTCAACACCACCGTTCCC

CAGGTTGTTTTTCTAGTTGTTTTTTTTGTTGTTCTTTGAGCTATACAACGTACAAGGGAA

ACAGTGTAAATCTGGTTCTCCTTCTCATTCATACAACCCTTATCCTAACTTCCAGGGAAT

GTAAGATTCAAGACCATAATCAGTCGCAAAGAAGGATCACATTTTTTCCTTACTCTAC

>c5164_g1_i1 len=468 path=[281:0-467]

AAGACCCTGCCTGATAAGGCTAAAGTACGCGCCACGTTGTCGTCCCAGTGAATGTATGCA

TTGTCCCTTATAGAATGTGCGGCAGTGACCCTTGACACGATGAAAAATGGGAGGAAGAAA

ACCACCCTGGATTTTTTGACAGTTGTCAGCATTGCAGTAGGTTGTTCTTGAAATCATGGA

GGGAAGCAATTAACTTAACGCGGTTTAGTCACAAATGACAGTGCAGGCTGCAAAGAGACA

AGGAGTTACAGAGAGGGTCAAGAGAAACGTCTCTGCCAGCTGAGGGAGCAGGGTCTGCAA

CTGTGTGGTGAAAGAAATGAGATCGCAAGCCGAAAAGTTGTCAGCCAGGTCCCCAGGAGT

CAAATAGTATCAAATAGTATCTCACAGACACCCTATGCTTCCCTTACATGTATATATATT

TAGAAGTCATCGTTCCTGTTTCTTTCCCTGCAGCCCAGTCTGAGGTTG

>c5172_g1_i1 len=249 path=[155:0-248]

TGAAGGAGATGTGAAATCTTGGAAGTACAGCTTTGCAATAGCCCTGGTCAGGCAGTAGAA

AATAGCCATAAAAAGCATCTCAGTGCATTTCTGTGATGGAATGCATTGTGTTACAATAGT

TAGAACCAAGCAACAGACAAAAATATAGGTTGAGTAGGCCTGAAAGCTAACTCAACACCT

CTTTTAACTTGCTGTGTGCTAGTCTTATCTACTGTTTTGTTCATTTCTAGTGGAAATGTA

GTCATAACA

>c5185_g1_i1 len=297 path=[414:0-261 42:262-296]

AAAAAAAAAAATGAAAAAGAATTTATATTTTTTTTAAAAAATTATGTTTATAGTTATAAT

TTATAGTTTTTGATTATTTTTTAAAAAAATACATTTTAGCTATACATATGAATAATGATA

GATATTCCAAGTCTCATTTAAAAGCATTTAAAAACAACTTAATTATTTTTTAATGATTAT

TTAAATATAATATTTTTTTATATCATTTCATTACAATTTTAAACATTTTACTTTAAAAAA

AATATTAAAATTATTTCATAAAATAGATTTATATTCTTACTTTAATGAAATCATTTA

>c5206_g1_i1 len=265 path=[1:0-264]

ATCTTTAAGTAGCATAACATGATTTTTCTCCAGTGTATTGTAGCACGTGATAGTGCCTGT

AAATAGGGTAGTTATCTGCAGTGAGTCCCTATGTCTGCAGGGTGAATTTTACCCTAGAGA

ATTTAAAAGTAAGTTAAAAATTCTGTCAGGTCTTAAAAACTCTTATAGAGAATATTATCT

TTAAGCAGCAGACAATGGATGCAAGTTTCACACTAGATTTTCAAATACTTAGGAAAGTTG

GATTGTCTGCTTTCTCTCCCAGATG

>c5209_g1_i1 len=251 path=[18:0-250]

AGGCCAGAGATCAGAGGTGACACACTTGAAGATGGTTTGCAACAACTTTTGATTAAATGT

CTTGCTCATCTTTTTGGAACAGAATCAAAATCTTGCTGTGTTAAAAACAACTTCTCAAAT

ATCAATAAAACATCCCCTTTCAAGCCTGTGTCATGAAGCTTAGAAAGTTTTTAAAGTGCT

CAAGAAGCAAGCATGCTGCAAAATCAGCATCTTCACAACTTTGAACAGTACTCAACCTTT

TAATGAATAAA

>c5211_g1_i1 len=250 path=[179:0-249]

GCCTTCTCGCTCGTCGCCTTTTTCTTTTTTTCCCCTAACCGACCGACACGGAGAGCTCCG

AGCTGCCGGGCTCGCTCAGACCGTCATGGCTACGAGAAGTGCGCTGCCGGTCAGAAACAC

GCGATCCCGGGGCTGCCGGCTGAGCGCCGAGCGGTGCGTGAGGCACCGCACGCTGATACC

GCGCCCGTTCGCGGCTGCTCGCGCCGAGGCTCCGAGGCTCGGGATCACTCGAGAGCCAAC

TCTTTTTTTT

>c5212_g1_i1 len=339 path=[279:0-338]

AGCTCAGGGCTCAATGCAATTGCAGCAGTAGCATCTGCCTGTCGTGGCTCTGCCAGTATT

GTGGTCTAGAGCGCCTTGACAGCTGTGCACCTCCTACACTTGGAAGTGCTCCAGCTAGCT

CTTTTGGCTGGCTGTGCGCCAGGCCAAAGAGCCCTTGATAGTGCTTTGCCTATTTGCAGT

GAAAGAAAAGCAGAATAATAGTAGATCTAGACTTGAAAATATGACTGGAGCCCCAAGTCC

AGCACCCTGCTACTCCTGACTGTTTACCATTTGTGTAGAGCTTTATGAAACCCAAATGTC

TTTCAATTATCTTCACTTTCCCATCTTTTTCTTTAAATT

>c5219_g1_i1 len=209 path=[399:0-208]

GCAAAATCTAATTTCTGTCTCTACAATTACGTGCATACGCATTTATATTTGCTTGATCAA

GGATATCATCGTCTTTAATGTAAAACAAAAGGATCACGCTGATCAGCCTTCCTTGGAGCA

CAGGTCTCTAGAGTTCAGCTACCTCAAAACCCCTGGAGTTCTGTCCTGTCCTTTGTCTAG

GGCATTTTTGATGGGATACAGTAAGACTG

>c5220_g1_i1 len=229 path=[207:0-228]

AAAAGTGCCACTCTCCAGCCAGTGTGGGCTAGGACTGAACAGCATTCTCATTTTGGTTCC

TTTGAGGTGGCTGCACATTGAAAAAGAAATGGTTTGAATAAAGGAAAAATGAAGTGCTCT

CTCCCTAATCACGAGGACTTAACAAACATTCAGTGGAAATTTCCATCCATTATTTTTTTC

CTGCTTAGGAAAGATGAGCTAGGAAGAAAATAAAAGAGGAAGCAAATGT

>c5221_g1_i1 len=404 path=[1:0-127 129:128-403]

GCTGGATGTAACTCAGAGCAGCTAGCTGGGGACCCTTGCATAGGAATTGTATATTAAACT

GTTTTGTTCTGGGGAGCTGCTGTGCGCTTTATTCTACCCCTGATGGTACTTCTACTGCTC

TGCCACCTTCCAGTATGATCCCAAACTCTGTGATACACAATCAGCTACTCCGTTGGTTCC

TTCTGTAGTGATTCTGGTCCTTAGCTGCTCCTGGAAACTTGTCCTAGTTGCTTTTTGCTC

TCATTATTCTGGAGATACTGTGAACACGTACACTGTGACACCACTAGCTGCAGAAAGCAC

TGTCCTCTGTCTTTCCCTCCCCAAGTTTCCCTGAACGTGGGCTGTTCTGAACCTTTGCTG

TGGGTGGGGCAGAGTTTTTCTGGGGGGGTTCGGGGGGGTTCTGT

>c5221_g1_i2 len=344 path=[259:0-67 129:68-343]

ACACGTACAAAGATTTACAAACTGTGTAGCCATTTAAAGCTCAGTAAAACTGAAACCTTG

TTCAAAAATCCAGTATGATCCCAAACTCTGTGATACACAATCAGCTACTCCGTTGGTTCC

TTCTGTAGTGATTCTGGTCCTTAGCTGCTCCTGGAAACTTGTCCTAGTTGCTTTTTGCTC

TCATTATTCTGGAGATACTGTGAACACGTACACTGTGACACCACTAGCTGCAGAAAGCAC

TGTCCTCTGTCTTTCCCTCCCCAAGTTTCCCTGAACGTGGGCTGTTCTGAACCTTTGCTG

TGGGTGGGGCAGAGTTTTTCTGGGGGGGTTCGGGGGGGTTCTGT

>c5224_g1_i1 len=331 path=[78:0-75 154:76-330]

GCCTCCTGGTGCAGTTTGCCCGCTGGGCTCCAGCTGGTGTGGTGGGCCCCCAGGGGTGCC

GAGGGAGCCGGGGCGGAGCCACCTCTAACCTGGACTTGGAGAGCAAGAAAGCCGCCCCCA

CCAAGCTGTCATGGCAGCAGCCAGTGAACGTCTTCCTGGCTGCCGGCCGCCCGCCCGGCA

TGGAGCAACTGCACCAGGAGGCTCAGCTCAACCTGCAGAGCCTGCTGCAAGAGGAGTATG

AGGAGCAGTATGCTGAGAGCAGAGTGACTGGGCAGACCTTCCGTGCCGCCGGCCACCCAT

CCCCCAGCACCCCCACGGAGCCCTCGCCCCG

>c5226_g1_i1 len=215 path=[193:0-214]

AAGGAGACAAACATGAGTATGAAATACAGGCTAGTTTTAGGTGCCTGCATGGCTGGATGT

CTTTAACGGTGTGACACTGAATCCTGCATGCCCCAAAGGCCAGCTGTGAATGGACTACCA

AACAGTTCAAGAGAGGAGGCAGAGCACAGGCGGTGCATCCAAATCCTAATTCACGCATGG

TTCCAGCTCTAATGTCTGAAAGCGTACTGTGTGGG

>c5235_g1_i1 len=299 path=[1:0-298]

AGGAACTGCCAAGTGTACATGCTGAAGAGCTCATTCAGAGCAAGAAGAAAATCCTATACT

CAAAATCTTCATGCTTGAATGAATATGTCCCATTGACTGTGGTGTGTTCATAGGTAGAAA

CTTTTGATCTGCTCAACTTCATTCTGAGTTCCTGCTTTAGGGAGCTCCCCCTTTGTCCCA

GGGGGCTCTGTGCTTCTAAATACAGTCTCATATTTGAATCTTGAGTTTTTTGTTCAGTGG

AACGTTTAGCAAATTTTGCAGAGAGGAAGAAGAGTGGCTCCATGCAGCGCTTTGAGTAT

>c5235_g2_i1 len=343 path=[873:0-342]

AAGCATGCAGAAAAGATTCTTAAAGTACTGTTTTCTGTTGTTCTACCAAACATGGGTAAA

ACAAATAATGTACAATTGGATGCAAATTTGTCTTGTGTAGAACACCAACAAAGGACATGG

ACTTGTGTGGCTTTCTTGTGACTGCTAACCTAGGAAAGGAAAACGGTCAGTATGCTGGGA

ATAAGGACAGTTTTGTAGCCTTTCAACCCATTAAGCTGTGAAAATAATTTCCTAGTCTGC

AACAGCCGCTTCTTAGGGATAAAACCATGTGCTATACATCAAAGTGCTGGAGAGAAATAA

GGTGTTGACTAAGTTTCAGTATAGGAACTGCCAAGTGTACATG

>c5238_g1_i1 len=242 path=[155:0-241]

AGCGTGGGAAGGATGTATGCTGAGGAGGGTAGGAAGGGCATGGAAAAAGGGATTTTCCTC

AAGTACTGTTGCTCAGAGCAATGTGATAGCCTGAGAAACTTAGTATACGGGAAAGAAGAT

AGATAAGAAAGGAAAGCCTCTAGGCCTTTTTATTTTGTGTGTGTGTGTTCTTATTCTAGT

TTATCACTGTGTGCAATTTCACATGATATAAAATCTAAATCTGAGCAAACGCAGTGGTAT

AT

>c5243_g1_i1 len=217 path=[297:0-216]

ACGCAGAGGCAAACCTGCTGTGTGGACACCGCAGTGCGGCCACACATCAGACAGCACCCG

CATCAATCAGATGGTTCCTGTAACTCATGACTTGAAGGGATGATGGTGAAGACCACTGCC

AGCACCCCTCTTTGCCCTTCTTAGCAGTTCTCTTTGGAAGCTCCCTCTTCAAACTGGTTC

TCTCGCTAGCAGACAAGAAGCATTGGAGAGCACTTGT

>c5247_g1_i1 len=264 path=[242:0-263]

GCCTTGGGGGACCCCGCTGTGCTCCTCGTGCTGCATCCTCACAAGGCAGAGGGTTTCTTC

CTCGTTGCCACAGATGGCACTGCTTAGTCTGGTGGGCATCATCACAGGGATGCTTCTGGG

AGCGGGGTGATGGCCAAGCCGGGCTGCAGTGGGTCAGTAAGGAAGCAATGCAGACCCGTG

CAGCCTTGCCAGCTCTGCACCTCACTCCTGCTTTTAGGTTCTCTATTGTCATCCCAAAGC

GGTTCTCCTCCTCCCCCTTCAGCG

>c5252_g1_i1 len=247 path=[225:0-246]

ATCACAGTGTGGTGCAGAACCTCGGATACCATACAGCACCAAATTTGTGCTACAAATAAT

ATTTACTTCTTTCACTCTTCCTTCAAGTTCTCCTGACGCTTTTCCTCAAGACCAAAGTCA

CTCTGGTTCTCTCCCGTGAGGAGTAAACATTTCATTTCTCAGATGTAGAAATCACAGCTA

AAACAAAATACTGCTCCCCAACTATTATTGTGTAAATTGGTCCTTCCTCTTCCATTCACT

CACTCCA

>c5255_g1_i1 len=229 path=[104:0-101 206:102-228]

AAGGCCGGTTATGAAGTTTGTTTCACTAGAGAAGTACGAGCCGGTGATCACAGAGACTCG

TTAAGCCTGAAGATGATACATATTTAAAGTACTTTGCAGCATGGCAGGGAGACCATAATG

TTATGACTCTGCATTTTCCTCCATGTGCTGCTGAACGCGTGCAAAGCAGGGCTTGGAGAA

GCAGAGCTGAGGTGGGGATTTACCCTTTGCTGTAAGAATGAAAAACGAT

>c5261_g1_i1 len=498 path=[1:0-497]

GACGTAGGTTATAGAAGGGCCCTTAACTTGTTACAATGCACAAAACAGCATGAAAAGAAC

TAATCAGTATATACAATGAATCGCAGTTCACTCAACTGATTGTAAAATGTTGTATTAAAA

TGGTTTCAGTTGTTACACCATGGGTAAATTCAAACTTTTTCAAGCAACATGCTAGTGCCA

TTGTAGGACTGTAGTACTTCTTTCCAAAAGCTGTTCCAGCTCTCACTCACATACCTTGAG

ACTATGATGAAGGACATAACTGCAACAGCGAAATATCGACACAGAAAACATTTTGTTAAG

GGAAATCCACACCCTATTTCCCTTCTACATGTGCAGTTGCTGTGCACTGCTAAGCGGTAG

AGGTTATCTCCCTGTAGCAGCAAACAACTAACAGATGACAGCGTGCGCTAGGACATACGC

TTTCCACTTATTGTGACTTACAGAAGCCTGCCTCACAGGCACTCTCAGCCATGCGCTAGC

CCCTGCATGGCTGAGAGT

>c5264_g1_i1 len=527 path=[602:0-421 1023:422-526]

TATCCTCCTCCTCCTCCACCACCGCCACCTTTTGAAGATGAGTGGGACGTTGTATCTTCA

TCCTTATCTACATCTTCGTGATCGATTTCAAAGGAATGTGATGGCAGTTTCTCTGGTCTA

CAGTCATTTTGTGGTAAATGTCGAAAGTCTTCTGAATACTGTTCATATTTGTAGTTCACA

ACATGCCACTGGGAACTGTAATATTTACAAGCCTCTTTAACAAACAGATTTTCTGCTTTT

TGTTCTGCATCTTCAGGTACAGAAGGGTACAGCGTCCTGATTTCCAGTGAAATCGTGTCT

ATTGAAAAGTCATCATCAGGGAACAATATAAGATCTTTAAACAGGCTATCTCCAATATTT

TTCTCAATCTCTGTGATAACAGCTTCGTAATCCAAAGGCTCCAAGAGTTTTGGTTTTTCC

TGGCAGCTCAGCCTCTGCATTTCTCCTCGATTCACTCTCTTAATGGTCTTTCTCTTCTTC

CGCAGCTCGCTGTGCTCCCTTCTGAAAACCCGTCCTCAAACACAAAG

>c5264_g1_i2 len=461 path=[602:0-421 65:422-460]

TATCCTCCTCCTCCTCCACCACCGCCACCTTTTGAAGATGAGTGGGACGTTGTATCTTCA

TCCTTATCTACATCTTCGTGATCGATTTCAAAGGAATGTGATGGCAGTTTCTCTGGTCTA

CAGTCATTTTGTGGTAAATGTCGAAAGTCTTCTGAATACTGTTCATATTTGTAGTTCACA

ACATGCCACTGGGAACTGTAATATTTACAAGCCTCTTTAACAAACAGATTTTCTGCTTTT

TGTTCTGCATCTTCAGGTACAGAAGGGTACAGCGTCCTGATTTCCAGTGAAATCGTGTCT

ATTGAAAAGTCATCATCAGGGAACAATATAAGATCTTTAAACAGGCTATCTCCAATATTT

TTCTCAATCTCTGTGATAACAGCTTCGTAATCCAAAGGCTCCAAGAGTTTTGGTTTTTCC

TGACGGCGCCTCGGCCCGGGCGGGGAGCGGCGGCAGCAGCG

>c5269_g1_i1 len=273 path=[181:0-178 360:179-272]

GAGGCATGAGATGCCAGGTGAGGGGGTGAAGAGCATTCTCGGGGTGCAGCCACCCACCCC

TACCACGACAAGGAAAGGTGAGGAGTGAGAAACCAAGGCTGTACCAGATCACAAAATAGA

GAAATGAAAAAAGTGAGTCTGGGAGCGAGATTGCAGGTGGGAGGACAGAGATGCACCAGG

CTGAACACAAAGCTGGCAAAGGGCTGGGAGTGTAGTGAGGGCAGGAGGAGCAAATGGTTT

GCCCCACGCTTCACTGCGGTGTCCCTAAGGCAG

>c5273_g1_i1 len=271 path=[37:0-30 35:31-31 36:32-270]

AAAAAAAAAATAAAACAAAATAAAATGAAATAAAAAAAAACTCTATTTTCGCTGTTTTAC

ACTTTCTTTTCTTTTTTTCTAACCATAATTGTTCTTTTTGTTCTATTTTATTTATTTTCA

TTTTTATTTTTGATCTTTTATTTATATGAAATTATGGAGATATTTAAAAAAGAATTTGAT

AATAATTTTTATTTGTTTTCATTGTAAGGAGATAAAATCACAGTATTTATTCAAGAAAAA

CCTTACATTTTAATAAATATAAATTATTTTT

>c5277_g1_i1 len=219 path=[222:0-191 413:192-218]

TATTTCATTTATTTCTTGCTGTTGTTTCAGGCTTTAATAGTTATCCTTCTGATCTTTTTC

ATTTTCTCTCAGTCCACGGTGCCTTGAGAAGGGAATGGTTCAGTGAGGAGATGAAATCTG

TGTAGCTCCACAATTCCCTGCCAGAGCTTTATTTTGCAGCATTTTATTCAGCCCCTGTCC

TCAGGGAAGGTTAAGTTGCTAATAGCAGTGTATGATCAT

>c5277_g1_i2 len=218 path=[222:0-191 25:192-217]

TATTTCATTTATTTCTTGCTGTTGTTTCAGGCTTTAATAGTTATCCTTCTGATCTTTTTC

ATTTTCTCTCAGTCCACGGTGCCTTGAGAAGGGAATGGTTCAGTGAGGAGATGAAATCTG

TGTAGCTCCACAATTCCCTGCCAGAGCTTTATTTTGCAGCATTTTATTCAGCCCCTGTCC

TCAGGGAAGGTTCATGATCATACACTGCTATTAGCAAC

>c5282_g1_i1 len=209 path=[1:0-64 182:65-208]

AGCAGATTACTATTGTTTGTTTTAGCTTATGAGATAACAAAAGAGGTCTTTCTACTGATT

TGCAGATCGTTAGGATTTTAGATTTAATTATTTGACAAGGATGTCTGTGTATCCCCTAAA

TATTGGAAAATAATATGTCATTTCTCCTTACATAAGATTTTTTTTTTTCATTGCTGTGGT

CCATTTCTGCTTGGACTTAATGTTTGGTT

>c5283_g1_i1 len=336 path=[330:0-335]

AAAAAAGAAATTAAAAAAAAAAAAATTAATGCATAATAATATCTCCATTGTGTAAATTAC

ATATTATTTACTATGCTAATCTATCTAAATATTTAAAAAAAAAAAGTGTAATAAAAAGGA

AATTATATAGATAAAATGAATTCTAATAGGAATCTTAAATAATTAAAAAAGGACAAAAAA

AAAATTTTATTTACTTATCAAGTTTATAATTAAATTTATTTATATATGCATATAACTTTT

ATTTTAAAAAAATAGAAAATTTGTACTTGTAATACAAAAAAAACTATTATTTTTATTATG

AGTATATAAATATATAAAGATTTTTTTTTTTTTTTT

>c5284_g1_i1 len=423 path=[53:0-422]

ATAAATTATTATAGATAATTTTTAAAAATTTAATTAAAATAATTCACTTTTACTATTGAT

CAAAAATGCTCTATTTACTATAATGTCGTTTAATTCTGAAAGTATCTTAGATCTTATATA

AATAATAGATTAAATTCCTTTTTTGTTAAATATCATTAAGGTATATCTTAATTTAGAATA

TATTTTTTTCATCTATATGAAGAAATATATATTTTAAAATGAAAGATATACCTCATTGTT

AACAAAAAGACATTTAATATGAATAATTTAAGACAAGAAGAAAAAGGATTAAACATAGTA

TTATTTCTAAAAGCTGTGTTTATCTTTAGAGGAAATTATTTATTAAATAAATTTAACATA

ATGAGAAATTAAAAAATATCATTTTTTTATATAATGTTTAATATATTAAAAAAAAAAAAA

AAA

>c5285_g1_i1 len=202 path=[222:0-44 267:45-159 381:160-201]

CTGTATTTTTGTTTCTGATGTTGTCCAGGGCTTCTAACAGCAGAAACAAAAATACAGACT

CTAAGGGTAATTTTACCATCATTTCCTAACAAACCTGCAGCTCTTCTATAAAAGGCCCTA

ATTCCAGCAATACTTTAAGACTTGAGAGAATCCATTTTTATTTTTTTTGCCCAGAATGTA

AGGAAAGGAGAAGGACTTCAGT

>c5285_g1_i2 len=203 path=[222:0-44 267:45-159 25:160-202]

CTGTATTTTTGTTTCTGATGTTGTCCAGGGCTTCTAACAGCAGAAACAAAAATACAGACT

CTAAGGGTAATTTTACCATCATTTCCTAACAAACCTGCAGCTCTTCTATAAAAGGCCCTA

ATTCCAGCAATACTTTAAGACTTGAGAGAATCCATTTTTAATTCTTTTTTTAGAAGTTAC

TGAAGTCCTTCTCCTTTCCTTAC

>c5285_g2_i1 len=309 path=[222:0-44 464:45-308]

CTGTATTTTTGTTTCTGATGTTGTCCAGGGCTTCTAACAGCAGAACTTGTCCCTGGTAAC

CAAGGACTTTATGTTATTGAGAGGAAGAGTAGTTGATAGGTAGAGAATCTTCCCCTTCTG

TTTTATAGATACAATGAAATCTTGTACAGTGAATTGGAGGACACAGAAATGTCTTTATTT

GACTACATTCTGGAATGCTTTGGCAAGAGAATTAGAATGACTCTGTGGTGGAAGTTGGAT

TAAAAGAAATTTCTGCACTCATGGTCAAACTAAGACAAGGGTAAACTTTAGTAGGTCTAA

AGAAGTTGG

>c5288_g1_i1 len=825 path=[3129:0-7 1563:8-551 2107:552-824]

TTTTCTTATTTTCTTATTTTCTTTTTAAGATCACAATATTTTTATTTTGTATTATCTTTT

AATTTCCCTATTAAATTATTCTATGTAATCATTTTTATTAAATTAAAACGTATGAAAACA

AAATGTTAAAAGGTGAAGAAACACAGATAAAATTAATATTTTTCTTTTTAGGTATACTTT

TAAGTTTACCTTCCAACATAATAATAAATGTGTCTTTTTTGATTAATAATATATACAATG

AAGAAATTTCCATAACAATATTGGGTTTTATTTCTGGATTTATGATAATCTCATCACTGA

TTCAACTGACTTTTGAATTTACTTCATTTAAATCTATTATAATTTGTAATTTTTTGAATG

CTGCTAATTTGTTTATTTTTTTAATATTAATATGTTTATGTAATGCATCAAAACATTATA

TATATATAATTTCTGCTTTTATTGGATTTTTTATTGGGTTTCTTTATTCTGCAAACACAA

AATATTCTTTATTATTTGGTATGCGAATTAATGGATATTTAGTTAGTGGAATAAGTTTTT

GTGCATTATTTTTTTTTATAATTAATTTGTTGATGTCATACTTAACTATTGAAGATGGAA

ATGTAAAATCATATTATAATGCAATAACATTATCAATTGGTACAGTTGTAGTATTAAAAG

TTTTAATCATTATATATATTATATTTATGCATTTAAGATCCCCATATTTTTTGGAGCAAA

AAGAAAGAATTGAATCGAGAAAAACTAAAGAAATAAATATTGATAGTGAATTAAATACAT

TTAACGAAGTAGAGAATGGAGAAAATAACTTATCAAAAGGTGTGT

>c5288_g1_i2 len=761 path=[1:0-487 2107:488-760]

AAAAAAAAAATATATGTATAATTTCATATGTATAAAATAATGAATAAAATATTTTTAAAG

TAGTTAATGTTATGATTTATTTTTTATGAATCTATTATTAAACCACAAAACCAAGGTGAT

AATGCAAAACTTATTAGAAAAGTAAGAGCACAAAAAGAAGATGAAACAGCAATATTCTTT

TCTTTTTGATCGGAATGTTTAAAACAATCACAAATTCTTCCATAACTTAAATTTATTAAA

GATCCATTTGTTGAACCTACTAGAAATATTACAAAGGCTATAAATTCATTTGTATATAAA

AAAGAATCTTCTTGTAAATTTTTTATTTTATAAGCCAAAACCAACAGTAATATTCTGCAT

ATACATAAAATTGTTACATACTGCTGTTTAAAAAAGGAAAATAAATTTAAATAAACTGTT

ACCAAAAAACTAAATATAAGATCACTACACTGAAATAGAAACATAAATAAATATTTATAG

TATACCCCTTTTTATAATTAATTTGTTGATGTCATACTTAACTATTGAAGATGGAAATGT

AAAATCATATTATAATGCAATAACATTATCAATTGGTACAGTTGTAGTATTAAAAGTTTT

AATCATTATATATATTATATTTATGCATTTAAGATCCCCATATTTTTTGGAGCAAAAAGA

AAGAATTGAATCGAGAAAAACTAAAGAAATAAATATTGATAGTGAATTAAATACATTTAA

CGAAGTAGAGAATGGAGAAAATAACTTATCAAAAGGTGTGT

>c5288_g1_i3 len=761 path=[1:0-487 489:488-760]

AAAAAAAAAATATATGTATAATTTCATATGTATAAAATAATGAATAAAATATTTTTAAAG

TAGTTAATGTTATGATTTATTTTTTATGAATCTATTATTAAACCACAAAACCAAGGTGAT

AATGCAAAACTTATTAGAAAAGTAAGAGCACAAAAAGAAGATGAAACAGCAATATTCTTT

TCTTTTTGATCGGAATGTTTAAAACAATCACAAATTCTTCCATAACTTAAATTTATTAAA

GATCCATTTGTTGAACCTACTAGAAATATTACAAAGGCTATAAATTCATTTGTATATAAA

AAAGAATCTTCTTGTAAATTTTTTATTTTATAAGCCAAAACCAACAGTAATATTCTGCAT

ATACATAAAATTGTTACATACTGCTGTTTAAAAAAGGAAAATAAATTTAAATAAACTGTT

ACCAAAAAACTAAATATAAGATCACTACACTGAAATAGAAACATAAATAAATATTTATAG

TATACCCCTTTTTTTAATTTATTTGGTATTATATGGGGATATATTACGAAGGTTATAAAA

ATGTTAAAAGATATTGGTATTAAACATAAATAATAATATTTAATTAGGCGGGCACCATCA

ATTATATTATTACAATTAAATATTTTTTTGGGAAATTTCAATTTTTTATTAATTTTACTT

ATTTTGGAAATATTATCTTTAACAGTAACCTCTATTTTTTTATCATCATTATCATTAGAA

TTTTTGTCATATGATGACACACCTTTTGATAAGTTATTTTC

>c5297_g1_i1 len=239 path=[1:0-238]

AGCATACTTAACCATTCCTAGACAATTATTGCTCTATTGGTATGAATATATACCAATAGA

GCAATATATTATAGCAGGTATAAAACCTGCTTCCATGGTTTTATTTATTTCCCATTTCTA

GGTTCTTCTTGGTCTATCCTACTTCCAAAGTCGTCTGTATGCGGTACTACACTGATCAGC

CTTTGATCTTGTGGCATTGTCTTTCAGCTCTTGAAGGATTCAGTAAGTACCACCTACTT

>c5303_g1_i1 len=283 path=[513:0-282]

GCTGTTGCTGACCTCATTTTCTTAGCTCCATAAAGCAACTACACACTACTGAATCCCTAC

CCCTGCCTTCTGGCAATACAGCACAGCCTGAAAAAAAGAGGCTTGCAAAGCTGCTTTTAG

GTACCAGTGAGCTTGCAGCCTTAAAGCACCATTTCTTGGTAGTGCTCCCTCCAACTGCCA

AATACCATCTTCAACTTACTGCTCAGCAACAACCTCATTCCCTGGCCAATTGCTAAAAAG

TATACTAGTAGGGGAGAAACACCTGCTTTTTTTCCACCACTGG

>c5304_g1_i1 len=312 path=[47:0-169 42:170-311]

CATATTTAATATTTTTGCTTTTAATATGTTATTTTTATATTTATTATTTTAATATGATCA

TAGTCAAATTTTTAAAAATAGAAACTTCAAAAAAATATTTACCTTCATAATGTTTACATA

GATTTATTAAATATAAATTTTATTTTAAAAATATAGAGCTATAAAAAATGCTAACTTTAA

AGAGCGTTTTATTATAAGTATCACATTTTTTTAAATGTATAACAATTTTAAATTATAAAA

TTAGGATTATGAATTTTTTCATTTTTTTTTAATTATTTAATTGTGTTATATTATTTGAAG

TATATTTTTTTT

>c5305_g1_i1 len=278 path=[301:0-277]

GAGAAAACAAGAGTTAAAATTGCTGGCAACACTGACCCGAAGGCCGAGGGGTGCGGGGTG

AAAGGCTGCTATGGAAAGCTCTTACATGATGATATACTGCTCTCTTAATACTGCTCCTGT

AGTTACAGAAACCGTTCGCAACAACAGACCCAGCAGTGTCTATGGAACTTGGAGGCTCAG

AGGAAGTGGAAAATTCTTTCAGTTTACACTTGAGCTTTTTCCACTTGGAATGTATGATTG

AGGAGTTTAAGCAAGGTTAGGAAAGCTGTGGCCAAGAT

>c5306_g1_i1 len=251 path=[263:0-120 25:121-250]

GTGAGGTCTAACACCATCAAAACAACTGCTGCTCAGTTCTGGGACGCAGATCGTGGCCCA

AACCCTTGCCACATAAAAGGTTAACACAGCCCTGGAACATTAAACTCAAGACAACTCTTC

AGTCCCAGTTTCCATTTTGTCATCTAGGCAGTTCCTGGTGCCGTCAGTCGATTGTGTTTT

CTTAGTTCTGCAGAATAACTTTTTGTTTTCTGCCTTAGCTTTCTGCTAGCTGCGAGCTGA

GTTGGCTCAGC

>c5308_g1_i1 len=262 path=[267:0-261]

GGGATGGGTGTCATTTTGGGGTTCAAAAAAGCTTTCTCCCTTTAGTACGCTTTTAAAACT

CTACCGTTAGCCTGCAGAAACGTTGCCAAGCAAGGAACTCTAAATACAGCCCATGTACTC

AAACAGCACTGTGCACCGTAGACAGCACGCAGAAGAAAGCAGTGGTAACTGAAGGACTGC

TCTCTGGATGTCGATTTTGACCCGAGACGCTCATTTTGAAGATGAGTATCAGCTGGAATT

CACTCAAGCGAAGTGCTTTGAA

>c5309_g1_i1 len=336 path=[1:0-199 201:200-335]

CCAATCCCTTATCCACTGAAAAGTCCACCCTTCAAATCTATATATTTCCAATTTAAAGAT

AAGGATGCGGTGAGGGACCACGTCAACGGCCTTGCACAAGTCCAGGTAGGTGACATCAGT

TGCCCTCTCTTTGTCCACTGATGCTGTTGCTCTATCACAGGAGGCCACCAGATTAGTCAG

GCACAACTTTTTCCTTGGTGAAGTCATGGCTGTCAGACAAAATCCCTGGGGATCGGAAAA

AGGGAAACATCACTCACACTTTCAAGAGAGGGAGGAAGGGGGACTCAGGGAGCTGCAAGC

TGGTGGGCCTCGCCTCTGTGCCTGGGATGATCATGG

>c5313_g1_i1 len=333 path=[1:0-203 205:204-332]

CGGAAGCTTTGGCCCCAACGCCCCCTCTGTTATGAGGACACTGTTCTGCTGGTGGCAGAA

ATTGTGTCCTCAGAGGGGTACAGCTGCGTTGTGTCCCCGGGGCTTCCCAGGAGCATTGCA

TTTTCGACGGGGCCGGTTCTGGTATGCACTTAAGAGGCCAACTGCTTACTGACAGTCGGA

GCTGATAGGGTTGATTTAAGGTCGTGGAGGAAGGACAATCCCTCTCTTCAGTGGTGGTAA

TGGGATTTTGTAACGAGGTAGAAATTGCATTAAGCAGGAAGCGCTTTCCACCAGGCAGGT

TGCAAGGTGAGGGGCTTTCCAGCTAAAAGCTGG

>c5326_g1_i1 len=206 path=[234:0-205]

AAGTATAGCACCAACTAACAGTAGCAGCAACCAGCAGTAGTAGAAAGATAACTTAAGTCA

GGAAAATCCATAATATAGCAGTTTGGGGATAATTACAAATTCAGCAAACCTAACTATATT

ATGACAGCGAATAGATTTACTATGAGTGACAAACTTACGTATATTTGCTGTTACATTACT

ATTACAGGTTTTCAAGTCAAGGTCAT

>c5339_g1_i1 len=341 path=[452:0-20 134:21-340]

CTTGAAATTGAGGAGATCTGGCTTAGTGCGTTTGTCGCTGCTGCTCCCGTGTGTGGGTCG

GCTTCCCGAGGAGCTCTGCTGTGCGCTGGAGCTCCCACCCAGCTGCGACTGCGGGGAGAG

GAAGGGAGAGCTCAGCCTGAGCCTCTCGTGCCCATCTCCTCCCCGTGTCTCCCAGTGCTG

GAGCTGAAACACTGCTTCTCAGACCTCGGGAAAAGGGCTGTGCTGGTTGCACACCTGGCA

GCCAGGTCCCTGCCGTTTCCAATTGCTGTTTCTGTTTTCACCCCCCCCCAGCCCCGACCT

ACGCCCCTCGCTGCAAGAAGACATGGAGGCCCTGGAGTGTG

>c5339_g1_i2 len=386 path=[68:0-65 134:66-385]

GGCCCGGCCGCCCCTTTCCCGCAGCCAATGGAGCCGGCGGGGCGGGCGGGGAGCTCCGGG

CCTTACCTTAGTGCGTTTGTCGCTGCTGCTCCCGTGTGTGGGTCGGCTTCCCGAGGAGCT

CTGCTGTGCGCTGGAGCTCCCACCCAGCTGCGACTGCGGGGAGAGGAAGGGAGAGCTCAG

CCTGAGCCTCTCGTGCCCATCTCCTCCCCGTGTCTCCCAGTGCTGGAGCTGAAACACTGC

TTCTCAGACCTCGGGAAAAGGGCTGTGCTGGTTGCACACCTGGCAGCCAGGTCCCTGCCG

TTTCCAATTGCTGTTTCTGTTTTCACCCCCCCCCAGCCCCGACCTACGCCCCTCGCTGCA

AGAAGACATGGAGGCCCTGGAGTGTG

>c5340_g1_i1 len=577 path=[1181:0-546 622:547-576]

AAACGAAAAAGAGAAAATAAAGAGAAAAAAATAAATTGAAAGAATATAAGTTCTCACTTA

CATGAGTTTCTTTTATAATAGTACTTTAGATGGATTAAAGTACTAATATGAATTCTATTA

TTATTTGCAGACTTTTTTAAAATATAATTAAAGTAAACATTTATCATTAAATTAATTAAT

TTAACATTTGATATACATATACAAGTAAAGGAAACCTTTTTGTTTATTATAAATATTTCA

GCAAATTTATATCTTTCATCTCCATATGTAATAATGTCTTTTTTTAAAAAGTATATTAAT

GTTAACATCTTTAAATTTATTTACTTCCTATATTCACTTATTAATAGGTTCTTCCTTTTC

ATTATCAGAACTGATTGTCTTTAATTTTATAAACTCTATTAAAACAACTATCAGATTTAG

CTACATAATTTGATAGGTATATCAAATCAACATAATAATCACCTCTTGGATAAATTTTAC

ATAATTTATTCTAATGATAATTTATAATAAATGTAGTAACAGTATTCGATTATCATATGT

TACTGTTAAAATCCCATTCGTAAAATAACTAGATGAA

>c5340_g2_i1 len=651 path=[1:0-620 622:621-650]

TTTTTTGATTATTCATTTTTTTTTCTTATTTTTAACTTTGTTTACATTTTCTTTTTGATA

TGTTACTTTTGTTTTCATTTTTGTGAAAAAATATATCTTTTTGTTTTAATCATAAATAGA

ACATTTGTAACATATTCATAGAATAAAAGATGCTTTTCTAAACTTAATAACAATAAATAT

AGTATATTTAAAAGAAAATATATAATACTATAATTTTTTTTAAGTGTAAATATTAAATCA

TATTTATTGAAAAAACATGTAAGACAAATAATGTCAAAAAAAATTAGATTTATTTTAACA

GTTAAGTCAGGTGATACCAATGAATATATATGCCCATATAAATTTGTTTGATAAATTAAA

GATGGGAAGGATAAAGATGGAGAATATATTGATAATGGAAATTTTTGTTTTGATTATGAT

GTTCTTAATAAAAATGTGTGTGAATTAAAGTAACATATTAGAGATTTGTATAATATAATG

AAAAAAGAAGATAATAATATTTATAGAGAAGAATTTTCATTACTATCAATAGTTTTAATA

AATATATTATTTGAATGTTATTTTTACATGTATTAAAATGGTGATCTAGTTAAAAAAAGT

ATTAAAGAGTAAATATTATACAAAATCCCATTCGTAAAATAACTAGATGAA

>c5340_g1_i2 len=577 path=[1181:0-546 1728:547-576]

AAACGAAAAAGAGAAAATAAAGAGAAAAAAATAAATTGAAAGAATATAAGTTCTCACTTA

CATGAGTTTCTTTTATAATAGTACTTTAGATGGATTAAAGTACTAATATGAATTCTATTA

TTATTTGCAGACTTTTTTAAAATATAATTAAAGTAAACATTTATCATTAAATTAATTAAT

TTAACATTTGATATACATATACAAGTAAAGGAAACCTTTTTGTTTATTATAAATATTTCA

GCAAATTTATATCTTTCATCTCCATATGTAATAATGTCTTTTTTTAAAAAGTATATTAAT

GTTAACATCTTTAAATTTATTTACTTCCTATATTCACTTATTAATAGGTTCTTCCTTTTC

ATTATCAGAACTGATTGTCTTTAATTTTATAAACTCTATTAAAACAACTATCAGATTTAG

CTACATAATTTGATAGGTATATCAAATCAACATAATAATCACCTCTTGGATAAATTTTAC

ATAATTTATTCTAATGATAATTTATAATAAATGTAGTAACAGTATTCGATTATCATATGT

TACTGTTAAAATTTCATCTAGTTATTTTACGAATGGG

>c5343_g1_i1 len=278 path=[256:0-277]

GCCCGGCAGCAGGAGCAGTCAGCGACGACGCAGAAGGCGGAGCGGGAGGTGACGAAGATG

GTGGTGGTGATGGTGCTGGGCTTCCTGGTGTGCTGGGCGCCATACACGGCCTTCGCGCTG

TGGGTGGTGACGCACCGCGGGCGCAGCTTCGAGGTGGGATTGGCCTCCATCCCCTCCGTC

TTCTCCAAGTCCTCCACCGTCTACAACCCCGTCATCTACGTCCTCATGAACAAGCAGTTC

CGCTCGTGCATGCTGAAGCTGCTGTTCTGCGGCCGCAG

>c5346_g1_i1 len=205 path=[43:0-152 343:153-154 198:155-204]

GATTATATCCGTTTTCATTAACTTAAAGAATAATGGTAAGCAGTAGCCCTCACTTTACAC

ATTTCCTTTCGAAATCTACCGTGCATTTAACTTATAATGCAGTGAAGAAAAATAGTTTTT

CACTGATGCAGTGCTTTCAGAATATTCCTACATTTCTTGCAAGCTTTGAGGAAAGAACCT

GTAAAAACTGCTGTAAGTTTTGACA

>c5353_g1_i1 len=596 path=[155:0-595]

AGGATGCGACAGCGGGGCTCTGGCAAAGATCTCTAACAACATGCAGGTAAATTGGTCACT

TCCATCACTTTCCCAGTCTGTTAAAACCCTTCAAGTCTGCTGAGGAAGTTCACTATCAAG

GGGACACATTCCTAAGCCTTTTTCAGCTACAGCCTGCCCTGAAGTGCAGGCAGCCAGCCC

AGATAACAAGAGTGACGACAATCTAGGTAATTAGAAGTGACTCCCATCCCACACAGCAAT

CAGCTTATTACCATACCACACTTCACTTTAGCCATGCTGCCTCCTAAGAACAGTTCCAAT

CTCTCCTCCAGTGTTACCCTGCAATAGCGAAACCTGTGCTCAAGTGTCACTGCGCAAAAC

ACCTTCCATGCTCTCTGCTGGTGATAGTCTTCCCTATCAGACTTCAAGCCGGTGCAGCTT

TAATGCTGATATTCAGTACATCCTCAAAACAGAATTTATTTAGAGCTTTAAACAACTTAA

AGAGTGCCACATGGGGAGCAGACAGGCTGGGTTTCTGGAATCCTCCTTGTGTGAGCTGAA

TCATGTTGCTATGTGGGTAGCAGGGCATGTTGCATCTTAGAAACTGGCAAGAAGAA

>c5354_g1_i1 len=634 path=[1109:0-288 179:289-633]

GTAACCTTGTGTTTGAGGGGGTTTGTTTACAGAGTTGTTCAAACAATAATACTGTTTGTG

CTTACTGCCCATGTGTAAAGATGAATATTACAGTGAGGTGGATGCAGTCAGAGCAGCAGA

ACAGAAGCAAGCTGTAATTTGTCTTGCCTAGGTTTCGGTAAGACCTAAGCAGCAGTGTGA

GTATTGGAGACAGAGGAAGCAGAACTGGAAACAAGCAGTGTACAAAACTGATCCCTGTCC

AAAACCAGCTGGGGAGGGCAGTGGTAGAAAGCTTTTGTTTCTAATCTGTGCTTTTCTGGT

TACCTCCCAGATGTCCCCAACTACTGAAGTGCAGCTTCATGCTGTGCTCCATCTGTTCTG

ACTGCAGGCTACTGCGACATGATGTGATCCCACGCTCACCCTTCCCGTATACCTGATGTG

GCAATCACGCAGCCAAGAGTTGAATTTGAGATTACTGATTTCCACAGGGATGGAAACTAG

AAAAGAAAACAAAATTGATCAGTCTTTCAGTGGCTCAGCAGGTGGTAACCACCATAAAAC

TGCACAACCTGTACTTTCTGTGCAAGGGAAGTGGTGAAATAGCAGCTGGTGGGGAACAGC

CCCACGTTGTGGATTAGGCACAGCTCAGACTGAG

>c5354_g1_i2 len=633 path=[1109:0-288 1397:289-632]

GTAACCTTGTGTTTGAGGGGGTTTGTTTACAGAGTTGTTCAAACAATAATACTGTTTGTG

CTTACTGCCCATGTGTAAAGATGAATATTACAGTGAGGTGGATGCAGTCAGAGCAGCAGA

ACAGAAGCAAGCTGTAATTTGTCTTGCCTAGGTTTCGGTAAGACCTAAGCAGCAGTGTGA

GTATTGGAGACAGAGGAAGCAGAACTGGAAACAAGCAGTGTACAAAACTGATCCCTGTCC

AAAACCAGCTGGGGAGGGCAGTGGTAGAAAGCTTTTGTTTCTAATCTGTTCGTTTCCCTC

ATGCAGGCGCCTGTGCAGCCCTGTGATTAACCAAGCACTCAAGTACTGCAACAACCAAGG

ATGTGGCCTTGGGGCTGCAGGAGCTTAAGGCTCTCCCTAAGCCTGTGCTGCTGCTGGAGA

AAGGCGTGGCCGTATGTCAGGTTAGGGTGGGAATCACGCAGAAGAATTCACACGTTATGT

GATGTTTTGTTGTAGGGTGAATCTGGAAACAGCACTGAAGTGTCAGTGCATTTTGAGTAC

ACAGTAGCTAAGTAAGTGAGCCTTCGTTTTGGAAGAGGTGGTCTTCATGTGATAACAGGC

ACATTTGACTCAGTCTGAGCTGTGCCTAATCCA

>c5358_g1_i1 len=216 path=[37:0-141 36:142-215]

CCCACACTGAGAAATAACGAGCAGTACCAGCGCTAATAAGGTTTTTATTTTTGGCACACT

GTATTCAGCAGCCAGTTACATAATGGGTTACAGGGCAGCAGCTCATTATTAGACAATTAG

GGGGAAAACAAAACAAAACAACAACCTCCTGCCTTTCAAATAACAACCACAAGAACATTG

AGAGCATAGAGGCTATAAGTAATTCTTGAGGAATTT

>c5359_g1_i1 len=240 path=[478:0-101 21:102-125 45:126-239]

GGCAGAGCTATGGGGAAGGACAGGTATCTATGGGGCAGAGCATGGATCTATGGGGCAGGG

CAGGGATCTATGGGGCAGGATGGTTCCCTATGGGTCAATCTATGGGTCAATCTGTGGGTC

CCTGTGTGTCCCTATGTATCCCTGTGGGTCGCTATGGGTCCCGGTGTGTCCCTGTGTGTC

CCTGTGGGTCGATCTGTGGGTCTCTGTGGATCTCTGTGGGGTCCCTGTGTGTCTCTGTGG

>c5363_g1_i1 len=275 path=[303:0-274]

GACTGGTTTGTCTTCATTTGTAGTTCATCTGAATGCATGTTTGGTGCTGTGAGCAATACT

ATTGTCAAGATCATCCTTTTAAAAACAAAAACCTCTGCACAGAAGGACACCCGTGCTTTG

TGCAGATGGAGATTGTTTCTAACCGTGACCCTGAGTGTTTTGTGGAGTTGAAATGATTGG

AGGCCCTGAAACCCAAGCCAAGGAGAGCAGGAGAGCGTGAGTGTGATCTTCGGGCGGCCT

TGGTAGGGGAGGACAGCAGAAAGCACGGGTGTCCT

>c5364_g1_i1 len=232 path=[155:0-231]

AATAAATGGTAGCCTGAAATCAGGACCAAAAGGTCAAAAATGTAGAAACATTCACAAGAA

ATGAAAGAACAAGAATAGCTTACTGGGAGAGAAAAGAAGTATCTTTTCACTTTGAACTCT

GACATCCAGCTCCCACAGGTGGCAAGCTCATAGCATCTATGCTATTTCACAATTCAAGTG

GACTTTGTAAGCTGAACTTTAATGCAGATCTGCTTGCTAAAAAAAAAGTGCA

>c5365_g1_i1 len=416 path=[923:0-15 546:16-415]

TGCGGTTGAGCCGTCACCATGGGCAACATCTTCGGCAACCTCCTGAAGAGCCTCATAGGC

AAGAAGGAGATGCGCATCCTCATGGTGGGGTTGGACGCCGCCGGCAAAACCACCATCCTC

TACAAGCTGAAACTGGGGGAGATCGTCACCACCATCCCCACCATCGGGTTCAACGTGGAG

ACGGTGGAATATAAGAACATCAGCTTCACGGTGTGGGACGTGGGCGGGCAGGACAAGATC

CGACCCCTCTGGAGGCATTACTTCCAGAACACACAGGGGCTGATCTTCGTGGTGGACAGC

AACGACCGGGAGCGCGTGAATGAGGCGCGGGAGGAGCTGATGCGGATGTTGGCAGAGGAC

GAACTGCGGGACGCCGTGCTGTTGGTGTTCGCCAACAAACAGGACCTCAAACACAA

>c5365_g1_i2 len=484 path=[462:0-83 546:84-483]

CCCAATCCCACAGCTGCAGCCCAATCCTGTGCCGCTGCCGGTGCCGGTGGTGCCCCCCCC

GCAGCTGACTCTGCACCGCTGTCCCCATGGGCAACATCTTCGGCAACCTCCTGAAGAGCC

TCATAGGCAAGAAGGAGATGCGCATCCTCATGGTGGGGTTGGACGCCGCCGGCAAAACCA

CCATCCTCTACAAGCTGAAACTGGGGGAGATCGTCACCACCATCCCCACCATCGGGTTCA

ACGTGGAGACGGTGGAATATAAGAACATCAGCTTCACGGTGTGGGACGTGGGCGGGCAGG

ACAAGATCCGACCCCTCTGGAGGCATTACTTCCAGAACACACAGGGGCTGATCTTCGTGG

TGGACAGCAACGACCGGGAGCGCGTGAATGAGGCGCGGGAGGAGCTGATGCGGATGTTGG

CAGAGGACGAACTGCGGGACGCCGTGCTGTTGGTGTTCGCCAACAAACAGGACCTCAAAC

ACAA

>c5367_g1_i1 len=1037 path=[1065:0-1036]

AAAAAAAAGGAATTTTAATTATATATACAAAATTAAAGAAAAAAGACTAAAAAATTTTAA

ATATAATTGAAAAAATATCAAAATTTTATTTTTATTTATTCACAAATAACCAAAAATTAC

AATAAATATAATTACCAAAATTAAAACAGAAATATTACAAATCACCTGAAATGTAGTGCA

TACAAAAAAATGTCTTGGATTGTTGATTAAAAAAAAGTTTCTTATTCTTTGTGTAAAGGT

TATATTTCCTCCAGTATCCAATAAATTATCTGTTCCAATATTTTCTAAATAAGAATGTGG

TGTGTCTATTAATCTCTCTGTGTAATTATCTAATATATATGAATTATTATTACTTCTGTT

ATTTCTACTTTGTAAATTATTTATTTCATAATAATATTGTTTAGAATATTCTTTATCCTT

TTCTATTTGTAATTGCTTATAAATATCATCATTTTTAATTATTTCTTTTGTTAATTCTTC

GTTTTTCAATTCTTCATTTAATTTATTTTCCTTTAATATTCTTTCTTCATAATTTTTTTT

ATGAATTTTTCTATATTCTTCTAATGCATCTTCTAATAATTTTTCTTTCATCTTTTAATA

TAATAATAAATATCATATATACACATATATGTTTTAAGTTAAATTTTTATATTAAAAAAA

TTATTTCATTAGAATGTGATTCTATAAACTTAAACCATTTAAATACTTTGAAAATAATAA

TTTACGATTCTTTTTATAATTTTATCTTTTTTTAAATATATAATGCATATTAAAAAGAAA

CGAATTTCCAACAACATTCAAATTAATGGAGAGTAAAAAATATATTTTATTTAGGAAAAA

GGTATAAATAATTTTTGTTTTTAAAAAAAATTATAGTAATTAAGAACTATAAATTTACTG

CAATGAAATAATTAAAATTATTAAGGAAAAAAAAAAAAAAATTGCAAAATAGAACGAAAA

CATAAATTTTTTTTTCTTCATTTATGAGTGGTATAAAGAAAAAAAAATGGTTATCACTCT

TATCACAAATATATAAA

>c5372_g1_i1 len=274 path=[296:0-198 519:199-208 530:209-240 38:241-273]

TTTTGTTTTTTTTTTTTTTTTTAACATAACAGGAGGCTTCAGTCTCCATTAAAAAAGTCC

AAATGTGCTTTAATGAATTAATTGAGAGACAGGTTTTTAAATAATTTCTCAAAAAGAAAA

ACAACTGAGGAAACTCCCAGTGGATTTGAATCAATAGAATGATCTCTGAATATAGAAAAA

GATGTCTCAGGAATGACACAATATTTCTTATTCATTATATGTAAGGGAAGAAAAAACAGG

TAATATTGGTCTTCACTGACATATAATGAATAAG

>c5372_g1_i2 len=232 path=[296:0-198 38:199-231]

TTTTGTTTTTTTTTTTTTTTTTAACATAACAGGAGGCTTCAGTCTCCATTAAAAAAGTCC

AAATGTGCTTTAATGAATTAATTGAGAGACAGGTTTTTAAATAATTTCTCAAAAAGAAAA

ACAACTGAGGAAACTCCCAGTGGATTTGAATCAATAGAATGATCTCTGAATATAGAAAAA

GATGTCTCAGGAATGACACAATATTGGTCTTCACTGACATATAATGAATAAG

>c5381_g1_i1 len=314 path=[1:0-313]

CCACATGCCAAGAGCAGAGCACAGCCCTGCGCAGCGCAGCCACCAGATGCATCCTCCCTG

CTGTGGGCTGGGACACCCCGAGTTCAGACATCCCCAGCAGGCAGAAAGCATGCAGCCACC

ACAAGCCCTGGGAACAACCCGTGACCACTGAGAAGGAGGAGTAAAGCACGAAGAAGAACC

ATCCATCTCCACCACAAAGCCAATGTCTGCACACTGTTGTCCATGTTCAAGTGAGGAGTG

TGAGCCAGGCCAGAGCTCCCCAGCAGAGCTCACATATGAGGACAGGAATCCATGCCCAGG

GTGAGCAGTACAGG

>c5386_g1_i1 len=431 path=[459:0-430]

ATATGTTACACTCAGACGTTTTGACTCACCAGTAAGAATTACTGAAAGTAAAGAAATTTT

TCCTGAAAATCCAGGAGGAACTCAGACTTGTCCTTGACAACATGTGGAAGTTCATTTTAT

GCAAATCTTGACAGAAGCCAAAGCAGGGACTTTGTGGAATAACTTGGTTCTTAAGGGCTT

TTCTACATGCACAGCAAGCAAAGGACACAAAGCATGAATCTGTATTTATACAGCACAAGT

GGAGAGCAAGTGTGAATAAGAGTGCTATGGCATCACAGAAAGCTGGTTAATGTTTAACGG

GGATGCTGACAAGCTTTGCAGAGGTTCATAGGTAGAATCACTGAGGTTGGAAGAGACCAC

TGAGATCAACTAGTCCAACCATCAGCCCATTGAGGCTGCATTTATGTGGGGCTTTGTGTG

AATACTGTTTA

>c5392_g1_i1 len=272 path=[155:0-271]

GTCAGGTGTCTTCTATTGCTTTTCATTGCCACAGTGACTGGCACAACCCTCGTTAACGAT

CTGAAGTTGCGGAGGTCAGCACCCATACTTGGTGCCCTATTAAGCAATAGTGAATTAATT

ATGGGAATGATTTCCTTGTATTATGTATGTTTTTGTATTTTGGGCTATAGAAGGTTCCAA

TTACTTCCAAGATCAAACCTGTTTCATTAAGAGCATATGGCATTGTCATAACTAATTAGC

TCACATTACTTGTGTTGATTCTGAGATATGAC

>c5398_g1_i1 len=217 path=[219:0-216]

GAAGGTAAGGATATAGGATTGAAAGATCCTATATCTGGGTGTATATATACAGTGGGTTCA

GGGCCAGGCAAACACAGTCACTAATTTTTCATCCTGGTATCACTCCTCTTTGTTGTCCTG

CAGCCCTTCTATCTTATCTCCACATGTTTGTTATCGTAGGTGCTTCTGGGAAGCTCTCAT

CTCTTATGGAGCTCATACAAAACATAACATTGAAATT

>c5399_g1_i1 len=273 path=[293:0-69 44:70-87 22:88-272]

GTCTTGGTTTGGTATGTTTTACAATGAGGATACGATGATATGTTCAGTCTGAGAATATAA

AATGCTTTTTTTAAAATGCATGCATGTCTAAGAGATATTTGTGTGAAACATTACTAGTGG

AAAGCTGACTGTAATTGCTTGTGCCCAGTGCTGTACAATGTGTTAGAAGCAGCAGCATTC

AACATTACAGCATTTAGTTTTAGCCAGCTACCTGCTTTGTATTTGCAGCCTTGGTGTAGG

TGCCTGGGCCAAGTATGCATGCTGAGGCGAGAG

>c5405_g1_i1 len=341 path=[1:0-94 96:95-119 312:120-340]

GTTCCTATAGCCTGTGTGCGGAGTGTGGGTGGAAAAAGGGAGGGGAACAATGGCTTCTTG

AATACCCGCTCCCTGTTCTCAGCTCCTTCAAGCCAGTGGCAGTGCTTCAGCTGGGTATGC

TCCCATCCTGGTGACCTTGCACAGGAAATACAGGCTCAATCTGCGAAAGTGAGACTGCTA

CAAGCTTTGTTTGCTAAATAGTAATACAGAACCTACACCAGAGCTTGGCTCGCGTTGGCC

ATTGGGCCACTGTGGTGAAAGAGCTGCTCTCAGCTTCGAGGATTCCATGTGTGGAATGGA

ATCCTAAGGGAAATCAGGCTGACAGCCAAATCTATGTTGAC

>c5405_g1_i2 len=256 path=[790:0-34 312:35-255]

GCACAGCCTTACTTAGCACTATGGGGGTCAAAGCATCCCATCCTGGTGACCTTGCACAGG

AAATACAGGCTCAATCTGCGAAAGTGAGACTGCTACAAGCTTTGTTTGCTAAATAGTAAT

ACAGAACCTACACCAGAGCTTGGCTCGCGTTGGCCATTGGGCCACTGTGGTGAAAGAGCT

GCTCTCAGCTTCGAGGATTCCATGTGTGGAATGGAATCCTAAGGGAAATCAGGCTGACAG

CCAAATCTATGTTGAC

>c5405_g1_i3 len=307 path=[227:0-60 96:61-85 312:86-306]

CCCATAGTGCTAAGTAAGGCTGTGCTCTCTGTGCTCCCAGCACCGTGTAGCTGAGGCTGC

CGTGGCAGTGCTTCAGCTGGGTATGCTCCCATCCTGGTGACCTTGCACAGGAAATACAGG

CTCAATCTGCGAAAGTGAGACTGCTACAAGCTTTGTTTGCTAAATAGTAATACAGAACCT

ACACCAGAGCTTGGCTCGCGTTGGCCATTGGGCCACTGTGGTGAAAGAGCTGCTCTCAGC

TTCGAGGATTCCATGTGTGGAATGGAATCCTAAGGGAAATCAGGCTGACAGCCAAATCTA

TGTTGAC

>c5406_g1_i1 len=290 path=[268:0-214 483:215-289]

AGAAAGAAGTAAAAAAAGAAAGCAAATACTTCTGTCTACAGAAACAATAAGCTGTTTTTG

AGACTTTCATCCATGAATTTACTAGGGTCCTGATGACAGACTGAGGTGATGAAACATTTT

TTCACCTCCTTTTTTTTTTAGGAGTGCAGGTTTATTAAAGTTTGCTGGGAAATCGATTTA

AAATATATATATATTCATTCCTTCGGGGTACTACTTTGCTTTTATCATCAAGTTGTAGTA

CATAATGAAGTTTAAGTTTATTAACCAGTGCAGAAGAGAGAGTAAGGTTA

>c5422_g1_i1 len=246 path=[224:0-245]

GAGATCATCCTTGTGGTGTTTTATGGACAAGATAAAAATGGGAAACCCCACCAGGACGTT

ATATGCCACTGGATAAAAGTTGATGCAGGTGGAAGCTCTGTATGGAAATACAGAAATATC

AAAGTTCTGCATTCATGTTATGGCTCCTCAGACCACTTCAACCCATGGTGGCTTATTATT

GGCACTTTGGTTATTGCTAATTTATTTTTCCAGTGCCATAAGCTGTCAGCCTGACACATC

CCCCAT

>c5424_g1_i1 len=245 path=[571:0-244]

GTAATTCATGCCTGTGTGCAAACTCAAGGCATTCCTTCAGCAACTGCGCTGGGTAAATGT

CCCCTAAGGGGTTGTTAGGGTTGAAATCTACATGCTGTCTGTTTATGATGATACCACCTT

CACCAGTGTTCTTAGCTTAGATAGTCTACCAGATCCCGAACGAACACATTTTATGTGGGG

TTTTAGCAAGGATTTTGGTATGAGCGGAATCAGAGTTGGGGTACTGTACACCAGAAATCA

TGAAG

>c5432_g1_i1 len=257 path=[1:0-256]

ATGGGGTGTGGGGGAGAGCTGTTCTAAACCAAAACAGCTGTTACATAGCCAGCAGATCAG

TGTGTGGTTCTCAATGTCTGGTTTTGGTAGTGTGCAAGACTGGAAAATAAGGTTTGGGGG

AGTCTTTGTGCATCCTTGGAACCTTGTAGGCATCCTGCTTGTTTTCTGGTTCCTTGACTG

GTCTCAGCCATGAATGTACAGTTTACAAATCAAATGTTCATTGTCTTTTGTTGCAGAAAC

AGAGGGGGAAGGAAAAT

>c5435_g1_i1 len=378 path=[171:0-377]

GGCTGGCTGTGATTTTCCAGCAGGGGTGATTGAGGGAATGTCCAAGGCAGTGTTTAACTG

AAGGCAGTCAGAGATAAGGCTCCTCGTGCTTTGTTGCAGAGCTCAGCAGGCAGATGTAGA

GAGCGTCTCTGTGCAGCAGTGTGGGAGGCAGGACCAGAACAGAGCCTGGCTCAGTGGTTG

GAGTGGTTGTGCAGAGGGGCAGGTAAGGCAATTTGAATAAAGCTTCCCCTTGGAAGAGGT

GAGGCGATGCAGCTGATCATGTTCTGGGCCTGGATCCAGCCCCAGGGTAGTGCTGTTTGA

ATTACCCCTTATCAGCCCCTCCGCTCTGTCCTGGGGATGCAGAGGCACAGCTGGCTGTTG

TCTCACAGCTTGTGCTCT

>c5439_g1_i1 len=250 path=[382:0-249]

ATTCCACCTAAACCCTACTACAGGCGGAGAGTATATCTCCACTCCCCTCTGATGCCTCCT

GCCCCAGAGACAGGGCTGGACCTTGTCTCCTCACCACAGCTAATGCTCAACACATGTGAA

AAGGCAAGGCCAATCTGCTTGGTGCTGGGAAGAAAATGAACCATGGGAATGAACTCATCC

CTAATTGCACTGCCTGAACTTTTAAAGCTCTTAGAGCCAAAACGTGACATCATCCTAATG

TGACATGATG

>c5451_g1_i1 len=441 path=[1:0-52 54:53-246 54:247-440]

CCCAAAATATCTCTCTTAGGCCCTACAGAGACTCACAGGGAAGAAAAATCCCCAAAATCC

CATATTCTCAGCCCCAAAAACCCCATAAAAAACCCTATAAACGGAAGGGCGGTGAGTGGG

TAGGAAAAGACCCCAAATGGACCATTTTTGACCCCAAAATTGCATATTTTCACCCCAAAA

TCGCCATGATTGACCCCAAAATTAACCCCCTAACCCTAAATGGATATATGGGGTGGAAAA

AACCCCAAAAATCCCATATTCTCAGCCCCAAAAACCCCATAAAAAACCCTATAAACGGAA

GGGCGGTGAGTGGGTAGGAAAAGACCCCAAATGGACCATTTTTGACCCCAAAATTGCATA

TTTTCACCCCAAAATCGCCATGATTGACCCCAAAATTAACCCCCTAACCCTAAATGGATA

TATGGGGTGGAAAAAACCCCA

>c5455_g1_i1 len=223 path=[489:0-222]

GATGTCATAGAAGAGTACCCTCTACAAAATGGGAAGAGCAACAAACTCCACACCAGAGAT

CAGAATATTCCAGATCCTACTTTGGAGTATGTTAAGTTTTCTAAGGAGCGGCAAGTTATG

GACAACAGCCCAGAAAAACTGAAGAAAGAGTTAGAGGAAGAGCTGCAGCTAAGCAGTGAA

GATCTGCGTAGCCATGCCTGGTACCACGGACGTATTCCTCGGC

>c5456_g1_i1 len=230 path=[49:0-40 120:41-229]

GATCATTGTGGTCCTTTTCAGCCCGGGCCATTCTGTGATTCTATTTGTCTAAACCAATCA

TGGTTTCAGTGGAGTAACCAACACAATGACGTTGTAAGGCACAAATGATCATTCCTATTT

TGCTTTCTTGCAGTGTCTGCAATCACAAAAGAAGCACTTCAGCACTTTTTCAGTGGTGTC

TTCCTGCTTTTTCCAAATGATATATATCAGTGATTCACTGATGTAGCATT

>c5457_g1_i1 len=206 path=[250:0-114 364:115-121 68:122-205]

GACTCTTGTAAGCATGCTGTTTTTCGTGTTATTTACAAGGGAAGATACAGTTCCTATTAT

GAAGGAGGCGCTGTTTGTTGCTTCACCTATTCAGCTGCAAACATAAAGTTCTGTAGCAAA

CAAAACAAGTCAGCATACATACTGAAACAAGTCACACATACTGAAATTTCTCTTTTTCCC

TGAGGGTCGTCCTCTTCATTTGTCTT

>c5458_g1_i1 len=513 path=[491:0-512]

AGGTTCTGGGAGCCGGACAGAAGGAAATAGCACCTTTGAAGCAGTTAGAACATGACCTAA

GCTGTGTTACAGAAATTAAGTCATGAAAGAATAGAAAACCACATACATTAAAAGAGAACA

AGAGAATCAAACAGGGAAGAAAGGAACGCAGTCTGAGAGCACCAGGCAAGCAGGTGCTGA

GAAGAAATGCAGAACTCTTGGAATATCCAGGATTCAACCACACAAAGAAATTGTCTTCCT

CCACTTCTTAAAAAATGCTGTTATTACACAAAATGAGATTTAGAGATTAGAATCTCAACA

GATAGGAAAAAGGCATTATAGACTAAAAGAACTGGTGATGATGATATGGAAGCTAGGATT

ATAAGGTTCAATAAGTAACCAAGATCCTCAAGAACTATACATAATCTGTTCTTTTAACAG

TTTTCATTGTCTGAGGGTCTTCTATCTGCTGTTCCTGAGCAGTTTTGTTTTATTAAGAGT

GATGAGAAAAATGTAGCAGCCACACTAGGCCCA

>c5458_g2_i1 len=243 path=[981:0-242]

GATTGGAAAAACAGTGGAAGAGAGAATATTAAGAATAATCCCTCTAACCCACAGCATGTT

CAGTGTGATTAAAAGGTGTGGAAGTAATTACTTCTGTCTTTTAATTGTAAAGATGTACGT

GGCTATAGAGTTTTAAATATAGTTGCTGTTAGGTTCTGCTCTGAAGTATTCTCATGTTGT

ATCAGCAGTGCTATCACAGGAAGAATTTCTTCTTGAGGTGTAGATTTTTCTGGTGTGCAG

TTG

>c5459_g1_i1 len=211 path=[477:0-210]

AGGACCAATCCCCAGAGATTTTCAGAACTGGCCAAGAGCTTGGCGTGCAGAGCAAGGGGG

AAGAAGAGAGGCCACCAGTGGATGCTGGATGATAGCCAGGAGGCATCCTACGATGACATT

TCTGTGTTTGTCATCCCACTACACAACAGGGTTGAGGACTGTTAGCATGACAGGCAGCAT

CTCAGTGCCCTCCTGCTATGGTGCTGTTGGG

>c5460_g1_i1 len=202 path=[502:0-201]

GATTCACTCTCTAACCAGAAGTTAGAGCTAACTGCTCTACTTGCTCCACGACACTTTCCT

AATCTCTTGGGTAGAGCACAGGAATCAGCTTCACAGACACTATCTCAGTTGTAACCTCAC

CACTAAACAAGGGACTCCCAGGGCAGAAGCTGCCGCACATTTCTACAGATTCATCCTCTT

CCTTTATTCATGTGAAGCAGAA

>c5461_g1_i1 len=207 path=[1:0-206]

AGTATTTTTCTGCCTTCAAATGCAAACTACCTTCCCAATACAACTGGGCTGAATGTGAAA

TGTTTGCATCTACTAATTACATTCAGACACTTATCCCCATGCTATGACTCCTGCAGGTCA

TCCTGTGATCATATGCATGAAGAATACATGTAATTAACTGTGAAGCAGTGTCTTAGCGTT

ATTGGCATCCAGTGTTTGGGCAGGTAG

>c5470_g1_i1 len=254 path=[570:0-253]

AGCACAGAGATGCAGCAGAGCTGTAACCCTGCTGGCAACTCACTGCAGGGGACATTCCCT

ATGGGATGTGGAGAGGAGCAGGAGGAGTTCAGAGCATCCCAGAAAAGCATGGGAAACTGC

TGATTTTGAAGAAAAACCTAACTGTGTTCTGCTACTCAGGGGCTGCCTTTTCTCCTCTAT

GAACCCATCTCTTTGAGTACTTTCCCAAGAGTATGGTTCTATCCATTTCCTGACCTTACA

TACAAAGGCAATGG

>c5471_g1_i1 len=336 path=[1:0-335]

GTTTTATCTAAGTAAAAGGTGTGCTATTTCTTTTGAAGTCTTTAAACATTTTTTTGTCAA

ACTCTATGGAGAAAAGTCTGCAGCACTCACCTACTTCTTCCATTATAGGATTTATGTGAT

AAGACCTTATCCCACATTTGTAGGCAAACACTGCCATGCACGATTATATGAAAAGTGATA

CAAACATATTTCACTTTCTACTTCTGCTGTGAAGGTCTCAAAGTTTTGAAAGTAAATATT

TCTTATGTTTGTAAAGATGTCTTTGCTTCCATTACCTCCACAAACTCTTAAAAGTTATGA

CCTCTGTCAAGTTTTATATGACAGTGTTTTTAGTTG

>c5473_g1_i1 len=206 path=[1:0-205]

AGTGTAGAGGATGCTCATTCATAAAGACAGAGGGTAAACTAGGAAAGCGTTCAATTATGC

TTTTTATTAAGACAGTTTCCCCATCTGTTACTGCAAGTAAGTGAAGAGAAAAGGCTCTCC

AAAAGGCTACTTTGCCTTCTAGCAGCGATCTTTACCTCTGATACTTGCATCACAGAAAAA

TTTATAGAAGCCTTTAGTATAATGAA

>c5476_g1_i1 len=237 path=[369:0-236]

AATGTCCCATCATTTTTTCGACCCTTCATTTTGTCTTTTAGTTTAGCAAAAGGGGATTTG

GTTTTGTCCTTCATTGACAAATCGAACATACTTGCTGTCATGTTATTCCTCATAAACTGA

ATGTTGACCTTTATTTCTCCTCTGTCTTTGGTTCTTTTCCCTTGTCTGGATTCTAAGTGA

AACCACTCTGTTTTCCTTCTTTGTGTTTGAGGAAAAGCAAAGAAGGAAAACAGAGTG

>c5477_g1_i1 len=202 path=[46:0-201]

AGTGCTACCTAGAACCCTGCAGTTAAGCACCAGCCCTACACCTTTAGTGTTACCTAGAAC

CCCGGTGCTCGGCTGGCCGCGCCGCGCTCCCGTTTTGCTGCAGCACGCTCCGGGCCGCGC

AGTCGGTGCTCTCCGTTCTGCCGTTGGATGTCTGACCTCTCCCAAGCCTTCGGTTTGTCC

ATTTTAGGGACGTTGGACCTTT

>c5481_g1_i1 len=317 path=[201:0-217 25:218-316]

GCAAAATCATTCACAGTGTGTGAGCTCAGGCCTTCCCAGTGCCTAGCTCTCCTATCTGAG

AATTCCCCAAGGTGTCAAGCCAAGGGGATGGTGTCTCTGAGTGTTGTTTTGGAACTGGGT

CACTGAAAAAGCAACGCAAGGCGCACTGAGGAAGTGACTACTTGCTTTGACTACTGCATT

TATTTTCAGGCCCCTCACTACAAGAATGACATCAAGGGGAGCAAAAGAGAAAATAAATGC

TAATTTTAAAAACAGTTATGGGTTAAATGTGTCTGCTGTGATAAAATGCTCATGTTGTAA

CTCACCAGCGTAATCAG

>c5481_g1_i2 len=250 path=[201:0-217 418:218-249]

GCAAAATCATTCACAGTGTGTGAGCTCAGGCCTTCCCAGTGCCTAGCTCTCCTATCTGAG

AATTCCCCAAGGTGTCAAGCCAAGGGGATGGTGTCTCTGAGTGTTGTTTTGGAACTGGGT

CACTGAAAAAGCAACGCAAGGCGCACTGAGGAAGTGACTACTTGCTTTGACTACTGCATT

TATTTTCAGGCCCCTCACTACAAGAATGACATCAAGGGCACTGGAACATGTTCAGAGAAG

GGCAATGAAG

>c5484_g1_i1 len=264 path=[384:0-142 47:143-263]

CCCCGCGGCTCCCGCAGTCCCTCCGGGGTGCGCGGAACCTCCGCCAGCCCCGCCGCCGCC

AGCGCTGCCACCGGGCGAGGAGGCGGTGAGACCATCCAGAACTTTGCGAAAATTGAATTT

CTTCATTTTGGAAAGAGCTCGAGGACGGTTCGTCATGGTTTTCCTTATCTACCCACTGCC

TTAGCTTTTGACCCAGTTCAGAAAATTCTGGCAATTGGAACAAGAACAGGAGCCATACGA

ATGTATCCTTTCTTATTCAGAGCG

>c5500_g1_i1 len=345 path=[339:0-233 18:234-251 18:252-269 589:270-344]

ATGACAGCATTGCCTGTGTAAGCATGGTTGGCATGAAGATTTTACTGAATACAATTAGTC

TTGAGACATGGTTGCTAGCCTAGGCCACATGCAAACCTTTTTTTATTCTGAAAACCCTTT

AAGTGTCCCAGGTAGTGACCTGTGTCCTCCCTGCTTGGATTATGCCAGTGAGAAAGATAC

TTTTTGCTGCAAACTTTTCCTCTAAGTGTTTTACCAGCAAATCAAAGGGATGAGCCACCT

TTTGCTGTCACACCACCTTTTGCTGTCACACCACCTTCCTGGTGTCTCTCAGAAGGCAAA

AGGATGTATGCCTTGTTGTCTTTTGCCCATTTCTGAGAGACACCA

>c5505_g1_i1 len=338 path=[728:0-337]

ATTGGCTTTCAGGGCTCAGAGGCAGGCAGAAGAATGCATCATTTAAATCCAGTACGGTAA

ACCAAGCCAATTCACTAGATAACTTGGTGAGCAATGAATAAGAGTTTGATACTAGTGGAT

ACAAATCCTCTATTTTATTGATTGCTCTCAAATCCTGTACTATATGGTATCTCCCATCAG

ATTTCTTAATCAAGAGTACCAGGGTGTTATACTACAATTTGCACTCCACTAGTAACCAGT

GTTTCATGAATAGGTCAATTATCAGCTGTATTCCTTCCCTATCCTCTGCCCTCAGGAGAC

AGTTTAATCTCTTATCTTTATGGCCTTCATCCCCCTTG

>c5505_g2_i1 len=229 path=[1:0-228]

GAAAGCCAATTACTATTTGCATTTGAATGGGAAAACCCCAAATCAGGAAGGAGAACACAG

CTTACATGGACAGTGCTTCCCCAGGGGTTCAAGAATAGCCTGACAATCTTTGGGAATCAG

CTAGCCAAGGATTTGGAGCAATGGGAGCGCCCCTCTGGGAAAGGAGTTTTATTGCAGTAT

GTAGATGACCTTTTAATAGCTACAGAAACAGAAGAATTTTGCATAGCAC

>c5506_g1_i1 len=216 path=[263:0-123 386:124-215]

GTTCAGAAAGCTTTTTACAACTGCCAGGTTGAGATCGAAAAGAAGTGGATTAGGCTTGAT

GGAGAAGATCCAGATAAGGCTGGCAACTGCTTAATGCAGTGAAAGAGAACAGCGGGCATC

ATTGTAGGGTATTTACACAGGAAATTCTATTGTTAGAAACAGCGCAATGACCTACTTTCA

GATTAAAAAAAAAAAGTCACTGAATATACTTTTAAG

>c5509_g1_i1 len=220 path=[155:0-219]

GTTAAGGTTGACCTCCTCAAGATCAGGGTCGTTGTTCTGTATTCGTTTCAGAGTTTCTTC

CACGTCCGTTGAGTTAGGCTCCTCATCAGGAACTGGTTTGTACTTTGTGGGCTTAATCAC

GCTGTTAAGTCCTTCTTTGTTCACGATGGTGCTGCTACCCAGTGCCTCGTAGTACTGCTG

GTTACTCATCAAGGTGTGCATGCCAAGGATGGCTAGAGAG

>c5513_g1_i1 len=252 path=[155:0-169 96:170-251]

GCCTCCTGACATCACCTGTGGCATCAGATGCTCACAAGAGACTCTGTCACCCATCTCGGT

GAAGTCTGCCAGCCGACCGAATCACTCCGTGCAGTTGTGCAGGAACTGATTATACATCTT

GCATGTGAGGGGGAGAGGGGAAAGGCTGACAGCTGTAACGAGATGGGGATGAGCTGCGTG

GTGACTGAGTCTTTGTTTCATGAAACTGGTGGATTCAAACACGCCCAGGGTCTGAGTGTT

GAAGTGTGTACT

>c5518_g1_i1 len=234 path=[185:0-233]

TTTGAAATCACTTTCTTTATACTATTTGGATGGTAGCTGACATTTGCTAAACTTAATATT

GTTACCACCTTTACAATTGATCAAACCCTAAATAATGTGGTTTCTCGGGGAGCCCAAAAA

TCAAATGCAATGGAACCTGCTCTTGACTAGGTGTTGTTGCAAGAAACTCTTCAACCTGCT

GGATGTTCATAGAGTAGATTTTGAAGTCTGCCCTGATACAAAGTTTAGCAAATG

>c5519_g1_i1 len=239 path=[1:0-190 192:191-202 192:203-214 192:215-226 192:227-238]

TTCTAATTTTTTTTTTTTTATTATTTAGAAGATCATAAATATAAGGAATTATATATATAT

AGGAAATTAAAATGAATTATTTTCTTTTTCACAAAATTAAAGAAAAGTGATATAATTAAA

AATATAAATAGCTTAAATTATCATTTTTTTGTAGATGTAAATTATGCGTGTTTTTTTAAT

TATTCGTGTAATTTTTCTTAATTTTTTTCTTAATTTTTTTCTTAATTTTTTTCTTAATT

>c5524_g1_i1 len=374 path=[506:0-373]

GAATGAAAATCTGAGGAACTTGGAGGACATTTAAACTTTGGGAGTCTCTTTTCCTGAAAA

TGAACACTAGATAAAGCGTATTAATTCCTTTCAGAAATAAACAGAAGTGACAAGTCAGCC

ATCAGGGCCCTCAGAAAATCAGTCCCTAATACATACTAATTTGTTGCTAGAAATGACTTG

ATTACAATCCTTATTCTAACAGATGTAACTCACTTCATATCAATTGTCACTCTGCAGCTA

AACAGAACAGCAGACATCAACCTTAGTGATGTTCTCACACCTATCTTAAACTCACTCATA

GAAAAGATCTATTTGTTTCATAGACAAATCCCAACAGCACAAATGAATTCACGTGTAAAT

CAACTTTACAGCAC

>c5525_g1_i1 len=426 path=[363:0-376 29:377-394 797:395-400 53:401-425]

TTTTAAATGAAATAAGTACTTTGGGAATTGTGTCATGTTGTTACTGGAAACAGAGCATTT

TCCTGGACAACTGGAGGACTTGCTCAAGCTGACAAGTGTTTCCTGACACTTTGTAGACTT

GACTTTTCTGTTGTCTTTAATTGCATAGAATAGGTAGTTATTAAAATGCCTTTGGTCTAT

AAGATCTCTTACCTTCATATGACCCAGGAAGTGAAAATGTGGTTCAGTGCAGAAACATTT

TCTTGAATTGATAAGAAGATACTGGTAGCTCTTCTCTATGAAGGTTGTTGAAAGTATAAT

TCAAAACCTAGCAATGAACTCTTGAGCTGGGAAGAAAAGGCAAAATGTAAGTAACATATA

TGTATGTTACTTACATTTTGCCTTATTGGTAAGGTGAAATCCATTACTGTGCTGCTACAG

ATGTTG

>c5527_g1_i1 len=394 path=[155:0-393]

TTTGTTGAGGGTGCAATCTGTCCCATCATCCAGGTCTCTAATGAAGATGTTAAATACTGC

TGGCCTTAGCAGCAATCCCAAGGGTACATCAAACGACTGGCCTTCAGTTGGACTTCAGCC

ACTGGTCACAACCACTGAAATTTAGCAATGCAATCAGTCCTCAGTCCACTTCACTGTCCA

CTTATATAGTACTTCATCAGCTGGTTGATAAGGACATCATAGGAGAATTTCAAAAGCATT

GCTAAAATCAAGATAAACATCATCCACTGCTCTCCACACATTCACTAAACTAGTCATCTC

ATCATAGAAAGTTATCTTCCTGAATGTCATTTCCAAATGTATTAAAGACTTGAGTGACTG

GGAGTAGTCAGCATGGATTTATGAATGGGAAATG

>c5528_g1_i1 len=300 path=[392:0-299]

ACTGCAAGGAATGACTACCTTTTATTGTGTTATTAATACACATTGTGTAAGATCCACTCA

GTGTCACTTATTAAGTTCTTGTGACTTTGGCCGTGTGATCAGAAGAGTTAAGTTGTTGTT

TAAGTACTGAAAAAACTTGTATAGCTTTTCTGTTACGGAAACCACACAGGACACAGTAAT

GTGATTTAGATTTTGCTTTCTTAGGGAGAGCATTTTAATGTTAACTCTTGGAGCCAAGGG

ACTTGACTGCTGACTTCTGTAGCTGTAGGCATTTCTGCTGCCTAGTTGCAGGTAAATCAG

>c5531_g1_i1 len=717 path=[736:0-716]

AAAAAAAAAAAAAAAAGAGAAAGATGAATAGAAATTTATTGTTTAAAATTGATGATTTAA

GAAAAAATAAATTTAATGAACTATTGAAGTATGGCAATAATGTAATTAACAAAAATGTTT

CAAATAATTTAAATGAAAGAAAATTAATATATGTTGGGAAACCAACACATGGAAGTGATT

TTTACTGGAAATGTAGAGAAATGATATCTTCTATTAAAAAAAAAACAAAAAGGTGTGCAA

GATATTTAGATTTAAATCATGATTTTTCAAGGGCTGCTTTAACAGGTGTTCCTAAAACAA

GTTTTGACCCCAAGAATAATATTTTTTATATTCAAATGGATAATGAAGAATGGGCAGGAC

TTGGTAGTAAAATGATGCTAAATATATCAATGAACATATTATTTTATTTACACATATCTT

TTTGGATATATTTTATATATTATAGATTATTAGTAAATAATAAATTAAATGTATTTAGTA

AATGGAAAAATAAAGATTAAATGTAACGAAATAAAAAAATATATATAATATTTTATGTTG

ATTATATATATTTTTTAGTGATTTAACAAAAATATAATTTTTTTCTTTAATATAAATATT

ATCTTTTATATAGTATTTATTTTTATTAGAGCTATTATGTTTTAACATAAAATTATATAT

TTTGTGTATTATACTTTTAAATAAAATTTATTTTACATTATATATATATATATTTTT

>c5536_g1_i1 len=472 path=[450:0-471]

GTCACTGCAGCCCTTCAGAAGCTTCGGCAGCTATCTGTACTGCTTCAACTCTCTGCCTTT

GCTCACACTACCACGCTGTGCCATATACCCGGTATGTCTTGCACTACATGATTTATTTCA

TGCTATCACCACAATCACACATTTTTAACCTCATTATCCTACAGAAACAGACCTACACAA

CTGTATGCTGGCTGCACACCCTTCTCTCTGTTCTCCTCTAATTTTCAGTCCGTGGACCAA

TTCCTACTAAATTCAGCAAAACAAAAATCCCTGCAGTATTGATGCGCCCTTTCTTACACT

GTCATTTATTTGCTATCTGCTGCTATGGTCCCCAGGATCATTTCTATTAGCAGGCTGTCA

CATCACGTTGTTGGATGGGGGAGTGGGCACAACATCACACAGCCCATAAGAGCCCACCAT

TTTGCATGGGAGAATTTTCAAAGGCTGTTTTCTTTTGAGTCACCATTGCGTG

>c5538_g1_i1 len=206 path=[155:0-205]

CAGCCGCCCTGTCCCATCCTGCCAAGCCCCCCATCACCATAGGCTCACCCTCTCTGGTTT

GTTCTGACCATGCTCCACACCATGCTCCATCCTTTGTGGGAGAGGCAAACCTCAGAGTTC

TGATTTGAAGGCTCCAGGTCTGAAAAAAGAAACCATCTGATGTCTTTGGGCTGCATGGGT

AGGGGATTTTCCTCCAGGGGCTGAAG

>c5543_g1_i1 len=548 path=[193:0-547]

ACAAAGCGCTGAGACACATGGCTGTTAATTCCCTTTTACCTCTGCGCAGCCCTGTACAGA

ATATTCAAAACTCTGACCACATCTGTGTTTTTGTGCTGTTATTCAAATCCCTCCATGTAT

TTTTTTTTAAGCAGCAAAAAAGAAAATTTCTAAAACGGCAAGGAAAAAAAAAGTAAGAAG

TGTTTGCTTATTTCTAACACAACTGCAGCCCTGGATAGGACCTGAGTGCCAGCCCAGCAT

CTCAGCACAGAGACCGAACCACTGGCTGCAGGGCAGTCGGAGGGGCCCATCTGAAGGAGA

GCAAAATGAGTTTCAGCGGAGGTTTTGCTCTGACAGCAGCCCTTCGGGAGTCCCATCCTC

GATCTGCTGGCTGCCAGGCCCTACTCGTTTGCTGAGGTATTGCTAAAATCCTTGGTGAGC

TCTCGTTGCTCAGAGCTGGGCCTCGCCGAGCACCGAGGTGCTTTCTGTGGGCCAGGGGAG

TTCTCGCCTCTGTGTGCCAGGACTGAAGCCTTGCTTAAATGTGCCGCAGCGCTTTGTTAG

TGGTGTAG

>c5550_g1_i1 len=204 path=[1:0-203]

GGCCAGGCTTCCCTTCTGCCCACTTCTAGGACTGCAGCATACTGTGAACTGTAACTCTGA

AAACTCAGTTTCCTCTTAAAGTTAAGTGAAAAGGCACTGAAGTTCGTACATTAACAACAT

AATCAACACGAGTATTTAATTGCCACCAAATTGGGTTCTTTTTTTTCCTTTGTCAATGTA

GGAAAGAGCACTAGAAGCCTATGT

>c5553_g1_i1 len=221 path=[489:0-220]

AGCGTCAGAGAACCGCAGCACAAATAACAACCAAAACAGGCAGTTGCAATGCAGCAGGCA

CCTACTGATCTCTGCACTCATAAAATCCCAACTACTCAAAGGCAGGGGAGATATTTCCCC

ACAACGCCAGGAACCAAAGTCAGCTGAATCAGGGACCCTTTGCAACAAAGGAATTGGGTC

ACAGCAGCAGTCAGGATCCAGAGCTGCCCTGTCTTTCAGAA

>c5556_g1_i1 len=301 path=[1:0-300]

GGAAACCGCTCCCCTTATCCTCGCTTGATCTTTGGGAGTTAAATATGTGCCCTTGTTGGC

AATAAGAGCATCGCAAGGGGTGGGGGGGAAGAAATGTTAACACGGATGTTTACCTTAATT

TCTTGCTTTGTGTGGACTGGGGAAGCTGGAGAAGAGAGGAGCTAAGGGACACAGGTCTGC

AGAGAAGGGAAAAAAGCTCTCTGAAAGCCTGCAGAGGTTGTGTCGGATTGAATCTGTAAC

TCCCTTGATTTTTGCAGTGGCTTTTCTTTGTCCTAGCATTTTTTTTTTAATTGTTATTTA

A

>c5561_g1_i1 len=264 path=[396:0-263]

AAAGATAAAGCTGCAACCTGGATGAGGCAATGACAAGGCCAAGGGTCTGACCTTAACAAC

AGCTCCAGAGTGAAGGACTGGGCCTGACCCTTACACTGCAAAGGGAAGTAATTCTACTAG

GAAAGCAATCCCACACTGCAACAGGTGATGTAAAAGGACAGCCGTAACAGAAGTATGGAT

TGAGGCAAACATTGACCTGTTGGCTGAGCCTGATGGCTGTCACTAGATTTGTTCTCCTTT

CCTGGTTTGGTGCTCTGCCCTTTG

>c5562_g1_i1 len=273 path=[251:0-272]

TTCGTCTTCAAAAAACACTCCAGGGCACGAAGCTCTGAGTACAGAAAATCCCATTTGAAT

TATTTAATAACGGTGTAAATAATCTCTAGCAACAAGAACAGGGCCTTCTCTCCCATCTGT

CACGCACGCTACTGTATCAACAACCCTACGACACTCCGAAAGCCTTCCATCCAAACAATC

TCTTGTCCTGAGAATGCACCATGGGGGAAGAGCCAGCCTGCACGCCCCTGGGTGGCGGCA

CTGCCCCCGGGCAGAGGATGGTGAGAGGGAGAG

>c5565_g1_i1 len=747 path=[758:0-280 17:281-746]

GTGTTTGATCCAGGACATCTCAAACGATGCACTATTTAAACCACACAGATATCCTTTACT

GTGCATCTGAAGACATCATCAGATTTGGTGGCACAAATGTGAAATATCTACATAAACTTT

CATGTAGAACTCATTGGAAGAAACAACACTAAATCAAAAAAGATGACCATAAAGGCAGAA

GGAATTACCAACGCAGAGGAGAAACATGCACTAACTTTAGAAAGACACAGAAGAGAGGGA

ATGCAAGGGTAAATAGCGGGGTTACTAGAACTGATGACACCGCACAGCAGTTTTGCCTAT

CACTTCTCCCCCTGTAAGGGGATACACCTATAAGGATCACTCAAACTTTCACGCTGATTA

CAACTTCTTCTCTACAGTCTCAACAGTTCTCTCTCTTACTTGCTTCCACTCCCAAATCTG

AGAAGACTTGCTATGGAAAGCAGATGCATTTTGTAAGTATTTAACTGATGTAAGCTTTCA

TAATTATTGTTTAAGGTTATGGCATATCATTTGAGAACCCTGGTGCAATTCAGAAGTCAC

ACAGTTATCCAGGTCAGGACTCAAGCCACCCAGACATCTTACTCAGGAAAGAGAGCAACA

AATCAATTACACAATCATGACCGATGGGTGTTTGTGCTACTCAGAACGGGAAATGAATCT

GTGCCTTGGCGTCATTTATACTACAGAAAAATGCTAAAAATGCTGTGCTGTAATCTGTTT

CAAAGACTGAATATTTGAACGTGGCTT

>c5568_g1_i1 len=213 path=[238:0-164 402:165-212]

ATTTGAATGGGGAAAAATAAGGGGGAAAATTGAACAAGTGACAAAGTATCCAGATGTCTC

CCCATTCAAATAGGTCTTCTTTCTCCTCCTCTTTCAGTTTATCTTGGGTTTCAATCTCAT

CCCTCCTTTCACAGATGTCCCTTTTCTCTTTTTCTGATGGTTCACTAGGTTCGGGCAGCT

CTGGTATGGGCTCCACATCACCACCAATCGTTA

>c5568_g1_i2 len=213 path=[238:0-164 25:165-212]

ATTTGAATGGGGAAAAATAAGGGGGAAAATTGAACAAGTGACAAAGTATCCAGATGTCTC

CCCATTCAAATAGGTCTTCTTTCTCCTCCTCTTTCAGTTTATCTTGGGTTTCAATCTCAT

CCCTCCTTTCACAGATGTCCCTTTTCTCTTTTTCTGATGGTTCACAGTTTCTACCAGCAA

TGGGAAGGTAACGATTGGTGGTGATGTGGAGCC

>c5572_g1_i1 len=209 path=[130:0-127 258:128-208]

CTCAGCTTCATACTGTTAGAAAGAGGCACAGCCACAAAACATGGGTGTGCTCTGTACTCA

GAGATGGCAGTGACTGTGAAGGTACAGTGCTTTTTCTTGCTGATATGAAACCTCAGCCCA

TAAGCAGGAGCATCTATTTTTATTATTAAACACATATTCTACTACCTAGTGTACGATGCA

AAGGCACTGAGCTAGTAAACTAGGTAGTA

>c5573_g1_i1 len=225 path=[31:0-105 15:106-224]

TAGCCTGTGAACAGCCTGAAATTGTGATCTACATTAATGGCCTTGACTTTCTCTGTGTGA

TTTGGTGGGAAGTAGGCAAATGGTTCTCAGTGATTTGTGTCGTATCCCTGAGGCGAAGTA

CCTGTGTGGTGTTGGACTGCCTAAGCTGGCTGGAATTGGACTGAGGTGGGAAAGTAGGAC

TCTCCTTTGACTGCCCTGGAGCTGCATGTAGCCAGGAGGCCACTG

>c5576_g1_i1 len=270 path=[297:0-123 25:124-269]

GTCAGTTTATGACCGTGGACTTAGATTTGGTGTGGTACAACAAATATGGACATATCTTCT

TAAGAGTATTTAAAATTTTCAGACACATTATCCAAATACGTATGTAACTGCATACATATG

CGCGCTGTACTCAAGTCAGTAGGGTTCTGGATATAGAAATATCCCACAGACATCTTACAG

AAGAATTAAAATCTTGGATTACATAGATGCTAATAAATTTTAATTCTTATACTAGCACAG

AAGTCCTCTTAAGAAATTAAGTCGCTCCTG

>c5585_g1_i1 len=351 path=[789:0-350]

GGAACAGAAACAAAAGATACTACAGGATAAATTAGACTGAGAAATCAGAACAAACTGAAA

AATGCCTTCCCCATCGCCCCCCCACCCAAAAACCCATGTAGTTCTGCACAATAGTAGTGT

TGGGTGGCTCCACATAAATGTGGTGTTTTTTTTTTTTTATTAGAGTATTTTCCTTTACTT

TGTTCACCAGATTTGTTTCAGGAGGAATACAGCTGCTACACAAAGAATAATGCCAGAAGT

TTTAAGAGGGTTAATGCGGTACTGTAGAACAAGGAATGTCCAAGCATGAAAGCAAGTATT

TCTTGTCCTGTTGTACCTTTGTGCTCTCTCTTGTGGTACTGTTGCTGGGTT

>c5585_g2_i1 len=253 path=[231:0-252]

TGCCACATCTTGCCTCAACAAAAGTATGGTAGTTCACTTACAGGTTGGAACTGCACACTG

ATCCTGAGGAACAGATTCCTAGAGCATCACTGGAAAGAAGTTATGTTACACCATGAAAGA

AGAAAAGTATTTTAGTGGACCTTAAAACAAACAAGAAGTATTTTATGCATATAGCAGATA

AAGATGACTCAGAGATTAAACTAGGGAACGATGTATTGCTCAATCTATTTTGGTTAAAAA

AAATAATAAGGAA

>c5586_g1_i1 len=335 path=[28:0-25 54:26-334]

GTAACCTTGTGTTTGAGTTGGATAGGTGCTGCAGCAGTTACTGGACAGTTCTTATGCTTC

TTTTTAGTGTATGTCTCACACCTTCATGTTATGAATCAAAGCTACGCTTGGCTCTGAGCA

AGCTGGATCTGCCTTTAAATGCAGTTAACAGTGAACTCACCAATTTCAAAGGCGTGTTGT

CACATGAAGAATTCTTAAATAATCTCAACGCTGTGAGAAAGTTGTGGAGCTTTTGGAACT

GGAAGATCGTGTAGAGGTTTTGAAATACTGGATTGGATTTTGTTAGCGCATATTTAGCAT

TTTTTTTCTTGAACACCTATGCTTGGTAAAGTAAG

>c5586_g1_i2 len=438 path=[55:0-128 54:129-437]

CACGGCACCATTATCCAGAAGGAAATATGAATAAAGTTGTGATGTAGTTATGAGGGTAGT

TTTGTGAGTGCCAGTGAAACGAGCACACTGTATTAGAAGTTCTTATGTGCTAACCATGGT

AGCTAAAAATGCTGCAGCAGTTACTGGACAGTTCTTATGCTTCTTTTTAGTGTATGTCTC

ACACCTTCATGTTATGAATCAAAGCTACGCTTGGCTCTGAGCAAGCTGGATCTGCCTTTA

AATGCAGTTAACAGTGAACTCACCAATTTCAAAGGCGTGTTGTCACATGAAGAATTCTTA

AATAATCTCAACGCTGTGAGAAAGTTGTGGAGCTTTTGGAACTGGAAGATCGTGTAGAGG

TTTTGAAATACTGGATTGGATTTTGTTAGCGCATATTTAGCATTTTTTTTCTTGAACACC

TATGCTTGGTAAAGTAAG

>c5587_g1_i1 len=249 path=[155:0-248]

GCTTAGTGTGCCACCTGCATTTGTTATCTTGCCACAGACAATTTAATCAGTTATAATTTT

TTTTTTTGTTTTGAGTTCATCTGAATTGTGAATTGATAGGCATGAGGAGAGTTCTTACAC

TCAGCATCAGTACAAGTCTATTGAATCTAGAAGTATCTAGGCACCTATGTAAAATCAAAA

TAAATGTCTGCACAATTCCCTTTGGACTGAGATTAGTGATTTGGCATGATATGAAATCCC

TATCCTCTA

>c5589_g1_i1 len=244 path=[176:0-82 258:83-243]

TAACTTTGTGTTTGATGGGGAAGAGTGCTTACAGTTTTGACATGTACAACATACACAGTT

GAACTTACCACTGTTCAGGGTGTATTTCAGCAAATTCGGTTTTCAGCTCAGAACTAAGTC

TTTCATTACCACTGCTAAACTCTGTTCTATGTAATGCTGAAGTCCTGTAATCTGTTCTTG

TGACCTCTATCATCATCCTGGTTTTCACTCAGCCTTTTCTTGTAAAGAACAGAATCACCT

GATA

>c5596_g1_i1 len=232 path=[155:0-231]

TAAAATCTCCCTGTTCGGTCCCCTCTTGCCTTCAGCCACCCGGCACGGGTCGCATGTAAC

CTGTCTGTGGTCCCAGTGACACACAGCTGCTGGAGCTGAACCGCCGTGTTCCAAACACAG

ACTGGGGCAGCTGCTTGCGCAGGAGTCATTGTGGTGACACTGTTGGTACAAGATCTGCAT

TTGTTTTAACTCTGCCGTCATGTACGGTAGAAACCACTTCGCTCTGCTACTG

>c5597_g1_i1 len=205 path=[183:0-204]

TGGACGCGGGGGGGTTGCCAACTCGCGGATGCCTTTTTATTCCTCTGGGCAGTGTCCGGA

GCCTAACTGGAGGAAGATTTTGGCGGGCTCGTTGCTCGCCGCCGCGTTGCACGTGTTGCG

TCTCTGGGGGGTTCCTCTCCGGACCGGAGGCCCTGGGTGGGCTTGGTCTGCCCCTCTGTG

GGACCTCCTCCCTATTTTGGCACTA

>c5601_g1_i1 len=221 path=[1:0-220]

ACCACCAATAAGTAGTTAATGTAGCTATTCTTCAAATTCTAAGAACCCAAAAAGCATGTA

TAAGAGCAAATACTTGTACAACTCAATAACCTTTAATAGGTTTGAAGAACTCAGGTACTA

AACTTCTAAGATTCAAAACACATTTGCAAATTCAATCTCAACTTAGACAATTGAATCTTA

GCATATCTGTACTGAAACATTTGAAATTGCTGTTAGTGGAG

>c5603_g1_i1 len=913 path=[25:0-41 25:42-83 25:84-125 66:126-912]

CTCATTTACTTCATTACAATTTTTTCTTTCTATTTCTAATTTCTCATTTACTTCATTACA

ATTTTTTCTTTCTATTTCTAATTTCTCATTTACTTCATTACAATTTTTTCTTTCTATTTC

TAATTTTTCATTTTCTTCATTACAATTTTTTCTTTCTTTTTCAAATTTTTCATTTTCTTC

ATACTTCATGTTTTTAATTCCTTGATTATCAATACTTTTTTCGCAATTATTACTCTTTTC

GTATTTATTATTATCTTTTTTTAACAATTTTTCCAATTCATTGTTTTTTTCTTTTTTTTC

TATTTCTTCTTTTACAATATCAATTATGCAATTTTTATCGAAATTATTTTTTTTATATAC

ATCAACTTTTCTATACTGATTATTATGTTTTCCTATATTATACTTAACTTTAGAATTATT

CTCAGATTTAGAATATAACTTTTTATGTAAAATGTTTTTTCTTCTTTTAATTAAATATTT

TTTCTTTATCATTCCTATATCTTCATCAGAAACAGCACTTTTAGACAAATTATAAATTTT

ATTCGTATTCATTTTTCTCTTTTCTTTTATTATCATTTCGTCTGTATATTCTCTTCCTTT

TCTTTTATTTTTTCCATAGTTAAAGTCATTTTTACAACTAATATTATGATTATTTTTATA

AAAATCTTTTAATTTTAAGAATACTTCATTACTATAATTTTCTTCTTTTAAAATATCAAA

ATTATTACACTTCAGTCTTTTTTTTATATTGTCATTTTCTTCATAATTTGATACAAAGTT

ATCACACAAATTGTTTTTTTCTTTACCAAAAGTATTTCCAACTAATTTTAAATTAATTGA

ATTATATGAACGTTTTTCCATATTAAAGATTATAATTATTTTTTAAATGTTTATACTTTT

TTTTTTTTTTTTT

>c5608_g1_i1 len=287 path=[330:0-106 64:107-130 88:131-196 64:197-220 88:221-286]

GTTGCATTGCACTGCATTGCGTTGCATTACATTGCATTGCATTGCGTTGCATTGCATCGA

GGCACGTTGCACCGCATTGAGTTGCCTCGAGGCGCGTCACGCCGCACTGCATTGCATTAC

GTTGCATTGCATTGCATTGCGTTGCCTCGAGGCGCGTTGCACCGCATTGCATTGCCTCGA

GGCGCATCGCACAGCATTGCATTGCATTACGTTGCATTGCATTGCATTGCGTTGCCTCGA

GGCGCGTTGCACCGCATTGCATTGCCTCGAGGCGCATCGCACAGCAT

>c5609_g1_i1 len=343 path=[65:0-217 64:218-342]

CACTTTGCTGGACCCGGAGCAGGAAGGGGTGGAAATATGGGGAAAGTTGGAAGTTGCCTG

GTGACTTTGGCCGGACTTTTGCTGGCGGCGGGAGCGCAGACGTTCACAGGATGCTGTACT

TTCGATGAACCGTTTAGCACCTGTGGCTATAGTCAGTCAGATGATGATGATCTTAACTGG

GACCAGGTGAACACGCTGACAAAACCCACCTCAGATCAATGGTTGCCTTCTGGTTCTTTC

ATGTTGGTGAATACATCTGGGAGATATGCAGGACAGAAAGCTCACCTCCTGATACCCCAT

CTAAAAGAGAATGATACGCACTGTATTGATTTCCACTATTATG

>c5638_g1_i1 len=219 path=[57:0-191 313:192-195 318:196-218]

GGCCTCACATTCAAGTCCCTCCTTTTCTCCATCACGCTCGGCAGCAGATGGTCGCAGTGA

CAACATATGCTGCTTCAGGTGTTGCCACTGCTGCCTCTCGTGCTCACAAGGAGTCTGGCA

GACAAGGGAGCAGAAAGGTGGGACAACCAGACCAGTGCAGACCCAGATCTCTCAGCCAAA

TTTCTTCAGCACAGCAGGGAAAACACAGGCCCACCGTGC

>c5640_g1_i1 len=480 path=[29:0-178 207:179-179 28:180-479]

AAAAAAAGAAAAAAAAAAATGTATAAAAGAGCTATATGTAATCGTGAATTAAAAGGGTAA

AGCATAAAAAGAATAAAATCTTAAATTGAAGTGTAGAATATTTAATCATTAAAAGGTTAT

AACATTGAAAATTAGAATTATAAAAATGTGTGTCATTGAAATGTTATCACTTTCACATGT

TTTTTTTTTTTGAAACTCGAAAATGATTTTGTAAACTCAAAAATTTTTTTTTTTTTTAGT

AATATTTAAGTTTGATTATTATTATATTGCATTTTTACAATTTTATTTTTAACTTCTTAT

TATTTGTAGCATTTTAATTATTGTGATTTATTTATTTTTTTTTCTATTATTTCTAAATAT

TATTTTATATTCATAGATTTTGTAATAATTTTTGGATTTTTGAAATTATAGAAACTCTTC

AACTATTTATCATATAAATATCACTATTATGCCTACTTTTTTTTTATTTTTATTTTTTTT

>c5642_g1_i1 len=361 path=[596:0-118 258:119-360]

AAGATAAATATGTAATATTCTTATCAATAGCTACATAGTGATAAATGTATAGATATGCAA

ATTCTTTAAAATATCAGTAACATACATAGAACAAATAATATATATCAAAATAATTAAACC

TTCATTAGCTAATGTAAGAATAATCATTGAAGCTTAATAGCTTGTCTATAAATAATATTA

CAGACCTTACTTTTATAATATTTAGAAAAATAAACAAAAAAAAAATACTTCATTTTGTTC

ATACTAAGTCGCAGGGATAATATTCAATCATTGGTTAATAAAAGGCATTATTAACCTTTT

TGGAAAATTAAAGGATTATTTAAATGAAGAATACGAAGTTAGTTATGCACACGCACACAA

A

>c5644_g1_i1 len=337 path=[247:0-336]

AGAACACATTGAAATTTCAACTTGTTCACCGGAAAGCCATTCCAGAACATGCCAAGACTT

GTAGTGTTGATGTCAGCCTGAAAAGTTTTCCAACACGACTCAAGAGAGAAACTGCAATGT

GTTCTCTGCCACACATCCACCTGAAGCACAGGGTAGTGCCGTCCTATCACCAGTGTTTTC

TGTGCCACTATTTTGCCAAAAGGCACCTCACCTTGTATTCAAAACAAGTCTTTGTCACTG

TACCATGAGTAAGAGTGAATATTAATTCAAAGCTGTACACAAACTTGGTTGGATCAGCAC

AGTGTTTGATTCAAGAAACTTTGAGGAGAGTTGTAAT

>c5645_g1_i1 len=458 path=[430:0-457]

AAAAAAAAAATTATAAAATAATATGAAAAACAATATATAAATATATATATGCTAATTATA

AATAGACTACAGAAATAAAAAATATCTTAAGAAAAAAAAAATAAAATAAACATACATTTT

CTAAAAAATATCAAGACAATTTTTTAAATAAATTTATTATTATAAAAAATATTTCCATAA

GAAACCTATTAAAATATTAATTAAGCTGGAAATGTATCAGTTGTATCGAAATATTAATCA

TTTTAAATAATTCATTTTTATTTTTAGCTAAAAGGTATATATTTGTTCTCTAATAGATTT

TAAAAAAAATTACAAATAAAATTAGTATTTCATAATATAAGGTTGCATTTACATTTAAGA

ATTTTGAAAAATATTCTTCAAAATAATAAGGAATAGATGCATATTAAGCAACATTTTAAC

TTTAATATAATGTCATCTTTTTAAAATATATTTTTTAT

>c5645_g2_i1 len=230 path=[208:0-229]

AAAAAAAAAAATAATATAAAATAAATTTAACAAAAAATTTATTATTTCATTATTCAAAAT

TTTCTAAGAGATATGTAAATATATGTATTCAAATTTTCTTATTTTTTTTTTTAAATAAAC

AAATATATATAAGTAGGTGCATAAGAAATATTTGTATTTCAAATCTTAAAAAAATAAAAA

TAATATCTTGCATATATTTTAGTAGCAAAATTATTTTTCTTTTATTTTTT

>c5647_g1_i1 len=382 path=[513:0-30 544:31-119 244:120-143 268:144-381]

TTTGTATTCCCTTGAATGACGCAGGAGGAACTAGGGATCTTTCTGTTCAGGTATATATGT

TGATTTGGGCTCAGGCAACGTGAAAGATGACAGAGGTTTATCCTAAGGACTGGTGCTAGC

AAAAAAAAAAAAAAAGCTTTGTGGTATGAGGCAGAGGATTGCATTTCAACCGTAGGGTTT

CCAGAAATTTTGTAGCAGTTTCATTATAATGTTTAATTGAGTTTGGATCCACATAAGTGT

ACTATTTGTGTTGCTTTCTTTCCCATAAAATTGCCAGTAAAAAATTAATCTCTTCAGTTC

ACCTATGTCTTTGGTTTGAAAGAATGACAAAAGCTGTCAAGTTGGTTTGGCAGCCAGCTT

CAGCTGCCCTGGTAAAGCAGTG

>c5657_g1_i1 len=238 path=[1:0-237]

AGGTCACTCCACCTGCCTGGATGAGCCCTGACCACTACCTAGATCTCTGCTGGGCTTACA

CTAAAATCCTTGAAGTACCAGAAGCAAAACGTCACTGATGTTACCAGCACTGCCTACCAG

CCTGAGTGTGTGTTCAGAAAACAAACAAGGATTCCCAAGAACAGCGATGCAGCTCCGATC

AAAGTTCTCCTCTGGGGGAAGGGTGGGCAGGAGCATCGGTAGATGCCGGATGCCTCCA

>c5657_g2_i1 len=222 path=[630:0-221]

AAACATTTGAACACACTGCTCATGCACTGAGGAAATCAAGCAACTCTTTCAGCTTAACAG

CATCAGGACAACTGTACCAGCACCGGTCACAGTGCTTGCACATACCAGCTGTGAGTATTT

TAAAGTAAGCTGTCCCAAAACTATTCTCTGTAGACCACAGTCTCTTTTTCCACCACAGCT

AAAAATCAGTAGCCCATATGACTGGTGGGTTTTGTTCTGCTG

>c5661_g1_i1 len=1628 path=[1685:0-1627]

AATAATAATAATAATGAAAGTAATTATGAATATAATATAAATTATAGTGATGATAATGCA

ATTAAATTAATATTAGAAACAAATTATTTAAAAGAAAATGTAAGAAATGAAAGTGTAGGG

AATAATATAAATTATGATGAAAATAATAATGTGGTAAATAATAATTTCAGTTTATTTTTA

AATAGTGATGAGTTAAGTTATAATAATTTATCTTCCAATAGTAAAATTATTGATATGAAT

AATAGTAATGGAAATCAAGTGAATGATTTTAATAGTGATACATATAAATGTATGAATGAA

ATGGATAATTTATTAGATAAAAATATAAGTAAAATGGAGAATAACTTTAACAATAATTGT

TATAATATAAATAAAAACACTGACTCATTTATGAAATATATAAAAAATTTTCTTGAAAAA

AATTATTTAAACGATCAAAAAAATTATATGAATTTATATTATAAAAGTAACATGAATAGT

AACATTACAACAAATAATACATCAGTTATAAATAACGAAGATTATTATAACCATATAGCA

AATAATATTTTTGATAATAAAAATGTGAATATATCTGATATTAATAATGTTAGAAATTTG

AATGCTTCTACTGGTTACAATAAGAAAACAAATGATAATGTTAATAAAAACATTTATAAC

TTTGATATGAATAATAATACGAACAATATTAATAATGTTAATTGGATAATAAATGTTAAT

AAAATTAACGATGCTAATAATAACAATATAGGTGACATTAATAAAAATAATGTTATTAAT

GATACTAATAATGTTCATAATGATAAAATGTATGATAACGAAAGAAATATTAACCATATT

AATATTACCGATGTAAACAATGTAAGTGTATTTATGAATAAAAAAGAGAATATTGAATAT

TCGAATTTTTATAATTCTTTGAATGATATAAAAAATGAAGATATGCATACTAAAAATAAA

TTAGATAAAAATAATATAAATGATAAACAAAATTACTGTTCAAATAGCATTAATTATCAT

GTAAATGATTATCAAAACTCTATTATAAATGATATGAAACATGAAAATAAAGATATTAAT

ATTATTGATTCTTTTAATATGGTTAATTTAAATAAAAGTTATAACAATAACAATGATCAT

ATAAATTCTGGATATTTATATGATAAATGTAGAAAAAATGAAACATATAATAACAAACGC

GAATACATTTTAAATGATGAAGCATCTTTACATTTAAATAAGAACACAGAAGCAAATATA

GAAAGGTGTTTTATGAATAATGTAAAGAATTTGGACAATAATTTTAAAAAAAAATTATTA

AATGATTATACTAATACTATGAATAATATAGAGTTAATAAATAAATTAAGTAAATCTGTA

GAAAATACTTTTTTGAAAGATTATAATAATTATAAAGAAAATATGAATTATTATAGTAAT

ATAAAAAATGAAAATCTAGATATTTTGCATAAATTGTGTGAAAATCCTTTATTAAAGGAA

AATAAAAACATAACAAATTCTAAGGAAACTGAGATAAGAAATGATAACATTTTATTTTTA

GATATAAATGAAAACACAAATATAGGTAATTATGTATATAATAAACAATATAATAATTTA

AAAATGAA

>c5661_g2_i1 len=584 path=[3351:0-583]

TAATGATGATGATGATGATAATGAGCAAAATAAGAATTTAAATGAAGATATATATAACAA

TGCAAACAATTTTTTAAATGAATATAATATATCTAGAAATGTAAATAGAAATGAAATTAT

TAATGATGACGAAAATGCTAATCATACAAATAATGATGCAGATGGAGATGATAATTTTGA

TTTAATAAATTGTGATATGTCTCTTTTATCAAATTTTAAACATAAAATAAAAGTGAATAA

AATTAATGATATACATGAAAAAAATTTATATAATTCTTTAGAAAACAATAAAGGGAATGA

CTTATTAAATAATGTGATAAATAATAATAATGCCTCTTTAAACAATACAAGGATTGTAAA

AAATTATTTAAGTAATTCAATAAATAAAAAAAATGAATCATCTCTAAAAAAAAAACTAGA

TGAAAAAATGATGAATTTGCTTATTATGAATAACAATGGAAAATCAAAAACTCAAATAAA

AGGAACTAATAATGAAAAATCGAAAATTTCTAGTAATAAAAATTCTAATTCTGTTAATTA

TTTTTTAAATGAAGTTTTATCTGATAATAATAGTAGTAATAATA

>c5663_g1_i1 len=206 path=[1:0-103 105:104-205]

CAGTTTTGTTCTCTTGCTGGTAGCACTTCATAGTGCAAAAGCTATTGTTGTTTGTCAATG

AAACAGCAGGCATACTTTAAAATTAAAAAAATAGAAAAGAAGAGAAACAGTTGTAACTTT

CACGTTGGGTTGATGTGACATCAATGACTGGCTGGGACTGCCAGCATTCCAAAAGGGATA

TCATCCTAATCTGCAGCCACTCAGGG

>c5672_g1_i1 len=277 path=[1:0-276]

TTCCAAAGATTTCCAAGTACATGTTTTAACTTCACTTAAATTTAAATCTGTGAGGCTTAT

CCATTTTCTTATTATGACAAAAACTTGGAAATACTTAAATTAATGTTTTCTAACTAATTT

TATTTTTTCTAAATTTTTGGCCTTAGTATCTTGGTGCTAGTGCATTCCTCTATTGAATGT

GCTGAAAAATAGTTTGTAGAATTTTCAGAACTTCCTTATCTTCTCTTGGTGTCCGTGTTT

TAGTAAGGAAGATGGCAAAAATTCTTTATTGTTAGTG

>c5680_g1_i1 len=247 path=[379:0-246]

GTTGGCCAGTGCCACTTCCATGGAATATAGTTGTGTCTCATTAAACAGGGCAAAAGTGTG

GGAAGGGCTCTGGTAACAAATGAGGCCAGCCAAGTACTCAATCCTTACCTTTTTTTGGAG

CTTGTCAGAACTTGATTTTCAATATGCAGGTTAGTATTTAGCCCTAACCTCATTTTACGG

ATGAAAAATTGCAACAAGCAGAGAGGCAGAGAAACATAGAACAGAGCAAACTCGCTGAAC

TCCATTA

>c5682_g1_i1 len=288 path=[257:0-287]

ATTACCAAGCAAAAAAATAACACGATGTTAAACTGTTTCAGTTGAAAATATGTAAGGAGT

TGTATGTGGATTACCACTACCATTCAGTTAATAGGTGATGCAAAGGGCGTGGGGTGGGAA

CATGTTTTAAATATTCCCTTTTAAAAAAAGAACTGTAAATAAGCAGTACTACGTTATTTT

AGACCTGAGCTCTGCTATCTGTAGTGGTTAGAACTAGGAAAACATGCTGCTTTCTAGTTA

TGCTACTGCTTCATTGTACCAGCATCATGGCGGGGGGGGAAGAATCAG

>c5684_g1_i1 len=222 path=[276:0-150 15:151-221]

TTCATCTTGGTTGATAAATGATCTTTTGAAAGCAGGAGTTAGCTCTAGACTTCGTCCAGG

TGTACAAGAGTCTCTTTAATAGTATTTCCCATACGTCCATACAAGAAAGATATAAAACTA

TTAGGGAGCATCCAAAACCAGGCTATGAAGATGATGGAGGGTCTGGTGGGCAAGGTGTAT

GAAGAGAAACTGAGGGCCCTTGGTCTTTTCTGATCAGAGCAG

>c5687_g1_i1 len=699 path=[51:0-268 319:269-675 818:676-698]

TCTGTAATTCCAGTTCACATACAGCATTGCTTCTCATGTGCTGCTTGAAGTTATCAGTGA

GCACCAATGCAGTGTAGATGGTTCTCAAGGTTTTACTCTAGGCTAGGTGTCTGTACCCTA

TGAAGTTTGTTGTGTTTAACCTTCAGAATGTCTTTGGCTGGAGCCCAAGTGGGTAATTTA

GTCTGTTAACTCCACAGCATCTTACCTGTGTGCACCACAGGTAAGATAAGTGCTGTAGTA

CTGTGGGGCACAGACAGCCAAGCAGCTGCCTGGAAGATCTGTGGAGATCCTGGAGATGAC

TTTTGTGGAGGAATGTTGGGCGCAAAGGGCAACGCTTGAAGAGCTCCAACCACTGGAGCT

ACTTGGAAGAATAGTGGCCAAAGTGGTATTTCTGTGGTGTTACCAACTCACCTTCTTAGG

GAATGAAAAGGGCCCTTTCCCAAACCTCATGTATGGGCTATTTCATGGTAGTAGGAAGTA

ATTCATCATCCTAGCTCTGGAAGTGGGTGAGCTCTACAATATTTCATGCTGCTTATAAGA

CCTGCGTGCTCGAAGACATCTCTGGAACATTGTTTGCTGAGGATCAGCATTTTGCTTTCC

CCATGGTGCACCTTTAATGGGCCTGGGGGCAAGGGTGCTTTGTTCATAGCGTATAGGGCC

ACAGGTCAATTTTGTTGTTCTTGAAAGCTGAAAGGAAAA

>c5691_g1_i1 len=228 path=[206:0-227]

TGTGCTGCCGCCCTTCACCCACACCAACAGCAAGTATCCTCCCCGTGGAGTCCATGCAAT

GGATGTAGCAATCACACTGCTACATTAAATCAATAGGTAGGCTTTTACTGCTTTCTGTCT

CCCATGCAGAGCACCATCCCCACTTTCTGAAGGCAGGTTTCTAACTAGCCACACTATGGT

GTGTTTAAATGCATCAGTCTGACGAATGCTAACAGCTTTTGTAACTTA

>c5695_g1_i1 len=207 path=[1:0-206]

ACAGATACAATGATAGGTGGCTTAGTTCAGAACAGATACTTTATTCTAGAAATAAATCCT

CCCCTCTTTCCTGTAAATCATATGACTGTACAAGCCAGGGTAGATAGGTATTTCATCTGT

TTCTTTTTAATGTGTACGTTACGCTGACGTATTGAGATTCTTCAGCAGTATGCTGAAATG

TCTGTTGAAAGACACTCAAACACAAAG

>c5695_g2_i1 len=221 path=[369:0-220]

TCCTAAGAACATCAGCTGCAGCCACAGTGTCCAAGATCTCTTGAAAATATTCCATACTCT

CACTGCACTTCTGTTTCACCAGAAATCCATGCCAGTACTCAGCATTTCCTGGAATCCTCA

TCTATTCATTGGGACATTAAATTAACCACCATTAAAGGAAATGAAAGCAGCAACAGTACA

GGGATCTTTTAGGAAAGCGAGAAATGCAGACCGTATAAAAG

>c5703_g1_i1 len=251 path=[229:0-250]

GAAAGGCTGTGGAGGTATCTGCTGTGGCAGAACTCTGGGGTAAATAAATAAACACCCCGT

GCGTCAGTACTCTTTTCAGTTCACAAGAAGCAACAAAATTATGTGTCAGGGCTTTCCCTT

TGTACCTTAATTAGTGTGTTTTCTGGGAAGTGACCATGTTCACCAGCATGAATTATCCCT

ATAATACGTTTTGTCTTCTTTCTTGATAAAATTTTGCAGCCCTCTCAGGAAATTTTGCAC

TGAAGTGCTGG

>c5717_g1_i1 len=211 path=[384:0-168 179:169-210]

GGAGGTGGCATCGCAGCCCACGGTGGGAAGGGGGGGAGGTCGTCAGGAATTGCCAACGTA

GATTCGTGGGGTGAAAACTGTGTTTGTCCGACCCGTGGGCTGCTGCGATGCACTGCCTGG

AGGCTGAGCTCAGCTTTGGAGGTCACGACCCAAATTTGGGGAGTTTTGGCGGGGAGAACA

GGAAGGAGCGACTCCTCAAGATTTCCGAGGT

>c5717_g1_i2 len=211 path=[384:0-168 552:169-210]

GGAGGTGGCATCGCAGCCCACGGTGGGAAGGGGGGGAGGTCGTCAGGAATTGCCAACGTA

GATTCGTGGGGTGAAAACTGTGTTTGTCCGACCCGTGGGCTGCTGCGATGCACTGCCTGG

AGGCTGAGCTCAGCTTTGGAGGTCACGACCCAAATTTGGGGAGTTTTGGTGGGTTGTCTT

TGGAGCACCTCGGAAATCTTGAGGAGTCGCT

>c5718_g1_i1 len=270 path=[33:0-269]

AAAAAAAAAAAAAAATATGAATTGAATCCTGATATTAAAAATCCTTTTTATATATTTGTA

TTACATAATACAAATATATATATTTATAGAAGATGTAATTTTTTTTTTTTTAAAAACAAA

AGTAAATGAATTAAAAATAACAATAAAATATTCAAAAATCTACTATATATTTTCAAATAG

AATCCTACAAAATTTTAAATGTTAAAAAATTTAAGTCTATATTTATTAAAAAATTTTACA

AAAGTTATAAAATTTTTAAATAAAAAATAA

>c5720_g1_i1 len=321 path=[323:0-149 484:150-320]

GGCATCTGACTCTTCCTTTGTACTCAGTTCAGTTTTTGTGTGAGAATCTCACTCTTTTGC

AGTGACTGTAATGTACATGCTAAGGAAGTACGTTTAACTGAAAGAGTGACTTGTATTTGA

ATTTTGCCCTCCCGCCTTTGAGGAGGTGATTAATTTTTTTTTAGCACCCTCTCTGGCATG

TTGTAAAGATTGAAGATGTATGTAAAGTGGTTTGAAAAATAAAACAATGCTTTGCTTGAG

CGTTTGGGGCTTTTACTTTCTTTGTCTGGTTTTCCTCACAGGACTGTCTTAAATGATCTG

TAGAAGGTTTTATTTAAGAAG

>c5728_g1_i1 len=265 path=[270:0-203 575:204-206 578:207-264]

TGTCTAGTGTTCAACGTCATAACAAAAACTGTTTCCAATATATATCATTTACCACATCAT

AACAACTTATACTAAATATTTCAAAATCTGAAAACTAAGGTAAGCTGCACAAAAATGCTT

CATGAATTGAAGAATCCAAAGAAATCTATAGATACAACTTCTACTAGATCTCTGAAAACA

GGATATAGTCCACAATAGCACAGAAAGGAAATGGTAGTTTGCTTTCCTCTCTGTATTCCC

CCCACTTCTCCCCCTTTTCCTTTCT

>c5729_g1_i1 len=1204 path=[39:0-1203]

AAAATGAAGAAAATTATATTTTTAAAACTACTTTTTCATAATTTGTATAAATAGTATATA

TATTTCAAAATTTTTAAAATAAAAAATAACAATTTATATGGAATAATTTATAGAGGTTAT

ACGAATTTTAAGTTATGTTTATTTTCAAAATTTAAAATATTTGAAAGAGTTATTAAAGAT

TTAATGCAGTTATATAAAAAAATAATTCATTTACCTCACTTCATAAAATATAAATTAAAT

TATTTAAATAATAATAATTACTTTATAATACAAAAAAAATGTTTTTATTGTTCTAAATTA

CTAAAATTAAAAAATGTAAGAAAAGTAAATGATGAAAATACCACTGAGCAAAATGATGTA

TATTATATATTAAAAAATAATGAATTACCTGTATGTAAAGAAAAAAATAGAATTGAACTG

ATATTATTAAATACAATTAAAACAAATAAACTGGATAATGTTGTAAAGGAAATATTATCA

ATAATCAATGAAAATCCTAAAAATAGAGATATTTATATAAATAAAAATTTAATGTTGGCA

TTTTACATTATTTTAATAAAAAGAAAATATTATGTAAATAATTTAAAAAATAGTATTTAT

GATAACTGTTCATTAAATATTTTTGATTTCATTCATTATAATATTAATCTACATTTGAAA

AATTACAACTTAAAAAATATATGTGAAGTACTTAATAATTTATCGAAATATATTTATAAA

AATAAATCTAACAATATCTCAAATGAACTTGTTAAAAACATATTTTATTTTTCCTTCTTT

AGTAATTTTAATAAATTTTATCCTAAAAAAAATTATAACAAATTAAATGATGAAAAAAAC

ATAAATAAACAAATTATAGATAACAAAATAAATGAGAAAAAATGTGATAAGGAAGAATGT

AATTTACATATATATAATTCATATAAATTAAAAAATTCTAATCAAACTAATTTAATAGAA

AGAAATATCATAAAAGAACAAAATTACTTAATCAAAGAAAATACTTCAAATAGTGAAATT

TCCATAAAAAAAGAAGACAGCAAAGATCTCTTAAATAATAATGAACTTAAAAAAATAACT

AACACAAATTATAAATATAAAGATTTCAATGAAGAAACATTTGCTTACTTAAATAGTTTT

ATTATATTTTTATCTAATCATCCTAATTTTATTAGTGAAAAAATTTTATATAATATTTCT

TTAT

>c5731_g1_i1 len=441 path=[167:0-440]

TATATATATCTATATGTATATATATGTGTATGTAGATATATGTAATTTATACATGTATAT

GAACATATGCTAATTATATATTCTTTTGATAATATGTAATTAAAGAACCTTAAATGAGAA

AAAAAAATGATTTATTACAATCTTTAAAATTAAATAATAAAAGAAATTTTATTATTAAAT

ATATATTCACATTACTGTTTTTTTATCTTTTCTGATAATAAAACTTTTTAACATTACATT

CTTTTTTTTAAGTACAAAAAATAAATAAAAAATATTTTTAGTGTGCTGTTTGTATTTTTT

TTTGAGTACAATAAACATTTTTAAAATGAAATATAATTATAAAAAAAATAATAACAAGAA

GTATGTCATATAAAAAGAATTATTATGTATATTTATTATTTTAGAGTTTTAAATAATTTG

ATTAAAAAAAAAAAAAAAAAA

>c5733_g1_i1 len=215 path=[407:0-214]

AATAAACCAGTAATGCTGCTGAGCTCCTCTCCAGTTGTTCTGCAAACACAAAATCATATA

CACTCCAGGAAGAGAGCTCAGGAGAAAAAGAATAACTTGATGACTCATCTATGAGGGATA

GCTTATTCCAAATGAAAACAAGAGGTACCCTACAGGAAGAAAGAAAACTTTTCCAGATTA

TTTGAAATGAGAACGCACTAAATATTTTCTAAATA

>c5738_g1_i1 len=293 path=[271:0-292]

AATCAGTTTCTTTCCCTCTGTCAGTACAGAACAAGTACACATTTAACAACACAACACGCT

CATAATCGCAACGAAGAGGAGCAAATGCCAACACCACAGAAATATTCAGTGCAAGGTAAC

ATAAGGTAACATGGCGCATGAAGTACAATCTCAATACAAACTCAAACACAGGAATATGAC

ACACACTCTACACATCATAACTACTGACTCCAAAATACATCAAGAAGGCACAACAGATGC

AAGTTGAAAATACATTCTGAGATAATGTAACAATGCACAGTGTTACCTTATGT

>c5742_g1_i1 len=204 path=[49:0-171 24:172-203]

TAACTACTTCAGAAGAAAGGGGAACAACACATGACCTCATTAGTTAGCTACTGCATGTCT

TCAAGAGCTCCATGAGATAGAAAAGCTCTGTGACTATTTGGTTTAAGAACATCTCTTCCT

GTTAGCATCCAGCCACCATTCTCACACTTTCCTGTATCACTTACTGTCCTGCTTCTACCT

GCAGCTATGCTAAAGTGAGATGCA

>c5746_g1_i1 len=210 path=[502:0-209]

GGATGTTCAGTGATTCTGTTCCAGGGGGGTGGCAAGCATGAAAGATCTGCTTGGCCCCTT

TCCCAAATGGAATCAAAATGAGTGCATGGAGTTTTCTCATCCATTCTTCTATTCACAAGA

CACAGATTCATGTGGAAGAGAGCTAGCAAAAACAACAGAACTACACGTAGGCAAAAACAC

TCCTTAGAGGAATTTGCTCATGCTCATTTT

>c5747_g1_i1 len=229 path=[309:0-75 21:76-154 56:155-178 486:179-228]

CATTTGTCCTATAGCTCAAGTGTTTGTTGCATTCCACCTCCTCCCCAAAACAAATACTTG

AGCTGTAGGACAAATGGCAACGTAACATAAAACAAGTGTAACTTTTGTAAACTACAATTT

GGGTGGTTTTTTTTGTTTTTCATTTTGGCCCATACATCAACAGGAAATTGGAATTCTTTA

TAGTCAGCCTGAATCTAATCAAGTACATCTTTCTGTCTGAAGGCACTGT

>c5748_g1_i1 len=245 path=[1:0-244]

GTACTCTGTAGACCTTCCAGTAGAGGGGCTGCCACTGACATAAACCTGGAAGACATGACA

CTACGTTCCTTACCTAATTAAGAATGCTCTGAATAGTTCTCTGATTAAAATAAAATATGT

TTGTCTAAATGGTCTACGAAGTTCAAGTGTGAACAGGGGTAAAGGTGGAGAAAGGAGTTG

AGAGGGAGAGAAATCCTTAAAATAGATCATAAAAGCATTTGAGCATAAACTGATTATTCT

AGAGA

>c5748_g2_i1 len=227 path=[649:0-226]

CTTATCTCCCTGCCTTGCCTTACATAAATAAAACTTAACATGCAGAGAGCTCAAACAAAC

TGCCCAGCACACAGGAGTTCCAGTGGAGAATTACACTTCACTTCTCTCTACAAAGCTATC

ACCTAACTCTACAGATGTGCACTTCACACATTCTTCAACACAATGTACACCAATTTCAGT

CAATTTCTGTTCAGATCAGATTTTTGCAGAAGAGTTCTATCATGTGG

>c5755_g1_i1 len=435 path=[587:0-434]

GGGGGCTCCCCCAGCCTGCGACGCTCCGTCCTGCTGCTGTTTCTGAGGCGGAGGTTTGAG

GCAGTCTCCTTGTGGAGGGAGCGACTGGTGGGAAAGTAGCTCAGCTCAAGATCACCCAAC

GTCTTCGTCTGGACCAGCCCAAGCTGGATGAGGTGAACCGCCGCATCAAGGTGGCGGGTC

CCAATGGCTACGCCATCCTTTTGGCCGTGCCTGGCACATCAGACAACCGCTCCGCAGCCG

GAGCTGGCGAGGCTGCCACCACCTCCACCCAGAGGCCGCTCAGGAATCTGGTTTCTTATC

TAAAGCAAAAGCAGGCTGCTGGGGTGATCAGCCTCCCTGTGGGGGGCAACAAAGACAAGG

AGAACAGCGGGGTCCTGCACGCCTTTCCGCCCTGCGACTTCTCCCAGCAGTTCCTGGACT

CCACGGCCAAGGCGC

>c5757_g1_i1 len=293 path=[434:0-46 480:47-292]

AAAAATGATGGGTTAAAAGTTACCACAGTGATTGATAACAATTTATTCCAGTAGGAAAGT

ATGAATGAAAAGAGAATATCAAAATCCAAATATGCATTCCTCTCCTACGTGGCAAAGCTT

TCCTATTTAGTTTACTAATGAAGAAAATGTCAAATTTTTTGTAATTTTAAAATTTTTAGA

AGTTCTTTAAATGCAAACAGTTTAAAGCAAAACCCTTTAGTTTCTGATTAAGCAGTTAGA

GTCTGCCTCGAGGATCCCTCTGATAATCTACATTTTGGTTTTCTCTTTTCATT

>c5758_g1_i1 len=256 path=[1:0-76 78:77-255]

ACATCTTTTGCACTTTTTTTCGAAGCAAAGCCGTGCCGTTTTAAACCACTGGATCTATCG

AAATGCCGGTTTGAGTTCGCGACACTATGTACTGCGTTTTTCATTCTCGTATTTGATATT

TAATCCTTTCTACTTGTCGCTAAATATAATTGTTTTAGTCTCCTATGGCATGATGATAGC

ATATGTATTCAGGTTATAGCTGTTGTGTTTAAGATGAAGAAAGTGAAAACATCTTTGTAC

ATTTAAGTCTGTATTA

>c5758_g1_i2 len=273 path=[515:0-93 78:94-272]

GCCGGAGCCCTCCCCGTCCCCTCCGGCCCCATCGTCCCGCTACGAGCGGGGCGCGGCGCG

GGATGCTGTGCCTTTAAGTGCCTGCAGATCGGAGCGCGACACTATGTACTGCGTTTTTCA

TTCTCGTATTTGATATTTAATCCTTTCTACTTGTCGCTAAATATAATTGTTTTAGTCTCC

TATGGCATGATGATAGCATATGTATTCAGGTTATAGCTGTTGTGTTTAAGATGAAGAAAG

TGAAAACATCTTTGTACATTTAAGTCTGTATTA

>c5762_g1_i1 len=284 path=[65:0-67 64:68-283]

CCTGCTTATTCAATACTTGCAGCTAGCCAGTTTTTTATATTTAAACACCCATTTCCTCCA

ACTCACACAGCAAGCTCTTGAATATGCCACAGGAACAGAACTGGGAGTGATAACAGTTTT

CTCAGTACCTTTCCTACTTTGTCAATTTGTGTACCTCTTGTATTGAGTCTGCCAAACAGG

TGGACAGCCAGAGGGCTGTGGTCTGGGAGGTGTATGAAGATGTTCTTAAGTCCTTTTACA

CATCCTTTTAGTTGCGAAGTTAAAAATGAGAAGTGGAAGAGATG

>c5762_g1_i2 len=247 path=[33:0-30 64:31-246]

TATTGAATAAGCAGGACACAACAACACACAAAGCAAGCTCTTGAATATGCCACAGGAACA

GAACTGGGAGTGATAACAGTTTTCTCAGTACCTTTCCTACTTTGTCAATTTGTGTACCTC

TTGTATTGAGTCTGCCAAACAGGTGGACAGCCAGAGGGCTGTGGTCTGGGAGGTGTATGA

AGATGTTCTTAAGTCCTTTTACACATCCTTTTAGTTGCGAAGTTAAAAATGAGAAGTGGA

AGAGATG

>c5765_g1_i1 len=282 path=[588:0-281]

TATTTTTTTTTTAATATTTCTTTTCAACATGGCAGATTAATTTAATTTCAAAGCAATAAA

AAGTAGCAGACATATAAAACTGTGACGGGTGGCTCCATGGAAAGTCACACTTACAAAGAT

TGATGCAATCCAGCTAACACAGAGCAACAGAGTAAACAGAACATATTACACCTCTGGGTA

TCCTTCCCCTCAACTCCACAGACTGTTTTTAGTAGATTTTCAGTCTCTGTTTCTGCATTT

TCTTTTAAAGCTAACGTCCTCATTTAATTTGAGCATGTGTAA

>c5768_g1_i1 len=214 path=[345:0-213]

GGTTTGCTGGCTTGTCCCAGGGCTTCCAGTGCACAAGGCTGCCATGAAGGAGCTCCTGCC

AGAGGTTTGGGCCACACTGCTGTGAGCCTCAGCAGAGCCCTTGTCTTCTTGTAGCTCCCT

TTCTTCTGAGCTTCCATTCACTTCTGGTTTTCTGTTGGAATCCCCAGTGCTGTAATAGTG

TCATAGAATCATTGACTCACCAAGGTTGGAAGAG

>c5773_g1_i1 len=223 path=[310:0-199 532:200-222]

TACAAAAACTGAGAATTTGTGCCGTGGCACCTCAGCTTTGGCAAAGCACAGAACACAATT

AACAGCTGGCTGCAGAAGCTTGGTTTAACAGAATCACTCAACCTCACACAAGAAGCTTGG

AGGCAGAGGCAAGAACAAAATGCAGCTCTCAAGGCAGTGCAGTCTGATCCTGTTCACTGC

TCAGGATGGCTGGGCCTCTTATGTCACAGGCAGTGGTCAAATG

>c5776_g1_i1 len=221 path=[249:0-220]

ACCACATTTTGCAGGGCATTAAACACAACATAGCCAGCCAGCTCCATGAGATGATTCTCC

TGCTGTATTTAGTACTGATGCAGCACAGACTTGAGTACCGCGTGTAGTTCTTGGCTCCAC

AGTATAAAAAGGATATAAAGGTACTCAAATACATTCAGAGGCAGGCAACAAAGATGACAG

AGGACTGCAGGATACATCTTGTGATACTGTTGTGTTTTTTG

>c5784_g1_i1 len=212 path=[1:0-211]

TAATAAGATGGGCATGGCAGAAAGAAATTTAATCCACATTAATTAAAATTACAAGTCAAC

CTTAGGACTAAGGCTACATAGAGCTGACTTAACCTTTCAGCACTAAAACTCTGAAGTTTC

TGTTTAAACTGATGAGATCATGCAGGCATAACTCCCAACTCCCATGATTAGCTTAATTTG

CATCTTCTGGTCATGTCTTAGGATCCTAATGG

>c5787_g1_i1 len=254 path=[27:0-253]

TGGTGTTGCTCAGGATGCCTGAAAGATTTCATCAGTTTAATCCCTTCTTCCTTCTCACGC

TGCACACGTATGCACTATGCCAACCAGCTTAGGTAACTGAAGAACATAGCGCCTACTTTT

GTAACAGAGCTATTTCTTCAATTACAACTTAAAATTTGATGGCACAAGAAAAGACCTTTC

TAAATGAATGCAGCCAAATATACATGAAAGGTAAATGTATCAATGACACTAAAAACAGAC

CAAGGCTTGCATTC

>c5789_g1_i1 len=209 path=[611:0-91 702:92-113 44:114-208]

TACAGTTAGCCTGAAGTGCTGAAGGTGGGGGCTTCATATAAATTTGGGTTCTAACAGACT

GAAATCATCATAACATAGCTTCAGGATTTGTGTTTTCCCCTTCTCCCTACCCTCAAACAC

AAATCCTGAAGCTATGTTATGATGATTTCAGTCTGTTAGAACCCAAATTTATATGAAGCC

CCCACCTTCAGCACTTCAGGCTAACTGTA

>c5789_g1_i2 len=474 path=[45:0-102 148:103-473]

GTATTATCTGTACAGGGGGAAACAGTGGTGTTTTTGTCCATGTTAGTGTTGTATTAAAAC

TTCTTTGAAATAGGTTTGCAGTCTCTTAATGCACTTTCAAATGCATTCATATTCATGAAT

ATGAATGAATTCATATTCATTTAAATGCTCTCATATTTCTGTTTGTGTCTCTTCAACAGC

ATGGGAACATGTTGAAGGAATTGTCTTTCAACAAAATCTCAACAATTTCCTGATTTTTGG

TTTGATGGTTTTTTTAATAATATGAAATCTCAGAGAAAGTAAATAAAGCATCTTAGTAAT

ACTACCTAAGAATATATTTGTCTGTTGTGTCCATCTCAAAGTTGGTATGTTGGGCATGGT

TTAGATTCTCCTGCAGTGACTGGCTAATACCATGATTTCAAAGGGACTTGCTAAACTCCT

ATGCTATTCTTTTATTTGAATACTCCTGATTTGTACTGCCTTGCAGACACTGCT

>c5789_g1_i3 len=952 path=[611:0-91 702:92-113 723:114-463 148:464-834 22:835-856 44:857-951]

TACAGTTAGCCTGAAGTGCTGAAGGTGGGGGCTTCATATAAATTTGGGTTCTAACAGACT

GAAATCATCATAACATAGCTTCAGGATTTGTGTTTTCCCCTTCTCCCTACCCTCAAAAGC

AGTGTCTGCAAGGCAGTACAAATCAGGAGTATTCAAATAAAAGAATAGCATAGGAGTTTA

GCAAGTCCCTTTGAAATCATGGTATTAGCCAGTCACTGCAGGAGAATCTAAACCATGCCC

AACATACCAACTTTGAGATGGACACAACAGACAAATATATTCTTAGGTAGTATTACTAAG

ATGCTTTATTTACTTTCTCTGAGATTTCATATTATTAAAAAAACCATCAAACCAAAAATC

AGGAAATTGTTGAGATTTTGTTGAAAGACAATTCCTTCAACATGTTCCCATGCTGTTGAA

GAGACACAAACAGAAATATGAGAGCATTTAAATGAATATGAATTCATTCATATTCATGAA

TATGAATGAATTCATATTCATTTAAATGCTCTCATATTTCTGTTTGTGTCTCTTCAACAG

CATGGGAACATGTTGAAGGAATTGTCTTTCAACAAAATCTCAACAATTTCCTGATTTTTG

GTTTGATGGTTTTTTTAATAATATGAAATCTCAGAGAAAGTAAATAAAGCATCTTAGTAA

TACTACCTAAGAATATATTTGTCTGTTGTGTCCATCTCAAAGTTGGTATGTTGGGCATGG

TTTAGATTCTCCTGCAGTGACTGGCTAATACCATGATTTCAAAGGGACTTGCTAAACTCC

TATGCTATTCTTTTATTTGAATACTCCTGATTTGTACTGCCTTGCAGACACTGCTTTTGA

GGGTAGGGAGAAGGGGAAAACACAAATCCTGAAGCTATGTTATGATGATTTCAGTCTGTT

AGAACCCAAATTTATATGAAGCCCCCACCTTCAGCACTTCAGGCTAACTGTA

>c5799_g1_i1 len=257 path=[407:0-151 173:152-256]

CTGTGCCCTCGCAGCGAGAGGTGCCCGGGGAGCCCCGGCCCGTGCTAACGGCCCGGCCTG

AGCCCTGGCCAAGTTTCTGCGACTTAGGGGAACTCTGGGGATGAGTAATCGCGCCCAGGC

GAGGGGCCGCGAGGGTGCTTTTTCTCCCCTCCCGCCGCCCTTCTCAGAACCGCGTTCTCG

GAGTGACTGGGTCCGCACATGATGACACGGGGAAAAAGGAGAAGAGAGCGAGCGAGTGAG

GAGAAAAAGCACCCTCG

>c5800_g1_i1 len=223 path=[33:0-189 316:190-190 224:191-222]

GGGGAAATCTCCCTTTGCTTTTGAATGCGTTAGTAGCTTTCCTCAGGGGAAGAGTGTGCC

TGCATTAACATCTGTTGTGGGTAGCACGAGCTGGGCTATGATTTCAAACCTATCGGGTAG

AAATCACATTGGCAGACTTAAAGATGAGAAGTCTCAGCTAGAATTGGTGTAGCTAACAGC

AGTTGGTGACTTTCTGATCTATATGTAGGATGCTCTTTTTTTT

>c5801_g1_i1 len=248 path=[296:0-200 502:201-247]

TATATATTTATTAAAAAAATATAACTCTTTAAAAATAATAGCATAATAAAATTAATATAT

TCTATTAAAAAATTGTCATCTATAATGAATTATTTAAATATAAATCCAAATTAATTTATT

TTAAAAGTTAAAAAAATATTTTTAATAAATCATATATACTTAATGTTTCTAAATATTTTA

TAAAGCACTCATTAAGAAATTTTTTAAAATTTATGGTTTATGTATACATTAAAAAATTTC

TTAATGAG

>c5803_g1_i1 len=295 path=[323:0-294]

TTCATTCTCCTGTAGTTTAGTATCATTTTGTTCTAGACAGGTAAGAACTGGCTTCACTTA

AAATTCAAGAGTAACAGAACAAGTGTAAGCAAAATAGCACACAATACCAAGTTGACAAGT

TGTGATTAGTGAGGGAAAAAAATGCTCAACATCAAATGGGAAAACACTGCAGTAAAACCA

AGAATTCCAGTTCTCTGACTGAAGCAGGTCAAGATCAGGATTAATGTCACAGGAAACTAT

TCAAGCTAGACAAAATGTTAATACTGCTCATGACCATGTATAATGTGATGGAAAG

>c5804_g1_i1 len=232 path=[155:0-231]

CACCACCCACCTCCAAGACCCCAGCCAACCCCCCGAAACACGCACGAAACGATGCACAAG

CACACCGAGAAACGCCAAGGCACAACACCCAGCACCTCCATCCGCAACACCAGCACAACC

TCCAGACATGAATACATACCAACAAATACACGCACTTCCCCTGGATCTCTGCCACTATCC

CCACGGACCTGTGCAACAAACCCGGCAACCAGCACTCTCCTCCCCCTGGAGG

>c5806_g1_i1 len=260 path=[303:0-259]

CAAAGCAAACAGCATGAATAGAACAGTGTTGATGAAGACAGAAGTGGCATTGAAGGCTGC

AATAACAAAGCGAGGCAGTGACATGAAGATAAGTGGTAAAGTAATTGCCAAGTGTTGGCC

AAGTGGTCTTTGCACCTTTCAGGAGTCTGTACCTTCTGCATTTATTTGTAAGGGAGTTAA

GAATCTGTGCTGCTGTACTGAGCTGTGTTTGCATTTGTGACCCTGCTAGCTACAGCTCTG

TGTTTCTTAATGGAGGGATG

>c5813_g1_i1 len=242 path=[289:0-241]

GACTGCAAAAAAATGCAGGGAGGCTTTCAAGATAAAGAATAACTTGTAATTGGTTTTGGC

AATTTCGGTGGAGTCAAGGGAGCTGACCAGTAATGACCATTTTTTCTTCTCACACCGTCA

TTTGTTTTCTGAATCGGCGAGCTGTGTCACAGATGCTATCGTAAGACTTTTCTGGACAGC

TCTCGCTCTCAGGAATATGCTTCCTGGAACTCTCCGCTTAAATGACTCATCATTGTGAAA

CA

>c5820_g1_i1 len=310 path=[1:0-309]

GGTGGTGGGGCGGAATGGGTCTGACTACAGTTCCACATTATACTCACGATGATTAATATG

TGGCTACTGCCAAGCACAAAGTTCTACTTCCCCCCTCCCCCCCCAGTCCTCCTGCTGCAA

GCTGATTTGTGATACCCTGTCAAGGTATTTTTCTCTTCCTCTGCTGTTGACCTACATAGT

AACTGATAGCAGGAGGTTGTCATCTTCTGTTAGTTCAAATGTTATGGATGTAAAGCTCCA

GCCTCACAGTTGTGAATTACTGAGATGTTAAATGATATAACTGTCATACTTTTTATTGAT

TGCTGCTGCT

>c5822_g1_i1 len=232 path=[197:0-174 163:175-179 380:180-231]

GACGGCCGCGGCGGTGCGTCGGGTCGGGGAGCGCGCCGTCCCCCGCCGCAAAGTTGGGAG

AAAGTTTCCTGCCGAGAGGGGATGCGCTGCTCGGGGCTCCCGCGGGCGCGGCACGGCTTT

CCCCTTCTGTTTGTTTGGCTTGGTTCTTTATTACACGCGACTTTGGTTTTTGTTGTTGTT

AATTCTTTTTTTTTTTTTTCCTTCCCTTTTTATTTATTTTACTTTATTTTAT

>c5827_g1_i1 len=206 path=[184:0-205]

CCCTGCACCACTGTGGCATTGGCTCTTCCCTGCCAAGCAGTCCGGCCCAGCCCTGAGTGT

ACAACCATCACTCAGCAAAAACCAAACATCGGTTCACCATCAGACTTCTCTGGGCTGAAA

CCAGGACATGTTTGTAGAACTTAGCTTAGTTTGAGCATGACTTCTTAAGAACTATTTGCC

CAGGTCTTAAGAACGTTCTTAAGACC

>c5829_g1_i1 len=204 path=[51:0-203]

AGTCTTCAGATAATAATAATGAAGTAAGGATAGAAGAATAAATACCTATTTACATTTATA

TACTTATGTTCTTTTCTTTTTTAATATGAATCCAAGTTTTGTTTTTTATTATTACATTTG

TGATTAAAATAAACTTTTAATTTAATTTTTTTTAGGGGAGTAATAATATTTTAAGTTCTG

CACAAAATAGTGAGGATCATGAAA

>c5835_g1_i1 len=209 path=[223:0-208]

GTGAGGCTACCTTATTGATGTGCAGAATCCATGTAATGTATGGATTCTAGTTTGTCACAG

AGGAGTGTCATAGAGTAAATGGATTAATCTGCCCCTTCAGTGTCCCATGTGCAGAATTTT

ATGAGAACCAGGCAGCTACCTTCCCTGAGTGATCTAAAAAAAAATCCAGAAAGAGGAGGG

GAAGGTAAAAGGATTAGGAGTTTGCTAGC

>c5836_g1_i1 len=291 path=[765:0-13 767:14-290]

ACCTCAAACACAAACAAATACCTCCTTACACAATGTTTAGGTTGCTAATTTACAGGATAA

TAAGAAAAAATAAGAGATAATTTTTTTTTATTTAATCTTAATTTATATCAAATTTCTTCA

ATTTTATGAGACTTACTTTTTTTAACGTCAATACTTGACATTTTAAGGTAGATTTTATTA

TATATTTTTCTTTATTTTTAATTCATTATATATTTATACAAAAATATAAGCTTTATTTTG

TTTTGTGTTGTTTTCTTTTTTTTTTTTTCCCTCAAAATTTCTGAACCATTC

>c5836_g1_i2 len=317 path=[428:0-23 38:24-39 767:40-316]

AATCATTTTTTTTTTTTAATTTTATCACGTTTAATTTGTACAAATACCTCCTTACACAAT

GTTTAGGTTGCTAATTTACAGGATAATAAGAAAAAATAAGAGATAATTTTTTTTTATTTA

ATCTTAATTTATATCAAATTTCTTCAATTTTATGAGACTTACTTTTTTTAACGTCAATAC

TTGACATTTTAAGGTAGATTTTATTATATATTTTTCTTTATTTTTAATTCATTATATATT

TATACAAAAATATAAGCTTTATTTTGTTTTGTGTTGTTTTCTTTTTTTTTTTTTCCCTCA

AAATTTCTGAACCATTC

>c5848_g1_i1 len=319 path=[403:0-236 25:237-318]

AGTAAGCAATTACAGAAACGGCTCGTGGTTGGATAGTTAAGGTCAGAATTTGTACTGGAA

ATGAGGCATGATCCACTTACACAATGCATGAATAATACATCTTGGAGCATTTACAAAGTA

ATGTGAGTTAAACATAGATGCAGAAGAAAATCACTTGGTCCCTAAACAATAAAAAATGTA

AATCCTTTATGATAGAGTTTGGGTTCTTGTAAACATGTTACTTTCTCACTGTTTACAGCT

GCCAGAGTCTGACACGTTCTGGAATAAAATTTCTGTGAACTCTTATCCTCTTTCGAAACG

GCCATCAAACACAAAGTTA

>c5848_g1_i2 len=260 path=[403:0-236 662:237-259]

AGTAAGCAATTACAGAAACGGCTCGTGGTTGGATAGTTAAGGTCAGAATTTGTACTGGAA

ATGAGGCATGATCCACTTACACAATGCATGAATAATACATCTTGGAGCATTTACAAAGTA

ATGTGAGTTAAACATAGATGCAGAAGAAAATCACTTGGTCCCTAAACAATAAAAAATGTA

AATCCTTTATGATAGAGTTTGGGTTCTTGTAAACATGTTACTTTCTCACTGTTTACAAGA

ACCCAAACTCAAACACAAAG

>c5851_g1_i1 len=212 path=[323:0-211]

AGCGGTGAAGTCTTTGTACACAAGTTGTGTGTCTTTGTGCCTGGCAGTACTGTAGCCCAG

CAAAACTTTCAGCAGAGTAGTGAAACTGGTTGGTGGTCTGAAACATCATCTTATTAAAGC

ATAGAATGAACTGCTAAAAGGGGGATTGGGCTGACTGCTCAAGTGCCTGAGAGTGTACTT

TTAAGTCCTTTTTAAATGTAGACCTGATTGTT

>c5852_g1_i1 len=582 path=[610:0-581]

GATGCCTGGAGTGAAAATGTAGCTAAAACAGTATTAATTTATAGTTAAATTTTAAGAGAT

GCACATCTAGCCCAGAGCTGGAGGCTGCTGCTGCAGTTATGTCCATGTTGGTTAAAGGAG

AAAGGGAGCACTCTATGTCTAAGGAATTAATAGTAATCCTAGAAAATTCCTCCATAGTGG

AATAGAAACTTAACGGTTTCTAAAGTCTAGGTGAAAGTGACAGTATTTTAAATTATTGCT

TTGAGGTAATCTTTCTCTATTACAGACCTATTATTCCTCTGTTCAAAGTAGAGTTTTGGA

GGGATTTTCATGAAGGCTTACTCTAGGCCTGGAAGATTCTTCCTAGGTTTTGGTCATAAG

GAGAAAGAAGCTCATCTGTAGAATGGTTTTGCAGGACGGTACCACTGAAATTACCAGTGA

AAACACTTTCTAGTCAGTTGAATGTCACTATAATAAGCTGTAGGTTCCTTTGGCCATCTT

ATCCCTATTTGCCATATGTCCTGCAGTTACTCTATCTCAAGACAAATGACTCTGGGAGGG

TGAAGCAAACCTTCTTAATCTCTTAATTTAGTATTGCCTTTT

>c5861_g1_i1 len=284 path=[418:0-283]

GAAGTCTATTTAGCAAAAGGAACTGAGGGACTTAAAACAGACAGCAGGATGCAGGACCTC

CCTGTCTCACCTGCCTTGTTTGAATTGAACCCCAAACCCATCACCTGCTGCCCAGCGTTA

TTTAGTGAGCAATGGGAGCCCAGCAGCCCAGCCTCACCGCCGTACCTTTGCACTGCTCAA

AGTCATCTTGGCACGGCACTGTTTGGCCACCTTGGGTGAGGTGGCTGCACTGTATCACCC

GGTGACAGACGGATCTCTTTGGTTATGATCAGGGTGCTGCAGCT

>c5863_g1_i1 len=237 path=[205:0-236]

GCAGATACAGCTCCAACTTCTAACATCTGGCAACGTCTTCATCCAACGTCCGTCTCCTCC

ACTGGAAGAATAAGATTCTTTCTCACAGCCTCGAATTCATGCTATCACTTTGCTAGCTGT

CAGTATTAGCTTGATGTTTTATACAAACATCTGCCATATCTCCCGTTCTCTAACACAGTT

CGTGTAAACTGTAGAAGTATAAAATTAAGCTTTACTTAAGACTTAATACATAATGTA

>c5865_g1_i1 len=239 path=[398:0-238]

ATTCAAACATGACCTGAGAGAAAGGGTGCTTAGGAATACCATGGCAGGAAGCTAGTATTC

CTGGTGTTTTTCTTTTGGATGCAGTACCTTTGGGGAAGGAAGCTTCTGGTTCTGGTAGAG

AGCTGGAAAACTGAGGCAGTGCTTAAACCAGTGAGATGCAGTGGTGGAGAGAAGTGAAGT

TGTGACTCACTTTCATCGCAGTTGTTCTGCGTTTTCATTGTGGTTGTTCTGAAGCAGTG

>c5871_g1_i1 len=1097 path=[485:0-1096]

TCTTTTTTTTTTTTTTTTTGTGTATAATATTATTCATATATATTTTAAAGAAAAAACTTC

ATTATTTTATAAATCATTGAATTATTTTTAGATATTCTTATAAAAATTAAACATTCATCT

GAAAAATGATAAATTCAAAAGAAAATGATACATTACCAAAAATATCTTTCAAAAATAAAA

ATATAAAGCTGATAAAAGTACCAAAATTTGTTTCAAATAAATGGTTAAAGAGAAAGCATA

GAGATGTTGTAGGTTTATTTGAACACAAAAATAATGAAATATCAAATTTATATATACAGA

AAGACGATAGTGATGAAGTAAAAAAATTAACATGTAATAAAAATTATACTATAAACACTT

ATATATTAAAACAAAATAAAATATCTTTAAAACCTAATAAAGGTACTAATCCATCTAATA

ATAATTTAAATAATAAAACAATTGAAAACTCAAATCAAAATAAATTAAAAATAAATATAA

ATAATCCTATAAACGAAAATTCCGAGCGAAATAAAGAGCTTGATTATGTTATTTGCGCAG

ATGTTATAAAAAATTGCGAACATACATATTCATTCTTACCACTTTTAGATGAAGATTATT

CTTTGATATTAAAAGAAAGGCATTATAATACAAATATAAAAAAAAATAGATATACTATTA

TAGAAACAAGAAATGAAGAGAATTTAGATTCTTCACAAACATTATTTAAATATTATACGT

CTGATGATAATAACAACTTAAATAAAGATAAAAATAATAAAAATAATAAAAGAGTTTTGT

TAGAAAATGAAAATAATTTTAACTCTTCATCTGCTGCTTCTAAGCAAAAAACAAAACAAG

CTAAAAAATTTCATATCTTTGATTTAGATAAAACTAAAATTAGCATGTTTAAAATATTTG

AAAGAGAAGGAAAAAAAGGAGTTCCTTTTTCTTTTTTTGCTAAAAGTTTTAATATTCCTT

CAAATCACATAAAAAATATATTAGATGAAATAGCTGTTAAAAGAAAAAGGGCTTCAGATA

AAAAATCAGTATATTTTTTAAAAGATTACATTAGTTAATATGTTTTTTTCTTTTACATTA

TATATATATATAATGTA

>c5871_g2_i1 len=265 path=[243:0-264]

AAAAAAAAAAAAAAAGTTTAGTTTATGTTATATTTGACGTTTTGTTAATAATTAATTTTT

CGTTTGTTTTCTATTTATTTAAAAATGTATTATAAATTGTAAGTCCTTTTACAAAAAAAA

AAAATAATTTTAATAAAAATAACGATGCATATATATATATATATATAGTTTTTTTCAAAA

ATTTGTGAATATGAAGATAAAATGAAGGGGAATAATAAATATTATTATAATAAAGATATA

TATAAAATATCTTTAAAACATTATA

>c5873_g1_i1 len=245 path=[1207:0-17 985:18-244]

GGTAACTTTGTGTTTGAGCCATTAAAAATAAAAAAAACTTTTACGAAAAAGAAGAAATTA

AGAGAGAAGATAATATAGAAGAAGGTGATTCAAATAAAGGTGACTACTTCAATAATATTA

ATAAGGAAAAGAAAAGTATACTTGTTGTTGATGATTTTAAAAACGAAAAAAAATCAAATG

ATAATTATAGAGAACAAAATGAAGAAAAAAAAAATAGTGATAAAAAATATGAAAATAAAA

GAAAT

>c5873_g1_i2 len=617 path=[595:0-389 985:390-616]

ATTTAAATAAAAATGAAGAAAATTATATATACAAAAATAAAAATTTATTATTAAAATTTA

TAAATGTAAGAGAAACAGATTTAATAGAAAAGATGTGTAATTATATTTTAAAAAATAATT

TATTTAGTGAAATAAATAGGAAAGATAATGGTTTTAATTATAACTGTTTTATTTTTAAAC

CAGATAACTTAAATAAAATTTCTCCTTATATTATTGATAAATTCCACTACATAAATATAA

GTCATACAATTCCTTATATATCTTTTATTATATATTTAGTTGATCAAATAAAAATATATA

AATTTGTAAAAAAAAAAATTATAAAATTATTTGAAGACCTTATAATTGAAACGCTAAATT

TTTTTAGAAAACAAAATAAAACCATTCGAACCATTAAAAATAAAAAAAACTTTTACGAAA

AAGAAGAAATTAAGAGAGAAGATAATATAGAAGAAGGTGATTCAAATAAAGGTGACTACT

TCAATAATATTAATAAGGAAAAGAAAAGTATACTTGTTGTTGATGATTTTAAAAACGAAA

AAAAATCAAATGATAATTATAGAGAACAAAATGAAGAAAAAAAAAATAGTGATAAAAAAT

ATGAAAATAAAAGAAAT

>c5874_g1_i1 len=243 path=[55:0-61 116:62-63 54:64-242]

GTAGCTCTCAGCCAGCTGACCCTGGTCTAACAGAACTACAATGAATGAGTGGTCCCCAAT

GACCAATGGACTAAGTCAGTTGTCTCCAAGTGGGCAACAGTCACCCCACTAAGGATGAGT

GTGAGTGGAAACATGTAAATCATCCTAAGGGGATGAAATGTAGGAGGATCGCTCCCTCCC

ATACACATTTTTTGAGCAAGAAAGGCGAATGCAAGAATTATAGCTGTGTCAGGTTGAAAA

AGG

>c5884_g1_i1 len=284 path=[536:0-283]

AAATATGGCAGGAATCCCTAAGAAGCCAATGGGGTTGAGAAAGAAAAAATAATCCCAGTT

TTTTGTTTGTAACACATTGTGGAAAAGATTAAGATTGCAATTATTTGTTCAAATGTTCAT

GTGAAGGAAATATTAGTGAAAAAAAAAAATCCTGATTATGATCCGTCAGCGAGTGATGTG

CAATATAATGCTTTTGGTTATATGAAGTATGATCAAGGCAGCAGTTGAAATATCTTTGCC

GTCTGAAAAGACTTTTTTTATAGATTAGAACTTGACACACTGAG

>c5886_g1_i1 len=230 path=[1:0-189 270:190-229]

ATTGTGCCATTACCTTGAAAACTTGGGGCACCTTGTGCTCACAGCTGCCTGAAGTCTAGA

CAGACGCCCGAGTACGCTACTGCCATCCTATTGCTTTGGCTGATCTTATAATCTCGGTGT

GTGATGGACTAATGTTTTCTTAGCAGAGTGTGTGTTTTTCACAGTGCACTCTCAGAAACG

CAGATTGTGTTTATCACTGTTTCTCTGGGACATACTTTACGTTGTGCTAG

>c5886_g1_i2 len=230 path=[1:0-189 191:190-229]

ATTGTGCCATTACCTTGAAAACTTGGGGCACCTTGTGCTCACAGCTGCCTGAAGTCTAGA

CAGACGCCCGAGTACGCTACTGCCATCCTATTGCTTTGGCTGATCTTATAATCTCGGTGT

GTGATGGACTAATGTTTTCTTAGCAGAGTGTGTGTTTTTCACAGTGCACTCTCAGAAACG

CAGATTGTGTCCAGCACTTTGTTATCTAGCACAACGTAAAGTATGTCCCA

>c5887_g1_i1 len=206 path=[315:0-205]

ACTCTCCATCTGTGGGAATTAATTACAGTCAATATATGTTCCTTGTGAGATGTTATTTTG

CAAACTAGGCATTGCTCCAGAAGCTTTCTTGGATAGAAATTAAATCCCATTGTAGCATGA

AACAAGCAAGGTAGGTATCAGGGAGGAGACAATACTGTGATTTACTGTAAGATAGAAATA

TTAAAATGTGGTGTGGAGGGGGGAAA

>c5889_g1_i1 len=271 path=[603:0-270]

GGAAAAAAAAAAAAGTCCTAAGAATAACTTACAAGAGGTTTCCACTGCAGAGAAAAGGAT

TTAAACAGGTCAGCAGGTGACCAGATGAACCCAGCTTTGTGTTTTGGCTACTGACTTCAT

TACAAAGATCTCCCTTTCTCCAGTTACAATACCAGAATGCCACAGCCAAGGCATGCCGCA

TGTACATAACAACATCAATAATGCTAGCAGCAATGCAAGAATCTAGCTTGTGGACTTGAA

GTTAGCCATGAAATGCAAGAAGGTTTACAAC

>c5890_g1_i1 len=1948 path=[37:0-1947]

AAAATTAATATAAAATAATATTAATTTAAAAATGTACATAAGAATATTATTTATCTACTC

TTCAATTCTTTTAATTTCGATTTTTCAAATAAACGGTCATCTTAAAGACAAAAATGTATA

TATCTTATATTTACAAAAAAAAATTGAATTTTTAAAAAAATATTATGATAGTTATTATTT

AGACAGTGAAGAATTAAATCTTACGCAAGTTTTCCGTTTTAAGAACCACATATATTTAGA

CTATACTGGAAGTGGTTTATATCAAGAAAAACAATTGAATGATTTTTATGAAGACATTAA

AGTAAATAGATATGGAAACTCACATAGTAATAGCCCTTCTGCCATGTTAACAGATGAAAA

ACTAGTAACCTCAAGGAACGCTGTATTAAAATTTTTTAATACAAATAGTAATGAATACTC

TGTAATTTTTACTAGTGGTGCGACAGGGGCATTAAAAATGATTGGAGAATATTTTTCTTG

GGAAAAAGAAAGTTTATATTATTATTTAAGAATCAATCATAATTCTGTTTTAGGTATTCG

AGAATATATAGCTGAGATAGGAGGATATTTTAAAGCATTAAATGAAAATGAAATTTCCAA

AATATTATATGATAGAAAAAAATCACAAAAAAAAAATGACGTAAATAATCCAATAAATTG

TTTATTTGCATTTCCAGCAAAAGATAATTTTGCTGGTGTTTTATACCCACTTGAATGGAT

AGAAGATATAAAAAAATATGGTTTGAGTGATAAATGTCATTGGTACGTGCTAGCTGATGT

AGCAGCATATGTGCCATCTCATAAATTTGATCTGTCAAAATATATCTATTTAGATTTTGC

AGTTGTCTCTTTTTATAAAATGTTTGGTTATCCTACAGGTATTGGGGCTTTAATTGTTAA

AAATAGTAGTTCAAGAGTTTTTAAAAAAATTTACTTTGGTGGAGGTAGCGTTGTAACAGC

TGTATGTGATTCACGAATATGCAAAAGGCAAGATAAAAATAGTTTTTCACATTATGAAGA

TGGAACACTCCCATTTTTAGGTATTAACGCATTATTATTTGGGTTTGATATACTATCAAA

AATATCATATGATATTATTACCAATCATATTAAAGCCTTATCAGAATTTACATTTTATGA

ACTGATAAATATTAGACACTCGAATTTTACAAAAGTTGCTAAAGTTTATCATGCTTATGA

TTCTCCATTAGATGGTATTATTGCGTTTAATTTATTTGATATTAACAATAAATATATACC

TTTTTCTCTAGTTGAATCTATTGCTGCATTTAATAATATCCATTTGCGTGGTGGGTGCTT

TTGCAACTCCGGTGCATGTCAAGATTATTTAAATTTGAATAATGAAGAAATAATCGAATC

ATACAAAGATAAAAACAGTTGTACTGAAAACGGTTCCAGTGATAATAAACCTTTCGGTGC

AATCAGAATTTCATTTGGATATTTGTCTACATTTAAAGATTCTTTTGTATTTATTCAGTT

TATAAAAGATAATTTTGTAAAATAAAACAAAAATATTCTATAATTTTTATGTACTATATG

CATCCATTTTCTAGTAATAAGAAATTAACAATTTTGCATTTTATTGTATATTCTAGTTAA

AAAAAAGAAAATTCTAACCTTTTTCTAAGAAAAATATAGGCTAAATATTATAATTTTATA

AATTGTATTCTTCATATATTAAATACGTTAGTTCAATTAAAATTTTTTTTTACAAAATCC

TACTTTAAAAAAAAAATTATTTTATTCCCATTCATTTCCTATTAATATAAAGAAATTAAA

AATAAGATTATTTATTATTATTTATTATTATTTATTTTTCTTTTAACAATTGTTTTATCT

CTTTTTCTTATCAATATTTATCAGTACAATTACTTTTTTCATATTTTTCGATTATAAAAT

TAATTTGTGATACATGTTCACAAAAATA

>c5892_g1_i1 len=228 path=[165:0-227]

AGCTATGAATGATACCCACAAGGCCTTAAGCCTTACAAAAATATCATTTTCTCCCTTAAC

TGTTAAGCAACACAACAATATTCTAAGTATACACTTGGTTTTGCAGCACAGCACTAACAA

AAGCTTGGCTTCTTGCACTGAACCAATGGACAATGCAATCTGACAGTCAGATCACTAGAA

GAGTGGCTGAAAAAAGTCCTGTCTGCAACACTGGCTAATACCGAGAGA

>c5894_g1_i1 len=421 path=[37:0-49 18:50-420]

AAAAAGGAATAAAAAAAAAATTTATTAGTATTTCTATTACGTTGATGTTATTTTACGTAT

AATGTTCTGATAATTAAGTATTTATATTGATGGAAAATATTTTGAGTAATTCCTATTTTA

CTATAAATTAAAAAACAAATAATTTTATATACATTAAAGAGAAAACTATTAAAAAATTTC

ATAATATTTATGAAGATATGATATTCTTAAATATAACAACATAAACCTTAATATTTTAAT

GAATTCCTTATTTATTTAAAACTAAAAAACTAAAAGTCATAAAAAAAAAATAGTGAATTA

TATTATTAAACAAATTTAAAAATATAGAAAAATAAATACTCATATTTTCATTTTCTATGT

ATAAATTAAAAATAAAAGCTATACAATATTTTTAGAAGATATAAAATTTATGTTAATATG

A

>c5903_g1_i1 len=207 path=[185:0-206]

TGCTAAGCAGGGGAATTACAGTATGTGTTAGGAATTGAAGATGAGCAGTGCTAACTGTGT

TATTTCTCAACAAATGCATTGTGCTCAACTGAACTCATCTCTGGAAATAATTAGTGGCAA

GAGTCTACCTACAGTTTGAATCAATGTTCCTGCGAGAATTTAGCAATAAATTGTGGATTT

GCCTTGAGAAAAAGATTCCTAATGGCA

>c5907_g1_i1 len=210 path=[476:0-209]

GGGCGGTGGGTCGCGCAGCCCTCGGTGGCCGCGTCCCGGCCTGCTGCCTTAGCGAGAGCC

GGGCACTGCAGCCTGTCAGCGCCGCCTCCGCCCTTCGGTTCTATGTTTGTAAACCTTACA

GGCATTTGTAACGTTGAAGGATTTGGGGCTTTATGACTTCTGAGCCTGCGAGCTAGATCA

ATGGACAGAATCTGTGTGCAAACATGAGAA

>c5910_g1_i1 len=262 path=[317:0-254 892:255-261]

CGTTTGCTGGTTTCTGCTTAGGAGTATTAGGGCTTCTCAGCCATCGGCGAGCTCAGCTTC

AGTCCTGGTGGTCCTGTTAGTCAGGGTAAAATGGGAGGCGAGAACCTGGAGATGAAAATA

TAACTGTTTATGATTCTGTGAAAATGGTCCTGCAGGAAGGTGGTAATGAAATCCGGGAGA

ACATATGGGAGAGTAAATGAGCTTATCTAGGTGTTCAGAATAGAATTATTGCATATCTAA

TGATGTTTTATTTTCTTTTGTT

>c5910_g2_i1 len=307 path=[631:0-243 578:244-306]

TATTTGGTGATATTGTATTTTAAGTATTCCTCATAGCTCTTTTGTAATGGTTTCATATAA

AATTCTTTCACTGAGAGGAAGCAACTGACACACTGCATTAACAGAAGCAAAGGAAAAGCA

ATCTGAATGCCACGGCATTTACAACAGGTGCCAGCCAGCTCCACGCAAAGGAAAATGGAT

TGCTGTTGAAATGAACTGCCTTCTAGAAGCAGAACTATTGATAAAAATTTAGTTGAGCAA

AAAAATATATGCCACTCACTGAGAGGCTGCCTAGCTCCCTGGTACGCTGGCCCAAACAAC

AAAAGAA

>c5911_g1_i1 len=486 path=[31:0-349 882:350-359 390:360-485]

CACCCCTCCCAGCAAGAGTTTGGAGAAAGGGGCACGCTGAGCAATGCAGTCTTCTTCCAC

TTGTACTCTCGCACCGCAGGAGAAGGGCAGGGGGAGGGGAAAGCCGCACAGACTCAGATT

CAGACTGCACACACACTCCCAGAAGCATCGCTGAGACAGAAAGGGTGACTCTAAGCCGCC

CCCCGCTCCAGGCACTCACGTGCTCATGTATTGGTTCAGGCTGTGACAAAGGAATTCCAC

TGCAGGCCCCCAGATGGGACCAGAGGCCCTATATTGAATTGACCATTTTTTGGTCCCTGA

AGAACATATAGAATGTGTTAAAACAATCATGATTTCAAATTGGCTGGGTATTTTTTTTTG

TTATATGTTTGTTTTGTTGTTTTTTGTTGTTTGTTTGGTTTTTTTTTTGTGGGGTTTTGT

TTGTTTCTGTTGTTTGGTTTTTTTTGTTTGTTGAGTCTAGCTTCAGAGCTCTGGGAGCAA

CAACAA

>c5911_g1_i2 len=486 path=[31:0-349 882:350-359 766:360-485]

CACCCCTCCCAGCAAGAGTTTGGAGAAAGGGGCACGCTGAGCAATGCAGTCTTCTTCCAC

TTGTACTCTCGCACCGCAGGAGAAGGGCAGGGGGAGGGGAAAGCCGCACAGACTCAGATT

CAGACTGCACACACACTCCCAGAAGCATCGCTGAGACAGAAAGGGTGACTCTAAGCCGCC

CCCCGCTCCAGGCACTCACGTGCTCATGTATTGGTTCAGGCTGTGACAAAGGAATTCCAC

TGCAGGCCCCCAGATGGGACCAGAGGCCCTATATTGAATTGACCATTTTTTGGTCCCTGA

AGAACATATAGAATGTGTTAAAACAATCATGATTTCAAATTGGCTGGGTATTTTTTTTTG

AATATTCAGTGTTTTATAAGTATTGTTCTCTGTTCTTCACAGAATATCTAGTTCAAGGAG

CAGGAATATCTGTACAGTTGTAGGAATGGTGTCTGAGCTCTTTGTTGTTGCTCCCAGAGC

TCTGAA

>c5914_g1_i1 len=224 path=[202:0-223]

AAACCGCTCCAAATACTCCCCACTTCATGGGTCCTATGAATGGAGAGGACGACCTGATTT

GCCAGAGCACAGCTATTCAGAGATGACCCTACCCTGTAGAATGAAAACATCCAAAAAACT

TGAGTCTAAAGCTCTATTTTATGAGGAAGAAGAGTTGCTGCTCCATAAATCTGACTCCTC

TCCAAAGCATGCAGATGTGAAGATGTCTCAAACATGCAGTCATG

>c5915_g1_i1 len=259 path=[391:0-258]

GAGAGATAGCAGCAACTCTTCATTCCTAGGTCCAGCTGGGTAGCAACTCAACTTCAAAAG

AGTTATATGTGCTGAATTGAACAAAGGCTTGACAGCAAGTTTTGGCAGTTTCTTTATTGG

TTAATATAACCAACTAGCAGTACTAGGATCCTAGTGTTTCATGCTCAACTTAAATAAAAC

TCAAGGAATCAACCTTTCTTTAATTCAGAGATACAATTTTATCTGTGAGTGTTTTTCAGC

AAATTACTTAGATCAAGAG

>c5917_g1_i1 len=591 path=[1239:0-379 671:380-590]

ATATATATATATATATATATTAAGATGTTTATATGTCGAATTAATTTATCTAAAAAAATT

AAAGAAAAAATACCTTATGGTGTTAAGCAGAGTCAAAACTATAAAGATGCAAAAAAACAA

GAAAGAATATCACTAGAAGGGGCAAGGAAATTAAAAGAAAGTAGAGGAATGATATTAGAA

GGAAAGAAAAGTTTATTTATGTGTTTACGTCAAAATACTGATATTAACTGGTATAGAGCT

GGTCAAATTTTAAAGCATTTAGAAATTCATCAAAGAGCAAAACCTGAAATAACTCCTAAA

CTTAGAGAGAAAATTACAAATATAGCAAATTTCGTAAAAAAAGGAAGATAATTTTGTTTG

TAATTTTAGCTAATCGCAACAATAAATTTAGAAAAAAAAAAAAATCTTTTTTCATGTACA

TATTCTTGAACATAAGACATTATATTATGATTAAAAATTATTAAAAATGATGTACATTAT

ATATTTTACTTATTTTTAGTATTAATTTTTTTACAGTTTTTTACAATAAATTTTTATTAT

TAAAAAAAGAAGAGAAAAAAAAAAATTAATTTAAATTAAAAAAAAAAAGAA

>c5917_g1_i2 len=444 path=[438:0-232 671:233-443]

TTTTTTTTTTTTTTTTCTTATTTTTTTGCTTGATGTAAATAAATATATCTTTTAATTTAT

TTAAAAATATGTTTGTTTCATAATTAATATACATATATAATTCTTTGATATAAATAAAAT

TTCAAATAAATGATATAAAAATTTTGAAATAAAATATAAAAAAATATTAAAGAAATGTAT

TTAATATGTTTATCGATGATATAACTCAAGATATTAAAAATGAAATAAATTTGAATAAAT

TTAGAAAAAAAAAAAAATCTTTTTTCATGTACATATTCTTGAACATAAGACATTATATTA

TGATTAAAAATTATTAAAAATGATGTACATTATATATTTTACTTATTTTTAGTATTAATT

TTTTTACAGTTTTTTACAATAAATTTTTATTATTAAAAAAAGAAGAGAAAAAAAAAAATT

AATTTAAATTAAAAAAAAAAAGAA

>c5918_g1_i1 len=465 path=[65:0-99 57:100-106 64:107-464]

GCTGCTCAGCAGTGTGGGCATGGTGTCCAGCTCCTTCTGCAAGTCCCTCAGCCAGCTTTA

CCTAACAGCTGGCCTCGTCACTGGTAAGTACAAGGAACAGCAGCAAAAATGGGAGAAAAA

GAGGGACTGTCCTTGAGGGGGACAATTTGAAAATTGCTAGAGAGGCATATGCATGTTTGC

ATTGGAATCAGTCTAAGCTTCAGGCGGTGATATCCAAGCTCCAGCTTATGGAGCTGTCTT

GAAAGTCATCTCTCCATGAAAACAGTACAATGTTGTTGTCAGTACAGCTCTTTCCAAATG

TCTGTCTTAGCATACCTGGCCAGAGTGGGTAGGCACAACAACCCAAGCCTCCATGCAGTC

TGTGCACATCCCTGCTCTTGCTCCAACCCCACAAGACCTAGGTGTTCCTGGTGGAGCAGA

AATGTGGGTCTTGTGCACGTGTGTTGTTTGCAGGCTTTTCTAAGT

>c5927_g1_i1 len=287 path=[1:0-286]

AACCGCTTAAAAGATCTCCTATCAAATACAAGCTGTAATTGGAGCTTAAGTTTTGCAAAC

AGATTCTGAGTATAGGCTTTCCAAGAGCTGTGCGTGTTCTTGTCTGAAATGCTTCAATCG

GTGCGACTTAAAAAGACGGAAGATCTGGCTGGAGTCACGCAGCCTGGTCTGGTTAGGGTA

GGCTTTCATTCTTCTGTTACATGCGTAGTTGGAACTTCCGCAGGCTCAGTTTTCTCGTAG

GTTACAGTTAAAGCTACTTCAAAGGCGTTGTTTCCTATTGTAAGCAT

>c5930_g1_i1 len=244 path=[39:0-88 279:89-243]

AAATAAAATAAAAATATCAAATATTTATGTTAAAAGTTTGTATATATAACAAGTTATATA

TGTTTTATTACTTCATTAGAGTACATTTTTTTTAATTTAAATTTTATATTTTATTTTATA

TATTTATATATTACATATATGTAAATAAATTATGAAAATAATTTAATTTATATATTTTAA

AAATAGAATAAAAGAAAAATTGAAATAATTATATACACATATATACATACATACATATAT

ATAT

>c5938_g1_i1 len=207 path=[185:0-206]

GGCTCTTGGCCATGTTTTGTGTGGCACTCGGGGCCACCTGGGTCCTGTGCTGTAGCTGAT

TGCCAGCCGGCACCAAACACTCCCAGAAGCCAGCGAGCAGCTCGCTAACAACCCTTCCGA

CTCAGCTGGACAGTGACTCAGTGGTTGAAGGGCTACATATACTCCATTGGGCCATCTGAT

GCTATCCTTCTCCTCAGGGCTGCTCTC

>c5940_g1_i1 len=428 path=[445:0-344 870:345-427]

AGAAATGATATGAAAAGGCAGTATGACAAGATGGATCAACTTGCACAGCTTCTGAATTAC

AGGAAGAAATCAGCTGAATTCCTGGAGTTGTACAATTCTGGAGAAGACATGAAGAAACGT

CACGTAATCAGGAATGACAAAGGAACTCTGAACCAGAGGTCTCTGACAGAGGAGGAGGCA

AAAGAGCTGGAAAACCTGGCTGCTATGGATTTGGAGTTGCAGAAAATAGCTGAGACATTT

AATAACAACAGGAGAGGCTGAAGATGATCTGCTTTGGTGTTTCAAAGCTAGGTGTAGCTG

TACTCACAATACCTGTATTTTGTGGTAAATTCACTGCCACTGGATTGTTGTTTGCCCTAA

AAACAAGAAATACTACTTACTGTCCACTATAGTGTAGTGATTTATAAAGCTGGAAATGTC

TTTAATTT

>c5944_g1_i1 len=336 path=[155:0-335]

AACACTTTTGAACTCCAAGATAAATTCACGTTTCCCTAAATAACGGATTCCTCCTTACCC

TGCTACACTTCATTTGAGGCTCAGCTCAGCAGTTTACCCAACCAAACAAACTCCTGGTGG

GTTCTGTTTGGTTGCTGTTTTAGTGGAGCTTTTTCTTTTGCTTTGTTTGTGTATATTTAT

GTATGTGTGTGTGTCTGAACTGGATTCTGAACAGGGTGTTGTGTCTGATAACTTCTACAT

CCTGAATTTATTTCAGTCCCAATGTTTGATTTCTGTTCTAATTTAAAATATTAAAAAATC

AGTTTTAGAATAAAACTTTTATTTACTTCCTTTTAT

>c5947_g1_i1 len=300 path=[63:0-299]

TATATATATATATATGTGTATATATATATTTTAATATAAAATTAACAAGCAAATTAGTTG

AAATTAATGAAACAAATTTTAAATAAGAAAGAAGTAACCTAATTAATATAATATGTAAAT

TATAGATATAAAATTATATTTTGTCTTTTATTTAAATACTATGTTAAAGAGGTAAATTCA

TATTAAAATAAATATTCACTTAGTATTTATTTTTATTTTTTTTCCCTTCCTGCTCTTCTA

AAATGTTTTGAAACATAAATTATGAAATTACACTTTTAGTAGTAGTAATTAAGAAATGTA

>c5949_g1_i1 len=318 path=[296:0-317]

AGCTGCCCCTCGGCTGCAGTGGGTTGGGGGGCAACGTGCTGCCCTTCTTCTGTGTCGATG

GCTGGCAGATCGAGGGCCGGCTGGAAATCCATGGTGCTCTCCATGGGAAACAGACAGGTC

GGGGGGAGACGGAGAGAGGGCTCCGGGGACACGGGACCCCCCCGCACCCCCAGCAGCGGC

ACTATGGAGCTGGATGAGGCGCCTGAGGAGGAGCCCTCAGAGATGCAGGGCCTGCTGGCC

CAGCTGGAGACCCTCAACCCCAGTCTGTGCGATGCCCCCAACACGGCCCCCGGGGTGGGC

GAGCGGGGCTATGCTGAG

>c5950_g1_i1 len=243 path=[1:0-242]

AACTCCTAATTTATGAGTGGTTGGATAAAACTAGCAATTACTTCTGGATTGATGTTATTA

AAGTGTGCATTGCAAATTCATTTATTGTAACAAGAACGTCTGATTTGGGGACGTCGAAAT

ATTATTGTGGTTTTTAATGTATTTACCAACAAAGTTTTAGAATTGTAACCTTCTGTTAGA

GGCAAAGTAAAATGCCAGTGTGTTCTGTATTTTCTTCTACAGACTTTATGTAAGCGTACT

ACA

>c5952_g1_i1 len=701 path=[479:0-610 1308:611-700]

AAAAAGAAAAAATAATGAAAAAATATATGGAAAGTATAGTAACACATTTTATTATAAAAA

TTAAAATCTTTTTTTATATTTATCTATATGGTTATTACATTTAATTTTTTTATTTTTTTA

TTTTTATATAATTAATAATGCGGATACATTTCTCCCTCATTTGGTTGTGGTAGTCTTTTA

TTTGCACTTGATAACATTTCTTGTCTTAAAGAAATTAAAATTGTTTCTATAGTGTAATTA

CGATTCCAATTTTTTAATATAGGTAAATTATTTTTAATAACCCTTCCGTTGTTATCTACA

CATGTCATTTCAATTTTTGTATCAAATTTCACGGTTGGTGGTGCATCAGGATAATTATCA

TCACAAAAAATAGTTAAAGAGTAAATTCTATTTTCAAATACAGTTCCAGGTTGTCCAAAA

ATGGTACATGACCAATTTGAAAGTGTTATATCATCAGCATTTTCTAAACCAAATGATACA

CCCTCACTTACATTTCCTTTTTGTCCTCTTTCTAATTCATCTAATAAACGAAAACTTCTT

GGAACAATTACCTCACTCATATTTTTATATACGAAAGTTATTTTTGAGTATAAAAAGATA

AGTACCAATAAATTACAACTAAAAAAATTCTTATACAATAATTAAATTTAAAAAAATATA

TTACTCTTTAGATGGGATAAAAAAAAATATATTTTTTATTA

>c5952_g1_i2 len=701 path=[479:0-610 1129:611-700]

AAAAAGAAAAAATAATGAAAAAATATATGGAAAGTATAGTAACACATTTTATTATAAAAA

TTAAAATCTTTTTTTATATTTATCTATATGGTTATTACATTTAATTTTTTTATTTTTTTA

TTTTTATATAATTAATAATGCGGATACATTTCTCCCTCATTTGGTTGTGGTAGTCTTTTA

TTTGCACTTGATAACATTTCTTGTCTTAAAGAAATTAAAATTGTTTCTATAGTGTAATTA

CGATTCCAATTTTTTAATATAGGTAAATTATTTTTAATAACCCTTCCGTTGTTATCTACA

CATGTCATTTCAATTTTTGTATCAAATTTCACGGTTGGTGGTGCATCAGGATAATTATCA

TCACAAAAAATAGTTAAAGAGTAAATTCTATTTTCAAATACAGTTCCAGGTTGTCCAAAA

ATGGTACATGACCAATTTGAAAGTGTTATATCATCAGCATTTTCTAAACCAAATGATACA

CCCTCACTTACATTTCCTTTTTGTCCTCTTTCTAATTCATCTAATAAACGAAAACTTCTT

GGAACAATTACCTCACTCATATTTTTATATACGAAAGTTATTTTTGAGTATAAAAAGATA

AGTACCAATAAGTACAAAATTAAAGGACTTCTTTTATATAAAAAAAATTAAGCAATTTTG

GGAAACAAACAAATTATAATAAAAAATATATTTTTTTTTAT

>c5974_g1_i1 len=334 path=[670:0-333]

ATGGCACGTGCAGGACTGCTTTTAGGACTATCAGCCTTAAGAGAATGTTGATGCAAAACA

CCAAGATAAAAAATAAGAAAGCAGTCTCCACCCATATAATGTCCAGGAGCTTTTTGTTGA

GCTTTGCTGGACCAGCAGATGTACAAGCTATTATTAATTTGGTTTAGTCCTTTTTACCTG

TGTTTAGATGAGCATTTAGATTACATGATTTTTTTAAGGTATTGTAATGATAAAAGGATG

AGAAGCAAGCAGACATTGCTGCTTATTTTTTTGGCACAAGTTTTTAAAATGCCCCCCAGG

AACTGTATGTAACAACCTCATTTTCCACATGTGG

>c5975_g1_i1 len=279 path=[279:0-278]

TGGGTTTGGCGACGCTGGCGCAGGCGTTGCCTCGCGCAGTTCTGCTGCGGCTGCTTCATG

AAGAGCAGCGCCGGGAGCAGCGGCACGTCCAGCGAGGTGGGCGGCACGATCTTGGAGATG

AGCAGCAGGAAGACGGTGAGGGCGAGCAGGACGGAGATGCAGAGCGTCATCTTCTCTCCG

CAGTCGGAGGGGAGGTAGAAGACGAGGATGGCCAGGGAGGTGATGAGGATGCAGGGGATG

ATGAGGTTGATGGTGTAGAAGAGCGGCTTGCGCCGGATG

>c5976_g1_i1 len=242 path=[220:0-241]

GCTATCTTCACCTATTCCTCTGCTACACTTTACAAGAAGCAAACTTCATCAGCATCAGTA

TTATGGGGCATTTCTGGGTGACACTGTTCTCTTCTGCTCAGTGCCAGCCCTTTCTGCAGG

AGGCTTTTGGCGTTCACCCAACACAGCCAGTGTAACACTTGTGCAGGGAAGCACCCTCAG

CAGCTGACCCCTACAGAGAAGTTGATGTGCATGCTTTATGAGGCCACACACTCTATGAAT

TT

>c5979_g1_i1 len=201 path=[155:0-200]

CTGAAGTTCCCTGCTTATCTATTGAAGATGTGCTGCCATACTAAAGGGCTATAAAGATGA

AAAGTCTGATCAGAGAAGCCCAGGCACAACTAAGCTCAGTGAACTTCTGATTGCCAGTGG

AAGCAAGGGTTCAGGTTTCTCTCTAGATGTGATCGATGGACCTATTACTCAAAGGGAGAA

CCTGGGGCTCTTTAAGGAAAG

>c5986_g1_i1 len=217 path=[349:0-216]

AGCAGCATTGCTCGGATTTAGCTGCTTGACATTTATTCAGCAAATTTAGATTTGAGACAT

GTGAAGTTTGGAAAGCGTTCAGGGAAGAACTATGCAAATAAAAGTAGATCTGGAAAACCA

CTTACACTTGTCTTGTTCAATCTGTTGAGTTCACTGAAAGGTACTGGCTTAGTCTCTGGA

ATTCAAAACAAGAAAGGAAATCCTATAAAAGAAGAAG

>c5987_g1_i1 len=214 path=[400:0-213]

GTGTCATAAAAACCCACCTCCCCCTACCCTACTTATTCAACTTCTGCTGTGTGATTGACA

TTCATCCTTCATGCTCCATTTTCTAGGCACCATGTCATCACTCAGAGCTGAAGCCGGGGC

AGCGGGTATCTGAGAGCTGGTCATGGGGAACGCCTCGGGCAGTGTGTGCGCCTGAAGCTG

GTGTTCCTGCCCTGTTTCTTGGGTTCTGCTGCTT

>c5997_g1_i1 len=305 path=[283:0-258 542:259-304]

CTATGAGAGTTTAGATCATTGTAACAAAAGGGTCTAGCAAAGCAATCCATACCAAAACTT

CAGTGGTTTGGGAATTCCATTGCCATAATGATATCAGTTAGAACAAGGAGCCTCAAATTT

TACACAAGTAATATGTAAATTGGCAAATGATTTCTGGACACTTGGTGTTTGTCATTTACC

AGCTCAAAAATGACAATATTCAGCTGAAGCACAGTTATTCTTTAGCTTCATGAGCCATAA

TTACCAACACTGCTGATTATTTCTAGTCTTGTGGTTTCTTTTTTTGCAAAAAGGACTGTT

TGGTG

>c6000_g1_i1 len=219 path=[351:0-218]

AAGGATCTTCTGCTTTCAAACCAGCCTTCTAAGTTGCATCACATAAAACCAGTAGCGTGT

GCTCCTAGCCATTTGTTTCTGCAGCTGGAGTAATCATGTCACGAGCCTGGTTTGTAAGTA

ATGCAAGTACTGCAAGGTAATTACAGGCAGAGGGAATAAAGTTCTGATTTATCTTATAAC

CAGCAAAGATAGCTTACAGAGTAGAGTACAGGAGAACAG

>c6001_g1_i1 len=351 path=[155:0-350]

TCTGTTCTGTTTCACTTCATACTGGAGAGAGAAAAAAAAAAAGACATGCACTGAAGTATA

ACTTAACAGAAATCACACATCAGCCCTACGTGTTCCTATCAACAGGTACATATCCTCTAC

TTTGATACCATGGGTTTGATTCAAATTAAGGGAAGCTGTTTCATATATTTATTTTTGCAT

TACCTTATTTCAGTATTTTGCAATTGAGTCCTTCAGTACAAGCCTGTGCTGGGTGCTGGT

TTGTAGATTTCTGCCCTCGTTTAATTTTTGTGTATGTGAAGCAATACAGCAGGAAGACTT

ATTCAGCCCAGAGCTCTTTTAAGAGTGATGGGGAAGATGTTTTCTGTGGAG

>c6003_g1_i1 len=221 path=[1:0-220]

AAGATTTAAAGTCAGTATTGTGCTGAAACTTCCCATCTCCTGGCTGTGGCAACTTAATAA

GCTGTAATGTTGTGGCTGACAGCTGGATACCTCCTGGAAAATACCTGTCTGGCGGGCTGC

AATGGAGATGGCTTAATTTTGAAACATCAGAAAAGATCCCAGAGATAGTGTATGTTCTCT

TACATAACTTACACATACAGGTGAGGAAAATGGTTGTCTGA

>c6007_g1_i1 len=208 path=[216:0-174 391:175-207]

AGTGTTTCTTCTACCTCAGCCTGCAAAAACAGAAAGCACAAATCAAAAGATCACAGCTGC

TGTTTGCCTTCAACTAAAGCTTCACAACAATGTGCCTGAAAGTAACTGCCTACACCTTTC

CATAAAGCACTGCATACTTTAGTACTATTTTCTCTGCCTGGTCTTTATAGTTGGTTTCCA

TCCATTTTGGATTTCAGGGGGAAGTTCC

>c6009_g1_i1 len=391 path=[37:0-332 36:333-390]

AAAAACAAAAAAAAAATAAAAAAAAAATAGATAATAGTATTATTAATAATAGCTTTAATA

ATGCTATTAATAATAAAAATAAGAATATAATTATTAAATGTTATGAACAAATAACTTAAA

AATATTTAATTTGTTTTTATTAATAAAAAAATGAAAAGTAATGTGTAATGATAAATTAAA

AATATATATCTTCATCCATTTTGAAAACATAAACAATGTACATAAATATATATACTACTA

AAAAATAACAATTATTTAATTTTTTTCTATGTCATTAAAAGTTTATTTTATTATTTATTT

TTTTTAAAACAACACACATTAAAAAAGGGTTGTAATACTAAGTACAAATAGCTATAAAGA

ATATCACAAATTTTACTCTAGAAAAAACATA

>c6012_g1_i1 len=245 path=[1:0-244]

AGTATGCAAGAAAAAAAAATATATAGTCTTGGGAGCAAAATAATTTCCAAGTAGTAAACA

TCCCCTTCACGAAGATTAACACATGAAAGGCGTATCTACTATCTTGTGCATTGATGGGGC

ACTACTCCATCTTTGCACAGTTCTAGTGAATAAAACGTGGTGCTGCACAAATACATGCTC

ACTGAGGCTTTTTGCAGACACTAAGCTCAGAACCAGTTCAGAATGCTTGAATCAAATGCC

ACTTT

>c6022_g1_i1 len=332 path=[451:0-233 71:234-331]

AGCCCAATAAACGAAGGATCTCTGTAAGGTGGAACTGCTGGGATTTAACCACAGGTGCAA

TTTCCCCCATGCACACTAGGTGGAAGTATGGAAAACCCTAATTTGTTCCGTCTTGACTTA

ACTGGAAGCGGTTGAAGTGTATAATAGCACCTGTCACCGCAGGAGGAGTCTTTTTTGTCC

TTCACCTGCAACTTCACCAATTGATGAGCCATAAGCAAGATGGCTCCCATTAGCACTGGG

TGTTTAAATGCTCTGGAAATAGAATGTGTTTTTAAAACCAGTCTGAATGATGATACAGTT

TAATATTTCTGATGTCTGTTTTTTCACATTTG

>c6027_g1_i1 len=274 path=[105:0-63 169:64-124 42:125-273]

AATTAGTTAATACATAAAGTCTACAGATTTTTAGTAATTATAATTTTCTTAATTTTTTTT

AAAATATTTATTCCTCTAAAATTTTTACATTTTTTTTTCCATTTTAGATAATTTTAATAT

TTTTACATATTTTAATAAAGTAAAACATTTTAAATCATAAGATTTACAATAATTAAACTA

CATATTTGTGCATTATATTTTAAATAAATATATATTATTAAGGTACAATTTTATTTTAAA

TTGTTTTATTTTTTTCCGTTTATTAACTTATATT

>c6041_g1_i1 len=620 path=[598:0-173 772:174-619]

AAAAATTGTGCAAAAAAAAAAAAAGAAAAATGATATTATGTAAAATGAAACAAGTTAGTA

TAAATATAAATATATAAACTATAATACAAATTAGTTAATAAATAAATGAATTTCATTGAT

TACATAAAAACATATTTTCTTGAATTCATTTTTTTGGCTATCATTTGAAATAATATTATA

TATTTCCTTTTTTTGTTATCAAATTGAAAAACATTGAATATTTTAACTTTTCTAGTTATT

TAAAATGAAAAGTTATAGTTAAAAAAAAAGATAAATGAGTATTAGCTTATATATACATAC

GTAAATACATACATAAAATCGATTGTATATTTTAAAAAAAATAATTATTAAATGTATTTT

AAGTGTATATTATTATTCATTTTATATATGTACATGTATCATAAAATAAAATAAGATTTA

TATATTATAAATTAAAAGCCTACTTAAGCAGTTTAATAATATTAAAAATTAAAACTGATT

AAATTAAGGTATATTATTTTTTGTATTATATTTTGCGGAAAATATATTTTTAGTTAATTT

TTATTAGCATATTTAATAAATGTAAAAATTCTTATATGTATTTATTTGAATTTTTATTTT

TTTTTTAATTAATTCAATTA

>c6041_g1_i2 len=516 path=[1195:0-69 772:70-515]

CTTTTTTTTTTTTTAAAATATGATATATTATTGAATATGATTTTTAATTACATAATTAAA

ACATGCTAACATTATATATTTCCTTTTTTTGTTATCAAATTGAAAAACATTGAATATTTT

AACTTTTCTAGTTATTTAAAATGAAAAGTTATAGTTAAAAAAAAAGATAAATGAGTATTA

GCTTATATATACATACGTAAATACATACATAAAATCGATTGTATATTTTAAAAAAAATAA

TTATTAAATGTATTTTAAGTGTATATTATTATTCATTTTATATATGTACATGTATCATAA

AATAAAATAAGATTTATATATTATAAATTAAAAGCCTACTTAAGCAGTTTAATAATATTA

AAAATTAAAACTGATTAAATTAAGGTATATTATTTTTTGTATTATATTTTGCGGAAAATA

TATTTTTAGTTAATTTTTATTAGCATATTTAATAAATGTAAAAATTCTTATATGTATTTA

TTTGAATTTTTATTTTTTTTTTAATTAATTCAATTA

>c6043_g1_i1 len=205 path=[282:0-157 21:158-204]

AAAAAAAAAAAAGCACAATGGATATTCCATCTTTGACAACAAGTATTTGGCTACCACCTT

GTGACCACAGCCATTTATGCTTTTGTAAGTAATGCCTTTACTTTTTGGAGAGCTCCTTCA

GAGAACAGGTCACATTAAATGCCATAGCAATCTATATGAGTTGACAAAAGCAATGCTTCT

TCCAAACTATTTAATTAATTAACAA

>c6051_g1_i1 len=242 path=[27:0-50 259:51-241]

AGGGAAGGGAAGGGGGGCCAGGGCTGTTCTTTCCATATGCCAGTACTAGTTACACATTTC

ATAACACAGGAAAGAGGGAGTGTATTTACATATACAGTGCATGGAATCTTTTACTAGGAC

AATGAATTCCATATCAAGATTCAGGTTGAAAGAGTTGGGACAAAAGAGTTCCAAAGAACA

AAGGGAAGTTAAAATTTACTTAGCATTTCCTCAGAGCTCCTTAGCTACAGATAAAAAAAA

AA

>c6051_g1_i2 len=203 path=[257:0-11 259:12-202]

AACACAAAGTTAACACATTTCATAACACAGGAAAGAGGGAGTGTATTTACATATACAGTG

CATGGAATCTTTTACTAGGACAATGAATTCCATATCAAGATTCAGGTTGAAAGAGTTGGG

ACAAAAGAGTTCCAAAGAACAAAGGGAAGTTAAAATTTACTTAGCATTTCCTCAGAGCTC

CTTAGCTACAGATAAAAAAAAAA

>c6055_g1_i1 len=344 path=[322:0-343]

TGAGATGATTTGTTTCGTTGCTTAAATCTATTAGAAGTTCCCAGTATCGTGGTCCCTTCA

CTCTGATCTGCTTCAGTAAGTGAAGCTCTGGAAGAAGCGTGGGTGCCATCATTTCCTCGG

TGGTTTCTGCATACCACTGTGTGCCCTTCAAAATGCCATCAGGATTCACAAATCAGCAAA

GCTCATTTTCATGTGAAAAGAAAGAAAATGTGCTTCTCACAACACAGTTGCAGAGGTCAG

TCAGTTTCTGTTGCTGAACTCTGAGTAAATACATAGCTTACCATTGCCTTTAACATTACA

AGACATAGAGAGATACTTGTCATGGAAGCATCACCATACGGGAG

>c6078_g1_i1 len=266 path=[285:0-222 25:223-265]

ACTAAGTATTTAGGGAATGTTGTAAACATAATCAATTCAAACTGCATTGTAAACAATGAT

ACAGAAAACTAATTTGGTGCTATCTTAGCTCAGTGGTGTTTATAGCTGTTAGAGGAAGTA

AGATGTCAGTGAAACTGTGATTGCTCGATTAGGGTGATTAGTTCTTTGTAGACTAATTGC

ATTAGAGCAGGATGTGAGACCTGAAGATCATAGCAGCAGTTGAGTGAATTACTATCCAAG

GCAATGTACTACTTTCAACAATAAAG

>c6078_g1_i2 len=265 path=[285:0-222 507:223-264]

ACTAAGTATTTAGGGAATGTTGTAAACATAATCAATTCAAACTGCATTGTAAACAATGAT

ACAGAAAACTAATTTGGTGCTATCTTAGCTCAGTGGTGTTTATAGCTGTTAGAGGAAGTA

AGATGTCAGTGAAACTGTGATTGCTCGATTAGGGTGATTAGTTCTTTGTAGACTAATTGC

ATTAGAGCAGGATGTGAGACCTGAAGATCATAGCAGCAGTTGAATGAATTAGTATCCACC

CTTTATTGTTGAAAGTAGTACATTG

>c6083_g1_i1 len=241 path=[563:0-240]

AAATGAGTCTTTTCTTGAAGTTACCCGCTGTGCAGTTAAAACTAAGCTGGAATATAAATG

GATGTTTTTGCAATCCGGGGAGTTATAACGTAGTATCACATAGCCTAGCAACAGCTCGGT

ATCGGCATAACTCACTGCCGTAACGAGCTGTGAGCACATAGGTGTGGCAGGAAAGTGTAT

AAGCTATTGCAAGCAAATCCCCAAATTTGCATATGTTGTTTTCATTTACAATGCTAATAG

A

>c6084_g1_i1 len=672 path=[57:0-582 56:583-671]

TGGAAAGGCAGCTCCTTGTTGAGAGGACAGGACTCGGCTCATCTCTCCAACATGTCATGT

GTAGAAGTAAGGTTCTGAGGGGGAAACTGGGTTGATCCAGTAGCATTCCTAAAAACATCC

AGGAAATTCACACTGCTTCCCTATGCTTGCCAAGAGCAGGAAGAACACATGGCAGGTCTG

CACCACGGAGATACCGGCCGTGCACAAGATCTCACTCTAAATGGATGAGCAAGCGCTCAA

ATTTAAACTCTGTTATATGGACCACCTTTTTACCCAGTCTTTACTGGGGCTTACTGGTTA

TCCCTGTGCTGCAGACCTCCCTAACAAGCTTCTCCTTAGGTAGTTCTTCTCTTGAGCTGT

GTCAGTAGCTCTCTGGGCAGAAGAAATTGTGCTGACAAGGATCATTTCTTGTTGGGGTGA

ATTCTGGCATAGGGCTTTGCAAGGAAAAACCAATTCAGGGTGCTATAGCTCTGCCAGCAG

CTCTCTTACTACAACGCAGGCCTTGGTCATGGTAGAACAGAAAATACTGAGTCATGTTCT

CAGCGACCTTGGTGTCAGTAAGAATTGTTCACGTTTTAATAATGTTTGATTAGCCCTTGT

TCTAATATCTGGCCCAAGTCATCCACATAAAGAAGATGTGCAGGAGGCTTTAAAGCATAA

GTAGCTGTAGAT

>c6086_g1_i1 len=265 path=[288:0-214 914:215-218 25:219-264]

GGTTATTTTCAGCTCTAAGATTGAGCACAACTGTTCTTTGCTTTCAAAATCAAACAGCAA

CCAAAGCCCCCTTGCAAACTTCAGCAAACCACCGATTGATGGAGTGAATAACAATAAACA

GAAAAATCATACCATTTCCTGTTAGAGCAGAGGATTGAGGCAGGATTCATTTCTGTGGGA

TACTCTGTCTGAGAGTGATCTTTTGGGCTTGCTTCCATGTGCAGCGACAATAAAAGGCAC

CATGGGGCATAGGCTCTTAAAGAGA

>c6091_g1_i1 len=286 path=[508:0-285]

GCTAAGATGCCTTGGAAAGGGAAAGAGCTGAGGATGTTGGATTTGAGCTGAGGAAATAAG

AAGTCATTGTCAGTGACACTTCTGCGAGGGACTTGCAAATACGCCATCACATACAAATTA

CTCTCTGATCATCCATCCCCTCCTCCGGATAAACACGCAGTCGTCAGGCTTTGTAACTCT

GGTCATTAGGCAGCAATTATTCAGCTCCTGCTAGCATTTCAGTTGCGCGGCCGACATGAA

TGGATGATTTCAGTGAGAGCTGTAAATACAGCCATCACAGCGCAGT

>c6105_g1_i1 len=284 path=[155:0-207 102:208-283]

TTATATTTTAACTTCCCATTCATAATAATAAAGCCATCGTTCAGAGACCCATGCTGAAGT

ATGCACTGTTAGAGAATTGTTGGGGAGAGCAGTAGTAATGCAATCTAAATGTTGTACAGA

AAATTTACACTGAAAGGTGTTTTATTAAAAACTGTGAGATTCAACGTTTGAATTTTATGC

CGAATGTTTCTAACTGAAAACTCAACTTCTCTTCTGGTATGAAAATTAAAATTTATGCAA

AATTGTGTCTATGCGGATTCTTTGTTATTGCAAAGAAACTGCAG

>c6110_g1_i1 len=234 path=[353:0-97 15:98-233]

ATTCAGAGTAGGAAAAGAAGAAAGCTACCATAGTTGTTTAGTCTTCAGTGAAGTATGCCT

CCAGTCCTTCCAGCTCTGCATCAGTAGTCGTATTTGAAGAGGTGGGAGCAGAATTTGAAT

TCACACTTTCCTGACTATTATTTTTTTGTTGAGAGTTTCCCAAATGATTTGGTTCCTTCT

TCACTTCTCTTCCACCACTTAATATCCTTTGACTTTCTGCAAAATACTGATTAG

>c6117_g1_i1 len=329 path=[155:0-328]

ACGCATCCCGCAGTCCGTATAAGCCTTAAAAACCACACGCAGGCACACCAAAGAAAGCTA

ACTTTTATTATCAGACAGCCCTGCTGTCTCCAATTGCAGCCGCACGTTCCTGCGCTGATA

CAACGTATTTTCTGAACACACAAGAAAGGATTTCCAGAGAAGTTGAGATTTCCAACGCTG

CCGGAGCCGCTTCCGACGACTGACAGACGAGACCGCCTCGCCTCACCCCGCACCGCCCGG

GGAGGAAATGGCGGCGGGCGCTGTCTCAGCGGGGAGCGGGGATCCCGGCCCCGCAGCGCC

ACCTCTCCGGCTCGGAGCGCCAGGGCCGG

>c6119_g1_i1 len=328 path=[330:0-302 25:303-327]

TATTTTTCTTTTGAGAATAAAATATTTGTTTGAATTAGTATAATTGTGCTATTAAGTGCA

CTTTTAATAAAATATGTGCGTTGATATCACATTAATATTTTCATTAAGAGTATTATTTAA

TATAATTTTTTCTTTTTTAATTATTTAAATTTTTTCCTAAGTGCTTTCTTAAAAAATAAT

AGGAAATTTGAATACATACTCTTTTCAAAGCACTAAAGACAATTAAAAAATGCATCTTTT

TTTTTTTAAAGATAACTATCATTATAAATTTTTTTTTCATAAGTGATTATTTTATTATTT

ACTTCTAAATTGTATAGGTTAATATTGT

>c6119_g1_i2 len=328 path=[330:0-302 632:303-327]

TATTTTTCTTTTGAGAATAAAATATTTGTTTGAATTAGTATAATTGTGCTATTAAGTGCA

CTTTTAATAAAATATGTGCGTTGATATCACATTAATATTTTCATTAAGAGTATTATTTAA

TATAATTTTTTCTTTTTTAATTATTTAAATTTTTTCCTAAGTGCTTTCTTAAAAAATAAT

AGGAAATTTGAATACATACTCTTTTCAAAGCACTAAAGACAATTAAAAAATGCATCTTTT

TTTTTTTAAAGATAACTATCATTATAAATTTTTTTTTCATAAGTGATTATTTTATTATTT

ACTACAATATTAACCTATACAATTTAGA

>c6126_g1_i1 len=241 path=[155:0-240]

ATTATGGGTTGCAGAGCCATGAGGGAAGAAGATGGACTAGGCATCAGAACTGCCCCAGCC

CTGCAGGAAGCCTGTGGTAGATGGTGGGCTGAGCTGAGCCCTGCTGGCACACTGAGCCAG

GAAGCCTGGCCCTCCTGTGAGCAAGTATTCTTGCACTCTTCGTGACATTTCAAAACCAAG

AACAAACGCCTCCTGAAAGCCTTGGGATGTGGGGAGAGCAGAAGGGCCACCAAGAAAAGC

A

>c6136_g1_i1 len=203 path=[1:0-202]

GGATTGCTTTGCAAGGAGGACACACTGCTTGTGGCTCGCCAGTGCGTTTGGGTAGTCCCC

GATGGCTGTGTACACATGGCCCAGACTGCTCAGTGCTGCGGATGCTGCCTCTCTGTCCTT

GAGTTTCATGGCGAGCACCAGCTGATGTCTGTGGTTAGTAAGGGCCTCCCGATAATTTCC

TTTGGAGAAGAATGCGGAGCCTA

>c6136_g2_i1 len=225 path=[563:0-224]

GCTGAGAAAGCCATTGAGATGCGAGCTTATGACGGCTTGGGCCACGCAGCTAGATGCATG

CAGGACCTGGAGAGGGCTAAGCAGTACCACGAGGAGCAGTTGCACATCGCTGAGAGCCTG

CAGGACCGAGCTGCTGAAGGCAGAGCCTCCTCCAATTTAGGAATCATTCACCAGATGAAA

GGTGACTATGACACCGCACTGCGGCTGCACAAGACACATCTGTCT

>c6141_g1_i1 len=261 path=[51:0-56 25:57-260]

TTTATAATTATTATTATCATTAAATTTTATAATTAAATAATATACGTGTAATATGCTTTT

ATATTTCCAATAAAACTATATATTGTATAGTGTTTTATTTTCACCTTTGTTAAACTATTC

ATTCATTTTATAAAATGCGTTTATATTTATAATATTTTAACATTTGTTATGAACAATATA

ATTATATTTCCATTGTATAAAATCTATAATATATAAATGATGTGAATTTTGTAAGTGTTA

AAGAAAAAAAAAATAAAATAA

>c6141_g1_i2 len=228 path=[1:0-23 25:24-227]

AACTTTGTGTTTGAGGTAGTATTATTTATATTTCCAATAAAACTATATATTGTATAGTGT

TTTATTTTCACCTTTGTTAAACTATTCATTCATTTTATAAAATGCGTTTATATTTATAAT

ATTTTAACATTTGTTATGAACAATATAATTATATTTCCATTGTATAAAATCTATAATATA

TAAATGATGTGAATTTTGTAAGTGTTAAAGAAAAAAAAAATAAAATAA

>c6145_g1_i1 len=207 path=[185:0-206]

TAAAAGCAGTATTTTGGGTTCAGGAATTTTACATCATTCACTATAATAATAAAGAGGTAA

TACAAAAGGGGATTTAAATCTGTCAGAGGAAGAATAAGTGCAAAGTCAGTCTGTTAAAAA

TTGTAAGCAGTTTTTCAGCAGACTGATGCATTATTTAACTGAAAGAAAAAAGTATCATAA

TAGTGTAATAACAGTACCACAACACTG

>c6149_g1_i1 len=424 path=[6:0-423]

TTTTTTTTTTTTTTTTTTATTCCTCTTTAAAATAAATTCTTTTATAAAAAGGTATTTAAA

TAATATATTATTTTGTATTTATCCAAAATAATTGTGAATCTTTATGTATTCAAAAAATAT

TTATTTCTTATAATCTTTGAATAATAATTTATATTCAAAATTACTGTTTGATTTATTCAT

TTAAATTATATTTTTATTGTATGATGTATCTTAATGCTAAGTACGTATATGGTATAGTAT

ATATAAATCTTATTTCTTAAAATTGCACTTCAAATTATTCGGCATAAATTTCATGTGAAA

ATGATTTAATAAAATTAACAATAAAGAATTTTTAGAAACTTAATTACTTAAATTAGTAAT

ATCTTTTATAGATATAAGTTTTTAAGTTACAATTTTCAACTTGTATTATTTTAATAAATC

ATTT

>c6150_g1_i1 len=225 path=[603:0-58 661:59-201 850:202-224]

TGTTTTTATATATTTTTAAAGTTTAAGTTAAGCCTTCATCTCCCACATTGCTTCAAAAAC

TTGGGAAATCTGCATAAAAGCACAACTAAGGTTTTACTTAAAAAGCACTGTAGACATAAA

TGCAACACAGGCAGACTCCAAAAAGAGCTTATTTGGAAGTTTCTGGAGTCAATGTCTGGT

GTAATCAACAAGGAATGTGGTTATCTGCTGCAAACTTCAGAAAAC

>c6150_g1_i2 len=224 path=[603:0-58 661:59-201 848:202-223]

TGTTTTTATATATTTTTAAAGTTTAAGTTAAGCCTTCATCTCCCACATTGCTTCAAAAAC

TTGGGAAATCTGCATAAAAGCACAACTAAGGTTTTACTTAAAAAGCACTGTAGACATAAA

TGCAACACAGGCAGACTCCAAAAAGAGCTTATTTGGAAGTTTCTGGAGTCAATGTCTGGT

GTAATCAACAAGGAATGTGGTTTTCTGAAGTTTGCAGCAGATAA

>c6150_g2_i1 len=202 path=[603:0-58 215:59-201]

TGTTTTTATATATTTTTAAAGTTTAAGTTAAGCCTTCATCTCCCACATTGCTTCAAAAAT

ATATAAAAACAGTGATATGGTATTAAGCAACTACACTGAGACTAGATTTTGAATAACTAA

TAGGAAAAAAACTTGAGTTGAATCCTAAACATGGTGTTAGGTGTTTTAAATTCTGTTTTT

TCTTATCAGCAAAATCATGCTG

>c6151_g1_i1 len=368 path=[53:0-367]

GACTGAAATCATTTCTACAGAACTGACGCCTGAGAGCTCATCTTGGTTTCACTGCCAGCA

TTACTGAAGTTATTCCAATGCTGAAGTTTGACCAAACGATGAATATTTCAGTCAGTAACA

CGACCACCTCTGAAAGTCAAGAAGTGTACTTCTCCCTTTCTGCTATGAGAAGGCATCCTC

TATTTGTTTTCACAATTACAGAGATCCCTGACAAAGAAATTACAGAGATGTAAAATCACC

AGGCAGTATCCATGAGAAACTACCGATGCCCACAGGATGAATTTTAACCTTACTGCATTC

AGTGTTTTATACTTGTGTCTGGGCTCCAACACACATACAAGGTAGAGGCCTATGACTCAA

GAAATGCA

>c6170_g1_i1 len=269 path=[247:0-268]

GCTGAAAAGAGCCAATTAATGGTGGTCCTTTCATGCCTTAATGATGACTCTAAACCTGAC

CTCCTGCAGCAAATCCTCTGGGATGGAAAAACCCATCAACACCAAAGGCTGTGTGGCTTC

TTCCTCTTTTTAATCCCTCTCTGTAAAGTATAGCAAGCAACACTCGAGCCCAACATAAAC

CACATTGTGACTTTCAGCTCCCAGTGTGATCCCAAAGCCATTCGGGGAGCAATAAAGCAA

CTTACAATAAATAATCTGGCATGTCCGAA

>c6173_g1_i1 len=206 path=[186:0-205]

TTTTTTTTTTTTTGTATTTTTTAATTATTTTGATTCTATGAATTTATTTTGCTATATTAA

AAGCTAAACTGTATTCAATGATATTTCTATAACTACCATGACGTTGCAATTTACCATCGT

ATAACGTTTAGCAAATACTTAAGTCAATCATTAATTTTTTAATATTTTTGTCTTTCATAA

ATGTACTTGTATATTCTTTTAGGAAT

>c6176_g1_i1 len=276 path=[155:0-275]

TGGGGTTTTGTCATTAATTTCCTGCTAAAGGTCCAATCTACAAGAATGCAACAAAGAAAA

CTAAAAGGGACTAGACACAACCTCCAAAATTCAATGCTAGGAGCTACCTTTAACCTTCAC

AGCAATTTAATCAAATGCACTTGGCACAGGCTAAAAATGACTTCATGGTCAGTGACTACA

GCTCAGCACACAGACAACCATTTATGAGGCACAGTGACTGCTCTGAGTGATGTGGCCACA

TCCTAACTGTGACACTACACTGTATGCCTTTATCAA

>c6186_g1_i1 len=384 path=[769:0-383]

AAATTTTAAGTAGATATAGTTCTACATTTTTTGCAAAGACATAAAATTTTATTTATTTTT

CAAAGATAGCAAAGTTTTTCTTATTATTCCTTTTAAAATAAAAATTTTTATATGTTGTTG

CATACACTGAAGATTAGTTAATCTTTAATTATTATGATAACAAAAATATGTTACAAAATT

ATAAGTTATGATATTTTTAAAAAAAAATAAAATAGCCAAAATATAAAAAGTAATTGAATA

ACAAATATCTAAATGGAAAACATGTGAATAGCTAATATCTTAACAGAAAATAAATAAATA

GCTATATAGCGAATAGCCAAATAGCAAATAGTTAGATGATAAATTGATAAATGTGAATCA

AATAAAAGAGCACAACGGACTAAT

>c6186_g2_i1 len=407 path=[385:0-406]

AACTATATCTACTTAAAATTTTTAAATAAAATTTATCCCTTGTTATTGAATAGTATTATC

TACCAATTTTTTATTATATCAAGAAAAGATATAAAATAGATAAAGATTTATTTGTTCATA

TAAGGTACTAAAGGAAAATGCGCATATTAAAATATGTCCAATAAAACATAGGAATCTTAA

AAGTTGTGCATATCAAAAATACATGAACATCATGGGATGTTCTAATTTGCTAATTTACCA

ACTGAAAATTCAAAAACTTTTATAATGCTTTTAATAGTCTGTAAAAAGGTTTCTACTCAT

TTTATCTTTTATAATATTGATTATTATAAATCATACTATTTAATTTAATTCAAAATTTAT

TGTACTTTTTAAATATGTATTATTATATTTATATAATTTTCTATAAT

>c6192_g1_i1 len=221 path=[199:0-220]

AAAATCTACATACGGAAAGTCTGGAGACTGTTTTTGGGGTATTGCATGATGGAAGTTAAA

AGCCAGCTTAGGCTGCTGATTTGCTAGTAAAGTAAGCTCTGAGCGGCAATTGCATTCCTC

TGCCTTTTCAGCTAATTTGAGGGATCAAGGGAGCTTGGTGAAAGGTTAGCCCATGCAGAG

AGTAGATGCACCGAAGTGGTAGTGCATAGGAGAGCAGGAGG

>c6206_g1_i1 len=249 path=[1:0-119 121:120-248]

ATAGGACCCCATAGGGCCCCATAGGGAGCCTTGACCCCATAAGAACCCCATAGGATCCCA

TAGAACCCCATACAACCCCATAGAACCACATAGGACCCCATAGGATCCCATACAACCTCA

TAAGACCCCATAGAGACCCTTGAACCAATATGACCCCATAAGACCCCATAGGACTCCATA

GGGCCCCATAGACACTCTTGACCCTATAATGACCCCATAGGATCCCATAGAAGCCCATAG

GACCCTATA

>c6214_g1_i1 len=208 path=[1:0-207]

GGAGTTCATCTCTGAATCTGTACTCGTACCTGGGATTGCTCCAACCCTGATTTCTGGGCA

GCAATAAACCAGATGAGGCTTAACAAAAGCAAGTGTAGTCTTGCACCTGGGAAAGAATAA

CCACAAGTATCAGTACAGTTTGGGGGCATGACATGCCGGAGAAGAGCTCTGCAGAGAAGA

ACCTGTGATCCACCAGGACCCCCAGGTT

>c6217_g1_i1 len=313 path=[35:0-248 34:249-312]

AAAAAAGGGAACGTGAGGAAAGTGTGGTTTCAATGTTAATAATAGTAATAGCATGTAGCA

GCAGCCAAAGATAATATGAAAAGTCCCATCACAGAGCTACAGCTTTAGGTGTCCTGGAGG

GGCATAGCTCAGAACTGTTTTGTGTCCAGCACTGACTGCAGTGGGCTCAGGGCATGTACA

GACCTTAGAAAGTAAACCATCCTGGGCCTGTCCAATAGAGACTGCTTGGTGATCTCAAAT

ATTGTAGGCAAAAAAAAACACAGGACTCCAGCCTGAAGAGCCATTGTATCCTAAGCACCA

GGAATCAAACACA

>c6221_g1_i1 len=223 path=[355:0-222]

GCTCCAAGTGAAGTAACCCAGTAGAAGGAACTACAGCCTCGGAAAGGCACCGACTCAGAA

CGCATTGTGGATTACCTTCACAGGAGCCAACACGTGCGGTACGTTCACGCCATGGTGCAC

ATAGCAATTATGAACAATAACGGTGCACCCAAGTTTTGCCACACGAGCAGTTGACAGCAT

GAGTCATCATAAGTCATTTGTTTTCCCAAGGAAAAGAAAAGCT

>c6230_g1_i1 len=234 path=[1:0-233]

CATGACTGTGCAAATCGACAGGTTGTCTGGGGAGATGACAGCTCTCCACAGCCAGCTGCA

GGAGCTGGTAGCACGCCGAAGGTCTGCCTGGTGTCAGAAGGCTGTAGCTGCAGGGCTCAC

CCTGTGCCTCCTGCCGGCATCACGATGGCCATTCAGGCAGCTTTGTGCCCACTGCAGCCA

CTGCTCACCAGCAAAGCTATCTCCATGTGAGGGCTAGCACTCATTTGCCCTGTG

>c6230_g2_i1 len=271 path=[423:0-270]

GAGCTTCGCCTGCTCAGAAAGCCCAAGTCCAAGCAGCTCTGTGTGTCCTGGCCTGGCTGC

TTTCGTGCTGTACAGAGCACTGCAGCTCTGCTCGTGTAGCACTGGAGCCCTCCAAGCCCA

ACGAGTCACCTACATCTGAGCAGGTATCTCCTATCAGCAGTGTTTGTCTGCCGCCCGAGA

TAAAAATTGGGAAGAACAAATTGAAAAATAAGTGAGCAATTTATTACACGGAGCAATACT

TGTCTGCTGCCTTCCTCTGTAATTTGTCGCT

>c6241_g1_i1 len=310 path=[510:0-309]

AAAACGTGTACAAGTGATCCACCAGACTAATCACAGAAGAATTCTTCATCCTGTCACAAT

AATGAAAAGGAGCAGTTACCGACAGCTTTGTCAATGATATCAGCAGCACGAGTACATTTT

CCTTAAAGAGATGTTGTTTGACAATAGCCATAACGAACAGAGGCAAAAAAATTATAACCA

AATAATTTATTTAGTATAATCCTTTAATCCAGATAGCTCTGAAGATCAAAATTAAAATGA

ACATATGCAAAGCCAAGCAGGAATATGTTAGTGGACCCACTGTCCGAGAAACTGAACGCA

CAGCAGAAAT

>c6243_g1_i1 len=226 path=[486:0-225]

AGAAACATCAGTGAACTGGAATCAGGGTCATGTACTGAGATTATTTAGTGGCAGAAAGAA

GAGCCTACTAAATTACTGCCCTTTCTATAATGATAGCATTTTAGGTGAGCATGGCTTCTA

ATAACGTATCTTTAAAGTTAGTAGCAGATATCAGACGCCTGACAACTGCCCCCTTAGCAG

TTTAAACTATGCAATAACCTCTTGGTACTTTTTCCTCTCAGAGGCT

>c6252_g1_i1 len=384 path=[433:0-335 906:336-383]

CTAACAAGGTGCTGCATGTAGATATGCAAGCAGAGAAGTGCAGCCCAAACGTAACAAAAC

AGAACATACTTTCTACCGCATTCAAGAAAAAAAAATTTGTGTTTAACAGCTGTTTTAAAT

TCAGATATACCCCGTTCAAGTAAGCATACTGTCAGTCAGTGAAATCTGCCCTCCTGGTAG

TTTTTGTCACCACTGACAGGCTCAAGGTGCCTTGTACCCCACCAGCAATTATTCTGCAAC

AGTGTTCGATGCTCCATGACAGATGCCCAGATAGGGAATTTCCACTGGCCTTAGTAACAT

CTGCTTTAGCGTATGACTACAGCACAGTGGTAAAATAAGCCAATTGTATAGCAAAGACTA

GACTTATTTTACCACTGTGCTGTA

>c6263_g1_i1 len=280 path=[258:0-279]

AACGGCCAGCAGCGCCGCGCTGTGCCGCCCCGTCCCGGAGGCAGATGGGCTGCTAAGGGC

CAGTCAGGGTGGGCCCTGGGGCTACTGAGTAACACCGAGACTTTTTTCTCTTCATTCTTT

CCCTCTACTCCTCCTGTCACGTTGCTGCTCCTCTAAAAGAGTCAGGAGCAAAGCTGAGGG

AGTCATACGCTTCTCAACGGACTGGGGGTGCTGGTGACCAAGAATGGAGAATGTACAAGA

AGATAATAATGAAAATTCCAACACTGGAGAAGATTCTGGG

>c6263_g2_i1 len=248 path=[740:0-247]

GCGGATAATACTGAGCAAAATGTCACTACAGAGGATTCTGTGTCAGGCCCTGTTGCACCA

GGGGATGCTGGAGCCAAATCTGTTCAGGATACTCAACAAGTAGCAAACTCTTCTCCTTCT

CAGGAGTCTGTTATTCCTGTACCCCGAAGGCTGTCTAGGCTTTATCGGAGCTCTGCTGGA

GTTCTATCCATGCAAGAAATTTTAAAAGAAAAACAGGCTCGATTCAGAGAAGCAAGAGAA

AACCGAAG

>c6274_g1_i1 len=563 path=[308:0-562]

CTTTGTGTTTGAGTAAGCTAACAGCTCAGTGTGCTTGTGTATATGCAGCATGCAAGTCTG

GTGCAAGATTCAGATGGTTCACATGTGCCTTGGAGCAGTCTTGTTAGCCGGGCACTCATC

GTCTTGCATGACTTGATTCCTTCATTTTGCTACTTTTTGCAACTAGTCTGACTGTTCCAC

GTTGTTCTTCTGGAGAGTGCCTCCAGATGTGTTCTGTGGGTAATCTCACTTCAGTGGCAG

GTCGTGGAATCACAGAATGGTTGAGATTGGAAGATCTGCAGGTTCATCTAGGCCAGCCCC

CCTGCTAAGAGCATGATCAGCCTGAACAGGCTGCTCTGGAGGAGGTTTTGAGTATCTCCA

CGGGTAGAGACTTTGCAGCTTCCCCAGTGTTCAGTATCTCCCTGAGTAAAGATGGATTTT

CCTGTGCTTGAGTAGAATTTTCTGCGTCTTACTTGTGCCCATTGCCTCTCACCCTGTCAG

TAGGCACTGCCAAGAAGAGCCTGGCTCCCTCTTCTTCATTCCCTCCCATTGGATAACTAT

ACAGGTTAAGCTGCCGCTACTCT

>c6275_g1_i1 len=225 path=[203:0-224]

CAAAATGAAAACAAGAATATCTATAGCCAGACTTCAAAACTGCATTTGCCAGCATCGTCA

CAAGGTACAATATGGATTGCATAAAGCACGTGTAAGCTACAAAACTTGATGCACACATTT

TAAATATTTTGTGAATAAAATACCTCCTGTGGAGCTGGGAAGCAAACAACTTGGCCTGTT

TTGCCTCATGAAAGCACAGCAACCTTCTTTCACCTTGTTTTGGTG

>c6276_g1_i1 len=232 path=[392:0-231]

TCAAACACAACATGGTAAAAGAGCAATTATTAATTGAATCTGCTCTTGAAAATGATTTGA

CGTCTGCTGATGAAAGGAGGTACAGGAGCCTAATGATGGTAAGATTTATAAAGTGCTTCT

CAGTGTAAGTTGCTGACACAAGAGTTTTATGAGAGCCTTGATTTGGGCTGCAATCTCTAG

ATGTTTCCGTGGTAAAATAAATGATGATGATGATCACAGCCCTCAAGGAAAG

>c6281_g1_i1 len=445 path=[1:0-215 217:216-444]

CAGGGGGGACAGGGGATGGAAAGAGGTGGTACCAGCCATCGGGGGGCACCTCCTCCACCC

CCACACCATGAGAAGCAGTTTTGGTGCTGTTGGACCTAAATCTGTGCCACCACCCCACGC

CCCTGGCTCCCCTCCATGGTGTCTCCTGCTGCTGAAGAGAGGAGGCATTTCTGGGAGCAC

TGGAGCCAGCGACCAAGCAGGATGAGGATGTGCTTCGGATCACTGCCAGCACTGCAATGA

CCTGCAAGGAGAGAAGACGTATCCCTGTCTCCTTTCTTGGAGGCCCCCGTGATCTGCAAA

ACTGGCTTTTATTTTGGCCAAATACCCTCCAAAGAGTATCGAAACATCTTGAGTCTTTCC

TCCTGATTCATAGGATCGTAGAATGGTTCGAGTTAGAGGGGATCCCTAGGGGCCATCTGA

TCCAAATCCCCTCCATGGTGTCAGC

>c6281_g1_i2 len=297 path=[845:0-67 217:68-296]

GATTTATCCGACAGATCCGGATCTCCAAATAAATAGATTTATCAAAACAGAATAAAACAA

GAAACCGGGGATCACTGCCAGCACTGCAATGACCTGCAAGGAGAGAAGACGTATCCCTGT

CTCCTTTCTTGGAGGCCCCCGTGATCTGCAAAACTGGCTTTTATTTTGGCCAAATACCCT

CCAAAGAGTATCGAAACATCTTGAGTCTTTCCTCCTGATTCATAGGATCGTAGAATGGTT

CGAGTTAGAGGGGATCCCTAGGGGCCATCTGATCCAAATCCCCTCCATGGTGTCAGC

>c6297_g1_i1 len=222 path=[1:0-221]

TGTGACAACTGTTAGCTCTCAAACAATTGCCTTGAGACTTTGCCTTACAAGGAAGGGCAG

ACAAGCATTAAGACTGCTGACAGATATGTCTGTAGAAGAGTAAGGTTGAAGCACATGGAG

CCTCAAGAAGAGATTGGGAGAGGGCACCTTACACACAAGGTGTTGACCAGGAAGCCTCTG

ACAGTTCCTAGGTGGGATACATATCCGCAAGTGAATTCATTA

>c6298_g1_i1 len=277 path=[203:0-126 330:127-201 330:202-276]

CCATTGCTGCCAAATAGTGACCCACAGGCCCCCACTGCACCCCGGCCTGCCTAATTACCC

CCTTAATTATGCACGCCATACGCAAATGAACCCTCCGGGTCTTAATTAGAGCCACGATTT

AGCGCCACATCTGCCAAATAGGGATCCATTCCTGCCCAATAGTGTTCCACTGCAGCCCGA

AATGCTGCCAAATAGTGATCTCCATCTGCCAAATAGGGATCCATTCCTGCCCAATAGTGT

TCCACTGCAGCCCGAAATGCTGCCAAATAGTGATCTC

>c6299_g1_i1 len=296 path=[274:0-295]

AGGCGGACCAGGCATCGGTGATGGTGCTTGTCCACCAGGATAGCCTACTGGAATTTGTGC

TGGTCCACCTCCAGCATAGCTTTGGGCAGGAGGTTGGGGATAGCCACCGAAGCCAGGGCC

GGTGGGAGGCACACCATACCCAGGGCCTCCAGGATAAGATGGCATTCCTCCAGCCTGGGG

AGCTCCAGGGAAGCCCCCCGGGGCAGGGTAACCCCCTGGAGCAGGGTATCCTCCTGCCTC

TGGATACCCAGTGCTGGGTGGTGCTGCAGGAAAGCTTCCTCCTCCTGCTGGAGGAT

>c6299_g2_i1 len=218 path=[742:0-217]

TTCCTCCTCCTGCTGGAGGATATCCTCCAGGAGCAGCAGGATAGGTGTACTGCCCAGCAG

GTGGGTAGACAGACTCCTGTCCTGTGGGAGGGTAGCCAGGGAAAGCAGGGTAGCCAGAAG

GGGGGTAACCTGGGTATGCCATTCTGAGGGAAAAGCGCGGAGCCTCCGGCCTCTCTCCCG

CAGCTACACCGCAAAATGACCAAAGGCAAAAAATCAAG

>c6303_g1_i1 len=548 path=[383:0-338 725:339-547]

AGTGTACTCACTTTTAGTACATGTATATATATTTTTTTTTTAGTGTACATGTTTATATAA

GCACATATGCCCAGTATATTATCAAACACAGAGAAAAATGTTTTTACTCATTTTATTCAT

TTAAATATTACTGAAAACATATATGTAATGCTATTTATAGACTAATAATAAATTGATTTA

ATGAGGATTTAAATTTTCAATAAAAATGAGAACAAATTTTTATTAAAGTTAGTAAATGAC

TTTAAAAATAATAAATGTTCAATTAAATAAACTATATGTGTGTAGTTTACTTATATGTAG

TATCCATACAATTTTACAGTTAAAATCCTTAAAGATTTCTCAAAATTCCCTTTTAATTTT

TTTGCTCAATATTTGATTTATTAATAGACTATTCAAGCAATTTCATGGTACTTTAAATAG

TCATATTAATAAAGAGCATATAGAGAGACCTCTTCCTATCTTATATATCATAAAATTAAT

TTAAATTGATAATTATATAAAGGAAATATAATTATTTATTTATAATAAATTTAACATATA

TATTATAT

>c6304_g1_i1 len=320 path=[542:0-319]

AGCCCAAAGCATCTCCCAGCCAGGTGAGCGGTGTGCTGGTGGGCACTGTGTGTGTTTGCC

TTGCTATTTTGATCCAGAAACTCATCTTCTCAAGGAGAACACTTAGGGTAGCACCTGCAT

CTTAAACGTCCTATGTACCGGCAGCAGCCTGAGAGAGATGGGAACCCCTCCCACCAACCC

CCAATTGCAGCTTGGCGTCCCCCTTGGTTAAGGCCGCCGTCTGCAGCACTCCCCGTGTAA

CAGGGAGAGGCCCCACCCGTGCCAACGGGTGCTCACATTCCCCTTTCTGCCTCCTTTAGG

CCAAAACGAGTGTCAAAAAG

>c6305_g1_i1 len=223 path=[1:0-137 309:138-222]

AGTGATGGCCTTGGTGGTTTCACAATCTCTGTTTCCCTGCAAGTCCATTTGATGTCCAGG

TGTTTCCTTGGTCACACAAGAAGTAAGTGAACTGGAATTTGGGAGTATGTTGTTTTTCTC

TCTGACCATTTAGGGCTTCTCGCTCCGAGTCCCAGCGGTCGCTGCAGGGGCCGCTCCTGA

CAGCGGCGAGCGGCACGGGCTGGCACGGCCTGAGCGGGAAACT

>c6305_g1_i2 len=224 path=[1:0-137 139:138-223]

AGTGATGGCCTTGGTGGTTTCACAATCTCTGTTTCCCTGCAAGTCCATTTGATGTCCAGG

TGTTTCCTTGGTCACACAAGAAGTAAGTGAACTGGAATTTGGGAGTATGTTGTTTTTCTC

TCTGACCATTTAGGGCTTTGAAGCTCCTGCGTTCCAGGTGTTTGTTGTTACACAGTAACA

GTTAAGCTACCTTTCTCAGAGTTTCCCGCTCAGGCCGTGCCAGC

>c6308_g1_i1 len=226 path=[254:0-225]

TCTCATCAAGAGACTCATAAATAAGGCGGTGATGTAGCATGGGTGAGCCCAGAAATCTGT

TCTGCTTTCAGGAAAAAAAATCTGTCTTTTGCATCTAATTCACATAGGGAGCAGGAGAAC

CCCTTCCAGCTTCCTCTGCAGCGCTAGAGGGATGATACCTCTAAAATATGTATGGTTATA

TGGTACTCTTGTCTTGTTAAAAAAAATGTGGTCAAATAATACAAAG

>c6314_g1_i1 len=269 path=[245:0-82 328:83-268]

TGTGGGTGTCACACCACCTGTGAGGATTGAGACTTCGTCTGCCTCCGTCACCGAGGGACA

GACCCTGGACCTCAACTGCATGGTGGCAGGGCAAGGCCACCCCCAAGTCACCTGGTACCG

ACGTGGTGGTGCTTTGCCCCCCAGCAGCCAGGTGTCGGGTACCCACCTGCGCCTCACCCA

GGTGTCAGTGGCTGATTCGGGTGAGTATGTGTGCCGGGTCACCCTGGGGACCGCCACGCA

GGAGGCTTCTGTCATCGTCACCGTGCCCA

>c6314_g1_i2 len=363 path=[952:0-129 1082:130-153 1318:154-176 328:177-362]

CCTCACAGGTGGTGTGACACCCACAGCATATGAGGAGCCAGCACCGGTCTGGATGGTGAC

AATGAGGGATGTCTCATGTGTGACGCTGCCTGTGGTCACCCGGCACACATACTCGCCCGA

ATCGGCTGCTGACACCTGGGGGATGCGCAGGCGGGTGCCCGACACCTGGTGCTTGGCTGG

CAGGGCAAGGCCACCCCCAAGTCACCTGGTACCGACGTGGTGGTGCTTTGCCCCCCAGCA

GCCAGGTGTCGGGTACCCACCTGCGCCTCACCCAGGTGTCAGTGGCTGATTCGGGTGAGT

ATGTGTGCCGGGTCACCCTGGGGACCGCCACGCAGGAGGCTTCTGTCATCGTCACCGTGC

CCA

>c6314_g1_i3 len=363 path=[952:0-129 1082:130-153 1106:154-244 615:245-362]

CCTCACAGGTGGTGTGACACCCACAGCATATGAGGAGCCAGCACCGGTCTGGATGGTGAC

AATGAGGGATGTCTCATGTGTGACGCTGCCTGTGGTCACCCGGCACACATACTCGCCCGA

ATCGGCTGCTGACACCTGGGGGATGCGCAGGCGGCTGCCTGACACCTGGTGGCCAGCAGG

CAGGGAGCCACCACGCTTGTACCAGGTGACAGTAGTGGGACCAGATCCTGCCACCACACA

GTTGAGATCAAGTGTCTGTCCCTCGGCGATGGCGGAGGATGAGGACTCAATGTGCACGGG

CTGGGAGGCTCCAGGGGAGTAGTAGGTGCCAGCACTGCTGGGCACGGTGACGATGACAGA

AGC

>c6321_g1_i1 len=238 path=[1:0-237]

AAGAGAGTTGTGTAACTTAACTCTTAAAAGCCCCTGCTGAAGATTAGCAAGATGAGGTGC

AGCTGGTGGCTGCTGGGTCCAAGTTACACACTGCTGTTTTCATTTTGTTTTTCTCTCATC

ATTTCGCAGTATCCCAATTGTTAAAATAAACCACTGCTCTTGATTTGTAGAAGCGATCTT

ACAGTTTAGGAGCCTGTATCTGAAATAACAAATTAACTGCTCACTGCAGAACACCAAA

>c6324_g1_i1 len=204 path=[1:0-41 384:42-203]

GTGGGGCTTCTCTGGGGCCCTTGTGGGGCTCTTATGGGTCTCATGGGGCTCTTGTGGAGC

TATTGGGTTCCTGTGGGGCTCTTGTGGGGCTGTGAGTCTCTTGTAGGGTTGTGGGGCTCC

CGTGGGTCCCTCGTGGGGTTGGGGAGGGCTCAGCATTGGGTGCCGCGGTTGGGATCTCCG

TGCTGTGGGGTGAGAGCTGCCGGG

>c6328_g1_i1 len=305 path=[157:0-304]

TGTACGACCGGCAGGGAGCCACGTACCTCTTCGACCTGGACTACGTGGAGGACGTCTACA

CCGTGGATGCCGCCCACTACGGCAACATCTCCCACTTCGTCAACCACAGCTGCGACCCCA

ACCTGCAGGTCTACAACGTCTTCATTGAGAACCTGGACGAACGCCTGCCCCGCATCGCCC

TCTTCGCCACCCGCCCCATCCGCGCCGGGGAGGAGCTCACCTTTGACTACAACATGCATG

TGGACCCAGTAGATGCAGAGAGCACCCGCATGGACTCCAACTTTGGACTGACCGGGGGGG

GCCTG

>c6331_g1_i1 len=210 path=[1:0-129 357:130-209]

AAAAAAGAAAAAAAAAAAAATTATTATATAAAATATGAGATTGCATAAGGTATTGAAAAG

TTATAAAGTATAAAATTTTAAATGAAAAATTAATTTTTAAAATTATAAATGGTATATTTT

TTTTTATACATCAAGCAATTGGGAAATTTTAAATAATTTTTCTTTATGCCCATTTTTAAA

TCAATAAAAAAAATAGAAAAATATACTTAT

>c6335_g1_i1 len=221 path=[1:0-220]

TGCCAGCACGGGCTCTAATTTGTTTGTTTGTTTGAATACACATAGTGCTACCAATTGTGG

TAGTATACTTCAAGGTGAGTTTGTGGTCTGTATTTTATTGCTTGTTTGTTACAACATTAG

TTAGAAAAAGTTAATAAATTGTACATGAATTCAGCGATGTGATCATTGACAAACTTGAAC

ATCTAGCTTTGCCAGGGTCTTGAAAATTGCCAGACAAACAG

>c6336_g1_i1 len=239 path=[217:0-238]

AACAGAAAGAGGGAGGCAGTGCTCTGAGCAGAGCAAGTCGGAGCACAGCTGGGCAGAGAA

CCCAGCGTCCCCACTCCCCATCTCGTTCTCTAACAAGCACTGTGAAACAGAAGAATGGCT

TTAAAGCAACAATGAGTTTCTCTCCTTCTTCTCTCCTCAGACAGCACTACATCTTGCTGC

AGAAAAGAACCAGCACCTGATGGTCAGCGACCTTATATCCCTGGGGGCAAACGTCAACG

>c6338_g1_i1 len=212 path=[45:0-19 44:20-211]

ATAATTAAAATAAAAAAAAATTTATGATTTAAAGCATTAAAAATTTCTTAAAGTCTATTC

TATGAATAACAAAAACACAAATTAAAAAATAAAAAGGCATAATCGAATTTTCAAATATTT

TAATATGTATTAAACAAAAACAAAAAAAAAAAAAATTCTTTTTATACATATATTGTAAAA

TTTAATCTCTTTATTTTCTTTTTTTTTTTTTT

>c6342_g1_i1 len=320 path=[500:0-319]

ACATGCTAGAAAAAAGATTAGAGTAGTGATATTGCAATACACAAAAAGAAACTACCCTAT

TTACCCATTCTTAATGACAAAGGACACCGTTTACTCTCTTGCTTTTTCTGAAATAATTAA

AAGCACGCTGCATTCCCCAGACAGACTACTCAGTTATCTGTGGTGCGAACATTTCACTTG

CACCGTGCTTGATAACAAAGAATTCCTAGCCCACTTAACTTGTTTTTACCATCCAGTTAT

TTCTTTAATGGCAAAGTAGGTTTCCTAACCTGTGGGAAAAGAGAAATCTGCTCCATTTTC

ATTTCGTTACAGGAGTCAGA

>c6348_g1_i1 len=223 path=[325:0-99 125:100-222]

TCGGTGCCCTTATTTGGTTGTGCGTGGGCGCAATTCCACAGCAGGAAATCATGACATTGG

TTATGGGCCGGTGACTGGCGGGGAACATGGTGGCTTTTGGTCCCTTATACAGCGCTCCTA

TAATAAAGAGCAAACTCAGCTGGTAACTGCTAAAGGGAGGTGAAAAAAAAACAAAAGGAA

ACCACTCGGTACAGTGTCCGGAGTTTGGCGGACTGCCCAGGCT

>c6354_g1_i1 len=269 path=[1:0-76 78:77-268]

GAACTGAATTTCCTGGGCAGGTCCATTCACTATGCTTCGCATAACAGTGAAACCAGCTTT

CAGAAGAATGTGGATTATTTGCTCTCCTTTCTTGAAAACTTCCCCTGCTGTATCGCCCCA

CACAGTATCATCAATGTACTGCAGGTACTCTGGAGCACTACCCTCTTCCAGTGCAGTCTG

GATAAACCCATGTCAAATGGCTGGGCTGTGTTGGGGCAAATGGGTAATCAATACCAAGTA

TGTAAAACGTAAAACGGGGTAATCAATAC

>c6354_g1_i2 len=366 path=[157:0-173 78:174-365]

CTGCTAGAGCATCACTAGTGGCATTGTTTTTCTTAGCCAGCCAGCATCCTAACTTAGTGA

GTGTGGCTAAAGCTTGAGCGGTACTGTTTCAGGGTACACTTGGATTTAAGAGACAGGATG

TCTCAGGAGCAGTTACCTTAATTGCAAAACCAGCTTGCGGAAGGATCTTGATTTTTTGCT

CTCCTTTCTTGAAAACTTCCCCTGCTGTATCGCCCCACACAGTATCATCAATGTACTGCA

GGTACTCTGGAGCACTACCCTCTTCCAGTGCAGTCTGGATAAACCCATGTCAAATGGCTG

GGCTGTGTTGGGGCAAATGGGTAATCAATACCAAGTATGTAAAACGTAAAACGGGGTAAT

CAATAC

>c6355_g1_i1 len=407 path=[75:0-257 37:258-263 74:264-406]

AAATGGTTTCCAAAGGCAAACCCTAGGGATAACAGATTAAAGATATATAATTACTCTTTC

CTATAAACTAATTAAAAACATTTCTTTCTAATTGTTTCTGCATTCTCACCACTGTTTAAC

ATACTTCATCAACATCATCATACAGTAGCCGATGACAGAATTATGCAAGAACTCAGGCAA

TGGTGAATCGGAGGTAAGAAAGAAACAGTTGTAAGAGATAAAAAGGAGAAACAGCAGTAA

GAAATAAAAGCCTCCAAAACAGGATTATCAATATTACCCTAACAGTAAGTATCCAGCTAT

ACTTTAGAAACTCACTCAGCCGACTGAGAGGAAAAGAACTAGCTAAACAAAACAGAAATA

TCATGAATTCCTATGCATTCCTCACTCTAGCCCATATAGATATATAA

>c6363_g1_i1 len=233 path=[1:0-117 428:118-198 320:199-232]

GGTCCTTCAAGATGTGTTTCAAGTTTAGTATAGATGTCTGTAAGGCTTGAATGTGAAGTA

CTGAGCAAATTGACAACATTTCCCAGGTGATACACAATTGCTGCTGTAGCAGACTTCAGG

AAATCTTGTTCAGAGGTCATATTCCTATTATATATATATTAAAAAAAAGCATGTTAAATT

GTATTTAGTATGTTGTGTTCAAGGTGGACAGACTTCTTTCTGGCAGCGTCGAT

>c6366_g1_i1 len=204 path=[359:0-17 178:18-203]

CTCACCACGACACGAGGATGGAGGACATGGCTAAAATGTGCAAAATTGTCAAGGCAGAAA

CTAAAGTGATTGGGAACTGCATCCATCTTTCTGAATGTCCAAGGCCAGAAGTCTTGCTGA

CAGTGAGCAGTGAGGTTTGCAGTGATACGGCTTTCAGGGGGCAAATGAGCAGTAGATGGA

TGGCAAAGTGGGCGTTTTCCAACT

>c6367_g1_i1 len=242 path=[1:0-24 26:25-241]

CACGTAGAACAGCCTCCGCTCAAACACATTTTTCTACTTCTGAGCTTTTTAGAAAGACTT

ATACAAATTCATCTCTTATACTTGAGCATTTTGTTTCGCTTAATATAACTATTTCTTTTA

AGCGTAAAGATGTACACAGTTTGCTATACACATTTTTTCAATACCTTCTCCACAACCTTC

TCATTTTTCAGTGAGACCTTTATGAACCAGTACAGCCAAAAGAAACAACTTCCTTAAAAA

GA

>c6367_g1_i2 len=418 path=[447:0-200 26:201-417]

GGTAACTTTGTGTTTGAGTTTTTCATATAAAAAATTAAGTACTTAGAACTGAAGTGGTTT

CAGAAAAAACTGTCTTTCAGTTTTTTGACCTGCTTACCTGATGCACATGATCGTTACAAT

CATTGATAAACTCCACTATCCACTCTTCTTTTTTTCTCCCCCAACTTTGAAGTAGACTTT

TCTGAAAAATACACCTCTAAAACATTTTTCTACTTCTGAGCTTTTTAGAAAGACTTATAC

AAATTCATCTCTTATACTTGAGCATTTTGTTTCGCTTAATATAACTATTTCTTTTAAGCG

TAAAGATGTACACAGTTTGCTATACACATTTTTTCAATACCTTCTCCACAACCTTCTCAT

TTTTCAGTGAGACCTTTATGAACCAGTACAGCCAAAAGAAACAACTTCCTTAAAAAGA

>c6368_g1_i1 len=321 path=[676:0-126 104:127-320]

GGCTCCCGGAGAACGAGACGGGCACTCGCACAAAATCCTGCGGCTTCAGAGCAGCCCTGG

CGGGCAGGGTTTTTCAAAGAGAGGTGTGCTGATTAGGTAAGATGTCATGGTCTTCTCCTG

TCGATGTAGGAAGATAGCAGATATGATCGCCTGAAAAAGCAGAGCTACCCAGTGCAAGCA

TGGCTTTCGCCCAGTCGCACATTATGGCAGCTAGAAGGCATCAGCACAGTAGACTCATAA

TTGAAGTGGATGAGTACAGCTCCAACCCCACACAGGCTTTCACGTTCTACAACATTAACC

AGGGACGTTTTCAGCACCCAC

>c6368_g1_i2 len=297 path=[1:0-102 104:103-296]

CGGTCCCCAGCCAGGGAAGGCAGCGCCGAGGCTCGAACCCCACCGCCTCCATGCCGCTCT

GCCGCCACTCGCCTTCCGCCGGGCCGGAGGAAGGGCTGCGCGAAGGAAGATAGCAGATAT

GATCGCCTGAAAAAGCAGAGCTACCCAGTGCAAGCATGGCTTTCGCCCAGTCGCACATTA

TGGCAGCTAGAAGGCATCAGCACAGTAGACTCATAATTGAAGTGGATGAGTACAGCTCCA

ACCCCACACAGGCTTTCACGTTCTACAACATTAACCAGGGACGTTTTCAGCACCCAC

>c6382_g1_i1 len=204 path=[440:0-203]

GCTCAGAATGATTTTCACAATAACTTAGAATAGCAGCTAACAAACAGATAATTGTTTTCA

GTGTGGGAAGTTTTCGGTAGTGCACAAATCCATTCAGCAAGATCAGGTATTTTGTGCAGA

ACTCCTAAGTTTATTCCATTGCACCCAAACCTTCAGGAGACTTTACAAAGTAACTGTAAG

TTACTTTTTTTCTTAACATGGCTT

>c6384_g1_i1 len=280 path=[2:0-106 109:107-279]

TTTTTTTTTTTTTTTTTTAATTGATTACTTTTATTGTGTCATTTAAAATTAGTGTATCAA

TCGTATTTATTATATTTTTCTTTTATTTTCTTAAATAATTCATATATTTATTTTTATATA

TTTTTATTTTATTATATTTTATCTTATTTCTTTTTTTTATATATACGTACATATGATATA

TAAATGATATAACATATATATTTATTTTTTAAACAATATGCATGTAATAGTTTATGTAAA

TTATAAAAATTATAAGAAAACTTATTCATAAAAAATTAAA

>c6384_g1_i2 len=306 path=[581:0-132 109:133-305]

GTAAATATATTATAATAACTTTATTCTAAGTAAATGATAAGACCGTGAAAAAATATATAT

GCATATAAATTTTTATACTGTGTTTATAGTAATATATATACGTAAATATATGTGTAATAT

ATGTTCACGGGAATTATTTTTATATATTTTTATTTTATTATATTTTATCTTATTTCTTTT

TTTTATATATACGTACATATGATATATAAATGATATAACATATATATTTATTTTTTAAAC

AATATGCATGTAATAGTTTATGTAAATTATAAAAATTATAAGAAAACTTATTCATAAAAA

ATTAAA

>c6385_g1_i1 len=219 path=[455:0-218]

GTGATCTTTTCCAACCTTAATGATCTTATGATCTTGGAGTGCCCGGAACCCCACTAATGG

GGTTTCTGCACATCAAAGGATGCTTTTGACAAGCTGGGCCAGGAGGGGGCCTGCACGGAG

ATGTTGGCATGGAGATCCAGAGCTGCCTCATGAATGTGAGCAACAGGAGAGAAATTAATT

TCCTTGCACACTGAGAGGAATCAAACACCTCCAGGGATG

>c6387_g1_i1 len=267 path=[1:0-266]

ATGTTCTGGGGTCAGAAACATTAAGGGAAAAGAATGATGAATTTTTTTGGTTCTGTGCAG

CAGCTACCCCAGTCTTGTGTCACATGTCTCAGGCTCTCTCTGCTTCTTTAGGATGAAACT

TAGCTTTCAAGGTTACGAACTTTCAGACTTGCATAGTGTGACTGGTGCACCCAGGCTCAA

AGCAACTCTACTGTGACTATCAAATAGCAAAACTCAAATAGCTTGGAGAGTTAGTGAGTT

TGGCCATACCATGTTTGGCTCAAAAGT

>c6391_g1_i1 len=304 path=[546:0-303]

CGTCCACCGGCTCCACCAGGCTGGAGAACAGCTGAGTTACAGCCCTGAGCAGCCCCAAAC

GCACTGGGAGCCCCGCAGTCACCCCCAGCCCTGCCCACTGTGGGGCAGCTGCTCCCAGGG

CCCCGGTGCACAACCCGCTGCCAGGTGCCTGCCGTCCATCTGAATGGAGTGCAGGTCAGT

GTGGGCAGTGCAGGGCCAGGAAAGAGCCACCATCCCCACTCCCACCGCAGCTCCCAGTCC

TCCCCAGCACCCACCACTCCAGCACCGTGCGGGTCCCGTCGTGGCCATCCAGGTGCTCAG

GCAG

>c6394_g1_i1 len=210 path=[41:0-40 40:41-209]

CTGGTGCTGGATGCGCTGCCTGAGGTGAGGAGGGAGCAGCCTTCCCCTGCTCTGGGCCGG

AGGTGGGGAGTTCCCATGCTGGGCCAGGACTCCTGGAGGCACAGAGAGGCCCCGTGTGGG

TCTGACCGGCAGCTCATCCCCCGCTCCATCCCTGCCTCCCCAGATCGCTGCCGGTGCTCG

GCTCACCTTTGTGAGGTCCACGCCGGTGAT

>c6409_g1_i1 len=240 path=[582:0-239]

TGAATAGCGATGGCTACCCAACACACCATCTATTCAGATACTGCAACACTGAATTTTAGC

TATGGACGTGTATTTGGTAGTGTCTGGTATCTAAATGTTTCCATATATTGGATGACTGAG

ATTTGTGGTTGCTATTCAGCTGTCATTCAGCTAAAATTTCTCAGGGAGTAATGTCAGCCA

CAGGAGAAAGCTGCTCTTAAAATAGTTTTCCTCTCCAAGAAAAAGTGCTTGTATTTTAAA

>c6418_g1_i1 len=267 path=[294:0-266]

AAAAAAAAAAAAAGAAAAATATTAAAAAAAATAACAAAAGAAAGTATTATTTTTATTAAA

ATATATATTGATAAATCATAAAACATGGTAATTAATAAAAATGTTAATAAAAGAGATTTT

TTTCTCCTTAATAACTTTTTTTCAGTAATTCTAAAAATCATTCTACACGTAATTTTTTTT

CATTATTTTACTTTATTCGAGCACTATGTAATATTTTAGAAAAATTCTTTTTTTCTAATA

CTATAAATATATGCATATAAGTAATTA

>c6419_g1_i1 len=317 path=[590:0-18 11:19-316]

GAAAAAAGCAGATCAAGTTAGTGTCATCAGTGATGGCTTTTGTCTTGTCTTTGTCTTGTA

AGGAGTTTAATTATTGTCATGATTGTTTTTTTTCCATCAAAAAGTAGAACCTTTGTAGGC

CGTTTTACCTATCTGTTGCTTTCTCTGAACAGCGATTCACTTAAAATACATGTACTGCAT

GCTTATAATTGCCTCAGGTGTTGGAGCATTTTTCCATCACATCTCTTTGTTGTCTCAGGC

AGCAGGGAGAGGTGCTGTGACTGAGTGCAGACATTGAGGCTCTGTTGGAGCTCTCAGCTG

TCACAGTTCAGGTGCTG

>c6419_g1_i2 len=308 path=[1:0-9 11:10-307]

AAATTAAAAAAGTGTCATCAGTGATGGCTTTTGTCTTGTCTTTGTCTTGTAAGGAGTTTA

ATTATTGTCATGATTGTTTTTTTTCCATCAAAAAGTAGAACCTTTGTAGGCCGTTTTACC

TATCTGTTGCTTTCTCTGAACAGCGATTCACTTAAAATACATGTACTGCATGCTTATAAT

TGCCTCAGGTGTTGGAGCATTTTTCCATCACATCTCTTTGTTGTCTCAGGCAGCAGGGAG

AGGTGCTGTGACTGAGTGCAGACATTGAGGCTCTGTTGGAGCTCTCAGCTGTCACAGTTC

AGGTGCTG

>c6422_g1_i1 len=242 path=[454:0-241]

TGTAATACATTCCTAGCTAGGTACAAAGTTATGGCCTGGGTCTTTTATATCAGATTTTCC

TGTAGCTACTTTGTTTTTAGGCTGCTTTGTCCAGACATGTCAGTGCAAAAAGAATAATCC

ATACCTCCTGCAGTCTGTTCACTAGGAGTTTGTTTTAAAAGGACTTGAGGGGATATTAAC

TCATAAGCTGTTAAAAAAAAAACAAATACAGCAGTAAGCAAAGGCACAGCTTCAGAAAGG

AA

>c6425_g1_i1 len=208 path=[528:0-207]

GGTTATTTTAATCTCAGTTTTAATTATCAAGCAAGCAGAATCTAGAAGGTGTTTTTTTCC

TTCTTTTTTATTTTTTCCTGTATGTGCATCTTTAATAAGTGTAAAAGCAGGTGTGATGTC

TGCAGAAGTAAAGAAGCACTGTGTGTGTCTTTATGGGTCTGGCGATGATGACACACTTGC

CCTCACTGAGAACACTGTTCAGTAATGA

>c6428_g1_i1 len=215 path=[1:0-121 123:122-125 440:126-214]

CTTTGTGTTTGAGACCGGTGTAGAAGAAAAAGGGTCCTTTTTAGGACTACTCCTGTTGTC

TGATGAGAGCTGATGAAAGGGAGAGTAATTCACCAAGATCTTTAAGTGCATGATGAAGTA

TAGAGACCTCCCCTCAGAACACCCTGTGTGTAGGTGTCATGGCTTTATGATTCTCGATCA

GTATTCCACATTATAATATCATGTAGTGCACTGGG

>c6429_g1_i1 len=232 path=[436:0-231]

CTAGTTAAATATTCCTCTTAGTCATAGCACCATTTGAAAGACAACAAAGTTTCCGTGCTC

TTAAGCTATTGTAAGCCTGCTAGAACTTAGCTGAATAACTGGTTCAGAGTTCTGGACTCA

ATTTCATTACAAAACTGCAGATGGAAGTAGGTTATAGCCTTCATAAGGCAAGCATTTGCA

GTGAAACTGCTTGTTTGTGACTAGGTTAGCTTTCTTTGTCTACTTTTAGTAG

>c6435_g1_i1 len=478 path=[1:0-477]

ATTACCGTAAATGAGCATCAAAAAGCTGATCCAAGAATACTAGGGGACTGCTGAGCCAAT

GAAATGAGAACTGGTGGTATCTCAAAGAGTTACAGTTAAACCTGGATGTCTCTCATACAA

CTATTTCCTGTCCATTTTCTGTGATGTGCTACAGACAGCACTTCTCAGACAAAAAGGTGG

CAGTCAGTTCTAACCTTCATCATATGTAGTCTTCCGAAGGAAAGAGTACTTTACCTACAG

GATAAAGAGTTCAAATAAGCGCTACATTTTTCTTCACAGGTTAAATGCATCCAGTGGTGT

GAATTGTGCCTTTTTATCAAGGATTTTATAACTGTCACCATTTACTTTCATTGATTTATC

TCTTATGTGAACACTATTCGTGGGCTTTCCCTGTTCAGTGCTGAATTGTGATGAACCGTA

TTCCATCACTGATGGTGTTAATACTTCCCAAGCAGTGAGGTGCAGCTCGCTTGCACAG

>c6435_g2_i1 len=208 path=[911:0-168 1080:169-207]

TAATAGTTACAGGTGAACTGTGAACATAGTTTTGTTTCTTAAAATGGACAGTCAACTGAC

TCCATTTCACGTAAGGAATAGAGGATAAGTTTTAGGTTCATAGTCAATGACAATGATTTT

TGAGAATGTATGAATACTAGTTTTTGTGAACAGAAGAATAACAGATTTTAGGTTTAGAGA

CTGGGGCTTAAAGGCTGTAGAGCAGCAG

>c6439_g1_i1 len=231 path=[373:0-230]

AAAACAACAACAAAGTAAACTTCAAAGACAGAAAATAAATATAATATATACTTTTATGAA

TGTATGCGGAAAGAGTGCAAAAAATTGCATCTGTAAGATGTTTAAATGCTGAATCATACA

ACCAAAATCTTACAAAAGCGCTTGTTTCTTTATACATCAGTTTTCTTATAAACACAAAAA

TATTGTAACAAATCTTACAAAGATCAAACCCTTTTGCCCTTCACCAGGATA

>c6442_g1_i1 len=208 path=[155:0-207]

TACAAAATTATTGAGTCTTTTTAATATAAATAGTCTATAAAACTACTTGGAACTCTGAGA

CAAGTCATTTTTTATTCAAATCATGGAAAATATTCTTTTTTGGTTCTACTTTTAAAGATC

TCATGGAAGAATGTTTGGAAGCATCTTCTGCTTTTTCTTAACTTTTGAGCTTTGTTTCAT

TTAGCTTAGCATGCTTTGCCAGGAGCAA

>c6448_g1_i1 len=234 path=[59:0-177 58:178-233]

ATTTTCTCTCTAGCCAAGCTCCATTTCACAGCGCTGGGAGTTCGTGGGTTCACGGCTTTG

GGCCCTTGGTCTGCTGAAGGATGGCCCTGCCCACTGCATGGGGCTGGGGGGGGCTTTGGG

ACCCTCCCGACCCCAGGCATCCTATGGCTGCAGACACCGCATGCACCTCCTGACACCGTG

GGGAGCTGGGAGGAGCCTGCAGGATGGGGCTTGGGGAATGTTGACAATGGTGGT

>c6453_g1_i1 len=327 path=[1:0-326]

GAGAGTGGGATCGCCCTTTGTGATTGTGTGTAGATACTTGGATTAATTAGTGAGGAAGCA

GGAAGACAGTTGGAAAATAATGCATAGAGAAAAATGATTCTCTTGATAAGGCCAATTAGA

AGACATGTTTATACGAACTAGACCACATCAGGTTTGACTGGCAAATGAGCAGAGCAATTG

ACTGTAGGTTACAGTCTGTGCATAACATTCAGCTGTTTTATGGCACCTGACTTGTGTTTA

AAACAACACGGGATGTTTCAAAAGCATCCCATCGGCGTGAGTAGTTGAGAAACCAGGGCC

AAAACCAATGTTCAGGTCTCACAGACT

>c6456_g1_i1 len=303 path=[59:0-45 58:46-302]

ATCACAGAAGTTCAGAAAAAGGAACCTCCAGTGACAGGCACAAGCTAAAAGTTGTTTAAC

GTTTAAATGTAAACAATGTTAAAAGTTGTTCTATAGTTGGGTCTTAAAATACAACACCAT

AAAACTGTGCAATGTATATTTTGTATGGAGTAGGAAATAATTTAATCAAGGAGAGCACAA

AAATGAGAAGCAGTTATGAAAAGCAGTAAATCAGTGTATACATGGAGAATGCAATTGCTG

AATGGTGGCACCTTGGAGCCAGGCTGTGATCATAGAATAGTTTGAGTTGGAAGGGACCTT

TAA

>c6456_g1_i2 len=285 path=[30:0-27 58:28-284]

TGTGATTTATTGAACCAGTTTGTAAGGAAAAAGTTGTTTAACGTTTAAATGTAAACAATG

TTAAAAGTTGTTCTATAGTTGGGTCTTAAAATACAACACCATAAAACTGTGCAATGTATA

TTTTGTATGGAGTAGGAAATAATTTAATCAAGGAGAGCACAAAAATGAGAAGCAGTTATG

AAAAGCAGTAAATCAGTGTATACATGGAGAATGCAATTGCTGAATGGTGGCACCTTGGAG

CCAGGCTGTGATCATAGAATAGTTTGAGTTGGAAGGGACCTTTAA

>c6457_g1_i1 len=341 path=[1:0-318 990:319-340]

GCTTACGCAGGAAAGGGCATCAAACACTAACAACAGGAACTGAGTCTTTCAGTTTATCAA

ACTACTTGGAAAAAAAACAACTCGAAAATAAGAAGTCATATGAAAAACAGTAACACAGGC

TTCAAACTGAGGACAAACTCTTAGGGATGTCACTGAATATTCAGCTACTCTAATAAAAGA

CAAAAAGGCACAAAACCAACCCACATACAAAATGTCACCACCAAAGTTCCCACCTGCTTT

TTCAGAATAAAGATTGTCAGAGGCTGCACTCCTCTGTCACAAAGAACTCACAAGGTTGCA

GTTTGGTGCTATACTTAGCACAGTACAGTAAGAGCCTGGCT

>c6457_g2_i1 len=247 path=[677:0-223 992:224-246]

GTAACCCCTAGAAGCTGAATAAATTAAAAGAGCCTTCTCCCCAGTGCCTGGATGTTCAAT

TACAACAGTCTCAAAGCTCTCAAACCCTTAAGTAAGTTTTACACCAGTAATACATTGATG

ATGGATCAATAGTACATAGAAGACATTGCCAAAGCTATAACAAAAAGGGTACGGCACAAA

CAAGGGCCTCTTCCACCTTGCTTTCCCAACACTCTATCCTCTCTGCATGTCCTTAGAGAC

AAGAAGA

>c6458_g1_i1 len=245 path=[223:0-129 353:130-158 382:159-244]

ATCTTAACTGAATTATTCTGAGCAGATGTTTCCTCTATCTAATTTGGAATGAAAACTTCG

ATGTACTGTTAGTGTGATTCTGCTGTACGCACAGACAGCGTGTGTTGTAAACTCAGTAAC

TGATTAATATAGTCCATAAATGTCACTGGCAGGCAGTGGCATCATTAGCACTAAGGGAAT

GCTTTAGAGTTTTCTCAGACCTCCTGTTCTCTCCTGCACATCTGGAAATGAGTAACTTCT

GTCGT

>c6463_g1_i1 len=249 path=[519:0-248]

ATATATGAGAAATTTCAATTTGTTCTTATGAATTTAATTTATATTTTATAAAGATTTCTT

TACATATAAAATGTACAAAATTGTTAATTTTAAAAGAGAAGCAATAAGATTAAAATATAG

ACAAGTATTTTTATATGGTTTATATTTTTTTCATCATATAAATTTTTTTTTATGTATAAT

TAAAAAGTTTTAAAAATAGTATTATGTAGGACAAGCATATAATAAAAACAAAAAATTAAA

CCTACTGCA

>c6465_g1_i1 len=212 path=[289:0-211]

CACATCAGGTTGACGAAGTCCTGTTTTCCATCTCTAGAAATGGACAGCGTGGCATGGAGA

GAAACCCACATCTTCTCCCAGGTTGGTTCACAGAGGGCAACATCTGTCCTCGCTTGGGCC

ACATCTGGATGAAAAGTGTAAGTCCACAGGTAAACAGCAGTGCTTCTCTGGTGGTTGGTG

ATGCAGGACCTGCACCTCTGATCCCATGTGGG

>c6468_g1_i1 len=203 path=[347:0-24 192:25-202]

AGCTCCCCAAAAAGCGTACAGAATGAATCCAGAAACTCTCCTGCTGCAGTCCCAGCAAGA

ATGCTGCACACTTGTACATAATAATAATAATAATAAGCATAACGTAATGCTTGTACATAA

TTTGCAAACCATAAATCACCAACCTTAAGACGTAGCTGTGTACATTGCTCTGTTAGTGTT

AATGCTTGCAGCTAATCTTAAAG

>c6469_g1_i1 len=295 path=[6:0-294]

CACAGGAGCCTACAGATACTGCAATTCCCATATAAACCAATGTAGTAATTAGGATGGCCA

ACAGTGTTCCTTTAGGTATGGCTGACTGAGGATCCGCAAGGTCACCTGAGATATTTGCGC

CAGCAAGTATGCCAGTTGCAGCAGGGAAGAAAATAGCAAATACAGAGAAGAAAGTTTCCT

CCTTTCGGAAATCTGGTCCAAAATTCTCCATGAATATTTCAGCTTTATAACCAAAGAAAC

CTTTAGCCTTCTTGCTGTCCAAAGGAATAAACGTTCCTATTACAAAGTCTCCAAT

>c6477_g1_i1 len=214 path=[155:0-213]

AGAGCAAGCATTCATCCATCAATGTCAGCTCTCTTTTTAAAATCCCTCTCGTGTTATTTT

CCTGACTACTTTTTTTTATTTTATGTGAAATCCTACAAGATCAAGATGTTTCTGTGTTCA

GATGTTATCTGGGAACTTTTCGGATGCAACGTTGTTAATGCAACTTGGTGCTTGATGACC

ACTTTTGGGAAACAATTCTGCACATGCTTTGACG

>c6482_g1_i1 len=210 path=[45:0-139 44:140-209]

CAATGTAGAAAAAGCTCAGCAAGGTAAACTCCTACAGTATGTTGTCTGAAGTTCATGAGC

AAGCTGTGTTAACCTTACTTAGGTGCTGTGAACTTGCAGATCATCAGTATTTCTGCAAAC

TATTTGTGTGGCATGTACCTGAATTTTTCTGCTTTCAAATCAGAAATAATAAACATAAAG

AGAGCTATAAACATATCTGCTAGTTCTTGT

>c6483_g1_i1 len=608 path=[155:0-607]

GGGTTACACAGGAGACTGTAGAAGTTGAAATATTTCAAGTAATCAATTCAGTAATAAATA

AAACAGCTGAATGAATGTTTCAGTCTCCACAGCCTAGACTTCTCGCATGAAGAACTGACA

AACATATGTTTAAACAACAGCACAAGACCAACGCTAACAAGAAAACACTAATAGCATCTT

TCAATTTAAAGGGAATCAATCATCATGCTACAGGGGTACAATATAACTGTGAAGGTTGTA

GCATACTGTGTTCATGCTGAAGTACACTCAACATGTAGGATATTTGCTGTGGAATATCTT

AAAAACAATATATAAAAATGAAAACAAAACAAACCACCAACAAAATATTTTGTTGTTTTC

AGGAATCTTAGGCTTTTTTCTTTCTTATCTTTATTTCTCTGCAGGATTTACTGATTTGAA

GCCGGTAAGTGTTGGCCTAGCAGTAGGATTGGCACAAGGATGGAAAGGGCAGTGGGATGG

GAGGAGTGCAGGGGAAGGAGCACTGCACCTGCAGCAGTGTGGAGCCACCCTCTGCTCCCA

TGATAACTCTGTTCCCATGATAACTGCTCCTTGCTCTGATGCTGCAGTCCCTCTAGTTCA

TGGAGAAA

>c6485_g1_i1 len=226 path=[204:0-225]

AAAGCCAGATCTGATCTGGCTTTCAAGTTAACGCTACGCTTCCCAAACCACGGAAAAGAA

AGATGGGAGACATACTCTGAGACCTGTCTTTAGGAAAGAAAACCAGAATCTCAGAGGCTG

GAAAAGGAGCCTGACTTTCTTGGTAAGATTTTTATTCTTCAGCATCCTTCAATCTCTACT

TTCAGACAGTGCAAGGACATGGCTTATAGAGCAATTCAGCAGTGAG

>c6493_g1_i1 len=361 path=[155:0-360]

TGTGCATGCACACACAGACATATATTTTTTGTGCAAATGTGTAACTGGCTGAGGTTTTTG

AGGTCTTCTACCCCAATGTTAGTTGACCAAAGTTGTTATATTTAGAGAAAAACAGATCTA

AACTAAGTAGGAAAACATGTATTTTTTAACTGAGTCTAAAGGCTGCCATCTTGATGAACA

TGTGCTATGGATAAAATGTCTAAAATGTAATCCATCTCATACAGTCAAAGGTTTTCTCTA

ACTGCTTTGTTTTTGTCATACCATGTTGTATTTTTCTGACCATTTGATGAATTTGTCTAA

AAATTATTGTTTGCCAACTTGTTTGTACTTAACTCTTTAGATCATACCTCACTCCAGTAC

G

>c6505_g1_i1 len=211 path=[17:0-14 32:15-210]

TCAGGTGCAGGGCTTAGCCAAACCTCACCTGGCAGCAGAATACATTTTGCTTTTTATCAG

AGCCACTTTCACACTCCCGCAACAGTCTCATTAGCCCTAATAGGCAAGCTGGGCTCCGAG

CATCAGCACAGCCCTGCCTTTGCACTGATGGAAAATTAAAGGAGTTTTCTTCCATATTCT

TCAGTGTGAGAGCGCAGCTGAGAATCTTTCA

>c6505_g1_i2 len=320 path=[329:0-123 32:124-319]

GGTTCTGATTTTGCCCTTTTACAGCCTAAGTAGCCCTAGGCAGGGACAACTGCCATAATA

AAGCTTCAAAATGCTGATCTCACTCTCTGGGAACTCAGGTTCTGCAATGCACAGAGCAGG

GCTGAGCCAAACCTCACCTGGCAGCAGAATACATTTTGCTTTTTATCAGAGCCACTTTCA

CACTCCCGCAACAGTCTCATTAGCCCTAATAGGCAAGCTGGGCTCCGAGCATCAGCACAG

CCCTGCCTTTGCACTGATGGAAAATTAAAGGAGTTTTCTTCCATATTCTTCAGTGTGAGA

GCGCAGCTGAGAATCTTTCA

>c6511_g1_i1 len=204 path=[182:0-203]

GTTTGATTTTTTTTTTTTTTGTTACCATAATTTTCAATTTAAAACATCTAATATTTATAT

AAACATATTTATATGATATTTAAAGTTTAATGTTGAAACACATTAAATGAATGTATTCAT

ATTTAATGTGTATATTATATTTTATGTGTTCGTAATAATGTTGTTTCAAATAAACATCCA

TCTTTTAGGTTTTTTTTTTTTTTT

>c6515_g1_i1 len=345 path=[645:0-344]

GTTAATCAGTCGATGGGGGGAAAAAGGAAAAAGATAAAAGCTTAGATGTAACCTCAACTC

TTCTGCTGCTATACATTGTTTTCGTTCATTTTAAAACACTTTTCTATGAATAAAGCTATA

CTTACCAGTGCAAAAACAATATAACAAACAAGCATATATCCCATATGCATTTGTGTGCAC

AGGTAATTTTATCTTCTAAAATATTACTATTGCATTAGAAAATAAATTAGCAGAGATAAA

CATATACTCTTTCAGGAGAGGGGTTTACACTACAATGAAGATTCCTGTATTCCTGCATCA

CACTACAGTGAAGATTCCTGCACTGGGATGTTTTGATGAGCGAGT

>c6516_g1_i1 len=204 path=[1:0-203]

GAGCTATGTGCTAAGCAATACAGGACCTTGAAGAAGTAGTTCATTGAGCATTATTGTTAC

AGATAGTACAACATGGACGTCTGTGATTCAACTCCAGATTCATAGAAGGCATCACCCACA

CAGTGCTGGTCTCATTAGAAAGCACTGAAGTAAACGTACTGCCAGAGCTAAAACACACTT

CAGCTTCTGATAACTGTGCCACAA

>c6518_g1_i1 len=217 path=[155:0-216]

GGTGGTAAAGAGCAGTAAAGGATGTTGGGCCAATTAAATCTACACTGATAGTGAAAAGCA

CACAGGCCTAAACCTGAGTAGTTTTGCTCCTAAATGGGAGCAAGGAGAGTTTGCAGGGTA

CTCTGCTACTGCTGTATGCAGCAGGCCCCCACCTATGGTACTTAAAACCTTGCTAACTCC

TGTTCCTGTCTCAGTAAGAGCCAGAGAAGTTTACTAA

>c6519_g1_i1 len=297 path=[273:0-296]

AAATCTACAGGAACTTTCTCTGTCTAGATCATGTGAAAGAAGTGCTGACGACAACCCGAT

CACCTTTGGCTGAGCTGACTGTCTTGAAGAAGCTGTGCGACCACCCCAGGCTTCTGTCTG

CAAGAGCATGTACCCAGCTTGACTTAGATGGACAGGAGTACTTGGAGCAGGATCATGAGA

GTGAAGCAGCTGTGCTTTCAGGTGCCAACAAAATAGATCATCTATCTGATGAGACCCTGA

TCCAGGAGTCAGGGAAAATGCTATTTCTTGTAGGACTCTTAGGGAGACTCCGGGAGG

>c6524_g1_i1 len=242 path=[1:0-241]

TTGGAATTGAAACATCAGCGCTTCATATTATTTGGATCATCAAGGACAGCAAGGCTGGTA

CCTTTTTGCTGTATGACGTAAAGCATGAGTATGCTGCTACACAAAGAACGGTGTTTCTGA

ACTTTCACCTTAAGTAATGTCTTCCCCACGTGATCAGAGCATTTCAGTTTCCCTACAGCA

AAATCCATGCTCCAGAATCATGAAGTTTTCATTCTATTTCAGCCATGAACTTCGCTCACC

TG

>c6525_g1_i1 len=203 path=[207:0-129 337:130-175 382:176-202]

AAACTCTGACTTTTTAACACTGTATCACATCGAGCACTTCACGGGTATGAAGCATTTCTC

ATCCCTTTGGCCACATTTGCTGACTCATGCACCTCATAGAAAGTATAACCTTAAGATGTG

TTTCTGACCAAGACCACTGAAGTTGTTCCAGAACAGAACTCCACAACGCAGGCCTTCTGT

GTGCCAAACATATCACTCAAACA

>c6528_g1_i1 len=209 path=[209:0-185 515:186-208]

ATGCTGGGAAAAATCTACAAACGTCATGAAAAGCTTTTGGCTCCTTTGCATGCAGCACCC

CCTGCCACGTGCACACAGCACCTCTCTTCTTTACAAAGATTACTTCTGCACTGATTCTTC

AACGGGAGGATCTTTAGCAGCCGCTGTTCTATGTGAGGTCTGCAAATACACGTGACAGAG

TCCAACTTTTGCTGCTGTAGGGCAACGGT

>c6528_g1_i2 len=209 path=[209:0-185 516:186-208]

ATGCTGGGAAAAATCTACAAACGTCATGAAAAGCTTTTGGCTCCTTTGCATGCAGCACCC

CCTGCCACGTGCACACAGCACCTCTCTTCTTTACAAAGATTACTTCTGCACTGATTCTTC

AACGGGAGGATCTTTAGCAGCCGCTGTTCTATGTGAGGTCTGCAAATACACGTGACAGAG

TCCAACCGTTGCCCTACAGCAGCAAAAGT

>c6536_g1_i1 len=252 path=[500:0-251]

TAATTTACTGATGCTTAAGCAAAAACAGAGAGATGGAGGGATATTTGCAGGTTCTAAGGA

AAATGAACATGACTTGCATTGTACTATCACTAAAATGCATTCTGCAGCAAGGCCTTCAAA

GCCTACATTGCATACTGGACTGAATTTTATGAGCTTTTAAATAAACTACTTTCTAGATTT

CATTACTAAATGAAGAACACTAAAAAACATTAAGCAATCCTGTTCAAAGAGTGGAAGGCA

AATCTTACTGTG

>c6544_g1_i1 len=207 path=[345:0-206]

GCTGGTTGATTTGCTAACATGGAACATATTACACTAAAATAAGTGACAGCAAAATACAGA

CTGCTGAGACATGGTGTGATATTTACTCAGTGCATGTATCAAGTGATATTTTGCAGGGCT

GCAAGCTCTGATTCTTCTTCTGCAACACTGGAGCCCAATGTCTCCACTTACACAATGTGC

TTGAGTTATGCTACAGTTGATGGTATA

>c6546_g1_i1 len=469 path=[601:0-468]

ATTAGGGTTAAAATCAAAATATGTCACCCTGTGGGATGCACGTAATTTATGGAGCTCTCC

CTCATTCAGCTAGGAAGACGGGCATAAACAAGGGAAGAGTTTAGTTCAAGCCTGACTATT

GTTCTTTTCACATTCTGGTCTCTGGGTAAGCGTTTATTAGCATCTCCTCAGCAGAATTAG

CATTCAAATAATTGTATCTGTGTTTTAAATTGAACACTGGAAAAGAACAAAATGTTTTGT

GACCTCAGTTACTATTGTTACTCTTACTGTTTGGACTGTTTCGAGTCCGTTTACCTCAAG

GCAGACTTGTAAGATCAACAACTCACTGTGGCAATCCACATCAGAGAGACCAGCAATAAC

ACAGGACAAAAACTATTTCACTTAATTTGTCCCCTGAAATGCATTAGGCCTTATGCATTA

CAGATTTTTTCAATCTTAAATGCTGAACCTATAGGCAAGAGATGTAAAA

>c6554_g1_i1 len=265 path=[503:0-264]

AGAGTCTTTTCATCATAGAATCATATAATGGTTGGGGTTGGAAGGGACCCAGGTCACCTG

GTTCAAACAACTGGTTGCCAACTGCTAGACCTGGTTGCCCAGGGCCCCAGAAAACCTGGC

CCTGAACACATGTGGTCCATAGGCTGCATGTGGCCTGGCAGTCTGTCCTGCAGCCCATCC

CCTGCCCTGCACTATCGTGATGGCAGTTCTATGCTGAGTGGCTCAGCCCAGCCCCGGGCT

CGCACCCATTGCTCAGCAACAACCA

>c6574_g1_i1 len=206 path=[1:0-205]

ATGCAAAAGATGCACTGATTAATATGTAGGTTTTGACTTTTCCAACTAGAGTATTGTAGT

TGCAGGAAAAATCTAAACCTGATGTGAAAAATACATGATTTCAGAAGTGTAAATAAGGAT

AATACACAATCTTGTTAGAGGTTTTTTGCCACTTTGAAACACTTACAGGCCTTATCTTTT

TTTTTCTTTTGCAAGTACTGAATATA

>c6576_g1_i1 len=227 path=[205:0-226]

CTGAGGTTTCATTAAGCATGGCAGACTTCTGATCAGGGGCAGTTTGTTACCCCTTGTTCA

GATGAGCCATCCAACCTTCTGTTTTCAGTCAGAGCAAGGCACACCAGCTTATCCAGCATA

GGCAATAGGTTAAGGAGCAAAGACCTGTGGTAGTCAATAGATCAGGTTAGCAGTTTTACA

AACAGCTCAGCTGTTAGATTTGGTGGAGAAAGTTGCGCACAGCTGGG

>c6579_g1_i1 len=262 path=[38:0-192 34:193-235 324:236-261]

TGATGCAGGCAGATTCCACATGACAGCTTCTGCTCCCCAGGCACATTCACCTCTTTCTTT

CACTGCAAGGTGAGTATAACTTTCTAAAGGAGGAAAATGTAAAACAAACAGACAACAACA

ACAAAAAAACCCCAAACTCAAGAAGTGGTAGTGTCTTCCAGAGACTTACATAACAAGGAA

AAGAACAACAGAATCATACAATGGATGGAGTAGGAAGGGACCTTAAATATCATCTGCTTG

AAACTAGATGAGGTGGTTGCAA

>c6579_g1_i2 len=262 path=[38:0-192 34:193-235 273:236-261]

TGATGCAGGCAGATTCCACATGACAGCTTCTGCTCCCCAGGCACATTCACCTCTTTCTTT

CACTGCAAGGTGAGTATAACTTTCTAAAGGAGGAAAATGTAAAACAAACAGACAACAACA

ACAAAAAAACCCCAAACTCAAGAAGTGGTAGTGTCTTCCAGAGACTTACATAACAAGGAA

AAGAACAACAGAATCATACAATGGATGGAGTAGGAAGGGACCTTAAATATCATCTGGTTG

CAACCACCTCATCTAGTTTCAA

>c6581_g1_i1 len=235 path=[355:0-115 24:116-234]

CGCGAGGGCAAGGGTGTCAAAAGTGAGTATCTACAGTTCCTGCTACAAGGGTGATGGTGT

CTGGGCTGGGCATGCAGCCTGTATGTGTGTGCTCATGGTTCCCTGGCTGAGTTTAGGCCA

CCAGCTCCTGCACACCTGCCTGAAGGGCAGCTGGGGCATGGCCACAATCACCAGCCTGCC

CTGCCTGCTGCTGACACCGGCTTCCCCTGCAGTTGAGAACCTGTACACAGCGCTG

>c6585_g1_i1 len=203 path=[53:0-31 85:32-52 301:53-202]

ATAGTTTATATAAAATAATATAAAAAAGAGAGAAAAAAAAAAAGATTAAATTAAAATTTC

AAGAAAAATTACATAAGAGATTTATATGTAATTCAATTACCATAAAGGATTGTTTAATAT

ATTTTAGTAAAGGAAATATTTGCATGCATATTATGCTAATGAGCAAAACTGAAATAATCC

GAGAAATTATGTTTCAAGTTGAT

>c6587_g1_i1 len=277 path=[1:0-212 214:213-276]

ATTGCCACAAAGTACTCAGCGTTTCCAGATGTACCGGAGAGCCGCCCACAACAAACACAC

AACAACCCTGCGGTCAAATCTGAATACATTCGTATTATTTATTGGCTGTATTAAATATAT

TATCTATTTGATAGTTACAATTTAGTAATACAATGCATGGCAGCTTTAACATAGAGATAC

CTCAAAATTACAATACAGGAAAAATGAAGATACCTAACAAGGGACGACTCCTAAAAAAGT

AAAAGTTCTTCTGTAATTGCCACAAAGTACTCAGCGT

>c6588_g1_i1 len=323 path=[1:0-79 81:80-322]

TTGATAACTGCCACACAGCTGAAGAATTCCGAGTCTGCCACCTTTAAAAACTCAAATGCC

AGAGCTGCACCAAAGCAGCGATGGTATGATGTGGCTTTATTGAATTCATAACTGAAGTAA

GAATTTAGTTCATAACCTCTTTTTAAGTAGTCTTGAGTGCTACTTTCTGTGCTAGTAAGA

GCAAGCTAAATTTAAAAGCCGTCCTCTTCTGTTATAACTACAGCTAAGATCAGAAACGTT

CAATGTGTAAGAGCCATAGAAGAATCAAGTACTTACATCATTTGCAATTTGGTTGTATCG

TCTCACTTTTAATGCATTCTTAT

>c6595_g1_i1 len=649 path=[1:0-648]

ATGTTGCTTGAAGCAGTGGCTAGAAGGCTAGAATACTTTGTAGACAGTGTAATCTTGAAT

GCAGTAGATGGCAAAAGACTGCAAAGATCAGCATTAAAGGGTTTTTTAGAAAAAATTAGC

ATTTTAGATCAAGTATTTTAGCTGTGAAGTAGATTTGGAAAAGCTGTGTATTGGAATACT

GTTCTGAAATGTGAAAGTGGGTTTGCTTAGATACGGAGATGTGAAAGAGGATGGTGAAAT

ACTGTGTCCCAGATACGGAGTCTTTGATGTGGCACATGATACAGAGTTTGTAAGTGAATG

TCTTTCATTCCTACAATTTTTTTTTGCTTCTGTGAACAAAGTCTCCAGCAAATGATTAAC

AAACCAACCAGGAAAACAACAGCGTGGATATGTTAAGTAAAGCAATCAGAATGTACTGCT

GCTCTATTACTTCTGTTCTCTTCAATACCTGAAGTGAGGATGTACAAAATTTAGGTGTCA

CTTAAGGCATTTGTGATTTGACACTGGTAAACGGTTAAACTTGCCAAAGTCCATGCAGCT

ATCTTTTTAGACTGGGAGAGCACTCTTTGTGCTTAATAATCACTGTGACACTAAAATATA

GTTGCAGCTTACATTTCTTTAAGTTATGTAGATTGAAAAGAAACAACAA

>c6604_g1_i1 len=484 path=[462:0-483]

ATCACATGGATTCTGACTTTTCTTCTGGCATACTTCCTTGCTCCTCTGGTCTTCATGCAC

TTTCGAACTGTACTGCTGTCCAGGGTGTTCCTCACTCTCTCTCCCGGATGGTTTCCCAGA

GGCTCCTTGAAGTGCTCTCCTTGCTCCAGGTCCCCATGGACATCTCTTCTGGTTTTCACC

ACAGAGACTCTACAGAGACTTTGGGCTACACCACTTTTCTGTGGATTTTCCTTTATATTT

GTGATCCCATCTCCTGCTGGAAGGCAGAGGAAATCTATAAATAGCATAGAGTGCTCAAAA

TATTGCACAGATGTGGCTTTTGCGGGCCCCAAACACCTTTTCCCCTCCACCACTTCACAT

GGGGGGAGGTCTCCTCTCAAAGCCACTGAGCTATGAGCATCTGGGATCCACAGGTATTCC

CCTCCATCAAGCAGGGAGATCACCTTTGGTATAGGGGCTGAAACAAACAAACAAACAAAC

ACCA

>c6608_g1_i1 len=215 path=[1:0-214]

GTGATGCTCCACAGTTGGTTTAGTACCAGCTGTTGAGCATCACAGCATTGCTAAGAAAAG

TCCTAGTGATGACCACCTCTGGCATATTGAGGAATGTGTTAATCAGGGACAGTATGAAAC

AGAAATCCATAAACTGACAGTGTTACTGGTGCAGACCATGCCCATGGCCCTGCTCAAGAC

TGTCTTGTTACTGCAACTACTTAGCAGCAGCCTTT

>c6616_g1_i1 len=267 path=[1:0-266]

GTTTCCAAAACAACTTTAAGGCAGCTGATGTCATAGGCTTACTGCCATCATAGCTGGTAC

TCAGCCTTTCCCTTATAGCTTTTTAATGGGTTTCAATATAGGCTTTTTCAAAGCCAACGA

GAACAGCTTACTCAGTGCACAGCTCAAGGGTTTCCCCCTTTTCCTACATTCCAACTTGGG

GCTGCGGGACTATCACATCAACCCCAGTGCTCCTCTGCTCTACGTTACACGGGCTAATGA

GGTGGAGCAAGGTCCCCTGCCTGGTGA

>c6616_g2_i1 len=263 path=[489:0-262]

GGAGTAGATGCTTTCTACAAAGACTAGCACCACAGGATCTGTGCGTGAGGTGTCCACAGA

TTTGGTAAGCCTCCAGCTGGCTTGCGGTGGGATCGAGATGCGGTGCTGGTTGCTGCAGTC

ACTGAAGGGCAGGGGTGGTGGCTCCTTGATTTTGGGACTGTTCGTGACGTAGTAAGCCAG

GATGCACATGGAGATCAGGCTGAAGGCAATGAGCAGCAGGATCAACCTGTGGAGCTCTAG

CTGTCGCACATGCCGCACTACTT

>c6621_g1_i1 len=222 path=[391:0-22 369:23-221]

TCTAAGCCACAGAGGATAACTTTGACTTCATAAATACTTTGGTTCATTATATAATTAAGG

TATCTTTAAGACAGAATGCCTTCCTATGTTTTTCTTTATAGCTTACTTTGGTAAGATTAG

AATAAGGTGCTTGGCACAGGCTCTGGTCTTGCATTTAGCTGGATGGAATGGATTTTTAAC

CAAATGCTTATGTTTTGTATTTTCATATCTAGTCTGTGGCTT

>c6621_g1_i2 len=279 path=[346:0-22 369:23-221 617:222-232 628:233-278]

TCAAAGTTATCCTCTGTGGCTTAGACTTCATAAATACTTTGGTTCATTATATAATTAAGG

TATCTTTAAGACAGAATGCCTTCCTATGTTTTTCTTTATAGCTTACTTTGGTAAGATTAG

AATAAGGTGCTTGGCACAGGCTCTGGTCTTGCATTTAGCTGGATGGAATGGATTTTTAAC

CAAATGCTTATGTTTTGTATTTTCATATCTAGTCTGTGGCTTAGACTTTCCTATTTCTTA

AGACTTGCTTGCTAACCAGAAGAAATAGGAAAGTCTAAG

>c6631_g1_i1 len=486 path=[939:0-435 1374:436-485]

TAACTTTGTGTTTGAGGGGGGTGCAAAAAACTGTAATGATGTCATTCATGTGGCTGCTCC

CCAACTGTTGCCCTTCTATAATGACAAAATCAATTTCTCCAAAGATGAACCGCCTCTTCT

TTTGTTGGCAGTGCATGGTACCTCTGATCTTAGAGTGTGTAGGAATATCGAAGTTTATCA

CCACGTCTACGTGTGGGATATCCAGACCTCTGCTTGCAACATCAGTAGCCAGCAGAATAG

ACCGTGCCTTGGCCTTGAACTTGTTTAAAGAGCCCAAGCGTTTGTTCTGACTCATCTGCC

CATGGAGAGGAATAGCAGTGAACCCAAGGTTACGGAGCAGTAGAGCAGTCCTCTGAGTAT

TGTTACATGTACTGCAGAATATCATGAAAGAATTTCCAGCTAGTTCATTCAATATATAAA

CCAGGTAGCTGTCCTTGAATTTGGATGGGATGAAAATGTAATACTGCTGTAGCTTTTCAA

CTGTCT

>c6631_g1_i2 len=486 path=[939:0-435 451:436-485]

TAACTTTGTGTTTGAGGGGGGTGCAAAAAACTGTAATGATGTCATTCATGTGGCTGCTCC

CCAACTGTTGCCCTTCTATAATGACAAAATCAATTTCTCCAAAGATGAACCGCCTCTTCT

TTTGTTGGCAGTGCATGGTACCTCTGATCTTAGAGTGTGTAGGAATATCGAAGTTTATCA

CCACGTCTACGTGTGGGATATCCAGACCTCTGCTTGCAACATCAGTAGCCAGCAGAATAG

ACCGTGCCTTGGCCTTGAACTTGTTTAAAGAGCCCAAGCGTTTGTTCTGACTCATCTGCC

CATGGAGAGGAATAGCAGTGAACCCAAGGTTACGGAGCAGTAGAGCAGTCCTCTGAGTAT

TGTTACATGTACTGCAGAATATCATGAAAGAATTTCCAGCTAGTTCATTCAATATATAAA

CCAGGTAGCTGTCCTTAAATGTGCTGTGTCTTCCAAATATCAGACAGTTGAAAAGCTACA

GCAGTA

>c6631_g2_i1 len=236 path=[214:0-235]

ATCGTGTTGGAAGAACAGCTCGAGCTGGAAGGTCTGGCAAATCGATCACCTTTGTCACAC

AGTATGACGTAGAGCTGTTTCAGCGCATTGAACACCTGATAGGCAAGAAGCTGCCAGCAT

TCCCCATGCAAGAGGAGGAAGTTATGATGCTAACAGAGCGTGTGGCTGAGGCCCAGAGGT

TTGCCCGAATGGAGCTGCGGGAACAGGGAGAGAAGAAGCGATCTCGGAACGCTGAT

>c6635_g1_i1 len=268 path=[316:0-57 25:58-66 34:67-267]

CCCAAGCCATTCTATGATTCTGTGTGTTCAGTATGCATGCTTACAACCCAGAAGAATCCT

AATTCAGCCAGAAGTGAAATTTCAGAAGATGAGAGAGGAAACAAACAGGCCTTTAACCAT

CTTTAAGAAGTATGTAGTCCTTTTTCTTGCTTGTTTTTTGTTTTTTTAATTAGGGGGAAA

AAGTGTTTTTTCTCCTCAGAGTTCCTCCTTACCTTTCCTTTGGTCCTCATTTCTCATGTT

TTTGCACCATAGACCCTCAAACACAAAG

>c6638_g1_i1 len=427 path=[31:0-426]

AGTATGTATATTTTTTTACTTCTACTTTCTGAGAAACACTGGCTCACTTAAGTAAAAGGC

ATTCAACTCTAATGAAGGCAGATTTGACATCCCCCGATTAAACATTTCGTAGGTCATTGT

AGTTCAAACAGATAATAAACCTGATGAATCAAACCCTTCACATAATACGTAATCTATTAA

CATAGAGCAATGGTCCAGTCTTGCAAAGTGTAGAGCAGTAGCAAAAAGCACAAAGTGCCT

TAAGCGTTCCTTGAGCTCTCTCAGACAGTTTGAAGAACTCCTCATTGCTCCACAGTCCTC

AGGGTTTTGCAGACAGATCCACTGTTCTGAATTGTGCAGGTAGGGAAGAAGTTAATGAAG

TTACTGGCTCTAATTTTGCAAAAGGGTTAATGGGATGCATTTCTGGGGGAAATCTTTCCA

AGAAAAG

>c6640_g1_i1 len=216 path=[163:0-47 81:48-215]

TCACCCAGGAGCCTGAAGTTCTCAAGGCCCACATACAGCTTCTGCCCCATAGATTGTGTG

GGGCTGCCCCACAGATCCGTCCTCGCCCCATAGATCCTATGGGGCTGCCCCACACATCCC

TTCACCCCATAGATTCTGTGTAGGACCGCATCGCACCCCATACACCCCCATACAACCCCT

ATAGGACGCCATGCAACCCCGTAAGATCCTATAAGG

>c6643_g1_i1 len=243 path=[357:0-242]

AGGAAAACTGTAGTGAGCCCAAAGGTGAGCTTTTGCATTTAGATGTTCTGCTCCTTAGAA

ATAGTTCAAAGGAACTAAAACTATCAGTACTGAAGTCTTATGTGAAACCAAATGGGAAGG

ATACAGAGCACAGTTCCTGTTAGCACCTGTGGTCTGAGTTAAATCTGCAGGGGAGGGAAA

GTGAAAGTGTTGGTCCTTTCATCTTAGTTCTTGCTCTGTAGAGAAAACTACTTTTTCCTT

TTT

>c6657_g1_i1 len=207 path=[223:0-206]

AGAAGACACACCAGGAGATCAGTGTGACCACGATATGATCCAGTTCAGCACACAGACCAC

TCCCTGGCAGCGCAGCTTGCACCTACCCAGGCACAGGTTCAGAAGCCCAGCCCTCCAGCA

CTGAAATGACTTTCAGAAATCATTTTTCCTCCCTGCAGTAATGTTTAAAGCAGGTACCTT

CATTCTTCCACACTCAAACACAAAGTT

>c6661_g1_i1 len=262 path=[394:0-261]

AGTCTTGTTTGGACTCCCAACTTGCTGTGCAGTCAAATTGCACTTGTGCTTTTCAAATGC

TATACTTACTTTAATGGCTTCTTAACAATTTTACTCAATTTCTAGGGGAAAGTAGGAAAA

CAGACTTTTTAAACATCTTATTGTGACTGCATTTAATAAAAATCTTAACTATTCAATATT

TACATGCTAAAACTTGTTCCCTGCAAGAGCTTTCACCTTTTTCCTGTTAAGATGAATAGT

TTTGCTTAATATATAAGCTTAG

>c6664_g1_i1 len=311 path=[289:0-310]

AAGTCTCGATATTTACAGTTGGAGGAGACAAATTTCACATCTTGGACCATAATATCTTCA

ACAAAGATATTGTATTTGCTTATATGATTCCAGCCCAGATCTGGCGACGGTGCCTCAACA

GACAAATGCAGCAGAAGCTGGCTGAGGCACCTTATGATGGACACAGCAAGGGATCAGGAG

CAACCCTGCCAAAGCTGAACCGTGAATCTTTCGTTTATGTTGATGAAGATGATGATACAG

ATGAGAAGGCAGAGGTGAGGGAGAAGAAAAGAGGCTGCATTATAGACAGCTTAAGAAGCA

GTAAGACGTTT

>c6666_g1_i1 len=240 path=[1:0-239]

TGAATAAATGTTGCTCCATAGCAGCAGAGCAACTGTATTTTCTTCAATGGTTTCCATCAT

TATGCATCTCTAGCTTTGTAAGCTGGTCCTCAAACTTTTGAATGTCTACTTGATGCTTCA

TTAGGAACCTTCCATTTAAATGAAAAACAGGTATGTCATATTTGTATTTATGATACCATG

CTGAGTTCTCCGGAAGGGTAATATCCACCTCCTGCAAAATAAACCTTGCCCTCTTTGTGA

>c6673_g1_i1 len=245 path=[671:0-244]

AATGGGAGCTACGCGAAGAGCATGGCTCTGTAACTCTGTGATGCTGGCACAAGCGTGACC

ATCTCTCAACTGCTCTCTCCATCCATCTGCACAAGTGTGTGATTGCATACTGAACGAGGT

CCGGGGCCTTATTTAGCCAGATAGAAACAATGGCTTTTGTCGCCCCATTTCCCAACGAGG

CCGGTTCCCCAGATCCAGTTTACCGTGCAGTGATAAACAGTTGTGCTGACCCAGTGAGAG

TGGAG

>c6673_g2_i1 len=247 path=[225:0-246]

AGCTCGCTATCCTAACAACTGCTGCTCTGCCTTCCCAGAACTGCCGGGCAATTCACAGAC

GCTGCCCAACCCCATCGCTCCTTGTAAATAAAGGCAGTTTTTTTCGTTCGTGAAAAGAAA

ATTGCAAGAAGAGAGCCAAGGTCCCTTACAAAAGGGTATAGGGCCATCTCCCTCACGAAC

TACAATTAATCTTTATTTAAAAACAGCCAAGCGATTACTGGATTTAACGCCGAGTGCTAC

AAAGGTG

>c6675_g1_i1 len=248 path=[1:0-247]

AAGAAGGGTTAGGCTTTCCTTTTGCAAAAAATGATGGAATTGTTCATTTCTGTCTACTCT

TTGGGAAGATAGGAAGATATTCTTCCTCTTTGGGGCTGGGGGTCCAAACTGAGGTCAAAT

AATTGACCTGCAGGTCCTAGCAGCACCAATGTGCTCTAGGAGAAGCTGAATGAAGATCTC

TGCAGTTGATACGTGGTGTCCATTTGCTGCCACATGGCACGGTGACTTTGCAGTGCTGAC

ATTGGTGG

>c6678_g1_i1 len=209 path=[487:0-208]

AATGCATTGTTTTACTACGTAATACTTCAGAGTTTTGGTGTAGAATACAGTAGTATTGTT

TCTTATTCAGTAAAGCTATGTAATGCTGTATCTAAATGGTTTAGATCTTTGAAATTAAAA

TCATCTTTCCATATCAAAGAGTGATCTTCAATCAAGCATTCTTTTAAGAATTCTGACTTT

CTTTTAAGGTGTTTGAAGTAGTAGAGCGT

>c6684_g1_i1 len=270 path=[15:0-269]

AATTAAAATAAATAAAAAAAAAAAATTAAAACTAGTAAACTTTAAAAAATTAAATGATAT

ATTTACCAAATGGTAAAAGATCAATTTTTTTTCATTTATATCCTTATAAAAAAATGAATG

ATAATTAATAAATTTCAATTTATTTTAAATGATTTCATTAATATATCAAATATGAATACG

ATACATATATTCATTCACTATTATTTAACTTTTATATTTCATTTTTAAATTTAATTTTGA

ATTTATCATTTCGTTTTTTTTTTTTTTTTT

>c6686_g1_i1 len=225 path=[203:0-224]

CCATCACAACTCACATCTGTAGAGCCCAAGAGCTCCAGGAGATGCCAAAAGCATGGGAGA

TTCTACAACAGCAAAATGGGGGGACACTGTTGTTAACTTCAGCCAGCAGTCCCTCAGTGA

AGGTATCCAGGCAGTTGTAACTAAATGAGATAAGACAGGAAACCCATCCAGAGAAATACT

TCAATGAATTCCGTGAACAAATTCGCCTAGAAACCACAAATGAGG

>c6686_g2_i1 len=225 path=[607:0-224]

CACGCAATACTCTAGACATGAAGCAGAAAACTTCCAAAGAATGTAGCTGCTTAACAAATG

GGGACGATAGCAGCTTTACAAGTAAGATTTTCCCTTCTTTTGAAGCTTACTGAGATGGGT

GTGTTGCACCAGAGCTGTGCACTAAAAGACACCTGTGTGACTGGGACCTCATCAATGTGC

TCTGACACAGACCTCACACAAAAGTCACAGATTTAAGAACTTCAG

>c6687_g1_i1 len=461 path=[483:0-362 988:363-460]

GGAGGGGACAGACGGACAGTAGGGACAGACGGTGGCAGAGGGACTAAGTGCTGTTTGGGG

ACAGCGCGGCACAGGCAGATTTGGGGACGGGAGCCCCGTTGTGTCCTTGGGGGGATTTGT

CCCCCATGTTCTTCCCTTCCGGTGCGGGTGACACCGCCGCTCAGCGTCCCCGTGGGCAAT

GGGGACACAGAACCGAGGGACCGGGGCCATTGTTCCCAACACAATGGGGAGGAAGAGGAG

GCCGCATTCCTCTGCCTCCGGACAGACCAGATGGACACACGGAACCGGACAGAACGGATG

CACAGGGGGGCAGAGGGCTTAACCCTTGGAGGCACGGGGTTGAAGGCACACGGCGGGATG

GGGTCACTGCTAGGACCAAAGAGTGCTGTCCCCCCGGGGAGGGGACACGGCGCCCCGGCG

ACCGTCACACACTAAAGGAGCAGCAGCTGCTGCACAACTGT

>c6687_g1_i2 len=466 path=[483:0-362 846:363-465]

GGAGGGGACAGACGGACAGTAGGGACAGACGGTGGCAGAGGGACTAAGTGCTGTTTGGGG

ACAGCGCGGCACAGGCAGATTTGGGGACGGGAGCCCCGTTGTGTCCTTGGGGGGATTTGT

CCCCCATGTTCTTCCCTTCCGGTGCGGGTGACACCGCCGCTCAGCGTCCCCGTGGGCAAT

GGGGACACAGAACCGAGGGACCGGGGCCATTGTTCCCAACACAATGGGGAGGAAGAGGAG

GCCGCATTCCTCTGCCTCCGGACAGACCAGATGGACACACGGAACCGGACAGAACGGATG

CACAGGGGGGCAGAGGGCTTAACCCTTGGAGGCACGGGGTTGAAGGCACACGGCGGGATG

GGGGACGCGGTGACACCCATCCCATGTGTCAAATGGTCGGCCTGGAGAATAAATATGTGA

AAAATAAACAAGATGGATGGATGGATGGGTGGACGGATGGATGGAT

>c6688_g1_i1 len=588 path=[285:0-587]

AATTAAATGTATATGTATATTTATTTTAGTAGAAAAGAAGCTTATATTCCTACATAATGC

ACTATTTTTTTTAATTTCACATTAACAATTTTCACTAATATATATATTATAACATAATTT

ACTATTAACAAAATAATCACATATTAGTGTTTTCGTAATTATTTTAATTATAGTAGACAT

CATAATTTTTTAATCATGATAATTTTAAAATTACTAACTCTACAAATCATGTCTAAAACA

ATAAACTATTATATAATATATTATTGTTATTTTAATTACTTCTTATTCAAAATTATGTTG

TTAATTTATAATACTAAATGTTTATTTATACATAAAGAGGAATCTTCATATATGAATTTT

ATTTCATAGTATTATTATTATATAACTACATTAACATAAAATTATTATATATCTTTATTA

AAATAAAGCTAAAATCAATTAAATAATCATAGAAATATACTCAATTTAATCACTTAAATT

TTTGCATTGTTATTCTTATTTCAGCTATAACATACTCCAATAATTTTTTTTTTAGTTAAT

TTTTATCTTGAATAATAGTGTATCTGCGATTTTTAAGGTTTAAGCTAT

>c6692_g1_i1 len=303 path=[365:0-302]

GTGTATGAGTTAAAATACTATGTCAGGAGAAGAATAAGCATTTGGTTTTGGTTTCTTCTG

GGTGTACAACTACCAGCTGTAATGAAGGAAGCATTACATTTTAGCAGTAATTATGGTTCT

GATTTCTGAGGACGGTTTACTGTTTTTCTCTAAGATCTGTGAGTTGATATCTGAGCATGA

CATTGTTTATTCACATATATAACCTCTGCTCCATCACTGTTAACTTTTAGTAAGTGCATG

TGTATGAACACGTAGAAGAAGCCTGTTTAGTAAAGAACTAATTAATACTAAGCAACCGTT

TAA

>c6692_g2_i1 len=205 path=[183:0-204]

ATTAACATTTTTGTATGCTAACTGCCTTTCTTATAAAGTGATCAGGCTCTGTGTTACTAG

CAGGGATATATGTTTTAATAGTAAAGTACCCTTATATGTGCAGAGAACACTACGTCTGTG

TTTTGGTTTTAGAGAGCAGTTGTGTTCCCATTCCATTTTGCAGTTGCTTTTTTACATTGC

TATGGGAACTGGTTCAACTTGTGTA

>c6694_g1_i1 len=244 path=[386:0-243]

AAACCTAAAATAATTAAATAGGAAAGAAAGAAAAAGACATAGTTCCAGAGTCTGAATGGG

TATCAGTCAGCTGATTTCCATCTCAGCCTCTGATCTGCAGCATTTTAATAGTTAAGATTT

GTCTATGGAAATCAGTTTGCTACAGCTACAGCACTTTACATGAGTATATTCTGCACATAT

TCAGGATACTTCTGATGTACTGCAATGATGATGCTATAAAGCTAATTTTAGGTAACTTTG

TGTT

>c6700_g1_i1 len=348 path=[517:0-308 179:309-347]

GCTGCGTTTTCTCACAGTATTGCAGGATTGTCCAAAACCTTCCAACAATCTATTTCACAA

AGTCTTACAGCTCACATGATTAGAATGTGATTATTGGAATGAAGAATCACAGTCTTTACA

CAGTGCCTCAGTTTTGCTTGGTAGAGAAGTTAATCATTTCCCGTGGTGGAAAATTAAAAA

CCAAAAAGGCATGTTTATGTCTTAACCTTCCTATCAAGCATGGTGAGTATTACCAACCTA

ACTTATGCCACAGCAAGATGGAACAATACTAAGAGAAAGGGAAAACCAGCACTTAGATTA

AGCAGATTTTTTATACAGCTCCTGTCAGTGTATTTAACACCTTTCTCC

>c6700_g1_i2 len=347 path=[517:0-308 825:309-346]

GCTGCGTTTTCTCACAGTATTGCAGGATTGTCCAAAACCTTCCAACAATCTATTTCACAA

AGTCTTACAGCTCACATGATTAGAATGTGATTATTGGAATGAAGAATCACAGTCTTTACA

CAGTGCCTCAGTTTTGCTTGGTAGAGAAGTTAATCATTTCCCGTGGTGGAAAATTAAAAA

CCAAAAAGGCATGTTTATGTCTTAACCTTCCTATCAAGCATGGTGAGTATTACCAACCTA

ACTTATGCCACAGCAAGATGGAACAATACTAAGAGAAAGGGAAAACCAGCACTTAGATTA

AGCAGATTTCTTATTTGGAGCAGGAGAAAGGTGTTAAATACACTGAC

>c6703_g1_i1 len=298 path=[1:0-297]

AACTTTGTGTTTGATTCTGCTCTGTGCCACTTACCAAATGCATGGGTTCAGTGTTTCCTG

ATAGGTAATCAGTGTTACCTTGAGGTATCTATCAACAATCAGGTCACTGTGGAAAGGTGA

AGATACTGCAGAGATTCCTTTCTTCCCTCAAAGAAGATGAAAACATTTATTTTAAGTGCA

TTCTGTATGTTCACAAGTGAGGACATGAGAGCCTTTCCCGACTTCATCTTCTGGAGATTA

TCTGCATAAGTTATTCCTATATCATCTCAGCCAGAATTTTCCCTGTACCAACAAGAAG

>c6705_g1_i1 len=247 path=[1:0-246]

TGCACAATTTATCTAGACTTGCTCCATGGTGTGGGCAGCAGCACTTCCCTCTCCTTTTGT

GAGTGTGCCATAGATCTAGCAGCTCCCTGCAGAGCTTCCCCATGCCTTCAGGTGGGAGTT

GCTTCACATTTCCCCAGTGTGTAGAGCTGGGTCACTCTTAGAAGTCAGAGCCAGTTCTAA

ATGTAGTTATTTGTGTGCAGAGCCCATGGAAATCTCACCTTCTTGGACAACCACCTCAAA

CACAAAG

>c6705_g2_i1 len=369 path=[449:0-368]

CAAAACCTGGAGGGCAAATCTGTTCTGGGAGGCAGTGCTTTGAAAAGAACTGAACACAGG

AGGCAGAGATATTTCCCATTTAAAATAGGTTATCATTTTTCTATCTGTGCAGTTAGCAAA

AGAAAACAAGAGAAAAAAAATCTTCCAACTGGCAGAGCTCACCTCCCAACACCTGAGCGT

GCCTCCAGCTTCCAGGCAATTAGTCAAGTCAAAGAGAATTAGTCAAAAACTTATGGAAGA

GCTTATTCTTTTGACACTTGCAGAAGCCCAACTCACGTAGCAAGCTGCCTGACTTCGACA

CATCTGCTGAAATACCTCTAGTGAAGAATTTGCTGCTGAAAGCTTAGACTGTCAAAGTGC

TGAAGTGGG

>c6706_g1_i1 len=238 path=[1:0-237]

AACTGAATAAGGCAGCCAGACCAGCTCCCTGTCTCATAACTACCACAACTAAGAAAAAAC

TATCACCTTCTGAGGGGTAGAGAGACATTTGTCATCCTGACCCAGTACCTAGAGAGGTCA

GCTGTTTGCCAGGGGCCTGCATGAAGGATTTGACAAAGTGGTTGCCATACCTTGTACATC

CCACTTGACTACTGCCTGCTGCTGCTGTTTCATGTAGACACCCATGACACCAGCGAGG

>c6707_g1_i1 len=202 path=[1:0-201]

CTTTCTCTGCAATATCAGACAGGCCATCAGTATTCAGTTGCTCCTCAAAGCAAAAGCAAC

AACAACTTTTGCAGCAGAACACAGATTTTTATTTACACAGCATTAAGAAGTATAACTAAT

AAAATCTGCCCAGATCTACACGGAGACTTGCACAACAGTAGTTTTGTTTGCTTAGGCACA

TTATTTCAATGAGAGGAGCAGG

>c6718_g1_i1 len=1448 path=[1:0-1447]

ATAAAAAAAATATAATAAATAATATTAATAATTTAGATATTCTTCATAATAACAAGTTTA

ATAATTTTAATAATACATATAATTCTTTTCTTAATTATAGGAATATAAGGGATAAAAATT

ACAATTCTAATTTTAATAATTTCATTTATAAAAATATATATCAATCTAATTCTCTTAATA

TTTATACTCTTAATGGATATAATTTAAAAAATAATATTATACATAGTGCTGGAATAAATC

ATAACGATTTATTAGTAAAAGAAAAATTAAGTGAATGTAATAGCATGATTTATTACAACA

TATATAGAACAAATTATAATAATAATTACTTTATTGATTATTTTAATAATAACAATAGGA

ACTATAATGAGAATTTTAACATGAATTATAATAATGGTTGTGGCATAAATTTTACTGATT

ATAATGATATTAACTATTGTAACAATTATTATACTACTAATAATGATTGTAATCAAAATA

ATATTATCACTAATAATAATTACAATATGAGTAATCAAATTAATGCTAACACGAATACTA

CTTGCAATAATAATACTAACAATTACACTAACAAAAGCAATGTTAATAATAATATTAACA

ATACTAATACAAATACTAATAGTAATGACAATTATATTCATTATACTAATAGCAGCATTC

TAAGTTATTCTATCAATCATAACATTAATTGTATTAATGGAGATAATTTCAATTATAATA

ACAGGAATATCATAAATGACTCTAAAAAAGTGAACTATGGTAGTATTATTAATAATCAGA

ATTATCATATAAATAAAATAAATGCTTTTAATCCATATTTACGAAGCAATTATTTAAACA

AGTATAAAATGAGTCATAAAGAAAAATGCATAAAAGAAAATTCACGATGGTATATGTATA

ATGTGGAAAACCAAAATAATGTGAAAAATTTTAATGAAAATAAAAAAATATGGAGAAACA

TATATCATCATAGAGGTAAAATAGCCAGCAAAAATAATTATCGTAATTCATTATATAATA

ATATAGCATATAATAAAAATTACTTTAATTTAAATAATTACTTATATTTAAAAAAAAAAA

AATATAATATGAATTACAATAATTACTACAATTACTATGATGTTACTGAAGATATGATAA

ATTCTTCATATTTTTCTAATAATGTTATTAATAATTTTTGTCATTTACAATTATATAACA

AGAAAAATAGAAAAGGTTATCATAAAAATATGAAATATAGAAATTTTTGTGAATCATATA

AAAAAAGCAATAACATACATTATAACAATTCCATAACAGGTTGTGATAAAAATGTGTTAA

ATAAAAAATATATAGATGTATTTTCAAACGGAAAGAAATTTAATAAATTTCAAAGAAATT

ATAAAACGAAATATATCAAAAAATTACATGATAAAAATAATAATGATAAATTTAAATCCA

CCAAATTT

>c6719_g1_i1 len=337 path=[61:0-224 285:225-230 400:231-336]

AGCAGCTCTGGTTGTTATTGTGCACGTAACAAAGTATTTCTGAGGGGAGATGGGAAAAGA

ATTGAAGGAAGTTTGTGGCAATCCCATTTCTGCTATTTGCATGGTATTTGTGAAGTCTTT

GTGGTCCTGACAGTATTCCCCTGTGTGAAGAAAATCAGATTTCATTTTGAAGTGTTCGTA

GCTCCCACCATTGTAGTGGGAAGTTTGAAACGTGTGAACTGTGTGGCTGAGCCAAAAGAC

TGAAAACCCAAACAAAACAACCACACGGGCAGAGCCCAGAATATTTCCTATCTTTAGGAA

ATTTGGAGATACGGCATTGTTCTAGGAAGCTGGCTCA

>c6719_g1_i2 len=253 path=[61:0-224 25:225-252]

AGCAGCTCTGGTTGTTATTGTGCACGTAACAAAGTATTTCTGAGGGGAGATGGGAAAAGA

ATTGAAGGAAGTTTGTGGCAATCCCATTTCTGCTATTTGCATGGTATTTGTGAAGTCTTT

GTGGTCCTGACAGTATTCCCCTGTGTGAAGAAAATCAGATTTCATTTTGAAGTGTTCGTA

GCTCCCACCATTGTAGTGGGAAGTTTGAAACGTGTGAACTGTGTGTTTGATTGGGTTTTC

AGTCTTTTGGCTC

>c6724_g1_i1 len=232 path=[1:0-160 162:161-231]

GGTGGCCACAGGCGGGGAGGGAGATCCCAGGGGCCGCGGGACTCATTCACAGGGGTCGGT

GTGGGAGGCAGCGCCAGGCCGCTGAGGGGCACTCAGTGAAGCCGCGGGGCGGGAGATCCC

ACCTCGGGTCAGCCGCCCGATCCCCGCTAAGCCCCCCGCCCCTGCGTTCCCTAGTGCTGC

TTGCCCCTCTGCACGGAGTGAAAAAAGAGTGAAAAAAAAGAAAAAAGAAAGA

>c6726_g1_i1 len=202 path=[41:0-77 240:78-201]

GGAGTGGGGAATAACGGACACGCGGGTCTCCCCTGAAATAGAGCCGCGTGGAGGAGCAGG

ACCTAAGAAGAGCGGGTGGTATTTTGGTGTGATTAGGAGATTAACATCCCTGGAACACCA

GGAGAGGAGGAGGAGGGGGGAAAGGAGAGAGAGACGGCACGAGTGAAGCTGTCCTCCGCT

GCCACCGAGGAGCCGAGGATGG

>c6728_g1_i1 len=293 path=[571:0-292]

GAACTGTAAAGATAAATTATTTGACAATGCTCATGCACCTGAGTTTCACAGAAATTAAAG

ATGTGCTAAAGTTCAATTTATGTAAAACTAAGATAAACGTAAGTAATTTCAGATAAAATG

AATTACGTACTTATGGAGTCCTAACAAAAGGAATCTGTACTGTTAGCTATTTTACAGCAA

TGAAAAAAACCTAGAGAAGGAATATCCATTTAGCTAGAAAAACACACTGTATGCTCTGAA

CAAGCTTCCCATGAACAAGCTTCCCAAGCTTCTCAAGAACTTCAGTCAGAAGA

>c6730_g1_i1 len=342 path=[480:0-302 50:303-341]

TTTCTCATGGGAAAATCTCTTCTAATTTTATAGTTTGGTTTCCAGTAGGCAGTTAAAATT

AGAAGAATCATAGGGAAAAATAATCTAAAGTTTTGTTTTGTTTTAAAATATATGCCTATT

GTTAAAAGTAGCTGCCAATTTTTATACAATGCCCAAAACTTTCTTGTCAACTCTGGTAAA

TGTGGAGGGAGAGGCTAATAGCAGATGGACAAGCTCTTTAGGAGTTGATAATTTTGTTAC

AGTATTTATAAGAATATCATACCTACATACATTTTAAGACTACATGATATTTACTTCCTT

TTTCATTAACAGAAGAGTATACAGACTAAAGCTTGACACTTA

>c6730_g1_i2 len=342 path=[480:0-302 782:303-341]

TTTCTCATGGGAAAATCTCTTCTAATTTTATAGTTTGGTTTCCAGTAGGCAGTTAAAATT

AGAAGAATCATAGGGAAAAATAATCTAAAGTTTTGTTTTGTTTTAAAATATATGCCTATT

GTTAAAAGTAGCTGCCAATTTTTATACAATGCCCAAAACTTTCTTGTCAACTCTGGTAAA

TGTGGAGGGAGAGGCTAATAGCAGATGGACAAGCTCTTTAGGAGTTGATAATTTTGTTAC

AGTATTTATAAGAATATCATACCTACATACATTTTAAGACTACATGATATTTACTTCCTT

TTTTCATTAATATTTTATAAGTGTCAAGCTTTAGTCTGTATA

>c6734_g1_i1 len=453 path=[1:0-452]

AAAAAAAAAAAAGAAATAAAAATATAATGAAATATAACACAAAGGCTTTCCGAATCAAAG

ACTTTTTTGGTGTGAAATGAAATTTTAATATAAATCTGTGTATAACTTTTTTTTTTAAAT

TATTGTATTATTTGCTTAATAAATACCATTATTTTTTCATATATACTTCATATAGTTAAA

ATTTGTTTTTTTTATATTTTAATTGCAGTTAATTGAAATTATAAATTTAAATTATTTTTT

TTTATTTGCTTATAAAACTTAATAATGTTTTTTTAAAAAAATATTTTTGTTTTTTGAAAC

AAAATATGCAAAATTTATTACTTCACTATTATACAATTTTATATAATGAAATTAAAAATT

AAAATGTAATTAGTATATATGGGAATATTTGACAAATGAATAAAAGTATTTATTAAAATT

TAAATTATTTTAAATTATAAAAAAAAAAAAAAA

>c6735_g1_i1 len=253 path=[263:0-56 509:57-57 510:58-252]

AAAAAAAAAAAAAATACATAAATACACGTATATATAAGTACATGTTTATTTAAGCTATTT

TTTAATTGTTTAAAAAACAAAATTGTCAGCGTAATACATATTTAATATAATTTAATATAT

AAAAGATATAACAAAGAATAAAAGAGATCAAAAATTTTTTTATTAAATTTTTTGCAATAT

ACCTGCTAAGATAATTTTTCTTAATAACTCTAAAAAAGAATGAGAGAAAAAACTTTAATA

CAATATTTAATAT

>c6735_g1_i2 len=210 path=[507:0-14 510:15-209]

ACAATTAAAAAATAGTTTTTAATTGTTTAAAAAACAAAATTGTCAGCGTAATACATATTT

AATATAATTTAATATATAAAAGATATAACAAAGAATAAAAGAGATCAAAAATTTTTTTAT

TAAATTTTTTGCAATATACCTGCTAAGATAATTTTTCTTAATAACTCTAAAAAAGAATGA

GAGAAAAAACTTTAATACAATATTTAATAT

>c6739_g1_i1 len=1303 path=[13121:0-1302]

ATGATAATAATAATAATAATATTAAATTAAAAAAAGAAAATCTTGATTTTTTAAAAAATA

ATTTTGATAAATTACTAATAGATGATTTTATTAAAATAAAAAGTTATTTAAATGGAAAGA

ATCATAATAATGATTTAAATAAAAATCATCAAAAAAAAATGATTAGAACTATATCTAATA

ATAATTCTATTTTAAATGATAATACTTTTAATAATTCTTCTAATGACTCTTTTGATAAAT

TATTAAAGTTTCCAACAAAAGAAGTTAATGATAAAAGTAATTATAGTAATTCAATTAATA

AAAATGAAAATATACCTTTAAATAATAATAATAATAATAATAAAAACATAAGTAGTAATA

ATGCTGTAAGCTTAAATTTAATAAGTCACAATAATAATAACAATAATGATAATAATTCTA

ATGAAAATTTAAATAATGATGTAGAGAATACGAATAAATCTGTTGAAAATTTTGGTATTT

TAACTTATTCTAATTCAACGAATAAAAACTTAAATTTCTCCAATGCGACAGGTTTTTTTA

GTAAAGGGAATAATATAATTAAAAAAGTGAATGATATATTTAATAATTCTTCTGTCTATT

CAGCTAGTTGTAATTTTAATAATAATAATAACAATATCCATTTAGATGAATGGAAGATCA

AAAAATACAAAAAGTATAATAATAATGATAGTCACAGAATTTTAGAAGAAAAAACAAAAA

AAGAAAATAAAAATGAATATTCAGATATATTTTTAAATAAAAATAATGATATGAATAAAT

TGAATATAAATTTTCCAAATAATGTACACGATATTAATCAATATAATTATAATCAGCAAC

CATTTGTAGGAAAAACAGAATATAATACTAATGATTTAAAAAAAAAAGTAAATTATAATG

ATGTAATAAATAAAAGTAATATAAATAGCAATGCAGAACATTTATCCAATAATATGAATA

AAAAAAGTGGAAAAATTAATAAGGTTCCTAATTCATATGAAAACGTAAAAAATCAATCAA

ATAATTTTACAAATTCTAGTTATTTTAATACAAGTAAAAATGTAAAATTAAATGAAAAGA

AAAATTCAAAAAGTGATAAAAATGTAATGGAAGATGATTGGAGTTCAATAAGAAAAAAAT

CAATCTTAGCTGAAAATAAAAAAGCAACAGATGATTTAATCTGGCATAAAATGAGTGAAC

AATTTGACAGAAATAATAAAAATTCAATGGATTCTTTAGATAGTAAAGTTGATAGTAGTA

ATATTGATAATAATAAGAATATTAATAATAATATAAATAATAA

>c6739_g2_i1 len=320 path=[14731:0-319]

GTTATTGTTATTATTATTATTATAATCATTAAAAATGTCTTCTCTTTTCACATTTATTAT

ATTTCTATCTTTAACATAATTGTTTTGATGAGAAATTTTACTAAAATCGTTAACATCATT

TTCATCATTGGAATCAATAGGAGATGAAATTTTTACATCATTATAATATTTATTATAACT

TCTTTTTACTTCATCTTTATTATCACTTAATTTATTGTTTCCTTTCGTATATCTCCATGA

TGAAGACACCTTATTATTTTCTCTCCACATTAATCTTTTATTATTATCAACATATATATT

ATTTTTTTTTAAACTATTTC

>c6739_g3_i1 len=5901 path=[7218:0-5900]

ATATATATTTATATATATATATATTTCTTTTTTTTTTCTTGTTAAAATGAGAATAAGAAA

ATCATGGTGCACATTATGTCGTGAGAATTTTGAAGAAGGAGATGAATGTATGCAATGCAA

ACAATGTAAGAAAAAATTTCATAGAGAATGCTTACAAGCAGAAAATTTAATCGATAATAA

TCAATTAAAAGATATAAAAAATTATTTATGTTATCATTGTATGAATGAAGAAAATGATAT

TCCAGAAAATGAAGAAAGATGTAAAATATGTAGGGAAAAATCATCAAACCTTGTTTTATT

ACTATGTGATGGATGCCCTAATAGTTATCATGTTACTTGTTTAGGTTTACAGTCAGAACC

AACTACAGAAAAATGGTTCTGCCCTGTATGTAAACCGGAAGAACATAAAAATTTAGATGT

TAGAAGAATGAGAAAAGGATTTGTAATTGATAACATGAATGGTGAACATGTTAATTCGTC

TACTTGCTATGTTTGTCAGAGACATGGAAAATTATTAGGTTGCGATTTTTGTCCTAATTC

TTTTCATCCAACATGTTTACCTGACTTAGATTTTGATAATATCTCAGATCAATGGGAATG

TCCTTGTTGTAAAAATGAAGACCCTTTATTAAATCAAGGGCATAAAAGGTGGACAAAAAA

TGAAATTCAAGAAATTATGAAAAAAAGGCAAAGAGAATTAAGTTTTTGGAGATCAAAAAT

TATAAAATATAGAAATAGATTCTTATTAGCTCATAGAAAGGATTTACAGCCTTTTGTAAA

TCCTAGAGTATTTGCAAATTTAACAAAAATATTCAAAAATGATTTTTATAATTTAAAAAA

TTATAGTGGAAATAAAAAATCAAACAAAAATAACAATGATTACGATAAAACAAATATGAA

AAAAGATTTAGATATTGATTCTAAGGAATTTGATGAATACATATCTTCTTTAGAAGATGA

AGCAAATGAAAACGCAAATAAATTTATTGAATCTGTGTTTTTGAGTGTTTATTATCCTAA

TGGAAGAAAAATTGAAAGAAGGCCATTAGTCGATAATGTTCAATTAAAACCACATCAAGA

AGATGGTGTGGAATGGTTACTAAAATCTTTTTTAACTGGTGGTGCTATTTTAGCGGATGA

AATGGGATTAGGAAAAACTATACAGACTTTATGCTTTCTTTCCTATTTAAAATGCAATAA

AATTGATGGACCCCATTTAATTGTTGTTCCTTTATCTACTGTTGGAAATTGGTTAAGAGA

AATACATAGGTTCACACCTCATCTTACTTGTATTAAAATTTGTGGATCAAAAAATGAAAG

AACTCATGCTAAAGAGGATAGATTAGCGGAAAAGGGATTATATGATTTATATGTAACTAC

TTACGAAACAGTTAAAAATGAAGAAGAATTTTTTGTAGAAACTATACCTAAATGGCAATG

TATAGTATTGGACGAAGCTCATAGAATAAAAAATCAAAGTGGTGCTATTAGACATTCTAT

GGATAGAGTAGTAGGAAATATGCGATTATTACTGACAGGTACTCCTTTACAAAATAATTC

TGCGGAATTATTTACTTTAATTAATTTTATGTTTCCAGATATATTTAAAAACTCTGAAAT

AATAGAGCAAGCATTTATGAATGCATCACAAAATAGTAGTAATCAGAAAAATAATAATTT

AAAAAATAAAAATAATAAAGATGAAAAAAATAATCAAAGTGTAAATAATTGCAGTAGTTC

TAAATTAAATGTAGACATTGGCAATATAGATTTAAAGTCTGCTATAAAAGAGGAAGATTT

AAAATCTATACGATGTTTATTAGATAAAATTATGTTAAGAAGATTAAAAGAGCAAGCAAT

TACACTTCCAAGAAAAATTTTCCATGATATATGGTTACCATTAGGAAGTTTATCAGCACA

TTGGTATAAAAGGCTCTTAGATATTAGATCAATGGTAGAAGAAAAAGTTAGCGTAAAAAA

ATTATTAGGTTTAGTTATAAAAATGAGAATTATATGTGGTCATCCCAAAGGAATTGTTAG

TAGGCCAACACAAATGGAAAAGTTATTTGCATTTTTTGAAGAGGAAAGTGAAGAAATTAA

AGAACAAGTTAAAAATGATGCAATGAAATTAAAAAATGTATCAGGGAAAGAACATATAGA

ATCTTCCTCAAAACTTATATTTATAGATAAACTATTGTGTCAATTGCATTATGAAAATTG

TAAATATGTTAAAAATTATTCCTCCAACTTTGAAAAACATAAGAAAGAAGTTGCTGCCTA

CACATATCATAAAATTCAAGAGCAAAATAATACACAAAAAAAAAAAAGAGAACATTCTGG

AAAATTTAAAAAAATTGAAGCATTAGAAAACAGTCCTTTATTTATGGAAATTTACAATAA

AGGAAAATTTGATTTGAAACCAACTTCAGAAAAGGAAGATTTATTAAAAAATTTTATGCA

TACTGTATCTGTTGATAATGAATGTCCATATAAAAAGAAAAGGTGTATATATGATAAAGT

GAAAGATCTTTTTAAAAATGCTAATAATCCTAATAACAAAAAAAACATTTTTTTTAAAGA

TGATAATAATGAAAATGATCTTTCAGATGATCGATATAGTTGTAACAATATGAATAATAA

TATAGATGATAATGAAGAAAATAAAAATGAAAGTGATAATTGTTTAAATTCTTCAAAGGA

AAATGAGAGAAATAACTTATTCTTTGATGATGAAGAAGATAATAGTGATTATAAAAAAAG

TAATAGAACAGGTAATGATGATAGTGATGATGGAGAATTTACAAAAAATTATGAAAGTTC

ATCTAATAAGAAAAAAAAAAAGAAAAGATTTATCTCAGAGAAAAATGTTAATAGTATGAA

TTCTAAAAAAAATAAAAAAGGAAATAGCAATAGCTTATTTGATAGCAATGATGAAAATTC

TGATGAGGAAGAAAAGAATGGAGAAGCAGATAACAGTGATCAGGATGAAGAAGAAGATGA

ATTGGGTGAAGAAGAGTATGAAGAAAATGATGATGCAAATATCAAAAAAAAAAAACATAA

CAGAAACAGTAATGAAGACAAAAAACTAAAAGGAAAGAATGTAAAAAGTATCAAGAAAGA

TGATAAAATTATTTCCTTAAATGAAAATTGTAAAATGATGAATATTAGAGAATTTTTTAA

AAGTGAAAAGGAGTTAAATAATTCACGGAAAAAAAGTGATACATTAAAGAGATTGGATAA

ATATGATATAAAGAAGGAAGAGTTAGACGAAGCAAAAACGGAAGAATTAAAGAAAATAAA

AGAAAATAAAAAAGAAGAAAATTATTATGAAAATAAAACAGTAGATGAAAATGAAGAAAT

AAAAATAGATTATAATAAAGATAAAAATGTGAATAATGATTCCTACAAAAAAGAAGAAAT

TAAACAAAAAGAAGAAAAGGAGGAAACTTATGAAGATGAAACTGTCAAATGTGATAATGA

AGAAAAAAAGATATCAACTGATAAAAATTGTTCTAATGAAGAAAATGTGGAAAATAATGA

AAGCATGAAAAAAGGAGAAATAAAAATGCATAAGGTGTTAATATTTACTCAATTTCAACT

GGTGTTAGATGAATTAGAAGAATATTGTAAGTATAGATGTTGGAAATATATGAGATTAGA

TGGGTCTACTAATAAATTAATAAGAGAATTAGACATACGTGAATTTAATTTAAATAATAG

CATATATTTTATTTACTTAATAAGCACAAGAGCTGGTGGATTAGGTATTAATTTAACTGC

AGCAAATCATGTTATTATGTATGATGAAGATTGGAATCCTTTCATAGATTTACAAGCCAT

TGATAGAGCACACAGAATAGGTCAGAGAAGGGAAGTAAATGTCTGGAAGTTGATGACTGA

ATGGACTGTTGAAGAAAGAATGGCATTTCGAAGAGAGCAAAAATTGAAATTAGACAAATT

AGTAGTTCAAACTCAAGATGATGAAGATTTGTTAGATAATTTTGAAGAAAAGTTGTCATC

TGATGAAATAAGAAAATTAATGTTACATGGAAAAGCAGCTATACAAAATATGAATATAGA

AAATACTAATGACTTTTCTTTAGAATTTTTTATCGAAAGAGGAAGAAGAAAATTGCCAAT

AATAAATGGAATAGATTTAGAAAAAGAAGAAAAATGCACCATAGAAGAGGAAATAAACAA

CATAAATGAAAATGATATTTATGTGGAAAAAGATGAAATTAATGATTCACAAAATAACGA

GGATGATAATACAATTGATATAGATGAAGTTATGATAGATGAAGCGGAAGAACCTGAAAT

TAGGCATAATAATACGAATTTAACAGATTGTAGTCCAACATATGCAGAAGGAAATGATCA

ATTATATTTAAGTAATCTTAATAAATCTAAAACATCTCTTTCAAAAACAAACACAGCCAC

CAATCAATTTGCGTCAAATAATTTGGGAAGTGATACGACATTGTGGAGATCTGTAAGAGA

AAGAAAAAAACCACAAAATATGTATACCCCTGAGTATTGGGGAAGAAAACAGGAGGTAAA

ACAAATAAGGCATGAATATAGATGCTTTATTTGTAATAATACGAAAAATTACAAAACTGT

TGTTAAAGATAGAAATGGAAATGATATAGAAATAGATTATGGAGATATGATAAATTGTTT

TCGTTGTCCTAAAACTTATCATAAGTTATGTGAAGGAATAAAAGATGAAAATGTTAAAAA

AACATGGACTTGTAGTTGGCACGAATGTTGTTTATGCTTTAGAAAATCATCTCAATGTGG

TAATTTATTAATACATTGTGCCACTTGTCCTACTAGTTTTTGTTATAATTGTTTTCCTCC

AGATTATGTAAGATATTATGTTGGAGAAGAATATTATCATAATCTACGTCAAAGAGGAGT

TAATTTTACACCTCAAAACTGGGTATGTTTTTTATGTTCTAAATGCAAAGCTGTAGAAGA

ACAAAAGAAAAGAAGGAAAATGACTAAAGAAGAAAGAGAAAATGAAAAACAATTACAAAA

AGAATTAAGAAGTCAATTGCAAGATAGCAAACAAGAAGAATTAGAAGCCAAAAAAAGAAA

GAGAGCTCAACAAATGGAAAGAGATAAATTTATTATAGAGAATAGGAAAAGAATAGATGC

TTTAGACCAAAAATATGAAACTCAACTAAGAAATGCATATGAAAATTTGTTTCCAGTTAA

TTTTGTAAAAGAATTAGTCAATAGAATAGAACATGCAAAAATGTTAAAACAAAAAGGAAT

TGATGAAGATAATAAAGAAGTCAATAAAAAAAAATCCACTACATTTCTACATACAAAATT

ACCACATAAACTTTTAGTCTTATGTGAAAATTGTAAATTGCCTTGTCATGCTAATTACAA

ATATCCAGGAAAATGCTGTTTTCCTTTAGAATTAGATAAATCTTATTTTTATGCCAATTC

ATCATTTAGTCAATCTAATAAAGAGAGTACCGATAAAAAAATAACTACAAAAAATGATAC

TTCCAATGAGCAAAAAGATAAGGAGGAAAAAAAAGAAGAGGAATGTAATAAAGTAAAGGA

AGAAAATATGAATACAGAAAAAGAATGTGAAAATAATATAAAAGAAATAAAAAAAGTAAA

AGAAGAAAATGATAGCAAAATAAATAATGTAGATGAAAAAGAAGATACAAATAAAAAATT

AGCATGTGATTCAAAAATAGAGAACAACAAAAATGACGAAGACATTACATTAAATAATTC

ATTAACCTACAAAAATAGTAAAGTTATCAATACCAATGGTGAAGAAAGCAAATACTTTAA

AGAAGCAATTACAGATGTTGCAAAAGACCAAAGTGATCATAACAATAATAAAAATGTGAA

ATGTGAAAAGGAAAATGAACA

>c6742_g1_i1 len=204 path=[182:0-203]

GGGCGCCGGCGGAAGGCTCGGCATCCTTTGCGGGAGCAGGACGAGCCTTCCCGGCGGCCG

GCAGCTCCAGCTCCGAGAGACTTCTCACAGTCAGCGTGGGCCGAATGCTCCTGAAGTGTT

TGTTTTTCCTCCGTAGAGCCATCTTGTACTCCTCCGTTGCAAAGCCTCTCTCGGTGAGCT

CCGAGGGGTTCCTGCCCCGTGCCA

>c6744_g1_i1 len=252 path=[428:0-228 702:229-251]

CACAATTCAGATACTCTGACTCCACGTTAGACAAAAGCAGGTCCATTTGTTAACCTTACC

AAAGAAGATACAAAGCATGACTTCAACAGAATTTCACAAAGCATTTTCTTCTAGCTATTT

CATGTTCCATTTCAATGTGCTTATTCTGGACATTTCCATTTGCTTCTCACCAAATTTAGC

TTAGTCACCTTTCAAAGCTGTCAAGATGCATGAGGGAAGTCACATTGTTTAATTGCATCC

AACCACCAAAAC

>c6746_g1_i1 len=352 path=[330:0-351]

CACTGAAACATTTGATACTCCGCTGTACATCTCAGAGGAAAAGCTGTTCAAAGCAATGTT

TGCTTTTGTCACTATAATAAAGCTCAACTCTGCCCAGTTGATGCAAAACTGGCAAAAAAA

ACACTCTAGAACCACATTTCTGGACCTCTGAAAAGAAAAATTCCTCACTGAAACAGGATT

TGCATTAAAAACCACCAAAAACAATATTCATGAGGTTAAGTCATCATGGCTTTGGACACT

GGCCCAATAAGGATAGCTGTGCTATTTAGCTGTCTGACATATCGAATATTAAAGTAGGTT

TTCATATCACAGATAAGGCGCTGTGGTTTCTTTTTCCTGTTTTGTTCCATAG

>c6748_g1_i1 len=262 path=[209:0-261]

GGGGGGGGAGGGGGGGCATGGATACCGGGAGGGGACCCCGCAAGGGAGACCTCCACAGCA

CACCACATTTCCGTCCCCCGTTTTAACAGCAGATTTGAAGCTCGGGAGCTTTAGAAACAC

ACCCGGAAAGAAAACGAGTTGCTCCCCAGGTGCCCAACAGAAAGAAAGAGAAGCAAAGAA

CAGCCCGGGATGGCGCTCCTGTCAGCGCGCGGCGGGGACAGCGCCGAACCAACGGGAGCG

CCGCGTTAAGAAACAGAGGAGC

>c6752_g1_i1 len=317 path=[53:0-316]

TTTACCCATTGGTCAAACAAACAGCAATATGGATAAGATAAGAAGGAAAGGGGACACCGA

AGACCCTGCGGGCCACCTGGTTGAGTTTGTGCTATGAGAAAGACATAGATGGAGCCTGAA

TCATGTCTGAGAATTCCTATCAGCAAAAGCTCAACCAGCACGGGGGACACAGCCAGGGTC

AGTGCAAGCCCCAGCACAGAAGACACGTGGGCACCCTGCCCTCTGCCGCTCATCCTGCGG

CTCCCGCGTGCTGCTGCTGGTATGGGAAGAGCTTTGGTCTCTTGTTCCGTAAGGATTATC

CCTGTTGAGTTTGTTTG

>c6755_g1_i1 len=226 path=[204:0-225]

CAGACTGACACCAGCTGAAGATGGAAGGCAGAGATGACCTTGGTTGGTTGGTAGGTATAT

ATGAGGGTGGTGGCCTACTGCCCTGCAGGGTGTGAGACAATATAGTGTTACTGCAGCCCT

CTGGTAAAACCAGAGCTCACCCTTCTCAGCCCTGAATAACGACAACGAGATCTACTCCAT

GCCATAAATACATGGAATATAAAGCAGCAGAGGCCAAAGCTGCGTG

>c6759_g1_i1 len=234 path=[37:0-103 18:104-233]

TCCTAGCTGCCTCTGCAAGTCTGGGGCAGCAGTCAGCTGGCTTGTCCCCTCTGGTCCTGT

TTTTGTGGCAGGAGATGGTTTACCAGAATGGCTGCCATCGCTGAAGGGTTGAGAATAATC

TGTTCATTTTAGAGAGCTTTCTCTGTTTTATTGGTAGGTGTCGAGTTAGCAGTAGGTAGT

AGATTTTCTCTGGAACATTTTGGAGTCAGATGCAAGGGCAAGACTGCACCAAAA

>c6760_g1_i1 len=337 path=[315:0-336]

TACTTCCAGTACATCAAATTACCTAAAGGATCAAAACATACTTCTATGTTTGAATGAACT

ACTTAGCATATTACAGAAGCAAAGGTGGAACAAACATTCCCTGGAGGGAGGTTGCAGATG

CCGGGCTGGTTAGTCTGGTACACAGCAGCTGGGCTCATAAAACCACCTCCCTCTGTCTAT

GTATCACACTCTTATCAGGATGCTATCTGAACACCTTTACAAACCAATTAAATCCAGACA

AATTGTATGCGAGAAATTGCTTAATCCAGAGAGATAAACAGCAAAATGATAGTATTTTGT

TGTCCTTCAACTCATTCAAAGCTCTATACCTACTAAA

>c6761_g1_i1 len=207 path=[313:0-206]

ATCTTCACTGTCATTCTCTTCCTCGGTGTCGCCATCATCTGAGGAGTCCTCACTTTCATC

ATCATCCTGCAGTTCTTAGAGCTCAAGATCTTGAATTCAGATGATGGTGCATCATGACCA

TGATTTGATCTGTGGCTGCAGAGAGGGCTGTGCTAATCTCAGGATGTAGGCAAATCTTCT

CAGGAAGTCTCACCGTGTCCTGTGCAG

>c6768_g1_i1 len=250 path=[228:0-249]

AGGCCTTACAGAGAAAGTCTTACATCTGGTGTGTGTGGAAAGAAAGCCAGAAAAGCAAAA

TCATACAAGCAAAAGAGAAACATTTTTAAGGACCTGAGAAAAAGAAAAAGCAGTTTACAG

CAGGGTTGTTCTCTCTATTCTAGCTACAGCCCAGACACAGAGAAGCATAAACAAGTAAGA

ATGAAGAGGAATGAACAGACATGACTGTTTACAAAGAATAAACAGGACTTATAAAGAGAC

CAGAGTCAGA

>c6773_g1_i1 len=321 path=[1:0-182 184:183-320]

TGAATACGGAGTCTCATTTTCATTCTACAAGACCAGAACGTTTTTATGAGATGCACAAAG

TAGGAAAGTGCAGAAAGCAACTTCAGCGTGAAAAAGTAGAGGAAGCGCTGTCCACGTCCC

TCCCTTCATCGAGGCCTCCCAACCCTCCTCAGGGAGTATCTCAATCTTATCTGTTCATCG

TTTCCAGTGTATTTCCTCTCACCCATAAATTTCTTATTCCCGCTTTCTACCACGCAGACT

TCAGGAAGTAAGGGGAGACTTATCGTGCCCTCCATCTGTGTATGCCCCTTCACAACGGAG

CTGGGTTTCAATTCTCACATA

>c6773_g1_i2 len=209 path=[1:0-182 620:183-208]

TGAATACGGAGTCTCATTTTCATTCTACAAGACCAGAACGTTTTTATGAGATGCACAAAG

TAGGAAAGTGCAGAAAGCAACTTCAGCGTGAAAAAGTAGAGGAAGCGCTGTCCACGTCCC

TCCCTTCATCGAGGCCTCCCAACCCTCCTCAGGGAGTATCTCAATCTTATCTGTTCATCG

TTTTTTCAGTGTTTTTGGCACCGCTGCAG

>c6775_g1_i1 len=321 path=[321:0-320]

GTGTCTCTCCATAAGAAAACCAAGTTATTACTAAGTTAGATTTTTATTGCAATGCCAAGT

ACTACAGTCAAATGGCATGCTGTGGCACACTGGCAGCAGCTGCAGATATCTGGTTCTTCT

CAGGAGCTTTCAGTGCTTTGAGTAATTCACAAGGCGTGTATGAAGTTATGCATTACTCTA

CGGAGCATCTTTCTACCTTATATGAGCAACCAGCAGGGCTGCTTTTGCGGTGTCCTAGAT

AGAAAGGACTTCTTTTTAATGAGGTCATTTAATTCCTCTGCAGTGAAGATTGTTCCTTAT

CCCAGGAAATTCCCCAGTGCT

>c6777_g1_i1 len=282 path=[1:0-259 837:260-281]

CTGGTCTGGGAAATACTTGCACATCAGATGTTAAGTGGCCACACTTGAGAACAGCATGGA

ATGGTGTATAAGCCAGGTCTATTAGCTTTCCAAACATAGACTGTACATTCTTCAGGCAAA

GGGGGCCATCAATGGTAAAAATTTGCCCTTCCCCATTGTTTAAGTCTATCAGGCGTTCCA

AGCAGTCTAAGGAATCCGTGCTCTGAAGCTCTTCAAGGTTTGCCATGCACATGACATACA

GCTTGGAAGGGAAAGGGAAAGGCAATCGATTTCCATTGCCTT

>c6778_g1_i1 len=360 path=[379:0-275 737:276-359]

AACGCAGCTATTTTAAAAAAGGTAAGGTACTGAGAAGTGGTCGTGTTGTGCATCAGTAAT

GAGATGTGAAATTAATGCTGGAGTTAAATATTACCATGAAAAATACTTCTCATGTGTTAG

CTTCAAGTGGTAGCACCGTTATGAGGTGTATGAGCACAGTTTATCTGAGAACTGGGAGGA

GGTTCACAGCTCATGGCTGAAGACTTACAGGAGTGACCTTCTGGAATAGCAGCTGGAGGC

CGGGCAGGGTTCCTAAGGCAGAGGGCTGGTGCTGGACATCTGTGCTCGCTAGCATCTCGT

GGTTTTAAAGCACTGATTGTAACTGGGTTTTGTGAGCTGGGGTGCCTCTTCATGAGTGCA

>c6781_g1_i1 len=206 path=[1:0-205]

ACATCTAAATGAACACACCCAACAACAACTGTTCAAGAAGCATGATTTGGATATTAAATG

AATTAAGGCTATCCACATGTACATTTACTGTGAAATGGATCTTTTCTAGATTGTTCACAC

TTTTTGTACTGGGCTATTTTGAGTACCTACACAAGTGCAGATCAGGAAACCCAAAGTGCT

AAGGATGGATCCTGGCAGCCTAGAAG

>c6784_g1_i1 len=218 path=[610:0-217]

TCAAATTTATGTTTTATCATTCCTGGCTGTTTATATTTAACTCAGCATCTGACATTGGAT

AAATGTTCAGTACTTAATTTAGCATACCATTCTAAGTGGTCATGACACAAAAAGAATTAA

AGGAATGAACCTGAATTTTGGGAAGAAGCAAGCAAGCAATGGCAAAGAACACTAACACCA

GAATATACCTTGGGAAGCAGTAGGAGAGAATAAACTTG

>c6784_g2_i1 len=230 path=[1:0-229]

GCTTCTGTTTCTTTTACAGCAAAGCATTAGGCCTGTCTTACAGGCAAGAAGTGAAAGCAT

CTTGTTCCGATCAAACTTAGGAAAAAAACAACACACTTGTTCTGAGGTATTCAGACTACA

AAACCAGAGTGCTCATTAGAATGTGTTGTACTTGAAACAATGAAGATGCAGTACCTGGGA

TGTTTAAGGAGCACAGCCTAAGCCTCACTGCTACTGCTCTTCTCTCACAG

>c6788_g1_i1 len=223 path=[167:0-105 272:106-222]

AGGCTAAGGCGAGACTGAAAATCAGTCACTAGACTGTGGAGGTACACAGCAACGCTGCTC

TGATCTGCTACCTCAACCACAGCAGAAGTAAGGACAATTTACAATCAAGCTCTGTGCCTG

AAACAGCCTTTCATCTCTCTCCTCAGCGCTTTGTTCAAATTTCTGCATTTCTCTGAACGT

AGCTCATTCATAACAACTCTATTGTAATGTCATGTCTGTTTGG

>c6789_g1_i1 len=214 path=[192:0-213]

AATTACAGATGTTATGAAAGTCCTGGACAGAAATGCTCCTGAGAGATACAGAAGCTGAAT

CTGTCATATAGAATATTTTACTAAGCTCATGTTGTCAGTTTCGAAGTGATTCAACAACAA

CACAACAAAAAAAACCGAACACCACCATAGGAAATATATACCAGAAATGTGTGTCCTTGT

ATCTAGTATATGTCAGTCATAGGAATAGGGCTGA

>c6790_g1_i1 len=526 path=[1:0-525]

GTGGCCCCGCTGGGACCCCCCCAACCCGCAGTACTCGGGGGTTCCACCTGAGCACGCAGT

GAAGATGACAGTTTGCTAGCACTGTGCTGATTTTTCACCATAATCCTCCTGAGTGCTCGG

GAGCCTCTATTCAACCTCCCGCCAGGGCGGCTGCAGCCATGGCCATGCAGGTGCAGTTCG

AGGCAAGCACTGACACATCTGCAGAGGAGGAGAGCTTTGGGCCAGAGCCCATATCTAGGC

TAGAGCAATGTGGTATAAATGCAAATGATGTGAAGAAATTAGAAGAAGCTGGATATCACA

CTGTTGAGTCTGTAGCACATGCACCCAAGAAGGAGCTGTTAGATATTAAAGGCATCAGTG

AAGCCAAAGCTGATAAAATCTTGGCTGAAGCAGCTAAACTGGTTCCGATGGGTTTTACCA

CAGCAACGGAATTCCATCAGCGGAGGTCTGAAATCATCCAGATCACCACTGGGTCCAAAG

AACTTGATAAACTTCTTCAAGGAGGAATAGAAACAGGGTCCATAAC

>c6794_g1_i1 len=225 path=[457:0-224]

CCCGTTGCAGTAATGGCAAACTCCCACTGAGTCACGAGGTGAGGGATACTGATTGTGTGC

CGGTCCCTGCTGGGGAGTTTGCTTTGGATGTGTTTTATTGCTGACTGCATATTCTGCTCC

GAGCACCATTCTGTCTATAGCACTACTGTTAATGGTCTGTCTGTTCCTTGCACCATTTTG

CATGTAGTACTACTCTTAATGATCTCTTTTCCATCAAACACAAAG

>c6798_g1_i1 len=344 path=[322:0-343]

AGGTCTGGCTGCCTTACTCAGTTCAGATGACTTACCTGTTATTGTTTGTTTTTCCTTCTC

AGCCTGGAGAACTGTGAAGCAGTTCTGCAGAGGCACCTCAGGCTTTGAAGCAGGCTTTTT

CCTCTTGTTAGTATTTCTCATGGCAATTTGCCATTTCTCATCCCTGTTTGTTTCCCCTCC

AACTGGATATGCCAGTGGGGGAGTTTGGGGCTGTTTGGTCCTGGATTGTGGATTCAATGC

AGATTGCATGTGAATTTGGCTGACTCTCTCTATCTCTGCCTCCCTGATAGTACACAGCTT

TTTGACTGTCTCCTGTGTGTCATTCAACTGTCACAGGAGACAGT

>c6799_g1_i1 len=208 path=[1:0-207]

GCTTTGTAGCGGTCGGTACCATTTCTCATGTGGGACATTCTTTAAAGGGGAAATTCAGCA

CACATCACCACTGTGTGTCTTCTGACCCGGTGCTTTTCCAGCCAGGTCTTGGCAAGTGTT

CTCTTCACCAGAGTGTGGAGAAGTGATTTACCCACATCTGCAGGCTGATAAATGGCGCAG

AGACACCAGTGCCTTTGTCTACGTGAAG

>c6801_g1_i1 len=289 path=[155:0-288]

GATGTTCTGGTGCCCAGTGGGAGAGGTCCTGAGCTACAGGTGTGAATACCTGAGTGCAGG

AGTGTGGGCAGACCTTTTCATTTCCCAAGGGATGGTGACCCCTGAGAAAACCAGAGGGAG

CGTGGCTGGGAGGAAGAAGTGTTTTGGGGGATATGGGGACTGGAGACACCTACTTGCAGA

CGCAAGGATCTGCTCTTGAAGGACGTTTGCATTTTCCTGCTGAGTGAAGCAGCCCTGACC

ATGGAGAATAGGCTGGCCTCTGCCACTGGGGAGCTGGGTGCCGCGGGAG

>c6808_g1_i1 len=202 path=[65:0-76 32:77-201]

AAAAAAAAAAAAAAAAACTTAAGTATGAATGGCTCTTTAAAATTGGAAAATGAGAATTTG

GATAAATATGCCCAAATATTGTTTAATGTTTGTGTAAAAGTAGTATTGAGTTGCAAGACA

GTGAAATATAAGCTGATAGCAGTGATGTGGAGCTGGCAAAATCTTCAGCTGACGCTAATT

GTAACTATAAATTTCTCCTGTG

>c6810_g1_i1 len=273 path=[61:0-143 30:144-272]

TAAAGGTCATAGTAAGTAACTTTCTTGATAATTAAAATGTTTTTTTTCTTTTTTAATTAC

ATTGATATTTTATGATTTTCTCAACCAAGAATACTCCCTTTTGATTGTCAAATTGTTTAG

GTAAATTATACTTCTTGTAGATTGAAGAATAGAACTTGTTTTAAGGCCATAATATAACAG

TTTTTTAAGTTCTATTAGTTTTTTACTTTTATTAGTTTTATTAGATTTTTTTTTTTCATT

TCAAAAATCTGACTTAAGCAATCGAGAACCACA

>c6812_g1_i1 len=288 path=[217:0-179 108:180-287]

GAAGCCACTCTGAAGTACTTCAGTACATACTCCATACATTTTTAATGACAAATATTCCAT

ATCCTTATCTAGTGGTTCAAATATGTTGCCTTAGCTTAATAAGGAACTTCTGCATGTCTG

CACACATGCAGATTTGACTGGAAATGTTTATTTCCTTGTGTGTTTTGCTTGGCTACTAAA

ATTTGGATTTTTTTCCTCTGTTCTTCCCTTCCAGAGCTCACAAGTACTCGCTGAAGATGA

AAGTCAGGCTACAGAAGATGAAAATGCTCTTCTAATCTAAATTTTAGT

>c6812_g1_i2 len=215 path=[1:0-106 108:107-214]

CACATTATTTTTTTCTTCATACTCAAACAAGTATTTTCTTGTACTGAGTTCTATATTACA

AGTAATCTGGTTGCTCTGTGGATAAATTGTGTTCACCCCTCTCGTCCATTTGGATTTTTT

TCCTCTGTTCTTCCCTTCCAGAGCTCACAAGTACTCGCTGAAGATGAAAGTCAGGCTACA

GAAGATGAAAATGCTCTTCTAATCTAAATTTTAGT

>c6823_g1_i1 len=283 path=[175:0-282]

AAAAAAAAGAAAAAAAAAAAAACAGAATAAAAATGTAAAAAATTGATAAAAGTAAAATAA

AATAAAAAAATTATTTAAAACGAATTTATATATTAAAACCATTATCCTAAGGAAATGTGT

AATTCTTTAATAAGATACAAGTAAAATATCTTTGTATCTCTATATTGATTCTTTACATTA

TTTATTACTTAATTCCTTTATTATATATGTGCAGTTAATATCTTCATAATTATATATTTA

CTATATTATTCCATGTTCATTTAATTATATAGTTAATTTTCAT

>c6826_g1_i1 len=261 path=[1:0-153 155:154-260]

CTGTGCTGGGTTTCTGGCCACCGAAGTCTAAGTAATATTTTCTTAAAATTGAATTATGTG

TGACAATGTGAAAGCCAGGGAGACCAGATGGTACCAGTGCCCAACGGCACCTACAGTTGC

AGGGTTTCACTATGTCCAGAGATCCTGCAGACAATCAATGAGCCACATTGAAGGAGCTAA

TTTTGTCAATTTGATCTAGGGAAAAATGTGACAGTAGAACCACTGGCAACAGTTTGTATG

TAAGGACAGGTGCTGTGCATT

>c6829_g1_i1 len=203 path=[1:0-166 168:167-202]

TGCTGACTGTGTCACTTGGATTCAGCATAGCAACAAAAGAAAAGAGAGAATCTACTCCAA

TACCAGGAAGATTGGGAAATTTATTAGCACCAAAGCAGTGGGCACCAACACCCGAGTGGA

TGTCACTAATAATTACAAATACCCAGAAGGTAAATTCTTTCTAATTTTAGCTCAGTTCTC

CTGCGTAAGCTGGGGCAAATGCC

>c6829_g1_i2 len=202 path=[1:0-166 238:167-201]

TGCTGACTGTGTCACTTGGATTCAGCATAGCAACAAAAGAAAAGAGAGAATCTACTCCAA

TACCAGGAAGATTGGGAAATTTATTAGCACCAAAGCAGTGGGCACCAACACCCGAGTGGA

TGTCACTAATAATTACAAATACCCAGAAGGTAAATTCTTTCTAATTTCAGCTGAGATGGC

ATTTGCCCCAGCTTACGCAGGA

>c6832_g1_i1 len=205 path=[183:0-204]

ACATTTATTTGATACTAAACTTAAGCGTGAGGGGAAGAAACTTTAGTTCTTTTCTACCAT

TTCTGTTATTGTGAAGCATTCCTTACAAAATAGGCCTTAAATTCTGTCAGGCTGCATCCA

GCTGTAGACCACATATGTTACAGCTGTGAAATTAACTTTTTTTTTTTAAAACCTATACCT

TGAATATTCTTTGTATTCAATTTAG

>c6836_g1_i1 len=201 path=[1:0-111 369:112-200]

AAACAACATTTATCTTTTTTTTAATTACCATAAATATTATTTTTTAAATGTTATTAATAT

GGTCAAGTTATTATGTTTTAATTATTTGTATAGAGTGTTTTAATGTTATAATGTGAGACA

TTATTTATGTTTTATATAAATTTAAATTTATATGTTTCTTTAAATCTATAGTATTGTTTA

TTTATTCTTTTATTTTGCATA

>c6837_g1_i1 len=419 path=[35:0-252 17:253-418]

ATAAAAAAAATAATTTTAAAAAAGTAAAAAAAAAAACGTATAAAATTATCAGGAAAAATT

ATATTTTTAATTTTAATTTTCCATATTATAAATTTGATAAAAAAATTAACTACAGAATTT

TTGATACTTCATATAAAAAAGTAAAACAAGAAGAATTACATAAAAAAAACTTATTTACAA

AAGAAAAAGATTTCAATAATATATGTAAAAAAAAATTAGATAATATTAGCAAAAAGGGAA

AATATTGTATCTTAAAAAAAAAGGAAATTAAGCAAAATTTTAATAAATTTTCAAAAGAGA

ATACTATTAAAGTAGAAAAAAAAATATATTATGAAGATTTGAATAATGAAAAAAAATTGA

AAAAAAAATGTGTATTGAACAGAATCATGACTTCTAAAAGAAAAAATAAAATTAAAAAA

>c6838_g1_i1 len=247 path=[1:0-246]

TGAAATATTAAAAAAAAATAAAAATGAATACGAAAATATATATAAAAAAAAGACGAATTA

TGAAAATGCAAATAAAATTTCGCAAATAAAGTATGTTAATAATTGTTTACCGCATAATAA

TATACATAATGAAACAGAAAAATCTGGTACTAATATATCAAAAGTAAAATTAGAAGTTGA

CTACTCAGAAAAAAGAAAATTCAATTCACACATAGAAAACTTAAGAAAAGAAAAATTAGA

AATCGAA

>c6838_g2_i1 len=452 path=[525:0-451]

TTTTTATTTATAATAAAGATACTTTAAAATATAAAATAAAGAAAGATTTAAAAGAGTATA

CTTCAAATTTAAACAATGAGGATCAATTTAATAAGTCTATATTAAATTCTACTGGTAAAC

ATACTTTTTCAGGTATTAATATAGAGTTAAATAAGATAAATGAGGGAATAAAGGAATATG

CTAATGAAGATGATAATAAGAAAACAAAAACAAAAAAAACAGATTTAAAAAAAAAAAATG

AAAGTATTTTAGGTTGTAATTCTAATAAAAGTTTAAAAGGTATTCTTTATAGTGATAATG

AAAAAGTTTACACAGATAATGATGTTACAAATAAATTTTGTGATACGATAAAAAAAAATA

ACTATAAAGAAAAAGTAGACAAATTTCTTGAAGAACATTCAAAAAATAATATATATACGT

ATAAGGGACATAATGATGAAATATTAAAAAAA

>c6850_g1_i1 len=239 path=[248:0-3 32:4-238]

GGGGAGTGACAACCAAAATAGAAAAACAGTAGTACAACAGTTGATGAAAATACTTCATGT

TTGTAAAACACCAATGTTTGAAACCAGGCGTAAAACATTGTAAGGTTTATTACTTAGACA

TGATTTTGAACATAAAAAGGCATTAATACACTAATGCAGTATACTAAATACATTTTCTAA

ACGTGTTTTTAAAGTACTGCTGCTGTCTAAACATACTGTTTAGATGAGGAAGTATACAT

>c6852_g1_i1 len=325 path=[303:0-324]

AGGTTAGAAACTCCAAACTGTCCCTCTTGTGTATTCACTGAGTGCACGTGGGCTGAGTGG

GGCTGGGATGTGGCAGCACAGGGGTGGTGCACAGCCTGGGCTCTGCTTCTCTCTCTTAGC

ATTGCTTTTTGGTGATGGGAGAGCAAAGCAGAGCATGCCAGGGGAGCAGATTTCAGTGGG

CTTTGGATGCGTGGGGCTCCCACAGCAACCTGGTGAGCAACGCACCGAGCATCCTACTCT

TACAACAGAGAAAGGAAAAGAGGGATTAAAACAAAAGGCATAAATGGCAGCAGCTTTAGT

TATTCCCTAAGTGACACAAGTCAGG

>c6864_g1_i1 len=294 path=[272:0-293]

GAAGTATTCTCCCCAGCTCCCTCCTCCCTTACCATCTCAGTGTTCTTTATTTCTATGAAA

CGGTTCCGAACTACCAAGCACTAGGACTATCTCAATAAACCAGATTTCCAGACAGATTCA

TAGGTAAAGTATAATTCACAGCCTAAGCCCAGCTGCTTCTTGTTGTGAGATATCAAGGAA

AACAGAATAGTAGCTCAAACAAAGTACATTGCAACTGGAAAAAGGAAGAAAACAGTTGCA

TAATAAAAGTATCAGTAGTCTCCAAGTCACACCTTCTGCACCAAGCCATTATGG

>c6870_g1_i1 len=221 path=[199:0-220]

TCCCTGTCTCTGCTTTTTTCTCTGAAGGAAGCAAGAAGTCCAAATTTATGTACAGAGCCC

TTCTTGTCCATTCAATGAAATGATGTACTTTTAAGTGTATAATGCACTACTTACACCATC

TTGCAAGTTGTCTTTCTTGTGCAATTATACAGGATGATTAGCCAATACAGCAGTGGCATA

AATCAGGACTTTGAGATAAATAGTTCTTGAATATATGTAAG

>c6872_g1_i1 len=203 path=[335:0-202]

GATGTCAACTAATAAGATATCAGCATGTATATTTACCATATGAGGAAATCCCGACTGAAG

AGCTCGTGGTTCAAATATTCTCAAGCCAAAGATTTAGCATCACTGCAGTGACAGCATTCC

CTATCCCAACAATACCTGCCAACTTGATTACTTTGAGAATTTGCCTTTTAAGACAGCATT

TATCTAAGCTCGTATACTTGGCT

>c6874_g1_i1 len=236 path=[1:0-235]

TGTAAGGATTATTTTCCTTACTTTAACATGAATGTAGCTGTTTGATCAGCACTATTTACT

ACGAGCAATCATTTGCATCTGTATCAAGCAGTTGATAAAGACAGATTCTAATCTTTCAAT

GCATATCTATTCCAATGATTTTTCTCACAAAAGTAGTGTTTTCCTCTGCAAAATATATTT

CCTTTCCCATTTTCATTGTAAACATCTTTGTTAATAATCTTTAAAATGTAGAAGCA

>c6875_g1_i1 len=265 path=[243:0-264]

TTTTTGGGGGAAGAGTGTATGTGTGGGAAGTAATCGGATACGAGCCAGGTAAATAGCTTA

TGAGCAGTTACTCGACTGGGTCTTCGAAGTGATAGCATAGATAACTGCATCAAGGCATAG

AAAAAAACTACATTAGGAAAGCAACATCGGCTGTACATGTCAGTATTTCCCTTCATTCTC

TTCTCCCAAAAAGGGTTTGTTTAGGGTGAGTTGTTTCATACACGTCTTATAAGGATCCTG

CAAGAGTAGGGTCAAAGGCTTTTTG

>c6876_g1_i1 len=209 path=[1:0-165 167:166-208]

TCAATCCTGTTCTTTAGATGGTTGTTCCAATTCTTTAGTTTCAACCACTGGAAAAGGAAG

AAAAATAACACATGATGAGGTGGGACAACTTGTTATCCCATCAGAAACCCCACATTGCTT

TGGTTTGGAGAAACAAACTGTAGAACAGGAACAAACTGAAGAATGGGCAGCAGAATCTAA

AAAATCGGGTGAGTCTTCCCCAAACCAAA

>c6878_g1_i1 len=243 path=[221:0-242]

AACATTGAACCAGCACAACAGATCAGTGGGCTCTCTCTTCCTCCAGTTATACTGCCCTGG

GTGAAGCGTTTGCAGTGATGAGACTACTTCCCTTCTGGTGATGACCTTGTTAAATTCTCA

GCTACTGACAGCATAGTAAACCAACAGGGTTAGTGCATATGGGGTGGGAAAAGACTGATA

GCCTCTTGAACAGGACAAGAGAGAACAGTTCCAAACTACAAGAGGGGAGATTTAGACTGG

ATA

>c6884_g1_i1 len=326 path=[468:0-325]

GCCAGATGGAGAACTTCCAGCAATAGGTTCTTGATTTCTGCATCGTGACTTTGATTCCCC

GTGTACAGCTGGAAACCTGGGCGCTCAAAGAAGGGCAGCACTTTGGACAGAGCCCGCAGC

TCAATCAGGTAGAGAAAATAGAGGTTCTTCAGTCTTCTGGGGCCTTCCCCTCTGGTGACA

ACCTCATCAAACCTCTGCTGGAACTCTGTAACGTTGGGGCCCCATTTCTTCTCTGACCAA

GTATCTTGTAAGAGGTACCTGGCGCTCAGGTGAATGTTGATGCTTGCATGGAATAAAAAG

GCAGCTGCTAAACTAAAGGAGGAAGT

>c6885_g1_i1 len=304 path=[425:0-223 36:224-303]

TATGTTCAAAGTATTGAAGCAAAGGTATGGAATAAAAAAAGAAAAATGAAATAATAATTA

ATACGTTTTATGATAAAATGAAATTAGTAAATAATATCAAAAAAAAAAAATAATTTGATA

TATATACTTATTGAAGTTAAGAGTCATGAAAATATAAATATGTATTAATATATATTCATG

TAGTTAAACTTCAAAAAATAAACGAGGTTTTAGTTTTATATATAAACAATATTATTTTTA

ATAAAAGTGAATGTTTTTATTTACTTTTTATTATGAATAATACTGTTTGCATATAAATAT

CTAT

>c6887_g1_i1 len=398 path=[263:0-264 153:265-397]

AATCCCTAGAACTCATCCACCGTGGAACACTGTGACCTGAACCTGTGGGTCTTGTACAGC

CATTAGCCAGGGGAACAACTGACCAGTGAAAGACCCTTGGTATGACCAGGGAAAGTGAAC

CATGGCCTTTGCTGGTGTGTTGCATGGGATTACAGGACTTGGACAAGGGAGGGCAGCACC

CTTTGTCAACTGCAGGTGTGGCTCTCCCTCGTATCATCCTTACATGTGGAGAGCAGGCTC

TCAGAAGAACATACATAAACACTGCTTGAAGTTACACTGTAAGTGAACAGTAAAACAATT

TATTACCATAACTTTAAACAAGATTTATAGTAATGCTTTCTTTCAAAGACACTTTGGAGC

AGTATCTGTTTGAAGGAAAGCATTACAATAAATCTTGT

>c6891_g1_i1 len=202 path=[1:0-22 24:23-201]

GGTCTAACAAATTTAAAAGCATTTAGTAGAATTAATGAAATTTGTTAAAAACACAAAACT

ATTAAAATAAGTAGTAATAATCATTGCATTTATAATGATATATTATATATTAGTAGTGTA

ATGTATATATGCTTTAATTTTCATTAGATTATTTATAACAATAATAATATAGAAAGAAAT

ATTTACAAAACAATGATATAAG

>c6891_g1_i2 len=230 path=[255:0-50 24:51-229]

AGCATTATATTAAAATATAAATATATCAGTATTTTTATTATAATTATTTAGTAGTAGAAT

TAATGAAATTTGTTAAAAACACAAAACTATTAAAATAAGTAGTAATAATCATTGCATTTA

TAATGATATATTATATATTAGTAGTGTAATGTATATATGCTTTAATTTTCATTAGATTAT

TTATAACAATAATAATATAGAAAGAAATATTTACAAAACAATGATATAAG

>c6908_g1_i1 len=637 path=[53:0-636]

GCGTGAAGGCGTTGGTGAAGGCGGACCCGGACGTCAGCCTGGCCAGCCAGGAGGCCGTGT

TCGTGCTGGCGCGGGCCGCGGAGCTGTTCGTGGAAACCATCGCTAAGGACGCGTTTGTGT

ACGCCCAGCAGGGCAAGAGGAAAACCCTGCAGAGGAAGGATCTGGATAACGCCATTGAAG

CTGTCGATGAGTTCGCCTTTTTGGAAGGTACTTTGGACTGAGTTGGGCAGGGATGGAGTT

CCCTGCAGAAGGAGCCTGAAGGCACCGGCGTGGGGTGGCAGAAGGGGTGCCAGGGGTTGG

ATGTGGTTCCTGGGGCTGAGACTCTGAGATGGGCAGTCGTAGTACTTTGTGTAACGTTTA

AGGCATTGTGAGTCCTGCATTTCAAGCGCGTGGTTTGTTTCTCTGTAAGTCTTCTGTATG

ACTTTGAAGGTCGGGTTCTGCTCCTGCACTGCAGGCTGCACCCCAACCCTCCCCCTCCGG

GCACTGTGGGGCAGAGAGCTCAGATCCCGGGGCAGAAAGCTGATCATTTATACACAGCTG

CTGCTTCATTCTTTAAATGGAAATGTGTTTTTCCCCTGTATGTGTTTTGTTGGGCTCCTT

TTGGCCGATGCTGTTAAAGGTGGTGGTGCACCGAGTG

>c6911_g1_i1 len=241 path=[217:0-240]

GTTTCCTTTTCAACTTTTCCAGCCTGCTTCAATGTGTTTGCTCTTACCATCAGGTGGGGC

TGACGTGGGGAGCTGATGGGGCAGAGCTTTGGTGCTGGGCAGCAGGTGGGGCTTTGGGGC

ATGCGCTAAGAAGCAATACTACAGTGCTATAATATGATGGAAAACACAGCAATAATGATG

GGGTGACAAAGTCCCAAATGAGAACAAACCCAAATGAAGCAGCCATGGATGCAGAAACAA

A

>c6913_g1_i1 len=373 path=[351:0-372]

CACTATGACTCAGAGATGATAAGAAGTGGCCTTGCATTCTTCATTACCTCGTAATGGCTT

TCAGTTTCATTTAGAGGAGAATTTCAGTTTCCAAGACCAAGGGAAGACAGTAAGATTTGA

TGTACTCAAGAATCTGCAAATATCAGGACTGCAGCTTAGGAAGTGGTTTCACTACTTTAT

AGAGTTTGAAGTACAGTAGACTTAGGAAAAATAATGCTGCCTGACCAAAAGGTAATATTG

TGCTTTGGCAGCTTCTGATCTCTGACATGTTTAGAATGCAGCAGTATAGAGAATTTACTT

GGGGTGGTGAAAATCACAATCCTTTTTTAATCTATAAAAAAAGGAAAAACAACAGCAAAT

CCTGAACTCTTAG

>c6913_g2_i1 len=206 path=[925:0-163 1088:164-205]

TGCTTCTGCAGCAAAATCAACATAACCATGTTTGTGCCTTAGCCATCACATGATTGCTGC

TGCTAAAAACAACACGATTTAAAAAAAACTTACGTCAAGATGTGAGAACAAAACAGCACT

GAAGAATTGCTGTTTTGCAATTGGACACTGCAAATGATCGATGACTTAAAACACAAATTG

CTGTAATAGAATGCTTTAAAAGAACA

>c6913_g2_i2 len=206 path=[925:0-163 725:164-205]

TGCTTCTGCAGCAAAATCAACATAACCATGTTTGTGCCTTAGCCATCACATGATTGCTGC

TGCTAAAAACAACACGATTTAAAAAAAACTTACGTCAAGATGTGAGAACAAAACAGCACT

GAAGAATTGCTGTTTTGCAATTGGACACTGCAAATGATCGATGAATTATCACCCATCTTT

CTGTTCTTTTAAAGCATTCTATTACA

>c6914_g1_i1 len=207 path=[1:0-206]

TGTTTGATGGGGGATTTTAGGAAGGTGAGCACAGAGAAAAGGCAAACAATTCCCAACCTT

TAGGGACAGGCAGAAACTCAGCCTCGTACACCACCAAGGGCAAGGCTCCTGCAAATCTTT

CACTTCACAGCAATTAAACACATCTGGTCCAATCAACGTGTAAAATGCAGCCTTATTCCA

GCAGGTATACAGAGAATTCCAGATGAA

>c6916_g1_i1 len=347 path=[325:0-346]

GATCTCTGTGGCTTGTTTGCAGGCATTACCTACATGCACACAATCAGACAGCTACAGAAA

CAAATACAGATGCCTTAAAAACAAATAGATCTGAAGAAATATCCTTGGCTTATGTTCTTT

TTTGCATTTGCAGCTTGAGTGTGTCTGGATTGGTTTATCAACTTTTACACTTGAGAGGCA

TTAAGCCTCTGTAATCTTGCCTCATCCTCCTTGTTTTGAAGTTAAAAGTATTTCTGGTCT

CAGACCTTTAGACTTCTAAGTATCTCACCATTAACATATCAGCATACTTGGGACTATGTG

TCTGGGTTTGGCTGCTTTCTATAATTGTCTTAGCTTTCTTAATCCGT

>c6917_g1_i1 len=214 path=[155:0-213]

AGAAAAAAAAAAAAAATCAATCCAAAACTGTATCTGTTTTATTGTTGAGATAACTTTTGA

TTTAGGAGACGCCTCTGCTTTCACCTTCCTCTGTGTTATTTGCAGGGTGTAAGAAACAGC

TTTTGTTTGATGATAAAGCAGATAAGACATGAATAAGAAAAGAGATATAGACCTGCAGAG

ATTAGAAATGCTCAGGTCTACAAACTGCCAAGAA

>c6920_g1_i1 len=219 path=[297:0-79 181:80-218]

TGGAGTAGAAAGACAGCCGAGCAAATTAAAGTCATACTGTCTCTGCTAACACTAACCTTC

CCCAGAGAAACACGCCAGCGTGAGTGATGGCATCAGCAAGCAGGGATCCTGCAGCAGCAC

AGACTCGTTTTCAGCACTGCTGCAGTTTCCCGAGCAGCACATTTAAATCAACCTTAAACC

TAAGTGCCAATCTACCTGCTTCTGCACCACTGAGCAGCA

>c6921_g1_i1 len=342 path=[39:0-55 377:56-341]

AGCAAAAAAATTTCTGTGTTACCTAAAAAAAATATATTGATACACATAAAAATATATAAA

TTAGAATTTTCTTTTGTTATAATATGAATTATATTTAATTTTCCTTTTTTATACTATAAA

AGTAAATATTTCAAAATATTCATTTTTCTTATTGAATTTTTTATTCTTTATTTATAATTT

TATTTTTGAATCAATTTTTTAATGAATTTAGCTATTTTTTATTTAATTATACTTTTTTTT

TTTTTTAAATGCTTTAATTTACATATATATACATCAGAAATATACTATTTTTTTATATTT

TTACAATATTTTATTTTTATATTTTTTTCTACTATAATAATT

>c6921_g1_i2 len=304 path=[375:0-17 377:18-303]

AAGTGCAGATATATCTCATAAATTAGAATTTTCTTTTGTTATAATATGAATTATATTTAA

TTTTCCTTTTTTATACTATAAAAGTAAATATTTCAAAATATTCATTTTTCTTATTGAATT

TTTTATTCTTTATTTATAATTTTATTTTTGAATCAATTTTTTAATGAATTTAGCTATTTT

TTATTTAATTATACTTTTTTTTTTTTTTAAATGCTTTAATTTACATATATATACATCAGA

AATATACTATTTTTTTATATTTTTACAATATTTTATTTTTATATTTTTTTCTACTATAAT

AATT

>c6935_g1_i1 len=404 path=[35:0-273 433:274-403]

GCCTTTTCTTTCCCAGGAAAAGAACCTGATGTGTATTCTAATTCACTGGATAATTTATAA

TGTATCGCAAGACAATAGGGCAGCTTCACAAACACTCCATACCTACTTTGTCCATAAGCC

CACAGCACTGAAATTTTGATCTTATTTTTCATCTTCTTCAAAACAAAATTTTAATGAAAA

TTTAAGTAGTTCTAACATCACAAATTACCTAATGATAGCTTAGCGGCCAGCCCGTAACAC

TGAATTCATTTCACTTACTATGAATCATTCCCTAGAGCTGTAAGGTTAAGTCTCCCCCTC

AAAATTCCTACAAATAGCAAAAATGAACTGTTACTTCTGATTTCCTCCAAATGAAGTGCT

AAAGTGTAGATAAAGTATGAATAACAGTTCATTTTTGCTATTTG

>c6943_g1_i1 len=229 path=[349:0-184 673:185-189 131:190-228]

GCATTAAAAAGTAAATAAAAACTGACACAGCTGGTGCATTAGTTCTCAGGCAAGACTTTT

TTCCACATGTGTTGCTGAAATATGCTCAAGAAGTTGTGGAAGAACTTGGATAAAAGTGGA

TCAGCAACAGAGTGAATCTCTCCCTAATCCCTAATTATAGTACAAGAAAAAAGCAGCAGA

TAGCTCAGAAATAAATTAATTGTCTTCATTTTCCAAAGGAAAAGATAAA

>c6945_g1_i1 len=201 path=[374:0-199 596:200-200]

AAAGAAACTGATGGTCTGAGGTGCCATCCCTCAGAACTGCCCAGTGGCCACTGGATGATG

TGACCTTTGCGTTTTGCCTCACAGGTGACAAAGCCAATGGAAATAGCTTCCTTTCCCACT

TTAAGTTATTTGATTACCTTGGTGTATGCTGGAAATTTGATACTCTGCCTTCTGAGCTTT

GTGAGCTGAGTATGTATCTGA

>c6950_g1_i1 len=249 path=[227:0-248]

AGAGTTCAGGAAGCGTTTGGACGGTGCTCTCAGACATGGGGTTTGGGTTTCGGGTGGTGC

TGTGCAGGGCTGGGGGTTGGACTCGGGTATCCTTGTGGGGTTTCTTCCAGCTTGGGATAT

TCTATGATTGTGTTTGGTCACGTTTGTCGCTCCTTCCCTCTGTGGATGGGAGTGGGAAAT

GCATTTGCATGTAGTTGTAGGGCTACTGCTGTGCTGGGATGCATTGCACAATCGAATGTG

TATTGCAAT

>c6951_g1_i1 len=207 path=[1:0-206]

TGCTTTGTTCTAGTAGTTCTTACATATTTTTGTTTCTGTTTAATGTTCATAGAGCACATG

TAGAAAGGCCCCGTAAGTAAAACGTGTGTAACTTTTCAGTAAAGCCTGCTCGTAAGCCAT

GAGTATGCATTTACACATGAGCCAGACTTGTTAAGAGCTTTGATAAATGCATAAGCAAGG

CAGAATAAAAAACACTCAAACACAAAG

>c6953_g1_i1 len=236 path=[214:0-235]

GTACCTGTCAAGATACAGGGCGAGCAGAAGAGGTTTATGAACACCATGCTGAAGGTGGTT

TTCTATAGCATCACGAGAAGTTGCAAAGGGCCAGTGCTACTCCATTCCAGGCACTATGCT

ATTTTCTGCTTATGAAACAGAAGAGATGTTTCAGGGCAGCTCACACTTCAGTGCAGGTTA

TTAACAGCAGGAGATCTATCCTCTGGTTCTGCACGGGCTTCAGGAAGGGAGGGAAA

>c6956_g1_i1 len=212 path=[190:0-211]

GGGCTGGAAACACACTGTCACAGAAGTGACAACCAGCCATAGGGGAAAAACAACCCCCCA

CCAACTGCACGAAGGAAGAACAAAGGTGGTAAGATGCAGGCTCCCTGTGGTTATGCAGAA

ACTGCACAGAGCAGTGTGCGTTCAGTCACAGGCAACAAGTTTCAGTGCTACCATAAGAGA

TAAGAGCTAAGTCAGCAATACAAGCCAAAAGG

>c6957_g1_i1 len=349 path=[327:0-348]

TAGCCGTTTTCCTGTTCTTAAGGGTTTACCTAAAGGAATGAAACATAGGTCCTAAAGAAT

TAATTTTTATCGGTATAACTGAAACACAAGTAATTGCTACACCATTTGTTTCACTTTTTG

GCATCCTAAAATAGAAGTGTTGATGCTGTTATGCCATGTACCTTTTGATTTACGGATGTT

GTATGCAAACAGTAGCGTTCAAGTCCTGTGCAATGTCCTGTGTAATGAGCAAATTGGCCT

CCAAGCAAGCTGATAGCTCTGTGCACCACTGTTTCACCTTAGTTTTTTAAGTTCTTCTAA

AAAAAAACAGAAAACAGCACATTAACTCATCATAACGCTTTTACTGTTA

>c6957_g2_i1 len=249 path=[879:0-248]

TACGTTGTGCTTTTATACTGCAGGGAAACTGATACATTTTAATAATCCCAGGATTGGCTC

TTACCCACCAAAGTCAATAAAGGTTATTAAGGCCAACTGCCATATAAATACATTGCTACG

TTGCTAAATGGACTGTCAAATCAGGAAATACAATTCATACCATACAGCACTACCAGGGTC

ATCATAAGAAAGAGGAAAAAAAAAAGTGACTTAGCTTATTTAGCAGCATAGCCGTTTTCC

TGTTCTTAA

>c6960_g1_i1 len=206 path=[728:0-205]

AGGCTAATAAATGGAGCAGAGAATAAACTCTAAATGCAGTGGTTTAAGAACCTTCAAAAC

AAAGCCAGAGGTCCTGGGCTTGCAAAATAGAAGACTATAAAGTAGACACCTGGGGAAAAA

TAACAGTTATTGAGAGAAGTATTAATACAGAGAAGCTGCGTACACATGTGGAGTCGTCAT

CATCTCACCACTGCCTCTTGCTGTTG

>c6960_g2_i1 len=275 path=[274:0-252 934:253-274]

CGTATGTGGGTTTCATACTTAGTGTCTACATTAGCTAATAGTGTGGCACTGGAGCTTTGT

ATAGCAACATGGCTGGTAGTGAGGATGCATCATCTCCATGGGGCAATTCTGAGAGGATTT

ACAGTGGGCTGATCCAGGCTGTGTACTTGAATGCCTGAGAAGGTTGGGAGTACTTCTGGA

GCGCATCTTCCTTTTGTAGCTCAGTCTGCAGAGGAAGTGATTCTTGCACTCCAAGACTGC

TCTACAGTTCATCTGCTACACAGTATGTGGGGGAT

>c6966_g1_i1 len=214 path=[346:0-213]

ACGAGGCTCACGAGCTGCAGGTGCTCCTGGGAAGCACAGGATTAAGTACACAACTTGAGA

TGCTGCACTTAGGAGTGCAGAGTCCAATAGAGAATGGCATGAGTGATGTATTGAGACATG

GGAAAATGCACTTGAGGGAGCGCTGAAAGAATGGAGTTTGAAATCTTTTGGTAGACTGGG

CTTAAATGGCGCCTAGTATAAAAAATGAGGGTAG

>c6970_g1_i1 len=207 path=[469:0-206]

ATAGTAGTAGGATCCTTCCTAGAAGTACCCATAAGCCACAGCACTTGTACCAAATGTAAA

TATTTTGGCAAGACGAAGTTTCTCAGTACACTATTCCTTTTTTTTTTTTAATCAGTAACC

TTATTCATCTGTGGTGAGTAAATATCACCATGTGGCAGACAGCTGCTGCCTATGACTGAC

AGCTTCTGTTCATTTGCAGGCTAGGGA

>c6972_g1_i1 len=244 path=[335:0-34 35:35-198 532:199-243]

CCAATTCTAAGAGGGACCAAAAGGTGCACATTTTCCTTCACATCCCTGTGAATTATATTA

TTATCATGACAGTAACGTAGAGCTTCCAATATCTGTCTCATGTAATGACTGGCTACAGCC

TCGCTGTACACAAATCCAGCATCTGCTCTCTTCACAATTTCAAAACACAGATCTGCTCCA

TCCATAAACTCAAAAACCACATGCTGAAGCATCCTCATATTGTTGAACTGTTGGAGACAT

ACAG

>c6972_g1_i2 len=209 path=[35:0-163 95:164-208]

CTTCACATCCCTGTGAATTATATTATTATCATGACAGTAACGTAGAGCTTCCAATATCTG

TCTCATGTAATGACTGGCTACAGCCTCGCTGTACACAAATCCAGCATCTGCTCTCTTCAC

AATTTCAAAACACAGATCTGCTCCATCCATAAACTCAAAAACCATATAAAGCATTCCATC

TGAGCTGTATGTCTCCAACAGTTCAACAA

>c6974_g1_i1 len=218 path=[196:0-80 277:81-217]

ACAGGCAATTAAAAATACATTTAGATACAGTCAGTCAGCAGGTAGAATTCTTCAGAACTA

CAAACAAAATAAAACTGTTTTTTTTTTTCCTTATTCTAGATTATGAGCACGAACTGATAA

ATGGCTTTATTTCCTCACTGTAATTATCAAGGGATGAAAAATTCTTTCTCCCCATGCACA

AATTTATTTCACTTGGTTTAGTGGACATCTGGTTACAG

>c6977_g1_i1 len=230 path=[208:0-229]

GATTTCTAAACAGAAAATGAACAAGCAACTCCTCTGCCTCTACCAGTGGCTACAGTACTA

GAAATGACTGCAGTAAAAAGTTAGGTAATAGTAGGAATCACTTGTTTTCCTCCAGAAAAT

TCAGACCTCCTGGTGCAATTAGCCCCAGTTTGGGTCACCCTGCTTTCCAGTTCTGAATCA

GAATATCCACGTCCAACCAGTAAGAAAAAGCATCAGTCTTAGATGAACAT

>c6978_g1_i1 len=254 path=[232:0-253]

GTGTTTATGGTTGATATGAAAAATTCCATCAGTCCTTTCCACTTCACTGATCCGAAGGAC

GGCAGGAGCTAATTCAGACGTTCATGGAAATAACAGTACCCAAGGCTAATTCGCAGTTAT

CCTGGGAACAAAGGTCCAAAGGGCTTGCCTGCATAGAGCGGGTGTACCACTGTGCTGGAC

AGAGGCAGCAGAGCTAGCCAGCAATGTGGAGCAGCTGAGAGCAGCTGACTGCTTTCATCC

TTTTGTTCTATTGC

>c6985_g1_i1 len=246 path=[287:0-245]

GAGCACATTCTGCAGCGTGGGCAGGAGAGCAGCGCTCCCATCCCATCGTGGGTCACAGCA

GAGCGTGGGGATGCTCTCCATATCAACCAGGGGGGTGTTTAACTGCTTCGCTTACGCTTT

GTCTTCGGGCACAGCCCGGCGCTATGTCGAACATCCGTAATCCAAAGGGCACAGTATGAG

GCTGTTAATAAGGAAGGTCCTCCACTGTGTTGTCCCTTGGCCTGGGAAGGTGCCTCAAAC

ACAAAG

>c6993_g1_i1 len=208 path=[63:0-25 31:26-207]

TTTTTTTTTTTTTTTTTTCTTAAGATACTCTGTGGAAGTAAATTTTGCTCTTTGTAGTAG

CTTTCATGGGTATAACAAACACTATCTCACGTTAAGTGGTATGCTAGACATTGGTTTGGT

TGTAATCACTATACTGGGCTCACATGGCCTGAGAAAGCTCAGGAAACAGTCTTTGGTATT

TCAATAGAATACCGGAGCAGTTTGTGTT

>c6993_g1_i2 len=212 path=[1:0-29 31:30-211]

AAGAACTTCAGAGAGTCTCTTTACTGTGAAACTCTGTGGAAGTAAATTTTGCTCTTTGTA

GTAGCTTTCATGGGTATAACAAACACTATCTCACGTTAAGTGGTATGCTAGACATTGGTT

TGGTTGTAATCACTATACTGGGCTCACATGGCCTGAGAAAGCTCAGGAAACAGTCTTTGG

TATTTCAATAGAATACCGGAGCAGTTTGTGTT

>c6998_g1_i1 len=279 path=[267:0-256 589:257-278]

TTTCCCTCCTGGCTGCAGGAGTGGAGCTCACTCTTACTGGTGTATGAAGCTGTATCAAAA

GGTGCACCCAGAAGAGAACCCACGTGGAGAACATGCTGTAGAACACAGTGAGATGTGAAA

CAAGTTCTGAGTTAACTGAGCATCTCGGTGCGCTCTGCGTAATTGCTGCAAGGAGAAATA

CATGGCCTCAGGGGCAAAGCACTTCAGAATATTCTTAAAGCAGATGTTCACAGGATGTTG

GGTAACACAGATAATTGTGGGTAGTTTGCTACCAGGCAA

>c7000_g1_i1 len=214 path=[1:0-146 148:147-213]

GTCGGGTTAAAAAGGCCAAAGGATGGATTGTCGGTGGGTGCTCAGCCCCATGGGAAGGAG

AACATGAGGACAGACCCCAACCAAGTGGTGGATCCAAAGGAAACACCTTCCAAGCACTGA

GATGTGATCAGGGCAGCCGAGAAGGGCGAGGTTTATTTTACACGTGACGAGAAGCGATTC

AGCTTTTCCATTTCTTTTTCTTTCTTTCTTTCTT

>c7002_g1_i1 len=251 path=[298:0-221 25:222-250]

AGTCCCATAGCTCAGAAAATAAGTTTGTTGACAAACATAGTTGACTACGGTATGAAAAAC

CAGTGTCCAGCAAACTCAGCTGAAAACAGTGATGCACAGCCTGCTTAAATTTAATAACTA

TGTTGGTTACTATTTTTTTTTTTACACCCTCAGATGCAAAATGTAAATAATCTCTAAGGT

ACTGTTTCCTTCACATTTACTTTCCAATCAGAGTGAATTTTCCATGTAGGAAAACAGTTT

CTCCTTGTATG

>c7002_g1_i2 len=261 path=[298:0-221 519:222-260]

AGTCCCATAGCTCAGAAAATAAGTTTGTTGACAAACATAGTTGACTACGGTATGAAAAAC

CAGTGTCCAGCAAACTCAGCTGAAAACAGTGATGCACAGCCTGCTTAAATTTAATAACTA

TGTTGGTTACTATTTTTTTTTTTACACCCTCAGATGCAAAATGTAAATAATCTCTAAGGT

ACTGTTTCCTTCACATTTACTTTCCAATCAGAGTGAATTTTCGTCTACGTTTTCCTTTGA

GTTAAGCAATTTCATCTGAGG

>c7007_g1_i1 len=337 path=[33:0-126 363:127-336]

AAGGGGCTAGCTGCAGTACCCAGAGAAGCCAGCTGAAGGCAGAGGATGGGTCGCAGGACA

GGAGGCAGTTGTTTGCACCCCAAGCTGGCGTTGGGTTGTGCGTGGTTACAGCAGATGTGA

ATTCAGCTTTAATGGTGTTGAACCCAGAAGTTGTTTATAAATGGTTGCGCACGATCATAA

ATCTATTTGGCTGAATTGGTTAATATTTGCTGATTAGGAAACGTATATATGTTTTTTTTT

CTTATTACAGAGCTGTTCCAACTTAATACATTTCCCTTGAATGGACAGTGATGACCTTTC

TGGGTTTTACCTCCCCCCCAGCAGTCCCTCTGTACAG

>c7008_g1_i1 len=223 path=[1:0-222]

ATTCCACCAGTGTCAGCTCCCCTGCATTAAACCAACGCTATTGCTGACCAGGCAGCTCAA

GAGCATGGTGGGGATGGTACAGACCATTACCTGGTGCGTGCCCGCTTGGCCATGCTAGAC

AAGAACTACAAGCTGGCAGAGATGATCTTCCTGGAACAGAATGCCACGGAAGAAGCCATG

GACATGTACCAGGAGCTGCACATGTGGGATGAATGCATTGTAG

>c7014_g1_i1 len=254 path=[746:0-253]

TTAAACAAAATAAGGTGGAACAAATAAACAGCGTTATAGAAACAAGCATAAATTTATTTA

AAATATAATAGTTCTTTTTTTCCTTCTTTATGTCTCTCCTTTTGTCGATGTAATTTTAAA

TAAAAATTATATTCTAATCATATAATACTGTTATTTTTTCTGTATAAAACGCATACGAAT

AATTCTAATTATTTAATGATAGAATTATGTGAATACAAATAAAAAAATTATTTGCTTAAA

ACATTTTTAAATAA

>c7014_g2_i1 len=223 path=[4:0-208 213:209-211 980:212-222]

TTTTTTTTTTTTTTTTTTATCCAATAATCTTTTTTTATTTGTTTTTTGTTTTAAAATCAA

AAATTCCATTAATTACTGTAATTGTAATAATGCATGAATAAAAACATGTTAACCTTTTTA

TATTTAACGTTTTAAGCAAATATTTTAATTTATTTTTTCGTCTTTATTTCTATTTTTCTT

TTATACTATATGTATTTTTAACTATACGGAAAAAAAAAAAAAA

>c7022_g1_i1 len=229 path=[1:0-228]

GGAAGGGCAGGAGGAGGTACAGAAAATAAATCATGAACACAGGTGGGAAACTATCAGTCA

GATCTGGAAAGGTGCACAGGGAGCTGAATACTTAAGAGTCTAGCGTTGAGAGTTGAACTG

ATAACTTCTTGCAATGCGGTTATTTTCTTCTGTGTTTTACTGTTATACCTGATCTCCTGT

TCACTATCCTTCTTCCTGTCCACTGGAGGAAAATGGCTGAACATCTTGC

>c7031_g1_i1 len=327 path=[305:0-326]

GAACAGCTGAAATTGCTGTCTGAGAACAGGCCAGACCCAGCAGAAGGGAAGCAGTGCAAA

AACATTATGCAGGGCAGCTCTCACAGAATCTCCTCTCCAGGCCCAAGGAGTTGAAATGGA

GATGAAGTGCCCATCCTTTCTAATAAGGAAAGCTCAGGATTTCCCTCTCTGCTCAAGTTG

GAAATCAGACTAGTTCTAATGTAAACAAGATGCAGGAGGGAAGAGAAACCAGCATCCCTT

GGAGAAGGGAGGAGCATCTCCCCAACACGTGGGTGAGAGGTCCCGGTGCCATCACTGCCT

GCTGCTTGTTCTTAGGGGAAGAACCAG

>c7035_g1_i1 len=269 path=[247:0-268]

TGCAGAATTGAGGAAAACAACTCAGAGGAGTGGGTTCACCCCATGTTTAGGTAAATCCCT

GTGTAATCTAAGCACAGCTCACAAATACTGTCTATACAGCTGCAGTGTTTAAGGAACAAA

GGCATGAAACAGGCTCTCCCACTCCTTGCCTGGTGCTGCATAAAGTACTAGTGAGTAGCT

ACTTCCAGGTGGGAGTCCTAGTTCAGGACAAAGACTGTTCAGATAGATAAGAGAGGTAAG

CACTGAAGTGACTCACTTGATCCCCGTAA

>c7037_g1_i1 len=265 path=[488:0-200 187:201-264]

AAAAAAAATTACAAAGAAGAAAAGGTAGAAAGTACAGCAAATCTATAAATGGTTTCTCAT

TGTTATCTTCATTTACATTTCTGTAGCTAAAAATCTATACAACAGGTTTACTTGACATGA

TGCTGTACATATGGTATCATGAAGCTTGTGTGGTGCTTTACTGTAATTTAGGTTCAAGTT

TATCCAGCTCATTTCGAGTGGACTACAGAATGAGAGAAGAAAGGAAGAAAGCAAATGGTA

ATGTTTCTCGAACACAATGGTTTTA

>c7049_g1_i1 len=315 path=[293:0-314]

CAGCAGACACTTTTATCAGCCTGAGAAGGAAAAATCTCCAGCAGTCTTTTACTAGTAATT

TTTGGGTGCAGTTTTTACAGTTTTATACGCTGGTTAAGTGCACTGACCTGGAATTCAGCA

AATCTGCTGAAGCTGCAGGGAATACATAAGCCAAACTCGACTCTTCTCACCTTAATTGCT

TTGCTCTTCTGCACCATCTGATACAATCACTTTTGAGCAGAAAATAATTTCCCTCAAAAA

GTTATTTTAAAAATATGCCTAAAACCAGATGTTTAGCTTTAAAAATGCACATAATTGTTA

CTTTATTCTGTAGAG

>c7053_g1_i1 len=323 path=[93:0-72 458:73-322]

AAGAAGTTCCTTTGCATATTGGTGTGGAACTTCCTATGCTCAGGTTTATGGCCATAAAAC

ATTTCTGGTTAATTTTCATTAAAGCTGTACTCAAATCCTTCCAGAGTATCAGGAAATTCA

AGTGGTGGCTCATCTTTTTTCATCAGTTCATCCAAGTCTATTCTAGACAATAAATCCTTT

AATGCAGTAGTCTTTTCTTTTTCATCAGGAACTCCTTGCTGGAGTTGAGCCATCATTACC

CAAACTGTCAGCAGATTTAAAACATTAAAATAAAAGTGCATCTATCTTCAAATGACAATC

AGCATTGCCCTGCCCTTGTATTG

>c7056_g1_i1 len=208 path=[1:0-151 @153@!:152-175 366:176-207]

CATCTATTGCGCAGCTGTCAAATGAGACTATCGCTAACAATATTGTTTTTGATGCAATAC

CAGTCTACAGGTAATAGACACAAAAATACAGAAGGAAGACACAGACCATCCTTCTTTTCA

GTTTAAATGCCTCCTTCCTAGTATTGTTTCCAAAAATGGTAATGACTTGTGTTCAGCAGG

GCTGTCGTTGAGTTACAGTTCAATGGAC

>c7060_g1_i1 len=258 path=[155:0-257]

GAAATCACTACCTCCACAGCAGAAGACAAAGATGGAAAGAAAACAGTTTACTACTCATTG

GGAAAACACTAAAAAACATTTAGGTACAGACTCAATATTGATTTTGGTCCCTTATCATTC

TTTTATTATCTATTTATTATTATTCTTACAACTTACCAACATTATTTTTAAATATTTGAA

TCACGTCACAAGTATCACATAGACAAGAGTGTGTTCTTTATACAGCTCAACACTCATTTT

CCCTCACGGTACTTGTTT

>c7063_g1_i1 len=229 path=[207:0-228]

GTGCTTATACACCATCTCCCACGCTGCTGCTGCTGCTAGGCAACGCTACGCAGTCCTACA

GCATCGGCTCAGGTCAACCAAAACCACAGGAGTAGATGGACACAGCGATGTGAGCCCGAG

CTGAGCCCTGCCCCAGCTGTGTGCTGGCAGCTGGGAGGCAGCGCTGTGGGAAGGGGCAGT

GGGAGCACACACACAGCCTGCACGGCCTCCTCCTCCTGCTGTGCGATGA

>c7064_g1_i1 len=560 path=[1:0-559]

AGGTATGGTAATTTCTTTGAAATGCTTTTCTATTTTATCCCAAAATTGGTATTTCATTGA

ATATTAAAATTTTGTGTTACTTTTCTTTTAGATGAATAGAATGATTTATTTTTTAATTCT

TAAAATAAAAAAAGTTCAATTATTTTATATTACTATAAATAATATTCGTATACATATAAA

ATATTATTTAAGTCTAAAACAGGTTGTAACTTTTGATACATGATAATGCGAATGATTCTT

GCTTATGATACTTAATTTAAAATTATAGGATTACAATTTAACTTTAAAGCAAAATAAAAA

AAAATAACTATTTAATAAACAGGAGGAATGAATAATTAATATAAATATGACTAATATTAG

TTTTTCTCCTTTTTAACATTCATTATTTTCATATAAATTAATTTTAAAATTAAAAATGCA

TATGGTGATATAGGTGTACATTTTTATAAGCATTCTTCTTTGTATGCAAAAAAATGAATA

AAAAATATATGAATTTAATTTTTTATTCCTTTTATTAAATACTTTAAAAACTATAATTAA

AAATCATATTTTTCATTATA

>c7066_g1_i1 len=242 path=[47:0-184 46:185-241]

TGTTTGAGTGTGTGTGTGTGTGTATGTGTTTATTCAGGTTTAAAAAATTACAAGTAATGG

TTTTATATTCCCAGCATTTATTTACAAATAAGAGATTCACATCTTCAGCATTACCAAGAA

GAAACTGTGAATAGCAAATGCAAAGACCACGAACCAGTCTTTCCGGATGACCAAAGAGCA

GGAAGTCTGTACAAATTGTAGTGTCTCTTGAAGCACAGAATATTGTATTGAGGACAAGAT

AC

>c7069_g1_i1 len=255 path=[291:0-96 156:97-254]

TCCCTCTAACAATCTTCACTTGCTTTTGAAACATACTGAGTTTTTCTGATTGATGGTGCA

TACTCATAGCAGGCCTCTAGTATGCAACTTTAACCACACTAGAATGCCTGGAAACATTTT

ATCCCTTGATTGTTTATTTTTCAATAGCTTCCCCACCTTCTCAGAGTCTCTTTTTTTGAA

GCTAAAGTTTCCCACTCTCCTCTTTACAGAGGGCAAACATTGGTGTGTTAATATTTGCAG

GAATACAGAAATATT

>c7082_g1_i1 len=257 path=[1:0-256]

GTTGAAACAGGCTGCCCAGGGAGGCTGTGAAGTTGTCTTTTTTGGAGATATTCAAAACCA

ATGCTTTCCAGTATAACCTACTTTACGGAAGCTGCTTCAGCAGGGAGTTGGACTTGATGA

CCTTCAGAGGTCCTTTCCAACTCTAAAGATTCTATGATTCTATGACAAGTGAGTTTGTTC

CTGGGTGAGCCTTGGGGCTGCTCTCCTGCAGCCCCATGGATGGATCTTTGGAGGATACAG

GGCAACTACTTTATTAG

>c7085_g1_i1 len=287 path=[265:0-184 450:185-286]

GAATTGGTGGTCAGAACCTATTAGGAGCCAAAGTGCATGTGGGACTACAGCTGCGTGTAC

ATCACAGTGCAGGCTTTGTTTTTTGTTACGACTGTTAACGAGGAGCACCGTGCACTTCCA

TTTTCAAACAGTTCCTATGGATGTAAACATCTGGAATAAAATGATATTTCAGCTACACTA

GCAACAGTGACATGCTTGAGGACAAGTTACCATGATATTAAAAAGTGCACTAATCCATAT

GAAGCTTTGCCTGTTGAAGATCTATCCCATGTGACTGTTGCTAGTGT

>c7086_g1_i1 len=359 path=[77:0-40 118:41-307 18:308-358]

TAAATTGTTTAAAAAAAATTAAACTGGTAGCTCATTGAAAAATTATGATTTTTTATTTCA

TTTCTATATAAGTTTACCATTAATAAATTTACTATTATGTAAAAAAGATAAAAAACAAGT

ATAAAATAAGGTTAATCAATTCATTCGTTTTTAAGAAAAAAAAAAATTTATATATTCTCA

TAACTATATATAAGACAATTATAGAATTTCATTTAATTAAATATATAAATATTAGAAGGG

TAAATTTTAAATTAAATATATAATAAATATTAAATCACTAATTTATTAAATAAGAATTCA

TATATATTAAAAATAGAGATAGCAAGAAAATTAATAAAACAGTAATGTAACAGAATATA

>c7088_g1_i1 len=237 path=[1:0-236]

AAGATTACTGTTTTCCTTTGCTTACCCAGGAGTATCTCCTCACTTAGTGGGAGTGTTTGT

AAATATGGCAGCGTTTCCCAAACTGTTTTGCTTCCTGTCTGTCTTTCAGCCAGGTTGGTT

CATTTGCTCTGTCTGCCCTCTGACCACACTTTCATTTACGGCAGCATAACCAGTGGTGTA

CTTTTACTTTGTGCTCCTCATGCCTGGGGGTTGGTGCACAGTGTCCACTCTCTCTAT

>c7088_g2_i1 len=274 path=[767:0-185 453:186-273]

AAAAGCACAGTGATAACATAAACCCAAAATGATACTTTTGAAAAGGAGCCATCAGAAGCA

TTCTGTTATCGGGAGAAGCAATTCAAGCTAATGTAACTATTTCATATCAAAGCTTTCACA

AACATTCTCTAATAGCTTTCACTATTCTTGATGTATTGCATCAAGTTGTTCAATATATCA

CTTTTTCCTTTCTTTTTTGCTGTCTCTTTCCCACTCCTGTTGAAAATAACCAAGGCAGGA

CTTCAGAACATCTGCATCCCTTTCATATAGCTGT

>c7088_g2_i2 len=274 path=[767:0-185 952:186-273]

AAAAGCACAGTGATAACATAAACCCAAAATGATACTTTTGAAAAGGAGCCATCAGAAGCA

TTCTGTTATCGGGAGAAGCAATTCAAGCTAATGTAACTATTTCATATCAAAGCTTTCACA

AACATTCTCTAATAGCTTTCACTATTCTTGATGTATTGCATCAAGTTGTTCAATATATCA

CTTTTTTCTTTTTTTTCAAAATGTAAGTTTAATTTTAACACCCTCCTTCTGAACTTCGTA

GCATAAAAAACAGCTATATGAAAGGGATGCAGAT

>c7095_g1_i1 len=267 path=[157:0-44 202:45-155 202:156-266]

TGGATTCCCTGTCCTACCATATGTAGCGAGCTCTGGATGTGTTTATGTTCTTCTTCTGTG

GAGCTGTAATTAGAAATGCTCTGAGCATATGACTGACCCGTTAACACAAGTACTGTTCTT

TGGGCTCTTATATGCATTTTAAGATAAAAATGGCTTTGTTCTTCTTCTGTGGAGCTGTAA

TTAGAAATGCTCTGAGCATATGACTGACCCGTTAACACAAGTACTGTTCTTTGGGCTCTT

ATATGCATTTTAAGATAAAAATGGCTT

>c7099_g1_i1 len=207 path=[237:0-111 348:112-113 27:114-206]

ACAGGCATCTTCTTCTACTTGGCTGTTTTGAAGGGAAGAGAGGAGGGAGTCACCAGCTGG

CTCTGGGCTGAGAGAGCCGTGTGGCCCCGTATCAGCATGCTGCCACCAGCCCCACTGTGA

GGAATGGGCAGAGCACAGCTCCTCCAACCCCCGCAGCACATCAGCCCCAGGGCTTCTTAC

CTCTCACCATGTCCACCCCAGAGAAGA

>c7109_g1_i1 len=264 path=[242:0-263]

GACTGATGAGAGGGTCTAGAGCCACAAGAAAAACCCGTGATATTTCATCTTGATGCACCT

CTGAGATCACCGGAGCATAAAGACTGGGAGGAAACTTGCTGTATATCTGAAGGAACAGTT

GATGCAACTGCGAGCATAAATCAGACAGGAAAGGAAGAAATTCCTGCAAAAGTCATTAAT

TTTGAGAGGTACTTTAATTATAGAACACACAATTAAGTATGTTTTCTTTCTCTTTTGCTG

CATTAGTGTGTGAAGTACTATGCA

>c7110_g1_i1 len=339 path=[369:0-178 548:179-338]

TTTTTTTTTTTTTTTTAATATCAATAAGTGATATTTGACCTTCAGTTTAACCTACAAAAC

AACTTTCAATCGATAATAAAACAGTCCATCAATCATTTTAGAGCTGAAAAATCCAGGAAT

AAAAATGCTGATAAGTAACATAAAGCCTCTTCCCATGTTTTTCACCTCCTATTGCTCCTA

CTTCAGGGAATGCGTCAGAGTATTTCCATAAGTCATCTCTCTGATTGTCTCAGCAATGAG

AACAACTGCATCTTGGGATAATAAAGGAGCTCCCCTACCAGGGTATATAATTGTTTCATC

AAATTCTGCAGTAAGCTTGATGCTTTACCTATTTCTACT

>c7115_g1_i1 len=301 path=[1:0-300]

TGTCGATCTTTGTCCTTTTCAGAGTAACTTCCTGCAGCTCTCTTTTCAGGTGAGCAATTT

CCTTCTCCGTTTCAGGATCCACCATGAAAATCTCATTGACCCTCTCTTTCACTTCCACCC

TTGGTTTTTGCTTCTCTGCAATGCTGCACTGGCTTTGCAGCTCTCCCAGCTCACCCGCGA

GCGCCAAGTTCTTGCTCCTCTCTTCTTCAATCAGAGCTTTAAGCTTGGAAGACTCTTTCA

GTAACTCCGGATCCTGCTCTACCTGCTTCACCTCCTTCACAATAATTTTGGGTTCTGCAG

C

>c7118_g1_i1 len=229 path=[340:0-24 365:25-140 607:141-149 537:150-228]

CCCTGGTTTGATCCCCTGTCAGGGAAAGAGAGATATCATCCATATTGACAAGAACACCTT

TTTCTTAATGAAATTATTATTGAATTAAGCTGTAATTTCTGATAGCTGATAATACACAAT

ACATTAACACAGATCATTCATGCACCTGCCTAGAAGTCAGCAGTGTATCAGTACAAACCT

CTGCTTGTGAGTCAACAGTTGGAGTTGGCCTCCCAGCTGTCACTGCAGC

>c7124_g1_i1 len=249 path=[1:0-248]

AGTAATCACCTGTAAAATAAAGGTATGTGGAGCAAATGAACGTCAACTATATTCTTCGAT

TATGGAGTCCAGCGTTGTCTTTACTTAGCTGAAGAAGTTATGGGAATCAGTATCAACAGG

ATGGTCCTGCAGAGTAAAGGCAGCTCTGTAGCACAGTGGTGGTCTCATTCTCCATGCTGT

GGAGAGAAGTGGAATGTTTGGTCTGGCTCTGATTGGTTTACTGAAGGAGCAGATTGCGGT

ACCTACGTG

>c7126_g1_i1 len=234 path=[273:0-233]

GCAAACAGTTGGGTGAGTTAAAAAAAATGCCTCATCATTGTGAAAGAATATTGCGTTTAA

ACATGGAAAGCTGCAACGTTCCTTTTTTGTTCCTGTCTTTGCTCAAATGAACTTCGGACA

ATTTCAAAATCCTGGGCACAACAAGAGTAATGCTCAGGCCTCAGAGCAAGAAAAATAATC

ATCCCCAGGGCAGATTGTTCTCAGCCATATTTCTCAACAGTGTTTACACAGAGC

>c7139_g1_i1 len=207 path=[185:0-206]

ATAGCATAGCATTGTAACTTCTCCTATATTCATCTTTGACAGGGGACTCATAGCCTTCAG

CTGTTTCTAATATTTTCACGCCGTGAGTCGGAATTTCCTGGCGGGCTCTCTGTTGCTAGC

AATGGACACACTGAAAACTCAGGTGCAAAGGAATTAATCAGTGCTGTCCACATGGAGATG

GTATCACCTAGTGGAGAAGCAGATATG

>c7143_g1_i1 len=226 path=[204:0-225]

GGTAACTCGTTTGGACCGAGGTCTGAAGAAAGGTGCTTTGGTTCTTTAAAACACCAGGTT

GAGTTGCTTTTTTGTATACACACACACACACATATCTGCCATGGAAAAGTTGAAGAAGAA

AGCCTTTTTTTTTTGCTTCCTGGATGAGAAGAAACCTGTGGGAAAAATGGGGTGCCAACA

GGTCTCATGGGGCCTAATGAGGGCTGAACACAAAAGGAATCCCAGG

>c7150_g1_i1 len=234 path=[155:0-233]

GTCCAATGCAGAGCCATGCTGTCCTCACTTGCTCTTCTGGATTCTTAACAGTGGAAAATC

ACTGGCATACAGAATCACCCTTGCCAAACTGCTCTGCCTGTGATGTTTCTGGAATGATCT

TTGCCTTGGTGCTGCTCAGTGGGAAGGAGACGGCATGCAGGGGACAACGTGCCCTTGTTG

GGTTCCCCATTGCTTTGGACCTACTGATGGATCACACAAACGCAGCCCAATGCA

>c7153_g1_i1 len=295 path=[273:0-294]

TTGTCTTTAAAAGACAGCCTTTTAAAACACACGCAAAACCATACCTCCTTCTTTTTCAAT

GTTCCAAAATTTCTGGAAGAGGACACAACAGAAACAAATCAGAAAACCACAGACTTACTG

GATTATGAAGATGAATCTGATGATGAAAAGCTGTTTCACAATAGCTCTTCTAGCCTCTTC

AGCCAAAGAGTCCATTCTTTTGTTGATAAAGCTGATCAAGGCCACATAGAATTTGCATCA

ATGTTTGATACTATTCATGCAAAAGACAATACTGAAGAGAAAGACAAGTTACCAG

>c7156_g1_i1 len=309 path=[337:0-308]

AGCCTTGCCATCCTTGTTGATGAAGAGGGAGCTGCCCTGCTCGTCAGTGTGATTGGTAAA

TCTGTGATTCATCAGTTCCATTTCCACAAGCCAGTGCACAGTGTCTCCTTTTCCCCTGAT

GGCAAGAGATTTGTGATTACAAAAGACAATGTTGCTCTTATGTACCACGCTCCTGGGAAG

AAACGAGAATTCAATGCGTTTGTTCTGGACAAAACTTACTATGGTCCATATGATGAAACA

ACTTGTATTGACTGGACTGATGATTCCAAGTGTTTTGCAATTGGAAGCAAGGACATGTCT

ACATTGTGT

>c7172_g1_i1 len=257 path=[119:0-114 233:115-144 59:145-256]

TGATGTTCAGTGCCCAGAAAACGCTAATGGTTCCCGTTTCCTACCTCTGGGGCAGGGAGT

AAATCAGTCATTGCTTTCACCTCAGTGCAACCCTAATGCTCACGCTTCTTTTTTTGCAGA

TGGATGTCCCGTTCCTAGATAGACATCCCAGTGAGACCCAGCTCAGATCAGCTGTTGTGA

TTTAAAAGAAATCTTTGGTGTAGACATGGAAGCTAAAAATGTGTTGACTTGCCCAGGGGT

GGAGAAAGGTCCGAGCA

>c7175_g1_i1 len=211 path=[155:0-202 401:203-210]

GAAGAGGCTGGTAGTTACTGCCTTTGCCACCCCTCACTGTAGATGGTTAAATGGGACCAG

CTGGATTTTGAGATCTCGCTTTGTCTATCTGCTGTTTGATCAAACTCTCTTTGGGTTGAA

TGATAAGAATCTATGGTAGAATCCACTTCTTTAGGTGTTCTTTCCTGTAATTTGTCTTGT

AGTTCCTTATTGTGTAATGTCAAAGAAGCAA

>c7179_g1_i1 len=204 path=[33:0-142 32:143-203]

GGGGTCTAGACAGGAAACTCAGCGTCTCTGATGCTCTTTCCTGGGGTGCAGTGCTGTGAG

CCAGTGGGGCTGACATATCCCCACCCATCCAGGCCACCGTGTGGGGCTGGCAGCATGGCT

CTGAAGTCATCCTTGAGCCTGTTCCCACAGGGAGGTGTTCCATCCCTGGGATAATTTTTG

GGCCCTCCTCTGGATGTGCTCCAA

>c7187_g1_i1 len=260 path=[1:0-259]

ATACATGCAAACTTGTTTGTTCCACTGCCAGTCTGCTAGGTGAGAGGACTAGCCCAAAAT

GCAGGGGCTGGGAAACCAGGAGAGCAGTCACTGTTTGGTATGGAAAGTGCTTAAATACAT

GCCATTCTCATACAGCCCTTCAGGCATGGTTCAGAGAAGCTGAGAAACAAAGACAGTCTG

GTCCTGAGATTCTGCTTGTTATGCAGCACATGAGTGATTTATAGAATCTGACTGCAAGTT

GTGAATTGATAGAATAGAGA

>c7192_g1_i1 len=240 path=[218:0-239]

GCTGTGGGTCTGTGCATGGTGAAGGCTGCAGTCACCCAGTGATGCTCTGGAGGATGTCCC

GTGCCCCAGGGGCACTGCCCAGCCTCCTGGCACCATTACTGTAAGGTGGCTGGCACTGCT

TGGGTTCCAGGTGGATGGAGAACAGAGGTCACCAAGGGGCACTGTGACTCGGTGGTTGAT

TTGGGAGAGCAGATCCAAGCAGCAGGACCCGGGTGCACAGCCCTGCCCGGGCACATCGGG

>c7196_g1_i1 len=213 path=[1:0-212]

AGGCGCAACGCGGCGACGTGGAAGGTAAGCCTTAGCCCGTGTGTGCCTCTCGCCTTTGAT

ATCTGACATGTACAGAATAGACGTTGCTGATGACATAACAAATTGTAACGACGTGTGGAC

ATGAAAATGTAGATGGGCAGTTGTGACACGCGCTGGGTGCCTTATCTGCAGGAACAATTT

GATATTTGGCAGAAAAACTCCTCTGCCTTTGAA

>c7199_g1_i1 len=236 path=[155:0-235]

GCATAAAGATAAGTGGTCTATTAGAAAGTGCAGAGATACTTCTGATTGTACTGTGCATGT

TGACCACTTTTTCAGTAAACACACTTACCAAAAGAAGCAGTTTCTGTAAAGGAGATTGCA

TGTTATCTTTTTATTGATGCTATGTTTAAATATTAAGCACCTTTAGGAAATTCAAAATGG

AACCCAGTGCTTTGGATGATTCCTGTTCCAGCATAAGAGGATTTTTTGTGTTTATT

>c7207_g1_i1 len=224 path=[291:0-223]

AGGCCTTTCCTATGGAGAACGCAGAGTTGTTCATTTAATTTGAGGTGACTGTTCTCAAGT

GCTGAGCAGATGGGAGTCCGTGGGGCTGCAGGATTGGGGCGCATTGGGGGATTTGGGTTC

TTCTCATTGGTTTTCCTTTGGTTTCAGGGCCGGTTGATGCCTGAGGAGGGGGTTCTGTGT

GATGCAGAGTGCACTGAATGAAGCAGTCGCCCCTCCAGAGGTTT

>c7215_g1_i1 len=205 path=[337:0-204]

GTATAGGAAATTAAATCAATGCAAATTTTGGGCCCGTGTTAGTGCTCACTATCCTTAAAC

TCTCTGTTAACTGTTTTGTAAAATGGTTAATTTTCTTTTTCCTCTCTCATCACTTATCCC

CCCAGTGCAACGTTAAATGTTGATGCGCAGCCTTAATTGGTTTAGTCACGAGAAGATAAT

CTTTTTTTCCTCCTTCCTCACTGTT

>c7219_g1_i1 len=258 path=[333:0-52 49:53-257]

TTTCTGGTGAAAACTGTTGCATAAATGGCATAGATTTGAGAAAAGACAATCTAAATCTTT

TCTGCTTGATTTCTGGCCATTTGCACACTTAAACTTGAATTTTTGGTGCAGTTGTCCAAA

AGACAAGAATCTGGAAGTGTGTGTGTGGAGGTTTCTTTAAATGGATCATTAACAAACTGA

AGTGGAAAAATTGAGAGGGAGAGGTGGAGAAAGTTTTGGATATTCAGCTTCATGTTGACT

GTTGATGAAAGATAATTA

>c7219_g1_i2 len=253 path=[1:0-47 49:48-252]

ACCAGAAAACGCCTAGCTTCCCTTTGCTTTTGTTCTCTATCCACCCTTAATCTTTTCTGC

TTGATTTCTGGCCATTTGCACACTTAAACTTGAATTTTTGGTGCAGTTGTCCAAAAGACA

AGAATCTGGAAGTGTGTGTGTGGAGGTTTCTTTAAATGGATCATTAACAAACTGAAGTGG

AAAAATTGAGAGGGAGAGGTGGAGAAAGTTTTGGATATTCAGCTTCATGTTGACTGTTGA

TGAAAGATAATTA

>c7220_g1_i1 len=210 path=[188:0-90 279:91-209]

GGGTTTTTTATGGGGTTTTTGGGGTCCAATGGGGGATTTTTGGGACATCCTCCACCCCAT

AGATTTGTGTAGGGTTAAGGTGAGATATTTTGGGGCAAATCTGGGAAATTTTGGGGTCAA

AATGTCCGATTTTGGGGCTGAAAATGGAGGATTTGGGGGTCTTTTCGCACCCATTCGCCG

CTGTTGCGTTTATAGGGTTTTTTATGGGGT

>c7225_g1_i1 len=325 path=[531:0-223 193:224-286 25:287-324]

CTTTGTGCGACTCACAGAATCCTTGGTTGAAATTATTACTGGCAAATAACTAGCTGAGAA

ACTGAAATTCAAGGAGAAAATGTCTGGGTGGATAATCTGGCTTTCTGACCTCCCGCAGTT

TGTGCACAACTTAAGAAGTTACATTGCTTTCAGAAAGTGTCTTTGACTGCACATATACCT

TGTCAGCTTCAAAGCAATGTGTTTTGTCAATAAGAGATGGTGCAAGGAAGGTGTGGTGTT

GTGGGTGGGAGGGGAGGTCAGCTCATGAACACCAATTTAAATGACCTTGATTTATTGCCT

TTATACTAACTCAGTGATTCTCCAG

>c7225_g1_i2 len=326 path=[531:0-223 193:224-286 255:287-325]

CTTTGTGCGACTCACAGAATCCTTGGTTGAAATTATTACTGGCAAATAACTAGCTGAGAA

ACTGAAATTCAAGGAGAAAATGTCTGGGTGGATAATCTGGCTTTCTGACCTCCCGCAGTT

TGTGCACAACTTAAGAAGTTACATTGCTTTCAGAAAGTGTCTTTGACTGCACATATACCT

TGTCAGCTTCAAAGCAATGTGTTTTGTCAATAAGAGATGGTGCAAGGAAGGTGTGGTGTT

GTGGGTGGGAGGGGAGGTCAGCTCATGAACACCAATTTAAATGACCTGGAATATGAAGAG

GCTGGAGAATCACTGAGTTAGTATAA

>c7226_g1_i1 len=225 path=[467:0-224]

AAATGATATGACTGAGCAGGGGAAGGCTACTGTTACAAAACCAGAAATGTCTGTGCTGCA

GAACAGGAGGGAGAAGCCTGCAGCCTGTGTGACCTGCTGAAAGTACTTGTCACTGGGATG

CAGCAAAGCACGTTTAGGAAACATCCCAAATGCCCAAGTAGACACCCTACGGTGCCAGGC

TGCTGAGAAGTGCCAATTCTGCAGCAGCTCAGGGCCCCTCCGGCA

>c7227_g1_i1 len=244 path=[222:0-243]

GCTGAGCTCTGCCCTTGCAGAAGATGGGCAGGAACAGGGATGGCGTGGTGACCTACGAGG

AGTTCTTGGAGACGTGCCAGCAGGATGTGGACATCATGAGCTCCATGCAGATCTTCGAGA

ACACCATTTAGAACACAGGGATCCAAGGATGGCCGTGGTGGGACACAGCATGAGAGACGT

TGAAAGTGAGGAGAAACCCTTAAAAACGAGGAGAAAACCCTTAATGATGAGGAGAAGCCC

TTAA

>c7227_g2_i1 len=447 path=[867:0-446]

AGTCCATCTTCTGGAAGAACTGCTCCACGTGCTCTGCGGGCGCGCTGTCCCGCGGGGCAG

GCAGTGTGCAGCGTCCCATCATATCATAGATGGATTTCATGATCTCCAGCATCTCCTCCT

TGGTGATGCAGCCGTCCTTATTGATGTCGTAGAGGTTGAAAGCCCACTTCAGCTTCTCCT

GCGCTGTGCCCCGCAGCAGGACGGACAGCCCGATGACAAAGTCCTCGAAGCACAGTGCCC

CACTGCCGTCCGCATCGAAGGCGTTGAACAAGAAGTGAGCGTAGGCACTGGAATCTCCCT

GCGGGAAGAACTGCGCGTAGATGAGCGTGAAGGTCTCCTCGTCCACGTGGCCGCTGGGAC

AGTCGTTCTTGAAGCCGCGGTAGAGGGACTGCAGCTCCTTCTTGGTGAACTTGGTGTGTG

CCTGCAGCTGCTCCAGCGCCTCGGGTT

>c7234_g1_i1 len=219 path=[1:0-132 431:133-218]

AATATCTTTATTGACAACATGGAAGAGGGCACTGAGTGCACCCCCAGTAAGCTTGCAGAT

AACCAGTTGGGAAGTATTGACCTTCCTGGGGGTAGGAAGGCCCTATGGAGGGATCTGGAC

AGGCTGAATCACTGGGCTGAGGCCAATGGAATGAGCTTCAACAAGACTGAGCGTCAGGTT

CTGCATTTTGGTCAGAACAATCCCATGCATTGCTACAGG

>c7237_g1_i1 len=391 path=[369:0-390]

ATGGATAAAATTAATAGAGGCGTATTTACATGCTCACTATTAATTGCATTATTCATTGAA

CTACAAAAATACTTGAACAGACATTTATAATTAATTGGTTAATAAAATAATTCGTCCAAC

CTTTTGTATATTGATTTGTTAATTGATCTTTGAAAGATTTTTGTAGTGAATTGAATAATA

AAATTAACAAATGACATGTAGAAAATTTTCTATTAATTTTATTCATTGTAATTACATTTA

ATATATATACTATATGTAATTCTCATATTTTTAACACTTTAATTATATATTCAATGATAT

ATATTTAAATCAAAGGAATAAACAAAAAGGCTTTAACTATTTAGGAGTTTTATTACTTCT

TTTATTAATTAGTTATACTTGAAATTAATTA

>c7246_g1_i1 len=213 path=[298:0-15 314:16-104 25:105-212]

AGGTAACTTTGTGTTTGAACCCATAGAAATCATCTGACATTCCAATACAGAGAAAGCCAA

AACTGGAGATTGGCACACTCAAAACTAATTCATATCATGTATCTCTACTGCTGTTGTCGT

ATTCTGTCCTGGAAAACGGCGATGACCAATCACACAGTATCTCAGTACTGACGCTGCTGC

ATAATAAACAGCTGTGGTACTGTGGCCCTGGGC

>c7246_g1_i2 len=321 path=[717:0-123 314:124-212 402:213-320]

TGTTTGAGAGGGGACTGGGAAACACACAAAAGCAATTTCACACCGAGCAAAAGGGCAAAG

AATACGAGGAGGTAGAAAATAATACTGACTTCAGGCTGAAATTGCATACCGGGTAACAGC

AACAGAACCCATAGAAATCATCTGACATTCCAATACAGAGAAAGCCAAAACTGGAGATTG

GCACACTCAAAACTAATTCATATCATGTATCTCAACCTACAAGTTGTCTCGGTATTTTCT

TCTATTGCCTTGCTGAAGGAGGGGTGTGAGTGTCACTTGATGGGCACCTGATGGGTGCCC

AGGGCCACAGTACCACAGCTG

>c7253_g1_i1 len=473 path=[451:0-472]

GAGGTTTAGGAAAAATCTTAGAAGTGTGGGGATCACTGGTGTCATGGGATTTCAACACCT

GTGGGATCCTGAGAAACTAAGCGAGTACCTGAGTCAGGGACGGTGCGGCTTAGAAAGATC

TGAGGAAGAACGGCTTATTTGGGGCTTGGCTCATGCTCACTGAACTCTATATAACACTAT

TCTGGAGAGAGAGTTTCCAAACCAAGATCCAAACCAAAGGTGGAAGCCTCCAAGTCAAAC

CCGGTCAATCACAGGAAGCACCAGTAACAATATCAGTTGCCCCTGTGGAGGGGAAGAAAT

GGAAATGGGTGTCCTTGCGTCAAGAACAGAAAAAAGAAGCAGCAAAGGCCCTCCCCAAAA

CCACCATCACCGAGAAAGTCAACATAGAAAACTAAGAGACGCAAAGAGGAGAGTGAGGAG

GAGGGAGTCACTGTCACCACTATTCATCGACTTCTTCATGTCTTCTCCATGCT

>c7254_g1_i1 len=272 path=[319:0-210 529:211-271]

TGTTAAAATTCCTTTGTGAAATTCCAGCTATGGGAATGCTTGGCCTTAAGCCCAAATGCT

CTAACAAGCCTTACATGGGACTACACGCTCACACCGATGCAAGACATGGCAATCCACTAA

TGGGGACTGGGAGAGGCACTCTTCAGCAAAGATGTTTACTTTCTGCACTGCTAGTGAAAA

ATCTTAAGTCAAGTCTAAGATAACTGCCTTTAATTCAATCATTTTCTGGGATCTACTGTC

AGAAAGAACATCCCACGGCCTACAGCATGCTG

>c7254_g1_i2 len=245 path=[319:0-210 25:211-244]

TGTTAAAATTCCTTTGTGAAATTCCAGCTATGGGAATGCTTGGCCTTAAGCCCAAATGCT

CTAACAAGCCTTACATGGGACTACACGCTCACACCGATGCAAGACATGGCAATCCACTAA

TGGGGACTGGGAGAGGCACTCTTCAGCAAAGATGTTTACTTTCTGCACTGCTAGTGAAAA

ATCTTAAGTCAAGTCTAAGATAACTGCCTTTGATTCTACCTTGTCCCTCTAGCAGCATGC

TGTAG

>c7256_g1_i1 len=264 path=[396:0-263]

TTGTGTTTGACGCGACTTTCTGCTCTCTCTGCAGTTTGGCACAGGCTCTCTCACTTTATC

CTGCTGATATCACAAGTTCTTAGTTACCTGATCATGAGGAGCACTCAAATGCACCGCTTG

TGTCACGATAGAAAGATGGGATTCACTCCAGTAAGGTAACATTGAATTTAACAGTAGTTG

AGATAAGATCTCTTCATATTTTTCAAAACAGAAGAGTGGAAGAATCATAGCAGGAATACA

GAAGTGCAGATACATTAGTTAAGG

>c7260_g1_i1 len=223 path=[201:0-222]

AGCACCTGGGAAGAGGAGGCACTCCCACCACACAGAGCTTTAGCTCTCTGCTGAGGTTCA

AGCAACAGAGTTTGGGATTTGGAAAGGGATCTTTGCTGGATATTCTCCCACATTGCTGCT

GCACACCCAGCCTGGCCTCGGCAGGATTTGCTTTCTGAGTCTTCTGCTTCCAAGTGAAGC

CTCTGCGATTTGGGATGTGCGGAGCTCAGCACTGGCCTGCAGA

>c7262_g1_i1 len=237 path=[123:0-176 25:177-236]

AGAGGATTTTATCCCCTGATCATCAAAAGACCATGAAAACTTCTTTGTCTCAGCCTGGAG

TTGACCATCGTTATTTGGAGCTGTGAACAAAATGCTTCATTCACTAAAACAATTAAACAA

GTTGTCTCCTGCTGCTGCTGTTGTAACACCAAGGTAAGGCCCTACTGCTACATGGGTGTG

TATGTAACAGAGAGGAGTATTTGGGTTGACTTTAGGGTGAAGGCTATCTCCTTAGCA

>c7262_g1_i2 len=207 path=[123:0-176 299:177-206]

AGAGGATTTTATCCCCTGATCATCAAAAGACCATGAAAACTTCTTTGTCTCAGCCTGGAG

TTGACCATCGTTATTTGGAGCTGTGAACAAAATGCTTCATTCACTAAAACAATTAAACAA

GTTGTCTCCTGCTGCTGCTGTTGTAACACCAAGGTAAGGCCCTACTGCTACATGGGTAGG

TAGGTAAGGTCTACCTGCTGTGGTGTG

>c7263_g1_i1 len=223 path=[201:0-222]

ACGGGAGCTGCACTTCTGGTTGTGGAGCCACATGGATGAACTTAGCAAACAGGACCCCCA

GCTCCCAAGGCTGGTTTCTGACTGGATCAGCACTGACCCAGAAAGGGTCTTTCAAAGGTG

TTTCCTTCCAGAGGGAGCACACCTGGATCAACTCTTACTGATCATTAAACCACAGCCTTC

GCTTCTGGCCACAGAGTCACTCCCCACAGGTATCTGTGTCAGT

>c7266_g1_i1 len=214 path=[1:0-213]

GGGTACAGCAGACGTATCTCTGAGTCCATGGGGAGACAAGTTGCCACACATTGATGTAAT

TAATTTAATGAGACGCAACAGGAAAAACAGCTATAGCAATAGGCTTTCTGCTTTCTCATT

GCTGTTGAAAGCTGCATTTTTCAGGAGCTGACCGTGCAAACAAGGTATGATACATGCAAT

TTCACCTAAAGACTTACACAAGAACTGCTTTCTC

>c7269_g1_i1 len=328 path=[351:0-327]

AAACACTGAAGGCTTAACTAGATTTAGGGTCCGGATTCTTGAAAGACACATAGCAAATCT

TATTAAGAGCTTCTCTGTGCCTTCAGGTAACTGCTAATGAAGACTTCTGAAACTGTTAAA

ACGTGATTTTGCAACTCAGTTCAGCAGGGCAATCTGTTACATAAAGCATTAATCTGTAGA

TTTTAGATATGGACTGCTGTGGTTGTCTGGTCTGCCCTTGTACACAGTGCTTGCCACAGG

ATTTCCCTGCTTTACGTCTAACAGCTCTGGCTGCGCTAAGGAGGCTGGTTTTGTTTGTTA

TTCCAGCACTGGCACTTGCTGCCTCCAG

>c7271_g1_i1 len=241 path=[449:0-67 25:68-240]

AGCGGTGACGGCGCAGCTGCCCCACGTCGGCCCCGAAGCGCTGCGCCGTCACCGCTGTGC

GCCGCCCGCCCCCCCGCAGCTCCTCCTGGCGGTCCCGCAGCTGCGCCAGATCTCGCGAGA

TTTCCACGTCGAGGCTGAGCAGCGCCAGCAGCAGCCCCGCCCACAGCAGCATGGAGCAGC

CCAGCGCCCCCCCCAGCAGTGGGGCTGCCCGGCCCCAATTCATGGTGCTTTGGGCGGGGC

T

>c7272_g1_i1 len=297 path=[269:0-296]

GGTAAATGGAATGTGTGTAATTGAATTTCTATGCATCTGGTGGGTATTGATACTTTTTTC

CATGCATCTATATAGGATATTTTCCTACAGTGTTGAAAGTGTCTTTCAAGTGAAATGCCA

TTTCCCCCTTTCTACAATTCCTGGAAGCCACTTCAAAAGTTAATACATGTGACAGTGGTG

TAAGAGAATATCCACACAGAAATGGGGAAGCAGGAGTTGATTTTAACCTTAGCTTTAATA

CTCACTGTCTTATCTTTGCACATCAGTCAACTGCTCTTTTCATTTCATATAAGAAAA

>c7284_g1_i1 len=318 path=[552:0-317]

AGGGTATATACCTACTGCAAAGACCACAGGAGGGTGTAAAGCAAGCAGTAGCCATGGCTT

CTGGATAAGTTTAGCTGCTGTGCATCAGTGGCTGCATTGTGGCACTGCAAGTAAAACAAC

ATCCTAACTCCACAGCATATTCCCACAGATGTCATTTGAAATGAGCTTCTTAAACATCAC

AGAACTTGTGGTGTGTAGATGGGCTAGTAGAGAGCAAATGCCAGTCTTCAGTTCTCCTGT

GGGAAGCTGTGACCCCCCCAGGTGAGGACCTCACCTGTGCAGCATGGCTGCTCCGGTGTG

CATGCTCAGATCAGGCTG

>c7286_g1_i1 len=214 path=[155:0-213]

AGACCATCTCTTCCAATAGCCTCATTGTTTGAGATGCAATATCCAACCTGAACCTTCCTT

GCTGCAGCTAAAGCTCAGATACCTTTCTGTCATACATGTCAACAGCCATGGAAAATAGCT

TATAAATGACCTTATTCAATTACAGTTAATTCCCTGGATCACAGCTTTTTATAAATTATG

TTTTCCTTCAGAAGAATTCTGTTCTCTTTTTAAG

>c7287_g1_i1 len=201 path=[305:0-200]

CAAAACATGTTTCAGGTTCCCAAAGGAATCAGCTTTTTGACTAGGGCTGGCTGCAGCAAA

CTCCCACCCTGTTTTGAGGGTGGAGCTGCAGCCTCCAGCACAGCTGCTCCAAAACCAAGC

TGAAGCAGCCGGCCCCAGCTCAGCGGTGCAGCACAGAGCTTCGTGTAACCGGCTGCTTCA

GCACAAGCAAGTGGTACGAGG

>c7292_g1_i1 len=244 path=[1:0-196 198:197-243]

GATCCTTAATTAAAAGGCAGGACCGTTCATTATGCAGTTCATTATCTTGTTGACACTCAG

AATATTGAAAATGAAGACTGCTTTTGTGCTTCTGAACACTAGAAAGCCTGAAGCGAGGCT

CCAAAGGCTAATTCTCCCTGCTGTTCGCTGAAGTAGCAGCAACAGAAAATACAACACCAA

GCAAAAAAGACAACAGCAGGCATCACCAACAATCTTGTCCTGCTCAGAGACCACCACAAT

GAGA

>c7296_g1_i1 len=243 path=[221:0-242]

GGTGGCTGACGAGAAGGCAAACGTGCAGCCTTATCTGCTTTTCAGCACCTTCGATGACTG

AGGAATAGACAGGTTGCTCCAGAAGTAACGCCTCCTCATTATTTATTATGGAAACTGGAC

TTTCTCTGTTGCAGGAAAGTATTGTGACTTGCTCTCTCAGCCCATGTTGGTTCATGCGTT

GGTAAAACCATCCTCAGTGCCATTAAACTCCTCCAGGCTGTGCCCGAGTGCCTCAGGAGG

CAC

>c7303_g1_i1 len=220 path=[53:0-219]

AAACTGTGGAGCAGTAAGCACCAGTCTGATACTCATGGCTGCTGAAGAGGGTATGAACCA

CATCAAGGAGAAAATGCAGCCTTCAGCCCTAAAAAATCAATCAGTGCTACCACCTTCTGC

CTTCAGTGGCTCCTCACCCTGTCCTGACAATGTTTCATCTTCTGAACAAAGCCAATCTTC

TGCCATTCTATTGAAATTGTTAGCAAAGGGACTGATGCTG

>c7306_g1_i1 len=417 path=[1:0-370 372:371-416]

GAGATTCACACATTGTTTTGACATTAACTCTTAACTGCACTGTCAGGCTTGGAGTGTCAT

TTAGAAATTCTGAGTTTGATATCCATCATCCAACGTAATACTGGCCTCTTGAGTTTTGCT

TCTAATCATTAGTGACATGCAGGTCACTAAAACACATGAGGTCAATAAACTCTAATAATG

CTTGCATGTGATATTCCTTAGTCTCAGGGTAAGTTATTAATTTTATAGGTGTTTTATGCC

AATAGTTGTGAAAGCAGAAGAGGTCCATAACCTGCCATGCTTCTTGGAGTTTATCCATTG

CTATTAGATAAGCTAGCTTTCAGCTCAGAAATATGGAGGATGATTTTTATTGTCTCAAGG

TGAAATAGTTATAAAAATGTCTTTTTCATGAGCTAATTCATCATTCTGGCACTAATG

>c7306_g1_i2 len=418 path=[1:0-370 464:371-417]

GAGATTCACACATTGTTTTGACATTAACTCTTAACTGCACTGTCAGGCTTGGAGTGTCAT

TTAGAAATTCTGAGTTTGATATCCATCATCCAACGTAATACTGGCCTCTTGAGTTTTGCT

TCTAATCATTAGTGACATGCAGGTCACTAAAACACATGAGGTCAATAAACTCTAATAATG

CTTGCATGTGATATTCCTTAGTCTCAGGGTAAGTTATTAATTTTATAGGTGTTTTATGCC

AATAGTTGTGAAAGCAGAAGAGGTCCATAACCTGCCATGCTTCTTGGAGTTTATCCATTG

CTATTAGATAAGCTAGCTTTCAGCTCAGAAATATGGAGGATGATTTTTATTGTCTCAAGG

TGAAATAGTTAAAAAAATGAAGAGTGCAACAAACATTAGTGCCAGAATGATGAATTAG

>c7306_g2_i1 len=281 path=[879:0-280]

TATTCATCTATAGTTCTGTATAGGAATTACATATTGGAGACATCTACAGCTCTGACATTG

CAAACACTCTGATGAGAAGACTTCATACATCCAGAGCTACTCATGTGCTTGGAGGTTCTG

CTCACATGTTAGAGATGAAGGCCTGAAAATATTTTTTTCTTGCACATGGCACAGTAAAGG

TGTATTACTCTTTTAAATTAACTCGGTCTCCTGAAATTAGTAGTACATCTTTGTATTTTA

TCATTAATGAAGCATTTTTTCCATTCACATGCTAAAGTAAA

>c7309_g1_i1 len=236 path=[1:0-235]

TGAATTCAGGCTCAGTGCTTTGCTCTGATGCAGAAAACCAAGCTATGGTGAGGGCAAACT

GCAGGAGTGCTCAGCTTATTTGCACAGCTCCTTTCCATCTGAGTACCCTTCAGGCATTCC

AAGCCCACGCTCCATCCTTCAGGAGCATACAACAGCTCTGTGAAGACTTCCTCAAATTTA

CATGTGATACTTGCCACAACTGCTGTTCTTCTGATGTTATGAGCTGGTGCTCAGCG

>c7316_g1_i1 len=285 path=[1:0-58 604:59-60 607:61-284]

GCAGCAATGGTTTCAGTAGCAAATCTAGCTGAGATAACTGTGTGTGAATCAGTGCCTTTT

TGTTTCCCCTCCCCCCCAGTGCTTCTTTTTTTTTTTGCCTCTCTGCCTTCTAAATGATTA

TCTCTAGCACCTCCAGAATTGTTTGGCCTACTAATGGGTATATTCTGAAGGAAAGCATTT

CATGAGGTTACAAGTCACAGGTTTTTCAGATTACACTTAAAACATGCATACCTCTCTTCA

ACATTACACACTTGACTTCAAGACATATTCTTTGCAACCCCACTT

>c7322_g1_i1 len=247 path=[155:0-246]

GCATTGTTCAGTGCTTCACTAATTTACTGAGAAAAGGTGTACTGCAAAAGTATATCTCCT

TTTGCTTGTTAATATTAATTTCCACAGCTCTCTGAGGTTCTAAAAATGGCAAGAAGTTAC

TTCAGGAAACATCTTTTTTGTTTAAGTAGACACCCTTGACTGGTGCCTACTGCATCTCAC

CAAAATTCTGTGATGGATCATTTGGCATCACCAGAAGAAAAAAAGCGTATAGCGATTTGG

GGTATTA

>c7323_g1_i1 len=217 path=[1:0-216]

TAGGGAAAATATTTTACTTAAGGTTTACTCAAAAACATAATGACTGTCAACAGACCTGCT

TTTCTCCATACATACTGGAGATTTTCTTCATCCATACTGATTCTGAAAGTACACTTTCTT

CTTGGCATCAATTATTCAACAGTATCAAGTCTATTTTATAAGCTGTGTTCACTACTCCCA

CTTTTTTTTGTTGTTATCATAGAATCACCAAGGTTGG

>c7329_g1_i1 len=315 path=[561:0-314]

AGTGGAATGGACTTTGGGTTTTCCAGAAACCCTCCAGGACTGTGTTTGCAAGAATAAAAA

GAAACTATTTTTAATGCTCTGTCTCAAATGAACGGTGTATTATTTGAATGAACCCAAGTA

GGCACCAGTCTATTTCCTCATTTAAAGAAAAAAGACAAGCTCACAGACAGAAAGATGTGA

AAAATGATAGTAAAGGAATTAGAAGTTACTTAGCATCTCACAGAAGGGTGTGTGAAATTC

TCCTTTACTTAAAGCCTTTCCATTTTTCATCAGTTATTGTATGAGGTGAGGACATATTGC

CCAAAACCTTTGAAT

>c7330_g1_i1 len=203 path=[1:0-202]

GAACTGCAAAATCCCTGGAGGCTGAATTTGTCCTTTTTCAAGGCTGGCTGTCTTCATCTT

AAATATTATTTGCTGAATATTGTGGGAGAAAAAAAAACATAACTTCAGCTGTGATGTGAA

CTTGTAGGTTGAATTTGGATGTAGGACTCTTTGAAAGCAAAAAATGAAACCTTTTTTCTT

TTCCCACTCTAATATTCACCACT

>c7331_g1_i1 len=444 path=[333:0-443]

ATGTAAAAAACAAAAACAAAAGAAACACTGCAGGAAACATGGGATTTCAGAAACAAACTG

GGAGACAACAGCTGGCATAGGACTTGAATCCTGCCCAGCAGATGCATATCTGTCCAGCAC

ACCTGTCTAGACAACTCCTAATGTCTACTCATAGCTAGATTACAATTTCACAACTTTAAT

AGTGAAACATTTCCCCCTGCCCCCAAAATCACAGGGGGATATAATCAACAAGAACATGAA

AAATCCTGTAAAATTACTTCAAAACAGGTCACTAATGTGACAGCATGTCATTTTTAAAGG

AGCTCTAAACCTAAGCAACACCACCATCATAGTGACAAAAGGGAAAGAAACATTATTACA

AAACACATCATTTCCGTATGTATCCGTGTCATCAAGCTGATAACTAAACTTTCTGCAGGT

CACAATTACTTTGCTAAATTGATG

>c7340_g1_i1 len=360 path=[1:0-359]

TGCAGAACTCTGATACCTCTTGTTTTGCCATAGTGCAGAGCTTTATTGGCAATACTTTTT

TTGCCACGATCCCACCAAAACAGCCCTTGGCAATCTCCCTGCAGCAGAATATTACTCCAT

TTTCCAAAACAAAGAGATAATTCAATCTCTATGGCAGCTCCTGTGAGTGGATGCAGCGGA

GAGAACAAAGAAGATGAAATGCAGTGTTTATTCACTATGGCTTAAACCTGCCAGTCCCAA

GGCACTTGGTGATGCACACCTTCCTCACCCTGTTGATGTTTTTCTGTGTTGCTGAGGTGA

TCCCTTCGTCCTATCACTGTTCAATCCAGATCAACACCATTTGTTCACTCCAGCCCCATA

>c7344_g1_i1 len=309 path=[1:0-308]

AGGCCATTCTGTGATTCTATGAAGTGCAGGTAAGTGTGATTTTTTCTTTTCTGAAGTACG

ATTTGTCACAAAGCTTACTCTAACACAAGTAGGCTTCCTGGCATTTTGTCATCTGTCATG

ACATGCCTAAGTAGATCTGTAAAGATAGTAAGAGATTTGGGTATCTGACTATCTCCAGAG

ATCACAGCTTAAGCATTATTTACACTGCAAAAGCACCTAAGAAAGTTGTCCTCCTAATCT

GGGCCAATATACCAAGGGCTGTACAGACACAGGTCTCCATTCCCGACACCTCGCAGGCAC

GGCTTGCGA

>c7344_g2_i1 len=260 path=[573:0-259]

ATGTGGCAGAACGTAACGCTATCTTAACATTAAACATTAACAATTGTCATTTTACATTCA

TATTCAAGTAGCAGAACATGTTTCTATTCCGGCATCTTTGTAGCCAGTACTTACCATGAG

TAGGATCTGAAAGCTATTAACTGTTACCTTACCATCATATATCTCAAAGGTAAAACTAAA

GAACAAAGCTGAGCATCATCTCCCCACACCATTTCATTCACACTGAGCAGCCTCAGCAGC

AATGCAGTATTTCATGTAAG

>c7349_g1_i1 len=209 path=[475:0-76 551:77-208]

ATCAAAACACCATCTCAAAAATAGGAGCAATGTTCAACATACCTGGTGTAAAAGTTAATA

AAAAAAGAAACCAGAAAAAGAGATGAGATGATATCCTTATGCCAACTGGACAAAAATAAC

AAATGAAAGATACAGATACAAAGAAATGCTCAGGCAGGAATCCTTAGCACAGTGGTAAAG

CAGTATGCCCACCCAATGTAGCCAATTTT

>c7349_g1_i2 len=208 path=[475:0-76 183:77-207]

ATCAAAACACCATCTCAAAAATAGGAGCAATGTTCAACATACCTGGTGTAAAAGTTAATA

AAAAAAGAAACCAGAAATTGCTTCTATATGGAGAAAATAAATATGCAAACTCTACTGGAA

AGACCTGATGTATTAACACGTACTGGTTACTCATCTTGTTCATTTTTACTATCTAACAAT

CATAAAATTGGCTACATTGGGTGGGCAT

>c7351_g1_i1 len=207 path=[185:0-206]

TGCGTGGCTTCCAGCCCTGCTTTGGGTGCAGGTGTCCCAACACATGTGCTGGAGCTGGCG

GCGTGCTGCTGGCAAACAGAACCTTTGGCTTGTATTCAGTGCCTTCATCAGCAGGGATTA

GTGTGCATTACATCCAGGAAGAAATTAACCCCAGAAGCCTTTCCAGAGTGAAAAGCTGGA

GGGCTTCTCTGTTCAAGGCAGTGAAAA

>c7352_g1_i1 len=226 path=[1:0-225]

TGACTTCCTGTGGCTCTGAGCTGCTCTTGAGGGTTTATGTTCAGCATCAGTCATCTATCA

TTACCCTGGTGGGACAGGAGGTAAAGTTGGATCACGTAGGGCACAAGGAGGCTCTCTTTC

TGCTGAGGCAGGCAACAGGCACAGGCCAGGTGCTCTGACTTCAAATGGGATAAATGCAGA

TACCAGTGCTGAGATAGGACGTGCCAAGAATTGCCTTAGTCCTGAA

>c7364_g1_i1 len=257 path=[268:0-224 519:225-256]

GCTTACTCCATTCAAAGCTAAAATAGCACCCACTCCAACTCAATTCACTTAACTTCCAGA

TTTGTTTTTAAACTGCTTTTGAATTCTCCCCTTACTAATGAAGCCCAACATAAAAGCCAT

TTCCTCAAAACAATGTTGGGATTTATGAACAGCTGTCCAAGATACTGATTTACAGATAAC

CAGTGATATACACATTTAATCTATTTCTCACATGCAAGAAGAAGAATAACCACTTAAATG

CCAATCTTGGGCATTAT

>c7366_g1_i1 len=204 path=[19:0-17 37:18-203]

ATTGTGTTTGACAATTCTCACAATGTGCAGGGTTTTGGAATTCAGTATGCTGAAGCGCAG

CAGTACCTTCCATGCTCAGGTCACAGGTGATGCTGTACTTACCTTAAGTGGTTCTAAAAT

TTTACTGAGAAACAGAACGAGCCTACATACCCAGACACATGTTACAAAGTACTGCCACAG

TGGGAAAGAGATCAACCGGTCAGA

>c7366_g2_i1 len=245 path=[695:0-208 933:209-214 909:215-244]

GTTTGTCTACAACAGCCACATTGCTACTAATCTAATCAAGGTAGGATTAGACAGCCATCA

TCATGCAGGTCTTAGAAAAATCAATTGTAGCCAGACCATTACACATTTTTGTACTAACTA

ATGGCAGCTCCCGCAGATTCTAAGATTATCTTAAAATAGTCAGAGCTTCTCTCCCTTCTA

CCAAAACTTCAATCCTGTTTGGTCTCCATCTGTCATTTCAGTTAATTCCATCCTCTCAAA

CACAC

>c7368_g1_i1 len=217 path=[1:0-216]

ATCTATGCTTTCCCCTTATGCTCTCTCTAGCACTAAGGCTATTTTTAAGCACCAAAGCAT

CCGTGAGCTGTGCAAGCTTCCCTTTATTTCTAACATCTATCCCCAGTGTTACGGGGGTAT

TTTAAGTGAATTTGTACTAATTCTCTTATTTAAGGACTAAAGCTTGGTGTGCCTCGTTTT

TCTGCTGGTTTTGATGCGCGTGCCTCATTTCTACTTA

>c7372_g1_i1 len=292 path=[197:0-291]

CATTCTGTCCATGTTTTGTCTAGGGCAGCAGCCTGAATTTATGCCTACGGAAAAATATAG

CAAGGCCGTGGCTTGCCGGAGGGAGCGCAGTTAATTGCCTTAATATTTGCTAGTTTGGTC

TGTAAGGCATTGACTGTTTTCTTTCTTGGTGCTGTATATTTGGCATTCTGCTGCACACGC

AATCATGTGAGCAAAGCACTTTTTCTTCCTCAGAACAGGAGCTGAAATTGTTTCAGTTTG

GTAGTCTGAGGAACTGTTTGTATGATCTGAAGACTGGCCCTCAAACACAAAG

>c7375_g1_i1 len=253 path=[147:0-138 516:139-252]

TAAGGATAACAAAAGATAAAATAGAAAACTGACAGCAGACATTTCATTAGCAGACTTGCA

GCACTCAAGTCAAGCAACACACAAGTTTATACTTTTCACTGTGAGGTTATAGCATAGATG

AATAATTTGAAAGTGCATTAGAATCAAAAGTTGATCATTCGAAATTACAAGCAAGTAAAA

TACACTCTAGAAGCAACTATTTCAGTGAATCCTTCAACATTATCACATAACTTATGGGAA

CCTCAGCAAAGCA

>c7377_g1_i1 len=375 path=[1:0-197 199:198-374]

CAGGCAAACATACCCCTCTGTGAGGTAGGTGCTTGTGTATGAATGCCTCTGTTTCCTCAT

TGCCATCTATTAAAGATTATCTCTCTATGAGAAAAGTGCACCATTTCCCATGTTTTCATG

TGTAATGCCTGTGATGTCTGCTATAAACATCATGAAAAATTTCTCTGCCCCTCCACTTAC

ATAGGTATATAAAAGCCACTGCTTTTCATCTTTTAAAGCACAGCAGCAGTGTTTAGGGCC

GTGTATCCTGCCATTGTTCTGGGCTGGGCATCTCACTGAAGTCACTGGGAGAAATGACTT

CAGGATACGGACCTATCTAGTCCATCTTTACAGCGGCCCCAGTAACATCCATATTTTACA

GATGGAAAAATGAGG

>c7387_g1_i1 len=273 path=[251:0-136 388:137-211 463:212-272]

TGAAAGCCCCAGATAAACGTTCCTGGAACTGGAAGCTCAATTTAAGATGACTCAGCGGTC

AGCTCGCATTCTAAATCCCCATCACATCGAAGGAGACATGATGTGTGCCTTGCTAAACAG

CATTTCAGATACCACAAAAGGTTCCGATAGTGAAGAGGATGACGATACCAAACTAACAAT

AAAAAGGAGTTTGGAAGAAACAAAGAAACAAGAAGAGGATGATGCAGAATTAGAAGAAGC

TATCAAGAGAAGCCTTGAGGAAATGTAAATGAA

>c7388_g1_i1 len=491 path=[47:0-379 23:380-468 580:469-490]

TCAGTGAAAGGGAAAGTAAAACCTGTAATACCAAGGCAAACAAGGAACACCAGAAACTAT

GGTGACAAGAAACATACGTTAGGAGGGTTAGACTTAAAACGCATCTCTATATGTTTGAAA

TCATCTAACTGGCATCTCTACTCTGACATTTTAGAAGGCCTGTGAACAGCAAGCTGCTGC

TCAAGTATGAAATACTTGTTTCAGAACCAAGAACTAAATATTTCTCTTGACAGTAGCTGC

CTGCCCAGCTTTTCCTTTGTTGTTCCCTGGAAATTGTTTTGATTAGTCCACACTGGAACA

TCAGCAGCAACATCCAGTTAATTCAGCATTCACTCTGCAGTGACATAATGTTGATTGCTT

ATATTGCACTCACAATTTTGACAGTATCTTTTGAAAAGAAGCTGTAAATAAGGGCTGATA

GCAGAATAGGGCTAACATTTATGTTTTACATGTTGCCCACCATTTAAGACCTCATGTGAA

AGAAGGTGATC

>c7398_g1_i1 len=210 path=[188:0-209]

GCTCTTTCTCCCTCCTGGTAACTTCTTCCTGCTCCCGTTCTGCAGCCTCTCTGAGAGGAT

GGCTCCCAGCACGTCCTCGGGCAGCACGCAGCTCATCAAGCCCACCTCTCCTGCTGCACT

GTCTCCAATGCTCAGCCACTGCTGCGTGGGGACGCAGGGAAGCACCAAGAAGCGATGAGA

GCTGGAGGAGCCCTGTAAGGGGTGCCCCAT

>c7399_g1_i1 len=355 path=[333:0-354]

AGAGTGGGAGTGTCTGATGCAGAAATGCTGGATAAAAGCACCATGATGAATCTTTCAGTG

TGTAAAACCTTGGGATAGAAAAGGAAACAATCATTCTCCATGGTTATCCAAAGCAGGCCT

GGAAGAAATAGGTTTCTTACAGTAGAAATACCAGACATTTATCATCACCGCTTCTAACCG

TAGAGAGTTTTCCACCCAAATGCCCAGTCTTGGCATATCATGAATTTGGGTCAAAGAGAA

CTTTTATAAACCTTGTTAGGCATCTCTGGGTGATGATCTAGCAATACTTGATCAGTAAGG

CCCAAACTCATTTCTTGAGTCCTCACAGTTTATTTGTTATTCCCGCAATGTTTTA

>c7400_g1_i1 len=243 path=[155:0-242]

GCTTTTATAGTAGAAGGAAACTACCTCTGAAGAAATCTGTGGTTTGGACACAAGGGTTTG

TCTGTGCTCAAGGTCCTGTCATTTCACTTTCACTTCAACGAAGCACGTGTATAACTTATT

GCAACAATTTGCATGTGCTTTTAATAAGGCAGTAAAGATGAGGTGTGCTTTACACAAACG

GTGTTAAGTGTACTCTTGTTCTGTGTGTATAAATATGCAAGCTGAGGTTGTCTCTACAAG

TGA

>c7402_g1_i1 len=266 path=[1:0-265]

TAGAAGAGGCAATGACGTATACCTGAGTCACTCTGGTGACCCGTACTTTTATAAGGGCAA

GGATTAAACTCTTATTTATGCTGCCAATCATCTAGAAATTCTGACTTAATTTTTGTCTTG

TATATTTTGTCCTCTATGTCAGGCTGATGGTACCCATAAAAGCAGGGTGGTTCTACTGCC

CTGTGCTGTGGTACCATGAGAAACCAGTAGAAGGAGCAGAGAAACTGAGAACTGGAGTGA

GATGGATGCCATAAGGATGCCAGCAG

>c7402_g2_i1 len=288 path=[488:0-287]

CCCAGGCCATTCTATGATTCTATCTATATATACATATGTAGATTAACATACATTTAAAAA

GCTATTCAGCAATCAAATACTCACTTTTGTATTTAGTGTGAGAGCAGGAGCATGTATATA

TCTACAATTCAGATACTGGCATGTAAAGATCATGTGAAGTTCACACATAGAAGAGGCAAT

GACAGCACCACTGTACTTCCTTACACCCAATAGCAAGCTTCTTGTTTATGAGCATCCTGC

TTTAACTTGTCACACAAAAGCACTTCTTGTGTCTGCTGGCATCCTTAT

>c7407_g1_i1 len=281 path=[259:0-280]

GTACTGCACATCACGATTCCACTTTCTAGCACACTTTGTTTTCTGAAGTGATTAATAATT

TAAAATACTGTGGAATTTTCTATCAGTATTTTACTGTAGATGTTTTCTTTCAGAAGTGAC

TGGCATCCAAAAGGTTGTAGAAATTCACTTTGCATTTTGAAAGACAAATACATTCCTATT

AGTGCACACTCACTTGCGAAATAGCCACTACCAGAAAGCAGCCTGAAGCAAAAATAAAGG

CAGTGAATATTAAAAGTAGTGGATGTCAGACTGTGACCTGC

>c7409_g1_i1 len=257 path=[389:0-256]

TGAGCTTTGTCCTTTGTTCTGGCTCCAGTGTTTGTGTACGACTGAGCCACAAGGCAGCGT

GCAAGCACTGAGCCAGTCCAGCTGGCCACCCTGCTTGGCTTGCTGGCTGGAGGCATGCAC

CAAACCCACAGCCTGCTGACTGCATTACAGCCAGCACTGCAGGACTTTGAAGCAGGCCTC

TCTTCCCTACACCCCTTCCTTCCCAGAGAAGCCAGCACGCGTCTGGCAAAAGGAGTGAGT

GCCTACAGCTCCATCGG

>c7412_g1_i1 len=284 path=[588:0-283]

AAAAAAAAAAAAAAAAATTCAATGATAGAATATATAAGAAAAATGATAAGGTTAATTATT

ACATTTATTATTTCAAAAGTATTATTAATATACAATTAAAAATACATTAATAGTATCTCA

TTTTCACTTATTATTTATCAATTCAAAATGAAATTTTATAATTATGTTATTTTCTTTTAA

TTTTTTTCTCTTTAAAAGTTTTTCATTAATAAAACGATTAAGTTTTTCCATTAAAGACTT

CGAATAAATTAAAATTGAATCAATTTTTTTTTTTTAACTTAAAA

>c7415_g1_i1 len=210 path=[1:0-209]

TACATGATCATAAAACTCTAGCTGGTAAATTTGCTCTTCCCTCACTTTTCCTCTTTCTTT

TGTGTGAAGTGCAGGGGGTTCTGTTACTCCTATCAACCTGGAATTTAAACTGTAAGAGTA

ATTAACAGTGACCCGAAGGAAATGTTATGGATGTTTAAAAAGCTGGAGGAATTCACCAGT

TCTTTCTCACCAGCTCTGCCTGGCATGTCG

>c7415_g2_i1 len=218 path=[570:0-217]

TGAATCTCTCAAGTTAGTTTGTATTGATTCTTGGTGGTCACCCTTCAAAGTTACACAAGA

TGTGAATAAGCTTTGCTCTTTTTCACCATTTTAGCAGATTTAAGAAGGAAGTCTGATGAG

ATCTGACAGAGCAGCTGAAACTAAAATCCAAACAGGAGAAAAACACTCAAAATAGATCTA

GAAATAAGAATTTAACTATATTTTTTAGACTCCTTTCG

>c7423_g1_i1 len=215 path=[1:0-214]

AGGTAATGTGATGAGGAAGGCAGTGGGACACATACACCGTGGTAATTTTACCCTGAGCAT

GCTCCTGATGTACTGCTGGCTCCCCCCTCGATCCCCCATCACAAACACCAGCTCTCCTTC

GCTTGTGGGCACAAGGCACTCCGCAGGCAAGATTGCATCAGCCAAGATTGCACTTCTTTC

ACTTCCCAATTTGCCAAAGGGTCTCTGAATGAAGG

>c7433_g1_i1 len=282 path=[260:0-281]

ATCCTGCCACACAAAGACCTTTGAGCCACTGAATCCTGCCTCTACAATGACCTTTAAAGC

CTGGGTGGAAGTTAAGCTGTTGGTTGTTTGCACTCATCTTCATACTTCCAACTGTTAAGA

CATAAAACACTATTATAGGAAGGCTGGAAGAGCCTTTCATCAAGCATTTGCTCACATAAA

GTGAGGAAGACTGAATTCCCAAGATAAGGACAGCTTAGTTAAGCAGCTCACTCTGGCAAT

CTTGTAAGAGACTTAACATCCAACCGTTTCTACATGGGTACG

>c7434_g1_i1 len=338 path=[530:0-337]

GGGGGGGGATTGACTATTGAGTGATAGAGAAGTTGAAGGGAGAGGGAGGGAGACAAGAAG

AGTGGGTTCCTATTGCAAAACATAGTGTTCAAGTGGGAACAGTTACCTCCATTTTGTTTA

AATACTGATGGACGCATGTCTTCTGAAGACAGCAGATGCCATACAGTTCTGGAGCATAGG

ATGCAGCTGAGATACCAAACATGTTCTTGTGTAAAAGAAGCATCAAGTGGATCACTGAGG

AGAGCCTCCTGCTCTTTCCAGTGGTTTGGCTTGCTCTTTGTCTCTCTGGGGTTTCTGAAC

CATAGCATCTTCTTACAAATTGAGAGGAATCTATTCTC

>c7437_g1_i1 len=248 path=[211:0-247]

GGGTACTATGTCCGTACTTACAAGCTGGAGGTCAGCACCAACGGGGAGGACTGGATGATG

TACCGACATGGTAAAAACCACAAGACGTTTCAGGCCAATGAGGACGCCACGGATGTGGTT

CTCAACAAGATCCACAGCCCAGTGCTCACACGCTTTGTGCGCATCCGTCCCCAGAGCTGG

CACAACGGCATCGCCCTGAGGCTGGAGCTGTATGGCTGCCGCATCACCGATTCGCCCTGC

TCCAACTT

>c7442_g1_i1 len=260 path=[225:0-259]

AGAAAGGAAGAAAGAAAGAAAGAACAAGAAAAACACTTTTGAAATAACCAGATTCACTTC

AATTCCTCGCCTCAGCAAGAGCCACACCGCCCTGGCCTCTGGTAGTTAACAGCTTCAGTC

AGGAAACCCGCAGCTATGGTGCCTGCCAGCCCTTACCCTTCATCTTTAATTGTTTCTCCT

ATCTACTCAGTTAATGTTGGTCCTGCCTCAGTTGGGTTTTGTAAAATAGTTTCTGTGCTT

TAAGTGACCTGTAGTTTATG

>c7446_g1_i1 len=328 path=[1:0-327]

AGACGCAATCCCACCTGGGATACAAAGCAGGCAGCTGCATGCTTTCCCACTGCAATCGCT

ATTACCAGGGCAATGTTATGAACAGTAGCTTCTGTAACAATGAAAAGACCTATGCAGCTA

GTTCATTTTGTCTCTATGGAACAGAACAGAAATCTTTCTCAGATTTGGCTCTAGTGACTC

AAAAGGGCATCATCAGAAAAGTAATGTCATTATAATTGCTAATGTTTTTTATTTTATTAT

CATGCTCAGTTCACTTAATGCTCAAGGGGCTGGCATCGGGAGAATGATTTTGAAAACAGT

TTGAAATATTTCTATTACAATGAAAAGA

>c7451_g1_i1 len=211 path=[303:0-96 212:97-210]

CCATCTTTCATATCAATAACTGCACAGTACATAAGAGCCATGGTGCACTGACTGCAGAGA

ATGGGAAGATGCTTTGTTAGAAGCAGAGCTGTACACTGGTATTTGCTACCATGAGCCATA

AGAGGCCTAGGCATCTAAGCACAAAGCTCAGGGCTGCTGTCCCTTGGGTGTGAGCAGCCC

CACTGTTGGAATCCAGGCTGCAGAGGAGGGA

>c7462_g1_i1 len=272 path=[250:0-271]

TTTCCTCATTCAGCTGAGGAAAATCTTGCAGGGTATGTTTTGTATATGTCAATGTTGTTT

TCAATTAGTAGGTTTTCTGTGACACTTCTTATGAAATAATAGGTCCTTATATCAAATACA

AGGTTAGGATGTGAGAGTTTAATTGTAATACTTAAGTAGCCTAGCATCCACTTTTAAGCT

TTTCCTTACATTTTGTAAAAAGCTAGATTTGAGTGTTAGAGTTCTGGCATGTTTCATTTT

TTAGAAAATGCTACACATAATTCTTGGAGGAA

>c7462_g2_i1 len=235 path=[499:0-234]

GTATTTATGTAACTGAGTATTGAAGTAGGGTGTGGGACTTCATCTGCAAGTTAACCTAAC

CAAAAGCATTTGTTTGCAACTGGATTAAATTATTTTGTTTCGTCACATTCTTGCATTTAA

GTTAATGCCAGCCATAAGAAGGCAGTGGGAGTGATGTACTGGGAAAAAGAGGGAGTTTGA

GATGTGTTTGTAACCAACAGTCTGGATTTTTCCTGTGTCATACTGATTCTGAAGT

>c7464_g1_i1 len=207 path=[1:0-16 18:17-206]

GTAGGCCTTTGGCTAAGCACGACACCAGCATTCCCCAGCAAGGAGTAACAGCAGTGTGTA

GCCTGGGCACCACAGCTAAAGCAGCTGTTGCTCTGCACAACTTCTCTGGAGCTGTTGTTA

CTTTGGAAATGGATTTCCAGGAGCCTGATGTGAACATCGTAGCCACTGAATTATGGTTCC

ACAGGCAGGCCAGCATCCCTTTCGAGT

>c7464_g1_i2 len=337 path=[350:0-146 18:147-336]

CCACTGTAGGCCTTTGGCTAAGCTGAACACAAGAATGAAAATAGTTTGTGCCTTGTCCCC

AGAGCAGCTCAGGACCAGTCACTCCATGCACAGCAAAGAGCAGTTCCCTCGCTGGTCCTG

AACCACAGATACTGAGCTCCTGCTCACCACGACACCAGCATTCCCCAGCAAGGAGTAACA

GCAGTGTGTAGCCTGGGCACCACAGCTAAAGCAGCTGTTGCTCTGCACAACTTCTCTGGA

GCTGTTGTTACTTTGGAAATGGATTTCCAGGAGCCTGATGTGAACATCGTAGCCACTGAA

TTATGGTTCCACAGGCAGGCCAGCATCCCTTTCGAGT

>c7465_g1_i1 len=246 path=[135:0-245]

GAAGTTGCTTATGCAGGAACCTCACTGATGTGAGGTGTCAGAGCAGAGGTTCTGTGGACC

TGGTGGCCTCTGAATTAGGGCTCCTAGCTAGGGGCTTTTTGTGAGGCACTTCAGTTGCAG

CTGAGCCCCCGGGCAGCACTGGCAGTGAGGAAGCAGAGGAACTGTCCATTGGTGAGCTGG

CTGAACGTGGAGGTGTGTGTGGGAGCATCCGTGTCAGAGCCTGTCTGTTTGAATGCACAG

TCTCTG

>c7466_g1_i1 len=211 path=[155:0-210]

TTTTCCTTATTACCACTTTCCAAAATGCAGCCGAAGCTTTGTAGCTGGCTGTCTTTGAAT

AGGAAGAACAATAGCAGTGCTCTGTAGTATTTTGAGGGGTTCTTGGGTAGCGGGTTTACA

TATGGACTACACCTGCTTGATGCTTCTCTTAGCTTTGCCTCCAAAAAGGAATAATACGAG

TCCCTCAGAATGAGCAGGTGTAGTCCATATG

>c7470_g1_i1 len=215 path=[303:0-157 510:158-214]

GTAACTTTGTGTTTGACTGGGAGGTAACTATTTTTACACTGCTGCATTGGGTAACTCAGC

TTACTTTGTGACTGAAGTTACAATGAATGATTTATCGAGCGAGACTTAAAATAACCCACT

TGACATAAATAACTTCCACCACTAAGCAAGAAGTTTTATCAAAGCATTTCACATTATTTT

AATATCACTGACTTCACGCAGTACCAGACAATGGG

>c7476_g1_i1 len=700 path=[241:0-580 25:581-699]

GTTGTGTCTTCTCTGCTACACCTACAGAGTGAAAGAATATGTCTTACTTCTAGCACTGAC

AATACATTTCTCAGAAAAAATACTACACAATTGGTAACTTCTGGGTAACTACTGGGTTTG

ATTATAGTTATCTTTGCGTATTCTGGAAAAGAAAATCATTAAGATGCACATGAAATTATC

TTCAGTGTGATTTTTTGTTTTCAGCCGAACAAGAGTTGTAAACGACAACATTTCTTAACA

GTACTCATTAGCACTTGAGCAACTGGCATCCATGTTTCTTTTGGGACAAAAAAATAATGG

TGTTTTTTTTTTCCTTCCAGAAGTGATCATTATGTGGCCCAGGAGTGGATGAAGACCCCC

ACAGTATATTTTGTGATTTTTCCATTATGCCATAGATGTATCAGTTACAAATTTAAGACA

TTTCCAACAAGCGTCATATTATGCAGTAAGTGCAAATAAGCATGACAAAGATCAGTTTAA

GACAAAAAAAAAAAATCTGCTAAGCCAATCAATAACCCAACTAACTCTAACATCAGCTGG

TCTCACTGCAAAGGTACCTTGTACATACCTACCATTACATCTGTTATTCGTCCGTTTTGA

TCTTGTATGCTGTTTCTCAGTTTGGTTAAAAGTCATTATTTCTCACACACTTGCAAAATT

TTCTTTTTATTCTAAAACATGACTGAATCTTGCCTACCAT

>c7476_g1_i2 len=635 path=[241:0-580 821:581-634]

GTTGTGTCTTCTCTGCTACACCTACAGAGTGAAAGAATATGTCTTACTTCTAGCACTGAC

AATACATTTCTCAGAAAAAATACTACACAATTGGTAACTTCTGGGTAACTACTGGGTTTG

ATTATAGTTATCTTTGCGTATTCTGGAAAAGAAAATCATTAAGATGCACATGAAATTATC

TTCAGTGTGATTTTTTGTTTTCAGCCGAACAAGAGTTGTAAACGACAACATTTCTTAACA

GTACTCATTAGCACTTGAGCAACTGGCATCCATGTTTCTTTTGGGACAAAAAAATAATGG

TGTTTTTTTTTTCCTTCCAGAAGTGATCATTATGTGGCCCAGGAGTGGATGAAGACCCCC

ACAGTATATTTTGTGATTTTTCCATTATGCCATAGATGTATCAGTTACAAATTTAAGACA

TTTCCAACAAGCGTCATATTATGCAGTAAGTGCAAATAAGCATGACAAAGATCAGTTTAA

GACAAAAAAAAAAAATCTGCTAAGCCAATCAATAACCCAACTAACTCTAACATCAGCTGG

TCTCACTGCAAAGGTACCTTGTACATACCTACCATTACATCCGTCATACATATGTTGTAA

TACAACTGGCATAAGGTGATCATCAGCTTTGGAGC

>c7478_g1_i1 len=613 path=[1:0-123 125:124-612]

TTATAAGCCTACTTCATGATAAGGTAACTATTAGTCATTCAAACTCATATTTCCCTTAAA

AGTATCTTAAATCCGTTATGGGATATAATGGTGTTTATTTCTAAGTGTGTGCCTGGAAGA

CTGGTTTTAAAAAAAATCATCGAGAAAAACAAACTTATTTTAATGTTCTGAAAAAAAATG

TTCCTTGTTTAAAATTGTCTAGTGTTTTTAGCGTTCTGTTAGATCTAATCTTAAACAGTT

TATTTAACACTTTCTAAATTACACTTTGAGGATATTAACTGCGTCCCATGGTAGATTTGC

TGTTTTCATTATTTGCAAGATGAAGCCGGTTGCCTGTAGACTAAATAATGAATATCAGTA

ATTCTTTTCAGTGTATGTAGCAGAGCAGCTACTTAGATGAATAGATAACTTCAAAAAGTA

AGAGACAAGCAACATCCTACACTATTCACATGTTTTTATGTAGCAAGTAGTTCTTCAAGG

AGTTGCAGTAAAATACCCTAGGAGCAGGAAAAGGTGTCTTAACATACCACATCTATATGT

GAATTATAATTGAATATATTTAGCATTTGAAATATGTTAAAAAGGAAATGATCTACGTGG

ATATGAGCTAGTT

>c7481_g1_i1 len=260 path=[265:0-223 495:224-259]

AGTGCAGAAGGTTACAGATTAGTTTACAATCGTTATCATCACAGGTGGTCACAGCTCAGC

TGGACAGGGTCCTCAGCAACACAATCCAAGGTTTTTGCCTTTCATTGCTTTAATTTCAGC

GTGAGCTGAATTAAATAAAGAAAAAAAAAATGATTCTACTTCCTCAAGTAGATATGAAAT

ACAAATGAACTGATGATAGAAATGTAAACCCCAACCACTGACCTTCCAGCCCTAGCTGCT

GTGCAATTCTTTTTTTCCTT

>c7484_g1_i1 len=210 path=[1:0-187 680:188-209]

AAAAAACACTTTTAAAAACCCTTCTTATTTTTTTTTTCCACTGAGATACTTTTAGCTATC

TTTTGAAGTTAGAAGAATATTAGAATACTGAAATACAGAATACAGAGCCACATTGTTTAT

GACAGAAAATGTCCTATTTATGAACACATAACTTTTAGTTTTCATATAAGTGTTTTTGAG

AGAAAGTAAAAAATGTAACTACATTTTTTA

>c7489_g1_i1 len=205 path=[317:0-42 178:43-204]

AAGTTGGAACAAAATCCACTCACAGAAGAGAGTTAACGTAAAAAAAAACATGTTGTATCA

TCATTTTTCTCAATGGAAAGTCGTTGCATGTTGAATAGGTGTTTTCTGGTTAAAAAGTCC

ATTTTTCAGATTTTACTGTTCACAAAAGTTTTGTTGGCTTGGGCTTTTTTGTTTGTTTGT

TTCAACAACAACAACAACAAAAAAA

>c7501_g1_i1 len=207 path=[164:0-161 326:162-206]

TTCTCATTTATTTCTTATTTATTTCTTTTTTTTTAAATATGATTGTATATTCAATTGAAT

TGTATTCCTTTATATTTAAAAATTATTTATATTTCTGTTATTGCATTTTGAAATATTATT

TATATAGTTAACATAAATATTTTAATATATCAATAAAATACACATTTATAATTTTTTCTT

CATCAGATATTCAAGGAAAAAATAAGT

>c7502_g1_i1 len=228 path=[1:0-227]

ATGTACAGTGGGTAAAAAAGGAGAACTGAAAAAAAGATTGAGTCAAGCTATTCTATCAAC

TGATAGTATGAGGTGATGTGACATTATAGTGCACCAGTATATGAAACTGCCTATGTTGTG

TTTCGCTACCGGTGCAATGGCCCTCTGGAGTGAGTATCAGCCTATGCTGCGGGCAGATAA

TGATGCAATTCTGGGCTACTTTGCCTTCCTGTGAGACATTCTGTTTGG

>c7505_g1_i1 len=489 path=[543:0-214 292:215-488]

GAAAAAGGAGTTTCAGAAGTCTAGCTAGATGGGAAGAGGAGGATGGCAAACCACCAGGTG

CTTGGGATGAAATGCTCACTATGATGGAGGGCCACCCTGGACCAGGCTGTGCAGTGCAGG

GCACAATATGCCTTAGGAATTACTTAAATAAAACCAACAAGTAAGTTACAAGGAAGTAAG

GTACTTCAAACATTTGCCAGCTTTTTGCTTCACACAACCTTAAATTCTTTCAAGCTTTCT

ACTTCACTTCTTTTAGCAAGGTCGTTCCTCAGTGTTCAGCACGTGTAGCTGGGTTGCACA

GCAGGGACAAGTTCCCCATGGGGTTATGGCAAGGCAGCAGGTCACCACAAGGAAAGGGGA

TCCTGCTCTTCTGTCACTCCCATGGCAATGCCATTCTGGTGCTTGCTGGAGCTTTACTGA

GCCAGTTCAACACAGCAAGGATGGAATGCCCCCTGCTTATTTTGTTTGTCCTGGGTTAGT

TTGTCAGCG

>c7505_g1_i2 len=294 path=[272:0-19 292:20-293]

CACACTCCTTGCAGTCAACAAACCTTAAATTCTTTCAAGCTTTCTACTTCACTTCTTTTA

GCAAGGTCGTTCCTCAGTGTTCAGCACGTGTAGCTGGGTTGCACAGCAGGGACAAGTTCC

CCATGGGGTTATGGCAAGGCAGCAGGTCACCACAAGGAAAGGGGATCCTGCTCTTCTGTC

ACTCCCATGGCAATGCCATTCTGGTGCTTGCTGGAGCTTTACTGAGCCAGTTCAACACAG

CAAGGATGGAATGCCCCCTGCTTATTTTGTTTGTCCTGGGTTAGTTTGTCAGCG

>c7506_g1_i1 len=218 path=[539:0-40 193:41-217]

CCTGTAAAATACCATCCTAACCACGCCACTCTTCTAACCATACATTTGTTAATTGTATAG

TAGCCAAGGTAAAAGTCACTTTCTCACTTCATATTAGTTATCGTCATGATCTAAATCTGA

TTTATTTCTTACACCAGAAAGTGAATTGCAAGTTGAGACTGCACTGTATGTCTGATTTAA

GTGAAAAACAGCAGTTTCTATTCACTTAGCAGAAAGCA

>c7506_g2_i1 len=217 path=[539:0-40 579:41-216]

CCTGTAAAATACCATCCTAACCACGCCACTCTTCTAACCATTCTTTTGTGATTAGTTTTG

TTCTTTATCCTGCTATTTCTATGGACCTTAATAAGCCATAACCTTTAACAACATAGTTCA

CAAATACCTCAAGGTAAATATTCACATTCACACACGTTACCTGCCCTACACGTCTCATCG

TGTAGCTTTGCTTGCTTTCTGCTAAGTGAATAGAAAC

>c7522_g1_i1 len=265 path=[401:0-264]

AATATTTCTTGATAGTGTTTTCCAGAAGTTTCAACTACCTAACATCCTTAAGTAGGGTTG

TGGTTTTCAGAACACGCTGATAGAGAAAAGCAGCCAACAATTATCTGTATTAGGGCAGCA

ATTGTTTTGGTACAAGTGTTAGTCTAAACATACATTCCAGTTAGTCTGCTACAACTGCAC

GTGGCCACCAATATCTGATATTGCTGTCTCCCAGGGATCATGAGTTGAGTTCCTGAAAGT

TAATTATGAAGCCAACATGACCTGA

>c7529_g1_i1 len=203 path=[219:0-202]

TGCCATCTAATCAAATTCACACATCAAGGGCACGGACGTGTTGTCCCCAGTGAACAGATG

CAGCTGTAGCAGAAGGAGGGACCCAAAATATAACCAAAATGAATGCGAAAGAAAGTGTGT

ATATGCTGTGAATTTCCAAAGCACTGAGATATATTTTTAGAGTGAATGGACTGGAACTGC

ATATATTGCTTCGTGTTTAATTA

>c7545_g1_i1 len=297 path=[155:0-296]

CATCCAACAATCAGCCGTCTGCCCCAAAGGGGGGGCTTGGATTGTTTTTCGTGTTCAGAC

AACAAAGTACCGCCACGACAGTTTATATCTGTTCCTACATATAATCTGCCATCCGTTTCT

ACAGTACAGGCACTGACTCGTGCCACTTCCACTCCTGGGCCAGAGGACAGCAGCTTCCTC

CGAGCGTGGTTGTGACGGTCCAGCAGATCCCGGGAGTATCTCAGCTGGCACACAGGACTT

CTCTGCAGGCCTGCTAGAGTCCCAAGTCCTGCACCAAGTGCCCGCTCAAACACAAAG

>c7547_g1_i1 len=271 path=[181:0-270]

GAGGCATACCAAAGGAAGAGGTAACAGGAGCACAGCTGTGCTGGTCAAGTATCCCATACT

GTGTTTCTTTAGCACGTGTTTTAGGGAGAGTCTTGAAAATGATCAGTGAACTTTGTGTTC

TTCTTTCCCATTGCTGAAGTAGCTTTGCTCCATCAGTAGCAGCTCTTTCAGTGGAATTCC

ATATGACTTTTGATGAAGTGGCAGAGGTTCTGTGACTGAAATTTGCAATTAGATTTGTTG

CTTTTGATTACCCTTGAATAATTTAACATTC

>c7563_g1_i1 len=226 path=[241:0-225]

AGTTTTCCAAAAGCATTACAGCATTGCTCCACTCCTACACTACAGGGCTAAAACAAAACC

CAGACTATTTAGAAATTATTTAATCAAGTACAATTCCTCCTCAATACCTGCACATATTTA

AACTCAAAATGTGGTGTGATTTGTATACAACTGTGAGATGCGTGTCAGTCCAACTTCTGT

AAGAAGTGACCAAACTTGTATATAGAGAGGGGAAAAAAGACTTGCA

>c7566_g1_i1 len=341 path=[345:0-312 25:313-340]

CCACTCATGCTCAGAACACCCCAAACCCCAGCTCTTCCCACTTCTTTTAAGGCTGTTGCA

CAGCAACCTGTCTCTCAGTTACACAAGCTCTGACTCGCATGAACTAAGGGTGTTTCTGCC

ATCATTAGATCAGGCTTGGGAGAACTGTGGCCCTATTCTCACTTCAGTTTTATCTACCAG

TCATCTGCAAAGGCTAAGCACGAGGCTTTTCAAAGCTTCATAAAGGATATGATTAATTTT

TAGTGTTGTTTCAGAACACACAGAATAATGAAGCCATAGTACATGAATCCCTCTGTAGAC

TGTACTGAAACAAAATTAAAAAGATAGTGCTTACTTTTTAG

>c7576_g1_i1 len=474 path=[47:0-339 46:340-473]

AGTACGAAGTCTGGAAAAATAATAGCGTCTTCAGGAACTGTTGAAGACGAGGACTGTCTC

TGTGCAAGCTGGGAAGCGTCAGGACTTGACTATTTGCCACTTGCTGATTGCACAGAACAG

TGCGCATTAAAATAAAATTCCCTACTTCAGTTCCTGTTGCTTGTCTTCTGCACAATTCCG

AGAGCTGTGAAGTTGTCAGAAAATGCACAGCCTTGCCTGTCAACAACTGCACATTTCTAA

ATTCCTACTGCAAAAGTTTCTCCTTCATGCAGGGGTTCCCGAGAATTGCTTCTCTTGACA

TGGGGCTCATTGAATGTAAAGATGCATACATACCTGCTTGTTTAAATTACAATAGAGAGA

TGGATGCCAATCGAGATCAGAGGATGCAGTGGTAGGTGTACAAAGACAGGCAAAGACATT

GTCTCTTCCCTGAAAGCTTGCAGTGTTTTCTTTCTATTGCCGTGGTACAAGTAT

>c7583_g1_i1 len=210 path=[209:0-187 764:188-209]

AGAGAAGCCTCACTACATTACACCAGCAAGACTGAACTTGAAGCTGTTCAGATACCTCAT

TTGAAATTGTCAGAATGAGATGTTCAACCTATAGATGAAAGCCTTTGAATGGAAGCAACC

CAATTATGGTTTACTACTAAACAATGAGCTTCATTCAGCGAGTACAGCATGCTAAGGAAA

TAACTGAATCACTCTTTCTAGTTGTCTATT

>c7588_g1_i1 len=222 path=[155:0-221]

AGAAAGAAGAGTCAGACTGACCCACCTGGTGAAGGGGCATAGCCAGCCCGGCAAGCACAG

GTGGAAGCAATTCACCTATGCAGCCAGAACCTCCCTAAACCCATTTAAGGACTGGCAGCC

CTTGGCACCCTCTGGATTATTCAGCAAGCCCTGATCCTTGAAAAGGGCTATTTGAATTCT

GTGAGCCAACCAAGTCAACACAGCAAAGCATTATTAAACACG

>c7592_g1_i1 len=383 path=[155:0-382]

GCCGATCTGCCCCTGACTGAGGCGGAGGAGGCGCCGCTCGCCGCCGAGAAGAAAGGGGCC

TCTAAGAAGGCGAAAGCCGGCGGCTCCTCGCTATCGCCGTCGAAGAAGAGAAAGAACAAC

AAGAAGAAGAACCAGCCGGGCAAGTACAGCCAGCTGGTGGTGGAGACCATCCGCAAACTG

GGCGAGCGCAACGGCTCCTCGCTGGCCAAGATCTACAACGAGGCCAAGAAGGTGGCGTGG

TTCGACCAGCAGAACGGGCGCACCTACCTGAAGTACTCCATCAAGGCGCTGGTGCAGAAC

GACACGCTGCTGCAGGTCAAGGGCACCGGCGCCAACGGCTCCTTCAAGCTCAACAGGAAG

AAGCTGGAGGGCGGCGGGGACGG

>c7597_g1_i1 len=342 path=[320:0-263 584:264-341]

CGAGGTGAGGCCTCACCAATGCCGGGGATGTGTCCATGTCACAGCACTCTGTCAGAAGCA

CAGCACAGTGTCTTTAATTTCACACAGGGAGGCACCAAGTCCCATGGTGCTTTGCTTGCA

TCCCCCAGAATCATACTGAATTCCAAGAGAGAAAAATGTAATGATGGGAAGACATTGCAG

GGGAGAGACATAGGCTTAGCTTTTTTGAATTTGGAATGAATACCACTTCAAATACTTGAG

CTTATATTTTACAAGTGCTTCCTTATTTTTTTCTACATTACAGATTTTTAAGGATATAGA

GACAAAGTACGAATAAATTAGTTGCAGATCCCTTAGTTTAAA

>c7604_g1_i1 len=238 path=[216:0-62 279:63-237]

CATCCTTAAGTGACCTTTGGCATGATTAAGGCAAGGAGTTCCCTAACACAGAGCCACAAA

GCCACATTATGCCAATGCTGCCAGAGAACAGCACCCATGCCTAAGAAGCTGTTGTTTTTT

CCAGCTCTCTGTTCATCTAGTAGAAGATCCTGCTCCTACTTAGAGACCACTCTTTATTTA

GGAGAGCAGGAGAAAGCAAAATTCATTGCATTCTCCCATGCATACCTTCTTTAAGCCG

>c7608_g1_i1 len=206 path=[428:0-205]

GCAGAGCCCAGCTGGGATTCCACGTGTCCCTGCCGTTGTACGCCCCTCAGGTGTGTTAGG

ACAAAGCTTCTGTTGTCACCTTGTCAGCGTTTGAAAAAGTAAAGGAAAAATAACGTGGAT

CGAGTAAGTTGTGCTGTTGGTTCTAATAAGTCACCCCCTCTGGGACTCTTCCGATTTCCA

GCATCCTGAGGACAGTTACAGTGACA

>c7614_g1_i1 len=514 path=[473:0-22 472:23-513]

ATAAAAATATAACTAATTTATATAAAAAAATATGTACAATTCAGGTAATTAAAAAAAAAA

AAGTTTTTATAAGATAAATATTAATGTGTAATAATTGTAGGATTTGGAACAATGAAAAAT

AATCATACTTTTTCCTTCTTTTTTATCTTATAAGATTTATTCTACAAAAAATATATGTAT

ATTATTAAATAAGTAATTGTAAAATTTTCAACATCAAAAATTATTTGCAATATGTTGGAA

TCTTGATTTTATTATTTCAACAGCTATTCTTATTTATTTCTGAATACGAAAGAAAAAATT

TATGATTGATAATTTAATATAAATTAAATTATAAAGGTGTAAGAAAATTTTCAATTATTA

AAAATAATAAATATTATATCTTTGTTTTTTAACATAAATATTCAATTTGTAAGTTAAAAA

TGTAAACAGCAAAAGATATTTATTTTGTTTTTCAATATTTGTTTTTTATTTTTTGTTAAT

CTTTAGTCTTCAATATTTTATATATATATATATA

>c7614_g1_i2 len=726 path=[237:0-234 472:235-725]

TTTTTTTTTTTTTTTTCTAAAAAGCGAAATGAACTTTAAATGTATTTTCCATTTCTATAT

ATAATAAATTCTAAAATTACAAATTTTCCTTAAAGTATAACGTGTAAAATTTTTTTTCCT

TTATACGTTGAAAGTGTAACTGCGTATATATAAATCCATGAATATATATAAAACGTGTAT

GTAACAGCAAAAATGTATAGTTTAAGTCTTTTCTTATAGTATACATTAATTTAAAAAAAA

AATATGTACAATTCAGGTAATTAAAAAAAAAAAAGTTTTTATAAGATAAATATTAATGTG

TAATAATTGTAGGATTTGGAACAATGAAAAATAATCATACTTTTTCCTTCTTTTTTATCT

TATAAGATTTATTCTACAAAAAATATATGTATATTATTAAATAAGTAATTGTAAAATTTT

CAACATCAAAAATTATTTGCAATATGTTGGAATCTTGATTTTATTATTTCAACAGCTATT

CTTATTTATTTCTGAATACGAAAGAAAAAATTTATGATTGATAATTTAATATAAATTAAA

TTATAAAGGTGTAAGAAAATTTTCAATTATTAAAAATAATAAATATTATATCTTTGTTTT

TTAACATAAATATTCAATTTGTAAGTTAAAAATGTAAACAGCAAAAGATATTTATTTTGT

TTTTCAATATTTGTTTTTTATTTTTTGTTAATCTTTAGTCTTCAATATTTTATATATATA

TATATA

>c7627_g1_i1 len=276 path=[1:0-220 222:221-275]

TGGGCTGCATCAAAAGAAGTGTGGTCAGCAGGGAGGTGATTGCTCCCCTCTACTCTGCCC

TCATGTGGTCCCACTGTCTATGACATTGATAAAGATGTTGAAGAGCATTGGTCCAGGTAG

ATAACATTAGTTACCCTTCCTTTGTCCACTAACGCTGTCACATGCATTGAGGTGTATCAC

TTGTGCTACAGGAGAGAAACATTCAAGAGCCTTTACACCTCTTAAGTACTAATATAATGC

AAAGAATAGGGAGCTAATGGCAAAATTATGAGGTGA

>c7634_g1_i1 len=334 path=[155:0-333]

AGAGAAAGGGTGTGACCTGCAAAAGGCATGGCAAGGAGAGCAAGTTTTACCATAATGGAA

AGCTCAGATATATATCAGCCTGTCTGCATACCTGTTTTTTAATACAGTTGGACGTGTAGC

AAACCAAATGCTTCTTTCTGAAATTGCAAGATGGGGTTGTGCTTTATTATTGTGTCATTG

CTTCATCCAGGATGAGATGGACTTGTGTCCACATACTTGAAGATGGCAGTGTTAGATAAC

TCATCCTGTATGACCAAGACTTCTGGATTGTAATTATTTTTGGATATTCAGACCGATAGA

GGAGAGAGGAGTCCTATAGATATTCTCCTGGCCA

>c7640_g1_i1 len=257 path=[235:0-256]

GGGGACCGGGTGTAGATGCCATTCTTTCTGCAGAAGGAGTTCTTGACGGAAAGGCGCCGG

TTGTTGAGCTCCAGGGCAGGGAAGCTGCTCTCATCCAGGCTCACCATCTCACCGTGGGTC

AGGGCGTTTGCCTTGGAGCCATTCCCCGAGTCTGACTTCTTGGCATACTTCTTGCTCTGC

CCACGGATGATGAGCACGAAGACAAGCAGGAGGATGAAGGTGAAGGACCTGGCTGAGGGC

ATCACCTACCGCTTCCG

>c7642_g1_i1 len=355 path=[136:0-135 718:136-354]

GGACCAGGTTGTCAGTTCGTTTACTCCATGTGTCAGTGCTGGGGACAGAGCAATACCCTT

GGTTTCCAGCGAACACACAGAATCTATGCAATGTACTCTTACTGAATGATCTGCCTTTAA

GAAATTCCTTCCTAATCCTTCTTAATTCAGTGATTAACTTGTGCCCTGAAACGTGTTTAT

ATCTTTCCTATAATATGTCAATATTATATTAGACACAAATGTCTAATATAGGACGTCTCA

TAGCACACCTCAGTAGATTCACATCATGAGTAAATTTTGCCTAGGTTGTGTCAAAGTCTC

AGGTTTAGACATTTGTGTAATTAATAAAAATACTTATAGTTACAGGAGGACTCCT

>c7642_g1_i2 len=236 path=[716:0-16 718:17-235]

AAATCCACGTCTCTCCACCTTCTTAATTCAGTGATTAACTTGTGCCCTGAAACGTGTTTA

TATCTTTCCTATAATATGTCAATATTATATTAGACACAAATGTCTAATATAGGACGTCTC

ATAGCACACCTCAGTAGATTCACATCATGAGTAAATTTTGCCTAGGTTGTGTCAAAGTCT

CAGGTTTAGACATTTGTGTAATTAATAAAAATACTTATAGTTACAGGAGGACTCCT

>c7648_g1_i1 len=319 path=[1:0-137 139:138-154 156:155-318]

AGAATTTGTAATGCAAAAATCTTACTCATGAAGTGTTGGAACAGCATAAATAACTCTAAA

AGGGAAGGATTATTGTGCAGGCTTAGCTATCTTCATGCATATTTGTGGGGACAGAAACAT

AATTTTTTTTCTTTGAGCTTCAGCAAGTTATGTTTCTGAAATGCATGCCCAAGTATATTG

AAAGGCATAAGTTGCAAATAATTAACTTACAAGGGCGTGCATACATTCAAAAAAATGACT

TTGGGAAACAACTGTGTCAGTACTTCCTATTTCCTAGTCAATAGTTTCTTCTCATTTTCA

GAGATGATGAAGTTTCTTG

>c7649_g1_i1 len=238 path=[45:0-237]

AAAAGTTCTTTTAATCAATCCAAGTAAAAAATATAAGATAATTTGCATTATTTTTAACAA

TTATATTAAAACTTTAACATTATTGTTATTTTGATATTTGTTTTCAATATTATTTTCATC

ATAATAATTAAGGAAGCTTTCATATATATAACTACTATTTTTATAATTCAAATTCTCACC

ATCATATTCATTTTATAATTCTACATTAAAATTAGGGAAAGTGAAGATGAAGAGATAA

>c7664_g1_i1 len=212 path=[179:0-211]

ACTGATGCAGATAAATAGTGGACATTTGGCACCGCCGCCACCGACCACTGCATGGGGACC

CCCATAGGGACCCCCATAGGGACACAATGAGACCCCATAGGGACCCCATAGACCCTATTA

AGACTCATAGCGACCCCATAGAGATCTCATAGGGTCCCATAGAGACCCCACAGAGCTCCA

TAGAAACCCCATAGAGCCCCTTAGAGACCCCA

>c7668_g1_i1 len=211 path=[189:0-83 273:84-210]

TTCTATTAAAATATTTAAACCGTTTCCTTTCCTTCTTCTTGATCTTAAAGATCACATCGA

TGCGGGGGGGCCGTGGGATCCCAGCACAAACGGAATGGGCAGGGAATGCCGTTCCCGTCA

CGCTTTGCTGTGAGGGCGCTCTGTACACTTCAAGAATATTTCCTTTTAGGTTTGTCAGAC

TGTTTTACCTTCCCCCCCCCCCATTGCCGCT

>c7694_g1_i1 len=214 path=[399:0-213]

GTGAATCTGGGGACAGAAACTGGTGTGTATTGAAATTATGAGAAATTCTGTTAAATGTTC

ATAGAGGCAGGACTGCAGATGATTAATATCTTCATGGCACTGTTTGTAATTTGAATGTTT

TGGAGTCTGCTTTTATATTTCAGACTAACAGCTTAATTTAATTCCAACTTCTCAAAGAGA

AAAACAAAGCAAAGCCTTGAAACCATTGAATCTT

>c7695_g1_i1 len=244 path=[27:0-24 @52@!:25-220 325:221-243]

GGATATTAATTCAGGATTAAGGGGAAAAAAAGAAGTTGCAGGAGGCCCTGCCGAGGTCCG

GCACGGCGCCCTGGACCAGGTCCTGTGCTGCTAGCTGCCAGCACAGGGCCTGGCACTGGG

TGAGGGGCTGCTGCTCAGCAGGCACAGGTGGTGGTGCTTTACCTGCCTCCTCCTGCAGAA

GGTGTGTGTTCAGCCTCTGCATCGCTTCCGCCCGCTCTGAGAAAACACTTGGAAGAGTTT

TCTC

>c7697_g1_i1 len=221 path=[199:0-220]

ACCCACTTGATAAGGTATTGAAGAAATGCAGAAACTGAGGCTGATTAGGCAAAGAGAATG

GCTCTGGGAGCTGAGGACAGGGCAGAATGCTCCCAGGGATAACAGCAAATCATAATGCAG

GAGTCAAAGTGAAGTTGTACAGTCAGCCATTTCGTAACAAGTTTTGAGCACTTGATTTTA

CATGTATGTACATACAGCTCAGATCTTTTTTTTCCTGCTCA

>c7701_g1_i1 len=220 path=[109:0-219]

GTGCATATACATGCACTGAGTAAGGTTAGTCACTCTACATGGCATGCCTGCTGTAACATG

GTCATTTGTTCCACAAAATGGACCACAGGAAAGTACACTGTATACTTTTATTTGAAAGTA

AAACATGGGAAGTTATATTTAGAAAGAGTGCTGAAAAGAAAAAGAGCAAGAATATTTCTG

AAGTTGCAGTAGAAACTTAAAAAAAAAAAAAAACACCACA

>c7707_g1_i1 len=265 path=[1:0-264]

AGAATCTAGCAGCACTCTGATCGGGCGAATGGGGAACTCCTGGCACAGAACAGCCCCCAG

TTGGGCAGGAGGAGGGGCAGAGCTCTGCACATGGGTCAGTGGTGAAGCAAAGCACCTTCT

GAAGAGCAGAGCAGCTCTTTCCTTGTTGTCCGCCTGTAGCAGATATGCTTTTTTTCTCCG

TTTTTTTTCTTAAATTTCTTTTTGTCTTTTCTAGATAGCATAGCTGTCCAGCACTGATAA

CTTTATTTATTTCTGCAAGATGGTG

>c7709_g1_i1 len=251 path=[455:0-250]

TGGAGGTCAAGCTTTTACATCTTCCGTTAGGTGAAAAGACTGCAACAGCTGTGCAAAACA

TACTGAAGCTTCAAAAGTGTAGTCTGGAGAAGTCAGACATCGAGTACTGCTTAGGGCAAA

CTCACAATGGCAGACAATATCACACAACCTGGAGCTGGTAAGAACAGATTAGTGATGGTG

CAAAGACGTTCCAAGCTGCAGCAGAGCAGCGCCCCTACTCCAAGCAGGTCAGTTGAATTT

AAAGCCTCAGA

>c7710_g1_i1 len=206 path=[255:0-133 25:134-205]

CTTTTAGAGTCTGCTAGAACCTCAAAGTCACTGAACCTTTCTCCCATGCAACCAGTGCAC

CCGAACCCCATATTTTACCAGTATGTCTTCCCACAACTCAACCCTTTGACAATATACTCA

ATTCTGTTGTTGTTGGTTGTTTTTACTGTGCTAGAACACTGTGGTACCTCTGCAGACTAG

GCTTTAGTTATAACGTAACCTGATTT

>c7710_g1_i2 len=206 path=[255:0-133 388:134-205]

CTTTTAGAGTCTGCTAGAACCTCAAAGTCACTGAACCTTTCTCCCATGCAACCAGTGCAC

CCGAACCCCATATTTTACCAGTATGTCTTCCCACAACTCAACCCTTTGACAATATACTCA

ATTCTGTTGTTGTTTGTTTGTTTCTTCAGTTTTGGAGAGAAAAATATGAAAGCCTGTTTC

CAAATCAGGTTACGTTATAACTAAAG

>c7711_g1_i1 len=250 path=[33:0-51 32:52-249]

ATGTGCAAGCAGAACAGCTAGCTACACAGCTAGGGATTTTTGGTTTTTTTAATAACTTAT

TTAGCATTAAGCTGAACACATAAAGAATTTAATGAGATGAATCATCCCAAAAACGCATGA

AATCTCATTAGAACACGATGTGGGAATGATGGTATCTGCAGCACATGCAGAACCACTGCT

CCTTGCTGCTCCTGAAACACAGGCTGCACCAGCTGCCCACCCCACCACCACGCTCGGCCC

TCAAACACAA

>c7718_g1_i1 len=224 path=[448:0-223]

GCTGTAGGTGTCCCTGTTTGTTGCGAGGAAGCTGGACTAGATGGCCTTTCGGGGCCTCTT

CCAACTCAAACGATTCTATAATTCTAAGGCACGTCCTTCCCTGTTCTGGTGCCTCAGTGC

ACGAGTTGCCGGCCACCTGAGAAACGTTCGTGCTCATAGAAAGGAAAATACAAAACAAAG

TAAAAGAAAATGAAATAATAAAATGAAATAAAAATAATAAAATA

>c7722_g1_i1 len=275 path=[1:0-274]

GTGATGAGCCCTTCTGGTCGTTACCCTGCCTGGCAGGAATTTCCTTAGGCTGAAGACATC

TGCTGGGGCTTTGGTCACCACAGATGTTACGTGGTGGTTGGGCCAAGAGAAACGTTCGAG

GTCTGCAGTATTAATAATTACAATTTGTAAATGTAGCAATAATTTGCCAGTAACTTGCAC

GGGGTGCTTTGAGAGGTTGTTGGGAGCCGGCTCATCTTGTGCAACGTTGGGTGTGTGTAT

TTTTGAAAGAGTCTTTTGTTACAGAGTTTGGTGGA

>c7728_g1_i1 len=240 path=[629:0-5 383:6-29 407:30-239]

ATATATATATATGTATATATGTGTATATATTTTTAGTATATAGTAGAAAAGTAAAACATC

TTTATAATTTGTTAACAGGATTCATAGAACATTTTCAAATATTACATTCTATTTATTCAT

TTTAATTTCTTATATATTGTGAATTTTCCATTATTATATAATAAAATATAGTTTTAATGT

TTCTATTTTATCTTTTTAATTTATGTAATTTTGGAAAAATATCTGTTTTCTAAACTATAT

>c7736_g1_i1 len=444 path=[779:0-420 1222:421-443]

CTTCTTCCCTCCTGCCAAGCCTTAAGAGCAATCATAACTTATTTCCAAAATTCCCTATCA

CAATATTCATTTTAAAGCTGCAAAGCAAACTGAACCAAGCATACTTTGTGTAAGTGGAAA

AAATAGCGAGAGCCATTGCTGTAACTTCAATCTTTTTCGACTCACTCTGGGAACCCCTCA

CTGTTAGTCACTCCTACTGATTGTAATTACATCATTTTGGCTAGTGGGTCATTCTCAAAC

ATATAAACACTTTACAAACCTAGATATTGTGAAATATCACTTATTTGTTTATTACTGGAG

GCACTGGCCAGTACCCCAACACGGCAACATCTGTAGGTTTACATTTCACGGTTACTTTCC

GGAAGAAAATCTTTCATCCCACTCTTCCTCCAATTATTGAAAAAATATATATATTATTGT

TGATATGCATAACAATATATAAAC

>c7748_g1_i1 len=406 path=[469:0-187 274:188-405]

GAGAGGAACATTGCCCGGAAATAGGGGCTGCAAGCTGCCAGCACCAGCCGATGGCAGGGA

AACTCCTTTCCTTTGACTTTCAAGACACAGTCCAGAAACTTATTGTGGTCCAACATGTCT

TTGAGTCCATCCTGGAGGAGGGTCTGCTGGTAGAGACGCAGTTCTTCCACTTGATCAAAA

GGCAAACCCATGGAAGAGAGCAAGAAGAGGGAGGTCAGCTTGGAAGATGTCGATCCAGAT

GTCATGGGCAAGATCCTTCATTACATCTACACCTCTGAGCTAGAGATCACAGAGCAGAAT

GTTCAGGACATCTTCTCTGTGGCCAACATGTTCCAGATCCCCTCCATCTTCACTGTCTGC

GTGTCTTTCTTGCAGAAGCGCCTTTGCCTCAGCAACTGCTTGGCTA

>c7750_g1_i1 len=210 path=[188:0-209]

GTATATTCTGTGATAAGTCAGCAGTCTCAGATCTTTGGGTAACAATGGCCGTAAATTACA

TATATATAGGTTGCCAAAGACAGTTCTGATTTTGAATTACTTATGTTCAAAAATCCTACA

AAACTATTAACTTCTGAAATTCTTCTGACTCTTGAGTATCCTACACTACGACTCGTTTGG

TGTTATAAGAAGCTTCCTTTTGCTTCCAAA

>c7775_g1_i1 len=337 path=[158:0-336]

AACTTTGTGTTTGAGTGTGTTTGGGTTTTGTTTCTTTTTCATTGAAGTTTAATTCTTAAG

TTCTATTTCAGTGTTTGTGTTTACTACTTGTGATCATGTAGTTGTGCCTTACATTGTGAA

AGAATTTAGTCCAGCGTGCACTGTAATTCCTAAACTCTGCGGTTTGAAAGGTGGCTGTGG

ATTTGGATGAGAAAATGACATCATGCATCAACCAACCAGTTCTGTATAAACTATGATGAA

ATGTTATTGAAATGTAATTTTTGTTTTCTAACCTGTTACAGGATCGATGAACGACAATAA

AAGAAAAAATTAGACCTTTCTAAAAAAAAAAAAAAAA

>c7779_g1_i1 len=317 path=[295:0-316]

AAGATGATCGTTTGAGCCATGCCTAGCACAACAGGGTCCTGGCTTGGGACCAGGGCCTCT

TAGGTAGTAATGAAACACAAGCAGCAAAGGACGTAACAGTTGGCACTGTAGACAACAGTA

CTGTTGTTACCTAATCATATTAGAATAGCTGTTTTTATTGCAACCAGAATAAATAAGGTT

ATCGGGATTTCTGTAATTTCGCTTATTTCTATGTAGAAATCTCCTAGAGTTAACGTCTGA

CTGAAGAACATAGTTACAATTTGCTATCTGCTAGAGAAATAACAAGTAATTCCAGTCCTG

CGTTTAATCAGTAAATA

>c7783_g1_i1 len=695 path=[1:0-522 1246:523-694]

ATTCTGCTTCTTGGCCTCTGCTTTAAAACAAAATAACAGTAACAATGACAACTGCAAACA

GAAACTAGATCACAGATCTGTTCATTTCCTGTCATTCACACTATCGAAGAATATTTTCTA

GCACATTTCTTTCATCTTTTCATTCTGCAAACCAAACTTAAAATTTCATTCAGTTCCTCC

CCTTCATCATGTCAATCGTTTTCCACCCTATACTCCATAATGTTTCATTCTATCATTATG

AAGTCTGTATTTCCGCATGATTTGAAAACCTGATTTTTTTCCACCGAGAAAGAAGGGGCC

TAAACATTAAAAATGGCTTAAACATTGCTGTCTTAAACTTACTTTTTTTAAGTGATCATT

CTTGTCTCCTTCTGGTGCTTATACCGTTTTATTTGCTGACTTTTCCTTTGTTTGATAAGA

TAGTCAGCCTATTTATTGCTGTTCTGAAACTTCATACTATTTTCTTAATCAAGGAAAACA

GCTTTATTATTAAAAAAATTTAGAAGATTTCAAATTTTGTTTTCTCACACTGTCAATGTG

ACTTACCCACTGACTGGGAGAAATAAGAAATACAGCACTTGTATTTGTATTTCACTATAG

TAATATTTTTCCTGATTTAGTTTATCTGCCCACTGTAACTCTAATCTAGCTTAAAAATAC

AGCTTCCCAGTTCTCCTTGTGGACAAAAGGTCTGT

>c7784_g1_i1 len=271 path=[1:0-270]

GTATGCAGCCGTATTTTTGCCTTTGCGTTCCAGTTTTAAGGAGTAAAAAAAATAATCTAA

CATTTGCTTTAGGCCAGTCTGGGGAATGACTAATCAAATGACAGGAAAAGGGTTTTGGTT

CTTGTAGACAGGCTTATTTGAGGTCACAGTAGATACAGCACTAGGACGCATCAATGTTAA

GTCTTTGCTGTCAGAACAGTGAGATATGTATTAGGTTGTTTATAAGTCTTCAGTTTGTAT

TTTGTATACTTTGTGCTTCCAGAAGAGTGGA

>c7790_g1_i1 len=238 path=[39:0-71 273:72-237]

GTAGGAAGAGCCTGGAAATGACCCAGAGGACGTGTTGGTAAACTGAGACTTGTATTGTGA

TGAAGAAGGTGTGGTGTGAGATGAATTAGAGGCTGTGGTAAAGTGAAGGTTGGCCTCTTC

TCTCGCATAACTAGTGATAGAACTAGAGGGAGTGGACTCAGATTGCACCAGGGGAGGTTC

AGCTTGGTTATCAGGAAAAATTTATTCTCAGAAGGAGTGGTCAGGCACTGGAATGGGC

>c7796_g1_i1 len=321 path=[47:0-212 259:213-320]

AGTGGGAGGAGGAGGAGGAGAAGATCGCTGTTTTCAAAGAGCAGTATATCTATCCTACTA

TTATTAACACAGAAAGGGAGGAGAAGTCCATGATGAGCTGGCTAAACACGCTCTCCATTC

ACGACTTCAACTCTTCCACTGCTGAGATGGAAGCTAACAACAAGAATTCAAAGAACAGTG

ATGTTGAAGGCAGTGATGGTTGTAATGATGATTCTGACTGAGTCAAAGCGTGCTTGGACC

TGGTACCTGTACTGTGAGCATCAAAAAGCATCACAGCTCGCTTTGTCAACGGAAAAGTGG

CCTCAAACACAAAGTTTAGGT

>c7803_g1_i1 len=205 path=[234:0-120 26:121-204]

CTCCTGGGAGGACGAGGATGACGAGACGGTGGAGCTGGGGGTGTTGGTCCCATAGCCAGA

TGAGGGGAGCGAAGCCAGTGACCACCTTCTGCCATCAGCCCTGTCGGTCCGAGGAGCATG

GCTGCGGGCGAAGGGGAAGTTGACGGAGGTGCTGGCGGAGAAGTTGCGAGGGCTGTCCAA

GGGGCTGCTCCCGGCTGTGGGCACT

>c7804_g1_i1 len=345 path=[354:0-306 692:307-344]

TAGGAGCATTTTCTCACAAACATACCATTCTTCTGCAAAAATATTCTATTTTCTTCCCAA

AATACACATTTTCTACTTAAAAATAATATTTTCTCACAAAAAAATATAACTTTTCTCCTG

AAAAGAGGACATTTTGTGAAAAGACATATTTTTGGGAGAAAAGTGGTATTTTATGGAGGA

AATGTCATAGATTTGGAGAAAAGCAACACCAGAAGCAACACCCCTGCATTCTGCATTTGC

ACACTGCAAGCCCCAGGCCTTCTGCTTTTTGCCCTAGTGTCATGGTTTTGTAACTCTGCT

ATTGGTATTCCACATCACAATACCATGGACAAAAGAGAAGAACTA

>c7806_g1_i1 len=437 path=[1:0-436]

GCAAGTCTTTGAAATCGAAGGAGATAAGAAGGTCTGTGGACAGTAAAAAAATGAGTTCAT

ATTATTCATCATGTAATATTACATTTTCAGGAGTCTACTAGCGTTCCTAAGAGTACCTTT

ACCATATAAAAGTTTACAGCAGCTTACTATCAGCCAGAGCAATCTCATAACAGTTTATGG

CCAATCCTCACCATTCACTTTCCAACCATAGCTTTGTTTCAGTCCCTTAGAAAGGCACAG

GAGCAGACGTTGCTCAGATGCAGCCCTCCCTCCTTCTTTGTATGCTTTGGATTCAGGAGC

ATCACACTGCTGCAATGGTTTGGCACCAACAAACCAGCAGGCACTGCTTTGCCTTCTCCT

GCTTAGCTATTTTTAATATTTGCTTACTCACATTTACTGTACTAAGCTCTTTACAGGAAA

GTAATTAAAAACTGTTA

>c7816_g1_i1 len=346 path=[1:0-345]

GACTGTGAGGCTGCACCAGTAGCTAATGGCACAGGTGCAGCCTCACAGTCTGGTGACTTT

GCTATGTGATCCTCTAAAACTGTTTCAGAACACATGAGGTACTTATATGCTGAAGATCCA

TTTCAGTAATGAAAACTGCAATCTAGAAATATATCCCAGTTACTCGAAGGAGAGAATTTT

ACTTTACAAGAGGCTGTGGTAGATACTAGACTTCAGAAATCTCTCAAACTTCTGAAAGCA

GAAGGAAGAATTGCATGCTTCTCAGCAGGGATTTGAATTTGGCTTCCTGCAGAACGAGGG

GCATAACTTTGTGCTGTGTGTATCTTCATTCAAATCCCTGCTGAGA

>c7822_g1_i1 len=221 path=[45:0-44 90:45-99 840:100-107 153:108-220]

AATATTTTAATAAAATTAATCATATAAAAAGTAATATTTCTAAAATTTAATACGAAAGTA

TCATTAATAAAAATTATTTATTTTTTTTTAAATGCAACTATTTTATTTATATTAAAAATG

CTTGTTAAATGTGGTATTATTGATGAATGAGCATATTATTATTATGTTGGTTATATTATT

TTAATTTCCTTTTCTAGTTTTATAATAAAAATTTATCAAGT

>c7822_g1_i2 len=253 path=[45:0-44 90:45-99 840:100-107 435:108-252]

AATATTTTAATAAAATTAATCATATAAAAAGTAATATTTCTAAAATTTAATACGAAAGTA

TCATTAATAAAAATTATTTATTTTTTTTTAAATGCAACTATTTTATTTTTATTGAAAGGA

CAGATTTATGTTTTTTATATATTTTTATTTAGTCAATTATTCATTTTAAATTCAACTTTT

TTTTAAATAAAACATAATGTAAAACAACTGTAAAACTTAATAATTTTAACTTGATAAATT

TTTATTATAAAAC

>c7824_g1_i1 len=311 path=[1:0-129 131:130-310]

CATAATCATAAGTAGCAAGAAATGCTGGGATTTTGCCTTTCCTTCTTTTAGTCACCAAAA

TCTAATTACTTGCCAGCCAGGTCAACTTTAAATGCCAACCCCATATACCATCAGTTGCAG

TGGTACTGCAGACAGCCTTGATTTCAGCATCAGCTCTCTAAGTCTACAGGGTGCGCAGCA

GATGCCAAGTACATACTGGTGATTAATCACCATTATTACCTGGGATTTGATGCACTACAG

TGCAGTACAGCTCCTGGTTTTGGTACTAAAAGCACTGAACAAACTTGCACTTAATATCAT

GCAAATATGAC

>c7831_g1_i1 len=222 path=[441:0-221]

AGACTTCTAACCTAGAATAGTTCTAAAGACTCTAAGCATAGCACCACCAAACTTAAAACT

TCTGAAGCACGCTCGTTTTTCATCCACTGTGTCGGTGATTTTTGGACAGATAAGTCACAG

TCCCATGGCCCCCATGACGGCATAAAGCAAGGGTGGGTAAACAGCTGGAAAAGGCCACCA

TGTGTGTGATTGATACGATGGTCCGGAAGCTCTACTGAGATA

>c7831_g2_i1 len=215 path=[222:0-207 658:208-214]

AAAGAAATGCAGACTGTTCTGCAGGTAAGCTTCCCCTTCAACACAGGATCTCTTCTGAAG

AGGACAACTCACTTCCCCAAGGTTTCCCTGGCTCACTTCAACTGAAAAACTCAGAGGTCT

GCACCAACTGAACAGGTGACATTAGTTCAAAAAAGCAAAGTTTTTAAACCATGCATGCAC

ATACACAGTAATATGTGATGGTACTGCACTGTTCA

>c7836_g1_i1 len=387 path=[574:0-354 928:355-386]

GGGAGGAGCGACTGCTGGCTGTCATCCAGGGCACTGTTATCTCCTAAGGGCAGCGCTGGG

ATTTGCTTGGTGTCACTCACTGGGCACGGAGTGGGACTGGGGTGAGGGCACTCGATGGAG

CAGATGGAGCCAGCAGAGCATGACTCTGGCCCTTCTCAGGGCTCACTTTGGGCACAGGGT

GGGACTTTGGCTGCAGGTGGCCATCCTCAGGGCCTTTCTGGACGTGCCTTAGCCAAAGGT

TGGCCCCATCAAGTGCCATCAGATGAGGGGCAGCGGTGCCTGTGCCTTTCCTGAGGGTCC

TTTGGGGGTTCACAGCCGGTGGCCATCACAGGGGAACAGCAGAAACCTCCCTGGGATTGG

GGAGCCCCGGGGCCCTGCAGCTTTGTG

>c7836_g1_i2 len=387 path=[574:0-354 203:355-386]

GGGAGGAGCGACTGCTGGCTGTCATCCAGGGCACTGTTATCTCCTAAGGGCAGCGCTGGG

ATTTGCTTGGTGTCACTCACTGGGCACGGAGTGGGACTGGGGTGAGGGCACTCGATGGAG

CAGATGGAGCCAGCAGAGCATGACTCTGGCCCTTCTCAGGGCTCACTTTGGGCACAGGGT

GGGACTTTGGCTGCAGGTGGCCATCCTCAGGGCCTTTCTGGACGTGCCTTAGCCAAAGGT

TGGCCCCATCAAGTGCCATCAGATGAGGGGCAGCGGTGCCTGTGCCTTTCCTGAGGGTCC

TTTGGGGGTTCACAGCCGGTGGCCATCACAGGGGAACAGCAGAAACCTCCCTGGGCAGGG

GACACAAAGCTGCAGGGCCCCGGGGCT

>c7840_g1_i1 len=209 path=[481:0-208]

AGCTCAAACTTATTTCAGTCTATTGTTAATGGCTGTACAGGCAGGACTACAGTCAACAGC

AGGCTGACAAAACAGTCTTGAGCTACTCATGGGAGTGACTTGAGTGGCTGCTTGCCCTAG

GGCTGGGAAGCCACCGCATCTGCAAGGAGACAGGACAAACACAAATATTTGCAGGCTTGC

CCAGCAGGAGAGCTGCGAAGGACACTTCA

>c7843_g1_i1 len=244 path=[1:0-208 210:209-243]

AACAAGCCTACAAGGAAGAGATAAAATAATTCAGCTTGTGAACTTCAACACTCTATTAAG

TTCACTGCAGAAAGCATGAGACCACCTCCCCATCCTCCACCTTCTTCTCTACCTTTCTGG

ACCTAGCTATGTTCTTAGCTTCCACTCCAGGCACTGACAGCTCCAGGAGGAAAGCAAAAC

ACTGAACCCCCAATGCAAGAACTTCTTCCTTCTAAAATGCTTGCCTCATGAAGTGAGCTG

AAGT

>c7849_g1_i1 len=215 path=[285:0-214]

TGCAGCTGTGCTCTCACTGGGAGCAATGTGTTAGGACACATGGTTGCTCTGTCCTCAGCT

GCCCCAGCACTGCTGCAGGACTGTCCTGACCCTGCTCCAGTGACCAAGTTACTGAGGTTG

GGCTCTTGGAGCCCCTGATCCACCTGCTGTTTCCTTGTCTGCACCCTCCACAGTGCCATC

TCTAGAGGCTCAGCTGTCTGTCCTGGGCACCCAGA

>c7851_g1_i1 len=312 path=[433:0-224 153:225-311]

TGTTCATTTCAAGGGCTGCAGTCAGGCAGCTCTTCTGAATCTCTGACTGCTGTCACCTTG

GGCCTCATTTGTATAATGAAAGGATAAATGATTTTAGCCTAGGCTGCCCAAAACAGGATA

AAAAGATCAGCTCTTGATTTTATCTCTAAAGAGACAAGAAGAGAAAAGGTTTTAGTTCTG

CAGCTGTAAGAAAGTGGCTTGCTAGAGAAATGGGACTGAACCTATGCAGGTGAGGAGGGA

AGGGCTGTATGGCTGAGGACCCCAGGTCCTCCCCCAAGGCTTTACCACCAGTGCTGTATG

GGAGTGACCATG

>c7851_g1_i2 len=239 path=[1:0-151 153:152-238]

GAGAAATGGGACAGCTGTGCCTCATTTAGTTAATTCTGAGAAGTTGTTTACAGTGGAAAC

TATTTGGTTAAAACGAATATGGAATGCTGAGCACTTTATGTCTGTAAACATCCATTGCTG

GGGGGCTGTAGAAGCTGTATGACTGAACCTACGCAGGTGAGGAGGGAAGGGCTGTATGGC

TGAGGACCCCAGGTCCTCCCCCAAGGCTTTACCACCAGTGCTGTATGGGAGTGACCATG

>c7854_g1_i1 len=207 path=[185:0-206]

GCATGTTGCTGTTTAAACTGTATCTGAACTAAGGAATTACAGGAAACACTGCATTCTCCT

TTTCATACTTTTATTTTTCTGCCCAGACTCCAAAGGTGTACACCACTTTGCCATAGTGGC

AGGAAGGGCCACTACTCAGTTGTTGCAGTCCCCAAGCAGGTGTTTGGTTCAGACTGACCT

TTTTTGGTTGAGGACTGAGGTGCAGGG

>c7857_g1_i1 len=216 path=[1:0-215]

ACTTGGTTTTCACAAATAATACAGTAGCTTCTTTATAACTTCTGACATGAAATTCTGCAA

TTTTTTGCAACTGATGCTAGGCATCTGGGGAGCACTCCTCCACTGTGCTATAAATACATT

TTATCTGCCTTGTTTGAGATCACTTTCAGCTGAAATATTTTGTGATGCTTCAGCGTAATG

CTAGGTGGAAGGTTGTGGTTGCTGTTTACTTGAAGA

>c7868_g1_i1 len=235 path=[300:0-77 @553@!:78-182 61:183-234]

TATACACATCTGCTCTAGTTAATATAAACCCAAGGCTGACAGTCCCTTTGCACGTTCATC

TAAGAGGACAGCCATGCTTTTACCAACCTCCATGCCCACCTACTGCAGCACCCGGCTGGG

GACAATGCTTCAAATCCCCCACGACACCTTGGGATACATTTAATAAGACACAACTTCAGG

CTCAGGCTCCGTTGCCAAACAGATGCAGACACATTCCTAAAGAGAACAGACACCA

>c7868_g1_i2 len=236 path=[300:0-77 @553@!:78-182 481:183-235]

TATACACATCTGCTCTAGTTAATATAAACCCAAGGCTGACAGTCCCTTTGCACGTTCATC

TAAGAGGACAGCCATGCTTTTACCAACCTCCATGCCCACCTACTGCAGCACCCGGCTGGG

GACAATGCTTCAAATCCCCCACGACACCTTGGGATACATTTAATAAGACACAACTTCAGG

CTCGTGTTCTTCTGGCAAACAGTTCCTGGAATGGTGTCTGTTCTCTTTAGGAATGT

>c7871_g1_i1 len=328 path=[306:0-327]

TGAGGTTCAGCTACTGGAACAGCTCCACAGCACCAGACTTAATTCCTTACCAGAACCTGC

CTACCCCCAAATGCTGCCTTATGGTAAACACTTGGAAGAAAGCTTTGCTAAAAGGAAGCA

GAAACACCACCAAGGGAATTTTACATGCTGACATTAAAGGGAAGTTTAACTTCACTGAGC

TGCTCATAGCACCAAAGGACAGCAGCAGCAGATGAAGCCTTTCATTAATATTTGCAGTAC

TTAATTTGATGTTTCTACAGACAGACCTGCCCGAACTCCAGGCTGTACCAATGCTAAATG

CAGTGGGGGAATCACCTCTTCTGGCTGG

>c7873_g1_i1 len=210 path=[357:0-44 17:45-82 147:83-209]

TTGCTGCAGTTTGTTTTACTATATTAGCGAAAAACCACTGCAGGGAAGGCAGTTATGCCA

GCAAAAGTTTTCATGCCATGATGGTAAAACAAACTGCAGCAAGATGATGACTTGTGAGAG

TGCCTTGGTCTAGCTGGAGACTGTAGGTATGTTTGTGTGTGAGTATAGGCTACAGCTCTA

AGGGCCTGCATGCATACAGGTATCAGTTTG

>c7880_g1_i1 len=303 path=[539:0-111 46:112-302]

GAACCTGCATTATATTTAAGTGAGCTCTCTTCTAGGAGGAGTTGCAATAAACTGTATGTT

CTGGACCTACCTGGATCCTTGTGTGTATTCAGTATTGCAGATAAATCACTCATGGAGTTG

AAAGGAAAGTACAGTTATTAAAGTACTTCCATGGGAACTAATCCCATTCAGAAATACAGA

CTGGCACAGTTGTTTCCCCGTTACCTGGGAGGACAGTGTCAACTGCCTACAACTGTAGGA

GTGTATTTGACAGTTAAATTCAAGTCTTTCAGAAATAAACTGTACCACATGAAGAACTTT

GTG

>c7888_g1_i1 len=220 path=[1:0-219]

GTTTTCTCTAAGATGTCCATTTGTGGAGAAGCAGCAATGAAGGCAGTTAACAAGGCCCAG

GCTGAGCTGCTGGAATGGATCATAGCAATCATTCTGCATGACCCATCAAATCCTCCATTT

CAAACCGAACGGGTACGCCACCTAAACAGAGAATTTATGAGAATCCCAAACTGGAACAAA

GAGGCAAATGCGATATCTTGGCTTTATGAGGATCTTCTAT

>c7890_g1_i1 len=224 path=[1:0-223]

GTGAGAACTAGGCCGCCTCCCTCCTTGTGCAATGTCATAACTAGGCCACAGCTGTTCTTC

CTGTTGTTCAGCTGCAGGGCTCTAGAATATCATAAGCTCTGGACTGTCTGTCTTGAATAT

GATTGGCTCTATCTGCTCTGTTAGACTCTGTGCTAAAAGCACACTTCTCTGCCTTTCTCC

AGCCCTTTAAACCCTGCTTCATCCTCTTCCTGGCTCCCAGTAGA

>c7894_g1_i1 len=320 path=[452:0-319]

AGGCTAATTAAATAATTATTTCAGTGAGGCCAGTGTGGTAATGAGCTCCAGATGTTTGGG

ATTCCACGGATAGTCTCTGAACTGGAGAAGTAATGCTGGGGTCAGGGACGGGGGACAGCA

ACTGGCTGGAAATCGCAGATAGCTCTCCTGAGCTCCTCAGCCTAGTGGAGAAGAGCCAGA

AGCCATCCTTTCACCTCAGTGTCCATTTCTTCACAGAAGGACTGGGAGTCTGGGACACAA

GAAGGACAGAATCAATGGGAAAAGACAGGAATTAAGGGGCTTGCAGCTGGCTGCAGGGGC

AGATCAGAGTTTTCTCCAGT

>c7897_g1_i1 len=217 path=[1:0-216]

GCAATGCAGTCAAAGATGCAGGGTTTCGGCTGAACAACCAGCTCTACGACATCATCACGA

TGCGCTATGCTGACAAGAACATGAACATCGACTTTGACAGCTTCATCTGCTGCTTCGTGA

GACTGGATGCTATGTTCAGGGCATTCCATGCCTTCGATAAAGATGGAGATGGAATCATAA

AGCTCAACGTCCTGGAGTGGCTGCAGCTCACCATGTA

>c7905_g1_i1 len=252 path=[37:0-251]

AAAGTCGAAAGTGGAACGCAAGAAACTAAGAATAGAAAAGCAGAACTTTATTTTGAAAGA

ATCAAATTCTAGAAGACAGAAAGAGTGGATAAAGAAGTGGGAAAGCTGTCCTGGCTGGTC

TTCACTCTACAGTCAGAATTTAGATCTGGCTGTGTTGCAAGCTGTATCAGAAGCAGAAAA

AGAATGTGAAGATGATGTATTTTGTCAAAATACAGGATCAGATTCAGATATTCCAGACCT

TTGGGACCTAAG

>c7907_g1_i1 len=251 path=[239:0-250]

GCTCCCTGGGCCAACGAAAACAAGAATATATTGCAACACAGATATAAGCGAGTTGGGTAG

CCAAGACCAACAATGCTGGCTGTATTGCTCTCAGAGCATATTCCTTGAACTACATCACTA

ATGTTGACTTTAGAGGCAGCTGTTTGATCCCTCCCCCAATTCATTTTCAAGAAAATCCTT

AGGCAGCAGTCTTTACAGAATTACAAATTCAAGCACAGTGAGAAGTATATTCTGTAAAGA

CTGCTGCCTAA

>c7911_g1_i1 len=233 path=[53:0-206 258:207-232]

GAGGCACTTTCCTCCCCCAGTAATCCACAGAGGACCAGCCACAACCTGACCCCAATTTAA

CACTTGGGCTGTAAACTTACTTAACACTTGTACAAGAATTTATTTTTTCAAACATACTGA

TGGCAGCATATGGCCCATCGACAGGTTTATTATTATAAGACACTTTTTCCACCAGGTTAT

AGTCTTTTTTATAAACATATGTTTCAGCACATAATAAAATCCCTAAGAACTCT

>c7911_g1_i2 len=234 path=[53:0-206 310:207-233]

GAGGCACTTTCCTCCCCCAGTAATCCACAGAGGACCAGCCACAACCTGACCCCAATTTAA

CACTTGGGCTGTAAACTTACTTAACACTTGTACAAGAATTTATTTTTTCAAACATACTGA

TGGCAGCATATGGCCCATCGACAGGTTTATTATTATAAGACACTTTTTCCACCAGGTTAT

AGTCTTTTTTATAAACATATGTTTCAGAAAGAGTTCTTAGGGATTTTATTATGT

>c7918_g1_i1 len=556 path=[49:0-555]

AATGGATAGGAGTTTTTGGTTCTTGTATGCAGTCTGGATATTCTTCATTCTTCCTATTGC

GGCTGATTGTAGAGCTCATTTCTGTGATGCCACCCTTGAATTGCTTGCGGGTTCACATGC

CAGTTGTTGAGGCACAACATGGGCACTCATTTATGCTCCAGTGGAGCTTCTGTGTGCTGA

GGGGAACTTTTCCTGGACAAGGTATGTTTTTTTCCTGTCCAAGATCTTTAAGTCTTCAGA

AGGAGTCCTTTGTGTGAGGTTTGGTGCTCCCACTTGAAAGCCGGGCATGGTGGAACTGCC

AGGGGGCAGGGCATGGGAGGAACAAACGTCAAATGAGGATTTTCTTGCTGCAGGCTGTTG

GGGAGCATAGCTGTGGAGACAGATGAATTCAGTTGGTGAGGCAACTTTGGAGTATGGGTT

TTCTATGTGTGGATGGGGGACAGTTTTTATATTTCAGTAGTGGCAGAGTGTTTTCTAGGA

AGCTGTGACCTTTGATGTCATTGTGTGTGAGTTTGTGTGTGTGAATATGACGTGCATGCG

AGTCCTACTGGTAATA

>c7920_g1_i1 len=316 path=[294:0-315]

GTCTGAGAGAGGGGGGACTGTCCCAGAGGGGAACGGAGAGGAAATCCTGAACGAGGAAAA

TCCAAGTGCTGAGCAGCCCCAGAAAGGAAAAGAGGACCCCAAAGTCACGTTTCTTGCTGG

GATGTGTGGGTGCCATCCCACTCCTTCCTCCCTTGGCTTCCTGAGCCCCTTAAATTGGGT

GTTTTCACTCTGAAAAAGAGCAGAGGGGGGACTCACTGCAGTGTCCCAGCACTGAAAGGT

GCCTACAAAGGGGACAGAAGCTCGAGATGTGGGGCAGTGGCTACAAGCCGCTCATTCCAT

GCTTATAACCCTAACT

>c7920_g2_i1 len=234 path=[798:0-233]

GGTGGGGAAGATCCTGTGTGATTAAACAACAGCCTAATTAATCCCTGTTTTATTACTTTC

TCTTGTCAAGTGGCTAAATAGGAGCTGTCAGCCGGCAGCCACCGCCGCTCACATTTGTTT

AGATCCCAAATTACTTCATCAGAACCATCTGCTCCGCGCGAGGGGCATTCAGTGGTTGTT

TGTTTCTCTCTCTGGACACAGCGTGGGGTACAAACAGTGTCTGAGAGAGGGGGG

>c7921_g1_i1 len=270 path=[66:0-269]

TTTTTTTTTCTAAAATTTAAATCTTTATTTTAATCTAATATTTTTATAATTTTTTTTTAT

AAATTTACATTTAATATATAATTTTCTTTGATATTTTAAGGAATAACTTTTATATACTCA

TAATATAAGCCTCTTTATAGTTCTGTTTAACATGGTAATATATGATTGAAATGATTTTTT

TTTTTCTATAAATATTCTCATAATTAAATGTTTTATTTAAAGAACTATTTATTCAAAAGG

AACGTAATAAAGCAACTAAAATATTAACTA

>c7927_g1_i1 len=265 path=[243:0-264]

GAAACATAATTCAAACTTAACCCACAAAGCTGTAACGTGAAACAGAAACCACCCCAAGCA

TCTTCCAGTGAATCAGTTTGTAGAATGCAAGAGGTTCAAGAGATTCTGTGCCTACAGCAG

TGCTAAGCACCTTCCTCAGCAAGTCATCCTCACTCAGGAGCATGTGCCAAAAAGTCACCA

AAGTTTACTTCAATGCATCCCATTCATTGCCATTTTCTGTGACCAACAGGCTGTTATATG

AGCTTCAACAAGGACTGTATTTTTA

>c7929_g1_i1 len=264 path=[520:0-263]

GGGGCAGAGCAGAACTTTCTGGGAGGAAGAGAGGGCCTGGCGGTCTTGTTTCTTCCAACT

TTTGCCTTGGCTGTCATGGGGTTGGAGAAAGATTGTGCAAAGGGTAGGTTTGAGATCATC

CTTGGGATTCGAAGCTCCTTCTGGGGGCAGGGCTGAGCCTTGTGGCTGGCTGTGAAACCC

AGTCATGGTGAGGGCAGGTAGAGGAAAGAAAGTGGTGCAAAGAGGCCTATGATATGCTCC

GCCCCACTTTCTTTCCTCTACCTG

>c7930_g1_i1 len=286 path=[264:0-285]

AGTACCTGCCTGTTAGTGCTTGTAAAGATTGAGTAATATATAAATAATTGGAGCTGATTT

CTTTCTAATGTGGCAGCCTCCATATATGTCCTTTCGCGTTATCTTGAATAATGTAAAAGT

ACTAAAAGTCTTTGTTATTTTATTCTTCCTATACTACTTGTAACATCATGAGGGAAAAAG

CATGATACCCATTGCAAAGCTTTAAAATGCAAAAGGTGACTTTAGAAATGGGTTTACATA

TTCTGCCCTTGATTCTTTTGCAAGTATATTCTAGTTATCTTGCACA

>c7934_g1_i1 len=210 path=[223:0-173 396:174-209]

TCAATGCGGTTACGCGCAGGGAAGCGAAGTCTCCTGAGGTACACAGCGCCTCGTGCAGCC

GGGGCTGCCTCACCGCACTGCGCAGCGCCAAGCTCCGAGCTGCTGAACCCCAAACGGGCT

TCCTGCTGTGCCTGGTCAGTGCCCACCTGAACCTCCAAGGCCTGGCTGGTGCTCCACCGC

TGCTCGCATTATTTTAACCTGCTGCCAGAA

>c7934_g1_i2 len=210 path=[223:0-173 25:174-209]

TCAATGCGGTTACGCGCAGGGAAGCGAAGTCTCCTGAGGTACACAGCGCCTCGTGCAGCC

GGGGCTGCCTCACCGCACTGCGCAGCGCCAAGCTCCGAGCTGCTGAACCCCAAACGGGCT

TCCTGCTGTGCCTGGTCAGTGCCCACCTGAACCTCCAAGGCCTGGCTGGTGCTCAGCGCT

CATCTTTCTGGCAGCAGGTTAAAATAATGC

>c7939_g1_i1 len=295 path=[539:0-294]

GCATCTGGGAAGCCAGCTGACATGAATGTAAGGTTAGCTGTTTCTGGTCTGGAGTTTATC

ATTGCTTTTCTCTAGGTGTGCTGTTAACAGATAAATATTTATGTTCTGTATTTGTGTTGT

GATAAGGATTTCATGCTTAAGCCTTTGGGAAAAGAAGTAACCTATGAGAAAACAGCGGGC

TGCAAACAAACTCCTGCCAAATACTCACAAAACAGACTAAAACAATGCCTGACTATGCAC

TGTAGAGGAAACTGAGTTATTCTGAAAGGCAGGGCAACGTTACTCAGAGCCTGTA

>c7943_g1_i1 len=308 path=[440:0-307]

ATGTATGACATGCTTGGGTCTGCAGCTGATTTGCTTCCGCTGTTCCTGATGGCACAGCCC

AAACACACTGCAAGCCAAAGGTGCTCCAGCAGTTTATAAACTCTGCAAAGAGGGCTGGGA

GGCAGTGGCAGAAGCTGTTATGGTCATTAGGACCATAAGGCACCACAAAGGACTGGGAGT

CCGTCCCTCTTCTATGCATCCCCCTTCAGGAAACACCAAACCCGTCTTTACTGTCAGTTC

ACCCCTTTTGAATTGGAGCTTTAATCGAGCAAGAAGAGCCCAGAAACGGAGAAATCAGGA

CTGATGAA

>c7944_g1_i1 len=298 path=[43:0-111 42:112-297]

TAGCTGAAGCTTGGTGACTGAACTAGTACTCTTCAAGATGCAATCTCTCCTTTTCAGAGT

CACTTCATCTTGATTGATTCTTGAATTAAAAAAGAAAACAAAAAGAAATTGCAACAGCCA

CCGCTGGTTCTTTAAAAAAACATTCAATAAAATAAGAAGCAGCCCCCTAATAAGTTAAAC

TTCATTGATATGTGTTGACACAGTTGCTAAAGTAATGGAGTTATCTCTTTGGACTTGAGA

AGACAGGATTGCACAATTCAGAAGCAGAATGTTTGTGACTATAATGTATTATAAAGGA

>c7945_g1_i1 len=219 path=[473:0-218]

ATGTGGTGTGAAAAACACAGTTGAATCCCTTAACAAAAATATCCAACTCTTTCTAAATTC

AGCCGGTCTCCATCTTCTGGTTAAAATAACAAACTGCAACTTCTCATTGTGCATAATAGG

CATCCTACAACAATGGAACTTCAAATATTTAGCATTATTGTTCAAAGAGTATGCCACAGG

ACTCACAAAGCAGTAAAATCACAGAACATTTTTTTTTAT

>c7961_g1_i1 len=214 path=[346:0-213]

TTAACACAATCAACTTTAGGTCTTTAACTACAAAACAAATGAAATGCATTTTTATTTAGC

TAATTCGTGCTCGTAGTTTAATCATGTTTCATATGCAGCGTTTTGTTACATCTTTGTAAT

TATCTACCCATTTCCTCTACTGTACTACAAGACTACCAAGGTTGGTTAGGAGAGGAAAGC

CTTCAAAAGAGCTCCAGGGATATTCACTCCCTTG

>c7965_g1_i1 len=246 path=[418:0-245]

CACGAAATGCAGCCTGGAGACGAAGCGTAGCGCTTCTCACAATCCTATAGTTGTGAACCT

GAGCATTTCGCTCCTTACAAGCACGATACCGCTGCTGAATTCGTCTGGTTGCCAACAACA

AGTTTCTGAAATCCCTCTGTTGTTTGTGCATTCGATAATAAGACTGTATCAGCGCTGCTG

ACCGATGCATCCTTTTTAAGTCTTGACGAACCTTCATGCCTCTAAAGGCAGCCTGGAGGA

CAACAG

>c7967_g1_i1 len=301 path=[41:0-156 40:157-300]

ACGACATCTGAGAAGTTAATTTTTAGAATAATTATAAGAAAGTTTGATTCTTAAGTTTTT

TCTTAACATAACCACACTGACTATACAGTGTGTATATTGTTCTTGCTCTTACACAAGGAG

AGAAAAACAACATTTAAAGCATCAATTGATGCTAATTACCAAAAGATAAAAATCAATCAG

GTTTTAAGCCTTCCAAGTTATCAGCTTCAATCTCTTATCTGGAGTAAGGCACACACTTCT

GCTTTCTCTTGAGAGACTCTGAAGTAGCCTGGTCTAGCAGTAATTCTCTTTGAAATGTTA

G

>c7970_g1_i1 len=1495 path=[295:0-1494]

AAAAAAAAATAAAATAAAATAATATTAAAATGTCATATATAAATATTCCAAGGGATCGAA

ATGATCCCAATTATAGATATAAAATGCCCAAATTAATTTCGAAGATTGAAGGTAGGGGGA

ATGGAATAAGAACGAACATTGCGAATATGGGGGAAATAGCTAGATCTTTAAAACGTCCAC

CTATGTATCCAACTAAATTTTTTGGTTGTGAATTAGGAACTATGGTGAAATTTGAAGAAA

ATGAAGAAAAAGCTATTGTTAATGGGGCACATAAAGAAAAGGATTTGGTTAATATTTTAG

ATAAATTTATTGAGATGTATGTTCTTTGTCCTCATTGTCTATTACCAGAAACAGATATTA

TTGTTAAAAAAGGTTTTTTAATTTGCAAATGTAATGCATGTGGAAATATAGGAGAACTAA

ATAATTCTCATAAAATTGCTACTTATATGATAAAAAATCCTCCTTTAATTAGCACTGTAG

GAAATAAAAAAAAGAAAATTAAAGAAAAGAAAGTAGAAAAGAATGGAAAAAATAAAAATG

AAAAAATTGAAAAAAATGAAGAAAATAAAAATGGGATAAATTCAGGAGCAGATATTGAAG

GATCTGAAGAGATAGACACAGATAAAAGTAATCAGAATACTAAAAAGGAGAAGAAAAATA

AGAAAAAAGATAAAAAAAAGAAAGCAGAAGATAACTTCGTTTTAGAAAAAGAAGCATTAA

ATTTTGAAAGTCCTGAAATAAAAGAAGTTATAGATAGGTTAAGAAGTTTATTTGAAGCTA

ACCCTAAAATTAGTGATAATACATTTTCGGAAGAACTAAGGGTTTTACAAGTATCTCAAT

GTTTTGATTCCAAATGTAGAATTTTTATTTGCTTATGTTCATTATTTGATGATAAAATAA

CTAAAGAATTGTTAGAAAAAAATATAAGATATTTAAAAAAAATTAACGATACTTCTGTAA

CAATTAAAGATATTTTTCTTGCCTTAGAATATTATGTAAATAAAATAGCTGTCAACTCTA

TTCATATTTATCCATATATATTACAAGTTTTATACAACAATGATATATTTGAAAGTAAAG

ATATTATTAAAAGATACGATGAAAATGATAATGAAATACAAGATCAAGTCAATGAAGATG

ATTATGTAAATAAATTTTATATTGAATGTTATAGCAAATGTAAAAATATGGCTAAACCTT

TTGTTAGTTGGTTAAAAGAAAATGATTCAGATACTGAGTCAGAAGAAGAAAATGTAAACA

ACAACGTTGTTAAAAATAATATTATAAATTATAAAGTAAAATCATTAGGTAATAATGAAG

GAAAAAGTAATAAGGAAAATAATAATTGTTCTAATGAAAATGACAAGGTTTTTGAAGATT

CTAAAAGTGAAAAATGTGATGATGAAATATTTTTAGATGCAAAAGATGGTATAAATTATA

CAGGGGATGAAGAAGAAATTGACATTGATGCCATTTAAGTATATATATATATATA

>c7987_g1_i1 len=213 path=[1:0-82 194:83-212]

GACTTGGAGGGCAGTGTTTGCAGCCACGGTACGCATACAAGTAGGTGACTTCTTTTAGAG

AGCAGAGCAGTGATTCGTGTTTGTGTAAGGGATGTGTGCTGCTCAGTGCACGGGATGGGG

GTTATAAATGCAGGAAGGTGTAGATAGTGACGTTTTGAATGAAGGGTCTTTCAGTTTTGC

TGTCCTCATAAAGCAACATGAAGCTGCATAAAA

>c7988_g1_i1 len=201 path=[397:0-200]

GGAGATCATGCTTCCACCTGCACCCTCACGTGTGGCTGTGATCAGCCCTTTTAAGGTGAG

CTTCACAGCTGCAGTAGGGAAACATCCCTTCAGAAAATAAAAGCAAGGACAAACCACAAA

CATCAACATAAAGTTTTAGGGGCAGGAGCAGCCTTTGTTTCTGTGCTACATTTTGTTTTG

ATGGCTGGTTGTTTTTTAAAC

>c7988_g2_i1 len=221 path=[199:0-220]

GAGTTTAATTAGATTATTTATTTTAAGCATTTACTGAAGTGAGCACAAAGCTAGAAAAAA

CTTTACATCAGATTCAACTACTTTTCAGTTGGAAGCAATACGAGAATAGATAACAGAGCA

CTAAAATCAGATCACCTTTATATTTGATCAGAAGACAATGGCAGCTGTATCAGCAAGCTG

GTAAGCAGCAATATTGAAAACTGGGGTCTAATTTATTGCTC

>c7997_g1_i1 len=362 path=[516:0-361]

AAAAAAAAAAAAAGAAAAAATTGAAAGAGTAATAAACGAAATTAAAAAAATTAATAATAA

CATTTTTTTTTTATGTCAAAATATTACAGTTTTAGATAATAAATTAATTACTAATTTATA

CAGCATAAAAAATTACATAAATGAAGAAATAAATAACTATCTAATAAAAAATGAAAAAAT

TAATGAAAATTCTATTTTTGTTACATGTTTAAGTATAATAACTAAAATAATAGAAAAACA

AAAATTAAAAAATGAAATAGTTCACATAAATTTCAATGTTAACAATAGCATACAGACAAC

TAAGAATAACAACTCACAAAAAAAAAATAGAGAAGCAAAATTAGAACAAGAATCAAACAC

AA

>c8004_g1_i1 len=242 path=[270:0-241]

AGCTTGTGCAATTCCCCCAGCAGGAGATCGAACGTAACATCTGGACATTGGGAGGGTGAG

TTGTTACAAGATCAGTGCTTGGCGCAGGGTGGCTGTGGCACTTTCCATATGTCACAAGTA

GCCCTACGGCCAGATTTACTCTATCTTTGAACCGTGGCTTTAAATGGCTGCTTAAACTTT

CTCCCAAAGCTTGGGTTGCCTTGGTTACAGTCCAATCCTTATGCACATCCCCAAGGCTAA

CA

>c8011_g1_i1 len=227 path=[1:0-167 169:168-226]

CTGCTCCTGAAATTTTACTTGGGGGTGAATATGATGCACCAGCAGTGGGTATGTCCTCTT

TGAATTCAACAAGTGATCTAAATGTGAAGTACCATGATGTTGTACCACTCTGTTTTCTGT

TTCAAGATAATGCTGTATGTGGGAGCCAGTTAACTGGCAGGAAATGTTATCGTGATGCTA

AAGTTACAGATGACTTAGTGTTGGTGCATCTCTATCTGAAAAAGTTA

>c8014_g1_i1 len=285 path=[263:0-284]

CAGAAGACTGCATCCTAACTACCAGATTTAGATGACAAGTTGGAAGGCTCAGGAGAGAAA

CAGTGACAAATAGAAACTGATATTCAAGGAGTTCCTCAACATAAGAAGCAGCAGCTAACT

TCTTCTAATTTTGTAAAATGGACTGAAAATAAATTCAGCTGTGTTTGAGAACTCATCTGG

TAGTCTATCACAGACGAGTACTTTTTGCCTTGATGGAAGGGTTGCAGACCTGGCCTTACC

CTCTTCTTCTCTGAGGTGAGTACAGTTCAGGTGGCCATGTATATG

>c8017_g1_i1 len=231 path=[262:0-184 447:185-230]

CTGGCCAAGACCAAGTCAGCCGAGTCAATCCCACATATGAGCGTGGATGCGGCGCTGCAC

ACCACAGTGATGCAGGATCTGGGCCTCCATGATGGCATCCAGAGGGTGCTTTTTGGCAAC

AACCTCAACTTCTGGCTGCACAAGTTGGTCTTCGTGGATGCTGTCTCCTTCCTGACAGGG

AAGAGGCTCTCCCTGCCCCTCGATCGTTACATCCTGGTGGACATTGATGAT

>c8029_g1_i1 len=384 path=[365:0-19 29:20-201 29:202-383]

GGCCCCATTACCACCTATACGGCCACGTATCAACACGAGCATCCCAACATGAAGACAACG

GAGGGCAATGGGTGGCGTTGAGTGACACCAATGGGTCCCAATGTCCCCAAGTAGTTCCCA

GGGACACCATGAGTGACAATGGACCCCATTGAGGGACACCCAACGCTGAAGGTCACCCAA

TGGGCCCCAATACCACCTATAGGGCCACGTATCAACACGAGCATCCCAACATGAAGACAA

CGGAGGGCAATGGGTGGCGTTGAGTGACACCAATGGGTCCCAATGTCCCCAAGTAGTTCC

CAGGGACACCATGAGTGACAATGGACCCCATTGAGGGACACCCAACGCTGAAGGTCACCC

AATGGGCCCCAATACCACCTATAG

>c8033_g1_i1 len=219 path=[1:0-218]

GAGCATCCATTGCCACCATTCCTGTAAAAAATGGCTCTGTCACATGCCAGCAGCAAGTAC

CTTTTGGGAAGCTGCCTCCTGTGTTACTCAACGACTGTCAGTGATGAAGCTGCGTTGCTC

AGCGGGTGCTCGATTAAGGAGATTGTTTCACAGTGTTTTATGCTACATCTGTTGCTGCTG

CTGCTGAATTTTATAAAGTCTTTATGGCAGACACATGCA

>c8036_g1_i1 len=228 path=[206:0-227]

TCCTGCTCTAACACAACCACCTCATAAATGAACTTTATAATCACGTAGAACTCAACATTT

CAAAATTAAACCACTAGACCATCAAACCCCAGAGCCAGAAGAGGTCACAAAGAAGAGTGA

ACAAATCAAAGTGTCACTGAGTCCATTTTCCTTTTCTATGGAAGGGTTTAAGATCTCTGA

GGAGCACAAGCCATAACACAATGGATTGATTTGCATTCACTTGCTCAG

>c8039_g1_i1 len=221 path=[199:0-220]

GATACTTCAGTGCTTTTGTAAATGTGGAGATCTGTCCTCATAGAGGCTCACCACAGATCT

TCAATCAGTTGCAACAGATGGTTCAGCGTTCTTGATACCTGCAGAAGTGTATTCCTGAGA

GGAGTCAAAATGTCTCAGTTATCCCACAAAATATATATGGTTAAAATTATGTATAAGTGC

ATGTTCTTAAGCAAAATAATCAGATGTGGATTTGTTAACAT

>c8052_g1_i1 len=481 path=[193:0-480]

TGCAGACCTCAAACTTCCCCATCGGTGTGTTTGTTTTCCAGCAAATCTTGAATTCAAGTT

GAGTAGGAGAAAGTACAAGTAATGGAACGGGCTGCAGACAGTTCATGCTTTCCTGGGAGT

GGATAAAGTAAAACACGGAGGAAGTTTCAAAGGACCGGGCTGAGTGGGTCCTGATGCTGT

ATCTGCTCCTATTTGGACACCAAAATGATGGATGTGCCATTCTGCTGTGCGAAGAGTGTG

GTGCTTTAGCGAGCTGTTTTTTGTGAGAGAGCCTCAAGATGTGACTGCCTCCAGGAAGGA

TGCGGTGGTGTTAGACTGCCAGGCCCGTGGGGAAGCTCCTATAACTATCACGTGGCTGAA

AAATGGAGGCAAAGTGTCTGAAAATGAAAGGATCTACACCTTATCCAATGGCTCTTTGTA

CATCAGTGAGGTGGAAGGAAGGAAAGGAGAGCAGTCTGATGAAGGATTTTACCAGTGCTT

G

>c8053_g1_i1 len=269 path=[1:0-268]

GAGAAAGTACAGATATGGACAAAATTGTCAACGTTTAAGCGACTCACTGAGCTCTTTTGG

CTTTTGTAGCTGTGACATCTGACACGTTGACTCAAAGAGTGACTAAGAGGTTCTGCCACT

GTTGGCAACCAGCACCTATCTTTTGCTTTAAATTTTACATTTTAACAGAGCGAAGTAGTT

GGCACCATTTTTCATAAGCTCTTGTTTACTTTGGTATGCTCTCGTTTGGGTGTCCTTCAC

ATCAACCTGCAAATTTATCATCTCTGGTT

>c8062_g1_i1 len=223 path=[427:0-222]

TGAAAGCCAGAAGCAGACTACCTTAGAACTCTGCTCATGCCTGTAGGACTCACTTCCAAA

ACGGCTTTTCACGTTGCCACTTTCTTTAAAATGATGAAACTTGCTCCTGTCACACAACTC

TTTGCTCACAGTCCAGCTAAGAGGTGGGTGCCAGCAGTCTCCAGGGAATCAGCTGGCAAC

GTACACCCAATTTAGAGAATGACCCCAGTAACCATGGACTATA

>c8063_g1_i1 len=214 path=[155:0-213]

AGAAGTGAGAAAATGTATAGAGAAATAATCCTGTATACAGTAAGGTTAGTGAAAAAGGAA

GGGGGAGAGGTACTCTAGGTGCCAGAGTGGAAGTCTCCCTGTAGCCCATGGAGAAGACCA

TAGTGGAGCAGACTGTCCCCCTGCAGCTCATGGTGTACCATGGTGGAGCAGGTATCCCCC

CGCAGCTCATGGAGGATCCCATATCATAGCAGGT

>c8066_g1_i1 len=322 path=[155:0-321]

AAACTGATTTCACCAGAAAGACACAACTCTGTCTAGTTCATGCTCAGGAAATCCGTTCAA

CAAGCAGGGATAGACCTGACCTTCTCTCAGTGCCAAGATCTTTGTTTGCACAAAGTAGGG

TTTGAGCATGCCTCATTGAGGACAGAAAACTGATGTTTTGCAAAAAAAAAAAAAAGCATT

TTTGGAATGCAATGCATACTGCTTTCCTATGTTCAAAGTTGGCAGTGGAAACACCAATAC

ATCACTGAAATGTTAAGCAGAACAGTGGTAAATAATGTGAAAATGTCAGGGGATAAGACA

GTAATTTTCCTCGTAATATAAT

>c8078_g1_i1 len=208 path=[456:0-207]

AGGTGAGCACAGTAACGCTGGAAGATCAGATGTTGCATGTTCAGAGAAACGATGATGTTG

TCATAAACAGAAGTATTGAGTATTAAGAGCAACAGCACTGCCAGAGCCAACCACACTTCA

GACACCGAAGTTAACCCTTGGTCCCTCAGCAATGAACACTGCATCAGAAACTGTTGTTCG

AGGAGCGATAGGACAACCTGTCACATTG

>c8091_g1_i1 len=212 path=[344:0-211]

CCTTGTTGGAGCTCCTACATATATGGGAACTGAAAAAGAGGAACAAGGGAAAGTGTATGT

GTACGCTCTGAACAAGACAAAGTTTGAGTATCAGATGAGTCTTGAACCTATAAAACAAAC

GTGCTGCTCACCGTTAAAACATGACACCTGCAAAGTTCTTAAGAATGAACCCTGCGGAGC

ACGCTTTGGGACTGCAATTGCTGCAGTGAAGG

>c8092_g1_i1 len=257 path=[235:0-256]

GCACAAGTGACCAAAAGCACTGTACCCTACTGACCAGCTTCTCACCCCGTCAATCTTTGT

GCCATCCAGAAATTCTATCAGAAATAATTCTATATTTACTTCCAGATCGCTGCCAAAAAT

GATGAATAGCATTGGGGCCCTGACTGATCCCTGCAGAGCTGCATTAAAAATGTTCCCATA

CAGCGACAATTCCCCATTGACAACTACTTTTTGAGATCTGTCAGTTACTCGGTTCTTAAT

ACATTTAACGTGCTTCA

>c8094_g1_i1 len=267 path=[245:0-266]

CAGTCCTGGCTGTGTATGGTCTGATCCACAAGCATGTTGTAGGTAATGGTGTTAGAGTTT

AGGCTCCCAGCTTTGGGTGGAGGCAGATGCTCAGCTGTCTGGCTGGGTCCTGTCCCCCTT

CTCCTTCTGGTCCTCCTGCCTCGGGCTACTCACACAGCACAGCTGGCCCCCAAGCCTGGG

GCTTCTCTGAGCCATTCCGTTCCTTGGAAGCTGCTTTGTCAGTGCTGTCCACATCTCAAG

GCACTATTAGGTTCTGTTGAGCTCGGG

>c8096_g1_i1 len=207 path=[119:0-133 118:134-206]

AAAAGTCAAACAGCAAGTAGCTGGCATGTTTCTGCATTCTTAGGGCTTACATCATCCTTC

ACTGAGAGGAGGCTGAAATTCAGCCTATGCATGCCCAGAGTGCAGAATAGCTGAAAACTG

TAGGCAAACCGAGCAACATATGGGAAGAGGAGAATCTGCTACATCTCCACACACAAATCA

CCCCCACGCAAACGCACGCATCATTTT

>c8104_g1_i1 len=291 path=[155:0-290]

ACTATATGGAGTTTGCATGCTGCTATTCCTGTGTAACTGTATGTTACATGTTAAAATCTA

CTTGTGATGGAGCAGGAGTATCTTTACCCCAGCCAACAAACATGTTACCTAGATCTATAT

ATCTGATGCACCCAGAACTTTAATTTGCAACTATAAAGGCAAGCCTTACAGCGTTGGCTA

TGTAAGAAGCAACATACTTCACTCCTACCACAAGCAACTGACACTTCAGAAGTGCCCTAA

ATAGCAATGTTCATGAATTAAAGATGATTTGAGGTGTATCACACATCTTTG

>c8105_g1_i1 len=224 path=[1:0-223]

AATCAAATATCAAACTGAGATGTTTGGTGATCTAAACTGTTGCTGATTTCTCATTCCTGT

ATTGCACTGGAACTGACCACACGAAGCCTCTGTGAGCTTTAAAAAAGCAATGCACTGCTG

TCTATCTCTGTTTGGGTTCTTTCTGTAAACTTGGACTTATCTTCCCCCTGTTTATCCTCT

GTATGCTATAAATAAAGATTTCCTGAACCCATAGAACAGGATCA

>c8112_g1_i1 len=291 path=[1:0-290]

TCTTTATGTTATTAGTTTGAACAACAAGCACTTGGTTGATCGCCCTATTAACATAAAGAA

TATATACCAACAAACATGCAAAAACTGAAAAACACAATAATCCGCATCACCTTTCCAACC

CACAGTCCCAAATCTTGATACTACCCATCAAAGTAAACCCATGAGTTTTCAAACGTAGCT

TTTGCCCTACAAATAAAAAATTTTCTGAGGTAGTTGTTGCATTGCTTGACGCTGAGAGAT

TGGCTGCAGTATCCACAGTCCAGTTGCCTCCATAGGCTTCAGTTAGGAGGA

>c8113_g1_i1 len=493 path=[471:0-210 682:211-236 708:237-492]

AAAATAATAAAAATTTTTATTTTTATAATAATTACACCAATACATATAATGGTCTCATGT

ATATATATATATAATTAAATTACTACTGTTTTTGTTAGAGAAATTTAATAATTTTGTTTA

ATTTTTCTTAGAGTACCATATAAATTAAATATTAATTATAATATATAGTTCTTTAAGCAT

TAATAGAATACTAATTAAAGAAAAATATAAATACTACTCAACAAAAAGTATTAATAGCAT

CTTTAAATAATATAACTTGTAATATGTATATAAAATAAAGAATCAATTACACACAAAGGA

TACCTATAGCAATGCTTTAATAGCAATAAAAAATTATAACATAAATTTTTATATAAAAAA

AAATGTTCTTTTTTTCTTTTACATTCTTAAAAAAATAAAAATGGGAAAATTTTTTGATAA

TCTATTTTGTATTATTATTATTTGATAAAAATGTTTAATTTTTTTTACAAATAATTAAAT

TTGAATTTTAAAA

>c8117_g1_i1 len=208 path=[211:0-173 210:174-207]

CTGTCTCAAACCACCAGAGGGGTATGAAGAAGCTTCAACAGTTCTTCTCTCTCCAGTCAT

CATCAGCAAATCAGCAAATGGTGGAGATGCAAAGTCCTCACAGCAGTGTAGATGAGGGAA

AAGGGCATGGTTTGACTCCCATGTGATCTTCACCTGTTGCTGATTTGCTGATGAGGACTG

GACATTAAGAACATTGTTTCATCCATAA

>c8122_g1_i1 len=370 path=[353:0-369]

TAGTCACAGACAACAAAAAAAAAAACCCCAACCATTCCAAGCTGGGATTGAGGAGATGGG

CAGCAGGAGCGGCCTGAGGGCGGTTTCCAACCGGTTGCTTTCCAGGGCCGGGTTCAGCTC

AGCACATTTTTTGCTTACCAAGACTGAGTTGAAACCCACCCACGATTCAGCTCCCCACGG

AGCTCAGTGCAGCACTCCTACCCCCACGGAACCCTCCCCATACAGAAGTTGGCTCCAAAC

AAACCCCCTTCGCACCGCAGCAGTCCCTGAGCACCAGCAGATGAACACAGCACACTGTAT

GGCTTATTTACAGGTTACACGGAAGACCAAGGAATGAAGAGAATGATATGTCAAAAAAAA

AAAAAAAAAA

>c8124_g1_i1 len=278 path=[346:0-223 620:224-226 66:227-277]

AGCTGAATAATACATTACAGGCATTCGTTTCCCCCAACACATGGGGTAACAAGCCCCACA

TAAACCATCACCAACATTTATCTTGGTGCTGCTATGAGAAAGCATCTGAAACTATCAGTC

CGGAGGTGGTTTAAAAACTAGATGAAACATGAGCAATTGAAGCACACTACATACACAAAA

AATAAATGACAGTTATGCCACTGTAATCTCCCATGCATCAGCACCTCACATCATCTTTTT

TTGTCCCCCTTCATTTGTTCTGTACTTCTTTGAGATGA

>c8124_g1_i2 len=278 path=[346:0-223 620:224-226 572:227-277]

AGCTGAATAATACATTACAGGCATTCGTTTCCCCCAACACATGGGGTAACAAGCCCCACA

TAAACCATCACCAACATTTATCTTGGTGCTGCTATGAGAAAGCATCTGAAACTATCAGTC

CGGAGGTGGTTTAAAAACTAGATGAAACATGAGCAATTGAAGCACACTACATACACAAAA

AATAAATGACAGTTATGCCACTGTAATCTCCCATGCATCAGCACCTCGAATCCTGTATAT

AGTCCTGGTCACCTCATCTCAAAGAAGTACAGAACAAA

>c8142_g1_i1 len=286 path=[340:0-228 582:229-231 585:232-285]

TTCAACAGAAAAAAAATGTTTAAATATACAGCCTAAGCTACTGTTGCAGCCCTCTTTCAT

GATTTCAATCTATTTCTACATCATGGCTCTCTTCCAGCATGAAAAACATGTCCTTTGCTA

GATCCATAAGGCTGCCTAACAATCTGTTTTCTACAGTTTTTCTGGAATTTGTCTGCATCT

TTGATCTCTCAATCTGTGATTTTACCTAAAGCTGCACCTTGGCACCAGGCCCCTTCCCCA

GTGAGGGGCTGCCAGCAGGGGGAACTCACTGGAAGGGAAGGGGCCT

>c8152_g1_i1 len=207 path=[545:0-142 25:143-206]

GAAGTTACTGTCAAAAGCTGGAGGATCCGATGTTTTTGAACACACCCAGCAGCTGGCAAC

TGCAGAAATGAAGCCTTACTGGCAACCCCAGTTCAGGGTCTAAAATCATGCCCTGCTCAG

GTTACAGTCCGCAGGAGCAGTTTTGGGGAGAAGAACATGTTAGTTTTGGTAAATGCTCCA

AAATGAATCGTCACATTACATGTTTTG

>c8152_g1_i2 len=399 path=[545:0-142 687:143-396 985:397-398]

GAAGTTACTGTCAAAAGCTGGAGGATCCGATGTTTTTGAACACACCCAGCAGCTGGCAAC

TGCAGAAATGAAGCCTTACTGGCAACCCCAGTTCAGGGTCTAAAATCATGCCCTGCTCAG

GTTACAGTCCGCAGGAGCAGTTTAAGGTAGAGGTGAAAGGAAGTGGCCTCCATTACAGTA

TGGATATTTAGAGTTTACTTTGATTCAGTGTAACTTTCTCTTTATTTCATTAATACCAGC

TGCACTTGTGCTTATTTTGAGTGCCAGAAGTATTGTGGCCCACTTACTACTTAAAGTATG

CAGCCCTTCACTGAAAAGATAGGAAGAAATTAGGCTTATCTTAACAGCAAATGTTACGTT

GGATGTCGAGGTGGGAGCAAATGGTTTCAGTCCTAATTA

>c8161_g1_i1 len=210 path=[1:0-209]

GGAGCTGGAGTTTAAGGTAAAGGCAGGTGCTTGAATAACTGAGGCAAAGGAATTCTTATG

GAACCAGAACATACCTAAATGTTTTGGTCCATCTGTTCTGTGGACTCTTCCATGTTAAGG

GAACCTGCCTAATCAAACTGGCTGTGCTGGTTCCAACTCATCCCAATTTATTGCTTTCAG

TTAAAAAAAAAACACACCAAAAAACAACAA

>c8167_g1_i1 len=234 path=[267:0-233]

GTGCTTTGCTACCAGAACTGCAGGAAATGCATCAAGGCTGCTGTGCAATGCTGGAGCAGT

CCTGTTCAACCAGTTGTGTTACAAGTGATAGCTATGCTATCAAGAAGGAATCAATATAGA

GCTGATATCCTCTTCTAATTCAGTATCTCTTCAAGATCTCTTCAGCAGCATTTGCTGCTG

CTTTTTCAAAAGGAGTCTCCTGCCAGTCCACAGAGCACACTTAGGAGCGAGGCA

>c8168_g1_i1 len=257 path=[213:0-256]

AGCTGATGACTTTGGGGGACTGCAGCTTCCTCAAGAGGGGGTCTATAACGCGTCCTGGCT

TTACTCGTGGTGCACGTAGGGTTTTGTGCAAGCAGCTTCTGGTTTCGGGTGGTCACAAAG

CCAAGAAAAGTGTCAGTCCCTCCAGCGTGGGGGAGATACTCCCCTCTCCCAGAGGACAAC

GCATTGCCACGTCTTTCCTTGCCTTGTCACCACAAGCAGCCATCCCATGTCCACCGTGGC

TCCCAAAGCTGCCAGCA

>c8169_g1_i1 len=262 path=[809:0-145 24:146-261]

AAAATAGTTTAGCGGCTTCATATCTTTTTCCTATTAACGCACCTACCTAAGAAGCTAATG

AAACTGGAAACTATTTTTACTGCTAAATAAAAACAGTAAGGTTTAGCTCTCTATTTGAGA

TCACTGTCTCAGTCCTTACCTGGATGTCTGATTCTCATTACTTATGTTAAAGCTGAATAA

CTGTTTATAGAAAGAAATTATCAAAGCATCTGTGGTTTTCTGAAATGTATCACTCTGTGA

GCTACTCTTTCTATTCTGAAGA

>c8169_g1_i2 len=241 path=[333:0-124 24:125-240]

GGTCTCCTTCTCTGGAGACATTCAAAACCCACCTGGATGTGTTCCTGTGTGACCTAGGTT

TTCCTGCTCTGGCAGGGAGATTGGACTACATGATCTTTTAAGGTCGCTTCCAAGCCCTGA

CATTCTCTGATTCTCATTACTTATGTTAAAGCTGAATAACTGTTTATAGAAAGAAATTAT

CAAAGCATCTGTGGTTTTCTGAAATGTATCACTCTGTGAGCTACTCTTTCTATTCTGAAG

A

>c8169_g1_i3 len=241 path=[333:0-124 457:125-240]

GGTCTCCTTCTCTGGAGACATTCAAAACCCACCTGGATGTGTTCCTGTGTGACCTAGGTT

TTCCTGCTCTGGCAGGGAGATTGGACTACATGATCTTTTAAGGTCGCTTCCAAGCCCTGA

CATTCTGTGATTCTTCTATTTCTATCTAGAAATACCAGATCTTGGACAACCTCTTCTTTA

CAGGTCCCCAAGTGTATGATATCCAAGCATTCCAATTCTTCAGAATAGAAAGAGTAGCTC

A

>c8174_g1_i1 len=208 path=[370:0-207]

ATCCTCAAGATGTCGACATATAGATTTTAACAGCTTGTAAGTTGTATCTCTGGAGAGCAA

GGAGACAAACATATATCTGTCTGTGACTGTTGCTATGATCAGAGCATTTGGCACAAGAAG

GGCAGTCTTGGTTTTCTTCAGAAGTGTCACTGAGAGCACAGGTATGCTGATCTTAGTGTC

TTTACCAAAAACCTTGGAATGGAAGCAA

>c8175_g1_i1 len=205 path=[183:0-204]

AGGCATTACAGTAGTAGAGTGACTACCACTGCATTAAAAAAAAACTCAGGTTATAAAACC

AGCACAAGGTCTCTGAAATAAGTGAAAGATGTTTTTATTTCTCTATTCTTTTTTTTTATA

GAACAGACTTTTGCTGAATAGGAGGATTGACACAACACAGGCAATGTTCTACCCAACTTA

TGCGAGAATATAAAATAACTATTTG

>c8182_g1_i1 len=377 path=[127:0-126 487:127-376]

GACCACCACACCTCTGCTAACCCTGGCTGAGATGAGGAGTTCCTCTAAGGCCATGATAAG

ATGCTACCATATGGACTGGGGTGCTAAAGGATGTGGTCAGTCCGTTGGGTCCTGGAAAGC

TTGTGTGAGAGAGGATGTGTCATGAATGTTCCCTGCATGGAAAATGCCATCCTGAGTACT

TAATTAAACTCAGATCCCAACAATATGGGAATGGGTCCAGTGAGGCCATGCCACTCATAA

ATTAAATTTCATTTTATTTGAGTTTGTCTGGTTTTGTTTGTATGTTTTTAATGTTCATTG

TGTAAAAGTAGCTGTATAGCAACATAAGTGAACCCCATTGCATGGGGCTGAGAGCAGAGG

AGCTTCTGGCTGGCATT

>c8185_g1_i1 len=490 path=[155:0-489]

ACATTATAAAGCTTGGAGAGGCCAGGTAGGTCCTTGCTATTAACAAAGAATGTCCATTGG

TGATCATGGAAGATTTTTACCTCTTACACCAACACAATTGTTGGCTGTTAATGACCACAA

GTTGACTTTCTTATTGTGGCCGTGGTAAACTCCTGGTGGACTTGGCCAACTCCTGCTTTT

CTCTCACTGGGGGTATAACACAGTCTGGGATTTCTGATTATTTTTCAGATAATGAAATGT

GTGTCTTCTAAAAGAATTACCCATTCCGTTTCAATTCGGCAGGGCGTTCGGGTATCATTT

GTGCAGCAGTCTGTATAAGGGTAACTCATGTAACAGCATGAGCCTTCAACATGAAACCTG

GGGACAGTGATGGGGTTTGTTTTGGGGTTTGTTTTACCCCTGCTCCACCTAACCCAGCTC

CCTGCCTTCTGCACCAGTCTGCACCGCTTGTGAGCTGTGCCCATAACTAAACAAGCTGGC

AGCAGAAGTG

>c8187_g1_i1 len=257 path=[133:0-86 107:87-226 358:227-256]

TGCTCCTGCTGGGAATTTTATACAGAAAGCAATTCTTCTTCAGAGTACTAGATAGATACC

CCAAACGTGTGGATGTGCACAACTTACTGAGAAACGGTCAGTTTGGACAAGGAACTTTGC

AGACTTCGTATATCTGGGCAAGCGTCTCAGAATTGCTTTGGATTATGGTTTCTTAGGCAG

TAGGGATGAAAGGGGAAAAAAAGTGTTTACAACTTCTCATTTCAGTACATTCGTAAGCGT

CTTCTTCAGCAACCTGG

>c8192_g1_i1 len=210 path=[188:0-106 295:107-209]

GGTATTTGTGGGGACCGGACGGTGGATGTTGGCGGGGCGGGGCAGTGAGTGGTGCATTTC

AGCGGTGGTGACAGCAAAGTTTAAGACAGGCCAAATTGCAAATGGCCCTGAACAGCTGCC

ACACCACGAAGCATCCCAGACCAGCCCATCTGCACGAACCACAGTATCATGATCAGGACA

CTCAGCACAGAACTGAATATCAGCTTCAGT

>c8193_g1_i1 len=201 path=[179:0-200]

AGCTCTGGGCCCGGGACAACAGCAGCCACATGGCACAGGGGAAAGATGAGGCCCCATCTA

GCAATGCACGCACACAAAAATAATAATAATTAAGCTTTTAAACGATTGCAAGGCAATTTA

AGCACCCCTCCTGACAGATGAATCTTCAGAGGCCTTGTTCTTATTGACTGAGGGAGAGGC

AGGTGCCAGGTGCAGGCAGCA

>c8194_g1_i1 len=211 path=[343:0-210]

ATTTTCATGCCATGTACAATACAGAGGTTCTAGAAGGTGTTCATTTTCCTTTGTAAAGAA

AAGATGAATGGTATTACTGAGCACCTTAATATTAACTATACCCATATTAACTATCGTTAT

TCAAGCTTAAAGCAATCTAGCAATTTTTAGACAACTGAAGAAAGCCAGCTACTACCCAGG

AGCATTTGGCTGTATACGGCGTACAGCCACA

>c8195_g1_i1 len=280 path=[155:0-279]

GTGGACACAACATCCCTTTTGCCTTTAATCTCCTGCAGGGAGATCTAGGAAACAGCAGGG

CACTGTGTCAGACACCTGTCCCTGCAGGTGAGCAGAAATCCTACGGGAGCAGAATCTGTG

GACACCTGTGTAAATATTATCAAAGCATTATCCTCCTGGGGAAGGGCAGGTGATGTGTAA

ATGGAAGTCTGCCTCTGGGAACAGCCAGCCTGGAAGGTGTCAGCAGCGAGGGAGGAAAGC

GTGGTGCATGTGGAAAGCTGTGCTGGTTGGAGGCAGCGTT

>c8206_g1_i1 len=238 path=[121:0-176 23:177-213 60:214-237]

CTCCACTCAAATCCCCTAACAAGCTAACAGTGGTGTCAGTGGCCTCCCAGCCTCCCAACT

CCCCCCAGAAGACGGTGGGCGTCCCACTGAATGTAGCGTTAGGACAGCAGATCCTCACAG

TGCAGCAGTCAGCACCCTCCTCCCCTGGGAAGGCCATAGTCAACCACACAACCACGCAGT

AAGAGTAACCTTATGCTCTCTTGGTTGGTTTAATAGGCTGTGAAATCAGCAGTACAGG

>c8206_g1_i2 len=201 path=[121:0-176 60:177-200]

CTCCACTCAAATCCCCTAACAAGCTAACAGTGGTGTCAGTGGCCTCCCAGCCTCCCAACT

CCCCCCAGAAGACGGTGGGCGTCCCACTGAATGTAGCGTTAGGACAGCAGATCCTCACAG

TGCAGCAGTCAGCACCCTCCTCCCCTGGGAAGGCCATAGTCAACCACACAACCACGCAGG

CTGTGAAATCAGCAGTACAGG

>c8208_g1_i1 len=231 path=[597:0-230]

GCTTTATTTTCTTATATAAAAAACCAAAACAAAACAGAAAATAGAAATTATCAGTGTCCT

GATCTCTGCTTCCTCTTGCAGTAAGAGTCATCCTTATATCCCATGAAAGAAATTGGTTGG

ATTGCTTAAGCTTGGCAGAGAAGCCATTGTGAATAAGAACGCCTCTACCTCAGAGAGATT

ATGTGTTCTGGAAGCACGCTATGCAATGTGTTGAATATTCATCTAGTGTTA

>c8208_g2_i1 len=217 path=[195:0-216]

GCTGTGAGTACACAATCATTTCTAATAGCTAACTTTCCTAGAGGACGTCAGACCTGAAAC

TATGAAAAAGTTTCCCAAGTTTGCAGTAATATGGGTCCAACAGCCTTTAAATGAAAAAGA

TGATACAGAAGTTCCCAGTCTCAAGCATGGTCTCTCATGTCAAACATGTTAAGACAGCAT

ACTCTAAAAGCTGAGTTCTTTGGATTACCCATCTTAA

>c8210_g1_i1 len=204 path=[1:0-203]

AGGCTATCAGTCCTTAAGAGCTCGCAGCAAAAGCAAGAGAAGCTCTTCACACTGTGAAGA

AATGATGAAATCCTAATTACACGCCCTCTTTATCCACAGTGGATGGACGGAGTATTTGCT

GAAAAGTGAATAAATCGCACAAATAGTGTTGCTCTGGGTGCGATGCGCAGCCTCGTGCGA

CTGAGCACAGTGATTTGAAGCAAT

>c8211_g1_i1 len=209 path=[187:0-208]

GCACGTTGAGCCCTCCACCAACCCCAGCCTGCTCTGCTTTCAATGCAAAGCAGAAGATAT

TGTTTTGACTGAAAGCATCTGTCTGCTCTTTACGCAGAAAAACATTTTGGGCAGAGCTGA

TAGGCAGCAACCTAGCACTGAGCAAAGGGAGCCACGGCCCTCAGCTCCTGCCATGCAACT

GGCACACGCTGTAGGTTTTTGGCAGGAGG

>c8212_g1_i1 len=206 path=[49:0-71 120:72-72 24:73-205]

GATATTTCTGTAAAAAAATATTCTAAAAGACATAGGGGTTCTGTGTACATTTTCTTTATT

GTATAATTTTGATTCAGTCCTACAAGACTGTACACTGAAAGGGGCAAAGATCTTGAGAAT

GACTTAATATTTACTGTAAGTACGACAGAAACTGGAGCTGGGCAAGGAACCTTCATTCTG

GTATTTTTTTAATACTTGGCAGCCTG

>c8231_g1_i1 len=248 path=[174:0-168 343:169-173 428:174-247]

AGAAAGATTTTTGCCACTGCCCAGTTTTCTTCTTGTAACTTCCATTTCAGTTCCTTTCAG

TGACTGAAGAGATGGCTTTCTTAAAAAACTTGAGGCAAAAGCAGAGATCATACAGAAATG

CCTTCTGTTGGTTTCTGTCTGAGGGAGAATTGTGCACCTGTAAAATGAACTTAGTGTATG

CAGATACACCTGAAGGTGATGAGATGCTTTAATTATATTGAAGCACAGGCTCTCTCTAGT

GCTTTGAA

>c8237_g1_i1 len=203 path=[51:0-57 25:58-202]

CACCGTCTGTAACAAGATCACCCTTGGAATATTACAGAACCATGCCACGAGGAGTTGGCC

AAATGACCTCTGGAGGTCTCTGCCAACCTCAACCATTCTGTGACTCTGTGAACAAAAATA

CTGGACAGACTTCAGTACCTACACAACTCCCAACTCACAGGGGCTAAATAATAAACTCTG

AGAAGTATGGTTTCATTATGCCT

>c8238_g1_i1 len=629 path=[281:0-278 560:279-545 1301:546-628]

GGACTTGCCACCTTCAGGAGTGTGATAAGAGATTTAAACAAGATGGTGGTTGGAGCCACT

GGTCACCGTGGTCATCATGTTCAGTCACCTGTGGTACAGGCATGATCACTAGAATTCGTC

TCTGCAACTCTCCAGTACCACAGCTGAATGGCAAGCCTTGTGAGGGTGAAGCAAGAGAAA

CCAAGTCTTGCCAGAAAGATCCTTGCCCAATTAATGGCAACTGGGGTCCATGGTCTCCGT

GGGATGCTTGCACAGTGACGTGTGGAGGAGGACTTCAGAAACGCAGCCGTCTCTGTAATA

ATCCTGAGCCTCAATATGGTGGGAAGGCTTGTGTAGGTGAAGCTAAAGGAACTCAGGTCT

GCAACAAACAGGACTGTCCAGTTGATGGGTGTCTGTCTAATCCCTGCTTTGCGGGAACTA

CATGTACAAGCTCCCCTGATGGTTCTTGGAAGTGCGGTGCCTGTCCTGCTGGCTACCATG

GTAATGGTATCCACTGCCAAGATATTGATGAGTGCAAAGAGGTTCCTGATGCCTGCTTTG

TCTTCAATGGAGTGCACAGGTGTGAGAATACTGAACCTGGATACAACTGTCTGCCTTGTC

CACCACATTTCACTGGGTCACAGCCATTT

>c8240_g1_i1 len=207 path=[1:0-206]

GTTTTGACTTTGTCTAGCTCAGGGGAGGCCTCATGGCAGCCTATAGCTCCTCATGAAGGG

AGCGGAGGGGCAGTGCTGAGCTCTTGAATCTTTCAGTAAGTACTCTGCAATCTGCTTATG

GGGTTAGTACTCTGCAATCTGCAATCCACAAGAAGCTACTTCTCATCTCCTTTCTCAGAT

ATGGACTATCTCTGAGGAACACCACTA

>c8241_g1_i1 len=232 path=[295:0-231]

AAGAGCGGGGATGTGGCTGGAGAGATGTGCATCTACATGTTTGTGGTGCCACAGTATATG

GGGGTATGAATATAAGCACAATTAGGCATAGCAGACCCTTAATATTAAATAAATCTTCCT

GTGCTATTTCCAACTTCGATTAAAATGTAACTCAAAATAGAGTACTATATGAGGGGTGCG

TTCTGATAAATGTGAAATAATGTTTTTTCATGAGTGCTCTGAGTGGATCGTG

>c8248_g1_i1 len=286 path=[1:0-285]

TACTTTTCATACCGTGTAAGTTGCAAAATTGCAAACAAGTTATATATATCTCAGCCAAGA

GAGTTACTGAATATGTACTCACTCCTGTGCACTATCGTTGTTCATCAAAACATATTTTCA

AATTATTAACTGCATAGTACTCATTACTTCAAAGCAAAATTCCTTGTTTTTGAAAAGTCT

ACCTGAAAATTTTATCTCATTTCACAATGGCTAAGATTTGTATTTTGGCAAGCAAGACAA

TAGAAATGTCAAAGGATAGAAAATAATCAATTTACTTAACCATTAA

>c8259_g1_i1 len=260 path=[238:0-259]

CATTGCTTGCTTTAACAGATTCTTCTTCTCTCTCTTTCTTTTCCTGGTTTTCTTCTGCTG

CAGTTTTATCTGCTCTGAAAAATATTCTTGCACTACTTAATGAAAAATAAAGGAGTTCAA

ATTCCTGTAAGCAAACTTCCAGTCGCTTGTGAGTAAGGTTCATAGTTTGTAGCAGCTTTT

CATCTTGATTAATTGACTGCACATTCTGTTGCTTAGCTACTGCTCTTATTTCCTTCACAT

ACTTCTCTCGAACAGACTGG

>c8259_g2_i1 len=209 path=[475:0-208]

ATCTGTTAAAGCAAGCAATGGAGATCTTTCCAACTCTACGTCTGCAGATCCTGTTGTGAA

ATGATGTGGCTGGTATGGGGTGATAATTACTTACTCGAATAACTATCACTAATGCTCTGG

AGGGGTAGAAGCTCTTCGTTTGACTTTTTCTATTAAAGCTAACAGAAAATGTACAATAGT

ATACTAGCATCCAGTTGGGCTAGGTCTGT

>c8274_g1_i1 len=251 path=[155:0-250]

GGCGGTCTGGTCCTCCATTGGTCTGTTGATCCATTGAAATGCTGAGCAGGGTGTTTTGCT

TCCCGGAGGCTGCGGGGACGGCGCTCCTAGGGCAGGTGAGGTGTCGCCCTTCGGGGGTCC

GGGGGAGTGACTCGGAGTGGTTTTGCTCTCTGCTCCCAACCCTCAATCTCTGTTCAGGTG

CAGGGGCGTGCAGTGGTGGCAGCTTGGTAATGTGTTTTACTTTGGAGTGAGGAAATCAGT

GGCTTTTGTGT

>c8277_g1_i1 len=252 path=[163:0-162 456:163-251]

TATATCCTAAAACAATCTGTGGATGAAAGTAAAACTGTGTTACGCAACACTAGTGAAAAT

TTATTATGTAATGTAGGCTCACAGACTCATGCAGCTATCAGTGGAAGAGAGCAATGTGTA

ATAAAGAAGCAAGAGGCTGAGAAGCAAAGTAATGTTTCTGTAATTGTTGAACAACAGATG

CCTTTGAATAATCCATGTAAATTGCTTGCTCCAATTAATTCCGTTCAGTGTTTTTCAGAA

TCTAGAGATTGT

>c8281_g1_i1 len=217 path=[349:0-216]

TTCTTCCTGGAACGTTATGGTAGTAGAGGGAATACCTTTGTAAAGTGGGTGTTGTGTGGG

ATACCAGCTTGTTGCCTACGTTCGCCTGGCTTTAAAACTGTTTGAAATCTGGTTATGCAA

CCTTGTATTTGGGATTTGGAGGTGGCAATTGCTTAACTGTTCTTGAATGCTTTGAAAGGA

AAAAATTAGTATATTTCCTTTACAGATAAAGAGTTTG

>c8292_g1_i1 len=281 path=[155:0-280]

TCCCAACACCCCTTCACCAGTGTGCGTGCTTCATGGATTTTATACCGTGCTCTGAAATCT

GAAGCAGTGCAGAAAGAGAACAGCTGCCCGAATTGAGCTGAGAAGCAGTGTTTCCTGCAA

ATGATGAAGTACCAATGTAGCCTGAAGGAAGGCTAATGGAGGAGGCAGAGGAACAGGTAG

CTGTGGAAGAACGGCGACTGTGCAGTGAAGCTGGAAATGTAGCAAGGAGAGAAAAATGTA

TGGGAGGCTGCATCTCAAATAGCGAAGAAAGGTATTTTCAG

>c8297_g1_i1 len=270 path=[45:0-155 22:156-174 44:175-269]

TGACTATAACAACTTAACTTTCCTCTCCGCTCTAGTAATGTTTAGCACTGAGCTTTCCCT

TCATGAGCTGTGATTTCCTCACTTCCTCATAACAGTCAGCAGTCTAAGTGTGTAATCTGT

GTATGTCAGCTGAGGTCTTGTTCTTTACCCATCTATTTTCACAGGTTATTGTATAAAAGG

GCTTAAACTATAGGAAGAGTGTTTTAGATGATAACTTTCTAAAATACAATAATATTTATT

CATTTGTAGATTTGTAATTATTAGAAGGGA

>c8302_g1_i1 len=287 path=[1:0-286]

AACTGGCCTCAGATTTAATATGCTATGGTGACATTCATTTTTACTTATGTTACTCATCTA

AAAGTTAGCCATATAATCTAAGTAAACTGTCTAGGATTCTTGAATAGTCAAGTAGACAAA

GAGAGAGGTAGTCTCAAAAACAGATTCGTGTTGTGTATTTTAAACTAGGTTGAGCTTCAC

TTGGAAAAAAGGATCTTTCTTTTATGCCTCCATTAATGGAGGCCTTGATTGCATTGGAAA

AGCCTAGAGTAAAAACAGAACACGAATCTCAAACACAAAGTTGCATG

>c8303_g1_i1 len=209 path=[411:0-29 35:30-208]

TTGTGTTTGATTTTCCACTGAGCTGTCCTCAAGTTGTTTCTTATTCCTAAATGAAGTATC

ATTGGGTACAATAGACACCTTGAGGACAGCTCAGTGGAAAAATCAAATCACTAGTTAGTA

CTATAGCACAAGCCCTAAGAAGGTTAAAGAAGATGGGTTTAGACTCAGAGTAATTATATG

TTAAGCATGCAAGAAGAAAAAGAAGAATG

>c8303_g1_i2 len=213 path=[1:0-33 35:34-212]

ACTTTGTGTTTGAGGGTGGTAATGAATTTTGGAAAAGTTGTTTCTTATTCCTAAATGAAG

TATCATTGGGTACAATAGACACCTTGAGGACAGCTCAGTGGAAAAATCAAATCACTAGTT

AGTACTATAGCACAAGCCCTAAGAAGGTTAAAGAAGATGGGTTTAGACTCAGAGTAATTA

TATGTTAAGCATGCAAGAAGAAAAAGAAGAATG

>c8307_g1_i1 len=212 path=[338:0-149 25:150-211]

AAAGGCCTACAGCAGATGGCTAGAAAACACAGTAAGAACAGGACATACATACAGTGATAC

TTCCCCCAATAAATACATTTCTCCACCTTCATTTGTGACTGAGAACCTTCCTAGGCCAGA

GACAGCATCCAGTTTTATAAAACTCAATGTACCCAACAAGCATCTATGTAAATGTATTTC

CAGATTACAGAAATTGCACAAGCAAGGCCATA

>c8307_g1_i2 len=235 path=[338:0-149 487:150-234]

AAAGGCCTACAGCAGATGGCTAGAAAACACAGTAAGAACAGGACATACATACAGTGATAC

TTCCCCCAATAAATACATTTCTCCACCTTCATTTGTGACTGAGAACCTTCCTAGGCCAGA

GACAGCATCCAGTTTTATAAAACTCAATGTCCCCAACAAGCATCTATGTAAATACAGCTT

TTAAGTACTAAAAGATGAAACACTTCTTTATTCTTAAAGGAAAATTCCAAGCAAA

>c8316_g1_i1 len=217 path=[411:0-216]

ATGTTCACATTTCTTAAAGGTTGTGTACTGTGGCTGTCAATTACAGAGGAGTGCTTGAGA

TTTGCAGGTAGGCTTCTGCAGCTTCAAGGAACCAAGGGTGCTGTTTCTTAGTGAATTACT

GAAAACGTTTCCAAAAAGCAAAGAATTTTTGCTCAGAAAGTGATGCTATATGAACTGAAA

TGATGACTTTTTAAAAACAGTGAGGCACTTGGACAGT

>c8320_g1_i1 len=379 path=[1:0-378]

ATAGAGAGGGTTTACAATCAGACTGTAATCTGGAATACACATTTGCTGGAAACACACAAC

ACCTAGGAAAGATTGTGTTTCCTGTTTGTTAGTTGGTGGAGACATAGTAGTTATTTTATT

GATCACATCCATGGGGATCTGATGATCCTACCATTTTATTCCTAAGAATTGGATTGCTTG

CACAGGGCTCCGTGTGAAATCTTTCTTGGTCACAGAATCACAGGATTTGGAAGGGACCTC

AAGAGATCCTTGAGTCCAACCACCCTGCTAAAGCAGATTACCTACAATAGTTCACGCAGT

TAGATGTCCCGTCAGGTCTATAAAAGGAGACTCCACAGCTTCTCTTTTTTGAACTGGGGA

GTCCAGAACTGGACACAGT

>c8328_g1_i1 len=242 path=[1:0-241]

AGCAGAGACTTTAGGGAGCACAGCAGCATTAGAGTTCGGCATCTTCCTTCAAAGAAAGGA

ACGCGCTGAGCCTTTGAAGAGCAATGCAAGCTGTGATTTGCATGCAAAACACTGCTTTGC

ACCGGTGAGCTGATGGTGTAATGGATGGAACAGCTGCATCCAGGAGCGACTCTGGCAAAG

GGGATGGAAGCTGCTCAAAGCCTTCTGGCTTTCTATAGTCCCATGCTTCAGAGTTACAGC

AT

>c8356_g1_i1 len=209 path=[11:0-16 721:17-23 323:24-208]

GTTGCAGAATTAGTCCTGCCAGCTCATAGCTAATCCTGAGTGAAAGCTGTGACAGCTGAT

GCTGTTTCCTCACAGCCAAGGCAGCCAATGTTCTGGGGCTGGTCGGGTGTGCTGCTGGAG

GGACAACCGAAATGAGGGGGAGGGCACAGATCAGTCACACCGGCACATCGCAGTGCCCCG

TCCTACCCAACCAGCCCCAGAACATTGGC

>c8375_g1_i1 len=259 path=[293:0-201 25:202-258]

GATATCTGTGGTACCAGGGTAAAGAATCCATTCCACTGGTACCACAGATATCCCACACAA

CTGCCAGGCCTAGAGCATAATTCACAGAATGGGGGACAGAAACTCCGCTGAAGTTGAGCA

ATTGCACACAAGAATGTTCACAACCTACATGACGCCCTCCTCCAGCATCCACTGAAGAAA

ATTCCCTTGGCGTATCAGATGCCACCACCTATGCCTGTGCTAGCCCTCAGGAGAGATCCC

AAGCACTCTTGGCAGCTTG

>c8388_g1_i1 len=209 path=[1:0-208]

TAAACCTGGAATAGGAGGTGAACCACTACCTGGACAGAAGCTGATACCTAAATCCAGCGT

GTTTCCTGCAGAGCTGGGATGTGGTGCTCCTGCCTGGTTAGCATTTGACAAACAGGTATT

GTCTTTTGATGCCTATTTTGAGGAAGAAGTGCCTGATAAAAATCAAGAACCATATAGAAT

AAGACACTGTAAAATATACTTCTACCTTG

>c8398_g1_i1 len=257 path=[356:0-51 407:52-256]

AAATTTACAGTTTTGAGAAGATTGGACAATAAGAATTCTCCCTCTCCCTCTAATCCATTA

TCTGATTCTTACTTATTACGTTATTGCCAAGCAATTACATAGGTAACTGTAAACCCAAGG

AAGCTGATGACTGAACCTAAAGTGAATGCTCTCCTCGTTGAGAGGTGGCATTCATTCTTA

CAATCTAGGTGTGTTCTCAGTACTAAAAACATTTTGCATAAATGGTGAAATGATGATTTG

TGTGGTGCAGAGGCCGT

>c8409_g1_i1 len=201 path=[333:0-200]

TGCGTTCTTTGGCAAAACTTTGCTTTAATGAACAGTTTTGCTTTCTTTCTTTAAATACTT

CAGCATACAGACGTGAAATATATCGTAACGATTGTGTATCTTCTTCTCTGTTGAGAAGCG

GAAGACGTGAAGCACATAATTAGAATACCTTTTCATTTTAAGGTTTATGGGGCACTGTTC

AGAGTAAGTATAGGTTGTTAG

>c8414_g1_i1 len=216 path=[372:0-158 179:159-215]

ATGTCCTATTGGCTAATTGTTTGTGGAAACACCAGTGTGGTGTACACACGCATAAATATC

AGCTTCCAACACCGTTTATAGATGCCAGTGTAAGCAGGTAGAAAACTTTTCTTGGCAGAA

CAAAACTAAGCTAGTGCACAGAATTAACAGGCGGGGAGATAAAAACCCCACCTTTTCATC

ACTTAATGCTTTCATATTTTGAAGAGTTCATATAAA

>c8415_g1_i1 len=221 path=[1:0-220]

TTTGGCTCCTTTCCGAGAATTCACCATTTAAAAAACAGTGTGAAGAAGGTTCAGTGTTCT

CAGAGTGATGGGGAACGTGTAGGTCTGACTGTGGTGTCTTGAGGCACCGGAGTCATTCAA

ATACTTCAGAACCATCTATTTTTAACTCTGACTTTGATTGCATTCCTTCACATGTATGTA

CGAGTATACAAGATTTGTCAGTGATGCCTTACATTGATGCG

>c8420_g1_i1 len=244 path=[436:0-243]

AAATGCGATTATGGCTTTTAAAGCCTGCAAAGGAAGAGAACATGGGACAGACAGGATCAG

AGTATGTAATGGCCACTGATAATACAGCTTCATATTCTAGCACAGAGTAAACATCTGGAT

TAAAAAGCATCCTATAACCTTTATGTCAAAGCAATCACTGTAAAAATGTGTTTTACGTAC

GGATGTGAGCTGCTAGCTGTACTGTGCTTTTAAATGCAGTCATGAGGAACTATGGAATAA

TAAT

>c8433_g1_i1 len=226 path=[1:0-124 126:125-148 150:149-225]

TGGAGTACTGCATCCAGGTCTGGGGCCTCAATACAGAAAAAATGTGGAGCTTTAGTGTGG

AGCTTTAGGAGAGGGTCCAGAGGAGGATCACAAGGACGATCAGAGGGCTGAAGCACCCCT

CCCACGAAGGCAGGCCGAGGGAGCTGGGCTTCCCCAGTCTAGAGAAGAGAAGGCTCCATG

GAGGCCTCATTAGGGCCTTCCAAGTACTTGAAGGGAGCTTATAAAC

>c8439_g1_i1 len=328 path=[341:0-198 18:199-327]

CCAGCTGGGTGCCATGCTCCCCTGCAAGGCAGCACAGGGTTCAGGGTGGGCCCACAAGGA

GGAAAAGGGGACCCGACACCGACAGCCTGACTGACCCCCGCGGAGTGATCAACCCCGGCC

TCCTCCCTCAGCCCTCCATGGCTTTCCAGCCCTGGGAGTCCCACCTCCATCCTCTCCTCC

AGCCCCCAGCAGAGCCAGGGCTCCGCGCCCACCAATCTCAGAGCTCAGAGCTGCCCAGAG

CAAAACCAGCCCTCCGACACCGCTCTCACCACCTGCCAACCATCTCGGCTATTTCTAGTG

CAGAAAACAGCAAAAAATGTAAACCCGG

>c8442_g1_i1 len=364 path=[641:0-316 998:317-322 285:323-363]

AAAGACCTAAGAATGTGCTTATTTTAGTTTTTGTAATAGTTTATATGATTATAAATGATT

AAATTTCACTTGATTTTATTTTCCAAAACTACTTCTAAAGTTCAACCCAATTTTTCTCAT

CATCACTTTTGTTATCATAAAAATATAGAGAAGACACTTTCACTTAATAAAATTCTGTAA

GCCCTGTGTTTTCTTTAGAACATTATAGTCATTGTTAATGTTATAACATTATAGTCACTG

ATAAAAGCTGATAAAAGCTGATAAAAGTAGCAAATTCCCAAAAAATCTGACTGGAATGTT

GTATTAAGAGCTTATTCATTTTTCAAATGTGGCTTCAGTAGGATAAGTTGCATTTTTAAT

GCAT

>c8442_g1_i2 len=364 path=[641:0-316 998:317-322 963:323-363]

AAAGACCTAAGAATGTGCTTATTTTAGTTTTTGTAATAGTTTATATGATTATAAATGATT

AAATTTCACTTGATTTTATTTTCCAAAACTACTTCTAAAGTTCAACCCAATTTTTCTCAT

CATCACTTTTGTTATCATAAAAATATAGAGAAGACACTTTCACTTAATAAAATTCTGTAA

GCCCTGTGTTTTCTTTAGAACATTATAGTCATTGTTAATGTTATAACATTATAGTCACTG

ATAAAAGCTGATAAAAGCTGATAAAAGTAGCAAATTCCCAAAAAATCTGACTGGAATGTT

GTATTAAGAGCTTATTCATTTTTTAATAATGCAGTTTAAATGCATTAAAAATGCAACTTA

TCCT

>c8442_g2_i1 len=288 path=[39:0-240 998:241-246 285:247-287]

TCTACTATGAATAGTTAATATTTTCTTGCCTCATTAATGTTTGGGGTTTTTTCCTGTGAT

ACAAGAGCAACATTATATTTTTGGTAGAGATTGGACTGAAAAATTCTGAAAAAATCAGAG

CTAACAGCCTTGATTGTTAGCTTTAATTCTAACAAACTGCAGCACTGGGCAGTTCATTTA

GGTTGAGGTGTCCAAGAAAATACTCAGGGTCCTCAGAGTACTCCAGATCCTTGAAAATTT

TATTTTTCAAATGTGGCTTCAGTAGGATAAGTTGCATTTTTAATGCAT

>c8448_g1_i1 len=215 path=[181:0-214]

AATCAAGTTGCCTATGCAAAGTGTAGGATCCGTGTCCTCTTGTTCCTCTATTAAAAAGCC

CACTGTTGCTTTGGGTTTGTTTGGGTTTTTTTTGTTTTTTGGAATGTGGCTTGGTTGCTA

GATTTTGCACGTGAGAAATGCAAATAGTCACAAAAGATGCTGCTTCAGGTTGAATCCACA

CAGTGCTGTGTACCACTCCTTCTTATTGTCCCTGT

>c8450_g1_i1 len=263 path=[297:0-111 573:112-231 527:232-262]

AGCCCATACTCAACCCCCTAGTTTCCCTCAATTCATCTTGTTAACACTGACGATAAAGCT

TCCTCTCCACTCACAGGGGATCCTCATATGCCACATGTTGCCTGTTAGTGACCACCACCT

CACATCCAAGTCTACATGAGCAGCCAGAGCTCTTTGCTAACCTGACCCTACACTGAAGGC

CAATATAGAAAAGTAGACATATCAGGAGCAAGATAGCAGTGTGATTTTTGAAGACTGGAA

TAAATCTGTAGAAAGGTTATTTC

>c8465_g1_i1 len=485 path=[243:0-484]

GCATGAGGAGTGTGGTCCTTCCAGCCTTCACAGTGCGTGCCCAGTACAGCTTTAAATGCC

AGTTTTGCTTCCTACTTATCTCTGGCCAGAAAGCCAGATGCTGCCTTCAGTTCAGCTGTG

CAGTCTGTCCCCACCACCACTCCCTGTCTCATGATTGGGCTGATGACACCCAGTGGTGCA

CACCCTCTTGAATTTCAGTGTAGAGCTGCGTGCCCTTCCTGCAGCCAAGGCCCCTCTTCC

CAGCACAGGAGGAACAGAATACCATGAAGTGTTAAACCAAGCACTGTATGCTTTATAGAA

ACCCATACACACATATAATAAAACAGGTGTGTGTGTGCATATATATACACATATAGTGCT

TGTATGCTCACGTGACTCTCCTAATAAAACAGTGTTTATGCTCCTTTATATAAAACTCAT

TTCTATGACTACCTGCTTAAAGAAAGTCAATCTCATTTAGGAGAACACATACAAGAAATT

AAATG

>c8466_g1_i1 len=214 path=[192:0-213]

AGCTGTTGAGTCTTGGTCACAGAAATCCCTCAGCAGTGACAAACAAGCCCCTCCAGTTAC

CCAGGTGCTGTCCTAACAGTGACACCTGGAGCTCCTCCAGCCCTGCACCTATTTACCATT

GCTGGCCAGGCAGGAATCAGACAAAGCTCTGCATGGCGGTGATGCCTGCAGTGTTGCTGA

TGCCAACAACTCACTAGAGTAGTCACTAGAACTG

>c8480_g1_i1 len=217 path=[373:0-216]

GCTGTGGGCTCCAGGAGAAGGCCCAGGGTATTGTTTTCCAGTTTCTCGATCTCTTCCTGA

CTTCCCTTCTCGACTATGCCAGTAGAAAAACACCACTCCCTCCTCTTATTCTTTTTCTTT

GTTTACTTAACTTTTTATCCTTCCAGTGCCTCCAGCAGGGAGAGAAGCAGAGCACAGTGC

TTTCCCCCTCCTTAGCAGGGATTTTACACCGACCACA

>c8480_g2_i1 len=209 path=[187:0-208]

CCATGGGATGGAGGAATGCAGTTGTTTCAGGGCTGCATGGCAATGAAGGCCTCAAGTTAG

GCATTAGAAACAGATCTTAGAAACAATGGCTCACAAAAATGGTTAACAAAACTGAACCCT

GAAAAATGTCTCCGAGGAACAGGAAGGGTTCACAGAGAAGGATTTTGACCATCAAGCTGT

ATGAAGAAAAATTCTACTCTGGGCTGTGG

>c8481_g1_i1 len=362 path=[402:0-361]

CCAGCACCAGCGGGGCTGGTGGCAGACACCGCTGTCCTGGGGACGTGGGAATGGTCTGGC

TTGGAAGGCCTGCTCTACTCCCACTGCTCCAGGAGACAGCATTGCAGGGGCCCATCTCTT

ACAACAATCTTGCCCATGAAAGGATCTATACCAACTTCGTCAGATAGAGTCATATTTCTC

CTGACCAACTCCTTGGGAGTTGTTCATTAAGAACCATGGACTCAGGAGGAGGGCGAGGAA

GGGATGTTGGAGGTCGCTCCACCAACCTGGCTCCCAGGTATGCACAGAGTACAATATAGT

GCACAGCAGCATCTGCCCCTGCTTTGCACACCTCAGGGACTCCCTCGTGCTCATGTCGGC

CT

>c8485_g1_i1 len=260 path=[1:0-163 165:164-259]

TGGAATGTTAGAAGAGATGTGATATTTTTGTCTTACTGCTTCCGTTGGAAAACTGGAAAT

CCAGAATAATCTTTGAAAGCTCTTTATAGTGAGGAGGAAGTAAAACAACCTCTATCTCTT

AATTACTACCTGTGGATAATACATTTTCATATATTTTTATATGTTACGATAAGGTTTAGT

GTTTCCAGAGGACTTCACATTAAAGTTCCAGGAAAAAAGTTGCAGCTGGTGATGAGAGAG

CATCGCTTTCATGCTGTGGA

>c8493_g1_i1 len=207 path=[185:0-206]

AGTAGCATAATGGATTTGTTTCAGATTATCCAGTGCTTCATTTTTAGCATATTTGAGAAG

ATCAGCTTGTCCCTTTAAAATTGTAATATTCCACGTAAAACTTTCTACTGATTTTTGCAG

TTTTCTTTCTTTTAAGCCTTCTCTCGCTCCTATATTGTGCAGGTTTTCATGAAACTCCAT

TGCTGCTTTCTGACATTGGACTATCTT

>c8493_g2_i1 len=294 path=[369:0-293]

GCTGAAATCTAAAAGCCCCGTCGAAGCCTGGAGGTCATACCTGAAAAAGCAGGTGATGAA

AATGGAGAATGAGAGAACTCTCATCTCATAATTATGGAGTTTATAACTCTATGACCAATT

GTAGCTGCATAGGACATTTGCTTTGCACTTACTGTTGAAAAATATTTGGAATCTTCAAAG

CACAACTGGAGAAGCTAATTACAATCTATTGAAACTGTGAATGTATGTAGCAACTCTGCT

TGTGAAGGCAATGATATTGTGTAACATGGAAATTACATTATTGGCCAAAACGTA

>c8496_g1_i1 len=243 path=[1:0-106 464:107-242]

CAAAGGCTCATGTCTTGATTGTATTCATTCAGTCAGGGTTGTGTAACAGGGCTGGTGCTG

TGAGAGTCAGAACCAAGCCAGCCAGCCATGCTGCCCTTTCTCATCTCTACTTTCTCTTGC

CATCAGCCAAAACCACTTTCACCCCGATAGCTGCTACCTTCACAAATGTTAAAACAATTA

ATCATCTTCATCTTCTTTCACTCAAAACACTTTTTTGTGTATCATAAAGAAATTTACATT

TTG

>c8501_g1_i1 len=201 path=[356:0-177 556:178-200]

TATACCTGCAACTGTGCAAGAGAAGCACACCAGACCGATTTCTGTGTTAAGCAGGCTATT

GTTGCCTAAGTAGGATTTGATAATTGTATTCAAGTTATTTCTCAGCTGTGGTGGACGTAA

CATCTTAGATAAAAACAGACTAACCTAAGTGCTTGGAGTTAAACGTGCATTTTTACAGAA

ATTCTGATTTTACAGAAATCC

>c8503_g1_i1 len=207 path=[1:0-206]

TGTAAGAGAAGAAACAGGTAATTAAAATATATTGCGACAAATTATTAATATAGAAAAAAA

AAAAACTCTGTGGAAGTCTGCCTAAGTGCTAAGATGGACTGGAATGCAGAAGCACATACG

TGTGTGAATGATTTTAATTCAGGGCCAGACAAAGTTCATTTTGAAAGGCCTGGATCCTGT

AGAATACATGGAGGGGGGGGGAGGGGG

>c8507_g1_i1 len=298 path=[427:0-85 238:86-297]

AAGGGCTTATTTAAGTATATCAATAGCAAAAGGAAGAATAGGCAAAATGGGAGTCTGAAT

GAGGTGGGTGCCCTGGTAACAGAAGACAGTGACAAAGTAGAATTACTGAACACATTCTTT

GCTTCATTCTTTACTGAAGAGACTGTCCCTCAGGAGTCCTAGACCATGGAGGTATGACAG

AGAGTCTGGAGAAAAGAAAAGACAGAGAGTCTGGAGAAAAGAAGACTTTGCCTTGGTCAA

GGAGGGTCTGGTTAGAGATCATGTCTGCAAACATAAATCCATGGGCCCTGACAGGATG

>c8513_g1_i1 len=234 path=[351:0-176 824:177-233]

CTTATTCTACTTTATGCATGAGGTTTCAATCTCATCATGTCAGGCACTGTCACTCCCAGT

TTTTCCCTTAGAAACAGTCTGGACTCTATTTTCCTGTAGCCTAAGCATTCTTTTGCCCCA

GTACTGTCCACCATGGTAGTTCATAATGTACCACAAGCTAGCAAAGAGGCTTACTTCCTT

ATCAGACAACCAGTTGGCAGGAGTCACTGCAGAAAAAAAAACATAGATAAGGAA

>c8514_g1_i1 len=308 path=[1:0-307]

GCATTTTAAGGTCTTTCTATCCAAGTACAGCGTGCAGGTCTTGCAGAAAGGTAGCATCAG

GTCACTTCTCTTTCCATCATGCTGAAAAGCAAACACTTCATTCAGTCTTCTGGACACAGT

GTGACACAAAACCAAAGGAAAATGGATGACCAAAGACATGGCCATCTGGGCAGTGGCTGT

AGCAGCCCCTCGTTCCCTTGGACCTGAGTTAAATTTCCCCTGGCTGTGCCAACTCCTGGC

AGCCAGGAGATTCGACCCACAGCACTGTTGGTGTGGCCTGAGCAGCACAGCATGTGCATG

GGATTTTT

>c8523_g1_i1 len=3331 path=[3365:0-3330]

ATTTTTTTTTTTTTTCTTTTTTTGGTAATTGATTAAAATATCCATATAGATATTATCCAT

ACTGTAATATATTTTTTATTATAATTTAGAAATACTAATTGTTTATATACATTCACTACA

TACATTTTGTTCATTAATTTTATTGTAATTATTTTAAAAAAAAAAAATCCTTTTATATGT

ATTGTATTGAAGAAATATATTTATTTATATGCTATAAAATTTATTATCACTTTTTTTTTA

TAACATTTATTAAAAGACTTTATTAAAAAAAAAATGTTAAAACCTATTATAATAAAGGAT

AAAATTCAAAAAAATATATTGAAAGATCAAAAATATGAAGATGATAAATCTTTTCTAAAT

CCACATGAAGGTGATAAGGCAAGTATACTGCAAGAAACAAGAGTTTTTTCAAGTTATCCA

TTGAATACACAAAAATGTTTACAAATTTTAACAAAAATTCTTTATTTAATAAATAAAGGT

GAAGATAGTTTAACATCTCAGGAATGCACAGATATATTTTTTAGTATAACAAAATTATTT

CAATCCAATAATGAAAGGTTAAGAAGGATGGTATACTTATTAATAAAAAACCTACCAGTT

AGCGAAAAAGAAATATTTATTGTAACAAGTTCATTAACAAAAGATATGAATTCAGCAAAT

GATTGTTATAGAGCAAATGCAATTAGAGTATTAAGTAAAATAATTGATTATTCAATGGCT

ACTCAGATTGAAAGATATTTAAAAACAGCAATTGTTGATAAAAATGCTTTTGTTTCGTGT

TCAGCGTTATTATGTGGATTAAATTTATTTTCAATTGCTTCTTGTGATATTGTAAAAAAA

TGGATAAATGAAGTTAGTGAATGTATTAATAGCAAAAACCCAATGATACAATTTCATGCA

TTAACTTTGTTATGCTCAATTAAACATCAAGATAAACTAGCCTTAGAAAAAATTATAAGT

TCATATAATAAAACATCAAATAATTTATCAAGTTCTTTAGCAAATTGTCTTTTAATAAAA

TATGCATCCTATTTAATATACTGTACAGAAGTAGAAAATGAATTAAATAATATGAGTAAA

TATAATCAAACGAATTATAAGGAAAAATATAATAATATACCTAATTCAATGCATAATATG

CAAGATTATACATTCAATAAAAACAACATGATTCATCCAACTACTAAAGTTTGTTTTGAT

TATTTAAAAATTTGCTTAAAAAGTAAAGATATCATGATTTTATTCGAATGCATCAAATGC

ATTTTTGATTTAGCTATTCATGATAAAGAAGGCAAAAATACTACTTCCGTATTTAATGTA

GACATATTAAATGAATGTATGAAAATTTGTCAGATTTTTTTATTATCATCAAAAACAGTA

GATAAATTTAGTATAATAAGACAACTTAATAAATTATCTCATCATAGACCACATGTAGCC

TCTAGATTAAATCAAGATATAGAAAATTTATTAATAGATAGTAACAAAAGTATATGCGTT

TTAGCATTTACAACTCTTCTAAAAACAGGAAATGAATCAAATATAGATAAATTATTAAAT

CAAATTAATAATTATATGAGTGGTGATAACAGTTTTTTTAAAATTCAGATTATTGAAGAA

GTAAAAAATTTATGTTTTATTTATCCAAATAAATGCAACATTATTTTAAATTTTTTATCA

AATAATTTGAGAGATGAAGAATCATATCAATTTAAATCAAATACAATAGATGCAATAATC

TTAATAATAAGTCAAATACCCAATTCAGAAGAAACAGCAATATTACAACTATGCGAATTT

ATAGAAGATTGTGAATATAACTCTTTATTATTGAGAGTTATCAGATTTTTATTATTACAT

ATTCCTAAAACAAAAAACCCATCTAAATATATTAGATATATTTATAATCGTTTAATTCTA

GAAAATCCAACTATAAGAGTTGATGGAATGTATGCTTTGTTTCATATTGCTCTAAAATGT

GCTGAAAATTCTAAAGATATTTTATTTTTGTTAAAATGTTTATTAGCAGATAATGATGAT

GAGGTAAGGGATAGAACTAACTTTTTTTATTATATTCTTAATGAAAAAATAAAGGAAATG

GAGTTAAACAAAAAAAATAATTTACTAGAAATGAACAATGATCAGAATATATCATTAATT

GATGATTTATTAACATTTGATAAATCGACAGATATTGATCATTTATTATATTTTATTACT

AATCATATAAAAGAAAATATAAGTGAAGAATTTCATTATGAAAAATTAAAAGATGAAATA

ATTAAGAGTAATGATATAAATAAAGATACACTTTATATAAAAAAAAAAAGTTCTTATACA

GAAATGTTAAATAATGTTAGTATGACAAAATTTATTGAAATAACAACTGATTCTATATAT

TCGGAAAATATTTCAAGTTTTATAGAAAAGTATAATATTGGCAATTTAAAAATTATTAAT

AAATCAATACCACTAACAGAAAGTGAAGCTGAATATACAGTTTTTGTAAAAAAATACATT

TATGAGAATCATATTATATTAGAATTTACTATTCACAATACGTTAAGTGAGCAAATATTA

TCTAGAGTGGCATTACAGATGAATACCTATGAAAATAGATGGTCTGTATTAGAGCAAACT

ACTATTGACAACTTACATTATAATACTCCTAAAAACTTATATGTTCTCTTAAAAAAGAAT

TCTACTTTTTCGAATAACGAACTAAATGAGGATTCTTATAATGTTAATCAAAATTTTCAA

ATTTCTTTGCATTTTTTAGCAAAAGAAAATGAAATGGATGATGGATTTCCTGACTCTTAT

TCTATTAATCCCATTAGTATACAAATAACTGATTTTATTATTCCCAAAATGTTAAGAAAT

GGAGAATTTAAACATATATGGGAGAGTATGAATAATTTGAATTCTGAATCAGTTAGTAAA

TTTAGCTTAAATTTTGAAAATATCCAATCTGCTGTTGTAGGATTATTAAACACACTAAAT

ATGATGGCATGTGATAATACAGATAATGTGGAAAGAAATGCAACAAGTCATAACATGTTA

TTATCAGCTAAATTTTTAAATGAATCAAATGTTTTATGTAAAGCTTCTTTAATTCTTTCA

CAACAGTATGGTTGTTTATTAAAAATTATATGTAGATCAAAAAATAAGTTTTTATCAGAT

TATATTTTAAAATCATTAGAATAATTTATTGAATGTTTATGTCTTTTAATTTATATAAAT

TACCACTCTTTTAACAGGATATATTTTAATATTATATCCTTATACACATATTTATATAAC

CTAATAAATTTAGTACTTTTTTATATATTTT

>c8525_g1_i1 len=218 path=[402:0-217]

AAAATTATATTTATATAAATAAATATAAATAAAAAAAATTAATAAAAAATAAAAAGGTAA

AGATATATTATAATAAACAAGCAAAACGTAACTTTAACATATGCTGCTTCCTTTTTTTTT

TTTAATTTGATTTACATAGAAGTATACTAATGAAAGATTTTATTTAAAGAAAAAATAAAA

TCATTGAAAATATTGATAAATATATTTTAAAAAAAAAA

>c8538_g1_i1 len=389 path=[367:0-388]

GCAAATGACCCGGGATCTAACTGATGAACAGAAGACTAAATAATTAGTTAATATTGATAA

ATATAGTTTATCATTATAGAAGATTGAATATTTTTTGCTAAAATATTCAATGCTAGATTG

CTCAGCTCCTCAAGAGGGAAAGTTTTAGGATAAAAAAGCAAAAGAGATCCTGGCTGCATT

CCTCTGCTGAAGTCTGTTAGTGTGGACCTACTTAGCCATACAAAACTTTTCAAATAAGAA

GAACCAGGTGCAGCAAGACAAGTCTGCACTCTCTAAATTGGGAGTATCTTATAATAACTC

TCTAATATATAATAATTCTCTACATTTGGAGTATCTTACTAATTATTCTGGTGTAAGTTT

TTATATTAATAATAGGCTTTTTCTCACAT

>c8541_g1_i1 len=248 path=[1:0-247]

GTAACTTTGTGTTTGATGGTAGGTATTGTGAAACTGTATACAGATTTTTTTTTAAAATGG

ACAGCTACTCGTTAGCACAAGAATATTGACTTAGTAGCTGCTGTTAGATATTTCTGTGGC

ATCTGTTGAGTTTATTTGATATGAAAAGTTAGCATCTTCTTAGAATGACTTCTTAAAATT

TTATTACTAAGATAATGGTTAATTAACTAGAGAATAAAGCTTAACATGAATTCTTCGTTT

CTCTACAA

>c8545_g1_i1 len=261 path=[1:0-260]

TTCTTCCTTTCTTCCTTTCTTTTTTCTGAAATGGAGTATACTAAGTGTGACATAAGCATC

TTTGAAAACCAAGGCATCAAGAAGTTCGTATTTCAAGAAGAAACTACAGACTACAACAGT

GGACAGAGTAAGAATTCTTTTTCTAGTACAGTTAAGAAATAAAGTTAAAAAGTTTAACTT

GGTAGACTGTTAAACTATATTGTAAAACTTCTGAAGAATCTCTTTTAGAAGGCCAAGTCT

CAGACCACCTCGTGATACTGA

>c8548_g1_i1 len=251 path=[79:0-250]

GGTGGACTGGATTTCAAAAACTGAAATGGCCCTGGGCTAGGAATGTTAAGTTTGAGCTGT

CTGAGGCTCGGTGGGCTCATGAAACATCAGGATATCTGTGGAGAGATGCCACACACAGAT

AGGCACCTGACTGAGGGATGGACCTGACCACTGAGGCCACCACACAGATAACCCAGCAGT

GCAAAACATGCTGCAATTAAGCGAGCTGTGCGATTAAAGTCCCCCTGGAACAGTAGCCTG

GATCAAAGAGG

>c8550_g1_i1 len=334 path=[590:0-333]

ACCCAGCAGGCCCTCACAAAGGGAACCACGCTAATACCGATACCAGCAAGTTCTTCCATG

CAAGAAAGTGGGAAGTACCAAAATAGATGAGTTCCCATAACAATGTCTGAGGAGCCACTG

AGCTGTGGCCAGCTCAGCACAGGACTGGCACTAACACATACACAAGAGGGGAAGGAACAG

AAAAAAACTCATCTGTAGTCACATAAGGCATTGAAATAGTTTGTTTAGAAGCTGTTTGAC

CCTGCTATTTAGTACAGAAATAACATGTCACTGTCAGAAAGAATGGCAACTGTCAAAGTG

TCAGTTAATACCCGTCCCAGTTGAGGTGGCCTTG

>c8551_g1_i1 len=234 path=[212:0-233]

GTAAATATCAAAATAGAAGGAAGGGAGCGAGAAGTAGCTTTGCATAAGAAGAATCTTCAG

TCACAGGTCAACATTCAGGAGGTTTTTTTGCTCTCTGTTGGTCTGCTTTCCTCTTCTGTT

TGCTGCTTGAGAAGGGTATTTGCAAATGTATGCTCTCCTAAGATTTGCCTCCTGGACATA

GAATGAATTGCATGTGATTCCCAGAGACTACCCAGCTCAAATCCCAGACAGGGC

>c8554_g1_i1 len=259 path=[53:0-258]

TCAGCTGCTCAGCCCACTCTCGTGCCTTCTTCCCCTCTCCTGAGCCCAGGAGGTTTTGCT

CCATGCTACATTCCCAAATGGCAGCAGCACCCTATGGCAGTTCCCAGAGGGAACCTCTTC

TCTCCATAGCAGGAGGGCTCAGGGTTCACCTCTGCATCTGGCCACCTCCACGATCCAAGC

AGTGCAGAGACCTTCCTTGCACTGTGATCTCTCTTCTCCTGCCCATCCATCTCTAGGCCC

TTCCAAGCAGGGGAGCGTG

>c8555_g1_i1 len=218 path=[249:0-163 25:164-217]

AAGAGAACAGTGACAGGAAGGAAAAAGAAATCAAATAAAGCCAGTCACTGAACCACTGTC

CTACCAGCAGAAGGAAACTTTTATTTTTGACTTCCTCTTAACTCATTAGAAGCATTTATA

CAGGAGTTAAACTAAGGTCAAACCAGAGCTGTATTAAAATAGAGAGCTGTCAATGCAAGG

TCTGTTGCTGTAACACTCACGAAGAGACAATTCCCATA

>c8555_g1_i2 len=218 path=[249:0-163 412:164-217]

AAGAGAACAGTGACAGGAAGGAAAAAGAAATCAAATAAAGCCAGTCACTGAACCACTGTC

CTACCAGCAGAAGGAAACTTTTATTTTTGACTTCCTCTTAACTCATTAGAAGCATTTATA

CAGGAGTTAAACTAAGGTCAAACCAGAGCTGTATTAAAATAGAGTGCTTTCCTATTCAGT

CGTGTGTTAACTGTATGGGAATTGTCTCTTCGTGAGTG

>c8558_g1_i1 len=226 path=[412:0-225]

TGGGTGGCCCTGTGTGGAGCCAGGAGATAGACTCAACGATCCCTAAGGGTCTCTTCCAAC

TTGGAAGGTTCTATAACTCTATATGCTTCAGCCCAGAGCTCATCTACCCCACACAGCCCA

TAAATGGGGACCCTGTGGTTGATGAGGCTTTTCCCAGAGCCCAGCTGGGGTCTGTGAAGG

GTGAAGCTGCTGGTGTTCCACTGCAGAAATCATCAGGAAGGCATGG

>c8564_g1_i1 len=411 path=[1:0-410]

ATACTACTTCTGGTTATTTTAGAATATGGATGTTGATCTGGGGATTAAGTTGGATTTACT

TATTATCTTTGTTGTGTACTCATAAAAAAGCAATAAGTGATATCCCTTCCTGTGGTCTTG

CTTTGTTAGCTGGGATGCTGGTGGGCATCTTAGAAGCATTCTTGATTTGAGAAGTAAAAA

GGTTGGATTTTCTTTTGCAAAGAGTATTGTTTTATTTTTGAAGCCAAAATACTCTGTTTG

CAGTATATCTTCATGCTTAATTTCTAGTGTTCAGTAAGTTGGGGATATGTGAGGGCTTTT

TGTTCTAAACAGTTTAATGCTGTAATCCACAGTCCATGCCCTCACAATTTCTCATGAACT

CCAAGGCTAACATGAACTGGAATTCCATTTTCCATACTGAAATTAGCTAAA

>c8573_g1_i1 len=319 path=[321:0-292 613:293-318]

CTCAGAATACAGGTTTGCTGATAAATAAGTTGCAAATGCTCTTGATGTTGTAGTGCGTGG

CTGCTCTCTTTGCAGCTTACGGATGTAGTCCACTGATGCTTTTAGAATAGTTCCCTTATT

CCAGCGCATATCCGGGTCGTTTGATTTGGGTATCAAGGTGCCCAGTTCTTTTATACGGTC

ATTAATATTAAATCTTCTTCTTCGTTCAATCAAGTTGTGATTGTCTTTCTTTTGCCTCTC

CTTAGCCAGTGCTCTCGCTTCTGACTCTGTGAGCTCCCTTTTTATATTAGGAAGATTAGC

TGGACAGGAGTTGCTGATG

>c8574_g1_i1 len=290 path=[155:0-213 154:214-289]

GCTCTCTGTGAGGAGCCCTGTGGCAGGACATACCTGCACAGCTCCTTCCTGCTCTGGGTG

GTGAATATAGATCTCTAGGTACAAGAAGCTCCGTAAAAGAAGCACGAATAAACACCTGTG

AGATGCTGTTCCTTACTTTTTGTTCAGCTTTGGGCACAAGTTCCCTGCTCAGTGAAATGC

CAGGCACGTCAGTGCCCTGTTTACCTGATCTTCCTGGTTGTTGCTCATGACGAGGTCATC

AAACTTGGGGAGGAAGAGTGCAAATACGTCACTTAAAGTCAAAACCAGGA

>c8575_g1_i1 len=208 path=[1:0-207]

GAAGGGGCCTCCTGCACCACACCAGTGCTGCTCTGCACCACCCACATCTCTGGGTTGCTG

TTGTCCTTCAGGGTGTACTTGTCACCTCTGGTCAGCTGCACCTCTCCTGCATCCCAGTCA

CACAGGGTGTCCAAGGTGAGGGGCTGGGAGGAACGCATCCTGCGCAGTTTCAGAGGACTG

ATCTCCTTGCTCCTCAAACACAAAGTTA

>c8576_g1_i1 len=292 path=[270:0-144 415:145-291]

ATTTTTTTTTTTTCTAAATTATATCCTAGTCATAATAATTTTAACTTAGATAAAAACTTT

CTTTTTCTTTCCTAACTTTTTCAAGTTATTTCTTAAAATAAAGTAACTTATATGTCTTAA

TATATTTCGAAATTTATATTTTGTACTTTATGGTTAAAATATATTAAAATTTTAGTAATT

GAAGAATATACATTTTAAATATGTTTAAAAAATTGGAGGATTACTGACAAAAAAGTAAAA

CATTTAAATGAATGTTATTTAAAAAAAATTATATTTATATATTATATATATA

>c8579_g1_i1 len=205 path=[183:0-204]

CAGCATGCCCCTTGCTTTTCCTGTGGAGTGAAGTTGCAGAAGCACATTTTCAGATCACTG

TATCGGAAGATCTCTTTTCTTCCCCCGCTCACACGTGGGGGTGATGGAAGCGGGTACAGA

AACTCCCTAATATTGATGGGCTGTGTTGGGTTTGGTAAATGTTATGGTGGAAAACGTACA

TTGATGTCTCTAAGTGATCTTACGG

>c8585_g1_i1 len=313 path=[189:0-270 141:271-312]

AGCCATACGTAGCCACCATTTCTACATGTTTTTTTTTCCCTTCAGCTCCAATCAATGAGG

AAAATCCACTACTTTAGTAGATTAGTAATAGAATCATCCATCTGTCAAACCAATGCTTAA

GTAGAAACTGTCAAAAGCAAAGCCCTATCATTAAGTCTTTACAAAAGAATATACTGCTGT

TGTGTGTTTTACAATGACTTAATATGACAGTGGCAGTGCTGACTGCCAGTGACTATTTCT

TGTTTGAAAAGTGGTTTGAACACAGATGTCTCTTTTTTTTCATCCTTACAGAAAACCTGG

GTTAAAAACTTAA

>c8588_g1_i1 len=229 path=[733:0-228]

AACCAATGCTGCAGACCAGAGTCAGTGTGAAAAAGGATATGTAAATACAATAAGAATACA

TAACTAAGACTGTCTGCTAGTGCGTAAGTGTCTTAGATTTCACAGCTTAAATCCTATTTA

AATTCTTGCATTGTAAGTGGGTGATTTTCTCCATCCGGTACAGTTATAGATCTTCCCTTA

AAGGCTTTTTCCTGTGGCAACTGCATACTGAGTCTCTGTCATCAGGCTG

>c8588_g2_i1 len=286 path=[264:0-285]

GAGGTACAAGTTCCATTCCATCCCGTATTTCAGGTTCGCTGGCTTGGCGTTCGGCATATT

TCTGGAGCACTTTGTAATAGTAGCAGTAAAGTCTGTGAGGCTGAAGCAATTCTCTTGCCA

CTGACTGTCCTTCTTTAGCAATTTTTTGTGCTTCTTCATCATTTTCCTTAGCCCATTTTA

TTTTCTCTAGCAAATCCTCCAAGCTTCTCTTAACTGGAACATAATGTTTCCATGGTTTCA

ATACAGTATAAAAATGTTCATAGTATTGGGAATCTTGCTTCAGTAC

>c8597_g1_i1 len=353 path=[1:0-352]

TGATCTATGTGGGGAGAATTGCTGTGACCTTATGACTACTGAGCGTAACAGCTACTTGTT

ATGTTAATACAGGGAACATCTGAGTTACTCCTTCAAGCATTTGAACACCAAAGAACGTGG

TGTCCACTTAAGTTCTTTAATTACTGATGCTTCCCCAGACAGTCTGGATGATTTTGTTTG

CTTCCTTATAGCAATTGCCTAGATATGTTTAGGCTGGAAACTGGGGAGTTAAGAACACTG

GGATGTTTCTGTCTTCTATCTAAATCAGATGTGGTCAAGCACATGCCTGTCTTGCAGAGG

GTGTACAGAGTAGGGAAGAACTAATCACTGAATGATGGAATGTGGGCCTTCTG

>c8600_g1_i1 len=284 path=[1:0-283]

AATAATATAAAAATCAAGTAAATTATATAGGATTAAATGAAAGCATTTATCGGCTTTATT

GTAAATATTCTCTGATCTATCTTTTGTGAAATGCTTCTTATTTTGCAAATAATAAATTTG

CATATAGTTAATTTTTGTATATATTAGCAAAAAACATTTAAAAATTTATAATTCCATATA

AAATTATATGTATACATATAAAAAGAGAGAAGAAAAGAGTTTTTGTATATAAACACGTTT

TTTATTTAGTATTTTGTTTATTGAATATGAATTAAAAATAAAAA

>c8601_g1_i1 len=222 path=[1:0-221]

AGGTGTGCAAGCAGGGTATGAAGATCTCTGCACAGCATGGAGGTCACCCCACTGCTGTTT

TATGCTAACAGCAGCACTCTGGCCAAGTTTTTGTATTACCGAAGGGCCAAACTCCTCCCT

GCCAATTATTCAGACAGTCACTCTTAAAATGCGAGAGAGCTTAGACGTTAGGAACCCGCT

GTGCCTTCTAAGTCACCTCAGGAGGTTGTTGTGGAAATTAAT

>c8605_g1_i1 len=236 path=[368:0-235]

TGTACAGGAAAAAGATCACTTGAAGAATGACAATAAAAACATTATGTTAATGGGCTATTA

AACAACAGTCCGGACATAGTGCAGGAACATCCTAAAACCACTGACTTAAGAAGTGAGAAG

ACACATCAGGGAGTGTCCATCTTACCTAAATGATTCCTACTGGCCACTCTGAAGCATAAG

GAGCTACCTGTCTCTGCTTAGACATGCTGTGTCTGTCAAGGCTTCCCTTCTGAAAG

>c8607_g1_i1 len=217 path=[1:0-216]

TCTCCTCCACAGCTTCGCTCCTCATTCCTTCCTTTTATCTTTGCCAAACAAATTTGCACC

AAATTGATTTCATGATGAAAAGTTTGATGTTAGATTTCTTCAGATTCCCATCACCTCTAA

TTTTTTCATTAGGAAGCATTCATTATCTTTTCATTACCTTCCTAATGGCTTACGTTTTTT

TTTTGCAGTGAAGTATATAAAAGTTAATAGAATACTG

>c8608_g1_i1 len=228 path=[206:0-174 381:175-227]

GAGATCGGTATTTCTGTCTTCTGTCAACTCTTGAATGTTCAAGAAAAAGTTAGGATTTTT

TCTCTCTAGAATTAAGCATAAAGTAAGCAATTATTCCCCATTATAGGTAATAGGAAAATA

CTGTTTGAAGAAAAATTGAACTTTGTTTTGCTCTCATTTATATATATTTTTCCAAGTAGT

TTCTTCAGACATTTAATCTGAATCTTTTTGGTGTGTGGTTGTTCTGGA

>c8613_g1_i1 len=216 path=[155:0-215]

GTGCAATTCTTGAGATCTCTGCCTTATTGCCTTTCCCACATCTGGTATCTCCTTTATAGT

TCCATGCTGATTTGTGCAGGGAAAATTGCAGTGGTTGCTCACAAGATGGCATTCTACCTA

CCAATGTGGTGTTCTGTACAGCGTTATGGATCCTCTGTTGTGACTTTTGTTTTGGGCTTG

GTGTTGTTCCTTACTTATATATGAGTTGCGGTTTTA

>c8630_g1_i1 len=340 path=[1:0-339]

TAAGCTACTGTAACATCAAGGATGTTCTAACAGCATGCAGTAGTTGCAGAATCAGGCCTG

TAGCCAGCTCTCTCTTGCCTTGCAGTTGCCTGTCTGTGCCAGTTGGGCTTTGGATGACAA

GCCCAGGGTTCAGAGCCTGCACCTAACTGTTCCCAGCATTTGTACCGAAGACTGGAGTGC

TTCTGTAATTGTGCCTATGAGCAGGAGGTGGTGCCTACAGGATGCTGATCTGTGTGGTCT

TTGCCCCTTGCCGTTCTGGTCTCACCCATTACTACCACAGTGAGTCTGGTGGACCAGCCT

TCTGAAGGCAAAATAAACGTATAAGCAGTTTGCCAGATTG

>c8630_g2_i1 len=207 path=[819:0-206]

CAGGAATATCTTAAGAGTGCCTCATATAGTGGATGTGAGGTAGTCAGTTACTGTAGAAAG

CTCTGTGACTGTCCTGTGGTATTCACCTCAGTCATACCCAGATAGTGAATGAGCCAACAG

CACTGTGCTGCAGCATGATAAATCAGGTTTGTTTTGCTTATTTTCACTGATGCTCGAGCA

AAACCAGTGCAAAGGGATAGTACTATG

>c8633_g1_i1 len=224 path=[536:0-223]

GCAGCTCTCAAAGTATTTTTAACGCTTCCCGGAGCTGTTTGGGGAGCTTAGTTCCCTTTC

AGGCCTTCACCCCTTAGCACGAATTCCAAGAGGCACTGAAGCCAGGCACGGAGCAGTGCC

GGCGGTGAGGCAAGGTGACCGTAACCTGCAAACACATCTCCAAGGAGGGCATTTCTCCTG

GCAGTCGGCACAGCGTGCGCTGACACCCGGGTTGTGATACGTAT

>c8637_g1_i1 len=303 path=[611:0-302]

TATCAAAAGACTGCATTTTTTCCAGATAAAAACATGCACGTAAGCGTACTATTCAGTAAC

TAACGCAGTATAAGCCTACACCCTAGGCTGCCTTGCTATAACTTGTCTACACAGCCATTC

TTCCTGGGCATGAACAGCTACAACTGGATGCAGGAGGTTCATGGTAGCAGTGAATCAGGG

GACCTGAAAGTTGTCTTTATTCTGCAGCTCAGAAATACACGAGTGTTGAATGATCCTCAT

CTTGCTCATCTAAGGGTTGTGTACATGAGACCACTCTTGAAAAATCCTAATCAGTGTTTG

AAA

>c8641_g1_i1 len=373 path=[400:0-108 508:109-372]

AGGTCCGCATACTTTTCCTTCAGAGACCAACTGTGTGACTCAGGATGATGACAGACCTGT

GGTGCACCATACACGCTTTGTGCGTTGATACTTCTTGATCTGTAGAAGGCCTGAAATGAT

CGAAGACTTCAGAACGCAGCTCAGGAGATGAGCATTTCTGCAAACTACAGACAGGGTCAA

TTAAAAAGAAAAAAGAAGTCCAAAATATTACCTTGTTTTAGGGTTGGAAACCTTACAAAA

TGCTTAGTGGGAATGTTATCCAACTATGCTCCATTCTGTGTTTTATGACAACTATGGCCA

ACATCAACTCTTTGGCAGCAAAGTTGCACCAAGTTGCTGTAGTGCCGAATCAGGCCCTAA

GTGTCATTCCTGT

>c8646_g1_i1 len=247 path=[225:0-246]

AAAGATAAAGTGCGTTTTTGATTTTGATTATTGAAAGTTTCATTAGAGTTTGGAAATCTT

CAAGTGTTCTGAAGAACATAGGGCAGAGCCTCAAAATATTTGCTTAACCGTATCTGGAAA

CGCCTGTGTACATTTCATAGTCCACGCTACCTTAAATTCCCGTTGCCTGGAATATATCTG

TATACCTCATTTGTTGCTGCTGTAGTCACTTTTACACATATTGCCTAAATTTTCAGGTAT

TGATAAT

>c8649_g1_i1 len=244 path=[496:0-243]

GAAAATCATGTTTCATATATTTCTGCAGTCTGCCAACAGCTGCTCAAGCAGAATTACTCC

GGCAGCAAATCCTACATTTGTGCTATCACCTGGTGGCACTGTTACACAATCTTCCAAACT

TCTGTTTTCGGTGCTTGACTCCTGGTAAGGGGAACAGTGCAGGTGTTACCCTGAAGGTTA

CATGGGGCCTCTGCAGGAATACTGAGGATGGAGAAGGCCAACTGCCTCTGACACTTGTTT

TTAC

>c8651_g1_i1 len=238 path=[216:0-237]

TCTTTCAAGGTTTTTGAGAATCTGAATTCAACTACAGAATGTGTCATTCCAGTTCTACAG

CACAGTTTATGGGTCTGAACTGGGTCATTCTAAAGGGAGGTTTGATGCTGAATGGCAAAA

TGTGAAGTATAGCTGAACTAAAGAGGTATTTAATAATACACATGCTTTAGCCTATCTTTG

TTCCTGATTGTCTCTGAGTCATCGTATTGTGTGTCAAACTAATTTTGCACAGTTTGTG

>c8653_g1_i1 len=357 path=[335:0-356]

GATCTTCCCTGTCAAGGAAACTGTACCCCTGCTGAGTTACAGCACACGGAGTTCAGTTTC

CTTGACAGGGAAGATCGATAAATGAGGTTTATCTAATGCTGCAGTGCTCAGGCTGTGTCT

GGCCTCTGCGGCCTTCCCCGTGCTGATGGCTCCTCTGCCTCAGGGTGACGTGGCAGTAGC

ATGGCCCCCTGGAGGGGCTGGTGGGGCCATGGGGAATAAGTGGATACCCCAACCTTGGAG

ACGCTTAAGGTAAGGCTGGTTGGGGCTCTGAGCACCTGATGGAACTGTAGGTGTCCGTGC

TTATTGCAGGGGGGTTGAACCAGATGGTCTGCAAGGGTCTCTTCCGACTCAAAAGAT

>c8665_g1_i1 len=449 path=[155:0-448]

GATTCCTTAATCAGATAAGCCCAATTAAAGCAATGCTCTAAAGAAAAAAACCAACACTGC

TGAAGCCAGGTTTCCAGCATACAGACCTGGACATAAGGAATATACTGGCACGAAGAATGC

ATGCATTTCAGTTCTTTTGATACCTCCCCTTCCCAATCTAAACACGGATTCAAACACGCA

ACTGTGAGCTTCCCAAAGGCAGCCCTGCACGGGAAGTGTTCTGTTCAGAACTAAGGGCTC

ACCCACCTGCCCGGAGCTGTGCAGCTCAGAACCACAGCCTGCTCAGGAGCAGACTATGCT

GATGCTGCTCAAGCTGAGCAGGCCTTTTGTGCCTTCCCAGGGCCACCGTGCATTAATCTC

ATAGAAGAAAAACAGGCTTTTATTTAGGGTGTTTTCCTGTAGATCTGGGGAAGAAAAAAA

AAAAAAGGTGGCCTGAAAACTGCTGGAAC

>c8669_g1_i1 len=204 path=[1:0-105 107:106-203]

ACAGAGGCAAAAATTGAGTAGCTGAAGTAGTTGGAACTATGACTATATTATTGTAAGTTA

CTGCTGGAAATGCTCGGTAGGATTAGTTTAGTTACAGCTTGTGCCTTGTATCCACGTGTC

AAACAGTGCTATGCACATCTTCCATTCTTGAGCTGTTTGTCCTTAACAAGCTGCTTGCAG

AGCTGTGGAAGGCTTCATTCGTGA

>c8672_g1_i1 len=384 path=[1:0-299 301:300-383]

ATATACAGCTGAGCCACAGCAGTTTTTATTTGTGCTAATTGCCATTATAATAACGACTGC

TGTGGCTCAGCTGTATATTTGAGCTCTGTGGGATCTACGTTCCGGGCAACGTTGTGTTTC

ACGATCCATGAATCATTCTGATTGCAATAAGGCTGTCAGCCCACAACCATCCCAATTTAT

GATCCTATCTCAGCTGTCCCAAGAAGCTCTGATGCCCTCTGGCAGTGTCCTTCCAGGGGG

TGGGGTCAGTGTGAGGAGCACACTGATGGAAATTCCTCCCTTGGTTCAGCTGGGAGTGCC

CTGCTGTGTTCATAGATCATAGCATCATAGAGTGGCCTGGGTTGAAAGGGACCACAGTGC

TCATCCGGTTCCAACCCCCTGCTA

>c8677_g1_i1 len=342 path=[14:0-51 66:52-341]

ACTGCAGGAGGTGTCCGCAGGGGGAGAAAAGGCCTGCTCGGCTGGTTGGGCTGCTTGAAC

TATTTTTTATGTCTAAGGAGAGAGGAAGAAAATGTTCATTAAAAATGTTCTTCTCTTGTA

CAGAATTTTGTTTTAAGGAATGAAAAAAAAAAATAGACCAGACAAATAGTGAAATTAACC

ACGAGGTACATTTCTTGCTGTTCTTCACGTAAGCCAAAATATTTTTGTTGCTTCAACTGC

AAACTGCCATACAGTGCTGTTCCGAAGATTAAAGGTCTGTATCGTGCCCTTACGTGCTGC

AGAGCTCGTTATTGATGAAAATGGCCCAAGTCAAACACAAAG

>c8681_g1_i1 len=267 path=[537:0-266]

CAATGTATAATGGTTGCTGTTAATGCATCAGCCTTGCAAATGGATGCACTAGACATCTCC

TTTCTGTATGCACTTGTTCTGATGTGGTTTTGGTGCTCTCCTCCAGGACTGTCAGCTCCT

GGCCACTGCTTAAGAGACCTGCATAGGGATGTCAGGGCTGTGCTACCCCAGCACCTCAGT

ATTACAGGGTCCAGCCTGTGCTCAATGAAGAATCACCTTTATTTTCATTACAACTGGCCT

TATCTACTGTGGGAGACAATCGAGCGG

>c8689_g1_i1 len=331 path=[179:0-330]

GCTGTCTACCTTGCCCGGTCTCTCGGGGGACCCGTCTGTTGTGGAGTTGCTAAGAATGGA

AGAGCAGCAGGTGTCAATCACTACCTCAACAGTACACCAGCAGCAAGGTCACACAAACCA

ATGCTGCAACAGCCAATTGCATTGCACATCACTTGTGTCATGGTTTTATGATTTTCGTTA

TCAGTGTTCCTCATCATGGCGTCGTGTACAGCGCGGGGAGTTGGAGGGTTGGTGCTCCAG

TTCCGGGGACTGGCAGCTTTCCGGATGCCTGGTTCTCTGGGGAGGGGGACTGCATGTCCC

GGAGGACTTTGCATTCGGAGGGGAGGATGAG

>c8697_g1_i1 len=205 path=[16:0-204]

GGCTGTGCCAGCAGCCAGGGCTTGAGAACATCAATGAGCTGCTCTTGAACCTGTGACCTC

CAGTACCAGAGGTGGATTTGTTTATTTCTCAACCATTGACCTGCTGTATGGAGCTGAGCA

AATTTCTTCATCTCTTTCATCTGCAGAATAGAAATGGCATCTTTGCCAGAAGCTGTAGGT

CAATATGGTGGTCATTTGGGAGCTA

>c8704_g1_i1 len=342 path=[402:0-295 697:296-341]

ACTCACTCCAACTGAGCCGTTTGCCAAAGCCTTCCCGCTTAGACGCCGCTTACACGCCGG

AGCCATTCGCAGCCGTCGGCGCCCTCAGAGATACAAAAAGGCGCTGAGCTCTTTGTCACG

TTGGAGGGCGCATCGTGTGTGTAAGGCCGCACTCCTACCGGAGCCGTTACCGGGAGAGCC

TCTCACGTTGCGCTCACGGAACCACAATCGAGCGGTTCAGCCGTGAGGAAAAACAAAAGG

GGAAGGCCCTGCAGAGGCAGCACGCCCACAAGCAGAGCCCCGCGATCAGCCATAGCCAGG

ACCCGATGCCAGCTCCCACCAGGCCCGACGCTCGGCCCCACT

>c8704_g1_i2 len=342 path=[402:0-295 63:296-341]

ACTCACTCCAACTGAGCCGTTTGCCAAAGCCTTCCCGCTTAGACGCCGCTTACACGCCGG

AGCCATTCGCAGCCGTCGGCGCCCTCAGAGATACAAAAAGGCGCTGAGCTCTTTGTCACG

TTGGAGGGCGCATCGTGTGTGTAAGGCCGCACTCCTACCGGAGCCGTTACCGGGAGAGCC

TCTCACGTTGCGCTCACGGAACCACAATCGAGCGGTTCAGCCGTGAGGAAAAACAAAAGG

GGAAGGCCCTGCAGAGGCAGCACGCCCACAAGCAGAGCCCCGCGATCAGCCATAGCGACG

TATTCGCTGGCAGCTGAAGTGGGGCCGAGCGTCGGGCCTGGT

>c8711_g1_i1 len=206 path=[1:0-205]

AGGCCTGTGTCTCGGCTGGGAATCTGACAGGGTGGTGCTCCCTCTTTTGGATGCAAGTTG

TTGAGTGGATGGGAGGGACTGGTCGGTTTGTTTGGCCCTCGTGCCTCCTGGGTGTGCGGT

GGTTCCTGCGTGTGTGTTTCGTGGCTGGGGCGGGGTGCCTTTGACGTGGCTTTGCTCTGT

TGTGGCTTGGGTCGCGTGCCCTTCGG

>c8714_g1_i1 len=290 path=[189:0-50 94:51-289]

AAATCACTCATTCCACAGCAAGTGAAACCTGCAAATGCCAACAAGCGTTAATATTTGTTA

AAGTGCCTTTTTTACTATCTCAGACAACTTATCTGTGAGTTTGTTTGATGGAGCGATGCT

GCTCTTCAGAACTGGGTATTCAGAGGCATTGCCTAATGGTCATTGTATTGCATAAGCCAT

ACTTTGTAATACTTGTATTGTTCACTCCCAGATTTTGGATGACAGTATACAGATGCAGCA

AAGCATGCTGCATCATCCGTTTTATTCATCCAAGTGGGAAATAAAGAAAA

>c8714_g1_i2 len=332 path=[1:0-92 94:93-331]

TTTTGTCGTGAATTCAGTGAACTTTGCTTTCATTTAGCACAGAGAAGCTAGGCTATCAAC

ATGTTTTGCTTCATTTATGCAACTTTACATTAGTATTTGTTAAAGTGCCTTTTTTACTAT

CTCAGACAACTTATCTGTGAGTTTGTTTGATGGAGCGATGCTGCTCTTCAGAACTGGGTA

TTCAGAGGCATTGCCTAATGGTCATTGTATTGCATAAGCCATACTTTGTAATACTTGTAT

TGTTCACTCCCAGATTTTGGATGACAGTATACAGATGCAGCAAAGCATGCTGCATCATCC

GTTTTATTCATCCAAGTGGGAAATAAAGAAAA

>c8721_g1_i1 len=336 path=[571:0-231 802:232-335]

AGGCACTACATGAAGTAATTTTCAGAAAAGACAGACAACAGATTAATTAGGTTCACAACA

GTATGGGTGCAGGGCAATAATTTTTAATGCACGCTTTGTATTACGTTGTTACAGCACACC

CAGCTGTTTTCCGTTTCCATTGCTGTTAGGAAGGCCTAGAATTTGAATCTGTGTTTGAAG

CTGAAAGTAATTTGTTACCTATTTTTTTTTTTACATCTAGAATTTATTCCCACAGTGAGC

ACCACATTGGCCCTAGTCAGACGGGCAAGGGGCCCATTTTGTTGGCTCAGAGCCTTGCAG

TGGCATAACCTGGAGCAGAATTTGGGCCTGTGGCCT

>c8721_g1_i2 len=336 path=[571:0-231 179:232-335]

AGGCACTACATGAAGTAATTTTCAGAAAAGACAGACAACAGATTAATTAGGTTCACAACA

GTATGGGTGCAGGGCAATAATTTTTAATGCACGCTTTGTATTACGTTGTTACAGCACACC

CAGCTGTTTTCCGTTTCCATTGCTGTTAGGAAGGCCTAGAATTTGAATCTGTGTTTGAAG

CTGAAAGTAATTTGTTACCTATTTTTTTTTTTACATCTAGAATTTATTCCCAGAAGAATT

AGAAGTTTCAGCTATGCTGCTAAGTATTGCCTAGGGAAAGATGTGACAGTGGGTAGCTTA

GCTCTCTGCTCAGGCCACAGGCCCAAATTCTGCTCC

>c8723_g1_i1 len=215 path=[386:0-135 521:136-214]

GAGGGGCAGAGCTGTGTCACAGGTTTCAGAGTCTCTTGCACCGACTCGGAGGTTTGAACC

AAATCAATCTTTGTTTCATAAGCAAGTTTTGTACATGCTGCATCGTGCATTTCACTTTCA

TATGCTTCTTGTACTAGATCAGGAGTGAGACCTTCAGGCATACTTGCTGCAGTGTCAGAT

AACAGTGTTGATATGCTACCTGTTTTAGCATCATC

>c8723_g1_i2 len=232 path=[386:0-135 122:136-231]

GAGGGGCAGAGCTGTGTCACAGGTTTCAGAGTCTCTTGCACCGACTCGGAGGTTTGAACC

AAATCAATCTTTGTTTCATAAGCAAGTTTTGTACATGCTGCATCGTGCATTTCACTTTCA

TATGCTTCTTGTACTAAATTTGAATCCTCTGTAACTCCTGATGGTAGCAAAGTCAAATCT

ATTTGCCCAATAGAGGAAGACACTTCAGAAAATAGAACTGATGATGAGAAAA

>c8735_g1_i1 len=238 path=[1:0-237]

ATCAAGAACAAAGTTAGCTGCCTAGAGATTTCAGCCACACTTAATATTCCTCAGTAGAGA

CACCATTTGAATTCTATCATCAGTACAGGCCGGTTCTCCAGAAACCCATCGCTAAGCCCC

TAGAGTTTATCCTCAGACCCCAGGAGGACCTGTAAACTTCACTTCACCTTCACTGTCAGT

GGCATCTGTGAGTTCCGGTCAACCCAGCAGCACCCACGGCAGGTAGCTCGGTGCGGAG

>c8741_g1_i1 len=361 path=[155:0-360]

ATCTGAAACCTGAAGAGTCTACAGCCTCACAAGTGCTACGCTAGACTTCCATCTTCAAAA

CTGGCAGTCCCAACCTGCAGCCCCACCATACCCCCACACCTCCTCTGCCAAAGTCCACCC

ACGCTTACACACAACTGGCCTACCAATGGACACCTGTGATGAGAAAACCAAGACCCAAAC

AAAAAAGCACAGAACTGGGTTTGTAGGAAAAGTTATCCGTGCACTTACTTGCCACCCTGA

TTTTTCCTCCTTCACTTCAAAATAAAAGGGTTGAACTAAATGGTACAAAAGGCACATTAA

GTAGCACTGAGCTGTTGGTTATCACAGCCATCCCACATTTAACAGCAAGCAGGAAACCAA

A

>c8744_g1_i1 len=255 path=[1:0-123 125:124-254]

AAGTCCCAGCTTCATGAAAAATAAGTGGTTCAATTAAAATGTACAGGAAGACTATGCAGC

TCTTCTCCTTCAGCAAGAGAATACAAATCTGAATTTTCAATATCAGTATTTAAGTTTGGT

GCTGTTTGTTTTCATTTATTTATTTGAGGTAAATACACAACACTTGCTTTGCTACAAAAG

GAGAGGTGAAGTTAGCTTAGGTTCCCTCATTTCTCAGACAAAAAAAGGACTCAATAGGGC

CAAAGTCATCAGTGG

>c8746_g1_i1 len=426 path=[53:0-425]

ACTCAGCCCTCTGGTAAACCAAACCATCTTAAAGAAGTGAAGCAGGGCTGCCAGCAGAGT

TCTCAGTGACGTCTGAAAACATATCAGCTGCTGCTGCTTAAAGGAGAGGGGCTGAGGAGC

TGGCCTCTGTGAGGGTCAGGGCTTAGGAACGATTCAGTAGGTGAAGATTCAACCAGTCAG

GCAAGAATACACTCCTGTTTTCCTCAGATGTGTCTGTGTCTTTTCCTCCTCATGATATGA

CCAAGATATATGTATATATCATGGAGGGTAGCATGCAGGAAATGCTTTTTTTCCCCCCAT

TCTAGATGATTCTTGTTTTTGCATACTACCATAAATGCTGCTTTTCCTATACATCCTTAT

TAATTTCTAGCAAAACATCGTTAGCAATAAAATTCATATTTAAGCGTGAAAAAAAAAAAA

AAAAAA

>c8747_g1_i1 len=230 path=[357:0-229]

TAAAGATATAACATTTTTCACAGAAGAATTGCCCAAGTTAGCATATTTCAAACTTGCAAG

CCCATAAAAAGTATCTGAATAGTCAAACACCACACAAGAGCAAAGATTAGGCTGCAGGAT

GCTTTCATAAAGCAACTAAAACAACCTTGTTGTTTTCCTTTCCTGCCAGTCCTTCCTAGC

GCATGGTGTGTCTTAAGCTGGTTACTGAATTAAACTCGAAGTGGCAGAAG

>c8747_g2_i1 len=201 path=[179:0-200]

GAGCTCCCAGGAAGAACACATGGGGCACCTCATCCCTTATCCTCTTTTAACAGGGAGCAG

ACAGTAATCCTGAAATTGCCTGCCCTACCTAGAGAAGAAGGCATGCAAGGGACCAACGCA

CTTCTCCTTTATCAATTCTCAAACAAGTAAAGAGATCTGACTTCTCTTGTCCTTCCCTCC

TCCTCTTCTGGGAAAAAAAGG

>c8749_g1_i1 len=202 path=[180:0-201]

GCACCCGAGCTGTGTTTGCTCGCCCAGGATCGGCTCACTCGTTACCATGAGGCACCCGGG

CTGCACCCCCTTTATGGTTCCTCTTGCAACATGTGCCTGGTGGCTTTGCTGGAGACGAGC

GTGGGGTTGGGCCAGGTGCTGCATTGTGCCTGAGAAACCTGAGGTTGCGTTCAGCTGCCA

GATTCTGCCAATCTCCCAAGGT

>c8751_g1_i1 len=450 path=[642:0-284 37:285-449]

ACCAGGAGCAGCATTACACGCACCTTGGAGCAGCTGGAGCAGAGCTGGGCAGGCCAGGCT

GTGCTTCCTATTCCTCATGTGTCCCGTTCTAATGTGCTGAGCACCAGGGCATTTCCCCAT

GGCTGACGTTTTGGATCATTCCTCGCTCCATGCACAGGGCATCTCTGTTTTGGGGCCTGA

CCAAAAATCACCCCTGTATGTGCCCTGGTGTCACCGCCGGTCATGAAACCTGAAAACTGG

GGCTCTCCTCTGCAGCACCGCTCCTGGGGATCGTTTCAGGGACATACATGACTAAAGCAA

GAGCTGATGAGCTACGAGCATCCAGGACGATCCTTTCCTCCGGCCAACGCGTGGCTCCAG

CACTCGGTTGACCCGCGGTTGCTTTTGCCAATGTTGCAAAACCAAACTGCAAATCCGGGC

AGTTTCATTGATTGATTTTTTCCCCCCCTG

>c8753_g1_i1 len=203 path=[181:0-202]

CATAAAGCAATACATCCCCACTTTTGACAACCCGACAACGCGTAACAGATTCTACAAACC

AGAAAAAGCCCTCACTTTCTTATTGTACCTCAAAGCCACTAACGCTGTCTAAAACCAACA

CTTTGCAGAAGTATATTCTTAAGGTACTATCTTTGTACACTTTCAGATGTCTAAATACAA

TCTGTTTAAAAAATATGTATGAG

>c8759_g1_i1 len=314 path=[1:0-70 180:71-313]

AAACTTGGCACATACATAGTGAGTGTCAACCCACTTCACAAAGTTAGGTTAAAGAAAAAT

ACAGAGTGCATTGGTGCAGGAACAGGAAATGGACCCTGTCTTACAGTGTTTGTGGACTTC

AGTCTTGCATAAACCTTTAGAAAATGAAATACCTGAAACATAATGACAGATTTCAAGTTT

GTGTGAAATTCTTAGTCGGGAGTGGTACATGATACATGGATGCGTACCTCCTGTATTTGG

AGTTTTGCCCATAAAATAGCATTTGGACATTGAGTTACTAATTTTGTGCTCCCAAGTGCC

CATTTGGCTTACCA

>c8760_g1_i1 len=307 path=[1:0-306]

AGCAAGTACAATTTTGAAAAGAAAAAACGGTATTCTGAGACAGTTATACTAATTATTTGT

TTAAATTTTTTGAAGCATAACTTATGTGGGGATCATTTTCTTCCCCTTTTTGTAAGGTAC

AATCATGTTCAGAGCACTTCCCTGTTTGACACTGACTGTGTAATGCACCCAATCAATGAA

AAGAGCCAAACTGAGCATATTTTCAGCAAATTTGTCTGCCAGCCAAAACTATAATTGATC

TTCTTCTAAGATTTGAGGCTTAATGCAATTTTGTGCCGGCTGCCCAGGTGAGGGGAGCAA

CTCCCAT

>c8766_g1_i1 len=242 path=[220:0-163 384:164-241]

AGCTACTGGCTGAAAGAGTGCTTTCTTTTCTCTGCTCTTCCTGGGGATCTGAAAGTTCCT

CCTAACAGGCACGTCTTCTGGGGGCTTAGGGAGAGGGGGAGGCCCCCCCGACACGTCGCT

GGGCGGCGGCTGTTGGTCGTGTGCCGTTGGGATGCTGCTCCTGCGCTGGCTGCTGCTAGT

CATGCTGGGGCTTGGGGGGACCTCGTCCTCTGCAGCTACTGCAGCCACCAGGAGCGCCTC

CT

>c8768_g1_i1 len=228 path=[225:0-59 285:60-205 505:206-227]

CTGCCCAAGTTTTTGCAATAGTGAACACAGCCATGTGCAATTCCATCAGTTTGAATTCAA

AGCTAAATCTTGGACACCTTAATATGCAGTGTAGTCATTTATAGTTTGTGGGTATACTTT

GTGATAAGGCTGTTGGCAGCACGCGTGGAAAACAAGAAGTGTTGGCTGTGTAGTTGTGTG

AGGAAGATGAGATTTTGGAATTTCTTCTGCCAGCATTCCAGCTCAGAA

>c8769_g1_i1 len=353 path=[402:0-277 975:278-280 25:281-352]

CTGGATGATCTTTAAGGTCCTTTCATGCCAACATAACACACCTCAGAGACCAAAAGGCCT

GCTTCAAATTTCATCTGTGAATGGAAGTTTACTGAAATTTGCATATACTCACATAGATTC

AAAGTAAAATAAGGAAGTTTTGCTTTCCAAACCCGTATATTGCAGTTTTCACAATTTTAA

AGGAAAGAAATGTTTAATAATGGCGTCTATTATTTCACAGGACTATCCTGAAACTAAGGC

TTTGTCTTAGCAAAAATTGATGCATTCAATCAAGATACCATAAACTCCTCTCAAGTACCT

CTGAATTAACAACCTCTTGAAGCTGGACATTTTACAGATACACGGCTAATGTT

>c8769_g1_i2 len=353 path=[402:0-277 975:278-280 682:281-352]

CTGGATGATCTTTAAGGTCCTTTCATGCCAACATAACACACCTCAGAGACCAAAAGGCCT

GCTTCAAATTTCATCTGTGAATGGAAGTTTACTGAAATTTGCATATACTCACATAGATTC

AAAGTAAAATAAGGAAGTTTTGCTTTCCAAACCCGTATATTGCAGTTTTCACAATTTTAA

AGGAAAGAAATGTTTAATAATGGCGTCTATTATTTCACAGGACTATCCTGAAACTAAGGC

TTTGTCTTAGCAAAAATTGATGCATTCAATCAAGATACCATTCTCTCCTCCATTCACTGT

GAAGATTAGGACTTTTTACTTAACTGCCAACATTAGCCGTGTATCTGTAAAAT

>c8771_g1_i1 len=300 path=[278:0-299]

AGATAGAACTCCCCATTCGATCCCAATGCCTGACTGGCTGCTGGTGATGGTGGTCTGGGC

TGCTGGAGGAGAGGGCTTCTCCACCCTCCCCTTTGTCAGAGCTGTTACAGTGTCAGTCTC

ATTAAAAATCCTTTCAAAATACTGCATTTGTTTTTATTTTTGCATTATGCTTTATGTTAG

ATTAGCATCAGCCATAGCTCTGGGTATATTCAGATGCACTCAATGCACTTATCACCTCAA

TTTGAAGCTGTGCCATTTCCAGAGCTTTATGCCAATGCCAATGTCTACAGGCACCTCGCT

>c8773_g1_i1 len=218 path=[447:0-16 46:17-217]

CAGTTAGAACAACCTACAGTTGCAGTTCTTTGTTCATCAAATTTGGTAAGCTTCTGTAGT

TCCTTGAAGACTTCTCCACGAGGTGCATACTCTAGAATAAGGTAGACTCGTGTAACATCA

TGGAAGTAGCCATACAGTCTGAGAATGTTGGGATGCCTGAGATGAGACTGTATTTCAACT

TCTCTTCGTAGTTGATGTTCTACCCCAGCATCCTCAAG

>c8773_g1_i2 len=246 path=[1:0-44 46:45-245]

CTTTGAATGACAGTATGATAGGGCATCTGCTAGTTCGGTAATGTAAGTTGCAGTTCTTTG

TTCATCAAATTTGGTAAGCTTCTGTAGTTCCTTGAAGACTTCTCCACGAGGTGCATACTC

TAGAATAAGGTAGACTCGTGTAACATCATGGAAGTAGCCATACAGTCTGAGAATGTTGGG

ATGCCTGAGATGAGACTGTATTTCAACTTCTCTTCGTAGTTGATGTTCTACCCCAGCATC

CTCAAG

>c8779_g1_i1 len=370 path=[32:0-369]

AAAAAAAAAATTAAATAAAAATGAGAAAAAAAATGAGTAAATGATATACAAATTTTAACA

AAATGTAAAAAACATATATAATAAATATATAAATATTAATTTGATCATTAATTCTTTATT

TATAAAAGAATATACACATTAAAAATAATGTGTTTTCACATTTGAAAAATTATAAATATA

TACATAAATGGTAAGAATATTTTTAAAAATATCTTTTATATAGTAATGAAATTATTGAAT

ATAAACATTAATATACACATGGAAATAATTTGTATTTTTTAAAAGTTGTTGATAAAAAAA

ATAAAAAATCAACAGCATTAAATTAATATAAAAATATATATATAACAGTATGTAAATATA

TATATTTTAG

>c8785_g1_i1 len=440 path=[418:0-439]

AGCAAGGCCCCAGGCATTCACCCTCCCAGTCCTGCAGGCTCTGGCACATGGCCCTGCCTG

GTGCACCAGTGCCTAGGAGGTCTGGTAGTACAGCAAGTTTGAAGGACGCAAGTCTTTCTG

AGCATCACTGCAAATCTCATTTAGATCTGAGAGCCAGGTCTGTCACCAGAGATGGGTGAG

CTGCAAGCTGCTGTGGGCTGACCGCTCATGGGTGGGAAGCGTAACATCTCCCACAGGGCA

TCCAACGCTTTCAGCAACACCTGACTGAACCCTGTTAACCTAAAGGCATAGACTTCACCC

TGTAAACCTTAAAAACATACAGCTCCTCCATGGTGGAGTCTTTTCTTTGTTACATTTCTC

CCATACTCCTCTGTTTAGGAAAGAAAGGTCTGTTCATGAAAAAATACTTGGCAAACTTCA

AATTTCTTTGTTTAACAGTG

>c8822_g1_i1 len=202 path=[1:0-201]

AATGTGTCATTCTGAGGTACCTGAGGCTCCAGCAGCGCAGTGCTTGGCACGGTGGGAGCC

TGGAGTTGCTCATGTATCAGCTGCATGAGGCCATGCCGACCCGAAGTCTCACCGTGCAGA

TGTTGGTTCACACCTTGCAGTGCCACTGACGGAGGGCAGTGGTGATGAAGCTGGCTTGGT

GCACTCGGGAAGAGCAACCAGT

>c8822_g2_i1 len=250 path=[586:0-249]

ATATCATCCCAAAACCAAGTGATTAAAACCAACAACAGCTACTATGAAAACGCGGTTTTG

TAGCACAGGACCGCATGGGATGCAGCAGGGAAAATCATTATGAGCGCACCAGCAGGGTGC

AATTATAAATAAACCCAGCTCTGTTCAGGCTACAAAAGCAGCAATAATCGGCCGGGTCTG

CGGTTTAGGAAGGACTAAAAACATTTACCAAAGGTGCTATTTATTACCAGAGACAGGGAC

GCAACTGGGT

>c8823_g1_i1 len=429 path=[561:0-428]

TATTCCTATATTCCTCAGCTTTTTTCACTAGGTACTAATGAGCCACTGATTGAACACAAT

AAGATCCATTAGTTAAATGCAAGGACAAGAGACAATAACATAGTATGGCTAATAAGAATA

ATGATTAATCTAGCTCAGGACAAATTAACAAATCTAGAATGACTGCTGAAATTACTGCTA

ACCACTGCCAATCCTAAATCAGTTTCTGAATCATAATACGTTACTTGTGGAAATTCCTTC

TCAAAAATACAGCTGCTGCAAAACACTTCTCCCCCACACCAGGTAATACAGTTACCCTTG

TTACTTGGCGTGTCATTATCTCACTTCCCATTTTCTTCCTGTTTCTACAGGCCAACATAT

GTTAGTTCAAAAGAAGAAACCTCAAGTTTCACCTTGCCAAGCGTACTTCTTCCTCATCTG

CCATCTCTG

>c8824_g1_i1 len=238 path=[392:0-237]

TTGTGTTTGATGGGGAAGGAAGGAAAAGGTTTATTACTGTTATGACTCTGTATTTTACCA

AAGGAAATTATAACTATCAGGTACCCAGCTGATTCTTATAATGCAATTTACTTGCTGTTG

GAAGCTGTCTGATGGTTAAAGTTAAATAAATATGATATACATGGACAAAATTGTACTTCC

CTGGCACAACAGCAATCTTAAAATATCTAAGATAGAAAGGTGAATTAGCAGAACACAG

>c8826_g1_i1 len=205 path=[15:0-57 390:58-113 219:114-204]

GGTTTCTCAGAGCTCTATTTTAGGGGCAGTTCTCTTTAAAGTTTCCGTACTTTACTTGCT

TGCTCTTCTTAAACTACTTGTGGCTGGTGATTGCTCAGAACCTTAGTTTGTCAAGGTTTC

TCAGCAAGACATCTCTACCCTCGAGGAAATCAACAGTTCTTCCCAATTTAGTGTCATCAG

CAAACTCAATTAGCATTCTTTTCAG

>c8832_g1_i1 len=255 path=[425:0-254]

GGACCATTGCAGAAAGTGTTGCTGTAAGTAGACTTTGCAGTGCAGTTTCTGAGGGGTGTA

GTGTTTGATGTGGCTGGTAGCAAATAGACACAGTTCTTAATGCAATATAGCAATTAAATA

CCACTGAATTCTCTAATTTCCTATACCCTACTGTCTGTGTGATGTCTAACAGTTTCATAT

TGAGAAGAGAATCTTTTTTGATGGCTCTACTCGTGGCAATTTCACTTTTTCCTCTCCTTT

TTTTTTTTTTTCCTA

>c8847_g1_i1 len=208 path=[239:0-152 391:153-207]

TATCATAGCCATTCCCACCACCATCCTGCATGTTTGTTTCGGCAGACATGGACTTGAATG

ACATTATCACGTATGTACAGCGCAGTATAGAGAAGATACCTGCACAAAGCTTCAAGTTAT

AAGCTTGATTTACTCAAGAAGAACCTGTTCCAGTACATCACCACCCTCTTTATGATATCT

GGACTCTGACATCGAGACTTGTGAGATA

>c8847_g2_i1 len=327 path=[475:0-326]

GAATCTGCTTTTCGTCTGAGAAGCCTTCACTTTTCAACTTGCCACCCAGGTGGCAATTAG

CAGTCCTTGCTTACAGCTGACGGTCAGATTCTGACAGTGATCCAGCAGATGGATAAGTAC

CTCCTGAGCTGGATTCAGCAGCAGTTTTGGAGGAGGTTGTATTCAATAAGGCAGTCGTTG

AGATGCTTTGAGCAGTGATGTTGTTTTCATCTGCTTTTAGAGAGCTGTGGGGCTGGCTGA

CTCTTAGAAGGAAGACTTTGGGGTTTATGAAAGTGCCCTTTATCATAGAATCACAGGGTG

GCATGGGTTGGGGGGGACCGCGGTGCT

>c8853_g1_i1 len=412 path=[1:0-411]

AATGTTTTTCACATAAATTCCTCAGAAACTTAGGGTATATTATTAACCCAAACGTTCTGC

AAGTACAAAAACTAACGTTCCATCACTTCAGTAACAGTGTCACTCTCCAAACCCTGGACA

GTTTTGCCTCCCTTAGATTAGGGTTTTTGTTTTTCATTTGGTTTTGTTTTTGTTTTGTTT

TTAATAAAAGACACATGGAGTATCTTACTTCTATACGTATGAAATGGAAAGAAACAGATT

TCTTCTCTCTAATGGTTTCAGAAAAATAAATGCCCTTAGATCGTATCCTGTTGTGCAAAT

TGCATACCCTGTCATAATGAATACATTACAAAGATGCATTCTAATGTGTAACACATGTTG

ACAAATTCAGTGTAAATTGATTTGCAAGGAGGTAAAAAAAAAAAAAAAAAAA

>c8870_g1_i1 len=262 path=[240:0-261]

TTTGACAGTCGCTGCTGACGGCTGAAGTCAATACAAAAACGTGCCTCTCCCAACCACCAG

CGCTCCCGAGCTCCTCTGAGCAGCTCTACAAACGCGCACCGAATCAGAGGGGAGAAGTTG

GGAAAAGCGGAGGGCGATCGCACGCGGCGCGGCAGACTCCGCCCGTCCGTACCGAGGCCG

CCCCGTCCCCGCGGCACCGCTTGCTTTGTAACGCCGTTAGCAGAGCCGCAAAACGCTCCG

AGCCCAAAGCACTGCACAGTGA

>c8876_g1_i1 len=246 path=[355:0-223 645:224-245]

AACACAGAATTCTGTTTGCGTGGGTCTTACTGGCAAAGCAATGAAACATCAGAAGGAATA

TATCTCAAATAATGCAGTATGGCAGCAGCCCTACACCAGGCTTTGGGCAGTGCTCTGTAT

ACAGCAGCCCTGCACCAGGCTCTGAGTAACACCCCACGTACTACACCGGGCACCGAGTAG

TGCCCTGCGTAGTACTGTCCCCTCCTCAACACAACCCGGGGCACTGAGCAGCGCTCACAG

CTCTGT

>c8877_g1_i1 len=263 path=[317:0-105 183:106-262]

AACTGTTTTTATTTCAGCTATGTGTAAGTGTCACATGAAGCTTTGATAGATGTACTTAAT

GAAATAAGACCACTTGTACTTTGAAACAGTGGAAAAATAAATAAAACAGAGCTCAGACTG

CTGCTTGAAGTTGACTTGCTGTTACAACGGACTGAAATGGAAGGTTAGGCTCTGGTGTGG

GGGTGCAGCCTTCCTATGGCTTGCCCTTTGTTGGGTGCCCTTCTCTTGGGTGTTGGTGGT

ATCTCCAGTATCTTCTGGCCATG

>c8883_g1_i1 len=403 path=[1:0-402]

AAAAAAAAGAAAATTATTAAAATAAAATGTAAGTTTTCTCTATTTTATTTAAAATTGCAA

GTAAAGAACCAACAGTTAATATATTTTAGAAAATTCTAACAATGTGTAAATAAATATGAA

CAGAATATGTACAACTTTAGTTGTTGTAAAAATTTTAGAATTTTTTCCATGGATAGATGA

TTTATTTAAATTATTTTATTTAAATAGATTATGGAGAAATACTATTTTAGATGTTATAAA

AAATACGAATTTTTTAATTGGAAAAACTACAATGCGTTTAAAAAATAAAAATTTTATTTT

ATTAATATTAAAATATATAAATGGTTCTCATAAAACTAATCAAAATATAATTGACCGTTT

TAGTTACTTAACTTGTGAAAATTTTAAAATATCTTCATTTCCA

>c8883_g2_i1 len=324 path=[1062:0-323]

AACATTTTTATCTTTTATAATAAAATATATAAATTTTAATTTCCCTTATATTTTATTAAT

TTTATTCTGTTATTAATTTTACATTAATATGCCAATGAAAAAGAAAATTGTTTATACTTT

TTTTTAACTTATCTTTGTATTCTTCATTTAATTCATTATTTACAAAACAAATAATAATAT

ATTTCAAATTCTTAGAATAAAAACAAAAATTTTTCAATAATGGTATACAAACAGGTGACT

TAATTATTAATTTAAGATAAATTAAATTCGGAAATATATTATGTATATAGTTGAAAAATG

GTGGATTTAAATTAGATGAAACAA

>c8888_g1_i1 len=261 path=[201:0-260]

GGGGTGGCTCCGCGGGGTCGGGCGCTGCTGCCGCTGTGGCGAGAGGCAGCTGAGCTGAGC

TGAGCTGAGGTATCGGTAGCTGTAAATGGGAAGCGGGGCTGGCTGCTTTCGTCGGGTTGG

CACTGGTATTTCTCAGAGCCGTTGCTCTACTTCGGAAAGGACAGCTAATCTATCGTTAGC

TGGTTTTGTTACTGCTGAGAAGTTTCCACATGGAGCTAGGCCGTTGCTGAGAGTTTTGAG

TCGGTTACTGCGACGCATCGG

>c8889_g1_i1 len=240 path=[372:0-239]

AAGCAGCAGCACCTGGCAGCCAGGTCAAGGCTACAATGCAGTTTTAGTCCACATGGCAGG

CGGGTTCCCTTACAGACAGACATCAGGATAGGCCAGTGGCTGCTCGCTGCAGTTAAGACC

TATGAATGCCCTTGGAACCCTAACAAAGCATTGCTTACATACAACATCCATTATACAATG

TACATATTTATGTTAATCAAAGTGCTGCTGGTATGAAAAGCCTTGCTAAAGACAACGATG

>c8904_g1_i1 len=306 path=[1309:0-51 380:52-62 391:63-305]

AGCGCTGCGCTGCGCTGCGCTGGGGGCGAGGCGGGCGGGGACTCGGCGCCATGGCTTCTG

GAGTAACAGTGAATGATGAAGTCATAAAGGTTTTTAATGACATGAAAGTAAGAAAATCTT

CAACCCCAGAAGAGATTAAAAAAAGAAAGAAAGCCGTTCTCTTCTGCTTAAGCGATGACA

AAAAGCAAATAATCGTAGAGGAGGCGAAGCAGATACTGGTCGGTGACATCGGAGATACCG

TGGAGGACCCCTATACGGCCTTTGTGAAGTTGCTACCGTTGAACGATTGCCGATACGCTT

TGTACG

>c8904_g1_i2 len=450 path=[184:0-195 380:196-206 391:207-449]

GCGGCGTCGGGGCTGACACAATGCAGAAAGCATCTCTCCTGCTTGTGGAGCCTTACATCA

CCCAGCAAAATGAGAGTCATGACAGTGCTGGCTGCGAACCGCTGCCGTAGCGTTTGTTCC

ATCACGGAAACCCACCCCGGCCCCAAAGTACGAAGTCCCGCGTCCCGCTCCCTCCCCGCC

CCCGGGTGCACCAGCAGGCTTCTGGAGTAACAGTGAATGATGAAGTCATAAAGGTTTTTA

ATGACATGAAAGTAAGAAAATCTTCAACCCCAGAAGAGATTAAAAAAAGAAAGAAAGCCG

TTCTCTTCTGCTTAAGCGATGACAAAAAGCAAATAATCGTAGAGGAGGCGAAGCAGATAC

TGGTCGGTGACATCGGAGATACCGTGGAGGACCCCTATACGGCCTTTGTGAAGTTGCTAC

CGTTGAACGATTGCCGATACGCTTTGTACG

>c8904_g1_i3 len=427 path=[1125:0-183 391:184-426]

TGCATTGTGTCAGCCCCGACGCCGCGCTTTAACAACTCCCGACTTCTATTTCGCACCGCC

AGTGTTCTCCGATCGCTATTTCTCCCCGTGCAAAGCGGGGTGACGACGCGCGCGGCTCCC

GTAACGCTACCGAGCCGTCAGACCTCAAACCCGTAGCAGTAAGGAAACAAAACGCTGACG

GGGCTAACAGTGAATGATGAAGTCATAAAGGTTTTTAATGACATGAAAGTAAGAAAATCT

TCAACCCCAGAAGAGATTAAAAAAAGAAAGAAAGCCGTTCTCTTCTGCTTAAGCGATGAC

AAAAAGCAAATAATCGTAGAGGAGGCGAAGCAGATACTGGTCGGTGACATCGGAGATACC

GTGGAGGACCCCTATACGGCCTTTGTGAAGTTGCTACCGTTGAACGATTGCCGATACGCT

TTGTACG

>c8905_g1_i1 len=208 path=[567:0-193 761:194-207]

AAAAAAGCTACATTCTAAAGATCACTGATCATAAGAATAACTTGAAAATTACATTAAATT

ATTGAAAATTACATTGCCTCAGCCAAGGCAACAGGAGGAACACTGTACAAAGAACTACAA

CCTAGAATTAAAGACCATCAAGCAAAATTAATTTCATTTGTTTTTGTTTTCCAGGCAGTA

TTCAAAATCATGTTCTCAACATGTAGTA

>c8905_g2_i1 len=202 path=[180:0-201]

AAAAAAAAAAAAAACGCCAGTCATTTGGAGACATAGTCCTTCTCACTGTGGTACTATGTG

GCCAATTCAAGTACTTTGACTCACACATTCACTTCTTATGAAATACAGTAACACTCCTGT

CAATATCTTGAATTTTTGTGGGTTCCATAATCCATTCACCTTTTAATCAAGCAGTAGTAG

TCTTAATGAAGTATTTACAGTA

>c8909_g1_i1 len=349 path=[615:0-348]

AGCACGTTCGATTTCCACATATATCTGAAATCAGAAGTAGCAATTTACATGTTGGAATTT

TCTAAAAAGGGACATAAACTATAAGACCACAACCCTATAAGACATCTACTTAACATTTTT

ATGCAACTAATTAATTCCACGTGCATCCATATACTGTAGTGATGCCATCCACAAAAATAC

ACTGATAAAATATAATGGGTAGGTGGAAAAATTGAACTATTTGGACCAAATTAATCTTAA

AAAAATGCCTAAATTCCTTTACCATTCCCTACACAAACATAAAAGAAACTTCATATCTAT

TTCAGTATATTTTAGCAGGCAAACTGTCTAATGCTTCATTTGCTATGCA

>c8923_g1_i1 len=254 path=[1:0-253]

AAAAAAAAAAAGGTAAATCTTAAATAATTATTTTCTAATAGGATTTTTTATTTAATTCCC

AATTATTTTACAGCACTTTTCTTTATTCTGTAAATATATGCTTATTATAAATCAATTAAT

TTTAACAAAAGCAACTTGATTATTACTCAAAACGATTAGCAAAATTATTTTTTCTATCAA

GTATATTTTTAAGTTAATATCATTTAATTTATAAAACTAAAAATTTTACATTAAAAAAAA

ATTTTTTTTTTTTT

>c8924_g1_i1 len=260 path=[1:0-14 16:15-232 234:233-259]

AAGCTAATTAAGAATTTAGTGGCAATTTAGCTTTCAGAAATCCAGAAAAAGTGACAAATG

TGGCATACTATTGGAGAAAAAAATCATAAAAGAATGATAAAAAAAAACCTGGAATGTAAG

TGAACCAAGTGATATTCTCCAAGAGGTTATTACCACCTTCAGGTTCTGTTTGACTTTATA

ACGCTTATGCTTCAGAACCAGGAAAGCATCAATAGTCCTGCCCTGATTTATAAAAAGCCT

TTCACTTGAATTTAAGGAAT

>c8928_g1_i1 len=213 path=[191:0-212]

GGATGAACAAATGATAGCTCCTGTAAGATGAACATTTCTGGAACTACATTACAAAAAACA

CATTTACTTGGGATAGGCAGTAGTTCTTTGGTTTGACAAGGAGTTTGGCTATTTATGAAA

AGTTAAATAGTGTTCTGCATGTTCTTCTGCTCTTTAATTGGTGTTAAAACACAAACTCTT

GGCAGATTTCTATACATGCATGTTAAAACCTTT

>c8934_g1_i1 len=202 path=[336:0-201]

AAAACGGGTTAATATAAATATTGTTAATAAAATTTCTGAAGTGTTTTGAATTGATTGTTG

TAATTCATGTTGATAATTTTATCTTACGATCTTTTTCTATATCAAGAAGTCCAGTGTTTT

CTAAATGCATATTTAATATAAAAATAAGATAATATAGTTTTTATTACCTAGTAAATAGTA

ATAAAAACATTAAGTTGATATA

>c8936_g1_i1 len=297 path=[323:0-49 372:50-58 50:59-296]

GCTGTCAACTGAGCCACAGCAGGTAAAGCAGAGGATCACATGCAGTGTGTTCACATGCAG

TTCAGAGCTTTGCACTTTGTGTGTACCTTCTTGTGACAATCTCAAAAAATCATGATTCTG

AGAAGTACTGTGAATTGTTCAGAGTTTCGCATACATTTCAGATCTTATTCCCATCTTGTT

TGCTATGTCAGAACCTGGGTATGGATGTCTCTCAGTACACGTGTTCTTTTTAGGAGCTTT

GCTGATTTGTGTTTATTGTGTATTACTTTTCAATAGGTTACCATCTGGAGTCCTTCT

>c8942_g1_i1 len=209 path=[457:0-208]

ATCCCCTGTCACCAGCTGCTCCTCTCGTGAAGCTCCTATCCTCCTTTCAGGGCTGTTCTG

TGTACCGTCAGGCACAGCCTTGAGGAGTCACATTTGCTGACATTAAGTTGACTACAGCTT

TATGGTCATTTTACACATGACAGCATGTCAGCTGTGTAAACCTCCCTGGAACAAGAGATT

GCCTACGACTAGCTGCCATGAAATTGAAA

>c8944_g1_i1 len=291 path=[423:0-290]

GGGAGTTTAATCCCTTTGTGCCTCAGTCATCTAATCCATAAAACAGGGTAATAATACCCA

CTAATCTCAGACGGCTTTAATTGATTATTTGTTAAGATCATCAAGAACCTTAGCAGGAAG

ACTTTAAAGACCTGTGAAGTCTACTAATTGTTTCATCATTATAATTGCTGACATATTATC

TGACTTCGCACCATTGCCTTTACCTGTATGTTAAAGTGAATGCAGAAAAGCTCGCTCTCT

GCTGCAGAGCTCTGAGATCTGATAGTGAAAAGCTACAGATAGAATCCCCCT

>c8945_g1_i1 len=218 path=[196:0-217]

CAGTGACACTTGGGCGAAGCTTGCTTAACGAGGGGGTGGGGCTGAGCTTTGAAGTGCAAA

TGTCACATGGCAGGAGTTTAGAAATGGCTTTGCAGGGGAAGCCAGGAGGTGAACAGGGCT

GTGGAGAAAGGACCTGCACTGGCATCAGTCTGTGTTTACATTTCTGCCTCTCCGTCCTTA

CACAAGCTGTGAAACCTCTGTGAGCAGCTTTGCATGTG

>c8946_g1_i1 len=314 path=[1:0-313]

CAGGATGCTTTCCTACAAATAAATACATATCAAATGAGAGATCCAAACTGGAGATCAGAG

CCAGAGCCATTGTAATGAATGTTGGAGGGTGTGATATGAGAAAGTTGTATGTGTGAACAC

TTCATTTACACTAAGAGCTCAGGCCAAAGGGCATTGAAGGATAGGTTCTTACAGGCTTGA

GTCCTACCCTGACCTCTGAAAGTTACGATGCAAAAAGTATCCTGCTGCACAACCATTGGC

ACCAAGCTGCTGATTGTCAGGCCTGCCTTTCATCTTGCACCCAGCAAGCAGCTGTGCAGG

CCTGACCTCGAGGG

>c8954_g1_i1 len=291 path=[155:0-290]

TTTTTTTTTTTTTTTTTGTGGATCTTGAGACCAGCACAGGCTTAAATCCTCAGCCGGAAA

CATCTAATACACCGCTTCTCTGTTCTGCTCTGCTAAATCAAGCTATTTGCATTGTCAAGC

TATCCCTGGCATTCCTTCCAGTGCTTATTCTGCACTAGGGATCCAGTTGTACTTACACTG

TATGCTCTGTCACATTCTTTGAGCCTGAGGGTGTACCACAAAAAATTAATCCACAGATTC

TTTTTGTTTTGTTTTGTTTTTAATTTAGCAGTTGACATGCCTGATAGAAAA

>c8957_g1_i1 len=352 path=[1:0-351]

GCACACAGAAAAAGAAAAGCAAAACCAAAATCTTGCCTTGTTTTCCTATGGTTGAGCCAG

AGGTTTCTCTCAGGCCAAATGAAACTTACAGCTCATTTTTTTGATGTAAGAATTTGGGTA

AAATGGAAAGCCTTCAGACCAGCAAAGGAAGCATTTCAATTATCACTACCTTACTGAAAA

GGGTGGATATCAATAAGCTTCTTTCATAAGCACAGAAAAATAAAACTGTAGACCAGAGCG

TCAAAAACAGCCTGAAAATTGCAACCAGAATCACACGACCCACTACTACTACAAGACCCG

CTGTGAGAATTTCCTAGGAATCAGTTACGATTTTCAGGCTGTTTTTGACGCT

>c8963_g1_i1 len=324 path=[302:0-323]

TGAAAGAGAATTATTTAAAACTTCACTGAATACTTGAATATACGATGGGTATGTGCAGCA

GTTTCACCAGTGCCAAAGAACTCCAGACTTTCTGAAAGACAGTAAACTCAAAATTGGCAG

GAAACCTCTCACAATGATGTCACAAATATTTGTTTGTTGCCACAGAATAATGTTTTAAAA

GAACACCTACTTCTGTGCTGCAAAGGCTTTGCGAATGTGTAATCACCCAGTTTTATCACG

GCAGAAATCAGCTGCTTCACGGTAAACGTATCTATCAGATCTCTCCCTTCTGCACAGATC

TACATTTAAGTAAACATATGCGTG

>c8971_g1_i1 len=220 path=[198:0-219]

CAGAGCTTGCACAATGCGGTGGGGCTGTAGGATTTTGGGGATGGTGGGAGGGAAGAGGTC

AAATCCACTTGATTCAAATCAAAATCACTACTGAAATACAGCATTTGAGAAGTGAACCTG

CTTTGCTACTGGACAGACCTGTACAAACCTCTTATATAATGCCTTCGCGAGGAAACTGTG

ACCTGCTGGGTCAGAAGCTAGGCAGAAGTTCTCAGTAGAG

>c8979_g1_i1 len=257 path=[1:0-256]

GCTTTCAAATATTCCCCTTGCTAGAGATGTAAAACTCCAATGAATTCAGAGTGTATGAAA

CCACTGAGGCTTTAATCCTCCCACTCCTGTTTTGCTATTTAGTCTATGAATGCGAAAAGG

ACAGATTTTCTACCGTGGTGTTATAGAAATTGCACAACATTTCACATCCAATCCAGAAAC

GTTTGCAGTGCCTCTGTCAAAAGCCAAAGCTGAATAACAACCATTTTGGCCTCACAAAGA

TGTAACCTTTCATTAAG

>c8979_g2_i1 len=230 path=[676:0-229]

ATGACCTGACTGTTTAGTCTGTGTGGGGGGGAAAAAAAAAAAATGAAAGTAAACTGTGTT

ATTTTGAGCCTGAGCAGTGAGTGAGTTCTCAGTATCTAAATGAGAGCTTTTAGAGTCAGC

TCCAGGTGTTGCAGGAAGTGGTGGAGGTGACTCAGTAAATGTCACACTTCCCTCCAAGTC

AGTTGTGCTGTCCAAAAATAACACTCCATGGGCATCTGGCTGCTCAGCAT

>c8980_g1_i1 len=304 path=[1:0-303]

ATCCAGCTGCATACAGCTCTTGACAGATATTCTTTTCAACAAATGCCCTGTGCATTCTTT

TTGATCATTGAGCATTTCATTCTCCTTCGTATGAAAGCTCCAAGTGGGTCAGAGGGTAAC

CTCGGATCAAAGAGACCTGCAGCTGGGGCAGACAGGCAGACAGTTTAGCCTCATCTGCTC

CAGAGTGATGTTAGCATAGTACAGAGCACACACTTGAGGCTGAACTCCCTGAGAGCACCC

TGCAGGCAGTATGGTAAAGCTCAAAGTAGCACGAGCACTGAGTAAGGCCATGACTGAGGA

CACA

>c8980_g2_i1 len=378 path=[947:0-377]

GTAAGGCCATGACTGAGGACACAGCCCAGTCAGAGGATGCAGTGCTGGGACTGGTCCATG

GTCTGAGAGGCACTCACAACAGAGGGAGAACTGGGGAATCTGTTTCCGAGGGATTTCAGT

CAACTGAGGATTTCTTGCCTTTTTCAAGTGCCATCAGCCTTCAGCCCAGACACAGGAAGT

TAGAATTACTGTGAATCATGTCTTCTCCTAAAAGTTAGTTGCAAACCCTATACAGTCTAG

AAGCGCTATCAGAAACTCTAAGTGCAATTGATCAAATTCCTTGCTGCTGCCAAGGATGGT

TTATTTGGAACATATTAAAGGTCATAGAACCATACAATAGCTTGGGTGGGAAGGGACCTA

CCTCAAAGATCACCTAGT

>c8981_g1_i1 len=238 path=[506:0-237]

GAAGGCACTTCCAAGTAGTCTCTAACATCCAGAGCACATTTTCTGCAAGAAATGCTGCAT

TGTGCCACAGTGTATCTTTAGTTTTGCTTGGGTCATGTGTGAAACACTATGTAACCTGGG

TCTGTTTCTAATGAGCACGAGGTCTTCTGCCTAAACTCTGCTTTAATGCTGCCTGGTATA

CACTTGCATTGAGCCTGGTGATGTGCAACTTGAAGGCTTTATATAATTAAGCAGGAAG

>c8985_g1_i1 len=228 path=[1:0-227]

ACAAACAATATAAAGTTCACTTATATGCTAGCAACCTATACAGGTTTAACCTAAAGGACT

GCCTACTTTGTAAGAATTCTCAAGAATGATCTAAATTCATTGTACTGTCTCTGCTTCTGG

ATTTCCTTAGAAGAATTTGTCTATAAAGATTACAGGTACATGAGATTTTTCAGACATTCC

TTTTGTTAGGTATACTGACATTTGAGATGATATCTATTCCAATCCTCA

>c8991_g1_i1 len=232 path=[1:0-231]

GTGCCATTGATAGTATAGGTCACAATGCCATCACTGTCCTCGTAGTCAACGTAGAGGCTG

TCGATGTGGGACCCCACAGAGTTGATTGGGTCTCCCTGGGCAAAGATGCTGTTCATGGTC

ATGTTGAGCACCAACTCCTTCGAGTCAGAGGTCTTGTCCAACAGCTTGTCCGTGATGGCA

TTCTCCGAAAGGAATTCCCTGGAGGTGAACGTGGGGTCGAAGTCTCTGTCAT

>c8992_g1_i1 len=201 path=[233:0-106 26:107-126 26:127-146 358:147-200]

TGGAGGGACAGCGGTCACACAGAGGGGACAACAGCAGGAGGATGGTGGGTACATGGAGGG

GACAGTGGGCACATGGATAGGGCAATGAGTACATGGTGGTGACACTGGGCACATGGAGGG

GACCAAAGGCACATGGAGGGGACCAAAGGCACATGGAGGTGCAGACAGCAGCCCATACCT

GCCGGAAGTGCCCTACTACAT

>c9002_g1_i1 len=257 path=[235:0-256]

ATCATTGCTATTTACAGGCAGCTATTAAGGGGTTTTTCATCGTTCTTGCCTATGTCTCTA

ACCCTACAAAAAACAGTGGGATGTCTACTCAGCTTACAATTAATATTCACAAATTAAAAA

CAGCACCATTTCTCTTAGCACGTATAGGTGCTCTTCTAGGCTACGAGTTCATTAAAAAAA

ATTCTGACAGCAATGTTCTTTCACATTTTCCTGGCTACATTTCAAAATATCCAAAAGCAT

CCAAGTTCCATTCAAGT

>c9002_g2_i1 len=213 path=[469:0-212]

ATCTCGCACATTTAATTTGAGGTAAAGGAATGTTTTCTATTTATTTCAAACAACATATTA

GTTTACATAGCCAACCCAAAGGAAGGTAAGTGACCTTCCTCTGAACTTTACTAGAGGAAG

GAAAGTAACAGCAGATGTTCATTTTAGCACAAGTAAATGAATTGGTATCACTTTCACAGT

TGTCTGTATAGGAAAAAAAAAGTTGAATATAGT

>c9004_g1_i1 len=228 path=[656:0-227]

ATGTAGGACAAGAGGAGCATCAGGCACGAGTACAGCAGTTACTTCTACCGCAAGAAGAAG

AGCTCCAGCATCGTGTCCTACAAAGCTTCTCGGTTCAACACCTGCTCCACCCTGGCAGAT

GCCCCAGCTGGGCTCTCGGATAAGATAGCTTTGCAAACCAGATTCTCTTAGCTGAACTTC

CCCTACAGGCTTGCTCTTGGGCCTGGATGTAGACGCTTGTGCTTGGCA

>c9004_g2_i1 len=248 path=[226:0-247]

GCAGAGGAGGAACATCCCAGGAGCTGCCTACCCGGGCCGAGACAAATGGAGCAAAACGAA

GCAGAAGACTTTCCGGGTGCTCACTGTCTCGGTGGTTTGCTTTGGGGTCTGCTGGCTGCC

CCTCCAGGTGGTCAACTTCATCAGAGACATTGATGAGGAGTTCACCATCCTGGACAAGAG

ATACGTGAACGTCATCCAGGTCTCATGCCACCTGATTGCCATGAGCTCCGCCTGCTACAA

CCCCTTCA

>c9008_g1_i1 len=218 path=[196:0-217]

TGCGTGTTTGACTTGCTCTTAAAAATACTTAAGATGTACTAACAGTATAGTTAATTCAGA

ACTGCTCCAACCTGGCCCTGAGTGAGCTTGATTTTTCCTTTTCCATATATGCCACATGGC

AGTGACTTTATTTCCGTTAGTCTCCTGGTTACCCCAGTGGATGTGACAAAAACGCATTGA

ATAACTTATTTTGAGATTATGGGCTCCTGGGCTCTGTA

>c9015_g1_i1 len=240 path=[271:0-239]

TACCGCTCCATTGTATCTGCTTGCTTCCTGGGCTGGGTGCGATGGACTCAAGCCTTGTAT

CCAAGCCTTACTGGCCAGATGATCTGTCTGCCTCCTCTACAGGCATGCCAGGGCATAGTG

CAGGCAAAGCTGTTTCTGCAACACCAAGGGATGGGATGAGGCAGACTCTGGACTGATGCC

TTGGACTCCTACCTGCTCTGCCTCACGCGAATGACTACTAATGGTGTCCAGCACTGTTTG

>c9016_g1_i1 len=298 path=[276:0-297]

ACCTTTATAGGTGGCCTGGATATTGAGATATTAAAAGCTGTCTCTGTGTGCACGCAGAGC

CTCATGTTACAGCACTGAGCCACACAACTGTGCCTTTGCAGCAGCAGGTGGGCTTCACAG

ACAGCGTACATATATGCAGGTCCATATATGGCTTTTACACTTCACTCACCCCGGCGCCTT

AAAACAAGCGGCAGTGATTCAGACTCGGTCTGCTCGGAGTGGGACACGGCGGGTTGGGGT

TGGGGTCGAGGTGCGGGTTGGGGCCGGGCGGTTTGGGGTTGTAACGGTTTTTACCTCA

>c9017_g1_i1 len=215 path=[551:0-214]

GGTTTGAAAGGCAGGTTATAGGTAAGCTTTAAGGCAGGAAGGGCATTAGCTGCAGCAGCC

CTCATAATTCCCTTTCATATAGCTCCATCCTCCTGTTTCAGCCCAGAGGGGCTCACATTG

TGCCAGACTCCTGGGGGCTCATTTCATCGTAGGGTTCATCATCACATTGAGTCACTGAGA

TGCCTGATGCATCTTAGTGCTGTTGCTGCTGTTTA

>c9018_g1_i1 len=204 path=[1:0-122 124:123-203]

ATAATTTATAAAAATAATAAAATAACTGTTCTTAAATGAAAATAATAATTTGTTATTTCC

AAAATAATAAAATGTATATAAAAATTTATATTCATTTATTTTATATTTTTCACGTAATTA

AATAATTATATTAATAAAAGTATGTAATATTTTTATAAGAGTTTCAATACATATTCCTTT

CATATAAAAACTGTTAAAAATAAA

>c9022_g1_i1 len=277 path=[304:0-124 579:125-276]

GGTGTGAGGTGAGCCCCTCGGCAGCCCTGCCCTGCCATCTTAGGGCAGCACGGCTTGGCA

GAGTAAAGGTGCTCAGTGCGAGCAGCGCCCGGCTAAACAGTGCCCGGCCGTGCAGAGGTG

TGCGGAGTTCTGAAAGCTATTCCTACAGTAGGTGAAATATATCGTGTGTACTACAGGCGG

CTGAGCCGCCACCCAGGCAGTTAGAGCAGCAGCGTGCCGGAGATACTGACACGTCTCAAA

GACGACAGTGTGCCCGAAGCGCAGCGAAACTGCGAGT

>c9030_g1_i1 len=453 path=[1:0-322 324:323-326 328:327-424 426:425-452]

ATGTGTGTCACATGGAGAACTCTGGTGAGCTGTAGAACCAAATCTAGGACAAAGATGAGC

GCTTGCATTTAATACTAACTCCATTCCTGCTTGCATCTTTTAATGCTCCAGTTCTTACTA

CCCCAGGAGGCTGAACCTGAAACTCATTGCTAATTTTTCAGCCTATCAGTGTGCCCGGTA

TATTTCTGCTGTTATAGTTCTGGAGTGTTTGCCATCTCTGATAGCTTCAGCCTAATGTCG

TGGAACAAACAAATTTCTTCTTTTGAGTAAAAGCCTTTTTTGTCTGCTTGCAACACAGTT

TTCATTGTTTGGAATCATTTCACGTTGGCTTCCAAATGTTTGAAAAGTAGTATGTTGTGC

TGATGATTGCTGAAGTAATGGCAAGCAGAGAGTGATTTGGGTCTGAGCTGTTACTTATTT

TATTGTTTTCTTCTATAAATATGATAGATGGCA

>c9046_g1_i1 len=352 path=[155:0-351]

TATAATAATTGAGACTATTTAATTACTTTTTTGTATAATTATATTATATATTAAGAATGT

TGATAGTTATATTGTGCTACTTGACATAATATATACTATAAAAATTTTAAAGACAAAAAT

CATCTTAATTTGTTGGCAATATATATATAAATTATATATAGATGCATATTAAATTAATAT

ATAAGTATGAAAGTAGAACTAAAAAAACTCACAATATTTAAAGATATTCATATACTGTAA

ACAACATTACGTTTGTGTAAAAATAGTATAATGCAAATTATAAAATTTTTCCTTTATTTA

ATTTAAAAATGTATTCTTTTTGTTTATCCAACAAGATTTTCTACAAAATAAA

>c9048_g1_i1 len=207 path=[165:0-206]

AAGGAAGGATGACAACTGGACTTGTTCACTAAAGACCAAGGCAACAAATCTTCACCATAA

GGCATTCTGTATTACTTTGCTTACATTACTTCCTTTGGTAGGCAGAATTTGCTTCTCAGA

ATTTCTAGGATTCCCTGAGAAATCGTTCCACTCAGGCTTTCAATCTTCTGTGAAAAAACA

CAGTGCAATAAAATCTGACCATAAATA

>c9049_g1_i1 len=296 path=[53:0-154 23:155-294 432:295-295]

TTTTTTTTTTTTTTTTTAATGGAATGGTTTGGAAATATATTAAGTGCTTGTTCGCAAACT

CAGAATTGCAAAGCAAGTGCATTTAACTATGGTGTTAGAGGAGAGGTGAATTTTTTTTCC

GAAGGTTGTGTTCCAGTGAGAGAAATTGCATTCACAATTGCCAGGATATCTTCCTATCCT

TTTCTATAGAAAAGTTGAAGTTTAGAAATCCTTTGGTGCCAACACACGGTTATAAATTAG

GGGCCTTAAAGGTTCACGTATAGGACGCATAGTTTAAGGACTGTCTCTAGATGTTT

>c9052_g1_i1 len=265 path=[1:0-264]

GCCCCCACCCCGCGCTATATACACCTATAGGGTCCAACCCTACACGTTCACCGTGGGCTC

GTGGTCCGGGGCTCCAATCCTGAGCCGCTCCGTCCCTGGGCGGGTGTTCCCCCCCCCGGC

CCCAGCTCAGTGCATGAAGACGGGTTTGTGGACGCGCTTGTGGCGTTTGAGCGTGGCGCT

TTTGGCGAAGCTGTCCCCGCACTCCACGCAGATGTAGGGCTCGTGGCCGTGCGCCTGCGC

CCGGTGCAGCTCCAGCTCGGCCCCG

>c9058_g1_i1 len=234 path=[212:0-233]

GCAGACAGCAGAGTAAACAGCACTCAGATTCTAAAGGGGTGGGATTAAGAAATGCTTTAA

AGCTGCACACTTTTTTCTGTTGCACTTTCGTGTGAAACTCCCTGGATCTGAAAGCAGTTA

GAAATACATCGAAATCTTACACACTTGTGCTAAGGGCACGTGCATTTGCCTGAATTTAAG

GTCATAGAAATATGGAAATGTTTGGGATCTGTTTGGACACCATAATATTTGGTA

>c9065_g1_i1 len=887 path=[2792:0-14 539:15-886]

TAACTTTGTGTTTGAGTCTTCATGCTTTTTTTCGTTTGTATTATCTTCTTCATGTTTTCT

TTCTGTTGAATTTTCTTTATTCTTTTTTTCACTTACATTATCTTCTTCATGTCTTTTTTC

ATTTGAACTATTTTCTTCATTTTTCTTATCATTTGAGCCATTTTCATCATTTTCTTTCTC

TTTTTTTGAATTACCATCTTTATTATCTTTTTCTTCATCATTTTCTTTTTTTGTCATAAG

AAAACTATAACTTCCATTTTCTTTATTATTTAAGTGATTTATATCAAGTGATGTTACATT

TTTTTTTCTTTTGAAACTTATAAATTTAGAAGCTTTAACTTTTTCAATCCATATTTTGAA

AATACAAATAATTAAAAAAACAAATAACTTTTTCATTATTAGACATATAAATATATTATA

TATTTTTAATTGTGCATTAATAAAATAATGGTAAATAATTATGAAAAATATATATTTTTT

TGTATTATCACAGGAATTCTTATTTTATAGAGAAATATTGATACTATATTTTTCTATTGT

TTTTTACAATATGAAAGAGTATTTTTGTATTTATATAAAGTTACATTTTATTAAGAATTT

TTTTATTTTAATTATCTAATTTTACAAAAAATTTTTTTCTCTTTTTTCAATAAGGATTTA

TATTGAAAGAAGTTATTTTAATATCTTTGACTTCATTTGAAAGTTCAAATATTAATGGAC

AATAGTCAATTTTTAAAACACAAGAAAAATCTTAAAACTAGATATATTCTATGTATCTTA

AAAAAAAAATTAAAAAAAGAAATAAAAATATTTTTATAATGTAACTGTTCTGTTGTGGTA

ATTATGTGTGTGTTATAAATTTAATATTTTGAAATTTTTTTTTTTTT

>c9065_g1_i2 len=1408 path=[3:0-535 539:536-1407]

ATTTTTTTTTTTTTTTTTTCTTGTAAAAACTACTTTTCTTGAACATAAATCAATTTGCTA

ATTTTTGAATTAAGTACAATTACTGTCATACCTAATTTTTGTAATGAGAAATATAAACTT

TGACATTCAGAACCAATATCTTTTAAGTAAACATTTTTTGATTCTATCTTTATATATTGA

GATGATAACTTTGATTCTGGGATAAATTCTAAAATTTTCAATATATGAGAACAAGCAATT

TTTAGGAAACTTAATGATTTTTCCAGTGAATTGTTTGTTGAAAGAATTTCATCAGTTAAT

GTTTTATATGAAGGAATGAAATTTCCATCTCTATCATGAGTACCATATTCCAACAAATTG

CTTTTAGTGATATTTTTCTCAACAGGTGTATTTTCATCTTTTTTTTTTATATTACTTTCA

TCATTATTTTTTTCGCTAGAATTCTCTTCTTCATTTTTTTTTTCGTTTGAATTATCTTCT

TCATTTTTTTTTTCGCTTGGATTCCCTTCTTCATGCTTTTTTTCTGTTGAATTATCGTCT

TCATGCTTTTTTTCGTTTGTATTATCTTCTTCATGTTTTCTTTCTGTTGAATTTTCTTTA

TTCTTTTTTTCACTTACATTATCTTCTTCATGTCTTTTTTCATTTGAACTATTTTCTTCA

TTTTTCTTATCATTTGAGCCATTTTCATCATTTTCTTTCTCTTTTTTTGAATTACCATCT

TTATTATCTTTTTCTTCATCATTTTCTTTTTTTGTCATAAGAAAACTATAACTTCCATTT

TCTTTATTATTTAAGTGATTTATATCAAGTGATGTTACATTTTTTTTTCTTTTGAAACTT

ATAAATTTAGAAGCTTTAACTTTTTCAATCCATATTTTGAAAATACAAATAATTAAAAAA

ACAAATAACTTTTTCATTATTAGACATATAAATATATTATATATTTTTAATTGTGCATTA

ATAAAATAATGGTAAATAATTATGAAAAATATATATTTTTTTGTATTATCACAGGAATTC

TTATTTTATAGAGAAATATTGATACTATATTTTTCTATTGTTTTTTACAATATGAAAGAG

TATTTTTGTATTTATATAAAGTTACATTTTATTAAGAATTTTTTTATTTTAATTATCTAA

TTTTACAAAAAATTTTTTTCTCTTTTTTCAATAAGGATTTATATTGAAAGAAGTTATTTT

AATATCTTTGACTTCATTTGAAAGTTCAAATATTAATGGACAATAGTCAATTTTTAAAAC

ACAAGAAAAATCTTAAAACTAGATATATTCTATGTATCTTAAAAAAAAAATTAAAAAAAG

AAATAAAAATATTTTTATAATGTAACTGTTCTGTTGTGGTAATTATGTGTGTGTTATAAA

TTTAATATTTTGAAATTTTTTTTTTTTT

>c9067_g1_i1 len=211 path=[126:0-123 250:124-210]

CATAGATGAGGCTCACTTCTTGCCTTTCTGTTGTGTGTCTGAGTATTTGAGCAACAATTG

AAAGAAGTTTTGCTCACAATAAAACTTTGAAAATCAAGCATTCAACCATATAATGAGAAG

CATTGTGATTGTGGTTGCAGATATTCGCTGTTGTCTTTGCTGCTGAATACGAATAAGGTG

ACATCTGCCACTGGGCATCTTTTGCATAGGG

>c9079_g1_i1 len=444 path=[834:0-443]

ACCAGTGCTGAGTACAGGGGGACGATTACCTCCCTGCTCCTGCTGGTCACACTATTTCTA

ATGCAGGCCAAGGATGGCATTGGCCCTCTTGGGGGGCTAATGTTTGAATCCCCAAACCTT

AGATGCAGGAAGAGGTGCTAGATGCTGACATGGTAGCCAGGGCTTCTGTCTCCCAGGAAG

CAGGTGTAGTCAGGTCTGCTTTGGAGCCTGCTGTTCTTTGCGCTGACCTCGGGAGATCCC

TCAGTATGCTGTTGTGTGCCACATGCACCAGCCCCAAAAGGGGATTCCCCTGTGGAAGGA

GGGAGAGGGTGAAGGAGGTTGAGGGCCCTTTTCTCGGGGATGGCTCTTCTGTGCCCTCTT

GCTTGCTGGAGGGGGAAGCACAGGGAACAGCTTTTACCTCCCATCCCGTTCTCTGTCCTC

CAGCCCTCTCCTCATCCTACGCAG

>c9085_g1_i1 len=235 path=[39:0-77 270:78-234]

GATTCTGTGATTCAAAATTAAATTGTCATCTCCTCTGGGCTGGAGATCTGAATGCATTCA

GTCAACTGCCTTGCCTGCTTTCAAACATGCCCAAACATGGAGTGTAAAGTCAAGCAAAAC

ACCTACTGACATTGTGTTCAGCAGTCTTGGTTCAAGGCTCATGCAGATCCTGCCCATCTC

TCTGAACCTGCATGCCCACTAGTGCTGATGGTAGCCTCCTCCCTGGGCAAGGTGG

>c9093_g1_i1 len=210 path=[188:0-136 325:137-209]

GGGTGAAGCACCCAGATACTACAAAACCTGAAAGGGCTTCGAATATTCTGTTCTTTTCAG

TACTGTGAAGGGAAAAACAGGGTGAGTGCCAAGAATGGTGATCAACTGCAGTTGTTCAAA

CCTTGCATTCTGAGCAGGGCATTGTGGATATACACAGAAGTTAATGTTAGTTCCTTCAGT

GGAAACTTTGTTTAGCATAAATCCTGAAAC

>c9098_g1_i1 len=255 path=[549:0-254]

ATGGTTCTGTCTCACCTGATACAGAGTTGAAAATTGATCCATTCTCTTTCAAAACAAGAG

CAAAATCCTGTGGAGAAAAAGATGGGAAGGGAATCCGGTCCCTTTTTCTGAGCATCCCAG

ATGAAAATTTTGAGGATCACAGTGCACCTCCTTCACCTGAAGAGAAAGATTCTGGATTTT

TCATGCTGAAGAAGGATAGTGAGAGGAGAGCCACCCTTCACAGGATACTGACTGAGGACC

AAGAGAAGGTGGTGA

>c9103_g1_i1 len=235 path=[377:0-234]

GCAGCTGCTGCAAACCGAGCTGCTGGCCTGTCCCCTCGTGCTGGGGGGCTGTGTGTGTTT

GCGTGCCGATGTCTCCTTTGCAGGCTGATGGGAAGCGAAGGCAGGCAGGCAGGCGGCGGA

GCTGGGCTCAGTCGGTGCCATCCAGAGTCTCGTGCTTGGAGGCTGTCGGTCAGCGTCACC

CAGGGACAGTGCTGGGGGAGCCTGGAGGCTGCAGGAGCTTTTAGTTAACTCTGGA

>c9107_g1_i1 len=209 path=[387:0-208]

TAGGCAGGGATCAAAGCAGCATCATTAGCAGGACAAGGCAGCACACACCAGCTCATGCAT

CTCAAGTGGGGCAACAGGTTTCGAAACAGTGGGATGAGGAAGGGAGGTGGGATAGAGATG

TGATGTTATGCTGTGCTGAGCTACACCACATCATGCCAATGGAACGTTTTGTGGACTACT

GAGAGCTTGTGTCCATCAAGCCTGGTCAG

>c9109_g1_i1 len=276 path=[254:0-275]

AGCCCAAAGCTGCTGCCTAACGACAGTGAGAAGCACAGCAGGCAGTGACCCCCAGCCCCC

ACCCGGCCTTCCACCACCCAGAGATGTCCTGAACCAAACAGGTCTACACAACACTCCAAG

AGTTCAGTTCTGTGAATTTACCCAGTATCTCTTACGAAGCCCCATACTTTCTGTCTGTTT

CAATTCTACCCACTTTTCTTTTATTTTACTGATGTATTCACATTTATTTGTACCGTGGAT

GTAGCTCTGTAACCTTTACTTTGAGTGATTCTGACA

>c9111_g1_i1 len=323 path=[201:0-322]

GTATTATAGTTAAATTTATTTTGAAGTACTTTTCTATTTAATTCTGTTCAGAGCGATAAG

GCTATCATATGAAAATGCCTAAATCTAAATGCAGTACTTCAACTTAAATTTAACTATAAT

ACTGGAAATTTGTAGCGCTGTGCCTATTTATAGCTTCTCATCCTCAGGAAAGTATCTGAC

TATTTATTCTGCTTTTCTAGTAGAGTAAGTGGCAGCTTCAGACCTGTCTTTCTGCAGCAT

CACTACATGTGACTTACCTAAATTTATGTCTTGCTGCCATAAACTATATTGTGTCATATA

GCCACCTCCTGTGAAATGCACGC

>c9114_g1_i1 len=246 path=[1:0-245]

TGTGTTTGTAATGACACATTAATTGGGATTCTTATGACTTAAAATTGTTATTTCTTATTT

ACTAACTCCAGTTCTGGAGGTGAAGATCCAAACCTTTGTAAGACTTAGGAAGAAACTATA

TATCATCAAAATGTATCCAATATATCAAAACGCATCAAAAGATATCCATAAAGGAGCCTA

ACTGCATTACATGAATAACTTAACGTTAAGAGCAGAATTATCCTGTTTTTTTTTTTCTTT

TCATTG

>c9119_g1_i1 len=212 path=[642:0-211]

GCAATGCCAGCACAGCAGATCCTCAGTGAGTGGGACTTTGCCTGGACTTCAGCTTGTGTG

GTGATTGGGATCAGGCTTATCCCACAGCGCACGGCCTTTGCCGTGAAACATTTAAATTCA

CAAATGCATTTTGGCAGCCGTAGGAAGGGGAAGTGAATGCTGGATAAACTCCTCGAATCA

AGCTGTGCCAAAAAAAAAGAAAAAAAAAAAAA

>c9121_g1_i1 len=227 path=[515:0-226]

TGTATGAAATATCTATTAATTACCCGAATTTTATGTTAAAAAATTTATAAAGTTAACAGA

TATTTTATATTACACTATAAAAGAATGAGATGAGAAAGATGTAATATTGATTTATTTTTT

AATATTGTTTTTCTTAAAATATAAAAATAAAATAAAAATTTTTTTTCGATAATAAATTAG

ACAATACTAGGATAAAATAGTTACATAAAAAAAAAAATATATATATA

>c9122_g1_i1 len=238 path=[463:0-33 69:34-95 131:96-237]

AAGCACTGGCAGCACAGTTTTTTTAAAGGAAGGATACAGTAACATTTTTTCAGCATGTCA

CTGATTCAGGGATCCAAATGCTTTGGGATGCCAACAGCTGTTTAACCTACGGTGGTCTTG

TTGGGTTGTGGTTGCTTTTTTTTAATCTAACTGAAGTATCTTAATGACAAATGTGATGAA

CAGATTGGTTTTACTCGACTTGAATCCTGCATATAGACCCATGTCCATATTTTTCATG

>c9122_g1_i2 len=275 path=[630:0-132 131:133-274]

AACATCTGCAAGGCTGTGACAGACAGAAGAATCCTCAGGTGATGAATACTGAGCCATAAA

TAATACCAAACAGCAGCAATCACTCTGGCCTTGAAAGCCAAAACCAACACGCAGAGTTGG

AAAGGCATTAAATGCTGTTTAACCTACGGTGGTCTTGTTGGGTTGTGGTTGCTTTTTTTT

AATCTAACTGAAGTATCTTAATGACAAATGTGATGAACAGATTGGTTTTACTCGACTTGA

ATCCTGCATATAGACCCATGTCCATATTTTTCATG

>c9123_g1_i1 len=201 path=[225:0-47 272:48-48 24:49-200]

AAGCCATTCTATGGTAAGTGCTTTCAACTGAGGATGTCAGGGAGGAGGAAGTTAAAGGAT

AACAATTTACCTTACAACAGTATTACAGACTGTTCACAGAATAATGCAATTGTTAAGGTT

GGAAGGATTCTACTGAGATCATCTGGTCCAAAAGCCCTGTGCTCCAGAAAGGTCATTTAG

AGGTCCTTGAGTATTTGGTGT

>c9125_g1_i1 len=739 path=[816:0-653 1660:654-738]

TTTGATTTTTTTTTTTTTGCTCTTTAGAAAGTGTTTTGCATATTTTATTTGCAGCAAACA

CTTAAAAAAAAAAAGATCTGGTTCATACATTCAGTAAATTCATAACAGATCTGCTGCTGT

AGCAATATGGCCACTTTCACACCAAGGAATCTTTTGAAAATGCAAAATGCAGGAATAGCT

CTAAGCAACATTTTGAACAGCTCCTGTTCTTTGTAACTTTTGGAAATAGAAGCTATCACA

TTCAGTAGCTTTGGTAACAGCCACAGGAATTTAATTCTTAAAGCAGCTCTGTGTTAGCAG

CCTTGGTAGAAAAATTAGGGGTTCATTAATCTCTTGGTTTGTCAGGTAGCATACATTATA

CAAAGACTGAAGAATAGACTCAAAATACGGCGTGAGTTCAAGGAAAGTGTCTTTCCACCA

CAATGCATTATTAAGAAGTATTTTTATCTTTAAATATCTGTCCATGTCTGTCCAAGCCTT

CAGATGGTACACACTGAAAAGAAATACAGTTTACACTCTTCTCTTTCTATAAGGCTATTA

ATCATAAGATGTTAAAGTATATATTTAGCTACAGAAAAACGTGGCTTTGTGTGTACATAT

ATACACATATCTTAGGTAACAGTAAAAATGTATGAAGCCTGTAACTGCTACCCAGGCAAT

CAAAGACTAATTTAAAGAAGCCATTGCCCCACAGTTGTTAACTAGCTTGGGCTCAAATTT

TCCATGACTGTATTTCAAT

>c9125_g1_i2 len=738 path=[816:0-653 25:654-737]

TTTGATTTTTTTTTTTTTGCTCTTTAGAAAGTGTTTTGCATATTTTATTTGCAGCAAACA

CTTAAAAAAAAAAAGATCTGGTTCATACATTCAGTAAATTCATAACAGATCTGCTGCTGT

AGCAATATGGCCACTTTCACACCAAGGAATCTTTTGAAAATGCAAAATGCAGGAATAGCT

CTAAGCAACATTTTGAACAGCTCCTGTTCTTTGTAACTTTTGGAAATAGAAGCTATCACA

TTCAGTAGCTTTGGTAACAGCCACAGGAATTTAATTCTTAAAGCAGCTCTGTGTTAGCAG

CCTTGGTAGAAAAATTAGGGGTTCATTAATCTCTTGGTTTGTCAGGTAGCATACATTATA

CAAAGACTGAAGAATAGACTCAAAATACGGCGTGAGTTCAAGGAAAGTGTCTTTCCACCA

CAATGCATTATTAAGAAGTATTTTTATCTTTAAATATCTGTCCATGTCTGTCCAAGCCTT

CAGATGGTACACACTGAAAAGAAATACAGTTTACACTCTTCTCTTTCTATAAGGCTATTA

ATCATAAGATGTTAAAGTATATATTTAGCTACAGAAAAACGTGGCTTTGTGTGTACATAT

ATACACATATCTTAGGTAACAGTAAAAATGTATGAAGCCTGTAACTGCTACCCAACTCTC

CTGTTTTAAATTCAGCTTTCTAATCTAAGGGCAAATGTTCATTGTTTTAGAGCATTGAAA

TACAGTCATGGAAAATTT

>c9140_g1_i1 len=234 path=[448:0-20 237:21-233]

AGGACAACATACAAGCAATGGTGAAAGTTAAAAGTATCAGCATGAATTACTAGTAACACA

CAGTCTTAAATACTTCCCTGTAGAACACAGAAGCTTCACAAAAGCCCTATTTCCAAGTTT

TGCAGTTCCACTACAGATATCCTGATAGAGATCTATAGAAATCCTAGGAAGCTGCTTAAA

ACAAGATAAATGCCACACACACCCCTTGACACTCACTCAAACACAAAGTTACCT

>c9142_g1_i1 len=221 path=[155:0-220]

GTTAGCCTGTACCTCCAAATAAGCAGACTTGGATAACAATAAACACTGGTAACTTCTCAA

GTATAGAAGAATTTTTCTCATCTTGTTTTCTCTTCTTCAATTAAAGACCAGTGAACCTTG

GTCAGTATCAGTTAGATCCAAATCTTTATGTGAAGAGTATCTTTCTCCCATTCTTGGACC

TTGACATCAAGCTTACTCAATGTTTCTCTCTCAGTTGTTGA

>c9143_g1_i1 len=463 path=[606:0-296 25:297-462]

CTATTTTAAATTAGCGCAGAATGACTTGTGTTCTGGTTTACTTTCAGTTAACTGTTATGG

TTATGAGATACGGTTTGGACAAGGGGGGATAAATACAATTACAGTAACAAATATCTGCAT

CAGATTAAATGTAGCACATGGGAAGACATAAGGGAGGTTGGCTGACACAGTTTTCCCTGC

TTATTTTTGAAGATACTTCATTCTGTAAATCTAACCAATCGTTTAACATCACTGAGATAG

CAATGCACGCTGAGGTGCCAGAAAGTAAAGCAAAGATACCACAACAAATGTATTTCAGAC

CATGAGAACCTTGAAAATAGGGCACCTTTACCTACAAAACTCCTTACTTTCCACTCACAC

CTTTATAAAAACCAATCCTTTTTCCACGTCCCCAAAGTATTTTTCCCCCTGAGCTCAGAA

CACGCCATATGCATACTACACAACACATTGATTTCTTACATTA

>c9143_g1_i2 len=463 path=[606:0-296 902:297-356 962:357-366 972:367-462]

CTATTTTAAATTAGCGCAGAATGACTTGTGTTCTGGTTTACTTTCAGTTAACTGTTATGG

TTATGAGATACGGTTTGGACAAGGGGGGATAAATACAATTACAGTAACAAATATCTGCAT

CAGATTAAATGTAGCACATGGGAAGACATAAGGGAGGTTGGCTGACACAGTTTTCCCTGC

TTATTTTTGAAGATACTTCATTCTGTAAATCTAACCAATCGTTTAACATCACTGAGATAG

CAATGCACGCTGAGGTGCCAGAAAGTAAAGCAAAGATACCACAACAAATGTATTTCAAGA

GAGGAAAAGAAGCAGTGTGTTGGTAGTTCACCTTCAAAGAAAATTCCAGGTCTGTTTACT

GTGGTCTCACAGTTTGGAATATGAGAATAAATCCTAGGAGAAGTGGATTACTGATTAGTA

TGCTGGAAGGAAACTGCTTAATGTAAGAAATCAATGTGTTGTG

>c9144_g1_i1 len=350 path=[1305:0-346 1652:347-349]

TGAAAACAATAAAAAAAATAATGATATAAATGAAAATTTTTCAGAAAAAGACTATTTAAG

AAATAATTCTGATAATTTTGAAAGTTGTGAATTCTCTGATAGTTATATAGATAAAATTAA

ATATGAAAATACTCCAGAATCTAGCGAAAATTATAAAATTAATATAAAAACTGAATCAAA

ACAAGATTTTGATAAAGAAAATTTTATGAATAACACAGATATAAGTGAAAATACAAAATG

TGACATGCAAATAGAATATTATAACACAATAAAAAAAAGAGAATTGAATAAAGAAGAAAA

TGACGCAAATGTAATGTTTTTAGTGGAAGAAAAAATAAAGAAAGAAGAAG

>c9144_g2_i1 len=493 path=[471:0-492]

AATAAAGATATTGTTACAAAGACTGAAGTGAAGGATGAAAAAAATAGTACTTTTCATGAG

AGTGAATACTATGAATATAAAAATAATAACATAGATTTAAAGGAGAAAAAAAAAATAAAA

AGTGTACTAGAGAGAAATTCACAAGAATATGAATTAAAAAATGAAAATGCAGAAATTTCA

AAAAATAATGAAATCCAAATAGATAGTATGAAAATTAATGGGCAATTATCGAATAGTGAT

TCATACGTTTCAGAACAAACTTATGATGATATAAAAGACAATTTAAAGGATTTTACAGAA

AATATGTATACTGAAAAATATTCTGATATTAAAGAAGAAAGTGAAAATGAAGATTTTGAT

CGTTACTTAGAAAAAAACAAAAGTGATATCATAGAATATGAAGATTTAAATGTAGATGAG

TATTTAAAGAATATAGAAAGTAAAAATGATATTAAAGAGGGGTATTCATCTGAAAACAAT

AAAAAAAATAATG

>c9145_g1_i1 len=276 path=[254:0-108 363:109-275]

ACTGGCTCTGGGAAGACACTCGCAGTATGTATTCTTCCCTTTCATCTCCATACTTCCTTT

CACTGTTGCCTTGTGGAAATTACCCTTGGCTGCTTGTTGCTAGTGTTTGGTCTTAGTACT

ACAAGCATAAAATGAGACGACTGCATGTTTTTAAAGCTTTCTTTATTGCATCACAGGTGA

GCCTCAGTTCATCTAACCTGTCTCGTTCACAGATGTGTTTTTTGCATGGTTCTTTTTTGA

TGTTGAAACGCTTCTCTTTTGGTCCGTGCTTATGGT

>c9150_g1_i1 len=264 path=[242:0-263]

GACTTGGATTAGTTTTTTTTAATTATACTCCCATCTGTTTAACAATAATTTAATTCTTTA

TAGGAGGAAAACACCTACCTTCAGAAATAAGCAAGCTACAGAGGAGAAAAAGTTACTACT

TACCAATTTCATAGCTACATCTCTAAGACAAAAAAAATGTTCTGTAAAGGTTTATCACAA

AGTGAATGCTGCTCTGCATAACCCACTATTAGGTTGAAGCCTATCTGGAAAGGACCCAAA

GGAGACTTAGTAAACCTGTATTTT

>c9150_g2_i1 len=288 path=[483:0-287]

TTAGTAAACCTGTATTTTTCTACTATCTTCCAGCTAATGAATTCGCTTAGGAGGGGAAAG

ATTGTCCATCTGGGCTCAGAAGGCCGCTCAAAATATCCCCATTCAGGAGGGGCAGACAAA

ATGGTAATGCTGTGGTTTCATTTCTTGATGCACAGAGTAGCTATAAACTAGAGGGAGAAA

ATGAACGTGGTATTTCATTTTCATCTGTTTCTCTAAAACACGTTAGCAGTGTGTGGCACT

GATGGTTGAAGATACTCTAGTCTGCTCATCTGCTACCCAGGTAATTAA

>c9152_g1_i1 len=237 path=[106:0-103 210:104-236]

ACCGGCACTGAGAGCAGCAGGTGATGTGCTGAGCCCTTCGGGGCTGGCAGCACCACTCCC

CACGGCACGGCACGGCTGCTCCTCCCAGCTGCAGGCGAACAGCAGGGCCCTGTGGGGTCA

GAGCCGGTGCCCGTGGGCTGCCCGGGACCCCCCGCCTCCGCCCCACGTGCAGAAGGGCCT

CCTGCTCCGGCAGATGGATTCGTTCCAGATAATGAGCTGTTCCAGAAGGAGCAAGTG

>c9152_g1_i2 len=288 path=[211:0-154 210:155-287]

GGGGACTTTGGCAGCGGCCCGGTGCTGCTGCTCGCGGCCTGGCAGGATGCAGGTGACTCC

GCCAGCATGGAGCAGCTGCTCAATAAGGCGCTGACTTTGGAGGATGAGCCCATTGCTGAG

GGGGCTGCTCTGGATGGGGCTGAGGGCTGCACGCCGGGCCCTGTGGGGTCAGAGCCGGTG

CCCGTGGGCTGCCCGGGACCCCCCGCCTCCGCCCCACGTGCAGAAGGGCCTCCTGCTCCG

GCAGATGGATTCGTTCCAGATAATGAGCTGTTCCAGAAGGAGCAAGTG

>c9163_g1_i1 len=281 path=[259:0-107 367:108-280]

AAAAAAAAAAAAAAAAAAACTACCGAAAAATTTATATTTTTGATTAAAAATAATGAAAAT

AATAATAATGAAATAAAATTATATCTTTTTTAAATTAATAAATATATAAGCAAAAATATA

AATGGTTCTCACTAAATTAAAAAAGTATGTACATAAAAAATTCACAGAACCTTTTTACTA

TTTTATGGTCATTTAAAATAAATTACTCGGAATGAAATTAAAATATAAAAAATATAGTAT

ACTTTTAAGATGTTTTTATTTCTTATATATATATATATATA

>c9168_g1_i1 len=206 path=[60:0-57 118:58-205]

TAAGTGTACATTTAACACTTCCTTTAATACTCTAGACTGATAAGAAATGATTGAGATATC

TGGAAATATAGATAGGAATATTGATATTCAAATGTTGTCCCAATTTGCTGCTGTCTGATA

TTCATGGAATGAATGTGGAAGGTAAGCAAGCAAGCTGCTAAGAATAGTATATTGCTGGGC

TAGTTTCTTTAGTAGTGAATTATTAG

>c9171_g1_i1 len=231 path=[47:0-230]

AGTGTTTGGAAAGTATGGCAGATATAATGAGTCTTGATTAAAAAAGGCATTTTTCTAAAA

TCAGTTTGTCTCACCAGACTGTGGCCAGGAGAACTGAAGAAACAGAAAAATCTTTCAAAA

GAAATTTGAGGAGTAGAGCTGCTAATTACAAAAGTTATGCTTTGGCGATAGATTAAAGTA

CTGATGCTACAGATAGAGCTCAACTTACCATTTTCACTGGAGATACTGATG

>c9175_g1_i1 len=1648 path=[1:0-1647]

TTTTTTTTTTTTTTTTTTTGTATCCTTAAAAAGAGAAATTTTTATTTAATGTTTTCAAAA

AATATCATTACGCATTTCTAAGTATTTAAATTAAGTATAGCATTTTTATATATGTATTTT

ATTTTTTTTTTGAATTTTAATTTTACACTTTTATTTATTATTACTTTTGTTATAATATTT

TTCTTTTTTCACTCCGAATAATAAAAATTGAAAAATTGTAAAAAAAAATAAATTGAAGAT

ATCCTTTAATCTTTAATATTTTAAAAAAAAAAATTACAGAGAAATTATTATAATTTAAAT

AAATTAAATTTTATATAAAATATACAAGCAAATTCCTATTGTAAATATTTTTGTAACATA

AAGAAGTTTTTGTTTTTAAAAAAAATATATTTTTAGTATAACTTATAAAATTTTCTTAAT

TGAATATGTAATTATATTAAGTATGATCAATTATAATGATTATATAAATACCTTATAAAC

ATTCAAATATTTTTGCATAATTTTATTCATATAATATGATTTAAAAATTAAGTATATATT

CATATTAAGATAAAAATGAAAAAATTAGGAATTAGTTCCAATCTAGACACTAATTTAAAT

CGTCTTAAAGAAAATGAAACCATTCTAAAAAAACCAATAGAAATAAACAAAAATATAAGA

AGATTGTCATTTTTAAATAATAAAAGTAATGAAAGTAAAAATGAACAGTTAAATAAGTTT

GATAAAACAAAAGTAAAAGAAATAAATGATGTTTTTAAAAATTGTATGGTAGCTTTATCT

CACAATAAGATATGCACTAGAAATGCTTTTGATATAAGAATAATTGATCATTTAGAAGAT

TTAGTAAATTTAAATGATGAAGAAATTAGTGATGAATTAAATGATGAACTATTAGAAAAT

GGAGATTTTAATTTATCTTTTACAAGAGCATCAAAAGCTATTGAAGGTGCTACCAAGGTT

TATGGTTATAGAGTTGAGGCAATATATGATCAGACATATAATTTTTTATCTAATATGAAC

ATAGCAAAACAAAATGAAATAAATGAAGAATTACAAGATGAAAAAAAGAATGTTAGTGAA

AATTCAACAAAAAAAATGAAAAAAAGAAAATTAGAATTTCTTCAAGAATCTTCTACCTTA

GCTAAGTCATCTGATATTACTATTGATTCTTTATCTTTATCAAATATATCTGTAGATACT

TTTTTTTTAAAATTAAATAGTTTATATGATCATTCTTCAGGTCAAAGTTATTTATTACCA

AATTTAATTTTAAATAATGATTTATCGATACAATTTGATGGTGATATAGATGCCTGTGAA

TATAAGAAAAGAAAAAAAATAGAAGAAATAAATTCAGAAGTTTTGAATGATAATAAAGAA

GAAAAAGAAAACATCTTATCCACAGATGCAGAAGAAAATATACATGATAATAATGATAAT

TTATTGATGAAACATTATAAAAAAAAATTATATTTGAATACAAATAAAATAAGAGATATT

CTTTTAAACAATGGGGAAGATTTTAACAATTTAAGCATATGCCCAGAATTAGAATATTTT

AAAGAAGAAATAAACAAACATAAATTAAAAAAATTAGATTCAAAAGGAGAAGAAAATGAA

GAAGGGGAAGGAGATGAAGGTAATAATA

>c9175_g2_i1 len=1730 path=[3313:0-1729]

AAAAAAAAAAAAAATAATTTTAATATTAAAATTTAAAATGCATTTGACAAATTTATATAA

TAATAAAAATTATATATATATCATCTAAATTTTAAAAAAGGGTGCAATTTAAAATAAAAT

ATTCCTTTTAATTTTTAAGCATTAAAGAATCATTAGGTTTAATATGGAATTCTACAGGTA

AACCATAACGAACATAAAAATCTTCCTCATTTGGTATCTTTTCTAAAAGTAATTCTTGAT

CATTACATGTATACAGTAAGCATACAAAAAGCATATGAATAGACATGCTATTAACTTCTG

AATCATTCAATTTAAATTTTGTCTCTTTAATAATGTTTGTAAAAGTTGTAGTTTTTTCTT

CATTGTAAACAACAATTTTATCATTTTTTTCTTCTTTTTGTTCTAAATTATTGGTATTTT

CTTGTTCATTTGGTTTAACAATATCACATAAAATTTGTTTGATTTTTGAAACATCTACAA

ATTTTGATATATTTTCAATATTCATATTTCCTAAACTATTAGCATGGGAAAAACTTTGAT

TAAATTGAATAATAGATGTATCATTAATTTGTGTATTTTGTTTTTCTTGATTATTTTCAT

TATCGTTTTCATTTTGCCATATATCCTCAAAATCCATATTATAAAATTTATCAATAGCAT

CATTTAATCCTTCTTGTATTTGACTTTCTTGTATTTCCTCAATATTTTCAGCATATAAAC

TTTGCATATTTTTAGATTCTGTAAAATTTATTTGATCAATATCATTTGTTATAAAATCAT

TATTAATCTCATTTAATACAATATTTAATTTAATATCAGTATTATCTTCATCTGAAGCAA

TAACAGGTAATTTAGAAAATTTCATTATTTTATCAGTCACTGTATTTACACAATTAATGC

AATTATCTTTTGTTTCAAAAATATAAGAAGTTTGATCAACAGAAAAAAGAACATTAGATA

TATACTTTTTTTTTTCTTTCTTCCATAAATCATAGGTTGATAGTTCTATATCATTAATTT

CAATGTGAAGATTTTCAAAATCAATACTTGAAATATTAAAAGAGGTTTTAACTCTCTTAT

GCGGAATTCCATTAACTAAAACAGTTTTATTTTTATATGTGTTTTTTGTAATATCCATTA

GATTTCTAAAGTTACCATTAACCATTATTTTACTAGGTATATAATATTGATAATTAAACT

GATTATTTACATCAATTGCATTTAATCTATTTTCAAAAGTCATAATACTACGATTCCATA

ACGTATCTTCGTCAGGAATAGATGATATCATACTTTTTTTAGTTGGTGTTCCACTTTGTT

GATGTTTAAATAAAACATTCTGAGAATTCAAATTAAAGTTATTTAATGTATTCATATTCA

TAATACTAAAATCTCTATTTTCACTCTTCATCATTTCTGGTAAAATTCCATTTAAGTTAC

TACTTTGATGAAAAGATAACATACTTTGAGAAAAATTTAAATTGTTTGATACGGAATTAT

TTAAATTTATATTATCAAAAGCCATTGATTCTTGCATTACATTTTCAATATTTAAATCTT

CTATTTTATATTCTTGATTATTAAAATTATTATTGCTACTACTCAAAATCTTATTATCTT

CCATATTGTTATTATTATCATCACATAAATTATCCATTGTCATATTATTACTTAAACTAT

TTTTAAAATTTTCTCCATAATCTTCAACCATTTTCATATTAATTTTTTTT

>c9182_g1_i1 len=342 path=[320:0-192 513:193-341]

GAAACGATCCCTAAAGGTAGAGATTAACTAAGTACTTCTGCTGAAAATGTATTAAAATCC

GCAGTTGCTAGGATACCATCTTACCTTGTTGAGAAATACAGGTCTCCGGCAACACAACAT

TCAGCAGACTCTTTGGCCTGCTGGAATCAGGAAACTGCTTACTATATACACATATAAAAT

CCTTTGGAGTTGGGCATTCTGAGAGACATCCATTTCCTGACATTTTGCAGTGCAACTCTG

CATTCCAACTCAGACAAGCTCCCATGCTGTATTTCAAAGCCATTTCTTGAATAGTTTACC

CAGACATCCTTGTGCAAATTGGGAATGAGGAAATGCAATGGT

>c9190_g1_i1 len=402 path=[1:0-401]

ATCTGAGCGTGTTTCTTAAGTATTTCACACTTTATAACTTTAGAGCATTTTAAAGACATT

AGTTCTACTTTTGTTGCACAAAACCACTCTATCACTAAGAACCACTCTATTTACCAGTGA

GTAATGCAGTACTTTTGTATTCCTGCTTAAACTTGCTATTAGCACTTTCTTTGAAGTAGT

TAAACTTGCAACCAACTAAGTAAAACAGCATACAGAAAGCAGCTAAGCAAGGACACTCCA

GTGGAGTTAATATTGTGGTTTATAATGTCCTCCTGTTACTCTAACACTAGGTTTGTCTTT

TCCACTTGCCAGAGATGAGATTGAGGGTAGGGACTCACACCTATGCTGTCCTGCAAACAC

TCAGAAGCAAAAATAGCTCCTTTTGCTTCTGTAGGTATGTGG

>c9192_g1_i1 len=369 path=[1:0-368]

TTTATGTAATAGATAAGGTCGCTGCTTCCCAGAACATGCAAGGGCAGCATTGGTGTTGGT

GTGGTGACTGAGGATGGCAAGTAGAGAGCAGGCTCTGAGATAAGCAGTTGTGTTTGGTTG

TTAAGTTTATTCACGAGCATCAGTAGATGTTAAGCTTTGAAAGAAGGCATCTCTCCTGCA

GCTGTGGGGATTGGGTGAAGATGGTGTGTTCCCTTGGCAGGTTTCTGGAAGCTCAGATGT

CAGAAAGTTCAGTTATTGTTCAGTCTACAATCATTAGGATTCATCTGCTGGAATGCACTG

TAGCCGGCCGGTATAGAGACTGCTAGCCCAGTCTTCATGGCAAAGCAGCGTGCGTGAATT

TGGTAATGA

>c9196_g1_i1 len=265 path=[34:0-31 66:32-264]

ACATCTGTAGTGTGGCAGGGAGCTGAGACTATTGCAACTTGACTACAGGGGTGATAAAAA

TAACTAGAACTAATTCTTAAAATAAATTCCCTTTTATTTTCATATTATTCTAGGTGGTTG

TGGACACAGCACAGATTGAAAATAAAGAAGCCATAATTACACAGTAGTCCCTGCTCCACT

TGTACTGCACTGTATTCCTTCCTATGTATTTCTGTCATACCTACTAGTATAATCTACAAT

GCAGTACTTTCACTTACCAGGTCGA

>c9198_g1_i1 len=226 path=[204:0-225]

CCAAAGCCCTCCCAAAACAAGACTGAGCCGTGTGGTGTCACCAGAAACCAGAACTGACCA

GACTGGATGTTTGTGTGACTGCAGCACGCAATGGAGATGGAAGCTGTACCTGTCCTGTCT

GTGCACACTGTGTGCTTAGAAAAAAGAGGATGGGAGTTACTGGGCCTTTGTGACTAGATG

CCTTTACAATGACTTCATTTATTGTATGTGATGCAGAAGAATAAGG

>c9198_g2_i1 len=538 path=[1222:0-236 1458:237-537]

GTAGCCAGCAGACAAGAAACACCATACCTTAAAGAAAGATCACAGAGCTGAGTGTGACAC

AGAACGTCCCATGGGGACTCTGAGCTCCAGAGCACATAGGCACTATTACAGAGCTGTGCT

ATTAAATGCATTCCTAGTGCGTCCCATGCAGCACAACAGGGTCAGAAACCCACGAGAGCA

TCCAGGGCAGCATCACTCCATAGGCAATGAGCTCGCAGGCACTGCATGCTTCACCCCAGG

ATGCCCGCGGTGCTGTGCTGTTTGTGCCGCACCATTTCTGCCTCGGGATGAGCTCACAGG

TCATAGGATGTGTTAAGAGCTGCTCATGGGCTCCACCTCAGAAAGACACCACGCTGTCTT

CTCTTAGAGCGCATTGTGAGGCGTTTGGGCTTTCTGTTCCTTCCTGTTTTCCCTGGACCA

CGCAGAACTGTTCTTCTAAGTGTGAAAATTAGCAGCTTCTGTTCATGTTTGGATTCATAC

CGGGCCCACGGAGGGGTTGGAACCGAGTGGTCCTTCAGGTCCTGCCTGGCCTGAGCCC

>c9198_g2_i2 len=538 path=[1222:0-236 431:237-537]

GTAGCCAGCAGACAAGAAACACCATACCTTAAAGAAAGATCACAGAGCTGAGTGTGACAC

AGAACGTCCCATGGGGACTCTGAGCTCCAGAGCACATAGGCACTATTACAGAGCTGTGCT

ATTAAATGCATTCCTAGTGCGTCCCATGCAGCACAACAGGGTCAGAAACCCACGAGAGCA

TCCAGGGCAGCATCACTCCATAGGCAATGAGCTCGCAGGCACTGCATGCTTCACCCCGGG

ATGCCCGCGGTGCTGAGCGGCTGTGGCACACAGCACTGCCTTCCATCCCACCCCACAACC

ACAGGACGTGCAGTGCAGGCGTGTGGCCGTTCCCAGGGTTCAGCCCAATGTTTCAGAACT

GCACCTCATTCCACTTCCTTATCCGAGCCCACTTCTTGTCTTTGCCCTCATACAAAGCGT

GCTGTAATAAAACCCCTTCAGCTCCCTGCATATGGGGAAAGGGAATGCTGGTGTGACCGT

GCTGCTCTGGGGGACACTGATGGCACAGACCACGGGCTCAGGCCAGGCAGGACCTGAA

>c9200_g1_i1 len=463 path=[163:0-462]

TTGCTGGGGAGGAGCCACTGTTTAAGCGCTGAATGCTGCCTAACCTGTTAAGCGCTCACT

CTGGCCTGCTGGAGCTTGTGTGGGCTGTTTGGCTGCCACTGCTGCTTCTGCAGGGACTGG

GGGGGGAGGGACGGGAGCTTTTAAAGAAGGTCCTAAAGCCGTTGTGTTCCCTGCAGCTGC

TCAACTGTACAGCACTCATTAACTGAGAACAGTCAGTAGTAGTATTTTGGCCTTGATCTG

TTTTTATAGTTACGTGGGAGGCAAAATAAAGGTCTCAAACACACAGTACACTTTTCAGTC

CTAATCATAAATGAATTGCGGGTGTTTCCAGAGCAGCATTCTCAGGAATTGCAAGAAAAG

CTACATACATAAGGCCATCATTTCTGTGAAGCAGCTGGCCTTCGCAAGCTGTGCTTTGGG

GGCTGGGTGTATGTAAAGTGGTGTGTATGCTGAAGACTATGAA

>c9201_g1_i1 len=225 path=[203:0-224]

GGTGGATTCTGATGAAGAAATACCATGTTTTGTAACCATGGGATTAATCAGTAGTACTCA

GGGATGCCAGAAACAAAGCTCATGAGATTAAAATCAGGGTTAGTGCTCTGAGTTGTCCAC

TTGGAATGTTACTGAATGGAACCCTTCGGCCTGAAGAAAGGGAGCTGGAGAGGCCCCAGA

ACTATGCTACATCAGATTCCACCATAATGTGACAAAATCTGGACA

>c9203_g1_i1 len=1270 path=[1380:0-1269]

AAAAAAAAAAAAAAAAATATAATTAGTACATTTAATATGCACTTTTATATTCTATTTGGT

TATTCAAAAGTAAATATTATTTATGAATTATCTGACTGACAATCTATGTTTTTATTAATA

TAAATATTGTAACCTTCACCACAAGGCATACTTGAAAATATTTCCTCCATTGATGAAAAA

TACTTTTTGTTTAAGGATACTGATGGTATATCAATATCCTTATTTAAAATGGATAACAAA

ACATAATTGAAAGCATTTGTTCTTTTGAAATAATCATAATATATATAATTATTTACATTG

AAAAAATGTATTTTAACACCCTTCTCAAACAATAGTTTACATTCATAAGGATTTAAATAA

TCCAATTCATAAGTGAATATTAAATTAGCATTTAAAGCACTTAATGTAAAAATGTTTTTT

TTTAAAATATTTTTGTCAATACTTAAATTCGGATAAACAATAATAATTTTAACCCCAAGT

CTATTTGTTATAACATGTATATGTTCAGGAATGTTCATATCTATACTGAATATAGAAACT

CGTTCTTTAAAACTATCGTTGCTTATATGATATTCTTCTAAACTATTTTTAATTCTATTT

TGAAGACAATTCTCCTTTGGTAAAAATATTATTATGAAAACTTGATTTTTTAATAACATT

TTTAGAAGAGGAAGTACTTGCAACACATTTTCAGCAAATATGGCTATATATTTTAACATT

TTATTATGGTTTATAACACTCAAGCATAAATAAGATTCATATAACGAACGAAATGAAGCA

CATAATGAATAATCATTTTTTAATTCATTACATGGATATTCAATAATATCATGATAAGGT

GCTATTATTTCATCATTCTTAATTTTACTATTAACCGTAAATATTCCTATTACATTTTTC

CCCTTTATTATATTATCCACATTTTCTCCTACATTTATGACTTTACCAATATATATATTT

CCGATTAAATATTTTAATTTTATATTATTATTTTCCTCACATTCATCTAGGTTCTTTTTT

TCTATGTCATTATTATTATAACCATTTTTCTTTACATTATTGGGAAAAAAAATGGAAGAG

CAAAAAGAGATACATTTTACCTGAATACAAATATTTAATTCATTCAATTTCATAGTTTAT

AGGTAAAAAAAAAAATAAAAAAAGACATAATTAAAATATAATTGAATAATATAAAAATTA

TATTATTTAAAATATATTAAACGATTGAAATTTTAATTTTTAAATTAAAATATTTTATTA

CGTGAAATTT

>c9207_g1_i1 len=226 path=[205:0-67 274:68-225]

TGTAATTTCTATGGCCACCCATCAGGGTCTCTGCCACAAGGATCCTTATAAGCACCAGAA

ACTCCAACAGCTGCTTCTTTTTGTGTACATTAGAAGGTTTTTCAAGGCGTGGGAAGATGT

GGTAACCGAAGGCCGTCAGAAGGAGCTCAGTGCTCCCGTTCTGCCCCAGCCCATGGAGCA

GCGCTCAGCCATGGGAACAACGCCCGGCCCACCTCACAGGGCCAGG

>c9208_g1_i1 len=223 path=[225:0-222]

AAGCTTAAATCATCTGCTGCTTATATCATGCAGCAGTTGGCAAATATCTGGGGGCGTTTC

ACAAATCACAATCACAAAATCATTAAGGTTGAAAAGACCTCCAAGATTACCCAGTCTAAC

CATCTCCCCCCAGCTATTTCCATTCATGTAACTATGGAATCATACAAATTACACCTTGAC

TTATTTCTGCAGTGGACTCGTGTTATCTTTTCTGACATTTTAG

>c9214_g1_i1 len=494 path=[472:0-493]

GATGAAGATAAAATTTCTAGCATTCATTACCAGTAATCACCTTCAGCAGAACAGCAATGA

TAAAACTTGCTGCACATGGACAAATCTGGTTATTCCAATGCATTTAACACTTAACATCAG

AACAAGCCATGATGTCACAGCTATGGCCTCAACAACAGTTTTACATCCACACTTTATGTG

CTGAGGGTGAGAGTAGGTTTTGTGTTTTCAGTGGGTCGTATGAGCCATATTGCAGTGGCC

TCTTTGGGAGAGCAGTGCACAGTGGGTGAGGATGTCCTCAGCTTTCTGGGCTTCTCTGTC

CTTAACGGGAAGATGTTCTCTGTGCAGTTTCTTGCTCACAAAATCGTCCACGTGCTTTTC

TGAGGCTGATGTGATGAGCCACTGAATTGAGCGCCTGATTAATGTTGTCTTTTTTTTTTT

CCTTTTGCTTTTTCTCTGTTTTCCTTCTTCCTTTGCCTGCTTAACCCAGAAAAACTGATT

GAGGGACTCAAATC

>c9216_g1_i1 len=225 path=[242:0-7 40:8-224]

TTATTTATTTTTTATTATATTTTCTCGATATATTGATTTCTTTTATCTAATGAAAGCATA

AATTGTGCATATTAATAAAAATATATGAATTTGTTTTTTTTTGAGTTGAATATATATAAT

TCAAGAAAAATATTTTTTTTTACTACATGTATCAATCAATTTTGATATTTTTTCCTTTCA

TGCTTAATATTCATTTTGTAAATATTCATTATTTTTTTTCAAAAT

>c9220_g1_i1 len=207 path=[185:0-206]

TGCTACTGCTGAGGCATTCTGTAAGTGGTTTGCGTTCCTTGTGGAAAATGAATTTATTGG

AAAATACGTATTTAGGTTTGCAGCATTTTTTTAAGTAGGTCATAAATTTTTACTGGCTGA

GCTGAACCAGGAAGTGATCCTTCAGCTCTGACCCATGCATGCGAGTCTGGTGCACTTTAT

AAAGATGTTTATCTGGCGTTGCTTAGA

>c9223_g1_i1 len=237 path=[155:0-236]

ACAAATTCACCTTTTTTCTCGGCTAAACATGCAGCTTCTCAAGTTGGTTACTCCGTTCCC

ATATTTTCACCTTCTCTGTACATCTGGTACATCTGGCCTGCGCTCACTGCCCTCCTGCAG

CACTTGTTGCTCACCTTCTGCAGTCTGTTGGCACTCACTTGTTTTTCTGCTCACGCCCTT

TAACCTCCGTGCAACTTAAATTAGCAAACAAGGAACACATTTTGAAAACAACAAGGG

>c9225_g1_i1 len=732 path=[710:0-731]

TTATTTTTTTGATAATTATATTTATAATATTATAAGCACCAATATATTTAAGAAGATCTC

TTATTTTTTTCACATATTTTTCTACTTTCAATAATTAATTTAAAAATAGACATAAGTTTC

AACATAATTTCTAACTATAATATAAGTTTAAAAGTAATAAAATTTTGAATTATATCAATT

TATTATTTATATTTGTTTTGTAATATTTTTTTTTAGATAAAGTTATAAGAAAAGATCGAA

TGTTCATTTTGTAAAAAAAAAAATGCAACAATTAAATGTAAAAGTGAAAATTGTAAAAAC

ATTTATCATTATCACTGTTTATATGAAACATATAATGAATTTTTCATAGTAACAAAAAAA

AAAAATTTTTAAATTGTGGATTTATATTAGCAAATCCCTTATATAATGAAATACTCTTTT

ACCCAAAGTCTTTTTTAGAAGAAAAAATCAAATAATGAAAACATATGTATGTTTTAAAAA

GCATAAATCTAAGAAAATGGATTTTTATGAATAAAAAAGAAGAGTATATAGACAATATAT

ATAATAATATGAGTTTATATTATGATGAAATAATGGGCTTAATTTATACATCACAAATAA

ATGATGAAATTATTTTTTTTTTCCAATATTTTTAAAAAAACTAGTTAACTCATTTTATAT

GTTGTAAAATATAAGTCATCCTTTTAGAGGTTAAAATTTTTTAATATGCATTATATATTT

CCTAAAAAAAAA

>c9229_g1_i1 len=224 path=[348:0-223]

GATGGGAGAGAGAAGGGGGAGGTGGGAGGAGGAGAGGGAAACACAAAGAAAAAACCTTGA

AAGATCTCTGAGCAGTATTTGTGAGCTAGACTCACCGCTTCAGTGGGGCACTGCTGCTAC

TACACTACGTCACCATGGAGTTCCACATCAAACATGAAATCATTTTAATGGAAACAAAAG

GATCCTTCAATTACCAATTCAGTATATAAAAGTAGATATATCAG

>c9241_g1_i1 len=225 path=[155:0-224]

GGTCATTTTTGTGTTAAAATCGCTTTTTTTGTTGTTAAAATTTCCCATTTTTGTATCCGG

AAATCCCATTTTGGGGTTAAAAAGTGCCATTTCAGGGCTTAAAAGTCCCATTTTTGGGTT

AAAAAGCCCCATTTTTGGGGCTGAAAGTCCCATTTTTTAGTCTCCAAATCCCATTTTTGG

GGTCAAAAAATCCCATTTTGGGCTCAAAAAGTGCCATTTTTTTTC

>c9245_g1_i1 len=388 path=[1:0-256 840:257-328 320:329-387]

CCTTCCAATCCAACTCAACCTACTCTATGATTCTATGACTGTTGCACTACATGACTTTGA

CTTGCCGTCTGCAAATTTTAAGAGATTTAGGGTAGCACAGTTGTTCCTGAAGACCCACCT

TGTTTTGCATGAACAAACCACTAACAGGTCTTACAGACTAAATTGGAAACTTCCACTGCA

AAAAGAAGCTGACCCAAGTGTTCTACTCAATTCACTTGTTCCAAAAAACTTCATTAAAGC

AGGCAACGTCTGGACTGCTGTTTGGAGTTCAGTCTGCTTTCAGTACTGCTTCCTTTCTGG

AGCTCCAGTATCTCTAGCCAAACTTAAGAATTTACCATGCTTTCACTTGTTTATAATTCC

TAGAGTTCTCTTCTGTGGCAATGTAACT

>c9245_g1_i2 len=378 path=[1:0-256 258:257-318 320:319-377]

CCTTCCAATCCAACTCAACCTACTCTATGATTCTATGACTGTTGCACTACATGACTTTGA

CTTGCCGTCTGCAAATTTTAAGAGATTTAGGGTAGCACAGTTGTTCCTGAAGACCCACCT

TGTTTTGCATGAACAAACCACTAACAGGTCTTACAGACTAAATTGGAAACTTCCACTGCA

AAAAGAAGCTGACCCAAGTGTTCTACTCAATTCACTTGTTCCAAAAAACTTCATTAAAGC

AGGCAACGTCTGGACTGGTGTAAACAGAGCCTTTATAAACCTCAATTAGTGACAGGTAAT

TGACAGTTCTTCCTGAAGTATTTACCATGCTTTCACTTGTTTATAATTCCTAGAGTTCTC

TTCTGTGGCAATGTAACT

>c9245_g1_i3 len=378 path=[1:0-256 258:257-318 429:319-377]

CCTTCCAATCCAACTCAACCTACTCTATGATTCTATGACTGTTGCACTACATGACTTTGA

CTTGCCGTCTGCAAATTTTAAGAGATTTAGGGTAGCACAGTTGTTCCTGAAGACCCACCT

TGTTTTGCATGAACAAACCACTAACAGGTCTTACAGACTAAATTGGAAACTTCCACTGCA

AAAAGAAGCTGACCCAAGTGTTCTACTCAATTCACTTGTTCCAAAAAACTTCATTAAAGC

AGGCAACGTCTGGACTGGTGTAAACAGAGCCTTTATAAACCTCAATTAGTGACAGGTAAT

TGACAGTTCTTCCTGAAGTATTTCTCCATGTGACTCATTCCCCTGTTGCATTCAGTTACA

TTGCCACAGAAGAGAACT

>c9247_g1_i1 len=212 path=[1:0-211]

GAAAAGAGAAAGGTCATGATTTTTACTCCAGATATTTTGCACCTTGGTATGGCGTGCTGG

AAGACCCTGTTACAGGATCTGCCCATGCTGTTTTAAGCAGCTACTGGTCAGAGCAGCTGG

GAAAGAAAGAAATGCTTGCTTTTCAATGTTCTCGACGTGGAGGAGAGTTGAAGATTTCAC

TTCATGATAATGGAAGAGTTGAAATTGCGGGG

>c9247_g2_i1 len=306 path=[379:0-305]

AAAAGAATTATTTAAGAGCAGAAAAATAACAGGGCTGTCCTTGGACGATTTCCGAGATGA

AGTCCTGAGATGAAAGTAAAGGAAACAATACCAGAGATCTTCAAGATTAAAAGTGAACAT

TTTCAAGCAATTTTTAAAAAATGGACAGTAAGTAATCAAAGATTTTTGAGGACAGTGAAC

GAAAACTAAGATATAATCAACACAGTATGAGTAATGTGAAGGTGTGATACATGACACTGT

TAGTGTCAGTGTCTTCATAAAGTCAAGTTTCCTTTCAATACAATAGCAAATTGCCCCGCA

ATTTCA

>c9253_g1_i1 len=372 path=[37:0-191 759:192-198 235:199-371]

TTTTTTTTTTTTTTTTTTTGAAGAGAGACACTGAGCAGTTTATTTGACATTGCTGCCGAC

CACAGTATAATACAACACATGTGGATAACTCTGGCCAAAGCATACAGTCTGCACGGCTGT

TTTACGCCAGAGGCAAACGACATCCAGCAGCAGCTTAGTCATCGTCCTTCAGCTTCTTGA

TCTTGTGCTTGTGGCGCTCGATCTCTTTCTGCAGCCGCTCGATCTCCTTCTTGTGGTGAT

CGATCTCCTCCTCGTGGTGTCTCCTTAAGGCCGCCAGCTGCTCCCGCTCCTTCTCCCTGA

AGTAGCGCTCCTCCTCGGCCGCCTGCTTCTTGCCGAAGGCCCCTCCGGCCTCGCGGATGG

AGCCGCCGCCAC

>c9253_g1_i2 len=372 path=[37:0-191 759:192-198 593:199-371]

TTTTTTTTTTTTTTTTTTTGAAGAGAGACACTGAGCAGTTTATTTGACATTGCTGCCGAC

CACAGTATAATACAACACATGTGGATAACTCTGGCCAAAGCATACAGTCTGCACGGCTGT

TTTACGCCAGAGGCAAACGACATCCAGCAGCAGCTTAGTCATCGTCCTTCAGCTTCTTGA

TCTTGTGCTTGTGGCGCTCCAAGGGCAGGCGGACATGGCGGCCGTGTTGGCGCTGCGGGG

CGGCCTGCGCGGGGCCCTCCTGGCGCAGCAGCAGCAGCGCGCGTGGAGCTCGGGCTCGGG

CGCCGACCAGCTGGGCGAGCTGGGCAAAGGCGCCGGGAAGGGCGGCGGTGGCGGCGGCTC

CATCCGCGAGGC

>c9258_g1_i1 len=696 path=[31:0-231 262:232-232 30:233-695]

AATAAAAAAATGAAAAAAAAAAAAGTTAAAGAAGAAGAAAAAATAGAAGTAGAAAGCTTT

ATGGATCATAATGAAAATGTTATAGGAGATAAAAAAGTAAAAAAAGAAAAGGTTAGGGAA

TATTTTAAAAAAGAAAAAAAATTATTGAAAAAATTGAATTTTATTAGCAAATTTAGTAAA

AACTCAATTAAAAAAAGTATGGTTGTAATGAATTATTCTGATGAATCTATGGAAAAAAAA

AAACTGTCCTTTTCATTAACGTTATTTAAGGAATTTAAAGATGATATATTGATAAAAATA

TTGGATGATTTATTAAATTATTATGAGAAAAATAAGTCAAAAATAAACATAAATGAATTT

ATGTATTTTTTATTTAATATATATTTAAATATATGCACATTAACAAAAAAAATCATAAAT

TATTTTATTGATTTCATATTTTCCTTCATTAAAAAAATAACTCAAACATCTCATAATATT

ATGTGTAGTTTATGGTTTTTATATATTTTATTTATAATAGAAAAAAACCATATATATGTT

TTTAACGATAAATTACAGAAAAAAATAATATTAGAACAAATATATATTCTAATACAAATA

TCTTTATATAGTTTTTATTCAAAAAATGTCAAAAATAATTATAATATTCAGACACCTTTA

CCAAAATTTGTACAGCCTTTTAGTATTTATTATATA

>c9261_g1_i1 len=269 path=[247:0-268]

AGCTTTCTGTTCTCTTCTGTATTGTTCTAATTGGATTCGGCTCCTGCTGACAAGGTGTAG

TCCAAACTGTAAGATCAAACAGCAGCTTTAAGGTAATTGCTGCGTTAATGTGCAGTTCAA

CCCTATTCACCGACTGGAAGCTCATGCAGCAGTTAGGTGTGTCTCCAGGTTTAAAGGGGT

TTTTCCTCTAAGTTGAACAAGTAGAAGCTGAAGTTGGCAGGTCTAGAGGAATGGCGAAAC

TCCAATTGTTTGCTGTCATTTTTAATTGT

>c9263_g1_i1 len=216 path=[212:0-215]

GAGTGCTCAGTACTTGTCAGCCAATCTCCCTACTGGCATAATATGTGGCTCTGATACACA

GGACACTGAAATTAGTAAAAATTCCACTTTGAAGACTAATGAAGCTAAATTTTGAAATTA

AAAATGTTTAAGAGATAAATAATAACACATTTCTGTGATCTCTGCTCAGAGTCAGTTGCA

GAGGATTACATTATGTGGGAGTCCCCAGCAGAGCTG

>c9265_g1_i1 len=1447 path=[1837:0-1413 3285:1414-1423 3295:1424-1446]

TATTATTATCATTATCATTATCATCATTATTGTTATCATTATTTATGTTATCTTCATAAT

CATAATCAGTATTACCATTTTCATGATTATCTTCATTTTTGTAATTACTATTATTATCAT

TATCATTATAATCATTATCATTTATACTATTCATATCATTATAATCATTATTTTTATTAT

TATTATTCATGTTATTGTTATTATCATCATGATAATCATTATCACTATTTTTTATGTTAA

TGTAATTATTTTCATTATCGTTATATACATTATTGTTATAATTGTTATTATTACCATCAC

TGTTTATAGTATCTTTATCATTATTGTAAATCATTTGATGAAAATTATTTATATTTTTTA

CATGTATCATATTACTTATAACATTATTCATTTTACTACTAAAACATTCACTGAGGGAGT

TTTTCATTTGGTTTAAATCTTTTAAAATGCTACTTTTTCTTAAAATGAAAGCATTTGTTA

CTTCACTAATATTATTATCACTCGAGATTTTGTTGTTATTATTGTTTATGTTATTTGTAT

TTTTTAAAAATGGTATGTTGGAATCATTCATGTAGTTTAAACTTACTTTTGCATTTTTAT

TACTGTTGTGTTCTTCATTTTTTGATAGTACATTATTTTTAAATTTCTTATAATCATTAT

TACTTTCAAAATTTTTATGAAATACATACATATCATCCATAAAATAATTTGTATTATTCA

TTTTTTTTAAAACATTTTTTGATTTATTTGTTAAATTATTCATATTAGTATTACTATGTG

ATTTTATTAAATATTTTTTTTCTTCATTTTTCTTTTTATAGAAATTATTTACGTTTTTAA

TACTACCTTTGGAATATATATATTTTTTATAGTTGTTCCATGATTCCATATTTTTTTCAC

TATGTAATGAATGATTTCTACTCATATTTTTTCCTTTTTCATTTTCAAAATAACCTTCAC

TATTATTTAATAACTTACTTGAAATTCTTTTAACATCCATTTTATTCAATGTTTTATTAT

GATGACAAATATTTTTTATATGTTTTGTGATATTACTTTCTTCTTTAATGGTTTTTTCTT

GATTACATTCAAAATCATTTGAACAATTACTTTCCACTTCATTATTTAATTTTGAAAGCA

TTTCATTTTTATTAAATTCATGTAAAACGGGAATATAAAAAGGTTCCTCTTTATTATTAT

AATTGTTTGTATTTTTGCGAAAATTCAAAAAATAATTTGTTTCATGAGGAAAGTTAGAGT

TATGTTCTTTTAAATATATCTCCTCTTCATTTCTATTAATCTGTTTTTCTAAATTACTTT

TTAAATGATTTTTTTCTTGATTAAGATGATTCTTAATTTCTTCATCAAAATAACCTACAT

ACCCCTCTTTTGTAAATAGTTCAGTTTCACTTTTATTCTTCATATAATTCATATTAAAAT

TATTTAA

>c9265_g2_i1 len=323 path=[3260:0-11 68:12-322]

AATTAAATAATAAATTAAATAATTATATAGATAAAAATAAATATTTCCAAAATTTACATG

AAAATAGAGAAAATATAATAAACTCTAGTGATTGTAATAAGAGAATATGTGACGGATTTG

AAATGGAACAAAAAGATACATTTTTAAAGAAGGAACAAAATAATAACAATAATAATAAAT

GTCTTATATATAATGAATTAAATATAAATAATAGTTTAAACAATTTTAAAATAGATGATG

ATGAATTGAATAATATATATAACCCTATATATAATTTTAATAAAGAAAATAATTTAATAA

ACAGTATTAATCACATAGATTCA

>c9265_g1_i2 len=1475 path=[1837:0-1413 3285:1414-1423 388:1424-1474]

TATTATTATCATTATCATTATCATCATTATTGTTATCATTATTTATGTTATCTTCATAAT

CATAATCAGTATTACCATTTTCATGATTATCTTCATTTTTGTAATTACTATTATTATCAT

TATCATTATAATCATTATCATTTATACTATTCATATCATTATAATCATTATTTTTATTAT

TATTATTCATGTTATTGTTATTATCATCATGATAATCATTATCACTATTTTTTATGTTAA

TGTAATTATTTTCATTATCGTTATATACATTATTGTTATAATTGTTATTATTACCATCAC

TGTTTATAGTATCTTTATCATTATTGTAAATCATTTGATGAAAATTATTTATATTTTTTA

CATGTATCATATTACTTATAACATTATTCATTTTACTACTAAAACATTCACTGAGGGAGT

TTTTCATTTGGTTTAAATCTTTTAAAATGCTACTTTTTCTTAAAATGAAAGCATTTGTTA

CTTCACTAATATTATTATCACTCGAGATTTTGTTGTTATTATTGTTTATGTTATTTGTAT

TTTTTAAAAATGGTATGTTGGAATCATTCATGTAGTTTAAACTTACTTTTGCATTTTTAT

TACTGTTGTGTTCTTCATTTTTTGATAGTACATTATTTTTAAATTTCTTATAATCATTAT

TACTTTCAAAATTTTTATGAAATACATACATATCATCCATAAAATAATTTGTATTATTCA

TTTTTTTTAAAACATTTTTTGATTTATTTGTTAAATTATTCATATTAGTATTACTATGTG

ATTTTATTAAATATTTTTTTTCTTCATTTTTCTTTTTATAGAAATTATTTACGTTTTTAA

TACTACCTTTGGAATATATATATTTTTTATAGTTGTTCCATGATTCCATATTTTTTTCAC

TATGTAATGAATGATTTCTACTCATATTTTTTCCTTTTTCATTTTCAAAATAACCTTCAC

TATTATTTAATAACTTACTTGAAATTCTTTTAACATCCATTTTATTCAATGTTTTATTAT

GATGACAAATATTTTTTATATGTTTTGTGATATTACTTTCTTCTTTAATGGTTTTTTCTT

GATTACATTCAAAATCATTTGAACAATTACTTTCCACTTCATTATTTAATTTTGAAAGCA

TTTCATTTTTATTAAATTCATGTAAAACGGGAATATAAAAAGGTTCCTCTTTATTATTAT

AATTGTTTGTATTTTTGCGAAAATTCAAAAAATAATTTGTTTCATGAGGAAAGTTAGAGT

TATGTTCTTTTAAATATATCTCCTCTTCATTTCTATTAATCTGTTTTTCTAAATTACTTT

TTAAATGATTTTTTTCTTGATTAAGATGATTCTTAATTTCTTCATCAAAATAACCTACAT

ACCCCTCTTTTGTAAATAGTTCAGTTTCACTTTTATTCTTCATAATAATACATTAAATGT

AGATTTTCTTTTAAATAATTTTAATATGAATTATA

>c9268_g1_i1 len=229 path=[234:0-66 14:67-228]

AACGCAGCACAGAAGTTGCATCTGCTGAACCAAGGTTTATTTAGACCACTCCTTAAAGAT

GCCCTGTTAGTTTTACAAAATTAATACAGCACGTAAAGTTAAGAGAAAGAGAAATGGCTA

AGGCTGGTCATCGCCCATGTTGTCTGACAGACAGTCCATATGCTGGGAGTACAGAGGTGT

AAGATCCTCGTTAGCCAACTAGTACTAACTAACTAGCAGGGCTGGAGTT

>c9280_g1_i1 len=343 path=[321:0-342]

AAGTCAAAACATAGAGCTACTCAAATGGAAGCACCATTGCTGAGTTCAGTGGATAAATAA

TAAAAGAGGAAGGGGACAGAGGTGCATTGGTGTGAAAATTAAGGAGGGACAGAAAGCTTG

CAGTTGAGACTCACTGGCTTTTAGCATTTTCAGAAAGTTAAAGAAACGTGAATGTGATAA

GCCAAGCTAGGAAGTTTCATGTAATTATGTTTCCTTTATTTTTTCAAGATAAGCACATTC

ATATTTGAGAATTATTCCATAAATCAGCACAAAATTCTTGTCAAATACTACTTCACAGGC

ACAACATAATTTTACCTACCAAAAGAAAATAATGGTCCTCGGG

>c9282_g1_i1 len=230 path=[155:0-229]

GCTACACATACAGCTGCTGCCAACTATTGAGGAGTTTTTACACTACCATTCCCAGGTTAT

GACCTGGAAGGAAAGGTCTAATATTGCACAAGGTGCAAGAAAAAAACCTCAGTCTAACCT

TATTAGCTATAAAGCTTGAATACTGACAAAGTACTAATTACAGAAGACTAAAGCAATTGA

AACAATTGCTCTGCACTGAGACTTAAGAAATTAATAAGCAGGTCCTGTGG

>c9284_g1_i1 len=220 path=[1:0-219]

AGCTGTCCTATCTACAAAGAATTAGTATTGCTACAGCTACACTGGGCAGCGATAGCCGGT

GTTAAGACTGACAGCAACAGGACTGGAGTAGGACTATCATTCAAAGAGACTCAAACAATT

TAGTACAAATCACATCACTACTCTCTTACTACATGGCATTTGGTACAGACTAAGTGTGCT

GCTACCTACAGGACTTACCAGCTCTTGTAGATAACTGAGT

>c9288_g1_i1 len=468 path=[541:0-369 1707:370-370 911:371-467]

GAGCAATTCTATGATTCTATGATTTCCCCCTTTGCATTTCCACTTATCACTGATTAAGGA

ATTGCTGAGAGAGGGCCCTCTTTAGTCAGGCTTTCAAAAATCATTAGATCAGATCCGAGC

TGGTAGCTGGTAAAGCTTCCCAGAGCAGAGGGAACACAGCATCTGACCTCTCCCATTGCT

TAATACTTGTTATCCTTGGAAAGTTGAACAGAGTGATATTCTGTCACCAATCCTATTCCC

TGTGCTCTGACCAAAGGCAGGTGTGAGCACAGCAGGCATCTATCACGCTGAAGCTACAGC

GCAGAGCATGGTTTATCTCACTCTTAAAAATAACAATAAAAAAATTCTGATTGTTCCTCC

CAAAGAATTCTGTCTCTTGGAACGGTCCAAAACTTTGGGTTTTATTTCCATAAATGGATT

CAGCCCAGAGGAGGTATCAAAAAGCAAGTCATTTAACTGGATAATTTT

>c9288_g2_i1 len=400 path=[1382:0-302 1707:303-303 25:304-399]

GTAAAGGGAACCACTGACTGGCACTGTCTGAAATACAAATGTGTACACTGCACCTGGCCT

TTGTTTTGCAAAAACTCTTTGCTGGAGCTTGATCCCCAAATCTCCAGGATTGACTGCGTA

TCGTTTAAGCTGAACTCAGGTATTCTCTTTGACATTAAGAACCTATCAAATGTTTCTGAC

AGCACTGGGGACAAAGGTTAATAACATTCAAGTGTACCTTTCCTCAACACTTTACTAAAC

AAGTACCTTAAAAAAATTCCAGTTTACTTCTATTTTTTTGCCTTTAGGCTGTGTTAATGT

TATTAGCTCTTAATACTGCTTTCTCAAGGATTATTCCCAGTGGTATTTAAAAGCAGCTTT

GTAACGGTTGGTCTTAAAATTATCCAGTTAAATGACTTGC

>c9288_g1_i2 len=467 path=[541:0-369 1707:370-370 25:371-466]

GAGCAATTCTATGATTCTATGATTTCCCCCTTTGCATTTCCACTTATCACTGATTAAGGA

ATTGCTGAGAGAGGGCCCTCTTTAGTCAGGCTTTCAAAAATCATTAGATCAGATCCGAGC

TGGTAGCTGGTAAAGCTTCCCAGAGCAGAGGGAACACAGCATCTGACCTCTCCCATTGCT

TAATACTTGTTATCCTTGGAAAGTTGAACAGAGTGATATTCTGTCACCAATCCTATTCCC

TGTGCTCTGACCAAAGGCAGGTGTGAGCACAGCAGGCATCTATCACGCTGAAGCTACAGC

GCAGAGCATGGTTTATCTCACTCTTAAAAATAACAATAAAAAAATTCTGATTGTTCCTCC

CAAAGAATTCTAGCTCTTAATACTGCTTTCTCAAGGATTATTCCCAGTGGTATTTAAAAG

CAGCTTTGTAACGGTTGGTCTTAAAATTATCCAGTTAAATGACTTGC

>c9289_g1_i1 len=218 path=[155:0-217]

AATTCCCTACAATATATCCAGCCTTCCTGATGTTACTGGTGGTTCCTCACAACTGCTATG

CAGCTTATAACAATAATTAATAACACCAGCTTAGTGTGAACAACAATAGTAATATCAGGT

TTGTATGAATTTCTCCTGTGTTCCATGGCTGCTGATGTATGATTAGATGATGCTGGAAAT

ACTACTACTACCTTCTAATACTTGCGTGGGTAAAATTA

>c9290_g1_i1 len=269 path=[317:0-268]

ATGTCAATCTCTGCATCTTGCTCAATGTTACTCTGGTGAAGGAAAGGGAGGAGGAGACTC

ACAAGCACTGAATTCTGACTTTTTTCTTGTATAAATTTGCTGTCCTGTCAGCCAAGAATC

TGTCTGAGGCAACATCCAGTCTTGTGATGGACATCGCCGACAGCCTTCTCAACAGCCCCA

ATTTTGAACCCACAGAGCAGGTTTCCAGTTTGAACGTGACTGACTGTGTTGTCCTAAACA

CTGGAGAAGTGACAGAAGGTATCTGTGTG

>c9293_g1_i1 len=215 path=[347:0-214]

AATTAAGACCCAAATTAAGACATCAGCTACTCAGATTACGAAGCAATGCCCGTTAGTATT

TCTGTGATCATTACAAACAATCTTTGGTTATCAGTCAACTGACCAGTTTCAACAACATGA

CTGGAAAACAAGATGAGACTCACTTCACAGTGATCATTACCTCTGCAGGACTGGAAAAAC

TGGCCCAGAAAGGAAAATCTCTGTCATCTCTTGCA

>c9294_g1_i1 len=1252 path=[1299:0-1251]

AATTTATTATAAATATATAAATATTTGTTTTTGTTTTAATATTTTATAAAATTACATAAT

GAAAGAAAAGAAAAGCTGTTATAAAAATAAGTCAAAAGCTGGTTATAATGTGATTCATGA

ACCTTGTAATATAATGCCAAATATTGAAAGAGAAATTGAAAATTTATTAAAAAATTTTAA

TATTAATAAATATGATTATAAAATAAATCATTTTTTGATAGATATCATACAAAATGAAAC

ACTTCAAGTTTTAAGAAATGCAAAATATATACAAAAGAAAAAGGAGAAGCAACTTTTTGA

AAATTCAAATGATAATGTTCATACAGGAAATAAAGATATTAATGAAGAAAATGAGTTAGG

TAAAAATATGAACAAAATAAATTGCAATACAAAAGAGGATAATATAGAAAGAGATAAAAA

TATCCATAATAGTATAAAGAATGAAAAAGACGAAAAGATGAATGAAATAAAATTTCCTAA

TAGTTTAATAGATAATTCAAATGAAGAAAGTTATAATGAAAAATTATTTCAAGAAACTTT

TGATAGTGTAAGTAATAATAAAGATAAAGAGACATCGAATGATAATAATTTAGAAATAGA

AAAAGATTATTCTTTATATAAAAATAATGATAATGATATAATAAATAAAAACAAAGTAGA

TAAAATTCATAGCAATGTGAAAATAAATACAAATGATAATTTTTTTAATAAAAAAAATGA

AAATGAGGAAATGAATGAAGAAACATGCAAATTAATCTATACAAATAAGTTAAACGAATG

TAAAATAAATGTCCAAGATTTTAATGAAGTTAATAATTATTCAAATAAATTATGTGATAC

TTCTGATAAATCAACAAATAATAATTTTGCTAATGGAAATATGAAGATTAAAGATGAAAA

AACTGATAAGGATGGAATAAGTGAGAAAATAAACAGTAGCTGTAATGAAGAAACTAGCAA

TCCAAAATATGTTGTTAATAACCATTATATTAGTAATAAAATTATTGATATTAATAATAC

TACTACTAATAATTATAACAATAATTTTAATAATGATAATGTAAATATTCATGATGTATG

TAAAAATACTAATGATAATGACAATATTAATACTGATAATTATAGTAGTAATAATAATAT

TACATGTACTAATAATAGTATAAATAATAATAATTCAATTAATAATATTAATACAGATAA

TAATAATCCATTTAATAATATTAGTACTAATACTAATAATATTAGTTTAAGT

>c9298_g1_i1 len=232 path=[210:0-231]

GGTAACTTTGTGTTTGAACGAGGGCAGTACTCGATGGAGGTAATGTCTATATTCTCAAAG

GGTGTTAACTAATTTCTATTAAATAACTTCAGAAAAAAAACTCCACACAAAACATTTTGG

CATTCTGGATACTGCAAATAAAATAGTAGCTTAATAAGACTCATACTTAACCCACTAATT

TTCCTATTTAAAAAGCCACATAAAAGCCATCAGCAGAGCACTGTCAAAAATG

>c9300_g1_i1 len=1434 path=[1414:0-747 36:748-1289 2702:1290-1307 2720:1308-1361 2702:1362-1379 2720:1380-1433]

TGATAAAAATAAGAAATATGTCCAAATCATACTACTCCAGGAAAGGCACAAGGAAGTTGT

CCTAATTATGGTAAAACTATTGTGGTTGATACCATTGAAAATTCAAAAGATAGTATGATT

GCTGATTTAAATTTTTTAAATGAAATACATACAGGTTATCTCAACAAAAAGATTAACAGT

AATGTAGAACTTTCTTATGAAAACAGCGGGATTGCCATGCATCATGGTGTAGATGATTCT

TGCCCAAAAAATAATGTGGATGAGGAGTTGTTTAAAAATAAGAGTGATTACAATTATGAC

ATGTGTAAATCCCATGTATTTTCTGAACGTGTAATTATGAAGGAATATGACAAAATATCA

AAAAAATTTAAGCATTATGGCCTTTATGGTTTAGGTGGGAGATTAGGTTCCCATATTAAA

AATGGAGAAAAAGTTGATGGAAACAATTTAACTTTACCAATGAAAAACCCATCGTTAATA

AAAAATTTGTTTGATTGTTCCATATATACATACTGTGTAGGACCTTGCTTAGAAAATTCA

TATGAAAATAGGTGTTTTCGTAATTTACCTGCCTTATATAATCATTCTACAAAGGAATGT

GTTATACTAGGTACACATGAACAAGAAAGAATTCATGAATGTAAGAAACATAAAGAGGAT

ACAAATGTCCCTAATTGTCAAACTGTTAGAAAAACAAAAAGTTTAAAAGATTGGACATTT

GTAACGTCATTTATTAGACCTGATTATGAAAAAAAATGTCCTCCAAGGTTCCCTCTAAAT

TCCAAAGTTTTTGGAATATATGATAAAAATACAGGGAAATGTAAAAGCTTAATGAAGGAG

AAATATATTGTTAATATTGAAAATTTTTATTCATGTTTAGAATATTTGTTTTTATCTTCG

TCAGATAGTCTATACCCTGATGTTAAATATAACTTTTGGGGAATATGGGTTTCAGACAAT

CCCATTAATAAAGAAAATATTACTATGGCTAAAGGAAGATGTTATCACATTAATGTAAAA

CCAACATGTGTTATACATAAAGACAATCATTTTTCTTTTACTCCACTTACAGCAAATGCA

ATTGATTTTGAACAAAATCTTGATATACCAAATGTTGAAGAATTATTAATTAACGAGACA

AAAAATGGTTTACTAAATCAAACAAAATATAATGCAGAAAATGGAGTTAAGAATCTTTTA

AGTTATGAAAAAATAGGAGCTCGAATTTCAAAGGAAGAAGAAGCTGAGAAAGCTGAAGAA

GCAAGAAAAGTTGAAGAAGCAAGAAAAGAGGAAGAAGCAAGAAAAGCTGAAGAAAAAAGA

AAGGCAGAAGAAGCAAGAAAAGCCGAAGAAAGAAAAAGGGCAGAAGAAGCAAGAAAAGCT

GAAGAAAAAAGAAAGGCAGAAGAAGCAAGAAAAGCCGAAGAAAGAAAAAGGGCA

>c9305_g1_i1 len=211 path=[1:0-210]

TCCCAGCTTCTGGAAAGGGCAAACCTCTCCCTGGGACTTTTTGCTGAGGGACCTAGATCC

ACTTGGTGAGTGATGCTTATGTCAGGCTTCCACCCCGATCTTTAGTACATTTATTTTTAT

TAGCCTTCTGTTCTTTCTAAGACTGTGTAGTGGAATTCCAAGGTCACAGCACAAACCAAT

GGGTGGACTACATGAGTGTGTGGGTGCCTGG

>c9310_g1_i1 len=484 path=[1:0-383 1573:384-384 1040:385-483]

CAACCCAAGCCATGATTCTATGATGAAAGCCCCTGAGAAGCAGGCATCACTTCAGTCTTC

CTCAGAAAACGCTGTGATTCTCTGAGACTTGTGAGGTATGATCCTGTGCAGCCTTCACAA

TGGCAAAGGTGACACAAGGTCCTCATCTCTGGTGGAAGGGGACAGGCAATGCAAGCAATG

CTGTGAGTGAAAATGGGAAAGGATATGTAACTAGTTTCCCAAAACCTGGGCAACTGTTCC

CACCCTTTGTCCATGTGCTGAGGGGATTTCAATTTTAGAAGAGGAGTGGCAGAGATTCCC

CTCAGTGACTTCTGGAGTGGCTATGAGTGCTCTGGCAAGTGGGTATTTCTTCTAGTGCCA

ACAAACATTTAAGGGCATTCTTTTTCTCTTTGTGTTCTTGGTAGATGCAGAACTTGAGAG

AGGTTCACCTTTGGATTTTTGCTCTGCTCTGGCTAGAGCAGCTGTAAACACTGACTCATT

TCTT

>c9310_g2_i1 len=374 path=[765:0-273 1573:274-274 1040:275-373]

AGGGAAGAGGTTGGAGTATTCTTTCTCTTTCAAACACTGAAAAAGTTTGCTTTAGACTAA

ATTTATACATATTGTGCTAGAAGGACCGTGTCAGTCATGCTGTATTTCTTTTTCCTTTCA

AAATTTAAACGTTCAGTTGGATAGAGGCTTGTCAATTTCATTCCTCTAGTGATGTCTTAA

AATAGTGAGCTTTTCTTCTTCTTCTGAAAAAAAAATAATAATAATCAAGCCATTATGAAT

AATCAGACTTTTCGCAGTGATGAAAAGACCTCCCTCTCTTTGTGTTCTTGGTAGATGCAG

AACTTGAGAGAGGTTCACCTTTGGATTTTTGCTCTGCTCTGGCTAGAGCAGCTGTAAACA

CTGACTCATTTCTT

>c9311_g1_i1 len=457 path=[313:0-456]

AAGATTTATCATGTATTTAGAAGGAAAGGAGAGAAGTAACTTCGGGGTCATGATTTATAT

GACTTTTTATATGACTTCTTGGCCTTGTAAGCCACTTTATTCACCTACAATCAAAATTAA

CATTCTTTCCATGGATGCACAGCCAAACTACATTGTTCCTGTTGTTTTGTTTCAGCTAAA

CTAATTTTATGCCACTAGAAACAGCAAAGTAGCCTGGAAAGACAGCAGCTGACTCTTGTT

CCACATGATATCACATCTTAAAATGCACTTATAACATCAACGTTGCATAAGCATGGTAGG

TGAAAGATAACTTAGACTATGAATGCTGAAAAGTGACAGATAATTGTGTGTATAATGTCT

AAAAACTCAACTTCTGAAGCACAAACCCGCTTGCTTATTACAACCAGCATGGGTCAAGCT

GCATGTTCCAGTAACACCTGGCCTAAACAGAAACAGT

>c9315_g1_i1 len=320 path=[298:0-319]

ATCTGCAGTTTCTCTTGTTGATATGATCAACCCATTAGGTCAGGGTGACAAGGGGACAGC

ATGAACACTGCCTATCATCCTGGGCTATGCCACCTGAAGGCCTAGGATGCCTCTGAGAGC

TAGTAAGTAATTTTCATAGCTGGCTGCAAGAGGCAGTTAGACAGCATAGCTTGGAGGACA

TGAACCCATCTGTAGCTTCCCTTCAGCCTGCACAAAGACACTTGGTGTCAGTTTTGCTGG

GAAACAACAGCTGTGATGGATATTGCTACAGTACTCCTATGAAGTAACCATGTCAGGGTG

CTGTTGCAAACAGAGGATTG

>c9319_g1_i1 len=1971 path=[1:0-1970]

AAAAAAAAAAAAAAAAAATTCATTATAAAGCAATCAAATTAATGCAATACTTTTTTATTA

ATTAAAAACACCTTTGTATATAAATAATTCAGTTTTTAAAGTAACAGAGATATAAATAAA

ATTTATTTTATATAAATAATTTGTATGCTAAATAATTAAAAAATAAAATTATTTTTCTTA

TTAAATTCAATGACTTTAAAAATTTTACATTATATAACATCTTTTTAATAGTAATGATAA

TTTTAATTTGTTAATTTTTTATTTTATTATATATGTATATATATTTATATTTGTAAATTC

TTATGTAATAATATTCTTCTTAATATTTTTTAAAAAATTATATATTTGTAAAAAACATTT

GAACTTTTTTACTAAAAGTTTAAATAAATTTTAAAATAAATATTAAGTTATTTAAAAGTT

AAAAACAATGGTTAAATTAAAGAAAACAAGCAGAAGGCAAACATTAAAACAGAAATATGC

CATTATAAAAAAAGTAGCTGCTCATAAAAAAAAGTTAAAGAAAATAATAAAGAAAACAAA

CATTCATAATAGAAGATCAACAAAAAATTCTTTAAAAATACCAGAGTGTATATTTAAGAA

AAGCATTTTAAAAAATATCAAAACCATTGCGGAAAGTAAAAAAAATAAAAATTTGGTAAA

ATGTACAGAAATTAAAATTGATGATTTTAATGAATCTACAATAAAAAATGTGAAATATTT

ATTAGAAAATAGTTTAAAGGATGATTCGGATGATGGTATTTCAAATGAATATTTAGAAAA

TATGAATTTTAAAGACTATGTTCATTTAAATTTTAATATAAAGATGAAATTATTTGAAAA

AGTGAAAAATGAAAATTATTCGAAATTAAATGATAAATATTTATTTATTGATAACTTAAT

AGAAGTAATAAGAAATAGTGATGCTCTTTTTTATATTGTAGATGTAAGAAACCCATTAAT

ATATTTAGATAAAGATATAATTGATTTTATTAAATTATGTAAAAAGGAAATTATTATTGT

TTTAAATAAATGTGATTTAGTTGATTCTGGTGTAATACAGCAGTGGCTTACATATTTTCG

TAATTACTTTATAACTCTACCTTTTATATGCTTTATTAAGAAGATTCCTAATTATCTGAA

TAAAAACAAATTACCCGAAAAGTTATATGATCATTTTATAAATAAAAGTTACGTAAAATA

TATTAAAGCTCTTTTTAAAGATACAAAAATAACATATGGTGTTATAGGGCATATATATAC

AGGAAGAAATAGTTTTATTCATACTTTGTTAAATGAAATGAATTGTAATAATAAAATAAA

AGGGACAGATATTAAAATTGATGAAAATATTAATATATATGGAAAAATTGGACTTATTTT

AAAAAAAGACTTGAAAGGGATGGAATTAATTAAAAAATTGCATTCATTAGATAAAAAAGA

AGTCATATCACTTTTAAATGAATTTTTATTATGCTTAAATGGGAAGAATTTAATAAAAGT

GTTAAGCATTCTAAAGGAAAATCATATTATTAATGAAAACCAGCTACATTTTTCATCTAA

TGATAACAAATTAGAAAAGAAAGAAAAAAAAAATAGAATAATTCATTCTTTTTTATATGA

AAATGGGGAAAATAATAACATAAACTCTAAGAATATGCAAAAATTATACAATAAATTATT

TCTGAATAAAATTCTGTATTATATAATTCCTAAAAGTAAAATAACATGCTTTAGTGAAAA

TTCTGATTTTCTATATCATTTTGGTAATTTGAATGATGATGTTTATCACAAAATTGATGA

ATTTATATTTTCACAGAAAAAAAAATATGGTAATTATATTATAATTAAAAGCGATAAGTT

TAATTTTTATACATAGTCTTAGTAAGATTTTTTTTTTTTTTATGTAACAATTTGACTGGG

ATCTATTGTTTTTCAATTACATAAAAGGAAGAAAAATTAATTATATATATA

>c9326_g1_i1 len=233 path=[1:0-163 484:164-178 240:179-232]

TCCCAAAAGGTTTTCCCATTAATATTAACAGATTTATGAATCTCATTATTCTGATTTATG

TCTTTTCAATATATTTAAGGCTATGTCAATATTAAGTTATTTGTCCCTTGTGAGCACCTC

CTACACAGTTTGGATTAGAAATATTTTTAAACATACACTGAGAAGTACTGCTTTGGACTA

AGAAGCAGTTGAGTTATGTAAGGATATTGCCTCTGAGTCCAAAGCAGTACTTC

>c9336_g1_i1 len=216 path=[728:0-215]

AAAATAATGTTATTATTAAAAAATTTTTAACAATAAGATTTATTTATAAAAAGAATAATT

TCACAATAAAATTTTTATCATAAAAATATTAAATACATAATTGTTGTGTGCATTATGCAA

CTTAGAATAGAATTATATATTTTTCCTAATTTATTTTACTGATAGAAATTTAAAGATATA

AAAATAAAAGCCATATAAAATTAAAAAAAAAAAAAA

>c9336_g2_i1 len=290 path=[268:0-289]

AAAAAAAAAAAAAAAAAAGGGCATGAAATCAATGTTTTTATTTTTTCCTCTCAAATATAT

TTTATCAATTATTATATATTTCCTTTCATCTAATAACTATATAATTAAAACACATAAAAA

GATACATATAAATTATATAAACATTTCAATGTTTTAATTATATATTTAAAAGGAATTATA

TTTATAACTCATCTATTCATCTTTCCATATTTTATAAAATATATATATAAATAATTACAA

AAAAAAAAGCATAACATATAATAGTACATTTTAGAACATTTCTTGTGTGA

>c9338_g1_i1 len=1727 path=[296:0-1726]

CATTATCATTATCATTATCATTATAATTATTATTAGTATTATCATTATTGTTATTTGTAT

TATTATCGATGTTAATTTTATTACAGTTATATATTTTATTAACATTGTTACAATCACTAT

TATATTCATTTAATATATGATCATCAATAAAATTATTATTTTTTTTAATTTCTTTTTCAT

CCATATTTTTATTCTGATTATAATAAAAATCTTCTTTTTGATTTTCTTTCTTAATAATGT

CTTTATAATATTCTTTGTTGCAATTTTCATCACTTAAACTATTTTCTTTATTAATTGATG

AAAAATAATTGGTATCTATATTATTATTAGTGAATACATTATTTAACTCACAATTTTTCT

TAAATTCTAATTCTTTTTCTTCCTTTATTATTATTTCGTTATCCTCTACATTATTTTTTT

TCTCTTCATCTTCTTTTTCTTCATTTATAATATTCATTTCATCATTTTCTTCTTTTATTT

TTTTATTTAAATTATCTAGTTGTTTTTCTTTATTAATATCTTCTTTTTCTATTTCGTTTT

TTTCTATTTCTCTTTTTTCTTTTTGATATTTTTCTAAATTTGTAATATTATTAATAACAT

CATTAGAAATATTCTGTTTTTCATATTCATTATTATCATAACCTTCTTCTTGTAAACAAC

TGCTGTTGTTGTTACTTAATGTTTGATTTTCTATCATTTTATTAGATTTTCTTTTCTTTT

TTTTTTTAAATTTTTCTATCTCTTTAATTTTGTTTTTTTTTAATTTTTCTTCTTTATTAT

ATATTTCCATACATTCATAAAAATCAATATTGTTATAAGTTTTTGCTAAAACAGTATAAT

GTAATAAATCAGAGATAATAGTAATAGCTGCTGAAATGGTATATGGACATTCTGAAAAAA

CACATTTGATTAAATAAGAAAAAGATAGTTGTGATGCTAAATCTATTAAAAAATAATTAA

AATAATAGATTTCAGCTTTTCTAACAAATATATCTCTAATTAGACATGTTATATTATCTT

CTCGATCACAAGGAATATAAATATGATCACAAAAATTTCCATCTAAATTGCTTAAAATTA

AATATATAATATCTTTATTATCAAAAATACATAAATATGGGACTTTAACAAACAAAAGAA

CTTTGAATAATTCTGTTATATTTCTAGAATAAAAATGATATAAAAATGCTTTTAAATATA

ATTCTCTTTTTTTTTTTAAAAAATGAACCACATCTTTCGTATTCTTATTATAAATAGCTA

TAGCACATCTACTAAAATATCCTGCTAAAACTTCATTTAATTGATTTTTATTCAGTATAA

ATTTCCAAAAATATTTCATTAATTTATCATTATATACAATTGAATCAGTAATTAATTTAT

TTTCTGAACTTATTATTTGATGACTTTTATAAGGAAACTTATATGATTTATCATAGTTAT

CTTTATCATTTGGTTCTTCAATAATATAACTTAAAATTTTTTTTAAATTTTCTTCTTTTA

TTAAGAAATTCAACAATTCTATATTTTCTGATTTGATTTCCTGAATTAAACCTTCATAAT

TTAATATGCTATCTAAATCATTCGAATTGATATATTTTTTTAAATCAAATGTAATATCGT

CATTTTTAAAACGCCAATATAGACCGTTTTCCATTTCATATAAATATTTTCAATAAAAAT

AAAATGTAACAAAAATAATATAAAAGGATAAAAAAAAAAAAAAAAAA

>c9348_g1_i1 len=219 path=[197:0-218]

ACTGCTTTAGAATAAAGCACAGTCGGCACCTGTCAGATGTAGACTGAAGACTTGTACAAA

AGATTCTTTTTCTTCCATCTTGAAAGAGTCTGTTTCCTTGTAAAGCATCATTTATTTGTA

TCCCCGTGTTGTTCTTTGTCTCTCGGTTAGGCATTAACTTCTTCCAGATCTGTGTGACTC

CTAGCCACCTGGGATTCTGATCTGACAGGACCTGCTAGG

>c9351_g1_i1 len=393 path=[407:0-42 449:43-267 38:268-392]

GGGTATACTCCCCTTTCGCTTTCTTCTTCTTCCAAACAGATATCTAAGGCTCAATCAAAC

ACGTGGAGGCACACTAATAAAAAGCCAAATTAAAAAAACCCAAACAATTGTATTTGCCCA

AAGGTAACTGCATCTAAAAGAAAAAGACTCAGCTCAGACTTTTCTTCTTTCCCCCTAAGA

GACACAGTTTGAAATTGACTATAATCTACCCCTGCTATGTGAGCAGTAAGATGGTGTTGT

ACTCCTTACCCCACCCTCCAATGCCCTCCTAAGGACCTTCACATTTTATAAATAAACTTT

TAGAGATCGTATTTAAATTTTCCAGTACAAAGTACAAACCCATTTAAAATTAGGAAATAC

AAACTTCTGGACCAAGGATAAATTAAAGATGTT

>c9354_g1_i1 len=223 path=[1:0-104 106:105-119 121:120-161 356:162-180 121:181-222]

CCTAAACTTTGTGTTTGATGGTGTCACACGGGGGCATTTGTTGTGTTGTGTTGGTGTCAC

ACGAGGGCTCTTTGTTGTGCTGTGTCCCTCCGGGTGTCACCCGCAGTCACCGCTGTGTTG

TGTTGGTGTCACACGGGGTCACACTCTGGTGTTGGTGTCACCCGGGATCACTGCTGTGTT

TTGTTGGTGTCACACGGGGTCACACTCTGGTGTTGGTGTCACC

>c9356_g1_i1 len=345 path=[95:0-200 47:201-344]

AATTTTCTTCCTTGAGATTTTGTTCTGAAGTATTTAGATGATTTAAAGCCATTAACAGAG

ACTATGCTACAGTAAATGCAGCAAGTTGCCTCTCTTTGCTTATGAAAATACCTTTTAGGA

GGATCTCAAAACAGGCAGCTATGTAGAGACTGGGGAACTAACAATGCTTTCCACTTTTTT

TTTTTTTTCTGGGAGTTTTTTAGTAAGTCCTTATCTTTAGAACAGGGTAAGTTATTTCTT

CCTTATTGTTGGCTTGTGACTAATAACTTTTTTGGGTTATCCTGAAACCGGTTAGAGACA

GATAAGTTTTTCTCCTTTGTACAATATATTTGGTCAGCCTGCTAG

>c9359_g1_i1 len=381 path=[443:0-345 786:346-380]

AGATTTTTCTTAAATATCTGTGTTATAATAAACATTTTTATATTAACATAAATAAAATTA

TGACAAAAAAATGTTTTTTTTTACGCAAATATTTCATAAATTAATATTTTCAAAAAATAA

AGAAAATATTTGTGAATATTCTATTTATAATGTTATTTTTCCCATTTTTGTAAATTCATA

TTTTCTTTACCGTGAATTAAAAAGTGTTCATGTTTTAAGAATAAATTTTTTAAAAATGTC

TCTCAATATTTTTTATGTATAATTTTATAAATTATATATCTTTAACTTAAATATAACATA

ATGAAAATTATTGAATTTACTTTTTTTTATTTTTATTTAGCTTTATATTATGATAATTCT

TAAATGTAATGTTTCTCTAAT

>c9360_g1_i1 len=210 path=[1:0-209]

AACCCAAAGCACTGATTCTGCTGTGTAGTTTGTAAGTACGAGAAGTGGTCTGTCTCACAG

TTTGCAGCTACGAAAGCTGATGTGTTTACACTGCCAGTCGGCAGATGAGCACAGCAGCGC

TGTCAGCTCAGGTTCCTCATAGGATGAGCTGGTGGGAAGGAGCAAAGCTCTGCAGACACA

GCACTGTTCTGGGGGAAGCTGCTTCAGAAA

>c9360_g2_i1 len=330 path=[682:0-329]

GGGGGAAGCTGCTTCAGAAACCTCTTCTGCTCAGTTTCATGATGGCAGAAAAAGAGAGGG

GGGGATAAAAAGGCAGACGTGGTAATTGGACTGGTTAATGTGCTAACTATTGCTAGAATG

AATCTGAATTAAAATTGACCTATTTAGCACAGGCCACCAGGATGATTTATTCCTTTGCTA

GTGCTGATACAGGTAGGGGATGACTTATTCAGTGTTAGGGATCATAAGACAGCCAAACGT

TTCCAGGTATGGTGCTTGTTAGCCCTTCCAGGGAACAGCTGCAGGAACCCATAAGGCAAA

ATGCAGTGAGAATCTTGCGTGAGGATCTTC

>c9364_g1_i1 len=202 path=[180:0-49 230:50-201]

GTGCAGTGAGGTCTCCCCTGAGTCTCCTCAGGACTAAACAGTCCCAGCTCCCTCAGCCGC

TCCTCATAAGACTTGTGCTCCACATCCCTCACTAGCTTCAATGCCCTTCTCTGGATACGT

TCCAGGGCCCTGACATCTTTCCCTCAGTGTTATACATATACATATATACATCGTATATTA

AATATATATATGTATGTATGTA

>c9365_g1_i1 len=300 path=[458:0-299]

GCTAGTGGCTATTGAGGTACACAGAACAGTCAATTTTAGAGCAGAATAATTAGTCAAGAA

TATGACTATTTTCAAGTACGAAACAGAAAACTACAGGATTATTTTTACAATGTGTCATTC

TTTCTTTGGTAAAGACATTTTTGGAAACAGCAAGTTACAGCACAGAATGCTTTAATCATC

AATAATTACTGGTACAACAACTGTCAGTAATAAATGAGATGGGTCAGAAATAAAAACATC

TAGGAAGTCTGAGCAAAGAAAGATTAGACAGGTATTATTTTAGAAGCACCCTGCTGACAT

>c9366_g1_i1 len=216 path=[194:0-215]

CAGCACTGGAGGGAGACAGCCCAGGGGCAGGGCCCCCTGTGGCAGAGCCAGACTGGAGCC

TTCTCTCCCCTCAAGGCCAGTGAAGATGCTACAGTAGATTACAGATCTGCTCTTGCCTCG

TGCTATGGCTCTACATGGCCTCAGCAGGACCACAGTGCAGTAACCTGGGCCCCAGACAGG

AGGAGAGCTTTGGCCTCAGGACAGACACTCTTCCAG

>c9368_g1_i1 len=566 path=[564:0-565]

ACTATTGTAAGCAAGATACTGGAAGTAATTTCAGACTTCTGTTGGGAGCCATTGCATGTT

TAGAATGCCTTTGCCTGTTGAACACCTGGTAAATACTGCTCCTGACTGCCAAAATAGCTT

ATACTCTGACATGGGAGATGTCATAGCTTAAGCGTTGCTATTAGCTTGTTCTCAGGACTG

TACTTCTACCAAGGAGCAGCTGCTATTTATGACTCAGTCTGTTGAGATGAAGTATATGTA

AATGGTTTCGATTCAGCTAACATTTTGAGCACACCGTAATTCTACTAATCCAGTGGTTTT

TATCTTAATATGCAAATTGCCTTAAACTCCTGAACAATTCATGTGCATACGTGTTCTTTC

TTAGCATCACAATGGAACTTGAGATCTGCTGTCAGCTTTATAAATAATACTAAGGCGAAC

TGTAAGTCTACATTTGCTGTGCTAAATCTCCTGAAGAGGTCTGTCTGCATGCTATGGCTT

TTTTTTTTAATACCATAGTGTATGCTTAAATAATCACTACAATAAATTAAGACTTTCTGA

ACCAGCTAAAAAAAAAAAAAAAAAAA

>c9371_g1_i1 len=220 path=[198:0-219]

GACTCAGTGCGGTCAGACAGCTGCTGTCTGCTCTGCTTGCTGGAGGAGCAGTACTTCCAG

CTGAGGTCAACGGCAGCCTTTCGGAGCTGTTTCCTGCTACTGCTGACAGCACGCCTTCTG

CTCCGAGCACAGCAGAGACAGCTTCCCATTGGTGCTGATTATGGCTGCGATTTATCAAGC

AGCATATAGAAGAGCTGCTCTCTTCTGCAGGTCACACTCT

>c9373_g1_i1 len=294 path=[155:0-293]

GCACCAAGTCCCACATTTGGATCAAATCTGGAGGCAACAGTTTATGAACCACCAACATAC

TCCGGAAAAAACACGGCTCCTTGTTCATCTTGCTGTTCTTGTTCCTCACGATGCCAAACG

TCTTGAAGCCCTCGTGCCCAACGGGTGATACCTTGAGGACCTCCAAGCACATCCCTAGGA

AGACGTCGTCAATGGGGTACAGCTCCAGCGTCTCGGAGGCCTTGTGCAGCCTCTTGGCCA

ATGGTCCATCCATGACGAAGCCTCCACCACCTGCATAGGGCGGGTAGATGCTTT

>c9374_g1_i1 len=291 path=[1:0-290]

AGATGTTGTTCAGAAAAAAAAAAACCAACACACTGATGCTCTTTGCATGTGGTAACAATT

TCGACTTAAAACTACAGGCAATGTTCCTTTCAGCTGAAGATTTCTAATGTGAATGAGTCG

CCCTTCTCCCAGACACTGAGCATGTCATTTATTTGTATTTCTTCTCATGTGCATTACTTA

TTCGGCAGCTTGCATGCCCGTATGTGTAATGTGTGTGGGATGAAGGAACTGGAATGGTTT

GGTCCTGTTCCCTTTAAAATCTGCGGAAGTTTTTCCCTTTGATTTTTATAA

>c9376_g1_i1 len=318 path=[1:0-317]

GCTCATATCTCTGTTTCAGAAGTTAGCTGTGGATTCACCTCTATCCACTTATTTGATCCT

TCTCCAGGCACGGAGCATGGCATCACAAACACTTCACGTGATAATGTTTCCACGACAACT

CCAACAGGCAAGAAGAATGCACTGACTCAGTGGGTAGATGCAGTAAAGGTGTTGAGAGGC

AACTGCTCAGTTTGTCCTGTTCATAGGCTCTCCAGCCCATAGTGAATCTAAATCACACCC

TCTACTCATCACAGCTCTCCTAAAATCTATTCTCTTGCATGAGTCCTGGGTTGTCTTAAC

CACCCTTGGCTGCCTCAA

>c9376_g2_i1 len=211 path=[591:0-210]

CCATCCATTTGCCAGCTTCCCAAACAGCTTCTACTACCCAGTTCCCACATGGCACAGCCT

CATGAAAGTTGGCCAGAAAGGCCTTCCTATCCACCTTTAAGTCCTTCTGGTTACACAGAA

ATGTGAAGGAAAAAAACAACTGTAAGGATATGTTCTTTCTATCGGCTGGCTAACCCAAGA

TAGCTGACTCAGGTTGAAGTAGGAATGAAGG

>c9377_g1_i1 len=249 path=[307:0-248]

GGAATTGTGCCTAGACACGTCAAACACAATTCCTCTATGTAGCTCCTTGAATAGGTACTA

CGGGTTAAATGAAAGGAATGAACAGAAGCTGAGCAATTTTATCCTTTGCTGCATACAGCA

GAAAGAACAGAACTTTGTTTTCCACTTTAGAATATTTAAATCAATTTGAGTGGAATGAAT

CAGTTTTTGCATTAATCACATTGGCCTTTGAAGGGAACACTCTAATCACTTGTTTATATA

GAAATTGCA

>c9384_g1_i1 len=205 path=[337:0-204]

GAAGAAAAACTGGTGCAAGGCAGGCAAGAGCTTTCCTGTGTGACAGTGCTGTGCTGTTGC

ATTGCTGGACGCCATGAAACCATCTCTTGAGATAAGTGCTTATTCATGAGAGCCTGGTTT

GTGCTCCTGCTCTGCCAGTCTTGGCCTGCGCTTTTCATTACTTTACTGGATGTTGTTGGG

AGGGGGTTTATTTTTGCTTAACTGC

>c9389_g1_i1 len=213 path=[155:0-212]

GTGGATGACCAGTATCTCAGCCTGAGGGAAAACATCTTGAGCTGCTATAAGTCAGTGTAT

TAGAGTTTCTTTATGCAACTTTGATTTTCTCTCAAAATCATAATTCTGAAAAGAATGAAA

CAATTTGTGTATAGCTCTTCCATCATGCAAGTTCTGCATTTTTTCAATCATCTCAAACAA

GTCCACAGTCTTGTTGGGAGCAGCAAAAGCAAG

>c9391_g1_i1 len=205 path=[296:0-30 25:31-64 359:65-151 445:152-204]

CTCATGGTGCTGGGTGGGCTGGGATGGCTGCATTAAATTGTCATCACAGTAATTCTGTGA

GCATAATACAAAAACCTATTGACTGCAAACTGAACTGGAAACCCATTGCAGGTACCGTGA

TAATAAATGTTTGTTATACAGTATTCTACAATGTTAAATTAGGAATCAGTTTTCCACACA

GAGGACTGTGAAGCACAAGGTTTGT

>c9396_g1_i1 len=585 path=[45:0-100 146:101-108 154:109-584]

GTGTTTTTTTTTTTTTTTTTCATTATGATAAAATGAGTGAATATATATTTGGATCTTTTC

TAAGTAGATTAGATAATATAAGGTATAATTTATTAAATATATTTAATGACATTAAACTTG

ATGATAATTATATAAGGAATATTAGATTAAATATAAGAAGAAAATTTGATATATTAATTG

ATAAATGCTTACCATCAAAAATAATAGAAGATAAACGATTTATAGTATTAATTGAAAAAA

AAAAGAATTACGATCATTTTCGTTGTCCTATATGTATGTTAATATTATATAAGCCAGTGA

AAACAAAATGCTCACATATATTTTGTAAGGAATGTATAGAAAAAGTTTTAAAGAAATTTG

ATTATTGCCCTATGTGTAGAAACAATATAAAATATGTTGAATTAGAAAATGTAAGTATAA

ATTTTTTAGGAAGTGAATATGAAAATATAAAGATAAGATGTGTAAATTGTAGAAATATTA

CAACAATAAAAAATTATGAAAATCATTTACTAAATGAATTAAGAAATAATTATAATAGTA

ATTACAATGATATAAATAATGATAATAATAATAATACTACTACTA

>c9405_g1_i1 len=247 path=[225:0-246]

GGATTTAATAATTGATTCAGGCCTGTATGCCCCCAGCAGAGACAGAAGAGCACCTCACAC

CTGCAGCAGAACTTCAAAGTTTGTCTCCTCAGAAAGGAATGTAATTCAAACACAGTGAAC

CAGTAGGTGCTAAAAGGAACTAGTTGAGAAATTCTGTCCATAACCGTGTCAATGACATAA

ATCAAAATGGTAATGCATATACTTCAATTGTATTCTTCCTTTCCAAAAAACAGGTATCAT

CTCACAA

>c9408_g1_i1 len=240 path=[231:0-239]

AGCAAGACTCATGCTTCCTTGTAGGGGTACTGCCCCCACTGATCTCACTGCAGGAACTTG

AGTTTCACGTTGTAGTGAAGACCAAAGCTGCCCTGTGATATGCACAGTTCTCTTACTTTT

GGAACCGAATCTTCTCATTCTGTGTCTTTACAGATGATGTTTGTAGGAGTAGAGGCAGTC

GCGATGCTAAAGATGCAGGGTCATGTATAATTCCACAGTGCTGCTCCCTTGGTGTCTTTG

>c9412_g1_i1 len=210 path=[279:0-209]

GTGTGTGTGTGTGTATATATATTTCTTTCCTACAAAGCATGTTGTTTCACTTTTTTCCCT

CCTCCAAGTGAAAAATGCTGAGTCTAGACAGTGTGAAATCTTGTGGGGACATCCTACCTG

ATGTTTATTGTTTCCTTCTCTTCGGTGTTAATGAAGAAAAATGATTATTCCTAAGTTGTA

ATTAGGGGCAGAAAGGCATCCTGGAAGAGA

>c9416_g1_i1 len=261 path=[497:0-260]

GGGCCCCCCCCCCCGCCTGGCCATCTTCTCCACGTTTACCAACCCCAACAGATCTTTCGT

CTGTATTTTGCTCAGTCTTCCCTCAGATTTAGGCTGGCGTTAAGCAGCCAATGTTCTTTG

GCACTGTTCATCTGCTCAGCTTTTCCCCTGCAGGCAACACCGACGCTGGAAGGAGCCTGG

GGGGCTCACAGAGCTTCCATCGATTCCTCCCAGGGAATGATCGATCCCATCCATCCATCC

ACAAGCAGCTTTGTAGACACA

>c9417_g1_i1 len=227 path=[34:0-12 47:13-33 534:34-226]

ACTTTGTGTTTGAGGGGGTTTAATGTCTTCTGAGAGGTATCAGGATATTGTCCCTTAGCC

TAAGCCTTTGGGTGCCGTGAGGTCGTATCAGCAGCTGTGGAATGCTGCTTTGCTGCTAAC

ATACGTCTTAAACACACACGAGGTATCTTCATCGGTAACTCTGGCTGAAATCTGTTTTTG

GGTACACCTTAAAGCAAAAACAGCTGCATGCCGTGGATTTTTTATTA

>c9417_g1_i2 len=212 path=[532:0-18 534:19-211]

GCCAGTGCAAAGCTTATAGAGGTATCAGGATATTGTCCCTTAGCCTAAGCCTTTGGGTGC

CGTGAGGTCGTATCAGCAGCTGTGGAATGCTGCTTTGCTGCTAACATACGTCTTAAACAC

ACACGAGGTATCTTCATCGGTAACTCTGGCTGAAATCTGTTTTTGGGTACACCTTAAAGC

AAAAACAGCTGCATGCCGTGGATTTTTTATTA

>c9425_g1_i1 len=256 path=[500:0-255]

GGTAGGAGGGACAATAAAGCAGAGAGATACTTCTCAGAGCCGTTGTTTCAAGTGGCCTGT

AAATTGGAAAGCGTTCTGTGGGTTTACGTTTCAGATGCAAGGCACATTTTTCACAACCGC

CGATGCTGAACTTTTTAACGAGTGTTTACGTTTTGTGTGATGAAACTGTACGATAATCGG

GGGAAAAAAATAAAATAAAACAAACGGAATAGGTGGTTGAGAGGGCTGGTACGTGCATTA

AAACGTTATCTTTAAG

>c9426_g1_i1 len=409 path=[1:0-371 918:372-375 442:376-408]

GAAGACTGGCACTGTTTCATCAAACAGGGCAAGAAAGGAAAATGAATGTTCTGGTGATAC

ACCTGCATTAAGCTGGTTCTAGTATTAGATCCGTGACAGAAGTTTCATGTGTAAATAGTT

GAGCATCGACATTCTCTCCACCAGAGAAAGAGAAATTATATTCAGGAACAGAATGTGTTT

TTACTCCTAAGAACTTTATAGGGGGCATGCAGAGGTCCCACAGAGATCCAAGAAAGCAAT

GGTCAGTTATGACTAATGATACATGATAAATCTTCATTCTGAAAGTGGGACTTCACATAT

CCTACAAGGAAATTTTTGATTATCATGTCATTTGGCACCTCTCCACCAAATCTACTGCAT

GACCATCATTTACCAGTTTCGCTCTGTCTGTCCCTGAGAAGACGCTGAA

>c9431_g1_i1 len=256 path=[185:0-255]

GTGCTGTAAGGCAGCTTTGGACTTGATCCAATCTAGTCTACTTATGTGGTGGGTGTTTTT

GTTTGTAACCAGGAACTTTTCTGGCACTGGCTGAGTTGAAAGTAGTTTTATTCATTATAT

TTTAGAGGGTAATAGTAGGCAGCTAAGGTAGGCTATGCGTTTTTGTGTTTATATATCTGG

TTACGTGAGTTTCTTGCTCCTTTATTATGTGATTTCAAATATATATTTTTCATCTGTAAG

GTGCCATGTAACTTTT

>c9433_g1_i1 len=226 path=[300:0-41 342:42-65 366:66-225]

ACATAATTAACTCTACAGAACTTGAGTGTTTGGGTTTTTTTATATCCTGTTTATCCCTAA

CGCCTTCAAACACAAATGCTCACCCCACCTCCAACCTCGCACACGCACTCAACAGGAATC

CAGGCATGAGACTGACTGGGCCAGGCTCTGCCTGCACCCAAAGCTGAGCAGTGAGGGTGC

TGCAGTCACAGCGCATTGCTCAGTGACATCACAGCCTGTTGCTTGG

>c9433_g2_i1 len=277 path=[703:0-41 342:42-65 768:66-170 24:171-276]

TCAAGTTCTGTAGAGTTAATTATGTGTTGTTGGGTTTTTTTTTATCCTGTTTATCCCTAA

CGCCTTTTAGACACTGGTCCTATGAACCCTTAATCCTCCAAAAGTGAAGTTGCTCACAAG

TAATTCTCAAAGAGATTTCTTCTTCCATGTAACACTTTCAGGATTGGAAATGCAGGTGAT

GGCTGTAGGGACGGGCAGCAAGAGCCGCTGCCAGCAGTGCCCAAAGCACAGCCCAGCACC

CAACACAAATGCTCACCCCAACTCAAACACAAAGTTA

>c9435_g1_i1 len=244 path=[376:0-243]

GTACTTCTGTCTCTTGTAACATAGACTCTCGCAGTAGGCTCTTTGTTTGCTATGAAAAGC

TGAAAGAATCTCATCAGTTTTCTACTTACAGGATGAATTAAGTAACAGCAAACTTCTATC

TTCCTGTTCCTCAAAACCTAAATATAGTATTGAAGTCCAATTTGATTATGCAAGGTCAAA

CCATAAGCAGCTACTGAATGCCTCTGAACTTTTTGACCCTTTTTTTCCAAAGATAATGCT

TCTG

>c9442_g1_i1 len=254 path=[1:0-253]

GCTCACTGCACTGAAGATGTCTGAGGTGACTTTCCTCCGTTGACTACAGTATGTTTTTTT

TTTCACCTTAATCCCTAGTAAATGTTACTTACATTTTCTATGGAATATCTTCTAGGCAAG

CCCTTACACTAAGACCAGAGGCTATACTTTCTGTGTAAAGGAGCAGAGATGGATACTGTT

GTTCTTCAATATATTTTTCCTGCAGTCTCTAACACCACTGTGTTTGTACTCTGAAAATTT

CCAAATGTGATACA

>c9442_g2_i1 len=209 path=[649:0-208]

AAGGATTGCTGCTCACAGCACAATGGCTCTTCAAAGTCAATTTCTCACAGAGACAGTGCA

CTTGTAAAAGCACACTAAGGATGTAGTAGAAGACTATACAAAAAAATGATTTACAAAGCC

AGTGACAGAAGTCTGTTCAAAAAATGTTAACCAAGCAAATGAAAGTAACCATGTCTCTCT

TTTATTATCTGGAAGACTGCCCAAATTGT

>c9445_g1_i1 len=215 path=[193:0-214]

TTCTGCACTATGGCATGTGAGTAGTGCTGATTGAACTGAAAACCAGTTCTGATGTAAAAT

GTTTGATGTTGAGGAGAAACATGCAGTGCTGGTCTGTTATTTGGGCAGTCCGTCTCATTG

CAGAGTCGCAGGTGATTTTGGAAGTAACATCCTGCAGTGGTTAGCTGGTGATCTTCAGGC

TCCTTCTGTCCTATGAGTTAAAATAAATGAATATG

>c9447_g1_i1 len=218 path=[282:0-138 420:139-157 44:158-217]

ATAAAGTTTTTAAACTTAGCCTGAGTCATATGTTCAGCCTCTGCTTGTCAGAGTTCACGG

ATCCTCATAGATTCAGTGGCTGATGTCACTGCAGCAGCTCCAGTGATGGGGAATCAAATG

CGCACAACCAGAGTGAGGATTGGGGAACATTTCTGTTGTAAGTGATGTGGAGTGTAAAAC

AAATAAAATACTCATGAAAGAAAAAAAAAAAAAATCAA

>c9466_g1_i1 len=472 path=[598:0-337 1239:338-338 40:339-471]

ATTCCAGCAACACCAAGCTTCTTCTTCCAGCTCTTTGGGTTTCTGCTGGGATATTTCCTC

CCCCAGGGAAGGAAATGTGGCAGGGTAGGGGCTGTTAAGAACTTTCACTGTGACCAGTGC

AGCAACACATAACTCTTAAAAGTGCTATTTTACATTCAACTTCAAATATTGCTCTTACAC

CCTGAGAATACTTGATTGAAATGAGTGAGGCATGTTGCTACTCCATGAGACCTTTTCCTA

GTACAAATAGGCATACAGAGCTGCTTATCCATATATCACCAGCCAGCTACAGTATAGAGA

TTCTTCACTTCTATCATCACAGATGTATTCTTCACAGCCCCATCATTCCTTCCTGTTCAC

TGTCCTGGTGGTAGAACCCCCTTCCCACCAGGTCAGTTTGTGCCACCATTCCTCCAGCTG

CTTTAATCTGCCATGCTGCTCTTGCACTGCCTACAAAGCCTAGCCTTTCCTC

>c9466_g1_i2 len=473 path=[598:0-337 1239:338-338 937:339-472]

ATTCCAGCAACACCAAGCTTCTTCTTCCAGCTCTTTGGGTTTCTGCTGGGATATTTCCTC

CCCCAGGGAAGGAAATGTGGCAGGGTAGGGGCTGTTAAGAACTTTCACTGTGACCAGTGC

AGCAACACATAACTCTTAAAAGTGCTATTTTACATTCAACTTCAAATATTGCTCTTACAC

CCTGAGAATACTTGATTGAAATGAGTGAGGCATGTTGCTACTCCATGAGACCTTTTCCTA

GTACAAATAGGCATACAGAGCTGCTTATCCATATATCACCAGCCAGCTACAGTATAGAGA

TTCTTCACTTCTATCATCACAGATGTATTCTTCACAGCCTCATCAGTTTCCTTCCTCCCT

CCAACTCCTCCAGTGTGATTGACATAGTACAGGAGTCGGTGTGTGGGAGCATCAGGCTTA

AACGCAGGTGATCAAAGCAAGGACTGATGAGGAAAGGCTAGGCTTTGTAGGCA

>c9472_g1_i1 len=276 path=[1:0-275]

CTGAGGGTACCAGGGCCAGATGAAGGTCTAAGGGTTAATCTAAAACACAATGCTTTAAAA

CCTGGTTCCTGGTGTCAGTAGGAGTTCTTCAGTATTTATTGAACATTCTTTCACGGTTTC

CATCCCATTTCATTGGCATTTTATTGCTCGCTGATACAAATTATCCAGGGTTCAGTAATC

AAATGAAAGATTGGGTGAAGCATTCAGGCTTCAAGCAATATATCTTTAGGAGATGTATTT

TCTAAAAGATTTTCCCATCTTAGACCCACAAACCGG

>c9477_g1_i1 len=288 path=[697:0-67 118:68-287]

ACTCCTGGCAGTTCCTCACACCGCCGTGCCCTATGAATGTACAGGCGACGTGGCAGCCGG

AGAGCCAACACGGAGGATGGAGACCCAGAACAAAACTCTCAGAACTGCAGCGAGAGGAGC

TTGCTGGCGTTGGAAAGAGGAATTTCTGGTCAACCATGTCAGCTTGTGCAAGGGACTTCT

GTAATGTGCATAAGGGTACCTGCGCCCTGCGTGTGACTTGAAGGAATCATATTAGCAAGT

TGGCATCCCCATAACCTACCCCTGTGAACCCAGGACGTGCACTTGCCA

>c9477_g1_i2 len=337 path=[1:0-116 118:117-336]

CTTTGTGTTTGAGTCCAGGGAGGGTCGGTGTGCCGCTGCCGCGCAGTGCTGGTGCTGAAC

ACAAGGAGTAGCAGGTGAGGAGCTCATTGAGAGGTCAGCTTCACAGGCAAACTGCCCCAC

GGAGGATGGAGACCCAGAACAAAACTCTCAGAACTGCAGCGAGAGGAGCTTGCTGGCGTT

GGAAAGAGGAATTTCTGGTCAACCATGTCAGCTTGTGCAAGGGACTTCTGTAATGTGCAT

AAGGGTACCTGCGCCCTGCGTGTGACTTGAAGGAATCATATTAGCAAGTTGGCATCCCCA

TAACCTACCCCTGTGAACCCAGGACGTGCACTTGCCA

>c9489_g1_i1 len=402 path=[1:0-401]

GGATGATGCTCGGTGCTGCTGCTGGGACTGCCCCAGGATCTCTGCTTCCATCCTCCCCCA

TGGCCTCCAGACTGCTACAGCGAGACAGAAGCTGAGGACCCTGATGATGAATCTCCCCGA

CACGGCTGTGACTCGCCAAAGAAAAGGCTGCAGAACACCCCAGAGAAAGCACAGCTCTTC

CAGACCAGTAGTGAATCCAGCAGCCCGCAGAGCAGCCCTCAGCCCCATTCACCTGTAGAA

CCCTGCGAGGAGGACCTTGAGAGCCTGATGTGGTGCCTGAGGCAGGGCGGGGTGTCCCTC

ATCGGGCGGCAGCGCTTCCTGACGCAGGAGCAGTGCCGTAAGTCCTTCATCCGGCGCAAC

AAGAACCCCCGCATCAACGAGAAGGTGCACGCAGTGCGGGCC

>c9496_g1_i1 len=337 path=[315:0-248 564:249-336]

GAGAAGATAAAAATTTAATGAGCAATTACTTTTTCTAATACTTAGTACAACATAGGCTGA

GTTAGGCTCTTGTAGATGGTGCTTCTGTAAATAGGGCTGTTGGTCTGAGGCCTGTTTGCT

GCTGCTTCTCCCATATATGCATTTTGTTTTTCATTTGTCTTCCCACGTAAGAGAGGCAGA

ACCATTATTCCTTCTTTAGTTCACATCTCAGAATATGAATTGATGAGGTAGTTTTCTTGG

AATTAAATAGCTGAAGATTGCCAAGCATCCTCTTCATCTCTAGTCTTTCAGCACTATGTA

CTTGAATAAAATATACATGATTAATGGATTCAAGCAG

>c9499_g1_i1 len=231 path=[1:0-195 266:196-230]

ACTTCACGTGGCCCCGAGAGCGGGCAATTGCCAGCGGCAGCCTCCCAAGGGAGTCAGGGA

TAGAGATGGCTCGGCGGTCCCACTTATACAGCACAACAGCTGCATCCATGTGGCCCAGGG

CACACGCCCACATCAGGGGGGTGCAGGAGAAATGATCCACATTCAAAGGGTCGACTTCCA

GCTCCAGATCAATGCTACGCTGATCCAGACCCTCATCAAATGGCGCACCAA

>c9499_g1_i2 len=231 path=[1:0-195 197:196-230]

ACTTCACGTGGCCCCGAGAGCGGGCAATTGCCAGCGGCAGCCTCCCAAGGGAGTCAGGGA

TAGAGATGGCTCGGCGGTCCCACTTATACAGCACAACAGCTGCATCCATGTGGCCCAGGG

CACACGCCCACATCAGGGGGGTGCAGGAGAAATGATCCACATTCAAAGGGTCGACTTCCA

GCTCCAGATCAATGCTGTCTGCGTGTTTGGTGCGCCATTTGATGAGGGTCT

>c9500_g1_i1 len=214 path=[229:0-84 439:85-213]

AAAAAAAAAAAAAAAAGAATATTTTTAATAAAATTAACAACATAATTGTTAAATGGTACC

AAAACAAATTCAAATTAAAATATTTATACTAAGATACAAACATAAATATGTTTCATTTAT

GAAAAAAAAGAATATATAAATATATATTTGCCATATTTTATTCCAAAATAAAATGTATAT

ATTTTACACCATTCTAAAAAAAAAAAAAAAAAAA

>c9502_g1_i1 len=212 path=[247:0-178 531:179-182 54:183-211]

GTATTATTTTTTTCTATGTTACGCCTAAAGTAGAAATTTATTTTTTTTTTATTTAAAAAA

ATATAACACATATATATAGTAATTTTTATATATACATTTATGTATAATATATTTACATGA

AATTAATGAAAATATAAATTTATTATTAAATTTACATAAAAAAATTAGTGTAGAATTTCA

TATTAAAGAAATAAACTTTTGCTTTTTAATTA

>c9502_g1_i2 len=212 path=[247:0-178 531:179-182 430:183-211]

GTATTATTTTTTTCTATGTTACGCCTAAAGTAGAAATTTATTTTTTTTTTATTTAAAAAA

ATATAACACATATATATAGTAATTTTTATATATACATTTATGTATAATATATTTACATGA

AATTAATGAAAATATAAATTTATTATTAAATTTACATAAAAAAATTAGTGTAGAATTTCA

TATAAAATAATTAAAAAGCAAAAGTTTATTTC

>c9503_g1_i1 len=203 path=[56:0-53 110:54-202]

CTACGGCTTTCACTCCCGATTTTGCTCCTTCCATTCAGCAGAAGGAAAAGGGGCCTGATT

GATCAGGAGATGCTCTGTGTTGTTGAATTGTCTCAATCACTGGAATCTCATACCAGAAAA

ATATACTCCATCATGCATGCTATTGCTAAATAGGTCAGAATTTGACTGGTAAGTAGGGAG

TTTCTTGCTCAAGGGAATGAGGG

>c9505_g1_i1 len=219 path=[1:0-218]

GGCGGCTTCGTGACTCCTTTCGGGACACGGCTGACCTTTCACCGTTACCGTCCTGCGAGC

CGAAAGCGACGCCTCAAAAGCAGCGCGCGCGGCAGCGCGACCGCCGAGGAGCGGCCACGT

GAAGAGCCGCGGGGCGCACCGCGCACGGAGGGGTTTCCGTGCCCGTTTAAGATGGGGACG

GAGCTGCGTTCCCTTTCCCACCGCCCCGCCCCGCTCGGG

>c9506_g1_i1 len=459 path=[296:0-220 572:221-458]

TCCCGTGGGGGCTGCAGCAGCGTGCCAAGCAGCCGCTGTCTCAGGCTGGAGGAGCTGATC

TTCTCCTCCTCATTGAGGCCATGCTGGGGGTCCTCCATCAGCTCGATCTCAAAGAGCTCC

AGCGCGGCGAGGCCGTTCTCGATCCTCTTCTTGTTCACGGCCTCCCCGCCCCTGCGCGTC

TCCGAGCTGACAACGATGCAGCGCAGCTCCGGGTCTGTGATTGCGGGGCCGTAGGGGTCG

CCCAGAGGCACGATGTCATACAGCACCGAGGGCTGTACATCCAGGAGGAACTCACGCAGT

TTGGCCGCCCGCAGTTCGTAGGGCTCTATCAGCTCCCGTAGGACTTTGTGGCGCAGCAGC

TCCCCGTCGGCCACCCCGACCAGCAGCCGCCGCCGTGCCAGCAGGCAGCAGGCGCTGAGC

AGCAGCCGGTGGGCGCCGTGCAGCCGATCGAAGGTGCCG

>c9506_g1_i2 len=276 path=[296:0-220 517:221-275]

TCCCGTGGGGGCTGCAGCAGCGTGCCAAGCAGCCGCTGTCTCAGGCTGGAGGAGCTGATC

TTCTCCTCCTCATTGAGGCCATGCTGGGGGTCCTCCATCAGCTCGATCTCAAAGAGCTCC

AGCGCGGCGAGGCCGTTCTCGATCCTCTTCTTGTTCACGGCCTCCCCGCCCCTGCGCGTC

TCCGAGCTGACAACGATGCAGCGCAGCTCCGGGTCTGTGATGGCGGCGCATCGGGGGTTG

GACGTGCGGGTGTTGCTGGGCCACGGACACCGCCTG

>c9511_g1_i1 len=278 path=[256:0-277]

AAAGTGTTTAGCTCTCTGTTAAAGAGAATTAGAGAACTCATTGCCAAATTTATTTCTTCA

TTTTGTAAGTGCCACGGCTTTTTTGTAGTTTCTCATAAGCCAACTTCCAAAGAAAAGCCC

TTCTTTTTATTAAAGGAAAGGGCAGTTTCTCTAAACAGCTTTGATTTTTTTTTAAGGTAG

TAATCTAAATTTTATAGTACCTCTTTCGAAAGTTCTCTATGCACTTAAATGTTAAGTAAA

AACTTTTTGTTTGTTTGTTTTGCTTCTGTTGATAGCAA

>c9512_g1_i1 len=1426 path=[1480:0-354 1835:355-355 1836:356-1425]

CTTTTTTTTTTTTTTTTTCTTGTTCATCAATTTTTAAATCTATTAATATTTTTAATGTTT

CAATATAATTTTCAAAAAGATTAGAAAAATCATTTATAGTATAACATTCAGAAAATCCTT

CTTCATATATATCCATTGCTTTATTCCATCTTCCTTCATAAACATAATTATTAGCTAAAA

GTATATATATAGATGTAATATTTTCATAATTTTTCAAAACATTTAAATTATTCTTTAATA

TTTCTACAACATCATTATTTAATACTTTAGTAGAATTAATTAAATTAAAAATTTCTTTAT

ATAAATCATACTTTGATTTATTTCCTAATTCTAAAGTTTCTTCACAATTTAGAATAAAAA

AAAAAGTGTTAATTGCATGTTTATGCATTTTATATTTAACATAATGATTAAATAGCAAAA

TTATTTGTTCAGGATAAATTGTTACATATCTTTTGATATATTCATAATTAATCAATTTGT

TGTTAATTTTTTCATTATATTTTAACTGGTAATACCATATATCTTCTTGTTGATTTAAAA

AAACATTCTGTAAAGATTGATTGAATATTTCTCTTATTTTTTGTATATTTCTTTGAATAA

ATAAAAATTGAATATACATAATGTAAATAGACTTAAAATGATACATATATAAAAGACATT

TTTCAAATATTGAATTGATTCTTTTATATTCTCTTTTATTATCATAATATATATCCCTTA

TCATTTCTATATTGTCTTTCAAATAATTATACCACAATTTAAAACTAAATGGAAAGTATT

TTAAGATAATTTCATATATTTTAAATAAATAATATTTAAACATATTAAAATTTTTTTCAT

TCTCTTCTTTTAAAAAATTATCAGAACTTTCATTCATATTTTTTTCTTTCTTTGAATTAT

TTTTATTTATTATTTCAGTAATGCAATATTCAATTATATTATTATTTGTCTCTAATATAT

TACTATTATCATTTCTTAAATGTATATCATAGAAATTTAAAACATATAAGCTATTTTCAT

TTTTTATGTTTTTTTCTTTAAATTTATTTATATATAAAAATAAATATTTAGTTGATATTT

TTTCTAGATTATTTTTTATATCTAATTCTATATTTTTTACATAATTATCATTTTCTGTAC

AAACGTTATCAATCAACCTTTTTTCAAGTAATTTATCTTCATCACATATATTTCCTTCAT

TAAAGAAGTCATCACAGTCATATAACTCTCCATATAATTCCATTATTATATTTTTCTTTT

CATGATCTTATTTATATTATCATCAGTCAATTAGTTAAAAAATACCAAAAAATATATCAA

AATATATTATACAATTTTTTTTTTTTTTTAAATATGTTTAAAAATTAAGTAGACATATAT

ATAAATTTGCAAAAACTACAAATAAATTTTTTTTTTTTTTTTTTTA

>c9523_g1_i1 len=256 path=[1:0-203 205:204-255]

GAAATACAAAGCAAGTTTTAGTATAGGCTTTGCTTCCTTGTAAGCCTAGAATATGGTGTC

TTTTTTAAAAAGCAGAGAAATTCTGGAAGGGTTTGGAGATCTGTTCTGTTGATGTATGGT

AAATGCCGTTGCGGTTATCATTCCAATATCATTTAGCAATGCATTTGTACACCATGAATG

TGTGGTGTACGTGTATATTGTATCTGTGCTTGTATTCTGAAACTCATGAGAAGATGGTTT

TCTTTTTTTCTTTTTG

>c9525_g1_i1 len=307 path=[1:0-306]

GTTACGTTTAAGTTTTTATAGTAATATACGAATATCCTGCTAGTTACCATGCTACATTTC

TGAAAGTTATACTGTACAAATATAGCTTAAAAGAAGCCAGAAAAGTATTTAACCGACCTC

TTTATTATCACCACAATACTGAACTGCCAAAAAATAACTGCTGGCATAGTTGTAGAAAAG

CATCCTTATAAGTATCACAACACACAGAAGGCATATGTATGGGGAACAAGGGACTTTATT

TTCCTCTTTGCCGCATTGTCTGTTCTGAAGAACATCCTGAAGGGAACAGAGATTTGTGTC

AGGCATA

>c9533_g1_i1 len=213 path=[191:0-212]

TTCAGCCACTGCAAGGTGTTTATTCATGAGGAGGAGGAGTGACAGATGTACCTGGTTCAA

CAGCTGTAGGCAATACATAAGCACAGGAGTACCTAAACTACTTCACTCTGATTTTCAGGA

AACAGCTTCTGAAAATCTACCCCAGTGTTCCTGATGTGTGGTATGCAGTGACAGTGCTGC

CATGCTCCTGTGCAGGTAGGAGAGGTACTGCAG

>c9543_g1_i1 len=344 path=[329:0-282 791:283-292 801:293-343]

CAATTGTTTTCCCTTCAGCTAATAAAGACATTATTAGAAGGAAAAACAATTGTTTCCATG

CAGTGTCTCAGCTTTAATGTTGGCTGACTAGTAGCACCAAGTTTTAGAATCTTATTTCAC

AGTGATTGGTTTCTCTAGAAACTTATTTTTGTTACATGTATTGAGTTGATGTAAATTAAT

TTTTAAACCTCACCCTGTGTATTTTGAAGATATGGTTCAAGCTTGAGGTTTTTTTATATC

ATAGATGAATTTAATTTGGCACAAATAACACTAAGGAAAGTCTCCCAGACTTTAAAATAG

CAGCAAACATTCCATCATTTATATTTTTTTTAAAGTCTGGGAGA

>c9553_g1_i1 len=215 path=[1:0-214]

ATTAAAATTTTTTTTTTTCATTTTATATTTTTATTTATCATATATAAAAATTAGGTATTT

AAGAGCTTTTTATACTCTTAACAAATATTAGTGAAATAAACCATCTGTATTTTCATTTTC

CATATGAATTTTTTTTTATATATTTTCTAAAAATATATACTTTGTTTTATTTTTTTTTTT

TAATGAGAGGTATGGCAAGGAAAAATGTACCAAAT

>c9554_g1_i1 len=1254 path=[1:0-1253]

AGAAATCTCCAAAGGAAGAGGAGGGTATGTATGTGCCAAAAGCATGTTGAGTGACATGAA

TCTTGTGTGTGCGTGTGAGGGATAGGAGCGGACAGCATGGGAAGGTAATGGTAATGCTTA

TCTCTGGGTACTGGAGGGTTGTTCCTGGAGCTCATCAACCTGTGGCTAAGAGCAGACTTG

AAGGGGTACGTCGTTTTAGCTGCTGGAATACTAACACAGCTTGTGAAACTGATTTGTGAA

AGGTGCCACTTTGCATTCTGTGCAAATAATTTCACTGATTTGGATTTTTTGCTTTAATGT

TGCCACTTTACAGGAGGGAATGGGATTTGCAGAGTTTCAGTCTGGAGGTTTTGGTGTGGA

GCACTGACTGAATGAGGAGCTGAATGTTAACGTGAATTGAAGCTGGAGTCTGTTTCTTGT

TTTGTGTACCTTCTTTGTGGATGTATCTGCTTCTAGTGGTTTACAGAGCTGGAATTTAAC

AATAGATTGCTTAAAGAGTTCTGAGCTGTGTTACTGGGGGCACTTGCTCAGATGTTTTCT

TTTTATAGAACAGGTGTTCCTAGATACTGGGCTGTTGTTTGCTTTGGGCTGTTTATGCCT

ATCTTGCACTGTCTAATCCCCAGGCATGCCAGCATAAAGGATATTCTGAAAATTAATGGT

ATCTCACTAAACATGTCCTGAAACCTATGGAGCTGTGTGGCATGCCAGGTTACATGGAGA

AGTCAGGGAGTGTGCCCTCTGTGCACCTTAAGCTTGTTGGGGAAGGAGAGAGGGGAAAGA

AATGGCACTAAAAAGGAGCAGGGGTGTGAAATAACCCTTGGGTTCTGTGCAAAAGCCTTG

CGGGACTTCCAGGCCAGTGTTTTGATCCTTGTGTCTCCCACCAGTTGTCTCATACCAGTA

AGTTCTCCCCAAATCCTTTTTAAATAGGAGCTGGTTTGTGGGTGTCCCTTCAAGCATTGG

CATTGTTGGCAACCTTTTTCTATTTGTAGATTGACAACAGCTGCATTCCATTTTAAAAGT

GTAATTTGACTCTTCTTTTCCAATTCTGTAAGGAAAATTCTCTGCTCATTTGTGAATGCA

TATGCTTTCCCTCTAGCCTCCCAAGAACCAAAGTGCATACAAGGCTTCTAGGATCTGCGT

TGCCTGTTTTCAGACTCCGTGGTGCAGTTTTTAGCTGGAAGTCCCATACTGAGGGCCTGC

TTTGTCTGCTGGCCTGGAGTTTACCTTTGTTCATTTATTTTTTTAAGAAAGGTG

>c9554_g2_i1 len=261 path=[2463:0-260]

GATTCTCCCACTGTGTTTAAGCACAACAAAAGAGAAGCCAAGCTATTCCATTGCCTGATG

ATACCCAGGTAAAACTCCACACCACAACGTGTGGAAGTGTGATGTGCCTTCCCACTTGCA

GCAGACATCTGGTGCTCTGTCTGCACAAGACCTACCCTAGCTCAGGAGTCCAGAACAAAG

CAGCACACAGACATCCAACAAGTCTCGCACACCTGCAGCCCCTGCAAGCATCACTAAAGC

CACAAAAAGAAAACACTCAGG

>c9555_g1_i1 len=205 path=[880:0-63 698:64-204]

CGAGGAAATTTTGGCCCATGTGGCCGCTGGCGTTGGAGCCGGGGTTGTGGTGCTGCTGCG

GCTGGGGCTGCTGCTGGGGCTGCTGCTGGCTGTGGCCGTGCTGGCAGCTGCTGCCCCTCT

GGTGCCACCGCTGAGCCCGTGGGTGCTGGTGGGTGTCCTGCTGGGCCCGGCGCTGCTCTG

CGCCCTGCTGGCCCTGCGTTGGCCC

>c9555_g1_i2 len=489 path=[350:0-347 698:348-488]

GGGGGACACAATGGGGACACGATGGGATGTGCCCATGGGGGAACATATCTGGCGTTGGGT

ACAGGACAGGATGCATCCATGGGGCGCAGCTGCGGGATGGCACTGGAGACACCACAGCGC

AGCCCCCCACTGAGCCCCACATCCCCCACAGGCCATCGTTGCTTCAAGGCCATCATGTTC

ACCTCGGGGCTGCTCCTGGGGTCCGGCACCATCTTCCTTCTGTGCTACAAGGAGCGCGTG

TTGGAGACACGGCTGAGCCTGGAGGTGAGCGCAGGCATCGCTTTGGCCATCGGCGCGCTC

TGCGGGTTGGTCACCGCTCTGCTGCGCAGCGTCGGGCTCTTCTCCACTGGGCTGCTGCTG

GGGCTGCTGCTGGCTGTGGCCGTGCTGGCAGCTGCTGCCCCTCTGGTGCCACCGCTGAGC

CCGTGGGTGCTGGTGGGTGTCCTGCTGGGCCCGGCGCTGCTCTGCGCCCTGCTGGCCCTG

CGTTGGCCC

>c9556_g1_i1 len=229 path=[505:0-228]

GAAAGTAATGGAGTCAGACCCACAGGTGGTGCAGCAACGGATTAACTTTCCTAAGACACT

TAGGTTCTGCTAAGTAAAATGTGCTAAAATATAAACATTTGCCTTTCAGTCACACCCATT

TTATTTACACAGCGAGAATGTATTTCCCGTTCCTTCTCAGATGCCCCCATTGTGGACAAA

GGTTTGTTGCACGGAGCAGAGAGTGCTCTGGGATCTGCCATAAAAATGA

>c9570_g1_i1 len=277 path=[1:0-276]

GTCCGGATTACAGATACATTCAAAGACATCTCCCTGTAATGGGAGATTTTGAAACAACAT

CACTACTTTAAAAATTAACATTTTGTGTACAATTATCCAAGTTTCAGACCCTTCACGCCT

TGCAAACCACCTGAAAACTCACATTTATTGACATGTTCTTTGCAGAGGTATGGAAAGGTA

GTATTGATCACCTATCAGATTTTGCAAATGCACTTGTGGAAATATTGGCACCAAACTCAT

CTCTGGTGCTCTGGTCTCTCCCGGCTCAAACACAAAG

>c9570_g2_i1 len=432 path=[918:0-431]

GGAGTAGAACAGAACGAGATGACATCTTTTCACCGAAGAAAAGGATGTACAGAAGAACCA

CAATGGGCCTACCGCTGCTTCTGAACTTTGGAAACTGAAATCTGATCCTCAAAATGGGGA

AGTTTCTGAAACCAGTTATAAGAGGTTGATTTGATTCTTGAAACGAACTCAGTTACTAAG

GATGAAGCCCGTTGTCTTTTGGTTGCAATACTTAATCAGTAGAACTGATTTGCTAATTAT

TTAGATGTTTTAAAAACTCTGTCATTTAACAATTTGGGTAGAATCTGATTTTTAAAGTAA

ATACACGTTATTACATTATATGTGCTTACATTTACAAGGCCACGTGAATTTTATGATACG

TGTGTTCTGCTCAACTAAATAGTTAGACCAGAAAGCTGTTAAAAATCTTCCAGCGGCAAA

AATTAATCACTT

>c9573_g1_i1 len=319 path=[790:0-12 106:13-318]

GGGAGGTTGGCTTTGCACCTTGCTGCCCAGCAGTGCGAGTGAGGCACCCAAAGATTGCTG

TCCTCAGCAGCAGCTGAGGTTAACCAGCTGAAGTTAAAGCAAATGACGATGAAAATGGCA

CAAAGTGATCCCATCTGGAGCAAGTAGTCACAAGGAGAATGCAGAGATCTGCCTCAAGAC

CTGCAGGATCCATCCTCTCTGGTTAGAGGTTTGAAGTCAAAGAACACTGTGAAAGCATTA

GCATTGCCTCTCTTAAGCTCAAACGTGTCTAAATTGTAGATTTTTCTTAGAGAACTTAAT

AAAGGAATAAATGCAGTGT

>c9573_g1_i2 len=411 path=[1:0-104 106:105-410]

TTAGGGGCACTGGCCACAAGCATTTGTTTCTTACACTAAATGGAATAAAGCGTTAGCAAG

GTTGAAGAAAACACATGCTTGTGGCAAGTGCCCCTAAAGCCAGACTGCACCTTGCTGCCC

AGCAGTGCGAGTGAGGCACCCAAAGATTGCTGTCCTCAGCAGCAGCTGAGGTTAACCAGC

TGAAGTTAAAGCAAATGACGATGAAAATGGCACAAAGTGATCCCATCTGGAGCAAGTAGT

CACAAGGAGAATGCAGAGATCTGCCTCAAGACCTGCAGGATCCATCCTCTCTGGTTAGAG

GTTTGAAGTCAAAGAACACTGTGAAAGCATTAGCATTGCCTCTCTTAAGCTCAAACGTGT

CTAAATTGTAGATTTTTCTTAGAGAACTTAATAAAGGAATAAATGCAGTGT

>c9579_g1_i1 len=217 path=[349:0-216]

ATCCCATTCCCGGCACACAGCTGCCTTACGGCCTTGGCCGCAGCTCACCCCCCGTCCCCA

CCTGTCCCCAGCCTGGAGCACGGCACCCTCTGAGCAAGCAGAGCAGCAGGGCACAGAACA

GCAGCACGGGGGTCGCTCGTCACGAGCAACGCAGAATGGTCAACGCGTTCCCTTTGCACT

GCAAGATCCAAGCTGCTGCTCCTCCTCCTCACACCCA

>c9580_g1_i1 len=364 path=[287:0-363]

GGAACATAGATTTGGGACTTAAAGATGGAAGCTATGAGCAGTACAGGCAGTTTCAGCGTC

GAAAGTGGCCAGATGTGAAGAGACCATCATCTTCTAAGTCACTCTGTGGGAAGGATGGGA

GGGCTAACTGCCTCAACTGCTGGACCTCAGCGAGGACATGAACTGGCCAGAACTTGAATT

CGTGTACAGACTAAGCAAAAATATGTATGATTTCAGAAGAATTTCAATGTTAGCTTGGAG

GACTGGATGAACTTGGAGGCCAAAATAGATAGTGTTTGCACTGGTGAGTGAATTAAATAG

ATGAAGTGAAATTACAGGAACCTGCTGCCAGGAATACAGGTCTCTTTCAGATCCACTCTT

GTAC

>c9585_g1_i1 len=271 path=[1:0-270]

AAAAAAAAAAAAAAAGAGAAACAAGAGGAATATAAAAGAGAAGGAAGCAAAAAGAATATT

AAAAAAAAAAAAAAACAGAAAAAGATTGAATATAATACTACTCATAGAATTAAATGAATT

TTATAAGCATAATTAAATACTTAATGATACATTAATATATATTTTAATTAAAAAGAAAAA

CTGAATAAAAATTCAGATAAGTTTTATCAAAGATAAATAATGAAAGAAAAACTAAAATTA

TGAAAAATTCTTTTAAATATATATATATATA

>c9587_g1_i1 len=235 path=[187:0-234]

CAGATTACAAACACTGCTCTAATGGCAATATTTTATGAACAGCAAAACCCTGGTTTTATG

ATGCTTTAACAAATCCAAATAACAATGACAGCTGTCTACACTTCCTTCTCCACCGAGGTT

ACTATTTCTGTAAGTTTCACAAATCTTCCCATTATTGCAAAGGAGGGTAAGGAAATCAGG

TTCAACATCACAGCTGAAAGCTACTTCTTAGTGAACACCAGTGCCACATTATCAG

>c9589_g1_i1 len=323 path=[33:0-225 32:226-322]

AATTATTATAAAAAAAAATTTTTTATTATATTGATTATTATTTTATTGATTGTTATTAAA

ATGAAAAAGAAACACCACTTCGTAAATAAAAATTATATAAAAAGTAAAAGTTATCATAGA

AAGAAAAAGAAGTACTTTATAGATCAAAAATTTCTACGTAAATATAAAAAAAGTATAATA

AATGAAAATAAAGTTGAAGTTACAAAAAAAGATCCTATAGAAAATTTCTTTTTTACACCA

ATAAGAAAAGAAAAAGATGTAGGAAAAGATGAATTAGAAGAAAAAGAAAAGAAACATAAA

GAAAGCTATAATTTAAATATAGA

>c9596_g1_i1 len=232 path=[8:0-119 128:120-231]

GATCCTGTTTGCATGGGAAGTGTTTATGGCAACGAGCTGTCGCTAGCTCTGGAGCGACCA

CGCAAGGACTGTCCTTCAGTCTGCCTTCTTCAGGCTTCTCTCCTGGGCTCACATCCTCCA

TGCTCCCGTGGGCTTCTGCCTCCTCTTCCCCTCAGGAGAGCTGCGGCGCCGCTCTGTGTG

GATCCGGGCAGAACGCGCACGTCCTGCGGGCTCAGTGAGCGCCCGGTGACCC

>c9596_g1_i2 len=295 path=[567:0-182 128:183-294]

GGAGACCGCGGCACCGCCGCCATGTTACGCCCCCCGCGCCCCACACTTGCCATGGCGTCG

CTGCGCGGCGGTGCCGCGGTCTCCGACCGCCGCGGGAGGGTCGGGTGCGCTGGAACAATC

ACCTCGGGCCATCGGAGCTCCCGCCCGGCTCTCCGTTTGGGTGGGTGGCAGAGGCAGGCC

TGGTGCTCCCGTGGGCTTCTGCCTCCTCTTCCCCTCAGGAGAGCTGCGGCGCCGCTCTGT

GTGGATCCGGGCAGAACGCGCACGTCCTGCGGGCTCAGTGAGCGCCCGGTGACCC

>c9597_g1_i1 len=294 path=[272:0-293]

TTGAAGCCACATTCAAGCAGTATCGCACCAAGAGTATTCCCATCATTAAAGCTAACAAAG

CGTCATCTGAAAGCACAACTCATCGCATTATAATATATGGAGTCCAAGATGAGAATGGAT

CCAATGTCCAGAATTTTGCCTTGGACATCACTCCTCCACCTGAAGTGATTTACAAATCAG

AGCCAGGCACTAGTGATGGCATGAATGTGCTAGGGATTGTGATATTCTCTGCCACAATGG

GTAGGTTTCAGACATGTTGGTTTGCAATAATCCAAGTCTCCATTTAAGAGAAAA

>c9604_g1_i1 len=234 path=[212:0-233]

ATAAGCTTTACTAATGCATCACTTGTGCTGGCATCTCCCCTTTTAGTCATATTTGCATGG

CAATTGATCAAAGACCACAGCCGTGTTTTATTCCAGCCCTGTAAGGCTGTTAGCACACAG

GCTGCAGCTCTACAGTTAATGCACGTCATTTGTTAGCCTGGGATATTTCTCAAGGAACTG

GCGCTTGAAGACATGTAGGAATTAAAAGCTTTCTGTGGCTGTCAGCTGGAAGGG

>c9605_g1_i1 len=602 path=[580:0-549 1130:550-601]

CTGCAGTCCATTCGCCGAAGCCGCGCACGCACTGCAGCCGGTGCAGCACCACCCCGTTGC

AGAAGATGAGCTTCCCTTCCTCCGGCTTGGAGCGGTAGGCGAGGCGCAGGATGAAGAGCT

CGAGGAAGGCGGACTCCAGCAGCAGATCCTGGTCCTCCCGCGGCAGCTCGGAGAAGCCCT

GAATCTTCTCGGCCCATTTGCGGATGACGTCCATGGAACCCGTGAGGAGGTCGTAGAACT

GCTGCACGTCCACTGAGTCCTCCTTCTCCAGGTGGCACGGCACCGAGTCCTGGAACTTGG

AGTAGTCCAGCTTGGTGGCGCTGGGGACAGAGTCGATGTGCGCCCGCACCAGAGAGGTGA

TGAGGCTGATGGGCGACGCGTCCGGCGGCTGTTTGGGTTTGGAGGGGAGGCGGCCGCGGC

GACCCTTCAGGCTGTCGGTCCGCACCACTTCTTTGACCATGCCCACGGCCAGGCACTTCT

GGAAGCGGCAGAACTGGCAGCGGTTCCTCCGCCTCTTATCCACGGGGCAGTCCTTGTTGG

CCAGGCAGATGTATTTGGCGTTCTTCTGCACCGTGCGCTTGAAGAAGCCTTTGCAGCCCT

CG

>c9606_g1_i1 len=430 path=[441:0-375 865:376-429]

ATATTTTTTATAAAAAGCAAAACATTATGAAATACTATAATTAATAATACATTCTAGTGT

TCAAAGTGCAGTAAAGAAAGTATCATTATAACAGTTATCTTTGAAGTATTTTTTATATGT

TATAAATAATAATAATGAAATAATACACATAAATATATATATATCTATAGAAGTTTTTTT

TTGCCGGTATCTTAAAATTCCATTTTTTTTTTTTTTACTTTGAAGCAATGCTAAAAGAAA

AAAAAATATATATTTAAGTATGAATTTGTAAATATAAATTTGTAAATAAAAATTTTCCTT

TCTATTTAAAACTTGAATATATTGTTCATTAAAGATAAAAAATTAAACTTTATGGTTTAA

AAATGAAAAAAACATTTTATTTGTTTAAATTTGCAATTCAAGTATTTTAGTTAATATATA

TATATATATA

>c9607_g1_i1 len=306 path=[35:0-36 71:37-186 34:187-305]

TGAAAGCAGAACTAAGTGGTTATGAAAGAGTCTGCCAAAAAAGAAAGATACGGGAGAATA

TTAAAACCCTAAATACTTAAGAATGAAATTGCACAAGTTAGATGAACGTTAGCTTATAAT

TTAACAAAGTATGGACTAAAAGAAAGTGAGCACGGAAACCATTTATGCACTTACAGAATG

CATTGCTAAAAAGAAGGCTTTTTTTCCCCTTAACTCTGTGTACTTAGTGTGAAAGTTTTG

ACACCCACAATTATTTAGCAAGTATAAGACGTTTAAGAATTTAATTATGAATATATATTG

AGAAAA

>c9619_g1_i1 len=296 path=[1:0-295]

GCTACCATGATGCTAAAATACTAAATCATTCCTTTGCTTATTCAATGAGTTCAACGCTAC

ATTTTTTCCCAGTTCTGCTGCATAAAGCTTTTTGCCAGGAAATGAAGCCTGACAAGTCAC

AATAACAAAGAACAGATTGAGCATTGATTGCTATAGAATCAAAAGATATGCTCAAATGTG

CTGTCACCCTGCAGAACTTGATCCCTTCAAGATTCTGAACCTGAGTGTATCTACCCTCTC

CATACTTAACTCACTTCCCTCAACTTTTAAAATTACATGAACACTTCGGATCTGAG

>c9631_g1_i1 len=237 path=[1:0-236]

AGAGCGTGCAATCATTTTCCACACTGCACACAGTCCGCGGGAGAGTGCTATTACAACCTC

TCTGAGGCGCGTTAGTATGACAGAGGCAGGACTCAGACTGTTTCTTTGGAAGAAGTAACC

CCTCCCTCAAACCGGGCTGCGACACCGACAGACGGCGGAGCGGGACGTACCCACGCGCTG

TAGAGCAGAGCGGAGACCTCAGAATTTTACCACAGTGTCACAGGTTTTAACTCACAG

>c9631_g2_i1 len=220 path=[626:0-219]

AGGGTGAGTGAGTGCACATGAGAGGAGCCTTCCGTGCATCCTCTAGGGAGGAGGGATCAC

CGAGGGGTGGAAGGTGGGCACCAAGTGGAGTCTGTGGAAATTTACCCCACAACACGTGAA

AAAGAAATATAAAGCTGTCAAACCAACCAATATTTAAACATCGCCACTTATTTCTACAAA

ACTTTATGAGAAGAAAACACAGTGCACGACTTTATGTAAT

>c9633_g1_i1 len=204 path=[1:0-203]

AATAAAAACATACCAGAATTTGACTGTGGACAGAGTGAAATCACAATTGTACCTGCAAAC

CTGACATTCTGATGCACTGTAACTGATTACTTCCCCTCTGCATAAAAGACAGACCCATGC

TGTAATTTCATATTGGGCCTCTACCTCCACTAGAGGTGGAGGTTGTCTCAGCTACCCAAT

AATGCCAGCAGGGTCTTTTAAACA

>c9634_g1_i1 len=446 path=[793:0-340 25:341-445]

AGAGGGACTTTCTAACCGGGCCTGTAGCAACAGGACAGGGAATCACAGTTCTTAACTTAA

ACAGGGCAGATTCAGATTTGTAATCAAGAAATACTTTCCAAGAGAATGGTAAGACAATGA

AACAGGCTGCCCAGAGAAGCCGTGGATGTCCCATCCCTGAAAGCATTCAAAGTCAGGTTG

GATGGGACCATGGGCAACCTGACCTAGCGGAAGATATTCTTGTCCCCATCAGGTGATGTC

CAACAGTCCCTTCCAACCCCAACCCAGTACATACAGACCGGTTTAATAGAGTGCTGTTTA

ATAATGTATTTGATCAAAAGATGAGAATATTAGCTCAGATAGCCATATTATTTGCCCTGA

GGAAACTAAGCAAGGTTTTTTGGTTAATTAGAAGTACAATTTGTGCATGTCTTGATAGAA

GTATCTTTTGCTCTGTTTGCAGTTGG

>c9634_g1_i2 len=604 path=[793:0-340 1133:341-603]

AGAGGGACTTTCTAACCGGGCCTGTAGCAACAGGACAGGGAATCACAGTTCTTAACTTAA

ACAGGGCAGATTCAGATTTGTAATCAAGAAATACTTTCCAAGAGAATGGTAAGACAATGA

AACAGGCTGCCCAGAGAAGCCGTGGATGTCCCATCCCTGAAAGCATTCAAAGTCAGGTTG

GATGGGACCATGGGCAACCTGACCTAGCGGAAGATATTCTTGTCCCCATCAGGTGATGTC

CAACAGTCCCTTCCAACCCCAACCCAGTACATACAGACCGGTTTAATAGAGTGCTGTTTA

ATAATGTATTTGATCAAAAGATGAGAATATTAGCTCAGATAACTATTTTATTTTTTAAAA

TGCTTTGCTTATTCAGACAAAGGTTTAGATATTTAGAGTTCTATTAAAAAAAAAACAAAA

CCACTTTCAATGTACTTCAGAGTAAATTAAAGAAAAACAGTTATTAAAATGAAGTAGCCC

ATGTAAGGAAAGTAGAATTATTGTAAAAGCTACCTATCATTACTTTTGTAGTTTAAGAGG

AGGCTGTAGGTGACAGAAATCTTAATTTGTGTTAAAATAAACTTAAGATATGTTTCACTC

TTTA

>c9635_g1_i1 len=322 path=[155:0-321]

AGAAAAAAAAATAACTCCATTTTTCTTTTAAATAGTTTAAAGTTTCTTACACTTTTTTTA

TCAAAAATAAATTGTTAAAAAATATATTAGTTTGTTGATAAGTATTTATAGATGTCTGTT

TTTTAACGCGTCATAATTAATGATTTTTCTAATTTTCTATACTTATGAATGAAACATGTT

ACTATGAATTAATGTATATCTAAGTAAAACTATACGTCTATATTTTTGTTATATTAGAAA

AACTTGTATACATATTTATATAAAGGAATTATTTTAATATTTATCTAATATTTCATGTAT

ATTTTTAAAAAAATTAGTATGT

>c9642_g1_i1 len=416 path=[394:0-241 831:242-415]

TGTTTGATCGGGGGGTGGTGGTACCCAACTGGTGCTGTAGGTAAAGGATGGGGGACAACG

ATACCCAACTGATCCCATGGATAAAGGATGGGAGGCACCCGTGGATAACGATGGGGAACG

TTGGTACCCAACTGGGCCCATAGATAATGATGGGGGATGCTGGTACCCAACATGGATAAG

AAAGGATGGGGGATATACGGGTACCCAACGGATCCCATGGATGAGGATGGGGGATGCTGG

CACCCAACTGGGCCCACGGATAAAGGACAGGGGACAGTGGTACCCAACCGGTGCTACGGG

TAACGATGGGGGATGCTGGTTCCCAACTGGGCCCACAGATAAAGGACAGGGGGCAGCAGT

ACCCAACTGCCCCATGGATAAAGATGGGGGATAGGGGTACCCAGCCGGGCCCGTGG

>c9643_g1_i1 len=298 path=[398:0-182 124:183-193 108:194-297]

CTCTAGCAAGCCTTGTGCACGTTCACCGTATGGACAAATCCTAACTTGCTCTGATGTTTC

ATCAAGTGGATTTAATATTGTGTCTCCACTTAATCTTGTGCAAATTTTATGTTCAAACAA

ACCCTACAGGGTATCTCTCATCTACGCCACTAACCCTGCTCCTGGTTTCAACTCCACACA

TCGGCTTCAGACTATCTGAAGCACTGTGGAGATGGAAGCGATAGCGTTTCCAAGGTGCTC

AGACGTGACATTGATGGAGGACATAGGGAGGCAGGTTCAAGAACCTCAGAGAAACAGG

>c9656_g1_i1 len=593 path=[1:0-592]

AATTATATTTTTACATTTACTACTTTGATAACCATCTATAATAATTTATATATGCATGAG

TGCTTAAACGGAATAAATAAATCAAAATCAATATAAAATATAAATTAGAAAAAAAAAATT

TCTTTTTATTGATATCTTTTATAATATTATAAATTTGTAATTTTATTTTAGTATAAGTTT

ATAACATAAAAGAAGTGGATGAGAAATTTTTTTTTTATTTTTTGACTTAATATTTTGTAA

ACATTTTTCTACCGATAACCTACATATAATTTCATTAATAATACTCTAAGAAAAATATTA

AATGAACAAAAATATTCGTTTTCAATTAATCACGAAAAATATAACAAAAAAAAAAAGAAA

AATTTATTAATAAATTATATGATAACTTATTAAAAGAACAAAGATATGATTTTGTTTTTT

AATAAATAACAGTGGAATAGTTCATGTATTTTCCTTTCTTATTTTACAAAATTAAATAAA

TAATTTTGATAATTTTTTTTTCTTTTAGAATATTGATTAATATATCCATTTCTCAAGATT

AATATTTTTAGTTTAATAATTATTTCAACTAAAAAAAAATTTATTTTCATCAA

>c9660_g1_i1 len=282 path=[504:0-236 225:237-281]

AAGTACAGCCCTTTGTGATGGTGGTCAGAAGTAATCCCTGTATCTCAAAGTCAGTGTAGT

CTTACAGCGTGTGTATAGATGGCTTCTGTGGGTCAACTGGCATTGGAAATGTGCTCGTAA

GTTTGAGCCTTGTTTTGCTTGTGGGCAGGCTGAATCTATATAGGTTAATATCGAGAGCTC

AGAGATTTCTTACCGACTTGAATATTCCAGGTAAATTTGCTTTGAAATTCTTTTCTTTCT

GAGTGTCTTTAGCTTGGGGGCTGTTGAAAACGATGTTTTTTG

>c9660_g1_i2 len=282 path=[504:0-236 740:237-281]

AAGTACAGCCCTTTGTGATGGTGGTCAGAAGTAATCCCTGTATCTCAAAGTCAGTGTAGT

CTTACAGCGTGTGTATAGATGGCTTCTGTGGGTCAACTGGCATTGGAAATGTGCTCGTAA

GTTTGAGCCTTGTTTTGCTTGTGGGCAGGCTGAATCTATATAGGTTAATATCGAGAGCTC

AGAGATTTCTTACCGACTTGAATATTCCAGGTAAATTTGCTTTGAAATTCTTTTCTTCCT

AAATCTGAGACAACAGACAAAAAACATCGTTTTCAACAGCCC

>c9662_g1_i1 len=229 path=[415:0-170 36:171-228]

CAACTACGCAGCTTCAGTACGTGATCTGTATCTGTGGGGGCCGTAGTGGTGCACAGCTCA

AAGCTCTGTTTTCAGTTTCTCATACAAGTCTTCCTGGTCGATTTTGTCTGTGTTGCTGTA

CCAGCGGTGGTAGGCGAGCAGCGCGGCGTTTTTGGCTGCAATGGAGCTCATCTCCATGAC

AACATCTACTACCTGAATGGCATCGAGTGGGCTGCGAGTGCCATGGAGA

>c9662_g2_i1 len=233 path=[37:0-174 36:175-232]

AGCTGCTATTTCGTGAGCACTTCCCTTGCAGTCAGCAGTCTGGAAGATATTCTCAAGGGA

AGTGCTCACGAAAGAGCAGCTGAACTTGCTTTTCTCCTCCTATGACTCTGTCAAGGTGAA

GAAGTGGCTGGCATACCCCCACTACAGCCCCCCGAATGAATTCCCACCTATCATCCTCCA

TGACAACATCTACTACCTGAATGGCATCGAGTGGGCTGCGAGTGCCATGGAGA

>c9663_g1_i1 len=313 path=[497:0-312]

TACAACACTTAAGCATTAGATTTGTCATTAAAATGAGCCACATCGGTTGGATTAAAAAGG

CCACAGTGTTTTAAAGGAAACACAACACCTTTCAGAAGGGCAAAAACAGGTGACAGTAAA

ATTACTAATTTGATGGGCAGCGATGTTGACCTGACTAGCACAAGACAACAAGGGGCTGAC

TACCTCTTTAGGAGTCCTGGAGTATTCACTCACTCTCTCCACAGAAACAATGTTGTTTTC

AATTTCTGTCCAAGAGCGCACCATCCAGTTCAGCACACCAGTTATCTAGCAGAAAGGAGC

AGAAACACTAAGG

>c9668_g1_i1 len=224 path=[374:0-223]

ACAAAATAATTCTGATCTAATCTGCTACACCAGTGTCTGCAAAGGAACATTCTTTAACAT

ACATGCAGCAATAATAATCCAGCTAAAGAAATTAACAACATTTTTCAATTATACAACATT

AGAGAAATCGTGACCTTGAGCCTACCGAGCAACATGTTTGAAGTTTGTATGTGAGTGATC

TCCCTAGGGCTGCCTGAAAGTTAAAGTGTATTGGCTTTCTCCCA

>c9670_g1_i1 len=251 path=[2:0-250]

CGCCGCTGGGCTGCGTCACCCGGAAGTGCGCGGGGGCGGAGGAGGTTCTAAGATGGCGTC

GCCTCCTCAGGGGGGCCCGATGGCGATCGCCATGCGGCTGCGGAACCAGCTGCAGGCCGT

CTACAAGATGGACCCGCTGCGGAACGAGGAGGAGGTGAAGGTGAAGATGAAGGAGCTGAA

CGAGCACATCGTGTGCTGTCTGTGCGCCGGGTACTTCATCGACGCCACCACCATCACTGA

GTGCCTGCACA

>c9670_g2_i1 len=708 path=[917:0-308 1226:309-311 1229:312-707]

ACTTTGTGTTTGAGGGGGTATTAAGTTAAAGGGAGCGGGGTGGGGGCAGGAGCAGGGCTG

TGCTGCGGGGGGGGAGGCAGCACCCACCCCCAGCCCCTACCTCCTCTTGTCCTTGATGCT

GTAGTGCAGCAGGAGCGGTGCGGGCTTGCCAAACCAACGTGAGAGCCACAGCTGCTTCAT

TGTCATGTGGTCAGGGAGCGCCTCGCCGTTGAACAGGATCTGCACGTGCTGCAGCGGCAG

CCCCAGGCGGTGGCAGAGGACCCTGCGCAGGTGCCGGATCTGCGCCCGCACGGAGCACCT

CACGTACTTCTGCTGCAGAACAGCCTTGCTCTTGTCCTTGCTGGAGCTCTGCTTCTCCAG

GCACAGCGAGACGTGCTCGTCGTAGCGGAAGTAGTGGGCGCGGGAGTGGTCGAAGGTGCT

GTAGGGCAGCCCCATGGGGTCACCCCCCACCGTGTCCTCGCCGCTGGGCTGCGTCACCCG

GTCGAGGCCGCGGGACTGGTAGAACTCCCGGATCCTCTTCTCTTCACTCTCCTGCAGGCC

GGGCACCAGCTTGTACACGATGTCCTGCATGACGCGGTCCAGCTTCAGGTTGAGCAGCGG

CTGCGTCTCGTGGATCTTGGTGTTGCACATGGGGCAGTACTTGCTGGTCTGCAGGTACTT

CACGATGCAGCTCTTGCAGAAGGTGTGCAGGCACTCAGTGATGGTGGT

>c9671_g1_i1 len=2983 path=[1877:0-2087 1005:2088-2111 1029:2112-2982]

TTTTTTTTTTTTTTTTGAAATAGTGAAAATAATTTATATTAAAATATATGTAAGAAAGAG

GATATTATTATAGTTTGAAGATTAATTGAAAACATTTATGAAACTGTTTTATTTCTTTTT

ATTTGTTTATCATTTTAATAAATTTTAAAGTATATATATAAAAATAAATATAATTGAATA

TCCTTTTTAACACACGTCATTTAGAAAAAATAGTATTAATTGATTTTATAGATATTTATT

ATAATTAAGAAAAATTATTTTCTATAGAGAAGAAATTTCTATTTATAAATATGTATTCCT

TATGGAAAGAAGTAAGTGAACAAATAAAAAAAAAAGCAGAGGATTTGAATACAACTTTTC

AAGAATTGAATATAAATAATATATCAAAAGATAATGGAAAACAAAAAGGTTGTAAATTAA

ATGAGAGAGAATCAGATAAAACAATAAAGGATAGCATACATGACAAATTAAACTTAATAA

ATAATAAATACCTAAATAAAGATTTTTGTGAATTACAATATTATAATAATAAATTTATAA

ATGGTTTTAATAATATAAAAAAAATAGTTGGTGATATATATAAAGATAAGTTAAATAATT

TAAGTAATGATGATTCAGATAAGAAGAAATTATATTTATCGAAAATTGTACCATGGAAAA

AAGCAGATATTATGATTTCTAAAATTTATAGAAAGAAATATGAAGAAGGGTTTCCTATTA

ATTTACCAAATAATAATTTAAATAATGAAGTATATGAAAAGATATTAAAATTAAATTTAG

ACAGAAATAGAATTTTGAATACTAATATTTTAGAAAATTATAATTTTAATTGGATAAAAA

AAAAAGAACAGAGTGAAAAAATAATGCAAGAAGATGAAAATTTAATAACTACTAAAAGTT

TTTTAGTTCCTTCTTATATGTCTGAAATAGATTTTTGGAAATCTTATTTTTTTAATATAG

ATATAATATATAATGAAATTGCTGATGAAATATATGAAAATAAAAAAAATATTTTAATGA

GTTATGAATCTGAATACACATCAAGTAGAATAGGAAATAAATTAGAGGATCAAAAAAATA

ACCATTCAATAAATGAAAATACCTTGAATAATTTGAATTGCGAACATACGAAAAATAAAT

CAGAGGATAAAAATGATTTTAAAATAGAAGATAAATTAAATAGCTTAAATGAAGTAAATT

ATACAAATAATATTAAGGAAGAAAAACATTTTAATTTTGAAAATGAAATCAATAAACAAC

ATCCAAAAATTGAAAATGATATAAATAATACTTGTAAAATAAATAATGAAATTAATATAA

ACAACTTAAACAGTTTAAATATATCAGATTATTCACAAAGTAATTCTCATTCTAATAAAA

AAAATTATAATATTGATATTATAACAAAAAATAATTTAGAAACGAATCTAATGGAAGCAA

AAGAAGATAACATCATTTCAGAGAAATTTATATCTACATCAGTCAATGAAGAAGAATATG

TAAAAAATGCAAAAATAAATGGTTTAAATATATATATGGATAATGATATTTATTTTAATT

TAAGTGAAAAATCTAATAAGTTTTCAAAATTTTTGAATAATTCTGATTACAATGATAAAA

AAGAAGATATTTTATTTTCTTCTAATAAAATAAAAAGTAATACGTTAAATGAAAATTATA

TAATTTCTGAAAAAAAATTAAAAGGAAATACTGAACAAAATGATAGTACTTATAAAAAAG

GAAAAGACGATAATAACATTAGTTTAAATGAGATGAAAGATAAATTATATTCACCTTTAG

ATATATCTGAATATAATTTAAAATTGTTAGAAGAAATTAACCAATCGAACAAAAAAGAAT

ATGAAAATGATTATCAAAATAATTCGTATGAATCTTTTTCTTCTAATAATAACATAAACG

AAGAAAAAGGAAATATATATTCTAATTTTGAATGTAATGATAAAAATTCGAACAACCCAA

GTGAAAATATTACGAATGAAGAAAATAACAATACTTCAAATTTTGTTAGTTGTAAAAATG

AAAGTATTCTAATTGAAAGTGATCCAAATAAAAAAACTAAAAATAATTATAAAAATAGTA

CAAAAAATGAAATAGAAAATGTACTTACTTCTGATAATAATAATTCTGTAAATATAGGAA

CGAAAAAATATGGATTTAATGAAAAGAAAGAAACACAAAAAGAATATGAAAATTGTTATA

TAGATTCATATAAGAACATGTTGGATGATACATGTAAAGAAAATAATATATTTCCCAGCA

ATATGGATTGTAATAAAATATATAAGGACTTTAAAAATAGTGAAAACAGTTTGAAATCTG

ATTGTATTATTATAAAAAATGATAAATCTAATTTTGAATTAAAAGAAAATGAGGTTACAC

AATCATATAATTTTCTCAATGAAATTAACAGTATAAATACAATGAATAATATGAATGTTA

ATAGTGAAAAAGTAAGTAGTGAAAATATGAGCAAAGAAAATTCTTATTTTGATATTTTAA

ATAATTCCACTCACTTCAAAAATATTGAAGAAACAAAAGAAAATATAAATTTTAATGTAG

ACAATTTAATAGATTTATCGTCAAATAAAATAAAAAATGACTCTGATAACTTTTTGAAAT

CAAGTGATATAAATATTGATGAAATAAATTTTGGAGATGATTTAGAATTTGATTTTTATT

CAAACAAATTTAATGCAGAAGAATTGGAGCAATTTGAAAAGGATCTTTTAAATGCGTGAA

AATCATTATTAAAATTTTAAAGATGTATTAGAAAAAATATTTCTTTATATTTCATTTGTA

AATTTTATTAAATAAAAACATATTAGTATAATGATTTACATCTATTTTATTACACACATG

AATATTTATTTATCAACATTAAATTTTATATAATTACTTATATTTTAATATAATTTTATA

ATTTTCTCTCTTTTTTTATATTATATTATATATATATATATAT

>c9676_g1_i1 len=232 path=[1:0-231]

GTAATTACAGTACTGTACTGAAGATAATATGCAACGTGGGATACAGTAAGGTAGAAACGT

GAAAATAAATTTCATCCTAAACTGACGTAAGTAGTTAGTTAAAAAAAAGCAACGAAAAGA

AGACAGCATTTTTAACCTGAAAAAGATTGTTTTCTTAAAATGGTATAGGAATGTCTACTG

TAAGTATAGCATAAAATTTTTAAAGAAGAAGAAAGCAAAAAAAAAAAAATCA

>c9681_g1_i1 len=206 path=[512:0-205]

AAGCAGCCAGTGGAACTCGCTGCCCTTAAAGTCCCTGTGACCTCTTGCTCTGCCAGCCCG

GTACTGAGCTGCAGCACTGAGCCCATGCAGCTGTCCCCATCCCCAGGCTTCAGCGATGCA

CCTGAGCAGCAGCTCCTCACCCCACGCTGCTCCACTGGGCAGGGTTGGAACAGAGCCACC

CACCGTGCAGCACCCACCGGCAGCAG

>c9691_g1_i1 len=252 path=[230:0-251]

GCAGTCTCATAGATCTGCCTAGCTATTCTAAAATTCCTAACTGAAGGCCTTGGGAGGAGT

TCCACCAATTGCTGTAAACAGCTCAAGAAAATATGAAGTTGACTCAAACATTTCAATCTG

GTGCAAAGGAATAACCTGGTTGTGAGTCTACAATGGTCTTTTAGAAAGGCTAAAAGAACA

GATAATAGCTACAGTCAAGCTGGACTCAAAACACCGTGGATTGCATTCAATTGCTATGAA

GTGGCCTGAAAT

>c9693_g1_i1 len=222 path=[440:0-221]

TTTTTTTTTTTTTTTTCCTTTTTAAGCAATTACACAAATTATTTTAACTTCAAGAACGTA

AGAAACTCACAGGCCAAATATTTCAGCTTTTGTTACCTTTTTTATCCTTTAAACAATATT

TGAAAGATTTTTGGCACCTATGAATTGGACCACAGATGGTAAAACAGAAACTAATCCAGA

AAACTGGTTATAGAAAGTCAGTCCTAACATCCACTTTTGGTG

>c9696_g1_i1 len=309 path=[1:0-308]

GCCCTTTAGACGAAGTTACAACTCTTCAGGAATATCTGATCTAAATAAAGACTCTTGCAA

TCAGCTGCACAGACCCAAACCTGAGGAGGCTTTGGCAAATCATTAACTGGCTTTAACGAG

CCCGTGCTGAAATAGCAGCCTGCTGGCATTCTCCGGATCTAACATCACATTTCTGGAATC

AAGCGCAAGAAAAATGAAAACTGGGATAAGCATTATCTGCTACGTTGTAAAATCCAATGC

AATGCAACTCTTAACCGTGCACCCAGTTCTAAGAGTGCAAATACACCACAGCTTATGTCC

TGCTCTGCA

>c9700_g1_i1 len=314 path=[1:0-123 1110:124-313]

AAAAAAAAAAGAAAAAGAAAACATATAAAGAAAAGAAAGAGGAAGAGTAAAAGGAGTGAA

AGAAAAAAGAAAATATAAAAAGAAAAGGAAGAGGAACAGTAAAAAAAAAAAAAAAACAGT

AAAAGGAAAAAGAAAAAGAACATATATAAAAAAATTCATAAATAATTTTAGATATATATT

AGTAAATATAAAAATAAAAAAATATAAATAAGAATAATGATAATTTTGTAAAGTGTAATA

ATAATAAAAAATGAATTAAAAAAAAATATCAAATATATTTAATATTGTATAGCTTATTTT

TTTATTTTTTTTTT

>c9700_g1_i2 len=471 path=[1:0-123 125:124-470]

AAAAAAAAAAGAAAAAGAAAACATATAAAGAAAAGAAAGAGGAAGAGTAAAAGGAGTGAA

AGAAAAAAGAAAATATAAAAAGAAAAGGAAGAGGAACAGTAAAAAAAAAAAAAAAACAGT

AAAAAACTGTCAAAAGGGAAGGAATAAACTATCCAAAGATGTATTGAGACAAGGCCAAGT

GAAGAAGAGAAAGGAGGCCGTGCTGGAGGGCGGTGGTACCGTTCCCTTCGCCTTCTCTCG

CGCCCTGGGAAAGCAGAGCTGTGATTGTTGGGAATGCTCTTTGCCTTCGAGGAGCTCCAG

GCACTGGGGGGGGACTGGCACTGCTCCTCCATCAGGACTCAGCACGGCTGAGCACAACAC

CCATCACAAACAGAACCCCCCCCTCCCTTTCCCACGGATCTCCTCCTCTCCCTGGAAACT

GCGCTCAGTTCCTCCAGTCAGCTGTCTATGCATCTCTCGTGAACTTCTGCA

>c9707_g1_i1 len=746 path=[1:0-270 272:271-313 315:314-745]

AAAAAAAAAAAAAAAAAAAAGGTGAAAAAAATATAAATATCTACAGGAACTACTTATATG

AAAAATTTTAAACAAATAAAAAAAGAAAAACGAATATACAAATATATATTTTTAAGACAA

TCTATAATGGATATATTTATCGAGGGTCTCATCGGAATATTTTAATTTCTCAAAACTATC

TTTTATATTCTTTAATTCTTCATCTGTGATTTTAAGAATTGGAATTTGTTTTGCAATTAG

ATCAAATAGTTTACTATGTTTAATTTTTACATTGGTATAAGAATTTATTATTTTTATAAT

AATTTTTGAGGGTATATTTATATCAGAATTTAAAGCGCAATGAATGTATAAAGAAGCTAT

TTTAAATAGTTCAATATTTTTATGTTTTATTTTGCTATAGGATCCAATTATATGCACATA

AGAAATAAAAATGTTATATAATTCTTTTTTACTAAGATTTTGTATATTATTTTTGTCGCG

TTCATATAATTTAATTATTGCATCAGCTAGTTTATTCATGAAATTTGTATTGATTTTATT

AAAATAAGTAAAAGATAAACATATTAATGATAAATCGTGAATAGAATATTTATCAATGGA

TGAAAGTATTATATCAATACTATTTTCATATATTTTACTATCATTGATTTTAATTTGTTT

AAAATAATTAATAATTAATGATATTTCTTTAGGTGTTAATTCTTTTTGTAATTCATAAAA

TTTATGTTCTATTAATCTCCAGATAT

>c9707_g1_i2 len=746 path=[1:0-270 1221:271-745]

AAAAAAAAAAAAAAAAAAAAGGTGAAAAAAATATAAATATCTACAGGAACTACTTATATG

AAAAATTTTAAACAAATAAAAAAAGAAAAACGAATATACAAATATATATTTTTAAGACAA

TCTATAATGGATATATTTATCGAGGGTCTCATCGGAATATTTTAATTTCTCAAAACTATC

TTTTATATTCTTTAATTCTTCATCTGTGATTTTAAGAATTGGAATTTGTTTTGCAATTAG

ATCAAATAGTTTACTATGTTTAATTTTTACACTAAAAATAAAATAAAACAAATAAAAATG

TTAATTTTTTCTTTATAAATAAACTTTCATTTATCTATTATATTTTACTATGTATAATTT

ATGTATATATATATATGCCTCTTTATATAGTAAAAAATTATTAAAATTCAAATGAACTAT

TTAATTCTGTAATTTTTTTTTAACATTTAAAATTATAATGTATTATTTAAAAGATAAATA

TTTAAAAAAAAAAAAATGATCCATTTATTTTATGATTCATATAAGAAATGTAAGATATTA

GAAAAATTTCATTATATATATTTAATGAAGAAAACAATTAGCTACACACAATATAAAATA

AAAAATAGTGATTCAAATTATTATCTTTATGAATTATATAAATCAGTTGAAAATTGTAAA

TCAGGAAATTTATTATTTAAATTAACTCATAAAGCATTAAATTATCAAATACATGACTTA

CATATCTGGAGATTAATAGAACATAA

>c9711_g1_i1 len=224 path=[1:0-181 420:182-223]

TTTTTGTTTGATTTTGGTTTTTTTCCCGGGTTTGTTGTTGTTGTTTTGTGTGTTCTTTTT

TTTGTTTGTATTTGCACAGAAATCAACAAAGTATTACATACAGAACAGAAATAAATACTT

TGAGTTCTTGAACAAGAAATATTAGCAGATATTATTTGAGACATAAGCAATTAACATATA

CCTATGTATTTCTGGGCTCCTTTGCCGAGTGCAGTGCTCTTCTG

>c9713_g1_i1 len=466 path=[1:0-465]

AAAATGTATAATGGTACACATCTACTGTCTTGTCCTTTGTGTTGCCAGAGCCTTGATTAG

AATTTTTTCACCTGGTAGTTGTTGCTACTACTTTGCAAATGCTTTTCAAGGTTTCTAACA

CATACTGTAAATTTCTTGGCCAATATTTATCCTCCAGGTTGATAAAACTGATTAAAATGA

CATTTAATACTTTCCTTCAAAAGCTTTCAACTTTGTGACTATGTAAGTATATAAGAAAGG

GTTTGTTTATATATTTTCTAATATATATGTGTACTTTAGCTTTCCTAGTGTAGTCCAAGA

AAGTATGACTTACAAGGAAAAAGGGAGAAAAAATAAATGGCTACTACAAAGCAGTTAGAG

ACATGGGGAATTAACTTTTGAGGAACAAGGTTGTAATGCTATAATACAATGGAAAGTACA

GCAATAATAGCAGTCTTTGGCTGCAGTACATGAGGACGGGCCCAAA

>c9715_g1_i1 len=255 path=[20:0-19 40:20-186 207:187-229 531:230-254]

GTTCTTAAGATAAACATCCCAGAACAGTAAAGAAGCTGAAGGCAATGAGGGACCAGAAGG

AAAGAAGGAAGCAGTAGGATCTGTACCCCGTCCTTTGCTTTTTGTGCACAGATTCTACGG

AGCTGGGTGGGAAGCTCCAAAGGCCTGGCCGTAGCCATGGATGAGGTGTCTGTGTGAATG

CTGTGACCTCTGTCGGCCTCAATCTGTGGACATATTTGCACAGCCGGTCAGTGCCCTGTT

TGGACTGGGTACATT

>c9715_g1_i2 len=255 path=[393:0-19 40:20-186 207:187-229 531:230-254]

GTTCTGGGATGTTTATCTTAAGAACAGTAAAGAAGCTGAAGGCAATGAGGGACCAGAAGG

AAAGAAGGAAGCAGTAGGATCTGTACCCCGTCCTTTGCTTTTTGTGCACAGATTCTACGG

AGCTGGGTGGGAAGCTCCAAAGGCCTGGCCGTAGCCATGGATGAGGTGTCTGTGTGAATG

CTGTGACCTCTGTCGGCCTCAATCTGTGGACATATTTGCACAGCCGGTCAGTGCCCTGTT

TGGACTGGGTACATT

>c9716_g1_i1 len=265 path=[410:0-120 168:121-264]

TTTTTTTTTTTTTTATATGTAAACAAATGTATTTCAACTGTAAAACAAAGTAAGTAGGAA

ATAGCCTAAAAGGTCACGGAAATATTAAGTGTACTGCACAGATTATGCTACAAGCTGTAA

TAAGCCAAATTAAGATCTACAAGTAATGAATAATTGGCTCAGAGGCTGGTGTCGAGGCAG

AAACTTTGGGTTCCTTGATCATGGCGCAGTTTACTCAGCCTTTGACCTGTTGTCTGTGCA

TGGAACCCACCTATCACAAAGGGGG

>c9716_g1_i2 len=227 path=[85:0-82 168:83-226]

TGTTCCTTTACATTTAGTTACAGTATTTGAAGGACGTCCTGGGTAGACAATCTGTTGGTA

TTTTTAAACATTAGCATTATGAAAAGCCAAATTAAGATCTACAAGTAATGAATAATTGGC

TCAGAGGCTGGTGTCGAGGCAGAAACTTTGGGTTCCTTGATCATGGCGCAGTTTACTCAG

CCTTTGACCTGTTGTCTGTGCATGGAACCCACCTATCACAAAGGGGG

>c9717_g1_i1 len=2129 path=[41:0-2128]

AAAAAAAAAAAAAATGAGAATAATAATATTATATATAAGTACATTATTAATTATGAAAAT

AATAATTGTATGATTATAAAATTTTATTATTTTACTTTATTCATATTATTTTACACTTAT

TAAAAAATAAATATTATATATTCTTATTATTTTATATTATTATTAAATTTTATTATGGTA

TCCATTGATTATAAAAATCATTCCCTTTATTATCTATAACAATAAAAGCAGGAAAATCAA

CTACATCAATTAAATGTACAGCTTCCATTCCAAGATCTTTAAAATCAATAACTTCCACTT

TCTTAATATTATTTTTAGCTAAAATTGCACCAGGGCCTCCTATACTTCCTAAATAAAAAC

CATTGTATTTTTTGCATGCATTTTTCACTACAGAAGATCTATTACCTTTAGCTAATGATA

TTAATGAACTTTTATTTTTCATCAAAATTTCAGCATATACATCCATACGACCAGCAGTTG

TTGGACCGAATGAACCACTTGCATAATTATCTGGTTTTTTTGCTGGTCCTGCATAATATA

TAGGATATTTTTTAAAATAATCTGGTATTGGTATATTGTCGTTAATAAATTGATCAACAA

TTTTTTTATGAGCTGTATCTCTAGCTACTACTAATTTACCCGTTAAAAGTAACAAAGTTG

ATATGGGATATTTGGATAAACATTCTAATATTTCTTCCATATCTTGATTTAAATTTATTT

TTACTCCATTATCTTGATTTAAATCTTTAAATGTAATTTCTGGTAAATACTTAATTGGTT

CATGTTCTAATTTTTCCATAAATACTCCATTTTTATTTATAAAACATTTTATTTGTCTAT

CAGCTGAACAAGAAACACCTATACCAATAGGACAAGATGCAGAATGTCTTGGTAATCTAA

TAACTCTAACATCATGAACAAAATATTTTCCACCAAATTGAGCACCTATACCTAATTTTT

GACTTTTTTCTAAAATTATTTTTTCACTTTTTAAATCTCTAAAAGCTTTTCCATATTCTC

CTCCATATTTTTTTATGTTGTCTAAATATCTACAAGATGCTAATTTGACCATTTTTAAGT

TCATTTCAGCTGATAATCCCCCAATAACTATTGCTAAATGATATGGAGGACAAGCAGATG

TACCTATTTCTTTTATTTTATCTAATAAAAAATTATACAAAGAATCTTCATTTAATATAC

TTTTTGTCTGCTGAAACAAAAAAGTTTTATTTGCACTTCCACCTCCTTTAGCTATAAATA

TTAATTCATATTTCGGGCCATCATATAAATAATTTCGATGATCTTTATTTAATTTTTCGT

TTATACATGAATTACTGTTTTCCTTTTTCTCCTTTTTAATGTTATTATATATCTCTATTT

GAGAAGGTAAATTATTCTTTGTGTTAACTTCGTTAAACATATTTAAAGGAGATAACTGAC

TATAACGGAAATTGTTATTTTTATAAGCATTATAAATGCCTAAATTGAGATATTTGTGGT

CATAAGTTGTTAATATCTCTTCATCTTTCTTTCCTAAAATTATTGCAGTACCTGTATCTT

GACAACCTGGTAAAAGCTTTTCTGAACTTATAACAGCATTTTTTATTAAAGTCATAGCAA

CATATTTATCATTTTTACTGGATTCTTTATCATATAATATATTCTGTAATTGATTTAAAT

GCTTTTTATTTAAAAAAAATAATATTTCTTTAAATGCATATTCACATAACTTTGTTAACA

CAAAAGGTGGAACATGAATGTATTCTTTTGCATCTTTTTTATTTGTATCTTGTTTAGAAT

ATATTTTTATATTACCATTCTTATCAAAAAAATTATTTGTATCTGTAAAATCATATCCAA

CATATTTAGTTTTATTAAAAATATCCTCGTTTATTTTAACTGTTTCAATATATTTACTGA

GCTCATCAATACGTGTATATTCAATATCTTCTTCTTCTTCAAAATCTAAAACATCTAAAA

ATTTATTTAAAGTATTAATATTTAATTTATTTATTTTATAAATGGAATTAATATTCACAG

TTTTAATTCCATAAATTTTTTTTGAAAACAATAATGGTATATTTTTAAAATTTAACATTT

TACTCTACTAAAAAAAAAAAAAAAAAAAA

>c9730_g1_i1 len=222 path=[200:0-159 360:160-221]

ATTGCCATACAACCCTAATGCCATTTCCCCACTGCTGCTGCCCGCTGTGCTCTACCTGCC

ACGTGAGTTTGCCACAGCATCCACGATTTCACTGCACTCAGATCCTTTTTTACACGTTTG

CAATAAACGTTTGTACCCACCTGATGACTTAACTACTGCATCTATAAACAAACAGCCAGA

ACAGAGCACAAACGACCTGACTGAGACTATATTGTGCAAATG

>c9736_g1_i1 len=1598 path=[1576:0-508 2085:509-1597]

AATAAAAAATATGAAGAAAAAAAAGATAATTTAAATTACAAAAATAATGATATAAATGAA

TTATTTAACAAAGATGAATTTATTTACACAAATACTTCAAATAAAAAATATGAACCTAAT

GAATTTATATGCAACGAATATATAAAGAATATATTTAATTTACTTTCTATTTTTTTTCTT

CAAAATATAAATATGGTAAATGATCATTATTTATGCCGACTTTTTTATGGATATAACAAG

AGTAAATTTTATAATGAAAGATATTTGAATAATTTATGTTTTGAAATAATTAAAAGAGTA

AAAAAAATTAGAGCTTATCATTTACATTTAATACTAGTTAACTGTTATTATTTAAATTAC

ATAGACACAATATTCGTTAAAGTTTTATTAATAAATTTAATCTCGAAATTTTCACAATTA

CCTTGTGAAGCAATGTGTAAAATTATACCTATAATCCCTTTATATATAAATTCGGAAAAA

CTAATTTATAAAGTTAACGTTATATATTCAAAAAAAATAGCTAGCTTTAATCAGATTAAT

CATATTATTAATTTATTTAAAAAGATGATACAGTATAATTTTATATCTCAAAAAAATGTA

TTTTTAACTTTTAAACATTTAAATAAATTTATAAATATTAAAAAGAATAATTTAAAAACA

AATATTCTGAAAAAAAGGGAAATAACAGAAGAAAACATGGAGCAAAATAATTTATTGGAA

AATGTTGGGACAAAAGACAATATAATAAATAATATAGTAGAAAACTTTGAAGAATTTGAA

TATCAAGATAAAAAAAAAAATATCATGATAAATTGTAGAAATGAAGATTTACCACTGGAA

GATAATATTTTATATAAAAATGAAAATAAAAATTTCGATAACAATGGTAATATAGATATA

ACTAATATTAAAAATTCTAAAGATGAAAATATTAGTGTAATTAAAAAGAAAATTTATAAA

AAAGATGAATCTTATAAAATTGAAGAACCAACATATATAAATAAAGATGATATTTCTAAA

AAAGATGAGATTATTCTTAAAAAGGAAGAAGATATTTTTAAAAAAGAGGACATACTGTTT

AATTTAAAAGTAATAGAAATGCACTTAAAACATGATTTAAAAAGCATTTATTGTCTTTTG

CCGAATGAATATAAGAACTTTCTTCAAAAAGTTAGAAACACATCATGTATTATTAATAAA

AATCTTCAAGGAGAAAAAGAAATTTATATTTTAAAAAAATATATGAAACTATTAAATTAT

AAATTTATTACTTTTACATATGGACCATATATTTTACACATTTGTGATCCATTTTATAAA

ATATATGTTGAATGGGAAAACGTATGGAAATTATACCCAATTTATGAACAAAATTCACAA

AAAAACTTTGAAATTAATAAGAATAAGCATATGAAAAAAGAAGGATTTGATGTAATTTAT

GTATCTCATGATTCTTTTTTAAATTATCAAAATGAAAATGAAAAAATGGAATATTTAAAT

TCAATTTTAAAGGATACAAAATTTTCAAGATGTAATACATTTGTGAATGAAAAAAATTCA

GCTGTGCCATTGGAAGAGAAACATATATATATGTAAAT

>c9740_g1_i1 len=238 path=[1:0-237]

GATGAACTCTGATTATAGAAAATAGCCGCTGTATTGAATTATTAAGCAGATGTATCTAAA

TAATTTTTTTATATTAACTACCTGAAAAATTGTACATGTACAAATTAGATCTTTTTCCAA

TTTGAGTAGATTTGACTTCCAGATTTTAATAATGGTGCCTTCTGTCTTATTCCTTGAAGA

TTATTCTCATCAGTCTTTTTCCAGTTAGAAGGACTAGGACTTAAATTACAGTAGGGAG

>c9744_g1_i1 len=301 path=[390:0-208 598:209-300]

GAGCTGCTGCTGACGGCTCAGTTCATCCTCTGCGTCTTCGCCAGCTTCGACGACCGCCAT

GACGGGCGCCCTGGCTCTGCTGCGCTGCCCGTTGGCTTCTCTCTCGCCCTCGGCCACCTC

TTTGGGATCCCATTCACTGGTGCTGGCATGAACCCCGCGCGGTCCTTTGCGCCCGCTGTC

ATCACCCGCAACTTCACCAACCACTGGGTAAGTGGGGAGCACTCTGTGGCCCGGCCCAGG

CCGTTCCAGCCCATCTCCGTCCTGCCCCAGTCCATCCCAGTCTGCCCCAGTCCATCCCAG

T

>c9744_g1_i2 len=264 path=[390:0-208 81:209-263]

GAGCTGCTGCTGACGGCTCAGTTCATCCTCTGCGTCTTCGCCAGCTTCGACGACCGCCAT

GACGGGCGCCCTGGCTCTGCTGCGCTGCCCGTTGGCTTCTCTCTCGCCCTCGGCCACCTC

TTTGGGATCCCATTCACTGGTGCTGGCATGAACCCCGCGCGGTCCTTTGCGCCCGCTGTC

ATCACCCGCAACTTCACCAACCACTGGGTGTTCTGGGCGGGTCCGCTGCTGGGCGCGGCG

CTGGCGGCTCTGCTGTACGAGCTG

>c9746_g1_i1 len=214 path=[147:0-213]

GATGCCTTGTCTGCTCCTTCTCTGCTCTCCCTTGAGCACAGCAAAGGTTAGGTTGCCAGA

CCCCGATCTCCCTGTGATGGCTGCAGGAGGGCCTTGGGCAGCAGTGCGGGGGCCTAGGGG

AGGGATGCCAGCAGGGATCCGGAGGGGAAGGTGCTTGTTGCTCTCTGCCTCGATTATTGG

TTTGCTTGTTGCCTCTGCAATCTCAAACACAAAG

>c9750_g1_i1 len=272 path=[479:0-249 785:250-271]

GCTTCTTGTAGCTTGAGCAAGCAAAATTGAACTTTCTTAAATGACAATAAAAATGAGAAG

TCTGATTTTTAGGTAAATAAAAACTGTCATGCTGTCCTTCTGACACTTAGAAGTCTCTAC

GACATAGAGAGGCTGTGTCAACAGTACATGTGGTGGGAGAATAAAGTTTTGTAGCAGTTA

ATTTACTGCCTCCTTTATTAACTACAACCACTCTTGTTTTATATGTAGACTCTTAGTATA

AATATAGGCACTACAAGCTGTAGCTTTTAGTG

>c9750_g2_i1 len=262 path=[240:0-261]

ATATTAAATCTCCATGCAAGTGTCCTCAGTCTGTTTCAGACAGCAAACAATAAGTACGTT

TTTTCTTGTATTGCTTCAGATTTTATCCATGTTGAAGTTGAATTTGCTAACAGTCCAAGC

TGATATTCTCTCCTTATTTCTATGTAGTTCCTCCTTTTTTTTATAGACATGGGGGCAGAT

GTTTTGAGGGTTTTTTACTTCACCTTTAGGGATTTTATAGTTTCAGTGGAGAGAAGCTCA

CTTTTAAACTCTTACAGATACA

>c9755_g1_i1 len=205 path=[186:0-72 394:73-204]

ATAAATTTGGAGACTGGGAGCTCACTTTGTTACTGCTTCAAATCCAAATCATGTCAGCCA

TAATGATATTTTTATTTCTTGTTTGCTTTTGAGAGTAGCAGAGCAAAGTTCTTACTTACA

CCTTAACAATTTTATTTTGTAGTACAGTTAACACTGTTTGCTTGTTCAGGTGTTAAGGCT

GTTGATTTTCCTTATTGATATACAA

>c9755_g1_i2 len=208 path=[186:0-72 259:73-207]

ATAAATTTGGAGACTGGGAGCTCACTTTGTTACTGCTTCAAATCCAAATCATGTCAGCCA

TAATGATATTTTTCCACACCATTCAGTACTCCCAACATGTTTCATTAAATATGAATTTAC

CAGGGAAGTCTAAAAATGTTGCACTGCCTACTCAGTCATTACCTAGTGCTGGAACACCGG

GCAACATTCAAATTTTATCCCCGTCTGT

>c9756_g1_i1 len=305 path=[2283:0-304]

AAAAAAAAAAAAAAAAAAGTTATTTCACTTACATCTAAACATTTCTTTTTTTAATTCACA

TGTACTTGTACATATAAATTATTATATGTTCATGTGAAATTGTAACAAAAAAGAAAAATA

TAATTATGTATTTTATTTTAATAAAATACTTGTACGTATTTTTTGTGTAATATTGTATTA

ATATTATATAATAATATGTACTAATAATATATAATAATATTGCAAAATTATAACTATTTT

TAAATGATAATTATTATTATCTTATCTTTCTAAAATACGGAGTCATCAAAAAAAAAAAAA

AAAAA

>c9756_g2_i1 len=1138 path=[1142:0-1137]

AAAAAAAAAAAAAAAAAAAGATGGCTGAGCACTTAGCTCGTATAATAGGAACAGAAGAAG

ATAGAGTGAACTGTCCATTTTTTTGGAAAATTGGAGCATGTCGTCATGGAGATCAATGCA

GTAGAAGTCATTACAAGCCAAATTGTTCACAAACTTTAGTTATAAGACATATGTATGATA

ATCCACCTATAGCTGTTGCAATTGCAGAAGGACAAATGGTTGACGATGAAGTACTGGATA

AAGCAGCTGATCATTTTGAAGAATTTTATGAAGAAGTTTTTGAGGAATTAATGAAGTATG

GAGAAATTGAAGATATGGTTGTTTGTGATAATATAGGGGATCACATTATTGGTAACGTTT

ATATTAAATATACACATGAAGATTATGCTGAGAAAGCTGTTAAGGAACTAAATGGACGAT

TTTATGCTGGTAAACCGTTACAGATTGAATACACTCCAGTTACCGATTTTAGAGAGGCAA

GATGCAGGCAGTTTGTAGATGGGCAATGTAGAAGAGGAGGATATTGCAATTTTATGCATA

TCAAACATGTACCTAGAAGTGTGAAAAGAAAGTTATATAGACGAATGTATAAAAAATTTC

CTGAATATAGAAAAAGAAGGAAACGAAGTGATGATTCGGATGATTATAATGATAGTTATA

GAGAAAGGGGTAGTAAAGATAAATATAAAAGAGATAGGTATAAAGAAGGTCATTATATTG

GTAGGCGGAGAAATAGAAGTAAAAGTAACGATTATGAGGATGATGCTGATAGAAGCTATA

AATATGCAAGAAGAGAAAATAGTGTAGAAAGAAGAGAAAAAATTGAAAGATGGAATAAGG

AAAGAGAGATGAAAAATATGCTAAAAAACAATAAAGATAATAAGAAGGAAGAAGATGATG

AAGATAATGATAATAATAATATAAAAGATAGTAAGGAATTAGAAATTAGAGATGATTAAA

TCCCCCCCAAAAAAAAAAAGAAAAGAAAATAGAAAAATGAAAAAGAAGAACAAAATAAAA

ATAAGAACTATAGTTATATAAAGTAATAATAATAAAGACAAAGTAATAATTTCCAAATGA

AATAATAATAATAATAAAATAAAGTATTAAAAAGCATTATAGTAAACAGCACATTTTA

>c9757_g1_i1 len=203 path=[433:0-202]

GGTGGTTAAAAGGCATATTTAGCAAGCACTGCGTTTTTCAGGCTGAATCACAACCATTTG

ACATTCAAAGCAGTCGGCAATGGCCATATTTCTTTGTCTTCAGGAGGAAAGAAGTAAAAA

GTGCTTTATTTTATTGCTTTTTACAGCACTACATTACTGTAAGGTATACTGAAAATATCT

TTGCTGTCCGTGGGCTGAGTGGC

>c9761_g1_i1 len=938 path=[945:0-937]

ATATTAAAAAAATATTAAATTTATTTAATGTTCTAGATTTTTATTTTTTCTTAAATATAT

ATTTTCTTTAAAAAAATAAGAATACTAATTGAATTTATTTTAAGGAATCCTATTTTTACA

TTTATTAAAATTTTTTTTTTATAAATATTTAACATTTTATTAATATTCTAAAATGGCATT

TAATAAAATTTTTGTATTATTCATATTCTTATCAATTTTCAAGTATTGTTATTCCCAAGA

TGTAATTGAATTAACTGATGCAAATTTTGAACAATTAACACAAATATCAACAGGGAATAC

TACAGGTTCATGGTTTATAAAATTTTATGCACCTTGGTGCTCTCATTGTAAAGCTATTAG

TAAAACATGGACACAATTAGCCGCTGAATTAAAAGATCAAATAAATGTGGCAAAAATTGA

TGTTACTACAAACTCAAAAACGAGAAAAAGATTTAAAATAGAAGGCTTTCCAACTTTAAT

ATTTTTTAAAAATGGAAAAATGTATGATTATAAAAACCACGATAGATCTTTAGAGGCATT

TAAAAATTTTGCTTTGGAAAATTATAAAAATATCAAAGCATTAGATCCTCCTTTACCTCT

TACTTTTATTGATATGCTTAAAGATTTTGTTTTTGAAACAATTGCAAATATAGACAGAAT

TTACAAATATGCCTTTCCATCTTTAGCTGTTATATCATGTATTTCATTTTTAATTGGATT

ATTTTTTGGTTTCATTTTACTAAGATGTTGCAGAAAAACAAAAACTACACCAACAAAAAC

TAGAGCATCACAAAAAAAAAAAGACTAAAATTATTAATATTTTCAATAAACGAGATATTG

AAAAAAATAAAATAACAATAATGATAAATTCAAATTGATAAATGCCAGTATAAAGAAATA

ATTTAAGTAAAATTAAGATATATAAACGAAGTTTAAAA

>c9770_g1_i1 len=379 path=[41:0-301 417:302-378]

ATTTACGAGCGTTCTTCAGGCCCATAAATGTTGTTTATAAGCAGATCAGGAAGGAGCTGA

GGTTGATCAAGCGCCTCAAACCCACCCCAATAGGCACAATGGCTTCCAGTCAAAGTCACT

CCTATGATGTTGGACAGTGTTTGCAGGCCAAAGGATTCCCCACATTTCATTTTATTTTTC

ATACGCACTTCTCAACACAACTAAAATTAAACAGCCCTTTGGGATACCTAAAAGGCTGCA

CTGATTTTGGCAACTACCTTCAGATAACAGTGCCAGCGCTTCCACCACGGATCTTGGATG

TAAGGTATTAAAGATTAATTAATTGTAAAGGTGTTAACACGCTCCTAAAAAGGCTGCCAT

CCTCATACGAAGAGTAGGA

>c9771_g1_i1 len=2345 path=[2708:0-2344]

TCATCATCATCTTCATCTTCATCCTCTTCATCATCTTCATCCTCTTCATCTTCATCATCT

TCATCCTCATCATCATCTAAATCGTCTTCATCTTCATCGTCATCTTCGTCTTCATCAAGT

TTATTTCTATTTCTTAAAAGTTTTCGTTGTTTATTTAAATAATTACGTGAATTTAACGAT

GGGTCTAATATACCTTTTTCTTTTTTTTCACTATCTACCATCATACATTCAGTAGTTAAA

ACCATACTAGCGATACTACAACTATTTTTAATAACACTTAAAATGACATTAGTACTATCA

ATAATACCATTTTCTACCATATTAACGAATTTATTAGTATTTACATCATAACCAAAACCA

TATTTATCTTTTGAATTTAAAATTATTTTAACAACATTTTCTCCATTTACACCTGCATTA

TCAGCTATTTGTTTAGTAATAACATCTAAACTACTAACAACTATGTTTGCACCCATTTTT

TGTAATTCTATTTCTGATTCTAAATTTCCTATTAAATCAAGATATTTTTTTTCTTCTTTA

TTATTATCAATTTGAAATTGTTCTTCAATTTTTTTATATATTTCTTGTATAAAATTAGAT

TTTATTATTTCTAAAAATGTAACTCCTCCACCTGGTACATAACCAATATCAATGGCACTT

TTAACTGCATTAGTGGCATCTTCATATTTGAATTTTCTTTCTTTTTGTTCTGTTTCTGAA

TTACCACCTATTAATATTTTAGCTATTCCACCTGATAATGCAGCTATACGTTCATTTAAT

TTTTCTTTATCATATTTAGATGTTGTTTCTTCATATTCTTTTTTTAAAACATTTATTCTT

TCATCAATTTGACTTTTATATTCTTCTTTTGTAATTAAACTTGTTCTATCCTTTTTAACT

ATTAAAGTATTAGCACTTCCTAATAGTGATAAATAATTATTATCAAAGCTATTCATATTA

TTACTTAAGTTGTTTAAGTCTAAACCAACATCAGCACTTATATATTTACTATTTGTAACA

ATAGATAAATCTTTTAGGTAATCTTTTCTTCTGTCTCCAAATGATGGGGCTCTTATAGGG

ACCACTTTGATTGCTCCTTTTAATTTATTTATAATTAATGTTTGTAGTACTTCATTACTA

AAATCATCAGCAATAATACATAATGGCTGTTTATTTTTGGCAAAAATTTCTAATATAGGT

AAAATTGATTGAATATTGTCAATATTTTGATCCGTAATTAAGGTTGAAACGTTACTATAT

TCAATATAATCTTTATTTTCATTATACAAAAGATATGGGTTAATTATACCTCTATCGAAA

TTATAACCTTCTGTAAATTCTAATTTATCATTAATATCTGCATTATCATCTAAAATTATA

GCAGCATTTTTACCTAATTTATCATAAGCATTTGCAATAATTGTTCCCATATGAACATCA

TTATTACTAGCAATTGTGGCAATATTTAAAATATCTTTGTAAGTTTTAATTGGTGTTGAT

AAGGATTTTATTTTTTCAATTATCATTTTTGAAGCTAATTGTATACCTCTTTGTATAGGT

ATTGGATTATGATTATTATTAACTTCTTCAATTCCTTTTTTTGTAATGGTTGCTGTCATT

AATGCTGTAGAACTAGTTCCATCTCCAGCTTTATCATTTGATATATTTGTACTTTCTTGC

ATTAACTTTACACCATTATTCTTTTTTCTATCTTTTAAAGAAATATTTTTTGCTATAGTT

ACACCGTCATTAATAATTAAAGGGCTACCATATTCCTTTTCTAACAAAACATTTCTACCT

CTAGGACCTAATGTTAATTTGACAACATCTGATACAGTTAATATTCCTTTTAATAATTCA

TTTCTACATTCATTTCCGTATATTATGTCTTTTCCTTTTATTTTATTTTCGCTCATCTTC

ATTTTTACATAATTTTCTTTTCTTCTACTTATTATTTTACTATTTATATATTTTAATCTC

TTATTTAAAAATATTCTACTTTTATTCATTCCTCTCTTTTTTTTTATACTATATCCATAT

CTCAAACAAATAAAATAAATTATAAATAAAAACACACTTATTTTCTTCATTTTATTTGTA

TTTCTCTATATAAATATATATATATTTTTTTTCAAAATTTTTTCTTTTCTATAATTTTTA

CTTTGTCAAATAATTAAAATAAATATAAATTAACATATGAAAAAAAAAAAATTAAAAAAA

AAAGTTAAAATAAAAAAAAAAAAAACGCGAACCATGGTATTTGGAGAAAAGAAAAAACAT

TGAAGTCAGAAGGAATGAATATTGTGATAGAAAACATAAAGTGTTAAAAAAAAAAAAAAA

AAAAA

>c9773_g1_i1 len=252 path=[230:0-251]

TAATATGATGCTAGCGTCATTAAAATTTAGACATGCCTTATTATTGAAACTTGAGCATCA

TCCAGATTTACCTTCCACTTTGCATTTCAAATAAACTGTTGTTTTTTTCTTCCCTCTGGT

GTAGGATCGCTACTATAGTAGTCAGTAACATAGATTAAAAAGGCATAATGTCTATAAATA

TTCTGGGTACTTGATGGGGTAAAAAAAATTTCCTGCTTATCCATTACATTCTGCTGGAAC

TGCAGTTCTGTG

>c9776_g1_i1 len=223 path=[343:0-222]

CTGGGGACATCAACATTACCAACTTTCTCTGCGCAAGAGAAAAGCTACATAAAAGGCACA

TTGGATTTCCTGGGAATAGGTCATTTCACGACTCGCTATGCCATACAGAAGAGTTCTCCC

TTCCTACAAGGGTCCAATTACAACACTGACCGTGATTTGGCAGAACTAGCGGGCCCTAAA

TGGCCAGCGCCAGGCTCTGAGTGGTTATATTCTGTGCCCTGGG

>c9782_g1_i1 len=2317 path=[45:0-2316]

GATTTTTTTTTTTTTTTCTTTTTAATATTATCATGATTAATTTATGACATTGAATATTAA

TTTTTTAAAGGGTATATCAAAGTTCAAAAATAATAATTTTTTAAAAATAATAAAAAAAAC

TATTTCATTTAAAAACATGAATACCAGCTGTGAAGTTAATATACCTGAAGAAGAGTTGAA

GAAGTCGGAAGTTTTATTTAAAGAACTTAAAGAATTAAATATAAATTACAGAGAAGTAAA

ACATGGAGTAGCTCATACTATTAAGGATTTATTAGAAATGAATTTAGATAATTCAACTAA

TATTATAAAAAATTTATTTTTAAAAGATAAAAAAAAAAAATTTTTTTTCATATGCACACT

AAATTCGAAAACAGTTGACTTAAAATATTTAGCTAATGCATTAAATACTTCTAACTTAAG

GTTTGTTGATGAAAAAAATTTAAAAAATATTTTAAATTTACTACCAGGATGCCTCACTCC

TCTTGCAATGAAATATGATAAAGATAATGTTGTAAAACTTTATTTTGACGCAGAGTTGAA

AAATATGGATGAAATAATAATTCACCCATTGCATAACTACAGTAGTTTGTACATGAAAAA

AATAGATGTAATAAAATTTTGCGATATACACAACCATTCTCCAGAATATATAAAAATTGA

AGAAGAAAAAATAAAAAAAGAAGACGGTCAAGAAGAAAAAATAAAAATAGATAATCAAAA

AAACAATGAAGAAACATGCTTAAATTTGAAAGTAAATAATAAATTTGATAATCAATCAAA

AGAGTACAATTTGCTAGGAATTACCTCAAAAAAACTGGAAAATTTCTCAGATTGGTACAC

TCAAGTAATAGTTAAAAGTGAATTAATAGAGTATTATGATATTTCAGGCTGTTATATATT

AAGACCTGGAGCTTACTATATATGGGAATGTGTACAATCATTTTTTGATAAGGAAATCAA

AAAACGAGGTGTGGAGAATTCATATTTTCCATTATTTGTAACAAAAGGTAAGTTAGAAAA

AGAAAAAAATCACATTGAAGGTTTTAGTCCAGAAGTTGCATGGGTAACTAAATATGGAGA

CTCAAATTTACCTGAAGAAATAGCAATTAGACCAACGAGTGAAACAATAATGTATTCTGC

CTTTTCTAAATGGATTAGATCTCATAGAGATTTGCCTTTGAAATTAAACCAATGGAACAC

TGTCGTTCGATGGGAATTTAAGCAACCAACACCTTTTATAAGGACAAGAGAATTTTTATG

GCAAGAAGGTCATACCGCTCATAAGAATGAAGAAGAAGCAGTGAAATTTGTTTTTGATAT

ATTAGATTTGTATAGAAGATGGTATGAAGAATACTTAGCTGTTCCAATTATAAAAGGATT

AAAAAGTGAAGGAGAAAAATTTGCAGGAGCAAATTTTACTTCAACTGCTGAAGCTTTTAT

AAGCGAAAATGGAAGAGCCATACAAGCGGCTACTTCTCATTATTTAGGTTCAAATTTTGC

AAAAATGTTTAAAATTGAATTTGAAGATGAAAATGAAGTAAAGAAATATGTTCATCAAAC

TTCATGGGGATGCACCACCAGATCGATAGGTATTATGATAATGACTCATGGAGATGATAA

AGGATTAGTATTGCCTCCAAACGTTTCTAAGTATAAAGCTGTTATCGTGCCAATACTCTA

TAAAAATACAGATGAAAATTTAGTACACTCATACTGTAAAGACATTGAAAAAGTCTTAAA

AAATGCACAAATTAATTGTATATTTGATGATAGAAGTTTATACTCCCCAGGATATAAATT

TAATCATTGGGAATTAAGAGGAATACCTATAAGAATTGAAGTAGGTCCAAAGGATATTCA

AAATAACTCCTGTGTGTTTGTAAGAAGGGATAATAATGAAAAAACGAACGTAAAAAAAGA

ATCAGTTTTATTAGAAGCACAACAAATGCTTGTTGATATTCATAAAAACTTATTTTTAAA

AGCAAAAAAAAAATTAGATGAATCTATTGTTAAAGTTACCAACTTTAGTGAAGTTATGGA

TGCTCTAAATAAAAAAAAAATGATTTTAGCTCCATGGTGTGAAGATATAACAACAGAAGA

TGAAATAAAAAAAGAAACTCAGAGATTATCTTTAAATCAAACAAATACAGAAACTACTTT

GTCAGGAGCTATGAAACCATTATGTATTCCTTTAGATCAACCTCCAATTTTACCAAATAC

AAAATGCTTTTGGTCAGGAAAACCAGCTAAAAGATGGTGCTTATTTGGTAGAAGTTATTA

AAATTTTTTTTAGAGTTTTTTTTTTTTTTTTTTTTTT

>c9783_g1_i1 len=289 path=[1:0-288]

AATATCATCAGCAGAAGAGGTGATGTGCGGAAGAGGCCCCTGCCATACAATGAATTACCA

GAGAGTGAAAAGAGATACACCTTATTCACTGATGAGTTTTGCCATATAGCGGGAAAGTAT

TGAAGAAGGAAAGCTGCTATGTGGAGTCCTTTAAGATGAGGAGACCACCACCACTATCTG

CGCTCTAAAAATGGATGAGATTCAAGATGTGAAAAGACTATACACAACACCAAAACGAGA

ACATTGCCACCTAGATCCACATTACTGGCCCCAGCATCCCAACATCAGA

>c9786_g1_i1 len=423 path=[445:0-370 409:371-422]

AGAAAAAGAAGACACGTGAGGGCTCAAGTCTCTAGTCTTTGCTCCCCGAGGACTCAAAGA

TTTTACTGTAACTAGCAGAGTGGTTGCATATGCAGCTGCATAGTGGTTCATGTTTTTCAG

CATCCCAGGGAGTTTTCCTTAGACCTTGTACCGGTTTACTCTGCATCTATGCTGAAGTAT

GACAACAAAATTCCCACAAAAGAGATAAGTGAGTACTTTTGACAGTTTATTTCTACTTCT

ACCTCAAAAGATGCAGATAATTCTGGCAAAAGAAGCATTCTATACGAGCATTTTGCTAGT

AGAGCTAATCTTTTTGAATTTTATATTTTAGCATTTCTCTTGGCACTCATAGCTATGCCA

GAACCTTCATAACACAAGTCTATAGAGCTTCACCAGAAAACTTTGGGTGTGTAATAAATC

TAG

>c9786_g2_i1 len=238 path=[224:0-177 827:178-185 409:186-237]

TTACTACTAAGAGGATGCTGATAGAAAATACAGCTTTGTTGGATTGCTTTGTTCCTTCCA

GAAGCATCTTAGTAGTAAATAATACCAGTATTCTGATAGCAGTGAGGTGCTGATAATGTA

GCTATACACTATTATTTACGTTATCTACAGTCTACTAATACATTTCAAAATTATTCTAGA

TTTATTACACAAGTCTATAGAGCTTCACCAGAAAACTTTGGGTGTGTAATAAATCTAG

>c9788_g1_i1 len=281 path=[284:0-179 13:180-280]

AAAAAAAAAAAATTCAATTACTAAAAATTAATATAAAGTGTATATATGATAAACCAAAAA

GAGTACACATTAAAAATAATGAGTTCGTATCGAATACATAATAAATTAATATATTTATAA

ACACATTCAAAAACGAAATGATAATAATAATTATTTATTAAAATAACATGTTCAATAATA

AAAATAATAGTTATATACAACGTGAATGAGCAATATAAATATTCATCTATGTTCTTAAAG

ATTTATAAAATATATACCTTTTCTTCACACAATTCTTTCAT

>c9792_g1_i1 len=234 path=[1:0-132 134:133-233]

ATTAACAGTAATTAATGCCTTGAATTACAGCGGGCTTCGAATGCTCACACAGAGATTCCC

AGCAGCAGGAAAAAGAACTGAAGAAAAATGCTGCATTAGAACAGAGGTTTGCAGTTGGGA

ACTGTCAATTTCCAGCAGTGACACCATCCTTGCCCTGGGGAATCTCCTCCCCACCAGAAA

GGGTAAAATACTTCCACATGTACTTTGCAAGAGACACAATAGTGACTGCAGTGC

>c9796_g1_i1 len=243 path=[276:0-134 410:135-242]

TTTTCTGAATAGAAGACACTCACTTAGAATAATTGCCTGAAACTCTGTTGATTTACAGGA

CAATTTCATTTACTTTGTGCTTAATTAAATACAAAGTATTGAGCAGAAAACAATATTTAC

AATATTTTTCCCTTACAAAAAAGTACAACCTTGTAAGCTTTCCATAGAACATTTGTTAAA

AGGCTATTTAAAATAACTTTTTTCATGCAGTAATTATCTCTATGTGGTATTATCTGAACA

CTG

>c9806_g1_i1 len=572 path=[776:0-571]

TACAAAACTATTATGGCTATTGCTAGGGTGCTCTGCGTTGTCACCACTACAGCCATTTCC

TGGGTTCCATGGGAACCTACTTAAATTTCCTGGAAGTGACAATTCAAAATTAAAATGATC

CTGCAAAATAAATCCAGAAAGCTGAGATTTCTTCTCATCCCAAGTTCCCTTCTGCATTGT

TTTACTGGCATTTATTACCTATTGGTGCTGTAAGTAGTAAATTTTCTACAGTCTTGAGCT

GCTGCTGCATTAAGAGAAGCTGCAATGGAGGCAGCGTGCCAAAAGGTGGATGGATGGGTT

TAGAAGAACCGTGTAATTCTGAACAACCTCTCTGTAGCTGGAGAAAAGACTCCAGTAGAG

ATAAGACCTTGAGGTTAGCATCTTGATCACCGTTACACCTCTTCATACAGCCTTCAACTC

TTGGCCAACTGTCAGAAGAGCCTCCTTGAGTAAATAGTCTCCCACTGAGCAGTTTGCTCC

CTTGCCACAGGGGCCTACGCTAGGGATCTGCCTTATGCTGAAGCCCAGCATGGGCACTGA

CAGCTGTGGTTTGCTTACTGGTTCATAGAAAG

>c9810_g1_i1 len=585 path=[1:0-584]

AGAGCCCTCCCCGCCCCCACAGAGCGAAGCACACGGCTGAGAGAAGACAAATTTAAAAGT

TTATTAAACATTGAGAATAACAGACGAAAAGAAAAGAAGATTTGGGCTGAACAGCATACA

GGATGCAATCCATTTCATGTCGTACAGGCACCGCTCCAAGAATGCTCGCAGCGATGCTGC

TGAGGAACTGCTGCCAGCTGCTGCTAGGGATGCACCCGTGGCAGTGGCTGCTCGGGGAGC

TCCACCACCGCCACGCAGCGCCGAAGGGAAAGCAAACGTTTGCTTCCCAGAGCACAGAGC

AGATGATGAAGGCTCAGCCGTGCCGACCGCTGCCTCCCGCTTCTCTCTTGGTGCACTCAG

TGCCTTACTGACCTCCAGAAAAGAACTTACTGCTCTCCAAGCTTTGCTGCAGCGCACGGA

AGTTCAGCTCACCGAGTTAGCGGTCCGCAGGCGAGCACTGCACGGTGAGGGACATGCTGG

GTCTGTCTGGGATACAGTACTGGGAAATGTCAGTGGTGCACGGATTTTTGTGATGCTGGA

CAAAAGGTTGGGTCCTTTGTAGCCATCTCCTGAAGCGTGAATCGG

>c9811_g1_i1 len=203 path=[189:0-202]

TTTTTTTTTTTTTTTTTTTCCGTAGCACTATATTAAAGAAATAATTACATAAAAAACATT

TATGCATGTTTAATATACAACTTTTTCTTTTACTATATAGATTATATATAATTTTATATA

TTTACTTGAAATAATACAAAAAAAATAAAATTTTCAATAAATATATAATATTTTTTATAG

GGAAAAAAAAAAAAAAAAAAAAA

>c9811_g2_i1 len=1523 path=[1869:0-1522]

AAAAAAAAAAAAAAAAAAAAGAGTTATAACAGAAAATAATAATGTAACTTATAGAGAAAA

TAAAGATAAAGTATTAATTTATGAATCTTTTTATTCTGTTAACATGGATAAGAAAGAACA

TATTGAGCATAGTAAAAGTAATAAGGACAGCAATGATGGTAATAAAAAGATAATTATCAA

AGATATCTATTATGATTATTTTAACAATAAGAATACAACCTCCCCTTTTCTAAATGATTT

AAATAAAAAGAATAATTCTTGTTATTGTATTTATAATAATAAAAACAATCATCATAAAAA

AATCAAAAATATGGATAATCACTATAAATGTATGTCACTACAAAAATATGAAATAAATAA

ATCATCAAAATATATGAATAAAAAATTGTTAAATAGAACAAAAAGAAACATCAATTATTC

ATTAGATAGACATTACTCTACTCTCATAATATATTATAGAATAAAAAAAATAATGAAATA

TATTACAAAATTGATATATTTAATTCATTTTTATATTTTAAGAAAAAAATATAAAAGAAA

TATTAGTTTATTACTCTGTAGTGATAATTACAAAGAACATAATAAAGAAAAAAAGAAAAT

GAAACATAAAAAAAAAAAAGATGAATATTTATGTTTATGTGAATTATCAGAATTTATATT

AATAAGTAATAGGTTGATCAATATTAATTACTTAAAAAAGAAGAAAAAAAAAAATCAAAC

AAAATGCTATTATAATAATTGTATTAAACAAACTCATATAAAACTCTCAAAAATCTTGAA

TATTAATTTTATAAAAAAGTACATAAGAATTGTGAAAATGATGAAAAAAAAAAGAATAAG

AAATCATATAATGAAAAAAACATATGTTTCAAATATTTATCATAGTAAAATTACTAAAAT

TATAAAAAATATAATTATAAGAAGTAAATATAAAAAGTATCTGAATTTTGTAAATAACAA

ATATATAGAGAATAGAAAAAACATATTTTGTAACAATGAAAAAATGAGAAGATTTAATAG

ATGTATTAATTGCAATACTTATTATAAAAAAAAAAGAAACGATCATTACAATAAAAACAA

CAATTGTGAAATGAGTAACAATATCATAGATAGCAATAACAACATTTATAATAAAAATAA

AAAGAATGTTAGTAGCAATAACAAGAGATGTAACAAAATCTGTATAAAGTATCATAAAAT

ATGCTATTTTTATAATAAAATTTATTTATATATTAATAATATTAATAAAGAAACTAAAAA

TCAACAGGGTTTAAATTATTTTTCATATATAAGAAATCATAAAAATATTAATTTAAGTAG

TACACTTACAAACTATAAAAATGAAATAAATATTAACTCTTTTAATTTAAGTGAGGAGAC

TGGTATAAAAATATCGTTAAATCGTAAAGAAGATACATATGATTTAAATGAAAAAATTAA

AAAAGAAAATATAGAAAAAAAAATATATATATATGAAAATATTAATAATAAACAGCAGTT

AAAAAAAAAAAAAAGGAAAAAAA

>c9814_g1_i1 len=815 path=[3987:0-765 43:766-814]

TTTATAAAAATTTAATATCTTTTTAAATGCAAATTGGTGCATTAAAAAATAATAACTAAC

AATTAATTTCAAAAAAATAATATATGGATTAATTGATATTGTTATTTTAAATATAAAAAT

AATGTTAAGGATAGGCTAAGTATTTTTATTATATTATATTTTTCCTAATTATAAATCAAT

TTGAATCATACTTATTTTTTATAATAAAATAGAAAATGAAATTACATAATATTGTATTTT

TATTTAAAGTGTTAATTGTAACATTTCTAAATTTTCAAAATGTTGATTATAATTTGAGTT

ACTTAGTTAATTATATAGAAAAAAATGAATGTGTAGGTAGAAAATTGCATGAGTTTTTTC

AACCTCTTTTTTTAGATAATGCATTAGATATAGAAAAAGTGACATTCCCAACACTACAAG

ATGAAATTGAAAAACTAGAAGATTTAAAATTAAAAAATTATGATAATTTTATGAAACTTC

ATAGAAACAATGAAGAAGAAAATGTAGATGAGATAGAAGGAAATTTAGATGGATTAAAAA

ATTATAACGATGAAGAAAATAATTATGCATATGAAGATGATAATAATGATATAGAATATA

GTAGAAATAATTTTAAAAGCATTAATAATATAACAAAAGATAATATTGATCAAATAGAAA

ATAATGAAAATGAAATAGAAAACTATGAAGATGAAATAGAAAATAACAAAAATGAAATAG

AAAATGATGAAGATGAAAAATATAACGATACAGAAGGAGATACAAACAATTCATTTTCTT

CTTCTCCGTTATTAGAAAGTGAATTGTTTGTATCT

>c9814_g2_i1 len=3026 path=[87:0-2976 43:2977-3025]

AAAAAAAAAAAAAAAAAAAAGATATACTTATTTATAAAACATAAAAATAGGAAAACTAAT

ATAAAAATCTATCATTAATAATATTATAAAGTGTAAAAATTTCCTTCTGATTTTTTTTAA

CATCTTCTTTATTTAAAACAAGAGGAACTTCTCCATTAGGTGTTAAAATATAAAAATCAA

TTAAATTTTTATCTGGTTGAACATGTTCTATATTTTCATATTTAACATAAGAACTACCTC

CGATATTCTTATTTTCTAATTTATTTATATTTTTATCTTCAACTATATCAACTAAATGTT

TTAAATTTCTTACTTCAATATTTTTAACTTTTAGAACAATGCAATCACTATAATAATATC

CAATAGTTAATTTAGATGGTAATATATTCTTTAATATTACTATTTCATCACCTTCATTTT

TTTTAAAAAAATTATACTGTATTAATCTATTAATTTCAGGATTATCAGTTTCATTTTCTG

TATATAAATTTTTAGATAATGTTGTGAAAACTAAACCACCATAAATAAAATATTTATTCC

GTATATCCCAATTATGTTGAGAGATTAAGTAATTAATTTTTGATAATTTAACAACTACAC

TTTTTATTTCTTTATTTCTTACTACTTTTACTACACATAAATCATCAATAAATTTATCAT

TAAAAAGGTATTGAAATCCAACAGATTCATCATTCCTTAATTTTGTAGTTCCATCATTAT

TTATATTTTTACCATCTACACTTAAAATTACATCATTTATTTTTAACCCATAACATTTAT

CTTCCTTATTAGAATAATTTATTATTTTTTCACTTAATTTTTGTTTTTTCATAGTCATTT

TTATATCTATATTATCTTTTTCATTTTTTTTGATATAATTGCAATAATCAATATCAGACT

TTTTATATTTCATTTGGTTTTCATCTACTTCAGTTACTAATATTCCTATATTCTCCTTAA

TAGCTTTTTTTTTTTCTAGATTATTTAAACCTAATGTTTCACGAATATGAGGGTTTTCTA

ATGGTTCGAATTTTACTCCTAAAAATGCATAGCCGTTATAATGATTTGTTTTATATATAT

CTAAAAGAAAATGTTTAATTATAGTAGTTGGTATAATATAAGAAATACTGTTTCCCATTT

TGTATGACTGAAAACAAATACCAACCACCTTTCCTTTTACTAAAGCTGGACCTCCACTAT

TTCCTGGATTCATTGGTGCATCTATTTGAGTTAATAATAACTTGTAATTTGAATGTTTAT

AATATTGTACATCTAATCTACTAATAATTCCTTTTGATATACTCAATTTGTCTCCACCTG

ACGGATATCCAACTGTTATTACGTCATCTCTCAAAGAAGGAAGAGAACCAAGTTCAAGTG

GTTTTATATCTTTATAAAAACGCTTATCTTGTACAACTAAAATAGCTATGTCAGCCTCAT

GTGCTACATATATAACTTCAGATTCATATTTTTTTGGGTTCCCGTGTTTTCTTATTAAAA

TTCTGGTGCTGTAAGAAATGTTATGAGCGTTTGTTAATATTAAACCTCCTTCAATAACAA

ACCCAGAACCAGTCAAATGTTTAGGTGAATTATTTTGCCATAACATTTCTATATTAGGTC

TAGTAACATCAACATATAATTTAACAACTCCTTTAAAAATCTTATTCAGAGAATCCGTTT

CTTCTAGTCCTTTTATATCAATGTTGTTGTTTTTATCTTTTTCGTTAAATTGAGAATTTA

ACTCATTACTCACAAATATTTTCTTTAAATTTTCTAAAAGTTTTTCACTTATTATGGAAT

TTTTTGAAATATTATTAGTTTTAAATTTGTTATTATTTCTTGAAATATCATTTTTTAATA

TATCACTTTTTCCTTTATTATTAAAAACCTTTATTTCATTAGTTGTCGAATAAGGATTAA

AATAGTTTGGAAAAAATCTTAAAAATCTTATAAATTTATTTTCTTTATTTTTTTTAGAAT

CATCAAAGATTGGATTATTACCTTGCTTAATAGATACGTAATATTTATCTGGTTCATCTT

CTAATATATTTTCTTTTTCATCAAATTTTTTGAAATTGTTTTTATTCTTAAATATTAAAT

TATCATGGAATAGTTTTTTTTTAATGAATTTTAAAAAGTTAGATACAAATGCATTTTTAA

TATCTTTTGCTTCATATTGATCATTAATTAATTTCAGTTTTCCTAATTTTTCTTCTTTAT

TTTTTGTATTATTTTGGAAAATTTGTCCATTACGATTTTTATTTTCCTCATAATTTCTTT

TTTCATTTATTAATGAATTATCGAAGATAATTTGTTTTATTGGATTTATAGTATTACCTT

TATTTTTTAATTTTGGTCTATTATTTTTTTTTTCTCCTAAAAATAAATCAATATTTCCAT

CTAAATGATTACTATTTTCATTTTCTTTTAAATTTAATATTTCTCTTACGTTTGCATTCT

TTTTCGTTTTTAATTCTAAATTAGGATGAAACGTAACTTTTTTTAGAACAAAGGGGTTGT

CTATTGTGTCTTTGTTACTATTTCCTTTATTACTAAAGAAAGGATTATTAAAATGAGAAA

AAACTAATTTATTTAAACCTGGATCTAATTTATGTATACTATAAGATTCATCATTCTTTA

TTTTATTATTGAACACATTTGTAAATGTATTTTCTTTTGTTTCATTTCTTTTTTTATTTA

TGTTACTATCTTTTATTAATTTATTATTATTTTGTTTCTCACTATTATTTACCAATTTAT

TATTATTATATTTTTCATCATTTTTTATTTTATTTTTTAATAAATTTAATATGTCTACAC

CATTATTATTTAATGCTCCTTTTTCTATATCGTTTTTTTTTGTATATGTTACAACTGGTA

TTTCCTTTTTTTTTTCTCTTAATTTTTCGTCTTCATTAATTAATTCTGTTTTTTCTTTTC

TATTATATAATTCATTTACTTCATTTAAATTATTTTTCAATTCATTTTCTTCTTCTCCGT

TATTAGAAAGTGAATTGTTTGTATCT

>c9816_g1_i1 len=216 path=[151:0-26 178:27-92 244:93-149 178:150-215]

GGGGTGTCCCTGCCCTCCTTCAGGGGTCCCAATCCCATTGTGGGGGTCCCTGCCCAGCTT

TAGGGTCCCCCTGCTCTCCTACAGGGGTCCCAGACACATCGTGGGGGTCCCCCAAAATCA

TTGGGGTCTCCCAAACTCATCATGGGGGTCCCCAATCCCATTGTGGGGGTCCCTGCCCAG

CTTTAGGGTCCCCCTGCTCTCCTACAGGGGTCCCAG

>c9818_g1_i1 len=202 path=[512:0-201]

AGTGGATTTTAAGATGTTTAGAAGGATGCTCAGCTACTCAGTTATTAAACACAGGGAGTG

TGTAGTTTTGAGAGTAGGAGGAATAATAAGATTTTCTATTTAAATAATGAATTTCCATCT

CCTTAAGGTTAACCCCCACGTAGTAGATTCACACCACCATTTCATATCATTCAAATGGTA

CAAACAACTGCATACAATCACT

>c9828_g1_i1 len=854 path=[1907:0-223 472:224-853]

ATCCTGAGTGCCAACAGTGCCAATGGAGTAGGTGCCCTCCTTCTTGTAGTGGAACTTGCC

GGTGCTGCGGTGCAGCAGGACGGTGTGCAGCACGGCCAGCATGGCCTCCTCCACCTGCCG

GCCCTCCACCGACACCTCCAGCACTTCAGCACGGCAGTTCATGGCGGCAGCGCGGCTGCG

GGGGCCGCTGGAGGTGCGCGCGGCGCCCACCATCCGCCGGGAGTTGTGGACCATCAAAGT

GAACGTGGTGAACCTGGCCAACGAGCAGGAGAGGCAGATCTGCCGCGAGAAGGTGGGCGA

GAAGCTGTGCGAGAAGATCATCAACATCGTGGAGGTGATGAACCGCCACGAGTACCTGCC

CAAAATGCCCACCCAGTCCGAGGTGGACAACGTCTTCGACACCAGCCTGAAGGACGTGCA

GCCATACCTGTACAAGATCTCCTACCAGATCACCGACACGCTGGGCACCTCCGTCACCAC

CACCATGCGGCGCCTCATCAAGGACACGCTGGCGCTGTAGGGCCCGTGGATGTGGGGCGC

CTCCCCTGGAGGTGCTTTCGGAGCCCTTTGGGATCTTCCATCCCTCCTCTCTGCCTCTGA

GAGCCTCTGTGAGGACCTGGGCTGGCTCTGCCCCCTAATTGTGGGGTGATGCTCCCGTGG

TGCTCGCCCGACCCAAGGAACCGGCTCGGCAGCCCCGAGGGTGGGTGAGTGGTTGCCCCA

CAGCCTGCCCCACACCCTGCCCCACGCCCTGCCTTCCTGTGGGGCAGAAATAAATTATTT

CCCCTTTCCCATCACGTCCCAACATCTGTGGGGTGGGAGGGGGATGGATAAGGGAGCCTG

GAGGGGGGAAGGGG

>c9828_g1_i2 len=854 path=[248:0-223 472:224-853]

CATTGGCACTGTTGGCACTCAGGATGTGGACTGCGACTTCATTGATTTTGCTTATGTCCG

TGTCTCCTCCGAGGAGCTCGACCGGGCCCTGCGCAAAGCTGTGGGGGAATTCAAGGACGC

GCTGCGCAGCTCAGGCAGCGACGGGATGGGGCAGATCTCCTTGGAGTTCTACCAGAAGAA

GAAATCCCGTTGGCCCTTCTCGGATGAGTGCATCCCCTGGGAGCTGTGGACCATCAAAGT

GAACGTGGTGAACCTGGCCAACGAGCAGGAGAGGCAGATCTGCCGCGAGAAGGTGGGCGA

GAAGCTGTGCGAGAAGATCATCAACATCGTGGAGGTGATGAACCGCCACGAGTACCTGCC

CAAAATGCCCACCCAGTCCGAGGTGGACAACGTCTTCGACACCAGCCTGAAGGACGTGCA

GCCATACCTGTACAAGATCTCCTACCAGATCACCGACACGCTGGGCACCTCCGTCACCAC

CACCATGCGGCGCCTCATCAAGGACACGCTGGCGCTGTAGGGCCCGTGGATGTGGGGCGC

CTCCCCTGGAGGTGCTTTCGGAGCCCTTTGGGATCTTCCATCCCTCCTCTCTGCCTCTGA

GAGCCTCTGTGAGGACCTGGGCTGGCTCTGCCCCCTAATTGTGGGGTGATGCTCCCGTGG

TGCTCGCCCGACCCAAGGAACCGGCTCGGCAGCCCCGAGGGTGGGTGAGTGGTTGCCCCA

CAGCCTGCCCCACACCCTGCCCCACGCCCTGCCTTCCTGTGGGGCAGAAATAAATTATTT

CCCCTTTCCCATCACGTCCCAACATCTGTGGGGTGGGAGGGGGATGGATAAGGGAGCCTG

GAGGGGGGAAGGGG

>c9834_g1_i1 len=240 path=[1:0-187 189:188-190 192:191-239]

GAACACTATTCATATAAAACCGATCTGTTACAAATGTTTGGGAGTTCATAGCTTACTGAG

AAAGCTAGTACCTTAAAATAAAGCATACATGAATGATTTCACTGCATTCCAAAAGAGGAA

ATTTTGATATATTTGAGACTAAAAAAGCTAGCCTGGCAGAAAAGAGTGATTCACTTCACC

TTGTATAAGTAAAACTAATTTATGCTGTTCACAATATTTGCAGAGAAAAGAGACAATAGG

>c9838_g1_i1 len=264 path=[1:0-173 175:174-263]

CGAAGTGTTTTTTTGTAACTGACTCCAGACAATAATGAGAGATGACTCGAGTTGGCTTGT

AATGACCCAAGGTTTTGTTTGGCAGCTCTAGGGTCTTCATCGCTCGAAGAAGATTCATCT

TTTATTCTATTGCGTGTGAAAATAAGCCTGGGAAGTGATAGGCTCCTGAACTTCCTGTTT

GCCATGGTCATCTGTGAAGAGTCTTCTCCTTTAAGTCTACTCCCACTCATACCTTGTTCT

GCCTTTGTTTCCTCTTTAATAGTG

>c9843_g1_i1 len=223 path=[201:0-70 272:71-222]

CATAGCACAAACATCCACCATAGATTCGCTCCATAGCTTCACCCATTATGTGAGCACTTG

AGTGCCAGTATACTGCTTGAGCCTCTTCATCCTCAAACTTGAGCAGCTCCAAGGTGCAAT

CCTCCTCCAAAGGACGGTCTAAATCCCAAACCATCTTATTCACTTTAGCAATAACAGTGT

TGTCAGCTAAGCCTTGACTAATTCCACAAGCAATTTGATAAGG

>c9843_g1_i2 len=339 path=[201:0-70 422:71-186 272:187-338]

CATAGCACAAACATCCACCATAGATTCGCTCCATAGCTTCACCCATTATGTGAGCACTTG

AGTGCCAGTATACACAGCTTTTTTCTCCTCCTCTCCTCTCTTGAGCACAGCTAAGATGCG

AGTATCTGCTGGAGGCCAGTTTTAATGCAAATCTAAAAATCAAAGAAATTTTAGTTAGCA

GATACTCACTGCTTGAGCCTCTTCATCCTCAAACTTGAGCAGCTCCAAGGTGCAATCCTC

CTCCAAAGGACGGTCTAAATCCCAAACCATCTTATTCACTTTAGCAATAACAGTGTTGTC

AGCTAAGCCTTGACTAATTCCACAAGCAATTTGATAAGG

>c9844_g1_i1 len=206 path=[266:0-116 467:117-119 384:120-205]

TTAACTGCAGGTCTGAATCGTTATTTTGAGTTCATTATGTTGGTTTTGTAACTGAATGTT

TAAAGCAGGTATACAGATGATATGATTTGCGCTTGCACTTGTTTGTTTTTGTAGCAGTCT

CCACAGTTACATGGCAAACTAAGAAACTATTAGAAGTTTGATGAGATCAGACATTTCTCA

ACAGTATTTTCAGGAATCTTCACCTG

>c9847_g1_i1 len=236 path=[2:0-235]

TAATTTTGAACTCAAAAAGTTTTTGGTTTTATTTTTCATTACTTCAAAGCATTCTGGATC

ATTAATTTTTAATAAATAAACAAAAATTACTTATGTTTATGTTTACTTAAAAAAAAAAAA

ATATATGAGAAGAAAAGAAATTAAATTAAACATATATTATTTTAATATTTTATATTTTAA

AAACTGCAATATTTATAATAGTATTATTTATTTGTAAAGATATTAATTACATTTAA

>c9847_g2_i1 len=432 path=[620:0-93 714:94-431]

AGACAAAAGTAATCAAATATATACAAAAAAAAAACAAAAAGACGATGATTTATATAAAGG

ACAAGGAATAAAATTAGGAGGAAATACATCAAATACAAATGAAGAGGAATTAAATAAATT

ACTAACATCTTCTCCAAGTGAAGTTAAGAAAATAAATATAGATGAAAATAAACCATTAAC

AACTTTACATGTAAGATTATATAATGGTAGAAAAATTTCTCAAAAATTTAATTATGATCA

TACAGTAGAAGATTTATTTCAATACGTTTATAGTGTTACACCCATTAATTTTTCACTATA

TTTTGATTTTCCTCTTAAATTAATTGAAAGAAATGAACAAACATTAGAAAATGCAAATCT

TTTAGATACTATTATAACGCAAAAACTAACTTAATAAAAATATGTAACCAATTTTTATAA

TAATAAAAAAAA

>c9847_g3_i1 len=432 path=[1436:0-431]

TGTATATATTTGATTACTTTTGTCTTTCAATGCAACATTAACTGTTTTATCTTTAGAGGC

CAATTCTTTAGGTAAAATACCTGCTTCAATATTCTCCATAAATTTTTTATTTTCTTCAAC

TTCTAAATCACGAAATTCACCATCATCTATTATAAAACCATTTTTGTATAAAGTGATATG

CCTACAGTTTTCTGATAATTTTGAACTCAAAAAGTTTTGAACTAAATCATCATCTGAATT

TTCAACTTCTAATCCACTGTTATGACCTCCTGTATAATGAGAAATTCGTTCTTTTTTTTT

TTTTTCATCTTTTCTTAAATCACTCAGTGATCTTATATTCGACATGATTCCCAAATATTA

TAATCTCCTATTTAATTTAAATATTTTATATAAAATATTTTTTTATGTTAAATGTAATTA

ATATCTTTACAA

>c9849_g1_i1 len=5627 path=[13730:0-245 1385:246-5626]

AAAAAAAAATAAAAATGAATAAAATTGCTGACAATCCTATCCCCTCATAGGTTGATAGTT

AATTCATGTTCAGTTGTGCTTATTTCTTTTGAAAAAGTATTGTCCTTTTATTATCTCAAA

AATTTAATTTTCATAAGATTATTTAAAGTTGTAATTTAAATTAATTTTAAAAATATTTAA

ATAATTCATTTTTTTTTAACCATACATATTAAAATAAATAGAAATAACACCCCCATATCC

CAAATCAAAAAAAAAAAAAAAAAAGTTTGATTAGGAATATTGTGATAATTCAGAATAAAC

ACAAAAAAAAAATATATACACAAATACAAAGGAGGAACTTATCTAAAGATAAAATGGGAA

GAAAAGCAAATAATCCGTCTACATTAGAAGAAAAAACCTTAAACAATAATGTTATTTTAG

GTAATAACATAAAAGAAAACATAGTAAGTGAAAAAAAACAAGGAGGATCAAAATTAAATG

AAAAAAAAACAAACAAAGGTACAGAGTTAACATCTAATATACAAATAAATGGAAATATTC

AGAAAAAAAAAGAAAATGATTTGGAAAATAAGAAAATAAATAATATAGAAGAAAATTTTG

TAAATAAAAGTAAAGAAAATAAAACAGAGGGAAGTAGTGGATTAGCAAAAAAGAAAAAAA

CAAATAAAAAAACAAAGAAGGATAATAATAATAAACCAAATGAAGATTTAATAAATGAAA

ATGTACAGGAAAATGAAACTAATGAAAAAGAAAAGGATGGACATATAAATGAGGAAAACA

AAAAGGAAAATGATAGTGATAATAAAGAAAAAACTAAAGATGGAAAGGAGAAAAAGGAAG

AAACAAAAAAGGATTCAAAAAAAGATATAAAATTGAAGGAGGACGAAAGAAAAAAAATAG

AAAATGAATTAAATAAGAAATTTTTAAAAATAATGAAAACGGATAATTATAAAAATATTA

ATGATAGAATGGATGCAGAAATGAAAAGAAAAACAGAAATTGAATTATTAATTAGTTCAA

TTAATACATCGGTATCAAAATCAAATGCCCAAACACCTAGACCAATTAACATGAAATTAT

CTTCAGTAGATATTGAAAATAAAATTAAGGAAGCCAAATTAAAAAAAAAGAAAATTAACC

CAAAAAATTTAGAAGAAGTTGAAGAAGTAAACATATATATTAATTATTTAGAAGAGCAAT

TGAATGTTACAAAAAATTATGAAAATTTCAAAAGTTTCCAAAATCGTCTAGTTAATTTAA

AGAAATCTTGTGAAGAAAAATTAAAAGAAAATTTAAAAAGTATAAATGAAATTAAGATAC

AAGAAAAAAAATTAAAATCAGTAGAAAAAATAATTAAATTGAAAAAGGAAAAGCAAAATA

TTCAAGTAAAGGAATCAGATGTAATAAATAAAGAATACATACTTCCAGCTGATAAATATA

CAACATTAATTAAACCATATAATTTTATGAATAGAATTCAAAGCAAACATTTCGTGTATG

TAGATAAAATAAACAATAACAATTTTGATAAAGTATGTTTATATATTACCGGATGTAAGG

ATGATGTAGAAAATTTTATCAATTATTTAAAAAACATTGATTTTACCGAAAAAAATTATG

TACATCTTAGTAATAAAGTATTTAAAATTATGCTTAATATTTGTGAAGGAAGTTTTAAAA

AAATGGAAGATGATACAAATGTTTTTGTTCATGTAGATAATGATACTCTTTATTATTGTG

GTATGAAAGCAGATGTAGAAAATTTAAAAGAACTAATAGAAAAAGCTATAAATGAAAAAA

ATTCTAACACAAAAAATGTCACAAAAAACATTAAATTAGATTCCGTTTTAGCTAGAGGAT

TTAACAAAGGTCTTTTGAAAGATATAGAAAATAAAACTAATACATTAATCAGAATGAACT

ATGATTCTAAAACTTTTGAAGCATCAGCTACCATCAAGGGAAGTAAGAATAGTGATATAG

AAGAGGCAGAAGGAAAATTAAATGATATTTTTAAAAATTTGGATTCAGAATTCATAGAAT

TTGAAGAAAGAGAATTATTTTCTTTATATAAAAAATGTTCTTATGAATTAAATGAAATAA

GAAAAAATTTAAATTTATTTATTATTCGTCATGATAACGGAATTAGTTTAGTAGGAAAAA

AAGAGAATATAAATAAAGCTTTAGAAATTTTAGATTATGCAAAAAATATTATATCCAATA

AGACAGTTAAAAAAAAATTAACAGAAGAAGAAGCCTTTTTGTTTAATGCTAATTATAGAA

GTTATATAAAATCAAAAACAGGTGCGGAGGTAAAAATATTTAACAAAAGTAATTATAAGG

AATTAAATATAAATGGAAATAAAAATGATATAGATAAAGCTTTGCAGATGATTGAAGATT

TATTAAAAAAGAAAAAGTGCACAGAAGTAGAAATAAATGAGAAGGTTATCGCTTTGTTAT

TATCATCCAAAGCACAAAAAATTAAAGAAATAGAAAAAGATACTTCTACAAGTATACAAA

TTAATAAAAGTAATCACATAGCTCAAATATATGGTCATGAAGACAAGATACATTTAGCAA

AAGAAATCTTAGAAAATTTAGTATTAACAGAAGGAAAAGAAAGTAAATTTATACCAAATA

ATTTATATGTTACAGTAGAAATGTCTGTTGAAACGGAACACATAGGAAGCATTATTGGAA

AAAAAGGAAGAACAATTAATAAAATTCAGGAGGAAACATTTGCTAAAAAAATACATATAG

ATAAAGAAAACAAAAAAATTTATATACATGGAACACAAAAAACAGTTGATGCTGCTCAAA

GAGAAATTTTAAGAATATTAAATAGAAGTAAAGATGATAATTCATATTATGATAGATATA

AACATTCTAGCAATTCTATTTCAGCTCATAGAAAATCCCATAAAAGTACAAGATCAGGAA

GATCTAATTATCGTTCTGAATCATCAAAGAACGGAGTTTACATAAACACTAATGATGAAA

AAGCTTTCCCCAGTCTCCATGATGTAACTAATATTCAATCGAAAAAAAATAAAAAATCTG

TAAATCATTCAAATACTAAAAGACAAGAAGAACAACGAGAAGTAAATGAAAATGAATAAA

AGATGGAAAATTTTATCCATTTTTTTTACATATATATATATACATTAATTTTTATCTATT

TTCAAATTAAAATATTTTCTCAATTTAATTCATACATGTGTATTTAATTATTGCATGTGT

TAAAAGAAATATAAATTAGAAAAAATATAAAATAAAAAAAAAAGATAATGTTATGAAAGA

TGTAAATATAGATAATATATTCAATTATGTAAATAATAAAAAAAATGAAGGGAAAAGCAA

AATTATTCTAATTCTTTATTTGTGTAAAAATCTCAAGAAAAAGCATATTGAGAATAGTAA

TAATGTATTAATTTTATATTTTAATTTACTACACATTTTTGAAGAGCATTTAATAAAGAT

TTTTAAAAGAAGAATTATTATTAATAGAAAAGAAAAATATTATGAAAAAAATGAATTAGA

AGAAAATTTTTCTATAAGGATTAATAAATTGCAGTTAAATTTTAGTTATATTATTTTACA

TATATCATATTTTTTGACTTTAAATAAAAACTATAGAAAAAGTTTCACATTACAACTTTT

TATTTATTATATACATATATTGACATTTATATATTTACAAAAATATATATTTCAAAAGGA

GAAAAAAATTAATGAAGTAATATTATATTTATGTTATATAAATATATTATTACTGATTCT

AATTAACATATATAAATTCAAATTACACGAAATATTAAACAAGAGAAATTTTTATATAAT

ATCAAAATATATGTTTGCAATTATTTATTTATTTAGTCATATAAAGAGAAATGGTAATGA

AAGAAAATTATCCATTTTCTTTTATGATTATATTTATAATGTGATGATAATTTTAATTGA

TAAATATTGTTTTTATCTTAAAAAAAAAAACAAAATTACATATGAAAGATTAACTACCTT

TTATAAGAATTTTATTATATTTATTTATAACTATATCAGTAAATTTAAAGTTTTTAAATA

TGTAGAAAGTTATCTATTAAGTAGTAATAAAAGTATTTTTTATAACTTAACCTTATTAAG

TATAATCAAAAATACGATAGATATACATTTTCTAAAATACAATCATAATGAAATTGATTT

AAAAAATTTTTATACTTTAAAATTTAAAAATAATCTAGAAGAAAAAATAGAAACATCGTT

AATTAAAGATCAATTAAACAATAATTTTGAGGATAATAAAATTGACATTATTACTAATTA

TATGAAATACAATGAAAGTAACAATTGTAATGATAATATAAATAAAAAAGATAATATCTT

AGTAAATGTAAATTATAAAACAATAAACTATATTATTTTTTTAAAATTATTAAAATTAGC

AGAAGTATTGTTAATAAAAAAGGATAGAATTTTTTTAACTTTATATATATATCGTTTTAT

TAATAAAATAGCTAATAATACAATAAAAGAAATTTATAAATGTATAAAAAAAAAAAAGAA

TGTATATTTGAATAAATGTGTCTATTATAGATTATTACTTTTATATGAATTTTATTTTAA

CATTCTTTTTTTTATAATTAACGAGGAAGGTAAAAATAGAAAAAAAGAAAGTAAAAAATA

TAATAAATTTCATATTTATTTTGTTGAAAATTCTTTCTTATATATATATGAAATATATAT

GTTATTGATCATTTTGTTATCCAGAATACGTAGTTTTTATAAAATATTTAATAAAAATAA

TTTTTCCTTATTAAAAAGAATTTTGTATATAAGAAAATGTAATAATTACATAATTATAAA

TAGTATTACAAAATTAATTTGTGATTGTTTTTTTAGTCAATCATTATTTTCATTTATAAT

TTTAAAATGTTTATTTAAAAATAATAAAAAATTAAACTATTTTTTACAAAAAAGAATCCA

TATATATACATCAAAAAGAAAATTTTTTGTTATTATAAATAATACCAATATATATATGAA

TAACAATAAAGGTAAAGATAAAAAATTTAAAATTAATAAAAATAAAATAAAAATTATTCA

AGATAATAATTTTAATAAATTAAAAATATTTAACACTAATTTGTATTTTGATGAGAATAA

AGGGTTTCATACATGTGTAAATAGTTATATAAAAGTAAATTTTTTGTTAAAAAAGGAAAA

TTATGAAGAAATATATTCTAATATCATAAAAGAAAATAAGAATTTAGAGTTTTATAATAA

CAATGTAACAACAGAAGAAGTTAAACAAGATAGTAAAGTTGATGGGAAAATAAATGAAGA

ATTAATTGAAAATTATTATAATGAAAATAAAATAATATATAATAAAAGATGTTCTGAAAA

CAAAGAAAAAATATATTCAGAAAATCTAAAAAAAAATGAATTGAAATATAATAATTTTAA

TAGAGAAATATATGATAATAAATTAATAACAGATTGTAATTTAATTTTTAAAAGGAGTTC

TAATAAAGTTGAAAGTGAACAGATTGAACAAACAAATTATAGAAAAATATATTTTCTTTT

ATTAATGAATAATTTTATATCAGATATAAAAAAAAAAAAAAAAAAAA

>c9849_g2_i1 len=1377 path=[1:0-1376]

TTTAAAGGCAGTTGGGCTTAGTCTTGATGTGTCGCGTCGTGACTGGCCCTTGAATGTGAT

GAGAGCTGTTCTAGAAGAACTGATGGAAGCAACTCCCCCAAATCTCCTAGCTAAGGAGCT

CTGGTCGTCATGTACTACACCAGATGAATGGTGGAGAGTTACACAGGGAAGAGCCTTAGG

GTACCAGAGAAGGTTCCATTCCGAATGACACACAATATTGAAACAGCACTTGGAGTGACA

GGAGTAGAGGGGGTTTTCAGGCTTTCTTGTGAACAGGTCCTTCATATCATGCGGCGTGGG

AGAGAAACTTTGCTGACGTTGCTTGAAGCTTTTGTTTATGATCCTCTGGTGGACTGGACA

GCAGGGGGTGAGGCAGGATTTGCAGGAGCTGTGTATGGTGGTGGCGGCCAGCAGGCTGAA

AACAAGCAAAGCAAAAGAGAAATGGAAAGAGATATTACACGTAGTCTCTTCTCTTCAAGA

GTGGCTGAGATAAAGGTTAACTGGTTTAAGAACAGAGATGAAATGTTGGCTGTGCTTCCA

AAGTTGGACAGTAGTTTAGAAGAATATCTCAACCTGCAAGAACAGTTGACAGATGTAGAA

AAATTGCAAGGAAAGCTACTGGAGGAGATAGAATTTCTTGAAGGTGCAGAAGGAGTGGAT

CATCCCTCTCACACACTTCAGCACAGGTATTCTGAGCACACACAGTTGCAGACTCAACAA

AGAGCTGTCCAGGAGGCGATCCAGGTGAAGCTGAATGAATTTGAGCAGTGGATAACACAC

TACCAAGCTGCTTTCAGTAATTTAGAGGCAACACAACTTGCAAGTCTCCTTCAAGAAATT

AGCACTCAAATGTGGCAGAAGTTTTGTTTTTAAAGTTACAAGTCTTGGTGCCTTTTTGTG

AAGCCTTGCTGATGTCATGCATTTGTGTGAATAGATGCTTTGCATCTTGCCTTAAAGCAA

AAGGGATGTTATGCAAAGAATGTAAGAGAAAGCAGGGAATGGGTGGGGAAGGGAGCACTA

GTATGCCCTGGGAGACAGAATGTAAGCAAAAAACCCCACAGAAACAAACAAGAAACAAAT

TAAAAACAACAACAACAACAAAACCCCACTTCTCAAAGTCTATTTTTGTTACAAAAATGT

AAATTTATAAATATATATATTCAGGAGTTGGAATGTTGTAGTTACCTACTGAGTAGGCAG

CAATTTTTGTATGTTATGAACATGCGGTTCATTATTTTGTGATTTATTTTTACTTTGTAT

TTGTGTTGGTTAAAATGAAAGTGACTGTTTGGCTTCTAAATACATTGAATGCGCTTTCCT

GCCAATGGGATATTTGGTGTACCAATACCCTTCAGAAAATGTAATAAAAACCAAAAT

>c9851_g1_i1 len=319 path=[339:0-318]

ATGCAGGGGATGGATGTGTCAGCCCTCAGCCAGGTGGGCAGGAGGAGAACAGAGACCCAA

TGCTGCCAGGATGGGACCCTGTGGGTGAGGTGGCTCTCACTGAGGTTCTGCAGGACCCGG

TCCCGCTCCAGCTGCGTGTCCCGGATCTTGTTCTGCAGCAGGATCAGCTTCTCCTCATAC

TGGTGCTTCAGTGTTTGCAGGCGCCGTTGGCTGTTCTCCAGCTCATCGATCAGCTTCTGC

TTGATCTCTATCTCGCAGGTCAAATCTGCAAGGTCAGCCTGGAAATTCACGGCCTTCTCC

TCTGCATCTGAGTCTGAGT

>c9864_g1_i1 len=344 path=[4:0-343]

AAAAAAAAAAAAAAATAATTTGAATATATTTTTATTTAATACTTTTCTCTTTCTTGAAAT

AAAATTTTAAAAAAATTATTAATTGAAAAAAAAAATTATTTTTTTTTAAAACATTATCCA

TTAAGTATTACATCTGCATACATTTTTTAGAATACTCAAAAGTTACCTATGTATTTAAAC

GTATTTAAAGAAAATGTCTGTTTTATTTAACGAAAGTGAATTATCTGGTTTAGATGCTCA

TAGTGCCTTTGGAAATAGTGAAGCTTCTAGTAATCAAAAAAAAAAAAGAAAATTAGATGA

AAATTCACATATAAATAAGAATAGTGTAGTAGAAGAGGAAGTGG

>c9864_g2_i1 len=2903 path=[3641:0-2902]

GAAGAAGAAGAAGAAGAAGATGAGCCATCATATGTTCAAGATGAAAATATGGCTAAAGTT

TTTGAAAAATTTTTAAAAACATTTTCTGAAAAAAAAAGTGAAGAAGATGATGATGATAAT

GTATGGAAGGATAATTTAAATTTTGATTTTCCAAGTAGTTTAGAAATAGCAAAAGATGCT

CATTATGTATTATTATTATTTTCAATTCTTCAAAATTCCTATTCGAGAAATAAAGTTTTA

GTAGTGGATATGAAACATGTACTGATGTGGGAACCAACTGACAAAAATCGTTTTGATATA

GGAAGCCAATTATATATGTATATAAAAAGGCATTTTTTAAGAATATTAGATATATTTGAG

CAAAAAGTACAAAATTTAGCGGAAAATATCAATCCTATAAAAACAAAAGAAGTTGGAAAA

GTATGCCTTAGATTTTATAATAAAAAAAATCCTATACATTCTTTAAGAAGTTTGAGATGT

GAAATGCTAGGGGAAATGATAAGTGTTCGAGGACAAGTAACAAGGACTTCTGATGTTAGA

CCTGAACTAACATTAGCAGCTTTTAAATGTAATGAATGTGGAAATATAATCAATGGTGTT

AAGCAGCAATTTAGATATACGCAACCAAGTAAATGTCCTTCAGCTAGCTGTAGTAATATG

TCTGACTGGTCTTTGGTTTTAGAACAATCATATTTTGTAGATTGGCAAAAAATAAGATTA

CAAGAAATTGCACAAGAAAGTCCACCAGGTTCTATGCCTAGAAACATGGACGTAATATTA

AGAAACGATATTGTAGATACTGTTCATGCAGGAGATCGAATTATAGTAACAGGATGCTTA

ATTGTAGTCCCTGATATTCCAACTTTAATGAAACCAGGAGATATACCTCGAAGTGTAGCT

AGACAAATGTTAAGAAAAAATGAAAATTCTCTGGTGTCTCAGGGATTAACAGGAATAAAA

GGTGTAGGAGTTCAAGATTTGAACCACAAATTATGTATATATGCATGTCAAATAGAAAAA

TTAAACAATTCTAAGAAAGAAAATTCTTTTGATGAACAAACTCAAGTAGATATTAATTGT

GAAGAAATTCTTAATTGTGATGATTTGAAATGGTTAAGAGAGATTGCTATGCATCCCAAT

ACAATAGATATATTAGCAGAATGCATTGCTCCGAAGATATGGGGAAACCTCGAAATAAAA

AAAGGAGCATTATTAATGATGACAGGAGGTGTTCAAAAAATTACATCTAATTGCAAATTA

AGAGGAGATATCAACATGTGTATTGTAGGTGACCCAGGTACAGCTAAAAGTGAAATTTTA

AAATATGTCGAGAGTTTTGCTCCAAGAGCCATTTTTACTTCAGGAAAAGGGTCAACTGCT

GCCGGTTTAACTGCAGCAGTTCATCGAGATCCGGATCAAGGGGATACAGTTTTAGAAGCA

GGAGCCTTGATGTATGCTGATCAGGGTATTTGTTGCATAGATGAATTTGATAAAATGGAT

GAAAAAGATAGAGTAGCTATTCATGAAGCTATGGAACAACAAACTATTTCTATTACAAAA

GCTGGTATACAAGCTACTCTTAATGCTAGAGCCTCTGTTCTTGCTGCTTGTAACCCCAAA

TATGGAAGATATGACTCTTTAAAAACATTTGCTCAAAATGTTAATATCCCTGCTCCTTTA

TTATCACGTTTTGATTTATTTTACACAATGCTTGACACTGTTGATATTGATAAAGATACT

AGTATCGCTAATCATTTAGTATCAATGCATTGTGGGGAAGAAGCAGAAAAACATCTTAAA

ACTCATGCAGGAAAATTAGATTCTGTAAAATTAGAAATATATTTAGAACTTAGCAAAAGA

GTCAAACCATTATTAACAGATGAAGCAAAATATAAATTAATACATTATTATGTTTCTTTC

AGAAATATTGAATATTCCCCAGGAGCTCAGAGATCTATGAGAATGACTGTTCGTCAATTG

GAATCCCTTATTAGACTTAGTGAAGCTGTTGCTAAATTAAAATTTTCTCATTTTGTTGAT

GTCAAACATGTTGAAATTGCATGTTCTATTTTTAAGGCTTCTATGAAAAAAATATCAAAT

GAAAAGGAAATCAATTTAGATGAAGAATATGACAAATCTAATAATTCATTGATTCATAAT

AAATCAAATAAACAAGATCAAGATAATGATAAATCTGAACAAACTAGAAAAATAACAACA

ATCAAAGCAAGTGAATATCAATGTATATCGGCTATAATCTTTGAAATTATTAAAGAATAC

GAATTTAATAATAACAATGAGAGTATTACACAAGATCAATTAATAGAACTATATTTGCAA

ACTTATGCTAAAGCAGAATCTAGTGAGCAAGTAGATGAATGGATTTATAAATTAAAAAAA

ATTATTAACAGATTAGTAAACCAAGATATGAAACTATTATCAGAAACCGATGAGCAAGAA

CCTACTAATATTATTTTAAGAATTCATCCGAATTATGCAGGTCCAATAATGGAAAATGTA

TCCAATAAAAATACGTATGGATATAATACATTCAAGAATTATCAAAGTAAAGATAAAATA

TCAGAGGATGATGTAGACTTTCAGGAAGATATTGACAACTTTTAAATATTTTTATTAATT

TATCAACTTATTTATCATATTTTATATTCTTTTAAACCATATGTATTGTTGAATGAAAAA

AATGGGAAATTAAATACAATAAAATGATTCTAAGAATGAGAATATTATTTTTAAAATTTA

TATTTATATTCGATTAATATAAAATTCTTTTTTTTAGATATGTATAATCTTTAAAAAAGA

AAATATAAAACTGTTTTTTATTTTGAAATATATGAACAAAAAGTTTTCATGATTACTATA

AATATAAAAAAAAAAAAAAAAAA

>c9865_g1_i1 len=264 path=[53:0-263]

AAAAAAAAAAAAAGTAGATTTATTTTTAAATTATTAAGTTACTCATAAATATTTATATTT

TATTACTTATAGCAATTTCTTCCATTTGTTTTTCCTCTTTAATGTATTTTACAGATTAAA

GATGATTTCCCTTTTTTTTTATTTTCTCCCAATTTCTTATTCATAACTTCAAATTTTATA

TATTTACGTGTACTCAACATTTCAAATTTTAGTACTTGCAAAATTACTATTTTTATAAAG

TTTATTATTAAAAAAAAAAAAAAA

>c9867_g1_i1 len=1979 path=[53:0-1978]

TGATTCCCTGATTGTTCTAATTTCTATAAATGTTAAAAAATAAGTAAAAATAAATATATA

TATATATATATCTTTAAAAATGATTTTGCATATTTTTTTAAAAGTTAATATTATAAAATT

AGTATGTGTAAAAATATATAAAAATAATAAAGTTTTAGTAATAAAAATGTATAAATATAA

ATGTTCTAGGTTAAGTGGGTTAATTATAAATTTCCAGCTATTACCATCTTTTCTACATTT

TTTTGTTCAGAAGATATATTTGGAATAGAGCATTTTGAGCTAAAATCGAGTGATTTTTCA

TCGTTTATATTTAAATTTTGATTCAAAGTAATAGGAGTATTAAAATTTTGTGAATGCGCT

AATGAGGAATTATAAAAATTTGCACGTTTAGAAAATTTAGAGCTATTTGATATTCCAGAT

GCAAAGTAGTCTTTCTCTTCATTTTGTTCAGTTGGTATATATTGTGCAGGTATAGGTACT

TCAATATAACATGGTACGTAAATAGGAACAGGAATTTCAACGTACTTTGGTTTGATTTTA

GGTACTTCTAATGTTTGTGTAATAACTAATTCTACTCCTCTTGGTATATTTTTGGAAATT

TGTTGATAACATACTTCTTGTCTTGGTTGAATAATTGGAACATCAACATATTTTGGTACT

TTTACTTCATTATATACAACTTTTTCTTTATTTTCAATTACTTCAACAACATTTTCAATC

TCTACTTCTTTAGTTATAACTTTTTTTTCTTTTTTCTCTTTTTTTGTGTCTAATGGTCTC

TCTTTAAAGATAATTTTAGGTATTTTTGTTACTTTTTCAATTGTGGTTGTTTTAACTGGA

CCAGGTATTACATTTGATTTTACATCTCTATATATATTTTTGGAATGAAATAATTCTTCA

GCACTTATGTTATCTTTTAATTTATATACCTCTCCTAAATGGTCATCATATATATTTGTT

CCATTTGGTTTAAATATTTGAGTATCCTTGTATGTACTTTCACTCATTTTTAATATGAAT

AAATTATTATTTTCAAGGAAAATCAAATTTATGTATTTTTTGTGTTTTATTATGAACCAA

CCTTTAAATAAGTATATTTTTGTGTGTAATTTACATAAAACAAATTTATAAAATATTATA

CTAAAAAAAAGACATTAAATATAATAAATATTACACTTATCAAATATTTTAAATATGTAA

ATTATATATATTTAAGTATATAAATTTGAACTTTCCTATTTTTTTTTTTAAACTATAAAA

ATAAATTTATATCATATTTCATATAAATAAAATTTATTATTCACATTAATTTAAAAAAAA

AGTTATTGTCTAAATATATAACTATTCCTTTAGTAATTTTTAATTTTAAGATTAAAACAT

AAATATTATTATTTTTTATAATAATAAAGAAAAATTTTACTACATATATATGGAATATTA

TTTTTACTTCTTACTATGTTTCTCACAGCTTCTATATTATTTTATTCTGACTTTTTCCTT

CTTTTATTTTTCTAAATATAAATAATAATAATTAAAAAAAATATTAAATACCACACACAA

CCAGCACGCACCGTGGGGGGCTTCTGGGGTGCCTTTATCCCTTTGGAAGGTCCTGGAGGA

TCTGTCCCCAGAATGCCCCCGTGGGCTGTCCCCTCCTTTGGGAGACGTTGGGTGACGTGG

GTTGGGTTGAGTTGGGTGGCGGCAATGGGGCTATGCTGTAGGAGATGCTCAAGGAGCGGG

GCTGGCACGTGGCTCCAGAGGGGTCCCCCAAGGGGTTGAATTGGGGTCTCCAAGGGTCCG

GGGTTGGTGGTGGGGTTGCAGCTCTTTGGCAAAGGATCGGGGCATGTGCTGGTCGTAGTG

TCCCCGCATCCCTCCCGGCACAGCTCCGGAGGCATTTCCGCTCCGTTCCAAATTGACAGA

AGAGGAAACAATAATTATTATAATAATAATAAAGAGAAAAAAAAAAAAAAAAAATTTTA

>c9871_g1_i1 len=298 path=[1:0-297]

TGTCTTTTTATTTGTCCTTTTATATCAAATATAACTTTTTCTTGTAAAAATGATGTTGAA

TCGTTTTGCAACGCACTAACTATTTCATTAGCAATAGTATCAGCCAAATCATTACCAATC

TTTTCTAAAGACTGGGGTTGTAAAATAAATGAAGATATATTATTATTTAAGTGATCATAA

ATTTGACTTTCATTCTGAAGCGATGATATTACATTAGTTCCATTGTTACCTCTAAAACAT

GATACATTCAATAGAGAAAAAAATAATAGAAAAGTAGATACAATGGATAATCTTTTTA

>c9880_g1_i1 len=453 path=[585:0-452]

GTGTTTGATACGACAGGGAAATGGGAGGAAACTGATGGATGGACGAGAAATGACTGCACC

ACAAAAACTTGTCTGAATGCTCCCATTAGAAAGTGAACTGTATTGCTTCCAGGCAGCATC

TTGAGATGGAAGGTGAAATCTATTTTTCCGTATAGAACATAGAAAACGTACAAGAAAACA

AAACACTCAGACAGGCAATCTGCACGAACAGAAGGAAATATTAAACTGATCCATAAGGGG

AAGTGCGTGCTTGGGGTGTCAACCAAGGGCTGATTAAATGTGAAAGGCTATGCTATAACC

CAGTCCTGAAGTGTTCTCCACAATCAGGAGAACTTCTGATTTCTGAGATAGCTTTTTTTT

TTCTTCTGTTGCCTTTGTTATTCATGAGAACTGTGTTTTCAGCAATGGAATTCCTCCCTT

GGGTTATATTTTGGGTCTATTATGCCTCTTTAA

>c9881_g1_i1 len=221 path=[191:0-220]

AGAAATACTCCTTGATTCCCAAGAATGAATTAATTTCCACCTAATCCAAAACACTGAAAA

GAACAGAAGAAATTCATCAAAGCTGTTCAATTCAAGAAGCTTGAGGACTTAATCCCCTGC

CTTGAAAAGGCCATTGTACAGCTCTTGACATCAAGGATTCCTGCATTCACACAGGTATCA

GGCAGAACTGTAGCAAATATCTGAGTTTTTGTCTAAGGATT

>c9882_g1_i1 len=1286 path=[6:0-239 246:240-1285]

AAAAAAAAAAAAGGAATTTTAAACAAGTAGAGAAGCATTTATGCAGCCATCTAGTTCAGG

ATTGTTAGTAACTTGTGCAAATTTTGCCCATATTACTTTTCCTGATTGTGTTACTAAGCC

TAATTCACTTACATTAACTTCTATTATTGTTCCTTTTGTTATAATTCCTAAATTAGTGTA

TAAATTTGATTGAGGATTAACTTTAACACTAATTATATCTAATGAAAATGTTGCTTTTAA

CTCGCTGTGATATACATTAGCTTTTTTAAATCGTAAACTACTTGGTCTTATATATCTTTC

AAACTTTGGATTTTTTCTAGTGAAGTCATTACCAACAAATGATATTTTATCAATTAATCT

TTTCCATGTTTTTTTTCTTCTTTTTCCTGATTTTACAACTCTTAACATTTCAGCTTCATT

AAGTGCTTGTATTTTAGGTATAGGTAGTTGCCATTTTCCTGCCTTATTTTTTCTTTTTTG

TTTAATTAAGTTAGTTAAAATTTGAGTATGTTTAATTTGATTTCTATCTAATAAATATGA

AGGTAATCCATTTTCATCACGAATTTTCTCAACACTTTTTACATCTTTTAGTTCATGTGA

TTTTATAGTTTTTTTAAGATTTATTTTTTCTGTATATCTTTTCTTATTATAAATTTTTGC

TTTTATTCCTCTTAATTTTTTTGCTTTCAAGGATTCTTTATGTACTTTTCTTGCTTCTTT

TTTTCTTAACTTTTCAAAGTAATCAAATCTATATCCATATCTTTTTCTATGTAATTCAAT

ATAATCATTTTGTGGCATCTTTACAGAAATAAAATAAAAAAATTAAAATAAAAAGAAAAT

GATATTATTTTGTATTGTTTTTCTAAGAGCATTTAAATTGAATATAAAAAAAAAAATTTA

CATCATTTTTTATTTTTTCTTATTAATAATTTTTTATAGTTTTACAATAATATTTTTATA

AAATTTGTAGAAAGAAAAAATTTAAGCAGCTTTTATAATTTTTTTTATAAACTTTTTTAT

TCTTATTATACTTTAAAAAATTTATTATAGTAACAGTTTAAAATTATTTAAAAAAATATA

ATATAATTTCTCCTTATAAATTTGAACTTTTTTTATTAATTATGCCAACAGTATTTTTTT

TTACATTGGAAAAAATTTTAAATGTCAACTATATGTTTATCTTCTATGAATGCGCAAAAT

AATTTTTTAAATCTATATAAGTATTATCTTATATTTTAAATAATTTAAAAATAACATTTA

AACATAATTTAAAAAAAAAAAATAAA

>c9882_g1_i2 len=1060 path=[2537:0-13 246:14-1059]

ACTTTGTGTTTGAGCTCGCTGTGATATACATTAGCTTTTTTAAATCGTAAACTACTTGGT

CTTATATATCTTTCAAACTTTGGATTTTTTCTAGTGAAGTCATTACCAACAAATGATATT

TTATCAATTAATCTTTTCCATGTTTTTTTTCTTCTTTTTCCTGATTTTACAACTCTTAAC

ATTTCAGCTTCATTAAGTGCTTGTATTTTAGGTATAGGTAGTTGCCATTTTCCTGCCTTA

TTTTTTCTTTTTTGTTTAATTAAGTTAGTTAAAATTTGAGTATGTTTAATTTGATTTCTA

TCTAATAAATATGAAGGTAATCCATTTTCATCACGAATTTTCTCAACACTTTTTACATCT

TTTAGTTCATGTGATTTTATAGTTTTTTTAAGATTTATTTTTTCTGTATATCTTTTCTTA

TTATAAATTTTTGCTTTTATTCCTCTTAATTTTTTTGCTTTCAAGGATTCTTTATGTACT

TTTCTTGCTTCTTTTTTTCTTAACTTTTCAAAGTAATCAAATCTATATCCATATCTTTTT

CTATGTAATTCAATATAATCATTTTGTGGCATCTTTACAGAAATAAAATAAAAAAATTAA

AATAAAAAGAAAATGATATTATTTTGTATTGTTTTTCTAAGAGCATTTAAATTGAATATA

AAAAAAAAAATTTACATCATTTTTTATTTTTTCTTATTAATAATTTTTTATAGTTTTACA

ATAATATTTTTATAAAATTTGTAGAAAGAAAAAATTTAAGCAGCTTTTATAATTTTTTTT

ATAAACTTTTTTATTCTTATTATACTTTAAAAAATTTATTATAGTAACAGTTTAAAATTA

TTTAAAAAAATATAATATAATTTCTCCTTATAAATTTGAACTTTTTTTATTAATTATGCC

AACAGTATTTTTTTTTACATTGGAAAAAATTTTAAATGTCAACTATATGTTTATCTTCTA

TGAATGCGCAAAATAATTTTTTAAATCTATATAAGTATTATCTTATATTTTAAATAATTT

AAAAATAACATTTAAACATAATTTAAAAAAAAAAAATAAA

>c9883_g1_i1 len=233 path=[1:0-232]

GTTAAGAATGTACTGCAAAAGGCATCTGTTTAAGTTCAGCACTGTTTCTCTTTAGATCAA

ACAGGCTACTGAGTAAGTTCATCAGCAATAAGGATTCATTTCATATAGGAACATGCTTTC

CTTCTGGAAAGTGTGCCAAACAAAGGATGAAATAAAACGTAAGATTAGAATGTTATCCAA

ATTGGGTAAATATCAACAGTTATTTTGTATTCTGACTTTTTTTTATTCTGATA

>c9888_g1_i1 len=352 path=[92:0-351]

AAAAAAAAAAAAATAGATAAAATAAAACCAAAAAGGAACTCCAAAGGGTTGGGTCTGGGG

GAAAAGCCAGCAGGTGGTGCCCCCAGGCAGCATCCTCTGCTCTGGTCTCTCTCCCTCGTC

GCTTTCATGCTGTCCTGGGTAAGCGATGCTGACGTTGGCACTCAAACACAAAATGAAAAA

TCCATTTGGTTTCTCGTGTGCTGCCTATGATCTATAAACATGGCTTTATTGGATGTTTTC

AGAAAGCTATCTGCTGGTAATAAGCATTTTATAGACGGCGGTGTCGGCTGCAACCCACAG

CATGAGCACACGGTGATGCCATGCAATAATATTAAGATTTATATGAAAATTG

>c9891_g1_i1 len=202 path=[208:0-61 270:62-201]

AGAGTGAGGAGAACGTGCGTAATGGTGAGCAGAAGCATCCCAGTCCTGCATGAGGCAAGA

GGGTCCGTTCTGTGGTGCTGGAGGGGGAGGAGGGAGTGCTAGAATTGTTTCTCCAGACTC

CCATCCACAGCTGCCAGCAAGGGCATAAATCCATTGCCTACTACACCATGTCTGTTCCCT

GCTTCTTACATGCTGTGCTCAC

>c9898_g1_i1 len=1128 path=[1090:0-742 106:743-774 78:775-802 106:803-834 78:835-862 106:863-894 1863:895-1127]

ATAATAATAATAATAATAATAATGGTTATAAAAATAATATGAATGACAGTATATATTACA

ATACTTTAAATGATAATCTAAGTAATTATTATCCACTTTCATATCATAACGACAACAATA

GAAATGATCTTAATTTTATGCATATAAAAAATAATGTAGCTAAAGATAAATATATCAAAA

TAAATGACAGAAATTATTATGACAATAAGATATATAACCAATCTATTAATAAACCAGATA

TATTTATAAAATTAGACAAAAATATAGCAAATATCAGAAATAGTAATATAAAAAGAAGTA

TACTTTCTCATAGAATAACAACAAGTAAAAATAATGGCAGTTATTACTGTGAGAAACTTT

TAAGAGAGCATAAAAATAAAATAAATAATGTAAATTACGATAATAATTCTAATTTAAACA

ATATAAATAATAATAAAGATTATATTATGTTTAAAAAGAGTAATTTATATCCTATTAATG

GTAATACTTCATACAATGAAGATAACATAAAATATATTATGAATTATGGTTTTAATTATA

ACAAAGATAATATTCCTTGTAGTAATATTAACAAGAATAGTATGAATATTATAAAATATA

ATAATAATAATAACATAATATATAAACAAAATGATATAAATAACAATAATAACAATATTG

CATATAAATCAAATTATATACATAATCATAAAAATATAGATAATATACAAACAAACATTA

ATTGCAATATTATTAACATGAAGAAAGACTTAAATATATTAAATAGCAAAGATAACAATA

ATTTTAATATTATTAACATGAAAAAAGACTTAAATATATTAAATAGCAAAGATAACAATA

ATTTTAATATTATTAACATGAAAAAAGACTTAAATATATTAAATAGCAAAGATAATAATA

ATTTCAATACAGTTAAAAGAAGTGAACAAATAGATTTACAATTAGGTGATAATTATAAAA

TAAAAGACAAAAAATTAAATGAACTTAACATATGCTATAAACGAGAAAATAGCAATGAAA

ACTACAGTAATGCAATAAAAAGTGATCTATTTAAAACAGCCCATTGTTATGAATTAGACA

AAAGTAATATAATAAAGATAATACCTAGTAATAATAATAATAATAATA

>c9899_g1_i1 len=251 path=[865:0-250]

TGTCATGAGAGTATTTGCAAACTCATTTGGAGACACACAAGGTCCTTCCCCTCCAGCTTC

TTCAGATCTGAGGAACTACTCGGTGGGGGCAGAACAAGAAGAGGCCTGGCGCAGCACCAG

GCAGCACAGTGAGATGCTGGTCACAGGGGCGTGGTAGGAAAGTCTGCAGGAGATCGCTGT

TTGCTGCAGATTCTTTGTCAAAACGCATCTGATTTTCATTTCTGCATCTGTGTTTTTTTT

TTTTCCCCTGA

>c9899_g2_i1 len=263 path=[332:0-195 527:196-262]

CAGCCCAGGCCGTTCTATGATTCTATGGTAAATTATCTTCTCCTGCTTTGCTGACTCAGT

AAGATACGGTTACATAGGATTTGTGGCTAGAAATAAGTATGTTGGCTTACCACTGGGTGA

ACTAAAAAGTGTAGTGATTTCCTTTGTAATGCTGGAACGATGAGCTGTTCTGCCCTCTCA

GTAGTGCTGTGCATGTAACATTGCTTTTGTGTATTAATAGCAGATAAACAGAAGAAAATG

ACTTTCAGGGTGTATTTATGGAA

>c9899_g2_i2 len=264 path=[332:0-195 25:196-263]

CAGCCCAGGCCGTTCTATGATTCTATGGTAAATTATCTTCTCCTGCTTTGCTGACTCAGT

AAGATACGGTTACATAGGATTTGTGGCTAGAAATAAGTATGTTGGCTTACCACTGGGTGA

ACTAAAAAGTGTAGTGATTTCCTTTGTAATGCTGGAACGATGAGCTGTTCTGCCCTCTCA

GTAGTGCTGTGCATGTGGTCCACTCTTTTTGTTCCCTCATTTTAACCGATCTCCCTTCTT

TCCATAAATACACCCTGAAAGTCA

>c9900_g1_i1 len=260 path=[328:0-135 729:136-163 19:164-259]

GAGCAGGACTTTCCTCTGCAGGGAGCTCTGAGCTCATCTGCACCAACAGGTGGCAGAACG

TACCGAGGAAAACGTGAAGGTTTCAGACCTGGGCTCTGCTGAGGAACCACTTTTTGCCCT

GAAATGTCAGATTTTACCTTGATCCTCAATTTGGGAAAAAAGTGGTTCCTCTGCACAATT

GCAGGAAAAGCACACAGACGTTTCAGCTGTGGCATTTAGTTAAACCTTCCCAGTAGTTCT

CCAACCTCCCAGCAGCCGCT

>c9900_g1_i2 len=261 path=[328:0-135 464:136-260]

GAGCAGGACTTTCCTCTGCAGGGAGCTCTGAGCTCATCTGCACCAACAGGTGGCAGAACG

TACCGAGGAAAACGTGAAGGTTTCAGACCTGGGCTCTGCTGAGGAACCACTTTTTGCCCT

GAAATGTCAGATTTTAACCCCAGTGATGAACTGTCCCAGCTGACCCTGACATCTGAAAGT

GCCTTTGGGACAGACTGAGCTGCAGCTGACCTCGGCTGGATGCCTGAAACTCAAGGAGCG

GCTGCTGGGAGGTTGGAGAAC

>c9903_g1_i1 len=273 path=[1:0-272]

GTATAAGAAATCTCTCCGTGCCCTCCACAAACAGAAGGAGAAGAACCAGAGGGAACAGCT

GGCTTCAGGCAAAGATAACTGCACATGCACTAGAAGGGACAGTGCCTCAGTGGAGAGTTT

ACCTTAATGTGCCCCTAGAGAAGGCACAGCTATTGAGGAAAAAACTATTCCTCCTCTGAA

GGGCTGAAAATAAATTGAGAAATCAAATCATGAGAGTCCCTTTTCAAAGTGGAAAAAGGT

AAGGGAGGAAAATGATGTCACAGCTGTTAAATG

>c9903_g2_i1 len=270 path=[806:0-269]

TGCGTGGCCAAACAGCAGTAAAAAAACCAATTGTTTCTAAATGCAAGGAAAGGAAGAAAA

TCCAAACAAAACCTAAATGATAGCTTTCCTATTTAGCATTCACACAGCCTTACCGAGGGT

AGCATTTCAGAGCAGGTGATGCTACCACTTACGGTACAACATTGGTCTTCAGCGAGAAAA

GTCACACCAACGTATAAGTGATGTTCCAGGCCCTCTGCCATCTTTGCGGCCCTCTGCTGG

ACTCTCTCCAGGAGATCCCTGTCTTTTTTG

>c9909_g1_i1 len=470 path=[498:0-469]

GTCTACAGAAGCCTTATTTTATACCCATGTCTGTGTAGAAGACAGCATCCTGTAGACGAA

ATTCTTTTAAGTGGCTGAGTTGTTTGAGATTAGGCTTGAAAGTGAGGAAAATGAGACCAG

TTTGACTGTAAATTGGAAAGACCATTGTCCTTTAAGATGATGCTTTCTGAGATGGTGTCA

GCTCTGAAACAGTGGCTCTTCCACTGGCAGCACACAGTCAGGACAGACAGGACGAGCAGG

AGCACCCAGTTGTTGTACTGTGGGGTAGGAAACAAGGACTTACAACAGGTCTTGAGCTAC

GTGCTGTGAGTACGCTTGTGTGGGAGTCTTCTTAGGAGTCTTTGTGGTCTGTGAAAATGA

AGGCACTTCTTAGATGTAGGGTATTGTAGTTGCTCTCGTTCCCAAAAAAAGGGAAAATGT

GTTATGTGTTCAGATGTCTTTTGCCCTTTTTTTGGGAACGACATCAAACA

>c9912_g1_i1 len=290 path=[480:0-289]

GCCCCAGCCACAGTAAATGAGCAGGGAAGAGTATGGGAACAGAGAAAGCACATACAAAAC

CTATCCTGACAAACTCCCACTGCCTGCAGCTCAGGGCATGGCCTCTTCGGTAGTAACACG

GTTGCCCAGCAAAGACAAAGCAGAAGTTGTCCTACCAGCTGCTCCTGTACTCTGGCTGGC

TCCCCGTTTCTGTTATGCCAAAAGGACACAGCTCTGATTGTCATGTCACTGCAGGAAAAG

CCCTGTGATTGACCCTGGTTTGTAGTTTGCAAGAGAATCCCAAGAGAGGT

>c9913_g1_i1 len=205 path=[183:0-130 314:131-131 315:132-204]

AGGAACAAAGCAGGATGTGTTTTCCAGAATTTGCATGTATAGCTTTAGAGTTGGAACTTT

ACTGACAAAATGGTCAACTGAGGAGAACAATTGAGATGCTTGTAGTGTTAGGCTTCATCA

TCCTATTAGCCTTTTTTTAGCCACAGACAGAAATACATAGAGTATGTGGGAACCTGCTTA

CCTGTCAATGGAGTATCTTCCTTCA

>c9916_g1_i1 len=264 path=[169:0-263]

GACAGATTTTTCCTTTTCACAAAGCAGCGCCTTTATTCCTGACAAAGGACCTCCAGCAAC

TATGGTGCCTCAGAAAAATCCAGTAGGTTGCAGACAAACACTTTAGACTCTTGCAGGTCC

CTTCGCACATTCACGTTTATAGCAGAATTTTTATAGCAGGTTTGCTGGGACTGCAGCTCA

GATTGCTCAAATCTCTCTTATCTCCAGCCTGCTATAAACTCAGTTTATGATCTGGTCAAT

TACTCTCTTGCCTTCTTTTTGTAT

>c9924_g1_i1 len=404 path=[307:0-276 160:277-300 607:301-403]

CTCCTCATTACAGGAAGAGGAGAAGAATGGACTCCTTTTTCCAAGCCCTATCCATATACA

AGGTTCTACTAACAGCAGTAACACTCCAGACTTCCATAAAAGAAGAAGAAAACCAGAAAG

TTAGAATTGTGGACTTAACAAGAGCATCAGAACTCAGTTTTGGCATGGGGGATTGCCATT

TACATTAGAATTACAATACCAGTGATGCTTTCCTATAGCACACACAGATGACAGACAGAC

CAGATAACCACACATCAGGTATAGGAAGGAAACAGCTGAGCAGCAGTGCTGCAAGCTGCT

CCTTCAAATCCACACATCCTGAATACAGGACCTCCTGGTCCTGGGGAACTCCTCACAGTC

CCATTGGCAATGCAAACAGCTTAGGCCTCCAGGTGTCTGTGGGA

>c9924_g1_i2 len=447 path=[307:0-276 160:277-300 184:301-446]

CTCCTCATTACAGGAAGAGGAGAAGAATGGACTCCTTTTTCCAAGCCCTATCCATATACA

AGGTTCTACTAACAGCAGTAACACTCCAGACTTCCATAAAAGAAGAAGAAAACCAGAAAG

TTAGAATTGTGGACTTAACAAGAGCATCAGAACTCAGTTTTGGCATGGGGGATTGCCATT

TACATTAGAATTACAATACCAGTGATGCTTTCCTATAGCACACACAGATGACAGACAGAC

CAGATAACCACACATCAGGTATAGGAAGGAAACAGCTGAGCAGCAGTGCTGCAAGCTGCT

CTGTGTGGTGACATCCCTGTGGATGATTCCCTCTGAAGCCAGGACTGCCAGAGTGTTAGG

TTAGGCTTATGCATGGGGAACTGAGAGACCCGTCTGCACTCCCCGCTAACAAAAAGTCCT

CTTAGTGCTGGACACTGTCGTTTTAGA

>c9926_g1_i1 len=366 path=[1:0-365]

TGATTAAACAAACACTAAAAGTGATATTAGAGATTTTTGAGAATGCTTTTAGTGTTTGTT

TAATCAGTGTTTGCTTTCATTCACTTGGCTGGGGTGCTGGCTCTGGGCAGATGATTCAAC

TTGTCTGTTTTTCCTGTCGCAGCAAAACGTGTTGTTTTTGCACAATCATTCAGGGTAGGT

CTAATTCTCCTTTCAGTGATGTTAGAAGAACCATTCCCAAAAATCTCCCCATTAATGTTC

AGCAGCTTTGTGAGCCCAGCTAACAATGCAGGCCAATTTAAAGGCAGCACTGCCAGCATT

CAGAGCTCCATCTTTGAACAAAAGAAAAATGAGGCAAAACAACCTGTCTTGGAACACTCG

AGCAAG

>c9928_g1_i1 len=1443 path=[1421:0-1442]

AAAAAAAAAAAAAAAAAAAACTCATTTTAATTTATTATTTTTTAAAATATATCCTCCTTT

CATGGAGAACATTTGTGTCATTTCCTAATTATGTTTTATTCTCTTATTCACATAGAGTTT

GATCAGTTTGGATAATAAATGGCCAATTTAAAAGTGCAGATTCGTATCCAGATAAATAAA

GGGAATTTATAAGTCTTTTATAAGTTTCTTGAAGGATTGTTCTATATTTAGACATAATTA

TCATTGGTTCGCTAGATTTGTTTCCACCTAATATAAAATGCTCAATTTCATTTTGATCAT

TAAATTCAGCATATATAATACTTATATGGTTACTTAGTAAAATCTGTTGATTATTTTCAA

CCAAAAACAAACTTGCATCAAATTCTTCTACAGTTGTTGTATTTTCATCTAAAGGCTTAT

TTAAAATTAATTTAAAATTTACATTTGATTTTTCTTCTTTAATTAAAAAATTCCTATCAC

ACGTAAAATTCATTAATATGTAAGCTTGATTGCCATCTCTTTTTAAACCTGAAAATTCAT

ATTTTGTGCATCTGCATGCTAAACCTAAAAATATTTTATCCGTAGTATTTATAGCTATTT

CATAAAATGTTCCTTGTAATGATTTCAAATCTATATTTTTAGTAATAACACGTAAGTTTC

CGTCATTTTTTCCTATGACTACATATGACATTAATAAAAAAATATAGAAAATTAATTTTA

TCATTTTTTCAAAATTATAATATATAAGGTATAAATATATTCTTGTTTTTTTTTTTAAAA

AATCAAATTAATTATTAATTAAATATATTAGATTTATAAATATAGTAAAATAGTTTAGAA

TTATAAATTTATTATAATGAGCTATGAAAAAAAAAAAAAATTAAAAGATTTGCATCTACT

ATATTATAAAAATATTTAGTATAATATTTCCTCTCATGTTTTTCTAAATTTTTTTTAAAA

GTTTCATCATTTTTTTTTATTAAATTTTTAGATTCCATTTCATAATCATCTACACTTACT

TCATTTATGATACTCTCATTTAAATTTTCATTCTTCAAAAATTTCTCAAATTCGTAACAT

TCGTGAATTAAATCATTATTCTCTTCATACCTAGAGGATTCTAATGTTTTAATCATTTCA

TTTAACCAACTAATATCCTTCTTTTCATAATTTTCACAAGTTTCATCAGTTATATTTTCT

GTTATTTCTGTTTCATCTAGTATTAGGTATTCAGGCATTGTTTCATAGATGTACGAAGTA

TTAATAACATTCCCAACATATTTTTCAACTAGATATAATGCTGTCTCCAAAATGACAATA

GAGTTATCTTTATCATTTGTTCCGAAATTTAAAACAGCTATATTCCTTTTATTACTATTC

ACAGAATTAGTCATATTTCTTATAAATGCTTTATATTTATTTTTTTTTTTTTTATATGTA

TAT

>c9928_g2_i1 len=369 path=[2841:0-368]

TTTTTATATATTGTGATCTTTATAATTTATTTATTTTTTTTATCACTATTAATTTATAAT

TTTTAAAAATTAAAAAAATTAATTAATGTTATTTAAGGATAAAAAATGTTTATAGTAATC

ACAAAAATAAAACTCTTTCATAGCATAAAATATATTAAAAAATTGTTTAAAAAAAAAGAA

TAATTCTAATAAATTAATTAAATTCCTTCAAACAATTACATACGTATATAACTTCTCCAT

TTTTTTATTTAGTGAAATTTATTAAATAAAAATATTAAAATGCATTAAACTATTTACAAA

AATTTTTTTATCAGAATAAAGGAGTTTAATTTCTTCTCTATATTAATCCTTAAAAAAAAA

AAAAAAAAA

>c9931_g1_i1 len=283 path=[261:0-282]

AAAAGAAACATAAACATTTGCAAAACAAGATATGAAAGATATTGTATTTATAAAAAACAG

GTTTTAAAAAGATCGGAAACATTCTTAAACACTTCTAACCTGTCTTAAAATGTTAAGTGC

TTTTGATTGGATGACAGACTACCACTTGAGATTTATGTTGTAAAACTGAAGTTACCTTTC

AGCCACCAGTTACAAATACTGGGAAATAAATAACATCTTACTCATTTTACTGACATAAAA

TAGTAGTTTCTCTTAAGCATGATTTATTTCATTGATGAGATGT

>c9932_g1_i1 len=644 path=[225:0-532 137:533-643]

AACAGATACTTTCCATCTGATAATAAGTATGCATTCTTTCTTGCTGGGAATCATTTGTTA

GTGATCTGGTGTCAAAAATAAGATTAAATTTCTACCTTGGTATGAGCAAAGACTGCTGTC

TGCTTAGCTGTGCTGCTTTCCCAACTGGGTTTTGCAAGTATGTTTTGTATAGCTCTATTC

CTGAATTGTGTAGTTCTGGGTTCAGTCTAATTTGTGATTAAGTGAAGAACTTAAAGGAAT

TTTCTTTGTTTTTGTCTGGTGAAGCTTTTTTGTTGTTCCAACCTACGTGGTTGTGTTGCT

GCAGTTGCCAGTGGCTTAATAAGAACAGATTAGAAGAAAGAACTGCTTAGTATCGATATT

TCTAAGAAGCAGATGGGACAGACCAATAGAAGAGACCAAGCACTGTATGTTCAGCCGGCA

GACCTTGCTGCTTTGCAGCAGGTGGCCGTGAGCTGCTGATGGTGAGCACCTCCCGGCCCC

CTGTCACCCTCATCGCTGAGCAGTGAAGGCTGGAGCCAAAGTCTTGCTGCTTGGAAAGTG

AATGCGCGCGTTGTCTCAGCGTGTAGGTGTGGAGGAATTGATATTTAACTCCTGTAACTG

TTCCTGCTGAGTATTTTTACAATACCCCATACTCAAACACAAAG

>c9935_g1_i1 len=826 path=[1:0-204 206:205-825]

AAAAAAAAAAAAAAAAAGTATATCAACGAATTCATAATTATTAAAAAGTTAAAAATTTTA

TATTGTTAATAATATAAATAAAGAAAAAATGACAATATTACATAATGAAGGGTGTGGATG

TAAAAGTTCTGATGAAGTATTAAAAGGAGGAGAATTTTTATTAAAATATATAAATCTTGA

AAAAGTTACAGCATTAAATGAAAAAATACATGGATCATGTAGAAAAATTTTAAAATCTTA

TGATAACAGGTTATCTGTAGATCATTGTGAAAGTGATGTTGATCATGAATTGATAATAAA

TATTCCTTTTACAAGTCCATGTAAAATTGTTAGTTTGTTCTTAATTGGTGGAGAAGAAGG

AAGTTATCCAAGAAAAATGAAAATTTTTTCTAATAGAGAAGACATTGATTTTGAAAATAT

TAATGATTTCAAATGTATTCAAGAATTAGAATTATCACAGGATTATCATGGATCAGTTGA

ATATCCATTAAAAGTAACTTCTTTATTTAATGTAAATTATTTGACTCTCTATTTTTCAGA

AAATTATGGATCGGAATCAACAAAAATATTTTATATAGGTCTTAAAGGTGTTGGAACTAA

TTATACAAGAAAACCAGTTGAAACTGTTTATGAAGCATCACCAAATTTAGCTGATCATAA

AATTGAAGGAAACGTAAATTCAACACATTTCTCATTCGATGCCTTTTAAGTGTGTTAATT

CAAATAATTAAAAAAAATAAATATATAAATAAGGTTAATTATTTATATATTCTTATTTGC

CTTACTTTTGTTAAAGAGTTTTTTAAGAAAAGTTTTTTTTTTTTTT

>c9936_g1_i1 len=423 path=[401:0-422]

AAAAAAAAAAAAAAAAAGACATAAGAAAACCGTGTTTATAATAGGATTGTGGAATAACCG

ATCCGTCCTATGTATTTCATCTTCCATGTGAAACTCAGTAGCACAGCAATGCCGAGCTGC

ATTTGCTTAACACCAATCTTTGGGTTTTTCTTTGCACTTTCACACTTTTAAATGCAATTT

TAAAAGAGCCCTGTACCTGTTGGGTTGATTAAAGGGGAGGTGAACTTTTTTTTTTATGTG

CTACAATGAAAATGAAAAATAAATAGGAAAAAGCCTATATCAACCAAAGCTTATGACATG

ATGTTAAAACGTATAACCTTGGGCAAAAGGGGCCAGTTATTGGAGTAGTTCTTGAGTAAC

CTAATTGTTTGATAAGGATCCTTGCCTGTTTAAGTTGTTGTTGGTTGTTGGAACTGCTTG

TGA

>c9937_g1_i1 len=271 path=[284:0-183 550:184-270]

GGTATTTTGGACACTTCTGGAGCAAGATCTCAGAACAGTGTACAGGAACAGAACATTAAT

GCCAACAGACAGTAATTCCAGGTTTAAGTCGGGAAGTGATTTCAGACTGCACCGATGTGC

AGAATTTGTTTTGGCAAATTCCGAACTATCATCAGTTACAGAAATTCACATGAATAATTC

CAATGTTTGACAAGAAATTCGAAAATGTACAAATACTACAGCAAATACGAAAACACTGAA

AATAACACTGAGGTGAAAAAAAAAAAAAAAA

>c9938_g1_i1 len=272 path=[189:0-271]

AAATGCAGAATCCTAGGGTTACTCTGACCAAGAGCCAAGCGTTAAAGGCAGGAATAAGCT

GAGCAGCTTGGCAGATTGAGGTAGACAAGAAGCTGAATGTGAGTCAGCAATGTGCTCTTG

CAGCAAAACAGTCTGTGTCCTGCCCTGTTAGCAGAAAGTGACCCATCAGCTCCATTAGGT

ATTTGTAAGAACATTTGGAGTGCTGTGCCCAGTGCTCCAGCTTGGGGCCAGTTTGGGCAC

CCGTGCAAGGGTGCAAAAGCATGCAGTGTATG

>c9943_g1_i1 len=202 path=[1:0-201]

TGAGCTTGCATGCAGCCTGGAGGGAAGCACTGCTGGGACAGGCGGGGTTCAGCCTTCTAG

GGGCGCGCCATTAGCAGGTAGAGGATGAATTATAACGTGGTACTGAAAACAACACTGGCA

ACAGGTTGCTATCAGGCACGTGTCAGACACCTTAAGTTCAGTTTCCCAACTAGCACAGCT

CACAGCGTACTAGAAAACAGAG

>c9946_g1_i1 len=452 path=[1:0-451]

GCTGGTACACCACAAACTGAGGATCTGCATGGCAAAAAACAGTGTGTCACTTTGCAAGAA

ATCCCCTTTAAGTGTCAAAAAATGCAATCAGAATCACAACCATAACCTACAGCATTTTGA

AAGTTCAGTATGTATTATCAGTCAAGCACTGCAAGGCAAAGAAAAAAGCTTCACAGGACA

TCACTTGAAATAACCTCATACATTTCTGTATTTTGTAGCAATTTGTTCACACAACTAGAG

GATTCCTAGAAATTAGCATGTTCTTTATAGCAGAGTACAAAACAACTACGTATATAGTAA

ATTCTTGATTTTTCCTCACACATTTCCTTTCATCTGCGTGTTAGATGGAAATTCTCTTGT

CTTTTCTCCATGCTGCAGAACAGGCAGTGATGTCAGAACAAACTGATTTCTACTCTTACA

CAATCAGAGTGCAAGTTCCGCCACTGAACATG

>c9946_g2_i1 len=274 path=[1110:0-273]

GGAATCTCAACTGACATCACCCACACTATCTTGCTGAGCAGCCTGTCTTTAACATCGTAC

TGATCGCTCTCAAATCCTCAGCAATGTGAAACCTGGTATCCTTTCTTTTCTGCCTGACAG

ACACAACTCAGCTCTGGGACTTTAAAACAGAAAACTACACTGTGGCTGGCACTCTGCTGG

TGGCAGTTTACAGGACAGGCTTACGGGCTTTGTCTGTATAGCCTGCTGTCAGTGCAGATT

GATTTATGTATTACTGTTAAATTTCGTCCTTGTT

>c9949_g1_i1 len=218 path=[179:0-217]

GAATATTTCCTTGCTGATATCATAGCATGTCAAGATGAAGAAAATCAAGCTGTTGTTTGT

GAGGATGGTGGCGCTGTAGTGGGATTCATGAGCTTATGCTCTCAAGTAGACGTTTCCTTG

TTACGTGAGTCTTTTGATCTGCGGCTTTTCCATGGACTCTGCAAACCACATCCTGAAGAT

GTTCTCCAAGTACCAAAGGAGCGAAGCATTCCAGAAGG

>c9952_g1_i1 len=261 path=[1:0-126 128:127-260]

TTTTTTTTTTTTATTTTTTTTTTAACATCTGAAAATATTTTAGTTCTTTTAAGATTTTTT

TATATTTTTATAAAACGTAACAAAAAATAAATTAATAAAAATTATAAATCATTTATTTTA

AAAATAACATTAAGAATTACATTATTTTTTTTTATTATGGAATTATGATATATTTAAAAA

AAAAATTGTTTATTCCTTTAATTAATATTCTAAAAGGATGAATTTTTTTAACATTCCTTT

TATATATATTTTTTTTTTTTT

>c9953_g1_i1 len=208 path=[186:0-207]

ATGGAGGCCCTGGCAGAGCAGGTAAGCCCTGGAGCTTTGCTCCAGCTTCGCTTTCCTTCT

TTATCCTCCCCACTGTTTCACGTTCCCCATCCATTCTGTGCTGGTGAATCCCTCAGAATG

GTTTTAGTCCTTTGATGAACCGCTTTTCTGATGTCTCTGAGGGAGCAAAGAGACACCACC

GTACCTCTGAGCTGCTGCTGTTGGGGTG

>c9954_g1_i1 len=231 path=[423:0-230]

AAAAAAAAAAAAAAAGGTATTTTTCTGTCAATATCTTTTAATACTTTTCTTTAGAATTAA

GAAGTTTTCTTGTATATATTATGATTATTTTTTCCTTCTTTAATTATTATTTTCATGTTA

GAGTTTTTTTATTATATCTAATTATCAGTTTTATCATTGAAAAGGGGATTTCATTGAACT

TTCAATGGATAATAGTTAATGTTCTTCTTTTTTTTAAGTATTTTTAGTTAA

>c9956_g1_i1 len=258 path=[1:0-257]

AAGCAAATCTGTCTGTGATCAGTGCTTTATGAAAGCTATGAGATACTGCAAAGGTTGGGT

TCAGCAGGCCTTGCTCAGACAAAATATGCAAACTACTTTTCTAACTGATTGCCCTCTGCA

ATACTGTGTGAGGCAAAACAGGACTCAAGCACAGCTACTTTGTTTGAGGGAGACCTGTGA

GGTGAAACAAAAAGATAAAGGCAGTAGGGATGCTCCAGCATCCATGTGAAAGCCAGGTAA

CATAGAACCAATAGCGTA

>c9960_g1_i1 len=207 path=[1:0-77 399:78-206]

GATGATACTAGTAATGGGTGATTTTTTAATAGTTCAACATTTTTGTCATGTCATTTCTTC

AATCCCTATGTTCCCTAGTTCATGCATTAGAATACCTCATTATGTTAGTCAAATATTTAC

TATATGTTAAACACAGAGCAAGTTTATACTTAAACACTCAGCATTACTCTAATTCTTTTT

CTGACAGTTACATCTCTTTAGAAAAGC

>c9965_g1_i1 len=428 path=[547:0-335 880:336-427]

CACCTACAATTATAGAAAAACTTAAACAAATGGATTAGTATTTCTTGAAGCCTGCAACTC

CCTGCTTATTGCTAGTGCACAAGAAAACATCTGCTAACTCTCATCCTTGCATCTTCAGAG

TAACTCGGAGCTCACGTACGTCAATACTATTACTTGAAAGATGTGGAACAGCGCAGCAAA

GTTATCTATTCCTCAAGAGCTTCAGAAGTCCATCTACTGCCAACACTGGGCAAGTTCATC

AACAAGGAGACAGGCAGAAAACTCAAAACTGAAAGATTAAAACTTCACTACATGAAAAAT

CCCATTCACGGCATTTTGTTTACATTTGCAGGCTCTGCTTCTCAGAAGATCACTGTCCCA

CAACACACATTCCTTTTGGAAACGAGATGGAACCGTCACAAGTTCCAAACAGAGAGGGGT

TCGCTGAT

>c9965_g1_i2 len=428 path=[547:0-335 77:336-427]

CACCTACAATTATAGAAAAACTTAAACAAATGGATTAGTATTTCTTGAAGCCTGCAACTC

CCTGCTTATTGCTAGTGCACAAGAAAACATCTGCTAACTCTCATCCTTGCATCTTCAGAG

TAACTCGGAGCTCACGTACGTCAATACTATTACTTGAAAGATGTGGAACAGCGCAGCAAA

GTTATCTATTCCTCAAGAGCTTCAGAAGTCCATCTACTGCCAACACTGGGCAAGTTCATC

AACAAGGAGACAGGCAGAAAACTCAAAACTGAAAGATTAAAACTTCACTACATGAAAAAT

CCCATTCACGGCATTTTGTTTACATTTGCAGGCTCTTTGCCTTCTGTGGCGCTCATTTCA

CACTTCACCTTTCGGGTCAGGCAGTGGGAGAGGCTGAGAGTCCATCAGCGAACCCCTCTC

TGTTTGGA

>c9969_g1_i1 len=219 path=[99:0-78 339:79-79 340:80-218]

AATTTACTTGCAAGTCATATAATATGAAGAGAAATTTAATTAGAGCAATTCTGTTTTGAA

GAAAATAATTACTCTAGCTACTTCCAAAGTGATGGGCGTGGAAAGAAACAAATGAGAAAA

AAAAACCAAAAAAACCTCACTGTACAAAATTCTTTATTCTTTAGAGACCTGTACAACTTT

AAGAAACAGTTAAATGAGTGCAGTTCTTATTAGGAAGAA

>c9972_g1_i1 len=259 path=[237:0-258]

TGAGCTGAGCCTGAACCCAAATACGGAACCACGCTGCTCCCGAAGGAAGCATTCTCTTGA

AGGTTACAAGCGACCTTGGGTGTTTTTCACAGCGCAGCATCAATGGACAAAATCCTTCCC

ACCACAGAGCCATCTCCGAATCCTAGAATCGCAGACTATTACAGAGTCATCACTGCTCTA

CATCTGATCAAAACAGGAAGTTTGCACAAGTATCCTGTGAGAAATCTGCTGCCTTCAAAG

GAAACAGGAGACCTGCAGA

>c9972_g2_i1 len=373 path=[473:0-372]

CAGCAATTTGCAGCGGAAATGAAATTAACTTTGTGCAGGTGGGTTCAGAATGGCAGTTGA

TGGTGAATGGGAGCTGCTTGAGGGACAGACTTCCAAAACTTAAAGCTTGTGGCTGACTGT

GTTGAAACCTTCCTCCAAAGAGATGCTCTTCACTTACTTCTTGTTTTCTCTCTGCCAGAA

GCTGACGCCAGTGGCCTTCCTCCGAAGAGCGGCCTTGCAGGAAGTCTGGGCAAGAAGCCT

GGGAAGGCACCAAAGAGAGGTACAGATACTCAACAGGCATCTGGGCTTGATGCACTGTCT

TTCTGCTTAGCTGTTCTGATTGTTCTTTCCTGGGACTGTTCCCTGCAGTTGACATCCCAA

CTTCCTCCCATGG

>c9973_g1_i1 len=331 path=[741:0-24 458:25-330]

CCTTCCACTCACTTCCATTCTTGCAAGAAAAAAAGGAAGTTTTATTGAGTTTCATAAATG

TGAGCTCTGTGATTCACAATCTTGAAATAATTTCATAGAATTTTGTAAGTACTTGGACTT

GGTTGCTTAATTACACTAGTCTCTGTTGATCTTGCTGCAGAACGTGCTTCTATACTGACA

GCTAGTGAACACTATTATTTGGGATCTCTTCATGGTAATGAAAATTCTCCAGAGGCATCC

AGATATCCCAAGATGTTTTTCTCCTTTGTCATGTAATGAGCAATATTTCCATGCTTTGCT

TTACTTTCTTAGCTGACCTAAAGCAGGGAAA

>c9973_g1_i2 len=393 path=[371:0-86 458:87-392]

AGTGGAAGGAGATAAACTGTTTGCTTTCAAGTTTTACAAGTTGCTGATGCTTCTGTAAGT

AGGCACAACTTAGTTCACTGATATGCCAGAAAAAAAGGAAGTTTTATTGAGTTTCATAAA

TGTGAGCTCTGTGATTCACAATCTTGAAATAATTTCATAGAATTTTGTAAGTACTTGGAC

TTGGTTGCTTAATTACACTAGTCTCTGTTGATCTTGCTGCAGAACGTGCTTCTATACTGA

CAGCTAGTGAACACTATTATTTGGGATCTCTTCATGGTAATGAAAATTCTCCAGAGGCAT

CCAGATATCCCAAGATGTTTTTCTCCTTTGTCATGTAATGAGCAATATTTCCATGCTTTG

CTTTACTTTCTTAGCTGACCTAAAGCAGGGAAA

>c9979_g1_i1 len=206 path=[391:0-90 48:91-114 142:115-205]

TCTCTCCTCGGTTTTGTGTTTGAGTCTGGAAAAGGATTAGCCTAAGGAACACAAAACCGA

GGAGAGACACGACAAAGAAAAAGCAATAGTTTTTTTCCACAGCCATTGTTGATAGAGCAA

GGAATCGTAGCAAGGAAAGCACAGTTGAGTTACAGAGGAGAGCAAAACTTTACGCTGACA

AATACAGCGAGGGGCAGTGATTGCCC

>c9979_g1_i2 len=206 path=[391:0-90 481:91-205]

TCTCTCCTCGGTTTTGTGTTTGAGTCTGGAAAAGGATTAGCCTAAGGAACACAAAACCGA

GGAGAGACACGACAAAGAAAAAGCAATAGTTGTTCCCTTGCCTTCAAAGGAAGATGTACT

GCTGTGCCAGCATGTTCCCTAACATGCTCATCCCCATGTACGGAGGGTCTGCAGCCTGCC

TGGGCAATCACTGCCCCTCGCTGTAT

>c9980_g1_i1 len=275 path=[253:0-274]

AGGCTCTTCGTACAGTAACAGGACTTATCAAGATATGCAGGTCAAAATAACAGGGATATG

TAACTGCTTTTGAAACGTCTGTTCAGTAAAGGGGTTGTGTATGTACTTTGAAAACTTCTG

TTTTAATTACTGATTTCTTTAAAGTGCTGTAGATGCTACAGTTGAAAACACTGAAGATGA

GATGGCTGCTCTCCATTCGTAAACTTCAGATGTGTTTTTAAAAGCTACTTGTGGATGTTG

GATGGGGAGGATGTGGATGGGGAGAAGACAGTCTG

>c9989_g1_i1 len=214 path=[1:0-213]

AAAAAGTTGTGTGTATTTCTAACATTCTTACCCGGCTTGCATTATCATTATGTACCACAA

ATCCTGAACGTACACGGTCTCTATGAAGCAAAAACAAAGCAGAAAATATTTCTTTAAAAG

ACACATTGCTCTTAAAAGTACACTGACCCCACTTGCTGCTATTCTCTGTGGGACTTTGGC

CTTGAGTATAGCAGAGAAAGCAAATGCTAGATAT

>c9996_g1_i1 len=273 path=[487:0-272]

AAACCATGTGCAGTCAGCCCAGGGGTGGACAAGAACCAGGGATATGACCTTTCCAGAGTG

TATAGGTAGATTTTACAGAACTTCCTAGAGTGGATCATTTTAAATATTTATTAGTCTTAG

TCAACCATCTGTCAGGATGGGTAGAAGCTTTCCCTTCAGTATCTGCTACAGCAAATGTGG

TAGCTAAGGTAATCCTGGAACAAATAATTCTCAGATATGGGATTGTTGAGACTCTGATCA

AGGTAGTCACTTTACATCACAAATATTGCAAAG

>c10000_g1_i1 len=220 path=[460:0-219]

AAGTGTAGAGACATCACCTGTGACACGTGTCACACTGCACTTACTGCTTGTGACAGTGGA

AGCCTTAACTGTCACTCTGTAAGGTGTTGGGCAATGGCCTCAAGAGCCTTGCTCCCAAAG

CTGCATTATTACTCTAAATGCAGTCTGGCATGTGTTCAGATTTTATTCTACTTTGGTTTA

GCACAAGTTGTGTTTATTGTTCTAAGGTACTTGAAAATGA

>c10003_g1_i1 len=239 path=[272:0-171 50:172-238]

AAAAGAAAAGAAAAGAAAAGAATGGCTGAACATCATCTTTTCTATTTTGGTAAAGCGCCC

TCCTCAGAAGGAAAGAGTTGATGTTTTCATTTGCCACTGGAATACTTTGGCTTTTAAAGA

CATTCTCAGGGGTTGTCAGCAGAAATGAAGTTTGAGCTTTTTGTTTGTTTTGCTTTCCTT

GGCCAGGACAGTTTCTTCATAACCTGTGTTATGGTAAAAGCCATCATAGAGGATGCTTG

>c10008_g1_i1 len=326 path=[458:0-325]

GTGTCACAGGAAGAGTTCTTGCTACTGGTACTTGCAGAGTCCTTAGACACTTGAAGATAG

TTTGGAGGACCCAACGGAGGTGAAGACAAGTTTTCTTCTTGAATATGCTGCATGTCTGGA

AGAGATGGAGGAGAAGCAAAACGGCAGGAAGGTGAGCTGCCACAGGAACTGCCAGAGACT

GAGCCTGCATATGTGGGCACTGGTACGGGACAGAATATTTTATTGTCTTATGCCAGCCGT

AGGAAGTCAAGTGTCAGTGGCATACGCATCAAAATAGCTGACTTTGGTTTTGCTCGTTAT

CTGCATAGCAACATGATGGCTGCAAC

>c10016_g1_i1 len=240 path=[1:0-117 582:118-205 207:206-239]

GAAGTGATACAAGTGTTAGATTTGTGACCTGTAACCTATCCTTTTGTTTCAAGTCTTTCT

CTACTCTTAAATACATTTACTTTGGGGACTTCTTTCTTCTCTAGCTTTTCAGCAAGGTGA

GCTTGCAGTCACCTGCTCTCATAAGTAAAGACAGAAGGGAGGGGTTTTAAGTAGCTTCTC

AGTCACTGTAAGATCTCCAAACTGTTAGAACATTCTGCTTCAAGGGGAAACTAAGGCAAA

>c10016_g1_i2 len=239 path=[1:0-117 582:118-205 273:206-238]

GAAGTGATACAAGTGTTAGATTTGTGACCTGTAACCTATCCTTTTGTTTCAAGTCTTTCT

CTACTCTTAAATACATTTACTTTGGGGACTTCTTTCTTCTCTAGCTTTTCAGCAAGGTGA

GCTTGCAGTCACCTGCTCTCATAAGTAAAGACAGAAGGGAGGGGTTTTAAGTAGCTTCTC

AGTCACTGTAAGATCTCCAAACTGTTTGCATATTTTTGCCTTAGTTTCCCCTTGAAGCA

>c10022_g1_i1 len=314 path=[1:0-313]

TAGCTGCCTTAGAAAAAGATTATGTTGTGCTAGCTGTTAGGCTATCTGCACCAGCTTCAA

ATAATTTAAGGAAAAACACTCAATAATCACAAATCAACACTATTTACACAGTGGCTGCAA

GCTCTAGAATGTAGCAAAATTTATAGAAACAGCAGCAAAGCAAAGAATGCTTTGAGAATA

ATCCTTCCAATATATATCATTACTAATAGATATATTTGTAAATGTATCATTACAATTGCC

TCCAAAGATTATTAGGCTACTGAAATTTTCTTTCAGGAAAGATCTTCAGTCTCCAGGTAA

GAATATTTAACATG

>c10022_g2_i1 len=233 path=[583:0-232]

GCTGTGCAGTTGTCCTTTATTGACTTGTGTAAGTTATTTTCATACAAAACAACACTGAGT

TACTGTAAGCTTCCCACCTACCTCTGCCCTCACGCAACCGAAAGTGAATTTTTAGCGCTG

ATAAGACAAATGTTTGAGGATGTAGAAGCTGTTTACTGCTCTTGTAATGAATTTGCAGTA

TGCTACTTTAACAAAAATAGACATTATTAGTGTACGTAAGCAGCATGCAGACT

>c10023_g1_i1 len=238 path=[1:0-237]

CGGATAGATGTGTTGGAGTAAAACTTCTGGAGAGGAAGGATGAAGACTGAAGCACTCTCT

ACTCTGAAGCTGCATGTCCTCATTGCTTGCTACTTCTAGCAAGCCTTTAAGTCAGACTGT

ATCCACATCTATCCGTTTGTTAGATACAGGCTAAAGAATCTAAATTTAGACATTTCCACG

TAAGAATTCAGGCACTGACTCTTTAACATATGGAAATATGACATACTACTGCACAGAG

>c10030_g1_i1 len=223 path=[1:0-222]

AAATAATTAGTTGCAGGGACCTAGCTTCCCCCTCCTTTTAAGGCCTAATTTAGGTTCTTT

ATCAAAATGATTTAAAATCCCTTTGGAAAGCCACAGCTCTTGAACCAGCACTGGGAAAGA

CTGAACTTTCATTTTTAGAGAAGGCTCTCACATTCCTACGATCTGTAACGAGAGGTACTC

AAAATACAGCCAGGTGAAGCTTCCTCTGCTGTAAATTTCCAGA

>c10033_g1_i1 len=232 path=[217:0-231]

GATTAACTTAATTTGAAAGTCTAAGAAATAGAAAACAGTAATCACAACATTGAAATTGAA

ACAAGGAGATACTAAGTCAAAATTAAGTAACCAGTGCGTTAACATTTTAATGTACTGGAA

CTTAAACTTGATACCGGCACCTTCATCAAACCACTTGAATTAAAGCGATTAAAACTAGCA

ATAAAATCAACCACTGCTGCTGACTACGTTGAAAAATGATGTTTTATAGCTG

>c10034_g1_i1 len=303 path=[155:0-302]

GAGTGAGGCCTCGTCCCTCTTGGATTCTTTCTATGTATGGAGGGAAAAACATCAGTTCAC

ACAGCAACATCATCTTCAGCAATGGCGGCCTGGATCCGTGGTCTGCAGGTGGGGTGACCC

AGAACGTCACTGATTCCCTCGTGGCAATTGTGATCCCAGATGGAGCTCATCACCTGGACC

TACGTAGCCGCAACCCTTGTGATCCCAAATCTGTGCAACAAGCTCGGGCTTTGGAAGTCC

ACTACATGAAGCAGTGGATTGCAGAGGCCAAGCACCAGCACTGAAGTGGTACTGTAACAA

CTG

>c10037_g1_i1 len=253 path=[401:0-252]

TTAACTCCATTAGTAGGGAAAGTTTCCTTCTGTGTAAGAAATGGCTTAGGCTGAAGCTGT

CACACGCAATAGGCTACCCTTCCTTTGAGTATTTTCACACTATGACTAGCACATCATCTG

CTTATGGAGTTAGCATTTGCTGTAGTGTGCTGCTACAACCTGGTGTGTAAACTCACTTTA

AATTTTGTAAGAAGGGGCTCTTCCAGCTTCTGTATTCTTTTACTAAAAGCTAAGAAGGCA

TGGCACAAAGCTG

>c10042_g1_i1 len=358 path=[1:0-357]

TCCACGCTGCTGAAGCTCAGCGCCACCGCCCTGCTCACCACCTGGAAAGCCGAAGCCGAG

AAGATCCTCCAGACCGTCTTCTTTGTGGCCAAGTGCCGGCAGCCCTCAGTGGTGCTCATC

ATGGAGGTGGAGTCCTTGCTGGTGGCCCAGGATGGCAGCCAGGCTGGAAACCTCAAGTCT

CAGCTCCTCTCCTACCTGGACAACGTAGCTACCTCGGCCGAGCAGAACGTGGTCATCATA

GGGACCACCTCACGGCCCGGCAGCATGGATGAAGCTTCCCACCGGCGCTTTGCCAAGCGC

TTCTACATCTCCCCACCAGACAGCATTGCCCGGCGGCAGATCCTCCACCATGCCCTCG

>c10046_g1_i1 len=237 path=[1:0-236]

TGAGGCATGAGCAGCAGTGGCTCACGTGTGGTGCTGTGCAAGGGCACACTGTACGAGGCT

GTGGCTCCACTCTGTATCCTAGGGCACCTGGGCCAGTTGAAGGAGGATATAACTAACTGG

AGAGCCTAGGGAGCAACAGCCCTAACCTGTTTATTCACAAGATGTTTTCTGCATTACTGG

TACGGGAAAAAAATAAGTGATTTCTTCTGCAGGATAGCGTAGGGAATTGTTGTGAGG

>c10049_g1_i1 len=953 path=[35:0-796 34:797-952]

TATTTTTTTTTTTTCCTCAAAATATTTTAACTAATATTTTAATAAGACTCTTTTATATTT

AATAAAAATACAAAGGAATATGGAAATCATTTTAAAATAATAATCATGAAAAAAAAAAAT

ATAAGAGAAATAAAGATTATCTTAACTTTTTATACTAAGTTATATTTTTATTATTCTTTT

TTTTTCTTAAAATATTTTTATCTAATTTTTTCTAATTACTAAAAATATTTATTTAAAATT

TAATTTTAGATTCAATTTTGGTTGAAATTTTAAAAAAGGAATTTTATCTCCTTGTTGTTG

AACTTCAGGTGTATTTCTATCATTTACAACTCCTAGTACAAATTTTAATATACCTAAATC

ATATATAAAATAGCTTTGGAAATTTCCATATCCACTTTTTCTAATTAATGATGTTAAAGT

TAGATTCATATTTAAAGATGGATTAAACATTTTTTTTTTTCTATTAAGAGCTTCTCTTAT

TTTTCGTTTATCAAATAATTTTTTTAATGGGCTTTGATTCAAATCATAATAATTATTTAA

TATTTTTTCTAATTTTTCTTCGAATTGATATAAAAATTCTTTAATTGTGTTATTAAGACT

ACTGTCTAATATAAATTGTTTGGTGATTAATTTATTTATTTCAAATGTAAATTTTTTTAT

TAAAGAATTTTTTAAATTTTCAATTTTATCTTTTTTTTCTAATAAATCTTTGTCATTCTT

TATATAGAATTTATTTAATATCATATCTTTTCCTTTGTTAATATAAGAGATTTCTAACAA

TAATAACTGCTTTAATATTTGTTTATAATATATATGTAATAGAGGAATCATATATAAAAT

GCTTAAATTATTTTGATAAAATATATTATGAGTATGAGAAAAATATTATAATGGGGAATG

TTATTGAAAATTATGGTGAATTAGCAGAAAACCTTATAAAATCTTCTTTGCTT

>c10049_g1_i2 len=922 path=[35:0-796 831:797-921]

TATTTTTTTTTTTTCCTCAAAATATTTTAACTAATATTTTAATAAGACTCTTTTATATTT

AATAAAAATACAAAGGAATATGGAAATCATTTTAAAATAATAATCATGAAAAAAAAAAAT

ATAAGAGAAATAAAGATTATCTTAACTTTTTATACTAAGTTATATTTTTATTATTCTTTT

TTTTTCTTAAAATATTTTTATCTAATTTTTTCTAATTACTAAAAATATTTATTTAAAATT

TAATTTTAGATTCAATTTTGGTTGAAATTTTAAAAAAGGAATTTTATCTCCTTGTTGTTG

AACTTCAGGTGTATTTCTATCATTTACAACTCCTAGTACAAATTTTAATATACCTAAATC

ATATATAAAATAGCTTTGGAAATTTCCATATCCACTTTTTCTAATTAATGATGTTAAAGT

TAGATTCATATTTAAAGATGGATTAAACATTTTTTTTTTTCTATTAAGAGCTTCTCTTAT

TTTTCGTTTATCAAATAATTTTTTTAATGGGCTTTGATTCAAATCATAATAATTATTTAA

TATTTTTTCTAATTTTTCTTCGAATTGATATAAAAATTCTTTAATTGTGTTATTAAGACT

ACTGTCTAATATAAATTGTTTGGTGATTAATTTATTTATTTCAAATGTAAATTTTTTTAT

TAAAGAATTTTTTAAATTTTCAATTTTATCTTTTTTTTCTAATAAATCTTTGTCATTCTT

TATATAGAATTTATTTAATATCATATCTTTTCCTTTGTTAATATAAGAGATTTCTAACAA

TAATAACTGCTTTAATATTTGTTTACGTATTAATGTTTGAAATCTTGCCATTAATTTCTT

AAAAATTGTATCCTTAAATTTTGTACCTGTTTGTTCAAATGTCAATAAATGAAACAAAAG

CAAAGAAGATTTTATAAGGTTT

>c10049_g2_i1 len=1308 path=[2215:0-1151 34:1152-1307]

AAAAAAAAAAAAATATAAACAATAATATTAGATATAAAAAAAAACAAAAGAAATTAATAC

AATATTTATTTGAAAAAAATTAGAAAAGATGTAATATGCTTTAAAACTAAATAAATTAAA

TTTAATATAAATATAGTTTACATTCATACTTTTTGTTAAATTAGTGTATACCTGTTGTAA

TTTTAACCTTTAAATTTTTACATAAAATTCACGTAGGAATATTCCATTTTATGTATGATT

ATTGTTTTGTGTGTATTAATTTCAATGTTGAAAATAAGAAAAGAAAATATATATTAGAGA

ATTGAAATGATAAATTTTTATTTAATTCTTTTTTATTTTATTTTAAGTGTAAAGAATACT

CATGAAAAAACAACAAAAGATTATGAATGCGTTAATTTATTATCACATAAGGATTTTCAT

GAAAAATTACAAACTTTAAGAAAATATCAAATAAAGGATAAAAATGTACAAAATAAAAAC

AAATATGTTTTTTCATATTTTGGAAGTTCTGGGAGTAATAAAAGAAGAATTTTAGAAAAT

TTATTTAATATATCATCAGAAAAAAGAAACTTTATTAATTTAGGATTTCAACAAAAATTA

GATCTATGGTTTGGATATGATAAAAATAATAATTTTAATGTTGTTCTCGATATAGATTTA

TTAGAAAATAAAGAATTTTTAGATAATTATGAAACTATATATAATTATGAAAAGTTAGTT

GATATTATTATTGAATCAACAAATAGTGTTATAATATCTTTATCGATGGAAGATATATAT

AAGGAAATATATGACGAAAAGGAAAAGAAGGATTGTGCAAAAAACATTACAAATAAAAAG

TATCTTATCAATTTACCTACAAAAATAGAAATTCTTTTAAATAGATTGAATCAGAAAAGT

AAAAATATGAATATTTATTTAATTATTACAGATAATAAAAAACTACATAGTGCTAATGTT

AATAAAGATATAATTAATCATTTAAAATTAAAGTATAACAATTTACATATTTATTTAATA

AAGCAGAGTAAAATGAATGTAGATTTATTACAAAAGAATAATATAAATAAATATGAACTA

TTAATAAATAATATAGAAGGTTCTATGAAAGAAATGAATACATTTAAGAATTTTGATATC

TTTAATGAAAATTTTGTTTATAATATATATGTAATAGAGGAATCATATATAAAATGCTTA

AATTATTTTGATAAAATATATTATGAGTATGAGAAAAATATTATAATGGGGAATGTTATT

GAAAATTATGGTGAATTAGCAGAAAACCTTATAAAATCTTCTTTGCTT

>c10050_g1_i1 len=299 path=[309:0-298]

AACCTTGGTGATTCTATGATTCATTAAAATATCTCAAATTTGTGTAGCTGTTGTAGTAGT

GCAAATGATTTAGGCATAGTCACATGAATCTAAAACTAGCACAGAGGTGACTTTAGATCT

GAATATCTATTTATGTTGATCTAGGATAGAAAATAAAAGCCTTTGGAGGCTTAATTGACA

GATTATTCAAAGACTGACTTTCAATCTGAAGTCCTTACCATAATTTCTGAATTTATGATC

AGCATCTGAGTTTAGGTCCTGTCAAGTAAAAATGAAAAATCATATTCGACATGCATCGG

>c10055_g1_i1 len=230 path=[366:0-229]

AAGGCTAACTGCGTTTTCCTGCTGATCTGACTCTTGTTCCTTGGTGAGCACAGTGTTGAG

GTGCAGTTCTTCCCAGAGCCCCCAGTAAGCAGTTTGTGCTCCCAGCGCCTTAACCACAGC

AAGCCCAAACCGGTGCTCCTCGTGCATCACATGGGTTGGGGAGTGGTGAGCTCACTGATC

TCTCTTTACACTGAAATAATGCCTGTCCCAGGAAGGCTGTCCCCTGTTGG

>c10058_g1_i1 len=274 path=[366:0-158 535:159-273]

GGAAGGCTTTTGTTGTTTATGAAAGTAAATATAAAAATTAAAATAATTTTAAAATACTAT

TAACCATGAAATCTACATATATACATACAATACATTTTTGTAAAAAATGAAACATTAATG

TATTTAGATAATAAATATATTATAAAATAAAAACTAGTTAAAAAAAAAAAATAAAATTGT

AGTATAAATCGAATGTTACATCAATTAGTTAATAATAACAAAATTGTATATACATAAAAA

ACAATATTTATTCTATTTAAAAATTATCATAATT

>c10070_g1_i1 len=370 path=[43:0-248 20:249-251 604:252-369]

AGCTCAGAAACCTTCAACTCTTGAGGTTTTACTTGTGGTATCGAGCAAGTTGCTTTCACC

AAGGAGGAGTCATAGCAAGCGTAGCTCTCCCTTTGCTTTTTATGCAATAGCTACCTGTCT

GCTTCTTTTACAAATGGTGTGTCAGATGGAGGGAGCAAAATGCCACAGGGTGGACTATGA

CTGGCTTCAGGCTCCCCGTCACTGTGTTTAGAAGACTGAATGTCTGGGTTGGACTGTGGG

AGAAATTCCCCTCTAATCAACTCTTTCCCTCTTGGAGAAGAAGCATATTCTGTAGCTGTC

AAGCAAGCACAAGAAATGACCGAAGATGGTTTAGCTTTTCCCCAGTAAAACTCTGTGCAC

TAAGACTTTC

>c10071_g1_i1 len=263 path=[1:0-262]

GGTGAGGAGGCTAACTGCCCTCTTTCAAGGAGTGGGTTAGGTAGATGGTTTATTTAGTTT

TCATGTCTTTGATTTTGAATCCAAAATGCCGGCACAGCCCATTTCTGCCACTTTTGGCAC

AAAATACTAAAACTTATTGTGAGGTAAGACTACACAAAGTAAGTTGATGCTCTAAAATAG

AGGCAAAGGAACTTCCATCTCCCTTACAGTCTCACACGTTTAATTGCAACATACAGTTCA

TAAGCCTGTGAGACTGTAAGGGA

>c10072_g1_i1 len=215 path=[193:0-214]

GAGGGAAACGAAGGTTTCTCTGCATGCTGGGATGGTGTGGGCTGGGAGGAGAAGGGGAGG

GAAGATGCTCCAGCTCAGCCTCAGGCAGGCCGGAGCACAAGGTGCGGTGTTGTACCTCAC

AGGGCAGGTATTTGGGTGCTCCTCAGCCGGTCCTGGGCAAGTCCTGCCCTGTTGGGAAGG

CACCCTCGGGGGTGGCAGAACTGATGCTAAGCAGA

>c10076_g1_i1 len=213 path=[224:0-174 492:175-178 402:179-212]

AGGTAACCTTGTGTTTGATGGGGGAGGGAGTGCAGAGGAAAAAAGAAAAGTCATTTTTCT

TTTCAGTCTAAATTGATTTTATTCTACAAAATGCTACTCAGTTAAAATTGTAAAAGCAAA

ACCATTTTAATATGTTCTTTCTTCCATAGCAGCAAGCTTTCTCTAACAAGCTTTTTGTTT

TAGTGCAAATACTGTAGGCCTGTATTACAGTAA

>c10077_g1_i1 len=535 path=[548:0-534]

AAAATGAAAAAGAAAAAAAAAAAACGAATTTCCTAAACAAAAAATATAAATACCTAAAAA

TATATGAAATTACAAATGAAATAAAGCATAATTTTTTAGAATTAAATATTCGAAGACAAT

TTTTATTTAAATCAATTATTGAAAAATTAACTAAATATAAAAACAGATCTTTACATTTTT

TCAATAAATACTTAAATATACAAAAAGAAATTAATTCATTATTTGAATTAATAAGTAATA

ATGCTTTAATTGATATTTTTGTACTTAGTAAAAATAGCTCGTTTTTTCAAAATCAACTAC

TAAAAAATTTTCAACGATTTTATTATCAAGTAGAAAATTCTTTAGTAGAGTAATAAATTT

TTAAATATATCCCTACTCATTTAATTTTATCATTTTTTTTTTTTAATAACAAATTATTTT

CATTAATTTTTTTTTAGTATCTACATTAAAATAACTTGTGAACTTTTTTCTTCTAAAAAA

AGTTTGAAGTGATATTTTATAGAATCATTTTTTTTTAATTTATCTTCAATCTCCT

>c10077_g2_i1 len=636 path=[1756:0-635]

TTTAATTGAAGTTCTTTTTTTTTTCTTTTTCATTTTTTAGCATCCTTAATTCAAAAAACA

TCTCTTTTTTTACATCTTTTATAAATTTCGTTCTTTTGCTACTTGAATTTTTTTCCTTAA

TTTTTTTTTGAAAAGATCTATTATTACATTCTATACATTTCATAATTTTCTTCTTATTTA

ATTCCTTTGTATTTATATCCAATAATTTAATTTCATTTTTTATCTTTTCAATTTTATATG

ATTCTTCCTTTTTCAATTTTTCAAATTCATCTTCAATTTTTTTTATTTTTTCTTCAAGTA

TCTCAATATCCATTATTTTAAATACATTATATATATGTGTATTTTTTATAAAATCCTAGT

GTCATTAGTTTTCTAAAAAATTACTATCATAAATACAAAATAATATAAGTTTCATATATA

AATCATAAGTTTATTTTTTTAAAGCAAATATGATATAAATAAATAATATTTTCCTTTAAA

AATAATAATTTTTTAAATGTACACATTAGTATTAAAATTATAAAAACAAAATAAACAAAT

TAATGTTCATTTATAAATATACTTATTTTTTTTAAAAGTAGGTTATACTTTGGGCTTATT

CAGAAATATATCATTAAGAAAAAAAAAAAAAAAAAA

>c10080_g1_i1 len=4084 path=[4290:0-4083]

AAAAAAAAAAAAAAAAAAAGTTATCACATTTCTGTATTTATTCCTGTTTGAACAAGTGTT

ATTTTGTCTCCTAAGCTAAGAGCTATTCCCTGTGTTCTACTTCGAACTCTTATACTTCCT

GATAATTTTCCATCAGAATTTTTTTGAACAGATAAGTTGGCTAAAGAATCCTCTCCAAAT

ATGCTTCTAGAAAATAAATTAACAGCAAAAAAAGCTGAGTTATTAATCAAACATTTTAAA

TCTTCTAAGGTCGAAATGTAAGATATATACACATCAATTGGACTAATATTAGCCTGTTCA

GGCTTATTTTTAGTTTCCAATTCATAATACTCTAAAGGCATAAATTTCTCAACTATGGTC

ATATTCGTATTTTTTATAATCAACTTTAGTAACTCAAATGCATCTCTGATAGAAGTGTTA

ATATTAATTTTATTTTCCCATTCAAATTCAGACCACATTATGCGAAAAAGATGAGAAGAT

ATAAAAGAGGCATTTATATAATCAGTCATGTTAACATTCAATTCATTTAATACAATGTAA

TTTTTATTACTATCATTTTTTTTTTCATAAAAAATATATCCAAATACAATTCCTGTTTCT

GTTGTGTGTACTTTTACACTTATCTTGAATTTTCGTTTTTCATTTGGAGATAAATTAAAT

ACAGGGATTTTATCTATAGGTTTTAAGTTTCCATGAGTTGATAAATTAATGTATATGTTT

TGCAAGTATACTCCTGATTGATTATATACATAAAATTCTATAATTAAATTAATATTAGAA

ATAATTGGAAATGCTTCAACAAATATATCATCATCTGTTCCCGTTAACCTTTGAGAGTTA

TATAATTTGGCCAAAAATAAGGAAGAGTAATTATGCTTAAATGAAGGATATTTAAACTCA

TTTTCTTCAAATAAAATATCATTATTTAATGTATACTTTAATTTAAGTTTTTCTAAATTT

TCATTAACTGAAACAGATTCTTCATCAAGCATGTTAAGTATATTTTTTTTTTCTTTTAAT

ACTCTGAAATAAATCTCATCGTCTACATTTGCTTTATCTTGTTCAATTAACATCTTATCA

TTAGGAATTTTGTTTAAATCAGTTAATTCTGTTGAGGCTTCTTTATTCATTTGATCTTTT

TTTTTAACATTTTTATAAGTAGAACCATATATACAGCAATATTTTTCTTCTTCTTTTTCT

AAAAATTTCTGAAAGTAATCATCTCCGTTCAAAAAAGTTCTTATTAAATTTTGAGTTTGT

TCATCTATAATATTCATGCCATTAGTTATTTTTAAAAATATTTTAAGGCATTGATTTATT

CTGATAACATTGCTATCATTTTCATAAATACTAATTGCACATTTTGCATTTTTCTGTACA

ATATATTTTATAAAACTTGCTAGTATATGTATAGATTTATTTCTAAATTCATTTATACGC

TTAATTAAAAATTCATTGTTAGAATTCTCTATATTATCAATTAAATTATTATTTTGAGTT

GTTTTATTTAGCTCTTTCGAATTGTGTGAAGACTTTCTAGAAAAACTACTTGATGATTGA

TTTAAGGAACTACTTAAAAACTCAAAAGAATCATTCACTTTTGTTAATAATTTTAAATAT

AATTTTGTTAGGCAAACACATATAACACATAAAAGTAAATCATCATTTTCACACAATAGA

TTATAAGAAAATGAATGTAAATTATCTTTATCATCACTAGAAGATAAATTATAATTCTTT

AAGAAAGCTTCAGTAGCATAAGTACCATCTTCTAATATTACAGTTTTTGTCTGAATATTT

GAACTTGATAAATTAAAATTCATCATTTTATTTTTTTTATATCTCTCGTTTTGTATTTTA

TTAATAATATCTAATTCAATATTACTATTTAAAAATGAAGATAAATTTTCATATAATTTG

TTCATAAAATCAATAATCATATTTTCATTATTCATATATTGTCCTAATACCCAGAAAAAA

ATTCTTAATATAGTTGACTTTTTAATATCAAAAATAGCATCAAATATCTTCTGAAGAATA

TTATTTTGAAAAATTCTATTATTTGCTAGCTTTCTAATGCATACAGCTGCTTCATAATTT

ATTTCTTTTTCATCGTCATTTACATATATTAGTAGTAAATCAACAATATTTAAGCATTCA

TTAGAATACATATTACATATATGTTGCAATGATTTAATTAAAATTTTTTTATAATTGTTC

ACCTCTTGATAACTAGTTGTTATACTATTTATTTGAAAAATATCATTCATATTTTTAAAA

TTGTTTAATTGATGATTTGTACCACTTTGCATATTTTTTTGAGTTAGATTTTTTGCATAT

ACGATTTGATCATTTAATTTTAATAGTTCTTTTTTTAAAACATTTAAAACTAAATGTACA

TTTCTTTTTGTTAATATATGTAATACTAAATTTAAAATTTTTATTTTTATATCTCTCGAG

GGAAAGCTTAAAGCCCTCAGTAAGTCCATGATATAATTTTCTAGTATATGTTTCCATTTA

CACATAATATAATATAATTTATCAATAACAATTAACTTAATATTATTATCATGTTGATTA

ATTAGTAACTTAATAAAACATTCACTAGCTGTTTTTATACTTAAAACAGAATTACTCATA

TATAGAAGACAACAAGCTCCTTCATATAGTACACTATTACTAACATTTTTACTTAACATA

TTTAATAGTATTTTAATAACATTATTTTTATATTGACTATAATCATTTTCATTTAATTTT

TTCTTTTTTAATTGAATATCTTCACTTAAACTATATAATGGACTATTTTTCTCATTTAAA

TTATCATAATTATAAATGTCATTTTTTTTATTATTAATAACCATATTTCTTTCGTTTTCT

ATTAATTTTTTTTTTCTTTTTATTCCTAAACCTTGTTCTTCTTCATCTCCATTTTCATAA

AACTCTTCTTCATCGGATCCATTTTCCTCCTTTTCTTCCTCATTTTCTTCATCTTCATCG

TTTATTAACAGATAAGAATCATCAAATACATGAGGAATATATAATTTTTTGAATAGATGT

ATAACTTCTAATAATATTACATCAGCAGTATCATATAATTGGTCATTAAGAGAAAGAATA

TATTTTAAAGTAGTTACAGGATCGACATCTGTCAACATAGATAATGCACTACGTTTAGTA

GATATATCAGTTTCAAGAAGTAACATTTTTTCTACTTCTTTAACAGAATTAGGCACTACA

TCTAATCCATGTTCTTTAATGATAGTATGAATACAACTTATTGCGTTTTTTCTTACGTAA

CTATGTCTATGAGTTAAATTTTTTGTAATAGCTTCAATTAAAGGATCTAATATTTTTAAA

TATTTTATTTTACTTAACAATCTTAAAGTAGACCCTCTTACATATTCATTAGGAGAAATA

ATATCATTCCTTAAAGCATTACATACTAATATCATTTCCTCTTTTAGGCTGCCATCATTA

TTGCATTTATCAACAATTTCAAAAAATATATGACTCATTTTTTTTAATCTATGATCTTTA

TGAGGAACTATAAATCTGATTACACACATCAAAAGGTTCCCATATGATTCACCTTGTATT

ATATTAAAAATCAAATTTTCCATTCCTTCAATTTTTTTCTCAACATTCTGACTCTCCAAT

TTTTTTTGAATTTCACTAATTGAAGGAATTTCATAATTATCTGTGCAAATGTAAAGGGTG

CAATTATTTTCTATTTCATAGCTACACATTTTTTCAACTTATATAGTGTGTATCTAAATA

TACTATACATAAATTTTGAAAAATAAAATCTTATAAAATTTTCTTATAATTAAAAAGTGT

GTAAATATTAAATTATGTATTATTCATTCAGCAATTTAACATTTATTTTTATAAATCCCT

ATTAAATAATATTTACTATAATAAAAATATTTTCATAAAAACCTAATTAAATGTAACTTA

TACTTACACATTTCAAAAATAAAAGTATAAATTACATCAATTATAAAAAATTTATGTGAA

ACAATACATATTACATATAAAAAGCTATTCAAATTCTTAATCAAAATATGTGTATATAAT

TCAT

>c10081_g1_i1 len=319 path=[1:0-132 134:133-318]

AGCTCCATGCCGTTCCCTCGGGCTCTGTCACAGAGAGCAGAGCTCAGCACTGCTCCCTGC

AAGGAGCCACCATGCAGCCACCATGAGCCTCCCCTCAGCTCCTCCGCTCTGGGCTGAGCA

AATGTAGAGACCTCAGCTGCCCCTCGTACGCCTAACACCTCTAAGATCGTAAAGTCCAAC

CATCAAGACAGCACCACCATTGGTTTAGCAGGAAAGATTTCTTGTAGAAATCAATGCTCA

GCAGTAGTTCTCGGAGAGCTCATACACTGCTTGGTGGCCATCAGCTCACTTGAGCAGAAG

AATTGCTCTGCATTTGGTA

>c10081_g1_i2 len=206 path=[593:0-19 134:20-205]

CTGCCCGCTCAGGGCGGCCCCAGCTGCCCCTCGTACGCCTAACACCTCTAAGATCGTAAA

GTCCAACCATCAAGACAGCACCACCATTGGTTTAGCAGGAAAGATTTCTTGTAGAAATCA

ATGCTCAGCAGTAGTTCTCGGAGAGCTCATACACTGCTTGGTGGCCATCAGCTCACTTGA

GCAGAAGAATTGCTCTGCATTTGGTA

>c10084_g1_i1 len=204 path=[61:0-163 680:164-167 228:168-203]

AAAATATCTAATCGGAATCTTTAACATGAGTAGATTTCTGTGCTCTCTTGGGTATATTTG

AGTTGCAGCAACAGCTGTGAGTGAATATTTATTGACGTAAACACACTTTCCTAGCAGAAA

TGTTGTCCAGCTGTACTGTTCAAATTCCAGCATAAGTCCCAGAGTCCTAAATACTCTGCA

GAAACTGGTCTGGAAGACTTTCTA

>c10084_g2_i1 len=235 path=[450:0-175 10:176-195 680:196-199 649:200-234]

GAATACTGAACAAAAACACTAATTTCCCCTGAAGTCCCGAGAAAACTTGAGAGTGTAATG

CATGTACTGTTTGCACTAGTAGCCCCACACAGCATTATCCTAGAACAGGAGAAAAATGGA

AACTACTTTATTACGCTTTTTTTGGTGTTCATTTCCAAAAAAGCAAAATAAAGCTCACCT

AAAGAAGGTTTCTAACTCCTTAGATGCTGCTAGAAAGTCTTCCAGACCAGTTTCT

>c10090_g1_i1 len=558 path=[313:0-557]

AGATTTCTTCTTCCTTACCCCTCCCAGCCCCATTATCCAGTTTGTTCAACGCTTTAAAGC

TCAGGAATGCCCTGGCCCTTTTGCAGCAGAGCCAGAGGCAATTCCCACTTTTGGCAAGCA

GTTGGGAGACTCAACAGAATTCGAATAGGAACAGAGGAGGACTAGATGCAGGGCACATCT

GTAAACATGTTGTACATCCGCAAGTCACACACACCCGCAAAGGTGATACAGGTTATAGGT

GAAACAGAACTATTTGCGACTGGCCAACCACAGATTTCAGACCTTGGACTGGTACAGAAC

ATCACTACTATATAGCACCATACCGGGTACGGCACTGTATAGTACTGTATACATAAATGG

AGTAGGCAATCCGTAGTCAAACTTGTAAAACTAAGCATTGCAGAAAGCTAGCGGTCTTAC

AAAAGGTACAGAATTCAGTTCCATCGTCCCACCGTGAACCTCAGCGCTTCATCCAGGAAC

AATCTCATTAAAAGAACAAAAGCAATTAAAAAAAAAAACAAAAACGCATGCTACATACTC

GTAGTTCAAAACAAAACG

>c10093_g1_i1 len=235 path=[1:0-234]

GGCCTCTACCCTCTACGGACGACGCTCTGTGCTTGCTTTGATGCTTCAGCTCTGCTCTGA

ACTGCTGTCCCGCGTTGCACAGCCCCTTTCCCACTCACACAGTGGGTTCCTTTCCTTCTC

TCCCAAAGCATCGGTCCCACTGCTGTGCTCGGGGGCAGAGCCGTCCTCCTGCCCTTCTTT

CAGTTTAGCCTAAGATGGTGAAATCCCCACAGATTGTTTGTTCCCCTATTTCCTT

>c10095_g1_i1 len=876 path=[888:0-875]

AATGTCAGTTATGCGCTTTTATTTTATATGTTTTGTATTATATTTTTATTTGTGCATTAT

TAATATAATTAAAATTATAAAAAGAAAAGATTTAAAATGCTTAAATATATATAAACATTG

TATTTTTAATACCTAAATTTTTTTTTATCATTGTTTAAAATTGTAGTTTTTATATTAATT

ATCTTAATTATGAATAGTATATTTTGTTTCTATATAAGATATTATTAAAATAAAATTGTA

AAAAAAAAGTTTAACATATAATATATTTCATGTTTCGTTGAATAAAAAAGAAAACATTAA

ACAAGTTTTCTATTAAAATATAATAAATAATATAATCCTTTTTAATTAAAATATAAATGA

AGTATTCCAATTGCTTTTATCATTATTTTTAAAAAGTGCGTCCAATAAATAAATATAGAA

TTGTCTTATATTCATTGTTTGAAAAGTGTTTTCACTAGAATTTATATAAATAATATTATG

TCTTGCATTTATAATTACATTTGTTTCATTTATTAGCAACGAGACCAAATTATTTTCGCC

TTGAAATTTATCTAATGCTTGTATATTGTTTTCATTGCTAGCATAATTACTTTCCAGAAT

ATCTTTTGAACAATTACCTATTTCAGATAGATTTATGAGAGATTTATCATCGTTTAAATC

AAATTGACAAAAATAATTTGAGCATTTAGAAAAATGATTTATCTCTGACTTTTTTCTAAA

TCTCTTAAAAAAGTTGAAAGAAAAAAATTTTTTTTTCTTTAATATTTTCATATTTTCTAC

ATACTAACGTAACTGTAACATTTAAAAAATCTGAAATGTAATAAACATAATAAAGAATAA

AATTCTTTATTAATATAGATATATTAATAATAATTA

>c10097_g1_i1 len=688 path=[1:0-382 384:383-687]

GATGAAATAAATAAAATGAATAATATAAATCAGTTAGATGAAATTAATAAAATGAATCAT

GTTAATAAATTAGATGAAATTAATAAAATGAGTGAAACTAAAAAAGTGAATGAGTTAAAT

AAAAGAGATGAAATAAATAAAAATGAAAATTTTAATTCAGATACAACTTCTATTAAGGAA

GAAAATATAATAGAAAAAAATTTAGTTGTGTTAAAAAATAAAGAGAAATATAATGAACAT

CAATTGAATGAGTATGGTTTAATAAAACCTTATTTTTTACCTTGTGAAAAAGATAACTTA

AATTTATTTGAAAATTATAATAATCCATTAATAAGTAAAAATACAAACGATCAAATTTCT

TCGAAAAAAAAGAATAGTTTAAGTGAAATCTTTGAAGAAAATATTAATTTGTTTTTAAAT

TTAAATTTGATTGACATAAATAAAAACAAAAAAATGAAGAAAATAATTATATCAATGGAA

GATTGTCTAAAAAATGATAAAAATATAATTTATGATAAAACAAAAAAAATAGAAACTTTA

TTTGATAAAATTTTCACAAATATAAAAGAATTTTTAAGTAAAGATATAAGTTATAGAAAA

AATGAAAAAACAGAAATATATTCTGATGAAAAGAAAGAAAAATCAAATGAGCAGTTAGAA

AAAAATTTATTTTCAAGAAAAAAAAGTA

>c10106_g1_i1 len=308 path=[315:0-307]

TGATGATTCTACGATTCTATATGTAAGTATAATAATGTAAGTTACGGTGACTCCTATCAG

GTTGAATTCAGTCTTACATACCTGGGATACAGCAAAAACATTCCCCAGCATCAGCATTTA

ATAGACATTCACATCACATAGCTTATTAGTTTCAGATACATTACTTAGAAACTTTAAGAT

AATTTTAAAAAATACCACATAAGACCTCATGTCATTAAAAACATTAAGAATAATGTTATG

TCCAGTCTAATGATATTTAGACTGGAAATCCATAATACCATGTACACTCCTACAAGAACA

TCTAAATA

>c10107_g1_i1 len=225 path=[161:0-224]

GGATGGAGGTGGGAAGGGGCACAAGGGCTGTTTTCCAAAATGACCCCTTGCCCTACATCA

TGGCACAGACTGTCCCCTCCCTGCCACCACTGAATCCTCCTCTCACCTCTCCCTTCAGCA

CACTGTTGTGCTTTGCAGCTTCTGGCTGCCTTGCTCCAGCATCATGCACAGCTGCTCCCC

GCCTGTGTGCTGTGGTTGCCAACACTGATGTAGAGGCTGGGCATG

>c10109_g1_i1 len=226 path=[204:0-225]

ACTGCTCGTGGGAAGGAGAAGTCCTGCAGGCTGAAAGTTTGTGCAGGTGAGCTGGGGATG

GTCCTGCAGCAGCCATCCTCCGGGGCCGGAGAGCTCTCCAACCTTCACCGTCTGTCTTGT

CCGTCAGGAGGCATCCGTCTGTCTGGGTTATTTATGGAGGAGAACTCTGGAGCTGTGTCA

GAGCCCTGGAGATGGCAGAAGCTCTGCAGACAGGTTTACCTCGTTG

>c10109_g2_i1 len=377 path=[407:0-376]

AAAGCCTGAGCTTCCCACGAGCAGTGAGTTTGCTGGTGGGAAGGAGAAGTCCTGCAGGAA

AGGTTGTGCCTGGGTGTGCTGTGTCGGAGCAGCAGTAATTAAGCTTTATTTATATGGGGT

TTCCCAGCTTACGGGGTGGGAAGTCTCTGTACGTGGGTTTGAGCGCTGCGTGCGAACTTC

TCGTCAAGTTGGGGGATTAGCAGCACAGGCCTGCGCGCCGCAGTCTTTTTTGGTCGTGGA

TCTGAAGCTGGTTTGCTGGCTGCAGGAGGAGATGTGGATGTGTGGATAGGTCAGCGGTAA

CCGAGCCCTGTAACCCAAGCTGCTGCCCTACCCAGCCCTGCTGGGAACGGTGGCGTTCTG

CTGGCGTTTTGGTGTCA

>c10113_g1_i1 len=306 path=[1:0-305]

TGTCACACTTATTTTGACTCATACAAGCACTCCTTTAGTAATTTCAAAGGAAACTCTCCC

TAGTTAACTTTAAGTCTTCATTCTGGTTAGATAGATTTATTTTTAATTGACTTTTCCTTC

TAATTTAATCTCTTTGATACTGAAAGCTAATTGAAAGAGCAGGTAGTACAGAGTAGCAGC

TGAGAAGACAACTATTGCCTCAGCTTGTGCATACGAATCTGTCTTTAAGTAAATAAGCAA

GCTTTTAGAACAGAAAATATAGCAACCTGCCTGCAACATGCTGACTAATTTCAGCTTACA

TTAAAA

>c10116_g1_i1 len=388 path=[136:0-90 591:91-387]

AAAAAAATACAAACATAGCACTATTATTGTTTCAGATCAGCAAGACATTTGTCTTCACAA

TGAACTTACACAATTCTTTAAACAAAAATCAGGGTAAACAAGTTTATTTGCCAACAAGTT

GTTAGTTTGGCTAGAACCCAAAAATTCTGGCATTACTAAGACAAACATCTGCTAATTCAC

TTGAAGTATAAATTATGGGAGTAGCCTGTCAATCTCCAGGCTTAGTTACTGAGAGAAGAC

AGTATACTTTCCTGGTGACTTAGATGTGCACTTGATAACCTGAAGTATTTTTGCTCTTTC

ATCTGACTACATAAACCTCAACCTCCATCCCTGAAAAAACTCACTTTTAAAATTTTTTAA

ACCCTTACAAATAAACATATGAGAAGTG

>c10119_g1_i1 len=245 path=[549:0-244]

TAAAGTGGTACTAGGGAGACAGTGAAGAATTTAAGAAAAAGACTAAAAGGCACTGGTCTG

TGGGATGCTGAAGTAGCCTGGAATGGAACCAAGGTCCATAGAAGTCTACAAGAGGTGCCC

AGTGCATGCATCCAGCTTTGGCCTGGGTCCTCAGATGAGTTTTCCAGAAGTGCTCAGTCT

TGTTACTCCTATCTGCAGTGGGAGCAGGGAAGCCAGCGCTGAGCCTTCCTGGGCTGCTGG

ATTTA

>c10120_g1_i1 len=251 path=[383:0-250]

GCCATCCTTTTCTGAAGATGAACAGTTATGCCATGAAATGCTGGCATCGCAGGAAGGTTT

GGTCTTAGCTTTCATGCACCCATCTGACATTTCAGGCAAAATAGGCTGGAGATAGACCAT

CCTACATGGTCACAGCTTGAGATTCAGTACCTTAATGTTAATGTATATTAACACTCAGCA

GCAGTTTTGTGTACCAACAACTGCTTTGACTGCTGAGCTCAGTGTTTCTAGATTTGTGAA

GACAGCATGTG

>c10127_g1_i1 len=305 path=[45:0-304]

GAAATATATTTTATTTTCTTTAAAGGAAAAAAATAAAATTATTAAAAGAGAGAAAGTTTT

TAGGAAGAAATATTTTAATGAGAATATATTATACTTTTGAGAGTAAAATTTTCTCTATTA

GTAACATTAATATAATTTAAATTTTTTTGCTCTTTTTAAGGAAAATGAACTGAATAAATG

AAAATTATTCATTAAATATTATATTTGAATATTTTGTACATAAAAATTAATGTTTAATGA

AAATTTCTTTTCTTAATAAAAACATAAAATTTTTATTAAAGAAAATAAATTCTCAATAAG

CTACA

>c10128_g1_i1 len=218 path=[843:0-217]

GTGTCTCTGTGATTTCTGGGGCCCTCCCAGGGCCGCACAAAATGGGGAGAAAACACCCAG

ATTTGGGTCAGCTGCACAGGACGTTGGCGCTGAACAACGCGGACTCATCACTGTGTGTGG

AGCTGCAGCTGCTCACGCTGGGTTTACCCGATTCTCCGTTTCTGTACCCAAAATGGGAGA

TAAAATTGGGCCTTTTTCAGTTGTTTGGGGTTTTTTTT

>c10128_g2_i1 len=444 path=[422:0-443]

GGTTTATTTCAGAGCAGCCTCGGAATTGGGGTCCAACTTGGGATGAGAAGTAGAGTTTTC

TAGCTGAAAATGGTCTTTTCCACCTAAAAACTTCCTTCTGCAAAGCGTTCTTTTCCACCT

AAAAATGGCCATTCTTCACCCCAAAACACCTATTTTCTACCTAAAAACAAAGCAGTCTTT

GGCTTGAGAAGTCATCTTCAACCTAAAAGCGGACATTTCCCTACCTATGAGTTTTCTACC

TAAAAGTGGTGGTTTTCTGCTTATGAACGCCAGTTCTCTACCCAAAACCAATCTTTTTCC

ACCCAAAAATGGTCTTCACTGACCTAAAATCTTTTTCCAACCCCAAAAACGGCCTTTTCT

AGTTAAAAAAAATCATCTTTTGCACCTAGAAACTCTCTCCAAGTCCACTTAGTTTTCTGT

CCACTCCAAAAATAGTCCTTTCTA

>c10132_g1_i1 len=812 path=[1:0-84 86:85-658 660:659-709 660:710-760 660:761-811]

ACCTTGCAAAAACAGTGTTACTTGACTCTTACCAAAAAGGACCTTTTTGAAACTCAGAAC

ACAGGAAACCAACGTGAGAAGAGGACTGGAATAATTTGTTTTCTGTGCACTTCCTGGCTG

AGCTGAGGGCAGAAGATTATTAAGGTTATTTAAAATGCTCTAGCAGATTTGTAGTTGCAT

CTCACCAGCACTGAAGGGGTTAAAAAGGATTTGTAATGTGATTATTGGAAAGCAATATGC

TGCTGTGTCAACGTAAAAGTCGCTAAGAGGATTCCTTAGATATTTGTTGCTAAAAGGTAC

AGTAATAAGGCATAAAAAGCAGTATGTGTGGTGGGAAGGAGATTAATAAAAAAGCATAGA

GGTTAAAAAATTTAGTCTGAACTGCATCAAATTTTAATGAGCTTATGAGCTGTGGGGTTT

GTTTGGATTTACAGGGCTGCTTTAATAGTTTTATTTACAGTAGCTTTTTCTCCAGCCTTT

GTTATTCACTTGAGCTTCCCACAGAAATCAAAGCTCATCGTCTTGCAGAATGCAGGTGGA

GCAATACCTGGGTGGAGCAGAGCCTCTGAGAGGCATCTGCAACAGGGACTCTTTCTCTAC

CAGAATTCACCCCCAACTGGCCAAATCCCAGATAAATAGAAATCCCAACTTAAAGAAAAA

AAAAATTAAAAATGAAATATATGAAAAAATAAAAAGTGAAATAGATGATGAAAAAATTAA

AAATGAAATATATGAAAAAATAAAAAGTGAAATAGATGATGAAAAAATTAAAAATGAAAT

ATATGAAAAAATAAAAAGTGAAATAGATGATG

>c10132_g1_i2 len=798 path=[1421:0-70 86:71-644 660:645-695 660:696-746 660:747-797]

GGTATTTTAAACTGGTTTTCAGTTTTGCACCATCTATGTGCACCTTTTATACCATCTCTG

ATGAAGAATAGCTGGAATAATTTGTTTTCTGTGCACTTCCTGGCTGAGCTGAGGGCAGAA

GATTATTAAGGTTATTTAAAATGCTCTAGCAGATTTGTAGTTGCATCTCACCAGCACTGA

AGGGGTTAAAAAGGATTTGTAATGTGATTATTGGAAAGCAATATGCTGCTGTGTCAACGT

AAAAGTCGCTAAGAGGATTCCTTAGATATTTGTTGCTAAAAGGTACAGTAATAAGGCATA

AAAAGCAGTATGTGTGGTGGGAAGGAGATTAATAAAAAAGCATAGAGGTTAAAAAATTTA

GTCTGAACTGCATCAAATTTTAATGAGCTTATGAGCTGTGGGGTTTGTTTGGATTTACAG

GGCTGCTTTAATAGTTTTATTTACAGTAGCTTTTTCTCCAGCCTTTGTTATTCACTTGAG

CTTCCCACAGAAATCAAAGCTCATCGTCTTGCAGAATGCAGGTGGAGCAATACCTGGGTG

GAGCAGAGCCTCTGAGAGGCATCTGCAACAGGGACTCTTTCTCTACCAGAATTCACCCCC

AACTGGCCAAATCCCAGATAAATAGAAATCCCAACTTAAAGAAAAAAAAAATTAAAAATG

AAATATATGAAAAAATAAAAAGTGAAATAGATGATGAAAAAATTAAAAATGAAATATATG

AAAAAATAAAAAGTGAAATAGATGATGAAAAAATTAAAAATGAAATATATGAAAAAATAA

AAAGTGAAATAGATGATG

>c10134_g1_i1 len=242 path=[355:0-79 216:80-241]

GTACTTCAATAGATGCCAGGACACAGAGCTCTTTGGTTGGCATTTGTTTTCTGAATCTAT

CTGCCTTTTTCACTGAAAATTGTTCAATCTGATTATTTTTTCTTTTGAAAAGCTTCTGAA

CTTCCTTGAACACATTCTGAGCACCATTGACATCACCAGTGGCTCCTTGGCACACTGCTG

TTAAATAAGCGTAATAGCACTGAGACCACCTGGATTCATTTTTAAGCCTTTCAAAGGATT

CA

>c10135_g1_i1 len=392 path=[470:0-365 836:366-391]

TGTGCGACCTGTTGTAGAAAGAACCGGTGGTACAGAAATCGTTACTTGAAATGTTCTGAA

TGGAACAAAGTGGCATAAATGTGTTATAAAAACAATATTCAAAGCCGAAATATTTTGCTC

CTAAGATGACGTTGAATTTATTGCTGTTATTTATTGTTGAATTGAGCCAAGTGAGGTCTG

TTAATTTGTGATCAGCAGTCCAATCTTACTGCAGACTTTGAAAGGGGTATGGACTCATCT

GTTCAGTTAACTACTTTTTTGACTTGGAAAAACTGGCACGGTTTTCTCACTAGCTTCCAA

TACAGAAGCATTCTATAAAATGACAAAGATTGGGATGCTGTAAATGATAGAATAGATGCA

AGTAAACAACACAGATCTCCTGTTTCAGTGTT

>c10140_g1_i1 len=208 path=[1:0-207]

GGGAATGCTCACTCTGCTGGGTTGAGCCGAGTTCTCTCTCTGGCTGAGCGCAGCAGGGTG

AGACGGAGCAGCTCTCCTTTCTGATCTCATTCCTTCCCTGGAGGCTTCTCCATAGGGAAA

GGCTAGAAAATTCTGAGTAATCCCTGTAGCACTGTAAGGGGAGTAATCAAACAAAAACCT

ACTGAAATCTGTCAAAACAAAAGCTGCT

>c10145_g1_i1 len=205 path=[71:0-55 35:56-204]

ATTAGCAATCAGGAGAAAAGGAAAAAAACTACAAAAGAGGATACTGAATTCTTCTGTCAG

GAGAAATAAGGATTATTAAACAACCTGTATCTGCTGAGTACCTTCTGGATTTTCTCTATT

CCATTTTGCACTGAGTTGTCGCTAGAAATCTTGTGCACATTCATACCAGTGGTTTGCCAT

TATCATATCAGTGTTGTAGGGAGGT

>c10147_g1_i1 len=220 path=[973:0-219]

GCACTTTCAGTGGAGGATGTTGATGACACCAGAACACAGTGCCAGCCCTGTGTACACTGA

GAACAACTTGTTGAAAATAAGTGTATATACAGTAGCTATAAGCATATTCTAGACTGTTGT

CAGAGTGATGTCATTTACTATCACAATTCTGGGGTCTTTGGTCTACAAAGACCAGTACGT

GCTCTTCTCAGTATGCCCAAGAGTTCAAGTAGAAGGGTAA

>c10147_g2_i1 len=509 path=[487:0-508]

AAGCCTGAAAAGCTTGATTATAACCTGCAGATAAAAGAGAGTGTTTGCATTGCCCTTCCC

AGGACACAGACTTGTGGTGAGATGGGTGTCAATTTTTACCCTAAAACTTCAAGTGACTCC

CTGATCCAGCTCATCTTACAGCTTGACAAAAACATTAAATGAAGAGATGATGCTGTACTT

ACGGTTGGGGACCAGAACAGGCAGCTGGGGAAAAACAAATATTCTTAAACTACTGTTCTC

ACCAAGGCCCACTGCCTGATCAAACCATGTTCTTGTTGGACAGCTGGAGAAAAGGCACAC

TGAGTCAGGTGTCCAAGCTTCATTCATCCCCTACTTTTCCATTAAGGCACGAAACATCTT

AGTACTGCAGAACTCTCAGTCTTTCTGCCAAATCTGACTTAAGTTGTCTAGGTAGAGTCA

AAACATGGAGAACTAAGGGAGTTCTAGAAGCCTGGTTTTGTCAGCAGAGCATGCCAGAGG

AGGAAGGTAGGAATTCAGTTTTACTACAA

>c10148_g1_i1 len=229 path=[207:0-182 390:183-228]

AGACTCATGAAATCACTCATAGGGTATTATAATTAAATAAATTTTTTATGTGATATATTA

TAATGATTTTATATATATGAGTAAAATATAAATAAATAAATTTTAAATTAACAGATTCAT

ACTTTAAAATGACTATTTTTATATATATATAAATTTTTATTTTTATATATCGTATTCCTA

ATTTTTAATACGTTATGAACAACACAGGCATATTTTGATATATCTGTAG

>c10152_g1_i1 len=205 path=[155:0-204]

GGGCAGTTCTGTTTCTTTCACAATGCACGGAGGCATTTTCAGATGACTTGGTTGGATTTT

TAACTCTTGAAATCATCAGGCCTCAATCTGAATAAATCACCATTAATGCTCAGCCATGAA

AAAATGACTTTTGTGGCCTTTCCTACTGCATGATATGTTGGCCTCCATATTCCTACTGTG

ACTGCTATTTTAATCAACTGTTCTT

>c10154_g1_i1 len=352 path=[1:0-351]

AAGAAATATACCATTTAAATTAATGTTATAGTAAAAATACAAAAAAAAATATTCTGTTGG

TGTTAAAAGTTATATTGAAAATTCCTCCAAAACTGTGATTCACACAGCTTAAGAACTTCT

TTCAAAGAGATTATCGAGCTATAATATTGAGTTAGAAAATATCACTATGCCAGCATCCGC

TTTTGTTATGAGAAAGAAAAGGTCAGATTATATAAATCATTTATATCTACTATTACAACA

TGTCATCAACTCTAAGACTTGTGGTGATGCAACCTTTCCCCCCACAATAATACACACTAT

TCAACCAGAAATCTGCATCCAAAATTTCATAATGGAAGAAAACGTGTCTTTG

>c10164_g1_i1 len=221 path=[1:0-220]

AAATCCTACAGCATTTTTAGTGCAGTTCATGTGTATACGCATATATTGAAGCTATGTAGA

CTAAGATCTGTCAGCTTTCTTGGAAGCGTGCTGTAGGAATTACTGCAAAGCAGCAAATGC

CTCCACAGAAATCCTGAAGGACTGAACTGGCCTGTGCTCACTGCAGAATAGGAGTTAGAA

ATGTAGATACTGTTTCTGAGCTCATTTGTGAAAAATTGCAT

>c10171_g1_i1 len=297 path=[183:0-296]

GTGCTGTGGCACAATGGACAGACTGCTAGAGAGGTGACTGTTTTTTGTGTTAAGAAGTAA

AATATTTACTTCCTTGAGAAAAAGGAAGAAGAATTATTTTTGTGGTTAATCTGTAAACAT

TTGGAGAACAAATTATGAATAGACTAAATTATGCAGACCACTGCAGTTTCCTCCCTAGTT

TGAAAATTACTTTCTGTGGTTTAATTGTGAAAACATGATAGAAGTTTATACTAAGAGAGA

CTGGTGAAGATTTGCTCTCTCTTAGGCAGAATATAACATGGTGAGTGAGAATTTGTG

>c10174_g1_i1 len=1607 path=[1630:0-156 42:157-1606]

TATATATTTATATATATATATTTAATTATTTATTTTGAAGTATATTAATATAAATTTGTC

TATTTGTCTTTTTCATTTTTTTTTTATTTTTTTCTTTCTATTTAAATTAAAAATAAATGT

TAAATTAATGGAAATATTACCGGCAGGATATTCTGATTTTAGAAACAAAAATTATTGGAA

TAGATTCTTCAATATATTAGATAAGAAAAATTTCGAATGGTATGGTAATTATGAAGATAT

AAAAAATATTATTTATGAATGTATAAGAAATAGGTTTAATTATTCTAATAATAGTGATGA

AAAAGATAATTTAAGTTTGAAAAGAATAAACAAAAATTGCCTATTGATAAATGCAGGTTG

TGGTAACTCCAATATAAGTTATGAATTTTTTGAAGATGGTTTTCAAAATATCATAAATTT

AGATTATTCAGAAGTAGTTATTGAAAAAATGTGTAAAAAATACGGAGATAAAATGAGATT

TATAAATATTGATATGAGTGACACAGAAAGTTTTGATAAAGTATTGGAGAACTTAGAAGA

AGAAAAAATAAAAAATAAGAATGACTATAAAATATTTTTTGATAAAGCATTTTTAGATGC

ATATTTATCTTGTGAAGAAAGTGAAGAAGAAGTGTGTAAAAAAAATGCTAAAAATTATTT

TTCACTTATATTTAAGTACTTAAATATTGGTGATGTTTTTATAATCATTACATTAGCGCA

GCACTATATAATTAAGGAAGTTGTTAGAAATATTTATAATCAATATATAAAATTAGAAAT

AATTCCTTTTCTAATTAAAAAAGATACAAATCAATTTAAATATCATCCTTATGTTTTTGC

ATTTTACAAAAGCAATGAAAAGAATGATTATACTGCAAAATTTATAAATTTGCAAAAAAA

TATGAAGGTTGTATCAATATGGAAATTACCACAAGAAATAAAACAGACACAAGAAAACTT

AAATCTTCATATTTTTAAAAAAGGAAAGAGAATAATTCTAGACATTTTTAACCAAAAAAT

AAACAAATGCGAGTATAATATAATTATCTATGACTCAAATAATTTAGATATAAAATATAA

TACAGTTCTTATTGTAATTCCAATTGGATATGAATTTCATTCTTTATATTCAACATCTGA

AGGAAATGAAGAATTAGCAAAAAAAGCTCAAACCAAAAGATTATTGTTAGTTATGAAATC

AAACTTTTTAGTAAAGGAAAACAAAGAAATAGACGAAAAAGAGGATATAAAAAAAAAAAA

TGATATATACCAAAATAATGTTACAGAAAATCCTAATTTCCAGGATGATAATAAAGTTAA

CATGAATAATCTTATAAAATGCAACTCAGACAAAAATACAAATATAGATTCAAATGATCA

TATAAAAATAAGCTCAGATAATAATAAAAATATTCCTAGTAACATTAGTCAAAGCAAATG

TTCTGAAAATAGTAAAGTTTATTCAGATGCTATGTTAAATAATAATGTTCATAATGAATT

ATATGAAGATAGAAATTCTATTAATATACTATTAGATTCAATCAAAAATGATCTTAAAAA

TATTTTAAATGATTTAGCTTTACCCAATTCAAACACAAAGTTACCTA

>c10174_g1_i2 len=1470 path=[22:0-19 42:20-1469]

TAGGCAATTTTTGTTTATTCTTTAGAAACAAAAATTATTGGAATAGATTCTTCAATATAT

TAGATAAGAAAAATTTCGAATGGTATGGTAATTATGAAGATATAAAAAATATTATTTATG

AATGTATAAGAAATAGGTTTAATTATTCTAATAATAGTGATGAAAAAGATAATTTAAGTT

TGAAAAGAATAAACAAAAATTGCCTATTGATAAATGCAGGTTGTGGTAACTCCAATATAA

GTTATGAATTTTTTGAAGATGGTTTTCAAAATATCATAAATTTAGATTATTCAGAAGTAG

TTATTGAAAAAATGTGTAAAAAATACGGAGATAAAATGAGATTTATAAATATTGATATGA

GTGACACAGAAAGTTTTGATAAAGTATTGGAGAACTTAGAAGAAGAAAAAATAAAAAATA

AGAATGACTATAAAATATTTTTTGATAAAGCATTTTTAGATGCATATTTATCTTGTGAAG

AAAGTGAAGAAGAAGTGTGTAAAAAAAATGCTAAAAATTATTTTTCACTTATATTTAAGT

ACTTAAATATTGGTGATGTTTTTATAATCATTACATTAGCGCAGCACTATATAATTAAGG

AAGTTGTTAGAAATATTTATAATCAATATATAAAATTAGAAATAATTCCTTTTCTAATTA

AAAAAGATACAAATCAATTTAAATATCATCCTTATGTTTTTGCATTTTACAAAAGCAATG

AAAAGAATGATTATACTGCAAAATTTATAAATTTGCAAAAAAATATGAAGGTTGTATCAA

TATGGAAATTACCACAAGAAATAAAACAGACACAAGAAAACTTAAATCTTCATATTTTTA

AAAAAGGAAAGAGAATAATTCTAGACATTTTTAACCAAAAAATAAACAAATGCGAGTATA

ATATAATTATCTATGACTCAAATAATTTAGATATAAAATATAATACAGTTCTTATTGTAA

TTCCAATTGGATATGAATTTCATTCTTTATATTCAACATCTGAAGGAAATGAAGAATTAG

CAAAAAAAGCTCAAACCAAAAGATTATTGTTAGTTATGAAATCAAACTTTTTAGTAAAGG

AAAACAAAGAAATAGACGAAAAAGAGGATATAAAAAAAAAAAATGATATATACCAAAATA

ATGTTACAGAAAATCCTAATTTCCAGGATGATAATAAAGTTAACATGAATAATCTTATAA

AATGCAACTCAGACAAAAATACAAATATAGATTCAAATGATCATATAAAAATAAGCTCAG

ATAATAATAAAAATATTCCTAGTAACATTAGTCAAAGCAAATGTTCTGAAAATAGTAAAG

TTTATTCAGATGCTATGTTAAATAATAATGTTCATAATGAATTATATGAAGATAGAAATT

CTATTAATATACTATTAGATTCAATCAAAAATGATCTTAAAAATATTTTAAATGATTTAG

CTTTACCCAATTCAAACACAAAGTTACCTA

>c10175_g1_i1 len=374 path=[408:0-345 55:346-373]

GTTAACAGAAAAAAATAAAGCATAGATTTATTGAGTCCTGCTCCCATCCTAGCAGCCCCT

TTCCCATTGAGGCTGATAGGGAGAAGGCTGCCATCCTAGACCACAATCAGAAACAGGGAA

GACCAAGAGGTAGGGACCCAGCAAGGAACAGCAGCCTTCCAGCCACCGTAGCTGAAGCCG

GGCTCACCCAAAGAAGGTCCCTGGACACCCCACCACACTCAGCCATGCACCCACACCTCA

AAAAGCAGCTATTGGTGGGCTGCATATCCAAGGGGTAGACCACATATCCCTTCTGTGGGC

AGACCTTGTCTCTGCTCAGGGTAGAGTTGCTGTCTCTGCTGGCCTGGGGACTGACCCTGA

CACTACGGTGCTGC

>c10175_g1_i2 len=374 path=[408:0-345 752:346-373]

GTTAACAGAAAAAAATAAAGCATAGATTTATTGAGTCCTGCTCCCATCCTAGCAGCCCCT

TTCCCATTGAGGCTGATAGGGAGAAGGCTGCCATCCTAGACCACAATCAGAAACAGGGAA

GACCAAGAGGTAGGGACCCAGCAAGGAACAGCAGCCTTCCAGCCACCGTAGCTGAAGCCG

GGCTCACCCAAAGAAGGTCCCTGGACACCCCACCACACTCAGCCATGCACCCACACCTCA

AAAAGCAGCTATTGGTGGGCTGCATATCCAAGGGGTAGACCACATATCCCTTCTGTGGGC

AGACCTTGTCTCTGCTCAGGGTAGAGTTGCTGTCTCTGCTGGCCTGCAAGCAGCACCGTA

GTGTCAGGGTCAGT

>c10182_g1_i1 len=277 path=[306:0-214 526:215-276]

GGAAGGTTCATGGGGGCATGCTGGGAAGGTTATAGGGTCCTGGAAGGCTCTTTGGGGGTC

CTGGAAGGTTCTTAAGGGGGGGTCCTGAGGCGGTTATAGGGTCCTGGAAGGTTCATGCGG

GCATCCTGGGAAGGTTATAGGGTCCTGGGAGGCTCTTTGGGGTCCTGGAAGGTTCAGGAC

CCCTCATGAACCTTCCAGCACCCTATGACCTCCCTAGGACCCCCCATGAACCCTACAGGA

CTCCAAACCTCCCCAGGACCCCCTCAAGAACCTTCCA

>c10183_g1_i1 len=251 path=[284:0-87 56:88-250]

TTAGGGAGTGGTAATGTGTTTCTGCAGTTAACTTCATCTGATTCCTGCCTCTCCAGGCAG

AATCCCCATTTCTGTACCTGTTTTCTCTTTCTCTTTTACGGATCCTTCAGCACTTTCTAT

GCAGTTTTTCCAGTATTCCTGCATGGAGGTTCATCAGTTACCATATCCACACCTCCAATT

GCTCCCAGGTTTTTGTACTGCCTGTTTTGAAGTTCTCTTTAACTAATTAAAAACGAAGTT

CTGCTTAAAAA

>c10189_g1_i1 len=225 path=[203:0-224]

AAAGATAAAAAATAAAAATAAAAATATTTAAAATAATAAAGAAGATGGAAAACAGAACAA

TCAATATTCAAAATATAGTGTACAGTAGTACAGTATCAAGTATTTTTGTTAGTCTGATTT

GTACACCACTCGATGTAATAAAAAATTATATACAATATAACAATAATATAAGTTTTGATA

AGAAGTATATTTTAAAAAAAATTACAAAAAAAAAAAAATCAAACA

>c10189_g2_i1 len=1074 path=[1488:0-1073]

GTTTAGGTAACCTTGTGTTTGAATAGGGAAGATATAATAAATTAATGTATTTTATATAAA

TATAATATAAAAAGTATGTAAAAATGAGTCAAAACATAATGATTATAATAATAAATTAGT

AATTAAAATAATGATATGTTGAAAAAAGGATTGCACAAGTAGGAACTATTTTTACTAGTC

TTGGAAATATTCCTATATAAAATCCTTTTATTCCATTTTTTTTAAAAATAAATTTAGAAA

CTGAAAAAATGTTATAATTAGAATTATTTGTATATTTAAAATAGTTATAATATTTATAAC

TATAGGTGTTACAACCATATTTATGTGATCCATATTTATAATAAATTGATCTATGATTAT

TGTGAGCATAATTATTTATTTTCACATCGCATATATATTTGTTATTATATAGATTGTTTT

GACATATATTATAAAAAGTGTTTGTTTTGTTTCTCGAATAGATATCATAGTTTTTTATTT

TTCTGTAATCAAATTCACTTTTGTGAATAATATCAATGCATCTTGCTTGCATATTTGTTT

TTATAATATCCAGTGGATGGGTTATAAAAGTAGTAATGGTACTACTTAAACATCCACATA

TAAATGGATACACAAATTTTTTAATAAATTTTTTTCTTTTTTCAAATTCAGTATCATTTT

TTTTTATATAGCTAACTAAATACTCATTAAGTGACCAATATAAAGCTGAAAATGGAATAT

CTCTAATAAGTGTTGATGTAATACCTTTATATATATTTATTTTAACTTTTCTATTTTTTT

TATCTTTAAATATATCAAATATTGTAATTTTATAATTATTACAAACATTCGCCTGAATAT

TTGTCTTATATAATTCTAATGGTGACGTAATTATTATTGAAATAAATCGTGAAATAGTTG

CACTAAGATAATAAGGAATTCCTTTTTCTTTTAATTCTTCATACACATAAAAAAATAAGG

TGTTATTTATTACATACAAATTAGTAGTTGAAACTAAACCTCTATAAATTGCTTTAATCC

CATAATTATTATAAATGTTTTTAAATGTTTGATAATAAAAAGAGTTAAATTTTA

>c10192_g1_i1 len=302 path=[280:0-245 526:246-301]

GTTTAGTGGGCAATATTGGTGGCAGCTGGATGGTTGGACTAGATGGACTAAAGGTCTTTT

CCAGCTTTAATGATTCTATGATCTCCATAAATCTCAGCATCTACTTCTGTCCCCTTATTC

AATTTTGCTGTTTTCTATTCATTTAATACCTTGTTTGGCCTCTTACTGTGAGCCACTCTG

TTGATAAAACAACTTATTTTGGATCCCTTGAACAACATAAGGACCACCTTTTACTCCTCC

CATTGCCATCTCAAACTCCTCATGATCACATCAGAGCTCTCAGGCTGTGCAGTGCATGCT

CG

>c10194_g1_i1 len=207 path=[377:0-206]

AATGTTGGTCAGGATTCAGTGTGTGTGAGAGAAACCTATGGACTAATCCTGGAGATTGAA

GTATTATCTACAGTAATAATCTACAGCAGTATTATCTACAGCAGTTCATCTAATGGCAAG

TTTTTGCTTTAACAGATAGCGAGCATCGCGTTTTCACGTGTCACATACCTTACTCTCTTT

GTGATGCTGCATGCAGAGGGAGGTGAT

>c10196_g1_i1 len=216 path=[1:0-215]

GGATGGATTTGCTCTCTTTGTGCAGTGCCCGAATTGTTTAGGGCCGTGGTCCTGCTCTGT

GCATATATCCTGTAAAATCCCTTTGTGAGGCCGGGGGGCTTCTGCACGTCCATGTGTTTA

ACAGTGAAAATAAATCCTGTGAAAATGAGAACTGGAAATTTAGGGCTACCAAAGCACAGC

CTGTACCTGCAGCTGTGTTGGGCAGTGCTGCTCCTG

>c10197_g1_i1 len=400 path=[593:0-99 20:100-399]

GTAGGCCGGGAGGGAATGAAGTCCTCGCGTTAGGGCAGGCGCAGGGGACCGAACAAAGCC

TGAGGCGTGCGGGGAGCGGGCGGATTCCCGGGGAGATCCCTGCTGTCAGCTGTACGGGAG

GGCTGGGGGCTGCTGGCCTGCTTTCTCACTCCTGGGATGAAAAATTCCCATGGGAAATCT

CAGCGGAGAAGGAAGCAGAGAGGTGTGCCCACCGTGTGTGCGAGGTAGCGGCTGAAGATG

ATCCCGGGATTACGGGCTGGGGTGACACGGACAGGGATGTGATTAACATCGATGGCAGCG

TGCAGCTGGGGCTGTGCAGAGGTTTGGAGGAGAGAAGGTGAAGTTACACAGAGAGGTGGT

GTGGAAGGCACACCAGGGTAACTCTTTGCTTGCTTCAGAA

>c10197_g1_i2 len=319 path=[1:0-18 20:19-318]

TTGTCCTCGGCAGTCAGCATGCTGTCAGCTGTACGGGAGGGCTGGGGGCTGCTGGCCTGC

TTTCTCACTCCTGGGATGAAAAATTCCCATGGGAAATCTCAGCGGAGAAGGAAGCAGAGA

GGTGTGCCCACCGTGTGTGCGAGGTAGCGGCTGAAGATGATCCCGGGATTACGGGCTGGG

GTGACACGGACAGGGATGTGATTAACATCGATGGCAGCGTGCAGCTGGGGCTGTGCAGAG

GTTTGGAGGAGAGAAGGTGAAGTTACACAGAGAGGTGGTGTGGAAGGCACACCAGGGTAA

CTCTTTGCTTGCTTCAGAA

>c10201_g1_i1 len=250 path=[490:0-249]

GGAGAAGCAGCAAGGGGTTAAAACCCCACCTGGAGAGGTTTAGGCTGGATATCAGGGAAA

GCTTTGTTCATCCAGCCCCAGACTGCATTATTTGAAATCAGAGCGCACAGTGTGAGATGG

ACAAACTTCCCCTTGCTAAATGACCTGGTTAGGAATGGAGCTGCTCTGGGGATGGGGAAT

AGGCAATGAATTTGCTGGAAAAAGGAAGCACTGCCCTCAGCACCAGGATTCTGTGCAGGG

TCTCAGCAGG

>c10205_g1_i1 len=221 path=[177:0-133 113:134-220]

AGTGTGGTCCCCCCAGAGCTCCTCTTCTCCAGATGGACCCATCCCATTCCCTCTGCTGCT

CCACACAGTGCTGTGTTCCAGAGCCCTCACAGCTCCACTCGTTCTCTGCACACACTCCAG

GGCCTCCACGTCTTTCTTGTACTGAGGGGCTCAAACCGAATACAGTACTCAAGGTGTGGC

ATCATCAGTGCCACATATAAGGAAATGATCACTTCCCTTGG

>c10206_g1_i1 len=217 path=[531:0-191 722:192-216]

ACTAATTAATTTTAAATGAGCTTCAGTGAGTATTTACTAATAATATAAATGTTAACTAAG

GAATTATTTTTATTTGTTGTTTTTGGGAAATTGTGTGCATATAATTATTTATATAATTGA

GAGGGAAAGGATTTAGTTTCTTTAATAAGCGTGTGCATATCTCTGCGTGCTATAAAAAAG

TACTAATCTTTAATTATATATTCTTGTGAATTATAAA

>c10210_g1_i1 len=217 path=[155:0-216]

AAATCCTAGGTTTGTTACCCACCTTGATTAGTGGACGACCAGCTGATCCGTGCTTCTTAC

GGCCTCCATTTGCATAGCCTCTTTAATGTGGTGTATCTGCTGGATTTTGAAATAAACGTT

TTCAGGAGCCTCTGAATTCTGAGTGATTTGGAGTATATTTTTCTGTAGGTATGAAGCATT

TCTTTCTGCTGATTTAAAGGGAATGCAAAACACTGGG

>c10211_g1_i1 len=217 path=[253:0-216]

ATCTATACCAGGCATCTTAAGGAACGGGACACTTCCCCTCCTAAACAGGACCCCCCTCCG

GTGGAGCAACCATCGTCCCTTCACTGATATGTCACCGGGCCTAGAATCCCCCGGAGCACC

TCCCCCAGTGGCGACGAAGACAGACTACTCCTCCGGCCGAGATAGGCGACCCACAGCGCC

ACCGTCCGTGCCCGGACCACCCATGCCTCAGGAAGGT

>c10224_g1_i1 len=202 path=[39:0-119 38:120-201]

GTGAGAGAAGAAGTGGACAGACGAAGGCTCTTTTTTCATGTGAGTTATGTTATAATTGTT

TTCCATGTCTACTCTGTTCAGGAGAAAAAACAATTGGCGTATGTTGGAGAAAGAAAACAC

TGAGAACAGGAAGAGGTGGTCCAAGAGTTACAGTAAGAAGGGCTTGGAATGAACTTAGGG

GGGTAACTGGGGGTCTGTCTGT

>c10227_g1_i1 len=321 path=[299:0-320]

GAAATAAGAGCCAGACAAGGCACTTCGTGAGATGCTTTCAACACATCAGCTTATTAGGTG

GCAAAGTCTTTATCAATTTTCTACTGTGAGAATTAAGTATAAGAGAGATGAGGTTGCATC

TGGTATTAGGAGGTTGCAAAAACATCAAGACAGCAAAGTCAATGGGTAACACAGCCTTCT

GATAATCGATAATGAAGATCAGAGGCTCAATTTCAGAAGACAAAATGACATCTGTGTTGT

TTATCACTTATTGATGTACTGAAGACTTGGCCTAGACTCTGGATTTTGCTGGATTACCTG

AACTTGAAGACTCTGTTAGGG

>c10230_g1_i1 len=217 path=[521:0-216]

ATTTTAAATTGTGATTAGCATCATTTTTGTACAGACACTTTTTCTTAATCCATAGTAAAA

ACAGGGTAGTGTAAAAATCTTGTTGGTGCTCATATATGTACCTAATTTTCTATTGCTACA

TTTGCTCTTAGGGCAAAGAATGTTAACTAGTTAGTACCTCTTGTAGCTATTTGACAGAGA

AACAAGCATCATATATCAGTATATAAAATATATATGT

>c10233_g1_i1 len=357 path=[373:0-318 25:319-356]

GGTACAGAGTTTGGGGTGAGGTTGGAGCAGCACCGGCTCTTCTTGCACTGTGATTTTTGG

GAGTACTATAGGAGAAGAAACTCCTCAGGAAGCAGAACTACCCCAATCCTCCAAAAGCGT

TCAGCACTTACGCTGCTGTGTGTCCACGGAAGCTGAGTTTTATATCCTAAACTCCTCATG

ATTATTCCAACCCTATATCAAAAGCTTTGGCACCCTTTTGTTCCTGTTCGGAAGGAGGGA

AGGAACCACCACTCCTCAACCCATGTGGAGATGACTCCGTTTTAGCTTCCTTTCTGCTTC

CATCAGTTGTGTTCATTAAATTAATTAACCCTCCCCTAAATTGAGGAGATCGTGCTG

>c10241_g1_i1 len=251 path=[1:0-250]

GGAAAATGCTCATAACAGACAAAAGTAAGTGCAGTCCTAACCTGATTCTAAATAAATGTG

ATTTGTCATGTGGGCACGTTTTGGATTTGTGCAATTCAGTGTTTTCTAGACCGGTGTTAA

CCAATGGTAATAATTGTAGGGTAGAGAAAGCACAGTATTCCATCATAGCTCTGAAAGAGA

GTGAGACAGTTGAGGATTCCTGTAATGATGCCGTGCAAGAAAATATGAACTATGTGAGGT

ATTGAATTAGT

>c10241_g2_i1 len=259 path=[693:0-258]

AGGAATGACTAAGCTCAGAGTTCAAAAGTCCTGGCATAACAATGCTCTCATGAGCACTCC

AAGCTATGCTTCAGGTGTTACTTTAACTGTACATCCACAAGTTCCCTCACACCACCTGAG

CCATTAGGTCGGTACTATCTTCTGAATTAAGTTGCAAGATCATGTGGTAATCATGGCTTC

TGGTTAATTCATATATATTACAAGAACAGTATCATCAAAACCTCAGTCCTAGTACGACGT

TCTGCTTCCTTCATGAACT

>c10247_g1_i1 len=233 path=[211:0-114 326:115-232]

AGGTAGGCATGCAAATAAAATACAGAACTTGAAACAGATAGAAAATGAAATTGACTGTCA

CTGTTATCAGTGTGGAGTTAGAATATTTGTATTGCAGCCAGCATGACATATGAAGAAGTC

TTTGTCTTGGTTACTAAGGACTGTAAACCACAAGGATTAGTAGGTGATATAGACAGCTTG

TGTCAGTGGTAGATCCCTTACAAGACAGAACAAAGAAGTTTGGGCTCAAGAAA

>c10249_g1_i1 len=509 path=[644:0-120 158:121-508]

GGAGGAGTTCACGGCGTCGAAGAGCTCTTCTCCCCCCGTCTCCTCCTTGGCCTCGCAGCC

ATCGTCTGTGTCACACTTGAGGTACCCACTCTCTTCAAAGAAGTATCCATTCCTTGACAT

CGCTGCTGAAAATGAAAGTGCTGGCTGCGTGTTACTACACACATCGAGGAAGTACCTGAA

ATTAAAGAATTTTGAGGAGGAAGTCAGAGCCCACAGAGACCTGGATGGGTTCTTGGCCAG

AGCCAGCATCATCCTTGATGAAACAGCCACCTCTTTGGATGATGTTCTTCGAGAGATGCT

GAAACATTTTGCTGAAGACCCCGAGAACACGGAGCCAAGCTGTAACTTTGAAAAAATCAT

GAGCACTTTATTCACGGATTCAGGGACCCCGCGAGAAGGGAATGGCATCCATGACGTTTC

AGAGCCAACAGGAATGAATATACAAACATGTACATCGCACCTCAAATTTCACCTCTTATC

AGATACAATTCAAGGGGTCACAGCGACAG

>c10249_g1_i2 len=466 path=[80:0-77 158:78-465]

GACTTTGGTGGCGGTTTCGGGGTCAGCGGCGGCGTTCTTTTTTAATTATTTCTTTTTAAC

CTTTTTCCACCCAACACTGCTGCTGAAAATGAAAGTGCTGGCTGCGTGTTACTACACACA

TCGAGGAAGTACCTGAAATTAAAGAATTTTGAGGAGGAAGTCAGAGCCCACAGAGACCTG

GATGGGTTCTTGGCCAGAGCCAGCATCATCCTTGATGAAACAGCCACCTCTTTGGATGAT

GTTCTTCGAGAGATGCTGAAACATTTTGCTGAAGACCCCGAGAACACGGAGCCAAGCTGT

AACTTTGAAAAAATCATGAGCACTTTATTCACGGATTCAGGGACCCCGCGAGAAGGGAAT

GGCATCCATGACGTTTCAGAGCCAACAGGAATGAATATACAAACATGTACATCGCACCTC

AAATTTCACCTCTTATCAGATACAATTCAAGGGGTCACAGCGACAG

>c10251_g1_i1 len=223 path=[201:0-222]

AAAATCAGGTGACAGATTGGAAAACCCACTTCTGCAGTTGCAGAAACGGAACATCCTGCA

ATAATCTGAGTTTGTTTTTTTTTTTTTAGATTGCTGCTTTCATAGCCGAATCCATGCAGA

GCTGTGGAGGCCAAGTAATTCCACCTGTGGGCTATTTCCAGAAAGTGGCAGAGTATGTAC

ATCACTTCAGAAGGGCATACCTGTAGGCCAAGAAGGCCACCGG

>c10254_g1_i1 len=220 path=[316:0-53 370:54-100 25:101-219]

TTAGTGTCAGATGCCATTTTGTTAAACTGCCCCTCTGCTGCCACCTGTCCGAGTGCTCAG

TTTTTGCCAATTATCTGAGTGTCACGTATCTTAAGAGCACATCCAATGAGACTATATTTA

GACCGTCTGCTCAATTCATCTGTGCCAGAAAAGAACCATTATATTATTTTCAATTACAAT

TACCATTGCCTGAATTTGAATTTGCAACAGTCAATGTAAG

>c10254_g1_i2 len=220 path=[316:0-53 370:54-100 416:101-219]

TTAGTGTCAGATGCCATTTTGTTAAACTGCCCCTCTGCTGCCACCTGTCCGAGTGCTCAG

TTTTTGCCAATTATCTGAGTGTCACGTATCTTAAGAGCACAACCAACCAGAAGTGCTTAT

AAACTCAAGTTCTGTTTCCTTAATAAGTAATTTTCAGTAAAAGCATTCCCGGAGGACTGG

GAAGAACTGTCTAGCCTTACATTGACTGTTGCAAATTCAA

>c10258_g1_i1 len=216 path=[1:0-215]

AAGGTGGTTCGTGGTGGGAATGTTCCAGTTTCAATGCTCTGGAGGGCAGTTTGGAGCTTG

GGGGGAAAAACGGAGCCTTGTTGTTTGGCACCAGGTGTGGGGAGGCTGCAGTGCATGGCC

AGGGCGGCCGGTCTGCATCTCTCGGTTGCCCTCTCTTTTCACGTTCCTGCTTCCTGCCCT

TCCTGTCCCTCTGTGGTTCCCGCTCTTGTTCCCAGT

>c10261_g1_i1 len=268 path=[45:0-188 22:189-267]

ACAGTTCTGACCACAAACAACCTGGTCAAGAAAAATACATAGAAGTTTTTATGGGTTTTT

TAATGTTTTTTTTTTTGTTGTTCTTTTTGTTTTAGAGGCAGCAATCTGTATTACAATTAC

AATGTATCATCTTATTTTTTCAACCACTACTTTCCTTTGTGATTCTACCAAGACCACAGA

CTCTTTAGTCACACTGCAAATACCTTCATGGCAATCCTATTAACATAAAACATCGCTTAC

AGCATGTTTAATGGACTTTTATTACCTA

>c10262_g1_i1 len=217 path=[1:0-216]

GACGGCCACGTCCTCGTCAAGGTCCTGGCAGGGCTGCGGGGTCCTGGCCGGGCGGGACAG

AGGGGTGGGTGTCCCCAGGGCCGCACCGCGGGGCTCCGTCCCCTTCCCCAAGAAACAAAG

GGCTCCGTGGTGCCCCGGTGTCCCATGAAGCAGAGTGATGTGAAATCCCCCTTTGGAGCT

CTGGGGCTTTTGCCCTCTAATTGCTGTGGGTCACTGA

>c10271_g1_i1 len=418 path=[396:0-195 592:196-417]

AATTTAGTGCAATTGTTCTTCTTCAGCATCCAGGAGTTTTAAACAGTGTCAGAGATTGAA

GGCTTGCTGAAATTGCTCGTATGTAACAGTGAATGTTGTTATCATGAATGTAGCTTGTTA

ATGCGTCCTTTAGAAGTGCCATTGTGTTCTGTTTGTTCCTATTGTATTGGTGTGAAAATA

TGTGGATGCTTTTATCTGTAGGAAAGATAAGAAAGAGAAGCCCCTATTGACGTGATTTTA

GTTACAGTATAGATGATGACTTAAATGACTTAAGTCGCGTTCTGAAAGTAAGAAATAAAG

CACTTCTGCTTATCAAGATAATGACTTGTCCTTTTAATTCTGTTGGAAAAAATGTTTTTA

ACTTGTATTAGAAAGGAATTTTGATGTATGTAAATTTGAAATCTAGATAACATGAAAT

>c10271_g1_i2 len=396 path=[965:0-173 592:174-395]

ACATACGAGCAATTTCAGAATATCTCTATATGTCATCTTGAAAAAGGCAATGTATGGAAG

AGCTTAAAGTTTGGTTTTCTTAAATAAGCCCACCAATAATCTTACTGCACCTGAATACTT

GGTTATTTTTATGAACCAAGAGTGAATTTAGTGCAATTGTTCTTTTTTTTTTTTTGTAGG

AAAGATAAGAAAGAGAAGCCCCTATTGACGTGATTTTAGTTACAGTATAGATGATGACTT

AAATGACTTAAGTCGCGTTCTGAAAGTAAGAAATAAAGCACTTCTGCTTATCAAGATAAT

GACTTGTCCTTTTAATTCTGTTGGAAAAAATGTTTTTAACTTGTATTAGAAAGGAATTTT

GATGTATGTAAATTTGAAATCTAGATAACATGAAAT

>c10272_g1_i1 len=260 path=[55:0-186 241:187-259]

GTTTGGTGGTCAGCAGATTGGAAAAATGAATACTGGTAGGGAATGATGATCGTTTTTTAC

TGAATCAGGTTGTTTTTGTGGAACATCACTGAATGTATTGTCAAGAAATTTAGCTTTACA

CTGAAATATTTTTTTCTTGTTTGTCTGTTTTAATACATGCAATGGTGAGATATTCTAGTG

CAGGTTTAATGTGTCATTATGATGTGAAAATAATCAGTTAAAAATCTGTACAGAAAATAG

TATTAAGTCTCTTTTGTAAT

>c10272_g1_i2 len=213 path=[55:0-186 52:187-212]

GTTTGGTGGTCAGCAGATTGGAAAAATGAATACTGGTAGGGAATGATGATCGTTTTTTAC

TGAATCAGGTTGTTTTTGTGGAACATCACTGAATGTATTGTCAAGAAATTTAGCTTTACA

CTGAAATATTTTTTTCTTGTTTGTCTGTTTTAATACATGCAATGGTGAGATATTCTAGTG

CAGGTTTGGCCAAAAGTGCTCTTAAACCTGAAA

>c10274_g1_i1 len=307 path=[439:0-149 155:150-306]

TCAATAGTAAACTACCTCCCCCTGTGAAATAAGACAAAAAAAAGGTTGTAGTCATGGAGC

AACAAGCTAATTTGTGCCAACAACAAAGCTGCCAATTGCCCGCCACGGCAAACTGACCAG

CCATAGTGTTTGCTGTCCGAACAAAGCTCTCCTTTTAGCTCTTTGGTTACCTTACTGAAG

CCCTTTTTCCACCTCACAGCACTTTTACTTAAATTGCTACCCAAGGACTGCCCTGTCACA

ACTTCTCACAGTGCATCCTTGGTAAAGCAAGGAATTAAACAGAAAAGAAGCACTAGATGA

AGAAGAA

>c10277_g1_i1 len=245 path=[71:0-99 70:100-244]

AAATTAGGGAACACACTCCCTTTACTCAGAACCCATACTGCCTGTAACTGTGCAGGTGGG

AAGGGGAGGGATTAGAACTTTCTATGATTTGTTGGAGCGGGCAGCCCGGTGGTACTGCAC

ATCTCCATCCTTGAAGATACAAGACTGGATCAGGGCCCTGTGCAACGTGGCACAGCTTGC

TTTGTTTTAAGCTAGGGCTTAGAGCAGGTGACTCTAGAGATCCTTTCCAATTGAAATTAG

TCCAT

>c10283_g1_i1 len=257 path=[687:0-256]

GAGGACTCTTAGCTTGAAAAAGGAGCTGAAGGGGAGCAGGACGAGCAGCATGACTGCAAG

CATCCAGGGATAGGGATGTGGAAGCTGCAGTGAGGTTTGCGAGGGCTGAGTTTCAAGGAA

GAAAAGAAATCCACCATGAGAATGGCATCCCAGCGCTCCCTGAGGCTCGGGCAGTTTGGG

GCTGTGCCCACTGCCCTCCAGTGCAGAACCTTTCCTCGGTCCCCCGCAGCACTGCTTGAT

GGCTGCAGCCCTGCACT

>c10283_g2_i1 len=249 path=[227:0-248]

GTTCTTCCGCGAGCTGAGCTCCCACTGCTGCAGGAGAGGGTGGATGGATTGAAATAATTC

AGCCCCCTGTGTGGAGCCACAGCCTCTGTGCTCTGCAGGGAGGGCTCAGCTCACAGAGAA

GCTTCCCCGGGACAGCCCAGAAGTGAAGCAATAGGGTAGGAAGGGCAGAATACACGGAGC

GCCAACGAGCGAGGACCAGGAGTACAAAGATGCTCAAATGAGGTGGGGTAAAGCAAATGA

GGACTCTTA

>c10289_g1_i1 len=212 path=[344:0-211]

GGCATGGGCACCAAGCTGAGCGTGATCTGCATTCACTGTGCTGAGAGGACCTCGATAAAA

GAAGAGACAGCAGGGAAGGATGAGAGAACAAGCTCTCTTTTTCCCCACAAGGCAGGAAAA

GAGCAGACAAGCAGTAAGACATCCACACCAGACAGAAGCAGGCCAGACTGAAATCCAAGA

TGCTAAACCCCAATTCAAGGAGAGCTCTATGG

>c10292_g1_i1 len=398 path=[775:0-13 20:14-397]

AGACCGCAGCACGTCGGCCGGGTCCCCGCAGGCTGCTCTGTAAATAGACGTCGCAAACAA

AGGGCGAGGGCAGAGACGGTGGCTCGGTGGAAGAAAAGGCTGCGGGCAGTTGGCAGAGCT

CGCAGGACGCAAAGCGGCAGCCCTCGGAGATCGCGGCGTGAACGGAACGGAAAAGGGAAA

ACTGGAAATGAGTCATTTGCGCGCCGGGGGGACGGCTTTGCACCACCGTCAGATCCCGGC

GATGGCTTTTACTCAACACAGGAAGACGTGGGGTGCGATAACCTCAAACCGCGCTGTGCC

AGCGGAGCTCTGCGGGCGGCACTGCGGGCGCAGCGCTGCGGCGGCTCCGGGCGGGGAGGG

CCGCGCCGAGATGGGTCACTGCGGTGCGTGTGGAGGAA

>c10292_g1_i2 len=403 path=[1:0-18 20:19-402]

GTCTGTGCGGGGCTGAAGGCGGCCGGGTCCCCGCAGGCTGCTCTGTAAATAGACGTCGCA

AACAAAGGGCGAGGGCAGAGACGGTGGCTCGGTGGAAGAAAAGGCTGCGGGCAGTTGGCA

GAGCTCGCAGGACGCAAAGCGGCAGCCCTCGGAGATCGCGGCGTGAACGGAACGGAAAAG

GGAAAACTGGAAATGAGTCATTTGCGCGCCGGGGGGACGGCTTTGCACCACCGTCAGATC

CCGGCGATGGCTTTTACTCAACACAGGAAGACGTGGGGTGCGATAACCTCAAACCGCGCT

GTGCCAGCGGAGCTCTGCGGGCGGCACTGCGGGCGCAGCGCTGCGGCGGCTCCGGGCGGG

GAGGGCCGCGCCGAGATGGGTCACTGCGGTGCGTGTGGAGGAA

>c10307_g1_i1 len=282 path=[1:0-281]

AGAGCTGCAGGTGGGAGCAGTGCGCTGTGGCCATTGCAGAGTTCAGGGCAACACCAGGAT

GTGTTTTGATGCATCAGAAGCTGAGGGGCTGTTTCCAGATGTGCAAAGTGCTGCCACCAC

TCGGGGTGTGCGGTGCACCTGCAGGTCTCTGGAGGGATTCCTCCATTAACTGCAGCTCCC

GCAGCACACGTCGAGGTTTCAAAAAGTGATATATTGCCCAAACTGGGGAAGGGATGCAGG

GCTGCCCCGTGCCACCCCATGTGCACGGCTGCTCATCATGCA

>c10308_g1_i1 len=253 path=[1:0-252]

ATAAAGAATCAAGTAAAACTACCTTTCTCTGTAATAAGAGAAAACTTAATTTACAAATTC

TTGTGCATTCTGTAACTTGGACTACTAAATAAAAAAATTACAGGAAACCATTCTTCAGAC

AATTCATACTGAAATTCTTATTTTTGTGCAGTACTTAAAAGATGCATCACTTCTGTAGCT

GCCGCAAAAGCAAAGTTCACACAATTGGTGAAACACAGAAGAATTGAAGGCAAAAAAAAA

AAAAAATCAAACA

>c10308_g2_i1 len=320 path=[461:0-319]

GGTGAAAGTATATGCATGTGATTTTACTGAGAGGACTAAGGAAGTTTTCCAGTGTGGCAG

CATTTTGTTGCTGCTGTTACTTCTCGATTTTGTTTTAGTTAGTTAATTTTGTTCATTTTG

TTATCCAAAAGTTTGGAAGGTCACAAAGTTAATTGTTATGGTAGGAACTAAGTAGCTGGT

CATAACTAATCAGGTACTGGCCAGAATTAACGTAGCGTTTTCTTAAACCTTAAGTTCAGG

ATTCTACTTTGCTGTCCAGAGAAACTTTTCTAAGCCACTTGTGCAAGTAGAGATACAGGT

GCTCAGTTTCATTATCTTTT

>c10309_g1_i1 len=227 path=[205:0-226]

AAGGCTGGAGTTAATGCCTCCCCCCTCCCTCTGTCAGCATTGTTTGGAAAACAGGGGAAG

GGCTCAGGGAGAGGCAGGTGTGCAAACAGCTCCTGCAGCTCCCCTCTGCTCTTGGCCTGC

AAGAAGATATTAAAACAATACATTCAGAGCTTTCTGGTAAAAGCTTTTCTCTATCCATGG

GTGGGATACATTTCCTTGTCCTGCCCTCACTCAGTGGGGTACCTCTG

>c10314_g1_i1 len=249 path=[1:0-248]

TTTAGCGGCTCAGGGGGACAGCCCATGACGCACGCCCGGCGCACAGCAGCGCACCGCTGG

CCACGTGTCGGCAGCTGCTATTGATCAGCCCAAACCCGGCCTGGAACCGTGCTTCAGAAA

CCAGCAGTGCCCTTGGGATGTGTGTGAGAAGCGCCCTGATGGAGGAACAGCAGGGATGGA

GCAGGGCAGAGCAGCTGGAGCTGGCCACACCACAGGTCCAGCAACCCCACTAGTCTGACT

GAAAGAGCG

>c10322_g1_i1 len=244 path=[1:0-243]

ATGAACTCCCTTCCCGGAAATCATTAACGTAAAAGCCAAAAGTAAGAGCTCTGTTGTTCA

CTTAAGACAATTAGCACAACCCTTATTTTAGGTAGCAGTCTGTAGAGAGACTCCGAGAGT

GTAAGTACTTTCTCCTGAAACAAGGTTGGATTACTTAATGCTGTTTGTTACATTTTAAAG

TAAACAATTAAGAAAAACTGAAACCTTTTTAAACCATTGATGCTGTGACAGTGCAAACAG

CATA

>c10322_g2_i1 len=328 path=[443:0-327]

ATACAACAACTGCAAACTGCAATAATGCAAAATGATGATGAAACTTTTTTTCAAGAACTA

GAGGCAGACAGACTGAGATCTAGACTTCAGATGGCTTCCTTTCAGTATAACAAAAGTGGT

TTCTCTTAAGACATGGTAAATAACACTGCAGCTGTCTGGCTGAGCTGGAGGGAGTCTCTG

AGAACTGCTGTGACAGGAAGGTGGTGTGTAGCTGTTGTTCTGTAAGATCAGATCTGTCAT

CTTGATGTGCCTGAGGCGTAGTTGTTGGTACCTGGAACTTTGAGCACTTTTATAATTAAT

AAAGTTTTAAACTCCTTTCAAAAAAAAA

>c10323_g1_i1 len=222 path=[43:0-221]

GTGGGCAACCAAGGTGATGATACCCTCCTCATCTGATTCCTAACTGTGTGGTGTTTGAAG

GGTTGGGCTCTGGCACAGATTGATAGAATGCCTTGTGTTCTTAATGGGCTTGCAGAGAGG

AATCTCCTAACACTGTATCAGCTCATGGGATCCAATATTATGCATGGTTCAGGTTTGTAA

ATTGAAATAGCACAGCAACTGGCTGGAAAGCAGCTTTGCAGC

>c10325_g1_i1 len=288 path=[155:0-287]

ATCAAAGAAAAGAGAAGCTACTTTTAGACTAGCTTAAAAATAACTCTAAGGAAAGACAAT

TGAGATCAACATGTTCAGGCTGCTACTGAACATGAAACAGCACTGGCATTGTCATTTCCT

CCCCTTGTATGCTACCAAGTACGTGAGAGGGGGGAGAAGAAAATGTCCCATCTGATTTGG

CCTACAAGTGATGCTCTGAGCCAGGAAAGGTACTCTGGCCCTGTTCATTTGTTCTGGCCA

CTAAGCTCTACTTGTAAGAAGTTTAGGAAAGTAAAGGATTAAAACAAG

>c10337_g1_i1 len=225 path=[1:0-224]

TAGCTCCTTTCAGAAGAACGTTAGTGACAGTGATGTAGCATCACGTCTTCTCTTACACGA

GGTCTCCTGGTAGTGCTCAGCTTCCCTTTTGCACAAATGAGCAGAACACCAACCCAGCAC

CCCCACAGCCCCGCTCAGGCCCTCACCTCTGCAGCCTGGGCACTTCCATCCTCTCCCCAG

CGCTGCAAAGGAGGTAAAGCACTTTCAGGAACCCCACATAAAGAG

>c10346_g1_i1 len=263 path=[531:0-262]

GCGCTCACAGGATGGAGGGTTCGCTTTCTCCACGTCGTCCTCCTCCACCTCCAGGACAGC

TCCGTCGTGGTCCAGCTTCACCTTCACCTTACCCTCGGGCAGGGCGCTGGGCTCCTCTGG

CCGCAGCTGGCTGCCCAAGGAGAAGCCGTCCTTGTGCACCAGCCACACCTTGTCGGTCTC

ATACCAAGCCTCCTCGGCAGCGATTTGCTCCTCTGTCTTGGCATCCCACGCGGCACGGCG

CGTCCTGCACACGCTGCGGCAGC

>c10348_g1_i1 len=216 path=[251:0-215]

ATGGACATACCCTGAAGACTGCATTGTACATGAGTTCAGATTATGTGACCGTAGTTGTTT

CTTCTGACTCTAAATCCACTTAACATTGCACTGCCAAACAAAAGAGCTGGGTCCACTCTC

TCGTTTAGCAATATCACCACAGGTGATCAGTGGCAGTCACTGAAGCTCACTGTGCTTCTG

AAGTTTAGAGTGAGTCATAGAATCATAGAATCGCTA

>c10349_g1_i1 len=216 path=[396:0-215]

TGCTTTCACAGTCCAGCAGCAGCTAGAGGTGCATTCACTGGTATGCTGTGAAAAGTTACT

GGTTGTGCAATCTATCTTCATGAATAGAGGTTGTGAGCTACTGGAGGTACAGAGCACTGT

GTAGGACACGGTTGTTTCTGTATTCCTGACATCACCATCCCATGCCTTAGTCAACAATGA

TGCTACTCCAGCAGCAGCAAGAGTTGCAGGATGGAG

>c10354_g1_i1 len=208 path=[450:0-207]

ACCGTTGCTGTCCTTGATGTCCACAGTGGCCTGTGCCTCCAGCAGCAGTGAGATGAGGTC

CAGGCTGCCACCCAAGGCCGCATGGTGCAGTGCTGAGAATCCATCTGCATCCTGGTAGTT

CACATTCAGGCGCTTGGCAGATCCCAGGAGCTCTCTAGTGATCACAGCAGGCTGGTGTGC

AACACTAGCACTTTTGTCATCAGACGCA

>c10360_g1_i1 len=347 path=[265:0-211 495:212-346]

GAAACTGGACCTATGACCGGAAGATGTTCAGATATTTCAGTCTTTGTGTATAATGGCACT

GTATTTTCACATGTCATTATTGCGCTATCAAGGCATTTTTTAGATTTGGGCTGAAGCATT

TCCATTTTTTTCCTCTCTTCAGCATTGGGCTTATTTTTGATGACAGTGGCAGAAAGAACT

GGGCGCTTTTTCGGTTGCTCTATCTGAGAAGAACATTCATCTGTGCCCTTTTTAAGTACA

CGGAGATTTTTAAGTCCAGATTCTTTTGTAAATGACAGTTATCAAGTTGATGGAGATATA

GTAGAGCCACCCGTTCCAATTCTGTCACCTGATCAATTTGTGAAAGA

>c10369_g1_i1 len=209 path=[187:0-208]

TGATGATTACCAGCAAGAGTATGAGGTTCACTTCATAAACTGGGACTAGATGATGAACAC

CCATGTCCTTGGCTCCTCACCAAAATTCTGAGTCCTCTCCTTACTAGGAATAACACAATT

ATCTAGGGGATATAAAATAGCAGTTCTAAGTAGTGCATACATTTCAACTGTGATGCATGT

CCCCTTTTTTTCAATCTGGCATTTCACTA

>c10369_g2_i1 len=238 path=[588:0-237]

GTCTTCACAGCATATTTATTTATTTTTTTACTTATTTTAGAGAAATATGCCTTTGATTTC

ACTAGAAACTTCTTCAGAGGAAGATGTTTTAGGCTGAGTATCTGAGATGCATGCATCTGG

ATGGTCAGAGATACAAGAAAAATAAAACAGAAGCTTAAAAGAGTAAGAATAATAAAACTG

AGGTTCCTCTTTAAATATACATCTATGGAGAAGTGTGGGGTACAGCTTGTATATGATA

>c10381_g1_i1 len=209 path=[187:0-208]

TCTGTCTGTGGCACCAGGAGAAAAAACCAACAAAACAAAACATGAGTCTGCTTTCAACGT

TCAGTTCTGGAGTTGCAACAAAGCAGTCCTGTTGGTGCCCACAGTCCCTGCCAAAGCTGT

GTCATTTCAGAGCTGCAGCACACAGTGAATTTCTATGGCACCAGTGCTGACATGAAGCAT

AGCGTTCAACCCCAGAGTGGTGCCTGTCG

>c10384_g1_i1 len=210 path=[300:0-88 395:89-209]

GGCAGGTCATACGGGATGGCAGGATGGGACAGGGCGGCTGGAGCAGGACAGGCACAAGAG

GGACAGGATGGGGTGACACAAAGGATGGCCCTGCCCTCCCCACTGGCAGCAGGACAGGCG

CTGCACTCTCTTGGGAACATCCTAACTGAGCACACTGCCTTCCTTACCAGGACAGTCAGA

TGAGAGAGCGTACTGAAAATGGTGAGACGG

>c10386_g1_i1 len=334 path=[426:0-191 617:192-253 925:254-256 682:257-333]

TTTTTTTTTTTTTTTTTTTTTAATCTTTTCTGGTTTTATTTAGGTAAAACTCATGTACAA

TTCACACCAGCAAAGAGCTCCAACAGATCTAATTGGCAGTGCAGATACTGAGAGCTACGT

GCTCAAATAAGCCGAGGTGGGATTACTGCTTCAAGTCAAAGATATTTTAACACAAGCTCC

AGCAATTGTTTATGGAGGACAGCTGTTACAGAGTAGGGTTTACTGATTTATCACTATTAA

TTACGTACCTTTCCTGGGGGATAGTTATGCACAACTTTCGAAGGCACGAATTTGCATTAC

AGATTTTCACAATTCCAATCCCAATCAACTTGCT

>c10386_g1_i2 len=376 path=[426:0-191 617:192-253 925:254-256 21:257-375]

TTTTTTTTTTTTTTTTTTTTTAATCTTTTCTGGTTTTATTTAGGTAAAACTCATGTACAA

TTCACACCAGCAAAGAGCTCCAACAGATCTAATTGGCAGTGCAGATACTGAGAGCTACGT

GCTCAAATAAGCCGAGGTGGGATTACTGCTTCAAGTCAAAGATATTTTAACACAAGCTCC

AGCAATTGTTTATGGAGGACAGCTGTTACAGAGTAGGGTTTACTGATTTATCACTATTAA

TTACGTACCTTTCCTGGAGGAAGGGGCTTGCAGCCTTTAGGAGTGTGGCAGATGTTTTGC

AAATACAGAGATGCTGTTGAAGATCCCCTGCAGGGATGCAGGGAGATGCAAAGCAAGTTG

ATTGGGATTGGAATTG

>c10386_g1_i3 len=314 path=[426:0-191 925:192-194 21:195-313]

TTTTTTTTTTTTTTTTTTTTTAATCTTTTCTGGTTTTATTTAGGTAAAACTCATGTACAA

TTCACACCAGCAAAGAGCTCCAACAGATCTAATTGGCAGTGCAGATACTGAGAGCTACGT

GCTCAAATAAGCCGAGGTGGGATTACTGCTTCAAGTCAAAGATATTTTAACACAAGCTCC

AGCAATTGTTTATGGAGGAAGGGGCTTGCAGCCTTTAGGAGTGTGGCAGATGTTTTGCAA

ATACAGAGATGCTGTTGAAGATCCCCTGCAGGGATGCAGGGAGATGCAAAGCAAGTTGAT

TGGGATTGGAATTG

>c10397_g1_i1 len=410 path=[1:0-75 77:76-409]

CCTTTTTTTTTAATACATGTTCTCTTCTTTGCATTCAAGTTTGGAAGAGATGTATTATTA

GCTCTGTGAGCATGTACGTCACTTGGACCACGCTTTCTCACCTGCATGTTTTCCATCATT

AATGAACCATATCGCTCTATTTAATTCCACAGTGGTTTAAGATTCTTGATTGCAAGACCT

GACAGCAAGGAGTATTTGCAGATTCCCAAGCTCTGTTCTTCATATAGAGGTGCTTGTTTT

AAATCAGGACTTGCTGTAAAACAGTGTCAACTCACTGACATGTTGTTTCCAAGGATATTC

ATGTTGAAGGAAGTGCTTGTGGTGGGTGGAGGAAGTTTCTTTCAATCATAAGGGTTGCTA

TGCAATTAGCAGAGCAAAAACCACCTGAGACCTCCAGATGTGTAATGCAG

>c10397_g1_i2 len=559 path=[690:0-224 77:225-558]

TAGGAGCCCATTATTATCCATTATTCTGCGATGCAGTATTTCCTTTTATTATTAATGTTC

TCCTGTGACTAATTCAAACAAGACCAGAAATCTTTCTGGTGAATAATTAACACTTCCTGT

CCTGTTCAGGTGGCTTGTAAAGACAGATTCAGGTGGTAAAAACACAATGTATATATCGTT

GTACTGTATATAGAATTGTATAGTAAATTAAGAAATGTGTTGCATCGTCACTTGGACCAC

GCTTTCTCACCTGCATGTTTTCCATCATTAATGAACCATATCGCTCTATTTAATTCCACA

GTGGTTTAAGATTCTTGATTGCAAGACCTGACAGCAAGGAGTATTTGCAGATTCCCAAGC

TCTGTTCTTCATATAGAGGTGCTTGTTTTAAATCAGGACTTGCTGTAAAACAGTGTCAAC

TCACTGACATGTTGTTTCCAAGGATATTCATGTTGAAGGAAGTGCTTGTGGTGGGTGGAG

GAAGTTTCTTTCAATCATAAGGGTTGCTATGCAATTAGCAGAGCAAAAACCACCTGAGAC

CTCCAGATGTGTAATGCAG

>c10404_g1_i1 len=308 path=[261:0-307]

GCGGCCCCATCCGCGCCGGGATGGAGGCGTCCGGCCGCTGCCGGCGGCACCTTCCCGTCC

TGCTGCTTTGGGTTAACGGTCTAATGATGCTTGGATTAGCAGGTGTGCATCAACAGATTA

ATCTCTGCAATGAATCTCTCATGCTAGAAAGGTTGCCTGCCTGTGGAAAGTTCTTTGAAG

AGATGATGAAGAAAGTGGACTCCAAAAAATGGTGCAACCTGACAGAATTTATCACGTATT

ATAATATCTTTACCCAGTGCACAGAACGTGAAGCCAATGATGCAAGTTGCTTCTGGCCAA

ACCCCCTT

>c10411_g1_i1 len=322 path=[498:0-321]

TTTGTTTGTTTGTTTTTCCTCCTTTATCACTTTCACCCCCCCGCTTTCACTCTCAAGTCA

CCTTGTCAGTAATCTCTGAGACCAGCCTGTCAGAACCAAATCTGTTTCAGTGGAAAAGAA

CCCTAATTTCCCAAGTCAAAGGTGACTTGCTACAACATCCTCGCTGCCAGCTCCTCAACA

AGACATTATCTTGAGATGTCTGTTTGAATTTGATGAAACTTCCATCAGAGCCACTGGGCA

CTTGATCCATAGACTTCAGTCATATAAAGTCCTATGTAAGGTCCACTCAATTCCCTGCAG

GAGAAAGTTTTTCATCTCTGTA

>c10414_g1_i1 len=223 path=[1:0-77 79:78-222]

GCAGGACTGGGCCTTAATTGAGCTCTGCCCACTGTTATGTGGAGCAAGTGGGTGATTTTT

AGTACATTTAGTGCTGAGAACAGGAGCTTTTTTGAGCTGAAAGAGGTGAAGTGGACCTCG

AGCGCCCAGTTACAGTGTTCAGTTTGCTCTGACGTGCTTAGGAGGGGGGAACGACAGCAT

CCGTGGGCCTCGATGACATGGAAATTCAGCAGGAACACTGGAA

>c10417_g1_i1 len=535 path=[749:0-534]

AATAGAAGCCGCTCACTTCTTTATGGAAGGACTCAGCTGCTCACAACAGCAAGCCATTGG

CTTATTCAACAGGCAAGTAGTAGACTTGTCCTTCTAGGAAAAGGGAAGATGGAAACCAGA

AGTTCTTGCTTGTACTTTCCAGAACACATCTTCTGAAGATATTTGAATATGATAGCCAAG

AAGACTGAGCAGTCTTTGAAGAAACAGAGGACCATGCAGCTGAAGAGATGCAATAGGTTT

TCAGAGAACTGAATCCTTGCACATCCTTCAGCTGAATAAGCCCACCCTTCTGAAGAGTTT

GGTCCAGGCTTTGTACATCAAACAACCTTCAGTGAAGTTATTCTTGCCTCATACAAGTGA

TCTTTTGCTTCACTGAGGGACTCGTCTTCACAGTCAACCATGGTGTGACTCCTCCAAGTG

GTCCTACTGGCTCTGAGAGGTGACCCCAGCAGTCTAGAAAGCCTGAAGACCCTTCCTAAA

AAGATGAACCCTGCGCTCTGTGCCTTTGACATATGGCAGCAGAGGAGCAGTCTGA

>c10419_g1_i1 len=373 path=[49:0-14 48:15-33 24:34-369 475:370-372]

AAAAAAAAAAAAAAAAAAACACACCTCAGTGTTCTAGACAAACATAGTGCTGACAGCGTA

ACAATGTGAGCTGTAAAGCAAATGTTTTCTCTTACAACCTGTGGTGTAGCCCTTCTGAGA

CTGGGAATGAAATCCATATTCCCAAAAAGAGGTCATCTGTGTTGCCACTGAGGTGGTGAT

TTTTTTACTGGCACATCTCTTACCAGTTCGCTATTTTTTAAAATGGAAACTGAAGAAGTG

CATTGCTTTTGAAAACTGATGTGATTTCTTCTTAGGGACTTGCAGATGGCATATTTTAAT

ATGCCCTTTCAATTTAAACAAATTATCTTACTCTATTTTTTTTCTCTTATACTGACAAGA

ACAAAACAGAAGA

>c10431_g1_i1 len=433 path=[411:0-432]

GTCTAGTCCTCTTTTTCTTCCACTAAAAATTTTGAGAGAACATAAAACATTTATAAAAAT

AAAATTACTTTATAACCATATACATATATAAATATGATTTAATAAAAATATAAAAAAAAT

GTGTAAAGAAATTTTTTGTATACATAAATAAATGGATATGATTGAGTAAATGTATAAATG

AAGTACAAATAATAGTATGATGAATTTGTAATTATAACTTACTACAATAAATTATATATA

CGTAATATATATATATATAATTACACAATCAAACAGTAGAATTAAAAATGCATTTCTTAT

ATAGAAATTAACAAAAAACATATATACATGGCAAATTATAGGTATTAACCTTTTTAAAAA

ATTTAATTTTAGAATACATATAATATTAAAAATAAGAATGTACACAAACGAAAAATAAAT

AAAAAAAAAAAAA

>c10438_g1_i1 len=287 path=[155:0-286]

GCACTTCAGATGAGCCGTGAATTGCCATGATGGTCTGACTAGTGATCTAGTGGGTAGCAC

GCTGCCTATGTCAGGGAGGTTAAAACTAGGTGATCATTAAGATCCCTTCCAACCCAAGCC

TTTCTAAGATCCTACACTGGAGGAGGCAAGAGGAACTGGAGGGTTGGTGCACACTGCTAT

TCCTAGAACCTGCCTCCCTTGGGAAGGTATTTCTGACATGCTTCTCTGTATGCTAGAAAT

ATCCATCTTTACATTCCCTAAATCATGCAGATGTCCCCATGCTCTTA

>c10449_g1_i1 len=223 path=[201:0-222]

ACTTAAAAATATTTTATCCTCATTGGTGCCAAAAGCTTTTATATAAACTTGCCACAAGAC

AAGGGTGCCAAGGAAGAAACATTCGATAGAAGAGCTGTTAAGAAGCTCAGTCTAGGCAAG

AAACCCCTGATCACAAGCACTGAAATAAGAGTCTATATACAGCTAAAGAAAGAAGTCTTT

CCCACTTCATTGTGTGATCACACAGAAGCTGGAAAGGATTTAG

>c10454_g1_i1 len=284 path=[436:0-283]

AACTGTGCTCTAATTCTGCATATTTTTTAAGGCTGTTGCAGTTCTAGATCAAAATTGAAT

CTAATTCATCGTGATGACACCCTTTGTCTACCTTGTACTTCTGATCAGTATTTAAAATAA

TGGTCTGTGAGCATAACTGTGTTATTACAACCGCTGTGATGTCAGTGTATTTATTTCTCG

TGGTAGATTATGGTTATAGCAAATCCTCATAAATGAAGGTCTATGACAGTGTCTGCTACT

GGGTCTTTATTGGCTTTTCGTCTGTCAAACACAAAGTTTAGGTA

>c10458_g1_i1 len=215 path=[549:0-41 104:42-214]

GCATTAAGTACAAACGCCATCTGAGAAACTGAGAAAGCTTTAACACTTAATGTATAAAGT

CTTAAGTTAATTCACATAGTAATAAAATTTATGTTCTGTAATTAGTTTTTTTGGCAGGCA

TCTGTTTCACAGTAATGAATTGGTAGCAGTTCCTAAAATGTCTGTTGACTGTCTAGAGAA

ATGTAGGAGAGCTGGCATTCTTAAAATGTTTCTTA

>c10458_g1_i2 len=276 path=[1:0-102 104:103-275]

GTAACTTTGTGTTTGAGCTTGTATACCTTTCTGCTTTTATTCTGCCTTGTTTATACACTG

GGCTGTTTATTGTACAATGTTCTGCGGTCCTAGAGTAGTTTTTACACTTAATGTATAAAG

TCTTAAGTTAATTCACATAGTAATAAAATTTATGTTCTGTAATTAGTTTTTTTGGCAGGC

ATCTGTTTCACAGTAATGAATTGGTAGCAGTTCCTAAAATGTCTGTTGACTGTCTAGAGA

AATGTAGGAGAGCTGGCATTCTTAAAATGTTTCTTA

>c10459_g1_i1 len=203 path=[437:0-107 222:108-202]

ATAAAAAAATAAATTTCACAACTAATGCCAGCTGAATGTTTACTGCAGTACCCAGCAGTC

AGTTTATCAACATGGATGTGATGCTCTGCCCAAAGTGATACTACACCATCTTCCTTCCTT

TCAGATCTTTGCCATATAATATCCACTTAATGGCAGCACAACTCTGCTCTCAAGTGTGAT

AATGACACTTGAACAGCATCTGT

>c10460_g1_i1 len=218 path=[232:0-175 756:176-179 25:180-217]

TAAAAACACATAATCTCAAAGTCAGAGGAACCCTCAAGCTCCATCCATTTGCCCAGGCAC

AAAATACTGCAGCAACATAAAGCCCCGTTTCCTCCCACTCTTTGCTAGTTCTGAATTCAT

CTCTGCACTTGTCCTGAGCAATTCTGACAGTCACTTATCTTCAGTCTATGGAAGCAGTAC

GCCAGCTGACAGCTGCAAAGCCATGTTTTGTTGTGCAG

>c10470_g1_i1 len=270 path=[1:0-269]

GCTGCACTGGCGGCTGCCTGCAGCATCCCCCTGGCACAGAGCAGCTGTCGGGAAGCCAAG

CAGCAGATCCTCTACACTTGCTGCTATGCCAGCCCGAGTCTGTGCCAGCACCTGGGGAGG

AGCCAGCAAGTATGGGATTTCCTTATGCAGCTCTCAAAGGGCAAGGTCCCCATGGCAGAG

GTGACTCGGAAGGTGGCCTGTGAGGCATCCCTATACCTGCTGGCCCTGCTTACCCTGCAG

CCCCAGGCCTCTCCTTCCAGGCTGGAGGAG

>c10471_g1_i1 len=348 path=[1:0-347]

GGTGAAAGCTGAAAAAACCTGAAAATTGAGAAGTCTCTGCAGGCCGTTAAAATAACTTTC

ACCGTATGTACTCCTGGACATTTAAATGCAATAGATTGCAGTCTTAAAGACCAAGTAATT

ACCGTTATCAATTAGAGAAGTGGTAACCTTGTTCTCAGCAGGTGCTCAAAGCTGTTGCCA

GCCTCAGTTCACCACGGGATGGCATTGCCCTGACCGTGGCCCTTTCCGGGCATCCCTTGC

TTCTTCCCAGCTCCTGGAAGCTCCTGTGTTCTGGAGGAGTTCTCACTGACCGTAGAGAGA

GGAGAGTGCCCAGAGCCACACAGGCAGCTGTGGGAGCAGCGCAGGAAA

>c10474_g1_i1 len=291 path=[1:0-290]

GAAATGGTTGCAGTTCTTCTCTTATGCTGTCTCACGTGCCAAAAAGGCAGAATAATGATG

TCCAGCACAGCTCCATCAACCAAAGATCCAAAGGACCAAAGATCCAAAGATCCAGAGGAC

TGCCATGCAGAAACATGCTGCAAAATCGAACCGCTGACATTTAGGACTGGAGAACTCTCT

GACTCAGGCCTCAGCTCCTCTAATTAAACTTAGTTTATCGCTCTTGCACAAATGCTAAAG

GACGAGGCATGATCTCTAGAAGTGAGTGGATATTTTCCATCATCAGCATTG

>c10486_g1_i1 len=201 path=[53:0-200]

GGCAAAATTGCTTTTCCACCAAACTTAAAGGTAGGGCACAGGTGGTTCTCTTCCACAGAA

GTCCACTGTAAATGTGTACCAAACACTTTATTTTCACTGAAAAGGGTAACAAATTAAGAA

AGTTGCGTATACAAACAGGTTGAACAAAATATTACAAGTGAACTTATCAATACATACAGA

ATCCCACCCCCCAAAACGTCG

>c10487_g1_i1 len=216 path=[194:0-215]

GCAAAAACGATCAGAGAAATAAGCTTCACGTATATTTCCAGACCCATTTACGTTGTTGCA

GCTCCTACCTCAGTCCTTCCAGTCCTCTGGCTTGGTAGATGGCCCAAACGTTGGGCAATG

CCATGGGTTAGAGGGGAAAGACGCAAAGATGACAGCACAGCTGCAGAGAGATTCAATGGA

TGGTGCTGCACCATAGCATATCATGCAATGCCAAAA

>c10488_g1_i1 len=281 path=[1:0-280]

GTAAACGTTCTTTAGTGATATGAATGTATCGTTCTTATGACATTATATCTGATAGTCTCT

GCAATACTATTAGTGAAACCTGACCATCCGTCAGTGATTCATTTGCTTATCTTTCGTAAA

GGGAAAATTGCTTTCAATCTGATTTGTTTTCTAAGAATGCTTGCCGGGCACAGAAGTGCT

GCTTTCCATTTAGCTGTTTTTCAAACGCAGTGAGAGCTCTACTTAACAAACCCCTTACAG

ACATCTATTCAGCTGTCCTCCAAGCTTCACACAGCCCCAGC

>c10489_g1_i1 len=228 path=[1:0-227]

TCAGATTCTCCAAGAAATGTCAGGAAGCTTCAGCAGGACTGCTGCTACAGACTTACTATC

TAAAATCTGTTTATAGGGCACAAATGATAGTTTCACCTAAAATGCTTTGGTTTAGTTTTA

AAATAAGGCAGCTTTACAGTAACTCAACAGGAATGCATAAAATCTCATAACAGCAATTAT

TCTTTTAGATCTGCTTTAAAAGAATGCAAATACCAGAGCAGTGGAAGG

>c10490_g1_i1 len=458 path=[436:0-324 761:325-457]

AATACCATGAGCAACACATTACAAAATGGGGATCATGAGATTAGTTGTTTTGAACTGGGA

TTCACTGCATGACCCTTACTATTCTGCACTTATTCTTCATTCATCTGATAAAGTTTTATG

CATGAGACTGTCTTCTGAGCAGAATTTCTTCTAGAAACAAGAACTAACAGAAACATACAC

AACCATGACTGCCAACCATACAGGATTAGGGTTTTGTATGTTTTCATTAGTTATGTTGAA

GTCCCAAGCGTAGAGAAGTTTGCTTGCATTGCCAGAGTACACCTGAGTGTTAATAAAGTT

TCTGTGGAAGGATTTTGCCATGCTGCAATGAAAATTACATTTGTGAGATGCGTAACAGGA

AAATGCATCAAGTTTGCACAGCAAAGAGTGGGGATTTGTTAGTTTTTCTCTCCCTTTTCT

GGACTAGCAGATCTATATCAGAGCATCTCACATAAGAA

>c10490_g1_i2 len=366 path=[436:0-324 894:325-365]

AATACCATGAGCAACACATTACAAAATGGGGATCATGAGATTAGTTGTTTTGAACTGGGA

TTCACTGCATGACCCTTACTATTCTGCACTTATTCTTCATTCATCTGATAAAGTTTTATG

CATGAGACTGTCTTCTGAGCAGAATTTCTTCTAGAAACAAGAACTAACAGAAACATACAC

AACCATGACTGCCAACCATACAGGATTAGGGTTTTGTATGTTTTCATTAGTTATGTTGAA

GTCCCAAGCGTAGAGAAGTTTGCTTGCATTGCCAGAGTACACCTGAGTGTTAATAAAGTT

TCTGTGGAAGGATTTTGCCATGCTGAACAACAAGATAAGCAAACAGATGACAAAATCCTT

CCACAG

>c10495_g1_i1 len=205 path=[205:0-204]

ACCTTCCTGCTCATCCAGCATCCTTGGCATCGTCCTGCGGGCCAGCAGTGACTGTCTCTC

TGATTCAGCGTGGAATTACTCAGCTGCAAGCAGTGGGTCTTACTCAGATGACACGTGAAA

CGGGTGAAGTGACTGCTGTCCCTCCTGGGAGGATGGCTGTGTGCTGCCAGGTTGGCAAAG

CTGAAATCCCGTGGCTTAAATGAGA

>c10496_g1_i1 len=360 path=[169:0-166 336:167-359]

CGGTAGAAGCTGAGGATCAAACCATGGCCAGTAGCACTGGAAACAGCTTTCACACTGGTG

CGGCCACACTTGGAGTGCTGTGTCCAGTCCTGGTGTCCGTAGTAGAAATGAGCTTACTGG

GATGGACTCCAGGAAGGATCAGTGAGGTGATAAAGTCACTGGAGTATCTCCCAATCTCTG

TCCCTCTGAGACTTGGGCATTGTAGGTTTGGATGGTGATTTGATTTACACAGGTCTTTTG

GTGCTCCACCGTGTCTGGCATCTTTCTCTTTTTCAACAAAGGCTTATTCTCCCTATTGGA

CTTTACTACCACAGAATACAAAAAGAACTGAACATATTTTTAAACCTCTGTATAAATCAG

>c10496_g1_i2 len=216 path=[529:0-22 336:23-215]

AAATCTTCAATCGATCACTAATCCTCCCAATCTCTGTCCCTCTGAGACTTGGGCATTGTA

GGTTTGGATGGTGATTTGATTTACACAGGTCTTTTGGTGCTCCACCGTGTCTGGCATCTT

TCTCTTTTTCAACAAAGGCTTATTCTCCCTATTGGACTTTACTACCACAGAATACAAAAA

GAACTGAACATATTTTTAAACCTCTGTATAAATCAG

>c10500_g1_i1 len=224 path=[202:0-223]

GGTCTTCTCGCGTCCCTGTGTGGTGGTTGCTCTTTCGGAAAGCAAAGGAACAGAGCAGGC

TAATCTTAGAGAAACTCTTAGTAGATGGGGGATCACACACTGTGGATGAGAGTTACTGGC

TCTAACAGGGAGCGTGAAAATGGCAGCAAACCATGCACCATGGAACAGCCCTCTTTCTGG

GTAGGTGTAGTGCCTTCTCCAACACTTTGCTACTCCCTCATTTG

>c10501_g1_i1 len=356 path=[544:0-355]

GTGGGATGCATGTAGAAGTATATATAATATGTCTGAATGGGATCTGCAATTCAGCTGACC

ACTTCTCTGTCCCAAAGCAAATCCAACTGTAGTGCTGTTTCTTGGCTGTGCAATTTGATT

TTTTCCTTCCTTCTCACTTGCATACGTGAAGGTTCTTTCATGCACTGGCAAACTCAGTGC

ATGGTTGCATCAAGGTCAGGGTTTCAGAGCACACAGCTGAGGTTGCCTCCTGCTGCTGAT

GGTTCTTTCTGAGTTTTCCTAGCAGATCTGCTTGTCTTTCTTTGCTCTGAGCTGCGGTGT

CTGTTCAGTCCAGATTTCTATGTCAGTGAGGAACTGTTGAAGCTACAAATATATGT

>c10502_g1_i1 len=246 path=[343:0-153 727:154-162 506:163-245]

TATACATATATATATATATAAAATAATCAATTTTTTTTATATAAATTCGAAATAAAATAA

TTAAAAAGAAAAAAGAAATGCAATATTCTATTAATATGCATTTTTATTATTGAATTATAA

TAATTATTGTCTAATTATTAATTATATGGATACTTATAAATATTATTTTTTTTCTATTTT

TTTTAAGATACTATATATTTCAGCTTTTTGAAAGTCTATTATTCAATGTACAGCTTTATT

TTTAGG

>c10502_g1_i2 len=276 path=[343:0-153 727:154-162 32:163-275]

TATACATATATATATATATAAAATAATCAATTTTTTTTATATAAATTCGAAATAAAATAA

TTAAAAAGAAAAAAGAAATGCAATATTCTATTAATATGCATTTTTATTATTGAATTATAA

TAATTATTGTCTAATTATTAATTATATGGATACTTATAAATATAAAAACAAGAATATGTT

AATACAGTATATAGATACACTTTTTTGCTTCTTTACAACCTACATTTATGGTTAGAATAA

GCAAATATATACCTAAAAATAAAGCTGTACATTGAA

>c10503_g1_i1 len=239 path=[375:0-238]

TTATGATACACACAAGCTACAAGACGATTATACTTTTGTTAAAAAAAAAAACAAACACCT

TTAATAAGCTTTACACCCAAAAGAACTTTAAAAGGTCTACAGCTGAATTAATGTGGGAAG

GGACTGGTACCCACTACTGTACTACAGTGGTGACAGAAGGCTATGTCCAAGTTTCTGCCC

CTAAAGACATTTTCCTTTGCATACGAAGTGGGATGGGACAAAGCCATTGTGACAGTGGA

>c10506_g1_i1 len=206 path=[184:0-205]

AGTCCAAGTTTCCGTAACTTCACAAGTTGGAAGAAAGGCTTGGGCAGCTCGCGGAGCTGG

TTGGCGTCCAACAGCAGCTCCTCCAGGCTGCGGCTGTAGCGGTAAATCTCCTCGGGCACG

GCGGCCAGCGAGCAGTGCCGCTTGTCGATGGACTCCACGTGACGGTTGCACCGCCACAGG

GGGATGCAGTGGAACATCGCCGCGGA

>c10506_g2_i1 len=342 path=[367:0-341]

TTCATTGTTTCCTAAATCAAGTTCTTCTAGACGCTGCAGCTGGGCAAGTGATTCGGGTAA

ATATGTCAGCAAATTCTCTCTAAGTTCCAGTGAAGCCAGGTTATAAAGATTCCCAATGTT

TTCAGGTAGTGCTTGCAGAGAGATATCATTTACTGAAAGACACGTTAGATTCTGTAATTC

AGGAAAGCTTTCGGGCAACCTAGTTAGTGGATTCCCACTGAAATCTGCTACCTGTAGTGC

CTTGCAGAATGAGATGCTTTCTGGTATTTCAGGAATATCATTTCGAGACAAATCGAGTTC

CACCAGCTGCATGAAATTCGCTATTTCTGGAGGGAGCCGCTG

>c10508_g1_i1 len=321 path=[366:0-247 832:248-252 618:253-320]

GCCAACCCTCCACTCATTTTCCTCAAAGCCACTGTTGGGATATGAAGAATAGGTTTTTGT

TTTGTTTTTCTACTAATAAACACCAATCATGATATAGCAGCTTCATGTTTACATCTATGA

TGTAAATAAACTCTAAACTCTCCAGATTATAAACTCTTCCATCACAAGGCTCAGAGCCCT

TTTACTTCAGGAACTCTCTAACACCATAAACAGTCCAATACAAGTTCAACATTCTTATTA

CATTCTTTCTCCTGTGGCAACTAGATAGCTCAGATGCTTGTTTTGATTTTCATATTTAAT

TTTTTTTGTGTGTGTGTGTTA

>c10508_g1_i2 len=321 path=[366:0-247 832:248-252 25:253-320]

GCCAACCCTCCACTCATTTTCCTCAAAGCCACTGTTGGGATATGAAGAATAGGTTTTTGT

TTTGTTTTTCTACTAATAAACACCAATCATGATATAGCAGCTTCATGTTTACATCTATGA

TGTAAATAAACTCTAAACTCTCCAGATTATAAACTCTTCCATCACAAGGCTCAGAGCCCT

TTTACTTCAGGAACTCTCTAACACCATAAACAGTCCAATACAAGTTCAACATTCTTATTA

CATTCTTTCTCCTTCATCACCAGCTTGTCTACTCTATAGGATTTCACTTCACAGGCTAAC

ACACACACACAAAAAAAATTA

>c10514_g1_i1 len=233 path=[211:0-232]

AAAAAAAAAATAAATAAAATAAATAAGTAAATAAATAAATAGATAAAACTCCTCAGTTTC

TGAAAGGCAACAAAGATTTCTTCTCACTTCATGTCCATTAGACCAGGTTGAATCTACAGA

CTTTGAAGATAGGCAAATGGCTGTTACAGTATGAGCACACAAGTTGAAAACATTACTCTG

ATGAGAACATTTATTGTGTAGTAGTATATCTATGATATCTGTGCCTTATTTTG

>c10520_g1_i1 len=225 path=[31:0-31 63:32-163 613:164-178 628:179-224]

GTGTCTTTTTAAGTTCCTTCATGTGATTTCATTGTAGGAGTGTGTATTCTATGTTTAGTA

AAACATAATAGGTATTGAACTTACTTGATGTCTTAAAGGATTTTCTCCTACTAGTGTATA

TATTTTAACATGAGTTATAAAAATTATTGTGGTCTACTACCCAGAACCATAATGTAATTA

GAATTAGCATTGTGTGTTGCTAGTTCTAATTACATTATGGTTCTG

>c10523_g1_i1 len=517 path=[7:0-224 232:225-516]

TTTTTTTTTTTTTTTTTGAAATAAGCATTTAAATAGTTTATTAAGAATTTAATAAAATTA

TAAGGATTTAAAAATACTATAACTTTTGACATTTTATAGTTATTTCTTATCTATAACTAA

AGTAATAAAATAAAAAAAAAATTTGTAGATGCATAAAATGAAAATAAATATCATCATAAG

AAAATAATAATATGAAAAAGGTAACATAAATATTTTTAAAATTAACAAAAAAAAAAAAAT

TAGGGATAGAAATTACTTTTGTAGTTCTTAAAATTTTATATCTTCTAGAAATTATTAAAA

AGATATTAATGTTCTATAATTTATTATTTTAAAACTATAAGAAATGTCAGATGTGTATAA

GTTTAGGAAAATTATGTGTAATTTCTTAAATATATATTCATGGACTATTTTAAAAAAACA

AAATGAAGATTTTTAGTTAAAAATGATATACAAGATGATTCAAAAAAGAATATAAAAAAA

TTTTTCCATCTATAATAAAAAAAATTTATTAAAAGAA

>c10532_g1_i1 len=418 path=[1:0-143 145:144-417]

ATGATAGTAAATATTATAAATCATTTCTTTAGATAATTATAAAATTTGATAATTGTAAGA

CTCATTTGTATCTAGTTTTGTAATTCCTTATAGATATATATTAATATTGTAAACTATACA

TTTATATATAGGTGTATTTTTCTTTTCTTTTTTCATTGTTATTTTTGATTAAATTACAAC

AATTTTTAAAAGTTTAAGAGAATAAATCTCATTATGAATGCTAATGTGTGTATAAACTAT

GTGAACATATAATTAAAAATAGAGAAAAAAGAATATATATAAAATTACATAGAAATAATT

AAAATAATTTTAATATATGAATTTAAAAAAAAGAATATTACATAAAGGAATTATTACTTT

TAGATGTAATAAAAGAGAGTAATATTCTTTTATAAATATGTAAAAAAAAAAAAAAAAA

>c10532_g1_i2 len=289 path=[806:0-14 145:15-288]

ACTTTGTGTTTGATATTCTTTTTTCATTGTTATTTTTGATTAAATTACAACAATTTTTAA

AAGTTTAAGAGAATAAATCTCATTATGAATGCTAATGTGTGTATAAACTATGTGAACATA

TAATTAAAAATAGAGAAAAAAGAATATATATAAAATTACATAGAAATAATTAAAATAATT

TTAATATATGAATTTAAAAAAAAGAATATTACATAAAGGAATTATTACTTTTAGATGTAA

TAAAAGAGAGTAATATTCTTTTATAAATATGTAAAAAAAAAAAAAAAAA

>c10535_g1_i1 len=202 path=[259:0-201]

GGGTGGGTGTACATTTGGTCACCGTTTTTAAGAGGAAACTCCTACAGCACCTTTTGCCCT

TTAAGCCGCGACTGCGCCCAAAGGAAGGTAAGCACAACAGCACATGTTGCCGCCTTCCAG

GCCATTCCTGCTGCGCTCCATCCTCTGCTGCTGGCTTATGACCAGAGCTGCTTGCCCGAG

CAGGGACATTCCTGCCCATCTG

>c10539_g1_i1 len=718 path=[1:0-717]

TGTCCAGATGCTTCTTGAACTCCATCATGTAGTTGTCAGTCTCCTACTCGACTCTGCAGA

CATGCATATATAAAAGTGTGTGTACATATATATTCTCGTTCTGTGCATAGTATGATTTTA

TCCATTTTTGAAAAGTCTACCATCAGTGCCAGTACTGACCATACAGGTTCATTTAGCGAG

TTATAATTATTGTGTTGAACTTGAATCAGCTGGACTGTAAGCCACAGAGAGACTTAACCA

TGAATTCGGATGCCCACAAACTAATTCTGGGGAATTTCTTGGAATACATCTTTAATCTGT

ATTTCTAATGTTGTTTTCCAGCCTCTTGTTCTTCCTAAGCAACTTCATTACACATGTATG

AGAGAAGGATTAATGTATTAATGGAATCAATAGTCAGTGGTGTCAAAAATGTGATGTTCA

GTGTTTGTAGACAGTATTGAGTATTAATGGCCTTTTGGGGTCTTTTTTATGCTTTTTTTT

CTTTTTCTAAAAAACTTTTTATATTTAAAAAAAATTATACAAAACAATCAGTTAAAAAAA

AAAAATGAAAAGTAAAAAAATTAATAAAATAAAAAACTGAAAATAGAAAACACAAAAAAT

TGATTATTTTTTATTTTATTTCATTTTTTCAACTTTTGTTCACACTTACACAAATTATTT

TTTTTGTATTAATTATATTTAAAAATTTTTAAAATGTATATATTTTATACATTTATAG

>c10543_g1_i1 len=307 path=[1:0-278 763:279-281 439:282-306]

GTGGGCTTTAGCCTTCCTCACTGCAATCCTGCATGCCCTGACAATGTTCCTACACTCCTT

CCAACTGACCAGACCCCCCTTCTACATTCCATAGACTATCTGCTTCCACTGGAATTTTTC

CATGAGCTCCTTGCTCATCCATATGTGTCCCTGCCACCTTTGCTTGATTTCTAATTCTTA

GGGATGCACTGATCTTCAGCTTGGAAGAACTGGTGCTAGAATGTCAACCAACTCTCTTGG

GCTCCCTTACCTTCCAGTGCTCTAACCCATGGCATACCTCCAACTATGACCTCTTCAAGG

ACCTATG

>c10543_g1_i2 len=307 path=[1:0-278 763:279-281 283:282-306]

GTGGGCTTTAGCCTTCCTCACTGCAATCCTGCATGCCCTGACAATGTTCCTACACTCCTT

CCAACTGACCAGACCCCCCTTCTACATTCCATAGACTATCTGCTTCCACTGGAATTTTTC

CATGAGCTCCTTGCTCATCCATATGTGTCCCTGCCACCTTTGCTTGATTTCTAATTCTTA

GGGATGCACTGATCTTCAGCTTGGAAGAACTGGTGCTAGAATGTCAACCAACTCTCTTGG

GCTCCCTTACCTTCCAGTGCTCTAACCCATGGCATACCTCCACATAGGTCCTTGAAGAGG

TCATAGT

>c10545_g1_i1 len=223 path=[461:0-222]

GTTTGATTGTTTGTTTCTGTTATTGATGATATTAAGCTTGCTTTATAAGCTACGCAGTGA

GTGAACTGTAACCATAGTGATTCCTTTTTCTTTTTCCCAGAAGGTAAGAACGTGGGGGGG

ACTACCCTGGTAAACACATACATTTCTTACAAGTCAAAAATAAACCTTCTGAATGGACAC

GTGGCCCTAGAATTTCTCACCGTTGTTCCAGTTTCTGTTAAGC

>c10548_g1_i1 len=348 path=[487:0-205 22:206-347]

AGCCTTTTCCTTTACACAAAAAGCGCTGCGATGAACACCCCCGGGTGCGGCTGTGGGTCT

GCCGCTGTCCGACTGCTGAAGGGCGATGTGAGACGGCTCCGGGTCCGGAGGCAGAAATTT

AAGTGCCTCAGAAGCTTGCCCTATGGGAACTTCAATACGTAGAGACCTCTGATGCCCCCC

CAGAGTGTGCTTGTTGTTGAATACAGCATTGGGGATTTGGGCTCACAGGTCCCACTCATC

AACTGACTGCTGAATGCTTCCTCTGGGTTTTGCGGTTCCTTTTTTGAAAACCGAAGAAGA

GAATGCAAGGTTCTTTATGTCTATTTGCATGACCTAACTGATAAAACA

>c10557_g1_i1 len=201 path=[425:0-200]

AGTGATGAACAAGAGCCTGCATGTCACGCTCATAAAAATCTACTTGACCTTCCAGAATGT

GGCTTGTTTTTCGTATCGCTGTTGCCACTGATGAAATGTACTACCCACCACCTCACTGTG

CTCATGTCCACTGTTTGGCCACCAAAAATGTTCAGCAAGCATGGATGAATGTAGGAGTGT

ACCATTTTTTTGGCATGGAGG

>c10559_g1_i1 len=657 path=[635:0-33 669:34-656]

GATTAAGATCCTCAATTAATTAAACATTTCCACTACAAGTCAGTTTACAGTTGGTTAGAC

ATACTGAAGAATTCAGGTTAGACTGAAACTGAAGAATTATGGGCATTAGGTTCACCATGT

TACTGATGGCATCAGAACTATAGCCATGTCATGCAGGTCAGGTGTTTGCTAAAATTACCA

TACTGTATATGAATCAAACAAGTGATTTTTATGGTATAAATAAAGTCTTAAGTATGCGTA

TTTTTGTAAAGAACAGCATACTGCATTACCTTTTAAGCAGTTAATTCTCACTATAAGCCC

ATTCCAAAGCTATGGGACATTGTCTTTGGCACAAGTGGAGGGAAGGTGTTGTGGCTTACA

CTGACATAAACAGGGTATTTTACTTCTTTTTTTTACTACTCATGGGCCTGCACCAATTAC

AATTTAAAAACAAAAACAAACCTGCAACTGCAAACAAGTTTAGATGCTACACTACAGAAT

GCCACGTTGTGCAGCTTTTTCACATTTTTCTTTAATGTAAAACCTGGTGAAAAAAGTCAT

GGAATCCAGGTACTACCCATTCACTGGGCTGAAATACCATATTCCCTTCAGCACAACAAA

ACAGAACAGCCTCAGCAGAACATATTCTGCCCCTGCAAATATCCTGGCCTTGTGATC

>c10559_g1_i2 len=790 path=[1436:0-166 669:167-789]

GAATCTGTTTAAATCAAAAAGGATTTGAAATACTTTTATTGCCATTTCACACTATTGAGT

CAAGAGAGTTAATACTGTTGAAAGAGTTAATAATACTGATGATGGAGTTAATACTGTTAA

CAAGTTCTGTAAAAGCAACAGTGGTGATGAAACATGAGACTACTGAAACAAGTCAGTTTA

CAGTTGGTTAGACATACTGAAGAATTCAGGTTAGACTGAAACTGAAGAATTATGGGCATT

AGGTTCACCATGTTACTGATGGCATCAGAACTATAGCCATGTCATGCAGGTCAGGTGTTT

GCTAAAATTACCATACTGTATATGAATCAAACAAGTGATTTTTATGGTATAAATAAAGTC

TTAAGTATGCGTATTTTTGTAAAGAACAGCATACTGCATTACCTTTTAAGCAGTTAATTC

TCACTATAAGCCCATTCCAAAGCTATGGGACATTGTCTTTGGCACAAGTGGAGGGAAGGT

GTTGTGGCTTACACTGACATAAACAGGGTATTTTACTTCTTTTTTTTACTACTCATGGGC

CTGCACCAATTACAATTTAAAAACAAAAACAAACCTGCAACTGCAAACAAGTTTAGATGC

TACACTACAGAATGCCACGTTGTGCAGCTTTTTCACATTTTTCTTTAATGTAAAACCTGG

TGAAAAAAGTCATGGAATCCAGGTACTACCCATTCACTGGGCTGAAATACCATATTCCCT

TCAGCACAACAAAACAGAACAGCCTCAGCAGAACATATTCTGCCCCTGCAAATATCCTGG

CCTTGTGATC

>c10564_g1_i1 len=684 path=[381:0-366 782:367-683]

TGAAAAAAAAGTTAAACCCGCAATAACGCTGCGCTACACATCTTGCAGCACTGTTTTAGG

TAATGCTATTTCACCATCTAGCAGAACACAGAAAGACAAGAGATGGAGGCAGAGGTGCTT

GCCAATTCCAGTCAGGCAAAAGCATAGCCACCTCAGCATCAGAGAAGCCTACTTTGCAGT

CCTGACCTCTTGTTACTCTCTACGCTACTTTTCTTTAAAATGTTAACAATATCAGGGATA

TATATAGCTGGATTACTTTTAATGTCCCCTTTCCACTGTGGAAGTGCAATTTAACGTAGA

GCTGGTTCTAAGTTATTTAATAATACAGTTTACTTATTGCACTGTCACAAATATATTGAT

TTTGGGTCTGAGAGTATTTAATAATGCAAATTATTTTTCACTAGTTGTTATAATTCAAGC

TCAGAAGTTGTATCCAACAGACGTGCAAGGCAAATAGTATAATTTCTCCAGAATGACAGT

CACAGTAGAATGACAATCGTTAACCATTTAAGTGATGCGATAGGACTCGGTCATTATTTC

ACATGCATCTTTTGATTTATTTTCCATTTCTGAACATGTTCATCTGATGACTCATTCTGC

AAATGACAAAAGCATCACTGAAGTATGTATTCAGCACAGTGCTTAAATGTGCTAACATAC

ATACATCTATCTGAAAGTAACATA

>c10564_g1_i2 len=390 path=[381:0-366 1100:367-367 1099:368-389]

TGAAAAAAAAGTTAAACCCGCAATAACGCTGCGCTACACATCTTGCAGCACTGTTTTAGG

TAATGCTATTTCACCATCTAGCAGAACACAGAAAGACAAGAGATGGAGGCAGAGGTGCTT

GCCAATTCCAGTCAGGCAAAAGCATAGCCACCTCAGCATCAGAGAAGCCTACTTTGCAGT

CCTGACCTCTTGTTACTCTCTACGCTACTTTTCTTTAAAATGTTAACAATATCAGGGATA

TATATAGCTGGATTACTTTTAATGTCCCCTTTCCACTGTGGAAGTGCAATTTAACGTAGA

GCTGGTTCTAAGTTATTTAATAATACAGTTTACTTATTGCACTGTCACAAATATATTGAT

TTTGGGTTTGTGCTTTTTTAAAAGCACAAA

>c10566_g1_i1 len=387 path=[801:0-21 59:22-386]

AACATCAGAGACAAGGAGTGGTCTGCTGGGCATGGGGTGCAGCTCCGAACACTGAGCAAG

GTTCAAAATGCAAGACAGGAAATGAAAACCCAAACAAACAGAAAAGAAACACACAGAAGA

AAAACACACCAAAAGAAAAAAAAAAGGAAAACAAACAAAAAAGGAGAAAAACAAACAGAG

AAACTGGGGGGAAAAGTCACATTCCAGCAGCAGCGTTTGTCACAGCACAGCCCTGGAGGA

CGCTGTGGGACTCAGCCCCATTTCAGACCCTGAGATCTGCTGTGCTTTGCAGACGTGGGG

TGGGTGCAGAAGTGCAACAAACCCAAAGCTCACTGTGAGCACAGCACGCAGAGGGGCTGC

ACGACAGGGAACATCAGCACAGCAGAA

>c10566_g1_i2 len=423 path=[1:0-57 59:58-422]

TAACTTTGTGTTTGAGGGTGTATGGAGCAGCTGTGGGTCCTGCATGGCAGTGGTAGTGCT

GCTGGGCATGGGGTGCAGCTCCGAACACTGAGCAAGGTTCAAAATGCAAGACAGGAAATG

AAAACCCAAACAAACAGAAAAGAAACACACAGAAGAAAAACACACCAAAAGAAAAAAAAA

AGGAAAACAAACAAAAAAGGAGAAAAACAAACAGAGAAACTGGGGGGAAAAGTCACATTC

CAGCAGCAGCGTTTGTCACAGCACAGCCCTGGAGGACGCTGTGGGACTCAGCCCCATTTC

AGACCCTGAGATCTGCTGTGCTTTGCAGACGTGGGGTGGGTGCAGAAGTGCAACAAACCC

AAAGCTCACTGTGAGCACAGCACGCAGAGGGGCTGCACGACAGGGAACATCAGCACAGCA

GAA

>c10569_g1_i1 len=255 path=[233:0-254]

GAATAGATAGTTCATTATCTTCAGACAACAGCATCCTGAAAGAATCTTCTATAACATCAA

GCCTCGTTCGATTAACAACTAACCTGATTCAGATCATAGACTGCAGCATGGCAGTCCAGG

AAGAACCCAAAGAACCATATGTCTCCTCAGTTCTTCCATGGATCATCCTACACAGGATCA

TCCAGCATGAGGAAGATGCTTTTCGATCCCTTTGCTGCCAGCAGCAGCAGAACCAGGGAG

AAGGAGTGAATCCTG

>c10570_g1_i1 len=242 path=[385:0-241]

CCGACGTGCCTGGCCCAGAGAAGCTGTTGGGGCCCTGTGGAGGAATGCAGTCATCCCTTC

AAAGCACAGGGCCTGTGTGCAGCTCAGACATTGTGTTTGTGACAGAACCGTGACAGAAAC

TTGAGTGGAATTAGCACTTACCCATCAGTCACTAAGAACACAAGATTTTCCAGTATTCCT

TTCATCTTTCCATCCATCCTACCTATCACAGAACACGTGAAATAACCCGCTGGGATGCCT

GG

>c10575_g1_i1 len=225 path=[254:0-75 329:76-224]

GGGAACTGTGACAGGTAAGTCATTACAGAGTGTTCATCCACATCAGGGTGGATAATTTCC

TCAGGAGTAATAACCTGCGGCACACCCAACCAGTCATCCGCCTGCTGCATGGCCTCGCGA

GCGTTGTCAACAGGCTTGCTTGGGTCCCAGGTCTCCCAGTCAGGGCACAGGCCTGGAGCA

CAGCTGTCAACGAGAGCCCCGAGTGCTTTGCCATCCTGCCAGTTT

>c10576_g1_i1 len=622 path=[1:0-621]

AAAAAAAAAAATAAATAAATAAAAATAAAAAAATGAATTAAGAAAATTATTTGAAATTAA

TAAAATAAATATATAAGATATATATATATATATGTTTACAATATATTTTAAAATAAAAAA

GAAAAAAAATTAAGAACAAAATAAGCATTTTAAAAGATGGCAACAAGATTTAAGAAGAAT

AGAAAGAAGAGAGGACATGTATCAGCTGGTCATGGTCGTGTAGGAAAACACAGAAAACAT

CCTGGAGGAAGAGGAAAAGCTGGTGGTTTGCATCACATGAGAATAAATTTTGATAAATAT

CATCCAGGATATTTTGGAAAAGTTGGTATGAGGCATTTTAACTTGTTGAAGAATCGTAAA

TATTGCCCAACTATAAATGTTGATAAATTATGGGGATTATTACCAGAAGAAAAAAAAAAA

GAATTTTGTGATAATAAAAATGTTGCTCCAGTAATAGATGTAACAAGAAAAGGTTACTTT

AAGGTTTTAGGAAATGGTAAGCTAAAACACAATCAACCAATAGTAGTTAAAGCTAGATAC

TTTTCTTCCTTAGCAGAAAAAAAAATAAAAGCAGTTGGAGGACAATGTATATTAGTTGCT

TGATTTAAAAAAAAAAAAAAAA

>c10577_g1_i1 len=210 path=[188:0-209]

GGAGCACTTCTCCTACAAAGACAGACTGAGGCAGCTGGGCTTTTTCGGCTTGGGGAGCAG

AAGGCTCCAGGGAAATCTCCTTGCAGCCTTCTGGTGTTTAAAGGGAGCTTATAAGGTGGA

GGGAAACCAACGTTCTATGTATGTGGATAGCAATAGGACAAAGGGGAACAGATTTTTAAA

CTAAAAAGGGGAGATTTAGTTTGGATGTCT

>c10584_g1_i1 len=402 path=[380:0-69 450:70-401]

CAGCTACATCTGAACCTGGCATCTGGAACAGAAAGGTATCTCTAATTAGCTTCTTTACTA

GTTTGTTGTAGGTATGCAGTGCACAGTACAAGTCCTGGTCTGGTCTGCCTCTTTGTACAT

TGATGAGTGACATCTTTTAGGCAACCCCTGTAAACACTCTCACTCAGCTGGAGCACTCAT

AAGTTTTGTTACCCACATGATTTTATTTGGTTAATCACCACACAGTCCATGTTCTGGGTA

AACAGCTCAAGATGAGCTGTCAGAAAGGTCACCAATTCCCAGCAAGTTGTGACTGCAGCA

TTGTGCACACTCCCCTTTCCAGCTCAGGTTTCTTTTGCACAAGACAGAAATTATACTTTC

TTTAAACAGTCAGAAGGAAAAAAAAAACCTGAGCTGGAAAGG

>c10588_g1_i1 len=231 path=[209:0-230]

CTGCGGAGGCTCTGCTCTCAGCTGCCCCATGGACCGTGCCTGGGGTCTGTGCTCAGCCAA

GGAGGGGGAGGAAAACTGCAAACCAAGGCACTGTGACACTGCGATACGCAATGGAACCCC

CCCAGCTTCCTTCCCCTGGCCTCTCCTGGCCTGCCCCCAGGCACAGGTCACTCGTGGGGA

CAACGGGAAGCGGCACTCCCCCACCGCCTTTCCTGAGCCGGTGTGGGGCAG

>c10589_g1_i1 len=368 path=[346:0-367]

ATCCTCAATCTGTGCTTTAAACAGCACTTTCAGTGCAAGAATAAATTTACTCTGCTTTTC

ACGTGCCAAGTATACATTTCCAAATTTCCCTTTCCCCAGAGGACGACCAATTTCAAAATC

ATCAAGAGACCACTGTCTTTTTTTAGTCTCCTCCTTTTTCTTTTTAGTAGACTCTTCGTT

TTTCTGTTTAGATACGCTGTCCGCTTCAGAACTCTTTGCTGTATATGGACAAGTAGAACA

ACTTTATAAAACAGACCAGGGAATGCAACACATTCACATGAGGAACAAGCCTGGGTGGTA

TCTACAACAATTTACAGTCTACCCTCCATGATATTTAAAACCTAGTGTAGCTTACCACTG

GCAAAGCA

>c10593_g1_i1 len=386 path=[1:0-94 96:95-385]

AGTCTGAATTTACAATTAATATGAACTTATCAGTATTAAAAAATGAAGCAAGAGGCACAC

CACAGCTTATGAATATATTTAGACATGTTAGTGCTCGGGGACTGTAACTGTATTAGCATC

AACAGGCAATATTCCTCATGGTGAGGATTTTAGCTTACTGGAGGTATAATCAATGTTTTC

TGCATATCTTCTGTAGGATTACCTTACAAAATGATAATGTTTTCTCATCTGTATGATAAA

TAACTATCAGAGATTTAATCCTATCACGCAATTTTGGGGAAAAAAAAGAAAGTTAATGCC

TACAATGGATGCATGCTCCCTGTGCAATTAGACCATAATTGAGTGCTTGGTAGTAGCAGA

GAGTCAGGGAAAAAAAAAGCTTGTAT

>c10593_g1_i2 len=301 path=[470:0-9 96:10-300]

TTGTGTTTGACGGGGACTGTAACTGTATTAGCATCAACAGGCAATATTCCTCATGGTGAG

GATTTTAGCTTACTGGAGGTATAATCAATGTTTTCTGCATATCTTCTGTAGGATTACCTT

ACAAAATGATAATGTTTTCTCATCTGTATGATAAATAACTATCAGAGATTTAATCCTATC

ACGCAATTTTGGGGAAAAAAAAGAAAGTTAATGCCTACAATGGATGCATGCTCCCTGTGC

AATTAGACCATAATTGAGTGCTTGGTAGTAGCAGAGAGTCAGGGAAAAAAAAAGCTTGTA

T

>c10598_g1_i1 len=204 path=[182:0-203]

GTCTATGTCCCTATTTGTGAGGCAACCATCCTCACCAAGTAACTAATTGGTGTTACCTCT

GGTCCTCCTTTTGCTGAAAACATACATTAAAGAAGTCCTTTTTGTTGTCTCCCACAGTAC

CTTTTTTTGTTCTCCACAGTGCTGGCCAACTTCAACCCTAAATAAGCTTTGGCTGCACAA

ATTTTCTCTGTACAGTTGTGAACA

>c10598_g2_i1 len=589 path=[929:0-588]

TGTGAACAGCACCTCTTGTAGTACCCTGACATCACCTGAACTTGTTTCCAGTGTTCAAAA

AGTTACCATCCAGGTGTTTCAGGAATTTTTAAATTGTTTGTTACAAAATTGTTTGTATCA

ACTGTGTGGTACTCCCAGGTAACACCTGACGAACTGATGTCACCCATGGGATGTCACCCA

TTAGCACAAGGATGGCTGATCTAGAAATATCTCTTTATTCTTTACATAATAATTAATTAG

TGTTGTCATCTGGGCTGGGTGGTGATAGCAGACTACCACAATGATATCTGTTTGCCTGCT

TGTCCCAAAATACTTAGCCAGAGGGCCCCAATCACGTCATTGCCAACTGTAAGCTCGATA

CAACCAAGCCGTTCCTTTACATACAGTGCCACACACTTCCACGCCTAACCTGCCTGTTCC

TCATGAAAAGCCTATAAGCATTCAACATGACACATCAATCACAGGACTCTTCTCACCAAG

TCTTGCTTATACCAACAATGTTGTAGGTCTGGAACAGAGTCAAGGCTTCTAGTTCCTCTT

GCTTATTCCTCATGCTGTATGCATTAATGCAAAAAAATTTTCAAATGTG

>c10600_g1_i1 len=231 path=[363:0-230]

GTGGGGCGGGCGGTACCTGCGAGGCAGCGGCGGCGCAGTGTGGGGCTGAGCCCCCCCTGG

ATGATGGCGAAGAGGTTCTGCTCTGTGGGGCGTCTGTGGGCCGCAATGCAGCGATCCAAC

CAACGCACCGACCTGAGCATGGCCTCCTCGACGCGCGGCCCCGTCTGTGTGCTGCTCACC

ACGCCGTCCAACTGCATCACAATGTCGGCTCCTGCGGGGATGTGGGGCAGC

>c10602_g1_i1 len=204 path=[182:0-203]

GATTATATCTCAACTTCAAAACAGGTTAGATCATCAGATCCTTTGTTTTGGTAGTTTTCC

TTCTTGTGTTCTTAACTGTGGACTTCTTTGTATATGGTGCTCTGTTGTTCAGAGAACATT

TCCCACATCTAAGGGGTTCTATCAATACAACTTTTAATAAATTTGTCGTCTTAATTAAAG

TAGATTTTGTGTGTGTGTGTGTGT

>c10603_g1_i1 len=333 path=[311:0-332]

GTGGGTCCAAGCAGTGCTAAAACAATAGACATCCTCCTGCCTTTTCTTTCTGTCAGGTAG

CAGAGGGGCTCACAGCTGTACTCTGATGTGTCACCGTAATTTCCCACAGCATTGCAGGCA

GCCTAGTAATAGAGGCAAGGATTCATAATCTGCTGGGATTCACTCCTCTGCAAACTGCTG

CAGGAAAGACAAAATGAATACTGTGTGATTGCAACCCAGCTTCTACAAGCCTCCCCACGG

GGCAGATTTTAGTGTGCAGAGAGAAGTACTGCGGAGGGGAACATGCCACAGCAGAGCTGG

GAAGAGAAACTGGAAGAGGCTTCTAATGCAGGG

>c10604_g1_i1 len=288 path=[199:0-287]

AAACATATGCATGCATGTGTACATGCATTATTATATGTATGTGTGCATACATATAATAAT

TTATGCCTACTTTTAACTTCCGGTATTTCATTCTTATGAGTTTATGATATTTCATGACAG

TAATTTCTTTAAGAACCTTTACTGGGAGAGAGAATTCAGCTGCCTGTGTTTTTCAAAAAC

TGGCTGCAGTGAATCAGTAGAACAGCTCTAGCCCTCATGTCTGACAACCTTCCAGAAATA

TCTGATGGATTTGTGAGGTATGGCGTGTCTTTATAAAAGCTATGTTGA

>c10612_g1_i1 len=271 path=[293:0-270]

AGTATAACTTGCTCATCATTGCCTATAAAAAGAAATACAAACAGTTAATATCATGTTTCA

GTGGTTAAAAAACCATCAGGGTGAGAAGAAAAATCTTAAAAATGGGAATTTAAATTTATT

TTTTCTATTTTGAAATCAAAACCAAAGCAGTGAAAAGACCAGTGCTGCTGCCAAGCGGTC

AGCCCTGCTCTCAGTTTCATGAAAAGCTCCATGTGACATTTCCAGTCACGATCAAACGCA

CCAATGCATTCCATTGTCTACTGCTTAACAA

>c10612_g2_i1 len=203 path=[585:0-202]

GAATGCACCTTTCTCGCTGCTCAGAGAATGCTTCCTAAGAAGGTAATGGAGGGTGCAAAA

AGTTGCCATAAGCAAAATGCATTGAGCTCACATGTCAAACGATGCCAGCTCATGCTGCCA

GCCTTCAGACTTTGGCAACCTTTGAGAAGGCACAAACGTGACAGCATGAATTGTAATTTC

TTTTGTATTTACAAAAACCAAGC

>c10613_g1_i1 len=223 path=[305:0-222]

TGCCTTCAACTGAGCAGTAATTTATCATCTCAGACAATTGCACTGGGGCACTGTGACATG

TTGATATATATATATGTAATGTTATATTGCTGTGTAACTATACAGTAGTTACACAATTGT

ACTGCAGCACTAGTAATACAGCTATTTCCTAAAATACCTCTCCGCCTACCTCTTCCCTGT

CTGTCCTGCAAAAGAGCACAACAACAAAAAATGTAGCCACAGA

>c10614_g1_i1 len=304 path=[282:0-204 487:205-303]

TGGGTCACCTCCACTGGTGCAGATCTTTTACAAAAGTAGTCTACAGGCTCTTGATCATCA

CTGACAACAATACATTGCTAACGGTTGTGACTACTTTGAAAAAACAGTATTTGGTAGCTG

AGATGTGGCTCTACCAAACAGTATTATTGTGCTCTTAGTATCTGTTGTATTTCTATGGAA

ACTAAACAGGAGACATTACTTTCAGAGTGACCTACATCTGTAATTACAAACCATGTCTTT

CATATGAAAAAAAGTAATACCTACGGGAAACTCTGCAAAGTCACTTCACTCAAAAAACTT

CTCA

>c10620_g1_i1 len=262 path=[330:0-175 25:176-196 46:197-261]

AGTAGAGCTAAATATAGGTTGTTTATGGGGAGGATCACTCTACTGTTGAACAACTTGCCA

TCTTAGTAATTAAAAGAAGACAAGTAATATCTTGAATGGTAAAAGTTTTAAGGTCTGAGA

AACCAGATATACAACTAGTGAACTTTAGAGGGAGGTATGAGTGTAGTATGCTATTTTCTT

ATCATGGCTTGTGGAAATAATAGCGTCATGAAAGAAATCTTCTGACATTTCCACCACAGC

TTCCCCATCTCTCTCTCACTGT

>c10621_g1_i1 len=332 path=[320:0-331]

GGACATGCTGAGGGCTGCCCAGCTGGAAGGCAGCCTGGCAGGAAAGGCCCTGCAGATTGT

GGTGGACAAAAAGCTGAACATTAACAGTGAGCTCTTGTTGCAGAGATGTCTAACAATATC

CTGTGCTGCTTCAGGAGAAGTTTTGACAGCAGGTGGAGGGAGGTGGTCCTTCCCCTTAGT

ACTGTTGAGGCTGCACCTGGAGTAGTGTGTCCAGTTCTGGCCTCCTCAGTTCAAGAAATA

CAGCAGAGGGCCACAAAGATGGTTAGGCACCTGGAGCTTGTCCTATATAAGGTAAGGCTG

GAGGACCCGGGACTGTTCAGCCTGGAGAACAG

>c10622_g1_i1 len=207 path=[453:0-206]

ATTTCCACAGCTACCATGAAAGCATCCAAGCCCGTGCAGGAGCCAGAAGCCAGTGTCACC

AGGCACAGCACTGCCTGGCATTCAGCTCACATCAGGCAGTGCTCAGATTGGTTTTCATAA

AAGCCATTCAGCAGCACTGTGGTTAGCATTAGGGCAAAATCCTGCTGATCCCCCCTGGGA

AGAGGCCGTTTGTAACAGTGATTGCTG

>c10624_g1_i1 len=244 path=[422:0-243]

GAACCTGTAGAACATAGTCTGCATGTAGGAGTGTATATATGTACATATATGCATCAGAAA

CCGATTCCTTTCTGTGAACTGACAGTCATCTGTTCAGAGCTGTCGCCTTCAAATAAGCAC

AACAGTAGGGGCAGGCTGCACCCGAAGTCCTTCATATTATTTGCAGCTTCCTTTGGAAAA

AAGAGGCTTTTGTGTGGGTAAGCAAAGCAAATTTGTATAACCTCTATGATGCAAATTGAA

GACT

>c10630_g1_i1 len=288 path=[516:0-265 798:266-287]

GTAACTTTGTGTTTGAATTTGTGAGAGGCAGCCCTGCAGACACCAAGGTCAGCACAGGAG

GATGGGAGGTGCTCCAGGTGTGGAGCATAAGTTCCCTGTAACCCCTGGAGAGGCCCATGG

TGGAGCAGCTGGATATGGCCTGATGGAGGCTACAGCCTATGGGGGACCCACTCTGGAGCA

GTCCATGAAGGAAGCATACCTATGCGAGAGACCCTAAGTTGGAGCAGGAGCAGAGAGTTA

CTATTAAGGAGGAGAGACAAAGTATTAGGGACTGACCACAGCCCCTAA

>c10631_g1_i1 len=287 path=[265:0-286]

GGAGGCATTGTGAGGTTGGCTGCTGTGCTTGAATACAGACAGAGGAGGCAGCTTTGGAAG

CCTTTTTAAAATATACATATATTTCTGCTATCTTACATCACTTGTACGAGGAAATTTGTC

TGTGTATAGACAGACATACAGCTGTAATGCTTTACAATCTTGCTCTAATATTTGCCTGCT

AAGGTGTATGTATTTCAGCTTGAGTGGCATATTCCTGCCTCCCTTGTCAGGCACCCTCAT

TGCCTGTTGTCCCTCTATGGCTCCATCTCTTCCAAACCTTGCCTTTT

>c10636_g1_i1 len=204 path=[530:0-203]

AGCGCATGCAGCCCAGCAGCCTTCAAATGGCTGGGAACCCAGTGCTTGGAAGTTCAGTCT

CCTGCTGACATGCAGAAGGGGTTAACCCCAGCAGGCACCCATCTGGAAGATCCCGGTGCT

GTGTCTCCCAGACCCGGTCAATACTCATTAAGGGGAAGCAATCCATACCTCCAAGGGGCT

GTCTGAACAAGAAAGAGTCAAAAG

>c10637_g1_i1 len=211 path=[60:0-56 117:57-59 361:60-210]

TACATTTACTACTTCCTTCATATTTCGTACTTTTTTCCCCCTACACCTGCTTAAAGTTGT

CTGAAAGTGCCTGATTTCTTAAGAGATGAGACTGTACATAGGAGAAAACAGTGAAATTTT

ACGCTGATTCACTAAGAATTGGCAACTTTGAAGTCCACAAGAAAGATGATCGTGATAGTT

AAATGGATTTTAAGTATGATGTATAGATATT

>c10637_g1_i2 len=229 path=[119:0-74 117:75-77 361:78-228]

AAAGAGGAAATGTTTAGTGAGGTAGCTTCAAATCATATTGAAAACAAGAATAATTGGGTT

CTAAAATTAAAGAGATGTCTGAAAGTGCCTGATTTCTTAAGAGATGAGACTGTACATAGG

AGAAAACAGTGAAATTTTACGCTGATTCACTAAGAATTGGCAACTTTGAAGTCCACAAGA

AAGATGATCGTGATAGTTAAATGGATTTTAAGTATGATGTATAGATATT

>c10638_g1_i1 len=308 path=[1:0-307]

AAAAAAAAAAAAATAAATAAATTAAATTAATCGAATATTATGAATTTATATTTTTATTAG

GAGTTAAAGTTTCAATAAAAATATTTATAAATTATTTAAAATGTTGTTAATATAACTATA

TATATTATATGAAATATTAAGTATTTAATACATACGTATACTTATATTTATACATACATA

TACTTATGTGCATATTAACATATGTAGAGATTTTAATACGTATATTTATATGTGTGTTTA

TTAATTATATATGTCTAAAATTCTTTAACTGTGCTTAAAACTTGCTGTTAAATAAAATTA

AAAATTTT

>c10639_g1_i1 len=205 path=[337:0-204]

GCTGCAAAGAATCCTGAGATCCTTTTGTAAGGAGGTCCAGCACATTCATAACAGCGCCTC

GGGAGTTGGTTGACTGATGCACAATACATTAAGATGAGTCAATATTATGAGATCAACCAA

AGATTGCTATTAAAGGAATAAAAACAAATAAAAATTGTTTTTTAAAAAAGGGAATCTCTA

AAAGCTTGAGCCGCTGAATTGATCT

>c10641_g1_i1 len=224 path=[139:0-68 207:69-223]

GGAAAACTACTAGCTTTTGGTGCCAGATCTTCTTTAATACGTTCTTCAGGCACTCCCTAA

GGCATGTCATGAATTGTCCTTTTAATTTGTGCAGGACGTCCAATGTGTTAGACATAATAG

ATATCTTTACAAATCAGAAACCCCTTGATGTTGGAAAACTACTAGCTTTTGGTGCTGGAT

AGACAGGACCTGAATAACAGAGAATTAAAGGAGGAGAAGTTAAA

>c10648_g1_i1 len=220 path=[352:0-219]

TGGAGGCTGTGGGTGCCTCAGCCAGATTTACTACAGGTAGCAAGACTGAAGTCTGATGTT

CTGATTTAGAACCAGGATTTTGGGGGTGTTTAATGCAGAGCCCTGACCCCAGGTGTGTTG

TGTTTTTTCTGTCAAAACCTTGTACAACACTTAGGGAGGGGTTAGCTATTTGAATCTATT

CTTCCGAAAGGAAAAACAAACCAACAGAAAAATAGATTCA

>c10651_g1_i1 len=507 path=[995:0-506]

AAAAAAAAAAAAACAACAACAAACCACCAACCCAACCTTTTCCGTGACACTAAGGGAGCA

CAGGCACGTCTGTAAGGCACTTCTGCATGGGCTGCTCCCGAAAGCCTGACAAGGAGCTGC

AGCACTGCGACCATTTCCAATGGAGAGATACGGGCTGAAACAAACCTCGAGGACCTCAAT

ACCTGGGACAGAGCCTTGGTCCCCATCATCACTGCCGGAAACCGACCTGCAGCAAGGAGC

GGTGCCAGGGGGCGGCCCCGGAGCATCGCCCCTGCAGAAGGATGAGTGGGGCCTGGGGCT

GCAGCTCAGCCCCGCCGTCTGTCAATAGCTGACCGTTAGCGAGCCAGCGCTGATGCGAGC

AAGCTGCTTAGTTCAAATGTTCCAGGAAGTCAAAAAATAGCCTCCGCCCTGAATAGGGAA

CTGCTTGTTTTCACAGGACTCGGAGCAGGGAGGCCGGACGCAGGCTGGGGAGCTCATGGC

CGAGAGCGGAGCCTGCAAGCGTATCAA

>c10656_g1_i1 len=431 path=[436:0-430]

AAAAAAAAAAAGAATAAAAAAAAGGAAAAAGAAAAAGAGAATTAATAAATTAATAATTTT

TTTTTATACAATTACGGAATGTATGCACATAAATAAGATTAAATATAATAAACATTTTAA

AAAATGGAAAAAAAAAAATAACTTCTCTTTGAACTTTATAAATTTATTCATAAATTTTTA

TAAAATTAAATATAAATTTTTTAAAAATTTTCATGAAATGCGTATATATAAAATAGTATG

GTAATAATATTTATTTAAATAACAATATAAATCATTTGCATAAATAATCATATTGGACAC

TTATATTAATTAAAAATATTAAATACTTAAATACATCAAAAATTCATTTAATTATTCACA

TATATATGTGGATTCGTGCATTTACTTTTTATTAAAACTATTAATTGATGGTGGTAGCGG

AATATTTTGTT

>c10657_g1_i1 len=293 path=[513:0-292]

CATGAAAGCTGCTTGTAATCTATACCATGCACACTATTCATTCTGAAACAGAAGAGCCAG

AACTGCACCCTGTACTCAATATGCGTGTGAAGCACAGAGCTGTACTGGGCCATAATGTCA

TTCTTTCTCTTTAACCTAGATAATAAGCCTTGATATTATCTATTTTTTTCCATTTTTCTA

AACCACTGCTAGGCAGATGTTTTCATAGATTACACAACTAACAAATCAAACATTACTGAG

TTTCTGGGTCTGCTTCTATTCACTCAAGTGCTCTAGGGGAATTGTGGTAGATG

>c10669_g1_i1 len=233 path=[203:0-232]

GTGAAGTAACAAAGGCATCTGGTGAAAATGTTCACTTAACAGGAAAGGAGTAGCTGACTT

GCTGGTATTTCAGCTGTATACATGAAGTAAGGCAGGTGCAAAAGGGGTTGCAACTGGTTA

TCCTTTCAAGGTGAAATCTCATCAGTCTCAGAAATTTGGCATGGTGGAGTCTTACGACTG

TAATTTCTTTTATCATGTCACAAGTGCATTAATATGAGTAGGTGTGTAATTTT

>c10678_g1_i1 len=399 path=[1:0-46 48:47-398]

TGCCCTGCTCCCCAAAGTTGGCACACTCGTACCGCTTGGCCACATCTTCCTCACACTCGG

TGTCCTCAAGGCAGAAGCTGGCTTTGTGGCCCTCAGCCACCTTGGTGCCGTTGGGAGTCA

GGATGTCGTAGTGGGTGAAGATGTCCATGCTGTGATAGTGCCTGCGAGAGAGCCCACAGC

GCCCGCTGCCCTAGAGCCAGCCCCTCAGGGAAGGCAGAGGGACGGAGGGGATGATGGAGG

GAACCCACAGAGCCACGTGCCCATCCCCACCGCTCAGGAAGGTCTGCATAGCCCCTGCTT

GGACACAGTGCAGCAAGGCCGAGGCAGAGCTCAGCCTCACCCAGGGATGGGATACGGCTG

TCCCCAGTCGGGCAGCCCGGGCAGGGGAGTCTTCCCCAG

>c10678_g1_i2 len=371 path=[753:0-18 48:19-370]

ATCTTCACCCACTACGACATCCTCACACTCGGTGTCCTCAAGGCAGAAGCTGGCTTTGTG

GCCCTCAGCCACCTTGGTGCCGTTGGGAGTCAGGATGTCGTAGTGGGTGAAGATGTCCAT

GCTGTGATAGTGCCTGCGAGAGAGCCCACAGCGCCCGCTGCCCTAGAGCCAGCCCCTCAG

GGAAGGCAGAGGGACGGAGGGGATGATGGAGGGAACCCACAGAGCCACGTGCCCATCCCC

ACCGCTCAGGAAGGTCTGCATAGCCCCTGCTTGGACACAGTGCAGCAAGGCCGAGGCAGA

GCTCAGCCTCACCCAGGGATGGGATACGGCTGTCCCCAGTCGGGCAGCCCGGGCAGGGGA

GTCTTCCCCAG

>c10679_g1_i1 len=282 path=[31:0-108 305:109-281]

TGGGGCAGCAGGTGGGAAAAGCGGTGAATTCGTACCATAAAAGTGTCCCATTCTCAGCCT

GGGCTGCACTGTGTGGCCATGCTCAGGTGTTTCTTGCAGCCTACGTTTGGTTAGACATTT

CAGAGCAAGCGGTTTGAGAAGGGTACGGGCAGCCTGCAGTGAGGCAATCCCTTCCTATAA

AGGCATGAGATCACACAGGCAGTGGCACTCCAGTGATTTATTTCTCAATGTTCCCTTCTT

CCTGCTGAAATCTCTTGCTAGAAGTCCCATTTCAATCTCTTA

>c10686_g1_i1 len=324 path=[584:0-300 904:301-323]

AGCAAGGAAGGCAGTAAGGAGAGGCCAGGGGCACCCGACCCAGGTGACGAGATGTCAGCA

CCTCCTCAGTGACCTTCCGTGCAAGGCATGAGGAGCCCTGAGAACTCAGCAGCCTCCCCT

CTGCTGCAGCCTCCCACGCTCCGGTGATGGCTGCAGAGTGACTTGGCAGCTCACGCCCAC

TTCAGACAACCCTTGGTTTCAGACCACTCAAAGGAACGTACATGGGAGAAGGAACCAAAG

CCTTTTATACAGTCAGAAAATATGGCATTTATGTAAGTGGCAAAATATGCTGTCTGTCAA

GGCTGCAGCTACCTGGAGAGCCTT

>c10686_g1_i2 len=323 path=[584:0-300 902:301-322]

AGCAAGGAAGGCAGTAAGGAGAGGCCAGGGGCACCCGACCCAGGTGACGAGATGTCAGCA

CCTCCTCAGTGACCTTCCGTGCAAGGCATGAGGAGCCCTGAGAACTCAGCAGCCTCCCCT

CTGCTGCAGCCTCCCACGCTCCGGTGATGGCTGCAGAGTGACTTGGCAGCTCACGCCCAC

TTCAGACAACCCTTGGTTTCAGACCACTCAAAGGAACGTACATGGGAGAAGGAACCAAAG

CCTTTTATACAGTCAGAAAATATGGCATTTATGTAAGTGGCAAAATATGCTGTCTGTCAA

GGCTCTCCAGGTAGCTGCAGCCT

>c10706_g1_i1 len=259 path=[259:0-252 561:253-258]

AGCTTAGCTGTGTGCAGAGCCCACTAAGGAAAATCTGAGCTCAAGGTACAGAGATGATGA

CTGCTCTGTGCAGTGACATCTGATGTCAGCAAAGGACGTGACCTATTCCTGGTATTGTGA

TTTAGCACTGGGAGCAGGTCAAGCTGCTGCTGTGATATGTTAGCCAGCTGTTTTGGGGGG

AAGCTTTGGCTTTGGGAACATCTGCACAATCTTTTGCAGACCAAAATGAGCCATTCTGCC

CATGTTCTGAGCTGTCTCA

>c10709_g1_i1 len=215 path=[155:0-214]

GTGCCCACTGCCTCTTGTCCTGTCGCCGAGCGCCACTGGGGGGAGCCTGGACCCATCCAC

TTTGCGGGTGCCCTTTGTGTACAGATGTTTGTATTGTGGAGACATCGGTGCTTGCGGGTG

GGGTTCTCTCTCGGTCTTCTCTTCTCCGGGCTGGGCGGTCCCAGGTCTCTCAGCCTTTTT

TCGTGGGGGGGATGCTTCGGGTCCCTGGTCACCCT

>c10710_g1_i1 len=256 path=[234:0-255]

CTACCAAATCCTCTTCCCCCTTTGCTTTGTGCGATGAGTTAGCACATGGGCTGTCGTCTG

TAGAAGCAATAGAGTGCAGGAGGCCACAAAAGAGCACAATCCATGGAACAAGACCAGGAG

AGCTTCGCCTCCGCCGATATCCTTCCTCTTACTGTGCAATCCAAGTCCTTCTGAAGCCAT

TCCGCGTGGGCACGCTCGGACCGGCGGGGCTCCGGGGCGGGCGAGGGCCGGGCACGTGTG

CCGGTGCCGGGGGGAG

>c10718_g1_i1 len=297 path=[557:0-272 283:273-296]

CAGCCCAGGGAACATTCATACTGGAGTGTTTGCTTCCCTGCATGCCCTTATCTCCACCTG

ACCTTGCCCAAGAAGGAAACACCTCTGAGGAAGATCAGAGGTCATGGCCTCATCCACGCT

CCCCAGCTCAGAGGGCAGCAATGAGCCCAGCATAGGGGTCTCAGGGATGACAGCCTTTTC

TCTAGCAGATCAAAGGGATCACTGCTGCACAGGGGTCAAGGAAAGCTCCTCTCACTCCTT

GCAACTCAACCCACAATCAAGACATCCACTCGCCCCTGCTCCTGGAATGGATCAGTA

>c10723_g1_i1 len=395 path=[1:0-394]

GACGTCTCCTTGGCCTCATAGATGGAGAAGATGTGCTCCTCACCGAACTCACCGAGCAGC

GGGCACTTGACCTCATACATCTTCAGGCGCCGTTTGAGCGTGGAGTTCTCCCTCTCCAGC

CCGCTCAGGCGGTTCTTGATGTCGTCGTATGCGGTGATGAGGGCGAAATGGGACGCAGAG

CACATCTCCCCCGAGAGGTCGGTGTTGGGGCTGTCCAGCCAGTCCTCGTCTGGCCCCAAA

GCCTCCGGCGTCAGGATGCTGATGTCGTCCTCGAACATGGAGTCCATGGCGCAGCGCGAG

TTCTGCCTTGCAGTGGGCCCCGTGAGCGGGGCGAGGCGGCGCAGGCTGGGCAGGGCTGCG

GCACAGTGCCGGGGCTCAGGTGAGGGTGGTGGGCA

>c10729_g1_i1 len=284 path=[486:0-283]

ACAGTGGATGTGAGCACAGTGAAAGCTGGACTGCATGGACAACATTTTTCTAGCAATGAC

CCTGTCATAGCAGCTGTGAAACAGTGGGTTGCGTCCACTGTGCAGATTTTTACAAGCATG

GTATGCAGGCTCTCACTCATTGCTGGCACAAATGCAAAGCCAATGGTAGTGACTGTGTTG

AAACATAGTGCTTCCCCCGATGGAGCACCACAACTTGGGATCAGGGCCGACTTTCCTGTT

GTCTCCCAGTTTGTCCGCTCCCACTGAAGTGGAGATGAGAAGAA

>c10735_g1_i1 len=231 path=[393:0-230]

CTTTGTGTTTGACGTGCGCTCAGGATGACTGCACTCTGGCCATTTCCCCCAGCTTCCAAT

CTTCATGGGAAAGAGCATGCTTCTTCACCCAGCCCCGCACAGAGCAGCACAGGAGACAGC

GTTATTCCAGCAGATGCTGCAGTGACAGACAAAATAAACTAAATAAAAAAATAAAGATTT

CAAGATCAAAGACGTAAGCAAGCGTCTTTTCTTCTATCAGGGCCTTTGAGG

>c10744_g1_i1 len=221 path=[199:0-220]

TGTGAAATCCTACGGGCCCTGCAGAAACTCAGCTGTGCCCTGGGGATTGTGAAAGAGCAC

GTTTCATCTCTGTGTTTGGATTAGCTCAACTATTTCTTTAGCCTGTTGTTAGAGCAGCCG

CTGGAAGGAGTCAGTCCTGCAGGTTCTGCTGGCTCCTTCCCGTGGTGAGGAGGCTTGGAT

GAACCTCCAGGTCCCATCTGCTGGGGCTGCAGTATCCTCTG

>c10745_g1_i1 len=326 path=[1:0-325]

GATGACTTCGTGTTCTGCCATTCTCACCCACTGACTGTGCTCTGTTGGTAGATTTATATT

GGAGCCATAATCCCATTGGGAGCGGCAGAAAAAGACGGCAGGCTTCGAGCTGCTGATGAA

CTGATGTGCATCGATGGAGTCCCTGTTAAGGGGAAGTCACACAAACAAGTTCTGGACTTA

ATGACCAGTGCTGCACGGAACGGTCAGGTGCTGCTCACGGTCCGGAGGAAGATCTTCTTT

GGAGGTATGTATGTGTCCACAGTGCTCAAACACATCAATGAATACAAAAAGTTTCGGGGG

ATTTTTAATCCTCATTTCAGTGAGTA

>c10746_g1_i1 len=237 path=[1:0-236]

TGCTAACCAGTAGCAAGCAAATCCCACTCTCTGGCTTTGTCACAAGTACACAGCAATGGT

TACAATTTAACACAAATTTTCTCCAAAAGAAAGGAGCACAAGCTGCATATATGATGAGAT

TTTGAATAAAATCCCCATGATGGCTGCAAGAAATTATGCAGTTATTTCTATTAATTTCAT

CTAAATATGATGATAAGGCTAGGTGTAGTCCAAATGCATAAAAAGAAACAAAAACAA

>c10750_g1_i1 len=201 path=[1:0-200]

GAAGACAGCCAGCTTCACAGTGAATAGTGGTAAGCTGAAATGTAGTAAAAGGATAGCACA

GTTTTACTTCTTGCTGTCAGAAATCTCCAGAACAGTTAAAGAACTTATGCTATACCAGTT

ACTTTAGTAGTGTTTTCCATGAAGAACTTGATTTTTCACAGGTGTCATGAACGAGCATAG

TGTGGAAAAGGAAGGGTGTGG

>c10752_g1_i1 len=331 path=[53:0-330]

TGAGAAATAACACCAACTTTGAGGTAAGTTTAAACTAGAGTATGTTTTGGATGTTACAGA

GGCCCAGTAATCTCTGGAATGTTGTGATAAGGCTAGTGGTGCATGAAAGTAGTTGTGTGT

TTTGTTGTGAGTCTTGCACTCCCAGCTGTTGGGAGAATAAAACTGTGTGCATGGAGCAAG

AATGACCTTTAATTCTTCAACTTTAGTCACTGGAAAACTTAATGTCATCTGTTTGTATGA

AGAAAAGACTTGGCCACAGTGGCTCAAAAAAACACGTTTTTTATTGCTTGGTATCTTCAC

ACAGTAAAAATCATAAAAGCTGAATCAGTGG

>c10754_g1_i1 len=418 path=[396:0-417]

GTTTCTGGAGCTTTTCATGACCGTAGAGACCTGGGCAGCATCTTCCCTTCATGCAAGCTG

GGACTTCTGATGGTTCAGCAAAGCACTTGGAACATCTTGTACTTCCTGAAAGCAAGGGAG

CTTGGATCTGCCAGGCTGACGTGTGCCGTGCGCTGCAGAAGAGCTTCTGCTTCCTCCAAA

CTGCTGCTCCTGAACAATATTTGTCTGGGGAACCTGGGAATGTCTCTGCCACTGCAGCAT

TCAGAGTGCCAAAGTAATTGAGGAGCCTGATGGATGCAGATACCTGGGTTGCTCAAGGAG

CTGCCTTTGAGAACCCCTCCAGTCTTCACTTTGTACCTTGGAGGACTGAAATATCTTTGA

AAATCCATCTGTAGTGCCTGGGAAGGACACACTGCTGTGCTTGTAAAGCAGTACTGCT

>c10755_g1_i1 len=283 path=[1:0-257 777:258-260 779:261-282]

GGATCTCTATCTGGAGATCACTCACCTTCGAGCCCTTCTATGAGCCCAGAATACAGGTAA

GATCTTTATTTTCATTACTCCTTTGTAACACAACAATCTCTCTGGTCCTATACCTGTTTG

CCATACACCATACTAGTAAGCCTTAGATTCTCTCATGCACATCTTTATTTTCTCAAAGTC

TAAGAAATAACTAAAATAATGCTTTTAGTACGTATGACCAAACAAAGGGAGTGGGAAGGA

TTTTCAGTAACATAACTATTACTTTATTATTATCACCAAATAA

>c10755_g1_i2 len=381 path=[1:0-257 777:258-260 562:261-380]

GGATCTCTATCTGGAGATCACTCACCTTCGAGCCCTTCTATGAGCCCAGAATACAGGTAA

GATCTTTATTTTCATTACTCCTTTGTAACACAACAATCTCTCTGGTCCTATACCTGTTTG

CCATACACCATACTAGTAAGCCTTAGATTCTCTCATGCACATCTTTATTTTCTCAAAGTC

TAAGAAATAACTAAAATAATGCTTTTAGTACGTATGACCAAACAAAGGGAGTGGGAAGGA

TTTTCAGTAACATAACTATTATTTGGTGATAATAATAAAGTAAATTTACCAGCTGTAAAA

ACATTAATTGAATTACATTCTCCCTTGTTTAGTCTTGGAAAGGTGGCAAGAACTTGTTGT

TTTGAATGCTTTTAAAGAGTA

>c10755_g1_i3 len=221 path=[659:0-97 777:98-100 562:101-220]

TACTCTTTAAAAGCATTCAAAACAACAAGTTCTTGCCACCTTTCCAAGACTAAACAAGGG

AGAATGTAATTCAATTAATGTTTTTACAGCTGGTAAATTTATTTGGTGATAATAATAAAG

TAAATTTACCAGCTGTAAAAACATTAATTGAATTACATTCTCCCTTGTTTAGTCTTGGAA

AGGTGGCAAGAACTTGTTGTTTTGAATGCTTTTAAAGAGTA

>c10756_g1_i1 len=222 path=[200:0-221]

ATGCAATATTGTTTTCCTCACCACAGAATACAGAAATGACACAAAATGCTGAAATAGTAC

TTAGCCCTATACAATAATCATAACACACTTTGAAATACCATGTTTTAAAAAGTATTGCAT

TTGCACTGTGTTTTTAGTTTATATTAAAATGCACTGAATACAGTTCAATTGTTAAAGCTT

TGTGTCCAAGGAGTGCAAAGTGGATGAACGTGGCGGGCTAAT

>c10757_g1_i1 len=361 path=[339:0-360]

AGTTTCCCATGCGGAACTTCAAATGCGATGGCAGAGAAATGGCAGATCTGACAGCCCACA

CTTCAAGCATTATTAAAAATTCAGAAGGCAACAAAAAATAGCACTGTCATTTTACTTTGT

AATGCCCACTTGTTTATGTAACAGTTTGAGGGCAACAGCAGGGCAAAAGCTGCATTTCTG

TTCTGCACTGCAGGCATGAAGAAGAAGTTGTAACCCTACAAACAGTTAGCAGAAACTATT

TCACACTTTTTCAGTTTGGCATGGTACTGTCATTGTTCAATTGTTATGGGTTGGAAGAAA

GTGAGATAGAGCTGATTGTCTTCTGATTAAGAGAGCAGCCCAAGAATTACGAGCTCTTTT

G

>c10764_g1_i1 len=207 path=[247:0-206]

AGACAGTATACAAGTTTATATATAAACACTCAACAGTTTATAAGTTAAAGCTGTGAAATC

TTCATTATCAGCTGCCTCTAAAGGGTGTTTCTTGGCATCCTTCATTTTAAGGAATAAAAG

ACTTAGGCACACTTCCCTAAGTACAAAATCATCAGAAAGCACCAGTTTGGAGTTAAAATC

TTCACCTCCCTCCCTGGCCCGGAGGGG

>c10765_g1_i1 len=216 path=[452:0-24 46:25-215]

GTCCAAGAGATGCTCGACCCCACAAGTGGACCAAGAGAAATCCGACCCTGCGGTTGCCCA

GGAGAATCCCAGCCCTATAGATGGCCCTAGAGAACTCCAATTCTACAGGCGGCCCAAGCA

AAGCCCAGCCCTACAGATGGCCCACGCAAAGCTTGACCCCACAGGTGGCCGAAGCAAAGC

CCAGCCCCACAGGCACCCCCGTCAAACACAAAGTTA

>c10769_g1_i1 len=213 path=[32:0-29 62:30-212]

GCTGATTCAATGTAACTTCTGTTCCATATCTTTGAGTGGTCTAGTTGATCTTTGCTGAAA

CACTTCCAACAACTGTATGCATTTAATTCTTCTGTGACACCAGGGAGGACAGAAGGGGAC

AAGGTGTAGGCACACAATTAACATAGTGTATTTTTCTCTACTCATTTCATACTCATTCCT

AGCATTTACTCATCCTTATAATAGAACTGTGAA

>c10773_g1_i1 len=259 path=[1:0-258]

GCAGAAATCTACACAGTGAGGTTGTTATATCATACAAAGTGTGAAAATGAGAGAGGTAGC

TGGGTGCCTCACCTCCTTAGGATCACTGGTGTTTTTCTGATAGTAAAATATGCAGTGGTG

GCAATAACACATATTCCACTCTGAATAACCTCACTGACAGCTTTTTTCCTCCCTCTCTCC

AAACTCTAAGGATTCTATCTAGAATTAATCTGAGAATAAATAAACTTTCCTACATTCACA

GCATTTCTTAATTGTTGGT

>c10773_g2_i1 len=244 path=[473:0-221 757:222-243]

CAAATATATATGTAAGTTTCCAGATTATGTTGGGTAACTTATTAAATACTACCGTACGGT

ACAAAGCTTATGTTCCATGGTATTAGCACTGGGGAGTTTTCATCTGACTTTTAGCATCAT

CTCCTGTAGTTTACAGTTGAGCTTTCTAAAAGGCCTCTGTTCTTCAAAAGAGACTGAGAA

TGGTTTAGTGGTTTTAAGTGTCAGTTACAGAAAATTGCTGATTATAATGTCTTCTCCTTA

CAAT

>c10773_g2_i2 len=245 path=[473:0-221 759:222-244]

CAAATATATATGTAAGTTTCCAGATTATGTTGGGTAACTTATTAAATACTACCGTACGGT

ACAAAGCTTATGTTCCATGGTATTAGCACTGGGGAGTTTTCATCTGACTTTTAGCATCAT

CTCCTGTAGTTTACAGTTGAGCTTTCTAAAAGGCCTCTGTTCTTCAAAAGAGACTGAGAA

TGGTTTAGTGGTTTTAAGTGTCAGTTACAGAAAATTGCTGATTGTAAGGAGAAGACATTA

TAATC

>c10774_g1_i1 len=235 path=[381:0-234]

GTTCATCAGAGAAATAGTTTAATCTTCTCTTAGACACTGAAGAATGTTCATGAATTTTAT

TTAACTTTAAAAGTAGCAGATGGTAATCCAGAAAAACTAAACAGGACGTTGGATTAAGGT

ATCTTTGATAGGTATTGGTTTAGGTTGTGCAAATGCAGACTACCAGCTTCTCACGAATGC

TTTTTTCCACCTTTAAGGAAGCCATCTAATATTCCTTAATTTATAAGCAAGGTAA

>c10781_g1_i1 len=320 path=[1:0-252 254:253-319]

TAATTGCTGACCTTCAGTTAGGAGATGGTTTTTCCTCTAAAAAGAGCTCTTAAAACTAAG

GGGTTTAAGCACTTGCAAACTATAGTATTCTATCATCTAGATAGCATTTTGGATGTATTC

AGTTATTAGTCTTTCTGAAAGTCTTTGAAAAGAACTGAGTGTTTCAAGCAAGCTGAAGTA

TAGCCAGGTCTTAAAATACAAGCTGAAGCTAACTGGAACCTTCTTGACCTGCGTGTATCC

TGATAATACTTACAGAATACATCAGAAATCAAAGGAAACAAATGAGATTAGAGTATTACA

TAATGTATTCTGTAAGTATT

>c10786_g1_i1 len=370 path=[406:0-284 691:285-310 716:311-369]

GAAGCTGAGTGTCTGCCCTCAGTTGCAGCAGCACTGTAGGTTATGGAGGAGAGGCACAGC

TCAGCCCGAAGTGGGACGAAGTTTCCAGCCATGTCTCCATATGAAAGCCATGCAGATACC

AAGGAGAGTGCAAGGGCAAATGCTGGGAGAAGAGGGAGAGCAGCAGTGTGTGATGGAGTG

ACAAGCAAGGAGTGTGGGGTGGGCACAGGACTGCAGGGGGTGGGAGGGACTTCTGTGTTC

CCCAAGCATTTCCCTACAGCAGTCACACCAGGTGGGCTTTGAGTATCTCCAGAAGAGACC

CCCACCTCTGGTCAGCCGTTGCAGTGTTCTGTGTGATCAGGAATGGACAAAATGGGGCTC

ACTGAGGTCA

>c10793_g1_i1 len=253 path=[231:0-142 374:143-252]

AGGGAAATAAAGTAAAAAAGGAATGGGAATGGGGATGGGGATGGGATGGGAGGGGAGGGG

GCGGCAGGGCCTGCACAGGCTAAGGTTGCCATGGGGGCTGGTCCCACCACCCCCACGCCC

GTCAGGCAGGGAGGGAGGGATCCGGGAGCTTTTGGATTCTGCACGTTAACCCCCAACTCG

ACTCTACAATACAGCTACATGCCAAGCTAAAAGGTGAAAGGTCATAGTACCAGGTCAAAG

GTTGCCTCGAGGG

>c10794_g1_i1 len=210 path=[229:0-209]

AGAAACTTTGAAATGTTTTGATCAGAAAAAATGAAGCTTCCATGATGTCTCTCCAAAATG

AAGCAGAGCAGCCACATCTTCCTATTACCAACACCTGCCAAAGGAGTCCCCCATCAGCTA

GAAGGAAAACCACAGCTCAGACTCCTGTGGGAGGCTTCAGGGTTGTGGTTCAATAGTTGG

AATACTGTCATTGACCTCAGAAGCTCATCA

>c10805_g1_i1 len=218 path=[224:0-217]

CAAAGAGTGAGTGTGGCCGTGATGAGCTGTGCAATGGCAGTGGTACCCCATAGGAAGCCC

TCCCAGGTCTCTGCAGTCCCTTCTGGGGCGTGGTGGTCCCCTCTGGTTCCCCGTGGGGTC

ACAGTGTTGCTGTGTCGGATGAGCTGCGGTGGCTGCATGGAGGATCCTGCCCATGTGTGT

GTCTCTCTCAAAAGGTGGTACCACGCCAACCTCACTCG

>c10812_g1_i1 len=230 path=[1:0-94 96:95-229]

ACCTCATGCTTTCCAGTTTAATGCCCAAAGCTTGTAATTTATCTACAGCAACCACTTCAA

TCCTATTAAAAAGTGCATGTGAAGAAGGGGAGGAGCTCCACTGAGGATAATAGAGATGGG

AGCAGAAATGTTCAGAGAAAAAAAAAGCATACAGAAGATAAAAGGGCATTAATCTCTTCC

ATTAAATAGATGTCTAAAAAGGCAGAGGAAAAAAAAAACAGAAAAAAGGA

>c10813_g1_i1 len=686 path=[263:0-685]

AATAATAATAATAATAATGATTACTTTATCAGATAACAAACATATTATTGAAAATCAAAA

AGAGATTGATTCTAATTCAGTTAAAAATAGAATTTATGAAAAAGGTTCAGATATAAATGA

TTCAGATATATATGAGTCAAATGATGATAATACAAAAAACAAAGGAGTGTGGTATAATGC

AAGAACAAAATGTTGGTTGGCATGTGTAAAAGGAAGTGGAAGACATTTGCGAGTATTTTC

TGTAAAAAAACATGGATATAAAAAAGCGAAAATGTTAGCTGTAGAATGTAAAAATGCAGC

TTTTTATAATGACAATAATAGTAATAATGAATATATAAATAAAAAAAATAATATTATTGT

AGATATAAAGAATGAATCTAACAGTAACAACACTGATTACTTAAATGATATGAAAGATTT

TGATTATAAGAAAAATATCAAAACAAATGAAGATAAAATAGAATCAGAAGAATCTATTGA

TAGTAAGAATTCAATTGATATGGATTATTCTAACAACCAAATTATTGTAAAAAATGAAAA

ATCAAATCTAAATACAAATTTTGAAAAATCAAATAAAAAAAGAAGATATAATACGCGAAG

ATGTAATTATAATTCTTCGAATGAATTAACTAAGGATAATAATAATAATCATCATCATTA

TCATAATAATAATAATTATAATAATA

>c10815_g1_i1 len=231 path=[1:0-132 @134@!:133-208 515:209-230]

TAGCGTTCTCTCAGTCATTATCTGGAAGGACAAAAGAACATTCACTGTGCACCGATTCTG

GCGTTAAGCTAAAGGCACAGCAATCTAAAACCTCTTAGATTGTAGCTACGTGACACATCA

AAGGAAAACTAATTGAAGTGTTTGCAGAACAATTCATTTTTGCATCAGCATTACTCAGTA

TAACAGGAGAACTGTTGATTTTATTTTTGCATCAGCTGAATCAGTTCCCAA

>c10817_g1_i1 len=312 path=[1:0-169 171:170-286 288:287-311]

TTAATGCTTTCTTTCACTGAACAGAAGGTTATAAAGCATAATGAAAGCAGAGCACTCATG

AAATACAACATTTTAAAGCTCAAGATCATTTCAGATGGAAGGTACACCCATATCAACAAT

ATCTCTGAATCTAAATTCTGAAATAAGGCATTGAATAGCTTTGCAAATGAAATGCTGTAC

TTCTCAAGAGGTCAAAGAAGCACCCACTGACTTACCTGGTGCAACAATATATTCCTACTC

ATAACATCATGACAAAACACATTTCAAATTCATTTTAGAACAATATTTAAATAACTATAT

ATTTTATAAACC

>c10820_g1_i1 len=213 path=[95:0-92 188:93-212]

ACACAGCTTTACTGACAAGCTGCTGCCTTCACCTCTTCACTGCGTGAGACTTCTGCTAAG

GGATGCAAAAGCCAGGAAGGCCTCTGCAGGCAGCTCTGAGCCCCGTGGGCCTTTGCTGCC

CTTCCCATTCCATTGGCTGCAGTCCCTGCTGGGTGTCTGGGATGGGATCCTGGGGCGCGG

TGCTGCCGGAATGCTGCTCGTTGGGTGCGTGGA

>c10823_g1_i1 len=212 path=[181:0-41 223:42-137 88:138-211]

ACAGAGTGCAACTGTGTGTGTAACATCACTTGGGATCTGAGCATCCTGACCAAGCTATGA

GCTGTACCACTATCAGTAATAACAGTAACTGCATATGCAGCTTTAAGATACCTTTGCTGA

GGTAAAACATACATAAACAACACACATTTCTATGTGTGTCTTTGTGGCATGGAATCATAG

AATGGTTTGAGCCGGAAGGTACCCCCAAAGAT

>c10823_g1_i2 len=213 path=[454:0-42 223:43-138 88:139-212]

TGTTACACACACAGTTGCACTCTGTTCTGAACCATACCAGCTGATCCTGACCAAGCTATG

AGCTGTACCACTATCAGTAATAACAGTAACTGCATATGCAGCTTTAAGATACCTTTGCTG

AGGTAAAACATACATAAACAACACACATTTCTATGTGTGTCTTTGTGGCATGGAATCATA

GAATGGTTTGAGCCGGAAGGTACCCCCAAAGAT

>c10827_g1_i1 len=461 path=[1:0-46 48:47-460]

GTGGGGTTCCTGGGGGCTGGCTGGGACTTGGCGGTTGGGTTGCCGAAGCAGAACATCGAG

AAGGGGGGCTGCCACACCAAACCCTAGGGGCACAGGCTGCCACAGCCCCGGGAAGGGTTG

GAAAGCCAGCCTCGGGGCAGGTGGAGACACAAATGAATGAGGAACTCACTTTGTTTAACA

CCAGTGCTGCTTCTCTACAGTTTTTATCCTGTACCTTCCCGGAAGAGTTCGACAGGTACA

AAGCAATCCAGGCCTCCTGGCCAACTGAAGGTAATAACAGACTGTATGCTTCCTTCAGAC

AGGTGGGAATAGCTGATATTTTAGACTGCTAATTGCACAGGAGCACATTACAATGTAGGG

AGACGGGAGGGAAAAACCGTACGCTGCAGGACGTAGAACAAAGCAAGGTGAAACTCACTA

AACCCAACATCTGGAAGTTGAGTTTAGTGAGTTTCACCTTG

>c10827_g1_i2 len=640 path=[877:0-225 48:226-639]

AGAGCGCTGCTTTCAAGGATGTGGCCCTGCTGCTGACCGTGCTGCTGGAGGAGGAGATGC

TGGAGACCCACATCAGACTCCAACAGGTGGAAGAGAGGGTTCGTGACTTCCTCTGGGCCA

AGTTCACCAGTGCCAGCGCTCCCAGCTCCTATGACCACATGGACTCTGACAAACTGAGCG

GGCTGTGGAGCCGCATCTCCCACCTGGTCCTACCCATCCACCCAGTGCAGAACATCGAGA

AGGGGGGCTGCCACACCAAACCCTAGGGGCACAGGCTGCCACAGCCCCGGGAAGGGTTGG

AAAGCCAGCCTCGGGGCAGGTGGAGACACAAATGAATGAGGAACTCACTTTGTTTAACAC

CAGTGCTGCTTCTCTACAGTTTTTATCCTGTACCTTCCCGGAAGAGTTCGACAGGTACAA

AGCAATCCAGGCCTCCTGGCCAACTGAAGGTAATAACAGACTGTATGCTTCCTTCAGACA

GGTGGGAATAGCTGATATTTTAGACTGCTAATTGCACAGGAGCACATTACAATGTAGGGA

GACGGGAGGGAAAAACCGTACGCTGCAGGACGTAGAACAAAGCAAGGTGAAACTCACTAA

ACCCAACATCTGGAAGTTGAGTTTAGTGAGTTTCACCTTG

>c10829_g1_i1 len=214 path=[346:0-213]

GCTCACTGCCTTACTATAACCCCACAGCATGGAGTTATTGAACCAGAGAGCCGTGCACTA

ATTCTGGTGAGTCCTAATCCATCACTTGCCACAAAGCCTTCTGTAATTCCTTGGAGTGGG

CTAATCTACATCCACTGTGATAATGGACAAAAATTTATTAGAGTGCAGATTCGTGAGGCA

CAGGATGTGTCTGGAACAAGCTCTCCGTCTAGAA

>c10833_g1_i1 len=281 path=[437:0-280]

ATCTCCTTGACAGGAAAGTGGGGTAATCCTGTTGTGACCAAGGCAGACGCACCATGCAAC

TCTTTGTGAATACCTCCAGAAACAAAGTTTTTTTCACTGTAATACAGCACAGCAGTTCAT

TAGAATAAGATATATTTCATGGTCATGCTGACATGAATGCATTATTTTAGATCAGCATAG

CTTTTGTATTCTAGCTCAGAAAAGAAGTTGTAACAATATAAGCATAGCTATGCTTATACT

AGCTGTGTGTTTACACATAATCATGAGCAGGAAGGAGAACG

>c10836_g1_i1 len=231 path=[1:0-151 153:152-230]

AATTCCAAAGCATGTATAGTTCACGTACATAACAGTACAAGCTTCTAAAGAACACGACTG

CAGTGGAGTGGAGAACAGAAAATAGCCCACCTAAACACCATGAAAACTCGACATAAACCT

TTAAAGCTGTTGTACTCCTTGGTCTCCATAGATGAGAGCCTGTAACTCTACACCAGGGCT

GACAGAATAAGTACGTGCTTTATATACATCTAGTCTAAACAGCAGAAGAAG

>c10838_g1_i1 len=405 path=[157:0-404]

AACTTTGTGTTTGAGGAGGTGCTCTCCGAAGCTGATTGATCGGATTTGTTTCCTTCCCCT

GAGCCCCGCGTGCTGCTGAATGCCAACATACATCGATACCCGCGTGCAGCTCCCACCTGT

CCCCAGGTCGGACCTGCCTGGACTTTTGGGGGGGGGGTAAACCCCTCATTTGGGGAGGCC

AGGCTGCACGTCACCGTGTGGGCTGGGCGCTGGGCAGGGATGGGGATGCTCTGCATCGGG

TCCATGAGGAGGACCAGAAGGCTTCCTGAGACGTTCGCCCTTCTCTAAGCACCAAAAATG

GATGGTGACAACAGCATCTGTCTGCAGGGACTGAAAACTGACTGCGTTAAGAGGTCATTT

CCAACCTGAGTGACTCCGCGAGTGGGCACGGTGGGGATGGGGGGG

>c10839_g1_i1 len=260 path=[295:0-202 25:203-259]

TTTTTTTTTTTTTTTTTAAAGTTGTAATTGTGTTTGTTAATTACATTTTTTTTAATGTTT

ATTAATGTCATAAACTTAAAAAAAAATAAATTCCTATTAGACCATATATATTTGTAATGA

CATTTTTTATAATGAATTATTCAATAAAATAATTAAAGAGAAAAAAAAAGTGATGCAAAT

ATTAACAGAAAAATAATTAGAATCTTATTATTTTATTATAATTATAAATAGATTATTTTA

TTGGTGAGATGTTTACAATG

>c10839_g1_i2 len=261 path=[295:0-202 497:203-260]

TTTTTTTTTTTTTTTTTAAAGTTGTAATTGTGTTTGTTAATTACATTTTTTTTAATGTTT

ATTAATGTCATAAACTTAAAAAAAAATAAATTCCTATTAGACCATATATATTTGTAATGA

CATTTTTTATAATGAATTATTCAATAAAATAATTAAAGAGAAAAAAAAAGTGATGCAAAT

ATTAACAGAAAAATAATTAGAATATTATTATTTTATTATAATTACAAAAAGAATAACATT

GTAAACATCTCACCAATAAAA

>c10840_g1_i1 len=249 path=[227:0-248]

AAAAAAAAAAAAAAACAAAAAAAGAAAGAGATTTTTTCTTTCAAAGAAGTAGGATTTAAT

TAATTTTGTATGAGAAAATTTTTCACGTATAATAATAAAATGTTTACGTAATTAATATTC

TTATAATTAATTTGTATTTTAATTAATTGTTTGTGTTTGTTAATTCAACAGCAATATGTT

TAAAATGCTGAATATAATTCATAACAAAGAAATAATTAATTTACATTTAATTAATAGTCA

ATATTCAAT

>c10842_g1_i1 len=204 path=[182:0-203]

AAAGAAAGCCACAGATACTTACCCGTTACTTTCCTTTCTGTGCAGAGGCAGTTCTCAGTG

TGTGCTGAATTGAGTACAGTTCCCATCACCTACTTTGGCCACTTGTTTTTCTCAAGAAGT

GCTTCTGACGATGTTATCTTCTGCATCGGTTTCAATTTCTTAGTTACCAGTGCAGTAAAA

GCTGAAATGGTATTAGATTTCAAA

>c10846_g1_i1 len=218 path=[1:0-217]

ATGAGGATGACGAAGCTCCCATGATAACTGGATTTTCAGATGATGTCCCCATGGTCATCG

CATGAAGCGTTTCTGACTGTAAAAACCAAATGGTGTAAATATTTTATTTGATAAAAATAG

TTGATTGTTTATTTTAAAAATGCACTTTGAGTTGCAATATGTTATTTTTCTATGGGCCAA

AAAAAAAATTTAAGAAACACTTTGTAGTAATCAACTGT

>c10846_g2_i1 len=380 path=[748:0-379]

GGAGCTTCGTCATCCTCATCTCCTAAGTCGTCCCCTGAGGAGAAGATGGCCGAGCCCAGG

CGGGAGCTGTAGTGGCTGTTGGCGAAGGCGGTGAAGCTGCTCTGCAGCCGGCGGTGCCTC

GTGTAGAGCACCACGAAGCCGGCCCCCAGCGCCACCAGCAGCAGGAACAGGATGGGGACC

ACGATGGCAGCCACATCAGTGGATCTGCCCAGTTTGGATTCAGAGGAGTCTTCACCAGCT

CCCAGCTCATCGTAGAGAAGCGTGGCTGGCTCCCCACACATCTGCCCGCCATACAGACAC

CTGGCCCGGACTGCAAAGGAGTAGTTGTGACCTATCTTCAGGTTGGAAACTTTGAAGAAG

TTCTCAGTGGTGTTCCCAAG

>c10848_g1_i1 len=398 path=[407:0-366 25:367-397]

CTCTCCAAGCCTCTGAGTAAAGAACTCCTCTAAAAGGGTCCTCCTACCCAATGCATCTAA

CTAAGCACTGGGGGAGGATCAATGGCTCACACTCGCAGCACAAGATCTGTATCTCATTCT

TTGCCCTGTCCCAGCTTCCAGGCCTCACCTTGCAGCTTCCCACATCCACATCTCGGCTGC

TCCCACTGAAGAACAGACACTGTAAGAACTTCAGGCCCTACACTCCTTCTCTACATACCA

GAGCAGAACTATTGCCTACAGTTTCAACAGGATTTCAGCCTTAAAGAAGGCACACACACT

CAGCATAGTGGAGATGACATTTAACTTATGATTGGAATGTTACTGGAATATTTCTGACAT

TAATTCACAGCTCAGTAAGTATTTCCACAATATAGGCA

>c10848_g1_i2 len=399 path=[407:0-366 773:367-398]

CTCTCCAAGCCTCTGAGTAAAGAACTCCTCTAAAAGGGTCCTCCTACCCAATGCATCTAA

CTAAGCACTGGGGGAGGATCAATGGCTCACACTCGCAGCACAAGATCTGTATCTCATTCT

TTGCCCTGTCCCAGCTTCCAGGCCTCACCTTGCAGCTTCCCACATCCACATCTCGGCTGC

TCCCACTGAAGAACAGACACTGTAAGAACTTCAGGCCCTACACTCCTTCTCTACATACCA

GAGCAGAACTATTGCCTACAGTTTCAACAGGATTTCAGCCTTAAAGAAGGCACACACACT

CAGCATAGTGGAGATGACATTTAACTTATGATTGGAATGTTACTGGAATATTTCTGACAT

TAATTCAAAGCTCATGCCTATATTGTGGAAATACTTACT

>c10856_g1_i1 len=302 path=[280:0-301]

GTGTTTGATCTGGCATTATAGATGATCTCACAATGAGGTAACAGCACCATATTTTTGCAA

AAACAGGCAGATGGAAAACGTAAATGAGAATTTTATCTCTAAGTGACACAACTCCTCTGC

ATAACAAATATGCAAGGATGTAGTATCATGTCCACTGCTGAACATCACATACTGGGAAAG

AGGTAGTCTGCCAAACAGTAGCAAAATCATCAAAGATGAAGAAGATGTATGTTAATAGAG

GAACAATGTAGAAACAGTTGTTGCCTTAATCTTTTCTTTGTTTAAAACAACAACTGTTTC

TA

>c10857_g1_i1 len=202 path=[1:0-201]

CAAAATATCTCATTATTAAATATGTAAAACTAAATGTGAACATGGGAGGCTGTGATTGAA

TTTTTAGTTATTTTTATTTGAAAACCAAAACCAAATGCTTTTGCTATGTAACATACAGAA

GCATGTCTTGTCGGACTGAGATTTTTGAAAAACAGGAATGGAAATGCCATACAAAGCACT

GTTTTTGGCCTTAGTAAGAATG

>c10860_g1_i1 len=338 path=[1:0-337]

GATCTCTTCAGTTATGATCCTTCGAATTTCATCCAGTTTGCTTTCCTTCTTTTCTATGGT

CTTATGGGCAGTTATTTTCTTGCATTCATACATCCTAGTACCAGCTGCTTCTTCAATCAT

AGCTAGAATCTCTGTCGGCTTCATATTTAGAACTTTAGTAATTTGCCCCTGCATAATAAG

GAAGTGAGGGTTATTGACATTGAGTCCAACAGAACCAAAGAGATCCTGTACACGGTTGTT

GGAAGCATTCATACCGTTGATAAGATACTTATTTCTACCTCCAACAATAACCTGCCTGGT

GATGGTGATCTCATCATTATTTTCAAATCCCAGTGGAC

>c10861_g1_i1 len=285 path=[263:0-94 358:95-284]

ATTGTGTGTGGTAGTCCAGTATTAAAGTAATATCTATTTATGAATTGGCACTCCTAGAAG

TACTTTTATACTCTTAAATACCATCACGTAGCAATAAAAACTTATGTCTCATTTCTGAAT

TGTTGCTGAAGAGCAGATCCTTCCCTCTGCTATCTGTTTGGGTTTGAATTTGCTCCCTGG

AGCAGTTGCCCTTCAATCCTTCTAGTTAGGACTGGAAGTGAAAAAAATGCAGCTACAGTT

TTTCAGTAGGAGTAAAAGCTGAGTTGTAGGAAAAAGCTGAATTGT

>c10861_g1_i2 len=297 path=[632:0-106 358:107-296]

AAGTCTTGTTTGCTGTCAGAACTACAGAGTGTTGTGCAGTGGAGCATGTAACTGCCTAAG

TGAAGTGAAATAGCACCAACCAATGGTAATAAAGGACAGGGGGAAAAAAAAACTTATGTC

TCATTTCTGAATTGTTGCTGAAGAGCAGATCCTTCCCTCTGCTATCTGTTTGGGTTTGAA

TTTGCTCCCTGGAGCAGTTGCCCTTCAATCCTTCTAGTTAGGACTGGAAGTGAAAAAAAT

GCAGCTACAGTTTTTCAGTAGGAGTAAAAGCTGAGTTGTAGGAAAAAGCTGAATTGT

>c10864_g1_i1 len=334 path=[312:0-196 673:197-333]

ATAGTCAAAACTGCACACCACTGAGATACATTACTTGAGAAATGATACAGTAGACTCAGT

CTGGGTCTGAACTTTTCTAATACAGTTGTAACAAAGAAGTCAGTCCACTACTGGAGAGTT

AAAAGGCAACAAACCCACTGCGTTCCAAGGGTCAGATTTCCCACACTGAAGCCCAAACCC

AAAGAAGAGTTTGCACTGACAGTAACCTTCAGCATCTCAGCAATTAAAACCCTCGAGCTA

CTACTACTACCATCTGCTCCAGCTTCCCAGGGAGCAGTGGATTAGCTATTCCTTGCTGCC

TCACATGTACTCACCCTGTCAAAGTCATGAACAA

>c10867_g1_i1 len=379 path=[700:0-134 304:135-378]

TTTTTTAAAAAACATGACAAAATTTTTTATTTTTACTCTATTAAATTTTCATAAATAGTT

GCAAATTATTTTAATATATGTAAAAACTATGAATTGAAAATAGACCAAGATTTAGTTAAT

ATATATATATATGCTACCCGATACTTGATAAATTTAAATACTATTTATTTTTGTATATAA

ATAATATTTACAAATAGGTTTTATTGAACTAAATTTTGTATATATGACAAGATAGTAAAT

TTACAAAAAAATAAATTTAGAAAGTTTAAAATTTGTCTACATAAATTGAAATTATCTATT

TATGAATAGGTATATTTTAATATAAAATTTATTTTCTTTTAGTTGTAATCAGAGTTAAAC

ACTATTAATTTTTATCAGA

>c10867_g1_i2 len=265 path=[283:0-20 304:21-264]

AAAACATTGGTTTTTTTTTGCACCCGATACTTGATAAATTTAAATACTATTTATTTTTGT

ATATAAATAATATTTACAAATAGGTTTTATTGAACTAAATTTTGTATATATGACAAGATA

GTAAATTTACAAAAAAATAAATTTAGAAAGTTTAAAATTTGTCTACATAAATTGAAATTA

TCTATTTATGAATAGGTATATTTTAATATAAAATTTATTTTCTTTTAGTTGTAATCAGAG

TTAAACACTATTAATTTTTATCAGA

>c10872_g1_i1 len=228 path=[1:0-220 1212:221-227]

TAGTATACACTCAAAACAAGCTTTAAACCTATGCCTAGGCTGACAGTCTTCTGTTAAAAT

GACCATATTCCCTAACCATGTTACTTAAAATCTGAGTAGTCAGCCTACAGTTTTGTCTAG

ATATAACACAGAAGTCCATGTATGAAGGTGTGACTTAACCTTGGAGATACAAAGTCTCAG

GAAAAAAATAAACTTCTAAAATAGTACTTAGCTATGCTACATTTTTCC

>c10872_g2_i1 len=368 path=[830:0-345 1210:346-367]

ATGGAGTGTGAATGTGACCTAAGGATAATTGCAAAGCTAAAGTATTTTCAGGAAACTGAT

TGTTTTGTCGTAGACATATGAGGAATTGAATATACATACAAACCGTGCATGTTAAAGAAG

TCCAAGTGGTTCTTCTAAGGTTGGTGTCAGTCTATTTCACGCATCTGCTTCTGACAAAAA

TACTGGTTAGTGCAGATGTCAATAGGGATTTCAGTGTTTTAATACTCTAATCTGAATTGT

TGAAGCACTGTGGCCAAGCTGTATGTGGCCATATGTGTGTGTAATGTTTGTTTTCAGGAG

GTTAATTGTTTTCCAGAGTCTAATAGATAAACCAGTGGAAGATAAAGCTTCAAATAGCAT

TCAAGCTT

>c10873_g1_i1 len=545 path=[771:0-544]

ACCCAGGCCATTCTATAATTCTATAATTCTATGATAAGTAAGGGGCCTCACTCAGAAAGA

TGGCTCTCATTCAGAGCTGGAGTGCTCATCTGCTGCCACTCTGCCTAAAGAACTCTGTCA

CTTCGTATTGCATACAGACAATGGTAAGCGTGACAAGCGTTGGTACCTGTCTGCTTCAAT

ACATCTGCTGGGACAGATTAATATGGAGAGTCCTTATGTAAACTAGGACAAGGACTTGAA

CTCAAACTGGGACAAAGGGATGGCAGATTTCACTCGACACCTCATGTTCCTAAGAGCCAT

TCTGAAATGCTGAAAACAGAAAAAAGTCTGATTTCTTAGTGTGACAATTACAGCAGTGAT

CACACACGGTCTCAAACACATCAATGATGGAATTAAAACATGGAATGCATGAGTTACAAT

TCTTCATTTAGGGAAGCAGATTTTAAAAAGAAAATGCATACCATGCAAAAAAAGACTGAG

TTGAGAAAAACCCGCCCATTGTCATCTACAGAGACCACAACTGAAAGGGGATTGAGTCCT

TCCAG

>c10876_g1_i1 len=407 path=[433:0-342 50:343-363 25:364-406]

TTATATGGCTTATAGATAAATAAAATATAATATTTTATAGAGTTAAAAAAAAAAATATTA

AATAAAATAAAATAAATTTAATATAAAAAAACGAAATGAAATTATAAAAATGAAAGTATG

ATTAGAAAACAAAGTTCATTAATAGATTGTAAGTAAATTTTTTTTAAAAAAAAACAAAAG

GAAAATTCCTAAAAATTAAATATAATAAAAACATGTTTCCTTAAAAAAGTTTGTAAGTTC

TAATAACTCAATTCTGATATTGGTTTATAATTATAAAAATAATTTGAATATTTTAGAAAA

TTTTTTAGGAGTACCAGTAGATATATGTATTATGAACAAAAACGAAATTATTATTTTTAA

TTTAAATTATAATGAAGTTATTAAAACGTTTGATATATTTATTAAAA

>c10880_g1_i1 len=212 path=[27:0-126 229:127-211]

GGAGGATTTCCTCTGTCTGTGTGACAACAGATCATAGCATATACATATACAGGGAAGAAG

CTCTTGCTTTTTATATTCCACAGGTGTATTTTTATTATTCTTTATTCTTTCCTGCTTTGG

CTTGTCAGTTTCCAGATCTGCGCATTAACATCTCTCTTCCTTCAGCTGCCTGAATGAACT

AATCTATTGCTGCTGACAGTGCCTCTGTTATA

>c10883_g1_i1 len=234 path=[212:0-233]

GCGTCGCTGTCCGTATACGGTGGCAAAACCAAACTGGGACCCATCCGTGAGCGCAGCGCG

CCCGCAGCGTCCCGCTGCCCGCAAGGGGCTCTGCTGGGAGCGGAGTGCTGCACGGACAGC

CTGTGTGATGGGGACAGGAGAGGGGACGGTCCCATCTGCGGGACGTACGCCGCGCGCTAC

GCACTGCGCCGCTCCGGACCCGCGCTCCTCCGCAGTGAGCGCCCAAAGGCAGCG

>c10896_g1_i1 len=392 path=[49:0-41 @15@!:42-73 120:74-232 120:233-391]

TGTCACTCAATGGCCCACTGATGTCACCCAATGGACCTCCAGTGTCACCCAACGGACCTC

CAATGTCACCCAGTGGGCCACTGATGTCACCCAATGGGCCACCGTCGCTCTTGGGACACG

TGCCACCCAACAGGCCACCAATGCCACCCAACGGGCCACCAACGTTACCCAATGGGACTC

CAACGCCACCCAACAGGCCAATGTCACCCAATGGGCCACCGATGTCACCCAACGGGCCAC

TGATGTCACCCAATGGGCCACCGTCGCTCTTGGGACACGTGCCACCCAACAGGCCACCAA

TGCCACCCAACGGGCCACCAACGTTACCCAATGGGACTCCAACGCCACCCAACAGGCCAA

TGTCACCCAATGGGCCACCGATGTCACCCAAC

>c10899_g1_i1 len=236 path=[321:0-235]

ATGGCAGATCCATCTGTTTTAACAGGAAAAAAAAAACAACCATCACGTTTTGATTCAAGT

CACGTGCCCAGCGCGCTGCGCTCCCCTGCGTGGAGGTTTACACGGTGCCTGGCTGCACGG

CTCGGTGACCGCCAGCTTAACACCGGTACTGGGGGGGGGACGGAGCAGTCCCCATTGCTC

AGTGGCCGTGCCGTGTACAGGACACTGATGGGTCTGGTGTGAATGGAGGGCGGTGG

>c10901_g1_i1 len=323 path=[293:0-322]

AGTAGTATATTTAAAATTAACTATATAAAAAGAATACAGTTATAACAAACAAAAGATAAA

CAAATTTTCATCATTTCAATAGCATAAAAAAGAATATTTCTTAAATACTCTTTTCTATTC

CAATAGTTACAACCTCAGTGTGTATATATGAAAAATACTTTTATATATAAATAACATTCT

CTATTTATGTTAAATACATTATATTTATTTTACATGTTAAACCTGAAATGAATGTAATTA

TTAAATGCATAATACTAAGATTTTGCATATGACTGTATTTCAACTTTCAAAAGTTTCTTA

TTCAAAATAAAAATAAAACAAAA

>c10904_g1_i1 len=228 path=[1:0-227]

GTAAAGCTTCTCACTGACATTCAGCAGCAAAGCAGCAGCCGGGCTGTGATTCCATGGCTG

CCTGCTCAGCAGTGTCTGCGGGCTGTGCTTTGCAGCAGGTGGCCCAGCCCGGTGACCTGA

CTAGACAGAAATTCACTGTGCTCCTGCGGCCGCCAATCACGCCGGGCTGACGGGATCGCC

TCGCGTGACCCCGGTGAGATTAGAAGGTTGTATGTGAGATGGGCAACT

>c10905_g1_i1 len=398 path=[177:0-291 6:292-397]

TAGCCACAACTTAGGATCTACAAGAAAACATTTACAAGACAGACACCAAGATATGAAAGC

CTCATCCATCCGGTCTGCTGTTGTGACAAGTAGTCCTGTAGTACATGCTATCAAACTCTA

AACCTGGGAGTCCAGGCTGCAATACTGGATAGGAATATCCAGCCATTCCTTGAGTCATAT

ACACAGCCTCTTACATACAGCCAAATATTCAAATGAAGTTATGTTCTATAAATATTAAAG

TATTTAAATAAAAGCATGTATATACTTCAACAGTACCCAAATAACAGCTTTTACAAGTAC

AAACCTGCTAAGCTTCTAATTTACAAATAACCATTTTCTGTATTGACAGATGCCCTCTCT

AACTCGTTTTGCCCCTCAGCAGCTAATGGGTGCAGCAG

>c10923_g1_i1 len=246 path=[277:0-245]

AAGGGCAAATGACTGTATTGTTTGCCCAAAGCGCCGAACTGCACCATTTCTTACAGGAGA

GATCAAGTTGGCCAAGGATGTGACCCGTGCTAAAGGCCGAACCCGTTTATTGCTTGGCTC

CTTAAAGTCAAGGCTGCAAAGACTGACAACATCATCATCTTTCTCTCGGCGTTTCCTCTT

CAGGGTGAAGTCCCATTGTGGTCCTGGAGTGAGAAATGTGAAAGAGCTGCCTCGTCTCAG

CGGGCA

>c10927_g1_i1 len=224 path=[202:0-223]

GAGGACTCACTGTAGGCTCATGCATGCTTCTCTGGTTTGATTTTGCTGTTTCGTTTTTTC

ATTCAACATTATGCAGTAGATCTGTATACCCAAACCGTACGTAATTTAAGCAGCAATGAA

GCTTCAGTTATATGAAAGCCAGCTTGTCCCCTTTTGGATGCAGTGGGACAAAAGGATGCC

TGCAGGGATGGGAGCCCCACAGACCAAGTTATAGCTAGAGGAAA

>c10927_g2_i1 len=255 path=[635:0-254]

GTACACTGTGTCCTGATGACCAATGCTCCATTGTTGATACAACAGGCCCTGTGTAGGCCG

AGACAAAGAGGCTGTGTATGTGCATGCCTGTACACGTGTGTGGAAGCAAAGCACACCCAT

GGGCACCCTGAGCTCCTGGCATGCTGGTCCCACCGACCAGCCCTGGCCAGCTCTGTTATC

ACTGTGGGGTCACGTCACTAATAAAGATCACCTTTTAGGGGCTAGTGGGATGCTCGAGCT

CAGTGAAGCAAAGCT

>c10928_g1_i1 len=259 path=[155:0-258]

AAGGGAAACGATTCATCTAACACCTTAGAATCTTGTGTAAATATTTTCCTTCTTCTCACT

GAACTGGCAGAGAGGAAAGTCACGCTATTGCTCAAAAAATATGAGAACATAAGTACATAA

CAAGATGCTATTTGCTATGATATTGTTGGCTGAGATAAAATTAGAGGAAAAAAAACTGTT

GGAAGTGAATGGACCATATTTGTTTTTTAAATTTCGGATGTTAGAAAATCTGCTCTGAGA

TTCTGTATAAAAACAAGGA

>c10930_g1_i1 len=410 path=[388:0-409]

AGCTTGGCTCATACCCGCAGTGTGTGCCCAGGGTGGTTCTGACAGCGGGGCACACACCTC

GCCCAGTCACATCTGGGCAGCCAGGCAGAGCACACGAGTCGAGAGCGACCGGGAAGCGGG

GAGAGGCAGAAGAATGGGTGATAAATGAGCGTGGAAGGTGTTAATGGAGCCAAATGTCCC

GAGTTTATTGGATTAGGAAAGTCAAACTAATTTTGAGATTACTCCTGGAGTGTGGGAACC

TAGATACTGCGCGCCATTTATCTGACACAACAGCAAAGTCTTAATGGCTGTCAGCTCATT

ACATTAGGAGAACACCGTCGAAGCTCTTGCTGACAAGATGCTTTCTCAGGGGCTATTAAT

TATATTGCGTTATTAGCTAACCGCGGTACGGGGACATATTTTGCATGTTA

>c10936_g1_i1 len=277 path=[255:0-276]

ACAACTTGAGGCCATTACCTCTCATGCAGACAAGCCAGACCGAGCAGGCCTTTGTCCCTT

ACAGCAGTTCTGCTGCTGGCTGGGGTGATAGAAGAGCCCAGCATGAGCTGCGTCAGCACC

GCGGTGAGACAGCAAGCACTGCCACCTGAGGTCAGCACTGCCACCACTGTGCTGCTCTTC

CCCTGAGACCACGGCCTGAGTTGCCTGTGGAGTCGCATGCAGTTCTTCTGGTTTGGCTTT

CCACAAAAGTACCAAATGCAGAGCCTTGCAGTCTTGG

>c10936_g2_i1 len=229 path=[753:0-228]

CTGTTGCAAACCTGCCAAGTTTCCTTAGCTGTTTCTTTTTTTTTTTTAATTAACACCACA

CTACTACATTGCTCCTTTTTGAATGCAAGGAAAGGACTTTGAAGGACCTCAGTGTGCCAG

CAGTGAGACCACCAGCCCTGCACTGACCCTACACGGAGGTTCTCTGAAACCACTATAAAT

GCCCACACTGAGCATTTGCCTTCCAGGGCCAGGCCATGCTGTGGGACCT

>c10937_g1_i1 len=402 path=[209:0-200 104:201-401]

ACTTCCTTCCCCCCTTACTCCATGGTCTTGTTCCTAGTTGACAGGATTCTGGAGTATCCT

ACATTTCCTATCAAAAACACTTCTGTGTGTGCCATTTAATAGAAGAATGGAGGTGTTAGC

AAAAATGGTGCAAACCTCAGGATACTGTATTCACATTGTGAATGGAGGCAGATGCCTTCT

GTCACCCTTCTTTTGTTTTCTTTTTGTATGATGTTCGTGTGACAAAGTGAAAGCTATTAG

ATGTTCTTAGCATCTGCTGATAGTTAAGATTAAGAAGGTTAGTCTGCAGCCAAGATTTGA

TGCAATTTTTGAATAAGGATCAGTTGTGTTTTTTCAATGTGAACAGAAAATATAAAAATT

GACGTTCTTATCTGTTTACAGAAAAACCTCCTAATAAAAGCA

>c10937_g1_i2 len=304 path=[1:0-102 104:103-303]

TACTGAAAGTATATCAATAAAAACCTAAGATTAAATACAAATATATATTGATATAATTAA

ACCCACATTCAAAACAACTGTTTTGACTTATCTTTCTTTCTCATTTTGTATGATGTTCGT

GTGACAAAGTGAAAGCTATTAGATGTTCTTAGCATCTGCTGATAGTTAAGATTAAGAAGG

TTAGTCTGCAGCCAAGATTTGATGCAATTTTTGAATAAGGATCAGTTGTGTTTTTTCAAT

GTGAACAGAAAATATAAAAATTGACGTTCTTATCTGTTTACAGAAAAACCTCCTAATAAA

AGCA

>c10938_g1_i1 len=205 path=[342:0-165 538:166-204]

TATATATATATATATATTTCAATAATTTAAAGAATGAAAATTTTCTTCATAAAATATTTA

ATTATCTATAGTAGATTAATTATATGAATGTATATATTTTAGCTAAAATAAGATTTATAT

TTCAAAAATTTAAAAAATAAATGAAATTTCTTTTTCCTTTATAAAGGATAATTCAATTTA

CCATAGTGTGGATTATTATAAATAT

>c10943_g1_i1 len=232 path=[1:0-231]

ATGAGTTAATGTGATGGTTTGAGGACTAGTTTGAAGCTATGTAACTCATAATGAAGTATT

TGGCTGAAGGCCTTTCAGGTGGGAATGTTGGATAGTTCTACTATTCTGCTGCATGTAGTC

CACTTTCAAAATAGACTTTCTGACAGAAATTTCCTCCAAAAATGGCTGCATAGAACAGTC

TTTTGTATGAAGATATTTAAGGAATGTTTTGTATAGAGTTCAAATTATCAGT

>c10944_g1_i1 len=397 path=[375:0-352 839:353-353 729:354-396]

AAAAAAAAAAAAATTTAACGAAATATAAAAATAAGAATAAAATTTTTTTTAAAACTTTAA

AACAAAAGGTTGGCTTTTTTTTATTCTATTTTATTATATTTTTCGTATAATTTAATTTTA

ATTTTTTCTAAGAATTTTACGTAAAAACACACGATAGCCAATTACATTTTTTGAATATAA

TTCTACATATCAAAGAGCGTAATATACACTTTTTTATTATATCAGAATAAATTTATTTTA

CATTGTTTTTAATTTTTGTAATTTAAGATAATTTTATTTATTACCAAATAAATAAAAATA

TATATAAACAACACTTAAATGTATTTTTATCATATTTGTTATAAATGTAATCAAATAATT

TTTTAAAAAAAATAACTAAAAAAACCTAAACATTTAT

>c10946_g1_i1 len=218 path=[196:0-217]

AAAAAAAAAAAAAATATAAATAAACTAAAATAAAATAAATTAAATTAAAAATAAAATATT

GTAACATCATGATAGAAATTACTAAAATATATATAAAAATAGAAAAACAAAGAATTTTTT

ATATAAAAATAATTTAAAAGGAAAATATATTTTTTTATATTTACACTAATAATTAGAGGA

AAATTTTATAATTTCAATTAATTTTAAAAAAAAAAAAA

>c10948_g1_i1 len=217 path=[141:0-156 104:157-216]

AGGAAGATAAGTTAATTAGTTTAAGTGTAATAACTTCAAGACCTAAGAAAACCTAAGTAT

TTGTCAAACATTAGATTTATTCACAGCAAATGATTTATTATTATTGTATGTTAGAAGATT

ACTTTGTGTCTGGAAGCAATTTGTAGTCATAACTTTCAAAAATTATTTAGAGAAAAAGTG

CTACAGTAATTAAAAAAAAATCCTTTTGTTTACTCCA

>c10959_g1_i1 len=263 path=[1:0-262]

AATCAATAAAGTACAATACTAAATTTATATTTGGCTTAAATGAAAATGAATACTTAAAAT

GTCATAAAAAGGTATTTAAAAAAAAAAAAAGGAATGATGCAACTATGGCACAGAAGCACT

GGGAAGAATTCATCTGAGAAGTAATTCTCGTGAACTTTCAGTGAATTCCTCTTTCTATTT

CTTGGTGCATGTTTCACATCATACACTGCTCTCTATCACTGGAGCCTGGTCAGTCTATCA

AAGTGTGCCGTTAGCTATTTTTA

>c10960_g1_i1 len=237 path=[1:0-140 142:141-236]

ATGTGGAGAGCCATAGCTGGGCAGCGGGGATGAAAGTTATGCCTGTCACATCCAGAAGTT

GTAAGCCTCTGTCAGTACAAAAGTGGAAAGCATTTGCTCTTAGAAGCGGATTGTTACGTG

TGCTGTCAGAGCCACCAGAGGGAGACTTAGTCCTGTGTAAAATGGCTTTGCGGCTCACAG

AACTGAATCAGGGTCTTAAGAGGGCACTGAGTCCTGTCCTTGGTGGTGGTACGTGGG

>c10967_g1_i1 len=507 path=[779:0-506]

GGAGAACTCCTTTCAACTGTGAAACTCCATAAATAAACAGAGATATTTCTGCTTTGTCAC

AGAGCAGTCCCACCGGGGAGGAAATGATGTAACCAACACAAACGGGACAGTGAGAGCCCT

GTGTCCAAAAATTACTCCTGATATCACATGAAAGTACGTAAGAGGGAATGGTTTCAACAC

CAGCATGCTTAAAGGAGGAAGGAGTGCTTTAACTGGACAAAATCTGAGGATGACATCTTT

CCATAAGCAGTAACAGCCACACGAGCACTGAGCAGCGAGGTGTTCCACCAGCTGAGACAC

GAAGCGCTCAGCAGAGGTCAGCACGTCCAATGCAGCAAAACATTCAGCAGCGCTTCAAAA

GAAATTTACAGCTGCTGCTGGAACGTGGCACACTGCACAGCGCTGCAGGGCGAGACAGGC

TCCAATGGGGCACTTCTGACAGTGGGCAGCTGGCACTTAAGTTTCACAGAATCACGCAAT

CATGGGGGCTGGAAGGGACCTCTGGAG

>c10970_g1_i1 len=266 path=[244:0-265]

GCAAATGGTTGATCCGGTGCCCCATGATGCTCCCAAACCTCCAGGGTATACTCGATTTGT

CTGCATTTCTGATACTCACTCGAGAACTGATCCCATCCAAATGCCATATGGAGATGTGTT

AATACATGCTGGTGATTTCACTGAGCTGGGACTTCCTAGTGAAGTAAAAAAATTCAACGA

GTGGCTAGGTAGCCTGCCCTATGAATACAAGATTGTGATAGCAGGAAATCATGAATTGAC

CTTTGACCAGGAATTCATGGCTGACT

>c10971_g1_i1 len=295 path=[53:0-294]

TACACCAGACATCCCAAAACATCTCATGCTGATGCTGACAGGATTCCTCCCCCAGAGACC

ACATGATAATTAAGATGTCTTTTCCCCACCCCACCTCCCCATAGTGCCTGCCTGGTATGT

GGGTCTGTGTGGGTCTGAAAAACTCAGAGCTGAGTGCTCCGCAGTGATCTGTCAGAGTTA

AAGATGTAATGGTGGTGGTACAGATTTCCACTCCAGACAGAATTGTTACTGTTTGTTTTG

GTCAAATGTAATGTTTCAATCTGTGCTTGTAGGAAGAAGAGTAAAACAGAGGGAG

>c10973_g1_i1 len=254 path=[7:0-253]

TTTTTTTTTTTTTTTTTCCTGCCATCTTTTGCTTTTCCAGCAGGGACCCTGGCAGCCGAT

GCCCCTGCCTGTTGGGTGCCATCTTCGCTCTCCCTTGAGAGCTGGGGATCGCTGCCAGGA

GCTGCAGGTCAGCGTGAACAGGAGAGAAGCAGCACGACACCTAACAGAGCAGGGACTGGG

GTCGTTATTATACAGAAGGGCCATTTTGGGGCCCTCAGGCAGGGTCGTGAGGGTGGAGCA

CGGTGGGGAACGAG

>c10974_g1_i1 len=209 path=[187:0-208]

AGGTTAAAAAACAAGGGAGAAGAATAGCGGCTGCCATCCACCAAGTGTGTGGGGAGCTTT

GGAAACCCATTTTTTCCTTGATCTGTGTACAGTTCTGACAGTGAAATTGCTCATAAGAGA

GCGTCATAAATTAGACTGCGGAGCCTGACACGCAGGAAGAGAATTTGGATTCCTTCTGGC

CATTACACTTTAAATTGATGGCAAGGGAG

>c10978_g1_i1 len=267 path=[350:0-18 106:19-266]

AAAACAAAAGGCTGCTGAGCCCAAGGTTTTGTTCTGAAGGTAGAAATGCATTTTCCTCCA

TGGACTTCATTGTGTGACAGAGATGTTGTCTATGAATAAATGTGTGATTTGGACATAATA

GCTTTTTCTGTCTTTGAAGTGGAAGCGTTTTTTATTCAGAGTAACTGAATGGTAACTGTC

ATGTGGAAAAACATAAGGTAACTCTGGCTGGTATTTCTTGTTTGTGGGTTGGTTTGGTTT

TTCTTGTTTATTTGTTTTTGTTCACTG

>c10978_g1_i2 len=300 path=[54:0-51 106:52-299]

GTTTTAAATAAAAGGGAGCTATCCTGATAAAGTTGGCTGGACCCTGCTTGTCCCCAAGGT

TTTGTTCTGAAGGTAGAAATGCATTTTCCTCCATGGACTTCATTGTGTGACAGAGATGTT

GTCTATGAATAAATGTGTGATTTGGACATAATAGCTTTTTCTGTCTTTGAAGTGGAAGCG

TTTTTTATTCAGAGTAACTGAATGGTAACTGTCATGTGGAAAAACATAAGGTAACTCTGG

CTGGTATTTCTTGTTTGTGGGTTGGTTTGGTTTTTCTTGTTTATTTGTTTTTGTTCACTG

>c10984_g1_i1 len=393 path=[409:0-352 761:353-392]

AACTCCCAAACATGTACCAGAAAATCGTGTCCACCTATAGACTTTCCAGTATGTCATTAC

ATTAATTGATTGTTGAATGTTGCAATAAGCATTCCTTAAACAAATAAATCACACAATCAA

TACTGTATTTCCTCTGCTACAGACAGCAAATTCCAACCACGTGGGTTTTCCAGAGTTCCC

CAAACACGGTCAAACTTCAACTTTTCAAGTAATTTTTGCAGATATTACCTCAGAGGAAAT

ACAGACCCAGTGTAAATTACCACAGCATTATCCAACACAGTAAAATGCCAGGAGATGCAA

AAAAATCAAGCAAAATCAAACAAATCTTTCAATGCTAAGCAAGAACAAAAATTGCTCTCG

GGATGCATTAGTAAGGTATTGCTCAAGCTAAGC

>c10991_g1_i1 len=209 path=[24:0-208]

GGGTTTTCAAGGACAGCGTGACACAATATGGACTACCTACAATGAAGACATATGAACTCT

GGAGCATACCAAACAAATTCCTTGCATAGTTGTTTTCCAGCACAGCATCAATCACCACAG

GGATGGGAGAGTGTCCAAAACAGCAGGAGAAAATGCTACAGCTTGCAGTTCTCGGATTGT

AATTCATTTAAAGCTGGGCACATTAGCCA

>c10993_g1_i1 len=263 path=[1:0-201 535:202-215 549:216-262]

CTGATTTTGATGAATCTTTAAATTCACTCAAATACGCCAACAGAGCCAAAAACATTAGAA

ATAAACCAGTTGTAAACTACAACCCTGATCAAGACCGGATTGATGAAATGGAACTTGAAA

TCAGATTGCTCCGAGAAGCTTTGCAGAACCAGCAAGTCAGTAATCAGTGTTCACATGACT

TAAATCAAGAAAGAAGTCGAATCAGTTCGCTAGAAGAGCACTGAACTTGAAGCTGCGTGA

GCTGCTCTTCTAGCGAACTGATT

>c11000_g1_i1 len=303 path=[155:0-302]

AGCCTTAAAAGGCTGATTACTGAACTTGAATTTCAGCTGAAGGTGGAACAGAACAATAAC

GAATCATTACTGGATAATATGAGGAAAGAAATTAAACACAAGTCAGATGAACTGGAAGAA

TTAACCCAGGAGAGGACGCAGCTGATTCACAGTTTGAGTCAAGTGCAAGAGGAGAACACT

CTTCTCCAGGAAACGGTGCGCAGAGAGTGCGAGGAACGCTATGAGCTGACTGCAGCTTTG

ACTCAAGCCAGGGAACAAGTACTGCAACTCAGAAAGCTCAGTGGAAACTTCCCGTTATCG

CCG

>c11001_g1_i1 len=405 path=[383:0-5 389:6-404]

GTTAGCAAAAAAAAAATATATCACTTTGAATTGTAATGGAATGTTTCGAGAAAATGTTAA

TAAACCCTTTACATTTTTTTAAATTGTTTTGATATTTTTATATATTTGTTATAATAAGCA

AAACATTTTCGAACTCAAAAAAATTTATATTATGCTTTTATTTAATAATTTTATCATAAT

TCCTCAAAATTTTCTCATATATATTTCTACTTATATTTACTTATATTACAATTAAATAAA

GTCATACTTTTAATAATATTAGTTTTGTTATTCTTTACTTTTTTATTCTTTAATAAAATT

TTCATCTACATATTCATTAACATTGCTTATCATACAATTTGCATTTTTATTATTATTCAC

ATTCTGTTTATATATTTATTAATATCTATATTCTTATTAGTGTCT

>c11002_g1_i1 len=581 path=[49:0-580]

ATATAATGATTTTAAAGAAAAATTTAAAAATTATAAAATTAGTATACCTAAATATAATGA

TATAGAAAAGACGAAGTTATATTTTTGTTATCAGCTTACGCATTTTTGTGAAATTAAAAT

GTTAAAAGCTCAAATAGTCGAATGTGCTATTTTATTATATAACAGATTTTATTTAAAAGA

AATTATTTTAGTTTATGATCCTAGAATTTTAATTTTCACTTGTATTATATTAGCTATAAA

ATTAGAAGGATATGGTAGAATGTATAAAATGAATGAATTTTTTAGTGATATAGATATAAA

TTTAGATAAAGTATTAGAACATGAGAATATAGTTTGTTCTTCCTTAGATTTTGAGTTGAA

TTTTTTATATACTAAAGAATGTATTTATTACTTAAAAAATAAATTTTTAAATTATATAAA

AAAATATATAAATAAAGATAATGAATTAAATAATTCTTTTGAAAATATATGTGATAAAAT

TTATATTAACACATCAAATGACTGTTTGAAAGTTATTGAGAATTTTTTTATTACCTTTAC

TTATACTCCCTCTCAAATAGCATTATACTGTTTTATAAATA

>c11002_g2_i1 len=480 path=[1060:0-479]

ATATTAAATATTACAACTATATTTCATTTTAATTAATATATAGAAAAAAAAATTCACTTA

AAAATTTTCTTTAATAATTTCCATTGACAAAAGAAAATGAATTAATATAATCATTTTTTT

GATATTTCTCTTTTAGTTTTCATTTTTTATATCAACTTTTTTATTAGATTCGTTTTGAGT

ATTATCTTCATCATTCAGTTTTTTTTTTCTAGATTTTTTACTATTTTTTTTTCTTAAAAG

CTCATAAATATCAATGCACATATCTAATGTCTCACCTATTTGCTTAGTCGTATTTTCATC

ATCAAATGTGTTTCTTAAATCTAGATGATTTTTATATTTGATATGAAGCTCATCAATTTT

ATTTTTTAATTTTTGAAAGAGAATTTGGTTATTATTAGTAATAAACTCTAAAATAAATTT

ATCTGCATTAACAATATTAAAATAAATTTTTATATTATTTATAAAACAGTATAATGCTAT

>c11006_g1_i1 len=243 path=[336:0-18 338:19-71 74:72-242]

CCCATGTCCCTTGGGAGGAGTCACCATGTCACCCTGTCACATGGTCACCCTGTCACATTG

TCACCCCCTCACATTGTCACACTGGCAGCCTGTCACCCCATCACATTGTCACCCTGTCCC

CTTGTCACCTTGTCACATTGTCACCCTGTCACAGTGTCACCCTGTCACCCCATCACCCTG

TCCCCATGTCCCCATGTCACCCTATCACACTGTCCCATGGTCACCCTGTCCCATTGTCAC

CCT

>c11006_g1_i2 len=431 path=[38:0-35 74:36-206 338:207-259 74:260-430]

ACATTCTAGCTCAGCGTTCGCTTGTCACCCCCTCAAATTGTCACACTGGCAGCCTGTCAC

CCCATCACATTGTCACCCTGTCCCCTTGTCACCTTGTCACATTGTCACCCTGTCACAGTG

TCACCCTGTCACCCCATCACCCTGTCCCCATGTCCCCATGTCACCCTATCACACTGTCCC

ATGGTCACCCTGTCCCATTGTCACCCTGTCACCATGTCACCCTGTCACATGGTCACCCTG

TCACATTGTCACCCCCTCACATTGTCACACTGGCAGCCTGTCACCCCATCACATTGTCAC

CCTGTCCCCTTGTCACCTTGTCACATTGTCACCCTGTCACAGTGTCACCCTGTCACCCCA

TCACCCTGTCCCCATGTCCCCATGTCACCCTATCACACTGTCCCATGGTCACCCTGTCCC

ATTGTCACCCT

>c11011_g1_i1 len=235 path=[142:0-139 282:140-234]

TAGGATAATGCCAAACCATAATTGCATCTAACTGGGAGCATTTGATGTATAAAATAATAC

GATTGTTTTACAAGTGGCTCTTGCCATGCTAAATTTCACATTGAGTTTTAGTGGTCAGTA

GTTTAGTAGTGAAGGAGTTCAAAGACGGACTACTTAAATGTTCTGCGTTTTAGTTGTCTT

ATGAAAATAATGAGAGCAGAGTTTTGGAGCCAAAGGTCTTTAGCCATAACTAACA

>c11012_g1_i1 len=228 path=[267:0-184 468:185-227]

GTAATGCTCATGCTGTTTGACACCATAAACATCTGAGATTTCATCACCTTGTTTTGAGAG

GAGAGCAACAGTTTCTTCCCTGCCTGTCTTCTGATTTCTGCTTCCACTTAAGCTGGAATA

TGGTAGTTGTAAGCTCATATTTCTGTTCTGTTGCTAATACTGTTACACGTTAGAGGTTGA

AAACCCTTGTGGTAGCAGCACTATGTTGAGAGCCTGTCAAACACAAAG

>c11019_g1_i1 len=270 path=[248:0-269]

GCCTGTCTGCTCCTTGATGGCTTTTACTCTTCGATCAATTCCATCAGTGTTAATTGGAAT

TTTTTTCCCCATCTTCTCTTGTGATAAGAATATATTTTCATGTAATCTGTTTCTTTGGGC

CCAGTGGTAAAAGGTCTGTCTTTGGGAATTCGGACATCTATTTTCCATTAAAATGAATGG

AAAGGATTTGAGCACCTTTGAGCTATTGTGAAGCAAATATTCTTGTTCCCCTGAAAGGGA

TCTCTTGTAGTGAATGACAACAGATAATCT

>c11023_g1_i1 len=357 path=[1:0-356]

TACAGGAAGCTCATCCATATGGAAGACGTGGCTTACACTTCCTGGAGAACTGTACTTTTA

GGACTAATTGATTCTGCCGGCCGCCCTTTCTCCTCCGGCCCCCAGCCCCGCTCGCTCCGT

TCCCCGCAATCCGACGCGGGGCCCTCCGAGATGAACCTCAACTTCACCTCTCCGGTACAC

CCGGTCCCCGCGCCCCGACCCACCTCCTTCTTCATCGAGGACATCCTGCTGCACAAGCCC

AAGCCCCTGCGGGAGGTGCCCCCCGAGCACTTCGCCGGCTCCTTGGCCTCCCGCGTCCCG

CTCTTGGACTATGGGTACCCCCTGATGCCCGCCCCGGCGCTGCTGGCTCCACACCCG

>c11023_g2_i1 len=202 path=[669:0-201]

AGCTTTGCGCAGCCCCGCATCTCTCCCCCAGCCCTGCGTGCTGCCGGGGTGCCGAGGTTT

GGTGTCGGTACGGAGGGGAACAAAACCCCCGGGCTCCTTTCGCCCCTTTGGCTGCATCCC

AGCGTCACCCCAAGCCTGAACTTTGCTTGGAAATCCAGCGGGGGGAGAGCCAAAAAATAA

AACCAAACCAACCAACAAAAAA

>c11026_g1_i1 len=238 path=[1:0-215 542:216-237]

GAAGATGGAAAAGGAACTATTTCTTTAATATTGAACAGGCATTAAATTGGCTTAGAGGCA

TCTATTGATATGAACAGGCTGTAGTGGGAGCTGACGTAGGTGGGGAGAGCACAGGGACTC

TTTGGCAGCCCAGTTCTCTTGAAGTTCCTAGTAACTTAAGTCTCAAATGTGAGCAGCCTT

GGAGGAAATCTCATAGATAAACCCAGGAGGAACTTTACTTCTCGTTTCCTGCAACCAA

>c11031_g1_i1 len=296 path=[274:0-295]

GACAAGTGATACAAGTCTACATCCTGCAAAACTCCCCGGGCGAGTTCCACAGGCCAATGA

AAAGGCCATAGTAAAGATGAAACTTCTGTCAGTTTTCACCATGCTGCTTACCTTCCTGCA

TGTCTTACCCTGGTCTACAGGAAGGGAATTCACATACAGTGCTCGTATCCTAGGTGAAGC

TGTCTGAGCATCTTCTCTATTTTTCTTTGCCATCTCTGTGCAAAGCATTACGCCCAACAG

CACCTATCATGAATGTCTGCCCTTACAGCATATTCCTACCCCGGTCAAACACAAAG

>c11031_g2_i1 len=300 path=[824:0-299]

CAGAAGATACTGTATGAAGACTGAGTCAGCCCCCAACCGGAAAATGACAGAAGAGAGACA

CTCTGCTACGTTGCATAAGATGACAGGACTAGTACAGCTCACTCCACAAATATCTGGGTC

ATCTGGTTGTCCCTATATCTGGGTCATCTGGTTGTCCATCTTCTGCCAGAAATAGGGACT

ATAAAGAAGTAATAGCATCACTCTTACAAGCTCTTGCCTGTGCATTGCTTTTCTTGGGAC

TGGGAACTGAAGACACAGGTTCTTATTTGCTCCTCACTCTGGGAAGTCTCAAGGCCTGGG

>c11032_g1_i1 len=264 path=[516:0-263]

GGACCATTTAGTGTGAGTTTGCTTTAGTGTTCAAAAGACAGGTAGTGAAGCATAGGGTTT

GATCAAAGCTTTTCTATTGTGTATATGTAACACTTTGTCTTTTGAAAACAAACCCAAACA

ACTACAAACCTTGAAATTCACATCAGAGATGGGCCCACTTTCTTAAGGTGGGGGGAGGAA

CACAAACTGATTTATTCTGACTTTGGAACTGGGAAAGAGACTTGCTTGAGAGGCCTGACT

TACGTGTTAGTATACAAGAGATGC

>c11035_g1_i1 len=224 path=[163:0-223]

GACGCACAGCTTCCCACAGAGGCACAGGACCGTCTCTGTGCAAGAGCCGAGCCTGCCCCA

GGCATGGTGTACTTGTTTGGAAGATGTCCTGTGATGGCACACAGAGCCGTGTCCAGTCCC

CCACCCCTCTTCAAGCCATAGTAGTTCACACTGGCTAGGGGAAATGCTTTACCATCTGTT

CGTCTTTAAGATCTGACTCATTATTTTTATTTCAGCTGTCGCCG

>c11038_g1_i1 len=242 path=[220:0-241]

TTGGATTCATTAGGTCTTCATGCAGAATTATATTCTCAATAAAAGAAACCAGTTCCATCC

AGTATCCACTATCTACACGCCTATGTTACAACATTTATATCAAATCTGGTATCTGAAGAA

AAGATACACATGTAGTATGTACATTTAAGTTACTTATTTTACAGAAAGATTAAAAATTCA

AGTCACATAAAACTCAAAATCTGTATTAAAATTGGAAATATGAAACTCCAATATTTACTG

GA

>c11039_g1_i1 len=586 path=[649:0-249 336:250-585]

GCAGATCAAGACCCTCAACAACCGATTTGCTTCCTTCATCGACAAGGTGAGGCTGACTGC

GCTGGGCTTGAATGCAGCCCTGACCCAAAGCTCACTTCATGGGGTGGGGGTTCGTGATTG

GGGTTGGGGTTTCACCAACCCTGATGTGATTTTCCTGCAGTTTAATAACCGTTATTAAAA

TCCCTGCTGTCAGTAGAGGTTTTTGCTCTACTCAGGCCAGCGTTGCACCCCCTTTACTTA

GCTGGCATTGCAGCCCCACACGCACCACCAAATGAGAGAAGGGGGCTCTTTGGAGAGGAG

CTGAGCCGGTAGAACAGACTTTTGATGTAGGTCAGGGCTTGCTGTGTTTGTTTGCACTGC

CCTGTACCTCTGCAGCTCTGTTATCTCCCACCAATGGACTGGGGACCCTGCGTTGTAAAC

CAGCTCCGGCATCCCCAAAATGTTAGGAATTCAGCTGTGGAGTTGGGTTTAATTGGCTGA

CACTGTGATCTCTGAATGGTTTCTCTTATGCGTCAGGCAAGCAGAATCTCCTTATCTTTA

ATTCAAACGCAGCCTCAATGCTGGAAGGCAAACACAGAGCAGCCCT

>c11039_g1_i2 len=347 path=[325:0-10 336:11-346]

CCCAGCGCAGTCAGCCCCACACGCACCACCAAATGAGAGAAGGGGGCTCTTTGGAGAGGA

GCTGAGCCGGTAGAACAGACTTTTGATGTAGGTCAGGGCTTGCTGTGTTTGTTTGCACTG

CCCTGTACCTCTGCAGCTCTGTTATCTCCCACCAATGGACTGGGGACCCTGCGTTGTAAA

CCAGCTCCGGCATCCCCAAAATGTTAGGAATTCAGCTGTGGAGTTGGGTTTAATTGGCTG

ACACTGTGATCTCTGAATGGTTTCTCTTATGCGTCAGGCAAGCAGAATCTCCTTATCTTT

AATTCAAACGCAGCCTCAATGCTGGAAGGCAAACACAGAGCAGCCCT

>c11042_g1_i1 len=259 path=[249:0-131 399:132-258]

ATTTGAGGATAAAAATAAATATTATCTAATAAAATTCTATTTAATAATATACATGTATAT

AATTTTTATTATTTGTATATTCAAAATAAAATAGGTTTTAATTCTTCTTGCATACATATT

AGATTCATGTGTAGAGAATAATTAACAATATCAAATAGATAAACTAATTATTAAAGGTAA

TTTTAAAGTCAAACTAGTAACCTTTGTTTGGAAAATTTTTTTTTCCAGATTTTTCTAGGA

AAAAAATGAAACAACTTAA

>c11048_g1_i1 len=303 path=[281:0-302]

GGATGATGAGGAGTCCTGGATCAAGTGTGTATTTTGGAAGTTTGAGGCTCTGGCATGAGG

GCATGATGTGCTTACTGTCAGCTTGCCAGCAGTGTTCGGTCTGTAGTATGGTTTTATAAC

ACAAGTATGGGTCTCCACAAGAAGTCACCTTTGCCTTCTAGTCAAGGAAAACTATATTCA

AAACGACTTCTGCTCCAGTTTGGGTGTGGCTTCTTATCTACAGTGCTCTGTGGTCTGACT

CCAGGAGGGAAAGGTCTGTGCTGGAAGAAGTAGCACTCTTTAGCCTCAAACACAAAGTTA

CCT

>c11051_g1_i1 len=280 path=[47:0-68 46:69-279]

CATCTGATGGCGTTTCCAAGTGGGTTCAAGTGTTGATGTGTGTGACTTCATGGCAGCCAA

GTGGCATCTAGGGACAATGAATGGCATTTGGTGGCTCCCAAGTGGCATCCAGGGTCAATG

AATGGCACCCAAGTGTCACTTAATGGCACCCAAGTGACATCCTGGGACAGTAAATGGCAT

TTGATGGCACCCAAGTGGCATTGGGGGTCAATGAGTGCCACCCAAGTGTGACTTCATGGC

ACCCGAGTGACATCTAGGGACAATAAATGACCTTTGGTGG

>c11052_g1_i1 len=274 path=[281:0-134 25:135-273]

AATTCAGATGAATGAAATGCCTGTCTAATGAGAAATCTCTGTCTCTCTATACATTCACCA

GCACCTATCAGCTTCCTGGAAAGGCATCTGCCTGCTTGTTTTCCAGCACCCACTTTTTGC

TCAGCTAGTTTTTGGTTTTGTCTCTTTATATGCTCAGGGCTGGCAGTGTGAATCCTTAAT

TTCTTCCTAGTTGTTATGATTTGGTATTTGCCACAGGAATTGTAGTATAACTGTGTAAAG

GGGATGGATAGAACAGGACAAAGTAGGACCTCAG

>c11057_g1_i1 len=226 path=[311:0-225]

TAATTTACTTTTTAACAGTTTAGGGATAAGGATAGTAGCTTGAAACACTTACTGGTGAGA

AACACCTAACAAAATCCATGTGCCAATAGTGGTACAAAAATGCAGAATAGAATTTTTATT

TAATTTGTAATTAGAATGCAACTTTATTAACACAGTTTCCTAAGTGTCATGCACTTGAGA

TTCTTCATACCGGTTTTCACTCAACCTTTGTAGCTACCTCTAAAGA

>c11060_g1_i1 len=278 path=[256:0-277]

GCACACAGTTTCACAAACGGTAGGCAAGTTGAAGGAAGAAATGAATTGCCCATGCCTAAA

GTGTTTATTTCTTATAACCTTTATAAGTAGCTACCTATAGCAAGGCTGAATGTAGTAGGA

AATGCTTAATGAATTCAAATGAGGTCTGAAACATAAATTAAGCAGTCTAACCATCCATAG

TAGCCATGTCATACTAGTCTCACTGAGAAATGGATATTTATTGCCCCCCCTGTGTCACAT

TGTCAAAATGAATGATATAATTATACCCTATCTGAGAT

>c11066_g1_i1 len=224 path=[246:0-172 564:173-177 25:178-223]

GTAACTTTGTGTTTGAGGGGCAGCGGGGAAGGCATTAGGGAGAAACTTTCAGAGAGCTGA

GTGGGGTTTGCTTTGTTTTGTGCACTGATGTATTTGTTAGTTTGTGGGGATAACTTTTTC

CTTTCCCTCCCTTTAATGTTCTTCCTTAACTCACCGAAAAACCATTCTTCAGTCAGTTAA

GAAGCAAACCAATGTTCTCAGTATCAATATAATACATTGGTGTA

>c11066_g1_i2 len=223 path=[246:0-172 564:173-177 423:178-222]

GTAACTTTGTGTTTGAGGGGCAGCGGGGAAGGCATTAGGGAGAAACTTTCAGAGAGCTGA

GTGGGGTTTGCTTTGTTTTGTGCACTGATGTATTTGTTAGTTTGTGGGGATAACTTTTTC

CTTTCCCTCCCTTTAATGTTCTTCCTTAACTCACCGAAAAACCATTCTTCAGTCAGTTCA

GGTACAACTGTATGTACTTACACCAATGTATTATATTGATACT

>c11067_g1_i1 len=245 path=[240:0-170 411:171-244]

TGTATTATACACAAGTTGCAAAAATCCTTGCTACAGTTAATTCTAACACTTCCATGACAA

TAGGCAGTATTTCAGGACAGAGTTGTTCTCAAACGCACATTAAAGGTTCCAGCATATGTT

GAAATACAAGTATGTAGAAGAAGAAAGTATACTTAAAAGACTAACTGCAAAGAAAGTCTA

TGCTTAGTTATCCAAAGCACATACTATCAGTACATCTCAACTGTACGTTTGTTTAAAACA

AAACA

>c11069_g1_i1 len=242 path=[374:0-241]

AGCTCCTGGTGCTGCTCACTGTGGCTGCTGAATGGCAGTGGTGTCCCGGACAGCCTAGGA

ACCCCATCTCACCACCCCCACTATGGCAATCCCACAGCAGCCCCTCTGGCAGAGTTCACA

TTGGGGAGGCACCGTGGCCATGGCCGAGGGCTCCATACAGCTATGGTTGGTGCAGGCTTG

CATACCACTGTGCCACAGCCCAGGGAGCACCTTGGGAGTCACCCCACCAGCCCCCAAGGC

CA

>c11073_g1_i1 len=364 path=[342:0-363]

AGGAAAATAGGAATTAATAGAAAAGGAGGCCAAGAGGTGATCTCATTGTTCCCACTGGTC

TCTTCTCCCTCATCACGGATGATGGGGATGTGTGGGCATGGCACAAAGCTGCACCAGGGG

ATGTTCAGAAGGAACATTAGGAGAAATTCCCTCACCAGGAGGCTAATCAAATACTGGGAC

AGGCTTCCTAGCGAGGTGGTTGATGTCCCATGCCTGCCAGTGTTCAAGAGGCATTTGGAT

AATACAGTCATTACCAGTGCTAGGATAAGCAAAGTCAAAAGACTTCTGCTCAAAATACTT

CTGCTGGAAGATAGAAGAGATGCTAGAGCGTAATAAATTGTCCCATTTGGCTGTAGTAAA

GAAG

>c11075_g1_i1 len=308 path=[377:0-243 638:244-245 640:246-307]

TTCCATGAACTCTCACACTAATCTGTCCAGCTCTTTATGGACACTGAGCAGGAACACTGT

TAGCTTTCATAAAGTGATAACCTCCCCCTAAAGCACTGAAGTTGCTCTGTACATGCATGC

TTAATCACCCCCACAGTCTGTGGACGAAACAGCATCCAAACATGAGATCTAGTCCTAGCA

GCTACACTGGCAAAATTAATAGATCTTCTTAAGTTAGGAGGCCCTCTTCTGTTACTTCAG

CTTTACAGAAAACCAACACCTGTAACTGGGGCTCAAATGACAGAAGAATTCTTTTGGAAG

TCTGTAAA

>c11078_g1_i1 len=234 path=[212:0-233]

GGAGAAATCATCCATGTTGTCAGAGAAGTGATCCAGGTTCTCGGAGAAATGCACATTGCT

GAGGAAATCCGCCTCGCTGAAGTCGCCCAGGTCGAGGAAGCCGGGGCCGGCGGGGGGGAA

GAGCCGCTCGGCGGCGAAGGGCTCCAGGATACTGTCCATGGTGCCCGCGGCGCGGGGCTC

CGCCGCTCCCCGGCCGGGCTCACAGCGGCGGCACGGTCCCGCCGCCCAACGACC

>c11082_g1_i1 len=249 path=[198:0-193 418:194-248]

AACTTCAGGCAGAGGAAGTTTGACTTCGAGGAAACTGACTGTATGCGTTTCCCATGCAAT

GAAGTCACTAGCATTGCCTGAAGCTGAACAAAGCTTCAGTGGGGTTTATAAAGTGCTATC

TTTTAGTATTTTATGCCTGTCTTTAGTAGTGTTTTTCAAACCAGCACCAGGGTTGTGAGA

TATAAAAAAATACTACCTCTGCCACTAAATACTGAGCCAACCCAGTGGGGGCTGTGTTTT

TGCATTCAG

>c11082_g1_i2 len=220 path=[198:0-193 392:194-219]

AACTTCAGGCAGAGGAAGTTTGACTTCGAGGAAACTGACTGTATGCGTTTCCCATGCAAT

GAAGTCACTAGCATTGCCTGAAGCTGAACAAAGCTTCAGTGGGGTTTATAAAGTGCTATC

TTTTAGTATTTTATGCCTGTCTTTAGTAGTGTTTTTCAAACCAGCACCAGGGTTGTGAGA

TATAAAAAAATACTGAAATAACAGACTGAATTTGAAAGGA

>c11086_g1_i1 len=214 path=[1:0-213]

GATTCTATGATTCCATAAGCAACAGCATACCTATGGCTCTTCCCAAGTTGTGTATCAATG

ACCATCTGACCTAAATTTTATTATCTATAGCAACAAAAGAAAATCCTTGTATGTTGCATA

TGTACCAACTAGAGAAAGCATTTCTGTAAGTAACTGGAGCTGCAGTAAATAGTACTCATA

GTCAAAAGCAACTTACAAGGATTTTCTTTTGTTG

>c11094_g1_i1 len=244 path=[1:0-95 501:96-96 502:97-243]

ATCAACATTGTTTTTCTCCTAGAAGCAAAACATGCATCATACCAGATACTCTGAACAACT

CTGTCCCAGCTGAAACTAAGACAAAGTTGAATGAGTTAAGGACCTATGGCTGCTTAAGTT

ATTGAGGAGCAAACTTCAAAAATTCAGGGAACCATTAAGAACTAGCTAACCTCTTTGAAG

TATGTTAGAAACCCCTTTTGCAGCACTTGTCAAAGTGGATGTGATTCTTTTAGTGGGAAA

GGAG

>c11107_g1_i1 len=296 path=[274:0-295]

GGGGGGGCTCAGACCTGCTCACCCAGCTCCACCTATCGCTACCGCAGCCTGGCCCAGCCG

GCCAGCGCCGGCACACGCCTCTCCAGCGTCTCCTCGCACGACTCCGGCTTCATCTCCCAG

GATGCTGCCTACTCCAAACCGCCCTCCCCGATGCCCTCAGATATCACCAGCCAGAAGTCC

TCCAGCTCAGCGTCCTCAGAGGCCTCAGAGACGTGCCAGTCGGTCAGCGAGTGCAGCTCC

CCCACCTCGGATTGGTCCAAGGCCAGCCCCTATGACCAGCCCGTGGTCAGCACCCT

>c11107_g2_i1 len=271 path=[547:0-270]

GGACCGTGTGGAGCACCTGCGGGAAGCTGAGATGGGCTCAGCCAGCAGCGTGTACCCAGC

CATGGGCAGCGAGGATGCTCCCAGACCCCGAATGTCACCGGCCACCATTGCAGCCAAGCA

TGGTGAGGAGGTGTCCCCTGCTGCTAGCGACCTGGCCATGGTGCTGACACGGGGGCTGAG

CCTGGAGCACCAGAAGAGCAGCCGGGACTCACTGCAGTACTCCAGCGGCTACAGCACGCA

GACCACCACCCCATCCTGCTCCGAGGACACC

>c11109_g1_i1 len=246 path=[263:0-245]

GTTCCTTCCAGTATATTGCAGCCAGCAGAGGGGGACACTAACAACATAATTAAGAACAGC

AATGCAGCTTTTAGCTTTAGCAGGCCTCATGGTGAGGACGCGGGGCTGGGAAATCCATCC

ACACAAACATGCAAGTGAAACGTTCTGCTGCAGATCACTGGCGTGTGGCCACGCACGCTG

GGACCTGCCTGTGCAACTCTTGTTCTGGCTTGGTGTGGTTCACAGAGGACACTTCATGGA

ACACAG

>c11111_g1_i1 len=224 path=[1023:0-223]

TGGCTGTGTGTCAACGCAAAAGCATTTTTCTTTATATCTGCATGATTATTTAGCAACTTA

AGTGATTTTTATTTTATTTGGAGCGCTTTTTCAGCAATATTAGCCGCTGAGGGGCTCAGG

ATCTATTTTAATAAAACAATCACTGTTCTATTTCACATCATGTAGATGTAAGCAGAGGAT

TCTGCAATTAGGAAGTTCTGTATCTGTCTCTTTGACTATACAAA

>c11111_g2_i1 len=498 path=[1:0-459 461:460-497]

CATCAACATTTGGATTTTTTACACAGAGATTTGTAAAAAATCCAAATGTTGATGGATACT

TTAAAAAAAAAGAAAAGCCTTCCTTTAAGTAAGAGCAGGCAGTTGTGGTTCACTGTGCAT

TTACTGCTGGCTGTGTAATTTCTTTTGAAATTAAAGTTTTGTGTTTTCATTGTATTTTTA

ACTTTCAGGTAATGGGGATATTTTAAAATAGTCTTTAGAAAAAGATGCAAACTGTCACTG

AACATCTTGCTGACACAAGTTTTAAAAACAGATTTGCTAGAAATCTGAAATAAAATGCCA

TCCTCAAGTCAGTGTTTAAGGCTCTGACGGTATAGCTTATTAGAGAATAATAAAGATTGT

TCTGCGTGAAACAATTTAGATTCTGAATCATGTGAAAAGTCGTTGAGCCTTCTTTTCATA

TGGCAAATTATGCAGAAGTTATGTCAAAGGCGCCAGACTCAGCTTCCATTGAGGTTGACA

GGAGATGCTGTTCTGTAC

>c11118_g1_i1 len=216 path=[1:0-215]

GCATGAGCAAACATAAATGTCACTTCAAAGGATATCCTAGATCTCAGGGAAGCTAATTGT

TCAATACCGGTTTGGGAGGCAGCAGTGGGAATATGTGATTAATTGCCATTCCGTTCCATG

TGCGTGCAGCTCAGGAAGAAGTGAAAACAAGAATTTCATGAGGAGATGAATGCAGGGCAA

CAGCTGTAATTCAGAATCAACTTTGCCTCGCTGAAT

>c11125_g1_i1 len=519 path=[497:0-518]

CTTTCAGATCTAGATTGTTTCCTGGGAACAGTGCTGTCTGTATAGCCAGAGATTCTTCTC

TTCATCAGTTAGATAAGTTTTCTTTCTGTATATGCTTATTGGCTTCCCTACAAGTATTTT

TACCTGGTATTGCTAGGAACCTGTCAGAAGCAGTTTTTCTTACTTATGAAAGAGAGCAGG

AAGAAGTAAAACAAGCCTAAAAGTTGTATGCAGCCTGTATTCTCTTTACTGGGATGAGAA

AATTGAACCTTTATTAGCAGCCCTGCACCTGGCTAGAGTTCAGAGTTTTTCTGTCTGTTT

TTCTTTTTTTCCTTTTTCCTAGCAACTGATGCAGTCACTACTATGGTGCTTGGAGGCTGT

TCAGAGATACCTGCCTTTTCTAAGCGCAAAATATTTCAACTTCACGTTGCAGTTTGTTTC

TCCTAGTCCATCCATCAGCATCAGGTGAGTATTAGAAGAACAATTCCACTGCCACCACCT

TCTCTGCCCTCCTTTCCTGCACCCTTACAAAGTAATAGG

>c11126_g1_i1 len=596 path=[17:0-248 17:249-497 772:498-595]

AGTTGGGAATTTGAGTGGGAAAGTCGGGATTTTCAGTGGGAATTTGGGATTTTGAGTGGG

AAAAATGCAATTTTGAGAGGAAAGTTGGCATTTTGAGTGGGGAAATGGGCGTTTTCAGGG

GGGTAGTTCGTATTTTCAGTGGGAAAAACCCTATTTTGAGTAGGAAAGTTCAGATGTTGA

ATGGGATGGTCGGTATTTTTAGTGGGAAGTTGAGTGTTTGAGTGACATAGTCGGTATTTT

GAGTGGGATAGTTGGGAATTTGAGTGGGAAAGTCGGGATTTTCAGTGGGAATTTGGGATT

TTGAGTGGGAAAAATGCAATTTTGAGAGGAAAGTTGGCATTTTGAGTGGGGAAATGGGCG

TTTTCAGGGGGGTAGTTCGTATTTTCAGTGGGAAAAACCCTATTTTGAGTAGGAAAGTTC

AGATGTTGAATGGGATGGTCGGTATTTTTAGTGGGAAGTTGAGTGTTTGAGTGACATAGT

CGGTATTTTGAGTGGGATAGTTGGGATTTTGAGTGGGCAAAATGCGATTTTGAGTGGGAT

AGTTGGTATTTTCAGCAGGCAAAGCCCTGTTTTGAATGGAATAGTCAAACACAAAG

>c11141_g1_i1 len=213 path=[1:0-212]

TGCAGATAATCCAGGACCAATGGTAGGCTGGCATTAACAACTGAGGCCCCATTACAGCCT

TGTTGTAGTAAGTGTGCTGTAATGTATATTTAGTGGATTCTCTTCACGGTTTTAGGAAGT

TTATATGTAATGATATTTAAAGTAACACACACTTTTTCTGCTGTTATTTTTGACATATAT

TTGAATTCCAGGTTGTGTTTGCAACCCCTGGTA

>c11146_g1_i1 len=228 path=[1:0-116 118:117-227]

TTCTTCTTTCTCAGTCAGACATTTTGATAACATAAACTATAAGTTAGAGGGGAGATGAAT

TGCTTTTATAAGACAGTGAGGAAAAGAAGACTGCATAAGAAAAAAAAGATGTTTGATGGT

GGGAGCAGGACTAAGGAAAATGAAAATGAAGGGGAAGCACTTTTGGAGAGAGAGAGAAAA

ATGAACAGAGAAGAGCGTGGAGAAGTGAGGAAGAAAATGGCCCTTTGA

>c11146_g1_i2 len=294 path=[411:0-182 118:183-293]

GAGAAAGAAGAAAAAAAGCTGCAGTTGTTTTTCTTCAAAGCTTGTGTATATTCATCTATA

GGCAGGTTGTGGTGTGGGCACAAAACTAACAGAAACAGAACATACATAATTCTGAGTAGT

AGCGGTAAAAATGAGTGAAGTGAAACAAATTATCAAGCTGTAGGTAGCTTTGAATGGAGA

ATAGGTGGGAGCAGGACTAAGGAAAATGAAAATGAAGGGGAAGCACTTTTGGAGAGAGAG

AGAAAAATGAACAGAGAAGAGCGTGGAGAAGTGAGGAAGAAAATGGCCCTTTGA

>c11148_g1_i1 len=249 path=[1:0-248]

AAAAAAATGTTAAATGTAAATCTTAACATTGAAAGTTTTTATAAAAATTTTAATTTTAAG

AATCATTCCAATTTTAAGAAGAGTAATTTCTTTATATTTCTAAGAGATGAGAATATACTT

GTAATACATATATTTTTGTTTCTCCTTTTTTGTTTTGAAGGAGTGTAAAATAATTAAAGA

AAACTTATGAATTATGTAAATATATTTTTACATAATATTTTTCCTACATATTTATTATTC

TTACAAAGT

>c11149_g1_i1 len=226 path=[1:0-225]

GGCAATGCAGTAAGACAAACTGGTCCAAAACAGTTAAAATATAACCACAGTGAACAATCT

AAATTAAGTTCTATCTTGAAGCTCTCAAACTTTGTCCTTGTATTCCTCCTAAATGACTTG

ATGCTGCTTCCATCTCTTGTATGCTTCCTTTTTTCAGGGGGCTCTTTGTACATTGATGTA

TGCCTCCTGTCACCCTTCCTTGACTTCCTGCATGTGTTAACAAACT

>c11152_g1_i1 len=369 path=[347:0-368]

TGTATTTGGAATTCCTAATGTACCTTTAAACAATATATTCTCACCATTTCTAACTCTTGG

TGAATTACGATCTGAGGACCATGTCTTTTTGTATAAAGCTAAGGCATATCTTAAAAATAG

AAAATACGATTCACTCAATTGTAAGCGTGTTTTAGACCATTTAGCTATGTATAATTTTGT

TAATCTTATACACTCATTAACTGAATATCCATCCCTTTCTGCTTTGTTAAAATCCTCTCT

TTCATCTTTTTCAATTATACTAATAATAGTATTTGAATAATCAGTCCATTTTCCCCATAT

GTCATGCTTTAAAGAACGAGGAAAAATTGATAATGAAGGACAAAGCATCAATTCATTCCT

CATACCAAA

>c11153_g1_i1 len=261 path=[43:0-260]

ATAATAATAATAATAGTATTAATAATAGTATTAATAATAGTAGTAACAATCAGAAGAATA

AAAAAAATGAAAATAATATTTTAATTATAACAGATGAACATAACAATTTAAAATCTAATC

ATATTTTAAATCAAAAAATGAAGATATATAAAAAAAGGAAGCTCAAAAATGATTTTCAAA

TGAGTAAGAAAAACACAAACACAACCGTATTAAAAAATAATAAACTTAATGAAAAAATAA

AAGGGAAAACAACTGTTACAA

>c11168_g1_i1 len=230 path=[1:0-229]

GCAGCCATTGTTCTAACTTGTTGCCTTGAATCAGAGCAGAGTAGTAGCTAATAAAACGCA

ACAGGTGGGAATTTATAAGTAAAAGCTAGCAAGCTGTTTTGATACTGACAGAGCGTGCAG

GTACCTAGGGGTAAATAATTGAAAGTAGTCTTGATGTGATAGCATTTTATCTTCTCTACT

GGACCCAGTAAATAAAGATTTTTGCTGGTAAAAGGAAAAAAAAAAAAAAA

>c11168_g2_i1 len=228 path=[620:0-227]

ATGGCTGCTTAGCTCATCTAAAATCAACCAGCCTTACCTGAAATAAGCTACCTTAGGACC

TTAAACACTTGCAGGGTAAAACAAGGTGATACCTCTGAAGCTTCTCCCTCCAAAGCAAAA

GGTAACAACAAAATACTAAAGTCACCAATCAGTCCCTCACGTTCCCACAACACTGACACA

GAAGGAACTGCATTATCAAACGTTCACAGCTGCAAACACCATGCCATA

>c11176_g1_i1 len=274 path=[1:0-127 526:128-273]

ATACAACTAACAGCCTCCTGTTGTGTCTATTAAATTCATCTTCATTCCCACCTATCGCAC

TGTTCCTATCAACAACCTCAACAGTGTGGCACTATGTTTATCCTCACTGGCTCACTATCA

CACACACATGCACACACACACACTGAAGCATTTTTTGTCTGCCTTCTCATCAACAAGGGC

ATTAATTTGGGTATTTCTTCTACATCCAGTTCACAGTTTTAGAAAACTACACAGAATTTT

GAATGAGGAGAGCATTTGCCTTGAAGTTTTCAAT

>c11180_g1_i1 len=255 path=[1:0-254]

AAAATATTTTTGTTTAAAATAAAAACAAGAATAACTTTTTAAAAATGGTGCTGCCTCTTT

CAATAGGTAGTGAAAACGTCCCTAGCTTTGGGCTGGTAATTGCAGGGTGATGAACTCGTC

GGCTTCATGTCTCTCCCTTGAAATTCAGCTGCTTTTCGCACGTTTCCCTGTTGGTCTTCC

CAACTCGTGGCCTTTCAGCCAGACTGTAACTGCTAGTGACAGGCTGTGATCATCCCCTTG

CTCTACAAGGACTGA

>c11189_g1_i1 len=268 path=[268:0-244 681:245-267]

TGCTGCTGACTTCTTTAACAACCTTCACTAGCGCGATGCTTAGTATAGCAGAAAACATAC

AGGTGCTGCTGGTGGAGGCTGACGCTGGTCTCACTGCCGCACGGGCACACGCATGCGAGA

AGTGAAAGTAATTGCTGCCACTCCCAGCACGTAGCGTGGGTGCAGCGGAGGAAACACGAC

TTCCTCCCACCTCTCGTCAAGAGGCCAGGCACAGCCAAAACAAATTGTGGGGAATGTTCT

GCCAGCCCCACCGCGAGGTGCGGGGTCT

>c11195_g1_i1 len=315 path=[1:0-314]

AACCTCCTCTTTAATGACCCTGAAGATGCTTGAGGGGGAGGGGAAGAATTCCCTCAGGAA

CTTTTGTGGCATTCCAAGGACTTGTCTGGACTTGTCTAAAGAAGCAGCATTATAAAAGAA

AACTGCCATACTGATATACACAGAAAAATTTTTTTGTTTTGTCAGTTATGGTGTCTTTGC

ACTGAAAGTAAAGGAACATTAACAGCAAAAAGGTACTTAGCAACAATAAAGGACATTCAC

AGTCAACTACATTAAAATTCCACTCACCAAATTATTATGTAGGAAGATTTCAGCAGGTGA

GGGGTGTTTGTCAAT

>c11196_g1_i1 len=202 path=[155:0-201]

GCACTGTAATGTGGCACAGAACTCAGCTCTACCAGTACCAGCACATGCACATCTTGCCTG

GGACAGTCTCAAAGGAATTAATACATGGCCCTTGTGTTGTCAGGTTTCTGATGTAACTCT

CATCAGTCTTCTTGTATCCTGGAAAAAGCCAAGCTTCTAGCTTTAACAGAGATAAGCTGA

AATGAGTTGCAAACAGCCATTA

>c11199_g1_i1 len=217 path=[349:0-216]

GGGGAAGAAATGGCTGAGAAGAATCTAAAATGATGCATGCTACAATGGTCTGTCCTATTC

AAAATGATCATAAGTGACCTAGTGAAAGGAGTAAAAAGTGAGATGACCAAGTCTGTGACA

ATATTGTCTTTCTCTAGAGTAGGAAAGAAAAGGGTTGACTGCAAGAAACTGTAGAAGAAC

ATCGCGATGTTGACAGCATAAGCTGTGAGTGGAAAAG

>c11205_g1_i1 len=358 path=[1:0-118 120:119-357]

CTCGTCCTTGGTTGCGTTGCCCACACCTGGTCCCGCAGCTATCAGGCTTCCTGCGAGCCC

AGCCCGGATTCCAGGCACTCCCCGAGCCACTGCAGAGCCGGGCGGTGCTGCGGCTGCTCC

TGCTGCACCCGGTGGACAATGGCACTGACAGCACGGAGCCGCTCCTGCAGTTTTTGCTGT

TCGTTTCTGCAGGCCTGCTCGGAGCTGCACAGGGGTGTGTACTTCCCTTCCTTCAGCGCC

TGCAGATGCTTCTGGCGTGTCTGATAGGCCACAATCTCCAAAAGGTTCCATCGCTTTTTG

TACCGAAGACATTCCGTGTGCGAATCGAGGCCATGGGACTCAGTCTGCAGCATGCACA

>c11205_g1_i2 len=257 path=[689:0-17 120:18-256]

GATGGGAAGCGGGTGGTGCTGCTGCACCCGGTGGACAATGGCACTGACAGCACGGAGCCG

CTCCTGCAGTTTTTGCTGTTCGTTTCTGCAGGCCTGCTCGGAGCTGCACAGGGGTGTGTA

CTTCCCTTCCTTCAGCGCCTGCAGATGCTTCTGGCGTGTCTGATAGGCCACAATCTCCAA

AAGGTTCCATCGCTTTTTGTACCGAAGACATTCCGTGTGCGAATCGAGGCCATGGGACTC

AGTCTGCAGCATGCACA

>c11213_g1_i1 len=204 path=[470:0-203]

ACATCACACTCCAGGAGTGGATTCAGACAGTTAAAGGGCGTTGCAAACTTTATGGAAGTT

TCAATACATAACACCCCTGACAAGAGTGGGCATTTTCAGGTAAAAACATTGATCACCCAT

GCTTCAAATTAAATGGGGATTTTTTCATTGAGGCCAAAGACACGTGAATAGGTTACCCTG

AAAAACACACTGCTGCCTCAAGAG

>c11215_g1_i1 len=202 path=[416:0-201]

TACCTGAAGCATCTGCTGCTTCAAATTAAGGACAGTGTAAAGGGAAAATGGGCTGTTGCT

ACAATGCTGGGAGTGAGAAAGTGAAAAACTGAGCAGAATCAGCGAGTTACAGAGATTTTT

GCTGTTTATTTGCAAGCAAACAACCTCTGAAGAATACATTTGGGGGACTCTCATATGAAT

GTACACCTAGAAACTCTTGAAA

>c11221_g1_i1 len=321 path=[1:0-320]

ATAATTACTACTTACCTTTTAATTTAATATGGTTTTTGAATGTAAATGAAAAATAATAAT

TTACTTAAATATTTTAGAAATAAAAATAGATTAGTTCCTAATTAAATACAAATTTATAAA

ACTAAAACTAATATGTCATTTTATAGCTTTAATTTAATTGTATATTCTTTTCAGAATATG

GTAAAATACTATTAATATAAATGAATTATGTTAAATTTAATAGTTTTTATTTTACTATTT

TTATTTATTTTAATAAGTAATTTGATTTTTTATAAGTATTTTAAAATAAAGTGAAATATA

ACTTTGATACATATATAAATA

>c11221_g2_i1 len=229 path=[811:0-228]

AAATATATTTTTTTCTTTTTTTTTAAATTGTAGAAAAATAACAATATTTTTTTTGGTTTT

ATTTTTTTTAAATACATATAGTTGTATATTATTTGTGATATTTATTGAAAATTTAATGAT

ATATAATTAAAAAACGACATAAAAAAAAATAAAAAGAGTACAAGTGAGATTTTTAATTAT

TACTAATCAATAAGGAGATTTTTAAGTTTATTTAATAATTACTAAAAAA

>c11222_g1_i1 len=252 path=[230:0-251]

ATGCTTACAAAAGCATGTAGCCATGCAGGTACACATGTAATTTACTCTGGATTCAAACAC

ACATGCTAGAATACTGCAGCCTAACAAGTTGCTGCACATCTGCTGAGCCAATTGAACTAT

ATGTGTATCTTTGGTTGGTGACAAATAGGAGGTGAACAAATTAAGCTTTTAGCCTTCTTT

TACTGTGGGCTAAATTAAGTCTAATTTTGCATAAGGTATGGATTGAAAGCATGTGTGTAT

ATATACGTTGTA

>c11227_g1_i1 len=323 path=[549:0-322]

GCTCTGCTGCATCTCTGTGCTCCTCCTCCTTCATCTCTCCCTGCTGAGGGCTCCTGGGAC

CCCTCACCCACCATCCCACCCAGTGCTGTGGGTTGCAATCCCCAGCCACCAGGACGCAGC

AGCACACGTAGCCACGGGCACACCAGGCGATGCCATCCATTGCATCACACCTTGGTCATA

GCATTGAGATTACAAAGCTCTTCACTGAGCATGGGAAAGGCTGAGGTCAGAATTTCTTCT

CCCCAGGCATCACTTTTTAACAAGATAGCAAGAATGAAGCGAAGTTTCCCATTGCACAAT

GTGCAGCTGTCTTGCTGTAGAGC

>c11230_g1_i1 len=245 path=[1:0-195 197:196-244]

GTGCTGCCTCTCCCCATGCTGGCCTGTGTAGCTGGTGCCAAAAGCATGCTGCAGGTAGCA

GGAGTTTGCAAATACACCTTGAGTGCATGGTGTGTGGGGAGGATGGCTGGGTTCAGTGGT

ATTTGCTCCTTCCTGAAGAGAGGATGATCTGTGCCTCTTAAGTTCTCTTGATTACAGAAT

GGAAAATACAGATACAGAAGAAAAGCTCAAACATTCCAGATTATAAGTGACAACAAGTAG

CTCCA

>c11232_g1_i1 len=229 path=[207:0-194 402:195-228]

GGAAAAGGTACCTTCCATAACATGGTATAGAGAAAGGTATGTTATCAGAGTGTACAAATA

AATGAGAAGGAAGTCTTGATCTTGGCCATTTCAATGCGTGATCCTAAAGGTTGTAGGTTC

ACTACTGCTGAAAGAAAAAGAGGGAAGAACGTTCTTGAGTGCCATGTATGTCTTAGAAGT

AATAAGCAGACAAGAGGTGTGCTGCTTCTACAGTACAGACTAGAGGAGA

>c11232_g1_i2 len=286 path=[207:0-194 436:195-285]

GGAAAAGGTACCTTCCATAACATGGTATAGAGAAAGGTATGTTATCAGAGTGTACAAATA

AATGAGAAGGAAGTCTTGATCTTGGCCATTTCAATGCGTGATCCTAAAGGTTGTAGGTTC

ACTACTGCTGAAAGAAAAAGAGGGAAGAACGTTCTTGAGTGCCATGTATGTCTTAGAAGT

AATAAGCAGACAAGAACGTTCTTCCCGCTTTTTAATAGTGCTTTGCAGTGTTTTGTAATG

GCACTTCCAGGTGATTGCCTCTGATTCTCAGCTTACTAAAGTATGT

>c11236_g1_i1 len=531 path=[681:0-530]

GGGGTTGCTGAGTTCGTCAGCCTGCAGCGGGGATAAGTGAGAGGACACGTAGGCATGTAG

GACAGCAGCAAACGCAACGCGGCTCCACTCAGCACTCCTTCCCACCACCCTCTGTTACAG

ACCAAGAGCGTTCTGCAGGACGGGGAGCCCCGGAGCTCAGCAGACACGGGTGACATGCAC

AGCCCTGCAGAGGCCGGGGCGGCCCCAGCCCCGATCTGCGTCCTGCAGGGCACTTCACAC

CATGCACGGGGCGCTGCTCCCAGAAAGCCAGCACGGGCTGAGCACGAACTGCAGGGCAGC

CGTGGCCCCACGGCACTTCCTCAGCCTCTAAGATCAGCCCCGGGCTGCCAGAGCCTCGAG

CAGCCCACGGCAACACGGGGCCTCCCTGACTCCTAAAGCCACCGTGTGCAGTGTGTGCGA

CGCAGCCGGGTCAGCGCAAGCTCGGACAACACAAACACGGCACACAGGTAGAGCGGAGGC

AGCGCACGAGAGTAAATGGGGTTTATACCATGTTGTACTGCTGGGGTGGAG

>c11239_g1_i1 len=335 path=[488:0-265 27:266-334]

GCAACAGGAGTAAGATGTTCAAATGCCTTTTTTCTTCACTTATCTATTAACAAGCTTTTA

CAGCTGCGGTCTGTTTATAGATCCTGTCCTGTTCCTAACAAGTTCAGTGGAAGTCTACAC

TTAATGTGAGTGGGAATTTGATTAGACCCGAGTAAATACATATTCAGTCCAGTCTTCTAT

TCCATAGACATAAGTAAGTTTTCTAATGGCTGTGTAATTGTTTTCACAAAAAGAACTACT

TTTTATCATCTTACTCCTGTTGCTACAAAAAATTAAAGCACTCTTATATACAGTTTAGAA

GAAGTGGTAGACCAGGCTTTGTGATCCATTGTCAT

>c11243_g1_i1 len=260 path=[1:0-259]

GCATATGTACCATGCACTGGAAAGGTATGAAGTAATTCTGCATGTGGTATGAAGCGATGC

TGTAATTCATATGACAAACTGACTCTGGGAATATAACTGGAATTTCTCTCCTCTTGCTAA

GTAGCTTTAATTTTCTTTAAAAACGTGTGTGATTTAATAAGGACAGGAAGTCATGCCACT

GTAAATAAGTTGTGTAATTGCATGTGTTATTGCAAAGCTGACAAAGTCAGCTTCATTGTC

AGAGGATACAACATTGGTTA

>c11243_g2_i1 len=236 path=[688:0-235]

TGAAGAATCAGGAGAACAGGGACTACAAAAGTAAGATGTAGATTTTACACACAAAGTATT

TTTCATCTGCATTTAAATGCTTACCAGAGTTTCACAAATTAATGGCATATGGGAAGATTT

GGTTTCTTTGGGGTATGATTCTTCAGTCATAATGGAGGAATACTTAAAAAAAAAAAAATG

GCTGAAATTTGGTAACCATTCCACTGTATTTTGAAAACAGAACCGAGTCACAGTCA

>c11256_g1_i1 len=405 path=[22:0-19 42:20-404]

TTATAACATTTTTATAAACCTTGTAAAATATTCAATTTCCTTCAAGAAAATCACTTTTTA

ATAACAATGTTATATATGTATACTATAAGAAAAAAAAATTATAAGGGGATTATAATTATA

CTAAAAAAAAATAAATTAGGCTAAAAACTCTATGTAACAACTTCAATTGTTGATGTTAGT

TTTTTAATTTTGCGTTTTATATTTTAATTCTTAAAAAAAAAGAACCATTGTAAAAAATAA

TCAAGGGATACTATAAATAAACTATATGAATTATCCTAAATATTTTAAAAAAAAAGGATG

TTAATTAAAACATCCTTACAGAACAATTAAAATTTTACACAAATATATAATTTTTTAATA

TAAAAGAAAGAAAATTTTTTTATTAACATTTATATTAATAATTCA

>c11256_g1_i2 len=511 path=[98:0-125 42:126-510]

TATTTTTTTTTTTTAAGAAAAATAAAGAAACATATATTTTTTTTCTACACATTTTATCCT

TTATCTAAATATAGAAGTCTTAAATGTAAATTCTATAAAAGCCTTTCTTTTATTCTTTAA

TGAGTATTGTAAAATATTCAATTTCCTTCAAGAAAATCACTTTTTAATAACAATGTTATA

TATGTATACTATAAGAAAAAAAAATTATAAGGGGATTATAATTATACTAAAAAAAAATAA

ATTAGGCTAAAAACTCTATGTAACAACTTCAATTGTTGATGTTAGTTTTTTAATTTTGCG

TTTTATATTTTAATTCTTAAAAAAAAAGAACCATTGTAAAAAATAATCAAGGGATACTAT

AAATAAACTATATGAATTATCCTAAATATTTTAAAAAAAAAGGATGTTAATTAAAACATC

CTTACAGAACAATTAAAATTTTACACAAATATATAATTTTTTAATATAAAAGAAAGAAAA

TTTTTTTATTAACATTTATATTAATAATTCA

>c11257_g1_i1 len=357 path=[621:0-356]

ATGGGCATCAAGACAATGTACATGTAACAGTTTGCAACCATGATTCTAGCAATGGAAATT

GATGGGCTCATGCTCTTAAGAAGCTGTTTGGGATAATGTATGTTATATGTACCTGTGTAT

CCGTTTATGTTTCAAAAAGCAGATGTACACCACTGCCAATTTTTTAGTAGTCATTCCCTC

TCTTCACTTATGACCTTTCTTGAAAAGAAAGAAAATATCCCAACACATAACACTTGATAT

AATAAGGAGCATACCGTAAGCAGTAACAGGTATTAGAGATAAGTCAATTATACTTACCAT

CTCAAAACATATGTTGTAATAACACAGCCACTGTAGCACTAAATAGTACTAGCTTAG

>c11260_g1_i1 len=283 path=[1:0-282]

TAAAGACCATCCAGTTCCAACCCCTCTGTCACATGCAGGGACACTTCCCACTCCATCACG

CTGTCCAAACCCCCATCCCAGGTCCACCCACCCACGCTCCTCTGGGCAGCCCTCATTTCA

AGCACGTGTGTTAAGTCTTTTTTTTTTCCCTCCCTCCAACTGGAATACTTGTAGTCGTTT

GACATCGTTTCAAACAATTTATGTTTTGCTACAGAAAGTTTTCTTCTAACAATATACACG

GCAAGTGGATGACTGTGCAAGCATGGTGTTACAGAATGCTTGG

>c11260_g2_i1 len=260 path=[521:0-259]

CACTTTTCCAGAAAGGCTGTTAATGAGGCATAACTGCATTGACAAAATAAGGCATTAACA

TTTAAATGGGCCTGCTTGTAGCAGGAAGGGTAGAGCACAGCCCTGATGCTGTTCAAGCAG

CTACCCTGCATTGCTCGTAGCTTTGGCTGTTCCCTGAGAAGACGGTGGAAGAACTGTTTT

CAGAGTTACGGGTTGCTGTCATCAGGGCTGCAGTACAGAGAGGAGAGACTGTTTGTCAGG

GAGCTCAGTGAAGGTAAAAG

>c11261_g1_i1 len=290 path=[1:0-289]

TTTTCTGTCTTCGTCACCATGCTGAGACCTAAGAGTCATGCCTGATGAATAACTTTTATC

ATTAGTCCCAACTGGGCTGAAATGAAGGCTGCTATGATCTCCTCAAAAAAAGAAAAGAGG

GGATACACCCAATGTGATCCCATCCATGACAACACCAGAAGACTCCTGTTAGTCTGTGAG

TCAGATTGCTGACCCGTGCGTTACTAAAATTTGTACTTCTGCCACCTTCGATGCCAGTTC

CACTGCAGCTATGGGAGGCACCTATGGCTTTCAGGTTAGTGTGAGTTTGG

>c11262_g1_i1 len=385 path=[604:0-171 25:172-384]

ATAAGACAAGCCTCCTGCACCACGGAAAGCAAGTCCAACCAGTTTGGAGCAGCTCTGTTT

AGATAACATCTTTCATGAGTCTAGTGCAGCAGGGAGCAATCTTCCACTTGTCACACCTGA

CAACACAGCGATGTAATCCCACACTTCTGATTTACAAGTGCCAAAGTTTCAAGGGCTGCA

ATTACTGGACGTGCATTTCTACACTATGGCTGCTGCACTTTAAATATTCTAATAATCTCA

TAATTAACGCAATCCATGTAGCTCGGTAATAGAGACATCCTAATTATGCTAGTGAAGCTA

CAGGTAGGGATTAAATAGTCACCAGAAGTATTAAAGCTTATTTGTTACTATTAGGGCTTT

GTAAAAATAGTATGCTGCAGTTCAG

>c11266_g1_i1 len=230 path=[208:0-68 277:69-229]

GTAAACAAGAATACGGATTAGCAGGGAATAAAAGCATGAAGAATAGCCAGAAGAGCCAGG

TATGGGTCTGATTTAATGCTTTTGGCCATGTCATCTTGCCCACAAGGATGAAATGCAATG

CTTTCCTGCTTCAGTCCTGCAGCAGCTCTCGGGTGGGGAGGAGGAACTGGGAATTTCAGG

ACTGGGAATTAGTTACTGACCACTTCCATGCAACTGAGAGAAAGCTGACT

>c11266_g1_i2 len=282 path=[683:0-27 471:28-120 277:121-281]

TCCCCAAAAACGCCATTTTCTCCTAAGCAGCAGTTACATCCCAGGACATAAGCATTTGTA

AGCCCAGGCAGACATGTCCCAGCAGCACAGCATCAAGCCCTCTCTGCTGTCTTGCAGCCA

AGATTTAATGCTTTTGGCCATGTCATCTTGCCCACAAGGATGAAATGCAATGCTTTCCTG

CTTCAGTCCTGCAGCAGCTCTCGGGTGGGGAGGAGGAACTGGGAATTTCAGGACTGGGAA

TTAGTTACTGACCACTTCCATGCAACTGAGAGAAAGCTGACT

>c11272_g1_i1 len=261 path=[274:0-260]

CATCGTGTTCTAAAGGACACTGATGTCTTGCTTTAGAGCAGACAGCAGATGGAGCAGCTA

CCTCGTGTAAAGCTCCTGGAGCAGTGCTGGTGGCCCGGCAGCACCCCAGGGGATGCTGTG

TGCCAAGTGGGACACGTCCTGGAGTGAGAATGGTTTGTTCTGTCCCTGGCTGGATTTTCC

AATAGTTGGGGCTTGTGATGCTTCCACTGGGGTTCACCCTGTTTGCCCTACTGAAAGGTG

TAAGCACCATCTTCTTTTGTG

>c11284_g1_i1 len=266 path=[355:0-234 202:235-265]

TTCTGGACTATATTTTTTTGTACACTGTAGCATGAAGATGTCTGTTTCTTATGTTTTTTA

TAAATTGCAGTTTATTCCAGATAAAACTTGTTAAAACCTGATGACTGTAATTGTAAGTTA

ATTGTGATATGACTTTAAGGTTATGAAGGGCTACTTTCTAGAGATTGGTACTGTATATCA

TTTGAACACTGAGACTCACTGCCATGGGAGATTGCATTTAAAGGAGATCACTTCTAAAAT

GAGTCAGCACATTAATACTTACATTG

>c11285_g1_i1 len=301 path=[279:0-300]

GATAGACTTGGGTCTTCATTATATTTGGTGCTGGATAATCCTGAGCCTGCAGCTGCACAT

TTGTCAACTCAGGCACACTGACTGCCCCAACAACTGGGCGTTTCTCAGCAGGATAGGGTT

AGGGGGATGGGCTATGAAAACTGTAGTTCAGGTTAAATGGATAATGGTTAGATTGTGGGG

AAGTGAAGTGGGCATGTCCTCTAACCCAAGCATTACTGGGAGTGATGTCCTCAGAACTGA

TTATGTCCCATCACACAGACATAGTGCACTAATTCCACCATCTTATCGGCCCACACCAGA

C

>c11287_g1_i1 len=248 path=[226:0-247]

GGGAAAAAAAAAAAGTTAATTATGAGGAAAGAAAATGAAGCAAATACCAGAATGACTTTG

TATTACCACTTCTTTGGAGTTTCTTAGACAAACTGTGGGATGGGTAACCTTGAGCCCCCT

TATATATCCTGAACATCACACATTGAGGGGTATCTGCTGCCTTCTAAATCTTGGACTGGA

AGCTTTGGTTTGTGTATTTCTGTCCTGGCAGTTTGCTGTAGTAATTAAATTAATCAGGTC

TCTTCCTT

>c11290_g1_i1 len=258 path=[297:0-155 19:156-156 20:157-232 526:233-257]

TGGAATAATGTTTTCATTGTGTTTGAGCTTGGTAACAAAAAAAATTGTCTAATTAAAATA

AATTGCAGTTAAAAAAAAAAAGGTTGCAAATATAATATTTCAACTAATTACAAGAAACAG

TTAAAATGGAAAAGTCTGTTGTATAACAGCTATCTTAAAAAAATACCATGCCAAGTTACC

ACAATGAAAACATTATTCCATAGTCAAGATTCATATTTTACATGCATTTTTTTCATTTCT

GTCTTATACTAAATTAGA

>c11293_g1_i1 len=265 path=[279:0-264]

TCATAGCAGAGGACCTTTGCTGGAGTCTCTCCAGTATATGTCCACACCTCTCCTGTAGTG

GGGAGCCCAGAACTGGAACCACCACTCCAGGTGTAGTCTCACCAGTAATGAGGTAGAGAG

GAAGGATCACCTCCCTCCACCTTCTGGTAATGCTTTGTCTATAATGCAGCCCAAGATACT

GTTAGCCTTCTTTGTAACGAGGCCATGTTGTTTAATCACGTTCAAGCTGGCATCCACGAA

GACCCACAGATGTTTTTCTGTAAAG

>c11301_g1_i1 len=407 path=[1:0-406]

AAAAAAACATATATAAAATCCTATACAAAATTATAAAAATTTTTAAAAAAATTTTTCAAT

GAACATAAAAAATGTTAAAAGGAATAAATTAATTTAAATATTTTACGATATTTTAAATAA

TTAAATTATTTTTTTGATAATATGCACTTTTAAAGAATAATTACATTTTGTAATTTAAAA

ATTTTAATTTTAAATAAATATTTCAATAAAATTCATTAGTTTATTAAAATGTATATATAA

TAATAATAATCTAATTAAATATATGTAATAATAATCATAATAATTTGTGCTTTTTAATTT

TAGCATACAACTAAATATATTAATAAAAATAATTCCTTTTAAAAATATTATGATTTTTGT

AAAATAAATATATTTATATATGTAGCAAACTTTTATAAAGAAGTAGA

>c11306_g1_i1 len=351 path=[329:0-350]

GCATCCAAGGACGCAGCCCAGCCGTCCACCTCCTTGCACTCTGCACTGATGGGTCCATGC

AGCAGATGGGAGCGATGCTCCAAGGCAAAGCTGCCAGCAGGAGGGATGGGGTGGGAGGAT

GGAGGTCTTGGCTCCCCAATAATGGGGTGAGAGGAATATGGGGCTGGAGAAGGTGTAGGG

AGCACTGCTGTCCCTGTGGCTGCAGCAGGTGGGGCTGATACAGGAACTTTAGGTGCGCAG

CTCTGTGCTTCTCCCATTGCCTGGAACACGGCTGGTCCGTATCTGCACCGAGAGTGAGCA

GAGCACATCAAACACAAAGCTCAGCTCAGGCTTCCCACAAGGATACACTCT

>c11311_g1_i1 len=453 path=[512:0-371 883:372-452]

TTTTTTTTTTTTTAAATTTTTAAGCCAGCTTTATTCAAAGTAGATGACTACGATTTAGGC

ATCAGCAATAGTAACTTCAACTTCCACTCCTGGCTCAATGCTGATGGAAGTGATCTGCTT

CACAATCTCAGAAGGGCTGTGCAAGTCAATGAGTCGCTTATGGATCCGCATTTGGAAACG

ATCCCAGGTCTTGGAGCCTTCACCACAAGGTGTCTTCCTGGTAGTGATTCGCAGAGTCTT

GGTGGGCATGCGCACAGGTCCTTTCACCTTCAGGTTTTTCTCCTTAGCACCTCTGATCAA

GTCAGCACAGACCTTCTCAAGTGATTTTACATTGCGGCTGGTCAAAGTAATTCTAATGCG

ATGAATTGCTACCTCTTGCTCCACAGGTGCCTTGCCAGTATCTTTAAATGCCATGGCGGC

AGCGGGGCCTTCCTGGCCGACTTATTCCCGGGC

>c11311_g1_i2 len=452 path=[512:0-371 21:372-451]

TTTTTTTTTTTTTAAATTTTTAAGCCAGCTTTATTCAAAGTAGATGACTACGATTTAGGC

ATCAGCAATAGTAACTTCAACTTCCACTCCTGGCTCAATGCTGATGGAAGTGATCTGCTT

CACAATCTCAGAAGGGCTGTGCAAGTCAATGAGTCGCTTATGGATCCGCATTTGGAAACG

ATCCCAGGTCTTGGAGCCTTCACCACAAGGTGTCTTCCTGGTAGTGATTCGCAGAGTCTT

GGTGGGCATGCGCACAGGTCCTTTCACCTTCAGGTTTTTCTCCTTAGCACCTCTGATCAA

GTCAGCACAGACCTTCTCAAGTGATTTTACATTGCGGCTGGTCAAAGTAATTCTAATGCG

ATGAATTGCTACCTCTCGCCGCCTCCTCCTGGCTCACCGCTGTTCGCTGGGCCTCGCGTC

GCGCCCCGCCCGGGAATAAGTCGGCCAGGAAG

>c11317_g1_i1 len=222 path=[193:0-221]

AAATCAACTTCTCGCAGTCAATCCTACAGAAAAAATAGATCCGTGTATTCGTGACATCCA

CTTTTACTGTTGGACTTGGTTTGGCTTTATTGATGCCTCAGGGACTTTAAACTTTAAGGA

GCAGGGAGCCGAAATGTCAAATATTTTACTCCTGTGATGTTCCATTTAAGTCATGGTATT

CCTGTGTCATTTCACACCAAGTTTCTCTGCATGGGTATCCTG

>c11319_g1_i1 len=210 path=[188:0-209]

GAGCTTAAAAAACACTAAAAAAGGGAAAAACTATCTTTCCGTTGGCTCCCTGAGCCCCGT

CCAGAAGAAAGAGGGCTGTCTGTTAGCAACAGTGAAAGATAATCCGGGTGTGAGTAATGA

TGGAAAAGGCCTCCAACATCCCCGGGTCCAATGGGGAACTCAACCCCACTGAGTCCACCG

GAAGGTCGTAGGGGGTGGAAGAGATCTCCA

>c11323_g1_i1 len=294 path=[272:0-293]

CAGCCAGGGGCTGGCAAACACCATGCGAGTGCAACAGCTCATTTCCTCCCAGGTGCGCTG

GTTACAGCCCTGTGACTGGGCTCTGAGTGCCTGTACCCTGTGAAGCCTGTGCTTGCTGCT

GCAATCACCTCCTTTTCCTGCCCTGTGACATTTGCACCACGGTTGCTGAAGCTTCTGCAC

TGGGATGGGTGTGGAGTGGGGTCCTGCTGGCAGCGGGGCAGTGCCCATCCCAGCTGCAGG

TGCTGTGAGCCCTAAGGATGCCCCAGTCCTGTGCTCACCTTCTTTGGCTCCTGG

>c11323_g2_i1 len=277 path=[797:0-276]

GGCTGTGCCATGGGCTCTGCCCACCACCTCTCTTCATGCCTTGGATCACCGCTGTGTTTC

TAGAGCCAATTATTTATTATTAACCGGAGAGCTGAGCTGGGAAGTGTCTAAATTCCTTCT

TGGTGTGAGGTGGCAGTCGGTATGAATATAGGTCTGTTATTTCTGAGTGGTTCTAATTTG

GCAACTGCTGAGCAGCAGTTACAGAGCAGCCCCATTCAGCCAAGCTTAACAGCTTAAAAT

GCTGCATGGATCTGCTTAGCGGGGGGCTGGAAGCCTG

>c11326_g1_i1 len=281 path=[503:0-280]

ACATCGCAGAATTTGAAACAGCTTAGAAGTGTTTGGATCAAGAGCTTCCATGTAGTTAAA

AGCCAATGAGAAGTTCATTCCTCAGTCACAAATAGGATACACAAAAACAACACAAGTCTT

TGAGATAAAATACAGCTATAACCTCAAACCGAAAAACTGAAACAACACAAAACCTCCATA

CTGTGGTATTAACAACTGATCCCTACCACTTTTTCTTATGACCGTGTTCATCATTCTGCT

TGATGAAGTTTAGAATGTAAGAACACATCTCTCTCTGAAAG

>c11327_g1_i1 len=288 path=[265:0-287]

GGTGACAAATGTGACTTCACAGGATCACTGATTGTTGTGCCTGATGTGGCTCAGCTGTCA

ACACCAGGAGTACGTGCAGAAACCGGCTCACGGGTTAGTGGGACGGAGGGCTATGAAACC

GAAGGAATCCGGGGACTTCGTGCCCTTGGAGTCAGAGAGCTGTCCTACAAACTGGTCTTT

TTAGCCTGTTACGTTGCACCAACAAATCCACGGTTTGGTGGAAAAGAGCTTCGAGATGAA

GAACAGACTGCAGAAAGCATTAAAAACCAAATGACGGTGAAAGAGTGG

>c11328_g1_i1 len=318 path=[296:0-185 482:186-317]

GAGTAAACATCTGAAAAATACATGCATTTTACTCCACGGTAAACAGAATCCAGGTAGAAT

TTAATATTAAATGACTGCAGATAAAAATCACAGGATTCTCTTCTGTAAAAATGAACTTGA

GGTCCTAAGCTTACTAAATTAGAACAATACTTGTTAAAAAAAGAAAGTAATTTATTTTAA

AACAACAATAAAAACCACTTAATTTCATTTTACGTGCATTAGCTGTGATTGGGTTTTTTT

TTTTCCAACAAATTATTTCCCTGATGATTTTAGTATCAGAAGCTTAGAAGAAATATAATG

AACAAATTCCTAGCAGTT

>c11329_g1_i1 len=218 path=[482:0-217]

GGGTTGCAAAGCCTACTTGGGAGCCAGTAACTAGCAGTCTACTGAAGAGGTCAATACTGG

GTCCAATCCTGTTCAACATCTTCATTAGTGATGTAGATGGGGTAGAGTACAACCTACAAC

CTAAATTAGCAAGTTTGCTAATAACACAGAACTGGGAGGAGTGGCGAATACACCAGAGGG

TTCTGCAACTATTCAGAGGGAGCTCAGCAAGCTGGAGA

>c11330_g1_i1 len=235 path=[473:0-234]

AAAAAAAAAAAAAAAATTATTCAAAGGAAATATCTTGTATATTTATAAAATTAATTTAAA

AAAAACACATTTCTATATATATATGTTATTTTTTCATTTTTTAAATATATATGTATAGAA

AATCCTGAGCTATCTACAATTATATATATAGGCGTAATATATAAGAAGATACAATACCAA

ATAAAAATTTTGAATAAGATTGTTAATTTTATACACCATTAAAAAAAAAAAAAAA

>c11336_g1_i1 len=236 path=[295:0-158 25:159-235]

AACTTTGTGTTTGATCTCTCTCCTCTTCTTATAGGTCCTCCAGCAGCCCTTATCCACATC

ATCACAGCAGTGTAAACATTCCTTATAAAATATTCCAATTTTTAAGAAGTTTCTTTTTTT

TTCCCCCCTAACCATGCAGATTTGTGGAAGAGCACAATTTAACTGCTTATATTTCCGTGT

AGTTGCTGCTGCTGCTCTCTGTCGTTTTCTATTTGGAAGAGAGACTTCTGGAAAAG

>c11336_g1_i2 len=237 path=[295:0-158 453:159-236]

AACTTTGTGTTTGATCTCTCTCCTCTTCTTATAGGTCCTCCAGCAGCCCTTATCCACATC

ATCACAGCAGTGTAAACATTCCTTATAAAATATTCCAATTTTTAAGAAGTTTCTTTTTTT

TTCCCCCCTAACCATGCAGATTTGTGGAAGAGCACAATTAAACTGCTTATATTTCCTTAA

TGTTTAACAATATTCTTGTGCAAGGTTTTCTTCTTTTCCAGAAGTCTCTCTTCCAAA

>c11336_g2_i1 len=223 path=[583:0-222]

ATCTGCCTGAATTTGTGTCGCTGTTTCTGTTGAAAGCGACTTGCCCTAGTTTGGAGAAGG

ACAACTCTGAAAGGGGTTATTTTTTCAGCTCCTGACTTTCTAAAGGAAACATTTGGCTCT

TGCAGTGAGCCTGGTGGCTTTTGTATGCATCTAGAGCTAAATGCATTTTTTTCTGGTCAT

TGTTTTGCACTAGCAGGTATTTTTTCAGAATGAGGCCGTGTAG

>c11339_g1_i1 len=210 path=[356:0-209]

TTAAAAAAAAAAAAAAAGGGAATAAAAAAAAAGCCACCATAAAGAGTCCCATGCCCAAAG

TGTTGGTAATATAACTGAGTGTTGATGACACTGAAGCTGCCTATGCAGCTGAATTTTGTA

CAAAAAACCAAGCCAGTAATGGTGTTTACCAAGGAGCAGGGTAAGAAGGTTGAAAGTGGA

AGAGGGAACAAAAAGGAAGATGGAACTACA

>c11342_g1_i1 len=601 path=[642:0-536 1178:537-600]

AGTTCAACAGTTTGTTGAACGCTATTTGTTGTGGAATTGACCTGAACATGACTATTTTGT

AAAGAAGCCCTTATGACTGGGTTATGCCTTCCTCGGCCTGGTGAAGAGAGATGAAGTCTT

GAATCCCTTTCCATAGACTTTTGTACTCTTATTCTCCCCTGGGACAAATGACTGGAACTT

CTGTATGTAAATCCAACTGGTTTGGGTTTGAGTAACAGTGGCCTGGCACAAGTATGCTGC

ATTTCTGAGACCTTCCGATGAAGCAAGTCAAGTTTTTTATCCAGCTTCTGAATGGAATCG

TACATGGCCTGACAATAACTTAGAACTTCCATGAGAGTTTTCTGCTGATCTCCAGTGTAG

GAAGCTTCAGATACAACACTCCCGCATTCATAATCTGGTTCATACTCATAAATTACACTC

TCACTGTCTTCATATTCTCCTTCACCGGTTCTCATCACATTCTGTCCCACTGGAGTTGGG

GCAAGTCGCTTTCTTTTATTACACAAAGACACAAAATCCATTTTTTCAGGGACATGACCA

TTTCCTCTCTGTGTGCTTGCAGAATGGCTCTTTTGTGCTTCCACTTGTTCAGCCAGGACC

A

>c11343_g1_i1 len=243 path=[1:0-67 69:68-242]

CTGCGTGGCCCCAGGCACACAGACATCTCAGATTGCACCCAAACCTGCCGGTCTCGCCAC

GTCTCAGGGCTTTTTTTGAGGAGGACCCCCCACAGATGGGCTCCCCACGTCCCTCCACCG

CCACCACTCAGGGCAGTGGCTGTCCCTGAAGTGCCATCTCACCCGGGCACCGCAGCCAAG

GGGCTGCCCCCAGGATGGGGACACACGTGGTTACACCTACCCCTCAGGGTGGCCAGCAGA

GAG

>c11355_g1_i1 len=208 path=[93:0-155 264:156-207]

GTGCAGCCGCCTGCCCAGAAGCTGCGCTGTGAGCTGCGCCGGCTGCTGGTGGAACGGGGA

GAAAGCTGGTCGGAAGAGCTGGAGTGACGTGCCCCGCACCTGGCAGCGGCATGGGGACTT

GGTCCTGCTGAGCGAGGACAGCTTCAGTGCTGCGCTGTGGGCAGGCGGCTGCACGCTGGC

GGCCTTTGAAGGGACGGGGTCCTGCGGG

>c11357_g1_i1 len=246 path=[1:0-245]

GTCTTTTCCAACCTTGGTGATTCATAGTTCTGAGGAAGAGGGAGGCAACTTCTCTACCAG

GCTAGGAGAGCTCTTTTGGCATTCACTACCCTGTTTTGTATAGCTGCTAAGAAGGGATGC

ATATGAACGGATAGACAAGCTAGGACAAAAGCTACAGAGAAAGTCTATGGACCCAACACA

GGCTCCAGGAAGATAGTTGCAAAACAATGGGCAGAAGACATGAGCAGGAGACCTATAGGA

AAGGCT

>c11362_g1_i1 len=234 path=[121:0-118 240:119-233]

AAGATTATATATGTATATTATACATATATACATAAAACACCAGGAAGGGAAACAGGCCTA

GTGATTATAGGAAGAACAGCACTGGATAACAGAAGTAGCAGTGCTTTTCTGCAAAGCTGG

TTAAGCTCTTATTAGAGTACATTCAATTTCAGTAGACAGTTCTATGTCTGAAGTGCTACA

GTATGATGATGAGACTACTCACAGTTTCACTAAACAAAACCTTGAACTGCCATG

>c11362_g1_i2 len=207 path=[241:0-91 240:92-206]

TCCTTTCAATCAGAGAGCCACAAGGACAGTTGCATGTCGAGTGAACGTATAATAGGAACA

GCCAAAGTAGGCTTTAAAAAGAATACAATATTGTTAAGCTCTTATTAGAGTACATTCAAT

TTCAGTAGACAGTTCTATGTCTGAAGTGCTACAGTATGATGATGAGACTACTCACAGTTT

CACTAAACAAAACCTTGAACTGCCATG

>c11365_g1_i1 len=218 path=[225:0-217]

TTTGTGCTTTTCTAGGGCTCTGAAATGAGGCTGAAAACTGATGAAGGTTCTGGAAGATGC

ACCAAAAAGGAGAGGAGGAGGAGGAGAGATGAGCAGAATGATCGGCTCTGTCACCCAGTG

GGAAGGAACATGACAGCGACAAATTTCAGGAGTGAAATTCTTACACTTTCTAGTTTAAGG

AGAGGGGTTCTGTTGAGGACTCTTCTAAAATGGAAGTG

>c11366_g1_i1 len=291 path=[11:0-290]

TGCTTGAAATCCTCCTGCTTTTTCAGCGTTCCACTCACCGGATTACAATTAATAATCCAG

TTTTACAAGCACTTTCATTCATTTCAAATTCTGCTCTTGTGCTAACTTTGTGTGAAGTCT

TTCAAAATCACCATAATGCTGTGGCACAGCACAAATGTCACTGAAAGTCAATGAAGAATT

ACTCCCTTAAATTACTCTCCAGAAGTTACAATCCATGCAGTTCTGCCGTGTCTTTCCTCA

ACTTCTTTCTTTTTTGAAACTGTATTACAGTTCAAATTCTTCCTCCAGTGC

>c11367_g1_i1 len=229 path=[431:0-228]

AAAAAATCGATGTATTTTTTTCCCGTCATCAAACCTGCCGCCCCTCCGTGTGTGCGCCGC

TGCCCCCGGGCTGCTCCGCCGTGCCCCGCTCCGTCCGTCGGCTTTTTCCCTTCTTTTTCC

CTATTCCTGCGGGTCGAAGGGGAGGTCTGAAGGCGCCGGGGGTGCTGCGGAGGAACACAG

CCCTTCGAGTTTCCTTCGAGTAGAAGGAAACGGCCCCGAAGACAATGGG

>c11373_g1_i1 len=283 path=[261:0-282]

GTTTTCTTCAGTGTCCTCCATAGTGTGCCATACTTCAGGAAAAGGGGACGGGATCAGATG

TAGGACTGGAACCCCTCTCAATAAAAATGGAACGTGGTCATCTTCAACCAAGCCTCGATG

TACATTATTCTGGAAATACTGTGTTTCATCAAGGTGATTCTTCAACAGATTCATGCTGTG

TAGCTTCTGCTCTGTAATTCAAGATGGAAGTGACTGTTACAGCTTTTGGACAATATGAGG

CACGTTAGCACACGTGAATTATTTTTCCTCTCTTGTGGAAGCT

>c11373_g2_i1 len=246 path=[521:0-245]

AAGAAAACCTCGACCAAACCACTATTGAAAACCTCAGCAAAATCCTCCAGGTGTTTGTAC

TGGAATATCTAAACCTGTGACACGTTGGCTGCAAATCTTACTTCATTTTAAATGTTTGCT

CACCTTCGCTTCTGTTATTTAATTACATCAGGGAGCACCGAAGCAAAGCAAAATATGGCA

GTAGCTCTTGTTACACTGATTACTGATTCGGTTGTTCCTGGCTCAACATTCCATAGAAAG

AAATAA

>c11378_g1_i1 len=212 path=[1:0-211]

AAGTGAAGGTAGGCTCTGTACATGGGAAGTCATGAAGCGGTTAAGCAAAACTGTACCACG

TTCTCCTGCATTGCATAAGCTGAAGTTATTAAGCAAGACTGCGTTTGTCATTGAGATTTG

CTACTGGTATGTGCATGAGCCCCATCCATAATGAAAGTTCACAGCTCGCTCAAGTGTAGG

ACGAATCACAGTTACAGCAAACAATGCTAAGA

>c11381_g1_i1 len=275 path=[269:0-274]

GCGCTTACCAAGCATCATGTGCTCAGATGGATGTCATACCAAAACCACACACATCCTGAC

TGTTAGCTGGAAGTCTGCTCAGTTTGGTTCTCAGTGTTTGTTAATTGCAAGCAGTGTTTC

AGCCTTCAGCAGGAAGTCGTTCAAAGGTAGCTAAAATGCGCTGTTCTTACAGTCTTTTTT

TCCTTTTCCTGAAGCAGTTGGCAGCGTAAGGCGGTTCCTGTAGGATGAGTGTGAATTACA

GCCACTCTGTGCCAGTGAACAGAACAGCTGCATTT

>c11386_g1_i1 len=209 path=[1:0-208]

AATAGAAGGTAACTTTCATATTAGCTGCCTCTTTTGATTACAGTGGCTATTGTGGAACAT

CTATCCATCTTTCAGAGTTCAATTTTTTTTTTCCGTTTTGTGCATAATAAAACATAAAAC

AGCTTGCATACCCTAATATATATATGCTTTATTCAGCTAAGAAAATTGTAGTTACTAATG

TGGGAAGTTTCTGTGGTTTTCTGATATAT

>c11387_g1_i1 len=220 path=[155:0-219]

ATCCTCCTCTTTCCTGTCCTGTTGGATTGCAAGGCCTTAGGGATAGGAAACATTTTCTCC

CCCTGTATTTGCACAGCAAATAACAGAGTCTTGATCCAAGGCAGGAGTCCTTAGTACTCT

GATAGTAGAAGTTGCATATATAGTAAAAGTAGTTACAAGGCCAGAGATCTGCTGGACTGC

ATTATTCACACAAGCTGCTTACATTTCCTATCCAGAGCTG

>c11388_g1_i1 len=285 path=[543:0-284]

GAAAAAAGCCGCCAAAAGGTATTTTTAATAGCAAATGAGAATAGATGAGAATGGAGTCAT

TTGCAGCTGCCTGTTTTATGTGGCTCTCTTGTTCCAACTGTAGGTCTAATGAGATGACAC

TGTTAAAGTACTCTGCAGTGTAATTTCATTACATCCTGAGCTGTTACACTATAAACACAG

TGGAGCTCTCTGTCATTCTTGGAAAAACTCCTAAATTCGTAAAGCCTGTCTCTGTAAATG

GGGATGTTGTTTGTTCCCCGTTCGGGTGGGTATCTTTTTCCTTCT

>c11396_g1_i1 len=413 path=[545:0-412]

CGATCTCGCTGTGATTCTCCCTGCAAAAGGCCGGGGAGGAAAGCAGGAAGATGGAGTTAC

CACCAGGCCAGGAGGCTCCGCTGCTTTCCCAACGCTCCCAGAGTGCAGAACACAGAGATG

CATTGGGGTCACTGGGAGTTAGAAGGACTGCAAGCACCTCGCATGCTGCTCGTGCACAAA

GGGACGCAGGAACGAGTGCAAGGAGAAGAGTCTACAGCTGCACCTTCCTTGCTTTGGATG

AGAGAGCCTCACTTGACGCCACGCTGCTTCCCTGCTATCTCATGAAGGCTACAAGCTAGG

CTATGCTCCTTGAAGAGACAAAGAAGCTTTTCCATCACCTCTGGAATGCTTCATGCCAGC

CAAAGGACTCCTTCCTTCACCACAGCCCCTGCTCTCAAGGGGGGATTCAGCAG

>c11400_g1_i1 len=329 path=[88:0-136 224:137-328]

TAACTTTGTGTTTGAAGGAGGAATTAGAGGAAGAACTGGGAATCAAAGCTGCTGTATCAA

TGGAGGGAGTACTTATGTCTGAGAACTGCACCTGCCAAATGTGAAGCTTGAAATGGAGTG

CTCCATGGTGCACTGTGTGTTTCCATCACACCTTCAGCTTGCCTAAATGTCACAGCAATC

ATTCCAGGATGGCAGTTCTGGTCGTTGTGCTTGCATGAACACACTTCTCTGCATGAACCA

TGAGGTCAAACAGAGTAGGTTTGAGTAGTGTTTGGCAATTCAGTAGTTAAAATGCTACTG

GAAGTTATAAAATGGGAACACAGGGGATT

>c11408_g1_i1 len=205 path=[1:0-204]

TAGGCTCTGAATCCTTAAAACACGTGTATGAAGTGCTTCTTTGAGGGCTGTGTTTCTGCA

TTTCAGTAGCCTCTTGTGGAAACATCTCTATATACCTCTCTATATGCACACCAGATGCAA

TTCAGTAGAGTGATAAAGGATACTGCTAAGACCATTTAAGGATGCATGTCACTATCAGAT

CAGTTAAGATCTTGTGGTCTGACAG

>c11414_g1_i1 len=220 path=[198:0-156 435:157-219]

AGGCCGACAGATTTGGGACAGCACTTGGAGCTGAAGTGAACAGTAACACTGTGCTGCCAC

TGTGAGACAGATTTTACTTCCTTATTTCAGAAGACCTTTATTTGCTAAAAAAAATGAAGA

AATTACAAAGTAGAAATACATTAACGTGAAATAAAGCTAAATAGTTTTTCAGTGTTTTAT

AGTAGGCTAATTCATATGCGACTGTTCTAACAGTCAAGCA

>c11418_g1_i1 len=250 path=[91:0-90 322:91-249]

GTGCCCTGGGTCGGTACTGCACTGAGTGAGTGGGGCCTGAGCTGGGCCTCTTCCTGCCCA

GCACTGCATCCTGCTTCCCCACACTGGGAGCGGGAAGTGCCCTGCTGCTGGGAACAGCCT

CCAGGGAATGTGCTTCTAGCAGTGCGAAAAATGCTGTGATTGTTTGGGGCTGTTCCCAGA

TTCACCTTGCTGGGGTTGCTGGGGCTTTGGGGAGCACGGGGCCATCCTCGGTCATGGCGG

GTAGTGATAC

>c11419_g1_i1 len=259 path=[22:0-176 787:177-183 465:184-258]

TGGATGTGGCACTGAGGGACACGGATTAATGGGCAGTGTGGAGATGGGTTGACAGCTGGA

CTAGATAATCTTAATGGTCTTTCCAACCTTAATGAGTCCATGATTGTATGAACAAGGAAC

AACAAGAAGCAGGTGATGTGTAGTCTGGAGGCTCCTGCTCACTGCAGCAACCTTACTAGC

TCCATCGAAGTAGAAGGGAAATTTTCTCTCGACTTTATCAAAGTTTACCTGAATAATTGT

GATTTTGTTTTAGTGTTAT

>c11419_g1_i2 len=226 path=[22:0-176 787:177-183 206:184-225]

TGGATGTGGCACTGAGGGACACGGATTAATGGGCAGTGTGGAGATGGGTTGACAGCTGGA

CTAGATAATCTTAATGGTCTTTCCAACCTTAATGAGTCCATGATTGTATGAACAAGGAAC

AACAAGAAGCAGGTGATGTGTAGTCTGGAGGCTCCTGCTCACTGCAGCAACCTTACTAGC

TCCAATTTTGTTCAGAACAGCATAACACTAAAACAAAATCACAATT

>c11428_g1_i1 len=215 path=[277:0-121 85:122-214]

GAAAGAAACCTGTAAGTGGTTAATAACAGTCATACAGCTCTGCCCCGACCTGTGGGATGT

TTCAGGTTCCAGGATCTGCCGTGCAGTTTGTTCGCATGATGCAGGTTTTTATCATTCTCT

ACATGGACCCCCAATTTCAGAGAGCTGTCTGGTAATACAGATGACCTCAAATCTTGGTTG

TGAGATGTACGTTCTGGTGTTAAGATCCCGAGGCA

>c11431_g1_i1 len=388 path=[155:0-387]

CACACCGCAGGGCGCTGTGCAATACCCCCTTCCAGTGCTGCGAGGGAGCCCAGCGAGCTT

GTGGCTCTTGGCAGAGCCCGTTTCTGCATCCCTTCCCCTGCTGGGAGCCATCTGCACGGC

TCTCACACACCCCACACCCCCCAGGTGCTGTGCTCATTCAGCTGAGATGCCTCACCTATA

GCTTGGGAGGAGTGGGACATCACCGAGCCATTCCTGTCCTGGGTTCAAGGGCAGAGCACA

CCATCACCACACTGACACCTCCCCATTTTATCTTAGCTAAGTACTGCTTGGTTGCTTCTC

TCCTCCAGGCTGGGGGTAATTTGGGTAAGGCATTCCATACAGGAGAACCCTACGTCTTCC

CTGGGTGTTGCCCCTATTTGGGCCAGGG

>c11432_g1_i1 len=299 path=[1:0-298]

AAAAGGAAGAAGCTTTAACAACCAGCGTAGCGTAAGAATGAAATTTAAAGGTAGTGATAA

TAATCAGTAGTCGTATTTTCAGTCCAAGTTAAGTTCACCTGCTGTCAAGAGCCAAATGAG

GTGCTGATTTCACAAGCATGGCTATGGTTTGCCCTGACCAGTCAGTATGGGGTTAACTCC

AACCAGTGCAGGTTTCTCTCCCCACTGCCTGGTGTGGGAAACTGAATTTCAGATCATTGT

ATTTCTAAATCTCAGCACAGCTGAATTTCAGTGAGGAAATTATATTATATATTACGAAT

>c11435_g1_i1 len=330 path=[308:0-329]

AAGAAAAAAAGTCGACAGCCGTATAAACTTGATAACATAGACAGGTCTTGCATTCCTCCC

CGAGGGTCATGGAAGGGCTAAGATTAGCAAAGGGGAGGTATCCAAAGCAGGCGGATCTGT

GCAGTTTTTAAGGCTGTCGACTTTTTTTCTTATTCATCCCATTCTAAATGAGTTTTTTTC

TACTCGCAAGCGGTCTTTTGAGACGCAATGTGAGTTAGTGTTGAAAACAACGCTTCAGCT

CTCGAATATTTCCAAGTAATTTTAGAGTAGAAAGGTCATGGTGCTTTTGATGTGTTCGTA

TTTAGAGAGCGATTACAATTGCAATCATAT

>c11438_g1_i1 len=214 path=[315:0-213]

AGTGGAGCCGAGACCACCGCGTGCACCACAAGTACTCGGAGACAGATGCGGACCCACACA

ATGCCCGCCGGGGCTTCTTCTTCTCCCACATTGGCTGGCTGTTTGTGCGCAAGCACAAAG

ACGTCATAGAAAAGGGAAGAAAGCTGGATTTCACCGATCTGCTGGATGACCCCGTCGTCA

GATTCCAAAGAAAGTACTACAAGAGTTCAGTCGT

>c11450_g1_i1 len=343 path=[264:0-261 526:262-342]

AATAAATGAAATCAATTTTCCCAAAGTCCAAACTCTTTTTCCTTTAATGCTAGCCAGTAG

GCCATCTCCCACCAGCTGTTCCATCTTGATTTCTCTTCCTGTCCTGTGCAGAAGAGGGAG

AGCCCAGGTAGTTGAATAGCCATGAGACAAATCTAATTCAGCAAACCTGGCTTCACGTTC

ACATTGTTCCTCAACCGTAGGTTGATTTTTCCTGAAGGAGGGAATAAAGATGACCATTAG

GCTCTTTCCTGTGGTGTCCAATGCCAGACAAGTGACAACAACCCCTGGACCTTTCCTGAC

ATTAGAAGGCTTCTCCTATCAGCAAATGAAGTCACAACTCAAA

>c11453_g1_i1 len=447 path=[1:0-446]

ATATGTAACTTCAAAAAAAAAAATATATAAAATTAAATGCTAATATATAATAAACAAAAA

AGAGGAAAAATATAAAGAATAATGTACATATATAAGCATAGTAATTACAAGTAATAAAAA

ATTTTATATGCTTAAATATATGAAAAGAGAAATACTCATAGAAATAGAATTAAATATCAC

TTTTTTAAAATTATAACTTTATGAGGAAAAAAAAAATTGTAATTTAAAAATTCATAATTT

ATAATTAAAGAAAATAAGAGAAAAAAAAATTCCATTTAACGAAACAATATACATATTGTA

AAAGCTTTATTATATATATAATAAATCCCTTAAAATTAATAATTAAAAGAATAAAAGTTA

AAATATTAATAAACTATTAAGATTTATTTTTGAAATATTTATACGAATACTTGTTCTTAT

AAAAAGTTATTTTTATATAATATTTTT

>c11454_g1_i1 len=1123 path=[2661:0-349 248:350-1122]

TTGTGTTTGAGAGTATTTCAAATATATCAGCAACTTATTTTTTTTTTCTCTATCATTAAA

AACAAATATAAATAAATAATGCAATAGTAAACATATTTTTTATGAAAAAAATTACTTAAA

TTACATTAATTGTAAAATAATAACATGGTAAAAACATTGTATAAATAAAAGATGTAAAGG

AAAAAAATGAATATATAAATAACAATAAAAATTTCACATTTCGCGTGTGATGATAAAATA

TTTATATATTTTATCACAAATTAAACATAAAATATATTAAACATATATAACATACACAAT

TAAAATATGTTATTATATTAGTATGCATTTTCCTTTTTTTTTTTAAATTTTACCATTGTC

TTCCAATAACTTTTACTGTCATTGTTGTGTGAACTATTTCATCTAACATATACAACATAG

TGAAACATGGAACAGCTATACTCATAATTATAAGAGTTGGAATCCAAAAAAATAAAAATT

CAACTTTTAAAACCATATTATAATTTCCCCATACATTTAATCTTGTTCTAGACCAAAAAG

GAATATTATGTTGGCTAACGTTAAATAAGTATTCAAATGGTTTTGTTTCTTCTCCTTTTA

TCATTTTTTGCCCATTTACAATTACAATATTTTGTTTTGGGGTACTTGACCAATAATCAT

CCCAATGATAATTATCAAAATCATATTCATACTTCCTTGTTCCATCTTCATTTAGATGAT

CACGTGGATTGCCATGATGATGATCTATATGATGATATAACCCCTTTGTATGCACTTCAT

GACCATGAGCATTTTCAATATGTATTTCTTCTTTATTTTTAATTTCGTTTGCATTACTAG

TCACTTTTTTATTTACTGTTGAAAAATTGTTACATATTAATTGGTTTTTTTTAATATAAT

ATGGCTTTAAGTAAGGAATTTTTTGATTAATTATTAAGTGATTTATATTTCTTAATCCGA

AATATAATTTGGAATTAAACTTAAACATTTTATTTATAAAGAAACAAACATTAAATATAA

ATAAAATATTTAAATTAAAAAAAAAAATAAAAATAGTACACTAAAAAAATTAACTAAGTT

TCCTTGTATTCTTTTAAAGAAAAGTAATAAAAAAAAAATTTTA

>c11454_g1_i2 len=1020 path=[1:0-246 248:247-1019]

AAAAAAAAAAAAAAATAAAAATATATAAATGTAAATATTTGTACATATATAAAACTGCTT

TTAAAATGCTGTATATTTGTTTAACAAAATTTGGAATAAAAATTTTAAACTGTATTTAAT

TATATATGTGGTGTGTACTATAGTACATTGAAAAAAAAAAAAAGAAAAGAAAGAGAAAAA

AGGAAAAAATATATCTATTAATTATTCATCGTCCTCTTCTGGTGGAGACTCTACTTCATA

AACCCAATACCATTGTCTTCCAATAACTTTTACTGTCATTGTTGTGTGAACTATTTCATC

TAACATATACAACATAGTGAAACATGGAACAGCTATACTCATAATTATAAGAGTTGGAAT

CCAAAAAAATAAAAATTCAACTTTTAAAACCATATTATAATTTCCCCATACATTTAATCT

TGTTCTAGACCAAAAAGGAATATTATGTTGGCTAACGTTAAATAAGTATTCAAATGGTTT

TGTTTCTTCTCCTTTTATCATTTTTTGCCCATTTACAATTACAATATTTTGTTTTGGGGT

ACTTGACCAATAATCATCCCAATGATAATTATCAAAATCATATTCATACTTCCTTGTTCC

ATCTTCATTTAGATGATCACGTGGATTGCCATGATGATGATCTATATGATGATATAACCC

CTTTGTATGCACTTCATGACCATGAGCATTTTCAATATGTATTTCTTCTTTATTTTTAAT

TTCGTTTGCATTACTAGTCACTTTTTTATTTACTGTTGAAAAATTGTTACATATTAATTG

GTTTTTTTTAATATAATATGGCTTTAAGTAAGGAATTTTTTGATTAATTATTAAGTGATT

TATATTTCTTAATCCGAAATATAATTTGGAATTAAACTTAAACATTTTATTTATAAAGAA

ACAAACATTAAATATAAATAAAATATTTAAATTAAAAAAAAAAATAAAAATAGTACACTA

AAAAAATTAACTAAGTTTCCTTGTATTCTTTTAAAGAAAAGTAATAAAAAAAAAATTTTA

>c11458_g1_i1 len=220 path=[1:0-219]

ACCAGTAGTATACTGTTATTTTACAGGTAAATAAATTAACAAAATGGTATTATTGAGTAA

CTTTCAGTTATGACACTTGTAATATTTTAATCCAATTGTGATCTGTGCTTTCCTTTATGG

CAGTATAATCTTTTATAACATTTAGCCTTCAGAGATTACTTTATCTTCTCCAAAGAGGTA

GACACAAATAGTTTTATATGTAGGATGACAGAATTCTGAA

>c11459_g1_i1 len=259 path=[155:0-258]

TTGTTTTCTGCTTGAGGAAAACATCAGGATCTCTGAGTCAGGTCCCTCAGGAGACCCTGA

GCTCTGGGGGCATTGGTAGCATTAGGAGAGCTGCAGTGCTGCAGGCATTGGCACCAGGGA

AAGAAAGAACTGAGCCCACACACAGCACCAAGACTCAAGGGTTGTGGTAGAGGCCATCTA

ACCTCTATCTTGGTACAAAGAGGAAATAAAGGTGGTTTCTGCAGAGCAGTTTTCATTACC

ATCTCTTCAGTGCTGCTGG

>c11460_g1_i1 len=430 path=[341:0-247 588:248-429]

AGCACGGTCCTACTAAATCCATCTCTTGATTTCTTTGGTGTCAGTGTTACATACCAAAAC

AAATAGAAACTGGATTACTGAAGTAGTGGGAATATGTTTTCAAGTCTGAACAATAGCAGT

ATTCCCAGTTTGCTCTTGTTTTTCAAGTACAAAGAGTAAGATTGCTGTAGAGCAGTAGAG

ACAATAATAGAAAACAATGTGGCTAATTTTGGAAGTAGGGTTTCTACAATTTATGTGTGA

AGAGAACTGCCTGCTAGTTTTTAGAGTAATGTGATGTGTCTGATGTCTGGCTTTGCTATT

AAGTTGCCTGAGTGTTGACAGTCTCCATATTCTTCAGAAACTGATGAACGAACTCTAATT

GCGTTCATTCTCCTGTATCTGCAGATTTCATCTGATTTTTGTACTTGTTTCTCAGCTGAA

GATTCTCCTG

>c11460_g1_i2 len=417 path=[341:0-247 25:248-416]

AGCACGGTCCTACTAAATCCATCTCTTGATTTCTTTGGTGTCAGTGTTACATACCAAAAC

AAATAGAAACTGGATTACTGAAGTAGTGGGAATATGTTTTCAAGTCTGAACAATAGCAGT

ATTCCCAGTTTGCTCTTGTTTTTCAAGTACAAAGAGTAAGATTGCTGTAGAGCAGTAGAG

ACAATAATAGAAAACAATGTGGCTAATTTTGGAAGTAGGGTTTCTACAATTTATGTGTGA

AGAGAACTACTGTCAGTCATTTCAAAAAGAAAAAATCTAGCTGGTGGTCCTTGCTGTGAT

CTCAAGGCTGACAAAGAACAAGCCATAAAAGGGCTCTGTAAAGTCTAAAGATGCAAGAAA

ACAAACTTTATAGCAACTGTTACTTACAATGTATTTAGTTATTTTAAGAGCTGCCTG

>c11461_g1_i1 len=230 path=[208:0-229]

GAGGCATTACTTTTGGAGTGACTTACACATATAAGCGTCTACACTATAGATGTCAGTAAT

TCCTAACATTTCCAGCATTGCAATTGCCACTGCTCAACAGCTAGTGCAACCCTATGCATT

TCAAATCAATGAAAGTTAGTTTTCAGACTCAAATTCAATCAAAAGTGACATTTGTTATGG

GAATAAAGGCATTTAGAGCATGCTTACCCTGTGTAGAATCCACTGTTGAT

>c11470_g1_i1 len=244 path=[410:0-243]

TTGTTTGTTTCATAGTTTTTTCATGAAATGGCCAGGAGAGTTAAAATCCCCAAACAGGTA

GTTTCAGTGGATGTTGAAACTTTAAGAAAGCTTGTTCTATCTCAGCTGAAAACCAGGTAT

GAGAAGACATTTGTCTGGTAGTTAATCTCCTAAAGTTTTGGCATTGAATGTTGACCTAGT

CAGACTTCAGATTCTAATAAAATCCTGTTATCTGTTTCTAGATGTGGAGGTGTCACAAAG

ATCT

>c11472_g1_i1 len=316 path=[1:0-315]

AAAAAAAAAAAAAAAATGAAAATAGAAGAAAATTAAGTGAATATATGAAGTATACAATTG

ACATTAAATAGTGCATTATATTAAAGGATAAAATTTTACAAAATTACAAAATTTCTAACA

ACTGTTGTTTAAATTTCCTCTTTAAATTTTTTTAAACTTTTTTTGAAATTAACCTTTTTA

TTTTATAATTATTATGTTAAAATTATTGAATGTTTAAGTCCATTAACACAAATTTTTTAC

AAATTTACATAAAATATTAAATTTTAAATTAAATGTCTTCTATATATATATTAAAAAAGA

GAAAATAAAAAAAAAA

>c11472_g2_i1 len=492 path=[1056:0-491]

AAATTTCTATATATTTTTCTATTTTTTTACATTTATTTTTCTTCTAATCTAAATAAAAGA

CAGTTATATATGCATGTTTTGAACCAATAAATAAGAAAAAAAGGTGTAAAATAAAGCAAA

ATTTTTAAAATATTAATATCTTAATTATATAAAATTTAATTTACAAAAATACATCAAATA

TGTCATATTAAAAAAATTCAACAATACATTAAATGTTAGTTTATTTATAACATTGACTTA

TTAATAAATTTACGCACACAAAAATTGAAAATTTAGGAAATTAAAATTTTTTGTATATTT

TTTTTTATATATATGTAAATTTACGATATCACCATATGGATATTAATTATGCGCAAATAT

TAAAAACAATGATTTAAAAAATTAATTTTAAAAATTCAGTATTTAAAAGATAATAAATTA

ATTTATTCATTTATATATGAATATTAATATAAAAAAAAGCAACTTGATAGAAAAAAGAAA

AAAAAAAAAAAA

>c11482_g1_i1 len=241 path=[155:0-240]

GCCTCTGCACTGTATTTTCCTTATTGTCTTTTTCTGACTTGCACTTCCATTCATATGTTA

GGGAAAGAAAGACTCCACAGCATCATATTCTGTTCTCATTCAGGAGTAGACTTTCTTCTA

GACTGTATTTAGAGTGTGCTTCTAGAAACATCTATCTCTTGGAGCAACTGTTTGTGGCTT

CTCCTTCCACCCCCCTTTTTTATTTAATAAAACTTCATAAATTTACATGCAAGTGGATTT

T

>c11483_g1_i1 len=209 path=[189:0-208]

ATGACTGAATTATGTTTGATAAGACTTTCAGTATAGGAAATTTCAGATCTAACAATTTCA

TCTTGACCAAGATGAAAGTGACCAAAACAGTTTTCTTGTTTGAAACACAGCCACAGAAGG

CCCTAACAAAACTGTAGGCAATATCACCTTTACTTGAATAGAAATAGGACTGTTTCCCCA

GTCTATACATTTGAAATTATCAGAGTTTA

>c11491_g1_i1 len=224 path=[1:0-138 140:139-223]

GAGCTCCTGTGTCACATCTGTGAGCATTGCTCACGTCCCAGCTCATGCCTCGTTACCAAA

ATAGTCTCAGCCCTGCGCTGGATACAGAGGAAATCAGATCCCCCTCAATTATGCAGCAGA

GGAAGCAGCAACGTCTCCTATCCAACACTCACTTTGGGTATAGGGTTCTCTATTCCTTAA

AAATAGAAACAACAAACGGTTCTGCAAAAAAAAAAAAATCAAAC

>c11495_g1_i1 len=249 path=[1:0-203 886:204-248]

GCTACCGCCGCGCCCGCCCCGCTGTCACAACGGTGACCCTATAGCAGCTCCCCAGGCGTA

AGGCTGCTCCGGACCTGACCCTGACAGCAGCAACCACCAATCCCTGCCACAGACCTCATC

GGCGTTGGCCAATGAAGACACTGTGGACAGGACAACGCCGGACCGGCTTTGCCCCGTTCT

TCCGGCTCTGCGTGGGGCCGCGGCTATCTCTTCGAGGAAGCAATGTTTTTGCTGAACCCC

TGCACAAAC

>c11495_g1_i2 len=454 path=[1:0-203 205:204-226 943:227-453]

GCTACCGCCGCGCCCGCCCCGCTGTCACAACGGTGACCCTATAGCAGCTCCCCAGGCGTA

AGGCTGCTCCGGACCTGACCCTGACAGCAGCAACCACCAATCCCTGCCACAGACCTCATC

GGCGTTGGCCAATGAAGACACTGTGGACAGGACAACGCCGGACCGGCTTTGCCCCGTTCT

TCCGGCTCTGCGTGGGGCCGCGGCAATCTCTTCGAGGAAGCAATGTTTGTGCAGGGGTTC

AGCAATTAGTGACCCAGGAAGGACAAGCATCCTGATACAAGTTGTAGTGATATTGTTAAG

CTTGTTAATGCTCATTAATGGATAGCCCTGTCGTTATGTTTAGAAAACAAGGAGAATCTG

TACCCTTGTACTAAGCAGGTAATGGGGGTATACTGAACTCCAGTAGATAAGGGAAGTTCT

GCTTCAATCACACAATATTTGCTTTCACAAAATT

>c11500_g1_i1 len=288 path=[173:0-287]

GGAGCAGTGGGGTGAGCAGGAGCTGGCAGGCACAGAGCAGCATGGAGCCAGGGGGAGGCC

CAGGGGTGCTGGCACCCAAAGGCAGGGGCCTGGCTCCGGGCAGGGTGGGAGGCAGGAGGC

AACCCCTGCATCAGGGTGCCAATGGCTCCCATCATGTGCAGTGTGGGCATGGTGACCCAG

GTGATGCCACCCAAGGAGACAGAGGGCTGACAGGACTAATGCCAGCCATGCTCATAAACA

AGCAGCCTCTCTCCACCTGGTTCTGCCCCCAGCCCTGCTCCTCTGAGT

>c11505_g1_i1 len=211 path=[1:0-18 20:19-210]

TATTGGCAAAGTATTTAATTTTTTTTTTCCCCTCTAGTTGTGCACTGATATCTGCTCCCT

GCCCCTGTAAGGGCTGAGTGGCAGCTGGCTGCTCCCTGGGCATTTTGGCAGGGCTGCTCT

CACTGCTGCCCTTCAGTGCAGCGACAGGAGCAAAATTCTGACAGCGACTGATGGAAGATG

GAAAGTAGAGGGGAGAGACTTACTGCTATCA

>c11507_g1_i1 len=346 path=[613:0-317 952:318-321 293:322-345]

GTGGATGCCCCCTCCCTAGAGACATTCAAGGCAAAGCTGAGCACCTGATCGAGCTGTAGG

TGTCCCCATTCACTGCAGGGGAGTTGGACTAGGTGGCCTTTAAAGGCCCCTTCCAACTCA

AGTGATTTTAGGATTCTATGACAAACACAAAAAGAAAACAAGCCAGCCTAGCCTCCAAAG

TTCAAGGTAAGAGAGAACCGACAGATGAATAATAAATAGCCATAAAGACTCAAGAAGATG

TGGTCTTGCAAAGGGAGCAAACGGATGCAAGCTGAGAAATGGCTTTGTGAGCCACAAAGA

GCAAGGGAAACCAGTGCTGTGTGAGGAGGAGGCTGGCCCCACTGAC

>c11513_g1_i1 len=214 path=[1:0-213]

AAGGTATCCAAATATCATTATTACAGTAAGAGAATTAAGAGTCTATTCCCAGAATATAGT

TTTTCAACATGTAAACAAATATTTGATTCTTTCGAAAAATACTGTAAAAATCCTATTATT

GAATTGGCGAGGGATGATTATATAAAAACATATTGCCAAGAATATAACTGTGAAAAAAAA

GAAATATCAGATATGTTAAAAGGAGACATTCATT

>c11513_g2_i1 len=225 path=[585:0-224]

TTCTGATGGTAATTCCCAATCATCCTCTACACCCATGCATACTCTTCTTAATTTATAAAA

CCAATAATGAATACTTCTATAAGAATAACATAGTTGACTTGAAAAGGTTCTAAGAGTTTT

ATCCATTTCATAATCATAATACTTTATAATATAAAAACATGATTCTACTTTTTCTCTTAG

AAGATTAAAAGCTTTATTTATTTTAAACAACTGATATATATAAGG

>c11538_g1_i1 len=211 path=[1:0-127 129:128-210]

GATTCAAGGTAGAATATGCACTGGAACACAGGAAATTCTCACAGCTGTAAACTTTTTTTC

CCTTAATTTTTACATAGTTTGTAAGAAAGAGAACTCCTCAAAGAATAGACAAACAAGCCT

TATCTGTTCAGATGGATATTTAACAGTCCAAGTTAATATCACCCAAGGAGTTTGATGGTG

GTGGTACCTGCTGGGACAATGACTCTCCTCT

>c11540_g1_i1 len=504 path=[1:0-503]

GCTGGGGGCAAGTTAGTATTTATTTATCTCTTGCTGATACTTCTGCCTTTACTGGGTTGT

TAATAACACGTGCTATCTGTCATCTGAAGAAACTGTGTGGGCAACTTCATAAGGTTCATG

TGAAGCTCTAAGAAAGCCGTGAAATTCCAACTTGGATAAAAAATACCATTAATTTTCGTA

GTTCCTAATAGAATTAGAACTAAATAGAACTAAAGAAAAACAAAAAATCAGGAATGTTGC

TTTCCTGATATTAACCTGAAAATATATTTTGTTTTATAAAATGTTTACGTTGCATCTCTT

TCTGTCTTGAAAACACACTGTTTTGCAGCAGTGATCTTGCTAATATGTTCACTATGTAAC

AAGGATGTATTTTCTTTGATAACCTTAATAGTATTTTTGGAGAAAATTCTTGGGTTTTGG

AGCTGTCTAGCAAAGAACTGGGCTTTGTGTTCTAGTCTTGGGTCAACTTTGATTTTGTTT

TCTCTCTACAAAATGTTGAGAGAA

>c11547_g1_i1 len=260 path=[1:0-259]

AGGAACCCAGAGAACCTGAGGAAGATGCTCTCTGATTGGAGCAGATAGAGAGGGATACAG

TGTAATTTGCTGTCTCAGTTTCCGTGAGAAACTTTGAATATGGTTCAGGCAATTCTCAGG

AGCGCTGCTTTCTTTCTTAAGTGCTCTGCAATGGACAGGTAGGTGCTTGCAGCTTTCTCC

AGCTCCTGATCTATAGCTCAGGCACAGCTACAGGCACCTCCTCCTGTGGTTCTGCTTCTC

CTGTGGAGCCGTTATGCACT

>c11550_g1_i1 len=250 path=[155:0-226 443:227-249]

ACAAACTGCAGCACAGCAAAAATATTGAGATGAAAATACTTGAAATCTTAAAACTACAAT

TTCATCACTACAAGCTGAAAATCTAACACATTCTTTTAGTCATTCTATTTATTGCATTTT

CTTAAGAATGTAAAGCTATCAGTTCCAAGCCATCTTTCAGCTTTGAGATGCTCGGATACC

TCATGTGATACTTCAACTTTGATTTTGCCACTTACACTTCAGGGCTTAAGCTGGTACAAA

CCTGTTTAAG

>c11554_g1_i1 len=421 path=[449:0-420]

GCAACCTGCTCCAGTGTGTCACCAGGTTAACAGATCTACATTCTTCTTCCAGCACTGGGC

CTAGGATGAAAGAGTTCTGTGCTGTTCAAAGCTACTAGTTTTGCCTGAGGTTGATGTGGC

AACTGCGATCACATTGACGCTCTCTTTCTCAAATAAATGTTGTACTTTGAGAAATGAAAC

AGAAACGTTTCTCTTCTCTTAGGCAAAACTGGAAGTTCAAGGAAGCAGCATTATTTATTT

TTTTTCCCTTGGAAGCAAACGCAATACGTAGAAGACGGCTTAGGCTGACTTCACAGGATT

AAAGGACTGCTTCATTGGTGTTGGTGCTCCAGAAGTATTCTCTTTATCATTTGCAGAATA

TGAAATATTTACATGAAAGTAGAGACGATTTTCACCATGAGTTTGGTCTGTGCTGAGATT

G

>c11569_g1_i1 len=232 path=[214:0-231]

AAATAAATAAATAAAAATTAAAGTATATTTTGTTACATTTACTGTAATATGTTTAACCTA

TTTTTTTACATTTATTATAATAACGTAAAAAAATAAAAAAATTTAAGATATGATATAATA

ATTTAATTTATTATTACACTTAACATGCAGAAGAAACTATAATTCTTCAATTATTTCCTT

TTAATTGATATTTAGAATAATCTATATATGTTAATTTTATAAAATAATTTTA

>c11577_g1_i1 len=357 path=[1:0-356]

AAGATCAGCTGGAAAAATCTGGAGTTAGATGCCGTCTAGTACAAGTGAGGAGAGTAGAAT

GTAGCTCTAAATAATACGAAAATGAAGTATTTTGGAATATGCACATCTGTGATTGGATTC

ATATCATATGTCAAGGGCTTGATTAAACAACATGTTCCTGCACTTCCTCACTGATTGAGC

ATTTTTTTCAGTTTGATTGCTTTGGAGTGTTGTAAAAACAGCTATCCCGGTTTTCTTCTT

AGTTTCCTGAGAAAAAAACAGGATTGTATAGTAGATCTAAACAACTTTTTTCAATTGACT

GATTGTTTTCAGTCAAATTTATTAAAATATTCGTTTGTTACTTCTGGTAATACTAAA

>c11581_g1_i1 len=212 path=[190:0-211]

ATTGTCTCTGTTAGCACGTTAGTGTCAAAACTGCCTCTTCCTGTAGAACTATTACTTTGC

AGGTTTTTCTTTACAGTGACTTTACAACCCCTCAGTGAACTCTCCAAAGTCACTTAATTC

AAGTGCTTGCAATATTTTACACATCAGTAAGCAGAAGCAAATATTGGTTGGGTGCCACTC

ACACAATTGCAGAAAGGACCCAAGAGATGACA

>c11582_g1_i1 len=249 path=[429:0-248]

GTGCTGTGCATTAGGTTGAATGATTGTCTCTTTCTTCAAAATCTACTTATAAAATTACTA

ATATATTTGAATAGTTATTCAATTCCAGTATGAAATGCAAAGTAATCTGTAAATCTGGCT

AGGAACAACAAGAGGGGCTTCTGCAAATACATCAGTAGTAAGAGGAAAACTAGCGGCACG

TAAGCAGCCCAATGAGAGCCCTCTGTCTGCCTGGTGTGGCATCTCAGCAGCTTCCTGTGA

TTTCTCCTC

>c11598_g1_i1 len=210 path=[342:0-209]

TAATAAAACGTGTAATTTTTATTCATATTCAAATAGTGAACTAGGTGCACTGTGTAACTC

AGTGCACCTAAGCAAGCAGATCAGTTGTTCCCTACTCAGCTTCGCTCAGAGCTATTTGTG

AGACATCAAATAGGACAGTCTGCCCTGCCATCACTTGAAAGATTCGGTGTTACTCTGAAT

TCTGGTGCTGAATTACGATTTTCAGTGATG

>c11601_g1_i1 len=219 path=[155:0-218]

GCAAGCAGTAGGAAGGTGCTAAGGCATACTTTTCAGTTCCAGCTGAGACAGTTGGACAAT

TCACTTTGACAAGGGAGCCCACAGCTTTACCTAGAGGCTTGTTGTTTGATACCCATTCAC

GGCAGTGTTTCTGTATCCACCATTGTATTTGTGGATCATTTCTAGCCTGATGCTGTATAT

TGTGCATGGAATCGATGAATGCCACAGAGAACACTTTGT

>c11609_g1_i1 len=225 path=[461:0-224]

TTAGGAAAAGAAACAAAATGCTTTCCAGGAAAGCAAATTTTACTCAAATATAAATACTGT

ACATCTTTAAAACTGAAAAACTAGTGGTGGGGCTCTAGAAGGAACACAGTTTTCTAGCAG

CTTTTATAACACTTGAGAAAGAAAAAAAGGAATGCAATATAGAAGCTGTAAGCTCATCAT

TCATACACACAGAAGTTTAATACTTGTTAAGACTTCAGTCCATAG

>c11610_g1_i1 len=410 path=[1:0-409]

ATAGCATTCCATAATTTTACATCTATACCATTAGAAAATAGAACAAAATTCTATTCAGCT

AATAAAATGTTTTCTTTATTTGAACATAGAATTCATTACTATAATGAATGGAATTCTCAA

ATTTTATTTAGCAAAAAAATATATCCTGAAGATATATTATTAACTAAAAGTTATAATGTA

TTTTCTTATGTAAAACCTAATAAAAATTATTGGTTCATTATATCATTAACAGTTATTCCT

TTTTTAATAATTTTATTGTTATTTGGTGTTTTTAAATATATGTTCCCAAAAAGGTTACAG

TTTCACTTTTTTTCTAAAAAGAAAACCAAAAAACATAAATATAGCTGCCTTAAATGCCAC

AAAAGTTGCAGAGATATTAGCAGAATAAGAAAAAGTCCCTCAAACACAAA

>c11610_g2_i1 len=240 path=[775:0-239]

GTAAAATTATGGAATGCTATCTGAACTAATTTTTTACCTGGTTGAATTAATTTTTCTATT

GATTCAATCTCTCTTTTGTTTATTCTAGAACCAAAATAATTTGTTCTTCTTCTTGTATTA

CTTTGAACATCATTAAATGATTCTCCTAACACTTCAATTAATTTTTCACTAATCAATTTA

ATATTATCATATTGAAATCCTTCTAATGATCTAGAATTTACAGGAATTTAAAAAAAATCA

>c11616_g1_i1 len=230 path=[297:0-33 331:34-229]

ATTAATATATATTTGTGTTATTGAATAAAGGAAAATAATAAAATATATATTTTATTATAT

GAGGAAAATATTAATAAAATAAAGGGTACTTTTTACTTTAATGTACATTTATGAAAATAA

TGATTTATAAAGTTTTTAGACGGAAGAAAACTATCTTAGTTCAATAAGATAAAGATGCAT

TTTAAAGAATTTTTTAGTAAAAAAAATTTACTTATATAAATGTATTTTTA

>c11616_g1_i2 len=368 path=[504:0-171 331:172-367]

TAAAAATACATTTATATAAGTAAATTTTTTTTACTAAAAAATTCTTTAAAATGCATCTTT

ATCTTATTGAACTAAGATAGTTTTCTTCCGTCTAAAAACTTTATAAATCATTATTTTCAT

AAATGTACATTAAAGTAAAAAGTACCCTTTATTTTATTAATATTTTCCTCATATAATAAA

ATATATATTTTATTATATGAGGAAAATATTAATAAAATAAAGGGTACTTTTTACTTTAAT

GTACATTTATGAAAATAATGATTTATAAAGTTTTTAGACGGAAGAAAACTATCTTAGTTC

AATAAGATAAAGATGCATTTTAAAGAATTTTTTAGTAAAAAAAATTTACTTATATAAATG

TATTTTTA

>c11617_g1_i1 len=214 path=[1:0-213]

AATAATAATAAAAATATTTTTATGTTTATTTAAAGGATTAAAAATTCTTTATGAAATAAA

GTATTCCTCTTATGGTATATAATATATATAAACTTATTCACTAAAGTCAAAAGATATATA

AATATATCCTACTTCCAACATAAGTTATAAAAGTATTTTCATAAAATATCAACTTGGTAG

AGATAACTATGCCATACTCTTTAATAGTTTTTTA

>c11622_g1_i1 len=216 path=[1:0-215]

ATATGAGCATGTAATTATTATGTCAAATATAGAATGATTATATATTGTTTATATGTAAAG

TAAATTTTTAATAAATACAATTAGTAAATGTTATTTTACACACTTTTTATTAATTTGAAC

TTTTAAAATGCTACAAAGTTCTTTGAATTTCCAATTAATAAAGCTATTGTTGATTTATTA

ATTGGCCGTTCTATGACTTTGCTAGAGTATTGAACA

>c11633_g1_i1 len=290 path=[306:0-289]

GTTAGTTATAGAATGTGAAAAATCTAAAAAAAAAATGAAAAATGGATAAAATTTATAAGA

GTAAATAAAAAAGAATGGAGAAAAATTTATAGGAATATATAAAAAAGGAGTTTAAAAAAT

TATATATTTTTATTGATTTTATAAAGTTTTATTTTAGTAAAAAAACTTTAAATTCTATTT

ATGTTATAATAAAAAAAAATTTTAACATAATTTATTAAAAATATATATATTTCAAAAATT

TGCTTTTTGTTGAAGGAAAAATTTTTAAAATATAGTTAAATAATAAAACT

>c11641_g1_i1 len=231 path=[1:0-230]

ACTTTGTGTTTGAGTGGGAGAGAGGGTCCAGCTGAGTTTATACAGTAGATGTATTAAGCG

GTCATTACTAAAACTCATTGGATATAAAAAAAATTCAGAATGAAGTTAAACCTCACAGAC

AAAACTGATACATATAAACTGATAAAGTAATCCAGAATTTAATCAAAGCCTACATAAACA

TCTGACAAATCTTTGCACAGAGAAGCAGCAGGGCTACCACTCCCTATCAAA

>c11643_g1_i1 len=207 path=[185:0-206]

GGATGATGTGTTAATGGAGAGCACAGCCAGCGTGGTGCCAGGTACTGGGGAGCTGTCGCG

TTGTGTCCCGGTGAATGGCAGTGCTGTGTAACTAACTCTGGCCGCGCTGCTCGGAGGCAT

CCCACTAAACCCTGTTGTGGTGCAAGGCTCGGGCGCTTCCCTGGTGGCGCACACGTCTCG

TTCTGCCCGCAGGTCTGCCGGCCTGGC

>c11649_g1_i1 len=305 path=[1:0-304]

GATATTCTTGCTAAACTCAGAATGCATTTGCCCACCACCACATGCAGACCTTATCAGTGC

TGCGGTCTTCGATGCTATCCTAGGCAGTAAGAAAAGTGGTAATAAAACTAAAAAGAGCAG

CACTTCAAGTCAGCTCGGGGCAGTGTTACGGTGTTACTTCTTCAGTCATGTAAGTCATGC

AGTCATACAGTGATCAGACTGAAAAAGGCACGAAAACACCACAGCAGAAAATACTCCATA

TGCAATGATAGCTGCACAGCAGATGGACTGTCGTTGTTCCAGCAACATTTTCAGAATAGT

CTTGA

>c11657_g1_i1 len=218 path=[1:0-217]

ATATATATGATAATTGAATAGATTTCCTATATTTTCTTTTTCATATTTTATGAAGGATAT

TTGATATAAATGTTTTTTCTTATATAATTTCTATAAAAAATAAATTTTTTTTAATTATAA

AGATTTTAAAATGATAATACGTTATTTTAAACTACTTATTATTTAAAAATGTTTAATAAT

AATATTCTAAATTTTTCTTCACAAAAAAAAAAAAAAAA

>c11664_g1_i1 len=211 path=[226:0-128 433:129-210]

TGTGTTTGAACATGGTACTACAGTGTGTCCTACACTGTCACTGGAAAGCAGCCAACTGCC

ATTGTATACAGAGAAGGGTGTATTGGCTTCCTGTAGCGCAACAGAAGTACTTCATGGAGG

GAACCTGCTCCCTGATATTTTGTTAGCAGCAAGAACTTGTTCTGTTTGTTTGTTTGTTTC

AACCTACTTTGGCTACTGTGATGAGAGGTAA

>c11676_g1_i1 len=286 path=[1:0-231 340:232-285]

TACAGCCACTTCCACTGCTCTCCTGCATCAACTATCCCTTCTAAAAAGCTCATAAAACTC

ATAACCCTCAATCATGATATTCCAGTTATGTGAGTCATCCCACTACATTTCCATGATAAA

TATACTAAAGAAGCTGGCAGAGCTCATTAACAGGGCTTTAAACTAAATTTGATGAGTGAA

GGGGATGTACTGCTGAGGAACAGAGAGGAGTAAGGGAACACTCTCACTTCAGGAACCAGC

AAGGGAAAACATCAGATTTTTCCCAGAGGTTTTGGGGAGGGCTTTT

>c11676_g1_i2 len=286 path=[1:0-231 233:232-285]

TACAGCCACTTCCACTGCTCTCCTGCATCAACTATCCCTTCTAAAAAGCTCATAAAACTC

ATAACCCTCAATCATGATATTCCAGTTATGTGAGTCATCCCACTACATTTCCATGATAAA

TATACTAAAGAAGCTGGCAGAGCTCATTAACAGGGCTTTAAACTAAATTTGATGAGTGAA

GGGGATGTACTGCTGAGGAACAGAGAGGAGTAAGGGAACACTCTCACTTCAGTTGGGCTT

TCAGTCACATTACCTTCTAAGAAAAGCCCTCCCCAAAACCTCTGGG

>c11681_g1_i1 len=244 path=[460:0-243]

CTACGTCAACAACGCGCCGTCCCACAGCAGAAAGATACAAATCCCAAAGCAGCTTCAAAG

AATCAGGAAACGACGCCCAAACAAACCACTGAAGTTCACCCCATGAGCAAAACAAATCCA

CGACGTTCCACACTGAACGCATCTATGGATTCAGGCCAAATTTCAGTGTCAAACGTTGGT

TTAGAAGTTTACCTCGATCCTCCAGGGTGCTGCAGACAGTACACATACAAAACACTGCTG

CTGG

>c11694_g1_i1 len=327 path=[305:0-326]

GATCCCCCAGCACTACTGAAGCCAGCTTTAAAGACATTGAGGCCATTTGATGAGACATCA

TTAAGTAAGAACAGCATATTGTTTATTAAGGCATGTTAGGAAAACAAACTTTTCTAATTG

CGTCCCTTGGATTTTAACACAGGGGAGGAATTAGCAAACATCAAATTAACCAACATCGTT

CATGTCAAAATAAATAACATTTTGCACTCACTGAGGTTATAATAAATTTTGGCTTCAAAA

TTACTCTCATTCTAGAATTCTCAGACGATTCCGTCACAAAGATCTTCTTTGTTTTATGAA

CCAAATTAGCCCAATTCCCTTGCAATT

>c11696_g1_i1 len=240 path=[1:0-239]

GGAGTCTCCTTCTCTGGAGACATTTGAAGCCTGCCTGCACGCTTTCCTGTGCAACCTATT

TTAGGGAACCCACTTTAGCAGGGAAGTTGGACCAGATAATCCCCAGAGGGATACCCTCTA

AACCCTACGATTCTGTGATACTATGTAATAAGAATGTAGCAGTGGTTTTACTGCCAAACG

CTTGTCATGTACTCAATAAAGACTCCAATTCAAGTGCTGCTTGCTTACTGTCTTACAGCA

>c11701_g1_i1 len=219 path=[222:0-192 414:193-218]

CCCAGCTGCACTGGGATCTGTACAAATATAATAGTAATGCCTGTGGAATAAATTCTCTGA

AGCTGGTTTTGGTTTCTCAGCAATGTGAATTAGCATAAACAGCTACACAAGGTAATTGTG

AACTGGGCCATCAATGTAGGCCTTTTCAAGTAAAGGAAAATAATACTCCAGACTAACTGA

GCATCCCATTTTTCTTATGGCAGTGTCATGCAGCCTTAA

>c11718_g1_i1 len=276 path=[1:0-275]

AGAGCACCGCAGGAATGATTACAGTAGGTGTGCATCTGAAACATGGTTTTAGATGTGTCT

CCTGTTCTGCCTGATATCTGTAAGATTGTGGCAGACATGCCATAAAGTAAATGAAAATAC

TAGACGGAAGGACTTTGGCATAACTAGGGGCACTGACTGGTGCAGGCTTTAAAATACTTT

ACAGAGCACACTGGCATAGTAGCCATCCATCTTCAGTGACTTTTCAAGCTCACACTAACT

CTAAGCATCATTTCCACAATTGCCCCTTTTTCCTTT

>c11719_g1_i1 len=284 path=[262:0-49 573:50-283]

AAAAAAAAAAAAAAAAAACACAGAATATAGAAAAATAAGTACAGGAATTAGTAAAATATA

AAATAATAATGTAAATTAATATATATACTAAAAATGAAATAATGAATATATTAAGTATAT

TAAAAATAATTTATACACATAAAATTAGAAAAGTATAAAATTTATTATATATTAATAACT

GAATGTTATTAAATATGTACATATGTATATATAATTAAAAATATAAATTTTCATAGAAAT

ACTAAATTTTTTAAAATTACTTGTTTATAAAAAGAATATAAATT

>c11725_g1_i1 len=474 path=[1:0-178 180:179-473]

TTTCTGTATTTTTTTTTATATTAATATTAACACATGTTATAATAGAAAAAAAATCAAAAA

ACCAATAATATAAAAAATAAAGAAAATATTCCAAATTATGATAAACAAAATTGATACATT

ATTTTGATTAAATAACTTCTACATGTGCTTGAATTTATGTAATTCTTAGGATCTACCTAT

ATGATATAACCTAAAAGCAAAATTATAATAATAAGAATACAATACATAAGAAAAGAAGTA

ATTAATTTTATTATGAATTATAGACTAATTCCATTAAATATATTATAAGACAGAATATAC

TTTAATTATCTGAAAAATAAATTGAATTATTATTTTATAGACATGTATTTTTTCTTTTTT

TTATATTTTGCTTAATATATTAATGAATATGGAATATTTATTATTAATTTTTCTTTCAAT

GTTTTCATTAGACTAAGAAAACAACTAAGTAGAGAACATGAGGAGAGAACTCGT

>c11727_g1_i1 len=230 path=[208:0-229]

AAAATTGCTCCATGGTGCAAAAAACACCAGTAAGCAGGGTGATGGGGAGGTGAATTTCAA

AGGTGTGCTCGTGATCCAATCCTGCACACACACACGCAGTGATGCAGGGGTGTTCCCGTG

TAATCATAACAGTTAATGTCCACATACAGCAAGCTCTTAAGATGAAACAGTGGACTGAGT

TTTGCTCAGCCTGCCAGGTTCTGTTTCTGATTCATCTTAAGAGCTTGCTG

>c11731_g1_i1 len=227 path=[155:0-226]

AAGGCAGATATTCTGTACTAAAAAAAAGTCCCTATTATTCCCAACAGCCAGTTTTACTAG

CAATTACACAAATGCAATTTTTACTTTAGCTATCTACTTATCACTAAAACCTGAACTGTA

ACCTTGCCTCTGTCTCTCAATTAAGTTCCTATTAACATTTAAAATCATAATTATTAAAAT

TTTAGTACACTTTTACCTTCACTACTTCATAACAGAGACCTGCTAGT

>c11739_g1_i1 len=339 path=[317:0-338]

AAACATGCTTATCTTCAGTTTTTGGAAGAAAACATTTTGTGAAGAATAATTAAGAACATT

TTTAAGAAATGCCACCTCTTAAGTTGTATGCCTGCACATCCAAAGGAAAAAAAACCTCTC

ATCAGCTATTTTGGGAATAAGCTGCATTTCACTTGACTCTTGTGCCACAAAGAAAAAAGA

TAATTAACAAAACTGTCTATTCTGTATTACATGCACCATTTGAACTGAAATTCTTGCACA

CTATATTTAGCTGTGGTATTTCTTTGTGAGACCAATGTTCAGTTCCAACAAGCATTTTTA

TTGAGTTTTTTGGTTTTTAAGTAAATAGCCTTCTTCCAG

>c11746_g1_i1 len=242 path=[241:0-219 615:220-241]

CTCTAAACTGTGATCAGACTGTCTGAAAAATGTCTGGATATCTACAAAGAGAGTAAAACT

CGCGAGTTGCCCTTTACTTTCCTTCAAATTTGATTTTTCAGCTGAAAGTGTGAAATGAGT

AAAATACCACTGTACTGCTCATACTTTCCTCATTTCACACTTTCCTTACAAGATTACACT

CTTACTGTAGTTCCTTAAGAAACTTTCTGCAATAACAACAATGTGCCCATAGGGATCCCT

TG

>c11752_g1_i1 len=350 path=[367:0-337 766:338-349]

GAGTAAGTCACTGAATTGGATGGTTAGTATTTTAAAGAATAAAATCTTGTTGCAGGTACT

ATTGCTTATGACAGGTAAAATATGGAACTGTGGATGTGCAAGTTAAATTAATTTTCATGT

AGCTGAACTTACCAGGGTATCTTTGCTTTGCCTTGGTTTTCTATTTGATTTTAAAAACTT

GTGTATGGTTCTTTGGGTACAGTGTTTTCTACAGAAATCTGTATGACGTAACTTTCAGTA

AGCTGTTTGATTACCTTTTTAAGGATGAAGAGATGGCCAATCTTTATGGACTATCTCCTT

GAAGAATTCACTGTAATCTTCCTCCTCCCACTAGCTTTTCTTACTCCTCT

>c11756_g1_i1 len=234 path=[2:0-146 149:147-233]

TAACTTTGTGTTTGAGTGGAGTTAGGTTTTTGAGAATTGATGAAAAAAGGCGCAGCTACC

CAAGAGCTTAAGGGTAACTGCAGTGGGAACTCAAAGTTATTCAGTTTTATTTGTCTGTAA

CTTCATACTTGAAATTCTACCTTTTTTAGACAAAGAAACAACCCCCCAAAAAAAACCAAG

CAACAAACAGCACATAAAATATGCACTATGGAAAAATGACAGTAAAAAAAAAAA

>c11757_g1_i1 len=239 path=[401:0-238]

AAAAAAAAAAAAGAAATGTATATTTATTTTATGTATGTTTATTTTTCTTTAGAGTAATAT

TTTTGATAACATAAAATGAATCAGGTTTATAATAAATACTAAATGATTTAAAGACAATTA

TATATAATTAAAAGATGAAAAGTAATATTAATTATTTGAAACACATATATTTCTTTTATC

AATGGACATTCTTAAAATATATTTAACAGAAAAAGAAATTATAAATAATTAATAAGATA

>c11762_g1_i1 len=233 path=[109:0-232]

ATTGACCCCCATTGAATCCCATTGAGCGCCATTGAATCCCATTCCATTTCAGCTCCGATT

CCACTCCACGTTTTCCCCCCCCATTGACTCCCATTGAACCCCATTGAGCCCCATTGAATC

CCACTGAACCCCATTAAACCCCATTGGATCCCATTGAACCCCCCTGAATCCCATTGAGCC

CCACTGAACCCCATTAAATCCCATTGAGCCCCATTGACCCCCATTGAATCCCA

>c11765_g1_i1 len=229 path=[14:0-125 492:126-228]

TATTTTTTTTTTATTTATTTTTATGAACACATTTTAAAATGTTGTAATATGAAGGATATA

TATATATTTCCAAAAATAATTTAATGCAACTTTTACATTAAAATTATCCATTTTTAATAA

AAATAAAGTATAATTTACTATTTATACAAGTATGAATAAATTTGATGATTTTATTTATCC

TTTTACATTTTAATAATTATAGCTACTAAATAAAAAAAAAATATACATA

>c11770_g1_i1 len=232 path=[363:0-93 154:94-231]

GGCATTCAGGAGTGAAGTCCTTCTCGTGCCCAATTTTATATTTAACATGTGTACATTGTA

CAGTTCTGTTTGTATAAGGCAGAGTCCAAAAATAAAAACAACATTTGCAAGGATTACTGA

ATTACAGAGATGAATAGAAAATGAGCAACCTATCAGACCTCTGACGTACTAACAGATCAT

AGTTTCTTGTTTTATGGCCGTGACTGAGGCACCAGTTTCTGTACGTGTTTGA

>c11771_g1_i1 len=285 path=[263:0-130 394:131-284]

CTTCTGTAGATGCTACATTAAAGAAAATGTGAGCCCAGTCTCTAAAGATTTCTGAAAACA

ATAGATGCTCTGCTTATTTGCTTTCCTTATTATTTCAGAGAAAGAGACAGGAAGGAGAGA

AAAGGGAAAGTAATGTGTTTTCTGTAAGTCTTTCTATTATTAAATATTGTATATTAATTT

CTTTCAGAGTAAATTCTATATTTGTATTTTGCATACAGTAGTTCATAACTACCATGTTGT

TTTATACAATTTAATGTTTGCAAATTTTTGTTTTATAGAATGCCA

>c11771_g1_i2 len=252 path=[623:0-97 394:98-251]

TGGAAACATATTGGTAGGTTATTTTTATTGCCTAGTTGATATGTGTACTGATTGCTGTTA

AATAGTTTTAATTCACTAGACATTTTTGGGTTGAAATCAATGTGTTTTCTGTAAGTCTTT

CTATTATTAAATATTGTATATTAATTTCTTTCAGAGTAAATTCTATATTTGTATTTTGCA

TACAGTAGTTCATAACTACCATGTTGTTTTATACAATTTAATGTTTGCAAATTTTTGTTT

TATAGAATGCCA

>c11772_g1_i1 len=240 path=[356:0-112 139:113-239]

ATGTAGTATCTGAAAAAAAATCTTAAATTTGAGATAATATGGAATATCAGCTACACAGCT

ATGTGTAGTAAAACTTCCCTGAGTTCTCCTGTTCAGGTTATACATTTAATGGAACGATGG

AAATATTCTGGGTATTTATAAAAGCTCCTTGGAAGTTTTATCAAAGTTTATGAATTAATG

CTTGTTATTTAATGCCGTGTACAAAAAAAAAAGTACAAAAAATGGTCAAATGCTTATAGA

>c11781_g1_i1 len=218 path=[53:0-217]

ACGGCTAGAAAGTGGACTGATTTGGATCCAGAAGAGGAAGAGAATAAAACTGTTTAGTGT

GTTATTCATTGGATAGTTGGCCCCTGATATCTGAAAGAAGTAGTTGCAGAAGTTTGGCAG

GGTTTCAGGGTTGTATGTTTCACAGTTGATTGAAGTAGCCTATAAGGTACTTCACTATAT

AGAGGAAGAGTAACACTTACAGGAGCTCAAACACAAAG

>c11795_g1_i1 len=245 path=[43:0-77 281:78-244]

AATTGTTTTTTTTAATTTTTTTTAATTCTTAATTGAAAATACAATTTAAAAATGTCAATA

TATTTATAAATATTACATCAATATAATTTAAGGCATCATGTTATTTATTTTTTTTTTCAT

AACATATTTTTAAAAATACAACTTAGAAATATGTATTTCACATATATAAATGGGGTATAT

AATTTTTTCTATAAATTTATGATTTCTTGTTTTTTTAATACTTTTGTTCTGCAAATGACT

CATCG

>c11796_g1_i1 len=476 path=[370:0-337 14:338-361 38:362-475]

AATAAAACATTGCTTTACACATATAAAATTAAATAAAAGGAAAAGAAAGTAAAAACATAA

AAATATTAAAAGTATAACAGATATATTTTTATTAAATTTAAAAATGTTTGAAGTAAAATG

ATATAGAATTGATTAAAAATAAAATGAAACAATTGAATAAATATTTATGTTATCAAAGAT

ATATAACTAATATTTTTTAAAATTTAAAATTATTCAACAATTGCATTTGAATAACTTTTG

AAAGCTCTTAATAAATATAGTATAATTGTTAAAAAAAAAAGAAAAGTTGGAAAATTTTAT

TAAATTATTATTAATTTGTGCTTTGTATAATTTGAAGAAGTATTTTTAAAACAAAAACTG

TATATGAAAATATATTACGAAATATAATTAACTTTTATTCTATAATTTGTACCAAGTTTA

TATTTGTATTAATATAGAAGAATTATAATATTAAATATATTATTAAAATAAATTTA

>c11804_g1_i1 len=314 path=[275:0-313]

TCTTACAAAATTCTCCTTTTTCCTTCACTTCCCTTCCTTGTATTATCTTTGTTAATCTCT

TGAAATAGGGGAGAGAGGGATTAGCATCATGAACTGATCTGAAGGCAAGTCCTGTCTCTT

TCCCCCACTCCAGCTCAAGTTTAAGAACTTGCATACGTCTTTATAGTTTACGTTGCCTAG

ACTATACACTGAATGTGGTAAAGCATTGAAACAACTCTTATTCCACTTTTGAATGAAGTC

TAGGTGTATATCAAGTTAAACAGAGCCAACCCTTTCTAGGAAAAGGCAGGTATGCTTTTG

TGAAAGGCGTTCAG

>c11810_g1_i1 len=565 path=[543:0-60 604:61-61 605:62-564]

ATTCAACAGTTCCCATACAGTGGGAAATGCCATTCTGCTTTTTTACTCCCAGCTATATAT

GAAAAAAAAAATAATCATCATTTTGTAGTAATGTTAGAGAAATCAGTTGGGGAAATAATG

CTATTTCTAGTATTTGTCTCCAGTGATTGATGTTTTGTGCCAGAAATGCTCCAAGATAGA

TGACTGCCCTAAGTCTCAACAAAACAAAATCCACTAAATCCTGTGGCATTTAAAGTGTTT

TCCTTTATTAGCCTATTATTGTCTAGGTTGAAGTAAACAGTCGCCTGGATAGCTGTAGTT

TACATTGCCAGAGATTTTGGTTAACTTCTATTTGGGGTTGAAGAAGCACAGACATTAGAA

GGAAACGCTGTTTTGTCAAAAGTTAATGAAAAATTAATGCATCACCTGTATGTAACAAGC

AAAGGTCTTCTGAATGTAGACTTCTAATGAAGTTCTTCATAGTATAAAGTCTAGGCTGAA

TTATTTGAAAGTGTGCTTTAACTATGTAGTACTTCTGCTTTGATACATGCGTTTAGATTT

ATAATACAGCAAAGCTTCCAAATGT

>c11815_g1_i1 len=214 path=[438:0-138 197:139-213]

CAGCACATGCTTTGCAGATGTGGCACTCCTCATGTCCAGGAGGATATTCCCAGGACATGC

TAACAGCCGAAAACATTAAGACATTGTAAACATCTCAACTGCTCAGTGATTTAACTACAC

TAAAAAGATGTTCTAATTTTGTAAGTTCGTGAAGATGTAAGGTTTGTTATATGTGTATTT

TATTTAAATATTTAAAGTTTATTTTAAGCATAGA

>c11815_g1_i2 len=214 path=[438:0-138 576:139-213]

CAGCACATGCTTTGCAGATGTGGCACTCCTCATGTCCAGGAGGATATTCCCAGGACATGC

TAACAGCCGAAAACATTAAGACATTGTAAACATCTCAACTGCTCAGTGATTTAACTACAC

TAAAAAGATGTTCTAATTTCATTACAAATCTGTTATATGAGAAACAAGCAGTTTTTAGGA

GCTGTTAAATCTATGCTTAAAATAAACTTTAAAT

>c11819_g1_i1 len=300 path=[278:0-299]

TGAAAGAAGAAACCTCACGCACATATATAAGGAGTAACATCTTGGGGTCTAAAACAAAGC

AAGAAACACCAAAGCAGTTTCCACACTGTTTTTCTGACACATTAAGCATGATTCAATAAC

AGTAAGTCCAAGTTGCAACAAAAAGTTAAAGAAAACTAATATTTCCTAGATAGGAGAAGA

AACCTGCAATTTTTAATGCAAGTTGCATTTAAGAAGCCTTGATAATAGGTCTTAGTGCTG

AAAAACAGCTTCCTTCTGTGGTTTCTTAAAACCTGTGGAATGCTGGAATCTGAAATGCAG

>c11820_g1_i1 len=276 path=[1:0-275]

ACCCTTCTGATTGCAGGGATGTTCAGTCTAGAAGTACAGCAATCACACAGCTGTCAGGGT

ACTGGGAAACCTCCTGCTGCCCTTCAGTCTGCTGGGTCGTTACCTGTCCTTCTGTTTGTG

CAGCTCTCACCATGGTTGCAATCTCCTGGCAGCAAGAAGGGCTCATTGGTAAGAGCCACA

GATGAGTTTCCTGCAGCCCTCAGGCAAACACGAGCATCTTATTGTCTCACTGGATCCAGA

CTGGATTCAGAGAAGGAATCCCCTCTACAGGAGACA

>c11838_g1_i1 len=230 path=[1:0-147 149:148-229]

GGGCAGATGATGAGAAATGACTTTCCAAAAGTACTGCTTTTATGCCTCTTGTTTCTTTTT

GTGGGAGAATATTTGGAAGAATGTATTTTCTTCTTGCTTCAAAGTTCTTAAATAACCACC

CAGCAGAGGTGAGACTTTCTTACCAACTTGGTGTAGCTTACAAAATTGGTGTAGCTAATG

GAAGAACTACTTCAAATGGGGGAAAGTGTCAGCTTTGTTGTGGAGATCAT

>c11839_g1_i1 len=261 path=[155:0-260]

GCCGATTGCTCTCATGAGGCATTTTGTTAGACTTAGTTAGGTGCTCCAATGTTAATTGCA

ACTCCTTTAAGAGCTGTGTGATCCTGAAATGCTGTAAACCATTTCTGTGATGTGCAACAA

AAATATGCTGGAGACTCATCATAGGATTTGTGAGCAGAGACTGTAAAACCATGCATACAA

CTCTGCTAAGAGAGACCCACCAAAGACATCTTTGTAGGTATGGGTAGCAGGACTGTACAA

TGGGGGTGTGTGTTCTGGTAA

>c11841_g1_i1 len=238 path=[384:0-237]

AAATTCAGGCTTTCTGAAACAAAAATAATACAGGAAATTCCCTGTTGGGGGTGGAGGTAG

CACAGAATAGCTGTTGTGCTTCAGCTCACACCCTAACTTATTTCGAAACAACTTCCCTGT

TTAGACAAGCCCAAAGATATTGAACATTTCCTGTGCTGGAAAAAGTTATAAGCCTTCTAT

TCCCACAGGCAACGGTCAATTTATAGCTGCCTTATTTTTGCCCTAAGGCTTGTAGTTT

>c11846_g1_i1 len=201 path=[5:0-200]

TTTTTTTTTTTTTTGTAATATTTCTATTTAATTTGTGCATGTTAAGTAAATTACTTTATA

TTTCAATACATAATTTAAAAAGTACTTTCTAATATGATACACATAACAAGTTATGTTCCG

TCTTCAGAACATTTTAAAAGTAAAATAAAAAATTGAATTTATGTAATTTATCAGTTTATA

ACAAAATTTTATATTAAATGT

>c11856_g1_i1 len=573 path=[577:0-545 1122:546-572]

CTGCTAACAGCATGGCACCCAACTCATCTATGTGGGGTCTTAACCTTTTCACACACATGC

ACAGTTTGCAGACGAGGAGAGCAGTATTATGCATCAGGTGCACGCCGTCAGCTTTGTAGA

AGATTCCATCTTCATGGAGTCATCTCTTGGTCCACGTTTGCTTGAAGACAGGTAACAGCA

GCCAAACTGAGGTGACCTTGCCACTGCCTTTTCCCTTTCTTTCCCTCAGCAATCTACTTG

ACATGTAGTTTGCAGAGAGTTTGTCAGAAGCAGCTGTAGAACTAATGCTTGTAGGAATTC

ATGAATCTGTTCTGTAACCCAGCAAGATATTCTTAACTAAGATTAGTAACTTCTAGCCAA

CAACTGTTTTGCTGTTAATGCACCAGAGAATTTGTGCCTGTTCTTTAGCCTGTGTTACTA

CAGTACACGGATTATCTGTATGGCTTATATTGCTTCTGCAAATGTCTACTAAATAATATC

TTGAACTGTTTTTGTGTATTTTGATAGTAAGTACAATTCCTGCTTTACTAAACAGCTTCT

AAATGACAGCTCTGCAGTTCACGCTACATCAAC

>c11863_g1_i1 len=207 path=[445:0-206]

AGGCTCAGTGCAAGGCAGGATGAGGACAGGATGATGCCCAGCACGGGGACGGTACAGGCA

GCAGCACGGGCCAGATGCAGCAAGGGCAGAGCTGCTCTGTTGGTTGGGCAGGGGGTCCTG

GCCCCATGGATCCCAGCCCAGGTGGTCCTGGCAGCTCTCTCCCTTTTGCATGCACTCCTG

GGGGCAAAATGGACTTCTCGATGGGGG

>c11871_g1_i1 len=239 path=[217:0-238]

TATATATATGTATATATATATATTTAACTTGTATTGTTTTTTAATACCTTGATAAAGTAT

TGTTCATAGAAAAAAAAAAATTTTTTTTAAATATTAATAGTAAAATATTTAATATTGAAA

ATAAAATATATATAATTATATTTATGGATAATAATAATATTAATTTATACTCTATTATGT

AGTAAAATGATTTATTAATTTAAATTTTTTAAATTATTTATGGAAATAATTTGATTTAG

>c11875_g1_i1 len=266 path=[89:0-127 88:128-265]

AGGTAAAGCAACTCCACAGACAGGTGGCCACGTTGTAGTTCTTGTCCAGAAGCACAGCAC

CAGGACATACTGCCCCCACAGGGGAGCAGGGAACATCAGCGCTTACCCAACTCTAATCCC

ACAGGCATGGGGCTCTTCCAAGCATGTTCTTGGCACAACACACACTGCTCTGCAGTATGG

GCCATCTGGGCAGCAAGAGGGGTGTCCTGGCACACCCACCTTAAGCACAGCAGTTGCAGC

TCCTGTCAATCAAGGAATCTCTCATG

>c11877_g1_i1 len=264 path=[242:0-263]

AGTGGAGCTCTGGAGGCTGCACCCGTTCTTGTCCCAGGAAACAGAACCCCTCGGTGAGGC

TGAGCCGCTCCCACGGCCATCAGCTGCTCTGCTGCCATTCTGGGTTTTATTCTGCAAGGT

GTGCTGTGTGCGCCAACAGAATCCTCAGTGCCATCCCCTGGGTTACAGTGGTGCTTCCTT

CCATGACGAGCACAGCAGGAGTGGAATGTCCTCATCCTCTGAATTTCTGTGGGCTCTTCA

GATACAACTTTTGCTTCTTTTGCT

>c11879_g1_i1 len=261 path=[239:0-260]

GAGAAAAAGGAAAACAAATTAGACAGTGATTAGACAACACTTAACTCCGAAGGTTAACAA

AAGTAACTCAGTTCAATGAGTATATCATATGTCCACTGATGGAAACATTTGACACACAGA

CACAAACTTGAATCATTTCTCTTTAGTGCTAATGAGACTCCAGAGTAACAGATCCAGGAG

CAAATTCATAAAATCAATGGGTTTTTTTCTGTAGATTACAGCTGTGGTTCTCCTTTAAAG

AAGGCATTTCCCTGCCCTATT

>c11882_g1_i1 len=277 path=[243:0-276]

AAAAAAAATTATATTGAAGTGAATTGTGATTCAATAGAGTATTTTCAAATTAAGTACAAC

AATATTATTTTTTAAAGTCTCAAATTACTTTAATTCTTAAGTATTCTTGTATAATAAAAT

AAGTTAAAGAATATTTAACCTAATAAAACTCATATAGAATAAATTCATTTACTTACCAGT

TTTAATTAAATAATTTTATAAATTTACATAAAAATAAAGATACTATTTAAAATTACAATT

TAATTTTCCTCTCTATATTTAAAAAAAAAGAAAAAAA

>c11894_g1_i1 len=255 path=[233:0-254]

GACTCAGGGAATTTTCACAGGCGGCACATGGAAATAGGCTTCCTTGCAAGTATCCCCTTA

TATGTATTTTCAGGTCCATATACCTTTTGTATTCAGCCTCCTGATGATCTGACTATCATA

TTTCCAAGCTGTTACTGAGCAGCATACCTTGGATTGTAACCGAGCAGGACTCCTCTTTGG

TTTTAGTCCAGTGTCTGACTGTGGCGAAGGTACTTTGGGTAAGTCACTCCCAAGATGGTC

ACAAATACCAGTGTG

>c11906_g1_i1 len=221 path=[302:0-116 418:117-220]

GACATCTAAGCTACCAGAGTACAGTGGAAGATCTTTTCTGTTGCCTAAACACCATCTTGA

TACTGAACTTAAATAAGAAACTGAACAAGAAAGGATCATCAATACTTTGAAATTTCCTGT

GTATCTTAAGAAACTACCTTTTTTTCCTTCTTTTTTTCTCACTCCCTTAATCAGAGATCT

ATTGTTGGTGCTGAGTGACCTGTATAAAGTTGGAGTAGTAT

>c11906_g1_i2 len=221 path=[302:0-116 25:117-220]

GACATCTAAGCTACCAGAGTACAGTGGAAGATCTTTTCTGTTGCCTAAACACCATCTTGA

TACTGAACTTAAATAAGAAACTGAACAAGAAAGGATCATCAATACTTTGAAATTTCCAAG

AAAAAAAGCAAGAATTGGGATTGAGTTGATCAAGAATCAAATACTGGACAATGTGGATTT

CTTCTTAGGACTGGGAATACTACTCCAACTTTATACAGGTC

>c11906_g2_i1 len=208 path=[601:0-207]

GTGGGAGCTTTGACATCAAACTAAATAGAAATGTCCTCTTGAAATTATTTTCTTCCCTGC

TGATCTCCACTGGGAAGTTTATAATGGGGCTTGAGTGAAAGCTGATGCCCTGCATTTGTT

ATATTCCCAGACTCTCAGCTGCATCAATACTCCCATCAATACACACACTTTGACAGAAAC

TGAGACAGACAGTGACTAATCTGATTGC

>c11908_g1_i1 len=285 path=[105:0-235 104:236-284]

AATTCACTTTTTCTTTGCAGTGGTAGCTTCAGTGACTTCTTCCCTGAGCATTTTATCCTG

TACAGTGATAAAACATGACCTGCATGTATGGCCAAACCATCTCTAAGTTACAATATTCAT

ATGATGAACAGCAATAACAAAGATCACCAGATAGCAATAGCCTCTAAGAACTGCAAAACA

GAAGTCTGTCAAGGGAATGCTATTAACTTGGGCACCTGCAGGCAAATTACACACACACAA

AAGCACTTTTGTCCAACAATTTTCCATTTTCTCACTTCCCTTCAG

>c11911_g1_i1 len=247 path=[291:0-246]

GTTCTCACAGTCTTTCAGGCTGATGGTAAGTGAGCAGATCTGATGTCCCTTGAGGATGTG

GTGGGGCATGCCTTGTGTTTTGTTTTGCACCTTGGCTTTGCCAAGGGGAAATTGGGTGTC

TTGCCATAAAATGACTGTACCCCTGTGGATTCAGGGCCCTTCTTTGACACTCTGCTTCTG

AAGAAACATGAGATTTTCTCACTCCTGTTTGCCATGGCACAGCTTTGCTTTGGTCACCTC

TTGACCA

>c11914_g1_i1 len=338 path=[1:0-337]

AAAAAAAAAAAAAAAAAACGAAATAAGAAACTTTACATAATATATAATCTTAAAAAGTTA

AAGATATAAATTAAAACAAAAAATATAATAAAAAAAAATTTTTAGTATGCTTTCTTTTTA

TCAATAGTGGTATAATAAATAGAAATGAGTAATCCCTTTTATGTAATTAGGCCCACCATA

AAAGTTAAAGCAATATAGAATTGAAAGATCTGAATATTAAACATACATATATTTCATTTT

TCATTATACTATTTACGTTTTATTACAAACTAGAAAAAAAATCTCTTTTTTTTTTCTTTG

AAAGAAATAAAAGAATAAAAAAATATATATATTTTTTT

>c11914_g2_i1 len=429 path=[631:0-428]

TATATTTAAGAAAGAATAAAAAAAGTTTCACAAAATCATATATTTTTTTTAGTAAATAAA

AAAATTTATTAATTATAAAACATATAGAATTTTTAGTAATAAAAAATTCGTTATCTTTTT

TATCATTTGTTCAGTTAAGCATTCTATAAATATTTAACAAAAAATTTTATAAAGATCATG

CAAAAAAATATTTATTACCTTTGTTAAAGTAAATAATATTTAATGACAAATTCCAAACTT

AAATTATTTAAATGATTAATAATTTTAAATATGTAGTTTTAACATGCTTATCTTTCTCAA

TGTGAAAAGTATAAGAATAAATATAATTAGTTTGTTAAACATATTATATCCTCATTTTTA

TTAATTTATATGTTATTTGCATAAGATATAGTATTCATATTTCTATTTTATATAAAAAAT

AACTTTCTC

>c11914_g3_i1 len=452 path=[1097:0-451]

AAAAAAATATATATATTTTTTTTGAGAAAGTTATTTTTTATATAAAATAGAAATATGAAT

ACTATATCTTATGCAAATAACATATAAATTAATAAAAATGAGGATATAATATGTTTAACA

AACTAATTATATTTATTCTTATACTTTTCACATTGAGAAAGATAAGCATGTTAAAACTAC

ATATTTAAAATTATTAATCATTTAAATAATTTAAGTTTGGAATTTGTCATTAAATATTAT

TTACTTTAACAAAGGTAATAAATATTTTTTTGCATGATCTTTATAAAATTTTTTGTTAAA

TATTTATAGAATGCTTAACTGAACAAATGATAAAAAAGATAACGAATTTTTTATTACTAA

AAATTCTATATGTTTTATAATTAATAAATTTTTTTATTTACTAAAAAAAATATATGATTT

TGTGAAACTTTTTTTATTCTTTCTTAAATATA

>c11915_g1_i1 len=221 path=[343:0-220]

GCTGAGCCACCAGCTGGGTTAAACCAGAACAATGGCAATGGGCTGGGGACCCTCAGAGTC

AGTCGTGTTGTTGGACAGCACGTCTGGAATTTTGTTTGTTTCTTATTATACCTGCATCTC

TTTTGTCTGTAGGATCACTCACTAGTAATTGCCCAACAGTAATGACTTTAGGTATTGATC

CTGTTGCTGGGAGTTGTGGTATCCTGTGGCTTAGTTACCTA

>c11918_g1_i1 len=473 path=[240:0-472]

AAAAAAAAAAAAAAATGAAACCAAAAAAGAGTTTAAAAACATGTTATATGAATTATTATA

TATGTTATAGATTCATAATTAGAAATTAAAGCAATGTTAATTAAAATACAATAAATTCAT

ATTAGTATGATAAATTATTAAAAAAAAAAATTTTTATATATTGTAGCATTTTTTCATTAA

TTAAAGTTAAGTTTTACGATCCCAAAAAAAAAAATAGAAAAAATTTTCAGGATGCGTTAT

TTTGTTAGTAAAAATTTATAATATATTTATACTATAAAAAAAGGAATAATTTGGACTAAC

ATATTTAACTTTTTAACATAAAAAAAATGTAAATAAAATATAAACCATCAGCTTCAACAT

TATTTTTTTTTTCCATTATATTTTTAATTTTTCATTTATCTTTGTTCATATTTTTATTTT

TTCATAATTTTACCTATTAAATATATTTATTTCTCCAAAAAAAAAAAAAAAAA

>c11921_g1_i1 len=371 path=[557:0-370]

AGTCCTTGGGGTTGCTTACTTCTTCTCAACACTCCTTGTGTCCCAGACATTGCTGTGGAA

TGCAAGGACAGTCAGAAGACTGCACACACAGATTGGGAAGACAAAGCAGAACATCTTGAT

GCAGTCACATTTTAGGTGTTCCATTGCTTCTTTTGCCTTGCTAAAGCAGACCAGCCTTAA

AACATGTCTCACCACAGCCAAGGCTTTCACTCAGCTGCTCCCCAAAACAAGCACATCTTG

CACTGGCAACGGTGAGTACATCCACTGCAAAGACAGTCTCAGTAATGAAAAAAAGCACCC

TCAACATGAAAAGCTGTAGGAGAGCAGGCTTCGTTAATTCTGTTCTTTGGGCATCCATTT

GCTTTAAAGCA

>c11923_g1_i1 len=203 path=[1:0-150 152:151-202]

GTGATTTTTACCATTTTAAAAGGAAAACACAGGAACTAATTTGTTATCTTTTTAGCTATG

TTAAAAGAAATTTTTCTGATGGGGGCTACTGATGTAATGAAAAAAGATATTGTCATTATT

TTGTAACACGAGCAAATTTTTTTATTATCTTATTTTTGTGAAATCCTTAGCTCTGATCAT

TTTGATTTATCAGTACATAGATA

>c11937_g1_i1 len=225 path=[203:0-224]

TGTTTTTCTGATCTTCAGCCTTGGTTTGGTGGCTGAGTGTGAAAGTGCTTTTTTCAAGCC

TTTTTATAAAAGCTATCTTCGAGGCAGAGAAGTATGTTATTTAGGAGCAGATCCATAAGA

TGCATTGTTCTCATTCGTGGGATTGACTTCAAAGAGTCCTAATCTTCCCTCATCTCAGCT

GCTCATAAATGTTGCTGGTCACTTTTATGTTATGTGGGAAGCTTG

>c11952_g1_i1 len=220 path=[1:0-219]

ATTTGCATTTTTTTTTTTTTTTCTTCAACATACTGTGTAATTACATTTAAATTTCCAATA

AAATGTTTTGTTCTATTTTTTAATTTTTCATATGTTGGTTTTAGGCATTATTTTTTATCT

TAATTATTTTTTTTTTAAAATAAAATTATTTTAGTTATTTTTACTAAAATTACATATACT

ATAAAAAAAAAAAAATGTTAAAATACAAGAAAAATTAACA

>c11952_g2_i1 len=272 path=[656:0-271]

TAGTGAAAAAAAAAAAAAAAAAGATCTAGAAAAAAAAAAAAAAAATGCAAATAGTAAAAT

TAATAACATGTTGCAATAAAAAATATATTTTTCATATATGAATATCTATAGTAATATAAA

GGTATTTTTAAAAACTATTTTAAAAAAATAAAGTTTAACTATATAAAAATAAATAAATAA

ACAACGCAAATATATCAAAATATTTGTTTATAAATATAAAAAAGAAAAGTAATATATTAA

TAAATATACTATTATTTTAATATATAGATTTA

>c11955_g1_i1 len=209 path=[199:0-185 487:186-208]

AAGGCTCTACACCTAACAACCAAAACGCCTTTATTTCACACACAAAACAGGTAGTTATTA

AAGGCTGTAGAAACAGAAATAAAGTGTTAGCTGTGTTTTCTGTATAGCTCCCAGCAGCCC

CTGATGGTGGCACTTCATCCTTTGGATCTGCTGTAAAGGTCAGCAGCGTGGCTGGGCTCA

GCAGTGCCAGGGGTGAGCACCCCTGGCAC

>c11983_g1_i1 len=297 path=[275:0-84 360:85-296]

AACAACTAGGGAAACATTAGGAAGACTTGTTCTAACAAGGTTCTGTTTAAATTCTTTTTG

ATGCCTACAATGTGCCCTAACTTATAGCCTGCTCTGTCTTCCTCCTTAAATGTCTTCCAT

GCTAACAGGCTTTTGTGTCTTGTGTCTCTGGTCTAACATGGATAATATTATAACAAAATT

GCACTAAATGTTTCCTGAGCTGTGAAGATCACCCTAGTTGTAGTGACATTAAATATTAGC

AACAAGAGCAGGACGGAAATTTTGCTGAGCAACAGCATATCCCTTAAGTAATGCTAT

>c11984_g1_i1 len=291 path=[269:0-290]

GATCTCACCTTCCATCACATTTATAACAGCTTCAGGAATGGAGTTCACAGAATATCTACA

TGAGTCAAATCACTGAGGGGCAGGGAAGGATGGATGTGGGGAAGGCATTCACAAGACATG

GAGGCTCAAGAGTTTGCGAGAGTTAGATGGTTCAGGCATCTGTCCTTTGTCAATTCTAGA

ACAGCATAGACTATAAAAACTTTTATTTGGCAAGTGCTGAATATGCATACAAAATGCAGA

ACATGCTATACAATTTAGAACTTTAATAGTGTTCTCTGGACATCAGGCATT

>c11993_g1_i1 len=240 path=[155:0-239]

GTGACAGATATGAAATGAAGAAAAGAAGAAAATAATTTCCGTTTTTCATCTGAAATCTCT

TCAGAGCACAAATTCAAACTGACAGAACAAAGATTAAAGAATACATTTCGCTGGAGAAGT

TATGCTTCCTCATAATTGTAACATCAAAATCTTTGACATCTGAGCCTACTTTATCTCATA

CAACTAAAACTGAGAAAAAAAGAGACACTCTCTAAGCCTGAGGTAAATAGATCGCAATAA

>c11996_g1_i1 len=257 path=[296:0-194 25:195-256]

AGAACTCCAGCCACTACAGAAGATGTGATAACGAACAGTCTCGCTATGGGTGTGAGCAGA

TCAGCCTGGGAACCACAGGCTTTATCATGCAACATGAAGAAAACACACCTACAAAGGTGC

TACTGAGCTGTAACTTTAAAACTCTGGACTAGCAACCACCTTGTAGCAACTAACTGTAAG

GCCCCTTTCGCCTGCAGCACTTTTGTGCCAGTAGTTGTAGTTGGTTTTGGAAAAAGCCAA

TCTTCTAATCCTTTCCT

>c11996_g1_i2 len=257 path=[296:0-194 490:195-256]

AGAACTCCAGCCACTACAGAAGATGTGATAACGAACAGTCTCGCTATGGGTGTGAGCAGA

TCAGCCTGGGAACCACAGGCTTTATCATGCAACATGAAGAAAACACACCTACAAAGGTGC

TACTGAGCTGTAACTTTAAAACTCTGGACTAGCAACCACCTTGTAGCAACTAACTGTAAG

GCCCCTTTCGCCTGCTGCTCAGCGTGGCCATACTTTAGCAATTTATGGCAAAAGGAAAGG

ATTAGAAGATTGGCTTT

>c11998_g1_i1 len=317 path=[447:0-159 153:160-316]

CCTGTGGTCTTACCTGGAGCTTATGTTTTTGGAAGTACTGCCTCAGCCACTATTCAAAAG

TGAGCAGCTCCACTAAGAAAACCATAGGGAAAGCAACTTCTCAGTCTCATCAGTTTTGTT

CTAACAAAACCTTTAGAACCTTCTGCTCCCTCTCCCACCCCTCTTCCAAATAGTGACCAG

TGCTTTGGGGAAAGTGTGTCCACACAGAAACCTGAGGCACAGCACAAGCATGCTGGGAGA

AACCTGGGCCAGGCCTCTGACCCAGCCTGCAGTGGTTCTTCACTCACCAGCTTAAGAAAC

CACACTCAAACACAAAG

>c12003_g1_i1 len=233 path=[124:0-123 344:124-232]

GATGTCCCCAATATCATCCATACCAGATAATTCAGTTTTAAAAAACTCCTCATCCTGCTG

AATACACTTTAGATCTTCAAAGTCAATACCCAGATTTTTCATGATGCTGTACAAATCATT

TGTTTTGTTATCTTGAAAGAGCGCTGCACTGTCTTCAGGAAGTTGCCCTTCATGTTTGCC

ACTGGTGAGGCTGTGTTATATGAGCTAACTTTCCCTATGTCCAGCTTATCGGA

>c12006_g1_i1 len=296 path=[313:0-19 20:20-295]

TTTTTTTTTTTTTTCTTTATTATGAAGAATCAATTATAATAACAATTGAATTTTGCATAT

TCAATTTTTAAATAACATGTTTTAATTAGAAAATTTCAATTTTTTAATATATCTCAAAAA

ATATTTTAAATTATATTTTTTAATAACCTTAATTTTTCAGTGTTACATTTTTTTATAATT

TTTATGATTCTAATATTCTTATTTGAATATAGAATATATTTAGATTAATATTCTTTTTTT

AAAAAGACAACTGTTTAAATAATTTGATATACATTTACTTTTTATATTAAAAAAAA

>c12012_g1_i1 len=276 path=[1:0-51 53:52-163 53:164-275]

AAATGGCTGCTAATCGGAAAAGCAGAAATGGGGCTATGATCCCACCTAGGTCACACAAAC

TTGAGCACAGGGAAACACCAAAGTTTCTCAGTGTTGTTGGGTACAACTCAGAGTTCACTA

TGGTAAATGTTGGAAGAAAAGGAAACACCAGAACACCAACTCAAACACAAACTTGAGCAC

AGGGAAACACCAAAGTTTCTCAGTGTTGTTGGGTACAACTCAGAGTTCACTATGGTAAAT

GTTGGAAGAAAAGGAAACACCAGAACACCAACTCAA

>c12032_g1_i1 len=458 path=[1:0-457]

AAAAAAAAAAAAGAAAAAAAAAACGACCAAAAAAAACAAAACCAACCCAAAATATGGCTA

TTTACATTTAATCTTCGTTAAAAAAAGAAAAGAAACAACTGAGAGCCCAAACAATACAAC

AGTGAAACAGATGGACAACTCAGGGATCTACAAAGTGGTAACGTCCCCTCACGTGCTGCA

GCCCAGACAGGGCTCACAGCACAGCACACAGTATCCACGCCAGCACAGTGCTTCCCCACC

ACCGTGGCATGCAGCTGGGCAGGCAATGCCCCGCAACGCCGAGCAGGTGACTGCTGTGCT

GCAAAGCAGCGCTCTTCCTATGGAAACCTGCTGATCGGGGCTGCACCCGGCGCTGCAGCG

GGACCACCACCCCTGCACGTCCAGCAGCAGCCGTCAGCTCGGCCCCTCTCAACAGGATCT

TCCCTTCAAGCTTTGAGCCACAGCTGGAGGTTTGCTGG

>c12039_g1_i1 len=330 path=[460:0-329]

ACCCTCCAATCCAGCTCCACATCTGGCTGTGAGCCCACCCACTGTGACCCCACCCGTCAG

GTGGCCAGGGATGCTGCTAATTGCCACAGATGCAGACATCACTTCTGTTAAAAGTTAAGA

AGCTGCTCTTTTATGACCTCATTATGAATCAGTGAATCTAAACTGACTAAAATGAGCTAA

CAACTTTTGGGATATAGAAGGAACACAAAGGCTGGTTATCCCATAGCTAGTTCATTTGCT

TCTCAGAAAGTAGAAAGCCCTCTGCTCAACATCTATCCGATAGAGATGACAATTTTGATC

AACGTGTGGAATACACTTATAAACGTTCCA

>c12043_g1_i1 len=284 path=[1:0-283]

GGAAATAGTTACTGGTAAATCATATAGATTATTTGTTTTGAAGGCAGGTATATCTTTGAG

ATGGGAGCCGTGAAGTTCTTTGTATTGCAGGACAGAAATCTGAAGAAGTTGAAATTTTTT

TGTCTTGAAAATAGTGTTTAAAAATTGAACGATTTGAGCTGAAATTGAGTTCAGAACAAA

TGTCTGAGTAGGCAGCAACAAAAAGTGAATGGCCGTAGTGGTCATATTTTAAAGGGACTT

CAGTAGGGCGTTTGTACAGGATCCGTGTCCTTTCTGAAAAATTG

>c12049_g1_i1 len=231 path=[95:0-230]

TAAAATAAAATAAATTAAAAAAAAGTACAAAATTATATTTATTTCTTTGAAGATTTATGT

ATTTTATATCTTAAATTTTATAAAATGCATATGTATAAATGAAAAATATTCTATTCCATT

CCTCAATTTTTAAATGAACATTAAAAAAATTATTTTATTTTTCTTTTCATTTAATTTTTA

TAATTTGAAATTAATATTAATATAAATAATTTTCGTTTCTTAATAATTAAA

>c12050_g1_i1 len=326 path=[160:0-325]

TATATATAAAAGAAAAATAAAAAATAAGACTAAATTGAAGGGAACAATTTTGGGTAAAAT

TTATAAGAAAAATTAAAAGAAACAGAAAATGAAGAAGGTGCAGGAATTATAAAATATAAA

AAAAAAAAAGGAATTAAAGGAAAATACTATAAAAACATCTTTTTATAAAGTAGCATACAA

TAAGGAGAATATAAAGAGGGTTATTAATGTTAAGAAGGAAAAAAAGCTATATGTAGATAT

AAATGGATAAAAATTGTAAAGAAAATAATAAGAATGAACTGAAAAGAAAAAATAAAATAA

ACTAAATAAAAAAAAAAAAAAAAATA

>c12051_g1_i1 len=335 path=[155:0-327 529:328-334]

GATCCATTATCACACACACACACTCAAAATTAAATTGTTCACAAAAGTTTACTGAATTCA

AGCTTAGGGGAATAAAGAAGAGTAACTTTTGCTTAGAAGGTATGGGTTTTCAAAAAAAGA

AGGAAACAAAAAAAAGAAGGAAAGAAGCCCCCAAACCTCCACATACTGCAAGAAAGGAAA

ATATGTTACATCAAATCCTGGAAATGAAAGCAAAAATGCCAGCAAGATGAATAATCGGAT

TGCTCCTCGCTGGAACCAGCCCTTGCATTTTGCTGCCTCTTTGCACGAACCCCCGAACAA

ACTGCATAGGAAAGTCAGTGCTTTTATCTGATTTT

>c12054_g1_i1 len=290 path=[268:0-238 507:239-289]

AGCTGAGTAAATGGGGCAGAAACTGAAATGCTTGCTTTCTGTTTCTGCCCCATTTACTCA

GCTGGGATGTATTAAGTTTCCCTTCAGTGTTTTCCAGTTCTATTATTTTATCTCAAGTAA

TCTTCAAATCGCAATATAATAAGTTGCTTATATCTTCAGCCAAGTCATGTCAGCATAATA

TTCTTTACTGACCGATAAAATAGAAGTCAACAAACCTGTGAGTTATCACAGAATGATTTA

GCTTAAAAGGGACATTAAAGATCATCCATTTCCAAAGTGCTGCCCATGGG

>c12061_g1_i1 len=306 path=[155:0-305]

GCATCATTTTATTAACCACTGGATCTGTACAGACAGTCACCCAAACACTCCTCGTATGTG

GCCAGCCCTGTCCACAGTGCACCTTCAGGAGCACAGGGGAGGCAGAAAAGAGGAGAGAAA

ATCAGAAACACTGAAGAGTATGAGAAAAGGGGATTAATTAAATGATGCTGATAATTTTTA

TTTTAACCCAAAGGTCTCTTCTATCCTCTGTGCTCTGATCAGCGGCCTCTTTACTGCGTT

TCCTGAGGAATATGCTCTCCATGTGGTTGGGTTTTTTTGCCTCTGTTATTTTCAGGGATG

CCTGAA

>c12062_g1_i1 len=1683 path=[2626:0-1682]

GTGTTTGATTTTTTTTTTTTTCGAGTTTGTAAAATATTTAACAGAGCAGTTTCCCAGCAG

ACTCCTGACCAGAATTGTAACCAGACTTCTAAAAAGTATATATATTTTTTAGATCTGAAA

ATTTGTCTTTATTTTTTACAAAATGGATTCCAGGAAGTTTAAAAGGCAATATCATAGAGG

ACTTAGGCGCAGAAATCAGGTGTAGTCATTCAAAGCATTCAAGTAGGAATATGGTAATAT

AAATATTTGAAAAGCATCTTTTTTTTTTCTCATAAATGTTCAAAACAAAATGTATAACTC

ATTCCAAACTAAAAATTGAGCTTTATAGCACGTATTTCCCTGAACAGAAAAAATAAGTAA

CTTTTGAAATACTTCTAAAGGCCTGAAAGCTTTTTCCTTGGTTAGTTACATTAGAAGACT

TGCCTTTGATTAAATTACTTCCTTTCCAAATATGGAAAGTATTACATCAGTCCACTGTTA

AAACAGACAATACGTTGCAAATTAATTCTGGTATAGTATGTTCCAACAAAGCCTAGTGTA

TAAAGGTGCTGAGGTGGCTTTGAGTTTAGTAGTCATCGCCTCGGCTGACAACCTCTTTGC

ACGTTGGGCGCAACAGTATGGTGTGCTCAAACTGTGCTGTGTACGAGCCTTTGATGTCGC

ACAGGGGAGGATAGGGATCCACGATGCCCAGGTCGCACAGGTTCTTCAATGCCATCAGGT

ATTTGCTCTCCCCCAGGCGATCCAGCCATCTGCGGCAGAAGGCAAGAGTACCGAAGTTTT

CATTGATGACGTTTAGCAAGTGTTTCGCCCTCGGAAGCCTTATCGGCACATGCCCCACAT

CGAAGTTCTTCATATAATGAGAACACTCCATATCATCGTGAACAACGCCTTTTCCTGTGC

TACCGAAGGTTTCTATAGCATACACTTCACCTTCCTCCATTCTCGTGGCTTCTCCTCCTT

TCACAATGGGCACAGTTTTTCCAGCATGTATCCTGTATGGCCCGATTGAGTGTCCATTCA

AATTGCGAATTGGTTTCACTTGGTATGTTTTGCCATCAATTTCAACTTCGTAAGACTCCA

TAACTTCTTGTATGGCCTCCCCCACATCACAGAGACGTACGTCTATTCCAGCACACTTTA

TTCCCGTATTAGTGGCATCCTTTACAGCTTCTAATAGCCTGTCATATTTCGGATTGAACG

TGACTGTAAAAGCACAGTCAATAATACGACCACTAATATGTGTCCCAAAATCTATTTTGC

AAATATCATCATAATGCAGCACTGTTGGATCTCCGGCATTGGGAGTGTAGTGGGCAGCAC

AATTATTCAGAGAGCACCCGGTAGGAAATGCAAGGCCTGCGTTTAAGCCATTTTCCTTTA

TCAGCTTACGTGAGCAGTCTTCTAGTTTTTCACAGATTTCTATCATTGTCATCCCAGGTT

TTATCCAGCTCATAACGTATTTCCTCACTTGTCTGTGTGCCTCTGCAGCCTCCCGAAAAT

CGTTCCAGATCTCCTCACTAGCTTGGTCTAGTGCTTTCTTCTCTTCACTAGTTATTCTCC

AAGCTGCAGTCCGCCCATCTTGCGTTGGTGGATATTGACATTCTTCTCCCTTCGGGTATA

TGTTGCTGGGAAATAGATCACATATTGGAATAGAAGGTGGATCTGTCTGAACCTTAGGTC

CTT

>c12062_g2_i1 len=882 path=[4352:0-881]

CTTCTTCTTCTTCTTCTTCATTTTCATTTTCTTCCTTCTCTTTTTTGTCTTCTTCATCTT

CTTCCTTCTCTTCATTTTTATTTTTTTCATCAATACTTCTCTTAATGTTTTTTTGAATTT

CCCTATTGTCTTTTTCAATAACTTTGTTACTTTCTTCAATATTTTCAAAATTCTCCTTCT

CATTTGAATTTATATTCAATAATTTCATTTTATAATCATTTTTTAAATTATCATTTTTAT

TTGGTTCTCTTATCACATTATTGTTATCAACATTACCTATTTTTGATTCACTATAATCTT

TCTCTTCATTCTCTTTTTTTTCCATCTCATCATCATCGCTGTAATCTTCATCTATAATTT

TTTTACTTTTTTTATTAAACTTATAATTATCTGAAATACTTTGGTCAATTATCTTATTTT

TAGAAAATACTTCTAAATTTTCTTGGCTATATATATTTGTTGCTTTATCATTTTCTTCCT

CACCATTTCCTTTGAATTTTTTTTTTAATCTTTTAAAATTTTTGTTTAAATTTTCATTTT

TTCTTTTTAAATTAATCGAATCTTCATTTTTAAGGTTTTCGTCAATATATTTTTCATTAC

TGTTTTTTTCTTGTTCTGGGATATCCATTTTTTTTCATTACTTTTTTCTAGTAACTTGAA

TAAATTATTAATTTTTTATTTTTCATATTAATTTAATAATATTAATGTATAATTATATAT

AAAAAGAAATTTTAATTAATTCATTTAAATTTTTTTTTATTATTATTTTACTATATTATA

TTAAATGGCACTCTTTGAAGAAATTCATTCAATAAATTGAAAAAAAAAAATTTTTGTTGA

ATTAAGAATAATGCATTTCATTAAAAAAAAAATTCAAAGGAA

>c12062_g3_i1 len=675 path=[43:0-674]

TTCCTTTGAATTTTTTTTTTAATGAAATGCATTATTCTTAATTCAACAAAAATTTTTTTT

TTTCAATTTATTGAATGAATTTCTTCAAAGAGTGCCATTTAATATAATATAGTAAAATAA

TAATAAAAAAAAATTTAAATGAATTAATTAAAATTTCTTTTTATATATAATTATACATTA

ATATTATTAAATTAATATGAAAAATAAAAAATTAATAATTTATTCAAGTTACTAGAAAAA

AGTAATGAAAAAAAATGGATATCCCAGAACAAGAAAAAAACAGTAATGAAAAATATATTG

ACGAAAACCTTAAAAATGAAGATTCGATTAATTTAAAAAGAAAAAATGAAAATTTAAACA

AAAATTTTAAAAGATTAAAAAAAAAATTCAAAGGAAATGGTGAGGAAGAAAATGATAAAG

CAACAAATATATATAGCCAAGAAAATTTAGAAGTATTTTCTAAAAATAAGATAATTGACC

AAAGTATTTCAGATAATTATAAGTTTAATAAAAAAAGTAAAAAAATTATAGATGAAGATT

ACAGCGATGATGATGAGATGGAAAAAAAAGAGAATGAAGAGAAAGATTATAGTGAATCAA

AAATAGGTAATGTTGATAACAATAATGTGATAAGAGAACCAAATAAAAATGATAATTTAA

AAAATGATTATAAAA

>c12065_g1_i1 len=211 path=[255:0-138 62:139-145 406:146-210]

TCCTTTTCCGTTTCCGTTTCATTTATTTTTTTTTTTCTTTAACAAATTCTTTAGTATATT

ATATTTTGTTTATGAATTTTTATTTTTTGTTATTTTATTTTTTTTTTAAGTTTAAATTTG

TTTTATTTTAAGAAAACCTAATTTTCTATAAAAAAAAAAAAAACGTAAAATTTAATATTT

GATTTAAATACATTCTATTAATATTTTTAAA

>c12075_g1_i1 len=202 path=[26:0-96 123:97-166 193:167-201]

GCCCCAAATTACTTAGTCACACTTGTATCTGAAACTAGTACCATGACTGAACACAGCAAG

TGTGCATTAAACTATTAAACATTGAGTATTATGTTTAGTCAAATTAACGTGCGTTGTAAG

AGCTTGTTGCATTGTGACAGCTGAGGTTTTCAGATCTGCCTGTGGCAGCAGACAAACTTT

AGAATCATAGAATGGCCTGGGT

>c12075_g1_i2 len=234 path=[536:0-128 123:129-198 193:199-233]

ACTAAGTAATTTGGGGCTGCAAATAAGATGTTTTCTAAGTGGGCTGTAAGAGCAAGGCAT

TCTCCATGGAGCTTCCAAAACCTCTCAGAAAAGCCTTCAGCTCTCAGGACATTCAGTGTG

GGTTGTTTTGTCAAATTAACGTGCGTTGTAAGAGCTTGTTGCATTGTGACAGCTGAGGTT

TTCAGATCTGCCTGTGGCAGCAGACAAACTTTAGAATCATAGAATGGCCTGGGT

>c12086_g1_i1 len=233 path=[1:0-232]

AAAGTAGTTCCTTAGAAGCTTTTGTAACACAAATCAATGGAACATTCTTCAGATTCGGAA

CTAGGGGGAGCTGTACCTCCAGATGGAACTAGTGTGCCCTTCCAACATCTGAACAAAGTT

TGCTCTCAGGAACAGCTAACTTTAACAGATTCACTCAATAATTCAGTTTTATTTTATTTA

TATAGATGGAGCTTTCTGATACCCTAAGCCTACCTTCACTTGTTTATCAAGAA

>c12098_g1_i1 len=400 path=[55:0-92 452:93-399]

TTTTTTTTTTTATTTTATTATTTTGTAATCAATAATAAAATTTAATTTATAAGAATATAT

ATGTTATGGATTATATTAAAAGTATTTAAATAAGGGGGGAAAAAAAAAATGAGATTTAAT

GAATAAAACTCTTTATATAGAAATACATAAAAAATTTAAGTATATCAATGCACAAAATGA

AACTAAAAAAGAAGAAATAATAATATGAAAGAACGATATATATATATATATACAAAAAGA

GTTTAAAATAATTAAAATGATTTTTATTATATATTTTTACAAAAATGTAATTTTGTTTTT

ATTTTTATATATTTTTTACTATATTTTTAGTATATATTTATTTGTTTTGTCAGTTTATTT

AATTTGAATAAATATTTTAACTCATTATTATATATATATA

>c12098_g1_i2 len=326 path=[450:0-18 452:19-325]

AGGAGAAATTTAGAGGAGAGGGGGGAAAAAAAAAATGAGATTTAATGAATAAAACTCTTT

ATATAGAAATACATAAAAAATTTAAGTATATCAATGCACAAAATGAAACTAAAAAAGAAG

AAATAATAATATGAAAGAACGATATATATATATATATACAAAAAGAGTTTAAAATAATTA

AAATGATTTTTATTATATATTTTTACAAAAATGTAATTTTGTTTTTATTTTTATATATTT

TTTACTATATTTTTAGTATATATTTATTTGTTTTGTCAGTTTATTTAATTTGAATAAATA

TTTTAACTCATTATTATATATATATA

>c12099_g1_i1 len=219 path=[477:0-162 48:163-218]

ATATAAATTTTTTTTTTTATGTTATTTTCAATATACAAGTTTTCCAGTTTTCGTCTTTTT

TTTCTTTAGGTATAAAAATATACTAAATTATATCCATTTTTTTATACACTTAAGTATTTC

ATATTAATATTTAACAATACAAAATATTCGATGAAATATGTTTAGAATTTATGGAGAAAA

ATATTTTCCTTTTAATATAAATTATAAACATATTTCATC

>c12099_g2_i1 len=298 path=[49:0-241 48:242-297]

AAAATTTTCTCTATTTTTGGGTGTGTAAGAGGCATAACTGCAATTAATGAAATTAAAAAT

TTTATAAATATATGAAAAAAAAGAGAATATAATTTTATTTATTTAGAACTTTAATTCTCA

TTAATACTTTTATGTTTTGATATTTTTTATGCTATATTAGTACACTTTTTTTTGTTTTAA

ATACAAATTGCTGATTAATTAAATAACTTTTTCTATATGAAGACTACTAAAATCACATAA

AAAGAATTTATGGAGAAAAATATTTTCCTTTTAATATAAATTATAAACATATTTCATC

>c12101_g1_i1 len=250 path=[1:0-249]

GTCCCTTCCAACTCAAATGATCCTACAGTTCTACGATCCCAACCCCAGGGTGTGCCCGGC

TATGGGAAGTGCAGCAGACAAGCCTGAATGGAGTGGCCTCCACAAAATCTGGTACTGCAG

GTGCGTCGTTATTATGGCCATTTTCCTTAAACTGTCTTCGGAGTGCAGGATGGCGTCCTC

TTGCATCACCATGTTCCTGGCGGCAAAATGGCGGCGGGGGGCGCGGCGGAGGCCCAGGCG

CGCTTCGGGC

>c12101_g2_i1 len=208 path=[455:0-207]

GACACACGTCTGAGCCCCAGGCCCAGGAGGAAAAGGATGGCTCAGTGCCAGCGGAGATGC

CCGATGCCTTCAGCTAGGCTTCATTTCAGCAAGTGACTCAGCTGGTCCCGCAGGTTTGAT

GACTGCTCCACGTTCTTCTGAATCGCTTCTTGCCCCATAACAGCCTCGTCCATATCAGTG

GATGTGGTGGGGCTGGAGGGTACTGAAG

>c12104_g1_i1 len=207 path=[339:0-206]

TACTCATTCTGAGTCAGGCAGTCAGATATTTCACCAGGAATTGTGTCCAAGATGTGCATT

TGGTTATTTGCTCTTTGTTATAGCTAACTTATTGGGTTATGTAAATATGAATACCTCTAA

AGAGAAACATGTTCAGAAACCTGAAATTATTTGTAACCAAGTTTTCTTAGCATCTCAGAT

GTTTATCAGCCATCTATTATTTAGTAA

>c12110_g1_i1 len=288 path=[89:0-236 340:237-287]

ATCCTGGTTAATGGTGAAAATGATGGCACCCTGGTGTGTCTGAAATGGAAGAAGATAAAG

GAAATTGAAGTTAAAGAAGCTGATTTTCACTGGCATTCTCTTCTTACTATACTGAACAAG

TCTATTTCGGATGAAAATGCTGTTTTAAGATTGATGGCAGAATGGCAGCTTGAGTCAGAA

TCCACATCGGAGTCGCTTCCAGAAGAGAAATCTAAGAAGTCACCTCTTGAGCCTTCCAGT

GCAGCAGTGACAGAAGATGGAAGCTTTACAAGTTTGCATTCAGACAGT

>c12115_g1_i1 len=244 path=[1:0-243]

GGGTGCGAGATGCTAAGTGACCTGGCTTATGGAACTAAGTCTATAGCAAAATTCAGGTAC

CCTAGCATCACAAAACAGTCTTCTGAATAAAGAAAACTACTCTATACTGCTCTTTTGTAC

TTTGGGTTTGGCATGCTATACTGACATTTGGTTGTGTGCATTTTTCCTGAGTGTGCTTTG

GCCCCCTTTTAACCAGGTTTCCACACCCCTCCAAGGGCTTCTTCTACTTCTACAGTTTCC

ATGG

>c12119_g1_i1 len=226 path=[204:0-225]

GTGTCCTCTGCACTGATTCTGTGACTTGATGGAAATGCTCAGCTAACCAAAAGGAGATTG

GTCTCCAAGCTGGTAATACATCAGGGTACAGGAATCATCATAAACCTTTAGGTTTGCTGT

TCATGGCTTTGAGAAATAGTGCAGTGAGGGATGCCCAAAATCCTAAAATATCTGACTGCC

ATACACTATTTTCCCCAAAGCCCTCGAGTTTCCTTCTACCAGGTAT

>c12174_g1_i1 len=212 path=[155:0-211]

GACTGCTGTGTTCTCCAGGGTTCTGCACTGTCCAGCTGGAAGTGAGCCAGAGTGGGGAAC

AGGTTGCCACGGCAGCACTGGCCTGGGTGCTGAGCACACTTCTCTACTGAGCTGTTGGCT

GAGTGCCTTCTACCTGCAGACCTTTTGCAGCAGTCTCTTGGAAAAGAGGTGCTACTGCTG

AAGAGAGGCAAGAGTTCTGTCCCTCTCTCTTT

>c12177_g1_i1 len=201 path=[179:0-200]

GATATATGCAAGTCTCCCCACCTAAGAGATGTTAATGAAACTGTCTGCCTCTTGTCTCCT

TGGGAGGAGGAAAGGGAGATCAAGAGGAAACAGTGAGCACCATAAAGAGCTGGAATAACA

CTTGAAACGGTGAAGCAGAAGGCAGTAAGCGAGGAGACCTCTGATGCAGAAATCAATCAT

TAAGTTAAAAGTTGTGCTGGC

>c12183_g1_i1 len=224 path=[429:0-200 651:201-223]

ATTCTTTGGTCATTCTGGCAACTCACGAGCTGCCAGCCTTCACTTCCCCACCTGTGAAAC

CATGAGTGATTGTGTTTCATAGGATGATTGTCCAACAACTATGGAAGAGAAAATGCATGT

CTTTAAAGGGAAGAGGCAACCCTGTACTCAGCCATTGCTATGCAAGGTATATATTCACTC

CTCCATAATGCTGAGTTTCTGTGACCACCTGGTGATCTGCACAG

>c12186_g1_i1 len=228 path=[1:0-227]

GAAAAAATATTGATGGTAAACATGTTTATCCCCTTTGGCTCCACGTTTACCATCACTGAA

ATAAAATTAAAAACTGGAACAATCAAACAGCATTCTGTTTATTTTTTTTCAATTGGATTT

TAAGGAAGTGGACCTACATAATGGGTTCTTATTTACAAGGCTGTGTGTTTGCACGTGTAA

GCATATTATTTTTAAGTTTAAAGAAAGTCAAACACAAAGCCTTGTAAA

>c12210_g1_i1 len=613 path=[591:0-225 817:226-612]

ACAAAAGCAACAACAACGACAACAAAATAACAGAAAAACATCGTTGGGAAGCATACCAAG

AAGTTCTCAGTCAGAAGACTGTACCTGAGAGCTACAAAATGATCAGCTGATTCAGCAAAG

GAGGTCCCAAACAGAGTGAGGAGGAAGAGAGCCCATCAACAGGCACAGCACCTGAACAGG

GGGCATGCCATTAAGATGAAATTCTACAATTTAAGAGTGGAGGTAAAGGGAGAAGCACAG

ACAGTATTGAGGCATTTTAACTCATCTGGTCTTGCTCAGTCTTGATGATGCTGCTTGTTG

TATCATTTTGAGTCCCTCTGTTCCAGACACTTAGATGTTGTGTTCTTGCTGATTTTTCAG

AGGCACTGAGAAAAACTTCCTGTGGAGGCCGTCTGCTTTATCAGGTCTTTTAACTTTGTA

TACAGAGGTCTTCAGTTCTTCTGATGTAAACATATAGCTATGGGACTCAGTCCTGCCTGG

TATAACTGTATTTCTGCTGTAAGACCTGACGTGGAGGAGCAGACCTGAGATCCAAAATGC

TGATTTTGGTAACAGTGCCTGTTTGTCATCAGTTTTCCTGTTCTCAAACTGGAAGCAGCC

ACCAAACAGCCAG

>c12210_g1_i2 len=478 path=[1181:0-90 817:91-477]

ACTCTGCTGCAGCTGGTTTTCATTCCTATTCCAATTGACTCAGATGGCCTATAGCTCCCT

CTTTCTCTTTACTGATCTTGTTGGAGGCATCAGGGAGAAGCACAGACAGTATTGAGGCAT

TTTAACTCATCTGGTCTTGCTCAGTCTTGATGATGCTGCTTGTTGTATCATTTTGAGTCC

CTCTGTTCCAGACACTTAGATGTTGTGTTCTTGCTGATTTTTCAGAGGCACTGAGAAAAA

CTTCCTGTGGAGGCCGTCTGCTTTATCAGGTCTTTTAACTTTGTATACAGAGGTCTTCAG

TTCTTCTGATGTAAACATATAGCTATGGGACTCAGTCCTGCCTGGTATAACTGTATTTCT

GCTGTAAGACCTGACGTGGAGGAGCAGACCTGAGATCCAAAATGCTGATTTTGGTAACAG

TGCCTGTTTGTCATCAGTTTTCCTGTTCTCAAACTGGAAGCAGCCACCAAACAGCCAG

>c12214_g1_i1 len=238 path=[51:0-75 126:76-237]

TGAACATCTTTGTGCAGCCCTTAAGACAAGAATTGGGAATAATACATTATTCAGTTGAAG

GTCTTTAAGGAAGCTTCTAAAGAGTGATAAGTTAAATACAGTTAGCAGCGTATAATCTAT

TTACAGCAAGACATTTTACTTATTGTCAAGAGAATATCAATACAAAATTCTGATCTACAA

ACTGCTACCTACTCAGAAGCTGAGCTCTTCACTTACTTTGTGAGGACAAGCTATCGTA

>c12218_g1_i1 len=247 path=[1:0-152 154:153-246]

CAAGTTCATGCCACCGAACACCAAAGGCCCAATGAACTGAGCTCTGATGTCTCCGTCAAG

GTAGACAAATATCGTAGGCAGATTCTTATCGGGATAGTTGGGTATGCAGGTGGTAGAGAT

AGCTTTGATGAATTTCACATCTCTGAACTTTTTTGAAAGCAGCTCAAATGAAGAATAAAT

TTGGAGAAGTTCTGGAGATTTCGGGAAAGGATTACGTTCAAGAGGTTACGAAGGCTGGGA

AAGGTAT

>c12220_g1_i1 len=213 path=[1:0-212]

GGGGAGAGCCTTCCAAATGGGACAGAAAGGAGAAGAGAGGGGGAGGGAGTTGTTGCCATT

TAAGCCAGAGGTCTTCAGCAAAACCTCAGTCATCTTTTAACCCCACGTGGGCACCCTGTG

GTGGTTTTAAGGGCCAGGGAATGGACTGGATTGCTGAGGTTACTGTTTTCCAAGCAAGGG

AATGTGTGTCTGTGGGTCAGCTGGGGAGTGTGG

>c12222_g1_i1 len=933 path=[1065:0-932]

AAAGAGAAAGAAGATAAAAATAATCTTAGAAAAAACAACAATGCAAGTGATCCATGTGAC

TTTGAAATTTGTTTTGAAATAAAAAAAGAAGAACAAATTGGTGTATCAGATAAATGTAGT

AGAAAAAAGTGTTATTTACACAAAGCAACTTTAATAAAAAATAAATCAGTAACTAATCTA

GAACCTCATGAATTATATGGAGAAGTAGAAAATAGTGATATTAGTAAAAGATGGAGGGAT

GGTAATTTTTCGAATAAAAAAAATGATTACAAATTTGAAGGAAACAACAATTCTGACAAA

GAAACAGGAATTTCGGAAGAAAATGGTGGAACTGATGATGATGAACATGGGCAATCTTGG

GAGGAATTTAATTTTGATTCATGGGATCCTTCTAATAATGAGTATAAACATAATTCTAAT

TCTAATTCTAATACTAATTCTAATTCTAACTATTTTTTACATAAATTTAATTCTTCACCT

GTATATAATAAAAGTGATAAACTTAATGAAGTTCATTTGATGAAAGAAAAATACTGTACT

CGTTTTACGAATAGTGACGAGAAAAATAACAAAGAAATTAGTATCTATGATAATATATTT

GAAATGCATCACAAACCTAAATGTCATAATTATTATGAATTAAAATGTAGTTGTTTTGAT

GAATCAGGAAAAGCGAAAACTGAGGATTTTGTCATATATATTAATCCAATATATAAAAGT

AAAAAAGAGGAGGAAGAAATTAAAGAACATGAGAAATCTAAGTCTAATTATTATGAAAAA

AATATTGTATTTATGAATGAAATTATTAATAAGTTAAGGTATTACAATTTTTGAAAATAA

AAATATAAATTTTAGGGTTACACCTAACTATGAAAAAATAAATTTTATAATTCAATAAAA

TATTTTTATATAAAATCAGTAAAACAATAAATA

>c12239_g1_i1 len=212 path=[1:0-98 100:99-211]

AGGAAATACGCACTGTGTTGCAAACTGTGTACTAGAATGGGAAAAAAAACCAACCACCTT

TGAGGCAGATTGATTATCTTGGTGTTAATAAAGAGGATCACAAGTAAAAGGCTTGGTGCT

GTAGGGAGGTAATTCTGCATTTACCAGGGATTTTATTCCAGCTTTTCAGTAACTTGTACA

ATAAACTAGTAGTTGTTAAAACTGAGGCTACT

>c12243_g1_i1 len=595 path=[573:0-394 968:395-594]

TCTATTTTTTTTTCATCTTTAATAAAATTTTTATTTTCGTTTACTACTTTTTGTTTTTCA

TTTTTTTCATTATTATCTAATGAATATAATTCTGTAGCTTGCTAACATTTTTTTTATAGT

GTATTTAAAATCTATAAAATAAATTATTATTTCTTAAAATATATACTTTATACTTCTATA

AATTATTTTTTCTTTACTTTTTATATTTTATATACACACATTTTTAAAAAGGAATAAAAA

TAACGATGTTTCTTTCTATAAATATTAAATATAAAATGTATACTTCGAAATTTAATTAAT

TATTTATTTAATTATTATATTTTATTATCGTCTTTTTTTAATGTTTTATTTAAAAAATAA

TAAAAATAATTGAAATACTATATTCTACTATTCATAAGAATATTAAGTACTCATTAAACA

TTATCAAAGCATATTTATAACAAAAAAAAATTTGAATAACATTAAAAATATATATCCTAT

ATATATGTTAATTTAAAAGGTCATATCATATTTTATGATTTTAAAAATGATTTTATAATA

TATTCTCAATGTATGCCTCATTTTAAAAAATTTTTTTTTTAAAATATATATATAT

>c12245_g1_i1 len=216 path=[241:0-100 149:101-215]

CCTGCCACCAGGGGCCAGTATCTGCCCGTGTAATCTCTGAAATTCACTCGTTTTCCAGCA

TTTAAAACCAAATCCCCGAAAGTGTTTTTATTTGAGAGACCTCAGCAGCATCTCTTTAAG

GCATTCATATCCTATTAAGAGTGTGCAATGCTTTCATTTCTGAAACGGAGGCTAAATGAG

ATTGGTTTGGGGAAAGTTTTATCTTTGCACCTTTTT

>c12246_g1_i1 len=238 path=[6:0-141 148:142-237]

AAAAAAAAAAAAATATCTTAAAAAGAAATAAAAAAAAAAATTGTTGATGAACTTCATGGA

AATAAAAATAATTGATTTTCAGAAATAATTTTGTAAGTTGATAAGATATTGATTATTACA

TCATGTATTATTTCATTTATAAATATGTAAAAAGTTATATTTTTAAAATTATGCAACATA

TCTTTATTATATTTATACACATTTTGTTTTTGTAACATTTAAAAAAAATGTATCATTA

>c12249_g1_i1 len=273 path=[1:0-272]

CATAAGTAGCATTTAGAGTTGGTTTGGAGTTGTTCTGAATTGCCTTTTGCAAACGAGTGA

CACATTCAGCTGTCATTCTTGTCTTCAGTGTTTAACGCTCACAAAGACGTCAGTAAATGA

CCCCTGCCTCTGATGCTGTGCGGTGTTTTATAAGGCATTTTTCAGTAACAAAAGTGAGCA

AACGAACCCCTACGGATAATGCAGTGCAGAGCGTGTGGCTTCTTCGTGATGCAGTCTGTC

TTCTGCTTTATGCTTATGCTTTCACCATCTGAG

>c12251_g1_i1 len=231 path=[375:0-230]

TGCATGAAGCGGGGAACAAATACAACTTCAACAAACAAAACAAAGCCAACAACAAACATT

CCCTTTGTCTTGTGTATGTCAAGATCTGGATAATCACTACACTGACAATTTAAGAATAAG

CATTTCTGATTGATTTTTGTTTGTCAGCTGCTCACAGCTTCTTCAGAGTGAAGGTGAAAT

CCTTCTAGGAAGCATTCAAATACTACCAAAAAGACAAAGCTCAAACACAAA

>c12260_g1_i1 len=212 path=[364:0-211]

GACATAGTTACAACAATGTAATTATCCCCCTATTTTTCTATCTGTTCCATTTATTCCAGT

TATTCTAATAGAATTCTAAAAGGATGAGAATTCAAAATTATGGAGCTGAAGTTCTCCAGA

TGTAATGAGAACAGCTAATGCTCAAAACTTCGTCAGACAAAAAAGCCAAATAACCACAGA

CAAAATATTAGTACACAAAACACAAGAGAAAA

>c12277_g1_i1 len=222 path=[126:0-123 250:124-221]

GTTTTTTGGCTCAGAGGCACCTGTTTGAACAGAAGTCACTGTCAGCAAAACCACAGCAGT

TGTAAATAGCATTTACTCTAGCACAGCGAATGCCTAGAAGGATGCTCGCTTGCCATCATG

TCTTACCAGACCCAGGGACCTTCCCAAGTACTGCCCTGGCATCTGCTCACACGGTGCCCC

TCCATGCTGACAGAAGCTCACCTGTGGCACTGGCGTAAACAA

>c12279_g1_i1 len=206 path=[184:0-205]

GTCAGAGCTTCTGGTTTCAAGCTGAGAAGGGTATCTCCAACTGAAAGCTTAGCGCTGTAA

AACAGACCAAGCAATCAACTTCATACTCTGGGGAAGAAACAGAAAGAGCTTCCTGCATTC

TCCAATTAAGTCCCTTATTGAAGTTAGGAAAGCTTTGGTTTTCCCCCCCCCTAAATCACT

CACCATCTCTCGGGGGATTTTACATG

>c12288_g1_i1 len=225 path=[242:0-142 385:143-224]

GCAAGCTAAAATTTAAGTTACTCCTGTACTTTATTTTAAGAAAAACAAATGTAATATAAT

TCAATGAATAAGCATAATCAGTTTCGACGATATATACATAAATAAATGAAATCGTAAACA

AAGATATTTGTATTTTTGGAATGGGTATGAAAAAGGTAATATAAAATGCAGTCTCAATTC

AACAAAATGTAGAAAAATAAAAATAAAAAATACGTTGTCAATTAA

>c12291_g1_i1 len=272 path=[250:0-271]

GCCATGCTGCGCTTCGTGCTGCTGTTCAGCCGGCAGGGGAAGCTGCGCCTGCAGCAATGG

TACCGGGCCGGGGCGGAGCGCGACAAGCGGAAGGCGGCGCGGGAGCTGATGCAGGCGGTG

CTGGCCCGGAAACCCAAGATGTGCAGCTTCCTGGAGTGGGGAGAGCTGAAGGTGGTCTAT

AAAAGGTACGCCAGCCTGTACTTCTGCTGCGCCATCGAGGGGGCTGACAATGAGCTGCTG

ACCCTGGAGCTCATCCACCGCTACGTGGAGCT

>c12309_g1_i1 len=214 path=[398:0-213]

CAGCAATAGCAAACGTACTTCAGAAGGAATTAGTTACACACAGGTAGGTTGCATTGTCAT

ATTTGGGTGTGATGCATTGTCACAGTCTAACAACCATTAAAGTTCAAAGAACTTAGTCAT

TCCACTTAGACAGTTTCACAGATAAAAATTAGGGTAGGTGAAGCTACAGTCCTGCAGTAC

CCAGTTGTCTACAGCCTAAATAATGGGTACAGTG

>c12318_g1_i1 len=346 path=[492:0-131 250:132-345]

AAAAAAAAAAAAAATTTATTATAAATATTACTATATAAATTGATATGAATATAGAAATAT

ATTAGAAAAAATATTTTTTTCCGTAATATATTGCATGAAATCTTATAAATTAAATACTTT

ACATTCAATTTAAAAAAAATGAATGTTATTAAAAAATACATTCAATAATAATAATAAAAT

GGACGTATATATATTATAATAATATTGGATAAACTAAATCTTAGTATTGTAACATAAGAA

ATAATAAAAATTGAAATAAAAAATACTTTTCTATAAATATTTCGTATCTTACTATAACCA

ATTTTCTTAACCATTAAAGAATATAAAAAAAAATAAAACAGAAAAA

>c12331_g1_i1 len=221 path=[1:0-220]

TATTTATGTCCGTTTTCATCAGACAAACTGTACTATTTTGACTGGGTTCTCATCTGACTT

ACACCAGCAAACGTAACAGCAGATTCAGGTGCTCTGGATTAAAAATTAAAAAAGAAAGCA

ATACCAAAAAACAGCTAAAAAATATCAAAGGAAGAAAATAAGGCATTTGCTAACCTGACT

AGCATATAGTGGGTTGATACTAGGGAGCTACATGACACCAA

>c12353_g1_i1 len=242 path=[39:0-163 277:164-241]

AAAAAAAATAATTTTTAGGTTTTAAAATTCATAAAAAGATATTTAATAAAAGTTTAATAT

TTTATTTTTTACATTATAATGGTAATACATGTAATGTGTAAAATATAACTTCAATAAATT

TGTAATAATTATTAAATAATGAAATAAGGTAATATAGAGATTATCATATTTATTTTTATA

TATCATTAATATATTTTATTATGAAATATCTATACATAATTATTAAATGTAGAACATCTT

CA

>c12354_g1_i1 len=215 path=[41:0-135 40:136-214]

GAGTCCTAGGAAGATTCGTGTTGGGAATGTTTGAACTGCTGTGACTGCACTTGAAAAATA

CCGAAAGGGCTAAAAAGCAATAAAGATTATGCCAATTTTTCAAAATTTTCTGGTTGTATT

TACAAACTTAGTTCTGTTTCTTGTGCGTTGGTAAAGGTTAGAATTAGTGCATGAGTGAGT

AAATGCGTATTGGTAGAGTTGTAGCCTCGTGTTCA

>c12361_g1_i1 len=276 path=[295:0-275]

AAAAAAGAAAAATGAAAAAAAATTTGCAAAAAAATTTAAAAAGAAAAATAATTTTAAGAA

AAAAAATTAAAAACAAAGAAAAATTATTTTATGCATAAATAAATGTCTTAAAAAATAGCA

CAACTTTTTTCCCAATAAAAAATTAAATGAAATTATTTCATTTAACCTGAGTTACCTAAT

ATAGAATGATTTTGAATTAAAAATTAACAAAATACAAGTTAAAAGTATAAAGTTATATTT

CATACATTTGACTAAAAAAAAAAAAAAAATAAGAAA

>c12369_g1_i1 len=1532 path=[1:0-1531]

AAAAAAAAAAAAAAAAAAATGTATGATATTTTAAAATATATTATTATAAAAGAAGAAAAT

TATAAAACAAATTATTATTTTCAAACTATTTTAATATATTTATCCTTATTACTATATAAT

ACTTTGAAAAAATCGTTTATCTATGAAATGAGAAAAATAGCTCAATTTTTTTTAGACTTT

GTTTTTTTTTTCATAGAAAATATAAAAAATAATTTAATTATTTTACAAATTATAGAATCT

TATGCAAATAATTTATATAATTTTAATGAAAATAAGAATAATAATACATTTTTCATTAAA

AAAAAAAAATATTTTTACTTACTAAAAGTTTATTTTAGAAAATTATTATCTTTATTTTAT

TTATATACAGATGAACTAATACTATATACAATTAAAAGTAACATAGATCAGGAAATTAGT

CAAAAAAATTATTATAGAAAAAACTTTAACAAAAATTTAAATATTGGCATTATTTGTAAT

TATGAATATATAAATCAAATGGATTTAAGTAATTTATGTTTTTATTGGGATTATTCAATA

GTATCAATGAGTCATGATAGAAGCGATTTATATATTGCAAGATGTATTAAGAACTTTCTT

CTATTATTAATTAAACTATTTAAAGAAAATAATATAGATAAAGAAATCTCTTTAAGTATT

GAAAATGAAGTAGATAAAGAAATCTTTTTAAATGAAGAAGAGAATTTAAATAAAGAAAAT

TGTTATTATTACTATGAAAAAATATTTAATTTTTTTAATATTCTAAATAATAAGCAAATG

CTAGAAAAGATGTTCAATGTTGATGATATACATTTGATTAAAGATAACATAATGGATTCT

TTAAATGATAACAAGCATAATTTGAAGGATTCTATCAATGATAAAAAAGTATTTGAAGAT

TCTGATTATAATACTGATTTTTATAAAAAAAATATTTATTTAAATAATGAATCATTACAT

GAAGAAAAAGAAGATTATGAGAACATTATTAAAAAAAACAAAATTTGTGATTCATCATAT

ATGTATAGAGAAAACGATGAGAAAAAAAGTGAAAGCAATATAGTACATTATAATTTCAAT

TTAGAAAAAAATATGAACAAAGAAAGATTAAATAATATGAATGAAGAAAATATATTTTTT

CATTTTAATTCAAAGATTATATATGATGATAAAAATAAAATCAAAGAAAAAAGTGAAGCA

AATTTAGATGTGCTATATTTTTTCTCATCTTTGTATAAAGACGCCAATAATTGTATCAAT

AAAAAGAAATATGAATATTTTCATGTTGATAAAATACATTTAAATATAGAAAAATATACT

TACAAAGAAAAATGGAATTATATAAAAAAATTAGAAAGAATTTTAAAACAGTACCAGCAT

ATGATAAAAATAATGTGTGAAATATTACATGAATCAAAAAATGTTGACAAAAATAGAAGT

AACTTAAAAATGTTTATAGATTTATGGTGGAATAATAGATATACGTTAGAACAACATTTG

AAAATACTAAATTTAGATATATCTAATAATTT

>c12369_g2_i1 len=477 path=[1968:0-476]

TATTATTATTATTATTTTCTTTTTTTATATTATAATTAAATTTCAAATACTTTTTCTTAA

CTTGAATAATTTGAACATTACAATTTTTTATAACAATAGAAAAAACAGTTAAATTTAAAA

GAATATTTATAAAATTTAATTCGTCCCAATATTTTACTGAAAGAAAAAAATTTTTCCACT

TGTTTATCCATGAAATAATATTTAACATATTAGAATAATAAAAAAGAGCTACAAAAATAT

CAAAATTGCTTTTTAAATAATATTTTTGATTATTAGCAGGTGATATAGTATCGAATATAT

ATGTATTTACATTTTTGTCATTTTCTTCATTTGTTATAAATTTTTTTTTTGAAATATCAT

TTGAATTATTATTATTTTCATTGATGAAATTTTTTTTTTTAGTGTTCAAGATAATTAAAG

GAATAGATATTAATTTATGTATAGAAAAACCTAAATTATTAGATATATCTAAATTTA

>c12374_g1_i1 len=207 path=[1:0-19 21:20-28 436:29-206]

CTTTGTGTTTGAGGGGGGCGTCCCCGGCGGCGAAAAAAAAAAAACGACGGGTGGGGAGAA

ATTAAAAGGAGGCGGCGATGAGAAAGGTGGAAAAACACGGTAATGGGGGAGGAAAAGCGG

GGGAAAAAATCCTCAAAAATAACAACAAAAACAACGCGGAAGCAAAAAACACACGCAAAA

AAAAAATCAAGACGCTCAAACACAAAG

>c12389_g1_i1 len=394 path=[394:0-393]

TTTTTTTTTTTTTTTAATTTCTTTAAAAAATATTATTTAGTTTTATTTTGAGCAAGTCCT

CAACTTTCTTACAATTTCTTAAATTTTCTCATAAATAAATACTTTAAATTTTTTATAGTC

AAGTTAAAAAACATATTTAACATAAATATTACTCTTTTTTTTTATTTTAACTTCTTTTCT

TAAATCATTACAAAGAAAGAATTTTCGAAAGTTCTTTTCCTTTTAGGGATTTTTTTCCAT

TTAATTAATTCACTATTTATCATTGTAAATAAAGGATGATAAATTTATTTATATAAAATT

TTGTATTAATTTTAATGTAATTTCTGTAAAGCTAATTATTATTTTGTTCTAATATATGTT

TATTATAAAATGAAGTACAAAAAAAAAAAAAAAA

>c12393_g1_i1 len=203 path=[181:0-202]

GGGATAGCTTGCATGCCATCCAAATAAATTATGTCAGTAGCTTCCTGCGTAAAGCTGAAC

ATCCACTCATATGCTCTTATCCCAGCCAGCTGAGGAAACAAACATAGCCCCTGTGCACAA

ATACAAGGGAAGCAATAAGGAAAAAGACTTGAATGATTCAAATTCTAAAATACAAGCCCC

AGAGAAAGGTCTAGAGATTCCAT

>c12403_g1_i1 len=219 path=[1:0-163 165:164-218]

AAAACTAATTTTTACAACTTTAACTAGGAGAAGAACTCATGTGACTGAGTAATTTGACCT

CTTCTACAAGAGATGTTACAATATGATGTGCATCTGGAAAGAACACTCCGTACTGTACTA

ACAGATTGTTCAAAGGAGGAGCGGTATGTGGCTAATGTACTTCAAGGTTTCATTCTCAAA

CCTGAACTTTTATTGTCAGGTGTAGGTGGTGGCAATGAA

>c12411_g1_i1 len=381 path=[359:0-380]

ATGTAAAAAAAAAGAATTCTTAGATATAATATATCAATGTATTTAAAAGTTTTACCATTA

GTTAATTTTTTATTTAATTTTTTTTCTTAAATTAGCATAAATAATTCGTTACATTCATAA

TAAGAATGAAAATTAATGAAGTAATTTTGTATTTCATAACAAAAGTTTTAAAGCAATAAT

TATAGAACCAATATAATGAAAAAATAAATAAATTATATACATGCTGATAAAATAAGAATA

AATTTAAATAAACAAAGTAACAATTAATATTTATATTTTTTTATTATAATTTGTTAAAAT

TTAAAATTATTCATATTTTGCTATATACTACTAAAAGTGTATCTGCAATAATATGAACTA

AAATCACATACGTCAAACACA

>c12415_g1_i1 len=219 path=[1:0-89 91:90-218]

TGATTACACTTGTGTTCATGTGATGCAACTGCGGATCCTCTTCATTTCAGTTTTGAAGCT

TATATTGTGTGGAGAAAAAAAAATGAATCTTTTGAGCAATGGCATATGAGTTGGTGGTTT

GCTCTCTTGAAAGGAAAAAGTATACCTCTCTTGAAGGACAGAGAAAATCATTTCTAAATG

CTAACTTTGTATCCGCAGAGTAATGGAGATTTGAAGAGA

>c12424_g1_i1 len=217 path=[1:0-216]

CGGATGGATTTGGGGTTTTGGGGGGGCTTTGGAGAGCATGTTGAAGCTGCACCACTGCAG

TTCAGCAATAGCCAAAACGCTGCTGTGGTGATTCTAGCTCCTGTAAACAGCGCTGCGGGC

AGCAGCAGCTGAGGGGCCCATGGAGCAGCACAAAGTGAGCTAGCAGAAGGATCTTCATGC

CAGAAAGTTACCTGACCTTACAGGCAGAGCCTGGTAG

>c12430_g1_i1 len=1107 path=[1:0-1106]

AAAAAAAAAAAATTTTTTTTTTAATAATATCAACATTATATTTTAAGTAAGTTTAGATAA

TATGGATAATCTCCCATGGGTTGAAAAATATCGTCCAAAAAAATTAGATGATATAGTTCA

TCAATCAAATGCAGTAAGAATGTTAAAAGAAGTAGTAAAAACAAAAAATATGCCTCATCT

AATTTTTCACGGTCCTCCAGGTACTGGAAAAACATCAGCTATAAATGCTTTAGCTCATGA

ATTATTTGGAAAAGATAATATAAAAGATCGCGTTTTAGAATTAAATGCATCTGATGATAG

AGGTATAAATATTGTAAGAGAAAAAATAAAAGCATATACAAGAATAAGTATTAGTAAAAA

TAAAATAAATAATGAAACAAATGAAACATTACCATCATGGAAATTAGTAGTTTTAGATGA

AGCAGATATGATGACAGATGATGCTCAATCAGCTTTAAGAAGAATAATAGAAATATATTC

AAATGTTACTAGATTTATACTTATATGCAATTATATCCATAAAATTTCTGATCCTATATT

TAGTAGATGTTCTTGTTATAGATTCCAATCAATCCCAATTAATATAAAAAAAGAAAAATT

ACTTTATATATGCAAAAGTGAAAATATTAATATTACAGATGATGCATTAGAAAAAATAAT

TGAAACAACTCAAGGTGATTTAAGAAGAGCTGTGTCTATTTTGCAATTATGCTCTTGTAT

TAATCCGCAAATAACATTAGAATCTGTTTTAGATGTATCTGGTTTACCAAATGATAATAT

TATTCTAAAAATTATAGATGCATGCAAAATGAAAGATTTAAAAAATGTAGAAAAAACTGT

ACAAGATATTATTGAAGATGGTTTTGATGTATCTTACATTTTTAAAGCGTTTAACAATTA

TTTTGTTACGAATTCTGATTATCAAGATTCATTAAAATATCAAATTTTATTAGAATTATC

TAGACATGATTATCGTTTACACAGTGGAGCAACACAATATATCCAACTACTAAGTTTTGC

TGTTAAAATACATTCATTATTAAATAGTATTTCATGAATTCTAAAAAAAATTCATTTCAT

TTTATTATAAAACATATATATATATAT

>c12436_g1_i1 len=212 path=[1:0-149 420:150-211]

AAAAAAAAAAAAAAAAGTTAAAAACCACAAAATTACATGTATATTAATTTCTAAAAATAT

ATGTATTTATATTTTCATAACTATTGTTTAATATAGAAAAATATTAATATAAATTATGTT

GCTTAAGTTTTTTTGGTTTTATGAAATTTTTTAAAAAGATAAGGTTTACTAATTAAGAAT

CTCTTTGAATATATTTATTTATTTTTTTTTTT

>c12447_g1_i1 len=236 path=[390:0-62 177:63-235]

TATGAGAAAAAAAATGTTTAGTTCCAAGTAGGTGTCTTTTTTTTAATCAACTCTGTGCAT

GAAGTCAGGTATCTTGCTAAGCAGTTGCACAGCAGATTACTGCTAGTTTTCCATATCTGT

AAACAAAAACCTATTTGTACATTCCAGTTTAATCTCATAGCAGGCATGTCAGAACTGGAT

AAGAAACTGAAGGTGTTTGACATATACCGTTTCAAGTTGTTAAACCTGAGCAGAAG

>c12457_g1_i1 len=211 path=[252:0-150 403:151-210]

AGCTGATGTACTTGATGGTGCACTTGAGGGTCTGTATTTTGGTGAGGGGCTGTCCCCGCT

GGCTGTAGGCAGGCGGCAGGTAGTTGCGCAGTGTGTGCAGCGCGTCGGCCAGGGTCCTCA

TCCGCAGCTTCTCCCTCTCACTGGCTTTCCTCCCTCCATCATAAAAAGGTCTCGGCAGCC

GGCAGCTCCAGCTTTTGCTGGCGGGCAGCAG

>c12464_g1_i1 len=291 path=[35:0-53 34:54-290]

CCCCCATGTGAGGCTGCCTTTCTGAAAGCTTGCACTTGTTCAGTTCTGTGTGTTTGCTTT

TGAATAGAAAAAGTGAGAATTATTTGCATTTTGATAGTTGTATGTAAAAATGTCAGGTGA

GTGAAACTGAGTTCTGCTTTCGTTGTGATTGTTAATGAGAGAGGTTAGTGAGTTACGTGG

CCTTTGTATCTTAGGTTAATTTGCATGAAGTTATCTCGTGCTTCTCTGTGCTAAAAGGAG

CTGTGAGTAGTGGCATTGAGGTGCTTTGGGAAAGAGGAAGACCATCAGTGC

>c12464_g1_i2 len=253 path=[18:0-15 34:16-252]

GAGCAGACTTGAAAAATGCTTTTGAATAGAAAAAGTGAGAATTATTTGCATTTTGATAGT

TGTATGTAAAAATGTCAGGTGAGTGAAACTGAGTTCTGCTTTCGTTGTGATTGTTAATGA

GAGAGGTTAGTGAGTTACGTGGCCTTTGTATCTTAGGTTAATTTGCATGAAGTTATCTCG

TGCTTCTCTGTGCTAAAAGGAGCTGTGAGTAGTGGCATTGAGGTGCTTTGGGAAAGAGGA

AGACCATCAGTGC

>c12467_g1_i1 len=318 path=[296:0-317]

GAAAGAAAAGGAACAATTCCTAAGGGAGCTTAAAGAGAGATTAGAAATGGAAAAGAAAAA

GGCTGCAGAGGAGCTAGAGAAACAGTTTGCAACTGAACTCCAGCAGCTGAAATCTGCAGC

AGAAGAGAAACATCGAAAGGTCACTTCTAGCCTGCAAACCCAGATAGCAGAGGCACAGAG

GAGTGAGGAGGCTTGGCTGCGTGAAGACTTACAGAGAGCAGAGGAAAAAGTGCAGCAGAA

AGCATACCGATTAGCAGAATATGAACGTGAGCTCAGTGAGCTTATGAGTGAGAAACGCCA

TGAAGTGGAAAAAGACCA

>c12480_g1_i1 len=237 path=[215:0-103 319:104-236]

TTTTTTTTTTTTTTTGTGTTTATAATTTGTTATAGAAAAGAAAAAAAAAAATGTATTTAT

TATTTTTTTCTATAATACTTCATAAGATTAACATTAAAAATATAAAAAAGTAGAAAAATA

AAGAAAATAAATATCAAAAATAAAAATTTTAAAACTATTTAGACTTAATCATTCTTAATA

GTTAAATCAAAATAATGAATATTAATTAAAAACTATTTTATTTTTATAAAAAATGAA

>c12482_g1_i1 len=219 path=[41:0-153 442:154-158 387:159-218]

CATAATTTGAATGAGAATAATATTCTGCAACTTTTATTTTAAAAACGCACTGCATAAGCT

TCTCAGTAAAGGGTACAATCGCTTGATGGAATTCCGGAGATCCTGAGGAGCTTTTAATGG

GTCACTTACATGGAGAGAAAACGCAGCAATTTCCTTATTAGACAGGTCTGCATCATCAGC

TTTGATGGTAAGTCAGCGTAATAGGATTGTCACACTGCA

>c12482_g1_i2 len=219 path=[41:0-153 442:154-158 199:159-218]

CATAATTTGAATGAGAATAATATTCTGCAACTTTTATTTTAAAAACGCACTGCATAAGCT

TCTCAGTAAAGGGTACAATCGCTTGATGGAATTCCGGAGATCCTGAGGAGCTTTTAATGG

GTCACTTACATGGAGAGAAAACGCAGCAATTTCCTTATTGGAGAGGGGTTTAAGTAGGGT

GCATGGAGGAGCAGTGCAGTGTGACAATCCTATTACGCT

>c12501_g1_i1 len=215 path=[155:0-214]

AGGACTGTGAGCTTCACAGCGAAGGGGGCCAGCTGACTTGTTAAGGATGGCTTCTGCAGT

TTGATTCCCATCAGTAGCCCGATGTTGTCCTGTAACTGGCAGAGCTGCTGAGTGGGAGGG

CACAGGGGGCGGCTCGCCCCTTCTCCTCCCTTCCCAACAGTCCTCTGAGTTTTCCGAGTC

ACATGTTGGATGATACGTGGAGAGAGAGAGAACAG

>c12509_g1_i1 len=410 path=[1:0-409]

TTGTGTTTGATGCTACGCAGTTAGAAAGGCTGTTGGACAACAGTGGCAGTACCAGGCAAC

TGCTAACGGTGAGCACAATGTGTGAAACTCACAACAGAAATTTCCAGAAACCATTTAGCA

ACAAACTTAGTGGACAGGGGACTGGTCTGGGCACTTAAAGGAAACTCAGCTACACAGAGT

TCTGGAGTCAGCCATAGCTTGTAGAAAAAGTGGGAAGTAATATTGGTCATTACAACTTCA

AAATGATCCTTACATCGTTGCTTGATAACTCCTTGCAAAAAGTGTACAGCTTCTATTGGA

TAAAAGTCTCCACGTGAGAAAATGAAAGTGTTCTTGTGGAGTAAAGAAAAGCAAAAGGAC

CGATATTCTCCTACAGTGACACAGAAACACAGCATGACTTGTCTATATAA

>c12513_g1_i1 len=241 path=[347:0-240]

AAATATTATTATAAAATATTTCATATTTTTGAATTTACATGAAATATAATACATATAAAC

TTATTTTTGGTATTTTTAATATTTTATTTTTTTCTGATTACACTTTAAATTTCAAACTTA

CAAAATATATTTTAAATCGTTAAGCATACAAATTTTTGTGTATACATAAAGTTATGTGAA

AAAATATTTCATAAAATTAACTTGAAAAAGAAATTATTATATTATTTTATTTTATTTTAT

T

>c12516_g1_i1 len=361 path=[357:0-360]

AAAAAAAAAAAAAAAAATTCACATAAAAAGATATATATTATAAAATAAAATGTTATATAG

AAAATATGAAAGAATATTAAAAGAACAATTAAATAAATTGACAATTTTAATTTTCCCTTT

CTCTTGTTTTTTTTTAAGAACTCTTTAAATAAAAAATTTTATTTATTATAGAAACCAATA

ACGTATCGTTTTTTTTATCATTTTTCATTGATGATTACTTTTGATAAACATTAAGCTATT

ACTTACTTTTTTTCTTGCTTATAGTAATTGGTGTGAAATAGGTATTGTTCTTTTATTGAG

TTTCAGAAAATTATAAAGATCTCACATAAAAAATCACCTCATTTAACCAAAGAAAAAAAA

A

>c12527_g1_i1 len=474 path=[452:0-473]

AAAAATAAAATAAAAAATATTTAGTTAAAACTAATTACAAAGAAGTAATTTTTATGTTAT

CCATTTTTTTTAATATATATAAAATTGTGAAAACTAAAATATTATGTATATTATATTTTC

ATTCTTTGACAATTTCTCTGTATTTAATTATTCGTATTTAGCTTTCTGAAAAAAATATCA

AATATGTAATTCATTTTATGTGTTATTAATAGATACATAAATTAATATAATTCCTTCTAT

ATTCAAACTATTAATTCTAACAAATAATGGTAAATCCGATGAAAAAAAATGAAGTTCTTA

ATAAAAATATTAAAATTTATAAGATAATTATTTTAGATATATATCTTCTTTATACCAAAT

TAATATTTTATTTATATAACGAAATTTTATTCTCATAAATAAATATTAGACATTCGTTAA

ATTTCCAATAACCATCTTGCATAGTTTAAATGAAAATAATAAATAAAAGAAAAT

>c12529_g1_i1 len=419 path=[1:0-378 460:379-418]

ACTCTACTGTGTATTACACAAAAAGGAAGGCTGCCAGTAGTACAAAGGACTGAGTCTAAG

GACAGACAAAAGGTAAGAAGGGGTACAGCATCAGATCTATTTCTCCAAGTGACAGATGCA

GGCTTTTTAACTAATGCTTTTAATGACTGCTTTTATACTCAGCAGCAGCTAAAGCAGAAA

AGCACAGTCTGTTTCAAAGTAAAGCCCACAAAGCAGAGAACCATAAAGATGACAATGTAG

GTTTTGTAAAGGAGAAAGTCAAATGTACAACTAAAAAAACCCCAAAACAAACACTTCAAA

AGCCCCCCCCATAAGCTATTACACTTGAAATGCAGACAACACAGATTACATATTTTCTAA

ATTAGTCAATTATTGATTTTAATACTTTTTAAATATGACCACCAACTTCTGGTTCACGA

>c12529_g1_i2 len=420 path=[1:0-378 380:379-419]

ACTCTACTGTGTATTACACAAAAAGGAAGGCTGCCAGTAGTACAAAGGACTGAGTCTAAG

GACAGACAAAAGGTAAGAAGGGGTACAGCATCAGATCTATTTCTCCAAGTGACAGATGCA

GGCTTTTTAACTAATGCTTTTAATGACTGCTTTTATACTCAGCAGCAGCTAAAGCAGAAA

AGCACAGTCTGTTTCAAAGTAAAGCCCACAAAGCAGAGAACCATAAAGATGACAATGTAG

GTTTTGTAAAGGAGAAAGTCAAATGTACAACTAAAAAAACCCCAAAACAAACACTTCAAA

AGCCCCCCCCATAAGCTATTACACTTGAAATGCAGACAACACAGATTACATATTTTCTAA

ATTAGTCAATTATTGATTTGAATAACTTTTCCCCATCGTGAACCAGAAGTTGGTGGTCAT

>c12532_g1_i1 len=240 path=[218:0-90 309:91-239]

AAAAAAAAAAAAAAATCAAACAGAATAAGTACAGAGCTTTATTTTTCTATTGGCCAGCAA

CTTAAATTGAGTTAAAATACAATAAAAAAAGCAATGAATATTATTATACAGAGGAAAAAG

TATAAAACAACTCAATGGACACAAGCCAGCTCTGTTCATATCTCATCCTTCCTGGCCTCT

CCCAGCCCTGCACAATGCACTTCAAGGAAACAGGCAAAGGCAACAAAGAAGCTACTGATG

>c12533_g1_i1 len=213 path=[35:0-123 17:124-212]

AGCTACTGCTGTTTAAGTGAAATGCATCTGTAAGCGTTAAAAATTTTTTGGGAAAGAGGA

CTTGCAAACCTCTTGCACAGTACACAAATTGATGAGATAGCTTTTCAGAAAACTCAAGTG

GCTTAATTTTTAGAGGATTCTATTACATGCAGTGCTCAGGAGCTTCACGGTGAGGGGTTT

CTTTCTTGAACATTTAAATTACATAAAAGCAAG

>c12539_g1_i1 len=207 path=[404:0-126 22:127-206]

AACATAGAAGTGGGGTTTAATATTAGGCAAAGAGCTGTTTTAATAGGTATTCTACAATGA

TGAGTGCTTCAGTATGACAGCATCTGTATTTCCAAAATGGTTGAAAGATAGGCTGTAGTG

ATAGGACTATTCAGATTATTAATCATTGGGATCTTCCCACTGTCTTGTGTAAGGTTATTC

AACTATTTGCAATAAGAAGTTGCTTTT

>c12540_g1_i1 len=261 path=[239:0-260]

GATGAAATATATATTCTAAAATTATAAATTTAAAATATTGTACTTTATTTTCTTTTTTCA

AATTTCTAGCGTTTCTTATTATTATGTTTAGAATTTTTTAATATAATTATTTAAAAATCC

ATACTATTAATATTTTTTTTCTCTTTTCCTTTTTAATTTTTAAATATTTTTATTATAAAA

TTTTAGCAATTTCTTAATGTTGCAATTGTTACAACTTATTTATAAAGGTTTCCTTTGTTT

TTTTTAAATAATATAAAATAT

>c12548_g1_i1 len=309 path=[441:0-308]

GTGCTGACCTGCAGGCTTCCCAGCCTTTGGGGGTCAGTTCTGCGTGGTGCCAGTTAGATA

GAACCGGAGCATTGCTTAATGCTTTTAGCGGAGATTAAAGCCGCGACTTAAAATTGAGAT

GCTCTGACTTGAGATTTCTGAGTATTGGTGCAAGGAAGTCGTACTCGTTAAGCAACTGCA

GCAGAGCACCGTGTGATCTGCAGTCAGGAGAGCGTTACCAAGGCACTGTGCTCTGCTGAT

GCTGACTTACTGACTGAGGGGATGTTCTGCTGCAAGCAGTGTTGGCCCTCGGACTGGAGG

TAACAGCAC

>c12550_g1_i1 len=498 path=[575:0-416 65:417-431 80:432-475 1092:476-497]

TAACTTTGTGTTTGACTTGCATTTTCATCCCAGTGTGTCTCTGGATCACACGTTACCGAT

GTATTCTTAAGCGTACTGTCAGATTCAGAGACATACGTTAATCTGCTCATGCTCGGGCTT

GAGTAAACTGACTGAGATACAGTACTCTCCTTGCAAGATGATTTTGGAGAATGTGATGTG

CAAATAAAACCAGCTTCAGATCCTGAAAATGCTGTTCCTGAAGCATCACTAGGAGAAGAG

TGAATCAGCGGTGAAGTTGAAGGTCTGAAGCTATACTGCTCATCCTCCAAAGGCTGCAAG

TCACACTCGGGGAGAACGGATTGATGCTCAGTACTATTCTGGGAAGCTGCAGAGAGCTTC

TCAAAAGTTACATGTTTCCCATTCCCGCTGCCAGCAGCACTGCTCTCTTTGGTTCCCTCC

TCCACCTTTACACTTTGCGTCTCTTCATCAGTGCTCAATTTCTGCACAGGCACTGCCTCA

GTAAGTCCTACAAAAGCA

>c12550_g1_i2 len=498 path=[575:0-416 991:417-431 80:432-475 1093:476-497]

TAACTTTGTGTTTGACTTGCATTTTCATCCCAGTGTGTCTCTGGATCACACGTTACCGAT

GTATTCTTAAGCGTACTGTCAGATTCAGAGACATACGTTAATCTGCTCATGCTCGGGCTT

GAGTAAACTGACTGAGATACAGTACTCTCCTTGCAAGATGATTTTGGAGAATGTGATGTG

CAAATAAAACCAGCTTCAGATCCTGAAAATGCTGTTCCTGAAGCATCACTAGGAGAAGAG

TGAATCAGCGGTGAAGTTGAAGGTCTGAAGCTATACTGCTCATCCTCCAAAGGCTGCAAG

TCACACTCGGGGAGAACGGATTGATGCTCAGTACTATTCTGGGAAGCTGCAGAGAGCTTC

TCAAAAGTTACATGTTTCCCATTCCCGCTGCCAGCAGCACTGCTCTCTTTGGTTCCCACC

TCCACCTTTGCGCTTTGCGTCTCTTCATCAGTGCTCAATTTCTGCACAGGCACTGCTTTT

GTAGGACTTACTGAGGCA

>c12552_g1_i1 len=241 path=[1:0-132 134:133-240]

GTTAGATCCCACTTTGTTTCAGCTCTATACTGTAACATAAAATAACTTGATTCTGTATTA

ACATACATAATTCTTCTTTAATTTCAGTGTATTTTCATCATTAGGCACCCAATGCTTATA

CCTTTATCTATGAGCTGAATGTAACAGTGGTGTCCACGGTGTCATTTCATTGTTTGTGTC

CATTTTCAGTTCTACATGCTAGCAGGGAGGCATCCCTACCATGAGAATATTACAAAAATA

A

>c12561_g1_i1 len=202 path=[217:0-94 312:95-201]

AAATCCAGCTTATTTCTTTAAAGGTAATGCATGCCCTAGAAATGCTAGGTTAGCTGAATC

AGTTGTTGCCACCAGTTTGGATTTTGCAGTGCCTCGCTCACTTTAGACTACTGCAAGAAA

CTTCCATATCATGGCCAGCTAATTAGTTCTAACCTTTATAATGAGTTTTCCTAGGCAGGA

TCATCTCCCAGATTAACCTTCT

>c12569_g1_i1 len=299 path=[1:0-298]

ACATCATGAATGGCTTTCATTGAGATCTCATATCTGAAAAATGGCTACTTGAATATATGA

AACGGGTGTGTTGTAAGTCCTAATATGTTAATCTTTGGTGCACTGAAATCCTTAAACAAG

AAACAGTTTCCAGTTTATTTGAGCTATAATAGCAATTATTATGAGCTGTTGCATTTTCTT

TTTTATTTTTAAGTGTAATCTTATTAAAATTCATCTCCCTTTGCCAGCTCCTCAGAGGAG

TAGGCTTTTAAAGAGATGTCTTCAGCAATTGAATATCATGATTCTGTGTTCTGAGGGAA

>c12570_g1_i1 len=228 path=[1:0-227]

AATAGGTAATTTGTCAACTTCGAGTTAAGACTACAACTTATCCCTCATTAAAGAAGTAAA

ATCTTTCAGCTGTTGGAAAGAACCGTGGGTATCATAGTACATATACATATAATAAAAATT

GTAGAACACCAATGAGATCAAGTAGAGTGTAGGCATATTCATCAGACTCTAGAAGAATTA

AGTGAAAGCCTTGCTCATTTTGTTAAACTCATGTCCAGCAACCAACTG

>c12576_g1_i1 len=399 path=[1:0-338 806:339-398]

AAAAAAAAAAAAAAAAATTTAAAGGCTTAAGTAAAAAATTGCAGTTTAAAAGCTTTAGCA

ATAATATTATTTAACTAGAATATAACTTTTTTTTTCTATAATTTTAATTAAAAATTATAG

CATGAAATATTTTAGCAAAAATAGAGAATCTCTTTTCCAGATTATAGAATAGTGTTTTCA

TATTTATTTTATTTTTTTGTTTCGGCTAAAATACATAAAGACTTTGGGAAAAAATAGCTT

TATTGCATATTTAACATATAAATTTTTTTTTAAAAAATTAAGATTTAAATAAAAAAGATT

TTAATTACTAAGTCTCTACATATTAAAGCAATGAAGTTATATAATTTAAAAATATATCAA

TATATAAAACTTTTTAAAATTTTATATATATTAAAAAAA

>c12582_g1_i1 len=413 path=[15:0-36 52:37-412]

GATTCTATGAGATGGGTTGAGTTTCGAAATATTTAGTCAAAAGGAAAAATACACACTGTG

GAGCATTGCTCTCCTGAGGCATGTTGAGTACATAGCACTGAGCAAAGGTAGGCTTATGAA

CACATTTCTACAAGCATCATTTCTTCAGCTCAGCATTGTCAATGTTTCAGATGAACTAGG

CAAGGCTTGAAAAATTCTCAAGCTCTTTTGCTTAACCCGAAGTACTGCAGCTGGACAACG

CGTTCTCTAGTGTGCCTGAAAGCAATGGACCAACAAAGCGGTTCACTAAACAAGTAGGGG

AGGGGCAAGGGGTTGGTAAAGGGAATTATCTATCTTGGATCCCTCTATGGAAACAAAATA

TTGGCCATATTTCATTCTTGGGGAAACTACTGGAGGAAAAGACAGTCCCAAAA

>c12587_g1_i1 len=289 path=[81:0-233 40:234-288]

GCAAAACAAAAGGAAGGTTTTGTGCAACCTCAGTAATAATTCTTCAGCTGGATGTTGTAT

AAATACACCATAAAATACAGCAGGGGAATGCAGACACATAATTAGTTTGATCACTTGCTG

TACCCCATGACCAAGCTATTCAGTGCCTGAATACACACAGTCTGCTGCCCGCCTGCGTGA

GCACCCCTGTTCTGGGACCACCCCAAACACGTCCTGCTCTGACAGCTTCCTTACTCAGCC

CTGCAGCAGCCATGCTTCCACACCTACTTGTACTTGGTTGCCTGTGTTG

>c12593_g1_i1 len=206 path=[185:0-183 407:184-205]

GATGGTAAAGTATTAGGGCAATTGGTGATGCATCTTCCTTAATCTATCGCTATCCCCATG

TAGTAATGGTTAGGTGCACTGTCTTGTTCTGTTTCATTCTTGCTGTATCATTTCACTTAT

TATCTCATTGCTTTGAACTTAAGATAAACACACTACTTTTGTCTAACTTTGTGTTTGAGC

TGGGCAAAACTTTACTTAGACTGGCA

>c12593_g1_i2 len=207 path=[185:0-183 409:184-206]

GATGGTAAAGTATTAGGGCAATTGGTGATGCATCTTCCTTAATCTATCGCTATCCCCATG

TAGTAATGGTTAGGTGCACTGTCTTGTTCTGTTTCATTCTTGCTGTATCATTTCACTTAT

TATCTCATTGCTTTGAACTTAAGATAAACACACTACTTTTGTCTAACTTTGTGTTTGAGC

TGGGCAACAAGCATGGAGCAGGGCCCA

>c12598_g1_i1 len=222 path=[1:0-221]

ATAAGAACAAACACATTTAAGTTACATTATTTCAAGATTAATTTCTTCTAAAACCTATTT

AGCAAAAATTTTCCGTGTGCATATATTAAGTATTCATATGACATTTCAATTTTGATTTAT

TTATTATTTTTGGTAGGTTATGTTATATTTATTAATTTTAAGTAAATTATAATGATATTA

TGAAATTAAATTATTTTTATGAATGAAGAAATCTCTTTTTTA

>c12605_g1_i1 len=227 path=[1:0-49 51:50-226]

ACTAGATGATCTTGGAGATCTTTTTCAATCTTGGTGATTCTATGAATCCATCCTATTTTT

TTCTCCTCTTGATATATACCCTCCCCAGTAATGCAGTCTGTAGACGGTGTTGTCTTGAAC

TGTAGGAAGCAGTGATATTCAGTAGAGGGTTCTTGGTGTAGACAGTCTTTCAACTGATAG

TTTTAGGGATTAGTAAAATTCATGGTTGATGCTATACAGAATATTAA

>c12608_g1_i1 len=202 path=[1:0-201]

TGATGCTTTCAGATAGAGCAGAAATGGGTCAATGGTTTCCAATGTTACCCATAAGGGACG

CAGACACGCAGTGTGGGTGGTACGTCTCAGCCCACCGATGGAGGTTTGCTTTTCCTGCAG

GAAGGTGGGCTTCATTCCCAAGGAAAAACGGAAGATCCCAAAGGCTCCTTAATTAATTGT

TACCATTACTTTGTGTCAGGGG

>c12628_g1_i1 len=248 path=[25:0-247]

TAAAAAAAAAAATATTATTAAAATTTTATATTCATATATGTGTTTTACATTATTTCTACT

AAATATTTTTATAAGTATTTATATATTCCTTTTATAAGAAACTTATATATTTAAAAATTA

TCATTTTTTAAATTGTTAAGAACAACATTTATATATTCCATTAATAATTTTAAACCAACT

TAATAAAAAGCAACGACTAATATAAAAATAGTAGTAATTAAACGTCTCTTATTTATTTTT

TTTTTTTT

>c12631_g1_i1 len=221 path=[199:0-220]

AAGGCTGATAACTGCTCTGGTCACAGTTTTTTGGAAAGTTTCATTTGGGAAAACAGCCCA

CAGCCTCCACCAATGCAATGAATCTGATCAGTCCAGAGGCAAACGGTGCAGGAGCAGCTG

TAGACTCTATCTGCTGAGTGGAGATTTCCTCACCCTTAAGATTGCTGACATGCTATTGAG

TAAAATACAGCACTGGCTTTCACCACAAGTCAAAAGGGAAG

>c12638_g1_i1 len=318 path=[296:0-317]

GCAGTGTTAGTGCAAAGGTCGTGGCACAGACCTGCAGTTACAAGTAAACTGCTGTAAGCT

TAGCCTTAGAAAAAGCACATGAGATTGTGTTGCTGCGTGGTGCCTCGGCTTATTTATTAT

CCACGCCATGCTAATGTTTAAAAAGGGAGAGAACAAGAGCTCCTGCTCGTTATGGCTCTT

GCAGTTCATGCATGGAGAAGGTTGCTGGTTTATTGACATGCCCTTAAGATGACTTTTCTC

TGCGTCCCCCTCCTGTAGGAACGTCTGCACTTCTGACCTTTCCTTTCACCCTACAGATAC

GTGGGGAACCTATCCAGA

>c12643_g1_i1 len=207 path=[185:0-114 300:115-206]

AGCTTAAGAACTGCCACAGACTGTACAGCGTGGTTGTGGATATATGTGGATAAACAAACT

CTATAAGAAACTTAATCATTTAGGTTACCTTTCCTTTTTCGTGATGCTTTTATATGTAAA

ATATTTTTTTACCACATTTTTTAAGTTTCCTAATTTCTTCGTATGTATATCACTTCTAAT

TATCTTAGTTTCTAGCAAGTGTGTAGG

>c12647_g1_i1 len=289 path=[1:0-288]

AAGTGTTAAAAAGTAAGCCAAACAAAACATTTTTAATCTATTTCAAGATTTTCTAAAGCA

TTTTTTAACACTTCATAAAGTATTCTGTAATCATATTAAATAATACAAAAATAAGTTTCT

TATTTTACTTTTAAAAATATACTTTTCATGAATGCTATTTCAAGTTTTAAAAAAAAATAT

TTAACTAAAAATAAAATTCTTAACTGTTCATATGAAAAATAAATATATTAGTATTTACAA

TTTCTAATATTCTGCTTATGTTAATAATATAATAACAAAAAAAAAAAAA

>c12662_g1_i1 len=202 path=[191:0-201]

TACGTAAAACAAAATATTTTAATTCTATTTATTTATCTATACATTATACAAAGGAGGTCT

ATAGACCAACATTTAAATGTTGGTTAATAGAAAATATTATATATCTTCTTGGAATAATAG

TCGATATATATGTTAAAAATATAAAATAGTCAGGTGATCAATAGATATTATTATTGACCT

TTATAATTGTAATAAGAATGAA

>c12663_g1_i1 len=349 path=[153:0-262 90:263-348]

CCTGAATTTCACCTAGAGTCTTACTGATACAAAAACTTTCCTAAATAAAAAAGCCAAAAG

GAACAGGATGGGGGAGAAAAGCATAAAACACCTCCAACAACAAAACCAACTCAATGTACT

TACTGAACACCAAACTTTAGCTTTAATCAAGCTCCCAAACAGTATCATCATACTAAATTC

TGTGGTGCGTTACCACCACCTCTGCTGACATTAAGCAACCACGTACAATTCATTTCTTTT

TATCATTCTACCTGTTTTATTACCTGTTCCGTCGTCACCTGCTGCCTGCTGCATACCCAA

CTGGTAAACACTGCCGTTCTTTTGCTGGAAATGAGGATGTAAAACATGT

>c12674_g1_i1 len=948 path=[976:0-947]

ATTGGAGGAATGGCACGTTCTATTTAAAGACTGAGACATAGAAAATATATGTACGTCAGT

ATGACTCCATGATAATCTTCCAGCCAACTGCACTGAGGTTGAGGACTTCAGTGACTGCAA

TGGTAATACTAGCAGCTTCTGTCCCGCCAATGTTCCTTGTCAATGTAAAAATGAAAAGCC

TTTTTGCAGATGTGATTACTACAGAGAGGGCTGGATGGAGTATTGGTACATGGGCCCCAA

ATGTAACCAGCTTTGGAACACACTGGATTTAATACTAGTAACTGTTCTTCCTGCAGTAGC

CCTGGTTTTCTTAGTTGTTGTAATATCTCAGTGTGTCCACTACTGTAAAGGTAAAAAAGC

CAGGAAGCAGACAAATTCTCCATACCTTGAAGTACACCATAACCCTACATTTAGTCAGGA

ACCGTATAGTAACCTGGGACGTGCTTCTCAGCAGCCACCAAGGGTTGAGTGGACTGGACA

ACTGCCAAAACCTGTTCTGAGAAGACAAGATTTTGATGATCTCCCAGGTCCAAGTCACTT

AGATAGTTCTAGCCCCAGGTATGCTCAGCCACAGAGGAGACCAGACTATCTTTCCAGCCA

GCAACCCCAACAATACGACGGCTTTGTCTATCCAGGCAACAATAGACCTTATGCAGGTTA

TGCAGAGGAGAGGCAGTATTCGAGATACTAATACCAGTGCTTCCTTGATCTGAAGTGTCT

CAGAACGCTGCACGATCAATGAATGAGCAATCCAAACGCTGAGAGATCAGTAAGGCCTTG

TGTGCCTGGATGCAGAGAAAATAGTAACTCAGAGAGAGCACTAAAAGTCATTAAAAAAAA

AGTGTAGAAGATAATTATAAAACAAAACATAATTATAAAACTATTATCCAATTTATTTAC

TGTATAATTTCTGAAGGTTTAACTTTTTTTGGATACCTGATTTCACGG

>c12681_g1_i1 len=229 path=[333:0-97 439:98-228]

GAATAAAAATATTTTTCAATGAATTAAATTTTCTTATCATTTACTTCATCAATGTTATTT

TTTTATCCTATATAAAGATTTATATAGAAGATAAACATAAAGAGAGAAATTTTTTTATAA

TGTTAAACTTAATAATTTTAACTACCTAATCTAAAACTAATAATACTTACTAATAGGACA

ATTTTTTTCTTTGTTTTATTTTCTCTCCTCTTTTTTCTTAATTTTTTCT

>c12684_g1_i1 len=223 path=[201:0-222]

TGGGTCGCTCGCGTCTGCCGCACTAAGCGGTTCGGAGACCTCCTGGGCAGACGCCGGCGT

AGCCACAGATAATTTCAGCGAGCTTAAGCTCTCGTCAGAATGCCGCGTTGAATAGGTGCC

GCCTAGCGATAACGCTCGTCTTGCCCTCCGCCCTCCGCCTGCCCTCAGACCCCATGGCAG

CCGCCGTGCCCCGCGCTCCACGGCTGACATAAAATCGATAAAG

>c12685_g1_i1 len=749 path=[67:0-420 33:421-748]

AGTTTTCTCATTTTTTTTCCTGTCTGTATTTTGAGAACTGTTTTTCTTTGACTGATTTTT

TTCTTATAAAGGAAAAAGAATAAGAAAACTGTCAATGGAAGTAGCATGCCCCATGATTGA

AACTGTCAGAAGTGGTTCAGAAATGAAATCTATGTCACCAGGAAACAAATGTCTGAGATC

AAAAGCTGCTCTAGTTTAGCTACTGGGCACCTCTCAAAGATGAACAGCACCTCTTGGACT

GTTATCCCTGCATTTCTTACCCAGGAAGGAAATCTGTGCAGAGGTGGTGTGGGAGGTTTA

CCTCCTGGTTCAAGGATGCCAGTCTTAGAACTCGAAACTTCTCTCTGCTCTGTAGATGGA

ACATGCTGTAACGGCAGATGCTGACACAACTCGGGGTGGCTCAGCAGTTTGGAGACGGCC

CTCAAAGATGCTCATTATTTCTCCTGAGAAAGAAGAATCCAAACAGTGGGAGTGGAACCT

GGCAGCCAGATCATCAGGGAAGCTGGCTCTGTCCAAAGTCACTCGTTCTAAGGTGCTCAC

AGTGCTATGGTTACCCTCTAAAGATGACTGGAAAGTCAAAGCCCCATGGAGTTTTGTGGG

CTCAGCTGGAGTGTCCAGTGACATGGGATCAGAGCACCGTAGCCTGTGTTCTGAGCTTAT

ATCCGGGAACATGTCTTTCTCTGTGGAGAGATTCCAGTTTGTTGAGTCAGTCAAGATCTT

CAGTGTGTTCTGGGAAAGCAGAGATCTAT

>c12685_g1_i2 len=360 path=[1:0-31 33:32-359]

AGGCAGTGGGCACAAACTGGAACATGGAAGATTCAAAGATGCTCATTATTTCTCCTGAGA

AAGAAGAATCCAAACAGTGGGAGTGGAACCTGGCAGCCAGATCATCAGGGAAGCTGGCTC

TGTCCAAAGTCACTCGTTCTAAGGTGCTCACAGTGCTATGGTTACCCTCTAAAGATGACT

GGAAAGTCAAAGCCCCATGGAGTTTTGTGGGCTCAGCTGGAGTGTCCAGTGACATGGGAT

CAGAGCACCGTAGCCTGTGTTCTGAGCTTATATCCGGGAACATGTCTTTCTCTGTGGAGA

GATTCCAGTTTGTTGAGTCAGTCAAGATCTTCAGTGTGTTCTGGGAAAGCAGAGATCTAT

>c12687_g1_i1 len=266 path=[155:0-265]

CGCCCCGCCGGGCAATCCGCTTTCATCGCGGAAAAGAAACATCGAAATCCGCAGCGCCGA

ACCTCCGCGCAACGAAATGAGCGGGGCGGGGGCTCGCTCTCTCTTTCTCTCTCCAGTCCG

GTGAAGGCAGCGAGAATCCCGGCGCTTTTCGGCCGTCGGCAAGGTACGTTCGGTTTGGTT

TTAAATAGGAATAATTAAAAGACAAAGGCGCTCGTCCGGCGCGCTGGGCGCGGCGCGGGG

CCGGGTGTCTCATGCAGGGAGCGCGG

>c12691_g1_i1 len=2023 path=[40:0-2022]

TATTAAAAAAAAAAAAATAAATAACAATAACCAATTTGAAAAAACTTTTAGTATATTCTT

AATCAATTAATACAAAATCTTCATTATTATTTTCCTCATCATCTATTATTATTTCATCAA

TTTCGTTTACTTTCTGGAATACCTTTTTATCATCTGTCTTTTTTTCATCACAAATAGTAT

GTCTCATTTTTTTTGCACTCTTGGTACTATCTTCCTCACTCATACTTCTTTCTTTTCTTT

TTTTGTTTATTTTCTGTTTTATAAAATATATACTTTCTAATGTAGGATCTTCTTTTAAAT

GAATTTCTAACTGATCATTACTATTTTGAAAATCAGTTAAAATCAAAATTTCATCATTTT

TAATATCCCACTCACTTAATGATTTATATAAACTTTTTATATAATCTTCATCATTTTCTA

GAAATAAATCATAATCAAATATGTTTCTATCTTGTTTATCTAAAAATGGATATAAGAAAG

ATAGTTCATTTGTGCATATATCTTTTATGAAATCATATAAGGTTATATCATTAAAATTTT

TAATATAAATATCTATCATTGGTTGCTGGCATATGTAACAGTTAGGATTAGGTTCTTCTA

ACTTTTCTGAATTTACTAAATTTCCTCTTGAAAAAATATTATTACCACTAACAATACTTT

TTACCCATACATGTTTTGCTTTGCTATTTCTTAAATTAATTTTTTCATCATTTTTATATT

TTAAGATTTCAAAGTATTCAATAACATGAATCAATTGAAATGCTTGAAGAGATGCAACTA

TTGCATTAGTTGATGAGATTGCTGGGATTATATTTCCTGCAATTGATTGTATATCAAACT

TACTTTTTTGATTAATTGAAAAATTCATCATTCTTAAATTAGATATAGCTGTTATAAAAT

TAATACAATCATCATCATCTTTATCAAATATTAAATATTCCTCTACATTTTTATTAATAT

TTAAATAACTATATAATTTTAAAAAGGTTTTTACATAGATTTCTATACATTTTTTTTTAT

CCCATATATTTTGAGCACATAATTGTATAGTATTTTCTTCTTTTTTATTTTTAAGTAGAA

TATTTTTATCATTTATTTTAATATTTTCATCAATATTTACATTATTTTTTGTTTTCTTAT

TCTTTTCAAATTCATTTTTTTTTTCTGATTTATCATAGCTTTTTATATTTTTTTCTATAT

TACAAGTATTATCATTAGAATTTTCTAATTCAAATAATATGGGTATAGGCATAATTGCAA

AATCCTTTTTTAAAGAAATAAGTTCACATATAGTATCATGAAATAAATAATTGAAAATAA

AAATAATTATTTCCATTTTTTCCATATTTCTTTTTTTTGATTCTTCTTCAATATGATTTT

TTATGTCAATTAATGTTTCATTATCATTTTTACAAAAAAACATTTCAAAAATTAATTTTC

CCCATGCAACACAATGTTCAGGTAAAGTTGGGGTTTGTCTTATTGTACATATAGCATAAG

TTTTATTTTTTGGTTTTTCTTCACAGTTGTAACATTTAGTTTCATTTGGATAAATTGGAT

ATACTTGACCATTATAACCAGTACTACCTGCTTCAATTAAAACTTTTTTTTCCATTACAC

ATAATTTATTCACATATTTTCTAGCTTTAATATTATCCAAAGCATTTATTATGTAATCAT

ATTTTTTTATATCACTACTTTTCATTATACACACATCAAAAGTATAAGCATTAATATTTA

AATTTTTGTTATATTTTAAAGCTCTTTCTTTAGCTACAACAGATTTGTATTTTTTTACAT

CTTTTTTTTTAAACAAAAATTGTCTATTTAAATTAGTTATATCAATTGTATCTATGTCTA

TTATATCTATATTTTTGCATCCTATTGTAATTATATTTTTTAAAAATTCACTACCTATTC

CTCCTGCACCTACTAATAATATTTTCATATTTTCTATTTTATCACATATTTTACTATCAA

AAATTTTTCTTATAGTTTTATGCATTTTAATTTTCAAAAATAA

>c12698_g1_i1 len=209 path=[687:0-208]

AATTAATGAAGTTCTAAAAAAAAAAATAATTTATTAAATTTTTGAAACAGTTAACAATAG

AACTAATTTTTTTAGTACTTTCACGAGCCTTTTTAATATAAAAATATTTTAAGAAAATTT

ATATTTTTAATTTTTTATAAATAAAATAAAAAAATTAGATTAAATATTTCTTTTAAAAAA

TAATTTTTTTGAACAAAAAAAAAAAAAAA

>c12698_g2_i1 len=359 path=[8:0-358]

TTCTTTTTTTTTTTTTTTCATTTATTTATTTCTGGTATTCCCATATAAATTTAAAGAACT

TATTATTAAAAGATTATTATATTATCTTACAAATTTGTAATTTTTAAGTAAATTTAAAAT

TATGTTCTCATAACAATATTCTACCAATCTTCATTAATGTTCTATTTTTAATAATAATCT

TCAAAATAGAAAACCGTCATATGAAAAATAACTTTTAATTGAAAATATTAAAAAATAAAC

TAAAAAATTAATATAAAAATAAAGAAATAATAAATCCTAAATTCTTGAAATTGAAAAAAA

TTGGAATTAAAAAAAATAAAGTGTGTGATGGAAAAAAAAAAAATATGCTATATAAATAT

>c12699_g1_i1 len=231 path=[308:0-152 23:153-230]

TCTGTGTTTTACCCAGTACATCAACTACTTTCATGGAACTGTATAGATGAAACTTAATGA

CATAGAATAATCAATTGTCTGTCAATAATAATCAAGGACAGAAAAATACAGTTAATGTGA

GCAAGTGCTTTTTGCTGAACAATTTCAAGCTCTGATCTACTCTGATCTGCAACAAAAATA

ACATAGACTGAATGTAGGCAGTTGGATAAAATCACTGTACTAACCGTTTTA

>c12709_g1_i1 len=214 path=[192:0-213]

AACGAATCAACATTTTAGCAAAAATAGGTCATCTTGTACAAAGCTATCATCATCAGTGCT

CCACTGACCTAATTACATGTTGATCCTGGAACTGTAAGCTCTTAAGATGTCCATGAAGTT

GTAAGAAAAGGGAGCTTTGTGCAGTAGCAGTAGTCATGTATTGCTCCTTGCTGTTGTAAT

GAATTCATGGTCTATATGAATGTAACTATGTAAT

>c12711_g1_i1 len=217 path=[43:0-6 42:7-216]

GGCCACAAAAGGACACACACGGGTAAGCATTAAATAGTTCTGTTCCTTCCATTGCTGCCT

GATAAGAATTAGTGGGAATTTGGAAGTGAACACTTCTTTTTCTAACACACAGAAGAGACC

TGAAGTACCAGGAGGGAATTACCAGGGATAGAGAGACTTGAAAAGTACAAAATTGGATGT

GTGGGGGTGGAATTGGAGTCTTATGGACTGAAATTAT

>c12711_g1_i2 len=230 path=[22:0-19 42:20-229]

TTTCCACACACATCACACTTAAAGGACACACACGGGTAAGCATTAAATAGTTCTGTTCCT

TCCATTGCTGCCTGATAAGAATTAGTGGGAATTTGGAAGTGAACACTTCTTTTTCTAACA

CACAGAAGAGACCTGAAGTACCAGGAGGGAATTACCAGGGATAGAGAGACTTGAAAAGTA

CAAAATTGGATGTGTGGGGGTGGAATTGGAGTCTTATGGACTGAAATTAT

>c12731_g1_i1 len=232 path=[210:0-231]

CCAGTGCATAGGTGCCCGTCTGACCCAAGGTTTGGTGCCGGAAGTAAATGTTCAGCAGCG

TCTGGACTTCATCATCAGAGCTGCTTCCAAAGATGTCATTGGGCCACAGAATCCTGCATT

CCCTTATTTGCCGGACGGCTTTAACCAGTTCGTTGTTTTTCAAACACTCCAGCATGGACT

GAATAGCTGCTTCTGTAGAATTGAAGGTGGGCTCAGATTCCACGCTCAGAGG

>c12732_g1_i1 len=242 path=[351:0-67 200:68-241]

GGATGGAGATTCTCCCTAGCCCTCTTCTTGCTATTGATGTATTTATAGAAATATTTATTG

TTGTCCTTTACCTTAGTGACCAAGTTCAGTTCTAGCTGGGCTTGGGCCTTTCTAATTTTC

TCCCTTCATGGCTTCACTACATACTTGAAATCATCATAAGTTGCTTGCCCACTCTTCCAA

TGACCATAAACTTTCCTTTTATTCTTGAGATCCAGCTGAAGGTCTCTGTTCAGCCAGTCC

AG

>c12736_g1_i1 len=233 path=[1:0-136 138:137-232]

AAATTATTAAAAAAAGAAAAAAATTAATTTACAATTTAACAAAAAAATATATAAATTCTA

TTTTTAAAAGAAAAATCAAAATAAATTTTATATTAATTTTAAATTAATATATTTAAAAAT

ATTTATAACTTAATAAAAGGAATGCATATGTGAATTTATAGATTTAAATATATTTATAAT

GAAAAGCTCATGTATTTTAATTTTGTATCATAAAATATTTAAACAAAAGAATA

>c12737_g1_i1 len=376 path=[570:0-375]

AAAAAAAATATAATTTTTTTTTAAATTCTAAAGGGTGAACCATGAAAAAAAATATTTTAA

TAAATAGAATTTGAAAATTTCAAATGGACAAATTTTGTGATTAAAAAGTTCCATTTAATA

AAATTTCAATTTTAGGAATTTTTAAAGCATAATATTTTATATATGTAAGAAACTTATTTG

AAATGTTTTTAAACCAATAAACAGATTTTTATAATTAATAATACTAGTACCTTAACTAAT

GTATTTTGTTTCCATTTTTATAAATCATTTAAACAAATAGATAAAATAATACATTATTAC

TAAAGCATATAAATACATGTAATTTAATATAACCATAATATTATATATTACATTTAATAA

AAAAATGAAAAATAAT

>c12738_g1_i1 len=327 path=[1:0-326]

AAAAAATAAAAAAAATAAATAAAAATTGATAAAAATTTGAGAATTTTAATAAATGAAGAA

AATGAAAATTTTAAAAATTAAAGTGAAAAATATTATCAAACTATAAGGAATTAAAAGGAA

GAAGACTAGAGAAATATTAACATGAATTTAGATCTTTTAAATTTAGGATTTTATCAACAC

AATTTCCCAATAGTATATTTATCTTTTTATATGTCTTATAACTCCCGCAAAACTTAATTT

TTAATAGAATTTTAGGTTATGTTTAATTGAACTGTTTTGAATTAATAAAGAAAAAGTGGA

AATATTTCAGTAGTATAAAAAAGGAGA

>c12748_g1_i1 len=289 path=[469:0-288]

GGGTGCAATGTTCTAGAAAGAAGAAAAAAAAAAAACTACTTTTTAGATATGTTCTTTCTA

GAACATTGCACACATTATTGTTTTTGTTGTTTAAGATTTTTTAAAAAAACTCTAAAGACT

AATTTTTACTCTTTAAAAAATGTATTTTTTCATTTACCTACTTACGAAATTATATACTAT

ATACATATTTATATAGATGAAGTATAATAGGTAACATATTAAACACATATTTAACTTGTA

TTCATTAACGTATTTCTTTAATTTATTTTGTGGTTTTTTTTTTTTTTTT

>c12751_g1_i1 len=247 path=[163:0-166 106:167-246]

GTGTGAAGTGACTACTAGACCTTGAGACTGAAGGTGTGTTCCAGTACTGTGTGGTGTTTG

GCTGGAAGGGCTGATGTTTTGGCTAGCTCTACAGCTCAGTTCCTATGCTGTGACCCAGGC

AGATGGGCTAAATAACACCATTGTGTTTTCAGCTGGAAGCTCATTAAGTAAGTAACTGAT

GTTCCACAAGGACTGACAGGATTTGGTAGTGGACTGTTACCATGAAGTGCTGGTGGAACA

TCAGTTA

>c12761_g1_i1 len=208 path=[225:0-207]

TACTTTGAGAAGTATGAAGTATTTGTATATTTTTTATATATTTTTTTAAAGCATAAGAAA

ATTTTATTAAATATATGTGAAACTTTCTTTTTCCTTTTATATTTTATTATATTCAATTTT

CTCTTACGCATTAATTTTTTTTTTTCATAATTTTTTTCAAAATTGTGTTCATATTTTTTT

AGAAAATTTCTTTCTAAACATACTTCTG

>c12766_g1_i1 len=310 path=[1:0-309]

ACGTAATTTTAAATAGAGAAAAGGTAAGATCATAAACATATAATCTATAATTTTTTTTTA

AATATAATTATTTTTAATAATAAAGATGTTTAACTTTATATAATTTTAACACATTATTTT

TTCACTACGAAAAGTTTTAAATATAAAAAAAAAATTCAAGTAAATTTTTTAAATTAATTT

GTATTTACGTTTATTATAATTTATTATTTTACATTTCTTAATATATCCTTCATAAGTAAG

AAAACATATTTGTAAAATACAAATGATAAAACTGTAAAATAATACATCAAAAAAAGAAAA

AAAAAAATTA

>c12774_g1_i1 len=201 path=[1:0-41 43:42-200]

CAGTATATACACAGAAACACAACAGTGCTGTTGACTCTGTTCTTTAATGAGCCAACAGCA

CTGTTGTGTTTCTCCAGAGGCGTTCTGGTTAACACCTATGATAATTTCTTTCTAGAAAAT

AAGCTCTGGGGTGGTTATGTTGCCTTGTGTTGTCTATTACATTTTTAATGGAAAACAGAT

TTATCTTTTAAAGTTTGCTGC

>c12775_g1_i1 len=331 path=[371:0-330]

TAAAAAAAAAATTAAAAGAACAAAGAATTGAGCAAGCTATTCATAAATTAAACGAAAAGA

GAAAAAGATATATTTATAAAATATGCACTGTCTTTTTGTTTTTTTAAGAAAAAATTTTAT

AAATAAGATTTTGAATGTTGTTTATATATATTATTAGAAAAAAATCCAAATAGAGTTTTT

TCTTGATTTTCATAAATTTTCTTTCAGAATTTTGAATTATTTATTTTCATGGCAAACTAG

TATTTCTTAATCCATCTACTTAAAAAATCAGGCTAAATTCTTAAATTTGAGTTACGAAAA

AATATTCATTATTATTTTTAGTTTTAAAATA

>c12786_g1_i1 len=279 path=[137:0-134 272:135-278]

CAGGCCATGACTCAAGAATTACCATACAGTGATCATGTTAGAATGCAATGAAAGTTTACT

GAGCCTCATCGGCAACTTCTTGTAAATACCTTACTTGACTGTATGCATGCCCTTCACCCT

TTGCGCTCTGCAATTCAGTGTAGGGGTTCCTTCCCCCGTGCTGTACTTCTGTGCTCCCCA

TCCCTCACACTGCTGGTGGTGTCCCTGCAGCCAAGCAGTCGGTCCACTCAGTGTCCAAAA

ATCCAGCCCAATGGAGAGATCTGCTGGTTGTCTCTCAGC

>c12793_g1_i1 len=241 path=[1:0-150 152:151-174 176:175-240]

AAAGCCACGTGAGAGACATGAAAACTTCCCTGATGAGTTAGGAAGCTCTTCTTAAGTCTC

GAGTTAGCTCTCGGCTAACTCGACTCTTTATATCTAAACGTATGGGTGGAGGAAAGCGCA

TTTCGAGAGTTACTCTCCTGTGCTGGGAAGACCACAAGATTTTATCTATGGTTCCTGAAA

CAGAATACAGAAGGGAATTGAGGTGATCCCCAAACACAACTCCATTGTGTTTAACACAGG

T

>c12808_g1_i1 len=258 path=[1:0-257]

GAGTCTGCTGTTAAAGAGGGAAGCAGGCAGAACCATCGAGCCTTTGGAAAGAAAACAGAG

GATTTATTTAACGTAATGTAAACTCTTATTTGTTTCAAAACTCATAAGCCCAGAACTTAT

TTTGAGGGAAGTAGAAACTAAGCTGTTGAAGAGAGTTCATGAATCTCAAATGCTAGAAAT

ACAGCTTAACTTGCTAGTCGTAGATTTCTATTCCCTTCAACTTTAGCAGTGCAGTCTTAA

GGCTCTAGAAGTTCTGGA

>c12810_g1_i1 len=231 path=[363:0-230]

AGCTTAGAAACAGAATTAGCCAAATGCTCATGTTGTCTAATTAAGGAACTAAGAGCTTTT

TAACAGGTCAAATAATTATTAGCATAACTTCAAGTTTCAGAAGGATCATTATGACAAACA

ACTTAAGGTTCTAATTTCAAAGAAAAGAAAAAAGCTATTTTGAAGTTTGTGTTTGAGCTC

CATGTCATGTGCTGTTTTAATATTAAGGGTTTGGGTATATTTTTTTTATTG

>c12817_g1_i1 len=204 path=[227:0-203]

TTTTTTTTTTTCTTTTTCTTAATATAAAATTCTATGAAAGTTAAATTTATATATTTAAAA

TCTTGTGTATTTTTAAAATATCTAGGGTGTGAAATATTCATAAAATTATTCGTAAAATAA

GGAATTTCTTTTATTCTTTAAAAAGCTACCTTTTGTATTTTTCCTTCTTAACAACATTAC

TAACTTTTCATTCAATAAGCCACA

>c12822_g1_i1 len=234 path=[422:0-233]

GACAAAATAAAAGATGAGAGGTATTCCCTAAGTCATTTATCCTTAATTACGCAGGCAGCC

TCAGCATGTAGCTCTGCCTCCATTTTTGTACCGTTCAGATTAGAAAAACAATTCAGATTA

AGGCATGGCAAAGCAGAGCTTGAATTTGCATTGAAATGCACGAGTGATTCCAGTAACTGC

TGGTGTGCAACATCTTATTCACCAGTAAATAAATGGAAAATAGCTTAGTATAAG

>c12824_g1_i1 len=297 path=[1:0-201 623:202-296]

GAATGTAACAGCTAACTACTTCCTTTCACCCACCCAGAGAACCTGAAAAAACTGTTAATC

TCCACAATCATGTTGGAGATGGATACAATTGTTACAAATTGTTGTTAAAGCACAAGCCAA

AAACAACTATCAGCACAGAATTCCAGTTCCAGCATCTCCTTTTTATGCTTGTTAAACCTA

TGTTCTCACATTCTTTTTTTCTCCTTCTGTGCGCGAAAGTCATAAAAGCTTGGAGAAGTA

GCCAGCGTATTTTCAGTTGCTGAATGTCGAAGGCGTCTTGAAAATCCCCCAAAGCCA

>c12824_g1_i2 len=275 path=[1:0-201 203:202-274]

GAATGTAACAGCTAACTACTTCCTTTCACCCACCCAGAGAACCTGAAAAAACTGTTAATC

TCCACAATCATGTTGGAGATGGATACAATTGTTACAAATTGTTGTTAAAGCACAAGCCAA

AAACAACTATCAGCACAGAATTCCAGTTCCAGCATCTCCTTTTTATGCTTGTTAAACCTA

TGTTCTCACATTCTTTTTTTCTTACTATTCATGGAGACTCGGTCATCTGTAAAGGTCAGT

TATCACTGCTGATCATCAGAGACCTCGAGGAAGAG

>c12826_g1_i1 len=414 path=[53:0-413]

TTTTTTTTTTTTTTTTAAAGATATGGGGCATTGCAAGTGGTAGATATTGAAACTCAAATT

CCCAGCAGTCAGCTGTGTCCAGGACAGGCATATGGGGCCCCTTCCTTAAGCGCTGAGTAC

TGTTGGCAGTGCCTTTTGGTTTGTATGTTGTGTTGCCTGGTTTGTGTGACTTTATTTCTC

ATGGCTCTTGTAAAACATGTAAAATAATGTGTACAGAATGGAATAGCAAAGCTTCTATGG

CAAAGTGAAATGCATTTTTTGCGATGCTGTAATTTATTTTTATTACTCCTTAGTAATTCT

ATGGCATTTTAAAAAGAAAAAAAATGCCTCACTTCCAGCTTTTTTTTGAAGGTTCTGAAA

CTACTTATTCATATGCAGACAGTGTAAAGGACAACAACAATAAAACTGTCAGTA

>c12828_g1_i1 len=255 path=[1:0-20 22:21-254]

GGGGAAGTAGGTCTTGGTTGGGGTTTGGTATATTTGGGTGTAAAGCATTAACATGAAGTG

CTGGACAAGATGTGGGAGCGTAATGGATAAAAACTGACTCCCATGACTGGGCGTGTGAGG

GGAAAAAGGGGGCAGCTTATGGCCCTCAGATATACAACTATCTGATTATAAATAAAGAGT

TGCTTGATATAAAAAACTTCTATTCTGGTAGTGGGTCTCCTCATTTTGTCACGCCTAACA

AAGGATGTTCAAATG

>c12834_g1_i1 len=418 path=[590:0-417]

GCATAGTTCCGTTTTGCAGTTTCAGATGATGTGCCAAAGAGTTGCTGTGCGCTCACTCTT

CTGTGGTGAGGGCAGGACTGACTTTTTCATCTTACTTTCTTCTTAAGAATGGGCCCCCGA

GTGTCTGTTCCAATCTTACAAGAAAGATGCATCAGTGGGGGGGTTTCATGGCTGTGTGTA

AATCAACTTGTCTTTTTTTTCTGTTGGATATTTATGCTTTTCAAATAAGTGCCAATAAAT

GGGAGAAAAAAAAAAAGCATGTTTTTCTGAAAAACAGTTTCCTAAATAACAAAGTATTTC

ACAGTTTTTGTTTTCATTGGTTTTGTGTGTTTTGTTTGTTTTTAAACCTTTCATACTCTT

GGAAAAATGTAATGTTTAACTACAAATTAGAGATTATTTATATCACTATTCAACTTCA

>c12846_g1_i1 len=234 path=[384:0-233]

GTAAAGGGTTCAGTTTGTTATACAATCTGTTTTTATCAAAGTGCCTTTGTTAGATGAGTT

TATGATTCTTTTCTTGGATTTTCTGTTCCTGTTTTGTCCTGGATTTTCATTCATGTTATT

TCTTATGCTGACATTCATAGTATATAAATCCTTGGTATTATGTAAGTTGTAAAACCTTCA

TTTTTGCTTATGAAGTAGCTGAGGATAATGAATAAATTATACAGGGCAGGGATT

>c12847_g1_i1 len=222 path=[309:0-221]

AGTTCTATTATGTACGCAGAAAACCTGTTATTAAAAGCATTCCTCTGTACTTCTGCATAC

TTCAAATCCAGAAGGCAAGTAAAACAGGAATCAAGAGAATTAATTTTGTCCTGAGACAGT

AGTTATTTTTATTAAAGCAACATTTCACAGCTCACTAGGAACTAGTATTAACCAGTCTCA

CAATACACTTCTACACAATCCTTACTTATTTTATTTTTGAAG

>c12848_g1_i1 len=207 path=[1:0-100 484:101-206]

AACACTGAAGTATAGGGGTACATTTTTAGGGGGCTGCATGTTTCTGAAGAAGTAGCCTAG

TTGTAAACACTTTTCAGATTATGGGAAGAGGAAGATTTTATTTTTTAATGTGTACCTAGC

TAAATGAGATCTAAGCATGTTTTTGTTGTGTTTGAGGAGTAGGAAAGGGGAAGCAGGGGA

AGGAAGCATCTCAACACACAACAAAAA

>c12859_g1_i1 len=281 path=[1:0-280]

GTTTTCTAATTTTTTTTTTTTTTTAATAGTAATTTAATATATTTTGTTTTAAGAAAGAAC

ATGAATTAAACTTTTTAATTTTTATATAATTAATAACTTTTTTTTTTATAGAAAAATTAA

CAAAAAAAAAGATATTTTTCAATGTTATTATGAAAAGATATTTTATATTTTATAAATTAA

TTTTTTTTTGACAAGATATAATAAAACATAAAATTGGAAAATAATTTTAAAATGTATAAA

ATTGGACAGAAAAAAAAATTGTAAATATATATATATATATA

>c12861_g1_i1 len=232 path=[125:0-231]

TGAGGCACTGGAAGAGGTTGCCTAGAGAGGTGGTGGAAGCCCTATTAGGCTCTGAGCGCC

TGATGTAGCTATCAATGACCTTGTTAAACGCAGGGGAGTTGGATTAGATGGCCTTTAAAG

GTCCTTTCCAACTCAAAAGATCATATGATTCTATGAAAATGAGAAGTGAGTCTATAGGAG

GGAGTGATACTGTTGTCAGTATGTACAAACAGATTGGTTTGTCTCTAGATAA

>c12863_g1_i1 len=218 path=[196:0-217]

ATAGTAATAATTTTAAAGACTATATAAAAAATATATTAACAAAATTTTTCATATCATTCA

CCTCATTCTTAGCTCATGTTCTTATATATATATATGTACTGTCTGAGAACATTTAATTTT

ACTATAGAAATAGAATAATATATAGTTAAATAAAGATGTTTCTATAAAAAACTATTGGAG

CATATAAAATATTTAATATATAAAGATACCATTCCATA

>c12868_g1_i1 len=250 path=[228:0-249]

AATACACAACTTACACTTATATTTTATATTTTGAAGGACTTAATATTGCTTTTACTTATT

CCTCTAAATACGAATCAAAAGTTTATTATGTTGACGATATATCAAATGATAATTTATATT

GGTTAATTCTTTTTACACCTGATGAAGCAATTAAAAAAAATGTTTTAAAAAAAATAGATC

TTAATAATTTATCTTTAAAGGATATAAGAATTTTGGATAATATTTGTAATAATTTAAAAA

AAAAAACTAA

>c12873_g1_i1 len=572 path=[1:0-488 490:489-571]

ATGCCCTGGGGGATGGAGGTGATGCCCTCAGCCCCAGCTGCCATCATGGTGGTGGTGCTG

CTCAGTCCGGTGTGGGGCTGGGATTGTTCTGTGGGGCCGTGACCACTCCACTGTGGCTGT

GACCCCTGTGTGGGGCCGTGACCTGTAGCTGTGAGCAGGGACCGTGACTACTATGGGGCT

GTAATGGGTATGTGGCTCTGTGATGGTTCTGTAGGGCCATGACCGCTCTGCGGGGCTGTG

ACTGTTGTGTGGGGGCTGTGACCATTCCATGGGGCTGTAACAACTCTGTGTGGCTATGAT

GGTTCCATAGGGTTGGGGTCGATCTGTGGTTGTTGTGTCCACTCTATGGGGCTGTGACTA

CTTCATGGAGCCATGATGACACCATGAGGCTGTGACGAGGGGCCATGGCCACTCTGTGGA

GCTGTAACGGTTCCATGGGGCTGCAACTATGCTGTGGGGCCATGACTGCTCTGTGGGGCC

ATAAAGCAGGGGCTGTGACCACTCCATGGGCTGTGATCACTCCATGGGGCCATTCCCTGC

CTCCCCCTACAGAGTACCTGAACTATGAGGAT

>c12880_g1_i1 len=229 path=[330:0-228]

AAAAAAAAAAAAAAAGAAAATTTTGTCATTACGAAGTATTAATAAAGTTTTGTTTTCTTT

TTGACGCCATTATTCTTAAGAAATTTCATTAAAACATTTCAAAATGCGTATGTATTACTA

TTCTCATATATTTAAAATGTAAAAAAATATAAATCCATACATCTGAAATTTTATAATATG

GAAAATCATAAAATATTTAAAAATATCATAGTTCTTGTTCTTGCATCCA

>c12883_g1_i1 len=209 path=[346:0-68 160:69-208]

AAAACAAATTGAGCACATAGAACGGTTGTGCTTGACTGAAAGATTGAGTGAATCAGCTCC

AAGCTGAGCAAGATACATTTTGATGGCTGGGACTGTAACTATGATTTCTGGGTCGATGCA

GACAGCCCTGATGTTCATCCAGTAGGCTGGTGTGCAAAAACTGGACACGCTTTGCAAGTC

CCTCTAGGTGCTGTTGATCCTGTGGGAAC

>c12884_g1_i1 len=554 path=[532:0-141 674:142-553]

TATTAAATATTTTTCCTTTCAATATAAAAATTTTTTATATTAAAATTAAAATTTATTTTT

AAGTTTTATTCCAATTATTCTAATAATTATATATTACTTTTTTTATCAGAAAATTTACAT

TTTCGTTTAAATAGTTGTAAAAATATAAGTGTTTCTAATTATTCAATTACCTATTCTATC

ATTTATACATGCATCTAAATATTTATTTAAATGAAACATCTTTAATTTAATAACTAATGT

ATGATACTAAAGCTACAAAAAAGGTATATTCTTCAATTATAAGTATCTTCCTAAAAGCGT

TAAATTTTAAAGGATAAAATGAAAATAATAATATTTATTTTTATATATCTTTTTTTCATG

AGACCACTAGTATAACTTCTAAAAAATGATACTTGAGAAAAATTAAAAAAAATTTGTAAA

TTTTTTTCTTATGTGAATCGTTTTTAAATGTTAAAAAATTTTTTTCGAATTATAAATCTA

ATATTTACTTATGATGTTTTATAAATATACATTCAATAGTTTTTAAGTACATGCATATAT

ATATATATATATTT

>c12885_g1_i1 len=229 path=[155:0-228]

AAAATTATTTCCAAGCTGGAGGGAAACAGAGAATAATAACACTCACTGCAGCTATAAGTA

GGAATGAGCAGTATAATTGTTTCTCTTACCTTGGTAAAAGGTGAAACTAGTTATGATTAG

CCGTACCTTTTGGCAAGCACTCCGGTTGTTTGAGTTGTTTTGTTTGTTTGTTTTTTTGTT

AAAACACCTTTCAGGAGTAAAAGTTCATCCACAAACCAACAAGCTTCTA

>c12887_g1_i1 len=285 path=[1:0-284]

AAAAAAAAAAAAATATACATTAAGGATAAAAATAATATGTATCTTATTTTAATTTATTAA

AATAAAAAAAAAAAATATACATGAAAATAAATTTATTTCTTTTTTGCTCCAGATACTTTT

GTCTTTTCTGTTCCACGAACTTTTTTTCTTCTATTTTTTAGTTCTTTTGCTGCTCTTCTT

CCTGTTTTAGTTTCTTTTGTTATTAATCCTTCTCTAATAAGTCTGTATTTTTTTTCAAAT

TTTTTAACTGCTTCCACATTTTTATAAATTAAGCCAAATCCTTTC

>c12887_g2_i1 len=985 path=[1223:0-984]

TTATTATTATTTTTTTTTTTTTTGTGTTAATTAACTCCTTTTTTATAGTTTATTTAGTAA

TAACATTTTAATCCTTTTAATAATTTTATTTGATATCTTAATTATTAACACTTTAAATAT

GATACATACTGATGAATCTGAAAACGAACAGGAAGTAAATTACAATAATATATTATATGA

TGACAATATAGGAGACGGAATAGAATACTATAATGATAATTTACCACATATTAATTTTTA

TTTAAAATTTCAAAAAAAACATTTTTTACAAAATTTTTTTAATGAAATAGAAAAAGAAAA

AGGTAAAAAGTGTATAGAAAAAGGTAAAGAAGAAAAAATATGTGAAGACAAAGAATCCGA

TGATAAGATAAAAGAAAAAGATAAGAATATAAAAATAAATGAATTAAATGTATTAAATGA

AGGTGGTTTTTATATAGATGATATTCCAGAAGAATGTTCAGATAATTCTACAGAAAATTT

TTATGAAAATCCTTTTATTTCACTTTCAGAAAATATTTTTCATTTAGATGAAAATGAAGA

ACAAACAAAAAAAATAAATTTAATTTTTCAAAATCATAAAAATAAGTTTACCTTTAGTAT

AGAACGTATTTTAGAAAATGATGAAACGTCTGAACAAAAATGTTTTTTATGTTATAAAAA

AAAAAAATTTAATAAAATAATAAAAAAATAAGTAAATATATAGAATAAAATAAAATATAA

AAAAGTTTAATAAAATAAAATAAAAAGAAAAATGGCAGATCAATTTACAATTCGCATAAA

AAAATTTATGCAGAATCCTCTTTTATGTAGAAAGCAATTTGCTTTAGAAATTTTACATCC

AAATAGAGGATCAGTATCAAAAAAGGAAGTTAAAGAAAAATTAGCTAAAATGTATAAATT

AAATGATGTAGGTACAATTATTTTATTTGGTTTTAAAACTTTATTTGGTGGTGGTAGAAC

GAAAGGATTTGGCTTAATTTATAAA

>c12896_g1_i1 len=224 path=[155:0-223]

TATGGATGATGCAAAGGCCCCGGGAAAAGTCCTGTCCAAACACAAGGACACGCTCTATGC

TCAAGCACTAGTGGCCCACCTTGCCACACATGTCCCGCATCCCCCCTCCGACAGGAGAAC

GCCACCACTCCCCCTCTCTGAAAGAGACACAAAAAACACAGACTTGCCCACAAACCTCCC

AATCCAGCGAGAAGGAATGCAGCACGCGAAGGCACGTCAAAAGG

>c12908_g1_i1 len=220 path=[198:0-219]

GAACTGAGACTAGCACTCTCACAACTACAAGTAAAGAGCTGTGCTACAGGAATTCTGAAC

AAAACTTTTTACAATCTTTAAACAAATTAAGTGAATTTTATCTTGGTTTCTTAACATACA

TAGGGTCCCTATGAAAGTAATGCCTCCTATTCATCTCCACGGAAACTGCAACAGATACAA

AGAGTACAAAAACACCGATAGAGAAAATTCTCAGCTACAA

>c12916_g1_i1 len=344 path=[1:0-343]

GTCAAACTGTGGGCTGTTATCATTCTCATCCAGGACCGTGATGGTTACCTTGGTGATGCC

GTTGATTTTGAAGAGGGAGAGCGCCTGGGCGTTGGTGTTGGGATCAAACTTGTAGGTGAT

CTGGTTGAGGTTGTCAATGGAGCGGGCCTGGACGCGGAGCACCTCAGATCCCAGCGGGAT

GTTCTCCATGATGACAGCCTCATAGCTGCTGTTGGAGAATTGCACGGCCTCATCCAGCTC

ATTCAGCAGGCTGACGTACACACTGCAGAAACCCTGGTTGTTGGGTGGGGCCTGGTCTGT

GGCTGAGACATTCATCAGGTAGCTCGTCTTGGTCTCGTAGTCCA

>c12921_g1_i1 len=274 path=[180:0-273]

ATATTTTTTATTTCATTATTTTTAATATTTTTGTATATATTTTTGTTTATCATTTATAAA

TGTATAATTTTTTTCAGGTATATTTTTTTATATAAGTGCATTAACTACACTTAAATAATT

TTAACATTTATATATTTTTTTGTTTTAAATTTTTTTAAATAATTAAATTTAGTAATTTTT

GTTTTGTTTTTATTTCATTATACTTTTATAACTTATAATTAGTTTTATGTAATTTAACCA

TTTATAATTAAAAATTTATGTAATTATATAGTAA

>c12933_g1_i1 len=254 path=[273:0-253]

AAAAAAAAAAAAAAATAGCTAAATAGAATTCATAATTTTTATTAAATATAAAGGAAACAA

CGTCATTAATATGTTCATAAGGGTGATTTACATGACCCTTTTTTATATTTATTTTTTTTT

TGTTAAGTGAAAACAGATGTATTTCTAGGAAAAAAAATTAATAATTAAAAATACTATTAA

TGTTATTTTTGAAGTTCATAAAATATATCTAGTTGTTCAGATTTTTTCTGTGTGCATAAA

AAAAAAAAAAAAAA

>c12945_g1_i1 len=309 path=[287:0-308]

TGGAAGAGAAGACCGAGAGGGGATCTAATCAATGTTTATAAATACCTAAACTACAGGAGA

TAACAGGATGAGGCCAGACTCTTTTCAGTGGTGAGTAGGACAAGGAGTAATGGCCAAAAA

CTAGAACATGGCAAGTTTCACACAAATATACAGAAGAACTTCCTTACTGTGAGGTTGACA

AAGCCCTGGAACAAGGTGCCCAGAGAGGTTGTACAGGCTCCTTTTCTTTGGAGATATTCA

AGACTCATCTAAATGCCTTCTTGTGTGATCTGCTGCAGGTTACCACTTCAGTAGTTTCAG

GAAATAGAA

>c12952_g1_i1 len=216 path=[197:0-182 424:183-215]

CCTATTTATTTCCATGGAAACTACCACAGATTCAAAGAGCACAATAATGCTGATGGAGCA

AAATCTCAGCTACAAAATGCTATTTTTTCCACACAGTCACCACCATTAGCTCTGCATTTT

CTCCTGTTAGGAACAAGAGGAAAACATCTGCACCAGCAGGGGTGGCCCACTGGTGCTGTC

ACCACAGAATCACACAGAGTCACAGAATGGCCTGGG

>c12954_g1_i1 len=352 path=[1:0-351]

AAAAAAAAAATGAACTATTATATTTTGTATACTTAAACATATGATGTAAAAATAATGTGT

AATATCATTAATGTGTTTATCTAACAAAGAAAAAGACTTATAATTGTAAAAATATTAGTG

TAAAAAATGGAATAAAAAAAAAATATAAAATAAATATATAATGAAATAAAAACATAATAA

AGTTTTGTTAACAAATATTTTTTTTTTTTAAGTTCAAAATAAATTATTTTTTTGATATAT

ACAAAATTTTACATTTTATAAAACTTTATACAATAAATAAAACAACAAATAGTTCAATAA

TTAATTACCAAAAAAAAAAATTGCTTTCATTGCATTATTTTTTTAAATTATA

>c12957_g1_i1 len=226 path=[155:0-225]

ATCCTGGCTCACCCCTGGATTTGCTTCAAGTTGCTCCATCTAGTTTACCTATGCCAGGTG

GAAATTCAACATTTAATCAGCAGGTTCGGGCAAAGATCTATGAGGTAGAACAGCAGATAA

AACAAAGAGGTCGTGCTGTGGAGGTGCGGTGGTCTTTTGACAAGTGCCAAGAATCAACTG

CAGGGGTGACCATCAGCCGAGTTCTGCATACCTTAGAAGTGTTGGA

>c12966_g1_i1 len=208 path=[253:0-138 391:139-207]

GCTGCATAAACTGTTTTTAAGTCAGTGAAAAAAATAGAATGGGGACTGTGCACGCCATTA

GAGCTGTGGAAACTTGAATTCATAGAAAGAATGGTAGCAGAGCACCAAGTGTCGTATACT

TGTATTTTAAAGTATAAAGACTGACAACAGTGTGTGCAATAGCAGTAGTCTTTTGTTTCA

ATGTGTACACTAAATTCTTTTTCTAATG

>c12970_g1_i1 len=207 path=[1:0-206]

AAAAGAAGAGAAGCATTTATAATAATAAAACCATGAATGAGTTTAGGTAAGTTGCAATGA

ATGAGATGCACCATTTGCAGAGGAATATGCCATGAGGATAGATGTATCTATACCACTCTT

TTCTCTGATTTTCTTCTGCTGCTTCTAACCTTGTGAATGTGAGCTGAGGAGCTTAGGCTT

GTATAGATGCAGTACTTCAGGAGAAAT

>c12974_g1_i1 len=272 path=[250:0-271]

ATTTCCAGCATCCCCGGTAAGCAGGGTCCCACCACTGGCTGTGGTCTGCACAAGGTCACC

AGCTGCTCAGCTAAGGAGTGACAAATGTAGCATCCTCACTTCAGAGTTAGCCTTTGGAAA

CTGCTGCATTCTGTAATTGTCCCTCAAAACTGTAACTCCAGAGCTTGCCTTCTTGTAAGT

GGAATCTTGTGGAGATTCTTCACTTCTGAGCATTTAGTAAGTGATTCCTTTGGCATGCAA

AGGATGACTCTTGGGAGGAAGAGTGAGCCTTG

>c12987_g1_i1 len=370 path=[383:0-64 36:65-369]

AAAAAAAAAAAAAAAGACTTTGTCTCTTTTTATATATAGAAATTATATTATTTTTTACAA

AAATTATTTTTTCTTTTTAAAGTGAAAATGCAGCTTTTCAAAATGTAAAAGGATAAGTAT

ATTACTAAACAAAAATAAAAATGTATTTTGAAAATAATTATTTAAAAAATATTAATTCTA

AAAGTGTTATTTTTTTAAAGGAAATTTTAATTTTTCAAGAGGAGAATTTTATTTTTTAAA

AACTAAGAATTTCATATTTTTTAGATAAATAAAAAAATACATATTTGCAAATTAGAACAA

TTATTTCTTTAAAAATTAAAGAAAAAAAAAAAGCACTGAAAAAATACAATACCAAAAAAA

AAAAAAAAAA

>c12987_g1_i2 len=322 path=[19:0-16 36:17-321]

CCTCACGGAAACAACAGATTTTTTCTTTTTAAAGTGAAAATGCAGCTTTTCAAAATGTAA

AAGGATAAGTATATTACTAAACAAAAATAAAAATGTATTTTGAAAATAATTATTTAAAAA

ATATTAATTCTAAAAGTGTTATTTTTTTAAAGGAAATTTTAATTTTTCAAGAGGAGAATT

TTATTTTTTAAAAACTAAGAATTTCATATTTTTTAGATAAATAAAAAAATACATATTTGC

AAATTAGAACAATTATTTCTTTAAAAATTAAAGAAAAAAAAAAAGCACTGAAAAAATACA

ATACCAAAAAAAAAAAAAAAAA

>c12988_g1_i1 len=307 path=[179:0-306]

GACATTCAGACTCTTAATTAAACATGCAGAAGGGTTGCCAATAGGTAATCTACTGTCACA

TCTACTCCTGGTGTAGCTAACAAGCTCATGAAATAATTCCGATTGGATAAGCACAGGAAG

ACCTATCAAACATGTCTGAAATGGGCTACTAACGGACTGTAGGCTAAGATAAAATTCAGC

TGGTTAACAGAATTGACCCATGTTACCCAGACATTCATCCGAGGCTTGATCATGTGAATA

ATTGCGCTTGTTTGACTCAGCAACAGAAGGCTGGTAAGGGCGAGTCTTGTGAACCGGGAC

CCACAGT

>c12994_g1_i1 len=268 path=[274:0-238 512:239-267]

TGTAATCATCTTCACATACCTCCACGTTACTTTGTTCGTGGTGCAGTACCATGTTTGAAG

CCTATGCTTGAACAAGGGCAAGGGAGGTATGTAAAGATGATTACAAGTCAAGGTAGAAGC

TTGTCTTTCATTCATTGAACACGTGGCCTCATAAATAACCAACATAATTGGAAAGCATGA

TTTGAAGACAGTGCTACCTAAGAAACAGAAATGTTTTTAAACTGCTACTTCTGCCACCTG

ATAGTGCAGACAATGGGATGTCCTGTCC

>c12997_g1_i1 len=355 path=[333:0-354]

GACTGCAGCGGGGAAAGGAAGTGGCCGGAGAGCTGCTGCCAGTTCTCATTTTGGGATAGA

GTTCCATGATGTCACCTCCCCATGTGACAACTGCTGTCCCCAAGGCGTTGGGGCTGTGGG

GCCCAGGATGGGGTGGCACACACATAACCGTACGCACATCTTTTTTACACTGCCCCACTC

CGGCTGTCACCTGTCAGCTCACACACGGTCCACAAAGAACTTCCAGCACTTTCTAGGTTG

GAAAGTCGCAGCCAGAGGGCTGTGGCCAACAGCTCCATGTCCAGGTGGAGGTGGTGATGG

GTGGTGACCCCCAGAGGTCCCTCCTGGGAGCGCTGCTCTTCACCACCTCTCACTG

>c13013_g1_i1 len=205 path=[235:0-141 64:142-159 425:160-165 431:166-204]

AGGTAATTACTATATGAATATGCAGAACTATTTTTGTACAAAAATTGTTTAAAAATTATT

TATGCAATGTTTGAATGCATTACCAGTGTTTCGGTTTTGATTGCCAATTTTTATCTTTAC

TTATGGTAGGAGAGAACTTAACCTATACTTCGTAGACAGTATCTATCTACTTTTCCTGAC

CTGGGCACGTTTGTAGATAGATACT

>c13018_g1_i1 len=211 path=[189:0-210]

TTGTATAAGTAAAACTGCAAGTGCATGCCCTGCTTCTTTCAACTTAAAATGGATTTCACA

CCATGACAGGTTTCGTTCTGCATTAAAATTGCAAATCTTCTGTGTGGCCCTGATATTTCA

TTTTGCTGCTTCATTCTGTTTAAAGGGAGAGTTGCTAATTTTTTATTGGCTACCCTAAAT

TAAGGCGGAGTGTCTTTTGGTAAACTGCTGG

>c13025_g1_i1 len=588 path=[1:0-587]

AGTACTGTCAGTTGCTGTGAGGAAGAAAAGCTGTTTAGAAATAACACCAGAATTGCAGGT

ATCTGAAACCTTCAACTTATTTGGGTAAATCTAGATTTTCTTTTTTTTAACCTTAGTTGC

TAATTAGAACTGTACTTCAGAAACCAGCGTTAAAGCTTTTAACAGTGATAACTGTTTTGC

ATAGAGTTCAACTAAACATCCTAAACTTCATGCAAGCATTCAGATGTAATGTAGAGAGAA

GCATAAAACTTAATGTCAGCATTTTCATATGAAACCCTTTACAGTTAAGGTACTGGCTAA

ATGCGTATTTCATATGCTTCCCCCGCCTTACTGTAGTTAAGCTATATAGTCTCATTATTA

ATATTTGAATCCTAAACCTTTTTTCTGTATTTGCATCTCTAATCTGCTATATTGTATCAG

ATTATTTTTTTTTTGTAGAGCAAACTTGGAATTACTTGACAATGCACTTAGTCTTTGATA

GATGTTCTAGAGTGCTACTACTTGAAAGAAATCTAAAAGTTGCTTGAAGTCTGTGAAGTG

TTATATTTTAAAACCAAGAAATGTTGTTTATGTATTTAGGATATCCTT

>c13028_g1_i1 len=209 path=[187:0-208]

GTAAAATAATCTCTACTTATTTCATATATAATAAAACTGATTAAATTAAATAACTATATA

ACCTTTATACAATTACCTATTTATAATAAATTTAACATATTTTTTATATATCTCATAGCT

TTTATTACCTTTCTCAATAATTTCATAAAACTTTACTATATTTCAGAAGATATGCAATTT

CTTGCTAACTACTTTGTAGTTTTTCTTTG

>c13029_g1_i1 len=247 path=[298:0-246]

AAAAAAAAAAGTAAATATAATCAAAATAAGAATATAAACTTTTTTATATATATCCAAAAA

ATAAAAATCTAAAAATATGAAAAATTAATATTTCTATAAATTATAAAAACAAATATAATA

ATAAAAGACTTAAGATTTTTTACTTTTGCACACAAAATATTTATGTTATATGTGCGCATT

TACAAAATAAAAGTTAGATTAATTTACATTATTTCTAGAAATCACTTTCAAAAAAAAAAA

AAAAAAA

>c13034_g1_i1 len=239 path=[455:0-238]

GGCATCTCCAGAGCACTCCAGGCAACAAGGAGCACGTCTGCATAGCAACAAGGGCTGCAT

GCTAATGTTGCTCACCACACAGCACCTTCACGTAGGCATGACTCTGACCAGCCCCCACAA

AGGCCTTCCGTCAGAACCAACTCTTTGTCTGCTGCCTTGGGGAGCTCATCAGATAGAAGA

GAAATAACAGAGGGATTTAGATGGAAAGGAACTTCAGGAAACCCAGATCAAACACAAAG

>c13039_g1_i1 len=203 path=[1:0-202]

GTGGGATGTAGCTCCTCCCTGATAAGAGTGGAGACTGGTCAGAGACACGTGGCCTCCCAA

ATGTCAGACAGAGGGGGTAAAGGGTAGGAAGGACAGATGCACCCATATTTACCCCTCCAT

ACTCAGATTTTCCTAGCAGAAATGTTTCATACTCCTACTTTTCTTCAGTCTAGTTGGGAT

GACGAGAAATGTATAATGCATTT

>c13041_g1_i1 len=316 path=[211:0-315]

ACCACAGTTGTTTGGTTAATTCAATAGCTTACTATCTCTGTTGATATCCAGGGCAACTTG

AAGTCTTCTGGTGGAAAACATATGGATCAGGGGCATAGAAAGGGAGCACTCTTGGCTCTG

GTCGTGTGACGGATTGAGAATTCTTTACATGGAGCGGAAGCAATAGCAGTAGCTGAGCTT

TTAGTCACACTTTTGTCCACTTGCATCTTTTTTTAAGCAGATGTAGCTTAATGCTAAATA

ATAATTATGCTGGTGGTGAATGTTAAAGTACATCTGATTCTGTGCGATTGCATCCATTAC

TGTTAGACAAGCATTG

>c13050_g1_i1 len=352 path=[589:0-328 939:329-351]

AGTGCCTCCTCACACCGTGCTGTCTGTACCTCGTGCTGCCAGCTTCAGAGATTGTAAGGC

CTGAAGTGACTGTTCAGCTATCCGGGGGCCCCGTGTAACTCCAAGTGAGAAATGCCATTA

TCTTCTTTAGCCGGTAATGAACTCGGTATCTCTCAGCTGATGTATTTTCCATGACAGGAC

GGAGGATGCTAGGAGGCAGAGCAGATGGCACTTCCCTGGTTGCGTGGCCCAGCGCCGTGT

CTCCTATTTGCACTTGCTTGACTACAAGCTCCAGCCTCTTTTCTTCACAGAGTTAAAGAG

CTCTTTCACACCAAGTATTTTCTCTCTGTGAAGTGCTTACAATCAAATCACA

>c13053_g1_i1 len=209 path=[415:0-208]

GGTCGCTTTTACATGACTGGAGGAAATATGCTTTTAAAAATTTCTCCTATTTCATTGATG

ACACAGATGAGAATATTTCAGCATCTGAAAGCTAAAATACTGTAATTAAGTAAATTCCCT

CAGCAGTTCAGCTTACAAGAACACCAAATCTTTTGTGGCTTTGAAGTCAGCATGTTTGTT

TATTAGCAATTAATAATAATCAAGAAGAA

>c13057_g1_i1 len=374 path=[1:0-373]

CAGCCATATTTGAGAAATGTGCTCAGGTAGTGCCATTAGCTCTGTGTTGTCTCACAGCCA

GCAAGTCATCATTCTGGTGTTAGTAGAGATTTTCCTCAAAGTCCACAAACAGTTGAAGTC

CATTACCCCAATACTGGCTGCGGCAGGAGTGTGTCCCCTGGGAGTGAAGGCAGCCTACAG

TCCATCCAGCACAGCAAGGAAACCAGCATGTGGTCCTGCGGGCTCTCATGGTTGCTGATC

CACTGGAGAAAACTCTGATCTGCCCTGTAGTTGCTCAGACTGTCTTGAAGACAGGTTGAT

GCCTGCTTGCTTTCAGTCCTTAGAGCCTCCTGCAGTCACCAAGACCTACAAGATAATAGC

ACCTGGTGTCACAG

>c13057_g2_i1 len=270 path=[703:0-269]

AGCAAGGTTAAGATACAACTAGGAATATACCAGGGTTTTTCCAGGTCCTGGACTCGGGCT

GTGAACACTGGATCATTTTTATGCTCTAACACAAAGCCTTTGAAGGCTTTGTGTGCTTCC

CAGAGTACAGCGCAATAGCTGAGGCTGTAGTCAGACTGTCTGCCAGAAGTACTTCTGCAT

TTCCATAGAAAGCCTCTGGAGCCCGTTATAAATCTGCTGAAGTGAAAAGAAATCTGCCCA

CTGTCTTCTGTTGGCTCAAGTAACACCAGT

>c13062_g1_i1 len=228 path=[206:0-227]

TGTAGGCAACCGACCAAGGAAGGAAGAAAACCAAATTCAAATTGCTTCCTTGGTCGGTTG

CCTACACCACTTGTTTCCTCTCTGGCTTCTCTCTGAAAGTTGCATTCTTTTCTCTTTCAG

GCCCTTAGCAAGATTGATTCAGTTATCAGTGCTGCATGTATAGTGAATCTGACGTGCTGT

GCAGGATTGTTCCAGCTCTTGATTTGTTCCATGTTTCACTTCTGTTTG

>c13069_g1_i1 len=307 path=[62:0-59 122:60-306]

AACTGCAATATTTAAAAAAAAACATATTTTATAAAAAAATATAATGTTAAAAAGTTCCTA

CCTTCGAATATTTCAATGCATGCTACTTTTTTCATTTTATCATTTTCATATTATCCAATT

TCATTTTAAATTATTTTATTTAAATTTAAAAAGTTTTACATTTTATTACTTTTAATATAT

TTAAATATGTTGTAAAAACTTAAAAAAAATATACATATATAATTTTATATAGTTAAATAG

GATCTTTTAAACACACACTATGTACTTTAAAAACGAATTCATCAAAATAAAAGCTTTCGC

TATGAAT

>c13069_g1_i2 len=268 path=[123:0-20 122:21-267]

TAGATTTTTTATTTAATGATTCCTTCGAATATTTCAATGCATGCTACTTTTTTCATTTTA

TCATTTTCATATTATCCAATTTCATTTTAAATTATTTTATTTAAATTTAAAAAGTTTTAC

ATTTTATTACTTTTAATATATTTAAATATGTTGTAAAAACTTAAAAAAAATATACATATA

TAATTTTATATAGTTAAATAGGATCTTTTAAACACACACTATGTACTTTAAAAACGAATT

CATCAAAATAAAAGCTTTCGCTATGAAT

>c13070_g1_i1 len=229 path=[264:0-58 104:59-80 344:81-228]

TGGATCCAATGGACTCGACACGTAGGTGATCGTTTCCACCGCTAGCACTGACCAAGCACG

GCACGGGGACCACGGCAGCAGGAGGGCCCTTGCTTGGCCACAGCCCCCCATAGCAGCTCA

GCCCCTCTCACAGGCAGAGATGGGGAACGCAGCAGTGCCCTTCGCACAGACCTGGAGACG

CACAGCACCATCAGCCATCTCACACAGTAACTCGCCCCGTGCTCGCATC

>c13076_g1_i1 len=216 path=[346:0-215]

TCACTAGTAGTATTATATAGGGCAAACAATAGTAGAAAAAAAGCATGAAAAATATCAGTT

AAATTCCACATTTAGAAAAGCAGGATTTCAAGAGCAGAGTGTGTGCAGACATAAAAAGAG

CAGTGACAGGAATCAAACTATGATCTGACACTCCTTAGAAGTGAAAGCAGGTTCTGAAGT

CTTTTGATTCCCTCTTGTATGCTAACAGCATGAAAG

>c13081_g1_i1 len=249 path=[155:0-248]

GACTATGACCCATGTACTTTGTACGTACCGGAGGACTACCTTAACAAATGTACGCCTGGA

ATGCGGAGGTGGTGGCAGCTGAAAAGTCAAAACTTTGATGCAGTGATTTGCTACAAAGTT

GGAAAATTCTATGAGTTGTATCACATGGATGCAGTCACTGGTGTGAATGAGCAGTGCCGG

TCATCTGAGAACTGGCCGACATAAAACTTCCCAACTGACGTGTCAACAAAACAGACCCCA

TAAACACGT

>c13082_g1_i1 len=264 path=[312:0-263]

AAAAAAAAAAAAAAGTAAATATATGAAATTAATTTGAAATATAAAAAATTAAATTTTTAT

TAGATGAAAACAAAGATGCATGTTAAGCCAATTATCTATATTAACGCAATAGAATTATGT

TTCTTTCTTTATATAACTAAATACAAAAAGGAATTTTTTATAATTTATTTATTTAAATTT

AAATATTATTTCTGTATGTATAAATAATTAATTCTCCTAGTAGAGAATTCTTTTGCATAT

AACTTAAAATTTTCGTATATATTA

>c13084_g1_i1 len=214 path=[686:0-213]

GGGTCGGATTCATCAGCAATTAATTACGGTTATTAGGGGCTATTAGAGGCTTTTAATTGG

GATTATTAGAGACTTCTAAGCGGATTTGGGGACTTTTAAGTGGATTTTATGATTCTTAAG

TGGATTTTGGGTGGATTTTACCGCTTTTGGCGAATTTTAATGGGGATTATTAGAAGTTAT

TAGTGGTTATTAGAAGTAATTAGAAGCCGTTAGG

>c13084_g2_i1 len=270 path=[248:0-269]

AGAAATTATTAGAAATGATTAGAAATAATGAGAAATAATGAGAAATAATTAGAAAAATGA

GAAATAAGAGGAATATTAAGTGAACATTTTGTGATTAATTACAAATAATTGGGAAATGAG

TAGAAATTATTAGAAAATATTAGAAATAATCAGAAAATTAAGTGAACATTTTGCGATTAA

TTAGTGGTAATTGGGAAATAATTAGAAATACTTAGAAATAATTAGGAATAAGAGAAATTA

TTAGAAATAATACAAATAATCAGAAAATAA

>c13087_g1_i1 len=264 path=[39:0-181 19:182-263]

TACGAAGATGACGCAGAGGAAGAGATAAGTACACAGACTTTAGAAGCTGACAGTATTGCA

AATATTCATTCATGCATTCAACAGCCAGAATACCCAAGGATGAAGTGCCTAGAAGCAATT

AACTTTACCACAAGAAGTACACAACAATAGATGGATGCAAATGCAGTCACCGTTATAAGT

TTCCAGCCTTCTTACCTGGTTACCTTGAACAAATAAACTAGGGTGAGGTGGGAGGAGAGG

TGGGGGGAGAACATCCTACCTTGA

>c13089_g1_i1 len=250 path=[1:0-249]

ACCATGGATTTATGATTTATTTGTATACAAATTAAAGCTCCAGTCGTGCTATTTGATCCA

TGTTTGATCTGTGGAGCTTCCTGAAAATCTTTTAAAGCCGCAGTGACCTACCCAGTTGAG

TGGTAAGAAGGTGGGAACTAAATGTCCAGTTCTATGAGTGCTTATATTGCTAACTTTGCA

AATAGCCTGATGATTCTGCAGAAACCAGAGGGTTCGTTCAGGTGATGGTAGGTGTGGCTG

GAGGATGGAG

>c13091_g1_i1 len=261 path=[358:0-181 60:182-260]

GGCTGGAATCACTTAAGAGCTTTTTTTCAGACCTCAGTTTTAGAAGTTCCCTGCAACTCA

GTTTTAGAAGTTCCCTGTAACTGAATGTCACAAATACTTTGGATAACTGAAAAAAGCAAG

ACATGATTTACAAAGTCAACTGGTCCTAGAGCATGGAATAAAATACACTGGTTATTCTTC

ATAAAAAAGTGTCATACTAGCAACCAAATAAAAGGTGTCTGATTCAACCTGTAAATATCC

TCTATTATCATTCACTTTTTT

>c13092_g1_i1 len=445 path=[983:0-24 532:25-444]

GAATTTGGTTCTGTTTCTCACCTCAAACACATGAATATTTAGTACAAAATGTTCTTTCAA

GGTGGGGCAACCATTTCTAATTGTGCCACGGAAAGGTGTTTTGTAACATTTTTACAACTT

GACTGTGAAAAAGCATTTCTGTGAAATATGTTTAGACAGACTACCTCCCATCACCCAGCA

TATCTCAAGGCTCTTTCTGAGTAGTCAATAAACTCATATTTCTTAAAAAAAGAAAGAAAG

AAAGAAAGCGCCTGGATGCGAACACTGAGTCACTTAATGTAAACAAATACCAGCTTTATG

ACAGATCAATAATGCAGCCACGCTTAATGCATTCACGCAAAGAGGCAATGACACTTCCCA

GGGAGCCGGGCTGTGCTGAGCAAACCGAGCCGTTCCCGCAGCCAGCGCGCATACATATCG

TTACGGAATCGTCACTTGCAAAACC

>c13092_g1_i2 len=685 path=[267:0-264 532:265-684]

GCTTTGCAACGAACGGTGCGTGGGGTTAAAATGAAGGCCAACCCAAATGGGGAGGAAAAA

TGAGCTACTGACGCTTTATCTTGCTTTGATACAGGCTCTCTCTTTGGTTTTCAGGCTGTT

TACTCAAAACAGCCCTTGAGAAAGATGCTGTGCCTCAAGCAGATGTTACATTCAACCGGT

TTAGCAATATTACGCCTATTTTTTCTCAAGCTAGTCAAGGAGGGGAGGGAGAATATAATA

TATGTTAGTGCAAGTTCACAGAGACAACACATGAATATTTAGTACAAAATGTTCTTTCAA

GGTGGGGCAACCATTTCTAATTGTGCCACGGAAAGGTGTTTTGTAACATTTTTACAACTT

GACTGTGAAAAAGCATTTCTGTGAAATATGTTTAGACAGACTACCTCCCATCACCCAGCA

TATCTCAAGGCTCTTTCTGAGTAGTCAATAAACTCATATTTCTTAAAAAAAGAAAGAAAG

AAAGAAAGCGCCTGGATGCGAACACTGAGTCACTTAATGTAAACAAATACCAGCTTTATG

ACAGATCAATAATGCAGCCACGCTTAATGCATTCACGCAAAGAGGCAATGACACTTCCCA

GGGAGCCGGGCTGTGCTGAGCAAACCGAGCCGTTCCCGCAGCCAGCGCGCATACATATCG

TTACGGAATCGTCACTTGCAAAACC

>c13093_g1_i1 len=280 path=[258:0-181 440:182-279]

TAAAAAAGAAAAAAAAAAAAAAATATTCAATTATTTTTTCAATGTCTTTATAAATTTATA

TTAACAGTATTTGTGACCATATATGTAATATTAACTATTAATTTCTGTTATTTTTTTATC

TTATAAAAATTAAATAGTTCACTAAATATTTCCTTATATTTCTATATTTTTTATATTCTG

AAGGACTGAAAATAATATTGCATTATGCAAACAGAATACATATGAATCTATATATTCAAA

TTTTTTATATTATCTAGGTAAAGCTCATTTGATAAATTCG

>c13099_g1_i1 len=231 path=[395:0-198 593:199-230]

AAAGTTAACCACAATGATATTAGCGTGGAAATATCTTTCTAAAATGAGACACCAGAAAGG

AAGAGTCAATTACTTCTAAATGCTTACAAAACCTAAAACGTTTTTTCCACCATTTTAGGA

AAAACTCATTAAAATGATTGGGAAAAGCCAATCTCATGGATATGGGAAGATACAACAAGG

TAAACAATTCCCAACTCCTCTTTTATGTTCCAGCTATTCCTTACATCTCAG

>c13099_g1_i2 len=232 path=[395:0-198 179:199-231]

AAAGTTAACCACAATGATATTAGCGTGGAAATATCTTTCTAAAATGAGACACCAGAAAGG

AAGAGTCAATTACTTCTAAATGCTTACAAAACCTAAAACGTTTTTTCCACCATTTTAGGA

AAAACTCATTAAAATGATTGGGAAAAGCCAATCTCATGGATATGGGAAGATACAACAAGG

TAAACAATTCCCAACTCCTAATGCCAACTGAGATGTAAGGAATAGCTGGAAC

>c13100_g1_i1 len=259 path=[237:0-101 507:102-258]

GAAACTTGCAAATGAGAAGACTTTTGTACAATATCGTTTCCAAAGCAATGTATTCACAAA

GCATAGAAGATATTTTTATAATGCCATTCAGGTGAGAAACCCCATTACAATAGTGGAGTT

CAATTTCAGAATCTCTCAAACTTGGATAAAATTGAAATAGAGAAATTACCTGGAACCAGT

GAAAAGGAAAACCGGGTTTAAAAAAATCCCACATTTCTAATTGATAATTGTCTCCTACAT

TTTAAATCTACTGTATTTA

>c13121_g1_i1 len=202 path=[1:0-201]

TGTTAGTCAGCCGGCTACTTAATTGGCTGAGAGTGTTTTTGTGTAGGGAACTGAAAGCCC

TTCTTGGAGCCAGGCTGCCCAGCTGTCCCCGGAGCCGCCCTGCCTCACCCCGGGCACCGC

ACATCCGCCACGGCCCCACGTCACTGACCACAAATCCTCTTCCTCACCACACATAATGAG

AAACATCCATACTATACATGCG

>c13131_g1_i1 len=206 path=[1:0-205]

AGACCGGGAGCCCTCCGGTGCTGCATGAACATCTCCCCGGCCCCGCTCCGTGCAGCCAAT

AAGGCACAAAGAAGAGTCTCACCACGCAGAGCTGCGTGCAGCACGGGCTAGGCTCCACAA

ACGTAATTGAGACAGCAGGAATTCAGTGCTAACTACAAAGCCTTGTTTTGATTACCGTGT

GGACTCCATCCCAACCTACTGTAGTT

>c13132_g1_i1 len=295 path=[335:0-294]

GCCTCTTCTTGTGGCCATCATAGGTCAACGATGAAAAAGAAATCCAAAAAGTTAAAAAAT

ATTGAGTTTCCTCCATCTCTCTGGAATTTGGTACACGTTATAACAACATTAGGATCCTGT

ATCTTTGGTAGGCACCACTTCAGCTGATGGTAAAGAGTGAAGGACTGATCTCTGTGGATA

TGAACTGACGGCACATGTGCAATGGCAAGCAAAACTGGACCAGTGTCCTTGCTGAAATCC

TTGTGCCAGAGGCAGTAATTTTTACCAGTGTTTCCTATCGTTAATGAAGATTCCA

>c13135_g1_i1 len=225 path=[155:0-224]

GCCGCTGCTTCTGAGAGAAGGCCTTCCTTCTGAGCACAGGTCACATGGAAATAAGCTCTG

CACATCCCAGCATCACAACTGATGCATACTCCAGTTCTGGCAAAACGAGGATCTTCACAA

AAACTGCATTCCTTAGCACCATACTTTGAATAATTCATTTCTGTAAGCGTTACTGGCCGC

AATTTGTCAATATCTCCAAATGCCACTCCTGGAACGTAGAGGGCA

>c13152_g1_i1 len=233 path=[49:0-127 176:128-232]

GTGGTGAGGTGGGGGTCAGCCTCTGCTCCCCAGTAACAGCTATAGAGAAAGAGCTAATGG

CCTCAAGTTGCACTAGGGAAGGTTCAGGTTAGATATTAGGAAATATTTATTCTCCAGAAG

AGTGGTAATACATTGGAAGAGGCTGCGCAGGGAAGTGGTGGAGTTACTGTCCCATGAGGT

GTTCAAGAACCACATAGACGTGGCAGAGGGGGACATGGTTTAATGAGAACCAT

>c13159_g1_i1 len=316 path=[427:0-201 649:202-315]

ATGGCTTAACCAGCTAAGATGAGTTCAAACAGCATTTCTCGTAGTGTTTCACTTGGCTAT

TTGCTATTGTAGTCACAATTTTACTCTTCATACATCATGAGAAAACAGAAAATAACAGAT

GGATTCCCAGGCTTAAGGAGAATTTTGATTCCTTGCCTTTCTAGCTCTTACTAAGCAAAA

ACGTAGGATACCAAAAGATGACGTCCGTGGGTTTTCTGGAATAGAACACAATTCCATCTT

CAGAAGACCTACAGAAATTGGCAAGCATTATAGCAAGAACAGCCCACTCCAAGAGTTCAT

TCCTCCATTATTTTTA

>c13163_g1_i1 len=349 path=[533:0-348]

ATAAAGAGTAATTTTTCCCCCCCCTCCCCTCTCCATCTCTCATGTAGCAGTAGACACTGC

AGAAAAATGAATTTTCCAGACAGGAGCTCACTAGCTTCAATCCAGTATCCAGAATAGTAA

TAGTCTATTACCTATTAAAAGCTTAGATTTCAATGGAATTAAATTTGTGCTGCTTGCTAG

CAGAAGAAATAGGTTGATAGTTGGAGAGGTACAAAGAGAGGTGGAAAAATTACTCTTTAT

CTGTTAAATCACTTTCTTCTTTAATGGCACACACGAATTCATATTTACTATAGAAGCATC

TTTTGCCTGATTCTCAGTCAGTGTGTTTTACTGTGGTTATTTATGTGGA

>c13164_g1_i1 len=218 path=[237:0-217]

TCCAGCTGAAGACCAGGCTTGCAACAACCACCACTTTTATGGAGATGTGAGTGGACTTTG

CTTCGGCAGCAATTCTTTGCAGCTGACTGGGAAGCTTGTGGATAAGGTTGTTGCTGTGAG

CGAGGAGGACGTCCTCATTTCAATGCTGAGATTGCTGGAGTACGAGCGAGCCACAGTTGA

TGCAGAAGGGGCCATTGGACTTGCAGCACTGGTTGCTG

>c13168_g1_i1 len=215 path=[1:0-56 58:57-214]

ACCTGCTGCTGTGGCTCAGTGTGGCTCAGAGTGGTGGGGCCCCAGGCTTTCTTCCTGCTG

GATGGTTGGTGAGGGTCAGGCTGAGCTCCCTGGTCCTGCAGCCCCGGCGTCTGCCACGCT

CATCCCCAGCCCACTCCTTCCTTCTCTCAGTCCTTCGCAACCCACATCCTGTGGCCACAC

GCTGGGAGGAGGACAGAGCGAGGTGGAGGCAGGGA

>c13168_g1_i2 len=237 path=[464:0-78 58:79-236]

CGTGCTGAGCCCCCCTGCAGCTCCCACCAAGCAGGGCTGTGATTAAACGGAACAGAACCC

CAGCTTTGCCCTGACCCACCTGGATGGTTGGTGAGGGTCAGGCTGAGCTCCCTGGTCCTG

CAGCCCCGGCGTCTGCCACGCTCATCCCCAGCCCACTCCTTCCTTCTCTCAGTCCTTCGC

AACCCACATCCTGTGGCCACACGCTGGGAGGAGGACAGAGCGAGGTGGAGGCAGGGA

>c13170_g1_i1 len=244 path=[346:0-243]

GGAAGCATTTTTCCATGATGTTGTCAACAGCTGTGAATGTGCCATTAGGACAGGGAAGAC

AGACAGGGGCCTGGCCAGACCATTCACAGTCTTTTAACTTGTAGGTACCTGCATGGCACC

TCATGCAGCAGTGGGTCCCTTTGGGATGTAGGTATTGTCCAAGTTGACACTGCACCTGCC

TCTTTTCTCTCCTCAAGCGGTTAAAGGGATCTCTTCCATCCAAAGTTGCCCGACGGAGCT

GCAG

>c13183_g1_i1 len=307 path=[1:0-306]

ATCCCCACTGGTTTCAGGAAACCAGTGGGGATGGCCCCACAAACTTCTTCTGCAGTATCA

TCTACGTCGCCATGGCCACTTCACGCCACAACTCTGTGGGATCCTTCGGTGTCCTTTGCC

TGATGATTGGGCAGTCTGTGAATCGGCACCTCCAGCTAGCGGGGTATGACGACAACACCG

ACTCTCTGCTGGTAGGCAATGCCACCTCCTCCGGTAACGGGACGGGTACCTGTGACAGGA

GCTGCTACGCCATCACTGTGGCCCTTTCCTTGAGCTTTCTGGTTGGTCTTTACCAGATCC

TGCTGGG

>c13187_g1_i1 len=202 path=[189:0-201]

ATAGGGAAGAACAACAGGACAGATGACTGCATGAGAGATGCTCTAGACAAAGACAAACGG

TAGAGCTGCCATACAGTGGTCAGCCTGTTAAGAGATAGGAATTCTGAGTTTTGGATAAAA

ATCATTCACAGCCCTCTTCATTTTGGGGAAAATAATTTCATTATTGCCACAGGATGGTCA

GGGGAAAAGGACAGTTCAGTTA

>c13190_g1_i1 len=228 path=[223:0-201 587:202-204 591:205-227]

AAAAATAATCGCAGTGGAACTGTTTACTGAGGATTGTATGCTTCAAAACATGCTGTACCT

GGACTCAGTTGTCTTATTTCAACAAAATGTTCCTATTCTTCCAGTTGCATTCCATTCAAT

TAACATTCCTCAAACAATACAGTAAACTGGAAGTGTATAGTTGGGGGTATTTGTTTACTT

TTCTCTAATGAATATTTCCTTTTTCCTTAAAACTTGAAAGGTAAGGAA

>c13194_g1_i1 len=257 path=[1:0-256]

GCAGCCTTTAGAGAATACACCATTCTCCCCAGTAGCTTCCACATGATATAACCCCCTGGT

GCTTGGACAACTACCTTTTCTATCAGCAATCTTATTTATGCATTGAGTACTTGTAGTACA

GATCCCTGTCAAAGCTGAATGTGAGTTCTTGAGATTTGAACACTCACTGAGTATTTCTCA

CACTGTGCCTCCTTCAAGTGCTGTTCTTGTTACCAGTAGCACATCATTAGCTTTGCAGAA

CCATTTGCTTCAGATAT

>c13195_g1_i1 len=353 path=[1:0-352]

AGTATTCAATTCCACTTCTCATACATATTTTTTTCTTTGTTTGAAAATCTGATTTTAACT

CGGGGGTTTTGAATACTTAGATGTCTGCTTTCTTTAGTAACAGCATAGTTTGGAAAGCGT

AGGGAAGAGATATTTGCTTTTCAAATAGAAGGGACAAAGAGTCTTCAAATATTACATATA

CATATTTATTTCTAGCGTTCTTCCTGTTACTTTGAGTTACTTTTCCTCCCTTCTCTAAAG

CCAAAAGAACAGACTGAATTTGGGATTTTTTGATGAGTTGACCTATCACATTTTAATACA

ACTTGAAAATTGTCGCTGTTGGTGGGGAGAAGGTAGGAAGTGTTTGTGTCATG

>c13200_g1_i1 len=298 path=[1:0-69 510:70-297]

ATTTCTCTCCCAGTATAACTTCACCCATCAATTTAACATACACTTCAACAGAGACACTGG

CACTCTTCCTACTTCTGCCATACGACTCAGCAATAACTGCAGAGGAAAAACCAAAGGCCT

CCAAACTGATTGCTTGTGGTTTAAGGTGACAGAGTAGATGGTCTGGAAATGGTGGTAAAT

ATTCTGATGGAAATTTGGCTTTCAGAAGAAAACATTTTTAAAGAGGAGATGCAGCAGGTG

GCTTCAAATGTAGCGGTACTATTTTTAGCTTACTGGATGGCCTCCGAATAATGAGAAG

>c13202_g1_i1 len=227 path=[1:0-42 44:43-226]

ATCGTGCAGCTTCTCCTTGATCTATGGAAAACAGGAATGGTGACTCATACAAAACACGCT

TGCCTCACTAAAACAAGTATTCTCCCAGCTAGTAGGCTGCATTCCCATTTGTCCAGTACA

ACATATGTATTGCCTTATATTAACAATCCCCATCTGGAAAGGTTACGGAGCTGGCAGGAA

TACAATCAGAATTCCCAAACACCAAAGCTTTATCTTAAAATAAAATA

>c13208_g1_i1 len=227 path=[297:0-226]

TGGAACTTGTGATGGATCAAAACATGGCCCAAAGGCATGAACATTACCTAAGACTGGGCA

GCAACATCTCGTGGCTTGTGAAGGGGAGTGACACAGAGGCTGAGGAGGTGCTAGTCTTTG

TCCAGATCTGGAACGTGAGAAACGTGAACCTCTCACACTGTGTTGCTGTGTGACCCCATC

ATCAACCGAGAGGTTATTCTTGATGTCTCCTCCTTTCCCATACAAGA

>c13211_g1_i1 len=396 path=[374:0-395]

CTGCGGACGTCGCTCAGCGCTTTGCCCCACGCCGAAGAGGAGCAGAGCCATAGAAATGCC

AGCACCGCCGTCACCACCAGGTCAGCCACGGGCAAAGCGCTGCCCGCCCCCCGGTAGAGG

TGCAGGAAGGCCACATAGAGGACGAGGGCAGCAGTGGAGTAAAGGAAGGAGAGGACGGCA

ATGGTGACGAAGAACTGAGAAGAGGAGGAGAAATCCCCCACCAGGTGAACGTCACCTCCG

GGCCACGCAGCCCCACACAGAGCCGGGAGGGAGGGGGAGAAGGCGGCCTGGTTCAACCGG

AATGGGTACCCGAAGGGCGCCCGCACGGTGGCGCTGCTGTTGTTGCAGGTGACGAGGAAG

GAAACGTCGCCGCTGTAACCCCCACAGGTGGCAAAG

>c13215_g1_i1 len=272 path=[181:0-178 360:179-271]

TAACAGAGATCTTTCTGGTCTTTCTTCAAGTGCAGTTGCAGATCCTCTTATGCTCTTTTA

TCACTTAAAATGGAAAGCGTTCTATAATTTTCCTCAGTTAAGTGTTCCTGGATTTTCCTT

CTACTTGCAACTTGCATTGCTAGGTGTATTCCAAAGTGCTAACTGTTCTGGTAGCTCGTT

TACGAAAATAAAATCTTAAGTGGAACTCCCAAAGTTATTTCACAATGGTGTGATCACTTC

TGCAACATTATGCCTAATTTTGGTATCCATAG

>c13229_g1_i1 len=262 path=[1:0-261]

AGATCTCGGAGCATCCTCTATTGTTTTTACAGCTCCTCTGTGACTAAACGCTATTTTCCA

ATTACTATACTTTATTTTTTTTCAGTTGGGCTCCTCATCCTTTCTTTGAAGCAACGTGAC

CGCATAGTAAACTGTTTTTGAGGAGTGTTTGCTGTTTGAGGTTAGGAGCTGAAACCACAG

TTTCTTATCTTGTCTTTCCGCGTCTGTTTTCGAATGAGCTAGAAGGGCAAGAGAGCAAAG

CACTAACAACCGAAATCTCCTT

>c13254_g1_i1 len=220 path=[286:0-133 419:134-219]

TTTTTGCCATCATTGCCTCTTTTAAGATACGCACTGTTTAAACTCACTGAAGAGCCAACA

ACAGTGTAATAAATGAAAGCTAACACAAATACTTTGCTAAATGTCTTTGTTTCGATATAC

GTATGTTATATTCAATATATGTATGTTGGTAAACCTATTTAATTTATGGGATTGCAGACT

CTTCTGAGGCTGACTTAACAAATGTTTATATAGTGCCTTG

>c13254_g1_i2 len=221 path=[286:0-133 25:134-220]

TTTTTGCCATCATTGCCTCTTTTAAGATACGCACTGTTTAAACTCACTGAAGAGCCAACA

ACAGTGTAATAAATGAAAGCTAACACAAATACTTTGCTAAATGTCTTTGTTTCGATATAC

GTATGTTATATTCAGTATATGTTATTTATGCTTTGGTGTTGGCTAGACAGCTTGACTTGG

ATTAGGCTGTATTGTACAAGGCACTATATAAACATTTGTTA

>c13264_g1_i1 len=228 path=[488:0-227]

TGGAGCAGAACCCCATCCCCCCACACCCTGCAACAAATGAGTTAGAATTTAAAAGCTGTG

ACACTGCATAATGCCTTTGACAGAGAGTTGTCATAGGTGCTGCTACTTAGGAGGTAATGC

CTCATTTTTCAAATTATGGTCTTCTTCGTTTTTTCCAATCTACCGTTGAGTTTAAAACAA

AAAAGTTTTTCGGATACGTTTTCTGTCTCTGCTCAAACTTTGTTTTCG

>c13268_g1_i1 len=251 path=[383:0-250]

GCGACTAGCTGCAGCTCACAACTATTTAAGTTTTCAAAAGTTCAAAACATCAGCAACCTT

AATATTACTGATTTACTGATAGCATCACCATTCTCTTGCATTTAGTTTGACAAAAGTCCA

CTCTGAATTTATGCTTGTTTAATGGCTTAATATTGTTTCTCCCCAAAAGTAAAAACTGAA

GTAAGGAATTCCTACCCAGTTATTACTTCAAGCTGACAGTCACTCACAACACAACAGTCT

GCCTAGGCCAT

>c13270_g1_i1 len=288 path=[71:0-86 60:87-96 70:97-287]

GGGCTGCCTTTTGCGCAAAGGTTAAGCTGATGAGAGGAGAGGGAGGGAGAAAAATAGCAA

CAACCCTCGACTCGAAACAAATCTCCTCTTCACCAAACACTCCTCTCTATTTCCTTGGCT

CTTTGGGGAGAAGGGAGGGAGCAATCCATCTTCCAGCAGGCGCACTCCCAGCAGACCTTG

CTATCAATGCAGTAACCAGGAGCATGCCCATGTCAGGGGGGTTGGATCTTTGAGGTCCCT

TCCAATCCGAGCCATTCTATGATTTTATGACTCTACCTTGTGTTGGAG

>c13270_g1_i2 len=297 path=[71:0-86 157:87-105 70:106-296]

GGGCTGCCTTTTGCGCAAAGGTTAAGCTGATGAGAGGAGAGGGAGGGAGAAAAATAGCAA

CAACCCTCGACTCGAAACAAATCTCCTATTGAAAAAGCGAGTCCCTCACTCCTCTCTATT

TCCTTGGCTCTTTGGGGAGAAGGGAGGGAGCAATCCATCTTCCAGCAGGCGCACTCCCAG

CAGACCTTGCTATCAATGCAGTAACCAGGAGCATGCCCATGTCAGGGGGGTTGGATCTTT

GAGGTCCCTTCCAATCCGAGCCATTCTATGATTTTATGACTCTACCTTGTGTTGGAG

>c13280_g1_i1 len=269 path=[290:0-110 22:111-268]

AATTGAAGGACTTCAATCTGCCCTTAAAAAAGCAATCAAAGAAACAACTCAGAAGAAGAA

AGAACATCGTATTCCTACTCACACCATAATACTAGGCAGTGATGAGCCCCACTGAAACCA

AAGCAAGTGCTTTTGCCTCTCAGCTTCTGAGAAGACAAGCACTGATCTTCAGGTTTCTAT

ATCAATGATATGACATGCTATCTCAAGGCACTGAGATATCAAAGATGGCTTCGGATGAAG

ACAACACTATATAAGCTATAATTTGCAAG

>c13282_g1_i1 len=267 path=[296:0-10 21:11-266]

ATTTTTTTTTTTTTCTATTATTTTTTAAAGTTATGTAGATAAAAAAGCACATTTTAATAT

AGTTTTTTTATGTGCATTTTAATGAATAATTTTTTTTTTTTATTTCTTTTAATATTAAAT

TTTATATGAAAAAAAAATTAAATTTCATATATATAAAACATTTTTTAAGATTCCAAAAAA

AAAAATTTTTTACAACTATTTATGAGGCAAATATAAATATTTTTATTAAGAGAATTTTTT

ATAAAAATATAATTTAAAAAAAAAAAA

>c13294_g1_i1 len=203 path=[226:0-202]

GTACATTTATAAACATTTGTTAAAGAATTTTCATTAAGAAAAAGAAGTTAATGAAATGAA

GTAGTTAAAAGAAAACATAATTGTACTTTTTTATTTATTTTTGAAATAATAAAAAATTAA

AAAAATATTATGTATATAATAAAATTTCATCATATTTTAATGATACAAATTAATCATAAA

AAAATTATATATATAGAGAGAGA

>c13300_g1_i1 len=228 path=[1:0-227]

GGCCATTGCACAGTTGCTGAAATACAAGGTCGCTAATTTGGGTGATGTTATGTAATGCTG

ACTGTTTGCCCAGTACATGGCAGGTGACTTAGGAAAAGTCAGGAAATAATTGTTTAAAGC

CTTGCCTGTTAACACGGCCATCAGCCCTGTTTTTAGAACAGTAATTCAGATGGGCGGATT

CCAGAGTTGGTTTATGAAACTTTGTCCACTTGAGAAAAAGCAGTTAAA

>c13307_g1_i1 len=311 path=[187:0-184 372:185-198 497:199-310]

AAAATCTTTATGTGAATATAAAAGAATCATTTCCAAAGAAATAAAACAAAAAAGTTTAAA

AATTAGTGCAACTTTTCAAGTTCTATGCAATACAGTGTTTACATTTGTTTTCAACTGACA

ATGTAGACACTGATACAACAGGAAATCTTACCTCTAGCTGGACTAATGGGGGAAAAGGAA

AAAAGCAAGGAAGCCTTAAGAAAGTTATTTTACTAGACATGATCAACTCAAACAATTATT

TTCATTTTCTAATTTCTAGACTATGGCTACTTAAATAAAACATTGGTATTGCTTATATTT

TTTACTTTCAA

>c13319_g1_i1 len=486 path=[464:0-485]

CTGTTGAAAATGCCAATTGCTTGGCAGGTCTGAACGATAACTAACAGTAGATACAGTTAG

ACACTACCATCAGAAATGTGCACAACTCAATATTCACAACATTTCTAACGTAAAATAAAC

TGAAAAGACTAATGTCACCACTACTTCCTTGGCGGCAGAAGGAAGGTTTAGTGAATGCAA

CAGAAGCGTGTGTGTATATTAAGACAAAAAACAATGGTGCATGTACACACACACAAATAC

ACACAAAGCACATAATCTCATGCCCAAGCACCTGCTAGTGTTACTGATAACAAAATCTAA

AGTGAATATTAACTTAAGAATGAAGGCAATTAAAAAGTACCCTCTGTTCCTATTAAAAAG

AAATACCAGTGTATTTTAAACAGAGAACTTCCTTATCATTTTTAAATTAGCATTAGGTTC

ATTTTATTTTTAAAACTTTGTCTTGGTATAAAATATTCTATTACGTTGGATTGCTGTGGT

TTTTGG

>c13320_g1_i1 len=223 path=[201:0-222]

TGACACCAAAGTCAAATTCTGGAAAAAAAAGACTGACATGACGAACTTTTCTACAGCCTG

CTCATATAACTTTGTGATTGGTATTGCCTTTGTCACACAGAATGCATTCTCCTGAAACAT

ACATTACCAGCCACAGAACTCCAGGGAAAAAAAAAACACCCAAGCAAAGCAATGCAGTTA

CAAGACTAGCATTTAATACACAACTCGTATGTTGCCTAGTGAG

>c13322_g1_i1 len=227 path=[169:0-226]

GCAGGAGATTCTGTGATCAGTAGAGAGGTATTTTCAATTTAAAAATGGCTTAATTTTTAA

ACATACCCTTTCATGCATAAAGGACTGTAAGCTTAATTGGCACTTAATCAATGTGTCTTT

GCCTTATAATAGTCAACTAAATGGTTGAAGGGTAGAGGTCTTTGCAATGAACATCCATAA

TGACTGTCAGAACAGAATTGATGTAGGGACTTCTTTTACAAAAATTA

>c13325_g1_i1 len=547 path=[1:0-546]

GATTTTATTCGATCATAAAGTAAAAAGGTTATTTTAGAAATATAAATAATCTTTTATAGA

TAAAAAGAAAATAAAAATTTTCTTTATTTTTAAAATATCTTAACACATTTTTAAAATTTC

TTGTAATTAAAATAATTTACTAACTATTTGATGACATGTGAATTCCTCAATGAAAAAATT

AAAAAGATTAGTTTATTTCTTATAATTTACAGGGAATTTTTTTTCTTGTTATTTTGAATT

ATAATTTTTTTTAACATAAAAAAAAAGTAAGATAAAATATTATTGAACTGCAACAATATA

TAAATAATTTATCTATAAATAAAAAAATTTTTACAACTATTTTTATATTTATATATTATT

ATCATTCTAACATAATATATAACTTATAAAAATCATTTTCTTATTTTTATAAAAAGTTAA

ATACACTCATATGTGCAGATATATGTATAAATTACAGATTCAAGCTTTTATTCTTTTTTA

TTAAAAATAAGATATATAAAAATATATATATTATATAAATTTTTGTCTCCATTTGTCATA

GTCTTAT

>c13338_g1_i1 len=240 path=[1:0-239]

GATTGTGGAAAGAGTCTTTGTTGGTGTACTTTTGCTATTGGACAAGCTGGTCCCAACCAA

AAATAACATGGTAGCACACTAAAATGCTTTCATTTTTCTGTTGCAGTTACCGTTTTGCAA

GAGTAGCAACGATGAGCAAATTGTTTCTCAAAACAGGGTACGCAGTAATTCTCATTGTCT

TTGGTGATCAGTGGTTTTGTTCCCAGTGGTTGCTGGCAATACTGACAAACAAAACAGGAT

>c13344_g1_i1 len=201 path=[1:0-200]

GAGGGTGGTTCTGCCAGGCTGGGCCTCTCTGCGAGGGCAGCACAGGGTGGGGAAGTGCTG

GGGAGGTCTGATTGCTCCTGCTGCGGAAATAAAGACCTGCTTTCACCTCCAGCAGCATCT

GATGCAGCTCATTTGCCTTTAGATAAGATTGAATCCCTTGTCTTGCACGCAGAACAGGCA

GAATGGCATGTGTAGATGACA

>c13346_g1_i1 len=226 path=[39:0-225]

TGGGCAAAAAGGAGGAAATCACTGTAAGTATTTAATAATTGATGCTATGGATGTTGCAGT

AAGGTGTTTTTTCACAGCAAGGCAATATAACTTAATAGGATTTTTCAGAGTTGAAAGCAA

GCTGCGTGGAAAATTGTTCTGGGTGAATTTTCATGCTGTTGATAATTTGCAGCCAAATTA

GTTTTGAAAGGCAAACCTCCATTTTATCTTTTGTTAGGGTTGTATG

>c13348_g1_i1 len=210 path=[188:0-101 438:102-141 44:142-209]

AAGATGGCAGAGGGCCTGGAACATAGCTTATAAGGAAAGGCTGAGCGGGCTGGGTCTGTT

CTGCCTTGAGACAAGGTGACTCAGAGGGGATCTGATCAATGCCTATAAATACCTTAGGTG

TGGCCGTTTCCTCTTGTCCTATCACTACACACAGCTGGAAGGAGTCTGTCCTCGTCCCTT

TGCCCCCCACACCTAAGGTATTTATAGGCA

>c13354_g1_i1 len=241 path=[219:0-240]

AAAAAAAAAAAAAATTTAATTAAAGGGAATAAAAAGTAAAATGAGTAAAAAAAAAAGATT

AGTTAATTGCAAATTTTAAATATTTCCATATTAATAGTATTATTTTTAAAGAATAAAATT

GAGGGAAATATATAATTTTTGTAAGAAAATAATGATATAAGAATTTTTTTTCAAATAAAA

ACACATCATAATTTACCATAGTATAATAAATATTTATATAATATAGTGTTCTGCAATGAC

A

>c13360_g1_i1 len=203 path=[481:0-202]

GGCCGAGTAGGCCTCGGGCCCGGAGAGACGAAGGCCGGGTAGGCCGCGGGGCCTGGCAGG

CTGCAGGCCTGGGTAGGCCGCAGGAGCGCTCGTGCTCACATTAAACCCGTAAGAAGCACC

AGACCTGCCTCACTGTGTCACTTCACTCAGCACCACGTGAAATAAACGTCAGGGTGTGGC

CTGGAGCTGGAGGCTCAGCCCTG

>c13362_g1_i1 len=212 path=[41:0-147 250:148-211]

GACGAAGCAGAACTGCAATACAATGAGCACACAGAACTACAGATCCACCTGCAAACATTT

CTTATAAAACCACTGTAATGGTGATCTGCACTCTGACTGGAGGGTTAGTTACTATTTTTA

AGAGCAAATGAAGAAGTTATCTCACTACTTTGATCTCAACAAATTGCAGAGCAAATTTTA

AAGGCACGGAGGGATGTGGGTTGGTGGGGATG

>c13363_g1_i1 len=304 path=[257:0-254 512:255-303]

ATGACTGCACTCACGTTGTAAGGGGGCTGGGGAGACGAAAGGAGTGGAAAGTATATGGAA

ATGATATATGGAAGGATGGCACGTGAAGGGGAGGTGTCAGGGAAGGCTGATGGATTTCAA

GTGAGAATACAACGAAGGGTAAATGGCTTCTGTTTAGCCTCACGCTGTAGGAACGCCCCC

AGAGATGGGCAGCCCTTCCAGTAAAGGGGGCACACTGGGATGCAAGGTGCACAGCAACTG

GTCCTGCTGGAGCTGAGCTCGCACTTCAGCTGCGCTGTGTGCTGCTGCTGCTCTACGTGC

TGCT

>c13413_g1_i1 len=279 path=[257:0-278]

AAAGACTGAAAGTTCCATAGAACAGCTTTTTTGGGTTCTTGCTATGTAGTTTCCAAACAC

AGAGAGGAGAAAATGGGTTTAACAAACAAGAAACACCTATCTGTCAAAAAACTTAAAAAG

TTCTGCTAGCATTCTTTCAATTTCTTTCTTCAGAAAGAAATCACACTCTAAACATTGTTT

GTAAGAAGTTAAATACACCAAACTTATGTTTAATACACACTTGTTTACTGAAATACCACC

TATTTTCTAAAAGGTAAGTAAACAATCTAGGAATCCCTA

>c13413_g2_i1 len=216 path=[706:0-215]

TACCAGTTACAAAAGAGAGAGTAGAACTAGAACACAAGTCTGTCATCAGTTAGATCCTTC

AAACGTAGCCTGGGTTACTACCTTCCAGAGAGTGCCCATTGCCTGAAAGTGTCAATAGTT

TCTATTCCCTCAGAGAAATGTTACTTATAGAAGCCTCCATTCAGTACAAAGTTCCTCAGC

CCTCTAGAATAAGTGTATAAGAGACTAAAGACTGAA

>c13423_g1_i1 len=269 path=[247:0-268]

AGCAGAGGTAAAAAATGAAGGAACCTACAGACACTTTATTTTTGGCTTTAAAGCATGACT

GAGGTGCTTTAAAAACCTCTACAGACAAGTAGAGAGTCTATACCAAAGCTAAAAACACAT

GATATGTGTGCAATGTCAAGTGTTGAAGATTTCATCAGAAGGGCTTTCTTACATCTTGTG

TTGATTTTGTTTCATACCCAATCACAACAGTGGTGTTGGAGTTTTAACTAAGGGATTATA

TTTGCTCACTACAGTAAAGCTCACAAAAA

>c13427_g1_i1 len=230 path=[367:0-72 11:73-79 452:80-229]

ATATATATATATATATTTGTATGCTTGCTACATGAAAAAATTCATGTATAAGAAAAAAAT

AAAAAATACATTAAAATAAAAAAAAAAAAGAAAATAAGTAAGAGTGAAAAAAGAGATGAA

AAATATATTTATAATTAAAAATTAAAAATACATTTATGGTTATGCAATTTTTAAAATTTT

TTTGGTAATTAAATTGCATATAGTTCTATATATATATAAAAAAAAAAAAA

>c13441_g1_i1 len=231 path=[1:0-230]

AGACGTCCTCTGGGTTTTTTGCATGTATGTTTTTCATTGTTATCTTTCACAGCAACAAAG

CTAATTCCAAAGCATTAGGATATGTTGCCTTCATAGATCAAAACTGACAGTTTCTCCACG

TTAAAAAATAAACCTTAGTGCTATTCTTGTGCTGTCATGAAGCATATTTAGGAATCCTTT

GGCTACATTTGTGCAGTCACTGTTGCTGTTGGCATTGCAGTGCATTTACAG

>c13447_g1_i1 len=267 path=[245:0-144 390:145-266]

AAATTTGACTTCATAACTAGAAAGGTAAGAGAAATCGACAGAATACAAAGCTTTAACTGT

TGCAACACGGCAGTATGGACTTCTGATATAAAATGTAGATGACTGAGTAGAATTTCATGC

CAAGAGTAATGGATGTTATCATAGTTTTTCTTCCTGTTCTGCCGTTTCTGGGTAGAGGAG

TAGTTTTTCTTAATAAGCTCCATGTCTGTGACGTTCCTTTAGCTCAGTTTGTAACACACA

GTGTGGGTCTTCCCAAAGTGCCAGGTG

>c13448_g1_i1 len=256 path=[412:0-255]

TTCCAGCTGTCTCGCTAGCCTTTCAAATGCTCTTTCCAAATTAACTTAGTAATCAACTTT

AAGCAGCTCTGTGATTTTTTTAATTATTATTTTTTCTACAGGCGAAAGACCTTTTTTCTG

GTGGCCATGAACGCTTCCAAGCGTAAACGGTCTCATTCTCTTTCAGCTCTGGTCATTGCC

CAGATGGAGTTTGAGATGGATTACACATTCCAACAGCACAAAAGGGGGTAATTTAACAAA

TTAAAACGTCAACAAG

>c13449_g1_i1 len=206 path=[371:0-123 145:124-160 25:161-205]

TACATTTCAGTACTGAATTCTCTATGATGAAGAGCAGTGGGCAGACTTGCACATTGGCAT

CTGCTACTAATGAAGCCTTCTATATAGAATTCTGGGTAATCCTTGAGAAACAATGCTAGA

CAACGTAAGGGATGTTGGTTGTCCCAATTTTAATATATGCTCAAAAGCACACTTAGGTAT

GATGGTGACCTAAGAAGCAAGGACTT

>c13451_g1_i1 len=212 path=[1:0-71 73:72-211]

GCTCTCAGCCTGTCCTTGCAGGAGTTTGTGGCTCAAATAATAAGGCTGAGGCCATTTGTA

GCTACATTACAGTAGAGCTGCTATTAAAACTTATGTGCCATAAATTTTGTTTGATTAGGA

GAAATTGTTTTCTCTCTTAAAATGTAGTTTGAATCTGTGGAAGTCCGTCTTAGTTGTTCT

GAAAAAGAACATTATTTCTTTGGTTGCCTTAA

>c13452_g1_i1 len=262 path=[240:0-261]

AATGGATAGCATATATACATTTATGTACATATATGTAGATATGTATAACGTAGAAGAGCA

ATTTTGGAGTAGAGCAGATGAAGAAGAGGAGTCAGATAACGGCCGTTTAGAGAAGTCAGA

TAACAGCCAGTTAGAGGAGATACCAGAGTTGAAATCCAAGCCTGACAGAGGGCAGGATAA

TGAGGAAAAGATAGAAGACAGTGAGAAAGAAGACGCTATGCATTCCAACTTGTGAGTGAG

ACTACAATGGATAGCATATATA

>c13454_g1_i1 len=594 path=[726:0-593]

ATTGCAATGTCCCATTATTGGGGTGTCCCGTTATTGGGGTGTCCTGGAGTTGAGATATCC

CTGTGTTGGGGTGTCCCATTACTGGGGTGTCCCAGCACTGCAGTGTCCTATTATTAGGGT

GTCCTGGCATTGCAGTGTCCCATTATTGGGGTGTCCCAGAGTTGGGATGTCCTGTTATTG

GGGATATCCCATTATTGGGATGTCCCATTATTGGGGTGCTCTGTTATTGGGGCATCCCAG

TGCTGGGACGTGCCATGGGTTTGGGTCTCCCGGCGTTGGGTGCATGGCGGTGTTGGGGTG

CCTTCAGTTTTGGGGTCTCCTGGTGTCGGGGTGTCTCTGTGTTGGCATCTCTCACGTTTT

GGGGTCTCGTGGCGTTGCGGCATCTGCTGTTTTGGGGGCATCTCCAGTTTTTGGGGTCTC

CTCCTGTTGGAGTCTTCCCCATTCGGAGCCGTGTCGCGTTCGGGGTTTCCCGGTGTCGCT

GTGCCCCATCTTTTGGGGTGTCCTCTTTTAGGCTCCCCTTAATGCTGGCCGCCCCTCACG

CTGCCCCCCACCCCTTTACCCCCCCCCCATAGGGGAGCCCCTCCGTGGCGCCTA

>c13455_g1_i1 len=236 path=[155:0-166 109:167-235]

TGTGCACGCTGGGACGCCGTCAGCACGCTGCTGCCAGCACGCTGGCTGCTCCCCCATCCC

CAGGGCTCGCGCTCAGGACCCGGCCCTGCTGCTGTTCACGCTCCCCTCCTGCCCTCAGCC

TGCTCCCCGACAGCTGCCGGCCCCTGTGTGTGCGTGTGTTGGTGCGCGGGTCCATGTGCT

GTGTGCAGGGCTCTGTGGCGTGGCCCGCACCTGGCTCTGCAGGGCTCAGTGCTTGG

>c13456_g1_i1 len=224 path=[202:0-223]

AATAGCATGGAACAGCGTGTATACATGTGTAACAGTGTAAACGTGTAACCTTGTGTATAC

ATGTGTGACCCTAACAGTGTGTAACCATGTATACACACGGTACTTGTGTGTTCGTGTGTA

ACTATGTGTGAACGCCTGTATACTCTGTTGCTGAATGTAAGGCTTTGAATGGCATCCTTC

ATGATGCTGTTGCTGCTGCCTGTGCTGCTCAGTGCGTACGGAGT

>c13468_g1_i1 len=227 path=[449:0-226]

ACTTTGTGTTTGAGCTCCCTGTAACAAAATCTTTCAAAAGCGATTTTACAAATTATTCTA

CATTAATGTAAAAAGGAACTCTTAAATTTAGCATCATGGATTCCACCCAACTCCCTTCTA

TCACAACATGAGTTTCGCCGGTTGTAGATGTTAGAATTTGTAATACATTTTTCTAACGTA

TCCTGTACATATTAAATAACCTGAAAGGCTCCTCTTGTAGTGCATTA

>c13474_g1_i1 len=237 path=[272:0-135 555:136-236]

AAATTTATTAAAAATATAATTTTTATTACAATTATTTTTAAAAATTATTGATGAAAAAGG

AATGTATATAAAATAAAAATGCATACATTGACAAATACGAAAAATTGAAAAAAACTTATA

AAATTGACAAATATGAAAATAAAAATTTCATACAACAATTGACAGATTTGAAAAATGAAG

AAATATTTATAATTTACTATTAAAATTTTCCCTTTAAAAATATCTATTTATAAAAAT

>c13488_g1_i1 len=239 path=[275:0-238]

AAAAAAAAAAAAAAATTATGTAACACTGTTTTTATTTTTCCACCCATAAAATTATACATT

TATTTTTATATATTTAATCTCACATAAACTTAATACATTCAAAAATAGTATGTATGAGTT

TTTTAAATTCTTTAATATGTTTCAACTTGTTAATTAATATATTATCTTTTATTTTAAATC

TTATTTTATATTACTTAAATTTATTTCATTTTAATTAAAAGTAGAATAATTCAGATTAT

>c13497_g1_i1 len=322 path=[1:0-321]

GTATCGATTCTGGATGTGCAGTGTGTGTAAGCACTGTATGAAAGCCAAAGAAAGAAGCAA

TTACAACTTCTGTCCTCTGTGCCACTGCTCAGCGTAGTGGAGGACAGAGGACTGCGTGTC

CTCCCATCTGCTGCATCTCAGAAAAGGACCTTCTAAAATGGTTATTTTTGTATGTATGAC

ATTTCTAACTACACTGCCAGTTCTGCACTGTCACAGATAGTGCCACTGTTACAGACATGT

TTCAAAATAACCACCTTTTTTCCTCTGTAATAAAGGCTTATAAGGAAACAGCATTCTTTT

CCAGTGGTGAATTGAGCCTAGT

>c13501_g1_i1 len=299 path=[167:0-298]

GTGCTCACCTGACGAGGCTTTTGTGCACTAACACCTTAACAGGGGATCTCAGCTTCTTTC

TTCATCTTAATGAATCAACACGTCTTTGCCTTCTGAGGCAATTGTAATCATCATTTTCTG

TGCCAGAAGTCCTTGCAGTTGATGGGTTGTAAAATCCAATGTGCTTTAATGGAAACCACA

AGGGTTTTATCCACAGAACGTGACTGAAATCTGAAGTTCTCTGTAGACTTGGTTTCTAAG

GCAAGGCTGCTTATCTGTAATTGAGAGTTTGATACCTATTTGTTACTCTTTGTCACTCA

>c13502_g1_i1 len=305 path=[1:0-242 606:243-304]

AGGTAATCTTCGAATTAAAAACACAGAACCCCAAGGCCTGTAATGCTGCGTTACTCAGCT

CTGAATTTTCACTAGAAATGTGTGCCTTAAATACTTTGCAAAGTCGAGGGAAGACTCTGC

ATCGTAGTGGCAGTTTCATCAATTCTTTGCTGCAGCTGGAGGAACTTGGATTCCGTAGTG

GCTCACCTGTGGTCAAGAAAATAGCTTTCATTGCATGGAAGAGTCTAATAGATAATTTTG

CTCTAAATCCAGGTAGATTAAAAAGAAGTGACTACTCAAAACAATTCAAGTTAACTTCTT

TTTAA

>c13510_g1_i1 len=204 path=[213:0-146 179:147-203]

TAGTGGAGGCCACGGGCTAGTGAGGTAAAAGTGAAATGACAATCTTATAGGTAAGTTAAA

GAAAATCTGATGGGAAACTGAGGAGGCTTTGGGGAGGTTTCTGTGCTAAATTTCTTGTTC

GGAAGACTGGATGGTTTTTTTGCCTCCTAACTTCACTCTGCAGACTACTCTGCAGGACAC

ATAAAGCAAACTGGCAGGTTTTAG

>c13511_g1_i1 len=269 path=[155:0-268]

TAAAGTGTGAAGTATTATTAAGGATGCATTTGCCTTTCTGGAGGCTCCACAAATCATCCT

AGCGATAGGTAGAGGTGGTTGGGAGCTCTGAAGACCTTGGTTGGGCTAGCCTCCATAACA

AATTCACAGCCTTCAGGAAGCACACGGTACTGGGACTTTTGCTAATGGTTTAGCATGAAT

CTTAAGTGGATTTCAAATTAACTTGATCGTTTTGGAAAAAGCTTGTGAATTCTTTAATAA

TGTGCAAAACTGAGTCAAACACAAGGTTA

>c13514_g1_i1 len=240 path=[282:0-214 547:215-239]

AGCACAGTGCAGACGTTGGCTACGTAATTAGTTCCAACCTCTTTGTTCTGCAAGTCAGGA

AATTCTAACAGATTTTGGGGGAAAAAAAATGGAGGGTAAGAAGAGAACTTTGCATGTTTT

CTTTGGTATGAGAATAGCAACTCTGTTAAAGAATACTAAAGCAATGCTGAAACAAGGTCA

AAAACATGGCATTGTTTCCAGTCTCCCTGTAGTAGCCCACCAGACAATTCCTGGATTCCC

>c13518_g1_i1 len=207 path=[185:0-206]

AGAGTATTGTACAAACAGATTAATAAAGACAGAACAGATTTTGCTTTACTAAAAGAATTA

ATGTGTAATTTTAATACTATAATCTAGTAAATTGTTTTATATATATCGTGTACTTCGAAC

ATTTATTTTTTTTAAGAAATTTTTACTTTTTTTAATAAAATTCTCCATTTTTTTTTTTAA

ATTTAAATCCTCATTGTTTGTAGATTT

>c13518_g2_i1 len=244 path=[369:0-243]

TACAATACTCTAGCAAAGTTAATGAATAGACAATTAATAAATCAAATGCTCTAGCAAATT

CATTGTACTGATAATTAATAAATAAAAAATTACGTTGAATAATGCGGAATGTTTTTATTT

TATGAAATATTAAAAATATAATGCTAAAATTTATTCATAAAGAGTATTAAGTGAATAAAA

TTATATAGTAATATTCTAATGAAATTAATGTTTAATATTAATTGATAAAAGTGTATACTT

TGTT

>c13525_g1_i1 len=312 path=[53:0-311]

GATGTAGTCCTGCCTCTCTTCCTATTCACACTCCCCACTATATTCTCTTTGCAGTTTTAC

TCAGATCTGTTGATTCTTTGCCAGCATTGTTCCCATGGCCATTGCTCCCATGTTCTCTCT

GCTTTCCTTTCTCGTACGTACATAATTCTGCTCATGTCCCCCTTCATTCTTCGTGTCCCA

TTTTTTCCTGCGTGCCTGTTGCAGATAGGTTTTTTCCCTCATATTCTATGTGAAACTCCC

CTACATGCCCTGGGACATGCCTGATTCTGTATCCCAGCTGCTCCTCAGATCCTGATAGTG

GGACTTTATTGG

>c13540_g1_i1 len=222 path=[47:0-154 46:155-221]

AGGTCTTTTTCAAATCTAGCAAACCTTTGCAAGATTTGAAAAAGACCTCACAGTCCTTAT

TCCCATGATGTAAAGCATATAAATATTTGCACAGAAGCTGATACTTTTGTGTCCTGCTGG

GTGTGCAGTAAGCAAGATGGAGCAGTGCTCTGAACAAGCCTGAAAAGAATTCCTCAATAA

GAGGGATTCCCACTGTGGGGAAAAAAAAATGATAACCTGTAT

>c13545_g1_i1 len=248 path=[364:0-247]

GTTATGTTAATAATATAGATAACACTTAAAAATTTTTTTTTTTTTTTCGAAAAATAAAGT

TTTATGAAATATATATAAGAATATTAGATTAAATTTATTTGTAACATTAATTAAATAATT

AATATAAATATATAAAGCATATAGAATAATATTGATAAAAATAAAACTCATGAAATATAT

TTGTGAAAATACAAGTATCATTTTACTTTATTTTTGAAGTATACAATTCTTTAAAAAAAA

AAAAACAA

>c13548_g1_i1 len=282 path=[1:0-281]

GTTTCCCAAAGCCACACCATCACCTGGAGCCTTCCCTGCAGCAGGACAGCTTTGCTAAGG

GCCGTGCAGGGAGCAATCCCAGGGCAGGCAGGGGAGAGCTGAGCCAGAGCTGACAGCAAC

ATGGAAAGGGCACTGCGGGGAGGAAAGCCGATGCCTTGGCAGCGATGTGTGTCCCGTCCC

CGCTGAGTGCCAGCAACTTAATCTGTGCTGATTGCATCTAAAGGCTGAGCGTGGGGCTGC

TTTCCTGCCTGCCTCAGTGGGGAAGTGAGCAGCGTGTGCCTG

>c13548_g2_i1 len=218 path=[714:0-217]

AGAAGGAGCTGAAGATTTCCTCCCAGCTGCGACCGTTGACAAGGTGTACAAACACGAGCA

GATAATCACACATTAAGGATGTGGCAAGCTGAAAGATTTCAGGAAGCAGCTCTTAAGCAC

AGCTCTGTTAATTTAGGGAGGAAATCGGTTTTCAGAAATTTGGCTTAACATCGGCTCCAA

AGCCAGAGGGCCGGCGCCAGCAGCCTTGTGCAATGGAG

>c13551_g1_i1 len=210 path=[188:0-209]

GCGTCTGCACTGCGATGGCGGCGTGGAATTTAAAGACTAAAAGGAATTCTTCCCACAAAA

TTAAAAGCCGTGACGCCGAACGACGCCGTGCCGGGTGGATCAGAGACCGCTCCGTGGGGC

ACACGCAGCCCCGGGGCTCCGCGTGGGGCAGAGCTGCTGCTGGGACAGCCGGCGCCGCCG

CGGGGATAACAGCACCAACGCTTTGGGGGG

>c13555_g1_i1 len=299 path=[1:0-298]

GGGAGGAGCTGTTACATCACCAGCTTGGCAGCTGGAAGTCAGATGGGTCTTACTCTCTGT

AACTGAGATTATTAGCTAATGGCTATAGCCAGTCCTGGGTGTGGTGCTTCCCATCATTAC

TGTCAATTATGTAGGTAAGATCCATACAGCAAAATCAGGACGACTGGGTGAAATTGCATG

TCAGCAGCACGCTACGACTTGAAAATGAAGCCTATAATGGAGATGCCTTTTGGCTATGGA

ACATGAGGGAACAGTGGGCATTTGTGCATGATTTGCAAATACAGGAGGTGAGAGAGGAA

>c13563_g1_i1 len=268 path=[340:0-172 25:173-267]

GGTAACTTTGTGTTTGAGCCAGAAGAACGTTTATTGACCACTTTACAAGTAATTTGCAAG

AGCAGGAATAGTTAAAAGTTCAGAGAATAAAATGAGTAGCTAGTATGTTTTAATTACTTA

TTGTTCCTCACATATAAATTCTTAATAACGCAGAAGGAAAAAAAATCCTGGAATACTCTG

AACATACACAACTTCCAGTACAGCTTTACTAAACAAAGCACACTGCATAAGATAGTCAGC

CAAAGAATTTGTAAGTCCTGCCCATTAC

>c13563_g1_i2 len=268 path=[340:0-172 512:173-267]

GGTAACTTTGTGTTTGAGCCAGAAGAACGTTTATTGACCACTTTACAAGTAATTTGCAAG

AGCAGGAATAGTTAAAAGTTCAGAGAATAAAATGAGTAGCTAGTATGTTTTAATTACTTA

TTGTTCCTCACATATAAATTCTTAATAACGCAGAAGGAAAAAAAATCCTGGAACACCTCA

AATAGGTAGAAGGGAGTCAGATTTGGACTGCAGATTGTATGTTTTTTTTATTGTCTTGTT

GCTGTAATGGGCAGGACTTACAAATTCT

>c13565_g1_i1 len=212 path=[400:0-211]

TTGTAGAGTTTACACTGACTGATGTACGTGCAAAAGACTTGCTTGAATGGTCAAGAGACA

CATACAGTCCGGATGAACACTACTGGGTCACACTGAACCGTCTGCATGGTAAGATGCATG

TTATTTGCACTAAGAATAACACCTGCAGTGACAGCACTGGGGTATGGGACAGAGAGCTGA

GTTCTTGTTCCTAAATCATCTGTTTGCCTGTG

>c13566_g1_i1 len=225 path=[1:0-224]

TGCTGCTGTCTGCTCAGCACCTGGCAGGGCTGTGCTCTGCTGTCAGAGAGCTGAAGTCAC

TGCTGGAACACTCGCAGTGCTTGCCTCCTTCCTGCCTACAGCTATGATTTTCCTTCTGCT

GCCCTCCTAAGGATCCCTGTTGATTAATACTTATTCTGTTTCCTAAAGAAATCTATTCAG

CCTCCACATACTACAGCTGAGAGCAATGTTTGAGAACCATGTTGG

>c13567_g1_i1 len=235 path=[401:0-209 610:210-234]

CCTCCATTTCATCTGAATTTAATAGAAATATACACTCTGGAGGATGGAACTAAAACTATC

CCTAAAAGCACCATTTTATCAGTATAAACTGCCTAGCCTGGACACAACTACACCAAATAA

GGATCAAATACCTACACTTTTAACATATCAGCACTAGTAGTAGGCATAAACAAACCCAAA

ACAGAATGTGAGATAAAACACTGACAGTTAACATCTTCTGATACTTCAGGGATTA

>c13571_g1_i1 len=202 path=[342:0-201]

TTTTTCTTTTTTTTCTTTTTTTTAACATTTTTTTTTCTTTTTCTTTACATTTTTTCACCC

ATTTCCGTCCGACAGCAAAACGGCGCCTTTTGTTGTTGTCGGTTTTTTGGGGGCGTTTTT

TTCCCACTTTTTGTTCTTTTTTACCGTTCCAAACGCTCTTTTTTGCCGCTCCCCCTCCGC

GTCCCTTCTCAGCTCCTCTTCC

>c13575_g1_i1 len=202 path=[1:0-201]

TCGCAATCATGAGACAACTATTAAGTCTACTTGTTTGTTTTTTTCTTTAGGCATATCTGG

TAATGGGTGTTAGAGGAGGGGGTCTCAGAAATACAAAGTGGCCTTGCATACTTGCTGCTC

GTAATTAGAAATTCAGGAACTGTATTGACAAGACAATGTATTGTTAATGAACTGTTGAGA

ACATTCAAACATGATCAGAAAG

>c13577_g1_i1 len=217 path=[195:0-67 419:68-216]

AAGTGTGGATGTAGTCTGAGTGGTAAACCCACACTGCAGCCTTACAAGGGTTAGCCTTTA

GCAAAGCCAAAATGCCTCACATTCCTTGACCTCTGCTAAAAGCACAAACCAAAATTACCT

TTGCAAGAATACTGTGTTTCCATACTTTTATTGTAACTGAAAGCACTGAAAAGTTGAACT

TCCTGTTCGGTGGAGTAGGACTGGCTGTTTTCACTCA

>c13589_g1_i1 len=215 path=[213:0-214]

GAAATCACATTCATTCTGCATAACGGGGGCACTTATCTACCAGGAAAAAGACCACCAGGA

TCTGGGGAAGGCTGTGCAGCTTCTCAGGCCTCACCATTTCTTATGAAAAGACTTAATTGC

ACAGGCCGGCTGGGGCACCTAGATTACATGCTGCTGTGTTGTGTATCCACTTAGTGCTGA

ATCATGGTGCAGATGCTTGCTAAATCAAATCTCAT

>c13599_g1_i1 len=234 path=[212:0-233]

AGAGCCCTGACTCCTATTTCATAAACACTGGCTTGGAAAATGAAACAAAATTCAGAGGAA

ACCCTAGACGTTTCTGCAGCAGCAGCAAGGCCTGCTCTCTGGCTGGAAGTGCATGGGGTG

CAGGGAAAAGTCTGTCAGGAGGCAGGGAGGGAAAAGCATGGGTCTGAACGTGGTGCTGGC

TCTGATTTACCTTACTGTGACACAGTTAAGCACTTCCAATCTCTGCTCTTTGGA

>c13602_g1_i1 len=362 path=[1:0-361]

GTTCAATAAGTGTTGAGGATTATTTGTTATCTGAACACGTCAGCTTACACATTAACAAAT

ACTAAGCTAAAATCTGTCTCTGAAGTATCAGCAATCACTGATAACATCAATATGTGAAAA

AAAGAATAAATATAAAAACAGGCCTTCACATTTGGATCCACTTGTTGTCACTCCGGAGGT

CCCTTGTGTCCAGTGTGTCAACTTTCATGTATTAATTTTAATTATTACACGATGACACAG

AACATTTCCAGAGTTTTATGTTTGCCAGAAAACTGCTTGGATGGGGACAAGGGATATTTC

CCCTTGGTCTAGGATACCTCCAGAACTGTATCTTTCTCACTCTCCATGATAACCCCAAGA

CA

>c13604_g1_i1 len=293 path=[1:0-292]

GTGCTGGGAATTGTATTGTCTTGAACATGGGATCCAGCCTGATGGTCAGATGCCCAGTGA

TAAAACTATTGGAGGCGGAGATGATTCATTTAACACTTTCTTCAGTGAGACGGGAGCTGG

TAAACATGTTCCCAGAGCAGTGTTTGTGGACTTGGAGCCAACTGTAGTTGGTACGTACTC

AAGTTGCACATCTTCAGTGTAAAACTAAACATTACCCATAGACCATCTTTTCATCTCTTT

CAAATAACAGTGTCCAAAAATGTCAAAACATCATCCAGCTATATACCTTTTAG

>c13605_g1_i1 len=266 path=[490:0-265]

TTCCACGTGGTGGTGAGCAGAGCCACAACGACACGCAGGTCTTCTTGAAGTTCACGCTGG

ATCTCAAGACCATCCTGGTGTCCACGGCCATGGGCTGCATCACCTTCCTCGGCGTGGTTC

TCTTCTGCTTCCTGCTCCTCTTCGTCTGGAGCCGGGGCCGAGGGCAGCACAAGAACAATT

TCTCAGTGGAGTACTCCTTCCGGAAGGTGGATGGACCCACCACCACCACGGGCCAAGGAG

GAGCCAGGAAGTTCAACATGAAGATG

>c13606_g1_i1 len=221 path=[199:0-220]

CATCCCTCTCAGTGCAAACCTGTAGCACATAGCACAGCAACAAAACCCTTACCCCTGGAC

GCATCCCAGTGTGCAGAGCTGACGACGGAGTGCTGGGAGCTGCTGCTACCAGGGAATTTA

TTGCAGCTCTTATCAGGAGAGGGTATGAGCATCTGTCTTCCATGGAGATGTGGTTGCAGC

CCTGCAGCTCCTCCTGCGTCAGAGCAGGATGAGGAACGGGG

>c13616_g1_i1 len=202 path=[53:0-201]

AAAAAAGAAAAGAAAAGAAGACATTACAATTATATTCATGAAACCTATTTTCATAACAAC

TATTTCGTTTATTATTTCTATTATTTTTTCTTAAAAGATTATCACTAATGTAACTTTATA

TTTACATATTAAATTTGTATATATAATATATACAAGAATATTTAATAAAAAACCAAATGA

CATATGTAAAAAAAAAAAAAAA

>c13625_g1_i1 len=215 path=[193:0-214]

TGCACTGCTCTTACTGTAGGTTGGATTTGGAGGTGATCCACTTTGCGTATGGTTATGGAT

TTGTTCTGGAAAGGATTTCACAGTGCTATCTGTGGAAAGCAGGGGAGGTTCAGTTGAAAA

AGAAAAGTTAAAGAAAGGATAGATTAACAAAGAAATACTTTCATTTTGCTGTAGCAGGAA

GTGTCATACCTCTCAAACATGTGAACTGTTAAGAA

>c13627_g1_i1 len=260 path=[1:0-259]

GCTGCAGTGTCCATGGCCACCACAGCATGCTGATCTCTTCATCATCAACAGGAAGCACTG

CCTGCAGCCAGCTCAAATCACACAGCAGCAGCTTCTTGAGGCATCCTCCACCTGCCCAGC

TGCATGCAGGGCACCAGTATTCAGCTCAGTGCCTGTCTGTTTCCCAGCTGTGAGGCCACA

TTCCTGCCCGTGGAGCACCACAATGCCCTGGAAGTGCACATCCGCTCCATGAAGTTGCTG

CATCTTGGGCACCAGCTCAT

>c13635_g1_i1 len=239 path=[533:0-238]

AATCAGGTCCAAGTGCTCGAATGCTTTTGATATAATCCACACTTTGATCTGATTCAAGAT

AACACACATGCTCTGTAATAGCTTGCCAACATACTTGTCCATTTTCAGACACAATATTAT

TAATATCATAGATGTCTTTTTTCTCAAATCCAAGTTCGCAAATCACATAATGCACAGCTG

GAGAAAGACAAGTAGTGACCGAGTCTGATGATGCCATCTCCGCTGTGCATTATGTGATT

>c13640_g1_i1 len=208 path=[186:0-39 226:40-207]

GGTCCTTATGGGGTCCTATGGGGTTCTACTGGGTTTTATGGGGTCCTGTGAGGTGCTATG

GGGTACTTTGGGGGATTTATAGGGTCCTATGGGCTCTTATGGGGTCCTATTGGGGCTTAT

GAGGGATCTATGGGGTCTTATGGGGGATTCTTGGAAGCCATCTGTGTCCGTTTTGAGGCC

AACTGGGGCCGTTTCAGTTCATTTTGAG

>c13642_g1_i1 len=260 path=[1:0-148 150:149-259]

AAGTGGGAGGCTCAAAGTTGAAACTATGCAGGATGCAGACGCTCCCTCTGCTTTCATGCT

GCCTCTCTGAGGTATCCCAAGACGTGCAAGGAAGCAGTGTTTCTCTTGTTTCTTTTGCGC

TTTTTCCGCAGTCAGCTCAGGAGGAGAATCATTCACCTACGCAGTGAGAAGATCTGATTT

CAGCTCTCTTGCTGCGTTAAAGAATTTGATGAGGTCTCTCCCACCTATCAAATATTGTAA

ATATCAGACTTCATTACTTA

>c13653_g1_i1 len=304 path=[244:0-241 486:242-303]

GAGTACAAGTGTGGGTTTGGTGCCTGTGTGTCTGCAGATTTTGCCTCGTGGCAGTGGTGG

GGCTGCAGCCCAGCAGTTCCTGCAGAGCATTCTCAGGGCTGTGCCGATGTTCTGCACAGA

GGTGTGGGTCCTGTGCTCAGCTCTGAGCTATGGCCATCGTGGGTTTTACTGTGGATCTGC

ACCATCACTACTCAGATACTTCTGCTAAATATGCAGCTATGCTAGAGGAGCTGTTAGGTA

TTTAGGGAAACAGTTTCTCCTTGGCACAGCTTAAACCAAGCAGTCATCTAATTTCCTCTA

GAGA

>c13654_g1_i1 len=237 path=[133:0-130 264:131-236]

CTGGGCAGCCTGTTCCATGCCCACCACTTTCTAGTGTAGAACCTTTTCCTGACATTCAAC

CTGACTCCCCCCTGACAGCTCCATGCCATTTCCTCAGGTTCTATCACTGTCACCAGAGAG

CAGAGATTAGCACTACCATAATTACTATAACAACTTGGCTGCGCTCAAGTGCTGGAACTT

TTGAATTGATGTGAAGCAATTGATTCTGGTTAGAAATTCTGTTATAATTTTATTAGG

>c13654_g1_i2 len=216 path=[265:0-109 264:110-215]

GGACCTGAGAGCGTTTGCACACCTCTAGCCCTGGCATCATCCATGCAACAGGTTACTAGG

GGACTTGACAATCGGATGTTTTAAATAAGGACCAGGCTTGTGAGTTTTCAACTACCATAA

TTACTATAACAACTTGGCTGCGCTCAAGTGCTGGAACTTTTGAATTGATGTGAAGCAATT

GATTCTGGTTAGAAATTCTGTTATAATTTTATTAGG

>c13658_g1_i1 len=208 path=[1:0-94 96:95-207]

CAGAGTTAGAGAGAGGGATGGTGGTGATTCTGCTGCAACTTCAGTGTGAGAAGATTGAAT

TTCCTTTCTTGAAGGCCTTTTGCAGATTCCCTGGAGTGTCTCCTGAGAAGTTCTGTTGAG

GAAAGATCACTTAAAAATGAGTTTGGTGAAAGAATACTGGCAAACTCCCACTGTGCTCAG

GTTAACAGGGACCAGTTACTCTCCACAA

>c13672_g1_i1 len=215 path=[244:0-19 26:20-214]

CTGTTCCACTGGAGGAGGGGAGACCTTACTGAATACGTGTAGAGCCTAGGGTGGGATCCC

TAACATTTCCAAATGTAAGTCGTTTATTCCAGCATCATTTTATCACCTTGGAAAATGCAA

TTCAGAAAAAAGTTACTGCTTTGACAGGATTTTGTAGTACTTGAACAGTAACACAACAGC

ACAAGCAAGCATCTAACAAAAGTTAGATGTGAAAT

>c13672_g1_i2 len=216 path=[5:0-20 26:21-215]

AGAGATTTTTAAAGAAAAGTAAGACCTTACTGAATACGTGTAGAGCCTAGGGTGGGATCC

CTAACATTTCCAAATGTAAGTCGTTTATTCCAGCATCATTTTATCACCTTGGAAAATGCA

ATTCAGAAAAAAGTTACTGCTTTGACAGGATTTTGTAGTACTTGAACAGTAACACAACAG

CACAAGCAAGCATCTAACAAAAGTTAGATGTGAAAT

>c13675_g1_i1 len=229 path=[1:0-21 23:22-228]

GGGATGACAATGATAGAAATCTGTTTATCTGGAGTGAATGTCTACAATCCAAGTACTACG

CAGCTCCAGAGCAGTTTAGCTACCAAAATCAGGTGCTTTCAGCCTGATATAACTGAGGAC

TGTGCTGACAAGAGTAAGATAGAAAGAAAGCTTATCAGAGCTGAGCCCTTAAGGTAACTG

TGAGGGTAAAGGAAAGAACGAATATGCCTAACTTAAGTCTTTTAACACG

>c13675_g1_i2 len=257 path=[463:0-49 23:50-256]

GCCCAGACTGATTCTCTTTACACCTCCACTGTTCCTTTCTCTATTCCTCAGTTTATCTGG

AGTGAATGTCTACAATCCAAGTACTACGCAGCTCCAGAGCAGTTTAGCTACCAAAATCAG

GTGCTTTCAGCCTGATATAACTGAGGACTGTGCTGACAAGAGTAAGATAGAAAGAAAGCT

TATCAGAGCTGAGCCCTTAAGGTAACTGTGAGGGTAAAGGAAAGAACGAATATGCCTAAC

TTAAGTCTTTTAACACG

>c13679_g1_i1 len=446 path=[1254:0-445]

AAAAAAAAAAAAGAAACTATATAGTAACTCCATTTACACATATATATTTATATGGGATTT

ATGTAAAAGTTTATTTTCATACATTAATATATAAGAAGAATTATTAATTCTTATTATATA

TATAATCGTTAAAAAAAAGAAAAATTCTAAATTATATTTTATTTTATTCTTTTTTAAATT

ATGATTAGATTAACATAAATGGAAAAAATTAGAAATATTAAAACTTTTGTTGTAATAATT

AAAAAAAGAAAATAAAAAATTAGTAATTATATATATGTATAGTTTTTAATAATAAGAAAA

TGAAAAAAATGAATAAAAATGTATTCAAATTTTGTATTGCTATTTTCCACCATAAGTTTA

TAGCATAAATATAATAAAACAAATATGATATGATAAAATACAAATTTTTTTTAGCATGTT

TCTAGAATTAAAAAAAAAAAAAAAAA

>c13679_g2_i1 len=339 path=[7:0-338]

TTTTTTTTTTTTTTTATACTTATCGAAAATATAAAAATATAACTATTTTATAATGACATT

CTTAAGTATTCCTTTTAATCATAGAAAACTTTACATTTTTATTAATTTGTAATTTTTTAT

ATCGGAAATTGTTTCAATATATATTAAAGTATTAAATATTTATATCGTTAAAATAAAAAT

CATTTATATTCATATTTTATTTTTTTGAATAGTTATTATATTGCTCCTTTTTTTCTCATT

TATAATAAAAAACAGAAATCTCACATTAGGTAATATTTAAATATTTAAATGTATTTATAT

AAATAAGATCTTTTTGATTATCTATAAAATTAAAATATA

>c13680_g1_i1 len=286 path=[359:0-232 643:233-233 70:234-285]

AGCTTCCCCCAAGAAATACCCAAAAAAACATAACCTATATATCACAGAGCACTACCACTA

GTCCCTTATTATTAAGGCTATGACCTTTTTAGATGTGCTTTTTATCTTTTATATCCTATA

TCTTTATCATCAAAAGAAAAACAGACAGAATCCTTTTCAATATAGAGAAAAATTGATTAA

CTACCATTTTTACATGTCCAGGAAAAGAAAATTACTTAAGTGCTTTGACAAATGTATGGT

GTATAATGGTTGGGTTGGAACCAGTGTGTTAACAGGTCTTCTGCCA

>c13680_g1_i2 len=286 path=[359:0-232 643:233-233 592:234-285]

AGCTTCCCCCAAGAAATACCCAAAAAAACATAACCTATATATCACAGAGCACTACCACTA

GTCCCTTATTATTAAGGCTATGACCTTTTTAGATGTGCTTTTTATCTTTTATATCCTATA

TCTTTATCATCAAAAGAAAAACAGACAGAATCCTTTTCAATATAGAGAAAAATTGATTAA

CTACCATTTTTACATGTCCAGGAAAAGAAAATTACTTAAGTGCTTTGACAAATGCATGGT

GGGGTAACAGGTAAATCAGTATGGCAGAAGACCTGTTAACACACTG

>c13683_g1_i1 len=201 path=[227:0-200]

GGTTCAACGGGGGGGCGGGGCGCTTCTGTGGGAAGTGCACAGCTGGGGTCGGGCAGGGCT

TGGAGAGGGGAGCGGCTGTCTGGGCTGTGACAGCCAGCGGGGTGCGTGCTGCAGCCCTCC

TCTGCCTGTTGGGGTGCGGTGTGATTTTGCAAACAATTTTGGGCTTTGTCAGGGAGGTGC

TTTGTGGACCCCGTGGGGCGG

>c13686_g1_i1 len=215 path=[155:0-115 79:116-214]

TTGAAGCTTTCCTAAGAGAAATTGGAAACACTGGTTACATTTAGCTATTTTACAGTTTTT

CTTACTTACATCATATGCACATAGACATGATTATTACATAGGTTTCCTTTTTTGTTTTCA

AGTAAAAAGTTATTGTGTATAAACCAGACTATTTCATTATGTACAATCAGCCCTTAAAGT

GCTATACAAATACTGCTTGACCAGCCCCATGTGTG

>c13695_g1_i1 len=228 path=[1:0-227]

ACAATAAGAATCCTCTGTCAACTACAGATTCTTTTTTTTTTTGTGGCAGATTATGAAAAA

ATTACAGAATTAATTTAGGCAAAATTATTCCAGGGAAAAAAAGAACAGCTTGTTGCCATT

TATACACAAAAGCACAGAAAGATGAGCTCTTGGGTGACATTCTGCTACACACCTGTATGT

TATAAATTTGGTCTTCTTCAAGTGACCGGGTAAGAGGTTTGGCAGTAA

>c13698_g1_i1 len=204 path=[1:0-203]

GCGGGTCGGTGGCTGCAGAGCAGGGGTAGGAGAGGAAGGGCAGAGATTGGAGGTGCCGAA

GTCCAGGGAAAGGTAAAGAGCTCCTGGCAGCCGGATGCAGGCCAAAGGTTCCCAGGTAGC

TGCATCTCACCCCGTGGTCCTGCAGTGCTGCTGGCTTTTGTCACGTCTGGGTTTTATTTC

CTTTATTCCTGTATTGTTTAGTCA

>c13701_g1_i1 len=205 path=[1:0-204]

ATTCTTGGTCTCTCCAAGGCTAAACATGTACACAGACAGTCAAGTGCAAACTTGTATAGT

GTCAACTGGAGAAAAAAAAAGTCTGTGTGTGATCTCGAACATACTCATTAGCTGCTTTAA

ATTTATAAGTAAGGTTTATTATAAATGAAATAGAGCACTATTGATTTTGTTTGGCATTGA

CTTGACCAACTGTCAGTGCTTTAAA

>c13712_g1_i1 len=229 path=[1:0-228]

GTTGGCTTGCCAAGCTGGCCTAGGAGATGAATGGTGGCACCAGTTAAGCGATCAGTCATT

TCCATTTCTGCAGTGCCATACTCAGCATAAAACGTGGAGCTGGGATCAGCACTTATATAA

GTCATCTTGAGGTCACTGCAGCTCATGCCCACCTCGTCTTGTAAGAGAGTCCTCAGTGTG

GCAATTGAGAGAAGAGCATCGATATCTTACCCACATTTACAGGGCATTT

>c13720_g1_i1 len=230 path=[9:0-79 89:80-229]

CTTTTAAGAAAAAAAAAAAAAAAACGCAGGACAGGCCAGCGGGGCTGCACCCTGACCAGT

GGCCCCCTGCCCAAAGCACACTACATGTAACAGCTCGCCCATACACGGCAGCTGAATTAA

TGGATTTAGTACAATGCTTTAGACAAAAACCGAGAGAGAGTGTGCCAGCATGGTTACTCA

GACTATATGACATGGGGGCAGAAAGCGTTGTGGTGAATGGGCCAGAAATT

>c13722_g1_i1 len=228 path=[1:0-227]

GCAACAACCTGTGCTCTCCAAAACATATTATCTTGTTCTGAAAAGGTTGCCCACAAAGCA

GTGCGTCAGGCTGTTCTCCTGGCTGCTCCTCCATCTCTGCACTGAGACTACTGTAAAGCG

TACCAAGAAGGTCTCTGATCTCACACCAGAGGAAACCTGATGATCCCATCCCCTCATTTC

AGAGCACCTGTGAGGATGCACAGAGGGTTGTACTTCACTGCAAGGATA

>c13722_g2_i1 len=254 path=[642:0-253]

AAAGAGATAACTGTCTATCTAGGACAGTGGGGAGCACTGAACGTAGCCTGAAATACAGAG

TTCCATTAGGAAAAATCTCTGACTGTTTGTTTTGCTGCATAGGCTGCTTTGTCTGGGGTA

GTGGTATGGTGAAGCCACAGGATCACAGAGTGATTGGGGTTGGAAGGGACTCTGGAGATC

ATCTAGTCAAACCCTCTGCGCAAGGAAGGTCCCCTGGAGCGCAGTACCCAGGAACGTGTC

CAGATGCTTTTGTG

>c13724_g1_i1 len=313 path=[559:0-312]

GAGAGACGAGCCGTGGATTGGAGCGTCAGCCCTTCTGCACGTTCCCAAAAGGGGGAGATA

ATTTCAGTCCAGTAAATGTAATGCATGCCAAGCGTGCTTGTAGCTCGAGGTTTCATCAAT

TATCTGGGATCTGATACAAAGATAATTGAATTATCCCGAGGCCTCTGACAGGTTTTGGAT

TAGTTTGTTGGAAAAGGAGCTGCTGCTTGAAAATTAATTAACGGAGTAATCAGCTGAAAT

ATGACTTCAGGACATGGGCTGGCCAACATCAGAGCACTTCGAGGACACCTCCTTAAATTA

AAGTATTCAAAGG

>c13725_g1_i1 len=258 path=[458:0-107 565:108-154 147:155-257]

AAGACAACTTAGCCTTGCAGAAAAACATCAGTTTCAGTCTCATGGATTTACAGCTCTTAG

CTCACTAGGCAGGAGCTTCCATTAATATCTTTCATTTTAGACTCATCAATAGTAACAGGT

AAACTAGCCACCATCATTTTAGCTTCCATGCTGTTAAAAATTGTCCAAAGATGCAAGTGG

GGCTATGATTAGAACTGTAGCTACTACTGCACAGAAGCATAACCAGCCCCGCTGCTATCA

GTAAAGACGTGCTTAGAG

>c13731_g1_i1 len=220 path=[232:0-140 373:141-184 416:185-219]

AATTAAACATCACAGGTAGTCTCCACTTCAAACAGCCTCACATTCTGAATAGAAAAAGCT

CCTTAGCACACAACTACTGAATAAAGATAGAGCTACAATACTAACTTTAGAGAATGCATG

ATTCTCATATCCTTGTGTTCATCTAGCATAAGACACGTAAGAAATATGACTGTACTAAAT

ACTTTTAAGTTACTAAGTTCTGTTCTGTAATGTTCATAAC

>c13731_g1_i2 len=220 path=[232:0-140 373:141-184 25:185-219]

AATTAAACATCACAGGTAGTCTCCACTTCAAACAGCCTCACATTCTGAATAGAAAAAGCT

CCTTAGCACACAACTACTGAATAAAGATAGAGCTACAATACTAACTTTAGAGAATGCATG

ATTCTCATATCCTTGTGTTCATCTAGCATAAGACACGTAAGAAATATGACTGTACTAAAT

ACTTTAAAATTCTAAGTTATGAACATTACAGAACAGAACT

>c13734_g1_i1 len=229 path=[53:0-228]

ATGTAACTGTGCCTATGGATAGACGTATTGTCAATGGACTGAAAAAGCCAAAGACTTACT

AGCATCGGGAGGAGCTATGGGTGGCTGTATGACTTAATTAAGTCCCCACAATCCATCTAC

AAAGTGACAGTGCTTCAGTCACACTGCCAAGCAAACAATTTGGCCAGTTTCTTTGTAATT

AAGAAACAAATCAGAAAAAGGATACTCTTCCTCCAGCAACATTTTCTCA

>c13736_g1_i1 len=211 path=[189:0-210]

GTTTTCGAAAGTGTATTTATTCAGCGTGTGGTTAAATTATACGGGGTTGTGCAACCCTTC

CCATTGATTGGCCTTGGTGCAGCTCAACACCTCTGAAGTCTTGGAGTCCTGCAACATTAT

GTTCCAGGCTGCGTTGAGCCTGGGAGCCAGGTCTGCAGATAAGCCTGCTACAGAGCCAGC

ATTGCACGTGTGTCCCTTTACTGGGCTCTGC

>c13742_g1_i1 len=224 path=[476:0-223]

AATTTCCTTTTGAAATATAGAATTCAGAAGTTCATATCATTACTGTAGGTGCTTTACATT

TTGGCTCCCAATTTTTAGCTGCCAGTTCCATATGCCAATTCACCTACTTTAATTTGGTTC

TGCAGGAGTAATAATAATTTTGTTTCCTAAAAGAAAAAATCCACAAAAAAAAAGTTTTTT

TTCTTTTAATGGACTGCTAAAACAGCTTCCTCCGATGTTCCTAG

>c13754_g1_i1 len=221 path=[107:0-220]

GAATCACGGTCCTGGGAAACCTGTTCTTTGTGTCCCTGCTTGAGTAGAGCTGTTGGACCA

GGGGACCTCCAAAGGTTCCTTCCAACCACAACCGTATCACTGTGATAACTATTCATATCA

TTATACAAGTCCAGATCCAACACTGGTGCATATAGGATTATCTTCATTATTTACCTGACC

AAAACACCATTGTGCAAAGTATTCTGAAACTGTCCAGAAGA

>c13763_g1_i1 len=240 path=[1:0-239]

AAAATATATTTTTTTTTTTTAAACATTTTAAGTAATTTCATATATAAAACTTAGATAAAA

TGTTTATCTTAATTGTTAATTTTTTAATTTCTATTGAGGTAAATGGTATCCATTTTGAAA

AAAATAATTTTTTATATCAAATAAAAAGAAACTCATATTCTCCAAAAAAAATATTTTTTC

AAATACTCATCTTAAAAATATAATTTTTTTAAAATGAATTATACAAAAAAAAAAAAAAAA

>c13766_g1_i1 len=250 path=[1:0-167 655:168-173 379:174-249]

TGGCAGTCAGGACATGACACTTTGAAACCTTAGCACTGCTGCTACAAAGATTTATGCTTT

ATCCTGATGGATTTTTTTTTTGGCTAAAGTTCACACTATGCAAAGACTTGCACATATTTC

ACACTACAAAAACCTCTGCAGTACTTTCATTGGAGATAACAACAAAACCTGTACTAATGA

AACTGCACATGAGGATCTTCCTCGCTATTTGTCCTGAAGTAGCAAGAAAGCCCAAACTGT

GACTCTCATT

>c13766_g1_i2 len=214 path=[1:0-167 655:168-173 175:174-213]

TGGCAGTCAGGACATGACACTTTGAAACCTTAGCACTGCTGCTACAAAGATTTATGCTTT

ATCCTGATGGATTTTTTTTTTGGCTAAAGTTCACACTATGCAAAGACTTGCACATATTTC

ACACTACAAAAACCTCTGCAGTACTTTCATTGGAGATAACAACAAAACCTGTACGCCATT

CAATAGCAAAATGAGAGTCACAGTTTGGGCTTTC

>c13775_g1_i1 len=207 path=[1744:0-206]

GGTAACTTTGTGTTTGATTTTATAATAATAAAAATTTTCTTTTTTTTAGAAAAAATCGAG

GTAATGAAATATTAGCATCACCTGAATTAATGATTAATAATGTAAAAAATGTTAATAAAA

AAAGAAGTAATTCATCCATTATTCCTAATTATAAGAATTACAAAAAGATTGCCTATAGAA

ATAATGCATTATCAATTTGTAAAAATA

>c13775_g2_i1 len=760 path=[1:0-759]

TTAAAATTTTATTAACTTTTTTTATGGAAAAAAAAATATTTGATGAATTATTGAGATTAA

ATAATTTTATTGATAAATTATTAAAACTGTTCTGATCAACTGAATAACTATTAAAATTAT

ATTTATTAAAAGAATAAATTTTTTTCTGTATGTAAGAAATGAAATATTTAAACATTTTTT

TTATAGAATAACAATGTTCAAACCTCTTTTTTCTTTTTTTTATATACAAAGTCATATTAT

TTTTTTCATTATGTGTAAAACTGTTATTAATTAAATAAACATCCTCACTTTTCATATCAT

ATAACGATTTTCTCTTTATTTTAGAATATTTACAAATTATATTATTGCTATATTTCTTTA

ATTTTTTTAACATTTTACTTTCTGCTATTAATTTTATCATATAAAAATTGTAGAGAATTT

TCGATTGCTTATATATAAATCTCAAATTTTTCCTTTTTTTTAATTCAAATTGAAAGAAAA

AATTCAATAATTCTCTTATCTTTTTAAATTGATTATTAAATGTTTTATCAATAATCCTAT

CCAATATATCTTTTTTTTCATATATTTTTATATATAAAAAATTATGTACATTCATTAAAT

TTTTATTAGTAATTATCTCAAATAATAACATACCCAATAGCCAAACATCACTTTTAAATA

TTTTTTCTTTTCTTTTTTGAAATTTATATTTTTTCTTTAACCTTTTTATTATCATAATTC

TTATTTTTGATACGTATTTATAAAATGAAAATATAGATAG

>c13777_g1_i1 len=246 path=[224:0-245]

AGCTCCTGAAGTCGATATGGGAAAGGGTTGCAGTTCTGTGCAGTGTTGGTGGCTGAAGGT

TTATACAAGAAAAAGAACAAACCTTGGGGTTCACCCCTCTCACTGCCATTCAGGCTGGGT

GGACACGGCCAGGTCTGAAGCACACAAAGGCTTTCAGGTCACTTGGCTACGTCCTTCTCC

CCAGTCCAGAAGTGCTTTGTTTGTGCAATGCCGTTTTGGTTCGGGGCTTGTTTTTGTTTT

ATTTTA

>c13781_g1_i1 len=251 path=[1:0-188 190:189-250]

GCCAGATTTTACCTATATTTCGCCGTCATTCTGAAGTTTGTCAAGAAGCCAGTGCTGGCA

TTGTGCTGAGTGACCAATGGAAGGGTGGAGGAGCTGCTTTGATCTCTCCAGCCTCAACAT

GCAGCATTTGGCCTCTGGACATTTCTAACCGTGCTTCTATATGGAGATGCAGTTTTCTCC

TCTGCAGAAGCAGCACTGTATTTTATGGCTGGGAAAAAAACAAAAAGAATTCTGACATCT

GGCTTTCTGAT

>c13781_g1_i2 len=251 path=[1:0-188 313:189-250]

GCCAGATTTTACCTATATTTCGCCGTCATTCTGAAGTTTGTCAAGAAGCCAGTGCTGGCA

TTGTGCTGAGTGACCAATGGAAGGGTGGAGGAGCTGCTTTGATCTCTCCAGCCTCAACAT

GCAGCATTTGGCCTCTGGACATTTCTAACCGTGCTTCTATATGGAGATGCAGTTTTCTCC

TCTGCAGAACCAGCACTTTGTATTAAATAGACAGTGAATTGTACACATCAGAAAGCCAGA

TGTCAGAATTC

>c13792_g1_i1 len=364 path=[574:0-363]

GCTGCTCTGTCACTACCAAGAGCCAGAAATAGCAGGGAAGGTCTGCTGTTGGCGGTGAAG

ATTGGGTTTGTAGCAGCCACTTCAGCACAGCAGATCTTCCAGGGGACAAATAGCACCTGG

CTGGAATAAGCCCTGTTTTCTCTAGCTCTGGGGATGTTTTCCTCAGCTCCAGGCGTTTAA

CAAAATGCTGCTTTACTGGAACTGCAGCAACTGGGAGATAAATGGACTGGCCATTATGTG

CTGTGCCACTCAGATAGCATTAGCTATTGCTGTCTGGCTGAGGTGTCTGGCCAGCACATG

ACAGTTTACCTTGTACTGAAAAGGGCTGTAGCACTGCTGAGCCCTCTGAAGCCCTCCGAA

AAGG

>c13808_g1_i1 len=260 path=[155:0-259]

AAAGCCTTTTCATGTCTAAAAAGGTGTCAAACTCTGCCATGTTGTATCAGTAGATAAAAG

GGAAACTGCAGTTAGAATATTACTGGGAGTTGTTGCAAACCGAAGTGCTGCTGGTGGAAC

CTAAACTCAGCGCTAGATTTAGCGCTTTTCCCCTCAGGAAAGAAAACAACTGTTTCAAGA

AAAACAAAATGATGACGGGTGGCAGAACCACTCTCACACAGTTCTCTTCCTCCTATCCAT

CACTGTGGCTACTGCCAGTA

>c13811_g1_i1 len=204 path=[182:0-203]

ATTGGACAGCGCACATTTTTCGCTAGCTCTGTGGAAACTAAAACATGTCAGCCACTGACA

GAGATTACTTGCACAGTTTATTTTCACAATATTTTTCTGCTCTGCATAACTGTGGCCTCT

GCCCAATGGGCGCCATGTTATCGGGAGCTTTTTCTAATCAGTTAATGGGCAGGATGATTA

ACCAGACAGATTGCGGCTCTTAGG

>c13813_g1_i1 len=280 path=[302:0-279]

AAAGATATAATAAAAAGTAAAAAAATTAAGTTACATTTTAAGAAGCGTTTCCATATATCC

TCTGAATGCAAGCTGGATTCTAAGAGGAAACCCCTTGAAGATCTGTCTTATATAGGGACA

GAGTTGTGATTGTTCATTCAGAAGTTTTACACTGTCAGTATTGGAAAAACCACTCACGCT

ATCACAATAGATATTAGTATTGTTATTTTCAGTAACAGCTAAGTCTATACAGCTTCCTAT

TCTGCATAGGCACTCTGTACAGATACTGATGTTTAATGGT

>c13814_g1_i1 len=296 path=[602:0-295]

TAAGGACCCTTCCAACCCAGCCACTCCACAGCTCTGTGACGCTTAAGAATCATTTCAACC

CAACGCATTCTATCATTCCATGGCTCTATGACCTTCCAGTACACACCCAATCCATTCTAT

GCTTCTATGACCTTTCAGGACCCTTCCAAACTAACCATCCCACCTCTCTATGATCTTTAA

GAACCCTTCCAAGCCAACCACTCTATGACTCCATAGAGACTACGACCCCCAGAGCTGCCT

GCTTGGCCAGAACCAAGCTTTCCAACCAACAGCACAAGCAGAAGGTGTCAATACAG

>c13820_g1_i1 len=202 path=[193:0-201]

CACTGAAGCTTTCCATAATATGCCCTCTGCCTTGTCATTTTGCACTTGGTGCTATTGATC

CAAGTTTCCCCTGCACACAGAGCTGAACGCTCTGCTGCAGAAATCAAAGGTAGAATGCTA

GCCTTATAGAAAGTAATGTGAATTAACATATAAAGCACAGGAGGTAAGGCAAAAATGGAG

TAACTGTAATGATGGGCTTGGG

>c13826_g1_i1 len=235 path=[1:0-234]

CATGCCTTCTGTCATTCATTAGGACCCCAGTGATCTGCTCTAGAAGTTCCAGCTGCTAGG

AGAGGAGCAGAAGAAGCTATGTGATCTTGACTAAGGAGAACACTTAAGATAATGACTCAT

AATGTGTTTTGAAGCCTTCCTGATCAGATTCAGGGACATGTGGTCAGCTCCAAACCTGGA

GCTGGTGTAGTTAACTTCTAACTTAGCGAGCTCAAGATGTGAAGTTTAATTTGTG

>c13833_g1_i1 len=216 path=[1:0-215]

GGAGTAGTAAAAAAAACACAATTTTTGTTTGAAATAACTTTATCACGGTGCCTTAAATTT

GGCCCTTGCAAGGGTTTCTGAGCTTGGGACACATGAAATATAAGGTTATCTTGAAGTTCA

GGGAAGGTAGATCAGATAGATATGGAACATGCTGAAGTAAACTGCTGTGAATGAGAAACA

CTTTACCAATATTAGTGTGGAATTGCGTGAGGGACC

>c13843_g1_i1 len=245 path=[1:0-63 500:64-74 75:75-244]

TGGCAGAGATGAGCAGGGCCTTGGGAGCCTTCCTGAAGCACAGGCTTGGTGATGTGCTTC

ACTTAGTTTGTGGAGGGCAGAGGCGTGGTGACTGTTGTAACATCTTTAATTCATTACCTG

TCTGTATCTAAGTGGATTTCAGGTGGTGTGTTACAAGAAATTGCTGCGTCAATAACATTT

CTATGCCATTTAGCTGCTGAGTTAGCAGTGGCCTCTAAACATGGAAGTGGAGAAAAATAA

AATTG

>c13855_g1_i1 len=204 path=[496:0-203]

CACCACCCCATCCTACAGCACCCACCGTAATCATAAGCTGGCAGAATTTGGCATTTTAGA

AGCCCCAGCAGCCCCTCAATGGGATGGAACCATTGCTGGGGTGCTGATCTTAAGGAAAAC

GCTTCTTGAAACATTTGCTGTATGCAAATGACTCTCTTTCTCCTTTGTCTTTCGAGACTT

CCCTCTGTTGACAAAGGAGCTGGG

>c13857_g1_i1 len=255 path=[427:0-254]

GAACCCGCACGCTGCCCATGCTGGCTCTGACCAGCCCGGTTTCATTAGAAACGAAGTTAA

ATAAATAGGGCTTGCACTCCCTGTCCTGCAGTTAGACGCCTGAAAGCTGTGCTTTCTCCC

TCCCTGCAGCCGAGCTCTGCTCACCAGGCGCAGTGTGCAAACACACAGCTCCTTGGCTTC

CCACCAGTGTATATAAATATATGCAGTTTTTTTTTTTCCTTCGCAGAACCTGGAGGCTAA

AGCAAGAGGGCAAAG

>c13861_g1_i1 len=407 path=[107:0-156 263:157-305 648:306-311 417:312-406]

CTAATAATAGAGAAGTGTAATTGTGTGCAGACAGCTAGTCCAGCCAAGGGGAAATCGCCT

TGCCTGTTGTGTAGCTTAGGCAAAATTGCCTTGAGTAATGAAGAAAAGCAAATCCAACAT

TGTGCCATCAGTCTTCAGTGACTGCTTGGGGAAACTATTAACCTAAGAAAGGCAGATAGG

ACATGAGGAAGCATCTTACAAAGAGCAGACTTGCAGCCCTGTACAAAAGGCAGCAAGTTA

TTCAGAAGCATCATGTGCCATAGCCAGCATTAGGAGGATCTAATGAGCTTCCACCATGGG

GCTGGGCTCCCATCTCCACATTCTGCCCTAAGCTTATTTTTATAAATACATTGATGAAGG

CTTTTTTCTTAGTCACTTCTTAGAACAGGAGCTGCTTCCTTGTAACT

>c13861_g1_i2 len=372 path=[107:0-156 263:157-305 648:306-311 571:312-371]

CTAATAATAGAGAAGTGTAATTGTGTGCAGACAGCTAGTCCAGCCAAGGGGAAATCGCCT

TGCCTGTTGTGTAGCTTAGGCAAAATTGCCTTGAGTAATGAAGAAAAGCAAATCCAACAT

TGTGCCATCAGTCTTCAGTGACTGCTTGGGGAAACTATTAACCTAAGAAAGGCAGATAGG

ACATGAGGAAGCATCTTACAAAGAGCAGACTTGCAGCCCTGTACAAAAGGCAGCAAGTTA

TTCAGAAGCATCATGTGCCATAGCCAGCATTAGGAGGATCTAATGAGCTTCCACCATGGG

GCTGGGCTCCCAAGCTCACCACAGCACTGCTGCTCTGTCCCACAATGAGTTACAAGGAAG

CAGCTCCTGTTC

>c13861_g1_i3 len=286 path=[107:0-156 25:157-184 648:185-190 417:191-285]

CTAATAATAGAGAAGTGTAATTGTGTGCAGACAGCTAGTCCAGCCAAGGGGAAATCGCCT

TGCCTGTTGTGTAGCTTAGGCAAAATTGCCTTGAGTAATGAAGAAAAGCAAATCCAACAT

TGTGCCATCAGTCTTCAGTGACTGCTTGGGGAAACTAGGAACTCTGTTGGCAGGAAGGCT

TTTCTCTCCCATCTCCACATTCTGCCCTAAGCTTATTTTTATAAATACATTGATGAAGGC

TTTTTTCTTAGTCACTTCTTAGAACAGGAGCTGCTTCCTTGTAACT

>c13871_g1_i1 len=280 path=[822:0-279]

TCTTTTTTTTTTTTATTTTTTTGCTTAAAAAAAAATCTTGAAAAGTAATTAAATGTTTTA

ATTAAGTATCCTAATTTTATTTTGTCATGTGGAAAACTGTCAGCCATTTTTTTAAAAAGC

GTTTTTTTCAATTTATATATAAAAAAAAAATGTATTTTACATTAGACATGTAAAAATAAT

GCAATTATAAAAAATTAATCTTATGGATTTCATAAAATTATATCTTTTTTTTTAGAATAA

TTAGAAAATGATAAAAATCAGAATTTAAACGCATATTTTA

>c13871_g2_i1 len=263 path=[5:0-262]

AAAAAAAATAAAAAAAAAAAAGTAAAACTAGTATAAAAATGCTATTATTTTTGTTTACAT

TGAAATGCTTTTCATATATCTATTTTTTGAAGTAATGTAGAAATTAATAAATTAAAAAAT

ACAAATTGTTGATTATTTATATAAATCAAAACGCATATTTTACTATGATTTATTTAAAAA

TGTATTTAGTTTTATTATGCTCTAATACATTCATACATTTAAATATGTATTAAGTACTAT

GTATTCTTAAATTAATATTAGTA

>c13874_g1_i1 len=208 path=[39:0-96 38:97-207]

ATTAAAAACAAATAAATTAAATCAAAAATAATAACCATACCTAATATATAAAAGAAATAT

GCTTTCTTCTTTATGTTTCTTTTTGATAAAAAAGAATAAAATTTTTTATTTTGAATAGAT

GCAGAGTATTATTTATTGTATAATTTTGCAAATATTTTATTGGCCATAGTATGTAAAAAT

TTAATTTTTAAATAAATATCAAAAAAAA

>c13876_g1_i1 len=272 path=[1:0-271]

GCTCCTCACCCTGAGCGTGACCAAGACCTTAACTTGCTGGGGGTCAAAGTGGGGGTTCTC

ACATCACGGTGCAGCCTCCCCATTGGCATCTCCAACAGCTCCAAGCTGCCTCCTGTGGCT

CCCTGATGGTGGCATCATTGTGTGGTGTTTTGTGCCCTTCTGTAGGGCCTGGATGAAAAT

ATCAGTCCGCAGCTTGCAGAGTTACTAGAAGCACCAGCTCTGACAGCGCTCTGCATCAAG

AAGGGCTATAAATTCATTTGGCATAAAATTGT

>c13899_g1_i1 len=229 path=[207:0-228]

GTCCTCGGGGGTCGTTTACTCCCTCCCAACACTCCTTTTATCCCAGAGATTGCTGTGGAA

TGCAAGGCAATCAGAATAGGCTCTGCACACACAGATAAGGAAGACAAACGAGAATGTATT

GACGCCTGTCACATTCTAGACCAATTAGGCCCTGTGTGAGGGTTCTGGCTTCTTTTGATG

TTGATAAGTCTAACTGTTGATAAGGGCTCCACAAAAACAGGTGCACAGT

>c13901_g1_i1 len=216 path=[1:0-215]

ACACCAGGTCAACTGGGCCTTCAGTGCATCCTATGAAATAAGCAGAAGCGTACAGATAAT

GAAAAAACTGCACAAAGCTGTAAATAGGACTGCATGAACACAGATATAACCTTGAGTACC

TTGTTCCTGGCTTGGCTAACCTTGCGGTGTATCTATAGCACTTTGTGTACAGTCTTTATC

AAAAGCTAAGAGCGTGCCAATGATCTAAAGCTTTTA

>c13907_g1_i1 len=214 path=[1:0-213]

TATTTTTATTAATACTGACTCTTTGAATATAGCTATCCATACAGATGAAGAGGTCTGCGG

AGTATTTCTGGGTGTCTGTGCCAGGACTCAGCCAACCCACCAGCACTGGGGCACCTGGAA

ACAGGGCTGTGTTATCCCCAGTTTGCAGTAGAGTGGTGTGTTTCTGTTCATGCATACACC

TGTGGTGGCTTCCAGAATAGGGTGAGAGCGGTGG

>c13920_g1_i1 len=233 path=[83:0-89 172:90-232]

TGCTGGCTGCAGGTGAAGGTATGGCCGGGGCATGCTGGGAGTCCTGGCGGAGGGCCAGAG

GAAGCTCAGAAGAGGATGGGAGAGGTGGTCCCCCTGCAGACAGGGTTGGGTGCTGAGGGC

GTGTCAAGATCGTGGCCACAGGGATCTGTCGCACAGATGACCATGTTCTTGATGGCACAT

TTCCTGATTTACAATTCCCAGTCATCCCAGGCCACGAAGGAGCTGGGATAGTG

>c13931_g1_i1 len=268 path=[155:0-177 11:178-267]

GCCAAACGTTGGTTGTAGTTGGGTTGTGAAACTGATGCTTTTTAACCGCAGATGTGGTGA

GAGTAGTTGGTTCTCTCCTGTCGTAATTTGTTTATCTTTTTCTATTTCTGGCAGTGCTTC

TCAGCTGTGTTTTGTCTCTGCTGTATGTTTTAGTCTTCTTTTCTCTCGAGAAACAAAGCG

CTTCATTGCTGAAAGCAGGTTTAAAAGCTGTCTGCATGCTAGCCTATAACTTTGTGTTTG

AGACTTACATAAAATTATAGGCTAGCAT

>c13932_g1_i1 len=208 path=[53:0-207]

ATCTGGCTCTGGCAGTGGGTGTAGACCTGTGTTGGCCACAGGTTTTACGAATGTGCGTTT

TCTGGGGGGATGTACTGAGAGCAGCGTGCCCTTGGGAGGTCCAGGTCGGAGCGGCTCGTG

ATTGCCTGTGTGTGGTGAAGAGGGGAGGGAGAAGAAGGAGAGGTGCTTTGGTGCTTGTTG

GGCTTGGGGGGCGGGAGAGGGGGGAGGT

>c13937_g1_i1 len=279 path=[159:0-185 352:186-278]

AGCTCAATTTGATACTGAATTAGTAAATTTTTACTTTTTTTTAAAAAAAATAAACAAAAA

ATAATTTTACATTTTCATAAAATTTAATGTCAACAATTAATATTATAATAATTATAATAA

CAAAAGAATCTCTAAAAGAACAAGAATTTTTTTCATTGAAAAAGATATATGTGTTTAATA

ATTCTGAAATTATAATTAATGTACAATTATTACATATTTTTTTAATCATATTTTATTATT

TTATTTTTTTATGAAGATTTCTCTTGTTGTCTATTTATA

>c13938_g1_i1 len=240 path=[282:0-216 536:217-239]

TGTTGAAAAAGATATCATGGAAGGTGAGCAAATGCTCTTTATGTGGTAGCACATACTGTG

GAGATCAGGCTTGGAGAAACAAGGAAAATAAACTACTAATATCAGGGCTGAAACTAACTC

AGCTGCCATGTGAAAAGTTGAGGGTTTCAAGGTTTATTTGTTGTATTGAAATGTGTGTTA

AGAATATTGGTTATTGTAGATTTTAAAAACCAGGTGGTATTTGAGAAAGTTCAAATACCA

>c13944_g1_i1 len=366 path=[1:0-365]

AGTCTGGGCAGAGCAAATGTAGCTAATAGCTCACTTTTTGTCAGGGTGCACATGCTACAG

CTTGCTTTCAAGAAAGTTACTTAGAGAACGTAGAATTACCCAAAACAAGATGACATCCTA

AATGAGCAAGCAAAGCTCACTGCTCATCAGAAAGGCAAAAATAAAGAGAAGAGCAGTAAT

GGGCATGCTTCAGATTCAGGTAAGATCTAATCAAGGCTGGACAGTTTTTTAATTCTGGTA

ACAAGCTATTTGTATATTACTGTACTTCTAAGTTACAAAGCACACGCTCCATCTTCAAGG

ATGAAGTGATATGCAAGAGCATTAGTGTAGCATGTAACGGGTTATTTCATCTGTTTCAAG

TCATTA

>c13944_g2_i1 len=279 path=[706:0-278]

TATTTTAGACTCTGACAGCTCAGCCAAAAAAGTCCTTTGGGTCTCATATCATGCAGTAGC

TATGTACTTGAAGTACTCAGATTTCCCACTTAGAGTATCAGCTTGCAGTGTGTGTGAAAC

CCATGTAGACAGCACTAGTTTTGTGCGTTAGTAGCACTAGCAAGTTTTTCATAAATTCTG

AAGGCGTGGGAAATTGATCTCTGTTCACATTGACCTTCCCTTCATTTTCTAAAATTCTAA

CAGCTAATTTGATATGCGAGTTCTAATGACTTGAAACAG

>c13962_g1_i1 len=228 path=[1:0-154 156:155-227]

AGATGGAAGGCATATGAGACCTTACGTTTTGTTCTGTGAAATTTGTGGAAGGCTTTCAGT

TGGTCTTCCCAGAGAAGGTAAGGAGAAAGTTGAAGAGAGGGTTAGAGTTGGGGCAGGAAA

AGTGTAATAGCTGATGGAGAAGGACTGGTTTTAAATAGAAATAGTATTTATACAGAGGAA

AGTTAAAATACTAATTCTACAAGAGTAAAGTTTTAGGATGTTGTAATA

>c13966_g1_i1 len=441 path=[1:0-332 334:333-386 334:387-440]

TCCCAAGGCACAAAGAACTGCTCCAAGCAAAACTGCATTCACAAACCCTGCTTGTAGCTG

TGGGGCTTACAAACATCCCTCCACCCACTGCAGGACTCTCAGGCACACTTTGTTACTGCT

CGCACCCTCTGGTCGTGAAGTGCTGGCCAATGTGGTTGGCATACTTTATAAGCTCAGAGC

TGATCTTCACGTCAAGGTGTTATGAAGAGTTGAAAGGTGCTCTGAAATACTGAGGTGGCT

CAAGTCCTGCACACACCAGGGTTCTCTTCCCATTGCACACACCACCAAGCAAAGGCTGTC

TGTCCCTCCTGTCCTGAATGCTTACGACAGAATGTGCTCCAGGACTCCTTGTCTGATTAA

CTGCTCACATTTCCAGCGGGGCAAGAAGTGCTCCAGGACTCCTTGTCTGATTAACTGCTC

ACATTTCCAGCGGGGCAAGAA

>c13973_g1_i1 len=307 path=[1:0-267 679:268-269 271:270-306]

GAATTAGCATCAGGATCACTCCTCATGAGTTGACCTCATTCAACATTCATATGGGCTAGA

TCCTGTTAGTCCACGTTACTCAGCACAGTGTATCTGAAATGCCAGATTGCTAATGCCAGA

GTAACCCAGTGCAGATCTGGTTAGCCAGCACACTGCAACACGGTCACCCCACAGGCACTG

CCTACATGCAGCCATGACAGTCCAGCACAATGCACAGCCTGGAACATTTTATACTATGAG

GCTGCAGCTGACACCCTTAGAAGAGTTTGCTGAGAGATACGAGAGCTTTTCCAGGCCTGA

CCAGCAC

>c13980_g1_i1 len=227 path=[205:0-226]

TGGACTGCCCCCAGGGCCTTGAGCCTAGCAGTGCTCTGCCATTTGCACTCAGTTTTGGTG

ACACCAGCATGGAACCACAACCTTGGGGGTGCTCAGGATGGCTGAGCTTGCACCAAGGTG

AGGTTTGGGTGGTGCTGGGGCTCTAATCCTGTCATTACACTTGTAGTCAAGGAGTCAAAC

ACAATTGCAGACAGAATCAGCCTTGAGAATGCAGCATGTGAACAGGG

>c13988_g1_i1 len=204 path=[95:0-47 142:48-180 347:181-203]

AACAAAGGCTTTTTTTTTTTTTGGTGGGAAGCTTTCCTTGCGGAAAAAAAAAAAGCCTTT

GTTAAGATATATACTGACTTCATTTTTGCTTCCATATTAATATTTCTTCCACTTGAATAT

ACTATTTCTTAATTTCAGAATATTTTCATTGTGCTTTTGATGTATTCAGTAACAGTAAAA

ACAGGTTGTGATCAGAACCTGTTT

>c13989_g1_i1 len=279 path=[1:0-278]

GCTATTCTGGCATTTTCAGGATCAGAGTTTTGAGTGTAAGTAAAATGTGGGCACCTTGGT

CCTGCCTCTTTGATATTGCTGTGGATCTCTACCAGGCATGTAGATATTGTTGCTGAATGC

TATTGTGGCTGAAAAATGCCTTCCTCATAATTTTAGTTGTTATTGTAAATGTATCTGAAG

CTATACATTAAAAAGCAGTCTAGTGTTGCTTTTGGAACCTAGTAATGTGACTTCATTGGA

GGAAACTGCAAAAGCTATCAGTTACAATTAATTGTTTTT

>c13992_g1_i1 len=271 path=[11:0-270]

TAGTTAAAAGGCTGGCCCCTCAACAAACAGACACATGGCAACACCAGCTCAGTTGCAGTC

AAGCACACAAGTTATTGTTAGAAATTATTCAAAAAGAAATAGAGAACATGATAAACCATT

ACTTGCTATATTAATCCCTGGTTCACCCGGATATCGAAGTGTAGATGGCTTTGGTTGTCT

CATCTTAAAAACCCATAAATGTTAGAAGTGACAAGGTTCGCAGAAGGATGACAGAAGTCA

TTTGGCAAAAGCTGTAAGAATACCTGGCTTG

>c13995_g1_i1 len=268 path=[246:0-267]

GCAGCCATGGAAGCCCAGATGCACTGCCTGGAGCTGTGCCACTCAGCAGAGAGCCGGTCC

TGTGAACAGCTCTTGGTTTACCACCAGCTGAGGATCACCCACAGCAGCTCTGTGACCCCC

TGATCCCCAGGCTCATGCCCAGCCTCCAGCAGCACTCCTGTGCATGAGCATCTACAGTCA

TTGAGTTTGTCATAGAGGGCCTACAACCAATGTTAATTTGTTGTAGATTCCAGAGCGTAG

CTTATTCTAGACTTCTACTGGCAGGAAT

>c13995_g2_i1 len=206 path=[674:0-205]

ATGGCTCACATATCAAGAGGAACCATCCCCCAGGTTTCTCACTATAACCATGCTGACCAG

GCAGCAGGAGCAAAACACGGGGCAGAAATCTGCAATGGTAACTTCACACACAGCCTTGGG

TGTGTCATGCACAGGAAAATATTTTTCCAGGACACCATCCAGGAAAGGGAGCGTATTTTT

TTCTGATTCTGGGCAGAGAATTCGCA

>c13998_g1_i1 len=211 path=[171:0-210]

CCGCTTTCCCTCTCCTCGCCGGCAGCGCAACCATTGCAGCGTGAAATGGGAAACTTCATC

TTCCCGTGGCTTTTTTCTTTTTTTCTCTTATGGTTGGGAATGGGTTGGAGCATCACCTCC

GTGCTGAGCTGGGAGGGGGGAAGGGAAAGGGAAACGTCCCCGTGCTGTGAGCAGCCGGCT

CTGCTGCCGTGCAATTGGGTTACTGATGGGG

>c14007_g1_i1 len=221 path=[227:0-190 25:191-220]

ACTTCTAAGACCTAATCAGGGCACAGTTCTCACTCAGTTATTGGTATCTGTGAACTTGAA

ACTACATCATACACTGGTAGAATGACAAGTCATGTCTTTCATGTAACTTTGTATAGACAT

TTGCTATTTCAGAGCTTAACTGATATTTTTTTCTCTTGCCATATAAGGCACTTTTTCCTT

ATGTAGTAACTACTGACAAGGTATTTTGTCATTGCATTGAT

>c14007_g1_i2 len=220 path=[227:0-190 417:191-219]

ACTTCTAAGACCTAATCAGGGCACAGTTCTCACTCAGTTATTGGTATCTGTGAACTTGAA

ACTACATCATACACTGGTAGAATGACAAGTCATGTCTTTCATGTAACTTTGTATAGACAT

TTGCTATTTCAGAGCTTAACTGATATTTTTTTCTCTTGCCATATAAGGCACTTTTTCCTT

ATGTAGTAACTTCTGATCAATGCAATGACAAAATACCTTG

>c14033_g1_i1 len=225 path=[155:0-224]

AAATTACATAAATCTGATGAGTTATTAGAAGTGGAAGAGAAGATTGTATTGTTTCAATTT

TGCTGTTTTGATCAGTATTGAAAGCTGACTTAGCAGAAAGCAATCCCTTTTTCTGGATAA

AACGGAAACAGCCTACAAGATAAGTTATTGATGAAATTAACTGCTGAAGTGGAGAGGAAA

TGTGACTGGCCAATACATGTCATAGTGTGAGCTCTATTAAATGAA

>c14037_g1_i1 len=298 path=[310:0-263 25:264-297]

ATAGAAAACGAGAGAAGAGAAGAAAATGGGGTGACAGAGAGGAGAGGGGACAGTGCTGGG

GAGGTGGCAGATAACAGAGCCCAGCAGTGTCTGTTGGACAGCTTTGGGTTTTCTGGGAGT

CCAACACTGCAACATTTGGTACAGAGCCGGAAAGAACGCCAAAAGCTATGGAAGTTCAGC

AGTTCTGCAATAGAAATACAAATAATTAAAGAAAAAGTCCACCGGGTGACTCAGAGCCAA

CGCTACAACCAAACCGTGTGCTGCTTTGTGCTCTGGAGAAGGCAGCTCTTCCTGGAAG

>c14037_g1_i2 len=299 path=[310:0-263 573:264-298]

ATAGAAAACGAGAGAAGAGAAGAAAATGGGGTGACAGAGAGGAGAGGGGACAGTGCTGGG

GAGGTGGCAGATAACAGAGCCCAGCAGTGTCTGTTGGACAGCTTTGGGTTTTCTGGGAGT

CCAACACTGCAACATTTGGTACAGAGCCGGAAAGAACGCCAAAAGCTATGGAAGTTCAGC

AGTTCTGCAATAGAAATACAAATAATTAAAGAAAAAGTCCACCGGGTGACTCAGAGCCAA

CGCTACAACCAAACCGTGTGCTGCGCCGTGAGCCCTTCCAGGAAGAGCTGCCTTCTCCA

>c14039_g1_i1 len=372 path=[360:0-371]

TTTCCTTGTAAGTTGCACCTATAAATTATTAATAAATAAAAAAAATATATATATTTATTT

ACTTTATAAGATAAACATACACAAAGTGTAATAATTAAATATTTACAAAAAGTTAGGCAA

TTATATGCAAATGTAAATAAATATACATCGGAAAATACATATAACGAAATGTATTAATAT

ATATATATTGTTATCTTATTTAAACACTGAATTATTTTCAAATAAATGAAATGATATATT

TCATTTATAATAAGACAGCTGACTTATAAGATAAAAAAATAAAGGACTTGAACAGAACGT

TCATAGTCATATTATTAATTTATTTTTATTATTTTATGCATCACTCTTAAATATATAAAA

TTAATTTTTTAA

>c14041_g1_i1 len=273 path=[1:0-103 300:104-272]

GCATCAACATCTTAAAAAATATATATATCCTACATCCATGTCACGAGTCAGTGACAGGTC

TACATCTTAAAATGCAGTTAAATGCACCAGTATGTACAGGGCTTCGTTGTTTTTTCTTCC

TGGATGACTTTGCAAGAACAGGAACTGTTGTCCCATCTATTTCAGCAACTGCTGCATAGA

AGAGCTCACCAGCAGTTGCCCACTGCCTTCCATGAGAAGCGCATGATATGGTCGGATCAG

CTATCAGCAAGTTCTCAAGGGGAGAAAGTCCTT

>c14047_g1_i1 len=211 path=[1:0-210]

TCTGTGTGGAAGAAAGTAAGCTGCATCTGTGAATTTTGAGGGAAATCTCAATTCCTAACT

GGCATTTATTCTACAGCTAAAAATCTCTACTCATGCCATTCTATAAATGCACCTGATTGT

CAGCACAGCTCTCTGGAATTCAGTGTGTATGCTACCTATGTAAAAACTACTTTAATTAAA

TGCTTATACTGAATTTGAAATTCACATCTAG

>c14052_g1_i1 len=312 path=[482:0-311]

AATATTTAAATAGCTCTACAAGCTGCAGTGTTTTTTTAGTGCATGAGAAAATATGGCTAT

TAATTAAAAAACTCAGTTGAAGTTGAGAACATAATACATTAGATAATCCTGAGGTCCAGA

AAATGGCTAGCCTATAAAGTTGTAGAACTCTGTAGGTTGTAAACAGGCAACCAAGGGAGT

ACACTTCCCACTGCTAATTTTACGGCTTAATGAGAAACAGGTTGCACACCAGCTTAATTT

TTATATATCATCTGGTCCTTTTCATTTTTACTTACTAAACTTTAAGACTATGTGTTTGTG

TTTGTTTGTTTG

>c14062_g1_i1 len=313 path=[1:0-312]

TGTTTGATCGGGGAGGGCACCTACGGCGTCGTGTACAAGGCCCGGAACAAGGTCACGGGC

GAGGTGGTGGCGCTGAAGAAGATCCGGCTGGACACGGAGACCGAGGGCGTCCCCAGCACG

GCCATCCGCGAGATCTCGCTGCTGAAGGAGCTCAACCACCCCAACATCGTCAAACTGCTG

GACGTGATCCACACGGAGAACAAGCTCTACCTGGTCTTTGAGTTCCTGCACCAGGACCTG

AAGAAGTTCATGGATGCATCATCCCTGGGCGGCATCGCGCTGCCCCTCATCAAGGTGGGT

CGGGGAGGGGGTG

>c14062_g2_i1 len=329 path=[581:0-328]

GTTGTGGTACCGTGCGCCTGAGATCCTGCTGGGCTGCAAGTACTATTCGACTGCTGTGGA

CATCTGGAGCCTGGGCTGCATTTTTGCTGAGATGGTGACGCGGCGCGCGCTCTTCCCCGG

GGATTCGGAGATCGATCAGCTCTTCCGTATCTTCCGCACGTTGGGGACACCGGATGAGGC

AGCCTGGCCCGGCGTCACCGCGCTGCCCGACTACAAGCCCAGCTTCCCCAAATGGGCCCG

GCAGGATCTGGGCAAGGTGGTGCCGCCGCTGGATGAGGAGGGCCGCAAGCTGCTGGCTCA

AATGCTGCACTACGATCCCAACAAGCGCA

>c14065_g1_i1 len=243 path=[375:0-242]

TTCCGGGGTCTCTAATTAGACAAGAAACAAGTTCTGAAGTGAGAAATAGCACAGTATGAT

AGACCAGTTGATAGAGCTAAAATACAGAATAAGCTCTCCGGCACACAGCCACTCCTCCAG

GTTATCAGATTAAATGATGCCCACATTGATTTGAGGAAAATCTTTCACACTGTACTTCAA

AGGAAATAAGACTCTCACATTCACTTCATATCAAGCAGTATAGAAAAATACCAACTAGGA

GGA

>c14073_g1_i1 len=229 path=[399:0-228]

GCAGGGATTTGGGGGGGTTGAGATGAGCCCCTGTAGGCACTATGGGGCTGAATCTGCCCC

ATGCTCCCCTCCTCCCTGTTCCCATATAAGGCAATGGGGCCACGAGAAACAGCCCCTGAG

ACAGACCTGCCCCGGGTCCCCCCGGGCCCCCTTATGTCCCTCCTTCAGTGCAGCAGCAGT

TTATGGCTTTCAGGATTTTGGTTCTGCTTTAAGGAGAGGAGAAAAAAGC

>c14077_g1_i1 len=205 path=[1:0-204]

GCTGAGCTGCTGTACCCTTTGCTTCTGCTGCTTTTGGTGTTTCTGCAACATGTTTTACTT

GATGCTGAAGACAACAAGTGGTAGCAGCAAAGTGTGAAGGTCTGTCTTAAGGAATACAAT

GCAGGAAGAAGTTCCTGGAATATCAGGAGAGCATTCTGAATGAATTTCAAGGCATTTTTA

ACACGAAAGGTATCTTTTCAGATAA

>c14077_g2_i1 len=210 path=[552:0-209]

GGATGTGACAGAATGAACATGAGAGAAACTTCTAGAGGTGGCCAGCTGTGGAAGATAATG

TTCTTGGTAATATTGAACCAGAACAACCCAGTGAGCACTCAACAGTTAACTTGTGACATT

GAATGATAATTATTTTGTCATTATGCAACTGATAAATCTGCAGTGCTTATGGTAACAAAA

AAATACATGTAGAAAGAGAATGGTAGCATT

>c14083_g1_i1 len=236 path=[167:0-235]

AATCCTGCATTAGAAAGGCTTAGGAAGGAATGGGCTTGCCTCCCCTCAGCTGCTTCAAAG

GACACACATCACGGTGCTCTGCATTGCATTGATCTGCCTCTACAGTTCTGAATTCCCAAG

AAGAGAAATGCATTTTGCAGCAGTTACATTTTCTTACCGACCTAAATTATCTTTCTTCCA

ATAACACCCGTTTTGTGTTTTATGTAGCTATAACCCTTCCACTTCACAAGTTAGCA

>c14092_g1_i1 len=201 path=[333:0-200]

TATCTTTCTATGTCTGGACATGGTGAACTTAAAATGTTCCACAGATATAGGGTTAAAAAT

TCCTTCTGTTTAGTGGGATAAAACATGTAAATAATTCACAGAACGCAGCAGGCATTTACG

GTTACACTCTGCCTCCTGTTACTGCACTGATAAGCCCAAAGTTGATCCAGTGTGAAAAAA

AACAATGTTCACATGTCGTAA

>c14100_g1_i1 len=203 path=[1:0-202]

AACAGGATAAAGGGAAACGGCCACAAACTGGAGCATAGGAAGTTCTGAACCAATATGCGT

AGGATCTTCTTCACAGCGAGGGTGACAGATCACTGGAATGGGATGCCCAGGGAGGTAGTG

GATTCTCCTTCTCCAGAAATATTCAAGGCCCTTCTGGACACCTGCCTGTATAGCCTACTG

CAGGGGGCTTGGAATTAGGTGAT

>c14106_g1_i1 len=203 path=[1:0-30 32:31-202]

ACTGATTCTGTCCCTTCGTGACTCGGGGGCGTTGGGGTGCGCTGTGCGCCGGTGTCCACC

GGTGCCTCGTGTTTTTGTGGTTCTGGTGTGCCGGGCCGTCGGGTCCACACGGTCCGGTGG

ACTCTGTTGTCCCTCTCCCTCCCCTGACTGGGGGCGGGGCCCCTCTATTCATTGCCTGTG

GTGACTGGGGCGGCTGCACTGGT

>c14112_g1_i1 len=380 path=[278:0-379]

GAATTTTTTTTTTTTTTTTATAATAGAACAAAAAAAAATTCAAAATGTTAACATAAATTT

ATAACACAAAATCTCTTTGAATACATGACTAAAATTCGTTAAATTCAGATGCCTTTACAA

TTATTCATTTTGAGTTGGAATATTTTTTTAAAGTTTATTCAATTTTATAATGCTTATTTT

TTTCATTATAATAAATTACTTTTTATTTTTTTCATTACAATATAAATATTAAAATTTTAG

TAAGATAGACTTAAGATTATATATTTTCTTGCTCTTTTTATTCTTTCAAAAAAAAAAACT

AATTCAAAAATGCAAAGACATATATTATAAATTTTAATTTTTAAGATTAATAAATCTTAA

TAAAAAAATTTTCATTAATA

>c14119_g1_i1 len=237 path=[1:0-236]

ACACACACAAAAAAATGCAGTAATTTCAAAATACCCCATTTTTAGAACACCTGATAGAAA

CATGAAGGTTAAGGAATATTCCCATCATTTAATCATTGGCTTCCAAATTTGGTGATATGA

ACTGTGCCATCAAGAGGATGAAAGTAGAAAGAAAAACAGAAAAGAAAAAAAAAGACCATC

TTCCAGCTGTTATTCCTTAGGTGTTCCGAATAGCTTATCAATTATAGTCCCAATGCA

>c14119_g2_i1 len=273 path=[429:0-272]

GTTTGATCTGGCACATTCAAACCAGTTTTCAGTTAAAGCACTAAAAAACGGTGTCTTTAA

TAAAATCGTTCTTGTGATTTCAGTAAAGCTTTGTTTTTTAAAAAGGAATGAAGATGTGGT

TAAATTCACAAAGTGCCAATTTAGTTCAACACGTTTTAATGAGTAACCCTAAAGGACAAA

TATGACACTGCATACAGTATGATGCATTACATTTTGCACACAGTGTTTTTCACACTCAAG

CTCTAGTTTTACACTGATGATACAAATGAATGT

>c14124_g1_i1 len=208 path=[1:0-77 530:78-207]

GCCCTGATGGGGATCAGGAAGGGATGTTACACCAAGGCTGTAGAAAGGAATGTGCCTCGC

TAGACCTGTCCCCTAGATTTACCTTTCCTTCTTGAGGCTGTCAGTGGTTTAGGTATGCCC

TCAGTGGGACATCGTCTGCAGAACGCTGCATTTGACAAGCATCTGTTAATGTAATCTCCA

AGCAACTGCCTAATTCCCTCTTTTTTGT

>c14128_g1_i1 len=202 path=[1:0-201]

AGTACTTTCAGAGATTCTTCTGTTATTCTGATTCTGAGGTGACACAAAAAAATGTGAAAG

CTGGAATACTGTGCAACCGAGATTGTTTAATGCTATGATGCTTCATGTGGATTCTATGGG

AATAAACATGTTCCCTCTTAGACTACCTACAGGGAATGGCATCCATTGCTAGCAATGTTA

GTTGGTCATAGTGCTGAAAAAA

>c14145_g1_i1 len=360 path=[338:0-156 495:157-180 519:181-359]

AAAACAGAAAGAGGCATATGTATTTCTAAGTAATTGAGCATCCTCTTTAAAATGTGAACT

TCAATGTTCTTACTCAGTTTGGTAATCTTACATCAAATATTGAGAGTTTTGCAAGAGCTT

AGAGTGAAACAGAATAAAGTTATTTAGTAAAAAACATATGCCTCTTTCTGTTTTCACTTA

GAAAAATAGCAATAAATGCCTCAAGTATTTAACGTTGAGGCCCTTGTGGAGGAAATTACT

TAGTACGTGCCTGATTTCCAACATGGCAGTAGTTGGAAACTATACCTCTAATAGTGACAC

AAGTGTGCGAGTCAGTATCCTGCCAAACCTGGAACTGCAGAAACCTCTCAACTCTGGGAG

>c14146_g1_i1 len=393 path=[371:0-392]

GGAGTTTTGTCTGGCGTGTCGCTGAGTGTTTTCTTCATGTGTTTGTTGATTTCAATGGTG

GTGCGGTGGTTCGGGTCGCGGGTGCAGTCACAGGTGCTTAGCGTGTTTGGATTTGTCGCC

ACTGGTTGGCGAGTGGTCGGAGTTGGGCCGTGCAGCGGATGAATGTGGCACTTTTTGTTC

CCGTTCCCATGCACCACCTTTGGATGGCCCGGGGGCACAGTTGCACTCCTGGAAGTTGGT

CTTTTGTTCTGTCCTTGGTGGCGGGGTCGGGGGGGTGTGTGGTTGGCCATCGGGATGTCT

CGTTTGCATCGCCCGGGGTTCGACTTCTTGGTTCCCTGGTTTGCTGCGCGCCGACCCAGG

GGGCAGTGTTAACATTCTTGCTTTGCGGGCTGG

>c14157_g1_i1 len=222 path=[422:0-221]

TGATATGCATCTTATGGTGTATTTTGCCGCAGTTGCCTTTTAAGCAGAATTGAAACTGCT

GAGAAGTCCTCATGTTCACACAGCGCTATCCCCCAAACTGCAAAACCAGGAAGGTCCACA

TACCCTACAGAGGAAGCTGCTCTTCTTTCCCAAGCTCTGAGGAAACGGGAAAGACTGAGA

CTGGGTCCTCTGAGCCTCTGCACCACCACAAGAAGAATCCAG

>c14158_g1_i1 len=222 path=[642:0-177 819:178-221]

AGGCTAAGTCATGGGCATCCAACCTTTTGTTTGCCTGGGCCTCAATGAGTGTAAGAGGAA

TTGTCTAGGGCCACACCTACGAAGGTTGCTCTGAAAGTAATTCCTCCTAATTATTTCCAT

GGAAACTAAGGCAGACAGAAAGACCACAATAACACTGTTTGTTAGAGCAAATTCTCAGTA

CTCTCTTTTCCAACACAGTTACCACCATTAGCTGTGCATTTA

>c14158_g1_i2 len=223 path=[642:0-177 423:178-222]

AGGCTAAGTCATGGGCATCCAACCTTTTGTTTGCCTGGGCCTCAATGAGTGTAAGAGGAA

TTGTCTAGGGCCACACCTACGAAGGTTGCTCTGAAAGTAATTCCTCCTAATTATTTCCAT

GGAAACTAAGGCAGACAGAAAGACCACAATAACACTGTTTGTTAGAGCAAATTCTCAGGC

TCTTGCTCACTGCTGGTGTAAATGCACAGCTAATGGTGGTAAC

>c14163_g1_i1 len=202 path=[1:0-201]

GTAACTTTGTGTTTGAGTGCCATACCTCTTGCTCTCTCTGCATAGCCTGGATTAAGCTCA

GAGAAAAGTTTTAGTGCCTTCCGTAAAAAGACATATTTTTCAATATTAAAGACTTAAATT

CATAGATCATAAATAATATAATTTCAAAATACTTAAGAATTCTAGGACTTCATTTGCATT

TCAGAGGTTTGGGACTGAATAG

>c14164_g1_i1 len=252 path=[1:0-251]

GTAACTTTGTGTTTGATGTCTTTTGTGCCAACTCATTACCTACCTTTTGTACTGTGATCT

TATATTTAAGCCTCCAAAAGGACTTCACCCATTACAAAGATAAAGGAAAAGTAAAGAAAG

AAGTGTCAGGTCGACTGTGGATTATGCAATCTGTGTAGGAATGTCAGTTTCTCCATTTGT

GTACCTTTTTGCCTTCATAACCTGGAAGTTGTGGCTGAATAGAGAAGATGCTAGTGACTG

TCAAGATATCTA

>c14167_g1_i1 len=209 path=[1:0-208]

ATATAATTACCAAATGAGTAACACCTGGCAAAGTAGAAAGACATAATAGAAAGGTTACAT

GTTCATATATACTGACAATTTTTAAAAAATATATTAATATGTTTTTAGAGCACAACAGTG

GAAATGGCAAGTAACAAGCACAGTCATTATGGAAGTATTATTTTTCTAAAAAATAAACAA

GAATCTAGTAGCAACAGCAGCAGTGATAG

>c14170_g1_i1 len=246 path=[1:0-119 456:120-245]

GCCTGATCCAGCCTTGCCTTGAAATTCTTCAGGGATGGGGCATCCTCCACATTTCTAGGC

AACGTGCCTCACTATCCTCACTGTAAAAGACTTTCTCCTTATATCTAACCTAAATCTACC

TTCCTTAAGCTCAAAACCACTTCTCCTTGCTCTCACAGAGAGCAGAGATCAGCACCTGCC

CCTCTGCTCCCCTCCTGAAGAAGCTGTAGGCTGTCATGAGGCCTCCCCTCAGTCTGCCCT

TCTCTG

>c14178_g1_i1 len=223 path=[357:0-222]

AATAAATATAAAAATAATGAAATATGCTAATGTTTTATAGTTATTTTTCCCTTTTTTTTA

TCTTAACATATTAATTAGAGTAATTCTTTTTTATTAAGAAATGTATATTACGAGATTATT

TGAATTTCTTTTATATTACTTATTTTAATACTTGGTTTCTCCTTATTTATGATCCGTTCA

TATTTTTATTTTTTATTTCAAGAAAAAGCGTTTTCCTGTTTTA

>c14183_g1_i1 len=234 path=[1:0-233]

GAGTCCTACAGAAAGTCTCTGGAGTACGGGTGTTCAGATCAGCATCCTGCAAATCACAAC

TTTGCATGGGTTCAGTCTCTGATTCTGGACGATGATTGTATGTTATTCCCACTAGCAAAG

TATGAGTATGATGGAAATACTCAATTTGTTTTAAATTAATATGAACTACATGAAATTTTA

AAGTAAATTAGTAAATTAAGTCTTATCTGAAGGCATCGTATAGAATCAGAGACT

>c14193_g1_i1 len=201 path=[1:0-78 80:79-200]

GTGGGCTGTATCACTGCCACTTGTGAGAAGGAGCAGATCTCAAGTACTCTGCCTTGCTTC

TGTCACAGCTCAAAGGCTCTAGCAAGGACTCAAACCCAACAGCACTGACGTGTTCTTTGC

CATATATGAAATCTTGTGTTATTAAGTGAAATTCTTGGTAAGTTAGTATATTTCTTCTGG

AGCAGTGTACTTAAGTACCAG

>c14199_g1_i1 len=225 path=[203:0-224]

GATTTTTTTCTTGGAGATAGTATGTACCACTGCATAGAAATTCCTACATAATACCACATA

TTCATATGAACGCATTCAAGTTGCATCTGTATTGCAAATATATACATGCAAAATAGGCAT

TTGCACAAGAAAAATAGATAAAGAATTGCACAAAATAATAGAAGTAGATTGGTTGCAAGG

TTATTTGTGACTCAGAAATCAGATAGAATATAGGCTGTGGAAGTG

>c14199_g2_i1 len=216 path=[405:0-215]

TGAAAATGAAGTGTGCTGCTATATTTTTATAAATTAGAATATGTATTTGGGTGTGTGCGC

AATACTGAAAATGTTAGAAACACCACAGTGGAAAATCTGTTAGGGCAGTTGTGCAGAATA

CACAGTGAAGTACAATGCTCAATATTTTGCTTCACCTAGTGAACCTGTCTAGGGTAAGGT

GTGATATTTGACACATCTCTGGGGGCAGATTCAATG

>c14212_g1_i1 len=203 path=[1:0-101 103:102-202]

TGTTGTGTGCTTCTACACCACGAGTCATGGCTTGCCTGAAAGCTGTGTTGTAGTTGAGCG

CAGTTATGGGGTGCCTCCATGTTGGTCTGATGGATGGTTTCATTTGCATTAGGTGATGCG

TTCTGGTAGTCCCTTCCAGCTCTCATAAACTTGCCTAGCTGTACACATTTAAGAAGCTGT

GAATCATAGAATGGCCTGGGTTG

>c14228_g1_i1 len=218 path=[233:0-217]

TGTTTAATTTTGTGGTTAGTGGCAGGAATCAAGTAGCTTAACATGGGGAAAATGGGTCCA

TCACAATGCTCAGATACTCTTATGATCCCATAAACAGATGGTTAAGTTTGTGAACTGCGA

ATATCCACCACCACACAAAACAGTTTTTATCTTAAGTTTTTCTCCTCATCTTTTAAAGTA

GAGCACTCCTATAAATATTAAAACAGCAACAAAAACAA

>c14231_g1_i1 len=280 path=[258:0-279]

AAAGTCTGCTTGATACTCCTGAGTAGGTCCTTAGCCTTGTGGGATTCCTGAAGTAGCAGA

TTAACATCACTACTATCAGAATTCTGTACATATAGAAACAAGAGAAAAAAAAATTAACTG

TTCAGGTACTTCGATAAGTGAAGTCTGAATATTATATATTGGCCATCTGTTGTAACAGCA

AAGTAAGGCCAAAATCAGCAAAGGAGACATTTTGCTAGACATTCTCCAAAATGTCTTTTC

TCCAAGAAAATAAATTCTAAGCGCTCTTGATGAAATTTGG

>c14240_g1_i1 len=320 path=[1:0-319]

ATTAAGCTTGAAATAGTGATAGATGCCAAGCTCAAATGGAGTGAGATAGGGCTCTGGAAG

CAAAGCTGATGGCAGTGAGTACTGAAGCTGTGACCCAGGCACTGCACTAGGAGCTGCCAA

GTTCCGTGTCCCTTAAGCCATCTGAGAGGTTCAAAGTCTGAGCACTTGAAGCAAATTGTA

CCAGGCCTTGTAGGCAAGGAACCAAGTGCTTGAGCACTGCAGAGCTTCTTCGGGTAGCCA

ACTGGGTGTGACTGAGGGTGGGCAAGGATTCTTATGCTGTGCCATTTATGGGATGTGTGC

TTTTAGTTGTGTTATGAAAA

>c14256_g1_i1 len=265 path=[427:0-264]

AATTCCACCCTACTCTGGAAATTACAGGCTTCATAACTGGAGCAGGAGATACTATAGGTC

CTTTGTTTCTGGTGGGTCCTGTATGGAGCCAGGAGTTGGACTAAGAAAGCTTTTTTTATC

TAAAACTAATAGTTCACTGTTCTAGGAGCATTAAATGGATTACAAGTTTTATATGGCTTA

CCAGCGTCTAGCCATATACTGATAATTTCCTCGCATATAGGGAACTGGTGACACTAGTGT

TATTAGCTCATAATTGTAAACTTGA

>c14260_g1_i1 len=422 path=[460:0-359 25:360-421]

CCCAGGCCCCCATTGGCTCCCCCAGCAGCGTCCCCACGAGCAAAGCCAGAGCTCTGAGTG

GAGCTAATGGGACTTGGAGAGCTAATGCTTACTAATAAGAGCGCGCAACTGAATGCATGG

GATTAAAAAATTAAAATGACAAAATAAGCTGCTTATGCGGTGCATCCAGCAACCACCTAC

CAGCACCTGGAAGTCACCCACCCAGGGCAGCCTAGGGCTGATATGAGACCCCAGCCCAGT

GGGCATCCCTGCTGCCAGCCAAAGCCCCCAGGCAGACAGACCCATCTCCATCCCAGCATC

TTCGTTAGCCAGCGTACTCATTAATATTTCACTGGGAGGCAGCACATACTTGTCCAAGCG

AGGCACCGTGGGTGGTTTAAGTGATTTGAATAAACGAGCTGCATCACTTAGGCACCTCTC

CC

>c14260_g1_i2 len=421 path=[460:0-359 819:360-420]

CCCAGGCCCCCATTGGCTCCCCCAGCAGCGTCCCCACGAGCAAAGCCAGAGCTCTGAGTG

GAGCTAATGGGACTTGGAGAGCTAATGCTTACTAATAAGAGCGCGCAACTGAATGCATGG

GATTAAAAAATTAAAATGACAAAATAAGCTGCTTATGCGGTGCATCCAGCAACCACCTAC

CAGCACCTGGAAGTCACCCACCCAGGGCAGCCTAGGGCTGATATGAGACCCCAGCCCAGT

GGGCATCCCTGCTGCCAGCCAAAGCCCCCAGGCAGACAGACCCATCTCCATCCCAGCATC

TTCGTTAGCCAGCGTACTCATTAATATTTCACTGGGAGGCAGCACATACTTGTCCAAGCG

TGGACATCTACAGCGCAGGGGTAGTAATTAGCACCTGGGAGAGGTGCCTAAGTGATGCAG

C

>c14268_g1_i1 len=311 path=[53:0-310]

GTAATAAAACACTTTAGCTTTTACTTTCCAAACACAGACAAGGGCAGAGAAGTGAGCAAA

ACAAAATTTACTTGGCTGAAAAGGATGAAGTGTTAGGATCCTTCAACATGTAAATTCTAA

TCTCTTACCATTTAGGAAAGAGTGGATTATTTCTGTTCACTATGCTCCTGAATTATCTGT

TGCAACAGGCTTTTTGATTTTCACACTGAACGTTTTGGCCACCTATTATAAAATTGTTAC

CATTTTGCAGTTCATTACTCTCTGAATACAGCCTCAGAACGGCCACTCGGATGACTAACA

GGAAGAGCAAG

>c14282_g1_i1 len=391 path=[177:0-390]

AATGAGTGGACTCTGAATTTGGATACTTCAACTTTAAGATGAAGAAATTTGCTTAAAAGG

AAAACCATTTCCAATCAATTGAGGCTTAAAAAAATATTAAAGTGCTTTTGCATAGGCTAG

TACACTTCCACAGGGTTTGAAAAGGAAATCACCAAAGGAAAAAATAGAGCTTGAATGCCT

CTTTTTAAGGAAAAAGATTAAATTTTCATGGATGTCTCCCATTTGTGCATCTTATGAGCT

CTTCAAAACTATTCTCCTAAGTTACTCATTCTGACCACTGTCAGGAAAAAGAACAGCAGG

CTATACAGATCAGTGACTGCATCTATATAACAATTCTTATGCTCCTCACTTCTTTGTGTG

ACAAGTTGTGTGATGCCTTCATTAATCTCAA

>c14283_g1_i1 len=298 path=[304:0-267 571:268-297]

GAAAACCTTTTCAGACTATTTTTTTGCTGTTTTTAAGTAGGACTTACGTTTGTGATCCAA

GATTCTGCAGCCAATTTTTTGTTGCGTCTTGACGTTCAGTGGATGCAGTGTCACATTTCC

ATACACATAAATTACAGCTGTTCAACTATGAGAAAGAGCACTGCGTCATAAATCATTGAG

CACGCTATGAAAAAAAGCCATCTGAGTTTTGGTCCCTGGCAGTGTCTCCAGCCTCCTTCC

TGCCTTTGGCATCTGTAAACTTTTGCAGATGCAATACTGAAAGTTATCTTTTTACATT

>c14287_g1_i1 len=306 path=[27:0-24 52:25-305]

GACCAAATGCCTTCCAACTTGATGACATTATTCTTAAAATCCTGTTAGTGCTGGTATTTT

CAGTTTCCTTGTTATACTATAGAATCACTTTTTGCTATTAAAATGATTTGGCTACAGTAG

AGTGCTATAGTATTGAAGAAGGAAAGATCCTTTGAAAATGCGTTGGTAGCCACAGGCATT

ATTGAAGTTTTACCAACCTGAATTCTTGAACTAAAATAAACCTGTCTTTTTTCAAAGCCT

GTGTAACTGAGGACATTTTATGTACCGTGTGGTTGTACTTGATGGAACTCCTTTTATCCT

ACTGAA

>c14294_g1_i1 len=293 path=[338:0-0 206:1-135 184:136-157 206:158-292]

AGTTTGGTCGGACCCAAAGGGACCCAAAGTCGGTTTGGTTGGACCCAAAGTTGTTTTGGT

TGGACCCAAAGTCTGTTTGGTTGGACCCAAAGTCGCTTTGGTCGGACCCAAAGTTGGTTT

GGTTGGACCCAAAGTCGGTTTGGTCGGACCCAACGTTGGTTTGGTCGGACCCAAAGGGAC

CCAAAGTCGGTTTGGTTGGACCCAAAGTTGTTTTGGTTGGACCCAAAGTCTGTTTGGTTG

GACCCAAAGTCGCTTTGGTCGGACCCAAAGTTGGTTTGGTTGGACCCAAAGTC

>c14302_g1_i1 len=207 path=[27:0-70 26:71-206]

TCTGTTGACAGGGCCTAGTGGGTTGCTCTAACTACGTTGCCGTTGCAACCTGAGTGGCTT

TGGGGAAGGAAAGGAGGGAAACGCAGCACACAGCTCAGCTGAAGGGCGCCCGTCCTTCCC

ACTGCGGTCACCCTTCCCCTGCGCTGTGAAACGATTTGTAAAGCCTCAAAAATGCCGAGG

AATGTGCTGTTCGAATCCCGACTGTTT

>c14311_g1_i1 len=278 path=[287:0-277]

TTTTTTTTTTTAATATATTCATTATTATTTATTAACAACTCATAAATTAATTTTTGTTTC

ACATAAATAATTTAAATTTATGAAATTTTATATAATCTAATTTTTAATTTATATGAGCAC

TATAAATATATTTCATAAGTTTTCATATATGGATATAGATATATATGTTGTAATTTTTCA

CCAAATACTTTATAAAATTTTTTTATTTTTCAGTTTTATCTTTTTTTTTAGTTTACTTCC

ATCTCCATCTTTATCGAATTTAAAGAAGCCTTTAGTTT

>c14317_g1_i1 len=315 path=[333:0-314]

AAAAAAAAAAAAAAAGGAATATTATGTTAATATTAATTTTCTATTTCGCTTAATTTTAAT

TAACTAATTTTTATTGTCAATATGAATTTTATTTTTGCTAATTTTAATGATTGTGTAACA

TATGCATGCACTTATGAAATAGGAATAAGTTATTATAACTAAAAGGAAATACATTTTATT

TTATACATATTCATTAATATGTTGTATTAAAAAAAAAATAAATTAATAAAGTATCATAAA

TAAGTGTTAAAAATAAAATTATATTAAAATATATTAATTATAATATTTTTTCGAATCGCA

TGCAAATATATAAAA

>c14318_g1_i1 len=202 path=[428:0-201]

ACTCATAGAAGTCTGCTTCTATTTAGTATGAAACAAATTCTTCTTGAATTTGGCTTTTTT

TTCAAATTATTTTACGTAATTCTTGTGCTAATTAGTGTCATTTAAATGTATTTGATAGAT

AATTAAAATTTTCTCATGTAATCTTATGCAAGTATGAATAGCTATGGTGATTTTATGTAT

GCATTTTTTTAACTGATTGGAA

>c14330_g1_i1 len=245 path=[223:0-178 402:179-244]

TCCTCCCAGGAATGTTCCAGATTTTTCTCTTTGGAATAGAAACAGCTGCTGCTGACTTCT

GCTTGATCCCAGGCCAATTTAAACATGGACAGAGAAGAGTGTGTACATTGGTTTGTTTTT

AGTTGAGGGGGTTGCAATGGGGGAATGTGTGATCTGTGTTGGTATGAAGAAGTAAGATGC

TGGGCTGGGGCTGTTCAGCAGGCTGCTCCATGCAGCTGTAACCGTCTATGTGTCCCCTGG

TGTGC

>c14332_g1_i1 len=252 path=[155:0-251]

GTGCTGTTTTCATGCAGAGCAATAGATGCATAGTGCAAAAGCCATTATGACATTTTCTTG

GCTTATTACAACTGGCATTGCCAGGTGTTTGTGCAATATTTTGCACAGAGGAGGAGCTCC

TTAGGACTCCAACTCATCCATGATATCCTACAACAAAAACAATGAAGTTATTCTATTTAG

GCTTATAATGGTTATGCAAGAATTATTTGGGGGAAACACGCTGGTACACTTAAAACTGCA

GAATGACTCTGG

>c14335_g1_i1 len=574 path=[1164:0-172 @587@!:173-544 25:545-573]

TGAACAAAATGAGCACTAAGTCTCTTTAGCTTTTGCTGCCATCCCTGCAAAAGCTAAAGC

GACTTAGTGCTCATTTTGTTCACTGTAATCAAGATTAGCTTGTTTTTGTTTTTTTTTTAT

AAAGTGCTTTAAAGGGAGTTTTACGTAGCCCTCACCCAAGGTTTAAGCATGGTAGACCTA

AAATCAAAATAGCTGAAATCAAGTATGGTCTCAGTATTTGAGGTGCCAAGATGAGTGAGG

CCATCCACATCTGCGAGGTTTGTTCTTGGCCACAGTGGCATCATTGCCCCTGCTCCACTT

CTTCCCCTAGAAATGGGGAAAGACCACAACTCCGCGCTCTCTGCAATGGAGGATGACTTA

CCTGTGACTGTCTAAAGGGCTTCTTTGGGGGCTGTCACATCGTTCTTTCAAATCTCAGCT

ATTCCACTGTGTGTGCAATGTGGTAAATAAGAAAACTCCAGAGCTGAGCTCATTTACGTA

CCATTGAACGTGCCCTCAGGCACTGCTGTAAAGAAAGCCATAGAAATCCTTCCATTACGT

CTGCAGAAAGCCTCAAAGCCAGACAGCACCTCGC

>c14335_g1_i2 len=491 path=[497:0-89 @587@!:90-461 25:462-490]

GAGAATGCTGGGGGGGAAAGGAAACTAAGTGCTGCTTTGGGCATGAAGGGAGGCAAAAGA

GAGGGTCCTCACTCCAGCCAGACACTTTCAAGACCTAAAATCAAAATAGCTGAAATCAAG

TATGGTCTCAGTATTTGAGGTGCCAAGATGAGTGAGGCCATCCACATCTGCGAGGTTTGT

TCTTGGCCACAGTGGCATCATTGCCCCTGCTCCACTTCTTCCCCTAGAAATGGGGAAAGA

CCACAACTCCGCGCTCTCTGCAATGGAGGATGACTTACCTGTGACTGTCTAAAGGGCTTC

TTTGGGGGCTGTCACATCGTTCTTTCAAATCTCAGCTATTCCACTGTGTGTGCAATGTGG

TAAATAAGAAAACTCCAGAGCTGAGCTCATTTACGTACCATTGAACGTGCCCTCAGGCAC

TGCTGTAAAGAAAGCCATAGAAATCCTTCCATTACGTCTGCAGAAAGCCTCAAAGCCAGA

CAGCACCTCGC

>c14337_g1_i1 len=225 path=[155:0-224]

TGCAGAAAAATGATTAAGGAAACTACCAAAAGAAAGGAGAAGGAAAAGAATAAAAGGAAA

CAAAAAGCAGCACCTTCTGAAGTATAGTAATGATTTATGATGCAATACAGCCATTGGACA

GCAATTGGTTCAGACTGCTGAGATGTTCTTCAACTTTCCCAATTTTGAGACCACGATGCA

CAAAGCAGCTCAGTTAGGCGTAAGAGAATATGTGATAGATGAGAA

>c14339_g1_i1 len=206 path=[1:0-205]

AATCCTACAGAGCTCATCTGTTTGGGAATTTTAACTATGACTGATATCCACTCCTTACTT

CGTTTCCAAAAAAGCTCATGGAATTACGCATACCAGTCTTTGTAGCCAGAGCTGTGGTGC

TTCCCATGGGCTGCCTGGCTGTGTGGAACGCTGCTCTGCTTGCTCCCCAGCCCTCCAGGA

GCAGGGAACGCTTCTTCCCTGAGTCA

>c14344_g1_i1 len=254 path=[1:0-77 592:78-250 598:251-253]

TCAGTTCTAACGAGTACTTGTTGAACTGAGGGCTTGACTAAAATATGGTATCTGGGGTTA

TGCTGAGCATTTTATGGGGAGTGCTCAAGCTGAGATGGTGGAAAAATTCTCACTTCTCCT

GGTCTTGTGCTGATACACCCTTATACTTAGTCAGAGGGAATTGGCCACAGTTTCATTGAA

GTCAAAGCTGTTTTCTTAAGGGAGAAATTGAGGGATTCTAAGTTAAATGGAACGCGTGCA

TTTTGTTAACAGCT

>c14345_g1_i1 len=211 path=[1:0-210]

AAGACAACTGGAAAAATGTCAGGACAGAACCTTCTACTGCCAGCTAGAACTCCCATCTGT

GCAGTACTACAAAACCTCATGGAGTGCAGTCAGTGCGAGTTAGCTCTGCGGCTAATGGTC

AGTTCGTTTGGATCCTGTCTTCAGCATGGTATCTCAAACAACATGGATTTATCCCTGAGT

GAGAAGGTATGATAAGGACTATATGCTGAAT

>c14348_g1_i1 len=212 path=[1:0-211]

ATATAATAATACACCTCATGGAGCGATAGGCGAAACACCCTCTTTTGCTATATTTGGAAT

AGACTGTATCTGTCCAGGGTTTGCAAAATTATTACCAGAAACAACGTATGAAGTGAGTAT

TATATACTTAAACGATCGATAGAAAAAAGTACATGAAAATATTAATAAAATCAAAAGAAA

ACCTATAAATGAGAATAAAAATATTAAGGTTG

>c14350_g1_i1 len=239 path=[499:0-238]

GTTAATCCCTCCTCTTTATTACAAGTGAACTGCTGTGCCCAGTGGTGCACAATATAATTG

ATGGTGACCATGTTGAGCTTCTTGGGAGCTCCTTTGTGTCATAAAAATAAACAATACCTG

AAAGCAGAAGGCTTTTATTGGACATGTCAGCACAGCTAAAAACAGTAGAGGAGCTTTCAC

ATGCTCAAGCATATTTGTGCTCATGCTACGAGATCCTTATTAGCCTCAAGATCCTTTCA

>c14376_g1_i1 len=233 path=[1:0-155 157:156-174 176:175-232]

GTGCTCAGCTGACTGATCCCAGTGAGGTAATAGTGATTTCCTGAGCCATGCAGTGTTACT

TTAGGCCAAAGTGACTCTTAATTGAGAGAAAACCAAAATGTGATGGTCTGCGGGATTCTC

CCTCAGACACTCATGTCTTCCTAAAGTAACAGTTGTGCTTTTTCAGGCTCTGAAGAAGCT

AAGTAAACTCAATTTTACATACTTCATACTATAGATACTGGCTATTATATGCT

>c14378_g1_i1 len=206 path=[1:0-205]

GAGGGCTTTCTTTCCTCTGCTTCATTCAATGGGTTTATTTGTTTTCAGTAGATTTCAGAT

AGGAGTACGCTAAGGTACTAATTCTGTTTTGCCACTTGGGTTAGAAACTGCCTTTGAAAA

ACTTTCTAGGCTCATTTGTTTGTGTAGTAAAACATGTCTGAAAATGATTCATTGATGATT

TTAACCTAGCAAGGCTGGTTGGAGAT

>c14396_g1_i1 len=203 path=[1:0-202]

AATATAATCAAAATTCTGTCTGTTCAACATACAGGTAAAGTTTGCTGAATTCAATAAAGA

CTACTGCTCACAGCTGAACTTAACTAGTGTTATGTCAGTGCTAGACGAGGACATCAGAAT

AATCTGTTCAGAACTCCACAGGCAATCCTTCTAATAAGATGCTAAAAATGATGCCTTCTG

AGAAGCTGGTTGTTACTAAAATT

>c14398_g1_i1 len=266 path=[244:0-265]

AACCACTAAAGGGTAGGAGGTAGATTTCTTTCTCCTCTGGAAGATTATTGCACTGTGAAC

CATGGTTTTAGCGTGTCCCCATTTTGACCTTTCATTTCAATCCCTTTCAGTACTTCTGTA

TTATAAGTAATGTAAGGCAATGAAAGGAAGACAAGAAATTGATTTGAGAAGTTAGTATAA

AACAAGCAGCTGTTCACCATTTAGGCCTTAAACAAAGGCTGAATGATAACAAGATTTTTG

GGAAAGAAAAATAAAAGAAGTATTAT

>c14398_g2_i1 len=208 path=[672:0-207]

AGTATCACAACAAAATAACAAATTTTGTTCTTGTATATTTTCATTCTTGTATATTTCTCT

TATACTGAATTATTTTTCCTCCTGCTACTATGAAATTTCTGTCTTTATCCCTGTAGAAAT

ATTGTATGTCATTTTACAGGTAATTTTTTGTGTACAGAAAAGATTCTTCCTGCCCTGGAT

AATATTTTGCATGGAGTGAACCACTAAA

>c14408_g1_i1 len=203 path=[255:0-202]

CAGCACTGCGATGCCGGCACACGTCTGCCCCCACTGCTGGAGCTGCTCACACAGCGTGCC

AAAGGCCAAGTCCAGGGCCAGGGGAGCGTTCATGACCCTAAACATGAACCCTGCTCCTTT

ACTGCTCTTTTTTTCAGCCTGTACATCTTTTAGAGAACCACCAGCAGATTTCAGAAGAGG

TCTTGAAAAAATGGGTTCAACAG

>c14411_g1_i1 len=202 path=[177:0-140 139:141-201]

GTCAAAATCAGTTCTTTACTTCACACTCCAAGAATGGCTGGACAATGCCCATAAATATGG

AAAAACATACCATCTACCTACCATCTCACTGTGTTCACAACCACTGTCAGGCCTCCATAA

ATGTTCAACAAGAATTGATAAGTGTCATTGAACTCCTCCTCACAGAAAAAAACGGCATCC

ACTGACACTTATCAATTCTTGT

>c14412_g1_i1 len=309 path=[145:0-258 72:259-308]

CTTCTACAGAAGGGAGTGAATACATGATTGCACCTGAACTCTTTACCCCCTACTGGGTAG

AATACTCTGCAGTTCAGCTACCTCTAGGTTTTTAAGGTTAGATAGTGCCACAGTCACTGC

CATTACAGCAAACAAAAGAGCTTGCTAGTTCCTTTCCTGCTGATCTGAATGCAGTTGCTC

GGTCAATTGTGGCTGAGAGATCTTCTCTTAAAGGTCTGTTAACAGTTAAGTACTAGTTTG

TGTCATAAATTGTGCAATGGTTGTTACAAAGTTGAGCAACGTTCCTGTAACAAATGCTTC

ATTTCTAGT

>c14418_g1_i1 len=240 path=[218:0-239]

TACTAAGAAAGATTCAGGGATGGGAAAATGGATGACGATGATGATGACATTCCCCAGCTT

TCATCCCATACATTGGCTGCCCTCCAGGAATTCTATTTGGAACAGCAGCAGAGAGAGGGC

ATGAAGACCTCCCAAGGGTTTAATCAATATTCTGTTGGCTCAATAGAAGAAGACTGGCAA

CTGAGCCAGTTCTGGTACAGTGATGAGACTGCATCATGCCTGGCTAATGAAGCAATTGTG

>c14422_g1_i1 len=222 path=[1:0-138 140:139-221]

TACAGCGGGGGCGTCCCCGGGGTTGCAGGGCTGACATTCGTGCACCCCCCAGGCGGCGCC

GCCCCCCCGCTGTAGAGCCGTGCCCCAAAGGCTTCCGCCGCGCCAATGGGAGCTGTGTGG

ATGTGGATGAGTGCCGGGAGGGGGGGCTGTGTCAGAACGGGGTGTGCAGTAACAGCCCCG

GCAGCTTCGCCTGTCTGTGCCATGAGGGGTTCATCCTCGACT

>c14426_g1_i1 len=223 path=[55:0-52 108:53-222]

GTGAAGGCAGAAGTGCAGAGGAGGCAGTTTGTGCTCTGTTCACCAGGACGTGGTGACGTG

GGGTGTGCGCTCTGCAAACGGAATGCCCAAATGGGTTCTAATTCCTCTGTAACTCCCAAA

GCGAATTCACAGCAGTGTGTTTCTGGAATACTTCTCCATGGGTGGTCTAAGAAGGCAAAG

CTGTGTTTTGTACTGAGCTGGTGGGATGTTAGGTTGTGTGTAC

>c14427_g1_i1 len=230 path=[208:0-229]

AAAGTCCTATTGCAGAAATACAGAAGGGCGCTACTGAGACCTGTTGCATTCTCTGTTAAC

TTATACTGCAGTATGTGCTACTACGCACTGTGTACTGGCTAAGCACATCCAGCAGTGGTA

CTATTCACCTGTACAGTTATGAAAGACACAAAATCAGCCTGTTTAGAGCCCTGACATACC

TAATAGCCTAAGTCAAGCCAGATTTCTGCAGCAGCAAAAACTCCCTCCTG

>c14428_g1_i1 len=258 path=[542:0-221 1059:222-222 1060:223-226 768:227-257]

ATGGAAGTATCACACTCCACGTTGTTAATGGGGTTAGTGGCGCCTGGCTGACAGCCGCCA

GCTAGCAGCAAGGAGAGCAGACCACAGGCTGTGCTGACAGCAGAGCACGCTGAACAAGCA

GCATGAAACTGCGGCGATGCTGCGGACCATGAATGGCTGGGGACATCCTACTGTGCTCAG

ACTAATATCCTGAGGTCCTACACATAGCCACCAAAAAAAGCCTGAAATGCATTAAGCACC

AGCTAAATTCTGACTTAC

>c14438_g1_i1 len=221 path=[199:0-220]

GCATCATTCCTGCATAGGAAACATGCAGTTCAGTTAGCCAAGCCTACCCAGAGCAGGTAT

GATTCAGTGCTACCAGCTGGGTTCTGTGTAAGCACTTGCAGGTGGTGCCTGTGTGCCTCT

AAAGAGGCATTGCATGTGGTGCAGTGTGCTGAAGGTCATGGAATAGGGTAGTGCCTGCTA

AGGGCAGGAATCGTTTCTCTGGCTGACTGACCGTCTCCTTA

>c14454_g1_i1 len=206 path=[516:0-205]

GAAAAATCATTTTTTTTTTTTCTTATTTAAGCTATATAATGTCCTTCTTTTTATGAAGTT

AAATTACATATTTGGAAAACATAAAAACTTATATAAAAAAAAATGATTCATATTTTATGA

AATTTATTATTTTGTAAAAATTTAATAAATGATGTAGAGTTTTATGTTTTAAATATAAAT

GTTTAAAAATAGCAATTTAATTAAAT

>c14461_g1_i1 len=276 path=[155:0-275]

GAAAGGCTTTGTCTTCTCAAACCACATCTTCCAGCCTCAGCCAGCTTCACAGAAAAAGCA

AAATAATTTGTCTCTGCTGCAGTTTTTAGAATTCCTTTTGCTACTGACAGTTTGTTTGTC

TTAGTCTTGCCGCTGCATCACAGTTCCCTAATGCAACACAGCCTACCAGGATTTGACACG

AGAGCAAACGATCCTGCAGAAGATCCTTCCGAGGTACAATTATTTAAACAGACGGAAAAC

AGGTAATGAAAAAGTGGGAATTCTCCCATCATCGCA

>c14463_g1_i1 len=294 path=[1:0-293]

GGCAAAAATGGAGGAGAGGAGACAGTGTTAGACCTACAGACATTCTTAGAAAAATGGGTG

AAAAGATGAGCACCATACTCCAAATACAGCGCATCAGACCACCACAATAGTTCATTGGAT

TTTATTTCTTTCTTTCTTCTTCAAAGTAGTGTCCCAGCAGTCTTCAGCAAGACCGAAAAC

TGCTTTTGCTGCTTTCCCTTTAAAAGCAGAAGGCTACAAAAGACAGCACAGGTAGATGTG

ACTATAGATACAGCAGCTATAAGGAGGAAAGGGCTCCTCTAGAAGTTATAGTTC

>c14466_g1_i1 len=241 path=[411:0-240]

GTTTGGGGTGGTAAAATCGGGCTTTTGTGAGGACAAAAACGGCGTTTGGGAAGGAAAAAT

TGCATTTTTTGGCACAAAAGTGGGAATTTCGGGCCAAATTGTGTACATTTCACAAAAAAT

GGCCTATTTTTTAGCAAAAATCAGCTGTTTTGGGGGGGGAAATGAGATTTCTGGGCTTTA

GAGCACGAAGGATCTCCAGTTTTGGCTCAAAACGTCCCTTTCTGCCCCCAAACTGCCTAT

T

>c14466_g2_i1 len=228 path=[206:0-227]

GCCTATTTTTGCCCTAATTATGCCCCAATTGCCCCCATTTTATGACTAAAAATGACTATT

TTTACCTCAAACGCCCCAATATTATCTTAAATACCACATATTTTTCTCATGGGGTAAAAA

TATCCTTTTTTTTTTACCCCAAAATGGGCAGTTTTATGGTGATGGAAGGGGAGATTTGGG

GGCAAGAAGGGCTATTTGAGGCCATGCTGTTCGTGTTTGAAGGGAAAA

>c14467_g1_i1 len=216 path=[217:0-215]

CTTGGGCCTCCTCAGGCTGGCTTGGGGGCAGAAAGGCTTCTAAGCTCACACCCGCAATAT

CTACTCCTTCCTGCCCTGTGCTAGCTAAGCATTAAGCCTGTGCCTAGAGGGGATTTTTCC

TAACATCGATCATTGCTGGGTGGTAAGGCAGGGGGGTGACATTCAGGCTGGCTCTGCGGG

GCTCCCGGCGTGGTTTGGCAGGCCCCAGGCTGCGGG

>c14488_g1_i1 len=334 path=[218:0-217 547:218-333]

ACTGACATTTCTTCTGATCCCAGCTGAGGAGTCAATACAGTAGCAAATGTACCAGCACGC

AGCTAGAAACTATCCTAGAAACGAACAATCTCAGAGAAAATCTTCCCCTAACAAGAAAGG

AAGAACTTAAGACCGTGAGCCTGTCATAGAAAATACCTCCATTCAATATGTAAACCCACT

TATACTGGAATCAGAGGCACTATTTTGCCTAACTGAAGAAAAAAAAAAATCATTTTGTTT

GCAACATGAATGCTCTCTGTCAGCCAGCCAGTTTGCAACCATGCAAACCCCAGTGTTTCC

AGGGGCAATAATTCTCCATTTCAAACACTGGTGA

>c14492_g1_i1 len=206 path=[171:0-205]

GGATATCAATTCAGCTTTCAAACCAAATTCTCTTTTAAGTCAGAACCTGGATGCCTACTG

GTGCATTTATCATCCCCAAACTATACACTGTACAAAGTAACAGTGCCAAAATTATTTCCT

TCACTGCTTTCACAATCCAGGAAGAATTTCACACCTTTCAAGTTGCAGGTGAAAATGTTA

CATTCCCACGTGAGCTGTACAAGCCA

>c14496_g1_i1 len=228 path=[380:0-227]

GTTTGGCAGCAACTAAAAGGAAAAAAAGGGAGAACAAAGTATCTTAGCAAAGTTAAAATT

AATGTTGTGCAAGCTTTAGCTTCACTATTTATTTCATTCAGAAATGAAATAATACTTTCC

CAAGTTTTCACATTCTCAAACTTGTTTTTTAATGAAAAGCAGCCATAAGAACATATTAAC

ATTTTCTGGCATATTTTGATAGCAACTGACAAGGTAGCAATCTGTTAT

>c14500_g1_i1 len=201 path=[405:0-200]

GGAGCTGGTGTATAACTTTTGCTAGTCAACAAGGGGTTCAGCTAGTCTCCACTTCCAGAT

TAGACTGTGTTTACAGCTAAATTAAACAGGATCTTAAAATTGCAAAGTCTTTTGTAGAAC

CATTCTGATTCTCTCATGGAAATTGAATCATTATAAACAATATCTTGAGAGCATCACTGT

GATATGTATTTTATCAGGCAG

>c14505_g1_i1 len=219 path=[1:0-113 115:114-120 471:121-218]

AGACACCTTCACAGATGTGACTGTGGCTGTTCTTACCTCTGCACCGGCTTTAGGTTGGTG

GATTCATTGTTTCTTTCTTCTGGATGGCCTCTGAAAAGCTATGGTAACTTGTAGAATCAT

TAGAGTTGGAAGGGACCTTCAAAAGTCATGATTCAACTCCCCCGCAATGAACAGGAACAC

CCACAGCCCAACCAGGGTGCTCAGAGCCCCTCCAGCCTG

>c14517_g1_i1 len=293 path=[271:0-292]

GACGGGTTTTTGCTTTGATTCCAGGTTGCTCATTATTTACTCTCACAAATGATTGAGGAA

GAAGATGGCAGTGTAGAAGGGCACTCCAAAACCTTACACGGGAGGAAACAATTATTAACC

TGGGACCAGATCAGGAGGAGATACGGAAGTTCAGCCTGGCACTGCGTGGCCTGCAAAGAC

ACAAGGACACCTGGTGGGCAGGGCCCCTCGCATGGTTGTGCTCATGAAGCTACCCCATTC

CAGAAGCTGAGAGCTCACTACCTTCCTAAGGTGGGGAAAGAGACATCTCTTCT

>c14520_g1_i1 len=208 path=[1:0-207]

GGACAATAAGGTGGACCCAAGAAAATACCTCGGTTCATTTTACACAGACTCTCTTGTCCA

TGATCGTGGTGCACTAAGGCTCCTAACAAGTGTAATCGGAGAGGTGAGTTTTCATGCTTC

CACAAGCAGGTGAGTCATTGTGGATTTAATTCCCTTATAATAAGGAGATCTTTAAGTGGT

GGTTTTAATCCAGGAAATGATCCACTGT

>c14524_g1_i1 len=262 path=[155:0-261]

TAACATGGTTTACTTTGATTCAGTAAGCCGAAGGTTAGTCCACAGTAGTGTTAGAAAGAT

GGTTTTTTAATCTTTGCTATTCAGCAGAACAAAATCTCACAGCAGGTATCACTGCAGATT

GTTTGATACCAGATACCAAAGACATATCTGATATCCTTAATTATTTGTGAAATTCATTGT

GTGCGGACAATTTTCTATCTAGTGCAGTACTTCCATGTAATTTTCTGCTTCTCTGTCATA

CAAATTTAACGTATTCTACTGA

>c14531_g1_i1 len=225 path=[333:0-224]

GCCGGCTCATCACAGAAGAAGCAGACAACGGTGCGGTCGGTGAGGCAGAAGAGCTTCACC

TTCTCGTGGTCCTTGCAGGGGAAGGGGCTGCGCTGAGCTCCCAGGATGGCATCCAGGGGG

AAGGCGCTGTAGCGCTCCACGATGTTGGCCAGCTTCAGGCTGGGGGCCAGCGTGGGCTCG

GTGAAGGTGCGGCGGCACTCGGGGCAGTCGCGGGCGCCCTGCGGC

>c14553_g1_i1 len=439 path=[1:0-438]

AAACCAAGCTGTGAAAAGCAGGGAGGTTCTCCTGGCCTCTGCACTCATGCAATGTGCTGG

GTTCAAGCTGTGCTATAATGCTCTTCCTGCCCTGCAAGGAGCATCATGAGTGCAAGGGTG

GAGTTGGAGCGGCACACGCTCTCTGCTGGGCTCCTGCTTCCATCCCCATGAGAAGTCTCT

GAGCTTGATTGTGGTGGAAAGGTGCTGTTCTCTATCTCCTAGAGGCATGTGGAGTGGTCC

AGAAAGTGCCAACCCCCAACACACCCCTTGGCAAAAGCCAGGCTGGTGCGTGTTCAGGAC

AAGAGCTACGGGGAGGGAGGTCTGTGAACAAGCAGGCACCAGCCATGACTTTCCTGGAGA

GAAACATGTAGGGAGAAATAGCCCCAGTGCTGCCAGCACACCCCTTCTCAACAATAGCAA

TGTCAGCACTGGGGCTATT

>c14569_g1_i1 len=215 path=[1:0-155 157:156-214]

GAAGCTAGTCTAGAGTGGAGAAGATGGAAGGGAGAACAAAAAATAATCTTCTGATACATA

AAAAGTGTATCACAGAACCATATGGCTAAAAGCAGCCTGAGAGCCTCTGAAAATGTAATG

CTCTCAGAATGTGTAGTACACCAGAAGTACACTCAAAGGCTTTGCTCATGCTTGGCTTCT

TCCTTCTGCATGGTTCGCATTCAACGCTAAAGAGA

>c14573_g1_i1 len=267 path=[1:0-266]

AAAGAAAACAGTCCACTTCCTATGACAGAACAGCAGAAATATGCCTGTACTGCCGAATGA

GCGCCGGCACTGGAATGAACGAGGCCAGTCTCTTCCTCAAGCCAGACTTCAGAGGAAAGG

GAGCCACCTGCTGCTCGAAGTGAGGCAGGCTGCAGCTTCTTTTCAGTTTCAATGCGTGAA

ACACCAGGGTGAGATTCAGCAAAAGAAGCTCAAAGCTTTGCAATTCTTCTTCCTCACGCT

GCAGATCCAGGTGGCAAGAGATGCGGG

>c14577_g1_i1 len=401 path=[1:0-372 822:373-400]

GGTGTTGCAGTGGCTGAGCAGCGTGGCGCGGCTGCCGCCCTGCAGGCGCTGCGCGATGTG

CCGCGCGCCGTGGCTGCCGATGCGCCGATTGTCCTCCACGTCCTTCTGCAGCAGCGCCTC

CAGGTGGGCGATGGCCCTGTCCCTCAGCTCCCCCTCGCTCACTGCCGGCTCTCTGTCCAT

CCACGCCCTCATCTCCCCCTTCAGCCTCTCGGTCTCCCTCCCCACGTTGACGGCGGTGGG

TCGGGCGTCGCGGACCCTCCCCATCCTTTCCTCCACGAACTCCACCAACGCCTCCGGATC

CCCCCGCGGGCCGCGGCCGCCTTTCAGCTCCAAGGCCACGGCCAGGCAGCCCAGCAGGGC

GATGGCCGGAGCTCCCCGCACCTCCATGGCCGTGGCCTTGG

>c14587_g1_i1 len=216 path=[203:0-215]

ATAAAGCACATGAAAGTCTCCAGTTCTTTTCTGACTTGTTTGATTACAAAAACAAAAAGC

ATTTAAATTCACCTTGGAATAAAACATCCTCTGATGTGCTCATCTCCTGTCTTCTGCAGT

GCTTCTTAGGACCATGTGTAGTCCCTGCAGGAGTCAGTGGATGATCGCCTGTGGCTTCAT

TGAATGGAAGCAGATCCCTGAATTGCTTTATCTCAA

>c14592_g1_i1 len=202 path=[1:0-201]

GCGGCGCTGGGGCCCGGGAATGGATCGGCCCGAAGGCGTGCAGGTGGGTTTGCGGCGAGG

GCGAAGGCTGCTCGAGAGCCCCGGGCGTTTTGCTCTTGTCCAGAGAGTTCTCCGAAGCCG

TCAGCGGCCACCGCGTAAATGTAAGGCTGAAAGGAGCTCAAGGAAGCGCTGACAACTTGG

CTGTCGTTTAATGTGGCCGTTT

>c14592_g2_i1 len=229 path=[369:0-228]

AGGAGATGTGTTGGTTGATAGCTCAGCACTGAGATGAGCTGTATGGGACAGCAGCGTTCA

ACAGAGTTTGCGTAAAGGGAAACTTATTGGCAAAACCATAGGTTTGCTTTGTATCTGTAG

CAGTTCACTGAAGTGGCTTCTTATACAGATGGCTTTCCAGAGGCATTGTGGATGGCATGG

CGAGAAAGGTGAAGATAACAGAAGAATGTGTAGTCATAGCTGATGTGAG

>c14600_g1_i1 len=210 path=[464:0-209]

AGACACAGTAATGCCTGTGGGTTTGTTTGGGGTTTTCTTGTAGTAGCTCTGAAAAGTTTC

TGCTCAGTTCTAGCATAGCAGGCTTTGTGAACTTTGCATGAAGTGCTGCACCCTGAGGCA

CTGGTAACCACTTGGTGGTCTTGTCTGTATATAAGTATATATAAATAAGCAATATTATAT

TTGTGTGTATAAGTACTTAAAAAGTACTTA

>c14606_g1_i1 len=304 path=[1:0-303]

GTGTCGTAGAGAGTCCCTGTTCTGCCACGGGAAAAGCACACATGAGCAGGGCCGCAAAGG

CTCGGGCGTCTCCCTGACCCGGCCACGTCTGACCCGGTCTCACAGTGCTCTGGGCCGATT

CCTGGCCTTCTTACAGCCCCTCGGGCCCACCCTCCTCCTGGACCCCCCAGCCCCGGCCGC

ACAGTGCCGTCTGGTCTCACCTTTCTCCTCCTCCCCCCCTGACCCCCTCTCAGCCTCCCC

CCACGAGCGCTTCCAGCCCTTTCCCAGTCTTGTCCAAGAGTTGGGCTGAGGGTGGGGGTA

GGAC

>c14609_g1_i1 len=275 path=[353:0-88 190:89-274]

GAAGAGCACAGGGGAATGTTAAGGTAATTACATAGAAAATGTTCCAAGGCCTGTAAGGGT

ATTTCTTATTATTATCAAGATGTGTTTTGGGTGGTGGTATTTTTGCTTGGAAACTTCTGC

TCTGGCTGGCATAAGCAGTTATCAAAAACTTCGATGGAAATTTGGATGAGAGTTGAATTT

CCATTTGGATTGTTGACTGTCTACGTGATTTCTGGCAAGACTGTCAACTGCTTTTGTCTG

CAGTTGACAGTCTTGCCATCCAACCCAATACAACA

>c14616_g1_i1 len=310 path=[17:0-14 32:15-309]

AGTCACTGATGGCGCATGTATACCCTACTGAAAACTAGGAACCTTCCTGGCCACTGCAGG

AAAGAGTTCCTTTCCCCTTTGCTCATACTGTTCTCCACACATTACCACAGAAAAAGTATA

TATTCTGCTGAACATCATAGAATTGCAGAATATCCTGAGTTAGAAGGGATCCATAAAGAT

CATAGAGTCCAACTCCTAGCTTGACACAGCACCACTCAAACTACGTGTCTGAGAGCACTG

TCTTGATTCCTCTTGAACTCTGGCAAGTTCAGTGCTGTGACTGCTGCCCTAGGAGCTCAA

CCACCCTCTG

>c14616_g1_i2 len=549 path=[33:0-253 32:254-548]

TCATCTCTTGGCTCCTTGAAGAGCGTACACATGCGCCATCAGTGACTATGGACAGACAGA

ACAAACTTAAGGCATTAGCCATCACAAACTAGAATAACGTTGCTGTACAGAAAGAAGCAG

ACATTCAGAAATGATGCAGAACGCAGACATTCTAAAAGCCTGAGAAAAAATAACATGCAT

GACAAATCTCTCCTAAGGAATAACTATGTCCTGTCATTTTTGGAGTACAGCCAACACACC

TCCGACTGTATTATATGTATACCCTACTGAAAACTAGGAACCTTCCTGGCCACTGCAGGA

AAGAGTTCCTTTCCCCTTTGCTCATACTGTTCTCCACACATTACCACAGAAAAAGTATAT

ATTCTGCTGAACATCATAGAATTGCAGAATATCCTGAGTTAGAAGGGATCCATAAAGATC

ATAGAGTCCAACTCCTAGCTTGACACAGCACCACTCAAACTACGTGTCTGAGAGCACTGT

CTTGATTCCTCTTGAACTCTGGCAAGTTCAGTGCTGTGACTGCTGCCCTAGGAGCTCAAC

CACCCTCTG

>c14625_g1_i1 len=365 path=[1:0-207 728:208-208 209:209-364]

GTATCTGACAGTAAAAGAGCTACTCCTTTCAGTTCTAATAGAAAAGACAGAAGATTACTG

CTTTTTGAGGTGGGCTTCTTGAGTTTTATGTATACATTTGGTAACTTGATTTATGCACAA

TGGAGAGAGTTCTTTGGAAGCTTCAAAATGACTAGTCTGCACTGTTGGTTTTGGAAACTT

GACATTAAGTGTGTTATGAAAACTGTAATTGCTCCTAAATCTGCTAAAAGATGTTTCATG

CTGCTTCATGAGTTTACTATCTTCTGTTTCATCTCTCCTAGATTCAGAGTTGTTCTTTTC

TGTGCTGCTTTGTCATCTAAACAAAAAGGAGAATCAAATAAATATACTTTTCTTATAAAA

AAAAA

>c14633_g1_i1 len=245 path=[339:0-76 194:77-244]

ATTAGCACTTCTATAAATAATAGCTAAAGCTGCCATAGCTAAGTATATGACTGCAATCAG

CACCATCAGGCCCTTCTTCGTCGCACACCCTCCAGGAGACAGAAGCTGATTGCTCTCAAC

AAGACGGCAGCCTCCGACGCGGCTGTTGATTCTTGTGGCCCACCTCGGGGCAGCCTCAAC

CACGCAGAAAACTCTCCCAAACAACCGTTTGGGATGTGTGAGACTAAAGTTTCTCCCCAG

TCTTG

>c14642_g1_i1 len=228 path=[1:0-227]

GATTCAGCTCCAAGAGTCAAATTATTCTGCTTTCTCAACAATCTAATTCCTAAAAATACA

AGATCTATCAAGGATGGTTTTGTTATATGTCATACTAAATCCGCATTGCACTTCTGACCA

TCCCCGGACTTGTGTGACTAGCAATATGGTACAGGGTAGAGCACAGGAGTTTTTGTTAAA

CCAATTGCCTAACCTACTATCTTTTCAGTATCACTGGAATGATTTAAA

>c14652_g1_i1 len=230 path=[208:0-44 477:45-229]

CTTGTCCTCCTTGGTGGAATACTTGTCAGCCTGGGCCTCCAGAGACTTCAAGTTGTTGGT

GACAATTTTCAGCTCCTCCTCTAGGTCACCACATTTACTACATCCCAAGCAGGGGCCACC

AGGCAGGGGAGGGGGTGATGAAGGGAAGAAGGGGGGAGCGGAAGGAGCACAGGGAAGGAG

GAAAGAAGGAGAGAGAACAAGTTGAGAGAGGAGAAAACTCAGCGTGGAGG

>c14652_g1_i2 len=205 path=[475:0-19 477:20-204]

CTTCCTCTGAGGCAATGAGGCTTCAAGTTGTTGGTGACAATTTTCAGCTCCTCCTCTAGG

TCACCACATTTACTACATCCCAAGCAGGGGCCACCAGGCAGGGGAGGGGGTGATGAAGGG

AAGAAGGGGGGAGCGGAAGGAGCACAGGGAAGGAGGAAAGAAGGAGAGAGAACAAGTTGA

GAGAGGAGAAAACTCAGCGTGGAGG

>c14662_g1_i1 len=211 path=[230:0-55 21:56-210]

TTGGTGATTTGTTTGTTTGTTACTAAATGATCTCTGACCGTGCAGTAATCATTTGTAGAA

AATCATCAAGCAGAATACTTAACTACATATTCTTGTCCCTGCTGTCACATGATTGCAGTC

ACCTTCACTCAGAAGGAATGTGGCTTTGCATATCTTGTGGAGAAAGCTCATAAAGATGAG

CTTGGTGAGGAAAAGAACCTTGCTGAAAGAG

>c14673_g1_i1 len=232 path=[291:0-169 22:170-231]

CAGCTCCATACTGGTGCAACTGAATAGAGGCTCTTCTAATATTGCTGGGGCTTCCACCAC

CTGGGAGGTTCACTAGCACAAAGAAGCACTGCCCAAAAGTCAACACAGCCAGTCCTTCCC

TGACCATCACTCATGGCAGACTATCCCTGGCCAAAGCAGAGGGGTTTTAATGCTGCCTCA

GACTTAGCTGACCTTAACTAAATCTGCCCTCCCCACCCCCAAAGCCTAAAGT

>c14680_g1_i1 len=219 path=[1:0-218]

AGACCAAACTTCCCCCTTTAGGACTCCCAGCGCTGACAGAATTCCCTCTTCCTCTTCCTC

CAGGAACTCCACGAGCCAATCAACTGCTCGATCTCGTGATTGACAACCTGCTTGGCGTAT

CAATACTCGCGATCCTCCCTCCCTCCGTCTGGACTGGGTGAGCTGCAGAGTGGCGGGCAT

TTTGTCCAATGAAAAAGAGAGCGGGAGGAGAGAGGCGGG

>c14680_g2_i1 len=210 path=[580:0-209]

TGGCTCGCTGTGTGGAGCGGCAGATGTGTCCACCAATGGGCCGACGGAAGGGACGTCGGA

GGAGGGGATGGGCGCGGCAGCGTCTCGCCTCAGCTGACAGCGGAGCGCCGCCTCCCATTG

GCCGTTAGCAGTGAGCGACGTAGATATGGACCAATAACAAGGGAGAGAAAGGGAACGGCG

GCTCCTTCCCACCCTCCGGTATGAGCGACG

>c14685_g1_i1 len=247 path=[225:0-246]

AGGATGGCTGCTGCATCTCAGCTCCCGAGTTACTGGGAGCAATTGCCTATGTGGCAATGC

TGAGCTCAGCTCTGCTGGTGGCTTCCCTGCTCCCCAAGGACCGACCATTTATTCCTACTC

TGGTTGCTCTCTTGTGACAGAAATTTCTTCTTAGCATCTGAGCGTGTCCCCTCCACGCAT

CACCCACAGAGCCTCTTGAGAAGGACGGATCCCACACGGAGCTGGGAGGAGACAGTGGAC

CCAGTGT

>c14688_g1_i1 len=850 path=[53:0-849]

GGGGGCTGCCAACACAGAGGTGCCACCAGCAGTAGGGATGGGGGCAGTGGGCTCTGCACG

GGCACAGAGCGCTGCTGGGAGGTGGCACTGCGTCCTCCTGCAGCCCCGGCGTGGGCTGTG

AGCATTTTGCAAGCACGGGGCCGGGTCGTCCCCACCCCAAAGCAGGGAGAGATGGGGACG

GAGCAGCTGTCACAGCATGTTCTGCCCCTGGGCACCTCCGGCTGCTGTGGCACAGCGCGT

TCCGTCCCCACCGAGCTCTGTGTCCCGGCCCCTGCTTGGGGTGACAGTGAGGGGCTCACT

TTGTCCCCAGGCTCAGGGTGGCACTGCTTGTATCCACCACTGTCCCATGTCCCTGCTTGC

ACCACACGCCCATCCTGCATCCCCTGTGCGGCTGATCTCGGGGTGCACCCCATGGGGAAC

CCCCCACGTGTGGGCCATCTCAGAGCATCCAACTCTTCCCCACACGCTGGCCCCACTTGG

CGAACGTGTGCAGCCTTTGTCTCTCCCCTGGTTCTGTTCTGAAGGTGCTCTGGGCGTTGG

TTTATGCAGCACACAGCACCCAGTGTGCCTCAGTTTCCCTCCCGGCCGCTCACCTGCACC

GGGACCTGCAGAGCCGGCCGGGAAGCTCGGCTGCACCGTGCCAGAGAGCAGCAAAAATTA

TGAATAAGCTCTCATTAATAAATACTTGATGATGAATCCAAATAGATTTCCTCCTCTGGA

GCAATTATTGTAAAATGGGCTCTAATATGTTGCAGGGACGGCCCCAAGAAAGGTTGGGTG

CATTTCAGGGACTAAGTGCCTTCCTTCAATACACTCTAATGAGCACTTTCAAATCATTTG

CATCTGTCTC

>c14706_g1_i1 len=202 path=[1:0-47 49:48-201]

AGAAAAAAACAGAATAAAAAAAAAAGAAAATGGAAGTACATTTATAACAAAAAGATATTC

CTAAAATAAAGAAAAACAAGAATATATGTTTTTACATATATAAGAAAAATATAAAAATAA

AATCAAAATCTATGCACCTATAATAAAATAAAAATAAGAAAAAAAAAATAATTTATAAAT

TATGATAAAAAAAAAAAAAAAG

>c14725_g1_i1 len=212 path=[159:0-211]

AACTTTGTGTTTGAGGGGCAGATTCTTTTTGGTGACGAGTAACAAGAGAGCATAGGGCAA

AGAGCAGGAACTGTAACACAGGGAGTTCCATACTAACACAAGGAAGAACGTCACAGAGTG

ACTGAGCACTGTAACAGGCTGCTCAGAGGCTGAGAGGTTTCCTTCTCTGAAAATAAATAG

CCTACTCATTCAGCATGAGAGCTTTGGAGGCT

>c14735_g1_i1 len=352 path=[330:0-351]

ATGCTTGGCTCTGAAATCAGAAGTGTTACTAGAAAAGCTTTCTAATCTACCCTTCTGTCA

CAAAAGGCTTCAAATAAACGATAGTTTATTCCCAACAGTAATTCACAAGTTACAGTTGAA

GGACACTCAGAAATCAAGGCTGGCAAAGCATAATAGGAATTATTTTCCTGGAAGCCAAGA

AATGCCATTTTCCTACGTGGACATAATTTAGCTTGAAAGACAAATGCTGAGCAATTTGGC

TGCAGTAAAGGAGAGCAATTACAATGGAAAAAGTAATGTTAGGGAAGGAAGTTAGCTGGG

AAGATGGAAGAAAAGATTCAAGGGCAAGGTGCAAGAGAATGTAATAGTTCTA

>c14743_g1_i1 len=208 path=[20:0-207]

CTGGTGTCCTGTCATGTGGGTGAGATGTTTGCTTTCTCCTACCGTTTCCTTCCACTTTAG

AACAATTACTTTTCTTAATAGATCAGTAGCTCTACTTTAATCTGTATTGGATCTCCTGCT

GTGCATTTCTGATAAAGGTCAGATTTAAAATGCGATCCTGTTTTGAGGCTCTTCTGCTGC

TATCTTGAACTTGTTTGTGTCAGATAAT

>c14762_g1_i1 len=358 path=[336:0-84 707:85-357]

ATTGCGGAGCGATGGGATTAATCTCTCCCCGCTATTTCGCAGCACTCCGTACGTGCCAGT

TGTGAGCAGAGTGCCATCTCGGCCCGCCTCATTCCCAGGCGTTCCTCTGGGCTGTTGGCA

TCGCTTTCCCAGCCGCCTCCATTGGGACACAAACCCCATCCCCGCTGCTTCGGACCCGCG

GCGCTCCCCGCACAAAATGGATGTGCGGAGCGTTGTGCCGCGCTGGCTGCCGTGGGAGAA

GTGAGCAAATGGGTCTGAGGCACAGCCAGGCTTATGTAACCGCTGCTTTGGCAAACTTGT

GCCAGCTGGGAAGGGAAAGTGTTTTGTTGCTGTCTTTTGATAAGGACGGAGGAGCGTG

>c14769_g1_i1 len=204 path=[1:0-129 131:130-203]

AATATAACTTGCATACCAGATACTGGGTTTTTTTACTGTCTGAATATTTTTAATTCAAAC

TTTGTATTTTTAAAGCTAAAGAAGGCTCTTGTGACCACAAGATCCCTTATTTGTATCAGA

ATCCAAGTAGGATGTTCTAGCCTCCTTGTGCTATCCAGGTGGGAAGCAGCACATGACTCA

GCCGTGTGTATTCAGATTTTGAAT

>c14779_g1_i1 len=210 path=[188:0-209]

AGACCTCTGGTGGCCACCTTCTCAAACACAAGCATTTACTCATCACATCTGGTTCAATTT

TTCCGTAGACTGATACAGGTTTGTCAGTTGTGCTATAACAACTGTGACTAACCCACAATG

ATTTCCAAACAGCTATGTGAGCATTAGCAACTTACAGCCATTTTTTTTTCCCTGTTTCTT

ATCTCTATTTCATGTTACCTTCTGGCTCAA

>c14779_g2_i1 len=234 path=[586:0-233]

AGAGCGTGGGTTAGCTGGAACAAATTCCATACGGTCACTTGAAACAGGTTAGGCAGCAGT

AGCGTGGGGAGAACTAAAAGCATCAGGCACTCTGTGGCTTTACAGCCTTAATTTTCCATA

ATTTATTTATTTTTCTCTGCAAGTTAAGTCCTGTTAGTCACTTGCAAACCAGTGTAATTG

CATCCACACATGAACCTTTCTGTTTAATAACTTTGAGTTTGGAGTACATTGGTG

>c14780_g1_i1 len=205 path=[451:0-204]

GGTGTGTTAGGATGTTACTTTACAAATGCATATTTCACTCCAAATTACGTTACAGATAGT

GAGAAACACTCAGTTCCTGATATCCTGGTGGAGTTACTCCCTTAAAGGATAACATTGGTG

TATCTGTAAGTGAACAAGTTGTTATAGTGGTGAAACATTTCTAATGTACAGTGAAGCAGT

CTAGCAAATACTATTCCTGTATGGA

>c14781_g1_i1 len=407 path=[474:0-406]

TTTTGTAAAGCGAGCAGAGAAAAACCAACACTACTGAAGCTTGGTTTTCAAAATGACCAA

GTTAAGCAAATATGAGTTTGCCTAACCAAGTAGCCTAAAATTATATACATACTGGAATCA

CACATCTACTGTTAATGAAAATCAGCTATTTAATATTCCAGATTTACCGCATGGGACAAA

TCTCCATGCAAGATGATCAACATAAATATCTGAGCAAGGGTTGGAATCTATAAGCAGGGA

GTTCTGCAAGGTGACCTCCAGATGTCATTCTAATCCATTATTACATAATTACACAAAAAC

TGTAATTGTTTTAGAGCAGCACACAAATACCACTAACTGCTTGACCTGATTTTCCATATC

AGAAAGTCTTTAAGTAACAACATAAAATAGCATCACTACATTAAAAA

>c14783_g1_i1 len=231 path=[73:0-230]

GGATTACACAGACTACAAGCCCTAAAAGATGAGGATCCTGTCTGCGAAACAGTTACCAGG

CTAGGCTCAGGAGAATGCAGAGGGACGGATCAAGTGAGGAACAGTCCTGAGGCTTTAGGT

GTCAGCTGAGAATATTACAGCAGTTTGTCAGTAACACAAGTCTGAGTAGCAGGATAAATA

GGCAGCCTACCCACAGGCAAGCCCAAGTTCCAACTCAAGCCATTCTGTGAT

>c14784_g1_i1 len=232 path=[1:0-111 113:112-231]

GGACGCCCTCGACATCAGGTCTGTTGGTACCAAAGCTGCCTGCTCGTGTTTGTGAGACTT

GTGGCAGTGATAAAGGATTATTTGGGCACATCTCCCATTTGTGTTTGTGGGAATTGAGCT

TGTTAAGAACCAGGTAGTTGAGCTGCATATCTTTCATACTATGAGTTTCCAAATCCTTCG

GAAAAGGTATTATAAAATGTTGACTGTAGAAGAACATGAATGTTTTAATGAT

>c14788_g1_i1 len=290 path=[303:0-289]

GCATTCCTGAAGATAAAAATGAAACCTTCCCACAAACAGCAGGAACACAAGATTGACAAC

AATAGTTTATCATAATCAGCTCCATTCAGTGGGCTGCACAAAAAAACAATTTTTACAAAA

TGACTATGTATAAAGGCTTTTGCAGAACTACTAAATCAGCATCAACCATTAGATTTTAAG

TCCAAATTAAGAACTGACAGTGTTTCAGACACTAGAATTACCCAGTAAGTTAACCCCAGA

GAACTAACCTAAATTGACCAAAAAAGGACCAAAAGATCTTTTCAGATAAT

>c14790_g1_i1 len=314 path=[51:0-140 50:141-313]

CATCCTGTAAGGCTGCAATTTTTCCAAAAACCTCCTCTTTGGCAGCTTGCACTTTACGAA

CTAGCTCTTGATCCTTTTCTACTAAAAGCACTTTATGACGCTCAACAGCTTCCTTAAGAA

CTTCTTTGTCTGACAGCAGTCTTTAGCAGCCAAGGAATTGACTTCTTGTTCAGATTTGTG

AAGTTTGCTAGTTAAAAGAAAGTTCTGCTCACGACACATCTGCAGTTCTTCCTCAATCCG

ACCAATGTGTTGTTCAGCTGATTTCTTTTCTGCCTCTGCCTCATATTTTATTTTCTCCTC

CTCAAACACAAAGT

>c14799_g1_i1 len=261 path=[1:0-42 44:43-260]

TGTTTGAGGGAGGCGGATGCTGTCTGTTCCTGGAGACCTGAATGAAAGGCTCTTTGGGGT

TTGTTGTGCTTCAGGTGGTGTGGTCAAGGGTTGTATGCAAGGAAGAAGCTCAGCAGGCTT

ATTTGAGGCAAGGCCTGGGGCGAAAACAATCGCTTTCCATATTTTCAGGCTTTCCTACAA

TGTTATCAGAGCTCTGCAGGTCCCGCTTCCTGCGGCGTAAATTGGGATGATAAAACAGGG

CTGCATCTCATGCTTAGCAGG

>c14799_g1_i2 len=365 path=[624:0-146 44:147-364]

CGAGAAGTGACATTTCAATTGCTGGTTTGAACAAGCTCAAGATCCACAAGATCCTAAAAA

TCACAGAATCCCATAGAATTGAACAAGCAAGATCCAGAAAGAGGAACTAAGGGGCACCCA

CCACCCAGCCTGGGCCAAGAGCTGAACGAAAGGCTCTTTGGGGTTTGTTGTGCTTCAGGT

GGTGTGGTCAAGGGTTGTATGCAAGGAAGAAGCTCAGCAGGCTTATTTGAGGCAAGGCCT

GGGGCGAAAACAATCGCTTTCCATATTTTCAGGCTTTCCTACAATGTTATCAGAGCTCTG

CAGGTCCCGCTTCCTGCGGCGTAAATTGGGATGATAAAACAGGGCTGCATCTCATGCTTA

GCAGG

>c14802_g1_i1 len=204 path=[159:0-203]

GATAACAGATTCTTAAGAGAAACCGTGGAGCAGGTACAGCACTATGTCAGAAATCCACAG

CTGTAAGGATGTAGGTGGTAGGAAAGCTTGGAAGAAGAACAGCATCAGCTATGAAGAATT

TTGTTTTCCTGGTCTGTAAGCGATGCTGCTGTAATTCTATCTGCATTTTTTTTTCTGCTC

ATTCACCACTCAGCAGCTGGTAGG

>c14809_g1_i1 len=321 path=[299:0-320]

ATGCTGGCAAGCTTTCCATTACCTGAACAGGAAAGAAGAGAACCTATTGAACTGCCCATG

GCCAGCACGGTCAGGCAAGAACAGAAATGATGGAAAACCAGATTCCATGTCAAATGAACT

GAACTGTTACTCCATCACTAACACTGCAAGCAACAAGAAGCTGCACCTCTAAGTATGAAA

CCTCAAAAAAAACACCCAACCATAAAAAAAGAAAACCCTTAAACCTCACATTGAAAACTT

ATTCTGAGAACACTGATGCATACGAATAAACTTTCTTCTGCATACAAAGCCTCCCATTTT

TTTTTCAAAATGGATCATAAA

>c14811_g1_i1 len=276 path=[1:0-275]

GTACTCTAAGATGGCTGCCTTCCCCATACTGCTGATTAATGGTCTCCTTCTGTGTCCACC

CTCAGCTGTGTGAGCCCTGACTTTAGTGAGCATACTTATGCGATCATGGCTGCAGATCAA

GCCCTGAGCTGTAAAGAGAAATAACACGTCTGTTTAAGCATACATAGGGCTGAAGTGTAA

GCTCCGTGCCTGTGACAAGTGTAGTGATGAGTTTCCAAAAGCAGAGCTGCTTAGCACAAC

TTGTCTGATATTCTTGTCCTGCAAATAATTTGTGGG

>c14813_g1_i1 len=428 path=[1:0-427]

AAAAGCTACCTGGTTATTTGAAGATTAGCCCCTCCATGCATACACTTTTCACCATCACAG

CACAACAGAGATTCTATTCCTAGAGGAACAGACTGTGTTTGCCATTTAGTATAGCCTAGG

GAATCTTTTAGCCACTTCATTTTCAACTAAATTAGCTCATATCTTTTTTTTTTTTAAAGA

ACAATTCTACAGTTGTTTAAAACAAATGTGCTGCCAAGCAGCTGGCATGTTCATCTTTTT

TAACTCATGCCACCTATGCAAGGATGAGTCTCTGAAACACGAAGGGGACCTGTGGTAGTT

CCCATCATTCCCCAATCCACAAATCAAATTAAGAGCTTGTCTAGTAACAATTAAGCTTGC

ACTCCAAGCCAAAGTAAAAATCTTTACTGAAGTCTTAATATAACACAGGTATAGAGATTA

AAGCTCCA

>c14824_g1_i1 len=226 path=[1:0-225]

GAAACAAAACAATCACTGACTCTTGTAAAACGTCTCGAGTTTCCCAGATGGAAACTCTGA

AGAGAAGCAAAACACCAGCAATTAAAGTCACCTGCTGTTTAATGACTCTCAAAAGTTACC

GTGCAGAACAGTTCCAACGTCAAAACTATCCCCAACTTGGAAGGCCAGTGGCACTACTCT

CCCTTCTACCACCCCCTGCTACTCGGGACAAAGAAGCCTTACGGAA

>c14824_g2_i1 len=241 path=[625:0-240]

AGTAATTTGATATTTTTGACCCTTAGTAATCATGTAATCATCTCCCAACATAGTACTTCT

TTTCTGCACACATGGAATGTAAATGGCCAGGATTTAAATTGATGACCTTTTCTTACTATG

CAAGTTATTCTTTAAGGTCTGGATTTACTTTAGTGCATTGTGGGAGGAAAAACAAAGATA

AGTGTTGTATATGGGATGGTTCTAACAGCACTTGAAACACATATTGGTGTGTTGTCAGTG

T

>c14828_g1_i1 len=209 path=[1:0-65 67:66-208]

TTGGGTTTTTAAAATGAGATTTTTTTTTTCCTTATTTATTTCTGCAGAGAAGAACTATTA

ATCTCCTCTGAACCTCTATTAGTGTTTTCCAAGCCTGATGGACAGGGGCAAATAAGATGC

TACGGGCTGTGTTTTGTACTGAAGAACAACAACCCCAAATGAAGGATGTCAAAACCTCCT

ATGTATGTAACTTTGTGTTTGAGGGGGGG

>c14833_g1_i1 len=277 path=[343:0-276]

GGAAAATAGGAAGTGCTTGTAATGTGGCTTGGCTGTGAAGTACCAGCTGTTGAGAACAAA

ACAGCTTTATTCAAAGGAAAGCATTTGGATCTTGAAATTGTTGCGTATTACATGAGTCTG

ATCAGAATCCAGGCAAACCAAACCAATGTTTGGTTGCATCTGAGTATTTGACCCACTTAC

TATGGTAAGTAGAGACTGTGTAAATCTTATTCCTCTGTACCATGTAACATTTGGGAATTT

GCAAACATTAGGACTTCCGGTATTAATATATTGTAAG

>c14834_g1_i1 len=211 path=[1:0-133 135:134-210]

CTTACAATAAAAGCATAGTTTACAAAGAAGTTGTAAGAAAAATACAATGCTTAAAAGATA

TGCAACATTGTTATCTTTTAACATTGTATTCATTTTTTTCATAGACCAAGCTTCAGAAGT

CTTGTGAGCATAAGATCTTTTAATGCAGTGGACAGATAGTGGAGTGACATGGTACACAAG

CTTTTCTGTGTCTGGTGCTTACAAGATCTTA

>c14837_g1_i1 len=314 path=[165:0-162 328:163-313]

GAAAACAGGCTGATGTTTATATTGCTGCTGAGCAGTGCTGAACACAGCCAAAGATCTGTC

TTTAGCTTCTGGCACTGTGCTGCCAGTGATGGGCGGGGGGGCACAGGGAGTGGGGAGGGG

ATGGAGCCAGGACGCCGACCTCAAAAGACCGCAGGGCATTCCATAATACATGACATTGTG

AAGTAAAAATGTGAAAAGTGGTGGGGAGTTGGCTGAGCAGCTGCTGTTTGGGAGCAGATG

GGCACTGGTTGACGAGTGGGCAGCAATTGCCTTGTGTGTCACTTGATCACTTGTTTTGTG

TGTGTGTGTGTGTG

>c14840_g1_i1 len=263 path=[1:0-262]

GTAGTGTAAGAGTGTAATTATTTGTAGGTGAAGATCCTGTTTTCCACTGGCATTAAAATA

TCCTGAACAGATAATAAATTCCACTGTAATAAGCTGCACAATTGCTTTAAGAAATAGACT

GCCCATGGTTTCAGAACCGTATATCCTACAGCGTATTTATTTTCAAGTAGGTTAACACTC

CTCAATGGCCGTTGAGCTGGCAACAGATGTTTAATCTAAAGGTCGTAATTTACAATGTGG

AACATGATTTCACTTACCACCTT

>c14841_g1_i1 len=213 path=[191:0-212]

AACTGAATTCCACAGAGGAAGCAGGGATGAGAGGATTCTGTCTTTAGGAAAGGGATACAA

TTATCACAGGAATCTGGAAGGCCTGTGATATCCATACTGCCAAGATAGAAAGCAACAAAC

CTCCAGTGGCAGAGACCACTTAAGAAGAGAAGACCTGCATCAGCTCACATTAGTGTGTGC

TTCTCATTTGGTAAAGAGGAATTTTCGCATGAA

>c14851_g1_i1 len=300 path=[1:0-299]

AGTTGCTGTTAAGCTGAAATTTTAAGCATGCTTATTAAACTAGGCTATTTCCTACTAAGC

AAAGTTTATAATTGCTGTAAAATATTTGTGTCTGGAAAAGGATCCAGTGTTGAAAAAAAT

GTGAACTACATTTTAATTAGCATAATGTATCATTAACTAGTTTTAGACAGCCACATGATT

TCTTTTAACACTTGTAAAGGTGTGGCTGGGTGGAATAAAGATTGGAAGACTACTTTTGTT

TGTTTATTTGTCCTTTCAAGCTTATCATTGAGTCTACTGTATACAGTCTTTCCATATTTT

>c14851_g2_i1 len=213 path=[745:0-212]

CTGCATGTTCTTCATTACCACACTAGCAAATCAACTTCCATAAAGACATCCAAGTACAGT

ACTGTAGTTCAAAGTGTATTTAAAGTCTGAATTTTATAGAAGTTACCCTAATATAAAACA

AGATCACTTTTTCTTCAGAAACCTTTATGCCTATTTCTTTTTACTAGTGTTTGCCAAAGA

GGTTGTTAGCAAAATCTTGATTTTGCATGGAAG

>c14869_g1_i1 len=211 path=[627:0-210]

AAGTGTGAGATTAGATGGTCTATCCAAAAACATTCAGTGTTAGGAGGGACCTCAAAGAAA

TCCATTGGGAATCTACCTGCATCTTCTGTTTTATGCAAGTCTTTCCTCTGAAGCTGGTTC

TCATTTGAGTGTTCCTCAGCAGCGTTTACTTCTATACCCATCCCCCTCCAGAGCCTGTCT

CACATAGATATCTGTGGAGCTAATTGGAATA

>c14869_g2_i1 len=336 path=[1:0-335]

TGTGGTTAACATTGCCGGCACAGCAGGAAGAGCAGCCATGGAGAAAGCCTGGACAACTGC

ACGCAGGCCAGCCTGGCACGTAGGATCACAGAACTAGCTGAAAATAATGACAGAAGGTAA

AATTAATGCTTCTGGAAGGTAAGCTGGACATGATAAGGGCTAGGAACGAAGCTGTATACG

GTGTAACTCACACGTGCTTCCAACGTGGCTCTATTTCTGGCATCCTGGAGCACTCTTGAG

TCCCACTGTGCTCACTGGGTGGTTTTTCTCCTGTTCTTGCCTGGTTGTATCCTTCTGCTG

CACAGAGGTGTGATGAGCGATGGCTTTGGAAGCTTG

>c14878_g1_i1 len=211 path=[374:0-204 596:205-210]

CTTATTAAAGTACTGCCAGCTACCATCTTCTGGTCCCTTTATAGAATATCTAAACATAGT

GAGTGCAGGATTTTTCCCTTTCAGCTTGTTTCTTAACATTTCTCTCTCTCTCCAGTCTTA

CAACCACTCCATTTCCTGTCTCACCAAAATACCATACAACAGTAAGAGCACTGTGATCTA

CTGGTGGTTCCATGTGCCAGAAAACATTCTG

>c14881_g1_i1 len=441 path=[1:0-440]

GTCTATATTCAGAAGTTCTGGAGGTAAGGCTGAAAAAAAGATTTTTTTTTTTATTTTTAT

CTCCCAGCAGATTTCAGCTCCTGGGATTTTTGTTAGAATAATTGGCCATTTCTCTTCCAG

AGTCCTTCCCCAAAAGCAAAGCCAGTGGCTCAGTCATTGGTGAATGCAAGCTAAACTGAA

TGTTACACATTAATAGATGGATGAGATGTCCACCAAGCTTGGAGACCATTCATGGTCATT

GATTACCACATACCAGCATGTGAGAGCTAAAGCTTGCAAATTCTTGAATATGCTCTCACT

CATTTCTTTCTATTGGATTGCTACCAGTTTTCTACCTTACCATTAGAAGTAGTGGACAGT

GCTTTTGTAATGGTTATGGATAAAGGAATAAGGAAAACTTTGCCTGCTTTGATTAATAAT

GTCTTTTAGCTTATTTCTTTG

>c14908_g1_i1 len=249 path=[250:0-223 25:224-248]

ATTTCTCCGCTCCTTCTCTCGACTTTATTCCAGGGGCCGGCGCCGGCTCGGCGGCACCAC

CTGCCGGGCACAGCGAGCAGACGGCTTTGTCTAAAAGCGCTTGCAAAAGCACCACCTAAT

CCTGCTGCTCCCCTCCCATGCTGAAGGGAAGGAGGGAGCCTGGGAGCCAGAACGCAGGGG

GAGCTGCTGGGGAGCGCAGCGGCCGGGGCAGCGCCCGAGTTCCCAGCGGCGGCCCCGAGC

GCTCCGCGG

>c14911_g1_i1 len=252 path=[337:0-28 310:29-140 67:141-195 540:196-198 544:199-251]

ACAGTATGAGAAAATGGGCATCTACTAAATTCCAATCCATAACAATGGGAGCATGGAAAT

GAAAGTTCTACAGACATTTTGGAAGGTGTTTCTGTAAAAATCCATGGCACTTCAGAGGAG

AATTTTATAAAAGCCTATTGAAGAAATATGATAGTGATGATGCTGAGGGGTGTGAAGTAG

CCAGACCATGATAGAGATGAATTGAAAATAGTGCAAATCATGAACCTTCTTTTTGTGATT

TCTCAATAGGCT

>c14913_g1_i1 len=499 path=[477:0-498]

AACAATATATTTATTTAAAGTAAATATTATTTAAAATTACTCTATCTATTTTTCTATTTC

CTAGATCTGTTGAATAAAAAATTGACAACAGTTGATGCTGATCTTACATTTGCAAGAGTC

AAAACTAAAGGTGCTAAGAGAATTAATTACAATCAATTTGTTGAAGCAATTAGAAGCTTA

GTAGATAAACACAGTTTAGACTATGATAAATTTGTAGAACAATTATGTAGCGAAGCTTCA

AATGGACCTATTTTATATGGTACAAAAACTGATCATGTAAGATTCTTCGATGATAAATCA

ACTTATACCGGTGTTCATAAACAAGGAGGTCCTACAATAGTTGATAAAAATAGAACTAAA

TTTTCTGATTTATCTGAAATTGTTGATCGTTCTGAATTTAATACTAGAGGAGTTAATGTT

AGTGTTGCTAAGAATGTTTAAGTAAAAAATAAAATGTAATTTAACTTTTATAATGCACTA

AAAAATAGTAAAAAAAATA

>c14914_g1_i1 len=428 path=[63:0-29 62:30-427]

GTTGTAGGGTGCCCTTTTTACCTCCGTTATCTATTTTTGCATGCTAAAACTATACTTTTA

GATAACCTACAACAAAAATATTATTGGTACAGGGCTGTCTGATCTTTTAAGACTTTCTCT

GTGTAACTTAGCATGATACTTTTCAGTTCAAATAAATACATATTTTGACTGTTTAAGCAG

TTTCATTGTGCTTGTCATATGCTTTAGTCATCCTTGGCTAGTACAGATATTAATAAGGAG

TACTTGATTTACAGAAGAGCTTTATCTGTACTTCTCAGTGTAAGCTGGACTCCTTCGAAA

TATTTGCAGTCAGCAGCAATAGATTTTGGTTTTCAAGCCAAGAGAAATTTCAGATTTGAT

GGTATATTCTTACAGCTTTGTCTGCTATGTTACTGGCTTCCTGTGATCAAATCCTGATGC

TCCCTCAT

>c14915_g1_i1 len=202 path=[1:0-74 76:75-201]

CCGATCTCCCTGAGGCATCTGTGAGAATATCATTAACACCAGAGAGAAGTTATTTACAAG

TAACTGGAAGTTGAGCTGTTACAGTTACTTGCACTTCATTAAAATCGCCAACCTAGAGCA

TTAAGGATAAAACATTAGAATAATACTTATGCATCTGACAAAAAGATTACTTGATAATTG

ACTTGTTGCCATTGTTTTTCTT

>c14921_g1_i1 len=231 path=[438:0-137 115:138-230]

AAATGGTTGCTAGCTATTTTTTCGTTTGGAATCTAAGATAGCAGTGCCAAAATGAAAAAA

TAGCTAGCAACCATTTTCAAGGCAGTTTTCAAGACGACTCATTGAGCTGAAAAGCACCAC

TGAAGAAACAGACTTTTTTATAATGTAAAGCTGTCTGTGACTTAGTGCGCAAAGCCTTAG

CTGGTGATACAAGGTTTAAACCTTAACTTTTTAAATAAAATCAGTTTGTGA

>c14921_g1_i2 len=207 path=[1:0-113 115:114-206]

TGAAATTCAAAGTAGATCGCAAGTCACGTTATGCTTAGACTTGATTCAATTTTCAACATG

TTTTCATGATGACTGTAAGCACAAAGTTTTCTATTACTGTTACTGGGACTTTTCTATAAT

GTAAAGCTGTCTGTGACTTAGTGCGCAAAGCCTTAGCTGGTGATACAAGGTTTAAACCTT

AACTTTTTAAATAAAATCAGTTTGTGA

>c14926_g1_i1 len=216 path=[226:0-215]

ATGGGCCTACTACGTGGATTGGGTTTGCTGGTACAGATCCATGCATGTGGGAGGGTGCAG

AAAAGGTTCAAGAGGACGTACTGCCTTTACTGTCCAGTCTCAAATTCACTTGAAATGTGT

TATGAGTTATCTTCTGTAGCAGCCAGATTAGGCGACAGAGATTTGGCTACTGAGGTCAGA

TTTGACTAAGGAGGTCTTTCTTTTCTATGAACAAAG

>c14927_g1_i1 len=252 path=[261:0-183 559:184-187 33:188-251]

CAGGTGGTGATGGCAGTGGGGTGAGTTTGCATTGCAGCCGAGGTTTGCAGCGTGAAGCAA

GGATGGAGAGTGCTGCTCTGGGAGCAGGGATGGAGCTCTTGGAGCTGCTGACAGTGCACG

TGTCCTGCTGAGTGGGCAGTACTCACAATCATACAGGTTGGAGAGAGCACATGCTAACAT

GGGGCACAGACACCTTAACGCTACTCAAAGCTGCTGCTATTTGGACTTCTTGGAGATACA

CATGCCACATTT

>c14927_g1_i2 len=212 path=[261:0-183 559:184-187 449:188-211]

CAGGTGGTGATGGCAGTGGGGTGAGTTTGCATTGCAGCCGAGGTTTGCAGCGTGAAGCAA

GGATGGAGAGTGCTGCTCTGGGAGCAGGGATGGAGCTCTTGGAGCTGCTGACAGTGCACG

TGTCCTGCTGAGTGGGCAGTACTCACAATCATACAGGTTGGAGAGAGCACATGCTAACAT

GGGGCACAAATGTGGCATGTGTATCTCCAAGA

>c14935_g1_i1 len=332 path=[1109:0-13 1111:14-331]

GAAGAATGCTATGTATGTGTCTCAATGCTATGTCAGTTTCCTCCTTCAGCTCTGAGCAAA

AGCCAGTTGTCTGGAAAAGAATATAGGAATAGAGCAACTATATAATGCCAGTTCTGTCAA

ATATTCTACCAGGTCTCAGTGGCTGTGGGGACATTGACATAACTTCTGCTCAGGTATCAT

GGCAGGATCAGTGGCTCTGAGATGCTGAAGCTTGTGGACATCCTGTTGGTGCAGGTTGTG

AGTGTGGGTGCAGATGGCAGCTCTGCAGTTGTGCAGTAGCTGCACAGAGAACCAATTCCT

TGCCACCTTTTCCCAGAATCCTCACTTAAAAA

>c14935_g1_i2 len=556 path=[1:0-237 1111:238-555]

AATCCAGTGTGGACAAACCTTTGTCTGATTTATATTTGGTGATCACTTGTGTTCTGTGCT

CACACACCAGTCTAATGTTGCAGCTGCTGGGGTTGCTAACATGTATTGTCTGTCTCACGA

CAAGGAGTCCCTATTTCACAACCAAACACTCAAAATTCATCAAGTTGGGGTGGGAGGAAG

AGCATTTCAGACGAGTAGTACCTGTGGTGTTCCTCAGCCTGCTGCCTTTGTCAGTGGGAT

GTGTCTCAATGCTATGTCAGTTTCCTCCTTCAGCTCTGAGCAAAAGCCAGTTGTCTGGAA

AAGAATATAGGAATAGAGCAACTATATAATGCCAGTTCTGTCAAATATTCTACCAGGTCT

CAGTGGCTGTGGGGACATTGACATAACTTCTGCTCAGGTATCATGGCAGGATCAGTGGCT

CTGAGATGCTGAAGCTTGTGGACATCCTGTTGGTGCAGGTTGTGAGTGTGGGTGCAGATG

GCAGCTCTGCAGTTGTGCAGTAGCTGCACAGAGAACCAATTCCTTGCCACCTTTTCCCAG

AATCCTCACTTAAAAA

>c14946_g1_i1 len=274 path=[445:0-87 16:88-273]

AAAATAGCACAGCAGTTCAGTTGTGCTCTTACATGATCTCTAATGTTAAACTCTGCTATT

AAAGTACAGTGGGAGGTGGCCCTGTGTGCTGTTTGCCCACTGGGAAGGTCTCCACGAGAG

CCTGGAGTGTGCTGTGAGCAGTAAGCATGGCGAGGTGTCTTGAGTTAGCAGTTTGCTGGT

GGAGGTGGAGCAGAAAGGCCAGGCGTTCAGCACCAACATGGGTGTGAGCATCACCAGCAC

TGGAAGAAACTGGGGAAGGGTTTGTCCTCCCCAG

>c14946_g1_i2 len=201 path=[1:0-14 16:15-200]

TACAAGCCAAAGGTTCTGTTTGCCCACTGGGAAGGTCTCCACGAGAGCCTGGAGTGTGCT

GTGAGCAGTAAGCATGGCGAGGTGTCTTGAGTTAGCAGTTTGCTGGTGGAGGTGGAGCAG

AAAGGCCAGGCGTTCAGCACCAACATGGGTGTGAGCATCACCAGCACTGGAAGAAACTGG

GGAAGGGTTTGTCCTCCCCAG

>c14950_g1_i1 len=241 path=[1:0-240]

GCAGGGGGATTTGGGGCAGACGGGGCAGCCCAGGAGGTTCTCCAAAGCCCTGCTCTGCCG

TCCGCTTCGACACCTCCAGGGACCTGAAGTCTTTGGGAGGAGGGGGGGTCTCCTTGGGCA

GGTCCAGGGAGCGTGGGGCGTCCAAGGCAGGGGTGGAGTTGGGCAGGGGCATTCGTGGAG

GCTCCGGAGGGATGTGTGTGGGCAGCGGTGGGGATGGTGAGGACCAGGAAGAGGAGCAGA

A

>c14952_g1_i1 len=314 path=[1:0-313]

CACTTGCAGGGAGCCATTATGCATGCCAGACTTTAATCCGCCCTATACACACGCTCTTTT

GTAGAATACCACATGCAAGCAGTGGGAGCAGATGGAAGAACTGTCCTTAAGGCAGTAAGA

ACCATATGCTGAAGGGAGCAGTGCCTGGGCACAGGGCTGCCAGCAGAGGCAGTGACAAAG

ATGAAATGGCAACACCTGAGGGAAAGAAGCAGGAAAAGTGGGCAGGGAGGACAGATTAGA

GTCAGAGAGGTTTGGGTGCAATAAAGTTGTCAGCTGACTACAGACACAAGTTTCACAACC

ATAGATCTTACCCA

>c14952_g2_i1 len=726 path=[1055:0-375 655:376-725]

TCTCCAAGATGCATTAAGAAACACAGAGACAGGAAGAGATTTTTGGCATGAGAGGAGAAT

GCAAGCAGGCATGTACAGCTGGAGGCGAGCGACGAAACGAGACTGAAAGATGGAGAAAAA

GATAACTTTATCCAAAAGCTTTCTCCATAGGCTTCCCTGAACCCTTACCCCGAAACTTCT

TGTTTCTAAATTGAAGGACTTGTGTTCTTCCAGTGCTGTCTGCTTTGTCCAACAAATGCA

TCAGGTGTCATAAAAAGTGATCCTTCCCTTTCACTTAATTTGCTCTCATGGAAGTATCAG

CTTAACAGATTTTTCCTTTATTTTTCCCTTCAAAATACTGGATTTATTCATAAACGAAAG

ATGCAAGCAATGTTTTCTTTGGTTAGGCAAATATACATATATTAAAAAAAATTGGGTAAG

ATCTATGGTTGTGAAACTTGTGTCTGTAGTCAGCTGACAACTTTATTGCACCCAAACCTC

TCTGACTCTAATCTGTCCTCCCTGCCCACTTTTCCTGCTTCTTTCCCTCAGGTGTTGCCA

TTTCATCTTTGTCACTGCCTCTGCTGGCAGCCCTGTGCCCAGGCACTGCTCCCTTCAGCA

TATGGTTCTTACTGCCTTAAGGACAGTTCTTCCATCTGCTCCCACTGCTTGCATGTGGTA

TTCTACAAAAGAGCGTGTGTATAGGGCGGATTAAAGTCTGGCATGCATAATGGCTCCCTG

CAAGTG

>c14952_g2_i2 len=413 path=[1055:0-375 1430:376-412]

TCTCCAAGATGCATTAAGAAACACAGAGACAGGAAGAGATTTTTGGCATGAGAGGAGAAT

GCAAGCAGGCATGTACAGCTGGAGGCGAGCGACGAAACGAGACTGAAAGATGGAGAAAAA

GATAACTTTATCCAAAAGCTTTCTCCATAGGCTTCCCTGAACCCTTACCCCGAAACTTCT

TGTTTCTAAATTGAAGGACTTGTGTTCTTCCAGTGCTGTCTGCTTTGTCCAACAAATGCA

TCAGGTGTCATAAAAAGTGATCCTTCCCTTTCACTTAATTTGCTCTCATGGAAGTATCAG

CTTAACAGATTTTTCCTTTATTTTTCCCTTCAAAATACTGGATTTATTCATAAACGAAAG

ATGCAAGCAATGTTTTTTTTAGTTGTTTATTTTTTTTAATATATGTATATTTG

>c14968_g1_i1 len=202 path=[432:0-201]

ATGGAAAACAATCCCATGCATTAAACTTCACAAAGATTTGTAGGAGTCATATTTAAGATT

GTTAAGCTCACCTGAAAGGGACTTTGAAAAAAAAAATCAATAAAAGCCAGTTCTAAAAGC

ATTGTCTCACAAACTCTGTGGAAAACTAGTAACAAGGATGTGTGCCAGTGATGGCAAAAC

AAGTCTTAGTTAACTACAGTAT

>c14972_g1_i1 len=231 path=[155:0-230]

TCTCCCCATAGTAAGCTGCAAAAAGTACCCTTTGGAGGGTTTGTTTTTTAAGAAAAACAC

CATTTTGTTTGTCTTCAGTTTTCTCTGGGAATATCTTTTCTCTGGTTTTGTCTCCTCTCC

CTTGTACGTCAGATGGGTCAGGAACACCAAATTCAGTAGCAAAGGCAGCTTACCAGCGCT

GCAGAGGCACCTGGTGTGAGCATACACAGTTAGATGTGCCTGAGCAGGGAG

>c14975_g1_i1 len=252 path=[153:0-102 477:103-251]

GGGCTGCTCCCTACCCACTGCCTTGTCCCATTCTTCCTGGCTTCAAGGAACAAAATCTTT

CTGCAATTCTTACAGCTTAAGGTCAACACACTTCTACTATTTAAAGCAGTAGCAAAAATA

ATAAAAATGCAGCTATGAGCATGTACATACAACTCTTAAGAAACACTCTGAGATATCTAA

GTCCCAGCTCACAAAAATGTTGGTGTTTTAAAAGTTAATTTGATTTTATTTTAAAAACTG

ATGGATGGAAGT

>c14998_g1_i1 len=243 path=[249:0-213 25:214-242]

GCCCAGAGAGGACATGGGATCTCCTCCCTGGAGGTCTCCAGAGCAACCTCTACATGGTGC

TGGGCACCCTGCTCTGGCTGTCCCTACTGGAGTAGGGTCTGAGTCCCTGACAGCCCCAGG

CATTCTGTGAGTGTGAAATTTCAGCATGTTTAAGTAAATCCACTGAACACTTACCTAGCT

CCTCCTCTAGCTGTAGCTCATGTATTGCTTTAATGTTGCTTTCTTTTAGGTGATGTCAGA

TAT

>c14998_g1_i2 len=243 path=[249:0-213 462:214-242]

GCCCAGAGAGGACATGGGATCTCCTCCCTGGAGGTCTCCAGAGCAACCTCTACATGGTGC

TGGGCACCCTGCTCTGGCTGTCCCTACTGGAGTAGGGTCTGAGTCCCTGACAGCCCCAGG

CATTCTGTGAGTGTGAAATTTCAGCATGTTTAAGTAAATCCACTGAACACTTACCTAGCT

CCTCCTCTAGCTGTAGCTCATGTATTGCTTTAATTTTGATATCTGACATCACCTAAAAGA

AAG

>c14999_g1_i1 len=261 path=[296:0-209 623:210-216 513:217-260]

TATTTAGGAAAAAAAAAATTTCCTAAATAAACTTTCATGACAAATCCTTATAAAAGACAG

CAGTTATCAATAAGATACACAGCTTTACAATTACAAGCAATGTAGTCCCGTGTAGTTGCA

TGAGAAATGGGGTGGTTGTACCAATTACACCTTTGTTTACTTCTAGGTATGTTTACTGAA

ATTATTTTAACTGTTGTTTAAATTGCTGTATTATATGCTTTGTTAGTGTTGTTATACAAT

TTATATACATTATAAAATTAT

>c15003_g1_i1 len=259 path=[237:0-258]

GCAGCCTGAGCTGGTGGGTGGCAGAGGACTGGAAGTGGGTGGGCTTTAAGATTCCTCCCA

AAACAAACCTTTCTGTCGTTCTCCAGTGGTAGAGCTACTAACTGAACTACAGTGATGCTG

CTTCTTGAGTACCTCTCTTGACAGTAAGATGGTTAGTTACTCTGTATTTCAATGATAATT

GAGCACAGATTAATAGTTTTCATTTCCTAAGTTCTTTACAGAAACTTACAACCATTTTGC

CTTTTGCTAAAACACACCA

>c15015_g1_i1 len=229 path=[1:0-109 111:110-112 114:113-228]

TAACTTAGTTGAATGTGGCTGTATGGATGGTGCACTTTTTCTGTTTGATTATATATGAAC

AGCATGTGCTGTTCAGTGACAGCAAAAGTATGTTAAAATCCAAAATTGGATTTTAACTCA

CAATCTTTAGTAGTTCACCTTTCTACTTTAGCTCTGTAGAATGTTTCTGAAATATTTTTC

ATTTAGGACTTTTTCATAGGATCACTAAGGTTGGAAAAGACCACTAAGA

>c15020_g1_i1 len=469 path=[447:0-468]

TGTCTCAGCATGCTCCTTGAGCATTAGGATCATCAATGGCACAGTGATGCCAGTGCCATG

TCCAGTTTGGTGTCATCAGTATGAGACTGGGATGTGCTGGGACAGGGACCGGGTTGTGGA

GGTGACAAAGAGTGGGGCAGCTGGCACAGGAGGGGTGACTGGGAGAGGGGGTGTGGGGCA

GCCACAAAGATGATTGGGGGCGAGGAACTTCTGTGCAGAAGTATGAGATGCCTGGGAGTG

TTCAGCTTGGAGAAGACCGAGAGAGGATCTTATCAGTGCTTATGAGTATGTAAAAGGTAG

GAGCCAATTGGATAGGGCCCAGGATCTTTTTGATGGTGCCCAGTGACAGGATGAGGAGCA

ATGATTTTAAACTGGAACAAGAAGTTTCATTTGAAGTTGAGGGGGAAAAAAAAATTCTTT

ACTGTGGGGATGACAGAGCACTGGAGAGATTGTGGAGCCTCATTCTCTG

>c15025_g1_i1 len=236 path=[1:0-235]

ATAGTTATGGTGCCTGAGTAGTTGTCTGCTTCAGGGAGCACTGGAGAAACAGAAATAATT

CTAAGATGCTAATAGCTGCAGATGAATCCCCACAGAAGTGGCAGTATATGATCAAGCTTA

ATTACAGGATGAGGAGATCAAGGCAGACAGTTCCCACTCTTTAATTGTCTCATCCTTGCA

TGCTGGACCAGAGATGTTGTGGTAGAGGCCCTGCTGAGGACACAGACTGCTCTGCA

>c15025_g2_i1 len=257 path=[427:0-256]

AATTGAATTAACCTAAGGACACACTTTTTGAGCAGTCTGCTCCTGCTGAGCTGGGAGCTG

AGCTCTGAGTCCTGAGAGAAGAGTCTTTCAGGATTCCACATGGAAGGCAGCAGGGAACAG

ATGTTACTCCTGTTATAACCCAGACAAGCATTAATTGTAGTAGCTCTTAATGTGCTATGC

CAGTCCTGATAAGCGCAGCGTCCCTTATGACACAAATGTTCCACTGTCTGTTGGCAAATT

TATAAGTTAAGGAATTA

>c15029_g1_i1 len=225 path=[155:0-224]

GCTGCTGCTCCCCTGCCGTCCTCAGCTCCTGGCATGGCTGCAGTGTGGGTGCTGTTTGCA

GCAGTGCAACCCCTGTGCTGGCTGGCAGTCGCTTTCTTTTCGTGCTGCTGACAATAGTTT

CCAATGCGCAGCCAAGTTAACTGAGCTGTGCGTTGGCACTGGAGCTGAGGAGCGAGGTTT

TGTCGCAGTTCTTGAGGCTGGAGGCTTTTTGGCAGAACTAGGTTA

>c15043_g1_i1 len=300 path=[278:0-299]

GCCAAAGAAAACTTTATACAGCAATGAGTTTGGTGTGTTCACTGAAGCAGGAAGCTAAGC

ATTGGGATGTACAGTCTTATTTCTGACATTTATGGAAATGCTGTTATTTGCAGTAAATAC

TCAGCTAGTCTGACAAAATATTTATGAAAGCAATGGTAATACACCAACAGACTGGTATAG

TTTGCCTCTGCATAAAATGCTGTAGCAATGTGGCTATAATTCATGTTACCTGGAATTCAG

AATTAAAGTAACTGATTATTTACTGATAGTCTTAGGACTATGTGAATTTTTTTTTTTTTT

>c15049_g1_i1 len=221 path=[183:0-220]

TATGTTATGTGTGTATGTGTCATATGAATGCTTTGCCAAAAATTAATAGAATTAATAACT

TTTGTAGTTGTCTCTAGAGACAGCGTGGTCTAAATGGTCTAAAGTACTTAAAATCCATCA

GAAAGAGTTAGTGGCAGCATTGAAATGACACATACACACATAACATAATACGTGTCCAGC

TGTCAGAATCTCTTCTGTACTGTGCAGTTAATCGAGTCCTG

>c15050_g1_i1 len=209 path=[1:0-208]

AGAAGATGGGGATTTCTAGGGCATGACTGAACTTCCTCGCGGGTTGCCCACTGGAAGAAA

GCCCCTTATAATCAGATTTATATACTATTTATCAGTTTAGGGAAGTTTACCTTTAGAACA

CTAGGTTAGGATGAGATGCAGAAAGATGTCACTGTCTGGGACTAACTTATGTGCAGATAA

TAGGTTTTGTAGATGCACAGGAACATATT

>c15051_g1_i1 len=212 path=[190:0-211]

TGTACCAGTAAGCATTTCAAACTGAAAAGAACAAATGTTAATCTAAATCAACACTTTTTC

CCCTCAGATGCTTAAAGTGCAAAGTACTTTTGTACTGTATTCTTCCCATTGCTACATTAG

GCAAAGTGGCATGAACTTTTAAAGATATTTTAAAAAAAACTACTAATCTAAAAATCGCTT

TTAATCAGAAGTTTACATGATAGTGCCAATTG

>c15055_g1_i1 len=230 path=[362:0-229]

TGCAGTAAATGTTCTTCTCTTCCACAAGGTTAATGAGCCCTTGGGACAGACTGGCTCTTT

GCAGAAAGGAGTGGCAGTGTTCAAACAGCTGTGCTTCTGTCACACTGCTGGATGTGCCCT

CTCAGCTCCGCTGTAAAATGAAGCACTGGTAAAAGCAGCAGCAATTAAAAATGTCACATT

TATTTTTCTTGATGACAGTGCTGTACAGCTCTGGTCTCACTTCAGCTCAG

>c15057_g1_i1 len=227 path=[227:0-226]

AGGTATGTCTGTTCCATTTGTGATCAAGGAAACTTCAGAAAACATGACCATGTACGGCAT

ATGATATCTCACTTATCAGCTGGAGAGACTATATGCCAGGTCTGCTTTCAGATATTCCCA

AATAATGAGCAACTGGAGCAGCACATGGATGTTCATCTGTATACATGTGGAGTATGTGGA

GCAAAATTTAATTTGAGAAAAGATATGAGATCTCACTATAATGCCAA

>c15060_g1_i1 len=247 path=[379:0-246]

AAAGGTCCGGACCATGAGCATAAAGCATAAATCGATCTAGTTCCAGCTTCTTAGCCAGCA

TGGCCAGATGCTGGTTCTGAACTATGGCAGTGCGGTACGTAGCTAATCCTCCTTCCTCCA

GAGTGGGAAACAGGTAGTACAAGTGGACACTTGTTAAGAACTCCACCACAGCATCTCCCA

GAAATTCTAAACGCTCATTGTGGTTAATCCTGGAAGGAGCTGGATCATCCTGTCCCAAAC

GGGACAT

>c15063_g1_i1 len=243 path=[330:0-194 568:195-198 528:199-242]

AAAAAAAAAAAAAAAAAAGTAACAGTGAAAAAAGCCCGGCTTCCCTTTTATGAGCAGTTT

TCAATTTTGGCCAGAAATTGCTGCGCCCACCACTCCTCCAGGTCAATGGGAACAAAGTGT

TTCAGCCGGGGGTTCGGGGTTTTCTCTACGTACTGCACAGGCCCACGGGTGCTCTCCCCC

CTCTGCCCCCCGTCCAGCTGCTGCTCCACCTCCTGCCAGGCTTCGTAGACGAAGCGGACG

TTC

>c15067_g1_i1 len=210 path=[112:0-109 222:110-209]

AAAAATAGCGAAGACAACTCCATGTTGAGCGAGCAGTCAGGGCACGGCGAAGTCTGGGGC

TACGGGGGGAGGGAGGAGAGGAGGGAGGGTCACAGCTCCATGCCTTACAAAAGGGAGCCA

AAAGCCCGACATTTTTTTAATATCCGACACGAACGCTGCCGTGCACACACACGTAACACA

CGTGCGTGGGGCGATGCGTGTGGCGCTAAT

>c15071_g1_i1 len=202 path=[1:0-72 74:73-201]

AACCTGAAATCGGCATCTTTGGGTTGAACTCCAGATGTTTTTCTCTGCATTCACTGTACA

GTGTAATAACTCAGTGATAACTGTTATAGCAGCACCATGAGTCTGCCTCTCTTATTCTGC

AGTGATGTTTGTGAACTATCCAGCAATTCTTAGAACAAGGGAAGATTTAAGGAGGGGGGT

TTTAGGTTGGATGTCAGGGGGG

>c15075_g1_i1 len=229 path=[1:0-228]

GCTGCATGCAGTGCACCTGAGTGCCTCCTTCTTGTCCTCCAGGCACACTTGCTTCCTTTG

GGAAGAAACAAAGCTCAAGCATCAGCAAATTAACAGCAGGCCCCCTTTGACCTTACTGAG

CATGCCGTAAGAGCTGAGGCTGGAGGCAGCCTGACTCCCCAGCCCCGTGCTGCTTTCAGG

GGAGTAGGAGGGGCTGGGACACAGTTTGCCAAAGCACGTTGTCAATGCG

>c15083_g1_i1 len=414 path=[1:0-177 522:178-194 539:195-413]

CAAGAGATTACCAACTTCTGGTATACAAAACGTTCTGGATGTTCAGGTTTTTTTCACCTG

TGTGTTATCAGCAATTCCTAACATTTCATCCTTATCTGCTGGTTGTAATTGTAGAATAAC

GAAACTGCAGGTGACTACGTATGGCATGTCTTTTCCCTCTAATACTTGTGTGTATGTGTG

GTGTGATTATGCGTGTGTACGTGCACAGCCACACATACACACGCATAATCACACCACACA

TACACACAAGTATTAGAGGGAAAAGACATGCCATACGTAGTCACCTGCAGTTTCGTTATT

CTACAATTACAACCAGCAGATAAGGATGAAATGTTAGGAATTGCTGATAACACACAGGTG

AAAAAAACCTGAACATCCAGAACGTTTTGTATACCAGAAGTTGGTAATCTCTTG

>c15083_g1_i2 len=307 path=[431:0-87 539:88-306]

TGTTGCTTTTCTGTACATCTTTAAAATGTTCTTAAATTAATGTACATAAAAAATTCGTTC

CCATTTGCTACAATGGGAGATGTGCACCTGTACGTGCACAGCCACACATACACACGCATA

ATCACACCACACATACACACAAGTATTAGAGGGAAAAGACATGCCATACGTAGTCACCTG

CAGTTTCGTTATTCTACAATTACAACCAGCAGATAAGGATGAAATGTTAGGAATTGCTGA

TAACACACAGGTGAAAAAAACCTGAACATCCAGAACGTTTTGTATACCAGAAGTTGGTAA

TCTCTTG

>c15088_g1_i1 len=213 path=[191:0-212]

GACCGGTAAAGCATACATTGACATTGCCATCAGCGCATACTACTGAATGTGCTTTTATTT

GGGTCTTCCTCCTTGAAGCTCTATTGCTTCTTTGATAGCTAAGTAGCTATCAAAGTAGTT

TAGTAGCTAAGCTACTACACAACCTACTACTACTGTAGGTTGTGTACACAACCTACTGTA

GGTTGTGTAGTAGCTTAGCTTGGAAATAATGCA

>c15088_g2_i1 len=254 path=[612:0-253]

AGGAAAGGGTGGGAGAAAATTTACCTTTTCTGCCTCGGTCCCGGCTTCCAGCTGCTGCTT

CTGCTTAATGCACATCTGTCCCACTTTAGCCAGGAGCTCATGCGGGTGAATGAAGACTCG

GGAGCTCAGCAGGAAAGTGAAGATGTAGGTCCTATCTGGATAATAGTCCATGCTTGGCTC

CAGCTGTAGTGGACAAGTGCAGCCTGATATGGGAGAGAGATGCGTGGACCCCGTTTATCA

GGATGGGAACATTG

>c15096_g1_i1 len=250 path=[179:0-249]

AAAAAAAAAAAAATATGTACATATAAATGTTTATGTATAAATAATATTAATATTTATTGA

AAAATTATGAACATACAATTTTTTTATTATTACAAATGAAAATAATATTTTAACAACTTG

AGTTAAAATTTTAAAAAATAAAAATGACTTCTATATTTTATAGAATAATTAAAAGCATAT

ACATACTTCATGAGCTATATATAAAAATAAAAAACAACCTGAAATTGTATTTGAACACTC

AAACACAAAG

>c15100_g1_i1 len=1292 path=[27:0-1291]

ATTCATAATAAAATATAAATAATAACAAATTAAATAAATATTTTAATAAAGAATATCCGT

ACTTCCCCCTTCCCCTCCCCAGAAAAAAAAGAAGTTAATATTTAAAAGTTTTTTTTATTT

TACATTTTATTTATATACTTTTTTTTTTCTGTAAATACATAGGTTTTTAATTTTTTAATT

ATTGTTATTATTATCATTTAATTATACTTTTTTTTAAAAACAATTTTTATTAACAAATGG

CAAAAATTTTGATGAAAAAATAAAAGATGCATAGGATGGGTAAAGTGGATCTTTCTTTAA

ATTTTCTTCTGTTAAGTTAAATATTTCATTTTTTTCGTGCTTATTTTTATCATATTCGTT

AATATTTTCGTTAATATTTTTATTCAAATTATTTGAATTTTTTTTTTTATAATAATCATA

AATAGTCATAGGTGGAAAATTAAAAGAAGACGGAATAAAAAAAATAAAAGGGGGATCTAA

TTTAAATTTTCTGTTCATTAATAATGGTGTTGTACATTTTGTTAGGCATATTTCACATTT

TCTTTTTTTATTTTTTTCTTTTAAAATTAATTCAACATATTTATTTTGAACATCATCAAA

AGTTCTTTTCATTTTCCATGAAATATATAACCAAGGATCACCAAAATTTACATCATAATA

ATTTAAATAATATTTTAATTGTTGTTCATCTTCTTTTGTCCATATTTTATTTTCATTTTC

TAATGGAGGTGCTTGAAAATTTTTAACAAAATCATCATATGAATCTTCAGGATATAAACC

ATAATTAAATTTATTTACATACTTCCATATCTTTCTTCTTTGCTGATTACTTAAGCATTC

TATATTATATAATTCTTTGGGTGTAATTGTAATAAATTCTTGCCAACTTTTAAAATATTT

TTCTATTTCATCAATATTTTCTCCTATATCAATTTTTTCTAGAAATGTTCTAATATCCCA

ATTTTTTGGTGCTTCTTTTGCACAACTTTTTAAAAGTTCTTTTTTTTCTATTTCGATTTC

TTCTGTTTTTTGTCTTCTTAAATGTTGTCTAAATAATTTCAAATCAAAGAATCTCCTTTT

CTTTTTTATCAATGGACCTGTATCATCAGAATTCCTACGAAATACTGAAGTGTGAAGTAA

TCTAATGTTTTTAAAAAATCTAATCAATGATTTATTCATTAAAAAAAAATTTTAAATATA

ATGAATTTGTATACAAAATTTTACTATAAATATATTTCTTTTATAGTTTTAATAAATACT

GTAAATTTTATATACTTTTCTTAATTCCTTGA

>c15111_g1_i1 len=352 path=[612:0-351]

GCCAGGGTTGCTTTGGCTTTGAAAAGCTGCACAGTATTTCCCAGTCAAAGAACACTTCAA

ACCGTATGCCTGTTTATGGTGGCAAGAGTTGAGGGATGTTCTAATCCTCATCATTTCTCG

CAGATTTCAGGGAAGGGGAAAGGAGAGAGAGTGACTCTGCTCTATGTGACTGCCTTGTTT

TCACCCCTCACCAGAGGGGTGTAGAGCAGAGCGCTGTCTCCTTGGGCCTGCAGCACAGCA

GTGAATTCCACTGGAGCTGAAGTAATGGCTGTCTTCAGCCATGAGAAATAATTGTCTGAC

TGGGAAGCAAAGAAACTCTGATTATGAATGGAAATAAGAGTGGAGGGGTGGC

>c15119_g1_i1 len=209 path=[322:0-129 25:130-208]

CCAGCGCCATTTCCTCTGTTTCCTCAGCCATACTGAGAGCAGAAATGTCACAGTGGTTTG

ATATCCCTGATATCAAAGGACACCATAAATTAGATTTTAGTAATTTTGTATATGGTGCAG

CCTAAATTTCATTTTCTTGTAAATAAAGAAAAAATGGGAAAAAAAAAAGAACTTTTTACC

CAGAATTTTCTCTGCTCCACTCCATGGGG

>c15127_g1_i1 len=212 path=[190:0-211]

AAATTATTTATAAGTAATAATAATTACATATATAGAAATAAGGCATTTCATTTAGATGAA

AATTATATTAATGAAGAACAGTTAAAAGTAACAAAAGAACAATCAAAAGAAATAAAAATG

GAGAAGGATGAATATAAAGAATATTTTTGTGAAAATACATGTGTACATAGTGATTATTAC

ATCAAATCAGATTATTATAATAATAATAATAA

>c15129_g1_i1 len=202 path=[155:0-201]

CACAAAAACTTGCATTTGTCTCAAACAGTATCTCCAAATCCATGTTAAATTCATTAGCAA

GACTAAGGGATTTGTTTCTGTATTGCTCAGAAAGTCATACTAAGGCAGCAGGTTTTCATA

AACAAGTGGTTGGGTACAATGATTGTTTCACTCAAACTCTGAGATACACTATTTGATCAT

TTATTATAAGAGTTGTAACAAG

>c15144_g1_i1 len=231 path=[161:0-158 320:159-230]

CAGGCCGTTTCATGACCACTAAGGCTCCATAAATAAGAGCACTTGATACAAATAAATTCT

TCGGCAATACATGAACCATCATCATCAATGGCAGACAAAACGTTTTGTGCAGCCATCTGC

ATTTTCCCTTCCCACCATAAACGTACAAAAATCAGTTAGAAAACCCAAGACCGAACAGAT

TCTCTGGAGCCATTTTCTCCATAGATTTATCTTACTTTCAAACACTCCTTT

>c15150_g1_i1 len=217 path=[1:0-49 441:50-216]

GGCCTTATGCAGAGAGGACAACTGTTGTCTTGAGCTTTTAAGTGGATTGATATGCAATGA

TGTGGTAGACCTTACTATTTCCAGTGATATTTCTGACTGTTCTGTTGCAGGTCTTTTTAT

TTTTTTTAATCAACTTTCCCAACTGTTGTGATAATATCAGAATGGAATTTCAGATTAAGT

CAAATGACCACATTATTAGGCAATTTTGATTAAGTAA

>c15151_g1_i1 len=317 path=[1:0-316]

AAAAAAAAAAAAAGAAAAGAATCCTGTGATTTGGAGTGCTCATGCATTGCCAGAGTTTCT

GTCAGGAAGGACAGTGAGGATTCCAGCGTTAGGGATTTTCTGAGGGCCAAGGTTCAGCTA

AAGTAGCAGAGCTGTCTGAGGCAAGCTTATAAAGCACCTGCTTGTTTTGTTTGTGACACT

AGAGCTTTTAATCTATTTTTCTGGACACTAAACATGTAAAACCTTTGGAAAATATATCCA

GTGGTATTAAACTTTAAAGCTTTTCAAAATCAGAATACTACGCAAATAGACTTAGCCCAG

ATTTTGAAAAGCTTTAA

>c15152_g1_i1 len=204 path=[155:0-203]

TAATCTGTGCTGCATCAAATTTCAAAGGTCTCCAGATGTATTTTGTCATCATTGTTTGTA

TCAGCAGACAAAATACCAAGACTGGTAAATTGTGTGGTCTAAATAGAAGGGCTGATAGTA

GAACCAATCCACTGTACACTTCCCATAGTCCTGTACTCTTCACACTGGAGTCTGCAGAAA

TTTGATGCAGCACAGATTACCATA

>c15157_g1_i1 len=212 path=[190:0-211]

GGGTCTTCTGACATCAAAATTAATAACACATGGTTTATTCTTCTGGTGCCTGCACATTCT

AAAGGCATTGGCTGTAGTGACTGATGCACGAGCACCGGAGGAATTTGTAAATGTCTGTTC

TCTATTTATGGGAGCATAACTCGATAAGTCAAATTTTGAGTTACTTTTGTTCTCCTCAAA

TCCTGTCTGTGGTTTTTTCCCATCGTGCTCTC

>c15159_g1_i1 len=204 path=[155:0-203]

AGATGCGTGGAATTGCCATAAATCAAGTATGACAACATGTGACTATGACTACCAGAGGAA

CCCTGAGAGCAGAGCTGATCTCCAGAGAGTTCATTCAGCATAAACTGCGTGTCTGAAGCT

GTACCATTAATCACCTTACCTTTATTGTTCCTACAGCAGCTAGTGAAGCAGTGTGTCACT

TATGAAAGTTCTGTAAGATAAATA

>c15177_g1_i1 len=296 path=[1:0-295]

CTAGGTGTTCAAAAAAATCAAAAGCCGTATGGAGTTGTCTTCTCTATCTTTAGTTAGGGG

ACTTGATGTGAAAAAAAAAATAAAGAACTGGTTACTCAAACTACAAGACTCCTAAGACCT

GAACTGCAGCTGATGTGACACTGAAATGAAACTACAGCACAGCAACACACTTGCTACGCT

TCTGCAGACAATCTGAAACAAACATTTCTAGCCTGAATTTATACTAGAGAATTCATGTTT

GACACCAAAGACATCTAATTCATGCTACCACTGGATCCCAGTACTCTCTCTAGAGG

>c15182_g1_i1 len=210 path=[1:0-165 544:166-169 251:170-209]

AGAGGGAATGGCCACTGTGCTCAGCACAGCTGCAGCCACTGAGTGGGCTGAAACATCTGC

ATCACACAGATGTGTGGGCAGAAGAAGGACGTTATATCACTCTTTTCTACTTTATTCTGC

CCTCCCTGATAACTATGTTGTCCATTTTAAAACTGAATTATTCAGTCAAATAAATCTTCG

GCAGGCTCAGGGTGGTTTATTTGGGTCTTT

>c15182_g1_i2 len=211 path=[1:0-165 167:166-210]

AGAGGGAATGGCCACTGTGCTCAGCACAGCTGCAGCCACTGAGTGGGCTGAAACATCTGC

ATCACACAGATGTGTGGGCAGAAGAAGGACGTTATATCACTCTTTTCTACTTTATTCTGC

CCTCCCTGATAACTATGTTGTCCATTTTAAAACTGAATTATTCAGTCAAAACAACCAACC

AACCAAAAAGACCCAAATAAACCACCCTGAG

>c15182_g1_i3 len=229 path=[1:0-165 544:166-169 511:170-183 167:184-228]

AGAGGGAATGGCCACTGTGCTCAGCACAGCTGCAGCCACTGAGTGGGCTGAAACATCTGC

ATCACACAGATGTGTGGGCAGAAGAAGGACGTTATATCACTCTTTTCTACTTTATTCTGC

CCTCCCTGATAACTATGTTGTCCATTTTAAAACTGAATTATTCAGTCAAAAAAACAACAG

CAAGCAAAACAACCAACCAACCAAAAAGACCCAAATAAACCACCCTGAG

>c15184_g1_i1 len=267 path=[245:0-242 488:243-266]

GAGGTTTTTCTAATATTAGAAGTGGCAACACGGTGTAACTGATGAGTATTATTGCAACAA

TGGCGTCAGATGTGGGTCTAGCTAAGAGAACGTCCAGTGTTTCTTTGGTGTGTGGTATCA

AGAGTAAAGAGCATCGTGCCTGGAGAGCACAACGTGGTACTTGTAGACCACCGACTGGTG

GAAACAGCATGGAGCAAGCAAGTCTCCTACAGCAAAACCGGGTCAAGATCCACAACCAAA

CCTGAAATAAGAGCAACCTGCTGTGTG

>c15198_g1_i1 len=219 path=[235:0-218]

GTACAGCAACACAAGAGGCAGGGACTCCACAGCTGCATGGTTCTACCACTTTCTAGAGGA

GCAGAAATCTTAATTCTGGAGTTGTTTCTTAATGCTTACTTCTGAAATCCATTCGACTTC

TCCTACGGTGTCAGAGATCTGGTGACCACGAATTTATTGGCCTCAATGAAATTTGCCTTA

GCGTTGTTAAGTCACAAGACCTGTCATGGCCCATACTGG

>c15208_g1_i1 len=202 path=[45:0-201]

AAAAAAAAAAAAAAGGAAAAAGAATATAATTGTATACACATTTATTAAAAAATAATAAAA

AACATTTAAATAAAAAAAATAAAATTTATTTATATCTTTTTAAATTAAATAAGTCTTTAT

CCTTGTAGGTATGTAAAAATGGGGTATTTATATTATTAATATTTAAATGAAACATAAAAA

TAATAAGTTTTTTTTTTTTTTT

>c15211_g1_i1 len=258 path=[274:0-83 529:84-257]

AACCACTGCAAAGTGCGGCTGCTGAAACCCTTAGAATACCCAGGGCAGAAGTACTAGTGA

TTTAAATTGCTTCTTTTGAAGTGCACAACAGCCACCTGTATGTGCCTTCACAGCAAAGGT

GGCAGTATGCATCTTCATCTTCTCCTTTATTGGACAAAGCTTGCAAGGCTAACTGAACCT

ACTTCTTCAAGTGTGTGGAAATGACATTTGCCAGTGCAGTTTATGGAAGCCCAGATGGCC

TCCAAATGATTCTCCCAT

>c15217_g1_i1 len=248 path=[134:0-165 301:166-247]

TTTTTTTTTTTTTTTTTCCAATAAATAATAATGATATTCTTTTTTTATTTTTTAAGAACC

ATGTAATTATTACAATTTGGGTAGTATAAAAATAAAATATATTAAAATTATTGTGAATAA

TTATCAACTGTTATGAATAACTAAAATCTTTCGTTCTAATTAAGAAAATTTATTTTATTT

ACAAAAATAATATATAAGTTTTATTTAAAAATATTACTAAAATAAAAAAAATTGAGTTTA

TTATCAGT

>c15219_g1_i1 len=298 path=[27:0-297]

AAAAAAAAAAAAAAAATGAACCATAAATTTTAAATGATAAAAAAGAATTTTTATAAAGGA

AAATCATTTAAAAATACATAATAACGTAAAATAAACACTAAAAAAATAAATAAAATAGTA

AAAAAATTTATTATATATATATTATAAAATCCATAAAGTTAATAATTATAGAAATTATTA

AATACTTCTCATATTTGATGGAGTTATATTTTATCATAACATATATAATAAGGTAAAAAA

TTTCTTTATGTAATTTTAATAAACAAAAAATAGAAATGAATAATTTACATATTTAAAA

>c15220_g1_i1 len=230 path=[241:0-102 465:103-229]

AGTTGATTCTTGTCTTCCAGTTTTCCAAGAAAGAAAGGAAGAGAATTGTCTCAATCAGTC

TCAGAATGTTCAAAATATATCTGTGAGTTCAAACACACTAAAACCAGAAGCCCTTGAATC

AAAAAGCTTAGAAGGGCAAGTAAACATTGTAAATACACTTAGTGTGCAGAATCAGGCAGG

ATACCGAAACAGTGTCCTCAATTTCAAGTTTGGAAATGGGAACACTGTTT

>c15223_g1_i1 len=240 path=[313:0-239]

ATAAATAAATAAATAAATATATATGTATATATAAGAAGTATATTTTATATTTAAATATAT

ATAGATAAATTAAGTAAATTTTTTTTCAATTTGCATCTTTTTTTTCCAAAAATTTGATTG

AAAAATAGGGTATAAACATTATTTGTTGTACATTTAAAATTTAATATACTTAAAAGAAAA

TTTTAATAATGTTTTTTTATGTAATCTTTATTTATTATTTTTAATGTTACATTTGGTTTT

>c15226_g1_i1 len=345 path=[45:0-222 388:223-344]

TTTCAAATTTTTTTTTTTTTAATTAATATTTTGATAAAAATATGCTAGCTTGCACGAATA

AAAATAATTTTTAATGTATAATCAAAGTACTTTTTATGAAGATTTAAGTTTTAAAAAAAA

ATATAGAAACTTCTATTAAATTAAATAAATACCCTTTAATGAATAATTATTATTTTAAGA

ATAAAAAAATTATTTCGTTAACATATTTTCACTAATATTATTATAAAAATTATATGAAGT

AAGTCATTTATTTCATATGATGAAATGTAATAAATAAAGAATACATATTCGTTATAAACG

TATTCTTATAATGAACGAAATGGAGTTTTCTCCACATTTTTGAGT

>c15228_g1_i1 len=250 path=[45:0-145 22:146-249]

GCCAGGATGCCATTGGCCTTCTTGACCACGTAAGCACATTGCTGGCTCATGTTCAACTGG

CTGTTGACTAATACCCCCAGATCCTTTTCCTTTGTGCAACTTGCCAACCACTCTGCCCTA

AGCCTGTAACGATGCATAGAGTTATTGTGGCCAAAGTGCTGGACCTAGCATTTGGTCTTG

TTGAAACTCATACAGTTGGCCTCAACCAATTAATCAAGCCTATCCAGATCTTTCTGTAGA

GCCTTCCTAC

>c15232_g1_i1 len=300 path=[33:0-214 32:215-299]

CAAGTGCTAACTGGATGCACATGGTGCATTATTCTTGTAGTGAAATGCTTGCAGTGGCAA

GACTGGCCATGATGGGCTGAAACAACTGATTTTTTTCCTTCACTATTTTTGATAAAACAG

AGCCAACTGGGAATCTCTGAAGCACATGCTATCCTCTGCTATTTCCTTCTGTTCCAGGAA

ATGGTTGAGTTGTGGGTGCTGATGAGGGGGAGGCAGAATTACATTCTCAAGCCAAGTTTC

CACTCTTTTCCAGCTCGTTATGCTGCCGTGAACCTTTTCCTAAAGAGGAAACCTGTCTCT

>c15234_g1_i1 len=583 path=[561:0-125 687:126-582]

GCATTACGTAAATCTTTTCAGTCCATTTTTGCCCAAACCTCTTAACTATAAATCTAAATT

TGTTTCCTCCAAATGAAAACTCTGTCTTAAATGGGGTGATATTTGTAGGTGGGGTTTGAT

CCCAGTGTTTGTCCTAAGCAAATAAAATTGTTAACATCAAATGGTTTTGTGGCCCAAACT

GCAAACAGAACTATCTGTGCTGTTAGGTTAAAACTAACATGGGTTTGAAGAGCTTAGGGG

GGAGGATCAGAGCAAATTATGTTCTAACAAAGGCTGTGCTGTGTTATGGAGGAAGGTTTT

AGCCCAAGATCACACTTCCTTTGCTTGTAACTGGACCTCTTCAAGAAGTTGTCGGGTATG

TTTTAGTTAAAAGAAGGGAAAATCTATGTAGTTAATGACTCTAAGATCATTTCATCTTAG

TTTGTTTCTCCTCCCCTCAGTAACAGTACAGGACTCTGCCATTTTCGCCTTTCAAAAGAG

GAGCACTTGGAATAAAACTTGGAATAAAAAAGTCTTTACGTGAAGCTTCATTTTTTGCTT

TGAAGCAGCATGTGGAGTGTATTTCATTTTGAAAATTCTTAAG

>c15238_g1_i1 len=220 path=[221:0-32 254:33-63 285:64-80 302:81-219]

AAACCCAAGAGAAACCCATCGAAGACTCAACAAAACCCCCCGAAACCCCCTAAAAACCCA

AAAAACCCATAAATAACCCCAAAAAGGACCCCAGAACTCACACGTTCTGGACCTGGAAGA

GACAAAGACCCCAAAACAAACCCAAAAAGAAGAAGAACCCAATGGGAACCCAACAGGAAC

TCATCAGAATCCCAACAGAAACCCACCGAAGACCCAACGG

>c15242_g1_i1 len=1258 path=[1236:0-1257]

AAAAAAAAATAAAAATAAAAAACAAAAGTATTAATGTGGACAAGGGTTTGAATAAGAAAA

TTCCCATTCAATTGTTTGAAAAAAAGTATAATGAAGGATAATATATATGAAATGTAAAAA

ATTAAATGTTGGCCAATGATCATTAATAAAATAAATTTCTGTAAATTCTGAAACTTGATA

TAACATAAAGTCAGACAATCTTTTCTCTCCTGATGTTCGTATTAAAATATTTGGTGGAGG

TAAGTCACTTGTTAGTAATTTTTTGTGATAATTGTATATATTTAATTGTTCTTCATTTAA

AAAAGAATATTTTCCACAAAAGCATTTTATTTCACAATTTGATGTTGATTCAATATGTTC

ATTTTTTATTATATTTTCTGAATGTTCGCAATTATTTTCATTGATATAATTTTTTAAAGA

ATAGGAAGAATAACAAATATTTTGTTCTTCTAATAATTTTTTATATGTGTCAAAATACAA

GTTAGGCATAAACTTGCATAAACTCATTTCATTTCTACTCGTATATGAGAAAAAGATATT

TAAAATTAAATTATCAAACTCTCTAGTTTTTTGTTCTATATCATTTATAATTTTTCTATA

GAAATCATTAATATAAGATAAGTTTCCTATTATTTTAATCCGTATTCTATTGTTTTTAAT

AAAATTATTAAAAAAATCTTCATTAGATAGACAATGTAAATTTAAATAAAATAAGAAATA

TATTTCTTGTGGGCTTCTATTATAATTGAATAGTGAAAGTGAAAACACTGATAAAATCTT

TATATTTAAACTTATGCATATTTCTATAATTTTAATTAATGCTTTAGATCCCATATAATG

ACCTAAAGAAGAATGAAAACCATTATCTTTCGCAAATCTTCTATTACCATCCATAATTAT

TGATATATGCTTAATATTAATTAATCTCCTTAATAATGAAGTTATTAATCTTTCTATTAA

ATTTAGAGCCATTTTTCAAGAGATTAATCCTTTTTTTTTTTTTCTTTGAAATTCAGAACT

TTCAATAATTAAAAAGAGTGGAGATACATAATATTTTTATTAAACGTAAGAATTTCCTCA

TTTATCTAAATTTAACAAGGTTTTTAAATGATAATAATTTTTAAAAGTATTTTAATTCTA

AGAATTAAATAAAGTGTCATAATAATAAACATAAATTAAAAAAATGTATCATTTCAATAG

GACATTATAATTTTTTTTAAATAAGATTTTTATTGTAAATTTACATTGTCTATCTAAT

>c15250_g1_i1 len=371 path=[1:0-165 167:166-312 314:313-329 1276:330-336 338:337-370]

AACACAGCTAATCCCCCCTCTCGTGCATACATACCCCTCAGGCCAATTTATGCTCAGTTT

CATACACCTGTGCATGGACTGCTATCCTGACAGCATGCTTGTCAGCTGAGATGCACGTTC

CAAAACTGTCTGGCTGTTCTCTTTCCATAAGAAGTGCTAAATATTTTTAGTTTAGGGTCT

GGTGTAATTTATAGCGGGATTTATATGATTTATCGGAGGGTTTTCAGAAATGTGCCTCTT

ACAGTCCTGAACAGGGAATACAGTAGGATTGATTACAGGAGGTTGACCCAGAGTGTTACC

ATCTGGAACAACATAAAAATAAGAAAATACTTGAAAACATTCTTCAATATCTAGTCATGC

TGATCAGAGTA

>c15250_g1_i2 len=315 path=[1:0-165 1130:166-256 314:257-273 1276:274-280 338:281-314]

AACACAGCTAATCCCCCCTCTCGTGCATACATACCCCTCAGGCCAATTTATGCTCAGTTT

CATACACCTGTGCATGGACTGCTATCCTGACAGCATGCTTGTCAGCTGAGATGCACGTTC

CAAAACTGTCTGGCTGTTCTCTTTCCATAAGAAGTGCTAAATATTTAGAAAGTCCTGGTA

ACAACTGAAACAATCTTTTTGACAGTGCAGTAGGAAGAATACAACAGTAAGGGTATCATT

AGCACTGCAAGACAACCTAAAAATAAGAAAATACTTGAAAACATTCTTCAATATCTAGTC

ATGCTGATCAGAGTA

>c15251_g1_i1 len=290 path=[174:0-173 567:174-289]

GTGTTTGAACTCCACTTCTTACAATTTAAGGATCTTCGCTGAAGATTGCTCCAGGTCCAC

CAGTACCAGCCCACTATAGAAGCTTACAACCACTTAGCAGCAGTGCTATTTTTTTTTCAC

AAAATAAGAGTATTTTCCAGATGCAAGTTCCAACCCTTTAACTGCTTTGAAAAAGTATGA

ACTCCAGATGGTCTAAGCTGGACCAATCCTTTCCCTTCTTTTTAGCTTGGTTTTGGATTT

GTCTTTGGTTTTTCTTCTTCTGTGGTACAGCTAATAATCTGTCATTCAGT

>c15252_g1_i1 len=465 path=[933:0-49 101:50-303 355:304-464]

TAGCATTTTAGTTTTGCCAGGCCGTTCATAGGATCATACTGCTTTGTGTATGGCTTACAT

TCAGTAACAATGCCATGTAGTAAAGTCAGAAGCAAATGGTAGGTTACGTGCTCTTTTCAG

CTTGATTTTTCAAATTAAGATCACTTCAAGTGGTGAATTCTGATGTGTGTCAGCATTAAA

AAGCAAGTAGAAGTATATGCATCCTATTAAAGAACCAGTTTGGGAATTAATTTTGCAAGT

TATTCTTTCTACTTTATTTACGCTTGGAAACTAAAGGATAGAAACAAGTATTTCATTTGG

TATTATTGACCTTTTCCAGTAATGGGCTTGAAATGATGGATTGAGTAATAAAACAGTAGT

GAGCTAGAAATGCATTTTTAAAAGCATTGTTTATGTAAACATGCATGTAATGCATGTTTA

ATTTTTGAAACTCTCCGATTTTAGAGAAACACCTTTCCATTCAAA

>c15252_g1_i2 len=305 path=[50:0-50 101:51-304]

ACGGCCTGGCAAAACTAAAATGCTAGCCAGAGGTGGACAGATTTTAAGTAGTGGCTTACA

TTCAGTAACAATGCCATGTAGTAAAGTCAGAAGCAAATGGTAGGTTACGTGCTCTTTTCA

GCTTGATTTTTCAAATTAAGATCACTTCAAGTGGTGAATTCTGATGTGTGTCAGCATTAA

AAAGCAAGTAGAAGTATATGCATCCTATTAAAGAACCAGTTTGGGAATTAATTTTGCAAG

TTATTCTTTCTACTTTATTTACGCTTGGAAACTAAAGGATAGAAACAAGTATTTCATTTG

GTATT

>c15253_g1_i1 len=1381 path=[1481:0-1261 3011:1262-1265 73:1266-1380]

TGCCGCTCGTGGCCTTCCATGGCCTGCAGCTCCTCCGGGGTCAGCCCCGCAAAGCCCGGG

GGGGGGACCGGCATCGGGGGGAGCCCAAAAATGGGCGGCCACGGCATTCCCAGCCACGGG

GGGGGCAGAGCGAAGCCCGGCGCTGCAGTGCCCTCCGACCCCGAAGCGGTGCCATCCCCG

GCCCTGGAGGCGGCTGGAGCTGCTGTGGGGCTCTCGGGGTTGGGGGGTCCCTGAGCGCCG

GGGATGGGGGGGAACGGAGCCACGGGCGGCCACAGCGGGAACATCCCGGGGGGGAACGGG

GGGAGCATCCCCTGTGGGAAGTTTTGGGGCTGAGCCCCCGGGGGGCTCTGAGGCGGCGCC

GGGTTCTGCTCTGCGGCTTCGGGGGGCGGCTGCGGGGGCAGAGAGGCCCGCAGGACGTCC

ATGCGGCACGTGGGGCACGTCTGCTGCCGCTGGAACCACGAGCGCAGGCAGCTGGTGTGG

AAGATGTGGTTGCAGGGCAGGCGTTTGGCCCCCGTCACCATTTCCTCGCGGCAGATGATG

CAGACGTTGTCCGTGGACTGCAGCTCCTCCGGGGTGGCGTCGGGGTAAAGCGTGTTCATG

TTGCGGATGGCGCGGCGCGACATGATGGCGTCCGTCACCGCCTTCTTGAACTGCCTCATG

GCCAGGTACATGGGGCGGATGGCGAAGAGGGGGAAGGTGTGCACCTTAATCATGATGGTC

ATGAAGGCCATGTAGAGGATGACTTTGATGAACCCGGTGAAGAGCTCCGTGTACAGCATG

TAGACGGCCTTGCTGTCCCAGGGGTTGTCGTTCTGCAGGTCGATGGAGTGCAGCACGTAC

TTGATGAAGATGGTGAGCACCATGGTCATGAGGATGGCGTACTCAAAGCCAAAGACCAGC

TGGACGGAGGCGCCGCGGGTGAGGATGCTGTGATAGGCGTGGCTGACAAAGAGGAAATCC

AGGATGGCCAGCAGGAACATGAGGGAGATGATGCGGAAATGGAAGAGCCAGGAGATGTTG

GGGCTCCTCTCCATAAAGTCCACGCGGTCGTCGGCCAACCAATGGAAGCATTTGAGGAAG

AGGAGGAGGGTGAAGAGGGCCACGAAACGGGGGCTGAAGTCGTCACGGAAGACGGTGAAG

GCCAAGCAGGTCTCGGTGACGGCGTACCAGGAGCGCTCCATCAGGTGCTCCATCTCAGCT

GCACGCAGCTGCCCAAAGAACACCTTGCCCATGAACTTCCCCAGCAGGAACACCAACACG

AAGGCCGGGCGGCCGTGCCGCGACCATGCTGTGCACGGCGGTGGTGATGCTGGGCAGTTT

GGCTCTGACCACGGCCGTGGTGGCCCACGCCTATTACCTGAAGCACCAGTTCTACCCCAC

C

>c15253_g1_i2 len=1341 path=[1481:0-1261 3011:1262-1265 2746:1266-1340]

TGCCGCTCGTGGCCTTCCATGGCCTGCAGCTCCTCCGGGGTCAGCCCCGCAAAGCCCGGG

GGGGGGACCGGCATCGGGGGGAGCCCAAAAATGGGCGGCCACGGCATTCCCAGCCACGGG

GGGGGCAGAGCGAAGCCCGGCGCTGCAGTGCCCTCCGACCCCGAAGCGGTGCCATCCCCG

GCCCTGGAGGCGGCTGGAGCTGCTGTGGGGCTCTCGGGGTTGGGGGGTCCCTGAGCGCCG

GGGATGGGGGGGAACGGAGCCACGGGCGGCCACAGCGGGAACATCCCGGGGGGGAACGGG

GGGAGCATCCCCTGTGGGAAGTTTTGGGGCTGAGCCCCCGGGGGGCTCTGAGGCGGCGCC

GGGTTCTGCTCTGCGGCTTCGGGGGGCGGCTGCGGGGGCAGAGAGGCCCGCAGGACGTCC

ATGCGGCACGTGGGGCACGTCTGCTGCCGCTGGAACCACGAGCGCAGGCAGCTGGTGTGG

AAGATGTGGTTGCAGGGCAGGCGTTTGGCCCCCGTCACCATTTCCTCGCGGCAGATGATG

CAGACGTTGTCCGTGGACTGCAGCTCCTCCGGGGTGGCGTCGGGGTAAAGCGTGTTCATG

TTGCGGATGGCGCGGCGCGACATGATGGCGTCCGTCACCGCCTTCTTGAACTGCCTCATG

GCCAGGTACATGGGGCGGATGGCGAAGAGGGGGAAGGTGTGCACCTTAATCATGATGGTC

ATGAAGGCCATGTAGAGGATGACTTTGATGAACCCGGTGAAGAGCTCCGTGTACAGCATG

TAGACGGCCTTGCTGTCCCAGGGGTTGTCGTTCTGCAGGTCGATGGAGTGCAGCACGTAC

TTGATGAAGATGGTGAGCACCATGGTCATGAGGATGGCGTACTCAAAGCCAAAGACCAGC

TGGACGGAGGCGCCGCGGGTGAGGATGCTGTGATAGGCGTGGCTGACAAAGAGGAAATCC

AGGATGGCCAGCAGGAACATGAGGGAGATGATGCGGAAATGGAAGAGCCAGGAGATGTTG

GGGCTCCTCTCCATAAAGTCCACGCGGTCGTCGGCCAACCAATGGAAGCATTTGAGGAAG

AGGAGGAGGGTGAAGAGGGCCACGAAACGGGGGCTGAAGTCGTCACGGAAGACGGTGAAG

GCCAAGCAGGTCTCGGTGACGGCGTACCAGGAGCGCTCCATCAGGTGCTCCATCTCAGCT

GCACGCAGCTGCCCAAAGAACACCTTGCCCATGAACTTCCCCAGCAGGAACACCAACACG

AAGGCCTGGATGTACAGAACGGCCATGCTGGGGCTGGATTTGGTGAGGTACACCACGGTG

GGGTAGAACTGGTGCTTCAGG

>c15256_g1_i1 len=1511 path=[1489:0-1510]

AAAAAAAAAAAAAAAAAAGATAATAAAAAAATTAAAAAGGTAAAATGGAAGTTAATATAA

ATGAACAAATTGATATTAATAAAAACAATTCAAGGGGTATTGGAAATAACAGAGGTAATA

TAAATTTAGTGAAAAATGGAGATTTATGTTATTCTCAAGATTTAAAAAATTATACTACTA

TTCAATTAAATAATGAAATAGAAATATCAAAAAATCCATTTCCATTACATGAAATAAATA

AAAAAAATGATATTCCATTATGTGCTCAAGAATTTTCTGATTTAACAGATCCACTACACA

TTAAAAAAATTTGTTTAGAAAAAGTACATGATTGGAATGATTTAACTGAAGATGATATAT

GTGTTAATCAAATATTATGTGGTTTAACAAATCAATTATTTGAAGTATCTTTGAAAGAAG

ATCTAGCGAAAAAAAGCAATTTAAAGAGAAAGCATGTTCTATTTCGAATATATGGAAAAG

ATGTACAAGAATTATATAATCCTATTTATGAATTAGAAGTATATAAAACTATGAGTAAAT

ATAAAATAGCACCATTACTTTTAAACACATTCAATGGTGGACGTATAGAGGAATGGTTAT

ATGGTGATCCTTTAAGTACTGATGATTTAAAAAATCCATCTATTTTAGTAGGTATTGCTA

ATGTATTAGGGAAATTTCATACTATAAGCCGTAAATATCCTTTACCAATTCATTGGGAAA

AAACTCCTTGTATTTATAAAAGGATGGAAAAGTGGAAAAATCAAGTTTTGAAATACAGTG

ATTTAGACAAATTGACATATGATATTAATAAATATATTAAGGAATCTGATAAATTTATTA

AATTTATAAAGGAATATACCCAATCAGATAATATTGCTAATCATATTGTTTTTTGTCATA

ATGACCTACAAGAAAATAACATTATTAACACAAATAAATGTTTACGATTAATTGATTTTG

AATATTCTGGTTATAACTATTTATCTAATGATATAGCAAATTTTTTTAATGAAACCACTA

TTGATTATTCTGTTAGCACATATCCATTTTTTACTATAGATAAAAAAAAATATATTCCTT

ATGAGTTAAGGAAGCTGTTTGTATCTACTTATTTATCTGCTTATTTAGGTAACTCTTACG

TAGCATCCGATGAAAAAGTAGTTAATTTATTTTTAGATGCAGTTGAAGTTCAAGCATTAG

GGGTTCATCTAATATGGGGATTCTGGTCCATCATAAGAGGATATCAAACAAAAAGCTATA

ATGAATTTGACTTTTTTTTATATGCAAAAGAAAGATTTAATATGTACGATGAACAAAAAG

AATACTTGATCTCAAATAATATAATTAAGGATTATTAATTAATATGATTCTAAAATAAAA

AATTTATTCAGAAATTTTTGTTCAAAAATATATATATATATATAAATTTACAACACACGA

AAATTAATATAAAAAATTATATGTATTTATTTAATTCTTTATGTTTATTAAATTATGATG

AACAAAAAAAA

>c15257_g1_i1 len=444 path=[2465:0-13 826:14-443]

GGTAACTTTGTGTTTGAGGAAAGTAAAATTGTACAAAAAAATTGTGATTTAAATCTAAAA

AATAAGGAAAAAAACATAACATTAAACAAATTGAATTATAATCATTTATTAAATAAAGAA

GAATATGAACAATCAAAACAAAATCAGGAATTAAAAATAGAAGAAAAAGAAAATGAAGAA

GAAAATATGAAAGATGATATTCAAAAAAGAGAAAAAAAAGAAGATAATGATGATAAAAGT

GAAAACATAGAAAATGAAGATGAAAAACAAGAACAAAGAGATATAGAAGAAAAAAAAAAA

GATAAAACCGTGTATGAAGAAGAATACAAAAATGAGATTAATAAACTTCTTCTAAAAAAA

ATTATTTTAAAAACACTAAAAAATGGGAAAAAGAAATTTAAATTAAGTAATAGTATTAAA

AAGAAAAAAAAATTAAAAAAAAAA

>c15257_g1_i2 len=1255 path=[1:0-824 826:825-1254]

AAAAAAAAAAAAAAAAAAAGAATTCTACAATTTAAATGATAATAAAAAAATAATAAAAAT

TCCTGTAAAAAAGGAAGAGAAAAAAAACAATTCAAAAAACATCTTAAAACAAAAGAAACA

AATTAAGGATCTACCTGATAAACAGAGAGAACGATATTCTTTAATTAATAAAGATAAATG

TAATTTATATATTTATAATAATTCAGATAATAATAAAAGTCAAGAAAATGAACAAAACAT

TGAAAATAACATTAAAAATAATTTAGAAAAAAATGAAGATCTTAATCAAACAGATAATAA

TTTAATGAAAAATAGTTCCATTTCTTGTTGTTCTGTTGAAAATAATCTTGATATGAAAAA

GGAAAAAAACAAATATACTTTTTATTCAGACTCCATTTACAACTTAGATAAAAAAGAAAA

GAAGAAATTTAGTAAGTCATTAATAAAATCTTATAATTATAAAAATAAACTCTCTGATAG

AATACTCACAAAGAATATATGTGGTGCTAATAATAAAAAAATTAATTACGTAAATAAAAA

TGATTTTAATTTCAGTTTTTCAAATTATATTTACAATAGAAAAAATTATATTTTTAATAA

AAATAAAATATTCAAACTAAGTAAAGATCTAAAAAAAGGAAAAATTAAAAAAGAAAAGAT

AAAAGATTCAGTAAAACAAAAGAGTGAATTAAATGGAAATAATAATAAAGAAAAATATAA

GAAAAATATTAGAAATAAAAATTTTAATAAAATTTTAGATAAAAAATATGAAAAATCATA

TCAGCTTAATAATAAAAATAAAGAATATAATGAAAAAAAATATAATGAGGAAAGTAAAAT

TGTACAAAAAAATTGTGATTTAAATCTAAAAAATAAGGAAAAAAACATAACATTAAACAA

ATTGAATTATAATCATTTATTAAATAAAGAAGAATATGAACAATCAAAACAAAATCAGGA

ATTAAAAATAGAAGAAAAAGAAAATGAAGAAGAAAATATGAAAGATGATATTCAAAAAAG

AGAAAAAAAAGAAGATAATGATGATAAAAGTGAAAACATAGAAAATGAAGATGAAAAACA

AGAACAAAGAGATATAGAAGAAAAAAAAAAAGATAAAACCGTGTATGAAGAAGAATACAA

AAATGAGATTAATAAACTTCTTCTAAAAAAAATTATTTTAAAAACACTAAAAAATGGGAA

AAAGAAATTTAAATTAAGTAATAGTATTAAAAAGAAAAAAAAATTAAAAAAAAAA

>c15263_g1_i1 len=3391 path=[1:0-3390]

ATATTTACATTAGCTTATTTTAAAATAACATTTTATTTTCTTTTTTTTTCTTTTTGTATT

ATTTATTATTATTTTATATAAGCAACTTCTAATATTCTTGAAAAATTTAACACAAAATGT

TTTAAACAGTTTTTGTAAAAAAAATAAAAATTAAAGATAAATATAATTGAATGTTTTATA

CATGATATTTAAAGAAAGCTTTGTCCATTATTTCTTAATTCATTTTATAATTATAAAAAC

CTTTTAGTTTTTTTGTTAAAATGGATTATAATTATATGTGCGATAAATTAATTAATAAAA

AAGAATTAATTTTTCATTTAGTAAAACAAAATGATGAAAAAATATATCACTTTTTAGAAG

AAGATAAAAATTTGATTAATTGTCAAGATGAAAATGGAAATAGTTTATTACATTGGGCAG

TTTTTTTAAATAATATATATTTAGTATGTTACCTATTGGAAAATAAAGCTAATGTAAATA

TTAAGTCAAACATACAACAAACTCCACTTTTTTGGGCGGTTTGCTCAAATAATATTTTTA

TGATTTATTTATTAAAGAAATATGGATGTAATTTAAATGAAGTAGATAACAAAGGATATA

ATTGCTTAATAATATGTGTTCAATATAATAGTATTTTATGTTTTTTTTATTTACTACATT

TGAATATTTCATTAACTGAAAAAGATTTTAACAATTGTTCTGTTTTTGATTGGTCTGCTT

ATAATAATAACATGTTTTTTCTTAGACTTTTTTCAATTTTTCTAAACAATTTCTATTCAT

TGCATCCAACTAATCCATCTTCCGTTTTACATAAAGCTATAATTGGGAATGCATATGAAG

CAGTTACATATTTAATTTTAAATAATCATCAAAATATATATGATATAGCAACTGATAATA

AAGAAACAATACTTGAGTTCATTGAAGAAAATAAAAGTAATATAAATTCAAAAATATATG

AGTTTCTGAAATGTAAAAAAACTCAAAAAATGTGTGAAAAAAGAAAAAGAATAACATATA

ATTTATACGAACCTAATGGTGATATAGGACCTTATACAATTAAAAAAAAGAAAAATTTAT

CTTTTTTATTAAACATTTATTCTAAATATCAAGAAAATAAGGCTCTTTTAATTTATCCAT

TCCTCCTTGTTTTAACTTACTTATACTTGAATCTAGTTTATTTTTGTTATATTAGAGTAA

AATTAGGATACAAGAACATAATGCAAGATATATTTCATCCTTTTCTATTTATTTTTTATT

ATTTTGCCATCTCAAGTAATCCTGGTTATTTAGAAAATTCAAAGGAGATATATAAGAGCA

AAAAAGAAGAAATGAAGGATGCAGAAGATAACGATAAAGATAGTTATGATAATGAAAAGG

AAATTAATGAAAATAATAGTTTATCAAAACAAAATTTCGTTTTTGAATATATTATTAAAA

ATATTAAGAAGGAAATAAAGAGGTATGAAAAAGAAAATAAATTGTGTGAATTTTCAAAAA

AAATTAAAAATGAAAACATTTTTGAATTGAAAGAAAGACTTAACATTGTATGGGATGTTA

AGCCATTTGAAATATCAGCATTTGATATTAACATGTTATGTCCAACATGTTTTTTATTTA

AAAATTTAAGAACCAAACATTGCCGTTTTTGTGATAAATGTATAGAGATATTTGATCATC

ACTGTGTATTTACTTTAAATTGTATGGCTATTGAAAACGCAAGAATATTTCTTATGTGGA

TTTTATCTAATATTCTTTTTTCATTATATATTTTATATATATATATATTTATATTAGTAC

CGTTTGAATAAAATGAAAACTGATCAAGATAATACAATGGAAAGTTCTATTGTGAAAGAA

AAAAAGGATAAATTTTTTAACACCTCATTTAAATATGTATTATCTGCATGCATTGCATCT

TTTATATTTGGATATCAAGTTAGTGTTTTGAATACTATAAAAAATTTTATAGTTGTAGAA

TTTGAATGGTGTAAAGGTGCAGATAGATTAAATTGTGATACAAACACAATACAGAGTTCC

TTTTTATTAGCATCAGTTTTTATTGGTGCAGTTATTGGTAGTGGGTTTTCCGGTTACTTA

GTACAGTTTGGTAGAAGATTTTCTTTATTAGTAATATATAATTTTTTCGTTATTGTAAGT

ATATTAACATCAATAACTCATCATTTCCATACAATCTTATTTGCACGTCTTCTAAGTGGA

TTTGGTGTTGGTTTAATTACCGTTAGTGTTCCTATGTATATATCAGAAATGACACATAAA

GATAAAAAAGGTGCATATGGTGTTTTACATCAATTATTTATTACATTTGGTATTTTTATC

GCTGTATTATTGGGATTAGCTTTAGGTAGTGGACCTAATGCAAAGTCAAAGGATCCCTTA

TCTGATTTTGAAAAAATATGGTGGAGGTTAATGTTTCTTTTTCCTTCTTTTATGTCATTA

TTTGGTATTTTCTTATTAGTTGTTTTTTTTAAGGAAGAAACTCCTTACTACTTATTTGAA

CATGGAAAAGTAGAAGAATCAAAAAAAATTTTAAAAAAAATATATGGAACTGAAGATGTT

GATGAGCCTTTAAAAGCAATTCAAGATGCTATTGAACAAAATGAAGCTGCAAAAAGAAAT

TCTTTATCTTTATTAGCCGCATTAAAAATACCATCTTATAGATATGTTATATTATTAGGT

TGTATTTTATCAGGATTTCAACAATTAACAGGTATTAATGTTTTAGTTGCCAACTCTAAT

GAATTATACAAAGAATTTTTAAATGATAATTTAATAACTATTTTAAGTGTTATCATGACA

GCAGTTAATTTCTTAATGACATTCCCAGCCATTTATATTGTTGAAAAGCTAGGAAGAAAA

ACTTTATTATTATGGGGATGTGTTGGTGTTATTATTGCATATCTTCCAACAGCAATAGCA

AACGAAATTGATAAAAACGCAAAATTTGTAAAAGTACTTTCTATAGTTGCAACATTTATT

ATGATTATTTCTTTTGCAGTCTCATATGGACCAGTATTATGGATTTATTTACATGAAATG

TTTCCATCCGAAATTAAAGATAGTGCAGCAAGTTTGGCTTCTTTAATAAACTGGTTGTGT

GCAATTATTGTTGTATTTCCATCAGATGTTATTATAAAAAAATCTCCTTCAGTTCTTTTT

ATTATTTTTGCAATTATGTCTATTATATCATTCTTGTTCATATTATTTTTTGTTAAAGAA

ACCAAAGGAAATGAAATAGGAACAAGTCCATATATTTCAATGGAAGAAAGACAAAAATAT

ATGGTTAAAGCCGCTGTTTAAATATATTTTTTTTATTAAAATTTATATAAAATTTATTAA

TGGAAATATATATATATAAAAAAAAAAAAAA

>c15266_g1_i1 len=293 path=[375:0-178 797:179-185 57:186-292]

ATGTTATGACTTCTGCTTTGTCCCAGTGGAGATGTTCTTAGATTTAGGAGTTGGACTCAA

CAATCCTTATGGACCCCTTCCAATTTGGGATATTCTATGATTTTACATAAAACATGTTAT

ATGCGTCTGATACTATGTATTATGTTTGTGTGTCCGAGAAGAAGCATAAAACGTGCTGGA

TTTAGCTGGAATGAACACATAGCACATCCAAATTGTCATCTGTTTCTTATGGACCTGCAT

GGAGCTGTGCTGGAGTTACAGAGGACAGACGACACGTCTACTTGCTTCTGGTG

>c15266_g1_i2 len=259 path=[375:0-178 797:179-185 561:186-258]

ATGTTATGACTTCTGCTTTGTCCCAGTGGAGATGTTCTTAGATTTAGGAGTTGGACTCAA

CAATCCTTATGGACCCCTTCCAATTTGGGATATTCTATGATTTTACATAAAACATGTTAT

ATGCGTCTGATACTATGTATTATGTTTGTGTGTCCGAGAAGAAGCATAAAACGTGCTGGA

TTTAGCAGGAAAACCAAAAGAGACTCAGCAGTGTGAAGACTGCCCTCCAGGTCACACCAG

AAGCAAGTAGACGTGTCGT

>c15271_g1_i1 len=294 path=[1:0-293]

ATTTCAAAATACTTTTCAATAGAGAAGTCTTGTTCCTGTGACAGAGATGTGAAAAATTGA

GATTAAAAATGATTCTTGATTAAATTCTCTTAAATCAATTCTCTTGATTAAAATCAAGAG

AAAAGCCTCTGCTTTCTGCAGTAGTGCAGGAATACAGGCATGACACATCTTTCATGTACA

ATCTTATACAGTATAGATTTCCGTACATTTCTAATTCATGTGAAGCACCTTTGTGGCTGA

TTCAATTTATGTCCTTGTTAACTTGTGCTTTCTCGCTTCCTTTTTTCATCTCTG

>c15272_g1_i1 len=219 path=[197:0-144 342:145-157 355:158-218]

AGGAGGCTGGTCTTCTGCCATCCCTTAGCCACATGCTCCTGCCACTTCCCTCTTGTTAGA

TCTAACAATTAAGAGAACGTGGAGACATTTATTTTGGCTGATGTAACAAGAAACTAACCG

TACAAAGAAAAGGAACAGTTTTCCACTGATGACGTGAATCATCAGCTGATTCTGTGATCA

GTCACTAAATTCAATGTAAGAGAGAGAGCAAGTCTGCAG

>c15280_g1_i1 len=224 path=[1:0-205 630:206-223]

CTACCTTAACCCAAAGACCTGAAACCCCACAACCAATCAAAAGCCAGCACCACCAGCCCA

CCACTTTGGGCTAGACAAGGCCAATTACAGGGATTTGGATGTTTGTGGAATAATGTTCTT

ATGATAGACTTTGCAGAGATGTGGTTTAATTAAGAGACTCATATGGAGAAATAATGCAAT

CACTGTTTTTGTGTCTTATAATGAGAAAATGAATTATGACCTAT

>c15281_g1_i1 len=225 path=[399:0-224]

GTGTTAAGAGCCAGGCAGAAGAAGAAAAAAATACACTTACTGAATGTCTTTCTGAAGACG

TCACAGAGCTTAATGCTAAGCTGCGTGGATGATATTCTTAGCTACATGCAACATTTGCCT

TTGGTGTGCAGACTCCATGACTTTGATTCTCTGTCTCTGAAGCAGGGCTGTCATTGCCGA

GAGGGGGTGGTTTTGGTAACAGTGCAGTGCTGGGTTTTGTAGGTG

>c15282_g1_i1 len=781 path=[1732:0-88 153:89-780]

GAGTTCTTGCACAGCTCTGCCCTGAGATGTCCGTGCTCCGTCCCGTCTCCTTGCCAAGAC

ACCAACGCGTTCCCCTCGCTCTGTTCCAGGGTAACCATCCCTCACCTCCCCGCTCCTCGG

ATGGGGTGAAGAGGAGCTGCCCATCTTCCCATCATCTTCCCAAGGAGATTTCGAGCACGG

CAATGGAGAAAGCCTTGTAGACGCCAAGGAGCAGCTAAAATACAGAATCTCCACTGGCCG

CAGGCAGCCAGCAGAGAAAAACGTTTCCACGCCTCTCCTGTGGTTTGTGCAGGAGCAAAG

CGCCGTGCACGACGTGTACGTGGAGATGCAGCCATGCGTCGTGGGGCTGAAGGAAGTATT

GATGGCTGGGAATCGATATCTCCAGCGGGAACCTCCTGACGGGCTGGTAATCAAGTCTGA

GAAAGCAACGCGAGTAGGACAAGAGGAGGAAGTGCCAGCTGGGCTGTGAGGGAGTGACAG

AGCGGTGATGAGAGCTTCAGCATTGCCACCAGTGCAGGAGCACCTCGTCTCCTTGGGCAG

AGCTGCTTCCTCCTCACGGCACCGCTGCAGCCCACGGCCCTGCGCCCACCATCGCATGCT

CTGAGCTCAGAGCTTGCATGTGGCACCCCCAGCTCTGCACCTCGCACCCTGCGGACCCCC

ATCCTCACCGTGGCACAGCCCAGCAGTCCCTCTGCCCGGCACCCTGCACCTCATGCACCT

TGCACCCATGCACCTTTCATCCCTTGTGCCCTACATCCTTCATTCCATAAACACTGCACC

T

>c15282_g1_i2 len=844 path=[1:0-151 153:152-843]

CTTCGGCTTCTGGGAAGCAGCTGCCCTTCCCCTGCACGAAGATCTTGTCATTAGTTTAAT

TGCAGGGCGAGCAGGGCTGATAGGAGCATATCAATGCCTCGGCAGCGCGCCAGCGCCGGC

CCTCAGTGCTGTTACCAGTCCCTGTGCCTCCAGGTAACCATCCCTCACCTCCCCGCTCCT

CGGATGGGGTGAAGAGGAGCTGCCCATCTTCCCATCATCTTCCCAAGGAGATTTCGAGCA

CGGCAATGGAGAAAGCCTTGTAGACGCCAAGGAGCAGCTAAAATACAGAATCTCCACTGG

CCGCAGGCAGCCAGCAGAGAAAAACGTTTCCACGCCTCTCCTGTGGTTTGTGCAGGAGCA

AAGCGCCGTGCACGACGTGTACGTGGAGATGCAGCCATGCGTCGTGGGGCTGAAGGAAGT

ATTGATGGCTGGGAATCGATATCTCCAGCGGGAACCTCCTGACGGGCTGGTAATCAAGTC

TGAGAAAGCAACGCGAGTAGGACAAGAGGAGGAAGTGCCAGCTGGGCTGTGAGGGAGTGA

CAGAGCGGTGATGAGAGCTTCAGCATTGCCACCAGTGCAGGAGCACCTCGTCTCCTTGGG

CAGAGCTGCTTCCTCCTCACGGCACCGCTGCAGCCCACGGCCCTGCGCCCACCATCGCAT

GCTCTGAGCTCAGAGCTTGCATGTGGCACCCCCAGCTCTGCACCTCGCACCCTGCGGACC

CCCATCCTCACCGTGGCACAGCCCAGCAGTCCCTCTGCCCGGCACCCTGCACCTCATGCA

CCTTGCACCCATGCACCTTTCATCCCTTGTGCCCTACATCCTTCATTCCATAAACACTGC

ACCT

>c15284_g1_i1 len=228 path=[206:0-16 223:17-227]

AGGAAATGGATCAAATGAAAAAAAAATTCAGTTGTTGCTTCCTTTAATAGTGTTGATTAA

TATGAATTAAAAATACCGCATACAGTGATGTGCCAGCAGTTCTGTCCTCTCATTCTCCAT

ATGTGGTGTTAACATAAGCGAAGGTTAGGATTAACAGTAGGACTGTTGTTGGGATAAGCC

TTATTCAGCGAGATCGCTTCAAAGTTGCATAAAAAACCCTCTCCGAAG

>c15292_g1_i1 len=441 path=[1:0-332 334:333-440]

TTTTTTGTCAGTGTGCGGCTTTATTGCGGCGCCGCGCGGGGGCCGCGCACGGTGAGGGCG

GAGGGTTTGTTGGTGAGGGGGTGCCACTGGCCCTGCACGCTGGGGTTGGCGGTGTGGAAG

GGTTTGCGGATACGCACGATGTCCAGGCCGGACTGGGAGGCCAGCTTGGCGGCCAGCTGC

ACGATCTCCTCGCTCGTCTTGCTGGCGATCAGCTCCTCCCGCACCGTGCCGTTCAGGTAC

TCGGCCTGCAGCACGGGCGCGGGGCACTCGCACGGCCGCACGTACACCACCACGTCCGGG

TGCCGCCGGGCGAAGTCCACCGCCTCCTCCTCCACGAACTGCCTGGCTCCGCGGGCGTCG

GGCGCGACGGGGCTGAACACCACCTGCAGCCGCTGCAGCTGCCGCACGTAGCGGCCCACG

CCGTTGTGCAGCACGGCCGCC

>c15292_g1_i2 len=441 path=[1:0-332 549:333-440]

TTTTTTGTCAGTGTGCGGCTTTATTGCGGCGCCGCGCGGGGGCCGCGCACGGTGAGGGCG

GAGGGTTTGTTGGTGAGGGGGTGCCACTGGCCCTGCACGCTGGGGTTGGCGGTGTGGAAG

GGTTTGCGGATACGCACGATGTCCAGGCCGGACTGGGAGGCCAGCTTGGCGGCCAGCTGC

ACGATCTCCTCGCTCGTCTTGCTGGCGATCAGCTCCTCCCGCACCGTGCCGTTCAGGTAC

TCGGCCTGCAGCACGGGCGCGGGGCACTCGCACGGCCGCACGTACACCACCACGTCCGGG

TGCCGCCGGGCGAAGTCCACCGCCTCCTCCTCCCTTCTCCCTCCCCCCTCCCCGCGGAGC

GCCGTTCCGCTTCCGGGCGGCGAGATGACGGGCCGCGGGACGGCCAGCCGCTTCTTGGCG

GCCGTGCTGCACAACGGCGTG

>c15293_g1_i1 len=327 path=[267:0-284 15:285-326]

CCTTCCTACTAAGCTTCTATGAATGTGGCTTTTGTTTTCTCTAGGCCCAGCATTAAGAAG

GTAAGTTGGTTTTTCTGGCTGAAATTGCAGAAGTTGTATTCTGTTCATGGATTTTTGTTC

TATTTTGATGCATGACACAGAGAACCAAACTTAAAATGGAAGATTATATCCAAGATATTT

GTATGAACCTCACTACAAGGATAGCCAAATACCCAATAAACTGGAATTTGTATTTCCATT

AAAGCAAATGAGTGAAAGTAGTTCCTAAAAATATCATCACATGTAAGCACAGAAGTTGAT

ACAGTTTTGTTTTAGTGTATTCTTTTA

>c15294_g1_i1 len=293 path=[271:0-292]

GAACACTGAGACTGCAGAGCAGATACCACGCATGTGCTTCAGCTAACAAACGAATCGAAC

GACACTACAGGCTTGACTCAGATTTCACTGCAGTTATGATCTATATTACAGAAATGTGAA

ATCAACAGATTCTTCCCCCCCCAAACATCTTCTGTTTTGGACTTTATTTTCTCATACTCA

AGGTTTTCTTGGGAATCTGCACAGCTGTGAGGAATTACAGTTGAATTTTAATCTACGTTA

AATGAGTAGGCTAGCAGAAAGTGAATTTACATCTTCTGTGCTTTTAAAAGAGA

>c15297_g1_i1 len=302 path=[1:0-301]

ATAATTACACTCCTGGGAGATTACAGACAAGGCTGTACCCCAGGGGTGTGATTATCCCAT

GCTAACCACATCCACTGAAATTGCCTTATTGCAGAAATAATCTCTATTCACCGGTGAGAT

ATTATTCAAAAGGCGCTTTTTTAATGGTAAAACCCCAACTTTCCAAGTTCAGACAAGATG

AAAGTATCTCTTGTGGTTCTGACTTTACGTGCCGCTTTGTATATATGTGTGTATATGCAC

ATACAGAGTACGTGATACAGACAGCAGAAAAGCATGTCACCAGCATCTCAGAGTTTGCCT

GT

>c15298_g1_i1 len=257 path=[449:0-256]

GCAAGGAGCACACACCCAAGGCAGTCAGAAGTTCTGGAATGGCATTCTTGTTGCCCAACA

CCTGGTTATGCAGATAGAGGAGAAAACAGAGTGACACCTCAGCCCGTAATGCAGATAAAG

GTGTGAGAATCCACTTGCTGGCTAGCACATGAAAGCATGATGACCTCGCCCCGTGTCCAG

GATTGCTCAAGCCAAAAGAGATGGCAGTGTTGATACACACATCTTCGTTTATAGTCAGAG

GGTAACACTCTTTCCAG

>c15310_g1_i1 len=237 path=[179:0-236]

GTGTTTGATGATGGAGTGCTCTGATTAAAGTTCGTGGTACCACCACCGGGAGGCAATCTT

GCACAGTGGCTCTACTGGGTTTGTCCGAAGCCGTAGATCATATAGCTGAATCGTGGGACT

TAACACTATGAGCGACATGCACCAGCTGTTGTTTAATTAAATTTGCAAGTACTGAAGAAT

AAGCTATTTCCTGGAAGGTCTGAAAGTGGTTGGATTCAAAGGGTCAAAGGTTCTTTT

>c15314_g1_i1 len=204 path=[182:0-149 332:150-203]

AGCAAAGGAGTAGAGGAAAGAGTACTGGACTTTGTCTTGTGATTAGAAGAGCAGATGGTA

AACTGTTACCAGTACGTCTGATCTGTTTTAACATGACGTATTTTTAGGCTCTAATAATAC

TTTTCACTTAACACTGTACAGCTTCTTACTTCAGTCTTGCATGCCTTATTTGAGTGCCTA

TGATATAGTTTACAAGCATTAATT

>c15316_g1_i1 len=410 path=[462:0-235 955:236-245 707:246-324 786:325-409]

TTTTTTTTTTTTTTTTTTAAATCACAGAATATTGACTTGTTTTATTAGTCACTTCATAAT

TTGTTTCAACCAAAAGACAATACACACAGCAGAAGTCTAATGCATTCCATGTAAGTGTTT

ACATCAGTTATGTTCCTCACTCACCTAAGACTTAGCAGTTAAGTTTTTCACATCTCAAAA

ATAGATTTGCAGAAGTTAAATGCCACCAGGCTCCCTGCTCAACAGGCAGGTTTTTATTTT

TAATCTAGTCTGGTGGTGCACTTTGTGTTTATTTTCTGCTTGGGGTCTGTGATGGAAGGA

CATGTTGCACTGCTTTCTTCAGAACTTTTTACTCTGTTCCTGTTTGTTCCTTTTCTTTTC

CTATGTTGTAATACTCATTTTACATAGAGCACCATCCCACCTCTTTGACA

>c15316_g1_i2 len=321 path=[462:0-235 786:236-320]

TTTTTTTTTTTTTTTTTTAAATCACAGAATATTGACTTGTTTTATTAGTCACTTCATAAT

TTGTTTCAACCAAAAGACAATACACACAGCAGAAGTCTAATGCATTCCATGTAAGTGTTT

ACATCAGTTATGTTCCTCACTCACCTAAGACTTAGCAGTTAAGTTTTTCACATCTCAAAA

ATAGATTTGCAGAAGTTAAATGCCACCAGGCTCCCTGCTCAACAGGCAGGTTTTTATTTT

TACTCTGTTCCTGTTTGTTCCTTTTCTTTTCCTATGTTGTAATACTCATTTTACATAGAG

CACCATCCCACCTCTTTGACA

>c15318_g1_i1 len=222 path=[199:0-101 3:102-221]

AGCCTCCCGTATCGCCCCAGTCTGCCTTCCCCTCCTGTCAGTGTCCCAGCGTGGCTCTTC

TCTGCATATCAGTGCACGTTTGCCCGGTCCTCAGGAAGGCAAGGGCACCGAGGTTCATAT

TTGCCCCTTCCCAAGCTCTCATTTTGCCCCGTTTGCTGCTGGTTTTCTTCCACCTCCGAC

TTAAGGCTGCAGCAGAACTGATGGTTGCAAAAGAATTTGGTT

>c15320_g1_i1 len=286 path=[1:0-285]

CTCTGGAGAAAGCTGCTTGGAGCAGGCTCGTGACTAAACCAATTCTGGACAAAAAGGAGT

TTGAGCCAGCCTTCATGTAAAAGTTCCTATATATCTCATATTGCACCCCTTGCAAGGTAT

TGCCGTGTAAAATTCATTTCAAAAGCATTTTCTTCCATTGTATTGAGAAATATGCAGATA

TTTAACCGAGGTGGACTGGAGATTCATCCAAGGAAAGGTGCCAAGCATTGGACTGGATGA

AACAGGACTTAGTTTGACGTGTTCAGTCCAACGTGGCAGAAGTTGG

>c15324_g1_i1 len=361 path=[1:0-202 713:203-360]

GTCACACAGTATATATATCACTGGAAGAATGCAGAGGATACAGCCTGAGTGTGCATTCAG

AGAACAAACTGCCTTAAATCCCCAGTACATTTTACCTTACAATGGACATCACAGTAGGAA

GGTGTATGGACTTCCAGAGCTTTATTTTTGTCCCTCTTCATGTACTTTGGTGTCTATAAA

TCTATCAACTCCAGGCGTGTTTCATCTGCACAATTTTTACAAGACACAGAGATGAGGAAC

CACTGAGAGAAAAGTGAGTGGGAGCTGCCTTGACATTCACATTAAATTATGAGACGTGTT

TGCAGATAATGTTGGGTTTTTGCTGTTAATAAAGGCTAAAACACTAAAATTTGTGAGAAG

C

>c15330_g1_i1 len=265 path=[247:0-59 516:60-264]

ACTACTGGCCTTGAGAATTTGACCACGAGATACTAAGTTGGGAAAAGGTTGGAAAAGAAA

GGTGACGTAGAGCTAGCACTTGGCTGATACCATTAGGGTGCTGTCACATGCACAACTGCT

CTCAGACATCGAGCAGTCCAGCTGCTTGATTTCTGGATTTAGAGGCTTGTAGTGCTGCAG

CTCTGAGTGTCACTTCAGACTTCCCAGACCTATAGGCTCTATTGTTTTCCCTATGAAATG

ATTTCTGATCGTTAACCCACTGCTA

>c15330_g2_i1 len=269 path=[247:0-59 307:60-268]

ACTACTGGCCTTGAGAATTTGACCACGAGATACTAAGTTGGGAAAAGGTTGGAAAAGAAA

AAGGAAACCAGATGTCTTCAAGTGGTGTACTGGAAGGGACAGCATGGTGTTAGCACCTTC

CTGGGACTGTGTGAAAGTGTACACTGAAGTAGTACTCCTTTATGAGTTGATTTCAAATGC

CTTCCCTGTTGCCTGGAATGAGTCAGGAGAGCAGCCATATGCTTGTGAGACCAACATATC

ATGGGATGCACAAAAAGCACACAAGAAAG

>c15331_g1_i1 len=225 path=[1:0-81 83:82-82 84:83-224]

AAATTTTATTAAAATTTTTATTTATGCAAATATATATATAATTTATGAATTATGTATAAT

AGCTCAATTATGCTGGAATATCTTTTTTTTTTCTTGCACATATTTCAATTATAGGTATTC

TAAATTTATATATTTAAAAAAAAAAGGGTTAATACATATAAGTAATTTTTTAAAACAAAC

AGAAAAAAATTTATCATATAAAATAAAAAAATTATGATATAAAAT

>c15332_g1_i1 len=257 path=[183:0-256]

GCTATGGCACTGAGGAGGTGGTGCTGCAGGGGCTGGGAGCACAGTCCCTGGGGTTCTGTG

CCCCGAGGTGAGGCTGTAAGCTGGGGGAGCAGTGCAGCAGGGCTGGGTGCTGCCCCGTGG

TGGCTTTGGCCCTGCCTGCCCCTCTGGGCTGCTGCAAGCTGCAGGTGCTGCTGATTGTGG

TTGATTGCAGCAGACTGGTGTAACTGGGAAGACGTGTGTTTGGAGGATGAGGACTCTGGG

TCAGTATGCCAGCAGGG

>c15333_g1_i1 len=513 path=[1:0-485 540:486-512]

GTGTTTGATTTTTTTTTTTTTGATTTTAACAGAGCTTTTATTTTGGAGAGTGAAAAATGC

TTTGTCTTTCATGTCAGGTACAGGGAGAGCAAAGAAGAGAAGAAGATGTCAAGTGAATCT

GTCTGTAAAGGATGAACAGTTTGCAGGACCTGCTCCTGGACCGATGCTTCTTTCTATTCA

GCGCCTTCCTCCTTTGCCACCCATTGAGATAGACTGGATAGTGGAAGTTGTGCCACCCTT

GGGAACTCCGCCTCCGCAAGAGGTGGCCTCATTGGACATCATCTGGGAGGCCCTGGAGCC

ACCGGAGATCCCACTGCTGGTAAAGCCTGAGGAGTGTGTTGAGCCTTTGGCACCCATTTC

TTGTAGAGTAGCAGTAGTCCCTCCAGAAGGAACGCCTCCCCCAAAGGATTTTGCCTCACC

AGACATGATCGATGAAGCAAGAGAACCTCTTGCAATTCCAGAGGAAGTGAAACCGGCTTT

GTGGACCTTCAGCAGGCAAAATGTCTGACCAGA

>c15333_g1_i2 len=513 path=[1:0-485 487:486-512]

GTGTTTGATTTTTTTTTTTTTGATTTTAACAGAGCTTTTATTTTGGAGAGTGAAAAATGC

TTTGTCTTTCATGTCAGGTACAGGGAGAGCAAAGAAGAGAAGAAGATGTCAAGTGAATCT

GTCTGTAAAGGATGAACAGTTTGCAGGACCTGCTCCTGGACCGATGCTTCTTTCTATTCA

GCGCCTTCCTCCTTTGCCACCCATTGAGATAGACTGGATAGTGGAAGTTGTGCCACCCTT

GGGAACTCCGCCTCCGCAAGAGGTGGCCTCATTGGACATCATCTGGGAGGCCCTGGAGCC

ACCGGAGATCCCACTGCTGGTAAAGCCTGAGGAGTGTGTTGAGCCTTTGGCACCCATTTC

TTGTAGAGTAGCAGTAGTCCCTCCAGAAGGAACGCCTCCCCCAAAGGATTTTGCCTCACC

AGACATGATCGATGAAGCAAGAGAACCTCTTGCAATTCCAGAGGAAGTGAAACCGGCTTT

GTGGACGTTCTGGTCAGACATTTTGCCTGCTGA

>c15335_g1_i1 len=249 path=[227:0-248]

TGCAAAGCTCAGCTGGGGAAACAAAAGAAATCGCAGCAGCAGTAGTGACTGTACCCCACT

ACGCAAAGGAGGGACTTTCACAGAGGAAAGTTTATCAAGTAATGGCAATTCTGACTCATT

TCCAAAGGAGGAAAGGTTTTAACATCCATGAAATTCAGCTGTCTCTTCCTAGACCACATA

GGTGTGTATCATACCAATAAAAATGTTTCTACGAGTGTCTGTTAGTAGACACCTAGGGAC

AGGAGCAGG

>c15336_g1_i1 len=311 path=[1193:0-13 905:14-310]

AAGGAAGAAAAAATAGAAAGTTAACAACCTTTTATTGTTTTACTTATAAGAAAACTACCA

ATAATCCTTAAAAAAAAACCTCTTATATGGGAAGTTTTTTTTTAATAAAGAACAAAATTT

TTAACAGGAGTATGACAACAGCTACATCTCATTGAGACCCGTGTCAGGCAAAAGACAACA

CTCCAGTGTCTTGTAAGACTTCATGGAGATGGCTCTATTCCCCTTCCTGCCCTATATTAG

CCTAGTTATCACAGGAGGCCTCAAATTAGCTCAGGACTCCAATCTTTATGAAGCATTTTG

ACTGTTTAAGG

>c15336_g1_i2 len=612 path=[590:0-314 905:315-611]

AGTAAATCCTTACGTTTTCTTTGAGAGACAGATGGCCAGGAAGGTCCTCAGTCAGCCCCC

CTTCTTGCTCTCCCCATTCATTGGTCTTGAGTGATTACTGGTTACTTGAGTGATGGTGGT

AGGCAACTCCTCAATATATACCAAAAGGGTCAGATACTTGTCTCTGAAGGAAAACTTCAA

AATAAAACGTATCACTGCATTCATGGTCGGGGCTGAGAGCTGTTGAAATAATTTCTCCAA

AATAATACCATCCTACAGTCAGCACCCTTTCTTCAGTTTTATAATATAGAATGCAACCAA

CCAGAGGTTACCTGCAGAAAGTTAACAACCTTTTATTGTTTTACTTATAAGAAAACTACC

AATAATCCTTAAAAAAAAACCTCTTATATGGGAAGTTTTTTTTTAATAAAGAACAAAATT

TTTAACAGGAGTATGACAACAGCTACATCTCATTGAGACCCGTGTCAGGCAAAAGACAAC

ACTCCAGTGTCTTGTAAGACTTCATGGAGATGGCTCTATTCCCCTTCCTGCCCTATATTA

GCCTAGTTATCACAGGAGGCCTCAAATTAGCTCAGGACTCCAATCTTTATGAAGCATTTT

GACTGTTTAAGG

>c15338_g1_i1 len=511 path=[489:0-510]

GATACCACACTTCCAGCATCAGGCGTCTGTAATTCAGCTTTGTTCTAAGATCCAAAATTA

CAAGCTTTCACCATCTGCTTTAGAAGTACCTGCATTGTATATACTATCAGAGACCTCTAG

TCTTCTAATGGAGTGATTTCCTTCTCTCCAATATTCTTCAGCTTCTCCTTTTCTCTACTG

AGTTCAGTGTCTGCCACCTCGATCATCTTCTCTTGCTGTTTCTTCCGACGATGTCGACAG

ATAATCACAAGTGTCACCACAATGATCATCAGACCACATGCAGACCCAATGGCTATGGCA

AGGAAGTGGATTTCTGAGAAATTCACTTTCTGCACAACTCTGAGTCGCACTTCTCCAATG

GTGCCATCGATGTCTCGTGGGTTCTTCACTTGGCAGGTGAATGTTCCATTGTCAGTGGGC

TGTAAATCCCAGATAACGATGGAAACATCATGACGCTCAATATGCCCATCCCAAGTGATT

CGCCCTTTAAATCGTCCAGCAGACGGCGTGT

>c15338_g2_i1 len=254 path=[977:0-253]

GATGCTGGAAGTGTGGTATCCCTGCTCGACCTGAAATTCTTGCAAGAAAAGAGAGTGACC

TTAACATAACTTGTAAAGTGTTATTTCCTTCACTGAAGAACAACAGGAGGACCTTGCCCC

ACGAACAGGGAGCCGTCCTTAATCTCCACAGCTGCCAAACGTGAACAAATTGGTTGTGAG

GATGCAATGTCAGCCGATTTGAACCAAACGCCGAGATGAGTGACTGGATAGATATTTGCT

GTGTCTGAGCAGGC

>c15348_g1_i1 len=255 path=[268:0-51 18:52-254]

AAAAAAAAAAAAGAAAGAAATAATGAAAAAGAAAAAATCATTCAAAATAATAATGAAAAA

AGTATAATTAATAACTTAAAAAAGGAAAATCATATTTGTAATATTATCAGAAATAATAAT

ATAATAAAAAATAATCAAAAATTGAGTAATTATTTGAACATTCATAGAAATAATCTAAGT

AATTACAAAAATTTAATTAAAACTAGAGAGTTATTAAAGAAAGATAAACAGCATATACAA

AAAAAAAAAAAAAAA

>c15348_g1_i2 len=220 path=[1:0-16 18:17-219]

ACTTTTTTTAAATGATTATGAAAAAAGTATAATTAATAACTTAAAAAAGGAAAATCATAT

TTGTAATATTATCAGAAATAATAATATAATAAAAAATAATCAAAAATTGAGTAATTATTT

GAACATTCATAGAAATAATCTAAGTAATTACAAAAATTTAATTAAAACTAGAGAGTTATT

AAAGAAAGATAAACAGCATATACAAAAAAAAAAAAAAAAA

>c15349_g1_i1 len=275 path=[1:0-274]

GGGATGGCTGCCCCCGCGCTCTGCTGCTCCCTGCGGTGCCACGGAGGCCCCATCGTCCAC

CCCGGCCCCACTGCAGTGCTCACCCAGCCCAGGGTTGGGCAGCGCCAGCCTGTCCCCATC

CCGGCTACGACAGCCAGACGATGGAGGACGACCTCCTTCTGCTGCAGCTGGAGGGGAAGG

TGAAGCTCTGGGCGTTCCTGCGCTCCTGCAGTCGGTGCAGCCCCACCACCACCATCATCC

TGGAGACATCGGCACTCTCTGGGATGCAGGGTGCA

>c15354_g1_i1 len=417 path=[471:0-416]

ATTTATATATTTATCTAATATAAACATTTATATATATACCTTCATCTTTATATATTAATA

AATTTACAAATGAATACAAGTAAGAAGCTTTATTTAAATATAATAAATAAATTTTCTAAT

TTTAATAATAAAATAGAATTTAATGAAATAAAAAACTTTGAATAATTAAAAACATATTTA

AAACATTACTATTATAATACACATTTATTATTATACTTTATTTTTATACATATATTTTAA

ATAATTTTTAAAATTAAATTCTTCTATAAATTATTTCTTTTTCTTAAATTTTACATATAA

TTCAAAATTAACATTTCTTATATATATATATATAAAATATCTATGCTTTGATAAAAAGAA

TAGTTATATAAATGTAAATAAATGGTTTTTTCATTATTAATATAATTTACATTATTT

>c15355_g1_i1 len=422 path=[87:0-421]

TATTTTTAAAATTTATTAAATTTTTGGAGTATAAATAAGACTTAAAGAAAATAGTATTTT

TATTATATACTTTATCATTAAAATAATTATTTTCATTTTTTTCAAAAAATAACTTCAGAT

TATTAATGACCATAAATTTATCATATGAATATTTTTTATAATTTTTCTTATTTAGTTCTT

TTATATTTTTTATATATGCAAAATTATTATCACTCTTATTATATGAAAAATAAAGTTCTA

AATCTCTTTTTTCAATTTTGCTAGAAGAAAAATTCAATTTATTAAATTCCTGTTTGTTAA

TTTCTGGAATAAAATTTTCATTTTCTACATTTAATTCGTAAAATTTTAATATTTTATTTT

TTATTATTTACATATATTATTATTATTAATTATACAACATAATTACTATTACGTAGGGAA

GG

>c15356_g1_i1 len=231 path=[49:0-230]

AACAAATATATTTTTTTTTTTTTTCCTATTTTATATATATTTTTTTTTATCTTTTCATTT

TTCTGCTGATCTATATAATGATTATTTACATTTTAATCTAAAAATTTTATATTACATTTT

AGTTAATTATACATAGGTTTTATATAATATTATTATTTTTTTTTAAGATTTATAATTTAT

TAATACCATAATTAATTTCTACTAATTTAATACTAAAGTGAAAATATAAAT

>c15363_g1_i1 len=490 path=[694:0-489]

AAAAAAAAAAAGAAAAAAAAAAAGAAGAAAAAATATGACTACTTTGAAAATAAAAGTTTA

GATGAAGAGGATTTAGAATTAATCGCAGAAAATACAGGAATACAAGTTATTAGAAATGAA

AAAGACACTAAACACAGAAGATTAAAAAAAATAAAAGATAGTGATGATGATGATTTTGAT

AACGAATATCATAAAAATGAGAATGAAAGTAAAATTGATGTATTAAAAGACAAAGATTAT

TTTATTGATGACAGTGAAGTTCCATTAAAAAAACAAAAAGGTGAATTAAAAAGTAATTTA

TTTAACTATGATGATAATTTATCAGATACAAGGGAAAATGAAAATGGTGAAGAAAGTTAT

CACATAACTTCCGATAACATTTACAAAGCAGATTCATATATACATGAAATAATTAGCCAA

ACTTTTGGGGACGTAAATACTGTTTTAGATATTATTAATGTTTCTCCAACAAAAAAAAAA

AAAAAAAAAA

>c15369_g1_i1 len=3208 path=[1:0-3125 6074:3126-3207]

AAAGAAAAAAAAGAAAAAAAGAACACGAGTTTGAGCGTTTTTAAAAAATAATATTCAGAT

ATAAATTTAATCTTGAACTTCTGTAAGTTCAAAAAAAAATTAAATGTACATGAATAAAAA

TTATATATATAAAAAAATTATATATATAAATGAGGATTTTTTTTTATTACTAATACTAAA

AAAAATTACTTTTTTATTTTATTAAAAAAATACATTTTTTTTTTGTAACTTCTTAAAAAA

CCGTTATAATTGACTAAGTTTATTTTTTTATATGGAATTTTATACAGCGCAGAAAAAACA

CTCATCGGTTTTTTTCAACTTAGTTAAACAATAACCAGGTGAATACTGAGATTTACATTT

ATCGAAATTTTGAACACAGAATTGTTTACATTCGGCATCCTTAGCTGGATCACTTGAGTA

TGTTCTAGAACATGAATGCTCTTTTCCTATATCGTCTTCATCAGCATAGGAAATTAGACT

TGATTTTATTCTCTTATTTTTAATATCTTTTAAAATATGTCTTATAGTTTCCTTCTGACT

AACTGCAGCTACTGCAGCAGCTTCAGCAGTTAACACATCATTATCTGTACCATATATTTG

AGGATTTTCACCTAAATCTGTCTTATCATTACCATTATCCTCATTATTTGTGAAGTTGGT

TTGATCTTCTTCATTATTCTCATTTTTTTTAGAAAGATTCTTAGATTTCAAAGGACTAAA

ATTTTTATAATAAAGCCTATGATAAAATTCAGGACCGTTTTTAAGGAAGTAATTAAAAGG

ATTAGGAGCTGACTTTTCATCAGGTAATTCAACCATACCTAAATTAACTTTGAAAACAAG

AGCTGTATGGATGAAATTATATTTACAAGTACTTGGTCCATGCATGTCAATTTTAAAGTT

TCCTTCATCTCCCCAATAATAACCCCAACTATTTCTAACTAACCAATAAGATCTTTTCTC

TCCTTTAATATTTATATATTTACCATATCCTATAATAGTAGCAGCATGATCAGGGTGTTT

ATAACCACATAAACTATGGACTCCTTTTCCATTCAGATCATAACCGAAAACATTTTCTGC

TTTTATATATGCAATAACAGAACCTTTATTTTGAATTTCACGTTTTACTATTCCTATAAA

TGTATCTATATTAGTATATTTTGCACTTTCATAGGCAATATATCCTTTAGAATATGTAAA

TTGTGAATAGTTAACGTATTCTAACAATTTCGTACTACCCCATAAATTGACCCATTTTTT

TTTTGGTTGAGGACAATCTTTACCTACATCTGTATATGAATATGGATGATCAGATTCAAG

AGGTAAAAATTTTGTTTCATCAATAATTTGGAGAAATTCTATTGGACTTGAACCTACAAT

ACATCTATATTTAGCTTTTCTTTTAGAACAATTAGCTACATATAATGCAGAACTTCTAAA

ATGACCATATCCCCTCATACATCTTATTGTTTCCAAGTGTAATTTAGATGCGAAAACCCA

GCAAACTCCACAATGACCTTGTTCTTCTACTTCAATTTTTGATATACAGCTTGTGTTATC

TTTCCATCTATCACAATATTCATCATTACAATATTTATTAGGTATAAAATATGGAGATAA

CAATTTTACTTCTTTGTCTTTTTTTGTTAAATCAATAATATCATTCTCATCTTTTTCATA

CATATCTTCAGTTACATTTATAATTTTTTCATATTCTTCCTCATTTTCAACTTCAGGCAT

ATCTTTAACATTATTAAAATATTCATTTGTTACTTCTGGTAATACTAAACCTCTTTTATT

TATAATCCATTCATCTACATTTTTAATACAAATTGCTGCATTTTTTAACTTATTTGGTAA

TGTAGTATCTCTTTGTTCATCATGAGCCCTTAACAATCTGGTTAAATTATTAAATACTTC

TGTTTCATTACCTAATTCATGATTTTCTAATGTTCCACTAACATCTACTTCTTTTAACAA

ATCACAATAATTCCTTATTTCCTCTCTTAATTCATCGTTAAAATCTTCCAATGTTATTAA

TTCTTTTTTCTTACTTATTGGATCTGTCTTATAAATTTTTTTCATTATATTATCTATAGT

TTCTTCAAGTTTAAATTCATTAGAGTCTTCATCACTTGACTCAGTTATGATAGATTGTTC

AAATTTTGCTTTATTATCAGGAGAAACATAATTAAAACACTCATTAGAAGTATCAGTATT

TCTCGCTAACAAAGTACAGTGAAAACATTTTGCAATATCTAAATTACCACTTAAAAAACA

TTCAGTTGTTATTGCATCACATTTTTCAGGAATAGCATTCTTTATAGCATAATTTTTACT

TTCAATTATGTTTGCAGTTGAATTTCCTGTGTGTACTTGTATTGAAGTGATTGGCTTCTC

AAATTTTGGTATCCTATATTTTTTCATTTCTTTTTTTGGTTCTGTAGAATCAGTTACTTC

CCATTTAAGAGTTAATATATCGTCTTTGATAACTGCAATAAATTTAAAAGTTTGTTCATT

TTGACATTTCTTTGTAAAATTTATATTTATTATTTCAGATGCTGCAAATTTTTGTTCTAA

TTCTATCTTTCCATCATCTGTTTCAAAATTAAGTGTCATATGTGGAACAAAACTCAACTG

AAAATAATTACCACATTTACCAGTTACTTTTACTCCTGTATTCTTTTTCAATAAAGCAGA

TTTAATTGGCATATTTACAGAAGTTGCATTTTTTAAAGCAGCTGCACTATCACCACTTGT

TCCTGCATCCCCATTTGCACCTCTTACTGTAACATTGAGTGCTTCTTCAGGTAAATCCGC

ACCCCCATTTTGATGATCTGCTTGATTAGGATTTGCTTCTCTAGAATCTAATGGTGCATC

TTGAGGTGCAGCTGGAGATGCAACCGCTGATGTAGTTACCCCATTCTCACTTTTACCTTC

AGGTTCAGCTGTAACTTCACGTTGAGTTGCAGATTGAACTTCTTGTTCTAAAGTTTGTCC

ATTTGCAGGTGTTGGTTCATTTTGTAACTCAGCTTTAGCAGCTTCTTTCTCACCTAAATC

ATTTGTTTGAGGTACTTCATCATTTGTACATTCTATTATATTTTTACTAAATATAACATA

TATTATCAAGAAGAAAAAAAAAGGAGACATCATTTTGTTACACTGATTAAAAATAAAATA

TTTTTAGACCTTTTTTTTATAATTATTA

>c15369_g1_i2 len=3209 path=[1:0-3125 6238:3126-3208]

AAAGAAAAAAAAGAAAAAAAGAACACGAGTTTGAGCGTTTTTAAAAAATAATATTCAGAT

ATAAATTTAATCTTGAACTTCTGTAAGTTCAAAAAAAAATTAAATGTACATGAATAAAAA

TTATATATATAAAAAAATTATATATATAAATGAGGATTTTTTTTTATTACTAATACTAAA

AAAAATTACTTTTTTATTTTATTAAAAAAATACATTTTTTTTTTGTAACTTCTTAAAAAA

CCGTTATAATTGACTAAGTTTATTTTTTTATATGGAATTTTATACAGCGCAGAAAAAACA

CTCATCGGTTTTTTTCAACTTAGTTAAACAATAACCAGGTGAATACTGAGATTTACATTT

ATCGAAATTTTGAACACAGAATTGTTTACATTCGGCATCCTTAGCTGGATCACTTGAGTA

TGTTCTAGAACATGAATGCTCTTTTCCTATATCGTCTTCATCAGCATAGGAAATTAGACT

TGATTTTATTCTCTTATTTTTAATATCTTTTAAAATATGTCTTATAGTTTCCTTCTGACT

AACTGCAGCTACTGCAGCAGCTTCAGCAGTTAACACATCATTATCTGTACCATATATTTG

AGGATTTTCACCTAAATCTGTCTTATCATTACCATTATCCTCATTATTTGTGAAGTTGGT

TTGATCTTCTTCATTATTCTCATTTTTTTTAGAAAGATTCTTAGATTTCAAAGGACTAAA

ATTTTTATAATAAAGCCTATGATAAAATTCAGGACCGTTTTTAAGGAAGTAATTAAAAGG

ATTAGGAGCTGACTTTTCATCAGGTAATTCAACCATACCTAAATTAACTTTGAAAACAAG

AGCTGTATGGATGAAATTATATTTACAAGTACTTGGTCCATGCATGTCAATTTTAAAGTT

TCCTTCATCTCCCCAATAATAACCCCAACTATTTCTAACTAACCAATAAGATCTTTTCTC

TCCTTTAATATTTATATATTTACCATATCCTATAATAGTAGCAGCATGATCAGGGTGTTT

ATAACCACATAAACTATGGACTCCTTTTCCATTCAGATCATAACCGAAAACATTTTCTGC

TTTTATATATGCAATAACAGAACCTTTATTTTGAATTTCACGTTTTACTATTCCTATAAA

TGTATCTATATTAGTATATTTTGCACTTTCATAGGCAATATATCCTTTAGAATATGTAAA

TTGTGAATAGTTAACGTATTCTAACAATTTCGTACTACCCCATAAATTGACCCATTTTTT

TTTTGGTTGAGGACAATCTTTACCTACATCTGTATATGAATATGGATGATCAGATTCAAG

AGGTAAAAATTTTGTTTCATCAATAATTTGGAGAAATTCTATTGGACTTGAACCTACAAT

ACATCTATATTTAGCTTTTCTTTTAGAACAATTAGCTACATATAATGCAGAACTTCTAAA

ATGACCATATCCCCTCATACATCTTATTGTTTCCAAGTGTAATTTAGATGCGAAAACCCA

GCAAACTCCACAATGACCTTGTTCTTCTACTTCAATTTTTGATATACAGCTTGTGTTATC

TTTCCATCTATCACAATATTCATCATTACAATATTTATTAGGTATAAAATATGGAGATAA

CAATTTTACTTCTTTGTCTTTTTTTGTTAAATCAATAATATCATTCTCATCTTTTTCATA

CATATCTTCAGTTACATTTATAATTTTTTCATATTCTTCCTCATTTTCAACTTCAGGCAT

ATCTTTAACATTATTAAAATATTCATTTGTTACTTCTGGTAATACTAAACCTCTTTTATT

TATAATCCATTCATCTACATTTTTAATACAAATTGCTGCATTTTTTAACTTATTTGGTAA

TGTAGTATCTCTTTGTTCATCATGAGCCCTTAACAATCTGGTTAAATTATTAAATACTTC

TGTTTCATTACCTAATTCATGATTTTCTAATGTTCCACTAACATCTACTTCTTTTAACAA

ATCACAATAATTCCTTATTTCCTCTCTTAATTCATCGTTAAAATCTTCCAATGTTATTAA

TTCTTTTTTCTTACTTATTGGATCTGTCTTATAAATTTTTTTCATTATATTATCTATAGT

TTCTTCAAGTTTAAATTCATTAGAGTCTTCATCACTTGACTCAGTTATGATAGATTGTTC

AAATTTTGCTTTATTATCAGGAGAAACATAATTAAAACACTCATTAGAAGTATCAGTATT

TCTCGCTAACAAAGTACAGTGAAAACATTTTGCAATATCTAAATTACCACTTAAAAAACA

TTCAGTTGTTATTGCATCACATTTTTCAGGAATAGCATTCTTTATAGCATAATTTTTACT

TTCAATTATGTTTGCAGTTGAATTTCCTGTGTGTACTTGTATTGAAGTGATTGGCTTCTC

AAATTTTGGTATCCTATATTTTTTCATTTCTTTTTTTGGTTCTGTAGAATCAGTTACTTC

CCATTTAAGAGTTAATATATCGTCTTTGATAACTGCAATAAATTTAAAAGTTTGTTCATT

TTGACATTTCTTTGTAAAATTTATATTTATTATTTCAGATGCTGCAAATTTTTGTTCTAA

TTCTATCTTTCCATCATCTGTTTCAAAATTAAGTGTCATATGTGGAACAAAACTCAACTG

AAAATAATTACCACATTTACCAGTTACTTTTACTCCTGTATTCTTTTTCAATAAAGCAGA

TTTAATTGGCATATTTACAGAAGTTGCATTTTTTAAAGCAGCTGCACTATCACCACTTGT

TCCTGCATCCCCATTTGCACCTCTTACTGTAACATTGAGTGCTTCTTCAGGTAAATCCGC

ACCCCCATTTTGATGATCTGCTTGATTAGGATTTGCTTCTCTAGAATCTAATGGTGCATC

TTGAGGTGCAGCTGGAGATGCAACCGCTGATGTAGTTACCCCATTCTCACTTTTACCTTC

AGGTTCAGCTGTAACTTCACGTTGAGTTGCAGATTGAACTTCTTGTTCTAAAGTTTGTCC

ATTTGCAGGTGTTGGTTCATTTTGTAACTCAGCTTTAGCAGCTTCTTTCTCACCTAAATC

ATTTGTTTGAGGTACTTCATCATTTGTACATTCTATTATATTTTTACTAAATATAACATA

TATTATAAAGAAAAAAAAGAAAAGAAGATATAAATAAAGAAAAAAAAAGAAAAAAAGAAA

TTAATAATAATTATAAAAAAAAGGTCTAA

>c15372_g1_i1 len=382 path=[1:0-314 316:315-381]

AGCAAGAAAATAACCACTGAGGACTTAACTGAAGTTTGGGTGATAGAAAGCTGCATCTTC

TGAATACATGAAAACGCGAAGGATGATACCATGCCAGCTACTTATAGGCAGACTCCCTCT

TTGATTTCACTTACACATTTTACTTACATATCAAGCCTTGATGATAACAGTTCTGTACCA

ATTAAAGTAAGGAGTCTGGAGATTTTCTTTCCCCTGGAGAGGACAGACTTCTGCCTTACA

GGTATTTTCCTAAGATTGTGACCCTTTTTTTTTGCAAGTCTGGAGGTTCAGTGTCATATG

TTGTTCATCAAATTGTAAAGTATTATTTCTTCTGTTTCCTTCAGTATGCACACACGGAGA

CATATAAGCTAACAGACCACTG

>c15377_g1_i1 len=240 path=[218:0-167 386:168-239]

ACACGCTGACAATTTGAAAGGAAATGTGGTGCTACGTGCACATACAAGTAGCTATGAGAC

TAGAGAAAGTGCTGTAATGAGTGGAGACAGCAGGGGCAGAACCTGACTCACGTGAAAAGT

TGTTAGTATCGTGAAATATAATTAGGTGTTGTGGCACTCTGAAGAAAACTGCCTGTTTAT

TATAATACATAGACTGCAGAATGAAAAAGAGCTATAACATACATATAATATTTCGTTTTA

>c15379_g1_i1 len=299 path=[583:0-298]

GCAATCACAGCAAGAGTAACATCTTCAGTGAACATTAACCAGCAGTAAGTCAACATGCTG

GTGAGTCAGTATAAATTACAGACTTCCCATACTCCCCACTCCATTTACAGTCACATATTT

GAACTAATTCATTTACAAGTTAACTCTTTCTACTCAACAGTGTACATTCTCTTCAAATAT

TTTGAAAGATCTCTCTTCAGAATACAGTTAAAAAAAAAAAACTGATCCCTAATAAGTTTT

TTCCATTAATTCAACAAGGAGTGGATTTAAGTTTAGATCTCTTGAAAAATCTGAAATAT

>c15380_g1_i1 len=544 path=[1:0-543]

GCACAGCCCCACTGCATGGCTGCACAGGTGATGTGTGGCTTCCTGAAGGTGCAGCAGGCG

CCGGGGGAGCCGCAGGGCTGGGCGCGGCTGTTCTGCGTGCTGCGCGGCACCAACCTGCTG

TGCTACCGGCGGGCGCAGGAGGCGGAGGGCGGCGTGCAGCCCGCGCTCACCATCGCCATC

AATAAGGAGACGCGGATCCGCGCCACGGAGCAGGAGGCGCGGGGCAAACTGCACAGCGTG

ACGGTCACCAACCGCTACGGGGCCGAGGAGGTGACCCACACGCTGCTGGCCGAGAGCCGC

GCCGAGACGCAGCGCTGGATGGAGGCGTTCTGGCAGCACTTCTACGACATGAGTCAATGG

AAGCAGTGCTGTGACGAACTGATGCGCATCGATGTGCCGCCGCCGCGCCGCCCTCCCGCC

CCGCTGCCGCGCCAGGGATCGCTGTACCACGAGATGGGTGAGCGCAGCCGCGTCCTCTTC

TTCCTCCTCCTCCTCCTCCTCTTCCTCTTCTTCATCTTCCTCTTCTTCCTCTTCTTCCTC

TTCT

>c15380_g2_i1 len=249 path=[1269:0-248]

GCAGCCATGCAGTGGGGCTGTGCAGCCAGGCGGCAGCACATGCTGCCATACAGCGGCAGC

CAGAAGGAGCTCTCCTCGTTGCAGGCGATGGCGAGGTCGTGCGTGCGGAATCCATCCTGC

ACCTCTGCCAGGGACAGCACCGTGTGTGCCAGGAGGTGGTATTTGGGGCCCGGCACGCTG

GGCGCAGGCAGCAGCAGAGCCCCCCCCCCGTTGGCAGTGGGGCTGCCGGGCCCCCCATCC

ATGGTGGCA

>c15383_g1_i1 len=2398 path=[2487:0-2308 49:2309-2397]

GGTAACTTTGTGTTTGAGGTGGGGGGGGGGATTCGGCTCCTCTCGCCCCGTTGCTGCCCC

ACTGCGTCCCCCGGGGCCTTCAGAAGCCGTTCCCGCTGATGTTGATGGACTGCAGGTCGG

CCACCTGCATGACGTTGATGCCGCTGCTGGCCAGGCGGGCGATGCCGTCGGGGCAGATGG

CTTCCATGGCCACCATGTTGGTGGCGATGAGGGCCGACGGCGTGGGGCCGCCGCTGCCTT

CCGACGAAGCCCCCGTGTCCATGGGGACGGCGGAGACGTTCATGGCCACGCTGTTGGCCG

CGCCGCCCTTCTTGTTCTGGTGGGTCTTGATGTGCTTGGAGAGGTGGTCGCTGCGCATGA

AGCGCTTGGGGCACTCCGGGCAGGCGAATTTCTTCTCCCCGGTGTGCGTGCGCTTGTGCC

GCTGCAGCTCATCAGAGCGGGTGAAGCGCTTCCCGCAGAGCATCCATCCGCAGATGAAGG

GCCGCTCCCCCGTGTGCCACCGCAGGTGGGCCCGCAGGTGGGAGGTCTTGCCGTACACCT

TCCCGCAGCCCGGGATGTGGCAGATGTGCTGCTTTTTTTTACCTGGATCCCCAGAGCTCC

TCCCCTCGCTGTCCTTGCAGTAGGGACAGGTGCAGGCTTCCCTCCGCACCTTCCGCCCCT

GCTGGGGCTGTGGGTCTGGGCTGGTCTCCCCTTCCTCCACCGCTGCGCTGTCGTCGCCCA

GACCGTCCCCGCTGGCTCCGTGCAGCCCCAGCTGGGTGCCATGCTCCCCGGTAGCGTTGG

CGATGGCCAGCGGCAGCCCTTGCAGCTGGTGCACTTGGATCCCAGACGCTCCCAAGGCAC

TGAGGTTAATAGTCTGGAGGCCTGGCATAGAGGAGAGCTGGGCGGCGTTGACCGTCACCG

TGCCGCTGGGGAGGGAGGCGATGGGGGTGAGCGTGGTGCTGGTGCTGCCCGTCTGCCCCA

GCGACACTCCCTGCATGGGGGCCAAGGTGATTGTCTGCGCCTGGGGGTTCTGCACCTGCA

GGTTCTGGAGCTGGATGGTCTGCCAGCTCACCTGCCCGTTCGGCCCCACCGTGGGCGTTC

GGATGATGATGGGCCCGGAGTTGGGCACGGCCTGGAGCTGGAGGTTCTGCAAGGCGTCCT

GTGAGATGGCTTGAGTGGCGAAGGTCTGGCCGGACAGCGGGGCGGTCTGCAGGGCTTGGA

TCGTTTGCCCCCCCTGGACTAACTGAGGTTGGATCAGGATCTGCTGCTGCTGCTGCTGCG

ACTGCTGGCTCTGATCCCCCTCTTTCTGCTGCCCCGGCTGCAGAGCCACCCCAGAGACCT

GGCTCTGCAGGGAGTCCGTGCTGGGGGCCCTCTGGGGCGTCTGCACTTGGACGTTTGTCC

CCACTGTGGAACTGCTGGAAAAATTCATGATGCCCATGTTACTGGTGGTGGTGGTGGTGG

AATAGCTGTTGGCGTTGGTAAAAAAAGAGCCGGAACTCGCGTGGGATGTGGCGATGTTGG

ACGAGCTGATGGCAGCACCTGAGGTGGTGGGCTGGGAGCTGCTGTCCGGGGAGCCGGAGC

CGCTCAGCGTGACGGTCTGAGAGGTGGGGGCCAGGCTGGCGGCCACGCTGTTGACAGGCA

GCAAGGTGATGTTGCCGTTGAGAGCCACGGGGACGTTGGCCACGTACTGGGTTTGCCCTG

AGAGGGTGTTGTTGGCCAAGCCCACCCCCTGGATGGGGACGGCCTGCTGCAGCAGGTTGG

GCATGGCGATGAGGTTGCCCGTCCCCGCTCGGCTCGTGATGATCTGCTGGTTCGTGCCGG

GGATGATCTGGAGCTGCCCAGAGGCATCCTGCTGCACGTTGACTTGGGCAGGGGTGGTGG

CAAACTGGAGCTGCTGCCCGTCAATGGTCTGGAACTGGGGAATGACCTGGTACTGGATGT

TGGGGATGACGCCCGGCAGGCCCGTGAGCACCTGCTGGTTCTGCAGGTTGGAAGCAGCCG

TGACCACGTACGGCCCCGTGCTCACGGGACGGCTTTTGGAGGGATCTCCGTTGCTGCCAT

TGCTGCCCTGCTCCTTGGAGGGCGTCGGCGTGGTGGAGCCGGTGGAGATGATCTGCCAGC

CGTTGGCCGACTGTGCGATCTGGGCAGCGGCGGCCGTCAGGTCCAACTCACCGCTGCCTC

CTTGCTGGCCCTGGGAACTGTTGGAGTTCTCGTTGGGAGACTCAATCCTGCTGCAGGTGG

CAGCCAGCAGAGCCAGCGGGGACGGCTGTGACTCCTGGCCCTCCCCAGCCGGCGAGCTGC

TGTCACTGCTGCCATCTCCTTCGCCCCTCGGCGGTCACCGGCGGGGTTAGGCCGCCGCCT

CAGCGCGCCGGGCGCGCCCGCCCCCTCCTCCCCGCCCCGCCGCCGCTCGGGCTCGGCC

>c15383_g1_i2 len=2397 path=[2487:0-2308 4795:2309-2396]

GGTAACTTTGTGTTTGAGGTGGGGGGGGGGATTCGGCTCCTCTCGCCCCGTTGCTGCCCC

ACTGCGTCCCCCGGGGCCTTCAGAAGCCGTTCCCGCTGATGTTGATGGACTGCAGGTCGG

CCACCTGCATGACGTTGATGCCGCTGCTGGCCAGGCGGGCGATGCCGTCGGGGCAGATGG

CTTCCATGGCCACCATGTTGGTGGCGATGAGGGCCGACGGCGTGGGGCCGCCGCTGCCTT

CCGACGAAGCCCCCGTGTCCATGGGGACGGCGGAGACGTTCATGGCCACGCTGTTGGCCG

CGCCGCCCTTCTTGTTCTGGTGGGTCTTGATGTGCTTGGAGAGGTGGTCGCTGCGCATGA

AGCGCTTGGGGCACTCCGGGCAGGCGAATTTCTTCTCCCCGGTGTGCGTGCGCTTGTGCC

GCTGCAGCTCATCAGAGCGGGTGAAGCGCTTCCCGCAGAGCATCCATCCGCAGATGAAGG

GCCGCTCCCCCGTGTGCCACCGCAGGTGGGCCCGCAGGTGGGAGGTCTTGCCGTACACCT

TCCCGCAGCCCGGGATGTGGCAGATGTGCTGCTTTTTTTTACCTGGATCCCCAGAGCTCC

TCCCCTCGCTGTCCTTGCAGTAGGGACAGGTGCAGGCTTCCCTCCGCACCTTCCGCCCCT

GCTGGGGCTGTGGGTCTGGGCTGGTCTCCCCTTCCTCCACCGCTGCGCTGTCGTCGCCCA

GACCGTCCCCGCTGGCTCCGTGCAGCCCCAGCTGGGTGCCATGCTCCCCGGTAGCGTTGG

CGATGGCCAGCGGCAGCCCTTGCAGCTGGTGCACTTGGATCCCAGACGCTCCCAAGGCAC

TGAGGTTAATAGTCTGGAGGCCTGGCATAGAGGAGAGCTGGGCGGCGTTGACCGTCACCG

TGCCGCTGGGGAGGGAGGCGATGGGGGTGAGCGTGGTGCTGGTGCTGCCCGTCTGCCCCA

GCGACACTCCCTGCATGGGGGCCAAGGTGATTGTCTGCGCCTGGGGGTTCTGCACCTGCA

GGTTCTGGAGCTGGATGGTCTGCCAGCTCACCTGCCCGTTCGGCCCCACCGTGGGCGTTC

GGATGATGATGGGCCCGGAGTTGGGCACGGCCTGGAGCTGGAGGTTCTGCAAGGCGTCCT

GTGAGATGGCTTGAGTGGCGAAGGTCTGGCCGGACAGCGGGGCGGTCTGCAGGGCTTGGA

TCGTTTGCCCCCCCTGGACTAACTGAGGTTGGATCAGGATCTGCTGCTGCTGCTGCTGCG

ACTGCTGGCTCTGATCCCCCTCTTTCTGCTGCCCCGGCTGCAGAGCCACCCCAGAGACCT

GGCTCTGCAGGGAGTCCGTGCTGGGGGCCCTCTGGGGCGTCTGCACTTGGACGTTTGTCC

CCACTGTGGAACTGCTGGAAAAATTCATGATGCCCATGTTACTGGTGGTGGTGGTGGTGG

AATAGCTGTTGGCGTTGGTAAAAAAAGAGCCGGAACTCGCGTGGGATGTGGCGATGTTGG

ACGAGCTGATGGCAGCACCTGAGGTGGTGGGCTGGGAGCTGCTGTCCGGGGAGCCGGAGC

CGCTCAGCGTGACGGTCTGAGAGGTGGGGGCCAGGCTGGCGGCCACGCTGTTGACAGGCA

GCAAGGTGATGTTGCCGTTGAGAGCCACGGGGACGTTGGCCACGTACTGGGTTTGCCCTG

AGAGGGTGTTGTTGGCCAAGCCCACCCCCTGGATGGGGACGGCCTGCTGCAGCAGGTTGG

GCATGGCGATGAGGTTGCCCGTCCCCGCTCGGCTCGTGATGATCTGCTGGTTCGTGCCGG

GGATGATCTGGAGCTGCCCAGAGGCATCCTGCTGCACGTTGACTTGGGCAGGGGTGGTGG

CAAACTGGAGCTGCTGCCCGTCAATGGTCTGGAACTGGGGAATGACCTGGTACTGGATGT

TGGGGATGACGCCCGGCAGGCCCGTGAGCACCTGCTGGTTCTGCAGGTTGGAAGCAGCCG

TGACCACGTACGGCCCCGTGCTCACGGGACGGCTTTTGGAGGGATCTCCGTTGCTGCCAT

TGCTGCCCTGCTCCTTGGAGGGCGTCGGCGTGGTGGAGCCGGTGGAGATGATCTGCCAGC

CGTTGGCCGACTGTGCGATCTGGGCAGCGGCGGCCGTCAGGTCCAACTCACCGCTGCCTC

CTTGCTGGCCCTGGGAACTGTTGGAGTTCTCGTTGGGAGACTCAATCCTGCTGCAGGTGG

CAGCCAGCAGAGCCAGCGGGGACGGCTGTGACTCCTGGCCCTCCCCAGCCGGCGAGCTGC

TGTCACTGCTGCCATCTCCTTCGCCCCTCTCGGGCGTTCCCTTCACCGCTGCCATCTCCT

CGGGACTCTCTGAATCGCTCATGGTGGCCGGGGGCCGAGCCCGAGCGGCGGCGGGGC

>c15387_g1_i1 len=267 path=[245:0-266]

GTCGCCCGCAACCCGCGCCAGGTGGGCCCGGAGCGGCGCCCGCTGGGGGATGCCCCCGTT

GTGCGGGACGCCCTGTGGGGAGATGTAGGGGTACAGCCCAAACTTGTTCTTGTAGAAGAT

GGTGATGTTCTGCCCTGCGAAGCGGCCACCCCGGTTCTCCACGATGCCGTAGTCGGCCAG

GGGCAGCCCCACACCAAAGCGGCGCTGGCAGCGGCTGCTGGGGATGTTCCAGAGCAGGGA

TCAGTGCTGCCTTTGCTGTGTCATGGG

>c15389_g1_i1 len=376 path=[2017:0-375]

ATTATAATTATTTCTTTTTATGTCATTTTCATTATATATATATGTATATGCATGAATGTA

TGTGTATAAAATGGCAATAAATAAAAGAGAATACATTAAAAACTATGATGAAATATATAA

TTTTAAAAATATAAATAAGGATAAAAAAATAAACTTCGAATATAGAGACAATGACATTGT

TTGTATAAATATTTCAAATAAAAAATATATTTTTTTATATAAATGTGCTAAAAAAAATAT

AAATAGTAGTAGTGGAATTATAAGCAACAAAATAATTTATACACCAAAAAAAGGTATAAA

TATTGAAGATTATTATAAAAAAAATATTTTTAAGAATACAATATTAAAACATAATTCAAT

GAAGTGTAAAAAGAAT

>c15389_g2_i1 len=1021 path=[1373:0-508 1882:509-1020]

AAAAAAAAAAAAGTTATAATAATTTAAAAATTTTTTAAATAAAATATAAAAGTAGTAATT

AAAGAATTTTCTTACATAATTTTTTACATATGAATAATAAGTATTATAATTAATAGGTAT

ATTATCAAAAATTATATGAATAATAAAATTATTATCTTTTCTTTTTTTCTTCTTTTTACT

TTTGTAGTTTTTAACACTTTTTCGTTTCAAGAATTTCTTGTTTAAATGACTTTTTATATA

ATTCTTTATATAGTTTTTAATCTTTCTTTTAAAATGAATATTTACATCACTTTTTTTCTT

ATTTTTCTCTTCATTTTTTATATATATTTCATTGTCATTACTTATATCTTTTATTATATT

ACAAGAATATTTATCAAATTCATCATTATAATGTTTATTATGAATATAATATTCTATATT

TTTTTTTATTAATAACTCTTTCAATTTTTTAATTTTCTTTTTTATTTGGCATCTCAAATG

ATAAATTTTTCGATCGATATATGAACCTATTTTTAATTCTTTGAATTTTGAATCTAATAT

TATTTTTTTTAATTTTTTATCTGAAACATTATTGGTGAATTTAATTAACTTAATAATATT

TTTTTCATTGTTTGCACTTGATAATTCTTTAATAACATTATATGCATTATAATTATTTCT

TTTTATGTTATTTTCATTATATATGTATTTTATTAACATCTCCTTTTTTGAGAGGTTTGA

TTTATCTCGTAGGTCTTTTTTATATATTATTATTTTTTTTTTTCTTCTATCTTGTAATAG

TTTGAGTATGTTACAAATATTTCTATTATCATCTTCATTATATAGATTGCAAAATTTCGT

ATGTTTAGAATGATTTAACTTTTTCTTTATTTTGCTTATTCCTGAACACATTTCATAATT

ATAATTTTTATATTTTTCACATATTTTAATATTGCTTAATTTCTTTTGGAGACTAAAATT

ATTATCATATTTTACTAAATAAATACTTATTTTATTATTCTTTTTACACTTCATTGAATT

A

>c15389_g2_i2 len=529 path=[2721:0-16 1882:17-528]

CTTTGTGTTTGAACCTGTTTTTAATTCTTTGAATTTTGAATCTAATATTATTTTTTTTAA

TTTTTTATCTGAAACATTATTGGTGAATTTAATTAACTTAATAATATTTTTTTCATTGTT

TGCACTTGATAATTCTTTAATAACATTATATGCATTATAATTATTTCTTTTTATGTTATT

TTCATTATATATGTATTTTATTAACATCTCCTTTTTTGAGAGGTTTGATTTATCTCGTAG

GTCTTTTTTATATATTATTATTTTTTTTTTTCTTCTATCTTGTAATAGTTTGAGTATGTT

ACAAATATTTCTATTATCATCTTCATTATATAGATTGCAAAATTTCGTATGTTTAGAATG

ATTTAACTTTTTCTTTATTTTGCTTATTCCTGAACACATTTCATAATTATAATTTTTATA

TTTTTCACATATTTTAATATTGCTTAATTTCTTTTGGAGACTAAAATTATTATCATATTT

TACTAAATAAATACTTATTTTATTATTCTTTTTACACTTCATTGAATTA

>c15392_g1_i1 len=1110 path=[1088:0-659 1748:660-1109]

GTGTGTGTGTGTGTGTGTGTGTTAGGAAGCATATTTATGTTTTTTTTTTTTATTTCAAAT

AATATATATAAACAAACATTATTTTCATAGTTATTTTTTTCTTCTTCTTTTTTATCTATA

TGTTCGATTACTTTTTGTATTTGAGTACTATTTTTTTTTTTTCCTTTAATAATAGTTGAT

TCACTTTTATTTTTCTTTATGCCTAAAATATAATCATTTTTAAGAGATTTACTTATCTTA

TTTCTTTCAATAACATTTTTATCATTTTCTAATAAATAGATAAGTTTATCAATTTCATTA

TTTATGCAATCTAAGTTTAATTCTTTTTTCATTTTAGTATCAATTGTATCACTGTTTTTT

GAAGTACTTAATTTCAGTAATTTCATAAATTTGTTCATTTTTTTTTTCAAATTAACTATA

TCATTGACACTTAAAATTTTAAGTGAATTTTCATTTATATGATCAAAAGACACATTTTCA

GCAAAATTAAATGGATTTTTATTTATTTTTGCATTTAAGTTACTCGTATAATCTGTTTTA

TCATTTAATTCCTTAATATCACTAAAGTGTAAATGAGCTTTTTCATAAATATTGAAAATA

ATATCACTTTGCTTAACAAACATATCCTTTTTTGATTTTTCTAAAATATTTTGTAAACTA

TTTTTTTTTGCATTCATTTCCATTTCCTCAATTTTTTTTTCCATTATTTTATTTTTTTCA

AATAATAATTTAATATCATTCTCTATTAAATTTACATGATTTTTTAGATTTTTTATTTGC

ATATCTTTTTCATTTATTTCTTCATTTACTAATTTCAAAATATATGATATATCTATTTTA

TTTTTCAATGATTGTTTTACATCTATGGTATTTTTGTAATTATTTATATTTGACAATAAA

TTATTTATCTGTGTTTGATTTTCACTATAAAATATTTTTTTATTCTTTTTTTCTTGTTTT

TGTTCTTTATCTTTTTCATGTTTTTGTTCTTTCTGTTTTTCATGTTTTTGTTCTTTCTCT

TTTTCATGTTTTTGTTCTTCCACTTTTTCATGTTTTTGTTCTACTTTTTCATCTCCTTCT

TCTTCCTCTTTTTCTTCTTCTTCTTCTTCT

>c15393_g1_i1 len=220 path=[1:0-219]

GATGCAAGCCGTGACACAGCATCAGTGTGGGAAGGCCTCCCCATGGCCCACACGTGGCTC

CAGCTGCCCTGTGCTGAATGTGGAGCTGCTACTGCAGAGCTGCTGGACCCGGCAGTTTCC

AGCCGACCTTTGCTCCTCAGTAATCGCAAAATGCTGAGAGGAGTGGCTGGGACAGGAACA

AGTCCAGCAGCAGGCCTTCACACCAGTCTCACTACTGTCT

>c15395_g1_i1 len=223 path=[239:0-222]

GGCGTTTCGAAGGGAGCAAAACGCTGCCCGGCGCCGTGAGTCCTTCCGAGCGCGGCCGCA

CGGGCGAGGGGAGGTCGCTCCCTCCCTCAAAAGAGGCGGTGCTGCGGCGGTGGAGGGGAG

AAACGCGTTGTTTACATAAATAAAGCGTGTCACGCGCAGCAACGCCGCCTTTCGCCCCGT

GAGCTCCGGCGGGCAGCGCTCGGTGCTGCGGCCGAGAGCAAAG

>c15396_g1_i1 len=298 path=[291:0-214 604:215-297]

AACTTCTTCCGCTTCACCCTTCCTCAAGTTAATTGTTACCAAAGTGTTTCCCCCTCTGGT

TTTCAACTTAGGAATTCCTTTTTCAAGACCTGTGCTCTGCTGTTTCCCAGTTACTGGGTT

AGAAGATGCAGGGAACAAACCTGGCTCCTCCCTCTGCACACCATCAATATTTCATCTTGG

TTTCAGTCTTGTGCAAGTGAGCACTTCCCATTACAGCATGCACCTTCCCCTGACTGCTTA

ACATGTACCAAGCACATTAATTAAAGCTGCTATCACTCCAGCTACAAAAGTATAAGAA

>c15396_g1_i2 len=313 path=[291:0-214 506:215-312]

AACTTCTTCCGCTTCACCCTTCCTCAAGTTAATTGTTACCAAAGTGTTTCCCCCTCTGGT

TTTCAACTTAGGAATTCCTTTTTCAAGACCTGTGCTCTGCTGTTTCCCAGTTACTGGGTT

AGAAGATGCAGGGAACAAACCTGGCTCCTCCCTCTGCACACCATCAATATTTCATCTTGG

TTTCAGTCTTGTGCAAGTGAGCACTTCCCATTACAAGATGTTTCCTTTCCAGCTGACTGC

TTTGCTGGCTCTCAAGCATGTTACACTCCATTAAGCAAGGCTGATTTTCCTTCCTTTTTA

GGCTGAATTTATA

>c15400_g1_i1 len=475 path=[1:0-474]

AAACGGGACTGTGGCGCCACAAGTGAGGATTGTGGCATCCCATACGGGGACAGTGACATC

ATATGCAGGGATGTGACGTCATAGACGAGGACTGTGACATCTCAGAAGGGGCAGCACCGT

CACAGGTGGAGATGTGTTGTGATAAGCGAGGACTGTGACGTCAGAGACGGGGACAGGCAT

GCCATAGATGGGGTTGTAACATCACGGGTGAGAAGTGTGATGTCATAGATGGGACCGTGA

CATCACATATGGGGACAGTGATGTCATAGGTGAGGACGCGACACCTCAAGAGAGAACTGC

GATGTCACACACAATGACTGTGATTACACAGATGGCGACTATGACATCGCAAACGAGGAC

CGTGACATCACACACGAGCCCTGCGACGTCACAACGGGGTCTCGTGACGTCACAGCTGCA

ATCAGCGATGTCACACGTGAGGTGTCTGCCATCACTGCTGACATCATAGGTCGTG

>c15402_g1_i1 len=262 path=[175:0-171 358:172-261]

ACAGGGAACCTGCAAGAAGATCAGACGGCCACCATTTGAGATAGTCAGCCACAGCAAACC

ATTAGACAGAACTGAGGAGACAGCACAGAGCACAGCTGCCCCTCTTCATGGGGATCCTTC

CAGTTTGACACTCATGTCAGACTGAACAGAGGGAAAACAAAATGTTATGTGAAGACAAAA

CACCGTGAACACCTGTGTCCTCTCACACTGCTCATTTTACTAGCAGAGAAAGAACGAGAA

AGCAATATAAACTATTTATTCT

>c15403_g1_i1 len=2333 path=[2384:0-1932 74:1933-2332]

GATTTTTTTTTTTTTCTTTTTAAACTGATCAAAATGTAGAAATAAAAATTTAGTTTAAAA

TCATAAAAAATAATAAGTAAAAACAAAATAAAGTGAAAGGAAAATTTATTTTATTTTTAT

AGAAAATGTATATGAAAATATAATCATTATAAAAATGAATAGAAACAAAAAAAATATCAC

TTTTTTAAATATAATTTTATTTAAATCAATTATATATATGTAAACTATGACTCTTCATAA

TTAAGACATTTTTTTTATTAAAATTTTTTTTTTTATTTATTTCATTCTATATTTCTCTTA

TATAATTAGTTATTTTCCCAATAACATTCTTTGTTTCTTTTAAAAAGGAAATAAATGAAT

CTACTATAATTATAAAGTTTTAATGGTGATAAAAATAAGAAGAAAATGAAGCTACGTAAA

CATAAAGGTTCATTAGATGAAGAAAAATTATTTGAGAATATCTCTAAAGATGATTGTGGT

TTGATAAAAAAGTTTACTTTGGGTGTGTGTGCTATGGAAAGTAAGGTAGAAAGTGCACCA

ATGGAATGTATATTAAAAAGATTAGCAAAGAGTGGAGATTTTAATATAATAAAATTTAAA

GAAGATATGATATTAAACCAAGATATCGATTCATGGCCAGTAGTTGATTGCTTAATAGCA

TTTTATTCTACTGGATTTCCTTTAAAAAAAGCAATTGAATACGTAAAAAAATATAAGCCA

ATAACATTGAATAATTTAGAAAAACAGTTGATATTACGTTCACGATTACAAATATATGAA

CAACTTAAGAAGTGGAGAGTTCCCCATGCTAATTATGTTGTAGTTGATCATGACACTGTA

AAAAAAGGTGAACATGTATTTGAAGAATATTATGATTACATAGTTTATGATAATATACGA

TTAAACAAACCATTCATAGAAAAGCCACTAAATGCGGATAATCACAATAATTGGATATAT

TATCCAAAAAATACAGGAGGTGGTTGTAAAAAATTGTTTCGAAAAATAAAAGATAGAAGT

AGTGAATACTGTCCAGATATACATAAAGTAAGAAATAATGGTACATATATATACGAAGAA

TTTATGTCCACATTTGGTACAGATATAAAAGTTTATACAGTTGGTCAAATGTTTGCTCAT

GCGGAAGCAAGAAAAGCTCCATCATTAGACGGAAAAGTTTGTAGAACTTCTGATGGTAAA

GAAGTTAGATATGCTGTTATTTTATCAGAAGCTGAAAAAATTATTGCTTATAGAATTGTT

GAAGCTTTTCAGCAAACTGTTTGTGGCTTTGATATATTAAGAACAACTATGGGTCCTTTT

GTTTGTGATGTAAATGGATGGTCATTTGTTAAAGGTAATGTAAAATATTATAATGATTGT

GCTCATATATTAAGAGCTATGTTTTTAGCAAAATTAGAAGAAAAATATAATATAATTCCT

CGAGATCTCGCTGATAACTGGTATAATATTGAAAATGAAGAAGAGGTGTTAAGAAAAACT

TTTAGGCAGCCAGATGATTTGCATTGTTCACATCACGAAGAATTATGTTCAGTTATCATT

GTTATGAGGCATGGAGACAGAAAACCAAAACAGAAAATGAAATTCATCACAGATAAACCA

CTATTATTAGATTATTTTAACTGTGAAGAAAATTTATATAATGTTATTGGAAATAGATCA

TCCATTGATGACAATATTAAAATAAATCAAGAATTAAATAGTAATATTGTATCTTTAAAT

AATGTTAAAAACAATAAAACAGATTCTTCTATATTTTCAAATGATACTTTAACCAAGATA

AACTCAAAACAAAATGATAATACTTCTAATACCCAAAAGAATGATCTCATTAATGATGAT

ACGACAAAAATAAATGAAATTAATAATGATAAAACATCTCAAGAAGATAAAAATATTAAT

AACTGTGAAAACGAAAAAAAAAACAATAGTGATAACAGTATAAATGAAAAAAATAACGAC

AAAATTGAAGTTCCAAACGAAAGTGATGCTACTAAAATGAACAATAAGGATTCCTCATAC

AGTAAGACATTAAAGGATAGTACTATTACGCAATCAGAAGAAATAAAAAAAAATAACTTG

TATAAAGCATATATGAGAAAAGAAATAAAATTTAAGTCACCAGAAGAATTACAAGACTTA

TTTTTAATAAATAATATTATATTGAACGATATTGAAAAGGAATACAAAACTATAAAAGAA

ACCATTATTAAAATTCAAGAAAGGAAAACAGAAGAAACAACAATAGAAAAAAGAAATGAA

AATGATAATGTTAGTGAAATTATTACTTTAAAAAAAGAAAATATAAATGATAA

>c15404_g1_i1 len=250 path=[205:0-52 259:53-112 14:113-249]

CTAGGAGTTACACTTGTAAGCAATAACATGAGAGTTGCATCAGCCTTTGAACCCCCTCTG

TTGAACTGCAAACTATTTCCTGTTCCTCCTGATCTGAGCAGACATCTCACAAAGCACTGC

AGCTGAAGATTGCTCCACATTCTAGAACACCCAAAAGATGTCAGTGAAATGGAAGAAGTT

TGCAGTTCAACAGAGGGGGTTCAAAGGCTGATGCAACTCTCATGTTATTGCTTACAAGTG

TAACTCCTAG

>c15408_g1_i1 len=405 path=[59:0-308 535:309-336 28:337-404]

AAGATCATCTAGTCCAACCATCAAATTGGTAGTGCCCTCCCAAATTTTCTTGCTGTGGTT

TGGCCTTTGCTGCAGTAGGGAGCTGCAAGGTTGGGCTGAACCATGTGTGTTCCATGTTGT

CTCAACTGCCTCAGACTGGTGGAAGTGCTTGCTCTGCTCCACCACCAAGCCAACGTACAC

AAACTGAGTGTATATAGGCACAAGGTGCTGTGGTGTTCCATCTATGAGCAGCAGCCAGGT

GCTAGGACCGTCTAGACAAAAGCTGCAAAAGTCTTCAGCCCTATAAGTGCTAATAAAAAC

ACTATACCTGCTTTGAAGGTTGTGTTTGACATTCCTTTCCTCTACCTGTTCCTTGGCTAA

TCCACAGGCTGTACAGGGAGTGTCTTTCACAACCTTCAAAGCAGG

>c15409_g1_i1 len=314 path=[342:0-313]

AAGGGCTCCACAGCTGCACTGCCTGCACAAAACAAATGCATCCATCAGGGCTGCCCACGC

TCACCACACTTGGTCACGCCCATTCCCCAAATGTCCTCGCCACAGTTGACACATCCATCC

CCCGGGCCTTGTGGTTCCAAGTACCCCCACCCAGCCCATGGGCCAGCACCCCAGATGACT

TTGCCAACACCCACATGGCCACACCCACTTCCCAGCGGCCATCACACACATGGCCATGCC

CACCCGAGGCCGTGCCTTTGCCAATACACCCCAACCCCGGTTCTCCCCCCACCTTTTGGG

GCACTGGGCTCCAG

>c15411_g1_i1 len=234 path=[45:0-137 44:138-233]

TTACTTATGCCATCAGATACTTGGTGCCCTCCGTTTATGGAGTTCTGAAATAATTTTTAC

TGTAATCACTTTAGAAACGCAGCAGTCACAGAAAATGCTTTGAGTAGCTTAAAAGCTAGC

AGTCAGGCTGCAGAAGATAAAGAAAGGTATAAAAAGTATCTTAATACGATATTGTGCTGA

ATGTCTTTGTGTTGTCTTCCATTTTATCATAGATCTGGAGTATGAAGCAAGACA

>c15413_g1_i1 len=1684 path=[33:0-1683]

ATATATATATATATATGTATTTGTACTTAAAATTTTATTTTATATTATCCCTTTATAACT

GCTATTGGTTTTAAACGAAAGCATTTATTTGATATTCCTGCATCATGACAAGTTTGAACA

ACTTCTGAAACATTTTTATATGACTCTGGTGCTTCTTCCATGATTAATTTAGGAGAAGCT

ACCCTTATTGATATGTTTTCTTCTTTTAATTTATTAAGAACATCTATATAATTTAATGTA

TTTCGGCTTTTATTTCTACTTAATGCTCTACCTGCACCATGGCAAGTTGATCCAAAAGTA

GCTTGCATTGCTTTTTCAGTTCCTGTTAATACATATGAATATGTACCCATTGTACCACCT

ATTAATATTGGTTGACCGCAATATTGATAATCTAAAGGTACTAATGGATGAAATGGAGGA

AAAGCTCTTGTAGAACCTTTTCTATGAACTAATAAGTTTTTTACTTTTCCATCAACAAAA

TGTTCTTCAATTTTGGCTATATTATGAGAAACATCATATATCACATGCATATCTAAATCA

TCTGGAGACTGATTAAACATTTTTGAAAAAGTCTGTCTTGCTAGAAAAGTCATAGAAGAC

CTATTAATCCATGCAAAATTACAAGCAGAACCCATAGCTTTTAAATAATTTTGTCCTTCT

TTTGAGTAAATAGGTGTACAAGCCAACTGTTTATCGATTACATTTATTTTATATTTGTTC

ATACTTTTTTCCATTTCTATTAATGCATCAGTGGCTATTTGATGTCCTAGTCCCCTGCTA

CCTGAATGAATCATAATACATACTTGGTTTTTTTTTTCAATACCCATTAATTTAGCACTT

CTTTTATCATAAATCTCATCTACTATTTGAATTTCTGCATAATGATTTCCTGCTCCTAAT

GTTCCCATTTGAGTTATTCCTCTTTTTTTTGCTCTAACGGATACGTAATTGCTATCTGCA

TATAAACTTCTTCCATTATCTTCACAGTTTAATTTATCTTCAATCCATGAATATCCTTCC

CTAACACACCAATCCATACCTAAACATAAAGCATCATCTAAATTCTCTTGATTACATAAA

ATAAAACCCTGTGAACCAACTCCTACAGGTATATGATTGAATAAAGATTGCGTTAATTCT

TCTTGTTTCGGTTTTATGTCATCATAAAATAAATTAGTTCTAATTAAACGAACACCGCAA

TTTATATCAAATCCTACTCCTCCTGGAGATACTATTGCTTCTTCTTTGTTCATATCAAAA

GCTGCTACATTCCCAATAGAGAAACCATAACCTGCATGAACGTCAGGTAATGCAATGGAT

GCTTTTACTATTCCTGGCAATGTAGACACATTAGCTATCTGCATTACTGCTGGTAAAAAA

GTAGAACATCTATTTTGTTCATATGTTTTTATTTCATCATCTATTAATAATTTTAATCTT

TCATTTACATAAATATGACCTTCTACATTCATATTTTGAACTAACCCTTTATTTATTTTA

TATAAATTTTTTTCCTTTGTTTTTTCAATATAACTAAACACATTGTCACCTCTTTTTTGT

GACTTCATTTTCTCTCTATTATTTAATTCATACGGTATATATTATTTGATATTTATTTTA

TTTCAATAAATTTGAAAATAAAATGTTATTTTAATTTTATTAATACTAAATTTTTTTTAA

TAGT

>c15414_g1_i1 len=482 path=[644:0-298 1117:299-305 948:306-481]

CACCCTTTCTCTCAGGTCATGTTTTCATTCATTCTTCTTGCTCACACCCCCTTGCTCATT

TCCCTTTAGAACGTTCTGTTCTTAAGCTATGTTGCCCTTTCTTGTGTCATGAATCATAAA

TCATAAACAACATCTTTCAGAGGTTTTCTTCGCGTTGAGTGACATCTTTCTCACACACGA

ACCAGAAGTACTCACAGTCACTGGACTGGAGTTCCCAAAAGGCACCCAGTTTTGCCAGAG

CCGCATCACACTCCCTGCTAAATCCTCAAGGCTCCAGATCACCTTGTACAACACTAATCT

TGTTTATGTAATGGAAGTTTTCATTTGGGTTTCTGTTAATGAGGCAGTCAAATTCAGCTG

TGCTGTGTGCTGGCTAACTGTTTGGGGCTTATTCTGTTTTTTTGGTAGTCTGGTACTAAT

TTCTTCTTTTAACTGATCAAATAAGTCTGTCTTCTGCCTGAAATCAGGGATGCTTAATCA

GT

>c15414_g1_i2 len=204 path=[1:0-20 1117:21-27 948:28-203]

GGTAGTAAAGGAGGCTTGTTTTTGTTTATGTAATGGAAGTTTTCATTTGGGTTTCTGTTA

ATGAGGCAGTCAAATTCAGCTGTGCTGTGTGCTGGCTAACTGTTTGGGGCTTATTCTGTT

TTTTTGGTAGTCTGGTACTAATTTCTTCTTTTAACTGATCAAATAAGTCTGTCTTCTGCC

TGAAATCAGGGATGCTTAATCAGT

>c15414_g1_i3 len=448 path=[644:0-298 1117:299-305 69:306-447]

CACCCTTTCTCTCAGGTCATGTTTTCATTCATTCTTCTTGCTCACACCCCCTTGCTCATT

TCCCTTTAGAACGTTCTGTTCTTAAGCTATGTTGCCCTTTCTTGTGTCATGAATCATAAA

TCATAAACAACATCTTTCAGAGGTTTTCTTCGCGTTGAGTGACATCTTTCTCACACACGA

ACCAGAAGTACTCACAGTCACTGGACTGGAGTTCCCAAAAGGCACCCAGTTTTGCCAGAG

CCGCATCACACTCCCTGCTAAATCCTCAAGGCTCCAGATCACCTTGTACAACACTAATCT

TGTTTACTGTTATCAACTTCAACATGGGATTATGTCAACTATTTCTCCTTTCAGATAGCT

CTACCTTTCAGAATTGAAGTTACCTTCGAAAAGATCTTTCAACTAATTCAAGCATTGCAG

TTGACTGATTAAGCATCCCTGATTTCAG

>c15417_g1_i1 len=216 path=[29:0-118 28:119-215]

ACCCACAAGGATTCACAGCATGCAAAGGAGGGCTAGAGATGGGGAAGGATCTGGAGGGCA

GGAGGTGTGAGGAGTGGCTGAGGTCCCTGGGTCTGTCTGGCTCAGAGCAGAGCAGGCTAA

GGGGAGGCCTTGTGGTGGCTGCAGCTCCTCACAGGGAGCGCAGGGGCAGCGCTGAGCTCT

GCCCTGTGTGACTGTGACAGTGACAGGGCCCGAGGG

>c15418_g1_i1 len=251 path=[262:0-9 34:10-250]

AAAAAAAAAAAAAAAAAGGACAGCATCAGGGATTTCCTCAAGATTTTGAGCCCAGTAACA

TCCAAAAAGGCTCTTTAGCTGCAAACTCTGGAATGTTAATGTATTTCTTGAGACTCTCTC

ATGCATCACCTGACTAAGAAAAATCCATAAAAAGGGAACACTCAAGATATGCAGAAACAA

TTAAAAAAACCCCAAACAACAACATCCCCTCTAGTAGGGTACAGCCCAGCCCAGTAGTAT

GGGGATTCCTG

>c15419_g1_i1 len=399 path=[416:0-237 20:238-398]

AATTAAAAGAAGAAGAAAAAAAAAATAAAGAAGGCAAAAGAGATGGTCAGAGATGAGAAG

AGGTCAGAAGATCAAAGTAAATCAAAGAGGGACATAGGTCAGAGGGTAAAGTAGGGTCAG

ACAAGATCAGAAAATTATGTAAGAGAAGGACAAAGAAGCCATGGAAGCTCAGAGGTATGT

CACAGCAGGACAGACTTGTCACAGAGGACAAGGTCAGAAAACAGGTGGTCAGGCATAACC

TTGCCAGGTCAAAAGAAGGTCCCACACACACTGATGTCCTCTCAGTCACTGCTTTCCAGT

GACCATGACAATGAACACTGGAGGCAGACGATATGAACAGAAAGGGCCAAGATGATTAGC

TGCTTATTCCCCTGTCAAGGAGGAAAAAAAAAAAAAAAA

>c15425_g1_i1 len=319 path=[45:0-254 624:255-271 641:272-318]

CGTGCAGAACGTGCACATCCCCAAGGTGCGCGTGGACCCCGACGGGCGCTGCGCCGTGAT

GCTCATCTACGGCACGCGCCTGGTGGTGCTGCCCTTCCGCCGCGACTCGCTGGCCGACGA

GCACGAAGGCCTGGTGGGAGAGGGGCAGAAGTCGAGCTTCCTGCCCAGCTACATCATCGA

CGTGCGGGAGCTGGACGAGAAGCTGCTCAACATCATCGACATGCAGTTCCTGTGCGGGTA

CTACGAGCCCACCCTCCTCATCCTCTTCGAGCCCACGCGCCCCGGCCACGTCTGGTTGGG

CTCGAAGAGGATGAGGAGG

>c15426_g1_i1 len=448 path=[54:0-447]

ATGATAATAATAATAATATTAAAAAACAGTTAGAAAATGAAGATTCTTTAAGTAAATATA

ATAATTATAAATTAAATTCTTCAGATAAAATAAAAAATAAATATGAAGCTGATTTAAATA

GTAGTTTAATTGGTAAACATGCTACTTATAGTATAATTAAATACAATTTAACAAAAAATA

ATTTAATAAATAATGTCTTAAGTGATATGAAATCTAATTTATCTCAAAATAATTTAATTA

AAAATTTGAATAATTGTAATTATTCTAACTCAGTTGATGAAATAAAAAATTTGCCAATTT

ATCAAAAAAAGAAAAAGATAGTAATTAAAAAAAATTGATTTAAATAATTTTTTGTGTTTA

TGTAATTTTTTATTTTTTAATATAATTTATTATTTACTTAAATGAAAAATATTGTGTTTT

TTTAATTCTTCTTTTTTTTTTTTTTTTT

>c15428_g1_i1 len=311 path=[339:0-310]

ATATAATGTGGAAAGTTTTGAACAGGTAATTATTATATAAAAAATATATATATTTGTAAA

TTTTTTTTTTATAAAAAAAAAAATTTGTCTGAGAAAGAAAAAAAAAATTATTTTGACATT

AATAATATTGATTAAATAAATGTAAAAAATGGTGAAAACATAATTAAACAAATTTTTTTT

TCATTAAAAAAAAATTTTTAAATAAACAAATATCCATTTACCTCATTAAAACAAAATAAA

GCGAAAATAAAAAATATCAAACAGAAAACTTCAAAGTAGTTATTAAATAAGAATTTAAAA

AAAAAAAAAAA

>c15432_g1_i1 len=319 path=[41:0-159 354:160-160 355:161-318]

GTAACTTTGTGTTTGAATGGCTGCCTCTTGAGAGGTTGAAGTCTTACGAATGAAGAAAGG

GACTCTCCCCCACTGAATCCATGTTGTCTTGACAATGCAAATGGTTCTTGTAACGTTCAT

CATTAATTGTATTACAGGAGTGGGTTACACTCAGAATTCTGGACAACGGTGCTGTTGTAA

CAGGTGTCATTGTTAGGAAAGAAGTACTATAGAGAAGGTGTAATGTTAGAGTAGGAAATC

AACAGGCTAAGAAAGAACAAATTTTTCAGTGTATTCAATTATTTAAGATAAAGAGTAGAA

GCAAGGAGGGTGTTGCTTT

>c15433_g1_i1 len=201 path=[53:0-13 52:14-200]

GGGGAGTTTGGTAGCTCTTGAACTATGTAACTATGTAAGCTACAGGATGGTAGCCTTGGT

ATATTAAACTTAGGCATGTGGTAGCATAGCACAGTTTTAATACCATCTTTTGTACAATAA

ACTTGCTTGTAAAATAAGTTAGTAAATTCTACTAATCTCTCCTTTGAATGTGGATTTTTG

TTTTTTTTTTTCATCCTTATG

>c15437_g1_i1 len=212 path=[48:0-65 42:66-211]

TTTATTTTTTTTTTTTATTTTAAAAATTATGTACTGAAGATAATATCATTTTTATTAATA

TAAATAAATATAATTTTATTAATGATGTCTCCATTAATAATTTTTTTAATTAGATACTTT

TAACATCTCACTACTTATTAATAATATTAAGTTTTATTAAAAAAAAAATTATCACTTTAT

TTTTCTTTTTTTTTACGATTTATTGTTGAAAG

>c15441_g1_i1 len=976 path=[31:0-273 304:274-274 15:275-975]

AAAAAAAAAAAAAAAAAAAGTTTTGAAATTTTTGTAATATTTTTGTCATATATAAAGTAA

ATTAAAAAAATAATTTTTTTTATTTTAATTTATTTTTAAACATTTTCATTTAATAAATTA

TCAAATGATACTCCCTTGCTTAAACATCTAATATAATGATAAATAAAATCTCCCATTAGA

GCTAATTGAACAACTTGCATAATAACAACCCATAAACCAACATAAGACTTAATAATATTA

TCTGATGAGTTCAGTTCTTTATGTGATACTATCCAAAAAAAAAAAGACAAAACTTTTGAA

AAAGCTTGTGAAGCTAAAAAGTGAGTTGTAAATGCTGCTACTTTTCCTTCCTTCTGAAAC

ATAGATAACTGTGGTAATACACAAACTGATTCTAGATATAAAGCAAATGACCATGATACG

TCAGCTGGTAGAAATGAATTCAAAGATGGATGAACAAAAACTGCAATTATAAATGCAGGT

ATAATTAAAAATAAGTTATTAAATATATCATTTACATTGTCATATGTGTTTTTATATTTA

TATCTACACAAATATACGATACAACAATTAATAAATAAAGAAATTGCTTCAACTAATTGA

TATAACCAATCTCCACTTTTATCATATGGTAGATATCCTTCAAAAGGTACTATAGATAAT

AATCTTGCAGTATTTAGAACAACATAACATTCCATCATTTTTAATGAAACACCTGCACAA

GATTTGTTCATTTCAATTTTAACAAATACCATTAGAAATGAAAACATACTAATTACAGAT

GATAATGTTAATAAAAATGAAAAATCTCCATCGGAAAATAAATGATAAAAAATAAAAACT

AAAAATAGAAAGAAACAATATATTTTTATCGAAGAGCTATGATCTGATAAATATCTCTTC

ATTTTTTCTGTATCATTAACTTTTCTAATAAAATCATTTACCCCTTTCATTTTTTTATAT

TATAGATAAATATATG

>c15443_g1_i1 len=252 path=[371:0-178 549:179-251]

CTTCTGAGTTCCTGTTGGCTCCCAACCAACACCTACTCCAGCTCAGTTTTTACATATCAT

TAATAATAAAAAACATATGATTTTACATATCATAAATAATAAAAAAATAAGCCTCCTCCG

AGATTGTACCATACTCATTATTTCCCACTTTTATCACTGACTGATCACAGAATGTGGTTA

GTTACTGACTGAAACAGAATGTGCACTGTTTCTTGGGCAATTTAGTGCATTTTCTTTGAG

GAAATTACTCCA

>c15443_g1_i2 len=225 path=[371:0-178 25:179-224]

CTTCTGAGTTCCTGTTGGCTCCCAACCAACACCTACTCCAGCTCAGTTTTTACATATCAT

TAATAATAAAAAACATATGATTTTACATATCATAAATAATAAAAAAATAAGCCTCCTCCG

AGATTGTACCATACTCATTATTTCCCACTTTTATCACTGACTGATCACAGAATGTGGTTG

TCTTCTGACTGAGCAATTCAATCTTTAAATAACCTTTTGATAATA

>c15445_g1_i1 len=240 path=[727:0-13 729:14-239]

AAGCAGCAGTGATACCTTCTCTCAGCAGCAGTGATAAACCTTTGGATATCCTGAGAAATA

AAACTGAAGTATAGTAAAGATCAATGTATTCTACCAACAGGGTTGTGGGCTCTTTTTCAA

GATAAATATGTTCTCCCAATCTATTTTGTCTTTCATATGAAGAGGGCAGAGGGAAAGGTT

AAGGAATACCAGTTCAACTGTTCCACCAATATCCTTAACCCAGTACAACACGGGTCCTAA

>c15445_g1_i2 len=365 path=[372:0-138 729:139-364]

TGTTTGAAGCATTTCATAGATGCACACAATAATCAGCAGAACACACGCACACACAGTGCT

GAGTTGAACACACACACACGCACACACACATCTGGAAAATGGGTTACAGTGGTTGAAGTT

TCAAAAAGGTGTACTGTCCCCTTCTCTCAGCAGCAGTGATAAACCTTTGGATATCCTGAG

AAATAAAACTGAAGTATAGTAAAGATCAATGTATTCTACCAACAGGGTTGTGGGCTCTTT

TTCAAGATAAATATGTTCTCCCAATCTATTTTGTCTTTCATATGAAGAGGGCAGAGGGAA

AGGTTAAGGAATACCAGTTCAACTGTTCCACCAATATCCTTAACCCAGTACAACACGGGT

CCTAA

>c15446_g1_i1 len=233 path=[35:0-122 533:123-130 165:131-232]

AGGTGTTTTGATATTGCAGTATTTACAGATACATAAATATGCTACAACAGTTATTTTCTC

CCACTTCACTGAAAATCTTGTTCAAGTGTCTTCTGTGATTACTGGGAGAACATTATGTAA

ACTCTGTTTGAGAACCCAGGAACACATTTTACAGGAAGTGCCTGATGATTCCTTTATCAA

AGCATAATTTCCTTGGTGAAACCCTGAGAAACTGGAAGAAAACTCGATCTGTT

>c15446_g1_i2 len=232 path=[35:0-122 533:123-130 440:131-231]

AGGTGTTTTGATATTGCAGTATTTACAGATACATAAATATGCTACAACAGTTATTTTCTC

CCACTTCACTGAAAATCTTGTTCAAGTGTCTTCTGTGATTACTGGGAGAACATTATGTAA

ACTCTGTTTGAAAATCAAGAGACCTTTTGCTTTATCTTTTTGCGCTGTTTTAAAATTGGA

TGTGTGTATCTGCAGCTGTCTCCTGATAACAGATCGAGTTTTCTTCCAGTTT

>c15448_g1_i1 len=251 path=[53:0-250]

ATAATAAAGCTGAGTTTAGCCATTATGAGCACAACACTGGTCTTTTTCCTTTTTTTCTTT

CTTCTGAGGGTTAATGCTGAGATAGCAAACGTCTGGATTTCTGGTTAGGTTGGAACTTCT

GGTACAGCTGAAACATGATACGCTGTTTCATATCTGGAGACTGCCATTCAGCAAACTATT

CTTGGTATTCTCTGTTAAATGAAGAGAGAGTCAGTCAGTTCTTACTTCCCTACAGAAACC

CAACTTCATTA

>c15450_g1_i1 len=213 path=[87:0-164 251:165-212]

TAACATACAAATATACTCTGCTGCACAGTGCCCTGCAGCACTCTTCTAGTCATTGCTTTA

TTAATATCCTCTTTGATTCATCCCTGATTAACTCAGATTGGTCTTGCTGCAGTAACTCTG

AAGACTTCCATAAGATTACTGGGTGGAGGCTGGTCACAAGTGGTGTCCCTCGGAGGTCTG

TCTTGGGAACAGTGCTCTTCAACATCTTCATCA

>c15452_g1_i1 len=232 path=[47:0-170 217:171-231]

GGGAGGACGTCGGAGCAAAGCTCTGCTGCCTCACCCCGATCTGGGGCTGCGCCCAAGGGA

CGCTCCGGGCCCCAACACCCACGGGAACGCGGCGACGGGCACTCAGCACCGGCACTGAGC

ACGCAACTCTCGTGCTGCAGGACGGGGAGAGCTGCATTTCCTCTCTCCAAGTGCGTTTTA

TCTGCCCAAAGCAGGCAGTTCTGGCTCAAAACGCTGTGATCCCCACGTGCCG

>c15453_g1_i1 len=226 path=[1:0-41 439:42-225]

AAGCACCTGCGTTCCTGCCCAATACACCAGAGATGCACAGCCACGAGCAGCTGATTTAAG

GCAAGCATTAAACTTCTGCTCATTGATTTCCAGCTTTGTTTTGCACGGGTACTTCCTAAA

GGGCTGTTAGCAACCAACGAGACTTCAGCACGCGCGGCGCTGCACTCAGAGCACAGCCTT

TTGCTAAGCAGCCGCACACGCCGTGCTTAAACACGTTTGTTTAAAT

>c15454_g1_i1 len=255 path=[1:0-254]

CTGTAGAAAGGTCAGTTTCTGCAAGACCTGTGGGCACTGCTTGAAGATACGTATGTTGTT

GGGGAAAACTGCCCATGAACAAGGCTGAGAGAATAGCAGCTGTTCTGGCAGGAAGGGCCC

TAAATAACCAGGTAGGGGAAAATACCTATCTGCCATGTATATGAGTGGGTCAGATCAGCA

GCCCAAATAGCTGCATCTGGTACAGGAAGGCACAGCACAGAGACCATCAAGCAAAGACAA

GGCAAGGAGGAGAGG

>c15455_g1_i1 len=340 path=[1:0-339]

TTCTCTGAGAGAGTAAGCAAAACATCCCATTTGCCTTCCCTGCCTTGTTCGCTGCTGTGC

ACAGCCTGTAAAAAGACACACCATAAACAGTTCCTATTTTGCCGTTCCCCTTGGCTGAAT

CTCTCATCTGACATATGTTCCAGTGTATTACCCATCTACAGCCTGCTTGCTTGCTTTTAC

AGCCCAGACTTCAGTTTTGATTGACACATCTGTTGCTTTGAGTAGCTATGATAAGCAAAA

AAGGCATATAGCTATAATTACCCATTGCTCCTTTTAAAAATGTGACTAGCCTTCAAAAGC

TATCCAACAGTGTGAGAAACTCACTGATGTAATGGTATCG

>c15458_g1_i1 len=226 path=[1:0-225]

GCATCTAGTAAAGGTGAGAAAAGTCCAAGAATCCAGGGGCATGAGTGCCTTCTGAATCAC

TTGCGTGCTTTTGAAACCTGTTTTTTAGCTCAGGGTTTGGAAGAGCTTGATCACGTGGAA

ACAGATGCAGGATTTTATCCCAGGGAAATATTTGTCATAAACTGTAGGTAATGATTTTCA

ATAGCTGTCACAGCAACTGAAGCATGCTCTTTTGGAGTTAACATAA

>c15460_g1_i1 len=311 path=[289:0-310]

CTTGAACTCACAGGGCACATTAAAGACATCCTGCGTTTGTCCCTCCAGTGTACCTCCACA

GACTGATTTGTATCAAGGATGTTTTAAGAAAAGGAGTTCTCAACCACACTTTCAAGAACA

GCCAGTCAGACAAATATAACTCTGGTCTACTGTGGTACAGGTATATTAACTCAACATATG

CAAAACAAATATAAAAAAATCACTGGTTTGCAATTATGGTTCTCAGAACATATTGTCACT

CTGTGTTCATACACTTGGAAGTCTACGATTGGGAATGTCCAACATCCAAAATCATTCACA

TGTGAATTAAG

>c15464_g1_i1 len=210 path=[1:0-209]

ATGAAGAAGATAAGAAGAGCCCTGTAAGACTTTGTTTTTCTTTTTTAAATGGTATAAGCA

AATCAATCATTAATAGTAGGTACAAAGATGGCCCAATTTTGATTACCCTATTGCCTGGAG

AATGGATTCGTCAATACAGTTGGTACAATAGGAAAAGAATATGCTTCCTAACATGAAAGT

GATGGCCAGAACAAAAGAACAGGATTGGGG

>c15464_g2_i1 len=223 path=[375:0-222]

AAGCCTTTCCCAAGCATCTGCCCCAACTCATTCAAGAACCTACAAAGATGCACAGGTAGC

TGCCTGCTTCAGAGGGGATATGCCTCTTTTAGGTCTGAATCCAGGTATTTTACTCTTTAA

TGATCTGCAGAAAGACAGGAAGGTTTTCCTAAGCTAAATATCAGAATGGTAGATCATGGA

TGAAGCAAGACATGAAGATAAGGGGAGCCTGACCAGCATCTCA

>c15466_g1_i1 len=278 path=[1:0-277]

TCAGAAACACTTAATTCCAGGATCGGGTTATTTCTGGGCAGGAAATCTAAATCTAGTGTA

AAGTATGACACTTAGTACTTGCTTAAAACATTGAAGTAGTTTCTGCTAGTATTGCATCAA

GTTCCACCTGTGGAATTAGGGGAATAACCCAGTGGATTCACTGACAAAATGCTTTCTTTG

GCAAAACACACAAAGGCTCAGAGAGCCTTTGAGCTGGGCAGGCTTCTTACAGGGAGTTGT

CATGGGAATTTTGAGTTACTTGTGTGCAGTGTGGATGG

>c15468_g1_i1 len=277 path=[343:0-187 89:188-276]

CATTGTGCAACAAGATGAACTGAAACAGCAAAAGGAACAAGAGTGGAACAAACTGGAAGA

GCAGAAAATAGACACAGAAGGGCAAAATCTTCATCGGAAGCAACAGGAAAAATCCTTGGA

GCAGCAAAATGACAAGAATGAGCTGTGGTCTGGGGGGGCTGATAGGAATCCAGCAAATGA

CAAGCTCCATGAGAGCCCTTGCTGGAGGCGTGCATCCTGCTGTCCTTGACGGATGGAGAA

GAGAGTCCTGCTCCTTTAGCAGGCATGACTGGACTCA

>c15473_g1_i1 len=206 path=[1:0-205]

ATGACTTGTAGTACTTCGTGGGAGAGTGAGACAAAGAGCATTTGCTGGAAGCACAAAACC

AGATGAAACCTATGGAAGCGTGACAGACAGAAATAGGCCAGAAAGTTTTGCAGTGGTGTA

TTACAAACTGAAGGTACCACTACTGAGGTCAATCTTGAATTCAAAACTCACAAGCTTCCA

AACAGCTCCATTGCCTTGAAGTCCAG

>c15480_g1_i1 len=211 path=[189:0-210]

GTATTTCTTGTCTGCATGCCATTGTGCTCTCCATGTAACTGAGCCTACAATATAAAGGTA

TTCCCAGCTGAAGTAAGCAGTGCTCAGAGGAACCTCATTCACACTCAAGTAGGTGATGTC

TAAATGTGACCAAGTACAGAAGAAAAGAACGATGTGCTTTTCATAGCAGTGAGTTTGATC

ACCACGCAGAGTGTAATGTATGCTTTCTGGC

>c15488_g1_i1 len=260 path=[681:0-115 220:116-259]

ACTTTGTTGCCCTTCTTTGAGCCTTTACATTCCTACACCATCATTTTTCGATGGACAAAA

CTGCTGAGAGATTTCCAGGCACGAGCACAGCCTGCATTTATCCCCACCTTTCCAATAACC

CTGCTCACCTTATGAGTTATCTGTCTCACCCCAATGACTTTCCTGCAACCACTCTGTCAT

TCATGCTGCTGCCCACATCTCCAAGATCTAGGTCCCAGTATTATCCTGACACCCTTCTCC

AGATTCTGTTGTGCTCAGGG

>c15488_g1_i2 len=363 path=[1:0-218 220:219-362]

TACCAACATGGCATTCCTACGCCTAACCATGATACATGTTGACTTCTTCACCATACTTTG

AGGCCAAGCTCATGCCCCCAGGTGGTATTGCACAGTTGTGTGCTGATGACACTGTCCAAT

CAGGAGCCCTGTGGGTAAGACACCCATCACTGGGCAGGCTGGCCATGCGGACTGATCCCT

TGGAGACTGTCCATGCTGTTTTGAAAGACAATACAGATCAACCCTGCTCACCTTATGAGT

TATCTGTCTCACCCCAATGACTTTCCTGCAACCACTCTGTCATTCATGCTGCTGCCCACA

TCTCCAAGATCTAGGTCCCAGTATTATCCTGACACCCTTCTCCAGATTCTGTTGTGCTCA

GGG

>c15489_g1_i1 len=421 path=[1:0-420]

ACAAAGCATCGCGTTCCCCTGTGCCCCCTGCTGTGTGAGCAGCAGCTCCCAGCCCTGCCA

GGAGGTGGGATGGGGCTGTGTGCTGCTGTGCTTCAGCCCGCGTTCCTCACAGTGGTGGCT

CCACTCATGTGAGTGGACACGGTTACAGCTGCTGCGGCGTGCGGCTGGGCACGGGCCTGA

GCTGTAACTCACACCCAGGCCGCTTGCTTTCTGTCCCCTAAAACATTTTCCAATCAAGTG

ATGCAGTGTGTGCAGAGCAAGGAGTTACGTAATATTTATGTTGAGCATGTGCTTGGAAAT

TCAATAAACTCGCATCTGCTGAGAGATTACGTAAGAAAGGTTTCTCCTGTGTTTTCTTAA

AGAATTTTTAAGTAACTTGAGAACACCGAGGAATATCAGAGCGGGAGTGCACGTGCTTGT

G

>c15489_g2_i1 len=236 path=[1010:0-235]

GCACTGCTGTCTGATCACGAGCCTGCAGCTTGCCTTCTTATTGACACAGCACTTATTTAG

CCAGCTACGGGCTCAATTTGTTGCAACCGAACGCATCCCATGTTCTGCTTCAAGTTGGAA

ATTATATACATTAGTAAAATTAACCTGAGGGATTCGTGATCCTCTTCACGACATCTTCAA

CTCGTACTGTAAAATACATGCTTAAATTACAGTTATTTATGAGTCAGCAAGAACAT

>c15504_g1_i1 len=237 path=[1:0-187 189:188-236]

ATCCCCAGCTACTAGGAATTCCTGTGAGTGTCTTAGCAAGATACTTTCCTGGCTTTAACT

CAGAACATCTACTTGAGACCAACTGGAGTCTGTCTTGCTTCCATTTCTGTGTTGATGCAT

TTGAAGAGGTTTGACTTAGACAGTGGAACATAGGATGTTTGAGTTAAGAAAATGTGCTTT

TTCAAAGGTTTGTTCAGTTCTGTGTACATTTTGATAAATAAGCACACGTACTACTCA

>c15504_g1_i2 len=210 path=[1:0-187 495:188-209]

ATCCCCAGCTACTAGGAATTCCTGTGAGTGTCTTAGCAAGATACTTTCCTGGCTTTAACT

CAGAACATCTACTTGAGACCAACTGGAGTCTGTCTTGCTTCCATTTCTGTGTTGATGCAT

TTGAAGAGGTTTGACTTAGACAGTGGAACATAGGATGTTTGAGTTAAGAAAATGTGCTTT

TTCAAAGGATTATTTTGACATAGTGAAGAA

>c15519_g1_i1 len=242 path=[427:0-143 67:144-241]

GGAGTCTTCTTAGAAAGCCCTTTCTCCAAATTTGCAAAAGAAGTCTCACTGGCCTGTTGT

AGGTGGTGGTACCTCATTTGCCTTTGATCAGAGAGTGTTACCCTTTTCTGTAGCCTCCTA

AACGCACAATGAGCATCTTCTGTGAAAGGCTGAGTTTGTGTGGTACGTCTTTGAGGTGTG

TTGATCCTGGGGCATCGTTCTCAGCTCTGCTGTGCTCTGTGTCTCTCCCTGCAGATTCCC

CA

>c15520_g1_i1 len=398 path=[31:0-220 15:221-397]

TCCGCTGTGGAGCTGGGGATGCTCCACCCCCCAGAACACGTGGGGGCTCAGCAGTGCAGG

CTGTGGGGCTGCTCGGGGTGAAGGCTGAGCGTGCCTTGCTCCCACCGCCGGGGGTGCCCT

GCTGGCCAGGCCCGATGTCCCCATGCTGGGTTGGGGGTCACACTGTGCCCTCACTGCCCG

CGCAGAGCCATCACTGCGTCCGACCGCCGCACACGGCGCCGTGCAGCTCTCACTTGGGGG

GCAGCCGGCGGGCACGGGGCTGCGCTCTGCCCCAGTGCTGCACTTCGCCCCGGTGCTGCC

TGTCCTGCGCCCGGCTCTCCTGACACAGGGGAGCCCTCCCTGCCCCACGTCCTTCACGCG

GCGCTCTGCTTGCAGGAGGAGCTCACCAGCACCAGCGT

>c15522_g1_i1 len=220 path=[1:0-219]

GGAAAATTTGTGTGACTTGCTCACAGAAATAACATGTTCACCATTTACTGGATTCCATGC

GAATAACCACGTGTTACTCTTTGCAGGTTCAGGCATGATACAAACACAGGTGCATACGAA

AACCAAAGTCCACCAATCTTTTCTGGTTGTCGTTTTTCCCCACTTCTCTCTCTCCCCGGA

GGGGCGCTCAGCTGAGCCGCAGCCTCCGGGGAGAGAGAGA

>c15528_g1_i1 len=469 path=[1:0-468]

GAAGAATGTTGACTGCCTCTAAAAACTCTACTAAACTTAAAGCTAAATACACAAGATAAA

TGAGTATGTCTAGACTTAGTACATTAGCTATAAACAGCTATATGATTTTTTTTTTTACAA

GTTTTAATAATATTTTTGAAAGAAATACTGCATCTTACATTCCACACTCTCTCTATAGGA

TTAAATCAGTACCTTTCCCTTGTTATCAAACATTTATCAAAGAACTTCCCAATAAGTGTA

TAATTAATAATTTGACTTCTCTTACTTTATTGAATCACTTTCCTATTAAAGTCAATTAAA

AAAATGGTATGATACTCACACTTGTAGGAGCCTTATTTATGAAAAGTAAAAGTGCATATA

GACATCTAACATATACTAGATTAAAAAGAACTTTTTTTTAGAGTCTGCAAATCATAATGC

GACCTCTTCTCCAAATCTACTTGCTTCTATCCAAGTGATTCACTTAAAT

>c15538_g1_i1 len=291 path=[269:0-252 522:253-290]

GGAGAGATCGTTGGTCGGAAGTCCAATGGCCTCCAAGATAATAGCGATGGTGAGGACTCC

TCCAGCTGGAACCCCAGCTGCACCCACACTGGATGCTGTTGCTGTGACAAGAATGGTGAA

GATTTGCCCAGCGTTGAGGTCAACGTTGTTCAGCTGAGCAATAAACACGGCTGCCATGCA

CTGGAAAATAGCAGCTCCATCCATGTTCACAGTTGCACCAATAGGAAGGATAAACCTGCT

GATCCTGTTGTCGACTCCATTGTTTTCCTCAATACACTTCATCATTGATGG

>c15538_g1_i2 len=280 path=[269:0-252 560:253-279]

GGAGAGATCGTTGGTCGGAAGTCCAATGGCCTCCAAGATAATAGCGATGGTGAGGACTCC

TCCAGCTGGAACCCCAGCTGCACCCACACTGGATGCTGTTGCTGTGACAAGAATGGTGAA

GATTTGCCCAGCGTTGAGGTCAACGTTGTTCAGCTGAGCAATAAACACGGCTGCCATGCA

CTGGAAAATAGCAGCTCCATCCATGTTCACAGTTGCACCAATAGGAAGGATAAACCTGCT

GATCCTGTTGTCGCCTCTATTCTGGGCCACTTCATCCATG

>c15546_g1_i1 len=264 path=[242:0-263]

GTCCTTTATTACCATGTGGAGCTATCAGTTTATGGCAGATTCTCTCATGCAGAGTTGAGA

ATGGTTGGCTTTGTCCCTGACATCTCAAGAACCAAAGGTGTTTCAGGGCACAGACTATAA

AGCTGGGTTGTCCCAATGGGTTGCTGATGCTGGAAGTGAGCGCATCTTCCATCCAGTTCT

CAGCCAGGGTTCTGGCTCCAGCATTTCTCAGCTCTGAAAGCTGAAGCTACCTGAACAGGC

TAATGTGGCAGAAATTCTTTTCTG

>c15548_g1_i1 len=497 path=[134:0-204 339:205-496]

CCTTGGATTGGCCCTCGTATGCCTGGAGGTGCTCCCACACGAAGACGTTCTCCATCTCAT

CCTGGATGGACAACACTGTGCCACCAACTTTCTGGCACCTCCGCATTGCATCCTTCTGCC

CCAGGGTGATCTCCATGTGGAAGGTGTAGCAGTGGTCCCTGAAGGGGATCCAGGATGAGT

CCTCCAGCGACTTGGGGCAGCTGCCGCTGAGCCAGAACAGCTGCTACTGGATCCAGAGCA

GCAATGGAGTCTGGCGCTTGGGCTCCTGCACCAATGTCACCATGGGTGTCATCTGCAAGA

TCCCCAGAGTGGAGGAGAGCAGCTTCTCCAGGGCAGCTCTGCCGGAGAACACGACGGCCA

TCGCGGTGGTGGTGCTGTCGGCGCTGGCGCTGAGCGCTCTGCTGGGCATCGCCATCTTCC

TGTACAAGCGGCGGAGGAGCTCCGAGCGTGGAGCCTTCGAGAGCGCCCGCTACAGCCGCA

CCACCTCAAACACAAAG

>c15548_g1_i2 len=426 path=[1079:0-133 339:134-425]

CAGGCATACGAGGGCCAATCCAAGGGTGCCTGGCTGGGCATGACGTTCAACCCCAAAGGA

GGCACCTTGGTTTGGCACGACAACACAGCTGTGAACTACTCCAACTGGGGCCAGCATGAC

ACGGGGCCCAGCATGCTGAGCCAGAACAGCTGCTACTGGATCCAGAGCAGCAATGGAGTC

TGGCGCTTGGGCTCCTGCACCAATGTCACCATGGGTGTCATCTGCAAGATCCCCAGAGTG

GAGGAGAGCAGCTTCTCCAGGGCAGCTCTGCCGGAGAACACGACGGCCATCGCGGTGGTG

GTGCTGTCGGCGCTGGCGCTGAGCGCTCTGCTGGGCATCGCCATCTTCCTGTACAAGCGG

CGGAGGAGCTCCGAGCGTGGAGCCTTCGAGAGCGCCCGCTACAGCCGCACCACCTCAAAC

ACAAAG

>c15554_g1_i1 len=431 path=[409:0-119 529:120-430]

TGTTTGAGTGGGGTTGGGAGGGTCCTTGGAGATCATAGAATGGATTGGTTTGGGTTGGAA

GCATCCTTAAAAGTCATAGAACTGTTTGGGTTGAGTGGAAGGATCCTTGAGGGTCATAGA

ATGGGTTGTGTTGGGTTTAAGGGTCCTTAAGGCCATGGAACAGGTTGGGTTGGGTTGGAA

GGGTCTTTCGAGGTCATAGAATGGATTGGTTTGTGTTGGAAGAGTCCTCAAATGTCATAG

AATGGGTTGGGTTGGGAATGGAGGATCCTTAAAGGTCTTAGAATGGATTGGCTTGAGTTG

GAAGGCTCCTGAAAGCTCCTTAGAGCCCTGGAATGAGTTCCAAGCTGTCCTCCACCATCC

CATCACCCTCCCCCCTGCGTGACACGTCCCCTCCTCGCCCCGTCCCGCAGACCTGGTGTC

GCAGAGCATCG

>c15556_g1_i1 len=203 path=[1:0-125 127:126-202]

CAGAATGCTGTTAGACTCCTTGGTGGAAGGTGTGGCATTAAAGACCATTGGTGATGGGCG

GTTTCCACAAGCAGGCTGTCCCATACTGTCCATCTATTTGTCCTTCGTAGGGGAAAAAAA

AAGAATGGCAAAACAGTGAGTCTGGAGGGAGGCAAAGCTTGAAATGATTGCACTTACTCC

CTAACCGCTCTGCCGAGGTCTGG

>c15568_g1_i1 len=300 path=[278:0-299]

CATCACCCAGCTCCACTAGGAAGTCTCCATGCCAAGCTCCTGTGTGTCGTTCAAGGGAAG

AAAACACCAACGCTCCCAGTCCATGGATGGAAACTGTCAACTTTTTAGGCTTGTGCCTTT

AAAGATCTCAGATATTTGTATGATCACTGGAAAAAAAGTCTGAGCTGCAGAAAATCTGAG

CCCAACAGAATGGCTGAGTGCTGAGATAGGGGCAAAAGGGGATGTGAAGTGTCCTTCGTT

GATGTTGCAGGGTAAAATAAAAATAATCTCTTACCTACTATCAAAACCTTTTGCCTTGTG

>c15571_g1_i1 len=216 path=[155:0-215]

TGGTGGTACTCTGTGGTGAATACTCGGGGAACTGTTTTCTAATTGTGCATATTTATGTAA

GCTGCAATTCTAATAGACCAGACGAATCCTTGAAGAAATAAGTGTTATTCTTCTAAAGTG

AAATTAAAACGAGAGCATGTGGACCTCCTTTGCTCTTGTTATGGTGTCAGGAGAAATAAA

TTTCAAAAATCAGAGCTGAGGAGGAAGTTAGTATTT

>c15576_g1_i1 len=237 path=[1:0-72 74:73-236]

ATCGTTCCTTGCAAGGAGCAGTTGAATGTCGAAGTAATTCTCGGGTTGTGCGTTTTGGGT

GGAGGGAGATCTGCACTGTGTATTTCTAGATTTGTTAAAAATCGGTTTGAGTCTCATTTT

GCTCAAGTTCTCATTTCTGTTGGAGAATTTCTTGTGTGTACAATCAGGCTGATTACCCTC

GATGTTCATCCCTCTGCTCTTTTCTCTGTGCAGCATAGTGTTGTTTGATACATTTGT

>c15576_g1_i2 len=206 path=[471:0-41 74:42-205]

ACTTCATCATAGGAACACACTGTAGCAACACCTGAATGAGATCACTGTGTATTTCTAGAT

TTGTTAAAAATCGGTTTGAGTCTCATTTTGCTCAAGTTCTCATTTCTGTTGGAGAATTTC

TTGTGTGTACAATCAGGCTGATTACCCTCGATGTTCATCCCTCTGCTCTTTTCTCTGTGC

AGCATAGTGTTGTTTGATACATTTGT

>c15578_g1_i1 len=244 path=[222:0-243]

CAAATTAAGATAAAAAGTTTCTAGCAGAAGGGCAGATACCCCCTCTACTGCAGTCAGTAA

CTATGAAATCTATTCACTAAAATAGGCTGTCCGGGAACACTCAGTGCTTCTACTGCCTGC

TACAAGCAATAGAGATGATGAACATGAGAATCCACTTAAACTTATGATATTCTGCTCCTG

ATTAAACTCTTTTAATTATCGTTATGAAAGTATGCATTTTGACTGGTTTAATAATTACTG

TTTG

>c15583_g1_i1 len=219 path=[221:0-218]

GTCCCAAAACGTATTAAGGACTATTTTAGAGCCTTTAACAACACTAATCAAAATTGAACC

TGCAGCAGTGTCAGCAGTCTTTGGAAAACATCTGTAATTGATACTTGGCTAGGACTGGGT

TCAGCATCTGTACCTCACCTAGTGGTGGACAAATAAACCCAAATTAAGCTTGCATGTTAC

AATTCCAATGAAACACCTCACTGAAGAGGATAAGAGAGT

>c15583_g2_i1 len=285 path=[441:0-284]

AGAAAAAAAAAAAAAACAAAAAACCCTATTCCTTGGGTGTCTTGTTCAGAATTTGTATGA

GGCACTTCTTAGTAAGTAGGGGCAGGTAGCATAGTGATTATCCTACCGCAAAAGGTAATA

GTTGTTTCAGGAGCTGTAAAAAGCAGTTCTGGATTTGTCCAATATAAACTCGTGGTCTGA

CAGTATTCAGTGTGATTCACTGCAGCCTTTCTGCCAACATCAGAAATACCAAGAAAAGCC

TGTTCTTTTGTGAAGAATCTGCTGTGTTAAGTTGCTTTTTATTGT

>c15586_g1_i1 len=236 path=[392:0-209 179:210-235]

AATAAGAAATGTAAGTTAAACATGAGAAAGGTATTTTTGCAAAGACAGGAGCATTTAAAT

AAGGTACGTTTATTTACTGTACTTGCATCTTTTTAGTTAATCTCACAGTGTGAAGAAAAA

GACTTAGGTTTTAGATCAAAGTGCACTGAGGAAATGTCATTATATTTTTAACAACATTTA

TTGGAAGTAAGAGGGTATGGCAGGAGACAGTGTGGGAGTCTGATGGGAATCCCTAG

>c15587_g1_i1 len=211 path=[189:0-72 262:73-210]

GTCAATCAGAGGTTCAGGCCATGAATCAACTGTTCCCATACAGGGTAGATTTAGACCAGG

TATTAGGAAGACATTCTTTAGTGTGAGGGTGGTGAGACGCTGGAACATGTTGCTCAGGGG

AGTTGGAACTAGATGATCCCCCTTAAGAGTGAGCCACTATATCATTCTGTGATGTCTTTT

GACCATTCTGATGTAAACCCAAGCTCAAACA

>c15588_g1_i1 len=269 path=[271:0-242 25:243-268]

CGTGCAGCCAGCCAGCCACGCCGCGACCCCGCGGGACTCGGAGGCTCACAGTGAATTATT

CACATCCCACGGTCACTGACAATGTGTTCTGAACAAAACCCTGAACAAAAAGTTCCCGAC

GCCCCGGGGACCGACCCTGCAGCACCCCGAGCAGCTGAAATGTCGGCCTGGGCCTGGGAG

GGCTGCGCTGGGGTGAGGAGGTTTGGATGGGAAGAGAGGACAGGAAAAGGAGCTGTGCTG

GGCGGGGGCGGCGGGCAGGGAGCGACCGG

>c15588_g1_i2 len=268 path=[271:0-242 513:243-267]

CGTGCAGCCAGCCAGCCACGCCGCGACCCCGCGGGACTCGGAGGCTCACAGTGAATTATT

CACATCCCACGGTCACTGACAATGTGTTCTGAACAAAACCCTGAACAAAAAGTTCCCGAC

GCCCCGGGGACCGACCCTGCAGCACCCCGAGCAGCTGAAATGTCGGCCTGGGCCTGGGAG

GGCTGCGCTGGGGTGAGGAGGTTTGGATGGGAAGAGAGGACAGGAAAAGGAGCTGTGCTG

GGCCCGGTCGCTCCCTGCCCGCCGCCCC

>c15591_g1_i1 len=307 path=[285:0-306]

ATAACTTTTATCACTTCAAAACTGCAAAATAAAGGGATGAAGTCACACATGCACTCCAAT

GCTTATCCTTAATACCCAATCAATACGCAAGCAATTAAATGCATATGTAGATTGCTGTGT

TAAAAATTACGTACAGTGGCAGAACTACAAAGATGCTGAAGTGTTACTGGTTTGTGTACA

ACAGACAAAATGGTCCCAGAGGCAGCAGCAAACTGTACTTTGCACATCGTTACAGCCCAC

CCAGTTCTGATACTTGCAGAAATAAACGTTACCCAGGACAGGTGCATCTAACACGCAGTG

CTAAAAG

>c15593_g1_i1 len=305 path=[1:0-171 173:172-304]

TTTGTGATTCTATGATTCTATGCATGTTTTCAGAATAGTCTGCCTGAAGATCTCTGATCT

TTCTTTCTTTTAGAAGAATCCTTTGTCAAAATGGCTGTTGCTCTGCAAAATGCTTGCCAC

CTTGGCCATGGCTGTATTGTGCATTTTCAGTTGTCTTCTGAGAAGTAGTTTGGGTTAAGT

AGCCATGATCTTGAATCACAAGAATGTTTTCTTCCTTAAACAATTTCATACTGCTGCTTG

CAGTTAAGGCATCTCCTTGCTTGGTTGATGCTTAAAAATTTTCAACATGAAAAATAAGAG

ATCTG

>c15600_g1_i1 len=219 path=[197:0-218]

CAGTTGGAAAGTTTTATCTTGTACCTACTTGACATAAACAAAGTCAGGTTGATGCTAGGG

AGTCATGTTACTAACGATAAATCTGAAGTTGATTGCTCTTTGCCAAAGTAGTAGGAATTC

TGGTGGTGGTGGCAGTGGTTGTTGGACACAACTAATGTGAGAGTGCTTCTGTCTGTATTG

TGATAACTCTGGGATTCAGAGTTATCACAATACAGACAG

>c15602_g1_i1 len=309 path=[287:0-308]

AAACAGCCAGCATAAAATTGGAAAGAAAGTATTCTAATGTTTACTTTCTTGGGAGCTCAC

GGGAGAGTTGCCTGTTTTTATATTGATCTGTGTAGTACGTATGTGTGTATTTATGCTCCT

CTTCAACAGGAGAGAGAGAGCTGAGCAGATGCTGATATGACTGTTGATGTTTCAGTATCA

TGTGGCTAGTGACACATTTGTCTCTTTGGATGACATGGAGCACTCTGTTCTTGATGCAGT

GTCAGATTTGGCCTGTCATGTCTTTGATACAGACTTCATTTATAGAACAAAAAATCAATG

TTTGTGTTT

>c15609_g1_i1 len=208 path=[186:0-20 207:21-207]

CCGCAAATGATCCATCACAGGAATGATCTTTTTACCAGTAAGCTGAAAATGTCTATAAAT

CTAAATGTGATTGCTTGTGAATTGCTACTTAATGTTTTCCAATATGCATATCCATAGGAA

AGCTGAATAGCTGCACCAGTGGAAAATCTCCAGACGCTTACTGTGTTTTCCAGCTCTGTT

TTAGTCAGAATGAGAATCAAACACAAAG

>c15612_g1_i1 len=233 path=[211:0-232]

GTGGTTTATATTTTATTCTCACTAGTGAACGCTGTATTTAGATGAGAGGTTTAATTACAG

CACTGGTGAGAAACTGGGTCATCTAAGATCTGATTAGCCCTCTGGAGATGCCCCTCCTCA

CTTAGTATCCAGGATGATTACTGCATTCCTAAACAACTATGTTCCGCACACACTACAAGG

AAACAACAGCATTACCACTGGACAGAACCATTCATTCCCACTCCCGCACTTCG

>c15614_g1_i1 len=210 path=[462:0-209]

TGCGCAGGAGTTTGCACCAGCTCCATTAAACCGGTACAGGTTTGCAGCAGTCAAGGCTCG

GTAAACAAAGCAAACCGCTAAGTCATATTTAAGCCGCCACGCAGAACATATTTCTTTTTT

CCTAGTGGGGAAAGATTTACACCAGATTATCTTAAATTGGTTTTTAAACAAATTCAGCTT

AAAGCGTTTAACTCGCGGGCGCAAATAATA

>c15616_g1_i1 len=239 path=[1:0-238]

GCATCCGTATCAAAGTATGTAAACTATGTCAAAGTAAGTTTTGGTATAGGTTACTTCAAC

TATGGCGATCACTGAGCTTTCTTTCCTATAAACTCCCATAAATGAGTTCTTGAAATTCAT

TGGAAAAATAACTAGTTTTGTATACTTGTAGGAGGGATTTCTGCCATCTGTGGTTTTGCT

CAAACTCAATGCTTCTAGTTCATGTTTTTTTCAGACCACCTATTACTGCTGTGTATGAT

>c15632_g1_i1 len=211 path=[189:0-210]

AAAGGCAGTAAAATCCCGTTCTGTCTACACTGTTGAGAGTCACGTCAGCTTGCAAAGGGT

CAAAGGCTTACGCAGCAATTTTTGCTAAACACAAAGTTGTTCCACAATACCATTTTACAC

ATGCTATTCTGGGAGTCTGCAGTTAATTACATTTCTGACAGTAAATAGGCAAAACGTCAG

GAAAAATTAGGTATTAGGAAGTGATTTTTTT

>c15633_g1_i1 len=252 path=[230:0-97 328:98-251]

TCTTATGAAAAGTTACCTTCTTAAAATATCCCATACTAACTATATCCAGTCTTCAAAAAC

TTACCAGCTGTAGTTACTGTTATGAGGAATACTCTGCCTTTTAGAAGCTTATTTTTTAAC

TTTTTACAGTTATTTACAATTGAAATGATGTGTTGTGAATGCATCACAAATACAGACAGA

ATGAAAAATAAACTTTCAATGGTACTGTAGTGTTCTGATAGATGAAGTTTTATTTTTAAA

ACAAGCTTGTTA

>c15634_g1_i1 len=319 path=[327:0-271 705:272-318]

TAATTTATTGCTAGACTGTAAAGAGACAAGTATTTCTAGTGTTCAGAAAACAAATGTGCA

GTGAGTTCATTCACAAATCCCACAACCATATGACTAAAGATTTGTTTCCATGAATAATTA

CCAGCTTGGGTACACAAATTACTGTTTGTAAGCAAATGTGAATTAGGCAGGGAAACAAGC

TCTAATATGTCCCCTTCTTTCCAGATAAATTTAAGAGTTACCCTTGAAAAGTTTGACCAA

TAAGTAACAGAGGACACCTAAAAATAGCAACCTTACTGCAGGATATATGGTACACTGAAC

AGTAAGGTTGCTATTTTTA

>c15640_g1_i1 len=540 path=[1:0-539]

AAATGTTTTTTTATTCTTTAAATTAATTATATCTCATTTTTTTCATTAAGATTTTATTGT

TTTTATTTTTTTTAAATAATTATGTATATGAGCAATTTTTTCTGGTTTTCATTTTTTCTT

TTTTTTTATGTTCTAATAAATCATAATCAGCAGTTCTAATATTTATATTTGCACTTGAAT

TGCTTTTTTGATTCTCTATATTTTGTAATATTTTCTAAATTTAGCTATTTTTTTATTTGA

TGATCCACTTAAATTTTGTATATTCATATATATTTTTACAATTTCTTTTTATATCATGAA

TTCTCTATTATAGCAATATATGAATAAAATAATAGCTTTATTTAGAATTCTTTTAAAATA

TTTTATATTTATATGGTACAATAATTTTTATACTTTCTGTATATTCTTGATTATTTTTTA

TCAACATTCTTTTTTATAAAAACTCATTTCATATAAGTATATTTTTTAAGGAAAAAACAT

ATTTCTTTTTATGAATGGCTGTACAAAATATTTTTTTTTAGCTTGAATAAATAACTATAT

>c15642_g1_i1 len=438 path=[445:0-65 511:66-408 853:409-437]

TTTCTGTGCTTCTGCAACACCGTGGGCGTCCTCTCTCTGAATACTGCCAATGGTTTGCTG

GGAATGGAGGGGATGATTTGCTTTGCCAACAGAGAGAAGCGTGTGAGAAGATGGAAGAAA

GGGTGTTGACTTTGCAATAGCTCCATAGTTGGAGTCCATTTTGCATTCCTTCTTCCTTTC

TCCTCTTCTTCCAGAATAGTGATGTTATAGGATGCAGAACAGAGCCTTGTTTTGGAGAAA

TCTCTGCATAGTGGTCAGCAAAACTCTTGAATCGAGTGTAAAAGTCCATCAACATGCATT

TGGAATGACTGTCAGGGAGGCTCAGCTGATGTGTCTTCCCAAGTAGATGCTGCAGATGGA

TTTGAGCCATGTCAAATGTTATTTCCTGCCATCAAAATAACTACTGGTGTGAAACTCTAG

AGCTCCTGACCCCATCGC

>c15642_g1_i2 len=356 path=[887:0-12 511:13-355]

TAACTTTGTGTTTGAGGGGATGATTTGCTTTGCCAACAGAGAGAAGCGTGTGAGAAGATG

GAAGAAAGGGTGTTGACTTTGCAATAGCTCCATAGTTGGAGTCCATTTTGCATTCCTTCT

TCCTTTCTCCTCTTCTTCCAGAATAGTGATGTTATAGGATGCAGAACAGAGCCTTGTTTT

GGAGAAATCTCTGCATAGTGGTCAGCAAAACTCTTGAATCGAGTGTAAAAGTCCATCAAC

ATGCATTTGGAATGACTGTCAGGGAGGCTCAGCTGATGTGTCTTCCCAAGTAGATGCTGC

AGATGGATTTGAGCCATGTCAAATGTTATTTCCTGCCATCAAAATAACTACTGGTG

>c15646_g1_i1 len=247 path=[1:0-144 146:145-246]

AGTAGAGAAGATGCTTTTCTTTTCCTACTGGTTTGCTGTCTGTGCTTCAGATGGGTTTCA

CACCTTTTTTCTTTCAATGATTCATGTCTCAAAATTTGAAACTGATTCACTGATTTCATT

TCTGTAGCTCCTATTATTCATGTGATGCAAAGCGGACAATCTGTGCCCCACAGTTCAAAC

CATCCCTTTGGGCAGTACGTAATTCTGACTCTTGGGTTTCTCAGCTTACTGAGACTGCGA

TACTGGA

>c15652_g1_i1 len=221 path=[236:0-58 295:59-220]

GCATTTTCATCAATACATAATATGCTAAAAAAAAAATAAAAAAAAGTTGTAATATAATTA

TGAATATAAATCTAAAACAAAAATAAATTGAAAATATATTAGTATTTCATAAAAATTTAA

ATTGATTATAAACAAAAATAAAAAATGAATTTAGATATAATTATGCAAATGTATAATTAA

ATAGAAAAGGAAAGACATTAAACAAATTAATAATTAAATGA

>c15653_g1_i1 len=607 path=[1:0-554 2391:555-559 561:560-606]

CCTTGTGTTTGATCTTCATCTCTTTTTTCTTCATTACTTTTATTGTTGTTTTCAATATGA

GCATTATTTTTTAATATGCTCTCTTTTTTCTGTTCACATTCTATTTTGCTAATTATAGTA

TTATATGGATATATATAATTATTTAAAATATTATTTGATTCATTATCTGTTATTTCATTT

TTTTTATTCAATTCTATATCTTCATATTTTTCATTTTGTTCATTTGTTATTTGTATATGT

TCCTTCAACAATTTATCAAAATCTTCTGAAAATTCTTTTGTGTACTCTTTTTGATTTTTA

CTTTTACTATCTTTTATTTCATTAGGGTCTTCTTCAATGTCTTTTTTTTTTTCTTCATTA

TTTTCGTTATTTTCGTTATTTTCGTCATTTTCTTTAATTTCTATTGAATCATTATTTTTG

TTTTGTAAGTTTGTTTTTTCATTTAGAGTTGAAGCATATGTATTATCAAAATTTCCATTT

AAGGAATCATTTTTATTAATTTCCTCATCAAACAAATTAATTTTCTTTTTGTCATTTTTA

CAATTAAATAATATTCTTTTATTTTTATTATACCATCTGTAAATTGTTTTTAAGCATTGC

AATCTAT

>c15653_g2_i1 len=608 path=[1170:0-517 2391:518-522 1693:523-607]

AAGAAAAAAAAAAAAAAAATGAAAGAATTGAATAATAATAATAATTTAGAAAATAAAGAT

GAAATTATAAAGTTAAAAAGGGAAAAATTGAAACCAATTCAAGTTAGAAGAAGCATAAAA

GACATTTTAATTGATAAAAACCCTTGTGATTATATTTATAATTACACTGGAAGAAATATA

AGTATATTTCCAAAAGATTTATTAAGTAATAATGAAAATATAAAAGATTCTTTTAATGAA

TCTGAAAATGATAAAAATAATTCAGAGAAAGAAGTAATAAGTACCATATCAATAAATGAA

CATGAACAAAAATTATTAGAAGAATTAAAAGAATTACTAAAAAAGGATTTAAGTAATGAA

ATGAAAGATAATATAAAATCACTTTTTATTGAAGTTCAAGAATTTTATTTGGAAATTAAA

AATGACTTAAAAATTAATTATCCATCAACTGAAGATCTATTAAAATTATATTCTACAAAT

GATTGTAGTGATAAAAGTAAAAATGCAATATGGAAAAGCTTTTGTTTTTATAAATTACTA

AGTGATCAATTAAACGATTTGCATATATCTACTATTTCTTCTTATAGATTGCAATGCTTA

AAAACAAT

>c15653_g1_i2 len=645 path=[1:0-554 2391:555-559 1693:560-644]

CCTTGTGTTTGATCTTCATCTCTTTTTTCTTCATTACTTTTATTGTTGTTTTCAATATGA

GCATTATTTTTTAATATGCTCTCTTTTTTCTGTTCACATTCTATTTTGCTAATTATAGTA

TTATATGGATATATATAATTATTTAAAATATTATTTGATTCATTATCTGTTATTTCATTT

TTTTTATTCAATTCTATATCTTCATATTTTTCATTTTGTTCATTTGTTATTTGTATATGT

TCCTTCAACAATTTATCAAAATCTTCTGAAAATTCTTTTGTGTACTCTTTTTGATTTTTA

CTTTTACTATCTTTTATTTCATTAGGGTCTTCTTCAATGTCTTTTTTTTTTTCTTCATTA

TTTTCGTTATTTTCGTTATTTTCGTCATTTTCTTTAATTTCTATTGAATCATTATTTTTG

TTTTGTAAGTTTGTTTTTTCATTTAGAGTTGAAGCATATGTATTATCAAAATTTCCATTT

AAGGAATCATTTTTATTAATTTCCTCATCAAACAAATTAATTTTCTTTTTGTCATTTTTA

CAATTAAATAATATTCTTTTGTTTTTATAAATTACTAAGTGATCAATTAAACGATTTGCA

TATATCTACTATTTCTTCTTATAGATTGCAATGCTTAAAAACAAT

>c15658_g1_i1 len=461 path=[439:0-460]

GTCCTAGAGAGAGTCAGAAAAGTAACTTCTCCAAGATGAATCCAGATAGGGCAGGGAAGA

AAAGACTTTGTGGAGAGACAAGCCCAGAACTCAGCATTTCCAGTGTAGTGGGTGGATGGC

ATTGGTATCGCTTTATTTTAATGTCCATCACCCCCAGATTGTTTTTACCGTCTCCTGCAA

CACTGCATTCAGAAATGGTGGTGCATCCCTTCATGGCAGCTTTCACAGTCAGTCCACCTG

TATTCATTAACCCAGCCAGGTCAACACACTGGTTTTCAGATCCAGTGCAGTTTACAGTTT

CGTTATGACACTGGAAAGAATCCACACTATAGCAGGCTGGACACTGGAATCCATTGGGCA

CATTGTTCTCTGGCGGCAATGAGACGGACGCTGTCCGACAGGCATCACCCACACAGCAGG

CCAAGTGGCTCCTTGCTTTCACCTTCCCGTAGTTCATGGTG

>c15659_g1_i1 len=280 path=[255:0-279]

AAAGAAAAAAAAAAATTGAAAATTTTAATTAAATTTTCCTTATATTTTTATTTTCAAGAT

ACCATTTTTTTTTGCTTTGTTTACTATTTCATATATTTACAAATTGAAATAAAAAATAGA

GAAATATTAATTTTTCTGTCTTATTTTACCTTATTGATATTATTATATTATTACCTATTT

AATATTCTCAAATTTAATTATTTAATAATACAACAGAATAACTCTAATTTTATCCTCCAT

TTTTAAAATAAATTAACTGGTTATTTTAAGGAAACTTGTA

>c15662_g1_i1 len=226 path=[279:0-225]

GTGGACGGCTCTGCTCCTGCCCGTGACAATAACTGCTTCGGAGATCATTGCTAATAACTG

CAAAGCTTCTTTACCTTCAGTACGTGTGCGGCTGGGAAGAAACCCGGCGTGATTTCGGAC

TGTGTCGACGTTCTGTTGGTTGTTCTGGTTGTTTTGTTGGCTACTTTGGCTTTGCGCTCT

GAGCACTCCTCTGCTCCTCCAATGGGAGCCCATTGCCGGGAGCGGT

>c15664_g1_i1 len=219 path=[337:0-77 414:78-218]

TGTTAGAACCATATAAACCAGTCCACAGAATAACAGTGAACTGGGTCCATACAGGAAAGC

AGGGACGCGAGACAGAAGACTCCTAAAGTCCAGCTTCCTACAAGAGAAGTACTGCGCTTT

CCCATCTCTCAGCCTGCTTTGTTCAATACAGCACAGACAGACAGTGGTGTGAAATGGTTT

TTATTTGAAGAGCACAGATCAGTTCCATTCAGGGGTCAG

>c15664_g1_i2 len=219 path=[337:0-77 25:78-218]

TGTTAGAACCATATAAACCAGTCCACAGAATAACAGTGAACTGGGTCCATACAGGAAAGC

AGGGACGCGAGACAGAAGCTTAAAGGCAGTGCTTTTGGAAAAACAGTAAATGCCTACAAA

ATAGCAGAAGAACAAAGCATGTGGGTTAGGAGCATAAACAGTGAAGACACCATCACCATT

AAAGGGCTGCTCTCCTGACCCCTGAATGGAACTGATCTG

>c15665_g1_i1 len=570 path=[493:0-52 492:53-569]

TTTTTTTTTTTTTTTTTTGTATTTTTTTTTTCTATTTTTAATCCGTTTAAAAGAAAAATG

GCGAATATAGCTAGAGAGCTTCTAAAAAAGCAATTTCTTGAATTAACAAGGGATCATAAT

GCTGGTTTTTCTGTTGGGTTAGTTGATGAATCTAATTTTTTTGAATGGAATGTATGTTTT

GAAGGACCCAAGAATACACTCTATGAAGGAGGTATTTATAATGCCACACTTTCTTTTCCA

TCAGACTTTCCTAATCATCCTCCCCAAATGAAATTTACCCAAGAAATGTGGCATCCAAAT

GTTTTTCCTGATGGAAGAGTATGTATTAGTATATTACACCCTCCTGGAGTTGATATTTAT

AATGAACAAGAAAAACCAGAAGAAAGGTGGAGACCAATATGGTCAGTAGAAGGTATTTTA

GTTAGTGTAATTTCTATGCTTAACGAACCAAATTTAGAATCACCTGCTAATGTAGATGCA

GCGGTACAATTAAAAAATAACCTACATGAATATAAAAGTAAAGTTAGAGCTCTTGCAAGA

ATGTGCTAACAAAAAAAAAAAAAAAAAAAA

>c15665_g1_i2 len=762 path=[247:0-244 492:245-761]

CATCAACTAACCCAACAGAAAAATTACCACATGATAAAATTATGAGTATAAAATAACAAA

AGAAATAAATTAAAAAATAAAAAAAAATGAATGAAAAAAAAAAAATTTGGACATTTTTTA

ATATTTATTTGGACTTTGTTTTTTTTAAATGATTAAATTTTTTCTCAAAGAAAATTTTCA

TATATTAAATGAATTTTAATAATATATAAAAAAATTAAATTTAAAAAACTTATTTATTTA

ATCAAAAAAATGGCGAATATAGCTAGAGAGCTTCTAAAAAAGCAATTTCTTGAATTAACA

AGGGATCATAATGCTGGTTTTTCTGTTGGGTTAGTTGATGAATCTAATTTTTTTGAATGG

AATGTATGTTTTGAAGGACCCAAGAATACACTCTATGAAGGAGGTATTTATAATGCCACA

CTTTCTTTTCCATCAGACTTTCCTAATCATCCTCCCCAAATGAAATTTACCCAAGAAATG

TGGCATCCAAATGTTTTTCCTGATGGAAGAGTATGTATTAGTATATTACACCCTCCTGGA

GTTGATATTTATAATGAACAAGAAAAACCAGAAGAAAGGTGGAGACCAATATGGTCAGTA

GAAGGTATTTTAGTTAGTGTAATTTCTATGCTTAACGAACCAAATTTAGAATCACCTGCT

AATGTAGATGCAGCGGTACAATTAAAAAATAACCTACATGAATATAAAAGTAAAGTTAGA

GCTCTTGCAAGAATGTGCTAACAAAAAAAAAAAAAAAAAAAA

>c15667_g1_i1 len=302 path=[1:0-292 294:293-301]

TTTCATTACCTCTTATACAAGTCAAACCCCTCCTGCAACTTGAGACTGTTTCCTCAGGTC

CTGTCTTTGTCACCTGCGAAAGGAGACTGACACCTCCTTGCTGCAACCTCCTTTCAGGTA

GCTGTAGAGATTGATGGTGTCTCCTCTCAGCCTCTTTTTCTCCAGACTAAATAATCTCCA

TAGCTGCTCCTCATAGGTCTTATTTTCTAGTCCCTTCACTCATTTTGTTGCTCTTCGCCC

CAAGAGTAAATAAGAAACCCTAGTCTTGTTCATTTTTTCAGTGTTTGTTTCCCTTCCTTC

CT

>c15669_g1_i1 len=245 path=[514:0-173 687:174-244]

GTCCTCGGTGACCAATGTGTGCAGCCAGGCTTCGTCCGCCACAGGAAACCCTCCCTGTCG

GTGCCCAGCATACGGGCCGTGCCCATCCTTCCATTGCAGCCCCTTCGCTTCCGTCGCGAC

GCCGACGTTAGGAGCACCAGCAGCGTCTGACGGAGGCTCTGGAGGTGAAGGGTTTGAATT

GTAACGGCTCTCCGCGGCGGGTCCGCCGTTCCTCAGTGCTATTGGTCGTCCGGGCGGTCG

TTAAT

>c15669_g1_i2 len=246 path=[514:0-173 245:174-245]

GTCCTCGGTGACCAATGTGTGCAGCCAGGCTTCGTCCGCCACAGGAAACCCTCCCTGTCG

GTGCCCAGCATACGGGCCGTGCCCATCCTTCCATTGCAGCCCCTTCGCTTCCGTCGCGAC

GCCGACGTTAGGAGCACCAGCAGCGTCTGACGGAGGCTCTGGAGGTGAAGGGTTAGGATT

AGAGTGACGCTGCGGGTCGTTTCACGTACCGTTAAGCCATCATTAACGACCGCCCGGACG

ACCAAT

>c15673_g1_i1 len=246 path=[472:0-245]

AATGCTTCAGTCCTCAATTCTCACTCAAGAACCCTGAATTTGGCCCCATAGCATATTGTA

AGTATGACTGTCAGGATGGAGTGAGTCAGCTATTCCTGTAGTAGACCAAACTATTTGCAC

AAAGGACTTCACTATTTACACATCAACCTGCAAATAATACAAGTTCAGCACCAGTCCTGA

CTTAAGGAGTAAAAACCTTCAAGTCAGAATATTAGTATTCCCTGTCACAAATGAATTCTG

ATGTAT

>c15684_g1_i1 len=241 path=[1:0-240]

ATAAAGGATTAATTCTTTAAAAATAAGTAAACATTGATGAGAAGTTTTCATTCATTACAA

AAAATTTGTATGCAATTATAAAGGTAGTACCTTTAATAAAATATTTCATTTCAATATATG

TATACTTCTATTAGAAAATGTACATAATAAAAAAAATTATTTTTCATCGCATGTATCTTC

ATTGGTAACGTATGCAATTTTATATTTTTCCTAATTTAAATGCAAAATTTCATTATTGTT

T

>c15684_g2_i1 len=276 path=[487:0-275]

TCATTCATTTTAAATTCTTTTGTAAATAATGACTACAGAAATAAAATTTTATATGTAATA

AAATCATTAATATCTAATAAAGTTTCATATACATTATATAATTGATTGTTATCATTATGA

TAACTTAAATTATCGTATACATTTGATTTATCAGTTTTCTGTCTAACAAATTTATAAAGT

TTTGTGTAGATAATTTCATAAATGGAATGTAGAAGAATATGTATTGTATTTATTTCATTC

ATCAATACTTTTGTAATAAACAAAAAACTTAAATTG

>c15689_g1_i1 len=242 path=[1:0-52 54:53-55 483:56-241]

ATTCGTAAAAATATTCAAAAAAGAAAATAGAAATAGCAATACAACTATTTTTTAATGCAA

ATTTTAAGATTAGTATTTTTACTCCTTTTTTATGTAAATGTAGCATAATTGTCTAAAAAT

ATAAAGAAAATTAAATCGTGTTTTCTGTATATTTAAGTTAATATTTTAAAACGTATTTTA

TTTAATTTTTTTTTTCTTCTTTAAATTGTTTATATGACAATATTAACTGTAAATGTTTAT

GT

>c15690_g1_i1 len=2506 path=[2484:0-2505]

GATAAGATGATACACTTCTTTTTCCATAAAATTTTTTAATAATAAATAATTAATACAAAA

AAGTCTTTTCATTTAATTATTATTTATTTTAATATTTTGATGTATTTATCATTTGTATGT

ACTAAAATTTTATTATTTATAACTTCATCTTCGTAAATTAATAAAAATATAATTTTTTTT

TTTATTTGATATTTCATAATATATTTTTATATTATTTCATCTCACTATTCAGTAAAAAAA

AAAAGGCATTTATTTTAATTCATTTATATTGCCTATATATATTAATTTATTTGTATATTT

ACTTTATTTTTTTTCTCTACCTGTTCACTTATATGTTTATAAAGTATCTTTTTTATATAA

ATTTTTAATTATCTATAAGAACCAATGGATATTGAAAAGGTATATGACGTAAACTTTAGT

GAGGAAAACATACTTTTGAGAAAAGAAAACGCAGAAGATAAAAATCTCCAACATAAGAAA

GATGTTAAAAACTTTTGTAGTTTATCAGAAAATTATAATTTTTTTAAAAGCAGTAATTTA

AATGATGAATTATATCATGAAAGAGATAAAAATAAAAACGAAAATACAAATAATGAGATT

ATGGACATCTATGAAGGGAAAAGTGAAAATTTTTATTATGAAAAGAGTGGATATTCGGAT

GTAGAAAAAAGTGATTTTTCTTTTGATGAAAAAAGTGAAAATAATTTAAATGAAAGAAAT

GAATACTCATTTACAGAAAAAAGTGATTTTTCATTAGAAGAATTGAATAAAAAAAGAAGT

GTATCATCTTATCCATTATATGATGAAAATATATTACAAACAAATGTAGATTTAGAAGAA

AATATATATAAGGCTATGGAAATAAACACTTATAATGAAAATTATGTAAATAATTATGAA

AAAAGTAAATATGTTCCATTAAATACAAATAAATTTATACAGAGAACAAAATATGATGAA

GAAAATAATATGAATAATAAAAAAATAAACGGTGAGGAAAAAATAAAATATATATATAAA

ACATATACAGCAGCTCCAATTTCTCAGACTGAGAAAACTGGACTTTGGTTAATTGACACA

GTTAATGAACTATTAGATAAATTATGTGACAATAAAAAAGATTATTATTTAAATAGTTTA

GACATAGTACAAAAGTATCCTAATGATGATTTGCTTTATACAAAATTGAATGAAAATATA

AAAGTGAAATGGCCAGATTTTTTAAAACTGAATAATTTAATATATTATAATCACATTTTA

AATATATATGAAAGTATGTATAAAAAGTTCATAAGTGACGAACAAACAAATTTTGTCCAA

AATAAAAAGTTAATGAAAAAAACTAATTCTATACCTTTACCAAAATTTCCATACAAAAAT

TTAAAAGATAGATGTATTTCAAAATATTATGAAAATTTACCAGAATTAAAAGTTATTAAT

GAAGAAAGTGAGATGTCACAACATAATAATACAAAAAATGATGCATTTCTAAATTTCTTT

TTTAAGAATTTTATTGATCAGATAAAGAATAAATATAATGAATTTTGTGATAATGTTGGA

GAAAAATTAGAAAAAGTAGAAAAAAAAAATTATAATTTTAAAGAGTATTGTATGGAAGTT

TCAAAAATTTTCGCCAGTAATATTCTAAATGTGATATCATTTTCTAAGTTCCCAGTATTA

TTAAACAATATTTTAGAATCTGTTCATGCTTGCATAAAATATCAAAATGAAGAAGAAATA

GAAGATAATGTTTTAAGTAGTTCATCCAATATACACAATAATAATAAGATGGAAACCATT

TATTCTTTACCATTGGATTCATGTAATACTACTTTTAATTTAAACACAACTATTCAATTA

AATAATATTCAAAATAAAACTACTCCGTTAAATTCTAATTATTTTGTATGTCCCACGAAA

GAAAAAAGGAATACATTATTTACATCAGCACAAAAAAAATATAATTACACTAGCAACTTT

AATTCTTTACCAAAATATACGAAGACATATGCAAATAACAGAAATAAATTAAGTAAATGT

TTCAGGACTGATGATATATTAAAATATAGAGAAATGAAACATAATGCTTATATTAACACA

AGTATGCCACTTGACAGTAAATTAAAATATTTTTCTTCAAAAAACAAAAATACAAATAAA

AATATACATGGAAATGAAAGTGAAAATGAGAAAGAAAACAATAAGTATTCAGAAAAAGAT

TCTAATATATCTACCCATGTTTTTAGTGGTCAATTTAAAATAAATAATGCGAACAATTAC

TATACTGTTAATTCATTGCCACTTGATGATTCTATAAAATTAGAAAATTCACCTTTTAAT

AAAAGAAGTCAAGTAGAATCATCTGAGTTTTTAGAATGCAAAAGTAATTGTACACCTAAC

TATTAATGCTAATAATTTATTTCTTTTTTTTTAAACCTTTTTATAATTTTTTAAATAATT

TTTTTTTTTTTTATCACAATGGGATCAAAAAAACCAGTAAAAGCCA

>c15699_g1_i1 len=218 path=[1:0-217]

GGATGGGGCCCTGGGCAGCCTGCAACAGCACCTATAAATCCTGCAAAATCACATGAAGTC

CTCTCCATCTCTCTTGCTTCATTTACAAATTCAGTGCACACCTACTAGATAAGGCCCTGC

AAAAAAATGTAGCAGAGTCCCAGTTGATTTCTTGTGTACTTTCCAGATTGAGAGTCTTAA

ATTCCTACCCAGAGAAAAGAGCATTGCTGCTGACTATA

>c15704_g1_i1 len=412 path=[277:0-72 25:73-185 138:186-411]

AGACACAGTGTGAGATGGAGTACTCACCGATGCTGCAGGGCAGACACAGTGTGAGATGGA

CGCTCCCTCCCCAAGGGCGCCAGGGTGGTCATCAACCTCTGGTCCGTGCACCACGATGAG

AAGGAGTGGGATAAACCTGAGGAGTTCAACCCTGGTGAGTTGTGAGTTCCCAGCCCAGTG

CCCAGGCTCTGCCCACACCCTGGGCAGCCTCACCTGGTGTCAGCAAGGGACACATGTGGG

ATGAGCAGGGGGGACACGGGCCGGATGCGCAGCACCTCGCTGATGGTAGCCTCCAGGTAG

GGCAGCAGTGGGCGGTCACTGAGGTGGGGGTGACGTGCCAGGCCAATCTTCTGGTCCATC

TCCTCCTGGATCTTCTTCTGGACCTGCAATAAGAGCAGGGAGGGAGCATGGG

>c15704_g1_i2 len=301 path=[277:0-72 349:73-74 138:75-300]

AGACACAGTGTGAGATGGAGTACTCACCGATGCTGCAGGGCAGACACAGTGTGAGATGGA

CGCTCCCTCCCCAGCCTCTGCCCACACCCTGGGCAGCCTCACCTGGTGTCAGCAAGGGAC

ACATGTGGGATGAGCAGGGGGGACACGGGCCGGATGCGCAGCACCTCGCTGATGGTAGCC

TCCAGGTAGGGCAGCAGTGGGCGGTCACTGAGGTGGGGGTGACGTGCCAGGCCAATCTTC

TGGTCCATCTCCTCCTGGATCTTCTTCTGGACCTGCAATAAGAGCAGGGAGGGAGCATGG

G

>c15708_g1_i1 len=267 path=[1:0-266]

GCGCATAGCTCCAAGAGTTGAAGAGATCTGTGTCCCATAATATGCAGAAATGCCTTTCTC

TACATCATTAAAAGACAGAGCTAAGAGTTAATTGAAAAGCATATGGACAATTACTGAAAG

GAAGCCTGCTAAATAGGACATCTGGATTAAGTTATTTTCTTTGACTTCTTGTGTTTAGCG

GTGATATAATACGATCCTGACGAGAGAATTGCTGCCCATCAAGCATTGCAGCATCCTTAT

TTTCAGGAGCTCTGGCAAGTGACTTCA

>c15708_g2_i1 len=231 path=[697:0-230]

TATCAGCTGCCCTAAAAAGTTTGGGGGAAAAAGAAGAGAAAAAGAGGAAAAAAGTCAGTT

GTGCTTTAAGTGAAGTTTGAACTCGGTGCAAAGAATTGCGTTGACTGATATTTCATTGTA

TTGCAAAAGCTACGTATCAGCACTGTAAGCAAACTTCACTTCAATCCACTCGAAATAATA

AGCACACAGTGTTCTTCCACTTCTTGATCTTAGTGTTCAAGATTGAAATGA

>c15716_g1_i1 len=236 path=[214:0-235]

AAAAAAAAAGTCTAATGCATTATAACATGTTGCATAAGATAAAAATTCCGTAGAGTATAT

CCTGAAATGGCAAACTTCCATGGAAAAAGCCTGTAAGCTTCTGCTTATTCATTTCAAACA

GGAACCAGTGAAGAGCACGCACTGAAACTTTCCTCTTGCACCTGTCGTTCCCTCTTGGGT

ACTCACAGACTTATTTATTTTTCCCCAAAAAATCAGACTCTGACCAAACCGTTATG

>c15720_g1_i1 len=387 path=[1:0-260 262:261-386]

GGATATCCCTCAGGACCCTACCGGGGGGTGGATGGGAAGGTGGAATGAAGGTTTGAACCC

TCAGCTTAGCAGGTCTTTTAATGGAAGCCTTTCCCAGACTCCTACCCTGCAGGTGGGGAT

GGGGATCAGCTCACAATTGTCTGCTGGGCTTTTCTGTGTCCTTAACACCATTCAGAATGG

ATTGGGATTCCCACTTAGGGGGAGAAAGGATTCCAAATCCTGCAGCGGATGCTCACCTGT

ACGTGTGTAGAAGTGTTGTATGAGGGATTGTAGTGATCCAGGCTCAAAACGTGCATTGTC

CATAGGAAGGCTTGCACAGTTTTCAGAGCTTTTTGTGTTCTAATGTTCTTTTGTTTCTTC

TTCAGTCTGAAAGGCACTTTATTATGC

>c15721_g1_i1 len=221 path=[1:0-220]

AAGCAGATGAGGAAGCCATCAACCAAGGGCCTGAACCTGCCAGCCATCCCCGGAGCCGGT

GAGACCTCCTGGGTGTGGGGCAGCCCATGGGTGCTGTGGTGTTGGCACACACACACACTG

GGAGCTGTGCCCCATCCTTTGCTTAAATCCATGTCCTGTCCTATAGAATCATATCAGAGT

ATCATAGAACGGCCTGGGTTGATAAGGATCACAATGATCAT

>c15724_g1_i1 len=271 path=[249:0-270]

GTGGGCTGCAGAGGTGGCAGGCAGTGGAGTGCCCGGGGCTGCCCGTTTCTGAGCTGGCAC

CTGAGCCTTTCCCTGCTGTTTACACGAGGCCTTGTGCATTTTGCTGCACGCTTTACGTCC

TCCCTGAGCGCACTGCGGGCTTCTGCTCAGTTAATTTCTAAGGGACTTCTGAAACATTCC

CACCCATATGGCTTGTATCACGGTCACTTTTATACATCCTGACATTCTGGATGTGTCCTG

GTACGCGGTCAGGAGAACCTGTGCTGGAGCT

>c15725_g1_i1 len=291 path=[431:0-83 247:84-290]

AGAGATGACATAAGTTATCAGTATATCTGATTTCCAAAGTCCTGATCCGTTTCCCAAATG

TGATTTCTGCTCTGGATTACTCACTCTGAAAGTAGTGAAAGATATCCTTTTGTTTTGTAT

GACATTTGCTTTCAACAGACAATATTAAATTTTAAAGGCAAATGTTTTATTTGGCTATGG

CCGGTTGACCGAAACCCATGGAGTAGGAGGTGGAGAAGGCTTGCAAACAAAGCTGTTAGA

AATAACAATATAGATCAGATCGTTACTGTACTGAATTGATTTTCAGACAGA

>c15725_g1_i2 len=238 path=[216:0-30 247:31-237]

TATGCTGATAACTTATGTCATCTCTGGTAATTCTGAAAGTAGTGAAAGATATCCTTTTGT

TTTGTATGACATTTGCTTTCAACAGACAATATTAAATTTTAAAGGCAAATGTTTTATTTG

GCTATGGCCGGTTGACCGAAACCCATGGAGTAGGAGGTGGAGAAGGCTTGCAAACAAAGC

TGTTAGAAATAACAATATAGATCAGATCGTTACTGTACTGAATTGATTTTCAGACAGA

>c15732_g1_i1 len=210 path=[188:0-209]

AAAGTACATAAACACTGAGTTAGACAGTAAAATAATCTGTCCAACACTTAGAGGGTACCT

AGAATATAGAGTTCTTCCTGTTCCGTTTGCAATAAAAACAATTGTGCAGAACTGATCAAG

CAGTTTCTTTCTTAGCAATTCCGACATGTCTGAGAAGAGCCCAGAAAGCTCGTGCTGGCT

GGGATCCTGACCCAAAGGGGAATGGTAGCA

>c15736_g1_i1 len=209 path=[1:0-208]

GGAAGACACATGTACTACATACCATCTCCTTTACTGGAAAAAAAGTCAACAGTTGTCCAG

TAAGGTTTTACCTTTTAAAAGTTTCAAGATGATTCGATCTCAGTCTTTGTCATTGCAAAT

TCCATCTCAGGAATGGAAAGCACCATCAGCCATTCCTACTGTGTCTCCTACAACACCAGT

TCTTCAAAGTGCTTTCCTGCCAGTCCAGG

>c15736_g2_i1 len=344 path=[694:0-343]

ACTGAGAAGAGATTGTCACAAAAGAAAAAAAAAAAACAACTCACCTTTGATTACAGAAGC

ATGTGGGTTTAATTCTCTATTTTAAAAAGGACAAAGATACTCACATGGACATACTTCATC

TTCAACCTTCCATTTTTCATGTTGCATGCTACACAGATACTGGGATGTCGTTCTTGGGAA

GCGATGGTATGCAAGGCGTGAATACTTTTCTCGAATGAGAAGAGCTTTCACCAGCCTCTT

GGCAGCTTGCTCATAATCATCAACTGTAACCCCTGCACAGTAGTCCCCGCTAATTGAAAC

TCTCTGAAACTCCGGTACATCAGGTGGCACTGCGGTAGCGGTAG

>c15746_g1_i1 len=480 path=[563:0-444 1155:445-452 1016:453-479]

CAGCCACTCGTCCTCCTTGCGCAGCCAACGCAGCAGCCGCGTCAGCGCAGCGATGGCCGC

CGCCTTCGGGAGCAGAGGGCTGAGGGACGCGTACAGCTCGAAGCGCGGCGTCACCCAGGC

CAGCAGGGTCTCGCAGGCGGTGACGCGGTAGAGCAGCCGCAGGGGGCGCTGTGCGCAGTG

CAGCCGTCCCTGCAGGCCGTGGTACAGAGCGAACAGCCGCTGCTGCTCCGTGGGGCGGCC

GTAAGGGGGGCAGCAGTCCGGGCTGGTAAACTGCGGCACCTGCAGCACGTCCTCCCCCCC

CTCCAACGGCTTATATAAGAAATGCCTGAGCTCGGGGGCTCCGGGCCGGGAGGGGTCGTA

GGCCGTATAACCCCCCCTGATGGCGGCCCCGAACTCTTCCATCCACCCCTGAGCCTTCAG

CTCCTCTTCCAACCTCCTCCTGCAGGCGGCGGCAGCGTAGAAAGCCTCGCGTTGGGTCAG

>c15748_g1_i1 len=309 path=[287:0-236 524:237-308]

AAAAAAATGCAAAACTAAATTACATGTAAGATAATTTTATAACCAGACATATCCGCTCTT

GTTTCCTTGGGAAATGCCAGCATTCAGAGCTGCCCACGTGCAGCATCTCTCCAGTGTGAG

GACATAAAGGTGGGGCCTAACCAGACCTTCCTCTTTTGAGCTGGAGGAATCATAGAATGG

CCTAGGCTGGAAGGGACCCCAAAGACCATCCATCTCCCTCAGCTGCCACAGGGCTCGCTG

CCCACCAGCTTTACCCTCACCATGGCAATGCTGCAAACCTTGCCATTTTCATGGGGCCGG

CTGCCCTTG

>c15748_g1_i2 len=207 path=[573:0-134 524:135-206]

GTTGGTAGGGATCTTAAAGATCCACCCATCAGGTTACCCAGCACTCCATCCAAAGGGCCC

CATCCAAGCCAGCCTTGAACACCTCCAGGGATAGGGCACCCACAGTTCTCTGGGCAGCAG

CTGCCACAGGGCTCACTGCCCACCAGCTTTACCCTCACCATGGCAATGCTGCAAACCTTG

CCATTTTCATGGGGCCGGCTGCCCTTG

>c15749_g1_i1 len=302 path=[333:0-301]

TCTCAGTGTTCTTTTCCAACCTTCGTGACTCCATGACTTCTCCAGGAGCCACAGCTCAAC

CGCTGTGCTCTGGTGCCATGGTCTGTTACTCATTGCTCATAAAGCCCCAGTTGGAAAAGC

AGTCACCTCATCAGGGCCGTTTTGCCCTGTGATGTATATTGTCTCCTCCGGATCATCTCC

GTCTCCAACTTGTTGATATGCAATGGGATTATTAGCAAGACATAATGCGAGTTAGAAAGA

GGACAACTCTTTCTTGCTGTGCTCAGCCCCGGAGCCTGGCCGTATCCCTTATTTAGGGAT

TT

>c15751_g1_i1 len=317 path=[449:0-316]

GCTGCAGGGAACCCCCATGCTGTACCCAGCACCTAAAACCTGCCGTGCTGGGGAGCACAG

CACAGACCCCTCCCCAGTAGAAGGGTTTCAAGCAGCAGCAAAATCCCAGTTATTGTCACC

GGATCTGTGAAACCCATCACACAACCACACAAGGGCTCAGCACACAGACACACCTACACG

CCAGGCTGACAAACAAAAAAAAGTATTGGAAGTGCCATTTTAACGATGATAACCTCATCT

GTGGAGTTAATGCGTTGTTCTCAAGGTCATTTCATCAGAAGGGTATTTCCTTTGGGTTGG

GGTGGTGCTGGCACCTG

>c15756_g1_i1 len=225 path=[457:0-17 36:18-224]

AACTATCTTGCTGGTGTACAGATGTGGATGGATGTGTTGAGGGCAGTGCTGATATGCAGG

CTCTGAGGATGAAGTTTCCTTGGCCAGCACCATCCTGGGCCGCCCTGGTGAGGGGGAGGC

CATGGAGGAGATGCCCAGATCTCTGGAAACACGAACTTGTCATGACCAGCAGCTCCTTGT

TGCTGCGGGTTCTGTCGTTGCTCTTTCTTCAAATGAGGCATGGGA

>c15756_g1_i2 len=242 path=[1:0-34 36:35-241]

CCTTGGGCAACCTGTTCCAGTGTCCTCCCAGGCGGCAGATGTGGATGGATGTGTTGAGGG

CAGTGCTGATATGCAGGCTCTGAGGATGAAGTTTCCTTGGCCAGCACCATCCTGGGCCGC

CCTGGTGAGGGGGAGGCCATGGAGGAGATGCCCAGATCTCTGGAAACACGAACTTGTCAT

GACCAGCAGCTCCTTGTTGCTGCGGGTTCTGTCGTTGCTCTTTCTTCAAATGAGGCATGG

GA

>c15758_g1_i1 len=625 path=[685:0-35 720:36-264 949:265-624]

CCAAGAAACAGTTTGCTAAATCTGTTTACTTTTTAACAGGTGGCAACTTCATCTCTGCTT

TAAAATTGTCAAAGACACCATGCTCTTTCTAGCTGCTATGGGTAACAGTAAGCAAGAGCA

CATATTTAGAGCTGTGAAGCACCATATAGTGAGGCAGTAGTCCTTCCAGCCTACTGGAGT

GTTGCATAAAGGTACTGGGGTAATTGAGCATGATACAGAAATCATTTGCTAGCAACTGAA

TGCTTTTAATCCAATGTTTACTTCTTCATGGAAAAGGTATCACAATACGTGGAAGGAGCA

ATACGTCACTTGGTCTGTCTTCTCATTTCCTTGTGCAGTGTTTGTGTCTGAAACCCAAGT

GGTATTCTGGGTTTTGATGTGCTCACACTCCTGTAAATTAGAGCACGTTTTGTTTTCAGG

AAGGCATAAACCAGTAATGTTTTGATCAAGTTGTCAGTGAAAACGATGCAAATTTGGGTA

CGCAGTTTCTCTGTTTCACAGAGAAGTGAACTGCATTCAAAAGCTTCTTTAAATAGAGGC

ACATAGCCTCACATAGTGCTGGTGAGCCTGTTGACCACTACCCAGAACTGCAGATTTCTA

CTCTTTAGAAAACTGCCAAGCACTT

>c15758_g1_i2 len=373 path=[1367:0-12 949:13-372]

AATGATTTCTGTATCATGGAAAAGGTATCACAATACGTGGAAGGAGCAATACGTCACTTG

GTCTGTCTTCTCATTTCCTTGTGCAGTGTTTGTGTCTGAAACCCAAGTGGTATTCTGGGT

TTTGATGTGCTCACACTCCTGTAAATTAGAGCACGTTTTGTTTTCAGGAAGGCATAAACC

AGTAATGTTTTGATCAAGTTGTCAGTGAAAACGATGCAAATTTGGGTACGCAGTTTCTCT

GTTTCACAGAGAAGTGAACTGCATTCAAAAGCTTCTTTAAATAGAGGCACATAGCCTCAC

ATAGTGCTGGTGAGCCTGTTGACCACTACCCAGAACTGCAGATTTCTACTCTTTAGAAAA

CTGCCAAGCACTT

>c15760_g1_i1 len=241 path=[1:0-176 487:177-193 504:194-240]

CGGCTTCTGCGGCACTGCTTCCTTTGTCCTGATCACCCATGAAAACAGCACTGTGCTGGG

GGGCACTGAGATGGTGACGGTGTAGTCAGTGAAGGGCTGCAGGGAAGTGCAGGTGAATGT

TCCCCTCTGGCCGTGCAGGAGCTCCACTCCAGAGACCTCCTCAGCCTCACAGGCAGGTGA

GGAAGGCCCTGCCAGCCAGCACACGGAGGCCACGTGCCGGCTGGCAGGGCCTTCCTCACC

T

>c15763_g1_i1 len=442 path=[1:0-441]

ACCAGATGCACTTTTGAGTGCTACACATTGGGTACAGGAGGTAGCTGAAAAGCAGAACTG

CTGCTTCACAGAATGACGCCAGCTCTCCTCCACATCTCACTGCAATTCTACGTCAAAACT

AAGCCTTCACCCTTCAAAAATGAACTCTGCTTTCAACATCAACTGCTTCATGACACCTCG

CCCTCATCCCTGGAATTGAGGCATAGCTGCATTAGCAGAACCCTTCAAAGAACTGCTTCA

AGGTGACAGCATCTGCTCCCAACAACTCTGATCCAAAGGGTCAGTTTTACCACAAAACTT

CTTAGCTGCATGAAGAGCATCCATTTGTTTCCCCTTCATCTACAACATCCAGATGAGGAT

GGCCATATATTCCAGCTCCACATCTTGCAAAGACTTACCCAAACAACAGGAATTCAAAGT

TAACCAAGCAGGGTAAGTTTGA

>c15765_g1_i1 len=233 path=[1:0-113 459:114-232]

TGTTTTCAAAGGAACCAAAGGGTTTAAACACCCAAGTCAGTAGAGATGAGCTCTTGACCT

TCTTTGGCAATTTTAGCCTTAGTTGGACATCTGAGTTTACTTCCCTCTCCTTTGTTTGTG

CATGATTATTTTTTTTTCCTGTCAACTGTTGGCAGAAATACGTATGGAACCTGTTTTGCA

GAGATAATCTAGGTTAGACATCCTTCTCTTAAAAATAATAAAATAAAAAATAA

>c15771_g1_i1 len=284 path=[262:0-283]

AAACTGAGACCAAGTTAGGAGAGGCACGTTCAGAACCAGTTAGAATGAAATTGATTGTTA

GAATTAAAGGAATCAGACAGATGTTAAATGATTAGTTGATTGCTCAGCTCGTCAGTACCA

ACAGAATGGCGTATTAAATGAAGGGTTTGCTTCAGGTAAAGAAATACCTGTGAGCATTTC

ACCATGCACTATTTTATACAGAAATGGCACAAACAGGTGAGAGGGACAAACATACATCGA

CTGAACAGTAGTGCTTGTACCTTTCGCTACCACAATGGGCCGTA

>c15773_g1_i1 len=303 path=[179:0-214 25:215-302]

AGACTTCATAAATATCTAGATTCATTTTTCCACCTTTTTCATTAGATGGAGATCATATCC

TGTGTCTTATCTTACATATGAAATACTAGAAGCACGGTATGAATGAGACTGCGTGGATGG

CCTCTGTCTTTCTGTATTAATATCACTGAGAACTCCATCTCCATGTGCATGGTTACTGTG

CCACCTGGGATTCAGGATGTGGTAGTGCATGACTGTATGGCTCAATTCTGTTGAAGCTGA

TTTTTTTTTCAAAATCAGAGACTTCATTTTGTAACTGATGTAAAAGAATTTAAGTGTGTT

ACT

>c15773_g1_i2 len=238 path=[179:0-214 416:215-237]

AGACTTCATAAATATCTAGATTCATTTTTCCACCTTTTTCATTAGATGGAGATCATATCC

TGTGTCTTATCTTACATATGAAATACTAGAAGCACGGTATGAATGAGACTGCGTGGATGG

CCTCTGTCTTTCTGTATTAATATCACTGAGAACTCCATCTCCATGTGCATGGTTACTGTG

CCACCTGGGATTCAGGATGTGGTAGTGCATGACTGAGTTACTGGTGGACCATCCTTCA

>c15774_g1_i1 len=308 path=[552:0-307]

TGTACACTACTACAGATGGTCACAATCCCAAAATACATCATATTTGCGCACAGATTATTT

TCTTCTTTACTTCTCTCTCCCTCATCTTTGGTTATTACACGTTTTTCATTTTTACTAACC

CTTTTTGTCCAGTCTGCATGAAATTCCCATCATCCACATATCCCCAAATGCATCACTTAT

CAGTGCCTGTGGCATGACAGATGTTACAAAAGATGTAGGCCAGGAAGGAGGTTTGGCTGG

AGGCTGCTGTTGGGACAGGGCAGCAGGAGAGCACCCGCTGCAAATTACAGTAAATGATGT

AATGTTAG

>c15777_g1_i1 len=325 path=[1:0-324]

GCAGTCTGCAGTCTGTAGCAGAGGTGACTGTTTTGGTTATTGCTCAGAAAGTTGGGGTTA

CGGAGGTCACACCTAGCCTGTAAAAGTGGAAAATGCTTAGTGACCAAACCAGTAACTGTT

CTGTAATGTATTAGGTAAAAGATCTCGAATATCGATGGCAGGAGAGCACCAGAATGTCCT

GAAAAGCTGTACTGAGACATATGAGAGAAACCTATTCACACATTGCACGTAGGGGATGTG

GAAAGAACCGTGAAGGTGGTTTGTTGAAGTTTCTGGGGTATGCCTCTTTCAGAGGCGTGC

TGCAATACTTGTAATGAAAGACACA

>c15779_g1_i1 len=503 path=[481:0-177 659:178-502]

GCTTTAATCTATAATTATTTTTCCTACCATGTTAAGAGCTATACCTTTTGTAGAACTCTA

AATTATTTCCAGGTAAGTTGCTTTCTTTTTCTTCCTAAAACCAGTTTTTCCATAGTGGAG

AAATGATGGGGGGGTGGAAGGCTTCCAGGTGATGCCAAGTGGTAGAGATAATGCAAAATA

AATAATGAAAGAGAACCTCAATGCAGAATTTTAGCTGGAAGGATGAGGGTAGGAACAATG

CTTGCCGATTTATATTTTAAAGTTGAATGTGTTTTAACTAGAAAGCAAGTGGGAAAACTC

AAGTTTGCCCACTAATCCAACAGTCAGGGAACTATATTTGGTTTCCAGAGGAATAGTAAG

GAAGTTGTTATTAAATAAAGGTCCATGAAGCACCGTATGTGCCAAAATGTACAAAATCCA

CCTGAAAGCTTAAAAGAAATGAAGAAAACATTCCACAGTGGTGCTTCATATGAAATACCC

AACTTTCATAGCAAAAGCAATCT

>c15779_g1_i2 len=384 path=[481:0-177 984:178-383]

GCTTTAATCTATAATTATTTTTCCTACCATGTTAAGAGCTATACCTTTTGTAGAACTCTA

AATTATTTCCAGGTAAGTTGCTTTCTTTTTCTTCCTAAAACCAGTTTTTCCATAGTGGAG

AAATGATGGGGGGGTGGAAGGCTTCCAGGTGATGCCAAGTGGTAGAGATAATGCAAAAAA

CCAGAAGCACACATGGACTCCTTGTATGCTATTCAACTATGACTGGTAGCAGGACGTGCT

CTGCAAACGGGCATGTAAGTAGCTGCTGTTATTACCCTAATGTTGGATGCTATGGTCCTT

ATGGTACTGCTTTTTTTTTCCTACAGCTGGTAGATGACAGAAGGTCCTTTAAAGGCCTTT

GTTACATCATTTCCTCAACTGTTT

>c15784_g1_i1 len=218 path=[196:0-217]

GAGGTTTGCGGGCAAACATCCGACGGCGGGGCCGCAGCCTTATCTATAGCTGCCGGCCCT

TATCTGCCGCTTATCTCGGGCGGGAGGGAGACGCGCTCCGCGCCCATCTGCGTCTCGGTC

CCTACGGGTGCCCCCTCTCAGAGACGCCGTCTCCTCTCGGAGATGCGGCGGCGGAGGGCT

CGGGGCCATCCCGGTCCCCGTCCCTGCGGTCGTCGGCT

>c15791_g1_i1 len=286 path=[574:0-12 31:13-285]

CACAAAGTTACCTAAACTAAATGGCCAACAAGAGATAACCAACAAGCATTGAGAAATTTC

TCCTGGTTTTTGTCCTGATGTTACATGTATCAATCATAGCTTTATTGGAGACAGCACTGG

GTAGTCTCAAAGTTTAAAAAGAGCTAACTTTGGGCTAAACATATGCAAGTTCCATGGTTG

AATAAACTTCTGAAATGTATGTGTGACAATTAGCAGTACAACTTTGCTGCTGAAGATCAA

GTGCATTCCTCACACTGTGATCACAAGGCATGTGTGGACAGATGAT

>c15791_g1_i2 len=303 path=[1:0-29 31:30-302]

GGTAACTTTGTGTTTGAGGTCGTGACAGCCAAACTAAATGGCCAACAAGAGATAACCAAC

AAGCATTGAGAAATTTCTCCTGGTTTTTGTCCTGATGTTACATGTATCAATCATAGCTTT

ATTGGAGACAGCACTGGGTAGTCTCAAAGTTTAAAAAGAGCTAACTTTGGGCTAAACATA

TGCAAGTTCCATGGTTGAATAAACTTCTGAAATGTATGTGTGACAATTAGCAGTACAACT

TTGCTGCTGAAGATCAAGTGCATTCCTCACACTGTGATCACAAGGCATGTGTGGACAGAT

GAT

>c15792_g1_i1 len=251 path=[445:0-250]

ATTCCTATTAGAAAACCAGTTTTACCGAACCACAGGAACATTTTTGCTACTGTTAAAGCC

AACGTTGTATTTTCAGTAGTTCTTGACATGACTGTTTACTGGAGAAACTTCTGGTTGTCT

GCGTGAATGTAGTGGATTTTTCTGTATGCAGATAGCATTATATAATTGCTTTCATTAAAA

CTGCTTAGCAAGGTCTAAGCTTCTCAAGAGAAATGCTAATGTACTGAATGGTTAATAAAA

GTTGTTATATA

>c15798_g1_i1 len=202 path=[1:0-201]

ACTGCACCCCTTGAACAACCAAGCAAACATAAGCCTCCGTGTCTTCCCAGAAACAGGCAG

AGCCCTCCTGCAGTTGCTGCCCTGTCACACTCCACTGTTGAGCAATCCTGATGGCACACA

GCAGAGAACAACTTGCTGCGTCGCACAGCTCAAGGAGGAAAAAATAGATGGCCACAGCAC

TGCTGGGGACACCGCGCAGATG

>c15799_g1_i1 len=299 path=[277:0-17 295:18-298]

AGTTGTTCTATCACTAAACATCAGCTGAGAGAATAAGAATCACTAAGTATCACAATCTAT

TCTCACACATGATGTTTAGTGATAGAACAACTCCACATGCTTGCCCTCAGAACACAGAGT

TCCACACCATCACTCCAATCACAACCTATGAAGAACAGCTCAAAAAAGTCAAAATTGCTT

CCTTCCACACTCCTACATCTCCCTCATTGTGGTACAAAAAACCCACCTCTTTAATATCAA

TAATGGTGGAAACGGTTTAAGTCATATGAATCCCCTACTTATAAAAAAAACACCAATAA

>c15800_g1_i1 len=218 path=[1:0-217]

AATTTACACACTTCTCTGTGCTTTCAATTTTTTGGCCCTGTCTCACTTAACCATGTGCAC

ATCAGACTGTAGAGCTACATCCAGTCCAGGCGTGAGCATGGGAGTACTGTATCATTTGAG

CTGCCTTTTTTAGCTTTTTTAGCTTTAATTCTGCCTTTCCTGTTGTACGGCTCTGTTGTA

CTTCTACAGTCCCAACATTCTGATTTACTGATCGCATA

>c15800_g2_i1 len=313 path=[681:0-312]

TTTGTGTCACAGCAAACAACCCAGGCAAGCACAAAGTATTTCTAAAAGCACTCAGATAAT

GAATTTGATTTTTTTCCATGAAAGAATGTATGTTTGCATGTATCTAAGGTCTACTAGATT

TCCATATATACTCATTTATAAATATATACTATATATACACACATATTTTAATTTTAAAAA

TGGGCAAATGTAGGTGTATGAATGTATTTTAATGTGGTGGATCTCAGAGTTGTGTAAATC

AAAACCTCTCAAATAAGTAGCACCCCCCGGTACGTCGTGGCAACGGTTTGTGTATTAACC

TGATCAAAGAATG

>c15802_g1_i1 len=234 path=[212:0-77 455:78-233]

GTGAAACAGCTCCTCCCAAACACCTCAGAGAGGGAAAAAAAAGCCATAAAAAGGGTGTGC

ATCCCATCACCTCTGCCCCTTGTTTCACCCCCAGGGTCTGCTGATCCCTTTCCCCTTTGG

AATTCTGTTAGCTTGGTGTTATTTTTGCTACTGGCACCAATTTGCACAGTGAACATCTTT

TCTGGCAAAAGGGGAGGACCTTTCCCCCAGAACAAGCAAAAGATGGTGGTGAAA

>c15804_g1_i1 len=235 path=[155:0-234]

AGGACAAGAAGGCCCCTGTAAATCTGTAATAACATTTCCAAAACCTCTCCATTAAAGAAT

TATATAGATACTTTAAGTATTTAAGTACTTAAGCATTAAAGATTTATACAGATTTTTAAG

TAGAACAATGTCTTGTTTTCCTTTGTGTTTTAGTAAAAGATGCAGTGTTTAAAATCACCA

TTGAAAAGACGGAGTAAATAAACTACAGCCCTTACAAATTCCAGCAGCACAGTAT

>c15805_g1_i1 len=214 path=[1:0-213]

ATGCAGAAATGGAATAATCTCAATATTATTACATAGTACATTTCAAGATTAATGCATCAC

TGGATATTCTTTGATCGTGTATACAGTGATAGCAGATTCTTGCTCACAGCAACGGATTTT

AGATTACAGCCAAAAAAAACAATGAGGAAATAGCTTCTGGCCTTTCTTGTAACAGTCAGG

CTTCTCAAATTGCATGTCTGAGACATTTATAGAT

>c15806_g1_i1 len=245 path=[589:0-244]

AACTAGGAGAGCAACAGATTTTAACAGTGGAATTTGCGTGGACTAAAACAGCCAAATTAA

CAAACCAACAAAAAGGAAACTAAACAGGATACATAGAGTATAAAACAAAGCAAAACAACG

AGATCCCTGAGTTTTACAGGCACCACTTCATTCTCCTAATTCATATTAGATTAGTAAGAA

CAGAAGACCCTATGAGATTATCTACTTTGGCCTCCTCAATTTCACAGGCTGTTACATTCC

ACCCA

>c15810_g1_i1 len=225 path=[389:0-224]

CGATCTTGCTGTGTCTTAAGGTGTTTTTTTGTGTTCTGTCACATAGGGGAAATGTTTTTA

GCTTATAGTGCTTTTTATAGTTAATGTAAGCACTTGGTCAGTGCTGCATAAAGAATTCCT

GTGCCCTTCTGAAAAACCCACTGACATTTACAAAAATACAAAGAATCCAAAGTATTGCAT

CATCATCTGTACCATATACAAGTTCTTGAAGATTGGCCTCCCAAG

>c15811_g1_i1 len=248 path=[1:0-139 426:140-247]

ATTCTACATAGCGGTCAAAAACGTGAAATTTGGCATAGCACATTCAAGAAATGAAACATG

AGAAATTATCCACCTAAGATTCCCATAAACCTGTACGGCAGAGCCCCACAAATTCTCCAT

AATTACACCTCCAAAACTTTTGCTTGAAGAACACCTTTTTCTTATACCAACAGCTAAACA

GCCACAGGAAAGGCAATAGACAAAACCAAGCTACAAGTGTAAAGGAAATAGCAAAATAGG

TACTGAGT

>c15812_g1_i1 len=240 path=[218:0-239]

AATTTAAATAAAAATAAAAAAATAGAATTAAAAATGCAAGAAATAGAAAAATTCATTAAT

AAATATATTAATATAAATAACGATGTGAATTTTATTAGTGATAGATATCATTTATTATTT

AAACAAAATGTTATGAATAAATCACATAATGATGGAATAAAGGATAATTTAAATTATATA

AATATACAAAATATTATTAATCATAAAAATATTGAAAATATATTAAAATGCTACTTTATA

>c15812_g2_i1 len=2173 path=[2585:0-2172]

AAAAAAAAAAAAAAAAAAATAAAGGAATTAAATAAGTAAAAGTATATATTATCTACATAT

AAAAAGATAAGGATCATCCATAATATAAATTTTTTTCCACAAGAAAAAAACCTTTTATTT

AATTAATTCATTCTACCTAAACATGTACTTGAACCACATAAACAAATCAATTTTTTTCCT

TCTGATTCAATACCAAATTGATAGTCATATGTTATTTCTTCATGAGCAACAATATCTCTT

TTTGCAAAAATAACAATATGTTTTAAATTTTGATCACAAGTTACAATTTTACAAAAGCAG

TTCGGCTCACAACTATGGTTTATAAATCTACTGACATTACCCCATTTAGTAGCATCAATA

ATTATATTTTCATTTAATCTAAACATATAACAACTACTTTCAATTTTATCATAATACTTT

TCTCTTTTATCACTAATTATATTTCTTATATATTCACCTATGTATTCAATAACCGGCTCC

CCTTCATTAATAAATTCACAGGTATATAGCCCATATCCATGTATATTGCTTTTTTTTACA

TACAATCGAGAATTTGATGAAATATTCATTAAATATCGATAGGACATTGCACTGTTAATA

TTATACTTATATTTTACTGTTTTCCTCATGCTTTTTTCCTTTTCTTTTTCATTTTCTTTA

AAAATGGATGCCTCAAAGTTTGAATATTTACATAACAAATTATCGTTATAAATAACTTCC

CCTGTATTATTCTTTATCTTTTTTTTACTTTCTTCTCGTTCTTTTTTTTTTTTAACATAA

TTTTTAATTTTATCACTTAAATTTTTCCATAAAAATTTGAAAAGTTTGTATTTTATTTGA

TATATTACACAAGGCAATGTTAAACCAAAAAAATCATGAACGTCAATTAATTTATTATAT

TCAACCAATTTATAAATATAATTTTGCAAATACTTATATATATGTTTATATTTCATAGTG

AATTTTATATATTCGTCAGTTGTGTATTTATCACAGCTACAATTAAAAAGAGATAAAAAT

TTCATATAAACTTCATTTATATCATAACCTTCTGCTAAAATAAATTTTTTTGCATCTTCT

TCTGAAAAATAATCTATACTTATTAAAGGAAATAAAATAACAGAGAAAAAGAAATTATTA

TTCTTTAATGTAATAGAAAAAATATAGGTTGCTCGTATCTGTAGAAATATTTCTTCTAAT

ATTTTTTTTTTTATAACTAAATCATTAATATTCACATTTTTATAAATATAAAATAAAAAG

AAAGAAGGTAGATTGAAAAATATTCTCATATTTAAAAAACCACATGGATATATCATTTTT

TCTCCATCATATTTTAATATTTCACCAATACTTAAAATGTTGTGACATCCAATCTTAATA

ATTTTTGATTTACATTTATTAACATTGTTATATGTATTCTTTTTATATTTGGAGAAATAT

ATATCATTAATTTTTTTTATATATACATCATTATTTTTATTTATGATCATATTTAAAAAG

TATTCTTTTTTCTCCTTCATATCATTTTTATTATTGATTTCTAGTATATTTTCATTTTTT

TCTGAATTTGGATTATTATATTTTGAAATAAAATTATTTTTTTTTTCTTCGTTTTTTTCA

TCAAAATTTTCATACTTTTTGCTTTTCTCACTTTTTTCATCGTATTTACAAACATCTTGA

AATTTTGTTATTCTTATTTTTTTAATTTTATCACTTAATTCAAAATAAAGATTTATATTT

TTTATTCGTTCCTCTTTTTTTTCTTGATTTGAATTATTTACTTTCATTTTTTCCAGAGGT

CTTTCAGTTGTATTATTATCATAATTCATATGATCAACTTCAATAATATCATCTATAAAT

GTTATAAATTTACTTTTGTTATTACTTTCATTTACATTTTCTTTATTAAATAAATTTTCT

TCATTTAACAAATTTCTGTTAGTATTTTCATCACATTTTTCTTTTAAATTTAATTCTTTC

TTAATCAAAATGGAATTAATTTTGTTTTCATTTAATACATTCGGTAAATATATATTGTAT

ACTTGATCATTATCATATATGTTTCTCAAAAAATAATTTTCACTTTTCTTTTGGAAAAAA

TATTCTTCATTTTTTAGAGTAATATTTTTTCCAATAAATAAGAAATTTTTTAGTATAAAG

TAGCATTTTAATA

>c15818_g1_i1 len=557 path=[1:0-556]

AGTGCTGGATTTCATCTTCCATGTCACCATTAGCAAAGATAGCTTGGAAATAGATGGGCA

ATTCAGCACAGCATCATGACCAGCCTCCACATTCACCCTTCCATAAGCTGACCCTCCAGG

CAGGTGGATCATCAAGAAAAGGACTGCAGCCAGAACTGTCCTTGTCTGAGTCATCTTCAG

CAAAAGGAATCGGCACTCTGCATTTCCTCAAGCTCTTCTTGGCCCTTTCATTGTTCTCTT

CTTCGTACCACCATTTGCATCACTTTCATCTCCCACACACAGCTAGAGATGACTGGAAAA

TGGTGTTGCCATCCCTCCACGCTGCAGCTGGGAACGGCACTGCTCATGCTTGGTTGCCTT

TCAGGAATGCTGAAGGGAAGGAAGTCGCTCAGTGTCAGCCCGGAGAAGGGACCTCCAGTT

CCAGGAGCACAGGAGGTAAAAGGCAGCCGTGCCAGCACACTCAGGTCCTCGGATACTCTT

AGTCCTGCCCCCTCCTGGTTGTCCTCATGAGAACAGAGAATTTAGAAGCGGCCCAGCTCA

GGACCATTTGTCCCAGA

>c15821_g1_i1 len=553 path=[531:0-552]

AAGTCATAAATAATAAAAAAAAAAATAATTTTAACTGTTAATTATTGTTCATTATTTTTT

AACTCTTGTTTTTCAGTATTAAAATATAATGTGCAGATAGATATAATAAATAACATTTAC

CAATTGTTTAAAAATATTTAATTATTTAATGATTTTATAATATTTTTAAGACTTTGCTCA

AATAATTCAATTTCAGATTCATCGAATTTATTTTCCAAAACAAAAAACTTTGCTATATTT

TTAATATTTTCACTGTCTAAAATAAAAAAATCTTCTTTATCTTCAATTACACTATTTTCT

ATTTTTTTACTTTGTTCTTTTTTTCTTAATAAATCCTTTTTGTCATTTTCTTCTGAATAA

TTACTTATAAATGAGTAATTATTATTTCTTTCATAATTTTCATAGTTTTCATCAGTAGTT

TCTATTTTTAGTTCATCTCCATTTTCTAATTTGATAGAATCTATTTCACCATTTAATAGT

TTTCTAATTGCTTTGGCTGCATTTTCTGTTTCATTAAAATTTGTATTGTTTCTTCCTGAT

GAAACACTAACAA

>c15822_g1_i1 len=317 path=[1:0-316]

GGTGTCACCGTTTAGTTCATGAAATCACTGACTGGAAGCAGGGGTCTGGAGGTGTACAGC

TGTACAGTGCATCACGTGGCAGGAGGACAGCCCGTGGGCATGGGGATAGAGCACTTGCAT

TATTAAAGCACTACTTAATTTATCAACCGGTTTGACTTCAGGTTGGGGCGCGACGCCTTT

TCCCTCCACTGACCTCTCTGGGGTTCAGCATACAGACAACTGGTGGACACTGATGGAGCG

AGAAGACAGGTCAGCACACAGATGCAAGCTGCTTTGTTTTTCTCAGTTTCAATGAGACCT

GAATGTTTTATAGTGTT

>c15829_g1_i1 len=309 path=[441:0-308]

GCTGTCTCCCATGAAGAGTTACAGTGACTATTGTTTACAGATGAACAGGAAATCAATCCC

ACGCTGTGCCACAGTTAGTGTTTTACAACAGTTTGTATTCACATGCAGGACTCTGTAGAG

CTCAACCACTAAAGGGTCAGGAGGCACGAGAAGAGAAGAGAGCGGACGTCTGGAAAGCAA

CACTTCCACAGATATTTCCTGCTTAAAACCTAGAGTTCCTTCAGACGTGTGCAGGAAGGC

AGTGGTGCTCACTAAGAACAGGAGGGCAGTGATGAATTGCGTGATGGCCACTGCTCATAA

CTGAAGTTT

>c15831_g1_i1 len=349 path=[704:0-186 1464:187-190 895:191-348]

GAAGACCTGAAGGCATAAGAAGAGTTAAAAGGAAATAAAATCATTATTTGTTACAAGTTG

GACACCTTCAACAGTTGTCTGACAGTCAGCTTTAGGAGCCTCATTACAGGGTAATTAGTT

GCATCTGCAAAATTCCTCAGGACCAAATTATCAAAATGTTATAAAAAGCAGAGCATGAGA

CTTGAATTCATCCCTTTTGGCAGCTGCATAATCATTAGCTGAGAAGCCATATCATTCTTT

TCTTGCACCTAAAATACACTCTTATGAATGGCAGCCAGTGAGCCAAATTTAAAATCTGTT

TGCATACAAGTGAACACGACAAAGAGAAACTGAGTATATTCATCAGCCA

>c15831_g1_i2 len=442 path=[1:0-279 1464:280-283 895:284-441]

CAATTAGTAAAGAGAGTTGACTTTCAGAAAAAAAAGGTTGTTACAGAGAAGAGATATTTT

GAGGCCATATAATTACATCTCAAAAGTGCAATAATAAGGAAAGAAGCTGCTCTGGGAATA

CAGGAAATGTTAGGATGCAAAAATACACCACTCAGTTTTACAGTATGCTTACCATGTGCT

ATGTAATTGCAGTGCATATTTGATTTATACTAATTACATTTCAGTGGGTTGATTTGCTTT

CTGACTGGAACAGAACTGCCACATTGCAAATGCAGCTTGCTCATCCCTTTTGGCAGCTGC

ATAATCATTAGCTGAGAAGCCATATCATTCTTTTCTTGCACCTAAAATACACTCTTATGA

ATGGCAGCCAGTGAGCCAAATTTAAAATCTGTTTGCATACAAGTGAACACGACAAAGAGA

AACTGAGTATATTCATCAGCCA

>c15831_g1_i3 len=402 path=[1:0-279 1464:280-283 285:284-401]

CAATTAGTAAAGAGAGTTGACTTTCAGAAAAAAAAGGTTGTTACAGAGAAGAGATATTTT

GAGGCCATATAATTACATCTCAAAAGTGCAATAATAAGGAAAGAAGCTGCTCTGGGAATA

CAGGAAATGTTAGGATGCAAAAATACACCACTCAGTTTTACAGTATGCTTACCATGTGCT

ATGTAATTGCAGTGCATATTTGATTTATACTAATTACATTTCAGTGGGTTGATTTGCTTT

CTGACTGGAACAGAACTGCCACATTGCAAATGCAGCTTGCTCATGCCTTTTGTGCACAGT

ATCACTAGCCTTGTTCTTTTAAAAACAGAATATAACCAAAACAAAACTATAAAAGCCAGC

ATCCTAGCTGCACATCATGGCTGATGAATATACTCAGTTTCT

>c15835_g1_i1 len=273 path=[15:0-272]

TTTTTTGAAAAAATAATGAATTTATTCTTTGCTAACTAGCTCTGCTATGATGTATCGAGC

AACATTTCAGAAATCCAAGTACCAAATACATGAACTGCAGTTGGCATATACAAATTATAA

GCACAATTGTAACACACATAAAGGTCGCTTTAATAGCACATATTTTTTAAAAGAAAAATC

AAAACATGTAATTTTGCTTCAAATATATTCTCTAAGTTTTCCACTCTTAGATGGAACAGC

CACCACTGTAATGAAAATGTTCATTTTTTTAAG

>c15839_g1_i1 len=225 path=[203:0-224]

AGAACCACTTTCCCAACACCTTCCTATCAGTCCTCCCTCAGTCACTCTGCATGGCGTGGG

AGAAGACAGAAGCAGATATGTGCCAACTGTCTCACTCTGTTCTTGAAAGTTTCTCCACGT

TGAAAGTAACTTCAGTCATCTCACACCTTCAGTGCAGAAGGGCTGTAAGTTAATCATCGC

AGAAGAGCAGATCTGACAGGTTTTGTAAGTTGTTTCTTTGGAGAT

>c15843_g1_i1 len=214 path=[145:0-121 425:122-191 538:192-213]

TTGAAGTCCACAATCCTTATGAGTCCCTTCCAACTTCCGATATTCGATGATCAGATGATT

CAGAGCAATGGTGTTTGTCTTTCCAAGGAGCCGGGCTGCATGATGAACCCTGCAATCCTG

CCGCCAAATGCAGCCTAGCTGCCTGAGGGGTCAGACCGCAGAAGAAATACACCAAGCAGA

ATTTCTATCTGCCCTGCATGCTGATTTTACTGGC

>c15850_g1_i1 len=268 path=[302:0-267]

GTGGACTTCTAAGGTCACCGCTTTGGAACAAGAACACAAGAAAGTCTTCACAGTCACATA

TATGAAGGTGCAATTATTTACATCCTTTCCAACTGTTGAGTCTGTGGATTTATGCTCACC

ATATCTGAGTCCATGAATTAGCAGGGGTTGGACCAGGTGGACCCTGAGATCCTCATCCAC

CTCAGCCAATCTGCACTCCCATTCAATATCACAGTGATTTATTATTTTAAATAAGCTTGG

ACATAATACGCACTATGAACAGAACCAC

>c15851_g1_i1 len=2917 path=[3071:0-2631 25:2632-2759 153:2760-2916]

TATATATATACATATATATATATCTCAAAATATGTAAAAAATATATAAAACATTAATTTT

AATAATTAGACAAGGAATATTATATGGAAGTAATAAATTTTACTGAAAATAAAGATACTA

ATGGAGTTATCATAAAGAATTTAATTGACATTATAAATTTAGCAATAAATTAAAATAAAA

TATAAAAAGAAAAAAGTTGTATATATTTACATAAATAATATATATCTATGTTTAAAAAAA

TAATCTCTATTAATATATTTTTTTTACTTTAAATATTTTATTTAAAATAAATGAAAACAT

ATCAAATACATTAAAAAAAATAGTATAACAAATTAAAATTAAAATTTTTTTTTTTTGTTA

TTAGACTTGGGAAGAAAAAGAATGTATGAAACAAATTTATTCAATTATTGATGATACTTT

GAGAAATAAAAGTTACTCCAATAATGAAATAAATCAATGGTCAAATGTGATTTGTGAAAA

TTGTATGACTTTTTTGTGCTCAAAGATGCTTCCTATGAAATACATAATAAGTTGCTATAT

TTTAAAAAAAGACGACAAAGAAACATCCATTCATTTGTCAACTTATTGGGATAAGGAAGA

TAAGCATTTTCAAATATGTTGGCCACAAGACTTAAAAAGCTGTAACATGCTTTGTTATGT

TAATATCTATATTATAAAAATATCAGATGAATGATGAAAGAATTTAATTTTTATTTAAAC

TCATATAAATTAATTTATCTATTTTAAAATAATGTAAATTTTTTTTTAAAATTTGTCAAT

CTTAGGTTCAAAAAATAAAAAAAAATAATATTTAACATATTTTAATAATTTTCTTTTTTT

TTTTATTCAAACACTTTAAAAAATATTTTAAGCTTTTAAATCTCTATTAATAAATATGTC

TGCAATTTTTTATTATTAATTTTTAAAATATATGACTTCAGCAAATAAATTTAATACTAA

AAAAAATATATATATTAGTAAAAATTAGATAGCACAATGCCACAAAAGATTTAAATATTT

TCCTCATCCTGTTTTTGACAAGCCCTTATACAAGCATCTTCGAACAACTTTTTAGTGTCC

ATAATTTCATTATTTTTTTTAAAATCATAAATTATTTTTTTCATAACTTTAGCTAATATA

ATAAGAGTTTCTGCCCTAGTTTTTCTCATTTTTTCATCTACAGACATATCTGTAAAAACC

TTTTTACATACTCCTTTTATAGTTTGGTCAATATCAATGAGGCAGATATTAAGCATAGTT

TCAACAATAGTTGGTAAGGAGTCTTCGATTTTTTTGTTAACTTTTGCAGTTTTTTCTAAA

GAAAAATTTTCTTCATCTTCTTTTTTTTTAATTTGATTTGCTAATGAACTAGTTTTAATT

AATGTTTTTACAAATTTAAATCCTGTTCCTATTACTCTTTTTTTCTGTTGCATTTTGTAA

TATTTTCCACTAATACCAAATAAACTGTATTTATCTCCTAAAAATTGATTAGCACAATTT

TCATAAGACCATGCAACATTTTCTAATATTACATGCCCAAAAGATGTCTGACATAATTCT

TTAATTTCATTTTTAAATTTAGTTATATATTCTTCTTGATTTCCATATATATAATTATTT

ATTATTTCTCTTAAATGTAGAGCTAATTGAACTTCTCTCTTATTTTGTTCTTTTACAATA

ATACGTTGCACATCTTCATCTTTATATAACTGTTCATATTCTACATACATAACCATCCTT

AATTTTCCAATGTATGGATCTAATTTTTCACTACCAAATAGTAAGGTAAAAAAAAATGTT

GAATCAATAAATTGCATATTATTTATAGCTGTTTTTCCTTCTTTGTCATATCTTCTTCTT

CTCTCAATATCTCCTAAAACTTGATATGCCTCTCCAATTTTCTGAAATTCTTCTTTTGCT

TTTAAATCTGGACATTTATCAGGATGAAATTCTTTTGCAAGTTTGTAATATTGCCTTTTT

ATTTCATTTTGACTAGCATCTATTGGTACTTTTAAAATTCTATAAAATTCATCATTTTTT

ACTTTTATGTTACCATTTTTATTATAATATTCATCATCATTAATTTCATCTTTCTTATCA

AATGTATTATTTTTATCATCAATTTCATTTAATACTTCATTAGCTTCTTCATCTAAATTA

TAATAAAAATCATACCATTCTCTTTTTTCTTCATCCCATATTTTTCCTGAGGCTTTTTTG

CTTATCGATTCAGGTGTATTTATAATTCCTCTTCCTATTTGATAACTAGCTACTCCCAAA

GACATTATTGGAATGATTATTGCACTTAACAATCCAAAAAACATTCCTTTAAAAAAACCA

TTAACACCCTCTGCCTTTGCACACAAATAAGGAGAAACAAATAAGCAACTAGTCCCAACA

ATTATTCCTTTTGTAACTGATTTTATACCTGAAACTAAACCAGCACCAGCATGTTTCGGC

TTTCTTGTTGAAAATATATTACTTATTAAACTTTTTTCTTCTTTTTCTTTTTCAAGATTA

TATTTTGGTATTATTTGAGTATTTTCATAAGGTTGTTTTTCATTCATATTGAAATTGCTA

CTTTGATTCGAAAATTCTTCCATTTCCTTAAATTTAAAAAATTTTTTTGCATCTACATTA

ATGATAAAAACAAAAGAGAAAAGTTATATTTATAAAATAATAAAGCAAAATTTCTTAAAA

GAAAAATAGCATTAATAAGAAAAAATAAGTGTATATTTGTAAAAATATTTTTTTATTTAC

TTCCTTTTGCCATTTCTTTTCCTAATATTATTCATTTAATCAGAAATATTATATATTAGT

TAATTTTAAAAAAAATAATATTAAATTAACATAAAACTTTTATTTATGTTTAACTATTAA

ATTATAATCAGCTACAGAACAAAAAAAAAAAAAAAAA

>c15851_g1_i2 len=2789 path=[3071:0-2631 153:2632-2788]

TATATATATACATATATATATATCTCAAAATATGTAAAAAATATATAAAACATTAATTTT

AATAATTAGACAAGGAATATTATATGGAAGTAATAAATTTTACTGAAAATAAAGATACTA

ATGGAGTTATCATAAAGAATTTAATTGACATTATAAATTTAGCAATAAATTAAAATAAAA

TATAAAAAGAAAAAAGTTGTATATATTTACATAAATAATATATATCTATGTTTAAAAAAA

TAATCTCTATTAATATATTTTTTTTACTTTAAATATTTTATTTAAAATAAATGAAAACAT

ATCAAATACATTAAAAAAAATAGTATAACAAATTAAAATTAAAATTTTTTTTTTTTGTTA

TTAGACTTGGGAAGAAAAAGAATGTATGAAACAAATTTATTCAATTATTGATGATACTTT

GAGAAATAAAAGTTACTCCAATAATGAAATAAATCAATGGTCAAATGTGATTTGTGAAAA

TTGTATGACTTTTTTGTGCTCAAAGATGCTTCCTATGAAATACATAATAAGTTGCTATAT

TTTAAAAAAAGACGACAAAGAAACATCCATTCATTTGTCAACTTATTGGGATAAGGAAGA

TAAGCATTTTCAAATATGTTGGCCACAAGACTTAAAAAGCTGTAACATGCTTTGTTATGT

TAATATCTATATTATAAAAATATCAGATGAATGATGAAAGAATTTAATTTTTATTTAAAC

TCATATAAATTAATTTATCTATTTTAAAATAATGTAAATTTTTTTTTAAAATTTGTCAAT

CTTAGGTTCAAAAAATAAAAAAAAATAATATTTAACATATTTTAATAATTTTCTTTTTTT

TTTTATTCAAACACTTTAAAAAATATTTTAAGCTTTTAAATCTCTATTAATAAATATGTC

TGCAATTTTTTATTATTAATTTTTAAAATATATGACTTCAGCAAATAAATTTAATACTAA

AAAAAATATATATATTAGTAAAAATTAGATAGCACAATGCCACAAAAGATTTAAATATTT

TCCTCATCCTGTTTTTGACAAGCCCTTATACAAGCATCTTCGAACAACTTTTTAGTGTCC

ATAATTTCATTATTTTTTTTAAAATCATAAATTATTTTTTTCATAACTTTAGCTAATATA

ATAAGAGTTTCTGCCCTAGTTTTTCTCATTTTTTCATCTACAGACATATCTGTAAAAACC

TTTTTACATACTCCTTTTATAGTTTGGTCAATATCAATGAGGCAGATATTAAGCATAGTT

TCAACAATAGTTGGTAAGGAGTCTTCGATTTTTTTGTTAACTTTTGCAGTTTTTTCTAAA

GAAAAATTTTCTTCATCTTCTTTTTTTTTAATTTGATTTGCTAATGAACTAGTTTTAATT

AATGTTTTTACAAATTTAAATCCTGTTCCTATTACTCTTTTTTTCTGTTGCATTTTGTAA

TATTTTCCACTAATACCAAATAAACTGTATTTATCTCCTAAAAATTGATTAGCACAATTT

TCATAAGACCATGCAACATTTTCTAATATTACATGCCCAAAAGATGTCTGACATAATTCT

TTAATTTCATTTTTAAATTTAGTTATATATTCTTCTTGATTTCCATATATATAATTATTT

ATTATTTCTCTTAAATGTAGAGCTAATTGAACTTCTCTCTTATTTTGTTCTTTTACAATA

ATACGTTGCACATCTTCATCTTTATATAACTGTTCATATTCTACATACATAACCATCCTT

AATTTTCCAATGTATGGATCTAATTTTTCACTACCAAATAGTAAGGTAAAAAAAAATGTT

GAATCAATAAATTGCATATTATTTATAGCTGTTTTTCCTTCTTTGTCATATCTTCTTCTT

CTCTCAATATCTCCTAAAACTTGATATGCCTCTCCAATTTTCTGAAATTCTTCTTTTGCT

TTTAAATCTGGACATTTATCAGGATGAAATTCTTTTGCAAGTTTGTAATATTGCCTTTTT

ATTTCATTTTGACTAGCATCTATTGGTACTTTTAAAATTCTATAAAATTCATCATTTTTT

ACTTTTATGTTACCATTTTTATTATAATATTCATCATCATTAATTTCATCTTTCTTATCA

AATGTATTATTTTTATCATCAATTTCATTTAATACTTCATTAGCTTCTTCATCTAAATTA

TAATAAAAATCATACCATTCTCTTTTTTCTTCATCCCATATTTTTCCTGAGGCTTTTTTG

CTTATCGATTCAGGTGTATTTATAATTCCTCTTCCTATTTGATAACTAGCTACTCCCAAA

GACATTATTGGAATGATTATTGCACTTAACAATCCAAAAAACATTCCTTTAAAAAAACCA

TTAACACCCTCTGCCTTTGCACACAAATAAGGAGAAACAAATAAGCAACTAGTCCCAACA

ATTATTCCTTTTGTAACTGATTTTATACCTGAAACTAAACCAGCACCAGCATGTTTCGGC

TTTCTTGTTGAAAATATATTACTTATTAAACTTTTTTCTTCTTTTTCTTTTTCAAGATTA

TATTTTGGTATTATTTGAGTATTTTCATAAGGTTGTTTTTCATTCATATTGAAATTGCTA

CTTTGATTCGAAAATTCTTCCATTTCCTTAAATTTAAAAAATTTTTTTGCATTTCCTTTT

GCCATTTCTTTTCCTAATATTATTCATTTAATCAGAAATATTATATATTAGTTAATTTTA

AAAAAAATAATATTAAATTAACATAAAACTTTTATTTATGTTTAACTATTAAATTATAAT

CAGCTACAGAACAAAAAAAAAAAAAAAAA

>c15856_g1_i1 len=252 path=[1:0-251]

AAGGTTAAAAATGTACCAGTGACTGAATGCCATGCATTGGTAATACTTTGTAGGTAATGT

TTTACGAAGTTGTAACAAAGTAAACAACATGGCTGCTTTTAATGGATCTTCCCAGCACTT

CCCAACATTTTCTGAGGAGGGGAGGGGGGAACACACAAAACCTCCAAGTCACACCTATGG

ATGATTTGTGTATGATCAAAAGAAACCCAACCTGTTAGAATAGGAAATAGTTGAACTACT

CCAGTTCTATAT

>c15863_g1_i1 len=234 path=[489:0-101 278:102-233]

AGCTTAAGTAGTTCCTGTTTCCACCTCCATCTCTTTCCAACATTGTGCCCTACTGAAAAA

GGATTACTGCCTACGCCCAGCTGTGTGCTTCCACTGTTCTTTTTGCCCTGGCTTTGGCAT

GCCTGTCACTGCATGGTGAGCTAGTGTCAATTTGAGTACCTGGAAAGTTGACGTCTCTTT

CCTATTTCACAGACTGTTGGGGTTGAAGTGGCTTATTAGGTGAGTTCTTGAGCT

>c15863_g1_i2 len=216 path=[194:0-83 278:84-215]

GAAAATGCTCCTAAACTATACCTTTCTCAACTGAGTCCTCTTAAAATTAGGAGTAGGCAG

CTTGAAGATGGAGACAACAGCTTATTGCCCTGGCTTTGGCATGCCTGTCACTGCATGGTG

AGCTAGTGTCAATTTGAGTACCTGGAAAGTTGACGTCTCTTTCCTATTTCACAGACTGTT

GGGGTTGAAGTGGCTTATTAGGTGAGTTCTTGAGCT

>c15870_g1_i1 len=269 path=[401:0-268]

AGTAAGAAATAAAGAATAAGCTTTGACAAAAAGCCAGATAACCAACCAGACTGCAGGCCT

TCCCAAACTAGCAGGCTCCGCGTTCTTGAAAAAAAACTTTCCTCCAACTGTTTTAGATGT

AAGCCATCTCAGTTAACAATAGAGAGAGCTCAGGTTTAAAGAGGCTGACTAAATACTCCT

TCCCTAGAGATCATTGTTTACTGTCCTCATTTTGATGCCATGGGGTGTTCTTCACTTTCT

CTTCAAGCTGCTCCCTGCTAATGGCACTA

>c15872_g1_i1 len=234 path=[239:0-233]

GCAAACCAACCAAACCATGTATGACCAGGCACTCCTGCTTTGCAAGCCTCTTCTGAGAGG

TCAAGGAAGTAGCATCTTTGAGAAGTGGCTAACACAAACACGCTGTGCCAGGTTTGCACA

AGTATTGTGCACATCTGTACTTTGTGAAAGCCACCAAATTCTGAAGTTACAACTGTGCAG

TTCATGGTTATTTTAAACTTTGGTTCAGATCGGTATATAAGGCCATTTCAAAAA

>c15880_g1_i1 len=317 path=[1:0-66 68:67-316]

TGGACACAAGCTGATATATTTTAATTAGTATTGAGTTGTTTCCTTTCGGCCTTTGAACAT

GAAAATTAAAAGCAAATTAGAGTTGAACAGTTTTGTGCAGAAGGCAGTATTGAGACTGTC

AAATTCTTGTGTTTTCTTCTGAAAATATGAAGGGAGTTCTTACAAGGTAGTTTGATTTAT

CAGATTTAAAATATGGCATGATCTTTATTTAGATTATCTTTAATTTTATACGTGGCTTGC

TTCAGCAGCACACTTCAGAATCACTTAGTTCTTAGCAAGCAGTAATGTATGCATCTGTCC

CAAAGTCAAACACAAAG

>c15886_g1_i1 len=386 path=[1:0-385]

CATAATTTAATGTTTCTAAATGCAAGACATAGGAATATACGTACTCAAGTTTTAAAATGC

ACATTTTTAAACCTCTTCAGGTTCAAATACATTCATACATACATTTTACTTTCCTATACC

TTCATTACCAGAACTCTCAAAAAAAATAAATGGAGTTCCATTCTAAGCATCCACACTATC

TACTCTTTCACAGTTACTTATGTTTCTAAATAAGAATGTATAATTTAGGAAAACAAGCTC

ATAATGAAGGAAATCTTAGAAATAAACCTGGCAGAATTCTGAAAAAATATGTATATTTCT

CAGCATCAGTTTAAGGCTCTGTTACTCTCTCTCTCTATATATATATATTTATAAATGATT

TTTTTTTTCTTTAAAATTAATAGACT

>c15893_g1_i1 len=221 path=[199:0-135 335:136-159 359:160-220]

ATTAAGGTTTTGCTGTCACATTTACATTGCCTATGCTTTTTAAGACCTCCCTCCTATTAG

AAATCTGTCACAAGTGTGCAGATCTGCTTAGGTCAGAGAGGTGCTTGGTAACTCTCAGGA

TGGACTGCACATTTGCTTTTTTGATTCTATTAAAATGTAGCCTCTCTGGGAGAGGCAGGT

GTGGAGGACTCTCACCTTGCTGGCACTGGGTTCATTTGCCG

>c15893_g1_i2 len=313 path=[199:0-135 335:136-159 471:160-312]

ATTAAGGTTTTGCTGTCACATTTACATTGCCTATGCTTTTTAAGACCTCCCTCCTATTAG

AAATCTGTCACAAGTGTGCAGATCTGCTTAGGTCAGAGAGGTGCTTGGTAACTCTCAGGA

TGGACTGCACATTTGCTTTTTTGATTCTATTAAAATGTAGAGCAAACAGTAGTCAGCATC

ATTCAGTGCCAAGGCACAGCTGTTATCAAAGGAACAAGATTCTTCCAGGAGAAGTGTGAA

AAGTCACCTCTGGTCATGGCAGAGGAAGAAGTAACAGAAAGCCCGTTCTTTCATCTTCTC

TCCTTGAGACAAG

>c15894_g1_i1 len=272 path=[337:0-271]

AGTCAGGTACAGCAGAAAGCTCAAAAACCTCAGCCCCCCTGCATTCCATTGCGATACCAG

GCAGGAAATTTGGCACATCTTCGCAAGTGGAAGTCTCAGTCTGCTCAGTGCAATCAGCTC

TCCAAGAACACTTCACCTTCCCCCAGGCTGCCTCCCTTTGTTCCCGTGGAGGGACACATT

AAATGTATCAATTACTCCAATAATTAACCCACTCTGCAGCTTCTCCATCATCTCGACGGA

TGCCCCCATCTACCCCTGCTGGCAGTGCTGCA

>c15895_g1_i1 len=1026 path=[1004:0-1025]

GGATTTAAATTTTTAATAAAAATATAGAATTCTTTTTAAAGAAAATAAAAGCCTCTTAAA

ATAATAAATGCTTAATGAAATAGATTATATATAATATAGTTAAGTGGTTTATAGCATTCA

AACTGTACTTAGATTTATTAAATTAGTAGAAGTAACTTCACAAGTTCTTTATTAATCTGT

TTGTTCAATACTCTAAAAAAGTCATTGGTGAGATAATTAATTAAACAATTAGTTTCTTTT

TCTGAGATATTCATTTTATATAGTTGCAAAAACCAGTTGTTTTTATTAAAAAATAAAATG

CTAGATAATATAACATGTAATTACGTAATGTTTTGGTTCTACAATTCCAAGATAAATCAG

CTATCCATTTTAAATAAACTTCTGACATTAGAGATTCCAATATCATACATATTTTCTTTT

TGAATAACCTTAAAAATTTCATTTTACATTCAAAACCTAAAAACCATCCAAAATAATTTT

TACTTAATGTCTCACTGAATTTTGCTAATTCTTATGGTATTTTTATCTTATTAATTTCTT

TATCTTTCTTTTGATCAAATTTATAAGAATTGTTAAAGAATACTATCTCTTCTAATTTAT

CTAAGTTTTCTAAATATTTTTTTCATTTTAATATAATGCTAATATAATAAGGGTTTTACT

AGCTAACGTTAGAATCTTGTCATTTTCATGGAATAGTTTATTAAATGAATGTTCGATCTT

TTTATTAATAATGAACACACTAATAAATATCCTATAGAAATTTTCTACTTATTTTACTTA

TTATTAAATTAATTATTACATTAAATTATTTTATAAATAATATTAATTATAAGCTATATT

AGTTATTTAGAAGTTAGTTTACATAATGCTCTATCAACATAATATATCATAATTTTCATT

TTTAAAATCTTCAAATACACACAAAAAGAATAGTGTAACTTTTTTTCTATTATTTATTAT

TCATAAAAAACAAAAAAAATATAATATTTTCTTTTAACTTACATACTGTTAATGTATTTA

TTTTTT

>c15907_g1_i1 len=224 path=[576:0-124 375:125-223]

GCAGGGGTACGAAGGACACTTCCAGAGCACGCTGGCCCCACAGCATCATCCCAGTGAGCA

CCTGTGAGTTGGATTTCTGTGAAGAAAAGCACTTCTGTTTTCTGGAGCACTGCTGGCCGG

AGAGCTGCAAAAATGAAGCAGATGTTTTATTTGTTGCTCTTCACGTCACTTACAGTGAAG

ATCCTATGAGACGTTATGAGATGAATTAAGCCTCACTGAGGAAT

>c15907_g1_i2 len=248 path=[226:0-148 375:149-247]

AGGAGAGCCAACACAATGGCTGCTACTGGGGAGTGGCACACATGCGATGCCACGGCACAG

GGCCTGCCCATCCCTCACTGCATCCAGGCACTGCAGGTACCCAGCACCAAGGAGGAGAGG

AACAGAGAGGAGCACAGATAGAGGGGAGATGCAAAAATGAAGCAGATGTTTTATTTGTTG

CTCTTCACGTCACTTACAGTGAAGATCCTATGAGACGTTATGAGATGAATTAAGCCTCAC

TGAGGAAT

>c15910_g1_i1 len=229 path=[207:0-228]

GCTGGCAGCAAAAAGTGATCTTTATATTCCATTTGTACAACTGCAACACGTCCTGATCCC

GAACCAGGGCAGCGCTGTCGAGGAAGCAGACAAACAGGTAAAGGATCTCATGCTCCAGAA

TGAGCAGTGGAGCTCACAAAGCCCTGCTGCCAGCAACACAGCAGGGCCTCGGCCAACAGC

TGAGCCAGAGCCCTGCAAATCTGCAAACAGCTGCTTCACATTCAGGGGT

>c15915_g1_i1 len=442 path=[1:0-441]

TCTCATCCTTCAGCCGTTGGTTGTCGGCCTTCAGGTCCTGCAGTGCCTTCAGCTCCTCCT

CCAACTCTGCCGCCTTCCGCTCCAGCGCCCGGCGCTCAAAGCGCTCCAGCTCCAGCAGCG

CCGGCCGCTCCGCGATGCGCTCCTGCCTCTGGGTCACCCTCTCCAGCTCCAACCGCAGCT

GCGTCACCTTCAGCTCCGCCTCCTGCAGCTGCTCCTTCAGCCGCTCGTTGTCCTCCCAGA

GGGCGTCATAGAGCTGCTGGTAGTCGATCGCTGGCGGCTGCGCTGGGGGGGGGCGCGGGG

CAGCCAAAATCCCGTTGCTTTTGGGGCTCTCCTCACTGCTCCCCTGCTCCTCGTTTTCTT

CCCCCTCAGACCCCATCTGTCCTTTCCGTCGCTGTCGGCTCTGCAGTTTGGGTTCTGCTT

CTTTGTTCAGCTGCAGGATGGG

>c15916_g1_i1 len=227 path=[579:0-16 600:17-226]

CGCCGCGCGATGGCGAGCTCTGGTTGAGACCAGATGGAGTGGAGTTAATAGCACACGGGG

CCAGGCCACTCGTTGGGCCAGCGCTGCTGCCAGAATCCTACTTACAGTTTACTATCTATC

CTACCAGCAAGCATCACACTTCACAGAAATTCCCTGAACACACCCTTTCAAACATCAGGC

ATACTTCCTATAGGCTTTACTTGCCACGTTTCATGTTCAAAATTGAT

>c15916_g1_i2 len=278 path=[274:0-45 600:46-255 598:256-277]

GCTTATTTGCCAGAACGTGAGACAGCATTGCATGTGCCTCTCTTTGCTCTGGTTGAGACC

AGATGGAGTGGAGTTAATAGCACACGGGGCCAGGCCACTCGTTGGGCCAGCGCTGCTGCC

AGAATCCTACTTACAGTTTACTATCTATCCTACCAGCAAGCATCACACTTCACAGAAATT

CCCTGAACACACCCTTTCAAACATCAGGCATACTTCCTATAGGCTTTACTTGCCACGTTT

CATGTTCAAAATTGATTGCTTTTTTTATCTCCAGCAAT

>c15922_g1_i1 len=413 path=[1:0-412]

ATGAAGCCTCCCAGAGGTTTGGGCCAATTTTGTCTCAAAGCCCATCAAAAGAAAACCCAG

TTATCAGAAACTCAGGGATATTTTGCTTATCCATTTTTAAAAAGTAGCTAATGCTGAGGT

AGATACAGGCCAATTCTGAAACTTTAAAACTATCTGTAATACTTTGAGATTTACTTTGCT

CCTTCGTCAGTGAACTCCCCGTAGTTTTCAACTGCTCACCTTCTGTCCTGCAGAACGGTG

TGGGCTCGCGCAAAACAATCTCTCCGTAGTTCAGTTGGGTGACACTACCGGATTGGGCTC

GCTCAGCCTTGTCTTGATTTGCTCCACATGGTGGGTGCCATTTGCAGCGTCTGATGCATG

GACTGAGATGTGAACGATCCAAATCGTTCATTACAGGTTCTAACATCCCTTAT

>c15926_g1_i1 len=268 path=[1:0-267]

GCTAACTTGTTTCCCAGCTGTCATGGCTTGGCTGCATCTCCAGCAGCATGAGGCAATAAG

ATAGGGCAAGACAAGACAGTGTTCCTCTAATCAGCTTTCTTTTCCTTGTAGTAAGGCAAT

ATTATGTCAAAGACTTCAGCATAAACTCATTCCTCTAGCTCAGCTTGTCCTATAATAAAC

ACCATGCCTTATTTTAAGGCAAAAATCAACCTCATTAGTTTCTTTAGAAAACTACTTAGG

CAAGTGAGTACCGTCATCCTCTATCTTT

>c15932_g1_i1 len=1412 path=[1761:0-1411]

AAAAAAAAAAAATTAAAAACAAAAACGTATGTGATGATGAAAAAGAAAATGGTAATCTCT

CATTTATAAAAAGAAAGAACAAAATAAAAAGTAAAGAAAATAATGATGAAAATAAGAATG

ATCAAAGCGCAGAAAAGAGTATAAAGAATGAAAAAGAAACTCAAGGTGAGAAAGAAAATG

AGAAAAATGAAATTTTCATAAATAATGAAGATAACGAAAGCAAAAATATAGTTGTAAAAC

ATAATATAAATGATGAAGATAATACAAAAGAACTTTTTAAAGATTATATTTTCTATATTC

ATAATGATATGCCATTTGATATTTTATCAATTATTATTTTATCATGTGGAGGACAAATAT

GCTGGAAAAATTTATACTCACCATTTAAATATGAAAGTAACAAAATTACACATGAAATTA

TAGAATCTCATGAGTCTATCAAATTAAATGAATTTAAATATAAAAGAAACATTATACAAC

CTCAATATATTTTTGACTGCTTAAATCAGAAAAAAATATTACCATGTAGTGACTATTTTA

TAGATAAAAACCTACCAGTTCATTTATCTCCTTTTATTGAAGATGATAATTATAAAAATT

TTGTAAAAAAAGAAGAGTATACAATAAATAAGATGCTAATTAAAAATGATTCTAATCAAG

AAAAAAATAATTCCAATGAAAATGATGATGAAGAAATTTTAAAATCAGAAGATGAAATGA

ATGATATAATTCTTGAAAGTCATAGAAATGCTGCTTTAAATAATCAAGTTGAATTAGAAA

ATGAAGATAAAAATATAGATGCTATGAAAAAGAAAAAAGATATAAGTATTTCTAATATCA

AAAATTTAAAAAACCAAGAAGAAATACAAAGAAACAAAATTGCTTTAAGTAAAAAAAAAA

GAAAGTTATATTCAAGAATTGAAAGAGCAGAGAAAAAACAAAAATTAAAAGTAGAAAAAT

TTTTAAAAAAAGCGAAAAATAAAAAAGGGAAAAATAAATAATATGTTTAATCCTATTTAT

CTGAAGAAATTTTTTTCTTCTTATATATTTTTTCGAAATATTACATCATTTTTTATATCT

ATCTTTTACCTATACATAAATTTTCTTTTCTTTCTATTTTTACATTTTTTTTTTTTTATT

ATATGTACATATACTAGCAAATATCCATAATAATTTAAAGTTAAATTTATCCATATAAGA

GGAAAAAATAAAAATAAAGTAAACATATAAAAAAGAACATACTTTGAAGTAAATTTATTA

TTACAAAAATGTATCTATAATTTTTAAAAAAAATGAAATTGCTTTTTTTAGCATATTTAA

AAAAGATACTTTTTTTTAAAATGTTATTGTTACAAAAATTACAATTTTCTATTTATTTTT

ATGTCATTTTTTCATAACATTTTATTTTATTT

>c15932_g2_i1 len=886 path=[18:0-885]

ATTTTTTTTTTTTTTTTTCTTTCTCATATTTGTTCTTATCTTTTTTATTTTCTAAATCCT

TTTTTTTTGTTTCATATATAGAAATAAAGTAAGGATCAAAAGATAAATGTTTAAATTTTT

CGTTTTTTAAAGTTTCGTTTTCTTCTGGTGGATATTGTAAATTATCTAATTTAAATAATT

TAAATAAAACAAATTTTAATAAAGAAATATAGTATTCAATAAAAGTTGTTATTAAACGAA

AATCAATTGATTTCGATAAATATGGTGTATAAATATGAGGTATAATCCAGGTAACTTTTT

TCTGCAAAATTTCAGCCTGTAAATAATAACCTTTAACACTTATAAATCCTTTTTTTATTT

TGTTTGTTTTATATACATAATAATGAAAATGATTTTTTAAATCTTCACACAATTTAACTA

AATTATTTTTTATTCCTAATTTCTCATTAGATGGTAATAAAGAATAAGCTACAATAGAAC

TTAAGGAATCATCTAACTGCTCAATTGCATAAGATAAAACAGGAAATCTTTCTTTTATAA

TATGATCTAATTTATATTTTGGAAAGTTGCTTATTATATTTTTACATCTTGTTTCATCCT

GTAATACTATTTTAAATTTCTTATATTTTTTTAAAAATATTTTAATTTTTCTAAAATCTT

GAATAATTTTTTCATGGGATAATTTTAAATAATCATTTCTTGTATAAAATATTTTGTGTT

TATAAAATTTTTTTCTATATTTTAAGGGGATTTCTTTAAAATCTTTAGGATAAATTCCTT

TAAATATACATAATTTTCTAAATTCATCTTCATTTATATATAATTTTTTTAATATACTTT

TTTTTGTTAAATACTTTCCTTCTTTTTTTTTTTTTATTTTCTTTCT

>c15940_g1_i1 len=234 path=[10:0-233]

AAAAAAAAAAAAAAAGTTAAAAGTCGTATTGTATTTTTAAAAAAATACTATAAAAATTAT

TAATGTATAAAAATAAAAATTCTATTTCTCTTTCGGAGGATTTTTAAGCATCAAGAACAT

ATAAAAATTCATAACCTTTTATAAAAAAAAAAAAAAGAATTGTTCCTTGCACCACCAAGG

TTTTACAAGTCAGCACTGCATCTTCACAGACCTGCGTCATGAATCACAGCATCA

>c15942_g1_i1 len=226 path=[193:0-191 384:192-225]

GTGTTAAAGGAGAGGATTTTTGTTCATTATTTTTTTACATTATTTTTTGAAACGCTTTGG

AGGTTTTTACTCAGTATTAACACCCAAATGAGGTGGCATAATCAGATTTCTATGTTGATG

AGAATCTAAGTGCTGAAGTAAGCATCACCATTTAGTGCCAGTTTGTCAAGGCAAAGATAT

ATTGGGATTTTGTGTTTGAGGAGGGAAAAAGGAAGGAAAAAGGAAG

>c15942_g1_i2 len=256 path=[193:0-191 121:192-255]

GTGTTAAAGGAGAGGATTTTTGTTCATTATTTTTTTACATTATTTTTTGAAACGCTTTGG

AGGTTTTTACTCAGTATTAACACCCAAATGAGGTGGCATAATCAGATTTCTATGTTGATG

AGAATCTAAGTGCTGAAGTAAGCATCACCATTTAGTGCCAGTTTGTCAAGGCAAAGATAT

ATTGGGATTTTGGTTTTGAATATGCTGGAAACAATTGTGATGAATTTCAACATCCATGTA

ATGCAATAAAGTGCTT

>c15944_g1_i1 len=318 path=[1:0-87 89:88-88 90:89-293 342:294-317]

TGATGGTGCACGTCGGGGCGCATCGGGCCGGTAGGCATGGCTAAGACCACCAAGACCAGT

GCTCATGAGGCCATTGCAGTACCGAGGTGGGTATGTGCCTCACTGTCACAAACACAGCAC

GCCATGTCCCCATAGCTGTGCTCGTTGCTGTGCGTATTGCCTAGTTCCAAGCAGAATGCT

AAAAGGCAGCGTCAGGAGGTGTCCTCCTTGTTTCTTCCAATCAGTGCTTTAAGTGCAAGC

AAAAGTACATGGAACAGAAGCAACTACCAACAGTGTAAGAAAAAATATCTTTGTGATTTT

ATGATGAATTCGTAATAA

>c15946_g1_i1 len=621 path=[27:0-426 26:427-620]

CCCCCACGTTTTCAAAGGGAAGCTCACCAAAAAAACCCCATCTTCAAACCAAGACGAGCA

TAACTGCCCATATCTGTTTTCTCCCCCATTGTTAACGGCAGAGTTTGTTTGTTATAGTTA

TTCGTAGGGGCTGCCCCAAAAAATACTGTTTCCCAGGTCCAGTAATTGCTCGAGGCGTTT

GGAAATTAATAGAGCAGCTCGGGGTGGGATGTGCTGGAGGAGCGGGATGGGTACGCAGCC

CAAATGCAGCTTTGGGGTGCTCTGGGGTGCTGCTGGTGGGGGTTGGAGGGGACCCACGAG

GACCGCTGAGTTCAGCCCCTGGCTCTGCACGGCATCACTGCTGAGCTGTCGGCTTTTTGT

CTTAGTGAAGCGGGCATCTTGTCGGGGACGGGCAGATGGCGTTCACCTGCTCGGGACCGA

CTCTCTCGCCCTTGCCTTTGGTGTTGGGGAATAATAACAGCCAGCTGCTAGCAGTAAAAT

AGGCTTAAGCAAATGTGATATTTTGGCATTCAAATGCACGACAAGAATGGGAGAATGTCC

ATAGTATTTGCATTAATTTCCCCTTTCCCCGTGGCAGGAGATTATGCACAAAGTCGGGTT

GGCACGGGGAAAGGGGAAATT

>c15946_g1_i2 len=206 path=[14:0-11 26:12-205]

GGCCGAGACTGAGCCCTTGCCTTTGGTGTTGGGGAATAATAACAGCCAGCTGCTAGCAGT

AAAATAGGCTTAAGCAAATGTGATATTTTGGCATTCAAATGCACGACAAGAATGGGAGAA

TGTCCATAGTATTTGCATTAATTTCCCCTTTCCCCGTGGCAGGAGATTATGCACAAAGTC

GGGTTGGCACGGGGAAAGGGGAAATT

>c15947_g1_i1 len=459 path=[437:0-458]

GTCCTCTATGTGGAAGAAACTTTCTTCTGTTCTGCTCCTTTATCCCAAAAGGATGACATC

CACACTGCATGCGTACAGAACAGTTTGCTGCTTCAAGTGCGTGGCTTTTTCTTTCATTTC

AATACAGGATTACTGTTCTGCTCATCTGATCAGGAGTCCAACCTGCGCCGGTCTGTTGAG

GGGGCTCTCCCTGTTAGGCTGTGTGCTGATCTCACAGGAGCAGTGGGGTGGGAGCTCGGA

GGTGTGCTGCATGCAGGCGGTGCGCATCCTCTTCAGAGCAGGGAGAGCCCCCACGGTATT

TCCTCCAGCAATCCCACAAACTCTGCTCTGAAGGCAGTGCAGGGAGCGTGCTGGCTGCCT

CTCCTCGCTGCTCCTCGCTTCATCGTTTCCATGTGTGCTGAAGTTGCATGAAGTTTTCAG

TTCTGATGACTAACTTCTTTTCTTCATGGGAAGAAGCCA

>c15954_g1_i1 len=221 path=[1:0-220]

AGCATGTTAGAAACAGTTCTAGAAGAGGTCTCAAAATGCAGACAGGAAAATAGAGTAGAG

AATTGAATGCATCAATTAGAGCTGATTGGCTTCAGAATGTGGTGGTGGGAAAAGGGGGTG

TACACAAGTGATAGACATTAGGTGCAAGAGATAAGTGAGCTCTATTTCATCCTTTGTCAC

AGTGATAATCCTTTAGGAGAGGCATGGAAGCAGTTTTGTCA

>c15955_g1_i1 len=407 path=[308:0-302 742:303-406]

GAGGTCGGCTGCCGTGTAGCCATCCTGGTCTCGGATGCTGAGGTCAGCGCCGTTCACCAC

CAGGATCTGGCAGCACTCCAGCTCGCCATTCTCAGCAGCGTCGTGCAGCGGTGTGCCGCC

CCAACCATCTGCTGTGATCTCACCGCCATGCAGCAGCAGCCAGCTCAGCACCTTGGCGTG

GCCGCGGCTGGCAGCAAAGTGCATGGCCGTGGCTCCGTCGTCGTCCCGCTCTGACAGGCT

CACTGTCGTGAAGCTCATCTGCGGAGCGCAGGGATGCTGTGCCGTGCCACGCCGTGCCGC

ACCGTGCCACCATGCCGTGCCCAGGCAGCACTTACCAGCCAGACGATGACGGTGTTGTGG

CCCATCTGGGCTGCAGCATGCAGTGGGGTCATGCCGTCGTTAGCGCG

>c15956_g1_i1 len=215 path=[795:0-55 151:56-214]

CCAATAGAAAGGTGGTGCCTGGAAACATTTCACCATCTATTCCTGTGCAGAATTACCAGA

CTAATGAGACGTGGCTCCTCAAAGGTGTGCATGTCATACTTCTTGCCCACAAAGCACGGG

GAGGAGGAGGTGATGTGTCATTACCTGGAGTGCTCTGCAGGGTGAGGGATGAGTTTATGG

AAAATCACAACCGTTAATCCTGCTGCAGGACTGGC

>c15956_g1_i2 len=376 path=[94:0-56 151:57-215 19:216-375]

TTTCCAGGCACCACCTTTCTATTGGGTTATTAAAGTAAACTAATGGGTAGATAATAGCAG

ACTAATGAGACGTGGCTCCTCAAAGGTGTGCATGTCATACTTCTTGCCCACAAAGCACGG

GGAGGAGGAGGTGATGTGTCATTACCTGGAGTGCTCTGCAGGGTGAGGGATGAGTTTATG

GAAAATCACAACCGTTAATCCTGCTGCAGGACTGGCAGAGCTCCTCCTGCTTGGTGCCCA

CCTGTAGGACCGTCCCACTTCTGTGCTGTTTCTTGCTGCCTTTTGCACTGCTCAGGGCAA

TCTGTGGACTGGATAAGCATCATGATAATCACAGAATGAATCATAGAAGCCTCGAATGGC

CTAGGTTGAAAAGGAC

>c15959_g1_i1 len=375 path=[403:0-324 25:325-340 880:341-345 46:346-374]

TAAGGAAGCACAGACTTTCAGTTGTGTGCACGCAACAAGCAGTTGCAGGGAGTGAACTTT

ACACAGCATGACTTCATCACATGAGAGCAGCCTGCCATTTTTCCTCTTTCTGCTCAGCAA

ACGTGCACAGCAACCTTCAAAATGCATTAAACATGCCGGAATAGGTTCTAGCTTAGTTTT

AAAACATTATCTACAATGAAAGCATGAATGTTCCCTACCCCGTACGTGACCTGAAAAAGC

CAAATCATTAAACAGAACTCAGTGTAAGAATCGTTTCGAGCCACGTGTCAAAAACTTTTA

TAAAATACAACTCGGATATTCCTTTCTTTTGAGCCCTTGATCAGCCAATCAGAATTTGAA

ACAAGATGGACAACG

>c15959_g1_i2 len=376 path=[403:0-324 727:325-346 880:347-351 754:352-375]

TAAGGAAGCACAGACTTTCAGTTGTGTGCACGCAACAAGCAGTTGCAGGGAGTGAACTTT

ACACAGCATGACTTCATCACATGAGAGCAGCCTGCCATTTTTCCTCTTTCTGCTCAGCAA

ACGTGCACAGCAACCTTCAAAATGCATTAAACATGCCGGAATAGGTTCTAGCTTAGTTTT

AAAACATTATCTACAATGAAAGCATGAATGTTCCCTACCCCGTACGTGACCTGAAAAAGC

CAAATCATTAAACAGAACTCAGTGTAAGAATCGTTTCGAGCCACGTGTCAAAAACTTTTA

TAAAATACAACTCGGATATTCCTTTTTTAATACATGATGCAAAAAAGCAGCCGTTGTCCA

TCTTGTTTCAAATTCT

>c15962_g1_i1 len=304 path=[1:0-211 213:212-235 693:236-279 501:280-303]

AGCTGTGAATGGTGGCACTCACTGACAGCAGCAGAGCTCTCCTATGATGGTAGATACCAG

CTAGCTTTTGTAAGAGATTTTTTTTTTATGAAAGCAAGAATATTCTACTATTTTCCCATT

TAAATTCTGTAACTAGGAGCTGAAACCTGAGCTATGCTGCTGTATTTTATTCCCTCCATG

GTAGTGATAGAGAAGGGAAGCATTTCAAGGGAAGGCGCAGGAAATGTGACACGCTAACTT

CTGGCTTGGTCTTTTTGTATGTATCAGTCTGTGTATACCATCTTTAATGAAACACAATGT

AAGT

>c15962_g1_i2 len=304 path=[1:0-211 213:212-235 693:236-279 736:280-303]

AGCTGTGAATGGTGGCACTCACTGACAGCAGCAGAGCTCTCCTATGATGGTAGATACCAG

CTAGCTTTTGTAAGAGATTTTTTTTTTATGAAAGCAAGAATATTCTACTATTTTCCCATT

TAAATTCTGTAACTAGGAGCTGAAACCTGAGCTATGCTGCTGTATTTTATTCCCTCCATG

GTAGTGATAGAGAAGGGAAGCATTTCAAGGGAAGGCGCAGGAAATGTGACACGCTAACTT

CTGGCTTGGTCTTTTTGTATGTATCAGTCTGTGTATACCACTTACATTGTGTTTCATTAA

AGAT

>c15964_g1_i1 len=213 path=[191:0-212]

AATGTGCACATGATAGGTGAATTGGAAAGAATTTGAACCTGAGTACTCAGCTAATGAGAG

ACAGAAGAGAGACTCAGTGTCAGAAACTAGGAAATATAAGATCCTGTTTTCCTTGTATCA

GCAGTTCCATTACTAATAGGGTGAGGGGGACCCTTTTCCCCCTTCCCCCTTTCCTGGAGC

ATGGGTCCATGGGTCCCCCTCACCCTATTAGTA

>c15977_g1_i1 len=226 path=[448:0-225]

GTAGGTCGCTCTGAAAGTAATGCCCCCTATTTATTTCCATGGAAACTACTATAGATACTA

AAAGCACAATGACGCCACTCGATAAAGCAAATTCTCAGCTACGAAACGCTATTTTTCAAC

GCGGTCACCACCATTAGCTACGTATTTCTGCCAGCGATGAACAGCAGCCCGTGTGCTGCG

ATGGTCAGTACCCGTGCCAGCAGAAGTGCCCCACTGCCCAAACAGC

>c15978_g1_i1 len=276 path=[448:0-76 21:77-275]

ATAAAATAAAACAGACATCCTCCAAGAGAAATCAAAGTCTCACTGTGCTAGCTACTTTCA

CTCTCACACCCTGTGTCTCTGGCTCTCAGTGAAATGGAAGAGCACAGCAATGGAAGAGCA

GCTTACTGGCCTGCCTGGATCCTCCTCAGCCCCCCCCCACAAAATATTATGGAACAACAG

ACAAATTTCCTGGCTAACAGCAAGCTGGCTCTGCTGACCACACCCAGGATAAAACAGAAG

GCAGAGCTGTCTTTTCCAGCCAAAGCATGATCTCAG

>c15978_g2_i1 len=276 path=[448:0-76 524:77-275]

ATAAAATAAAACAGACATCCTCCAAGAGAAATCAAAGTCTCACTGTGCTAGCTACTTTCA

CTCTCACACCCTGTGTCTCTGATCTCTTATTGGAGCTTTCAGCTGAAGCAGAACTTGAAA

TAAATAAAAAGTGAAAAGCATTTCTCTTGTAGACTTAAGTATTCTTTTCCAAGTCAGATG

ACAGAAGTGTTGAGGACACCAGAATTGCTCACGTGACACAAAGATGGAAAGGCTGCATGA

ACTCTTTAAACCTGAGATCATGCTTTGGCTGGAAAA

>c15978_g2_i2 len=335 path=[1066:0-19 1047:20-135 524:136-334]

ACTTTAAAGTACTGTTGATAAAAGTGTTTTGGTTTCTGTTTGATTTTTAAATACTTTAAA

AACATACTTTGTTCTCTTTTGTTTAGTCACGTGTACAGCTTAGTACATGTGACTGTAACT

CATGGTGAAGGCTTGTTCTGATCTCTTATTGGAGCTTTCAGCTGAAGCAGAACTTGAAAT

AAATAAAAAGTGAAAAGCATTTCTCTTGTAGACTTAAGTATTCTTTTCCAAGTCAGATGA

CAGAAGTGTTGAGGACACCAGAATTGCTCACGTGACACAAAGATGGAAAGGCTGCATGAA

CTCTTTAAACCTGAGATCATGCTTTGGCTGGAAAA

>c15981_g1_i1 len=439 path=[1:0-438]

AGCGTTACAGTTCTGTCTGCTGATGATGACAAGAAGGAAAGATCTTGTGTATGCCATTTG

CATTAATACTCTAGAAGACACACAAGAGGAAGCTGCTTTGTAAGGTCTCCTTGCAAGTCT

GTCACCTTTATTTTCATATCATAAGATCCTGTGAGCAAGTAGTGAGCCCCTGGAGAGAAG

CGCACAGAACGAACATCGCTGGCATGAGGATGATAGCTCTGGACCATTCGCCCTCCTCGA

ATATCATACAACATGCAACTGGAGTCCTCTTGTCCTGTAGCCAGGAGACGACCGCTAGGA

TCAACAGCTACTGATGCTACTGCACTTCCTGTTCCATGAAACGTTGTTCCCACGACACGA

ACACAACTTGGCACTCTCAGATCCCAGAATCTTACAGTCTTGTCCTGGGATCCAGATGCG

ATCATCCAACCACTCCATG

>c15984_g1_i1 len=203 path=[197:0-202]

GGTTATAATTATAACTTTCAGAGTTAATGACTTTACAAGCTCAGAAATAAGCCTTTAAAG

CAATACTGCATAATTTACCTCAAACATGAATGTTATTTAAATGCACTGCAGAACTTAATT

AGACTAAATTCTGACAGTTTCTACTAACATAAGTGAAAGTACCTTGAATAATGGCCAGTA

GGATGACAATGACAAAGAAGAAA

>c15995_g1_i1 len=242 path=[220:0-241]

GGGGTTTCTGGTGTGGATTCTTTTTGGATTCCTGGGGAGGAGGGGTTCCAGAGGGATGGG

GTCCGGGGGGACTCGCGAGACGGGATGTCCAGGGAGGGAGGCTCCGGGAAAGGGGGAGCT

CCCTGGGGTTGGGCTTTCGGGGGGGAGGTTCTGGGACCACCCGCTCTGTGCCGCGGAGGG

GGACCCGCCTGGCGCGTGTGACCGCATTGTTGCCCCGCAGGCATCCAGAAGGAAGAGCGT

CC

>c15996_g1_i1 len=257 path=[1:0-256]

GTGAGCTCCCACCACAGGGTTGTACCCAGAGTCACTTTGCTCTGTATTCAGGAGCTGGCT

GAAGCTTCACGGCTGAGCAGCTTCTAAACCCCATGAAGAATTTTCTCTGAATTAAATCCT

CAGTACGATGCTTTCTGAGTTAGCAGAACACACAGGTGCTGCTGAAAGAGCTACCTGAAA

CCCTCAGGCTTTCAGCAGCCTCTTTCCTGTGCATATTAGATGATGCTGACAACTGCCTGT

CATGTTTTCACCCAACA

>c15997_g1_i1 len=447 path=[271:0-446]

AAAAGAAAAAAAAAAAAAATTGAAGCCAAATAGATTCTTACATGGAAGAAAATAGGCAAT

TATGTGTTCAATCCTCAGGCAAAACTGAAGTGGCTTATTGATTAACTTGGACAGGGCCCT

CCGTTGTTAATACATGTAGTATTTAGAAGAGTTAACAAAAAAAAACAAACAAGAAAGTAC

ATCACCGAACAGTTGAAAGTCAAGAGATTTTCAGACATTTTGATTGTTATCTTGAATAAA

AATTATTTGACATTATATAGCCATCGGGGGAAGTTACTAAACACTTCAGTGTTCCTATGT

GAGTAAGAAGTATTTCCAGGAAAATGTAGGAGATGTACAGGTGACTCATATGCTTCGTTC

TCCCACACTGTCACCTTCAGAAGCAGTTTAGAGAGAAACTGAAGGATATTCCATTCAGTT

TTCCCCAGTTGTCCTTTAACTAGCATG

>c15998_g1_i1 len=234 path=[578:0-233]

GTGCTGCACAATAAACCATCCTGTGTTTTTCTGTAGAAAATTTTACTCTGTGTGAGCCTG

TGAACAGAAGTAATTAATTGCAAGTCTTAATCACATGGCTGAATCACTTGAACTAACATC

AGGTCTTAAAACTTGCTGACATGTTTCCCACAGAAAGCAGGTGATATCCTCAAATTGTTT

CTATGCAGTGTTTAGCTGATGATCAGAACATCTCCCTAGGCAGCTTTTGACAAA

>c16001_g1_i1 len=738 path=[716:0-20 737:21-737]

AAAGGCCATCTATCCCTAGTTTTTTTTTGTGTGTGAAAGACTCTCTTAAGTCAGTAGAAG

ACTTCAGCAGGCTTTCTAAGCTTCCTTTAGGTTAAGTTAGAATTTGTCAAGAATACTGAA

GAAACGACACAGTGGCACTGTATTTTGAAGACCTATGATGACTGAAAGTTGTGTAGGTAC

ATTTAGGATAGCTTTGAGTAGATCTGCTGGACTTACCTTGTACACAGCAAAAAAGAACTA

GGGATAGATGGCCTTTGGTTAACTCAGGGTAGACTCGCATGCATATTTGTTTAAAAAATA

ACATGCAAGCTTCCAAATTATTCTTAAAAATCATTTGACTCTAGGCTTACTTGCAACTTT

ACATTTTGTTTAGATATTTGAATACATTTCTTCTGACACTTAGCTGAGGTACTACTTTAT

TCCTAATACTAAAAGGGAAAAACTGAAAAGCAGTTATGTGCGTGCTTAAGAATTTTTTTT

TAAATCTGTCGTGGTAGTGTCCTACAAATCAGACACCTCTCTTGAACTTAATTTTGTTTG

GTTTGGTGTTACATTCAACTGGAAAATCTTTGATAACTCTATGGTTCTGCATTGGATAAT

TTTCCTGCAGGAAATATACTGAAGGAAGAATTAACAGATTTATGTTCATTCATATTATCC

TTTCTGTAACTGTTTTACATCCAGCTCGGGAAATCCTTTTCTGTCTCTGGCTCACCTTTG

CCCCCAAAGGAAAGAACA

>c16001_g1_i2 len=776 path=[1431:0-58 737:59-775]

ACAGTGTATAATTAACTGTTTCTGTTGTGTATGAAGCTTATTATGGTCTCATCAAGGCCT

TTTTTTGTGTGTGAAAGACTCTCTTAAGTCAGTAGAAGACTTCAGCAGGCTTTCTAAGCT

TCCTTTAGGTTAAGTTAGAATTTGTCAAGAATACTGAAGAAACGACACAGTGGCACTGTA

TTTTGAAGACCTATGATGACTGAAAGTTGTGTAGGTACATTTAGGATAGCTTTGAGTAGA

TCTGCTGGACTTACCTTGTACACAGCAAAAAAGAACTAGGGATAGATGGCCTTTGGTTAA

CTCAGGGTAGACTCGCATGCATATTTGTTTAAAAAATAACATGCAAGCTTCCAAATTATT

CTTAAAAATCATTTGACTCTAGGCTTACTTGCAACTTTACATTTTGTTTAGATATTTGAA

TACATTTCTTCTGACACTTAGCTGAGGTACTACTTTATTCCTAATACTAAAAGGGAAAAA

CTGAAAAGCAGTTATGTGCGTGCTTAAGAATTTTTTTTTAAATCTGTCGTGGTAGTGTCC

TACAAATCAGACACCTCTCTTGAACTTAATTTTGTTTGGTTTGGTGTTACATTCAACTGG

AAAATCTTTGATAACTCTATGGTTCTGCATTGGATAATTTTCCTGCAGGAAATATACTGA

AGGAAGAATTAACAGATTTATGTTCATTCATATTATCCTTTCTGTAACTGTTTTACATCC

AGCTCGGGAAATCCTTTTCTGTCTCTGGCTCACCTTTGCCCCCAAAGGAAAGAACA

>c16003_g1_i1 len=241 path=[203:0-240]

AACCTCAACAGGGTTATTTTTCCACCTGTACCAACTAAGACCATGCCCTTCAGTTAACTA

CAGACATCTCAGAACCATTACGGAGGTTATTAGACATTGTCCTTGGTGATCAGTTCCAGT

TGTTGTGAGCATAGGCTGTCTGGGAGGTCAAGCTTCCTGCTCCCTTTTCAGTGCTCCTTC

CCTGAATTGAAAGTCTTTGAAGTATTTCTCAATTCCAGGAATTTTCTTCAACAATACTAA

G

>c16007_g1_i1 len=271 path=[1:0-207 209:208-270]

TCAAGTATGCAAGAGGAGCTCAACAAAGGAATGACTGACATAGATGTTCATGATGATACT

ATCTCCTTTTCTTCTAAACGTTTAGAAATGGCGAGACTTGAATGTCATAAGCAGTTTGCT

GAAGTCCTTCTTCAGAAAGCTCCCAGAACCCCTTTTCACCAATGACAAATATGCAGATTT

TATAGATGCCAATAGAAAAGAAGATCCAGTTGAACGTTTGAAAACACTGAAGAGGCTGAT

TCATGATTTACCTGAACATCATTATGAGACG

>c16008_g1_i1 len=321 path=[451:0-320]

CTTTGTGTTTGATGTTGGTCTTTCATTTAAGCTTGATTACAATACCATCATTTTTGTGTA

AATTGCAGCTTCAAAACAAAAAAATCTCACCAAAATGCCAGAAGCCACGAAGCACAGAAA

CAGTGCTGTCAAAAAGCCTGCGCTACTCACAGATTGACAACTATTTCATTAATTTGGTGT

ACTAAATCCCAAAATATTTCTGAACTTCCATTTTGACAGGTAAATCTGACATGGTCTTAC

AGTGTTCAAGTTACAGTCCCCAGGCTAATGTGCGCTTAGAATTGTGTCAACTTTTCTAGC

AGTTTCAAAAAAACCTTTTTG

>c16010_g1_i1 len=347 path=[1:0-192 194:193-346]

TTCACAACAGTGGGCTGAATGCTGCTCTGTTCCTGTCTGTAACTGCTCCTTTTGAACACA

GACCTTCAGCACATTGTAGGGTGAGTGTTTACTGTGCCTGCCAGTTGTCACTGTCATTGC

CTGGGGCAGGAGGGAGAAACGTGGCCTTATATTAAAGGCGTTTCCATTTGATGTAGCAAC

AGTAGCATTTGATGCTAATATTAGGCAGAGAGAATTTTGCCTGGTTACATTTTGGTAACA

CTTAAGTTTTTATGCTTACAGAGAACTGTTTTCATTAAAACATCACTTCCCTCCCTTGCA

TGAAAGGCAATTCTGAAAGTGAAATCAGGATCTTAGCAACTAAAGTG

>c16012_g1_i1 len=563 path=[546:0-282 1113:283-403 130:404-562]

TGAATAAAAATGGGGTGAAGGGCACCCCAAACCACACATCCCCCCTCTCATAAATTATTT

TGGAGGGAACTACTAGCAAGGCCACCAGACTTCAACCATTAAAAAGGCATCAGGGAAATA

CTGTTGTCTCTACATTCTAATTAATTGCAGTATCATACAAAAGTCTCAAAGATAGTTCTG

CACTTGAACAGCCTCTTTCGATGGGCAAAAGCATTAAGAATTTAATAAGGATAATGGCAA

TGCATCAATAGTGAAAAATATGCTATAAGACTGACCTTCTCACCTCAACACTGGCACAGT

AAAACTGCCTCAGTTAAAACAATTCTGTGTGGGCTGTGTGTGCTGTCTGGATGGCTTCAG

CTGAGAGGCACGTTTTACCTGTTCTCACAAAACAGTGTTGTACTGAAAGAGGAATATCGC

TTTGCTGTCAGCTGTTTTGAGTGCGCTCAGTTTGTCTGGTTTTCTTTGCTTTCCCAGCAA

ATAGGAGCACAGAAGCAGGTAAAACTTGTTGCATTTCTCTAAAAATGTTTTGTAAGGAGT

ATTTCTGTGCCAGTGTTGAGGTG

>c16013_g1_i1 len=329 path=[415:0-293 5:294-328]

AATTAGAAACTTTATTTTGAAATACATTACACAGTTTACATGTGAAGTGCTTTCGCTGTA

AGATACTTCAAAACATTGCACTGAGCAAAATGGCTGACCGGTTTCCTTGAAGCTCTTCCA

GATATGTTTTGTTTTTATTAACAATATCTTTCAGAACTAAGATGCAACTCAGGCATTTGT

ATTCAGAAGCTGTTACTAAGTTTACCTTAGGGAGAAAGTACACGCTTAACTGTTCCAGTT

AACTATGTGCTTCAGTAATGATGTTAAGGATTTGGCATTGAGACCGAAAGAAACCTGAGT

CCTAATTAAAACTCGTATTCACCAAAGAT

>c16018_g1_i1 len=206 path=[1:0-160 162:161-205]

TGTGACGATGGCAAATCTACAGTGTGAAGCCTCCTTAGAAGTGTGTGAATTCACAGAGCC

TGCTGTGACCCCGTGTTGAATACAGAACTGTCTGGGCAACTATTTCAACTGACTGATGTA

ATACAGTGCCTGTTTTCGGATTGACTCAATAGTTTCAGGTGTCTTTCAAATACTGACTTA

AATGAATCTGTGGATTTTCAGGTTTG

>c16023_g1_i1 len=214 path=[14:0-53 68:54-213]

TGCCTGCAACAACTAACAGCTGTGTAATGACAAGAAAGTAGAAATTAAGCTCTAGGGACC

CACAGTATTACTGCTTGGAGGAACTTTAGGAAGAAATTAAAGTAAGGTTCCCACTTACAT

ACGTCTGAAAAATCAATGCCTAACACTAACATAAGGTAAGTCACGCTGCAAGAGACCAGG

CATTGAATCTCTTCATTTCTCTGTAACAACTAAT

>c16023_g1_i2 len=205 path=[454:0-44 68:45-204]

ATGCTGCAAAGGTATGTGATTTCAAGAAATTTGTGGATGGAAGGGGGGACCCACAGTATT

ACTGCTTGGAGGAACTTTAGGAAGAAATTAAAGTAAGGTTCCCACTTACATACGTCTGAA

AAATCAATGCCTAACACTAACATAAGGTAAGTCACGCTGCAAGAGACCAGGCATTGAATC

TCTTCATTTCTCTGTAACAACTAAT

>c16030_g1_i1 len=243 path=[78:0-77 308:78-242]

GCATGTTTTAAGAATCACACTTGTAGGAACCTGTGATGACCTACTTTGTCTGCAGACCAC

TGGAGCAGTTTGACATACAGATCTGAGGGAGGTGGCCTTGACCATATCTTACCTTCTCTG

CTTAGGACAGTGTAATTTACTCTTCCTGATTCCTAACAGATGGAGCCTGACTTGCACTTT

TCCATGGTTTCTGTGTAAGGTCTATGCTAAATATATTGAAGTTACCTGAAGCACTATTGT

TGT

>c16034_g1_i1 len=222 path=[200:0-221]

ATCATTTGCATGAAGTCTCCACCATGAATAAATATTTACAGAGCAAGTAACAGACACAGC

AGCTGCTCCATTATTTACCAAAGACACCTTAATTTTCAAAATTAGATCAACTTATGCCTT

TTAATTTCCTCTCCCCAAGTGAACTTTGCCTCACACTTCGATTTTAGTGCTAGCAGGTTC

AGGGAGGTGTTTTGAGAAATGAGATAAAATCTAATGAAATAG

>c16040_g1_i1 len=297 path=[549:0-296]

CTTTGTGTTTGAGGGTTCTGCAGAGCTGCTATAGTTTGGCTTTTCTCTTAGGGAGGTTAT

CCTTAACCAGTACCATCTATATATTTATTTCTGTTTAGAAAATATAGAAATGAGATACCT

GGATACTTTTCATGTTGGTTTTGAATAGGAAGTCCTGCTGGAGTAACTGTTACTAGTTGC

TCCCAAAGGAGATACCAGTGTCAGTAGATACTATCAACATTTTTTACCTTAACATTCTAT

TGTTTGGAGATGTCCACACCATAGCCTCATAACCCACACTCCTCATCAAACACAAAG

>c16049_g1_i1 len=203 path=[1:0-134 136:135-202]

TACAAGGCCTATTGTTCTCAACACAAGAACAATTAGAAATTACTTGGAATAAAGTAATAC

CAGTAGACATTTGTCCAAGTGCCATCAGCACGCTATTGTGATGTACTGTATTTGAGCTTA

TAGATATTATCTTACCTCAACTTTTCATGCAGTCATTTTGAGGGTAAAGGAAACACCACT

CAAAGCAAAAGAATCCCAGCAGT

>c16050_g1_i1 len=428 path=[1172:0-427]

AGCTAAGCACCTACATCTGTACTGAAGTAACTGTATCCTGAAGAATTTATAACCCTTAAA

GTGAATACAGTATTTACTACTACACTGAAAAAAACCCGAACATAGTAGTGCAAAAGAATC

ATTCAGAAAGCTACACTCACCCTGTATGTTTCAGGGGCCTTGTGTTTATTTCTACTGTGC

ACAATGGAATAAGAATCACACAATGGTTTAATATATAAAAATATATCTAACACAGGTGGT

CTGATTTTATAAGCTTCCGTGTTAAGATTTTCTATTCAGATCACAGTACAACTGAACCAA

TAAAGACTTCTCTAAATGTCCTATATTTCACTCGGCATCTTATTTTCAACTGAACATTTG

TATGTGGCTTGTCCTCTGGCCTTTAAAACCAAACAACAATCCCTCAGGAGCGAGCACTTT

GTGTTCTG

>c16050_g2_i1 len=406 path=[384:0-405]

ATGTAGGTGCTTAGCTTTGCATATGTATAGGGTTTCCATACACTCAGGAAAGTGAAGTTT

TGCATTTGTAGGTGCCAGTTTTATAAAAAGCAGGACCAAGTTCTCCATGGTCTATCTGCA

AAGTGTTAAACATGTAAGCCAGTGTGTTTACTTAAATACACACAGTTATTACTAGCTTAA

GCCTCCTTTGACTTCAGTTTTTCTGACATAAAAATACTTTCATTTTTGGGCTAATAGTTT

GCTTGAGAATGAGCTCTAAGCTCAGCAGCGAGCTGCTATGGGACAAATGCAGATACAGAA

CCTAGCCAGTATAAGAGGTAGAGCTTGAGTCAGTTGAGCCTGACCGCTTTCTATTGCCAG

CTATATTAACTGCTCTTGTTAATGTTTTTTACAGTTTGATTTGTAT

>c16052_g1_i1 len=400 path=[1:0-399]

AGCCCTTGGAGAGGCACATGGGCACCCTGGGGGGCCTCTCAGGCACCTCTGTGGTTTAAT

GGCAAAACACTTTCACTTCTTCACCCACTGCAGTGAATGACAAAGGTTGGGCAGTTCCAG

AAATAGAGGCCCAGCCCTTGGTGCCTTCAGGTGGATTTCTGGCCTCAGTGAACTCAGTCT

GAGTTTTGTCTTTCAGTGTAATGAGGGCAGGAACTCTGATCTGGTGTGTAGAAATAGGCA

ATAATGATAACACAGATTAATTTGCTTTTGTTGTTCAGTATATTCAGTGAGAAGTGATTG

ATGTCTGTGCAATGCTCTGAAGGCAGAGAAAGCCTGCCCTATATAGTAGACGTGGAGAAG

TCACTTCTGTCCTAACTTGTGGAGAAAGATGAGCTTGCAT

>c16053_g1_i1 len=821 path=[874:0-546 1421:547-820]

AAAAAAAAATAAAATAAGAAAAAACAAAAGATAAAAAATAAGAAAAATAAAGAAGCTAAG

AAATTATTATAAATTTTAAGGAATAAACGAAAAAACATAAAAAGGAAAAGAAAAAAATGT

GAAATGCTCGTAAAAAAAAGATATTTCAAAATAATTTATTTATAAAATGGGAAAAGATAG

TCAAGAGGAATCTTTAATAAATGGAGAAAAATTATGTGAAAGAAAAATAGAAGAATGTGG

AATATATAAAATTAAGAACAAATTAAACAAATTAGACTTTTTGGAGGAAGAATTTATGTT

TAAAAATTATTTTATTAATAATTACAATTTTCATGATTATAAAAAGAAAACTATTTCTAA

TGAGACATGTAATGAATCTCTAGAAAAAACTAAGATTAGAAATAATGAAAATACAAACAC

ATATAATTTTTTTGATACTATTTCCACTGAATACCAAATATTAAATAAAAATGATGATAT

ATTAATTAATAATGATTACAAAGTTGATCAAAATAGTAGTAACTACAACAATGTTCCCAT

TACAGGGAAAAAAAATAGTACTAATGCATTCGAACATGAAAATATATTTTGTTTTCAACT

AATTTTAAAAGAAATTATAAGAGAAAATACAAATATAAAATATAAAATAAACTTTTGTGA

ATTTTATAATTTTTTTCTTAATCTAAAAAAATGTAAAGTTATTGGTCTTATCCCTTCTGA

TGATCAACATGATGAACAACATTCAGATTCAACAGTTTTACATTTTATTAACTTACTCTT

TTACGAAAGAATTCAATTTATAGAATTAAATGATTTGACTA

>c16056_g1_i1 len=375 path=[697:0-374]

GAGCAGACGGCCTCTTATGAAGGTAATTTTTATGTTCTGACAACACAGTGAAGCTCCATT

AAATACAACTTAATGTAAATATTACAAAGGATTTCACTTTTGAAAACATGTAATCTCTTT

CTAAAATTAATTATTCTTGTCATCATACTGCTGTTGTCAGTGGTTATTTACAGTGTTAGA

GCAGGCTCTGCAAGGTACATAAGGACCAACTGGTTTGTTGAAGGCCATAAAGATCACACG

AAACAGAACTTTTAAAACTGAATAAAGGTGTGTTAAATAGCATAGGGAAGTGCCCGAACA

TCTGAACTACACATTCATTAGTTTATTATTCTATATTCTTGATACTGTTGTGGTAAATAT

ATATGCTTAACGATT

>c16056_g2_i1 len=371 path=[349:0-370]

AATACAAGAAGAAAGATATTACTTTCATGTTCTGATTATTCATATTACTAACATTTTTCA

TTAATTGCCATTTCATATGTTAGTAAGACCATTCTGTAATGCAAAATGACTTCTTAATAA

AACTCTGCTGCTTTTTCAATGTCATTCAACTGAAACTTTCAGTGAATACTTGTGGTCAGT

TATCTCGTCCCTAATCTGAGCATAAGGACTTGATGAAAAATAGTAAGGACCAAGACACAA

AGGCTCAGTAAAATACTCTTCAATTTTGTTTGCAATGATTCAGCAAGAAAACAATTGTGG

TTATTTTATAAACCTATCATAGTGAAATGGAACAATTTTCATCTCACCAACAGATTCTGA

AATTTCCATGT

>c16060_g1_i1 len=309 path=[1:0-308]

AACAGCATGAGCCAGAACAGAAAGCCTTTGAAGCAGCAGCTCTAGGAAAGTGGAACGGTT

GTTCAGCCCTGAAATGGGAACAGCCTGCAACAGCTCTGCTACATGCAAAACATGCAGTTA

CCCAAGGGAGTGGTAAAGGGGGTGGAGTAGGGATTGCCTAATCTTAATTTAAAAAAAGCC

TGTCTTACTGAGGACATTAATTGCTTTTTTAAGAGGAACAGGGGGCACAAAAATTAAGTT

TTCGAGGAATTTAATCCTGGTTTCTCTCTGCACAAACATTCAGAAAACACAAATGCTTGT

GTATATGGA

>c16060_g2_i1 len=225 path=[573:0-141 715:142-224]

GTGTATATGGATTATTAGGAGTTGCAATACTAAAGCAAACTTTTCAATCATATCCCTGCC

AATCCCGAATCTAAACCAGTCCATACCTCTTCTGCTGGATCCCCTTTGTCTGTTACCGTC

ATTCTCTGATTAAACTCCTTCTTCTACAGATCACACTATAGTTTGTTCAATTTTTACGTG

TGCAAAAGGGAGGCATTGCTTTTACCTCAACACTCGTGAACAATA

>c16060_g2_i2 len=226 path=[573:0-141 881:142-225]

GTGTATATGGATTATTAGGAGTTGCAATACTAAAGCAAACTTTTCAATCATATCCCTGCC

AATCCCGAATCTAAACCAGTCCATACCTCTTCTGCTGGATCCCCTTTGTCTGTTACCGTC

ATTCTCTGATTAAACTCCTTCTGACACACAGTGATTTCTGAAATCTCTTAGTGAAATCCT

AAAACAAGTGGGTTTCAAAAATATTGTTCACGAGTGTTGAGGTAAA

>c16061_g1_i1 len=229 path=[687:0-13 141:14-228]

GCACCACTGATATAAATGTGAATAGGAAACTGCATTCATCTAGGTCTGATAGTGTAAAAT

CAGAGCATCAGTATTTATGGTATGTCTGCACCTTTATTCCTATGCTTGACTATTAGCAAT

ATTACATGTATATTATATATCTAAAGTTATACCAAGCACCTTTAAAGAAATCAGAGCGTG

TGCTCCGTGCAGTCCCCATATCCCCATACCCACATCCTTACATCCCCAT

>c16061_g1_i2 len=337 path=[19:0-121 141:122-336]

TCTTCTTTTTCTTTTTCTTTTTTCCCAAGGAAAACAGATGGAGAGAATCAGATCATAACC

AACTCCTTCCTCCTCTTTGTTTACGACACTATTTTAAATTATAGAATTCGGGGCTGCTTT

ATAATGTGAATAGGAAACTGCATTCATCTAGGTCTGATAGTGTAAAATCAGAGCATCAGT

ATTTATGGTATGTCTGCACCTTTATTCCTATGCTTGACTATTAGCAATATTACATGTATA

TTATATATCTAAAGTTATACCAAGCACCTTTAAAGAAATCAGAGCGTGTGCTCCGTGCAG

TCCCCATATCCCCATACCCACATCCTTACATCCCCAT

>c16062_g1_i1 len=222 path=[1:0-68 70:69-221]

CAAACCTAGAATCTTCTGATACAGGGAGAAGGGGCTCATGGATGCAGCAGACCACCAGGA

GTTATGTGGAAGGTTTAACAGATGAAAAAGTGAAGGCCTATCTTTCCCTTCATCCCCAGG

TACTGGATGAGTTTGTGTCAGAGAGTGTAAGTGCGGAAACTGTTGAAAAATGGCTAAAAA

GAAAAAACAACAGACAGGAAGGTGGTTGAAGATGGATACGAA

>c16063_g1_i1 len=216 path=[1:0-100 330:101-215]

TTAGTTTAAAAAACAAAACAATTTATTATTTGTAGTGCAAATCAACTCATCCTAATGCCT

AAAGTAATACTCGAAATATATAAGTATTATGTATTTATTAGCACATTATTTAATTACATT

TTGGACTTTGCCGTTTTTAGTCTGTTTTCCTAAAAATGAAGGCTTCCCTCTAATCACTTG

CGTGTCTGTAAGTACTACTTCCTGGCCACAGACTTG

>c16064_g1_i1 len=391 path=[1:0-390]

GAAAATATCTGGAGAAAATTTACCACCAATGGATGCTAAACCTAATAGTCTGTAACAGGG

CTTGAGTAGGAGCTGTCCGGTGAAGTGGCATCCACTTGAGTTCACTTCTTCCAAATATGA

GATATTAAGGCCTGTATTCTCCTCACGTTTGTGCTGCTGTAACTCCAAACAAAGAAGTGA

GCTGGTGAAAAGCAGTTGATTCTTCTGATCTCAGAGCCTGTAGGAACAGCTGCCCTGCAT

TGGTCAGGATGACAAATGCAGCTGTATGTGGCCTCTTAGAAAACCAACAGAAATTAGTGG

GGATGGGCTTGTATTTGCAGTTTCTATTTTGCTTATTAGAAAATAAGGCTACAGTTTAAT

GTGTGTGCACAATAAAGATGATAGCCAACAA

>c16067_g1_i1 len=233 path=[1:0-75 77:76-232]

AAGCATGTTTTGTTTCAGCCATCTGTTGCTTAAAACTGCAAATCTCTGTAGTATTTTCTT

TCTCTTAAAGTAGAGCTTCCTCTGAAATATTGACACTGTATTAGAACCTCTGTAATGCAC

AGGTCTACTTTGTGTTTCCCATCAGTTCCTTGATTAAGGCATCGTTTTGTGAAATCTTGC

AAGTAGCAGAAGGATGTGAAAGAAGTTTTCTTTCTTCTTTTCTATTATCAGTG

>c16070_g1_i1 len=224 path=[90:0-86 331:87-198 177:199-223]

AAAGTATACCTCAGACAAAACAGAAGCAGTTGAATCCAGCAGTCTCCAGGCTGTGGGGAT

GTGACTGACCAGCATGCCTCTGCAGTGGAGTGTAGCCAAGTCCTCTGTAAGGCACAGGAG

GTGACTGCCCAGTCCTGTGGATGTGACTGCTGCTTCTCTTTGCAGTTTTGGCTGGTAGAG

AGGCTGCATGTGTCTTGAAGAGATTTTTAGATGCGCTCAATCAG

>c16071_g1_i1 len=253 path=[455:0-252]

AGTTCTCATGCAAACTTCCTTTTCCAATATAGACAAGTACTGTTTTAAGGGCTTCATTAG

GGTGAACTTCAAAATGGCAAATATTTTGCTCAGTATCAAGACAAATGTCTTGAGCATTGA

GGGTATTACTTTCATAGAGAAATCCTTGTTCCCATATAGCACACGAGCTAACATCAACAG

TTTGCCATCTGTTTCCATCCCTTTGGGCCCACACCCTATGTTCTATTGGGTAGAGTATAG

CCCCATTATGATT

>c16071_g2_i1 len=250 path=[228:0-249]

GCATGAGAACTTCTTGTGATTGTATACTCATTGGTAGTACCATTGTAGACAGGAGTAATC

ATTCAAATATCTGTATTTGTAACTTTACTAGCATTATGGGATGTGATTTTCATTATTCAG

CTCCAGCTACGTCACATCAGTTGTTGCAATCTAATTATATGTTAATCCAAGATTTGTTAC

CTGCCCCAATTGGAATGAATCTAGATTTAGCAAAGAAACTTGTACAACATGACGAGATAA

TGGAAAAAGA

>c16078_g1_i1 len=1776 path=[1:0-1461 1463:1462-1775]

AAAAAAAAAAAAAACAAATATATAAATTCATATAATGATAATATATCTTTAAAAAGAAAA

AGTATGTCTAATGTAAAAAAAAAATACCTTAAATAATATATAAAAACATATTTACTTCTA

GAATTTAATAATTTCCTTTCTTATATATATCCATAGCTTTTTTCTTTGACCAATTATGTA

TTTCAAAAATTTGATTCATTAAATCTTCGCTATTTTCTGAGACTTTTTGTATTTGAAAGG

ACTCTAAAACTTGTGATATAATAGCTGATATATCAAAATATTTTATTTTATCATTTAAAA

ATAAATTATTACATATTTCGTTAGATGCATTAAGAACTGTTGGAAAGAAGTTTCCCTTTT

TCCCTGCATGATAAGCTAATTTAATACATGGAAATTTATCTAAAGAAGGTTTATAAAATG

TAAGCGGAGATGTTTCAACTAAATTTAATGGTTTCAAATTCGTTTTTATTCTATTAGGCC

ATGTTAAAGCATATAAAATAGGAATCTTCATATCCGGATAATACATCTGACTTATAACAG

ATTTATCAACAAATTCAACACAAGAATGAATTATACATTCTTTATGAACTATAACTTCAA

TATTATCATAATTTACATCAAACAGAAAATGAGTTTCAATAACTTCTAATCCTTTATTCA

TCATTGTTGCAGAATCAATGGTTATTTTTTTCCCCATAGTCCATTTAGGATGTTTTAAGG

CATTTGCTGAAGTAACATTTTTTAATTCATTAAGAGAAATATTTTGAAAGGGTCCTCCAG

ATGAACATAAAAAAATTTTATTTATATTATTTATTTTCGAAAAATTATCTTGCAAACATT

TAGTTTTTAGTACCTTATTGTTATCTAGACATTGGAATATAGCACTATGTTCAGAATCAA

CTGGGATGACAAATGAATTTTTATTAACATTTAGTAATTTCTTTAAAAAAAATCCAGCTG

ATACAATAGATTCTTTATTAGCTAATGCAACAATTTTATTATTTTTAATTGCATATATTG

TTGAATAAATCCCTTGAAAAGAATCAATTCCAATTACTATTTTATCTATAGTATTATCAT

TACATATTGTTTTCATGCCTTCATCACCAATTAATATTGTTGGATGGTAATTTTTTATAT

TAAGTAACAAATTTTTTAATTCTTCATATTTAGTTTCATCATGTATACATATGTATTTTG

GTAAAAATTCTTTTGCTTGTTCATATAAATCTTTTACATTTTTATTAACATATAAAGCTT

CTACATTAAAAATTTTTTCAATTTGATTACATTCCTTTATTATATCAAGAGTGTTTCTTC

CTATACTACCAGTGCTACCAAAAATACCTATATTTATTTGTTTCTTACTATTAGTTATAT

CTATGAAATTACTTTTTAATAATTTTACATTTATATAACTTTTAATATTCTTTAACTTTT

CTGTTTTCCTTTTGCCTAGTCCTTTTAATGCATTATTTATATAAGTTATTTTGTATAGTG

TCTTTTTACTTGCATTTTTTGAGAATAAGTAATTAATTTCTAATACTAATAAAATATACA

AATATATAAATATTACCATTTATTTTGTTTTATTATTTTATATATTTTTATTATTTTTCA

TCCTGTGTTATTTTACACATAGTTTATTTTTTTATATGAATGAAATATATTCTGTTTAAT

TTTAAAAATATATGAAATATGTTAATAAAAAATAAAGATTAAAATTTTCATAAATGAAAT

GAGCACACAATTAAAAAAATAAAATAATTTTTTTTT

>c16081_g1_i1 len=271 path=[249:0-61 311:62-270]

GTCTTTATAAGAGGAGGCTGTGAGGAACTTCATGGAAACAGAAGTAGACAAATTGGCAGC

ATAATGCTTGATGACAGTCAGACATTGACAAATGTAGCCTAGTGCACATAAAAATGAGAA

TTCTGAACTGCTTGCATGCACAGCCCTGTTCTAACTTATCACTAATTCCAAAAGGCAAAA

GGAACATCCAGCACAGTATAGAAATTCTTCTCACTGCTCAGTGGCAGTTAAAGTCTACAT

AAGAGGATGTGTTTGATGGTGCACTTGAATG

>c16081_g2_i1 len=210 path=[1:0-184 186:185-209]

CATTCAAGTGCACCATCAAACACATCCTCTTATGTAGACTTTAACTGCCACTGAGCAGTG

AGAAGAATTTCTATACTGTGCTGGATGTTCCTTTTGCCTTTTGGAATTAGTGATAAGTTA

GAACAGGGCTGTGCATGCAAGCAGTTCAGAATTCTCATTTTTATGTGCACTAGGCTACAT

TTGTCAATGTCTGACTGTCATCAAGCATTA

>c16083_g1_i1 len=1088 path=[1066:0-1087]

AAAAAAAAAAAAAAGATAGTGAAGGAGAAATTGCTAGTGATAAGGAGAAAGAATATATAT

TAAAAAAAAATAAAAAAAAAGATAAAATGGAATCTTATAGTGAAATGTCATTTAATAACA

ACACACATATTTTAAATATTGAATGTGATAGAGAACTTAGAAAAAATAAAGGGTCTAATG

AAGAAGAAAAAAAAAAAGAATTCAAAATGAACAATAAAAAAATAGAAATTATTACAGAGG

AAAATAAAAGAAAAAAAATAAATAGTGTTTTTAATGAGTTTAATGACATAGAAGAAAATA

AAAGGAGAAATGAGGAAAAAGAAGAAATAGAACAAATAAAGGAGCTAGAGGAACTAGAAA

AAACAGAAAAAAGAAAAGAAAAAAGGTCAAGTGAAGATGTACATAAAATAATGAAAAGGG

AAGAAGAGAAAAGTAAAAAATGTGATCATGAAATGAAAAATGAGGAAAAAAAAGAAAGTA

ACAAAAATCAAGAAGATTATAAAGAAAAAATGAAAAAATTAAAGATTCATGAAAATGAAA

TTAGTGAATTTGATGATGAGAATAGTAATATAAAAAAGATTTTTATTGATACAAAAAAAG

AAAATAATATGAGTAAAAATAAGGATGTAATTGGTAATAATAATAATTTGGGAATTGAAA

GTAATAAATGTGAAAATGGTGCTAAAAAAGAATATATTAAATATAATGAAGAGAATTTAG

AAGAAAAAAAGTTATTTCAAGATATAAATACGTATGCACAGTATAAAACTTGTAATCAAC

AAGATATAATATTAAAAAATTTTAATAAAGCAAATAATTTAAATGATAAAAAAGTAGCAG

AAAATAAAGCTATTAAAAAAACTATATACGAAAATTTGTTAGAAAGTAATATATCATTTA

ATAGAAGAAAAAGAAAAAATGTAGAAGAAGAAAATTTACAATATGAAAATAATAATAGCA

GTAGTAATTTCAATAATAATATTGAGAGTAATATGAGTGACGTTATTAGTGATGTTAATA

ATAATAACAATAGTAATAACCTTCATTTTAATAATAATAATTATGTAAATAATGATGATA

ATAATAAA

>c16083_g2_i1 len=440 path=[2548:0-439]

TTTTTTTTTTTTTTTTTTTTTGTTCTTTTTCTAGTAATTCTTTAATTTGTTTTACACTTA

TTTGTTTATTATTTAATTCTTTTAATTTTACTAGTACACCAAAATCATCAAAATTAATTT

TATCATCTTTAAAACTAGCAAAATTCACAAAATTTTTTCTACTTTCCACTTTATCACTGA

TTGCATTTTTACTAATTTTAAAATTTTCTCTTCTAATATCAATATATTCTCTTCCCAAAC

TTATTGATCTAGTTATTACACAACCTTTTGATTTTTTTAAATCTTCAGAAATATAAAAAA

CCATTATATAATTTTTTTGAGTAAAAAAGGAGAAAAAGAGAAAAAAAAGAAAATAAAAAG

AAAATATGGAAAAGTAAAATGAAAAATTAAAATAAAAATAAAAGAAAATAGAAAAAGAAA

AAAAAAAAAAAAAGAAAAAA

>c16088_g1_i1 len=270 path=[248:0-269]

TTAGTTTTGAAAACCAGAAATTGTTTGCTGACCAATGAGCCAGCTGGAGAAGGGACCCAA

GCTAGCAGTGAAGATGCACCTTGCTCATACACTGGGAAACCAAGTTCCACAAATGAAAAT

GCACTAGCTTCCAGAAAACAAAGTTTCTCTGAGCCTATAAGTGCCACCAACCATTCTGCT

TATTTTTACTGCCTGCTAACACTGGATAGAAAAGCCTACAGAGGAAAACATGAAGATTTC

CTAAAGTCTGTTAGCATAATGCAATGTATG

>c16090_g1_i1 len=213 path=[191:0-212]

ATATTGTAAACTGCCAGGAATAACTGATGCAGCCCTTTGCAGGCTGCGTGCAGACATCCT

TCTCCAGCTCTGCTCGTGCCAGCGCAAGGGGGAACAGCCCTGACCTGCACTCAGGGGTGG

TGGGAGTGGGCAGCAGGCAGGAAGAGCCACTTGCTCCATTGTTTGTCTGTCCACAGCTTG

AAATGTCCACTAAAGCCTCCAGAGGGGCCTCAG

>c16091_g1_i1 len=331 path=[248:0-53 248:54-107 248:108-161 301:162-330]

CAACCATTTCCAGAAATGCTGCACCTAAATCCACTTCAAACCAAAAAGGCACTTCAACCA

TTTCCAGAAATGCTGCACCTAAATCCACTTCAAACCAAAAAGGCACTTCAACCATTTCCA

GAAATGCTGCACCTAAATCCACTTCAAACCAAAAAGGCACTTTAACCATTTCCAGAAATG

CTTCAACTGCAGCACTATAAACGTGTCTTTATCGTGCTCTACTTGCAGAACACACACAGT

CAAATTCAAAGGCAATTAAAGCTCCTTTTTGGAACTGCTCAGGATGTTGGCCGTTATACC

GAGGTGCTACAGATGCAACTTTAGTTGCAGG

>c16097_g1_i1 len=1221 path=[1:0-1220]

ATACATATATATATATATATATACCTTAAAACAATATTTAAATAATATTAAATTTATATA

AAGATTATTAAAACAAAACAAATATTAATTATAGGTGTGTATAATAAATTATATTATCAA

GCATTTAAAATATTATTTAAACTCAATAATTTTATGGCATTTTCTTTTACAGTTAATTTC

ATAATGTTACCTAAATGAATACAACATTCAATACATGTTTTTACCATAGATTCAAATATA

TCAATAGGAACTTCTGCATCAAATTCTAATAAAATAATTTTATGTGTATTCAGTTCAATA

ACTAGAGTCAATTCAGGAGAACCTGAATTTATTTCTAATTGATTAGCATCTACTATCATA

GTGTTTTGTAGATACAGAACTGAACAGGATGATATAAAATATTTAATTGCTATACCTGCA

TCAATTAATGCTAATAAACAAGTATTTATAGCAGCATGTTTTAAACCTCCATCTCTTTCT

ATGATATATAAAAAAATATTTATTTCTGAGTTTTTATACAAATCTAATAATATAATATTT

TCGCATATATTTCTTACATATGCGCTTATTTCATTTGTTATATTATCTTTTGTTTTTTTT

TTTCTTTTTTCATAAACACTAAAAGGAGATAAAAATACTTCACATTTTATGGAACATTTA

TCATCTGATTTTTTTAATTCAGTTGGTCCTTGTACATAGGACAATATTTTTGTATTACCT

ATTTCAAAAAAAGCAAATCCATCTTCATCTGTAAATATATTTTCATTACCTAAATGAATT

TTTATTAATCTACATTCATTTGATTTCCTTCCATCAATTCTATATCCCTCTTCATTTATA

TATTCTTGAATTGACATAATTAGAAAGAAAAATTTTATTTATATGTATATTCTTAAATAT

GAATATTTATGGGATTAAAATTTTTTTATTCTTATAATTTTTAATATTTATGAATTATTT

CAAAAAATAGTTAACAAATGGGTGCTTAAAATCACTATAAAAAAGGAAACTTACATTGGA

TAAATTATAAAAAATATCGAAGTTATAAGAATTAATTTTTTGTAACATTGTTCAAATTTA

TTTAAATTCATTAAATAAAGGTAGATATATCCATTATATCATTTAAAAAATTGTAATCAT

TTACATGTTTAAATAAACATAATTATATTAATAGAATATAAAATTTTCATTATGCATTAA

AATATATTTACAGATGAAGAT

>c16101_g1_i1 len=450 path=[1:0-449]

TAGTTGTGGGAATGTCCCCTCACTGGTTCATGGCTCAGCAGTGGCCCAGCAGCATAGAAG

GTGAGCACTTCTGTATTTCTATACTTAATTTTGCAAATCTTGCATCAGGTTTGTTCCTTT

TGGTTGTTTTGTGTTAAAAAGAAATCCTCGATTGGAAAACTCAGACAAGCATCTTTGCAG

CCCTGAATTACTCACTGGTAGCAGCAGGAGCTGCTGTTGTGCAGGGTGAGTGCATGAGTG

CTGCTGAGGGCAGTCGTGGCTCAAGGCACCCCTGCCTGTACCTGTCCCCCTCCCTGTGCA

TTCTGCAGGCAGGAAGCGGTCCCACACCACCTCCACATGTCACTGTGCCTTTGTGGTCTG

CAACCCAGCAAGTCTGGGTTCAGCCTCTCCACCTCCACACATAAAATGATGATGATGCAA

AATTTTTAAAGCATTTAAAAAATAAAAAAA

>c16111_g1_i1 len=356 path=[377:0-283 732:284-355]

ATATCAGCAAAGAAAGCAAAAATGGGAGTGCCAGCCTTGACTTGTCAGACAAAGATAGTA

GTGCACAGCCAAACCAGAAGGTTGGAAAAGCTTCAGAACACCAAAGAAGCATGTCTTGTG

TAAACAGGAATAAGTTTGAGTTTGATGATGAGAGAACATGGAGTGATCTTGATGAAAACT

ATGTTCATGATGATTTACCTGAAAAATACACTAAAATGCCCTCACAGACTGACTTTTTCA

GTAAGAATGATACACCTTTCCCAGATAAAACATTAAAGAGAAAAGTTGCCTCAAAGAGGG

GGGAAGAACAGTCTAAGCAGTCTGTAGTGGACAGCGATTCAAATGGACCTCCATCA

>c16113_g1_i1 len=488 path=[1:0-247 249:248-487]

GAACAGGACTTTTTATTCTGCAGGTGTTCCTGCACATATGGCGGCAGCCTGGTGCTCACT

TGATCTTCATCCACATCTTCTTGCAGGAGAAGCCGCTGGGAGGACGAAGGCAACAACGGA

CGTTAAAGAAGAGCTTCCTGTTTCTTGTTGCATTTGCAGGTCCACTGTCTCCTGCATAGC

TGACGTGGCCTTGGTGCAATGTAGGGTCTGTCTCTGCTCTCGTCTTCATGCCCATGTTCT

GTCTGACGGCCAGGTATCCGAGGCTGAGCTTCTGGCTGGCACTGCGCTTTGTCTCAAGGA

TCTCCTCTTTCACAAGCTCCCTCGGTTCCATTTTCTCATTTCTCCTAGCGAGCCGTCCGT

CGGAGATCGCCGGCAAGCGAGCCGCCTCACGGGAGCGAGCGTGGTCGAGTTAGCGGCGCT

CCTCCCCGTGCCAAAGGGAGCTGCTTTGCTCTGAGTCCGCATTTGCGAGGACGACGAGGA

CGACGAGG

>c16113_g1_i2 len=253 path=[944:0-12 249:13-252]

CTTTGTGTTTGACGCCAGGTATCCGAGGCTGAGCTTCTGGCTGGCACTGCGCTTTGTCTC

AAGGATCTCCTCTTTCACAAGCTCCCTCGGTTCCATTTTCTCATTTCTCCTAGCGAGCCG

TCCGTCGGAGATCGCCGGCAAGCGAGCCGCCTCACGGGAGCGAGCGTGGTCGAGTTAGCG

GCGCTCCTCCCCGTGCCAAAGGGAGCTGCTTTGCTCTGAGTCCGCATTTGCGAGGACGAC

GAGGACGACGAGG

>c16114_g1_i1 len=265 path=[397:0-264]

AGAGGGGAGCGATGACGGGGCCGGCGACCTCCTCCTCATTGCTGGAATCCGTTTGCCTCT

CCTGAGTAGTGGCTCTCAGTAGACATGAGCTTATCTCCAGAGACTCAACACTCTTTCAGG

ATTGCCTCTGTATTGCTGAATCCAGTCTGTAAAATGCTTCTTTATTCCATCAGACCAACA

TGCACTATCCTGTATAGAAGTGGCTAAGGAGGAATAGGGGTAAGACCAAAGAAAATACAC

TACAGAGTTACTGTAGTTTTCTTGA

>c16117_g1_i1 len=204 path=[182:0-203]

GTCTTAAAAATACGTAAGTAAATGAGTCATTACTTCAGCTTAACACATTCATCCTTCACC

AAAGGAAGCCTATAGAATTGTAGATCACCTGAGTTCCAAATGAGAAGAAAATGCTCTTCC

CCACCATCACAACCCCCACCATTCACAACCGCCAGTGTAGTCACAGCCGTTCTGTAACAG

ATTCATACCAGCCTTGCATCAAGA

>c16118_g1_i1 len=1895 path=[1:0-1841 1843:1842-1894]

TTTATTTTTTTTTTTTTTTTTTATGTACTCATTTTATATATATACATATATTATTCTGAT

AAAAAAGAAAAAAAAGATACGAACTGTTGTTGTGAGGGAAGTTTTTCATTATTATCCTTC

ATTTCCAATGTAGAGCTAACATTATTTTTATTTAAATTATAATATTCTAAAAAATAATTA

TCTTTAAAATTTGGGATTTTATTATCCAAATTATCAGTTTCCTTTTCATTATTATCATTA

TTAGAAGTTACTGGACTCGTTTGAAATTTCCTTTTTATAGATAATAAATCTTTTTCATTT

TTTTCATCCAAATAATTTTCTTTAACTTGCTGGTGATAAAAATTCACTATACTATTTTCT

TCTGTATCCATTGCACCATTTATATTAATATCTTTTTTTTTGTCTAATTCATTATTTATT

TTATTTATCATTTTATCTTTTTCAGAATCGTATTCCATGAATTCATTAGTATAATCTTTC

TTATTTTCTATTAAAAATGCATGATCTTTTCTTTTTATTTCTCCATTTTCTTTTTCTCTT

TCCTTTTCATTTTTTTTTAATTCATCAAATAAAAATTCCTTATTTTTATTATAAACGTCA

TTTTTTATTTTGTTGGCAGTTTTTTTTAATTTCAAAACTCTGTTAAATTCAACTACTCCT

ATAATTTCATAAGCAATTTGTGCAATATTATCCCATTGCTCTTGAGTAAAAACTAAATTA

CCTTTGATTATTTTATCATATAAAGTTTTTAAGCATTTGATAACTTCTAACAAATTAATT

TCACTGTTTTTATCATTTTCTGTACTATTTAAATTACAGTTTAATTTACTATCACTATTT

AATGATTCCGAATCGCATTTTTTATTTTGAATTAATGTATTTAAATTTTCTTCATTATCT

AAATAATCATCTTCATCATCATTTATATCATCACTGGTAGCTTCTTGTTCATTGAAATTG

ATTTCATGTGCGGCTGTACTACAACTTGAATGATTTTGTTTTAATTTTAAAGTATTGAAA

TTATTAAAGTTCTGATTTGCACTTAAAGAATCTATAATATTATTAGAAATATTTGAACTA

ATAGAAAAATTGTTCCCATTTTTTGCAGTAAATCCATTCATTTTATTAGAAATAAGTTCA

TTATAATTTGAGGAATAAATATTTGAATTATTTGTATTATTACTGTTTGCATCAAAATTA

GCAAATGAATTAATAGAATTTTTTATTCTACTTCTTGTATAATTGTTTAAGTTTTCAAAT

GGAGTATTATTAAATAAATTTAATTTTAGATCTTCTTCAGAATAGTTTAATAAATTATCA

TTAAATGATAAAGCAATTTTTCCTATATTAATTTTCGGTATATCATTTTTATTAGAAATG

GAATCAACAGAAGAATAGGCGGTTGAAGTCATAGCATTCATTTTATTACTATGTACATCA

TTATCATTCATATCTTCTAATTTTAAATCACTTTTTTTTTTTAATGAAAAACTTTTAACA

TATTTATCATTAATTAAACCATTACTGTTCTTATTGCATATATCTATTTTGTCAACAATA

ATGACATTAGGAATACTTGGAATATTTATACCATTAGTAAAAGGTAACTTATAATTTCCT

GGTACTCTCGTTTCTGCTAAACCATGTATAAAAGGACAATAGTAAGTATTACATGATCCT

TTTTTATAATCTTCACATCTTTTTGTTTTATAAAAAAGAGGATGGTATAAAATTTCTTCA

GAGGAATGAGCAAAAGGGCATTCACCTCCCCTTAAACATAAACTATTAATAGAACCATCA

CTTTTTGTTTCTACATCTGGACACATTATTGTTATATAACGGATAAAAGAGGAATCACTT

AAATAAAATGGACATCTTCTATTCCAAAATTCATT

>c16118_g2_i1 len=213 path=[2020:0-159 1843:160-212]

TTTTTTTGTTTTTTTTTTTTTTTGGTAAAAATGGTATATGCGACTTTATTAAGTGAAGAA

GATTTGAGTCGATTTAGAACAAAACAGTGTAAAAGATTATTAAATGGTGGTTGTAATTTT

GGGTTAGATAGATGTCAATATAGCCATAATGAATTTTGGAATAAAAGAGGAATCACTTAA

ATAAAATGGACATCTTCTATTCCAAAATTCATT

>c16122_g1_i1 len=395 path=[1:0-394]

AAAAAAAAAGAAAGAAAACAAAAGAAAAACCACAAAACAATGACTCCATTTTTTGTCCCT

GGTAGGTAAACTGGGAACCTGCACTATTTTTAAAGCAAAAAAATCACCACAACTGTACAT

ACTGTCACTCTCCATAGTCAGTCTTTAGAGGAATTTTTTTTCGTATTTCTACGCTGTAGT

AGATCTTAGGGAATGCATTTTATACAGATAAACCTGTAGGGAAGGGAGAGGAGCTTTTTG

CCATTAAAAGAGGGCTGATAGGCAATGGTTGGAAGAGCCATAGTAAAAATTATGTACAAA

GATGTTGTGATTCTGACAATTTAAACCTAATGAAGTCATACGTGTCGTATGTAATCTTGT

AGATTTGTACTTTAAAAAGAAAAATCAACTGGTAG

>c16126_g1_i1 len=255 path=[1:0-254]

GAGTGGAGTTTAAGCCAGGTGAACTGTTCTGCTCGGAACAGGATCTTCAACATAGCAAAG

ACACCTGGTGCAATGGGACCTGCCTGTGTCTGGGCTGTGTGGGTTCTGTGACAGGAGGTG

CTGTGCTGTGTGTGGGGAAAACATAACTGAAACCACCTCCTCCTGCAGATGAGACTTGTG

GTTGCTTCAGAATAATAATAAAATAACCCAGATGTGGGCTGAAGGTAAAATAGCCCTCCT

CATCCAACCTGCAAC

>c16129_g1_i1 len=222 path=[1:0-221]

TGCCTGGATAAATCTCCAGTTCTTGCAGCTTAGTCTTATTGATGTCTGTAGCAATGACTT

TGGCTCCTTCTTTAGCAAAAGCGATAGCAGTGGCTCGTCCAATCCCTTGTGCTGCAGCAG

ACAGAAGTATTACTTTCCCATCAAGCCGCCCCATGCTGCCGACCAAAAAATCTCTGCCAG

CTTGGTACGCAAAATAACCTTTGTAACAAAGGAAGTCATATG

>c16133_g1_i1 len=214 path=[1:0-161 163:162-213]

CAAGGCATTCCAATTGTAGGCTTTTCTTTTTAATATTCTACAATGGGTGACTTAAGCTGG

TGCTGCAGCCTCTTTGTTGTTTGAAGCATTATTATAGTAGTAAGAGCTGACTTCTTTGGG

AATTGTGGAGGTAGAAATAGAGACGTATTTCTTGCCCGGTCGTGCCAAACCACAGCACAC

ACATTACGTTGGGGGCAGGGTGAGACTTTTGAAC

>c16134_g1_i1 len=290 path=[510:0-289]

AATGGCAGTATTCTCTTAGATGACACCAAAAGCATTTACGTAAAGAAACGTATTTTCCAT

CTCATTTTCAACTCTTCTGAATAATGTTAGAGAGAGAAGTTTCTTAAAAAAATAAAGTAA

CATGCCTGAAATTGGAATTACTTTTGTTCCAGCCTGAGATCAGAATAGAATCTAGTAGTA

ATAAGCACATCCTGGGAGCACAGTAAAGCTCAGGATCAAGCTGCTTATCTCACATGGAAA

TGCATCTCTGGAAACAGTAATGATTGTAGTATTAGACACACCTCACGAAG

>c16136_g1_i1 len=381 path=[1:0-96 98:97-112 114:113-380]

TATAAATGAAATAAAAAAAAAAAATAATGTAAAAAAAAGGAATGACTATAATTATAATAA

GATTAATAGTAAATATAATAGAGAAGAAAATGTAATAGAGAATAATTCAGAGAAAAAAAA

AATAATTTCTGTAAAAAATAAAAAAATTACAAAAAAAATGAAATATATGAAGAGAAAAAA

AAAAGAAAGAAAAATAGATAAAATGTATAATCAATTATCTATGGTGAATTTAAATTTTTT

TTCGTTGAAAGGAAAAAAGGAAAAATCAAAATCATTCATTTCGAAAAATGAGGAAAAAAA

AAAAACAGAAAAGGAATTAAAAAGTGAAATTAAAAAAAAAAATTTATACCAAGAAAAAAA

AAAAAAAAGGAAAAAATTGAA

>c16136_g1_i2 len=298 path=[718:0-13 98:14-29 114:30-297]

GTAACTTTGTGTTTGAGAATAATTCAGAGAAAAAAAAAATAATTTCTGTAAAAAATAAAA

AAATTACAAAAAAAATGAAATATATGAAGAGAAAAAAAAAAGAAAGAAAAATAGATAAAA

TGTATAATCAATTATCTATGGTGAATTTAAATTTTTTTTCGTTGAAAGGAAAAAAGGAAA

AATCAAAATCATTCATTTCGAAAAATGAGGAAAAAAAAAAAACAGAAAAGGAATTAAAAA

GTGAAATTAAAAAAAAAAATTTATACCAAGAAAAAAAAAAAAAAAGGAAAAAATTGAA

>c16141_g1_i1 len=921 path=[1385:0-920]

TTCTTCTTCTTCTTCTTCTTCTCCTTCCTCTTCTTCATCATCATCTTCATCTTCTTCTTT

ATTTCTGAGTCTTTGTCGTTTTCTTTCTCTATTCTTTCTTTTTTTCCTTCTTCGAGATTC

ACGTTTTTTTTTTTTTTTTCTTGCCTTTTTTGATTTACCTTTTCTTTTTCTTTTTTTCTT

TTTACTTTTTCTACGAGATTTTGATTTCTTTTTTTTAGAAGAATTTCTCTTACTTACTTT

ATTACCTCTACGTCTTCTTGATAAAGATAATAATATACGGCTTTTAATTAATTGCTTTTT

TTTTGAAGTCTCTTTATCAATTATACAGCTTTTTAATGAATTATTTTCAGTTAATATTAT

TTGTAATCCAAAATTCTCATTTTTCTTTATGAGGATATTATGAGTTATAATCCTAAGAAA

TAGCATAAAATTGTATACTTTATTTTTTTCTATATATACTTTTTTTTTTAAAATCTCAAA

AATTTGATTATCCTTTATAAAATTTATTGCACCATTTCTATAATTCATTAAATATAAGTT

AAATCTATTTCTACTTTCATTTGTTTTTTTAACTACTTTAATAATAGGGATGTTATCATT

TTTTTTATCCAAATAATATATTTGATTTAAATAATTTTTAAAATTATATATGTCTTTTGT

TGTCTTTTTATTAACATATAAAATTATAATTAAATTAATTAAAAATACAACAAGGTGCTT

TCTCCTCAATAAAACTTTTATAAATTTAAAATTATTTGCATTTATTTTTAACATTTTTTT

TTAACTAATTAAAATTTTTTTTCATATTTATAAAAATGTTCACTAATTTTTTTTTCTTTT

TTTTTCATTTTACAAATTTTTAAAACAAATTTTGAAATATTTAAAAAAATTATATTTCCA

CAAAAAAAAAAAAAAAAAAAA

>c16141_g2_i1 len=215 path=[268:0-214]

ATAATCAAACACAAAGTTACCTATATTTCTACTTCTTTTATTTTCCTCTTCATCTGTTTC

TCTAGCAGTACTTTTTTTTATTCTATTTCTAGGAGGGCGATCTTCATCTTCATCTTGCTT

AGAACGACTATTATTTGATTCTTTCATATTTACCTTTTCTACACTTTTCATGTTTCTCCT

TCCGTTTTCTTCATCACTATTTCTTAATTGTAAAT

>c16145_g1_i1 len=244 path=[237:0-180 539:181-183 542:184-243]

CAGCATTATGAAAGGTATGAAGTGTGCCCAGAACTCTTTCAGTTAATTAGACTTCAACAT

CACAAAATAACAGTAAGGCCAAGTTCTGCAGAACGTATCCTTCCTTCAGACATTGGCAAC

CGGAAGAGTTGTATAATATTAATATATCAGCCACAGCACACCAAACCCCCAGGAACTTGG

GGGCTTGGCACCCAGCATCCCTCCTGCCACGGGCATGAGGCAGAGCCTGCGCTGTCAAGC

CCCC

>c16148_g1_i1 len=226 path=[233:0-182 523:183-195 25:196-225]

GAACCAAGCCTTGCCACGTGTGGAATACACTGATCAATTCCAATCGATCTGCTACAGTCC

TTTCTGTCAGCCACATGTCCCCTACCAAGTCAAATGCACAGCCACAGAATCAAACTCTAG

GTGACTCAGAAGAACTGAGACAACATACATGGACCGATCTATTTTAATCACACCAACCTT

CCTGCCACATTTCAATTGTTTATCTCATTGCTAGACTAGCCAGGAA

>c16150_g1_i1 len=469 path=[447:0-169 617:170-252 700:253-468]

TATTTCTATTTAATTGTCAAAAATACTTATAATTTCTATACAGAAGACAATAATGTCATA

GAGCAATGACTTTTCTTTATGAAAACTAGTAACAGATTTTGAAAAGGTAGAGGTTAACTA

CTATACAGATTTCACAAAAGAAGATAAAAGAAAATAAACAGTACAACATTGATTTTTTTT

TTTTTAAAAAGTTAGAAGGCCTACAAATACGTATTGTGTTTGTGCTTGGCCTCATTCTTG

ACCTTTTCACATAGATTTTAAATGGGCTTAATTAAACTTGATTGTACACTGGTAATGCAA

GGCATTCCTATCCACATGACTGATAGCTATAGTTCTGTACTGTATTTGCTTAATTGTTCT

GATGTTGATGGAATAACAAAAAAAGGTTTGTTGTTTTCTACTTGATTCCATATACTTTGT

TTTCTCCATATTGGTTTTAAATGAGTTTTTGGCGGAGAACAAATGCGGG

>c16150_g1_i2 len=386 path=[447:0-169 700:170-385]

TATTTCTATTTAATTGTCAAAAATACTTATAATTTCTATACAGAAGACAATAATGTCATA

GAGCAATGACTTTTCTTTATGAAAACTAGTAACAGATTTTGAAAAGGTAGAGGTTAACTA

CTATACAGATTTCACAAAAGAAGATAAAAGAAAATAAACAGTACAACATTGATTTTAAAT

GGGCTTAATTAAACTTGATTGTACACTGGTAATGCAAGGCATTCCTATCCACATGACTGA

TAGCTATAGTTCTGTACTGTATTTGCTTAATTGTTCTGATGTTGATGGAATAACAAAAAA

AGGTTTGTTGTTTTCTACTTGATTCCATATACTTTGTTTTCTCCATATTGGTTTTAAATG

AGTTTTTGGCGGAGAACAAATGCGGG

>c16156_g1_i1 len=257 path=[431:0-256]

ACTGTATATGTTTTCAGAGAAGCCAGGCTAGACACTTCCTCTCAGCTACAATCATCCAAC

CCATTGATTCTGCAGTGAGCGCACCATCTACCAAATCCACATCTCTCCAGAGAATGAAAG

TCCCCCTTCTTGACCTCACCTTATCCCCTGGCAACAAAGATTGCCTTTCTTCCTTGGCTA

CATGGAACACCCGTTCCCTCATGGAGACTTCTTCTGAGTGCTGGGGTTGAGAGGCCCCAG

GACATCTTGGCCAGGAA

>c16160_g1_i1 len=205 path=[183:0-204]

ATAAAGGCTATTATTCTGAAAGCAGATAGTCAAAAACCTAAATCAAGTTCTGCCACTCAG

GCAAAAGCTTACTTTAAGCTTAGTACTTATATCAACTAATTCTCACATCACTGTATTACA

AAAACCCACAAATATGTTAAGACAGAAGAATCACCCTTGAGTCTTCACATATTTGTGGGT

TTTTGAAATACAGTGATGTGAGAAT

>c16166_g1_i1 len=233 path=[211:0-232]

GAGCCCTTGCTATTAGTCGTGGGAGTGTTTGCACATCTTTAAAAAAAAAATTAATGACAA

ATCTGGCGTGAGGAGTTAATGACTGTATTTGCATGTTTTTTCAGTGCTGAGTTCCCAGTC

AGGCAGTTGGTTCACAACTGTGACATCACCAGAAATCGGTACACTGGCTTGGACAAGTCC

AAACTTATACTGTAGCTTTTCTTTTAAACAGACCTGAAAAGGGATGTTCGATC

>c16168_g1_i1 len=207 path=[185:0-206]

TCCTTGTGCTAATATAAAACCGTCTATTGGCTACATATGCAGTATTTTTATTACCACACT

GAGTTCTATATTTAGATAAATTGTCCAGGTCCAGTACTTTTTACAAGTTTTAGACTTGTA

TGCTTCTTATCTGTGGAAATTGTTCTAATGTCATTGAAAATCATCAGTGTTCCTCACCAG

CTGATGGTGAGAATTGAATCCTGTTTG

>c16171_g1_i1 len=464 path=[442:0-463]

ATCTAATCAGATTTCGCCCCGATGTATGCAGCATGTCATCTGCAAAGGGTCTGGATCAAT

CTTTTAAAAAAGAGAATAACAACTTCCCAACTGCTGCCAATGTTCTTCCATTATGTGGGC

CAGCAAGAAGTAAAGCATTCATTACCTCAAAGAAAATATAGGCTCATTTACGTTGAGTTT

GTTCTCATGTAGGGATTTGTAGGAATTAAGCATGTTAGCTTTCCAGACTAGGCCTACTGC

ACATAAAATACACAGTGCTCGAGCTAAAAGTCTCTTCGAATTAAAACTCAAATTAGTTAG

TGAGAAGATTATCGTACTGCTCTTTCTGAAGCATCTGTGAAGCAAGCTGAGAGGAATGGG

GCATACTGTGTCAGCTATTGATGTGGGAAAAATGCAATTAGGGGGGAAAGATCTTGTCTA

AAATTAGACAAGGCAACTTTCAGAAATATGTTCCACAGTTCACA

>c16172_g1_i1 len=360 path=[486:0-359]

GAGCCTACCAGCAAATACTAAATTACTTACAGGAGTAAATTGAAAAGGACTAATCCAGAC

TGTTTTAGCTTCCCTCTTGTCAAAAGAGCTCATTCACTGGAACCAATTTCATCCCTTAAA

GTATCTCCTTCTGAGGCCACTCTTGCTATTGGCATTTTCTCAGTCAGATGTTCCAGACTC

TGGAAAAATACATTATTAGGCCATGCCAAGCATTTTGGCAGTAACTTTCCACTAGGGAAA

GGAAGTGTGGTAGAGAAAGTTCAGAGATGCTCCTTCCTTCTGCCAGGAGATGCTGCTAGT

ATGTGTCAAAGCTGACTCAAATGTCAGGAACAAGTGAACCACAAAGAATGGAAAAAGAGC

>c16173_g1_i1 len=267 path=[547:0-266]

AGGCGCACTGTGCTCATTCCGGGCTCCCAGCACAAAACAGACAGCGATCTCCTGGAGGGA

GCCCAGCGGAAGGCTCAGAGGTGATGCAGGGCCTGGAGCATCTCCCTGCGAGGAGAGGCT

GAGCGACCCGGGGCTGTTCAGCCCTGAGAAAGGGAGGCTGAGAGGGGATCTGCTCGGGGT

CCATCCATCTCTGTGGGAGCCAAAGGGACGCGGCCAACCTCTTTTCGGGCCGTGGGTTCT

CCTTGGCTGGAGGTATTGCAGACCTTG

>c16180_g1_i1 len=666 path=[644:0-665]

GGAACTGGCAACAGACCGGGAAGGATCTTTTATTCTGTCTAGAAAATCTACATTCAAGAC

TTAAGAAAGGAAAGCAGCAGACACACTGCTTGGGCTTGTGTGCCATCTGGCTTAACCCTG

ATCGCTGACAATGGGATGGCCACTGAGTGTGAAAAGTCAGACCGCTGTGATCTACTTCAG

CTTAAAAACTGCGCCTGATTGAAGACAGCACAAGGAACAGTACAGGAGAGAGACCAAGCA

CACATGTGAACGTCACATTCGTTTGCTCTGCAGTTCCAGAAACACGCTGAGAAGAATGAA

TCCCAGCAGTGGTATATAAATCAGTCCACGGTGTGGAGAAAAGAAATGTGAAACTGAAGA

CATTGTGAAAGGAAAGAACAACATATCTTTTCAAGAGATTGATGATTATTTTGTGAGATG

CTGGGATGCTGGCAACATATGGACCAGGCAGCATATCTGTCAGGCTTTGGTCAAAAACAT

CAAGAAGGACTTTCTGACCTTCTGTATCAGAAAATCAGAATGGCCATCTCCACCGCTGGT

TCTAGTTTTATCATCAGTGGCAATTTCTGAGGTCAGCCTGGCTTAATCTTTAAAGACTAT

GCTAGCAGTCAAACAGGATAGTTGCTTGCGTGAGCTGGTGTCATTCAGGATGAACCATAT

GAGCAA

>c16188_g1_i1 len=217 path=[195:0-216]

GAAAATTTTCTACACCACAGTAACAAAAGGAACAACAAGATAAAAGCAACCCAGCACCAA

TGAGCTTTTCACTCTGCCAAACATAACTGGAGTTTTTTATCTGTCATGATTGTATTTAAT

TAACTGGCCCTATTGTAATCATCAATTAATTGCCAGTATTAGGGGTTGCACTTCAGCATC

TTAGGCCAGGGCAATGCTGATTTTTGCGCTGCAAGCG

>c16193_g1_i1 len=277 path=[1:0-25 27:26-276]

TGTTTTCATGGGTACGTGATGCATTCCTTGGCACCAGGCAGATCAGCTCTCTGCGCTGAC

TGCAAGTCCCCAAGGGGATTTGGGCTCTGCCCCTTTGGCTGGTCCTTCAATCACCTTGAA

ACACACTGTCCAGTATCACTGCGAGAGACAGACATTTGGAGGTGACTTTATCCATGGAAA

GGGAGAATTAGGAGGGGAAGGAGGCTCAACCCAGCTTAACAAAACAAAATCGTGCACAGC

ACAGCATGATCCAGGAGGACCTTTTAGAGAGGTCCAG

>c16194_g1_i1 len=282 path=[1:0-256 557:257-281]

GATGGAGACAGCACTCCTTACTCCTTGGTTATAGAGGTTTTTCACAGCTGTTTTCAGAAG

ACAGGCTTCTCATAAAACTTGCTGGGTTTCTTCCAAGCAGCATTAGCTAAAATGATGTTT

GTTATTTTTTCCAAGCTATACATTTGACTAGTCCAGAGTTAGCAATTAGGCTCCTCAAGT

GAGGTTTGATTGTGCCTGATGAAAGAGCAAACATTTTACATTACCCTTATGGATGCCTGT

GTCTTCTTGCAACCTTTCAGATGCTGTTGAATATTCCAGTTT

>c16197_g1_i1 len=258 path=[236:0-257]

ACAGTAAATTTAAAATGGAAATCCCAAGTCAACCTCTGCCTATTTGTAACACACCTACAG

TAAGTACCGATCAGATTTAGGTTGCATTGACTAACCAAGAATAACTATATGACATGACAT

CCTCTTTACACAGATGCCAGCTGAGCACAGCAGAGACTGAAGACAGCTGAAAGTAACAGG

ATGGCTGTTGTAAAGAGTTGGAATTTTGTTCACTTAGAAAGCTCCCTGGTGTTTCCTGAA

CTTGAACAGAGATGTACA

>c16200_g1_i1 len=294 path=[479:0-197 208:198-293]

ATCAGCAGTTTGCACTTATCTTTCTCAAATGTCAAGTATTGTGCATCTAGATGTTCAGCA

TGTGGCACTCTTGGATGCTCTCATGTTTCCAGCTAGGAATACAGGGAAGTCTTCTGACCT

TCTGACTAGGATGGTTTGGAGTAACCCAGCTGGAAATGAGCATTAAGGTGCTGAAAGGGG

TCATATTTTCCCTGCTAACCTGGGCACCTCCTCGGGTTCTGTTCCACTGCGCTGATCAGT

ACCATCTCACATTGAAAGCAGAGGTGGTTTCCGATGCCTTTACACTACCTGGCT

>c16201_g1_i1 len=356 path=[1:0-355]

TATACAAAATGCTCAAAATAAAGTAGCAAAAGGTTTCTTTACATAACATTACAATCTAGC

CTACAAAACAAACAAACAAATAATGGGATGGATTCTGCATGGAGGTACGGATTCATAAAT

GAATTAGCGGATTCATTAATGACCTAGGAAGTGGTGTGGATACACAGTTTGCTGACAACA

CTCAGTTATTACGAACCATACAGAAGATGGCTATGAGAGAAGAGCTGCAGGCTTTCAGAG

TCCTGAGTCAATGGCAGGGCCAGTGGAATTCCATATATATAAATAAGAAGCACTCCAGCT

TGCATACAAAAGATAAACTCTAACCTAACCTTTATTGCTGAGGAGCAGTCCTTGGG

>c16202_g1_i1 len=206 path=[337:0-205]

CTGTTATGATGAATATGAAATCTAAGGGTTGTGCGCCAGCCGTATAGAGCTCAAACTGGT

AGCCAGGCATTCACAGGAGTTAACTGGGGATGGTTCAGATTTCGGACTCATCAGTAAGAG

ATCAGGTCTAGAAAGATAAAATTGCAATCTCTATGGAAAATGCTTCCTGAAACTTGTGGG

CAATGTTATCCAGCTGTGGAAAGAGG

>c16205_g1_i1 len=296 path=[571:0-18 422:19-295]

TGTCAATTATATGTAATACATGGACAAATAACACAGAAAAATGTGTAGGCTGAATGGTGT

CTTCAGTGTTAACCATAAAAATTAATCATTCACTTTTCAGAAGGTTTTATTTCTTCAGCC

ATAAATAGCAAATTACATTTTAAGCTAGGACACTATGCTAGTTCTTGAATAGTTACTCCT

TTTTTGTGTTACAGACATTACCTTGCCTTTACTTATCTCTTTGGAATGAAAAACACTGAA

CACTAACTAGGTAGTCTGTTCTGTAGCTTGCATTGAAACCAAAAGTTGTAAAGAAT

>c16205_g1_i2 len=427 path=[272:0-84 357:85-149 422:150-426]

GTCCATGTATTACATATAATTGACACAGATATTTTACTGGTGTTGTGTGTTTAGGATTTG

GGGAGGAGGGGGGGAGAAGAATGTTGAGAGGTTATAATTGAAGAAAGAATTTAAGGGTAA

ATTTTGCTTCTTTTTACATGATCTAATTTAATGGACAAATAACACAGAAAAATGTGTAGG

CTGAATGGTGTCTTCAGTGTTAACCATAAAAATTAATCATTCACTTTTCAGAAGGTTTTA

TTTCTTCAGCCATAAATAGCAAATTACATTTTAAGCTAGGACACTATGCTAGTTCTTGAA

TAGTTACTCCTTTTTTGTGTTACAGACATTACCTTGCCTTTACTTATCTCTTTGGAATGA

AAAACACTGAACACTAACTAGGTAGTCTGTTCTGTAGCTTGCATTGAAACCAAAAGTTGT

AAAGAAT

>c16208_g1_i1 len=207 path=[339:0-206]

AGTCTTATAACAGCTGTGTTGTGTTGTTTTTCTCCTAGTGTCTCTCTTTGCTTCACCAGA

GCATAGTAATGCTTACCAGAACAGAACACAGCTGTTATAAGACTTGAAGAACTGTGTCCT

TTCCCACTGGAAGCTCTGCAGCAAGAGCTGAGCAAATACAGCCATGCCAAAGTCTTCATC

TGGAGTCAGGAAGAACCACAGAATATG

>c16213_g1_i1 len=246 path=[390:0-245]

CTATAAAAAAAAAGTTCTCTTAATTTGGCCCCTCTTCAGGGAGTAGAAAAAAGATGAATA

AAATTCAGTTTCTAACTCCTTTAACTTCAGGTATTCACAGTCTTCCTCCCCTGCTCCAAG

AACTACAACCCTACAGCCTTTGAAATATCACAACGAACATCACAGCTTCTTAAAGCTAAC

CTAGAAAAACACATATTTACTGCAGCTAATCCTGTGAAAATTACTAAAGGGAGGATATAA

GAAAGG

>c16219_g1_i1 len=360 path=[1:0-106 108:107-359]

AGACTAAAAAAGGGCAGAAAACAATCTTGCATCTGAACTGATTTAATTGTGTGATAGAGT

TGAGAAACTAAGATTGAATTACAGCCTTTTATTTGATTATGGTATTATGGGGGAGGAATA

ATATTCATATACCAGGGCACATTATGCTGTACGACATCCACAGAAACATTCTGAAGCATT

AAGGTTCTCAGATGGACACTGTGGTTCTGTGACATTCACTGCTGAGCAATATTAAGGGCT

GGATACTGCAATAATTTTAAAGTACCCAACTAACTACTTTTCTGCAAAAAGAAAGAAAAA

ACTAAGGAATGCTTTAAGCAGCCACCCTCACTACATATAAAGTGCCCTGGTATATGAATA

>c16224_g1_i1 len=236 path=[177:0-235]

AGCTAATTTCTCACACAGCACTGCTTAGTCATTCGAGCTTGTGCTGTTCTGCTCACGCAG

CACTGATCGAACTTCTGTGTTTCTGGTGTGACTACTTAAGGCAATCAGTGCTCTAAAGTA

CTCTTGAGGAATAGGTATTACTCTAAGGAATTTGAAAATTAGCTATAATTTAAGTCAAAA

TTAAAAATCTTTATCTAAGCAAAGGCCCTGCTGCCAAGTCTTCTTGTCATGGTTGC

>c16225_g1_i1 len=229 path=[1:0-228]

ATTAAACTATGAAAAACGAGAGTGCTGAGTATGGTACGATTTTGTGTTGACTCTTCATTT

CATCCACCGGTGCACTGACTGAATGGTGCCTTGCTTTGCTGTAGGCTCAGAGCAGATGGA

TTTCCCAAGATATACACAGAAATACGGAAGAAAGTCACGTCATATGTTACTGAGTAACTG

TGAAGAGGGTTTTGAGGCCATCCGTTACGCACTGGAACAGGTTGCCCAA

>c16226_g1_i1 len=246 path=[1:0-68 70:69-85 87:86-245]

GCATGGAACACGGGCACAGCCCAGTGCAGGGCCTGGGTTGTTGCATGGGACATGACACAG

GGCACAACCCAGTATGCGACATGAAGCAGAGCCCAGGCTGCATACTGGGCCATGTACAGC

CCCCAGTGCAATGCAGCCAGGAGGAGGCAGAGACCAGTTTACATGCAGTGCCACATGCAG

CCCCCTGTGCAATTCTGTTGCATGGAGGCTGTGTGCTGTTCTGGGTGCTGCATGCAAGAC

TTGGGT

>c16228_g1_i1 len=203 path=[181:0-202]

TGGTCTTGGGCTGCGTATCTATTCCAAATGTGATTTAGAAGATGAGAGTTGATGAAAAGG

GTGCTACAGCTGCCTTAAATTGGTTCCCACATGCAGAACTCGTGGTTATGCCTTCTTTTT

TAACATGACATCCAGATGCTCAGTGTAAATCACAGACCAACGTAAGATGAGCTTTGTTTT

AGCAATGCCAGCTGATACAGAGA

>c16229_g1_i1 len=820 path=[1403:0-819]

AAAAAAAAAAAAAAAGAGAAATATAATATAAAGTGGAGTTAAAAAAGGGATAAAAATAAT

GAAGAAGTTAATTACTATACAAAAAATTTCTAATTTTGATTTTTAATTAGCTTTATATGT

ATATATATATATTTCACTTATATATAAGACATTGACACTATAAAGAAATAAGAAGAGACG

GAACAAAAGATTAATATAAAGTTATATTTAGATTTTCATATTATAAGAATATCTTTCATT

CTATATTGTAATTTATATACTATTTTATTTATTTATTATTGTAACATAATGAAGATAAAA

TGTACAACAAATACTAAAAATAAAAAGCTAAAATTCAATTTTATTATGCACTTTATATGA

TTCTTTGGTTTCAGTAACCCAATATTCTGTACCATAATCTTGGTAAAAAATAACTTGTTT

ATCAGAAATATTCTTAATACATTTTTTTTTCATGTTTTTATATTTCTCTTCTATTTTGTA

TGTTGGATACTCTTGCAAATAAAAATCTTTCATTTTGTTATAACTTTCTAAGTAATCTTC

ATCATTCTGTATATCATACCTAAAATATATAAAATAAATTTTAGAAACATAATGAAATAT

ATTTAATGAGTCAAATGTAAATATGTGAGAGAAAATTTTATTATGTAAGCATAATGAATA

TAGTATAGGAATATAGAATTAAGATATTTTTTTAGTATATAGTTCTACAGAATTTTCAGA

TATTAAGAAAAAATATTTCTAAATTATTAAAAGGAAAAAATTAAGAGAATAATTTTTAAA

TAAAGAAAATAATACAGAAATAAAACAATATTATTGATAT

>c16229_g2_i1 len=689 path=[737:0-688]

AAAAAAAAAAAAAAAAAACTTGCAAAATATTATTACAAGAATAATTCTTGTGAACACGTT

ATAAAAAATAGCATTTGATTCCTTTAACGTGCATAAAATTAAAAAAAAATAATTATTATA

TTTCTTTTTAAGAATAATCTTTCAGTAAATATAAAAAAAATATAAACATACATATACATA

TATTTTAATTATACTTTATTTAATGATAGCTTAGCTAGCTATAAATCAAGCCATGCATGA

AAAAAAAAAGATAAAACAAAAATAAAAATTAAAATAATTATTCAAGTCCTGTTGTTCTTT

GATAAGGTATTTAAAAATATAGATTCAACAATATATTGATTTAATGAAATAAAAAAAAAT

GAAACTTGCAGATCTTTATAAAAGTTTAATATTTTCTTTATATTTTTCCTTTTTTATTTG

ATAAAAAAAAAAAATAATAACTTTACTCTTTAAATAAAATATATTATTTCTTAATTTTTT

AATATTTTATTCTATATTTATAATTAATTAAAAGCATAAAGTCCTATATTTTCAAAAATC

TAAAAAAATGTCGAAATCCAATACTTGCAGATGTGATATGTCGGCTTATGCAAGGAATAG

ATTTTACAACCCAAAAGTTCACGTGTCCGGATGGTAAGAAATTCTGTTAATTACAATATA

AAATTTAGATTAAAAAAAAAAAAAAAAAA

>c16231_g1_i1 len=261 path=[343:0-14 358:15-206 577:207-260]

ATATATTTATATATGTATAAATATATAATACAATCCAAAATCCTAGACCTAACACTTCTT

AAAGGATAATTCTCATCAACTAGGCAAGGATCTAGAGACTTTGGGTTCTCCTCTGTTCCG

GTGGTTGTAGTGCTGTGGGTTTGCTGCTGTTCTGTACGCCTCAAACATTGCAAACAGAAA

ATTGTGAATGGTCTGGCTTCCCCCAGTGGGTTTACACAGAGACATTTGATCCACAGTTAA

AGGAAAGGACCAGTGAGCTCA

>c16232_g1_i1 len=477 path=[543:0-414 1136:415-421 1143:422-476]

AGGATGAGTCATTAACAGAAAATCTACAGATATGAAAATGCAAGGAAGAGTGTTAATTAT

TATATTTCAGTGTGGCAAACAGCAAGCAACTACCTGTGCATCATTAAACTGGGACGTGCC

TTCGCCTCATCCTAGCAGCTCAAAGGAAAACAAACAAGAAAATACACACCAGAAGACACA

CTCAAGAAAACATTCCCTAAAAAGATAATGGAAAAGCAGACTCCTTGACTTATTTGCTAT

AAACATGCTTGAGATACAATTGCAGACTAAAGGAAACAAAGTATGACAGCTACAATAAAA

TTGCAGTGCTAAAAGGCCAAGCAGAAAAAGTAACTGTTACTATCAGTTTTTGTTCTAATA

ACCAGAAGCATTCTGCATGACTAGGGGAGCTAAAAGCTGAGCCCTATACAAAACAGATCT

TTCACTTCATGTGTTAGGGAGATTTGAGTACGGTTTTTTGATGAGTGAAAGATCTGT

>c16234_g1_i1 len=336 path=[1:0-335]

TTGTGTTTGATTTTTTTTTTTTTAATTAAAAGAAAGCAATGTTTTTATAGTCAAAATGTA

AGTATAGATTTATATACATTTTAAAGAGAATTTCTTATTATATCCGATATAAATTCAAAT

GAAATATTTGTACAATATGAAAAAATTATATGATGAAATTATCTGCCTTATTGCTTAAAG

CTAGTTTTTTATTAATTATAAAATCCATAATTCATTATAAAAAATTTTTTTAAACAATTG

AAACATAATAAAGTTTGAAATGTTTTATTACGAGAAAATTTTTTAGGAAATTATAATAAT

TCCTTTAATAGTTTACTTAAAATACTTTTTTATGTA

>c16238_g1_i1 len=242 path=[374:0-241]

GGAACAACCAACAGATTTCTTAGGATTGAAATTTAGTACGACCCGCAGTTATCAGAGAAC

ATTTAAACCAAGAAGTCAGACAGAAAAAAGAAAAACCACAGGAGGGATATACATTTACAC

CCTTCAGGATAATTACCTCAGTACACAATTGCCAGGCTGTTTAAATAAGAGCAATAGTCT

TGTTCTTTTGGTTTCAGTTCCAATTAAGTGTGCATTAGCGTATTTGCTACAGTCTTTTTC

CA

>c16242_g1_i1 len=262 path=[327:0-148 74:149-261]

CCAAACAACTCCCAGGCATGAATGAAAGCCTGGTGTGGTGCTACACTGTTTTATGATAAT

GGACTGTTTCTTATTTCCTTGTTCTATTTTCTGACATTTTCCGAAGAAGTGTTATAAACT

AAACATATTTTCCCAACCATACCATTGTCTGTTGATTTTTGCTTTCTTATCAACTTCTGT

GGGAACCAAGAAAATACTTTCATGCAGGCTACATCTTGTAAAGTTATTTTTGTTTTTTTT

TACAAAAGGATGTGGAGTTCAG

>c16253_g1_i1 len=562 path=[950:0-130 2:131-561]

AGCCGAATTAATTAGACTTGAAATTGACTGAAATATAACAGTGTGAAAAGTACATTTCTT

TCTTTCTTTTTTTCTTACATGACACTACATGCATTGCAGATGGGAGCACTTTCAGTTCCT

ATATGTCGGAGGACCACGGCGGGATCTTTTTACCATAATGGTACTTGGATCGTTTCAAAG

GAGGCTTTTCATCAGCAGTTCTCCTTTGTGCAGGTCGGAAGATGGCAGAGTTCTAGCTCC

ATTTGTGTTCTGTTAATACTCAGCGCACATCCTTGGACCTTAATTCCATTTTTCCCGAGC

TGGTTTTCCTGCAGACAGCAGAAGGCTGCTTCTCGCTCACATACGCACTTAATGGATGTG

TCAACTTCCACTGCCGTGCCAGGATAAGCTTGCCTTTAGGTGTGCAGAGCTGCAGGGAAT

GTCACCTGCAAGTCAGTCCTATGTTGTCTGTCTGTATTTACATTTTCAGGTGCATGTTGT

CATCTTGACACGTGGTAGAATCGTTCTGTTCAAAGGCCCTCAGATGCAATAGGCTCCTTT

TGCAGTGATCGTGGAGCAGCAG

>c16254_g1_i1 len=249 path=[227:0-248]

GATGCTTCTTCCCCTGGGTATGTAAAGCTCGGCGGTAGCTCTCCATGTATAATTTATTTG

GATTTTTTTATAAAACAAAGTGCACCCTCTTGCTTCTGTTTCACAGTAGTGATGTATGAA

CGGCTTTGGGCACTGCACTGCGATCCTTACAGAAACTGTACACGGGAACGAGAAGTGTGA

TTTGTTGTCCACAACCCGATCTCGAAATGTTTATGCTGTAGTTGTGTCTGAAATACACCA

CGTCCGTGC

>c16256_g1_i1 len=288 path=[584:0-287]

TGATGTGCTTCGCCTTCAGCCCGTGCACAAAGACGGCCTCCAGGGCACAGCACAGCGTGT

TGGCGTTCCCATCATCGCTGGTCACCACCGCATCCGAGGAGACGTACTGCTTCTGCAGGG

CCTTGATGGAGCTCACCAGCTGCTTCTTGATCAGCTGGATGACTTCCTTGGGGTCATCGG

CGTGGCTCGAATGCATCTCAGCTGCTGCTCCCACCCGCGGTCACATGTCACCGCCTGCGC

GCCCCGGCCGGGAAACCCGGAAGTGGCGGCCGCGCGCCCCTCCCCCAG

>c16262_g1_i1 len=237 path=[747:0-236]

AGAGAAAAGCCAGGCAGGTTCTTTTCTCTCATGCTCTGGTCTTGCTCTGCAGTGAAACAG

GGTTTGGCCGACTTCCCAGAGCAGTGCAATGCTGAGCAGGAGCGGGAAGGCACTGCTTTC

CCAATAGGTGCTGCTCCTGCCAGCAGCTCTTTGCCAGCCTTCTATGTTCAATTTGCAGTG

CAGGAGCTGTGTTCCAGCACTCAATTCCTTCCACCCAGCGGCACGTCCTGCGGCTCT

>c16262_g2_i1 len=289 path=[267:0-288]

GCGGCACGTCCTGCGGCTCTGCTCATCTGCTTTGTGTGACTGGAAGCATCAGTGACCACT

GCAGCTTGGAACATTTACCCACAAAGCAAACCCAACCCAGAAGTGCAAGTCCCAGAATCA

GAAAAGCTCATTTCGAAGCCAGAGGTGCTGAGTGCTTGCGCAGCCTGACTGACTGCAGGA

GCTGGCACTAAGAGCACTGTGTGCTTAATGTCCATAAGTACTCCCCAAACAGCAAAGGAA

TGCTGTGCTTTCTCTTTGCTGCCCTGAGCTGAGGGAGCTTTAATATACA

>c16263_g1_i1 len=234 path=[1:0-233]

GAGCAAAGCATGGTGCAGTGCTCAGAGTGCCCAAGGTCCCTCTGCACACCATCCTCAGGT

CTGGGCACCACCCCCCTGAATAGCGCAGGGGAAGACCAAGCCAGGAGGTGACTGCTGGGC

TCCTGCTTCCTTGTTGCAAGACCCCAGTGGGGCCAGGGAGCTCCCCACCCTGGGCCCATC

CCTGGGGCTGCTAGCAGACCTCTAGGGGTGCAACCAAGCTCCAGCCCCTCACCT

>c16267_g1_i1 len=205 path=[1:0-204]

CAGAAGTGCAGCACAGCACTGCAGAGCAGCAGTGGGCTCGGCTGCTCGCCGCCCAGCGCT

GGCTGAACCCACCATCCAAGCGCATCTCCTCGCTGTGCCTTCCAGTCAGCACGTTTTACA

TCGCTCTGCTTTCACCTGTCCCTTTCCCTCTGGAGACCTCTGCACAGGATTTACAGCCAC

TGCTCCCAAAGGGACCGGTGCTCAG

>c16269_g1_i1 len=766 path=[167:0-765]

AAAAAAAAAAAAAAAAAAAAACACTAATATTGTCTAAAACCTAAAGTCTTCTTATTCTAA

AGAGACTAATTATTAATTTTTTTTAAAAATAGTTGTCCATCTATGCTAATAACTATTTAG

CATATATCATATTAATATACGGATTACTTATATATATATATATATATGAAAATTAATCTT

CAACTTTTACTTTCAATAATTTTAGTTTTCATCTCCTTGTTCACTATTTTTTGTTTCTTC

ATTAGAATTTTTTTCTGCAGGTTCTTCTTCTTCACTTTCACCTCTCTTTTTAGTATATAT

TCCAAGTCCAACTGCACTAGCTATAAGTACTGCTAATCCAGTAGCAATTGATGAAATTAA

CAATAATTTTTCGTTTCTCTTCTTCTTTTCTATATCTAAATCAACTTTTGCCAATGAAGC

TTCATCTAATGCACTGCTTGGTACTTGAATAATTTGTTCACTAGGAGCATTTTCATTATC

ACAGTGGCATGGATTAAAATATTTAACAGCGAGAAAAACGGAGAAAATGAATAAAACCTT

TGAAAATTTCATTTTAAATTAAAAGAAATATTTAAAAATAAAGTTTTTCTAGAAAACAAA

TGCAAAAAAATAATAAATGTTATGAACGAATTTTAAAAGTATTTTTATTTTGACAAAGAA

AATATTTAAAATAAAATTTTTTTATGACAATGTTTTTTTTTTTTTTCTGTATATTTTACT

TTTTAATTTATTTTTTTATTTATAATGAAATTTTCAAAGGTTTTAT

>c16273_g1_i1 len=230 path=[544:0-229]

GGAGCCGTTGCTCCCCACCGACCTGCGCTCCTCCTCCTCCCACCGCCTCTCAGGCAGAAG

ATGAGTGGAGCCCTTCAGAACAACAAGCCAAGCAACACGGCAATGAACCTGATTGCAAAC

GAGCGCACAGAGATGTGGAGCGCCGTGTTTGTCTGGCAAGAGAGAAAGAAGTGACCTGGT

GAGACACAGGTTCCTGAGCAGTTCTCGTGGTGTGAGCAAAGAGAAGCCAT

>c16274_g1_i1 len=326 path=[1:0-325]

AAAAAAAAAAAAAAAAAGATAAATTTATTAATGCACATTAAAAATTCCTAAACTTACAAA

TGTATAAATAATTCATTAATTATTCAAAATAGTTATATTGAAATAAAAATACAAAAAAAT

TAGATGAAATATTTTGGAAAATTATGCAAAATTGTGTAAAATTTTATAACAATTTAGTGA

AATAAGTTTAAAAAAGAAAAAAAAATTATACTAATAAATATGATATCATTTCATTAAGAC

TATACATAATAAAAATAATGTATTTGAATAGTTCATATTGTTTTTATTAACGAAATGAAA

AAATAATGGAAAAAAAAAAAAATCAA

>c16285_g1_i1 len=206 path=[1:0-205]

AAACTAGGAGAAGTTATTACAGGAAAATCAATGAAGTCTTCTCTCCAAATAGTTACTTGA

GTATTTTCTGCGGGATGCATAGGTAACATTTATGAATGGACATCACTAGGCACTGAATAG

TGGAAATTGGTGATTTGTAAACTGAATACCAGTTTTTGAGAGATGGATTTTAGCAGTAGC

CCACCTTTGAGGCATTGTTTTTATAT

>c16286_g1_i1 len=328 path=[1:0-327]

ATCTTGACTCTGGGGCGGCACCTACGAGAAGGCGCATCCGCCCCATTACATCGCCCACCG

CAAGGGCTGGCTGTCCCTGCACACCGGCAACCTGGACGGGGAGCGGTGCGCGGCGCAGCG

GGCGGTGGAGGACTCCTTCGTGCGGCGGTTCCTCCACGGGACCTTTCCGGGCTGTTTGGC

CGACGAGGCGGTGTTGAAGCGCCGTTCCAACTCTCTGCTCGTCTGCCTTCTGCTGCTGCG

CCGACTGCCGCCCTCAAAGCTCTGCTTCCTGCTGGGCTACGCAGAAACCCTCCTGAGCCA

CCTCTACAAATGCCCCGTGCGCATCGAG

>c16288_g1_i1 len=250 path=[161:0-177 89:178-222 25:223-249]

TAAAATAGTGCTTAAAAAAATGTAAGATACAAACTCAAAATTTTGTTTGAGAGTTTATAA

GTTGATGAGAATGAGGGTATGTTCAAGAAAGGCATCCTTGGAAAAGTGACTGCAATAAAT

ACAATTTGAGCAGAATAGTGGGAAGTGAACAGATAGCCTCTAAGGTCTGGCAACTGATTT

TTTTTTGAAAGTTCACTTCAGAATGACAATGGTGTATTATTAAGGACTGTAGTAGGAGAA

GTAGACAGCA

>c16289_g1_i1 len=201 path=[237:0-163 401:164-200]

ACATTAATGGCTGAAAATTAACACTGTGTATCAAACTGGAAATCTCTATCCTTACATTAG

CACACAAATGCTAATAACTGTGGCAGATACTTTAGATGTCAGGGTAAGCAATCCACCTGT

GATCCAGTAACAACTTTTGCCTGCCTCAGTTTGAGTAAGTTTAACCAACGGTAGGGCTTG

TTGTGAGCTGTCACATTGTAG

>c16289_g1_i2 len=201 path=[237:0-163 474:164-200]

ACATTAATGGCTGAAAATTAACACTGTGTATCAAACTGGAAATCTCTATCCTTACATTAG

CACACAAATGCTAATAACTGTGGCAGATACTTTAGATGTCAGGGTAAGCAATCCACCTGT

GATCCAGTAACAACTTTTGCCTGCCTCAGTTTGAGTAAGTTTAAGCAGAACTCCAGCTAC

AATGTGACAGCTCACAACAAG

>c16290_g1_i1 len=238 path=[216:0-237]

AGAAGGAAAGAAAATCTATGGCAAGGGAGAAGGGGAGGTTTCTTGGAAGGGGAGAGAGGG

ACACTTTATACACCACAGTAACGAAGAGGTAGCTATTTTAATTGTTCCCTAACAAAATGT

TTTGAAGAAAGTGCAGTTCTTGACCTCTGCCCATAGGACAGGGAAATCATGATCAAATGA

TCAAATGGAGTTCATTTAAACAGATCATCTCATTAAGTAAAGCAGAAATGATAATGGA

>c16298_g1_i1 len=296 path=[1:0-295]

CTTCATGGCACGCTGCAGAAGATCGATGTGTTTTTGAAGTGAAACTCGGAGTTTTTACCC

GGAAACAACGCGGACGCCTGCTCTGAATGCTAAGCAGCTCCTTGGGAGGACGTCCCCCAG

CACAAAGGGCAGCGCTGTGTGCCAGGTCCGTCCTGTCCCCGCGCCGGGCTGTGAGCTGGG

TCACCTGCTGTGAGCCTGCGCCCCCCTCTCTGCCACTGGGGAGCTGTTCTCTGTTCTCCC

GGGTGGTTTTTCAGATGAAAGGAGGTGAAAAGCACTTCCATTCACTGTGCAGGAGG

>c16308_g1_i1 len=323 path=[301:0-322]

GTACTAGGGGTGTTAAAGGGTACAGACAGTATTAACAGTATTTCCTACTGCCATCATTTG

AAATATAGAGCAGGCTGGGTGAACGATCACAATCCAGACTGCAACATTTCTTATTTGACA

GAGTGTACTTTAAATTCACTTAAGCAAGAGCACTGAAACCAGTCTTATTTCCAGTCACAG

GTTTCTTTCGTGAGATTTTTTCCCCCCTTCTGTCATGAATCTTCTGCTCTTGAATTTCAT

CCTCTAAGAAACCAAGTAATGGATTTGTTAAAATTTCATAATTGTGGCAAAGTCTGCCTT

TCTATAATGACCTTTAAACTTCA

>c16314_g1_i1 len=312 path=[1:0-311]

ACCAAGTCACCAGCTAGCAGCCCTCAGAACACCAACAGCCAGGGCCCAGCATTCCACGTT

GGCTTTCCCACAGCTAATCCTCTCAAGACTTTTCAAACCAGCACCAGCTCTTCTTTGCTC

CTACCTAATGGCAGACACCTCTTTGATTTCAGCAGCATTTGGCTCAGGCACCAGCTTGTT

TGCGACATCCGTGTTTGTGATATCTTGACTTTCCCAGGTAAATTTGCGCCTAACAGGACT

CCCATTTTGATTCAAAGGAGTCCTAAATCTCTTCTGCAGAAGCAGAAGTTCCACTACCAG

TTTCAGTCAGTA

>c16321_g1_i1 len=208 path=[186:0-207]

GGGAGGTTTTGGTGCAGGGACTTTCAGATGCCAGCATCACAGACACACTGCAGTACAACC

AGAGGCAGTTGGAGGCTCACACCTCACATGCCACCAGAGCAGGGCCGTGGCTTGACACCC

AGGAACATCATCTTCACACGGAGGCAGGACTGAGAGCTGTGTGGTCCCACAGGGAAATGT

ATTTCTTCCAGGAAGCTTTCATTTCAGG

>c16325_g1_i1 len=353 path=[331:0-352]

TTTGTTTTGCTTTTCTGCATTATAGTTAATTCCAGACACTGCTTCCCTTCTTCTGCAGAG

CTGTCTTTCCATACCCATGCAATTATCTAGGGGATTTTTCTCTCCAGCCTATTACTGACG

TAGTACTTAAATACACACAGTGAAAAATTAGCAAACATACATTGAAGTGTAACTGGCGTT

TGGCCTGAATGATGTGCGTGAGGCAGTTCCTGCTCTTGGTGCCTTACACATTCTCCTCTG

CTAGGGCTGGTAATGAGGGGAATTTCATCTAGAAGGCAGTGGAAAGTTTAAGGATTTGTT

TGGTCCTGGGACCCATTCACATGTTTGTGTCACAGGAGGCAAAATCAGGCAGA

>c16328_g1_i1 len=240 path=[218:0-239]

AATGTCTTTTTTTCCATTCTATTAAGACCTTAAGCAAAATATTTGTCAAAATCACACTCA

GAAGTAAACTTCAGTCCACCTTGCAATGCCTTCATCATTGTGTATTTATTTATTTAGAGA

GCCCTTTATCAAGAAAACCATGATTAGACAAAGTCTAGATTTCCTCAGCTTATATTCCAC

AGACCTATGGTACAAAACATCAGCACACACCACTCCTTCCTATTTATCCACAAAGCCCAG

>c16330_g1_i1 len=201 path=[333:0-200]

ATAAGAAAAAGTGGTCTTTGCTGTAGAGAATGCAGCATCTCAAAAAATACGTGGAGCTTT

TTTAAGTAGCTGAAGATTGTGTTTAAGAAAAGGTGTCTGTTGCGTTAGGAAAGTTTGTAA

TGCAAATAAGGGTAAGCGAATTATTAACTACATGGCATACACTTAAATGGGTGAAATAAA

ATATCCTGTAGCTACAGCTTG

>c16332_g1_i1 len=202 path=[1:0-201]

GAAGATGCTGGAGATTACACGTGTGTGTGTGGAGACAAGACAACTACAGCATCTCTGACA

GTGCATGCCCTCCCTCCACATTTTAAAAAGGAGTTGAAGAACGTGGAAGCCACAGAAAAT

GGCGTGGCCACTTTCTGCTGTGAGCTGAACAAGCCTGCAGCTTCTGTGGAATGGAGGAAA

GGGGACAGAGCCCTGGAGCCAA

>c16332_g2_i1 len=208 path=[544:0-207]

GGGCACAACCGCAGAGCTGGTGATCCGTGATTTAGATCTGGCAGATGCTGGTGATTACAC

ATGCTGTTATGGAGATCAGAAAACCACAGCTGCTCTAAAAGTGAATGCCTTGCCTGCACA

TTTCAAGGAAGAAATGAAGAACGAGGAAGCCACAGAAGGTGGAACAGCCACGCTGCAGTG

TGAGCTATCTAGGGTCGTCCCCGTGGAG

>c16336_g1_i1 len=211 path=[189:0-210]

GGCCAGCAGGTTGAGGGAGGTGATTCCGCCCCTCTGCTATGTTCTTGTGAGACTCCACCC

AGTGTACTGCATCCACTTCAGGGGCCCCCAGCAAAAGAAGGACATGGAGCCGTTGGAGCA

GGGCCACAACGATAAACAGAAAACTCAAGCACCTCCCCTATGAGGACAGGATGAGAGAGA

TGGGGCTTTTCAGCCTTGAGAAGGCTCTAGG

>c16338_g1_i1 len=210 path=[188:0-137 326:138-209]

TGCACTTACTCTCTTCTTTGCATCCTCTGCCGGTCCAAATGGATCTTGAATTCCTTGCAA

TCAATAAGGTAATTTTAAAGCCTAGAGTTTGAAGTGCCTCTTGTTTCTTTTCTTTCACCT

GTTTTGAAATCAAATGAAAGATGTGGTACAGTAGCATACCTCTTTGAACTCACTGTGGAA

AGAGGAAGGTGGAATTCCAAGTATTCTCTA

>c16343_g1_i1 len=402 path=[36:0-35 72:36-151 188:152-401]

AAAAGGAGATTTTGAATCTCCTTTTAAAAACGTGGCAGGCAGTACAGAAGAAAATAGTGG

TACCCCCAAAGCACAGCTCTTAGCTGGGGGGGTTATAACAGATTGCCTCTTGGTGGGGCA

GTGTGGAATGGGAGGGGAACCTGCAGGTAGCACAAGCACTGAGTAACCAGCCTCAGGAAT

GGCATTTATTTGTATGGACTTTACAGCTGGCCGCCTGCATTTGATTCGGTTGCTTTTTAT

ATTCAGTGTTAACGTAGCTCTGTGTTAAAGGGAACTTCAGCACACTCATGTTCTCCCAGC

TGGCGGCTGCCAGGTAACGACACCTGCCCGTTCTGGTGGATGGGAGCGTGTCCCTGTGAC

TGGGGCACCTGCCCTGGCACGAGCCTCCCTGCTGCAGGTGCT

>c16343_g1_i2 len=271 path=[848:0-20 188:21-270]

GCAGTGTGGAGGCTGGTAGTGCAAGCACTGAGTAACCAGCCTCAGGAATGGCATTTATTT

GTATGGACTTTACAGCTGGCCGCCTGCATTTGATTCGGTTGCTTTTTATATTCAGTGTTA

ACGTAGCTCTGTGTTAAAGGGAACTTCAGCACACTCATGTTCTCCCAGCTGGCGGCTGCC

AGGTAACGACACCTGCCCGTTCTGGTGGATGGGAGCGTGTCCCTGTGACTGGGGCACCTG

CCCTGGCACGAGCCTCCCTGCTGCAGGTGCT

>c16344_g1_i1 len=203 path=[33:0-34 68:35-36 70:37-202]

CCTGGAGCATTCACTGCCCCACTCCCTTACAGGATTTCCCAGGATTGCTTATGTAAATCA

TGCGTATATTTTCCTTCATTGAGGAAGGAAGGGGTTGTAATATTGACCATAGATTTTATT

CAACTGCCAGAAACTGTGTTAAAGTAACATTAAGGGATTGACTTACAGCCATAACGGTGA

GGAAATTTTGAGTTAATAGTTAA

>c16347_g1_i1 len=337 path=[13:0-336]

AAAAAAAAAAAAATTAAATGACAATATAAAATTTATTTATTTATAATGTAATTTTTGTTG

AAGCTATTGTGAAAAAGAGGTTAAATCCAAGTTATATATGATTAAATCTTAATCTTGACA

TTTAACTAATAAAGAATATTATTGAGCTATTTGAATTTATAATACGATGCATAAAAATGC

AAAAATGAGTTTTGTGTGCATGTAAAATATTCTAAAATATATGTATGTTAAAATACCTAC

CTGGATAATAGATGATATCAACCTTTTATGATTTAATAGTAAATTATGAGGAAATAAGCT

ATTCGTTATAATTGACATTTAAAAAAACAGATAACTG

>c16350_g1_i1 len=212 path=[1:0-58 60:59-211]

AAATGCTACATGCTACTTCCAGAACACTTCTTTCATAACTACAGTTCTGTGTGCTGTGCT

TTTTTTTTAAGTACCCACCCAACCCTGTGTACACTGTGGGAGTCATGCTGTGTGCCCACT

GAAGTGACACAAATACATATACTGCAGCGAGCACTGCAACTTCCAGATGATGTGCATACA

AGTCATATTCTACAGATAAAAATCCGATTTCA

>c16360_g1_i1 len=583 path=[561:0-582]

GCCTAGGGACCAGGGCAGGGAGCGGGCGCAGCAGCTGGCACAGTCAACACACAGCCAGGG

AAATCCTGACACGGGCACTCGTGTCGCAGTGCTTGGGCTTCTTGAAGGATGTCAACTTTG

CTTCTTGAAGCTTTTTTTTTGCTGTAGTAGATTGTAGCAAATGTGACCTTTTGAAAATGA

CGGTTCAGTGTATTGATGTGTCTGTATGTATGTATTACTTTACATAGGTGATGCCAGATG

CATCTCCCGCAGAATAATTGATGCGAGTTGCGTAGTGGCACCAAGCATGCTGTAGAGGTC

TAGAATGAACCCAGCTACGACAGAAACAGTTTCTTTACACACACAAATACACAACCACAT

GCAGAAAAACTTGCGTATGTGAGACTTTGCACAGATAACTATTCCAGCGTAACAACACGT

AGAATGAACTGCTATATATAAAGTTCTATACTTCATACGCCTTTTTTTCCGTTAACTTAT

AGTGTGCACATTTATTTTAAAAATAAGCGTGGACACATATAGCCTGATATACATATGTCC

CTGCACTTACAGATCTCTGTACGCAAAGAAACTGTTTCTGTCG

>c16361_g1_i1 len=302 path=[1:0-301]

GTCATGTCTTACCTATTTGCACAGGTATGATTTGCTTGATAATTTCTTTAAAATGTAGTC

TGATAAAGGTTGTTGATATTTCTCCCTTTCACTTTTAATCGTAGTTATGCAAGTTCTTTT

GACACCTTGAAGCTTTTAAACAGAAAAATTAAAGCTTGTGCTACATCATATTTCTGCAGA

GGTGAAGTTAAATGGCGGTTGTAGGGAGAACGGGGAGAGCAGGGAGAGTGTGCAGTTCCC

ATCTAGTGCCACCATGAGAAAATCTTTCACTGTGACTTTCATTTTTGAACTGTTGCAGTG

TG

>c16362_g1_i1 len=207 path=[185:0-206]

AAGAAATAAGCTAATAAGTATAAAAAAAAAAATGAAAAAGAAATAAATAAATTTAAAAAT

AAAATAGATGGTAATAATCAAATTGAAAAAAAATATAGAAAATATCAAAAAACAGAAGAT

AATGATGGTAGCATTTTTAGAACAAATAAATTTACAAAAGAAAACCATGATAAAAAATTA

GAGAAAAAAAAAGAAATTAATATTTTT

>c16369_g1_i1 len=711 path=[1:0-356 358:357-710]

TAACTTTGTGTTTGAGTCATGTAGGACGGTAGGTTTATATTAAACTACTTTGCTCTCAGT

CTGGTTTTCTCTACGTTATGCACTTCAATTGAATTAAGTATATTTAATTACAAATATTGG

CAAAAATTAAAACCCTTAACTGTACCATTTTGCATATGTGTTAATAATACTATTGAAAGC

CATTACAGGTGGCTGGCTTAACTCAGTGATAGACAATGCTTTAATTTCTTCTAGGGGAGG

GGGAAAATAATGGTTCATCTGTTGTTATTAGTTTGGACACAGTGCACAAAAACATGAATG

TTAAGTTTGCATGATTTAAGCATGAGTATAGAACAATAAAGAAACACTTTATGCTGAATT

GTTTTTAAATGGTCATGGCTCATCTATTCAATTTTTGTAGTGGAATGACTCTTGTGATTA

GAGATATGCTCAGTATTAACATATTAATAACATTTGGCAGTTTTTCTTGAATTTAATTTG

TTTACACTTCTGTACTTAGCATCAGCTACAATTCCAGCATTTTTTTTTTCAAGCAAGACT

GTATTCAACACAATATTTGGTAATATATCATTGCAGAATCAGTTCCAACTAAGTTCATGA

CCTATGGTGACTAGAATTGAAAACTTAAGTTTTAATGGTCAGGAATCATTCATATGCTCA

CTGACTTTGTCAGCTAATGTTTTTGTTCCATTGCTTTTAAGTAAATAAATA

>c16378_g1_i1 len=220 path=[297:0-219]

AGGGATTTAATTTACAGCCTGACAGTGGGATGTGAACTGTATGGGTGATTGAATCATCCA

GAAGATGTCTGAGGTGGTAGAGGAAATGGAGGGAACAGAATTCATTGGAAGTGTTGCCAG

CAGGAGTGGAGTGTGCACACAGAGATCGATGCATTTCGGTGGGAGCAGCGTGAGCTCAGA

GCCAGGAGCGAGGGCATTCAATTAGAAATGCAGGTAAGCA

>c16379_g1_i1 len=368 path=[346:0-153 500:154-367]

GCTGCGGAGCCATCGGGGGTCCAGGGGGTGTCGTGGGGGGGGGCGTTGCGGCGGATGACG

GCCAATCGGCGCTGCCGGGGGACGCGGAGGGCCCCGAAAAAGGACTCCTCCCCCCCCAAA

ACCTCGTGGGTGAACCCATAGCGCGCCTCACCCCTTGGTGCTGTCGATGTGGGGCCGCAC

GCAGCCGTGGGGCGCGAGGTCCAGGATGTGGCTGTGGGGCAGTGGGGGCCGCGCGGGGGG

AAACGCGGAGGAGATCCGCTGCAGCACCGCCCCCGACAGCGCCCCCCAACGGCTGCGCTC

CGCCTCCCGGAACCCGGAAATGGCCCCATCCCAGTGCCCGTCCTCGTAGCGCCGCCTCCG

CAGCGCCT

>c16379_g1_i2 len=289 path=[346:0-153 714:154-288]

GCTGCGGAGCCATCGGGGGTCCAGGGGGTGTCGTGGGGGGGGGCGTTGCGGCGGATGACG

GCCAATCGGCGCTGCCGGGGGACGCGGAGGGCCCCGAAAAAGGACTCCTCCCCCCCCAAA

ACCTCGTGGGTGAACCCATAGCGCGCCTCACCCCGCAGGACGTAAAGGGAGAGCGGCTCC

AGCAGCAGCTCTGCCCACTCCTGGGGGTCCCCAACGCTGCGCAGCCGCATCACCGCCGCC

GACAGCAGAGAGAGCCCCGCGATGGTGCAGCCACAGAACTTGGTGCTGT

>c16390_g1_i1 len=314 path=[1390:0-313]

ACCACCCACTTCCTCACTGTACTCACATCTACTGTTTGGTCTTCATAATTTTTTCACATG

AAGGAATTCAGTGATGCACCTTTGTTTCTTACGGACTTCAGTGCGAGAAGCTGTTTTGTC

AGACTGTCCCACTGCTGCTCTCTACTACATGGCAACAAAATGTAATGAAAGATTGGTGGG

AAAACTTAATCCATACTACCATACCACCAACGTTCACCTCTGACATTGTGATCAGACATA

ATAACATAGGATACATTACATTCAGAGCAGGCCTGGAAAAAATATTAAAATAGAACATTC

TGTACTTGCTTAAA

>c16390_g2_i1 len=481 path=[537:0-480]

AACGTGGATTTGAAGATGTGTTTTAGGTTGAATTACTTACATATCTCTCGCTATGAAAAC

ACAGCTTTGTAACCTTAACTGAGTCTGCTTGATTTTTGGCATTACTTTCTGTTGTATTGT

TTCACAGATGTTTTCATAGTTATATTTGGGAAACATTGTCTTTTTAAAGTGAGATTACAG

AATAAACAATAGAGTTTTCTTTGGCAAATACTTAGGTTGCTCCAAAAGTAATGCTTCCTA

TATATGTCCATGGAAACTCCAACAGACGCAAAGAGCGCAGTAACACTGTTTGATAGAGCT

AATTCTCAGCTACAAAACACTGCCACCACAGTGTAGTGTAGTTGCCACCCCAGGCTGATT

TGCGTGGATGAGCTGATTGAGATGTTCTTCATTTTGTGATGTGACAGCTGTGCATTGTTG

TCTGGAATGTGGCTTCTCATTTGCTTCTGTGTCACCACTGCTGAAATGTACCACCCACTT

C

>c16393_g1_i1 len=201 path=[1:0-200]

GAGCTGCTGTACCTCCATGTGAACATCTGGACGGTCTTTGAATAGGCCTGGATGGGGCCA

GTCAGCCTTGCCTTGGTTTTCCCTCAGCTAGCATCTGTCCCATACTGGCACCCAATTGCC

TTGTTGGCATGTGCTATCCAAAATCGTGCCATCTGATGAAATAAGCTATATTTAGCAAAA

GTAAATGAAGCTTATTTCATC

>c16398_g1_i1 len=324 path=[302:0-323]

ATTTTATTTTTTTTTTTTTTTGTTTATGACAAGAATAATTCTTTATAGCTCCTTGTCTCC

TTATAAATTTTGTTTTTAAAAATTAATAAAATAAAATTGCAATATATAATTTAAATAAAA

GCAATATGAAAAATAATCATATGTATTTTTTTATCAAGAAAAATCTAAAGAAAATACTCA

CATTAATTATATATATTTCATCTAATAGAAATTAAATTGATGTTTACATTGTGATTTTTA

GATAATTCTTGAGTATTATCTCAGAACTTTAAGAGATCATTTAAAGATATTATAAAGAAT

GTTTGTGAAAAAAATCAAAAAAAA

>c16411_g1_i1 len=232 path=[210:0-231]

GCCTTTGGGGGCAGTGAAACTACAGCACTCCGTGATCTGAATGAATTGGAAGTGGGATGT

TGTTTGTGAAAACGCATATGTTCAGAGAGCTCAGGTGGTGTACAGGAGCATGACTTGTCC

AGGTTCACAGCCCCTCAGCCTGGGGGTCTTTGTTCTGTAGAGGGTTGCCTTTCTTCCTAA

AAGATGCCGTGGAAGGCAGTAAGAGCCAAAATGTTCTTCAACTGAAATGTGT

>c16413_g1_i1 len=204 path=[177:0-203]

AATCCCTTTCTAAAACCTACTAAGCTATTTGACTCGCTGTCCTGTTCTTGTGAATAAGTT

AAAGCCTTCCCTGCTGTAACTGGAAACCAACTATTGTGCGATAGGCTATTAGCTGTATGC

ATGCTGAAAACCAGGGGCTGCTTGTTTGTCAAATACCAGTGCAGGCTAAAAGCGGTCTGT

TTGCTCAGCAGAGATCTGGAAACG

>c16418_g1_i1 len=205 path=[255:0-204]

AAGGAAGACTTCAGAAAAGCCAGGCCCTGTCCCTGTCCCCTTCTCTGTAGGAAAAAACAC

AATCCTACCCCCTTGCTAAGAGGGTTTTGATGAGAGGCTTTCCAGGGTGAAGTGGGTGGA

TCAGTATTCACTGCAACACTGAGCTGTACTGGTCTGAAGCCATCAGCTTGCCCTTGGCAC

AAGGAACCCTATTGCAGGGGAGCAG

>c16421_g1_i1 len=375 path=[633:0-374]

ATCAGTTAAAATGGCAACCTTATCATCAGCTGCCATGGCTTACCACAAGCATCCAATACT

TGGTGCACTCTCAAGGAACCAGGGCTATCCTGAGTCCCGTCAGGAAATGGTTTCTAGGAG

CTCACATTTTAATTTATAGTAGAGCTTCTTGGCTGGTTTCTTTCCTGAACCAGGGCTGTT

CTCTGAAATCAGGAATTTCCAGTTGGCACCAAATATCCATCTGTCCTTCAGCAGCTTTTC

ACCAGCTCATTAATAAATTCATTGGATTCCTCCCCAGCAATGTTTCTTTTCCAAACCTGG

AAACTAAATTGTTGACAGTGAAGAAAAACAACATATGTTTAGTCCTCAACAGAATGTTAA

TTTAGGTTTAAAGAA

>c16422_g1_i1 len=214 path=[181:0-213]

TAAGGCTCTGTAACAAGCTCAGTATGCACAAGTTATTGGAATAACTTACAGGGCAGAAGG

ATGAGCAGGTGAAACAATGACAGTGGAACAGTGAAATTGCTTTGGGAATAAAACCACATT

CTCTTGCTTGATGAAGAGTCTCATCAATGCCAATGATAAGAGGTTCAAACTTCCAACACT

GAGATTTTCAAGAGGACAGTCCTGGATTTAATAA

>c16423_g1_i1 len=224 path=[1:0-223]

GGTTGCGTGGCAGTTTCAGCCACTCCATTAGTTTGGCAATTTGAGAGGTTCTTCTCATTT

GGTTGGGGTTTTTCACTTTCCTGCACTTTTGCCTTCTTAATGCCTTTCATGTTATGAGGC

TTTTCTATACAGGCTGTTTATAAGAATGCACCTCTTATTTTATGTCTGCTTCTTTCACAG

GCAGTTTTGCTCATCAATCTAGCAGCAAACAAGCACAGGAGATG

>c16423_g2_i1 len=202 path=[582:0-201]

GTTCTTTGTGGACAGCCACATGCTCTGGCTTCTTTCTCTTGGAGCTGAGGAATTTCATGT

GCCCACAGAGCACTGCCAATGCAGGATGGTAAGAACTGGACTCAAGGGCAATGGGCTTTG

ATGCTTCCATCCGGCTACATGCACGGCATTGCCTTGAAAACTTGGGCTTGGGCTTTGAGA

CGATAGAGATTGCAAGCTGTGG

>c16426_g1_i1 len=213 path=[1:0-160 162:161-212]

AAGCTGCTTTTAACCTACTGTAGTGCCTGATTTTGAACAAATAAAGGAAGTGAGTCTTTT

TATTGACTTAAGGGGTAGTTTAGACTCCAGGCTTTGAATCCTGTATAGGGCCTTTTTAAG

ATATTTACTACTAGCTGTCATTTATTGCAAAATACCAGAGTATGAGTCTTTATATGGTGT

CCTCAAAGGTTCAAGGCTCTGTGTGAAGCAATG

>c16427_g1_i1 len=237 path=[1:0-236]

TGAAGACACTTTGTTATAGCAACCGCTTTAAACTGGAAAGCTTAGAAAGCCTACCAGTAT

AGGCTGGGAGGATCGAGACCCATTTAATGTTCTCTGTTATTAAGCACTGAATATTTACTG

TCATTGTGGTTTTTTACAAGTTTGTCTGCAAGTGTGATTGTTGTTTTAATAGTGCTTGTT

AGCAATAATTCTGTCTTTTTTTTTTTCAATGTGTGCAGATAAAATGCAGAATTTGAA

>c16430_g1_i1 len=270 path=[248:0-269]

AACTTACAGCAACCCTTATAACAGAATTTCTGAATATATGGTACAGACAAGCAACTCAAA

AAACCCAACAAAGTTCAGTTTAGACCCCAAGCTAACTTTCTCAACCCATACAATAAATTC

TTGAGCATGTTACCCCCAAATGAAAACAACCTGCATGCAAATGACAACATATGTAACATT

CTTTGGAATAGGGTTTAAATTTCTCTCATTCCACGCCGGGCACAGCAGAGAGACGATTCC

CACCGCCACGTGGAGCTGCTGGTGTGTTGT

>c16438_g1_i1 len=293 path=[271:0-183 455:184-292]

TGTTAGAGAAAGAATTGAAAAACTGGTGTTCCACTTTCTAATTTTCAAGAACAGAAGGTT

TTTGGTTTTTATTTTCTTCTTAGTTAAATACCAGCTAGCTAAAACCAGTTTGAAATCAGT

TTTTCGTTTTGCCTAACGTACAATAAGCAAAACTGCATGAAAGAATGTATCTTGTAGGAA

AGAAACAGCACAGAATTGGGTCCTACTCATGTAAATGCTATTTATACAGCATTCTTGTAC

CCACACAAAGCCCACACTGAGTACCACTGAAGTCACCGGGATCCTGCTTTTCT

>c16441_g1_i1 len=260 path=[155:0-259]

GATGCTTACTCATTTTCAGGCACTGTAAAGATGTTCCATAGTATAACGTTGTCATTCTGT

CAGTATTATAGTAATAGGATGCTACTCTGTGATCTCAGCTTTGTGAGAGGGCTTTGGAAC

CCAAATCAAATCTGTCTCCAGATAGATGTGGAGAGGAATTAGATTCCAGGAGCAGAAATG

CCCATCTTCTGTAAGATGTATTAAAATGCAGCAGATGTGTGCAGCAGTATGTCTGTAAGC

GAGAAAGTTTGACTAAGAAA

>c16442_g1_i1 len=555 path=[910:0-192 378:193-554]

CACTTGACTAAAGCAACTTTTGCTGCAACCCAACACAAAAAAAGAGGTATCAGAATAAAT

GAAAGCTTTCCATGAGTCACTTTCATATGAACATACACTCTTAATGCTACAGATGAGAAA

ACTCCAAGGTGATTGAGCCTGTAGTGATAGCAAACCCTTACACAAAACAGCATCAGCTGT

TACATGAAGGCCACCTCCCACCACCTGAGCTGCTGCAGCCCCTCCTCCCAAATGCCAGAA

TCATTACCACCACCTGAACACCCTCACTAAGGATTTGGGATCAATGGATCTCTCTGTGCT

CAACTATTTTCTTTGTCTCATAAGAAGCACTGACTTCAAGCACACAGTTGGTGAGGCCCC

CATGAAGTTCTCTGCAGTTTGCTGAATAAGACTGTGCACATTTTGAAACAAATTAGAAAC

TTGGTCTGTTTGATTTGTCACTGAGAGAGGCTCCAAAACATTTTAGATTTTAATGCTCTT

CTCTCATGTACTCGGAAATCTTCAAGCTTAAGCTGTGGAACATACAGTTACCTTTGTGAG

TATGAACTCTCTGCA

>c16442_g1_i2 len=381 path=[359:0-18 378:19-380]

GTAATTCCACAGTCTGCTCCCTCCCACCACCTGAGCTGCTGCAGCCCCTCCTCCCAAATG

CCAGAATCATTACCACCACCTGAACACCCTCACTAAGGATTTGGGATCAATGGATCTCTC

TGTGCTCAACTATTTTCTTTGTCTCATAAGAAGCACTGACTTCAAGCACACAGTTGGTGA

GGCCCCCATGAAGTTCTCTGCAGTTTGCTGAATAAGACTGTGCACATTTTGAAACAAATT

AGAAACTTGGTCTGTTTGATTTGTCACTGAGAGAGGCTCCAAAACATTTTAGATTTTAAT

GCTCTTCTCTCATGTACTCGGAAATCTTCAAGCTTAAGCTGTGGAACATACAGTTACCTT

TGTGAGTATGAACTCTCTGCA

>c16446_g1_i1 len=292 path=[526:0-291]

AAGCTATTTGGGAGGACAACGGATAGCTAAAGTTTTGATTTTAGACAAGCGTAACATTAA

AACTATGAAAGAGGTTCCTAGAAAAACAGGGCACAAACAGAAATAGATCTTAAAGTAAAG

CAAGTGAAAGCTGATATTCACATACTCAGGTGTGTTGGGGGGAACAATTTAAAATGGAGT

ACGTTCCTTATTTGATAGGATTGATGATAAATGCTTTGTGACAATTTAGAAAAGCACTTT

AATTCTGTAATGTATTTGCTCCCTCTCTAAGGAACCTTGGTCATCGTTTCTA

>c16448_g1_i1 len=240 path=[155:0-239]

ATATGAAACTATAAAACTGGTCAACCACGCTCTGAATGGTCTGTGGATGTGATAGGACTG

ACATGCTACTCTACTTGTGAAAGTGTTCTCTTGAATTTTTGTAATCTTGTCTGTGTTCAA

TTTCCTCAGACACTTTCATGACAAAACAAATGAAAACTAAGTTATTGCTACTCCTCTGAT

TATTACTGAACTTCTCTCAAATTTCTACCTAAAAGGGGTACTACTAGCACAAGCTAAGAT

>c16450_g1_i1 len=413 path=[903:0-155 15:156-183 43:184-412]

GGTGTAGTTTCCTCCTTCGGATCCAAGGAGCATTTCTGGTGTGGAGCGGGGAATAGAAGG

TGTCCCCATGGCAGCGTCAGTGCAGTGACCGCGTCCCGTTGTCCCCTTTGTAGTCCCTTC

AGAAGCAGCCCTGCAGGAGCTGTGCTTCGGCTCACGCAGGGCAGAGCCCGGCCTCGCGTG

CCTTCCGTCCTCACTGCACACGAGGCTAAAAGGAGTTTAACTGTAAAAAGAAAAGACAGC

CTTGAGCTGTGCAGAGAATAGGGCATCCCGGCCCCTCCAGGAGCAAAGAGCTGCTGTTGG

AAAGCGTGAAGCGGGGCAAAGGGTGGCCACGGCGGTGGGGAGAGCTTTTGCTGACTTGAT

GGATTTAATTAACCAAAAATAATTAAAAGGTTCAGTGGGAGCTCAAAAAAAAA

>c16450_g1_i2 len=356 path=[651:0-126 43:127-355]

TGGATCCGAAGGAGGAAACTACACCCGGTTGGGTCTCCTCCTCTCTGAAGGACCCTCCCA

GGCTCTGAAGGACGGCAGTGTTCCTGCAGTGACACCTCACAGCCTCCTTATGCCTCTGGC

TTTGCTGCCGTCCTCACTGCACACGAGGCTAAAAGGAGTTTAACTGTAAAAAGAAAAGAC

AGCCTTGAGCTGTGCAGAGAATAGGGCATCCCGGCCCCTCCAGGAGCAAAGAGCTGCTGT

TGGAAAGCGTGAAGCGGGGCAAAGGGTGGCCACGGCGGTGGGGAGAGCTTTTGCTGACTT

GATGGATTTAATTAACCAAAAATAATTAAAAGGTTCAGTGGGAGCTCAAAAAAAAA

>c16451_g1_i1 len=241 path=[1:0-240]

GTGTTTGATCACCTGGTACCTGGTCTCTGTTGATAGTTATAGATGATTTTATGACTAAGT

TTGAAAGTATTACACGTAGGAGAGAATAGAAAAGTTGTGGACTTTACAACAGAAGCTCAA

GATGTTGTTTGTCTAGTGATAGGAGACTGTTTTTGCCAAAATTATACATTTTCTTTTGCC

AAAATGGGGATGTCAAACTCAGATTATTTGGCTATTGAAAGGTACCAGCTGTTACCAAAT

G

>c16452_g1_i1 len=234 path=[212:0-233]

AATAAAACCTGAATCTTTTCACCTATTTATACGAGGATTATATTAAATACACACTATCAG

AATTAAGTGAGGAACTCTATAATTCTGTCCACAGTGAAAACCCTGAATAAAACTATTTTT

CAAAAGGCATGTATGTGGTTTTTGAACTGTATCATTTCACGTCTCCTGTCAGAGTGAACA

TATGTATTTCTCACACTGTGTGTGTATACAGACATACATACACACACACACACA

>c16454_g1_i1 len=530 path=[1:0-529]

ATCAAGAAGCTGACCTACGTGCACGTGGGGCTGTGGCTGCTGCTGCTGGCACAGATGTGC

GTGGGGCACCTCAAGCTGCTGCCCCACGACCGCGTGGCCATGCCCTACCAGTGGGAGTAC

CCCTACCTGCTCAGCATCCTGCCCTCGCTACTGGGCCTGCTCTCCTTCCCCCGCAACAAC

ATCAGCTACCTGGTGCTGTCCATGATCAGCGCCGGGCTCTTCTCTGTGGCGCCGCTCATC

TACGGGGCCATGGAGATGTTCCCTATGGCTCAGCAGCTCTATCGGCACGGCAAGGCGTAC

CGCTTCATCTTCGGCTTCTCCGCCGTCTCTGTCATGTACCTGGTGGTGGTGGTGGCCGCC

CAGGTGCACGGCTGGCAGCTCTACTACAGCAAGAAGCTGCTGGACTCCTGGTTCACCAGC

ATGCAGGAGAAGAAGAAGAAATGAGGGGGGTTGGGCGGAGGCTCCGCGCGCCACGCGTGG

AGCGTTCGGGGGCCGCTGGCGGTGCTGCAGGGCGGCTGCTGCGGGTGCGG

>c16458_g1_i1 len=207 path=[613:0-206]

AAACAAATAAAAAACCCCTATGCTGTTAAAAACATACTTTCTATGCAAAGCATTTATTAC

ACAATGAGAGGCCTGGACAAATAATTTCCATAATAAGATCTCCCCCCCCCGAGAAGATTC

TGAATATTCAAACTGAAAGAGCAAATATTTTCCTGCAGAAGGTTGCATGAAGACCCTGAC

TGTCATCAAACAAGCACTGCTAACATA

>c16458_g2_i1 len=237 path=[215:0-236]

AATACCCAAAACCACCAGCCTAATTCTACAGTATTTGTTGCTTTAATTATTCTGACATCG

CTGAAATAATGTATTTCAGGAGGGGATGCTGAAGAGAAATATATCCCGATGAACAAATAC

TCTAATGTCCAAAAACCGTTTGCCTTTTACACAAATCTGAGAACTCTGGAAGGTTTATAT

TGCTTATTCGATTAGATGGATCACAATGTCACCAGACTACTACTGTGGAAAAACAAA

>c16460_g1_i1 len=493 path=[1:0-492]

ATCCACAGTCTTTATCTTGGGGCTCTGCAGCTTTGCACCTTGGAAAATGAGATATTTAAC

TTTACACAGGTGAAGGCAATTTAACAAGCACCATTTAATACACATCACTTTTAAAGCACA

GCTATTTTAGTCAATTAATAACACTGTGCTTTAATAACTGTACCATGGTTTTGCAGTCGT

TATCAGATGACATATCCTTATGAGACTAGAACCATAGCTTCTGAAAATTGCTGTCCTCTT

GCTAAGGCCAAAGAATGGTTAGGAACTTACTGCAAGTACATGTTGTGGTACACAGCCCAT

GGAACTCAGGAGGAGGCAGGCAGAAGGTAGGTGAAATGCCCTCTGGAAATGCTTATTTTC

CTTCCCAGGAGTATCTGGGCTATTAATATAGACCAGACATTGAGTTTTGAGGCAATGAAT

ATCAAAGGAGGTAAGTCTCTCCCAGAGGGATTAATGGTCTCTGGTGGCCCTGAGCCATAA

ACTTGTAATCCTG

>c16461_g1_i1 len=206 path=[184:0-205]

GCTGAGCTGCATACAGTTGCTGGATGCACTGCTTGCATGAGCTCGCCAGCTGCTCTCACC

CTCACCCTCCACCTCAGGAATGGGGAGAAAATAAGAAGTTGATTTTCACAGACGAAGACA

GGAAGGTCATTAACCAGACACCCACACTGGCAAAACAGACTCAACTTAGGGAAAAATTAT

TTAATTCAGTGTCAATGACAATGGAG

>c16462_g1_i1 len=1373 path=[1942:0-1181 3865:1182-1192 438:1193-1215 461:1216-1372]

TAATGGTGAAAATGAGTAAAGAGAAAGAAAAATGTTTTATGAATAATTTAATGTGGTATA

AAAAAATATTTAATAAAAAATATGTACGGAAGTAAAAATAATAAAATTTATATTTAAAAA

AAAAATAAAAAACATACAAATATCAATGGTAAATCTAATTAAAACTTAATAAAAATATTT

AAATAATTTTATTCATTTTATATATACATTAAATTACACAATTTCTTGTTTAAAATTACG

ATTATGTATTTTATGTGTGTAAATTTTTAAAGATCTTCTGGAATATTTGCAATAATATCT

TTTAAGTATAATGATAATCTATAACCTGCTAGAACCATTTGTTTGTTTAATATTCCTTTT

AAATTAATAACAAAATGTTGGTCAACCTTAATTTGCATATCTTTATTCACTAGTCTAACA

GGTATGTGCGAATAAATATGCTCAATAGCTAATTGATGGGATTCATTTGCAATACTATCT

ATAAAATCAATTTTATTAGATGGAATTGTTAATTTACTACCAAAATGTTCAGGGGGATAC

ATATTCATCAATACATGTGCTTCCTTTTGCAAAATACTAGGATTTATATGAGGCCAGTGT

TTTTTTCTTGAGTAGAAAATGTTATCACATAAATGATGTAGCTTTCCTATTTGTCCATGG

TAATTTAAAGTAATGTCTGATCCTCCTTTATCTCCATTAGGGAAATGCTCATTATAAAAT

GATATTGTATGTAATGGCTGATGGATATCAGCAAATATGTGTATAAAAAATCTTAGGTAA

AAATTGTAAGAAAAATAAGTACCATGTCTAGGTTTTTTCTGAACTTGTATTAAAATCTTA

TAAATATATTTTAATACTCCCATAGCATTATATTTTCCTTTATGAGCATTTAAATAATAA

TGAGGAAGGAACATTTTCGTAGGATTATATGGAATCTGTACAAAATGCCATTCATTAAAT

ATATCTAATATCTCATATCTTCTAATATCACTTGGGTTAACAGTGCTATGCTCATTTGAT

TTTATATGATCAGCCCATAATGCACCAGTTATAGGATCATTAAAATCTCTATCTTCTTTA

TAATTCAAAAAAATTTTATTTAAAATCCTCTTTTCTTTATCATTTAAATTATAATATGCT

ATTGCACTAACAATCATATGTGGTTCATCTGACCAACAAATATTTTTTTTAATTATTCCT

TCTCACAATTTGTCTATTTATATTCGTTTATATTTTAAGATTTAAATTTTTAGACTTTAA

ATAAGTTGTATGTTTAAGATATATATACATTAGTATTTTAAAATTTTCAAATATTGTATG

TGTTTAAATGATTGAAACTTTTTTATTAATAATAAGTACACTTTTAAGTTTAT

>c16462_g1_i2 len=1373 path=[1942:0-1181 3865:1182-1192 3134:1193-1372]

TAATGGTGAAAATGAGTAAAGAGAAAGAAAAATGTTTTATGAATAATTTAATGTGGTATA

AAAAAATATTTAATAAAAAATATGTACGGAAGTAAAAATAATAAAATTTATATTTAAAAA

AAAAATAAAAAACATACAAATATCAATGGTAAATCTAATTAAAACTTAATAAAAATATTT

AAATAATTTTATTCATTTTATATATACATTAAATTACACAATTTCTTGTTTAAAATTACG

ATTATGTATTTTATGTGTGTAAATTTTTAAAGATCTTCTGGAATATTTGCAATAATATCT

TTTAAGTATAATGATAATCTATAACCTGCTAGAACCATTTGTTTGTTTAATATTCCTTTT

AAATTAATAACAAAATGTTGGTCAACCTTAATTTGCATATCTTTATTCACTAGTCTAACA

GGTATGTGCGAATAAATATGCTCAATAGCTAATTGATGGGATTCATTTGCAATACTATCT

ATAAAATCAATTTTATTAGATGGAATTGTTAATTTACTACCAAAATGTTCAGGGGGATAC

ATATTCATCAATACATGTGCTTCCTTTTGCAAAATACTAGGATTTATATGAGGCCAGTGT

TTTTTTCTTGAGTAGAAAATGTTATCACATAAATGATGTAGCTTTCCTATTTGTCCATGG

TAATTTAAAGTAATGTCTGATCCTCCTTTATCTCCATTAGGGAAATGCTCATTATAAAAT

GATATTGTATGTAATGGCTGATGGATATCAGCAAATATGTGTATAAAAAATCTTAGGTAA

AAATTGTAAGAAAAATAAGTACCATGTCTAGGTTTTTTCTGAACTTGTATTAAAATCTTA

TAAATATATTTTAATACTCCCATAGCATTATATTTTCCTTTATGAGCATTTAAATAATAA

TGAGGAAGGAACATTTTCGTAGGATTATATGGAATCTGTACAAAATGCCATTCATTAAAT

ATATCTAATATCTCATATCTTCTAATATCACTTGGGTTAACAGTGCTATGCTCATTTGAT

TTTATATGATCAGCCCATAATGCACCAGTTATAGGATCATTAAAATCTCTATCTTCTTTA

TAATTCAAAAAAATTTTATTTAAAATCCTCTTTTCTTTATCATTTAAATTATAATATGCT

ATTGCACTAACAATCATATGTGGTTCATCTGACCAACAAATATTTTTTTTAATAAAAATA

ATAAAAAAAAGTAATAATAATGTTAACTTGATCATAATTCAAATTAAAAAAATTAAATAA

AAAATATTAATAAGAAAGTGGGTATATTTTCTTACATTAAGAACCTAAATTAATAATAAA

AAAACAAATTATAAAGCTCAAGGATACGATAAACTTAAAAGTGTACTTATTAT

>c16463_g1_i1 len=302 path=[1:0-144 146:145-301]

TTGATTTTTTTTTTTTTAGTTTTTGTTATGGGCTAAAGTAAACACAGTATGGAGGTTCAG

GTATTCCAATATCTAGTGAGGAAAAGTAAGATTATGGTAGTTGTGTTGGTTTTTTTTAGT

TATTTTTTCCCTGGTGAGGAGATCTAATGAACAGTAATGTTGAAACGCAGAAGCAGCAGA

CAGATTCTGGCTGTTACACAGAAAGGCAATAAAATATCATGTGATCATAAAACCTAATTC

TGAATAAGAAGTTGTGGACCTTAACTTTCCTTAGTCCCATGTGTGTCCTTGAAAACCAGT

CG

>c16463_g1_i2 len=256 path=[658:0-98 146:99-255]

GTAAGGGATCAAGCGCTCTGAAACACATAATTTGGTGTATTCAGGACATCCTCCCAACGC

ACACGTTCTGAACTCCATAATTTCACTAATACCAAATGGAATGAACAGTAATGTTGAAAC

GCAGAAGCAGCAGACAGATTCTGGCTGTTACACAGAAAGGCAATAAAATATCATGTGATC

ATAAAACCTAATTCTGAATAAGAAGTTGTGGACCTTAACTTTCCTTAGTCCCATGTGTGT

CCTTGAAAACCAGTCG

>c16464_g1_i1 len=317 path=[1:0-261 263:262-316]

TTATCTTCCTTTTGCTCCCCACGAAGAAAGCACGACCAACACTTCCATAATTTTATAGAA

CTTGACATTTACTCAGTATTTAAAGGTCAGGATGTGAAAGATGCTATAAGTGCAGAATTA

AATGCCTTTAACAGATGAATGTGGAGGCTTTCTTCAAAGTCACTCCATAATAAGTTTAAT

TTAAGGCAGCTATTTCTGGCACACTGCAGCCAGTCGCCCACACAGCTGTGAGCTCCAGAT

GTGTTCTCAGGTTCCCCTTTTGAAAGATTACATAGCCTTTTAAAGTACTAACTATTGCCA

GCATAGCGTGTTCTGGG

>c16471_g1_i1 len=431 path=[409:0-302 855:303-430]

AAGGCTCTCAGCCTCAGCTCTGCCTCCTCCTCCACGGCTCCGTGACACCATGAGCGCCTG

GCGGCTGTGGGCCGCCGCTCTGCTGCTCCAGGTGTCGGCAGCGGAGGCACTGGCAGAGCC

GGAGCTGTGCTACGTCCTCGATGGGATCCTCTTCCTCTACGGCATCGTCCTCACCATCCT

CTACTGTCGCCTCAAGTTCATGACCCGCAGAGCACTGCAGGAGAAGGCCAAGAAGCCAGA

AGAAGGCATCTACACAGGGCTCAGCACTGAGCACCAGGAGACGTACGAAATGCTGCAGCA

CAAAGCCTGAGTTCGGACCCCCCCCCACCACCACCGCTGCGCCCCCTCCCCAAATTACTG

CTTTTGGGGCTACGATGCAGAATTGGAGCTTTTCCCATCCAATAAATGGAGGTTTTCAGC

CCCACGTGGGT

>c16477_g1_i1 len=215 path=[193:0-214]

TGGAGGAGGAGAACGAGGACCTGCGCCAGCGACTGATCGAGACGGAGCTGGCCAAGCAGG

TGGTACAGAATGAGATGGACAAGCTCCGAGAGAATTCCCTGAAGAAAAGGGGGTCTCGCT

CCATTGGGAAGACCGAGAAGAAGCCATCAGTGCAGGAGGACAGTGCAGACCTGAAGTGCC

AGCTGCACTTTGCCAAGGAGGAGTCGGCCCTGATG

>c16482_g1_i1 len=313 path=[277:0-198 18:199-306 495:307-312]

TCCTACTGTTCCACTGCCTCTGTCCTGGAATTTTATGTCCCTGTAGCTAGGAGGATGGAG

GAAGGAAAGAAGTACAGTGATTAACATACAGGATAAAACACTGTTCTTTGAAGGCATAGT

TGCTAGTCTGTGGGGAAGCTGCAGTGTGCAGTCAAGGACAAGTACATTTGGTTGAACAAG

GGAGAATTGTAGGATGGGGAAAGAGGCAACAATGAGGGCTTTCTTTAGAGATTTGGACCA

AAACCATTTTGTTGGGCTGTGTTCAAGCCTATAATTAGAAAGTTTAAAGTTCTACTCTAC

ATGAATAAATAAA

>c16485_g1_i1 len=3138 path=[3139:0-1709 4849:1710-3137]

TTTTTTTTTTTCTTTTTTTTGAAAAATTAATAAATAGTGATAAAAAGAATTAAATTTTAT

TACATATAAATTTTTGACTAAAGAATGTATGAGCATTATAATATTATATATTTGTAAATA

TCGAACATTTAAATTTTAGTAATAGTAAATAAGGATATTTACTAAATTTATGAACATTAT

AAATTTTTGCTGCATTAAAAATAATAAAACATTTATTTAGCATAACAATATTTAAAAGAT

ATAATTTTTATATAAAATAACTCGTTCGTTTTATTTTTTTTAATAGAAGAGTTATATAAT

AGTTATACAATTATATTTAATAAATAAATTAAATATACAGTACTCCACATCTTCTATATT

AATTTTATTGCCTAGTGTATAACAATTTTTTTTTAACAGCATATTAAACCAAATAATAAT

AACCACATCAATCAAACCTTTACGGTTATAATCTATTGGGTTAGCAAATTCAATTATGGA

AGAAAAATAACAAATCTTAAATTAAACATGTTGGGTGGCATAATAAACTTTGACAAGTAA

TTTGAGTTTATAGTGAAAAAAGGTAATTATTTTACAAAAATAAAGATATCAGGAGGAATT

CCAAATTTCAATTAGAGATCTTTAAAATAGTACTTCAAATTGCTAATTATTTTCTAAAGA

AAAAAATCAAGAATTATTCAAAGCAGATACTTATATAATATTAGAATTGTTATTATCTAA

TAATTATATGCAATGAATAACTGATTTATCTTTTGGAAGAAATATAACCTACACCATTCA

CAAATTCAATTAGCTGAACATATACTTCGCTATGCAAATAAAACAATTATTCAGATAAGA

ATAAAATTTAACACAAAAATTATGTTTATCATGTAAATTAATGTGAACTTATTTATGACA

GATAGTTCTATATATACTTATAACAGTTTGTGCTTTAACTAATATTATGATCACACTACA

ACATTTCTCGTATTATATTTTGATGTGATGATCCTGGAAAAATATCTAACATAAGAGGAT

AAAAAAACTCCAATAAATAAATAAATTTAAGGCTTTTATGATATCTATGTTAAAGTTCGA

AAATAAATATATTATAAGATATTACAAACAAAATATGTGCATTTATGTTATAGATCAAGT

TGTTAAAAGGGATAGCCTTGGCAATAATATTAACTAATAAATGTTATTATTGCAGTGAAA

AACATATAAAAAGGTACTATATGCATCATAAAAATATATGAAGTCATTTATACATGATTT

ATTATTTATTATTTATTATTTATTAAAATTAAACATTACTACATGATGCAACAACAGCTG

CTGTAATAGCTGCACCTTTTCCTGATCCATCATCTGCAGGAATAATAATCAAATTCTCAG

ATTTATCGGCTAAAATTACTTTCAAATGTTCTTGTAATCTTTTACAATACCAAGCATTTT

TAACAAATAATGATCCATCAACTCCACATGAAAATTTAGAATGTTCTATAATTTTTATTC

TTTTTGCTATGGCAGCAATTGCACCAGCAGCTAAACCAGCTGAGCGATTATAAACAGCTT

CGCAAATTTTTCTTAGAGCATATACTTGTTCATCGGTTAAATCAATATCCCATGCATCTT

TTGCAACTTTTCTTGTTTCTTCAAAATTTTTTGAAGTATCGTTCAATACAACACTACCTG

ATTCAGAATTAAATGTATCAGTTATCCACATTTTTTTTGAAGATGCACTTTGCAAAACAT

TAACCATGTATCTCCTAACCATTTCTCCTAAATAAGCACCTGAAATCATTTTCTCAAATA

ATTGTCTACTTCTATTAGAAGAATACCAATCCATTACTAAATCAATTGGTGCTAATGGTA

AATCTTTATCAAAATTACCAAATTCAATATTTATAATTTTTCCTGAATATTTATATTTTT

TCCATTCTGGCTCATAATAACAACCATTTGATCCAGTTCCCAATATTATTCCTATATAAC

AAGGTGGAGTGCTTTTTCCTTTTTGATATGCACATGACATTAATGTACCAACAGCGTCAT

TAACTACACAGCAAACTTTTGCTGGTACACTTGACCTTGTAAATGCTTCATTCATTAATT

TACATACATCACGACCTTCTACTGGATCATTTGTAGCTCTTCCTGTTTCAAATCCTTTCG

TCCAATCAATAAGAATTGCACAATTAATTGATGGAGAAGTGCAAGGAAAAGAGAAAGTAA

ATCCAACACTTTTAACTTCTTTACTATCTAAATCATTGAATTCTCCCATTATATATTTTA

TCCTTTCTGCAAAATGATCAAATAATTGGGAAGCTGTTGCATTTTTATCTAATAATCCTT

TTTCATGGGAAAAAGTTCCTGTAAATTTCAAACTGTAAGTTTCCTGATCTCTCTTAATTT

TACCATTTCCATCTAATGAAGCTCTAACAGCTCTAAAATTCGTTCCTCCAAAATCAATTG

CATAGTAGGTTCCTTTTTCTTTACCTGAAGGAATTTCAGATATACATGAATCCAACATTT

TAAAACTGCATTCATGTGGAATCCACAAATTGGGATGCTTGCGATGTGCTTCTAACCCTT

TTTTTAATTCATATACAAAATTATCTACAAATTCTTCTAAAAGATAATATGGAAGTCTTA

AATGATCAACAAATTTATTTATTCTTTCTAATAATTCTTCATTTATTGGGATATCACATT

CAATGGTATCTAATTTTGAATATACAACGTTATCCTGTACTAAATTGTATTCTCCCATTT

TTATTTATTTTATATATAAAATGTAGATTAATAAAAAAAACTGATAAGAAAATAAATATA

ATATATTGTAATAAAACTTTTTTCTAATAATACTTATATATATATAAACGTAAAATGTAT

AAATAAAAATAAATAAATAGTTAAATCATAATATCAATAATATTATAACTATATAACGCT

TCAAAAATTATATAATATAAACTGCAGTGAAATTAATATATACAACTAAAATAAAAATAA

AAAAAGGATAAATGTGAAAAAATAAAAATAATATAAATATATATAAAATTTAGTATATAC

ATAGAATATATACATGAAATTTAACGTATACAAATTGAATAAATATAAAATTCAATATAT

AAAAATGATATTAAAAGA

>c16487_g1_i1 len=271 path=[1:0-39 41:40-270]

CTGGGTGAGCAGATCCCATTTCCTGATGGGGATTTCCCTAGGTATGTCTACCGTCCAAGT

GAGATGCTCCAGATCCACCTGAAACTTCTCTAGGGTTTCTGTGTCTGTGTGGGGTTTCGT

GCCTTGCGCTGCTGGAAGATGACAGAGCAGCTTGTGCAACTTTGGAGCACTCTCTTGCTT

CCTGACCACCCTGCCCCTGCAGCTTTGTGATGGTGAGACCTCAGCAAAGCCCACGCTGGG

AGGAAGAATGCAGAAAAAAAGAGACATGTGG

>c16487_g1_i2 len=252 path=[497:0-20 41:21-251]

CGCTGGTTCAGCTCGCACAGCGGTATGTCTACCGTCCAAGTGAGATGCTCCAGATCCACC

TGAAACTTCTCTAGGGTTTCTGTGTCTGTGTGGGGTTTCGTGCCTTGCGCTGCTGGAAGA

TGACAGAGCAGCTTGTGCAACTTTGGAGCACTCTCTTGCTTCCTGACCACCCTGCCCCTG

CAGCTTTGTGATGGTGAGACCTCAGCAAAGCCCACGCTGGGAGGAAGAATGCAGAAAAAA

AGAGACATGTGG

>c16491_g1_i1 len=417 path=[618:0-192 25:193-416]

ATAGCTGTTGCTCAAGATTTTGTCGTTGTTCCGCACTTTAAAGCACTTCAAGGTTTCCAG

TGAGAGGGAAAATATAAAAGGGACCCACGTCTTATCTACGTCCGCGTACAGAACGGACTC

CTTGATGGCATCCAGCTCTGCTTTGTGGGATAAAGTCCAGTCCAAGCCGTTCCCAGGTGC

TTCATACTGGAGCAGAGCAGAAGGGCTTCAGCGTCGTGCATCGCCGGCAGATGGGTCTCT

CCAACCCATTTCGGGGACTCCTGAAGCTGGGCAGCCTGGAGCGGCGCGGGGCCATGGGCA

TCTGGAAGGAGTTCTCCTGCGAGTTGTCGCCGCTGGAGCTCCGGCTCTTCCTGGACCACG

AGGACCGCATCTGCGTGGAGAGCTTTTCCCTGCTGCGCTGCGAGTCGCTGGCGCTGA

>c16491_g1_i2 len=417 path=[618:0-192 810:193-416]

ATAGCTGTTGCTCAAGATTTTGTCGTTGTTCCGCACTTTAAAGCACTTCAAGGTTTCCAG

TGAGAGGGAAAATATAAAAGGGACCCACGTCTTATCTACGTCCGCGTACAGAACGGACTC

CTTGATGGCATCCAGCTCTGCTTTGTGGGATAAAGTCCAGTCCAAGCCGTTCCCAGGTGC

TTCATACTGGAGCGGAGCAGAGGGCTCACAGCCATCCCCACCGTCCTCGGGGTACTCCAG

CGTCTCCCACTCCTCCTCCTCCAGCTGCGGGCGGACCTTGCGCAGCGCCTCGCGGATCCG

ATCCAGCCAGTCCTCAGCCTCGTCCCGGGAAGGAGCCCTCAGGCAGAGCCTTTTCCCCAG

GAACACCAGCTCGAAGCGTCCATCCGAATGCGTCAGCGCCAGCGACTCGCAGCGCAG

>c16492_g1_i1 len=307 path=[285:0-306]

ATTTTACTTGGAATGGATGTAAGTAGCAGTAATCCATCTGCTGATTTGTTTATCCCTTGA

TTCAACAAAATTGTTGCATTTACAACTAAACACACTAATGGGTAATATAAGAAGTGCTGT

GTTTTTCATGGGAAAAGAAGGTGAAATAATGTTCAGGAACTAATGAAAGAAAACCCAGAC

ATGTAAAGCTACAGATTAGTAATAATTTCATAAAAATAAAGAAATATATTTACTTCTCTA

CTCAATGAACATTCATTTTAAGTATCTACACAAAGGTGAAAATATGGTAACTTGAAACTT

CTCTATA

>c16494_g1_i1 len=671 path=[955:0-23 1488:24-25 981:26-469 514:470-646 691:647-670]

CAAAAACACACACACTTCTATTTCTAAACTGTCTGTTCTGCCTTTTCCTCTTTCACTATC

TGTGGCACTTCCATTTTGAGTAGTATTTAAAATAAGATCCTGGGAAGGAACTGCTGCACT

GTAGTTGCTGGAAGGGTTCATCAGCATGTTTTTTGACACTCAGCAGTAATGTAAATTCCT

AGACCTCTATCCTCAAATCTATTTCTTGGGGTTATTTAATCTGGAGAAAAAGATGACTGA

GAAGATGCACAATTACGATATTAAGGGCTGCTCAAGTAAGTAAGCTGCTCTTTTTGTCTG

GTACAGACAGCATGGGAGTCACACATTCAAATTGTCAGAGAGGTTTAGATGAGGTACTAG

GATTTCTCTGACAAAGGGTAAACATTTGTAATCTCCACCTGGGCTGGTCAACCTGGGCTC

TCTTTGTAGTCAACTACAGGAGAGCGGTCCTTTTTGTCAGATGGATTTGCTCTTGTCTTT

GAGGACTACACAGAATGGGATGAATTAGGTCCCAGAAATGACTGTTTCTTTCCATTAACT

TCAGAGGTCATGAGCACACATGTAGATGTGTCCATTGCAAACACCCACCCTTGGGCAGGA

CAGGAAAAAATTTTTAACACTGAAGATGAAGGGAAGGATTTGATTGCCAAACTTCTTTGC

TGGAAGTTTAG

>c16494_g1_i2 len=218 path=[497:0-16 514:17-193 738:194-217]

GGTATGGCATGTGCTATTCTTGTCTTTGAGGACTACACAGAATGGGATGAATTAGGTCCC

AGAAATGACTGTTTCTTTCCATTAACTTCAGAGGTCATGAGCACACATGTAGATGTGTCC

ATTGCAAACACCCACCCTTGGGCAGGACAGGAAAAAATTTTTAACACTGAAGATGAAGGG

AAGGATTTGATTGCTAAACTTCCAGCAAAGAAGTTTGG

>c16495_g1_i1 len=277 path=[255:0-276]

GGAGCCGGGTGAGACGCTGCCGCTGAAGCTCAAGTGGATCCTGCGTCGGGAGTTCTGGCC

TCAGTTCCAAAACCTGCTGAAACAAGGCACCGAGGTACGGGCACACAGAGCTCCTGGTGC

TCTCAGAGTGCTGGTGATGGCCCTGCTGATGCAGCAGTCCTTCCCTGTGCAGATACACAA

GGGGTACCTCGATGACCCCCGCAACACAGATAACGCCTGGGTTGAGACCGTTGCCATCAG

TGTGCACTTTGACAATCAGAACGATGTGGAGATGAAG

>c16499_g1_i1 len=536 path=[1:0-535]

AACAACCATTATAATTCTTAGAATTTTTTTGTTCAATATACGAATCTTTGAAATATAAAA

AAGTTTCTACACTAAATATATCATTTATTACCATTATAATCTTATTATATAGTTAAATCC

CTTATATAGGTAATAATTTTTTTTTCAGATATTAGTTTACTTTTATGCCCAATTTTTCTA

ATTATGTATGTTTAGGTATTAATAATTTGTTCATTTAATGATTTAGTAAAGATTTTGCAA

CAATTATATCTTGAATTTCTTCTTTTAAATATTTTTCATATATCCAGAATTTATTTTTCA

TCTGTTTTTGATTCTGTGGGATCTATTTTAATATTACCCAATGATATATCCCTGAACTTT

AAAATCTCACAACAAGATATATAACAAGGTTTACTTTCATCATGTGATTTTTTCTCACAT

GTATCAGAATCACCTATTAGCATAGAATGTCTTATATTTTCTTCATTATCATTTATCTCA

TTATCATTTATCTCATTTTCATATGTTTCATCTTCATATGTATCATTTTCATATGT

>c16499_g2_i1 len=431 path=[1027:0-430]

GTATCATTTTCATATGTATCATTTCCTTTATATCCTTCATTTGTTAACCATTTTAAAATA

TTCCTTGTAGATATTTTATTCAATGATTTTTTTTTCTTATTTGAAAATCTTAAGGTTAAA

TATAGTAATCCACTAATTAGTGTTCCTAGTGAAAATGCTAATGCTATATCTGTAAATGAT

AATAATAGTTTTTTCTTTTTTTTATAATTTTCTAATTTTGAGTCAAGTTCTTTCAATGAA

CTTAAATTTTGAAAAGAAATAAAACTTGCGAAAGATAAATTAAAACCTAATAAAATGAAA

ATTAATAGTACATAAAAAAAAAAAATTATTAAGTATCTCATTTTAATATATAAAATAAAT

AAATATAATATCCCTTTTATATATTAATGTTTAAAAAATATTTCATATGTGTATTTTCAT

TTAAAAACTTT

>c16501_g1_i1 len=251 path=[1:0-175 177:176-250]

AAGTGTCTGGGCCTATCCCTAATGTTATTAGGGAAAATAATAGCCCTGCTCTGCAACAGG

GACTTGAAATGTTATTTTCCCAGATGTCCTGATTAAAACATGACAAAAGTTAATGGTCAG

TAGAGACGGAGATTTTTTTTCGATTAAAAGGGTAAAGGACATGACCATTTCAAATATGTT

GTTGGTACCCTGTTAACAAAACTGAAGCAACTGCCATTTGCTATTTCTAGATGGTTTTTT

TTTTCCATTAA

>c16503_g1_i1 len=245 path=[248:0-203 452:204-244]

ACCACAGCCTCCCCAGGCACACCATTCCAGGCCTTAGCATTTTCCCAAGGAAAATGATTT

GCTCACGTCCAGTTTCCCCACACTCGTGGCCATTGCCTTTTGACCTGTTGGGCTCATGGA

CAACACACGTAATGGCTCAGGAAATTAAGCCCCCAGCAAACCTTAAGGGAGGTGACGGCC

TCAAGTTGAGCCAGAGGAGGTTCAGGTTGGGTATGAGGAGCAATTTCTCCTCCCAAAGAG

CAGTG

>c16512_g1_i1 len=213 path=[353:0-59 17:60-212]

ACACATGCTCAAGAACTCAATGGAGAGGCAGGATCCAAGAACAGCTCAGAGCCGGAAGGG

AAAAATGGATGAATATGGATCCACACCATCTCTGTGCTGATCCTGTCTGTAGAAGCATTT

GGCAGAAGGGAGAGGCCTGGTGAAAATCGGAAAGCAATGGCATGGAAGAGAAGAAAAGAG

CCACTTGAATGCATTTCTGAGCACTGGTTTGGT

>c16513_g1_i1 len=461 path=[1:0-408 1184:409-460]

GTCATCTTCTGTCATGCCCATTTTCATGGGCTGCTGAACTTCCACCCGGATGATATCTTC

GTAGATATCATCGCCTTCATCTTCACAGGGGACGCAGTCATAAATATCCTCCCCCAGATC

GTGCTCATCAGCCAACTCCTCCAGGCTCCGGTACACATCGTCATCATTCTCTGTGGTCTC

CTCTGAAGGGAATGGCCTAATTCCTTTGGTTTGAGCAATACTGTGGAAGGAGAGCCTGGA

TACTGCAGAGATCACCTTGCCGAAGTCCCGCACATCAAACAGGTCAAAGGGGTCGAACAG

GTCGCTGTTTCTTAACCCAAACTTGTCGTGACACACTTTGAGGAAGGTTCGGATGTTCTT

CAAGCAGAGGAACTGGGACATCTGCGGCCTGAAGTTGATGTCCTTGAGGTCGATGGAGCC

GGGGGAGAGGTTGTGCAGCAGTTGGCACAGTTCCTCTGCTT

>c16513_g1_i2 len=577 path=[1:0-408 410:409-576]

GTCATCTTCTGTCATGCCCATTTTCATGGGCTGCTGAACTTCCACCCGGATGATATCTTC

GTAGATATCATCGCCTTCATCTTCACAGGGGACGCAGTCATAAATATCCTCCCCCAGATC

GTGCTCATCAGCCAACTCCTCCAGGCTCCGGTACACATCGTCATCATTCTCTGTGGTCTC

CTCTGAAGGGAATGGCCTAATTCCTTTGGTTTGAGCAATACTGTGGAAGGAGAGCCTGGA

TACTGCAGAGATCACCTTGCCGAAGTCCCGCACATCAAACAGGTCAAAGGGGTCGAACAG

GTCGCTGTTTCTTAACCCAAACTTGTCGTGACACACTTTGAGGAAGGTTCGGATGTTCTT

CAAGCAGAGGAACTGGGACATCTGCGGCCTGAAGTTGATGTCCTTGAGGAGGAAGGAGGA

AGGCCGAGAGCGCGAGCGGGGCCGCGGCCATGGAGGAGTGGAGGCAGTGCGGGCGGTGGT

TGATCGACTGCAAAGTTTTGCCCCCCAACCACCGCGTGGTTTGGCCCTCGGCGGTGGTCT

TCGACCTGGCGCAGGCGCTGCGGGACGGGGTGCTGCT

>c16514_g1_i1 len=502 path=[870:0-408 1279:409-501]

AAATTTTATTTAAAGGATTGTATATTCATGTGAAATATATTTATTTTTTCTTATGTAATG

CAATTATATAATAATTATGTACTGTTAATAAAAATATTTTTATAAAAAATGAGTTGACTT

TATATACTCTTTATTAATTTGTTTGTTTATTATTATACAGAATTCCATGAATAGGCAATT

TAATAAAACTATAAAAAGTTACCATGTTAACATTATATTTGTATACTTTTTAATATTTTA

ATGAATGATAGATATGCTTCCATAACATAAATTTAAATAATCAAAAGTTGTAATCTTAGG

GGATAATTTTTATGTTAATTGCAAATATGAAGATTATGCATACAAAAAAAAAATTGCATT

CATTTAATATTTTTATTACATAGAACATCTTTAAAAGACAACATAGGGTAAACAAAACTC

TTAAGAAAACAAAAATACATTATTGGTATCAATATTATAGTATAGAAAAAGAAAATTATG

TCAAAATCTCTTTAGTTTTTCA

>c16514_g2_i1 len=457 path=[1:0-363 1279:364-456]

ATATATGCTTTTGTGAGATTTTTTATTAATACCATTTCCTCTAAGAAAACTTGGACTAAT

GATAAAACTTTATTTTTAAGTCTTTAATTATATAATGTTGAAAATAATGTCTAATATAAA

TCCTATTAAAATAAGTTAAAATTAATTTTAGAAATCATAAAATATGAACTCATATTTTCC

TAAATATATCAGTGGCATTGAAATAATAGGTTATTTGTTTGTTACAACCATTACTAGGCA

ATATAATTATTTCTCATTATCTACCTGTATAAAATATTCATTTTTATTTATAATATAGGA

CTTATAACATGTAAAGTAATAAGTTCAAATACTTTCGGTTCAGATAATTCCTGATTTTTC

TTAGAAACAAAACTCTTAAGAAAACAAAAATACATTATTGGTATCAATATTATAGTATAG

AAAAAGAAAATTATGTCAAAATCTCTTTAGTTTTTCA

>c16515_g1_i1 len=226 path=[1:0-188 190:189-225]

CTGTGCATCTCCTGGGTATGACCTCTAATGCTGCTGACCCTTCCCTCCATCGCTTTCATT

TACTGTGCTCAGCGCCCTCGGGCTCCTCCCGCTCCTCGTGGGTCGGGGGCCGAACTCCCC

CCGCGGCCCCCGTGCGGGTGGGGCCGGGGCGCTCCTCCTCTGCAGCGGAGAGGGAAAGGG

AAAGCGAAAGGGAAAGAGCAGGCGATGTCCCCGCAGTGGGGCCGGG

>c16517_g1_i1 len=463 path=[441:0-462]

AACATGGAAAGAAAAGCAGGGGCAAAGAACAACTATGTACATGCACTATGAATGTAAAAG

CCAGGACGAGTATGCTTTCTAGTAGTCTTCACACGCAAATGTCTCAGAAAAATACGAAAG

AATTTTTAAAAGCAGACTGTTGGTGCAAAAGTAGTGCTGGTTTCGATATTTAGTGGTGCT

AATTTCTACCACTGTCTCAACACCATGCTGATTTACATCTTCGTGTCATGATTATTGAAT

GTTTTTAACTAAAAGGCTCTATCTATTCACAGCAAGTATAAACTAGTTCTGCCCACCTTC

TGTTTACAAGAAAGCAGTCTGAGATTGTTCTGCTGCACTCACGTTAGGAGCCATGCTTTT

TTTTTATCCATGTCTCAAATCATGTATTTCTCTTCTTTATCTACCTGTCTGCATGTGACT

CCATTTTGTTACTCAACAGTCTTTTCACTGGTGCTTCAAAAAT

>c16520_g1_i1 len=970 path=[1:0-969]

ATTAATGAAATTGAAAAAAAAAATATAAATTTTAATAAGTCAAAATTTGAAGAGAATACT

AAAATGATCAATAAAATGTTACATATAAATAAAATTACAAATGAAAAAAACAATAATAAC

GAAGAAGATTTTCACATCGACTATTCTACTGTTAAAAATAATTTAAATTTTAATTCTTCA

ATCAAAAGAAATTGTAATGATTTATTTCAAATAGAAAATACAAAAGAGAACAATCATTTT

ATCAACGAAGAATTAAAGGAAAAAAATATAGTTAATAATTCATCATTTGTAAAAAACTAT

TTAAAAAATAATAAATTAGATAATATCAATTATAGTACTTTTTATAATAATTCTAAGAAA

ATGTTAGTCCATAGAGATAATATTCCTTATAATTTTCAGAATATCATTAAAACAGTAGAT

AAACAGACAGACAAAAAATATAATCTAATAAATTATTTTTGTGATACGAATAATGCTAGC

AATATAAATAATAATCATACAAATAATGTGAATTATGTTGATATAAATGATAATAATGAA

AAGAACTATATTAGCAGGGATGATAGTTTTAATAGAAAATACAATGATAAAGAGAATATT

TATATTGATAATGAAGGAGATTATATAAATAACAAGGATTATTTTTACTTTAACAATGAT

AATAATTATACAAACATAGACGAAAATTTTTACAGAAATAATAATGAAGTTAACTATATA

AATAACAATGAAAATTTATATTTTAATAACTATAATTATAAAAATAATGACGATAAATTT

TATAATAATGTAGATGAAAGCAATTGTAATAATAAGGAAGTTTATAATATTACTGATAAT

GCAAATAATAATATTGAAACAAAAGATATTAATAATTATAACTATGTTAAAAATTTTAGT

TACCAAAAATACAATATAAATTCTATTTGTAGCAAGAATAATTACAATAAACGTAGAAGT

AACAATAACA

>c16520_g2_i1 len=691 path=[1964:0-690]

ATTTTTTTTTTTCAATTTCATTAATTGAGTTATTATAATTTTTTTTATTCCTGTTATTAT

AATTTGATGAAAAATAAATTATATCATCTGTGAAAATATTATTATCTACATTATCATCAT

CACCTTTATTATCAATATAATTTATATTATTACTAGTAATAATACAATTATTATCGTTAA

TGTTATCTTTTTTATCTATATCATTAGTATTATATTCATTTATGTTTATAATATTACTAC

TATTATTATTATTATTTTCTGAACTACCAACAAAGTCATTATTATCGTTCGTTTTTTTTA

AATTTTCTAATTTTTCAAATTTATTATTATATTTTTTTAAAATAGTATATTCATTTTCTC

TTATAATATTATTCTTCCCCTCTATGTAAAAATTATTTTGTTTCTTTGTATTATCTATTG

ATAAATTTTTACAATATAATTCTTTTTCATTATTTACATTATTTATATCACATAAGTTGT

TATCGTTAATTTTATTATTACTACAATTATTTTTCGAAAAATATAAATTATTCCTTTCTT

TTGTATCATTTAAATTATTTATTATTTCTCCATTTATAATTTTTATTATGTTGGAGATAT

TTTTATTTGTACTTATTTTAGTATCATTATCAAAATTGATATTTTCATTTATAACATTAT

TATTACTAGAAGTGGTAGAATTATTATTACT

>c16527_g1_i1 len=211 path=[523:0-210]

ATGCGAGCGGGCGCTGAGAAGAGCCTCCAGTGATCCTGTGGAGCTGAGGGACAGAGCAGC

ACTCCTGAGGGCCCTCTTCAGAGCTCTGGCTGCCCTACTTCACGGCATGAGTCCTGCAAG

AATTACGGGCTATGAAAAACAGGGAGGAAAAGGCTCAAGTGCCACTTGAGGATTATAAGC

AGCTGCTAGAAAACAGGACAGCATTTGAAGG

>c16528_g1_i1 len=325 path=[1:0-324]

AAGATGCCTTCAAAAACATTGACTGTTTAGTGGTAAATCCTTAACTGTGCAGACTGTAGC

TTTACGCATCAGCTGTCTTTGGAAAGTTACATTACACAATTATATTACTTTTTAAAGCCT

GTAGACAGAAACCACCATTTCAAACCCAGCACAGAAGTTCTGAACTGGTTGAAGTGGCTT

TTTAGTATAAGTTACTGTTATTCCACTGTATTCATCACAGCAAAAGTGTTTACAGGTACT

AATCCTGACCCCTGCACAAGGTAGCATTTTTTTCTTACAAAGGCAGGGATTGTTTTCTTT

AATTTAAACTGCTCAAACACAAAGT

>c16536_g1_i1 len=232 path=[335:0-231]

ACAGAAATCTGAACATACTCTGAATTCAAAGTGGTAGTGCTGTTTCTGTTTTTCTAATAG

TAATGCTTCAACCAAAAGTACAACGTGACTTACTTAATATTTGGATTTCTGCAGAAGAAG

ATGGTGAAGGGATAGTTCTAACTAGTAGAAATTAGTCTTTTTGTTTGCTTTTTACTGAAG

GTATTTTTAGTAATCTTTGTCTACAAGTGTGGTGAGATGCTGGAACAGATTG

>c16537_g1_i1 len=422 path=[644:0-421]

AAAAGTTTTGTCAATACTTCACACTGGCCATAGATTCTCTCATAGGAAGAATGCCAGGGA

GTGGATCTGGACAGAGTATGGTATAAGCATGGCTGATTCACGGGCTCACAGCCAGAGCCA

GGGGAAGAATGTGGAACTCCATGCAGAGAAGGCTGTGGCACAAGGTCGGTCATTCAGGAG

CACATGCACCACCCAGAACTACAGGGACAGAAGGCTTTCGGCTTCTAACCTGATCTGACG

CTTAGAACTGTACTAATGGTACAGAAAGAGGAGCCAGAGGAGCCTTTCAAAGATCTCCTG

AGTGAACAGAGAAAAAGGTGCCACTGCAGCCTCAGCTCATCCAGATTTTACATTGCATTT

CTGTCCATTTTGCACCCATTTATGATAAGCGATTGAAAAGTGGGGAAGATGCTCATAATG

AG

>c16541_g1_i1 len=230 path=[460:0-229]

GCATTAAACTGAGGGCTCCTCATATATGAACAGAAGGAGGAGGGAGCACCGCTCGTGCTT

TCTGCAGAGCATGAAGGGGAGGCAGGGCTCACCGTTTGCTTTGGGGGTCAGTGGAGCCTC

TTCCTCCTATTGCCCATCACCTGCTCATGCAGGATCTCGGGGCGGATGCTGGACTCAAGC

AGTTTCCTGCTGCTCCCAGCTCCAGCTATGGGGTCCAGGCCCGGCATCCT

>c16542_g1_i1 len=203 path=[439:0-202]

CCTTGTGTCTTCTCTCCATCCTTTCCATCCTCTGCCCATGTCCCTCTCCCAGGCAGGAGC

AGTCCCTTGCTCTGCGCGTACTCTCAAATAGAGCCTCCGAGGTGAGAGCTCAAGATCCAG

TTCGTCTCTCACAAGGAGCGCTTTCCAGACAATTCTGGCTGCTGTTTTTAAAAGGAGAGC

ATAGAAAGCAGGAGGAGAAAAGG

>c16545_g1_i1 len=219 path=[1:0-218]

CTTTTCTCTCCTCCCCTTCATTCAAGCTGGCAAATTCCTTCACCTCCTCCTCCACATCGC

CTGACTTCCAGCAGAAATGCTGAGAGCGCTGGAGAGTTTCCTGCTTTGCGTCCCGATGTG

CCTGGTGCCATAGCAATCCCTCGTCACTCAGAGGAATGATCAAAGGGGAAAGTCCTACTT

AGCAGCCTCTGCCTTGTGTCTGGGGGTGAGCTGCCTTGG

>c16548_g1_i1 len=388 path=[380:0-387]

TTTTCTTAACTGCTGGATTTTCATCATGACATCCAGTAGTGCTGTTACTGGAACCAGAGG

TAAAAAAATATTTTACTGAACACACTATGTACAAATAACACACTTGGGATAAAGCCCATA

AAATTCCTCTGAATGTTCTCTTCAAGCAATGTAAGCATAACGAAGAGGAGCAGAGCAGTT

GTGTTATGATTAAATATCAGGCAGAATGGAAGCAAGAACCCAATTCGGCTTATAGTCGCT

TGTAGAGTCCTAAAAGTGAATGACGGTAAAAGCAATTAAGCAACTTCTTTATGAAAGAGC

AAATACATATTGTTAGCATAACAGCAATTAGAGAAAGTATTACTGAAAAGTAATTCATTA

CTTACAACTGCTTTGGATGGAGAATCAT

>c16554_g1_i1 len=203 path=[1:0-202]

TGGTATGAGGTTTTGCATAGCCTGTTTCTTCATACCTGAGATGCCACGTGGCTGAGGCAT

CTGCTTTACAGCAATAAGCACCTGTCTCAGTTGGAATTCAGAAAGGGGATGCCTGACTGC

TATGCTTCACGTGCTGCTGTATCAGCCATTCACATCTCTGCCAGATGTATTTAAACTCCC

TGACAGTGCCAGGGGCCACACAA

>c16554_g2_i1 len=382 path=[361:0-381]

AAGACCACTGTCTGCTTGGAAGAGACTGCAGAACCAGACCTGGAATCAGACCAGCACTGA

AACAAGGCACAGTAAGTACTTTTAAACAACTTCCCCAGGCATGGCAAGCACTACCAACCC

AACAATCAGCCTGGGGTCTATAGAAATATTACAAACAGACCGTCTGTCTTACAGAGTTAC

AGGAAGGAATGGAAGCTGCCAGTGAGGGTAGGGATGGAGGAGCAGACACGCTGCTTGCCA

TAACCACCAGCTTAGCACTGCATTGATTGAAGTCACTTCATCAAGTGGCCCTTTTCTCCT

CTGGAAGACCAGCGTACAGGAGGTACTGGGAAACTGTGTCAGACACTTTATGTCATCTTG

GTGATACCTCATAGTATGAGTT

>c16559_g1_i1 len=339 path=[561:0-338]

ACTTAACGAAAGCAGGTCTTCTAAGGAATCTTCTGAAGTTTTCTATTTTAAAAAACAAAC

AGTTTAGAGTTACTCCACAGAGCAATGGAAGAGTGTTATTTCTGTCTGTGTATCTTTATG

GTCAGAAAGTGCCACACCTGGTTCCCACACAATCCAGAGATCCCGTGCTTCCAGCATCCT

GCTTCACTGGCAGTCAGTGTGCAGAGAAGTGAGCTGGATGCAGTGCCCAGCTGTTGGTAT

CAGTGTGAGTTTGCTTTGCATGCCCAGGGCTTTGGTTCCATTTGCAATGGGATTGTCTTG

CTAAGATCACACCAATCCCTAAATGTGAGCTTTCTCCAG

>c16559_g2_i1 len=303 path=[281:0-302]

GAGACACGGCCAGCTTTCGCTCCAGGCCGTTAAACCCTTGCAATGAAAACCTTGCACACC

ACACAAGTAAAAATAAAAAAGTTGATCTGGTCCTCGTGTGGACCTGCCCATAGCACAGGA

CCACATCAGTCACCTTCCGCTTTGGAACGAGCTCTGTCTGTGCTCAGGGAGGCTCAGGAA

AGCCGCAGGAAAGGAGACGGCTGCAGACAATGAGCGCGGTGCCTAGGCAGGGGTAACCTT

CATCCAAACAATTGGCAGGGCCTTCTCTCTCTTTGTTTCTCCACTGCCCTTCTTCCTCTC

TGT

>c16560_g1_i1 len=228 path=[155:0-227]

GTTAGACAAAACATTTTGTTCAGTGCATTTTCTGTTCATTACTTTAGATATGTCACCAAT

AATGAAAGTTAACGATTCAAAGTTTTTTGCTAAAATCTCTGTGCCAAATAACCAACAAAG

CAGCATGCTTTTATCACAGGAAAACAGTGAAAGTGAAATACTGAACCCCTCACTTACCAG

ACAAAGAAAGCATTATTACTTCTCCCCTGCTCAACTTTACCATAACGT

>c16569_g1_i1 len=207 path=[155:0-206]

ACATACAAGAAAATAGTAATGATATTATTGATGATGATAGTAATGAGAATATACATGATT

TTGATGAGGTTGTGTTAAACTCAAAAAATAAATATTACGATAAAAATAAAATAGAAGACA

ACTCTAATTCAAATAAAAGTAATAATGATTTAAATATATATAATAAAATTAAAACTCAAA

TAAATATGGATACGAAAAAAATAGAAG

>c16572_g1_i1 len=231 path=[1:0-230]

TTGGAAATGATTTCGTTTGTGACGATGAATAATGAAAAAATTATCAGCAGCCTAAATTCT

GTGTTCAAGGTACAGACAGAACACGTTTCACCGAATAGCCTGTGTGATGTACTTTTGTGT

CTCTAGAGCAGCTGGAATGAAGTGACAATGGGGTTTCACAATAGACCTAAAAATAACTTG

GGTCCGCTGCATTTGGTTACCATCTAGCAACTGTACATTCATGTGAATATG

>c16585_g1_i1 len=205 path=[230:0-157 24:158-204]

TTTTTTTTTTTTTTTGTTTTACACACCTTTATTTTGTTTAATTTTCATTATAATGGAATA

TATATTGCTATGAATTTAATTTTTATTAAATTTTTTTTTTAAATAATTAATTCTAAAAGC

ATAAATATGCAGTTATTTATTTTAAAATAAATATATATATTTCCTTTTGATTATTTGTAT

AGGAAGAATATATCAAAGAGGAGCA

>c16586_g1_i1 len=282 path=[191:0-110 303:111-128 321:129-281]

TCAGAAAAGGCTCGGCAGCCACCTCCCCTTGCTCGGGAGTAGCATAGGACAGCTGTGCTG

CTTCCTGTGCTTCGAAGGAATTGATAGCACTTCAGGAAAAGTTTCTGTCCCCTTTGCAAA

TGGATGAGGGCTTTGGTGTGGCAGCACCACAGCCCTCATCCATTTGCAAAGGGGACAGAA

ACTTTTCCTGAAGTGCTATCAATTCCTTCGAAGCACAGGAAGCAGCACAGCTGTCCTATG

CTACTCCCGAGCAAGGGGAGGTGGCTGCCGAGCCTTTTCTGA

>c16587_g1_i1 len=210 path=[230:0-56 436:57-209]

AAGTCTACCTGAAGTCAGTGAGAGACCTCTAATCTATTTCAGGGGACGAAGCATTTGCTG

AAGACACTAGAGGCCTTCCAGACTGTCCAACACACAATGTTTAATATTCCTTAAGTGCTC

GAAAATATTTTGTAACCACTGTGAAAATTTCACTGGTATTTAGGCTGTTCTGGGGAAGAA

TGGGCACTTGGTTTTGTTTCTTTCTTCCTA

>c16587_g1_i2 len=282 path=[45:0-110 418:111-128 436:129-281]

TAGGAAGAAAGAAACAAAACCAAGTGCCCATTCTTCCCCAGAACAGCCTAAATACCAGTG

AAATTTTCACAGTGGTTACAAAATATTTTCGAGCACTTAAGGAATATTAAACATTGTGTG

TTGGACAGTCTGAAGACACTAGAGGCCTTCCAGACTGTCCAACACACAATGTTTAATATT

CCTTAAGTGCTCGAAAATATTTTGTAACCACTGTGAAAATTTCACTGGTATTTAGGCTGT

TCTGGGGAAGAATGGGCACTTGGTTTTGTTTCTTTCTTCCTA

>c16589_g1_i1 len=457 path=[478:0-79 934:80-456]

GAGTGTCTTCCTCCGGCTCGGCGGATGAGACCCTTTTTGCTCTTCCTGCTACATCTGAGC

CTCTGATGCACTCCTCTGCAGAGGGAAATAAGAGAGACATTTCCGAGATGAAATACTCAG

AAATGGAACCCTCATTTACTAAAGCAGAAGCAGCCACTATATTTCAAGCTGAAGAAATGC

AGCTAGAGGGCCCTAAAGAATTATGTGATCTGGAGAAAATGCCTGCACAGCCGCTGATTA

TGTCTCCAGAAACTCCTGAAGATTTGTTTGAGAAGAATGAGGAAGATATCTTTTCTAGCA

AGGACAAAGGTAGAGATGGTGATTCTGGGGACAAAAAGGCATTGAAAGAAGATGCTATTC

GAAGGGAAGAGTATGTAGACTTCAAACAGTATGAGCCAGTATGGGAAGTAGAAGGTGCTG

GTCATGGACTGAGCGGCTTGAGAGAGGACATTGAAAG

>c16589_g1_i2 len=398 path=[932:0-20 934:21-397]

TTGTTGACCTCCTTTACTGGCGAGGGAAATAAGAGAGACATTTCCGAGATGAAATACTCA

GAAATGGAACCCTCATTTACTAAAGCAGAAGCAGCCACTATATTTCAAGCTGAAGAAATG

CAGCTAGAGGGCCCTAAAGAATTATGTGATCTGGAGAAAATGCCTGCACAGCCGCTGATT

ATGTCTCCAGAAACTCCTGAAGATTTGTTTGAGAAGAATGAGGAAGATATCTTTTCTAGC

AAGGACAAAGGTAGAGATGGTGATTCTGGGGACAAAAAGGCATTGAAAGAAGATGCTATT

CGAAGGGAAGAGTATGTAGACTTCAAACAGTATGAGCCAGTATGGGAAGTAGAAGGTGCT

GGTCATGGACTGAGCGGCTTGAGAGAGGACATTGAAAG

>c16600_g1_i1 len=262 path=[43:0-201 42:202-261]

GCAAACAGAAGATAAGAAACACAGGTTTGTGAGGAGTAATGCCTTTTGAGTTTTTATTAA

TTTCATCTTGTTTTCACTTGTATTCTAAAGATACTTTAAAAGGGAATGGCCTTCCAGGTT

ACATGTAATTAGAGGAATATCTGCAGGAGGGTTCAAATATTTGCTGACTGCTTTTTGGGC

TGAGTAACTACTAGCTCAAATTTTCAAAATAGCGTATCTTTTAAGTATATGGAATGTATC

GTAAAAATGCAGAGTGAATATT

>c16604_g1_i1 len=312 path=[1:0-18 20:19-311]

TGGCAGGGAGATGCTTCCTGCTGCTTTTTGCTAATGCCCTTGGATATTTCCTTGGCTGGT

TACAGTGCTGCATGCAGGGAGGTCTTGGCAAGTTTATGGGCATAAGAGTGAGCCATGCTC

CTCAAAGAGACAGCTCTGGAGAGAAATTTAGGGGCAGAAGGAGGGTGCCAGCCTTCAAGA

GGTTGATTGGCCCAGTTCCCCTTTGCAGTTCCTAGGCAGCTGGTGCTGGGATTGAGGGCT

GGCTGCTGTGACAGGCCAGGGGGATGCAACTGAGGCAGCAAACAGGGCTCCACACAGCTG

CTTTAGAGTTTA

>c16609_g1_i1 len=224 path=[33:0-138 32:139-223]

AATTAGCTAATAAAGGTTCCAGATGTAAATAATACAGTAAACATATGCATTCAAACACAC

TAAGCTATTTCCAACAAACAAAGCATGGAACATTCACTCAATATTCTTTGAAAGGAACCT

GGACGAAGGGATTTTGTGTATGTGTGGTACACTGGACTAAGCTTAAAAGACAAAAGGATA

CTTCTGAAGAAGGAAAACATGAGGTGTTATGGATGTTCCATGCT

>c16611_g1_i1 len=232 path=[49:0-158 403:159-159 332:160-231]

TATAGATATATTTTTAAAAAAACACATATAACAATTAATCCCACAAGGCTATTTTCTGTA

CAGATAATATTCAGTGAATATATTTAAGGTAGTCATAGAAGCATTGATCACGAGACTGTT

CTGAGTCAGTTATTCTCATGTATAAATCGGACTGGATGAACATAATTTGTTAATGTATAC

AGCAGTAGGGAATTTTAAGAGATTCCTTTCATGCGATGTAAGAACAAATTAT

>c16619_g1_i1 len=343 path=[362:0-225 21:226-342]

GAAAGCTTTCGGCTGTAAAGCTCTGGGATTCAACCCTCTAAGTTGGTTGAAACTTGGCTA

GATAAAGCCCTGGTCACCTCTCTGCAATAAAGAAGACTCCACAGGCTCTCTGACGAACTG

TCTCTTGTTTACAATGAAAATCCCTCAGCACATTTTGTTCCTTTTATTCAACTGGAACAT

CCCTTGTTGCAGATCTACGCCTTTTATTGTGCAAGACCAAGTTCATAGTGAGAAAGTAGT

AAGAAAGGAAAAACAGGATTTTGTGTGACTATAAACTCAATTATAGCTATTTTATTTTAA

TAAGGAGACCAAAAAGTTGTAAAAAAAAATAGCTATAATTGAG

>c16620_g1_i1 len=262 path=[293:0-143 436:144-261]

AGTTACACGAGAGAAGAGGCTGACGCCCAGCTCACTACAACCACCCTTCAGGTAATTATA

GTTATTCTGTTAATTTCAGTTCTCAAAGCCTGTAAGTATTACTTGTCAATTCCAGGAATA

ATTACACACATTTTTAGTTGGAGAAGCATATTTAAAATCACAGATAAACATGAACAGCTT

TTTTACAGGCATATGAATTACAATACTGCAGAAAAAAGAAAGAAAGAAAGCTTCATCTGA

CTTGGTTCCCGGACAGGAAAGA

>c16630_g1_i1 len=229 path=[68:0-228]

CTTCTCTGAAAGAGTGGTCAGGTGCTGGAAGGAGCTATCCAGGGAGTGGTGGAGTAACCA

AGCCTGGAGGAGTTCAAAAAACACTTAGATGTAGTACAGAGAGACATGGTTTTTCCTGTC

TTCCAACCTGAATCTCCCTTAGTGCAACTTGAGTCCACTACCTCTAGTCCTATCACTAAT

ACACAAGAGAAGAGGTTCACCACAGCCTCCCTTCAGGTAGTTGTAGAGA

>c16634_g1_i1 len=1349 path=[1498:0-1348]

TTTTTTTTTTTCTTTTTTCATTATATCATAATATCAAAAAAACTAGAATTTTTTCTATCT

TCAATTGTGTATTTTTTTTTTTCATAAATTTTCCCTTCTTGTGTTATTGGACCGTTTATT

TCATTCGGAAAATTATCTTGATTGTTTATTTTATGTTCTGTTGGATCAAAAGAATTTTTT

TTTTTTTCATCAATACATGGCCAAGGAGCATTGCAATTTGAAGCTACTTCTCTTTTTATA

TATTCGTTCGAATTATTTGTTAAGAAAATATTATTACATTTTCCTTTATATAAAGAAGGA

GATTTGCATATATATTGTTCTTTTTGATTTTTTAGATATTCTTTTTCCCAATTTTCAGGG

CATTCAAAATTCCAATCCATTTCACAAAAATTTTTTTTTTTACAAGGCCAATTTACTGAG

CATTCTTTTTCAAAAAATTGTTTATCTTTAGTTGTTAAATTTTCAATAGATAACTTTTTT

TTATTGCATAAATCAAATGTATGTGGAGCCAAGCAGTATCCTTTTTCTGCTAGCCAATTC

ATTGGGCAATCCTGTTCATAATTTTTTTCACAGGAATTTGAACACAAAAATCTTGTTTTA

CATTTTTCTTCAAAACTTTTTTTCATTGATATTGTAAAATATTTAAAGTTTTTTTTTCCT

TTACAAATTCCTCGATAGTTTTGTGTTGGAATACATTCATATAAATTTATTTCATTCCAA

TCTTTTGGACAGTTTGAAAGAAATTGTTCAGAAGAGCATTCTTTATAACAAGGCCAATAA

GCCTCACATGAAATACTCCAGTTTTCTTTTTCTTCATCAGACATATGTGTAAATTTAATA

TTATTACTATTACACATACCTCTATAAACAGTAATATCTGCCTTACAACTATTATCATCT

TGTACTAAAAAACCTTCTGGACATGGATTATTATAATCTTTTTTTCCATCTTCACATGTA

TCTTTTGATATTGTTTTACATGGCCATTCAACATTACAACTGGATTCAAAATTCATTTTA

TCATTTTCTGTAAAATTTTCAAATGATTGATTTGTTATGCAAAATCCATTATATTCAGAA

TCAGCTACACATGTAATTTTTCCATTCTCAGTAGTATTTTTTGTCCAATATTTTGGACAA

GATAATGTATAATCTTTTATACATTTTCCTAATTTTGTTAATTTTTTATTTTCTTCTTCA

TGAGCTTTTTTTATTATTTTAATTTCACTTTTGTTGATTTCCTCTTCACTTATTTTTGGT

GAATGTTTGACAACATTTTTTAAAAGTCCATTTGAACTTAATGAACTTTCAACTTTTTTA

TTTTCATTTTGATTGAAAGGAATACAAAA

>c16643_g1_i1 len=338 path=[53:0-337]

TGGAGAGACGTATGAATTGATCAGGTAAAAAACAGGATGCACTGAAGATCAGAAGGTGGT

GTTTGATGTACGAATGAATGTTTGATTAGATCTGTGAGGTGGCTTGCAAGCTGCACCTTG

GCTTGCAAGACGCTGTCTGTTCTCATAGCTGCCAGAGGTGTGTGCATTGTGTTGGTGTTC

AGCCGTGTGCCCCTCTGTAATAAGAAAGCACTGAAATTTTCATCTCAGGATAAGATTATA

AAAATTGCTAGTAAAAGCAAGTTTTTGTTCCCTTTTTATATACAAAAAAATGACTTTTTA

CTGAAATCTTCTAAATGAAAAGACATATATTACATATA

>c16649_g1_i1 len=256 path=[33:0-255]

ATTTCTTCTGTTTTTATTTCTTCTATTTTTATTTCTTCATTCAGTTTTTCATCTTTTTCT

TCTGTATCTTTTTGGTTTTCTTTGTCTAAAATATCATCGTTTTTACTCTTTAAATGGGGT

TCATTATTTTTTTTTATATCTTTAATAAAATTGATGAAAAAAAAAAAAATAAAAAAAGAC

ATTTATTTAATTTATTCATAAAATACCCTATGTTTTGTATTAATTGTTATATACAAGAAC

AAAAAAAAAAAAAAAA

>c16652_g1_i1 len=566 path=[144:0-491 2867:492-492 636:493-565]

CAATTTTATTATTAATTATACAATATTTGAAATAATCAGAGGTTCAATATTGATATAAAT

AATCGTAAATGGTGTATTTAAATTATTTTTTTTCAATTAAGAACATAATGCATAAAAAAT

AAACAAAGAAAAAAAATAATATATAGCTAAATAAAAAATAGAATTCTCATAAAATTTTAT

AATTAAAAAATAAAATTTTAACTTTTCATTAGTTTATTTAAATGCAACATATAAATAAAG

TGCATATTTAAGAATAAAAAAAAAATAAAAAGATAAAATTTTTTCTTTTTTTAATATTAG

TAAAATACATTGAAATATGTTAATATATAAATATATTTTATTTTTTTTGAAATGAATAAT

TATTCGGAAAACGATTCGGATTCTACTGTTGAAGAAACATTGGTGATGCTAAATTTTCCA

CAATTAAATAATGATGAAAATAAATTAAAAAATTTAAATATTTTTCGTAAGAAGAAAAGT

GAACATCCTGAAAAAATACCAAATTCAAATAACTTACCAAACCAGTATACAATTAATGAT

ATAAATGGAATCGAAATTGATTTAAC

>c16652_g2_i1 len=788 path=[1448:0-714 2867:715-715 2163:716-787]

AAAAAAAAAGAAAATATAATAATTTTAATTTATATTATTAATGCTTTGTGTATCTTCAAG

TCTTTTGATAATTTTCTTTCAAATTTAAAAAATATACAAAAATTAATAGAAAAAGATAAA

AATGTAATTATATATATATTTGTGTCCTAAAAAAAAGCTTTATCATAATAGTGCTTAATT

TTAAATAAAATAAAAAAATATATTATATTAATAATCTATTTTGAAAGAAATTATTTTTGT

AGAATAACCTTCGTAAATTGTAAAATCATCTCTAGTCATTTTATTTTCATTTTTTATATT

TCTGTTATAATCATCATATGATATCTCATAATTTTTTAAGTCATTTTTATTTTTTAAGCT

TTCAGGCTCTTTAAAATATAAATTTGTTCCAATGCTAGAAGTCTGAATACCTTTAAATTT

ATATTTATCATTTATTACACATTGAGGGTTTTCAGTGAATATATTTTTTAAAGTTATACT

ATTAACTTCAAATGGTATATTATCTATAATATTATTATCTAAACAGCTTTCACTTTTTTT

AAGAGTGCATTTATCGTTATCCACTAATTCTTTATTAGTTTCATTAACCATATAAGAATC

AGAATTTATAACATTTTCATTCATTTTATATTGAACCTCTCTATTATTACATTGTTTATT

ATTTTCTTGAATATCATAACTATTACAATTTACGTTATTGTCGTTTTTATTAAGTATAAT

ATCATTATTCAAAGTTTCTTCTGTATTATTATTATTATCTTTAGTTAAATCAATTTCGAT

TCCATTTA

>c16653_g1_i1 len=210 path=[32:0-29 62:30-209]

GATGAGGGCCAATGTGTTAAATCAAAATTATTTTAAATTTCCTACTTCTAGTTTAGCCTG

GAAGCACTGAGGTTCAGTACAGCTTCCCGAAAGGAGAGAGCAGAAAAGCTGTTGAGCTTT

CCTTGAGTTTTAGGAAGTGCCTGTTAAAGTGTTTTGTCAGAATGTTTGTTTTGGCAAACA

AGAGCTTCATTTAGAAAACACACAATAAGC

>c16655_g1_i1 len=442 path=[39:0-222 19:223-441]

TTTTTTTTTTTATTACTATTTTACCAACTCATTGAAATGTACTATATGCTAAAAAACATA

AAATTCTAATTTTGAAATACTGTGGACATAAATTATTTCAAAATTGTAAGTATTTCTATA

GATTTTTTTATAATTTTTACAATAAATTGTTGAATTATACAAATAATAATTTTCCTATTA

ATATTATTACTAACTTTAGATGATATCATTTTGTAATATATTAACCCATTAACATTATAT

CAAATTATAAATTACTCTTTTTAATCATATATGTTTATTACATTTTTATTTAAAGGAAAA

AAATATAAAAATAATAATTTTAATTTGAGAATGTTAACTTTTAATATAATTACTTTAACA

TTATTTCATAAATTTATTGAAAAAAAAAAAACTAGTAGACTGAAATGTTTACATTTATAT

ATAAGATTCTTTTTATAGTTAG

>c16661_g1_i1 len=290 path=[399:0-16 66:17-289]

GTATTTCTATCGTTGGAGGGGGTGGTTCATGGGGTGGAGTCTTCTGAAAGAGTTTTGTAC

AGCTGTTTTTGATGAAGACTGGGGGCTTCCTACTATGGCTCAGAAATAAATTTAAAAAAA

GAATTAAAAAATGTTGGAAGGATTTATAGCGACATTCTTACCATCAAGTTTTACCTTCTG

GTTGTTAAATTAACATTAATTAAAGATTAACTAATACTTTTTTTAAAACAAAAACAAAAA

AATAACCCTTCCCAAAAAAACAGCAGACAACTTAACTCAGAGTAAAGATG

>c16661_g1_i2 len=338 path=[1:0-64 66:65-337]

GCAACAAAAATCAGAAGTATTCATACAAACTTTTTCAGGGGACTGAGAATGTGTTAAATT

TGGTGGGGGGTGGTTCATGGGGTGGAGTCTTCTGAAAGAGTTTTGTACAGCTGTTTTTGA

TGAAGACTGGGGGCTTCCTACTATGGCTCAGAAATAAATTTAAAAAAAGAATTAAAAAAT

GTTGGAAGGATTTATAGCGACATTCTTACCATCAAGTTTTACCTTCTGGTTGTTAAATTA

ACATTAATTAAAGATTAACTAATACTTTTTTTAAAACAAAAACAAAAAAATAACCCTTCC

CAAAAAAACAGCAGACAACTTAACTCAGAGTAAAGATG

>c16666_g1_i1 len=250 path=[155:0-249]

AATTGATAATATCTGCTTCTTCTCTATTACCTGAGAGAAGACATCACCAAATTGCAGACA

AAACAGGAGTTTGCGGTTACCTTTATAACAGCTGGCACATGAAAACATGAAACAAACTAG

CAAATGACCTTAGTGTTCTATGCTGTCCTTTCTGTATATCACCTGGAGATTACTACAGTT

TTATGAAGAACCCTAAGAGACTTGGGATACAGGAATGTAAAAGCTGTGTTTCACTTCATA

CTTAATATGG

>c16671_g1_i1 len=202 path=[223:0-60 44:61-201]

GTTCTGAAAAATGCAGAACTACCTAAAATAATCAATTTGCAGTAGAAAATAAACTGCAAC

CAAGACAACTGTAGGAAGAGATGGTAGTAGTTCATTTTATTTTGGAGGATGCCTTCATGT

GAGGAATTTTTGATGTCTCAGAGATCACACACTGGCAACGCACAGCAATTTTCTTATAAG

ATAGACAGTAATGACTAGATAA

>c16674_g1_i1 len=275 path=[294:0-194 42:195-274]

TGACTTCTTTTTTTTTTTTTGGATTCCTTCCTAAGCAGATCCCACGCTGATGATATTGAT

GTACGTGGGGGAGGGAATGGAGGAATGACTGCCCTAAGCAAAAGGAAGCACGGAGAGCTA

GAGCTTGGTAATGCAGCAGGATCACCTTCAGCCTTTATTAGAGCAGCCCACAAAAGAGCT

GAATACCTCACTGGTGTATTACTCCATCTGAAGATACTACTAGCTAAAAAATAGCAAAAT

AATGCAGATGCATTGCTCAAAATAAAATATTACGA

>c16680_g1_i1 len=351 path=[356:0-101 14:102-350]

AGATTTAGAAAAATAAAGAGAAGCAATACAGTAACGCCTCAAGTATTCTTCAAATGGGGT

GGGGGGAAGGAAGGGAAATGCAGGAAACTTTTTTTGTTTAATTTGGAGGATATTCCAGGT

AAAATATTTTAATCCTGAAAATTGTACTTCTAAACGCTTCACTTTTGTGCTGTAATTGCA

AATTTAAAATATTGAAGTATAGTCTATATTAAGTAAGGTCAGTGGATCACTTCAATGAGT

ACAGAATTTTCTGTAGAGGATGGACTTCTGCTGCAGCCTGGTATATAGACAAAGGCCAAC

ATTTGGGTGTTGTTAGTACTTCATCATCTTCATACTTGTAACTGTGTACTT

>c16693_g1_i1 len=234 path=[154:0-124 75:125-233]

TAATAATAATAAAAATAAAAATCCTGAATGGCTTGAGAATGGGTGGCAAAGTGGTGAGGA

CACTTTGACCATCTGGAACTTGAGGCTAAGCACACTCAAACCAGAATTTTGGCTTAGAGT

TTGAGGTGGCACATTGCTAAAGAAAAACTTCCTAATAACTCTTGATATACTCTTCCTTAA

GATAAGTCCAAAAAACTTTTGAAATGACACATTATATAAACTATTTAGTTAATA

>c16694_g1_i1 len=224 path=[201:0-223]

ATAATTATAATAATCATAATATCTAAACTATGAATATTTATATAAAGAATGAGAAAAATA

AAATTAAGATTAAATTTTGTTTAAGATGTTCATAACAACATTAATGATAATTACAATAGC

AATGATAATGTCTAGTTTTATGATAGTTCTTTCAAATAAAGCAATTAAACTATGTTAAAA

TAAATAAGAATAGTATATGGTAAATAAAGATTATTATGAAAAAG

>c16696_g1_i1 len=439 path=[49:0-214 263:215-438]

AGCGTATGCACATCAACTTCTGTAAGCAGCTGCAGAAGAGAAATCTCAAAAGATTGTTTC

CTGCCCAGCACGCTGCCTCCAGTTAAGTGTTACTACAGCTTTTTAAACTAAGTGACATTC

TTCCTAAAATATTACGTAAAATGTAAACGATGTCCAATCTTTGCCCCCGTTATCAGCCCA

ACCAATGTACCTCTCAAACATACACATACACAAGCTTCAAGTCAGGTTATTTAAGAAAAT

CCTATTCAGCATTTTTCTCTGGTCACACTGTGAAACATAGTTCCTACCCAAACCACTTCC

CAGATGACTAACATCTAACTCCTCTACAACACCTGTGTCCAGCACACAGACCTCCTGGAA

GCTGGCACACAGGTATGTACACGGCTTGCAAGCCACTCTTGCTTACAGCTTCGTTCGCTC

TAAGCAAGAGTGGCTTGCA

>c16706_g1_i1 len=296 path=[39:0-225 38:226-295]

AAACAGTGCAAAATCTTTTCACTGTGCAAGAGCTTTTTATCCAAACATTCCTGGGTTACT

ATGAAATATACTTATGTATAACATGCTGCAGTTACTTTTTCTGTGAATCGGGTTGGTTTC

CTTTTTAGTTCTCAAATCTAGAAGATCAAATTTAACTTTTGAAAGGGTAAAACAAAACAA

ACAACAACAACAAAAAACAACCAGATGTGATCCAGTCCAACACTGAAATACGTGCATTTG

GCAGAGATATAGGTCTGTGCATCCACTTCATATACAGTCCACTGTGGTTGCAGAAA

>c16709_g1_i1 len=216 path=[77:0-152 38:153-215]

ACTGGGATGGGCCATTGGTTCTATGGGCCGTTGGTCCCATGGGCCGTTGGTCCCGTGGGC

TGTTGGTGCTGTGGGCCGTTGGTCCCGTGGGCCATTGGTCCCGTGGGCCATTGGTTCTAT

GGGCCTTTGGTCCCATGGGCCATTGGTACCACTGCTGTTGGTTCTATGGGCCGTTGGTTC

CGTGGGCCATTGGTTCCGTGGCCCATTGGTCCCATG

>c16732_g1_i1 len=275 path=[49:0-72 48:73-274]

TATATATATATATATATAAGTCAATACATATAGAAAAAACTAATACAAAAATTATTGATT

TGAAATAGGAGAATAAAACTATAACTGAAACGTAATAGTATATATAAAATTTATTGAATC

ATATTTTCATCTTTATCATATAAAAATTAAAATGTAGAATGACGAACATAAGAATAAAAT

TTATATTAGTTAATATTTAATTACGAAAAATTGTTTTAAATTAATGGAGGTATATATAAA

TTTATTTTAATTTTTATATTAAAAAAAATTAAAAT

>c16736_g1_i1 len=328 path=[271:0-327]

AACACTGCTTTTGAATGAAGGCAAAGGATTTGAAATGGAGGGCAGAGAATTCATGTAGCA

TAGTGGAGCTGGGAATGTTAGAAGTATATTAATGTAATAAGAATTAGACAGATCCATGGC

AGATTGTGCATTCTGCAAGTCCAGGTGAGTCTGAGTCCCAGGAAATCTCTGATCCTTCCT

TACTGAGCTGTGTAGTGCCTGATACAAATTCCACTTTTTCCCTGGTGCATCACGTATTGA

CTGTCTTCAAGGGCAGTAGTCTATGAATAGGCAGGATTTTATTTCTTCTAATTTTTACAA

GGAAAAGGAGATGAATGAGAATAGCTTA

>c16747_g1_i1 len=255 path=[153:0-99 251:100-254]

AGCAGCAGAGTTCAGTGTCCTGCAGATGGGATGGGTGAGGCTGCTTTGTTGGGTGCCACC

TGTGAGGAGTTGAAATAGGGTTGGAGTCTGCACCTGGAGTGAGGAAGGCAACATTGCTCT

TACCTTTGCTGTTACCACGACCAGGAAGGATGTAGGAGACATTACTGGATAGGCAGGTGA

TGTACTGTGCTGGGGTTAAGCCGTGGTTGAGGAGTGTGGAAGAGTGTGATGGGTGATGCT

GGAGAAGAAAGCACA

>c16747_g1_i2 len=286 path=[726:0-100 251:101-255 465:256-285]

CTGCAGGACACTGAACTCTGCTGCTGCCCACACTGGGACCAGAAGGCAGGGAGGTTTCAC

CAACACCAAATAAATGACAGAAATAACACCACCAGGAAACAGAGGAAGGCAACATTGCTC

TTACCTTTGCTGTTACCACGACCAGGAAGGATGTAGGAGACATTACTGGATAGGCAGGTG

ATGTACTGTGCTGGGGTTAAGCCGTGGTTGAGGAGTGTGGAAGAGTGTGATGGGTGATGC

TGGAGAAGAAAGCACATACTGAAACGTGGCTGATGCAGAGCAGGTT

>c16748_g1_i1 len=230 path=[155:0-229]

AGGTTAAACAGCCTATTCTCATTCACAGCTAATTCAGGAGGATAAGAGAAGTGGTATATT

GTTCATGCTGTTTCCATGAGAGTTATTTCCTAGAACGGACAGATATTATCTTGCCAAGCT

AGGACAGCCTTAGTAAGAATTGGTCCTTAATATTAAGGTTTTTTTTGACTTTGCAAACTA

ATTTTTATCTACAGCAACAGGCTTACCTTGACTTGTGCGTGTGCCCACCG

>c16760_g1_i1 len=2358 path=[29:0-2357]

TATATATATATATTAGAAATTTTAATAATTAACAGAAAGAAAATATTACAAAAAAATGTA

AAAATATATAAACTCTCATAAATATAAAAGACAAAAATATCAGTTTTAAACATCATAATT

TAAATAATTTATATAAATACGAAATTTTTGTAATTTTTGAAATGGCATATTTGACAGAGA

AAATAAATAATAATATAAAAATTGAAGAACATAATATGAGTGATAATAAAAATAGTTTAA

AAAAAAATATTAGTGAAGATAAAGAAATTACATTCACATTAGAAGAGAATAAAGATTTAA

TAAATAATAAAGATACATATACAAACGAAAACAACAAAGTAGAAGATAAAAAATACATAA

ATAAAGAAAACATAATAAATAACGAAAACATGTTAAATGAAAAAAGTGAAGAAAAAGTAA

AAATTAATAATGATAAAAACATTGAAAGTGAAAATTATAAATCACCTGAATCATTAATAA

GCGATAATAATTCTTCTGATAATTTAAAAAAAGATATGAAAGATATTGATATAGGAGATG

GAACAAAAGATAAAAATGAAGAAAAGGAGAATAAAGAAGATGATACATCAAAAAAAAATA

TTAGTATTGAATTGCTAAAAATTTGTGATGCTTTAAAAAATATAGGAAATAAGTATTTTA

AGGAAAATAACTATATTGTTTCTTTAAAATATTATACAGAAGCCATTAATTTAATAAAAA

AATTTTTTGATGATACTCATGTAGAACTATTAAAACAAATAAGTATTGATAATTTACAGA

ATGAAATAAATGTAGATGAATCAGATAAAGATATACTTAAAGAATTTTACACCAAAAGTG

TTGTTTCTAAAAAAAATGATTTTATAAGTATAAAAGAAACTGATCTTTACATTTATTACA

CAAATAGATCTTTTTGCCACATGAAATTAGAAAGTTATGGTTTATCCATTCAAGATATTG

ATGAAGCAATAAAACTTAATCCTTTTTATGCAAAAGCTTATTATAGAAAAGGGTGCTCTT

ATTTATTACTATCTGATTTAAAAAGTGCTTCAGAATGTTTTCAAAAGGTACTAAAATTAA

CTAAGGATAAAAATTCAGAAATAAAATTAAAGCAATGTAAAAAGCTTTTATTTGAACAAC

ATTTTCAGAAAGCCATAGAATTAGAACAAAAAATGCCATACTATCAGACATTAGTATTAG

ATTCTCTTAAAATAGAAAATAATGATGCTCCTATATATGACAGAAATAATTTAAATATAG

ATTTTCTGAAAAAGGTAGTGGACTATATAAGTGTACCAAATCAAAAATTAAATAAAAAAT

GTGTATGTGCAATAGTTTTAGATGTAATTAAACTCCTAAAAGAACTACCAACTTTAGTTT

ATTTAAATTTACAACCAGATGAAACTTTAACTATATGTGGAGATATACATGGGCAATTTT

ATGATTTATTAAATATCATGAATATAAATGGTTATCCATCAGAGAAGAACTCTTATTTAT

TTAATGGAGATTTTGTAGATAGAGGAAGCTTTTCTGTAGAGGTAATTATCTTTTTATATT

TGGCGAAACTAATATTTCCAAATAATGTTCATTTAACTAGAGGAAATCATGAAACAGATA

ATATGAATAAATTATATGGATTTTTAGGAGAATTACAAGAAAAATATGATGATAAATTAC

ATGCATTATTTTCTGATTCATTTAAATTTCTACCATTGGCATATGTATTAAACAAAACGA

TTTTTATATGTCATGGTGGAATACCAAGTAAAACAGATACAACTCTGGAAGATATTCAAA

AAATTGATAGAAATACAGAACCTTTAGATGAAGGAGTAATGACAGATTTACTTTGGTCAG

ATCCAAATGAAGAGAAAGGATTTAAACCATCTAAAAGAGGAATAGGGTTTTCTTTTGGTA

CTGATATTACTGAAAATTTTTTAAAAAAAAATAATTTAAGTCTTATTATTCGATCTCATG

AGGTAAGAGACGAAGGATATTCAATTGAGCAAAATGGAATGTTATATACAGTTTTCAGTG

CCCCTAATTATTGTGATATTATGAAAAACAAAGGAGCATTTTTAAAATTCAAAGGCAATT

CAATTAAACCCGAATGTGTAACATTTACTGAAGTAAAGCATCCTGATGTTCCATCTCTTA

AATATGCCCATAATTTATATCAAAATATTTAAAAAAGAGAATATTATATGAACTGTAAAA

AAATAATTATACTTTTTGATATATGTTAATTACAAAACTTTTGGAATTAAAATTCTTTTT

AATAAAGTACTACATATTTTATTAAAAAATTAACCTTAATATTCATAAACTATTCATATT

AAAAAAAAAAAAAAAAAA

>c16773_g1_i1 len=224 path=[290:0-173 504:174-182 472:183-223]

CAAACACAAAGTTACTTTTTTTTTCATATATTTTAATTTTTTCATTTTTTATATTTTTGT

AAGACATTAAATTTAAAATTTTTTTATATAAGTATGTAAATATAAAAAATTTTGTTTAAT

TAGAATAAGATAATATTATTAATATTCTTTTTTCTTTTTTTTAATATTTGCTTTTATATA

TATAATTTTTGGTTTTTATATTTGTTTTTCTTTTTTCTCCTTTA

>c16773_g1_i2 len=254 path=[290:0-173 504:174-182 44:183-253]

CAAACACAAAGTTACTTTTTTTTTCATATATTTTAATTTTTTCATTTTTTATATTTTTGT

AAGACATTAAATTTAAAATTTTTTTATATAAGTATGTAAATATAAAAAATTTTGTTTAAT

TAGAATAAGATAATATTATTAATATTCTTTTTTCTTTTTTTTAATATTTGCTTTTATATA

TATGTATATGTATATGTAAACACCAAAAATTACCATATAAATGTTAAAATAAAGGAGAAA

AAAGAAAAACAAAT

>c16774_g1_i1 len=297 path=[37:0-181 394:182-290 400:291-296]

GTCAGACTGCCCCTCTGCTGCCATTCGTTGCACACCAATAAAATGTAATGGAATAGTGGT

GGGAAAGTTCAACTTTTACTGCCATACTACCAGCATCTGCCTCTGATGTCATGGGCCAAC

ATAGAAAAATATGAAGCATTTTTTTGGAGCAGCCCTCATAGCACTTATAGCATACAATAA

TTCAATGAGACTTCCTATAATTTAGATGCTACTTTCATATTCCTAATCCCAGAAGTCTAT

TTTTTGCAAACTCAGGGTCTCAGCTTTTAATAAGCTCCCTGCTATTATTCAGCTTCT

>c16791_g1_i1 len=298 path=[199:0-199 25:200-297]

GCCACCTATTTCTACTTCATATAGTTCTGTTAACCTGGGAAATAAAATGAAACAATCCCT

ACTGGAAACACTACAAGAAGAGGAACTATAGCAGCCTTTCTACCCAAACTACGAAGGCAA

GAGCAGGGATTTTACTGCCTCAAGAACACATGTAGGTCAGACAGAGATACTTTAAAAGAT

CAAAGATCTACAGAAAGATTATTTTCCAGCAGAATTGCTGTGGTAAATATTTAGCTTGAC

TGCTGAATAGCTAACTTTTTCTTCCTGTAAATATTTATAAGCTCCAAGCAAAAACAAT

>c16791_g1_i2 len=245 path=[199:0-199 398:200-244]

GCCACCTATTTCTACTTCATATAGTTCTGTTAACCTGGGAAATAAAATGAAACAATCCCT

ACTGGAAACACTACAAGAAGAGGAACTATAGCAGCCTTTCTACCCAAACTACGAAGGCAA

GAGCAGGGATTTTACTGCCTCAAGAACACATGTAGGTCAGACAGAGATACTTTAAAAGAT

CAAAGATCTACAGAAAGATTTTTTTCTTTTGCTGTAGTGTTCATGCTTTTGTAATTCTTT

CCCAA

>c16792_g1_i1 len=241 path=[423:0-240]

GTATATATAATATCAGAGAAAACCTGTGAATGGTAACAGTAAAAAGTTACTTTGACTTCA

CTGAATTACAAAGGTGGATCATCAATAATTTCAAATTGTTCTCATCTAAACTACTGCTAA

TATGGTTTTTAAATTTAGAGAAGATTAAATCTTATTTGGTTTCCAAATATTTCTGTAAAC

AGAGAAACAAATAGAAACAAATAAACAAAAAAAAAGGCTTGTCTGCTAAAGCTTCTTAGC

A

>c16797_g1_i1 len=313 path=[155:0-312]

AACAGAATAAGCAGCACAAGAGATGGGTTCAAGCCCCAATTTCTTATTTTAACAGATTTC

TTCAGAGAAGCTCTGCTATCCATATTTCTGCAGCCTCGTTGTTCACTCATGGCCATCTTT

TGATCACATTGCTTGTAGATGTCATGAAAAATATTGATGACTTGTAGAATAGAAACCCAA

GATAAACAGTAACAGAAATGTTGCCATGACAATATTATGGTAATACTCAAAACATTTTTG

GCAGCGTTAAAGTATACAAAATAATTATGTCAGTTATTGCTCGAGGTAGAGTCCCCAGCA

TGAGCTTAAAGAG

>c16809_g1_i1 len=340 path=[522:0-339]

ACTGGCCATGCCCCCCTGGGCATTGCGTGGGGCAGCCCATGCTGTAAGCTGCAGTCAGCA

CATACAGCTCACCGTGTGGTTCTGCAGGAATGAAACGCACACCTGTTACAATCAGCACAT

GTGGCCATAGCCAAAGCCAACAAAATGCACTCTGATGGCCAGTGCCCTAAAAAACTGGCA

TTACACTACATGGCACTACATTTCCATACTCCTTACCATGAGGTAGTTAAAATGCATATC

AACATCTAGCATATTACAGGCAGTGATACCTAAAGAGCATTTCTATCAGCAGGGTCTGAG

GAAGGCAGAAATCTTTAACCAGTATTTACATTTAAAGCAG

>c16817_g1_i1 len=291 path=[487:0-290]

AGTCCCTCACTGCACTGTGTTCTTCTCGCTCCCTGTGCTTTTTCTCTTAGCAAGCAGCAC

GAGGCGTGATGCTGCCACCTGCTCCTCGAGCTGGAGTCCTGAATATTCTTCAACCTGTAA

AGTTCTGCACGGTTCAAAGTGTGCCCATCCAGTAGGGGAGGGGAAATGGAGTTATTTCCC

ACTGTCTGAAAGGCCACCTCCAGCCTTTCTGTGCCGGAGCTTTCGGGAAATTGGATGAAT

CAGATTTCCCCATCTTTGTGCATTAGTGTGTAGGTGACACCACCGCTCGTT

>c16822_g1_i1 len=327 path=[346:0-239 42:240-326]

ACAAAGGCTCCCGTCCTCTGGCAGCATTGGAGAGCTGCTGCTTCAAGGGGGAGGTGGTGT

AGGGGATGACCCTCAGAGATGGAAGCGCTGAATAGCTGCAGTGAAGGTCTGAAACAAATT

GCCAAACAGTAAAGGTTTGAGCTGGAGCTGCGCTGGGGTGAGGAAGGAACAAAGTAATTC

CTTTTGTATAATACTTGTACAGAAAGGAGGTTCTTTTAAAGTGTAACTGAGAACCAGATT

TTGATTTTAAAACGTTCCTTCACCTTTCTTCCCTGAGTGCATACAATTCAAAGCTTTCCT

GGTCACACCAATATTTGGCACAAGTCA

>c16824_g1_i1 len=517 path=[155:0-516]

GGTAACTTTGTGTTTGATTTGCTGGTGTTTGAAGGCTGCTATAGAAATGAAAATTAGCAT

TTGGTTCCTAATCAAATCCTGCTCAGCTGTGAGGTATGTAAACTAATGAAGTACCAATAC

CCATGAAGCTGGGTGAATAAACTGATAGCACAGTGTGAGTTCCCCAGGTTCACAGTTCTC

ATTTCTTTCTTCTGCTTTGACTGTGTCAGGTGTAGAGGTGTGTGACTAAGGAGCAGGTAT

GGAACTTGCCTGAGCCATGGTGCTTAGGAGGAAAGCACAGTGATTTCTAGTATGAGGAGC

CACAACTATACGACAGATTTGTATGTACTGTCCATACACCCAAATTATACTGCTTGAAAC

AGTTCCGTTTTCATGAAGCAGGAAAATTTCCACAAGAAACAAAATGTGTATTTTGATGTG

TGCTTTAGTATAGGCTTAATACCAGTTAGCTCTTTCCTTTTACAATGCTATTTCAAAAAA

GGGTGATGAGCAGCCCACAAATAGCAGAGCTGGAACT

>c16835_g1_i1 len=341 path=[207:0-340]

AACCCATATGATTTTAAGTCCAGGATTATGAATGTTTCTGGTGTGCTGTATTGTACCAAT

AGATGCATTTTCCACGTAACTGCCCACGCAACCTCCTAGTCCTACATTACTGTGCTTTTT

CTCAAAATGTTTTCTTCTTGTGGATACCAGCCATTGTCTTTACACCAAGAATGGCTCATG

GCTTGCATTTGTATGGAAAGGTGCTTGCAAAATGTGTCTTAGTGCACCGACACTAATCCG

TATTAAATAGCACATGTTAAATAACACAGCCACAGCACTTGGCCCCAAGTCCTGCTAGCT

GAGGAGCTGGCATACGAATTAATACTGTACTCTCAAAAAGT

>c16839_g1_i1 len=473 path=[501:0-472]

TGACCGCTTGTCTCCCAGTGATATGTGCACTACTAGGTCACTCGGTGGAAGAGCTGCTGC

CATTGTAGTGCAGCAGCAGGGAATTGACAGAGCCAGAATTAGGCCGGCAGTTATTCCACA

TATGGAAGGCCATAAAGCTGTGGGCATTTGGCCAGGGGCAGGCTACCGCTCTGAGGGTTG

CAGGACATCAGTGAGCAGGTGAGATACTGCATTCTTAGCTGGGCCCTTGTTAGCAGGCCA

TTGGTTGGTAAACATTTGATGTGTTTCTGTGTTGTTGGTGAACTTTTCTCAGTTGTTTAA

TTACCTCATTTGGTGTGCTGCAGTTCGAGTTGAACTTCTTTCCTTTGCAAGAACAAGCAG

CTCACTTGGACACAAGTAAACAAATGACTTAAACAGATCTTTCATGGTACTACAAGACTG

GTAAATGAAAACAAAGTGTACTCACAGTGCAATTTTGTGCTTTGTACTGCCTG

>c16858_g1_i1 len=203 path=[281:0-19 283:20-202]

GACCAGAAGATCAACATCCTAAAAACAGGGTGATGCACGTGGACTACTCCAATTTCTTGG

CTTCGGAAAGTTTTTAAGCCGGCTTGAATGACTTTGTTGAAAAGGTCGACGTCTTCGAGG

CCCCAGCCTTGGATGGAGACGTCGAAGCCCCCCGCTCGAAGAAGGTCACCTTTGTAAATG

CAGCAATACTAATGGCTCAGTGA

>c16858_g1_i2 len=242 path=[43:0-58 283:59-241]

GTGGAAGCTTTGGACCCCAAGCACATTTTATATTGTTTTGGATCAAGATTAGGGTCACAA

AAAACAGGGTGATGCACGTGGACTACTCCAATTTCTTGGCTTCGGAAAGTTTTTAAGCCG

GCTTGAATGACTTTGTTGAAAAGGTCGACGTCTTCGAGGCCCCAGCCTTGGATGGAGACG

TCGAAGCCCCCCGCTCGAAGAAGGTCACCTTTGTAAATGCAGCAATACTAATGGCTCAGT

GA

>c16862_g1_i1 len=344 path=[249:0-343]

AATTAACATTTCACAGAATCATAGCATAGGTGAGGTTGACGGGCCCAGGCCCATGTGTGC

ATCACTTCTGAAGGTCTCACAAACCAAGACCCCTCTCTGGAAGTCTGTTTTCATGACCTT

CAACCAAAAGTACTAAACATCTTCCTATGACACTGCAGGCACACCGCTCCAAGTGTGGAC

ATCACCCGTGGTGTCTGACACAAGTTGTACAGAAGTGCAACTGTGTTTGATGACCATATG

AGATCAGGCTGAGCACTGAGCTAAGGCAGCATTTCTGCAGCCTCTCAGTGGGAGATGTCT

GTCCTTGCTGTGACTCAGCTGCAGAGGTGCTGCCTTAGCTCAGT

>c16874_g1_i1 len=295 path=[22:0-19 42:20-294]

GAATGGCCTGCTGTAAACCCAGGTCGGAATGTTAAAAAAGGCTGCTGTTTAAAACTGACC

AAAATGAACAGAAAAGTGCATAAAGGTACGCATTCCAGAATGACACCAATGCAAATAAGG

AAATGCAAGCCCTGTGCTGCACCAAGACCTCTCTTTGCTCCCGGACCCCTTCCGCAGCTA

AAATGAGCAGACAGAGCATCTGTCTGCCAACGTGTCCCAGTGCTTTACTTGGTTGAGGGT

CCTCTTTTCACAAATAGTGCATGAATGTCTGCTACTTTGGTCAATATTTATGCAT

>c16875_g1_i1 len=210 path=[41:0-120 280:121-209]

GTCTCCTGGAAGTTACAGAGGTAGGAAAAGTTCTGTTCCTCCATCTCCGCGAAAACAGAC

AGCAGTTGTTGGGGGTCAGTAAAATACAGCTCAGGCTCCTGGAATGGAAAATGTGTGATA

TCTACAAATAACTGCATGTGTAGTGTTGCAGGAAGGGTATAAGCAAGGTAAGGGCCTCGC

CTGTGCTCCCGATCCCAGCTGTTCTTTCTC

>c16878_g1_i1 len=203 path=[326:0-61 489:62-70 70:71-202]

ATATATAAAATCTTTAAGATTATAAAAATATTACACTAAAAAAAATGACTAGAAAAAATA

TAAAGGTAATTTTAATTTTGGGTATTAATTATCGTCCTATTTTTTTTTAATAAAAAACTA

AAATTATGAGAACTATTAAGAATTAGATCAATTTAATATTTCATTTTGATATAATTTCTT

TCAATGCAATTTTCTTTTTCTCA

>c16879_g1_i1 len=212 path=[426:0-211]

TTTCATAACATGTCCTGTGTGTAAGTGTCTGAAGTAGTGCCATAATGTTGTCAAGTGTTT

TAAGCAAAGTTGAATGAAGAATTAACAACTGAACTTGCAGAGAGTATTACTTACATGTTT

TAGGCTATTTCTCACTTAATACAACATGGTAATTGTCTAAGCAGAATGTTTATGTGAGGC

AGTAGTTGTGGGAAAGGGACTCAAACACAAAG

>c16885_g1_i1 len=259 path=[389:0-258]

GCAAAGGTTTGATTTGTGCTTTTTATTTTTATTTCTGTCTTTAGTATTTATTCCATAAAG

GGTAATGTAGTGGAAGGCATGTGTTGAAGATGTGTTTAGTATTGATAAGAGAGAAGGAGA

AAGAGCTGGAGTAATATGATAGGAATGCTCTCATTTACATTTACTGAAGTTGCAGGTCAC

CCATACAAGTGGTGTACCTTGAAAGCAACTGCTGTGTTAAAAAAGCAAGGTATTCTGTTT

GATATAAAGTAAAAAACAT

>c16890_g1_i1 len=1034 path=[53:0-1033]

TTTATATTTAGTTAGATATAGATATTTTTATATATATTATTCACTTGAATTCTTAAATAT

CTACACATAAAATTTTTAATATAATATTGTCAATTTATATCTGTAATATGGATAATAAGG

TATTTCGTAAAGAAGATAATTTTTTTTTATATTTTCATTAATGAAAAACTATTTTTAATT

AAAATTAAATCATATGTTATCTATTTAATTTACCTATGAGATTTAAATTTTTGCCATATT

GAAGATAAACAAGAAAAGATGATGAAAAATAAAATTAAAAAAAAGGATAGAAGAGAAATA

TAAAGATAATACTATAAATTTATTGATAAAAAAAATATGATTAATAAGAATGATAAAAAA

TATCCACAACAATTTTTTTATAGCTATGAATGTTTTACATTTAAACAATATAGGCTTAAG

TGCATAGTGAAAATTGTTTAGAAAATCTCTTTAAAATGAAATTAAACTATTTATAATATA

ACATAATTCATAATTTATGTAAAGCTGTAAACTCGTTAAATAAAAAAGGTATTTTATTTA

CAATCAAGGTCAATGTTAATATATAGCTGATAATGTTACATATAAACAATGGAATACAAA

CAACTCATGAAAGAAATTCAGTTCCTTTTAATGCTTGTTATAAATAATATCTTATATAAA

ATATTGAATTACATTAAATATATTATATTTATATCATTTAAATTTAAAATAAATTTACCA

CGGTTAAAATTGATAAATTATGAAATAGTGTAACCTTATTGAAATTATTTAAAATTATTA

TAAATAGCTGTAATTTTTTCATAAAATATTTAATGATGCATTTATTATTGTGAAGAATGT

CATATGTAAAATCAATCATATATTTCTACAAACGCATTAACAATGCATAAAAGTTTAATC

ATATGCTTTAATATTAAATGAATACTTAGGTGATTGTTTTATTAAATGGCTGTACATTGA

CTTTGTTGTTAATAAAAGGATTAGTTATATGAAGAATATGTAGTTGGTCATGAACACACA

AAAAAAAAAAAAAA

>c16894_g1_i1 len=231 path=[117:0-154 275:155-230]

GTTATTGTTATCAATTTAAAAAATTTTATTCTAAATTTTTTTATATAGACTAATAACTAT

AATATGTATAGAGTATTTAAATTTACTTTTAAGCAATGTAATAAATATGAAAATTAATTT

CATAAATTACAATTATTTTCACTACGTTGAATTTATGAAGTTAATAAAAAGGAAGAATAT

CATTTAAAGTTTATCTGATTATTTGTTATATTATCCAATTTTTTTTTTTTT

>c16895_g1_i1 len=263 path=[308:0-28 21:29-262]

AAAAAAAAAAAAATTTTAATAAAGTCTTAAGTATAACTGATATATTAAATACTAATGAAA

AAGAAAAATTAACAAATTTATAATAATATTTTGATAATTATTAGAAGTAAAAAAAATTAT

TACGAAATATCTTTAGAAAAGATATCTTGTAATCTTTAAAAGAAAATGTTTCTATTTTCT

GATTACTATTTTTTTTCATTTAATTAGAACTAGCATCTTTTTATTTATTTATGTTTTAGA

TGATTTATATGTAAGCTTATTTA

>c16898_g1_i1 len=201 path=[391:0-200]

GAAGCTGAGCCCACGGAAGTCCTCGGCACCCGTGGTGAACATCAGCAGGATCTGCAGGTT

GCCCTCTATGATGGAGCAGTTCTCCAGCTTCTGCAGCTGCGACACGTCGTTGCGGATGTC

CATGCTGCCGCAGATTTCAGAGGGGGCCTGCACCGGGGGCAGCAGGAGGGGGCCGAGGAG

CAGCAGGGCAAGCAGCAGCCG

>c16906_g1_i1 len=203 path=[191:0-202]

AAATAAAATATACATAAATGTTAATATATTAAATATTTTTTGTACTAAATTAAAAATTCA

AACAACATAAATAAATTTAAAAAAGAGATAAATAATAAGCAAATGTAATGTGAAAAAATA

TAAATGAAATAAAATAAAAAACAAAAAACATTCATAGATCTTTTTATATGTTAAAATACA

ATTACAATTTAAAAAAAAAAAAA

>c16908_g1_i1 len=345 path=[1:0-21 23:22-344]

TTTTTTTTTTTTTATTTTATAGATTATTATCCGTATTCGTATAATTTTAGGAATTATATA

GTTACATAAAAGATTTTTATATTATTAATAGTTACATTTTGGAAAAAAAAATATAAAATA

TTTCAGCTAAATCTTATATTTTAAATTTGAAATGATTAAGAATATTTTCATTATACTATA

TTTACATTTTTTATGATAAAAATGATATTATCTTCTATCTATCATTTATATAATTTTTTT

TAAATAAATACATGTTACGGATTTAATAGAATTAAGATACACCATATAATAATATTTTAT

TACAATTAATACTACATTTATTCAGACTTCTTTTTTTATTTAAAG

>c16908_g1_i2 len=500 path=[523:0-176 23:177-499]

ATGTTTTACTAATTATGTTTTCATTTGTTTCTTTAACTTAATAACAGTAATAATAATTTT

TTTTAAAATCATAAAGAAAAAAGAGGAACTAAAAATATAATTATTTATTTTTAAAACTTC

TTATGTTAAACTACTTTATTTCAATTATAAAAAAAAAACACATGTATACAAGTGCTAATT

ATTATCCGTATTCGTATAATTTTAGGAATTATATAGTTACATAAAAGATTTTTATATTAT

TAATAGTTACATTTTGGAAAAAAAAATATAAAATATTTCAGCTAAATCTTATATTTTAAA

TTTGAAATGATTAAGAATATTTTCATTATACTATATTTACATTTTTTATGATAAAAATGA

TATTATCTTCTATCTATCATTTATATAATTTTTTTTAAATAAATACATGTTACGGATTTA

ATAGAATTAAGATACACCATATAATAATATTTTATTACAATTAATACTACATTTATTCAG

ACTTCTTTTTTTATTTAAAG

>c16913_g1_i1 len=231 path=[489:0-230]

AGCATGGCTCAAGTTTGAACAAAAAATAACTTGAAGTGATCTGACAACAGACCTCAGGCA

ATCGAGGCTCCTTAGAAGTATTTGAACAATATAACCCATACTATGTGAAAATAGTTTTCA

AATTTATTCCTGAAGCCCAGTAGAGGATGTAGTCAAATCCAGCAGGTTTACGTCTGCACT

TTCCATTACCCAGCTAACAAATACTGAGTCTCGAACAGCACCACCCCCAAA

>c16917_g1_i1 len=237 path=[439:0-85 667:86-93 107:94-236]

TGGATTTTGGGTGGTGCTGTGTGGGGCCAGGAGCTGGACTCAATGATTTCTTCCAACTCA

GGATATTCTAGGATTCTGTGATAATTTATACAGCTGTGCCATCAGATATTATACCAATAC

AGCTGGGTAAGAAAGAGCAATCCTAAGCATTACTGCCACTTACTGAAATCTTTCTTGGCT

CAACAGCAAAATGTTGAAAGGAAAGGATCTTTAGAAGTAAGTATGGATGAGTATCTA

>c16919_g1_i1 len=299 path=[35:0-176 17:177-298]

AGCCAGCACTTACTGACCTGCTCAGGATCCGAAGCTGTGAAATGACCAAGCCAGTGTTGC

AGCTGCACCTTCTCCAAAAGAACAGCTAGGGGGTTTTGCTGCATAGAGGTCCCCATGTTC

CATATTTGCTGGAGAACACCATGCTGGGAACTGATCATACGTTGTGATGAACGAGCAATA

TTAGCAGGTAGACCAAAAAATTCAGGTTTATCATCTTCTGGAAGGCTTTCAATAATATGA

CGATAATCCAAGATACTGCAAGAATTTGGTAAAGAGATCGAATGCAGGAAACTAGTCAT

>c16923_g1_i1 len=578 path=[51:0-577]

AAAAAAAAAAAAAAAAAAATTCCATATTTTTTTATTTGTGTAAATAAATGCGATTTAAAA

TTGTCAAAATCATATAAAAATATAAGAAAAAATATAAAAAAAATACTACTTAAATATTTA

GATTTACGTGTTTTAAAAAAAGTTAGTAAAAAAATATTTTTTATTTCATCTAAGAAGGGA

TATAACATTGATAAAATGTTAAAATATTTTAATAAAAGAATGATGAAAAAAAGAAATATC

AAATATTCAAAAAAGGAAAAAATTTTCTTTTTACCATTTGAAAGACATAAAATTTATCTA

AAAAAATCACTGAAGCATTTATTTTTTATAAAAAAAAATAAACAAAACTTAACTTTTGAT

ATAATAGCTGAAGAAATCAAATTAGCTGTTAATTCTCTAAATCATATCATTGGAGAATTT

AAAAAGGAGCAAATTTTAAATAAAATATTAGAGAAATTTTGCATTGGTAAATGAATTTTA

AACAATAAAAAATTAATAAAAGAAAATATGTAAAAATACAAATATACTAAAAATAAAAAT

ATAGAAGTTTAATAAAAAGGTGAAAAAGAAAAAAAAAG

>c16927_g1_i1 len=223 path=[236:0-68 36:69-222]

CCTCTCCGTGCCCGTGCCGGGGCTGCTCCCTCCCCTGCAGCCTTTCTCTGCACCCGCGGA

CCCGCAGCCGTTGGGAAGGAAGCGGCAGGGAGGAATACATTGACCTCGTGGCAGCTGCTG

GAGTTCTGCGGGGATGCACTTCACCTGCAGAGCCCATCGCAGCATTGCAGAGGGACTTTC

CTGCCTGGTGACTTGGAGCAGGAGCAGGATCGCCTCATTGGCT

>c16932_g1_i1 len=245 path=[145:0-212 144:213-244]

CAGTGCTAATACATGAAACTTCTCCAGAATTTCAGAAAGCTGCCCTTTATCACTGCATGC

TTTTTCTGCTTGCTTCGCTCCCAGTATGTTTCTCATGTGAATTCATATTTGCATTAAGAC

AAGCATTTTTCCAGCTTCAGAGAACACAGAACAACTGGCAGACATCACATTCCAGAACCA

AACCGGTAGCCTTTCAGAATGATCACAGAGGACAAGGAGGTCTGGGGCGATGCTGGCGGG

ACTGG

>c16933_g1_i1 len=737 path=[43:0-700 1067:701-704 747:705-736]

AAAAATAAAAATATTTAATAAATAAGATAAAAATGAAAAAATAAAAACTAATAAATTTAT

ATAATAAAGTTAAACTTATTTTTTGTAGAAAATTTAATACATACATACATATATATAAAT

GATACTAATTGAAATAAATTTTAAAAATGAAATATAAATTTTTTTTGTTTTAATTTGTAA

ATCAAAATGAAAATACCCAACCCGCGCGTTTATTTAGATGTAGCTATAGGAGGAAGAAAT

GCTGGAAGAATGATATTTGAACTATTCATGGATAAATTACCAATAACTTGTGAAAATTTT

CGTTGCTTATGTACAGGAGAAACGGGTTTAGGATATTATTTAAAACCAAGATGGTATAAA

AATACCCCCATTCACAGAATTGTTACGGATTTTATGTTTCAAGGAGGAGATTTTAACTTT

GGAAATGGTTTTGGTGGAGAATCAATTTATGGACAATATTTCAGAAACGAAAAATTCATA

TATAAGCATTCAAAAAGAGGAGTATTATCTATGTGTCAAACGAGAATTAAACATACAAAC

AATTCTCAATTTTTTGTTACATTCAAAAGTTGTCCATGGCTAGATAAAAAACATGTTGTA

TTTGGTCACCTGGAATATGGTTTTGAAACCTTATCTTTTATTGAAGAACAATCTACATTA

ATAGGAAAACCTAAGAAACAAGTTTACATATATAACTGTGGTGAAATTCCATTAGATAAA

ATTAAATATAAATCTTA

>c16935_g1_i1 len=273 path=[288:0-262 564:263-272]

ACAATCAGTAGGACCTGTGTCACGAGCTCAGCAATGATCAGCCTGTCATAGACTGTTACA

AAGGACGTGACACAGTGTCATTTTCTGCCTACTCCTCATTCCCAAAGGTTGTTCTTTTTC

CTTTGGGACCAAATGATCTTATTGTGTGTATCTGTGTTCCTACCAGAACACCCTGAAGGG

GTGTGATTGCTCGGGAATTACTTTTTTTTAGTTCTGTATGGAAATCCATTAATAAGATAC

AACGATCACAAAGGCCTTTTGAAAAACTGTTGG

>c16937_g1_i1 len=218 path=[279:0-3 283:4-217]

TTGAATTACAGAATACAATTCATCTGATCCACTTCAACACAAACTGATATATACACAAAG

AAGAGACAGTAAATTCTCTGAATAAATCAGGTTTTGATGATCATTTGCTCCTTTCTTTAT

TAAAGCATTATGTCCTGCCAAAGACTAACCATCTGCCTTCCCCTTCTTTTCCTCTCTCTC

TCTCATCTCTTCCCTCTCTCCCCCAGAAATGTCAAACA

>c16938_g1_i1 len=299 path=[39:0-298]

TATATATATATGTATATTAGGATAATTTAATTATTAATTCTAAATTATTTTCCTTTTTTT

TATATTTCATTATTTTTCTTATACAAAATTTCAACATTTTTTTTGTTTTTTATTAAAATA

TATACAAAATTATATAAATTTCAATTTTCTAGTAATTTTAATAAATAAATATAAATGCAA

ATATTTGGCATACAATCCCAAAATATTTTTTCATACAACTTTGTTCTGATGATTAATTAA

ATCTTGTTAGAAACAAAAAATTTTGTTATTGTGTAAATTTCATCAACATATTAGTATGC

>c16938_g2_i1 len=371 path=[837:0-316 1167:317-370]

TATTTATATATAAAGTATATATATATTTTTTGTATGTATGTAATATATTTGTTAAGTTAT

ATTAAGTCAAACAAAACAAATATAAATATATTTCTCTTGGAAAATTTAAAAATTTTTTGA

AAATAACTGTATTCTATTAATAAATGTAATAAAACAAGTAATAAATAAATGAAATACAGA

AATGTAAAACATGGTGGAAATGAGATAATGGAAAAATGATATATGTCAGAAAAAACTACA

AAAAATAGGAGGAAAGATTGAGAAAAAGAAGGATGAAAGGAATTGTTATTAAAGAAAAAC

AAATTAATGAATTATACTTCCTTCTTTTTAATGTAATTTTCATTAGATGAACTAAAAATA

AGGAAGTATAA

>c16938_g3_i1 len=212 path=[339:0-157 1167:158-211]

TATAAATATATATATATATATACAAGTTATTAATTTACATTTATTTCTGGGAGACTTATA

ACATTTACATCCATATATAAATAAGAAAGAGAAAAATCTACACACATTCATTTTCACTCT

TCCTTTTTTTTGTAGAATAATTTTATGAAATATATTAATTCCTTCTTTTTAATGTAATTT

TCATTAGATGAACTAAAAATAAGGAAGTATAA

>c16939_g1_i1 len=277 path=[311:0-83 54:84-87 58:88-276]

AGGAGAGGTTTGAGAGAGGAAGATAATGGGGTTTCTCTCTCGTGTTTGTGTCACTTCTGA

GTATTCTCCCTTTCAGCACAATCTTATAAATCAAATCCATTCCACAGTCACTTTTTCGGT

TCAATTTTATCAATTTTACCTACCTTTTCAAGAACTGTCCAAGAGAAGGGCAGAAAAGGA

GATTTTGCAATAAGTCCCCATTTTCAGGCAAAATGATTACATGTTCAAGGAGAGAGGTAA

CCTGTAACTTGTTTTCCCTAAAATACATTCCATGCAT

>c16943_g1_i1 len=268 path=[276:0-50 326:51-51 32:52-267]

TTTTTTTTTTTTTTTTTAATTCAAAATTATTTTATATTATTTAGCATCTCTAAAAAAAAA

ATTGTCATATAAATTAGACTTTGGTGATGAAATTTTTCTTAATTTATCTTCATATGAATA

CACTTATTTTTAATTTCATATATCTTCATTTATTCAAATAATTTTTTTAAAAGTGGACGT

TTTTTTTAATATTTTTTTTGAATAATTATTTATCAATTTCAAGATTAATTTATTAAAAAT

TACATTGAGAAAAAAAAAAAAAAATTGA

>c16944_g1_i1 len=501 path=[53:0-500]

GTGTGCACCCAGTAGCAACACTACATACATTACACTGCCTGTCTCATGAGTAATAGAGCT

TAGCCTGGGATGCTTCTACATAGTCCAAACAAGTTTCTCAGAAAGAGGATGACTTCTTTC

AGTTGAAGGTGATGTCATTGAGCACAGCCTCTCAAGAAGGCCTTGAGAGTGTTCAATTCC

TTTGCCAGAACCCTTGCAACACAGCACTCATTGCCTTCCACACCATCAGGCAGCTTCAGA

TCAGGAAACAAACAAACAAAAACCTGCAGTGAGAGATGATGTCTGGCAGCAAAACATATT

ATTTTAGTTGAAGAGTAACTTTGCTGCAACTTGCTCCTGCTCTCAGTGTCCCCCTACCCC

ATCATCAGAAGCTGTTCCCTGGTATGGATGAGTCTCAGCACCTCTTCTGCAGGACTGCGT

TACCCATAACACAACACAATTCAATGGCTGCATAAAACAGCTGTGTTTTGGAAATCATCT

TTATATTGATGGCATACATTT

>c16947_g1_i1 len=225 path=[241:0-173 461:174-174 462:175-224]

GTCTTTGTGAATAGCCAGCTATGTAACAAGATTACAAAAATAAAAATAAAAAAATAGAAA

TTCTTGTGCTTATCTTCACAGTGAAAAGATTTTTTCCGACCTGCAGTTACATTCAATTAC

ATAGAGAAACACAGATTTTTTTTTTCTTTTCCTTCAGGTTAGTACAATGGAGTGAGCAAA

TAAACCTGTTTGCAATAGCAGCACTTAATAGATCCAGAACCATAA

>c16949_g1_i1 len=307 path=[218:0-306]

AAAAAAAAAAAAAAAAAGTAACTAGAATTTTATATGTACTTAATTTAGAATAAAGAGTTC

ATTAATTCCTCATGTTTTGTTTTTTGTTCTAAAAACATATGTATTTAAAATTCAAAAAAC

GAGCATATAATTATATTTTTAATTATTTAAACTAAAAAGAAAAAAAAAATTAACTTTTTT

TCTCTTTCATTTAAATTAAATGTCTCTCTGATATTAAGTATATATATTTAATAAATTTAC

ATTGTCATATGCAGAATTCTAAAATATTGAAAGTTTACTTATTAAAAATTTATATAAAAT

ATTTAGA

>c16950_g1_i1 len=218 path=[53:0-217]

TGTATTGTTAGTCTCATCTTAACCTACATCCTTCTGGTAGCTGCAGGTGGTCAGTGCTTA

CTTGGCCACCTTTAAGCAAGTAGGCAACCATAAGACAAACAATACAGTGTGCTGAATGTT

GCTGTGTACTGATAAGCGTTTGGTGGCTGGCTGCAACACAAATCTCACAAATCCTACTTT

TCTTTCCTTTGCTCAGCTGCTCTTCAGGCCCTAAAGCG

>c16953_g1_i1 len=271 path=[35:0-108 17:109-270]

GCTACGCGGGGCGGCAGGGCCGCCGCCTCCCCCGAACATTCTGGGCATAACGACGAGCAG

GCAGAGCACCGACACCACCGACACCACCGCCTGCTGCGGCACAGAGGTAGTCCTCAGACT

GTTCATCAAGTTCACCCAAATCCAGGAAATCCTGAAGGTAGTGTTTACCAGAGTATTCAG

CAGATGAGAAATGCAGTGGCAAAAGAGATAAAGAGTGAGAGGACAAGAGGAAATGGCAGA

GACTTAGCATTCACACTCTTTATCTCTTTTG

>c16957_g1_i1 len=202 path=[44:0-131 241:132-201]

AATAGAAGGGCAGGCTGAGAGCAACGTGTTTGGTTCAGCCTTTAGAGGCCGCTGTCTCCG

TCTCCATAGGTACAGAGAAAAGTTTGGAAAGGAGGAATTCAGAGCCTTCACATTGATTAG

AAGCAAAGGATGCAAAAAACAGATGAAATTCTATACAGAAATACAAAATTACCACCTTAC

TGTAATACTAGTATCTGAAATA

>c16960_g1_i1 len=266 path=[163:0-139 303:140-162 667:163-265]

AATGGAATGGAATGGAATAGAACGGAATGGAATTGAATGGAATGGAATGCAATGGAATCA

ACAAGAGTGAAATGCAATGGAATGGAATGGAGTGGAATGGAATCAACACGAGAGGAATGC

AATGGAATGTAATACAATTGAATGGAATGGAATGGAATGGAAACTACCCAAATGGAATGG

AATGTAATGAAGTGTAAGGGAATTGAATAGAATCAATCCGAATGGAATGGAATGGAATGG

AACAACCCGAATGGAATGGAATATAA

>c16961_g1_i1 len=228 path=[29:0-52 14:53-227]

CCCTCCCCTGCTCCGTGCTGGCTGCCCCCCGCCTCACAGCGCCCGCAGCGCCCGCAGTCC

GTTCTGAGGCACCCCGGGTTATCAGCAAGGCCGCGGCGCTGCCCTCCCCACAGCACCCGT

GAGGGGTGTGGGGTGGATGAGTGGGGGGCTGCTGGGCTGAGAACGGGCTGGGAGGGCTCA

CAGGGCGGTGATGGGTGTTCCTCAGGGTCAGTGCTGGGTCCGGGCCTG

>c16962_g1_i1 len=202 path=[209:0-115 15:116-201]

CTGCTTATACACACATCGTGCTCCTCCACGGCAGAGATCAGAAGCTACCAGAAACCCAGA

TACAGAAACAACAGGCTCAAAGATCAGCAGGAGGTTCACCACCAAACCACCACCTACCTC

TTCTGACCACCAGGAAGCAGAACAAATAGCATGAATTTCTTACATACAAATGCTTGACCC

TGCTTCCTGGTGGTCAGAAGAG

>c16963_g1_i1 len=224 path=[229:0-77 444:78-223]

AGAGGTAAGAGGTTGGCAAAAATGGTAAAGATTATAGCATATCTAAATAGGAAAATAGGT

TCTGAATTTGATTTGTGAGAGGAAGAGTGCAGAGAGAGATCTGTGTCGGGACAGCATAAA

GGTTATAGCAGTACAATTTTTGGTGAGCTTGGGAGAGTGAGGTGAAGGCTGCAAGATTTA

AGGAAAGAAAGAAGGCAAAGAGCTGGATGAATTTTCAAGTCTGT

>c16973_g1_i1 len=380 path=[393:0-203 768:204-379]

GTTCTGCCGGTGCATTTAATAACTGAGATTTCTCAGCATTTAAGCTTATTCTACAAATAT

CTCACTGCAGCCCAAACCCAATTTCACGAACTGCTGCAATCAATGCTCCAATTCCATTTT

ACATTTCTTTCTTATTTTTACCCTACAAAACAATCCCTCTTTATGATAACTAAAGATCAA

ACGAGACCAGCAGCCTCCTGTCACCACAGGCAGCGATCTCTCAATCTGTTCAGATGCTCC

ATGACACTTAGTTTTTCACCCCTACTCTTCCATTAGAAGCTGTTCCAGAATCAAGCTTCT

CAGGTATCTAAATCATTCTTCTGAAGTTAAAGATGGGGAGAATTATTATTATTTTTTAAT

ATAATTCTCCCCATCTTTAA

>c16980_g1_i1 len=286 path=[53:0-285]

AAGCTCATATTCTCGTTTGCTTTTGCAGTACTTCCAACAGAACTATTAGAAAATTGAGGA

GAGGCCCTTCAGTGTTCTACTACACAATAAACTGATATACCCATAAAAGATTCAGGCATT

TAATCATCTGCTTGGCAATGTTAGGTTCCTAGTAAAAAAGAAAAATGAAAAAAATCTATG

TATTTATAGACAAAAAAGCTCAACTATTCATGTTGAGCTTCAGAATTCACTTTTCAGGGC

AAAACTGCATTCAGATCCACTGTTCTCTGCAGACTTACCTTTGATT

>c16982_g1_i1 len=250 path=[94:0-39 82:40-249]

TTTTTTTTTTTTAAATTTATCTAAGAGTTAATTAATCATAAAATTGAGCATGCCGTATAT

CATTTAAAATCATTGAAAAAAAATTTATATTTCATATATTTATAACTTTAAGATATTTGA

ATTATTATTTCAAAATTTGATTTTTTATAATATTATAGAGTAAAAATATAATTAAAATAA

AAAAATATATAGAGAGAGAATTCTTTATATTTGCGTGCAAGGTTGAGTTAATGCAAAAAA

AAAAAAAAAA

>c16982_g1_i2 len=250 path=[42:0-39 82:40-249]

TTTAAAAAAAAGAAAAATGTATATGGGAAGAAGAAATCATAAATTGAGCATGCCGTATAT

CATTTAAAATCATTGAAAAAAAATTTATATTTCATATATTTATAACTTTAAGATATTTGA

ATTATTATTTCAAAATTTGATTTTTTATAATATTATAGAGTAAAAATATAATTAAAATAA

AAAAATATATAGAGAGAGAATTCTTTATATTTGCGTGCAAGGTTGAGTTAATGCAAAAAA

AAAAAAAAAA

>c16986_g1_i1 len=246 path=[43:0-206 303:207-245]

AACAACTGACCCACTACAGGTTTTGGGTGTAGAATTAATCTGTACAGCAAACAATCTGCT

ATGCCTACAACATTCTTGCAGTCAGGATTTTAGCTGAACTATTTACGTGCACGAAGCTGG

TTGAGCTTGAGTAGGGCCAGGAGAGCAGTCTAGATGTTCCAACAGCTGTGGCTGCCACAA

ATGCTTGAGAGAAAAGCTCTATCCCTTCAACCTCATTAGTTTACAGGCTGCCCAGAGAGG

TTGTAG

>c16988_g1_i1 len=244 path=[265:0-164 508:165-243]

AAAACCCCAGATGCGGAGCTTCAGGGAGGGGCAGTGTGCTCCTTTAACAACAGTTAGTCA

GAGTTCTGAGAATGTTATATAAATAGAGTTTATGGAAACTTTATTTTAAATGCTTTGGGG

AAAGGAGAAAAGCTGTGGCAAAATACTGTTGGCAGGGAGCTTCAGTTGTAATTTCTAATT

GCTCTTAAGGATTTACTGATTGTGTGTGATCTTTGTAAAAGCTGTTGATGTTCTGAATGA

GGAA

>c16992_g1_i1 len=221 path=[47:0-42 46:43-220]

CTGGTTAGCCGTTTTAGACTGACACAAACAGTCATTATACACGTATATACGCAACACTGT

AAGCCTCAAATGCTGACAAAAGTCATCAAGTAATTCAGGTAGAAATTTCTAAAGTCCTCC

TAGACCACGGACCTGCTGAGAGAGAGGGGTGGCAGAAAGTCCTGTAGTTGTGCACTCATC

AATTCAGTGGCACTTCGCCCGCTTCAGAAAACTCACTGCAG

>c16993_g1_i1 len=283 path=[41:0-187 321:188-282]

AGCCCGCCCGCGGCGGGGCTCGGCGGCGGCGGGCGGGCTGCGGGGCAGCTCGGTGTCCCG

CGTGGATGACGACGATGAGGAGGAGGACGAAGAGGTGGCCCGGCGGGGCAGGAAGAGGCG

TTTGAAACGAGAAGAGTCAGGCAGGAGGAAAAGGGCCCCGAAACACAGCGTGAGCAGGCC

CGAGAGGAAGAGCAGGAAAAGGAACTTCTGCGGCAGCCGCAGCCCCAACGCCGCCGGCCA

GCCCGGCATCCCCGGCAGCTTCCGCGGCAGCACCATGGGGCGG

>c16999_g1_i1 len=229 path=[43:0-134 21:135-228]

ATATGCTTTACCCTGGATTCATAGAAGTTATTGCCACTGAATGCTCAAAACAATTTTGAG

CTTAAGAATTAAAGGAATTAGGCCTCTTTTGACAGCCACTGGAAGTAGCCTAGAAATGAT

TGTGTTCTGTTACTGAATACTTTTGTTACTAGAAATTGTAATGGTTGTCTTTTCTGCAGT

TTTTCCTTAAAATTGTAAGTACAGACTGTTAAGGGAGAAGTTCTTTATG

>c17000_g1_i1 len=238 path=[262:0-94 356:95-95 24:96-237]

AGTAGTCTATCTTCAATTAGACAAATACTTGATGGATTATCAGCTGGAATAAGGTTGTAT

GGTGTTAAGGTGCACTTCGAACAGCTTTACAAAAACCACCATTACAAGAAGTACAATTCT

ATACACATACTTAGTACCTTTCAGCAAACCTATTTAACCTGCTTAGTTAACACTGTTCTC

TAGAGACCCATTTAGAATCAGAGTGAACCCTTACCTTCTCCATGTTTATCTGTAAACT

>c17001_g1_i1 len=280 path=[430:0-219 649:220-279]

AAAAAAAAAAAAAAAAAAAAGATTGATCACACATTCACATTTTAATTAACGTAATGTTAT

GAAATATGCATATCAAGTACAACACAATAATTTTATTTTTTCGTTCTTTTAATTAACACT

GATTAGAGAATATTCTACAATAGTAATATAGAATGTAATACTTTTATTTAATATTGTTTA

TTTTTTTCTTTCTTTGTTTAACTGAAAAATAATTTGTGTGCAATTTTACTGAATTTTCTT

CAATATTTCTGTTTACTTTGTTTATCCCTTCACGTAAGGA

>c17008_g1_i1 len=227 path=[79:0-188 25:189-226]

GCACTGCCATGCACCTGAGCTCCAGGAAATCACACACCATTAAATAAAGAGCTGCTGAGA

GGCCTCTGCAGTTCAGCTCACCAAAGGAGCCCACAGCAAATGAGAAAAGTGGTGGAACAC

AAGCATGCTGCCTGCTTAAGGTCCAACGAGGAGCAGCCAAGCTTGGAGATGTGCAAGAAG

GATTGCAAAGCTGACGTTAAGGTTGTTCCAGCATACCTCCTCTAATG

>c17008_g1_i2 len=339 path=[79:0-188 267:189-338]

GCACTGCCATGCACCTGAGCTCCAGGAAATCACACACCATTAAATAAAGAGCTGCTGAGA

GGCCTCTGCAGTTCAGCTCACCAAAGGAGCCCACAGCAAATGAGAAAAGTGGTGGAACAC

AAGCATGCTGCCTGCTTAAGGTCCAACGAGGAGCAGCCAAGCTTGGAGATGTGCAAGAAG

GATTGCAAAAGACAAAGCAGATAATAACGTATGCAACCTTAATAGGGTAGCCTAAATTTG

GGGCAGGTGCACAATGCTGCATCTCTGCTTATTAAGGAGTTAATGTGGGTAGCATGGCTT

TGGTTGCTGGCTGATGAATGATGCCCTGATGTTTCTTTT

>c17013_g1_i1 len=211 path=[245:0-185 451:186-186 452:187-188 453:189-210]

CCACAGCCTCCTTGGGCAACCTGACCCTTCTTAAAATAAGTGACAAAAAAAACCAAACCA

ACACCTTCATTGAGTGTCGTACTGACCTAAACAAGACTGCTCTGAGATTGCTTGTGCTGC

TGTGCCATACCCTGGTAGCACCAGCAGCGTATTTAAGAACAGAGGTCTTTATTTATTTCC

TCCACATTTATTTCAGTTTTAAAGAAAAAAA

>c17015_g1_i1 len=269 path=[286:0-120 20:121-268]

AGACGCCAAGAAAGGATATTGTGCAATGAAAATAGTAGGGAAAGAAGAAACAACACTACG

TATTGCAGGCAATCTGCTCAGGAGCAGCTCTGAGATCAGAGGGGGGAAGTTTGAGAGGTC

TAACTTTAAGGTCTTGTGGTTTGTGTCAGTAGTCACCGGTTCTGCCAGCCCCACGAGTGC

TTGAGCCCTGCTGGGGCCATAGGGAGACCAAGGCTTTGCTTTTGGATCCCTGGACCCCAA

GATCTCACTACTTAGGGTGGTATCAGTGG

>c17019_g1_i1 len=297 path=[91:0-43 18:44-67 158:68-70 45:71-296]

AGAAATCTTAGCAAATTTCCATTAAATGTTACACAAACATATGGAGTTTGTGTGTTACTT

GTTTACTTTGTGATGATTTCAGCCTTGAGAGATACCGCACAGTTTTAGTCATTAAGTACA

CTATTAGCTGGAGAAAAGCTAATTGCTGAATAAAGAAAGCTGAGTCTTACTCAGCAAAAT

TAGAAAATGTTACTGTTGAACAAAGCAGTGAATTAAAAATAGCTCCTTGGTGCTAACCAA

GCAGTAAGATAAATAAAAAACACAGGATCATAGATTGAATGAAGTTGGAAGGGCTCT

>c17020_g1_i1 len=217 path=[153:0-216]

TAGCTTGAGGAAGAAATGTCATCCTAAAATCCCACTGGTACTAAACCACGCTTAATCACT

CCTGCAGATAGAATGCTGCTTCTGAGATGCCTTCCAGATGAGCCTTATCTTTAGCATTTA

AGCCTGTCTGCTCCTATATAATTCGTGAACTTGTTGGTTTTCCCTCCATAAAGGCCATGT

CTGATTTTCCTCACAGACCTATAGCCAAGAGATTGAA

>c17022_g1_i1 len=260 path=[101:0-200 353:201-212 365:213-259]

CATTCCACTTCACCTCTATTAACTGGGAGCCGAAACACAAGCATCACCAAAAGCCACGGG

ACACAGACACCTCGTGCATTAAGCTGGTTCTGGGGATGTGAAGAACCGACATAACCCTCA

CCTCCCCCCGGCACCGCGCGCCGTTCCTCAGGGAACGTTCACCGAGCCTCAGGGGCGGGT

AGCGGGGCACAACAGACGGCCCCCGGCCATCTCCGGCCAAGCACTGTGAGTGCCGCGCAG

GGCCGGAGATGGCCGGGGGC

>c17026_g1_i1 len=248 path=[53:0-247]

AGCTTTAGAAATGTCTTCCGAGCCTAGAAAATGCATTTAATGATTAATCTGAGATTCTCC

TAATTAGCTGGGATGAACATCTTCAGAGCTTGACATAAATAAAAACAATGGTAAAATATT

ATCAGTGCCCCCAGGATGTTACTGATCGTAAAGTTACACGTTTTCTTGTATGCATTTCTT

GTTTTGCTCCAGAAATATACTGTGCAAAGTGATGATCACTTACATCAGTTAGAAAGCCTA

GGATTGAT

>c17029_g1_i1 len=208 path=[37:0-124 36:125-207]

AGTTCCAAGGTGTACACCTCTGCTTTTCCCTTCTGGTGTGCGTTAGCTTTGAAGAACTGA

TAGTGGCAGCTTTAGGGATATGTTGGAGGTTTTGCCTTCGTGCTGCCAGGTTTTTTTCTC

TGTTCGGCCATACGCAGTCATTAGCAAGACAAAACACATTTTTGGGCTGGATAATATTTC

AAAACATGTAGGAATATGAAGCACGCTT

>c17031_g1_i1 len=217 path=[75:0-28 104:29-139 598:140-141 352:142-216]

TTCTTGTAGCTTATGGGCCTGATGCTGATTTTAAAGTAGCTTGTGTATTGACTTCTGATA

AGTAAGATTTTGGTTTTGGCTGTTGAGATTAGAAATACAAACCTTATAGCCTCTGCTCTA

CTGTGTAATGCTTATTTGAAGACGTGTGCCAGAATATGTCAGTTAATTAGAGCAGCTCAG

CTAGGGACTTGTCATTAACAGAGATCACTGGAGCTAC

>c17039_g1_i1 len=213 path=[259:0-11 277:12-93 64:94-212]

CAGCACAAAGAGCACTTCATTTTTCTTGTAGTACAGAAGTTGTTAAATGTGGAGCAAGAA

CGTATCAATCAGTGGAAATGCATTTAGACAAAAATATCATTAAGTTGGGTGGCATTCAGA

AGGCCGAGCTGCTACTTGAGAATGCATTATATGTGGTGCTGCAGATTGTAGTTTGAGCCT

TCTATGGTGTAAACCAAGAAAAATGAAGTGCTC

>c17045_g1_i1 len=265 path=[43:0-202 553:203-205 556:206-264]

TGTGGACAAATGTGTTTTCTCTTACCCTCCGACTGACTTAGAGGAGCCACAGTTCAGTCT

CTGAGATCTAGAAGTGCATAATACTAGTAACTGATTTATTGTTTTTGCATCCACCCTATT

CCATGATATCTGGTAACTAGGACAATCATTGAAACCAGAGACTGGTTTCCTTCCTTAGCA

AAAGCATGATACAGAGATGTTTCTGGGAGATTTTTATTTTCTTTTCTCTTTGTAGCAGGA

AAAAGCAAACAACTTTCTCCCAGAA

>c17048_g1_i1 len=300 path=[339:0-41 62:42-299]

GGCTTTTGAATATTTACTCTGGAAATGGAAGTCAGCCTTGTAACTAGACGTACGTACAGC

AGCAACATAAACTAGTTCAGAAGTGCCTATTGCTTTAATTCTAGAACAAGCTATCATGTA

TATATCTATATAATCTTTTGGTTAAGTACAATACTGTCTTACTCTACACATAATATCTTC

ATATTGAAAACTACTACTATGCAACATAAACACACAAGAAAATGAAGCTTCCCCGAAATG

AACAGATTCCGATTTCATTTCAGAGATGGAGGACAATGCTGACTTCCATTTCCAGAGTAA

>c17049_g1_i1 len=311 path=[328:0-20 349:21-141 42:142-310]

CTTCTCTGGAGAGTCTCTAGTGAAGTAAACTTACAGTTTCCTAGAACTTTGATTGTTATT

TTTTTTTATTGACATGATAGTGAAAGCAGGTGTATGATTTCCAAAGCTTCTAAATAAAGT

TCTTTTTGCTTTAGATGTGGGGCCAATGCATTGTTCCCACCTCATTTTCTTCATCCTTCC

TGACAACTCTATGGCTTACACTGGAGACCTTTCAGGTAGCTGCTATTACTTACTGTAAAG

CAATGGAAAGGGCAACAAGAGAGCAGAGCAACAAGATCCCAGACCACAGCAAGTGACAAC

ACACTCAAACA

>c17050_g1_i1 len=227 path=[246:0-152 42:153-226]

GTTTGAGAGGGGAAAAAAAAAAAAGAAAAACGTTTACTGCTCAAATGTTTTTCAGTGTTG

GAACAGCAGGGGGAGATGGAATGGATAGTTATGTTTGTATTTGAGTCTTTTCTATGTGGA

AGCTTAGAAAACCTATTTTGTCTAATGAACATACTTTGGGAAAATAATTTCATAGCTACA

TTATTGTTATGTTATCTTAGTAGATAGGTAATTAAAAAAAAATCATA

>c17058_g1_i1 len=1482 path=[1534:0-1481]

TCATAACCTAAATATGTCAATTTTATAATTTATTAATATAATATACATAAAGGATATATA

ATGAAGTTAGATATATCGTGTTTTAGTTTTCTATCAAAAAATGAATATCGTGTTCTGACG

GCTATTGAGATGGGAATGAAAAATCATGAATATTTGCCAGTTTCCTTAATAGCAACGATA

GCCAATTTGAGAAAAGAAGGAATAACAAATGTTTTAAAAAAATTATTAAAAAATAAATTA

ATAAGTCATCAAAATAAAAAATATGATGGATACAAATTGACATATTTAGGTTATGATTTT

CTGGCACTCCGAACCTTTTTAAAAAGAGGAATATTAAAAAGTATAGGTAATCAAATTGGG

GTTGGAAAGGAATCAGATATATATATATGTAAAGATATAAATGAAAATTTATTATGTTTA

AAAATTCATAGATTAGGTAGAATATCATTTAGAACTATTAAAAATAATAGGGATTATTAT

GGAAAAAAAACTTTTAGAAATTGGTTATATTTATCAAAAATTGCAGCAACTAAAGAATAT

GCTTATTTAAAAGCATTATATGAGAATGACTTTCCTGTCCCCAAACCATATGATATCAAT

AGACATATGATTATAATGTCATATGTAAATGGATATCCATTATCTCATGTTAAATTAAGT

AATCCTTTTAAAGTTATAGATTTTTTAATAAATACAATCGTAAAATTTGCAAAAGCAGAT

ATTATTCATGGTGATTTTAATGAATTCAATATCTTAATTGATGATTATGAAAATGTAACT

ATTATTGATTTTCCACAAATTGTGTCACTACATCATGAAAACGCTAAAATGTATTTTGAA

AGGGATCTTAAGTGTGTTATAAACCATTTTTTTAAAAAGTATAAAATAAAAATTATAGAA

TATCCTTTTTATGAAGATGTTATTTCATTAAATAATGATAAATTAATCATTGACGAAAAT

AACTTATCAAAAAATGAAGATAAAATGCTTTTAGAAATTTTACAAAATGATAAATCAGAT

TATGATTCTGATTATTCAAACATATCTAACCAATCAAATATTAATCAAATAGTACATTCA

GAAGGTGACGAAATGAATATCTCATATAATAATAGAGAAATAGACGAATATAATGAGGAA

GAAACAGAAATAAAAAAAAATAATGTAATTGAAAAAAAGAAAGAAAAAGTTGAAAATATG

AAAAAAGAAAGTAATGAAGTAGATCATAATAATATTATATTCTATGATAGTCATGAAACA

TTAGAGAATAATTTTAAAGATGCTTATGAAAATGATGAAAATAAAAAAAAGAAAAATATA

AAAATAGAGAAAAGAGAAGATTTAATGGAAAATATTATAAAAGAAGATTTTCAAAATGAA

AAAATAATGAAAATGATGAATGATGAAAGTAAAAAAAAGGATCTAAAAAGTGATGAAAAA

AATATTATTTGTGAAAAAGAAATAGTTAATTGTGAAAAAAGT

>c17062_g1_i1 len=206 path=[338:0-205]

TGTCAAGCAGCCTCCTCGTGTTCCCAATGCCAGCTCCACCTGTAAATGTCCCTACACACA

CATACATGCACACACACACACAGTGTAAGTGACTTGTTTGTACATCACGTTTAGTTTAGA

TACCGGTGTATGTATAGATGACAAAGTATATTTCTATAAACTTGGCAGTTAGGTGCATGT

GTGCGTTTCCATCTCTGTATTTCAGG

>c17063_g1_i1 len=714 path=[35:0-713]

AATAAAATAAAATAAAATAATAATTTTAATTTTTTACTTATATTTATTTTGATATTGTTA

TATTAAGTATTGCTTTATATACCTTTTTAATGAACATAACTAAATTCATATTTATTTTAT

TGTATTATTATTTTATAATATTTAGTTTTATTTTATTCATTTCATCATGTTTTATTATTT

TATATTTTATTAAATTTAACTTTTTTATAAAAAAAGAAAAGTTCTTAAAGTAGAAAAAAA

AAAAACAATTTTTTGTAAAACACACAAATTTATATATTAACAATTTTTTTAAGAAAATGT

CGAATATAAGTAATGCTCTTATATGGGAATTAACAAAGAAAAACAGTTGTTTCCTTAAAA

AAAATAAAAGAGGAGGAAGTAGAGTATTCTCATGTGATCCTTATAATGTTTATTGTAAAA

ACACTCCATCTAATAGTGGTTTAGTAAAAGATAATTGTATAAATGTGAGAATATTGAAAA

GAAAACCAGTATTACTTGTTAAAAAATTAAATGAAAAAAATGGTGTTGTTACTAAAGAAT

GTAAAACAAAAAATATTAAGAATGTAGAAAAATTAATTGAAGAATATGGGAAGAATGAAA

AAGAAAAAAATAAAAGAAAATTATTGAAAAAATACAAGCGTTTATTCAAAGTATATTGTA

ACACTCGTAAAAGAAATAAATGAAGAATTTAAAAATTATATATATATATATATA

>c17067_g1_i1 len=228 path=[235:0-34 15:35-227]

GAGGAGGTTGGTTCTGAACAGAGGAGATCTGCTTAGCCATTCCCCCATGCCGTGGGTGAG

CCACGCTCTGGCTTATCAGGAGGTGAGCAAACTTCTGCCAGATCAGTGAGCCAGAGGTGC

ACAGAAGGGGCTGATGAGCACTGGAGCTGCGGTGAGGGAGTAGGCCAGGCTGAGTGGCTG

GGAGGGGAAAGCTGGGGAGAAGGGGGAAGAAACTTGTCCCTTGTGCTG

>c17073_g1_i1 len=274 path=[406:0-273]

GTTATGTCTAATGATGAAAAGAGAACCCCAAGTAAATATATAAAAATTTACTTTTTCTTT

TTAAAAGAAAGATAGTGTTTTCCACGTTCAGCATTAAAGCAGAAATATTATTGGTATTCA

AGGTTACTTGACACTGAAGAATAAGAATGCAGAATAGTTCTAACAGCAGTTTAAATAATG

TTGGTATTTCTGATAACTACCACATTTTGTGACTTTTCAACAGTTAAACATCCATACAGC

AGCAAATTTCCAGAAAAGATTCATGTTATTAGAC

>c17075_g1_i1 len=202 path=[180:0-201]

ATTCTCTGCTGTCTCATAGGAGGCGGTGCGCCCCGATCCCTTGGGTTGGCCGAAGGCGTC

GGGGGCTTTCTTCCCCTTTCCTTCCCATCCCCTGGTGGGGAAAAGGATGGCTTTGAGCAG

CCTAATTGTTCCCCGCGCATTAGATACATTAATGAGGTCTCTGATCAACCGGCCGCCTCT

CCGGCGAGGAGGCTTCAAGTAG

>c17076_g1_i1 len=401 path=[449:0-89 30:90-399 870:400-400]

TTCACAAACGCTGAACTCAACCTGTTGAAGAGGTACGTTATTAATACCACAGACAGAAAA

AAAGCACAACCACCAAAAGGAATAAACAACAACCCAAGTAACTTATTTCAAAGCTGCACT

GCTGCAATCAACATCTAGGCTCTGCTGGAAAGGCAGCTTTTAGCCACTCCTCTCCCAAAA

ACATCTGTCACTTGCTGGCATTTAGGACATTCAAAGAGTGGCAGAATCGCAGCTGTAACA

CCTGGAAAGGCAAGTCAGCATCTAGCACTCTGCTTCTTCTACCTATCAAATAGATAATCT

CTTACCTTTATGATTTACCTCTGCACTATACATTACTTAGTTTGGCTTGCTCACTTTAGA

TTTTTAATAACAATAATCACAGCATTCATAGCACAGCACCT

>c17076_g1_i2 len=325 path=[16:0-13 30:14-323 870:324-324]

AGCACCTCAACACTAACCCAAGTAACTTATTTCAAAGCTGCACTGCTGCAATCAACATCT

AGGCTCTGCTGGAAAGGCAGCTTTTAGCCACTCCTCTCCCAAAAACATCTGTCACTTGCT

GGCATTTAGGACATTCAAAGAGTGGCAGAATCGCAGCTGTAACACCTGGAAAGGCAAGTC

AGCATCTAGCACTCTGCTTCTTCTACCTATCAAATAGATAATCTCTTACCTTTATGATTT

ACCTCTGCACTATACATTACTTAGTTTGGCTTGCTCACTTTAGATTTTTAATAACAATAA

TCACAGCATTCATAGCACAGCACCT

>c17078_g1_i1 len=371 path=[598:0-313 1169:314-316 914:317-370]

TAATTAGGAGTTATTCCTAGGACTTCAGGGCGCCACTTGCTTCTCTGCTGCTATGTCTCT

GAGCAGTTTTCCTCGCAACAGAGAGATTTTTGATGGTTAAAAACAAACAAAGAAATCACA

GCAATTGAGTTAAGCCAGTTAAACAAAGAAATCTTGAAAATCGGTCAACGGCAAAGAGTA

TCCTCTAAGGTCAATGAGATAACCATGCCTCTCCTGGCTGCTGGATTCCAAATAGCATAC

AGGCATGGAAATCCAAACTGTTTTTTAAAAGCTAAATATCACAACCCCTTTAGAAAAATG

TTGTGTTGGACGCACAAGCATTTATTTCATTCTTACTGTTCTTCATTTTCATATCATGAG

AAAGAGGTGAC

>c17079_g1_i1 len=217 path=[29:0-152 14:153-216]

ATTTTTTTTTATTTTTTATTATATTATGAAAGAAGTTTTTTTTAATATTTACTAGACAAA

GATTCATAAAAAAAAAAAAAAAGAATAATTATTGAATTGAAACTTAAACAAAATAAAAAT

TCGTAGTAAACATAATATTAAAAAATTATAAAATTAATTTTTCTTATTATCTATGTAATA

ATAATAGTGTAGTCGTATTAACAAAAAAAAAAAAAAA

>c17081_g1_i1 len=212 path=[341:0-17 343:18-211]

CCTGTGCAGCCCGTGAGTGGTTGGGTTTGGGTAGGAAGGTGTTCTCAGCTCCTCCCTGCC

CCGTGGGCTCTCCTGGCCGTGCTGCCCTGATGGGAGCAGTCAGGGATCCAGGGAGCAGCA

TGGCCCGCTGCCTGCGGGGCTGTCCTGCTCTGTGCTGCTGTGCTCAGTGCTTACACTCAT

CCCTGCTCCCAGGTTCCTGTGCAGCCCGTGAG

>c17081_g1_i2 len=308 path=[39:0-113 343:114-307]

GCCGGGTACGACCACGCGCAGGTAAATGCAGGGGGCTTTGCTTGCCCCCCTCGTCTTGTT

CAGATCATCCCCTTCTCCTGGGCCTTCAGAAGCAGCTGGCTGAGTTTGAGCTGAGGTTGG

GTTTGGGTAGGAAGGTGTTCTCAGCTCCTCCCTGCCCCGTGGGCTCTCCTGGCCGTGCTG

CCCTGATGGGAGCAGTCAGGGATCCAGGGAGCAGCATGGCCCGCTGCCTGCGGGGCTGTC

CTGCTCTGTGCTGCTGTGCTCAGTGCTTACACTCATCCCTGCTCCCAGGTTCCTGTGCAG

CCCGTGAG

>c17082_g1_i1 len=548 path=[576:0-547]

GCCAGACAGACCTGCAAATAGAACGGCAAGTCAGACACCCACCCCGTGAGTCAGGCTTCC

ACTCTCCATCCTCACAGCATCAGCTTATTAAATTAAAGCACTGAGTAGTGGTGACACTCT

GTGGGCATTAATGTCACCAGCAGTAACAACCCTCATGACAGCTGATGAGCCCTTTTTAAC

GTTATTAACCATTTACATCGAGCTGTGGAACAGCTGAGCACACATCACCAATGGGGAGGG

CAGGAGGTCCCAGTGCCAGCAATAAGAACCAATGCTGCCCAGCTCAGCAGCTGGGAGGGC

CCAACACCCACCCTGCAGCAAGCACCACAGCTGGCAGCAACCTCTCTCCTCATCAAGAGC

AACTCCCAAGCCACCACTGTGAAAATCCAGGTGTAAAGCCAATGAAGGCCTTCTGCTGAA

TTAAGAGAGCCTGAATGTGTGTGTGCAGGCAGTAATTAGAGAGAAATCCACGGGACTCCG

ATTTGCACTTCACCACCAAAAGCTGTGGTGTTTTACAGATGCACACCTGCTGAACTCAGC

ACCTGAGA

>c17089_g1_i1 len=350 path=[1:0-301 303:302-349]

GCCAACAGGGCTGATGTGCTGGAAGATCTCATGGGGGAGTGAGGGGTAAAGGGGCATTCG

CCACACACACTAAACTACATGCTTTAAAAAGTAAATACATCTGTTAGCATGAGTGGGATC

ACAAGTCTGAACTTTGTGTGCCTCTGAGTGGTTTGTTTTTTGTCTGCAGGACATAATTTG

TTTGCTTAACTTCAGTTTGCCTCACGTTTAACAGGTTTAGGCAGGAGTAGCCATGTAAAA

ACAGCAAACATTCTGACTGAGCATCCACTCCCTGCTTACTTCAGGGCTGGGAATGCTGTA

GCTCAGGTTATTACTTGTTATAATGATGCTCTCACACAACTTCTGTGGTT

>c17090_g1_i1 len=204 path=[182:0-94 277:95-98 281:99-203]

AATAAATACCTTCAGCAACCAAAAATCACAATTCTTTCTGAACAAATGAACATTCTTAAA

ATTCTCTCTACATACCAGTGATAATAGGTATGAAGTTTTTTTTTGCTGTTTTTATTTTTT

TTTATAAGAAAAATTCTGTACAATATGTTCAAGAGTAACAAATTGGAAAATAATGATGAA

CGTTAATGCCTTTTCTGAAAGAAT

>c17093_g1_i1 len=247 path=[225:0-169 395:170-246]

ACCTTGGTGATTCTATGATTCTTGGACCTCCCTGTCAAATGCCAAACTGATTATTTCACA

ATTCTTTACACTTGTCAAACAATGCTTTCACGGCATCCCCTTCAATTCAGATAAGGAGGA

GAGACAACTATCTGAAGGATACATCATCCCATCTAGCCTGTGTCAATCTCTACAACATCC

TTTTGCATCCACGCATGATTAATAAATTTCTGTTAATATTCTTCCCTTCTGCCACTGTTT

CTCAAAA

>c17094_g1_i1 len=285 path=[1:0-40 42:41-284]

AGGCCATTCTATGATTCTGTGACTTGTGTTTGCTCTTCTCAGGCTTCTTAAACATCTGTG

CTGCAGGTGTATTGACAAAGAGGTTTCCTCAAGTAGCAGGAAACCTCTTGGGCAGGAAGT

ATGATGAAGCTTTTAAAGGAATCTTTCCCTTCAGGAACTGGAACACCAGGGTTTCCTTCT

GAACACCAGGAAGCACTTCCATGCTGTGTGGGTGGTGGAGCACTGGCTTGGGCTGCCCAG

AGAGGCTGTGGAGAAATTCCTCCTGACGTCCAATCAAACACAAAG

>c17095_g1_i1 len=258 path=[41:0-69 40:70-87 20:88-257]

ATGTTCTTTCTGGTCTTTAACATTCATACTCTGGTGAAACATATTTCATGTTTTATTTAA

CAAAATGTGTATGTCCCTTGAGCGTGCTGACATTTAATGTTCTGTAACTATCATAAACCT

GAACTCTAACATCCTTCTACCATACAGTGGCTTTCTTTGTGCATTTATCTAGCTATGGTA

ATAATCAGTAATTTTGAACTGCATCTAGGTGAAGCCTAGATTCCTGCTTGAATTGGTTTT

CATATCAACTGTGATGTA

>c17095_g1_i2 len=353 path=[274:0-164 40:165-182 20:183-352]

TACATCACAGTTGATATGAAAACCAATTCAAGCAGGAATCTAGGCTTCACCTAGATGCAG

TTCAAAATTACTGATTATTACCATAGCTAGATAAATGCACAAAGAAAGCCACTGTATGGT

AGAAGGATGTTAGAGTTCAGGTTTATGATAGTTACAGAACATTAAATGTCCCTTGAGCGT

GCTGACATTTAATGTTCTGTAACTATCATAAACCTGAACTCTAACATCCTTCTACCATAC

AGTGGCTTTCTTTGTGCATTTATCTAGCTATGGTAATAATCAGTAATTTTGAACTGCATC

TAGGTGAAGCCTAGATTCCTGCTTGAATTGGTTTTCATATCAACTGTGATGTA

>c17097_g1_i1 len=229 path=[207:0-228]

TAGATATTTGACAGATGACATTTAGAACTGCAAGCAAATTCCAAGTTCTCTTCGACAACA

CAGATTACATATTTTGTTTGCAGTAAGATCTCATTTTCTCTTTACATGCAATTAAAATCC

TTACTGTTACACCATTTTGTATATTCAATGGATATCTACTTAAAAAAAAAAATTGCTTTC

TAGAAGGTAAGAGGTGGGGTCATGGATTTTTTTCAAGACCAGATCAAGA

>c17098_g1_i1 len=353 path=[61:0-320 401:321-352]

GCCATGGGCTAAAGGAGGTGGCCACCATGTGGAGTGGAGGTCATGTGCTGCAGGGGGTGG

CCATGGGCTGCATGGGGTGGCCACTGCATGGAGGTGGTGGCCACATTGTGTAGGTGATGG

TCACATCATGTAAATGGTGGCCAAGAGCAGAAGAGAGCGGCCACAGTGTGGAAGTGGTGG

CCATGGGCTGCAGGGGGTGCTCACAGCATGGGAGTGGTGGCCAAAGCACAGAGGTGGTAG

CCATGGGCTAAAGGGGGTGGCCATCACATGGAGGTGGTGGTCATGTGCTGCAGGAGGTGG

CCAAGAGCCGCAGGGGGTGGCCACCCCCTGCAGCATGTACGCACCACCTCCAT

>c17099_g1_i1 len=211 path=[189:0-210]

GTTTAGGACGCTGTTGGTACACTTGTTCTTTTCAAGGCTATGTGTATTTTACTTCATGTG

GAAGTTGGCATACATTTGCAGTGCAGCAATTTAATGATAGAGGGAAAAAATAAATATACT

CAGGGCAAATGAACTGCTGTGTACAAAACTGAACCAGAAGAAGTGTCTGATATCATGTGG

GTAATAATATCTGAAAACTTTGTTTTAGGTA

>c17099_g2_i1 len=217 path=[377:0-216]

AGGAAGTAAGCCCATTATTTCATGTAATGGATTAAAGTTCAGGAATGCTTCCATATAATA

CTGCGTAAAACGAGTAAAATGGAGGAAGATTTATTTAGATAGAATTCTAGGAAATACTGA

AAGGGATATTGTCAGCTTTGTATCTACTTAATTCCAAATGAGCTTCTCTATTAACAACAG

GTATTACATCAGCCTCAAATCACTTTCACCTGTTTTA

>c17107_g1_i1 len=306 path=[38:0-305]

CTTGCAAAAAAAAGAAGGGAAGTGACCTGGTATTTGTTGTACGATTAACAAATTGTGTCT

TCTGTTCTCATCGAAGGGAACAAACAAGTGTGGAGCTGCTCCTTCTGGGTGAGCTTTGGT

CTTCCTTGTTATAGTTGGCAGTGAAACAGGAGCAGGCAGATTTCTCTCTTTGCAGATGTT

AGGGACTGAGTTCTTCTCACACTAGTTTTGTGGAAAGAGCTTTTGGTCTTTTCCTTACAT

TCAGGTGAACGTGTTCTTGAATGTGTTCTGCCCAAGGATGAAGCACTGTTCCCAGGTTGC

CCAAGG

>c17110_g1_i1 len=218 path=[1:0-99 101:100-217]

CTCCAGTGGTTGAAGGCACCTCTGCAACACAAAGCAAAGGCTAAATAAAGCATCAGCAGG

ATGACCAGCTATCAAATCTCTACAGTACAATCCCTCCTACCTAGAGGGTTTTTGTAAGAA

AAGGGTATGTAACAAAACAGCTGCTTTCACAAGTGTTTATTCTACAGCCCTTTTAGTGCC

CGCATCTCTTCAGCAGTTTTAAACTGCACCGTGTAGGA

>c17113_g1_i1 len=202 path=[235:0-201]

GAAGGGCAACAAAGCTGGTGAGGGCCCAGGACCTCTTCAGAGGGGCTGGGGCAGAGTTAG

CAGCAAGATGCACTTCTAAAACCAAGTCTGCTGCGTTTACATCATCTGTACCAATCAGTA

AAGGACAAAGATAACAGCAACTAGTTTTTAGGCTGCAAGAGATAACTATGAAATGACTAG

GTGAGCCCCAAGTTAAATGTGG

>c17115_g1_i1 len=216 path=[492:0-215]

TAACCTTGTGTTTGATGATGAATCTTAGGGGGAAAAAAAATATTTACACACTAGATAGCT

CTTCTCAAGATGTATTACAGAGGAACACTTATCCCTTTCTAAGGATCTTTTCAGTTAATA

AATAGCACTTCATTCCCAATTACATTTTTGTTCTTAGCAGAGCAAGAGGAGGAAGCTTGA

GTACAGAAGAGAATGCTCATAAATACAGGCACAAAG

>c17116_g1_i1 len=339 path=[317:0-338]

AAATTAGAGGTGAAAGGAAGGAAGGACAAAAAAATATAAACAGTCTTTGGAAATAGGTGT

GTTCACAGGAGCCTTATTGTGTAGAGACAGCATGAGCCAACATAAAGCTTCTTCACAGGG

TGTCAGTCCAGCCCTATGTCACAACAGTGAAACGTTAGCAAAATGAGCCCAGTGTGTAAT

TGAGGTGCTGCAAAATGATAGAAGAGATTGAGAGACAGGGAATGAAACTGAAATAGAGGC

ATAAAAACTGGAAACAACATAACGCTTGAGTAGGTCAAGAGTCAGTGGCAACATGGTTGA

TACAACAGCTGGGTTAGCACGTAATGGAAGAGAGCAAAT

>c17123_g1_i1 len=214 path=[1:0-207 750:208-213]

CTCTCTATATATAAATATTCTAAAATATTTTTATTATCTTATAATGGATAACTTATTCTA

CATCTATTTATATAGTTTGAAAAAAGTTCATTTTTCTTTAAACTTAAATCTTCCGTAAAA

TAAAACTTTTGTTCAAATTTAACATAATTTTTTCTTTAAAAATTTATTGTTCATCTTAAC

AATAAATTATCATCTAAAATAAAATGATTTATTC

>c17124_g1_i1 len=201 path=[413:0-200]

AAAGCGCTGCCGGGAAGGGGGCCGGGGCCGAGCCACCACAGTGGGGCACTAGTAAGCAGC

CTAAGTAGAACCGGCCTGGGGCTCGTTGGTCTGGCTCTGGCAGAAATCAGTGTGGTTTGT

GCCTAGCTTTCCATAGGAGGTCGAAGGTTTCATGTCCTCATTCCTTCTCTTTCCCGCAGG

CAATGGAGCATGCAAACGGAA

>c17132_g1_i1 len=275 path=[1:0-71 73:72-74 76:75-274]

GACAGACAGAACAATTAGTATGGACTGTTTTCTCAAAACTAAAAAGAAGAAAACCAACCA

GCATGGCCCACCGAGTTTAGGAAATTTGGGAAACAGGTATTGCAGGTTTTAAATCCAAAA

TACACCCAGTGAAGCATGGAGTACGAATGTTCAGAATGAGTCTCATCTACAGCATCTATT

TCAGTATTGTACTTGCAAAACATTGTCTTCTGAAATAAATGTGTTTTCCACTAAAAGAAG

TCTTCTACTATTAAGTTTCTTTGAGGTGTTTGGCA

>c17133_g1_i1 len=224 path=[202:0-223]

TTGCCTATGTCTCATCCAACCTCTATGCTAATTTACCTATACAAAATTTGCAAGGGCATT

CATGCAATCTGTTCGTTACTCTAAGCTATAAAAAAGTCAGCTCCCTGCTTACAGTTACTT

TGGAACGCAAGAAGGGCAGCTTCTTACTTATTGTGGCATATTTTCAAATCAAGGAACTAT

AACCATGTTTCATGGTCACTCTCATAGAATGGCTTGGGTTGGAA

>c17138_g1_i1 len=215 path=[413:0-214]

AGTGTTCCAGGCTTTTTGGGTACTGTATGGTGTTTCATTTTTTGCTCACCCACCTCTTTC

TTCTTCCTCTTCCCACACTGAAGACCTCAGTTAATTCACTGCAGTGAATTACACAGCCTC

TCTGTGTCAGCTCATGTTCTGCAGTTCACATGCAGTTGTCCTCCTCAGCCTTGCTCTCAG

GCCATAGAGAGAAGCTGGGACCTCAGACCTCACAG

>c17151_g1_i1 len=428 path=[1:0-427]

GAGAACTAGCACCTGTAGTATTGCTTTTCACAAAAAAATATACTAGTAGTTGCAACTATT

AGTAGCCTTTGTGTATGTTGGAAACATACTAGTCTATGTACTACTGCTACTTCAATGTCA

TAGAGCTTAGATGTTTAAAACAACTTGGCAGTCACTGGAAACATTAATTCCATTGGCAAT

ACCTCATGGTTTTGTTTCAGGCCCTTAATGCCTGCAAAATAGCAGTTAATGGACAGTTGA

GCATATAGAAATTAAGTGACATTAAAATAAAAAGTACTGGCATCCAAAGTAATCTCTGGT

TTACAAAAATCCCTGATGAGATAAATATGTATGTGCTGGTGCTTTTTATTTGGCTTTAAT

ATCACCTGATTTTAGTGTATACATATATGTACTTAAGATATTATGGGGATATTATTATAA

GTATAGTA

>c17151_g2_i1 len=228 path=[811:0-227]

TGCTAGTTCTCATTTAATACCATGGAAACCCTTCTATAATCTATGATTGTATTAACTAAA

CTGCAGTGATATCCAAGTATCTCCTAACTTTAGAGAAATACTGCTTTCAAACAACAGTTG

CAGCGTTCCCAAAGTGAATTGCAAACTGTAAGCAATGTACTATTAACCTAGTGGTTGCAT

GTGAGAGAATTGCATTAGCTCTCTGGTAATTGCAGGATTCTTCAGTTA

>c17153_g1_i1 len=240 path=[1:0-239]

TGTGATCTTCAACACTGCCTAAAGGAAGGGAAGCCTTCTGATTTTGTAGTCTATGTAAAG

GGGGATTATTCAACAATACTCGAAGTGCATGCCAAGAGATAGCCACATACCCTTCTGCAC

ACAGAACATCTTTTAGGGCTAAGGATTCCAAATAATACAAAGAAGAAAACCAAAGTAGTT

GACAACTGGACTGCAGCTGTCAGCCAGAAAAACTGTCCTGTTTGAGGAACACATGGTGTG

>c17154_g1_i1 len=220 path=[448:0-219]

AATTACTACTATATCCTTCCCCCCCAAAATGTTTACTTTGTGATAGAAAAGGACAATGAC

TTTTTGTATTTATCACAGCCCAGGGATACACAGGCATTCATTTCTTACACAAGTTATTTC

AGCGGGAGGCAGTTAAAACACAGGCAATTAAATAATTGCATTTACTTGGTGGGATTTCAG

TATAGAAGTATAACATAGGACAAGAATGTAATTATTTAAT

>c17159_g1_i1 len=227 path=[1:0-115 117:116-226]

ATTTATTACGCAATAAAGGAACTGTATGTCAGGTTTTCTGCTCTTGCTTGCTCACTTCAA

TTACCTTGGAGCTTGGTGTGGCTATTTTCTTCTAGGAAGTAGCTAGCCAAGTGCTCATGC

ACTTAGACTTTTACTTGTATTGCAGTTTGCAGTCTTTAAACTGTAAAACTAGAGCTTGTT

CTCTTATAGGTCACAAGTGCTTCGTTCTCTGTGCACTTCTGTGCCAG

>c17163_g1_i1 len=219 path=[385:0-218]

GCAATCTCCTCCTGCCCCACTCGTCGTACTCCACCTCGCTCAGCTCCTTGCTGGCCAGGC

ATAGCGTCCTCAGGGTCTCCTCCAACACTGACATCCTCTTGCGGTCGCTGTTGAAGTCCA

GCATGGCCAGCACTTGGTACGTCCTCTTCACCCCCAGCTCACTGATGGTGATGGTGTCCT

GCGTTCGGGACAGGAAGACATAGCCCAAGCTCCTGGCTG

>c17166_g1_i1 len=255 path=[397:0-87 484:88-254]

ACAGATGGGCACTCACCAGATGAGCATTAGCTAACCTGGGTATCCAGCACAAGAAATGTG

CAAAAATGCTGTATGGCAGACATAGCAAGGAAAGGCAGAGCACCCTGCCATCCAGCCATG

GGTGAATGCCCATAATGAGCCATTTGCTTCATCAGCTCTCCCTCAAGCATTTCAACAAGG

ACAACATTAAGATGCTCTGCTAACCACCCAGTGCCCCTACTCCTCGCCCATTACACAGTG

ACTGCAGCTGCAATA

>c17172_g1_i1 len=231 path=[1:0-15 17:16-230]

AACAAGCTCAGAATGCTGTGGTAGTGAAATCTACAGTTCTGAAGTATAAGATGGCTATTT

TTACCAATTAACTTTTTGTAATTCAGTGTACTTTCTGAAGGTTCTGGCTAGCACAACCTG

TATACCATATATTCTGCATAGCATGCCTGAAGAGCTAATAATGACAAGTGATTTAAAATA

TGATTAATTTGAACACTGAGTGCTTCACTGAATAAAGAATCAAACACAAAG

>c17172_g1_i2 len=283 path=[294:0-67 17:68-282]

GCTCGATATAGATGGTCATTTGCTGTCACAACTGGTGAATTGTGCCTGTCTAAATAAATG

AATAGGCTTGTGGTAGTGAAATCTACAGTTCTGAAGTATAAGATGGCTATTTTTACCAAT

TAACTTTTTGTAATTCAGTGTACTTTCTGAAGGTTCTGGCTAGCACAACCTGTATACCAT

ATATTCTGCATAGCATGCCTGAAGAGCTAATAATGACAAGTGATTTAAAATATGATTAAT

TTGAACACTGAGTGCTTCACTGAATAAAGAATCAAACACAAAG

>c17183_g1_i1 len=214 path=[344:0-213]

ATTCTTCCCAGTGCACTCCTGGTCAGCTGGAGAAGATCAACAAGACCCCAGCCTTGGTCA

GCAATGCCACAAACAGATATAACTATTATTCCCCCAATCCAAGAACAGCTGCAGATGAGT

AAACTAACATTTGTGGGAGGATTACTGCGTGGTGCTTGCTCACCCTGTGTTCTCCCAGCA

GGAAAAGTTAGGATCTCACCTGGGAACAAATCAA

>c17189_g1_i1 len=570 path=[1:0-50 52:51-569]

GCTTGGGTGTCTCTGTGTGGTAACACCACAGAGGAGGCTCTGTGAAGATAAGAAACTTTG

GTCATCACTGTGGAATTCCTGGTATGAGAATGCAGAGCGTCAGAGTAGAGAGTGGATCCT

TGCTGAACAACTGTAATCATGAAGGCAGTTCTTATAATTGTCCTAATCATCTTCACTTCG

CTGTCTTCAGGGAAAAAGTTTCGGTGGTGCACTCTCTCCGATCTAGAACAGAGGAAGTGT

GCTGAGCTGTCCAAAGCACTAACAGCTGTGCTGCCTGTCACTAGCATCGATTCATTTGCC

AGGATTTCTTGCATCAGAGCCCACAATACATATGACTGCATTGATAAAATCAGAGTCAAT

AAAGCAGATGCTGCTTCTCTGGATGCAGGAGATGTTTACTCTGCTGTAAAGCTATATGGT

TTGACCGTCGTGGCAAAGGAGATCTATGAGCAGGGGGAATTGAACCCAGTGCACGGACAG

AAGCTGCAGAGTTGGGATTTAGTTATTTCTGTTACATATTTTATCCTATCACAGGAAATT

GTGTTTTTGCTGTGGCTGTTGCCAAGCGAG

>c17189_g1_i2 len=703 path=[1095:0-183 52:184-702]

CACAAACACAAAAGAAGCAGGTGGGGAATAAGGGCTGTGAATTGCTCAGGTAAGGTATCT

AATGTAGGAACAGAAATGTGGGAGAAGTGTTGCCGAAGTACATGAAGCCAAGGAAGTGCT

AGGAGGAAAGCAAAACTGGGAAGAAAATTTTCTTTCTGAAGTGTGATCAAGTCTCTCCTG

AATTGAAACTTTGGTCATCACTGTGGAATTCCTGGTATGAGAATGCAGAGCGTCAGAGTA

GAGAGTGGATCCTTGCTGAACAACTGTAATCATGAAGGCAGTTCTTATAATTGTCCTAAT

CATCTTCACTTCGCTGTCTTCAGGGAAAAAGTTTCGGTGGTGCACTCTCTCCGATCTAGA

ACAGAGGAAGTGTGCTGAGCTGTCCAAAGCACTAACAGCTGTGCTGCCTGTCACTAGCAT

CGATTCATTTGCCAGGATTTCTTGCATCAGAGCCCACAATACATATGACTGCATTGATAA

AATCAGAGTCAATAAAGCAGATGCTGCTTCTCTGGATGCAGGAGATGTTTACTCTGCTGT

AAAGCTATATGGTTTGACCGTCGTGGCAAAGGAGATCTATGAGCAGGGGGAATTGAACCC

AGTGCACGGACAGAAGCTGCAGAGTTGGGATTTAGTTATTTCTGTTACATATTTTATCCT

ATCACAGGAAATTGTGTTTTTGCTGTGGCTGTTGCCAAGCGAG

>c17193_g1_i1 len=211 path=[216:0-28 245:29-130 347:131-154 371:155-170 347:171-194 371:195-210]

ATAGAGACCCATAGAGAACCCATAGGACACATAGAGACCCATAGAGACTCCATAGAACCC

TCATAGAGACCCATAGGGAACCATAAGGAACCCATAGGGAACCATAGAGACACGCTCAGA

TCCATAGGGCCCCCATAGGGACACCCTCAGACCCATAGGAACACCCTCAGACCCATAGGG

ACACCCTCAGACCCATAGGAACACCCTCAGA

>c17193_g1_i2 len=227 path=[476:0-44 245:45-146 347:147-170 371:171-186 347:187-210 371:211-226]

AGACTCATAGGGACCCATAGGGACTCATAAAGACTTGTAGCACCCCATAGAGACCCATAG

AGACTCCATAGAACCCTCATAGAGACCCATAGGGAACCATAAGGAACCCATAGGGAACCA

TAGAGACACGCTCAGATCCATAGGGCCCCCATAGGGACACCCTCAGACCCATAGGAACAC

CCTCAGACCCATAGGGACACCCTCAGACCCATAGGAACACCCTCAGA

>c17215_g1_i1 len=249 path=[415:0-188 86:189-248]

AATTCCATCATTTCAATGACACTATGAGAAAGTCAGTCCTTTTTTGCCTGAGGTATATTT

GCCTGCTGAGAACAGCACTGCTATACAGGCACTGACCATCAGGTATCCTATTTCTATCTA

TTACACATGCATCGTTTAATCACATTACCCACAGAGATCTGGCAGAAAAAAACAACAGAC

CACTGCTGCTTTCTCTTCATTCATGGGACATGAAATACTGGTTGACTTTCTCATGGTTAT

ATTCAGTAA

>c17215_g1_i2 len=316 path=[415:0-188 603:189-315]

AATTCCATCATTTCAATGACACTATGAGAAAGTCAGTCCTTTTTTGCCTGAGGTATATTT

GCCTGCTGAGAACAGCACTGCTATACAGGCACTGACCATCAGGTATCCTATTTCTATCTA

TTACACATGCATCGTTTAATCACATTACCCACAGAGATCTGGCAGAAAAAAACAACAGAC

CACTGCTGCACATCCTGAAACATGAACTTTCTAAGTAAGGTTGGAGTAGTTTATACTACT

AAGTGAGGTTATACTCAATGTTGCCCAAGAAGTCTGGTAATTCCACTCATACCGTAATAA

ATCCATATCCTTTGGA

>c17221_g1_i1 len=294 path=[502:0-214 36:215-293]

TATATTACTGTTGACATAAATATTTTTTCCATTTTATATATTTATAAATTAAAAGAGAAA

AAATAAAAATTAACAAGGTTATTAATAAACTATATATTAAAGAATATTATTTTTAATCTA

CAAAAAAGATAGTAATAAAATAGAGAAATAAGGAATTTCAATAATATTTAGCAAATAAAA

TAGTAACGATAACTTTATATAAAAGTGTAAAAATAAGTTTGCATTTGTTCGTTAGACTAA

TTTATACTCTTTAAAAAAATAAAAAAAAAATTTAGTCATTATGTCATAATATTA

>c17225_g1_i1 len=408 path=[769:0-267 1036:268-407]

AATAAATCTGATATTGATTTCAGAGTTCTGGTTGTGGTGTGATGCAGGCTGTGCGAGGGT

TACGTGTGCATGCCAGTTTTACAACAAGTGCGTCGTCTTGAGAGATTAGTTTAAGTGGCT

CCTGGATTTCAGATTGGCTATGTGGAGTATGCAGAGCAGTTGTTAATGGATGTCTACCAG

TTTTCTTCTAATTGCATGCCTGCTCCTGATGCCAGCAAGAAGCTAATTCAGACCTCCAGG

AATGTTTTGAAATCATCTCCTTACCAAACCTGTTCTTTGCTTCCAAAAATCTCCACTGAT

TTAGAAATATATTAGCACGTTCCATATTTGCTTTACGGTTTCTTTAGTCAATTTGTTCAT

CATTATAGGCTCTCATCAGACTATGATATCAGAAGTCACAGTGTTGTA

>c17225_g1_i2 len=459 path=[769:0-267 25:268-458]

AATAAATCTGATATTGATTTCAGAGTTCTGGTTGTGGTGTGATGCAGGCTGTGCGAGGGT

TACGTGTGCATGCCAGTTTTACAACAAGTGCGTCGTCTTGAGAGATTAGTTTAAGTGGCT

CCTGGATTTCAGATTGGCTATGTGGAGTATGCAGAGCAGTTGTTAATGGATGTCTACCAG

TTTTCTTCTAATTGCATGCCTGCTCCTGATGCCAGCAAGAAGCTAATTCAGACCTCCAGG

AATGTTTTGAAATCATCTCCTTACCAAATACTTTTTATTTCAGAAAATCCTTTAAATGTT

CAAGCTTAACTAAGTTTGGTTTGATGGTCCTTAGTCAGCATTCTTGCACTCAGCTCTCTC

ATTTTCGTCATGATATCCTTGCCTCGGCCTCTTAATAATGGCTCTAATAATTCCTAGATT

TGAAAACAGCACATCTGTTTTAAGTAAGAAATTGCTCAA

>c17230_g1_i1 len=300 path=[546:0-299]

AGCCCTCTAGGGAAAGAACTGACCTCCACAAATCGCACCCAAAACTAACTTGGTAGGCGC

CTTACAGGGGTAAGAGGGACAACATCACCACAATTCCGTCCCAGATTCAGTAGTGTACTG

TTGACACGCACACACAAAAGCAAACAACCTTCAGTCTTCAGATACCAAAGAATGGTACCT

TGCCTATTCCTTGACTTGCAACAATTCTGCTATATTTAGTAGCCTTGAAGCCTTTAACTT

CACCTATTCAATAGGGAAGAACAATTCATATGTTGTATTTCTCTATCCTCCCCAAACAGG

>c17232_g1_i1 len=216 path=[194:0-215]

AAGCCTATTTACAGGTGGTGTTGATGAGGCTGGAAAGAGGTTCTCTGAGCATGAAGTCTT

AAGACAGCAAATGACAACGAAAAGTAAGGTCAACTCTAGTGCTTTATGCAACACTGACAA

AGGACTGCTCCACCACTGCTCTTCAACAATTGCAGGTCAGCACAGTGCGTACGGAGCAAG

CCCTCGAGCCCCCATCAGCACAATGGCTTTCTCATA

>c17233_g1_i1 len=425 path=[373:0-239 211:240-424]

TGAAAATGAGGCATTAGTGTCAGTGCGTGGGACTGCAAAACATTTGCTATTGTCTGGTAA

AGCAGCAAGTGTTCTTTTGCCTTTGTATTCCATTACAGCAATCGACCTCTTCTCCTTTCA

CTGGATCTGATGCAGTGTATGATCTACAGAATTAAGATGATTTGCAGCATTAGGCTCCCA

GCCTACTCTAAGTAGAACACCATATAATATGTTGCCTGACTAGAACATGAGCTTTTTAAG

ACTAGTTTGGTGCATCATTCATCCATGACACATCTCCCAGGAGAAGAAACAGCAAGAAAC

AGGCAATGGGTGTGAGGAAGGAGCCACTCATCCTTTAGACATCTGCATGCACTGATCACA

AAGTACAGGCAGCAGCAGAGTTCACACAGCACCAGAAGGAGGGAGCCATCAGAGAGCTTC

CAGTG

>c17233_g1_i2 len=302 path=[373:0-239 612:240-301]

TGAAAATGAGGCATTAGTGTCAGTGCGTGGGACTGCAAAACATTTGCTATTGTCTGGTAA

AGCAGCAAGTGTTCTTTTGCCTTTGTATTCCATTACAGCAATCGACCTCTTCTCCTTTCA

CTGGATCTGATGCAGTGTATGATCTACAGAATTAAGATGATTTGCAGCATTAGGCTCCCA

GCCTACTCTAAGTAGAACACCATATAATATGTTGCCTGACTAGAACATGAGCTTTTTAAG

CTCTTGAATCTCTGTACTTTTGTGGGAAGTCAGAATATAATAGTTCTTTTTTTTTTCTTA

AG

>c17234_g1_i1 len=220 path=[352:0-219]

GATGGTGTTTCCTGTGCATGTGCTGTGTTCTTTGTTCTTGCCACTTAAGCAAGCAGTAGG

AACATGCTGCCATATGGGAGCTGTATGGGGCATACTAGGTCTTAAGAGATGTCCATCTCC

TGAGCACAAATGTTACATAAATGTAAACCCAAGGAAGCCTGTAGATGGGGTTTCAAATAC

TTCTTAATACTTTTCCATGTAACTGAAATGGAAAAAAAAT

>c17237_g1_i1 len=205 path=[455:0-204]

AAGTTGCCCAGAGCCACCTTGTAATCGTTCACTCTTTATTTATTTGCTTGCTTGCTTGCA

TAGACTACATCCTCTTCCAGGACTCTGTGTAGACAGTGGTGACTCAAAATCAAAACCTTA

AGATGAAGATCAGTAGTACTAAATTCAGAAGTCCAATTTAGTATCACAAGGGTATGGTTT

CTAAGAACACTTAACATAAGGGAGT

>c17238_g1_i1 len=273 path=[1:0-218 220:219-272]

GGGTTCTGCCCCAAAAAGAGTCAAAACCAGACTCCAGCAGAACCTCTGGATGAGAAATAA

CTCCCTTGTCGATCTTCATCTGCTTTCCTGTGGCAGATTTACCTGTTTTCCTTGAGCTAT

TTTTGGTTGCTCTTTAGACATATATGTGGCACAGGATGATTGAGAGTAATTAAAAAGGAA

TTACAAGGTGCTGTGCTGGTGGTAATGGAAGAAAGGTGTCATCGTAGCGTGAGCAATATG

CAAAACATGCTAGCTGTTACACACCCACACGAT

>c17240_g1_i1 len=279 path=[1:0-278]

TGTGTGTGTCACCAAGGAGTGTTTCCAGGCTGCCCCTTCCACCACCATCTGCTGTCTGGG

TGTGATTAAAGCAGACAGGGAATGAGGCTGAGAGACTTTTACCTGTTCTGCACGCTCACT

GCTGGGACTGCAGCATGCTTACACTGGGATGGTTTGTTATTCTGCTGGCTGATTTTCATC

CCAGGGTAGCATCACTGCCATCATTCTGCTGGTGCGCATCTGTCCTGGAGCTGCTGCTTT

GCTTTCCTGCCAGTACAGCCCCAGCAGCCAGGAGATAAA

>c17257_g1_i1 len=388 path=[155:0-387]

TTTCCCTCCTTTGCCTGCTAATTGCATCCATGACTGCCGTAGCTGTTGGAGGTAGAATTT

ACTTCCCTTCGTGTCTTAAATTTTAGGAAAAATTGGAAGCTAGGCCAAGAAAATAGAGTG

GGACCATAACAGAATGAAGTGGGGACAGAAGCTGTGCTCCTTCCTCTCCACATATTTGCA

GGCTTATCTGTTCAGACAGGCACGAATTGCCCAATAAGACTTGTATGCAAATCGTTTTAA

GTTCTTTCTGGAAGGATTTGACACAACTCCAAAGCAAAAAGAGAGCAAACAAGACAAGTG

TACAAAGCTGTACTGTTGTTTTCACTATTTTATGCTACACTTCAATAATGCTTATTTCTC

CTCACAGGCGGAAAGAAAATGCAATTTG

>c17258_g1_i1 len=214 path=[460:0-213]

ACTTCCTCCGCCGCAGGTACTTCTCCACCCGCAGGATGTCATCCGCACAGATGTCCTCCA

CCTTCAGCTCCCCGTGCTCCGCCAGCTTGGTCTCCAGCTCCAGCAGCACCCGGCCGCGGT

ACGCGACGCCCTCGCCCTTGGGGTAGAGCAGGACAGGGTCAGCAGCCACCGCCCCGTCCT

CCGCCTCCGCTCTCGTTGGCTTCAGGGCAGAGAG

>c17264_g1_i1 len=273 path=[251:0-272]

CAGGGGATACCCTTCATACTAACACACACAGGTAAAACAGTGCAATCTGAAAAACAGCAT

GCAGCTTTGTTAAGCAAATTTCCATCGCCCGTAATTAGTGAGCCTGCAGAACCATGCAGC

ATGACAGAAATACGTTTCCAGTGACTTGACAACACAACAGAAGTGACTTACTTGTGACCT

GCACGTTTTAAGGACCGTTAGCAATCCCCTAAGCCACCTCACGGTGCCCGGGTGAGGCAG

CGCCCACCTCCCGTGCCCACATCAAACACAAAG

>c17271_g1_i1 len=258 path=[1:0-257]

TCTCGATAACTGTTTGCTGACTCTGGCACATTCTGTGACTCTCTTAGGACCTGTCTGGAA

ATACTCTCAAATCCTTGTGTTTTTCTGTCTGCTTAAAGTTCCAGCTCCTTGGTTCTACAT

TTTCAGTTAGGACATCCATTGGATTTGTGTACATCAGGTGACTTTTTAGAGGGAGGCTCT

GTATTCCTGCGGAACTGTGCTTCAGAACGTACGTTTCTTTAAGAGACAGTTTCGCAGGAA

TACAGTTCTTTGTATTTG

>c17278_g1_i1 len=316 path=[19:0-118 138:119-315]

TTTGTTATTATTTTTTTTATTTAACAAAGTAACTCTTAGTATACATCATACTTACGACTG

TCAAATCATTTAGAAAGGCCAATAATATTTCCACTGCAGGATATAACTTCCATTAAAAAA

ATAATAACAAAAGTTTTTAGTGCTTAATTTGTATAGTAAGATTCATGCTGTTTGTGGAGC

TTCTCTTTCCTATATAATTTGACTGCTTGCATATACTTTCAAAATCAATTATGTGTATGT

ATATATATATATTTCCATAACACTGTAGAGTCTTCATTTTCAAGTAGTTTAGATGGCTGC

AAGGCTAGTTATGGAT

>c17283_g1_i1 len=205 path=[155:0-204]

TTTAGAGCATAGGAATGATTTTTGATATTGGTTGTTACTCTGACAAATTCCATTTCTTCT

ATATAAAATAGACATGGCACCCTGTCTCAGTGGTGGGACTGTGCATTTTTTTCATTCTTT

TATTTTTAGCTTTAGCTGCTTCTCCCCTCAATGAAGAACTGTGGCTGTGCTCCTGTCGGA

CTGCTAAAATCTCTATTCACATATG

>c17290_g1_i1 len=252 path=[986:0-251]

TGGTGCTAACACAAGGACAGGTAAGCAGAAGGACTTTTGTGGCTTCAGTTCCTGTTGCAG

TCTGGTGGTGCCACGTGTATAGGCTAACCCAAAGGAACAGCTGGCATTGCTGTAGCAAAT

CCTGTGTGTTCCTTCACATCCCTAGTTGTAATTCTCCAACAGCATCAGAAATTGCCTCAA

CATGACAAGATCAAAGATGGAAAAAATTCTTTTATGTTCCAAGAAAATACCACAGAGTAA

TAGACTCCTTAA

>c17290_g2_i1 len=400 path=[380:0-399]

AACACAAAGTTACCTAAAAACAAACAAGGCAGGTAAAGCACGTTAGGAGAAGCCAGCAGA

AGAAGGATTCCCATTCACACCCCCTTGGACTTGTCACGTGGCAGTACAGAAGCCAAAACT

TGGCAGTCCTTAAATGAGACAACAGACAGCGAAAGCATCTGCCTTTATGCACACATGCTA

TACACAGGGATGCTTTGAACAACATTACACAGATGCTGGGAGAACCCAGCAGGCAGCAAC

CTCTGGTGGCTCTGGCAAACAAACTGAGGCAGATGCTGTGGGTTTTTGCTTTCTCTACCT

TCAGGAGTGTTTCCAAATGTTCCTACATCTGACTTTTTAAGTGTTTATAGCTTCCAGGTG

GAAGCCTGAAAAGCAGTTATCTATCACATGTCAGTTCAGT

>c17291_g1_i1 len=262 path=[277:0-261]

GAACAGGAATTAGGCTTTTATCAGTGCTAAAAAACAACCTTGAAAGTACTAAAAATAAAT

AAAGCCTTTTTGTTCAGTAATTAGCTCAACTACTTCCGCGAGAAAAAGTATGTGCAAAAC

CCTTTGCTTTAATTAAATCTCTATTCCATTTAGTGTCTGTCTGCTTTCCCTTATTGTGGT

AAGCACTGCAGCTAAGAATTTCCTTGTATGGCTTGCTGTCTCCCCTGAGAAGGGATAGGT

TGTGAAGTTAAAAGCTTTTTTG

>c17291_g2_i1 len=336 path=[553:0-335]

AATCACAGCAGTATTTGGCCTCAGCCACAAAATCGTTCATGTACGAGAAAGGCTGTGAAG

ACTCTGGAGGTTTGAAATTCACTCAAACCCAGCCCAGATCGGTCCCAGTCTGTTGACCTT

TTGGACATGTAGGAGGGCCCTTTTGTCATTAGCTGAGAAAAGAATCTGCAGGATATTGTT

CAAAGGGCCAAGGAGGGACTTGGAGGTTTTTCCATGGCTTCTCCAGTGAGGACCCCAAAA

CTGCCTCCTGCAGGAGTTAAGGGCCATCACAAATCTCACTGCTCTCTACTTTGGGTGGAA

GGTTCAGTTTTTTAACTTGACATCTCTGCTTCAGCA

>c17297_g1_i1 len=346 path=[597:0-40 315:41-345]

AGACTAAGCAGTACAACACAGGGACGTACAGCAGGCTTTCCAAAGAGCCAGCAGAAGTCT

GATTTCTTAGGCCACACTTCCTTTCAGCGAAAGGAAGCTTTCACTGTGCAAGTCAGCTTT

GGCTCAAGCATGAGCAGAAAGTAGTTGGACCAGTTTTTCTTATCCATTGTTATCCTTACA

AACTATTTTATAGAGAAAGACTGAGGTCAAGAATGAGAGCAAAGCAAGATAGCATGAATA

CTTTTCATCCCCACCTTTCTTATGGACAGGGTTCGATAAGACAGCAAGTGAGGTAGAATA

TGCAAGAATTTTCACTGCCTTCCATCCACCTTTCATCTCACTGTAG

>c17297_g1_i2 len=321 path=[299:0-15 315:16-320]

CCCCCGCCTGCTTTTTAAAGAGCCAGCAGAAGTCTGATTTCTTAGGCCACACTTCCTTTC

AGCGAAAGGAAGCTTTCACTGTGCAAGTCAGCTTTGGCTCAAGCATGAGCAGAAAGTAGT

TGGACCAGTTTTTCTTATCCATTGTTATCCTTACAAACTATTTTATAGAGAAAGACTGAG

GTCAAGAATGAGAGCAAAGCAAGATAGCATGAATACTTTTCATCCCCACCTTTCTTATGG

ACAGGGTTCGATAAGACAGCAAGTGAGGTAGAATATGCAAGAATTTTCACTGCCTTCCAT

CCACCTTTCATCTCACTGTAG

>c17298_g1_i1 len=395 path=[1:0-324 929:325-345 555:346-394]

CATCTCCTGCTGCTTATCTACCCAGCCTTTTTAAGTCAGGGTCCCAAGCACAGCTCTACC

ACAAGTTTGGCCAAAAAGAGAAAAGCAAACAGAAGCTGAACATGGAGATACGAAATCTGG

AACTAAATGCGGCAGCAGCAGAAAGTCTCAAAAATAAGAGCAGAAATTTTCCCATAGCTG

AGATATGTGCCACAGCCATTGTGTCACACTCCAGTAACAGCCTGCTCTGAGATCACACCT

TTTCTCTCACACTGTATGGGGTGTGATTTAACACACCCTCTGCCCCAGGACAATCTGAAG

AGTTAAAAATCAGTGATACTTCTCCAAACAGAGGAGGATGTTGTGTTTGAGGTCCATTTT

CAGCATTTGACACACAACATCCTCCTCTGTTTGGA

>c17300_g1_i1 len=229 path=[1:0-228]

GTCCTACAAAATCGGCAACTGGAACTCCACAGCTGGTGCAGGCACTTACATCTATGCGGC

CACTAAGAGCCACTGAGACTATTACAATGTTACACACAGTCATGAACAGAAGCCTCAGCA

AGCCCAAGTCTCTGGACTGTATCTCTAGCCAAGAGAAAGGCAACATAGGCCTCAGTTAAT

GTTAACAGACTTGTGAGATAAGCAGGTTCTTGTCCAGTATCCATACAGC

>c17304_g1_i1 len=353 path=[466:0-234 21:235-352]

TATTCCAGATAGGTGAAGTCAGCTAATCTACTCAGCTTCATGTGACGTGAATAATTCTTA

TTGGCAAGAAACCTAAACAGTTCATTAATGCACTTTTTTTTTTGTAAGACCACAAGGGAT

AACCTTGAAATGTTAAGTAATTAAAATGTAGTTATTTTATGTAACAACAGAATGAATCTC

CACCTATGCTTAGTATGAAGTAGGTGGTTTTGGTTTAGGTCTTTTTTCTTTTTTCCCCAT

TTAAGTTAAAGAATGGCTGTTTTAACTGAGTAGTTCTGTGGATTCATTCTTCTTTTATTC

CCAGTGCTTTCTTGTCACGCGCCTGTAGATTTTCAGTTATACTCTAAATATTT

>c17308_g1_i1 len=207 path=[185:0-206]

GCCAATGAGTATGGAAGAGCAGGAAACAACTGCGGGCAGAACAGGCTGACGGCAAGATGA

GCACATGGTATCTGATAGACCCAGCGAATCCAGAGCACATCAGGGAGGATGAATTAGGAT

TTGAGACCATGAGAACAAAGGACTGTAATAAAGGAGGGAGTGAAGGAAGAAGAACAGATG

GTCCCTTCTTTATCTCAGAGTTAGATG

>c17309_g1_i1 len=288 path=[1:0-87 89:88-287]

AGTCAAACTAGTTATCTCAGATCTCTCAGCATAGAGAAATGGAAGATGTGTAATCAGTGT

CTGTCAAACTGGATAGCAGTCAGTCCCAAGATGCTTAGGGACAGTTAGGGATTTTTCTTA

AGTCTATCATTGGAGCAATGTGTTTTATTTTGTACACTGTAATTTGAAACCCCCTCCTTT

TTTTTCTTCTTGTGTAACTGCAATAAGATAATTTCTTTAAAATAGCTTTCTACAATGTGT

TACTGTGCACGCTCATTAAAAATGGTGATTACACCTTGTTTCCTTTAT

>c17314_g1_i1 len=274 path=[1:0-273]

TTTCTACATGACTGAGTTATAGGCAATTCCACGATGAATATTACATCCTCTTTATACAAA

GAAGATGAAAGGCTCTCAAGCCAACTGTTGGTGGTATCTACTTCTAGAAGGCAAATACTG

CATTCTGTTTTCTAAACAGAAAAATGCAGAGATCAGGAATGGAAACCAGGGTAAAGGTAA

TGAAAAATGAAGAGTTTATTCAAACCAGTTAGCTGGGAAGTGACCTTAATCTTACTGCTT

TTCCTTGTTGCAGTTGCTCATTGTGCGTCTTCGA

>c17316_g1_i1 len=228 path=[1:0-227]

TGTTGCTTTTCCTACGGAGAGCTGATGTGCATTTCTGAAGCAATCATAAAGCCGGAGGTG

CTGAGGTTTAGTCTGCGTTCTGCCATTTTATTGATTAAAGTGGCACATCTAAAAATCTTC

CTAGTGCTCTGTAAACAAAGTGGTAGTGGCTTATCTGTGATTCTTGTGAAGCAGCGTGCT

AGCTGAACGTGTGCTGCTGCAGAATGTCCCCAAACCCGGTCAAGGAGG

>c17317_g1_i1 len=317 path=[375:0-316]

GACTGGTAAGTGAGGAGGCAGAGGGGTGCTGCCAGCCCTCCTCTCCCAGAGCTCAAGGCA

GGGTGTAGCTGGGGCAGGCAGAGCAGGGAAGGACACATGCCTTTTCCTGAATCTCAGATC

ACTTGTCTTCTCTGCCTCTCCCTGTGTACGAGGCTTGGAGCTGAGGTAGCAATGCCAAAA

CAGTCAATGTAATCTTAATGCATGTCTTTAAAAGCTGCTCAGTAGCAGCAGCTCACGCTG

CGCTCACCAGCTTTCAAAAGGAGAATGATCCTGAACTTTACCATGGTCCCAGCTGGTGCA

TTAGCACCACTCTTGGC

>c17317_g2_i1 len=210 path=[188:0-209]

TCCCAGGGTGTCGAGTGTGCACCCAGCTGCACGTTTTATAGACAACGTGTGTCCGCTCAG

CGCGATTCCTCCTTTGATTTCTCCGAAGTGGGCAAATATTGACAGTCCTCACCGTATCGG

TTCCGTTACATGTGCAAGCTGCTCCTGGCATCTTGGAGGCCTGCTCAGGTGCAATTCCCT

TTCTTGGAAATGTCGGGATGCCCTTTGATG

>c17321_g1_i1 len=230 path=[514:0-229]

TCATGGGAACAACACCGTGCTTTCAAGACCGGGATCTTTGTGTTCATAGACTGTTTCTAC

TTAGCCTCTGTCTTAACTATTAGTAAGCAATCTCTGTGGTGCTCTTGAGTTTGAAAGATA

GCATGATGTGTGTGATCTGCACTGTACTGTGTGTGCATGCATAGATATTGGCTGGATGAC

TCTTAACAGCCAAATAACTTTTTGAGAAGCTGCCCATCTCTTTGTGTGTG

>c17323_g1_i1 len=297 path=[300:0-270 570:271-296]

TACTCTATGATTTTATGATTCTAAGATGCTAGACGTAAATCTGAAAGCAGCACCAGAACC

AGGCTGCCCTCCTGACATTACACTACTGGATCTAGTATAGGTTCCCAGAAACCAGAGCAC

ACATTGGGATAGTGCTCTGCAGGAGCGAGTCTTGGTCTGATATGAGAGCTGCTACCAAAG

ATTGAAATCCAGTGGACCCCAAGCTGACCCTGTACTGTGCTCCTCACTGCAGTGAGATTT

TTCCATTATCCAGGGTAACCCACTAAGCACTACTTCAAGCAATAGACAGGTGAGGAG

>c17323_g1_i2 len=297 path=[300:0-270 25:271-296]

TACTCTATGATTTTATGATTCTAAGATGCTAGACGTAAATCTGAAAGCAGCACCAGAACC

AGGCTGCCCTCCTGACATTACACTACTGGATCTAGTATAGGTTCCCAGAAACCAGAGCAC

ACATTGGGATAGTGCTCTGCAGGAGCGAGTCTTGGTCTGATATGAGAGCTGCTACCAAAG

ATTGAAATCCAGTGGACCCCAAGCTGACCCTGTACTGTGCTCCTCACTGCAGTGAGATTT

TTCCATTATCCAGGGTAACCCACTAAGCACTTCTCCTCACCTGTCTATTGCTTGAAG

>c17324_g1_i1 len=236 path=[7:0-235]

TATAATTTTTATTTTTTTAAATTATTTATATGGGTTTTTTAATAAATTATATATATTTGA

TATGTTCTGAAAATATATTATGTGAAAATATCATCGTTATTTATTTGTAAGATATTTTTA

AAAAATATAAAAACATTTTATATTAGGGATTTTAAAAAATAGATATATATTATTATTGTT

TTATTTGACGTATTGCTGATAACTAAATTAAGTATGTATAAGCTTTTTAAATATGT

>c17325_g1_i1 len=399 path=[377:0-398]

ATCACCCAGTTCCCACACCATACCTGAGCCCTGCTGCTGCTTTGCTCCAGTGAGGTGAGG

CTGGGGGTGAGCACCCCTAACAGTACCCTCCTGAGCCGGGGCCATGCTGGGAGCAGCGCA

GCTCTTAGTGGCAGTGGGATTTGGAGGTCATTCACATCGTACTGGGTTCCCTCTGCCTGT

GGCTGGAAGGCGATTTGATGGAGATGTGGTCTCACAGTCCAGGGAGCAAGCCATGCCCTG

GGTGCATCCAAACCAGTGTGGCCAGCAGCGTGAGGGAGGGGATCTGTCCCTCTACTCTGT

GCTATGAGACCTCACCTGGAGTGCTGCATCCAGTCAGGGAGTCCTCAGCACAGGAGAGAC

ATGGACCTATTAGACTGCATCCAGAGGAGGGCCACAAAA

>c17327_g1_i1 len=262 path=[486:0-261]

GAACACTCATGTACCAAATGTGACTTTACCACCCAGGTGGAAGAAGAGATTTCCCGACAC

TACAGGAGAGTACACAACTGCTACAAGTGCCGTCAGTGCAGCTTCACGGCTGTGGACACA

CAGTCACTACTCGAGCACTTCAACACCGTGCACTGTCAGGAGACAGACGTCACTACAGCC

AATGGGGAGGACAGTCACGGTGTTTCGGCTATCAAGGAGGAGCCAAAAATTGATCTCAAG

GTCTACAGTCTGATTAATCCAG

>c17330_g1_i1 len=211 path=[285:0-210]

AGCAGCCTGCTAAGGACTTCCAGGACTTGAGGAGCGATACTGACTTGTTAGCGCTTTCCC

CCACATACTCAAGTAGGGATTGGGATTTTCCATTCTTAACTGCATTTGCAAATGCAGGTC

ATGAGAATCCTCTGGGACAGCATGCCCTGTTGCTGCTGCTGGGACGTACCCGCATGAAGC

ACTTGCTCCAAAATGGGCCCAGCAGCAGCAA

>c17341_g1_i1 len=274 path=[252:0-98 351:99-273]

AAAAAAGCAGTTTTGGTTTTACCCACACCAACTAGACCCAAAGAACAAAAAGTTAAATAC

ATTTGAAGACTCAACTTGGCAACATTTAAGAGCTCAATCCTCATCAAGAGTTTAATTAAT

TTATTGGCAGTCTAGCACATCAGGCTGTGCTATTCCTAACAAAGTCCTCTGTTTCAGTTA

AAAAGTCTTTGCTTTCAAGATTTTCCACCAATCTAACTGGTACTCATAGACATCAAGAGT

CAGAGGAACAAGTTAAGCTAGTGAATGACCAAAA

>c17343_g1_i1 len=261 path=[1:0-260]

GATCACTGGAAGTGCAAAGTAGGGTGAGTGCTAGAATGACGGAGATTTTATTCGCTCAGC

TCCCACGCTTGTCCTGCTATATCTGTGCTCTGCCTCAACAGATGGGCTGTGTAGTCTGTT

CCCTTCCTGAGCTGTGCCCAGCACTCCAAATTGGGAGTAACAAACGTGAACCAGCAGATC

TGCACCAAGTTCCACTGTGCTGTGGTCAAAGTCAGTTATTTTAAGTTAGGGAGAGCACTG

GAAGTGATTGGTGAAATGCTT

>c17352_g1_i1 len=203 path=[1:0-202]

GAGATAAGATTTAATCTTAAGTCCTCAGGCTAATCAAAACACAACAGATATGCTATCAAC

AGTAAGTAACAGGGCTTTCCTTTTTCTCTAATAGGAAAGCTTTAACAAAGCTCATAAATA

ACCTTCTATGTCAACATTAGCTATAGCAAAACTAGTTAAAGCACAGAGACCTAGCCAAGT

TTCTCCATACTGCCCTAACAAGG

>c17363_g1_i1 len=276 path=[254:0-24 279:25-275]

GATTTAAACCTTCCAACAAATCTAGTTCCAGGGGAACAGGAAGGGTATATTAGAGAAAGT

GTGTCAAGATTTAAAAAATTACTTTAATCCTCTCGTATCGTATTTTATTCTCAATCATTC

TCATTATTCATTATAGGCATGAAATTCCTGAGATAAAATATTCAAGATAAAGCTATCAAA

ACCAATTTCCCTATCTTTTTCATATCGGTGCACTTATCGCAATTACGAACAAAAACGCTT

TCAAGTCATTGGCAACACTGTTCTCTCTTGTACAGC

>c17364_g1_i1 len=204 path=[182:0-141 324:142-203]

GTGCACCTCAATTTTAAGTTTTCTTCCTCCAGTCTCACAGTCAGTATTTTAGTTCTTGGA

AGAACACATGAAATAAAATACATTTTATGTTTAGAAAACAAAGAGATGAACCACAAACTG

AAAGTAGACTACTACTGAAATGACCATTAATCTTGTTACCTGAAAGAACAGTAGACACAT

ACCTGTCAAATGATCCAAGTAATA

>c17371_g1_i1 len=284 path=[599:0-251 850:252-283]

CTTGTCCAAGCACCTGATGGCATCCTTCCTGGCCAACAGATGTTGCTATCTTCCTTCCAT

TTGTTGCAGGGCCAAAGAATTAGACGCTCCTCCTTGTTGCTCTTTACAATCAAGTGAATG

CTTTCAGCCATGGTTTTATTTCTGTTGGTAAAGACAGCAAAACTGACGTACAACCACAGA

CACACAGTACAGCATAATGTCATGATACGCCCTGCATCAGCAGTCTCCCAGTGCTCAATT

TGGAATGCCATCGATGACCTATCTGTCACCAAGAGCAGTGGCAG

>c17372_g1_i1 len=246 path=[299:0-245]

GTGCTGACCTTGTTTGAACTCCCCTTTTTAGCTATTCTGGGCCCCTCTGTTTAAGCCATT

ACTCCAAAAGGAGCCTCCGATTAATCTAAGTAAATGCATCATTACAGTCCTAATCCATGT

CCTTCTGAAGTAATGGCTTAAACAAAGAACAAAAGGAAATTGTTATGAAACAGCCTTTTA

TCCCAGGCAAAGGTCACATTATGAGGTTTTTAATTTTGTCAGCCTCCGTTATATTGGGCC

TTGCCA

>c17375_g1_i1 len=259 path=[1:0-113 115:114-258]

GTGTTGGTGTGAGCTGTGTGTGGTGTTCTGAGTTCCCTTTTGCTGTTTGCCCTCTGGGCC

ACCGTGTGCCTCCTTAGCAGTCCGTGTCCCTCAGTGTGTGAGGGGCTGCTAAGGTGCACA

TACACACAAAGTTAGACAAAGCCATAAGTGATGGGAACAAGGGGAAAGAACACACCTGTG

AAGATCCTACAGTCATTCTGAGCAAGCTTACAGAAATTTTGCAATGCTGTTTTTGAATGT

GACTGCACTATGTGAAACA

>c17375_g1_i2 len=248 path=[455:0-102 115:103-247]

AGTCAGAAAGCCCTACATAGTTTTAGCTAACACCAGAACCCAACCGTTCATACATTTTGG

GAATAAAACTAGTCTCTTCGTTATCCTGAGGCTTCAAATGAAATGCACATACACACAAAG

TTAGACAAAGCCATAAGTGATGGGAACAAGGGGAAAGAACACACCTGTGAAGATCCTACA

GTCATTCTGAGCAAGCTTACAGAAATTTTGCAATGCTGTTTTTGAATGTGACTGCACTAT

GTGAAACA

>c17377_g1_i1 len=502 path=[480:0-288 769:289-501]

GGCAGCCTGAGCTGGTGAGGGGCACCCAGCCCATGGCAGGGAGTTGGAATTCCTTGACTG

AGGACCTTCCAACCTCAGCTGTTCTATGATCCTGCCTAACATAGAATGCAATGTTATAAG

ACAAGGTAGAAGTTTGCGTTCCCTTTGCTTCAGTTACCTCTGGGAAGAGGAGTAACCAGG

GACCAGAATAATGCAGACTCAGCATCTTCAGACAGCTGCCCATAGCTGGAGCTCGGGGCC

ATGATGCACAAATCAGTAGTAATCACTATTCTACTTGTGGAGCCTCTCTCAAAACAAAGT

GCACTGCGGAGAGTGTCCAAGTGTAGCATATCCAAGTAGGATACTGGGATACTCCACTTG

GCCCACTATTACTGCTTACTGTCTCCAAGTCCATCTCATCTTTCTGCACAAACTCTTCTT

TTCTTTCCATTTTTCTTCCCAGTGATAATGGCCCCATGCTGGTTTATATTCTCAGGTGTT

CACTATGAAACCCTGCTTGCAC

>c17379_g1_i1 len=365 path=[1:0-277 279:278-364]

GTGGATTTACAAACAGTAATTATTTCAGGGAAGCTCATTCTTCATTGTTGTTCTGCTAAA

GTGGAAACCATACAAACAACTCATTAAGAGGTGAGAATAACTAAATTAAAGACTGCTGAT

TTACTGAACTGGTATCGTGTAGTCAGAAACAGAGGTCAAAACAGGAACACAGATCACTGA

ATGGAACTTAAAGACTAAACCTGTGCATCATGGTAGACAGAAACCCCTCTCAGGAGAGAA

GCTGAATTTGACCTGATTCTAGCTCTAGTAGAAAAGTTAAAGCTTGTTAATTTACTCAAC

ATGCTGTACAGTGCTGTTAAGAGTTTACAGTATTTCTTACTACTGCTTATTTTCTCTTTG

CTACA

>c17379_g1_i2 len=241 path=[776:0-153 279:154-240]

TGCTGGCTTTGATTCAGAAATGCTCAAAATAAGAATAAAAAATACTCATCAATAAGTTGT

ACGTTGAGAAGAATATTTGAAAGAACAGGAATTACAGGCATGTGCCTCTTTTTTAAACAT

TCATTATGAATATGTTTTTTCCAATATATAAGTAAAAGCTTGTTAATTTACTCAACATGC

TGTACAGTGCTGTTAAGAGTTTACAGTATTTCTTACTACTGCTTATTTTCTCTTTGCTAC

A

>c17380_g1_i1 len=362 path=[804:0-31 464:32-34 840:35-361]

CCTCACCTAAATATCCCCCCTCCCAGTTCAAAGCCGTTCCCCCTTGTCCTATCACTAGTA

AACCACGGTCTCCCTTCCACACCTCCAAAGCACTTGGGCTTATTGCTGGGTGGCTATTGG

CTATTTGTGCTCCTCCTGTGTGCTGCTGTCGCTTCCTTGCGCCCTGTCCAGATCCGTACA

GCTAAATCTCCAAAACCAAACAAACACATTTAATTAGGCTGGGGCACTACTGAGGAGCTG

GAAGTTTCTTGGAGTCGTTATTACCTCACCCCGTGATTTATGTGCGCACGTATTTTGATA

AGAGCTACTCATAATCCTACAGACACTGCCAGCAAATAATTTTTCAGTTAGGCAATAATG

AC

>c17380_g1_i2 len=398 path=[396:0-67 464:68-70 840:71-397]

GTCGCCGCCCTCTGTGTGGAGGGCTTCTTCCTGATACCTGGCCTGGGCCTCCCCTGTCTC

AGTTTGAGGCCGTTCCCCCTTGTCCTATCACTAGTAAACCACGGTCTCCCTTCCACACCT

CCAAAGCACTTGGGCTTATTGCTGGGTGGCTATTGGCTATTTGTGCTCCTCCTGTGTGCT

GCTGTCGCTTCCTTGCGCCCTGTCCAGATCCGTACAGCTAAATCTCCAAAACCAAACAAA

CACATTTAATTAGGCTGGGGCACTACTGAGGAGCTGGAAGTTTCTTGGAGTCGTTATTAC

CTCACCCCGTGATTTATGTGCGCACGTATTTTGATAAGAGCTACTCATAATCCTACAGAC

ACTGCCAGCAAATAATTTTTCAGTTAGGCAATAATGAC

>c17384_g1_i1 len=211 path=[1:0-210]

GCTCTGTGTTACCTGCAGTGCTGTGCAGGGTCTGCGTGGAGGCATTTTTCGGTCACTGTT

GTGTTGGTGAAAGCTTATTTCCCCTTACACCCAACATCTGTATTGCCTCTTTATGTCTGG

CTCTCAAGTGTGGAATGTAAAGCAGGTGCAATATAAATAAACTCGGCGCTTCTGGTGTCA

TCAGAGCCATACGAGTCATGTGATGGGTTTA

>c17385_g1_i1 len=301 path=[24:0-217 242:218-300]

GAACCAGTTGTTATGTTCAACAAGCACCCTACTTGCAACGACAGATCTGTTTCTTCCAAT

GCAACTCCTGTGTTCTTTTCTGTGCATTCTCACGCACATGCCTGCATGTGTGCGTGTGTA

AATGCTCAAATTCAATATGAATTACTATGCCTTTCTGCATACAGAATCGGATGCTTCCAG

AGAATTGGTGACAAGATGTGAGCCTTTCATACCGCTGCCATTATTTTGCTCATTTTAGTA

ATGAAAAGATGTTGCTATCTGAAGGCTGTTTGTCAGAAATGAATAGGCAGCCTCATTCCA

G

>c17386_g1_i1 len=378 path=[356:0-377]

GAAAAGGAAAAGGAAAAGGAAAATGAAAAAGGAAAAGAAATTATGTATGAAGAATTAGTG

GATGACTATGATGAACTAAATTCATTTTTAGATTTTAGCTTAGACAAGTCATCTGGTATA

TTAATAGAAAAAACAAATATTAAAAAAGATCTCCTTAACCCAACAAATAATATTAATCAA

ATAAATGAATATTTAATAGATGAAGATTGGCTTATGTATATTCGATCATTAATATAATGA

ATATAATAGCTTTAAAAATATAAACAACATATGCTTATATATTTTTAAGTTAATTATTTT

TATAAACATCTATTGTTCTGCCTTATTTTCACATATTTATTCGTTTTATTTTATCTTTCC

TTTTTTTTCTTTTTTTTT

>c17386_g2_i1 len=2161 path=[711:0-2160]

AAAAAAAAAAAAAAAAAAAGACTTATATATATAAACCATTTATACCTTTTGGAAGTTTGG

TTATGACAAAAAAAGAAATACTAAAATTAAACTTATTAGAGAATAATGAATTAAAAAAGG

AAAAAATATATATAAGCAAAAATATATTAAATAAGAAGAATAAAATGAGAAATTTAAATA

CAAAAGATAGTATAAAAATAGATAAAGATACGATACAGTTATCTTTAATATCAATTATAA

TGCTTTTGAATGTTTTAAGAAAAAACTCCAATTATTCATTAATGATAAATTTCTACATAA

ATTTATTAATGGATAAATTTTTAGAAAAAAAAAAATATATTCATATAGATGATTGTTATT

TAATAAATAGTTACAAGGTTAAATTAATAAATCCTTTACACAATAACCATAAAAAAGAGT

TTGTTATAAATGATAACTATTCTATGAAAAAAAAAATGGAAATAATAAATAATGGATATA

AATTTTATGCATCTTCATGTAAGTTACAAATATTTGAAATAATATTTTACGAAACATTAA

AAGTATATTTAAGCTTCATTAACGGCCGTTACTTGCGCACTTTTATTTCAAAAATTGCAC

AACTAAATAATATATACACAAATAAAAATGTTCACTTTAAAATTAAAATAAATAGATTGT

TATGCAGCAAACTTAATAATCTATTATTAGAACAACTATTGAATATGAAGTTAAACAAAA

TTGTTTTAAGTAACATAATATCTGCTTGTAAATTTTTTTTTATTTCATTACTTGATGAAA

ATGAAAATTGCAATTTTTTTCTGAACAATCCAGGTGAAAAATCATTTTTTTTTATAAATT

CTAGTAATTTTTTTGGAAATGAAAAAGAAAATGAGATAAAATATATTCAGCACATGAATA

CTGATAATATTAAATATGAAAAAAAATATGAATTAATTCTTCAAAAAAAAGAAAAATATG

AAGAATGTTATGAAATTAAAAAAAATGATAATATACAAAATCATTATTCGTATGATTCTT

TTAATAAAACACTATATACAAAAATCTTAAATGTAGGAGAAAGAAATTCTTCTGAGAAAA

ATAATGAACCTTACATTGAACAAACAAAAAACGAAAATAGGATAAATGATGAAAAAGAAA

ATAAAATTTTAATGAATACTAAATTAAAAATGGATGAAAATCAAGAAATAGAAAAAAATA

ATAATCAAAACAAAAAAGAAAGTTATACAAATAATTTAATAAATAAAAATTATATTTTTA

ATGAGTATGAAAATTATTATACGAAAAATAACAAAAATGATTTATCAATTAATAAAAGCA

GTAATTTAAAATATAACGAAGATAAATATATAGATAAGAAAAATAATTCTGATGATTTCA

ATTGCTTGGATGCTTCATCTGTTAAAAATTCGGATTTTAGTGACATAAAAAGAAGTTATT

TAAATATAGATAGCATGAACAGATTTTCACAAAACATTGATGTACTAATGGAAAAAGGTA

ATAGTAGCAATAATTCAGTTTATAAATATGATAATTCTACTTTAGATATTAACACAATAG

TTAAAAGTGAAGAAAAATCTAAAGTAAGAATAATTTATAAAAGAAAATATGATATAAATT

TTAATGAATATGATAAAACTAACAAGATGAAAAGTACTATAATATGTATGAAACCAAATG

ATATAAATAAATTAATTGATTGTTTTCTGAAGTTAAATATAACAATTTTTGAGTGCGCCC

AATATATATACAACAATATTTTTAAAAATAAAAAGGAAAAATTATGTGAAGATATAAAAC

AAGACAAAGATATATATATGTGGTTTCAAAACTCCTTAAATGTTCTAAATATAGAAATAT

CTGCTCTTGGTCAAATTTATAATAAATTTCAAAACACTACAATATATATTAAAAAAAAAA

AAAAATTTGTAAGTCACACTATAAAAATAAACACAAATAAATTAAAAGAAATGGACGAAA

TAAAGAAATATTTAGAAATGTATTTGTTAGAAACAAAAAATAAATATGAATCAACAAAAA

TGATCATTGAAAAAAGAGAGAGATTGAATATGTTAGGTTGTAACGAATCTTTAAATGAAA

ATGGAAACATTAATGAAAAAGAAAAGGAAAGGGAAAACGAAAAGGAAAAGGAAAAGGAAA

A

>c17395_g1_i1 len=482 path=[1:0-481]

GTTTAGTTTTGTGAGCATGGTAGTGAAAGATTGATGGTTGGATAGTTGATCTTAGTGGTC

TTTCCTACCTTAATTCATTCTATGATTCTATGCTCCTCCAGAGTGTCTTCCACTGTCGTT

TTCTGTTTTTTGTATGGATATGATCCTCTGCATAAAGCACAGATTGTCACACTCTGGAAA

GTTGAATGTTGGCATTTTTGTTCTACAACCATAATCCACTAGGATCTTCAGTTCAGCAGA

TCAAAGCCAGGAAGATTGTTGGGCCATCTTTCTCTTGAACTGCAATTATTGCATGGATAA

CACAGAGAATAATGAAGGAATGGTGTTTTCCTGTAAGTAACATGGAGATGAAAGGAAGGC

TATCCCACAGATGTCACACTCCATTTCCACTTCTTTTCCTCTTCATTGGTTTGTTTGATT

GATGCTCCACATGTAGAAGAGACTTGTTTTTTCTAAGTTGATTTCATTTCTAAAATGTAA

AC

>c17401_g1_i1 len=261 path=[239:0-260]

TGTGGTGGTAAGCATGTAACTTTGGGTGCAGGAATCTGCTGAACAGAACAGCTTACAAAA

AAGATGGGGCTATACCAGCTGCAGCTGTTTAGATCAAAGAATCTTGTAACAAAAGGGTGA

GACCTTGGACTCCGCTCCCATAAATATTGTTCTTCATCTATGAAGGACAAGACCTTCCAG

CTGTAGAAGCTTAATCAGGAGTAAGCAGACACACCGCAGTTTTTCTTCACTGGCTTCACA

TTTTCACACTGATTCTCTAAG

>c17402_g1_i1 len=217 path=[155:0-123 8:124-216]

TTGTTGTAAATTATTGCCATTTGCCTCCCATGTAACTTATCAGTGGATTAGATTTATATT

TTGACTACTGTTAGATATATTGAAAATAAAATTTTAATGTTTCTGTGTCTAAAAGTGCAA

AGGTAATGCTTAGCTCAAGTGTTATTTTTGAAGAATGTGTAGGGGGAAACTACTTATGCC

ACAGAAGTGGTTCTGCACCATAGTCCTTTTATAGTTA

>c17409_g1_i1 len=259 path=[1:0-258]

TCTTTCACTTTCATTCTCAATGATGGCAAGATCACCGCCATTCTTCTTGCAGTATTCCCT

TGCTTTTTCCATAGGCATTTCTTCCTTGCTAAAGTAATATTCCTTGTGATTATATATTAT

CCAGTCATCTTCACTAACAATGTATTCATAATCAAATGTGGATGTAGGTTCTGGTTTCAG

TGTTGCTCCTTTTTTTATTTGACAAACATAGTCCTGCATATGTTCACAAAACATATCATT

CCATTTCATATCATTATAG

>c17411_g1_i1 len=250 path=[263:0-249]

GTCATCTCCTTCACGCTTCCTCCTCATTCCGTTTTCCTTTGGTTCCTGATAGCGAATTTT

GTCCATTTTTTTTCTTGATAGTTGGTAGCAGTGTTCATGCACCCAGCCAGAGGCAAAGAC

CACTCCTGTCTCTGACGGACCTCAAGCCTGACAACTGGTTTTCAAAAAAAAGCTGACTTC

CATTCTTCAAAAATAAATCACGTAGTCTCAGCTGAGACTCGTTCTTCCCTTGGGAGACTC

AGCTGAGACT

>c17421_g1_i1 len=253 path=[265:0-252]

TGTTTGATCCCGCCAAGAAGTTCTCCCTTTATGTTATTTATTTATGTATATATATTCTTA

CAGTGTTGTTTTACGGACAGTTCTGTTCTGTATATGTTACTGACAGGCTGAATGTTAATG

AAGATCACTGTACTTTGTGTTCATGTGTCAATTTGTTATTTAAGAGCCAAAAGCCAGTTC

TTAAATTTATGTACTTACTTTTGGACTTGGTATGCCTCTCTTTCGTCTTGGTCTTTTATC

TAGACACTAATAG

>c17440_g1_i1 len=352 path=[721:0-351]

AGTTGAACTTGATGAAGTAATGAGGGAAAGAAGTTAACTGGGAAATATGCCAGACAAATA

CTATCAGGACAAGTTCAGGCTGTCATTGCCTGGCATTTTATGAAGCCTCTTATACATGGG

AGCAAGTTAGAGAGAGAGCAGACGGCCATTGCTCAGTTGTCATACAGATTGGATTCCCTC

TGCATTCAGATGAATGTTGAACTGTAAAGCATAGGTGCCTCTGATTTACAAAAACGGTGA

TTGTAGAGTGCAAAATCTGTTTTATAAATAATTATTACCATTGAAAAGGGAAACTAACTG

AAGCAATACTCGTTGACTCTAAGCAGGAATTACTAAATCCTTCAGCTGTATG

>c17440_g2_i1 len=383 path=[361:0-382]

AGGCTAGGCCTTGGTTTCACTTACCAAAGAAATCATGTAGATATAGAACTACATTCCATT

CTTTAAGAAACAAACGAGTCAAGAAAGTACACTTGGAAGAGGAATGACACTAGCAGTGTA

AAACCAATTATTCTTCAAGATGCCTTGGAAATCCACGTTCGTATCTGAGTATACATGGCA

AAAGTTCCATTAATTTCGCTAGATACCAAGCTAAGCAGGAGTGGGTATAGTATTAAGCTG

GCATTGTTTCCCAGCTGTACTCTTTCCCTGTTTAAACCCAGGGTCACACAGTACTAAATG

GATGCTTCTGTGTCTCTTCTGGGGCAGTGCCATTTCAGGGCATTCTTGCCTCCCAGCTTC

TGACTTCTGTGCCATCTGTCCAG

>c17450_g1_i1 len=211 path=[323:0-210]

CACATGAACAGCTGAACTGTCCAGCTCTACTGCACACGTAATCTTAAGTCTTGCTAAAGT

CCTGGAGGACCATTTTGGCAGATGCTTCTCCTTGTTCCTTCGTTGTGCCACTTGGAGTCT

GTTCTGAAGTCAGAAAGTCAGTCTGAACGTTGTATGTGTTTGATAAATGTGTTTCCATCA

GCTAGATACATGAGTGAACAGAACAGCACAG

>c17451_g1_i1 len=207 path=[373:0-206]

GGATTCCTACATGGGGGAGTGTTCCTATTCCAGCTCCTTCTTTACCAGCTTGAGGATGCT

GTGGGGGTGTTTGCTCTGTCAGACTGAGTCTCAGTGAGCAGCCAGCAGTGAAATCCACCC

TGTGGAGATGCCAAATGTCAGTGGCGCAGACATCTACCCACCACTAATGAAGCTTACTGA

TGAATAGTTAGCTTTGTGGCAGCCTGC

>c17452_g1_i1 len=275 path=[185:0-274]

GCTCAGCTCCTCGGTGAAGGCATTCCTACATCCACCAGCATGGAGAACGCGCTGGGAAAC

GGCCACGTTGCTCCTCATCCACGCAGGAGACCTCCATAAAGGCTTCAGGTCTCCGGAGGA

AGGCGGCAGGGACTGTCCCCAGGCGTTAAGGCAGGAGATTCAGCATGAGCTGCGCCCGCT

CGGTCCGTCCTGTGATCGCATATCTTTCTTGGACGGAGTTCAAAGGCGGCCGTGGAAACA

AAGCGTTGGCAAACACACCTTGGGAAGGGCTTGGG

>c17456_g1_i1 len=216 path=[348:0-215]

AGATGCTACAAGTAGCTTTGCAATAAAATATGCCATCTAAGATATCTATTCAGCCAAGAT

TTCACATCAACAAACTATTCAACAGGTATGACTACACTGCAAATAAAAAGTAGACATAAA

ACTGGTCATAAGGTTAGCATTAGGGACTGGCAGTCTTCTCACCATATGACTGCCTCGTGA

GTCATTTGTCCAGATGTGCTCAGTCACCTGAGGAGT

>c17461_g1_i1 len=219 path=[197:0-218]

TGTTAATCAGTGACAAATTGAAGAGGCTCATCTACACTGCACTCGCACAGTTTCAAAATA

CCATGCTGTAGCTAATTATATCTGCCACTGTTCTCTCGGATGATTTCATCCTGATCAGAT

ACAGTGTGATTCAGCTTATTAACTCAAAGCATTAATCTTTCCATCACTTCCAAACTTGTG

TGAGCCGTGCTATTCAGGGATGGGACCTCAAGGAATAGG

>c17463_g1_i1 len=320 path=[251:0-319]

AGTTGAATTCTCTACCAAGATTTGGAAGTGAATGGGCAGAAGGATTTGCCTATACTAATG

GTTTGCACTCATCCCAAAGGCTGCTGAAGGGAAACATGACTCCAAGATGCACTGCGCTGC

ATTTTTCAGACACTGGAAAGTGTCAGTGCTGCTTCACAGCCCTCAAAAATGCTCACTCAC

TATCCAAAGTTTTGTAGAAGTATTGACAATTGGAAAGATAAAGTATGAAATAAAATCAAA

TTTAACATTTTGCGATGATGGGAGGAACTTAAGATGAAAGAAAGACAAAGTTATCGGTAC

AAATCATAAAGTATGGATAT

>c17470_g1_i1 len=413 path=[10:0-412]

TTTTTTTTTTTTTTTGTTATTATATTTAAAATAATGATATTAAATATTATAAAAATAAAA

ATTATATTATTTGCTTTTTATTTATAATAAAACTTAAAAAAAACTATGTGTATATATTAA

TGAATTTTATATATTTTCAACAATTTTGTCTAGGTGGTGTAAATCTTTAAAAAATAAAAA

AGTATTCCTTATGGATAAAATTTTTTTAATTTTTTTACATTATAAAATAATATTACATAA

ATTTAAGCAATTTTTTAAAAATTCATTTTAATTAAATTTTGTTTAATTTACACTTATTTT

TCACTTTCACTAAAATACTTATTTTTTTTTGTCATACTTATATAAATTATTTTGTGTTTT

TTTCTTATAAATATCTTATATAATTTTATGAAAAATTAGATTTTTTTTTTTCT

>c17479_g1_i1 len=295 path=[273:0-141 415:142-294]

AACACTTTCCTGAGCGCTATTTATAACTGGATACCAATTTCGTATTTCCAGATATAACAT

GGGATTCCAAACGAATTTGTCTTGAATTTTTATCCTTAATGGTCTTAATAACAGGCACAA

TTCATAAACAAATGTCATATGTAAATAGATTTTACTTGGTTTTAACAAAACAGCTTTCAA

ACCTGAATATCTTAGCTTTGATTTTTACTCTCCACAAGAATTGTTATCTTTCATAGACAG

AAGTACTATTTTCTCCTTCAGACCATTCTACAGAAGGCTTTTTCTTAGATACAAG

>c17480_g1_i1 len=214 path=[470:0-213]

TGGTTGCCAGGAGTATAAAAGCTCCCATAAATTATATTCCTTACAAAGCTGAGAAACTGA

ATATCACTGTGAGCAAAGCAAGTAATTAACTGCACAAGTTTTGTACCTGGACAGGTGTGA

AGCTGCTTTTAGCCCTCTCCATCCGCACAATTCAGCTGAAAACCACATGGCAGCTGTGCA

AAGCACTTTTCAGCCGGTGAGAACTCTTATTTAG

>c17483_g1_i1 len=319 path=[451:0-318]

AAAGGAAGTGGAATCTGACATAAATAGAGGGAGTACAACTTATTTACTTGTGGACATGGA

GAATGCAACACCTGGTAGAACAAAGCCCACAGAAGCAACTGGGCTCATCTGTTGCTGTGC

AGTACAATGATATCACTCTTCAATTTTTCCTATTTTCAGTGTGTATTTGTGGGATTGATA

GGGGTTTGGAACTCCGGAAACATCACTTTGGGTAGGAGTAGAAAAGGTTTGTGGTAATAG

CTTGTTACTGGAAGGGAACGGAGTGTTGCTTCTTCATACTGTCAGGCACAGAATCTGTGT

GTCTATCCAGAAGTGGATG

>c17486_g1_i1 len=216 path=[1:0-215]

GTAATTCCTCTCAAATGACTTTTGGCAAAGACAATCAATCAAGGTCTCTAGTAGAAAATA

TAGAGCCATACAGATGTCTGACCTTGTTAGGGGCAATATTTGCACTTAGTTGTGTTATGG

CCATGTGCAACATTTTAATTTGAGAATTATGTGATTTTAGGTGTAGATCACTTGAGACAG

CAGCTTATTTTGTCCTCTGATCTCTTTCTTCTGTAA

>c17488_g1_i1 len=201 path=[405:0-127 532:128-200]

AATCAAGCCAGGGCAAGAACTACACTCTGAATTCCCACTCATTTTATCAGTATTCCAGGT

AAGAAAGTAACTGCTGCCATGTGTAGTCCTTCTGGGTGAGGAAGCAGATATTCTGTCTGG

AAAAAATTGTTATGATTTTTCTGTAGTCAGAAGAAGAAAATCAATGATCAAATTTTGCAT

AGGAAAGTTCTGTCAGTGGGG

>c17488_g1_i2 len=201 path=[405:0-127 179:128-200]

AATCAAGCCAGGGCAAGAACTACACTCTGAATTCCCACTCATTTTATCAGTATTCCAGGT

AAGAAAGTAACTGCTGCCATGTGTAGTCCTTCTGGGTGAGGAAGCAGATATTCTGTCTGG

AAAAAATTTTTATGTTAAATTGTAATGCACAGGAAGAAAATTGGAAAATTAGATTGCCCC

ACTGACAGAACTTTCCTATGC

>c17493_g1_i1 len=530 path=[790:0-529]

GCCATTCTATGATTCTATGAACATATAGGTTTTCAGAAGTCTGAGAGTCATATTCCTCCT

TCAAAGACTTACACACGCCTAGACCACCTTGGAGCTGTTGTTGGGCTAATAATTCACTAC

TGTGGGGTGGTCTGGAATCCTTCTGAGGTCTCTTGAGCTCTGCAGACCTTTCTCCATAGC

TATGGGCTTGAAGCTTGGCCAGTAGCATCTCTGTTGAAAAGGAAAGTCCAAGAGCATCTT

TGCTAGTATCTTCTTCCTCTCCCCTGTTAAATGTTTGCTGTTTCTTTGTGTGAAGCTATA

TGTTGCCATCTCAACATCTCTGGGTTTTTTTTTTTTCTTGCCAGCTGTCTAATCTCTTGG

AAAGGTAATTTTCCATCAATTTCATGTGCAGCTGCCCAAAGTGTGATAGAAAATGCCCTT

CAGCAATTTTCATACTTCAGCCAGTTAGTTGTGGTTCATGATACTCCAAGCCACACGAAC

TCAAACCTTGTAGTCTCCATTGTCCACCAAACCTCCTTCTCTGTTCTGCT

>c17494_g1_i1 len=282 path=[592:0-281]

AGGAATTGCTCTCTGAGGCAAGTCTCACACACAAGCAGAGCCAGCCACCAATCTCACTTC

CCATTCACATGCTCTGTTTTTCTTCTCTCTAGAAGAAATTCCCAAATCATAATGTTTACT

GATCTTCAACTTACTTGAGGGCCATACTTTCTCTGGTACCAAAAAACAGATGGATTAAAC

TGCCAAGGAAGAACAGGATTGGACTTCCAGTCCCACTGAGCAGATGGACTGGACACGAAG

ACTTAATGGGCAGTGAGTAGTGGTTGGCTGAGACCTGTTCTG

>c17496_g1_i1 len=442 path=[574:0-441]

AGCTGCTGCCGGAGGAGCCCGGTGAGGAGGAGCTCAGCTGAGAGGAGGGGGAGTTGAGCC

CCCAACGCCGGCCCCCACCGCCCCGAATCCTCTGGGCAGGAGATGACCAGTCTGCGGGGC

CCATAAGTGCAGGGCATGGTGGGCAGGAAAGTGCCACCACTGCAGGTGATGATGTCCCGC

ATGTGCTCAGGTTCGGGGCGGACGCTGGGGGTGACGTGGATCTCATAGCCCTGGAGCAGA

GGGCGGCGGCGGGCCCGGCTGAGGGCCTGGGACAGACTGAAGCCGAAGTGCTGCTCCTGC

TGGCTGTCCCGCACCAGGAAGGGACCCGGCGGAAGGATACGGCCGCTGCGGGAGCTCTCA

TGGAGCCACGTGGGGGTGACGATGGCGATGCCACGCGCCACCGCGCACAGGAATTTGATG

GTGCGGCGGACGCGGTCGGTCA

>c17501_g1_i1 len=307 path=[10:0-306]

TAAAAAAATAAAATAAAATAAATAGGATATATTTTTAGGTGTCATAAAGCCATTGATGCC

TTGTATTTATATATATGTATATATCAGCTATCAAAAAAAAAATATTTATATATATGCATA

TGTAAATTTTTTTCATTAATGTTATTTCTGTATATAATAAAAGAAAATGCAAAAAATAAT

GTTAACGTATCTTCGATATATTATTTAAAATTCAGGGTTTATATAATACTTTTACACGTT

TACTAAAATATTACTTTGATTATTTTTTGTTAAATTAAAGAAATATATTTTATAAATAAT

TCATTTA

>c17503_g1_i1 len=228 path=[1:0-227]

GAGGGATGGGAAAGAGCAGGGACCGCCTGTCCCTGGGGGTCCCTGAGGGTCTGGGCACAT

CCCTGCTGGCACCACACTGAGATGTCTGTCTGTGTGCGTGTGTCTGTGCTTGGCGTGGTG

TGCGTGGGTCCGGCTGTGCCTGGGTGTGCTTCTACTGTGTCTGCGTGTGCATCCTGCTGT

GTGGCTGGGTGTCCCTTGTGTTCACAGAGAGTCTGGCCAGTGCCAAAG

>c17503_g2_i1 len=287 path=[675:0-286]

GAGCTAATGAGGCTGCAGGGATGAGCTCACTGCCGGAGCAGCCTCCTTCAGTGCTCTCTT

GCAGATGGTATCCTCCTTCCGTGAGAGACAGGGATGGGACAAGGGCTCTGCCCACTCTGC

TGCGACGGCATCCCTGTGCTCAATTTGGGGTGACCAGAGGGATGGGAAAGAGCTGGCTGC

CCGGGGAAGGGGAGGAAGGCAGCTACACAAGGCACCGTGTCCCTCCCCTGTGGGCCAGGC

TGATGCCAGGGGACAGCCACCATCTCACAGACATTCCGGGACACGGC

>c17514_g1_i1 len=233 path=[225:0-232]

GGAATGATGAATTGGGTCTTTGTGAAGCACTGGGAGCAAAACACGGGTTCTGTTTCATCC

CTGCTGCAAGGTTTCCTCGAGCTCAGAGCTGGCTTTTGTGATTTTGCTGGATTTGGGCTC

AGGTTCCTGATAAGTAGGTGCATGTGCTGAGCCCACCACTGACCTGGAGCTCTGGGAAGT

GTTTCTAGGTTCCTAACCAGGTGCTTTTTCACTGTATGGGGGAACCTGGGTCT

>c17515_g1_i1 len=290 path=[268:0-289]

AAGTCAACCCAAGGCCGGTCGGGGAGTAAACCCCACATAGGCAAGAGGGGGAAGCAAGCA

GCAGGGAGCCACTGATGGGCATGGACAGGATGGGGATTTGGGGAGCAGCAACCCTGGCGT

CAACAATACAGGCACACCGTGAACGCAACCCCTACACCACAAAGCTGACTCTGAGCACAT

CCCTCCCTGCTGAGCAAGGGCTGCACACCCCATGGAGAAAGCAAGGGACTCTGCTGTCCC

CACAGTGCTTGGCACGGCCACCCATGGCTCAGCCCCACTGCCAGTACCAT

>c17517_g1_i1 len=214 path=[192:0-115 308:116-213]

GAGTACAAAAAGAGAAACCAAGGCAGCTCTAAGACTGTTTTCAAATGCACTACAACCGCA

TATTGGATTTATGTGGCAAAGTTTTCACAGCTGGGGGCTGCAGGATTAGTCTCAGTGAGA

AGAGTCCAGAAGCTGCTTGAGATCAGACAGGAGTCGGTGTCAGTACGATTTCCTACTGGT

TTCATTTGGACTGTGACATCCTGGACAGCAGCAC

>c17519_g1_i1 len=217 path=[229:0-216]

TCGTTTTGTTGTAATAGAATGTAAGGTTCTGTGTGGAAGTGAGTTCTTGTGGAGCTGTCT

GACCCTCAGTGGCTCAGTGACAGGTGCTTGCTGCACAGCGCACCCTCAGCAGTCTGTGTC

ATCAGCCTTCACCTTTGGTGTGAACCTGCTTTCTCAGGGGGGTAGACTGGATGCTCCAAC

CCCCATGGCTCTGTGTAAGGGCAAAAAGTTACCCACG

>c17529_g1_i1 len=209 path=[1:0-208]

GGGACCAGAAGTATAAGATTTCCACATTTTAATCTGTTTTTGGCTCACCTTCATATGTAG

CCACACATAAACAGATTAATCTTTGTATGCCTCAGTTTTTCTATTTGTTAATGACACAGC

TTCACGGATTCATCTGTTTGTGTAAATCTGACTGTGAAATGTTTATGTTAGATATTTTAA

AGTTTCTCAATTGTCCCAATGAGTGTCTA

>c17530_g1_i1 len=229 path=[275:0-228]

GCTTCTACCATGAGGATTCTTAGGAAGAGCTGTACAGATCATGCACCAGCAGTTTCAGCA

GTATACTTTTCAGCCTCACTCAGATGCAGATTTAGACATGACAATCATGAAGTGGCTTTG

CTGAGCTGACCTTTTTCTTGTTTGTAGAAACTGACCAAAGCCACATGAGGCAGCATGCTA

AGGAAATGTTAAGGAAAGTACGAATGCAGATAAAGTCTGATTACACACA

>c17545_g1_i1 len=445 path=[2:0-60 63:61-444]

AAAAAAAAAAAAAAAAAATACAGGTTTTCTGTTTTTATTTCTTTTTTATAATTTTCTCTA

TGTTAGATTATCACAGAAAAAAATATACATTTTCTCTTACGTAAATGTTTATGAATATTT

ACTAAAAATATCAGAAAAGATAAAATAGTATCAAAATAGTATTCAAATAGTATTCAATTT

TTATGAAAAGTTATTGAAAAGTTGTCTAAATTTAACATTTACTGAGTTCTTAAATTATAA

CTTTAATTGTTTGTGTAGTTAAAAATTACAAAAAATGTTTTTCATTTATTACATCTACTA

AATGAATTAAATGTTTTTTTTTCTCATGTAAAAATAAATAATACTTTAAGTTGTAAACCA

CATTTAAAATTCATCTTAAAATATAAATTGTAATTTTTAATTTTTTTTCTATATTTTCTT

TGCATATATTTATTCTCTGTTAATA

>c17548_g1_i1 len=248 path=[414:0-247]

GATCAACAAGTTATAATTTCTCTAGGCCAATCCAGCAATGCGAACGCAATAGGTTCATAA

CATATCCTTGAGAAGGGAAAAAAGCAAGCATACAGCTATTTAACAGCAGAATGCAGGTAA

TAAAGTCTGTGTTATAAAGTGAAGATCTTCCTCAAGGAATTCCTTAAGACGTATTCCATG

ACAATAAGCAGTAAAACAAAGATGAGGCAGCTACCACTTGAGAACAGAAAGGCTTTTGGA

GACACTAC

>c17553_g1_i1 len=240 path=[183:0-239]

GGTGAGTAATTTCTAAAATGAGTAAATTTGGCTTTGTTTCTGTTCTGTTTTGCTTGTATA

TGTGAGTTTCACCATCTCACTTGGTTTACCACTACCTCAGTTTCCTGGCAACACAGACTA

GCCTGGTCCACCAGGCTGTGGTCTACGTAGTGTTCTTCATAAGGTTTACTGTTTTGGAAA

TAGCATCTGAGATCATCCAGTAGAAAATACTGAAATGTAAAGCATTACTATCACACATCT

>c17568_g1_i1 len=366 path=[669:0-20 648:21-292 22:293-365]

CAACTTCTGCAGCTGTATGTTTTGTTCCCTTTGGACACTGAAGGGAGAAACTGATCTGCA

GAATCATGACTATTCACAAATAATGAATGAAAAAGTGCAGTGAAAGTCTGAGCCTGGGCT

TAGTCCTGAAGGTATAGTCAGAAGAGCTACGTGAAATAGTAAATTCTGTTTCAGAAGATC

ACCCTTGGCTTGGTTGTGGTATGTTAATACTTGGGTACTCAATCTACTCCATTTTAGTAC

AACACTAACTAAAGAGCTAAAAATGTCACTGGTATTAGAACTCTTCTGCCATTCTACTAA

TAATTTCATAGCAAACAGTTAAAGTTATAAGAAACACAGCTGAGAACGTGCTCACTCCTT

TAATTA

>c17574_g1_i1 len=269 path=[401:0-268]

GAAAATAGAAAAAAATAGAGGAGATGATTTTGAACTAATTTAGTGTAGAATCTTGGCAGT

GCCAAAAAGGAGATTCTGGTTTGGAAGGTCCATCTTTTCACTAATTCTGGAATTCAGAAG

GTCTCTTTTTCCACTAATTCTGGCATACATACTGTTTCAGCTCAGTAACATGGAAGAAAG

CTAATCAAGTGAAACGAGAAAACATAGCAGTAGTCTTCTGCTCAATTAGTGAGTTGAACT

GGCTCCCTAAAGCAGAGCTGATGTGAGAG

>c17576_g1_i1 len=532 path=[1:0-315 833:316-531]

TCATTCAGAAGCTGCTGTTGAGGCCCAGCTCCCAGCATGCATGGGACGATGCAAACCTGC

ACATTCCAAAGCATCCCACAGCCATGCAGCTCCACAGCAGTACCAATGGAAAAGTAAGAA

AGGACACAGTTTTCTACCGTGGGAGTCAATGTAAAGGTTGGTTATGACACTATTGCTCTA

TCTGCTAATGTCTTATGTCTCATTACCTTACTCCTTGATTACTGAAAACTGAAAGCTGGC

ATTTATGAAGTCAGGCTTTAACCATACAATTGGGTCAAACAGTCACACCCCTAAACTCAG

AATAGTCCTAAAAGACGTCATATCTGAAAACAGTTTTGATTTAATCCTCTTTTATCAACA

GCAACAAAGAAAGTTGCTTCTCATTTTTCAATGGTAAAATTTCAGAGCAGTCAGAACTTC

AGATTTGAAATGAGAAGTGTGTGTGGAGCAAAACAAATTAATGAACAGAGCATGCAGCAT

TTCTGTTCAAATCGTATTTCTGCCTTCTTTCTTTTATCAGTGAAGTGAATGG

>c17581_g1_i1 len=387 path=[365:0-386]

ATTTTTTTGCTGTTGACATTAAAGAAATCCTTCCATTCTGCTAAGCAGCTCCTCTTAGAA

ATCCCTCTTGATCAGCTCTCTTAATCTTGGAGCCTCTCACATAGTCTCAATTTTCTTCTT

CAAAGCAGTTCCTTTTGTAGTGAGAGAGATTAGTATCATCTTTCTTTGCATAAACTCATG

TTTCATGTGTACTTCTAGTTTGAGGCTAAACAGTTGTCTTGATAAAAGAAAAACCTCATT

CTAACATCTAGTTTAGATACCAGACAACTTGAAAACTGTATATATCTATGCACAGCATGG

AAATAAGTTGTAGTTATTCAAATTGCAATTCCTTCTCATTGACTCTATCCACCTGATGGA

ATCTGCATACTTCCCAGTTACTGAAGA

>c17584_g1_i1 len=203 path=[821:0-202]

TTCCAGATCTGCTCAGTGTTAGAAGCACTGAATGAGCAGCATTGATCAGGCCCAGTCATT

CAAGCTCATCTTCCTTCATTCTGACAAAGACACGTTCCCATAGAACGTTGCTAATATGGG

AACAAATGCTTCCTCTGTCCCTCTTTCATGTTAATCTTTCTGTGTTTGCTCAAACCGCAG

CCCTGAAAGGCAGCGTCAACTGG

>c17584_g2_i1 len=343 path=[1:0-342]

ACATAAAGAAATAAAGCACTCCTTGCAAAGTGGGTAGGCCTTCACCGAGTTCACTGCTAC

AGAACTGACAGCATTATTTCACTACAGAGAGGTAATGGGTTGGTTTACGGCAATAAAATA

CTGGAACACAGGACATACCCTGCATAAATGTTTATTTTAGAAGATAAGTGTAAGCTTTGT

TTGTAGGTTACGCGCTATTAAAAGGAATAGCCACAGAAGTTAGGTGCTATAAGAGCTCAG

ATAAGCAGCTTGACAGGCTTTGACAACTGCCAAGGGAACACACACCTCTGCACTGCTCTG

CCAATTCACCACACATCCACCACGCAGCTCAAACACAAAGTTA

>c17585_g1_i1 len=233 path=[1:0-90 92:91-232]

AGTTAATAGACCCATTTCAGGGAACATTTGCAGATCTCGTCCCTTGAAAATAACAAGTGT

GGGAGACTATAGCTCTTGTTGTACCCTTACAGCTGACTTGCTGTACGCTGGTGGATGGAT

CACACTAGAAAATGGAATGCCAGCTCTGAGCAAGAAAAGCACCACTTTTGTCATGGTAAA

GATGTTCTGCAGCAGAAAATTGGAATAGGGGAATGTTTATGGTCAGCTCATGG

>c17590_g1_i1 len=342 path=[155:0-341]

GCTAGTGCAACAATTGATGATTAGAATTCAAAAATTCATATCCCACATGAATTCTTCAAT

TTTTGTTTATAATTTGCCCTCCCAGCAGATTGTCCTTACAGTAAGCTTATTTATCAGAGT

ATTCAACAAAAGAAGGGAACATTGTTTTACACAGTATATCTTAGACATCGGAAATGTAAT

TAATTTTCATCTTTAAAAATTTCTTATGAAAATTAATTTCCATCCCATTTTAGTCCTGAA

TGTTGACTGGAGCTAGGATAATACATCACACAAATGTTGATTTAAATATTTAAACAAATT

CACTTACTTTGGATTTACATCCCCTTTGGGCTCTACTTTCCA

>c17602_g1_i1 len=234 path=[691:0-233]

GCACTGACCTGAACCCTCTCTCCTCGTCCAGGATTTGGGGTCCCCAGCATCACAGCCACC

CGGATCCTAAAGGGGCAGTTGAAGGGAAACCTGGGACCTGAGACCCCCCTTGCTCTGGAT

TCTTTCCCCTATGTGGCTCTCTCCAAGACATACACTGTGGACCGGGCGGTCCCTGACAGC

GCGGGCACAGCCACTGCCTACCTGTGTGGGGTGAAGGGCAACTACAAGACAGTG

>c17602_g2_i1 len=231 path=[347:0-92 439:93-230]

GGAGCATGAAGCCAAACATCCCATGTTTTATTGCATCCTGTGATCCTGCCCACAACAGAG

GGCTAGAGGAGGGCTTGGTAGGGTCTCTCAGCCCACCACCATGAAGGCAGCCATGCAGAG

GGCCAGGAGAGCGAGTGGGGGCCGTGGGGAGTGCTGGGTGCTGCCAGAGGCCCTACGGTG

TCCACCACAGCGGGGTCTGGCATCATAAGGCTTGAGGCACGCGGCATAAGC

>c17602_g2_i2 len=232 path=[347:0-92 25:93-231]

GGAGCATGAAGCCAAACATCCCATGTTTTATTGCATCCTGTGATCCTGCCCACAACAGAG

GGCTAGAGGAGGGCTTGGTAGGGTCTCTCAGCCGTCCCCCTTGACTTGGAGACCCACAGT

GGGGAGGACGTGGTGGTGCTGGCCCAGGGCCCCATGGCCCACCTCTTCCATGGGGTGCAG

GAGCAGCACTATGTTGCCCATGCCATTGCTTATGCCGCGTGCCTCAAGCCTT

>c17614_g1_i1 len=339 path=[636:0-269 905:270-338]

TCAATCCTGCAGAAATTTTTCTTTAGCACTTTGAGGTCAATTTTGGCCAAAACAAGTTAA

CACAGCTTTTACAGTTTCTCTTTCAAGTTAATGAATTCAAAGCTATAATTTGGTTCTATT

TTGCTGGTAGTGAAACTGAAATGCGTGAATTCAAGTAGACTGTGGAAAACCTGTTTAATA

CGTGAGACTAGAAATAGGTAGAAATTAGGGGAAGTATTGGAAAAGCATGAATGTTGGAAA

TTACCAAATGACTTGTCTGTTGAATCCTCTAAACCTCATCCATCATTTTTAATGCATATT

TCATAGATACGTACATACATACGCGCGCGCACCTGCTTG

>c17616_g1_i1 len=237 path=[215:0-176 392:177-236]

CAGCCCGGTGAATTACAGCTCTTCTGAAGCAACTCTTTAAGCACTACCTGCTAATCCATC

ACAACCATGTTGGGGAGAAAGCACTGTCCCCATTGGACAGATGAAAGAACTGAGGTTGCC

CCTCAGTCCTCATCCACCTGAGGAGATTCAGGAGGTTGCGGCTCATCAAACAGACAGAGA

CAGCTCTCACCCTGAAGCTCATCCAGGAATGCTGGATCTTCACACTTGTCTCTTCGG

>c17619_g1_i1 len=236 path=[576:0-14 578:15-235]

TCTTCCATTACATGGCTGAGCAGCACAGGCAGAGCAGCACAGGGATGTGCATGCGAATGC

ACACATACGTACCAAGGTGGCCATCTGCACCATGGTGGGGATGATGGCAATGAGAACATG

GGTTGAGCCGGTGGTCGCAGGCTCCCAGGTGGCCAGTGCCTTCTACGACACGGCGCTGCT

GCTGGTGGTGAAGAACTTCTACAACCAGACTAATTCAACTGCCCCTTCCCATGTAA

>c17619_g1_i2 len=353 path=[431:0-131 578:132-352]

AAGAAGTGAGACACAGATGGGGATCTTCCGATGTACCTTGTCCCCCAGCTTGGACAAGCC

ATAGGCTGACACCAGCGGACTCAGACCCAGGACAAGGTTGTAGATGACATAAAAATTAGA

GACAGCCTTCTGCTGAGCAGCACAGGCAGAGCAGCACAGGGATGTGCATGCGAATGCACA

CATACGTACCAAGGTGGCCATCTGCACCATGGTGGGGATGATGGCAATGAGAACATGGGT

TGAGCCGGTGGTCGCAGGCTCCCAGGTGGCCAGTGCCTTCTACGACACGGCGCTGCTGCT

GGTGGTGAAGAACTTCTACAACCAGACTAATTCAACTGCCCCTTCCCATGTAA

>c17628_g1_i1 len=260 path=[295:0-259]

AAAAAAAAAAATTAATTTAAAATCTATTTAAAGAATATATTAAAATTTTTAACATAAAAT

AACTCTTTTTTTTTGAAAAACAATTAGATATAGTTTAACTAAATATACTGTATGAAAAAA

TAAAAATATTTATTTTATTCCATTATATATAATAAAATAGAATTAATAATATACAATAAA

AATGTTTAATAATTTCATAATTTTCAAATTTATATTTCTAATTAATAGTGTATTTACAAT

AAAAGTTCTTTGGCCAAACG

>c17636_g1_i1 len=259 path=[237:0-258]

TGAGGATTGTACTCTTGTGGGACTTAAAGGATTATGAGGCACGTGGGGGGAGAGAGAGAA

GGCAAACTGATCACTGACTCCACTGGGGACCCCTACTCCTCTAAACCTGAACTTGTAGTC

TCAGAACTTGGCTTTATTAACTTCAGTTAGTGTTTCTGCTGTTACTAGAACATCTTCATG

CTGCAGAGGTGTTGGGTTTTTCTTGCTGCTTTTCATTTGTCTGATTAATTTCAACATGGA

CCCTCTTTAAGTGGCTCTG

>c17639_g1_i1 len=257 path=[195:0-256]

GTAAAAGTGCTTCCTCTTTTTCTAACCGATGAGATCTCAGGGTGTTACATTAGAACACCT

ACAGCACTAACAGTGCTGCCAGAAAAGCGAGGACACTTCAGATGATGTGTTCTTCAAATG

CATGTTTGTTCCCTCTGCGTCAGTCATAATTGTAACCAGGCTGCCCTCCCCATTTACCAT

GGAAACCAGCAGATCTCTTCAAACACGCTGGTGGTAGCTGACAGGTATCCTTTCTTTTCT

ACTTAACCTCCCAAGAG

>c17641_g1_i1 len=284 path=[155:0-283]

ATCTAAATGGTTGGGTATCGGATGCTATGGAAGACAATGCAGAAAGCTACGTAGTCAAGA

TGTGAAACATCTGCTGCTCTCCCCTTATCCATAGAGCCAGCTGTGTCATCACAAAAGGCA

ATTACGTTGTTGGGCAGTCTATGTCCATGGTATATCTATGTTGGCAGTTAGCAGACTCCT

TGTCTTTTATATGTTTAGAGAGTGTTTCAGGAGGAATTGCTGTATAATTTTCCCAAGGAC

TGAGGTTAGGTTCTGTGACATTTCTTTTTTTTTCCAGTCACTGG

>c17645_g1_i1 len=207 path=[1:0-206]

GTTTGCCAGTGTCTTCATTTGTCCACTAAAAGCAAAAAGAATGAGGAGTGTGAAATTATT

AACAGCAAGTTTGGTCAGAAACTTTCTTGCTGACCTACCATCTGTATTGAATCCATCGAA

ATTCATCCTGAAGGTCAAAAGTTTATTATCAAAGATAACTGCGTTTCCTCAACTCACGTT

AGTACGAAAGTTTACACTGTCATGATG

>c17645_g2_i1 len=304 path=[650:0-303]

GCTCCTGCCTAAGTTAGAGAGCATGTGGGACAGCAGCCTTTGCTGCAGATGGGCAACAGG

CGCTGAAGCTTTTATTTTAGCTCAGCCCAGAATGCAACTCATGTGAAGCTGAAATGAAAC

AATTCACTACTACTCTTAAAATTCTGGAGTACAGATACAGTATCTGAATCCCAGGCTTTG

CAGTCTGGCAATTTTGTCAAGCTGTGCTTAGTTTGTATAAATATGGAGGAAGTTGCAACC

AGAAACTGAAAGGTTTTGGAGTTGTTGGGAAACAGTCATTTCCAAATGGATGTTTGCCAG

ATCA

>c17646_g1_i1 len=234 path=[241:0-233]

AAAATAAAACTCGTATTTTGGAGTGTATTAATTTTTTCACATAATTATAAAAATTTAGGA

TATATTTTATTTATTTTTATTGTTAAAAAAGTATATTTATATATTTCAAATAAATTAACA

TATGAAAGAAATTTTAAGACAACACTTATCAAAATATATAAAAATTTATATGAAAAAATA

TTTATTACATTCTGATTAATGAAAAATAAATAGAGGTGTAATTAAAATATATAA

>c17650_g1_i1 len=212 path=[264:0-136 25:137-211]

TTTTTTTTTTTTTTTTTAAGATGAAAACTTGACTTTTGTGTAAGGGAAAGAAATCAGATC

AAGGGCAACAGGACAGCCACCCTAACAGTTCTGACAACCTGGGTCTGTTGTACAGCTTTG

TGGAGCATTCAGAAGGTCACCACCACCAAAAATAGGCCCCAGACAAGAAAATACCTAAAG

AAACAGCAACATAAATCCCTTCCCTACTTCTT

>c17650_g1_i2 len=212 path=[264:0-136 400:137-211]

TTTTTTTTTTTTTTTTTAAGATGAAAACTTGACTTTTGTGTAAGGGAAAGAAATCAGATC

AAGGGCAACAGGACAGCCACCCTAACAGTTCTGACAACCTGGGTCTGTTGTACAGCTTTG

TGGAGCATTCAGAAGGTTACCATCCCTGAAGACATATGAAGGACACAGGGAAAGAAATTC

AGTGGGGAAGAAGTAGGGAAGGGATTTATGTT

>c17656_g1_i1 len=210 path=[223:0-209]

ACCTGGGCTCTGTGTACTGGGAGCGCTGTACAAGGTGATTAACCTCATCCTTACTGCTGT

CTTCCATCAAGGAATACAAATGGAATATACGCAGCGCTGCAGTGTGCTGGTATGGCATCG

CTTCTTTGGTTGTTAGATCTGAAAATAGGATTGGAAGTGCTTAGTGGTTCTGACTTGCTC

TAAGTCAGTTTTGTACAATTAATTCGTTGG

>c17664_g1_i1 len=214 path=[242:0-213]

AGAGGGGAAGGAGAAAAGAGGATGATGGAAGGAGGTGCGCTTGTTTGGTGCATTCAGAGA

GCACTCAGACTCATTGCCTGCCCTAATTCCAACACGTGCACTGCTGTGGGAGGGTGGTTC

TGCTGCCCCAGGGCCTGGATGCTGCATCATGGCCCCAGCGTGCACCTGACCAGTCCCTCC

CCCCCCCACAGGCCGCTCCACATCGCTGCACGGA

>c17674_g1_i1 len=280 path=[191:0-188 380:189-279]

GCTTGTGAATGTTTTATCTGGGAGACAGATTATCTGAACTGCCTTTTGAACCTCGTGGGT

GGACACGAGCATGGCAGTAGTGTGCTGTGCATGTAGTGGGGGCTGTTTTCTTCCTTCTCG

TTTTCAGTCACAATTCTGTGTGCTAACAGTAGCTTCTCACATCTGAGATGTTTTGTATTT

GGTGAAGGGGCTGGAGGGTGTAGAAAAGCAATTGTAGCTGAAGTTTTCTGGGGTGTCTCA

GTTATCAGTGCTTGCCGTTTGGTTTCATGGGGTTAGTAAT

>c17681_g1_i1 len=407 path=[551:0-406]

CATGTGCTGATAAGGACGAATGAGTTTGTATATTTCCTCAAAAACACCACATTCCATTAA

TGAGCACCTGGGAGGAATGACAGGTCTAGCAATAACCCTGATATCCCACTCCATGCAGTG

TTTTAAAGTACATATTCTAGAGAACCCTCTAATTATCCCATAAAGATTTGTAATATTTAT

GGGGCTTGCAACCCACCTAGCCTTGAAATAGCTAAAAACTGAGATCCATCGCAAAACAGA

TGAGAGCTGGCAGTATTATGGACTGTGTTTTTAGATTATATTCAGGTTTTACTAAACGCA

GGCTGCATGCCTATGGAGTACTAACATTAGCTGCTTATGTGTGTTATGTTTATATGTAAC

TGACAAAAAAGAATTAGGGTCATTCAAGTGTTCTACTTGCATGTGTG

>c17691_g1_i1 len=215 path=[193:0-56 250:57-214]

GATCTCCATTAAGAAGTGGCAAAAGCCTTATTGTTTGAGGATTTACAATGGATCATAAAA

CACATTAGAAAATATAAAATAAGAATTCTTCATTGGCATGGAAATGACCAAATTAACCTC

TCTTTCTAATTTCACATCATCAGCATAAATTGCAATGATTCCAGAAAGTGCCCAAATCCT

AAACATGGATCTGTATTCCTGCGTAACTTCATGCT

>c17710_g1_i1 len=203 path=[335:0-202]

ATCTTTATTGTTCAATAATTAAACTGCATAAAATTACTTGGAACAAATGTTTCATTGGTA

AATATATTTCCTGAGTTTTAAGCCATTGGAGAAATTATATTTTCAGTCATTACAGTTTAG

CTGTACCCAAAGGTTCAGTTACTTTGGAGACAGTATTCTGATTACTGTTCATTCGCTTGC

AGAGGGAGACTCTGATAACTACA

>c17711_g1_i1 len=226 path=[1:0-225]

AGCCAACATTTCCCACTTCTGGCCTTGGTTTGGATGGGGGTTCTGGTGAGCTCTTGCCCA

GCTTCCCACTCTGGGAGCCTGGTTTTTACAGAGCCCACAGCTGGGAGCCTGGAGCAGGAG

CCTTAATTCATCCCCAGGCGAGGCAGCCCCAGGGTGAGCTCTAAGCCTCTCTCTGTGCTG

CTGGATCAGGCCTGAATTAGGCGCCTGCCCTTGTCCATCATAGGTA

>c17719_g1_i1 len=251 path=[227:0-250]

AGGCAGTTCTTCCCTTTGGGGACACTACCTCTTCAGCATTTGCACCCATGCTCCCCAAGT

TATCCCCCTTTTCCCCTTTGGGCCACTGGAAACTGTCTCCTCCCCACCTGTATCTTTCAG

ATACGAAAATTCTAAGAGGTTATTTGCCAAAGCAGGGTGGATTTTTTTTTATTACTGTTT

GTTTTGTTGTTTTCTTTCCTACAGCATCCTGAGTACAGCAAGGTTCTTAGGTGTGCTTTG

GAAATCCAGCA

>c17725_g1_i1 len=255 path=[233:0-210 444:211-254]

GCACCCCAAGGATCCCAAGGGGCACCGACGGGCCCCCGGAACAGCCGCTGCAGACACAGA

CAACACCAAGCAGCCACCTGCTCTGCCCGGCCTGCCAGAAGATCCGTCCGCCGCGGGGCC

GCCGCAAGCAAAAGAGCAGCAGGCACCAACCGCCACAAAAGCAGCGCACAGACAGCAGCA

CCAAACCAACAGGGATTCCCCGCTCCTCACCCATAAGTTGATTCGTCCACTAGAGAGTCA

AGGAGTGATCAGCAG

>c17727_g1_i1 len=233 path=[425:0-232]

AAACAGGATGAAGAAAGCCAGTAACAAAAACAGAAAAATGCACCTCACGTAAGGGGAAGC

TGTAGAATATCAGGGAGAAACGTCTGAATGCTAATTTTGCTCACAGGGATAGAAATAACA

TCAGAGTAACAGCAAAGTTCATCTGGGATTGTAGATCAGACAAAATAGCTATGTAGCCAG

AAAAAGAAAAAAAGATCAAGTTAGAAGTGGAAAGTGCCTGTAAAACTTCTGAT

>c17731_g1_i1 len=354 path=[485:0-351 858:352-353]

GCCTTCGCCAGCCGGGCGGGCGGCAAGAGGCACGGCAAGAAGGCGCGGCTGCGCTGCAGC

AGGAAGCCGCTGCACGTCAACTTCAAGGAGCTGGGCTGGGACGACTGGATCATCGCCCCG

CTGGAGTACGAGGCCTACCACTGCGAGGGGGTGTGCGACTTCCCGCTGCGCTCCCACCTG

GAGCCCACCAACCACGCCATCATCCAGACCCTCATGAACTCCATGGACCCCGGCTCCACG

CCGCCCAGCTGCTGCGTGCCCACCAAGCTGACCCCCATCAGCATCCTCTACATCGACGCG

GGCAACAACGTGGTGTACAAGCAGTACGAGGACATGGTGGTGGAGTCGTGCGGC

>c17741_g1_i1 len=306 path=[1:0-84 623:85-305]

GATCTTGTGACAAAGCTTCTTTCTAATGGGGCCTGGGGGAAACAAATTGCATTCCTAGTC

CTGGCTATCGCTGCTGCCTACTTAGGCCAGATCATTAAACATTTTTACCACTTCCACTGA

TTTCATGTGAATTTAAATCCAAACTGATTCAGATTCCCTGTGTGTATCCTATGTAGCTTC

ACTGGGGATGGAGAATTGTGCTTGCTATATCTAAAAAATACAAGAATTGTTTGCTTAAAT

ACTTCCTTGTAAAGTAAATGTTGAAACTTGTGGTGAATTTAGTCTTGGTAGTCCTTGTTA

GCAACA

>c17741_g1_i2 len=239 path=[621:0-17 623:18-238]

AATCAAACACAAAGTTACGCCAGATCATTAAACATTTTTACCACTTCCACTGATTTCATG

TGAATTTAAATCCAAACTGATTCAGATTCCCTGTGTGTATCCTATGTAGCTTCACTGGGG

ATGGAGAATTGTGCTTGCTATATCTAAAAAATACAAGAATTGTTTGCTTAAATACTTCCT

TGTAAAGTAAATGTTGAAACTTGTGGTGAATTTAGTCTTGGTAGTCCTTGTTAGCAACA

>c17742_g1_i1 len=248 path=[67:0-154 66:155-247]

CATCATGGAGGAAACAGGGGTATATCATTCCAGAAAATTCTCCAAGTGCAGCATAGCAGA

ATACAAAGAATTTTTACTTCGTGGGGGAGGTGCCTGTCTTTTCAACAGGCCAACAAAGCT

CTTTGAAACTACTGAGTGTGGAAATGGATATGTAGAAGCAGGAGAAGAATGCGATTGCGG

TTTCCGAATGGAATGCTACGCAGACTGTTGTAAGAAGTGCTCTCTTTCTAATGGAGCTCA

CTGTAGTG

>c17745_g1_i1 len=289 path=[1:0-247 249:248-288]

GTCAGACTCTTGATCCTTCCCATTGCTGTTCTGCAAAATTCTTAATTATCCCTCCTTCAC

TTGGTTATGGTTTTTTGTTGCTAGAAGAAACTTTCTGATCTGCTGTGCTAAATTTCATGA

TCAGTCCTATTGTGAAGAATGTATTTTTTTAACTTGGGGTGAAGCTGCATTTCTTATACA

ATCATTAGGTGGTAAATACTACTTCCCATTATTTTTGTTATTCACTCTGATTTGTGGTCT

TGCTAAGCTATTTCAGTTGTCTTGTAAATGTTGTTAGTGTAGTTACATT

>c17749_g1_i1 len=262 path=[1:0-261]

AAACTTTGTGTTTGAGTCGTGTATAATTAGCACAGGCATTTATGCTTCTCTCTCAGAGCG

AAGCACCAGAGCTCCCACTGCTGTTTGTTTGTATTTAAGACAAGATGACGCTGTCACTTC

TTTCTTTAACAGAAACATGAAACGGGGGGAAGGGAACGCAACAGGAAGAAGCAAAGCTAA

ATTACTCACAGGGCTTTTAAACTCCTTCCACTCAGTTCATTTTCCTGCAGTCCCAATACC

TGGCAGCTGCACCCAAATGCTG

>c17756_g1_i1 len=229 path=[281:0-228]

AAAAAAAAAAAAAAAATGTTAAATGATGTATATTATAAATAAAAGCAAATACGCGTTAAT

AATTACATAATATAAATGTAATAATTTTTACATAATATATTATTGGAAAGAAATACTTTT

CATGTCCTTGAAAACATGAACTGAAAATATTTTTTTTTTTAAAAAAATCCATATATATAT

ATAATAAGTATTGTATAAATAAAATGTTAAAATAATAAAAAAAAAAAGA

>c17760_g1_i1 len=287 path=[217:0-286]

TGAAGAAAAAGCTTAATAATGTGAGACCTAACAAGCACTACATTCTTAAAAACATCTCTT

CATTTAATGTGCTGTTTTGAAGGATGCCCCTTTGCTGTAAATCAGGAGTTCAGCCAGCAT

CAACTGGTGCCTCTCCCACAGCCAGCACGCACCTCTCCACGCACTCAGGCTCTGGTCCGA

CCACGGGACTCGGCCATTTGCAGCATAGCAGCCCTGCTGCCCTCACCACACGCACTCTCA

CTGCCTTTCATCAAACTGTATCAGATTATTACAGTTCAACATCCTTG

>c17763_g1_i1 len=580 path=[1:0-579]

AGATGCCCTTGCCTGTGCTCCTACCCACCTAAATAATTCACCATGCCAGAGTCCTTCCTA

TCCACTAGGAGACGTGCAGTCGGGTACAAATGTGCCTCCCAAGTTATTTGCACTAAACAG

AGCCTATTTCCCCCATCTTACGTAGTGCTGCGAAGCGCAATGAAACCTCCAGTTGCTCTC

CATCAATCTTTTCCTGTATTGTATCTACATAGCAAGCAAGATGTTTTCCTTCACCGCCCT

CGATGATGGATTTCCTCCATGCAAAACTTCTGGACACAGGGCAGCTCTGTGAGCTGCTCT

GGGATTATACAAGCTGATCTGAAGATGAATTAAGCCTCTCTATGTTTCTTGCAATCTGCA

TAAGCAGGCGTTCCTCTTTCTTGCAGCGCAGCAGGTTTCTGTTGATGCTGACATTTCACT

GCACGTGGGAACATTGCAAGGCAGCAACACCAAAACGGGGTGTTGGACTCTGATCTGGAG

AGAGGCAGTTTGTACCCCGTGAGTTCTGGTGATGGCTGTGATACGCAGTCATGTTACGAC

TCTCCGTATCAGTGATCAGGTTCTGCCTGCACCACTCCGG

>c17763_g2_i1 len=411 path=[1503:0-410]

AAGGCTCGTTTTCCCCCTCATGCATTTAAGAGAGGGAAAAAAAGGGATAAACTGCAAGAG

TCCAGAGTGCTTAGAGTAATTAAACTGCAGGTTGCACTCCGCTCTTGAGATGGATTCTAT

CATACTGCACCACACCGGGATCTGCACAGCCCTCCATTGGCCCCCTGAGAGGAGACCAAA

AGTTAAGCTTATAGCTTCGATTTGTATCACATGCCTGCTGAAAAAGCAGCTCTGAATTTG

CTTTTAGACAGCACTCTGTCACTCTTACAGGCTACAGCTGCAGGAGCTGTGAGATGCACT

GCTGTGTGGTGCAGCCACGGGCACTGCACTGAGGTTTGTGTAAGGCCATCTCGGTTCCAG

CAGAGCTGAGTTGCTGCCTGGAAGTGTTTGTTTTCTGTCTTCTCTTCTTGT

>c17765_g1_i1 len=234 path=[1:0-233]

TTACATTCAAGAGACCACCCATACATCTAGGCACTAATTTCCAACCCTGAGCTTCTCTGA

TGACAAGTTTCACAAGCCCATTAGGACCAGGGCAGCTGCCAATAAAGACAGAGTGCAGAC

AAAGGGAAAAGCAAGTTCAAAAATTGAAGAGCAAACCTTTCATGTCTTTGTGTTCTCATA

AGCAAAAGCAGTATAGTTGTAGTATGTGAAACAGGCCCAACTCAGGGTGTTTTG

>c17773_g1_i1 len=279 path=[155:0-278]

GAGAAGCTTATCCGAGAAGAACTGCTTCTAAAAGGTTACTGCAAACGATGCTACAAAATA

CAGCTGTGGGTCATTGCAAGAGAAGAGGCATCAATGCATGGAGAATGTGATACGCAGGGA

AAGAAATAATTGCTTGTCTGAGCATGCTAAATTAACTATCTTCCTGCTGCCACAAACTAA

TTTGATCTCCCAGGACTCATTTCCTCAGCTAATTGGAACTTCAGTCGCCACACATGTAAA

TTCTGCCTTGCAGCTGTATTGTTTTAGGCTAGTTGAACA

>c17775_g1_i1 len=380 path=[1:0-245 659:246-379]

TTTTAAAAAAAAAAAAAAAATTGACATATAACTAGAAAAATATATACGTATATATATATT

GATATTTATATTTTTTTCATTTAAAATTTTTTTTTTTTATTATTGACACGTTAAATGGCA

ATATATGAATACATGAATTCATGTGTAATTATCCTTGCAATACAATGCATAAAAACTGTT

TTTAGATTTTACATACATAACAATTAAAGTACTTTTTTTAAATATCTTATTTTAAATAAA

AAGAAAAAAAAAAGATAAAATATATATATTTAAGATAAAGATTATTTTATAAAATAAATA

ACAATATTTTTTAAAAACCGTGTATATGTATTGATATTTAAATATTAAAAACTTTCATAG

TGATTCTGCTTAGTGTAATA

>c17776_g1_i1 len=231 path=[257:0-125 382:126-206 25:207-230]

GAACAATTGGAAAATGCTACAAAGTAATTTCGAAGAATTTTTTTTTTTTTCTTATATGTG

AAAATTTAAAATACACCGTTTATTCTTTTACTAAATTAATTTTTAGTTAAATTTTTAGCA

TTCAATGTATAACTTTCTTCTACCTTCTTCAAATTTCTAAATATTAAACTTTATTTCTTT

TCATTTATTTTTTATTTGTAAACGTAGATCATTTGTTTCAATTGAATCTGC

>c17786_g1_i1 len=304 path=[273:0-303]

GGTGAACATCTTAACATATTTCCATTGCCCATATACCTTATTTATTGTCTCCATTTGTTC

CAAGAGGCAATACAGGCCCTAAAGCTTAGCTACCTTACGTAAACGTTTTGGTTGTTATTT

TGAGGATGATTGTATGATAGTGATGGAAACTTCTTCACTTGTTTGTTCCAGACATCATTT

CACAACCTCAGTTCCAGAGATGCATTGTTATTTGTTTTGTTTTTTTTTTAAAAAAGTTGC

AGATGGTGTCATCCAATTGGAAACTTTAACACCACAATCCTTGCTGCTTGTTTGCCTAAA

GGTG

>c17793_g1_i1 len=637 path=[911:0-636]

TGAGGTGGTTTCAGAATTTATAATGATTATAGATAGATATATTAATTTAGGAGTTTTATA

AAAGGTATATAATTTAAGGAATTTTATATTGATGTTGTTATCTAAAAAAGATTGTGCAAG

TTTATGCAGCATAATTTGTTTGTGTGCATTTTTATGAATCTAAGATATAAAAATATATAT

GCATGAACTGTTTTTTAGAAAAAGAAAGTAAGAACTTTTAAGTTTTTGTTTGTCTATTAT

ATGAATATATAGAAACGTTTTTATCACAGTTTTCAATAATGTAGAATAAAACTATATGTA

TCCAAAATATAAATGATTACATACCCTTTCATAAAAAAGTTATATTTTTGTTGCTACATC

ATTACAAGGTTTATTCTTATAAAGTTCATTTTAACTTGTAGAAATGTAGAAATGACAATA

TCAAATAATGTAATATATTTATATTTACTACTTCTTTTAAAATTACTAGAAAAACTTTTT

CTTTATAATTCTTCCCAGTATTCTTTTAATGACTTTTCATTATAAAATAACTTTATATAA

ATTACAATTAATATACTACAATTAAAATTTCCTCTTTTAATACTTAATGAACATTCAAAA

ATAAAAAATCATTTCCTTTTTATTTATTTTTCCATTA

>c17805_g1_i1 len=292 path=[270:0-291]

AGCAAGATTACCTTAAAGAAAAATACCTGTAGATTTAAGAATCTTTGGTGCTCTATTGTA

AAATAAACAGAGGTACCCATCCATTACCTCTCTTGTCTTCAGGGGAAGATTTTCACCTTC

CTTGCCACAAATGGCTCCATGTTACCCTCTCATGCTCTTCAACAGACATAGGTTTCCCCT

CAGGGAGATGCCAAGCCCCAGCCTTATTTTTGTTTCACGAGTAATGGTTCAGAATTACTC

ACCCATACGCGGTGAGCTCTTGGCTGCTGAACCCTGAACATCCCCCATAATA

>c17807_g1_i1 len=270 path=[699:0-20 109:21-269]

TGGAGTGAAAGCTGGCTCAAACACAAAGCAATGGTTGTAAGTCACAGACAAAGATTCTGC

TGTAGTTTACCTCTCAAAATGACATTTTGTTTCACTTCCCAATGAAATGAAAGCTAAAGC

TGGAGGAGCAGCTGGTAACCCCCACACAGGGCATCACAGGTCTCAAACACATGCATGCCT

ACCCGCAGGCTCTGAAACAGAGCTAGGTGTGCAAACCTCCTATCTGTTACCACATAAAGA

GGGGGGAAAAAGGGCAGAAATACTCACCCA

>c17807_g1_i2 len=357 path=[1:0-107 109:108-356]

GAGCTAGAATTTAAATTCTAGCTCCTCTATGAGGACGAGGAATGACGAAAGTAATTGTGG

AACTTGGGTACAGTGAGTACAGAAGTAATTTCTGTCACCATACACATCCACAAAGCAATG

GTTGTAAGTCACAGACAAAGATTCTGCTGTAGTTTACCTCTCAAAATGACATTTTGTTTC

ACTTCCCAATGAAATGAAAGCTAAAGCTGGAGGAGCAGCTGGTAACCCCCACACAGGGCA

TCACAGGTCTCAAACACATGCATGCCTACCCGCAGGCTCTGAAACAGAGCTAGGTGTGCA

AACCTCCTATCTGTTACCACATAAAGAGGGGGGAAAAAGGGCAGAAATACTCACCCA

>c17821_g1_i1 len=235 path=[1:0-234]

AATAACCATTAAAAATAATATATTTTGTATTACTTTTATGTAAATTGAGTTGATTTTCAT

AATACAAATAGAAAGTGAAGATAATTTGAATTTCCAGAAGCATGGGTTCAGTCTTAGGAG

AGGGAAATGTCTTAAGTCTTTGTACTTTTATAGAAGACCAGAGAAGGCAACTAGCATTTC

CCATAGTAAAGTGTTCAGAATAATTACATGTGACTTCCTTTTCTTCTTTCAGTTA

>c17827_g1_i1 len=207 path=[155:0-206]

GAAACTGTTATCAGCATTCCCTTATCTGGCTCCTCCAGTCAGCTGGAGTTGAGAAGGCTG

TGGACTGGGGCAGGAGGAAGGAAACATCCTGTGCTTCATGCTGCAGTGGGGCTGCAAATC

CTCGTAGGCACCACTCCTTGTGCAATGCTGAGGAGCAGCTTCTGCCTCAAACCTGCTGAT

CTCTGGCTAGATTCTGCAGTGATGGAG

>c17834_g1_i1 len=233 path=[315:0-232]

CCCGGGCTGTGCGGTGCCGAGTCACGGCGCTCGCGATAGGCACCGGCCGGGAAAGGGAAT

TAAAAGAACGGTTTGATTTAAAAAATCGCGATCCGTGCCCGATCGGGCGAGCTGAGGGCT

GGGTCTTTGCGGGCGAGATGAGAGGTTCGTGTCTTCAACTGGCCTACAAAGTCCCAGTTC

TCGGCTCCCAGGACCTGCCGGGCTCGCAGCGGGCGAAGACGGAGCGGTTGGGG

>c17838_g1_i1 len=284 path=[1:0-283]

GTAACTTTGTGTTTGATTTGTTTTGTTTTTCAGTGCTTTATTAGCCCTACGTATCTAGTC

TAAGAGGTTTTGAAAAGATACCATTCTCCAGAAAATAGCCCCACTGCTGGAAGTTATAGA

CTGACCTGGACGTCAGATACATACTGCATCCCCCAAACTGCTCGAGTAACAGAGTTTTCA

AAGGGCAACAGAAACCCTTTCTGTGACATGCCCACCTTCATTCTACATGGCTTCTGTAAG

CACAGATGTTTTTGGCTGCCAAAAATATCACCTGCAAATGATAC

>c17838_g2_i1 len=327 path=[523:0-326]

GTGCCCAATCCCTTTTCTGCAATGAGGTACGTATCTGCAGTGCCTGTACTTCTAAATCTA

CTTTTTTGAAATTATTTACGTACATTTTTCTGAAAGAAAGTTTTGGTATCTGGACAGATC

GAGCAGAATGTACGCTTAGAAATAACCGCACGTCTTCCTGAATGCCTCCTTCTTTATACG

AAAGTTTAGGAAAGCTGTAGATATTTCTATGGGGTTCAAAAGAGCCATTGGTGGTAATGA

TGATCGTTTCTTTGAGTCAGTCAGCCTGCGTGGGGAGCTTCTGCTCCCTCTTGTGGTGAA

ACTCCAGAGCATAATTTGAGGCTGAGC

>c17840_g1_i1 len=258 path=[236:0-257]

AAAAAATTTTATATAAATAATTTCTTCATTGTATATAATTAACAAAATTTGTTTTCTATC

TATGAAATTGCATTAATAAATTTTTTTTCAAATATAATTTTTATATGGTTAGTTTACAGG

ATCATAATTTATATCCATTTCATTTTTATTTTTATCTTTATTCCCATTTATTTTTTTTAA

AGTATAACATAACACACAGTATATAATATACAAAATCTTTTAATAAAGATATACCAAAAT

TATTTAAATCATCTTTAT

>c17840_g2_i1 len=351 path=[803:0-350]

AAAAAAAAAAAAAAAGAAGAATTATTATATGAACAAGAAATGTCAAATAAGAAACTAATT

TAAGTAAAAAAAATAAAGATATAAGAATATTATAATAAATAAAAAAAAATAGTGAACTTG

AAAAAAGGGTAAAGTTTTTAAGAGTTGAATTGAAAAAATAAAAAATAAATTATATAAAAT

GAAATAGAAATATCAAAGATGAAAGATAATATGTAAAATTAAATAAGAAACCTGCAATTA

AAAGGTAAAAAAAGCTATAAAAAAGAAACAATTAAAAGAAGCTTTATAATCATCATATTC

AAAAATCTAAAATTAAAAAATAAATGGTTATAAAGATGATTTAAATAATTT

>c17843_g1_i1 len=216 path=[1:0-215]

GTTTTCACAACTGACACATGAATTCCATGGAGGGAAAACCTGGTGCCTTAAGCTGATTGT

CTCAGTAATGCTATTCTGTGACTACTAGTGAATGCTACTGTACAAAAATCTGTTCATTGC

TGCTTTCAGAACAGCTGTGCTTGATGATAGAAAGCAGTAGCAAAGGGAAAATTTGGAAAA

CTTAGACATATATACTACAGTGAAAAAAGATTTATT

>c17845_g1_i1 len=369 path=[1:0-368]

AGATGCTCCTCCCCCTTTTCCTCCCCTATTTCGATCACGTTCTGTCTTTGAAATTAATAG

CCACCCTTCTCAGATGATGTTATCTGTGAGTTAAATAACACTGTGTCCTTCATTGCTTAA

AATGAAGTATGAAATAAAGCAAAATTTTTTATAAGCAATCACTGCATGTTTGTGCAACAT

ACGGCATTGTTCTCAGAAATGGCTAAAAACAATGCACAAACGGAGAAAAACCACACACCC

ACAGGATGCTGAGACTTTAAAATAAAGAATATCTTTACATTACCTACTGTGCATCCTAAG

TAAGTCTTTTGTAATGACCAGCTAAACAAAACATTTCTTATAACATTTTAATTTCTTTCA

CGCTATAGA

>c17847_g1_i1 len=245 path=[277:0-244]

AAATATCCCTTTCAGTTATTCTTGACAACTTGTATTGGCTGAAGCAGGTAATTAAACGAT

GTCACTAATTTTAAAGCATCATCACAATATGACACAAAGCTACAATACGGCAATGACCAT

TTTTAAACAGCAGAAGGGAAGAGGTATGTTGATTGGATATAAACTGATTAAGAAGCTGTC

TTGGAATTGGTTGTTCAGGCCATCCTTGTTGTCCTATCATGAAAGGAGGAGAGTTCAGTA

ATGGT

>c17848_g1_i1 len=294 path=[1:0-266 689:267-270 694:271-293]

GTCTGCATTGCCGTGCTAGGCACTGCTCTTTGCTTCAGTTTTCCCGTTGCATTTTATAAA

TGGTAGCTGTGGGAACTGTCGCTAGGCAGTAGGAGGGTTTTTTTCTTCCTCTAAGGCTGT

TAGTGTTTGTAAACAATGCTTTGTCAGCAGTGAAATTCAGAATAGGAAAGGTGGTGTGAG

GACTAAAAGAACTGCTTAACTCTCTGTGTTTCTTAAGAAGTGAAACACCTCTCTTATGTA

ACACGAGACCATTTTTTTTCAAGAAGACTGTTGCTTTGAGCCATCTCAAGAACA

>c17850_g1_i1 len=265 path=[243:0-264]

CGGCCTCGCCGTGCTCCGAGGCTTTGGGCAGAGCTTTCTGCAGCCGGGGGTCCGATGGGG

GTTTGCGCACCCGGGGGTCGCTGGGGCGGTCGGTGCCGCTCGCTTTGTGCTCGGCGGGAG

TCGGGAAGGCGTCGGCGGAGAGCGCATCGTTGGAGACCGCCGCGGTTCGGTCGGCCGTCG

GGTCGGTTTTGTCGCCTCCGCCGGCGCTCACCGTCTGGAGGATGCGGGATAGGAGCTCGA

AATCGGGCAGCTTGGAGGGGGGGTG

>c17852_g1_i1 len=218 path=[145:0-217]

ACTAATAAGAAACAGTTTAACTGCAAGAATGAGACTGCAATCCCTGTGCAGCTGTTCCAG

ATGTTGGGGAATGGTACACTCAGTGTGTAGGGCCTGGAAGGGAGAGTGTCATGGTCAAGG

AGACCTGGCTGTATTTTTAATTGCATTTTTTCTTATTGGAAAGTCTTTGGGACAGAGTAG

CTCCCTTTGTTCTGCACTTACACAGAGTTCTGTTGTTA

>c17861_g1_i1 len=280 path=[478:0-279]

GAACTATTTGGAAATGCATAAAGAAGATCTTTATATATATTTAATGCCACGCTGAACTCT

CCTCACAAGACAGTCTTATTTGGAAGTGTTGTTGAGCTCTTCTGTTGGTCTGTGTCCGAA

TAGAAAGTGTTCCACGTGAACTTTTAGGATAGAAGTGAGTAGCTTGCGAAAAGTCTAACC

AACGTGTGTTTGTTTGTATATCTTTCAGTGACTTTAAGGAAGGAGGAACTAGCTCGTTTT

GCTCTTTCTGTTGGTCTCAGTGCTATTTGAATGGAGCGTT

>c17863_g1_i1 len=287 path=[211:0-286]

AAGGAACTTTTAATATTTACTTTTCATTTTTAAACATTTCATAGTTTTTTTTTTTTAATT

TCTTAATTACCTATAAAATTTAAATCTGATTTTAAATACTTTAAATTTATTAATGTTGAT

TTATTGTTTATTGAAAAATATATTATCTTTTTTAAAATTGATTTTACAATATTTAAAAAA

AAGATATAAACATTTATAAAAAATTAAAAAAGAAAATCTTAAATAATTATAAATGAAAGC

AACTTAAAACGGAAATAATACTTTAAATATAACACAAAAAAAAAAAA

>c17866_g1_i1 len=259 path=[237:0-258]

ATCTCACCCTTCTCCAAAAGCTGCCTGGACACGTGCCTGGGCGCCCTGCTCTGGGTGTCC

CTGCTTAAGGAGGATTCATACCAGATGAACCCAGAGGTTTTGCCAGCTTCAGCCGTTCCA

TGATTCTGTGAAGACCTTTAGAAAGTTCAACTGAATGCCAAACTAAATTGCCTACTCATA

GACATCTAACATGAATTGAAAGTGAAAACAACTCCAAGATTTCATTTCTGTAATACCATT

CATTGTAGTAAATGTGGTA

>c17866_g2_i1 len=249 path=[473:0-248]

ATCCTGTGAGAGAGAAAAAAAACACTAATCCTTGATTCTAGAAGTGAGACACAGTGATTG

ACACGGTTTTGATGAAAACTTTGAGCATGAATGACAAGCCTAAAGAAATTGGTCACCTGG

AAATAAATATATCAGTGGTCTTAAGGGCATAATAAAGTTGCATATTGGATGATGCAGTCC

AATATTGGATGATAAAGTCCAATAAATTTGGACAGAATTCATGCCAATCCATTAAGTTCT

GGCTTGTGG

>c17868_g1_i1 len=215 path=[349:0-214]

TGTGTTTGATGGTGAGCTCCTTGCTGGGCTCGCTGCGCCCGATCTTGTTGAAGGAGTACA

TGCGGATGCTGTACACGGACGCAGGGTGCAGGTCCACGATGTTTGCCTGGGAATTTTTGT

GGTAATGTGTCATTTTCCCCCCCCACAGCTCCTGATGGGCCTCCGATGGATGTCACCTTG

CAGCCGATGACATCGCAGAGCATCCAGGTCACCTG

>c17869_g1_i1 len=325 path=[341:0-285 626:286-324]

CAGCACCTCACCACTCTCCTGGTGAAGAACTTTTTCCTAACATCCAACCTAAACCTCTCA

TCTTTCAGTTTAAAGCCATTCCCTCATGTCCTGTCACTAACAGACTGTGTAAAAAGGTGG

TCCACCACCTGCTTGTAAGCTCTCTTCATGTACTGGCAGGCAGCAACGAAATATCCCCAC

AGTCTTTGGTTCTCCAAGCTAATAGTATAATCTTCTAAAAAAAAATTAACATAAACACAG

TCCAGATATAACATTGAAATATCTTCATTTTCTGAAACATTCTTGTATCTGTAGTTCTAT

CCAAAATTTAATACAGATTTTCTTA

>c17869_g1_i2 len=325 path=[341:0-285 25:286-324]

CAGCACCTCACCACTCTCCTGGTGAAGAACTTTTTCCTAACATCCAACCTAAACCTCTCA

TCTTTCAGTTTAAAGCCATTCCCTCATGTCCTGTCACTAACAGACTGTGTAAAAAGGTGG

TCCACCACCTGCTTGTAAGCTCTCTTCATGTACTGGCAGGCAGCAACGAAATATCCCCAC

AGTCTTTGGTTCTCCAAGCTAATAGTATAATCTTCTAAAAAAAAATTAACATAAACACAG

TCCAGATATAACATTGAAATATCTTCATTTTCTGAAACATTCTTGTTACTGTAGAACAAA

TAAGAAAATCTGTATTAAATTTTGG

>c17873_g1_i1 len=224 path=[225:0-13 @239@!:14-199 424:200-223]

TTTTTTTTTTTTTTGTCATTCATGTGTTTTAATCAAAGGCAGATAATTGATTTTTCAATC

TGAAGTTAACTGGGAATTGGTCTCTGAACATGGTACAAAAAATCATCATGAAAACAACAA

ACCCAAACCTCTTTCATATACCCAGTATTTCCATCCTAATGCTTACAGGAACAGTTAGTT

ATATTTGGTGCCTTCTTCCCCCTCTAAAAGAACCTTTTAGAGAG

>c17873_g1_i2 len=247 path=[447:0-36 @239@!:37-222 424:223-246]

TTACTAATCAGGGCTCAAATTGACAAAATACAAGTCAGTCATTCATGTGTTTTAATCAAA

GGCAGATAATTGATTTTTCAATCTGAAGTTAACTGGGAATTGGTCTCTGAACATGGTACA

AAAAATCATCATGAAAACAACAAACCCAAACCTCTTTCATATACCCAGTATTTCCATCCT

AATGCTTACAGGAACAGTTAGTTATATTTGGTGCCTTCTTCCCCCTCTAAAAGAACCTTT

TAGAGAG

>c17887_g1_i1 len=220 path=[1:0-219]

AGGGGAAACTTAATTGAATATTTCAGTTTGATTAATAATAGAGAAGCAGGAACATCGTGT

TTGATTTTAGGCATAAGCCATGTAAGGGCTGTTGAAATATTGCTGTTGGTGAGTGAAATG

AGCTAGAATGCTAAGGTGTGCTTTCCTTACTGTGTCACTGTGTTTCTGGTGACTGAGTAT

GGGCTGTGACTTTGCAGTTTGAGTTCCCCACTATGAGGTG

>c17890_g1_i1 len=203 path=[155:0-202]

ATTTGAATACCCGCCGATGATGCATGCTTACTTAGGTAAACTTTTATTAAGTTTGTGTCA

TTAATAATATCCATTTTACACTTATTATCTTTTTTTTATGTATATCAAGAAAAACTTCTT

CAAATGCTTGTTGTTCTATAGAGAAAATATTCTTTAATTTTAAACAATATTCATCTGTCA

ATGCCACATCTAAACAGTAATCG

>c17892_g1_i1 len=224 path=[31:0-223]

AGTCTGAATGCAGAACTACACAGATGAAATGTGCTGGTCAGATACTGCCTGCTTGTTTAG

TTTAATGATGTTTGTCAACCAGCAACAGATGGTTTCTAGTGTTGGAGAGAAGATGCGAAA

CTCTCATGTAGCTGCCAAGCTTATTGGCTGAATCCTGGTCCTTAATAGGGACCAAGAGAA

GATGGGAGGTCTGGGGATGAGGAGACAAGGCAAGTATAGGAGGA

>c17897_g1_i1 len=345 path=[305:0-344]

AGGAAGTCTTTAAGCATGGCACGCCAAAGACCGCTGAAGAAAACAACACGTGGCATTTCA

CTGTGTCCCCATCAGGCCTGGGAGCAGGTAATACCACACTTTGGAATGGTCACTCACAAC

TCTTGCTCCTGACTGACCTGATCACCACTCAGCATCACACCAGCACTTCCCAGAAGGCAG

CTTCCTGGCTCAGGTATACAGCAACTGCTGCAGACAGCTTAGCTGTGGTTCCCATGTATG

TTACTATTAAAGAGGACAGCTTTTCAGTTTTTCTTGGCCACCAGCATGTTAATTAATTGC

CCTTTGAAGGCCAAATATGGTTCTCAGCTCAAAAGTTGAAGCAAA

>c17904_g1_i1 len=221 path=[545:0-220]

AACTGGAAGACATATGTACACAAAAGCCTGTTATGAACAGCCTTCTCACAAGCTCAGAAA

ACTTTCTGATGACTCATTTCTTCCAAATAGTAGATAAGCTTCACTGGAAGCTTATGGAAG

AAAACGGGAACTTGCTACACACAGAGTTCAAGTTTTTTTAATTTTGAAAGTGTATTGCTA

TGTGTTAGATATCTGAAAATAAATTTAATGTTGGTACTTAA

>c17906_g1_i1 len=292 path=[488:0-251 776:252-252 207:253-291]

AAATAGAGTAGGATGGTGTGAAGCATTCAGAACTAAAAACGCCTTCCATCTACCCTCAGC

TTGTTTCCAGGCTGGAATTCCTTCCTTCCTGACTCTCATACTTCCCCCTGTCAAGTGGTG

CAGAATGATAATGAAGGGTGCAGTCAGTCCATAATGCTTCATCTTTGCTGCTTCTTCCTC

CTCACACTTTTCCTGTGCTCCAGCATGGGGTCTGTTCCTCAGGGTGCAGTCCTACATTAA

TTGCTCCGACTTGGATCCTTCCCCCAGGCTGCAGTTCTTCAAGAACAGTTTT

>c17911_g1_i1 len=264 path=[1:0-263]

GATCCATGGAAGCAAATTCTGGAATTTTCTGCTGCAGAGCATCCTTCACGTTGTGTTCTT

TAATGCTCTCCATTCCAATTAATAGCTGAGAGGCAACCACTGCTTCACTGACCTGTCTGG

CTGCTCTTACTGCAATCCAAGCTCGCACTTTGTAGCCTTCTGATTTCAGAGTCTCGCCAG

GAGAGGAAGAGAGTTCTCCAGCAGAGATCTCTGATTTTGAAGGTACCGTCTTATCTCTTT

GTGACAAATTCTCATTATTTGTGG

>c17919_g1_i1 len=250 path=[1:0-165 167:166-249]

GCAAGAGCACACTGCTGGCTCATGCTAAGTTTTTCATCCACCAGGAGTTAGGAGAAGGGC

TGGCAGCAATAACCACCTCAAACACAATGTCAGCTCTTGCTAGCCTGAAGCAAGGAAGAC

CTTAATAGTCTGCAGTCAGTATTCTCTTTTTCACCACTATAGCAACTAGATGTCTGAAGA

AAATCTGCACTATAGAGGTACCTGTTTACAGCAGCATGTCTAGAGGGTGTGCTCTTGCAG

CTTGGAAAGC

>c17921_g1_i1 len=213 path=[383:0-17 211:18-212]

GTAACTTTGTGTTTGAATGCTTGATGATTTTCTAAATGGAAAATTAATACGATGTAATTA

AATTAGTAGGTGGCAGGCAGGCAAGTGAGGCTTCCAAGGAACATAATTTCACAAACCTTT

GTAAAAATATTTTTAATACACATGACCTTGGGGATCTACATCTGTCTGGATTCTAACATC

TGCCTGCGCTGCTGAAGTCATATAAAAGAATAG

>c17935_g1_i1 len=247 path=[1:0-141 508:142-246]

TAAGTGCTGAAAATACACTTGGTGAGATTACAGGGGAACAAACACATGGGAGAGCCACGT

GCCCCACCGAGTGCACCTCCTTTGGCGGTGACCCACTGTAAGTGGCCATGGCAGGTGCAT

GGACTCACTGCTCTCATCTACAGCAGCAGCCAGATCAGAGCAAAGGCTCCACATCGTTCT

GCCTTGTGAATTCAGCCCTGAATAACTGAAGAACTCCAGACAGCGGGGAAAACACGATCT

GGGATTG

>c17936_g1_i1 len=295 path=[427:0-294]

GTCTAGGCGGCTCCATGATTCTCGTTGGACCGGAGAACCTCACGCTGACGGTGCATCAGA

CCGCAGTGCTGGAGTGCATCGCGACAGGCAACCCCCGGCCCATCGTCTCCTGGAGCCGCC

TAGACGGCCGCCCAATTGGTGTGGAGGGAATCCAGGTGCTGGGGACAGGAAATCTCATGA

TCTCAGACCTCAGTGTGCAGCATTCCGGCATCTACGTGTGTGCTGCAAACAAACCTGGCA

CTCGGGTGCGAAGGACTGCTCAGGGCAGGCTGGTTGTGCAAGCCCCTGCAGAGTT

>c17937_g1_i1 len=234 path=[212:0-107 320:108-233]

AAAAAGCGAAATCCAGCTTGGGACATCTACAAGCAAAGAAATCACTCCCCCCCTACCAAA

AATTTTAGAGGTTGGAGAGCTCTCTGACATTGCATATATGATAAACAACAAACCACATTA

CTGGTAATACATATATATTCCAGCACATCATTAGAGAAGAGCTTGTAGCTCTTCCGAAAT

AGCACAAAATATTACACACTGCAGCACAGCACAAAACAAGACACAGCTAGGTGT

>c17941_g1_i1 len=218 path=[508:0-217]

AACTTGTAATCATTGTCTTCTTCTGGTGCCCTCTGCACCCCTGTTGGGATGTGCCTCTGC

TTGCTGCATTCTCACTGCTTGTTAACAAACTTCTCAGGTTTCTGTTGTTTATTAGCAGCT

GAGAAGCACTGAGCACCTGTTCAGGATTAGGCCTCAATTGCTGCTCTGTGTTGAGGCAGT

GTAGTCATTTATCTTGGGCTCTGTCGTAGAATCACAGA

>c17943_g1_i1 len=222 path=[285:0-221]

ACTATGGAGTTGGTCTCTCCCTTTTCCTTTAGCTCCCATACACAGTTCTCTTTCCTTTCA

CAAAAGGAAATACCCTACGTTCAGAAACACATTATTAAGAATCAGTATTTTAATAGCACA

ACAGTTAGCTTTTGTACCATTCAATAACAATATTCTAGGAAAAAAAGTGGGAAAAACTGA

AGTGCAGTATTTATAGTCACGAAGCTTGTCCAAAAGATAAAA

>c17945_g1_i1 len=314 path=[273:0-313]

GCAGAGCTCTGACAGCCAAGGGTGCCACGGGGATAGGGGTCCTCCAGCGGTGGGACTTCA

GAGCTGAGTGAGGCTGTGGCAGAGATGAGTGCTCCCAAGCAGGCACACCCGGCCCACGTC

CTCACCCCTTTGGTCCCCTCCAGCATCACCACAAAGCGAGTGTGGCAACAAGGGTGATGC

CAGGCCCTGCAGGCGCCAGGCTGCCAATTAACCTCATTTAGCTTAGATGCAAATGAAGAT

GGAGAGCATCCCTGCAGAGAAACACCAGCAGGGTGCAGGTCTGACCCCTGCCAAGTGTTT

CTCTGCAGGGATGC

>c17946_g1_i1 len=221 path=[737:0-220]

GACTTAAGTGAAATTTTGATATCGCGTGATTTCGTGCTAGTAAAGCTTTCTTAAAAAAAA

GTGTCCTGTTCTTATACTTTTACTCAGTAGTGCTTATCTTCATCTTAGCTTTTCAATTGG

AAATCTGTACTTGATAATAGTTTAAACCCCAAATATCTGATTAAGAATAGAAGACCAAAC

ATAAATTAAGATGATGCTGAGACAGCCTTTGAGAAAGTTGC

>c17946_g2_i1 len=292 path=[270:0-291]

AAGTTAATTTCTATAATTTCAACATGAATTTGAAGTACTCAGTGACAGACTTGCATATAC

TGAGATAAATGAATCAAAAGTATTCTTGAAATCTATTTAACCTCAATCCCAACAGCGTTC

ACATTCTCTTATTGTTAGGAAAAGTCAACATTGCATCCTAGTTGGTTATTTTTATATTCC

TACATAAATATGTGACAGTCAGGTTAGTCTGGTTTTGTATTAAATTAGGCATTCATCCCT

TGTGTATTCTTATACAACAGTTCTGTTGATAAATAAACCTGTGTGGTTGGAT

>c17954_g1_i1 len=278 path=[500:0-277]

TTGTGTTTGAGGTGTGTAGAAGATGAGATTGCTGTGGTCTTACCTGAAAGGAAGTCAGGA

GGTGTCATGGGAAAGATGAGGGAGATTTTGGGCATGGGGAATGGCCTTTAATGTGGTGGA

ATGAGTAAGAGAATTAAGAATGTATTCCGAGGCTGAAGGGTACTGTTTAGTGGGGTGGAG

TAAGGCTTGCTGTTGTCTCATGCATGGGAAGAAAGTGTGTGGGTGGTAGGGCAGACTTGA

GGTTCTTGTGGTAAATTTACAGAGGGGACGTTTCTGGA

>c17960_g1_i1 len=239 path=[1:0-151 625:152-156 283:157-238]

TATATTTATATTGAATAGATTTCATTAGACAAAAATATAAACCTAAATATCTTGTTCAGT

AATATTTTGTGTGCAAGTGTTTCATTTTTACTTGTCCTTTCTCAATCAGATTCCATAAAG

AAAAAAAAAACTATGAACTCAAGTATTAAAACCTTACCTCAAGGGAAAACTTCTTGATCA

GAATTCTGTTGCTTCTTTATGATGAGTCTTATAAAACATAATTCAACGGTAATTTTGAT

>c17960_g1_i2 len=201 path=[1:0-151 625:152-156 158:157-200]

TATATTTATATTGAATAGATTTCATTAGACAAAAATATAAACCTAAATATCTTGTTCAGT

AATATTTTGTGTGCAAGTGTTTCATTTTTACTTGTCCTTTCTCAATCAGATTCCATAAAG

AAAAAAAAAACTATGAACTCAAGTATTAAAACCTTACTTCAATCTTCAAAACATGCATCA

AAATTACCGTTGAATTATGTT

>c17980_g1_i1 len=371 path=[1:0-370]

ACAGCTCAGTCCTAGGTGTTGCTTTGTGAGCGTAGCGTAGCCTCCATTCAGTGGCACGGA

TCCCAGAAGCGCAGGCTGCATGGAGAGGGCAGCCCCAGGGACGTCCCTAAGCCCGAACGT

TGGCTGTCCGTCCCGTTCTCATCCGCCTGTCTGGCCTGGATCAGTGCCCTGCCTGCATCA

CCTAGCTGTAGCACCACCTAGGACTGAGCTGTAGCGACTTCCTTGTAGTTAAGAAGATAC

GAGCGTGACTTAGAGCATCACGTCTTGACAGCGTTCTTGCTTCTTAGGAACAGATTAAAC

GTCCTGTGTATTAGTTTGACTTCCATGGTGCAAAACCTCATGTTGGGAACAGTGCAATCT

ACCTTCAATCA

>c17984_g1_i1 len=205 path=[1:0-204]

AATAGCTTATGATGAGATGGTCCTGCCCCCAGTTGCCCAGTCCACTCTTTTGCCTATACT

CGACCCCAGGGCAGAGATACTAGTTCTTAAATGGTTCCTTGCTGTAGATTTATCACAGCT

CATATCCTCATTTTCTTTGTGCAAAAGGACATAATGGCCATACGGTGGCCATAAGACAAT

AGCGGTCATAAGCAGATGCTTCAGT

>c17987_g1_i1 len=257 path=[205:0-256]

GCAATTAAATCCAGTTAATAAAACTGACGTGGTACACAGCACTTGAATTCTATCACTGTT

CTTCAAGTCAGGCAGTAGCATTTTCAGTTTGTAAGCTAACACTTTTACTAATATTTTTGG

CACAAAATCTAATTCCTCATGCAATTTCTGTCCTGAATTCCCTGTACGAATTAACTGCAT

GCAAGCTCTACAAGTCTTCTGTTCCCAACAGTACCCACAGCATCCCCCAACATGCCACCC

CCGTCAAACACAAAGTT

>c17992_g1_i1 len=229 path=[199:0-228]

AGGAACGTCTCAAAGCGCACAGCTACTGCTATTACACCACATTCAGCCAAACAAGAAACT

AAGTGCAAGTCCTGGCCCAAGTAGTACCTCTGCCCTCAGCAGAAAAGAAGCACAGTGACA

CCAGAAAGCCTAACGAAATATCCCTACCGCTCAAGGAGCAAACTCACAACACAGACATGA

CTAAGCAACCAGAACTATGTCCTTTCAAGCCGATTAATACATCTGGGAA

>c17993_g1_i1 len=265 path=[243:0-264]

AACTTTGTGTTTGAGTGTGGACGTATTGCTTAAAAAATAATAATAGGGTATAATAATAGA

GTATTAAAGTCCTAAAATGACTAGCAATATTAGCTTATTGTCTTGTTTAAAAAACAATGG

ATTTATCTGTACCTGCAAGTAGCAATAGCTGGGGTGTAATACCTAATATATGTAAGGTGA

TGCGTGTGTATATAGTGCCTGTTAGCTGATACTGTCCTAGGCCCCCATGTCTTGTAAAAA

GGAAGGGCTAGGAGAAATTTTGGGA

>c17996_g1_i1 len=251 path=[1:0-250]

GAAGCTGAGCCTGATGAAGAAGAAGTAAGCTTGCTTTTTTTTTCTCATATTTATTTCTTA

AAATGAAATGGTTTTATTTTTTAAATGAAAATGTTCTATTGATTCATACCTCTTAAATCT

GACTCACTTTTTAGTATCAGGAAGTGGATTTATTTATTTAAAAAAATCTTGTTTTCTAGG

CTACTGACAGTCTAAATACTTCACTTTTCATTGCAGGCATGTTTGATTTCTAGCGTGTAT

ACCTAGAAAAT

>c17997_g1_i1 len=217 path=[397:0-216]

GCTCTTACATGGCCGGTAGAACTCAGGGCCATTTGAGAAACCATTGCTGATAACCAAAAG

GTTCAGGAGGTTTTAATACGTTTAAGAGGGAAGGAAGGGATAAGAGCTAAATCTGGAAAT

ACGTAAAAGCTTGGTTCAACTGTGTGGCTGTAACTGCAGCTATCAGAAAACCTACCTTTA

TGAAGAAAATGAAATGCTGTTGCTGCTATGTAAAAAT

>c18000_g1_i1 len=233 path=[211:0-232]

ACTGGTTAAAATACATAGCTCTTTGAGTAGCATGCTGCTGTGCCTTTTGCTGTAGGATGG

GTGTCTGGCAAGGTACAAACAAGGGAATGGTAACAAAGTGTCTTTGAGCAGTTAGGTTAG

AATGATAGTTAATGGTGATAGGACAAGGGGGAGTGGTTCAAGCTTAAGGAGGGATGGTTT

AGATTTGATGTCAAGGGGAAGTTTTTTATCAAGAGAGTGGTGAGGTGCTGGAA

>c18005_g1_i1 len=318 path=[296:0-317]

TGTGATGTTCTGAACGTCACCCTGTTCCCAGTGGTGTGCAAAAGTTTGCCATTGCACTTG

GAGTTTGGGCACGGGCCTCTGCCCCAGCACATATCGGAGATGTCCTTCTCTCGTGCTACC

CGGTAGGAGCAAGGCAATGTGACAGGTTGTCCTATCACTCCTTGAACAACAGTTTCTGAC

ATGGTGTGCACTATGAAGGTCTGTATGACAATCCAGTGAAACAACACAAAGTGGGACATT

TTGGCTGACGGCGACCCACAATTGCTTCTGTCTTCCGGATAGGTGCCCTCTTCCCCTCCC

CTTCCAAGATCCTGAGCG

>c18006_g1_i1 len=287 path=[265:0-286]

GTGCAGCAATCCACCTCGGAGTTAACACAGGCTGCCTTGTCCTCGCCGCACTGTGACACC

AAATACTTCAACTCAGGTGTAGCCCTGGAGCTCATGCACCTTCCTCTCCCCTGTTGGAAC

AGTGGGACAGAGCTGTCGTCTCCCTCTGCAGGCAGCAGGTACTGTCACACCTCCTCATCC

TCCCCGCGGGTCACCGGGTGCTGCAGCTGCGGGGTGGGACGTGCCCTGTTGGTGGGGCTG

AGCCCTGGAATGCAGGGGATCCAGGTCAGAGCAGCAGGACTTCTGGT

>c18009_g1_i1 len=209 path=[54:0-208]

GGAGGGCTTCTATGCTGCTCTTTGTGTTTGAGGTGGAGAGAAGGGCCCCGCCGGGTGGGA

GCGGGCCGTGCTGAGTGGGGATTGCCGCGGTGGCATTGCTGTTTTCCCAATGAAACACGG

GGGAGGGAATCGAAATGTTAGTTGGTTGCATGCCTGAATATTATGCATGTCACGTTTTAG

TCCTTTCTTTTTTTTTCTTTTTTCTTTTT

>c18016_g1_i1 len=246 path=[1:0-197 199:198-245]

GTGTTGCTGAGAGTACTGACAGTTGGGCCCCACATGTGAGCGGTGGGCTATGGAAAGGAG

ATGTGCCTGATGAATGATGGGAATTTGCAATAGCTGATTCTTATCATCTGAAGATTTTAT

GCTCAATGAATTTAATGCATTTGGTTTTCTTTTTAACTTAATTTATCTGAGAACAAGACT

GACTTTTTTTTGGCATTGCCTTGTATTTCATCATTGTGCACAAGAACACACACACAGATG

AAATAC

>c18018_g1_i1 len=214 path=[192:0-213]

GTTTAAAATAAACCAAAATCATCTGGGATCTAATCCTACAAGCCTTAACCCTCACCGACA

CAAGAGGTTCCATGGCCTGTAGTGATCTGGTTATACAGATGGCTGTCAGGAGTAAGATGT

GGACAAGGCTTTTTCCGTCCTTGTCAGGATTGCTTCCTTTTGCCAGATGATTCAGAGGGA

AAAATTATGATTGCAGCTACTTTTATTTTATGTT

>c18034_g1_i1 len=374 path=[352:0-373]

AAGTCCAAAGCTGTTAATCTCATCAATACTTTTGCAACATTATTAAATGGTTTTTGATAA

AGGACTTAGATAATGAGGGATCTTACTTGCTTTTTCAGTAAAATTGTTTTTACAGTGTCT

CTCACTTGGTTGTATTTATTGTTGTTCTTTGGGAATTTTTGGAACCACCCAGTCCTGTTG

TTCTGTAATGTCTGTTCATTTTCTGCATAATGCTTCATTATGTGACAAAGTCATCCTAGT

TCACCGTGCTCTCTTCTCTTTTATCCCCCTGCCCCAATTTGTAGGAAAGATTGGAATTAC

AATTTAGCCTTTGTGATCATATTTAAATATGAATCCTTTTCTCCTACACAGTCAAGAGAG

GAAAGCAGAAAACA

>c18041_g1_i1 len=223 path=[1:0-222]

GCGAAGTGCTGAAGGACATCGAGACGGCGTGCAAGCTGCTCAACATCGCCGCAGACCCAG

CAGATTGGAGCCCCGGCAACGTGCAGAAGTGGATCCTATGGACGGAGCATCAGTACCGGC

TGCCGCAGCTCGGGAAGTCCTTCCAGGAGCTGTCGGGGAAGGAGCTGTGCGCGATGTCGG

AGGAGCAGTTCTGCCAACGCTCGCCCCTCTGCGGGGATGTCCT

>c18048_g1_i1 len=216 path=[364:0-62 22:63-66 26:67-215]

TCCTTGTAGGTACAGCGTGTACTGGTACTTCCCTGCTTCTCTGCTTATTTACTTGTGGCT

TTATTAATAAACTACTGCGATAAAGAGTACAGCAGTGGTTTGAAAGATGCTATAAAAGTG

CAAAATATTATTGTCTCTGTAATGCTGTCATGATGAGTAAAACTTTCCAGTATTGCATCA

GCGTGATTACCCTGCCACTCCCCATCAAACACAAAG

>c18053_g1_i1 len=205 path=[469:0-19 307:20-204]

ATGAAGTCTTCTTGGCATTTTTCCCTAGAATAAACTTTGATGCAGAGAAACAATGTTCTT

CTCCATTTTGACAATAAACATGTAAAGAAAGTATTTCAAAGTCCCCCTGGAACCTACATG

AATGGAAGATAAACCTCAATATGAGGAAAAACAACAAAGCCAAGAGACCCCACACCCACA

CACAAAACAACAACAACAAAAAAAA

>c18053_g1_i2 len=257 path=[235:0-71 307:72-256]

ATTGTCCAAAACATCTGCACAGCAATTCCTTTACTTATTTGCTCTGTCAGGTATCTGCCT

TCAGGTCAGAAATTCCCTAGAATAAACTTTGATGCAGAGAAACAATGTTCTTCTCCATTT

TGACAATAAACATGTAAAGAAAGTATTTCAAAGTCCCCCTGGAACCTACATGAATGGAAG

ATAAACCTCAATATGAGGAAAAACAACAAAGCCAAGAGACCCCACACCCACACACAAAAC

AACAACAACAAAAAAAA

>c18054_g1_i1 len=262 path=[240:0-261]

AATGTCAGCATTGGTAGGATTGATGCAGGAAGCTGTTAGGAAAACGTAGTCTTTTACTTG

TGAAAGAAACAGGCCGACCCTATTTTGTAACTGTGTTGTGGTGCTTTATCAATACATCGA

GTGGCGTTCCTTTCATTTTTTAAAAGAACAGATGCTATAAACCTAATTTGTGTTTGTTTA

TTGTCTTAATGATAAATCTGGAAGAAAAAGATACAGAGACCCTCTTGTCCTTAATTATAT

TTACAATTTTGAAATCGTGTGA

>c18055_g1_i1 len=369 path=[195:0-368]

AAGTTAAAATGGCAAGATAGAGCTGTCCTTGCCTGTTTCCTGTGTGCTGAGGAGGATGGC

TCTGGGTGTACTCACCCCTTCTCAGTGCTGTGGGGTCCCTCAGCGTTCCTGCAAGCTTCC

AAACCTTTCTGAGCTTCTTTCTGTGCTGCCTGTGGGCAGCTGCCTGCCTTGCCCAGTAGC

CCTGCTTTGTTCAAGTGAGCTGGGTGTGTTGTGGGGTTGTCAGATGGCATTTGCTCATGG

CAGCATGGGTCTGGGAAAGATGTTTTGTTGTGCTGTTCCAGGTATCCCCTGCCAGACTGA

AAAATGCCCAAAAAGGTACTCAGGAGAGCCCTGCCAATGTCAGCATGTCATGCTTTGTGT

CTGTTAAAT

>c18058_g1_i1 len=219 path=[351:0-218]

ATTTCTTAAAGTTATAAGCCATACTGTGGACTCTTACATTCAGAATTAGGTCTGCAAACA

AGTAAATTATTCACATAATAATAGTTCTGCCATTTAAATGTATCCCTTGTAAACTTGCAC

ATGGAGACAAGTGCTTTTTTTATTTTTTAGAATAACTTTTAAGCCTTTAAAGGATAGAAT

ATACGTTTCTGGATTTTTAGATGTTGTCTTATAAAAGCA

>c18066_g1_i1 len=219 path=[29:0-62 28:63-218]

GGAGGGCTGGGGGGGGGAATGGGGTTTGCTGCAGAGCCGTTGGCTGTGCAGGATGAGTTC

CCTGGGCTGCCTCTGTGTTCTGGTTGTGCTCTGCAGGGGGCTGAAGCCCAGACATGTGTC

TTGTGATGGTGGGAGATGCTGTTGGTAGGATATTAGGGTGCTCTGGAAACGTTGGTAGTA

GAAGTAGTTATGGTAGAATCATAGGATGGCTTGGGTTTG

>c18069_g1_i1 len=211 path=[234:0-21 23:22-210]

ACAAGACATCACACTAGATGATGGAACTACTTCAGTAAAGTACAGTTCTCTGCTTCCTCA

GTTTCTCACATTGCCTCCACTAGAGCCACCTCCACAGACAGGAAAACAAAGTGTTATCTC

TACACTCCAAACTTTGTGACCCAGCTGACTGAAGAGAAATACCTTGAGCAAAACACTCAG

TCTGACAAACAGTTTTATTTCTGACCTGGAA

>c18085_g1_i1 len=221 path=[378:0-16 90:17-220]

AAAATACAAAAGACAAGAACCAAAATACACCGAGAAATAGGACAACGTGATTAATCGACG

TGGGTCTGTGGTGGGACATGAGCAAATGAGCAATTCATGAATCACTTCTGAACAGACTTG

AACACACCCTCTGTGCTGTGGGCATGTGATGACATGGTGTCCTTTGAAACAGCAGTGCTG

CATGCTTTTGGAGCTCAAACGAAAGCTGAGCTATGTGTGAA

>c18085_g1_i2 len=293 path=[1:0-88 90:89-292]

GAAAATAAATCAAATGGAAAAACATTTTTCTGTACTTACTCTGAAATGTTTTTGGATTCT

TATGGTGTTTTGATGTTCGTTATGTGTATAACCAAAATACACCGAGAAATAGGACAACGT

GATTAATCGACGTGGGTCTGTGGTGGGACATGAGCAAATGAGCAATTCATGAATCACTTC

TGAACAGACTTGAACACACCCTCTGTGCTGTGGGCATGTGATGACATGGTGTCCTTTGAA

ACAGCAGTGCTGCATGCTTTTGGAGCTCAAACGAAAGCTGAGCTATGTGTGAA

>c18090_g1_i1 len=254 path=[458:0-34 493:35-88 70:89-253]

AGGCACCCTGGAGATAGTGCCCATCTGCTATCAACAGATTTTTTTTTCCAAATTTTTCTC

TTTTTCTTCCATTTCTACCTTTTCTCCTTTTTTTTCTCTTTTAAGGAATTATCTTTTTGC

CTTCTTTTCCACTGGAGAAGTTGGCTGGTATTAGCCAATGTGAACATTTTGAAAAACCTC

TTCACATGTGCTCTATTGAGACTTGTTAAGGTTTGGCAGCTAAATTCTCAGAAAATAGGG

AAAAGGGACAAAGG

>c18090_g1_i2 len=234 path=[1:0-68 70:69-233]

CTCAATGAGTTGGTCGGGGAAGTATTGGGCCTCTGTAGCTCTGAAATGGCATCCTGAAGC

ATAGTATGCTTTTTTCTCTTTTAAGGAATTATCTTTTTGCCTTCTTTTCCACTGGAGAAG

TTGGCTGGTATTAGCCAATGTGAACATTTTGAAAAACCTCTTCACATGTGCTCTATTGAG

ACTTGTTAAGGTTTGGCAGCTAAATTCTCAGAAAATAGGGAAAAGGGACAAAGG

>c18091_g1_i1 len=260 path=[238:0-259]

GCTCTGCTCCTTAAAGGAGAAATAAGCAGCTTGGGGAAGGTCAAAGAAGAAATTCTTGTT

CAAACCCAGAGTCCCTGAGCCCCAAACTGAAATACAAGCCACCGCCTCCCAGACGATGAA

TGGCGAGCCGTGTATCCAGCTGATAAGCACGATGTTCCTTGGGATGCAGCAGGGCTGCTG

CCCATGGACCCTGCCCAGTAGACATATATGATGGTTTTGGACCTAACAGATGCTATATCC

TGCGATATGTCCCCATGATG

>c18092_g1_i1 len=350 path=[1:0-349]

GTATTTCGTTAGGATATATTATTACCTAAAAAAAAAATTATTTATTTTTAAAGATTGTAA

TTGCTTATTTAATAGAATTTGTTATACTTTTTTTTTTATATGCTATTTTGAAAATTTTAT

AATTTTTTTTCTAATTCTTTAGTGGTAGTGTTTTTTTTTAAAAAAAAATATTTTAACACT

TTAATAAATATATTTATTAAATGTAGTTATATTTAAAAGTGTTTTTTTCATATTAAATAT

AGCTTTTCAATAATAGTATCTTAATGTAAAATAAAATTTTTGAAAGTAATAAAAAAAATC

ATAGAAAAACCAACAATGTAGTTTCTTAAATTTAGGTTTTCATTTTTTTT

>c18094_g1_i1 len=212 path=[1:0-211]

ACATGTGTAGTAATGTATGCGCATCACAGGCTTGTGTTGGAATGTGGCAGCAGCTGATGC

TGTGCAGTGCGTTATCTCCCCTTGTATGGCCTATCTTCACTCTAGTTTCATAATGGTGTC

AGTTTTAGCCTAGCCAAAAGATCTTTAACTGACAGAGGGGACCAAATCTTGGCTGTAAAC

TTTCAAAACCGTGTTAAAGCGAGATTTGTAAA

>c18095_g1_i1 len=236 path=[214:0-235]

CAGTAGGAATGAGAGGAGAATGTGTGCAAACCCCAGAGCCTGGAAATGGACTGACTCCCT

GCTAAGGTGGTGTGCAGTTCCATCCAGCACGAGTGTGGTCTGGTGCTTTTAGTTCTTGGA

AATCTATATTCTTCTCCAGCTTCCATGCTACACTTTGGGCAAATGATTTGGAAGGCAGAG

TCTTAAATAGAGTACACATCTGTGGATGACCTTTCTGAAGAAATGCTTGTTTGGGG

>c18098_g1_i1 len=245 path=[279:0-244]

ACTATGTTTAACACAGGAATACTAGAGATCCCATAAGTTCTTTCTATTAACAAAAGTGAG

ATTTGCTCTCTTATACAGCATTCCTCAAGAACTTAGTTTGTTCTACCCCATCACTTTAAA

TCTCTTGCTAGCATTGATTTCAGAAGCTATATGCTTTGAATAGGATTGTTTTTTAACTCC

AGGATGTTAGTGATCAAATCCCATAAACTAACATAGTACAATCCCAGAATCAGACAATGC

GTAGG

>c18099_g1_i1 len=268 path=[246:0-94 341:95-267]

CAGATTTCAGAAGGAAGGGACTGTCAGGCCAGGTAGTCCAAACATCTGCTCCAGGCAAGT

GTGACTTTCTAAGCTGCATCAATACTGAAAGCCTTACCTAGCTTGTTCTTTCCCCAGTGG

TTTGTTGCCCAAATTCACGCAGCTCCCTCAGCAGCTGTCTTTGGAGTAAACTATCACTGA

AGTAATAACCGTGGCTATTGTCATCAAGAGCATCAATTAAAAGGAGTGCGAAACAGAGCC

ACGGAGTGAGCTAGCAGTTAGGCAGCCT

>c18105_g1_i1 len=255 path=[1:0-254]

ATGTGAGCAAGGGTGGATTTCAAGACTTCCTCCACACTGCCATATCCTAAATGAAAACTC

AAGGTTTTCTCCTCCCCAAACTTCCATTCCTGTGTCATATGAACCAAGATAATCAAAATA

CTTCTTACTCACAGAAAACAATCCACCTGCCATGGTGGGAGACCTGATCACATCGATCTT

GGACTTCCTTCGTTTCTGTTCTCTCTCAGGGGTAGTGTGCCATGTGAAGACCAATCTCCA

GTCAAATCCACCAAT

>c18108_g1_i1 len=247 path=[1:0-33 @35@!:34-224 526:225-246]

CAGTGCTGTGGTGCAGCCCTGGGCCATGGTTTGTAGCTGTGTGTGCACTGCAGTGAGGCA

GTACTGCAGTGTGGGGTAAGAGGGCACGCTGGACCTTGCAGGGCTGTGTCCTAATGTTAG

CCCTAGCAGCTTGCCATTCAGGATTCATGGGAATTCTTGTGTTAGATGAATCCTGGCCAA

AATTCAACTCGAGTATAATTGAATCTTCTGTTCAAGTTTCTTTTCTGCCACTGTCACTTG

TGACAGA

>c18110_g1_i1 len=261 path=[1:0-15 17:16-260]

AGTCTACCCAATGAAGGTCAGCTCTGGGCTTGAGCTGAAGAATATGCCATAAATCACTAG

GGGTGGCTGTTCCCTGTTCCTCATGCCTCTTGGTGTTTCTCATGGTGAAGGTGCATGCTA

GCACATGTGCATGAGCTGTTCTCAGTCTGAGAGGTAGCTAACAGCATCTGATTTCTGCCA

GCGGTGCGTGTGAGTGGTGTCTCAAGGCAGGCTGGACTGCATGCTGTCTTCTCTTCCAGT

TTGGATGGACCCATGTGACGT

>c18112_g1_i1 len=221 path=[22:0-220]

TGGTTGGACTTGATGATCTTGGGGATCTTTTCCAGCCTCAAGGATTCTGTGATCTGTGCC

ATATAGGAAGCTGTATCTCTAGTCTTTTAGCTGGTGTTTCTTCAAGTTGCTGGTAATGCA

GAAATCATCCCTCAGTGCCATGGGGCAGCCTTTGTAAATGGGGAGACAAATCACCTGCTT

GTGGACTTTCCGTTTACCCTCCTGGGAAAAGCAGGAGAGGG

>c18116_g1_i1 len=503 path=[1:0-502]

GGTGAATGTTGAGCTGTCTGCCATGATGTGCATGCCTATGCTCCTTCTCTTGCCTTGATG

AAGTTCCTGGTCTGGTTGCTTAGAGAGTGACTTTTTGTACATCCCCCCAGCCAGCACGCA

ATACTTTTTATTTTAACATAGATGGGAAAAAGCTGGATGGAGGGATGTACAAAAAGAACC

CCCCTCTGATGTTGAATTTTAGAGGTCTGCATCAGGTTGCACTACACCTGGAGAAGGTGG

TGATGGCTCCCCATCCCAGTTGTACGCTGGATGCAGTAGGCTAAGGTTCTCCAGTTCTGG

GAGTCTGCTTCCACCTGCTGTTGTGGGACTTAGGATTTGCTTTCAAGGGAACCAAGAAGA

TGCCACTAAGCATTTGGTTTGTGAGGGACCTTCACCTCTGTCACTTCTCCTGTCACCTTG

TTTGTGCATCCAGTCCCTCACTGTGTCCCACCACAACCCATATGGGGTCAGGGGAGGTGG

AGGTCCCTCACAAACCAAATGCT

>c18122_g1_i1 len=299 path=[25:0-298]

AGGGGGGAAGGGTTTTTGGTTGGAGATCTGTCTCTGCTGGAGCTTTCCCCTGTATGCCTG

GGTCTTTGGAGATGGGTGAGCAGTCCTTTTTTCCTTCTCTTGTAACACCTTTTTATTGTA

TTAGTCCCTTTGTCATTACGTCTCTTGTATCACCATTTCATCACATTGATCTTTCTGATT

GTTACAACCACGCAAGCCTGACTGGTCATGGCCAGGAGGAGATGGCAGTCTTCTCTTCCC

CTTTGTGAGAGTTCACTGATGAAATGAACACAGGCAAAAAGTTGATAACAAGAGATAAA

>c18123_g1_i1 len=267 path=[367:0-266]

TTATTTATAATATTTTGGAAATTTTTATATTATTAAATATATATACGAAATTTACTTAAA

CGCAAATTTAATTTTATGTTATCTTTTTGTGATTTTTATTAATATTAATAAAATATTACA

AATAAAAAAAATACATTAAAAAAAAAGAAAGTCATTTTAATGAAAATATGTTAAGTATTT

TAGAGATGACATATGAAAAGAAGTTTATATAAATTTACAACTATTTTAAAATAGATATTT

ATTTATATAAAAATTTAAATAATAATG

>c18131_g1_i1 len=457 path=[997:0-406 1404:407-456]

GCTGCAAGAGTACACAGCTGAATGGCTCTTCATGTGGTAAATGAGCCTTTTGTTTTGCTT

TTAAACACACACCTCTACTTCAGCAATAAGAGTGAACACTGCTGCATATGTGCACCAGAT

TTTGGTGTACATTTGGTGTACAGTTGTATGGATAGATGGCTGGAAAGCTGTATTTCCAGA

AGTCACCCCAGAAAAAAAAATCTGCAATGTGATTTGTGAAGAAGAGTGAAAAAAACTGAG

AAAAGTCAAGAAAGGGACCCTGTCAGAACACAGTCACTTACCATATCAAAACTAAATTCC

AGTGAGTTGAACATCTTCCAGTAAAATTAAAATTATATTATTAAGAGAAAGTAGTTAAAT

TTCCATATGAGTATAGTAACAAGCATCCCAGAAATTCAATTTGGGCATAAACATTTTTTT

CTCCTTCTGTCAACTATATTTCTAAGGAACATAACGT

>c18131_g2_i1 len=521 path=[499:0-520]

AGCACCTACTTTTCATTGTCTTCATCACTCTTTCTGTTGGGAACCTTTTCGGTTTTACTT

GAAAATTCACAGCATCATTCACAGGCACCGGTGTTGTAAAAGTAAAGGAATAGCCATTTA

TTCTTTTATTTCCTTGATAGGAATGGAGTTGAAACTTGGAGTTTTAACTGGAAAGTTCAT

CTTTCTTTGTCTCTAATTTCTATTTGCAGAGTTGTAAGACAAAAGTGAGTAAGTTGTAGC

CTAATTGTGGCTTTTTTGGTAGCTGAAAGTCCTCCTTGGACAATGTGAATTTAAGGAATT

AATTCTTCATGAAGCAAAATTACATAAGTGACTGTGTTTCCTTGTAAAAGAGCTCGTAAA

TGTTGGAATAGAAATGCATATTAAAAAAAAGAAAATCAGCTGCCTCTGGAGGCCTTACAG

CATTGATCATAAATCCTATGGTAATAAGAAGTTTTCATGTAAACTAGCAGAATTGGTTAG

GATTGTTTGACTTGAAAATACCTCTCCCAGTTCACCTCACA

>c18139_g1_i1 len=289 path=[509:0-288]

GATTAGTGTCAGGGAGAAGCTTTGCTGTAGTTGTGGAGATGGTCCCTGGGGTCACTCACC

TCTGACTGTTGTTCTGTGCTGATGGCTCTGTAATGCAAACTGCTTGATATGTGCTGTTCA

TTATCGGAAATGGTGATTAGAAGAGGTATGTCATAAGGAGTTCCTTAAAAATAAATAAAA

TGGCAAACTAGTGATCGTAACAAAGGTTGGGCTCTGCAGATGTCGCCAAGGATGCTGCAA

ATGTCAGGGATGGTTACTGTGCCTTGTGAGGCAAGCGGGATTTCATGGC

>c18144_g1_i1 len=391 path=[195:0-315 551:316-341 5:342-365 29:366-390]

TTTACAGCAACTACTGCCTAGTCTTCCATTATTTGATTTCATATATAACATAGATGCAAG

ACTTTGAAGCAATTAAGAATGAAGTAATCTTTCAGCACACTGTCAATAACGAGACTCATA

AATGAAGATTGATAGCACAGTCCTGGCAGCGAGGGGGAAGAGAAAGGAGAGGCTGGAGGG

AATTCCATGTTTATGTTTGGTTGTATTAGCTGCATGTGCTTTGGATGGTCCATTGCACAG

ACAAATTTTAAGAGTTAATATTGAACCAGAGGAAAAAACTCACTCTCCTCCTTTTTCTAT

TGATGAGGGCTGCGCCAAAAGCAATGCCCCCCACTCCATGGTGTTGGCCCACAACACCAG

AGGCGGACGGTGGCGGCGCAGCAGCAGCGGC

>c18150_g1_i1 len=260 path=[615:0-169 784:170-259]

TGACAAGATCCTTCTGACTTCAGAAGTGATTCTCAGCACAGAGTTGCGCTGCACTGCAGT

TAATCCACCATCAGTCCCAGCATGCTTTACTGAACAGCCACAGTAAGCTGCAACCACTCA

GGTCCTGCTTCATACAGCCTTTTATAGCCAACACAAAACGGGCAAGCAGCCTCATGCAAC

CAAAGGCAGCAATTCACTGGAGTCACAGCATCAGCTTGCTGCCTTGGATCCTTTCTGTGT

CTTTGCTTTGGCTCCCCACA

>c18150_g1_i2 len=260 path=[615:0-169 313:170-259]

TGACAAGATCCTTCTGACTTCAGAAGTGATTCTCAGCACAGAGTTGCGCTGCACTGCAGT

TAATCCACCATCAGTCCCAGCATGCTTTACTGAACAGCCACAGTAAGCTGCAACCACTCA

GGTCCTGCTTCATACAGCCTTTTATAGCCAACACAAAACGGGCAAGCAGCAGCTTTGTGA

AGAGCCAGGCATTCCAGAAAAACTTCTGGAGCCAGGTGGGATCTTCCCAGTGAAATGTGG

GGAGCCAAAGCAAAGACACA

>c18152_g1_i1 len=206 path=[184:0-205]

ACTGGTGGACAAGTGGTCGGGCTCCATCGAGATCGGGGTGACAGCACACAACCCCAACAG

CCTGGAGTACCCGGCCACCATGACCAACCTGCGCTCCGGCACGTGGATGATGACGGGCAA

CGGGGTGATGCACAACGGGACCACCGTCCTCGATGAGTACGGCCACAACCTCGACCGGCT

GAAGGCAGGTGACACGGTGGGGGTGG

>c18153_g1_i1 len=383 path=[49:0-314 531:315-328 545:329-382]

TTTTTTTTTTTTTATATGTTTATTTTTTTTAGATTTTTTTAAAATTGTGTATTATTAATA

TTTAAAAAATTAATAATCACATTTTAATGTTGCACAATTTATTTTAAAAAAAAATAAAAT

GACAAAAAAACAAAAATAGAGAAAGCTTTTAAATAAAAATATAAATTATAATTATAAATT

ATTATTTTATAAAAAACTTTTTGTATTTATTTTTTGTTATTTTTATTTTTAATATTTAAC

CTAATGTATTTTTAAGATTTTTTTTGTATAATTTATAATTTTGCTCATATCAAAGTTATA

TTTCCCTTTTATTATTTTTAAAGAAAATAATAAAAATGTGTTTGCAAAATATATACACTT

TTTTGTTATTTTCTTTAAAAATA

>c18158_g1_i1 len=213 path=[187:0-212]

GCTCTTGGCAGGTTCTGAGTCCCCGTGACATGGAACAAAAGAGATGCAAGAGAAACTACA

ACTTTTGGAAGCCAAGATAGAAGAACTAGAAAAAGAAAACCAGGTGCTAAATAGGCAGAA

TGTTTCCTATGAAGAATACGCGCGCCTCCAGAAGAGACTGAAGGACTTGCAGCGCAGACA

CAACGAATTCCGGAGTTTGATTTTGAACCCCGG

>c18160_g1_i1 len=370 path=[1:0-369]

TGACAGTAAACAAAAAATACTTTGCTTTCAGATTTGCCTATGACGTGCAACACTTTTTGT

ATCAAGTCAGCTGCTAGAAGAAGACAGCCACTGTTTGGCAAGGGAAGGACCATCCAAGTT

TCTGTACAAGTTCTGTTACACGGTAGCCAAGAAGATGAAGGTTTGTGTGTGGGATGAGTG

TCATTATTGGATCCCTAAATTGGAGTCCAGTGGTTAACAGGCTCGGAACCATCTCCCTTC

CTATGCAGAGGATGATTTTCACAGTTGTTCAGTTGCTGTTTTTTATTTTTAATGCTAAAA

TGGGGAAGTGAGGAGTTTAATATTTTAAGGTGGTTATTCAAGTAGCTGTAGCTCCCAAAA

TACATGAGAA

>c18179_g1_i1 len=606 path=[584:0-605]

AGACCCCATTTAAGGGCTGACTGGCAGTGAAAAAGGATTTCTTTCTGATGATCTCTCCTT

GTTTGAGTTTTTCCTTGTGATCCTAGAATCTTCTGATACAGGTAAGTAATTTTCTTACCT

TCCTTTATAGCACCTTTGTATTGTGCTGACCCTTCTGTCATTATATCTCTGGTGAAATAC

TTTTCCCGTTGTATTGACCTGTTTAATTGTTACATAGCTGAGGATCCGTCCACGTAGGAG

AGGTTGCCTGGGGCTAGTTAGATTGCTCCCTGTATTGCTACAACATTGATCAGGGAAAAA

AAGAATGACTGTTATTATAGCGGACTCCCTTCTGAGGGGAACTGAGGACCCAATATGCTG

CTCGGACCTGTCCTGCAAAGAGGTATGTTGCCTGTCTGGAGCCCATGTTAGAGATGTTGC

TAGGAACATAACTCGCCTGGTCAAGTTCTCTGATTTTTATCCTTTACTAGTTTTCCACTC

TGGAAATGAAGAAATAGGCAAAAGAAGTTCTCAAGCAAATAAAAGGGACTTCAGGGCTTT

AGGAAAGCTGCTGAAGGGTTTGGGAGTGCAAGTAGTGTTCTCTGTCCACTCAGTTGGTGA

GTGGGA

>c18197_g1_i1 len=400 path=[199:0-399]

TGTAGTTATGAAATCTGGCTGTGAAAAGCAAGTGGAGGGAGAATTGCTTACATTCCTGCT

TGTGTGCTGGATTAAACAGGGAGAAATGGAAGGCTCTGCAAAGTACTTGGAACTGTTGCG

TATGGCAGAACATTACTTCCAGCAGTCTGTTAACAAGCCAGAAAGCTCTGTTGAAATGAG

TCCCGGCGATGAAATCTTGACATTGCTACGGACAACAACGTTTGATGTGAAGGAGTTTGA

GAGAGAAGCAACTTGCCTTCCTCCGGAGGATGATGACAGTTGGCTGGAGATTACTCCTGA

TGCTCTGGATCAGATGCTGAAGGAGGCAACCGGTGGATCCCAGCCTTCCTCAAATGAAGA

AGAGCAGAAATATGACTTGGAAGCAGTTGCTGAAAGTATG

>c18199_g1_i1 len=254 path=[155:0-253]

AATAATGCAAGGTATGGGGTAAGCAGTGCTTCCAAGGCACGGTGCTCCAAAGGAGTGCTG

TGTCACCACAGAATAACTCATTCCCCGCTCTCTTTTGCAGTGAATAGTTCATTTACACAT

TCAATGTGGCAAAAAACAAGTCAGAGCACAGAAACCTCCGTTCATGGGGCTTCGGACAAC

CTTAATTCTCATACTTCTCCCTCAATGCCTTAATTTGCAATCTTCATCCTTTAAGTGGAG

GTAAATTCTCAGAT

>c18212_g1_i1 len=236 path=[1:0-235]

GTCGCATTTGGGGCTGATTCATCACATTTTGGGGCCAATCTCCCCGATTTTGGGGCCCAT

TTGCTCCGTTTTGGGCCTCTGCAGCTTTCAGGGCGCTGTCACTTTAAGAGCAGGAAACAT

AGAGCTTCAGAAGCCCCGAAAACTTCCAAAAAGACCCCAAAATTTGTGCCCCCTCCCCAG

TTTTACAGAAACCCCACAAATATGGGGGAAATTCCATTCATTGGGGTCGTTTTCCT

>c18214_g1_i1 len=239 path=[244:0-238]

ATCAAGGTTTTAAATGGCATATTGGATGCACGCTGGGCAGTGGTAAGCTGAGGAAAAAGG

GACGGCGACGGTTGAGAACAAAGTAAAAAGGCAGCGCGAACAAGGCTGGCATTATCCCAC

GGCTCCTGGCCCGAGATGCGCTGTGAGCCTGCGCCGTGTGCTTTGTAGGGAGCTGCATCT

CCCACCCTTGAAAAGGGCTTCAGTAAATGAATGGGCAAAAGACCTAAGCGAGGAAGTCA

>c18219_g1_i1 len=229 path=[293:0-73 88:74-111 126:112-228]

GGTCTTCCAGCCAGCTGTACTGTTGTCACCGCCCCGGCAGAAGAACAGTCCGGAATTGTC

TCCCCTCCAGAACAGTGAGATCTCTCTGGCTGAAGTAGACTTGGAAGTCTGTTATATACA

ACCATTTCTCAGTCTACTGAAAACGCTGAGAGAGAACTGTGCAAGGAGAGGAGCTCACCT

TCCCCCTTGAGAACGTGGAGCGTGGCTGAAGAAAACTGTCTCATGTCTC

>c18222_g1_i1 len=208 path=[186:0-207]

CTTAATATACTCCAGCTTGGCTATTGATGCTATCAATAACTTGTCAACAAAAAACTACCC

ATCTACTTAGTTACATGTAATGGCCCACATCCTACAAAAACAAAGGGGTCATATTTACTC

AAGTTTATGAATGCATTCCTGACAATAATCTTCTAATAGACAAATCCACCAAAATAAGTC

TATTTAAAATGACCCTTGGCAGAAGGAG

>c18228_g1_i1 len=246 path=[246:0-58 305:59-245]

ATAATTCACTCTCAAACACAAAGTGGATCATAATAAAAAAAAGAAACTAGGGAGAGGAAA

GTGCAGGTCGTTGTGTGTTCACCTGTGGGACCAAAGATGCTCATACGTGCTCAGAGAATG

AGGAGCACAGAAGGAGCATCCCTGGTAGAGCAGGGTTCAGACAGGTAACGGCCTCCAGTG

TAAATGGGTGGTTGGGAGTGGGTGCACGCATGTGCAGAAGAGGGCAGAGCGAGAGAGAAG

GAAAGA

>c18246_g1_i1 len=245 path=[223:0-244]

GACCACTAGAGGGCGCTCGAGCACCGCTTTCTAAATCTCCTTTTTAACCCTGTAACCACG

GCCGGGCTGCGCTGCGAGTGGGGAGAAGTGTTGGGGGGTCGAGTTCCGAACAGTCATCCC

CTCTTCTATGCGGGAGCATGAAACGGCCCTTCTGACCTACACAGGCCAGTACAGCATATG

CAGAGGGAATAGAGGTGATTTTTGACTTTTCAGCGTTTTTTCACTGCACAAGTACAAGCA

CTGAG

>c18246_g2_i1 len=302 path=[445:0-301]

GGCAAGGAATGAAAGCATTCAAATGACACATGTCCTTAAACCAGGGTGCTTGTTTTTGCA

GGACCGGTTGCGATTAACATTTGAAAAGGTTTGTAAATAGTGTTTCATGCAAATGTTCAC

TTGGACACAGGCTTGGAAGCTATTAGTTATTCAAAAACAGCTCAAGCATTTTGTCATTGC

TGTCTGCTGCATGTTCCTTGCTTTAATCTGTCCTTTGGTCTCGGGGCTGCCAGATTTCCA

TAACTGGGTCACCCAGTTCTTTCTCAACTTTCATTTCACAGCAGAAAGCAAAGTTGAGAA

AG

>c18248_g1_i1 len=486 path=[514:0-485]

AGAACAGCAATCTAAGTCACAATTACTACATTTGAAACGCAAAACCCATGAGAGGAGGAT

CTCTGGTTCTTGTTTGGCTTATAAGCCCTGAGATGAGCCTCTCTTATGCAGCAATTAGAA

TCTTTTCTTTCTTGTATCTACTGGGACATGCAGCTGGAGTTACCAAACACTGGGAATATC

ATTAACTTACACTTAAGTCAGAGCTCATCTTTTTGCTGCTAGCACTGCTACACAGGCAGC

ACACCCTTCCTCAGATCAAGGCCTGCATACAAACAGCTCTGGTCAATGATTATCCCTTGC

AAAAAGCCTGTGACATCGTCCCTCCGGTCACCTGTTTACATCCCAAACGTTCTCAAGTTC

TGCTATTTCAAGCTTAACATTTCTGACCTTAGGCAACGATATTCAATCCAAGCCCTCCTT

CCTATATCCTGCAAGGCAAACAACTTCAGGTCAGTTTTAGCAACCAGCGGCTTCACGCCG

ACGTGG

>c18251_g1_i1 len=222 path=[155:0-221]

ACCAGGAATACTTTCTCAATAGCTCCTTTGCTGTTACACCTTTGCAGTATAATATTTCAT

GCTTTTGAAGAACTGAGAGCATTTTTTAAAGCAATTTATGACTACTGTCTCCTATAAACT

CAGAACTGGGGGAGGAATACTCATCTCTGTGCAGTGTACAATCCCTTCAAGAAACAGACA

ACTGAATAGAACATAAAAATTTGTAATTTCATTCATTTTTGG

>c18254_g1_i1 len=212 path=[223:0-211]

GCACTTCATTCCTACAGCAATCTCAGAAGGGTTAGCTTCCCACTTCTCTCTTTATGTAGC

GCGCTTATTGAGCATTGTATTCTTTGGCAGAAGCTAACTGAACAGACTATGAAGATCAGC

TTATTTTAACATGCTTTGGGAGTGGGTGCCAACAGCTGATAATGCATTTATTTGTTTTTT

GGGGGAAAAAAAATAAAAACACTTAAATATTA

>c18262_g1_i1 len=215 path=[45:0-189 590:190-192 592:193-214]

TAAACATACTTATCTCACTTTAAATATCAGTGTTTCACTTCTGAATTAATAAAGGGCTTT

TAAACTTCTATTTACGAACGGTTCTAATTGTTTATAACTAGACAACCTGTCACTTCTTAC

TGAGTGTATGAGTAATAGTAATAGTACTGAGGGAGTGGTTAACTGAAATAAAATCTTCAT

CTGTTTTAAATGACCAGCAGCTTGCATTCTAATCA

>c18262_g1_i2 len=382 path=[45:0-189 590:190-192 236:193-381]

TAAACATACTTATCTCACTTTAAATATCAGTGTTTCACTTCTGAATTAATAAAGGGCTTT

TAAACTTCTATTTACGAACGGTTCTAATTGTTTATAACTAGACAACCTGTCACTTCTTAC

TGAGTGTATGAGTAATAGTAATAGTACTGAGGGAGTGGTTAACTGAAATAAAATCTTCAT

CTGTTTTAAATGATTAGAATGCAAGCTGCTGGTCAGTCACAGTCAGCAGCTCCTCAAATG

AAGTCTGAAGCTAAATCCAGATGGATTTTTTTGATGGCTTACCAGCTTTGAGGTAATCTT

GCTTTGATGTAACCATTAATGAAAGCATTCATAACTTGTTCTAAAGCGGCACTCAGATCC

ATGCTTTGGAGGCAGCTGCTGG

>c18262_g1_i3 len=359 path=[402:0-166 590:167-169 236:170-358]

CCAGCAGCTGCCTCCAAAGCATGGATCTGAGTGCCGCTTTAGAACAAGTTATGAATGCTT

TCATTAATGGTTACATCAAAGCAAGATTACCTCAAAGCTGGTAAGCCATCAAAAAAATCC

ATCTGGATTTAGCTTCAGACTTCATTTGAGGAGCTGCTGACTGTGACTGATTAGAATGCA

AGCTGCTGGTCAGTCACAGTCAGCAGCTCCTCAAATGAAGTCTGAAGCTAAATCCAGATG

GATTTTTTTGATGGCTTACCAGCTTTGAGGTAATCTTGCTTTGATGTAACCATTAATGAA

AGCATTCATAACTTGTTCTAAAGCGGCACTCAGATCCATGCTTTGGAGGCAGCTGCTGG

>c18272_g1_i1 len=221 path=[273:0-220]

AAGTTTTAATTCCCTGCATAATGCTCATTTTTACAGCACTCATCTTTCATCCGCTGATAA

GAATGCATCTGAAAATCTGTGCTTGAAGGCAGATGTGTTGTCAGGAGTGACATTCTCTCT

GACCATATAATACAGTTACATGTTAATGTAAGCCAGTACATAACCCCTTCACTGTAGAGC

ACATTTGAGATAAACATCACCTACAACAGAGAGGGATGTTG

>c18276_g1_i1 len=278 path=[536:0-277]

GCCGGCCCCGGCGCAGCCTCCCGGGCCCTGCGGCCGTGTCCCGAGGCCGACGTGACCGAA

GGCGGTGGGCGCCCCTACTTCTTCACTTTGGCATCGTAGGTGCCCGCGTTCTTGTAGGCG

CTGACGTAGCCGCTGTTGTCCACGATGTTCTCCCGGCCGCTCTTCCCTTTGCCTTTGCCC

GTCTCGTCGAAGCGCTCCTTGTGGGACCCCGTGTACTTGGAGGTGTCCGTCAGCCGCTCC

ACGGCGCCCACGTTCTTGGCTTTCGTGACGCCCACGTT

>c18285_g1_i1 len=247 path=[345:0-246]

AAAGTAAAATAAAAATTCAGTATTTTCAACTGTGTTATGTATTAGCTTGCAGAAGTCACT

GATACTGATACGAAGGAAGAGTGAAATTTCAGGGCCAATTAAAGATGCATAGGGTTGTAG

GCACATTACTTCACACAGTATTTCTTGCTCTAAGCTGTACAAATATATAAAGACTAGACT

GTAATTCTATTTTTGCTTAAACTGGTTTATTTCTTTTTATTTCAGACTTGGATCGTGAAG

TTCAAGG

>c18286_g1_i1 len=208 path=[214:0-179 25:180-207]

TGGATTTAAAGAAAAAGGAAAAAAAATGTCACCTTGTTCCTTGTGATAGTAAGCTTTCTT

TGAAGGTATCTTCTTTCTTGCAATATCTGATGTTTTTGTATGTTCTTCATAGATTTGAAG

GTGAAAAAATCAAACCCATGAAATGCCTACTTAATGTATCCAGTGGGCCCATATATGTGT

TAATAAATGTGGTCATCAGAATGTATTT

>c18286_g1_i2 len=209 path=[214:0-179 393:180-208]

TGGATTTAAAGAAAAAGGAAAAAAAATGTCACCTTGTTCCTTGTGATAGTAAGCTTTCTT

TGAAGGTATCTTCTTTCTTGCAATATCTGATGTTTTTGTATGTTCTTCATAGATTTGAAG

GTGAAAAAATCAAACCCATGAAATGCCTACTTAATGTATCCAGTGGGCCCATATATGTGT

AAATAAATACATTCTGATGACCACATTTA

>c18287_g1_i1 len=270 path=[1:0-246 707:247-269]

AAAATGCATAACACTGATTTTCATTCCAGTGATTTCTTTTTATGAAGTCAGGTGTTAAGA

TAAAGTCTTTGTAAAGCTTCTGTTGTTTCCAATAGGCACAGTACTTACTCTGCTCAGCAA

AAGTTCAGTTTGATAACTACCACAATGTCACTGATGTGAAATGTTAAGGAGGAAATTGGT

CATGAATTATCCTTTGAGCATGCGTCTTCAGTACTCATGTCAGAAAGGAATACAGTACTG

CCCAGTGAAAGACAGGTGAACCTCATTCAC

>c18293_g1_i1 len=204 path=[1:0-203]

GTTCAATGTTGCCTTGTTTAATATTTAAAGAAAACTGATGAATCTTAAGGCAAACAGAAG

TAGCCTAGCTTATGCTGCTTCTCATCTCATCCTGTAGCCATCCCAGTTTCTAAGCCCAGT

TCTACGTCCCACATTCAAAGCTAAAGCTGAGGGCAGAAAGCAGAAAACAGGACAGTGAGG

AACCAGAAGTCACTACCAAGCCAT

>c18295_g1_i1 len=218 path=[196:0-217]

TGATGGCTTTCCAGTTCTGGTGGGATGAGCCAAAAGAGAAACCATTGGACGCAAAGTATG

TGTACATGCCATCATATCCTGCAGACAGGATATCCTCCTTGTGCCCTTCCTCCACCAGCA

GTGCTATGAACACAGCATCATAGGCGGTGTTGCGGAGAGAATGGGAACCTGATGGTGTGA

GAAGGTTGGCCCAGGATTCAGCAGGTGTCAAGTAGGAA

>c18303_g1_i1 len=243 path=[265:0-242]

ACTGACATAAGAGTTTTCATCTCTCACAACTGTGGGTTAGAACACACTAGACAAATACAT

GTTATTTTTATTTGGGACCAGAAGAAGCTAAGCAACACCCCATGTTACTACAGCTCAGAT

AAGCAGTAGATAATGCTTTTAACTCATGTTCATAATGCTTAATCCACCTGACAGTTGCAG

AAGCAGCAGTGTGATCTCCCCTGACAAGGCTCTTGCCAGAAGAGCCGAAGAGAGGAAAAC

ACA

>c18304_g1_i1 len=260 path=[1:0-259]

TGAGGGCGTTCTCAGCCAACACCAAAGCTGAAAGGACATCCAGGCGTTGCTCGCTGTACT

TTTTATCAGAAATGACTCTTCGCACGGCCTTTGCAAATGTTGAAACCAAACTTGGGGAAC

CTTCAGAAATTGACCAGAGAGATAAATATGTAGGCATCTGTGGCCTCTTTGTACTGCATT

TTCAGATTTTTCGGACCGTAGACAAGAAATTTTACAAGTCATTGTTGGATGTCTGCAAAA

AGGTACCAGCCATCACATTA

>c18304_g2_i1 len=220 path=[672:0-219]

TGTACATTGTCCAGTGATCTTTGAGAGTGGCGTGATTGTCAATTATTTCATCCAGAGTGA

TTAAGACTGTCAGTAGCTCTCCCAGATGCTCGTACATTGCCTGAAAATGAACTCCTGACG

TCTCTATAATTTTAGGAGCATTCTTGTTGCTGGTGTAGAGAACAGCCAACTGATGCACTA

CATTCACCACCACTTCGTAACATCGGGTAACAAAGCAAGA

>c18313_g1_i1 len=228 path=[314:0-150 28:151-227]

ATTACTTTGATTATTAATGATACAATCAGTGCTGATAAGAGGGGTGAATGTGTGAACATT

CAGCTTGAAGTAATTTTCAATCTTCCTCTCTCCACATACTTTTCTATATGTTACAAGTTT

TTCTATAAACTCTTAACAGAGGAAGAGTTGATTGTCTTGTTTTCTGCTTTACGCTTTAGG

GTCAGTCAAATATGTCTTGATTTTTAATTAGCATCTCCTATTTGTCAG

>c18316_g1_i1 len=282 path=[414:0-281]

TGATTTGTAGGCATCAGAAGACCTGCTACAAGTCTTCAGATCACTGGCAATTCATTTAAT

AAAGAAATAAGCTGAAATAATGCTGTTGGATGGCCTGATTATCACTGAGTGTTGAGATGA

AGCAGCGTGGACAAGCACAGCAGCTTGGCATTGGCCAGAGGGTGCCCACAACACATGCAC

TGCATTCCCAGGCCATGGGCAACTTGACCACTACCATCCTGGGCAGGATTAATTGCAGCA

GCCAACTGGAAAGGCTGGGAGCAAGCTCAGAGCTGCTCCAAG

>c18325_g1_i1 len=206 path=[184:0-62 247:63-205]

TTTGCTGGAGTTGGTGGGAAGCACTTCCATGAGCAAATGGGGAATGCTTCCAGAGCCTGA

TTGTTACACAGAAAGGCTATGCTTTAACTTACCAAGGTGAGACATTCATTTATGCTGTCT

CCCCTTGGTTTGTCTATATGAGTGGCTGAGAGAAAATGTTAAATCATTCTTAAAGCATAC

AATGTAAGAACAATCTTTGAAAAAAA

>c18329_g1_i1 len=351 path=[329:0-350]

AAATGATAACCTTTTAAAATATTGATGCTAACAAACGAACAGATTTTCTTCACAAGCTCG

TTCACATCTACCTGCACCACAACGCCACGCAGAGCTACATACATGCCTACCTATCCGTCT

CCTTTCGTTCTGCTGACGTTATAGGTTGGGTTTTAAATCGCTCAGAAGTCTTTCTATTTT

CACCAGGAGTGAAATAGCGCCTCGGCCTTCGCGGCCCTCTTTCACGGTCACCACCGGCAG

ACAAAGCGCTGCCTGACGTTGACATCTCAAACCGAGATTCGCTTTTAAACCACACGATAG

AAAGTGCATAGAAAAGAAAATAGAAGAGAGAAGTAAAGCATTCATGATAGA

>c18333_g1_i1 len=213 path=[1:0-212]

AAGTGGTTAAGAGGCAAATGAGATGGCATTTTTTCACACTTGGACAATTTGAGCTCTGTC

TGCATGGGCTATATTTGAATCTCCTCAAACTTGAAACTGAATGAAGATTTAATTTTACAG

GAAGCTACTTTGGTCATTCATTCCCTATTGTCATCTGCAGCAATGCTTTGCTTTGTGTTG

TGCAGCTCCTGCCTGCTCATTTATGGGTCCCTG

>c18341_g1_i1 len=264 path=[1:0-263]

GCAGACACCCCCACTTTACCCCTTCAAGCTTCGCCTTGTCTTTGCTGAAAACTGATCTCA

GGTCTCCCCTGTTCAATGTTTCAATCATGAGACCACCCAGCCCACAGGCAGAATGTTCCA

TCTGTGCTTTTAACCCAGCTGCGGGCGGCCTGCGCTCAGTTTTAATTCAGTGATCTGGTC

TTGGTTCTTATTGAATACAGGGAACAGACACCTACTCCTAGGGTTTGTCAGCTCTTCAGA

AACAGCTGCACAGAAACCAAAGAA

>c18341_g2_i1 len=325 path=[785:0-324]

GGCTTCCTCATTACAACATGAGTCTTCATATTAATCTCTAATGTGATACCGAGACCTCAG

TCTGACTTTGCATCTTTGACTGAGGGTAACTGGACACTACTCCCATTTTACGTGGAAAAA

ATGGAGACACAGAGAGGAAGGACTCAAGTCTGGAGATGAATAAAAAGCATCAACACAGCA

AATGGTGGTATTTGCTTTTAATCCCACACAAATCTTGACCAGACTCCTATTGATGGACAC

AATTTCACCGGATGAAGAGAAGAGCTTGAGAAAAATCCAATGGGAAGAGCATTTTTTAGT

TGTGTCCAAAACTTCAAGTGATTTC

>c18346_g1_i1 len=223 path=[201:0-222]

GCTGCGGGAACCCATCGGGGCGGTGAGCCCTGCAGCCGCCCCACTCCTGTGGGAACGGGT

GAGAGGATGCCGGGTGCCGGTGCGTGGTGCTGGCAGGGGATGATGCTGCCGGCTGGGACA

GAAGCAGGCTGAGCCTTCGTGTGGCGTCGTGGATGCTTAGCGGGACTCCGGGTGCTGTGA

GCCCCAGTGAATGCCAGAGAGACACCCCAAGCTGCACACTCTG

>c18352_g1_i1 len=219 path=[295:0-218]

GTTTAGAGAAGGTAAGGTCCAATATGGAGACACAGTAAATGCTCAACTATGGCTAATAAG

TCCAGCTGAGTTGGAGCTGCCCTGTTAAGAACCACCAGGATCCCTGCAATGCACTTCTCA

TCACAGTATGTTCTGGCTCAGGCATAATCCTATCCTAGAAGCAGCTATACTCATTTTCAA

CCTACTGAAATTCCTGTACAGTGGAGAGCTGTGACTGTA

>c18355_g1_i1 len=223 path=[1:0-222]

AACATTACGGATATTTTGTTCAGTGACAACAACTACACACAGTCAGCATTCTATGACCAT

ATGTTTGTCCAGACTGGTGTTGTGTCATTTTGCTTTAAACAAGCTCACTGACACAACTAT

TTTTTAAATACCGTGACATGTCATAACTTTAAAGTTCTAGCATATCCTGAGACAAACCTG

CACTGGTATACTTGAAAGGCAGAATTTATTGCTCTGGCCATTA

>c18356_g1_i1 len=247 path=[225:0-246]

AACAGGAAGGGGAATGGCTGTTTATGCATTTTTGATACTTTTGTTTTTATTGTTCATGTG

TATTCCTTTCTGGCTGGTGTGATCAGTTTAAGTCTTGCTTGATCAGGAACAGTGGGTAGA

TTAAAAACAGAACTTTTTGTTGTTTTCACTCCGGAGGAGCAGAGAAGGTCCAGTGTTGTA

CAAAGTGTTCTGCTAGAGGTAATGAGCCCAAGAAAATTCTAAAGAAACTGCTAAGTGGTT

AAAAAAA

>c18374_g1_i1 len=211 path=[189:0-210]

GGTCTTCAAAGGGGTACTGTGCAATGATCAGATGAGGAATGCTGCAGCAAAGATTCCCAG

CATGGTTTGGTCTACGTTGGGAACAAACACTCAGAATTTGCTCAAAGGGATTTGGAAAAA

AAAAAAAAGAATGGTAATTTCCATACTAAAAATGGTAATCAAAAAGATATCTCACGCAAG

GCACCTGTCATCTGATTTGGCAGGTCATGCA

>c18377_g1_i1 len=202 path=[180:0-201]

AATACGAGAGAAGCAGCTGATTAGCTTTAGCTTACTTGAGTTTTCAACTTCCACTGAATG

CATGGATAAAATTCCCAGGCTGAACATGATTTCAAGTCTCTCCATTGTTTAACTAACGTG

CAAATCAGAACACTAAGTTTAAAGAAACCATACACAAACATTTCAGAATGAAATTAGATA

TGAAATTGGATAGGAAAAAGGT

>c18378_g1_i1 len=229 path=[10:0-228]

AGGAAGATTTGGGAGCCTATGATTTAAAAAGTGCTTGTAAATATTAGTTTACACAGCCAA

AAACTATGGCTATGACACTTTATGTTTGGACTCTTAGCACACGGCACATACCAAACATCT

CAATCTTTTAAAATGATCAAATTCTTTTTAAGTTTGTATGGATGTCAACATAATTTGATT

TCTGAACTGGATTTACAGCATTTGGGCTACATCTCCTGTACATCTGTTG

>c18382_g1_i1 len=330 path=[341:0-89 34:90-329]

TCCTTGGATGGAGGCAAAGCCAGGTATGATCCAGTTAGTACCACACCTTCAGGTTTTTCA

TAGCAATTGCAGACAGAAAAACATTGGTCTCAGTGCAGCAGACCTTGGAATGGGTGCTAC

TGCAGACTCCCACACAGACGTAGTTGGTGCCACCCTTTGCTTCCTTTCCCAGGGCAGCTA

CGATGTGGCAGACCAGCCATGGCACATTTCAAGTAGGATAAAGCCCTTACTCACCCACAC

CTGTAAACCCCCTCCATGGCCAAGTATTCTGATCCCTCTTCACTTAGAACTTTCCTGGTT

TTCCTGTGCCAGCTGCTGCTGCCTAACGTC

>c18391_g1_i1 len=270 path=[1:0-269]

TACCTGAAGAATGAGGGCATTTTGGACCTGTACCATAGAATGTCATTAAATATCTACAGA

CAAAAGGTGTTTATACTGGTTCTTGTCCAAAATCATCCAAACCTTCAGAGACAGCATCAT

AGGTGTAAGAGAACTGGTATTTTGTAGACGGTATCAGAGCCTTTGGTGAGGAACAGGCAA

GACTGACCAGACCAGAAGTTAACCTTCCAGCAACAGACTGAAAGGCAGCTCAGCTACCAA

AATCAGAAAGAGAGCACATCATTGCTGGTG

>c18391_g2_i1 len=207 path=[679:0-206]

TTTGCTGTGCTGGAAGTGTTTAGAAACACTTAGCTGACAGGATGGGTTTTGGGCTAAGAT

GAGGCATGATGTCCTTGTTAGACACTCTAACGAGTTCTTTGACAAGGGGGCTGGTTCTGC

TCCGCTAATTGTCACGGCTGCTTCTGGAAACCTGTCTATTAGAAACCACATTTCACAGGT

GACTGAACAGCCATCAGATGGAAACAG

>c18392_g1_i1 len=208 path=[303:0-207]

AAAACCCCTTTGCTGACTTTGCCACCTCCTGGTGATGTGACAGCGTTTCCCAACAGGAAA

ACAGCTGAGTTATGGGACCACAAACCCTGAACAAGGAAGGTGTGAAGCCCACCCAAAGCC

TGCCTGTAGCTTCCCTAGCGTCCAGTTGGCTGCTGTGTCCTTCCTGTGGCTATGAGATGC

TATGGGGGGGGATGTATCCCAGCAGTTA

>c18406_g1_i1 len=216 path=[430:0-215]

CTGCACCGCCAACTTTGCTTGTCTCTAAAATGACACCATCACTTACTGCAATTGCATCCC

CACAGCTTCTGTTGCTGAGAAGCACATCGTGGTCACTTTCAAAAGGCTGGAATCAGATGC

TTAGAATAACAATTAACTATTCCACTGCCAGGCACGGTAGCAGCAATGACCCTTTGAAGA

GTGGCTTCATCTTGAGCTCCTACCCTGAATGAGGAG

>c18413_g1_i1 len=279 path=[465:0-278]

ATCTTCATAGATGTTGTACGCACTCAAACACAACATCTATGAAGATTTGAATTGCAGAAA

TAAAAGCTCCAGTTGTGTCTATTTTTACCAGGACAAGAATCCTGCCATCTGCCTGCAAAG

GCCTCTTATTAAAAGAACGTACTGCATTAAGCATTATTCAGCATCTAAGCACGTGGCCCA

TTCCAGAGACAATTCAACTCCTCATCAACACCGTATAAATAAATTAACCCACATCAATTA

TGCATATGGAGTCTCCAAAAGAAGCAAGACCAACCACCA

>c18419_g1_i1 len=237 path=[57:0-158 292:159-159 293:160-236]

TGCTGGTACCTACAAGGTGCAAAGCATCAGTTGCTGCCCAAGGCCAGAGGCACCCAGTAA

ACAGACTGCAGAACCAGGGACTGACCCCGTGCACACTACAGGACCCCAGGCTACTTTCTC

ACTTGTTCGGTCCACCCACTCTTCCCTGGCTAGAAGGAATGCTTGAAGGCTGCTGGGCAT

CTTCTTTCCTGTTTCCTCAAGCAGCTCTGCAGAAGCAGTACCAACGGATGTTGGCAG

>c18420_g1_i1 len=206 path=[213:0-77 411:78-205]

ATCATTCATTTGACATTAAAACGAGGTGGTTCAGCACAGAAAAAAATGAGTTATGTTTGA

ATGGACAGATATATCTTTCCTGCAGAAGAGATGCCAAACATTCTTTATGTGCCAAATTGG

CTGATGTATCTGGTATTTAAAATGGTATTCTGCCTTATGAGCTTCACTTCGAATTGCAGC

AGTTCTACTCCTTCTCCATCTGCGGA

>c18421_g1_i1 len=376 path=[335:0-345 681:346-375]

GGATTTGGAACTTGCCTTACTCAAGCGCTGGTTTCTATCTGATACCTCGAGAGGAGCATG

AAAGCAGTGTGATAGGCTGCATACTTATAGAACTTTTAGTGGTTTGGGGACTTGAGAAGT

AGAGGGCTTTGGGTAAAAGGACTCATTTGTTTGCTTTTCTTCCCTGCCTGCCCCATTTCT

CCCCAGACTACCAGCACGATCCTTATGCTGAACGCAATCCCTGCAACACCATTTGCTGCC

GAGAAGACCTGAATCCTTCCTTCCCAGTGCCTGCAGGCTGCTATGACTCCAAGGTGTCAG

ATTTCCGCCTGGCCTCAGCATTCACCGCCACAGCCATCAATGGTCCCCCAGTGCAGGGCG

GTCTCCCGGTCTTCAC

>c18421_g1_i2 len=376 path=[335:0-345 740:346-375]

GGATTTGGAACTTGCCTTACTCAAGCGCTGGTTTCTATCTGATACCTCGAGAGGAGCATG

AAAGCAGTGTGATAGGCTGCATACTTATAGAACTTTTAGTGGTTTGGGGACTTGAGAAGT

AGAGGGCTTTGGGTAAAAGGACTCATTTGTTTGCTTTTCTTCCCTGCCTGCCCCATTTCT

CCCCAGACTACCAGCACGATCCTTATGCTGAACGCAATCCCTGCAACACCATTTGCTGCC

GAGAAGACCTGAATCCTTCCTTCCCAGTGCCTGCAGGCTGCTATGACTCCAAGGTGTCAG

ATTTCCGCCTGGCCTCAGCATTCACCGCCACAGCCATCAATGGTCCTCCAGGTGAAGACC

GGGAGACCGCCCTGCA

>c18423_g1_i1 len=312 path=[1:0-311]

GGCTTCGGTGACAGTTCTCTTGGCTTCAGTTTATTGAAGAGTAAAACTAATTTACAGCAG

CCATCCAGCCTGTAAGAGCCGGACGTGGGGCGCTGGGGGTGGGCCAACCTGTGACACAAC

GCCTTCACCCAGCTACAAAACCGGGGTCCAATTTGCAGTACAAATTGGACAAATGTTGTA

ACAGTGTAACGGGATCCTGTGAAATGGATGCATTCAGTTAATGTAGGCAGGCTCTTGGCT

CGTTCCCTTTCTTCGGATCCAAACGCATCAGAATGTGCCTGATTGTTTGCCTCTGCAGCC

TGCTGTGAAATA

>c18425_g1_i1 len=213 path=[191:0-131 415:132-212]

GCAGGACGGCAGCAGGCAGGGACACGCGAAGTCCTGATTTCCTAGAGGGCTGGCAGGTCT

GGGGGAGAAAGGAAATGAACTGGATAGGAGGGAAGTGAGTTCACGGTCAAAGGCAGCTGC

TAGTTCCAAATTTGAGGATGTGTTCTTGGCCTCGTGAATCCTTACCGGAATCAGGGAGAA

AAACATTCTCCAGAAATTCAATTTAACACAAAG

>c18427_g1_i1 len=231 path=[1:0-230]

AAATATTAATAGATTGAGCTGCTCCTGGCTTTTTAAGCACTCGCTTTCCTTGCCGGGACG

GTGCAGGGCTGCGATAGCAAAGCTGTTTACTGCAAGGATGGATATCCTGCAACACAACAA

GCCCCGGGGAGCAACGTGCCTTAAAAGTTGAAGGGAACTTGGTGGCGTTACCATACAACG

TTGCGCAGAAGCATGGCAAAAAAAACCACCAGAACATGCGCCAAATAAAAG

>c18433_g1_i1 len=241 path=[1:0-93 262:94-239 495:240-240]

GGCCGGGAGAAAGGCAGTCCGACACGCACCTCCGCACGCCCGTTAGGCTGCGTAGCGTGT

TAGAGGTGAAAAAGATCACAGCATCCCGTCAAACCCTCGGATGAACCTTTCCTGTCCGTG

CTACAAAAGAGACTCCAGGCCAATCAGTCCCCACGGCACGGACGAAACAAATGACTCTGG

TGAGGGCTGGCCAGCTGTACGTCAGCCCGGGCTATTTACAGCTCCTGCAGGGCGCAGTGA

T

>c18435_g1_i1 len=436 path=[518:0-435]

TTATATTCATTTTTTTTTTTTTACGTAATTATATTTTTTTCTTATTTCATTTCTGCTTTT

TCAACTACTTTAATGACTACATTATTTAATTTTTACATACTTTTTTAATAATTAACTAAA

AAAATTAATTACTAAACTTATCAAAATTGTATTTATAAAATTTTTAGAATTTACATGCGT

AAATAAAAAATAATGTGTTTAAGCTTATGAAAACTAGTATAAAATCTGTGTATAAAAAAA

AAAAAAAACATTCCATATAGAATGTATTATATTAACAGTATATAATTTTTTTTTATTAAA

CTTTAATTAATTTTATTAAATCATTAAAATATAAATAAATATATAATACACAAAAATTTA

TAAAATTTAGTGAAATATATTTTAATTATTTTTAATTATAATAAAATAAAAAAAGTAGTT

ACATATTCATAATAAA

>c18445_g1_i1 len=203 path=[1:0-202]

AATGGAGTTTCTCATAGCACAACTGCTTCTCCATACTGGGCTTCTACATTTGTCCACTCC

TGACTCAGACAATATTGCCTTTTTTATTGCTAACGCTTTCTGCAGCGTCCTGGGTTTTCT

TGGCAAGTGTCTCACCCAAATCACGACTGATTTTTGGACCCTGCTTCATTCCTTCATTTC

ACAAACTGCTGCCTATGGTGACA

>c18458_g1_i1 len=286 path=[264:0-285]

ACTCTTATAGACAGGACTCTCGAGGCACCACAGCTTGCAAAAGCATGAGCAGAGAAAGGT

CCACAGCCTTATTTCCACCGTGCTCTGCACCTTGCAGCAGTGCAACACCAACTGACGGTG

GCTGGGCTTTTGGCAGCAGGCAAGGTGCTCTCCCCGCCTAACTCAAAGCAATTAACGTGC

AGCTGTAGCAGCAGAAGACAGCCATGCTGGAGCCTCTGCCTGCCCACATCTAATCCTTTC

CATCTGTCCCAGTAACTAACAACTAAACCATTCCTCAGCTACACTA

>c18459_g1_i1 len=240 path=[1:0-239]

TACGCTGTGTCAGACAGCAGAGGTTCAGTGCATGATTGATTAAAGTGTGTACAGAAAAAA

TGAGATTACGCTTACTAGTATCTGGTAAATTTTTGATGTAGATAGAGTTTATATAAGATG

AGTTGTACCTAATCAAAATAGAATCGGTGCAGCTGACATTTAAGATTAAAAGTGCTACGA

GATAATTTTAAACTGAAGACCAAGGAAAACATGCCAAGTGTGGAAAGTAAATTGTTCAGA

>c18465_g1_i1 len=510 path=[488:0-509]

GAGAAGGTAACTTTTTATATTATCTATATTTTACTAATTTTCATTCTATAAAAGGTTATT

AATATTAATATTAATATTTATTGATCAATGTCAAAAAAGTATTCCCTGTCATATAATCTA

TTCAACAGAAAATTCTCGTATATCTGCAGGAAACTGTTTTTTATTTTTATTCATGAATTA

CAATCATTAAAATGTATTTTTAATTTTTATCTTGATAATATAAATAATTCTTAAAGATGA

AAAAAATATACTATGCATACACAAAATCTTCAATAAAAATAGAAAATCAATTCCCCTAAT

CAAAATTTCAAAAATATAAATTGTCCTAATAGTGAATTCATACTTTGGTTTCTTTACTAT

TCAATCTATATTTATACTATAATTAATATAAAAAGGTATAAAAAACAGTAACAATGAATA

CAAATTATTTTATATCTTTTATAATAATAAGCAAATATAGTATTTTCTATTCAGAAAAAT

GAAATTTAATTTTTATTTTTTTATTTAATA

>c18473_g1_i1 len=232 path=[1:0-231]

GGGTTGCAATAATTTTTACATGCACAATTTTTATATGCACAAGTTTTAAAGACAAGTTCT

AAATCTTCAGATGTCTCTATTTCTTAACTGATCAAGTAACACAGAAGATGCAAAGCTGTT

AAACAATTTCCTGACAGGAATGAAAAGTGAATTAGGTGCAAGCCTACGATACTGAAAGCT

GAGTGGTGGTTACCAAGAGACAGACAATTTCGATAAATTATTATATTTTAAT

>c18484_g1_i1 len=335 path=[866:0-18 869:19-334]

CACAGAATTGCATGAAAGATTCATGCAATTCTGTGGAAAGTTAATGGGAGCCTACTGGGA

TTCCTTCCTCCCAGCCATTTCCATGTGTTTTTAAGGGGTGCATTGTGCACGGGAGGCTGT

GCCAGCTGTTGATTGTCATCTTCCTCATGTCTGTGATAAAAAGCCACTCAAGAAGGCTGA

AATATGAGAACGTCATTAGCTTTCCTATTGGCAGAAGTGCTTAAAAAAGCCACAACAGAA

AGAAAATAGATTGCATATTGAAATTAAACAACAAACAAAAAGCACAGCAGCTACCGCAAG

GTTTTGTGTAGCCTCTGGAAAAAGCAAAAACATGG

>c18484_g1_i2 len=428 path=[1:0-111 869:112-427]

GAAGCTCAGATGGAAGGGGATGCTTTGCTGAATCTGATGGTGTGATTTTTGATAAATGCC

TGTATTAAAAGCCATACGTTTTTATGGGGAGTTAGGAAGGTCATTTAATTCTTTCATGCA

ATTCTGTGGAAAGTTAATGGGAGCCTACTGGGATTCCTTCCTCCCAGCCATTTCCATGTG

TTTTTAAGGGGTGCATTGTGCACGGGAGGCTGTGCCAGCTGTTGATTGTCATCTTCCTCA

TGTCTGTGATAAAAAGCCACTCAAGAAGGCTGAAATATGAGAACGTCATTAGCTTTCCTA

TTGGCAGAAGTGCTTAAAAAAGCCACAACAGAAAGAAAATAGATTGCATATTGAAATTAA

ACAACAAACAAAAAGCACAGCAGCTACCGCAAGGTTTTGTGTAGCCTCTGGAAAAAGCAA

AAACATGG

>c18487_g1_i1 len=388 path=[1:0-387]

AAATTCTCCCCGATCTTTACATAGGACAGGAAAGCTTAGATGAGCTGCACAAGAGCACTA

CCAGGAAGATACTGGCTGCTGCCCCATCACTCCTTGCATTGCCTCAGCGTCCTTCCCATG

GACATGTGGCACTAAGGAGACTTTGCTCACTGAAAGCCACAAGCCAAGCAGTAGGTACTG

TGAGTAAAGACTGCATGAATAAAAGCAAGCATATCATCATTTTGTAGGAATTTATTTGAT

GGTGGGGTTTGTAGAGTCTCTTGCTGTAAATGTTTCCTGACACAGTATGCTAGCAAGCCG

TCTTACTTCTTTATATTGTTATTCGGTCAGGCTTCGAGCATTGACTTTAATTTGCCATTC

AGGGCTCTGCCTCAGGGGTAGCATGTGT

>c18492_g1_i1 len=259 path=[1:0-258]

GCTCGCCATGTCACAATTCAGCGGCTGTGCGGCACATGAAGCAGTCTATTCAGAGGATGC

AAGTCAGACAGCCCCTGGCTGCCTGTGGTTTGATTCTGGGACATCACAGCCAGCCTTTTC

TCCAGCTAATACAGAAACCGCTTGTCTGGGGAGAGGAAGGTCTACTTCATACCTCAAGCA

AAGGGCATTCTCACAAGTCAGACAATGCCCCAGTGCACCTCTGATTAGAAGTCATTGCTC

CCCCCCAGCTGAAAAGAGA

>c18495_g1_i1 len=201 path=[5:0-200]

CCTAAACTTTGTGTTTGACTGTGTAAGATATACAGGGGGTAGAGGAGGAGGAGCCCCTTT

TCCCAGAGTGAGAAAGGAATGGAGGTTGCGTTTCTGCTACAAACATCTTTTTTCCTCCTT

TCCACTGTTGGTATTGACTTATGCAGCTCCTTTCTGTGCAGACTCATTTTGCTGCTCTGT

GTTTGAGGTGCTTAACAGATT

>c18500_g1_i1 len=286 path=[522:0-285]

TTTCTCTGTATGAGTAGAGCTTTCTGATTCTCCTTATTTAGTGGCATTTCAGCTTTTGCA

ATTTGCCAGCATCCTTCTCGTTGTCATGCTGCACTCCAGCATAGCTGGAGCCTCTGCCTG

TAGATACAGCCTTTGCATGCGAATAAGTGTATTCTGTTTAGTCATCCAAGATGTTGATGG

AAATACCAAATAGCTCTGGATTCAAGACAGATCTCAGTGAAGTCCCATTCAATACATCCT

TTCAACTTAGGAGTACCAATTATTTCAATACAATTACGCACTCATG

>c18501_g1_i1 len=306 path=[323:0-305]

AAAAAAAAAAAAAAAAAGTAATCTGTGTGATGGTGGTAAATCAAATGATGATCCTTGGTC

ATCAGAAGTTAGTTCTTAATTAACAAGTGAACCTATTCTAATTGAACTACGATAAAGAGA

CTACAGGTAAGCATGGTAGAATTTTAAACAAAAATCTATGCTATATGTAAAAACATACAC

AAATACTGAGATTTTTTTTTATATTAGCTAGCACATGTAGGAAACTTAAAGCTAGGAGGA

AAGGCAGTAGTTGATTTTTAAGGTACTTATAAGATACTTATAGATCTGATGGATTAAAGT

GAACAG

>c18510_g1_i1 len=277 path=[495:0-217 763:218-225 216:226-276]

ATTTAAAATATAACTTTTTTTTTTACATGTATATAAAGCAAAATTAGTTAATAAAAATTT

TAGGACATGAAAAAAAAAATTTCTATTATATAAAATTATTTAATATTTTAAAACTAAATT

TAAAAAATTTCCCAAATAAATAATACTGCTATTAATTTTAAGTTTATTATTTAGATACAT

TTTTTTTTATATTCTGTTATTTTAGAAAACGTGAAATGTAAAAATTAGATGAGATATTTA

TATACATTTTTTATTGTTATACAAATGGAAATGGCTA

>c18510_g2_i1 len=233 path=[35:0-173 763:174-181 216:182-232]

TTTTTTTTATTTATTTGTATTTTTATGTATATAATTTATGTTCTCTTATATATATGCTTA

TATCTTATTTCTTTATATTGGATATTTATAATACCTTTTATGATAATAACATGAACAAAA

GTAATGTATATTAGCAATAAAGTGATTTTAAATCTTTCTTTTTAAAATAAAATATAAAAA

TTAGATGAGATATTTATATACATTTTTTATTGTTATACAAATGGAAATGGCTA

>c18510_g1_i2 len=277 path=[495:0-217 763:218-225 720:226-276]

ATTTAAAATATAACTTTTTTTTTTACATGTATATAAAGCAAAATTAGTTAATAAAAATTT

TAGGACATGAAAAAAAAAATTTCTATTATATAAAATTATTTAATATTTTAAAACTAAATT

TAAAAAATTTCCCAAATAAATAATACTGCTATTAATTTTAAGTTTATTATTTAGATACAT

TTTTTTTTATATTCTGTTATTTTAGAAAACGTGAAATGTAAAAATTTTCACCTTATTTAT

TTATATTATTAATAGCCATTTCCATTTGTATAACAAT

>c18512_g1_i1 len=245 path=[521:0-244]

GTAACTTTGTGTTTGAGCTGGGAGGTGGTAGCAGCGGATGTTGGGATGTGTATCAGTTAT

CCCAGTTCTCTCACTCATGTGCTATGACCTTCACCCTGTGTAGGAGGTGGAGAGGTGTTG

CCTGATAGAGTGGTTACCTCAAAAGAAAGCGTGACTTATTCACGTTTTCTAATTCCCTCC

ATCCCCTTAAGAATGCTGCTTATATCATAAACTAAGACATAAAGAACTCTGTTTCATTAA

ATACT

>c18523_g1_i1 len=267 path=[125:0-194 62:195-266]

GGCATTCTGGATTTTTTTTTTTTACAAAAGCAAGGGTTGGGTTTTTCCCTCTTGTTTATA

TTCATCTCTGAAAGTTTTCTTTTTTTAATTATTTAGTCAAAACCTTAACTGTAATGCAGT

GGGAAAACTGGGCTTTTTTTTTTTCTGTAACTTGAAGTTGGATAACCCTTTTTGTTAGCA

TTTGCTAGCTGGAGGCTAATGTACTGGACAAACTATCTTGGTAGTTAAATAGAAATGAAT

AGCTAAAATTGACATTTATATTTTCTT

>c18528_g1_i1 len=226 path=[204:0-225]

ACTACGTACATTGCAGAAAACAGTGAGACTTCGGAAAGGTCATCTGAACAATCACCACAG

AAAACAAAACCCAAGCAACTGGGATAAATGTGCCTTAGATATGCTTTCAGCATGCAAAAA

TAACAAACTTGATGAGCAGTATTCCGAGTTGACAATACTTCCTTCTGTCTATATTGCAAG

ATTTCTTGTTTCTGCAAGTACGTGGGAATCAGAGCTGCACAACTTG

>c18528_g2_i1 len=215 path=[622:0-190 431:191-214]

CAGTGAGGTTTGCAAGAATTATGGTAGCTACGGCTATTTTAAATGTCTGGGTGGGAAAGG

GAGGGCCAGGAGGAGAGGTTGTGTAACAGACTTAACTCTGCTTTCATATGCTAGGGCTGC

TTTATTTTGTTGATTCATTAAAATTAATGAAGCAGATCAGCTTAAGCATGCATCTCTTCT

TCTGCGGTTCTCGAAGAAGAAGCTGACAACTACCA

>c18528_g2_i2 len=215 path=[622:0-190 812:191-214]

CAGTGAGGTTTGCAAGAATTATGGTAGCTACGGCTATTTTAAATGTCTGGGTGGGAAAGG

GAGGGCCAGGAGGAGAGGTTGTGTAACAGACTTAACTCTGCTTTCATATGCTAGGGCTGC

TTTATTTTGTTGATTCATTAAAATTAATGAAGCAGATCAGCTTAAGCATGCATCTCTTCT

TCTGCGGTTCTGGTAGTTGTCAGCTTCTTCTTCGA

>c18537_g1_i1 len=274 path=[493:0-40 283:41-273]

GAGTGCTCACTTACTACCTCTGAACGATACTCACGCTGTAGTTTGTCTCTTTCTTATCTT

TTTGCATCTTGTAATTAACTCTTTGTTTCCCTCATAAATTGTAATGTACATTGTAATCTT

TTAAAAGAATCAGGGTTGCGTTTGCAACTTTTAAAAGAGCTGAAGTGGAAACATTGGGTC

TTAGGTTTAACAGAACAGTGAAACTTGTAAATACTGAGAAACATTTTGAATGTTGTTCAT

CTTGTTACTAATCCACGCAAAAAATACAACTAAT

>c18537_g1_i2 len=269 path=[247:0-35 283:36-268]

GTTCGTATGAGTTAGCATGAGGTAATAAAGCTGTGTTTTGTCTCTTTCTTATCTTTTTGC

ATCTTGTAATTAACTCTTTGTTTCCCTCATAAATTGTAATGTACATTGTAATCTTTTAAA

AGAATCAGGGTTGCGTTTGCAACTTTTAAAAGAGCTGAAGTGGAAACATTGGGTCTTAGG

TTTAACAGAACAGTGAAACTTGTAAATACTGAGAAACATTTTGAATGTTGTTCATCTTGT

TACTAATCCACGCAAAAAATACAACTAAT

>c18550_g1_i1 len=226 path=[155:0-225]

GAGCTCTTTTGGGCAGGTAAGGACAAGTAAAAGACATTGCACCTCACTCAATACAAGGCT

GAAGTTCTCATCTCCAAAACCAACTTCAAGCTCATCACTTCAGCTTTTTAATACCATTGC

CAATATCAGAAAGTCAGTAGCTTTACAGGTTTTGCTAAAGATAAATTTACTTTTGTTAAT

GCAGCTGATCTCAAGTGAGATTTTTCTTTCCAGAAAATATGCTCTG

>c18554_g1_i1 len=237 path=[49:0-213 338:214-236]

GGATGCGTGGGACCAGAGAGTTGTGTTGGCATTTGGTGACTCACCAGGGCCTGTGTGCTT

GGTAGCAATTAGGGTCCTTGGAGAACACCACCCCATCTTCACTTCTAGCCTTTATCCAAC

ACTGAGCGAGTAGAAGAGCAGAAGAATTGGGCATCAGTGCTGTGATCCAGAGAGGAGATG

CCTCCTGAAGTAGAACCCTGAGAGTTATGAACAGCAGATGCTGTAGGCTGCAGATAA

>c18554_g1_i2 len=290 path=[49:0-213 262:214-289]

GGATGCGTGGGACCAGAGAGTTGTGTTGGCATTTGGTGACTCACCAGGGCCTGTGTGCTT

GGTAGCAATTAGGGTCCTTGGAGAACACCACCCCATCTTCACTTCTAGCCTTTATCCAAC

ACTGAGCGAGTAGAAGAGCAGAAGAATTGGGCATCAGTGCTGTGATCCAGAGAGGAGATG

CCTCCTGAAGTAGAACCCTGAGAGTTATGAACAGTAAGGTTTTTTTACTTCATCAACAAC

TGGCATGGCTTAATGTAAAGCAATCTGAATATTTTATCTACACTCTCTGT

>c18558_g1_i1 len=281 path=[1:0-280]

TAATGTTATACCTTTCCTTTACTATCTCCTTATTTGAATTATCTTTTAAATGATGCTACT

TTCCTCACATTGAACGCCCTTGCTTTGGCTAAACCAAGAAATTAGTAGAAGGTCCTAAGT

AGAGACAATCAGTAGCAAATTAAATGAAATTTAGTAGTATTACAGTGAAGATCTGCGTGT

GCTACTGACATTTTTCTACAGCTGTACAAGGTAATGCTACTGTCAGCTCCTGACTTGATT

TTCTACATCAGCCTTTTGTATTAATTTCAGCCAGTAACAGA

>c18564_g1_i1 len=239 path=[489:0-238]

ATTTAGAAAAATACGATTGTTTGACAATACATTTTTTTTCACCTCTTCTAGTACTACAAG

AAGGTTGCAGGAGAATGCACTGCAAACCTATGCTGGATTCCCTACTAATGGTTAAAATTT

ACTGTCTTTCAAATACAAGGAGTAAAATAGAGAATGCGGGCCCGTTGTCCATAGCATGGT

GAAGTTTGCACAGAGATCATGCATAACTTCATTGGTGGCTGTCCTGACAGATAGGATTG

>c18565_g1_i1 len=377 path=[515:0-376]

AAATTACTGAATGCTGCAGAAGCAAGAAGGGGAAGGAAGATGATGGAGAGAAGAGATACA

AATTGAGAAGTTCTCAGTAAAAAACCTGTTCAGCATCACCTTAAAGATGAATAACAGAAG

ATACCGCTTTTAATGCAAACGAGTACAGGGATTCACATGCAGTTGTGTCTGCATTCAAGA

ATGAAACAGGTCACTGTAGTACACTAACACTGGAATGACATATAAAAAGCAACACCCAAC

ATGGAAGATTGTAAGACAATGAAAAGAGTTTCTGAATGGCACAGCAGACACCTTTTAAAA

TACTGGCTGAAGACTTCTAATTGTAGCAGTCTAGACAGGACAAAAAAGCTAAAAGCCTCT

TTAAATGGCTGATGTTA

>c18571_g1_i1 len=2126 path=[2265:0-1999 4265:2000-2020 4265:2021-2041 4265:2042-2062 4265:2063-2083 4265:2084-2104 4265:2105-2125]

AAAAAAAAAAAAAAAAAAAAATGGGAAAATATGATATAGCTAGAAGGGCAGAAGAAGGAG

ATAAATACTATTATAGAGAAGAAAGATATGAAAAATTAGATAATAATAGTTTTACAAATA

AATCAGATATTTATATGAAAGAAAAAGAATCATATTATATGGGTGAAAATAAAAAATATA

TATCAAGAAGTAGGGAAAAAATGGTTGTAGGAATGGGAAGAGAAAATTACAAAAATGAAA

ATATCTACAATAGTTATAAAAAATCATCACTTGAAAATCATTATGGAAAGAGGAATGAAT

TGTTAAATGCAGATAAAAATAGAAATTATTCAAAAAACAAAAGAGATTATCATCAAAAAT

TTTCTCGTGATAGATATTATAAAAGATCTCCGAGGAGAAATTTTTCGAAAGATAGAAGAA

AAAATATTATTTATGAAAGGAATAATGAAAAGAAATTTAAAGAAAAATATAAGTATGAAA

AAGAAAATAATAAATGGAATAATAACAATAATAATGATAATAGATCTGGTTTATATAATA

ATCATAGAATAAGAAATGAAAAAATTAATATGAAAAGATATTCACGAGATGTTGATAGAA

AATCATATGATAATAATTCACATAAAATGAATATAGGAATGATGAGAAGAGAAAGACAAA

AAAATATACGAAGTCACGTTTATAAAAAACCAGATCCATGTAAAATATTTGTCGGAAATA

TTTCTACAAATGCAAGAGAAGAAGACGTAAGAAGAAAATTTTTACAATATGGAGATATTA

TGAATATGCAGTGGAAGAGAAGATTTGCATTTATAGAATATTTTAAGTCTATTCATGCAA

AAAATGCAATTGAAGAAGAAAATGGAAAAATTTTTTTTGGTGAAGAATTAAGTGTTCAAC

CTCACCATCCACTTCCTTATGGAAATAAAAATAATGAATCTAGAAAATTTCCTTCAAAAT

TTCAAAGAAATTTTTCTCCTCAAAAAAATGAATCTAGAGAAAAAAAAAATTCATTAAGAA

TTGTTGTAAAAAATATTGATGAAAATGCTAGTTGGCAAGATTTAAAAGATTTTGGTAGAG

AAGTAGGATCTGTAAATTATGCTAATGTTGTTCAAGGTTATGATAAAGAAAAATATGGAA

TAATTGAATATTATAATTATGAGACCATGAAGAAAGCTATTGATATCTTAAATGGAAAAA

AATTTAATGGAAAAATTGTGGAAGTTATGAAATATAATGAATCATTATTAGATTTGAGGT

ATAGAAATAAAGAAAGAGAATATAACAATGTAAAAGAAAATCATTTTGCAGATAGTGATA

AATATTATTATAAAGATAAAATATATCATGATCATTTAGATGACAAAGAAAAATACAGAA

GAGACAAATTTTATGATAGAGAAAAAAATTATGAGGGAAATAAATTTAATAGGGAAAAAA

ATGATGAATTTGATAGAAGTGATAAAAATTATAGAAAAGGAATGAATGAAAAGAGAAGGA

GTATCTTATATAATGAAATAAACAGAGAAAGAGATAAAAGTCATAGAAGCAAACAAGAAA

GTAAAAATAATGAAATAGAAAGAGAAGACGTAGAAAGAAGCCAAAGAAGTGATAGAGAAT

ATGGAAAAAATGAAGATGAAAGAGAAAGAGCAAGAAGGAGAGAAAGAGAAATGGAAAAAG

GTAGAAAGAGAGAGAGAAGAGAAAGAGAAAAACATAACAGTGAATCAAAAATAGAAAGAA

ATTTGAGTGTAGATGATAGAGAAGTAGAAAGGGAAAGGAAAAGAGAGAGAATAGAAAGAG

GAGATAAAAGTGAATCAAAAAATGAAAGAAATTTAAGTTTGGATAGAAATGAAATGGAAA

GAGAAGGAAAACATGAAAAAGAAAGATTAAAAAGTGAAGCAAAAAACGAAAGAATCATAA

GTGAAGATGAAAGAAGAAGAGATAAAAGTGAAAGAAGTGAAGAAGGAAGAGAAAGAAGCG

AAGAAAGGTATGAAAGAAGGGAAAGAAGTGAAGAAAGAAGAGAAAGAAGTGAAGAAAGAA

GAGAAAGAAGTGAAGAAAGAAGAGAAAGAAGTGAAGAAAGAAGAGAAAGAAGTGAAGAAA

GAAGAGAAAGAAGTGAAGAAAGAAGA

>c18573_g1_i1 len=229 path=[525:0-228]

GTTGGGGTTCCAATTTTTTTCTGGATTTTTGTATTCAAACCGTAGATAAGGATGCTTTGC

TTCTATACTTTCACACAGTTCTCTCAGACTACTGATCTCAGAACTCAATTGCTTCTTTTT

TGCTAGATGGGCTTCTTGTTCTGCTTCTTCATATTTCAGCTTTTTCATTTCATCCTGTAG

TTTTTCTTTCGTTTTTCTGATAGCTTCAAATGCCTCTTGGTCTTCCTTG

>c18574_g1_i1 len=235 path=[285:0-86 371:87-97 523:98-234]

TAAAGGTCCTTTCCAACCCAAACCCTTCTCTGGTTCTGTGATATTTCTGATAATGCACAA

ACACATGAATTGGGAGGAATTTCAGCTCTCACATGCTGCTGTTATAAGGCTATGTGCCAA

CATTGTGAAAGGGGGAAAAGTTCAGGGATACTCTTCAACAGCTCCTCTTCCCCTGCCACA

GAAACCCGATACAACATACCAGCACACACAAGTCCATTATTCATCACAGTATTAA

>c18578_g1_i1 len=413 path=[545:0-412]

TAAAATCAGAGATATCTAGGATCTCACTTGTAAATACTGTTTCTCTCATGTAAATATTTG

TGCTGTCTATGCTAGAGAGTATGGGTTAGGTTGTTTATTAGCTAGTAGAATCCACAGATG

ATCGAATAAGATCTTATAATGCATCTGAACATAATGCTTTACCAATTGGCTTCTAAGCTC

TTAAATGTTTAGAACTCTCATCTCCTTGCAGAACTGTCTCTAGTTGCAGAACTCTCTAGC

TGGTTACCGACATTTTCTGACATTGCTATATATCACACTATGTATCTAATATAACTAAAA

GATGTTAAAGGAGGAAATGCAAGTAAAAAAGAGATACTCATGAAGCCTCCTAATAATTTG

GTGCAGACTTCGCCAGAATGTTTGGTACCCACTAGAATTCATAGTTTGGATTG

>c18579_g1_i1 len=349 path=[687:0-310 997:311-348]

GGCAAGATGCCTAAAGATGAGATTATGAACTTGTATACGGTAAGGTCTTGGCTAACACAT

CACTTCAAAGAAAGCTTCTGAAAACCAAGCTTGTCTGGTCCGGTTTTATAACTATCATTA

TAATCAGGTAGTAGATGGTATGAAGCTAGCAAGTTAATACCCTATTGGGAAATAATGAGG

CAAACTCCCATGGATGGGACGTGATTATTCCGGAAGGGTTTCCTAACAAATATGTTGAAA

AACAGCCAAGTACTGAGAAACAGTGAGGAGAGAAAGAGATGGGAGTCTCCCTTCGAGTCC

CGACAACTACTTTCAAGTTGGGCTTCTATCTAAATCATTTAAATGACCA

>c18588_g1_i1 len=230 path=[235:0-171 14:172-229]

TATGTATTATGTATTCTTTCACTTTAATTATCACATATTTATATTTAGAAAATCGATTTT

ATATAAGAATTTTATAGAACAAATCTTTTCCTATTAAAAAAACAAGTCTTTAAATTTTAA

GATTATAAAAAAAGGTTTATATTCACATAAAGGTTAAAGCCTCGTAAGAGATTGATGCTC

AAGATTTTTTAGAAATTTAAGATGCGTAATAAAGTAAAAAAAAAAAAAAA

>c18592_g1_i1 len=255 path=[192:0-254]

TTTGTAATTATCACTATCATTACTATAATAAATAATATTATATTTAGCTTTTTTCTCGTT

ACATATTTTTTTTTTTAATTTGCACATAAAAACTTGTGCGCGTATTTATTTTATTTTTTC

CATTTATGTTTAAAATAAGAAAGTGATATATATTTTTATGTTTTTTTTTTCTTTTTTAAA

GGAAAGATGTTTTAAGAATGATATACATTCTAATTTCACTTATTTTCCTAAATATTAAAA

ATATATATATATATA

>c18594_g1_i1 len=362 path=[1:0-361]

AGAGGAAGGATCTTCCTATGAGAGGGGAAAGCTAAACTGCTAAGGCTTGTTTTAAAAATA

TAATGCTTGGGATACTACCCTGGCATTCTGGAAACTGAGCGTCGTATTTATCCTCAAAAT

AAGTATGTAGGTACAGATGTGGTTCAGATGCCTTCATTCTGTTACTCTGCTGATGAACCT

TGGCCCAGACTGGGAGTGAACAGGATGGCCTTCAGATTTACTTACCTCAGCTAAGCTTTT

TCATTTGAATTTTGGAAAGGGCTTTGTGTTTTTGTAGTATGAATAACTTCAGGGCACTGG

CTTTCAACCTTCTGATTTTTAAACCCCAGCTGCTCCTCAGTGGTTGGGCAGACCCTGTCA

GA

>c18599_g1_i1 len=241 path=[1:0-240]

GGCTCACTGCTTGTGTTTACCACTCCCTGGGCATCCAGCACCATCACGTTTTCCTCCTTC

AGTTTTGCCGCAGATTGAGGAATCACCTCCACTTTTGTTGCACTCTGACATTGCTCAGAG

CTGCCCAGGACTTCGTTCTGCTTGGTTTCACCCTGTGTGTGACAGAGCAAAGGTAACTGG

CTGCTTCCTGCCTCGTGGACAAGACACTGCACGTTGTTCTCAGTTCCAGCAGCACGAAGT

C

>c18601_g1_i1 len=202 path=[1:0-201]

TAATTCTTATTGACCGCTGCCTGTGTTAGGTAAGTAATACCGTAAAACAGTAGGATGAGA

CTTAACGTAAGACAGCGTTTGATTTTGTATGTAAAGGATAAGTTGCTGGCAGATTCTGCT

TCAGAACGTAAGAGTCTGTTTTTCGGTTGTTCTGGTAAACTAATGGTGAGCGTTTTTATA

CGGCTGTCCTGCACTGTGTGAG

>c18616_g1_i1 len=248 path=[979:0-97 754:98-247]

TAAAGAGGGAGGGAGCCGGCTCTGGAGAGCCATTTTTCTCTTTCCTCTGCCCCTTCAATT

AAAGCGAGACACTTCCCAAAGGCCTCTTGGCCCTTCTCCTTCATTTTTGTCTCTCTCTTC

CCCTTCCTTCCCAAAGTTCATGCTTTCCCTAGAAGTAAGAGTTTCTTTGCCGAAGGTTTG

TGCCATGTAGGCCAACCTGCATGCTGCTTAACGCTGTGGGAGCGCTGACGTGAGTGGAGA

GAGCAGGA

>c18616_g1_i2 len=463 path=[441:0-312 754:313-462]

CTCATGTACCAATTAGATGCACATCCTTTCCAACAACGGCTTTGCAAACACTCAGACAGT

GTATCTTTGGATCTTAACTGTCTTCCCATTCCAGAAGTGAGGCTTTTGGAAACACCAGTG

AAGACACCTTCCACTCCATGCCTTTCCCCTACCGTACAAAAATATAAAGAGGCAAAGCTA

AAATAACGTTGTAAGTACAAGGGAAAATCTTCAGGTCGTCTGGTTTTATGTGCAGCAGTA

CTCCAGCATTCTTTCACTCTCCTTAGGCCTACCTCTGCTCACTTTAAGATCAAACTGGCA

GTTTTAGCCAGCACTTCATTTTTGTCTCTCTCTTCCCCTTCCTTCCCAAAGTTCATGCTT

TCCCTAGAAGTAAGAGTTTCTTTGCCGAAGGTTTGTGCCATGTAGGCCAACCTGCATGCT

GCTTAACGCTGTGGGAGCGCTGACGTGAGTGGAGAGAGCAGGA

>c18618_g1_i1 len=381 path=[241:0-380]

AGGTCTGTGTACAGGTGATTCTTTCAAGTAATTTTATTTGATACTTCTGAGACCATCTTA

GCAGACTCTCCTTGTGAAGACATCATTAGTGTAAAAGCCATGTCCAGCCTGCCTTGTTGC

CGTGTCCAGCAGTATAATGGGTGCTGGGCAAAATGCTGTGCTGTCTTTTGGTTCAGTTTT

TCTTCCATTCTGACTATTTATCTCTAGGGTTGGTATTAAGTACCTCCAGATGGGGTCTTG

TGGAAGACCTAATGTTGCATTCCCTTGTTGTTCAGGAGGGCAGGGATTATCTATCTGAAG

AAAGATGTGGGTGTCCCACACCTTGGGCTTGACTGCTCGAGGTCACAGATGGGACAGTTC

TGCAATTGTGCTTGTGTTTGA

>c18626_g1_i1 len=247 path=[225:0-246]

GTGCTAGGGAAGCAGTGACAGCAGAGCTAACCTAGTGGCAGAACAGCACAAAGGCTGCAA

AGCTGCCTGAACTTGAGCACTGCCTGATTACAGTAGTTAATAAACGTAGTGTATGAGATA

AGACTGTAGCAGTTTAGAAATAAGTACTTTCCACAGAAGTTCAAAATGCTCTAAGTATGG

TACACTACTCTTAGGCCAATCTTAATCCACTTATTTTAGTAAAAAACAGAGCCCTTTTCT

AAGCATA

>c18630_g1_i1 len=206 path=[411:0-149 197:150-205]

GGTTTCAAACTTTCAGCGCTTACAGACAATCAAAACCTGCATGCAATGCTCTCAAGTATT

TGAAGAATAAAATGAAGATTTATCGAGTCTGAAAAAGGAGAAACTGAACAACTGGTCTAA

CTAAGAACTGTCACATTCTGGCCAGATAAATAGAGCAGAACGTAAACTTCTCTCAAACGT

GCTCCGATGTTTTGCTAAACATCTGT

>c18639_g1_i1 len=270 path=[148:0-145 294:146-269]

AATAAAAAAAAAGAAACCTAATGGGAAAAAAACCATTGTAAATACAATGCCACAAAGAGT

TATGCAAGGAAGATTTGTGAAGACGTTAATACTACTTATTCTCTCATGTGAGAGAGAAAA

GTAGAAGGAAACCATGCAAGCCTTTCTACTCTTAGCCTAACTTTTATTAAATTGTATATA

CAAACTTCTTTTGTGCTTCCAATAGAGAACATATTTCCTTATTTGTCTTAAGAAAAAAAA

AAAAATCCACCCCAAACCAATAGGACTTGG

>c18647_g1_i1 len=226 path=[204:0-112 317:113-113 318:114-225]

GAGCAGAGCTCCAACTCCTCATCTGAAAGATGTATAAGGAGAAAATTTTTCTTTGGAAAA

ATCCTTTATAAAGCAGCACTATCTAAATGAAAGATGTAACCTGTAAAAGGTATAAAAAAA

ATGAGACAGGCAGATTCCTATCATGTTCTTCATAGACACTATCTTGGGTAGAAAATTTGA

ACTGTATCTGTCTTTGCAAAGACCACATATACAATTTTGGGGTATT

>c18649_g1_i1 len=223 path=[201:0-63 265:64-222]

TACTTGGGTTTATTAACTACCTTACCTCCCTAGCAATCTCAGATCATCTAGGAAACCTCA

CCTAGCCTGTACAGAATGACTGTTACCCTTCTTATTTTTTTTTACACAAAGGGACAGCAT

TTAATATATATAATTTCATTACCCAACCATTCTTTGCCTTTTTATTTTAAAGGAAGAAGA

TATTTTTTTTCTCTCAAAAAGTTCTAAATGGAGACATTGGAGA

>c18651_g1_i1 len=216 path=[1:0-101 103:102-215]

AGCAAAGCCTGCCTGCAAAACGGTTGGGAAGTGATGCACAGCAGCTCCTCAGGCCTGCAG

CCTGCGTGGTGCTATGACTGCGGTATCCAGCTGCACCAACACTCCTGCCTACAGCTCATA

ATTGATGAATACTATTTAACAGCAAAGCATTTGCTTCATTTTTCCCTCTGACATCTGCAG

CAGACAAAGAGAAATGCATCGTGTCATCTCCTGAAG

>c18652_g1_i1 len=771 path=[774:0-770]

ATGAATTTAATGGTGTATCCTTAATTAATAAATTTCCTTTTAGCTAAAATTTAATTCTAC

AAAACATGTGGTTATATAAAAGCGTATATATGAAGATTTAAATAACAATTTGTCTAATTT

TTGGTAATTAAGCTCTCTTCACGGATATCTATTTATTCCTACGTATTTTTTCTTTTCAAA

TATAAAACAATGTAAACAGATAAGAAATTTAATTTAAACATTTTATATATAAATATATGT

GATTTTCTTTAAAATTATTAATAAAAATTGCTTTTAATTGCTTGATAAAACATATATTTT

TTTTTGTAAGCAATAAAAATAAACAGTTTATGGTAATAAACGACTGTCTATTTTTAAATA

AAGCAGAAATAAAAGTGAATTATAAAAAATTTAAAAGTAATATTAAAAAAAAAAAATGTA

ATATAAAGATATTTTTATAAAATTCCCCATAAATAATAAAATAGAATATTTAATAAAAGA

AATAAAAAACAAAAAAATAAATGAGTAAATAATCTTTTGTTTTATATGCATAGTAATATA

AACAACATAAATATATAAATTAACATAAATTTTATCTTTTTATAAACTAAACCATTTTCG

AAATGTTAACTTCATGCACATATTAAAACACTTATAATACTATGTATATGAAAATTAAGC

GTTTAAAATTAAAAATCTTAAAATATCCTATACTATGCTTTCCCTAATTATTTTTAAAAA

TATACTTGTATCAATTATGTATATATAATACATTTATAAATAAAATGAATA

>c18659_g1_i1 len=250 path=[228:0-249]

TTAGGGCTCACTGATTTCCAAGTGACAAGAGTGGAAATTACTGGGTGTAGGTGACTGTTA

CTTGTATGTTCATGCTCAGAATTGCTGCATCTGGAACTGCTTGCACTCTGCTTCCACAAA

TGGAGGGAAAAAAACCAAAGCTTTATGTGTGGACAGATTTTGGTTTCTCAAAATTACAGT

AAGTGTCTATTTTAGTCTCTTGAGTATCTATTTTGTAAATGGGGAAAAATGCACTTGGTT

AGAATTTTTG

>c18669_g1_i1 len=296 path=[307:0-295]

AGCCTGTTTGAGGCTCAGCAGCAGTTATCTCACCTGGAGATCACCAAGAGTCAGCTTGAA

ATCCAGCTTCATACAGTCGTTCAGGCTAAGGAAGTGATACAAGGGGAAGTGAAGTGTCTT

CAATATGAGCTGGAAACAGAGAGATGTCTAATGAAGCAGGAACAGAAAAACATGGCACAA

CGGCTCATTCAGATAGAAGAGCAGCATAACAATACCCTGAAACTTCAGCAAACTGATCAT

GAAGTGGAAATAAACAAACTCCTACAAGACCTGGCAAGTGAACGGGAGGGGCGCCA

>c18674_g1_i1 len=303 path=[361:0-205 4:206-302]

TAGCTAGGCCATCTGAAGACGAGACATTTTACTGCGAAGTAATGTGGGAGGCTGCTTTTG

ACTGATACAGGTCAGAAGTTGATGCTTTTTCAGGCAGAAAACTTACTCATTGTACTATGC

TGAAGAGCATACAGTACTAGAGAGCAGGCAGGTAGGATGTAGAACAACCCACCACTGTGT

ACTAAGATCAGTATTCCCCAATGAAGAACACTGCAATTCAATTCAAGCACAGCTAATGCA

CTCCTTTCAGGTAACGTGGGTTTTTTGTCAATATGTTTTTATGTAGATTAATATGAATGT

GAG

>c18677_g1_i1 len=646 path=[655:0-645]

ATTTAATACTATGAAAGTTGCTGCTAGCTTAATCATTTTCGCTGTTTAAATCTCCAAACT

TGAATGAGTATAAAATTAAAAAGTTTTTATTGCTTTTAGTATAAGCACACTTATGTTTTA

TTATTATTACATACATAATTTATATAAAATGAAAAACTTATTTATATATTGCTTTATATC

CTATAATCAACAACATACTAAAATATTGTACAATAATAAAGATTACTCTTAATTTTCTTA

AAACCTCCACTAGGAAAGTGGTACAAGGTGAATAATTATTTGTACGACTACAATATTTAA

AAAAATATCTCAATAGTTCATATAAAATTATTAAATAGGTATATTAATAAAAAATAAAAA

ATTTCTTAAAAACAGTTGTATTTTTTTTATTAATTACAAAAATAATATTAAATATTAAAA

AAATTCACCTCATTAAATTTAGAAACCTATGCTAAATTTTATAAATTATCTAAAAAAACA

AAAAGTAACAAAAGAATTAAAATTATAAATCTTAATTAAAATTACATTAAAACATAAAAT

ATGGAAAAAAAATTTAGATAAAAACATTTAATAAAAAATATAAAAATTTTTTAAAGTACA

TTAGTTTTAATATTTGTATTTTTGTATATTCTTAAAGTAGTTAAGA

>c18680_g1_i1 len=324 path=[482:0-296 778:297-323]

AGCATACCACATCTCAGTGAATGAGGAGCTGCAGCATCTGAGCGGTTCAAGTCTTCTGAA

GATATGACTTCAATACTACCCTTTTGTTGCAAAAAAAGAGAATGCTTCATCACCTTTGCA

AGCACAGCAAACAGGTTAAGACGAGGAAAGCTTCCAACAACTATCATGCCTCCACACTGC

TTCCTCATTCCCTCCATTTTCTCACTCTCCACCCATTTCCACGCCATCACCTCTATGGCT

CCCTGACCCCGATGCTCCCCACTTGCCTGGCGTTGGCACATCCCCTCTGCAGCATCACCA

CAGGCTCCAGATGGCACCTTGAAG

>c18681_g1_i1 len=204 path=[336:0-203]

CTGCCGGCAGTTTCTCTGGCTCGTGCAAGACTGATACCAGGAATGGAGACTATCAGACAC

CCCAGAGCGTAATAAACTACGACAAACCTAAAGCCAATGGTATTCTGACCTCTGTGAGCT

ATAGTACGGAATACCAGAAACCTCAGGCAGTCTCTGGGTGTTGAGTAACCCTCACTCCCT

AGCGCGTTCGCCTGAGGTTTCTGG

>c18682_g1_i1 len=203 path=[155:0-202]

AGTGAGGAGGAGGAATTCTAACAGGTCAACATTAGAAATTTTACACATACTGAATTTCTT

CGTTTTTTTTTATTGTGGTTTCTACTCAATTAGAGACTTCTTTTAAAGTAAGAAATTTAA

GGTAGCGTTGTACAGGCTTGGATATAGGAAATACCATAGCTTACTGAATCACTTCAAAGC

CATTGACTGAAGATGCCGTAGTA

>c18688_g1_i1 len=224 path=[1:0-223]

TTGGGTGAGGGAGCGCAGGAACAGGTTATCCAGGGAGGTGGTGGAGTCTCCTTCAATGGA

GAGATTCAAGACCCAGCTGGACGCTGACCTGTGAGAGCCATCGCAGGGAGCCTGCTTCAG

CAGGGATGGACTTGGTGTTGTCTGGAGGTCCCTTCCAACCCATTGCCATTTGGTGATCCT

GGATCAGGACAGATGTGTACTGCACTCATCCACAGCTAACTCAG

>c18691_g1_i1 len=262 path=[53:0-8 52:9-261]

TTGTTGGTGTCAGATCTTGTTCAAGTAAATATATAAAACATATAGAAATATTAACAAAAA

AATTTTCTCTAATATATAAAAAGAAAAATGTATAGATAAAATAAATATATGTTAATAAAA

TATATACTTATAAGTGAATTGATTAATTTTAAAAGACAAAATGTGTGCTCATAATAAATG

ATATATTTTTATATAAAAAAATATGTACCATTTTTTAAAAAATTATTGAATTTATTTAGA

GGCAGTTAAAATTATTGATTGA

>c18695_g1_i1 len=427 path=[41:0-362 40:363-426]

AGAAGCAAGACATCACCAGCTGCTACTTATCAAGGGGCTGCAGCCATGCTAGCTCAGATG

AGCTGCACATTATCAGCCAGCCAAATTTGTTTCATTTGCAACGAGATCAGCCCTTGCTGT

GCCATTGCTGGGGAGGGATACACCACAAACAAGCGTTGTAAAATTAAAGCTCCTACATTG

CGTAGGCTACACTCTAGCCTACGCAATGTAGTCACCTCCCAGTAATTAAGTTTCCTAATG

ATGTGCTGCATAGTAGCAGAAGTAACAAGCTTTTACAACTGCTCAAAGACTGCAGGTCAA

GGATCCATCTTCTTATCACACTGTGTCTGTTCACTTCTATTGCGTCTGCCACAGCGGTTT

CTACAGCCTCCTCTCCTCACACAGAGTTGACCAACCACACCACTGGTTAACTTCCCTCAG

CTTTCAG

>c18702_g1_i1 len=202 path=[155:0-201]

GGCAGTTTTAAAGACTTTTATAAGTCATAATATTAATCACCAGAGTTCACTAGAAGGTCC

CTGTAGCCCCTTGAATAACTATTACCTCTTCTTTATGCATCAATATTGCTTAAATTAATA

CAAACATGGAAAACATTAAAATCTCAATATGTATGCAGAAAAAAATACCAAGTTTTAAAA

GAAAGACTCCTAACATTTCAAG

>c18705_g1_i1 len=207 path=[1:0-206]

ATCTTCTGAAAGCTGAAGCTGGACAGAGCATTTTTCCTGAAATTTCAGATAGCTGGCACA

AAATCACTTTTTGTTAGTGGCATATTTATTTCTAATGCTCATCATAGTGACTTTCTGTAT

TGGTCACTTGAATTTCTATGATGCAACTAAGAATAGCGTAATATATATATTATAATTTCT

ACATTCTCAATTTTGGAAGCTAAATTA

>c18710_g1_i1 len=262 path=[567:0-261]

TCGTGTGTTGTGTGAGTGCTGTGGGTCTGATGTTGGCGGAGCGGAGTTTTCTTCCGGCTG

TGTGTGGGGATGCGGTGGTGCCCAGAATTGGATGTCTTTGAAGAGTTGGGGATGCAATGA

ACACTGCATGGTGGTCGCCTTGTTGCTTTCGATGCAGTTCGGGTCTTTGGGAGGTATGTG

CTTAATGTTTCCTGCTGCGTGGGAGAGTTTGCGATCGGCCGTGGCGCGGAGGGGTGGGGT

GGCTGATGCCACGTTCCTAGGG

>c18710_g2_i1 len=306 path=[284:0-305]

ACACCGGCCCAGCCGCCAGCTGCCATCCCAACTCCACAGAAATGCCAACACCGAACAACA

CTGGCTCCAAAAGCAACAACGCAGCATCAACGGTTCAACGACCAACAACACACGCCCAGG

ATCCCCGAGCACAGACAGCCACGCTGCACCAACAGCCTCCGCCGCAAGAAACCCATCCAC

CAGCCGCACTCTCCACTGCTAGCTCCCCACCTCAGGAACACAGCCCAAGAAACCCCCCGC

AAAAGAAAACCACCTCAAGGGCCCGGGAAAAGCCCCCATGCATCCGCACCCCCCAATGTT

CAGCCA

>c18721_g1_i1 len=270 path=[248:0-269]

CAAGGCTGAGTTCAGCTGGGTGGATGGATGTCTCTCCATAGATGGGGAACCACGCTGTGT

TTCTGCTGGTCACCCAGAAGCCAGTTAATCACAACAAGCAGAGAAATGCAGAAGCAGACT

CGTACTAACTTCTAAGTCAACAAGATTTCTTAGACAACCAATGAATCTATGCACAAATCT

TTCAGTGCTTCTAAAAGCCCTGCAATAACAGCCTGGTAAACAGTGAGCCACAAAGTCAGC

AGGGAAGCACTTGGTGGGGGTTAGCACCAG

>c18725_g1_i1 len=254 path=[97:0-201 316:202-253]

GGATCCTTCAGAATATCAACATTCTGTTGAAAAATTGAGTTAATTCCATTTAGTCAAAAA

TGCCATTTATCTTCTAGTACAAAAATGGTCACTTTCAAGAATTTATCTTACACACTGTTA

AGGAATGTTCTGTAAAGTTCTTTAAACCGGATTTAAATTGCTCTTAAGTCATTTAAGTCT

TCTCTCTCCAAACACTTTCACCGTCACATCAACCACTGCTTTATTTAAGTTCATTCCAGC

TAAGTTTACTTTTG

>c18727_g1_i1 len=243 path=[221:0-242]

GTAAGCACTTGTATGTCTTGTTAAGTAAAGCCCGTTACTACAGTCCTACTGATAGGATGC

TCAACTTCTAATTTATTTTTAGCCTTATTGTGAAAAAAAAAAAGCTGTAGAGTCCAAAAC

TGACATACATATTTACTTGACAACTCCCATACCTATCAAAGGTGATCTGCCAAAGTTCGA

GGAAAAGGTTGAAAAGTAGAAAGATTTGCCTGCTCTTTAATGAGGACATTTCTTTGTTTA

CTT

>c18730_g1_i1 len=287 path=[1:0-286]

GGGCAATTAATTAACCCCCGCCACCGCTAACCCTGCCAGGCCACCACTTCTGTGCTGATT

AATAGGCTAATTAATATTCATTAATGCTAATTATGCTGGCGTATGAAGCCTCGCAGTGTC

ACCATTGAAAGAGGAATCTAATTAACAGCGATTCATTGAGCCGTTAAGCTCTCATCAATA

TTATTTAAGGTTAGTTAGTGGTCTAATTAAGGTTAATTAACAGTTAATTAACAGTTAATT

ACTTGCATAAACCCCTGAGGTGCTTTTATGCCCTATTTAAGAGCAAC

>c18730_g2_i1 len=381 path=[887:0-380]

TCCTTAATTAGCCATTTTCCTCATTATTTGCTACCACTGGGGGGGGGTGGTCTGAAGGGG

TAATTAACCCCCACGTAATCATTCACAGTTAATTAAGAGCAATTAGGGCTCGGTGTGCAT

CATTAGGACGCATTAGGGCTAATTGTGGGTATAACGATTCCAAGGGCTCCAACGGGACTT

AATTAAAGGGATTAATTAAGGATTAATGCAAAGATAACACAGGGCAATTAGAGTAGAACA

AGCTGCGATTATCAGGATTAATTAAGACTAATTAATTAGGAGCCACCACGTGTAATTAAG

GCTAATTAGCATCTAATCACCATCAATGCCATCAGCTCTTCTTTCGGCTCAATCAGACTG

TGATGAAGCCCTAACGAAAAG

>c18731_g1_i1 len=281 path=[209:0-280]

GAAGTACAAGTGTTTGCTTTTTGTTACATTTTGAAAGATAATACAAGTCAAGTCAAACTT

GCACCTTTTGCAGAGCAATATTATGGTGGTAGTAGTTGTTACTACTATTACTACGTTTTC

CAAACTGAAAATGTCATCCTGAGCTGCAGAAGTTTTAAAGTTTGGATTGAACTCCTCGCC

AATCAAATGCCTGAATTTGAATGTAGAGACTCAGTGTAATTTTTGCTTAAAATAATCATA

GGAAAATTTTAAAAATTTACCAAAAAGTAACTTCCTATTAC

>c18733_g1_i1 len=202 path=[1:0-201]

AGGCATCAGCTCTGCCACTGTCAGAATCTATTTCTTCCCTATGCATTTTGAGCTCCCAGA

AGCCCAAACACATCTTGAAACAGGTTCTATAATAGCAGAGTATAGCACACACACGTATAA

AATATGTCAAATCCCAACAAAAAATGAAATGTTTCCATCCATTACAGGACGGATTATATC

CATTACAGGATGCTTCACTTAA

>c18746_g1_i1 len=285 path=[417:0-284]

ATAACATTTTAAGAGATGTTTTTGTATTATTTTAACACCCCAAGTATTTGAGCATAACAT

TTCCTTATACCAATGCTAGGTGTTTGAACCAATTTAGGGCTGGAATCACTCTTTATTTCT

CTTGAGCAGAATCAGTGCCTGATCAGGAAATAATGGGGAGCGTTTCAGAATTTCCCCTGG

CACTGTCAGCCCCACAATTTAAGCTGCTGTTAGCACTTTCTGCCAGCATCAGTGCCTTGT

CCCCACAGTTTAAATCCACAAAACAGAATGGAAATGTATTGTCGG

>c18757_g1_i1 len=271 path=[354:0-26 328:27-198 15:199-244 596:245-270]

TATTTGAAATAGAACCCATATAATAACTTTATTATAATCTGTTCATTACGGTGATCTGGG

GATGGACCAGGATCTGGCTGGTAGCAAGCAAAGCACCACAGAGCAAGAAACTGGTCCACA

AAGTCTATCTTTGCACTTATAAGACTATGTTCAGGCAGCAGGATCCTGGAAAGTGAAATG

TGAATTAGTGTGCATGATAGGGGGTTAAAAGCTCTTCAGAAAGTAGCATTAGCCTAGTGG

TCAGACTTCCGTCCTGTGAGCTAGGGCAGAA

>c18775_g1_i1 len=264 path=[100:0-97 198:98-263]

AGGCCCTGGAACATGTCCAGAAAAGGGCAACAAAAATGAGAATTGGGGGGAAAAAGAAAT

TTGGAGGACTTGATTCTGGGCACAGGCTATGAGACTTGTTAATGCTTCATGCTTATCTCT

GAGCCCTAAAATTCCTGCTGTACTTGGTGCTTGTTCTTCGTGAAGAATTCTTATGCCTGT

CCTTGTGTTCTGAGATCAGCGATATGGAAATAGAGGGAAGCAATTCATAGTCTCAGTCTT

GATAGGAAAAAAAAAAAAAAAAAA

>c18775_g1_i2 len=217 path=[199:0-50 198:51-216]

TTTTTTTTTTGTCCTAAAATTCCTTACATGAAATGACTTAAAAAGAGACTTTTAATGCTT

CATGCTTATCTCTGAGCCCTAAAATTCCTGCTGTACTTGGTGCTTGTTCTTCGTGAAGAA

TTCTTATGCCTGTCCTTGTGTTCTGAGATCAGCGATATGGAAATAGAGGGAAGCAATTCA

TAGTCTCAGTCTTGATAGGAAAAAAAAAAAAAAAAAA

>c18776_g1_i1 len=204 path=[1:0-203]

AAGACAGACTGTCTCCTAAAGAAATTTTGGATGTTCATTGAACTGCAACCTAAAATCACT

TTTCTTCAGATGTATACGGTGTGCAGGTTATCATAGTACCATGCCAGCTATTTCCAGTAG

GGGGAGAAAATGATGATGGAGTCATATCCTTACAGCTACCCTACTTATTTGTAGAACATG

TAATAAGTAGGGTAGCTGTAAGGA

>c18777_g1_i1 len=206 path=[189:0-205]

GACAATGGATAATCCTGAGTGCTGAAAGTTAATTCTCATCAAATGTGATCAGTTTACCTT

CCAGCTTTTCTTTCTAGAATAAGGATAAGTGTACTTGTGCACCAACCTTGCCATTGTTGA

CATTGGAGTCATCAGAATGTTCTTGGTAAGAAGTTGGTCTACAAGCCAAACTATGTTTTT

TTTTTATTTAAAAATTAACAAGTTAC

>c18786_g1_i1 len=243 path=[1:0-242]

GTGTGTGATTGCTATGTATGTTGTGTTGTTTCATTAGATTCTTTTGGCCTGTGGAAGTAT

TTGTTTGCTTCATTGATGAAGTCGTTTATTTAGGCTGTTTTTATCACTGCAGTAGCTGGA

GTGGCACTTGTGATTTTCCAGATAATTGCTTAAAGTGTTTTGTTTGCAGTTTAATTATTA

CTCGTGATGCTTGTGTAGTATTTCTTCAGAAATTCAGCCTCAATCTTTTGTTACAGAACT

AAA

>c18797_g1_i1 len=218 path=[196:0-217]

GCTTAGAGTGCTTTCTAGTTAGCAGCTGTTAGCACTCATATGGCTAGAAAAGAAGTGCTA

TGTAAGGAGATGCGATATGCTTTCTATGCTTCTGCATGCTGCAGAATTGGCTGCCATTCA

TTAATTTCCCAATCAGCAGCAGCAGCTGAATGGCTTGCATGAAGAATGCTGCTTCTCTCC

ACGTATCCAACCAATACTGACTATTTGCATTGAACCTG

>c18797_g2_i1 len=247 path=[391:0-246]

GAGGAGCTTCTGTGAAGAACCACCCCAGTGTTAGCACTGCCACTATCCTGTGCACTTCCT

CCAGCTTCTCACATGCTGCTATTCCACAATGAGCACAGCCCATGTTTACATGGCAAAAAA

TGCACAGAATGAGCAATGCACTTTCAAAGCACTTTCCTGTCCATGTCAGCAGCAGAGAAG

TCAGCTGCAGCTTCAGATGTTACATTGATTCACATATTATCAGGGTAAGCATGAGCTGAT

CAATGAG

>c18805_g1_i1 len=401 path=[155:0-400]

GGATTTCATTGAGAATTGTTCCCAGTAAATGTGTATGATGCCACTTGAGAGCTATGGGTG

AAGTATTTCCTTACCTCATTGTAGAGGCTATGGATCCTTTTCTTCCTTTAGGGAATCGCA

TTGCTTATGAAAATACTTTGCAATGCATCTTTGTTCCCAGCTGTTCTGATGTGCAGTTTC

TACTGTTGTGGGTTTGTTGCTGTTGATTCACTTTACAAAACTACACTTCTTATAATTTTT

ATATATCTGCCTGTTTGCAGCTGTGGTATTTTAAACACCAAGACTAAAGGGAGTAACCAT

TTGTTTCACATCACTTAAAAGGAGATTGTCAAATCACAGTGAAATAGACGTGCTCCACAA

AGCACTTTTCTCCTGACAAAAAATAGCAATTCAAACACAAA

>c18812_g1_i1 len=210 path=[1:0-7 9:8-209]

ACTTTGTGTTTGAGGGGACATGGTGGTGGTAGGTAGATAGTTGGACTTAATGATCTTGGA

GATCTTTTCCAGCCCTTATGATTCCAAAAAAAGAAAAAGCAACACATCGACTCCTATAAA

AGCAGGAAAACCTTTACATGCTAGAATTCATACAGACAGCTTTAACAGAAAACAAAACTT

GAGAACTGTATTGCTCACTTAGATGATACA

>c18818_g1_i1 len=263 path=[1:0-262]

CACAGTTGATGGCTGGTGATAATTACTTGGGCTTAATATTTACCCTACATAAGAAATTAT

CTAACCTTTCTTCATCTTGGCTATTCTCCACACTTGTTATGAAGACCTGATGGGCTCATC

ATCTTTATCCCCAGGAGAGACGAAGACCAAACTGTTCTTTCAGCAGGTCTTTCCTATTCA

GAGCCTTTCAGGTTGGACATCCTTGGACAAAACAAGCACGAGGCACTCTTCCTCTTATTT

TTAAGTATGATGCAGACCGGTGG

>c18826_g1_i1 len=455 path=[1:0-454]

ACCAAGAATCAATTCTAGAAACTCAGCTGTTACGATCACAGAACAAGCGCAGAAGTCAAT

CTGTCTCCCTCTTCTCTAAGATTAATTGCAACGGTGTTTTTCCACCCTCTCCAACCCAGA

GGCAAACCCACCTGGGCAACCAACTGGATCTCAGTCATTAATAAAAATACATTTTTTAAT

CTCATAATTCCAATATATTTGTTACAAATTTGAGTAAAACTTGAGAAAGTTATTCTTTCA

GAAGTTTTAAAATTGAATAAAATTTACTTCAGGCCTTATGCACTTGAAGGTAATTACAGC

TACAATCTTTGTGTTTTATTGTACCTTTCTACAGTATCTTCTAAGGGAGCAACTGCTCCT

CAGAAGCTTATTTCAGAAACAGCTCTTCATTTTAAGATATGATTTGAAAATGCTGTATCT

GGCATAAGATACCTTAAATCTCATAAGAGATCTAG

>c18828_g1_i1 len=257 path=[235:0-256]

AAGTTTCAGAAGCAGTTTTGCTCCCTAGGAGGTCTCAATCCACACCTGAAAACATTTAGG

GAAGCGTTCTTTGAACAGAAAGAGAAGAATGCTGCTGTTACAGGTGTGGTAGTAACAAGG

CTGCCTAGCAGCCATCCCCTAAAAATACACAGTCTCAGGGCAGGCTGTGCTTCAACGGAA

GCCAAGCTGTTCCCTTTGGGTCTGCACATCAGACAGCAGCAGTCCCCACAGCACACCACT

AGCAGTGACCCACTAAA

>c18829_g1_i1 len=252 path=[1:0-133 135:134-251]

AGGCCTGGACGGAATCACCAACTTCTCTGAGATGCTTTTGAAGTTCAAGCAGTGGAATGT

GCTGGTTCGTGCATTACTAAGGCTTGCTCCTGTGTTGCTTAAAAATCCCCAGTCCATGGT

GCTGTAGTGTTCTGGCATGGAAGTCTGCAGTGTGCTCATTTTACATGAGGTTACTGAGCT

ACAGAGCCAGTGTTGTTCCATGCTGTTCAAAAACATCCAGGTAAAAAATGTACTTTATTT

TGGAACTTCACA

>c18836_g1_i1 len=327 path=[585:0-279 867:280-326]

ACAGGGTGTCTCAAATTGATGTGCATGTACAGATGCAAAGCACACTTTTACAGATGTACT

GAAATATGTGGCTCCTGTGAGATATCTCAGCACCCGTGCTTGTGAAACGTGTCTCATTAA

AGCATATATAAGAAATGAGGTGGCTGGGAGAAGAATGAGCTTAGCTTATAGTTAAAACTT

CCTTCTACTCAAATTTAGAGCAGATTCTTGCAATATACTGTTATTCTTCTACAGGCACTA

ATTGAGGCCGGCTACAGTAGGATGTCTGGCATCCATCTATGGTCCTCCAGATCCTCAAGG

CCGTCCATAGATGGATGCCAGACATCC

>c18838_g1_i1 len=231 path=[209:0-230]

TTTTACTCCTCAGTACGGTAAAAGCTAAGCAAACCACTTCAGAACAGAGTTTAGCAGGTC

AAAGAGCTGTTTTGCGAGAAATCCAGACAACTGTATAAAAGTTAATGCCAAACGGCAGTA

CCAAGAGAGTGAATTAGATCAACAGAAAAAAAAAACCTGCTGATAGCATTCAGTATCACA

CGTACAGCTTGGAAGCTGGGTATAAGGAAGTCAAAATTAGCTTAAGAAAAA

>c18842_g1_i1 len=1311 path=[27:0-1310]

TGTTTTTTTAAAAAAAGAAAAAAATAAATAATGATATTATTGTCCATATAATAAATTATT

TAAAAATCAAAATTTATGTAAAAGTATAAAAAAATTGTATTTTTTCCTGTATGTCTTTTT

AATTCTATAAACATTCGTAATATTTTTTTTTTATTGTAATTTAAATTTTCTTAAAAGAGA

AACATTTTATTTTAATTTCTTAACAAAATTTTTAAATGGAAAAATATATTTGTTAAAATA

ATAATAAAAATAAACAAAAAATTATGGCTTAAAAATATAATGATAATCAAATCAAAATAT

ATGAATTAAAAGAAAGAAAAAAAAAATTTAACAATGCATTTAATTCAATAGGATGTTTCA

AGAATATATTATACCAAAATAGTTAAAGGTTCTTCTATTATTAAAATTAAGAGTTTTCTA

AAAGAGTGTTTAACGCTAATGTTTCATTCCAGTTTGCTTTTATCAAAGCATTCTTTGCTT

GATCTTCTGTAAATCCCATTTCTGTAATTTTTTTAATGATGACTTCTCTTGGTTCTTTTT

TAGGAACAGTTGCAAAAGTTTTTGTCCATACACTAGCTGTTTTTAAAAAAAGAGAATAAT

TTTCTTTATACATCTTCGCTACTTCCGCATCTTGAGGATCATCTGGTTGTGGATCTGATA

AAAGTGCTTGAATTGATAAAAGAGCTGTTCTAATTGTTAATGCAGGACTCCATTCATTTT

TTAAAACATCCAAACATATAGCACCTGTTTGACTTGAAATGTTTGGATGCCAAATTTTTG

TTGAAAATTTTATTTTTGGTGGATTATATGGATAATCATTAGGAATATTTATATCTAAAA

TAAAATGTCCATTCTCATATGGTGTTCCAGATGGACCTTTTATAAAACCTACCCACTCGA

ATATATTAGAATCCTTTATATGTGCATCTATTTCTTCTACGTTTTCATTTTCAATATCTT

TTAATTCTTTTTGTAATCTCAATAACTCTTTTGAACTTCTCGATGTTGAATTCATTTTCT

ATATATATACAAATAATATACTTTATATTTTTCCAAAAAAATCTCATATATATTTTTTTA

GATTTTAAGTGAAAATACTGTACACACAAAAAATAATAAAAAGTTTAAAATAAAAAGTCA

ATTTTATCTATTATTTAATGTATTCTATTTTATGTAATTTATATTAATGCAATTTATATG

TGTACAGTCCTTTCATTTATAGTAATCAATTAAACAAAAAAAAAAAATAAAATAAAGCAG

TACAAAACATATAAAAAAATATATACATATTCTTAAAATAAAAACTAATAA

>c18846_g1_i1 len=205 path=[183:0-204]

ACTTATGGACAAATGAAAGGACCTTTAGAGCTTGAAGAATGCAAGCAGCTAATCTAGCCT

TGAGAAGAGCAACTTCTAAATTTTTTTAGTCATTTTAAATGAGCTGAACTTTAATGAAGA

CATCTGTTTTAAAACAAGGCAATGGCTGAAAATGCTTGAAGTACAATTTGAATCAAGTTT

TCAATCAATGATATGTTCTAGAATG

>c18859_g1_i1 len=216 path=[1:0-215]

ACAAATAGTTGCATTGGTAATTACACTTCTAATATATATTTAAAGCATAATTTTACATGT

AATTAGAAAAAAAATCCCTTCTATTAGCATTCTTTTCAGTACTGTACAGAATAGACTTAG

AATATCAGGGACAAGTTTTTCTACACTTAATTTAATCACAATGCTGAAATTTGTCTGAGT

CTTCTGAATGAAGCTCTGATGCTAATACGTGAAGTG

>c18865_g1_i1 len=213 path=[439:0-212]

GATTCTGAATTCTTTAAAGAAAATCTTTTGGCTTTCTTAGCAAGTCATTCTTCCACATTT

TTACATGCTTTTATGCTGAATTCTTTAAGTTGCCATTAACAGAAGTCTCAGATATTGAGC

TTATAAGGCCTGTACGATCCCTTCTCTTCTGACAGCAATTTCTGATGGCTTTGTAGATGG

CGAAGACAGGGATGATCAAACTAGGCACACCAG

>c18867_g1_i1 len=212 path=[155:0-211]

GCTTCTCCTCCTGTCCTCAGTTTCACATTTTGCAGTTGTCCCTCTGTGGGATAAATATTT

TTCTTTAACTTTTCAATTTCTGTGTTCTGTAACTTTTCCTGAAGCTGTACATAAAGCTCT

TGAGTCTTCTAGTAAATTAACATCTCTTAGACAGGATGGAATAAAATTCTTTTTGTTTTT

GATGTTTCAGAAAAGGGTTTCTGTATGAGAGG

>c18877_g1_i1 len=202 path=[1:0-98 100:99-201]

GCAAACCGTCTGGGATAAGGAAAACTCTCTCTTTTGCTCCGTAAGCAGCAAAATAACACA

GCTAGACTTCATGGCTCATTCAACAAAAATTTCTCTGTACCTTAGTATCTGCCTTGAATT

AAATGGAACCGTTTCAGAATCTCTGTTTTCTTTTCAGGAAAACATTCCAAAAGGTAAACA

GCATGAGGAAAAACATGGAGAT

>c18892_g1_i1 len=252 path=[552:0-251]

AAAACTCCAAACATTTCCACACATTTAGGTACTGTCAAATTATACTCCACTACATTTGGA

GTAGATTCTTAAAATTTTAACTCTGGAGTGGCAAGCTACCCTTATGATTTAAATGCAAAA

ACACTGAAAATTCAGGATGTTTAATTTGAAGGAAGCAATTAGAGTTTTTTACTCCTTAGT

CTTTTTTTACAAATGGCTTGCCATGCTTCCTTAAGAGCTAATATGTATTTTCGAGTTTTG

ATTGCCTTGCTA

>c18895_g1_i1 len=272 path=[1:0-201 528:202-271]

AATCCAGCTACAAAGATCAGAGTTGTGTATTAGTTCAAGACAAAACCCAAAAGCACATCA

CCACAAACCGTGGTGCTTCGTACCACATGGCAACAATTTCCACTGGTCTGTTCAAAGGAT

GCTACTTCCTTTATTACATTGGTTATTGCTACACGGAAAAATACTCTCTTATTCTCAACT

GTCAAAATTGAGAAGTCTCACTCATTCCTACAGTTGATGCAGGAAACAGCACTTCGTATC

TTCAATGGAAACACCACTTCTCTATTCAGAGA

>c18897_g1_i1 len=316 path=[29:0-190 336:191-315]

ATCGTGGAGATGAGCCTGAAATGCAGTTCAGCACTGCTGGAACCCCTCGCTGCCTCCCAG

AAGAATAGCAGCCTTTTCTGTATGTAGTTCTGATTTCTACACAGCCTTTTTTGCAGACCC

CGTTCTCCCACTGTGTATAACCATTTTGTTCAAAAATTCTCAAAAGAAAAAAATTGGCTG

TTAGGAAAAGCCACCAAAACTGATTTTCAAATTCAAAGTGAACTTGACCTAAGAATGAAC

ACATCTGCCTGACATCCCTTCGTAAGAACATACAGCACAATCACAGTGAAATTGTTTATA

GCACAAAATATCCTGT

>c18900_g1_i1 len=206 path=[195:0-205]

AGAGTTATTGCATTTATTTGTACAGACATCATTCTTTCAGGCAGGACATTTTCTTTCACT

AGTGTTCATACTTGCTATTAGCCAGGCCAACTTACTCCAAGGTGGTCACTCCTTTGCAAA

GTCAGATACACATGCCCATATTTGTTATTTGAAAATGTGGCCCAAAATACAGATGGCAGC

ATCTTGTGCCTGTAGTTAAACATGAA

>c18903_g1_i1 len=256 path=[31:0-21 53:22-255]

GAGTAACTGAGCAGCTGACGTGATTTTATGCAGAAATGAGGATAAGGCCTTGTATGTTTT

TATGCTGTGCTAAAGTGACACTTTTTCAGAAATTAATAGAGCTAATCATGTAGGTGTGAA

GGGGTAATTGGATATTTATTTATTTATTTACCCATTTATTTAGGTTGCTAGTTTCTATAT

GATTTCACTCTGGTTTTCCTCGTTTTTTTTCCTTAGTTTCTTAACAAGCCTGCTATTTTT

TTATTATTATTATTAT

>c18922_g1_i1 len=204 path=[202:0-181 539:182-203]

AGAATCATACTTGTGCCATTCAAACACAAGTATGATTCTTTTAGAAACAGACTGCGTAGA

TGAATTCCCACAAAGGTGAAAAAATACATTGTGTCTGTTTGTTTGTTTGTTTTGGCCCAT

AGGATTCTCCTTGCTTTGCAGGTATGACCTATTTTATAGTGCCTTAAAATTGTCTCTGCT

TACATTATGATCTGATCATTTGTA

>c18931_g1_i1 len=237 path=[369:0-236]

AGGTCAGACAGAGAACATGTGTTTCATATTTGCAAATCTGAATGATTTATATTAGAGGAG

CAGGGCTGGACCGTGTGCAGCCTCTAGGTTGTTAAAATTTAATTTACTAATCAATTACTA

TCAGATGCGAGACTTCTTCAGCTGCCCCATACGAGGAGCATAGCCTCTCACAGAACCCTG

TCTTGATTTCAGTGAGGTATCTCCTTCCAGGGGCTGAAGGGAGGCAAGGTGGAGCGA

>c18934_g1_i1 len=288 path=[1:0-44 46:45-287]

ACCATTCTAGAGCAGCAATACGTATTTTAAATACAAAGACGCAGAATCATTTCAGATTTA

AAAATGAAAAGGAAGCTGAGCTTCTGGAGAGTGCTGTAATGATGATTCTTTTGATCTGTA

TTAGGATTGAGGAATTCATGATGAACGGAGTAATTTCCGGCTCACGTAATTATCAAAGAA

GATGCAGTTTGTTGAAAATAGATCTAAAAACCTGAAGCTTTACTGTATAAAATCAACATA

TTCAAGACTTAAAAACTGTCAAAACCCCACAGCTTTCCCTCAGATATA

>c18934_g1_i2 len=276 path=[564:0-32 46:33-275]

CACTCTCCCCTCCAAATTCAGATCAAAAGAATCATCATTTCAGATTTAAAAATGAAAAGG

AAGCTGAGCTTCTGGAGAGTGCTGTAATGATGATTCTTTTGATCTGTATTAGGATTGAGG

AATTCATGATGAACGGAGTAATTTCCGGCTCACGTAATTATCAAAGAAGATGCAGTTTGT

TGAAAATAGATCTAAAAACCTGAAGCTTTACTGTATAAAATCAACATATTCAAGACTTAA

AAACTGTCAAAACCCCACAGCTTTCCCTCAGATATA

>c18948_g1_i1 len=201 path=[1:0-200]

AGTATTCTAGTTAATGGTTAACTTTAAGATTATCTTTTTCTTTTCTTCCCCTTACTCATT

TTAGTTTCAGTATATTCTAAGCAGCTTTCTTTTGCATAATTGCCTGTTGCAAATAAGATT

ATTGTCATCATATCAATGAATATATGAGTTCAATTTGTAATTATATTAGTAAGTTGCAGT

ATACTTGCAAAAAGCCAAACT

>c18952_g1_i1 len=209 path=[53:0-144 322:145-208]

GCAACGTTTTATTTTTCAGAGCCTTGTAATACCCCAGCTGAAAAGAATGGTTGGACTGCT

TCTTATCTTGGAAAGATCTGGTTCCCAACACAGCAGTCCCTTATTGTTGAATATCCTTTC

ATCTTGCTTAAGATCTCTTGCTGGAAACCATGAAGACGTGGTAAGCGGCCTTTAGACACG

TACCCAGATGATTACACTTACACGTACGT

>c18952_g1_i2 len=208 path=[53:0-144 196:145-207]

GCAACGTTTTATTTTTCAGAGCCTTGTAATACCCCAGCTGAAAAGAATGGTTGGACTGCT

TCTTATCTTGGAAAGATCTGGTTCCCAACACAGCAGTCCCTTATTGTTGAATATCCTTTC

ATCTTGCTTAAGATCTCTTGCTGGACGTTGTCTCTGTTTTTCACCCCAAAAAAGCAGTGT

GGAACGTACGTGTAAGTGTAATCATCTG

>c18962_g1_i1 len=214 path=[279:0-213]

TGAACTTCACCCCATTGGCCTCAGTCCAGTGATCCAGTCTCTCCAGATCAACAGCGTACA

CTGACTGCCCTCTGTTCTCCATCCAGAAATACTGCAGCCCCACAGTTGCTGTCCTTCAAA

GCATCTCTGCAGGCTCCTGTGGCAAAGCCCTGTCCCCACCAGCTTTCAAAGACCAATTAT

GGGAATGCTGAGAAATCAGTGAGGCTGGAGGGAA

>c18963_g1_i1 len=308 path=[263:0-260 524:261-307]

TTTTTTCCTTCTGGTACCTCAGGAGTTTGTGGCCCTGTTTTGTTGTAGAGTCTCTTTGTT

TCAAAATTAACTGTAATCTCACTCAGTTTTTCACTGTGGAGGTTCTTTCTGAAACACAGC

CTTTTAAAAATTGATTTTCTTCAGTGCTGGTTTTGCAGTTTACCTATGAAACTATGCTTT

GTGGAACATAACCAACAGGTTTCTGAAGTTTCCAAGGCCTCTGCTCATACAGCCTTCTCT

TTGCCTGCAGCTTTGTGCTACTGCTGCTATTTTGTTAGTCACAGAATCATAGAACCCTTA

GAGTTGGA

>c18974_g1_i1 len=210 path=[512:0-185 327:186-209]

GCTGACACTACTTTTAAAATTAAGATTAGGGTACGTATCCTTTATCAATAATGTACTCAT

ATATAAATGTGTCAAAACCACCTTCTCAGTTACTTAAGTCCTAGCTGATACTGTAGATAT

CTGAGTTTTAAAATAAACATAATTCTTCTGCCTTCAAACCTGCAAAATTTCAGTAGTCTT

TTCAGAAACAGGCAATTAAAATGTAGAATC

>c18974_g1_i2 len=209 path=[512:0-185 720:186-208]

GCTGACACTACTTTTAAAATTAAGATTAGGGTACGTATCCTTTATCAATAATGTACTCAT

ATATAAATGTGTCAAAACCACCTTCTCAGTTACTTAAGTCCTAGCTGATACTGTAGATAT

CTGAGTTTTAAAATAAACATAATTCTTCTGCCTTCAAACCTGCAAAATTTCAGTAGTCTT

TTCAGATTCTACATTTTAATTGCCTGTTT

>c18986_g1_i1 len=523 path=[155:0-522]

TGCAGCAGGCTGCGGGCAGAGCTCTGCCTGCCTGTGCCGAGGGCCCCCAGCCGTGGGGCA

GCAGCTGTGGCACGTGCAAAGCGCGGGGTGCCCGGAGGGCCTGGCAGCACACGCTCCTGC

CGGGCCGGGGCAAGGGCTGTAGCGGCAGCGGTGTGTGACATTGAGACGTGCTGCCCTTGG

CTGAGCGAGGCTCGGGCTGCTCGGGAGTGTTTGGGTTTGAGAGGTGACTTCAGAGCCCTG

TGACTTCCTTTTCCATCTCCGATGCCGTCCTGCCGGCAGTGCCCTTGGCTGTCTCAGGAA

GGATGCTCAGTGGGAACGGCCGGGGCCCCATCGGCGCTGGGGGGTTCGAGTGGGGCAGCG

CAGCCCCCGGAGCCGCCCCACAGCCCGTCCCCGTCGTGCGGTGGTGCGGCCGAGCCCTCT

TCCCTGCACACGGGTCCCAGCCCGGTGCGCCCCGCCGTCGGGCCGTGCCTGCCCTGGAAC

AGTCGCCTCATTGTCTGCGGTCTGGGGCCCCCACTCGCCCACG

>c18992_g1_i1 len=290 path=[1:0-289]

TAACTGCAAATTCACTTCAAGTTAAACAGCATTTAACAATCACCTGAAGGGCTTCACTCT

GGTGGGGGAGATGTGACTCACCTGGCCAGGCTGAATTAGTGATATCTGAGCAAGTTCTCT

CACTCATTTCAAGTGAAGGAGAGAAAACAGTGCTGATCCTAAAGTCCATCCCTTCTAGAA

CACGCTGCTGCAATCCATCATGTCTTTTCCCATAATGGTCCTGTATCTCTTCATTGAACA

GAAGCTGAGCCTAGGAAAGAAGCCTCAAACACAGGCATGTTGACAACTGG

>c18997_g1_i1 len=283 path=[313:0-254 591:255-258 571:259-282]

GCAGCCTGAGCTGGTGAGGGGCAGCCAGGCAAGGAGTTAGAACCGGGTGGGCTTTAAGGT

TTCTTAGAACCCAAACCATTCTGTGATTCTACGATTAAGAAAAACAAACAAGGTAAACAG

CAAGTGTAACTCTAGAATAATAGATACATCTACCATAAAACACACACACGCACACAATGA

ACTAATTGAAATCATCAATAGCAAAAAGAATTTATAGTTTAAGATGACTCTTCATTCTTC

TTCATTCTCTTGCTTTATGACTGCTAAAGACAACTTTAACCAC

>c19001_g1_i1 len=211 path=[1:0-141 143:142-155 157:156-210]

GGCCTGAACCAATGAATGAGCATCAGGTGAGAAGGTGTGGATAACGCAGGGAGCTCAGGT

GCAAGCAGTGCACCTGAGCAACCAAAGTGGAACCTGGGCCCACCCCTCCTTACTCTCATT

TAAGGGTTAGTAGCTGATGGAGCAATCTCTCTCTTCAGAGATTGAGCATTTCTTGAGACA

TCACCAGTCTTCAGAGAGGGTGAGCTACCTT

>c19003_g1_i1 len=225 path=[435:0-224]

TGTGATCTGTGTGCTGAGTGCAGCCGTGGGTAGCGCTGAGGTACCATGCTGCAGGTGGGA

GCCGATGCTGGAGCCAAGGGAGGCTCAGCACTGCGCAGGACGCAGCCCGGGGATGGGAGC

ACTGACAGCAGCAGTCGGGGCGGACCTTTTCCTCTCCACACATTTTTTCCTCCTAGGAAC

AGGAATGGATGAGATCACAGCTGGGCTACCTCTACCAAGAGAAAA

>c19004_g1_i1 len=284 path=[518:0-283]

GACTCTCTGCATAGGGTGATCATAAACATCAGCTGAATCACACTTAAATTTGCTGGGTGC

ACCTCTAAATGGGAATGCTCGACACATGAACACGGACACCTGCGTAACTGGAAGAAGATG

TTGGAATCTCACAATGAACACAGACGTGTGCACTGATGCATTTCCATTGCTGATCTGAGG

GTACCTCAAAGGAGACCCCACTGTAGGAGACACAGGAGCCCTCCTGCCCTGATCCTGGCC

CTATCAGCATAATCCCATTGCACCATGCTGTGAATGTGGAAGGG

>c19007_g1_i1 len=218 path=[1:0-217]

GCAAGCAAAGGGACAAAGGCTCCCTTGGAGGAAAGACAAACCCCAGAGAAGCATTTGTGG

CTCGTTAAAGCCATTCCTCCTTTAAGCACAGCCAAACTCAAGAACAAAGTGGAAGAGAAG

GTGCGATGCGTCCCAGCAAAAGGCCCTTCTCTCCCTCCTAGAAGCCCACGTTCAGGCAGG

CACCTGGTAGATGAGGAGCAGTGTGAATTAAAAGGAAA

>c19009_g1_i1 len=215 path=[1:0-214]

GTAATTATCCACTTCCTTATATTTATCCTGTTATCTATAGCTGTGGAAACTGTTGCCACA

TACGAGATCTGTATCCATTTTGCTGAACCGCCCATGATTACACTAGGATAAAGAAAAGGA

AGTGGATAATTACAACCCTTATTATATAGAAAAATGTCCAGCATATTTTTAACCTTTGAT

ATCTAGAAAGCATTACTTGGTTTTTGTCTTAAATT

>c19019_g1_i1 len=237 path=[1:0-236]

AACTTTGTGCTGTGGAATTTTAGTCCTAGAGTGTTCTAGTATATATTGCCCACAAGGTGT

GTGTTTCCTTATTTTTAGTCTTGTTTACATTTGTCTTGTGGTGTGTCAGGCTGTCTGTGA

CATAATCCTGCAATAATACATGTTAATTATTTTAGCGTTAGGCGTATTGGACATTCCCTA

TTGTCAAAGTTTGTGGGAGACTGTAATCTGTACTCAGAGGGACCTCACTGCTCGGAG

>c19022_g1_i1 len=213 path=[277:0-212]

GTGACCCATGCAGTCTACATAGCACGAACTGCACGATGCGTGGAGGAACATCCAGCCCAG

CTCAGGCAGTCAGGTGCCGCAAGGGGTCGTGCTTCAATACCAACCACCTCCAAAGCTGCT

TGGACCACTTCATATCCTGCTAAGAACTCTTTCAGTTGGAGTATAGCAGGCCTCAGATCC

CCTGTATCCTCAACAACAAAACCCCCCTCAGGG

>c19024_g1_i1 len=319 path=[101:0-256 466:257-264 474:265-318]

AGTATATTCAACAAGATGGGCTCCTCTGATCTACCACAATGACACTGCAGTAGTTGACTA

GAGCTTCTGTGTTCTATGGTTGCTAATGATACTCCAAACACAGTTTTTAAAATGCTACTG

TGGGGGAAAATCTGATGGATAACATTAAAAATAAATTACTGTCAACACAACCAATCATCC

TTCAAACCAAGCAACCTCAGTTACTTGAGAGCTCAAACTCAAATTATTAACACAAGTTTG

AAAATGTTATTTGGGGTGGTTTTAACATTTTGCATTCATTCTTAGTTGAGTAAATGAATT

GAAGAATGTTAAAACCACC

>c19027_g1_i1 len=333 path=[1:0-137 695:138-332]

GAGAACCCACAAGGACAGCCTAGTATTTCTGTCTCTCACCCCTCACTACAAAATTCTCTT

CTTAATTCTATCACTTCTGCCAAACAAGCAGAGCTGAGTTTTACTTTATTGCATCCCTTC

AGGGGACACTTCCATAATACAGTCTTGTTTTTATTCTTTTTTTTCACAGTGCTGCTTTGT

AGGTGGACCAGACTCTTTTGAAGGGGAAGTGGGGGTGGAGGGCGAAGTTGAAAAAGCAAA

ACCTTATTCTTGTGTGGCTGCTTCTCCTGCTTTTCCAAGTCATAGTATAACTACTGTAAA

GAGGGCAACATGCATTTCTTCATGCTTATGAGG

>c19034_g1_i1 len=347 path=[479:0-346]

ATTAAAAAATAAATGTAAAAGTAAAGTGAATAGTAAGAATTTACATTTAGAAAGATTATC

ATTATCAAAAAATATAAGCTCAGAAAAAGAGAAAGAAATAAAAGCAAAAAGAAAAAGAAA

AGAATCAGATTTGAGTAGTGTTCAAAAAGATAATACAGATAATTCATTAGTAATTAGTAA

AGAAAATCAAAAAAGGGATCTTCAAATATTTATTGATAAATTTAGTTCATTATTTAATGA

AGAGCATTTTCATTCTGTATATGAAGTTATCAATGTTATGAAATTAATATTATACGAATT

ACAAGTAATACATAATGAAAATTATAAATCTCTTATCAAAGGATTTA

>c19049_g1_i1 len=203 path=[1:0-202]

GGGCCACCAATATACCTACGACTGCCTCATTGGCAGCGCTAAGTGCAAGGCAATCTGCTA

GTTCTAAAAGTGCATATACTGCTGGGGCTCTCCATACAGGAGATACCCCTCTGTGTCCTG

TACCCGCTTCAGGTCTCGACTTGTCACCTCCTTGTACGCACGCATAGACCAGTTGTACAG

AGCAAGTGGACTCTACAGAGCAA

>c19051_g1_i1 len=310 path=[241:0-309]

AGAGTTTACTAGTTCATTGTAAAATGAGTGTGGATAATTACAGGTTAGTAATTACCATAC

TGCTGGTGTTTGTAATACCCTATGCAGGGATTAGTATTGTGATCCCTCAACACTAACTGT

AGGATTGCAAAGACTTCAGAGATCTCTGTGATGATAATAAGAGGTGGTGCTGATTGTTAT

AAAACAAAACCAAGATTGTGACTGTCCTGCAGAAGCCAGCTAGTACAGAAGTGTACTGGT

CTTCACTTCATGGGTTTTGTCCCACTCCACAGCAGACATTCTCATTGGGGTATCAAAACC

TACTTCAGCT

>c19052_g1_i1 len=275 path=[334:0-149 41:150-274]

TCTCCTCAGTCTTTAGTCTGCCTCTTTCTCCTATTTGCTGTCTGCACTGCTTATTTCTGT

CTCCTTGAGTACTTCTCGCAAGAGTTCTTAGCTCATTTGTGTTTTAATCATCCTGATTTG

TCTACAGCCCTGGTTGCTGTCATCTCTGCAGGTCACTGTGCAGTCTGTGTGTTTCTTACT

CCGTGGCTGTTTGCTGCGATCAGCACATTTGATCATGGTTGAATTTACATCAAAGAAGCA

CCTGACAAAGAAGCGGGCCTGATAATAGGCTCTGG

>c19064_g1_i1 len=219 path=[1:0-218]

GTTTGTTTGTTTGTTTTGTTGTTGTTCGTTTGTTTTTGTTGTTGTTTGGTGGGGTTTTTT

TTTTAACCTTTACGTTTGCAGAAGTCTGTTTGAATGCAGGCAACTTCACTTGTTGGTGAC

ATGGATATGCATCTGACTTCCTAATTTTTTTCCTTTTCCCATCTCCATGTGGGAACTTCC

ATGACCCAACAAAAGGGGTCACTTGAGCCAGCTCAAACA

>c19067_g1_i1 len=316 path=[1:0-233 235:234-315]

TCCAGACACCCTCTCATGTCACTGTTACATGGGAGAAGAAGCTGATCCCCACTTTGCTAC

AGCTCCCTGTCAGGCAGCTGTAGTGGTGATATGTGCTTTATATAGAGAAATTGAAATGTG

TATATATTCATATATATGTAACAAAGTTCACCTTAATCAGAACCGAGTGCTTAACTGCTG

AGCCCTAGAGACTGAGAGAGGTCATTTAAAATGTGGCAGAAACAGAAGTTGAAGATCATT

AGCAGGATTAGAATAGAAGTGTAGGCAGGAAAATAAAGAGACTGGAAAGATAATATGAAA

TATCTTGGAAAGAGAT

>c19081_g1_i1 len=233 path=[1:0-232]

GTAACTAATTATTAGAACTCTTCTAGGGATGTGATATGTATTCCATCGTATTCAATTCTT

AAAATGACTTAGAGCTATAGCTTGTCCTTAATTAAAATAAATAAATAGTAAGGAGATTTT

TAATGCTTTAAATATTTTCATTGTACTTTGTCTTTATGCCAAGATTAGTGCTGACTTTCT

TGGTTCTTAAACTTTTGTGCTGAGGATAGGTGTACTTTGTGAGTTACTGGCTT

>c19088_g1_i1 len=201 path=[361:0-200]

AGTATTTAAACATATCAACAATCAGAGGTTAAGCGTTAAGCCAGTGGTGTGTTGATCATT

TCAACTGGATAAGTCATGCAGAATGGAGTGCCTACAGTCTGCTGGATAAAGCCGCTAAAG

TAGGAGCCTGCTGCACTACCCAACACGTTCAGTTCACAGCTCTTATTCCCACAAATGACA

GAGCCAGCAAGGAGCAGAGCA

>c19088_g2_i1 len=203 path=[181:0-202]

GATGAAAAAACTCCAAGATACCTGAAATCAACACAGAAGCAAACAAAGAAAAGAAAAGAA

AAATCAAACTCAGGAGCCAGAAAGAAATCTAGCGATGATTGCACCAGTACAGCTCTCCAT

GTATTTGCACATGAGATTGTTTCCAATTTATGGACATGCTTCCTAGAGGAAACTGAAAGG

AGCAGTATTTAAACATATCAACA

>c19091_g1_i1 len=234 path=[212:0-233]

GGAAGTCTGCAACCCCTGAGTACTTCAGTTGATGAGAAAAGGAGAGGGGACAATGCAGTT

GGGAAGTGGGCATAAGAGGAGGTTGTGCTGCTGGTGCTTTAAGACACCCTTCCAGGGAAA

TGCCCCCAGTAGAGTCTCCCATTATTAAATAAAGAAAGCTGAAGCTCTGTCTCTTTTTGG

GACAAAGACAATTGGACCTATCCATTATTAATTTCCTCTGATTCTTTTGCTGCA

>c19109_g1_i1 len=286 path=[501:0-49 238:50-285]

TAGCAACGGAAATAACTACGGTACATCTCACATTGTGCTGTGGCACAGACAGAAAAAGAC

AGCTATTACAATTATTTTGCCTGTCTAACCACTTTTCAGCGGTTTTTAATGAGAAATAAG

GTTGCTGACCTCAGATGTTTTGTGACTTGTCTGCATATAATACTAGAAAATTACAAGCTA

ATACATTTCAAATTGAAATGTTTGCATATGGCAGAAATTCCTGGGCTTTCCATTTGGAAG

CCTTTCTGGTAGCCCTGCTTTATCGACACAGGTAAACCAGGAGCAG

>c19109_g1_i2 len=354 path=[120:0-117 238:118-353]

CTCTTTGTCATGATCTGCTTTGAAGTAGTATCAGAAATAGTGCAAGTGTTTCCAGTGCCC

TACATTGTAAGTGCCTCTCACTTATCAAACTCCCCAGTGACATTCTTGGTACCCAGAAAG

AAAAAGACAGCTATTACAATTATTTTGCCTGTCTAACCACTTTTCAGCGGTTTTTAATGA

GAAATAAGGTTGCTGACCTCAGATGTTTTGTGACTTGTCTGCATATAATACTAGAAAATT

ACAAGCTAATACATTTCAAATTGAAATGTTTGCATATGGCAGAAATTCCTGGGCTTTCCA

TTTGGAAGCCTTTCTGGTAGCCCTGCTTTATCGACACAGGTAAACCAGGAGCAG

>c19113_g1_i1 len=206 path=[184:0-205]

GAGATTTATCGTTGGGCTGCATAATAGCATTTTTTCAAGGAGCATTTGAACAGCTGCCAT

CCATCCCAACTTCCAAATGCAATCATCTTGATATGCCTCGTGACTAAAGGCAGCCAGCGA

CAAGAAGCAGTTTCAAAAGCCAATGGCCCCACTGATTGGCACTAACATACTGGACGGAGT

GCTGAGCAGTTTTATGGCTACAAAGG

>c19117_g1_i1 len=317 path=[381:0-316]

GACTTGAAGACGTCTGTACTGGCACAAAGGATGAGGTAGAAGCAAGTAGTTTGGAATAAG

AAATGCAGAACTTCTTCGGCTGGCTTAGTGATTGTGTAGCTCTGGTCTTCATTGCAGTTC

TTTCTTCATGCATAACAGTGTTCGTTCTGTTTACTGTCACTATGTGTTACAACATAATAT

ACATTTATTGGGTGTAAGAATGTATGTTTGCATACCTCTCATTATTTAAAATCATTAATG

TGCTGTTACTTAAGTGTGTACATGGTGCAATTCAGTTGAATTGCACTCAGCTGTGGCCAA

GAGGTCTAAACGTTCTG

>c19124_g1_i1 len=326 path=[400:0-110 56:111-325]

GTGTTTCATAAATTCCACTATGGGGGAGTGAAAAGAATGCAAGTTTTCCAGAGGAAAACT

CAATAGGATCAGCAGTTCTTTCCAACAGGTAGATTTTCCACCTCAAAAAAAAAAAAAATC

ATAAGCCAACTGAGAAGTAACAGAAAAACTGCTATGTGGGACTCCTCATACTTCACGTAC

TGTCAAAATGCTGTTTTACTGCTACATTTGGCAGATCGTATGCAGAAATCCTCAGCTCTA

TTTGTTTCTTCAGCACTAACAAGAGACCCATTAAGGAGCCTAATATAAATATTTCGATCA

AAAAACAAGAGCTGCCGAAATACCAA

>c19124_g1_i2 len=242 path=[29:0-26 56:27-241]

GATGGAAAAACTTGGTGCCTCAAACACAAAAAAATCATAAGCCAACTGAGAAGTAACAGA

AAAACTGCTATGTGGGACTCCTCATACTTCACGTACTGTCAAAATGCTGTTTTACTGCTA

CATTTGGCAGATCGTATGCAGAAATCCTCAGCTCTATTTGTTTCTTCAGCACTAACAAGA

GACCCATTAAGGAGCCTAATATAAATATTTCGATCAAAAAACAAGAGCTGCCGAAATACC

AA

>c19129_g1_i1 len=486 path=[881:0-39 458:40-485]

GCAGCCCCCGGGGCGCGTGCCGCATAATAGTACGTTCGGAAGTTTGTGGGGAAGGGGGGG

AATCAACCGCACCGACTGCACGGCGCGCGCCGGGGGCTGCGGGGAGTGGAACGCTGCGGC

GCTGGGGGGGTTCAGCGCACGGCAACGAGAGCGGGATGTCGAGGGACCCCTCGCTTCGGC

CCGGCTCTTTCCCACGCGCCCTCGGATCTTTTGTCACCATAAAAGTCAGTGAATGCCACG

AAACGACGAACGGGAGCTGAGGTGAGGCGGCGGCCGGCGGTGGGGATCCGGGAGCCGGCG

TGCGGCAGCCCCTTCGGGTGCCCCCATGAACGAGCGTCGCGGCCGGGGCTGCCGGGACAC

CGTGGGGCACCATGAGAGCGGGCCACGAGCGGAGCCAGGAGTGCCTCCCGCCAAAGAAGC

GGGACCTTCCCGCCGCCAGCGCCGGCACGGAGGCGGGACGGGCGGGGGGGGCCCCGGCTT

CGACCG

>c19129_g1_i2 len=463 path=[441:0-16 458:17-462]

TTTTGTTTTTGTTTTACAGTTTGTGGGGAAGGGGGGGAATCAACCGCACCGACTGCACGG

CGCGCGCCGGGGGCTGCGGGGAGTGGAACGCTGCGGCGCTGGGGGGGTTCAGCGCACGGC

AACGAGAGCGGGATGTCGAGGGACCCCTCGCTTCGGCCCGGCTCTTTCCCACGCGCCCTC

GGATCTTTTGTCACCATAAAAGTCAGTGAATGCCACGAAACGACGAACGGGAGCTGAGGT

GAGGCGGCGGCCGGCGGTGGGGATCCGGGAGCCGGCGTGCGGCAGCCCCTTCGGGTGCCC

CCATGAACGAGCGTCGCGGCCGGGGCTGCCGGGACACCGTGGGGCACCATGAGAGCGGGC

CACGAGCGGAGCCAGGAGTGCCTCCCGCCAAAGAAGCGGGACCTTCCCGCCGCCAGCGCC

GGCACGGAGGCGGGACGGGCGGGGGGGGCCCCGGCTTCGACCG

>c19131_g1_i1 len=242 path=[261:0-199 25:200-241]

ACATATACAGACTGGGAGAAGAACCCACTCCAACAGCTCCACATCCCTGTACTCAGGTCC

CCCTGGATGCAGCACTCCAGATAGGGCCTCATGAGGGCAGAGCACAGGGGACAATCACCT

CCGTCTCCCTGCTGTCACCTCTCTTGAGATGCAGCGCGGGATACAGCTGGCCTTCTGGGC

TGTGTGTACACTGCCGGCTGCGAGGGCAGCTCTGCTGAGAAGGGCCGGGGGATCCTGATG

GA

>c19131_g1_i2 len=242 path=[261:0-199 460:200-241]

ACATATACAGACTGGGAGAAGAACCCACTCCAACAGCTCCACATCCCTGTACTCAGGTCC

CCCTGGATGCAGCACTCCAGATAGGGCCTCATGAGGGCAGAGCACAGGGGACAATCACCT

CCGTCTCCCTGCTGTCACCTCTCTTGAGATGCAGCGCGGGATACAGCTGGCCTTCTGGGC

TGTGTGTACACTGCCGGCTGGGGGGGCAGCTTTTTCATCCATCAGGATCCCCCGGCCCTT

CT

>c19137_g1_i1 len=335 path=[1:0-139 141:140-334]

ATACAAATTTGCTACATGTATTTTAATAATGCACAAAATAATATTTAGTTTGGATGATTT

TTGTTATACAAAAAAAAAAATAAATACATTAAAATGTAAGAAAACTTTTAATTTTTCATA

TTCAAAAAAGTTAATGTTACTTTTTTTTTTTTTATTCTTTCTGAGTGTAAAATTAGTAAA

TGAAAAATTAGAGAATAGATAAAAACAATCTTTATATCCTTGTTTTTTACGCTACAACCT

TTTATTATGAAAACTTTAATATAATCAATGAATCTTTTTAGTAATTTAAATATTTTGTTT

TTTCTTGTAATCGTAGGTTTTTTTTTTTTTTAAAT

>c19137_g1_i2 len=215 path=[283:0-19 141:20-214]

AATATATCAAAGCATCACAATTTTTTTTTTTTTATTCTTTCTGAGTGTAAAATTAGTAAA

TGAAAAATTAGAGAATAGATAAAAACAATCTTTATATCCTTGTTTTTTACGCTACAACCT

TTTATTATGAAAACTTTAATATAATCAATGAATCTTTTTAGTAATTTAAATATTTTGTTT

TTTCTTGTAATCGTAGGTTTTTTTTTTTTTTAAAT

>c19146_g1_i1 len=203 path=[1:0-202]

GTATCAGTTTCCAGCTTGACCATGCTCTTACACTGAAAAGCTGAGACGTGCATATTTAAG

TTCAAAGCCTTTTTTCCAAGATCTGCACAAATGTAGATCTGAAGTTGTACTGCTCTTACA

GCACAAAATACACATGGTTAATATTCCTCCCAGGGCTACGAGATTTCTATAAAGCTGACT

TACACCAGCAGACACCTGCTAGA

>c19150_g1_i1 len=257 path=[339:0-256]

GCTGGAGAAAACACCCAGCCCTCGACGCTGACACAGCACACACAGCTCCCTCTTTGCTTG

TCCCTTTCTGCCAACGCCTGTCCTGGTACAGCCACAGGCCCTGTCACCCCTGGCCCGTGG

GGACGTGGCTCTGCACCGTGCTGGTTGCCCATTGACCCAGATAAGGAAAAGCAGAGTTTG

CAAACTGAGCAAGAGCGGAGCCTGATCCCAGCGCTGTCCCCGTTCCTTGGGGATGAGTCC

CATACTGCTCTGTTGGC

>c19155_g1_i1 len=206 path=[1:0-97 99:98-205]

GCAAATCTATCCGAGCAGATCTGAGTCCCTGTGCCGGCCTGTGCTGCACACGTGTGCTCC

GTGCTGATCCCGGCACCACGTATTCCTGATTCCCTGAGGCAGGAGCTGCAGGCTGCAGAC

CAACGCCTGGCACAGCAGCTGATGCGTCTGCGGGCACAGCTGAACCGGCTGAAGGTGGAG

CAGGTCTGCCACCGGCACAAGGAGAT

>c19160_g1_i1 len=219 path=[361:0-218]

TTTTCTCGTTCCAGAGCACCAATTGAAAGCAGGGATGCAGAAATCAGGCAGGGGCAGTTC

TGCAATCCAGTTTGGCAGGACTCTGAAACCGGCGACTCTCTGAAATTTAAGGAACATTTG

GCTGTTTCTCCACAGACATTAATTCACAACAAAGGTCCTTTAACGGCATCAGATCAAAAC

GGTGCTCTTTGCTGTGACCGTGCCCAGACCTCTGCTTTG

>c19169_g1_i1 len=389 path=[398:0-388]

GGGAGATTGAACTTCTTTCATCTATTTTTATATGCAAATTGAAGGCAAAATAGGAAAAAT

TTTTTATTGAATTTTTTCTTGCAAAATTAATAAGTAAATAACTACAAAAGATAAAATTTT

ATCACTTATAAAAGTTATATTCTTGATGAATAATAGTAGATGTATAATCACCTTTTAGTA

TTTATAGGAATTATGTTTTAAGGTTTCAATGAGAGTAGATACTAGTTTATATAAAAACCA

TGTGAAGTTAAAATATTTTAACATAATAAAAATAGTTATCTATTTATTATATATATTACT

AATTCAAGGATTTTAAATATTATGTAAAATAAATGTTTATTTTAAGTTATAAGTAGTAAT

TACATAAAAATAATTTATTGTCTACAATG

>c19176_g1_i1 len=257 path=[275:0-203 21:204-256]

ATTTCGGTCTTGAGTCAACACCTCCACCTGTATCGGGGCACGTCCTTGTCACAGAGTTCT

CTCTAGATCTGAGTCCGTGACGGGGGCAAAAAAGCACAGAGGGGAAAAAAAAAGAAACAA

GGAGAGAGGGGGAAGAAAAAACTGCTCCACCTTTTGCAACGCAGCTGGCCCCGGGGCGGG

CCCTGCGCTCCGGGAGAGGGGGACGGGGGAAGGGTACGGAGATAGCAGGTGGGTCTAACA

AAAAAAAAAAAAAAAAA

>c19182_g1_i1 len=430 path=[1:0-429]

GACAGGGTTGGAAGTGCGGGACATAGAGTTGGTCATGGCCCAGGCCAATGTCTCACGTCC

CAAAGCTGTGCGGGCACTCAGGCACAACAATAACGACATCGTCAATGCCATCATGGTGAG

TAGGGGTGTCCTCATCCCTTATGTTTTTGGGGGTACAGTCCAGCCCTGCATTGACGCTGT

GCTCTCTCCTGCAGGAGCTGACCATGTAGCGGCCGGCCCCGGCCCCCTGTATAGGTGCAC

AGTTCTCCTCGCCCTGATGTCCCACCTAACTCCACATCCCCAATTACCTGGTCCCCATCT

TGCAGGCCATACAGGGGCTTTAGGACCCTTCCAACCCCCAGGGAGAGGACCCCCAATCAC

CACGCAGGGAGGCAGGGTGCGATCCAAGTGATACATGAGGCCATTTATTGAGCATTAGGA

TGCGGGCAAG

>c19191_g1_i1 len=204 path=[213:0-172 25:173-203]

ACGCTCAGCTGCCTACTGCTATTCCCACACAGCTGCTGCACTTCAGCCTGAGCTGCTGCA

GGGGTGCTTTCTTCCCAAGGGTAACGCTTTGCATTTCTCCTCTTTAACTCTTGTAAGGTT

CCCATTGGTCCATTCCTCCAGCACGTCTATGTCCCCCTGAAAGACAGACAGATCACACCA

ACATAAAGGAACAAAGGGAAATAC

>c19191_g1_i2 len=205 path=[213:0-172 385:173-204]

ACGCTCAGCTGCCTACTGCTATTCCCACACAGCTGCTGCACTTCAGCCTGAGCTGCTGCA

GGGGTGCTTTCTTCCCAAGGGTAACGCTTTGCATTTCTCCTCTTTAACTCTTGTAAGGTT

CCCATTGGTCCATTCCTCCAGCACGTCTATGTCCCCCTGAAAGACAGACAGATTCCCCCA

GTATTTCCCTTTGTTCCTTTATGTT

>c19197_g1_i1 len=344 path=[476:0-343]

ACTCCAGCTTTTTATAGCACATCCGGTATGGACATTAAACGCATTCAGGAAATGCTCTTT

CAGCCCCAAACATCCCTTCCCCTGGACCAGTAAGCTGCAGGGGTTTGCAGTGCAGAGATG

CAGGACCGGATGTTTGGTTTTGTGACTGCTGCTTTCCTGGATTACTTCTATCTGTTTAGA

GAAAGGAAATTGAATAACAAAAGTAAATAAGTCTGTCTGCCAGGTGTATGAGCAGCTTGG

AGTGGCCTTTCCATTTGGCTTTTCTTTCTTTACCTTGCAAGCAGCCTGCATTATTTCTGG

ATGCAGCCAGCCTACCCTGTATTTATTTAATTTCCTTTCTCTAA

>c19202_g1_i1 len=201 path=[179:0-200]

CATATTTTGCCTCTGGTAAATCCATGTTAAGTATTCCCAACTACTTTCATGCTCACAGAT

GGCTTCCACAATGACTCACTTCTCATGGAATCATTAAAGTTGGACAAGAACTCGAAGATG

ATCAAGCCAGAATAAGGGGAAATGGCTTTAATCCAGAAGAGGGTAGATATCAGAAAGTAA

TTCTTTACTGTGAGGGTGGTG

>c19205_g1_i1 len=271 path=[297:0-270]

CTCCCTCAGAAGAGGCCAGCAGTGCTGTCTCCCTGGGTCTGAGTCCCCTCCTTCACTACC

TCCACTCAGACCGTGGATCTGCTGGTATGGTGACCAACAGCACCACTGGGAAGTGATTTT

GTGGGTGCATCAGAAGCCTTGTTCACCCTGTTGTTTTGTTTGAGTCATGTCTGGGAGACA

GCGCTGTCATAGCTGTCATGCTTTGGGTGCTGCCTGGGGTCTCTTTCTGTCTTCCAACAT

CAGTTTTGCCCTTCTGGTGGCTTTTTCTGGT

>c19208_g1_i1 len=245 path=[155:0-244]

TAAGAGTCACTTCAGTAAAGAATTTATGATCTTGCTATGGGGTGGAGTAGGTGATCTTGG

TGTTCAAGGTTACAATGTTAATGAATTTACTTATGACAAGCTCATTTTTTGTCTTTTTGA

TTAGGTGTGGAGACAGTCTTTATAATTCAAGAGATTTTTTTACTCAGAAGCCTCTAAGGT

TAATCTGGAACATCCCTTTTCTAGCTCTTCTTAATTACTCAATTAGCCTTTACAGCCTTT

TAAAA

>c19209_g1_i1 len=271 path=[1:0-270]

GAGCGGGCGTGGGGATGACAGCAGGAAGGCATGTCTGGGTGACAGGACAGAGGTGGGACG

GGAGAGAGGATGGCCCCAGGGTGAAAATGTGTGTTCATACAGCTGTGCTGGGAGCGGGAA

GGGTTTGCAGTGAGCTGAGACGTGGCTCTCTGTACGTTCCAGTTGTGCTCTCACCACCAG

GTATTTGCCTCCCTCCTTTGGGCGTTCCCATCCCAGGAGCCTGTGGAGTCACTCTTACCT

TGGCACACACGTGTGTAATTGCAGAGCTGGC

>c19214_g1_i1 len=224 path=[1:0-223]

GATTATTTCTATATTCTCATTTCCCTACACCATCATTTGTTGATCTATCTTTACAGTAAC

ACAGAGAAAAATCAAGCGAGCTCGAAGGTATTTTGTTAAATATTTGAAAATACATATACT

GGCTAAAGAACAGGAATTAGGACTTTAATTTGTAAAGAAGTGAAGTGCAATTTCAAAGCA

CTTGGATAGAATAAGAAGCAGTTCTTTTGTTGTTGTTTGTTGTT

>c19224_g1_i1 len=240 path=[372:0-239]

GTTGTGGTATTATATCTTGCTCTTCCCAGTGCTTCTCCAGTACAGAGACATAAGTGGAAG

CAAGTGGTGTTACACAGTGTGACATGCTTAATTGAAATAATTAAGAGACTTTTTGAGACT

TCTTGATCAGGACTCCTTCAGAACTCCAAGAAGGTGTTAAAACTAAACAGTACTTTTCTT

TCATAGCAAGCACACATCCATTTTGTATCTTTCTAACAGAAAAGGGAACACACAAATATA

>c19232_g1_i1 len=242 path=[390:0-241]

TAGGGCCCTAGCACTTCATGCAGTGAAGGGTTGAGGTTACGCAGTTATTCCAGGTTTTGC

CTCCCATGCGGCTTTGTGACTCATGAGGTTGTCCTGTCTTTGTGAGGAAAAAGTACATCC

TTCTGCTTACTGTGCTAAAACACCGATATTCTGCCCAGATTTTACCTTATTGGCAATCCC

CGTTCACGCTTTCCTACACTGTAGGCTATTCCTATGCTCAGGTGCTATCCTTCCACACCA

GG

>c19233_g1_i1 len=225 path=[1:0-202 522:203-224]

AAGAAAAGCTGAAGGTGCGTTCCTGAAAAGTAAGCGAACTGCTGTGTCCTCCCAGGTGAG

AACCTCCTGCTTAGCTCTCCCCATTGCCATTTTTCTTATAGCACCTTCCACTTCCAGTAC

TTCTAATGATCAAAGACATCCACCAAACTAATGATATTATTTGTGGTAAACCCCTGTGAC

AACCTGTGACCCTTTTCTTTACCTCCACATTTAACAATGTGGAGG

>c19248_g1_i1 len=266 path=[1:0-180 182:181-265]

AACATTTGAGGTAGCTCACACCTTTGTTAGACAGGAAACAATGTTGAATTCCTATTGTAC

TTAATGGAGAACATTCGATAAACAGTAATTTCATTATCATCCTCTAAACGTTTAGGATAC

ACACTTGCTTTCCAGTTACTGAAAATACTCTTTGAGAACCAGCAAGTATGAACTTCCAAC

ATTTTTTTTGGTAGGACTTTGAGATTACAATATGAGATTCCTCAAGTTACTACAATAAAA

AACAACCAAATTGAACTGACTTTGCT

>c19250_g1_i1 len=269 path=[1:0-268]

TGGTTTCTCCAGAGGTAGAAAAAAGGCTCTCAAAATACAGTGGCTTCTATACTAAAATCC

CTTAAAGAAAGCTATTCACAGGCTCATATTCATAAGCTGTCAATAGAGACTATGAGCGTA

GCCCTCAATTTTCCATACGTAAATATTCCAATATTACTGTAATGCATCTATCTGAAAAAA

TAGTGCTATTTACGAGAAGGGCACTGATCATACCACAGAATTCCTACTTCAAATGCAAAT

ATTTTGTGTAAGAGTCTAAGTGTGAGAAA

>c19254_g1_i1 len=202 path=[426:0-201]

GAGTTGCTCTGAAGAAGGACAGGAGGAAACCCAAGGTATCCCTGCTGACAGAACTCCCAA

CTTCTCCAGCCACCACCATTGCCCCTCAGTGGGGGACCCTCCCTTCACTCTGTTCCTCGG

TGCACCCCACGTAGGTACCCAGCAGGCCCAGCTCCAAGGACCGCCGCCACAACCAGGCCG

GGCTCTGTCAGCCGCAGCCCCG

>c19255_g1_i1 len=205 path=[1:0-113 115:114-124 126:125-204]

GTGTTTGAGGGGTCTGTCAATATGCAAAGCTGGAATTCCAGCACGGGGAGAAATCTCAAC

CTAAGGAAGTCAGAAATAGGTTCACAGGGCTCCCCTCAGCCCCACCTGCAACAAAGCACT

TTCTCAGGCAGAACAACTTTATATACATCTGCTAAGAAGACCATATGACGGCTGAGCTTA

CCAACGGAGCCTCCAGCAGAGAGCT

>c19256_g1_i1 len=883 path=[897:0-813 1711:814-846 1743:847-882]

TTTTCATATTTTTAAGTAAATAAGCAGTTTTGTATTAGAGATAAACATTACCACATGTAG

TAAGAATTGAGATTTCTTTGTTTTAAATAGTATCATTATGAGGGGGAAGGTGCAATCATT

AGAGATGCAGCAAAATCCATTTTGCTGTATGTAGGTATGTTCTAGTTCCTTCTTGTTCCA

GGGGTTTTCTTCAGGCTTTTGTTAATGTTTTAACTTAAATGTAGCTGCACCATGATGCCA

TTATGACTCTGTAGGAACACCTAACTACTTGCTAAGACCTTTACAATAGTGTGAATCAAT

CCGTTTGTTATGACAACTCTTGAAATTTCTTTGAAGAGCTTTTAAAGAGAACCCTTTGAG

CTTTTCTGAAATTTGGATATGTATTAATGGACATCAGTGCAGTGTTGGAATATACAAAAC

TACTCATACCTGTGTGAATTTCATTCAGGATGTCATCTTTTGTAGTTCTGAAAAATTTCT

TTTCAAACAACACCAGTAGTATGAGTTATTCATAAAAATCATCTTATACCACTGGGATTA

TATAGTTAGGGTTCAGTAATTGCGTATGGCCTTCTTGACTTAAGTGTAACGTAAGAGAGA

GTGAGAGCAAGCACAAGTACCACCACTGAAGGGCTTAAGTGTGTTAGGTGTCTCTGTAAC

TATTAGTATTGGAAATTAATGTAAATGATTCTTTGAGACCTTGAAATGAATTAAGCTGCT

CAAGTTGTTTTAAGTCTGATAGTTTAAAGATAGAGAACTGCAAGAAATTTTTGATTGCAA

AACCAAGTAAGTAGTGAACAGATACACACACATACTGTAGACAGGAAATATCTATTACTT

GCATAGTCCATTACTAATTGTAGTTTTCTACTATGGCACCGCT

>c19264_g1_i1 len=333 path=[1:0-332]

ACTGATACAAAGTCTTTGTTTAAATGATCTACAAAATGCCAGCGTAATACAGGTATGATA

AAGAGAAGTTCTTAACACAACTTTTGTATTTACAGCATTATCAACAGCACTTAAATCTGC

CTTGGAAATACAAAGAGCATGTTTCTATTTTGAACTAAGCATGTTTGACAGCATGCTTAG

TTCTCTCATTTACACCACACTGCCGTAACATTAAGGGTTTACCATTAATCCAGGTTTTAC

TACAAAGCATCTCTGTCAAACACCGGCAAGACTGCCCAACATCTGTCATCCAACTCAGAG

GATTAAGTCTTTTTTTATAGTGAGATTCTTGTA

>c19264_g2_i1 len=237 path=[835:0-236]

TTTGGACTGAATTCATGCCACAGAGGTTGAATATGACAGCTGGCAGGAAGCGGAGGGGAG

AATACATCCTCTCCCTTTCCAGTCACCACATTTCTGTTGCCATGGTGATTAATTCTCAAT

TGAATGGCTGTCTATACATTTCTTTTCAGTAAAAACTAAGTAAACTTAAATTGAAAATAT

GCATTTGACTAAAATAACAGACTCCACATATTCAACAGGTTCAGTAAAAGTTCTATG

>c19268_g1_i1 len=323 path=[252:0-249 502:250-322]

TTAATATAATATTTCTTAAAATTTTAATGCTTTATATGTTAAATTAACATTATTAGAAGT

ATACATTTTTAATATTTCTATATATGAAACATATTTATTTAATGCTTTTTATTGATATAA

AAACAAAATAAAGTTATTACATATTTTATGTTTGTAAAGTTATATTAATATTAATTCTTT

TATATTTAATTTTATACATAGAATGTAAAAGATTTATGTTCTATTTTGTTTTAACAAAAA

ATGAATAAACAAAAAAAAAAAAATTTGTCATATATTATACAGAATATATTTAAACTAAAT

AATTTTTCATTTTTTTTTTTTTT

>c19279_g1_i1 len=296 path=[456:0-267 723:268-295]

AGCATAGACCATATAAATAACTCCCTTCATTAACACAGAATCAAAAACTGTTGCTGGGTT

TTCAGAACTATCACTGTTATGAACTGGCTTTAAAGGCTTTTTTTCTTTATCTGCAGCTAC

AACTAACCAGTGTTCAGCACCAGTGTATTTTTATTCAGTGCTGACACATGCACCTTGTGT

CATCTCAGATGCTCTGGAGTATTCCACTTGGCTTGCCCATGAAGCTCCAGCAGGAACAGA

TCTCTTCTAGGTTTTGTGCCAGCTTGTGCAGATGTCTCTCATACTCTACAGCCACT

>c19279_g1_i2 len=297 path=[456:0-267 179:268-296]

AGCATAGACCATATAAATAACTCCCTTCATTAACACAGAATCAAAAACTGTTGCTGGGTT

TTCAGAACTATCACTGTTATGAACTGGCTTTAAAGGCTTTTTTTCTTTATCTGCAGCTAC

AACTAACCAGTGTTCAGCACCAGTGTATTTTTATTCAGTGCTGACACATGCACCTTGTGT

CATCTCAGATGCTCTGGAGTATTCCACTTGGCTTGCCCATGAAGCTCCAGCAGGAACAGA

TCTCTTCTAGGTTTTGTGCCAGCTTGTGTTTGAGTGGCTGTAGAGTATGAGAGACAT

>c19280_g1_i1 len=414 path=[255:0-413]

TTTTTGTTTTCTTTTACCCTAACAGCCAAGTTGAATTCAAGCTGGGCTTTTGTCTGTCTA

ATTTTTTCCCTGCAAAAATGATTTAACCAGGGGTAGATTATCTTCTGTGACTTGCATAAG

GCTGTTCATATAGGAAATGCCAGGCTTACTGCAAATCTCTTTTTTTTCCACTGCAAGAAG

TAAAATCCCACAGTCCAGTGAGCAGGGCTTAGGGAAACAGGAATTAACCCAGAATTTCCT

CCAAAGAAGCAAAAAGAAAGATGAATGGCACTAAAACTCAGACCTCCCATCTCTATTATG

GTTGGAAAACGTGGTACAATAATGCCACGACTCATGGCAGGATCAGAAGTGTAGACAGTT

ACTCCGGTCTTCAGCAAAATGGAACTGGCATGCATCGTTCCAGCTCCTACTCTG

>c19285_g1_i1 len=266 path=[447:0-85 532:86-265]

GTAGGGTAAGCCAACCCTACACAGAACACCTTTTATAAGAATACTGGAATCACAAACTAT

CTGAGGTTAGAAACGTTTCTTGTCATTGCCTAATACTGTCAAGTCCAGCCTTTCCTGTAC

AAGGAATACTGCACAGTAAGCTAGAACAGATTGCTCAGGACCCTGTCTGATCTCATTTTG

AACACCTCCATCAGGGAATGCTATAAATCTTTTAATGATCAGCCACAGTTCAGGAATAAT

GGTCTTGTGTTCAAGGTAGCTCTTCT

>c19287_g1_i1 len=234 path=[181:0-233]

AGTTTCTCAACAGAAAGCCCAACTGATCAAAAGAAGAAAGCAATTTGCTGAACGCCCAGA

ACATCAACACGGATCTACCTCTGAGCTGGCAGTGTGGGACCGATTGGAAACTGTCTATGC

CAGTCAGCCAGACCTAAAGGAGGAACCATTGAATTCCTGGATTCATTGACAAAAGCAGGT

TTGTGAGACAAGGAAACCGTAAGGCAAGGGATGCAGTAACTGCTACTGACAAGG

>c19288_g1_i1 len=211 path=[1:0-42 44:43-210]

TCTCTATCCTCACAGCACTCCTGTAACACTATAAAACCCGATTCACAACAGAAAACAAAG

GTGTGCATATGCATCCAGTTAACTTACTTATTTTCTAGGTCAGCTTTAGTTGCAGACACT

GAACTGACTCGTACAGTCAGACATGTCTGGATCAGTAGCAGAATTGTATTCATGTTTTTT

CCTATAAGGTCTTTGTAGCCCGTTAAAACAG

>c19293_g1_i1 len=227 path=[291:0-226]

GAGAATAATTCATCTTTATTAATGCCTGTAGAACATAGTAATTTAAATAAAGAATGTTTT

TGGTTTTTCTGTGATCCAAAAAATTTTATTGCTTTATATATTCCGCTTGTTGTTGTTGGA

AGGTTATTGTTTTTTATATACCTTTACAAACAGAATACTCATACCAGACCAGTTTCAGGA

AAAAGTAATCCAAAAAAAGATATAGGAAAAAGGAGAACGCATAAAAT

>c19296_g1_i1 len=213 path=[43:0-15 21:16-212]

TTCTCATGATTTGCTTCAGCCTCCAGCAGGGACCTCTACGAGGAGTGCATTTGCTGTGCC

TTTGCTGACACCCTCTTCCCTCCCCCCTTCTATGTCGTGTGTTCCAGGCAGAAATGCGCC

TGCAGGACCAGCAGCTGGCCAGGCAGCTGATGCGCCTCCGCAGCGACATCAACAAGCTGA

AGATAGAGCTGCAAAGAGCTGGGAAGGGTATTA

>c19302_g1_i1 len=316 path=[315:0-315]

GCGTCTTCAAATCAAACATACGCAGCTAGGTGCTGTTCTCTAGAGCTCACAGTTGTGCTA

TTCCTGAATTTGCTCCTGTGATTATAATCACTTTTCCACTCAAGTCACGACCCTGAAGTA

TTTCCATCGCAGTACTGTTTCCATCATATTTTTGTCTAGTAGGTGGCTTTGCTGGATTAT

CTTCAACTGTAAAGGCCAATCTTGGATCCAGATAGGTAGTTCTTTTGTTTATGTGGTCTA

CAAAATAGACCTGTCCATTTTCATCAGTCTCCTGCTCCCATCCATATGGCAGACCTCCTG

CAACACGTTTCCTCTT

>c19311_g1_i1 len=238 path=[1:0-237]

TAAGTGTGAGACAGTGTGTTTCATCAGTCACGCTTCTCACTGTAGGAAAATACCATATTT

ACCTATTTCATTTTGCCTTTAAGTGTATGAACCTGAGGAGTGTCTGTGGGATATATTGTA

GGCAATTGAAGGTATGCATCCTAAAAGATATGCCTGTGAGAAGGTTTAGGAAAACAGCAA

CTCTGCAGTCGGTTCCATAAATGTCACAAGTCATTACTAACAAAATAGACTTTCTTGG

>c19312_g1_i1 len=215 path=[236:0-143 22:144-214]

GGGCTGTGGGGTGGGGGGACTGTGGGGCAGTGGGGACCCCAGTTCCCCGCCTGACCCCCT

CCTCGCTGCCAGGCGAACCCTCCATCCTGTGAGCGCGCGGAGGACCTCGCCAGCCTCCTC

TACCTCAACGAGTCCAGCGTCCTGCACACGCTGCGGCAGCGCTACGGCAGCAACCTCATC

CACACCTACGCCGGCCCCGCCATGGTGGTCATCAA

>c19317_g1_i1 len=265 path=[1:0-176 178:177-264]

CCTAAACTTTGTGTTTGAGTGATTAGAATATGTATATACGTGTACATTTATTTTTCTATC

AGTGAGTACATACAGCTCATCTGTGGAAGATGACTTGTGTGTGTACACAAAGCTATTCTG

ATTAAAAATATATATATCAACAAATATTTCCCTGCTGGTACAGGTGTACGGCACTGCTAC

CTATCCGAGTGGGCTGCACCCAGAGCTCTCACCTAACAGCGAGCGTTGCTGCTACAGCAG

CCTGCTGCTTTTTAGTCGCACCGGG

>c19324_g1_i1 len=273 path=[251:0-181 433:182-272]

TGAAGGATCTCCTCCAGTCTATGTATTCAATATAATTTTCAGGAATAATACATTAAAATT

GTCAACAAAATACAAGATTAATTTTTAAGATTTTGTGAATATCACTGTCTCCTCCTCTGA

CAGCTGCACCTACAGAAGAAAGCATCAGAAACAAAAACCAGACTATGCCATTAACAACAT

CTCAAGCAAACAGGTTACCCCGTGCTGGCTGGCTAAAAGCACGCTGGAAATCCTCGATGA

AGTGTTCATTCAAGGACCACAATTGCTGAAGAG

>c19324_g1_i2 len=210 path=[251:0-181 645:182-186 650:187-209]

TGAAGGATCTCCTCCAGTCTATGTATTCAATATAATTTTCAGGAATAATACATTAAAATT

GTCAACAAAATACAAGATTAATTTTTAAGATTTTGTGAATATCACTGTCTCCTCCTCTGA

CAGCTGCACCTACAGAAGAAAGCATCAGAAACAAAAACCAGACTATGCCATTAACAACAT

CTGCAGGGTTTGTGTGCCCGCACAAAAGCC

>c19328_g1_i1 len=346 path=[324:0-345]

CACTTGTCTGACGAAGAATCAGCAATCTTACCAGTGCAAGGAGACCTTAATTCACTAAGG

ACTACTTTCCTCAGCAGGAAGAAGTGTACTAAGTTTAACCAATCAAGTGGTAACCACATG

ATAATCTGCTTAGAGTCAAAATGCCAAAGTATGAGTATTGAGTGTTTTTCTTGGTAATGT

TCTCTTTTTTTTAATGAAGCATTCTCAAGTTGTTTTTAAACTTTCTCCTGAAATAATTCC

TTGTCTATTTGTCTCTTGAAGCATTTCTCCTGAAGTATTCCTAACGCCAGTTACTCAGAG

AAATGAATGCACATAAACAGCTCATATTCAAAACACTTTTGGAAAG

>c19328_g2_i1 len=205 path=[647:0-204]

TCTTTGGGAACTAATAACATGTTTTATATTGCTTCTACCAGCATTAATGAGAGACTGGTT

TGAGGTTTCATAAAGGTTAGTGGTGGTCAGGCATTCTGTATGTGTAGGATATGAACAGCT

GAGATTTACTGTTGTCAGAGATGTTTTATCCAGGGAAATGCAAGATGTCTCTTACTGCAG

TCACTACAGCAGCGGGAACCGCGCG

>c19346_g1_i1 len=339 path=[611:0-338]

TGAGATGCAGATGTGCCGGGTACGACAGAGTGAATTGTCGAAGTGGGTTCAGACCGAGCA

CGTTTGATGCATAGAGAACCTGATATACAGCACTGGTATTCTCATAGCTGTGACTACGCT

GTTATTTGTACTGAAATTGCTGGTAAATTTTCATCTACTTGCACGATACTGAATTAAGGC

CCACAGATGGCACTGAGCAGGAAGATGATTGGTGATTCTGCAAGCGCAAAACAAAGGTGG

CTGGGAGGACCATATTTACAGCTGCAAAATGCCAGGTACGTTTTCACCTCCTGGTAAAGC

TTCAGAAGGCGTTGGAAGTGCTAGCAAGGCAGTCCCCAA

>c19350_g1_i1 len=202 path=[155:0-201]

GCTCATTTGAGCTTGTCCACAGAGCCTGTCCTGCCTGCGTTTAACAGCAGTTTTGTCACT

TGTATTCTTTCCTGCTTTAAACCCAGGAAAGACATCAGAAAGACAGAGATATACTACCCT

CACAGCACACATTTCAAGAAACTATAACCAGCTATTTCGTTGTGCACAAACAACTACTTT

CTCTTGATAGTTGGTTAGCAAA

>c19352_g1_i1 len=249 path=[271:0-61 23:62-80 46:81-248]

TCTGCGCAATGGACAACAGGAGACCTTTGCTTCTTTCTCATCATATGGAATCAGCTGTTT

TACATCAGCTTAAAGGTACTGTGACAGAGGAGAGAAAAAGACATCCTTGTGCTTCTGAGA

CAGAATTCCCATTAGATGAGTAGTATATGCAATGCATTAAAACATCCCAAAAGGTCTTCG

AAACAGGAAGAAATCAAAGTCACAAAGACAGAATATACAATGACCAATTTATATTTCCTG

TGAAAAATG

>c19355_g1_i1 len=660 path=[163:0-659]

GCCAGTGGGGTCAGTAGAATCATTGGGGTTAATGGGGTCAATGGGGTTAATGGGATCAAT

GGGGTCAATGGGGCCACGAGGTGGGCGTGGCCTGACGTGGCCCCGCCCCCAGGAAGCACG

CCATGATCGCCACCAACGCCTTGGCCTTTGTGGGTGGGGCCATGATGGGCGCGGCCAAGT

GGGGCCCCTCCTACGTCCTCATCATCATTGGTCGGTTCCTGCTCGGCGCCTACTCAGGTA

TCGGCCAATAGGAAGCGAGTTTGGCCCTGCACAGCCCCGCCCCTTAAAAGGGCGAGGTCT

GCTCTTCGAGGGGTCATGCTTGATCTTAAAAGGGCAATGCTGTCCCTCAAAGGAATGGTT

ACATCTTAAACGGGTGATGTCGCCTTTCTAAGGCATAATGCCGTCTCTTAGTGACAGCAC

AGCTTCTTAAAAGGGCTATGCCACCTCTTAAAGGGGCGATGTCATCTCTTAAAGTGACAG

CACCACTTCTCAAAGGGGCGATGTCACTTCTTAAAGGGATGATGTCTCCTTTCAAAAGGA

TGATGTCATCCCTTAAAGCGACAGTATCACTTCTTAAAGGGGTGATGCCACCTCTTAAAA

GGGCGATGTCACCTCTTAAAGGGCCAACACCATCTCTTAAGAGATTACATTGCACCTTTA

>c19358_g1_i1 len=309 path=[1003:0-308]

AGGCAAGAAAGGAGGTACAGAAGGCAGAGGAAGGCTGGAATGAGCTGAAGATGAGATCAA

GGGGAAAAGGCTTTGTTGGCATTTGTGAAAGCTATTAGTTGCTGAGCCACTGCCTTCATT

GTAGATGAATAAAAATCCTCTTCAAGTATTCACCCAAGGTAGTAAATATCATACTAGAAA

CCCTTTCTTTTTCCCCACCCAACTCACAGCATTAAAAGTGATTAATTTCTATCACAATAA

CGAGTCCTTTCCCTGCACTGACTTCCCCACACACCAGACCTTACTGAAGGTAAGGTCTGG

TGTGTGGGG

>c19358_g2_i1 len=524 path=[502:0-523]

GTGTTTGATTTTTTTTTTTTTCGGAAAAAAAGCTCCTTAAGACGCTTTAATTCAGTTTCC

TGGATCCCATGACTGAGCTCGGCCTGTGAGCGGGGTCCCATGACCACAGCACGCAGGCCC

CGGCCATCAAAAGGCTCCTTTATGCCCCAAAGACTTAAACCCCTCTTGGCTGAGACCCCC

AGGTTCCCCATGCCCAGCCTGGGCTGCATCCCCCCACACCTGGGGATGTGTCCCATTGCC

CCGCTTGTGGGGATGCTGTGCCCGGGACCCCTGAGGTGGGTCTGGCTCCTGCACTGTGGG

GACCCCACATCTCCCTCCTCCCATCACTGCGAGCAGCAACGGGCCCAGACCCACAGCCCT

CTGCTGGGACTCCCTCTGAAGGAGCACAGCCCACACCTCCCGGTTGGCTCTGGACCTCAG

GGGGCTGGGCTGAACTTTTCTGCCTTTTTGCTCTTCTTTTTTAACTCCAGACACCTCGCA

CCAGCCTGTGGGGCTCCAAATCCTCCTGTGGGGCTCCCAACCAT

>c19361_g1_i1 len=222 path=[292:0-136 16:137-221]

TAGAGCTCTCTTCTTTTCTATGGGCCTGTGACTGAAGAGGGCATCCTTCCAGCTTGTTGA

TCCCCAGGTCACCTGACTAGATTTGACTGGCAGTGAAAACAGGACAAGTTTAGCCCCCCC

CATTTTTTTTTGTATCTAGTGGCAGATTAAACCAGGATGTTATTTCACTGCTGGCTCCAG

CCTTCTGTATCCCTAAGTATGTGCAGCCAAATCCTATTGGAA

>c19362_g1_i1 len=273 path=[1:0-188 190:189-230 405:231-272]

GGCCTTGAATGCCTACAGGGATGGAGCATCCACACACTGTGCAACCTGTTCCAGTGACTC

ACCACTATCCAAGGAAAAAATTTCCTCCTAAAATTAATGACTCTAACACTCTGCTCAACA

TTTTAGTTTTACCTCCCGAAATTTTGAAATTTTCCAGCTGAGAACTGAACACATTAGTGA

AAAAAACAGAGCTTTAGACTTCAAGTAGAAACTGATATCAATTCCTTCTCATTTTGCAAA

ACATTTAAAATGCTAACAGCTTGTTCAAATTAA

>c19380_g1_i1 len=252 path=[1:0-251]

GCTTCTCCCTGCGGGGGTTGGGTCGGGCCCTTTGCGGGGGGTGGTTGGTGCCCTGCTGGG

CTCGCCCTGTGGGGGGCGCTGAGGCCCCGCCGGGCTGAGGGTTGGGACGCGGACCTGGGG

AGTCTGTGGTGTGTGGGGGCTGCGGAGCCTTCCCTGGGCCGGCGTCCTGGCTTCTGCCCT

GGCCCCTTTTTGACCACGGGGATCAGAGGGGGAACGCCCAGAGGGGGAGGGACCTTTGGC

CGCTGGTTCGGC

>c19394_g1_i1 len=849 path=[1:0-848]

TTTTTTTTTTTTATAAATTTTTTGATTAACAGTTTTATTATTGTCTTTACTTTTTTTTAT

AAACTCTCTTTTTATTTTATTTTCTATTTTGTTACTTTCTAAATTTTCATCAATTTTCCT

TATAATACTTATTAATGGCTCATTTTCTTTTTTTTCTCCCTTAGAATTTATTAATGTTCC

ATTTTTATATGTTTTACTTTTTTCTTCAACTCTTTTAATTTTAAGAATATCATTATTTAT

ACTTACTTTTGAATTATTTAAAAATTTATGAGAAAAATTAAAATTAGGATTTTTCCACAA

ACACGAGATATTGTGTTTAATTTTATTCATATTTCTTTCATTAAATGTAGACTTATTATT

ATAAGAACATTTGGCATATTTTATATTAGCCTTATTTATCTTTAAATTTTTATGAAAATC

AAAATATGGATTATTTATCTTTTTAATTTTTTTATAGCGATTTATGTAACATTTACCATC

AATACTTGTAAAAAATTTAAATTCATCAATATTGGAATCATAATTTTTTTTTTTAATGTT

TTCTCTGATTAGAAATTTTCTTGAAATATTATTATCAAAGTTGTTACAAGACAATGTGTT

TAAGAAACTGAACTTATTGTGTTTAACGCTTTTTGATATATTATCTAGTAAAATATTATT

TGAATTATAGAAACTTTTGTAATTTTCATTAGAATGCCTTTTTTTATAAATAAAATGCTC

CTTAAAAAATTCATTTAAAGTTTTATTTAAATAATTATTACAAAAATTAAAGGTTACTTT

ATCAATATATTTTTCATTTAGTAAATTATTAAATTTTTTTTTTACTTTTATTAAATTATA

ATAGTAATG

>c19394_g2_i1 len=214 path=[1653:0-213]

AAAAAAAAAAAATTTTATAAAGAATGTAGAAAAAAGGGGGAAAAATACTGCATGTATAAT

TCTAAAAGTGTTGATATAGTAGATGAAAAAGCTGATATTATTAAAGAAAATATTAAAACG

GAAAATGATATATGTAAAAAAACTTTAAGTAAGGTAAAAATATATAACTCTATAAAAGAA

AGAGAATTAATTGATAAAAAAATGAAACTAAACA

>c19395_g1_i1 len=269 path=[401:0-268]

CTGCAGCTCATTGCTTCCTACTTCCCAAGCCCAAATCCTGGAGCGTGACCCTGGTAATTG

AACGCTAATTAAGGAGGGAAAACAAGCAGCACCCTGCATTTGCAATCCGCCGAAGTAACA

TAAATGAGATCTAAATGGTGACCTTTTTACCGAAGCAGGGGTGTTAACCAAGCATGACAA

ATGGAAGGGAAAAGGGAAAAAAAAAAATAAAAGACAAAACACTGCTGCATTTGGTAATCC

CAGCTCCTCTGTGCACAGCTGTGGTGGGG

>c19401_g1_i1 len=235 path=[213:0-234]

AACAAGAAAAACACCTGTCCTGGCACCTGTGCCAGCACAGAAAACCGTCTGTTTGCAGTC

TGGGAGTGCAGGGAGCTGTTCTCAGAGTGCTGCTAACACTGGAGGCTGTATAACACTGTA

TTGGGTTGTCTGAGCTTTGTGTAACTGTGTTCACTAATCATAGCCCTGTACGCATCTGTA

TGTTTTGGAGTGTGTATTAAATGTAAGCCACTGCCTTTTTCTCGTGAGTTTATAT

>c19402_g1_i1 len=350 path=[319:0-349]

AAAAAAAAAAAGAAAAAAAAGATTTCCACTAGCAGAACATTGCATGGAGTGAATTTCCTT

ACAACTAAATGGCCAAATGCAATGCTGATCTCCAGTCTCCAACTAACCAATACGGTGTGT

TTGGTCAACAACCTTTTCCTACCAATGGCAATTTTTTTTCCTTATTCTTCATTGTACTTC

ACCATTAAAAGATGAGCCCCAAATTCTCTGGTGAGATTCCTGTTGGCTGAGACCCAGACC

CCCAAGCTACATCTCAGCATACCTGCACTGAGATCGACTCCCTGTTAATTGCTTATTTTT

GAAGTGTGGTTTGTCTCTGTCATCTCACAGAAGATGAATAGTACTGATGT

>c19403_g1_i1 len=201 path=[425:0-200]

AAATTAGAAAGGGAGGTTGACATGCTGGTTTGAACAACTTCAATTCAGAGACTCCTGGAG

AAACTTAGGGAGGGAACAGGCTGGCTGATCAACAACCATGCTGGAAAGAACCTGGGGGCT

CCATGAACAGTAAGTTGAATGTGAGCCAAACATGAGCTCTCTTTAAAAGTAAAGCAAGCC

ATATGCTGAGCAACGTCAGGT

>c19412_g1_i1 len=208 path=[348:0-207]

ATATTTGAATGAAAGTATGCCCTGAGCTCTAGAGCCACTACAAAAACAGAAGAGAGAAAT

TGTATGCTTCATTCTCTGCTGGATCCTGTCACGTTGCTGGAATCCATTAGAAGTAGAAAG

CTGCTGCATGGAGTCCAACATGATGAGATGAGGAAGGCACTGAAGGAGAAGGCAAGTTAA

ACCTATTGGCAGAGGTAAAAGACATCCA

>c19416_g1_i1 len=220 path=[398:0-219]

GCCAAGAGGGCGGAAAGCACACTCAGCCTTCCATGAAAAGTGTTCTGATGTGTGAGGAGA

GGCAGACAGGGGGATGCAGAAAATAAATACTTTGAATTAGGTCTTTTGGAATATCCTTGC

TCCAGGAATAGTGTCAGCCTAATTCTAGTACACAAACCTCTCTATTTGCTTCCAATTTTC

TCCACTGGTAACGCTATATATTATCGAGGACCGCACACTT

>c19427_g1_i1 len=257 path=[16:0-256]

AATAAAGGAAGGTACATTTCTACTGAGATGGTAGTGAACCTCTGGAATCTGTTGCCACAG

CAATGACCAGTAACACCTGCCACAGCACCAGCAGGGTCAGAAAGGGATTAAACAAATCCA

GGCACAAACAGGGAACAAGTAAGAATGTTCCTACTAACATCTCAAATACAAGAACTGTGT

CTGCAGGAGACAGCATCGCAGCAGCCTGGCACTTGCACTCTGTCTGAATAACAATACATA

TAGGGCTAGAGAAGAAC

>c19444_g1_i1 len=267 path=[245:0-266]

GGGGTCAGCCTCTTCTTGCAGGTAACAGTGACAGGATGAGAGGTAGCAGCTTTAAGTTGT

GCCAGGGGGAGGGTTCAGGTTGGATATTAGAACTGAATTACTAACATCTTGTGGTAGTGG

TTGAGCTATGAGACACCTGGAAGGGGAATGCCTGGGAATGGGCTTGTCATCCAGCACAGC

ATTTAACTTTTAGGTGCTTAATAGTAGGGAGCAGCATGCTAAAGTCTTTTAAGATTTTTA

CCTACAGTGTTAGTATTACATTGTTAA

>c19445_g1_i1 len=477 path=[155:0-476]

CGACGTCAGCGCCACCGTGCGGGGGCAGCGCGCCGTCATCGTCACGTTGGGAACGCGCGG

AGACATCGGTCCCACCACCGTCCTATCAGACAGCACCCGCAACATCGTGGCCGCCATGAA

GGAGCACGGCGTGCGCAAAGTGGTGGCGTGTCTGTCCGCCTTCCTCTTATGGGATCCTGA

GAAGGTCCCCACGCGGCTGCGGGCGCTGACGGAGGACCACGCGCGGATGCACGCCGTGCT

GAGCGGGGCCGGGCTGGATTACGTGGCCGTCATGCCGCCCCACATCGCCGACGACAAGCC

GCTGACGGAGGCCTACGAGGTGACGGTCGGTGGCACCGGCGGTGGCTCGCGGGTCATCTC

CACGCCGGACCTGGCCCATTTCCTCGTGCGCTGCCTCAGCACCACCGAGTTCGACGGGAA

GAGCGTCTACGTCTGCGGGCACTACGGCTGAGGGGGGGCGGCGGGGGGCATTGGAGG

>c19447_g1_i1 len=204 path=[1:0-129 446:130-165 389:166-203]

TTTAAACCATTACGATGCTTTATCAGAATTACTCCATCCACACGTATCCCAGAAAGGAAA

TCGCGTACTGCATCTTCCGTTGCAGAGTAAGGTATACCACGTAGAAATAAGTAAAGATCA

TCAGGGTTAAATGCATGAGAATCCTTTCTTGACTGATAGCCTGATTTAGGCATACCTACA

TCACCTTGTCTTGTGCCATTGGTG

>c19448_g1_i1 len=222 path=[306:0-221]

GATGTAGGAACTTTCTGGTAGGTACTTTTCTACACTAATATCTTAGACAGCTTCTTGTTT

CAGAACTGCAATTAAACGGTCTGCATCAAAATAACTTCCTTTTGAAGTTAATCCTCTATG

ATGATATTCATCCAGAAGTTCTCAGTCACAGAAAGAATTTTCAGAGGACAGAACGTTTGA

AATTTTAATGTAATAAAAAGTTATTCTTAAAGGCAAATAGCT

>c19451_g1_i1 len=211 path=[1:0-210]

AGTAGCAGCTATTGGGCTTCGTCCTGAGAAGGGACTGAGATAAGTAGGTGTAGCATAGGA

TTGCTATGATACCCCAACAGTCCTGGGTTCCCTTAATTCTTAACTTAGTTTGGACAAAAT

CTGCCTTAGAATGAGCGTAAAAGAAAGGAGTAGAGTCCAATTCAGTGGTTCCAGTGGGTT

TGAAAATAGCACACAGCCCCCCTCAAACACA

>c19457_g1_i1 len=212 path=[277:0-211]

AACTGCTACAGATCAACAACAGCCCCAGTGTCCCATCCCTCCTGTGCTATTCAGTGGAGG

GCAAGAGTTAGAGAAGTTGGGAATGAAGAAGTGAAGTCGATCTGGGAATAAATGGAGAGG

TGAAGGAAAGGTGTTTTCATTAGTCTTTGTTTCATGCTATCCAACTTAATTAGCAGTAAG

TTAAATTTTCCCCAAGTTGAATCTGTTTTGCA

>c19459_g1_i1 len=246 path=[224:0-245]

AACATCAGTGATATGTTGACTTGCTAACTCATGACTAGCATTTAAAAGAGAGCAAATCAA

AGTTGTAGTAGGCATCCTTATTTGTCTGCAGTCACAGTACACTCTATCTTAGAGAAAGAC

TTGTATTTTCTAGGAGCAGACCCAAAGCAACGCAGGGTGATGTCAGGCTGTTAATAGGGC

TGAGTGTCAGTAACAGGTAAAGAAAAAACTAGGTTTCCATATGTAAATAGGAGGCATTAC

TTTTGG

>c19464_g1_i1 len=220 path=[1:0-219]

GAGAACTGCAGCAGTGGAAAGCTGTCTTGGTCTCATTAGTTATCCTACAGGAGATTATGA

GCCAATGAAGTGCTTTAATGAGGTGAAAGAAAAATGTTTTCATACTTCTAGGGAACGGGG

AGGGAGATAGTCATTATTTGAACCTTACTGCCGTCACATCTCTGTCACGTAACTTTTGTC

ACCTGGCTTCCTTTTAGGATACAAGGCTACAATCAGTGCG

>c19485_g1_i1 len=207 path=[1:0-163 165:164-206]

GACTTTTCTCCTCCAAAGTCTCAGTTTTCACCCAGACCTACATGCTGCCTGGTTTCCAGC

CCACTGGGTGACCTGCAGTGCTGCAGCCAGGCAGGAAGAGCATTTTGGTGACTTGGGCTC

AGCAGGTCCAGCTATTGGAGATCTGCCCTGCAAAAGTACCACTGTGCATTAAGTGTGAGA

AAAGAATGCATATGCATATTAACAGCT

>c19488_g1_i1 len=315 path=[318:0-222 623:223-314]

ATGCAGACTGTGGCACTGGGGCTGCTATGGGGAAGCTGGGGTTACGTACGAGGCAGCTGG

GATCGCATTGGGAGGGGGAGGGATTGGGTTAAGTGTGGGTTAGCTGGGGGCTTCTGGGGA

GCACTGGGATCGGTGTGGGGCAGCTGGGGGTTCTGGGTTAAGTATAGGGCACCTAGGAAG

GAATGGGGTTGCTATGGAGCATCTGGAATTAAGTGTGGGGCATCTAAGGGAGACTGAGTG

GGTATGTGGGACTTGGGTCAAGTAGGGGAGCAAAGGGGAGCAAAGCACGTGTGGGGCGGC

AGAAGGATCTTGGGT

>c19494_g1_i1 len=226 path=[1:0-225]

ACTCAGTACACACTGCAGCTCATGAGCTGAAGTAAGGTCCGAAATAAAATTGCCAGCAAG

TGCCTCCTTTGTACATTTCAGAAAGGAGAAGTACCAGAGAGGTTTGGTACCTTGTTTCTG

TGCACGATGTTATTGGAGGCTGCAGGATTTGAGCTGAACCCATTGGGCCAATATTTGATC

CGTCAGGAGGTGACCGTCAGCCCTGAGCTGAGCTGTGAGCTGCATG

>c19499_g1_i1 len=347 path=[358:0-228 34:229-346]

AACAAGTTGGTCTCCATCCTTTTTATCAGCTCCCTTTTCTTTGCTGTTAAGGAAGTGAGG

TCATTCCAGCAAGCTGATCAGAGGCTCAGTACCTATCAAGTCTATCATCGGTAATGAGGT

TTACAGGTATGGAATTTCCTTCCCCTTGAATCACTTTCCCTGTCCCAGAAGCTGGTGACG

TTTAAGTAACAGCTATATTTGTAGTCTAGCAGGTTAGGATTTTTTAACTTGCAAGATTTA

ACTCCAGTTGGCTGAAAAATTAATTGTGACATAAATTGTGACATTCATGTGATCCATAAG

AATCCAGCATGTCCCAGCAGTTGGTAATGTGAGCCAGCAACCAGCCA

>c19507_g1_i1 len=206 path=[281:0-131 412:132-205]

GTCTTGATACTATGACATCTAAATACACCGCATTGTTGTTTTTAGGGAGTTCAAATGTTG

ACCTCCGTATATTTTCTTACATACTAATAAAATGAATTTAAAACTTAAAATAACCTGTTA

TTTCAAGTGCTTCATTTCCCTCATAATTTCTGAAGCTGGGAGCTAAAATAATGTTTTTCT

GGGCCTTAGCACATTGCTTGATCTTG

>c19523_g1_i1 len=211 path=[191:0-210]

TAACTTTGTGTTTGACAGCTGTATCTATTTTGTTTTTTAGATTATTAATCGAGGACTGGC

ATCAGGGAAACTGTTCAGGTCTTTTGTTTTTGTTCAGAAATGTGTGAGTTGCCCTATGAT

AGCTTTTCAACAAACAAGTTGATAAGACAAGCTTAATGAGAAAATAAAAAAAATACAGCT

GTTTTCTGATACTTGAAGCCTACAGTTGCAT

>c19527_g1_i1 len=209 path=[261:0-208]

GTCCACAGAGAATGTGTACTTCAAGCCTTGCAGAATCTCTGGAGCAATGTAGTCTGGGGT

CCCACAGAAAGTGCTTGCCTTGTTCTCACCAACTACATTTTCTTTGCACATTCCAAAATC

AGCTATTTTGATGTGGCCTTCTTTATCGAGCATAACATTGTCCAGTTTTAGGTCTCTATA

AATAATGCCTTTGCTGTGAAGAAACTGTA

>c19538_g1_i1 len=210 path=[235:0-209]

ATTAACGCTTTCCTTGTCCCCTTCTGTCTGTTTCTTATGGGATTGGAAAACAAATTTGCT

CTCTCAAGCAAATGATGATTTTCAGCATGTACTTGAAATGTTTTTAATATATGTCTGCTG

GACAAAAAGTTCTTATAAGTGCCTCTCAATTATTAATTGTGTCATAATACTTACGTACTC

TGAAATTATATTTTAATTAATAAGGAGGAT

>c19541_g1_i1 len=272 path=[187:0-271]

TATTATTACGATTATTTTCTTTTTTATTTTTAGTTTTATATATTTATAGCACACTTTTTT

ATCTTTCATTTTACTTAAGAAATTTAGATAACTCATTTATATAGTATAAATGAATTCAAC

AATTGTTCCTTCCTAATATAACTTTTTTCTTAAAAGATTAAATTTTTTAAGAAATCCTTG

TAAATATTAATGGAGAATTTTAATAAGAATTTTTATTTTTTTTAATTTAATTTGTTAGAA

TCTTTGATGTATTATTAATAAAAAGTTGTGTG

>c19547_g1_i1 len=382 path=[555:0-175 342:176-381]

AAAAAAAAAAAAAAGTAAAATTCATTAAATTAATATTATAGATATATATGTATATATTAA

TTACTTTTAATAAATAACGTGTATATATAAAAAATATAATAAATTCACATATATGTTACT

AGACATTTTGGCATTTTAATATTTAGAAAAATATATTTTTTTTCTTTTATAATTAGATAA

TTGTAATATATTTATTAAAAATGTGGATAGAAGAAAAATAAATAATATTTTAATTTATTT

AATATGTACAAAATTAAAGATTTTTTATATGTTAAAATATTATAAATTATACACTTTTAA

AAATAAAGCATTTGAAGAAAAAATTTTAATTACAAAAATAATTTTAAAAAGGACAAATGT

AAAATTATATGAAGTATAAAAT

>c19547_g1_i2 len=270 path=[278:0-63 342:64-269]

TACTACTGAAAAAGATTTACATAGGTTTATATTTTTTTGTAATTTTATTGAAACATTAAT

AAAAATAATTGTAATATATTTATTAAAAATGTGGATAGAAGAAAAATAAATAATATTTTA

ATTTATTTAATATGTACAAAATTAAAGATTTTTTATATGTTAAAATATTATAAATTATAC

ACTTTTAAAAATAAAGCATTTGAAGAAAAAATTTTAATTACAAAAATAATTTTAAAAAGG

ACAAATGTAAAATTATATGAAGTATAAAAT

>c19558_g1_i1 len=240 path=[155:0-239]

AAATTGCTGCAGCAGTTACATTTTCATGCTCAAGTTTCATTTTCCTGAAATGGCCATCAA

AATTTGAAGTTCTCGTTTTCACTGGCGCTGAATGCTCTTTGTGTTTTAACCTATTCATTA

AGGAAGCAAGTTAAAGTAAGAGTTCTTTTGCAAGGACTCCATGTCTGAAGCGGATAGTAT

AACCTGTCTTAATCCTTAAAGAGCAGACGATCTGCTTTGGGATCCAAAGTTTGAAGTATA

>c19583_g1_i1 len=232 path=[123:0-120 244:121-231]

TATATATATATTTTTTTTTTCATATTTTTACCTATTTTAATTTTAAAATTTCATTAATTA

CTTAAAATCATTGTTTATTAAATATTATTTCAAAAAAAAATTTTTTTCATGAAACTAGTT

TTTTGTTTTATAATTTTAGTACAAATAAAAATTTAATTTAAAGTAAAAAATGTGTTAAAG

ATAATAATTAAATAAAGAATGATGTAAAAAACTTCAACAAGCTAAGTGTTAA

>c19596_g1_i1 len=531 path=[155:0-530]

CTCCAGCACGCCAGGGAGGGCAGAGGCGTCCTGCAGCGGGGGCCCACAGCTGCCCCACTA

ATTGAAAACTAATTATATTTGGCAACAAGGGCCAATGCTGATAACTGACTATTTTCTGCA

GCGGTGTGTAACCATTCTGTACTTTTTTCCTGTACTACACTGGCAATAAGAGTCTCAGAG

TATTCCTAGCTTTAGGAGATGGCTCTGCGTGGTTTATTGGCCTCACAATTACAATCCAGC

GGTTTCTACAATGGCTTTCATTGCTTTAAAAACACTTCTGGCAAAAAGAGCAGCAAATAG

TTTTCCTACAGAAACCGCTGGTTCAGCAGACAGTTTATTTATCATGAACGGATAAAAGCA

CCGTTACGCTGAAGCTTTGATTCAGTGTGGGATTAAGGGAGCATCGGAAATACTGCTTCC

TGCGTGGCTGCGTTCTGAGCCACCAACAGCAGCACTCACTGTCAGGGATAGTATTTCCAC

TTAAAGTACTTGCAGAATGTGTTGCTTTACAGAACAGAGTTTCTAACCTGG

>c19601_g1_i1 len=220 path=[189:0-65 254:66-219]

AGGCTTCTCTCACTGGAAGCTGCACCTGCACACCAGCTGTTGTGATACTCCACTACCAAG

CTATTTTTCATTGAATTTCCCCTTAAAAATGTGTTATATTTGGCCATCCTTGCTCCAGTT

TTTTCCTGCCAACTTGATTCTTAGCATCTCATAAAATGAAGAATTTACAAAATATATAAG

AAGCAGCACAGTCCAGGTTGAAAAGTGTTGGGGTGCCTAA

>c19602_g1_i1 len=222 path=[513:0-14 515:15-221]

CCCTCAAACACAAAGCCAACATTTCTTGAATAGCTTTAACAGTGGCTTTGTAACTAGGAA

TATGTAGTGGTAACTACAGGTATTGATATTTGAAAGATACCTTATACTGCCAACGCAGTT

AATGTAGAAATCATAATTTCAAATGAGTAATTTGTTTTACTTCTGTCAGCAGATTATAAG

GACTACGTTGTGATTAAGTTTAAATACCAAAGATTTGTGAGA

>c19602_g1_i2 len=264 path=[242:0-56 515:57-263]

AAAAATTGGAGAATCTTATTTTAATTCTTGATCAATTTTGTGATCAATGAACATAAACCA

ACATTTCTTGAATAGCTTTAACAGTGGCTTTGTAACTAGGAATATGTAGTGGTAACTACA

GGTATTGATATTTGAAAGATACCTTATACTGCCAACGCAGTTAATGTAGAAATCATAATT

TCAAATGAGTAATTTGTTTTACTTCTGTCAGCAGATTATAAGGACTACGTTGTGATTAAG

TTTAAATACCAAAGATTTGTGAGA

>c19603_g1_i1 len=254 path=[53:0-52 106:53-54 108:55-253]

AGTTGCTCCAAAACGTAAAGTCATAAAATACTTGGAGGAGATTGGCCTTTTGATCTGCTA

AAACTTGGCTTAGTGACTTAGGCAATGATGTTTTGAGGCTTATACAGTACATAACAGGCA

ATTGTCTCCTGGAAGTTCTCTTTGTTTTCCTTTTACAGTCATGCACAACTGCAACAGTAT

GAATGTGAATTCCATGAAGAATCTTGACTTTACTGTTCACAGTCAAGGTGCTCATCTGCT

GAAGGCCAGATAAT

>c19603_g1_i2 len=270 path=[215:0-70 108:71-269]

GAAATCCTGTCTATCTACTAAGTGCTCATTAAGAGAGTGAATGGGGAATTTTCATGGAAA

TTTGTGTGATGTGCTAAAACTTGGCTTAGTGACTTAGGCAATGATGTTTTGAGGCTTATA

CAGTACATAACAGGCAATTGTCTCCTGGAAGTTCTCTTTGTTTTCCTTTTACAGTCATGC

ACAACTGCAACAGTATGAATGTGAATTCCATGAAGAATCTTGACTTTACTGTTCACAGTC

AAGGTGCTCATCTGCTGAAGGCCAGATAAT

>c19607_g1_i1 len=357 path=[511:0-257 51:258-356]

AGTTCTTCCAAGTAATCAACAGTCTTGATATGAGCCTTGTAATAATGTGGAAGACCCTCG

CATTTTCTCTTTGGATTCCCACTCCACATTTCTTTTTAAGGTTACAGAGAAGAAGAAAAA

AAGTGAATTATGTGGTTACATTTGGCAGTTAATGTTTCTCTTCTAACTTCCAGCTGACAT

ATTTCTATACACTCAGCTGAACAACCTTTTCTAGTTGATTTCACTGACACAACGCAGACC

TCAATCCTGAAAATGTCCATTTTTTTTATTTTTTACTTTTTGCTGAATATTCTTAGGGCT

TCTCACGTAAACAGCATTCTTGAGTATTTAAATTTCATATTCTTACATCTGTTCCAA

>c19608_g1_i1 len=211 path=[155:0-210]

GCTGACTTTTTATGAGTTTGAAATGAGTTGTATAATGATGAGTCTAGCCTGCAGTAACAT

TTAAAGCTCTTCATCTGACCACTCATCAAAGCTTGTTTGTGTGTGTGTGTACACCTTTTG

TGAAGTCCTTTAAAGTGTGTTCCAAAGACTCTGCGTTCTTTAACATAAAGTGGGTTGCTT

TTAAAGGCCTTCAAATGAGGGCAAAAGTGAG

>c19609_g1_i1 len=268 path=[251:0-60 125:61-267]

AATGCAAGCACTGAGACAGCTGGCAGAATCAAAGTGGGACGTGCTGTAGAGACTTAGCTG

GTTCCTGATACATGTATTGATCTGTGATGTGTAGTCAGATGTCTCCTGTGATATCTGGGG

GAGGGGGGACGGACAAATTCCTTAGTTTTCCTTAGTTTTCCTTAGCGAAGGCAGGACATC

ACAGCATGAGTGACCAGACTGCCTCTTCTAATATTACTACTAACTATGCTTCTCTCACTA

GCAGATTGAAATACTGCGTTATTGAGTG

>c19609_g1_i2 len=331 path=[1:0-123 125:124-330]

GCATTGCAGCAGCTCCCTCAGTTGACGTATTCAGGGTAACAATAGAAATGAGACCATTTC

ACTCACAGGCTGATTTTGCCATGAGAAGTTGACAGTCTGAGCGCTGATAGTAGTGAAAAG

CTGCTTCCTGATACATGTATTGATCTGTGATGTGTAGTCAGATGTCTCCTGTGATATCTG

GGGGAGGGGGGACGGACAAATTCCTTAGTTTTCCTTAGTTTTCCTTAGCGAAGGCAGGAC

ATCACAGCATGAGTGACCAGACTGCCTCTTCTAATATTACTACTAACTATGCTTCTCTCA

CTAGCAGATTGAAATACTGCGTTATTGAGTG

>c19617_g1_i1 len=227 path=[1:0-226]

CGAGGAGATCGTCGAGCTGAAAGGGAACGGAGAAAGCCCTGTGCTGTTTGAGGAGTGAGG

GTTTGCGGTGCCTGAAGGGAATGGCTGTGCTGGGAGATGACGTTGTGGTGTCAGGGTGAG

ATTTTTGGGGTGCCTGAGGGAACGGTGGTGCTGGAAGGGAGCAGGGTGGTGGTGAGGAGT

TCTGGGGTGCCTTCGGGGAATGGCTGTGCTAGGAGGTGACGTTGTGG

>c19620_g1_i1 len=234 path=[1:0-233]

GACAGCTTGCACTGCTGAGCTTCTCTGTGACTGGAACACATCAGCATAAGACCAAAAAAT

ACTTCTTGTGCTTAACTTCAAGGGAAATGAAAAGCCACTCTCTGGTAGAAAGAGGGTAAT

TTGGTAACTGGACAGACTTTACAGCATCCGTGCGTCGTTAACAGGGGGTCTTGTGATCAC

CTAGAGAAAATCCATCTGAGATGATATCAAGTGTAGCTGTGTGTTACTGTGTGG

>c19624_g1_i1 len=210 path=[4:0-209]

AAAAAAAATATTAAAAAAAAATAAAATAAACTTTTTAAAAAAGAAGATAAAATTTATGTA

AAATTATTTATTAGATAACTTTTTATTCTATCTGCAAGGCTACATTTATCCATTTTAAGA

ACTTTTTGCTGATCTTTATGAGAACCATGTTCAATTGATTCATTTGGAAAATAAATATTA

TGAACATATAAATTATATTTATTTATATAA

>c19635_g1_i1 len=292 path=[506:0-291]

GGATCAGATAGAATAAATGCTCTGTAGCTGTGTGTAGCTGTTTAGAACGAATGCTCCAAG

CAACAGCTGATAGCTGCTCATTACCTGTTGATGGTACTAAATGCAATATGTAACACGTAT

TCTTTGAGATTTTTCTTTTGTTTTTTTGTTTCAAATTAAAAAGCTGTGTGATGTTTATGT

TCAGCTGTACAATCATGGTGATGGTAGAAAATAGAGCATGGAGTTTTGTGTTTTAATGAT

GAGTGTAGTCTTCTACTTTTTAGATGGATTAAATACTTCTGACAAACACTTA

>c19639_g1_i1 len=267 path=[288:0-208 11:209-232 35:233-266]

GCAAAGGTCACTTGCCAAAGGCAACTTAACAGCTCACTCCTTCCAAAAATCAGATGCATC

TCACCTTTGCCCTTCTTTTCCAACCTACATCCCACAGCCCATTCAGAGGCAGATCTAGGA

TGTGTGACCCTCAGTCGGCTCAGCTCACTAAATTAAGACAAAACCAATACACAGTGCAGC

ATTCCACTGTTTGACTGATCTAACTTCTGGATGCCAGAAAATTATAGCTTGCTCAGCTTT

CTTACCCATCCCTTGCACTGGCTGTAG

>c19645_g1_i1 len=287 path=[1:0-286]

GATTTTACTTTTGGGTTTAATGATAGAGAAGTGAAAGTGTTTCAGTCTTTTGGCTGAAAT

GGATGAAGCTGTTGAAATTCAGTATTGCTTCCAAAAGCAGGCATAGTGTTTAAGCTGAGA

ATTTTGCTTCTGTTCAGTATACCTCAATCTTCTACCCAAATAAGCTATTAAGCTATGGCT

GATTAGTCTTCCCTTGGCATGTGTGGTACCATAAAGCAATTGGGAATTATCCCATCTCCT

TTTTTTTTGTTGTTCTAAACTGCACTGATCCCAGATCAAACACAAAG

>c19652_g1_i1 len=232 path=[210:0-231]

AGTATATGGCTGCAAATGTCAAACGCAGTATTTAGAAAAACATTGACAATAGATTTTTGC

ATGTTAATTTTTTAGACAAAATATAGTTGCATATTAATTGATACTGACCGTGGAAACAAC

ATGTAGAATTAATAATATTGATTTTTCTTTTCTTGAAGCAAAAGTTGCTTTTATACCACA

ACTTCATTATCTAGAAATCAGCATGATGAAATCTTACAGTAGAGCACATGAA

>c19662_g1_i1 len=226 path=[53:0-225]

GAAACCCAGCAGCAGGTAAGAGAACAAGGAGAAATCAGAAACCAAAAACTCATCTACAGT

GAAGGAAAGGAAGGGATGAGAGGAAGGTTGGCATGTGCGTGGAACCTGGAACACAAGCAT

CCCAAAGGACATCTCCTGAAACTCCTGAAAGAACAGTAACTGTCAGAGATGGTCCCATGG

CCACCCACAACCATACTACCCACACCATGATGGCAGGAGAGCAGCA

>c19663_g1_i1 len=284 path=[299:0-283]

ATTTATATTTCACTCATCACCACAGACTAAACATCTGTTTTCTAAGAGAGCTCTCTGTAG

CTGAGATGCTATCACACCACATCCTGGGGACGTCACTTTTTGTGGCTTCTCAGGTACTTA

TCCATGAATAATATGTGAAAATCTGACAATAAATTCTCACAGCCTGAATGCAGGGATAGA

TGCTAGCTAGGAGTATTAACTTTTATTAGGCTACCTCATATTGTTGGAGAACATGCAAAT

GAAGTTTCAGGCAAACAAGCCCTTCTTCAAATAAAAATAAAAAT

>c19668_g1_i1 len=233 path=[211:0-110 322:111-232]

AGTGGAGAGTCATTAAGTGCATGAGTGAGAGAGAGGAGTCATGTCCTCTCTGCAGAGCTC

CTGCTCTCTCACCACGAGGTGAGGCCTTCTTCCCCCTTGCCCACACCCAGACCCTGCGAG

CTGGCTGCTGGAAGAAGCCATCAGGTCTGGGAAGGTGACCAACCGTGGCACATGTCCAAA

TGAGTCACAGAATCCCAGAATTGTTTGAGTTGGAAGGGATGTTTAAAGGTCAT

>c19669_g1_i1 len=201 path=[179:0-200]

ATGTTTTAATCGATACGTAATAAGTGCTGTAGAGAACTCTTGCAATCCTGGCAAGCTACA

TTTGTATTTTTTCATGAGTTGTGTGGGACAGTCGCTATTATCTGTATTTTAAAATGCCAA

ATACAATTCATTTAACTTTTTCATATGGAGTGGAAGAAATGTGAGAATACCTGAAAGCTT

CTTAGAACAAAGAATTGAAAA

>c19676_g1_i1 len=228 path=[360:0-227]

AGAGGGCGCGCGTCGCCACCAGCACTTCCGGTTCCGTTCCCATGGCGACCGCAGCCTCCA

GCTCCGGCTCCACCTCCACCGGCCCCGGAAGTGGAGGCGGGACAGGAAGTGCCGCCCTCA

GCCGCTTCCCCTCTCTGTTCGCTGCTTCCGCCAACAGCCGCAGGCGCAGTGCCCGGTTAA

GCCCCGCCCCCCGGCGCCGAAGCCCCGCCCCAACTCCGCCTCCAGGAG

>c19678_g1_i1 len=623 path=[712:0-561 1271:562-622]

TGCCGCAAGACAGCCACGGCGGCCGTGACGTCAGCGCGGGCTGCCGTGACGTCAGCGCGG

GCGCCATGACGCCCCGCCGGGGCGCTAGTTGAGCGTGCCGCGGCAGCTGTCGGTGCGGCA

GAGGCAGGGGATCTTGGTGTCCTCGATGGGGAACTTGTAGTCGTAGGTGATCTCCTCGTT

GACGCCGATGGGCTGCTTGGAGTAGATGACGATCTTCTTCTGAGCCTCCAGCGTGATCAC

CTTGGCGTAGCAGTTGGGCGTGCAGCAGTGGTTGATGAAGCGCGCCAGGTTGCCGCATTT

GGTGGCGTCGATGATGGTGTCGTGGTCCACCCGGAACAGATAACTGCTGCCGATGCCCTG

CTGGGCATAGCGCTTCTCACGCATGTCTGCCACCACCTGTCGGATGTTCTGCCCCACATA

CTCGATGACCATCTCGTCGGCCGCGATGGGCTCCATGGCAAACAGCCCCCATTCGTGGAT

GCGGCTCCGGCCGAACCTCAGCCGCTTCTTACGGAACTTGAGCTGGTTGAGCTTGAGCAG

CTCGCTGTCCAGCAGCGCCGCGCCGCCCCCCGACCCCGACGGCCCCGACACGCAGGGCCC

GAACCGCGTGCTGTCGGAGCGGC

>c19678_g1_i2 len=624 path=[712:0-561 77:562-623]

TGCCGCAAGACAGCCACGGCGGCCGTGACGTCAGCGCGGGCTGCCGTGACGTCAGCGCGG

GCGCCATGACGCCCCGCCGGGGCGCTAGTTGAGCGTGCCGCGGCAGCTGTCGGTGCGGCA

GAGGCAGGGGATCTTGGTGTCCTCGATGGGGAACTTGTAGTCGTAGGTGATCTCCTCGTT

GACGCCGATGGGCTGCTTGGAGTAGATGACGATCTTCTTCTGAGCCTCCAGCGTGATCAC

CTTGGCGTAGCAGTTGGGCGTGCAGCAGTGGTTGATGAAGCGCGCCAGGTTGCCGCATTT

GGTGGCGTCGATGATGGTGTCGTGGTCCACCCGGAACAGATAACTGCTGCCGATGCCCTG

CTGGGCATAGCGCTTCTCACGCATGTCTGCCACCACCTGTCGGATGTTCTGCCCCACATA

CTCGATGACCATCTCGTCGGCCGCGATGGGCTCCATGGCAAACAGCCCCCATTCGTGGAT

GCGGCTCCGGCCGAACCTCAGCCGCTTCTTACGGAACTTGAGCTGGTTGAGCTTGAGCAG

CTCGCTGTCCAGCAGCGCCGCGGAGCCGATGGCGCTCAGCAGCCGCCGCTGCTCCGAACG

CCGCTCCGACAGCACGCGGTTCGG

>c19683_g1_i1 len=291 path=[155:0-290]

ATAAATAACAGATCACCGTCCTGAGCTCTGAATTGCCCGGCTTGAGTGTGTCTTAAATCA

GACCTTTATCATTGCTTATTGTGAGGCTGCTTTTAATGGAAGTGCGGAAATCTGAATAAC

AAAACGCGCCGGGCCCCGGCCGGCGTCACGGCTCTGCCGTCAGCGGAGCAGCTCCGAACC

GCGAACTTGGATCGCTGCTCTCGGGCCGCGCGGAGCCCCCCCGGCTGCGGGGGGGTCACG

CTTTGCTGGCACCGCGTCAGCGCCGGGAGCGCTGCGGACCCGTCACGCCGG

>c19690_g1_i1 len=255 path=[61:0-144 60:145-254]

GGGGCTGGTGGATGCTGTGGTGTACACATCCCGAGTGACAGAGAAGGCAGAGCAGCTGCT

CAAGGTGCTGGTGCCAGGGCAGGGCATCCTGTATGAGAACGATTCGCACAGTACAGAGGA

TGAGTCCCTGAGCCGCTGCCACAGCCTGAGTGCTAGACTGCAGAGCAGCTTCCAGGTGAC

TGTGATGACACCACTCAGGGAGAATGCCTGCAGCAGCCCCTTCGGACCGTCCCCACCCAC

CATCCGCATCAACGA

>c19705_g1_i1 len=208 path=[186:0-207]

GTCAGGAAGTCTGGCTGCAGCTGGCAGGCGCAGGGACGGCACAGGAGCTTGCTGTTGTTC

CTCTCTGTAGGAAGTTTCCAGCTAAACACGGAGTCCAGTAGTATCTGCTAAATTGGACAC

AAGCTGCAGGTCAAGGAGGTTCCTTAGGCTGAATCTCTAGATGTGCTGCAAGGTGATTTT

TGTTTATTTTGTTTTCACTTGCAGTGCA

>c19712_g1_i1 len=298 path=[201:0-297]

CTCTATGATAATGTACTAGAAATAGGAATTCAAGAAGCTGCAAATCATCAATTCAATTCT

TGAAATAGAATTTTGCGTAAAAAACTTGAAAGGAACTGGTAGGAGCAAGTGTATGAGATG

CTCACAGATGTTTACCTGGAGCCTCCAGTGCATTCATATGAACTAGTAAAAATAAAAAGA

TGATTGCAATTCTTTCTCATCATTAAGCATTCTTTTTATTTGCTGACATGACTTCTCATT

TTAAAGGATGTTTTATCAGCACTAGGGAGAGTGTTTAGAGAAAAAAATCTGATAGTGG

>c19713_g1_i1 len=227 path=[217:0-186 461:187-226]

TCTAAGGAGGGATGACCTAGAAAACAAAGCATACAGGAGAAATACTATTTTTAAATATAC

TGGCCACAGAGAGACATGAAAAAATTGCATGTGGACAAAAAGACCACCACGATATAAAAC

GAAATATGGAAAAATCTAGAATGTTTTCTGTTTCTAGGAACTGAGGGGAACCACAGGAAA

ACAAGTTGATGGGGTGCGGGTTGCATTCTCCCTCCCCCCTTTTTTAT

>c19717_g1_i1 len=360 path=[610:0-359]

GGCCAACTGTTGGCCAACTGTTGGCCAACCGTTGGTCAACTGTCAGTCAACTGTTGACCA

ACCGTTGGCCAACCATTCATCAACTGTTCGTCAACTGCTGGTCAGCCATTGTTTTGTCAG

CCATTAGTCAACTGTCAGTCAGCTGTTGGCCAACCGTTGGTCAGCCGCAGGGCTGTCCCT

TGCCCTGTGGGGTGGTTGTGGGTCCCACCAGGTCCTGCGGGAGCCCCCAGAGAGCAGCTG

GGTTCAGAGCGAGGGGGGCAGAGGCTCAGTAGGACTGGGCAGGAGGGCAATGAGTGTAGA

GGATGTGGCCATGATGCAGGGCTCCTGTCCCATCCCAGATCACCGGCGTGTCCATCCTGA

>c19729_g1_i1 len=228 path=[425:0-227]

TCACAGCACTACACCTGATACACTAAGACACGGACTCACTGTATTAGTGAGGAACGGAGC

TTGATTACAGAATAACCCTGACATATATAACACAGGTCTAAATCCTGACTCCACAGCAGT

GCAAGAAGACGATACCACCCATCTTTTAGGACAGTAACCCGAATTAAGACCCCTTCTAAC

ACGTTGGGTTGGTTTTGCTTCTGCTTTTAAAACCATCTAGGGTTGCTG

>c19729_g2_i1 len=235 path=[213:0-234]

TCAACAGTTCCTGATTAACATTGTGCACTTTCCTACGTGCACATCTGTTCGTGTGCAGCA

CGGAATAATCAGAAGCAAGGAATTAGTCAGAGCAGTCTATATTCTGCATGACAAAGAGGA

GCACAGGATCCTGCAGGAGTGCTAAGGGAGAGCAGGAGTGGGGCTGGTGCAGGCAGTGTG

TGCAAGCACCCATCGCCATGCGGCAGGCACCTGCCCTGCTGCTCTCTGCCCCCTG

>c19732_g1_i1 len=207 path=[185:0-206]

AGAACTTAAAACCAAGAGGGCAGTGCAGTGATGAGAGAAATACATTTGAAGAGCTCTACG

TTTTTCCTTTATTACCAAGATATTGTTTTATGTATATATTACCTGCTAAACAGATAAACA

TGAGCATAATTTTTAAAAAGGTTTTTAGCCTTGATAGGACTTTAAAGCAGTGAGAAAAAT

TGTAAGTGTAAATCCTTGAAAGCTTAG

>c19769_g1_i1 len=342 path=[623:0-14 605:15-341]

GGTAACTTTGTGTTTGAGAGGTTAGAATTTATGAAGAAAATTTAGAAGAAATGAAAAATA

AGTTTTTTTATGATAAATGCAATTCATATACAAATAGTGCGAATTCGTATAAAGAAAATA

AAATAGACAAATATTTAGAAACATATAGGAAAATTGAAAATATGGATAAAAATAAAGAAA

ATGAAACAGATGAATTTTTAGAAATTTATGATCAAATTAATAATGAAAAGAAAAATAAGA

TAATAAATAATTCATTGTTTATAAATGATTTAAGAAAAGAAGTTGAAATAAATAAAGAAA

ATTTAAATGAAATGAAAGATAAATTATTTGATGATAATGATT

>c19769_g1_i2 len=620 path=[312:0-292 605:293-619]

TATAAATAAAAATATGGAAAATGAATTAGACGAATTTTTAGAGATATATAATAAAACTAT

AAATATGAATAAAAATAAAGAAAATGACATTTTGAAAATTTATAGTACAAATAAGAACAA

TTTTACAAATAATTCTCTATTTATGTATACTTCAAGGAAAGAAGATAAAATAGATGATGA

AATATCAAATGAAATGAAAAATGTTTTATCTAATAATAAAAGTAACTTATCTGCAAAAAA

TTTAAACGGAGGTAACTCACAAAATAATTCAGAATTTATCTATTCTTCAAGAGGAGAGGT

TAGAATTTATGAAGAAAATTTAGAAGAAATGAAAAATAAGTTTTTTTATGATAAATGCAA

TTCATATACAAATAGTGCGAATTCGTATAAAGAAAATAAAATAGACAAATATTTAGAAAC

ATATAGGAAAATTGAAAATATGGATAAAAATAAAGAAAATGAAACAGATGAATTTTTAGA

AATTTATGATCAAATTAATAATGAAAAGAAAAATAAGATAATAAATAATTCATTGTTTAT

AAATGATTTAAGAAAAGAAGTTGAAATAAATAAAGAAAATTTAAATGAAATGAAAGATAA

ATTATTTGATGATAATGATT

>c19774_g1_i1 len=274 path=[207:0-273]

ATGGTTATGCAGTCCTTTCTCTATTCCATTACAGATGAGCGCTGGATGACAGCCACTCAC

TGAGGCTCAGGGCTTACTGTAGGGCAGAGGGTAACAGACTGGACTGGCCGGGTAGTGAAA

ATGTGCTGAAGAACAGCTGTGAATTTCACAGGAAGGGGAAGTTCCCCAACACGTGACTGA

GGGTTACAGTGCACATTGAGCAGTACGTGTTCCCCAGTGACATGCACTTCCTCATTGCAC

TGATGAGACTGCAATCCCTTGATAGGATGCAAAG

>c19776_g1_i1 len=1374 path=[1:0-903 905:904-1373]

TTTTATTTTATTTTTTTTTTTTATGTGTGTGTTTGTGTAAATAAAAGTTAAAATTATTAT

AAAATGATCCTTCTTTCAAAAATGTTATTAAGAATAAATGTTTTTCTATTTTTTAATAAT

GATAAATATATATATGAATTAACGTACTTTCTTGTTTTACAAATAAATAACATATTTTTT

TAATGTATAGAAAAATGCCATTTTTCCATATTATTTTTAAGGATATAATCACTCATTTTT

TTCTTCAATATATAAATTGATTTCATTTATTAATTTATTTAACATTTGAATTTTTGTTAA

ATTTTCTTCCACACAACTTATAGAATTAAATTTATTTGACATTTTGTCATCACCATTATT

TTCTTCTTTTATGTTTTTCTGGTTTTCCGAATTATTAATATGAATTTTGTCGTCTTTTAA

TGAAGGATTTTCATTAGTTTCATTTAACTGGTGTGTGTATTTTTTGAGAATTTGTTCAAT

AAAAAACTTACATAGAAAGAATATTTCTCTAGATGATGGACTATATGGATCTATATCACA

ACAACCTATACCTTTTTGGCATGCCTCAATGAGATTTTGATGAAAAGTTACTTTTCCTGA

ATAGGGTACACTCATTTCTGAACACATTTTTTCAACGGTATAATTTGATATAAAAATAGA

TTGATACATATTTTCTATAATTCCTAGTATTGGAATATTAGTTTTTTTACAAAATTCTAT

TTCTTTTTTAACATCACAAATAGATAGAATATGAGGAGTCGTAACTATCAAACAACCATC

TAAATTATCTTTCAAGTAGGAACAAATAGTCAAATGTTCATCACTAGTTCCAGGTGGAGT

ATCAATAATTAAAAAGTCTAAATTTTTCCAATAAACATCACATAAAAATTGTTTAATTAA

ACCATTTTTTTTTGGCCCTCTCCATATGACTGGGTCATCAAAATTTGGTAATAAATAGCC

AACGGACATAATAGATAAATTATTTTTGTAAATTGGTATCCATCCATTCATACTATAATT

TACATCGTAATTTATTGTTTTTGTTAAAATAGGAATAGAAGGTCCACATATATCAATATC

TAATAGACCAACATCATAATTTAAATAAGATAATGCAAAAGCTATTTGAGCAGCAACAGT

AGACTTTCCTACACCACCTTTACCAGATAATATGAGAATCTTACACTTTACATTTTTTAA

ATTTTCTTTGATCTCATTGAAAGCTTTGTTTTTTTCTTTTTCCTTTTCTTTCTTCAATTC

AGGATCATTGCAAATCTTCCTATTTGGACATCCTTCACAAATTTTAGACTTCCCTGCATT

TTCATTATCTATACCTGGACATTCTTCTGGAATAGCAACTGCAGAATTTTTTTT

>c19784_g1_i1 len=234 path=[351:0-58 424:59-233]

AAATATGGAGATAGAAAAGTATTTTATTTTATGAAAAGTGTACCTTTCCAATCGATGTCT

CTCTCAACAATTCAACAAACACAGAACTGTTTTTACATCCATCTGCATGCAAATTTGTAA

GAAAATACATTATTTGTGTATTTCCTCTATGCTTGTAAGTTAGCTAAAATAACCATGTGA

TAGAATTATGTCAGTAAACCACTAGTTTTGCCAGCTTCTCACCTGCATTTCATT

>c19790_g1_i1 len=462 path=[155:0-461]

TACTTGGAGGAGAGAACCAGGGAGGGTGCGTGTTTGCTTCCTCCACCCAACATAGCTGCG

CTGGCTCATCCCTGAGTCACGTTCTTGCTCCTCTCCTTCAAAGCCAAGATCCCTAAAGAG

GACAACTTTTACCTTTTTCCTCCTCGGATTCAGAGTTTTTCTGCATTCATGGTGAGAGCC

TTCCACACAGTTGTCTTTGACATTTCATCTGAGATGTTGATCTCAGAAGGGTCAGTCCTG

TCCTTGCTGTTCTGCTTGGGGACACCCACGTAGCTCGTCTTCCCCAGGCTCAGCTGGACT

GAGTTTGCAGCAGCAGCTTCCATCATCTGCTGAGACTCCTCTTCTTCTTTGGCTTCATTT

TTGGCATCGTCCAACTTCTTCTTCTTGCTTTCTGGCATGAGGTCATCCAGCCAGCTGAGC

TCTCGCTTCGAGAAGCAGACATCCAGGACTTTCCGAACAAAA

>c19792_g1_i1 len=213 path=[670:0-22 157:23-212]

ACCATGTAGCTATCCCAGCCCCTTTTAAAGATTTAGTCGGAATATAACCATAGGAATCTG

TTATCCAACAAAATCATGCTACATAGAGTAAATTATCTCAAGTGGGATGAGATCAAAGCA

ATTAATCTTCCTGAAGATTTCAAATTCCTTAAAAGTGGGAAAGACATTAAATGAACAGAA

TCATTCTTACTACGCCATCAATAACACACTGGG

>c19792_g1_i2 len=346 path=[1:0-155 157:156-345]

ACCATGTAGCTATCCCAGCCCCTCTGCACAAACAAGAGAAAATCTGGTTACAAAATGAAC

TTGTTATTGTCATCAGAAACTCTTCATACCCAATGCCTTAAAAAATTGATTTTGATAGAT

TCTACAGAAGCACAGGACCCTTTGCTAGCAACTTACTTTAAAGATTTAGTCGGAATATAA

CCATAGGAATCTGTTATCCAACAAAATCATGCTACATAGAGTAAATTATCTCAAGTGGGA

TGAGATCAAAGCAATTAATCTTCCTGAAGATTTCAAATTCCTTAAAAGTGGGAAAGACAT

TAAATGAACAGAATCATTCTTACTACGCCATCAATAACACACTGGG

>c19798_g1_i1 len=210 path=[342:0-209]

GCATATGTAGTTCTCTACTTGTCCAAATAATCTTCAGAATAGCTTCTAGAAAGGCTGAAA

AGAATGGATGTGACTTCCTGGAGAAATGTTATATGTGTGCTGCCAGGGGCAAGGGACCAG

AAAGCAGATAGCACAGTCTGCACATACGAATTAGTCCAGCAGCATTTGTTTCCCTTTCAG

AAATAGCAGCTTTGAGATTTATAACGGATT

>c19802_g1_i1 len=310 path=[288:0-309]

AAAAATTGCTTCTCGGATTTTCTACAGATCATTTATTTTGTTCCTGAATTCTGACCAAGA

ATTCTTACGTTCTTAAATGTCTGTAGCTTTGCTCTTTTAATGTTGTGAAAATTACCTTGA

AAATTACACAAACTGTGGTAAAATTTGTTTAATAGGTAATACAATGCTAATGGTCTCTGG

GACATAAAGTTCTTACGGATTCTATTATGGGTTCTGTCAGTTTGGCTTAAATGTAATTAC

TTGACGTTCTCCTGGCTCAGCTTGAGACTTACAAGGCTGCAGAAATAATTTCATCCCTTA

ATGTGATCGA

>c19809_g1_i1 len=238 path=[1:0-237]

GGGATTGTTTTACATAGATACAAGACAAAGAAACATCAAGTTCTTCAGCCCTAGCAGCAG

TTGAAGTCAAAAGTTCATCCTTGTGTATGAGGATGTTTTGAGTACAGAGAATGTCTGTCC

CTTCCCACCAGGCAGGAGATGCTCCACTGCACAGATCATCTTCCTACTTCAGCCAGGTTT

GGGATGAGGCGTGCTTTTTCATCTTGAGGGAAAATTAATGCCTTGTATCAACTGATAT

>c19809_g2_i1 len=223 path=[631:0-222]

AAAGAAGACATAAGCAGAGTAGCTTGGGAAAGGGAAGGAAGAGAACTCCTGAAAAAAATT

CCATGATGAAGGATCAAGAAGAAAAGGCCATCTACTTGGTGCCTGTGCTGAAGAATGTTA

TGTTGTCCAGGAAGAAAGCAGAGAAGAGGAAAAAAAATAGCATAAAATTGATGAAAACAA

GGAGAGAAGTTGGCAGTTCAGAGTGAGCTTTGATGATGTCAGG

>c19839_g1_i1 len=221 path=[1:0-220]

ATTTTTTTCATCTCTACAAATACTGATACATTTGTTAAAATATTGGAAAGCTCCTTATGT

GGTCCAAACATAATTTCAGTGATAAAAGAGTAACTTTTTACAAACTTAATTGTAGAACAG

AGTTGACTTTCTTCTATCTCTATTGATACACGTTAAATTCAGGAGCATTTCTTTCAGTTT

TATTCAGGAATTGAAAAATAATGTTTTGTGGCAGTATTAGT

>c19841_g1_i1 len=293 path=[283:0-228 826:229-234 517:235-292]

CCTCATTGGGACCCGAAGAGTAGGGCTGAGCCCAAAGGTACGCACCCAACAAAGCCAGTC

CTCAATGGGACTGGAAAATGAATAGGATTGAACTCAAAGATATGTACCCAACGGAGCCGG

TCCTCAACGGGACTCCAAAATGAGCGGGGCTGAACTCAGATACATACCCAACAACAAAAC

CAACGCTCAGTGGAACTCTGAAGGGAACAGGACCCAACCCAAAGATGCATGCACAGTGTC

AGCCCTGTTCCCTTTGGAATTCCATTGAGGGTTGGCTTTGTCACTGTGCATGC

>c19841_g2_i1 len=306 path=[563:0-247 517:248-305]

TCCAACCCCCTGCTAAAGCAGGTTGAGCAGGAAAGCATCCAGGTGGGCCTTGAATATCTC

CAGAGGTGACTCCACAACCTCCCTGAGCAGCCTGTTCCAGCGCTCCGTCACCCTGGCAGT

AAATAAGCTCTTCAGTCCTCTTCCCTTTGAGTCCCACTGAGGATTGATTTGGTTGGGCAC

GCAGCTTTGGGTTCAGTCCATTTCGCTTCTCAGCCCCGATGAGGACCGTATTTGGGTGTG

CAGGTCTGGTGTCAGCCCTGTTCCCTTTGGAATTCCATTGAGGGTTGGCTTTGTCACTGT

GCATGC

>c19842_g1_i1 len=215 path=[542:0-22 47:23-214]

TTTCACTGTCTGGCAATCTATTTAAATAGAAGAAATGGTGTATAACGTTCTTACAGAAGT

TTCTGTGCAGGGGATCCCCTTTCTGCAATGCAGAAGCCCAAGACAAAAAGGCAAGTTTTC

ACTTAGTAGGATATTTTCCTTTCTTGAAGTAAAAACTAATTTTCTGAACATCTTTTAGAT

TTTAAAATGGATTATTTTTAAAAATAAAGTACAAC

>c19842_g1_i2 len=284 path=[23:0-23 47:24-215 239:216-283]

TTAAATAGATTGCCAGACAGTGAAAAATAGAAGAAATGGTGTATAACGTTCTTACAGAAG

TTTCTGTGCAGGGGATCCCCTTTCTGCAATGCAGAAGCCCAAGACAAAAAGGCAAGTTTT

CACTTAGTAGGATATTTTCCTTTCTTGAAGTAAAAACTAATTTTCTGAACATCTTTTAGA

TTTTAAAATGGATTATTTTTAAAAATAAAGTACAACACAAAGCCCTGACAACCTGTTGTC

TCCAGGGACACACCTGTGCAGTGCTGTCTAAACGTGGCTGGTGG

>c19848_g1_i1 len=342 path=[320:0-266 695:267-341]

GATCCAGTAATCAGATAGTGCAGTCAGTATACAAAGCAGCAAGTGTGAGATCAAGCATGT

CAGAACCCAAGGGCCGATCAGAGGATATCTGCAGTACTGATATCTCCACCTCTTCTGCGG

ACCATGAAGGCATCCCAACTAGAAATGAACTAAAGCTTCAAAGACCAGATCACAGCCTAT

GTTCCCCCACATTCAATGTCTAAACCAGAAGAGTTTGGAAATGGCTGATCTGTAGAGGCA

GTGAAGAGCCATTGTATCCAGAAATACAGCAGTCATCGACAGAAATGTTACCGGGAGTAA

AATGATGCTTTTGGACAGCTGATCTTAACAAGAATGATGAAG

>c19850_g1_i1 len=290 path=[442:0-289]

GAGGTCTTTTCCAAGGTGAGCAATTCTATGATCCTATGATTCTACGAAAACATCCATTGC

AAAGCCATGAGTTGCTAGGGAAAGCCATGTTAGAAGTAAGATCTGATACCAGATGATAAA

GCTCGGCATCTTTATGAAAACTGTCATGTAAACAGAATGTGAAGAGAAGGTCAAAATCAC

ACTCAGGCTATAGGCACAGTTCAATAGGCAGACACCAGGGTGCCACCAACGTGGCACAGC

CTTTGGACTTCTCAGCCGTAGCTCAGCAGCTGTGGTTCGGAATTTCTCTT

>c19857_g1_i1 len=225 path=[1:0-224]

AAGGGAAATCTATTCGTTTGACTTAGTGCTCTGACGATTAATTGCTTGCCAGATTAAATT

GCTCCATAAAACGCACACTACTTTATTCGATCTCTTCGTGATGAGATTAGCTAGGCCATG

CTGGTTCTTTGTATGTTAATTACAATGGTATTTTCTACTTCTATTTAATTATTAAAATGT

TCAGTGCAGCATGCTTTCTCCTAGCCTCAGGAATGATGTATTTTG

>c19858_g1_i1 len=204 path=[1:0-203]

ACCAACTCTCAGTTCTCCAGATAAAAGAGAAGTCCTGAAAGGCAGAAGCCATGGAGGGCT

ACCGTCACGTGAATAATACCCTTCTCAAGTGAAAAGCAGTTTGATACCTTGTGAACTGTT

GCTAAACCTTGCTTGAGCGGTGCTATTCTGAAGAACAAAAGTCTTGCACTTGATTTTTCT

TATTTGAAAATTGTTAGGCAGGTG

>c19860_g1_i1 len=392 path=[370:0-64 435:65-391]

AAAAAAAAAAAAAAAAAGCCTAAAATTTATTTGAAGAGTTCTTTTTTAAAGTTTTTCAAT

TCAGTAATTATATATACATATATATATTTTCTTTTAATTAAATCTTCTATTATCATTAAA

TAAATTATCCTTTAAATAGATTTTTATTAAATATTTTTTATTATAAGTGCAAAATTTTTA

GCATGAACACTCACATAAGCATTTAGCATTATAAAATTATTTAGCTACATAACTTTATAA

TAGTTTAACAAAATTTTTTGAGTGTAATAAAAAAAATGTAAAGAAAAAATGAAATAAAAT

AAAATTTCGTGAAATATAGTAATTTATTATTTAATAAGAACAGACTAAATAATACATTTT

TTTTTAATAATATACTTAAAAAAAAAAAAAAA

>c19860_g2_i1 len=328 path=[1:0-302 304:303-327]

TTTTTTTTTTTTTTTAAGTATATTATTAAAAAAAAATGTATTATTTAGTCTGTTCTTATT

AAATAATAAATTACTATATTTCACGAAATTTTATTTTATTTCATTTTTTCTTTACATTTT

TTTTATTACACTCAAAAAATTTTGTTAAACTATTATAAAGTTATGTAGCTAAATAATTTT

ATAATGCTAAATGCTTATGTGAGTGTTCATGCTAAAAATTTTGCACTTATAATAAAAAAT

ATTTAATAAAAATCTATTTAAAGGATAATTTATTTAATGATAATAGAAGATTTAATTAAA

AGAAAATATATATATGTATATATAATTA

>c19872_g1_i1 len=213 path=[191:0-212]

TCTCTCACTGTTAAGTAACTTTCCCTTTCACCAAGATTTTAAATCACTGTTTAAAGCTCT

GTGCAAACATAATTAATCCATGGTCTAAATATTTAGCCACAGGTCACAGAAATTTGTTGA

AATAGGCAATGTGACAAGGTAGGTGGTCTTCTAATTAATATTTCTTTACAGATCTGTAAA

CAGTACAAGGCTTGTTTAACAACACAGAAATCG

>c19873_g1_i1 len=308 path=[1:0-307]

CAGTGTGAGATTTTGGGATACTTCGCCTGGTATTAAAAGCAAACAAGTGCTGAGAAACGC

TTCGGTAACAAAACCAAATGTTTTCATTTTTTGGTTTCAATTGTCCCTCAGTACCAACGT

CTGACTCTTTAGCTTCAGCTCACACACAGCTCAACCAGAAAAGGAGCCGTTTATTGAGCA

GCATTGCTTCTGCTGGGGCTCGGATGCTGTGTGGATCTGCCCTCCTTGAGCGGAGGCTGA

AGCTCTGCTGAGAGCGGCTGCGGCGCTGTACCTTTCTGTGCTGAGGATGCATTGTCTCAT

TGCTGTAG

>c19874_g1_i1 len=264 path=[43:0-196 21:197-263]

GGCCCAGATGGGAGATTATAAGGTGAACATGTTGCCAGTCACTAATTCTGCCTTCAGTGC

ATCTGCCTGGTCTCTTTCTCCCGCTGATTTTTCTGTCTCCTTGCTATTTGTTCCTTAGAT

CTTGGCTGCTGGAAGGAAAAATTATAGGGAAAGTTGTGTCCTGTTCTGGGATACAGAAAT

GTTCAGCACAGATGAAACATTCATATAGTGTACCTCCCAAATATTTACAAGCCTTTAGAA

GGTATTAAAAATAACATTTATTAA

>c19878_g1_i1 len=256 path=[745:0-16 747:17-255]

CCCATCCCTTACCCCATGTGCCTCCATCACCCGTGCCAACCCCCCGCGCTGTCCCTTCCG

CCGCAGATTCTGTGCCACCTCACAGCACTGCAGGAAGGCGTCTCGGCAGCCCCCGGCCGT

CGTCACCCGCTGTCCCCTCTGCTGGCACGTGGCACGGATGGGCAGCGCCGTCGCTCCGTC

CCGGCAGCAGCGCCACATCACAGTGTTATTGCGCCACAGCATCAACGTGACGGTGACACT

GTCGGCAGTGGAGGGG

>c19878_g1_i2 len=378 path=[356:0-138 747:139-377]

GGGGACATGGGGACATGGGGACACACGGGGACACACCGTGCAGTGCCAGCAACATGGATG

CGTCGCCACAGCCAGCTCTCAGGGAAGAAGCTCCTCGTGGGGACGTCCTCATCATCATCC

AACAGCTGCTCTGCCAGCTGTGCCTCCATCACCCGTGCCAACCCCCCGCGCTGTCCCTTC

CGCCGCAGATTCTGTGCCACCTCACAGCACTGCAGGAAGGCGTCTCGGCAGCCCCCGGCC

GTCGTCACCCGCTGTCCCCTCTGCTGGCACGTGGCACGGATGGGCAGCGCCGTCGCTCCG

TCCCGGCAGCAGCGCCACATCACAGTGTTATTGCGCCACAGCATCAACGTGACGGTGACA

CTGTCGGCAGTGGAGGGG

>c19879_g1_i1 len=228 path=[206:0-227]

AAAGTGCACCGAGAACGTAGTTTAAATATAATTCCTAACACACTGCTTATTAGAAAAGGG

CCACGCTTACCTGTGAGCAGTAAGAACTTGCTTAAATAAGCAACTGCCTCAGGCTTCTAA

AGGGTCCACAAACTCCCAATCAAAAATTACTGCTGCCCCATCAATGTGTCATATAAATGT

TGGCATGTTAATTGTGTATTTATAAGATTGTTAATGGTTAGTTAATGT

>c19886_g1_i1 len=213 path=[1:0-212]

CATAAGGACCGTGGCTTTTCTAAATACAGGCACTCAAACTCCTCAGAACAGGGCCCAAGC

AGTGCCTGCAGCACAGGGCTGGGCACTGTGTGGAGGGCCCTACCATTGGGAGCTGTAGGT

AATCACATATTAAATGTCTCTGCCATGATGTTGTGTGAAGGATGCTGCCTTAGCTCAGTC

TTGCTATGACTGAAGAGCTTTGCACAGGCTGTG

>c19886_g2_i1 len=213 path=[381:0-212]

TGCAGGCACACTGAGCCCTGCCTGGCTCAGGTCCGCTGTAGACAGCCTGCACCCAGGGCA

CAGCTGGAACAAGGGATTTAGCTGCAGCTGGTAACTCAGCTAGAACATTCAGCTGTCATA

AGATATACACGGCAATTGTGGAAACGACAGCATGCATGAGCAGGTACTCCCTCACAAATC

AGACTTCTCAATGTCACTGATGCATCTATAAGA

>c19906_g1_i1 len=268 path=[175:0-267]

GGCTTCGGGATGTAAGAAACTGCCCTAACTCAGGTGCCCACTCAGGTGCTCACAAAGTTA

CAGCTTCTTGCATCCCGAAGCCTTCTGTTTGTAGAGAGTAAAATTAAAAATCAAGTCCTC

TTTATTTTATAATCCTAAATTCTGTGTTTCTTCTGCTTGGAAGAAACTTCTTTAATATTT

AGGAAACTATGCAAGTGCAGTGGTGGGGATTTCTGCTTCCCATCGCAGTACACGGGATGG

GCAGTGGAACAAAGATGAACTATACATC

>c19909_g1_i1 len=279 path=[623:0-278]

GCTCCTTCTTCAGCTCTTGACTTGATTCAGAGCGTTCTCTGTCAGAGCTCAAGTACACTC

CAAAGGAAGCATCCGTGGCACTCCTCAACATGAGCCAGTGACCAGCAGCGCCTGGCGTGG

TGTCACCAGAGCCTTCTGGAGCCACAGCTCACCCCAGCTGCAGACAGAGGAGGGAGGAGG

AAAGCACAGCCTGGTCAGGAATGTTTACCCTTGCAGAAGTTGCATCGCTCAATGACATCC

AGCCAACCTATCGTATCTTGAAACCATGGTGGGATGTAT

>c19910_g1_i1 len=309 path=[287:0-308]

AAGCTTCTCACCATTCGCTCATGCCCTTAGTTACCAAGTCCACCTATGTTTCCATTCTTT

TCATAAGGAACAGCCCAAAAGGGAGAGAGAAACAGTCAGGGTGCCTGCTTTGTATCTGAG

CACAGGATCTTCCCAAACTGCTGCCTGGGATCTCTACAACGTGACCTGTCCCACAGTGAC

TGGGTCATTACACAGTTTCACTGCCATTAACATATCATTAACAGAACATCTCCTTTGGAA

GTCCTTCAGTGGTATTTCCATAGTACATGGAGTTCCTGACTTCAGTATACTAGTTACAGA

ATTCAATAA

>c19911_g1_i1 len=428 path=[363:0-228 595:229-427]

CGGCTTCCAATTAGAGCCTCGTTAGAAAGGAAAGCTGGAGAGGTATTAGAGAGCAGGCCG

GGAGTGAGCCCAGCCTTGGGAGCTGGAAGGGACATCCCCACCGCCTCGCCTCACTGGTGG

GCACAGTGGGGCCGGGGGGTGGCAGGGTCCCAGCTCTGGGCACCGAGCAGAAGGATGGGA

GATACCCTTTGGGGATTGCTGCAGGAGCAGGGGTCAAAGCCCCTGGGATGCAGTGAGACT

GTATGGCGCAGGGTTTGGGAATGCTCAAGGGGGAGCTGGGAACAGAGAAGAGGGAGAAGA

TGGGGAACTGGGGGCTGTAGGCTTTGCTGTATCCTCCCACCTGCAGAAACACAAGGATCA

GAGATTGCACATCCACATGACACATGGTCTGCAAAGCCACATCTTTGCCCAGCCCTGGCT

GGCAGCTG

>c19919_g1_i1 len=527 path=[51:0-288 50:289-526]

AGGTAACTTTGTGTTTGAGGACGTAATTTCTTAATATATTTTTATTTTAATATTAATTTA

AGGAAAAATATTAAATGAAGTTAAAATATTATTAGAATTTTTTCTGCAGATTGAATATTC

TTTTCCAACTTAACTATCCATCAATGAACAGATGTTTTTTTCAAATAAAAATAGAAGATA

CATATATTAAAGAACTTTTTATTATTGTGTAATCTGTGAAATTAAAAGAAGAATATTTTT

ATTTTTTATTAAAAATTGAACAATTACAATAATCATAAAGAAATATATATTTTTAAAATA

ATTTCAGTTAATTTTTTTCATTGCATTATTAGATTAAAAAAAGAAAAGACAAAATTAGTT

TTTCAAAATTGAAGTAGTACAATAGAAAAAATTGAAGTTTCAATATGAAAATGATGAACT

ACATTTGTTTATTTACGAAATGAAATAAGAAATATAAATTCTAAAGTAATATTGAATCTT

CTTATATGATAGTTATAAATATATATTTTTTTTATTTTTATCATTAT

>c19920_g1_i1 len=303 path=[281:0-302]

CGTGCAATGCTGTCCAGCCTGCAAGCAAACACACGTTCAGAACCAGGCATCACCTTCGTG

GAGCTGGAGGCAGTGAGGAGGAACATCTGTCTGCCTCTGCTGCCTGTGCCATGCAGCTCT

GTGGGACAGCCAGCCCTGTGCCAGGACCTGCAGCTCTGCCCCACAACTGCAGCCCTGCAG

GAGCCCCTGCAGCCCCACCTCTCCTCAGCTGCACCCAGATCGTGTGATGGAAACAGTACA

GACCCAGCAAAACCAGTGGTGAAATTTGGGGTACGCAATTAGGAAAGAGACTAAAGCGAA

CAG

>c19923_g1_i1 len=232 path=[192:0-170 473:171-173 478:174-231]

AGCATGCTCAACCGTTTCTCTGACCTTTTCTCCAAATTCAGGAAATGAGCAATCAAAGTG

CCAACTTCCCCTTCCCCATGACAACCCGTCTGCCACTTGCACAACTACTCCCTGACTACC

AAAAGCACACAATAATGCTTCAAATACACTCACCCACAGCCCCTATACAGCGCAGAGGTA

GCTGGGCGTTTCTGCACTGCTATTCAGAGCCTGCGAGGTGGTAGAGCTAAGT

>c19928_g1_i1 len=215 path=[1:0-54 56:55-214]

TAAGAGAACGAGAGAAAGAAGGGGAAATAAATAAGAGGCAGTGTGCGGAGTAACACCTGA

ATCGGCACAGAACGAAACGGGCCGTTCCACGTAGCGCTATGACAGGGCTCAGGATCTCCT

CTGCCCGTTCACCTGACGAAGCCAGGCGCTGCCCTGCTCAAACAGCTCAGCGGTTTCTAA

GCTACCCCACTGAGGGAGCAGAAAGCCGCCTCAGC

>c19929_g1_i1 len=270 path=[1:0-197 284:198-269]

GACTCGTGAGGTCAGCAGCGTTCTCCTATCAGTGGTTCACTTCCAACCAAACCAGCAGGC

ACTGACCCAGCACTGCGCTTTTTGTTTCACGGGACTCGGGTTCACCTGAACCTTTGCTGA

GAAACACTGATGTTTGAAAACACAAAATCTGCAACTGAATGCATGATTTTCAGGTGACAA

AGCAGAAGAAAATTAACTGCAAATATTCAGTGCTTCCTTCTATGTCAATGTTGACAGTTC

CTTTTGCTTAAATGTTTCCTGACCATTTTT

>c19929_g1_i2 len=355 path=[1:0-197 199:198-282 284:283-354]

GACTCGTGAGGTCAGCAGCGTTCTCCTATCAGTGGTTCACTTCCAACCAAACCAGCAGGC

ACTGACCCAGCACTGCGCTTTTTGTTTCACGGGACTCGGGTTCACCTGAACCTTTGCTGA

GAAACACTGATGTTTGAAAACACAAAATCTGCAACTGAATGCATGATTTTCAGGTGACAA

AGCAGAAGAAAATTAACTGCAAATGAGTTAAAATTGCGTGAATGATTTCGTATTAGGCTT

CAATAGTTAAAAATGGTGACCTTAAAGCCAAAGCAAACGAAACGCAAATATTCAGTGCTT

CCTTCTATGTCAATGTTGACAGTTCCTTTTGCTTAAATGTTTCCTGACCATTTTT

>c19934_g1_i1 len=206 path=[184:0-205]

GCAGCACTGGGCAGCGAGGCTCAGTCCTGCCAGATGCAGACAGAGCAGCATCTATGATAA

AATTCCCAAAGAAAAGACACAGTTACACAGGAGCTCTCTAGCAGTTTAAAAAAATAAATG

CAAACTCATTTAAAGGGTAGTCTGTGTCTGGATATCACTCTATTAAAAATAAAAGTAAAG

AAAAAAACAGAATGGAGGATTATGAG

>c19936_g1_i1 len=1213 path=[155:0-1212]

GAAATTATAAACCAGAAGATGGAAGTATAACTGATTGATTTACTGGTTTATAATTTCTTC

CTTTTTTCTTTGTTTTTGCATCATTTTCATCTATTATTCTTGTGTTTTCATCATTTTTTG

TAATTATGTCCTTATTATTAATGGTTTCTTTATCCTTTTGTAACACATTTCTTTCTATAT

CTGGGTATGTTATATTTTGATTAGGATCAAAAGAATTTACTGTTAAGAAAGTAAAAGAGT

AATGATTTTTTTTTTTTAATAAGCATGTTGGCATTTCATCAAAAAGGGAACATGTTCCAA

AATCATTCATTGAATTTAGTTTTTCATTTGTGGAATCTAAAATCCAAACTCCCCATAAAT

ATTGTTTCTTCCTTCTATCATCATATTCTGGAGAAGAATAGAATAATCGTTCAATACATT

CTTTAAAGGAAGTTCTATAGTTTTCATGACGTTTTCTAGCACTTTCACACTCTCCAGTAC

TGTAGTTAAAATAACCAAATTCTGTATTATTTAATGGAAATCTCGGAGGACATTTTGTCT

TATAGTCTGGACGTAAAAAGGAAGATGCATAAGTCCATTCTTTTCCTTGTTCTTGTTTCG

TTGGTAAAAAACATCTTTCACGACTCCTTGAATTATTATGTAGACAATTGTTAACTCTGT

ATTGTTCGTGAGTTCCTAATATAACACATTCTTTTGTTTTATGATTAAAAATAACAGGTA

AACTACGAAAGCATTTATTATCTAAATCTTTTTCAAGGCATGGACCTATGCAATGCGAAT

AAATGGAACAATCAGACAAAGTATTTATTATATGTGGATTTAACATTTTTTTAGTTACAT

AATTTTCTGTTGGATCATAATTGTGTAAGGGATATTCAATATTAGAACCTAATCGACCCC

CTAGTCCATGAGAACTAAAATATAAAAAGTTATTCCTTAAATTTTTTGTTTTAACTTTTG

TAGATAAAAGACGGTTATAGAAACTTTGAAAACACATATCATATTCTTTTTCCTCTTTTA

TAAGCAAACTATGATTTTTGTTATTAGGACAACGCACAAAAGAACCCTGGTACATTGCTA

ATCCACTCTTTGAAGGTGGGATTTGAATCTCTCGTTTTAATTTTTTATTCAAAATTCCAA

AACTAATTTCATTTAAAAAATCTTCGTTATATGTATTTTTTTGGAAATCCATTAAAAAGG

TTTTTCCATAATT

>c19947_g1_i1 len=385 path=[565:0-91 13:92-384]

AGCTGTCCTCCCTCCCCTGCTGCAGGCTCCATCTTTCTCAGCAACATTAACCATTACTCT

TTCGTGCAGTCTTGCACTCGCCACTTGCTTAATGCTCAGAATGGCCCTTGGGTGGCTGTA

AGGGTACGTATGACAGTGGAGCAACTTAAAGCAGGCATTTTGTTTTGAAAGAAAAAAAAA

ATCCCAAAGCAAGTCTAAACTGAAGTTTGTAACCTACTTTTTATTGTAATAAGAGCTTCA

TGGTTATGCTTGTAATAAATTATTTACAAAGTAATTGGACAGAAACAGCGCTAGTGGGGA

AAGTAGTGTGGTGTCTTTCCCTTCACCTGTAGGCAGTGTGTTCTGCCCAGCCTGCTCCCT

GCAACGGTGTCACTGTCACTCAGAA

>c19947_g1_i2 len=305 path=[1:0-11 13:12-304]

CAAGCTGCTGCCTGCTCAGAATGGCCCTTGGGTGGCTGTAAGGGTACGTATGACAGTGGA

GCAACTTAAAGCAGGCATTTTGTTTTGAAAGAAAAAAAAAATCCCAAAGCAAGTCTAAAC

TGAAGTTTGTAACCTACTTTTTATTGTAATAAGAGCTTCATGGTTATGCTTGTAATAAAT

TATTTACAAAGTAATTGGACAGAAACAGCGCTAGTGGGGAAAGTAGTGTGGTGTCTTTCC

CTTCACCTGTAGGCAGTGTGTTCTGCCCAGCCTGCTCCCTGCAACGGTGTCACTGTCACT

CAGAA

>c19948_g1_i1 len=243 path=[1:0-98 100:99-242]

TTGAATTCCTCCGTGTGGGGAAAAAATGGCACCCACTTGCATTCATGAACTCTTGCTGAA

CATTTATGGAAACCTAATAGTGAATTTGAGCACTGTGAGGTGATGGTGAGTGGTGCATTT

CAGCAGTGACAACTACGACAGTGGGTCACCTCAACTTGACATGCAGGCTCTTGTTCAGCA

CAGATTAACATAAATTGCTAATGGCGGCAAATATGTTGAAAGATAGAACAAATTTGCAGC

TAC

>c19952_g1_i1 len=299 path=[308:0-242 16:243-298]

GCTACTCAAGCTAGATCAAAAAGCAAAAAATTTCCCATTTTTAATTTACGTAATAGTAAT

ATTTTTAGAATTTTAGTTTATGAAGATATCCTTTTATCCAAGAAAATAAATGGTTGCAAT

GGAAAAAAAATAACTAATATTAATAAATTCCTCAAGGGCATTAATATTAATATATATTAT

ACTTAAAGGTACAACAAATGAATAAATTGTTTTACGTAATACATCCAAAAGCTTAATTCT

ACTGTGCATATATATACACATTTGCATTTGTTTATTTTTATTTTAATAAAATTATATAT

>c19956_g1_i1 len=249 path=[1:0-248]

GCGAATATCACAAAGCCAAGGCTTCCCACGAGCGAACACCTCTACAGCGGTACAGCCCTG

AGACACCTCAGGGAGCGCCCAGCTCCACACAGCACGGCTGTTATAGAGCCTCAGCGAGGC

TGGGGGCTGCTGGCAGCACGGCAGGGAGACAGGCGCTTTGGGTTACGTTATGGATGACGA

GGTGGTGTTCCACCAGCGAGCTCCCACGCTGCGCTGTTTTCTTCACTTACACAAAAAGAA

AAAACGTTT

>c19956_g2_i1 len=600 path=[1030:0-599]

TATTTTGAACTTTATCTCCTCTCCTCTGCTTTGTTTTTCTTCCTTTCTTGTTTTGAAGAA

TACTCTTTCTGGTCTTTCAGTAAAAATGTAGTTCTATTTAGGTTTTCTCTTTTCTCTCTA

ACATTCAGAGTATTTCTGTCTCTGTACTATTGCCTACCTGATCCTCCTATCGTACTGCTG

GGTTCCATATCAGTGGGTGTAAGAGCTGTTTTTGGTCTTGATAATTGCAGTGCTTTTTAA

CTCTATTATCGGTGTGCATTTGCTGAAAGCTACGTTTGTTCTTGAAGCCACTTTCATATA

TTTTTGTAGATCTGCTTTGTCTCGTCATTCCTGTTATCTCATACTGTGATTTTAGTACTT

CATGTAGCTAGCTGAAAGGATTTTCTTTCTTAAATGAATGATCAGAAACTCTGAGGCCTT

ATACTAGGCCAATTTGGAGGGAAAAGCAACCAAGCAAAGGAAAAAAATGCCTTATTATCT

AGATAAAATGTCAAATCTGTTCTTAACTAATAGCTTGATGCAGTTAAGTAAGGTATGCAC

TAACTTAGCTACTCACTTAATCTCTTGAAATAACTACATAAGACAAACTCACTCTAAGAA

>c19958_g1_i1 len=210 path=[1:0-133 135:134-209]

CGTCTGCCTGGCAGCTGTGACATCCATTGGGCAGCGCCAACAAATGGCCATAGGCTCGAC

ACTTCTCCATGTCGAGATTCCTGACTCTTCTGTCAAACTGGCGAGGTAGTTTGGGGAGGG

TTAGGGCTAGGATTGGGTTGTGTCAGAGTTAGGTTTTGGGTTAGCGTTAGGATTACGTTT

AGAACTAGGGTTGGGATGGGACTGGGCTTG

>c19962_g1_i1 len=311 path=[289:0-310]

GTGAATTGAACCAGCCTGCCAAAGAAGGGACACTGAGTAGTGTTTCTAGAGGCAGCATGC

TGGTAGATTAGCCAAACCAGTTTGTTTGTCCCTTGTGATCTAAATATAGGGAACCTCCTT

AATTAGGGAAGGCATCTGAGCTCTGTGCAACTCTTAATACACGCAGGGAGATGAAGTTCT

TCCCTTAGCGCCCAGCTGAAGGGCACTGCTAGTGCAAGGCTTCCCTCCCTCTGCATGGAG

CTCGGCTGTTAGCAGAGCATAGCACTGAAAGTTCACAGAAGTTGAAACTGAAAATGGATT

GCTGCAGTGAG

>c19969_g1_i1 len=222 path=[1:0-221]

GAACCTTCTGCTGTTCCACCACCACACCGTGCTGCCCGACTACGGCCGGATCCGCGCCCT

GTCGGGGGCCCTGGATGAACTCTACGAGGACTTCAGCGCCCTCAAGGAGCAGCTGGGGCG

GCTCTCGGGGCGCTTCGTTGAGGTGGAGGCCTTCGTGGACCAACTGAGCCGCAGCCCCGG

GCCCCGCACTGCCCCCGGGCCCCGCACTGCCCCCAGGCCCGG

>c19969_g2_i1 len=413 path=[789:0-412]

GGTGGTGGAACAGCAGAAGGTTCCGCAGCTGCGAGGTGATGGCGAACCTCCAGTTGTCCA

AGATGAGCGTCAGGTTGGCGCAGCCCCGCGGCGCCCTCCCGGCGTCCGCTCGCTCGTCCC

ACGCTGCCGGCTGGCGCTTCAGCTTGGCCGCAGCGCCCAGAGCCGCGAGGAGCAGGAGGG

GCAGAAGCCGCGCCATGGCTGGGGGTGGTGCGGTGAGACGGAGCCCGGCTCTCGGGGAGC

AGCTCCGCCGTGACCTCAGCCCCGCACGAGGACATCCCTGCCGGGCCCCGCGTGCCCCTC

CGGGCCCTTAATTGGAGGAGAGGCCCCAGCCCCGCTGCGGGGACGCATCGCCAACAGCAC

CGCAACGGGAAGCCGGGGCGGGGGGACGCGCTGGGCCCCGTGGCTGCCGGCTG

>c19974_g1_i1 len=252 path=[625:0-251]

GGCCTTTCTCTGTCGGGTGAGAGCCAGCCCATAGGCTACGTCTGTTTCTCCTCAATAATG

TCTGAAAACATTCTTGCATGTTTGTTTGCAGATTTAGTGTTGACCACAAAGTTCAAATGC

TCCGGCATTTCAGTTTGGGGATCACAGCAGAAGCAAATTTGGCCACGCAGAAGCTCCAGA

AAAGCAATTTGGGATTCATGTATGAGTGACAGCATATTTCTATTGCCTGTTTCAAAGAGC

TCTTAGAAACCA

>c19974_g2_i1 len=335 path=[313:0-334]

ATGCGGGCTGATGTTAGGGGCTGTGGTAGCTCCTTTCACTCAGTATGTTTGCTGCATACA

GTACTTCAAACTACAGATCTCTCCAGGTCCTCAGATGAACGTATCAGCCCACAATTATCT

CAGCTAACACCGCCCCTAACATCAGCCCGCATTCCTGTACTGACAGTCTTCGTCTGCAGT

TCTCTTTGTGAGAGAAGGTGGGTGGTGGAAGAAGGGGCAAGTGAGAAATTCCAGTGAAAG

GAGACTGATGTATCGTTGCAGGTCGCTGTTATGGGTAGAATTAGTCACTGGGGGGCAGTG

CTTAGTTGTATATTTTCCTAATTGCCACAAGTGAT

>c19977_g1_i1 len=301 path=[1:0-244 246:245-300]

GTTCACCCCCTGACCCCTTTAGCTGGTCTTTTATTTGAGAGTATTCATGAAATGTAGTGA

AATCTAGGAAGACCTAGCTGAGGGCTCAGTCTCTTCTACACTTAAAATAAAAAAGTGCAG

TAATAATTAAAAACAAAAAAATGCAAAAACATAAAAGAGAGGTGATAGGTAATCCTTTAG

GTGGTTGTTCTGCATTTTTTCTAAAAGCAAATGGTGAGCTTCTAAAACAAAACTTTGTTT

CAGAGGAATGGGGGATAAAATCTAACAGTGTATCTTTTGAATATGATGTGAATTAAGCCC

A

>c19985_g1_i1 len=314 path=[292:0-167 460:168-313]

AAAAAAAAAAAAAAAAATAATGAACGCATAAGAATTTTTTTGTTTGGTTTTTAATTATAA

CTGGTACAAAATTTAAAAAAAGATTATAAATTCCATAAAAAAATTAATAAACATATAGAA

CCTTTTTTTTGATTAATTTTAACTTTAATTTTTTTTTTTTCCCAAGAGTTCTTATTTATT

AAGAGCAGAAAGGAAATTTGGCTTAAGTTCCATACATAAAAAAGTAAACAGTAAAATCTT

CTTTTTAAAGTTCCTTTTTTTTTTTCCACTGAAGAAAATTGAGAAATAAGTTGTCTTTTA

GATCATGTTTATTT

>c19995_g1_i1 len=234 path=[155:0-233]

ATTTGCTACAACTCACTGCTGTATGTCACTTTTATAGAGGTTATTAGGTGTGCTGTACAC

CAAATTGAAAGTCTTTGGGGCCTCTGTATTTTGGCAGGCTGTAACTATTTTCCTTGTTTA

ATACTAGATGAGGTGAAGCAAGTAAGGCAATAGGAAAAAAAAGTGCCTGCAGAGGTAATT

AAACTTGGATGCTCTAGGCTAAATTAGGTGAAACCAAATTGTAGCTGATAGACA

>c19996_g1_i1 len=269 path=[247:0-268]

GTGCTCCAGGCTTTAAAGATTAAGAAATCCTGATCTACCATCAGCATGCATTAGTGTAAC

ACGGACAACGTGGGTACAGCTGGAAGCGTGTGACCTGGGAGAGCAAATGTCCAGCCCCAA

ACCCAGCACGTACTGCTGCGGGGCTGGGACAGCTGCCTTCTGCCAGCCTTGCATTCGTAG

TGGAGTACACCCACTGATAATGCTGGCAAATGATTGCGTTGCTTTTAGTGTAAGCCTGTA

GGGGTCACTGACATGAAGCTGTAGGTCAA

>c19999_g1_i1 len=221 path=[148:0-56 318:57-220]

TAAAACACTTTATTGGGGCCCGGGGCTGACTCAGTCACTCCCGCCCGCAGCGCTCCGCCT

TAACCCCTTCCTGCCCGGCCCCCCCAGTGTGTCACTTGAACGCACGTCGCTCCTCATGGC

AGTCCTGGGGATGGGAACAGCACTGGGGAAGGGGACACCTCTGGACAGAACTATCCTTGG

GGACAAGGCCACCTTTGGGTACAGGGCCATCCCCAGGGTTA

>c20003_g1_i1 len=230 path=[208:0-229]

GTATTTCTCCATTCACTGCAACAGCTGAAGTTGTCTGCATATCATGCCAGCTTTTGCTGA

AGTTCTGCTTCAGCTCATTCATCAGGCTCATGCCAAGTTTTTGTTTAATTTCAATCAGGT

GCTTTTCTTTGGCTGCTAGTACTTGCCGCAAAGTTGAAATTTCATCCTCTAGCTTGGCCA

GCTCAGCCTTTAACTCCTCTTTTTCTTCTTCAGAAATCATACTGGTGAAA

>c20012_g1_i1 len=242 path=[220:0-241]

GTCAGAAGTTGGTTACTGTAAATACAGCTTTTCTCTGACTACATGTGGGGGGAAACAATG

GGAAAAGCACAGAACAAAAAAAGACATTAATGAAAAGAGAAAACAGAATATATGCTTCCA

AGTAGCACCAATTTTTCCCAAGTGACAAGTTATTTACTTTTTAAAGTTAATGATGGCAGC

TTGAACTCAGCTTGTCCTAAACTTAAGGCTCCCAACAGTTCTTCCTTTTCCACGGGCTTC

TT

>c20018_g1_i1 len=251 path=[1:0-36 38:37-250]

GATGGCTTCTATTTATCGCTACAGGTAAGGTTCTTGGCTCTTGCCTATGAGTGTGTTCTG

CAAGGTTTGTGTTGGAGCTAACCAAATAAGCTGTCATCTCTGAAAAATAATGTTTGAATC

AACCACTGTTCAGTTTTGAGAAATGTTCACTCTTCAAGATTAATGTTGATTTTTATTTCC

AGCTCTAAGTATGAAGCACTCACTTTTAATTGCTCCTGGATATCTGCTTATAGGCTGTGA

GCCTAATTGAA

>c20021_g1_i1 len=210 path=[1:0-209]

AAGGAGCAGCTGTGTGCACATGACAGGTGTAGGCCTGGAAACTAAACCACCGTGGCATGT

TTCAGGGATAAAATTGGCCTTTGAAGACTGCCTGGTGACAGAGCGGGTGAAATTAATTTT

GCAGACATATCGAGGTAGAAATCTCTCTCGCTGCATAACCAAGGAGCCACAGTCCTGCCT

GCAGCAGGAGTTTGGTGCTGGCAAATAGGG

>c20037_g1_i1 len=247 path=[289:0-174 627:175-181 470:182-246]

TATCAAGAACTGCTTAGCACTTGGCTTATTCCCTTAAGATTTATCATTCGTTCATGTCTA

GAGGAATTTTTAGTGCAAAGTTCAGGGGTGCATTTCATGCTTTTTGCTTCTTTTGTCTAC

AGGATTTTAAGTGTCTCCATTTTTAGAACTTTTTTCCCCCTCTGACTTGCTCATATATAA

ATACACTTATTTGGGGTTTGGATAATTCAATATCCTTATAGAGATCATGCATACAGGGTA

GTTCAAA

>c20037_g1_i2 len=247 path=[289:0-174 627:175-181 25:182-246]

TATCAAGAACTGCTTAGCACTTGGCTTATTCCCTTAAGATTTATCATTCGTTCATGTCTA

GAGGAATTTTTAGTGCAAAGTTCAGGGGTGCATTTCATGCTTTTTGCTTCTTTTGTCTAC

AGGATTTTAAGTGTCTCCATTTTTAGAACTTTTTTCCCCCTCTGACTTGCTCATATATAA

ATCTGCTGCATCATGTATAAATTGAAGAACAGAGTACACTGTTTTGAACTACCCTGTATG

CATGATC

>c20038_g1_i1 len=258 path=[241:0-257]

TAACAGAAAGCAGAGCCACACAACCTGCTCAGCTATCAAATGTAAGGTTGAGCTGAAGAG

TTCTGGCTGTGCTGACTGAATCTCCATTATTTGTAATGGGACCTGGGACGCTCGGCTGAT

ATGGGGAGACGCCTCACTGAGTGCCTTACCCTGTGACCTAAATTTCACTGTGCATTTCTG

AGAGTTCTTGCAAACATAAATGCAAGTATGCAGGAAGACAGTACAGCGTTTCACTTTCTT

TGTAAAATCAGCCTTGTG

>c20039_g1_i1 len=281 path=[1:0-207 209:208-280]

ATGGAAAAGTACTAGGAAGTTTGGAGTGCATTCCCCACGCAGCTTGGCACAAGAGGTAGC

GGTCTACATGCCCGTAACTATAGATATAGAACTTCCATATGCATATTCTCATACTGTTTC

AATGATTGAAATTGCATGCTGGAATTCAGCTGTGGTCAACAAAGACAGAGCCATAAACAC

ATGAAATGGTCCTCCTGGTTCAACTGCATCTCTTTGTGAGTCTGCTTGTAAAATGGCAGA

TGCCCTCCAAGTACAGGAAGTTCTACCTCCACTACACTGTA

>c20039_g1_i2 len=409 path=[1:0-207 540:208-408]

ATGGAAAAGTACTAGGAAGTTTGGAGTGCATTCCCCACGCAGCTTGGCACAAGAGGTAGC

GGTCTACATGCCCGTAACTATAGATATAGAACTTCCATATGCATATTCTCATACTGTTTC

AATGATTGAAATTGCATGCTGGAATTCAGCTGTGGTCAACAAAGACAGAGCCATAAACAC

ATGAAATGGTCCTCCTGGTTCAACTGCAGCTGGTTGGTCTGCTCTAGGACAAGGTAACAC

TATCCAATACTGCCAATTCCTCTTGAGCACTGCTGTGCTGCAGAAGTTCTTGACAACCAG

CTTCCAAGCCAGCTCTCCAGTAAGCACACAGGAGATAGATGGATAAAGTTCCAGCCAAAA

TCTGCTATGAGAGAAATTGAAATAATACTGGTCCAGAAATGATGTACAT

>c20040_g1_i1 len=209 path=[187:0-208]

GGCCAGCGTATCAAAAGGTAAGATTACACATAAGCACTGCTGTCTCCCTGCCACAAAGCT

AACAGTCCTTCTAGTCAAGAGAGATCGGTGGGGGGGTTGCAGTGTGTCAGTTCTTGCACC

TCAGGAGACCCAGATGTGCAAATCTTCCTCAGTCCTCAGCACTGCAGAGAGGCAGGAGTG

CTGGAAGCAATTGGTCCAACTTTCTTAGT

>c20042_g1_i1 len=393 path=[259:0-392]

ATCACGGTGATCATTTCAGCAAGCTGAATATTTTTTGGCTTGCGTGTACAAACAGCTACA

GTTGTTTCAAGCACAATGTTGACTGCTGCCAGTATCTAAGCAAGAGGAAAAACAGAGCTC

TCACACAGAGGCAGAGTTTGCAAAGCAATAAACTGGAGTTCACACAGAGGTGGATTTCAT

AATACTAGACAGAGGTTTGGAATTTAAATCCCATCTCTTACTGGACAAGAAATTATGGCA

AGTTTTAAACATGTTCTGTACACACAGAACTGGCAAATGTAATATGAAACCTTTAGTTTG

CCTACCACTTTTGAAGGATTCGCTAAGGGAAGCAAGTTCATTGATACAGCACTTTAGTGC

CACCTAAATTACAACACCCACTGCCTCACTGTG

>c20047_g1_i1 len=213 path=[191:0-212]

ATGGGCAAAAAAGCATTGTCCACAGTTGCAGCATGTGAAAAGGGTGGACGTAAACACTGT

GAATCCTGCAGTCCTGCACAGCCTCACCTCCGGCTCCTCTATCGCCCCGTGTTACTCATC

CCTGAAACCTTTGGGCTTTTGATGTTGAGGTTCTCATAGACGGAAGCTGACTCATCATCA

GCCACAGGCTGATGATGAGTCAGCTGGCCAGAT

>c20048_g1_i1 len=266 path=[398:0-265]

AAACTTACATTTACAAACACTTTAGTGAGTTTCACGTATGCGAGAGTTGCACTGAAGTGT

TTGTAAATGTAAGTTTCAAGTACAGGGTTGAAATGCTGAAACTTTATGTCTCCAATCAGA

GAAATAATAAATACCTCTTCAGATTCATCGTTCAGTCGAGAATATTGTACCTGAGGTTTT

ATGGGAAAAGTGAGGATGGTGATGAATTTAATGATGCAATACGCAAGCTGTTCTTCTCCT

TCAATGTCCTCATGGACCGTCCTTTA

>c20049_g1_i1 len=334 path=[312:0-333]

GGACTCCAAATGGGACGTGTGCCATCACGCTCAGTGGAAGGGTCCGAGGTGGCTGGGGAC

GTGTCTGAAGACCTCTGGTGACCCACTGAGGTCACGTCTCCATCACTGAGACTGGTGACA

CCAGGATCTGTTCCACGGGAAGAGCCAAATCTCAACAGCTGGCACTTGGGGACCATCTTG

GAGCGGGTCTGATGTTGGGGGCCCACCGTGTGCCCCGAAAGCACTCACAGGCAGCGCGTT

GGGTTGCAGCTGTGTGTGTTTTGTGTCACCTCGGGAGCTCAGGATGCACCTGGGCAGCGC

TGAGCCCCTCTCACTGCAGCAAACCCAACTTTCG

>c20060_g1_i1 len=263 path=[275:0-262]

ACCAAAATGACTCCAGCTACCTCCAAATTTTTGGGGCAGAAGGATAACAAAAATGGTTTC

ATGGAAACTAAAGTCTGTCAGTTCATTGGTGAAAGCACTTAAATAGAACTTATTAGCTAT

TCAGGAGGAAGGTTTCGCTAAAGGTACAATGCATTTATGTATAAAAGTACAGCTCAACTC

CTGAAAACTGCCTACGTAAATAAGCACTTGTCACATCCTATTTTTGTTGATCTTTCTTTT

TCTCCTTCTAAACTTGTTTACTG

>c20071_g1_i1 len=363 path=[1:0-340 726:341-362]

GTTTGGGTGCCCGCTATCAAACACAAACTTTTGTCATGTTGAAACCTGAAAGTGAACCAA

TTCAGGTTCTCTTCTACAAGATTTTGAAAAATAAGATTACAAGATTCAGAATAAATTTTG

CCTGCCCTGTGGAGTTCCTTTATTCCCTTCAGCTCAGGCATGTCCTACCAACGGCCCGTC

TGAGGCCCAGCATAGCTCAGAACGCAGCCATTCCCAGCCCCACACTATAAAAGCAGCAGC

TCTCACCACAGAGGGGCCCCAGGCACACACGCATCATCCACAGCAACCCAACTGTCAGTG

CACGAAGTGATGGCAAATAATTGCATACATCTATGTTGTCCCACAACATCAGAGGCAGAT

GTT

>c20075_g1_i1 len=279 path=[223:0-278]

AAAACATTGTATTTTCTCTACCAGTTTTACAGCATTGTTCCATAAAATGTGAATTCTGTG

ACTCCTTTTAGATCTGCCTGCCCAAGTCTCTCAGTATTTTTGGCTTTGACAGAAAGTACA

ACAGATAGAAATGCTGAACAGTCTCCTAGGCTTCAATATTGAAAGAAATTAGAACTATCC

TCTCTGAAAGGCTGAGATACATCTAAGCCTAAAATTGCTTTATTAGAGTGCCCTCTCCAG

TGCCATGTGAGATTTCTTCCTATCCAGATGCCAGGCATT

>c20076_g1_i1 len=220 path=[382:0-219]

GTTATGTAAATAGCATCCATTTTCTAGCTTATGCTTAGGCTATAGAGGGTGTTTTTCCCT

CTTTACAGATGTCAGTCCAGTCTTTGTTATCCCACCTAGCTCAGTGTGTCACTCACAACT

GAATTTGGCTCCTTCCTATTTCCGTGTAGCTGCCAGATCCTCCATAGATGGTGTGCTGGT

CTTGTGTATAAAGCCTTTATGTATACATTTTCTTAAAAAA

>c20089_g1_i1 len=269 path=[247:0-268]

TATTAATATACTAATTTTGAAGAAAAGATACAAATAAAAATAATTTTTATTATATGCTTT

TAAGCTTTTCTACTTATTAAAGAGCTAAAATAATATGTAAATCTCATAAATAATCTTCTA

CATTTTAATGAATTTAAACAAAATTAAAAATTTTTTATTTTAATTAAAATTTTTTTTCAC

ATTTAATTAGATATATTTAATTTTGCATAAGAATAGCAATATATCATTGTAAAGGACACC

TTTAATTTTCCAAAAAAAAAAAAATCAAA

>c20091_g1_i1 len=323 path=[301:0-322]

GCGAAAAATTCCATCTCTATTTCAGCTAGCACACCTTAAGAAAACTCAATTAAAGCACGA

AGAATCCTGCTGAACCTGATGTAAGTTTCATTAAAGAAAGAGCAAAGCTGTACAGTCAAA

AGGACTTGCATCTGTACAAATAACATTGATATTACCAGTGTGCTTTGTGCCACTCTCAGC

AAACTGTTCGGGGTTGAGACAGAAATGTATTGTACAAATATTGTACTGAAGAGAGGCAAA

TCACATCTTCAAGCACAGGAGTTACATTTTAGAGCATAACCTTAATCAAATCGGTCGTTG

TGTTTTAGTGATTTCTACATCAG

>c20093_g1_i1 len=234 path=[364:0-118 19:119-233]

TTGGGAAATCTCGAATGTATGGAATTACAAAGATGAACTGTCTTTTTTATCCAATCCAGT

TGTTTCCTGTGTACGTCGAATGGCTGGCAAGATGACTGTTTGCTCTCTCCAGTGATCACA

GACAGTGTGGTGGCTAGGTGGGGCAAGCTCTGGCAGCTCATAAAAATCAGGTCTGTAGGA

GCCACGTGCTTTTTGCGAGGTGCTTCTGTTTGCTTGTGCTATGCAAGTAAAACT

>c20098_g1_i1 len=212 path=[190:0-211]

GTGGTTGCCTTTGTTTGTTGATTTGAAACTTGGATCTACAGCTACGTGATTTAGAGAGAG

CTGTGCTGTCCCTTCATAACTCTAGCTGCAGTCTGCAATAGCTCTTGGCACTCAAAAGCA

TAAATCCAGTATTAGTAATGCAGTGATATGCAGTTACCAGCCAGTTTATGGTGAGATTTA

GGGAGATGATATAAGCATTTTAACTTTGTGTT

>c20101_g1_i1 len=231 path=[455:0-230]

CACTCGCCGTCCTCACAGCTCGCGGGTCGCGTTACGCCCCACCGTTGTGCAAACAGCAGG

TGTGCGAGGTGCCGCAGGCGTGACAACGGGGCGGCGTTGCCGGGCTTAACGTCGGTGCGT

CCGTCCATCCGTGCCGCAGCGAGCGGTGCGTGCGCCGTGGGGCCCTGCGGCCGGCAGCAG

CGGTGCCGGGGCCATCAATATTTCACGAGGAGAGAGCGCCGTGACCTGGAG

>c20102_g1_i1 len=361 path=[574:0-324 207:325-360]

TGTTTGAGGGGGTTTGGCTGCTATCAGTTGTGACAAACAAACATAGCATGGCCAGAATAA

TTTGCTGTTGTGCCTTGACTCCTCTCAGCAGCACCTGACCAGACTGACGCCTTCTTTCCT

AGGCGTGCTTTGACATTCATACTCAGTAGATTTACTGCTTCTCGTCACACTTCTCTTTCC

TTCGTAAATGCCATTGCTGACAAAGACAGAATCCAGCTTCCTTTCAAATCCTCAAGGAGG

GGTGCTTTTATTGATGGTTGTTGGAAGAATGTTGTGGACGCTGTTGTTGTACAACCTTGA

CCTCTGTTATAAGTGTCCCAGGAAACCAGCTGCTACATAAACAGTAAAGCAGCACTTTTT

T

>c20107_g1_i1 len=243 path=[221:0-242]

GTAGTAGGTGCCAAGATGGGAAGGCATATGAGCAGCTGGCAGCCTGGTATTGGGGTAGAT

GCCTCCAGTTGGTGAATTCCAGTATGGATTAGGGGCAGCAAAAAAGCTGGATGAGGTTAC

GGGCAAAGCAGGGGGATGGGGAGCTACAAAGTTCATCTTCTGGGGGTGTGCATGGTAGGA

ACTCATGTAGGGATTTAAGGGAGATATTCAAGAAGTATTAAGATACTAAAATGTAATGTA

TAT

>c20111_g1_i1 len=220 path=[392:0-219]

AAAGCTATGTTTTCTTTTGGACTCTCCAGCATAGGTTAATCTTGAGAAATAAAAAGTAAA

TAATATGCAATCAAGAGCAATAACCTGTTGAGAAGTATGGATGGAGTGCTAGCTAAATTG

TCTGTGACATTTATTTTTAAAAGTGCTTAAGATGTTATTGAATGCAATGAAAAAAAAGCT

TCTGTTCATGTGCCATTTAAAAATAAAAATTGAATGGCTA

>c20112_g1_i1 len=217 path=[465:0-216]

GGCAGCTGAAAAGGTCAAGGAACAGGAAAAAGGAGGACAAGCTACATCAGTGACACATCA

CCCATGCTTGTGTACACTGGGACAATCCAAGGGAGGCAAAGCAGGGCTGAAGGAAGCACA

AGCTTTCAGACACCTCCCTGAGACATAAGCCACACAGTTTTGGAGGGTAACATTCAGCTC

TGGTAGGGGAGGTCCATGGGTGATGTGTCACTGATGT

>c20115_g1_i1 len=280 path=[26:0-52 79:53-279]

CTACAGACACTTTCAGCATATGTGTGAGTCTCTCCCCCCCAAATTAAAAACATTGGGGTC

TTCCTGTATGAGCTGTGGGATGAGATTTTCCTCTCATCATTTACTGTTTCTGCTTTTTCT

TTCCAGGTTTACGTGCTGCAGAGCTGAGTAAGTTTCCCTCCATACAATGAAGGAATTTGG

GGTCATCCCATGGGATCAGCCATGAGATGATCATCTGACCCTTTTCATCATGCGTTTCCT

ATTTGTTTCTTTTGCAGAAAAAAACGTTGCAGAACTGAGT

>c20117_g1_i1 len=265 path=[1:0-264]

AAACACCTCGGAGAGCTGGCAGCTCCCGAATTTCGGGATACATCGTTCAGAAACGCAAGA

TGGGCAGCATGACCTGGGTGCCAGTCAACAGTGTGCCCGTAGCAGACAAGAAGCTGAAAA

TAACCGATCTCAAGAAGGGTCTGCAGTATGAGTTTTGTGTGGCAGCTGTCAATGCTTCTG

GCATAGGAGATATGAGTGCACCGTCACAATCTGTCTTTGCTCGGGACTCCACAACACCTC

CAGGTAAAGTGAGGGACCTTAAAAT

>c20118_g1_i1 len=203 path=[1:0-87 89:88-202]

CCTGCCCCGCCTCGGCTGAGAGCGGCGGGGACGGCCCCGACTCCTGCGTGGGAAAAAGCG

TGCGTGTTTAGAAAGCATTAGTAATAATAATAATACTAAAAGAAAGGAAAACGAGCAAAC

AACGTGCACGGCTGGCAGCCGTACGAACGGAGCTGTTCGCTCGGGCTGTGCGCATTACAG

CTGTCCCGGCGTGCGGGAGCAGC

>c20120_g1_i1 len=269 path=[1:0-268]

GTTGAGTGACCCTGCAACGCAGACCGCTTCCTTGCCCCAGGAAGGGCACCGGGGAGGTAA

GCTCTTGGTGTGCCCCATGTCGGGATGACTGCCCGGCACATCTCACGTCCTGCCAAGTGG

GACTTCTCCATTTTGCAGCACTGGCAGCAATCCCACCTTCAGACACCCCTTCAACCCACC

CAAGAGAAGACATCCCCACCCCATTACACACAGCCCGGGATCCGCCACGCGCTGACCCCG

ACCGTGCACACACATTTTAGAGCGGAGAA

>c20121_g1_i1 len=465 path=[223:0-464]

GCTGCTGCTGCTGCTGCTGCACGGCCGCTGGTTCCCCTTCCTCTGGGCTGACCTGGGAGC

CTTCGTGGCTTTGGTGGGCTCATCCCTGCGGTGTCGGTGGCGCCTATCCCGCCGGCCCCC

CATCACCCTCCTGCAGGTTTTCCAAAGCCACGCTCGACGCCGACCCCACCACCCGCTGCT

GCTGTTCCAGGATGAGGTGTACACCTTCAGTGACATGGAACGGAGGAGCAACCGCGCTGC

CCGCGCCTTCGCTCTGCGCCTGGGCTTGCAGCCGGGCCAAACCGTGGCTGTTTTCCTCCC

CAACGTGCCCGCCTATGTCTGGACATGGCTGGCTCTGGCCAAGCTGGGCTGTGCCATGGC

GTGCCTCAACTGCAACGTGCGGGGCCGTGCGCTGCTGCATGCACGGAGCGCTGCGCAGGC

AGACATCGTGCTCAGCTGTAGTGAGCTCCAGGCCGCCGTGGAGGA

>c20123_g1_i1 len=456 path=[477:0-125 22:126-455]

AAGAAGATGATGATGAAGAAGAAATACTAAAAAAAAAGAAAAATCAAAAAGCAAAAAGGG

CATCTAAAAAAAGAGTTAATGAAAAAAAGGATATAACAAATAAAAAGAAAAAAAAAAGAA

AAGGGCAAACTGAATCTGATGATGTTGAGAATAACATGATAGATGATATACAAAAAAACA

ATAAAAAAACTACACCTTTAGATGTTAAAGAAATAGAAAGATTAATTGTTGAAAATGTAA

CATTAAATGATCCTGGTGATGGACTAAAAGATGTTGAACTTTTAGATTTGATTATATTAG

GAAATAAAGATAAAATGCCAGAATTGGCAAATCTTGATATTAATCAGTTACGTAAAATAA

TAAATTCATTAAATGAGATGGATGGAGCACCTATTTACTATGTAAAAAAGGATAAAATAG

TATATAAATGTTAAACTACTTTATTTAATTTTTTTT

>c20131_g1_i1 len=417 path=[1:0-416]

GAGGCACGGCCGCGTGCAGGACGCAGAAAAACGCAGCAAAAGGTCTTCAGCAAAACCTCC

CCGCCCTCCTCCACAAAAACAAACAGCTCAGAGAGCCCTGGGGAGCTGCTGCAGCGGCGG

AAGCTCTCGGCCGGGTGTGTTTGTGCTGAAGGGACGCCGCGTTCCCATCGCGGCCCGGGC

TGCTCCCGGCGGCTGCCCCAAAAGTGCCACGTGGGAGCAGGGATCGCTGCGGCGCGGAGC

TCCGCTCGCTGCGCGCCGCTGGCTTCCCTTGTTTGCCTCGTTCGTAACGTCCCGGCTGCA

GCTAAAAGGGCCGCTGCTGCACGGAGAGGCAGCCGAGAGGCAGCGCAGGCGTTGCTCAAC

CCCACTCGCTTCCTTCTCGCGAGAGGACGTGGCCGTTCGCCCGCGGTTGTTTTGCAG

>c20133_g1_i1 len=307 path=[253:0-306]

ACGATTTGCTTAGGAAAATTAAGGAAGGAGCATTGTGCCATTGTTTTCATTACCTGGCAG

CTGTGAGCAAACTGCAGGAGAAGCAAAGGGGAGATGACAAGTCAGGATCCAACCTCGTCC

TTCACCCTTCGTGTGTCACATGGCCAAGAGGACCTTCCTTTGTCTTCAAAGCTCTTCACC

GTGGTGGCATTTGCACTGGATGATGACCAAGCTGAGAAGAACAGAAATCACAGATCAGCA

GTGCTTGTGCTCCTAAGTCCAAAGTAGTTGTGCTCTTTTGCCCAAACCCTGCCATGGGCT

GAGTGCC

>c20146_g1_i1 len=221 path=[1:0-220]

GCAATGGCACAGCTGGATTTAGTAGCCCAGGGCATGGTGCATCACACTCAGCCTGCTCCC

ACCACCACCTTGCTCATCAGAAAGCAACACGTTCCACAGAGCGAGCAGGAATGCAACCTG

GCACGGACAGCACTGTGTGATGCTCACCAAGGGTTTGCTGGTGAAAATTTAAGTTCATTA

CAGGGCTGATGCTTCCACAAAGAGGTTGTGACTGCAAGGAG

>c20149_g1_i1 len=241 path=[363:0-105 70:106-240]

TCTGCTGATATCATTGATATCAAAAGATTGAGCACCATTGACAAGTTACAAGACCTACAG

ACATTGATGGTATCTGATTGCCCTTCTGGGTCAAATAAAGTATGGGTTTTGTTTTCCTTA

GAAACCAGTTGTTAAGTGCTGTTACTTCAAAGCAACATCAAAATCAGAAAAGTTCACACA

GTTCAGGCAGTAAACAAACCAAATCTGCTAAGATACAGTAGCAGTAAAGCCTGACAAAAT

G

>c20149_g1_i2 len=204 path=[1:0-68 70:69-203]

GTTACCTATTGCGTGTAGGTCCCATTTAGCTTGCATCTGTTAACTTCTAAGGGACTCTAA

AATATTGGTTTTTGTTTTCCTTAGAAACCAGTTGTTAAGTGCTGTTACTTCAAAGCAACA

TCAAAATCAGAAAAGTTCACACAGTTCAGGCAGTAAACAAACCAAATCTGCTAAGATACA

GTAGCAGTAAAGCCTGACAAAATG

>c20154_g1_i1 len=206 path=[404:0-205]

GGTAATTAGATATCCTTTATCCCAGCCATGGGTGGAGAAAAACAGGAGAAAATCACTTTC

AATAGAATTAGTTTAAAGATGTTGACAAAAAAATTTCCCATTAGAAACCCTTAAGTAAGA

CAGAGGTATTTAGTAAACAAAATCTTTCCAACCTTTAGTTATTGGCTTAAAAAAGTGGCT

GCAAATCAGTGAATTAAGTTGTTCAT

>c20156_g1_i1 len=241 path=[219:0-240]

GTTTCTTGATACAGAAACAATTCCAGAACCCAGCTTTTTCAATAGGAAACTCATATAAAA

TGCAAAGACAGAAGTATCTATACTTAATGTTATATATTCTATTCACATGGAGCTACAGAA

ACTTAATGAAAGAATGACTTATACTAAGACCACAACTCTTAAAGTTGTGCAAGCGTACCA

AATGGACAAATATAAAAAACAAACATAAGGATAATTAAAAAAAGCAACACTCGAGAATAA

G

>c20159_g1_i1 len=222 path=[1:0-136 138:137-221]

AAAAAAAAGCGAGGACAATCTTAATTCTGCTGATAAGAGTGGGAGGAAGACTACTGCTTT

TTCCCCACTCTTTCTTCAACCAACTTGCAATGCTAGTTAGGAACATCTGTTAAACTCAGC

CTTCAGAATGGATGTAAATGACCTGGGATAACCTAGTGCCATATTCTTTAATCCCTGCTT

GTGCTGTGGGTCTTAATTATTTCATGAATACTGATTTAAAGA

>c20166_g1_i1 len=270 path=[248:0-269]

TTAAATAAAAATAGTCATAAAGGTATATATATATTTTAAAAAGTAATATATATACTTTGA

ATTAAATATCAATAAGAATTTCATATGTTGAATAAATATTAAAAAAGTATATTTAAAATT

TAGTAAAATTAAATAAATTTTATTAGATTAAAATAAAGAATAGAAGAATCAAATGGAGTT

AAAATAAAAATGCTTAGAATACCTGAGAATATAAAAATTTAAATAAATATTAAATTTAAA

TTCATTCATTTAAATATAAAATTAGATAAA

>c20167_g1_i1 len=299 path=[525:0-298]

AACTACACATATGTGGCTTCTCGGCCAGACCACTGTCTATGCACGAGGAGCTGGCTGTAG

CAACCCTGAGTGGGTCCTAGGCTGGAGGTGTCATCCGAGAACGTACTTATTTTTGTCTCT

GAAGCCTTTCTAAAGCAAGACAGGAATGAGGAATGTTAGGTATTCTTACTGAAATCTGTG

TTTTTGTGAACATGTGGACTCAGGGAAAAACATAAATAAAACAATCTTGTTAATACATGT

ACATACAAGAGGGCAAATCCTTTTGCTGGACCACAGTCTAATAATAAAAAGAGAAAATG

>c20172_g1_i1 len=223 path=[1:0-222]

ACTGCTCTCATTTCCCCGGCAGGCTGCAGGCAGCAGCACGGGAGGCTGCACTGAGCCAGC

GTGCGGGGAGCGCGCGTCAGCCCCCTGAGTCACGGTGACTCCACCAAAACAAGCAGCAGC

TGCTGCCAAGCAAGCAGCAAATTAACCGTGGCCTGCCAGGGATAGATTTGATTAAAATGG

ATACCAGAGCCGCTCGTGCCACTGAGTGAGCTGTGCTGGGATG

>c20175_g1_i1 len=242 path=[1:0-163 165:164-241]

TGACTTACTGCAAAAACAGATCAGTAGTGGATCTACACTCACTGGAGAGAAACTGAACAT

TATGCTGGCAGTAAGCACTTTCAAAAGTCCTTGGTTTTGTAGTGTAACATAGTTTTCACT

TTTCTGATACCCATTGTCAGTCTGTTTGTAAGGGAGAATGTTAATTTTTTTTTACCATCC

CATAATAAGTAATTGATTTAAGTTCTTGATGTGTCTACTCTTTGTTGTGCTAATCCTTGG

TA

>c20183_g1_i1 len=231 path=[1:0-167 169:168-230]

GTGTTTGAGGTGGTTTGTTTGTTTTTGGCACAGCGCCTTTGTTTACTTAAGGTTTGTTTG

TTATAAAAGCATTTCTTTTCCTGTAGTAGCAGCAATTAACAATAAGCAATTACTAACACA

TGCAGTTACGTGAAGAATGACGCTGCATCCTCACATTGTGTTCTCTGAAAATTCTACCTG

GAAACATTTGGTCACTTCTGAAGGGATGTCCAAACCCTGAACATAAATAAT

>c20184_g1_i1 len=224 path=[246:0-178 25:179-223]

CCTGGGGCAGAGCTGTGTGAAAGGATGGCCTTGTTACCACCAGCTGAGCCCAGCCCTGGC

TTCGCTGGGTCGGTGTTGCTGTCAGCAGATGCCTCTTTAGCAGAAGTAGTAAAAGAGAGA

AATCCACACGGAAAGTAGGTTAATTTTTAAAAAGGCAACACATTTAAAGAGGTTTGCAGG

TTCCCCTGGGAAGCCCTCGCGAGGACTCTGCCAGGCAGGAGCTG

>c20184_g1_i2 len=224 path=[246:0-178 424:179-223]

CCTGGGGCAGAGCTGTGTGAAAGGATGGCCTTGTTACCACCAGCTGAGCCCAGCCCTGGC

TTCGCTGGGTCGGTGTTGCTGTCAGCAGATGCCTCTTTAGCAGAAGTAGTAAAAGAGAGA

AATCCACACGGAAAGTAGGTTAATTTTTAAAAAGGCAACACATTTAAAGAGGTTTGCAGA

TCCCACCACGCAGCCCCATCAGCTCCTGCCTGGCAGAGTCCTCG

>c20184_g2_i1 len=223 path=[689:0-222]

GTGGATTTCTGGCTTCTTTTCCCTACTGTTTGCATGATTAACTCTGCACCGCTCCCTTTC

AAACACGACTCCCACTTGTAAATCCTGCTGTGCGGAACCAAGTCGGTAGCAGTGAGGATT

TGTCTTTTTGAGTGAAAGTTTGTTTGACCTGAGCTTTCACTGAGTGCTTTGTCTGCTCTG

GATACAGCCTGCTTTTTCCTTTTTAAACTAATTTTAGATTTAC

>c20185_g1_i1 len=223 path=[1:0-222]

ATAGGGGTGAAAGGCATAACTTCTCTGGTTGGCAAGAGGGCCTTTCTGTCTTCTGTACCT

TCCTGAAGGTGGGAAAGGTCTCACCTGGCAGGGTGAGCAGTAGCCACCAGTGCGAGGCTG

CGTGCCACTTCATCTGGGTTACTGGGGCACTGATGTATAACGGAGTCTGATGCAGCTTCC

TAACCCTCTGAAGCCATTGAAATAGATGTCTGTGGCAGGGAAG

>c20189_g1_i1 len=204 path=[293:0-203]

GCAGGAACCGTCCCATTGGGTACCTCCGGTCACGTGATAGCAGACGTCCGCCGGGTCGTC

TTTCGCTCCTCATTGGTTCATTGTCCATCTCCCCAAGCTCCTCGTGCAGTAGTTCAAACG

AGCCTTTTCCTTTTTCTGGTTGGAACAAAGGTTTGGTTCCTTCCAGCAGCGCTTTGCTCG

TGCACATCTTCCACAGTCCTGCAG

>c20194_g1_i1 len=385 path=[1:0-275 277:276-286 288:287-384]

ATTTCTCTATTAACTCTTCTAGGAAATTCTTCATAGAGAGTGCTCTCGTTCTTGCTTTGT

TCTTGATTTTCCACCACTGCCTGCTTAGCATCTCAAAATCCTGCCCAGCTGTCTCGCTGT

ATAGGAAAATGTAACTAATTTTAAAGTTCTAATCAATGATGAGGACTAGGAAACAGAGCA

AATAGCAAATCAGCATCAGAAGAAGAGAGAAAATGAACGGACTCCTGATAGCAGGTTTTC

TTTCCAGTTTTCAAATTGTTACCAGTTTGAATGTCCCCTACTTTGTTGGATACAGTCTGT

GAATTTGTTCAATTTGGGTGAATGTAGCTGGTGTGGCTAATTTGTGCTTTCTCTCTTTTC

AGTGCTTGCACCTCCTCCACCGTTG

>c20195_g1_i1 len=318 path=[296:0-317]

GCTAAAAGCACCCTCTGCCAAACCTAGCTCATCCCCCTGCCTTCAATTACACCTTCTATC

TAGCTCCTCATCCCCCTCAAAAAATCTGGCATCACACTTGAGCACAGAGATGCACACAGA

GGCACTGCCATTTACTCCTTGCCAGGCTCCCACTTAACTAAACCCATTTTCATCCTTCCC

TCAGAATTGCTCCTGTTCTCTCATGTATCTGGATTTTGTTTATGCATTGGTTGTTGCTGT

AAGCTTCTGTGCTGTAATGTCTTGCGTTAGCTGCTGTTGAATGAAGTCCAAATAACAGTG

TGCTTTGAGAAGGATGGG

>c20207_g1_i1 len=423 path=[307:0-422]

GCAATGGCTTGACTCTGGAACAGTGACTTATATACCAGCAGTCTCTGATTTGCTTTGACT

TTGTCTCTCAGTACAGTACTGTGATTCCTGCTACTTCAATCTTTACTAAGATACAGGGAC

CTTACACAATGAAACCTACTGTAAAATCATTTTGCTACTTCACCATTAGGTGCAATCCAA

AACAAATTCTGATGTTTCCACAAATATCACTCCCATAAAAATATGCCATTTCTATAAATA

CTAAACAAATGACAGCCTAACAGCATATGATGAAAAGTCATAATACTACCATATGGTTTA

AACTTCCTAAGTGTCCTTTACATGCATTATTTCTAATTAGAAGTGCAGTGTTACAGCACA

TAGATAGGAATACTAGGCATTTTCCTTTTTTTTTTTTCCAAAATATACTTTTTCAGTACA

ATT

>c20216_g1_i1 len=261 path=[1:0-260]

AACCAAAATAGTGGTGGGTGCTGGGCTCCACACACCGCACTGAACGCTGCCATGCGCCAG

CACTGAGCGCTCCTCATCCCACTGAGATACAGAGGTTTGGAGTGCAGGCACGGCTGTGAG

CTATTTGTGCTTAACAGCTGATCTTTGGAAGAGAAGTGCTGTGGGATAATTTCTTTCGAT

TTGAGTTTGCACGTTGTGTGACTATTAAAAACTTCAAGACGACTGTTAAGTAAAGACAGC

TCCTTGAAGTGCCAGATGGAA

>c20217_g1_i1 len=212 path=[239:0-211]

TTCAGCAAGGTTTCGAGAAAGTTTATCTCAGCTGTGTGTTTTTCTGTCCGGGTCTGTTTG

TCCTGTCGCTCCCCATGAATGTCAGATACCCACAGTTTTTCTGTGTTGTTCCGGAGAGGA

GAATGTCACTGTTGTCAGAGGAGACGTTTGAAGGGCAGAATGAAGCCGACTGTGTTATGT

CATGGTTTTAAAGAACCACGTTACGATTTCAG

>c20219_g1_i1 len=247 path=[1:0-246]

TGAACCAATGTAATAAAGCAATGTTTGCTGGTGTGTTTTGGTACAATGTGTGCATGCTCC

TGGTTTATTACATTGGTTCAGTTATCCAGACTAACCATAAAAGCTGATAAAACGGAAGTA

GACGAACACAAAGTCAGTGTTTTATGGATTAAGTCAAAGCTCCGGAACTGTTACCGGGGA

ACAGGGATTCTTCAGCAGTTGTTTCTGAAGAACCTCAGCTTGTCTTTGGCGGGGTGGGGA

GGAGGAG

>c20231_g1_i1 len=215 path=[213:0-214]

GAAAAAGTACTTCATGTTTACTGTAGGCCCCATCCAGGCAGTAACTTAAGATCTAACTTG

ACACCTGGAAAAAACTGAAAGGGATTGGACAGATTTCCAGCAGTTATACTGCTTTAAGAC

ACCAGAAAAGAGACTGGAGTGCAGGCATCTCAGTTCTCAGGTGGTTGATGCTAGTGGGAA

AAAACACATTCCTGTGCCTTATGTTGTTTCCTCCA

>c20233_g1_i1 len=217 path=[195:0-216]

TTATATTATTTATTAGAACTTTTTTTTATTATATGTAAATGTTACATTTTTGAACAATTT

TTGCACATCTTCAATCCAGTGAATTAATAATTTTTCTAGTAATTGGAGATAATTTTTATC

ATTTGCAAATAATTGAATTTGATTAACCGTAATTTTCGGATAGTAAATTAATTTATTTTT

TGAAAATATTTGAGTTATATATGACATGAAATCTATG

>c20233_g2_i1 len=422 path=[389:0-421]

GTTTTGTAACTTTTTAATAAATTCATTTAATAGTAATGTTATTAAACTATTTAATTCATC

CGAATTTTTATAATATTTTGAAAAGTTAAAAATTAATTTTAATCTATTCACAATAATAGG

TAAAATATTATATACATTTTCTAAAGATACATTATCTTGCAAATTTCTAAAAGGTTCTTC

TAAAATTTTTAATGCTTTTAATTTTTCTTCTAGTCTTTCACATTTATCTTTTATTTTTAT

TATTAAAGAATTAAATTTTTTTACATTTACACAATTATTTTTATCTAAAACATTTAAAAT

ACAACCTATTATATCTAAATTTAATTCTTCTAAAAGATCTTTCAAAATGTTATGTTTTTT

CTCTACATCAGAAATATATTCACATAAAGATAAATAATTTATATCTTTATCATAGATATT

TT

>c20243_g1_i1 len=230 path=[208:0-229]

GCTTTCCAAAATGACATTTTTAAGCCCAAAAAGTGCCATTTTCAGCCTCAAAATGAGATT

TTTCAGCCACAGAAGTGCAATTTTTTGCCCCCCAAAATGGGATTTTCAGCCCCAAAATGG

AATTTTCAACCCTGAAATTGGGTCCCAGAAGCCCAAAATTGAGACTTTCAGCCCTAAAAT

GGAGTCTCGATCCCAAAAGTGCCGTTTTCAGCCCCAAAACGGGCTCCATG

>c20245_g1_i1 len=446 path=[227:0-445]

ACCGCACTGAACCAGAACCTACTAAGATTAGTGAAACATGGTTTTCTCCTCCTCCAACAG

AGAAGTCATCACAGTGTTAACTTGACGTTAAAAAGGCCGCTTAAATTTGAGAGACGAAAG

CGTCCCTTTTTTGATGGCATTATGTAAATCCTGCCTGTCGATGAGCAGTCCGGCTTACAG

CACCTTTAATGGGATAGCGCGGTGCGGCTCAGACCTGGGCTTTGCGTGTTGCCGCATCCT

GGTGTTCTCCCGGGATGGAATTCCACACAGATCAGTAGGGGTCTGCCTGCATCCGTGCTT

CTTGCGCATCGGAGCCTGTGTTGTGTGCAGCAAATGGTAGTTGGGAAGCACGGGGTCATT

TATTTTTAGCTTGGCCAAGGGTGTTGTGCATTAATTCATCTATTTAATGTGGAAAACACC

CCTGACGTGTAGTGAAATACTTGATA

>c20258_g1_i1 len=270 path=[248:0-144 525:145-269]

GACAGACTGAGAATAACATGCTCTGCCTGTCACTAGTTCAAAGGTCTACAAACATCTCCT

GATTTGGAACCTCCCCTCTACATCTGTGCTACGGGAGAATTGGAGTTCAGATCTGTATCC

CAGCACCAAACATTTATACGTATTGTGTAGCTCAAGTGCAGAGAAAAAAGAGTGACTTGC

CTCTCTCACTGACAGTCACTGAAGTGAGCTCTCAGAGCCACAGCTGCAGGAAAGCAGGGG

GCTGTCAGAAAAACAGCCCCCTGCTTTCCT

>c20261_g1_i1 len=230 path=[331:0-229]

CACAGTAAAAATATTAAGTTATATTTTTCTTTTCCTCCCATAGCCACTGTTCTTGTCTTT

CGGTCTGTCCAAACACAGCAACATCTTAACACAGAGCTATTTGAACAAGAAATAATTGTA

ATTACATGCATAAATTCAGTAATTAAAGTGCAGCTTTACCTTTTGAGGCAAACTGCAGCT

ATACCGTAGCCCAAGCAGCCTTTTCTCTACTCAAGCAATACCTACGATAG

>c20263_g1_i1 len=276 path=[434:0-275]

AGTGGCACAGAAGAGCCAATGTGGAAACTGAAGGCTAACTGAGATTGGCGTTCTCCACCT

GTGGGTAGGGTGAAAATAACATGAGCAGATCAGTGGACTCCCAGACTACGGCTAAACTTC

TTTATCATCTCTACAACCAAGTGACAAATCAAGAAGAGCTAAACCAGAACGCAGGGTAAT

CTGTAATCACACGGTCACACAATTAAACATTAGCTTTATTTCTAAAACTCACTAAGAAGA

AATAAATACACGAGCAGAAAAGAAACCCAAAAGCAT

>c20275_g1_i1 len=280 path=[1:0-279]

GCTCTCAGAGCAGAAAAGCAAAGCAAACCAAAAGCAGAATCCAGTCACTAACAAATAGTC

TTCACTTTAACGGTGACAGCATTTGTACAATCACAGTTGTACAAATCCAATGTCTCTCAA

AGTGAAAATATGCTTGTAAAAACACTAAGAAATTTTTTATATACTTCTACCTATTTTAGT

AATTTGTTAGAGGCAATGAAAGCCAGTAGCTTGAAAAGCTTTTCCTGCATAGAATTACAA

GCAGTAGAAGGACCAGCACAAGCATCTTCACTAAAAATTG

>c20284_g1_i1 len=318 path=[165:0-96 469:97-191 164:192-317]

ATGTCGGATGGGAACTCTTTGTTAGGTCGAGCCCAGACATGGCACACGTGCTTCCTGCAG

GGATCCAATCCCTTCCAGGGCTGCATGTCCTACCCAGGAATACACAAGTGTAGCAGAGAG

ACCTTCTCACCCCAGCATACCGCACAGTTTGTATCCCACGTTCTCTTCCTCCCCTGCATG

AGAGAGAAAAATACAGTGAGGAAGACCCAAAGAATTCCCCAGCTTATGGAGGCAGACACC

TCCAATCCTCTAATCCTGTTTGATGCAGGCTGCTTGCTGTGAAGCCCAAGGTCTCCCTGC

TACACTTGTGTATTCCTG

>c20284_g1_i2 len=207 path=[83:0-80 164:81-206]

GATTCCTCAGAGAGCACATCACCCCATTTCAAGCTACTGCCTGCACTCAGGAGCAGGGAC

ACAGTGCCTAAGAGCAAAAACACAGTGAGGAAGACCCAAAGAATTCCCCAGCTTATGGAG

GCAGACACCTCCAATCCTCTAATCCTGTTTGATGCAGGCTGCTTGCTGTGAAGCCCAAGG

TCTCCCTGCTACACTTGTGTATTCCTG

>c20289_g1_i1 len=212 path=[1:0-211]

GGCTGGCGCTGCTTGTACAGATGTGCATGACTCATCCGGAGAGGGCAGAACATCTGCATA

GAGAAACAGTCCTGCGAGCTATCTGCCTACATACTTCATTTGAAGGAGGATGTCCACTGA

AGAATGCTGAATTTGATACTTCAGGAAATAAGCTACCTAAAAGAAAATCTGCATTACTGA

GGAGATGAAGAATACAGTGGCAGATCTACAGA

>c20293_g1_i1 len=266 path=[244:0-265]

TGTGCACATATGCCAGATATTGAAGCTCCGCTGCCAGGGGAAATGCTTGTCATGTTGCAC

CGCGGGGAGGTTGGGGGTGCAGTCGGGTGGGGTGTCTGGTTTGGCGGTTCGCTTTGCCTT

GGCTGCCTTCTGTTCGGTGTGGAGTGTGGAGGTTGGGGATGGGCCTGTCGCAGTCTTGGG

GGCGGAGAGGTGTTGTCCTTGTGACTGGGGGCGGCTTTTGTTCGCCCCTGCCTTGTGGCG

GGCACTTTGTTCCTGTCGGGCATTTG

>c20297_g1_i1 len=254 path=[361:0-230 759:231-253]

ACTGCGCTCCTGAGGTGTAGTAATTGTGTTCCAGCGTTCCCTGAGACAAAGACATAGATG

TGGGACATATTGCTTTAATGTAGTTCTCGGAGCCCTTACTCTCAGCTTAAGTATGTAGTC

CTGAAGGAGGTGAGAAGTGTATAGTTATTGGTGTTTCCTCCTCCTTCTTGTTCTTTCAGA

TAAGCCAGGTCAAAAAAGGAATTAGAAGTGATATACAAAGGTCTTTGTAAGTCAAGTCAA

AGCCATAACTTCTC

>c20301_g1_i1 len=322 path=[1:0-321]

CAGAGAACACACATTTGAGAGGAGACAGTGAGCATCAGAACATGAAATAAGCATCTCCTG

TGGAATTCCTAGGAGTAGTGCTGTCAGTTCTAGGAGGCCTTGTGCACTGGGCAGGCAGCA

GAAACACAAATCCATGCTTCTCCTTGGTCCTTCAAAGACCAACTTCCTTCTTTTATTGTA

GCCTGTTTTCTTCCTTATCTTTTACTCTGTGACCTCAGATAAGCGTTTTGTTCTCTGCTG

CTGGTTCTTTGATACTGGTATTCAGTTTTCTCTGGTTTGGAAGTTATTGTCCCGTAATCA

CTAACACAAAAAAGGCAACAGG

>c20310_g1_i1 len=317 path=[449:0-316]

GGAAGGCATTTTCTCACTGGGCTAACAGAAATAAAGTGCAAGCAGAGATTTGCTTTCCCT

TACTTCTTCCTGAACACATCTGACAACAGATTTTAAAAGAGCCATTTAAGTTTCCCAGAC

AGTTTCGGTAATTGATTCAGAATTTTTACAGTCCAGGAGACATACTTGTTTATTTTAGCA

GGCTGGGGAATTGTTCCCTCGAAGCCTTTAAAACAAAGACATAGAGCTCTCAGTATATCA

GCAGGAGCAGACAGCGCAGAGGAAACACTACACACAGTGTTAGATGATACCCCTTGATCC

CCCTCAAACACAAAGTT

>c20313_g1_i1 len=211 path=[401:0-210]

AGAAAACATGTCAACAACTTGTCTGGAGCAATACTTTTATTGTTTTGGAGTATTGTCTGA

AACATTCTTCCACTCAATTTTTATATAGCCCTCAGCATCACTATACTTCACCAGTGGTTT

TTTATTGCTTCCAATGCAGTCCATGCATCAAAGACAGTGTGAAAAGATGTGAAAAATGAA

GAGCTTTGGAGTAAGCATGCAGAAGCACCTG

>c20314_g1_i1 len=276 path=[255:0-248 557:249-275]

CTATGACTTCAGTTGCACATCACTTGTTAGGTGACTGCCATCTAACGTACAAATAGGTTC

TTTGAACAACATCTACTACACAACACCAGACACCCTTAACTCCTTACACTAAGGGGATCA

AAACAATCACTTGTCCATTCAGGAAGACCCCGTGCCATCTACATCTAATATTCTAAAATA

CGCACACCTCCACAACCACTTAGATGTCTCAGTCAAGAATAACTGTGCAGTTCTATGGTT

TGGGGCTTTTTTTTTCCACTCATCTCCTAGGGAAGA

>c20314_g1_i2 len=276 path=[255:0-248 504:249-275]

CTATGACTTCAGTTGCACATCACTTGTTAGGTGACTGCCATCTAACGTACAAATAGGTTC

TTTGAACAACATCTACTACACAACACCAGACACCCTTAACTCCTTACACTAAGGGGATCA

AAACAATCACTTGTCCATTCAGGAAGACCCCGTGCCATCTACATCTAATATTCTAAAATA

CGCACACCTCCACAACCACTTAGATGTCTCAGTCAAGAATAACTGTGCAGTTCTATGGTT

TGGGGCTTTCTTCTTCCCTAGGAGATGAGTGGAAAA

>c20317_g1_i1 len=281 path=[173:0-280]

TTTGTGTTTGAGGGGGTTACTTAATATTTTTGTGAGACCTTTTTATGTGGATGGTGCTTT

TGCCTGTAGTCTGGGAGTTCACAGGCTTCAGCCTGATCTTCCACCCCTGGGACTGTGCTG

ACCCACTGTAATGGGGTCAGTGTGGTTTATACGATGACTGTACAGCAGTATCTCAGTAAT

CAGTTCTCAGATGCCAATCTCTGGGCTTTGGTCAGTGACCCTTCGCTGCTGGGGCTGTGC

AGCAGTAAGCTTTGCCCTGCTCTGGAGGAGTCTGGGAGCAG

>c20318_g1_i1 len=273 path=[1:0-272]

AAAAGCTAAGCACTAGCCATTGATGGATTTCTCCTCAGACTCGCTGCTGTCTGCTCACCA

CTCCTTCAATCCGTACTTGCCTTATTTTTCCAGAGAATATCCCTATAACTCTCAAGTTCC

CCTCTTCTCTGTGGATTACTGATTCAATCTACTCTCCATTTCAATAGCAATTGCAAATTC

TTTGCTGTGATCTGTGAGCTTCTTTTGCAGCTTATTTCCTCTTTCTGCTCCACTCCCTCC

GTCTCCAAGGGCCTTGCTTTCATTGAGCGTGAT

>c20319_g1_i1 len=2241 path=[2286:0-2173 25:2174-2240]

GGTGGCAGGGCCCCCCCCGGCCGTAGGGGCAGCCCTGGCTTCACCTTGAGGGACAGCGTG

GGGCGGCGGGCGGCCCGGCGCAGCTCCAGGTGGGTCAGCGCCTTCCGTAGGGCCCTGGTG

TCCTTGGTGGCCACGAAGACCACGGGGTTGGGGGCGTCGGCGGGGTTCAGTTTCTGCCGC

TTGCTGGCGGGGGGAGGACCGGCGCGCAGCCCCGCCGGTAGGGGGCGCCCGTTGGCCGAG

AACGAGACGCTCGACGCTCCCACCCGTACCCGGGGGGGGCGGTGACCCTCGGCACCCCCC

TCGTAGCCACGCTTGCCCACGAGGCCCGCCAGCCCCCGGCTCTGGCTCAGCTGGTTCCGG

TTGATGGGGGTCTTCGTACCCCGCGGCGTCTTCGGCTTCAGGGGGGCCTGTGCCGCCAGC

TCCTTGACGATGGCAGCCAGCGGCAGCCGCGCCAGCGGGTGGATCTTCAGCGGCGCCTTG

GCAGCCTTAGGCAGGCGGATGGAACACTCTGCTTTGATGGCGTTGGCGTTGATGGGGGCG

TAGGGGCGGCGGGCGGCGCGGCGGGGCTGCAGGACGTCCCGGCACAGGCGGCGGGCCAGG

CGGGTGAGGCGGGTGGTCTGCAGCAGGAAGGTCTGCCGGTTCTTGGCCAGCAGTTTGGCC

CGGCTGGCGCTGGTGGTGTAGGGGGAGAGGCTCTGAGCTTCGGGACGGGAGAGGTGACCC

CGAGAGTGGGGCTCCGAGGCGCTGCGTGCTGCTCCCTCCAGCTGGGTGGGGGTCTTCAGC

CCCCCGTATTTCTTCCAGTAGATCCAGCAGGAGGCGCAGAGGCGGCACTGCATGTTGGGG

GGGCCCCAGGCGTACCACTGGGCTGACTGAGTGGTGTGGCAGCTCTCGCACGTCAGCCCC

TTCTGGAAGCCGGCGCCGTTCATGCCGGGTTTGGAGCCCACCGAGATGATCTGGTTGGGG

TTTGGCTTCGTGTAGGTGGGGATGTACACCTGCTTCAGCTTGCTGTCCGCTTCTGCCGCC

TTCAGCCTCTTCTGCTGGATGTAGCGGTCGGTGGTCTTCCACATGTAGTAGAACTGCACG

ATGCTGGCTAAAGACTTCCAGGGCAGAAAATCCTGCCGGATGTCATTGAAATCCTTGCCA

TACTTCTCCAAGGCCTCCTCGAACAGCATGGCCTCGGAAGCCGACCACTCCTCCATCTCA

TCCCGGCACAGCACCGGCCCCCCCTGCGGCACCAACGTGGACATGGCCTTGGCCAGGTCG

TACCCGTTCCTCTGCAGCGTGTCCATGGCGTGGAACAAGGTGATGTCCCGGGATGCAGCA

GCTGCACTCATGTGCAGGCTGGGCTGGCGGATGGAGCTGCTGCAATCCAACGCGCGGGCG

AACGTCCCAACGGCTCGTGCCACCACCAGGAACTGGTCGATCTGTCGGTCTGTCAGGGGG

TTGTCGGGGTCCCACACTTTCATCTCCATTTTCTGTTGGTTCCGGTTGTCTGATTCGCCT

TCTGCCAGCCGGTCTGGGATCTCTGCTTGGTATTTGCAGCCCACCCGGATCTCCCCCTGA

TCGGCCAAAAGCGTTTTCTGGACGGGGTCGAACACCAACGAGTAGAAGAAGCAGTCCTCC

TTTTCCAAGTACTGCCCCAGGATATCGGTCTCATTGAGGAGCGTCACGCTGCACTTCCCC

CTTATATGGGTGGCTGGCAGCGATTCGAACTGCCGGGAGAGGAAAAGCTCGCGGTGCTTC

AGCTGATGCCGCTGCTGCTCCGTCATGGTGGGCTGCTTGGATTCCTCCTCAAATTCGCGG

GCATTGCTGTCTGCCAAGCTGTTGAGGCTGCTGGAGATGTCCCGGCGGCGGAACAGGCAA

ACCACCTTGGCCTCCACGTTGCCATTGGCAGTCTTGTTGAGCTCCTCGATGCGGCGGACG

AGGTAGGGGTTGCTGGAGGAGTTCTCAAAGTAGACGTAATCGCCGACGCGGTACATGTTG

GCCGCCATGGCCGCTCTCTCGGCCGCGTTACCCCCGGGCCGGACTCCGGCCGCGCTTCAT

TGCCTCCCGTTCCCGCCGCCTCCGCACCGGCACGACCCGTTCGGGAACCTCCGGAACGGC

TTCGGAAACTTTCGGAACGGCTTCGGAAGCTTTCGGAGTGACCTCGGAAATCTTCGGACC

AATTCGGAGAGCTCTCCTCGAAGCTCCCGAGTCGCTTTCCGGAAGTTCCCGAGGTTGTGA

CGGAACTTGCCGAGGATCTTC

>c20319_g1_i2 len=2242 path=[2286:0-2173 4459:2174-2241]

GGTGGCAGGGCCCCCCCCGGCCGTAGGGGCAGCCCTGGCTTCACCTTGAGGGACAGCGTG

GGGCGGCGGGCGGCCCGGCGCAGCTCCAGGTGGGTCAGCGCCTTCCGTAGGGCCCTGGTG

TCCTTGGTGGCCACGAAGACCACGGGGTTGGGGGCGTCGGCGGGGTTCAGTTTCTGCCGC

TTGCTGGCGGGGGGAGGACCGGCGCGCAGCCCCGCCGGTAGGGGGCGCCCGTTGGCCGAG

AACGAGACGCTCGACGCTCCCACCCGTACCCGGGGGGGGCGGTGACCCTCGGCACCCCCC

TCGTAGCCACGCTTGCCCACGAGGCCCGCCAGCCCCCGGCTCTGGCTCAGCTGGTTCCGG

TTGATGGGGGTCTTCGTACCCCGCGGCGTCTTCGGCTTCAGGGGGGCCTGTGCCGCCAGC

TCCTTGACGATGGCAGCCAGCGGCAGCCGCGCCAGCGGGTGGATCTTCAGCGGCGCCTTG

GCAGCCTTAGGCAGGCGGATGGAACACTCTGCTTTGATGGCGTTGGCGTTGATGGGGGCG

TAGGGGCGGCGGGCGGCGCGGCGGGGCTGCAGGACGTCCCGGCACAGGCGGCGGGCCAGG

CGGGTGAGGCGGGTGGTCTGCAGCAGGAAGGTCTGCCGGTTCTTGGCCAGCAGTTTGGCC

CGGCTGGCGCTGGTGGTGTAGGGGGAGAGGCTCTGAGCTTCGGGACGGGAGAGGTGACCC

CGAGAGTGGGGCTCCGAGGCGCTGCGTGCTGCTCCCTCCAGCTGGGTGGGGGTCTTCAGC

CCCCCGTATTTCTTCCAGTAGATCCAGCAGGAGGCGCAGAGGCGGCACTGCATGTTGGGG

GGGCCCCAGGCGTACCACTGGGCTGACTGAGTGGTGTGGCAGCTCTCGCACGTCAGCCCC

TTCTGGAAGCCGGCGCCGTTCATGCCGGGTTTGGAGCCCACCGAGATGATCTGGTTGGGG

TTTGGCTTCGTGTAGGTGGGGATGTACACCTGCTTCAGCTTGCTGTCCGCTTCTGCCGCC

TTCAGCCTCTTCTGCTGGATGTAGCGGTCGGTGGTCTTCCACATGTAGTAGAACTGCACG

ATGCTGGCTAAAGACTTCCAGGGCAGAAAATCCTGCCGGATGTCATTGAAATCCTTGCCA

TACTTCTCCAAGGCCTCCTCGAACAGCATGGCCTCGGAAGCCGACCACTCCTCCATCTCA

TCCCGGCACAGCACCGGCCCCCCCTGCGGCACCAACGTGGACATGGCCTTGGCCAGGTCG

TACCCGTTCCTCTGCAGCGTGTCCATGGCGTGGAACAAGGTGATGTCCCGGGATGCAGCA

GCTGCACTCATGTGCAGGCTGGGCTGGCGGATGGAGCTGCTGCAATCCAACGCGCGGGCG

AACGTCCCAACGGCTCGTGCCACCACCAGGAACTGGTCGATCTGTCGGTCTGTCAGGGGG

TTGTCGGGGTCCCACACTTTCATCTCCATTTTCTGTTGGTTCCGGTTGTCTGATTCGCCT

TCTGCCAGCCGGTCTGGGATCTCTGCTTGGTATTTGCAGCCCACCCGGATCTCCCCCTGA

TCGGCCAAAAGCGTTTTCTGGACGGGGTCGAACACCAACGAGTAGAAGAAGCAGTCCTCC

TTTTCCAAGTACTGCCCCAGGATATCGGTCTCATTGAGGAGCGTCACGCTGCACTTCCCC

CTTATATGGGTGGCTGGCAGCGATTCGAACTGCCGGGAGAGGAAAAGCTCGCGGTGCTTC

AGCTGATGCCGCTGCTGCTCCGTCATGGTGGGCTGCTTGGATTCCTCCTCAAATTCGCGG

GCATTGCTGTCTGCCAAGCTGTTGAGGCTGCTGGAGATGTCCCGGCGGCGGAACAGGCAA

ACCACCTTGGCCTCCACGTTGCCATTGGCAGTCTTGTTGAGCTCCTCGATGCGGCGGACG

AGGTAGGGGTTGCTGGAGGAGTTCTCAAAGTAGACGTAATCGCCGACGCGGTACATGTTG

GCCGCCATGGCCGCTCTCTCGGCCGCGTTACCCCCGGGCCGGACTCCGGCCGCGCTTCAT

TGCCTCCCGTTCCCGCCGCCTCCGCACCGGCACGACCCGTTCGGGAACCTCCGGAACGGC

TTCGGAAACTTTCGGAACGGCTTCGGAAGCTTTCGGAGTGACCTCGGAAATCTTCGGACC

AATTCGGAGAGCTCGGCCTGAGCTCGGGCTTTTCCGGAAGAGCCTCGTTAAATTCCGGAA

GATCCTCGGCAAGTTCCGTCAC

>c20321_g1_i1 len=203 path=[355:0-202]

ATATATTTTTATTCCTTTTGGTCAGTTCAAAGAAATAACTGAAGTAGAAATACATTAAAA

AAGGAAGAAGTTGAGATGTGCATGTACATACTTCTAGAATGATTTTTATTATTATTTTTA

CTGGCAGTACTCTTAACATGTTGCTTGGGGATCTCCCATTACAGATGGTACTGTGATCAG

TAACTTCTGCACATAAAACCTTG

>c20324_g1_i1 len=349 path=[327:0-348]

GCCATTCTATGATTCTATGATGATCCAGAAACAAACCCGGCTACTTCATTTGATCTGCAT

TAAATCTGATTTTAAAGATGTGCTGGTTTCTTCAGCAGCAGCATGGCTTAGTACTGCTGA

TTAGAACAGGCACTGCAAGTCTTCTGTCAGACTCCAATGAAAAGTAAGCAATTACACAAG

AGCATTCAGTAAAAAACAAGTTTAGAAAACGTCATGATCAATTTTTATTTTACTGCTTAT

TACTGTATCACCTTAAAGTATATGAACACTCAAGATTGCTTTCAAGTACATCAGCTGTTA

ACTGGAAGCTAGTGAACAGGACCAGCTTTTGTTTCTCTTTTTTGCGATA

>c20329_g1_i1 len=317 path=[295:0-316]

GCTCTCCAGCCTCTCATCCCCTGGTCTAAACACACAGCCAGGGCTGCCCCATCCCAGGTG

TAGAATCTGGCACCTACTCTTGCTAAACTTCATTTGTCAAGGTCTCTTTGAAAGGCCTCT

CTGCCCTAGAGGGAGTCAACAGCCCTTCCCAATTTACTGTCTTCCACAGACTCACATAAT

GTGAATTCAAGTCCCGTATCCAGATCTTTTATAAAAACACTTTAGAGAACTGATCCTAAA

ATGGAGCCCTGGGGAACCCCACCAGTGACTGGTCACCAGCTTCATGTAACTCCCGTTACC

ATCTTCATTCTTTACTA

>c20330_g1_i1 len=248 path=[1:0-247]

GCTTACATAGATAATCATATCTGTCTTCTTTTAAAACACAAAGCAAAAACACTTCATCTA

GTATTGAAAAATTGTGGTGGATGAGATGTTGGAGAAAAAAATGTGTATGCATTCTGGCAG

CAGGAGTGGTTGGTGCCTCTGGTAATTAAGTGTAAACATCTGCAGATTGTACGCTGCAAA

GGCAATGTGTTAGATTTACATCTGCGTGTCTAAAAATATTCCTAATTGCTCAAAAATATG

AATGCTTT

>c20333_g1_i1 len=211 path=[327:0-210]

GCCTTTTCCTGGCGCTTCCATTTTCTGACATTAATGCAATCCCTTTGTTGTTTGGTTTTT

GAGGGCCTGAAAGGCCTGGATCTTCTGGTAGGGAGAAAATAGGGCTAAGACGTTTCTAAA

AAATTATTTAGTGTCTTGAAAAGAGTTTGACTGTACCATTGTAGCATTGTTTCAAACCAC

ACTGAAAAAAAAAAAACCTTATGAAAATTTA

>c20336_g1_i1 len=238 path=[239:0-214 641:215-237]

ACACAAAGCTGTGTCATCTTCACACATTCCTTAGGTTCAGTAAATTGTTTCTTTCCCAGT

GTAGTTGTCGTTACGGTTGTGAGCCATCCAGAGAAGTTCCCTAAGAGGGGCTCTTGTGCA

CCTGCACCAGAGGGACCAGAACCTGCTCTCGTTTGCTCAAACATCCACATCTCTGCGTAT

GAGCAACAGAGCAGGGAAAGGAACGGGGCTCTGTACCTTTACTTCGTAAGCCATATCT

>c20336_g1_i2 len=239 path=[239:0-214 453:215-238]

ACACAAAGCTGTGTCATCTTCACACATTCCTTAGGTTCAGTAAATTGTTTCTTTCCCAGT

GTAGTTGTCGTTACGGTTGTGAGCCATCCAGAGAAGTTCCCTAAGAGGGGCTCTTGTGCA

CCTGCACCAGAGGGACCAGAACCTGCTCTCGTTTGCTCAAACATCCACATCTCTGCGTAT

GAGCAACAGAGCAGGGAAAGGAACGGGGCTCTGTAGATATGGCTTACGAAGTAAAGGTA

>c20337_g1_i1 len=231 path=[1:0-230]

CTGGACAGGTACGGATGTCACCATGCCAGCCTGGGTTTGGTGAGGCCTGGAGTATGATCA

AAATTTTGGGAACGCAGTGACAGGGGCAGACACCTCTCGGAGTGTGATTCCCAACTTCCT

GCCTTAATGCAAATCTCAAGACCACGAGCCATGTCACCAAACCACTGCTCACGGCTGTTC

GCTGAACCTGACACATTCTACTCCCCAGGCCTTATGAATTGTAATTGAAGG

>c20338_g1_i1 len=202 path=[180:0-112 293:113-201]

ACACTCATTTTGGTAGCATTAACTTTTCTGTAACTTACTGGTCTATTAACCAGAAACAAG

TCCAAAGTATCTCATAAAAAATGAAATGCTGTTTTAATGTTTCCTCCTAATCCTTGGCTC

TTTCAGAGACACACAGCATTATCTGTGGAATGGGCATGTTAGATTAGCTGGATGAGCATA

TGAGAGTTTATAAAGTATCATA

>c20340_g1_i1 len=207 path=[1:0-206]

GGTGTAGGGGTTGCACAAGGCTCTGTTCTATTCTCAATTATACAGAGATGATCTGAGGCA

ATTTTTAATGATGTCTGTTCAATACGGATAACTCTTCAACGTTGCATCAAAGTAAAATCC

AAAAATATCCAAAACCAAGATCAATATATTTCAGTATTCTTAATATTTAGCTGGTGTTGC

TGATAGTCTGCACCAGTTTCCTGGAGA

>c20340_g2_i1 len=240 path=[369:0-239]

GGCATCTGAAAAAGCTGTAGCTCATAATCTTTCAAGTCTTGCGTATCAAAATTAAAATAG

CATCTATTTTAGCCCTACCAGCAAACAGCTTGCTGGTAAGTACTTGCTGAAACTCTTGGT

GCTTCTAGAGCTAAATTTGGCCATAAGTAGCTGGAAGAAATCATTGTAAAATGCAGGGAA

AGCAATGCAAGTGCTTCTGCTGTATCGTTTAGCAGGAGACACCGGGATGCATCGCAGAAA

>c20348_g1_i1 len=210 path=[188:0-67 431:68-209]

GGTTAAATTCTAAGGATGTTCCCACAATGTTCTTACCCCAAGTTCATAGTAAAGTCAGGG

GAAGGCAAGATTTCATATTCCAGTTCAAACTTAAGTGTAAACCGTAAGCCAAATTATAAA

CAACATCTACAAGCCTCATTCATACACTATAAAAAGACTAGAAGAGTACTTCTGTGCACT

TCTGTGTTCTTTTCATACTGTCTTGTACTA

>c20353_g1_i1 len=265 path=[243:0-264]

AAACTCATTCTCTATTTAGTAAAAATTAAAACCAAAAATTTCAGGACATAAATTTATGGC

ATATAAATATATGCATAATTCTGCATGCAATTTGCAATAAAGCGATAATGTTAGACACGT

GAGATGAAACACAGTCACAGATACGTCAAGCAGTTTCACTGAAGCCTGATCAGCAGTTCA

TAGGAAATTATGCATTTAACAATTTTTAGTCTTCCTCATGTATCATGAAATGTCTCTTAA

GGACTCAAACACAAGGTTACCTAAA

>c20355_g1_i1 len=286 path=[264:0-78 343:79-213 478:214-285]

GCCCGCCCTCACCAGCAGCCCCAGAACACTGCTCTGACACAGCCTCTGCCCCAAGCAGCA

GGTGCTAAAGCTACACTAGGAAGCAAGGCAAGGACACTGTAGCAGCAACCAGTGGAAGAC

TGGGTTCTGGTGCTGCTGGTGAGGGCGGGCAGAGAAGAGCAAGGCATTGTCTGTGAGGGA

TCAGAGTGGTTCAGCAGTGTGCTGTGTTAGTATTGAAGCACCGTCTGTTTTGTCTTGATC

GAGAAGGAGAACAGAGCTTGAGGGTTTTTTTTACCAAATATTACAT

>c20361_g1_i1 len=263 path=[241:0-262]

GAAATGACCTCGTTCTGTGACTGGAAATTGCTGCAGCGATTGCTAGGAGGAAAGGTCAGT

CATGCCTCCCAAAAAATCCAGCCTTACTAAAATGGGTATAGAAGGGTTAAAGAAGCCTGG

TCATTTGCCAGGTGTTGGCTTCGATTTTAGAGTATTTACTACAGAAAAGCCCTTATAATG

TAGATTTTATCAGCCAGATGCCATGATATAACCCAAATCCAGGAACTTATGCGTGACTCC

TTAGTTTCCTGCCAGCTTGGTCT

>c20364_g1_i1 len=322 path=[1:0-321]

AAATAAAAAAGGGGGGTGGGGAACTGATTTTAAAAAACTACCACTCTTAGTATTACTTGT

GCCACATGAAGAACAAGACAGCTGCATTTCTGAAAATCAGCACTGCAAAACTACACGCAG

TCCCTCCGTAAAATGACAGCAAATAAACAGAATCTCATGTAAGAGAACAGCTTATTACTG

TATAGAGCCTCCTAACTGCTTAACCAAGTGAGCCTTCAATAGTCATTTCTTAACAAGTAC

GTACATAAAACAAGGTCAGGGATCTACAATAGAAAGGATTTTGCAATGATATTTCAATCA

GTTCAGAAAGGGAAGGAGTTGG

>c20365_g1_i1 len=268 path=[1:0-267]

TGTCCAACATCCAGATCTCCATAAGACCACTCTTATTTCACTGCTGTGCCCAGAGGAAAA

CTTAACCTCTAGATAAGTGCATTTGTCTCACTTGGAATTGACTTTGCAGCAGAAAATCTT

CAGGGACTTTTATATTCCAGAACTTTAGTGGGAGTTGGAAGGCGTCCCTCAGAAGCCTGC

AAGTACCAGTGCTGCTTCACTGCCCTCTTCCTAGTGATGCCAGAGAAGTTTAACCCCTGG

GAAGATGTTTGTCACTTGAAGAATTGGA

>c20366_g1_i1 len=266 path=[456:0-265]

ATCAGCCTGGCTGAGCTTTGGTCACCTCTGATGATCCGAGTTTGGTATCTGAAGATGGAT

CTGGCTTCACGTAGATGCCAGGTAGTCCGTGAGACATCAGCTTCGTGATGCTAGCAGAGG

GCAGTTTATTATCACTTACGTGCTTCAACCAACCCTTAGGATAAACTGAACAGCAGCATC

TCAATGACACCAATTGATAAAGCCTTTAAACGCAAACACTCAGGTGATTCAGAGAGTGGT

CAGAACCCAGAATCTCTTGCCTGAAG

>c20367_g1_i1 len=242 path=[220:0-241]

AAATGGACCAAGAAACCCTTTTTCCTCACATTTACACCTAAAATAGGAACAGAAATCCCA

TTTTTCCACCAATTTTCCCTACGATGGCCAAGGGATGCCTCTTTCTCCCTTATTTTAACC

CCAAAAACGGACGACGGATCCCTTTTCCCCCATCTCTGCCCCTAAGATGGCTCCAGGAAC

CCCTTTCCCCTCCATTTTAGCCCCAAAATTGACCGAGAACACTTTCCCCCCCCCCTCAAA

CA

>c20380_g1_i1 len=221 path=[267:0-220]

AACAACTAGCTCAGGTACTGTGAAATAGAAATAGAGGCATGGAAGAACTACCAAACTTAT

TTTTTTTATCCAGATACCTATATTTTCATCATTTAGATTTTTAACTGCCCATTTCTAGGA

AAACACCTGCATATACACTGAAAGATTTACATGCAACTTCACAACGGTATTTTAAATTAG

AATGTATGAGGACCCAAATAGCTAAGAGCTGCAATTAAGAT

>c20383_g1_i1 len=261 path=[249:0-260]

GCTGGCAGAGCGAGCCTCTTTTGAAACTATTTGTGATCAAAATATGTCAGAGCCATATCT

TCAAAATACAGTAGGAAAGACTTTTTGTCAGCGGATTGCTTAATTCATGTTAACAGTAAA

CTATCAACTGTATCTTTGATTATTCATCCTTCCTGTCACACCATATTCCTATCAGGGAGT

TACAGTGCTGCTCTTTTTTCTGCTTTCCAGATTTTGTTTCCTTTGTTGTAAACACACTGC

TCTCAAAAAAAAAAAAAAAAA

>c20392_g1_i1 len=286 path=[264:0-285]

GACTGGGCTGAAGCACATTCGTGGTCCTTGGAAGCTGGCGGTTCCCATTAGCTAACGCTC

TTGTTCAAGGGCAGTGCTACCCAGGCACAGAATGGAATGGCAATGTCAGGGCTGGTTGTG

GAGCGGCTCGGCTGCCACGTATGCTGTTAGTTCTGAATAATTTGTAGGAACGTTCTGAAT

TTTGTAAACTTTTTAATGTGCTAAAACTTGGTTATGAGGTAACATTTACAAATAAGGGAT

AACTTGTGTACTTGTTTTTGTATTTTGGTCTCTTTTTTTTTTTTTT

>c20396_g1_i1 len=305 path=[1:0-274 276:275-304]

CTTCACCCTTCCATGAAGAAGCAGGCGCCTTCTGCTAATGGCAGCCCATACAGACCGGTG

CCTTGCTGTCACACTCTGTAAGAGGGACCCCAAGAGGCTGTTTTAAGACATGCTTTAAGT

TGCATGTTGAAAACAGGCCAGCAAGTAGAAGGCTGGAGGTTGGGGTGGGCAAAAGAGTGT

GCTACAACTACTACCTTCTGTTCTGCCCAGAAAGCTGCAGCTAAACAGAGCAGTTCTGAG

AAGGCTGTGTACTTCCCAGAGCTCATAGCCTAAACTGGAACCCCACAGGTCTGTCACAGT

CTGTT

>c20403_g1_i1 len=287 path=[1:0-286]

CAGTCAGAGGCACATCTGTCCTGGCTGCGGGGCATGGGCTGCGTGTGGACATCCCCTGGG

CAGTGGTCTCTTGCATGAATTCATGTGCATTCAGACTGTGAGGAGGATGTCCAACAAATG

TGGATGTGAATCCTGTTTTCATACAGAAATGCACTTGCTCCTTTCTTCTCATGAACGTAT

TGTTTCATCTCTGTCACTATTGGAAAGTTAAGCTCAAAAAATAAAGTTGATTAGAAGTTC

AAGTGCTGTTCAGCAAACCAGATGAGCGTATTATTACAGTTCAGATG

>c20404_g1_i1 len=579 path=[15:0-578]

TTATTTAAAGTAATAAAAAAAAAACAAAAATAAAACATGTCAAAAAAATAACAATATAAA

TAACATAAAATATGAAAATAAATTAAAATTAAAAAATAAAAATAAAACTTACATAAAAAA

TTAAAATAAAACATAAAATATAATAAGTGTGTGCTACATCACATTTAAATTTTGTGTTCA

TAAATACTTCAAATTTAAATTAAAATTTACATAAAAACTAGAAAATGGTGCATGCTGTAT

TTCGCTAATATCTGTAATAACAAATAAATAATACACAAAATTTAGTATATTCATAATAAA

AAAAGTAATGTAAAGATGGTAAACATCTAAAGGACAATTATAAAAAAAAGGAAAAATCGA

ATTTTTAATTTATTTTAATAATATATTATATATACATGCATAAACATATACATAAATATA

TGTATATATGTAACATGTATATGTAGTAGAGTTATATCAGTAGTAATAGAAAAAACTGAA

ATATAAATTATTTTCATGCAATTATAAAGTGCTTATTTTTATAACAAAAAAAATGATAGA

AAAAAGGAAAAAATAAAATAATTAAAAAAAAATATAAAT

>c20409_g1_i1 len=311 path=[289:0-310]

AAGATAGCACTTGTGAGGAATATCCATCCTTTCATAAAATCTGGCGTCTTGCTGGATCGC

GAGTCTTGTGGTATAGCCAGCAATGGTGCTGACCTGGACTAGGTTCAGAGGGAACTGGAG

ACAGCTTGGTTATTTTCTTTGCTTCATCCTTTCTCTTGTTTCTTTCTGTTCTCCTAAACT

CAGCAAATGTTTGTGCTTGTGTGGTTTTTGCTTCCCTCTGCACTTTAAAACAGCTTCTCT

AACTCCACCTAATCTGGGTGGAAGGAAGGATGGAGAAGGAGGAGAGAAAGGAAGGAAGGA

AGAAAGGAAGG

>c20411_g1_i1 len=233 path=[211:0-232]

GAAGTGAGGAACTAGCAGCCCACTAAGGAGAACATGCCAAGAAAGAAGCTGTGACTTTGC

CAGGTGTATCCATCAGCAGGATGAGGAAGCTGCTATCCAACTATTTGCTGCAATTCAGGA

ACACGCTGTAGTAACGTTTTCAAGCCTGGGAAGATAAAAATCCATTCTGATGAACAGCTA

TAAGCATGGCTGCCTACTACTGCAGTCTACCAAGAAGGAATCAGTCTGCAACT

>c20415_g1_i1 len=334 path=[1:0-333]

GCATCAGCTCTGATGGCTGAGTAGGTGACTCTTTCACTCTATGTGTGTGAGTTTTTTAGT

TCATAACAACCTGACAGAATCATGGAAGTAAGGTTGGTGGGACAGTAACTCCACAAGAGA

AATCAATCCTTTAAAACACGAATCTAGTGACAGTTTGTGGTGAGCACAATGCTGTTTTTC

TAAGGGTTGGATTCTCATTGTTGTACATTTTTATTTAAAAATAAAATTTTTAAATAAAAG

AGAAGTGCCAGACAATTTTGTAGCATTTTGTAATGCAGCATGAGGAGAACTTGGTAAAAG

AAAGGAAATCTATTAACCTTGCTTTTGCTATGAA

>c20419_g1_i1 len=211 path=[1:0-102 104:103-210]

TATTTGCAAAATATATTTTTAGAAACAGATAAAATTCATTAATAATATTACTGGAAATGA

AAGCGTCTAAGTTAGTAAAGAAAGCTATACTGTGTTTTCCTACATAGTCACCAAGGCCAT

CCAGGTTCTGCATCTCCACAGACACATCATACATCTCTGCACGCAGAACACACAGGTGTA

TGATACTGCATCCAGCTCAGTACTTGGGGGT

>c20427_g1_i1 len=225 path=[203:0-224]

AAATACCACGCAGCAAACACCAAGGGTCAGAAGCCTGAGCTTCTACCTCAGAAAGGAAAA

GAACAACTAGTTCAGGACTAGAGAATGTGTGACACGGTAATGTTCTTTGGTACCTATCCT

GCCCTAATGATTTTCTCTGTAATGGTTGAATCGAAGCTCCATGAATGAGTTCTGGAACAC

AGTAAGAGCTTGATTTTGTTCTGCAACATCAGTTTGCCCCAGCAG

>c20429_g1_i1 len=202 path=[334:0-201]

TTCACTGTTACTACACTGAAATTCCTTGTTAATGGAATGCATTTGAGAGAGAGAGAGAGA

TGGAAAATAATTTGTTTTCGTAGCTCCATGTCCCAGTGCTGGGTAACACGAATATCCCCA

AAGCATGTGCCAAGCAGTCATGAAAAATATTTGGAAGTGTACCACCTGAAGACAGAAGAG

AGAGTAATGACCTGTGCTATGG

>c20439_g1_i1 len=236 path=[1:0-21 23:22-86 88:87-235]

GCCGCTCGCAGCGCCGCCGGACACGCCCCATGCACGGCGCTTAGTCCTGCAGGATGCTTC

AGACTAAATCCGGAATCTTTCTGTGCAATTCATTGTGATTCCTAACCTGGAGCACCAGGA

TGCATACGTATTTCAAGCAGTCTGAATTTGATCCCTGGCGTACCTATTTTCTTTTGCAGT

CAGAGCTTTTTTGGCACTGATTTCCTTGCCTATCAGGAGGTGAACTCAGGATATAA

>c20439_g1_i2 len=307 path=[572:0-92 23:93-157 88:158-306]

ACTCGCTGGAGCCCTTGTCTGGGGACAGTTCAATGTGCAGTGTCTCCGTGTTGCTGTGTG

TCTGTGTGATCCCAGTAGTGATGTGCATCCATAACGCCCCATGCACGGCGCTTAGTCCTG

CAGGATGCTTCAGACTAAATCCGGAATCTTTCTGTGCAATTCATTGTGATTCCTAACCTG

GAGCACCAGGATGCATACGTATTTCAAGCAGTCTGAATTTGATCCCTGGCGTACCTATTT

TCTTTTGCAGTCAGAGCTTTTTTGGCACTGATTTCCTTGCCTATCAGGAGGTGAACTCAG

GATATAA

>c20441_g1_i1 len=209 path=[165:0-208]

GGGTGTCACTCGTGGGCTGTGTATTGGTGGATGAAGTTGACTCTGAAGTTTCTGCCTTCA

GAGGATAAGATACGAAAAGTCTTGCCAGAGCCCTCAATTTGTTTTTTATTATAAGTATTA

AAAATAACTTCTTCAGTAGCTTTCAAATGCTTTTTCTCCTTCTTAATTGCAAAACATCTG

GAGCGTAAAATTGGAAATAGTTAATAATC

>c20451_g1_i1 len=202 path=[180:0-201]

TTCATTTTGCCATCGGATCGGAAAATTTTTGAACCCTCAATTGAAAAGGTTTCAGGGAAA

TAAAACATTTTAGAATGAAAGATAAACCGATTCCTCCCTCTTTCAGAACTTAGCAAAGCA

TCCTCACATGCTCCATATTCATTTCTGCTCATTGCAGAATCTCAGCTGAATTTTTCTTAT

GTCAAGACAGAGTTCTGCTTGA

>c20462_g1_i1 len=350 path=[328:0-349]

AAAAAAAAAGGAAATTAAAAAATATATATGTTTATATATTAATATTAAAATTTTGAACAC

ATACTGATGAACAGTTAAAAATAAAATAATGAAATTGGCAAATAAAAAAATGTATATGAA

GATTAGAAAATTATTAACATAATATTTAAACAAAATACTGATATATAGTAAAAAATTATA

GAAAATAATTTATTCAATAAATTTAAGTAGTACTGTAACTACACTAAAACAATAAGCATT

AAAAATATTTGCAAATATTTTAAAATATAAATATAAATGGAATATAATTTTATAGCAAAA

AATATGATAATGAAAATAACTGAATGATAAAAAAAAAAAAAAATATGAAA

>c20463_g1_i1 len=385 path=[1:0-325 800:326-329 804:330-384]

TTTTTTTTGCTTTTGTAATTCCTCCTTTGTTACAACGAACCAGAAAGAGGGGATTCCATG

GAAACCAGGAGGTCACAACAGAACCGCGGAGGTGGGGATGGGGGGAGCGGAGCCCCCTCC

TCACCTCCCGGCGGCGGTGGGATGGGAGGGCCGACCCGGTGCTGCTTACGCCGTAAGGAA

CGGCACGGAGGCGGAGCTTTGGGCTCTGTGCTCCGCCAAGCGCTGCTCGGTGGGTTCGAC

AGCACCGGGGGTGTGCGGGGGGGTTGGGGGGGGGAACGTTTCCCATCCCAAAACGCTGCT

GTGGAGCCCTGCAGGCACGCGGCCAGCCAGCCCCAACTGTTCGGATCTGACGGCTCTGCT

GCCGTTCGTGCTATGGGGCTGGCTG

>c20470_g1_i1 len=212 path=[327:0-74 25:75-211]

GGGTGACACTGCTTCTGCACCAGCGGGATCCAGGTGGAGGCCTGCTCCAGCACCTCCTGC

ACCGTCTCATGCCCCAGCAGGTTGGGCAGCCGCATGCTCTGGTACTCGATGCCTCGGCAC

AGCAGCATCGGGGCTGGGATGGGCTTGCAGTTGGAGCGCTTGTAGGAGAAGTCGGGCTCC

CCGAAAGGGAAGAGCCCGGCGGTCGAACCCAG

>c20470_g1_i2 len=213 path=[327:0-74 401:75-212]

GGGTGACACTGCTTCTGCACCAGCGGGATCCAGGTGGAGGCCTGCTCCAGCACCTCCTGC

ACCGTCTCATGCCCCGTTGGTCACGCAGCAGAGCAGCCTGAGCTGGAGCCCGCTCGGCAC

TCCGCTCAGCCCACCGCCCCGCCATGCCGCGCCGCCTCTGCGCCCTGCTCCTGCTGGCCT

CCCAGTGCCTGGGTTCGACCGCCGGGCTCTTCC

>c20472_g1_i1 len=421 path=[399:0-420]

GCCGCGCTATCAGTGCCAAGTGTTTGAATCCCCACGAACCACCTGAGCTCTCCCAGCCCC

AACCTCCTCCCATCGGACCACATCCCCATCTCTTCCCAGGCACAAAAGGACACAGCCACG

CAGGGATCACCCCAGCAGGTGCCTGCATGTCCCCAGGGCACTGGGGCAGCTCCTTTGCCC

TCAAACCCCTCTCCCAGCCCCGGCTGTCTCAGCACATCATCGTAATGAACAAAGCCAAAG

CTCCGGCTCCCATGCACCTGCCCGTGTTTGCTTCGAACCGTGCCTGTTACTATATCCTAG

AGCAAATATACCAAAAGCTGCATCCCTTGTAGCGTCTGCTTTCTATAGAGTGGTTCCCAA

ATCCAGGCACTGACACCAGAGATGCAGCTCAATCCTCAGGCTCAAAGCATCCCCAACCCA

G

>c20473_g1_i1 len=320 path=[255:0-319]

GGGGAGAGCAGCCACAGCACGAAGGCCGTCTTCAGCTCGTAGTAAAAGGGCAGCCAGGGC

AGGAAGATGTCGGTGATGGTCTCCGCGGCCATAAAGAGGGCGAATACGATCCAATACATC

ATCCAGCGGACGTATTCCCGGATGTTTTTGGTCTTCACTGCTTTGTAGGAGGCGTAGGCG

GGGTACAGCATCCCGAACAGCAGCCTCTCCACGTATTTACGGATCTACCCGCGTAGTTCT

GCACCGCTCGTATCTTTATTTCTCCCTTTATCCGCTCATCCGCCTCCTTTGCCATCCGCC

CGTATCCCCGGCGGCGCCCC

>c20478_g1_i1 len=217 path=[297:0-216]

TAATTGCTCTGCTAATTTAATGAATCTCTTCATGCTGCTTCAAATTCCGTTCTGGGGTGT

GAGTGTTGTAGCTGGCAGACTGTGTGCTCCAAGCTAAATGAGAAGCAAAGATGAGTTCTC

ATCTTCAGTTTCTGGATTTTTCTTTTGCGATGTAGTTCACGGGAGGGTCTGACAAAGTTG

CTGTGAATATTGATGCTATACTCAGTTCTAACTCATA

>c20479_g1_i1 len=211 path=[155:0-210]

ATCTGTTGCAGATGTATTTTCTGGTCTTGTATTGTTTTTCCTGTAGCCGACAGTAAGTAA

GCCTGTTCTCGGTGGGTAGGTTGTTCTGTCTGTGAGAACAGTCTTAGACACATAGAGAAT

TAGAGGCCTCCACTAGTTCTAGCTCTGCTGTAAGCTCCTATAGAGTGCTGGGCAACTGGT

CACTGTGTTTTGAGGGCCTTAGGGGCTGGCA

>c20490_g1_i1 len=203 path=[173:0-202]

GTAAGGAGCGGACCATTGCCTTGGTCTCCCTTTGATCCTAACTCATAAGGTCTCATCCGG

CTAATCATCAGTCCTTAGGTAAAGCTTCATGAGTTGTTGCTGCAGCTCTCTCCTCCTTGC

TGGCCCATCCCTTCTGACCTAAAGGGCCTGTATCAGATCCATTACAGTGCTCCAGCAATG

TAAGCTATCAGAATATATTTCTG

>c20496_g1_i1 len=207 path=[411:0-206]

GGAAAAGTCTTAAGTGAGCTGTAACTTTCCTCTTTGCTTTTCCTCCCTCCTCTGTTTTTG

ATGTAGGCTTTAGAATTCCAGGCCAGTTTGGCTGGAGCTCAGATGATCCATTCTGCCTAA

TAAGTGTGTTTGTTTAGGGCTTTAGGTGAATGGTACCTAGTTATTCTGAGCATAAGGAAG

GGAAAATAAATAATTTTGCTATTAAAC

>c20497_g1_i1 len=274 path=[634:0-273]

TGCTTCCACTTTCTTCCTGCTGCCCTCCCAGGAGGGGTCTGAGTGCCGTATGCCGTCGTG

CCCACCTGGAGTGAGTGCAGACCAGGGCAAGTCTTCACGAGCAGCCTGAGGAAATAACAC

TATGGGAAATGAAATGAAGAGCGTATCGCTTGTACTGAATGGGCAAAATGCAAAACCAAG

CCAGTGTGATGTCTCCACACCTATCACCTTTTGGCTTTTACCTAAAATAGGAGAAATATA

AATTTCCTGCATATGAAAAGCACCTTATTATGAG

>c20497_g2_i1 len=214 path=[192:0-213]

ACCTCCCCTCACCAGCAGCAGTACCAGGTGCTGTGTGATACTGCTACCCTTGAACTGATC

CCTGCATGAGTCATCTCCGAGGCTGCCGAAGCTGACAGCTCACGTAAAATCACCGTCCAC

AGATTACATTCAAAGGGAGCTTTAAAATGTTTTCTAATTATTTTTACTTCCACTCCAGTG

AGCACTGTTAATCAAGAGCTGTTTCCAAAACACA

>c20501_g1_i1 len=215 path=[1:0-214]

GGTAACTTTGTGTTTGACGGTGGTGGGAGGGGCAGCGCCCGCGGGTGGCTCCGGGTGCCC

ATCGGGGGAGAGACAGTGTGTGGAGTAAAAGTACAGTGAAATAACGCAAGGAAATAATAA

AATGCGATGATGAAAATAAGAGAATAAAAAAGAACAAGAGAATAAAAAGAACGAGAATAA

AAAACAAAAAACCAAAACAAGAAAACAGAAACAAA

>c20506_g1_i1 len=260 path=[167:0-101 354:102-117 370:118-259]

TTATGCTAATCACTTTGAGGTAAAGTGAAAAAGTTTATGTTGTTTAGCACTAGGAGGGAT

TAGCAGGGACAAAGATAGTTCCTAGGCTTTTTATTTAAAAACATTTTGTATTTATGAACC

AATAACTGCGCGAAGTAATTGGTTCATAAATACAAAATGTTTTTAAATAAAAAGCCTAGG

AACTATCTTTGTCCCTGCTAATCCCTCCTAGTGCTAAACAACATAAACTTTTTCACTTTA

CCTCAAAGTGATTAGCATAA

>c20509_g1_i1 len=218 path=[196:0-217]

TATGGATTCCAGACAAAACACCCCCAGATGAAGCCACTGGAAAACTCACTCCAAGGCCTT

CCAAGACTAACAGCTTCACAGCTATTCTTTAGGGCTGGAGAGGTGTCAGCCCTTTGGAAG

AGATTCTGCTCCCTCGACTCAGAGCCTGAACTCCCAGCTGCCTTGGGCAGGAGCTCTGTA

ATGCGTAATTCAAGGCACTGGGCATACTGAGCATGCTG

>c20510_g1_i1 len=455 path=[559:0-414 17:415-454]

AAAAAAAAAAAAAAAAATTTTAACCATAATTTTTGCTTTAATTACATTTCATATTTATTT

TTTACTTATTGAATATATTGATTTGCTAATAAAACTATATATATATACAGTAAAATAATG

TTAACTCTTTTTGTTATATTTTATAAATAAAACATCTAAAAAAGAGTATAACAATTTAGT

AAACATGTTTTAATTATATAGAACTTAAATCCTTTTTATTTTTTTTAATTTTATGCATTT

GAAATTAAAGATTTTTAACAATTTTCTTTAATTATACACTCATTTCAAGCATTTTTTTAT

TTTTAGAAAGTACATTTTTTTTTAATTTAATTTTATTATATACATAATTACATTAGAAGA

AAATAAATTTAAAATGAATAAAAGCATATATAAATTATTAGAAGTAGGTGTATTAACAGG

TGGCTTTTTTTTTATTTGGTATTTAACAAGTGAAA

>c20514_g1_i1 len=351 path=[1:0-350]

AACCCTAAAGGGCCACTGCTGGTGCCTGTGAGCAGTGGCCCTTTAGGGTTGCCTTAAATA

TCTGCACACTGAAACACCTCAGCACTGACATGCAGCTGCTTATGCTCTTCTGAAAACCCA

GAACTCTGCCTTCAAATTGAAAATCCTTTGGTTTTATCTTACCTTCCTCAGGACTTTGTT

GTCCCTAATGCTAGCAGTAGGGCACAGTGTGAGGGCTGCCACCAGAAGGCAGCCTGCTTT

AGAGAGCAGCTAGTGCACAACTTCTCCCAGCAGCTACAACTCCCACAGCTTTGCAGGAGC

TCAAGCTGTGTTCAGGGCACCCACTGGGCTGTGGCCTCACAGAGCAGCACG

>c20522_g1_i1 len=295 path=[163:0-294]

GTAATTATCCCTTTTTCAACTTCTGAGCCATCCAGATTTCTGAAGTGAGTGTGCAAGAAA

GTATTTCGGTAATTTCTCCAGGCTTCCCTTTATGATTTAGGTGCAATGGTGAGATAAGTG

TTGGCTTCTGATTTACAGAAGCAAAGTTATCGTGCAGTTTACTGAATTGTCTTGTAAGCC

AGGCAATGGTTTTGAATGATTAATATGGCATCATCTTCCTCTTCCCACCTCTGGTTGTTT

CATTTTCAAGGCTGAGAACAACTTGGGGTATTGCTGGGGAGTTCAGATGTTCAGT

>c20529_g1_i1 len=231 path=[375:0-230]
[truncated: 53,146,366 more chars]
